
JOURNAL INFORMATION
==============================
NLM Title Abbreviation: Innov Aging
Journal ID: Innov Aging
NLM Journal ID: 101703706
Journal ID: innovateage

Innovation in Aging

EISSN: 2399-5300
Publisher: Oxford University Press

ARTICLE INFORMATION
==============================
PMCID: PMC7741497
DOI: 10.1093/geroni/igaa057
Article ID: igaa057
Article version: 1
Subjects: Full GSA 2020 Abstract Book Pdf, AcademicSubjects/SOC02600

Program Abstracts from The GSA 2020 Annual Scientific Meeting “Turning 75: Why Age Matters”

Publication date: 2020
Electronic publication date: 2020 Dec 16
Volume: 4
Issue: Suppl 1
Issue title: Program Abstracts from The GSA 2020 Annual Scientific Meeting “Turning 75: Why Age Matters”
First page: NP
Last page: NP
Copyright: © The Author(s) 2020. Published by Oxford University Press on behalf of The Gerontological Society of America.
Copyright year: 2020
License: This is an Open Access article distributed under the terms of the Creative Commons Attribution License (http://creativecommons.org/licenses/by/4.0/), which permits unrestricted reuse, distribution, and reproduction in any medium, provided the original work is properly cited.
License URL: https://creativecommons.org/licenses/by/4.0/

Page count: 969

==============================
ISSN 2399-5300 | ONLINE
Vol. 4 | No. S1

### INNOVATION IN AGING®

An Open Access Journal of
The Gerontological Society of America

**Program Abstracts from
The GSA 2020 Annual
Scientific Meeting
"Turning 75: Why Age Matters"**

OXFORD
UNIVERSITY PRESS

THE GERONTOLOGICAL SOCIETY OF AMERICA®

### INNOVATION IN AGING
An Open Access Journal of The Gerontological Society of America

**Laura P. Sands, PhD, FGSA**
**Editor-in-Chief**
Virginia Tech, USA
230 Grove Lane (0555)
Blacksburg, VA 24061
E-mail: lsands@vt.edu

**Karen Jung, MSW**
**Managing Editor**
The Gerontological Society of America, USA
1220 L Street, NW, Suite 901
Washington, DC 20005
E-mail: ia@geron.org

**Steven M. Albert, PhD, FGSA**
**Deputy Editor-in-Chief**
University of Pittsburgh, USA

**J. Jill Suitor, PhD, FGSA**
**Deputy Editor-in-Chief**
Purdue University, USA

**Christine E. Bishop, PhD, FGSA**
**Associate Editor, Economics of Aging**
Brandeis University, USA

**Howard B. Degenholtz, PhD, FGSA**
**Associate Editor, Long-Term Services and Supports**
University of Pittsburgh, USA

**Min-Ah Lee, PhD**
**Associate Editor, Relationships & Well-Being**
Chung-Ang University, South Korea

**Richard Pak, PhD**
**Associate Editor, Technology**
Clemson University, USA

**Alison Phinney, PhD, RN**
**Associate Editor, Qualitative Research**
University of British Columbia, Canada

**J. Tina Savla, PhD, FGSA**
**Associate Editor, Methodology**
Virginia Tech, USA

**Jennifer Tehan Stanley, PhD, FGSA**
**Associate Editor, Psychological Sciences**
University of Akron, USA

**Roland J. Thorpe, Jr., PhD, FGSA**
**Associate Editor, Minority Health/Health Disparities**
Johns Hopkins Bloomberg School of Public Health, USA

**Elaine C. Wiersma, PhD**
**Deputy Associate Editor, Qualitative Research**
Lakehead University, Canada

### ADVISORY BOARD

**Jacqueline L. Angel, PhD, FGSA**
The University of Texas at Austin, USA

**Toni C. Antonucci, PhD, FGSA**
University of Michigan, USA

**Kenneth Covinsky, MD**
University of California, San Francisco, USA

**Kenneth F. Ferraro, PhD, FGSA**
Purdue University, USA

**Karl Pillemer, PhD, FGSA**
Cornell University, USA

**Wendy A. Rogers, PhD, FGSA**
University of Illinois Urbana-Champaign, USA

**Richard Schulz, PhD, FGSA**
University of Pittsburgh, USA

### PROGRAM, PUBLICATIONS, AND PRODUCTS COMMITTEE

**Ishan Williams, PhD, FGSA, Chair**
University of Virginia,USA

**Tamara Baker, PhD, FGSA**
University of North Carolina at Chapel Hill, USA

**Dana Bradley, FAGHE, FGSA, PhD**
University of Maryland Baltimore County, USA

**Cynthia Brown, MD, FGSA**
The University of Alabama, Birmingham, USA

**Marilyn Gugliucci, PhD, FGSA, FAGHE**
University of New England, USA

**Stephen Helfand, MD, FGSA**
Brown University, USA

**Judith Howe, PhD, FGSA, FAGHE**
Ichan School of Medicine at Mount Sinai, USA

**Judie Lieu**
The Gerontological Society of America, USA

**Robert T. Mankowski, PhD**
University of Florida, USA

**Megan McCutcheon**
The Gerontological Society of America, USA

**Christina Mueller, FAAN, FGSA, PhD, RN**
University of Minnesota, USA

**Viviana Perez, PhD, FGSA**
Oregon State University, USA
Linus Pauling Institute, USA

**Darina Petrovsky, PhD**
University of Pennsylvania, USA

**James Powers, MD, FGSA**
Vanderbilt University, USA

**Philip Rozario, PhD, FGSA**
Adelphi University, USA

**Debra Umberson, PhD, FGSA**
University of Texas, USA

**Danielle Waldron, MS**
University of Massachusetts, Boston, USA

**Deborah Waldrop, PhD, FGSA**
University of Buffalo, USA

**Fellow of The Gerontological Society of America (FGSA)**

### OUP PRODUCTION DEPARTMENT

**Alexandra Reinhart**, *Journal Manager - Production*

### INNOVATION IN AGING
An Open Access Journal of The Gerontological Society of America

### EDITORIAL BOARD

**Kathleen Abrahamson, RN, PhD, FGSA**
Purdue University, USA

**Tamara A. Baker, PhD, FGSA**
University of North Carolina at Chapel Hill, USA

**Scott R. Beach, PhD, FGSA**
University of Pittsburgh, USA

**Sara J. Czaja, PhD, FGSA**
Weill Cornell Medicine, USA

**Adam Davey, PhD, FGSA**
University of Delaware, Newark, USA

**Nancy R. Gee, PhD**
Virginia Commonwealth University, USA

**Megan Gilligan, PhD, FGSA**
Iowa State University, USA

**Ellen L. Idler, PhD, FGSA**
Emory University, USA

**Mary Ann Johnson, PhD**
University of Nebraska Lincoln, USA

**Yoshinori Kamo, PhD**
Louisiana State University, USA

**Giyeon Kim, PhD, FGSA**
Chung-Ang University, South Korea

**Hui Liu, PhD, FGSA**
Michigan State University, USA

**Mariana López-Ortega, PhD, MPP**
National Institute of Geriatrics, National Institutes of Health, Mexico

**Anne Collins McLaughlin, PhD**
North Carolina State University, USA

**David A. Nace, MD, MPH**
University of Pittsburgh, USA

**Marcia Ory, PhD, FGSA**
Texas A&M, USA

**Karen A. Roberto, PhD, FGSA**
Virginia Tech, USA

**Miles G. Taylor, PhD, FGSA**
Florida State University, USA

**Robert Joseph Taylor, MSW, PhD**
University of Michigan, USA

**Noah J. Webster, PhD**
University of Michigan, USA

**Richard Winett, PhD**
Virginia Tech, USA

**Jeremy B. Yorgason, PhD, FGSA**
Brigham Young University, USA

**Anna Zajacova, PhD, FGSA**
Western University, Canada

**Fellow of The Gerontological Society of America (FGSA)**

### Abstracts

GSA Annual Scientific Meeting 2020

Abstracts are arranged numerically by session and in the order of presentation within each session.

Abstracts are published as received.

### SESSION 2801 (PAPER)

### INNOVATIONS IN GERIATRICS AND GERONTOLOGY EDUCATION: NOTES FROM THE FIELD

### ENHANCING BEHAVIORAL HEALTH COMPETENCIES FOR SENIOR CENTER STAFF: LESSONS LEARNED FROM WORKFORCE TRAINING EFFORTS

Bronwyn Keefe,[1] and Jennifer Tripken,[2] *1. Boston University, Boston, Massachusetts, United States, 2. National Council on Aging, Arlington, Virginia, United States*

Increases in the numbers of older adults with mental health and substance use concerns compel us to identify best practices in training to address these issues. Senior Centers are an ideal location for behavioral health education programs as they are the go-to place for many older adults. This session will describe a program funded by The Retirement Research Foundation and offered in collaboration with Center for Aging and Disability Education and Research at Boston University and NCOA to increase senior center staff knowledge and skills. Approximately 250 senior center staff in Illinois, Florida, and Wisconsin completed an online certificate in Behavioral Health and Aging. Results show that 100% of respondents felt that the training was useful for their job; 93% felt that they will be a more effective worker as a result of the training; and 97% felt that the information they learned in the training will make a difference with the people they serve. We held key informant interviews to assess the impact of training and participants stated that their knowledge, skills, and behaviors were influenced by the program. At the organizational level, leaders reported new programming related to behavioral health and revised practices and protocols. This presentation will cover: (1) the extent to which training participants mastered the competencies needed for effective practice; (2) knowledge and skills gained from the training program; (3) Senior Centers' capacity to identify and refer older adults to mental health services; and (4) organizational changes related to behavioral health programming with older adults.

### MY HEART HAS ALWAYS BEEN SET ON PEDIATRICS, AND OTHER INSIGHTS FROM FIRST-YEAR MEDICAL STUDENTS

Jennifer Inker, Sarah Marrs, Madeline McIntyre, Leland Waters, and Tracey Gendron, *Virginia Commonwealth University, Richmond, Virginia, United States*

Senior mentoring programs provide medical students exposure to a community-dwelling older adult mentor with whom they meet multiple times throughout the program. The goal of these programs is to expose students to healthy older adults, increase their knowledge of topics in geriatrics and aging, and increase the likelihood that students will pursue geriatric specialties. Though research findings show that senior mentoring programs have the potential to increase medical students' attitudes towards older adults (Samra et al., 2013) and their willingness to consider working with older patients in the future (McManus et al., 2017), a critical shortage of doctors who specialize in geriatrics still exists. Moreover, the demand for geriatrically-trained physicians is expected to continue to increase (American Geriatrics Society, 2018). In order to develop avenues for successfully fostering interest in and pursuit of geriatrics specialties, we need to fully understand students' perceptions of working with older adults. The purpose of this qualitative content analysis was to explore first-year medical students' (n = 216) perceptions of working with older adults. We asked students to respond to the following reflection at the beginning and end of their Senior Mentoring program: How do you feel about working with older patients after you complete your training? Our findings suggest that while students feel more comfortable with and open to caring for older adults, they do not feel compelled to pursue geriatrics. Themes and sub-categories emerging from the data provide insight into why students continue to dismiss pursuing geriatrics.

### PROFESSIONAL TRAINING AND PERSONAL DEVELOPMENT: LESSONS FROM 10 YEARS OF INTERGENERATIONAL LEARNING

David Steitz, *Nazareth College, Rochester, New York, United States*

This presentation will highlight a ten-year collaboration between the Nazareth College Gerontology Program and St. John's Senior Services – the St. John's Collaborative for Intergenerational Learning (SCIL). Specifically, semester-long intergenerational coursework (Adulthood & Late Life, Issues in Aging, Aging & Community Service) and community-based service projects will be showcased with a focus on design, implementation, assessment, and impact. Emphasis will be placed on the reciprocity of these collaborations, the personal and professional benefits of these exchanges for our students, and the subsequent impact on the community partners and the individuals they serve. Various models of intergenerational learning as well as our new intergenerational residency program will also be discussed.

© The Author(s) 2020. Published by Oxford University Press on behalf of The Gerontological Society of America.
This is an Open Access article distributed under the terms of the Creative Commons Attribution License (http://creativecommons.org/licenses/by/4.0/), which permits unrestricted reuse, distribution, and reproduction in any medium, provided the original work is properly cited.

### USING STORIES TO INTEGRATE GERONTOLOGY CONTENT INTO AN INTRODUCTORY DESIGN (ARCHITECTURE) COURSE

Gail Towsley, Jim Agutter, Antonius Tsai, Jareth Archer, Sarah Sovereen, and Molly Hanrahan, *University of Utah, Salt Lake City, Utah, United States*

We describe how a partnership between design and gerontology introduces students to concepts of legacy, meaning making and empathy in an undergraduate design course. Introduction to Design Thinking is offered through the Multi-disciplinary Design Program at the University of Utah and is a class that explores concepts such as the design process, human-centered design, rapid prototyping and multidisciplinary team dynamics. In collaboration with faculty from design, medicine and gerontology, the course addresses real-world problems by navigating across disciplines such as art, business, engineering, science, and gerontology. Five phases of the design process are introduced: Observation (collecting material), Analysis (finding patterns and insight), Ideation (solution exploration), Refinement, and Implementation (communication). To apply these processes, students engage in a group project where they interact with older adults residing in long term care (e.g., assisted living). Students undergo HIPAA training and didactic lectures covering narrative interviewing and aging content such as long term care and generativity prepare students for interacting with residents. Throughout the semester, students meet with the older adult to understand, gather, construct and then develop a designed artifact that reflects the stories and the memories of the participating residents. The designed artifacts (e.g., memory maps, videos, small books) are presented and given to the participating residents and their families. This project results in rich stories for both the residents and students. Students also gain interviewing and empathy skills necessary for designing compelling artifacts and potentially age-friendly long term care environments where 2 million older adults live.

### SESSION 2802 (PAPER)

### WHAT'S NEW IN DEMENTIA EDUCATION?

### EFFECTS OF DEMENTIA SIMULATION ON NURSING STUDENT'S KNOWLEDGE AND EMPATHY

Michelle Kimzey, and Jodi Patterson, *Texas Christian University, Fort Worth, Texas, United States*

The numbers of people living with dementia are overwhelming. Dementia education is important to prepare nursing students to care for this population. Care for people with dementia requires a better understanding of the reality of dementia. Dementia education must empower nursing students to appreciate, provide care, and support the needs of the caregiver and people with dementia. The purpose of this study was (1) to determine the effects of the dementia simulation on nursing students' knowledge of dementia and empathy for people living with dementia, (2) to explore the level of empathic concern, shared affect, perspective-taking self and perspective-taking other of undergraduate nursing students towards individuals with dementia. A convergent mixed method design was used with pretest and posttest serving as the quantitative arm of the study. A focus group discussion with themes extracted served as the qualitative piece of the study. The convenience sample of 65 undergraduate nursing students. Students completed pre and post-test surveys which included Dementia Knowledge Assessment Tool Version 2, Comprehensive State Empathy Scale, and demographic questionnaire. Content analysis was conducted on focus group responses to qualitative interview questions. Quantitative results reported a significant increase in empathy. Qualitative findings supported the quantitative results. Results revealed the dementia simulation positively impacted students' empathy for people with dementia. This learning activity was innovative and created excitement and intrigue about caring for people with dementia. The dementia simulation experience created an awareness of dementia and ignited nursing students' passion to care for this population.

### MOBILIZING COLLECTIVE ACTION FOR PUBLIC HEALTH APPROACHES TO DEMENTIA EDUCATION

Jennifer Severance,[1] Susanna Luk-Jones,[1] Griffin Melissa,[2] and Glenda Redeemer,[3] *1. UNT Health Science Center, Fort Worth, Texas, United States, 2. Alzheimer's Association, North Central Texas Chapter, Fort Worth, Texas, United States, 3. Tarrant County Public Health, Fort Worth, Texas, United States*

Addressing increasing rates of Alzheimer's disease and related dementias (ADRD) requires public health approaches including prevention, early detection and diagnosis, and outreach to low-income and minority communities facing higher risk and adverse health and economic outcomes. Communities are seeking ways to enhance cross-sector collaboration and overcome underdeveloped relationships and fragmentation that are barriers to effective public health responses. In this exploratory study, we evaluated outcomes of a community-wide effort to mobilize systems-level changes, build public awareness, and increase access to early detection services. A community-based organization, public health department, and academic institution in North Texas partnered to expand ADRD education programs and outreach for underserved communities. Nineteen community health workers were trained to provide brain health and ADRD education programs and refer to financial, legal, and social resources. Through collective action, 371 participants attended 26 education sessions delivered in English and Spanish. Forty-five percent of participants identified as non-white and 61% reported low educational attainment. Participants (n=314) completed post-surveys. As a result of training, 89% of trainees could recognize common warning signs of Alzheimer's disease, 86% understood the importance of early detection and diagnosis, and 96% knew activities promoting cognitive health. Findings revealed strategies to increase collective action such as sharing data, establishing referral methods, and adopting dementia-friendly and age-friendly frameworks. Results show that collective action has the potential to build a community's capacity for targeted ADRD education and improve access to early detection and brain health education for at-risk populations.

### PREPARING STUDENTS TO INTERACT WITH PERSONS WITH DEMENTIA IN A RESEARCH CONTEXT

Roberta Peterson,[1] Elizabeth Avent,[2] Zachary Gassoumis,[1] Jeanine Yonashiro Cho,[1] and Bonnie Olsen,[1] *1. Keck School of Medicine of USC, Los Angeles, California, United States, 2. University of Southern California, Los Angeles, California, United States*

By U.S. Centers for Disease Control and Prevention estimates, the number of older adults with Alzheimer's Disease and related dementias (ADRD) is expected to increase 278% by 2060 to affect approximately 13.9 million individuals. Research is needed to not only improve understanding and treatment of ADRD but to also study its effect on the physical, emotional, and psychological well-being of persons with dementia (PWD) and their care partners (CP). However, due to the diminishing cognitive and functional capacities of PWDs associated with the progression of ADRD over time, research efforts are sometimes hampered by a plethora of potential scientific, logistical, ethical, and emotional barriers. This session will introduce an educational approach used to train students who are interested in conducting in-home research among PWDs and their CPs and share lessons learned through the program's pilot training of undergraduate and graduate students (N=6). Through didactic training, role-playing exercises, and experiential learning processes, trainees are equipped to accompany research project interviewers into the homes of PWD and assist in implementing research protocols. Students receive extensive training in the disease trajectories of ADRD, the impacts of disease on PWDs and their CPs, ways to communicate and interact with PWDs, best practices in promoting the protection and autonomy of human subjects with dementia, and approaches to obtaining quality data for research.

### TEACHING WITH TOONS: DESIGNING A NOVEL BLENDED-LEARNING CURRICULUM FOR COGNITION AND DEMENTIA IN NURSING EDUCATION

Bryan Brown,[1] Gina Kang,[1] Anna Schwartz,[2] Richard Marottoli,[3] and Andrea Rink,[3] *1. Yale School of Medicine, New Haven, Connecticut, United States, 2. Yale School of Public Health, Longmeadow, Massachusetts, United States, 3. Yale University, New Haven, Connecticut, United States*

There is a shortage of learners completing nursing training and pursuing roles in geriatrics and dementia care, possibly caused by ageism, misconceptions, and personal experiences such as with family members, along with uncertainty and discomfort and providing care to this population. Using Kern's six-steps of curriculum design, we set out to design a novel, blended-learning intervention to improve dementia education at a local nursing school. After reviewing the literature, local needs assessments were carried out in the form of stakeholder discussions and semi-structured interviews with a subset of nursing students. Interview results, themes from the literature, and incorporation of the current learning objectives in the existing curriculum, were integrated to create a new lesson plan, including a "flipped classroom" component using 2D vector animation, as well as animation-assisted, interactive, case-based lecture and discussion format. Media was designed through an iterative process including review of content outline, objectives, storyboards, and concept art by stakeholders and content experts throughout the design process. This novel approach to interdisciplinary, blended-learning curriculum design has the potential to improve nursing student attitudes and foundational knowledge about dementia and cognition.

### TRAINING PROGRAM IN POPULATION NEUROSCIENCE OF ALZHEIMER'S DISEASE AND AGE-RELATED DEMENTIAS

Caterina Rosano, *University of Pittsburgh, Pittsburgh, Pennsylvania, United States*

The recent successes of medical science in extending lifespan, with marginal improvements in healthspan, have increased the number of adults reaching very old ages, but also their burden of age-related comorbidities. For these "new" populations of older adults, cumulative exposure to chronic conditions, biological and chronological aging as well as lifelong environmental factors, interact with each other in ways that are both very complex and not yet well understood. Understanding these complex pathways and their contribution to brain aging is fundamentally important to conduct rigorous etiological research into the causes of ADRD. We are also seeing great technological advances in measuring health factors in general and brain characteristics in particular, the application of which is providing ever more precise phenotypes but also very large and complex datasets. Such "big" data require careful sampling designs and analytical approaches infused with an understanding of the condition being studied to effectively produce new knowledge to move research to treatment and prevention. We propose that the successful clinical neuroepidemiological investigators of the future must be able to link comorbidities, environmental exposures, lifestyles, genomics, e.g. host susceptibility, with knowledge of modern technology of neurosciences and measurement of brain disease and data science. We will describe our experience at the University of Pittsburgh in leading a new training program in Population Neuroscience of ADRD. Our curriculum responds to the changing landscape of career pathways, technological innovations, and demographic shifts in the aging population.

### SESSION 2803 (PAPER)

### UPDATES ON INTERPROFESSIONAL LEARNING STRATEGIES

### EXPANDING WORKFORCE CAPACITY TO CARE FOR OLDER VETERANS: THE VA GRECC INTERPROFESSIONAL TRAINING EXPERIENCE

Kathryn Nearing,[1] Sumathi Misra,[2] and Katharina Echt,[3] *1. University of Colorado Anschutz Medical Campus, Aurora, Colorado, United States, 2. Veterans Administration, Nashville, Nashville, Tennessee, United States, 3. Department of Veterans Affairs, Atlanta, Georgia, United States*

The Veterans Health Administration (VHA) contributes more to training healthcare professionals in geriatrics/gerontology than any other entity nationally, with the Geriatric Research Education and Clinical Center (GRECC) network serving as a leader in geriatric-/gerontology-specific interprofessional education. The Associated Health Training (AHT) Program is supported by all GRECCs, training ~427 trainees annually (FY17). Each AHT program brings together a diverse array of trainees – the specific constellation of disciplines unique to each GRECC based on local capacity/expertise. Common to all programs is intentional interprofessional training, aligned with Interprofessional Education Collaborative (IPEC) competencies. In 2016, the VA Office of Academic Affiliations (OAA) administered a 22-item survey to characterize the depth and breadth of geriatric-/gerontologic-specific interprofessional education across GRECCs. Questions explored how AHT programs addressed each of the four IPEC competency domains. Responses were de-identified; at least 2 coders independently applied directed coding to responses. Across GRECCs (n=18), 323 interprofessional training activities were coded, of which 9% were didactic; 27%, clinical; and, 63%, combination. Interprofessional education activities were integrated with profession-specific curricula (65.3%) or featured as part of GRECC-specific core curricula (5.4%). GRECC AHT interprofessional programs involved an average of 11 disciplines. GRECC AHT programs provide a vital infrastructure for building workforce capacity through robust, interprofessional training that engages diverse disciplines across a variety of care settings representing the continuum of care for older Veterans. GRECC and OAA efforts are critical to enhancing the quality, and expanding the capacity, of this workforce to meet increasing needs for patient-directed, team-based care for older adults.

### INTERPROFESSIONAL EDUCATION ACTIVITY TO IMPROVE COMMUNICATION WITH OLDER ADULTS WITH HEARING LOSS

Laura Gaeta, and Tara Sharpp, *California State University, Sacramento, Sacramento, California, United States*

The purpose of this study was to determine how an interprofessional education (IPE) intervention with Communication Sciences and Disorders (CSAD) and Nursing students can affect their ability to communicate effectively with older adults who have hearing loss. As the older adult demographic increases, healthcare professionals must provide competent care, which includes effectively managing hearing-related communication difficulties in an increasingly diverse population. Faculty received IRB approval to conduct a descriptive mixed-methods study to determine knowledge and satisfaction of students completing an IPE activity. Students were divided into teams of CSAD and Nursing students. Students listened to a brief presentation on IPE before they were introduced to a complex case study of an 84-year-old male with age-related hearing loss. We administered a knowledge assessment questionnaire (KAQ) we created regarding communication with older adults before and after the activity. A total of 92 participants in the two programs (n=36 CSAD, n=56 Nursing) completed the KAQ before and after the activity and an evaluation with a Likert-type scale and open-ended questions. CSAD students scored significantly higher than Nursing students on the KAQ at baseline ($F=25.69$, $p<0.001$) and KAQ scores increased significantly ($F=57.04$, $p<0.001$) among both groups from pretest to posttest. The evaluation data indicated students were able to learn other perspectives and found the experience valuable. Based on the improvement in scores on the KAQ and evaluation data, this IPE activity increased knowledge related to communication with older adults with hearing loss and awareness of the roles of other professions.

### INTERPROFESSIONAL EDUCATION: A MODEL FOR ACADEMIC AND COMMUNITY COLLABORATION FOR PROFESSIONAL EDUCATION

Elaine Jurkowski, *Southern Illinois University at Carbondale, Saint Charles, Missouri, United States*

Teamwork and collaboration across disciplines is becoming critically important as we meet the health and human service needs of people growing older and their families. The myriad of competencies, language and tasks specific to each discipline are not easily or intuitively mastered within discipline specific curricula. This presentation aims to provide a model that addresses the curricular needs for course preparation through inter-professional educational strategies. While the traditional IPE components are address, this model also integrates in the training process, education through the lens of the social determinants of health, community collaboration through health and human service networks, population health and public policy. This presentation will lay out the model and articulate specific educational strategies to address each of the dimensions of the model, to include the flip classroom, experiential activities, assessment and intervention tools, panel discussions with community and agency partners and epidemiologic/population health data. This model identifies a unique approach to teaching students and professionals about collaboration across disciplines for the benefits of addressing the needs of an older adult target group. This model also moves the process of teaching interprofessional collaboration and education beyond understanding values and ethics, roles and responsibilities, team care and communication.

### USING A VIRTUAL CURRICULUM TO PROVIDE INTERPROFESSIONAL STUDENT EDUCATION IN ALZHEIMER'S DISEASE

Alyssa Indelicato,[1] Lauren Germain,[2] Liliana Barros Una,[1] Alice Krueger,[3] Ann Ludwig,[3] Sharon Brangman,[1] and Telisa Stewart,[2] *1. SUNY Upstate Medical University, Syracuse, New York, United States, 2. SUNY Upstate Medical University, Syracuse, United States, 3. Virtual Ability, Inc., Aurora, Colorado, United States*

As Alzheimer's disease increases in prevalence among older adults, there is an increased need for health professionals to effectively communicate with patients and their caregivers. Interactions between providers and Alzheimer's patients differ from a typical patient-physician encounter. The project created a virtual learning environment (VLE) to better prepare students to engage with Alzheimer's patients and their caregivers. The VLE includes a clinical space, clinical office, and a patient's home environment for students to practice their health communication skills and to better understand the lived experience. Nursing, MD, and Physician Assistants students were recruited and assigned a virtual provider (avatar). Students engage with a virtual patient (bot) in an exam room and then are directed to engage with a virtual caregiver (bot) in a clinical office. The virtual patient and caregiver have been programmed to respond to questions respectively and provide detailed insight into their lived experiences. After these encounters, the student is asked to react on the information obtained. The student is asked to explore the virtual home environment and triangulate the home experience, the caregiver's perspective, and the patient's responses. We are enrolling students to participate in the project and will complete the evaluation data. Students will be assessed for knowledge, self-efficacy, and their overall satisfaction with the virtual learning environment. We anticipate students will have an increase in each of these areas and will have improved communication skills with this at-risk, older adult population.

### UTILIZING AN INTERPROFESSIONAL VALUE SCALE TO OPTIMIZE TEAM ASSESSMENT

Jennifer Mendez,[1] Atta Illahi,[2] David Cicala,[2] Martha Schiller,[3] and Shanique Brown,[1] *1. Wayne State University, Detroit, Michigan, United States, 2. Wayne State University - School of Medicine, Detroit, Michigan, United States, 3. Wayne State University, Pharmacy & Health Science, Detroit, United States*

Wayne State University Interprofessional Team Visit (IPTV) is a program that introduces medical, nursing, occupational therapy, pharmacy, physical therapy, physician assistant, dental, and social work students to working in a collaborative team during an older adult home visit assessment. This 60-minute visit, students ask questions about the older adult's daily activities, nutrition, medications, family health, and social supports. Interprofessional education is crucial for health care delivery, there is a need to evaluate the engagement of students in teams. The Interprofessional Socialization and Valuing Scale (ISVS), measures self-perceived experiences with collaborative teamwork, including the ability, value, and comfort in working with others. The ISVS tool was used to evaluate educational experiences of students. There are 18 questions pertaining to perceptions of what students have learned from other disciplines. Students respond to each statement using a 7-point scale with 1 = "Not at All" and 7 = "To a Very Great Extent." The Pre and Post data discuss the differences between groups of students in feeling comfortable working in teams. The feedback ensure better care, value, and education of future healthcare practitioners.

### SESSION 2804 (POSTER)

### BUILDING ALLIANCES ACROSS THE GENERATIONS

### "NOT AS BAD AS I THOUGHT": UNDERGRADUATE STUDENTS' PERSPECTIVES ON AGING

Nasreen Sadeq, *University of South Florida, Tampa, Florida, United States*

Ageism remains prevalent in our society and negatively affects older adults. Undergraduate education could be a potential avenue for combating ageism among individuals moving into the workforce. The current investigation is a pilot study in which students enrolled in an upper-level undergraduate course were asked to reflect on how their perspectives on aging had changed following the completion of the course. The course, Psychology of Aging, provides students with an overview of the psychological aspects of aging, including cognitive, physical, and social changes. Consensual qualitative methods were used to explore student responses (N=150) and analyzed using Atlas.ti 7. The results indicated that majority of students had a more positive view of aging following course completion. The three most common themes that emerged were 1) positive aging, 2) living arrangements, and 3) social relationships. For the positive aging theme, students recognized the positive aspects of aging, with many commenting that aging was not as bad as they originally thought. Nearly all the responses in the living arrangements theme reflected students' previous assumptions that most older adults live in nursing homes. In the social relationships theme, students realized that aging does not make people value social connection any less. The findings suggest that providing students with accurate information about aging has the potential to correct their negative views of aging, and highlight the importance of gerontology coursework in higher education, particularly for those pursuing careers in which they will regularly interact with older adults.

### A THEMATIC ANALYSIS OF MENTAL HEALTH STUDENT PERCEPTIONS OF WORKING WITH OLDER PERSONS WITH SERIOUS MENTAL ILLNESS

Stephanie Santos,[1] and Michelle Zechner,[2] *1. Rutgers University, Middlesex, New Jersey, United States, 2. Rutgers University, Piscataway, New Jersey, United States*

Older people with serious mental illness (SMI) are a rapidly growing population that is expected to double in the US by 2030 (Pratt, Mueser, Bartels, & Wolfe, 2013). Negative attitudes towards older adults and people with SMI are reported for medical, nursing, and mental health students, but little research has examined mental health student attitudes about older adults living with SMI (Nochajski, et al., 2009; Smith & Cashwell, 2010). Education can improve attitudes towards vulnerable populations (Frailing & Slate, 2016). This retrospective qualitative study aimed to identify mental health profession student's perceptions of working with older adults with SMI throughout a fifteen-week, online, mental health course, that educated students on the unique needs of older adults with SMI, available community services and resources, barriers to adequate services, and the effects of ageism. Undergraduate (n=13) and graduate (n=3) forum responses were gathered at three time points (Week 1, Week 5, and Week 15) to examine their attitudes about working with older adults with SMI. De-identified responses were aggregated. Conventional content analysis was used to identify themes. Two researchers manually coded aggregated information, and met to reach consensus regarding underlying themes. Content analysis suggested the following themes: negative feelings towards older adults, personal experiences with older adults, generational differences, cultural views of aging, attitudes about aging, and generational differences. Suggestions for future work with mental health students to reduce stigma and encourage them to work with older adults with SMI will be presented.

### A THEORETICAL FRAMEWORK FOR THE DEVELOPMENT OF VIEWS OF ONE'S OWN AGING

Sarah Marrs, Jennifer Inker, Madeline McIntyre, Leland Waters, and Tracey Gendron, *Virginia Commonwealth University, Richmond, Virginia, United States*

Senior mentoring programs have been established that provide medical students exposure to a community-dwelling older adult mentor. The goal of these programs is to expose students to healthy older adults, increase knowledge of geriatrics, and prepare them to care for an aging population. However, even while participating in a senior mentoring program, health professions students still demonstrate some discriminatory language towards older adults (e.g., Gendron, Inker, & Welleford, 2018). In fact, research suggests ageist practices occur, intentionally or not, among all health professions and within assisted living and long-term care facilities (e.g., Bowling, 1999; Dobbs et al., 2008; Kane & Kane, 2005). There is reason to believe that how we feel about other older adults is a reflection of how we feel about ourselves as aging individuals. As part of an evaluation of a Senior Mentoring program, we found that students' attitudes towards older adults were not significantly improved ($t$ (92) = .38, $p$ = .70). To further explore this, we collected subsequent qualitative data. Specifically, we asked students to respond to the open-ended prompt before and after completing their senior mentoring program: How do you feel about your own aging? Our findings have revealed just how complex students' views towards aging and elderhood are, pointing to a need to develop a theoretical framework for how these views are formed. Thus, the results of this qualitative grounded theory study illustrate the stages of development medical students' progress through as they come to accept themselves as aging humans.

### ACROSS GENERATIONS: A UNIVERSITY COURSE PROMOTING INTERGENERATIONAL RELATIONSHIPS

Megan Foti, *Stockton University, Galloway, New Jersey, United States*

In order to support University students in meeting general studies requirements, while also promoting knowledge and understanding related to aging, Across Generations was developed. Across Generations provides students with a basic understanding of intergenerational relationships and the importance of fostering intergenerational collaborations in order to positively influence our communities. Beyond topics in older adulthood, this course helps students learn more about different generations, diverse cultures, and how to interact and form meaningful relationships with those different from them. Students also have an opportunity to interact with older adults and reflect on their experiences. Additionally, they complete a needs assessment and program development activities in order to identify relevant needs and identify ways to address them. In some cases, actual programs are developed and continue after the course ends. Across Generations topics include: introduction to aging and intergenerational relationships, learning from each other and the benefits of fostering intergenerational relationships, public policy, grandfamilies, older adult isolation, cross-cultural considerations, conflict resolution from an older adult viewpoint, and identifying needs and creating opportunities. This presentation shares the course development process, course learning outcomes and topic outline, student experiences and feedback, and recommendations for similar course development.

### ACTIVE AGING FOR UNDERGRADUATE UNIVERSITY STUDENTS: LESSONS FROM STUDENT SEE ME AT 70 COURSE REFLECTIONS

Emily Roberts, *Oklahoma State University, Stillwater, Oklahoma, United States*

Demographic factors tell us that the number of adults age 65 and older will nearly double by 2030. A holistic and proactive approach to providing positive outcomes in aging requires integrated strategies focusing on providing organizational structures to support this demographic shift. Active aging is a framework first developed by the World Health Organization optimizing opportunities for health, participation and security in order to enhance quality of life as people age. Active aging allows people to realize their potential for physical, social and mental wellbeing throughout the life course. This presentation shares outcomes from a university honors active aging seminar entitled Active Aging for L.I.F.E. in which students were provided with information on a wide range of topics in order to build their personal understanding of the relationships between Longevity, Independence, Fitness and Engagement. Student were then asked to develop a final paper entitled See Me at 70, in which they were to use the course content to develop a narrative of power and inspiration about their future self at age 70. What tactics would be used through their lifespan to develop a behavior and belief system which would enable them to live their life to the fullest both physically and cognitively? How would they respond to the challenges of aging and turn them in to opportunities? This presentation shares key themes of the student work, including the relationships between independence and interdependence, the connections between physical and cognitive health, maintaining purpose and facing unforeseen challenges with a positive attitude.

### AGE-FRIENDLY UNIVERSITY CAMPUS INVENTORY

Maria Claver,[1] Adriana Weathersby,[2] Alexandra Wilkinson,[2] and Nicole Smith,[2] *1. California State University, Long Beach, Whittier, California, United States, 2. California State University, Long Beach, Long Beach, California, United States*

An increasing number of older adults are turning to educational institutions to provide career training, intellectual stimulation and social interaction. Response to the educational needs and interests of this emerging population calls for new opportunities and innovative practices of teaching, research, and community engagement that colleges and universities are poised to offer. In Summer 2018, CSULB became the third university in California to be designated an Age-Friendly University. This study aimed to identify strengths and opportunities for growth at CSULB as related to each of the 10 Age-Friendly University principles. This study is part of a larger, national study conducted by researchers from

UMass, Boston. Interviewees were asked to indicate on the Campus Inventory survey tool the activities and programs offered by the university. Data were collected from CSULB administrators through email after a phone introduction and entered into an Excel document for analysis using qualitative methods. Results provided a clear overview of the types of activities and programs available to older adult students and guided the identification of gaps in services, from the perspective of administrators on campus. The results of this CSULB survey will be compared with results from several AFU campuses across the United States. Additionally, results will be compared with a future Campus Climate survey, which will assess the age-friendliness of CSULB from the perspectives of faculty, students and community members.

### AGEISM ON CAMPUS: OPPORTUNITIES TO INCLUDE AGE IN DIVERSITY AND INCLUSION EFFORTS

Cassandra Barragan, and Stephanie Wladkowski, *Eastern Michigan University, Ypsilanti, Michigan, United States*

Diversity and inclusion are essential perspectives on university campuses. In recent years, there has been a nationwide decline in admissions resulting in changes to traditionally FTIAC driven college campuses. An environmental scan was completed at a mid-sized midwestern university to explore age-inclusive barriers and opportunities for change. In-depth interviews were held with 28 EMU stakeholders representing a wide variety of ages in leadership positions across campus. Students aged 40 and above (N=248) were also surveyed about their experiences on campus. Qualitative analysis revealed ageist attitudes about older adults and older students from at all levels of the university. Results demonstrate that initial responses to 'age-friendly' focused on stereotypes of older adults, but attitudes adjusted when reframed as older learners and further refined when older learners were defined as 40 and above. Additionally, there was a distinct disconnect between ageist perceptions towards older adults and older students which highlights the importance of intergenerational opportunities as an approach to combat ageist attitudes on campus. While these barriers require long-term and complicated solutions, participants described the many benefits that older learners bring to enrich the campus. Results of this research revealed opportunities to reframe aging in the context of diversity and inclusion efforts on campus. Adopting diversity efforts to include age can benefit universities in not only admissions, classroom experiences, and connections to surrounding communities.

### DANCE AND MUSIC IN CHILDREN'S LITERATURE: A QUALITATIVE STUDY OF INTERGENERATIONAL SOLIDARITY THEMES

Stephen Fogle, *University of Nebraska Omaha, Omaha, Nebraska, United States*

Intergenerational Solidarity is consistently recognized by the United Nations as a primary focus for work being undertaken to build a society for all ages. This study utilized a qualitative methodology to examine themes of intergenerational solidarity contained within children's literature. Specifically, this study explored intergenerational examples of dance and music shared by older adults and children. McGuire's (2016) Growing Up and Growing Older annotated bibliography for preschool-to-third grade children, which contains over seventy pages of non-ageist children's literature references (N= 411), served as the sample frame for this study. A sample of six story and picture books was selected after inclusion criteria and availability from two public children's libraries considerations were met. Inclusiveness of the present sample is manifested through geographic origin of dance and music traditions as well as the age range, gender, primary spoken language, and kin relationships of the older adult and children characters. Results revealed three intergenerational solidarity themes: 1) a humanizing portrayal of an older adult, 2) common cause, 3) continuity of tradition. This study demonstrates the efficacy of the arts, specifically dance and music, for facilitating intergenerational solidarity. This study identifies three themes that primary school teachers and children's librarians can utilize when selecting reading material about intergenerational solidarity. Finally, this study contributes to decades of pioneering educational gerontology literature focused on combating ageism through development of curricula that stimulate discovery of the elder within.

### HANDS AND HEALTH AT HOME: AN INNOVATIVE INTERGENERATIONAL PROGRAM FOR MEALS ON WHEELS CLIENTS

Meghan Gatward,[1] Rachel Logue,[1] Courtney Vanderlaan,[2] and Susan Brown,[1] *1. University of Michigan, Ann Arbor, Michigan, United States, 2. Michigan Medicine, Ann Arbor, Michigan, United States*

Homebound older adults represent an understudied population who are at greater risk of losing hand strength and manipulation skills that, in turn, can lead to increased disability and cognitive declines (Dayanidhi and Valero-Cuevas, 2014). The Hands and Health at Home program was developed through a partnership with the University of Michigan's School of Kinesiology and Michigan Medicine's Ann Arbor Meals on Wheels program to demonstrate the feasibility of an intergenerational approach to address unmet needs of Meals on Wheels recipients. Undergraduate movement science student trainers were paired with a client who they visited twice weekly for 5 weeks. Students received training, including mock training scenarios, from an interprofessional team with backgrounds in social work, nursing, and neurorehabilitation. Home training protocols were developed using commercially available games and occupational therapy tools with the aim of improving hand function and facilitating socialization. Pre- and post-assessments included hand strength and dexterity, and client-reported measures of physical function and self-efficacy. Feedback from clients and students was overwhelmingly positive with several students indicating that the experience had stimulated interest in pursuing gerontology careers. Changes in quantitative assessments were variable across clients although pinch strength increased significantly in the non-dominant hand ($p<0.02$) and was predictive of measures of self-efficacy ($r=.78$, $p<0.02$). To our knowledge, this pilot program is the first of its kind and demonstrates the value of an intergenerational approach aimed at improving quality of life in Meals on Wheels clients, and may be of benefit for other underserved older members of the community.

### REDUCING AGEISM IN A LIFESPAN DEVELOPMENT COURSE USING SERVICE LEARNING: DOES AN AGING FOCUS MATTER?

Allyson Graf, and Callie Bolling, *Northern Kentucky University, Highland Heights, Kentucky, United States*

A rapidly growing older adult population underlies the importance of reducing ageism. Research shows that college students typically hold negative views of older adults. While education and demonstrations within aging-focused courses contribute to reducing ageist beliefs, attitudes, and behavior toward older adults, contact with older adults amplifies these effects. This study investigated whether integrating contact with older adults into a lifespan development course was effective in decreasing college students' ageism. The sample (N = 104; Mage = 19.94, SD = 3.27) were enrolled in the psychology course, largely as a major requirement for social science majors (51.9%) and health science majors (38.5%). As part of the course curriculum, a portion of the students (n = 57) interacted with older adults to fulfill a service-learning requirement; a control group (n = 47) were not given this option. Knowledge (Facts on Aging), behavior (Relating to Old People Evaluation; aging-related career intentions), and attitudes (Anxiety about Aging and Ambivalent Ageism) were assessed at the beginning and end of the semester. Quantity and quality of contact with older adults was also measured at baseline as a covariate. In a series of ANCOVA analyses, students with aging-related experiences across the term did not differ significantly on any measure compared to those without these experiences, controlling for experience with older adults and baseline assessments. The implications of this finding in the context of research may signal that focus of the course content may be an important moderator of the effectiveness of service-learning experiences with older adults.

### SENIORS AND UNDERGRADUATES MUTUALLY BENEFIT FROM AN INTERPROFESSIONAL SERVICE-LEARNING HEALTH PROMOTION PROGRAM

Britteny Howell, *University of Alaska Anchorage, Anchorage, Alaska, United States*

Although benefits of service-learning and interprofessional education (IPE) have been separately well documented to be effective for students in gerontology and geriatrics courses, few curricula appear to integrate both aspects into a single course for undergraduate students in public health. This poster discusses the development and implementation of a service-learning health promotion program utilizing IPE embedded within two courses in two different departments at a mid-sized university. Students worked in interdisciplinary teams and acquired interprofessional educational learning outcomes while they engaged in their first experiences working with diverse older adults at a low-income, independent-living housing community. Twenty-five students (N=25) each team-taught 2 sessions on nutrition, physical activity, and stress reduction techniques in a 10-week program. Qualitative and quantitative results are presented which demonstrate significant learning outcomes from the students about the health needs of the aging population and increased comfort in working with older adults. Older participants in the program also reported positive health and psychological outcomes from their participation. Limitations, challenges, and next steps are also presented.

### STARTING THEM YOUNG: INTRODUCING HIGH SCHOOL STUDENTS TO COMMUNITY GERONTOLOGY

Marjorie Getz,[1] and Sherri Morris,[2] *1. Methodist College, Peoria, Illinois, United States, 2. Bradley University, Peoria, Illinois, United States*

The Clinical Research Experience Internship Program (CREST) provides participants with foundations in scientific research appropriate for high school students interested in clinical careers in health-related disciplines (for example, nursing). The overall program goal is to provide research and career experiences to individuals from disadvantaged backgrounds, racial/ethnic minorities, and others who are underrepresented in these fields. The focus of this presentation is that part of the CREST program that has been ongoing since 2013. One program mentor has provided an internship experience to 22 high school students training in and work experience with community-based programs designed to improve health for older adults (identified as 'community gerontology'). This poster presentation describes some of these experiences (e.g., preparation of caregiver support materials, preparation of nutrition based materials for congregate meal sites for older adults, coaches' training and program implementation of several evidence-based community programs). Students have worked with older adults in senior housing facilities, supported housing complexes for veterans, and congregate meal sites for older adults. Because of the program experience, the CREST program helps dispel common stereotypes about older adults and encourages students exploring possible clinical career options to consider focusing on older adults as client populations. Program components are described which can allow conference participants to decide on the applicability of this type of programming for their own communities. Qualitative data are presented that provide insights into these experiences as these influence choice of college major and projected career paths and attitudes about working in community-based healthcare with older people.

### THE AGE-FRIENDLY UNIVERSITY: THEORY AND PRACTICE IN INSTITUTIONAL TRANSFORMATION

Phillip Clark, and Skye Leedahl, *University of Rhode Island, Kingston, Rhode Island, United States*

Growth in the network of Age-Friendly Universities (AFU) signals the importance of making higher education more age inclusive. Commitment to AFU principles creates opportunities for universities to develop new initiatives and activities that embody them. However, changing academic culture can present unique challenges as well as opportunities. Theories of organizational change help in developing strategies promoting greater involvement of older adults in communities traditionally focused on younger adults. The University of Rhode Island's experience in continuing to develop as an AFU illustrates the complex challenge of transformation in institutions typically slow to change. This presentation focuses on the following three elements. First, uncovering and confronting ageism in academic settings is a critical first step in opening up the campus community to students of all ages. Second, identifying champions who can advocate for change in different institutional segments is an essential element in expanding the AFU movement. Third, promoting intergenerational programs helps to build bridges between traditional age and older students, particularly emphasizing the contributions that older adults can make to the academic enterprise. Implications for expanding the AFU network while focusing on individual institutions include the following: (1) using theory to drive practice in an intentional and strategic fashion; (2) identifying factors opposing change, particularly since they are usually hidden and not widely recognized; (3) developing a strategy to address these barriers, especially one tailored to the unique institutional context; and (4) recognizing the larger social, economic, and political forces generally in higher education that establish the context for AFUs.

### USING THE AGE-FRIENDLY 4MS FRAMEWORK TO REDESIGN A COMMUNITY CLINICAL EXPERIENCE

Margaret Avallone, and Staci Pacetti, *Rutgers University-Camden, Camden, New Jersey, United States*

The Age-Friendly 4Ms Framework (IHI/Hartford Foundation) was used to redesign an existing undergraduate nursing community experience, teaching students how to evaluate what matters to the individual, medications, mentation, and mobility. As part of the NJ Geriatric Workforce Enhancement Program (NJGWEP), a 5-year grant supported by DHHS-HRSA, ten senior nursing students joined a team of bilingual social workers, APNs, and a PharmD in an affordable housing urban highrise. This paper will describe the implementation and evaluation of a redesigned clinical experience using the 4Ms framework. Students visited older residents with bilingual social workers, performed health assessments and developed person-centered plans of care. Students presented resident cases during weekly interprofessional conferences to promote team collaboration and planning. Residents who screened positive for dementia were referred to an interprofessional Memory Assessment Program. Medications were reviewed using the Beers criteria, reconciled, and referred to primary care providers if appropriate. Fall risk was assessed and managed using the STEADI toolkit (CDC). Students were evaluated on attainment of geriatric-specific competencies, including medication review, cognition and depression screening, and fall risk assessments, by direct observations and interview. Following the semester-long experience, students completed a retrospective pretest/posttest survey to evaluate achievement of objectives based on the 4Ms. Mean scores for achievement of learning objectives ranged from 4.7-4.9 on a Likert scale of 1-5. Students identified barriers that older diverse individuals face when managing chronic health problems in the community. Students also valued the partnership with the social workers, stating, "We learned from each other."

### SESSION 2805 (POSTER)

### DEMENTIA EDUCATION AND TRAINING STRATEGIES

### A LONGITUDINAL AGING AND END-OF-LIFE CARE CURRICULUM FOR MEDICAL STUDENTS USING THE GERIATRIC 5MS FRAMEWORK

Andrea Schwartz,[1] and Kristen Schaefer,[2] *1. Harvard Medical School, VA Boston, New England GRECC, Boston, Massachusetts, United States, 2. Dana Farber Cancer Institute, Brigham & Women's Hospital, Harvard Medical School, Boston, Massachusetts, United States*

Medical student training in geriatrics and palliative care is critical to prepare them to care for older adults and those facing serious illness. We created a longitudinal Aging and End of Life Care Curriculum at Harvard Medical School, using Kern's Curriculum Design Model. We conducted a focused needs assessment survey with course and clerkship directors, then implemented curricula based on the AAMC and Hartford Foundation's 26 learning objectives in Geriatrics (Leipzig et al, Acad Med 2009), and "Raising the Bar for the care of seriously ill patients" which established competencies for medical students in palliative care (Schaefer at al, Acad Med 2014). We structured the curricular content to enable spaced learning, using the Geriatric 5Ms framework of Mobility, Mind, Medications, Multi-complexity and Matters Most (Tinetti at al, JAGS 2017), which aligns with the Age Friendly Health Systems Initiative priorities. Students participate in trainings on Delivering Serious News and Goals of Care Conversations, structured home visits with older adults, and clinical reasoning sessions focused on falls, delirium and polypharmacy risk reduction. The curriculum includes interactive, case based and jigsaw learning, as well as flipped classroom learning. Students are evaluated using a three part longitudinal Objective Structured Clinical Examination with an aging patient, which demonstrates an increase in medical student clinical skills in geriatrics. Individual sessions of the curriculum demonstrate increases in student knowledge of and attitude to geriatrics; longitudinal assessment is ongoing to ensure that students graduate ready to care for an aging society with competence, knowledge and compassion.

### AN ALZHEIMER'S CURRICULUM TO EDUCATE THE CURRENT AND FUTURE PUBLIC HEALTH WORKFORCE

Taylor Kennedy,[1] Molly French,[2] Linelle Blais,[1] and Nia Reed,[3] *1. Emory University, Atlanta, Georgia, United States, 2. Alzheimer's Association, Washington, District of Columbia, United States, 3. Centers for Disease Control and Prevention, Atlanta, Georgia, United States*

Alzheimer's disease is the 6th leading cause of death among adults in the United States and the 5th leading cause for those aged 65 and older. Nearly 14 million Americans will be diagnosed with Alzheimer's dementia by 2060, but the public health workforce is struggling to meet current demands. As the older adult population continues to grow, the public health sector will need to ensure a sizable and competent workforce is prepared to meet the needs of those living with dementia as well as their caregivers. In support of national efforts to promote and ensure a competent workforce, the Alzheimer's Association, Centers for Disease Control and Prevention, and Emory University developed "A Public Health Approach to Alzheimer's and Other Dementias" (ADOD) curriculum. The free, introductory curricular resource was first piloted by faculty and students at undergraduate schools of public health across the country; however, due to its broad applicability the curriculum has since been updated and expanded to educate graduate students in schools of public health, students in related disciplines, and practicing public health professionals. The curriculum provides an introduction to ADOD as a public health crisis, basics of dementia, the role of public health in addressing the epidemic, and the creation of dementia-friendly communities. The purpose of the curriculum is to educate future public health workforces about ADOD; encourage the current public health workforce to apply knowledge to practice; and seek to improve health outcomes for those living with dementia, as well as their caregivers.

### AN INNOVATING TOOL TO ENHANCE THE ORAL HEALTH OF OLDER ADULTS WITH OR WITHOUT DEMENTIA THROUGH THE 4MS MODEL

Maryam Tabrizi, and Margo Melchor, *The University of Texas School of Dentistry at Houston, Houston, Texas, United States*

Oral health of the elderly is the most neglected area of healthcare by many providers across healthcare disciplines. Often due to lack of Medicare funding and affordability of the elderly that leads to a constant high cost of non-traumatic dental related emergency department visits. By 2030, the number of adults in the USA will increase to 74 million. Over 22 million (30%) of older Americans will need specialized geriatric care. Simultaneously, in 2025, a national shortage of medical geriatricians will be close to 27,000 full time positions. This shortage has a greater impact on oral health often due to limited access, affordability and shortage of trained dentists to manage patients with special needs, particularly those with cognitive declines. There is an urgent need for an innovation for workforce enhancement nationwide. Innovation that can create a network of age-friendly dental clinics could be based on the 4Ms model-Mentation, Mobility, Medication, and Matters most. Project ECHO is a telementoring guided practice model that revolutionizes health education and increases workforce to provide best-practice specialty care by reducing health disparities. By utilization of the Project ECHO among community dentists, this makes transformation richly effective. Training oral health providers for utilizing the project ECHO model is a logical response for the shortage and to increase oral health access. Project ECHO trains general dentists & dental hygienists to provide specialty care services. Meaning the elderly can obtain the care they need in the right place, at the right time, with better treatment outcomes and reduces costs.

### EFFECTS OF ANIMAL-ASSISTED THERAPY ON VERBAL COMMUNICATION AND AGITATION IN ADULTS WITH DEMENTIA IN LONG-TERM CARE

Jenilee Woltman, and Ladan Ghazi Saidi, *University of Nebraska Kearney, Kearney, Nebraska, United States*

The effects of Animal-Assisted Therapy (AAT) on verbal communication and agitated behaviors of older adults with mild to severe dementia were examined. We conducted a pilot study at a long-term nursing facility in rural Nebraska on five residents with various stages of dementia and agitation (over two reported incidents of agitation within two months). The study consisted of four sessions of assessment with and four sessions without the presence of a certified therapy dog. The assessment battery included a Mean Length of Utterance (MLU), and responding to a Picture Recognition Activity (PRA) task to assess the production and comprehension in verbal communication respectively. Agitation Behavioral Scale (ABS; Corrigan, 1989), and a General Care Survey (GCS) were used to measure agitation and agitated behavior. The results showed statistically significant differences in the performance of all five participants when the dog was present as compared to when it was absent. We observed an increase in verbal communication (57.4% MLU; 28% PRA), and a decrease in agitated behaviors. This pilot study brings evidence that AAT can be a cost-effective, non-pharmaceutical therapy approach for individuals with dementia to decrease agitation and increase communication. References: Corrigan, J. D. 1989. Development of a scale for assessment of agitation following traumatic brain injury. Journal of Clinical and Experimental Neuropsychology, 11: 261–277.

### MANAGING OLDER ADULTS WITH COGNITIVE IMPAIRMENT: AN INTERPROFESSIONAL, STANDARDIZED PATIENT APPROACH

Shaun Varrecchia, Carol Maritz, Colleen Maher, and Megan Strauss, *University of the Sciences, Philadelphia, Pennsylvania, United States*

Several professional organizations have called for increased preparation of health professionals capable of working with older adults, including those with cognitive impairment. Standardized patients (SP) are often used in interprofessional education (IPE) in the health professions, but limited data exists to support their use when teaching about the care and management of older adults with cognitive impairment. The purposes of this project were to: 1) develop, implement, and assess an interprofessional standardized patient exercise involving physical and occupational therapy students and 2) to evaluate students' perceptions of a SP encounter on relevance and utility to patients with cognitive impairment. 88 students representing physical therapy (DPT) and occupational therapy (DrOT) were assigned to interprofessional teams to evaluate an SP portraying an older adult with cognitive impairment. At the conclusion of the session the SP provided the group formative feedback. Student teams then completed an assignment to develop a collaborative intervention plan and addressed questions about roles and responsibilities and communication/teamwork. Pre-/post- surveys focusing on the knowledge of roles and responsibilities, communication, and teamwork were completed by all students. Students also completed an evaluation about the SP experience. Results demonstrated student agreement to understanding the role of the other's profession improved 28.67%; being comfortable communicating with the geriatric population improved 27.31%; and working in interprofessional teams can improve geriatric patient care improved 32.11%. These findings demonstrate that use of SPs has several advantages in teaching students how to work and communicate with individuals with cognitive impairments as an interprofessional team.

### TEACHING FAMILY NURSE PRACTITIONER STUDENTS TO PROVIDE MENTAL HEALTH CARE TO OLDER ADULTS

Victoria Grando, and Roy Grando, *Saint Luke's College of Health Sciences, Columbia, Missouri, United States*

In recent years, FNPs have been challenged to deliver mental health services in the primary care setting. Over half of mental health services are provided in primary care, and one-quarter of all primary care patients have a mental disorder. Moreover, 20% of older adults have a mental or neurological disorder often not diagnosed. Nationally, it is estimated that 17% of older adults commit suicide, 15% have a mental condition, 11% have dementia, and 5% have a serious mental condition. There is a paucity of adequately prepared primary care providers trained in geropsychiatric treatment. A didactic course was developed to instruct FNP students in the skills needed to provide mental health treatment in primary care. We discuss mental illness in the context of culture to ensure that treatment is congruent with a patient's unique cultural background and experiences. This shapes the patients' beliefs and behaviors that influence the way they view their condition and what they perceive as acceptable solutions. We then go into detail about the common mental conditions that older adults exhibit. Through the case study method, students learn to identify the presenting problem, protocols for analyzing the case, which includes making differential diagnoses and a treatment plan including initial medications, non-medical treatments, and referral. Students are introduced to the DMS-5 to learn the criteria for mental health diagnosis with an emphasis on suicide, depressive disorders, anxiety disorders, bipolar disorders, substance use disorders, and neurocognitive disorders. We have found that students most often misdiagnose neurocognitive disorders.

### THE DEVELOPMENT AND EFFECTIVENESS OF A SELF-EFFICACY ENHANCEMENT PROGRAM FOR OLDER ADULTS WITH MCI

Jungeun Lee, *Cheju Halla University, Jeju, Republic of Korea*

Purpose: This study aimed to develop a self-efficacy enhancement program and examine its effectiveness in enhancing dementia preventive behaviors by improving cognitive function, dementia knowledge, and self-efficacy, and reducing depression of older adults with mild cognitive impairment (MCI). Methods: An equivalent control group pretest-posttest design was conducted at an advanced general hospital in Seoul. Participants of older adults with MCI visiting clinics were randomly allocated to an experimental group (EG, n=16) and a control group (CG, n=16). The EG was provided with a 8-week intervention (60-minute, weekly) utilizing self-efficacy enhancement strategies and the CG received the usual care. The intervention was an integrated configuration making up of physical, cognitive, and emotional activity and followed by a 4-week maintenance during which both groups engaged in self-learning at home with a dementia preventive guidebook. Outcomes were evaluated at pretest, 1st (8th week), 2nd (10th week) and 3rd posttest(12th week). Results: There were significant differenes in cognitive functions, dementia knowledge, self-efficacy, and dementia preventive behaviors, but not in depression between two groups over the time. Regarding cognitive function' subdomains, significant differences were observed for visuospatial/executive, attention, language, and delayed recall. Conclusions. The integrated intervention consisting of physical, cognitive, and emotional activities, not a simple merger of single intervention focused on cognitive reinforcement, was effective in improving their cognitive functions, dementia knowledge, self-efficacy, and dementia preventive behaviors. It suggests that this program can be utilized as an education program to prevent dementia for MCI in dementia support centers, public health centers, clinics, and hospitals.

### THE PREVENTING DEMENTIA MOOC: CONTRIBUTION TO FIRST NATIONS' HEALTH AND WELL-BEING

Lynette Goldberg,[1] and Dianne Baldock,[2] *1. University of Tasmania, Hobart, Tasmania, Australia, 2. CHAC, Smithton, Tasmania, Australia, United States*

Dementia is a global public health issue. First Nations people are at increased risk due to complex intergenerational factors grounded in inequalities in health services and economic and educational opportunities. While there is yet no drug-related cure for this progressive and terminal neurological condition, evidence confirms that increased understanding of dementia and modification of lifestyle factors can reduce risk. The primary potentially modifiable risk factors are not completing secondary school, midlife hypertension, obesity, type II diabetes, depression, physical inactivity, smoking, hearing loss acquired after the age of 55 years, and social isolation. Inherent in these factors is stress, affecting mental health. Addressing these factors globally could prevent or delay over 40 million cases of dementia. The free Preventing Dementia Massive Open Online Course (PD MOOC) is a globally recognized 4-week course that aims to build self-efficacy in knowledge and management of modifiable risk factors. The course has reached over 68,000 people world-wide and is rated highly; however, its contribution to First Nations communities has not yet been investigated. We describe the content of the PD MOOC, report on its impact in a cohort of older Aboriginal people (≥ 50 years of age) in Circular Head, Tasmania, Australia six months after course completion, and emphasize the importance of including traditional approaches to healing. We describe a protocol in which cultural determinants of health can be infused into the PD MOOC and evaluated to promote health and well-being globally for older First Nations people.

### SESSION 2806 (POSTER)

### RESEARCH, EDUCATION, AND TRAINING INNOVATIONS

### ASSESSMENT OF AGING AND DISABILITY CONCENTRATION IN A MASTER'S PROGRAM

Kristine Mulhorn, *Drexel University, Philadelphia, Pennsylvania, United States*

A concentration in aging and disability is an alternative concentration in our Master of Health Administration (MHA) program. Students to take both of their electives in this area as well as completing their residency in a post-acute care setting. To ensure students meet the learning objectives, the following assessment plan. First, a curricular map of the areas"" of competency within our program are outlined and then the content and areas of assessment are considered based on whether the competencies are "Introduced", "Reinforced," and "Mastered." Next, the key assignments in each course within the concentration were aligned with the Program Objectives for the MHA Program. Another assessment strategy involves the competencies of the on-campus residency. A comparison of the competencies of the residency for those students who conducted their residency in the acute care setting to those competencies of the residency for the students who completed their residency in a post-acute setting. A final method for assessment is a review of the evaluations and the final paper for the residency. The residency is a capstone project in which students conduct analysis based on the operations management factors within the facility.

### CONTINUING EDUCATION FOR GERONTOLOGICAL SOCIAL WORK: FINDINGS FROM POST-GRADUATE ADVANCED PRACTICE TRAINING

Raza Mirza,[1] Alison Kilbourn,[1] Samir Sinha,[2] and Jessica Hsieh,[3] *1. National Initiative for the Care of the Elderly, Toronto, Ontario, Canada, 2. Sinai Health, Toronto, Canada, 3. University of Toronto, Toronto, Ontario, Canada*

The social work profession aims to help all individuals, families, and communities enhance their overall well-being. While gerontological social workers primarily work with seniors, they are often tasked with addressing the needs of not only their senior client, but the client's network as well. Social workers are also asked to stay current with respect to new legislation, policy, and systemic changes to help their clients. Thus, gerontological social workers often need to obtain advanced practice education in a number of areas, as related to gerontology. Responding to this gap, the National Initiative for the Care of the Elderly (NICE) and Sinai Health offered a series of innovative advanced practice gerontological social work courses - hybrid online and in-class - to those looking to improve their knowledge and competencies in the following five identified 'high impact' areas: (1) Medical Assistance in Dying (MAiD); (2) cultural competence and LGBTQI2S; (3) mental health; (4) legal issues and aging; (5) dementia. Participants in the courses completed pre- and post-surveys assessing knowledge, attitudes and competencies with the subject matter, with responses helping to improve understanding of how to provide the most appropriate resources for those who care for older adults and how to better shape future education/training programs. Findings suggest that gerontological social workers may benefit from 'tailored refresher courses' that bridge knowledge and practice gaps, that the optimal update time may be every three years, and that clinicians benefit from being trained by interdisciplinary teams, rather than by someone of the same profession.

### DEVELOPMENT OF SYNTHETIC HEALTH RECORDS TO SUPPORT URBAN PLANNING FOR HEALTHY AGING

Yuezhong Liu, and YIn Leng Theng, *Nanyang Technological University, Singapore, Singapore*

Urban planning for healthy ageing is about planning for ageing population, which considers the needs of older adults and communities during the planning process and the implications of decisions for human health and well-being. However, access to real electronic health record (EHR) data is hindered by legal, privacy, security, and intellectual property restrictions. The lack of freely distributable health records become one important issue for healthy ageing urban planning. This research develops a source of synthetic health records based on reviewed and meta-analysed evidence on the association between built environmental characteristics related to lifestyle chronic diseases for urban planning. Type 2 Diabetes Mellitus (T2DM) is used as a case study for proof of concept. This research methodology includes three steps: 1) Review and meta-analyse of the individual and built environmental variables related to the prevalence of T2DM. 2) Develop agent-based modelling and simulation for synthetic health records. 3) Evaluate the simulation result with standard healthcare file format in Geographic Information System (GIS) application. The pilot validation compares the annual prevalence of T2DM by age group and ethnicity with the public available health data. The simulation results roughly approximate age, gender and racial group at diagnosis curves (R2 = 0.876), it correctly generated more than 90% of patients for the all age group in Singapore. As a summary, these pilot validated synthetic records could be used as a risk-free (no privacy & security issues) data for supporting urban planning for healthy ageing.

### EXAMINING THE ROLE OF AGING EDUCATION: EDUCATIONAL THEMES ACROSS 2013-2019 GSA CONFERENCE PRESENTATIONS

Lisa Hollis-Sawyer, *Northeastern Illinois University, Chicago, Illinois, United States*

Gerontology education and the goal of "geriatric competence" are considered invaluable within the field but barriers exist in communicating ideas and training needs across areas of specialization. The main challenge of higher education institutions throughout the world is to develop professionals capable of understanding and responding to the current issues of diverse aging populations. The specific focus of the presentation will be the examination of aging education "themes" within and across different conference sections' presentations in GSA conference proceedings across the last six years. Specifically, this presentation will review the outcomes of qualitative content analyses from several GSA conferences' research presentations regarding the role of education across different disciplines in the gerontology field. Thematic analysis of several past years GSA conference programs of 2013 through 2019 yielded some of the following education-related themes (sample): theme #1: community education for clients, practitioners and applied service providers, theme #2: intergenerational educational mentoring/programming among researchers and practitioners in the field, and theme #3: professional knowledge updating for geriatric professionals. The implications for increasing emphasis on ongoing geriatric education for all professionals in the field and those they serve (older adults, families, and community members) will be discussed. This presentation offers insights regarding trends in educational issues within the field as well as the unique valuation of gerontology education within different areas of field specialization. The importance of examining different areas of specialization exists because different aging-related disciplines can share knowledge and resources in providing an integrated array of educational and training options.

### FOCUSED ETHNOGRAPHY IN GERONTOLOGICAL RESEARCH AND EDUCATION

Susan Patton, *University of Arkansas, Fayetteville, Arkansas, United States*

Gerontological researchers often use qualitative methods to understand beliefs, values, and shared practices. Focused ethnography (FE) has emerged as a form of qualitative research focused on a specific issue or shared experiences in sub-cultures in specific settings, such as older adults experiencing chronic illness. However, examples of FE in gerontological research literature are few and there is limited methodological guidance on using FE. This presentation provides descriptions of FE based on existing literature and experience in ethnographic research. A systematic review of extant literature was conducted using CINAHL Complete, MEDLINE Complete (Ebsco), and PubMed. Published studies using FE are summarized to demonstrate the methodological foundation and the versatility of FE in exploring health issues in different populations and specific groups of older adults. Nursing students completing a course in chronic illness were trained to conduct an FE on one of the patients in their care. Students were provided a semi-structured interview guide based on the eight questions in Kleinman's Patient's Explanatory Model of Illness. The focused ethnographies were then analyzed by qualitative content analysis. Beliefs older adults hold about their chronic illness fell into three categories: regretting lifestyle choices, grieving loss of self, and finding a "new normal". Focused ethnography is a relevant research method that can be used to understand health behavior of older adults and can be used to teach and evaluate cultural competency in nursing students.

### HUDDLE UP! A COMPREHENSIVE APPROACH TO IMPROVING INTERDISCIPLINARY HUDDLE IN A VA GERIATRIC PRIMARY CARE CLINIC.

Juliana Wilking-Johnson,[1] and Joleen Sussman,[2]
*1. University of Colorado School of Medicine, Aurora, Colorado, United States, 2. Rocky Mountain Regional VAMC, Aurora, Colorado, United States*

Spending time in brief meetings or "huddles" is associated with greater job satisfaction and less burnout, especially when team members have mutually agreed upon goals and can participate in decision-making. However, huddles are often unproductive and may have opposite the intended effect if not tailored to the specific team involved. We sent a survey to all members of our VA Geriatric primary care team (including geriatric medicine fellows, social work, psychology, audiology, pharmacy, faculty and support staff) asking them to rate our huddle's impact on their stress level, efficiency, learning, preparedness and feeling supported at work. Responders indicated if they received needed information and if they understood what information was needed from them. We then held a team meeting to establish mutually agreed upon goals, expectations and organization of huddle, which were then reinforced with visual and timer reminders. After 6 weeks utilizing the new format, we administered a post intervention survey assessing the impact of the change. The initial survey revealed that the geriatric medicine fellows had worse ratings than other trainees, staff, and faculty. Fellows were more likely to say that they did not know what information was needed from them; and they did not receive information needed from others. The follow up survey showed improvement in all scores among geriatrics medicine fellows and allied health professionals, including 100% of respondents indicating they receive needed information. Overall, comments regarding the intervention were positive, demonstrating that a structured, organized huddle tailored to a specific team, can be beneficial.

### IDENTIFICATION OF SARCOPENIA IN AMAZONIAN ELDERLY

Myrian Faber, *University of the State of Amazonas, MANAUS, Amazonas, Brazil*

This research has applied the SARCF to identify the presence of sarcopenia in 236 women aged 66.27 ± 5.76 year from Amazonas, who have attended activities aimed at the elderly. The SARCF analyzes five objective variables for assessing sarcopenia, related to the functional pattern (strength, walking, sitting and getting up from a chair, climbing stairs and falls). Functional patterns are scored to independence = 0, some dependence = 1 and a lot of difficulty or functional disability = 2. Regarding falls during the year; none = 0, ≥ 1 ≤ 3 = 1 and ≥ 4 = 2. Values ≥0≤4, suggest absence of sarcopenia, ≥4≤10 sarcopenia. Results has revealed 100 (42.37%) elderly women without difficulties to carry objects weighing 5 kilos, 130 (55.31%) have presented some difficulty and 6 (2.55%) couldn't do it so. As for walking, 108 (45.76%) haven't needed help, 128 (54.23%) have needed some help. Regarding to sit and get up from a chair, 17 (7.20%) haven't needed help, 218 (92.37%) have needed help and 1 (0.42%) couldn't do it. Climb stairs 17 (7.20%) haven't needed help, 173 (73.30%) have needed help and 46 (19.49%) couldn't do it so. Concerning about falls, 150 (63.55%) elderly women haven't fallen, 57 (24.15%) have fallen between one and three times and 29 (12.29%) have fallen more times. The final SARCF score has revealed a sarcopenia prevalence in 54% of the surveied population; CI = 0.30, CORREL = 0.90 (54.05%) sarcopenic; 6 (2.55%) pre-sarcopenia, 24 (18.9%) sarcopenia, 24 (18.9%) severe sarcopenia.

### INSPIRING THE STUDY OF GERONTOLOGY AMONG MILLENNIAL STUDENTS: A RURAL PERSPECTIVE

Elaine Jurkowski, *Southern Illinois University at Carbondale, Saint Charles, Missouri, United States*

The study of gerontology across different generational cohorts is not static but rather dynamic. As we consider teaching gerontology to different age cohorts, what similarities and differences do exist? How do the educational needs within higher education differ across the different cohorts of the workforce? This presentation aims to identify and present specific learning needs and styles of the millennial cohort, as compared to other generational cohorts with the effort of building educational strategies for this workforce cohort. The presentation will also address the unique needs that a rural workforce faces and how to address these within the millennial cohort. Strategies such as online learning, flip classroom, experiential strategies and research projects/practicum will be addressed with a specific focus on the engagement of the millennial student. These strategies are all described within the context of teaching gerontology through a specific Certificate in Gerontology program located on a rural campus setting, but meeting the educational needs of people across the country.

### INTERPROFESSIONAL HEALTHCARE EDUCATION ON AGING INCREASES STUDENTS' CONFIDENCE IN MEDICATION MANAGEMENT SKILLS

Laura Finn, and Deborah Summers, *University of the Sciences, Philadelphia, Pennsylvania, United States*

Students studying for health care professions have limited opportunities to learn about medication use and aging in an interprofessional experience. Health Care students who interact in a simulation of age-related sensory changes can identify adaptations for safe medication use and counseling necessary to promote healthy aging. This research assessed the impact of a simulated team experience on pharmacy and physician assistant students' confidence in understanding age related changes and in learning adaptations to promote safe medication use for older adults who may experience those changes. 63 pharmacy and 113 Physician Assistant students participated in 2-hour Interprofessional Education (IPE) sessions. The teams of pharmacy/physician assistant students utilized glasses to simulate changes in vision and gloves to simulate conditions of arthritis and neuropathy which increase in prevalence with age. Teams practiced skills of medication counseling and empathy towards their peers experiencing the simulations and learned medication administration adaptations for aging well. Pre survey results show a deficit of Pharmacist-Physician Assistant IPE with less than 20% of students reporting a strong understanding of the other profession's role in developing an older adult's care plan. Post survey results demonstrate an increase in students' confidence in both understanding how sensory impairments may affect a patient's ability to properly administer medication and confidence in counseling older adults on safe medication use. Descriptive data on learning in Interprofessional teams, Pre/Post comparison data and application to students studying other majors will be presented.

### MEDICAL STUDENTS' LONGITUDINAL LEARNING WITH OLDER ADULTS: GERIATRICS EDUCATION MENTOR PROGRAM

Marilyn Gugliucci, and Victoria Thieme, *University of New England College of Osteopathic Medicine, Biddeford, Maine, United States*

The University of New England College of Osteopathic Medicine (UNECOM) Geriatrics Education Mentor (GEM) Program is in its sixth year. It's a joint project between older community living adults and 2nd year medical students within the Osteopathic Clinical Skills course. The GEM program goal is to foster understanding and importance of the person/patient-provider relationship. Pairs of osteopathic medical students (N=87 +/- 5) were assigned to an older adult GEM (mentor) volunteer (N=87 +/-5) living within a 50 mile radius of UNECOM. Snowball sampling was used to recruit the GEMs; student participation is required. Four (4) home visits were conducted over 9 months with an assigned GEM; each visit included a new assignment. Students observed, summarized, and recorded experiences communicated by the GEM for each cumulative assignment. Data from assignments were "graded" and content analyses of open ended evaluation/summary questions were conducted. This program is UNE IRB approved. Student pairs completed all assignments. Assignments analysis on Blackboard revealed that students developed respect and awareness of life and medical experiences of the GEM; expressed understanding of the GEM's life experiences and goals for what "matters most". Relationships with older adults were established while students maintained professionalism and succeeded in competent and confident interactions. Documentation review from the 4 assignments aided in building students' geriatrics knowledge, attitudes and skills. Pre-clinical medical education tends to be episodic; providing an opportunity to establish longitudinal assignments over the course of the academic year with the same older adult augments relationship skills and learning in geriatrics.

### STARTING AN OLDER ADULT LECTURE FOR FIRST SEMESTER NURSING STUDENTS WITH A TRUTH OR MYTH GAME

Lynn Brown, and Pao-feng Tsai, *Auburn University, Auburn, Alabama, United States*

False ideas about the physical and psychosocial characteristics of older adults exist in America. It is especially important that nurses are not susceptible to myths and stereotypes as these myths can affect the quality of patient care. For example, some people stereotype older adults as forgetful, disabled, ill, and unable to understand new information. Misconceptions and negative stereotypes are also present in first year nursing students. It is vital that students assess their own attitudes about older adults to form positive attitudes and gain knowledge about aging and health care needs. To achieve this goal, the older adult lecture in a first semester theory and fundamental course begins with a PowerPoint slide presentation asking students to distinguish truths and myths. The truth or myth topics include a) developmental tasks; b) common physiological changes; c) a comparison of delirium, dementia, and depression; and d) addressing health concerns of older adults. Active discussion follows the activity. Seventy to ninety percent of students correctly answered nine of ten questions related to older adult content on the final exam. Considering the increasing number of older adults in the health care setting, nurse educators must dismantle negative stereotypes with creative teaching strategies.

### WANT TO ENGAGE MORE PHYSICAL THERAPY STUDENTS IN GERIATRICS? TEACH THE 4MS

Tiffany Shubert,[1] and Cristine Henage,[2] *1. Shubert Consulting, Chapel Hill, North Carolina, United States, 2. UNC School of Medicine, Chapel Hill, North Carolina, United States*

In 2019, 1.2% of the 209,000 licensed physical therapists in the United States had completed sufficient training to be Geriatric Certified Specialists. The dramatic demographic shift in the population will require all physical therapists to have foundational knowledge of evidence-based management of older adults. Our purpose was to pilot the impact of an 8-week curriculum for physical therapy students that integrated key concepts of rehabilitation for older adults with the Age-Friendly Health System's 4Ms (Mentation, Mobility,

Medications, What Matters). The curriculum included guest speakers from medicine, social work, nutrition, pharmacy, community providers (YMCA) and older adults. Every class modeled how to assess the Ms and integrate information into a plan aligned with what matters to the client. Students completed a pre-post survey to evaluate their understanding of the 4Ms, and self-assess confidence in applying concepts to practice. Results supported the value of integrating the 4Ms into the curriculum. Over 89% of respondents reported assessing medications and mentation was very important to patient care compared to 11% and 33% pre-course, and 78% of students reporting they were highly-likely to advocate for the 4Ms as part of their practice. The 4Ms provided a framework that made geriatric care more appealing. Several students commented they had no interest in geriatrics prior to the course, but were more confident in their abilities and more interested in caring for older adults. Findings from this pilot support the value of the 4Ms as a framework for graduate-level allied health programs curriculum development.

### SESSION 2807 (POSTER)

### CAREGIVER EDUCATION AND TRAINING

### A PILOT STUDY ON THE EFFECT OF A SELF-TRAINING PROGRAM ON ASIAN AMERICAN CAREGIVERS

Tongtong Li, Yufu Jiang, and Iris Chi, *University of Southern California, Los Angeles, California, United States*

This presentation reports an exploratory study evaluating the effect of Asian Care Training (ACT) Manual on Asian American (AA) caregivers' care capacity and knowledge growth. A total of 65 AA caregivers were recruited from community-based organizations in Los Angeles and passed the screening questionnaire, in which 44 (37 females and 7 males with an average age of 61) completed the evaluation process. Participants were given autonomy to choose topics they desired to learn out of 25 topics in ACT manual and completed one-month learning. The data were collected via structured questionnaires before and after the self-learning and were analyzed with paired t-test on 5 questions about care capacity and 4 about caregiving knowledge. One-month self-learning of ACT manual enhanced participants' care capacity in 4 out of 5 sectors: confidence in caregiving (t=-2.2, p=0.015); ability to deal with emergency (t=-1.88, p=0.032); to solve concerns of the care recipients (t=-4.54, p<0.001); to communicate more smoothly (t=-2.85, p=0.0028). Improvements were found in 3 out of 4 sectors in knowledge: knowledge of Alzheimer's disease (t=-4.43, p<0.001); appropriated approach to displacing care recipients (t=-5.03, p<0.001); and information about caregiver support resources (t=-7.25, p<0.001). This self-learning method of ACT manual was feasible for AA caregivers to broaden knowledge and care capacity while adapting with their intensive workload. This study constitutes an important step towards establishing culturally tailored self-training programs for Asian American caregivers for frail older adults via multiple platforms such as online curriculums and mHealth app.

### CAREGIVER-CENTERED CARE HEALTH WORKFORCE COMPETENCIES: DEVELOPING CONSISTENT SUPPORT FOR FAMILY CAREGIVERS

Jasneet Parmar,[1] Sharon Anderson,[1] Lisa Poole,[2] Wendy Duggleby,[1] Jayna Holyroyd-Leduc,[3] Suzette Brémault-Phillips,[1] Pollard Cheryl,[4] and Anwar Haq,[5] *1. University of Alberta, Edmonton, Alberta, Canada, 2. Dementia Connections Magazine, Calgary, Alberta, Canada, 3. University of Calgary, Calgary, Alberta, Canada, 4. McEwan University, Edmonton, Alberta, Canada, 5. Covenant Health Network of Excellence in Seniors' Health and Wellness, Edmonton, Alberta, Canada*

Family caregivers [FCGs] are the backbone of the health system. They provide over 80% of the care for people with dementia, chronic illnesses and impairments. Despite evidence of their contributions and consequences of caregiving, support for FCGs has not been a health system priority. Education to prepare health providers to effectively identify, engage, assess, and support FCGs throughout the care trajectory is an innovative approach in addressing inconsistent system of supports for FCGs. We report on development and validation of the Caregiver-Centered Care Competency Framework to help with curricular design and subsequent evaluation of effectiveness of care providers working within healthcare settings to engage and support FCGs. We used a three round modified Delphi approach. An expert panel of 42 international, national, and provincial stakeholders agreed to participate. In the first 2 rounds, multi-level, interdisciplinary participants, rated the indicators in terms of importance and relevance. In the 3rd round consensus meeting, participants validated the six competency domains, including indicators in small group sessions. Thirty-four experts (81%) participated in the round 1, 36 (85.7%) in round 2, and 42 people (100%) in round 3. There was stable consensus across all three rounds, 96.07% of participants rated the indicators as essential or important (Round 1, 95.81%; Round 2, 94.15; Round 3, 98.23%). FCG research has been primarily focussed on educating FCGs to provide care. These competencies will shape the design of educational curricula and interdisciplinary training programs aimed at supporting the health and social care workforce to provide caregiver-centered care.

### CO-DESIGNING CAREGIVER-CENTERED CARE: TRAINING THE HEALTH WORKFORCE TO SUPPORT FAMILY CAREGIVERS

Jasneet Parmar,[1] Lisa Poole,[2] Sharon Anderson,[1] Pollard Cheryl,[3] Wendy Duggleby,[1] Lesley Charles,[1] Suzette Brémault-Phillips,[1] and Jayna Holyroyd-Leduc,[5] *1. University of Alberta, Edmonton, Alberta, Canada, 2. Dementia Connections Magazine, Calgary, Alberta, Canada, 3. McEwan University, Edmonton, Alberta, Canada, 5. University of Calgary, Calgary, Alberta, Canada*

Family caregivers [FCGs] provide over 80% of the care for people with dementia, chronic illness and impairments. Despite evidence of their contributions and consequences of caregiving, support for FCGs has not been a health system priority. Our innovative solution, to reduce caregiver distress and support caregivers' wellbeing, is to educate the health workforce in a meaningful manner based on evidence. We validated Caregiver-Centered Care Core Competencies required to address the gap between what FCGs report they need and preparation of healthcare providers to meet those needs. This competency-based education will prepare healthcare providers to effectively identify, engage, assess, and support FCGs, and address the inconsistent system of supports for FCGs. We co-designed our Caregiver Centered Care Education using effective practices for dementia education for health workforce and co-design. We engaged over 60 multi-level, interdisciplinary stakeholders with expertise in health workforce education, frontline healthcare, dementia care, health policy, and family caregiving. We ensured that we included FCGs of people living with dementia. The teaching/learning resources include competency-aligned educational modules, multimedia resources, and facilitators guide. As the hallmark of effective education is content relevant to learners' needs and contexts, our education is designed flexibly, to be tailored to settings and learners. We are pilot testing the Caregiver-Centered Care Education, for acceptability and effectiveness, in five contexts: primary care, acute care, homecare, supportive living, and long-term care. Our education will support Caregiver-Centered Care in all settings providing dementia-related care. Health workforce support will be essential to maintain FCG wellbeing and sustain family caregiving.

### RECOGNITION OF ELDER ABUSE BY CHINESE NURSING UNDERGRADUATES: EVALUATION BY THE CAREGIVING SCENARIO QUESTIONNAIRE

Zhuzhu Wang,[1] Jiayuan Zhuang,[1] Qiaoxian Zhang,[2] Xiaolan Lai,[1] Qixi Liu,[3] Baoyuan Xie,[4] and Qinmei Tang,[5] *1. Fujian Medical University, Fuzhou, Fujian, China, 2. The First Affiliated Hospital of Fujian Medical University, Fuzhou, Fujian, China, 3. Ningde Mindong Hospital, Fu'an City, Fujian, China, 4. The Second Affiliated Hospital of Fujian Medical University, Fuzhou, Fujian, China, 5. Xiamen University affiliated second Hospital of Fuzhou, Fuzhou, Fujian, China*

This study aimed to evaluate the recognition of elder abuse (EA) among nursing undergraduates in China and determine whether recognition is related to sociodemographic factors and education. We conducted a cross-sectional study with stratified random sampling, using the Caregiving Scenario Questionnaire of Chinese version (CSQ-CV). Questionnaires were disseminated to 343 nursing undergraduates ranged from 1st to 4th year at Fujian Medical University, China. The content validity of CSQ-CV is 0.97. 340 students (99.1%) effectively responded. 223(65.6%) of them identified trapping someone in an armchair; 108(31.8%) locking someone alone; and 3(0.9%) accepting someone was not clean as abusive. The majority correctly identified four out of five non-abusive (NA) items, while 210(61.8%) incorrectly identified camouflaging the door to prevent wondering outdoors. With respect to potential-abusive (PA) items, less than half of the students made right judgements. Only 30(8.8%) correctly identified not taking her to family gatherings; 46(13.5%) telling her only having breakfast after bathing; and 50(14.7%) hiding tablets in someone's cereal or tea. Mann-Whitney and Kruskal-Wallis tests indicated no significance related to correct recognition of the three abusive items with sociodemographic factors and education. The students who were female, caring older adults at home or had detected EA cases were more likely to correctly recognize NA items (Z=-2.428,P=0.015;Z=-2.028,P=0.043;Z=-2.534,P=0.011). Besides, female students got higher scores of CSQ-CV (Z=-2.000, P=0.045). Nursing undergraduates' recognition of EA, especially in neglect and PA are still at a low level in China. Education about EA in nursing undergraduates' curriculum and training program should be encouraged.

### SUPPORTING CAREGIVERS OF OLDER ADULTS THROUGH EDUCATION IN MINDFUL GRATITUDE PRACTICE

Sungsim Lee, *Loyola University Chicago, Chicago, Illinois, United States*

This presentation describes a supportive mindfulness practice for caregivers of older adults based on the principles of Won Buddhism (an integrative, a modernized Buddhism). As the aging population grows, there is a significant increase in recognition of the negative impact of caregiver stress on older adults' quality of life. The ability for caregivers to deal compassionately with stress is essential, as caring for older adults can awaken feelings about one's own vulnerability and mortality. The 'Mindful Gratitude Practice' offers a way to cope with stress, cultivate self-care, and improve the care of others. Relevant research will be summarized, which shows mindfulness and gratitude practice respectively benefit positive influence in both physical and emotional well-being. Mindful Gratitude Practice as a spiritual approach that fosters caregivers' emotional stability, reduces their stress and improves the relationship between older adults and their caregivers. In this presentation, three processes of Mindful Gratitude Practice will be described: 1. Understanding a mindfulness practice by establishing intention, attention, and attitude, 2. Learning the principles of a gratitude practice and implementation, and 3. Incorporating mindfulness into a gratitude practice. Research results have demonstrated that through this learning process, caregivers have acquired the concept of interconnectedness, experience grateful moments, and a deep feeling of appreciation in their caregiving relationships. The presenter will guide participants in a short experience of Mindfulness Gratitude Practice. Further readings and resources will be provided for those who are interested.

### SESSION 2809 (PAPER)

### END OF LIFE | PAIN MANAGEMENT

### CHALLENGES RELATED TO INFORMAL CARE PARTNERS' INVOLVEMENT IN END-OF-LIFE-CARE IN ASSISTED LIVING

Molly Perkins,[1] Ann Vandenberg,[2] Candace Kemp,[3] Mary Ball,[3] Joanna Jungerman,[1] Elise Abken,[2] and Alexis Bender,[1] *1. Emory University, Atlanta, Georgia, United States, 2. Emory University School of Medicine, Atlanta, Georgia, United States, 3. Georgia State University, Atlanta, Georgia, United States*

Limited empirical evidence suggests that caregiver burden is greater for informal care partners (family and friends) in assisted living (AL) compared with other long-term care settings, particularly within context of end of life. Using qualitative data from a larger 5-year, 7-site study of end-of-life care in AL funded by the National Institute on Aging (R01AG047408), we investigate informal care partners' involvement in end-of-life care and identify challenges related to informal caregiving that might contribute to care burden. Grounded theory analysis of ethnographic data and in-depth interviews (average interview length = 97 minutes) with 59 racially and ethnically diverse informal care partners (mean age = 60) shows that informal care partner involvement in end-of-life care varies across participants and over time and is shaped by multiple intersecting social and structural determinants. At individual levels, these include many personal, situational, and relational factors. Personal factors include but are not limited to care partners' own physical and mental health and material resources (e.g., ability to pay for supplementary care). Situational and relational factors include care partners' awareness (or lack thereof) of residents' impending death and the quality of the caregiving relationship. AL and wider community-level factors include understaffing, staff turnover, inadequate hospice support, and lack of access to these services. We find that informal care partners navigate these caregiving challenges through a basic social process we conceptualize as "negotiating risks." Strategies for easing caregiver burden and improving informal care partner and resident quality of life at end of life are implicated.

### DEVELOPMENT AND EVALUATION OF THE CARING AHEAD: PREPARING FOR END OF LIFE IN A DEMENTIA QUESTIONNAIRE

Pamela Durepos,[1] Sharon Kaasalainen,[1] Jenny Ploeg,[1] Tamara Sussman,[2] and Noori Akhtar-Danesh,[1] *1. McMaster University, Hamilton, Ontario, Canada, 2. McGill University, Montreal, Ontario, Canada*

A palliative approach is recommended in long-term care to support persons with dementia and help families prepare for end-of-life. Despite this, 50% of family caregivers of persons with dementia report feeling unprepared for death. A questionnaire is needed to assess caregiver death preparedness as an outcome measure for strategies within palliative care. A mixed methods design with qualitative and quantitative phases was used to develop and evaluate the 'Caring Ahead: Preparing for End-of-Life in Dementia' questionnaire. The questionnaire has 30 items organized into Medical, Relationship/Personal, Spiritual and Practical subscales with a 7-pt Likert response scale. To date, the questionnaire has been tested with 117 participants who are 61 years old on average, female (86%), adult children (77%) caring for a person with dementia in long-term care. The mean item score is 5.61 (SD 0.71). Participants report limitations in preparedness related to: 1) communication with healthcare providers about traditions and preferences for end-of-life care; 2) knowledge of the dying process and; 3) life purpose after death. A test-retest with 32 participants demonstrates a high degree of reliability; Intraclass Correlation Coefficient 0.91 (CI95%: 0.31-0.97). A moderate positive correlation between participant total scores and a single global preparedness item suggests concurrent validity, r=.66 (CI95%: 0.51-0.80). These findings will be used to refine the questionnaire and contribute a valuable measurement tool for clinicians, researchers and policy-makers working in palliative care.

### LAST PLACE OF CARE AND END-OF-LIFE QUALITY OF LIFE IN THE UNITED STATES: EVIDENCE FROM A NATIONAL REPRESENTATIVE DATA SET

Yifan Lou,[1] Nan Jiang,[2] and Katherine Ornstein,[3] *1. Columbia University, New York, New York, United States, 2. National University of Singapore, Singapore, New York, Singapore, 3. Icahn School of Medicine at Mount Sinai, New York, New York, United States*

Background: Quality of life (QoL) during last stage of life has raised expanded interests as an important aspect of person-centered care. Last place of care (LPC), refer to the last place decedents received their formal end-of-life care (EOLC), has been identified as a key indicator of older adults' end-of-life QoL, but the relationship was understudied. This study explores the association between LPC and end-of-life QoL among American older adults. Methods: Data used seven waves of Last Month of Life data with a total sample of 3068 Medicare decedents in NHATS. Outcome is end-of-life QoL assessed by eleven measures on four domains: pain and symptoms management (SP), quality of healthcare encounter (HE), person-centered care (PC), and overall quality of care (QC). LPC was categorized into home, hospital, nursing home, and residential hospice. Multivariate logistic regression analyses were used to examine the relationship with covariates. Results: LPC varied by most demographic characteristics, except immigration status and education. Older adults whose LPC is hospital, compared to those who had home-care, were less likely to have great experiences on HE, PC, and QC. People dying at nursing homes are more likely to receive care meeting their dyspnea and spiritual needs. Residential hospice is negatively related to respected care, clear coordination, and keeping family informed, but are more likely to provide PS and spiritual care. Discussion: Home-based end-of-life care has certain advantages but still has room to improve on SP and religious concerns. Hospitals should keep reforming their service delivery structure to improve patients' QoL.

### RATES OF DIAGNOSES INDICATING OPIOID DEPENDENCE AFTER HOSPICE LIVE DISCHARGE: A NATIONAL STUDY

Thomas Christian,[1] Ian Breunig,[1] Joan Teno,[2] Pedro Gozalo,[3] and Michael Plotzke,[1] *1. Abt Associates, Cambridge, Massachusetts, United States, 2. Oregon Health & Science University, Portland, Oregon, United States, 3. Brown University, Providence, Rhode Island, United States*

Opioids are an important tool for managing Medicare Hospice beneficiaries' pain and symptoms. Little is known about the prevalence of opioid dependence among patients discharged alive from hospice. Using 100% Medicare hospice, acute inpatient, and Part B claims from Federal Fiscal Years (FY) 2017-2018, we identified hospice beneficiaries that were discharged alive during FY2017-2018 and associated with diagnosis codes in subsequent health care incidents indicating opioid dependence. We used a crosswalk from the Agency for Healthcare Research and Quality to determine which codes represented opioid dependence. We characterized beneficiaries and their hospice providers using information from the Medicare Enrollment Database and Provider of Services file. There were 468,204 live hospice discharges during FY2017-2018, among which 9,282 (2.0%) were associated with subsequent health care events including a diagnosis code signifying opioid dependence. Post-hospice opioid diagnoses were more frequent among beneficiaries who were younger (for ages <65 relative to 85-89; Adjusted Odds Ratio [AOR]=6.23, 95% Confidence Intervals [CI] 5.73-6.77); dual-eligible (AOR=1.40, 95% CI 1.34-1.47); and relative to cancer, diagnosed with lung (AOR=1.83, 95% CI 1.72-1.95), heart (AOR=1.23, 95% CI 1.14-1.34), or liver diseases (AOR=1.25, 95% CI 1.10-1.42). Beneficiaries with opioid incidents tended to have survived much longer after hospice (150.9 days with opioid incidents vs. 13.6 days no opioid-related incidents). The states with the highest rates of post-hospice opioid-incidents per 10,000 live discharges were Kentucky (435.2), Wyoming (386.0), Tennessee (359.3), Washington (345.6), and Idaho (342.9). Further monitoring can ensure that hospice beneficiaries receive appropriate care during and after hospice enrollment.

### SESSION 2810 (PAPER)

### SOCIAL SERVICES: ORGANIZATIONAL AND SERVICE USER ISSUES

### COVID-19 INFECTIONS IN LONG-TERM CARE FACILITIES IN WUHAN, CHINA: CHALLENGES AND LESSONS

YI CAI,[1] Jing Wang,[2] and Bei Wu,[3] *1. Wuhan University, Wuhan, China, 2. Fudan University, Nantong, Shanghai, China, 3. New York University, New York, New York, United States*

The impact of the COVID-19 infection on older residents and the direct frontline workers in long-term care (LTC) facilities in Wuhan, China deserves close attention. The fatality rate for older residents infected by the COVID-19 is among the highest in China Viral outbreaks are likely to occur in LTC facilities due to group-living arrangements, lack of precautionary measures, and older residents' vulnerability to diseases. In this study, we aimed to explore different stakeholders' experiences and challenges in the midst of the spread of the virus in LTC facilities. We conducted telephone interviews with four groups from two LTC facilities and two hospitals: twelve older residents (Four suspected cases and two infected), ten family members, four direct frontline workers (two infected), two nursing home managers, and four health care professionals working in the two hospitals that infected older residents were transferred to. We found that the gap in the transition of care quality between LTC facilities and local hospitals was widened during the COVID-19 outbreak. LTC facilities were slow to take precautionary measures and underprepared to handle the crisis after the infections occurred. The wellbeing of older residents was significantly impacted during the transition, particularly for those with dementia. Health care professionals in local hospitals were under tremendous stress providing treatments for older residents while ensuring their safety. There is an urgent need to improve transitional care and the capacity in preventing and handling this type of crisis for older residents in LTC.

### DOES THE STRUCTURE OF AGING SERVICES MATTER? A COMPARATIVE CASE STUDY OF CALIFORNIA COMMUNITIES

Haley Gallo,[1] Kelly Marnfeldt,[1] Adria Navarro,[2] and Kathleen Wilber,[1] *1. University of Southern California, Los Angeles, California, United States, 2. Azusa Pacific University, Azusa, California, United States*

As the older adult population grows and Federal funding remains stagnant, coordination of services at the local level becomes more critical. Building on the Federal Administration for Community Living model, California's Master Plan for Aging creates opportunities for innovative restructuring of the way aging services are delivered through the Area Agencies on Aging (AAA). We conducted a comparative case study of California AAAs (N=5) representing different levels of integration, from standalone departments of aging (Los Angeles City, Riverside County), to partial integration (Los Angeles County), to full integration with aging and disability programs (San Diego County, San Francisco County). We examined the impact of departmental organization and integration on the AAAs' service delivery for older adults. Interviews with leaders of the AAAs were coded by two researchers using constant comparative analysis to identify themes within and between the AAAs. Emerging themes revealed the role that "structure," "politics," "funding," and "visibility" play in service delivery for AAAs with varying levels of integration. Findings suggest that integrating the AAA with other departments (i.e., Health and Human Services) and programs (e.g., Adult Protective Services, In-Home Supportive Services) improves coordination and allows for greater visibility of the AAA. Key stakeholders in standalone AAAs, however, fear that integration would hinder their visibility and "agility" in service provision. Findings shed light on best practices for locally coordinated aging service delivery during a window of opportunity for California AAAs, yet they can also inform how aging services are provided for local governments nationwide.

### OLDER ADULTS SEEK OUT CONGREGATE NUTRITION PROGRAMS FOR SOCIALIZATION

Kristen Robinson,[1] and Susan Jenkins,[2] *1. Social & Scientific Systems, Inc., Frederick, Maryland, United States, 2. Administration for Community Living, Washington, District of Columbia, United States*

Social connectedness is vital for healthy aging. Older adults often have fewer opportunities to socialize due to reasons such as illness, death of spouse, and mobility limitations. The Older Americans Act Congregate Nutrition Program provides meals and related nutrition services in group settings to people age 60 and over. Until now, the contribution of congregate meals to the socialization of older adults has been primarily anecdotal. However, according to data from the 2019 National Survey of Older Americans Act Participants, over 40% of the 1.5 million people receiving congregate meals said that they started attending to socialize with other people. According to the data, two-thirds report their social opportunities have increased since becoming involved with these services. To measure the impact of program participation on socialization outcomes, an evaluation compared congregate program participants to non-participants. Findings from regression-adjusted socialization outcomes found that congregate meal participants were less likely to screen positively for depression (18% vs. 24% p<.05) and have greater satisfaction with their socialization opportunities (94% vs. 86% p<.01), yet there was no significant difference in a measure of perceived loneliness. Based on these results, we used weighted, bivariate tests to detect differences between congregate meal participants who were satisfied with the socialization opportunities to those who were not. Our findings suggest a significant relationship between number of days per week participants attended congregate meals and satisfaction with socialization, X2 (2, N = 1,072) = 7.5, p = < .05.

### POST-ACUTE CARE FOR MEDICARE BENEFICIARIES IN ACCOUNTABLE CARE ORGANIZATIONS

Erin Colligan, and Brittany Branand, *NORC at the University of Chicago, Bethesda, Maryland, United States*

Post-acute care (PAC) is a component of health-care utilization and spending that is subject to the discretion of providers. Prior research has demonstrated that Accountable Care Organizations (ACOs) recognize PAC as a logical target for increased efficiency and cost savings. As part of the evaluation of the Centers for Medicare & Medicaid Services (CMS) Next Generation ACO (NGACO) Model, we investigated NGACOs' approaches to PAC services and the impact of these efforts on utilization and cost using a mixed-methods study design. We conducted interviews and surveys with NGACO leadership and providers and performed a difference-in-differences analysis of utilization and spending based on Medicare claims data. We found that NGACOs focused specifically on establishing partnerships with skilled nursing facilities (SNF) to facilitate transitions in care by establishing new channels of communication, sharing performance data, embedding staff in SNFs, and (in some cases) sharing financial risk. We observed a statistically significant decrease in SNF spending, a trend toward fewer SNF days, and statistically significantly lower expenditures for other PAC settings (e.g., inpatient rehabilitation and long-term acute care facilities). These findings suggest that NGACOs have contributed to improving transitions in care and diverting beneficiaries from intensive PAC settings. Nonetheless, the reduction in PAC spending alone did not translate to a decline in total cost of care. Future ACOs may need to expand their focus to the inpatient utilization and spending that precedes PAC in order to impact total cost of care.

### SUPERVISORY LEADERS IN AGING: ONE-YEAR PRACTICE CHANGE OUTCOMES OF INNOVATIVE TRAINING PROGRAM

Daniel Kaplan,[1] Barbara Silverstone,[2] Keith Chan,[3] and Amanda Spishak-Thomas,[4] *1. Adelphi University, Cortlandt Manor, New York, United States, 2. SBW Partners, Inc., New York, New York, United States, 3. State University of New York at Albany, Albany, New York, United States, 4. Columbia University, New York, New York, United States*

Social services for older adults are instrumental in addressing vulnerabilities associated with aging. Yet, practitioners report needing expanded geriatric knowledge and enhanced supervision. Agency-based supervision is essential to skilled practice and staff retention, directly impacting the quality of services delivered by the teams they support. The Supervisory Leaders in Aging (SLA) program of the National Association of Social Workers (NASW) was designed to strengthen supervision of the social service workforce. The SLA program, adopted in four states (IL, FL, MD, and NY), trained 134 MSW supervisors who support 1,200 social service staff, aimed at enhancing the well-being of 264,000 clients annually. This paper reports newly available final outcomes data from the 3-year implementation study of SLA. Trainees self-rated use of relevant supervisory best-practices was measured with novel 30-item instrument which captured frequency in use of supervisory best practices. The measure was administered prior to the first session and at three and twelve months after the final session. Comparisons of ratings across time periods demonstrate a range of positive and significant increases at the end of program workshops (0.12–0.56; mean of 0.30 points) and after one year (0.18–0.53; mean of 0.34 points). Supervisory best practices were maintained by those who already engaged in these behaviors, and participants who previously underutilized best practices adopted and maintained these behaviors as a result of the workshops. Implications of this tested model for enhancing workforce capacity will be discussed, including variation of impacts by supervisor characteristics and retention of learning gains over time.

### SESSION 2811 (PAPER)

### ETHICAL AND LEGAL ISSUES | CHALLENGES

### HUMAN RIGHTS IN OLDER AGE: A CRITICAL REFLECTION OF THE DEBATE AROUND A UN CONVENTION ON THE RIGHTS OF OLDER PERSONS

Stefan Hopf,[1] Federica Previtali,[2] and Nena Georgantzi,[3]
*1. National University of Ireland Galway, Galway, Ireland, 2. Tampere University, Tampere University, Finland, 3. Age Platform Europe, Brussels, Brussels Hoofdstedelijk Gewest, Belgium*

In recent years ageism has received increase international attention. In 2016 the UN dedicated the International Day of Older Persons to the fight against ageism and the World Heath Organization launched a campaign to combat ageism. This growing interest is also illustrated by the establishment of the UN Open-Ended Working Group on Ageing, and the related work on a UN convention on the rights of older persons, which, among other things, aims to provide better protection against discrimination. The ongoing discussions about a convention is accompanied by tensions between views assuming an older persons' specific convention may reproduce age-related group differences and could perpetuate ageism, and those who argue that it will help reducing it. This article critically reflects on these discussions and some aspects of a potential convention that could provide basis for ageism critique. We refer to central sociological and legal arguments of the debate around ageism and age-based distinctions, which show clear intersections, e.g. the legal discussion one the justifiability of the general use of "age limits" and the socio-scientific debate on the relationship between age categorization and ageism. These intersections serve as central starting point for the question whether and to what extent age group differentiation and targeted human rights protection may (re-)produce ageism. Finally, we argue in favor of re-framing the debate about a convention on the rights of older persons towards a more universalist approach, which addresses possible age inequalities and critically reflects on the connection between chronological age and targeted human rights provisions.

### MEETING THE PHYSICAL AND MENTAL HEALTH NEEDS OF OLDER OFFENDERS: DOES AGING IN PLACE WORK IN PRISON?

Eddy Elmer,[1] and Heather Campbell Pope,[2] *1. Vrije Universiteit Amsterdam, Vancouver, British Columbia, Canada, 2. Dementia Justice Canada, Kingston, Ontario, Canada*

In many countries, the proportion of older people in prison is growing due to longer sentences, increases in convictions for historical offences, and longevity. Moreover, harsh conditions of confinement coupled with the negative effects of a criminal lifestyle may contribute to 'accelerated aging' in this population. Indeed, many prisoners develop health problems that are more commonly seen among people who are up to ten years older. Correctional institutions are increasingly struggling to meet the complex and expensive healthcare needs of these offenders, especially at end-of-life. Some institutions have taken the position that prisons were never intended to be nursing homes, nor can they be adequately adapted to fulfill this role. As a result, these institutions attempt to place some aging offenders in healthcare institutions within the community, provided that their risk to the public can be adequately managed. Other institutions have argued that the needs of aging offenders can be successfully met behind prison walls and have taken steps to allow prisoners to 'age in place.' After summarizing the research on the physical and mental health needs of aging offenders, this presentation considers the advantages and disadvantages of meeting older offenders' healthcare needs both within and outside the prison setting and provides relevant examples of both. Special attention is paid to the issues of social isolation and loneliness: both may contribute to accelerated aging, and perhaps even the risk for re-offending, raising questions about which correctional settings are most beneficial for minimizing these problems.

### NAVIGATING CONFLICTING RIGHTS AMONG RESIDENTS, VISITORS, AND FAMILIES IN LONG-TERM CARE FACILITIES

Angela Perone, *University of Michigan, Ypsilanti, Michigan, United States*

Background: An abundance of long-term care regulations creates a bevy of rights for nursing facility residents, staff, and families. Front-line workers and managers have significant discretion and responsibilities for interpreting these rights. Building on street-level-bureaucracy theory (Lipsky, 2010), which focuses on how front-line workers implement policy, this study examines how staff at various levels (direct care, mid-level professional, top management) resolve conflicting rights. Methods: This study employs a novel advanced multi-method qualitative design with semi-structured staff interviews (n=90), content analysis of long-term care facility policies (n=75), and participant observation of two facilities for a multi-layered comparative case study. Findings: Data analysis revealed variations in staff responses to conflicting rights regarding autonomy and safety (e.g. fall prevention, dementia, coronavirus) and discrimination (i.e. sexual/racial harassment). While harassment was rampant, direct care workers responded more deferentially to residents and often justified harassment as part of a customer service job in one's home. Staff at all levels relied on teams to develop creative problem-solving approaches, but team composition and discretion varied significantly between facilities and staff levels. While staff included few social workers, staff heavily relied on them to adjudicate conflicting rights. Implications: Conflicting rights impact resident care and relationships among residents, staff, and families. This research provides policymakers and practitioners with new data about how staff resolve conflicting rights, which can facilitate stronger policies to support an overburdened and underpaid long-term care workforce. This research also expands street-level-bureaucracy theory to include managers and reveals how various team approaches can produce diverse solutions.

### OVERCOMING ETHICAL CHALLENGES IN CONDUCTING RESEARCH AMONG PERSONS WITH DEMENTIA

Jeanine Yonashiro Cho,[1] Elizabeth Avent,[2] Roberta Peterson,[1] Laura Mosqueda,[2] and Zachary Gassoumis,[2] *1. Keck School of Medicine of USC, Los Angeles, California, United States, 2. University of Southern California, Los Angeles, California, United States*

By 2060, the number of older adults with Alzhemier's Disease or related dementias (ADRD) is expected to encompass approximately 13.9 million Americans. Recognizing this, the federal government has prioritized research on ADRD and their effect on the physical, emotional, and psychological well-being of persons with dementia (PWD). Such research is complicated by disease-associated decline in cognitive and functional capacity among PWDs which can impair participant ability to process and communicate information, potentially increasing their exposure to negative research-related experiences and compromising the accuracy and reliability of provided data. Nevertheless, as a key stakeholder group, PWD should have the right to participate in research on ADRD. This session will present a case study of ethical issues that emerged in an NIH-funded study utilizing mixed-methods to examine caregiving and care dyad relationships between PWD and their care partners over an 18-month follow-up period. Ethical issues examined will include: (1) PWD capacity to consent to research at baseline and (2) during follow-up visits, (3) Obtaining accurate and reliable data from persons with mild to moderate dementia, (4) Assessing PWD distress while engaging in data collection processes, and (5) Reporting of negative caregiving and life experiences, such as elder mistreatment. The discussion of these topics and presentation of lessons learned holds promise for improving research methodology in studies involving PWD and enabling researchers to increase the involvement of persons with mild to moderate dementia in research on ADRD.

### PROSECUTION OF ELDER NEGLECT: A CLOSE EXAMINATION OF CASES FROM KING COUNTY, WASHINGTON

Kriti Gogia,[1] Alyssa Elman,[1] Sunday Clark,[1] Page Ulrey,[2] Marie-Therese Connolly,[3] Elizabeth Bloemen,[4] Billie Johnsson,[5] and Tony Rosen,[5] *1. Weill Cornell Medicine, New York, New York, United States, 2. King County Prosecuting Attorney's Office, Seattle, Washington, United States, 3. University of Southern California Leonard Davis School of Gerontology, Los Angeles, California, United States, 4. University of Colorado School of Medicine, Aurora, Colorado, United States, 5. NewYork-Presbyterian Hospital, New York, New York, United States*

Elder neglect is common and can have catastrophic consequences. Cases may benefit from integrated responses from multiple sectors. Little research exists describing prosecutorial involvement and its impact, but existing evidence suggests neglect is seldom criminally prosecuted. Our goal was to closely examine neglect prosecution in a jurisdiction that has been a leader in using prosecution to attempt to address it. We quantitatively and qualitatively analyzed legal case files of felony elder neglect prosecuted in King County, Washington from 2008-2011. 13 cases were prosecuted, with a total of 10 victims. 90% of victims were female, with a median age of 88. 90% were unable to ambulate, and 90% had dementia. Defendants were commonly the victim's adult child (38%). 23% had previous criminal citations/convictions. 46% of cases occurred in an Adult Family Home. 15% of cases went to trial, and all trial cases ended in conviction of some charge. Themes identified included: (1) perpetrators were either professional caregivers receiving compensation or non-professional caregivers financially dependent on the victim, (2) victims were malnourished and severely injured at time of reporting, and (3) medical expert contribution is imperative given complexity of these cases. Victims were unable to participate in prosecution in any case. This research shows that these cases are seldom prosecuted, even in a jurisdiction focusing on this phenomenon, but highlights characteristics of cases and demonstrates they may be prosecuted without victim participation. Future research is needed to examine prosecution's impact on elder neglect to better understand how it may be optimally used.

### SESSION 2812 (PAPER)

### LONG-TERM CARE ISSUES: RESIDENTS, WORKFORCE, AND ORGANIZATIONAL

### ORGANIZATIONAL COMMITMENT AMONG RESIDENTIAL SENIOR CARE WORKERS

Tina Kilaberia, *UC Davis Health, Sacramento, California, United States*

Despite growing evidence of the increase in the aging population nationally, there continues to be a shortage of health and social care professionals who work with older adults. Some studies examine this phenomenon by looking at motivations that underlie commitment to geriatric careers. Others study commitment among those who are already geriatric professionals. Both the volume and diversity of the aging population challenge organizations to provide care. Drawing on 44 interviews, observations of 62 meetings, and a 5-year immersion, this organizational ethnography looks at commitment factors at a large, urban, faith-based residential senior care organization. Commitment factors are examined on three levels: daily tensions and rewards; value tensions and rewards; and deal breakers and clinchers. Findings show that intrinsic identity-based factors such as affective bonds with older persons and sharing in faith values sustain commitment on the person level. Interprofessional tensions may detract from commitment. Implications pertain to the role of leadership in equity-related and ethical tensions as well as the improved uptake of allied health professional expertises such as social work and chaplaincy. This study extends the extant knowledge by incorporating perspectives of social workers, chaplains, rehabilitation, recreational, diet and environmental services workers in addition to the more commonly examined groups such as nurses and certified nursing assistants, and in a setting that includes Assisted Living in addition to long-term care.

### REGIONAL VARIATION IN HEALTHCARE USE AMONG MEDICARE BENEFICIARIES WITH ALZHEIMER'S DISEASE AND RELATED DEMENTIAS

Elizabeth White, and Momotazur Rahman, *Brown University School of Public Health, Providence, Rhode Island, United States*

In this national prospective study we describe regional variation in healthcare utilization among Medicare beneficiaries with Alzheimer's disease and related dementias (ADRD) in the six years after diagnosis. We use 2008-2015 Medicare claims and other administrative data to map nursing home, home health, hospital, and hospice use across hospital referral regions; and examine the relationship of state and county supply-side factors to time beneficiaries spend in different settings. The sample includes 1,158,655 Medicare fee-for-service beneficiaries diagnosed with ADRD in 2008 and 2009. Nationally, beneficiaries spent a mean of 70.6% of survived days in the community, 23.9% of days in nursing home, and 5.4% of days in hospital. 37.2% of beneficiaries who died within six years had received hospice. Distinct regional patterns emerged. Adjusting for beneficiary and local characteristics, beneficiaries in Midwestern states spent the most time in nursing homes, while beneficiaries in Western states spent the most time in community. The probability of receiving hospice was generally highest in Western and Southern states, and lowest in the Midwest and Northeast. Controlling for beneficiary, local, and state characteristics, we found the following factors to be associated with beneficiaries spending less time in nursing homes: fewer nursing home beds in the county, higher state Medicaid long-term care spending for home and community-based services (HCBS), and state use of Certificate of Need laws. These findings illustrate that state investment in HCBS, and state and local regulation of provider supply are important factors influencing where individuals with ADRD receive care.

### THE IMPACT OF LEADERSHIP STYLES ON QUALITY AND FINANCIAL PERFORMANCE IN HIGH MEDICAID NURSING HOMES

Justin Lord,[1] Ganisher Davlyatov,[2] Akbar Ghiasi,[3] and Robert Weech-Maldonado,[2] *1. LSU-Shreveport, Shreveport, Louisiana, United States, 2. University of Alabama at Birmingham, Birmingham, Alabama, United States, 3. University of Incarnate Word, San Antonio, Texas, United States*

This study examines the association between leadership styles on resident quality and financial performance in under resourced nursing homes (70% or higher Medicaid census). The Bonoma/Slevin leadership model was used to classify managers into four categories, autocrat, consultative autocrat, consensus manager, and shareholder manager. Survey data from 391 nursing home directors (response rate of 37%) from 2017- 2018, were merged with secondary data from LTCFocus, Area Health Resource File, Medicare Cost Reports, and Nursing Home Compare. Two models were ran to examine the effect of leadership styles on the dependent variable(s) nursing home STAR data (quality) and operating margin (financial performance). The independent variables were composite scores for leadership styles, with autocrat as the reference group. Control variables included organizational (ownership, chain affiliation, size, occupancy, payer mix, staffing, and race/ethnicity), and county factors (Medicare Advantage penetration, per capita income, poverty, education, unemployment, and competition). Multivariate regression was used to model the relationship between leadership styles and nursing home quality and financial performance. The consultative autocrat was associated with lower quality ($p < 0.05$), while the consensus manager was associated with lower profit margin ($p < 0.05$), as compared to autocratic leadership. The consultative autocrat, who solicits information from the staff yet still makes all significant decisions, is associated in lower quality; however, a consensus manager, who delegates their authority to the group, is associated with lower financial performance. Under-resourced nursing homes who face dual pressures need to recognize trade-offs of different decision making styles for quality and financial performance.

### THE RELATIONSHIP BETWEEN DEMENTIA, RACE, AND SCHIZOPHRENIA LABELING IN NURSING HOMES FOLLOWING THE CMS PARTNERSHIP

Shekinah Fashaw,[1] Theresa Shireman,[1] Ellen McCreedy,[1] and Julie Bynum,[2] *1. Brown University, Providence, Rhode Island, United States, 2. University of Michigan, Ann Arbor, Michigan, United States*

Preliminary research demonstrated an increase in the reporting of schizophrenia diagnoses among nursing home (NH) residents subsequent to the Centers for Medicare & Medicaid Services (CMS) National Partnership to Improve Dementia Care. Given known health disparities and higher antipsychotic use for Black NH residents, we examined how race and dementia influence the rate of schizophrenia diagnoses among NH residents. Using a quasi-experimental study design, we examined changes in schizophrenia reporting among long-stay NH residents' quarterly and/or yearly Minimum Data Set 3.0 assessments between 2011-2015. Employing a difference-in-difference analysis adjusting for independent variables (e.g. demographics, diabetes, heart conditions, and functional status), we examined the relationship before and after the partnership. There were over 4 million MDS assessments annually. Schizophrenia reporting increased 12.3% in the dementia group as compared to 9.3% in the non-dementia group ($p<0.0001$). Black residents had a significantly higher likelihood of schizophrenia reporting (4%, $p<0.0001$). After controlling for covariates, there was a 16.5% increase in schizophrenia reporting for Blacks with dementia and 14.9% increase for non-Blacks with dementia ($p<0.0001$). There were no racial disparities identified among the non-dementia group after controlling for covariates. NH residents were more likely to have schizophrenia documented on their MDS assessments, and following the partnership, schizophrenia reporting rates increased faster for Blacks with dementia than their non-Black peers and their peers without dementia. Further work is needed to determine if schizophrenia diagnoses are appropriately employed in NH practice, particularly for Black Americans and persons with dementia.

### SESSION 2813 (PAPER)

### ENVIRONMENTAL AGING AND AGING IN PLACE

### CULTURE CHANGE INITIATIVES IN HIGH MEDICAID NURSING HOMES: DOES TIME OF ADOPTION MAKE A DIFFERENCE?

Latarsha Chisholm,[1] Akbar Ghiasi,[2] Justin Lord,[3] and Robert Weech-Maldonado,[4] *1. University of Central Florida, Orlando, Florida, United States, 2. University of Incarnate Word, San Antonio, Texas, United States, 3. LSU-Shreveport, Shreveport, Louisiana, United States, 4. UAB, Birmingham, Alabama, United States*

Racial/ethnic disparities have been well documented in long-term care literature. As the population ages and becomes more diverse over time, it is essential to identify mechanisms that may eliminate or mitigate racial/ethnic disparities. Culture change is a movement to transition nursing homes to more home-like environments. The literature on culture change initiatives and quality has been mixed, with little to no literature on the use of culture change initiatives in high Medicaid nursing homes and quality. The purpose of this study was to examine how the involvement of culture change initiatives among high Medicaid facilities was associated with nursing home quality. The study relied on both survey and secondary nursing home data for the years 2017-2018. The sample included high Medicaid (85% or higher) nursing homes. The outcome of interest was the overall nursing home star rating obtained from the Nursing Home Compare Five-Star Quality Rating System. The primary independent variable of interest was the years of involvement in culture change initiatives among nursing homes, which was obtained from the nursing home administrator survey. The final model consisted of an ordinal logistic regression with state-level fixed effects. High-Medicaid nursing homes with six or more years in culture change initiatives had higher odds of having a higher star rating, while facilities with one year or less had significantly lower odds of having a higher star rating. Culture change initiatives may require some time to effectively implement, but these initiatives are potential mechanisms to improve quality in high Medicaid nursing homes.

### IMPLICATIONS OF EXCEEDING THE AGE AND INTERVENTIONS TO EXTEND LIFE PURPOSE: PERSPECTIVES FROM ASIAN CENTENARIANS

Ad Maulod, Normala Manap, Sasha Rouse, and Angelique WM Chan, *Duke-NUS Medical School, Singapore, Singapore*

Centenarians have often been regarded as living paradigms of exceptional longevity yet little is known from their perspective about the purpose, meaning and quality of living longer lives. In Singapore, the number of centenarians has multiplied 30-fold from 50 in 1990 to about 1500 in 2020. Although centenarians are respected as 'national treasures' – having witnessed Singapore's transformation from British colony to global city state, their needs remain invisible in both the healthcare and social sectors. The tendency to romanticize exceptional longevity neglects a deeper understanding of (i) its consequence on the oldest old (85+ years) who may be impacted by severe functional and sensory deficits and (ii) their experience of social isolation in the family and communities. This paper discusses findings based on interviews with 15 Singapore centenarians (100 to 111 years old) and their family carers. Diverse experiences of longevity are shaped by these factors: health status; personal disposition; strength of family and social networks; exposure to adversity and coping resources; spiritual beliefs; role loss; and changes in the lived environment. Appropriate health and psychosocial interventions could have been delivered earlier in the life trajectory to enable better quality of life and continued social engagement. Learning from the challenges (eg. social withdrawal; extensive functional and sensory losses) of existing centenarians contributes to a more precise understanding of how best to harness the productive capacities of our oldest old.

### SENSE OF COMMUNITY AND QUALITY OF LIFE FOR OLDER ADULTS IN PUERTO RICO 2 YEARS AFTER HURRICANE MARIA

Tommy Buckley, Denise Burnette, Humberto Fabelo, and Mauricio Yabar, *Virginia Commonwealth University, Richmond, Virginia, United States*

Psychological sense of community plays an important role in the wellbeing of older adults, especially in cultures that revolve around community involvement and support. In 2017, Hurricane Maria devastated Puerto Rico, deeply altering community structure and social life. This study draws on the Ecological Theory of Aging to test the proposition that older adults' sense of community is associated with self-assessed quality of life two years later. We conducted face-to-face interviews with a non-probability sample of 154 community-dwelling adults aged 60+ in Puerto Rico. We measured sense of community with the Brief Sense of Community Scale (BSCS) (range 0-32, mean= 24.75, SD= 6.04) and quality of life (QOL) with the EUROHIS-QOL 8-item index (range 0-32, mean= 21.61, SD=5.92). We used multiple linear regression to test the association of sense of community and quality of life while controlling for relevant covariates. Higher levels of sense of community were associated with better quality of life ($\beta$=0.270, $p<0.001$), while increased mental health symptoms ($\beta$=-0.557, $p<0.001$) and poor self-rated health ($\beta$=2.964, SE=7.17, $p<0.001$) were associated with lower quality of life. Findings indicate that sense of community is an important contributor to older adult's quality of life in Puerto Rico, perhaps in part due to cultural and age-related factors. Moreover, sense of community may serve as a protective factor against adverse outcomes after large scale natural disasters. Researchers should continue to examine this association while advocating for and developing policies and programs that promote older adults' sense of community in post-disaster contexts.

### THEY DO THEIR BEST, BUT THEIR BEST ISN'T GOOD ENOUGH: HOW SOCIAL HOUSING CAN SUPPORT OLDER TENANTS AGING IN PLACE

Christine Sheppard,[1] Andrea Austen,[2] and Sander Hitzig,[3] *1. Sunnybrook Research Institute, Toronto, Ontario, Canada, 2. City of Toronto, Toronto, Ontario, Canada, 3. Sunnybrook Hospital, North York, Ontario, Canada*

Toronto Community Housing (TCH) is the second largest social housing landlord in North America, and is home to over 27,000 older adults, half of whom live in 83 "seniors-designated" buildings. There is inadequate and inconsistent delivery of services in these buildings, negatively impacting tenants' ability to age in place. We conducted two half-day consultations with service providers (n=74) and tenants (n=100) to identify strategies to improve unit condition, promote stable tenancies (i.e., prevent evictions) and enhance access to health and support services for older adults living in TCH. Through facilitated discussion, participants identified their top two recommendations for each priority area and reflected on the strategies that were hardest and easiest to implement, as well as the ones that would have the most and least impact on quality of life for older tenants. Participants recognized the need for more education as a way to empower older tenants and reduce stigma associated with unit condition issues (e.g., pest problems) and arrears. More frequent touch points with tenants was also recommended as a way to identify older adults at-risk of eviction and work proactively (instead of reactively) to support them. Service providers and tenants believed that system navigators working directly in the buildings would be a key facilitator to building trust and helping older tenants access needed services. Outcomes of the have several program and policy implications for TCH, as they partner with the City of Toronto to design a new integrated service model for the seniors-designated buildings.

### WHICH SEAT AT THE TABLE? THE WAYS THAT SENIOR SERVICE ORGANIZATIONS ARE ENGAGED IN AGE-FRIENDLY COMMUNITY EFFORTS

Caitlin Coyle, and Shayna Gleason, *University of Massachusetts Boston, Boston, Massachusetts, United States*

Senior centers across the nation continue to serve as important access and focal points for older adults to voice their desires, get basic needs met, and to engage in opportunities that support many of the key concepts of Age-Friendliness (i.e. social participation, respect and social inclusion, civic engagement, transportation, and community supports and health services). Senior centers are the front line of aging services and thus in a position to implement programs and raise public awareness about age-friendly initiatives. The purpose of this presentation is to present and discuss the involvement of senior centers or other senior service agencies, as well as to characterize the mobilization of a community after joining the movement. We will present 5 case studies of age-friendly communities who are in the implementation phase of their initiative, to illustrate the challenges, opportunities, and outcomes associated with placing a senior center at the forefront of the movement. Based on the results of this work, we will present a typology of age-friendly community initiatives as a mechanism for supporting other communities make this transition. We conclude with a discussion of how age-friendly communities are part of the paradigm shift of aging in community and the ways in which this work intersects with other health policy initiatives with which cities and towns engage.

### SESSION 2814 (PAPER)

### SOCIAL ISOLATION AND SOCIAL SUPPORT

### DEPRESSIVE SYMPTOMS AND LONELINESS AMONG BLACK AND WHITE OLDER ADULTS: THE MODERATING EFFECTS OF RACE

Harry Taylor,[1] and Ann Nguyen,[2] *1. Duke University Medical Center, Durham, North Carolina, United States, 2. Case Western Reserve University, Cleveland, Ohio, United States*

Loneliness is consistently linked to worse depression/depressive symptoms; however, few studies examined if this relationship varies by race. The purpose of this study was to determine if race moderated the relationship between loneliness and depressive symptoms among a nationally representative sample of older Black and White adults. Data come from the 2014 wave of the Health and Retirement Study (HRS) Core survey and Psychosocial Leave Behind Questionnaire; only Black and White older adults were included in the analysis (N=6,469). Depressive symptoms were operationalized by the CESD; however, the 'felt lonely' item was removed given concerns with collinearity. Loneliness was operationalized using the Hughes 3-Item Loneliness Scale. Sociodemographic variables included gender, age, education, household income, employment status, marital status, and living alone or with others. Furthermore, social support and negative interactions from family members and friends, and religious service attendance were included in the analysis. Lastly, we created an interaction term with race and loneliness. All analyses used survey weights to account for the complex multistage sampling design of the HRS. Missing data were multiply imputed. Older Blacks had higher rates of loneliness and depressive symptoms compared to older Whites. In multivariate analysis, we found race significantly moderated the relationship between loneliness and depressive symptoms while controlling for sociodemographic, social support, negative interaction, and religious attendance covariates. For both older Blacks and Whites, greater loneliness affected depressive symptoms; however, the effect was stronger among Whites than it was for Blacks. Findings can be used to create racially sensitive depression interventions.

### GEOGRAPHIC DISTANCE AND SOCIAL ISOLATION AMONG FAMILY CAREGIVERS PROVIDING CARE TO OLDER ADULTS

Lun Li, and Andrew Wister, *Simon Fraser University, Vancouver, British Columbia, Canada*

Family caregiving is associated with social isolation, but the role of geographic distance between caregiver and receiver in caregiving experience is unclear with mixed research findings. This study examined the relationship between geographic distance and caregiver social isolation (CSI), and explored the interaction between geographic distance and caregiving intensity in association with CSI. Based on the Ecological Model of Caregiver Isolation, hierarchical linear regression and ANCOVA analyses were applied to conduct data analysis with the 2012 Canadian General Social Survey (N=2,881). Caregivers living a short distance from receivers reported the lowest CSI than coresident, moderate and long distance caregivers. Being involved in higher intensity caregiving as the primary caregiver, undertaking more caregiving tasks, and providing care more frequently resulted in higher CSI scores. Additionally, long and moderate distance caregivers reported greater CSI than coresident and short distance caregivers only when providing higher intensity caregiving. Geographic distance is a salient contextual factor affecting CSI, and longer distance creates environmental barriers for caregiving provision. Employing a granulated measure of geographic distance positioned within an ecological framework facilitates an understanding of the nuanced association between geographic proximity and CSI. Furthermore, the identified interactive effects between geographic distance and caregiving intensity on CSI further reveal the complexity of caregiving experience. The findings are relevant for programs supporting caregivers in different contexts, especially physical distance.

### HOW LONELINESS, DEPRESSED MOOD, AND DIET AFFECT EATING HABITS AND GROCERY SHOPPING

Erica Solway,[1] Dianne Singer,[1] Preeti Malani,[2] Jeffrey Kullgren,[1] Matthias Kirch,[1] Cindy Leung,[1] and Julia Wolfson,[3] *1. University of Michigan, Ann Arbor, Michigan, United States, 2. University of Michigan Medical School, Ann Arbor, Michigan, United States, 3. University of Michigan School of Public Health, Ann Arbor, Michigan, United States*

This study examined the prevalence of loneliness and relationship with cooking and eating habits among older adults using data collected in December 2019 from a nationally representative sample age 50-80 through the National Poll on Healthy Aging. Older adults who live alone were more likely those who lived with others (19% vs. 8%) and those with a high PHQ-2 score were more like than those with a low PHQ-2 score (20% vs. 11%) to report that they do not cook dinner most days (0-2 times/week). Those who rated their diet as fair/poor were more likely to indicate they cook few meals a week (0-2) compared to those who reported a good or very good/excellent diet (18% vs. 11 % vs 8%).

Those with a high PHQ-2 score were less likely than those with a low PHQ-2 score to say they do major food shopping less than once a week (31% vs 47%). Those with a high PHQ-2 score were more likely to say that that always ate alone in the last week compared to those with a low PHQ-2 score (17% vs 7%). These findings demonstrate that older adults who lived alone, had a higher PHQ-2 score, and had a poorer diet were more likely to cook and do grocery shopping less often. Strategies and policies to support older adults to address depressive symptoms and to increase cooking and improve diet may have many health and social benefits and will be explored through this session.

### I AM NOT INVISIBLE: THE IMPACT OF AGE DISCRIMINATION IN THE WORKPLACE

Jasmin Tahmaseb McConatha, Frauke Schnell, Lauren Stricker, and Jacqueline Magnarelli, *West Chester University, West Chester, Pennsylvania, United States*

Ageism and age stereotypes are widespread. They shape the lived experiences of older workers. This presentation focuses on the results of responses to an online survey exploring the impact of ageist treatment in the workplace. The results of online surveys from 113 teachers over the age of 50 indicated that ageist treatment is widespread. An analysis of open ended questions addressing the stressful impact of being victimized by ageism indicated that feeling invisible, isolated, and helpless are the three most common responses to ageist treatment in the workplace. Being victimized by ageism presents a threat to older workers sense of self and feelings of competence. The cultivation hypothesis suggests that in technologically advanced societies such as the United States, people often rely on the media as a primary source of cultural information. Media images tend to depict older adults in ways that maintain and create ageist stereotypes. Our research suggests that the framing of media content significantly influences the self-worth of older workers. In this presentation, we discuss examples of ageism in the workplace, the family, and the media, and discuss ways of combating biased and discriminatory treatment. Based on our ongoing research, we make suggestions for ways of responding to and coping with ageist treatment.

### MEASURING CHILDLESSNESS AMONG MIDDLE-AGED AND OLDER AMERICANS

Xiao Xu,[1] Jersey Liang,[2] BoRin Kim,[3] James Raymo,[4] and Mary Beth Ofstedal,[2] *1. Yale University, New Haven, Connecticut, United States, 2. University of Michigan, Ann Arbor, Michigan, United States, 3. University of New Hampshire, Durham, New Hampshire, United States, 4. Princeton University, Princeton, New Jersey, United States*

Existing literature on childlessness among middle-aged and older Americans is sparse, and measuring childlessness is not straightforward for those with complex family histories. To address this knowledge gap, we examined data on 19,929 respondents age ≥50 from the 2016 Health and Retirement Study. All analyses accounted for complex sample design to generate nationally representative estimates. The proportion of respondents without children differed significantly depending on how "childless" was defined: 1) 14.9% (95% confidence interval [CI]: 13.9-15.9%) having no biological children, versus 2) 10.4% (95% CI: 9.5-11.3%) having no children/step-children that were living and in-contact. When measured based on absence of biological children, the prevalence of childlessness was higher in younger cohorts (17.7%, 13.2%, and 9.0% for age 50-64, 65-74, and ≥75 years, respectively, $p<0.001$) and among more educated individuals (17.4%, 12.3%, and 9.6% for more than high school, high school, and less than high school education, respectively, $p<0.001$). The prevalence of childlessness was also higher among men (16.7%) than women (13.2%) ($p<0.001$) and among non-Hispanic whites (16.0%) than Hispanics (9.8%) ($p<0.001$). Similar patterns, but lower prevalence, were observed when measuring childlessness based on absence of children/step-children that were living and in-contact. Although non-Hispanic whites (16.0%) were more likely than non-Hispanic blacks (13.0%) to have no biological children ($p=0.007$), a similar proportion of them had no children/step-children that were living and in-contact (10.8% versus 10.6%, $p=0.06$). Given fertility decline and growing family complexity, these findings help inform the structure of social support and long-term care needs of middle-aged and older Americans.

### SESSION 2817 (PAPER)

### INFORMAL AND FORMAL CAREGIVING ISSUES

### CAREGIVER MENTAL HEALTH OUTCOMES: ARE THERE DIFFERENCES ACROSS GENERATIONS?

Susanna Mage,[1] Laura Rath,[2] Kathleen Wilber,[3] and Zachary Gassoumis,[3] *1. University of Southern California, Leonard Davis School of Gerontology, Los Angeles, California, United States, 2. Archstone Foundation, Long Beach, California, United States, 3. University of Southern California, Chatsworth, California, United States*

Informal caregivers are a critical component of support for the rapidly aging population. Previous studies have addressed the effects of caregiving on mental health. However, they have not focused on differences among generational cohorts of caregivers of older adults, i.e., Millennial (born 1981-1996), Generation X (born 1965-1980), Baby Boomer (born 1946-1964), and Silent Generation (born 1928-1945). As the Millennial caregiver population grows in parallel with older adults and their increased needs, we must better understand Millennial responses to caregiving. Millennial caregivers provide a similar intensity of care as Baby Boomers in terms of hours per week but are more likely to be fully employed (40+ hours per week or more). We used caregiver data from the nationally representative Centers for Disease Control's Behavioral Risk Factor Surveillance System (BRFSS) survey from 2015-2017 to conduct negative binomial regression (n=50,745). Data analysis indicates that Millennial caregivers have an incidence rate ratio of 1.22 times more self-reported days of "stress, depression, and/or problems with emotions" compared to Generation X caregivers ($p<0.01$); 1.64 times compared to Baby Boomers ($p<0.001$); and 2.38 times compared to Silent Generation caregivers ($p<0.001$). Generational differences show that Millennial caregivers may have different needs than older generations of caregivers. Rather than assuming that the policies and interventions designed for older generations of caregivers will fit younger generations, implications of this work can help inform: 1) the design of programs to support caregivers' mental health, and 2) policy considerations that address the unique needs of a younger caregiver population.

### DIVERSE FAMILY CAREGIVERS, DIVERSE EXPERIENCES IN CARING

Heather Young,[1] Janice Bell,[1] Rita Choula,[2] Tina Kilaberia,[3] and Susan Reinhard,[2] *1. University of California Davis, Sacramento, California, United States, 2. AARP, Washington, District of Columbia, United States, 3. UC Davis Health, Sacramento, California, United States*

The Home Alone Revisited Study examined the experience of family caregivers including demands of caring for someone with physical, mental or behavioral health care needs, revealing a large proportion of caregivers managing medical/nursing tasks. The aim of this paper was to explicate racial/ethnic differences in the prevalence of tasks and the perceptions of caregivers regarding their responsibilities. The study was a nationally representative, population-based online survey that oversampled underrepresented groups. The sample included 2,089 diverse family caregivers (Hispanic, n=307; Black/African American, n = 231; Asian/Pacific Islander, n=196; White, n=1,284; multi-racial, n= 71), 57% women, ranging from 18 to 91 years of age. Overall, fifty-two percent of caregivers reported performing at least one medical/nursing task for their care recipient, with a greater percentage ($p<0.01$) engaged in these tasks in two groups: Asian/Pacific Islander (62%) and multiracial (62%). Across the five racial/ethnic groups, we found significant differences in having a choice, feeling pressure, and the influence of culture and religion on the decision to take on medical/nursing tasks. There were also significant differences in the prevalence of performing 17 specific tasks, reports of difficulty with the tasks, the extent to which respondents felt prepared, and the strain that they experienced in providing care. The findings reveal both commonalities and significant heterogeneity in the caregiving experience, suggesting the importance of advancing the dialogue among health and social service providers about providing culturally and linguistically appropriate care and developing responsive tools and resources to support family caregivers as they engage in complex care.

### GRANDPARENTING AND HEALTH IN LATER LIFE: INTENSITY AND AGE, GENDER, AND URBANICITY VARIATIONS

Yue Zeng, and Yu-Chih Chen, *The University of Hong Kong, Hong Kong, China*

Grandparents play increasingly indispensable roles in providing family care. Although prior cross-sectional studies have shown a positive link between grandparenting and health, we know little about the optimal engagement level of grandparenting, its longitudinal implications, and variations on health outcomes. Guided by the role theory and social model of health promotion, we used propensity score analysis and multilevel analysis with three biennial waves of China Health and Retirement Longitudinal Study (2011-2015) to examine the longitudinal impacts of grandparenting intensity (no, low-, moderate-, and high-intensity) on health (mobility limitations, depressive symptoms, cognition, and self-rated health) among 4,925 older adults aged 45 and older, and how these impacts vary by age (45-59/60+), gender (male/female), and urbanicity (urban/rural). Controlling for the baseline sociodemographics (e.g., education and income), health limitations (e.g., ADLs and IADLs), and health behaviors (e.g., drinking and smoking), our results showed that, compared to no grandparenting, grandparenting provided at a moderate level was associated with fewer mobility limitations, lower depressive symptoms, and better cognition. Furthermore, grandparenting had a positive impact on physical, mental and cognitive health for 60+ older adults but not for the young-old. Both older males and females showed better physical health if they provided care at a low level, but older females showed better self-rated health. Older adults in the rural area showed better physical health; for the urban area older adults, better cognition. Findings suggest that policies aimed at supporting grandparents should consider the optimal threshold and variations by age, gender, and urbanicity.

### RURAL-URBAN DIFFERENCES IN SOCIAL CONNECTEDNESS AMONG ADULT FOSTER HOME RESIDENTS IN OREGON

Ozcan Tunalilar, *Portland State University, Portland, Oregon, United States*

Social isolation has been linked to negative health outcomes, especially among older adults. Although ability to maintain social contact and existing ties to one's community is a primary benefit of receiving long-term supports and services in a community-based setting, few studies explored how geography might shape these residents' access to family members and friends. The current study explores this question in the context of adult foster homes (AFH), a type of family-style residential care licensed for five or fewer unrelated adults. Using cross-sectional data collected annually from 1,500 AFHs between 2015 and 2020, the study examines whether older adults residing in rural and urban AFHs in Oregon differ in terms of levels of distinct types of contact with their existing social networks. AFHs were designated as rural/urban at the zip code level using the definitions provided by the state coordinating organization for rural health. Results from negative binomial regression models show that rural residents were significantly less likely to receive help from their family members and friends in getting to medical appointments or outside activities (e.g., meals, walks, shopping) or receive social visits or phone calls compared to their urban counterparts. Rural and urban residents had similar levels of help with personal care and taking medications. These results remained unchanged after accounting for a set of home (e.g., Medicaid contract) and resident characteristics (e.g., acuity). These findings suggest important public health implications for improving rural residents' social connectedness and interventions aiming at improving social participation in long-term care residents.

### THE CAREGIVER TRANSPORTATION SCALE MEASURES THE IMPACT OF DRIVING CESSATION ON CAREGIVERS

Michel Bedard, Shauna Fossum, Dwight Mazmanian, Hello Møller, and Jessica Lowey, *Lakehead University, Thunder Bay, Ontario, Canada*

Driving cessation can impact retiring drivers, but we often neglect to consider its effect on caregivers. Caregivers may have to deal with important changes when someone they care for ceases driving, but we have few means to quantify these changes. Hence, we aimed to develop the Caregiver Transportation Scale (CTS) to measure this impact. We developed a bank of positive and negative questions, then pretested it with a small sample of caregivers (N = 11), leading to reduction and refinement of the questions. We pilot-tested this set of questions with a larger caregiver sample (phase 2; N = 73). Preliminary validation of the tool relied on correlation analyses with the Zarit Burden Interview (ZBI) and relevant demographic questions. For phase 2, the mean caregiver age was 61.9 (SD = 10.23, range 20-83); most caregivers were female (80.8%) and were adult-child caregivers (61.7%). The final version of the CTS contains 24 items. Internal consistency (Cronbach's alpha) was .90. The mean caregiver score was 64.75 (SD = 16.76, range 24-98); about 1/3 of caregivers' scores fell above the middle possible score of 72. The scores were positively correlated with the Zarit Burden Interview ($r = .74$, $p < .001$) and negatively correlated with the availability of others to help with driving responsibilities ($r = -.36$, $p = .002$). The CTS has the potential to help inform, develop, and evaluate services for caregivers who provide transportation support for older adults who ceased driving. However, further validation is required before we recommend its use.

### SESSION 2818 (PAPER)

### DIVERSITY IN AGING: RACE AND ETHNICITY

### AGE AT IMMIGRATION AND DEPRESSION IN OLD AGE: THE MEDIATING ROLE OF CONTEMPORARY RELATIONSHIPS WITH ADULT CHILDREN

Heejung Jang, *University of Michigan, Ann Arbor, Michigan, United States*

For the growing population of older immigrants in the United States, both age at immigration and familial relationships are important factors affecting psychological well-being. This study explores how age at immigration and contemporary relationships with adult children combine to explain older immigrants' depressive symptoms. This study uses 2014 Health and Retirement Study data from a sample of 759 immigrants age 65 and older who have at least one adult child age 21 or older. A series of ordinary least squares regressions and mediational analyses were conducted. Findings indicate that two aspects of familial relationships, associational solidarity and structural solidarity, significantly mediate the association between age at immigration and depressive symptoms. Specifically, immigrating in later-life was associated with a lower level of depressive symptoms through its relationship with structural solidarity. Immigrating in later-life was also associated with a higher level of depressive symptoms through its relationship with associational solidarity. In addition, giving monetary support to children and providing care for grandchildren may alleviate depressive symptoms for older immigrants. This study suggests that relationships with adult children may differ with age at immigration. The types of support that older immigrants provide to their adult children may be crucial because such support may instill a sense of obligation and reciprocity that may be beneficial to the psychological well-being of older immigrants.

### DISCERNING UNMET SELF-CARE NEED AND CO-DESIGN OF A MOBILE APP WITH CHINESE IMMIGRANT CAREGIVERS TO PROMOTE SELF-CARE

Kexin Yu,[1] Haojun Jiang,[2] Mandong Liu,[3] Shinyi Wu,[3] Maryalice Jordan-Marsh,[3] and Iris Chi,[3] *1. University of Southern California, Palo Alto, California, United States, 2. Nanjing Agricultural University, Nanjing, China, 3. University of Southern California, Los Angeles, California, United States*

Immigrant caregivers are the backbone for supporting frail older adults in the United States. However, caregivers' own self-care need is often neglected, especially among racial/ethnic minorities. In the current study, a co-design approach was employed to develop a mobile app for raising awareness and promoting behaviors of self-care among Chinese immigrant caregivers. Individual in-depth interviews conducted through two co-design phases, i.e., conceptual design and prototype design. Twelve caregiver participants voiced their unmet self-care need and expressed their opinions about adopting the co-designed mobile App to access self-care information. The unmet self-care need includes both self-care barriers and supportive resources, and App features the caregivers wish to have. Three self-care barriers were identified in the co-design interviews, including culture-specific stressors, immigrant-status related challenges, and work-related restrictions. Caregiver participants expressed that they wish the care recipients could have education on the boundaries between them and a caregiver. Additionally, the participants reported wanted to learn about handling care emergencies with the co-design app and would like to see short videos and stories included in the App. Participants expressed mixed opinions towards adopting mobile technology – while most of the participants appreciated the content provided, some were concerned that learning with mobile technology could add to their burdens, and technical difficulties could prevent them from using the App. Designing the App to be engaging and fun emerged as highly desirable. The co-design process appears to be beneficial in having participants to articulate both current self-care barriers and preference for a mobile technology tool.

### EXAMINING RACIAL AND ETHNIC DISPARITIES AMONG OLDER ADULTS IN LONG-TERM CARE FACILITIES

Kathy Lee,[1] Rebecca Mauldin,[1] John Connolly,[1] and Weizhou Tang,[2] *1. University of Texas at Arlington, Arlington, Texas, United States, 2. University of Southern California, Los Angeles, California, United States*

Background: Nearly half-million older adults from minority racial and ethnic groups in long term care face disparities in quality of life and quality of care. However, there is little information about the associations between a resident's race/ethnicity and the types of official complaints lodged. Methods: This project was a mixed methods study using a sequential explanatory design to examine ethnic and racial differences in types of complaints and rates of complaint resolution in a local Ombudsman Program. First, resident race/ethnicity and complaint data were collected from the Ombudsman Program and analyzed. Then, we conducted focus groups with Ombudsman Program staff and volunteers to provide a more complete interpretation of findings from the first phase. Results: Residents from ethnic/racial minority groups were less likely to generate Resident Care complaints and more likely to generate Resident Rights complaints, compared to non-Hispanic White residents (p<.05). Resident Rights, Quality of Life, and Administrative complaints were less likely to be disposed satisfactorily, compared to Resident Care complaints (p<.05). Themes emerged from our qualitative findings include language barriers and more efforts required for residents' rights due to concerns raised more frequently among minority residents. Implications: Cultural competence training for Ombudsmen as well as care professionals should focus on skills and knowledge that value diversity, understand and respond to their unique concerns. Ombudsmen play an important role as they create an avenue for the residents to discuss their concerns. Implementation research may improve our understanding of the development and delivery of the Ombudsman Program.

### MITIGATING THE IMPACT OF LONG-TERM CONSTRUCTION ON THE HEALTH OF OLDER ADULT RESIDENTS IN NEW YORK CITY'S CHINATOWN

Simona Kwon, Yi-Ling Tan, Jennifer Wong, and Janet Pan, *NYU School of Medicine, New York, New York, United States*

Introduction: Recent proposed major construction projects in New York City's Chinatown often last multiple years. Little is known about the health impact of construction on vulnerable populations such as older adults. In Chinatown, approximately 20% of residents are older adults, live below the poverty level (34%), have a disability (47%), and nearly half report limited English proficiency. Objectives: We are conducting a mixed methods study to describe possible health and psychosocial outcomes of construction on older adults in Chinatown. Methods: We used a community-engaged modified Delphi process to identify priority areas related to construction and older adults which included: 1) a scoping review of the health impact of long-term construction; 2) key informant interviews of academic experts; and 3) convened community stakeholder leaders to review key focus areas and evidence-informed, culturally-relevant mitigation strategies. Five priority topics were identified: 1) Construction site emissions; 2) Noise; 3) Outdoor nocturnal lighting; 4) Neighborhood changes; and 5) Relocation. Results: Long-term construction contributes to adverse effects of air pollution, noise, and changes in the environment, with exposure to particulate matter and unwanted noise associated with higher morbidity and mortality. Unsafe sidewalk due to construction increase the risk of falling, the leading cause of death among NYC seniors. Construction-related stressors may isolate older adults from vital services and social networks. Conclusion: Long-term construction poses serious health implications for older adults. Stakeholders should adopt a community-engaged approach and identify meaningful community priorities to inform practical solutions to mitigate the impact of construction on vulnerable Chinatown older adults.

### OLDER ADULTS' REFLECTIONS ON AGEISM AND RACISM

Janna Heyman,[1] Colette Phipps,[2] Peggy Kelly,[1] and Linda White-Ryan,[1] *1. Fordham University, West Harrison, New York, United States, 2. Westchester County, West Harrison, New York, United States*

The older population is becoming more racially and ethnically diverse. By the year 2050, 39% of those 65+ will be from minority groups, up from 21% in 2012 (Ortman et al., 2014). These figures have significant implications for aging policy, including concerns over ageism and racism. Discrimination can take many forms, and can be present in legislation, advertising, attitudes, the workplace, and the health care system (Snaedal, 2015). The present study examines perceptions of racial and age discrimination of older adults living in the community and its impact on their quality of life. Using a cross-sectional design, 134 participants over the age of 60 were surveyed at three senior centers with ethnically diverse populations. The Attitudes to Aging Questionnaire (AAQ-24) was used to assess participants' perceptions and experiences with aging and perceptions of racism were assessed using an adaptation of the Modern Racism Scale. Findings from the AAQ-24 revealed an average score of 27.1 (SD=6.66) for psychosocial loss, 28.3 (SD=5.34) for physical change, and 30.5 (SD=4.65) for psychological growth, indicating moderately high levels of ageism. For the racism scale, the average total score for all respondents was 34.4 (SD=7.05), also moderate. This study helps shed some light on what older adults feel about the aging, as well as their concerns with racial discrimination. The insights gained from older adults' experiences and perceptions can help shape policies for future generations.

### SESSION 2819 (PAPER)

### SOCIAL SUPPORTS IN DIVERSE AGING EXPERIENCES

### AN EVALUATION OF THE DEMENTIA FRIENDS USA PROGRAM IN NEVADA: CHANGES IN KNOWLEDGE AND BEHAVIORAL INTENT

Jennifer Carson,[1] Peter Reed,[2] and Zebbedia Gibb,[2] *1. University of Nevada, Reno, Reno, Nevada, United States, 2. University of Nevada, Reno School of Medicine, Reno, Nevada, United States*

Dementia Friendly Nevada (DFNV) aims to develop and promote communities in becoming more respectful, educated, supportive and inclusive of people living with dementia and their care partners. To date, six communities are engaged, representing urban, rural and tribal communities. Each community convened an action group comprised of volunteers from a range of sectors, including people living with dementia as key participants. Each group used a participatory action research process to assess community needs, develop specific goals and actions, and document community change. As part of this process, five of the six communities adopted the Dementia Friends program for community education and awareness raising. This program is licensed nationally by Dementia Friends USA. From June 2018 – September 2019, these communities achieved widespread dissemination of this program, training 68 Dementia Friends Champions (i.e., trainers), who in turn delivered 55 training sessions to 607 new Dementia Friends (i.e., completers) across Nevada. To evaluate the impact of the program, DFNV partnered with the Sanford Center for Aging to conduct a pre-post survey (n = 504) to assess program-related knowledge change as well as commitments to take action. Results showed a statistically-significant (p <.001) increase in participant knowledge, with 17% higher scores at the post-assessment. In addition, given 10 options for specific actions, responses ranged from 12% of participants committing to “start a dementia friendly effort” to 70% committing to “support dementia friendly efforts.” Overall, the early impact of this ongoing effort to educate the public about dementia across Nevada has been very successful.

### EXPLORING THE EFFECTS OF ARTS INTERVENTION GROUPS ON WELL-BEING AMONG OLDER ADULTS

Emily Ihara,[1] Kathryn McNeil,[1] Adriana Lopez-Piper,[1] Maxine Eber,[2] Catherine Tompkins,[1] Holly Matto,[1] Niyati Dhokai,[3] and Jatin Ambegaonkar,[3] *1. George Mason University, Fairfax, Virginia, United States, 2. George Mason University, Vienna, Virginia, United States, 3. George Mason University, Manassas, Virginia, United States*

Engaging in the arts reportedly improves well-being, but research is limited on the specific effects for community-dwelling older adults. The purpose of this randomized controlled trial was to examine how taking part in different arts interventions (dance & music), affects older adults’ overall well-being compared to a social conversation control group. Sixty-four participants (mean = 71 years old) participated twice weekly in a 10-week intervention that included ballroom dancing (n=23), ukulele playing (n=17), and social conversation (n=24). At the conclusion, three focus groups were held to assess participants’ experiences and subjective evaluation of the interventions’ impact. Twenty-two out of the sixty-four participants (dance= 8, ukulele = 6, social conversation = 8) took part in focus groups. Transcripts of the recorded focus groups were independently coded and compared. Common themes were agreed-upon by two researchers. Focus groups revealed positive outcomes for participants in all three groups. Several themes emerged across the intervention groups compared to the control group, including participants feeling challenged as they crossed their comfort zones, reporting increased confidence, enhanced social connections, and a sense of accomplishment when learning new skills. Community-dwelling older adults reported improved health-related outcomes after taking part in arts and social conversation sessions. Implementation of community-engaged arts intervention programs for older adults in the future may examine motivators which attract participants, foster positive social connections during sessions, and use participant-empowering pedagogical adaptations to retain participants. These factors can increase the efficacy of arts-engaged programs and help improve well-being in older adults.

### FINANCIAL EXPLOITATION VULNERABILITY AND SOCIAL ISOLATION IN OLDER ADULTS: RESULTS FROM A LONGITUDINAL SURVEY

Gilbert Gimm,[1] and Scott Beach,[2] *1. George Mason University, Vienna, Virginia, United States, 2. University of Pittsburgh, Pittsburgh, Pennsylvania, United States*

OBJECTIVE: This study examined the prevalence and characteristics of socially isolated older adult participants in a survey funded by National Institute of Aging (NIA), and the association of social isolation with financial exploitation (FE) vulnerability. METHODS: Baseline data were collected from 528 participants (age 60+ years) and recruited from two regions. Assessments included a detailed cognitive battery and an array of self-report measures including socio-demographic and psychosocial variables. Social isolation was measured with the Friendship Scale (Hawthorne, 2006). Participants also read six randomly ordered “scam” scenarios (investment opportunity; Medicare phishing and prescription drug fraud; health product; sweepstakes; telemarketing) and provided credibility ratings for each (1 = Not at all credible; 10 = Extremely credible). Ratings are coded “0” (credibility rating 1); “1” (ratings 2-4); “2” (ratings 5-10) and summed, with total FE “vulnerability” scores ranging from 0-12. RESULTS: Preliminary results showed the prevalence of social isolation was 12.5% overall. Social isolation was higher for males (17.4% vs. 9.8%, p<.05); single / never married (28.7% vs. 12.5%, p<.01); and those in the lowest household income (under $10,000) category (28.3% vs. 12.5%, p<.05). However, we did not find evidence that social isolation varied significantly by race or educational attainment. FE vulnerability risk was higher for socially isolated older adults (2.44 vs. 1.44, p<.001) compared to non-isolated participants. CONCLUSION: Social isolation is associated with FE vulnerability. Therefore, identifying the prevalence and characteristics of socially isolated older adults is needed to improve the targeting of interventions for those at greater risk of FE vulnerability.

### THE ASSOCIATION BETWEEN SPOUSAL CAREGIVER STATUS AND PSYCHOLOGICAL WELL-BEING AMONG OLDER ADULTS

Yang Wang, *University of Illinois at Urbana-Champaign, Urbana, Illinois, United States*

Older adults often bear the responsibility of taking care of their spouses who have physical or cognitive impairments. Although previous studies suggested various caregiving-related negative experiences might be related to older spousal caregivers’ mental health status, the components of psychological well-being (PSW) among older spousal caregivers have not been fully explored. This study examined the association between spousal caregiver status and PSW. Data were drawn from wave 2014 of the Health and Retirement Study. The sample consisted of 3,857 adults who were above 50, and 376 of the participants provided care in activities in daily life (ADL) or instrumental activities in daily life (IADL) to their spouses/partners. Three hundred and thirty-one of them had a spouse/partner who needed care, but did not provide the care to their spouse/partner. The majority of participants,

3,150 of them, did not provide care, and their spouse/partner did not need care. Four domains of PSW were assessed: purpose of life, life satisfaction, positive affect, and negative affect. Univariate analyses and multivariable linear regression were used for analyses. Spousal caregivers and non-caregivers with need had a worse PS status, compared to non-caregivers without need. But after adjusting for socio-economic resources, health status, or social support factor, the difference in their four domains of PSW was insignificant. Older spousal caregivers' PSW could be protected by promoting that population's socio-economic resources and health status. Future practice needs to address caregivers' emotional need. Future research need to examine the long-term effect of caregiving on older spousal caregivers' PSW.

### WHAT ROLE DO DIFFERENT TYPES OF SOCIAL SUPPORT PLAY IN LIFE SATISFACTION AMONG KOREAN OLDER ADULTS IN RETIREMENT?

Jihee Woo,[1] and Hyojin Choi,[2] *1. University of Pittsburgh, Pittsburgh, Pennsylvania, United States, 2. Sungkyunkwan University, Seoul, Republic of Korea*

Individuals are taking more responsibility for their retirement. However, economic stress generated by inadequate planning may pose a grave threat to well-being in retirement. Drawing on stress theory, this study examined how different types of social support mediate the relationship between economic stress and life satisfaction. We used the data from the 2013 and 2014 Korean Retirement and Income Study. Our sample was restricted to older Korean adults in retirement 55 to 96 years of age who were head of household (N=1,672). Confirmatory factor analysis was used to evaluate the measurement model for six latent constructs: 1) economic stress; 2) emotional support; 3) informational support; 4) instrumental support; 5) appraisal support; 6) life satisfaction. Structural equation modeling (SEM) was used to test this hypothesized model. The results revealed that both measurement model (CFI=0.985, TLI=0.983, RMSEA=0.036, SRMR=0.035) and structural model (CFI=0.978, TLI=0.984, RMSEA=0.030, SRMR=0.039) fit the data well. Standardized results from the SEM model adjusting for sociodemographic variables showed that economic stress directly predicted life satisfaction ($\beta$=-0.39, p<0.001) and two of four social support types directly predicted life satisfaction (instrumental support $\beta$=0.29, p<0.001; appraisal support $\beta$=0.25, p=0.004). Analyses demonstrated that economic stress may lead to lower levels of life satisfaction directly and indirectly through its effect on instrumental ($\beta$=-0.07, p<0.001) and appraisal support ($\beta$=-0.04, p=0.013). These findings will help inform policymakers and institutions of the need to alleviate economic stress and increase particular types of support with potentially more serious impact on the well-being of older adults in retirement.

### SESSION 2820 (PAPER)

### ECONOMICS OF AGING

### CHILDREN'S EDUCATION AND PARENTS' COGNITIVE AGING: EVIDENCE FROM THE COMPULSORY SCHOOLING LAW IN CHINA

Zhuoer Lin, and Xi Chen, *Yale University, New Haven, Connecticut, United States*

This paper investigates the effect of promoting children's education on level and trajectory of parents' cognition. Using three waves of China Health and Retirement Longitudinal Study (2011, 2013, 2015), we decompose the longitudinal measures of parents' cognition into baseline level of cognitive deficit and rate of cognitive decline through a standard linear mix-effect model (aged 45-100, N=9,736). Exploiting the temporal and geographic variations in the Chinese compulsory schooling law enforcement, we identify the causal effects of children's education on parents' baseline level of cognitive deficit and rate of cognitive decline using instrumental variable (IV/2SLS) approach. Our estimates suggest that children's education not only reduces parents' level of cognitive deficit (0.17 SD/year less), but also decreases their rate of cognitive decline (0.08 SD/year less). These novel evidence offers new insights into the intergenerational effects of children's education on parents' cognition, stresses the importance of lifting literacy in improving accuracy of cognitive diagnosing, and provides another policy option to delay cognitive aging and related diseases.

### DOES LENGTH OF TIME CAREGIVING AFFECT NET WORTH? CAREGIVERS APPROACHING RETIREMENT AGE

Shirley Porterfield,[1] Linda Quinn,[2] Patricia Stoddard Dare,[2] LeaAnne DeRigne,[3] Miyuki Fukushima Tedor,[2] and Cyleste Collins,[2] *1. University of Missouri–St. Louis, St Louis, Missouri, United States, 2. Cleveland State University, Cleveland, Ohio, United States, 3. Florida Atlantic University, Boca Raton, Florida, United States*

Using a nationally representative sample of N=3,659 adults (mean age 55.6 in 2016) from the 2008, 2012, and 2016 rounds of the National Longitudinal Survey of Youth 1979, we examine the impact of work-limiting health conditions, caregiving, and the length of time spent in a caregiving role inside and outside the home on total family net worth. Repeated measures analysis finds having a chronically ill or disabled household member (CIOD), or caring for a household member with a CIOD inside the home was associated with reduced total family net worth (TFNW; -$146.7, p<0.002), on average, across the three time periods. In-home caregiving across time periods does not have an additive effect, but rather appears to result in a one-time drop in TFNW. Respondents who provide care for out-of-home family or friends report higher TFNW on average over time ($73.3K, p<0.001). Respondents with a work-limiting health condition report a lower mean TFNW over this time span (-$53.1K, p<0.005). Caregiving inside the home has 2-3 times the impact on TFNW as having a work-limiting health condition, though in the overall model, the effect of the two variables together on TFNW is additive. An adult is at the greatest disadvantage with respect to financial preparedness for retirement if they are both a caregiver (or have a CIOD in their household) and have a work-limiting health condition than if they have only one of these characteristics or have neither characteristic. Even a single time period of caregiving reduces total family net worth over time.

### EFFECTS OF PAID LEAVE POLICIES ON WORKING CAREGIVERS' LABOR SUPPLY

Soohyun Kim, *Columbia University, New York, New York, United States*

Approximately two in three caregivers are in the labor force. However, paid family leave is the only policy support for working caregivers to date, which helps to balance their work and care responsibilities. I analyze the data from the 1998-2014 Health and Retirement Study to examine 1) how paid leave policies affect labor market outcomes for workers in need of caring for a spouse/partner or an older parent/-in-law and 2) how the effects differ by gender. Paid leave policy is distinguished between employer-provided leave and state government-provided leave. Using the first-difference approach, I compare the short-term and long-term changes in the extensive and intensive margins of labor with and without access to paid leave policies when a health deterioration of the older family member occurs. My preliminary results show that, without paid leave policy, the health event of a spouse/partner or an older parent/-in-law affects women's labor supply but not men's. Paid leave provided by an employer increases the labor supply for both women and men, with the more noticeable long-term effects for men. State paid family leave increases women's wage and salary both in the short run and in the long run. My findings underline the importance of paid leave policy for retaining the workers in need of providing care for a family member, particularly for women.

### IDENTITY THEFT AMONG OLDER ADULTS: RISK AND PROTECTIVE FACTORS

Marguerite DeLiema,[1] Lynn Langton,[2] and David Burnes,[3] *1. University of Minnesota, Twin Cities, Saint Paul, Minnesota, United States, 2. RTI International, Washington, District of Columbia, United States, 3. University of Toronto, Toronto, Ontario, Canada*

Although financial exploitation and fraud targeting older adults have been the focus of increasing academic attention, research on identity theft among older adults is virtually nonexistent. Identity theft refers to an intentional, unauthorized transfer or use of a person's identifying information for unlawful purposes (Federal Trade Commission 1998). Society's growing reliance on technology to transfer and store private information has created increased opportunities for financial predators to access and misuse personal data. Results from the most recent Bureau of Justice Statistics' Identity Theft Supplement show that nearly 1 in 10 adults aged 65 or older experienced identity theft in the past year, with financial losses totaling $2.5 billion. Given the high frequency and cost of identity theft among older Americans, more research is needed to guide prevention efforts and interventions that support recovery. This paper examines the risk factors, protective factors, costs, and consequences of identity theft victimization among older adults, focusing on differences between those aged 65-74 and those 75 or older. Findings suggest that the prevalence of identity theft is lower among those 75 or older (6.6% versus 10.3%), but those 75 or older experienced higher average losses per identity theft incident ($155 vs $96). Compared to those aged 65-74, a lower percentage of adults aged 75 or older engaged in online shopping, thereby reducing their risk of identity exposure (48% versus 24%). However, they were also less likely to engage in protective behaviors such as checking credit reports, changing passwords, checking account statements, and using security software.

### LONG-TERM CARE FINANCING FOR AN AGING POPULATION: THE EXPERIENCE OF SINGAPORE

Ngee Choon CHIA, and Huijun, Cynthia Chen, *National University of Singapore, Singapore, Singapore*

Singapore has a rapidly aging population. Long-term care (LTC) is one of the largest financial risks facing elderly in Singapore. Singapore implemented Eldershield, a long-term care insurance scheme which provided defined cash benefit payouts in the event of severe disability; but capped at a maximum of six years. Eldershield enrolled people at age 40, but offered an opt-out option. As of 2015, 65% of those aged 40 to 83 opted to be covered by Eldershield, making Singapore as having the highest voluntary LTC insurance rate in the world. This paper uses an actuarial multi-state disability model and calibrates the transition probabilities and duration-of-stay at various health (disability) states to assess the adequacy and comprehensiveness of Eldershield. The time-limited cash benefit design in Eldershield helped defray about 13% of LTC costs. Removing the time cap will help defray 23% and 26% of the LTC costs for elderly male and female respectively. Furthermore, the simulation results demonstrate that relaxing the trigger benefit and having staggered payouts will improve the adequacy of long-term care insurance. The experience of Singapore's LTC insurance offers insights into the challenges of designing an insurance that tends to occur at higher age and insuring against a cost that could range from zero to a significantly large sum over a long period. Even with the enhanced Careshield Life, which provides cash payouts for life, other policy designs, for example caregiver grants, may be needed to ensure more adequate financing of long-term care.

### SESSION 2821 (PAPER)

### HOUSING ISSUES IN LATER LIFE

### A SCOPING REVIEW OF INNOVATIVE HOUSING FOR OLDER ADULTS: FOCUS ON HOMESHARING, COHOUSING, NORC-SSPS, AND VILLAGES

Kishore Seetharaman, Atiya Mahmood, and Habib Chaudhury, *Simon Fraser University, Vancouver, British Columbia, Canada*

This scoping review explores the benefits, and challenges of major innovative housing and service models for older adults. Several countries are prioritizing housing and service innovation for older adults to address the limitations of traditional market housing in meeting their housing needs and challenges. This study contributes to this issue by reviewing peer-reviewed and grey literature on four well-established innovative housing and service models: cohousing, homesharing, Naturally Occurring Retirement Community Supportive Services Programs (NORC-SSPs), and Villages. Based on the findings synthesized from 65 sources, a pairwise comparison of (i) cohousing and homesharing; and (ii) NORC-SSPs and Villages was conducted on the basis of: (a) financial aspects (e.g., affordability); and (b) psychosocial aspects (e.g., social interaction and engagement, intergenerationality, autonomy and interdependence, safety, diversity and inclusivity); and (c) long-term sustainability. While there were several financial and psychosocial benefits related to each model, the following challenges were identified that need to be addressed to further improve the relevance and effectiveness of these models: (i) lack of funding and resources to support NORC-SSPs and Villages; (ii) inter-resident conflict in homesharing and cohousing; (iii) limitations of informal support provided by fellow-residents in meeting the needs of older adults with complex needs in all four models; and (iv) lack of inclusivity and sociocultural diversity in cohousing and Villages. By integrating research on older adults' housing needs and innovative solutions, the findings of this study could guide future housing initiatives that seek to adopt these innovative models by highlighting their strengths, while recognizing areas for improvement.

### BARRIERS AND FACILITATORS TO HOUSING WITH SERVICES: LESSONS FROM THE RIGHT CARE, RIGHT PLACE, RIGHT TIME PROGRAM

Edward Miller, Elizabeth Simpson, Pamela Nadash, Natalie Shellito, Taylor Jansen, Yan Lin, and Marc Cohen, *University of Massachusetts Boston, Boston, Massachusetts, United States*

The Right Care, Right Place, Right Time initiative (R3) was developed to enable seniors to remain at home as long as possible, while reducing health care costs. It was implemented in four senior housing communities in the Greater Boston area, and consists of two on-site wellness teams (wellness nurse, wellness coordinator), each responsible for about 200 participants across two housing sites. This study aimed to understand barriers and facilitators to implementing R3. Data derived from 31 semi-structured interviews with R3 staff, housing personnel, and community partners (e.g., first responders), as well as 150 key program documents. Facilitating factors in implementing R3 included: top-level management support; formal and informal mechanisms of communication between wellness team members and building staff; substantial discretion, flexibility, and creativity provided to wellness team members; and daily ambulance reports from first responders. Barriers to implementing R3 included: impediments to resident recruitment/engagement; initial role confusion between wellness team members and existing building staff; limited wellness team time at individual intervention sites; challenges establishing systematic relationships with case management staff from the hospitals, AAAs, and insurance companies; and the decentralized approach to data tracking and information exchange. This study suggests several lessons for implementing housing with services initiatives such as R3. Top-level support and buy-in at the organizational level is essential to program development and implementation. Despite early challenges, key program elements can improve over time (communication, data processes, role clarity). Establishing trust with both R3 participants and housing staff is key to building relationships that promote program success.

### DISTINCTIONS ON THE IMPORTANCE OF AGE-FRIENDLY SERVICES BY OLD AGE GROUPS: A COMPARATIVE STUDY BETWEEN THE USA AND THE EU

David Bogataj,[1] Marija Bogataj,[2] Kathryn Hyer,[3] and Kathy Black,[4] *1. University of Ljubljana, Ljubljana, Slovenia, 2. University of Ljubljana, Ljubljana, Ljubljana, Slovenia, 3. University of South Florida, Tampa, Florida, United States, 4. University of South Florida, Sarasota-Manatee, Sarasota, Florida, United States*

Based on the initiative of the World Health Organization in 2007, when describing the global age-friendly cities by a guide to enhance active aging and the age-friendliness of communities, Black and Hyer (2019) presented generational distinctions on the importance of age-friendly community features by focusing efforts on the built, social, and service environment in USA. Their study aimed to examine the differential salience of community features by older generational age groups including Baby Boomers and younger persons 50 years and older, and older cohorts, born before and during WWII. They found that the Chi-square results indicate significant differences across the generational age groups in all domains. We wanted to compare the preference of the same age cohorts in Europe. The sizes of samples were half of the size of samples considered by Black and Hyer, therefore we used z+4 tests which also have shown the distinctions pertaining to preferences on housing and participation in social activities. The differences between ranking in importance of Age-Friendly Community Features by Older Age cohorts in USA and EU are presented and discussed.

### SOCIAL DETERMINANTS OF HEALTH FOR PEOPLE WITH SERIOUS MENTAL ILLNESS AFTER TRANSITIONING TO THE COMMUNITY

Kathy Kellett,[1] Kaleigh Ligus,[1] Kristin Baker,[1] and Julie Robison,[2] *1. UConn Health, Farmington, Connecticut, United States, 2. University of Connecticut, Farmington, Connecticut, United States*

Approximately 10 million, or 6 percent, of the U.S. population experience serious mental illness (SMI) (NAMI, 2019). Social determinants of health (SDOH) associated with this population can provide important information for targeted innovations with the potential to reduce disease burden and improve quality of life. Using secondary data from Connecticut's Money Follows the Person Rebalancing Demonstration, this research compares people age 50+ who transitioned out of an institution onto the Medicaid HCBS Mental Health Waiver (MHW) (n= 271) to those receiving Mental Health services through the Medicaid State Plan (MHSP) (n=278). Analyses examine SDOH in both groups and are organized around five broad domains: Finances; education; social/community context, health/health care, and neighborhood/built environment. MHSP participants were significantly more likely to report not having enough money at the end of the month at 6 (42% vs. 21%), 12 (37% vs. 20%), and 24 (37% vs. 17%) months. Significantly more MHSP than MHW participants did not like where they lived at 6 (12% vs. 1%) and 24 (24% vs. 5%) months. Significantly more MHSP than MHW participants were unhappy with the help they received in the community at 6 (22% vs. 8%), 12 (23% vs. 7%), and 24 (19% vs. 5%) months. Groups did not differ by education, social/community context, health/health care, feelings of safety where they live, or on post-transition hospitalizations, ED use or reinstitutionalization. To improve quality of life in the community, MHSP participants could benefit from greater assistance with finances, housing, and community services.

### SESSION 2822 (PAPER)

### CARE VALUES AND PREFERENCES

### DO OLDER PEOPLE EXPECT RECIPROCITY WHEN PROVIDING CARE? EVIDENCE FROM A NATIONAL COHORT

Cheng Shi,[1] Yajing Zhu,[2] and Gloria Hoi Yan Wong,[1]
*1. The University of Hong Kong, Hong Kong, China,*
*2. University of Cambridge, Cambridge, England, United Kingdom*

Background: The debate on whether caregiving is selfless or self-serving is unresolved. From the perspective of caregiving as a moral responsibility, the motivation to provide care is altruistic. The exchange or reciprocity perspective suggests that care is provided with an expectation to be cared for in the future. The context of filial obligation and rapid socio-economic transformation in recent decades in urban and rural China provided a unique opportunity to investigate these two driving forces. Methods: We tested the effects of caregiving experience in older persons on their care expectation from their children using data from the China Longitudinal Aging Social Survey (n=8,308). Using a multiple-indicator multiple-cause model, a special form of structural equation modelling, we examined the effect of caring for one's older parents, helping adult children with housework, and caring for grandchildren on older persons' care expectation from their children. Results: Older persons in urban areas had higher expectation for future care from their children if they have helped them with housework or took care of their grandchildren, whereas their experience of taking care of their own parents is related to a lower care expectation from their children. Although a similar pattern was observed in rural areas, only the experience of helping adult children with housework significantly increased care expectation. Discussion: Both altruistic and exchange motivations are involved in caregiving. Caring for older parents was more likely altruistic. Caring for an adult child in both urban and rural areas, and grandparenting in urban areas, were motivated by reciprocity.

### PROMOTING THE POSITIVE EXPERIENCE OF CAREGIVING: A SYSTEMATIC REVIEW OF THE POSITIVE ASPECTS OF CAREGIVING SCALE

Yeonjung Lee,[1] and Lun Li,[2] *1. University of Calgary, Calgary, Alberta, Canada, 2. Simon Fraser University, Vancouver, British Columbia, Canada*

While caregivers endure considerable challenges, including potentially deleterious effects on their own well-being, the possibility of positive outcomes for caregivers, such as feeling rewarded and satisfied, has been recognized in scholarly research as well. Caregiving outcomes can be complicated by different aspects of caregiving experiences. Particularly, the Positive Aspect of Caregiving (PAC) scale covers four main positive aspects including caregiving personal gains, motivation for caregiving role, caregiver satisfaction, as well as self-esteem and social aspect of caring. The objective of this study is to summarize the research findings based on the PAC scale and further improve the understanding of the positive experience perceived by caregivers. A systematic literature review was conducted to identify the existing evidences. This systematic review identified empirical research studies written in English focusing on a PAC scale that were published in a peer-reviewed journal between 2004 and 2018. Systematic search was carried out within 10 databases. After careful review of 193 abstracts yielded from the databases, 38 journal articles were identified to have used the PAC scale. Analysis of the selected literature provides four themes that how PAC has been used. The themes are to measure caregiving outcomes, test the effect of caregiver program intervention, complement other caregiving outcome scales, and test the validity in other languages and countries. This systematic review highlights the importance of taking into account of the positive experience of caregiving and further to promote it in a way of buffering the negative outcomes of caregiving.

### THE INFLUENCE OF WORK STATUS PREFERENCE, HEALTH, AND GENDER ON INFORMAL CAREGIVING STATUS

Shanika Koreshi, *Massey University, Palmerston North, Manawatu-Wanganui, New Zealand*

Current, literature has found a negative relationship between caregiving responsibilities and paid employment. There are two possible causal explanations; people with low employment prospects self-select into care and caregiving is time-consuming therefore, it require carers to accommodate this by reducing work. While there is considerable work on the influences of caregiving on work, there is limited research investigating how employment influences informal caregiving decisions. We investigated longitudinal associations between work status preferences (over-employed, underemployed, satisfied) and informal caregiving among older adults in New Zealand. The present study also explored whether gender and perceived health moderated the relationship between work status preference and caregiving. The study used data provided by three waves of the New Zealand Health, Work and Retirement Study. The findings suggest that underemployed and overemployed participants were more prone to taking up caregiving compared to participants who were satisfied with their work status. Regression analyses revealed that females with poor self-reported health were most vulnerable to experience incongruent work status preferences. The direct effects of work status preference on willingness to care may imply conflict between policies promoting full time labour force participation and social welfare policies that are continuing to rely on family carers to support the community. This evidence has implications for care policy given the importance of informal care in sustaining ageing in place policies.

### WICKED PROBLEMS: AN EXPLORATION OF ELDER MISTREATMENT COMPLEXITY

Julia Rowan,[1] Kelly Marnfeldt,[2] Kathleen Wilber,[3] and Susan Enguidanos,[4] *1. University of Southern California, Oxnard, California, United States, 2. University of Southern California, Santa Monica, California, United States, 3. University of Southern California, Chatsworth, California, United States, 4. University of Southern California, Los Angeles, California, United States*

Elder mistreatment (EM) complexity, while described anecdotally, lacks an empirical foundation for measurement. Improved knowledge on the range and nature of concurrent issues that complicate EM intervention would inform the development of more effective solutions and enable greater precision of evaluation research. The purpose of this qualitative study was to explore factors contributing to complexity in a sample of EM cases that was selected based on difficulty reaching resolution. The sample was drawn from those reviewed by an experienced EM Multidisciplinary team (MDT) and determined to require long-term case management (n=39) beyond the capacity of the MDT's usual response. Case manager narrative documentation of ongoing assessment and social service records were qualitatively coded by two researchers. Inductive content analysis, with iterative code reconciliation, was used to identify issues and problems both related and concurrent to EM. Eighteen themes and 74 sub-themes emerged, with 93% initial coding agreement between researchers. The most frequent themes were problems with Caregiving (80%), Cognition (80%), Physical Health (80%), Behavioral Health (69%), Socialization (64%), and Finances (62%). Refusal of formal services was common (90%), yet all accepted visitation by the case manager, suggesting informal support may be effective. Diversity, interconnectedness, and emergence of issues along the duration of case management indicates a system approach to intervention design and evaluation is warranted. This research underscores the need for holistic intervention for highly complex EM, and lays the foundation for objective measure of complexity to standardize selection for specialized intervention.

### SESSION 2823 (PAPER)

### SERVICES AND INTERVENTION

### CREATIVITY AND DEMENTIA: NEW DIRECTIONS FROM EMBODIMENT, RELATIONALITY, AND CITIZENSHIP DISCOURSE

Pia Kontos, Alisa Grigorovich, and Romeo Colobong,
*The Kite Research Institute - University Health Network, Toronto, Ontario, Canada*

There have been important advances in research on creativity that have provided a more inclusive view of everyday and ordinary creativity, including that of persons living with dementia. However, these developments are limited by a lack of engagement with scholarship on embodiment, relationality, and citizenship. We address these limitations by drawing on a relational model of citizenship that offers a critical rethinking of the nature of creativity and the imperative that these be supported in long-term dementia care. We draw on transcribed video-recorded interactions between elder-clowns and residents living with dementia in one long-term care home in central Canada. These are analyzed with reference to key theoretical tenets of the relational model of citizenship. Embodied selfhood (i.e., the primordial and socio-cultural dispositions of the body that are fundamental sources of self-expression and relationality), are identified as key to the creativity of persons living with dementia. We argue that creativity is not an individual cognitive trait but rather emerges from the complex intersection of enabling environments and the embodied intentionality of all involved. We conclude that creativity must be supported in everyday life through organizational practices and socio-political institutions that more fully support the relational, interpersonal, and affective dimensions of care.

### EVALUATION FINDINGS OF A COMMUNITY-BASED INTERVENTION FOR OLDER ADULTS WITH A HISTORY OF TRAUMA

Carmel Rabin, Karen Edell Yoskowitz, and
Barbara Bedney, *The Jewish Federations of North America, Washington, District of Columbia, United States*

Between 70% and 90% of Americans aged 65 and older have experienced at least one traumatic event such as a sexual or physical assault, disaster, illness, or terrorism. Trauma exposure in older adult populations is linked to physical, mental, and cognitive decline. A new approach to improve outcomes of trauma-affected older adults is Person-Centered, Trauma-Informed (PCTI) Care, which promotes the dignity, strength, and empowerment of trauma-affected individuals by incorporating knowledge about trauma into agency programs, policies, and procedures. The Administration for Community Living/Administration on Aging has awarded The Jewish Federations of North America (JFNA) a grant to develop innovative PCTI interventions for Holocaust survivors. This includes a community-based intervention whereby local leadership councils are developed to identify Holocaust survivor needs, distribute grant funding, train caregivers in PCTI care, and forge partnerships to advance community-led Holocaust survivor care. This program has been implemented in eight major US cities where 168 community leaders dispersed 25 grants serving approximately 500 Holocaust survivors. JFNA conducted an evaluation of the first six of the eight cities to determine the impact of this community-based model on participants and Holocaust survivors and investigate the process by which a community-based model can be replicated. This evaluation used surveys and semi-structured interviews to collect data on variables including understanding of PCTI care, awareness of Holocaust survivor needs, strength of community partnerships, and leadership council sustainability. This session will review evaluation findings including best practices for community-based models of PCTI care, and applicability of findings to other older populations.

### MACHINE-LEARNING MODELING TO PREDICT HOSPITAL READMISSION FOLLOWING DISCHARGE TO POST-ACUTE CARE SETTINGS

Elizabeth Howard,[1] John N Morris,[2] and Erez Schachter,[3]
*1. Boston College, Dover, Massachusetts, United States,*
*2. Hebrew SeniorLife, Boston, Massachusetts, United States,*
*3. Profility, Boston, Massachusetts, United States*

Increased attention to post-acute care (PAC) settings and available services to meet patients' needs following acute hospital discharge is needed as these settings are being utilized increasingly in models of care delivery. The primary purpose was to generate a model to identify the most predictive factors relevant to hospital readmission within 90 days following discharge to one of three types of PAC sites: home with home care services (HC), skilled nursing facility (SNF), in-patient rehabilitation facility (IRF). Specific aims were to (1) examine number and characteristics of older adults discharged to the 3 PAC sites; (2) compare 90 day hospital readmission rate across sites and acuity level; and (3) examine assessment items across population and subgroups to identify variables most predictive of hospital readmission. 2015 assessment data from 3,592,995 Medicare beneficiaries were analyzed representing 1,536,908 from SNFs, 306,878 from IRFs, and 1,749,209 receiving HC services. Total sample 90-day readmission was 25.8 % . Patients discharged to IRF had lowest readmission rate (23.34%), and those receiving HC services had highest readmission rate (29.34%). Creation of risk subgroups however, revealed alternative outcomes. Among all patients in the low, intermediate and high risk groups, the lowest readmission rates occurred among SNF patients. Factor analysis of assessment variables indicated bladder and bowel incontinence and functional limitations were the most distinguishing factors between the very low and very high risk subgroups.

### OREGON'S BEHAVIORAL HEALTH INITIATIVE FOR OLDER ADULTS AND PEOPLE WITH DISABILITIES AT 5 YEARS: WHERE TO FROM HERE?

Walter Dawson,[1] Allyson Stodola,[2] Serena Hasworth,[2] Jason Kyler-Yano,[2] Jaclyn Winfree,[2] and Linda Dreyer,[2] *1. Oregon Health & Science University, Portland, Oregon, United States, 2. Portland State University, Portland, Oregon, United States*

Oregon's Behavioral Health Initiative for Older Adults and People with Disabilities is entering its fifth year. This novel state-level Initiative seeks to better coordinate services and resources for older adults and people with disabilities who have behavioral health needs by assigning a Behavioral Health Specialist (BHS) for every 60,000 adults 65 + and embedding them within local service agencies around Oregon. BHS primary job functions include improving coordination and collaboration between local service agencies, providing complex case consultations (CCC), and delivering workforce development training and community education. Five years of data from Portland State University's Institute on Aging's ongoing evaluation of the Initiative suggests significant impact in terms of workforce development trainings, community education, and new community partnerships. Data are collected from BHS and Initiative stakeholders (e.g., aging services agencies). Data collection tools include quarterly reports from the BHS, including a CCC reporting instrument; semi-structured interviews with stakeholders assessing Initiative involvement; and an electronic post-training survey (and two-month follow-up survey) for stakeholders attending BHS trainings. After five years, the evaluation appears to show the Initiative has delivered an abundance of innovative collaborations, workforce trainings, and educational opportunities aimed at better supporting the behavioral health of older Oregonians. It also highlights several persistent systemic barriers including a need for additional public funding of behavioral health, the challenges of accessing Medicare for behavioral health, and siloed agencies and organizations. Future evaluative efforts could explore adding outcomes-based assessments of the Initiative, including local-level quality improvement projects initiated by BHS within their communities.

### UNDERSTANDING NURSING TURNOVER: THE CASE OF HOME HEALTH CARE

Joanne Spetz,[1] Alon Bergman,[2] Hummy Song,[2] Amber Rose,[1] and Guy David,[2] *1. University of California, San Francisco, San Francisco, California, United States, 2. University of Pennsylvania, Philadelphia, Pennsylvania, United States*

Only a few studies of nursing turnover have examined post-acute home health care. This study examines factors that are associated with home health licensed nurse turnover using linked employee-level and patient-level data from one of the five largest home health companies in the US. The data include variables from human resources and payroll systems, visit logs, discharge records, physical and mental health assessments, care plans, and patient encounters and is organized at the employee-day level. We measured turnover using human resources data, including measures of voluntary and involuntary job separation, and from exit interviews that allow classification of whether turnover was associated with agency-related factors (e.g., pay, schedule, supervisor, coworkers) versus personal factors (e.g., family needs, relocation). In bivariate and multivariate analyses, explanatory variables included nurse demographics, patient population characteristics, and the degree to which nurses can delegate tasks to home care aides. We found a downward trend in turnover for licensed nurses between 2016 and 2019. Attrition in the first year was 34% for full-time nurses and 45% for part-time nurses, most of it occurring in the first 180 days of employment. The rate of voluntary turnover was nearly four times as great as involuntary turnover. We found that agency factors accounted for 26% of monthly turnover on average, while personal factors accounted for 74%. In states in which licensed nurses could delegate more tasks to home care aides, turnover rates were slightly higher than in states with little delegation.

### SESSION 2824 (PAPER)

### CIVIC ENGAGEMENT | VOLUNTEERING | SUCCESSFUL AGING

### CONSIDERING LTSS THROUGH THE LENSES OF SOCIAL CONSTRUCTION OF TARGET POPULATIONS: THEORY AND NEOLIBERALISM

Tommy Buckley,[1] Carole Cox,[2] and Israel (Issi) Doron,[3] *1. Virginia Commonwealth University, Richmond, Virginia, United States, 2. Fordham University, New York, New York, United States, 3. University of Haifa, Haifa, Israel*

The global increase of the older population has led to a greater demand for long term support services (LTSS) that address their rights and needs for care. However, policies among countries remain diverse with varying options, services, and recognition of human rights. This study applies the Social Construction of Target Population (SCTP) theory which relates to the perception of older adults and Neoliberalism, a political theory associated with policies of economic privatization, deregulation, and free market activity to the analysis of LTSS systems. As an example, in the United States, the concept of Successful Aging, conflicts with the need for LTSS while present Neoliberal policies that stress minimal government intervention and regulation contribute to little support for any comprehensive LTSS policy. This paper reviews existing policies in 7 countries, representing diverse regions, cultures, and political systems as a means of understanding the relationship of SCTP and Neoliberalism to LTSS. Highlighting the roles that these theories may play can assist in understanding and developing systems that meet the rights and health care needs of older adults.

### EVALUATION OF SENIOR COMMUNITY CENTERS IN RURAL AND URBAN PENNSYLVANIA

Janet Melnick,[1] and Heather Shanks-McElroy,[2]
*1. Pennsylvania State University, Dunmore, Pennsylvania, United States, 2. Keystone College, LaPlume, Pennsylvania, United States*

This is a state-wide study of rural and urban Senior Community Care Centers in PA. The study focused on five key outcomes: To create an inventory of PA's rural and urban SC locations, To analyze SC attendance and program participation, To analyze the challenges and opportunities that SCC's face in providing services in rural and urban PA for a growing senior population. To identify innovative and successful models of senior community care centers within the state. To formulate policy recommendations for the state. Particular attention was placed on innovative programs who are attracting and serving the needs of Baby Boomers,

### IS VOLUNTEERING AN EQUALIZER? A QUASI-EXPERIMENTAL STUDY USING PROPENSITY SCORE ANALYSIS

Seoyoun Kim,[1] and Cal Halvorsen,[2] *1. Texas State University, San Marcos, Texas, United States, 2. Boston College, Chestnut Hill, Massachusetts, United States*

Formal volunteering in later life has been extolled as beneficial for both physical and mental health. However, research points to potential selection bias, in that older adults with key advantages, such as higher wealth, are more likely to volunteer and reap its benefits. As such, we test two competing propositions: Volunteering may act as an equalizer if it benefits the health of the least wealthy the most, or it may further exacerbate disparities if it benefits the health of the wealthiest the most. To that end, we analyzed data from the 2012 and 2014 waves of the Health and Retirement Study ($N \approx 15{,}000$). First, we used relevant covariates (e.g., sociodemographic characteristics, informal volunteering, and health) in 2012 to predict volunteering in 2014, developing the propensity score weights from these results. We then performed several regression analyses to assess the influence of volunteering on self-reported health and depressive symptoms among the general population (ATE) and volunteers themselves (ATT), while comparing the findings for the highest and lowest wealth quintiles. We found that volunteering enhanced self-reported health and reduced depressive symptoms. Further, those in the highest quintile experienced significantly fewer depressive symptoms from volunteering while those in the lowest quintile did not, albeit with no significant differences between the two coefficients. The study enhances the nuanced understanding of volunteering and health while suggesting that unmeasured factors felt strongest among the least wealthy—such as financial distress, discrimination, or lack of organizational support—may attenuate the benefits of voluntary activity.

### RETIREMENT PLANNING: REIMAGINED AND REVAMPED FOR SUCCESSFUL AGING

Rajiv Nagaich,[1] Carol Redfield,[2] Ben Harvill,[1] and Scott Schill,[1] *1. Aging Options, Federal Way, Washington, United States, 2. Aging Options, Seattle, Washington, United States*

What grade should current retirement planning programs receive in which thousands of Americans participated in for their third segment of life? If success is measured by basic goal achievements that most desire (i.e. financial security, not be a burden, and avoiding institutional living), then we have failed significantly. Ninety percent of the population want to age in their own home, yet, only 30% get to. Sixty-six percent report that their employers did "nothing" to help with retirement transition; another 16% state "not sure" to what employers actually did. Seventy percent will travel through Long Term Care services in their lifetime, yet only 8% have LTC insurance. Only one-fourth of Baby Boomers are confident that they will have enough money to last through retirement. Retirement contribution starts with the first paycheck followed by 3-4 decades of employment with possible access to retirement preparedness counsel. Why, then, has planning fall short of being adequate? Are individuals too trusting of the programs from their employers or financial advisors that they don't question completeness? Are professionals practicing in siloed spaces that they fail to realize their services may impact client's other areas of life, especially, last years of life? Why is there a lack in a multidisciplinary team approach which can ensure better predictability of outcomes? We propose a multi-pronged, integrated program: LifePlanning Model, which includes a coordinated, multi-disciplinary approach with a pull-it-together focus for the emerging retiree. The wholistic-based framework guides people through issues in the health, housing, financial, legal and family domains.

### THE INFLUENCE OF SOCIAL ROLES ON THE WELL-BEING OF OLDER CAREGIVERS: SOCIAL ISOLATION AS A POTENTIAL MEDIATOR

Sol Baik, *University of Maryland, Baltimore, Baltimore, Maryland, United States*

Engagement in productive roles has been associated with better health in later life. According to identity accumulation theory, more social roles help individuals be more socially integrated, leading to enhanced psychological well-being by diminishing the risk of focusing their energy into a single role. However, less is known about the mechanisms behind the relationship between productive roles and mental health of older caregivers. The aims of this study are to examine: (1) the association between productive social roles and psychological well-being for caregivers and non-caregivers, and (2) the mediating effect of social isolation on this relationship. The study analyzed 3,951 community-dwelling Medicare beneficiaries ages 65 or above from Rounds 1 to 3 of the National Health and Aging Trends Study (210 caregivers, 3,741 non-caregivers). Multiple-group analysis for structural equation modeling was conducted to compare caregivers to non-caregivers. For both caregivers and non-caregivers, having a volunteer role increased well-being, while paid employment was not significantly associated with well-being. Social isolation mediated neither employee nor volunteer role with well-being for both caregivers and non-caregivers. Assuming that volunteering for others without monetary compensation brings meaning in life, this finding supports the provision of services (e.g., respite care) that could supplement caregiving time, allowing caregivers to have more freedom to use the time in such community-engaging roles. Even though self-rated health was controlled, there is a chance that healthier individuals engage in productive roles. Thus, reciprocal relationships between productive roles and health should be considered in future investigations.

### SESSION 2825 (PAPER)

### SOCIAL DETERMINANTS OF HEALTH

### A CONFLUENCE OF POLICY INEQUALITY: HEALTH DISPARITIES IN OLD AGE AMONG AMERICAN INDIAN AND ALASKA NATIVE POPULATIONS

Sadie Giles, *Virginia Tech, Blacksburg, Virginia, United States*

Racial health disparities in old age are well established, and new conceptualizations and methodologies continue to advance our understanding of health inequality across the life course. One group that is overlooked in many of these analyses, however, is the aging American Indian/Native Alaskan (AI/NA) population. While scholars have attended to the unique health inequities faced by the AI/NA population as a whole due to its discordant political history with the US government, little attention has been paid to unique patterns of disparity that might exist in old age. I propose to draw critical gerontology into the conversation in order to establish a framework through which we can uncover barriers to health, both from the political context of the AI/NA people as well as the political history of old age policy in the United States. Health disparities in old age are often described through a cumulative (dis)advantage framework that offers the benefit of appreciating that different groups enter old age with different resources and health statuses as a result of cumulative inequalities across the life course. Adding a framework of age relations, appreciating age as a system of inequality where people also gain or lose access to resources and status upon entering old age offers a path for understanding the intersection of race and old age. This paper will show how policy history for this group in particular as well as old age policy in the United States all create a unique and unequal circumstance for the aging AI/NA population.

### A SCREENING AND REFERRAL INTERVENTION FOR FOOD INSECURITY AMONG OLDER EMERGENCY DEPARTMENT PATIENTS

Aileen Aylward,[1] Timothy Platts-Mills,[1] Liane Wardlow,[2] Conor Sullivan,[1] Jessa Engelberg Anderson,[3] Andrea Morris,[4] and Rayad Shams,[1] *1. University of North Carolina at Chapel Hill, Chapel Hill, North Carolina, United States, 2. West Health Institute, La Jolla, California, United States, 3. ServiceNow, San Diego, California, United States, 4. West Health Institute, San Diego, California, United States*

Food insecurity is prevalent among older adults, negatively impacts health, and may increase healthcare utilization. Risk factors include poverty, lack of transportation, and social isolation. Community-based services may mitigate food insecurity and other social risk factors. However, identifying those at risk and connecting them to services can be challenging. We implemented a screening and referral program in an Emergency Department (ED) to identify older adults facing food insecurity and connect them to a local Area Agency on Aging (AAA), which arranged and tracked delivery of community-based services. ED nursing assistants used the Hunger Vital Sign screener to assess food insecurity in patients aged 60 years and older. ED Care Managers (CMs) saw all who screened positive and made referrals to the AAA. The AAA conducted an intake assessment and arranged services. Patients were contacted three months after their ED visit to evaluate health, quality of life, and satisfaction with services. Of 423 patients screened over 7 months, 45 (11%) reported food insecurity. Of those, 25 were referred to the AAA. Patients were not referred to the AAA due to CM inability to make a referral (7), declining services (4), or other reasons (11). The AAA reached 21 patients and 9 received at least one service. Of those, 5 were reached for follow-up and reported satisfaction with services. The most frequently requested service was Meals on Wheels (10). Food insecurity is common among older ED patients. An ED-AAA partnership is feasible and connects older adults to beneficial services in their communities.

### FOOD INSECURITY AMONG OLDER ADULTS: A TRANSPROFESSIONAL, COMMUNITY-BASED COLLABORATION

Shirley Girouard, and Michele Solloway, *SUNY Downstate Health Sciences University, Brooklyn, New York, United States*

To address inequities related to food insecurity among older adults, a better understanding of the phenomenon was needed. An innovative screening tool to distinguish among high, medium and low risk and that considers cultural preferences related to food acquisition and related behaviors was developed and piloted. Screenings and healthy eating education were offered at health fairs and other community events. Information about food insecurity and healthy eating as well as resources, such as food maps, guidelines, and food preparation materials were disseminated. Preliminary findings suggest that over half of those screened have high levels of food insecurity. Approximately 37% have five or more comorbidities that combined with food insecurity, represent a significant threat to health and well-being. Elected officials and community leaders soon learned about this initiative and sought education and screening for their constituencies. The research and project evaluation will be used in collaboration with these leaders to identify polices at the local, state and federal level to promote health equity and reduce food insecurity disparities. Efforts are under development to integrate the new screening and referral mechanisms in community-based primary care practices.

### IMPLEMENTATION APPROACHES TO STRENGTHEN A FOOD INSECURITY INTERVENTION FOR OLDER ADULTS IN AN EMERGENCY DEPARTMENT

Aileen Aylward,[1] Timothy Platts-Mills,[1] Liane Wardlow,[2] Conor Sullivan,[1] Jessa Engelberg Anderson,[3] and Andrea Morris,[4] *1. University of North Carolina at Chapel Hill, Chapel Hill, North Carolina, United States, 2. West Health Institute, La Jolla, California, United States, 3. ServiceNow, San Diego, California, United States, 4. West Health Institute, San Diego, California, United States*

Food insecurity is prevalent among older adults, negatively impacts health, and may increase healthcare utilization. Emergency Departments (ED) are an important site of care for older adults. However, the feasibility of screening for food insecurity in EDs is unknown. We assessed the feasibility of implementing a screening and referral process to identify and address food insecurity among older adults in the ED and then monitored progress to overcome barriers to implementation. We developed a semi-structured interview (SSI) guide using the Consolidated Framework for Implementation Research. Prior to implementation, ED staff with diverse clinical backgrounds participated in SSIs. SSIs were analyzed using rapid analysis. Before and during implementation, we engaged hospital leadership to refine the screening and referral process. During implementation, we identified barriers through periodic reflections with staff, observing screenings, and reviewing Electronic Medical Record (EMR) data. Staff agreed that food insecure older adults would benefit from community services. Nursing Assistants (NA) were identified as key implementers. ED leaders expressed concerns about regulatory compliance, EMR integration, and NA scope of work, which were addressed. During implementation, barriers included competing priorities, lack of knowledge, and discomfort with the topic of food insecurity. Stakeholder input and reviewing EMR data led to adaptations including modifying criteria for referral and embedding training into NA orientation. Leadership and staff supported food insecurity interventions but identified several concerns. Steps to facilitate implementation included identifying staff to screen, EMR integration, and building staff efficacy. Reviewing screening data and soliciting stakeholder feedback enabled ongoing adaptations that strengthened implementation.

### RISK PROFILES OF OLDER RURAL RESIDENTS WITH FUNCTIONAL, NUTRITIONAL, AND SOCIAL NEEDS

Raven Weaver, *Washington State University, Pullman, Washington, United States*

Community-based service organizations are well positioned to address social determinants of health by offering a range of services/supports to community residents. To identify health needs and service delivery gaps among a geographically and economically diverse eight-county region, a needs assessment was conducted to support community-based agencies efforts to better support aging residents. A random sample of adults responded to the survey, with 1,280 respondents aged 60+ (mean age=71); the majority of participants were White, female, retired, reported at least some college education, and lived with at least one person. Cluster analysis distinguished three groups of residents, informed by typical enrollment-type data and a social engagement index. A series of one-way ANCOVA and chi-square analyses were conducted to examine how low-, moderate-, and high-risk groups differed on social, nutritional, and functional health needs. High-risk respondents were significantly more likely to report needing social, nutritional, and functional health services, compared to moderate- and low-risk respondents. High-risk respondents were more likely to experience barriers to seeing a physician (X2=34.054, p<.001), a non-emergency ED visit (X2=22.799, p<.001), and an unplanned hospital visit (X2=14.484, p=.001) compared to members of either low- or moderate-risk groups. Ongoing efforts to identify high-risk residents and proactively target moderate-risk residents support low-cost community interventions (i.e., assessing residents for services in locations regularly attended, such as senior meal centers), rather than high-cost interventions (e.g., emergency care, hospitalizations) are essential. Findings inform community-based outreach approaches that target social, economic, and environmental factors essential in improving health and achieving health equity.

### SESSION 2826 (PAPER)

### RESEARCH METHODS I MEASUREMENT ISSUES

### A PEACOCK THAT DOES NOT SHOW ITS FEATHERS IS NOT MUCH DIFFERENT FROM A TURKEY: ON BEING A PHYSICIAN EXECUTIVE FOR 25 YEARS

Saraswathy Battar, *Michael E. DeBakey VA Medical Center, Houston, Texas, United States*

Compelling common threads in the fabric of being an academic and clinical geriatrician, VA health care executive and physician champion for VIONE – a national medication deprescribing project that was launched at 59 VA programs across the nation will be presented in a thought provoking, objective, evidence based and legacy oriented format. Highlights include a)success factors b)networking c) effective presentations d) lessons learned e)implementing elements of High Performance Delivery Model (HPDM), f) transforming ideas into actions that help rise and shine g) creating a legacy h)survey readiness h) keeping the main thing the main thing i) understanding others and helping others understand you. Making significant contributions in career is important. How the work is captured, projected, made visible as a viable, actionable solution to a critical and significant mission critical problem, strategic engagement with stakeholders, implementation with measurable outcomes, passionate engagement while being an affirmative, non-controversial leader who is eager, willing, available, capable and knowledgeable etc., are some of the other non-negotiable aspects of getting work recognized. Experiences shared include creating VIONE – a medication deprescribing project that won VA USH Shark Tank competition as a Gold Status Fellow with outcomes such as over 95,000 veterans impacted, over 194,000 non-essential medications deprescribed with accomplished cost savings of over 9.6 million US dollars in 4 years. This profound journey taught me the importance of effectively displaying worthy work with global relevance with dignity, credibility, pride, persuasion, leadership, efficiency, teamwork, art of persuasion, ponder, recharge, rejuvenate and start fresh – time and again.

### ARCHITECTURE OF A SMART HOME FOR AGING IN PLACE

Neda Norouzi, *University of Texas at San Antonio, San Antonio, Texas, United States*

The United States Department of Health and Human Services (2017) estimates that there are 65 million people age 60+ residing across the fifty states. A national survey conducted by the American Association of Retired Persons (AARP) indicates that 76% of people ages 55+ prefer to age-in-place and live independently (2018). The Census Bureau American Community Survey (2015) estimates that 13 million adults have difficulties living independently, 80% of which receive assistance in their private homes. However, only 50% of these homes meet the physical needs of people who choose to age-in-place (AARP, 2018). Recent advancements in technology have led to the development of smart homes. Technology can support aging-in-place and independent living by offering necessary tools for building systems that identify behavioral patterns and offer automated decision-making. However, not all older adults are customed to using technology or comfortable with being monitored with artificial intelligence (Wang et al., 2019). In response to this concern, the current study used grounded theory framework to analyze 62 interviews of people ages 55-93 to indicate if and how older adults prefer to utilize technology in their homes. The results of the study presented that while some older adults felt they might be too old to learn and use technology, nearly 85% of the interviewers agreed that incorporating technology in the built environment could benefit them. They are especially willing to learn and use technology in their homes when the benefits are related to their health, social and emotional connection, entertainment, safety, and daily chores.

### INTERSECTIONALITY: MAPPING CRITICAL RELATIONS FOR QUALITY IN LONG-TERM CARE RESEARCH

Katie Aubrecht,[1] Ivy Bourgeault,[2] and Tamara Daly,[3]
*1. St. Francis Xavier University, Antigonish, Nova Scotia, Canada, 2. University of Ottawa, Ottawa, Ontario, Canada, 3. York University, Toronto, Ontario, Canada*

Intersectionality is a useful method (Lutz, 2015) for interdisciplinary long-term care (LTC) research to advance a more critical understanding of how experiences of quality are shaped by mutually reproducing social divisions, identities and relations of power that shape LTC. This paper discusses insights from the “Mapping Care Relationships” stream of the Seniors – Adding Life to Years (SALTY) project, a pan-Canadian program of research examining clinical, social and policy perspectives on quality in LTC. “Mapping Care Relationships” mapped how promising approaches to care relationships are organized and experienced in LTC. From January 2018-August 2019 our team of nine researchers conducted rapid ethnographies in eight nursing homes, two in each of four provinces across Canada. We purposively observed and interviewed workers from a wide variety of positions and backgrounds, informed by an intersectionality approach. We traced how promising approaches in person-centred dementia care (PCDC) in particular may reify the subordinated status of care workers (some more than others) and reinforce inequities within LTC systems. In multiple LTC homes, front-line care workers described experiencing physical and emotional harm in care relationships with residents which caused them distress. However, consistent with a PCDC approach, the harm was attributed to ‘behaviours’ clinically symptomatic of dementia. In framing power differentials from a medical perspective, PCDC makes it possible to interpret harmful experiences as 'part of the job’ and something workers should know to expect, prevent, avoid, redirect, or ignore. Lutz, H. (2015). Intersectionality as method. DiGeSt. Journal of diversity and gender studies, 2(1-2), 39-44.

### SESSION 2827 (PAPER)

### LONG-TERM CARE RESEARCH

### INFLUENCE OF CONTEXT AS A KEY INGREDIENT FOR HIGH PERFORMANCE ON SKILLED NURSING FACILITY READMISSIONS

Roman Ayele,[1] Kirstin Manges,[2] Chelsea Leonard,[1] Marcie Lee,[1] Emily Galenbeck,[1] Mithu Molla,[3] Cari Levy,[1] and Robert Burke,[4] *1. VA Eastern Colorado Health Care System, Aurora, Colorado, United States, 2. University of Pennsylvania Perelman School of Medicine, Philadelphia, Pennsylvania, United States, 3. UC Davis Health, Sacramento, California, United States, 4. Center for Health Equity Research and Promotion, Corporal Crescenz VA Medical Center, Philadelphia, Pennsylvania, United States*

Improving hospital discharge processes and reducing adverse outcomes post discharge has become a topic of national importance, especially for older patients transitioning to skilled nursing facilities (SNFs). The substantial variation in rehospitalization outcomes suggests contextual factors may play a role in performance. We conducted rapid ethnography with opportunistic interviews (148 hours of observations, 30 clinicians) at hospitals that were high (n=2) or low (n=2)- performing in terms of their readmission rates from SNF, as well as 5 associated SNFs, to understand how contextual differences influenced performance variation. We used thematic analysis to categorize contextual factors and compare differences across high and low perfoming sites. We found three main contextual factors that differed across high- and low-performing hospitals and SNFs: team dynamics, patient characteristics, and organizational context. First, we observed high-quality communication, situational awareness, and shared mental models among team members in high-performing sites. Second, patients at high-performing hospitals had better insurance coverage that made it feasible for clinicians to place patients in SNFs based on their needs instead of financial abilities. Third, staff at high-performing hospitals were more engaged in the transitions process and rapport building with SNFs thereby facilitating smooth transitions of care from hospitals to SNFs. For example, clinicians at high- performing sites were actively engaged in directing the discharge process, but this was not observed in low-performing sites where a lack of clinician engagement lead to delays. Understanding how context affects transitions could be used to target organizational, policy, and staff level interventions to reduce adverse outcomes.

### NH COMPARE'S HEALTH DEFICIENCY 5-STAR RATING: DO WE LEARN DIFFERENT THINGS FROM SURVEY AND COMPLAINT DEFICIENCIES?

Lindsay Peterson,[1] John Bowblis,[2] and Kathryn Hyer,[1]
*1. University of South Florida, Tampa, Florida, United States, 2. Miami University, Oxford, Ohio, United States*

The Centers for Medicare and Medicaid Services (CMS) publishes a 5-star rating system for nursing homes (NH). Currently, the 5-star rating for health deficiencies weights deficiencies from annual recertification surveys and complaints equally. Complaint deficiencies may contain different information than survey deficiencies because complaint deficiencies originate with consumers and complaint inspections are less predictable than surveys. The objective of this study is to construct separate 5-star ratings for survey and complaint deficiencies, and to compare them to CMS' 5-star rating for health deficiencies. Using CASPER and ASPEN Complaints/Incident Tracking System for all NHs in 2017 (N=15,373), we calculated the 5-star rating for health deficiencies as reported by CMS, and then decompose CMS' rating into separate 5-star ratings for survey and complaint deficiencies. The overall distributions of the CMS' deficiency rating and survey deficiency rating are similar. The distribution of the complaint deficiency rating is different from CMS' deficiency rating. Using complaint deficiencies, more NHs have 5-stars (26.5% vs. 10.5%) and fewer facilities have 4-stars (11.2% vs. 23.3%). Comparing the ratings for each facility relative to CMS' rating, 35.3% of NHs have a different survey deficiency rating while 54.4% have a different complaint deficiency rating. A 5-star rating based on survey and complaint deficiencies results in different ratings for NHs, indicating that complaint deficiency ratings contain different information from survey deficiency ratings. CMS should publish separate ratings based on survey and complaint deficiencies to provide different information.

### PROFILING NURSING HOME CARE SPECIALIZATION GROUPS

Xiao (Joyce) Wang,[1] Jeffrey Burr,[2] Jennifer Hefele,[3] and Joyce Wang,[2] *1. University of Massachusetts, Boston, Massachusetts, United States, 2. University of Massachusetts Boston, Boston, Massachusetts, United States, 3. Booz Allen Hamilton, Arlington, Virginia, United States*

The nursing home (NH) industry has experienced a shift toward care specialization. This study used NH-level panel data from 2011 to 2017 to describe unique care specialization groups in urban areas using latent profile analysis (LPA) (N= 64695, with 12,143 unique NHs). We focused on urban NHs because NHs specialize in care due to competition and memetic pressure, more likely to be the case for urban NHs. To identify care specialization profiles, LPA was applied using different types of specialist staffing levels (physical therapist, occupational therapist, physicians, and dietitians) and the share of special care units aimed at chronic conditions like Alzheimer's Disease and AIDs. Model diagnostics and information criterion guided selection of the best fitting model. Model stability over time, interpretability of results, and parsimony were also taken into consideration. The final results indicated a 4-profile model fit the underlying data best and the patterns remained comparatively stable over seven years. The 4-classes are uniquely identified as: high use of specialists of all types (3%), moderate use of specialists of all types (7%), mixed use of specialists and special care units (26%), and low specialization use (64%). From 2011 to 2017, the size of the 'low specialization' group became smaller, whereas the high and moderate groups grew larger. In addition to describing a clear trend towards increased care specialization, our findings indicated great heterogeneity in NHs' care specialization patterns in urban areas. Future studies should examine market and organizational characteristics, as well as performance outcomes for different specialization groups.

### WHAT'S DRIVING SNF READMISSION RATES? EXPLORING DIFFERENCES IN PROCESSES BETWEEN HIGH AND LOW PERFORMING HOSPITALS

Kirstin Manges,[1] Roman Ayele,[2] Marcie Lee,[2] Chelsea Leonard,[2] Emily Galenbeck,[2] and Robert Burke,[3]
*1. University of Pennsylvania Perelman School of Medicine, Philadelphia, Pennsylvania, United States, 2. VA Eastern Colorado Health Care System, Aurora, Colorado, United States, 3. Center for Health Equity Research and Promotion, Corporal Crescenz VA Medical Center, Philadelphia, Pennsylvania, United States*

Despite the increasing national focus on improving post-acute care outcomes, best practices for reducing readmissions from skilled nursing facilities (SNFs) are unclear. The objective of this rapid ethnographic study was to observe processes used to prepare older patients for post-acute care in SNFs, and to explore differences between hospital-SNF pairs with high or low thirty-day readmission rates. We stratified hospitals according to readmission rates from SNF and used convenience sampling to identify two high and two low performing sites and associated SNFs (n=5). We conducted intensive multi-day observations (n=148 hours) and key informant interviews (n=30 clinicians) to describe hospital processes for discharging patients to SNF. We used thematic analysis of interviews and fieldnotes to identify differences in transitional care processes of hospitals discharging patients to SNFs. Hospitals used five major processes prior to SNF discharge that could affect care transitions for older adults: recognizing the need for post-acute care, deciding level of care, selecting SNF facility, negotiating patient fit, and coordinating care with SNF. During each stage, high-performing sites differed from low-performing sites by focusing on: 1) earlier, ongoing, systematic identification of high-risk patients; 2) discussing the decision to go to a SNF as an iterative team-based process; and 3) anticipating barriers with knowledge of transitional and SNF care processes. Identifying variations in processes used to prepare patients for SNF provides critical insight into the best-practices for transitioning patients to SNFs and areas to target for improving care of older adults.

### SESSION 2828 (PAPER)

### INTERNATIONAL FOCUS

### ARE THE SELF-EMPLOYED MENTALLY HEALTHIER THAN SALARIED WORKERS? EVIDENCE FROM KOREA, MEXICO, AND THE UNITED STATES

Hankyung Jun, *University of Southern California, Los Angeles, California, United States*

Self-employed workers are often reported to have better health than salaried workers. Whether this is because self-employment has health benefits or healthier workers are self-employed is not clear. Self-employed workers may have higher job satisfaction due to higher levels of self-efficacy and autonomy, but may also experience higher job stress, uncertainty, and lack of health insurance leading to mental health problems. Self-employed workers in the U.S. may have different characteristics than those in Mexico and Korea given different working and living environments as well as different institutional arrangements. This study will examine the association between self-employment and mental and cognitive health for older adults in the U.S., Mexico, and South Korea. It uses harmonized panel data from the Health and Retirement Study, the Korean Longitudinal Study of Aging, and the Mexican Health and Aging Study. We compare the health and selection effect of self-employment using a pooled logistic model, fixed-effects model, and a bivariate probit model. In addition to comparing self-employed and salaried workers, we analyze differences between self-employed with and without employees. By using rich data and various models, we address reverse causality and estimate the relationship between self-employment and health. We show that the positive health effects of self-employed workers in the U.S. disappear once controlled for unobserved heterogeneity, indicating the possibility of healthier workers selecting into self-employment. Interestingly, for Korea and Mexico, healthier individuals seem to select into wage work which reflects the difference in working conditions across countries. Further analysis will show effects by business size.

### FACTORS INFLUENCING PRACTITIONER CHOICE IN NONPARTICIPATION IN MEDICAL ASSISTANCE IN DYING

Janine Brown,[1] Donna Goodridge,[2] and Lilian Thorpe,[2]
*1. University of Regina, Saskatoon, Saskatchewan, Canada, 2. University of Saskatchewan, Saskatoon, Saskatchewan, Canada*

Canadians aged 65 and older compromised 79% of the individuals who chose a medically assisted death between January 1 – October 31, 2018. Despite public approval for MAID, and positive professional reception, few practitioners are participating in MAID care and the underlying factors influencing this require exploration. What are the factors considered by practitioners when contemplating MAID participation? Interpretive Description guided a qualitative, exploratory project. Data included semi-structured interviews with 35 practitioners who currently did not participate in MAID, interviewer field notes and reflective content. Data was analyzed through open coding, a constant comparative approach and thematic analysis. Participants are contemplating numerous endogenous and exogenous factors in determining care participation. The endogenous factors included philosophy of care, MAID/faith/spirituality congruence, the conceptualization of duty, comfort with death and previous life experiences. Within these factors, participants described knowing if MAID participation was, or was not, possible through a process of self-reconciliation. Those who thought MAID care participation may be possible, but yet were not participating, intentionally contemplated numerous exogenous factors including risk, time and practice factors, patient, family and community considerations, system structures, and visibility. Non-participation in MAID care is important to understand to support the self-determination of older adults who may consider MAID. Contemplating involvement in MAID care is complex with both numerous factors influencing practitioner choice. Practitioners require care options, safe passage, respect, model of care clarity, removal of practice barriers, open conversations, enhanced education opportunities and time to facilitate safe, supportive work environments and client care.

### FACTORS RELATED TO WORKPLACE WELL-BEING: OCCUPATIONAL HEALTH AND SAFETY OF OLDER WORKERS IN HONG KONG

Vincent Lee, Daniel W. L. Lai, Kin Cheung, Calvin Luk, Xue Bai, Jessica J. Li, and Joan Wu, *The Hong Kong Polytechnic University, Kowloon, China*

As Hong Kong is undergoing rapid population ageing over the past few decades, and this trend is expected to persist due to low fertility rates and extended life expectancy. This trend has significant implications for Hong Kong's labor market and workforce. This study aims to gain a better understanding of the experiences, needs, and definitions of older workers in Hong Kong. It helps to draw attention to issues of occupational safety and health (OSH) risks and help identifying and promoting strategies and interventions for effectively supporting the ageing workforce in the workplace. The major objective of this research is to identify the OSH problems and challenges perceived by the older workers, employers and other stakeholders. Both quantitative and qualitative research methods are applied. A qualitative study with focus groups and individual in-depth interviews have been conducted with a representative sample of 70 stakeholders, including employees, employers, representative of labour unions, policymakers, NGO representatives and researchers in the related disciplines. We obtained their views on age-related changes within the older workforce and how such changes would affect their work ability, the OSH issues facing the older workers in different industries, and the facilitators and barriers associated with remaining in or joining the labour force. Feasible intervention strategies for effectively supporting the ageing workforce in the workplace are also being identified.

### LIVING WITH DEMENTIA: FLIPPING STIGMA ON ITS EAR

Deborah O'Connor,[1] Alison Phinney,[1] Jim Mann,[2] Habib Chaudhury,[3] Kishore Seetharaman,[3] Ania Landy,[4] and Malcolm Paulina,[5] *1. University of British Columbia, Vancouver, British Columbia, Canada, 2. University of Victoria, Surrey, British Columbia, Canada, 3. Simon Fraser University, Vancouver, British Columbia, Canada, 4. University of British Columbia, Vancouver, United States, 5. University of British Columbia, Vancouver, Canada*

The language of social citizenship has emerged in the academic literature as one way of shifting the discourse to counter persistent problems of stigma and social exclusion for people with dementia. What this means and how it is experienced however from the perspective of those with dementia remains unclear. As part of a larger Participatory Action Research (PAR) study, an Action group of people with dementia began meeting in June 2019. The group now consists of ten members and meets monthly. The first task of the

Action group was to assist in developing a more refined and practical understanding of the construct of social citizenship. Facilitated discussions were guided by the following questions: What are experiences of social citizenship by people with dementia? What kinds of practices and relationships promote the capacity of people with dementia to experience themselves as social citizens? Emerging findings indicate that the stigma is readily identified as a dominant aspect of the experience of living with dementia which needs to be 'flipped on its ears'. Strategies for countering stigma include recognizing how language can both facilitate and block change, acknowledging dementia as a time of both loss and significant growth, remaining visible as a whole person – equal and also different - and maintaining active participation in one's own life. These themes tie directly to the components identified in the academic literature of citizenship. However, members of the Action group were clear that the language of social citizenship is neither empowering nor strategic.

### QUALITATIVE INTERVIEW ANALYSIS: UNPACKING PACKED INTERVIEWS

Michelle Silver, *University of Toronto, Toronto, Ontario, Canada*

Retirement is an ever-evolving, dynamic, and complex social construct we associate with the end of one's career. Exploring what retirement means to different people can contribute to a better understanding of the implications of this important transition at the individual and societal level. However, sifting through participants stories is not always a straightforward endeavor, particularly in the case when participants have something to hide. This paper examines the value of qualitative research methods in unpacking complex personal narratives. As the landscape surrounding mature workers' experiences continues to change, this paper extends policy debates about retirement, as well as scholarly conversations about the richness and complexity of qualitative research.

### SESSION 2829 (PAPER)

### ALZHEIMER'S DISEASE

### ASSOCIATION BETWEEN FRAILTY AND DEVELOPMENT OF ALZHEIMER'S DISEASE–RELATED DEMENTIAS

Chih-Ying Cynthia Li, Brian Downer, Lin-Na Chou, Kenneth Ottenbacher, Kyriakos S. Markides, and Soham Al Snih, *The University of Texas Medical Branch at Galveston, Galveston, Texas, United States*

Frailty is associated with an increased risk for Alzheimer's disease and related dementias (ADRD). However, this association has not been investigated in older Mexican Americans; a population that is high-risk for frailty and ADRD. This study investigated the association between frailty and the development of ADRD over 9-year period. We analyzed 860 Mexican Americans ≥76 years old of the Hispanic Established Populations for the Epidemiological Study of the Elderly (Hispanic-EPESE) who have been linked with Medicare claims data. Survey data from Wave 6 (2007/08) was used to categorize participants as frail (either pre-frail or frail) or non-frail according to the Fried phenotype. The main outcome was ADRD diagnosis after Wave 6 interview. ADRD status was determined using the Chronic Conditions Segment of the Master Beneficiary Summary File. We estimated ADRD disease-free probability during 2007-2016 using midpoint of interval data method stratified by frailty status. Mean age of the study sample was 83.2 years (SD=4.4) and 59.3% were female. We found individuals who were frail had less ADRD-free months (46.5; SD= 36.5-52) compared to those who were non-frail (66.0; SD= 47.5-120). Individuals with a score of less than 21 points on the Mini Mental Status Exam had greater risks of ADRD development (Odds Ratio=1.35, 95% CI= 1.05-1.74) compared to their counterpart, after controlling mortality as a competing risk. Our results suggest being pre-frail, frail or cognitively impaired are risk factors for ADRD in community-dwelling older Mexican Americans.

### OVERLAP OF PAIN AND DEPRESSION AMONG NURSING HOME RESIDENTS WITH ADVANCED ALZHEIMER'S DISEASE AND RELATED DEMENTIA

Emmanuelle Belanger,[1] Richard Jones,[2] Gary Epstein-Lubow,[2] and Kate Lapane,[3] *1. Brown University School of Public Health, Providence, Rhode Island, United States, 2. Brown University, Providence, Rhode Island, United States, 3. UMass Medical School, Worcester, Massachusetts, United States*

Physical and psychological suffering are interrelated and should be assessed together as part of palliative care delivery. We aimed to describe the overlap of pain and depressive symptoms among long-stay nursing home (NH) residents with advanced Alzheimer's disease and related dementia (ADRD), and to determine the incidence of pain and depressive symptoms. We conducted a retrospective study of a US national sample of fee-for-Service Medicare beneficiaries who became long-stay NH residents in 2014-2015, had two consecutive quarterly Minimum Dataset assessments (90 and 180 days +/- 30 days), and had a diagnosis of ADRD in the Chronic Condition Warehouse and moderate to severe cognitive impairment (N= 92,682). We used descriptive statistics and Poisson regression models to examine the incidence of each symptom controlling for age, sex, and concurrent hospice care. Sub-groups with self-reported and observer-rated symptoms (pain/PHQ-9) were modelled separately, as were those switching between the two. The prevalence of depressive symptoms was low (5.7%), while pain was more common (18.2%). Across various subgroups, 2% to 4% had both pain and depression, but between 20% and 25% were treated with both antidepressants and scheduled analgesia. Depressed residents at baseline had an incidence rate ratio (IRR) of pain of 1.2 at the second assessment, while the residents with pain at baseline had an IRR of depressive symptoms of 1.3 at the second assessment. Our results support the expected relationship between pain and depressive symptoms in a national sample of long-stay NH residents with advanced ADRD, suggesting the need for simultaneous clinical management.

### PRE-LOSS GROUP THERAPY FOR FAMILY CAREGIVERS OF PERSONS WITH DEMENTIA: TRANSLATION INTO PRACTICE

Katherine Supiano, Troy Andersen, Marilyn Luptak, Cynthia Beynon, Eli Iacob, and Hui Li, *University of Utah, Salt Lake City, Utah, United States*

We developed Pre-Loss Group Therapy (PLGT) for dementia caregivers at risk for Complicated Grief (CG). PLGT is a manualized ten-session multi-modal group therapy that includes elements of cognitive behavior therapy, motivational interviewing, exposure therapy, memory revisiting, meaning-making, and self-care. We implemented and evaluated three PLGT cohorts in three long-term care facilities with family caregivers at-risk for CG whose care recipient had a life expectancy of 6 months or less and resided in a long-term care facility (NT = 24). Evaluation of participant preparedness for the death of the persons with dementia (PWD), self-care and grief outcomes showed significant improvement across multiple domains between pre and post-group, notably a statistically significant decrease in grief as measured by the Inventory of Complicated Grief score from baseline (M = 25.67, SE=1.80) to post-group (M = 14.41, SE=1.65) $t(21)= 6.280$, $p<0.001$. Clinician-rated grief severity declined (N=22, $\beta = -0.472$, SE = 0.018, $p < 0.001$) per week and grief improvement increased (N=22, $\beta = 0.259$, SE = 0.023, $p < 0.001$) per week, as assessed on the Clinician Global Impressions Scale. We subsequently trained two LCSWs to conduct PLGT, and both clinical outcomes and treatment fidelity and skills measures achieved performance levels of master clinician-trainers. Family caregivers at risk for CG may benefit from group therapy targeting preparedness and pre-loss grief experience, as we provide with PLGT. Manualized PLGT is suitable for implementation by LCSWs in the settings of hospice and long-term care.

### RURAL-URBAN DIFFERENCES IN SURVIVAL AND NURSING HOME USE AMONG MEDICARE BENEFICIARIES DIAGNOSED WITH DEMENTIA

Momotazur Rahman,[1] Elizabeth White,[1] Kali Thomas,[2] and Eric Jutkowitz,[2] *1. Brown University School of Public Health, Providence, Rhode Island, United States, 2. Brown University, Providence, Rhode Island, United States*

There is poor understanding as to how survival and healthcare utilization vary among older adults living with Alzheimer's disease and related dementias (ADRD) in rural versus urban areas of the United States. This prospective cohort study used 2008-2015 Medicare claims linked with nursing home and home health assessment data to describe differences in survival and healthcare utilization in the six years following a new ADRD diagnosis between rural and urban populations. The sample consisted of 1,203,897 Medicare fee-for-service beneficiaries who were diagnosed with ADRD in 2008 or 2009. 77% (n=921,853) resided in metropolitan counties, 14% (n=162,857) in micropolitan counties, and 10% (n=119,187) in rural counties. Rural residents were on average about six months younger than metropolitan residents at diagnosis. Metropolitan residents survived a mean of 1211 days after diagnosis. Adjusting for individual characteristics, beneficiaries in rural and micropolitan counties survived 29.2 fewer days (95% CI -34.0,-24.4) and 31.9 fewer days (95% CI -36.1,-27.7) than metropolitan residents, respectively. Compared to metropolitan residents, rural residents spent 59.8 more days (95% CI 56.7, 63.0) in nursing homes. We found similar patterns in nursing home use for micropolitan vs. metropolitan residents, though the magnitude of the differences was smaller. Differences between groups became more pronounced the greater the time from diagnosis. These findings demonstrate that urban-dwelling older adults with ADRD are significantly more likely to remain in the community and less likely to use nursing homes than individuals in rural and micropolitan counties, particularly in later disease stages.

### THE IMPACT OF CHANGING THE CMS NURSING HOME COMPARE 5-STAR RATINGS ON THE USE OF ANTIPSYCHOTICS

Shekinah Fashaw,[1] Theresa Shireman,[1] Ellen McCreedy,[1] and Jessica Ogarek,[2] *1. Brown University, Providence, Rhode Island, United States, 2. Brown University, PROVIDENCE, Rhode Island, United States*

Due to their potential to increase falls and death in people with dementia, antipsychotic medications (APMs) have been the subject of several federal efforts to reduce their use in nursing homes (NHs). In 2015, the Centers for Medicare & Medicaid Services added inappropriate APM use to their NH 5-star quality ratings. We examined the impact of this policy decision on NH residents with dementia by race/ethnicity. Using a quasi-experimental study design and Minimum Data Set (MDS) 3.0 assessments, we examined long-stay NH residents with dementia. We examined changes in APM use quarterly (2013-2016) using interrupted time series analyses, stratified by race/ethnicity. There were about 1 million NH residents per quarter. Baseline use of APMs among persons with dementia was 29.1% for Whites, 29.2% for Blacks, and 33.7% for Hispanics. All three races experienced significant declines in APM use prior to the addition of AP use into the quality rating ($p<0.001$). During the first quarter of rating system changes, there were significant declines in APM use for all three races: Blacks, 0.48%; Hispanics, 1.0%; Whites, 0.49%. Subsequent rates of decrease in APM use did not differ from the baseline rate of decline ($p> 0.5$). The policy change did result in a one-time, significant drop in APM use, but did not alter the rate of decline already in place, presumably stemming from the National Partnership instituted in 2012. Hispanics started with the highest rate of APM use and experienced the greatest decreases over time and with the new star rating measure.

### SESSION 2830 (PAPER)

### LONG TERM CARE: RESIDENTS, STAFFING AND ORGANIZATIONAL ISSUES

### ADVERSE CONSEQUENCES OF UNMET IN-HOME MOBILITY CARE NEEDS AND RISK OF HOSPITAL STAY AMONG OLDER ADULTS IN THE UNITED STATES

Shalini Sahoo, *University of Maryland, Baltimore, Elkridge, Maryland, United States*

Mobility limitations are the most prevalent late life disability and are strongly associated with negative health outcomes. Research suggests that 1 in 5 older adults with limitations in activities of daily living report needing more help than is received. The objective of this study is to address a gap in the literature by directly examining the relationship between adverse consequences (e.g. home-bound, bedridden) of unmet in-home mobility care needs and hospital stay for a national sample of community-dwelling older adults. Data was analyzed from round eight (2018) of the National Health and Aging Trends Study (NHATS), an epidemiologic panel study of nationally representative Medicare beneficiaries ages 65 and older living in the communities (n = 4,344). Community dwelling adults with one or more adverse consequence due to in-home mobility limitation had 1.931 times odds of hospital stay in the last 12 months, compared to the counterpart with no in-home mobility limitation (OR = 1.931, SE = 0.153, $p < 0.05$), after adjusting for the covariates. Community-dwelling older adults who have adverse consequence due to unmet in-home mobility care needs are more likely to be immobile and are more likely to have hospital stays. By addressing the needs of this population, the rate of hospitalization can be decreased resulting in fewer stressful events and better quality of life. Policies to improve long-term services and supports and reduce unmet need could benefit both older adults and those who care for them.

### CHARACTERIZATION OF SKILLED NURSING FACILITY RESIDENTS ADMITTED WITH SUBSTANCE USE DISORDERS

Rhea Sindvani,[1] and Lisa Barry,[2] *1. UConn Health, Farmington, Connecticut, United States, 2. UConn Center on Aging, Farmington, Connecticut, United States*

For reasons including the opioid epidemic and more widespread substance use across all ages, the prevalence of individuals meeting preadmission criteria for skilled nursing facility (SNF)-level care and who have substance use disorders (SUDs) is growing. However, little is known about this population. We characterized a sample of residents with SUDs in two SNFs that target admission of difficult-to-place individuals in Hartford, Connecticut. Residents admitted between June 1, 2018 and May 31, 2019 and had an SUD per Pre-Admission Screening and Resident Review (PASRR) were included. Using retrospective chart review, we collected data including demographics, physical and mental health conditions, psychiatric medications, and participation in SNF-provided SUD counseling. Of 163 residents admitted with an SUD, all were admitted following an acute hospitalization. Residents' average age was 49.9(SD=11.7) years (range 21-79). They were 61% male and racially diverse; 56% Caucasian, 27% Hispanic, 16% Black. SUDs on admission included opioid use disorder (48%), alcohol use disorder (33%), unspecified psychoactive SUD (26%), cocaine use disorder (25%), and Other (20%). Of these, 18% and 16% were taking methadone or suboxone, respectively and 25% were taking an antipsychotic medication. Comorbidities such as bipolar disorder (15%) and viral hepatitis (26%) were prevalent. A total of 40 (25%) residents participated in SUD counseling; none of the aforementioned factors was associated with participation. This is the first study to characterize a sample of residents from SNFs that target individuals with SUDs. Improved understanding of this unique and growing subset of the SNF population may help optimize their treatment.

### DEVELOPING LIFE STORIES FOR NURSING HOME RESIDENTS AND EXAMINING THE IMPACT ON RESIDENTS AND STAFF

Miriam Rose, Farida Ejaz, and Brian Polk, *Benjamin Rose Institute on Aging, Cleveland, Ohio, United States*

A life story program was implemented in 16 nursing homes (NHs) in Ohio with partners including a company specializing in life story work and a gerontological institute. The aim was to evaluate the impact of the life story program on residents and staff. NH sites were selected from an urban/suburban and a rural county using sampling procedures ensuring variation in auspice, quality star ratings and bed size. A longitudinal design was used to conduct in-person interviews with residents at baseline (prior to the life story interview), immediately after the interview, and approximately a month after most life story books were delivered to a NH. Resident eligibility criteria included being age 60 or older, Medicaid-eligible, long-stay and having no to moderate cognitive impairment. Residents' (n=238) average age was 77 years, 66% were female, and 52% had resided in the NH for 1-5 years. Cognitive scores declined over time, but depressive symptomatology improved significantly. Residents had very high levels of satisfaction with care, enjoyed telling their life stories and would recommend the program; these findings did not change. A pre-post study design was used with staff (n=198), who included nurse aides, nurses, administrators, social workers and activity staff. Their average age was 44 years. Although staff job satisfaction did not change significantly, the vast majority enjoyed learning about residents' life stories and used them in care planning. The findings demonstrate that life story work may be useful in promoting person-centered care, although further testing is needed with a more generalizable sample.

### VALIDATION OF THE MEDSAIL TOOL TO SCREEN FOR CAPACITY TO LIVE SAFELY AND INDEPENDENTLY IN NURSING HOME RESIDENTS

Whitney Mills,[1] Mark Kunik,[2] P. Adam Kelly,[3] Nancy Wilson,[4] Steven Starks,[5] Ali Asghar-Ali,[4] and Aanand Naik,[2] *1. Providence VA Medical Center, Providence, Rhode Island, United States, 2. Center for Innovations in Quality, Effectiveness, and Safety (iQuest), Houston, Texas, United States, 3. Tulane University, New Orleans, Louisiana, United States, 4. Baylor College of Medicine, Houston, Texas, United States, 5. University of Houston, Houston, Texas, United States*

Capacity for safe and independent living (SAIL) refers to an individual's ability to solve problems associated with everyday life and perform activities necessary to live independently. Little guidance exists on the assessment of capacity for SAIL among nursing home residents. As a result, capacity for SAIL is not fully considered in the development of discharge plans to ensure safety and independence in the community. The Making and Executing Decisions for Safe and Independent Living (MEDSAIL) tool was developed to screen for capacity for SAIL among community-dwelling older adults. In this cross sectional pilot study, we tested the validity of MEDSAIL for use with nursing home residents. Participants were twenty-four residents of a Veterans Health Affairs nursing home. Exclusion criteria were cognitive impairment too severe to complete the protocol, diagnosis of serious mental illness or developmental disability, inability to hear, and inability to communicate verbally. Participants completed two assessments: the MEDSAIL interview administered by a research assistant and the criterion standard capacity interview administered by a geriatric psychiatrist. We examined internal consistency, convergent validity, divergent validity, and criterion-based validity. Five of seven MEDSAIL scenarios approximated acceptable levels of internal consistency ($\alpha$>0.70). MEDSAIL scores were positively correlated with the criterion standard (0.88, p=0.001), and the Wilcoxon Rank Sum Test statistic was also statistically significant (p=0.001). MEDSAIL has promise as a user-friendly brief screening tool in nursing homes to understand resident capacity for SAIL and to inform development of discharge plans to keep the resident safe and independent in the community.

### VALUE-BASED PURCHASING FINANCIAL OUTCOMES AMONG MINORITY-SERVING SKILLED NURSING FACILITIES

Jennifer Gaudet Hefele,[1] Hari Sharma,[2] Bryant Conkling,[3] Lili Xu,[4] and Xiao (Joyce) Wang,[5] *1. Booz Allen Hamilton, Hollis, New Hampshire, United States, 2. The University of Iowa, Iowa City, Iowa, United States, 3. Booz Allen Hamilton, Bethesda, Maryland, United States, 4. University of Iowa, University City, Iowa, United States, 5. University of Massachusetts, Boston, Massachusetts, United States*

Skilled Nursing Facility (SNF) Value-Based Purchasing (VBP) ties post-acute payments to readmissions performance. Minority-serving SNFs tend to be poorly resourced and understanding the financial implications of SNF-VBP is important. Our study examined VBP payments and penalties among minority-serving SNFs. We conducted cross-sectional analysis using public data sources. We defined minority-serving as SNFs with >50% of residents who are African American (AA)/Black (n=764) or with >50% of residents who are Hispanic/Latino (n=164). Majority-White SNFs (>50% residents who are White) were the reference group (n=11,002). Outcomes examined were: being in top 20% of performance rankings; receiving a bonus; receiving a bonus that was above the median bonus dollar amount; being in bottom 20% of performance rankings; receiving a penalty; receiving a penalty that was below the median penalty dollar amount. Logistic models estimated the likelihood of experiencing each performance outcome for AA/Black-serving and Hispanic/Latino-serving SNFs in reference to majority-White SNFs. Results show that minority-serving SNFs not only perform worse but also experience greater negative financial impacts. Among those penalized, Hispanic/Latino-serving SNFs had the largest average penalty amounts: $32,575 compared to approximately $26,000 for both AA/Black- and White-serving SNFs. Hispanic/Latino-serving SNFs had 1.69 times the odds of receiving larger than median penalties and AA/Black-serving SNFs had 27% lower odds of receiving higher than median bonus payments. Average penalties approximate the average salary for a certified nursing assistant, the primary direct care worker in this setting. Results from this study raise concerns over long-term impacts. Alternative approaches to encouraging quality should be considered.

### SESSION 2849 (POSTER)

### ELDER MISTREATMENT: PREVENTION, PROGRAMS, PRACTICE, AND POLICY

### AN ORPHAN NEEDING A CHAMPION–BARRIERS TO EVALUATIONS OF DECISION-MAKING CAPACITY

Ronan Factora, and Saket Saxena, *Cleveland Clinic, Cleveland, Ohio, United States*

Impaired decision making capacity is common in medical and community settings, often coexistent with functional impairment and dementia. Though the legal system determines competence, deciding if guardianship is appropriate often relies on a medical assessment of decision making capacity. Physicians conduct cognitive evaluations but are often unaware of a person's functional impairments and are unfamiliar with their role in pursuing guardianship. Despite the individual roles of the legal system, medical system, and community agencies (e.g. Adult Protective Services (APS)) in such cases, no agency claims responsibility for ensuring this process's fidelity. As numbers of older citizens in the US increase along with associated increasing numbers of cases involving impaired decision making capacity, ensuring this processes' fidelity is essential. We sought to identify the barriers (recognized by each of these parties) in determining decision making capacity of persons for whom guardianship is being considered. Surveys were conducted with professionals in the legal, medical, and APS arenas, to identify barriers to obtaining capacity determinations. Common themes identified amongst these entities included needing to include functional status in this evaluation, and the need for clear communication between the legal system, medical system, and APS. Unique concerns identified in the survey included APS caseworkers challenges in finding physicians willing to complete capacity evaluations, judges' concerns about evaluations' failure to address safety issues and need for immediate action , and physician's concerns about liability and compromising the physician-patient relationship. Continued collaboration between these systems could overcome these barriers.

### APPLYING EVIDENCE-BASED VIOLENCE PREVENTION STRATEGIES TO ELDER ABUSE IN PUBLIC HEALTH

Carolyn Ham,[1] and Cory Bolkan,[2] *1. Washington State Department of Health, Olympia, Washington, United States, 2. Washington State University, Vancouver, Washington, United States*

Elder abuse is a growing problem with significant public health implications. Because elder abuse shares root causes with other types of violence (e.g., suicidal behavior, intimate partner violence), awareness of elder abuse as a violence prevention priority is rising among public health professionals. Major limitations, however, affect delivery of effective population-level primary prevention for elder abuse, necessitating increased community partnerships. In Washington State, the Department of Health's Injury and

Violence Prevention Section and the Department of Social and Health Services Adult Protective Services Division are leveraging existing strategies to increase identification and reporting of potential elder abuse from falls and injury prevention partners (i.e., opioids, suicide). We describe: (1) challenges and opportunities in creating unique cross-program collaborations, (2) the combined education and outreach efforts of this partnership, and (3) strategies for sustained collaboration. Additionally, we share results of a scoping literature review on evidence-based violence prevention strategies applicable to elder abuse between 2015 – 2019. In the Pubmed and Academic Search Complete databases, the following terms were searched: elder abuse prevention, primary prevention, shared risk and protective factors. Only six articles were identified that addressed primary prevention efforts. Researchers note that primary prevention of elder abuse is poorly understood and challenges exist in applying methods from other types of violence. Education for key community members on identification of abuse is a promising intervention targeting shared risk and protective factors for public health to pursue. Cross-sector community partnerships and rigorous evaluation of primary prevention approaches are needed.

### CAREGIVER RESILIENCE AS A PROTECTIVE FACTOR FOR ELDER ABUSE

Elsie Yan, *The Hong Kong Polytechnic University, Hong Kong, China*

This study examines the protective function of caregiving resilience and caregiving self-efficacy for elder abuse. A convenient sample of 600 family caregivers of community dwelling older Chinese in Hong Kong were individually interviewed. Participants were assessed on the caregiving context, care recipient physical functioning and behavioral problems, perceived caregiver stress, neurotic personality, caregiving self-efficacy and resilience. Past year elder abuse was assessed using Revised Conflict Tactic Scale and Older Adult Financial Exploitation. Elder abuse is common in this sample: 7.5, 11.5, 24 per cent reported physical, psychological, and financial abuse respectively. Injuries were reported in 2.3% of the sample. Hierarchical linear regression analysis was conducted. Number of co-residing days and hours of care provided per week were entered into Model One; Care recipient physical functioning, behavioral problems, and caregiver stress were entered into Model Two; Caregiver resilience and caregiving self-efficacy were entered into Model Three. Care recipient behavioral problems and caregiver stress were prominent factors associated with abuse across all models. With the exception of physical abuse, caregiver resilience buffered the effects of care recipient behavioral problems and caregiver stress on all forms of elder abuse. No such effect was observed for caregiving self-efficacy. Intervention efforts aiming at fostering caregiver resilience could potentially prevent elder abuse.

### COMPARING ELDER AND CHILD ABUSE PREVENTION APPROACHES IN JAPAN: STRATEGIES FOR REDUCING ELDER ABUSE

Asako Katsumata,[1] and Noriko Tsukada,[2] *1. Rehabilitation Institution, Chiba, Tokyo, Japan, 2. Nihon University, Tokyo, Tokyo, Japan*

This paper examines trends in elder abuse cases (types of abuse, traits of abusers, victims and their relationships, levels of disabilities and dementia of victims, etc.) by using longitudinal data (2012-2018) collected by Japan's Ministry of Health, Labour and Welfare since its enforcement. Considering these trends, this paper then compares the Elder Abuse Prevention Law to the Child Abuse Prevention Law to assess differences in policy and program provisions and how those relate to successful prevention outcomes. For example, while reports for both elder abuse and child abuse cases have been increasing, governmental actions taken in response have varied. The Child Abuse Prevention Law has been modified 6 times since its enactment based on abuse cases, but no amendments have been made for Elder Abuse Prevention Law based on case or evaluation data. Moreover, there have been many public awareness campaigns for child abuse prevention, but none for elder abuse prevention. These efforts appear to have positive outcomes including increased reporting of child abuse to police. This analysis aims to compare abuse data, abuse laws and public health efforts for children and older adults in Japan. Findings seek to identify disparities and areas where the public approach to child abuse can inform and strengthen elder abuse policies and programs.

### ELDER ABUSE AND NEGLECT: TRAINING FIRST RESPONDERS IN RURAL ARKANSAS TO RECOGNIZE, RESPOND, AND REPORT

Robin McAtee,[1] Valerie Claar,[2] Laura Spradley,[1] and Whitney Thomasson,[3] *1. UAMS, Little Rock, Arkansas, United States, 2. Oaklawn Center on Aging, Hot Springs, Arkansas, United States, 3. University of Arkansas for Medical Sciences, Little Rock, Arkansas, United States*

As the incidence of elder abuse and neglect continue to rise and plague our country's older adults, it is imperative that their plight is recognized, reported and elicits an appropriate response. At least 1 out of 10 older adults suffer from at least one type of abuse each year (DOJ, 2020) and only 1 in 24 cases of elder abuse is ever reported to authorities (National Center on Elder Abuse, 2019). Since 41% of Arkansas' population live in rural areas, reaching and educating first responders who work in these areas is a priority, yet has been a challenge. It has been ascertained that virtually no elder abuse or neglect related training for first responders occurs in Arkansas. In 2015, the Arkansas Geriatric Education Collaborative (a HRSA Geriatric Workforce Enhancement Program) developed an education program and mobilized it to multiple first responder groups including the AR State Police, multiple city and county paramedics' organizations, EMTs, local police officers and fire fighters. The program was further enhance late in 2019 when the training was made available on-line in conjunction with dementia training. The content and methods of training and test results revealing knowledge gained will be reviewed. Follow-up stories from first responders who have put their training into action in the field will be told as they reveal how they have used their training to identify potential abuse, neglect and self-neglect cases and how they have recognized, reported and addressed specific cases.

### INCONSISTENT CAPACITY ASSESSMENT ACCESS FOR ADULT PROTECTIVE SERVICES: NATIONAL SURVEY RESULTS

Theresa Sivers-Teixeira, Kelly Sadamitsu, Gregory Stevens, Christina Penate, and Bonnie Olsen, *Keck School of Medicine of USC, Alhambra, California, United States*

Throughout the country, access to professional capacity assessments is inconsistent. Capacity assessments are often a critical factor in case management of elder abuse investigations. This unmet need for many Adult Protective Services (APS) programs hampers their ability to protect older adults from mistreatment and preserve self-determination when appropriate. In June 2019, electronic surveys regarding access to capacity assessments were sent to APS leadership in all 58 California counties. A similar survey is planned for each US state in June 2020. In California, with 100% response rate, 53% of counties had no access to assessments and 56.1% had no funding for assessments. Most assessments were completed by physicians or psychologists. Seventy-three percent of counties reported that primary care physicians complete requests for capacity declarations less than half the time. Physicians decline to complete capacity declarations because they don't know how to do the assessments (22.4%) and are concerned about being called into court (28.5%). Findings from the national survey will be presented along with maps illustrating capacity assessment accessibility. Factors that appear to influence accessibility positively (forensic centers) and negatively (lack of funding and lack of trained evaluators) will be discussed along with policy implications.

### PILOTING THE ADULT PROTECTIVE SERVICES' IDENTIFICATION, SERVICES, AND OUTCOMES MATRIX: FOCUS GROUP FINDINGS

Jarmin Yeh,[1] Pi-Ju Liu,[2] Jacques Perkins,[1] Andrew Butler,[1] Sara Stratton,[3] Kendon Conrad,[4] Karen Conrad,[5] and Madelyn Iris,[6] *1. University of California, San Francisco, San Francisco, California, United States, 2. Purdue University, West Lafayette, Indiana, United States, 3. San Francisco Adult Protective Services, San Francisco, California, United States, 4. University of Illinois at Chicago, Chicago, Illinois, United States, 5. University of Illinos at Chicago, Chicago, Illinois, United States, 6. Northwestern University, Chicago, Illinois, United States*

The historical lack of outcomes-related data in Adult Protective Services (APS) has affected funding coming to the program. Without data quantifying the effectiveness of APS services, policymakers have been unable to justify budget increases to improve wages for workers or professionalize the field. For the first time in APS history, the U.S. Administration for Community Living sponsored a pilot project to implement a novel evidence-based assessment tool into APS electronic systems, called the Identification, Services, and Outcomes (ISO) Matrix. The goal was to improve APS' ability to reduce harm of abuse and neglect and maintain client's independence to live in the community. APS workers in San Francisco and Napa Counties were trained and phased into using the ISO Matrix over a six-month implementation period. This poster presents findings from six focus groups conducted between February 13 and March 28, 2019, with 34 San Francisco and Napa County APS workers and supervisors. Utilizing semi-structured, in-depth interviewing techniques, APS workers and supervisors expressed their views and experiences. Facilitators and barriers of implementing the ISO Matrix were assessed and opportunities for improvements were identified. Findings revealed a tension between their hopefulness that the ISO Matrix could modernize APS data-tracking and yield evidence of improved client outcome to bolster the field, and their frustrations about new burdens the ISO Matrix placed on their labor and workflow. Understanding frontline perspectives of APS workers and supervisors has practical and policy implications for adopting the ISO Matrix in other counties and states across the U.S.

### THE IMPACT OF RELATIONSHIP SATISFACTION WITH ADULT CHILDREN ON DEPRESSION AMONG OLDER ADULTS WITH ABUSIVE SPOUSES

Sukyung Yoon, *University of Wyoming, Laramie, Wyoming, United States*

Adults (65 and older) comprised about 15% of the 2019 South Korean population (hereafter Korea), but are estimated to be 20% in 2025 and 40% in 2050 (StatisticsKorea, 2019). Good relationships with spouses impact mental health during later life (Santini et al., 2015) but 10.2% of women and 7.6% men 65 and older reported they experienced spousal violence (The Domestic Violence Survey, 2016) Moreover, violent behavior in baby-boomer marriages was significantly higher than their counterparts (Suh, 2015). Previous research investigated how relationships with adult-children impacted older Koreans' mental health(Kim & Ko, 2013) but few examined the influence on older-adults with abusive spouses. This study investigates depression among older-adults with abusive spouses, and the impact of relationship-satisfaction with their adult-children on depression. This study utilizes the 13th wave of the nationally representative Korea Welfare Panel (2018). The sample consisted of 353 older adults 65 and older with abusive spouses over the past year. The dependent variable was depression, measured using the CES-D-11. The relationship-satisfaction with adult-children was measured on a seven-point Likert scale. Education, health, religion, sex, and age were included, and multiple regression analysis was conducted. The relationship-satisfaction with adult-children and good health status were significantly reduced depression among the population. Health care professionals and practitioners should screen for elder abuse and depression. Additionally, programs are needed to help older - adults develop good relationships with family members are needed.

### TURNING 75: WHY AGE MATTERS FOR VICTIMS OF ASSAULTIVE VIOLENCE REPORTED TO POLICE

Lynn Addington, *American University, Washington, District of Columbia, United States*

Americans are able to age in more active and engaged ways than previous generations. These changes bring many positive opportunities, but also might affect the risk of criminal victimization for older adults. In considering these risks, an initial question is how best to identify older adults. One common default is to use age 65 and older, which suggests older individuals are part of a homogenous group. The Census Bureau's multiple category approach illustrates another option, which captures variations as Americans age. This study explores the risk and characteristics of non-fatal assaultive violence using a multiple-category age definition and uses police data from the Federal Bureau of Investigation's 2016 National Incident-Based Reporting System (NIBRS). These data were collected from 34 states and cover a range of crimes including aggravated and simple assaults. NIBRS data are well suited for this study as they collect details about the crime including victim and offender demographics and incident details such as victim-offender relationship, weapons, location and arrest. Preliminary results indicate that 34,689 assault victims were adults over the age of 65. Using a generic measure of older adult (age 65 and above) masks important variations in these assaults. Distinct patterns are observed between those aged 65 to 74, 75 to 84 and 85 and above. Within these age categories, differences also occur across racial and sex groups. The patterns observed can provide more nuanced guidance to challenge traditional assumptions about older adult crime victims and inform policies tailored to support these victims.

### USING LATENT CLASS ANALYSIS TO IDENTIFY TYPES OF ELDER ABUSE: MULTIDISCIPLINARY TEAM INTERVENTIONS

Gerson Galdamez, Zachary Gassoumis, and Kathleen Wilber, *University of Southern California, Los Angeles, California, United States*

Elder abuse is a complex, multifaceted problem that can devastate the lives of older adults, and overwhelm medical, legal, and social service providers. To address this problem, the Elder Abuse Multidisciplinary Team (MDT) is an oft-utilized intervention that brings individuals from various professional disciplines together to address elder abuse. With the passage of the Elder Justice Act of 2010, the Federal Government included budgetary provisions in support of a particularly promising model of MDT: The Elder Abuse Forensic Center (EAFC). Although the EAFC model has been implemented at four sites in California with promising outcomes, the specific structures, processes, and practices that currently define this model have not been validated with rigorous research of other MDTs in the United States. In this study, we surveyed 81 elder abuse MDT key informants across the country, using instruments guided by the most current EAFC model (Yonashiro-Cho et al., 2019). We then used Latent Class Analysis (LCA) to group these teams based on EAFC model characteristics. We hypothesized that elder abuse MDTs would fall on a spectrum: those that were identical or nearly identical to the EAFC model, those that had some EAFC qualities, and those that were not identical. Results of the LCA supported our hypothesis and revealed three types of elder abuse MDTs: EAFCs (n=26), Semi-EAFCs (n=24), and Non-EAFCs (n=31). Policy makers, advocates, and professionals seeking to form new teams or support existing teams can draw on these findings to make decisions about MDT creation and sustainability.

### SESSION 2850 (POSTER)

### AGING IN PLACE: SUPPORTIVE INDIVIDUAL, FAMILY, COMMUNITY, AND ENVIRONMENTAL FACTORS

### A QUALITATIVE STUDY OF HEALTHCARE EXPERIENCE AMONG CHINESE HOMEBOUND ADULTS RECEIVING HOME-BASED MEDICAL CARE

Rui Zhou,[1] Joyce Cheng,[2] Shuangshuang Wang,[3] and Nengliang (Aaron) Yao,[3] *1. Shandong Uniiversity, Jinan, China, 2. University of Virginia, Springfield, Virginia, United States, 3. Shandong University, Jinan, Shandong, China*

Home-based medical care (HBMC) is emerging in China, but research understanding the efficiency and effectiveness of this new care model is rare. In this study, researchers interviewed 17 Chinese homebound adults aged 45 and older (53% females, mean age=76) who have received HBMC, and collected detailed information regarding their experiences and attitudes toward HBMC. Participants were recruited from healthcare institutions in Shanghai, Jinan, and Zhangqiu of China. The evaluation of patients' experiences with HBMC yielded both positive and negative aspects. Positive experiences included 1) the delivery method was convenient for homebound patients; 2) health problems could be detected timely because doctors visited patients regularly; 3) home care providers had better bedside manners and professional skills than hospital-based providers; 4) the medical insurance covered the cost of home care services. Negative experiences related to the supply and quality of care, including 1) the scope of current HBMC services was too limited to meet the needs of homebound patients; 2) the visit time was too short; 3) healthcare providers' professional skills varied greatly. Findings from this study suggest that the HBMC model benefited Chinese older adults, primarily homebound adults, in terms of convenience and affordability. There are opportunities to expand the scope of home care services and improve the quality of care. Policymakers may consider providing more resources and incentives to enhance HBMC in China. Educational programs may be created to train more HBMC providers and improve their professional skills.

### A SCOPING REVIEW OF AGING IN PLACE: IS THE CURRENT LITERATURE INCLUSIVE OF AFRICAN AMERICAN WOMEN?

Reginald Tucker-Seeley,[1] Carlene Davis,[2] Jubilee Ahazie,[3] Matti Robi,[1] and Leora Steinberg,[1] *1. University of Southern California, Los Angeles, California, United States, 2. Sistahs Aging with Grace & Elegance, Los Angeles, California, United States, 3. University of Southern Califonia, Los Angeles, California, United States*

Approximately 80% of adults aged 50+ report a desire to stay in their homes as long as possible, or to "age in place." Yet, as the US aging population becomes more racially and ethnically diverse, the frameworks used to describe "aging in place" will require explicit recognition of the issues specific to racial/ethnic minorities. For example, given the intersection of historical discrimination related to race, gender, age, and lower socioeconomic status, older African American women are at increased risk for poor health outcomes as they attempt to “age in place.” We conducted a scoping review of “aging in place” studies in the US (N=479), to determine whether current “aging in place” frameworks included issues relevant to racial/ethnic minorities generally, and African American women, specifically. Our inclusion criteria for articles were as follows: 1) a definition of “aging in place”; 2) a conceptual framework related to “aging in place” and/or 3) the incorporation of racial/ethnic minorities generally and African-American women specifically in the research sample. We adapted the PRISMA (Preferred Reporting Items for Systematic reviews and Meta-Analyses) framework for our review to search the PubMed database for “aging in place” studies. After applying our inclusion/exclusion criteria (N=244), the findings from our review showed that approximately 40% of studies included an explicit definition of “aging in place” (N=100), but few studies focused on African American women (N=20). Future studies on “aging in place” should consider the unique challenges that African American women face as they navigate the challenges of “aging in place.”

### CREATING AN AGE-FRIENDLY COMMUNITY IN AFFORDABLE HOUSING USING A RESIDENT HEALTH RISK ASSESSMENT FOCUSED ON THE 4MS

Jennifer DeGennaro,[1] Sherry Pomerantz,[1] Margaret Avallone,[2] Melonie Handberry,[3] and Elyse Perweiler,[4] *1. RowanSOM, Stratford, New Jersey, United States, 2. Rutgers University-Camden, Camden, New Jersey, United States, 3. Fair Share Housing, Camden, New Jersey, United States, 4. Rowan University School of Osteopathic Medicine, Stratford, New Jersey, United States*

The NJGWEP team in partnership with Fair Share Housing/Northgate II (NGII), an affordable housing complex in Camden, NJ, employed an iterative quality improvement process to collaboratively develop a Resident Health Risk Assessment (RHRA) to meet the needs of the housing facility and incorporate the essential elements of the 4Ms framework (Mentation, Medication, Mobility, and What Matters). Using the RHRA, NG II social services staff and Rutgers School of Nursing (RSoN) students were trained to collect health information and administer several evidence-based screening tools (i.e., MiniCog, TUG, PHQ-2). A final element of the RHRA still in development is the documentation process of referral and follow-up based on personalized care plans. Since July 2019, 43 RHRAs have been completed (60% female, mean age 66, age range=43 to 88). Almost all residents (94%) have at least 1 chronic condition (HTN, DM, COPD, CHF), although only 26% have an advance care plan. Most (81%) were screened for future fall risk; function (ADLs/IADLs) was assessed for all (100%). Every resident who was able or did not refuse (88%) was screened for cognitive impairment. Just 7% were taking a high-risk medication (i.e., an opioid or benzodiazepine). The NJGWEP team has initiated an age-friendly community at NGII by providing education on geriatric-focused topics and implementing the 4Ms-focused RHRA to detect issues impacting the resident’s well-being. Establishing a follow-up process to track referrals to available resources will enable NGII to allow residents to age in place with appropriate supports.

### ENHANCING ACCESS TO HOME MODIFICATION FOR OLDER ADULTS: RESEARCH RESULTS OF A NATIONAL SURVEY OF STATE UNITS ON AGING

Julie Overton,[1] Jon Pynoos,[1] Emily Nabors,[1] Damon Terzaghi,[2] and Bernard Steinman,[3] *1. University of Southern California, Los Angeles, California, United States, 2. ADvancing States, Arlington, Virginia, United States, 3. University of Wyoming, Laramie, Wyoming, United States*

With aging populations and cost constraints, state home and community-based care (HCBC) systems are increasingly being challenged to support older adults at home. Home modifications (HMs), changing the home environment to support health, safety and independence, has been found to be cost effective, improve quality of life, and help prevent falls. While the passage of the Older Americans Act in 1965 made State Units on Aging (SUAs) the designated state-level agencies responsible for developing and administering assistance to older adults, little is understood about the extent to which they prioritize, implement, and fund HM services. In the last decade, new developments have elevated HM on the public agenda, yet the creation of policies and programs depends on a solid knowledge base. Funded by the Administration for Community Living, the University of Southern California and ADVancing States conducted a survey of the 56 SUAs to ascertain HM efforts, targeting, Older Americans Act fund allocation for HMs, collaboration with other state entities, needs and challenges, legislation, and accomplishments. With a response rate of 91% SUAs (N = 51), the survey revealed: 88% of SUAs engage in HM efforts with most (61%) integrating HMs within HCBC and long term care programs; 74% work with their State Medicaid Office on including and delivering HMs via waivers; and the most pressing needs as funding and more HM providers. Analysis showed great variation of SUA involvement in HMs based on the state size, SUA location within the state government, and connections with other state agencies.

### FACTORS THAT INFLUENCE LENGTH OF MEMBERSHIP IN A CUSTOMER-DRIVEN ORGANIZATION: A VILLAGE

Joonyoung Cho, Ruth Dunkle, and Karen Harlow-Rosentraub, *University of Michigan, Ann Arbor, Michigan, United States*

Membership is a critical feature of the survival of customer-driven organizations. As a membership-driven organization based on neighbors helping neighbors, many Villages express difficulty in having enough members and lack confidence in sustainability. This is the first study examining the association between length of membership and motivation for becoming a Village member. ShareCare, the first Village, was founded in 1994. We used an open-ended questionnaire to gather information from a representative sample of current Sharecare members (N=100). Three researchers were involved in coding responses with discrepancies resolved via collaborative discussion. Length of membership was categorized as: less than 10-years, and more than a 10-year membership. Motivations to join membership in ShareCare were categorized as: instrumental, social, and altruistic. We conducted three separate logistic regressions with covariates controlled to examine associations between length of membership and various motivations to become a ShareCare member. While the most frequent reason for joining was instrumental where the member would receive service (e.g., care coordination, and home visit), the least motivation for joining was altruism, where the member could help other members (e.g., running errand, and lawn care). More than a 10-year membership was not associated with social or instrumental motivation to join ([OR] 0.50, p = 0.27, [OR] 0.94, p = 0.95) whereas more than a 10-year membership was associated with altruistic motivation to join ([OR] 5.31, p = 0.02). Our findings provide guidance regarding motivating members to join and maintain membership in a consumer-driven organization.

### FINANCIAL RESOURCES AND LATER-YEAR RELOCATION FROM THE ANNUITIZED ASSESSMENT OF INCOME AND ASSETS

Jun-Hong Chen,[1] and Sojung Park,[2] *1. Washington University in St Louis, Saint Louis, Missouri, United States, 2. Washington University in St. Louis, St. Louis, Missouri, United States*

Solid evidence has shown financial resources play important roles in housing decisions among older adults. Despite the growing research on the joint assessment of income and assets as valid economic well-being, little attention is paid to its role in relocation in old age. Drawing from the Behavioral Model of Elderly Migration, this study examined to what extent financial resources are associated with the likelihood of moving in later years. The data came from the 2017 Panel Study of Income Dynamic (PSID). A sample of 1354 people, 65 years and older, was used in the analyses. We used the annuitized approach, which is different from conventional approaches that assume people draw down all available assets to satisfy daily needs and leave no assets for use in later years. We (1) assessed annuitized assets based on the 2019 IRS Mortality Table, (2) assessed yearly income using supplementary income (i.e. income plus non-discretionary expense). A final indicator of the summed score was used in a logistic regression to predict the likelihood of moving. A set of covariates known to affect later- year relocation at an individual level (e.g. health condition, living arrangement change), environmental level (e.g. rural, non-metro area) are controlled for. In clear conflict with previous studies, we found annual financial resources did not significantly influence relocation among older adults. The notable absence of the well-known role of the economic factor provides critical initial evidence about the importance of simultaneous assessment of financial resources for the literature on later year relocation.

### IDENTIFICATION OF RESEARCH TRENDS IN AGING IN PLACE USING TEXT MINING ANALYSIS

Ha Neul Kim, and Seok In Nam, *1. Yonsei University, Seoul, Republic of Korea*

Since 1980s professionals and social service providers have focused on aging at the place where people lived. This is the initial concept of the Aging in Place (AIP). Over 40 years, the topics have developed and extended to other disciplines welcoming different perspectives in the study of AIP. Therefore, this study aims to understand the overall research trends in Aging in Place (AIP) studies using text mining analysis to track the evolvement of AIP subtopics not only in Gerontology but also in various fields. To identify the topic trends, we collected the titles, abstracts, and keywords from 1,372 international articles that were published from 1981 to 2019. Then, keywords were extracted and cleaned based on precedent literature and discussions. We analyzed the keywords based on the degree of centrality and visualized the keyword-networks using VOSviewer and Pajek. Top-most popular keywords are "independent living", "housing", "older adults", "home care", "daily life activity" and "quality of life." The change in topic trends shows that in the 1980s to early-2000s, research focused on organization and management level of intervention, home(housing) for the older adults, long term care. In the mid-2010s, health-related topics such as daily life activity, health service, health care delivery and quality of life have emerged. Recently, the topics have extended further to technology, caregiver, well-being, and environment design, environmental planning that support independent living of oneself. The research result shows that the interdisciplinary approach regarding AIP is not only inevitable but also encouraged for an in-depth discussion of the field.

### INFORMAL VOLUNTEERING HELPS OLDER ADULTS STAY PUT TOO! BUT, DOES GENDER MATTER?

Huei-wern Shen,[1] and Tam Perry,[2] *1. National Taiwan Normal University, Sintien, New Taipei, Taiwan (Republic of China), 2. Wayne State University, Detroit, Michigan, United States*

Many older adults desire to remain in one's home for as long as possible, and many factors have been identified to be helpful, such as formal volunteering (doing unpaid work for religious, educational, health-related or other charitable organizations). While many older adults volunteer formally, many others volunteer informally (providing unpaid help to friends, neighbors, or relatives who did not co-reside). However, less is known about the relationship between informal volunteering and relocation. Guided by the social and material convoy framework, the present study explores the intersection of gender, informal volunteering, and relocation (no move, move within area, and move out of area). Utilizing data from 2008 and 2010 Health and Retirement Study, 8,361 older adults who were 65 and above in 2008 were included. When older people's financial resources, health, environment, and demographics were controlled, findings from multinomial logistic regression showed that older adults who volunteered informally were less likely to move within area two years later. When stratified by gender, it was found that female (n=4,832) volunteered informally in 2008 were less likely to move within area within two years, too; whereas for male (n=3,529), those who informal volunteered in 2008 were less likely to move out of area in 2010. According to the findings, informal volunteering helps older adults stay put. Future research is needed to understand why informal volunteering helps reduce short distance moves for women but helps reduce long distance moves for men.

### ISLAND AGING IN THE NINTH DECADE AND BEYOND

Elaine Eliopoulos, *PhD student, Weston, Massachusetts, United States*

This novel research was designed to explore the lived experience of the aging body and its impact on social exclusion in remote island environments. Twenty three participants, aged 80 to 102 years old, in the Pacific Northwest, USA, were interviewed to explore the role their bodies played in everyday life. Despite presenting limited choices and lifestyle options, the island communities appeared to foster rather than impede a sense of competency and autonomy in the lives of these later life participants. Their ability to navigate their worlds, despite their physical limitations, was apparent in their reports of inclusion in the manner they desired. The island community context, characterized by low technology and mostly face to face encounters for goods and services, presented the participants with the possibility of accessible and meaningful engagement. Their sense of self and inclusion casts doubt on the dominant narrative of decline typically used to describe these late years. New narratives of corporeal being emerged from the data, demonstrating a complexity not captured by the singularity of the decline narrative. The visual methodology utilized enriched the depth and range of the semi-structured interviews, encouraging participants to think critically about their bodies. Possibilities for further research could explore the nuances of these new narratives and whether a different understanding of the older body may be useful.

### MEASUREMENT INDICATORS OF AGE-FRIENDLY COMMUNITIES: FINDINGS FROM THE AARP AGE-FRIENDLY COMMUNITY SURVEY

Kyeongmo Kim,[1] Tommy Buckley,[1] Denise Burnette,[2] and Sunghwan Cho,[1] *1. Virginia Commonwealth University, Richmond, Virginia, United States, 2. VCU, Richmond, Virginia, United States*

Cities and counties worldwide have adopted the concept of “age-friendly communities” to promote the well-being of older adults. An age-friendly community is a place that provides a safe and affordable built environment and a social environment that encourages older adults’ participation. A major limitation in this field is the lack of valid and reliable measures of age-friendly communities. This study used data from the AARP 2016 Age-Friendly Community Surveys (N=3,652 adults ages 65 and older). This study included 57 indicators of age-friendliness (e.g., housing, transportation, public space, civic engagement, volunteering, community, and health services); socio-demographic characteristics; and health-related characteristics. We randomly split the sample into two subsamples for confirmatory factor analysis (CFA) (n=1,682) and structural equation modeling (SEM) (n=1,682). The CFA resulted in a three-factor structure to measure age-friendly communities: built environment, transportation, and social environment. Model fit indices were acceptable ($\chi^2(44)=14204.09$; $p<.001$; RMSEA=.067; CFI=.912; TLI=.909; SRMR=.05). Internal reliability of the three-factor structure was excellent ranging from .93 to .96. The SEM model showed that older adults living in a community with a greater built environment ($\beta=.119$; $p=.001$) and the social environment ($\beta=.199$; $p<.001$) had higher levels of physical health, after adjusting for all other variables. The findings highlight that the measures of age-friendly communities are reliable and valid. Practitioners and policymakers should work on improving both the built and the social environment to promote the well-being of older adults. The findings also suggested that researchers can use the measures as an evaluation tool for an age-friendly community initiative.

### MEASURING AGE-FRIENDLY COMMUNITIES IN NEW ENGLAND: PROMISING PILOT FROM THE NEW HAMPSHIRE ALLIANCE FOR HEALTHY AGING

Laura Davie,[1] Alison Rataj,[2] Beth Dugan,[3] Renee Pepin,[4] Josephine Porter,[1] Jennifer Rabalais,[5] and Matha Tecca,[1] *1. University of New Hampshire, Durham, New Hampshire, United States, 2. University of Massachusetts Boston, Kingston, New Hampshire, United States, 3. University of Massachusetts Boston, Boston, Massachusetts, United States, 4. Dartmouth College, Lebanon, New Hampshire, United States, 5. University of New Hampshire, Concord, New Hampshire, United States*

The New Hampshire Alliance for Healthy Aging is a statewide coalition building partnerships that support and promote healthy aging throughout the state. Through a collective impact approach, six domains (fundamental needs, living arrangements, caregiver support, social and civic engagement, physical and mental wellbeing, and advocacy) were defined to characterize and support the ongoing evaluation of age friendly communities. This poster describes a measurement framework and the development of a strategy to support gathering data across northern New England. A committee of state and national experts has convened to identify the best available indicators and measures for each of the domains and to expose gaps in available data. Representation includes individuals representing the University of New Hampshire, Tri-State Learning Collaborative on Aging (TSLCA), UMass Boston’s Department of Gerontology, and the 100 Million Healthier Lives Initiative (Institute of Healthcare Improvement). Researchers scanned national and state level sources for credibility, consistency, and availability of comparison information. Across the six domains, 43 indicators were selected. 26 did not have available data. Factors measuring social determinants of health are central and especially difficult to quantify, demanding new strategies and data collection approaches. Funding is essential for efforts to define and pilot a new data module to capture a broader set of meaningful data to measure and evaluate age friendly communities. Comprised of grassroots efforts across the fastest aging region of the country, Northern New England, under the Tri-State Learning Collaborative on Aging, is a prime location to use as a pilot project for this module.

### MORE THAN JUST A ROOM: RESULTS FROM AN INTERGENERATIONAL HOME SHARING PROGRAM IN TORONTO

Laura Martinez,[1] Raza Mirza,[2] Andrea Austen,[3] Lynn McDonald,[4] Christopher Klinger,[4] Jessica Hsieh,[4] and Tonya Salomons,[5] *1. National Initiative for the Care of the Elderly, Toronto, Canada, 2. National Initiative for the Care of the Elderly, Toronto, Ontario, Canada, 3. City of Toronto, Toronto, Ontario, Canada, 4. University of Toronto, Toronto, Ontario, Canada, 5. Services and Housing in the Province, Mississauga, Ontario, Canada*

Older adults prefer to live in their own homes for as long as possible — to 'age in place' — but for myriad reasons may be unable to do so. To address this, a number of housing alternatives have been explored, including homesharing, or homeshare, an exchange-based shared housing approach with the potential to empower older adults to age in place by enabling them to obtain additional income, companionship, and assistance with completing household tasks in exchange for renting out a room in their home. An intergenerational homesharing pilot program was launched in Toronto, matching older adults (55+) with postsecondary students. With limited research in the area, a mixed methods research study was embedded within the pilot project with the goals of: 1) conducting a scoping review to map and synthesize the literature related to outcomes of homeshare participation for this population, 2) conducting in-depth interviews with homeshare participants (N=22) to learn about their experiences, and 3) conduct a full evaluation and exit survey to better understand the implications of the project. Results were organized around the following themes: (1) benefits and challenges of participating in homeshare for older adults; (2) intergenerational engagement as social exchange; and (3) the key role of agency facilitation as a determinant of the experience of homesharing for older adults. Results spoke to the unique benefits and challenges of participating in homeshare for this population. Findings were used to derive implications for policy and practice, as well as highlight areas for future research.

### NO LONGER AGING IN PLACE: HOUSING DECISIONS AFTER 100

Susan Bowers, *Northern Illinois University, sycamore, Illinois, United States*

The number of aging individuals is growing and, along with it, a subset of the oldest-old (those over 85 years), including centenarians. Although researchers have begun identifying issues and needs related to this population (Dunkle & Jeon, 2016), still little is known about decision-making processes as they relate to housing. In rural areas, in specific, centenarians are limited by few residential choices and lack of geographic mobility. In this study, decision-making processes are examined, with an emphasis on interactions between aging individuals and their rural family caregivers. In addition, since family caregivers typically experience a pattern of burnout over time (Yilmaz, Turan & Gundogar, 2009; Yikikan, Aypak, & Görpelioğlu, 2015), a second focus of the study is caregiver stress. Data for the study are drawn from semi-structured interviews with a sample of family caregivers in the Midwest. All caregivers had a 100-plus family member recently placed, or in process of placement, at a residential long-term care facility. To meet criteria, all facilities were in towns of 4000 individuals or less. Data consisted of qualitative interviews with the primary family contact (female in all cases), and were analyzed according to Strauss & Corbin (1990). Decision-making themes centered on health and family pressure. Stress themes centered primarily around work. Data are discussed in terms of family strengths, health and wellness, and the need for continued programming for family caregivers, particularly in rural areas.

### PATTERNS AND PREDICTORS OF RESIDENTIAL CARE TRANSITIONS OVER TIME AMONG MEDICARE BENEFICIARIES

Linda Chyr,[1] Chanee Fabius,[1] and Emmanuel Drabo,[2] *1. Johns Hopkins Bloomberg School of Public Health, Baltimore, Maryland, United States, 2. Johns Hopkins Bloomberg School of Public Health, 21205, Maryland, United States*

Older adults prefer to age in place, but sociodemographic characteristics, health factors, and socioeconomic resources may influence their decision to move into other residential care settings (e.g., assisted living) or nursing homes. The characterization of residential care transitions and factors contributing to these transitions is limited. This study describes patterns and identifies predictors of transitions across community, residential care settings, and nursing homes among N=7076 Medicare beneficiaries in the National Health and Aging Trends Study, from 2011-2018. A discrete-time, multi-state Markov model was used to estimate the annual probabilities and hazards of transitioning across settings, adjusting for sociodemographic, health, and socioeconomic factors, mortality risk, as well as censoring from loss to follow-up. Most beneficiaries did not experience transitions: annual probabilities of remaining in the community, residential care settings, and nursing homes were 93%, 78%, and 73%, respectively. Being older, having dementia, being hospitalized in the last year, living alone, having multimorbidity, and having some or any functional limitations were associated with higher hazards of transition from the community to residential care settings and nursing homes. Being on Medicaid was associated with a reduced hazard of transitioning from the community to residential care settings (hazard ratio [HR]: 0.57; 95% CI: 0.36-0.91), but a higher hazard of transitioning from the community to nursing homes (HR: 1.37; 95%: CI: 0.98-1.91). As long-term services and supports increasingly shift from institutional to home and community-based care, our results can inform the design of federal and state policies targeting transitions across the care continuum.

### WHAT DOES IT MEAN TO AGE IN PLACE AS AN OLDER HOMELESS WOMAN? FACING AN ALTERED SENSE OF PLACE, BELONGING, AND IDENTITY

Judith Gonyea,[1] and Kelly Melekis,[2] *1. Boston University, Boston, Massachusetts, United States, 2. Skidmore College, Saratoga Springs, New York, United States*

The emergence of "aging in place" as social policy in the U.S and globally reflects a deepening understanding that a home is more than a physical domicile, it also represents a source of personal and social identity and offers one a sense of place and belonging. In this qualitative study we explore the question, What does "aging in place" mean to older homeless women navigating the shelter system and streets? Using a phenomenological approach, we conducted semi-structured interviews with fifteen chronically homeless women in their fifties using the shelter system. Our analysis process was inductive and iterative with the culminating phases being the generation and interpretation of themes. Our analysis revealed the links between place, sense of belonging, and identity. To be displaced from a physical home can present challenges to defining one's very existence. Specific themes emerging from the women's narratives included the ways in which shelter and street life impacted their sense of personal control, privacy, security, health, and comfort as well as underscored that shelters are dehumanizing places that further diminish one's sense of self and self-worth. The interviewed women sought to construct a positive sense of self through speaking about their past, present, and future roles as well as identities gained through social relations and place identity connections. Based on the findings, we suggest strategies by which shelters might better respond to unique needs of older women, including adopting ways that do not further disempower or stigmatize them but rather promote pathways out of homelessness.

### SESSION 2851 (POSTER)

### ALCOHOL AND ADDICTIONS

### DIAGNOSED OPIOID USE DISORDER AMONG OLDER ADULTS: COMPLEX COMORBIDITY AND MANAGEMENT CHALLENGES

Peter Treitler, Stephen Crystal, and Richard Hermida, *Rutgers University, New Brunswick, New Jersey, United States*

In the face of a widespread opioid epidemic and many policy changes affecting opioid access and management, it is important to understand the prevalence and characteristics of diagnosed opioid use disorder in older people and their implications for effective management of this high-risk population. We examined these issues in an ~40% random sample of Medicare beneficiaries with Part D coverage. In 2017, .8% of beneficiaries ages 65+ were diagnosed with OUD (opioid abuse or dependence diagnoses), an increase from .5% in 2015. The late-2015 transition from ICD-9 to ICD-10 may have contributed to this change, but the rate also increased post-ICD-10 by 9.1% from 2016-2017. The profile of individuals diagnosed with OUD reveals a population with complex comorbidity and multiple health challenges: 45% were diagnosed with major depression, 7% with alcohol disorders, 45% with anxiety, 8% with hepatitis C, 26% with cancer, 38% with COPD and 19% with pneumonia (risk factors for opioid overdose), 56% with diabetes and 27% with heart failure. 97% were diagnosed with pain conditions, 85% received opioid prescriptions, and 38% received benzodiazepine prescriptions. These patients represent complex and potentially competing challenges in concurrent management of pain, opioid use disorder, multi-substance use and opioid use disorder. Development of effective, integrated care models to simultaneously address these interrelated problems in this high-risk population should be informed by a closer focus on their multiple needs and monitoring of the adequacy of health system response.

### FOSTERING CONTINUITY OF CARE FOR MASSACHUSETTS LONG-TERM CARE RESIDENTS ON MEDICATION FOR OPIOID USE DISORDER

Rosanna Bertrand,[1] Chiara Moore,[2] Katherine Fillo,[2] Katherine Saunders,[2] Stephanie Baker,[3] Amy Berninger,[4] Teresa Mota,[4] and Nicole Keane,[4] *1. Abt Associates Inc., Cambridge, Massachusetts, United States, 2. MA Department of Public Health, Boston, Massachusetts, United States, 3. Healthcentric Advisors, Woburn, Massachusetts, United States, 4. Abt Associates, Inc., Cambridge, Massachusetts, United States*

In 2016, the CDC estimated that 2.1 million Americans had Opioid Use Disorder (OUD); about 1.8 million related to prescribed painkillers. Older adults are especially susceptible; SAMHSA estimates that 2.7 million older adults will misuse prescription drugs by 2020. The Massachusetts Department of Public Health (MDPH) issued a 2016 Circular Letter advising long-term care facility (LTCF) administrators that, if otherwise eligible for admission, facilities are expected to admit individuals diagnosed with OUD, and provide medication for OUD (MOUD) as prescribed. Yet, many facilities express concern for admitting residents with OUD. The MDPH and their partners are conducting a multi-faceted training/technical support (TS) program to foster best practices across the continuum of care, targeting LTCF. The 15-month program consists of in-person learning sessions, a comprehensive toolkit, on-site TS, weekly contact, and a peer-to-peer webinar. Pre-training data indicated that 24 of 42 recruited LTCFs had not admitted residents with OUD. Although licensed LTCF practitioners can obtain a waiver to prescribe certain MOUD, only 4 of the 28 LTCF medical directors interviewed had done so. Subject matter experts led topic-specific discussions in the first learning session to educate on OUD/MOUD, dispel myths, make community connections, and provide resources. Almost all participants agreed that the session met the objectives of understanding OUD as a chronic disease, recognizing the stigma of OUD, gaining knowledge of MOUD treatments, and obtaining strategies to enhance best practices across the continuum of care. All items on the pre/post-session assessments indicated a significant increase in understanding (37% versus 60%, respectively).

### HAVING A CASINO IN THE COMMUNITY: IMPLICATIONS FOR OLDER RESIDENTS

Cindy Bui, and Caitlin Coyle, *University of Massachusetts Boston, Boston, Massachusetts, United States*

Casino-going has been acknowledged as a common leisure activity for older adults, but what having a casino in the community means for the local older population has been understudied. Previous research has focused on problem gambling among older adults, but little is understood about how older residents perceive having a casino nearby and further how it impacts relevant senior services. This mixed-methods study gathered perspectives from 14 senior center directors and older residents (N = 411) of communities in Massachusetts that surround Plainridge Park Casino, the first casino that opened in the state in 2015. We conducted qualitative interviews with senior center directors and distributed a quantitative survey to older residents of the surrounding communities and those who visited the casino during the study period. We found that while most senior centers did not engage in trips to this "hometown" casino, many had other creative interactions with the casino, such as using casino space to host senior center events or seeking funding support from the casino. Older residents exhibited low rates of problem gambling risk, preferred to go to casinos outside of the state as an excursion, and attributed their reasoning to go to casinos to socializing rather than gambling aspects. While we must be aware of risks for problem gambling, this study also highlights the possibility of reframing a local casino as a potential asset that could contribute back to the community by providing resources for senior service providers and expanding social engagement opportunities for the older population.

### MEDICALLY ASSISTED TREATMENT OF OPIOID USE DISORDER AMONG ELDERLY AND NON-ELDERLY MEDICARE BENEFICIARIES

Jennifer Miles, Stephen Crystal, Peter Treitler, and Richard Hermida, *Rutgers University, New Brunswick, New Jersey, United States*

Although medication for addiction treatment (MAT) is known to be the most effective treatment for opioid use disorder (OUD), these medications are widely underutilized, especially among older adults and racial/ethnic minorities. Of the three main MAT modalities, Medicare covered buprenorphine and naltrexone in 2017; methadone was not covered until 2020. We examined MAT prescribing among elderly compared with non-elderly Medicare beneficiaries. Our sample was drawn from a ~40% random sample of 2017 Medicare beneficiaries with Part D coverage and was comprised of elderly beneficiaries (age 65+) with OUD (N=112,314) or who experienced opioid poisoning (N=9,657), and non-elderly Medicare beneficiaries (the Medicare disability population, age 0-64) with OUD (N=161,423) or opioid poisoning (N=13,591). MAT was underutilized in both Medicare populations, but especially in the elderly population. Of elderly beneficiaries with OUD, 5.1% and 0.8% were prescribed buprenorphine and naltrexone, respectively, compared to 15.5% and 2.3% among non-elderly. Among elderly beneficiaries with opioid poisoning, 3.1% and 0.8% were prescribed buprenorphine and naltrexone, respectively, compared to 10.1% and 3.2% in the non-elderly population. Sharp racial/ethnic disparities were identified within each age group. These findings highlight the need to expand access to MAT for Medicare beneficiaries, particularly older adults among whom underutilization is pronounced. Several recent Medicare policy changes have sought to address this issue, but continuing efforts and close monitoring are warranted in an effort to dramatically increase rates of treatment for elderly with opioid use disorder.

### MEDICALLY TREATED OPIOID OVERDOSES AMONG THE U.S. ELDERLY: TRENDS AND CORRELATES

Peter Treitler, Stephen Crystal, Richard Hermida, and Jennifer Miles, *Rutgers University, New Brunswick, New Jersey, United States*

High rates of opioid prescribing and comorbid medical conditions increase risk of overdose among older adults. As the US population ages and the rates of opioid use disorder (OUD) increase in the elderly population, there is a need to characterize trends and correlates of overdose in order to more effectively target policy and practice. Using a ~40% random sample of 2015-2017 Medicare beneficiaries ages 65 and older with Part D pharmacy coverage, this study examined medically treated opioid overdoses among US older adults. The sample included 13-14 million beneficiaries per year. The rate of medically treated opioid overdoses among elderly Medicare beneficiaries increased by 15% from 6 per 10,000 in 2015 to 6.9 per 10,000 in 2017. Those with overdose were disproportionately female (63%), non-Hispanic white (83%), with diagnoses of pain conditions (96%), with diagnoses of major depression (63%), and with high rates of conditions that decrease respiratory reserve such as chronic obstructive pulmonary disease. 13% had co-occurring diagnosed alcohol use disorder, 36% were diagnosed with opioid dependence or abuse, and 12% were diagnosed with hepatitis C. Older individuals with overdose represent a complex mix of risk factors; identifying those most at risk (as well as those who have very low risk, whose pain management may be compromised by overly-rigid interpretation of opioid use guidelines) is key in order to address multiple risks, balancing risk reduction with appropriate pain management.

### OLDER ADULTS AND OPIOID EDUCATION: LESSONS LEARNED FROM EXPERIENCES IN RURAL ARKANSAS

Robin McAtee,[1] Leah Tobey,[2] Corey Hayes,[1] Laura Spradley,[1] and Sajni Kumpuris,[3] *1. UAMS, Little Rock, Arkansas, United States, 2. University of Arkansas for Medical Sciences, Little Rock, Arkansas, United States, 3. University of Arkansas, Little Rock, United States*

Nearly one-third of all Medicare participants were prescribed an opioid by their physician in 2015 (AARP, 2017) and in 2017, Arkansas had the 2nd highest opioid prescribing rate in the nation (CDC, 2019). Approaching older adults (OA) about opioids and pain management can be a sensitive topic. Educating and altering long-term treatment with opioids is especially challenging in rural areas where literacy, especially health literacy, is suboptimal. The Arkansas Geriatric Education Collaborative (AGEC) is a HRSA Geriatric Workforce Enhancement Program with an objective to improve health outcomes including an emphasis to decrease the misuse and abuse of opioids among older Arkansans. To address this crisis, the AGEC partnered with local leaders such as the AR Drug Director, academia, Department of Health and Human Services, and multiple community based organizations to create age-tailored educational programs. Unique aspects of approaching and educating rural OA about opioids and pain management will be reviewed. Outcomes will be discussed such as their lack of knowledge about: what is an opioid, why they were prescribed, and what are viable alternatives. Also discussed will be lessons learned that resulted in more effective methods of reaching and teaching rural OA. Partnering with the AR Farm Bureau helped the AGEC reach 100's of farmers in the extremely rural and mostly agricultural areas. Learning to not use the word opioid resulted in more participants and in a more positive attitude and outlook on attempts to change the culture of opioid use, misuse and abuse among older Arkansans.

### PRESCRIPTION PAIN RELIEVER USE AND MISUSE AMONG MARIJUANA USERS AGE 50+ YEARS

Namkee Choi,[1] and Diana DiNitto,[2] *1. University of Texas, Austin, Texas, United States, 2. University of Texas at Austin, Austin, Texas, United States*

Marijuana use among individuals aged 50+ has steadily increased over the past decade, with 8% reporting past-year use in 2018. National epidemiologic data also showed a

36% rate of past-year prescription pain reliever (PPNR) use in the 50+ age group in 2018, a decrease from 40% in 2015, but still significantly higher than for younger age groups. Little research has, however, focused on older adults' dual recreational and/or medical marijuana and PPNR use. This study, based on the 2015-2018 National Survey of Drug Use and Health, examined rates and correlates of dual marijuana and PPNR use and misuse among those aged 50+ who reported past-year marijuana use (N=2,632). Our findings showed that 43.6% of past-year marijuana users did not use any PPNR, 47.1% used PPNR but did not misuse, and 9.4% misused PPNR in the past year, showing that one in six dual marijuana and PPNR users reported misusing PPNR. The risks of PPNR use/no misuse and PPNR misuse were higher among those who had more chronic medical conditions and major depressive episode. Additionally, the risk of PPNR use/no misuse was associated with high frequency and medical marijuana use; and the risk of PPNR misuse was associated with younger marijuana initiation age and marijuana and other illicit drug use disorders. Thus, correlates of dual marijuana and PPNR use/misuse among older adults are poor physical and mental health problems and problematic marijuana use. Older adults with marijuana and PPNR misuse need access to evidence-based treatments for pain management and substance misuse.

### SESSION 2852 (POSTER)

### ALZHEIMER'S DISEASE AND RELATED DEMENTIAS

### ADAPTING RESOURCES FOR ENHANCING ALZHEIMER'S CAREGIVER HEALTH FOR DEMENTIA-CAPABLE SERVICES

Mindi Spencer,[1] Maggi Miller,[2] Diana Jahries,[3] James Davis,[3] and S. Melinda Spencer, *1. University of South Carolina, Columbia, South Carolina, United States, 2. University Of South Carolina, Columbia, South Carolina, United States, 3. Center for Success in Aging, PRISMA Health, Greenville, South Carolina, United States*

The overall goal of the PRISMA Health - REACH (PH-REACH) project was to reduce caregiver burden and improve caregiving skills among caregivers of community-living Alzheimer's disease and related dementia patients and, as a result, improve care and quality of life for both the patients and their caregivers. This evidence-based, person-centered program emphasizes positive aspects of caregiving and provides tools to improve stress management, caregiver self-care, and coping skills for managing problem behaviors. PH-REACH provided in-home caregiver training, support, and service referral to 126 caregivers in the Greenville area. Trained coaches delivered the program to caregivers of persons with moderate to severe dementia in its original format but later adapted it to better fit the caregivers' needs. Analysis of pre- and post-test data showed that both the standard and adapted interventions provided benefits across multiple caregiver outcomes, including reduced caregiver burden, ability to manage disruptive behaviors of the care recipient, increased caregiver self-efficacy, reduced depression, and a slight improvement in the number of chronic health conditions. This supports and expands on previous research that has demonstrated the ability of this program to translate across different community-based and clinical settings. The tailored version of PH-REACH succeeded in assisting these caregivers, meeting them where they were in their caregiving journey, and provided measurable benefits to both their mental and physical health. Overall, this project provided evidence of the utility of the PH-REACH intervention and laid the groundwork to extend caregiver training and support to other institutions, both inside and outside the health system.

### ADVOCACY TRAINING AS A WAY TO INCREASE DEMENTIA LITERACY AMONG COLLEGE STUDENTS

Tsuann Kuo, *Chung Shan Medical University, Taichung City, Taiwan (Republic of China)*

In 2017, WHO passed the Global Action Plan on Dementia and declared the effort to increase dementia literacy around the world. Although dementia is mostly associated with older adults, the prevalent rate of the early on-set and the need to take care of people with dementia has mostly fallen on younger family members. Therefore, the purpose of this paper is to develop an intervention aiming to increase dementia awareness among college students. Through training and action plans, 85 university students from 9 different departments formed 12 groups to develop creatively and to conduct advocacy or public education in various communities. A questionnaire of pre- and post-test was conducted after students had completed their action plans. The response rate was 86% and the results were three-folds: (1) 31% of the students had someone in the family with dementia; (2) 35% of the students indicated that they were familiar with dementia; and (3) the pre- and post-scores on dementia awareness ($p<0.001$) and dementia attitudes ($p<0.001$) had significant improvement. This study demonstrated that there is a need to start the effort to increase dementia literacy because one-third of college-age students might be potential caregivers for their family loved ones with dementia. Preparing the students before graduating from college is a good entry because they can become health professionals to take better care of dementia patients and their family members. In conclusion, policy and practice implications will be discussed so the communities can become more dementia friendly in the future.

### ARE QUALITY-OF-LIFE SCORES STABLE AND SENSITIVE OVER TIME FOR NURSING HOME RESIDENTS WITH AND WITHOUT DEMENTIA?

Tetyana Shippee, Xuanzi Qin, Zachary Baker, Stephanie Jarosek, and Mark Woodhouse, *University of Minnesota, Minneapolis, Minnesota, United States*

The proportion of older adults with Alzheimer's Disease and Related Dementias (ADRD) in nursing homes (NHs) has been increasing over time and creates a mandate to meaningfully examine their care. There is also a growing recognition that person-centered measures are important for dementia care, and consensus about the need to maximize residents' quality of life (QoL). Yet, because QoL is fundamentally subjective, and residents with ADRD experience declines in cognitive function, their ability to make complex judgements about QoL has been questioned. This presentation will longitudinally assess whether QoL scores for residents with ADRD are stable and sensitive over time compared to those without ADRD. We use 2012-2015 Minnesota Resident Quality of Life and Satisfaction with Care Survey data, which contain in-person resident responses from a random sample of residents of all Medicare/Medicaid certified NHs in the state, about 40% of whom have AD/ADRD. These data were linked to the Minimum Data Set (MDS) 3.0. and facility characteristics data. The final sample contained 12,949 cohort-resident pairs, 8,803 unique residents, and 3,120 residents participated in more than two surveys. QoL scores of residents with and without ADRD were similarly stable over time and sensitive to health status change. We also found that stability of QoL scores may be driven by cognitive impairment as opposed to ADRD diagnoses. Thus, self-report QoL scores can also represent the QoL status for nursing home residents with ADRD diagnoses, and residents with ADRD diagnoses shouldn't be excluded from quality of life surveys based on ADRD diagnoses.

### BUILDING COMMUNITY SPACE FOR SUPPORTING RESIDENTS LIVING WITH DEMENTIA IN A HOUSING COMPLEX DISTRICT IN TOKYO, JAPAN

Mika Sugiyama,[1] Tsuyoshi Okamura,[2] Fumiko Miyamae,[2] Ayako Edahiro,[2] Madoka Ogawa,[3] Hiroki Inagaki,[3] Chiaki Ura,[2] and Shuichi Awata,[2] *1. Tokyo Metropolitan Institute of Gerontology, Tokyo, Tokyo, Japan, 2. Tokyo Metropolitan Institute of Gerontology, itabasi-ku, Tokyo, Japan, 3. Tokyo Metropolitan Institute of Gerontology, Itabashi-ku, Tokyo, Japan*

It is estimated that by 2025 the number of people with dementia will reach around 600 thousand, approximately one out of five in the older population in Tokyo, Japan. At the same time, the number of older people living in a single, couple household is expected to increase. We built a community space for older people in the largest housing complex district in Tokyo, and with the goal of creating a dementia friendly community (DFCs). In this study, we used the community-based participatory research approach to create a model of an inclusive community space with a human-rights-based approach, which is embodied in the PANEL framework by the Alzheimer Scotland organization. The community space where everyone, regardless of with or without dementia, can freely spend their time, and seek consultation on healthcare and older care. It also serves as a Dementia Café, where people with dementia can get together and chat. Places open 3 days a week. Those users can casually seek consultation by physicians, health nurses and psychologists. From April 1, 2017 to March 30, 2018, the average number of visitors was 11.6. Number of consultation was 182 times (female 81.3%, 80s' =31.3%; 70s' =23.1%). Historically, service delivery for the people with dementia was hospital-based in japan, but our community space established a new method to provide consultation to people with dementia, from a professional perspective, and to cooperate with appropriate social resources and related organizations as needed.

### DEMENTIA CAREGIVING, CARE RECIPIENT HEALTH, AND FINANCIAL BURDENS

Rashmita Basu, *East Carolina University, Greenville, North Carolina, United States*

Objective: While about 75% of people with ADRD receive care informally by their family members, relatively little is known about the effect of the quality of caregiving on maintaining carerecipient's health and financial burden of out-of-pocket (OOP) healthcare costs. The goal of this study is to examine the quality of caregiving on the out-of-pocket healthcare costs among ADRD patients and if caregiving prevents deterioration of physical health of carerecipients. Data and Sample: We used a nationally representative sample of people diagnosed with ADRD from the Aging Demographic and Memory Study, subsample of the Health and Retirement Study. The study sample includes carerecipients whose caregivers participated in the survey (N=261). Outcome measures: Primary outcomes were deterioration of carerecipients' health (1=yes, 0=no) and annual OOP healthcare costs. The quality of caregiving is captured by if caregiving made them feel good, feel useful and fee closer to carerecipients. More than 70% caregivers reported that caregiving make them feel good or useful. About 60% of carerecipients' physical health was maintained, and average out-of-pocket costs was $3,701/year ($0-$31,051). Multivariable logit for binary health outcome and OLS regression for OOP cost were estimated. Results: The likelihood of health deterioration was significantly lower for carerecipients whose caregivers reported that caregiving made them feel useful (AOR=5.1, 95% CI: 1.9- 14.5) and lower OOP remained significantly associated with presence of usefulness of caregiving (cost decrease, $3000 [95% CI: $6309-$918). Positive feeling of caregiving is independently associated with lower OOP cost and deterioration of physical health among ADRD patients.

### EVOLUTION OF PUBLIC KNOWLEDGE ABOUT DEMENTIA CAUSES AND SYMPTOMS: A GENDER PERSPECTIVE

Pedro Santos,[1] Constanca Paul,[2] and Cláudia Silva,[3] *1. Biomedical Sciences, Abel Salazar Biomedical Sciences Institute - University of Oporto, Porto, Portugal, 2. University of Porto, Porto, Portugal, 3. ESSA, Lisbon, Portugal*

The research objective is to monitor the evolution of public knowledge about dementia causes and symptoms, over a three-year period and by gender. The survey was made available at the Directorate-General of Health website and disseminated by email to relevant health and social stakeholders and through social networks, in 2015 and 2018. Respondents (n=1478 and 1716, respectively), included mostly women (79.4% and 83.3%). In both years, respondents showed a higher knowledge on symptoms than on causes. Total knowledge about symptoms and combined knowledge scores were higher in 2018 compared to 2015 (p=.012 and p=.0.2), respectively). "Neurological brain changes" were considered the main causes of dementia, by both genders in 2015 and in 2018 (>80% of respondents), with an increase in relative frequency being observed only for women (p=.039). "Psychiatric disease" and "drug consumption" are now less regarded as causes of dementia by both genders, with significant change over time also among women (p=.006 p=.001). On the contrary, in the last survey more women (+3.7%; p=.049) and men (+9.3%; p=.022) considered "stress" as main cause of dementia. "Confusion and disorientation", "wandering and getting lost", "difficulty managing and paying bills", "difficulty remembering things from the day before", and "difficulty managing daily tasks", were considered the most common symptoms, but only the last two significantly increased in 2018 (p=.018 and p=.000). Women knowledge increased regarding more causes and more symptoms compared to men. These findings will help to inform public debate and decision-making on gender-based policies to address awareness and stigma about dementia.

### FROM INFANTILIZING TO COLLABORATING: INTERACTIONS IN AN ADULT DAY CENTER IN TAIWAN

Shumin Lin, *National Chiao Tung University, Hsinchu 300 ROC, Taiwan (Republic of China)*

Many studies have found that interactions in long-term care settings are characterized by infantilizing speech towards older adults, which was principally interpreted as detrimental to older adults' health and self-esteem. These studies, however, focused on how caregivers talked to older adults and were conducted primarily in Western countries. How older adults respond to and make sense of such speech has received little empirical investigation (Marsden & Holmes, 2014). In this paper, I re-examined issues related to infantilizing speech based on 6 months of fieldwork in an ADC in Taiwan, which serves 33 older adults (aged 66-94), including 16 diagnosed with dementia. My data (including observational fieldnotes, 72 hours of video-recordings of naturally-occurring interactions, and conversations/interviews with caregivers, older adults and their family members) show that the ADC was discursively co-constructed as a learning place with frequent didactic interactions that occurred both ways. Many older adults (those with dementia included), with little or no education before, cherished the opportunity to be "students" for the first time. Caregivers also appreciated learning various things from the older adults. Furthermore, didactic interactions co-occurred or were interspersed with relational interactions, including teasing, humor, and bodily interactions that show mutual friendliness and care. By taking into account the wide variety of interactions, attending to the contributions of all parties, and situating these interactions in the personal as well as social histories, this study demonstrated that even didactic or so-called infantilizing interactions were used by caregivers and older adults as they collaborated to create strong positive relationships.

### GO LOCAL: INNOVATING LOCAL HEALTH DEPARTMENTS' ENGAGEMENT ON ALZHEIMER'S

John Shean, and Molly French, *Alzheimer's Association, Washington, District of Columbia, United States*

The aging Baby Boom generation is a major force behind projected increases in the prevalence of Alzheimer's, which is expected to grow from 5.8 million (2020) to 13.8 million (2050). Local health departments play a major role connecting people living with dementia (and their caregivers) to services, supports, and education, and to ensure safe, accessible environments where they can flourish. From September 2019-July 2020, two local health departments (from San Diego County and the City of Boston) participated in a yearlong collaborative pilot project with the Alzheimer's Association to advance cognitive health, dementia, and caregiving issues in their local jurisdictions. The National Association of County and City Health Officials (NACCHO) provided expert guidance and input throughout the collaboration. As part of the project, the local health departments: scanned their current work on healthy aging, identified existing data sources, and examined how existing healthy equity initiatives can apply to cognitive health, dementia, and caregiving issues. Action plans were developed, with a focus on policy mechanisms to initiate and sustain these projects and workforce development initiatives. Plans corresponded to actions of the Healthy Brain Initiative Road Map, helping elevate recommendations to change systems, policies, and environments. In fall 2020, LHDs will be able to use best practices, case studies, and tools developed from San Diego's and Boston's pilots to address Alzheimer's as a chronic condition. The tools will help them engage health officials, develop action plans, and train the public health

### IMPROVING DEMENTIA CLINICAL RESEARCH PARTICIPATION: STRATEGIES FROM A PORTLAND, OREGON, PILOT STUDY

Nicole Bouranis,[1] Sherril Gelmon,[1] Elizabeth Needham Waddell,[1] Dawn Richardson,[1] Hyeyoung Woo,[2] and Allison Lindauer,[3] *1. Oregon Health & Science University-Portland State University School of Public Health, Portland, Oregon, United States, 2. Portland State University, Portland, Oregon, United States, 3. Oregon Health & Sciences University, Portland, Oregon, United States*

The NIA's strategy to improve ADRD clinical research participation emphasizes local community collaboration. Literature that focuses on a person with dementia's decision to participate in research does not speak to specific state or local factors nor the effects of local efforts. This study aimed to develop strategies to improve dementia research participation in the Portland, OR metropolitan area. A community advisory board comprised of clinicians, researchers, advocates, people with dementia, family caregivers, and older African Americans was established for this project. Thirty-three interviews were conducted with clinicians, researchers, advocates, people with ADRD, and family caregivers. The Robert Wood Johnson Foundation's Culture of Health Action Framework was used to conceptualize motivation strategies and reflect elements that describe research participation among people with dementia. Strategies were identified to improve dementia clinical research participation: 1) Identify and promote local champions for ADRD clinical research participation; 2) Promote policies and processes that incentive cross-sector collaboration; 3) Recognize caregivers as full research participants; 4) Include people with ADRD and caregivers in the research design process; 5) Offer alternative options to reduce participation burden; 6) Evaluate and improve relationships between healthcare/research staff and patients/participants. These strategies can be used in conjunction with the Culture of Health Action Framework as a roadmap to form organization-community partnerships, facilitate motivation and empowerment, give decision-making power to people with ADRD and promote a local culture of research. Studies should be conducted in a larger context or as pilots in other communities to determine contextual relevance and generalizability for other areas.

### LONGITUDINAL ENGAGEMENT IN MODIFIABLE LIFESTYLE BEHAVIORS AND DEMENTIA RISK

Roger Wong, *State University of New York Upstate Medical University, St. Louis, Missouri, United States*

Dementia is a major public health threat as there are currently no definitive methods to prevent, treat, and cure the disorder. Recent efforts have focused on modifiable lifestyle behaviors to reduce dementia risk. Yet, the majority of these studies have utilized cross-sectional data or are limited to specific geographic areas. The purpose of this study was to explore how longitudinal engagement in modifiable lifestyle behaviors (physical activity, smoking, and social contacts) influence dementia risk. This study analyzed eight annual waves (2011-2018) of prospective data from the National Health and Aging Trends Study, a large nationally representative U.S. sample of older adults. Each wave, physical activity was measured as engagement in vigorous physical activities; smoking was measured as current engagement in cigarette smoking; and social contacts was measured as visiting friends/family outside of their home. The dependent variable was number of years to a new dementia diagnosis. Multivariate analyses were conducted using the Cox proportional hazards model with survey sampling weights applied for a national sample of 6,800 community-dwelling older adults dementia-free at baseline. After controlling for sociodemographics (age, sex, race, etc.) and health (physical health, chronic disease, etc.), longitudinal engagement in physical activity significantly decreased dementia risk (Hazard Ratio [HR]=0.60, p<.05), however, there was no significant relationship with smoking (HR=1.12, p=.58) and social contacts (1.06, p=.83). Our findings indicate physical activity is a promising modifiable lifestyle behavior for prevention. Future research should explore physical activity interventions that are most effective in reducing dementia risk, such as strength-based or aerobic-based activities.

### LOUISIANA GERIATRIC WORKFORCE ENHANCEMENT PROGRAM: ANALYSIS OF LEARNING OUTCOMES FROM DEMENTIA TRAINING

Katherine Kirsch,[1] Catherine Lemieux,[1] Laura Ainsworth,[1] Sarah Choate,[2] Ashleigh Borgmeyer,[1] Cassie Slaton,[3] and Scott Wilks,[1] *1. Louisiana State University, Baton Rouge, Louisiana, United States, 2. Louisiana State University, Hattiesburg, Mississippi, United States, 3. Louisiana State University, Ocean Springs, Mississippi, United States*

A recent Alzheimer's Association report noted that by year 2050, the number of Americans diagnosed with Alzheimer's disease and related dementias (ADRD) will triple to over 15 million. The report referred to primary care as the front line for meeting this demand, yet the nation faces a severe shortage of ADRD trained, primary care professionals (PCPs). Louisiana Geriatric Workforce Enhancement Program (LA-GWEP) addresses this demand. The purpose of this study was to examine preliminary data respective to LA-GWEP effectiveness with interdisciplinary education and training seminars, primarily aimed at medical, nursing, and social work PCPs. Three seminars were conducted in south Louisiana: Seminars 1 and 2 addressed effective communication, verbal and nonverbal, among persons with ADRD and caregivers; Seminar 3 offered basic overview of dementia symptomology, stages, and behaviors. Pre- and post-training session data were collected on-site. Participants completed questionnaires that included a 10-item knowledge assessment and 20-item Dementia Attitudes Scale (DAS). These measures contained Likert response formats; higher scores indicating greater levels of ADRD knowledge, in patient and caregiver contexts. Paired sample t-tests were conducted to observe any significant pre-to-post improvement, Cohen's d for effect size. Seminar 1 revealed no significant pre-to-post difference: t = -1.019, p = 0.320. Adjusting content from audience feedback, Seminar 2 revealed significant pre-to-post difference: t = -7.516, p < .001, Cohen's d = 1.2. Seminar 3 yielded significant improvement on DAS scores: t = -2.96, p < .01, Cohen's d = 0.34. Implications for seminars in future years of LA-GWEP are discussed.

### PREVALENCE OF DIAGNOSED ALZHEIMER'S DISEASE AND RELATED DEMENTIAS IN MEDICARE ADVANTAGE

Eric Jutkowitz,[1] Julie Bynum,[2] Susan Mitchell,[3] Noelle Cocoros,[4] Oren Shapira,[5] Richard Platt,[6] and Ellen McCarthy,[7] *1. Brown University, Providence, Rhode Island, United States, 2. University of Michigan, Ann Arbor, Michigan, United States, 3. Hinda and Arthur Marcus Institute for Aging Research, Boston, Massachusetts, United States, 4. Harvard Medical School Department of Population Medicine, Boston, United States, 5. Harvard Pilgrim, Boston, United States, 6. Harvardpilgrim, Boston, United States, 7. Hebrew SeniorLife, Harvard Medical School, roslindale, Massachusetts, United States*

A third of Medicare beneficiaries are enrolled in Medicare Advantage (MA); however, little is known about MA beneficiaries diagnosed with Alzheimer's disease and related dementias (AD/ADRD). MA plans have incentives that may influence the type of beneficiaries who enroll/disenroll from plans and the documentation of diagnoses. We calculated the prevalence of diagnosed AD/ADRD in 2014 and 2016 in three MA plans representing ~30% of the MA market. We identified beneficiaries ≥65 years of age enrolled in the MA plans in 2014 and 2016. Among eligible beneficiaries, we identified individuals with AD/ADRD using ICD-9 (2014) and ICD-10 (2016) codes included in the Medicare Chronic Conditions Warehouse algorithms for AD/ADRD. We determined the age and sex of beneficiaries diagnosed with AD/ADRD, and whether they disenrolled from the MA plan for any reason (e.g., death, enrollment in a different MA plan, enrollment in traditional Medicare or discontinuation of a plan) within 364 days from the date they were first identified has having AD/ADRD (i.e., index date). In 2014 and 2016 the prevalence of AD/ADRD diagnoses was 5.7% and 6.5%, respectively. In 2016, AD/ADRD beneficiaries were on average 82.4 (SD=7.3) years of age, 61.8% female, and had multiple comorbidities. By 364 days post-index, 32% of beneficiaries with diagnosed AD/ADRD had disenrolled from their plan. The characteristics of 2014 beneficiaries with diagnosed AD/ADRD were similar to their 2016 counterparts. In conclusion, MA beneficiaries with AD/ADRD are predominately female, have multimorbidity, and the age-stratified prevalence of AD/ADRD diagnoses is lower than rates reported in traditional Medicare.

### PROFILES OF FAMILY AND FRIEND CAREGIVERS OF COMMUNITY-DWELLING PEOPLE WITH DEMENTIA: A LATENT CLASS ANALYSIS

Eric Jutkowitz,[1] Lauren Mitchell,[2] and Joseph Gaugler,[3]
*1. Brown University, Providence, Rhode Island, United States, 2. Minneapolis VA, Minneapolis, Minnesota, United States, 3. University of Minnesota, Minneapolis, Minnesota, United States*

People living with Alzheimer's disease and related dementias (ADRD) receive most of their care from family/friends, but little is known about the organization of this care. We used data from the Health and Retirement Study and latent class analysis to determine variation in the hours of care received by community-dwelling people with ADRD from disease onset up to 6-years post onset. At incidence (n=1,158), the latent class analysis identified two groups of caregiving patterns. In the first group, 10% (n=109) of people with ADRD received 481 hours (SD=177) of care. Most care was provided by a spouse (411 hours) with less from children (28 hours), other family/friends (17 hours), and paid individuals (25 hours). In the second latent class, the remaining 90% (n=1,049) of people with ADRD received 114 hours (SD=202) of care which was distributed between spouses (12 hours), children (51 hours), other relatives/friends (22 hours), and paid individuals (29 hours). By 6-years post incidence, 7% (n=76) of the original ADRD cohort remained in the community, and we identified two latent classes independent of those identified at incidence. Almost 15% (n=11) of people with ADRD received a majority of care from a spouse (376 hours) with care supplemented by children (10 hours) and paid individuals (54 hours). The remaining 85% (n=65) of people with ADRD received 294 (SD=314) hours of care from spouses (13 hours), children (104 hours), other family/friends (83 hours), and paid individuals (67 hours). Policies/interventions supporting caregivers must account for the heterogeneity in the organization caregivers.

### RACIAL DIFFERENCES IN POST-ACUTE UTILIZATION AFTER HIP FRACTURE IN MEDICARE BENEFICIARIES WITH ADRD

Indrakshi Roy,[1] Amol Karmarkar,[2] Amit Kumar,[3] Meghan Warren,[3] Patricia Pohl,[4] and Maricruz Rivera-Hernandez,[5] *1. Northern Arizona University, flagstaff, Arizona, United States, 2. Virginia Commonwealth University Health, Richmond, Virginia, United States, 3. Northern Arizona University, Flagstaff, Arizona, United States, 4. Northern Arizona University, Phoenix, Arizona, United States, 5. Brown University School of Public Health, Providence, Rhode Island, United States*

BACKGROUND: The incidence of hip fracture in patients with Alzheimer's disease and related dementias (ADRD) is 2.7 times higher than it is in those without ADRD. Care complexity, including extensive post-acute rehabilitation, increases substantially in patients with ADRD after hip fracture. However, there are no standardized post-acute care utilization models for patients with ADRD after hip fracture. Additionally, there is a lack of knowledge on how post-acute utilization varies by race/ethnicity, in this population. OBJECTIVES: To investigate racial differences in post-acute care utilization following hip fracture related hospitalization in patients with ADRD. METHODS: A secondary analysis was conducted on 120,179 older adults with ADRD with incident hip fracture, using 100% Medicare data (2016-2017). The primary outcome was post-acute discharge dispositions (skilled nursing facility [SNF], inpatient rehabilitation facility [IRF], and Home Health Care [HHC]) across various racial groups. Multinomial logistic regression examined the association between race and post-acute discharge dispositions after accounting for patient-level covariates. RESULTS: Compared to non-Hispanic Whites, minority racial groups have significantly lower odds of being discharged to SNF, IRF, or HHC, as compared to home. Adjusted odds ratio for Hispanics discharged to SNF was 0.28 (CI=0.24-0.31), to IRF was 0.46 (CI=0.39-0.52) and HHC was 0.64 (95% CI =0.54-0.75), as compared to home. CONCLUSION: ADRD patients have higher risk of hip fracture. Findings from this study will provide insight on how to reduce racial and ethnic disparities in post-acute care utilization in vulnerable populations and improve quality of care and health outcomes.

### SLEEP DISTURBANCE AND DEMENTIA RISK: FINDINGS FROM 8 YEARS OF PROSPECTIVE DATA

Roger Wong, *State University of New York Upstate Medical University, St. Louis, Missouri, United States*

Recent evidence indicates sleep disturbances increase dementia risk. Despite extensive support for this finding, numerous studies are based on cross-sectional data and no research has examined this relationship using a national sample. The purpose of this study was to analyze how sleep disturbances are associated with dementia risk. This study used eight annual waves (2011-2018) of prospective data from the National Health and Aging Trends Study, a large nationally representative U.S. sample of older adults. At each wave, sleep disturbances were measured as: 1) trouble falling asleep in 30 minutes, 2) trouble falling asleep after waking up early, and 3) taking medication to help sleep. The dependent variable was number of years to a new dementia diagnosis. Multivariate analyses were conducted using the Cox proportional hazards model with survey sampling weights applied for a national sample of 6,800 community-dwelling older adults dementia-free at baseline. After controlling for sociodemographics (age, sex, race, education, etc.) and health (mental health, physical health, chronic disease, etc.), trouble falling asleep in 30 minutes was not associated with dementia risk, (Hazard Ratio [HR]=1.01, p=.98), however, trouble falling asleep after waking up significantly decreased risk (HR=0.40, p<.01), and taking sleep medications significantly increased risk (HR=1.72, p<.01). Our findings suggest mixed evidence on how sleep disturbances are associated with dementia risk, but needing sleep medications may be predictive of elevated dementia risk. Future research should explore pathways or behaviors that may decrease dementia risk among individuals who wake up at night, but have trouble falling back to sleep.

### STATEWIDE IMPLEMENTATION OF THE MUSIC & MEMORY PROGRAM: FACILITATORS, BARRIERS, AND LESSONS LEARNED

Jung Kwak,[1] Jung-Hwa Ha,[2] and Katherine Britt[1],
*1. University of Texas at Austin, Austin, Texas, United States, 2. Seoul National University, Seoul, Republic of Korea*

The movement of evidence-based interventions into routine institutional settings like nursing homes is challenging.

Among non-pharmacological interventions to address behavioral problems of residents with dementia, Music and Memory (M&M), a popular individualized music listening program, has been shown to have potential to improve quality of life among residents. To examine facilitators and barriers to implementation and sustainability of the M&M program in nursing facilities, a statewide (online and mail) survey of nursing homes was conducted in Wisconsin where the statewide implementation of the program occurred. The response rate was 41% (N=161). Descriptive statistics and content analysis were conducted. Over 80% of facilities provided the M&M program, and 86% of them planned to continue the program. The majority of respondents found the M&M to be beneficial to residents but also reported that the program was not equally effective for everyone, and M&M was time and labor intensive. Barriers to sustainability were: lack of buy-in by direct care staff, use of technology, costs of equipment, inconsistent volunteers, and families not supportive or helpful. Facilitators were: support of facility personnel, family, and volunteers; observing positive effects of program, M&M training provision and support, family involvement, and accessibility of equipment. Targeted resident selection is needed to identify the residents most likely to benefit from the program to avoid possibility of increased agitation or discomfort. Careful consideration is needed for facilities to identify realistic costs, labor, and staff buy-in to promote success.

### THE VOICES OF PERSONS LIVING WITH DEMENTIA: EXPLORING THEIR INFORMATION NEEDS TO LIVE WELL

Michelle Kimzey, Ramona Baucham, Chelsea Martin, and Carol Howe, *Texas Christian University, Fort Worth, Texas, United States*

There are unique challenges and considerations when receiving the diagnosis of dementia. There are interventions, services, and supports for people with dementia and their care partners, yet they are often unknown, disconnected, and may not be widely available or easily accessible. Health literacy was defined as the degree to which individuals have the capacity to obtain, process, and understand basic health information and services needed to make appropriate health decisions. Using a descriptive qualitative design, the purpose of this study was to describe how persons living with dementia and their care partners obtain, understand, and use information to make health decisions to live well with dementia. The convenience sample consisted of 28 care partners and 15 people living with dementia participating in 6 separate focus groups. To illuminate findings, data was analyzed using a hybrid approach (deductive followed by inductive). Four themes emerged deductively as persons gain health literacy in dementia (access, understand, appraise, and understand). The notable finding is the trend at diagnosis where they first are "seeking the expert" ,and as they move from dependence and gain understanding they are "becoming the expert", and finally as they apply information they are "acting as the expert" for themselves and others. Engaging them in research not only gave them a voice but more importantly it influenced the health information that will be developed and implemented by them. These findings suggest there is a wealth of knowledge to be gained by persons living with dementia and their care partners.

### TRIPLE JEOPARDY? STRESS AMONG DEMENTIA CAREGIVERS THROUGH THE LENS OF INTERSECTIONALITY

Ruotong Liu, and Iris Chi, *University of Southern California, Los Angeles, California, United States*

Despite the benefits to economy and public health, caregivers are negatively affected by caregiving activities. Dementia caregivers, compared to other caregivers, are experiencing higher levels of stress, due to reasons such as the care recipients' changes in personalities and behaviors. Previous studies have documented differences in caregiver stress across gender and racial/ethnic groups. However, few studies have looked into caregiving differences within both gender and race/ethnicity through an intersectionality framework. This paper seeks to explore what are the differences in caregiving stress across the intersectionality of race and gender. Using Round 5 and Round 7 of NSOC and NHATS data, we examined differences in caregiver stress across and within different gender and racial/ethnic groups in terms of financial, emotional, and physical stress. 1,206 caregivers were included with 61% female, 50% White, and 32% Black caregivers. Logistic regression results indicate that female is less likely to have financial stress, but more likely to experience emotional stress. Compared to White, Black caregivers are worse off financially but better off physically. Both Black and other racial/ethnic caregivers are less likely to have emotional stress. Within the intersectionality framework, compared to White female, Black male are 3.4 times more likely to experience financial stress, all the other groups are 38%-71% less likely to have emotional stress, and Black female are 53% less likely to have physical stress compared to White female. The findings highlighted that in order to develop more effective interventions or policies, unique areas are to be focused for different population subgroups.

### SESSION 2853 (POSTER)

### COGNITION AND COGNITIVE ASSESSMENT

### A MULTIPLE STAKEHOLDER PERSPECTIVE FOR EVALUATING COMMUNITY-BASED DEMENTIA CARE

Johan Suen, *Duke-NUS Medical School, Singapore, Singapore*

For holistic interventions and research on dementia, it is fundamental to understand care experiences from the perspectives of carers, care recipients, and care professionals. While research on care dyads and triads have highlighted the effects of communication and interactional aspects on care relationships, there is a lack of knowledge on how individual-contextual and relational factors shape the provision and receipt of care in terms of decision-making processes, resource allocation, and expectations of care outcomes. Thus, this paper sheds light on (i) how carers negotiate care provision with other important life domains such as employment, household/family roles and conflicts, as well as their own health problems, life goals, values, and aspirations for ageing; (ii) how older adults with dementia perceive support and those who provide it; (iii) the structural constraints faced by care professionals in delivering a team-based mode of dementia care; and, taken together, (iv) how community-based dementia care is impeded by barriers at the individual, relational, and institutional levels. Findings were derived from semi-structured interviews and observational data from fieldwork conducted with 20 persons with dementia (median age = 82), 20 of their carers (median age = 60), and 4 professional care providers. All respondents were clients and staff of a multidisciplinary and community-based dementia care system in Singapore. Our analysis indicates the impact of dementia care is strongly mediated by the interplay between institutional/familial contexts of care provision and the various 'orientations' to cognitive impairment and seeking support, which we characterised as 'denial/acceptance', 'obligated', 'overprotective', and 'precariously vulnerable'.

### ADAPTIVE REUSE OF DISTRESSED MALLS FOR DEMENTIA-FRIENDLY CITY CENTERS: OUTCOMES FROM COMMUNITY FOCUS GROUPS

Emily Roberts, *Oklahoma State University, Stillwater, Oklahoma, United States*

It is estimated that 5.4 million Americans have some form of dementia and these numbers are expected to rise in the coming decades, leading to an unprecedented demand for memory care housing and services. In searching for innovative options to create more autonomy and better quality of life in dementia care settings, repurposing existing structures, in particular vacant urban malls, may be one option for the large sites needed for the European model of dementia villages. These settings may become sustainable Dementia Friendly City Centers (DFCC), because in the case of enclosed mall construction, the internal infrastructure is in place for lighting, HVAC, with varied spatial configuration of public spaces. This presentation describes the community engagement research being conducted by a research team at a Midwestern university, laying groundwork for the DFCC model for centralized dementia programs, services and attached housing. Focus group outcomes from four disciplines (caregiver, physician, designer, community development) detailed four principle themes including : community revitalization, building sustainability, urban regreening and the nurturing of innovation to further a culture of dementia care which is inclusive, progressive and convergent with the needs of an aging. The DFCC model can be seen as one opportunity to make life better not only for those with needs associated with dementia now, but also for ourselves in the future, therefore educating and updating future stakeholders about the value of this model of care will be critical in transforming current hurdles into future opportunities.

### AGE COHORT DIFFERENCES AND DEPRESSIVE SYMPTOMS AMONG COMMUNITY-DWELLING OLDER AMERICANS

Ethan Siu Leung Cheung,[1] and Ada Mui,[2] *1. Columbia University, Bronx, New York, United States, 2. Columbia University, New York, New York, United States*

This study uses Wave 3 National Social Life, Health and Aging Project to examine the correlation between age cohorts [60s (n=1204); 70s (n=1176); 80 and older (n= 724)], cognitive status, and depression symptoms. In the total sample, 53.90% were females, 76.15% Whites, 15.29% Blacks, and 8.56% Asians. Compared to the 60s and 70s cohorts, 80+ cohort was cognitively more impaired [Mean (SD) of MoCA Short Form were 10.7(2.9), 10.0(3.2), and 8.1(3.6)]. There were no age cohorts' differences in depressive symptoms experienced (Mean of CESD Short Form = 21.03; SD = 4.06). In order to identify predictors of depression, multiple hierarchical regressions were performed. The 60s sample was the reference group to compare with 70s and 80s cohorts. Results showed that age cohort variables had a significant independent effect as well as a joint effect with cognitive status in explaining depression scores. For each age cohort group, parallel regression analyses were conducted and all models were significant. Findings suggest that ADL impairment was the only common predictor for depressive symptoms for the three cohort groups, and the association was the strongest for the 60s cohort (b = .31). Other unique predictors for 60s cohort were lower-income, more IADLs impairment, higher stress and cognitive impairment. For the 70s cohort, unique predictors of depressive symptoms were female gender, unmarried, and less socialization. For the 80 and above group, correlates of depression are female, White, and high stress level. Findings highlight the necessity of age-sensitive programs on depression support for community-dwelling older Americans.

### COGNITIVE STIMULATION THERAPY OUTCOMES ACROSS HEALTHCARE AND COMMUNITY SETTINGS FOR INDIVIDUALS WITH DEMENTIA

Randy Gallamore, *Saint Louis University, St. Louis, Missouri, United States*

Cognitive Stimulation Therapy (CST) is an evidenced-based intervention for individuals with mild to moderate dementia. Originally developed in the U.K., CST has been adapted in several areas of the United States as a meaningful group intervention to help aid in recall and reminiscence for this population. Adaptations of CST have now been developed, including the application of these groups in medical and healthcare settings. However, no study to date has compared memory and mood outcomes of community CST groups to healthcare CST groups. This study will examine the differences in memory, mood and physical mobility scores across both rural and urban settings where CST is used. Two-hundred and sixty-six total participants who have completed all 14 sessions were analyzed, with 150 who participated in a rural hospital and 116 who were in community or university settings. Preliminary data shows that CST is an intervention that can be used effectively in both environments. The results from this study show that improvements in scores were seen in both community (SLUM = +1.75; Cornell = -1.41) and healthcare settings (SLUM = +2.59; Cornell = -2.63). CST might be a meaningful intervention to also help in decrease depression and loneliness in this population. Continued group interventions should be developed in medical and healthcare settings as a resource for patients and family members with dementia-related disorders. There should also be further consideration on the factors that impacted the difference between the two settings.

### DID THE 2008 FINANCIAL CRISIS (LITERALLY) CHANGE MINDS?

Cliff Whetung,[1] and Abhinav Bhattacharyya,[2] *1. NYU, New York, New York, United States, 2. New York University, New York, New York, United States*

This paper investigated whether experiencing a major asset shock (30% or greater decrease in total assets) changed the cognitive health trajectories of older adults (aged 65+ at baseline)? There is a robust literature supporting a relationship between potentially modifiable environmental and individual risk factors and cognitive health and emerging literature supporting a relationship between physical health shocks and later-life financial stability in both the US and internationally. This analysis employed six waves of Health and Retirement Study Core Data (2006-2014) to estimate the causal effect of major asset losses in the period of the 2008 financial crisis. We matched respondents using a rich variety of covariates including education, race/ethnicity, income, total assets, chronic health conditions, depressive symptoms, frequency of physical activity engagement, and engagement with religious activities. Using both propensity score-matched growth curve and difference-in-differences models, we found a small, but significant, negative relationship between major asset shocks and total cognition scores. We also identified a significant overall negative effect on total cognition on all older adults in this sample regardless of exposure to asset shocks. Additionally, both the frequency of asset shock exposure and magnitude of effect on total cognition scores was larger for low-income African-American and Hispanic older adults. These findings suggest in part that recovery from large economic crises may be especially difficult for older adults occupying vulnerable socioeconomic positions and that such events may accelerate the cognitive decline of those who are at or near retirement when asset shocks occur.

### EARLY LIFE CIRCUMSTANCES AND COGNITIVE AGING: LONGITUDINAL EVIDENCE FROM CHINA HEALTH AND RETIREMENT STUDY

Zhuoer Lin, and Xi Chen, *Yale University, New Haven, Connecticut, United States*

Objectives: This study examines the long-term relationship between early life circumstances and later life cognitive aging. In particular, we differentiate the long-term effects of early life circumstances on level of cognitive deficit and rate of cognitive decline. Methods: Cognitive trajectories were measured using three waves of China Health and Retirement Longitudinal Surveys (CHARLS 2011-2015). Linear mixed-effect model was used to decompose the individual level of cognitive deficit and rate of cognitive change in a sample of Chinese middle-aged and older adults 45-90 years of age (N=6,700). These two dimensions of cognition were matched to four domains of early life circumstances using CHARLS Life History Survey (2014), including childhood socioeconomic status, neighborhood environment, social relationships and health conditions. Their associations were examined by linear regressions. Stratification analysis was further conducted to investigate the mediating effect of education on early life circumstances and cognitive aging. Results: Childhood socioeconomic status, childhood friendship and early life health conditions were significantly associated with both the level of cognitive deficit and rate of decline. In contrast, the community environment, including childhood neighborhood safety and social cohesion, only affected the baseline level of cognitive deficit; and childhood relationship with parents only affected the rate of cognitive decline. Moreover, education was found to be a mediating factor of these relationships. Conclusion: Exposure to disadvantaged early life circumstances have significant negative effects on later life cognitive deficit as well as rate of cognitive decline. Nevertheless, these long-term impacts can be partially ameliorated by higher educational attainment.

### GENDER, COGNITIVE STATUS, AND DEPRESSIVE SYMPTOMS: FINDINGS FROM THE NATIONAL SOCIAL LIFE, HEALTH, AND AGING PROJECT

Ethan Siu Leung Cheung,[1] and Ada Mui,[2] *1. Columbia University, Bronx, New York, United States, 2. Columbia University, New York, New York, United States*

This secondary research is based on the Wave 3 National Social Life, Health and Aging Project (n = 3,104). The association between cognition, gender, and depressive symptomatology were examined. Findings indicate that 54% of the sample were women and the mean age was 72.95 (SD=8.29). Bivariate analyses suggest that there were no gender differences in cognitive status (Mean of MoCA Short Form = 9.73; SD = 3.26), age, and stress (Mean of PSS = 7.69; SD = 3.90). There were significant gender differences in terms of marital status, income, education, stressors, social participation, and social support. Compared to older men, older women reported a significantly lower level of both education and income. Multiple regression results show that gender has an independent effect and a joint effect with stressors in explaining depressive symptoms. Parallel regression analyses for each gender group were conducted and models were significant ($P < .0001$). The only common predictor for depressive symptoms was ADL impairment, and the impact of this was stronger for males (b=.32) than for females (b=.17). For older men, unique correlates of depressive symptoms were being not married, more ADL and cognitive impairments, and higher stress. For older women, a higher level of depressive symptoms was associated with being younger, lower-income, a higher level of ADL and IADL impairments. In addition, white elderly women reported a higher level of depressive symptoms than Asian elderly women. Findings suggest gender and racial differences in depressive symptoms experienced among older Americans living in the community.

### HEALTH PROFESSIONALS' KNOWLEDGE OF HOW TO REPORT A MISSING PERSON WITH DEMENTIA: A NATIONAL SURVEY

Margaret MacAndrew,[1] Elizabeth Beattie,[2] Dubhglas Taylor,[3] Jim Whitehead,[4] and John Quinn,[3] *1. Queensland University of Technology, Camp Hill, Queensland, Australia, 2. Queensland University of Technology, Brisbane, Australia, 3. Brisbane, Australia, 4. Queensland Police, Brisbane, Australia*

In Australia one in five land searches conducted by Police involve a person with dementia. Over a third of these people go missing from a health care service and 15% are not found alive. Delays in commencing a specialised search for the missing person with dementia contributes to the risk of death. Delays in Police searching may result from ambiguity in current policies about how to report a missing patient/client. This study aimed to explore health professional's knowledge about how to report a missing person with dementia and reasons for delayed reports to Police. 246 Australian health professionals completed an online survey. Most were registered nurses (n=124), allied health professionals (n=69) and medical practitioners

(n=22) who worked in a range of settings including acute care (n=111), community care (n=59) and residential aged care (n=44). Over a third (n=81) did not know their care service policy for reporting a missing patient/client and did not know if their health service had a policy specific to reporting a missing person with dementia. 20% did not know how long they needed to wait before reporting a missing person to Police and fear of calling Police too soon or wasting their time were common reasons for delaying a report. These findings confirm a degree of misunderstanding about current policy and procedures for reporting a missing person with dementia. Addressing knowledge deficits and standardising approaches to reporting a missing person with dementia in Australia would be recommended as a step toward improving their health outcomes.

### HOW DOES NEIGHBORHOOD ENVIRONMENT INFLUENCE DEMENTIA ONSET?: EVIDENCE FROM THE NATIONAL HEALTH AND AGING TRENDS STUDY

Yi Wang,[1] and Weidi Qin,[2] *1. University of Iowa, Iowa City, Iowa, United States, 2. Case Western Reserve University, Cleveland, Ohio, United States*

Existing research has established significant associations between older individuals' social participation (e.g., visiting friends and family, going out for enjoyment, etc.) and their risk of developing dementia. However, little was known about the role of neighborhood environment (i.e., neighborhood social cohesion, neighborhood physical disorder). This study aims to investigate the moderating effects of neighborhood environment on the relationship between social participation and dementia incidence using data obtained from 8 waves (Rounds 1 through 8) of the National Aging and Trends Study. 7,416 Medicare beneficiaries aged 65 or above who were community-dwelling and free from dementia at Round 1 (2011) were selected. Among them, 1,005 participants developed dementia in the followed-up years from 2012 to 2018. We conducted survival analysis using the accelerated failure time (AFT) models to examine the moderating effects of neighborhood social cohesion and neighborhood physical disorder on the onset of dementia. The unit of time-to-event data was in years. Findings showed that the interaction term of neighborhood physical disorder and individual social participation was significant, indicating neighborhood physical disorder moderates the protective effects of social participation on dementia incidence. Specifically, among older adults who participate in social activities at the same level, the progression to the onset of dementia is 1.06 times faster for those living in neighborhoods with higher level (i.e., one unit increase in the score) of physical disorder. The findings recommend modification of physical environment at the neighborhood level to facilitate social participation for the betterment of cognitive health in later life.

### THE CORRELATES OF COGNITIVE STATUS AMONG OLDER AMERICANS LIVING IN THE COMMUNITY: DOES GENDER MAKE A DIFFERENCE?

Ethan Siu Leung Cheung,[1] Ada Mui,[2] and Seth Hoffman,[3]
*1. Columbia University, Bronx, New York, United States, 2. Columbia University, New York, New York, United States, 3. Columbia University, Rego Park, New York, United States*

Utilizing the data in National Social Life, Health and Aging Project (n = 3,104; 54% female), the study examined the predictors of cognitive impairments in terms of community harmony, community safety, frequency of neighbor contacts, depression, and demographic factors. Bivariate analyses suggest that there were no gender differences in cognitive status (Mean of MoCA Short Form = 9.89; SD = 3.33); nor were there gender differences in age (mean age = 72.95; SD=8.29), ethnic composition (76.1% whites; 15.3% Blacks, 8.6% Asian), community harmony, community safety, frequency of neighbor contacts. On the other hand, men had more education and income than women. Psychologically, older women reported higher level of stress and depression scores than older men. Multiple regression results show that gender has a significant independent effect and joint effects with stressors and community factors in explaining cognitive impairments. Parallel regression analyses for each gender group were conducted and models were significant ($P < .0001$). There were common predictors of cognitive impairments for the two groups but variables had differential impacts on older men and older women. Specifically, IADL had stronger effect on older men than on older women in predicting cognitive impairments ($b = -.23$ vs. $b=-.10$); perceived community harmony had stronger impact on older women in explaining their cognitive status ($b = .26$ vs. $b=.22$); older women's cognitive status benefitted more from perceived community safety than older men ($b = .61$ vs. $b=.43$). Regardless of gender, older Whites scored higher than Black and Asian elders in their cognition scores.

### SESSION 2856 (POSTER)

### EMPLOYMENT, TRAINING, AND EDUCATION: WORKFORCE

### A FOLLOW-UP CONTENT ANALYSIS OF AGING NETWORK CONFERENCE PROCEEDINGS: ALIGNING RESEARCH AND PRACTICE

Todd Becker,[1] Rajean Moone,[2] and Joan Davitt,[1]
*1. University of Maryland, Baltimore, Baltimore, Maryland, United States, 2. University of Minnesota, Woodbury, Minnesota, United States*

The federal government established a collective of agencies tasked with providing support and services to older adults and their caregivers known as the Aging Network. In order to effectively provide these services, the Aging Network must identify and disseminate current best practices. To this end, the Aging Network hosts three annual conferences: the National Association of Area Agencies on Aging (n4a), the Alliance of Information and Referral Systems (AIRS), and the National Association of States United for Aging and Disabilities (NASUAD). This content analysis of key themes emerging from Aging Network conference abstracts (N = 2,392) from 2009 to 2019 expanded upon a similar study analyzing the preceding decade of conference materials (Moone & Cagle, 2011). Reflexivity, analyst triangulation, and confirmability and dependability audits were used to enhance trustworthiness. The most common themes included planning and program development (n = 260), general policy (n = 166), and long-term services and supports system reform (n = 162). Although some themes, such as consumer-directed support (n = 122) and advocacy (n = 111) were consistent with the original article (Moone & Cagle, 2011), others, such as managed care (n = 124) and financial exploitation (n = 50), demonstrated a societal shift in older adult supports and services. These results offer an additional effort towards disseminating current best practices and emerging issues to advance translation between research and practice. Researchers can use these results to better align their research agendas with the needs of the Aging Network for evidence-based interventions.

### BARRIERS TO ENGAGE LOW-SKILLED ADULTS IN EDUCATIONAL OPPORTUNITIES: A GLOBAL PERSPECTIVE

Abigail Helsinger,[1] Nytasia Hicks,[1] Meghan Young,[1] Oksana Dikhtyar,[1] Phyllis Cummins,[1] and Taka Yamashita,[2] *1. Miami University, Oxford, Ohio, United States, 2. University of Maryland, Baltimore, Maryland, United States*

The demand for adult education and training (AET) opportunities is substantial as older adults are remaining in the labor force at older ages, and are facing substantial technological changes in the workplace. Strategies to engage middle-aged and older adult workers in AET often exclude low-skilled and sub-populations. The engagement of these sub-populations in AET is challenging as access, awareness, and program costs associated with AET opportunities often target highly skilled populations. The inequality in AET participation warrants specific programs and strategies to address challenges low-skilled adult workers face in pursuing AET. The purpose of this study is to identify AET opportunities for low-skilled middle-aged and older adults, as well as highlight major barriers to engage and retain these sub-population in AET. Data were collected from 36 key informants through semi-structured interviews and through document reviews. Key informants represented Australia, Canada, Italy, Norway, the Netherlands, the U.K., and the U.S. Descriptive methods were used to identify barriers in recruiting and retaining low-skilled middle-aged and older adults. We particularly focused on the barriers related to cost, language, access, and awareness. Results highlighted opportunities tailored to support adult workers in the pursuit of adult learning opportunities both domestically and internationally. Barriers including learning histories, lack of long-term person-centered support, as well as the role of multiple forms of learning, such as formal and informal learning, were identified. Last, we provide recommendations for recruiting and retaining middle-aged and older adult workers in AET programs.

### CREATING AN AGE-FRIENDLY WORKPLACE FOR OLDER WORKERS AND EMPLOYERS

Lisa Hollis-Sawyer, *Northeastern Illinois University, Chicago, Illinois, United States*

Despite the clear aging trends in the U.S. and global population (e.g., World Health Organization, 2015), there has been a lack of "age audit" tools to evaluate the age-friendliness of workplace environments to facilitate older adults' positive mental/cognitive health, physical health, social/interpersonal health, and general aging adaptation. The present study did a review of the literature and audit tools across several disciplines (psychology, gerontology, kinesiology, anthropometry, audiology, vision science, human resources management, architecture, and social factors engineering) regarding the assessment and design issues underlying "age-friendliness" in the workplace. Further, the present research pilot-tested a new audit tool in two organizations (educational, industrial). The researcher, in coordination with two independent raters, conducted a content analysis of the different peer-reviewed articles and books across several disciplines and available age audit tools/approaches to identify: (1) current practices in age-friendliness assessments (e.g., "user-friendliness" of audit tools for practitioners), (2) potential biases/limitations in age assessments (e.g., "decline/decrement" aging perspective), and (3) "gaps" in evaluations to create a more holistic evaluation approaches. The following conclusions were made: (1) most assessments focused on one factor of functioning (e.g., psychomotor capability), (2) existing tools are limited in options and functionality for daily assessments, (3) most focus on decline and limitations in functioning, and (4) need to design multi-sensory, multi-function assessments reflecting an integrated and coordinated system of sensory, psychomotor, social, and cognitive performance. A holistic model of the outcomes for workplace design "fit" interventions to create more aging-friendly workplaces based upon pilot test results will be presented.

### DEVELOPMENT AND VALIDATION OF AGE-FRIENDLY EMPLOYMENT MEASUREMENT

Mi Sun Choi,[1] Holly Dabelko-Schoeny,[1] Katie White,[2] and Marisa Sheldon,[2] *1. The Ohio State University, Columbus, Ohio, United States, 2. Age Friendly Columbus, Columbus, Ohio, United States*

Scholars have paid attention to the concept of age-friendly to address aging workforce issues. Although prior research has been conducted to conceptualize age-friendly work environments by investigating older workers' perspectives, little is known about the practices and conditions that promote organizations to employ older employees longer and theory driving measurement of age-friendly employment (AFE). To address these knowledge gaps, we developed the AFE measurement tool based on the existing measures, focus group data, two-round Delphi study and a pilot test. We collected primary data from human resource professionals working in a large city in the midwestern state and evaluated the reliability and validity of the AFE measurement using exploratory factor analysis and confirmatory factor analysis. Results supported the hypothesized five-factor structure. We obtained 16 indicators of AFE: 1) accommodation: fewer physical work demands, reduced work hours, mobility and transportation support; 2) development: career advice, a training needs assessment, and training opportunities to employees all ages; 3) maintenance: financial and medical benefits for full-time workers of all ages, and wellness programs benefits for full-time and part-time workers; 4) utilization: move into a different position, involvement in decision making, knowledge or skills transfer). Accommodation, maintenance, and inclusion factors were predicted by organizational size. A major strength of this study was that the AFE measurement was constructed using a lifespan theory (Selection Optimization Compensation model). The findings of the current study enable employers to self-monitor their ability to employ and retain older employees, especially for small organizations with less than 20 employees.

### EXCLUSION AND INEQUALITY IN LATE WORKING LIFE–ON THE GENDERED RISKS FOR OLD-AGE EXCLUSION IN SWEDEN AND POLAND

Andreas Motel-Klingebiel,[1] Jolanta Perek-Białas,[2] Indre Genelyte,[3] and Susanne Kelfve,[4] *1. Linkoping University, Norrkoping, Ostergotlands Lan, Sweden, 2. Jagiellonian University, Cracow, Malopolskie, Poland, 3. Linköping University, Norrköping, Sweden, 4. Linköping University, Norrköping, Ostergotlands Lan, Sweden*

The labour market activity of older workers and their ability and disposition to maintain it depend on institutional conditions, age norms, labour demand and shifting overall economic conditions. The paper discusses exclusion and inequality in later working life from a European comparative perspective and emphasises shifts in late work and retirement patterns as well as later-life outcomes in Sweden and Poland. An emphasis is on changing institutional conditions on the national and branch level. Gendered risks for economic exclusion and later life precarity are stressed. Analyses for the two countries are contrasted with Germany and the UK. The analyses are part of the research program 'Exclusion and Inequality in Late Working Life: Evidence for Policy Innovation Towards Inclusive Extended Work and Sustainable Working Conditions in Sweden and Europe – EIWO' (2019-24). Analyses use data from SHARE and EU-SILC and address older workers of age 60 and older in Sweden, Poland, German and the UK. They find increasingly heterogeneous preretirement and transition patterns, new gender gaps and increasing risks of economic exclusion in retirement. Situations differ between countries with the prolongation of late working life in Sweden having a mostly positive effect on gender inequalities with low education and specific migrant groups as an exception. Poland is specific case due to unequally low retirement age for woman (60) and for men (65) with consequently large structural gender differences and increases in the process of increasing labour force participation of older workers and increasingly gendered risks for old-age economic exclusion.

### EXPLORING EMPLOYMENT TREND AND RELATIONSHIP WITH INDIVIDUAL AND FAMILY-RELATED FACTORS IN 65 OR OLDER KOREANS

Yeonji Ryou, and Ryou Yeonji, *Iowa State University, Ames, Iowa, United States*

The purpose of this study is to identify the trend of the employment status in 65 years or older adults who reside in South Korea and to explore the relationship between the status of employment and individual and family-related factors. This study utilized 10-year and 6-wave secondary data from the Korean Longitudinal Study of Ageing (KLoSA). The original panel sample is a random sample of 10,254 adults who are 45 or older, but for the aim of this study, the participants younger than 65 years were excluded. The number of samples in each wave is different, ranging from 4,013 to 4,335 due to the death of the participant, the rejection of additional interviews, and the refreshment participant collected in Wave 5. The findings indicate that the absolute employment of the people aged 65 or older and the proportion of working people among those have increased over the past decade. In this study, it is also found that there is a close relationship between employment status and individual factors such as gender, educational background, health condition, region, etc. Moreover, the results suggest that there are various facets of the relationship between employment status and family-related factors including whether living with children, the number of the member whom I help with daily activities, the total amount of financial support from/to children/parents/other family or whether participating social activities, etc. The implications of the need for employing the older population and the consideration family-related factors in the policy-making process in Korea are discussed.

### LIFE EMPLOYMENT HISTORIES IN MIDDLE AGE AND THEIR ASSOCIATION WITH HEALTH OUTCOMES IN LATER LIFE: A SEQUENCE ANALYSIS

Oejin Shin,[1] BoRin Kim,[2] Sojung Park,[3] JiYoung Kang,[4] ilan kwon,[5] and Seoyeon Ahn,[6] *1. University of Illinois at Urbana Champaign, Savoy, Illinois, United States, 2. University of New Hampshire, Durham, New Hampshire, United States, 3. Washington University in St. Louis, St. Louis, Missouri, United States, 4. Hannam University, Daejeon, Republic of Korea, 5. Michigan State University, East Lansing, Michigan, United States, 6. National Pension Research Institute, Jeonjusi, Republic of Korea*

Despite consistent evidence on the negative effect of precarious employment on health, little is known about the dynamic pattern of employment over the life course on later year health. Using the longitudinal data (1-18 waves) from the Korean Labor & Income Panel Study (KLIPS) (n=1,705), this study aimed to (1) identify long term change patterns of employment in two middle-age groups (early middle-aged: 40-49 / late middle-aged: 50-59), (2) examine the association between the patterns and self-rated health in old age. We apply sequence analysis with 18 years of working status and conducted regression health outcomes in the 18th wave. The result of sequence analysis found the differential employment patterns: among the early middle-aged, five patterns were identified consistently full-time, consistently not- working, transit to self-employed, mixed pattern, retired. Among the late middle-aged, five patterns were identified: consistently full time, mixed pattern, consistently retired, transit to not- working, consistently not working. Regression results indicated a negative association between precarious employment history and health in later years: Among the early middle-aged, members in the "consistently full-time" were likely to have better health compared to "transit to self-employed". Among the later middle-aged, "retired" and "transit to not –working" were likely to have better health compared to "mixed pattern group". The findings suggest the importance of employment history in middle age to predict the health outcome in later life. Policies to support experiencing precarious or self-employment are needed to prevent health disparity in later life.

### RISING ABOVE THE FLOOD: EMERGENCY MANAGEMENT AND GERONTOLOGICAL SOCIAL WORK

Susanny Beltran,[1] Paola Luigi,[2] and Nancy Kusmaul,[3] *1. University of Central FLorida, Orlando, Florida, United States, 2. University of Central Florida, Orlando, Florida, United States, 3. UMBC, Baltimore, Maryland, United States*

Older adults, particularly those from low-income communities, are disproportionately negatively affected by natural disasters. As the older adult population grows and natural disasters increase in frequency and intensity, social workers must consider their role in supporting the needs and safety of this population. Social workers practice in varied roles including policy advocate, service broker, and educator, all of which are crucial in disaster management. This systematic review summarizes the literature on the social work profession's involvement with emergency management with older adults, and identifies gaps. Using the Preferred Reporting Items for Systematic Reviews and Meta-Analyses guidelines, authors searched AgeLine, CINAHL Complete, MEDLINE, PsycINFO, and Social Work Abstracts for peer-reviewed publications between January 1, 2009 and March 1, 2020. Examples of the terms searched include social work*, respon*, prepar*, disaster, crisis, emergency, geriatrics and older adults. The initial searches yielded 298 publications. After removing duplicates and screening articles for relevance based on titles and abstracts, 21 publications were retained for full review. A total of 11 articles met inclusion criteria. The body of literature identified was small. The majority of the publications constituted conceptual papers, textbook reviews, and letters to editors requesting greater emphasis on emergency management. Only three empirical studies were identified. Broadly, the publications discussed: (1) policies and resources; (2) needs; (3) capacity across practice settings; and (4) interventions. Findings reveal an underdeveloped area of social work practice, and highlight opportunities for researchers and practitioners to define gerontological social workers' role in emergency management and detail best practice guidelines.

### THE INTERPLAY OF SENIORITY AND SUPERIORITY TOWARD PERCEIVED EMPLOYABILITY OF OLDER WORKERS THROUGH A CULTURAL LENS

Clio Y.M. Cheng, and Vivian Lou, *The University of Hong Kong, Hong Kong, China*

Background: Hong Kong will become a super-aged society comprising more than 21% of its citizens aged 65 or above by 2024. With longer life expectancy and better health conditions of the elderly, the mentality of embracing "hidden gems" - older workers was under the spotlight. Extended working life provided a golden chance for employers and/or human resources (HR) personnel to manage this demographic change. Sau Po Centre on Ageing was commissioned to initiate a study in 2017 to 2018 on how employers and/or HR personnel perceived employability of older workers in Hong Kong, and to consolidate good practices of elder-friendly employment. Methods: Aiming to garner opinions from a wide variety of employers and/or HR personnel, 33 in-depth interviews were conducted with participants from various industries and company sizes, including both large corporations and small and medium enterprises (SMEs). Among the participants, nearly half (n=15) possessed managerial position, other including employers (n=1), directors (n=6), officers (n=8) and others (n=3). Among these companies, 19 did not have any mandatory retirement age policies, while seven had their retirement age set as 60 years old, and another seven set their retirement age at aged 65. Results: Participants tended to link seniority, in terms of both chronological age and loyalty, with superiority, affecting their perceived employability of older workers. This study has multi-level implications on a multi-generational age-inclusive workforce management strategy on recruitment, retainment and retraining. Suggestions on good practices of an age-inclusive and age-diverse workforce were also made at individual, corporation and societal level.

### SESSION 2857 (POSTER)

### END-OF-LIFE CARE AND BEREAVEMENT

### ASSISTED LIVING ADMINISTRATORS' APPROACHES TO ADVANCE CARE PLANNING

Elise Abken,[1] Alexis Bender,[2] Ann Vandenberg,[1] Candace Kemp,[3] and Molly Perkins,[2] *1. Emory University School of Medicine, Atlanta, Georgia, United States, 2. Emory University, Atlanta, Georgia, United States, 3. Georgia State University, Atlanta, Georgia, United States*

Assisted living (AL) communities are increasingly home to frail, chronically ill older adults who remain until death. State laws mandate that AL facilities request copies of any advance care planning documents residents have and make forms available upon request. Using secondary data from a larger study funded by the National Institute on Aging (R01AG047408) that focuses on end-of-life (EOL) care in AL, this project investigated barriers and facilitators to conducting advance care planning in AL. Data included in-depth interviews (of 86 minute average length) with 20 administrators from 7 facilities around the Atlanta metropolitan area and aggregate data collected from each facility regarding facility, staff, and resident characteristics. Findings from thematic analysis of qualitative data showed that key barriers to planning in AL included lack of staff training and reluctance among administrators and families to discuss advance care planning and EOL care. Important facilitators included periodic follow-up discussions of residents' wishes, often during care plan meetings, educating families about the importance of planning, and external support for staff training and family education from agencies such as hospice and home health. Three study facilities exceeded state requirements to request and store documents by systematically encouraging residents to complete documentation. These facilities, whose administrators discuss advance care planning and residents' EOL wishes with residents and families during regular care plan meetings, were more likely to have planning documents on file, demonstrating the potential of long-term care communities, such as AL, to successfully promote advance care planning among residents and their family members.

### EXAMINING ASSOCIATIONS BETWEEN LEGAL FRAMEWORKS FOR MEDICAL PLANNING AND INTENSITY OF END-OF-LIFE CARE

Melissa Berkowitz,[1] Allison Hoffman,[2] Anthony Sacco,[3] and Norma Coe,[1] *1. University of Pennsylvania, Philadelphia, Pennsylvania, United States, 2. University of Pennsylvania, University of Pennsylvania, United States, 3. University of Pennsylvania, University of Pennsylvania, Pennsylvania, United States*

This study aims to understand the correlation between the legal framework around planning for medical treatment and the intensity of care received at the end of life. Advance directives (AD) allow an individual to legally document their wishes for medical treatment and end-of-life care, and durable powers of attorney (DPOA) allow them to identify an individual to make medical decisions on their behalf in the event they are no longer able. These laws vary greatly across time, place, and in their complexity. We estimated fixed effects model, which controls for time-invariant and state-specific factors affecting end-of-life care. Our primary outcome variable is the number of inpatient hospital days during the last six months of life, sourced from the Dartmouth Atlas Project. Our explanatory variables are hand-collected state-level legal statutes including whether the state (a) has default surrogate laws, (b) recognizes the ability to create a DPOA and/or AD, (c) permits oral directives, (d) authorizes a combined AD/DPOA, and (e) provides an official registry for ADs. Preliminary findings show recognizing DPOA and AD are negatively correlated with our outcome variable (-2.6 days; Std err 0.272) as are having default surrogate laws (-0.36 days; std err 0.157). However, allowing oral directives had an opposite effect (-.46 days; std err 0.157). Neither authorized combined AD/DPOAs nor AD registries resulted with significant correlations. These findings indicate that the existence of a basic framework such as default surrogate laws and a law governing ADs are key policy features correlated with reduced intensity in end-of-life care.

### EXPERIENCES OF LGBT PATIENTS AND FAMILIES WITH HOSPICE AND PALLIATIVE CARE: PERSPECTIVES OF THE PALLIATIVE CARE TEAM

Cathy Berkman,[1] and Gary Stein,[2] *1. Fordham University, New York, New York, United States, 2. Yeshiva University, New York, New York, United States*

The lesbian, gay, bisexual, and transgender (LGBT) community experiences discrimination and stigma in accessing health care and social services – including palliative, hospice, and long-term care. Healthcare providers not recognize or address disparities in care. Providers and institutions may be uncomfortable with sexual orientation and gender identity and expression issues, and often don’t inquire about these. LGBT patients fear being open about their identities, not receiving equal or safe treatment, and having their family of choice and designated surrogates disrespected or ignored by healthcare staff. This study examines the degree to which hospice and palliative care providers report inadequate, disrespectful, or abusive care to LGBT patients and family members. A cross-sectional study using an online survey was completed by 865 providers, including social workers, physicians, nurses, and chaplains. Among respondents, 55% reported that LGB patients were more likely than non-LGB patients to experience discrimination at their institution; 24% observed discriminatory care; 65% reported that transgender patients were more likely than non-transgender patients to experience discrimination; 20% observed discrimination to transgender patients; 14% observed the spouse/partner of LGBT patients having their treatment decisions disregarded or minimized; and 13% observed the spouse/partner being treated disrespectfully. Qualitative data are presented to illustrate discomfort with LGBT patients and spouses/partners, disrespectful care, gossip and ridicule, inadequate care, and denial of care. Implications and suggestions for implementing non-discriminatory and respectful institutional and public policy, and for staff education and training to provide competent and respectiful care to this population are presented.

### FAMILY CAREGIVERS’ DECISION-SUPPORT NEEDS BEYOND THE DECISION AID

Katherine Britt,[1] and Karen Schlag,[2] *1. The University of Texas at Austin, Austin, Texas, United States, 2. University of Texas at Austin, Austin, Texas, United States*

Of the estimated 16 million U.S. family members currently providing essential yet unpaid caregiving for persons with dementia (PWD), many will also make end-of-life (EOL) care decisions as surrogates, a process that can be fraught with uncertainty. Even with dementia death rates rising, many families delay advanced care planning (ACP) discussions, and surrogate decision makers often lack crucial information and support, implicating the need to further study this topic in aging. While decision aids (DA) serve as a support tool for caregivers, they can be less effective when failing to address unresolved decisional needs. Utilizing the Ottawa Decision Support Framework (ODSF), which asserts caregiver decision needs affect decision quality, this study sought to identify surrogate decision-support needs extending beyond general ACP. This mixed study used cognitive interviews and focus groups with family caregivers (N=13) and healthcare professionals (n=14) to assess their knowledge and understanding of hospice and artificial hydration and nutrition. Data were audio-recorded, transcribed verbatim, and analyzed with thematic content analysis. Three main themes were identified: DAs alone aren’t enough to foster quality decision making for surrogates; individualized communication is necessary to clarify PWD and caregiver value priorities and disease trajectories; and clarification of the impact of care choices within situational contexts is quintessential. Further development is needed to create a practice protocol from these themes to inform professionals assisting surrogates in ACP at EOL. Practical implications from this study include highlighting the importance of individualized communication between PWD, providers, and caregivers in addressing EOL care decisional needs.

### IDENTIFYING GAPS IN KNOWLEDGE AND BARRIERS TO UTILIZATION OF HOSPICE AND END-OF-LIFE CARE SERVICES

Lenard Kaye, Nicholas Silver, Carli Hutchison, and David Wihry, *University of Maine, Bangor, Maine, United States*

Hospice and end-of-life (HEOL) services have traditionally been underutilized, especially in rural areas. A community assessment, performed in eastern Maine, sheds light on the extent to which public and health care professional attitudes and knowledge explain HEOL service access and utilization levels. Structured surveys were distributed in the fall of 2019 targeting community residents (18 years and older) and hospice/health care professionals. Residents (n=213) displayed significant levels of ignorance concerning the purpose and availability of HEOL services. Although a range of HEOL services were rated as highly important, residents were unable to confirm their actual availability. Overall, one in every two residents did not know if particular HEOL services existed locally (ranging from a low of 27% that were unsure whether grief support services were available to 67% unaware of the presence of a community hospice house). Hospice/health professionals (n=22) were aligned in their belief that the public did not have a clear understanding of the intent of HEOL services. Interestingly, community residents lacked equivalent measures of confidence in the HEOL knowledge levels of health care professionals. Significant barriers to the timely utilization of HEOL services included: stigma/fear, lack of education as to the purpose of these services, financial barriers to access, and scarcity of selected HEOL services. For both respondent groups, the top ranking suggestions for increasing HEOL services utilization were earlier discussions between providers and patients concerning the need for such services and increased public and professional education around death and dying.

### IT'S LIKE WE SPEAK A DIFFERENT LANGUAGE: SUPPORT NEEDS AND PREFERENCES OF OLDER LGB WOMEN WHO HAVE LOST A SPOUSE OR PARTNER

Korijna Valenti, *Miami University, Oxford, Ohio, United States*

Needs during and following end-of-life (EOL) experiences are distinctive for sexual and gender minority (SGM) people and, in particular, older lesbian, gay, and bisexual women (LGB) women; however, access to supportive services is limited. This poster presents findings from a qualitative study of older (60+) LGB women who have lost a spouse or partner. This work draws on queer gerontological theory, addressing issues of individual agency, systemic silence, and marginalization by invisibility of older SGM women in order to illuminate specific areas in which policy may be changed and improved. The study analyzed participants' (n = 16) reflections on their experiences of losing a loved one, including how they sought out and received essential grief support and the type of support they would have preferred and from whom, particularly immediately following their spouse or partner's death. Thematic analysis revealed three main findings: 1) having a women-identifying support presence at the time of death for both themselves and their spouse or partner; 2) needing LGB women (or women allies) during EOL for support; and 3) preferring grief groups comprised of other LGB women (or women allies) based on their feelings of difference from gay men and heterosexual/non-SGM women and men. Findings reveal the need for a better understanding among healthcare and social work support professionals regarding LGB women's grief needs and preferences; grief options (e.g. lesbian and non-monosexual (bi+) grief groups); and how to implement policy changes reflecting these needs and preferences.

### MEASUREMENT OF INEQUALITY AND COMPRESSION OF MORTALITY IN INDIA FOR 4 DECADES: 1970–2015

Mukesh Parmar, *Jawaharlal Nehru University, New Delhi, India, New Delhi, Delhi, India*

The studies relating to measurement of compression of Mortality in India is scarce. Most of the studies relating to mortality in India are focused on either life expectancy, or adult, and child mortality. We have used methods suggested by Kannisto (2000) and Canudos (2008) to measure the compression of mortality phenomenon for India for four decades viz. 1970-2015. Dispersion measures like simple mean, median, modal age at death; and some complicated measures like life disparity, standard deviation above mode, standard deviation in highest quartile, Interquartile range, Gini coefficient, AID and C-family were calculated for India from 1970-2015. We used the age specific death rates from abridged Life tables given by Sample Registration System published by Govt. of India. Our results show that inequality in mortality is decreasing in general but the gap between male and female is increasing. There was an average of three years difference in mean and modal age at death between male females in 2011-15. Overall, mean, median and modal age at death has increased in four decades but other inequality measures like Gini coefficient, AID, Standard deviation (SD) and coefficient of variation has decreased in four decades in India. C50 indicator, which indicates that 50 percent of deaths are happening in that age interval, declined from 26 years to 20 years for males and 27 years to 17 years for females, thus indicating the rate of compression of mortality is higher for females than males in India during 1970-75 till 2011-15.

### OLDER ADULTS' PERSISTING BELIEFS ABOUT THE DEATH PANEL MYTH AND ADVANCE DIRECTIVES

Allyson Coldiron,[1] Lauren Bennett-Leleux,[1] Diamond Lee,[1] Lucas Childers,[1] Rachel Donnell,[2] Alexandra Sandlin,[3] Arthur Marsden,[4] and Michael Barnett,[1] *1. The University of Texas at Tyler, Tyler, Texas, United States, 2. The Memory Assessment and Research Center, The University of Texas at Tyler, Tyler, Texas, United States, 3. University of Texas at Tyler, Tool, Texas, United States, 4. Syracuse University, Syracuse, New York, United States*

The political debate preceding passage of the Affordable Care Act included controversy over a bill that some claimed would establish a "death panel" to judge if older adults were worthy of receiving medical care. This claim was false, as the bill would instead incentivize physicians to inform Medicare patients about advanced directives: legal documentation of one's end-of-life preferences. However, the death panel myth led to the removal of this bill from the Affordable Care Act, and a poll five years later found 41% of Americans still believed in the death panel myth. We investigated the effects believing in this myth had on older adults, hypothesizing that those who believed in the myth would have lower advance directive completion rates and more negative attitudes towards advanced directives. Community-dwelling older adults aged 65 to 102 years (N = 182) in a large city in the southern United States completed an interview survey. No relationship was found between belief in the death panel myth and advanced directive completion; however, older adults who believed in the myth had lower perceived need for advanced directives than those who did not. Surprisingly, 47.1% of older adults who believed in the myth also supported incentivizing doctors to inform patients about advanced directives, suggesting that many older adults who believe in the myth do not know that the controversial bill was about advanced directives. Results suggest that the death panel myth may have long-lasting effects, specifically persistent distrust about policies promoting advance directives.

### REFRAMING THE END STAGE OF RENAL DISEASE: HOW AGE MATTERS

Deborah Waldrop,[1] Patricia Denny,[2] Sandra Lauer,[2] and Kathleen Grimm,[2] *1. University at Buffalo, Buffalo, New York, United States, 2. Erie County Medical Center, Buffalo, New York, United States*

End Stage Renal Disease (ESRD) conveys high symptom burden, multimorbidity and the greater likelihood of hospital death than other serious illnesses. Increases in people with ESRD occurred most sharply among adults age 75+. Despite high mortality risk, few with ESRD consider end-of-life preferences or discuss with a physician. The purpose of this study was to explore the nature of advance care planning in ESRD. The study utilized mixed methods and both qualitative and quantitative data was collected during in-depth chairside interviews with 31 people while they were on hemodialysis. Participants ranged in age from 29-85; Mage=60; (N=13 [40%/)60). The data was divided above and below the Mage, and distinct differences were found in the nature of advanced care planning by age group. Greater numbers of people >60 (N=11[61%]) were not considering a transplant while (N=9[69%]) of those under 60 had a failed transplant and were again on the waiting list. Although the majority of participants had a health care proxy (N=27[87%]), more who were >60 had a proxy who knew their wishes (N=14[78%]) compared with (N=9[69%]) who were 60 compared with none <60. The qualitative data illuminated these age-differentiated responses in themes: Older age and (1) Multimorbidity; (2) Frequency/intensity of hospitalization; (3) Diminished hope of transplantation; and (4) More acute death awareness. The need for disease-specific advance care planning—with hopes and expectations about transplant--and attention to the influence of age and decline cannot be overstated.

### SOCIAL EXCLUSION AND SUICIDAL IDEATION: ANALYSIS OF THE BEREAVED LIVING ALONE

Bomi Choi, Hey Jung Jun, and Sun Ah Lee, *Yonsei University, Seoul, Republic of Korea*

The aim of this study is to explore the relationship between social exclusion and suicidal ideation among bereaved older people living alone. When people with a significant loss in the familial relationship are exposed to social exclusion, they likely experience poor mental health and suicidal ideation. Using the Korean Community Health Survey 2017, logistic regression model was applied to the bereaved older people living alone, 65 to 110 of age (N=14,659). Social exclusion was comprised of three network-based components: exclusion from relationships, social participation, and community. Relationship exclusion means the lack of contact with family, friends, and neighbors, respectively, at least once a week. Social participation exclusion refers to the lack of participation in a religious, socializing, and leisure activity, respectively, at least once a month. Community exclusion covers two indicators of trust in neighbors and perception of neighborhood reciprocity. Results showed that indicators of relationship, social participation, and community exclusion were associated with suicidal ideation. Bereaved, living-alone older people were likely to have suicidal ideation when they lack contact with neighbors (OR=1.13, p<.05), participation in the religious activity (OR=1.12, p<.05) and socializing activity (OR=1.20, p<.05), and trust in neighbors (OR=1.29, p<.001). The moderation analysis showed that exclusion from socializing activity was associated with suicidal ideation only among females. The results of the analyses imply that interventions that promote social participation could improve the mental health of the bereaved older adults living alone.

### SOCIAL SUPPORT AND DEPRESSIVE OUTCOMES IN OLDER ADULTS: AN ANALYSIS OF THE HEALTH AND RETIREMENT STUDY

Julia Tucker, and Nicholas Bishop, *1. Texas State University San Marcos, San Marcos, Texas, United States*

Given population aging and impact of both spousal and social support on the health of older adults, the protective role of social support amongst recently bereaved older adults represents an important area of research. The aim of this study is to identify the relationship between recent widowhood and change in depressive symptoms in older adults, and how social support moderates this association. Utilizing observations from the nationally representative Health and Retirement Study, the analytic sample consisted of 2,890 adults age 50 and over who were partnered or married in 2012. Depression was measured using the Center for Epidemiological Studies Depression scale short form (CESD-8). Positive social support was measured as perceived social support from family, friends, and children. Widowhood was a dichotomous measure indicating mortality of spouse between 2012 and 2014. Autoregressive multiple regression was used to determine if widowhood was associated with change in depression from 2012-2104 and whether positive social support moderated this relationship. Widowhood was associated with an increase in depressive symptoms from 2012-2014 (b=0.967, SE=0.145, p <.001) and social support was negatively associated with change in depression (b=-0.021, SE=0.004, p <.001). Social support appeared to moderate the association between widowhood and change in depression (b=0.068, SE=0.026, p =.010), though widowed older adults with higher social support appear to have more rapid increase in depression than those with lower social support. These preliminary findings and implications for supporting bereaved older adults will be discussed.

### THE ROLE OF HEALTH, SOCIAL NETWORK, AND RACE IN ADVANCE CARE PLANNING AMONG MEDICARE BENEFICIARIES

Jung Kwak,[1] Heehyul Moon,[2] and Soonhee Roh,[3] *1. University of Texas at Austin, Austin, Texas, United States, 2. University of Louisville, Louisville, Kentucky, United States, 3. University of South Dakota, Sioux Falls, South Dakota, United States*

Advance care planning (ACP) is linked with high-quality end-of-life outcomes. However, ACP engagement level among older adults varies significantly by demographic, social, and health characteristics. In this study, we sought to identify characteristics associated with informal and formal ACP, in order to inform development of targeted education and outreach efforts that are tailored to diverse groups of older adults. The data came from a nationally representative study of Medicare beneficiaries living in communities, the National Health and Aging Trends Study (Round 8, N= 5,547). Multivariable logistic regressions were conducted to identify individual characteristics (i.e., race/ethnicity, age, gender, income, functional disability, cognitive function, perceived health, and numbers of people in social networks) associated with ACP engagement. Rates of informal ACP (talking to someone), and formal ACP, completing a healthcare power of attorney (HPOA) and a living will (LW), were 56%, 60.5%, and 56% accordingly. Logistic regression showed that individuals who were married or had a larger social network, and had higher functional impairment and health needs were significantly more likely to engage in both informal and formal ACP. However, individuals with memory problems (only informal ACP) and African Americans and Hispanics were significantly less likely to engage in both informal and formal ACP. African Americans without dementia were more likely to have completed HPOA compared with Whites. Findings suggest an important role of social network, and functional and cognitive health in ACP

with implications for developing targeted outreach efforts in faith-based or social group settings, and healthcare settings.

### TRENDS IN THE RECEIPT OF CONSISTENT HOSPICE PROFESSIONAL VISITS AT THE END OF LIFE AND RATINGS OF HOSPICE CARE QUALITY

Thomas Christian,[1] Joan Teno,[2] Kyle Cobb,[3] Sara Galantowicz,[3] Alan Levitt,[4] and Cindy Massuda,[4]
*1. Abt Associates, Cambridge, Massachusetts, United States, 2. Oregon Health & Science University, Portland, Oregon, United States, 3. Abt Associates, Cambridge, United States, 4. Centers for Medicare & Medicaid Services, Baltimore, United States*

Caregivers have identified consistent visits by professional hospice staff at the end of life as positively affecting experiences of care quality. Little is known about the prevalence of such visits. Using 100% Medicare hospice claims with discharge dates in Federal Fiscal Year 2018, we identified the rates of providing skilled nurse or social worker visits to hospice beneficiaries in at least two of the last three days of life and compared these rates to percentages of caregivers indicating they would "definitely" recommend the hospice and caregivers rating the hospice a 9 or 10 on a 10-scale from CAHPS Hospice scores. Among our analytic cohort of 762,238 hospice discharges, 509,585 individuals (66.9%) were visited by a nurse or social worker in at least two of the last three days of life. Beneficiaries lacking these visits were more likely to be black (black 39.6% vs. white 32.2%; AOR 1.32 95% CI 1.29-1.34) or resided in a nursing facility (nursing facility 37.7% vs. patient's home 32.1%, AOR 1.39 95% CI 1.36-1.40). The mean hospice-level score for achieving these visits was 64.8% (median 70.2%; IQF 53.0%-80.9%). The Pearson's correlation coefficients between hospice-level rates of visits at the end of life and the caregiver percentages for "definitely" recommending the hospice was 0.2418 and for rating the hospice a 9 or 10 on a 10-scale was 0.2587. These findings demonstrate significant variability across hospice providers and signal a positive correlation with caregivers' quality ratings. Future work is needed to monitor the provision of these visits.

### VIDEO INTERVENTION TO INCREASE KNOWLEDGE ABOUT AND INTEREST IN PALLIATIVE CARE AMONG MSW STUDENTS

Cathy Berkman, *Fordham University, New York, New York, United States*

As the population ages and more people live longer with chronic and life-limiting illnesses, more healthcare professionals with palliative care skills are needed. Social workers are part of the palliative care team, but there is little, if any, content on palliative and end-of-life care in MSW programs. A 24-minute video featuring nine palliative and hospice social workers was produced with two goals: 1) increase knowledge of social work students about palliative and end-of-life care; and 2) interest social work students in a career in palliative social work. MSW students from three schools, in NY and Alabama, viewed the video. After viewing the video, 94 students participated in the mixed methods study, completing the brief, anonymous, online survey. The mean level of understanding about what palliative social workers do, rated from 1 (no understanding) to 5 (very good understanding), was 2.96 (SD=.99) before viewing the video and 4.31 (SD=.61) after, for an increase of 1.35 points (95% CI=1.14, 1.55) (p<.001). The mean level of interest in a career in palliative care social work and working with seriously ill persons and their family members, rated from 1 (Not at all interested) to 5 (Extremely interested), was 2.52 (SD=.99) before viewing the video and 3.45 SD=.80) after, for an increase of .91 points (95% CI=.79, 1.07) (p<.001). Qualitative data supporting the quantitative findings will be presented. This study suggests that a video intervention may be an effective tool to increase knowledge and interest in palliative and end-of-life care among social work students.

### WHAT IS IMPORTANT AT THE END OF LIFE? PERSPECTIVES FROM EXPERIENCED HOME CARE WORKERS

Emma Tsui,[1] Emily Franzosa,[2] and Kathrin Boerner,[3]
*1. CUNY Graduate School of Public Health & Health Policy, New York, New York, United States, 2. James J. Peters VA Medical Center, Bronx, New York, United States, 3. University of Massachusetts Boston, Boston, Massachusetts, United States*

Home care workers (HCWs) make up a large and rapidly growing sector of the American health care workforce serving older adults. This study focuses on a common but understudied feature of home care labor: workers' thoughts around what makes a "good" or "bad" patient death. While researchers have investigated patients', families', physicians', and other care providers' perspectives on this issue, the perspectives of HCWs, who contribute substantially to home-based care at the end of life, have yet to be explored. We conducted 40 in-depth interviews with HCWs in New York City on their experiences with and reflections on patient death. We used a inductive, iterative approach to analyze data on what HCWs believe is important for dying patients. HCWs described EOL values that align well with the views held in common by patients, families, and other care providers, like the importance of not being alone when dying and being physically comfortable (not in pain and not suffering). In particular, HCWs conceptualized a detailed role for themselves when providing EOL care near the time of death. HCWs' sustained presence and relationships with patients may uniquely position them to assist in the attainment of patients' EOL goals, particularly when HCWs understand what these goals are. HCWs' potential for playing this role, however, is jeopardized by a lack of training in EOL care and by the limited information they receive about a patient's health status.

### SESSION 2858 (POSTER)

### FAMILY CAREGIVING

### AN EXPLORATION OF LEARNING RESULTS AND CURRICULA GAPS WITHIN AN ONLINE GRANDFAMILIES CERTIFICATE PROGRAM

Jennifer Crittenden, *University of Maine, Bangor, Maine, United States*

Professionals who work with grandparents raising grandchildren have cross-cutting training needs that span content in gerontology, social services, child welfare and program development. To address these needs, a unique, asynchronous, online continuing education program was launched by the UMaine Center on Aging. To-date the program has 177 individual program completers with learners from across the U.S. and Hong Kong that are affiliated with a diverse set of organizations and perform a wide range of professional and lay functions. Participant data indicate that the program has appealed to a wide variety of learners including participants who serve caregivers generally (60.8%) and grandparents raising grandchildren specifically (81%). A small majority (55.6%) of the agency-based learners reported serving, on average, more than 40 grandfamilies annually. Self-reported learning levels were notable ranging from a mean low of 3.46 out of 4 points (N = 157, SD = 0.59) for the volunteer recruitment and mentorship programming module to a mean high of 3.79 (N = 167 ,SD = 0.45) for the caregiver self-care module. Evaluation results from the first seven learner cohorts underscore the efficacy of program content as well as the utility of performing an initial program needs assessment to guide curriculum development. Practice implications for future continuing education efforts targeting grandfamilies professionals and lay leaders include: the need for easily accessible online education in combination with supplemental training opportunities addressing topics such as the long-term impact of substance use disorder and trauma combined with locally relevant content on grandfamilies and legal resources.

### ASSOCIATION BETWEEN SELF-REPORTED HEALTH AND DEMENTIA SYMPTOMS AMONG PEOPLE WITH DEMENTIA AND THEIR CAREGIVERS

Eric Jutkowitz,[1] Mauricio Lopez Mendez,[1] Rowan Iskandar,[2] and Laura Pizzi,[3] *1. Brown University, Providence, Rhode Island, United States, 2. Brown University, Providence, United States, 3. Rutgers Ernest Mario School of Pharmacy, Piscataway, New Jersey, United States*

People living with dementia (PwD) can often reliably self-report their health; yet, there are limited data on their and their primary caregiver's self-reported overall health (excellent, very good, good, fair, and poor). We used data from the Aging, Demographics, and Memory Study (2001-2009) to quantify the association between PwD's cognitive impairment (Mini-mental State Exam), physical limitations (scale [0-10] of activities of daily living), and behaviors (scale [0-12] of behavioral symptoms on the Neuropsychiatric Inventory Questionnaire) and self-reported health. We estimated two ordered logistic regressions estimating: 1) PwD's self-reported health (analyzed n=308); 2) primary caregiver's self-reported health (analyzed n=135; 173 PwD did not have primary caregiver in the survey). We controlled for the PwD demographics, chronic conditions, and if they lived in the community. The regression estimating caregiver's self-reported health also controlled for the caregiver's relationship to the PwD, and whether the caregiver lived with the PwD. PwD's self-reported health was lower (4% excellent; 16% very good; 22% good; 30% fair; 30% poor) than caregivers (14% excellent; 27% very good; 32% good; 23% fair; 4% poor). For PwD, one-additional physical limitation, but not cognition or behavior, was associated with 1.15 (95%CI: 1.01,1.30) times greater odds of self-reporting poor health compared to all other categories. For caregivers, one-additional behavior, but not cognition or physical limitations, was associated with 1.17 (95%CI: 1.01,1.37) times greater odds of self-reporting poor health. For PwD, interventions targeting physical limitations may increase self-reported health, but for caregivers, interventions targeting behavioral symptoms may increase self-reported health.

### CAREGIVER-PROVIDER COMMUNICATION ABOUT PAIN IN PERSONS WITH DEMENTIA

Catherine Riffin,[1] Sylvia Lee,[2] M. Cary Reid,[3] Keela Herr,[4] and Karl Pillemer,[5] *1. Weill Cornell Medical College, London, England, United Kingdom, 2. Weill Cornell Medicine, New York, New York, United States, 3. Weill Cornell Medical College, New York City, New York, United States, 4. University of Iowa, Iowa City, Iowa, United States, 5. Cornell University, Ithaca, New York, United States*

Pain in older persons with dementia (PWD) is severely under-detected and under-managed. Family caregivers can play an important role in addressing these disparities by acquiring the requisite skills to communicate PWD's pain symptoms and behaviors to health care providers, but little is known about how caregivers of dementia patients and their providers approach such pain-related discussions. We employed qualitative methods to explore the perspectives of family caregivers of PWD (n=18) and health care providers (geriatricians, general internists, neurologists, emergency room physicians) involved in PWD's treatment (n=16) regarding pain communication. We specifically focused on participants': 1) priorities and expectations for communicating about pain and dementia, 2) challenges to communicating about pain and dementia, and 3) strategies and recommendations for optimizing communication about pain and dementia. Analyses revealed that caregivers and health care providers expected to receive accurate, detailed information from one another, but uncertainty in both groups around differentiating pain behaviors from dementia symptoms acted as a barrier to effective information exchange. Additional challenges to productive pain-related discussions were identified by caregivers, including provider fatalism and lack of interpersonal skills, and by providers, including patient-caregiver disagreement about pain symptoms and unreliable caregiver reporting. Participants endorsed using practical approaches, such as pain scales and logs, as well as rapport-building strategies, such as affirmation of caregivers' input, to facilitate collaborative discussions.

### CAREGIVING INTENSITY AND CAREGIVER BURDEN AMONG DEMENTIA CAREGIVERS: THE MODERATING ROLES OF SOCIAL SUPPORT

Ling Xu,[1] Yiwei Liu,[2] Hui He,[3] Noelle Fields,[1] Chen Kan,[4] and Dorothea Ivey,[5] *1. The University of Texas at Arlington, Arlington, Texas, United States, 2. Central University of Finance and Economics, Beijing, China, 3. Xiangtan University, Xiangtan, China, 4. UTA, Arlington, Texas, United States, 5. University of Southern Maine, Portland, Maine, United States*

Objective: Using the stress-coping theory, the aims of the present study were to test what levels of caregiving intensity (hours actually spent on caregiving every day) posed the most negative influence on caregiver burden as well as how social support moderated such associations among dementia caregivers. Methods: Data from the baseline assessment of the Resources for Enhancing Alzheimer's Caregiver Health (REACH II) (N = 637) were used. Caregiver burden (12-item Zarit caregiver burden scale), caregiving intensity (caregiving hours), and social support (Lubben social network, received support, satisfaction with support, and negative interactions) were the main measurements. Separate multivariate regression models were conducted with Stata 16. Results: The results showed that the relationships between caregiving hours and caregiver burden were nonlinear after controlling all of the socio-demographic variables. Further analyses showed that when caregiving hours reached 13.50 hours per day, the levels of burden were the highest. In addition, received social support, satisfaction with social support, and social network significantly moderated the relationship between caregiving hours and caregiver burden among dementia caregivers when they were examined separately. However, only social network played a significant moderator role when examining the four social support indicators simultaneously. Discussion and conclusion: These findings suggest the need for programs and practices on educating caregivers regarding how to identify, approach, and gain social support/s, especially in how to broaden the caregivers' social network while caring for a family member with dementia.

### CHILDREN RAISED BY GRANDPARENTS OR NONGRANDPARENTS: WHICH HAVE GREATER ODDS OF HIGH ACADEMIC PERFORMANCE?

Tamar Shovali,[1] Melissa Bright,[2] and Kerstin Emerson,[3]
*1. Eckerd College, St Petersburg, Florida, United States,*
*2. University of Florida, Gainesville, Florida, United States,*
*3. University of Georgia, Athens, Georgia, United States*

There is a 19-fold greater likelihood that children removed from parental care will be raised by a grandparent than any other caregiver. Theorists and practitioners highlight the importance of monitoring academic progress to understand the benefits of at-risk youth living in grandfamilies. Using a nationally representative dataset we examined academic performance for children in three caregiver (N = 814) categories: Grandparent (73.1%), Foster parent (12.7%), Other (nonfoster, nonkin/nonfoster; 14.2%), with significance testing across groups. Children were between 6-17 years with grandfamilies and foster families caring for significantly younger children compared to the "other" group. Overall, 76% of children were reported to have high academic performance in math and 79.6% had high academic performance in reading/writing. Grandparents were caring for a significantly higher proportion of non-Hispanic White children with statistically significantly higher reported academic performance in math and reading/writing compared to nongrandparents. Logistic regression model A showed for both foster parent (AOR 0.57, CI: .35-.91) and other (AOR 0.55, CI: .35-.86) caregiver groups were significantly negatively correlated with high math performance compared to grandparents. Model B showed the same statistically significant and negative relationship to reading/writing performance outcomes for foster parent (AOR 0.56, CI: .02-.35) and other (AOR 0.51, CI: .01-.32) caregivers compared to grandparents. Controlling for relevant caregiver and child variables both models suggest that children living with grandparents have 55% greater odds of high academic performance compared to children raised by nongrandparents. Findings support placement of children with grandparents. Supporting grandfamilies with appropriate social services will be reviewed.

### EMOTIONAL EXPERIENCES OF DEMENTIA CAREGIVING TRANSITIONS

Emily Ihara, Catherine Tompkins, William Kennedy, Rhea Vance-Cheng, Bianca Kwan, and Kendall Barrett,
*George Mason University, Fairfax, Virginia, United States*

Research indicates that family caregivers of individuals living with dementia are at risk for high levels of stress, depression, physical health declines, and illness. The health and well-being of family caregivers is critically important to a long-term care system that is dependent on them to continue their caregiving role. In-depth individual and focus group interviews of 16 dementia caregivers were conducted to explore the emotional experiences of caregiving stress during transitions of individuals living with dementia to a higher level of care. Data were transcribed verbatim, checked for accuracy, and analyzed by at least two members of the research team. Line-by-line coding, memo writing, and constant comparative analyses were conducted until redundancy, when no new themes were discovered. Caregivers described various levels of feeling overwhelmed and symptom progression leading to the move to a nursing facility. Social isolation featured prominently, with caregivers describing a gradual erosion of their social network and socializing opportunities because of their caregiving responsibilities and the care recipient's deteriorating symptoms. Caregivers described feeling isolated and stigmatized. One caregiver said, "…you're being less invited, you're being less involved. People don't know how to deal with you…I don't know if they become the pariah or I become the pariah." At the same time, maintaining social connections and having help with caregiving featured prominently in the coping mechanisms described. The health of caregivers is equally as important as the person living with dementia, and programs, interventions, and resources should be a priority for supporting families through transitions.

### EXPLORING THE STRESS AND STRENGTHS ARISING FROM THE COMPLEXITIES EXISTING WITHIN GRANDFAMILIES

Catherine Tompkins,[1] Alice Zic,[1] Ellie Carlson,[1]
Ali Purvis,[1] Loriena Yancura,[2] and Danielle Nadorff,[3]
*1. George Mason University, Fairfax, Virginia, United States, 2. University of Hawai'i, Honolulu, Hawaii, United States, 3. Mississippi State University, Mississippi State, Mississippi, United States*

The opioid crisis and other social problems continue to increase the number of grandparent-headed households in the U.S. There are challenges and benefits that result from grandparents parenting their grandchildren. Grandparents often report the joy in watching their grandchildren grow but also report on the complexities that may lead to stress. Two-hundred forty-one grandparents were recruited using Qualtrics' Online Panel Service. In addition to a standardized perceived stress scale and demographic questions, participants responded to open-ended questions related to the benefits and challenges of residing within a grandparent-headed household. This presentation focuses on comparing demographics, perceived stress, benefits and challenges of 108 current grandparent caregivers to 133 grandparents who were no longer the head of household at the time of the survey. Grandparents who currently were raising their grandchildren had a higher perceived stress score (p=.03) compared to grandparents who had raised their grandchildren in the past. An exploration of the demographic variances and responses to the open-ended questions, will begin to explain this statistically significant difference in reported stress. An additional complexity arising for a subsample of 10 current grandparents raising grandchildren, included simultaneously caregiving for an older adult relative. It is imperative to study the complexities existing within grandfamilies, from the perspectives and experiences of the grandparent caregiver, and develop interventions to reduce stress and increase the grandparents' ability to cope with situational, emotional and relationship changes.

### FACTORS RELATED TO FAMILY CAREGIVERS QUITTING THEIR WORK

Masakazu Shirasawa,[1] Yoshihito Takemoto,[2] Kazutaka Masuda,[3] and Ryousuke Hata,[4] *1. International university of Health and Welfare, Nabari, Mie, Japan, 2. Okayama Prefectural University,, Soujya, Okayama, Japan, 3. Mukogawa Women's University, Nishinomiya, Hyogo, Japan, 4. Hokusei Gakuen University,, Sapporo, Hokkaido, Japan*

As the working population declines in Japan, preventing family caregivers from quitting their work has become a government priority. Approximately 100,000 people leave their jobs annually because of caregiving obligations. The present study examines the reasons behind caregivers' resignation and the factors that prevent caregivers from quitting. In January 2018, 3,000 sites were randomly selected from care management institutions nationwide. Surveys were conducted by mail, first with one care manager from each institution, then with an elderly person requiring long-term care and who had a family caregiver overseen by that same manager. The second survey was contingent on the response to the first. A total of 1,719 valid responses were received in the first survey (response rate: 57.3%), and 594 in the second survey (response rate: 34.6%). The surveys found that 21.2% of family caregivers quit their jobs. Caregivers also quit their hobbies (23.6%), neighborhood associations (7.2%), and stopped volunteer activities (5.4% ). Eight items from the survey of people requiring long-term care and who were supported by their family caregivers, and 5 items from the survey of care managers were analyzed in binomial logistic regression analysis with continuation of work (yes/no) as the dependent variable. Caregivers are less likely to continue working if they are older and their dependents require extended care, and more likely to continue working if they and their dependents are satisfied with the care manager. Care managers could therefore play a crucial role in allowing caregivers to find a better balance between caregiving and work.

### FAMILY CAREGIVER ASSESSMENT IN PRIMARY CARE: A NATIONWIDE SURVEY

Catherine Riffin,[1] Jennifer Wolff,[2] Julianna Maisano,[3] Sylvia Lee,[4] and Karl Pillemer,[5] *1. Weill Cornell Medical College, London, England, United Kingdom, 2. Johns Hopkins Bloomberg School of Public Health, Baltimore, Maryland, United States, 3. Weill Cornell Medicine, New York City, New York, United States, 4. Weill Cornell Medicine, New York, New York, United States, 5. Cornell University, Ithaca, New York, United States*

Family caregivers play an important role in the healthcare of older adults, but their circumstances, needs, and risks are often unknown to medical professionals. Standardizing how caregivers' needs are assessed in healthcare delivery can help clinicians design care plans that take caregivers' capabilities into account and provide targeted recommendations for caregiver support. Despite the potential of caregiver assessment, little is known about its use in primary care practice. The present study surveyed a national random sample of 1,000 U.S. primary care clinicians (physicians, nurses, social workers) to characterize current practices, barriers, and facilitators of caregiver assessment. A total of 231 completed responses were received. A minority of respondents (11%) reported that their practice or clinic had a standardized procedure for caregiver assessment; one in ten (10%) reported that they had personally conducted a caregiver assessment using a standardized instrument in the past year. The most common barriers to caregiver assessment were lack of time (65%), inability to have private discussions with caregivers (36%), lack of access to referral options (30%), inadequate reimbursement (30%), and reluctance of caregivers to discuss their needs (30%). The most frequently endorsed facilitators to aid future implementation included better availability of referral options (77%), easier referral mechanisms (67%), co-location of mental health specialists, care managers, or social workers (65%), and training in how to address caregiver issues (61%). Findings are discussed within the context of emerging healthcare policies and practice initiatives designed to promote caregiver assessment in health care settings.

### GRANDPARENTS CARING FOR GRANDCHILDREN, FAMILY STRUCTURE, AND DEPRESSIVE SYMPTOMS IN CHINA

Fengyan Tang, and Ke Li, *University of Pittsburgh, Pittsburgh, Pennsylvania, United States*

It is a cultural norm for Chinese older adults to engage in co-parenting and caring for grandchildren. Previous research documented health advantages for grandparents who provide occasional, extensive, or even custodial care to grandchildren in China. Yet there is little information regarding the impacts of living arrangement and its interaction with grandchild care on grandparents' psychological well-being. Using three waves of the 2011-2015 China Health and Retirement Longitudinal Study (CHARLS) data, this study examined the longitudinal association of depressive symptoms with grandchild care intensity and living arrangement among adults aged 40 and above (N=5,037). Mixed effects regression models were applied to examine changes in depressive symptoms and the associations with explanatory variables. At baseline, about half of respondents reported caring for their grandchild (ren). And nine percent lived with grandchildren only, that is, in a skipped-generation household and taking a custodial grandparent role. Overall, depressive symptoms did not change over time. After controlling for sociodemographic and health covariates, we found that providing medium level of care (i.e., between three to 10 hours per day) was associated with fewer baseline depressive symptoms, whereas grandparents living with grandchildren had more symptoms at baseline relative to those living with others. Further, an increased level of caregiving in the skipped-generation households was associated with more depressive symptoms. Given that custodial grandparenting is a growing phenomenon in China, further research needs to investigate how to reduce caregiving burden and associated adversary effects and how to promote overall well-being in this population.

### IDENTIFYING CARE-RELATED NEEDS OF FAMILY CAREGIVERS OF PEOPLE WITH DEMENTIA: BEYOND THE STRESS PROCESS MODEL

Sara Moss,[1] Lauren Gebhardt-Kram,[1] Holly Dabelko-Schoeny,[2] and Jennifer Cheavens,[1] *1. Ohio State University, Columbus, Ohio, United States, 2. The Ohio State University, Columbus, Ohio, United States*

The psychosocial stress process model (Pearlin et al., 1990) remains a dominant theoretical framework characterizing the transactions between factors affecting outcomes of informal caregivers of people with dementia (PWD). Despite widespread agreement that the model provides an important framework for understanding caregiver experiences and predicting caregiver outcomes, it is not sufficient to clarify the needs of caregivers. Needs are conceptualized as the skills and resources that could be used to ameliorate the negative impacts of caregiving and promote quality of life (Gitlin & Hodgson, 2015). Determining caregiver needs requires appreciation of the complex background, contextual, and stress-related variables described in the stress process model and requires empirically- and theoretically-driven understanding of the diverse resources, materials, and skills that individuals require for global health and functioning. In this study, we conducted a content analysis of existing measures of dementia caregiver needs (N = 54), content analysis of materials related to evidence-based dementia caregiver interventions and government reports and documents (N = 28), and semi-structured in-depth interviews with current caregivers of PWD (N = 12) to identify the personal and care-related needs of family caregivers of PWD. We propose a framework of five inter-related need categories (Health-related needs, environmental needs, psychological needs, social needs, and needs related to the care and functioning of the PWD) that transact with the factors described in the stress process model, ultimately influencing functioning. In the future, we plan to test this model empirically with a nationally representative sample of caregivers.

### MENTAL HEALTH AMONG EMPLOYED FAMILY CAREGIVERS: A GENDER-BASED ANALYSIS ON THE ROLE OF WORKPLACE SUPPORT

Lun Li,[1] Yeonjung Lee,[2] and Daniel W. L. Lai,[3] *1. Simon Fraser University, Vancouver, British Columbia, Canada, 2. University of Calgary, Calgary, Alberta, Canada, 3. The Hong Kong Polytechnic University, Kowloon, China*

Compared to men, women undertake more family caregiving responsibilities, and thus take more toll in health and wellbeing when they are employed. The current study examined the gender difference in mental health among employed family caregivers, focusing on the role of workplace support in balancing work and caregiving roles. Guided by the social role theory and the moderated-mediation model of employment adjustment and mental health, we analyzed a nationally representative data from the 2012 Canada General Social Survey - Caregiving and Care Receiving with a sample of 2,426 participants selected. Moderated-mediation analysis based on the SPSS macro PROCESS 3.3 was used. Women employed family caregivers are more likely to undertake higher intensive caregiving, make more employment adjustment, and further report worse mental health status than their men counterparts. Gender difference was apparent in regards to the workplace support. For women, the moderating effect of workplace support is significant only when there are at least 5 different types of workplace support available at their workplaces, while for men, the moderating effect is significant when there are at least 2-3 different types of workplace support available. Women employed family caregivers experience worse mental health when employment adjustment is needed for their care responsibility. Findings have implications for providing workplace support for family caregivers given that women benefit less from workplace support compared to men. Further study is needed to explore the impact of various types of workplace support for women employed family caregivers, and to provide tailored support to them.

### OLDER CAREGIVERS' POSITIVE AND NEGATIVE EXPERIENCES WITH CAREGIVING

Latarsha Chisholm,[1] Lynn Unruh,[2] and jacqueline Lamanna,[3] *1. University of Central Florida, Orlando, Florida, United States, 2. university of central florida, Orlando, Florida, United States, 3. UCF, Orlando, Florida, United States*

U.S. population growth is greatest amongst the oldest-old who are most likely to need temporary or permanent reliance on formal or informal care-giving. With co-survival of older spouses, and greater longevity, informal care-giving is increasingly provided by older adults for other older adults. Little is known about the positive and negative care-giving experiences of older informal caregivers. This observational study examines the relationship between the care-giving situation and the positive and negative perceptions of care-giving in caregivers age 65 and older. A survey of 108 older informal caregivers utilizes well-validated instruments to measure perceptions of two positive (positive self image, positive life) and two negative (stress, uncertainty) experiences due to care-giving. Regressions statistically assess the relationship between care-giving situations and these positive and negative perceptions. We find that while some care-giving situations, such as higher amounts of unpaid support, are related to positive care-giving experiences, others, such as care-giving a recipient with a greater number of ADL deficits, having more care-giving responsibilities, and having increased hours of work due to care-giving, are related to more negative experiences. Some situations, such as lower income and greater involvement in care-giving, are related to both positive and negative perceptions. Higher levels of emotional support were related to mixed positive and less negative perceptions of care-giving. Result indicate that healthcare system and community support is needed to provide opportunities for unpaid support and assistance with caregivers of recipients with a high number of ADL deficits and caregivers who have more care-giving responsibilities.

### QUANTITATIVE STUDY OF INDIVIDUAL CREATIVE CAREGIVING SESSIONS ON WELL-BEING OF CAREGIVERS AND CARE RECIPIENTS

Felicia Wheaton,[1] Safiyyah Cole,[1] Sai Raj Kappari,[2] and Matilda Johnson,[3] *1. Bethune-Cookman University, Daytona Beach, Florida, United States, 2. Westcliff University, Daytona Beach, Florida, United States, 3. Bethune-Cookman University, Bethune-Cookman University, Florida, United States*

There were approximately 34.2 million unpaid caregivers of adults age 50+ in the United States in the last 12 months (NAC & AARP, 2015). These individuals provide important care for older adults with physical, psychological and cognitive problems. There is a growing awareness that caregivers are also at risk for physical and mental health problems and therefore also require support to reduce stress and maintain optimal health. Research suggests that engaging in creative and artistic activities may reduce stress and improve physical and mental health among caregivers. Researchers from Bethune-Cookman University partnered with the Atlantic Center for the Arts in New Smyrna Beach, FL to evaluate their Creative Caregiving program. Approximately 10 informal caregivers and their care partners met for 2 hours each week over the course of 6 weeks from February 3-March 9, 2020. Participants learned how to connect mind, body and spirit by using the arts as a tool of self-care, social interaction, and learning. At each session, participants were asked to rate their overall wellbeing on a scale from 1-10. Pre- and post-session data was analyzed using paired samples t-tests. Results indicate that there was significant improvement in wellbeing and participants reported improvements in their stress, mood and relationship with their caregiver or care partner. These findings were observed for both caregivers and care partners, suggesting that arts programs can benefit both. Such programs are a fun and cost-effective way to improve wellbeing, at least in the short-term.

### RELATIONSHIP QUALITY WITH ADULT CHILDREN AMONG GRANDPARENT CAREGIVERS

Heejung Jang, *University of Michigan, Ann Arbor, Michigan, United States*

For the increasing number of grandparent caregivers, relationship quality with adult children has important implications for the well-being of grandparents. Based on solidarity, conflict, and ambivalence, the present study examines how parent-adult children's relationships differ by grandparent caregiving status on depressive symptoms and psychological well-being. This study uses The 2014 Health and Retirement Study from a sample of 1,197 grandparent caregivers age 51 and older. Latent class analysis is applied to measure affection and conflict in older grandparents-adult children relationships. Results from the latent class analysis identified four clusters: amicable, ambivalent, detached, and disharmonious. OLS regression models are estimated the association between relationship types and depressive symptoms and psychological well-being by grandparent caregiving. For the depressive symptoms, disharmonious relationships with adult children increase depressive symptoms among co-parenting and custodial grandparents. Also, ambivalent and disharmonious relationships with adult children reduce the psychological well-being of older grandparents. The study discusses the variances of the relationships with adult children and their effects on grandparents' well-being. The results will shed light on the importance of familial relationships and will be beneficial for the development and maintenance of policies and practices that support the families of grandparent caregivers.

### SELF-EFFICACY OF FAMILY CAREGIVERS OF OLDER ADULTS WITH COGNITIVE IMPAIRMENT: A CONCEPT ANALYSIS

Tarik KHAN,[1] Karen Hirschman,[2] Matthew D. McHugh,[3] Mary Naylor,[2] and Tarik S Khan, *1. University of Pennsylvania, PHILADELPHIA, Pennsylvania, United States, 2. NewCourtland Center for Transitions and Health, Philadelphia, Pennsylvania, United States, 3. University of Pennsylvania, Philadelphia, Pennsylvania, United States*

The purpose of this concept analysis is to address fundamental gaps in the understanding of self-efficacy in family caregivers of older adults with cognitive impairment, including updating the 26-year-old concept analysis with a contemporary definition. With the first of the baby boomers set to turn 75 in 2021, the growing number of Americans with Alzheimer's disease is predicted to more than double over the next 30 years, while the pool of potential family caregivers is likely to diminish by half. Research demonstrates that increased self-efficacy can help family caregivers of older adults with Alzheimer's and other types of cognitive impairment experience lower burden and depressive symptom severity. This study utilized Walker and Avant's method of concept analysis, an eight-step iterative process that helps to clarify the meaning of ambiguous concepts. A literature review was conducted from July 1993 through March 2019 using PubMed/MEDLINE, Scopus, CINAHL, and Embase. Eight defining attributes of this concept were identified. The revised definition of self-efficacy in this population is "a family caregiver's confidence in their ability to: manage behaviors and other caregiving stresses, control upsetting thoughts, acquire medical information, manage medical issues, obtain self-care, access community supports, assist with activities of daily living and other care, and maintain a good relationship with a relative, friend, or neighbor of an older adult with cognitive impairment." Practice implications include tailoring interventions to improve family caregiver self-efficacy. Policy implications include fostering evidence-based health strategies through payment and delivery incentives that further support caregiver self-efficacy.

### SERVICE-SPECIFIC SURVEYING TO SUPPORT PERSON-CENTERED HCBS FOR OLDER ADULTS AND THEIR CAREGIVERS

Caitlyn Walsh, and Jessica Tice, *Florida Department of Elder Affairs, Tallahassee, Florida, United States*

The Florida Department of Elder Affairs (DOEA) annually surveys clients receiving state funded home and community-based services (HCBS) to measure their satisfaction with services. Historically, the same survey instrument was used each year, to afford question-level comparisons across time. However, in 2015 internal contradictions were identified between individual-level satisfaction ratings and qualitative statements made by the respondents later in the survey. The high rates of satisfaction typical in survey responses were also contradicted by findings from a comprehensive program evaluation which revealed high percentages of clients who terminated their services and many caregivers reporting strain and varying types of personal crisis. To address these issues, the annual Client Satisfaction Survey and methodology was redesigned to be more specific regarding details about the delivery of direct services, and the sampling methodology was revised to constrain to the recipients of discreet service types. The results from these new service-level surveys will be presented for each of three direct services: case management, personal care, and homemaker. Findings revealed differences across regions in the state, and highlighted the frustration experienced by HCBS clients with high worker turnover and low training for special conditions, such as Alzheimer's or related dementia. Complaints and suggestions collected from clients and caregivers were shared with program managers for consideration in changes to policies, training, and other areas of service improvement toward becoming more person-centered. Overall, this service-oriented approach to surveying has yielded more actionable results and has been adopted by DOEA as the preferred method for all client-level surveying.

### STORIES OF THE EXPERIENCE OF SEARCHING FOR LONG-TERM CARE AND ELDERCARE: CASE STUDY AND NARRATIVE PERSPECTIVES

Connie Corley,[1] and Ryan McCarty,[2] *1. Fielding Graduate University, San Gabriel, California, United States, 2. Aging Simplified, Royse City, Texas, United States*

With over 40 million individuals aged 65 years and older in the US, and by 2050 rising to an estimated 89 million, age matters, driving an increased need for long-term care/eldercare. Coupled with the higher costs of care, the search for long-term care/eldercare services can be a difficult prospect for adult children decision-makers. We present the experiences of adult children decision-makers in the US, using two methodological approaches: narrative and case study using autoethnography. In a narrative inquiry of 9 caregivers responsible for making long-term care/eldercare decisions for their parent(s) the zoom model was applied to conduct the analysis. Findings suggest that decision-makers have a strong sense of duty towards helping their loved ones find long-term care solutions. Decision-makers searched for many types of care solutions ranging from home health care to nursing homes. The experiential response most consistently stated by the participants was stress. These results are augmented by an autoethnographic case study in "six acts" illustrating how sense of agency in the caregiving journey can be enhanced. Participants with industry experience had a minimal advantage over those with no experience when it came to navigating the search for long-term/eldercare. We highlight why stories of family search for long-term care/eldercare matter, and how they can be leveraged for fundraising, advocacy, and healing. Implications for policy, research, education and practice are highlighted.

### THE DIFFERENTIAL EFFECTS OF CAREGIVING INTENSITY ON OVERNIGHT HOSPITALIZATION IN THE PREVIOUS 2 YEARS

Kylie Meyer,[1] Zachary Gassoumis,[2] and Kathleen Wilber,[2] *1. UT Health Sciences at San Antonio, San Antonio, Texas, United States, 2. University of Southern California, Los Angeles, California, United States*

Caregiving for a spouse is considered a major stressor many Americans will encounter during their lifetimes. Although most studies indicate caregiving is associated with experiencing diminished health outcomes, little is known about how this role affects caregivers' use of acute health services. To understand how spousal caregiving affects the use of acute health services, we use data from the Health and Retirement Study. We apply fixed effects (FE) logistic regression models to examine odds of experiencing an overnight hospitalization in the previous two years according to caregiving status, intensity, and changes in caregiving status and intensity. Models controlled for caregiver gender, age, race, ethnicity, educational attainment, health insurance status, the number of household residents, and self-assessed health. Overall, caregivers were no more likely to experience an overnight hospitalization compared to non-caregivers (OR 0.92; CI 0.84 to 1.00; p-value=0.057). However, effects varied according to the intensity of caregiving and the time spent in this role. Compared to non-caregivers, for example, spouses who provided care to someone with no need for assistance with activities of daily living had lower odds of experiencing a hospitalization (OR 0.77; CI 0.66 to 0.89). In contrast, caregivers who provided care to someone with dementia for 4 to <6 years had 3.29 times the odds of experiencing an overnight hospitalization (CI 1.04 to 10.38; p-value=0.042). Findings indicate that, although caregivers overall appear to use acute health services about as much as non-caregivers, large differences exist between caregivers. Results emphasize the importance of recognizing diversity within caregiving experiences.

### THE PATH TO CAREGIVING: ASSESSING CAREGIVERS AND DEVELOPING A CAREGIVER PLAN OF CARE IN THE ACUTE CARE SETTING

Michelle Camicia,[1] and Barbara Lutz,[2] *1. Kaiser Permanente, Novato, California, United States, 2. University of North Carolina-Wilmington, Wilmington, North Carolina, United States*

Family caregivers of older adults report lack of preparation for their role, particularly upon acute hospital discharge following a medical event. Addressing the needs of family caregivers in the acute care setting prior to hospital discharge requires the identification of the caregiver, an assessment of caregiver preparedness, and a plan of care to address gaps in preparedness. The Preparedness Assessment for the Transition Home 7-item (PATH-7) is a valid and reliable instrument developed to assess family caregivers readiness for the caregiving role during acute care. The PATH-7 paper-pencil self-administered assessment was implemented in clinical care in medical-surgical nursing units in 2 acute care hospitals. Interventions to address gaps in preparedness were selected from a catalogue of interventions to develop a caregiver plan of care. The most frequent challenge identified by family caregivers was fulfilling the caregiving role on top of their other roles and responsibilities. This illustrated the need to assist family caregivers with exploring options for recruiting others to help with their roles and responsibilities and identify solutions soliciting and organizing help. This novel program promotes addressing the needs of the family unit, moving to a family-integrated are delivery model. Implementation challenges included in-person contact with caregiver to administer assessment, resources to respond to identified gaps in readiness, and lack of technology-enabled assessment administration. Positive staff experience with identifying and addressing needs of caregivers was a facilitator of staff engagement. Identifying, assessing, and addressing the needs of family caregivers of older adults is feasible in the acute care setting.

### SESSION 2859 (POSTER)

### HEALTH CARE, PROMOTION, AND SOCIAL SERVICE DELIVERY

### BARRIERS AND CHALLENGES FACED BY SOCIAL WORKERS CARING FOR DEMENTIA PATIENTS IN ACUTE CARE SETTINGS

Ruth Dunkle,[1] Katherine Cavagnini,[1] Joonyoung Cho,[1] Laura Sutherland,[2] Helen Kales,[3] Cathleen Connell,[1] and Amanda Leggett,[4] *1. University of Michigan, Ann*

*Arbor, Michigan, United States, 2. Wayne State University, Detroit, Michigan, United States, 3. UC Davis, Sacramento, California, United States, 4. The University of Michigan, Ypsilanti, Michigan, United States*

The nature of dementia care provided by social workers across various hospital settings is unexplored. This study utilized the "rigorous and accelerated data reduction" (RADaR) qualitative analysis technique to explore the process of care among social workers for persons with dementia (PWDs) across a Midwestern tertiary care system with two aims: 1) to identify environmental barriers and supports to quality dementia care in two hospital settings (medical and psychiatric emergency departments (ED), and the main inpatient hospital (IP)), and 2) to identify existing strengths and challenges to high quality social work dementia care within these settings. Twelve qualitative interviews were conducted with a purposive, snowball sample of social workers in dementia care in a large, academic health care system in 2016. Results identify environmental barriers in both settings (physical space design, patient-environment interactions, safety, and discharge disposition). Environmental aspects that promote quality care include supportive staff and family in the patient environment in the IP and ED hospital sections while the discharge disposition is more relevant in the IP. While there are some areas of social work involvement (discharge, psycho-social needs, treatment/management issues) that promote quality of care across locales, the pattern of performing roles varied, e.g. there is more focus on discharge planning and less management of competing demands in the IP than in the ED. Also, social workers were more involved in the diagnosis of dementia in the ED than other settings. We offer policy and practice recommendations to improve care for PWDs in academic hospital settings.

### DEMENTIA SEVERITY IS ASSOCIATED WITH EARLY POTENTIALLY AVOIDABLE READMISSIONS IN AN ACUTE CARE HOSPITAL

Seigo Mitsutake,[1] Tatsuro Ishizaki,[1] Rumiko Tsuchiya-Ito,[2] Akira Hatakeyama,[3] Mika Sugiyama,[4] Ko Furuta,[5] Kenji Toba,[6] and Hideo Ito,[5] *1. Tokyo Metropolitan Institute of Gerontology, Itabashi-ku, Tokyo, Japan, 2. Dia foundation for research on Ageing Societies, Tokyo, Japan, 3. Tokyo Metropolitan Geriatric Hospital, Itabashi-ku, Japan, 4. Tokyo Metropolitan Institute of Gerontology, Tokyo, Tokyo, Japan, 5. Tokyo Metropolitan Geriatric Hospital, Itabashi-ku, Tokyo, Japan, 6. Tokyo Metropolitan Geriatric Hospital and Institute of Gerontology, Itabashi-ku, Tokyo, Japan*

Understanding the association of dementia severity with early potentially avoidable readmissions (PAR) could encourage the identification of the target patients for the health care providers to provide transitional care (i.e. follow-up and coordination care) to prevent early readmissions. This study examined whether dementia severity before admission was associated with PAR within 90 days (90-day PAR). This retrospective cohort study was conducted using a Diagnosis Procedure Combination database linked with routinely collected dementia assessment data from a large acute general hospital in Tokyo, Japan. Patients aged 65 or older who were discharged to home or facilities (n=8,910; mean age: 79.8 years, standard deviation: 7.4 years) between July 2016 and September 2018. The dementia severity was classified as normal, slight, moderate, severe dementia based on the Dementia Assessment Sheet for Community-based Integrated Care System 21-items (DASC-21) from the patient or their family at admission. We conducted a multivariable logistic regression adjusted for covariates (sex, age, insurance copayment rate, diagnosis at admission, Charlson Comorbidity Index, unscheduled admission, ICU utilization, surgical treatment, length of hospital stay, discharge place) to examine the association of severity of dementia with 90-day PAR. Among the patients, 225 (2.5%) experienced 90-day PAR. The adjusted odds of 90-day PAR among patients with moderate dementia were 1.571 times (95% confidence interval [CI]: 1.102-2.240) and patients with severe dementia were 2.386 times (95% CI: 1.294-4.398) higher than the odds among patients without dementia. Patients with moderate and severe dementia before admission would be the target with high priority for providing transitional care.

### DISPARITIES IN COMMUNITY IMMUNIZATION RATES IN NEW ENGLAND: FINDINGS FROM THE HEALTHY AGING DATA REPORTS

Alison Rataj,[1] Nina Silverstein,[2] Chae Man Lee,[3] Haowei Wang,[4] Richard Chunga,[4] Taylor Jansen,[4] Beth Dugan,[4] and Shuangshuang Wang,[5] *1. University of Massachusetts Boston, Kingston, New Hampshire, United States, 2. University of Massachusetts Boston, Needham, Massachusetts, United States, 3. University of Massachusetts Boston, Boston, United States, 4. University of Massachusetts Boston, Boston, Massachusetts, United States, 5. Shandong University, Jinan, Shandong, China*

Vaccinations are effective preventive tools yet are underutilized in the older adult population. The purpose of this study was to describe community rates of three vaccines (flu, pneumonia, and shingles) in MA, NH, and RI. State and community rates were compared to identify disparities. Data sources were the CMS Medicare Current Beneficiary Summary file (2013-2015) for the vaccine rates and the American Community Survey (2012-2016) for population characteristics. Small area estimation techniques were used to calculate age-sex adjusted community rates in each state and are reported at the community level and statewide. Results showed comparable rates of flu immunization (MA 60.8%; RI 59.1%; NH 59.3%). NH had the best statewide rate of pneumonia immunization (77.8%) and MA had the worst (72.0%). There was variation in shingles vaccination rate: 39.7% in MA and 30.3% in RI, perhaps reflecting differences in access. Within state disparities were observed. MA shingles vaccine rates ranged 57.80% - 21.17%, pneumonia ranged 79.78% - 61.21%, and flu ranged 71.09% - 51.46%. In RI shingles vaccination rates ranged 42.1% - 21.1%, pneumonia ranged 82.1% - 61.8% and flu ranged 68.4% - 52.1%. In NH pneumonia rates ranged 84.18% - 71.87% and flu ranged 67.14% - 52.11%. Strategies to address within and between state disparities are needed. Greater awareness of the benefits of preventive measures like vaccines may also help improve rates. Materials like the GSA guideline about aging and immunizations could be useful in educating providers and policymakers. This research is funded by the Tufts Health Plan Foundation.

### ECHO-CT: AN INTERDISCIPLINARY VIDEO-CONFERENCE MODEL FOR IDENTIFYING POST-DISCHARGE TRANSITION-OF-CARE EVENTS

Mariana Gonzalez,[1] Lauren Junge-maughan,[2] Lewis Lipsitz,[3] and Amber Moore,[4] *1. University of Pennsylvania, Philadelphia, Pennsylvania, United States, 2. Beth Israel Deaconess Medical Center, Boston, Massachusetts, United States, 3. Hebrew SeniorLife and Harvard Medical School, Boston, Massachusetts, United States, 4. Massachusetts General Hospital, Boston, Massachusetts, United States*

Introduction: Discharge from the hospital to a post-acute care setting can be complex and potentially dangerous, with opportunities for errors and lapses in communication between providers. Data collected through the Extension for Community Health Outcomes-Care Transitions (ECHO-CT) model were used to identify and classify transitional care events (TCEs.) Methods: The ECHO-CT model employs multidisciplinary teleconferences between a hospital-based team and providers in post-acute settings; during this conference, concerns arising in the patient's care transition were identified and recorded. Results: 675 patients were discussed during interdisciplinary videoconferences. A total of 139 TCEs were identified; 52 (37.4%) were classified as medication issues, and 58 (41.7%) involved discharge communication or coordination errors. Conclusions: These identified TCEs highlight areas in which providers can work to reduce issues arising in the course of discharge to post-acute facilities. Standardized processes to identify, record, and report transition of care events are necessary to provide high-quality, safe care for patients as they move across care settings.

### EXTRA HEALTH AND HEALTH-RELATED COSTS OF LIVING WITH FRAILTY AMONG RURAL AND URBAN ELDERS IN CHINA

Yalu Zhang,[1] and Ada Mui,[2] *1. Columbia University, Cliffside Park, New York, United States, 2. Columbia University, NEW YORK, New York, United States*

Growing attention has been focused on how to improve the affordability and accessibility of healthcare services, especially for elders (aged 55 and above) who have higher levels of medical needs. Following the standard of living approach, which assumes that people's standard of living would be negatively affected if additional needs (i.e., healthcare) arise at a given level of household income, this secondary research examines elders' extra health and health-related costs of having chronic diseases and disabilities in rural (n=5,509) and urban (n=3,225) areas of China. Bivariate analyses show there were no significant differences between rural and urban groups in terms of the prevalence of having one or more chronic diseases (56% vs. 58%) and at least one type of disability (15% vs. 13%). Multivariate analyses indicate that living with chronic diseases incurred more extra costs for rural elders than their urban peers, after controlling for individual and household characteristics. On average, rural elders who had at least three chronic medical conditions would spend 108.3% more on medical services than those who had no chronic disease; elders with at least two types of disabilities would spend 59.8% more than those with no disability. The extra health-related costs were boosted when people had at least one type of disability (63.6%), but this was not the case for those who had chronic diseases. Statistical significance was not found among urban elders in China regarding both health and health-related expenditures. The results suggest that rural elders need support to manage their chronic health conditions.

### HEALTH, QUALITY OF LIFE, AND ECONOMIC IMPACTS OF HOME CARE VOUCHERS FOR MIDDLE-INCOME ADULTS WITH DISABILITIES

Joanne Spetz, Jacqueline Miller, Connie Kwong, and Laura Wagner, *University of California, San Francisco, San Francisco, California, United States*

The Support at Home pilot program provided financial support for the purchase of home care services by middle-income adults with disabilities in San Francisco to support aging in place. Enrollees had income below the area median and made copayments based on household income. The mixed-methods evaluation of the program incorporated administrative records, surveys of clients and comparison group members, surveys of unpaid caregivers, surveys of paid care providers, and focus groups with clients and unpaid caregivers. Outcome measures included the Older People's Quality of Life Questionnaire, Patient Health Questionnaire-2, an adapted Burden Scale for Family Caregivers, and self-reported falls, emergency department visits, and hospitalizations. Analyses included pre-post chi-squared and t-test comparisons between client and comparison groups and multivariate regressions. An economic analysis was conducted to learn whether changes in costs associated with reduced health care utilization were greater than the costs of the program. Results indicated statistically significant positive changes in client ratings of personal and financial stress, but not in the composite quality of life score. There were statistically significant reductions in attendance at medical appointments, falls, emergency department visits, and hospitalizations. Similar changes were not found in the comparison group. The focus group data supported the findings regarding personal and financial stress and indicated that clients and their caregivers perceived quality of life benefits. The economic analysis indicated substantial cost savings from the program due to reduced use of medical services. Due to its positive impacts, San Francisco has made Support at Home a permanent program.

### HEALTHCARE EXPERIENCES OF LGBTQ+ ELDERS: TWO FOCUS GROUPS

Kierra Foley,[1] Nancy Hodgson,[2] and José Bauermeister,[1] *1. School of Nursing, University of Pennsylvania, Philadelphia, Pennsylvania, United States, 2. University of Pennsylvania, Cherry Hill, New Jersey, United States*

LGBTQ+ elders experience significantly higher rates of disability, poor general health, and behavioral health problems than heterosexual and cisgender older adults; additionally, LGBTQ+ elders are less likely to seek medical attention or have a primary care provider than other older adults. In previous qualitative studies, LGBTQ+ elders have reported difficulties navigating healthcare systems due to provider incompetence in treating this population, financial barriers, systemic homophobia/transphobia, and high disease burdens. However, few qualitative studies have been conducted with this population from a nursing perspective, and little is known about the role of nurses in the health and wellness of LGBTQ+ elders. Nurses are uniquely positioned to help address both the physiological and psychosocial needs of older LGBTQ+ adults, and garnering an understanding of these needs directly from this community can help better direct future research. Accordingly, two focus groups were conducted with LGBTQ+ elders at different queer community centers in Philadelphia by a nurse scientist. This community engaged research aims to learn more about how nurses can serve this population through identifying their priorities. Through thematic analysis, it was found that LGBTQ+ elders reported being concerned about housing access, discrimination from healthcare providers, and challenges in accessing mental health care. Additionally, the researchers' hypotheses were triangulated with the participants' responses to demonstrate the utility of continued community engagement in addressing specific population needs.

### LEVERAGING NOVEL LABEL DESIGN TO IMPROVE OLDER CONSUMERS' USE OF MEDICATIONS

Alyssa Harben, Andrew Nathan, Kaitlin Anderson, Deborah Kashy, Laura Bix, and Mark Becker, *Michigan State University, East Lansing, Michigan, United States*

Over-the-counter (OTC) medication is a convenient, affordable way for older adults to treat a variety of conditions; older consumers use more OTCs per capita than other people for varied reasons. As with any medication, OTCs carry risks. Because the labels of OTCs are frequently the only information accessed by consumers, labels designed to communicate safety information are paramount for these products. Yet, available studies suggest that consumers do not regularly access the comprehensive information in the Drug Facts Label (DFL) when making purchase or use decisions, tending to rely on information found on the front of the package. Herein, we evaluate how four OTC label formats (standard; standard/highlight; critical warnings on front/highlighted; critical warnings on front not highlighted) affect how aging participants attend to critical information. Sixty-eight participants (65+) engaged a computer-based task answering yes/no questions that required use of labeling information about the warnings or active ingredients (AI). Results indicate that AI information is found accurately and relatively quickly compared to warning information and highlighting and placement on the front of the package had little effect. By contrast, warning information was found slowly and with low accuracy with the standard label, and highlighting and front of pack placement both significantly improved performance. These results suggest that novel labelling strategies could result in more effective, safer use of OTC medicines among older consumers and provide insights that could be used by regulators as they work to improve policy focused on improving mandates for OTC labeling.

### LONGITUDINAL TRENDS AND PREDICTORS OF MEDICAID UTILIZATION AMONG MIDDLE-AGED AND OLDER ADULTS

Jane Tavares,[1] and Marc Cohen,[2] *1. University of Massachusetts Boston, Florence, Massachusetts, United States, 2. University of Massachusetts Boston, Boston, Massachusetts, United States*

Over fifteen million older adults in the United States rely on the means-tested Medicaid program for healthcare coverage (accounting for approximately 20% of total Medicaid beneficiaries according to the Centers for Medicare and Medicaid Services). With the older adult population growing exponentially, there has been concern that steadily rising Medicaid spending will skyrocket among this group and that there may be a need to reconfigure coverage of the program. However, few studies have longitudinally examined which factors are related to utilization of the program over time among older adults in order to better understand how any future coverage changes might impact this group. This study used the 1998 to 2014 waves of the Health and Retirement Study (N=8,162) to analyze a representative sample of those 50 and older regarding demographic, health, and economic trends associated with Medicaid utilization over a sixteen-year period. Descriptive analyses showed stable longitudinal patterns such that those who utilized Medicaid had significantly poorer health and fewer financial resources compared to those who never accessed Medicaid. Multivariate analyses further revealed those who were older, female, minority race/ethnicity, less educated, in poorer health, below the federal poverty line, and with lower net wealth had a higher proportional risk of utilizing Medicaid during the observed time period than their counterparts. The findings from this study highlight the importance of monitoring changes in the documented risk factors over time in terms of their impact on Medicaid utilization and underscore the need to consider how these factors may be interrelated.

### NARRATIVE GROUP INTERVENTIONS TO REDISCOVER LIFE WISDOM AMONG HONG KONG CHINESE OLDER ADULTS: A WAITLIST RCT STUDY

Esther Chow,[1] and Sai-fu FUNG,[2] *1. City University of Hong Kong, Kowloon, China, 2. City University of Hong Kong, Kowloon Tong, China*

Objectives: To recognize, and rejuvenate the life wisdom of Hong Kong Chinese older adults, a new strength- and meaning-based Narrative Therapy (NT) in practice is developed, with two objectives: to examine its effectiveness in enhancing wisdom; and to test the longer-term effects at 2 and 8 months respectively. Method: A waitlist randomised controlled trial (RCT) design was used. A total of 157 older adults were recruited, 82 of which was randomly assigned to 12 intervention groups to receive four 2-hour NT sessions using the 'Tree of Life' metaphor, to assess perceived wisdom at baseline (T1), at the end of treatment (T2), and at two (T3) and 8 months after treatment (T4). Results: Participants in the NT group showed significant improvements in the wisdom outcome measure [$F(2.726, p = 0.041)$]. As such, the results of latent growth curve models with time-invariant covariates for impact of NT on wisdom scores suggested significant effects two months after treatment (T3) with controlled the effects of age, gender and educational level [$TML(11) = 17.306$, $p = 0.098$, $RMSEA = 0.079$, $CFI = 0.960$]. Most improvements were sustained at 2- and 8-months post intervention. No adverse reaction was recorded in any of the cases mentioned at all study sites. Conclusion: The findings have significant theoretical contributions for professional social work practice to ground a new theory in understanding wisdom in older adulthood, develop a new clinical practice that appreciate and celebrate life wisdom, and a practice guide to be disseminated among health and social care practitioners.

### OLDER AFRICAN AMERICANS' EXPERIENCE OF THE OPIOID CRISIS

Sharon Rainer, *Thomas Jefferson University, Moorestown, New Jersey, United States*

In 2017, over 1200 Philadelphians died from opioid overdoses and an estimated 50,000 -70,000 people in the city are addicted to opioids. The opioid crisis is a family crisis that touches all communities affecting those using opioids, their family members, and the community. As policymakers develop responses to the opioid crisis, a multigenerational perspective is critical. With much published on the opioid crisis, there remains little understanding of older adult's perceptions or experiences to this public health emergency. Older adults bring a unique voice. They are grandparents, parents, opioid users, and people living with chronic pain and/or addictions. Center in the Park (CIP), a nationally accredited senior center in Philadelphia, Pennsylvania sought to understand older African Americans' perceptions of the opioid crisis and how their experiences inform senior center programming. CIP leadership sought a community partnership with Thomas Jefferson University College of Nursing to respond to what some in their community called "a domino effect" of the opioid crisis. Using a Community-Based Participatory Research Model rooted in humanistic research theory, a qualitative study was designed. Three focus groups (n29) were convened. All focus group recordings were transcribed and checked for accuracy. An iterative axial coding process was used. All transcripts were coded using the qualitative software program NVivo12. Findings suggest older adults are concerned about younger generations and addictions. In addition, older adults lack knowledge of the use and safety of opioids and have a general mistrust of the medical community to adequately limit the opioid epidemic.

### ORAL HEALTH DISPARITIES AMONG CHINESE OLDER ADULTS: EVIDENCE FROM CHARLS

Chengming Han, *Case Western Reserve University, Cleveland, Ohio, United States*

This paper explored the effect of the type of health insurance on dentist visits among older adults in China. The data were drawn from the CHARLS-II (2013). The sample included older adults aged 60 and older (N= 6767, n(urban)=3272, n(rural)=3495). Multivariate logistic regression models indicated that in urban and rural places, respondents with a governmental/civil servants' insurance and those with an urban-employee insurance are more likely to visit a dentist in the survey year. Household registration status (hukou) does not play a significant role in dentist visits when insurance types are adjusted for. In other words, employment status, and the coverage of health insurance presented more significant effects on dentist visits. Differing from previous studies about urban-rural health disparities, this study disclosed substantial institutional influences on dental care access among older adults.

### OUTPATIENT AND INPATIENT SERVICE USE BY CHINESE ADULTS LIVING IN RURAL LOW-INCOME HOUSEHOLDS

Peiyi Lu,[1] JUN YAO,[2] Chunyu Yang,[3] Mack Shelley,[1] and Li zhang,[4] *1. Iowa State University, Ames, Iowa, United States, 2. NANJING MEDICAL UNIVERSITY, NANJING, Jiangsu, China, 3. Nanjing University of Information Science and Technology, NANJING, Jiangsu, China, 4. Nanjing University of Chinese Medicine, NANJING, Jiangsu, China*

Background: This study applied the Andersen Model of Health Care Utilization to explore the variables associated with health service use among Chinese adults living in rural low-income households. Method: A survey of 2,429 adults living in 787 low-income households in Jiangsu, China was conducted. Respondents were asked the presence of outpatient service in the past one month and the amount of hospitalization in the past one year. Mixed effect logistic and negative binomial models were used to examine the relationship of individual-level and household-level characteristics with health service use. Results: Health condition was the predominant determinant of both outpatient and inpatient service use (Odds Ratio [OR] >1, p<0.001). Individuals living in a poor household were less likely to use outpatient service (OR=0.05, 95% confidence interval CI: 0.00, 0.71), and the longer in poverty status the less likely to use outpatient service (OR=0.92, CI: 0.86, 0.99). Age was associated with lower likelihood to use outpatient service (OR=0.93, CI: 0.93, 1.00), and this relationship was stronger for larger households (OR=1.01, CI: 1.00, 1.01). For inpatient service use, most household-level measures were insignificant. Conclusion: Rural Chinese health service use was influenced primarily by needs variables. Outpatient service use was constrained by household enabling variables. Older adults were at a disadvantage for using outpatient service when the family prioritized younger members in allocating resources. These results suggest the need for policy advocacy to expand insurance reimbursement and improve benefits for poor older adults.

### PHYSICAL IMPAIRMENT, INSURANCE COVERAGE, AND HEALTHCARE UTILIZATION AMONG U.S. CHINESE OLDER ADULTS

Jonas Attilus,[1] Mengting Li,[1] Qun Le,[2] and XinQi Dong,[1] *1. Rutgers University, New Brunswick, New Jersey, United States, 2. Rutgers University, New Brunswick,, New Jersey, United States*

The relationship between physical impairment and healthcare utilization is well studied. However, few studies examined this relationship among immigrant older adults whose health insurance status may represent a barrier to healthcare use. This study aims to examine the relationship between physical impairment, health insurance, and types of healthcare utilization. The PINE Study provided data of 3,157 Chinese older adults age 60 and over. Most (70.67%) of them had insurance. Physical function was assessed by Activities of Daily Living and Instrumental Activities of Daily Living. Healthcare utilization was evaluated by the times of physician visit (PV), ER, and hospitalization, separately, in the past two years. Logistic regression was used. After adjusting for covariates, among the insured patients, every one unit increase in ADL impairment was associated with higher odds of ER visit (OR:1.32 [95%CI 1.21-1.45]) and hospitalization (OR: 1.37, [95%CI 1.25-1.50]). Every one greater IADL impairment was associated with higher odds of PV (OR: 1.26, [95%CI 1.12-1.43]), ER visit (OR: 1.21, [95%CI 1.16-1.26]), and hospitalization (OR: 1.23, [95%CI 1.18-1.28]). Among the non-insured, every one unit increase in ADL impairment was associated with higher odds of ER visit (OR: 1.82, [95%CI 1.19-2.78]) and hospitalization (OR: 3.05, [95%CI 1.51-6.16]). Every one unit increase in IADL impairment was associated with higher odds of PV (OR: 1.24, [95% CI 1.09-1.42]), ER visit (OR: 1.33, [95% CI 1.17-1.52]), and hospitalization (OR: 1.53, [95%CI 1.32-1.76]). These findings highlight disparities in healthcare utilization. Longitudinal studies are needed to strengthen causality between physical impairment, health insurance, and healthcare utilization.

### RACIAL AND ETHNIC DIFFERENCES IN PERCEPTIONS OF, AND BARRIERS TO, COST-OF-CARE CONVERSATIONS AMONG OLDER ADULTS IN FLORIDA

Kyaien Conner,[1] Jacqueline Wiltshire,[1] Erica Anderson,[1] Edlin Garcia Colato,[2] Iraida Carrion,[1] and Barbara Orban,[3] *1. University of South Florida, Tampa, Florida, United States, 2. University of South Florida, Garden Grove, California, United States, 3. University of South Florida, Tampa, United States*

Cost of care conversations (CoC) between patients and doctors have been shown to lower overall healthcare and patients' costs. How, it is unclear why CoC are not occurring more frequently among high cost patients such as older adults. To address this important question, we conducted three race-stratified focus groups (n=10 Whites, n=9 African Americans, and n=8 Latino/Hispanics) to assess perceptions about, and barriers to, CoC in a convenient sample of adults ages ≥65 from Adult Centers in Tampa Bay, Florida. An inductive content analysis approach was utilized by research team members to analyze qualitative data. Findings indicated that CoC are not occurring. White participants perceived that CoC were not occurring because they did not have issues paying for care. African Americans perceived that CoC were not occurring because doctors are not trained to understand finances, insurance, and medical billing. Latinos/Hispanics perceived that doctors are meant to take care of patients, and receptionists, administrators and billing departments should handle CoC. Wait time and perceived stress/rush of doctors were identified as CoC barriers for whites, while doctors' attitude was a barrier for Blacks/African Americans, and perceptions about CoC being "taboo" was a major barrier for Latinos/Hispanics. Overall, participants indicate that it is easier to have CoC if they had developed a good rapport with the doctor, had confidence in the doctor, and felt the doctor was interested in and cared about them. The findings suggest that promoting CoC among older adults will require addressing social and cultural concerns of racial/ethnic minority groups.

### SUPPORTING THE TRANSITION FROM HOSPITAL TO HOME FOR OLDER ADULTS: CASE STUDY RESULTS

Lori Weeks,[1] Brittany Barber,[1] Eileigh Storey MacDougall,[1] Elaine Moody,[1] Lexi Steeves-Dorey,[2] Grace Warner,[1] Marilyn Macdonald,[1] and Ruth Martin Misener,[1]
*1. Dalhousie University, Halifax, Nova Scotia, Canada, 2. Nova Scotia Health Authority, Halifax, Nova Scotia, Canada*

It is often during transitions between health services that many issues arise for older adults, such as care that is poorly coordinated, additional burden placed on family and friend caregivers, and inappropriate placement in nursing homes. The Home Again program delivered by the provincial health authority in Nova Scotia, Canada, provides transitional care through providing additional support beyond what is normally provided through home care services to help older adults transition home after a hospital admission. The purpose of this research was to identify what factors contribute to older adults being placed unnecessarily in a nursing home when they could receive care through the Home Again program. Through using a retrospective multiple case study design, we analyzed interviews for five cases including older adult patients, their family or friend caregivers, and healthcare professionals in each case. Results indicate all hospitalized patients experienced a major health event or rapidly declining health. All family and friend caregivers experienced burnout and frustration from the lack of sufficient home care supports and quality of services available, such as services provided throughout the night. Healthcare professionals discussed that patients were placed on a waiting list for nursing homes due to lack of home care supports and resources for caregivers. This study contributes to our knowledge about better processes to ensure that hospitalized older adults are not unnecessarily admitted to nursing homes which can result in reduced healthcare costs and improved delivery and quality of care to older adults and their family and friend caregivers.

### THE EFFECTS OF ORGANIZATIONAL-LEVEL FACTORS ON THE NATIONAL DISABILITY PREVENTION PROGRAMS FOR OLDER ADULTS

Ya-Mei Chen,[1] Wan-Yu Chiu,[1] Hui-Fen Mao,[1] and Ling-Hui Chang,[2] *1. National Taiwan University, Taipei, Taiwan (Republic of China), 2. National Cheng Kung University, Tainan, Taiwan (Republic of China)*

In 2017, Taiwan implemented nationwide disability preventive programs that aimed to maintain older adults' function and prevent disability. This study aimed to evaluate the impact of organizational-level factors on older adults' improvement through participating in the 2019 preventive disability programs. There were 202 disability preventive programs, which included a 2-hour sessions per week for 12 weeks, in Taiwan in 2019. Participants' physical, mental, and social functions were assessed before and after participating the programs and reported to the government. A total of 7194 older adults' assessments were received. This study administered survey by the end of 2019 in the 202 programs in Taiwan and 8 organizational-level factors were assessed, including effectiveness, efficiency, professional, support from the upper organization, and cooperation with community, adequacy, responsiveness, and sustainability. Multilevel analysis was conducted to assess the impacts of organizational-level factors on disability prevention programs. A total of 170 programs (84%) has responded to our survey. About 48.2% of the programs were implemented by local public health centers. The domains of responsiveness and sustainability are rated highest among these programs. Programs with the following characteristics were associated with better program outcomes among older adults: better ability to provide outreach services was associated with improved depression; higher level in profession domain was associated with improved f mobility; better program responsiveness was associated with improved in nutritional status and oral function, and depression risk. Our results shed lights on how to improve a national disability prevention programs from programs' characteristics point of view.

### WHEN DO MEDICAL DOCTORS RECOMMEND LESS EXPENSIVE MEDICATION PRESCRIPTIONS IN U.S. ADULTS 50 YEARS OR OLDER?

Jose Aravena, *School of Public Health, Yale University, New Haven, Connecticut, United States*

The US is the country with the greatest average spending for prescription drugs per person (US$ 1,200). 85.0% of older people consume prescription medications, with high rates of polypharmacy. The aim was to analyze the factors related to the recommendation of a less expensive prescription by a medical doctor in US older adults. A cross-sectional analysis using the data from The National Poll on Healthy Aging (NPHA) 2017 was conducted, with a total sample of 1666 adults age 50 to 80 residing in the US. People were asked if they have received a less expensive prescription by a medical doctor in the last two years (yes/no). Sociodemographic and health variables, active patient medication-cost behaviors, and doctor active medication-costs actions were measured as covariates. Weighted and stratified by region logistic regression model was conducted in a 70% random sample. The model was validated in the 30% remaining using ROC curve and AUC. In the parsimonious model, ≥4 visits to the doctor (OR=2.06, 1.33 - 3.18), perception of medication costs as a burden (OR=1.76, 1.25 - 2.47), the doctor talked about medication costs (OR=5.54, 3.90 - 7.88), doctor awareness of medication costs (OR=1.81, 1.34 - 2.46), and being Non-Hispanic Black (OR=1.90, 1.20 - 3.03) were linked to a higher odd to receive a less expensive prescription. The model presented a moderate-high fit (AUC:0.71; sensitivity:84.4%, specificity:49.8%). Awareness and training in the active prescription of less expensive medications by the medical doctor seem fundamental to reduce drug costs burden in older adults.

### SESSION 2860 (POSTER)

### LONG TERM CARE I: POLICY AND ECONOMICS

### A COMPARISON OF HOME HEALTH CARE BETWEEN FINLAND AND SOUTH KOREA IN LONG-TERM CARE SERVICES

Jihyun Park,[1] and suhyun kim,[2] *1. Yonsei University, Seoul, Republic of Korea, 2. Kyungpook National University, Daegu, Republic of Korea*

Population aging is one of the significant global issues. Long-term care is emphasized as “aging in place,” and it is known that home and community-based service is a cost-effective way to achieve this. Over ten years have passed since the introduction of long-term care insurance in Korea, and it is necessary to improve home health care in long-term care. The aim of this study was to identify the measures that must be undertaken for enhancing home health care in Korea by comparing it to the home health care in Finland. The data were collected via a literature review, expert interview, and field survey in Kuopio, Eastern Finland, from March 16 to 23, 2018. Based on the comparison between Korean and Finnish home health care, some issues related to home health care in Korea that need to be resolved were identified: the complex process involved in availing home health care, low utilization rate, higher cost than home health aide services in long-term care, and undifferentiated roles in home health care between registered nurses and nurse assistants. Several strategies could be utilized to enhance home health care in Korea, such as a simplified procedure to use home health care, clarification of roles between registered nurses and nurse assistants in home care, supervision of the integration of home care services by registered nurses, and an expansion of home health care into comprehensive assessment and nursing activities for chronic illness care and health promotion.

### ALZHEIMER’S STAFFING, SERVICES, AND OUTCOMES IN ADULT DAY HEALTH CENTERS

Joanne Spetz,[1] and Jason Flatt,[2] *1. University of California, San Francisco, San Francisco, California, United States, 2. University of Nevada, Las Vegas, School of Public Health, Las Vegas, California, United States*

Growing demand for care for Alzheimer’s Disease and related Dementia (ADRD) has resulted in rising use of adult day health centers (ADHCs), which employ teams of professionals including licensed nurses, nursing aides, social workers, and activity directors. This study evaluates the scope of services and staffing models of ADHCs that provide care to persons with ADRD compared to ADHCs that do not, and examines whether there is an association between staffing and client outcomes, measured as rates of hospitalizations, falls, and emergency department visits. We used facility-level data from the 2014 National Study of Long-Term Care Providers (NSLTCP) Adult Day Services Center module. We conducted bivariate comparisons and estimated multivariate regressions to identify ADHC characteristics associated with staffing and client outcomes. ADHCs that offered ADRD services had higher average daily attendance, greater shares of revenue from Medicaid and self-payment, and greater proportions of Blacks and females. They also had greater percentages of enrollees with depression, cardiovascular disease, diabetes, and needing assistance with activities of daily living. There were also greater numbers of registered nurse, licensed practical nurse, and social worker hours per enrollee day, but fewer activity staff hours per enrollee day. Multivariate regressions focused on ADHCs that offered skilled nursing services and revealed that total staff hours per enrollee day were not higher in ADHCs that provided ADRD services, controlling for other characteristics. However, staffing was greater in chain-affiliated ADHCs. Higher staffing levels were associated with lower rates of falls and emergency department visits.

### ANALYZING NURSING HOME COMPLAINTS: FROM SUBSTANTIATED ALLEGATION TO DEFICIENCY CITATIONS

Kallol Kumar Bhattacharyya,[1] Lindsay Peterson,[1] John Bowblis,[2] and Kathryn Hyer,[1] *1. University of South Florida, Tampa, Florida, United States, 2. Miami University, Oxford, Ohio, United States*

Complaints provide important information to consumers about nursing homes (NHs). Complaints that are substantiated often lead to an investigation and potentially a deficiency citation. The purpose of this study is to understand the relationship between substantiated complaints and deficiency citations. Because a complaint may contain multiple allegations, and the data do not identify which allegation(s) lead to a complaint’s substantiation, we identified all substantiated single allegation complaints for NHs in 2017. Our data were drawn from federally collected NH complaint and inspection records. Among the 369 substantiated single-allegation complaints, we found most were categorized as quality of care (31.7%), resident abuse (17.3%), or resident neglect (14.1%). Of the deficiency citations resulting from complaints in our sample, 27.9% were categorized as quality of care and 19.5% were in the category of resident behavior and facility practices, which includes abuse and neglect. While two-thirds (N=239) of the substantiated complaints generated from 1 to 19 deficiency citations, nearly one third had no citations. Surprisingly, 28% of substantiated abuse and neglect allegations resulted in no deficiency citations. More surprisingly, a fifth of complaints that were categorized as "immediate jeopardy" at intake did not result in any deficiency citations. We also found a number of asymmetries in the allegation categories suggesting different processes by Centers for Medicare and Medicaid Services (CMS) region. These results suggest that the compliant investigation process warrants further investigation. Other policy and practice implications, including the need for better and more uniform investigation processes and staff training, will be discussed.

### AVAILABILITY OF AUDIOLOGY SERVICES IN ASSISTED LIVING COMMUNITIES IN FLORIDA

Daniel Pupo, Hillary Rouse, Lindsay Peterson, and Kathryn Hyer, *University of South Florida, Tampa, Florida, United States*

Florida has one of the largest populations of older adults in the U.S., and as a result the state also has a high prevalence of hearing loss. Given the growth of assisted living as a housing option for older adults, the purpose of this study was to determine the availability of audiology services in assisted living communities (ALCs) across Florida. Data on ALC location, characteristics and audiology service availability were collected from the Florida Agency for Health Care Administration (AHCA). County socioeconomic data were collected from the U.S. Department of Labor. Logistic regression and chi2 tests were used to examine the relationship between county socioeconomics and whether an ALC provided audiology services. We found that of the 3090 ALCs in Florida, audiology services were present in only 57 (3.2%). ALCs with audiology services were significantly more likely to be located in counties with a higher education level and a higher average income. This suggests a shortage of ALCs with audiology services in counties where residents have fewer resources. The results are concerning, given that individuals with fewer resources are less able to pay for audiology services on their own and evidence showing that poor hearing health late in life impacts individuals' health and quality of life. Policy implications will be discussed, including the need for more ALCs to provide audiology services in counties with fewer resources. One possible solution is teleaudiology, which would enable a single audiologist to diagnose and prescribe hearing aids to patients in underserved areas.

### CORRELATES OF SKILLED NURSING FACILITY (SNF) PERFORMANCE IN THE SNF VALUE-BASED PURCHASING PROGRAM

Dan Andersen,[1] Sherly Binu,[2] and Mike Sacca,[3] *1. RELI Group, Sykesville, Maryland, United States, 2. RELI Group, Baltimore, Maryland, United States, 3. RELI Group, Baltimore, United States*

We examined the results of the 2020 skilled nursing facility (SNF) value-based purchasing (SNF VBP) program to identify correlates and potential drivers of SNF performance in this program. The SNF VBP program provides incentive payments to SNFs based on their performance on a risk-adjusted hospital readmission measure (i.e., the rate at which SNF residents are admitted back to the hospital within 30-days of being admitted to the SNF). SNFs are assessed on this measure for both improvement compared to their historical baseline and overall achievement compared to their peers. All SNFs that are covered under Medicare's prospective payment system are included in the SNF VBP program. We performed analyses to assess the correlation between individual SNFs' performance in the 2020 SNF VBP (n=15,201), which is based on actual performance in fiscal year 2018, with contemporaneous matched data related to SNF health inspection results, staffing, and performance on quality measures (these data form the basis of the five-star quality rating system on the Nursing Home Compare website). We also examined longitudinal trends in these non-SNF VBP program variables and their association with changes in SNF performance in the SNF VBP program. We controlled for important SNF-specific factors (e.g., for-profit status, connected to a hospital). We found strong contemporaneous and longitudinal associations between SNF VBP program performance and some, but not all, of these factors. Our findings are supported by decades of empirical research in SNF quality and highlight potential policy alternatives that could further incentivize high quality care in SNFs.

### DEPRESCRIBING AND POLYPHARMACY FOR MEDICARE BENEFICIARIES UNDER GUARDIANSHIP IN LONG-TERM CARE FACILITIES

Tami Swenson, *Des Moines University, Des Moines, Iowa, United States*

Local judicial courts vary in the amount of supervision they provide guardians, which makes the practice of guardianship uneven. To begin to address the evidence gap to inform best practices for persons under guardianship care, this study examines the issues of polypharmacy and prescribing patterns for four therapeutic classes most commonly targeted for deprescribing for older adults. The Medicare Current Beneficiary Survey (MCBS) for 2015 and 2016 is used to examine facility-dwelling Medicare beneficiaries under guardianship compared with those that are not. Logistic regression is used to examine association of polypharmacy outcomes and guardianship care controlling for patient and facility characteristics. Statistical models are adjusted using Fay's Method with replicate weights for the MCBS complex survey design. Approximately 12% of the facility-dwelling Medicare population in 2015 and 2016 are persons under guardianship care. Persons under guardianship were more likely to have polypharmacy or to be prescribed 5 or more medications (Odd Ratio (OR)=1.168, 95% Confidence Interval (CI)=1.156 to 1.180, p<0.001) than facility-dwelling Medicare beneficiaries not under guardianship care. Medicare beneficiaries under guardianship were more likely to be prescribed PPIs (OR=1.229, 95% CI=1.222 to 1.237, p<0.001) or antipsychotic medications (OR=1.240, 95% CI=1.232 to 1.247, p<0.001) but less likely to be prescribed benzodiazepines (OR=0.920, 95% CI=0.913 to 0.927, p<0.001) or antihyperglycemics (OR-0.726, 95% CI=0.721 to 0.731, p<0.001). Medical decision support services, such as guardianship care, are increasing in importance as shared decision making between patients and physicians evolves to address polypharmacy and deprescribing for older adults.

### DOES LONG-TERM CARE SUBSTITUTE FOR HOSPITAL CARE? EVIDENCE FROM THE CHINA HEALTH AND RETIREMENT LONGITUDINAL SURVEY

Wei Yang, *King's College London, London, United Kingdom*

Publicly funded long-term care (LTC) support is shrinking in many countries despite continuing increases in the number of older people who need care. Evidence has shown that the LTC services have an impact on the efficient use of the resources in the health care sector by reducing rates of admission and associated costs through assisting older people with daily living. This paper seeks to examine whether and to what extent these services are substitutes. We use a fixed-effect instrumental variable GMM model to predict the effect of long-term care services on the utilisation of outpatient and inpatient care services. Data are drawn from China Health and Retirement Longitudinal Survey 2011, 2013 and 2015. Our findings suggest that LTC significantly reduces the use of outpatient care but not inpatient care. We have also found LTC use is concentrated among the rich, but the substitution effects are stronger among the poor compared to the rich. This indicates that the poor would benefit more from subsided LTC services. We urge the Chinese government to take action to develop its formal LTC system and to channel more resources to its LTC system, which will benefit the older population in general, and the poor in particular.

### EFFECTS OF NURSE STAFFING, WORK ENVIRONMENT, EDUCATION ON ADVERSE EVENTS IN NURSING HOMES

Seonhwa Choi,[1] Eunhee Cho,[1] Eunkyo Kim,[1] Kyoungeun Lee,[2] and Soo Jung Chang[3], *1. Yonsei University, Seoul, Republic of Korea,*
*2. Tongmyong University, Pusan, Republic of Korea,*
*3. Gangneung-Wonju National University, Wonju, Republic of Korea*

This study examined the effect of registered nurse (RN) staffing level, work environment, and education on adverse events experienced by residents in nursing homes. A cross-sectional study was conducted with 216 RNs working in nursing homes who were selected using random stratified sampling by location and bed size. Self-reported questionnaires regarding staffing level, work environment, education level, adverse events, and nurse characteristics were administered. Data from the National Health Insurance Service were used to describe nursing home characteristics. Both multiple and multinomial logistic regressions were used to control for the characteristics of nurses and nursing homes, and investigate the effects of nursing staffing level (number of older adults assigned to a nurse), work environment (Practice Environment Scale of the Nursing Working Index), and level of nursing education on the adverse events experienced by residents. An increase of one resident per RN was significantly associated with a higher incidence of pressure ulcers (OR= 1.019, 95% CI=1.004-1.035). Poor work environment increased the incidence of adverse events such as pressure ulcers (OR= 3.732, 95% CI=1.155-12.056) and sepsis (OR=3.871, 95%CI=1.086-13.800). Compared to RNs with a baccalaureate or higher, RNs with diplomas reported increased incidence rates of pressure ulcers (OR=2.772, 95%CI= 1.173-6.549). RN staffing, work environment, and education level affect the incidence of pressure ulcers, and the work environment affects the incidence of sepsis among residents in nursing homes. Policy-wise, improving the level of nurse staffing, nursing work environment, and nursing education will improve health outcomes of residents.

### EXPLORING THE COMPLEXITIES OF QUALITY MEASUREMENT IN ASSISTED LIVING IN WASHINGTON STATE

Carolyn Ham,[1] Cathy McAvoy,[2] Roger Gantz,[2] and Lindsay Schwartz,[3] *1. Washington State Department of Health, Olympia, Washington, United States, 2. Washington State Department of Social and Health Services, Olympia, Washington, United States, 3. American Health Care Association/ National Center for Assisted Living, Pittsboro, North Carolina, United States*

In Assisted Living (AL) choice and quality of life are highly valued by residents. Prior research has found many interrelated components contribute to AL residents' experiences of quality. There are 543 licensed AL facilities in Washington state, with 33,830 licensed beds; in 2019, resident cost per year ranged from $44,000 to $78,000. In 2018, the Washington Legislature identified a need for consumer access to unbiased information on AL quality and directed the Department of Social and Health Services (DSHS) to form a workgroup on quality measures. The workgroup consists of representatives from state agencies, AL provider associations, advocacy organizations, AL providers and AL residents. To inform the workgroup, DSHS conducted a study of AL quality measures. Five statewide programs were identified and analyzed in 10 categories. The WA workgroup then assessed a series of measures across six domains: community participation and quality of life; consumer satisfaction; equity, diversity and inclusivity; informed choice and decision making; person-centered planning; and, resident safety. The workgroup assessed and chose to adopt CoreQ, a satisfaction measure developed by the American Health Care Association/National Center for Assisted Living (AHCA/NCAL) and endorsed by the National Quality Forum (NQF). In evaluating potential measures of quality, workgroup members weighed regulatory, industry, provider, consumer and family perspectives. Outcomes of this process include assessment of AL quality measurement in other states, selected measures for Washington and key insights into relative prioritization of quality domains by different stakeholder types.

### LONG-TERM CARE MARKET TREND AND PATTERNS OF CAREGIVING IN THE UNITED STATES

Rashmita Basu, *East Carolina University, Greenville, North Carolina, United States*

Objectives: The current study aims to: 1) identify patterns of the use of long-term care services and supports (LTSS) among community-dwelling individuals; 2) examine if the changes in supply of formal care predict the use of informal care (IC). Methods: Linking the market supply of formal LTSS to individual level Health and Retirement Survey data from (N=7,781), descriptive and regression analysis were performed. Results: Supply of formal home and residential LTSS indicates a stronger upward trend. More than 90% of people used IC and 40% received both informal and formal help. People aged under 60 years, IC from spouse dominates but then falls sharply and an adult child becomes the primary source. More than 20% reported unmet needs. Regression analysis indicates that the formal home care supply significantly predicts the likelihood of using IC. But the rate and intensity of unpaid IC use among individuals aged 85 or older is low and that of paid formal care use are highest. We find that about 20% of care recipients experienced at least one unmet need with ADL assistance in our sample. The prevalence of an unmet need sharply decreases as individuals receive care from multiple caregivers (including paid professionals) but receiving care from too many caregivers contributes to higher unmet ADL needs. Discussion: The findings suggest opportunities to create a holistic system of care for people needing LTSS.

### LONG-TERM CARE NURSES' INTENT TO STAY, JOB CHARACTERISTICS, AND ORGANIZATIONAL JUSTICE : A STRUCTURAL EQUATION MODEL

LIN WANG,[1] Miao Miao,[1] and Xiaoping Yu,[2] *1. Shanghai Jiao Tong University, Shanghai, China, 2. Ruijin Hospital, Shanghai, China*

Background : Gratifying the elderly health-care demands and promoting high-quality of nursing services are based on the effort of long-term care(LTC) nurses. However, the high turnover rate of LTC nurses has become very serious in China. What remains unclear is whether the organizational justice and job characteristics affect the LTC nurses' intention to stay. Objective: The aim was to investigate intention to stay among LTC nurses in relation to organizational justice and job characteristics. Method: A cross-sectional study was conducted with a convenience sample of 545 LTC nurses. Data collection was performed between July and November 2019. Data were analyzed using structural equation modeling. Results: Most of LTC nurses reported to stay in nursing or current work place, however they still had strong desire to leave if there were other job opportunities. Organizational justice and job characteristics were significant predictors of LTC nurses intent to stay. LTC nurses job characteristics partially mediates the relationship between organizational justice and intent to stay. Conclusion: This would suggest the importance of administrators/ managers understanding how to promote organizational justice, foster a justice climate and increase LTC nurses' perceived job characteristics. The organizational justice culture programs should be develop as LTC nurses retention strategy.

### STAKEHOLDER PERSPECTIVES ON REDUCING HOSPITALIZATIONS AMONG NURSING HOME RESIDENTS

Denise Tyler,[1] Cleanthe Kordomenos,[2] and Melvin Ingber,[3] *1. RTI International, Waltham, Massachusetts, United States, 2. RTI International, Research Triangle Park, North Carolina, United States, 3. RTI International, Rockville, Maryland, United States*

Organizations in seven states have been participating in the Center for Medicare and Medicaid Innovation (CMMI) initiative aimed at reducing potentially avoidable hospitalizations among long-stay nursing home (NH) residents. The purpose of this study was to identify market and policy factors that may have affected the initiative in those states. Forty-seven interviews were conducted with key stakeholders in the seven states (e.g., representatives from state departments of health, state Medicaid offices, and nursing, hospital and nursing home associations) and qualitatively analyzed to identify themes across states. Few policies or programs were found that may have affected the initiative; only New York (NY) was found to have state policies or programs specifically aimed at reducing hospitalizations. Market pressures reported in most states were similar. For example, stakeholders reported that the increased availability of home and community-based services and the growing presence of managed care are contributing to higher acuity among both long and short stay residents and that reimbursement rates and staffing have not kept up. Stakeholders suggested greater presence of physicians and nurse practitioners in NHs, better training around behavioral health issues for frontline staff, and more advance care planning and education of families about end of life may help further reduce NH hospitalizations. We also found that all states, except NY, had regional coalitions of health care related organizations focused on improving some aspect of care, such as reducing hospital readmissions. These coalitions may suggest ways that organizations can work together to reduce hospitalizations among NH residents.

### THE EFFECTS OF OLD AGE CARE SERVICES AND LONG-TERM CARE INSURANCE: EVIDENCE FROM KOREA

Hoolda Kim,[1] and Sophie Mitra,[2] *1. Black Hills State University, Spearfish, South Dakota, United States, 2. Fordham University, Bronx, New York, United States*

Along with a rapidly aging population globally, the need for homecare is on the rise. Such care can be a heavy financial burden on older people and their households. To address this issue, the Korean government initiated two programs: care services for low-income individuals and voluntary long-term care insurance for people age 65+. Although it has been a decade since the implementation of the programs, there is limited evidence on how they affect the economic lives of older people, with and without disabilities. We use the 2008-2018 Korean Longitudinal Study of Aging (KLoSa) that provides detailed information about healthcare and homecare utilization and out of pocket costs for people aged 45 or older. We investigate the economic effects of old age public assistance programs using multivariate linear regression models and a dynamic panel model. The preliminary results indicate that older people with disabilities are more likely to utilize homecare services but do not experience any significant extra costs associated with homecare, implying that old age public assistance programs mitigate the financial burden of homecare for older people.

### THE IMPLICATIONS OF AN AGING UNDOCUMENTED POPULATION IN ILLINOIS

Padraic Stanley,[1] Brittney Lange-Maia,[1] and Raj Shah,[2] *1. Rush University Medical Center, Chicago, Illinois, United States, 2. Rush University, Chicago, Illinois, United States*

Both academic and public access literature regarding undocumented immigrant older adults is incredibly sparse, and there is no known literature forecasting its growth or the impact of that growth. Therefore, this two-part project commissioned a demographic report to analyze the current undocumented population in Illinois and its projected growth by 2030. We then convened a cross-sector collaborative of leaders in aging, healthcare, immigrant services, and their intersections to discuss the direct practice and structural policy implications of an aging undocumented community. According to this report, the number of undocumented immigrants who are over the age of 55 in Illinois will grow drastically by the year 2030. In fact, the population aged 65 to 74 will increase from 3,392 to 47,271 (more than a twelve-fold increase), and those aged 75-84 will increase from 594 to 7,621 (an eleven-fold increase). Under the current immigration and healthcare systems, without access to Medicaid, Medicare, or any public benefits, these individuals have extremely limited access to services necessary for older adults to age successfully in place—rehab, nursing homes, home health, homemaker services, long-term care, etc. The study also analyzed demographics from the current undocumented older adult population. This poster will summarize the findings of the demographic portion of the report, the themes and findings of the structural and practice implications of an aging undocumented community in Illinois, and provide policy recommendations to prepare for the wave of undocumented older adults by 2030.

### TRENDS IN DEFICIENCY CITATIONS IN FLORIDA ASSISTED LIVING FACILITIES

Hari Sharma, *The University of Iowa, Iowa City, Iowa, United States*

Despite numerous anecdotal reports of poor quality and residential safety concerns in Assisted Living Facilities (ALFs), there is limited federal oversight of ALFs. Usually, state surveyors conduct inspections of ALFs for compliance with regulations and issue deficiency citations and/or fine non-compliant facilities. Florida is one of the few states that publicly releases inspections data. The aim of this study is to fill the gap in our understanding of ALF quality by examining the trends in deficiency citations in Florida. We obtain data on 1,047 ALFs with 25 or more beds operating in Florida between 2012-2018. We use descriptive methods to examine the trends in citations over time and further stratify by profit status. We also evaluate whether facilities get cited for the same deficiencies repeatedly. Every year, approximately, one third of the facilities were free of any deficiency citations. From 2012 to 2018, fewer facilities were cited for resident care and medication but more facilities were cited for training and staffing. Approximately 45.8% of not-for-profit and 35.1% of for-profit facilities were free of deficiency citations in 2018. A majority of facilities cited for a given deficiency were cited at least once again for that deficiency within the study period. Florida ALFs appear to be improving only in some deficiencies but getting worse in some other deficiencies. Furthermore, repeat citations are common suggesting that facilities fail to improve their care/service patterns to avoid repeat citations. More stringent regulations and stricter enforcements may deter facilities from repeat citations.

### TURNOVER INTENTION AMONG DIRECT CARE WORKERS OF OLDER ADULTS IN CHINESE HOSPITALS AND NURSING HOMES

Zhenzhen Zhang, Shuangshuang Wang, Nengliang (Aaron) Yao, and Zhang Zhenzhen, *Shandong University, Jinan, Shandong, China*

High retention rates among direct care workers (DCWs) affect the quality of aged care. However, limited research has explored factors associated with retention in the Chinese aged care industry. This study compared turnover intention and job satisfaction among DCWs in Chinese hospitals and nursing homes. A total 370 DCWs from 7 hospitals (297 contractual, 73 non-contractual) and 311 DCWs from 7 nursing homes (27 contractual, 284 non-contractual) located in Fujian, China were recruited to fill out a questionnaire. Overall, DCWs from hospitals reported lower turnover intention (20.5 % vs 37.0%) and higher levels of job satisfaction (31.1% vs 16.4%) than DCWs from nursing homes. Specifically, contractual DCWs from hospitals indicated lower turnover intention (14.8%) than non-contractual DCWs from hospitals (43.8%) and both types of DCWs from nursing homes (36.3% and 44.4%). Higher job satisfaction was associated with lower turnover intention, but did not mediate the association between DCW types and turnover intention. Findings suggested that the government and institutions should help DCWs complete the identity transformation from non-contractual DCWs to contractual DCWs to enhance job security and benefits. For nursing home DCWs, licensing and registration requirements shall meet the standards for hospital DCWs. Attention is also to be paid to working conditions and staff welfare of DCWs, including social insurance, pensions, and trainings, to improve job satisfaction and reduce turnover intention.

### UNDERSTANDING NURSING HOME RESPONSES TO MINNESOTA'S NEW VALUE BASED REIMBURSEMENT SYSTEM

Zachary Hass,[1] Kathleen Abrahamson,[1] Dongjuan Xu,[1] and Valerie Cooke,[2] *1. Purdue University, West Lafayette, Indiana, United States, 2. Minnesota Department of Human Services, St. Paul, Minnesota, United States*

Even though Value Based Reimbursement (VBR) systems for nursing homes (NH) continue to expand, we have little understanding of how NH respond to VBR. In 2016, Minnesota passed VBR legislation for NHs that increased care-related funding and tied increases to a facility's composite quality score. While care-related expenditures increased with VBR, the incentive for quality did not work as intended. We investigated the differential responses of facilities in their care-related expenditures and quality scores. Data were derived from cost reports and quality measures for the years 2013-2017 from 300 free-standing Minnesota NHs. Latent Class Growth Analysis was used to cluster facilities by their joint care-related cost and quality score trajectories over the period. Three interpretable trajectory clusters emerged: medium-to-high cost and medium-to-high quality (n=172), low cost and medium-to-high quality (n=54), and low cost and low quality (n=74), all during the pre-VBR period. In all three clusters cost rose significantly with VBR, but only in the low cost and low quality cluster did quality also rise significantly. The quality improving cluster had the highest percentage of government-owned and rural facilities as well as the largest annual increase in care related spending. The medium-to-high cost and medium-to-high quality cluster had the highest concentration of urban facilities (Twin City Metro Area) and were the most likely to be non-profit and chain owned. Although the new VBR system appeared effective in achieving its goals for a subset of facilities with lowest cost and quality, the majority of facilities increased care-related costs without improved quality.

### SESSION 2862 (POSTER)

### LONG-TERM CARE II: CHALLENGES IN CARING

### A SYSTEMATIZED REVIEW OF UTI MANAGEMENT PROTOCOL FOR LONG-TERM CARE RESIDENTS

Akanksha Arora and N. Ruth Gaskins Little, *East Carolina University, Greenville, North Carolina, United States*

Clinical acuity for the elderly population has significantly intensified. This population is at heightened risk of infection, especially urinary tract infections (UTIs). Urinary tract infections exist as a clinical leviathan in the long-term care (LTC) industry and, despite its high prevalence rates, there exists a dearth of research on management protocol. The following review aims to summarize the current literature, identify, and comparatively analyze the current UTI management guidelines among LTC residents to guide provider leadership development of standards to prevent UTIs. The Joanna Briggs Institute's approach to systematic reviews was implemented to search the following databases: ProQuest, PubMed, CINAHL, and MEDLINE. 538 citations were assessed, with 32 articles included in the review. Inclusion criteria comprised of clinical trial studies, a time frame of 2005 to current, and no restriction on the study country/region. Key results were collected and analyzed using a data extraction tool. Study findings show that consistent protocols are not followed by licensed staff to prevent, diagnose, and treat UTI's among the elderly residing in long term care facilities. Inappropriate use of antibiotics is problematic due to the lack of specific practice guidelines for testing, diagnosis and, treatment. Studies implementing (1) successful clinical management strategies (i.e. antibiotic initiation, urinalysis frequency) and (2) facility administrative strategies (i.e. incontinence nurse specialist, DON/nurse leadership education) are needed to establish gold standard practice guidelines for the LTC industry.

### ADVANCED PRACTICE CLINICIANS PROVIDING AN INCREASED SHARE OF PRIMARY CARE IN SKILLED NURSING FACILITIES, 2008-2016

Elizabeth White, Cyrus Kosar, and Momotazur Rahman, *Brown University School of Public Health, Providence, Rhode Island, United States*

Rising care complexity in skilled nursing facilities (SNFs), coupled with growing shortages of geriatricians and other primary care physicians able to see SNF patients, have increased demand for nurse practitioners and physician assistants (NP/PAs). We used 2008-2016 Medicare Part A and B claims and nursing home assessment data to describe longitudinal trends in NP/PA practice in SNFs. We identified 8,877,094 SNF post-acute primary care visits for 1,494,113 Medicare beneficiaries. The total number of visits increased from 850,285 in 2008 to 1,189,553 in 2016. The share of visits by NP/PAs rose significantly over time, from 24% of visits in 2008 to 43% in 2016. 71% of SNFs (n=10,139) used NP/PAs in 2016, up from 46% (n=6,696) in 2008. The number of NP/PAs practicing in SNFs more than doubled, from 4,472 clinicians in 2008 to 10,000 in 2016. The number of physicians practicing in SNFs declined from 26,297 in 2008 to 19,745 in 2016. NP/PAs represented 14% of all SNF medical providers in 2008 and 34% of providers in 2016. In 2016, 48% of NP/PAs were SNFists (i.e. >90% of visits billed in SNF), vs. only 11% of physicians. SNFs with NP/PAs are on average larger, more likely urban, for profit, and care for larger populations of racial minorities, than SNFs without NP/PAs. SNFs with NP/PAs also have more short-stay Medicare residents, more admissions, higher nurse and rehab staffing levels, and higher case mix. These findings show that NP/PAs are taking on increasingly prominent roles as medical providers in SNFs.

### AGE MATTERS: BUILDING BLOCKS NEEDED TO INFORM NURSE STAFFING HOURS REQUIREMENTS IN RESIDENTIAL CARE FOR OLDER ADULTS

Heng Wu, Christopher Kelly, and Lyn Holley, *The University of Nebraska at Omaha, Omaha, Nebraska, United States*

This study addresses the need for more complete information about the impact of nurse staffing hours (NSH) on nursing home quality of care. We used national data to examine the relationship between three types (Registered Nurse, Licensed Practical Nurse, and Nurse Aide) of hours, and long-stay quality of care measures over time, taking into account the possible confounding influence of regional differences. Data analyzed were from U.S. Nursing Home Compare datasets which reflect quarterly reports, July 1, 2018 - June 30, 2019 (14,768 facilities). The hours for each staff type in each facility were compared with the facility's four-quarter quality average scores for each of the 12 measures. Results showed only one strong and statistically significant relationship (Beta= .548; p< .001) between Nurse Aide hours and the quality measure used in data sets to exemplify facilities that serve "lower-risk" residents. Analyzes using multiple R (.517) indicate that the linear combination of the three NSH types strongly and significantly (p< .001) predicted the four-quarter average scores and explained 27% of the variance in the scores. Holding the other two NSH types constant, the scores for that measure increased by 63 for each additional increase in the Nurse Aide nurse staffing hours per resident per day. There was no multicollinearity among the three types of staffing hours. This research adds information to the foundation needed for future research about process indicators to assess their efficacy as measures of actual quality of care, and will be submitted as a Technical Note to journals.

### AVOIDABLE DEATHS--NURSING PRACTICE FAILURES TO HONOR ADVANCED DIRECTIVES IN SNFS--A NATIONAL SAMPLE

Mary Dellefield,[1] Byron Batz,[2] Catherine Verkaaik,[3] Diane Carter,[4] and Caroline Madrigal,[5] *1. VA San Diego*

*Healthcare System, San Diego, California, United States, 2. Hahn School of Nursing, Temecula, California, United States, 3. VA San Diego Healthcare System, San Diego, California, United States, 4. self-employed, Denver, Colorado, United States, 5. Providence VA Medical Center, Providence, Rhode Island, United States*

Ongoing evidence of failures to provide cardiopulmonary resuscitation in skilled nursing facilities (SNFs) has resulted in the federal regulation - F678 Cardio-Pulmonary Resuscitation (CPR). Descriptions of CPR-related non-compliance and nursing practice failures are contained in Statements of Deficiencies (CMS-2567). These data provide a unique opportunity to describe practice failures at the point-of-care. A mixed methods case study using content analysis and descriptive statistics was used for a purposeful sample of SODs from 11 SNFs in six states derived from a 2012 first quarter national CMS report of 42 avoidable deaths associated with immediate jeopardy citations. A codebook was developed and tested, based on empirical evidence reported in Office of Inspector General (OIG) 2014 Adverse Events and the Institute of Medicine 2004 nursing surveillance framework. Two trained and independent coders analyzed data. Analysis of SOD quality was conducted. Patterns of practice failures were identified. Ownership included 3 not-for-profit; 1 governmental; and 7 for-profit facilities. The 2012-star ratings ranged from 1.0 – 2.8. The practices of 5 RNs and 5 DONs were described. OIG categories included abuse and neglect, care transitions, and medications. Practice failures were associated with inadequate initiation of CPR resulting from improper processing of orders, poor identification of resident status, poor RN and DON surveillance, absence of CPR certified staff, and a lack of urgency in nursing's response. The quality of SODs, based on 5 parameters, ranged from 18% - 100%. SODs are useful as data sources. Identified practice failures are useful in developing best practice protocols.

### BETA TEST OF A PAC DEMENTIA KNOWLEDGE TRAINING MODULE INCLUDED IN A NURSING HOME ORIENTATION CURRICULUM

Jerry Brown,[1] Peter Baylie,[1] Candidus Nwakasi,[1] Pranay Reddy,[1] Katie Ehlman,[1] Beth Nolan,[2] and Teepa Snow,[3] *1. University of Southern Indiana, Evansville, Indiana, United States, 2. Positive Approach to Care, Ada, Michigan, United States, 3. Teepa Snow, Efland, North Carolina, United States*

Providing quality care for older adults in long-term care can be challenging, and this issue appears to be more pressing with people living with dementia. Using the Positive Approach® to Care (PAC) model, a 2-hour module for new staff nursing home orientation curriculum was designed to help introduce the concept of working with people living with dementia. Twelve undergraduate students participated in a beta-test of the nursing home orientation. A pre-and-post 38-item survey was administered to measure knowledge level and improvement. Participants also responded to qualitative semi-structured questions after the orientation. Descriptive statistics and bivariate analysis were conducted. Results indicated an improvement on dementia-related knowledge in most of the survey items (21 of 34 items). Examples of statistically significant differences in the pretest and post-test identified are knowledge on the effect of pressure in the palm to comfort a person with dementia (p-0.039), vision as the most powerful sensory input during dementia caregiving (p-0.001), and functionalities lost when the left temporal lobe shrinks (p-0.014). The qualitative evaluation showed that most of the participants indicated a change in dementia caregiving views — including how to pause if permission is not given to engage, and to respect personal space. These findings prove important because the PAC orientation curriculum was successful in improving the students' knowledge and perspectives on dementia. This training program could be a useful tool if implemented into nursing home employee orientation.

### CALCULATING THE TRUE COSTS OF FOOD SERVICE IN LONG-TERM CARE: DEVELOPMENT OF A COSTING METHODOLOGY

Mikaela Wheeler,[1] Karen Abbey,[2] and Sandra Capra,[2] *1. University of Queensland, Queensland, Australia, 2. University of Queensland, Brisbane, Queensland, Australia*

As population's age and the need for long term care (LTC) increases, so too does the focus on the costs to provide that care. Providing food, oral nutrition supplements and meals, can be a considerable expense to a home. The objective of this research was to develop a valid foodservice costing tool (FCT), to calculate the real cost of providing foods and meals in LTC. Current costing methodologies are not specific to LTC and do not account for all costs of a foodservice, including staff, procurement and nutrition supplements. An initial tool was developed using the systems approach in conjunction with literature and professional knowledge. This was piloted in real world contexts, using volunteer LTC homes. Four iterations of the tool were completed to assess its feasibility in calculating costs and useability. Managers were interviewed after completing the tool to gather an understanding of how the tool was interpreted and to refine completion. Following feedback, the resulting tool consists of nine sections, measuring both costs incurred in meal production and service as well as analysis of staff workloads. Preliminary results show consistency between homes within Australia, indicating that the true cost is much higher than that reported in the literature to date. The development of a comprehensive, usable tool which captures the total cost of foodservice allows homes to accurately report and understand costs from a systems level. This information can be used to demonstrate cost effectiveness of a foodservice and the potential to justify and plan future system changes.

### DEFICIENCY CITATIONS IN NURSING HOMES THAT PREDOMINANTLY SERVE RESIDENTS WITH SERIOUS MENTAL ILLNESS

Dylan Jester,[1] Kathryn Hyer,[1] and John Bowblis,[2] *1. University of South Florida, Tampa, Florida, United States, 2. Miami University, Oxford, Ohio, United States*

Studies suggest that nursing homes (NHs) that predominantly serve residents with serious mental illness (SMI) are of worse quality due to poor resources (i.e., high Medicaid-paying census) and lower staffing. We used national Certification and Survey Provider Enhanced Reports (CASPER) data to examine the deficiencies issued to NHs from 37,800 recertification inspections of 14,582 unique

NHs from 2014 to 2017. NHs were categorized into "low-SMI" and "high-SMI" facilities using the lowest and highest quartiles, respectively, of the proportion of residents in the NH with SMI. Bivariate analyses were used to assess for differences between low-SMI and high-SMI NHs in the number of deficiencies, the deficiency score (a point-based metric developed by the Centers for Medicare & Medicaid Services), and the scope and severity of deficiencies. In total, there were 245,178 deficiencies issued. In comparison to low-SMI NHs, high-SMI NHs received a greater deficiency score and more deficiencies per survey ($p<.001$). Deficiencies given to high-SMI NHs were associated with greater risk of harm ($p<.001$) and were of wider scope ($p<.001$). High-SMI NHs were cited 215% more often for resident abuse or neglect and 61% more often for the policies that prohibit and monitor for risk of abuse and neglect in comparison to low-SMI NHs. In conclusion, high-SMI NHs were documented for providing worse care to residents, with one particular area of concern being the heightened risk of resident abuse and neglect. Implications for policy and practice will be discussed.

### DEMENTIA KNOWLEDGE AND CAREGIVING SKILLS IMPROVEMENT FROM USING THE PAC MODEL: A PILOT COMPARATIVE EVALUATION

Jerry Brown,[1] Pranay Reddy,[1] Candidus Nwakasi,[1] Beth Nolan,[2] Teepa Snow,[3] and Katie Ehlman,[1]
*1. University of Southern Indiana, Evansville, Indiana, United States, 2. Positive Approach to Care, Ada, Michigan, United States, 3. Teepa Snow, Efland, North Carolina, United States*

The diverse needs of persons living with dementia in nursing home settings presents challenges for Certified Nursing Assistants (CNAs) to provide quality care. There is a lack of educational preparedness among nursing home CNAs regarding dementia knowledge and skills required to care for a person living with dementia. As direct caregivers for persons living with dementia, CNAs play an important role in long-term care. This pilot study evaluated the dementia knowledge and caregiving skills of newly trained CNA students. The students were trained by an instructor certified using Teepa Snow's Positive Approach to Care (PAC) curriculum. Conducted in a rural southwestern Indiana community, this study evaluated CNA students' knowledge and perception of dementia, as well as their skill performing the Positive Physical Approach™ (PPA™) technique to approach and connect. A 38-item knowledge and perception survey and a 12-step observed skills assessment using a standardized patient encounter were administered to CNA students. Data were analyzed using descriptive statistics and bivariate analysis. Preliminary results indicate that 100% of students correctly answered the survey item regarding non-confrontational body language, while 29% of students correctly performed the corresponding PPA skill. There is a statistically significant association between the knowledge that people find pressure in their palm comforting and the ability to perform the corresponding Hand-under-Hand® and PPA techniques. Incorporation of PAC into current CNA curriculum may equip CNAs with the knowledge and skills required to provide better care, with the potential to improve the overall quality of life for persons living with dementia.

### DOES NURSING HOME RESIDENTS' RIGHT TO SELF-DETERMINATION IMPROVE THEIR QUALITY OF LIFE IN SOUTH KOREA?

Minhong Lee,[1] Kyeongmo Kim,[2] and Sok An,[3]
*1. Dong-eui University, Busan, Pusan-jikhalsi, Republic of Korea, 2. Virginia Commonwealth University, Richmond, Virginia, United States, 3. Korea Rural Economic Institute, Austin, Texas, United States*

Background and Purpose: Addressing issues of quality of life of nursing home residents based on the human rights-based approach has been a top priority in the long-term care system in Korea but no study has yet examined the relationship between self-determination of nursing home residents and their quality of life. This study aimed to examine whether greater levels of self-determination in the provision of daily care were associated with higher levels of quality of life of the residents. Methods: We collected data from 332 residents (+65) at 20 nursing homes in a metropolitan city. We measured residents' right to self-determination using the autonomy scale of the Client-centered Care Questionnaire. We also included quality of life, socio-economic characteristics, ADLs, depressive symptoms, and social networks. We ran multiple regression analysis using SPSS 26.0. Results: The findings of this study revealed that greater levels of residents' right to self-determination were associated with higher levels of quality of life ($\beta =-.425$, $p <.0001$). Older residents who were higher levels of depressive symptoms were likely to have lower levels of quality of life ($\beta = -.265$, $p < .001$). Conclusions and Implications: This study adds to the growing literature on the ways nursing home residents' self-determination contributes to their quality of life. More opportunities for self-determination in their treatment should be given to promote recovery and to encourage participation in the decision-making process. Nursing practitioners and policymakers in Korea should develop programs and/or services that enhance residents' self-determination to improve their quality of life.

### ELECTRONIC ASSISTED LIVING TECHNOLOGY IN CCRC, ASSISTED LIVING, AND IN-HOME CARE

John Hall,[1] Linh Lee,[2] and Joshua Littlejohn,[3]
*1. envoyatHome, LLC, envoyatHome, Pennsylvania, United States, 2. University of Pennsylvania School of Medicine, University of Pennsylvania, Pennsylvania, United States, 3. University of Pennsylvania Health System, Philadelphia, Pennsylvania, United States*

A qualitative study based on structured interviews with 21 healthcare leaders from CCRC's, In-Home Care Agencies, or Medicare PACE facilities was conducted. Implications of electronic assisted living technologies on caregiver workforce were assessed. The use of assisted living technology was shown to have implications for workforce support and the alleviation of demands on caregivers. Communication and assessment tools were also found to be useful in the reduction of caregiver stress. There is optimism regarding the effectiveness of high-tech platforms in easing caregiver burden but there is skepticism about the return on investment given the initial cost and time needed for onboarding and data organization. The lack of user-friendliness and the required time to train to use tech are also barriers. The use of technology for remote check-ins and to monitor vitals is desirable. Also predicting dementia risk and monitoring for wandering are other opportunities for tech adoption. The enthusiasm for technology is tempered with caution for who will keep tabs on remote monitoring and who bears the responsibility to respond to the information gathered. Labor shortages, time constraints, and disorganized documentation are incentives for tech adoption. High-tech solutions for would ideally be user-friendly and help reduce staff demands. Except for the prevention of re-hospitalization by monitoring vitals the benefits of adopting new tech are not perceived as cost-effective.

### EXAMINING CONSEQUENCES RELATED TO UNMET CARE NEEDS ACROSS THE LONG-TERM CARE CONTINUUM

Meghan Jenkins Morales,[1] and Stephanie Robert,[2]
*1. University of Wisconsin, Madison, Wisconsin, United States, 2. UW-Madison, Madison, Wisconsin, United States*

At some point in our lives, approximately 70% of us will need support to help with daily care. Without adequate assistance we may experience unmet care need consequences (UCNC) – such as skipping meals, going without clean clothes, or taking the wrong medication. This study examines the likelihood of experiencing UCNC related to gaps in assistance with activities of daily living (ADL) and instrumental activities of daily living (IADL) across long-term care arrangements: informal community care, paid community care, residential care, and nursing homes. We examine a sample of older adults receiving assistance in a care arrangement (N=2,499) from the nationally representative 2015 National Health and Aging Trends Study. Cross-sectional and longitudinal regression models, adjusting for differences in demographic and health/functioning characteristics, examine if type of care arrangement in 2015 is associated with UCNC in 2015 and change in UCNC by 2017. Holding all else constant, there were no significant differences in UCNC related to ADLs in 2015 across care arrangements. However, those receiving paid community care were more likely to experience UCNC related to IADLs (going without clean clothes, groceries, or a hot meal and making medication errors) compared to those receiving only informal care (OR=1.64, $p<.05$) or residential care (OR=2.19, $p<.01$). By 2017, paid care was also significantly associated with continued UCNC, but older adults in informal care arrangements were most likely to experience a new UCNC. Results suggest improving/expanding assistance with IADLs among community-dwelling older adults, and promoting equitable access to residential care, to reduce UCNC.

### FAMILY MEMBERS' EXPERIENCES OF MAINTAINING AN OLDER RELATIVE'S FUNCTIONAL ABILITY IN LONG-TERM CARE

Vilhelmiina Lehto-Niskala, Outi Jolanki, and Marja Jylhä, *Tampere University, Tampere, Finland*

Family members have important role in care of older people. In residential long-term settings family members can find themselves in an ambiguous situation: officially, responsibility for provision and quality of care rests with the care provider and staff members, but in practice family members participate in caring. This study explores the role of family members in residential long-term care settings, particularly in supporting their older relatives' functional ability. Developing and maintaining functional ability lies at the very core of healthy ageing policies and long-term care. The data consist of semi-structured interviews with family members (n=16) from eight long-term care facilities in Finland. Thematic analysis yielded three themes: maintaining personhood, engaging in everyday life and monitoring care. Family members in our study were actively involved in care that supported the functional ability of their older relative. However, family members had also conflicting views about who was responsible for care provision. Some participants willingly accepted their caregiver responsibilities even in residential care, while others described their involvement in care not as a matter of choice but rather as one of necessity in order to ensure good quality care. It is important to see the family members' viewpoint which, based on the results of this study, emphasizes personhood and continuity of care. If they are willing to participate, family should be able to take part in caregiving together with the care staff and their role should be recognized.

### HOW CHARACTERISTICS OF FLORIDA'S ASSISTED LIVING COMMUNITIES IMPACT SERVICES AND RECREATIONAL ACTIVITIES OFFERED

Jocelyn Brown, and Daniel Pupo, *University of South Florida, Tampa, Florida, United States*

There are currently 3,090 assisted living communities (ALCs) serving older adults throughout the state of Florida. The services (e.g. physical therapy) and recreational activities (e.g. cooking classes) offered within these communities likely differ depending on a variety of characteristics such as location, licensure type, and profit status. The goal of this work is to determine how these characteristics influence the number and types of services and activities are offered within Florida's ALCs. Data on the services and recreational activities, location, and characteristics of ALCs were collected from the state Agency for Health Care Administration (AHCA) website. Counties were classified as rural or urban and based on data from the 2010 U.S. Census. Linear regressions were used to model the associations. The results indicated that rural-based ALCs provided significantly fewer services and activities, compared to urban-based ALCs. ALCs that were for-profit, with more licensed beds and with a limited nursing service license showed increasing numbers of services and activities, while ALCs with limited mental health licenses showed significant decreases. ALCs that are rural, non-profit or that hold limited mental health licenses to provide fewer services and recreational activities for residents than ALCs without these characteristics. Policymakers and administrators should work to ensure that residents living within these communities have adequate access to services and activities by addressing administrative, logistical, and financial barriers.

### MULTIPLE CHRONIC CONDITIONS AND PROVISION OF SERVICES IN RESIDENTIAL CARE COMMUNITIES

Christine Caffrey, and Jessica Lendon, *National Center for Health Statistics, Hyattsville, Maryland, United States*

This study describes the relationships between the number of selected chronic conditions among residents and the number and provision methods of services provided by residential care communities (RCCs). Estimates are from the 2016 wave of the National Study of Long-Term Care Providers conducted by the National Center for Health Statistics. Chronic conditions were measured by whether the RCC reported having at least one resident with any of the most common chronic conditions: dementia, diabetes, depression, or heart disease. Services included were mental health, social work, therapeutic, dietary, and skilled nursing. Each service type was categorized by provision method (provided by employees, arrangement or referral only, or not provided). Among RCCs, 63% had all four conditions among residents, 23% had three, 12% had one to two, and 1% had none. About 66% of RCCs provided all five services, 16% provided four, 15% provided 1-3, and 3% provided none. Of the 63% of RCCs that had all four conditions among residents, 69% provided all five services, 29% provided 1-4, and 2% provided none. In these RCCs, a greater percentage provided dietary (69%) and skilled nursing services (33%) with employees compared to the other methods; a greater percentage provided therapeutic (85%) and mental health services (83%) solely through arrangement or referral compared to the other methods. This study found that, in 2016, RCCs with multiple selected conditions among their residents tended to provide a greater number of services for managing chronic conditions. How these RCCs provide services varied based on service type.

### PREDICTORS OF EVERYDAY CARE PREFERENCE IMPORTANCE RATINGS FOR VETERANS LIVING IN THE NURSING HOME SETTING

Caroline Madrigal,[1] Lan Jiang,[1] Whitney Mills,[1] Wen-Chih Wu,[1] Diane Berish,[2] Tonya Roberts,[3] Kimberly Van Haitsma,[4] and James Rudolph,[1] *1. Providence VA Medical Center, Providence, Rhode Island, United States, 2. Pennsylvania State University, University Park, Pennsylvania, United States, 3. University of Wisconsin-Madison, Madison, Wisconsin, United States, 4. Penn State University, University Park, Pennsylvania, United States*

Preference-based care is required by the Centers for Medicare and Medicaid Services and is linked to improved quality of nursing home care. The federally mandated Minimum Data Set (3.0) Preference Assessment Tool (PAT) is a 16-item standardized measure used to facilitate preference-based care through rating how important residents' daily and activity preferences are. Little work has explored how Veterans' unique demographic backgrounds and functional/cognitive abilities may influence how they rate their preferences (compared to general nursing home residents). Therefore, the purpose of this retrospective study was to explore the relationships between Veterans' demographic/clinical characteristics and number of preference importance ratings. Our sample (n=194,068) consisted of Veterans admitted to community nursing homes after hospitalization at a Veterans Affairs facility for heart failure between 2010-2015. We used ordinal regression to explore predictors of preference importance ratings. Veterans were, on average, 78-years-old (SD=10.42) and mostly male (95%), white (81%), married (46%), cognitively intact (74%) with extensive functional impairment (60%) and minimal depressive symptoms (74%). Veterans rated an average of 12.47 preferences as important (SD=2.86; range=0-16). Veterans living with cognitive impairment, depression, and extensive functional impairment who were not married or separated had a lower number of important preferences (all p<0.0001). Veterans that were female, under the age of 85, and any race but white had a higher number of important preferences (all p<0.0001). Discussion will include implications for planning and delivering preference-based care for Veterans as well as next steps in research and practice to better understand and fulfill Veterans' everyday care preferences.

### REHABILITATION THERAPY STAFFING COMPOSITION AND POST-ACUTE CARE QUALITY IN SKILLED NURSING FACILITIES

Xiao (Joyce) Wang,[1] Jennifer Hefele,[2] Laura Driscol,[3] and Joyce Wang,[4] *1. University of Massachusetts, Boston, Massachusetts, United States, 2. Booz Allen Hamilton, Arlington, Virginia, United States, 3. Boston University College of Health and Rehabilitation Sciences: Sargent College, Boston, Massachusetts, United States, 4. University of Massachusetts Boston, Boston, Massachusetts, United States*

Rehabilitation therapy staffing is crucial in achieving high quality of post-acute care (PAC) in skilled nursing facilities (SNFs), but few studies have explored therapy staffing composition and how it relates to SNFs' PAC quality. This study describes SNFs' therapy staffing composition and its association with facility-level PAC fall rates. Our study was a cross-sectional study using facility-level data (Q3 2017-Q2 2018). Data sources include Nursing Home Compare, Payroll-Based Journal data, Area Health Resource File and LTCFocUS.org. The first independent variable was the share of total therapy staff in the direct care team, a ratio between total therapy staffing hours and total direct care hours (includes nursing).To further understand the composition of therapy staff with different qualifications within the therapy team, two variables were generated: 1) proportion of higher skilled therapy staff (i.e. assistant, therapist) hours of total therapy hours; and 2) assistant to therapist ratio. Multivariate linear regression modeling was used, controlling for other characteristics and state fixed effects. Our results show SNF therapy staffing compositions varied significantly by profit status, chain affiliation, and urban/rural location. Further, SNFs with higher shares of therapy staff and higher skilled staff had significantly lower fall rates. However, SNFs with higher assistant-to-therapist ratios had higher fall rates. Our results demonstrated the value of having a multidisciplinary team with higher skilled staff. The results also supported researchers' concerns that recent Medicare payment change (i.e. the Patient Driven Payment Model) may negatively impact quality by reinforcing providers' incentives of reducing rehabilitation staffing and/or using lower-skilled staff.

### SAVE-CLC: AN INTERVENTION TO REDUCE SUICIDE RISK IN VETERANS WHO DISCHARGE FROM VA NURSING HOMES

Kelsey Simons,[1] Katherine Luci,[2] Lauren Hagemann,[3] Lindsey Jacobs,[4] Emily Bower,[5] Morgan Eichorst,[6] and Michelle Hilgeman,[7], *1. VA Medical Center--Canandaigua, Canandaigua, New York, United States, 2. Salem VA Medical Center, Salem, Virginia, United States, 3. Salem*

*VAMC, Salem, Virginia, United States, 4. VA Boston Healthcare System, Brockton, Massachusetts, United States, 5. Veterans Health Administration, Canandaigua, New York, United States, 6. VA Northern Indiana, Mishawaka, Indiana, United States, 7. VA Medical Center--Tuscaloosa, Tuscaloosa, Alabama, United States*

Prior research has established transitions into and out of nursing homes as periods of suicide risk for older adults. Deaths by suicide were found to be 2.4 times as likely among Veterans within six months of discharge from US Veterans Health Administration (VA) nursing homes when compared with gender and age-matched Veterans from the general VA patient population (McCarthy, Szymanski, Karlin, & Katz, 2013). Despite these trends, suicide prevention interventions implemented during nursing home and post-acute care transitions, including those taking place from Centers for Medicare and Medicaid Services regulated nursing homes, are lacking. Suicide Awareness for Veterans Exiting the Community Living Center (SAVE-CLC) was piloted as a quality improvement intervention to reduce suicide risk for older Veterans discharging from VA nursing homes. VA clinicians from three sites provided a friendly contact by phone after discharge ($n = 66$) to screen for depression, facilitate a strengths-based discussion about service needs, and provide service referrals. Compared to a group of patients discharged prior to the start of the intervention (matched on location, age range, and Care Assessment Need scores), SAVE-CLC patients received more depression screening within 30 days after discharge (chi square = 38.7, $p < .001$) and were seen more quickly for mental health care ($t = 3.1$, $p = .005$) when indicated. Implications for suicide prevention with older Veterans and for the general population of older adults receiving short stay services in US nursing homes will be addressed.

### WORK OF ART, ART OF WORK: ARTISTIC LITERACY AND QUALITY IN LONG-TERM DEMENTIA CARE

Katie Aubrecht, *St. Francis Xavier University, Antigonish, Nova Scotia, Canada*

This paper shares results from a thematic analysis (Braun & Clarke, 2006) of semi-structured interviews with a purposive snowball sample of 15 leaders in dementia arts education and praxis from Canada, the United States and United Kingdom. Interviews were conducted as part of a multi-phased collaborative, interdisciplinary arts-informed research project that aimed to operationalize quality mental health and dementia care in long-term care (LTC) from a relational perspective, with a focus on LTC staff literacy. Artistic literacy that is cultivated through creative arts-making and public exhibiting was described by participants as crucial to supporting and promoting quality within long-term care. Quality was imagined as a work of art and operationalized in terms of artist competencies, capacities and conditions. Artists included LTC staff, residents and their family and friends. Our analysis identified five themes related to artistic literacy: space-making, validation, fostering community, means of engagement, vulnerability and resilience. Drawing on cultural sociology (Bourdieu, 1993, 1984) and aging studies theory (Basting, 2018), we consider and discuss the role of the arts in disrupting unexamined assumptions about quality in LTC and advancing innovation in LTC staff mental health and dementia care.

### SESSION 2863 (POSTER)

### MENTAL HEALTH

### “ME CUIDO ACTIVO MANIZALES”: A COMMUNITY CARE MODEL BASED ON AN ACTIVE AGING FRAMEWORK FOR ELDERLY PEOPLE IN COLOMBIA

Lina Gonzalez Ballesteros,[1] Santiago López Zuluaga,[2] Ana María Ortiz Hoyos,[3] Oscar Gómez,[4] and Lina GonzalezBallesteros, *1. Physician, Bogotá, Colombia, 2. Sociologist, Bogotá, Colombia, 3. Psicologist, Bogotá, Distrito Capital de Bogota, Colombia, 4. Physician, Bogotá, Distrito Capital de Bogota, Colombia*

Colombia, as other countries in the Latin American region has aged at a higher rate than countries from developed ones. This challenges the way these countries decide how to attend the ageing of their population. “Me cuido activo Manizales” is a community care model based on an active ageing framework developed in Manizales, Colombia in 2017. It is based on collaborative and experiential learning activities for elderly people and their caregivers in the areas of care and self-care, writing and memory, healthy habits, physical activity, resilience and rights, and economic productivity. A before and after analysis of the 88% of enrolled subjects that participated through 2017-2019 (Median age = 70; IQR = 76 years) show stable results in independency (Barthel before = 97,23 Vs Barthel after = 94,68; $t = 1,48$; $p = 0,14$), instrumental activities of daily living (Lawton-Brody before = 3,63 Vs Lawton-Brody after = 3,58; $t = 0,748$; $p = 0,47$), resilience (CD-RISC10 before = 28,61 Vs CD-RISC10 after = 28,28; $t = 0,40$; $p = 0,69$) and MMSE scores (before = 25,72 Vs after = 24,62; $t = 1,67$; $p = 0,10$). Qualitative analysis evidenced increased awareness of self-care lifestyles and active ageing, and the need for institutional presence providing logistic and personnel support and better ways to engage groups rather than the individual. We believe this is a holistic approach that focuses on key aspects of the current perception of elderly functionality and that it engages individuals and communities along in a self-aware healthy ageing.

### ADULT CHILDREN’S MIGRATION AND HEALTH-RELATED QUALITY OF LIFE AMONG OLDER NEPALESE ADULTS

Ghimire Ghimire,[1] Devendra Singh,[2] Sara McLaughlin,[1] Dhirendra Nath,[3] Hannah McCarren,[4] Janardan Subedi,[5] and Saruna Ghimire, *1. Miami University, Oxford, Ohio, United States, 2. Asian College for Advanced Studies, Lalitpur, Nepal, 3. Grande International Hospital, Kathmandu, Nepal, 4. Miami University, Oxford, United States, 5. Miami University, Miami University, Ohio, United States*

Traditionally, adult children have served as primary caretakers and providers for older Nepalese adults. However, out-migration of adult children for employment and other opportunities is increasing. Health-related quality of life (HRQOL) in older Nepalese adults in general and in the context of adult children’s migration is poorly understood.

This study aims to assess HRQOL of older Nepali adults and its relationship with adult children's migration. We used existing cross-sectional survey data on 260 older adults from the Krishnapur municipality, which has witnessed a high rate of adult migration. HRQOL was assessed using the SF-12, which provides a physical (PCS) and mental (MCS) health component. Scores for PCS and MCS range from 0-100; a higher score indicates better HRQOL. Simple and multiple linear regression were used to assess correlates of HRQOL. Participants had suboptimal HRQOL [mean (±SD): PCS =40.4±9.2 and MCS=45.2±7.7]. After adjusting for covariates, adult children's migration was associated with lower MCS scores (β: -2.33, 95%CI: -4.21, -0.44). Individuals with more than one child had higher MCS scores (β: 2.14, 95%CI: 0.19, 4.09). Females (β: -3.64, 95%CI: -7.21, -0.06) and those with a history of unemployment (β: -6.36, 95%CI: -10.57, -2.15) had lower PCS scores than their respective counterparts. The presence of one or more chronic conditions was associated with significantly lower PCS and MCS. Our findings suggest that out-migration of adult children may negatively effect HRQOL among older Nepali adults, specifically their psychological well-being. Additional research is needed to investigate potential moderating factors that may serve as important buffers.

### AN INNOVATIVE MENTAL HEALTH MODEL FOR TREATING CULTURALLY DIVERSE OLDER ADULTS

Tobi Abramson,[1] Jacquelin Berman,[1] and Madison Gates,[2] *1. NYC Department for the Aging, NYC, New York, United States, 2. NYC Department for the Aging, New York city, New York, United States*

The mental health needs of older adults are largely unmet, a finding even more prevalent within culturally diverse older adult populations. Added to this is the high rate of social isolation. Research has indicated increased connection to mental health services when services are embedded within physical health care settings. For those attending community centers, 85% indicate that they are socially isolated, 68% indicate they are lonely, and 53% have a mental health need (compared to 20% nationally). The need for innovative programming is evident. When examining the needs of diverse older adults, it is increasingly important that new and innovative approaches address social isolation, loneliness, and mental health problems experienced by this cohort. Utilizing this knowledge an innovative model of embedding and integrating mental health services, provided by bilingual and bicultural clinicians, into congregate sites (older adult centers) was implemented. Those that participated were mainly female (72.1%), 68.5% English-speaking, 14.5% Spanish-speaking, 13.6% Chinese-speaking and 3.4% other. Spanish-speakers had more depression than English-speakers and both had more depression than Chinese-speakers. English and Spanish-speakers reported more social isolation and Chinese-speakers compared were more likely to participate in engagement. Chinese-speakers were less likely to be in clinical services with a positive screen compared to English-speakers. Overall, 75% engaged in treatment; 37.3% and 41% showed a 3-month improvement of depression and anxiety, respectively. This presentation focuses on the innovative components of this model, how to engage diverse older adults to utilize treatment, steps needed for replication, and policy implications around integrated mental health treatment.

### COGNITIVE HEALTH IN MASSACHUSETTS, NEW HAMPSHIRE, AND RHODE ISLAND: FINDINGS FROM THE HEALTHY AGING DATA REPORTS

Richard Chunga,[1] Taylor Jansen,[1] Chae Man Lee,[2] Shuangshuang Wang,[3] Haowei Wang,[1] Nina Silverstein,[4] Frank Porell,[1] and Beth Dugan,[1] *1. University of Massachusetts Boston, Boston, Massachusetts, United States, 2. University of Massachusetts Boston, Boston, United States, 3. Shandong University, Jinan, Shandong, China, 4. University of Massachusetts Boston, Needham, Massachusetts, United States*

Over time persons with Alzheimer's disease (AD) have impaired health, lower quality of life, and increased mortality compared to those without AD. This study describes state and community rates of Alzheimer's disease, self-rated cognitive difficulty, and the % of the population age 85+ in three New England states (MA, NH, RI). Data sources were the American Community Survey (2009-2013 RI, 2012-2016 MA/NH) and the CMS Medicare Current Beneficiary Summary File (2012-2013 RI, 2015 MA/NH). Small area estimation techniques were used to calculate age-sex adjusted community rates for Alzheimer's disease and related dementias (ADRD), self-reported cognitive difficulties, percentage of older adults 85 years or older, and the percentage of adults age 65+ living alone. State rates (range) were: AD: RI 14.4% (8-23%), MA 13.6% (6-19.31%), and NH 12% (5.49-33.51%). Self-reported cognitive difficulty: MA 8.3% (0-25.16%), RI 7.8% (2-18%), and NH were 6.9% (0-34.21%). Adults 85 years and older: RI 17.6% (6-24%), MA 15.2% (0-32.23%), and NH 12.9% (0-27.91%). Living alone: RI 30.4% (12-45%), MA 30.2% (6.25-50%), and NH 26.1% (6.13-72.55%). While there was significant variation across states, Rhode Island had the highest state rate of ADRD, older adults 85 and older, and percentage of older adults living alone. Within-state disparities among AD rates, cognitive difficulties, and living alone was highest in NH, but MA had the largest variation for community rates of adults 85+. Understanding the prevalence of brain health is important to policy and practice efforts to promote age-friendly communities. This research was supported by the Tufts Health Plan Foundation.

### CROSS-LAGGED PANEL ANALYSES OF RECIPROCAL EFFECTS OF SOCIAL ISOLATION, PERCEIVED LONELINESS, AND SOLITARY ACTIVITY

Ke Li, and Fengyan Tang, *University of Pittsburgh, Pittsburgh, Pennsylvania, United States*

Social isolation and perceived loneliness are major issues as they may place older adults at greater risks for health problems. The objective status of social isolation and the subjective perception of loneliness may have distinct meanings, and their longitudinal reciprocal relationship remains unclear. The purposes of this study were to examine the reciprocal effects of social isolation and loneliness among U.S. adults aged 50 and above and to explore the moderating effect of solitary activities by using the data from three waves of the Health and Retirement Study (HRS) collected in the year 2008, 2012 and 2016. The index of social isolation was created by summing five commonly used indicators, including marital status, living arrangement, and three types of social contact. Loneliness was assessed by a summary score of 11 items. Solitary activities included 13 activities with limited or no social interaction. The results estimated by the cross-lagged effects model showed positive reciprocal relationship of social isolation and perceived loneliness across waves: respondents with a higher level of social isolation were predicted to have increased loneliness, and more perceived loneliness was significantly associated with a higher level of social isolation in the following waves. The results also indicated that solitary activity had a direct effect on decreasing loneliness. This study improves the understanding of reciprocal effects of social isolation and perceived loneliness over years and indicates that practice needs to address the issues of social isolation and perceived loneliness at the early stage and provide more opportunities of solitary activities.

### DENIALS, SURPRISE CHARGES, STARTING OVER: HOW MEDICARE RECIPIENTS NAVIGATE THE MEDICARE MENTAL HEALTH COVERAGE GAP

Matthew Fullen,[1] Megan Dolbin-MacNab,[1] Nancy Brossoie,[1] Jonathan Wiley,[2] and Gerard Lawson,[1]
*1. Virginia Tech, Blacksburg, Virginia, United States, 2. Marietta College, Radford, Virginia, United States*

Medicare is the primary insurance provider for approximately 51 million older adults, including those who seek mental health care. Medicare provider eligibility was last updated in 1989, and approximately one-third of the graduate-level mental health workforce (i.e., Licensed Professional Counselors and Licensed Marriage and Family Therapists) is excluded from Medicare, despite these professionals participating in Medicaid, TRICARE, the Veterans Administration, and private insurance plans. This Medicare mental health coverage gap (MMHCG) raises concerns about older adults' access to mental health care, resulting in a policy misalignment between Medicare's provider regulations and a growing number of older adults seeking mental health care. However, little is known about the precise impact of the MMHCG. To better understand how the MMHCG impacts older adults, we interviewed 17 Medicare-insured individuals about their experiences accessing mental health services. Using a phenomenological framework to analyze our data, we found that Medicare recipients described several consequences, such as: 1) a detrimental impact on their mental health and well-being; 2) concerns about having to start over with new providers due to commencing mental health treatment only to have services interrupted once the provider is no longer Medicare-reimbursable; and 3) relying on pro bono services from Medicare-excluded providers with uncertainty about the long-term sustainability of these arrangements. The presenters will describe how these findings fit within the current Medicare mental health service context, including the direct impact on older adults' mental health. Discussion will also focus on policy implications of the findings and possible solutions for addressing the MMHCG.

### GENERATIONAL DIFFERENCES IN SUPPORT AND CARE OF INDIVIDUALS DIAGNOSED WITH SEVERE MENTAL ILLNESS

Deborah Finkel,[1] Per Bulöw,[2] Pia Bulöw,[2] Monika Wilińska,[2] Cristina Joy Torgé,[2] Marie Ernsth Bravell,[2] and Magnus Jegermalm,[2] *1. Indiana University Southeast, New Albany, Indiana, United States, 2. Jönköping University, Jönköping, Sweden*

As part of the process of de-institutionalizing the Swedish mental health care system, a reform was implemented in 1995 moving responsibility for social support for people with severe mental illness (SMI) from the county to social services in the municipalities. In many ways, older people with SMI were neglected in this changing landscape of psychiatric care. To investigate possible generational differences in support experiences, two nonoverlapping cohorts were created from surveys conducted every fifth year between 1996 and 2011 in one middle-sized municipality in the south of Sweden, aiming to detect the needs for social support. Cohort 1 includes everyone detected at the 1996 survey aged 65 and 79 years (N = 92). Cohort 2 includes individuals first detected at the 2011 survey who were aged 65 to 79 (N = 104). Results indicates significant differences between the two cohorts in diagnosis, reflecting changes over time in diagnostic tendencies. Cohort 1 was on average 10 years older than Cohort 2, even within the restricted age range. After correcting for age, there were no differences between the two cohorts in education, functioning (CAN and GAF), or marital status. Although Cohort 1 experienced more days of institutionalization than Cohort 2 (median = 424.5 days vs. 382 days), the difference was not statistically significant. Cohort 2 had significantly higher additional subsidies and disposable income, as well as significantly higher income from other sources after retirement. Results indicate the changing demands that older adults with SMI will place on care systems.

### LONGITUDINAL CHANGES IN FUNCTIONING AND SUPPORT AFTER INITIAL SURVEY FOR INDIVIDUALS WITH SEVERE MENTAL ILLNESS

Deborah Finkel,[1] Per Bulöw,[2] Pia Bulöw,[2] Cristina Joy Torgé,[2] Monika Wilińska,[2] Magnus Jegermalm,[2] and Marie Ernsth Bravell,[2]
*1. Indiana University Southeast, New Albany, Indiana, United States, 2. Jönköping University, Jönköping, Sweden*

After the Swedish Mental Health Care reform was enacted in 1995, many individuals with severe mental illness were released from long-term care institutions. As a result, they had new living and support situations. Following 236 individuals over time supported investigation of the effectiveness of social support services in caring for these individuals. Surveys were conducted in 1996, 2001, 2006, and 2011 and annual data were available from national registries. Primary diagnosis was psychosis (over 60%); depression was the second highest diagnosis (20%). Mean age in 1996 was 60.8 (range = 45 to 86) and 47% of the sample were women. Only 36% of the group had more than nine-year compulsory education. Compared with other groups in the larger study, this group was more likely to have never married (66%) and 18% had accumulated more than 10 years in an institution over their lifetime (mean = 6 years). Even after correcting for age, functioning as measured by the Global Assessment of Functioning and a 9-item measure assessing hygiene, economy, food preparation, etc. declined over time. As a result, although 49% were living independently (with or without support) at the first wave, only 34% were doing so at the last wave; by 2011 66% were living in a special home or institution. Examination of longitudinal trends in income indicated that disposable income and total income from all sources was constant between 1990 and 1995 but increased significantly over time after reform was enacted. Overall, results suggest mixed success of social support services.

### MENTAL HEALTH ISSUES IN MASSACHUSETTS, NEW HAMPSHIRE, AND RHODE ISLAND: FINDINGS FROM THE HEALTHY AGING DATA REPORTS

Taylor Jansen,[1] Richard Chunga,[1] Chae Man Lee,[2] Shuangshuang Wang,[3] Haowei Wang,[1] Frank Porell,[1] Nina Silverstein,[4] and Beth Dugan,[1] *1. University of Massachusetts Boston, Boston, Massachusetts, United States, 2. University of Massachusetts Boston, Boston, United States, 3. Shandong University, Jinan, Shandong, China, 4. University of Massachusetts Boston, Needham, Massachusetts, United States*

Mental health issues in older adults are prevalent, yet often undetected or untreated and can contribute to poor physical health, increased disability, and higher mortality rates. The current study describes state and local community rates of mental health indicators of older adults 65+ in MA, NH, and RI. Data sources used to calculate rates were: the American Community Survey (2009-2013 RI, 2012-2016 MA and NH), the Medicare Current Beneficiary Summary File (2012-2013 RI, 2015 MA and NH), and the Behavioral Risk Factor Surveillance System (2012-2014 RI, 2013-2015 MA, and 2014-2016 NH). Small area estimation techniques were used to calculate age-sex adjusted community rates for more than 150 health indicators. This research examines disparities in rates for 3 mental health indicators depression, self-reported poor mental health, and self-reported poor/fair health status. Depression rates: MA 31.5% (19.91-48.82%), RI 30% (19.7-38.5%), and NH 28.8% (18.26-40.56%). Self-reported poor mental health: RI 7.5% (4.8-12.5%), MA 7.0% (2.10-16.59%), and NH 6.9% (3.42-10.13%). Self-reported fair/poor health: RI 20.4% (8.6-38.8%), MA 18.0%, (7.2-34.38%), and NH 16.5% (13.31-21.60%). Results showed variability in rates across states. MA had the highest rates of depression, the greatest differences in rates, and access to the most mental health providers. RI had the highest community rates for poor physical and mental health, and the highest percentage of residents age 85+. Understanding the distribution of community rates makes disparities evident, and may help practitioners and policymakers to allocate resources to areas of highest need. Research funded by the Tufts Health Plan Foundation.

### MENTAL HEALTH OF HOMEBOUND OLDER ADULTS IN CHINA: THE MODERATING EFFECT OF LONELINESS

Tao Chen, Shuangshuang Wang, and Nengliang (Aaron) Yao, *Shandong University, Jinan, Shandong, China*

Homebound older adults are confined to their homes due to physical, mental, or social limitations, which contributes to elevated levels of depression. However, the mental health status of the homebound population in China is relatively overlooked. This study compares mental health status between homebound and non-homebound older adults, and examines the moderation effect of loneliness. The sample consists of 1,301 older adults aged 60 and over (39% homebound, 49% females, mean age = 69) from Shandong Aging and Health Survey, conducted by Shandong Provincial Government in 2019. Mental health status was measured by feelings of depression, not cheerful, bored, not calm or peaceful, and not happy. Compared to non-homebound older adults, homebound older adults tend to be older, lower educated, live in rural areas, and in worse health conditions. Results from generalized linear regression models show that controlling for demographic and physical health status, homebound population were more likely to have worse mental health status than other Chinese older adults. Feeling lonely, isolated, or lack of companionship intensifies the adverse effects of being homebound on older adults' mental health. Findings from this study suggest that homebound older adults in China had both physical and psychological sufferings. Social programs and interventions may be designed to improve homebound older adults' mental health. As the number of homebound older adults increases in China, medical care models may be tailored to improve the accessibility of healthcare services among people who are confined to their homes.

### SOCIAL ISOLATION AND HOSPITALIZATION RISK FACTORS AMONG COMMUNITY-DWELLING OLDER ADULTS

J. Mary Louise Pomeroy, and Gilbert Gimm, *George Mason University, Fairfax, Virginia, United States*

PURPOSE: This study examines psychosocial risk factors associated with hospitalization among community-dwelling older adults in the United States. METHODS: Using two waves of the National Health and Aging Trends Study from 2011 and 2015, we conducted descriptive and multivariate analyses of individual-level data from a nationally representative sample of 8,003 Medicare beneficiaries ages 65 and older. Associations between hospitalization and risk factors including social isolation, depression, and anxiety were assessed. Covariates included gender, race/ethnicity, age, region, insurance type, falls, and comorbidities. RESULTS: Overall, about 20.9% of older adults reported a hospitalization within the past year and 22.2% were socially isolated. The odds of hospitalization were higher for socially isolated adults (OR 1.17; $p = .02$), for depressed adults (OR 1.25; $p = .01$), and for individuals with anxiety (OR 1.25; $p = .02$). Individuals living in the Western region had lower odds of hospitalization (OR 0.71; $p = .001$), whereas men (OR 1.13; $p = .03$), those requiring assistance with activities of daily living (OR 1.48; $p < .001$), and those having one (OR 1.41; $p = .03$) or more (OR 3.05; $p < .001$) chronic health conditions had higher odds of hospitalization. CONCLUSION: Social isolation, depression, and anxiety represent significant psychosocial risk factors for hospitalization among community-dwelling older adults in the United States. Efforts to reduce health care costs and improve health outcomes for older adults should explore ways to strengthen social integration and improve mental health.

### THE ASSOCIATION BETWEEN HEALTH, SOCIAL ENGAGEMENT, AND PSYCHOLOGICAL WELL-BEING AMONG OLDER ADULTS IN CHINA

Yuekang Li, *Brown School, Washington University in St. Louis, St. Louis, Missouri, United States*

Psychological health and health-related quality of life of older adults have been long minimized by caregivers, service providers and the society in developing countries, such as China. According to the Stress Process Model, the stress of physical disadvantages influences mental health outcomes directly and indirectly. However, being socially engaged has the potential to reduce disease burden and improve psychological wellbeing of older adults. The purpose of this study is to examine the role played by social engagement in the pathway through which physical health predicts mental health. Using the WHO Study on Global AGEing and Adult Health China wave 2010, 6,276 individuals ages 60 years and older were included for analyses. Structural equation modeling was used to construct a directional path leading from the functioning and chronic disease, impacting the social engagement, in turn impacting the psychological wellbeing. All variables in this model are latent constructs. Functioning and chronic diseases in later life were associated with social engagement and psychological wellbeing, and the link between social engagement and psychological wellbeing was also significant. The effect of function was greater than that of chronic diseases. Though the significant indirect effect of physical health on psychological wellbeing was not supported in this study, this study suggests the multiple roles of social engagement as coping resources in the stress process of physical impairment and illness of older adults. This present study also adds to the existing literature by exploring how SEM methods can be applied to studies of social engagement.

### URBAN-RURAL MENTAL HEALTH DISPARITIES IN CHINA: THE NEIGHBORHOOD EFFECTS

Chengming Han, *Case Western Reserve University, Cleveland, Ohio, United States*

This paper intended to explore the neighborhood effects on mental health disparities of urban and rural residents in Mainland China. Data were drawn from the CHARLS baseline (2011). The sample included 450 neighborhoods, with 3907 urban residents and 13,391 rural residents older than 45 years old. Multilevel model was used to determine the neighborhoods' effects and individual effects on depressive symptom scores (CES-D). Independent variables included social activities, health status, and demographic characteristics. The result reveals three context effects of urban-rural neighborhoods: first, people living in urban communities reported better physical health, higher educational levels, and lower depressive symptoms than their rural counterparts. Second, people living in urban communities are more engaged in social activities than people living in rural villages. Third, the urban neighborhoods present more variations in depressive symptoms and social activities than the rural neighborhoods.

### SESSION 2864 (POSTER)

### DIVERSE POPULATIONS

### CHALLENGES AND PERSPECTIVES ON BREAST CANCER SURVIVORSHIP: THE JOURNEY CONTINUES

Victoria Raveis,[1] and Simona Kwon,[2] *1. New York University, Maplewood, New Jersey, United States, 2. NYU School of Medicine, New York, New York, United States*

Women have a 1-in-8 lifetime risk of breast cancer. Earlier diagnosis and treatment advances have improved 15- and 20-year survival rates. Increased survival can mean coping with the effects of cancer and its treatment over an extended period of time, while experiencing age-related changes in functioning and the emergence of other health issues. To explore breast cancer survivors' perspectives on their issues and concerns across the life-course, focus groups were conducted with a culturally diverse sample (N=18) of survivors (72% white, 28% Black, 11% Hispanic). Participants were 44-82 years old. Most, 83% were 50 and older, 56% were 60 and older. The majority (83%) were diagnosed in their 40's and 50's. Two were diagnosed in their early 30's and one at age 68. Participants reaffirmed the necessity, as a breast cancer survivor, of being a life-long health advocate on their own behalf, and the importance of being self-informed. As one woman commented: "Knowledge is power". Survivors shared that their emergent health issues were complicated by their cancer history, and, that, as a cancer survivor, "I never stop worrying". A widespread concern was not knowing if the health issues and co-morbidities they experienced (such as joint pain, neuropathy, tendinitis, heart disease), were age-related, a consequence of their cancer, or a late treatment effect. An overriding sentiment expressed was that clinicians have not recognized the importance of quality of life in cancer survival. As a survivor succinctly stated: "We are living longer, but we need to live long with quality of life."

### COPING WITH HURRICANE MATTHEW: LESSONS LEARNED ON THE IMPORTANCE OF RELATIONSHIPS

Allison Gibson,[1] Ethan Engelhardt,[1] and Erin Murphy,[2] *1. University of Kentucky, College of Social Work, Lexington, Kentucky, United States, 2. UT Arlington, School of Social Work, Arlington, Texas, United States*

The effects of natural disasters are daunting among older populations, especially those with intersecting vulnerable social locations, such as low-income aging racial/ethnic minorities. Yet, there is a paucity of literature on these experiences. The purpose of this study was to explore the experiences of this population before and during the 2016's Hurricane Matthew. Using a modified grounded theory approach, study participants were recruited through a snowball method and through flyers that were posted throughout affected communities. Semi-structured interviews were used in this study. The analysis focused on the identification of themes. The study aimed to gain knowledge of the experiences of 15 aging minorities with Hurricane Matthew (n=15). The sample was primarily 73.3% females, with a mean age of 69, and of racial/ethnic minorities (53.3% Black and 46.7% Hispanic). All participants self-identified as low-income. The main theme that emerged from this study on how low-income aging minorities cope with natural disasters was the reliance on social capital and the importance of human relationships. The results suggest that in the context of having low-income, aging minority rely on a range of support. Some support came through information and tangible help they received from family, friends, church community, and neighbors. Bridging social capital came from federal and non-profit agencies as well as help received from their employers. Pre-disaster planning should focus on supporting older populations in building social capital. This may be especially beneficial for low-income, aging minority, who may lack adequate financial resources on which to rely.

### DEVELOPMENT AND VALIDATION OF A COMMUNITY ASSESSMENT TOOL FOR DIVERSE RURAL FAMILY CAREGIVERS

Michael McCarthy,[1] Morgan Lee-Regalado Hustead,[2] Rachel Bacon,[2] Yolanda Evie Garcia,[1] Dorothy Dunn,[1] Heather Williamson,[2] and Julie Baldwin,[2] *1. Northern Arizona University, Flagstaff, Arizona, United States, 2. Northern Arizona University, Flagstaff, United States*

The aging of the United States population has led to an increase in the prevalence of Alzheimer's Disease and Related Dementias (ADRD). Many individuals with ADRD receive care from family members and friends who, themselves, experience poor health outcomes. ADRD family caregivers from diverse backgrounds are frequently underserved by health and social service systems. However, research suggests that diverse caregivers possess culturally-based strengths that serve as protective factors against poor outcomes. This presentation reports findings from a study to develop and validate a community assessment of the needs, assets, and resources of rural Latinx and Native American ADRD family caregivers. Initial assessment items were based upon validated measures developed for diverse ADRD and comparable caregiver populations. A Delphi process was used to obtain ratings from a 9-member expert panel (ADRD policy makers, providers, and family caregivers) about the degree to which the assessment was: (1) inclusive of different cultural groups; (2) respectful of cultural values and norms; (3) comprehensive with respect to needs, assets, and resources, and; (4) relevant to the lived experiences of diverse rural caregivers. Experts also provided qualitative feedback about modifying phrases to make the assessment more relevant to diverse respondents, removing sections, and adding items and response options to better reflect rural life. After two formal rounds of review and multiple iterations, between 77% and 100% of experts agreed that all sections of the assessment met criteria. Findings highlight the benefits of a systematic process for developing and validating community assessments for diverse, rural populations.

### ESTIMATING THE RISK OF POVERTY TRANSITIONS AT OLD AGE IN THE UNITED STATES: A SURVIVAL ANALYSIS FOCUSED ON THE HISPANIC POPULATION

Antonia Diaz-Valdes Iriarte,[1] and Fidel Bennett Ramos,[2] *1. Universidad Mayor, La Reina, Santiago, Region Metropolitana, Chile, 2. Universidad de Chile, Santiago, Chile*

Many Americans work well past the Social Security full retirement age. Moreover, the high labor participation rate of aged workers does not translate into better living conditions and decreased poverty at old age. According to the OECD's relative poverty threshold, the poverty rate at old age is 23.1% (14 points higher than the absolute measure), showing important increases as age progresses. In the context of financial pressure faced by SS, understanding the factors that could help people ensure themselves against poverty is crucial at old age, especially for groups such as Hispanics. Hispanics tend to rely mostly on SS, and tend to retire earlier than non-Hispanics due to limited working opportunities. Using data drawn from the Health and Retirement Study (2008-2016), cox survival models, were conducted to estimate the risk of transitioning into poverty during retirement on determinants of long-term accumulation of disadvantages over the life course (i.e.: wealth accumulation, medical care access, unemployment history). Results suggest that being foreign born and Hispanic are strongly associated with an increase on the probability of experiencing poverty at old age, conditional on health, education and employment status. Furthermore, delaying claims of SS by one year reduces the probability of falling into poverty by 3%, supporting that socioeconomic conditions faced by older adults, especially, the interactions between such factors and ethnicity are key in explaining poverty at old age. This would help policy makers to implement policies could be targeting those factors earlier on, beneficiating the individuals and society as a whole

### HEALTHCARE AFFORDABILITY AND ASSOCIATED CONCERNS AMONG OLDER ADULTS IN FLORIDA

Jacqueline Wiltshire, Barbara Orban, Kyaien Conner, Edlin Garcia Colato, Erica Anderson, and Iraida Carrion, *University of South Florida, Tampa, Florida, United States*

Rising healthcare costs create significant financial burden for Americans and is a threat to the well-being of our growing, racially/ethnically diverse, older population. In a mixed method study, we assessed ability to afford care and ascertain concerns about healthcare cost in a racially diverse sample of Floridians ages ≥ 65. We surveyed 170 adults (40.4% White, 27.6% African Americans/Black and 31.8% Latino/Hispanic) and conducted three race-stratified focus groups (n=27). Most participants had Medicare coverage (97.1%) and 27% also had Medicaid. Approximately 11.6% of Whites had problems paying medical bills in the past 12 months versus 14.9% of African Americans/Blacks and 24.1% of Latino/Hispanics. Additionally, 13% of Whites, 19.2% of African Americans/Blacks and 20.4% of Hispanics reported not getting needed prescription drugs because they could not afford them. Approximately 45.7% either perceived that their doctor "never" takes into account costs for treatment or did not know whether costs were considered. Multiple regression analyses showed no statistically significant racial/ethnic differences in affordability problems. From the focus groups, healthcare cost concerns most frequently identified by participants were the high cost of prescriptions drug, rising co-pays and out of pocket expenses, and medical billing. Participants' concerns about medical billing included understanding their bills, transparency in billing, timely posting of charges, and uncertainty about who to talk to about billing problems. Our findings suggest that routine discussions about healthcare costs with doctors or designated healthcare personnel should help ease financial burden and healthcare costs concerns among older adults.

### PARTNERING WITH TITLE VI DIRECTORS TO PUT SURVEY DATA TO USE IN IMPROVING THE HEALTH AND WELL-BEING OF NATIVE ELDERS

Collette Adamsen,[1] and Ramona Danielson,[2] *1. University of North Dakota, Grand Forks, North Dakota, United States, 2. North Dakota State University, Fargo, North Dakota, United States*

Through Title VI of the Older Americans Act, federally recognized American Indian tribes, Alaska Native villages, and Native Hawaiians are eligible for grant funding to promote elders' access to nutritious food and address other community-specific needs, such as transportation. Title VI directors are often short-staffed and work flexibly and creatively to accomplish administrative tasks as well as direct care for elders. The National Resource Center on Native American Aging (NRCNAA) developed the "Identify our Needs: A Survey of Elders", conducted with adults ages 55+ every three years since 1999, to help directors fulfill grant-required requirements to report on community needs assessment data about elders' needed services and health status. Directors administer the survey; responses are then scanned and analyzed by NRCNAA, with a site-specific summary returned to directors, at no cost to the site. To learn more about how survey data are utilized, NRCNAA staff interviewed four Title VI directors from across the US in 2018. In addition to meeting the needs assessment requirement, directors use the data to seek resources and support, build collaborative relationships, and evaluate and build their programs. Conducting the survey has allowed for discussions with elders about dimensions of elder health, advanced medical directives, and medical power of attorney. Additionally, specific interventions have been designed to address needs identified by the survey, including diabetic foot care, yoga, and falls prevention. Through the survey, the NRCNAA is able to support many dedicated Title VI directors working to improve the health and well-being of Native elders.

### RACE DIFFERENCES AND DEPRESSIVE SYMPTOMS AMONG COMMUNITY-DWELLING OLDER AMERICANS

Ethan Siu Leung Cheung, and Ada Mui, *Columbia University, New York, New York, United States*

Using data from the Wave 3 National Social Life, Health and Aging Project, this study examines cognition, stress and social support factors associated with depressive symptomatology among older White (n=2356) and Black/African Americans (n=473) living in the community. Bivariate analyses suggest that Whites were slightly older than Blacks [(M(SD) = 73 .24(8.18) and 72.52(8.69); 71)]; and had higher unmarried status (66.58% vs. 43.76%). In terms of cognitive functioning, Whites scored significantly higher than Blacks [Mean (SD) of MoCA Short Form were 10.44(3.06) and 7.75.0(3.33)]. There was race difference in depressive symptoms experienced (CESD Short Form: M(SD) = 20.99(4.01) for Whites; M(SD) = 21.35(4.33) for Blacks). In order to identify predictors of depression, multiple hierarchical regressions were performed. Results showed that race had significant independent effect and multiplicative effect with IADL impairment in explaining depression scores. To identify predicators for each racial group, parallel regression analyses were conducted and two models were significant. Findings show that unmarried status and IADL impairment were common predictors of depressive symptoms for the two groups, and the impact of both variables were stronger for Blacks (for unmarried status; b =-1.42 vs. -.52; for IADL impairment b = .23 vs. .13). For Whites, other unique predictors of depression were male gender, lower income, more ADL impairment, higher stress, less socialization and poor friendship quality. For Blacks, the only unique predictor of depressive symptoms was being younger age. The different correlates of depression for White and Black elders provide new insight into the design of race-sensitive interventions.

### RACIAL AND ETHNIC DIFFERENCES IN PREVENTIVE SERVICES USE AFTER THE AFFORDABLE CARE ACT'S ENHANCEMENT OF MEDICARE BENEFITS

Mohammad Usama Toseef,[1] and Wassim Tarraf,[2] *1. Wayne State University, Farmington Hills, Michigan, United States, 2. Wayne State University, Detroit, Michigan, United States*

As a consequence of the Affordable Care Act's enhancements of Medicare benefits, certain recommended clinical preventive services became available to Medicare beneficiaries without cost-sharing. We study the impact of these mandates on racial/ethnic disparities in the use of preventive services among traditional Medicare beneficiaries. We analyze nationally representative data on non-institutionalized Medicare seniors from the 2006-2016 Medical Expenditure Panel Survey (N=27,124). Our preventive services of interest include yearly receipt of cholesterol check, blood pressure test, flu shot, endoscopy, blood stool test, clinical breast examination, mammography and prostate exam. We estimate propensity score weighted difference-in-difference (DID) models to test for differences in preventive services utilization by race/ethnicity. Among traditional Medicare beneficiaries, we do not observe significant change in the use of most preventive services for Blacks and Hispanics compared to their White counterparts. However, Hispanics have significantly increased their use of blood stool tests relative to whites. Overall, we do not find major evidence to support a differential effect of reforms on race/ethnic minorities' uptake of preventive services following the mandates. Our results suggest that despite an overall benefit trough services expansion and cost-sharing elimination race/ethnic group differences persist. As such, disparities might continue and would require additional interventions. Reduction in disparities is a stated goal of US policy for many decades and achieving equity might require additional work and more varied and targeted interventions.

### SYSTEMATIC REVIEW OF INTERVENTIONS FOR LATINX AND AMERICAN INDIAN FAMILY DYADS COPING WITH CHRONIC ILLNESS

Michael McCarthy,[1] Angelica Sanchez,[1] Yolanda Evie Garcia,[1] Dorothy Dunn,[1] Heather Williamson,[1] Julie Baldwin,[1] Morgan Lee-Regalado Hustead,[1] and Tamilyn Bakas,[2]
*1. Northern Arizona University, Flagstaff, Arizona, United States, 2. University of Cincinnati, Cincinnati, United States*

The United States is experiencing growth in populations from culturally diverse backgrounds. Studies suggest that Latinx and American Indians experience chronic conditions such as cancer, heart disease, and diabetes in greater numbers than whites. Literature also suggests that Latinx and American Indian families play a significant role as informal caregivers for loved ones with chronic illness. However, little information is available about interventions to assist these patient-family caregiver dyads cope. The purpose of this systematic review is to synthesize published studies about psychosocial interventions developed or adapted for Latinx and American Indian care dyads in order to determine: (1) the benefits of these interventions; (2) their distinguishing features or adaptations, and; (3) recommendations for future intervention development. The protocol for this review was registered in advance with the International Prospective Register of Systematic Reviews (PROSPERO). We searched the databases CINAHL, PsycINFO, MEDLINE, and PubMeb using MeSH-derived keywords developed in consultation with a research librarian. Studies were included/excluded based upon pre-specified criteria. Three-hundred thirty-five records were identified, screened by the research team, and tracked according to PRISMA guidelines. After removing duplicates (n=9), studies that did not pertain to the conditions of interest (n=13), and studies that did not meet inclusion criteria (n=305), eight studies remained. Relevant information was abstracted from the final studies and synthesized by the research team. The majority of interventions for these populations are in cancer. Findings about benefits are largely inconclusive. Adaptations include a focus on cultural contexts, as well as culturally-based strengths, caregiving norms, and values.

### THE ROLE OF AGE, POLITICAL AFFILIATION, AND FRAMING IN ATTITUDES TOWARD HISPANIC AND LATINX IMMIGRANTS

Mengzhao Yan, Zachary Gassoumis, Kathleen Wilber, and Sheila Salinas Navarro, *University of Southern California, Los Angeles, California, United States*

The United States is experiencing rapid aging and increasing racial and ethnic diversity. Nevertheless, political rhetoric about immigrants has stoked negative assumptions and beliefs adding to fear and cultural misperceptions. Among those most affected are people of Hispanic/LatinX ethnicity, who comprise approximately 18% of the population. To address negative stereotypes, we sought to test how framing affected attitudes about Hispanic/LatinX immigrants and how people in different generations across the political spectrum respond to framing. As part of the "Latinos and Economic Security (LES)," a national research project funded by the Ford Foundation, we launched the "Well Being 501 Latino Economic Security" survey through the American Life Panel of RAND Corporation in 2018. Before answering the survey questions, participants (n=739) were randomly assigned to three different conditions: a 100-word priming statement focused on Hispanic/LatinX work ethic/religiosity/patriotism (33.29%), a 100-word priming statement focused on justice/equity/fairness (32.75%), and a control group with no priming statement (33.96%). We used multiple linear regression to examine relationships among demographic variables, age, political affiliation, and priming statements and attitudes toward Hispanic/LatinX immigrants. Key findings include: 1) age, political affiliation, education level, race and ethnicity, and gender explained 47.5% of the variance in attitudes; 2) baby boomers and generation X were significantly less tolerant of Hispanic/LatinX; 3) priming statements played a salient mediating role in neutralizing negative attitudes. By employing a developmental perspective, we proposed six recommendations from the aspects of reframing policy narrative and developing educational programs targeted at improving attitudes toward Hispanic/LatinX immigrants.

### TOGETHER, WE CAN MAKE THIS PLACE OUR HOME: CIVIC ENGAGEMENT AMONG ASIAN IMMIGRANTS

Cindy Bui,[1] Kyungmin Kim,[1] Qian Song,[1] and Yuri Jang,[2] *1. University of Massachusetts Boston, Boston, Massachusetts, United States, 2. University of Southern California, Los Angeles, California, United States*

Civic engagement is an important dimension of age-friendly communities but has been understudied among Asian immigrant groups. While research has attributed greater civic engagement among immigrants to acculturation factors, the influence of acculturation may be conditioned upon Asian immigrants' social network and place attachment to their city. We used data from the Asian American Quality of Life survey to analyze civic engagement activity (e.g., City council meeting, voting in a City election) among a diverse sample of middle-aged and older Asian immigrants in Austin, Texas (N = 994). 34.5% of the sample had participated in at least one civic engagement activity in the past 12 months. We examined how such civic engagement is associated with acculturation factors, and further examined whether one's friend network and perception of their city moderated the association. We found that number of years lived in the U.S., familiarity with mainstream American culture, and number of friends in one's social network were positively related to civic engagement activity. Furthermore, we found that the association between years lived in the U.S. and civic engagement was more pronounced for immigrants with larger friend networks; the association between familiarity with American culture and civic engagement was more pronounced for immigrants with more positive perceptions of the city. These findings highlight that acculturation may not operate alone in civic engagement among Asian immigrants. Rather, it may also be important to create opportunities for Asian immigrants to feel connected to their community and build meaningful friend networks to encourage civic engagement.

### SESSION 2865 (POSTER)

### TECHNOLOGY AND OLDER ADULT INTERFACE

### #DEMENTIA: AN EVALUATION OF THE WORLDWIDE DEMENTIA TWITTER NETWORK

Varun Ayyaswami,[1] Divya Padmanabhan,[2] and Arpan Prabhu,[3] *1. University of Maryland Baltimore, BALTIMORE, Maryland, United States, 2. University of New England College of Osteopathic Medicine, Biddeford, Maine, United States, 3. University of Arkansas for Medical Sciences, Little Rock, Arkansas, United States*

Social media engages an international network of healthcare stakeholders. Our study characterized the #dementia community on Twitter, which we hypothesized has increased substantially in the last six years. Symplur Signals, a healthcare social media analytics platform, was used to analyze public #dementia tweets between 1/1/2014 and 12/31/2019. Spam or users unclassified by the platform were excluded. Tweet activity, content, user characteristics, engagement, associated hashtags, sentiment, and network analysis metrics were obtained. Exactly 2,149,494 tweets made by 105,938 users resulted in 21,460,413,770 impressions. There was a 44.6% and 29.1% compound annual growth rate of tweets and users, respectively. The five most frequently tweeted words were people, care, help, living, and support. Among users with identifiable locations, the United Kingdom, United States, and Canada had the greatest number of users. Healthcare associated individuals (17.8%), health advocacy organizations (17.6%), non-healthcare associated individuals (17.0%), and non-healthcare associated organizations (13.8%) were among the top 500 influencers based on tweet number. Hashtags related to caregiving (#Caregiving, #carers, #care, #caregivers, #caregiver, #dementiacare) and Alzheimer's disease (#alzheimers, #Alzheimer, #Alz, #EndAlz) were among the top 15 associated hashtags. Analysis of the 1,000 most recent tweets shows more tweets with positive (71.0%) than negative (29.0%) sentiments. Network analysis mapping connections between users shows advocacy organizations (i.e. @ASAging) and other health individuals (i.e. @LEAD_Coalition) were central conversation hubs in 2019. The #dementia network discusses themes important to older adults. The network's rapid growth has enabled increased dissemination of dementia-related information by both healthcare and non-healthcare associated individuals and organizations.

### CARE ME TOO: A MOBILE APP FOR ENGAGING CHINESE IMMIGRANT CAREGIVERS IN SELF-CARE AND ITS USER-EXPERIENCE TESTING

Mandong Liu, Tongge Jiang, Kexin Yu, Shinyi Wu, Maryalice Jordan-Marsh, and Iris Chi, *University of Southern California, Los Angeles, California, United States*

Caregiving and self-care are challenging for Chinese immigrants in the U.S. due to limited accessible support and resources. The team developed a Care Me Too app for engaging Chinese immigrant caregivers in self-care and conducted a user-experience test to assess its usability and acceptability. The aims of this paper are to report the testing results and develop guidelines to support mHealth app design for immigrant caregiver populations. Twenty-two Chinese caregivers participated in the test, which consisted of two parts: in-lab testing and one-week at-home testing. In-depth face-to-face interviews and follow-up phone interviews were used to test the app's usability and acceptability and solicit participants' feedback for app design and functions. Directed content analysis was used to analyze testing transcripts. Participants reported uniformly positive ratings of usability and acceptability of the app and provided detailed suggestions for app improvement. We generated some mHealth app design guidelines, including weighing flexibility vs. targeting majority preferences, increasing text sizes, using colors effectively, providing engaging and playful visual designs and functions, simplifying navigation, simplifying login process, design functions to cater to the population's context, etc. We concluded that culturally and linguistically appropriate mHealth apps are appealing to immigrant caregivers for health promotion.

### LEVERAGING ASSISTIVE TECHNOLOGY RESOURCES TO SUPPORT AGING IN PLACE: A SCOPING STUDY

George Mois,[1] and Jenay Beer,[2] *1. University of Georgia, Lawrenceville, Georgia, United States, 2. Institute of Gerontology, Athens, Georgia, United States*

Aging in place is the preferred living arrangement for most older adults. However, the challenges that often accompany longevity coupled with housing which lacks proper modifications presents concerns about older adults' safety and wellbeing. Advancements in assistive technologies have promising potential in helping address many of these challenges and support aging in place. The purpose of this scoping review was to survey the current literature to understand why, how, and what assistive technologies are adopted and utilized to help support aging in place. We followed the Arksey and O'Malley (2005) methodological framework for scoping studies, searching seven databases and systematically assessed 611 titles/articles. Findings were organized using frequencies and themes. Following the inclusion/exclusion criteria, 12 articles were included. Upon thematic analysis, three main themes emerged: 1.) challenges experienced in the context of aging in place, 2.) technology adoption, and 3.) technology types and applications. Findings indicate technology can serve an important role in helping support aging in place and can serve as a medium to deliver and increase access to resources to support physical, social, and psychological wellbeing. The technologies most frequently utilized include personal devices and smart home technologies. The adoption and use of technologies can be impacted by the perceived ease of use, perceived usability, family/caregiver, self-selection, involvement in technology development, policies supporting access, and environment factors. Our findings indicate that there is a current gap in the understanding of how older adults are interacting with technology and how long term use impacts wellbeing and aging in place.

### LIVING ARRANGEMENTS, ELECTRONIC COMMUNICATION USE, AND PSYCHOLOGICAL FUNCTIONING

Hyunju Shim, Jennifer Ailshire, and Eileen Crimmins, *University of Southern California, Los Angeles, California, United States*

Older people who live alone may benefit more from using electronic communication than those who live with others. Although living alone has been linked to a higher risk of depression and social isolation, few studies examined the effect of using electronic communication separately by living arrangements. The current study examines the effect of electronic communication use by living arrangements for people aged 65 and older. Using the 2011-2018 National Health and Aging Trends Study (NHATS), we examine how the frequency of emails/texts is associated with changes in psychological well-being and depressive symptoms accounting for sociodemographic, health, social network characteristics (N=6,897). Multilevel growth curve models showed that those living alone or with others were more likely to have fewer depressive symptoms at baseline if they used electronic communication, but the use did not affect their trajectory of depression. Those living alone or with others who used electronic communication did not have higher psychological well-being at baseline, nor did it affect their trajectory. The overall findings raise a question on the effectiveness of promoting electronic communication technology as a substitute for in person interaction for older adults living alone in the community.

### MEDIA IMAGE LANDSCAPE: AGE REPRESENTATION IN ONLINE IMAGES

Colette Thayer, and Laura Skufca, *AARP, Washington, District of Columbia, United States*

This study looked at the extent to which the 50-plus population is portrayed in media images online. A random sample of images was drawn from 2.7 million images downloaded from professional and semiprofessional domains and social distributions for brands and thought leaders. Natural language processing technology was employed to find images using topical guides chosen to be reflective of online images. Results of this study showed that while some media has moved toward more positive visual representation of older people, the 50-plus population is still not accurately portrayed in the media. For example, while nearly half of the U.S. adult population is age 50-plus, only 15% of images containing adults include people this age. In addition, when the 50-plus are shown, they are more likely to be portrayed negatively than those under age 50. The 50-plus population is often portrayed as dependent and disconnected from the rest of world although most are actively engaged in their communities. They are rarely shown with technology and in work settings. Furthermore, while a myriad of vibrant personalities come across in images of adults under age 50, the representation of people 50-plus starts to homogenize and exaggerate stereotypical and outdated physical appearance characteristics. This study demonstrates the need for visual representations that reflect greater diversity and authenticity of the 50-plus population as these images affect the attitudes, expectations, and behaviors of older and younger people alike. Keywords: ageism, reframing aging, media image representation

### MODERATING EFFECT OF RACE AND ETHNICITY BETWEEN TECHNOLOGY USE AND SOCIAL ISOLATION

Sunghwan Cho, Tommy Buckley, and Kyeongmo Kim, *Virginia Commonwealth University, Richmond, Virginia, United States*

Social isolation among older adults has brought about poor outcomes of their health and well-being. Information and Communication technology (ICT) is known to alleviate social isolation of older adults. However, it is unknown how ICT access and use are associated with social isolation by race. This study examined the association of ICT and social isolation from the National Health and Aging Trends Study (NHATS), estimating moderating effects of race and ethnicity. The sample for this study was community dwelling Medicare beneficiaries aged 65+ (n=5,567). An index for ICT was formed from five domains: social network websites, email and texting, working cellphone, tablet devices and online computer use (range 1-5, mean=3.96, SD=0.927), and social isolation was derived from responses to five areas: living arrangement, attending religious activities, numbers of important people to talk with, attending other activities such as club participation and volunteer work (range=0-2, mean=1.74, SD=0.927). Race and ethnicity included White (69.6%), African-American (20.7%), Hispanic (5.5%) and Asian/Pacific Islander (2.5%). Multiple linear regression was used using a moderating effect of race/ethnicity, including relevant covariates. Findings revealed increased ICT use was associated with lower social isolation ($b=0.05$, $p<0.05$), and race/ethnicity was a significant moderator in the association between ICT and social isolation for African-Americans ($b=0.08$, $p<0.05$) and Hispanics ($b=0.15$, $p<0.05$) compared to White older adults. The findings indicate that racial differences should be considered when applying technology use to reduce older adults' social isolation. Practitioners can provide racially competent ICT services for older adults interested in tech-based communication.

### ROLE OF HEALTH INFORMATION TECHNOLOGY IN THE ENGAGEMENT PROCESS OF OLDER ADULTS' CANCER TREATMENT DECISION MAKING

Maryum Zaidi,[1] Priscilla Gazarian,[2] Heather Mattie,[3] Lisa Kennedy Sheldon,[4] and C. Ann Gakumo,[5] *1. UMASS, Boston, Shrewsbury, Massachusetts, United States, 2. University of Massachusetts Boston, Boston, Massachusetts, United States, 3. Harvard T.H. Chan School of Public Health, Boston, Massachusetts, United States, 4. Oncology Nursing Society, Pittsburgh, Pennsylvania, United States, 5. College of Nursing and Health Sciences, Boston, Massachusetts, United States*

This study investigates the role of Health Information Technology (HIT) in the process of patient engagement in treatment decision making in older adults in cancer care. Despite the role of HIT in patient engagement processes and government incentives for HIT development, research regarding HIT is lacking among older adults. The following study is a secondary data analysis of a subset of the Health Information National Trend Survey (HINTS 4, Cycle 3), including individuals 65 years old and above. Chi-square tests, logistic regression, and linear regression models were fit to study several sociodemographic, socioeconomic, and psychosocial variables in this study. The results show that education, poverty status, and self-management domain of the patient activation (which is a precursor of the engagement process) were significantly associated with access to and utilization of HIT. No significant differences between access to and utilization of HIT and the diagnosis of cancer were found. However, fatalistic beliefs about the diagnosis of cancer significantly impacted the use of HIT in all models, including those controlling for cancer diagnosis and access to HIT. Specifically, a one-point increase in cancer fatalism score is associated with a 59% decrease in the utilization of HIT, giving evidence that fatalistic beliefs about cancer can drive engagement behaviors regardless of a diagnosis of cancer. Our study provides vital information for providers and policy researchers to take into account for future implementation and development strategies of HIT in cancer care for older adults.

### THE CONNECTIONS OF ELDERLY CHARACTERS AND TAIWANESE SOCIETY IN EDWARD YANG'S MOVIE "YI YI" (A ONE AND A TWO)

Sheng-Chin HSU, *Ming Chuan University, Taipei, Taiwan (Republic of China)*

As an aging society, Taiwanese is facing the low birthrate and low death rate, and many policies and social systems are facing difficulties. According to the social atmosphere, young and senior groups have many conflicts in between. Finding an alternative approach to reveal the social value of aged people becomes an important mission. The Taiwanese' movie "Yi-Yi"(A one and a two, 2000) is the final masterpiece of Director Edward Yang, and he won the best director in Festival de Cannes. The story is taken place in a traditional Taiwanese wedding party, and there is a grandma who was invited to this party before her pass out. The grandma did not weak up until the end of this story. The director Yang filmed this sick character in the story and he showed the family members were gathering around their grandma. This study adopts the narrative analysis on elders in "Yi Yi". There are three findings. First, the elder character is speechless but her sickness drives family members coming home. Second, the long term care is a heavy duty for family, but it reflects the preparations of individual physically and emotionally. Third, the meaning of image of elders is not image itself but family solidarity and social connection. The narrative theory and gerontology build a perspective to understand the social values and narrative functions of elderly people in "Yi-Yi". It shows the conflict between different age groups and enlarges the spectrum of understanding elders in both Taiwanese' movie and Taiwanese society.

### SESSION 2866 (POSTER)

### AGING POLICY AND ECONOMICS

### ADDING LIFE TO YEARS: ADOPTING AGE-FRIENDLY PUBLIC HEALTH IN WASHINGTON STATE

Carolyn Ham, Marci Getz, and Janna Bardi, *Washington State Department of Health, Olympia, Washington, United States*

Age-Friendly Public Health is an emerging concept and practice aiming to equip the public health infrastructure to more readily address aging issues. In 2019, the Washington State Department of Health (WA DOH), began operationalizing Age-Friendly Public Health within the agency. Work entailed bringing together a cross-agency workgroup on healthy aging and drawing on expertise from agency programs including environmental health, immunizations, falls prevention, emergency preparedness, chronic disease and many others. The workgroup conducted an internal assessment of 'touch points' regarding aging work within WA DOH. The Life Course Perspective was used as a theoretical framework to categorize current efforts, and existing research on social determinants of health in aging were used to identify potential areas for future impact. In addition, the Healthy Brain Initiative (HBI) Road Map for public health was used to identify current and potential approaches to addressing Alzheimer's Disease and Related Dementias. This workgroup inventoried WA DOH's current efforts that align with the HBI agenda, and identified 88 opportunities to promote healthy aging and brain health. This work contributes to the emerging field of Age-Friendly Public Health by demonstrating strengths and challenges of advancing healthy aging through state public health agencies.

### BRINGING OLDER ADULTS TO THE TABLE: OPPORTUNITIES FOR CONSUMER ENGAGEMENT IN PLANNING AND POLICY MAKING

Alice Prendergast, and Kristi Fuller, *Georgia State University, Atlanta, Georgia, United States*

Efforts to include community voice in health policy and service planning are gaining recognition and support in the United States. Findings suggest community involvement can contribute to a better understanding of systems and factors that impact health, and, subsequently, more effective and sustainable policy and program design. Additionally, engagement can increase community buy-in, and community members can gain a greater awareness of services; increased confidence navigating systems; feelings of social connectedness; and capacity to advocate around issues through participation. Despite these findings, the extent to which community members are engaged in planning and decision-making varies considerably. Researchers from Georgia State University conducted a review of state plans on aging using the Person-Centered Outcomes Research Initiative (PCORI) Engagement Principles and the Health Research & Educational Trust's Community and Patient Engagement Spectrum as frameworks to assess evidence of community engagement. The frameworks recognize engagement throughout the planning process, including design, data collection and interpretation, and dissemination. The review revealed that few planning processes described significant engagement, but rather met the minimal requirements established by federal policy. Federal guidance on community-informed planning practices is sparse, as are resources to support states in adopting these processes. To address this gap, the research team drew on the frameworks and other promising practices to design two community engagement projects, both in partnership with Georgia's Division of Aging Services. Methods for participant engagement, data collection, interpretation and application of results, and lessons learned through both projects will be discussed, as well as potential implications.

### ECONOMICS RISKS OF AND GROWTH OF THE HIGH DEDUCTIBLE HEALTH PLAN AMONG PATIENTS WITH COGNITIVE IMPAIRMENT

Kevin Lu,[1] Sam Li,[2] Jing Yuan,[3] and Minghui Li, *1. University of South Carolina, Columbia, South Carolina, United States, 2. University of Tennessee Health Science Center, Memphis, Tennessee, United States, 3. Rutgers University, Piscataway, New Jersey, United States*

OBJECTIVES: High Deductible Health Plan (HDHP) is characterized by higher deductibles and lower monthly premiums. Nevertheless, health economists are concerned that HDHPs may reduce or delay needed care, which will ultimately lead to poorer access to care for chronically affected participants. The objectives of this research are 1) to investigate the HDHP enrollment trend over the past decade; and, 2) to determine the effects of HDHP on risks of financial access risks among adults with cognitive impairment (CI). METHODS: Data were obtained between 2010-2018 from National Health Interview Survey (NHIS). Financial access to healthcare was assessed based on 6 survey questions by CDC. For data analysis, simple T tests and Chisq tests were used where appropriate, with multi-variable logistic regressions implemented to evaluate the effects of HDHP on risks of financial access. RESULTS: Of the 103,649 enrollments, 1,148 were with cognitive impairment and 102,501 were without CI diagnosis. A 55% increase in HDHP registers with cognitive impairment was observed from 2010 (30.50%) to 2018 (47.24%). After controlling for confounding variables, patients with HDPHs were more likely to have risks of financial access compared to those without HDHP (OR= 1.313, 95% CI, 1.002-1.719, p=0.0483). CONCLUSIONS: HDHPs are intended to support effective care options and reduce health care costs. Our research among CI patients with HDHP experienced more financial access risks than those without HDHP, indicating that HDHPs might have unintended consequences of healthcare usage. Employers and health care decision-makers may need to consider providing compensation to those HDHP enrollers with CI.

### LOCAL INITIATIVES TO FUND SERVICES FOR ELDERS: INCREASED COMMUNITY RECOGNITION OF THE IMPORTANCE OF SOCIAL CARE

Athena Koumoutzis, Sara Stemen, Renusha Maharjan, Jennifer Heston-Mullins, Pamela Mayberry, and Robert Applebaum, *Miami University, Oxford, Ohio, United States*

Despite the growing number of older adults in the U.S., federal and state funding for non-medical supportive services remains limited. Recent work reports that states with a more generous supply of supportive services, including home delivered meals and personal care, have fewer low care residents in nursing homes. To boost this supply, some local communities across the nation are exploring alternative funding sources. Our review found 400 local communities across 15 states using voter-approved local revenue streams to fund aging services, such as property tax levies and payroll and sales taxes, and that this strategy has been politically popular. In this paper we provide results from the first national survey of these local communities. Study results found considerable variation by state in number and scope of local initiatives, with Ohio and Michigan each reporting about 70 communities with local property tax levies, while California and Washington had only one community each using this approach. Local programs ranged in size from generating less than $25,000 in annual revenue to more than $35 million. The organizational structure for these programs, and the administrative approaches, such as the use of care managers, varied by state and community. Programs provided an array of services, but typically included traditional social care services such as home delivered meals, homemaker/personal care, transportation, and home emergency response systems. Criteria for program participation also varied, but most were targeted to serve older adults with disability who did not meet Medicaid financial or functional eligibility criteria.

### MONEY FOLLOWS THE PERSON AND INFORMAL CAREGIVERS: INSIGHTS INTO A NEW STAGE OF THE CAREGIVING CAREER

Julie Robison,[1] Noreen Shugrue,[1] Chanee Fabius,[2] Richard Fortinsky,[1] Martha Porter,[1] and Kristin Baker,[3] *1. University of Connecticut, Farmington, Connecticut, United States, 2. Johns Hopkins Bloomberg School of Public Health, Baltimore, Maryland, United States, 3. UConn Health, Farmington, Connecticut, United States*

The Money Follows the Person (MFP) program transitions people to the community after extended institutional stays. This study examines effects of this transition on informal caregivers in this new caregiving career stage. Analyses explore whether and how MFP affects caregivers according to caregiver race/ethnicity, and care recipient age and disability type. Data come from surveys with 686 caregivers of persons in Connecticut's MFP from November 2014-November 2018. Using Pearlin's Caregiver Stress Process Model, bivariate and multivariate analyses examine predictors of multiple caregiver well-being indicators. Care recipients: older adults (50%), and younger persons with physical (35%), mental health (8%) or developmental (7%) disabilities. Caregivers: non-Hispanic White (62%), non-Hispanic Black (24%), and Hispanic (14%). Caregivers' average assistance is 5 days/week, 6 hours/day, with 3 activities of daily living and 5 instrumental activities; 11% are paid for caregiving. Compared to other community-based samples, they report low mean levels of burden (4.7 of 16), anxiety (2.2 of 18) and depressive symptoms (31%), and high positive feelings about caregiving (9.5 of 12). A majority feel less stressed (60%) or no change in stress (20%) compared to before and during the institutional stay. Caregivers across the four care recipient groups don't differ on most outcomes, although more caregivers of people with developmental disabilities (82% vs. 55-61%) report less stress once the person transitions. Black and Hispanic caregivers report more intensive caregiving, but White caregivers report more burden and subjective stress. Findings illustrate the benefits of programmatic support during a newly defined post-institutionalization caregiving career stage.

### PATTERNS OF COURT MONITORING OF GUARDIANSHIP PRACTICE IN IOWA

Tami Swenson, *Des Moines University, Des Moines, Iowa, United States*

Guardianship, as a best practice, should provide persons with medical incapacity support for better medical care decision making, but the examination of health outcomes for those under guardianship has a dearth of evidence in practice and within the research literature. This study addresses this gap by using the publicly-available Iowa Courts online database to collect data elements useful for the investigation of the process of guardianship practice. A systematic random sample using the 99 counties within Iowa as strata was extracted for all open guardianship cases. Most of the sampled cases (96%) had private appointed guardians. Approximately 28% had case histories over 10 years, 26% 5 to 10 years, 12% 3 to 5 years, 22% 1 to 2 years, and 12% were open cases for fewer than 12 months. Court monitoring of medical conditions varied with annual, biannual, triennial, or 5 year requirements. Delinquencies in filing required medical condition documentation was common and over half of all cases had at least one delinquency in their case history and 33% had a filing delinquency within the past 2 years. Local judicial courts vary in the amount of supervision they provide guardians, and the findings from this research provide needed research to inform guardianship practices as demand increases for these services with the demographic imperative of an aging population and increasing prevalence of dementia-related health conditions.

### PROMOTING HEALTHY AGING THROUGH POLICY CHANGE ON THE FEDERAL LEVEL: STRATEGIES FOR SCHOLARS AND CLINICIANS

Alison Hernandez,[1] Lieke van Heumen,[2] Luming Li,[3] Marie Gualtieri,[4] Ivorie Stanley,[1] Lisa O'Neill,[5] and

Nancy Kusmaul,[6] *1. Northwestern University, Washington, District of Columbia, United States, 2. University of Illinois at Chicago, Chicago, Illinois, United States, 3. Yale School of Medicine, New Haven, United States, 4. Washington, District of Columbia, United States, 5. University of Arizona, Tucson, Arizona, United States, 6. UMBC, Baltimore, Maryland, United States*

Influencing health policy change is a significant focus for scholars and clinicians in the aging field. Nevertheless, they seldom receive formal training on how to influence the policymaking process effectively or how to translate their clinical and research experiences to inform policymakers best. Exposing scholars and clinicians to the policymaking process can advance their effectiveness as they seek to realize meaningful change to promote healthy aging of our population. This poster presentation focuses on providing scholars and clinicians with strategies to understand and influence the federal policymaking process. The presentation addresses the current policy environment in which federal health and aging policy is made and describes challenges to this process. Four strategies are identified to help scholars and clinicians influence the policymaking process: 1) identify a problem and any relevant policy that corresponds to the issue, 2) identify evidence-based solutions that relate to quality improvement, population health, or reducing per capita cost of healthcare, 3) grow relevant networks, meet experts, build relationships and connect with key stakeholders, 4) identify potential unintended consequences or barriers to the implementation of policy. By providing examples, this how-to aging and health policy road map provides context and guidance to stakeholder engagement, frameworks, and methods that can be used to engage in the policymaking process. The final part of this presentation explores ways to integrate health policy training and experience into scholars' and clinicians' professional development.

### SIMULATING THE EFFECTS OF CONSTRUCTION IN NYC'S CHINATOWN ON FRUIT AND VEGETABLE CONSUMPTION IN LOCAL RESIDENTS

Stella Yi,[1] Yan Li,[2] Valerie Imbruce,[3] Yi-Ling Tan,[1] Victoria Foster,[1] Vivian Wang,[4] and Simona Kwon,[1] *1. NYU School of Medicine, New York, New York, United States, 2. Icahn School of Medicine at Mount Sinai, New York, New York, United States, 3. Binghamton University, Binghamton, United States, 4. NYU School of Global Public Health, New York, New York, United States*

In 2017, the mayor of New York City (NYC) unveiled a 10-year plan to close the city's largest jail complex and to build four satellite detention centers – including one in Manhattan's Chinatown. Chinatown is a destination for affordable produce and its retail produce sector is comprised of street vendors and small stores, a style of fresh fruit and vegetable (FV) marketing the city promotes to achieve its goal of equitable access to healthy foods. The objective of this study was to project the impact of the proposed construction activity on FV consumption among residents in Chinatown. We developed an agent-based model that accounts for individual and neighborhood-level factors (e.g., age, gender, education, food environment) to predict FV consumption at the neighborhood level in NYC. We assumed that long-term construction will lead to the closure/migration of fresh produce vendors and therefore a reduction of FV access. We simulated three scenarios in which the number of fresh produce vendors is reduced by 5%, 10%, and 15% due to construction. Results suggest that planned construction could decrease the consumption of FV by 2.1%, 4.4%, and 6.8% among residents in Chinatown if the construction would reduce the number of fresh produce vendors by 5%, 10%, and 15%, respectively. Preliminary sensitivity analyses demonstrate the negative impact of the construction on FV consumption could be greater among older (65+ years) vs. young adults. The planned construction of a detention center in Chinatown may decrease the consumption of FV among its residents, particularly older adults.

### THE FAMILY AND MEDICAL LEAVE ACT: A POLICY ANALYSIS AND RECOMMENDATIONS TO ADDRESS EMPLOYED CAREGIVER BURDEN

Geunhye Park, and Erin Robinson, *University of Missouri-Columbia, Columbia, Missouri, United States*

Family caregiving plays a pivotal role in the long-term care system in the U.S, as there are over 40.4 million people providing unpaid care to individuals aged 65+ (U.S. Bureau of Labor Statistics, 2019). The majority are women providing supports to a parent/grandparent and provide an average of three hours of care each day. This places greater demands on family caregivers in balancing their dual caregiver/employment roles. The Family and Medical Leave Act (FMLA) of 1993 enables family caregivers to take unpaid leave to provide supports to immediate family. While FMLA was intended to provide flexibility to employed caregivers, many struggle with family-work conflicts and caregiver burden is high. Therefore, this conceptual paper offers a critical examination of FMLA and how family caregivers of older adults are impacted. Results of this analysis revealed three themes. First, FMLA is largely inadequate for employed caregivers, as only 60% of the workforce are eligible and unpaid leave restrictions create considerable financial hardship. Second, employer discrimination is high and family caregiving discrimination claims have dramatically increased since FMLA was enacted. And third, many employed caregivers are unaware of FMLA policies and eligibility requirements, which results in underutilization of benefits. Based upon these results, several policy and employer recommendations can be made, such as expanding FMLA coverage to include paid leave and non-immediate family caregivers. Additional recommendations will also be addressed. As it has been nearly 30 years since FMLA was enacted, updated policy is vital to continue supporting employed caregivers in their roles.

### THE LONGITUDINAL IMPACT OF PRIVATE HEALTH INSURANCE ON THE USAGE OF OUTPATIENT SERVICES AMONG AGING KOREANS

Narae Kim, *University of Southern California, Los Angeles, California, United States*

South Korea has a universal national health insurance system; however, its coverage is only about 65%. Consequently, many Koreans have supplemental private health insurance. This study aims to examine 1) how utilization of health care services and out-of-pocket expenditure of outpatient services varies by private health insurance status and 2) whether this relationship changes for these individuals over time. We analyze six waves of the Korean Longitudinal Study of Aging (KLoSA) from 2006 to 2016. Our primary outcomes of interest are the total number of outpatient visits and total out-of-pocket spending on outpatient services per year. Our independent variable of interest is private health insurance holding status. We use simple OLS regressions for each year to test if differences exist and change over time, controlling for age, sex, education, income, and various indicators of health conditions. The difference between those who never had private health insurance and those who always or sometimes had private health insurance becomes larger over time. When comparing total out-of-pocket expenditure, those who always had private health insurance have the lowest spending at the beginning of the study period (2006); however, their spending increases sharply by 2010 and remains higher than the other groups for the rest of the study period. Findings suggest that those who always had supplemental insurance use outpatient services more frequently and spend more out-of-pocket for services as a result. In addition, discrepancies become larger over time. As private health insurance holders age, the risk of higher utilization of outpatient services grows.

### TRENDS IN HEARING AID USE AMONG OLDER ADULTS IN THE UNITED STATES, 2011-2018

Nicholas Reed,[1] Emmanuel Garcia Morales,[2] and Amber Willink,[3] *1. Johns Hopkins University, Baltimore, Maryland, United States, 2. Johns Hopkins Bloomberg School of Public Health, Baltimore, Maryland, United States, 3. The University of Sydney, Sydney, New South Wales, Australia*

Hearing loss among older adults is prevalent and associated with dementia and health care utilization. However, cross-sectional data suggest less than 20% of adults with hearing loss use hearing aids. There is a paucity of studies examining trends in hearing aid ownership over time. This study analyzed data from the 2011, 2015, and 2018 cycles of the National Health Aging and Trends Study (NHATS), a nationally-representative longitudinal study of Medicare Beneficiaries. Participants were asked "in the last month, [have you/has [he/she]] use a hearing aid or other hearing device?" ("yes" or "no"). Among a weighted sample of Medicare Beneficiaries 70 years and older (26.47 million in 2011; 29.70 million in 2015; and 33.28 in 2018), the overall proportion who own and use hearing aids rose from 14.96% in 2011 to 16.90% in 2015 to 18.45% in 2018. As age increased so did the proportion of older adults who used hearing aids. A smaller proportion of Black Americans used hearing aids across time and experienced a smaller overall increase in the proportion in hearing aid ownership over the 8-year period compared to White Americans (+0.78% vs. +4.30%). Black women had the lowest rates of hearing aids use across the 8-year period. Notably, older adults at less than 100% of the federal poverty level experienced an overall decrease in proportion of hearing aid ownership and use. This study lays the groundwork to examine the impact of the Over-the-Counter Hearing Aid Act of 2017 across subpopulations when it takes effect in 2021.

### TRENDS IN MORTALITY IN PUERTO RICO BETWEEN 1979 AND 2018: AN ANALYSIS OF THE PUERTO RICO HEALTHCARE REFORM

Alexis Santos, *Pennsylvania State University, University Park, Pennsylvania, United States*

Between 1993 and 2000, the Government of Puerto Rico decided to transform the role of the government from a provider of healthcare to an insurer. Despite claims about the success of the reform, no study has assessed whether it improved the health of the population or reduced mortality. The aim of this study is to assess whether the implementation of the Puerto Rico Healthcare Reform of 1993 reduced mortality and infant mortality in Puerto Rico in a significant way. I calculated crude death rates (CDR), age-standardized death rates, infant mortality rates, total deaths and life expectancy between 1980 and 2018. I used a quasi-experimental design to study the effect of the implementation of the Puerto Rico Healthcare Reform on these indicators. The primary objective was to estimate changes in trends after 2000. The Age-Specific Mortality Rates have reduced since 1980. The least pronounced change for 2018, in comparison to 1980, was for young adults (20-24 years, 25-29 years, and 30-34 years). The CDR was affected based on the implementation of the reform, but the Infant Mortality Rates was not. The Standardized Death Rate and deaths indicate that there was a small reduction in these indicators. I also found that the gains in life expectancy were concentrated in older adults (aged 65 and older). Analysis of all-cause mortality indicators allows for the evaluation of this healthcare reform. The reduction in mortality in the post-2000 period was not entirely due to the trend that existed before the healthcare reform was implemented.

### SESSION 2867 (POSTER)

### SOCIAL DETERMINANTS OF HEALTH AND AGING

### A SCOPING REVIEW OF HOW FINANCIAL HARDSHIP IS MEASURED AMONG OLDER ADULTS IN THE UNITED STATES

Reginald Tucker-Seeley,[1] Ryan DoyLoo,[2] and Leora Steinberg,[1] *1. University of Southern California, Los Angeles, California, United States, 2. Tucker-Seeley Research Laboratory, Malibu, California, United States*

The association between socioeconomic status and health/healthcare related outcomes across the life course is well established; however, the specific mechanisms that underlie this complex association are not well understood. There have been calls in the health disparities literature for greater explication of the socioeconomic factors associated with differential outcomes for racial/ethnic minorities and socioeconomic groups. Recent research offers an expanded notion of socioeconomic circumstances by including indicators of financial hardship; however, there has been little conceptual and measurement clarity for gerontology research. To fill this gap, we conducted a scoping review of how financial hardship has been defined and measured in research with older adults. Using an adapted version of the Preferred Reporting Items for Systematic reviews and Meta-Analyses

(PRISMA) protocol, N=25 articles were identified through the following indexes: PubMed, CINAHL, PsycInfo, Sociological Abstracts, and Embase. Articles were included if they were published after January 1, 2000, published in the United States, and conducted with adults aged 50 and older. Our study found neither a consistently used term for nor a definition of the financial hardship experience; however, two key domains were consistently measured across studies with older adults: food insecurity and medical expenses were measured in N=9 and N=13 studies, respectively. Greater conceptual and measurement clarity in research on financial hardship among older adults helps to unpack the complex ways in which socioeconomic circumstance are experienced, make comparisons across studies measuring the financial hardship experience, and to identify the specific aspects of financial hardship for intervention.

### ACCESS TO INFORMATION ON NEEDED SERVICES MEDIATES THE ASSOCIATION BETWEEN INCOME AND SELF-RATED HEALTH

Sara Moss,[1] Katie White,[2] and Holly Dabelko-Schoeny,[1]
*1. Ohio State University, Columbus, Ohio, United States,*
*2. Age Friendly Columbus, Columbus, Ohio, United States*

Prior research has documented that individuals with fewer financial resources report lower self-rated health. The relationship between financial status and health is important to understand among older adults, for whom income is often fixed, and who face unique health stressors. In this study, we examined whether physical activity, connection to community, and access to information, variables which have demonstrated associations with financial resources and health in previous studies, accounted for unique variance in the association between income and self-rated health. We conducted a secondary data analysis of a community sample of 184 adults over the age of 50 in a large, Mid-western city. We tested a mediation model, entering income as a predictor of self-rated health with frequency of physical activity, feelings of being disconnectedness, and ability to find information about needed services as hypothesized mediators, controlling for the effects of race, education, and gender. Access to information about needed services, ab = .08, 95% CI [03, .15] accounted for unique variance in self-rated health scores beyond the direct effect of income, c' = .38, $p < .0001$, 95% CI [.22, .53]. These findings suggest that higher perceived access to information about needed services may partially explain the association between income and self-rated health. Future researchers should track these variables across time to establish causal associations between access to information and health. Additionally, further study of perceived access to information and objective measures of access to information is warranted, as the role of health literacy in this study is unclear.

### AGE AND SOCIOECONOMIC GRADIENTS IN FRAILTY AMONG OLDER ADULTS IN INDIA

Ravindra Chowdhary, *Tata Institute of Social Sciences, Mumbai, Maharashtra, India*

Although individuals with frailty and lower socioeconomic status (SES) are vulnerable to morbidity and early mortality, few studies have investigated this association. We intend to fill this gap with a study using older adults aged ≥ 50 years from the SAGE WAVE II in India. The Aim of study is to examine the association of frailty with SES and how this association varies across different age groups. A modified Fried phenotype approach with five frailty indicators was used to categorize 6560 older adults as frail, pre-frail or robust who had more than two, one or zero indicators, respectively: grip strength, exhaustion, weight loss, walking speed and physical activity. Multinomial logistic regression estimated the likelihood of being pre-frail and frail for various levels of SES, controlling and not controlling for confounders. This study also shows the overall socioeconomic gradients and age patterns of socioeconomic gradients of frailty indicators using predicted probabilities. Approximately 26%, 55% and 20% participants were robust, pre-frail and frail, respectively. The number of frailty indicators was positively associated with lower income and education levels in the case of controlling and not controlling for confounders. Also, among the higher age groups, individuals with low SES had higher chances of being frail.Overall, the results in this research indicated a negative low SES and frailty association as found in previous studies worldwide. This highlights the need for comprehensive and centered public health interventions for older adults with low SES.

### HEALTH ACCESS AND OUTCOME DIFFERENTIALS AMONG OLDER ADULTS AGE 50 YEARS OR ABOVE IN GHANA

Kodwo-Nyameazea Yale,[1] and Nana Akua Amponsah,[2]
*1. University of North Carolina at Pembroke, Pembroke, North Carolina, United States, 2. Coastal Carolina Neuropsychiatric Center, Fayetteville, North Carolina, United States*

Health outcomes are substantially influenced by demographic and socioeconomic indicators. Their presence or absence can act to boost or curtail health opportunities and outcomes. These health indicators are often not evenly distributed across geographic regions. The focus of this study is to explore how Ghana's north-south regional divide manifests in the health outcomes of its people. The study was based on a data from the Research on Early Life and Aging Trends and Effects (RELATE), a harmonized cross-sectional dataset with 147,278 respondents from 14 countries including Ghana. This study pooled 4724 respondents from Ghana aged 50 years or above. The study found that several demographic and socioeconomic indicators had significant associations with optimal health. However, most of the associations were attenuated after other variables in the model were controlled. Specifically, the study found that being a male gender and doing rigorous work were risk factors for living below optimal health in both the North and South regions. However, using healthcare services and being single or unmarried were protective factors but only in the South region. The results of the study also showed that indicators for good health generally favored the South region but the North region reported higher self-rated health score, smaller proportions of overweight and obese residents, fewer numbers of chronic health conditions, and a smaller proportion of resident living below optimal health. This finding demonstrates that the link between positive health indicators and healthiness has subtle nuances that could be attributed to how people conceptualize health and healthiness.

### INNOVATIVE PARTNERSHIP BETWEEN PRIMARY CARE, AAA, AND ACADEMIA CONNECTS 4MS AND SOCIAL DETERMINANTS OF HEALTH

Suzanne Leahy,[1] Katie Ehlman,[1] Lisa Maish,[2] Brad Conrad,[2] Jillian Hall,[3] Ron Wells,[3] and Laura Holscher,[4] *1. University of Southern Indiana, Evansville, United States, 2. Deaconess Clinic, Evansville, United States, 3. SWIRCA & More, Evanville, Indiana, United States, 4. Generations Area Agency on Aging, Vincennes, Indiana, United States*

Nationally, there is a growing focus on addressing geriatric care in primary care settings. HRSA's Geriatric Workforce Enhancement Program (GWEP) has called for academic and health system partners to develop a reciprocal, innovative, cross-sector partnership that includes primary care sites and community-based agencies serving older adults. Through the University of Southern Indiana's GWEP, the College of Nursing and Health Professions, the Deaconess Health System, three primary care clinics, and two Area Agencies on Aging (AAA) have joined to transform the healthcare of older adults regionally, including rural residents in the 12-county area. Core to the project is a value-based care model that "embeds" AAA care managers in primary care clinics. Preliminary evaluation indicates early success in improving the healthcare of older adults at one primary clinic, where clinical teams have referred 64 older adult patients to the AAA care manager. Among these 64 patients, 80% were connected to supplemental, community-based health services; 22% to programs addressing housing and transportation; and, nearly 10% to a range of other services (e.g., job training; language and literacy; and technology). In addition to presenting limited data on referred patients and referral outcomes, the presentation will share copies of the AAA referral log, to illustrate how resources were categorized by SDOH and added to support integration of the 4Ms.

### POSITIVE SUBJECTIVE HEALTH OF OLDER HOMELESS AND OLDER IRISH TRAVELERS: A PARTICIPANT-LED APPROACH

Bridin Carroll and Kieran Walsh, *National University of Ireland Galway, Galway, Galway, Ireland*

Older people experiencing homelessness and older Irish Travellers (OTOH) are both over-represented in the cohort who use acute health services. Impending health care reform in Ireland will be based on primary care models, meaning home and community care will be, for the first time, underpinned by a regulatory framework. For these reasons, this study aims to gain a nuanced understanding of how OTOH, as marginalised older people, might be best served by new home care and community care models. Using a qualitative, voice-led approach, a life course and structural determinants lens is employed to probe the health conditions, experiences and expectations of OTOH, as well as their perceptions and values around the concept of 'home'. The research processes and outcomes of one of five phases of research are presented in this paper: participant-led research. In this phase, five OTOH were trained and assisted to complete a short research project which fed into the goals of the wider study. Emergent findings suggest that social connections underpin health and well-being for OTOH, throughout the life course, and presently. This was also seen as a fundamental element for healthy and positive ageing. In addition, 'home' was defined with reference to the presence (or absence) of familial or other social connections. This study represents an important contribution to scholarship on old age social exclusion. It is entirely novel in its approach to focusing on OTOH health and wellbeing. The outputs of this study also have important implications for upcoming health reform policies in Ireland.

### PREDICTORS OF BREAST CANCER SCREENING BEHAVIORS AMONG AMERICAN INDIAN WOMEN IN THE NORTHERN PLAINS

Soonhee Roh,[1] Yeon-Shim Lee,[2] and Heehyul Moon,[3] *1. University of South Dakota, Sioux Falls, South Dakota, United States, 2. San Francisco State University, San Francisco, California, United States, 3. University of Louisville, Louisville, Kentucky, United States*

Purpose: This study examined predictive models of the utilization of mammograms among American Indian women adapting Andersen's behavioral model. Using a sample of 143 American Indian women residing in the Northern Plains. Methods: Data were collected using a self-administered survey completed by 143American Indian women over the age of 45 in the Midwest. Logistic regression analyses were conducted to assess predisposing (age and marital status), need (personal and family cancer history), and enabling factors (education, monthly household income, mammogram screening awareness, breast cancer knowledge, self-rated health, and cultural practice to breast cancer screening). Results: Nested logistic regression analyses indicated that only 55.5% of participants reported having had a breast cancer screening within the past 2 years, whereas 21.0% never had a mammogram test. After controlling for predisposing and need factors, higher education, greater awareness of mammogram, and higher utilization of traditional Native American approaches were significant predictors of mammogram uptake. Conclusions: The findings highlight important implications for intervention strategies aimed at improving breast cancer screening and service use among American Indian women. Educating health professionals and American Indian community members about the importance of breast cancer screening is highly needed. It is critical to assess a woman's level of traditional beliefs and practices and its possible influence on screening participation and future screening intention. Given the findings, prevention and intervention strategies, including public awareness and education about breast cancer screening are promising avenues to reduce screening disparities among American Indian women.

### THE NH ALLIANCE FOR HEALTHY AGING: USING A COLLECTIVE IMPACT APPROACH TO ADVANCE HEALTHY AGING

Jennifer Rabalais,[1] Laura Davie,[1] and Alison Rataj,[2] *1. University of New Hampshire, Concord, New Hampshire, United States, 2. University of Massachusetts Boston, Boston, Massachusetts, United States*

The NH Alliance for Healthy Aging (NH AHA) is a statewide coalition of stakeholders building cross sector partnerships that support and promote healthy aging throughout the state. Formed in 2016, NH AHA works to promote its shared vision to create communities in New Hampshire that advance culture, policies and services which support older adults and their families. As the largest coalition focused on the health and well being of older people in the state, NH AHA currently engages over 300 participants representing more than 185 organizations and/or groups statewide. Participants will hear how this collective power has led to early successes in advancing AHA's five strategic priorities, including early statewide policy successes. A review of the collective impact model will be provided with discussion on how NH uses this model intentionally to align the work in the aging field of NH. NH AHA's theory of change starts with changing the conversation around aging across NH's communities and is the foundation for the efforts to advance Reframing Aging principles and recommendations through the work of NH AHA and statewide. NH AHA's strategic plans to become a resource and hub of Reframing Aging activity in the state will be reviewed. As a result of the presentation audience members will be able to 1) describe the 5 conditions of collective impact 2) give 2 examples of how NH is utilizing these conditions intentionally and 3) describe NH's efforts to create common measures for the aging field in NH.

### SESSION 2868 (POSTER)

### ENVIRONMENT AND AGING (SRPP)

### A CONFIRMATORY FACTOR ANALYSIS OF THE BRIEF SENSE OF COMMUNITY SCALE FOR SPANISH-SPEAKING OLDER ADULTS IN PUERTO RICO

Tommy Buckley, Kyeongmo Kim, and Denise Burnette,
*Virginia Commonwealth University, Richmond, Virginia, United States*

Psychological sense of community is a concept used to describe how individuals feel about their community. The Brief Sense of Community Scale (BSCS) is an 8-item scale that includes these four domains: membership, needs fulfillment, emotional connection, and influence. It has been used in various contexts and was validated with young adults in Puerto Rico. The purpose of this study was to validate the BSCS for use with Spanish-speaking older adults in Puerto Rico. We conducted face-to-face interviews with a non-probability sample of 154 community- dwelling adults aged 60+ in Puerto Rico. BSCS is comprised of a 5-point likert-type scale with score values ranging from 0 (strongly agree) to 4 (strongly disagree) (total score range 0-32, mean= 24.75, SD= 6.04), and it showed good reliability in our sample (a=.85) and acceptable subscale reliability (membership, a=.85; needs fulfillment, a=.85; influence, a=.66; and emotional connection, a=.69). Five competing factor structures were tested based on prior research using confirmatory factor analysis (CFA). The CFA indicated that a four factor structure from the original scale was the best fit ($\chi^2$ (16) =25.9; p=.06; RMSEA=.06; CFI=.98; TLI=.97; SRMR=.04). The BSCS showed significant correlations in the expected direction with quality of life (r=.41), social isolation (r=.34), loneliness (r=.27) and self-rated health (r=.17). We conclude that the BSCS is a valid and reliable scale for measuring psychological sense of community with community-dwelling Spanish-speaking older adults in Puerto Rico. Future research should confirm and extend our findings with other Spanish-speaking older adult populations.

### ASSESSING HOUSING ACCESSIBILITY FOR OLDER ADULTS IN JAPAN: A CONTENT VALIDITY STUDY

Rumiko Tsuchiya-Ito,[1] Björn Slaug,[2] Tomonori Sano,[3] Miki Tajima,[1] Sakiko Itoh,[4] Kazuaki Uda,[5] Takashi Yamanaka,[5] and Susanne Iwarsson[2], *1. Dia Foundation for Research on Ageing Societies, Tokyo, Japan, 2. Lund University, Lund, Sweden, 3. Waseda University, Saitama, Japan, 4. Tokyo Medical and Dental University, Tokyo, Japan, 5. The University of Tokyo, Tokyo, Japan*

Scientifically validated tools to assess housing accessibility for older adults in Japan have been lacking. To address this, a rigorous procedure of adapting an existing housing assessment tool—the Housing Enabler, developed in Sweden—for valid use in Japan was conducted. The original tool was translated into the Japanese language, using established translation procedures. In the process, researchers checked the appropriateness of technical terms and adjusted specifications to be in accordance with Japanese standards. An expert panel approach was used to validate the content of the Japanese Housing Enabler. Thirteen certified occupational therapists, architects and care-managers (average experience=14.5 years) participated as experts in the content validity study. They rated each item with regard to relevance for assessing housing accessibility in Japan, on a scale from 1(=Not relevant) to 4(= Highly relevant). They suggested adjustments and additions that they found to be relevant to capture particularities of Japanese housing and building design. After individual ratings, the experts gathered for consensus discussions on suggested revisions of the item list. As a result, the number of items was substantially increased (from 161 to 283). A content validity index (CVI) was calculated for each item (i.e., the proportion of experts rating the relevance as at least 3). Using a recommended threshold of CVI ≥0.78, more than 90% of the items were considered relevant, thus supporting the content validity. However, the large amount of items might jeopardize the feasibility of the instrument. Further studies are needed to evaluate feasibility, criterion-related validity and aspects of reliability.

### COMMUNITY CANCER RATES IN MASSACHUSETTS, NEW HAMPSHIRE, AND RHODE ISLAND: FINDINGS FROM HEALTHY AGING DATA REPORTS

Shuangshuang Wang,[1] Chae Man Lee,[2] Bon Kim,[3] Nina Silverstein,[4] Frank Porell,[3] and Beth Dugan,[3]
*1. Shandong University, Jinan, Shandong, China, 2. University of Massachusetts Boston, Boston, United States, 3. University of Massachusetts Boston, Boston, Massachusetts, United States, 4. University of Massachusetts Boston, Needham, Massachusetts, United States*

Cancer is one major health condition that affect people's later life quality, which could be intervened from the community level. This study compares rates of lung cancer, colon cancer, breast cancer (in women), and prostate cancer (in men) among adults 65+ in 3 New England states (MA, NH, and RI). Data were from the Healthy Aging Data Report (see www.healthyagingdatareports.org), which reported on 150+ health indicators at the local community and state level. Data sources were the Current Medicare Beneficiary Summary File (years) and the American Community Survey (years). Small area estimation techniques were used to calculate age-sex adjusted community rates. Average state rates of cancers (range)

are: Lung cancer MA 2.1 (1.0 – 4.4) NH 1.6 (0.9 – 2.9) RI 2.1 (1.3 – 2.9); Colon cancer MA 2.9 (1.8 – 4.1) NH 2.4 (1.8 – 3.7) RI 3.2 (1.6 – 4.5); Breast cancer MA 10.9 (5.3 – 16.4) NH 9.8 (5.4 – 14.8) RI 10.7 (7.2 – 13.9); Prostate cancer MA 13.8 (7.4 – 24.0) NH 11.5 (5.9 – 17.3) RI 13.8 (9.5 – 17.7). NH has the lowest rates on all four types of cancer; MA and NH were similar regarding average rates, but MA communities had the widest disparities for lung, breast and prostate cancer. Findings suggest within and between state variations in cancer rates. Policies and programs may target geographic areas/communities with high rates of cancers, examine environmental effects on cancer rates and develop strategies in reducing cancer rates.

### LONG-TERM SEQUENCES OF INTERGENERATIONAL LIVING ARRANGEMENTS AMONG OLDER AMERICANS

BoRin Kim,[1] Jersey Liang,[2] Xiao Xu,[3] Mary Beth Ofstedal,[2] and James Raymo,[4] *1. University of New Hampshire, Durham, New Hampshire, United States, 2. University of Michigan, Ann Arbor, Michigan, United States, 3. Yale University, New Haven, Connecticut, United States, 4. Princeton University, Princeton, New Jersey, United States*

Living arrangements are critical for intra-family exchanges such as physical, financial, and emotional supports influencing older adults' health and well-being. Existing research is largely based on short-term observations of living arrangements. This study aims to explore longer term dynamic patterns of intergenerational living arrangements among older Americans and their sociodemographic and health determinants. Data came from the 1998-2016 Health and Retirement Study. Sequence analysis was employed to identify long-term patterns of intergenerational living arrangements for 3,025 individuals who were age of 51-64 at the baseline (ages of 69-82 at the last wave), have at least one child, and were observed 10 consecutive times (Obs.=30,250). Living arrangements were categorized into co-residence, proximate residence (i.e., 10miles from children), and nursing home. Multinomial logistic regressions were used to evaluate the associations of individual characteristics with the different living arrangements sequences. Four patterns of eighteen-year living arrangement trajectories were identified: Transition to proximate residence (17%), stable in distant residence (24%), stable in proximate residence (38%), and stable in co-residence (22%). Younger age and working (vs. retired) status were associated with stable coresidence rather than proximate or distant residence. Respondents who retired during the study period were more likely to move close to their children. Contrary to expectations, changes in self-rated health and functional status had no significant effect in long-term living arrangement sequence patterns. These findings suggest that intergenerational living arrangements among older Americans tend to be stable and not to be significantly affected by their caregiving needs.

### MULTILEVEL FACTORS FOR LIFE SATISFACTION AMONG RESIDENTS IN NON-URBAN SUBSIDIZED SENIOR HOUSING

BoRin Kim,[1] Sojung Park,[2] Casey Golomsky,[1] Marguerite Corvini,[1] Allison Wilder,[3] John Wilcox,[3] and Allysha Winburn,[4] *1. University of New Hampshire, Durham, New Hampshire, United States, 2. Washington University in St. Louis, St. Louis, Missouri, United States, 3. University of New Hampshire, Durham, United States, 4. University of West Florida, Pensacola, Florida, United States*

Although a quarter of HUD-assisted properties for older adults are located in rural/non-metropolitan areas, there is limited understanding of the population living in these locations. Advanced age and low income are known risk factors for poor physical and mental health. Older adults in rural subsidized housings may be at increased risk for poor health and social isolation due to their isolated locations and small-scale housing complexes. This presents the additional challenge of service provision for the residents' needs. This study aims to explore multi-level factors affecting life satisfaction among residents in subsidized senior housing. Data were collected for five subsidized senior housings in New Hampshire: two in Coos county (rural, population=33,055) and three in Strafford county (suburban, population=128,613). Mixed-methods approaches were used: Community/organizational-level data were collected using semi-structured interviews conducted with the directors of senior housings. At the individual level, quantitative survey data were collected from 82 residents of five senior housings. Contrary to expectations, we found that residents in rural senior housings were likely to report better life satisfaction (Coef.=0.597, $p<.01$) than those in suburban areas despite controlling for individual-level factors such as age, gender, education, marital status, health, social relations, and service use. The most salient terms used in the interviews with directors of rural senior housings include limited resources, tight community, and emotional support. The last two may be protective factors positively influencing life satisfaction among their residents. Our results contribute to development strategies to improve quality of life among residents in rural/non-metropolitan subsidized senior housing.

### OLDER ADULT COHOUSING COMMUNITIES: THE LIVED EXPERIENCE

Sherry Cummings,[1] and Nancy Kropf,[2] *1. University of Tennessee Knoxville, Nashville, Tennessee, United States, 2. Georgia State University, Atlanta, United States*

"We're on the leading edge of the baby boomers so we don't do anything like anybody has ever done before and that includes aging." (Tammy, SS). This quote embodies the perspective of older adults engaged in a new housing phenomenon – older adult cohousing communities (OACCs). OACCs are designed, managed, governed and maintained by the older residents themselves. Seventeen such communities currently exist, and more are being developed by active baby boomers who are searching for more meaningful, relational and eco-friendly options for aging-in-place. The older adult cohousing movement in the U.S., which began in 2005, is small but growing quickly. However, a dearth of literature exists on this phenomenon. This qualitative study examined older adult cohousers' lived experiences. The study employed an existential-phenomenological research method. Maximum variation purposive sampling was employed. Twelve communities were identified that represented the full range of geographic environments, structures, missions, cost and size. Interviews were conducted individually or in focus groups. In all, 73 older adult cohousers participated in the study. The Gerotranscendence Theory of Aging was used to guide the development of the structured interview questions and to organize data analysis and interpretation. Digital recordings were transcribed, and an inductive method was used to allow codes and themes to arise from the data itself. Patton's (1990) criteria for judging themes was employed. This poster will clarify the nature, principles and structure of OACCs. Themes that emerged from the study - benefits, challenges, aging-in-place, interpersonal relationships and personal growth - will be described .

### TRENDS IN DEATHS OF DESPAIR AMONG OLDER ADULTS, 1999-2018

Peter Sun,[1] Edward Lawlor,[1] Nancy Morrow-Howell,[2] Sojung Park,[2] and Timothy McBride,[1] *1. Washington University in St. Louis, Saint Louis, Missouri, United States, 2. Washington University in St. Louis, St. Louis, Missouri, United States*

"Deaths of despair" (DOD) is a new phenomenon that includes deaths attributable to suicides, drug overdoses, and alcohol-related liver diseases. While many studies have focused on the rise of DOD in midlife, there is little research that focuses on the trends of DOD in later life. To address this gap, we used publicly available data from the Centers for Disease Control and Prevention to carry out descriptive and spatial analyses of DOD among older adults (aged 65 and over) from 1999 to 2018. Nationwide, we found that DOD rates of older adults increased from 2009 (44.7 deaths per 100,000) to 2018 (57.4). This increase was notable among male older adults who were Black non-Hispanics and White non-Hispanics (WNH); however, Hispanic populations saw a 4.8% decrease in DOD between 1999 and 2018. Of the WNH decedents in 2018, alcohol-related liver diseases were the most common cause of death (55%), followed by suicides (36%) and drug overdoses (9%). Additionally, 55% had a high school degree or less, 22% had some college but no bachelor's degree (BA), and 22% had a BA or more. Geographically, we found that DOD was concentrated in the West in 1999 but has since then spread nationwide. In 2018, the highest rates of DOD among WNH were in the western and southern states. These findings suggest that attention to deaths of despair cannot be limited to midlife; policies and program interventions aimed at slowing this epidemic must include people in later life as well.

### TYPE OF COOKING FUEL AND MORTALITY AMONG OLDEST-OLD IN CHINA

Yao Yao,[1] Danan Gu,[2] Huashuai Chen,[3] and Yi Zeng,[1]
*1. Peking University, Durham, North Carolina, United States, 2. United Nations, New York, New York, United States, 3. Duke University, Durham, North Carolina, United States*

Studies have reported that elder adults are susceptible to the indoor pollution. However, evidences on the mortality risk of household air pollution (HAP) on the elderly, especially on vulnerable oldest-old population, are scarce. We aimed to estimate the association between HAP exposure from solid fuel combustion and 7-years all-cause mortality using a nationally representative dataset of oldest-old population in China. We used data from wave 2011 to wave 2018 of Chinese Longitudinal Healthy Longevity Survey. Household cooking fuel were dichotomized as solid fuel (including kerosene, charcoal, coal, wood, and biomass) and clean fuel (including electricity, solar, natural gas, and coal gas). The cohort contained 6,167 participants, totaling 21,357 person-years. There were 3,836 deaths between 2011 and 2014. About half of the participants (53.8%) used solid fuel for cooking. In the fully adjusted model, the mortality hazard ratio (HR) for solid fuel users was 1.10 (95% CI: 1.03-1.18). We observed significant interaction between HAP and urban/rural residency but not between HAP and gender. Our study showed exposure to HAP from solid fuel combustion was associated with higher risk of all-cause mortality in the male and female oldest-old population. This adverse effect was more pronounced in urban residence who use solid cooking fuels, suggesting attention should be paid on reducing HAP, particularly on susceptible population.

### UNDERSTANDING THE PATHWAYS OF NEIGHBORHOOD ENVIRONMENTAL INFLUENCES ON HEALTH AMONG U.S. CHINESE OLDER IMMIGRANTS

Yi Wang,[1] Man Guo,[1] Jinyu Liu,[2] and Kara Carter,[1]
*1. University of Iowa, Iowa City, Iowa, United States, 2. Weatherhead East Asian Institute, New York City, New York, United States*

Neighborhood environment has proven to be consequential for older Americans' physical, mental and cognitive health. However, this topic is much less studied among older Asian immigrants, a fast growing immigrant group who embrace values of collectivism and community connections. The current study used the first wave data (N=2920) of the Population Study of Chinese Elderly (PINE), the largest population-based sample of Chinese older adults in the U.S., 1) to examine the direct associations between neighborhood environment (social cohesion, physical disorder) and health outcomes (self-rated health, depression, and cognitive health); and 2) to identify possible mediators at intrapersonal (sense of hopelessness, sense of mastery) and interpersonal levels (social engagement, cognitive engagement) through which neighborhood environment influences health. The results of Sobel tests from path analysis showed that neighborhood social cohesion was associated with better health outcomes on all the domains: self-rated health (b= 0.050, p<.01), depression (b= -0.202 p<.001), and cognitive health (b=0.092, p<.001), whereas neighborhood physical disorder was associated with poorer self-rated health (b= -0.069, p<.01) and more depressive symptoms (b=0.174, p<.001). Full and partial mediations were detected. For example, neighborhood physical disorder influences depression completely through intrapersonal traits, higher sense of hopelessness (b=1.879, p<0.001) and reduced sense of mastery (b= -2.656, p<0.001). Neighborhood social cohesion contributes to better cognitive health partially through increased social engagement (b=1.696, p<0.001) as well as cognitive activities (b=1.392, p<0.001). The findings identified the ecological component in resilience building processes, and provide evidence for mezzo-level intervention to improve health among aging U.S. Chinese immigrants.

### USING IMPLEMENTATION SCIENCE TO SUPPORT A RESEARCH AND PUBLIC POLICY SECTOR OLDER ADULT SOCIAL HOUSING PARTNERSHIP

Sander Hitzig,[1] Christine Sheppard,[2] and Andrea Austen,[3]
*1. Sunnybrook Hospital, North York, Ontario, Canada, 2. Sunnybrook Research Institute, Toronto, Ontario, Canada, 3. City of Toronto, Toronto, Ontario, Canada*

One quarter of the residents in the City of Toronto is comprised of older adults, and this number is expected to continue to grow dramatically over the next few decades. The development of evidence-based interventions to meet the health and social care needs of Toronto's aging population can be hampered by failing to account for broader implementation considerations that can adversely affect successful uptake. The present initiative provides a case-example of a research and public policy sector partnership that used an implementation approach to co-design an older adult social housing model for low-income older adult groups. Implementation science is the study of the uptake of research evidence into practice. Our team used the Consolidated Framework for Implementation Research (CFIR) to support the planning, implementation and evaluation process of a new social housing model for older adults by: 1) identifying all relevant stakeholders; 2) generating evidence via qualitative interviews/focus groups, a scoping review, secondary data analysis, and an environmental scan; 3) facilitating large scale stakeholder consultation events with older adults, front-line practitioners and other community agencies; 4) supporting the development of an evaluation framework; and 5) providing opportunities for knowledge exchange and transfer across each phase of the initiative. An implementation science approach has augmented the ability of the City of Toronto to optimize the co-creation of housing strategies aimed at improving the overall wellness of vulnerable older adults living in social housing. Further, a number of valuable lessons were learned on how to foster successful research and public policy relationships.

### SESSION 2869 (POSTER)

### RETIREMENT (SRPP)

### FINANCIAL WELL-BEING ACROSS AGE COHORTS AND ITS IMPACT ON RETIREMENT SECURITY

Stephanie Skees,[1] and Stephen Roll,[2] *1. Washington University in St. Louis, Saint Louis, Missouri, United States, 2. Washington University in St. Louis, St. Louis, Missouri, United States*

The lack of retirement savings in the United States has been well-documented. For example, in 2013 roughly one-third of households near retirement lacked either a defined benefit or defined contribution retirement plan (U.S. Government Accountability Office, 2015), and the median retirement account balance is $0 (Oakley, Brown, & Saad-Lesler, 2018). While this lack of savings is concerning, less clear is the relationship between retirement security and a household's sense of financial well-being. To that end, this study uses the nationally representative 2017 Survey of Household Economics and Decisionmaking to investigate the relationship between both retirement security indicators and subjective financial well-being—as measured through the Consumer Financial Protection Bureau's financial well-being scale—across the life course. Specifically, we use multiple regression approaches to compare how financial and knowledge-based indicators of retirement security contribute to the financial well-being of young, mid-career, and pre-retirement cohorts. Preliminary results indicate that feeling "on track" for retirement savings and comfort in making retirement savings decisions were among the strongest contributes to a sense of financial well-being across age cohorts. However, these indicators were particularly important to the financial well-being of older households. Surprisingly, having a 401(k), IRA or pension had no significant impact on savings when controlling for other factors. This study speaks to the importance of both providing effective retirement savings tools, including both educational and financial resources, at multiple points across the life course. It also contributes to a small but growing literature on the intersections between subjective financial well-being and financial decisionmaking.

### GROUP DIFFERENCES IN PATTERNS OF RETIREMENT AND SOCIAL SECURITY BENEFITS RECEIPT IN THE UNITED STATES

Hyesu Yeo, *University of Georgia, Athens, Georgia, United States*

Extended working lives are a clear phenomenon in the U.S. However, the Social Security benefits and old-fashioned retirement concept has not changed by the new trend. This study is to examine 1) whether retirement and Social Security benefits simultaneously happen as Social Security was planned, and 2) differences in groups based on the trajectories in retirement and Social Security benefits. This study used data from the Health and Retirement Survey (HRS) 2000-2016. The sample consisted of respondents the ages of 54 or older who worked for pay without receiving Social Security benefits in 2000 (N=1,924). A Latent Class Growth Analysis was conducted to classify the number of groups based on individuals' trajectories in retirement and Social Security. The bivariate analysis tested group members' characteristics. The final model included four groups: 1) 'delayed retirement and Social Security,' 2) 'early Social Security but re-entry of the labor market after early retirement,' 3) 'delayed retirement but early Social Security,' and 4) 'retirement and Social Security at the same time point.' The bivariate analysis demonstrated significant differences between groups in retirement age, Social Security age, education, health, and income. The result indicated that Group 2, with lower health status, education level, and income, was vulnerable in retirement. The group retired and received Social Security comparatively earlier, whereas they finally retired at the oldest age after reentering the labor market. This finding suggests that Social Security and retirement do not happen simultaneously, and Social Security is not enough for some people's living.

### LIFEPLANNING: A BETTER RETIREMENT PLANNING OPTION FOR OUR TIMES

Rajiv Nagaich,[1] Carol Redfield,[2] and Ben Harvill,[1],
*1. Aging Options, Federal Way, Washington, United States, 2. Aging Options, Seattle, Washington, United States*

Ten thousand turn 65 daily. Majority look forward to retiring in the beginning and then become afraid of outcomes they often hear about- dealing with institutional care, becoming a burden, or running out of money. This is not because retirees do not plan, but despite of having planned their entire life for retirement. Many employers provide financial retirement planning such as a 401K plan. Individuals have relied on employee benefit plans to ready themselves, yet few are "very confident" about it. Two-thirds of retirees say their most recent employers did "nothing" to help them transition into retirement; 16% are "not sure" what their employers did. Many may be overlooking important factors in their strategies. Among retirees who currently have a retirement strategy, 85% have factored Social Security and Medicare benefits into their strategy. Most have included on-going living expenses (79%), total savings and income needs (57%) into their plan. Fewer than half have considered other critical factors (e.g., investment returns, ongoing healthcare costs, inflation, long-term care needs, tax planning, etc.). Only 9% have contingency plans for retiring sooner than expected and/or savings shortfalls. The truth is that education offered by employers tends to be traditional planning advice, which may not be enough to address the concerns retirees will have in retirement. To this, we introduce a multi-disciplinary LifePlanning Framework which takes a wholistic, integrated approach in addressing the many complex issues of retirement found in health, housing, finance, legal, and family. Our results may impact future practice, research, and policy.

### MEANING OF LIFE AND WELL-BEING: PRELIMINARY RESULTS OF THE SUCCESSFUL AGING STUDY

Zvi Gellis,[1] Kim McClive-Reed,[2] Bonnie Kenaley,[3] and Eunhae Kim,[4] *1. University of Pennsylvania, Philadelphia, Pennsylvania, United States, 2. University of Pennsylvania, Menands, New York, United States, 3. Boise State University, Boise, Idaho, United States, 4. Texas State University, San Marcos, Texas, United States*

Meaning in life for older persons has become a focal research point, with findings that a greater sense of meaning is associated with better outcomes on a range of health and well-being factors. Our study examined relationships between scores on several personality scales, including the Meaning in Life Questionnaire (Steger et al., 2009) and the WHO-5 Well-Being Index, a proxy measure of mood/depression. Community-dwelling members (N=535) of Osher Lifelong Learning Institutes aged 50 and up (mean age 71.4, SD = 6.93) at 3 U.S. sites completed surveys. Higher wellness levels were significantly correlated with increased resilience, optimism, life satisfaction, and presence of meaning in life, while lower levels were associated with greater searching for meaning in life. A multivariate linear regression model (F = 55.597, df = 4, p = .000, R = .566, R2 = .320) showed that wellness scores increased with higher scores in optimism (ß = .348, p =.000), resilience (ß = .183, p = .000), and presence of meaning in life (ß = .106, p = .019). However, searching for meaning in life significantly predicted decreases in wellness scores (ß = -.084, p=.019). These results support those of previous studies, suggesting that for older persons, an ongoing search for meaning in life is linked to negative outcomes than a perception of existing meaning in life. A variety of available interventions aimed at increasing meaning and purpose in life (Guerrero-Torelles et al., 2017) may contribute to better health and well-being in older adults.

### PRODUCTIVE AGING IN INDIA: PRE-RETIREMENT PERCEPTIONS, PRIORITIES, AND GENDERED PERSPECTIVES

Reema Sen,[1] and Eva Kahana,[2] *1. case western reserve university, Cleveland, Ohio, United States, 2. Case Western Reserve University, Cleveland, Ohio, United States*

This study explores the perceptions of urban, middle class, white collar employees in India approaching retirement, to unpack 'productive aging' through a specific sociocultural lens. India, is aging fast with limited social security. Projected growth in the 60+ age group is 326% (2000 -2050) and 700% for the 80+. Prior studies largely focused on rural and blue collar adults. This study involves a sample of 76 people's (41 men, 35 women of relatively high SES) attitudes to aging. Key findings show, self-perceived health status was different between men and women (ordinal regression analysis showed p value 0.034 at 95% CI). 51.22% men rated their health excellent/very good compared with 28.57% women. 12.2% men rated their health fair/poor compared with 25.72% women. Despite this, results of men and women's perceptions of aging were remarkably similar though living in a country with entrenched gender inequality. Cultural influence was apparent from the gendered difference (p value 0.036, 95% CI) in response to the question "our society frowns upon paid work after retirement" (20% women agreed compared with 4.77% men). Interestingly, despite social constraints 68.58% women agreed that they prefer a paid alternate career after retirement compared with 53.66% men. Another difference (p value 0.006, 95% CI) on the question "I need to be gainfully occupied for my own personal satisfaction" had 100% women reporting they agree or strongly agree as compared with 85.37% men. Findings will be discussed in the context of gender and changing family structures among adults in late middle age.

### SESSION 2870 (POSTER)

### TRANSPORTATION

### AIR TRAVEL CONCERNS AND COMPLAINTS OF PERSONS LIVING WITH DEMENTIA AND THEIR TRAVEL COMPANIONS

Colleen Peterson,[1] Tamara Statz,[2] Sara Barsel,[3] Robyn Birkeland,[2] Joseph Gaugler,[2] and Jessica Finlay,[4] *1. University of Minnesota, Saint Paul, Minnesota, United States, 2. University of Minnesota, Minneapolis, Minnesota, United States, 3. Roseville Alzheimer's & Dementia Community Action Team, Minneapolis, Minnesota, United States, 4. University of Michigan, Ann Arbor, Michigan, United States*

Air travel is increasingly accessible and rising numbers of older adults choose to fly. The prevalence of Alzheimer's disease and related dementias (ADRD) is also expected to increase due to advances in longevity and the aging Baby Boomer population. An ADRD diagnosis does not necessarily end the desire to travel for leisure, and air travel may be necessary for family and/or care reasons. Taken together, more persons living with ADRD are expected to use air travel. This is concerning since adverse outcomes, including confusion and delirium, can be exacerbated by unfamiliar environments, such as airports and in-flight situations. Yet there is little research on air travel with this population. The current study seeks to 1) understand air travel experiences, issues, and needs of persons living with ADRD and their travel companions; and 2) identify facilitators and barriers to safe and comfortable air travel for persons living with ADRD. Persons living with ADRD (n=49)and ADRD travel companions (n=176) provided information on their air travel experiences through a nationally distributed web-survey. They traveled for family visits (80.0%) and leisure (38.0%). Both persons living with ADRD and travel companions indicated anxiety (50% and 60.7%, respectively), difficulty understanding announcements and signage (39.1%, 55.2%) and getting lost or separated (37.5%, 53.2%) as primary travel concerns. Qualitative comments identified themes of frustration with locating family restrooms and quiet spaces. Results from this study will inform ongoing efforts to develop dementia-friendly airports and improve older adult travel experiences, independence, and quality of life.

### FATAL CRASHES AMONG DRIVERS AGE 60+ IN CONNECTICUT, MASSACHUSETTS, NEW HAMPSHIRE, AND RHODE ISLAND

Chae Man Lee,[1] and Beth Dugan,[2] *1. University of Massachusetts Boston, Boston, United States, 2. University of Massachusetts Boston, Boston, Massachusetts, United States*

Fatal crashes are related with the spatial components of physical environments (e.g. roadways, land use, and political boundaries). This study compares rates of fatal crashes among drivers age 60+ by counties in 4 states of CT, MA, NH, and RI. The GIS application is used to visualize the location of fatal crashes and to identify whether it is clustered as hotspots. This study pooled data related to fatal crashes in CT, MA, NH, and RI from the Fatal Accident Recording System (2008-2018). Sample (n=2,373) inclusion criteria were subjects (driver age 60+) had to have complete data on variables of interest and be involved in a crash with at least one fatality. More than half (n=1,387, 58.5%) of drivers had a fatal injury. Results showed that the county with the highest incidents of fatal crashes was New Haven, CT (n=183 involved, 53% fatality), Worcester, MA (n=179, 61.5% respectively), Hillsborough, NH (n=75, 65.3% respectively), and Providence, RI (n=94, 59.6% respectively). The GIS spatial analysis showed that crashes were clustered along roads with the highest speed limits (interstate highways or multilane state routes) and found that the hotspots of clustered fatal crashes were located in counties with big cities with high population densities (New Haven CT, Hartford CT, Springfield MA, Worcester MA, Boston MA, Concord NH, and Providence RI). Identification of these crash hotspots will be beneficial for drivers and policy makers. The findings may alert drivers to high risk areas and policy makers can implement countermeasures.

### RISK FACTORS FOR OLDER PEDESTRIAN INJURIES AND FATALITIES AMONG COMMUNITIES IN MASSACHUSETTS

Shuangshuang Wang,[1] Nina Silverstein,[2] Chae Man Lee,[3] Frank Porell,[4] and Beth Dugan,[4] *1. Shandong University, Jinan, Shandong, China, 2. University of Massachusetts Boston, Needham, Massachusetts, United States, 3. University of Massachusetts Boston, Boston, United States, 4. University of Massachusetts Boston, Boston, Massachusetts, United States*

The number of pedestrian crashes in the United States has increased by 35 percent from 2008 to 2017. Among all pedestrian fatalities in 2017, 48% were pedestrians aged 50 and older, which suggests a disproportionate threat to older residents' health and safety. Massachusetts has a large older population and is experiencing increased numbers of older pedestrian crashes. This research identified risk factors and community characteristics contributing to older pedestrian crashes and suggests leveraging the state's age-friendly efforts to speed the implementation of countermeasures. Based on ten-year statewide crash data (2006-2015) and community indicators from the 2018 Massachusetts Healthy Aging Data Report, this study examined 4,472 crashes across Massachusetts that involved pedestrians age 55 and over. The leading reasons for crashes were driver's inattention, driver's failure to yield right of way, and driver's issues with visibility. Older pedestrians were hit while walking in the road, often in crosswalks at intersections. Many factors were found to contribute to older pedestrian crashes: time of day (rush hour), time of year (winter), and community factors (higher rates of disabilities, higher percentage of racial minority residents, higher number of cultural amenities, and lack of dementia-friendly community efforts. Greater awareness of older pedestrian safety risks is needed. Communities highlighted in this research warrant priority attention from planning, health, aging services, and transportation authorities to improve older pedestrian safety.

### SPATIAL CLUSTER ANALYSIS OF FATAL ROAD ACCIDENTS FROM NON-USE OF SEAT BELTS AMONG OLDER DRIVERS

Oluwaseun Adeyemi, Rajib Paul, and Ahmed Arif, *University of North Carolina at Charlotte, Charlotte, North Carolina, United States*

Identifying county-level spatial clusters of fatal accidents due to non-use of seatbelt among drivers 65 years and older can help with injury prevention policies and targeted place-based interventions. We estimated the odds and identified hotspots of fatal accidents among drivers 65 years and older (n=57,715) based on a cross-sectional analysis of data from 2010 to 2018 from the Fatality Analysis Reporting System. The outcome variable was fatality status (fatal/non-fatal), and the main independent variable was seatbelt use (not used/used). Other covariates were drunk driving, distracted driving, and speeding while age, gender, and airbag deployment were used as confounders. Rural-urban status of accident location was used as an effect modifier. Odds ratios were calculated from logistic regression. The age-adjusted fatality rate was computed as the crude fatality rate per 100,000 population weighted by the average population composition by age-groups. Spatial autocorrelation was assessed by local Moran's I, and cluster analysis was performed using the Moran's I index-derived Z-scores. The median age-adjusted seatbelt-related fatality rate per county was 2.35 per 100,000 population (IQR: 5.60). Not wearing a seatbelt was associated with an 11-fold (Adjusted OR: 11.37; 95% CI: 10.18-12.70) increased odds of a fatal event in metropolitan counties and a 7-fold (Adjusted OR: 7.43; 95% CI: 6.10-9.04) increased odds in rural counties and small towns. Hot spots for seatbelt-related fatal road accidents were found in multiple counties in Texas, South Dakota, and Mississippi. Study findings can be used for county-specific interventions tailored to 65 and older for preventing fatal road accidents.

### VISITATION ACCESS TO U.S. NURSING HOMES: AN ANALYSIS OF FACILITY LOCATIONS, RATINGS, AND DISPARITIES

Mary Helander, *Syracuse University, Syracuse, New York, United States*

Research findings suggest that family visits to nursing home residents are important for countering depression, increasing residents' well-being and quality of life (Miller, 2019; Durkin et al., 2014), maintaining physical function (Shankar et al., 2017), and improving general health (Parmenter et al., 2012). Presence by family can directly impact a resident's care quality, since family members may take a role in monitoring their older relative's status (Miller, 2019). Unfortunately, regular family visits to nursing homes may be difficult, or impossible, due to challenges that include distance, travel time, lack of transportation, and cost (Fields et al., 2019) (Miller, 2019). These same challenges may translate to socio-economic barriers for families, eliminating long-term-care as an option for older relatives (Ferraro et al., 2017), (Angel and Berlinger, 2018). This paper considers the issue of nursing home visitation access and examines related disparities through spatial and demographic analysis of 15,000+ US facilities monitored by the Centers for Medicare the Medicaid Services. Mathematical models are used to analyze facility and population data, using access measures adapted from the geography discipline (Lou and Wang, 2003; Paez et al., 2019). Analysis explores whether higher rated nursing homes are more likely to be closer to affluent populations, and whether socioeconomic status is a significant factor in overall access. Analysis reveals patterns of access disparity with respect to nursing home ratings and geographies. For example, proximity to higher rated facilities increases monotonically with median household income. Specific policy recommendations are discussed.

### SESSION 2871 (POSTER)

### SOCIAL NETWORKS AND AGING

### COMMITMENT AND ATTACHMENT TO PET AND EMOTIONAL WELL-BEING AMONG LOW-INCOME PET OWNERS

Ke Li,[1] Fengyan Tang,[1] and Mary Rauktis,[2] *1. University of Pittsburgh, Pittsburgh, Pennsylvania, United States, 2. social work, Pittsburgh, Pennsylvania, United States*

Pets play an important role in older adults' lives, as people treat pets as their companion and family members. Owning a pet has been believed to be beneficial; however, previous literature demonstrated mixed results of the effects of pet on people's well-being. Using data collected from 392 food pantry users in Pittsburgh, this study examined the relationships of pet ownership, attachment and commitment with emotional well-being, and investigated whether sociodemographic profiles conditioned those relationships. emotional well-being was assessed by global mental health, positive functioning, perceived negative feelings, and perceived positive feelings. Commitment to pet was measured by a 10-item scale adapted from the Miller-Rada Scale about the likelihood of giving up pet under various difficult circumstances. Attachment to pet was measured by the 23-item Lexington Attachment to Pets Scale about emotional ties to pet. About two-thirds of respondents (66%) were pet owners. Multiple regression analysis showed that pet owners perceived fewer positive feelings (e.g. happy, joyful) than non-pet owners. However, among pet owners, a higher level of pet attachment was associated with more positive feelings. Gender and education significantly moderated the effects of pet ownership on emotional well-being, as male and employed respondents were more likely to benefit from owning a pet. Moreover, the positive effects of attachment and commitment to pet were stronger among respondents with higher levels of education or in the labor force. future studies need to investigate how to promote the benefits of pet companion and address the challenges faced by low-income pet owners.

### EFFECTS OF HOUSEHOLD SIZE AND PARTNER STATUS ON FINANCIAL WELL-BEING AND FINANCIAL EXPLOITATION

Jessica King McLaughlin, Elizabeth Anderson, Ashley Taeckens-Seabaugh, Jennifer Greenfield, Eric Chess, and Jessica King, *University of Denver, Denver, Colorado, United States*

Social isolation and loneliness are significant risk factors for older adults (adults over the age of 50) for a multitude of reasons. This study explored the connections between social isolation, vulnerability to financial exploitation, and financial wellbeing in a sample of older adults. Using the concept of precarity as a guiding framework, this study assessed behaviors that expose individuals to financial exploitation as well as indicators of financial wellbeing in individuals with varying degrees of social isolation. One-hundred-eight community-dwelling older adults completed a pen-and-paper survey containing measures of risk to financial exploitation and financial wellbeing. Participants were predominantly female (75.9%), White (90.7%), and highly educated. Fifty-eight participants were unpartnered (53.7%) and 67 participants (62%) lived with at least one other person. Chi-square analyses resulted in a significant association between partner status and being a victim of financial exploitation ($\chi2(1) = 10.842$, $p \leq .001$). Furthermore, there was an association between social isolation and engaging in certain behaviors that put someone at risk for financial exploitation (such as reading junk mail). As for measures of financial wellbeing, partner status was significantly associated with feelings of financial freedom ($\chi2(3) = 8.608$, $p = .035$). These findings indicate that socially isolated individuals may be at greater risk of financial exploitation and have lower levels of financial wellbeing, such as feeling unprepared for retirement ($\chi2(2) = 7.358$, $p = .025$). The results of this study highlight the risks involved with social isolation and indicate further research is needed into ways to mitigate these negative effects.

### FACTORS ASSOCIATED WITH POSITIVE AGING AND HAPPINESS OF THE OLDER PEOPLE IN HONG KONG

Daniel W. L. Lai,[1] Vincent Lee,[1] and Elsie Yan,[2] *1. The Hong Kong Polytechnic University, Kowloon, China, 2. The Hong Kong Polytechnic University, Hong Kong, China*

Happiness is essential to one's well-being and impact on every aspect of our lives. Happier people are living longer, they are healthier. Happier people are more likely to be physically active and enjoy better sleep habits and practices. While few existing research studies had examined the determinants of happiness of older people, especially in Chinese society. Understanding happiness in the context of social unrest and political instability is thus limited. This study tested the correlates of happiness at interpersonal, psychological and environmental levels at a time when there were extended scale of violence, destructions, and clashes in the community between police and protesters in late 2019 during the anti-extradition campaign. In social unrest, older people, due to their function and mobility, could be emotionally and physically vulnerable. A total of 1,209 older persons aged 55 and above from Hong Kong answered the questionnaire by stratified random sampling. Our findings show that their overall resilience was strongly and positively associated with levels of happiness. Due to the recent political instability Hong Kong, their satisfaction toward social and political situation of Hong Kong also correlated positively to levels of happiness. We suggest that future interventions and policy initiatives should put extra emotional and tangible support to older adults, particularly during social unrests and unstable political conditions, in addition to strategies for the enhancement of resilience and mental capital.

### SOCIAL ISOLATION, HEALTH, AND HEALTH CARE: PERSPECTIVES AMONG OLDER ADULTS RESIDING IN PUBLIC HOUSING

Ruth Sanchez, Brooke Wagen, Whitney Williams, and Elizabeth Jacobs, *University of Texas at Austin Dell Medical School, Austin, Texas, United States*

By 2035, U.S. adults > 65 will outnumber children. The growing lack of affordable housing combined with fixed incomes will lead to more older adults residing in public housing. Public housing authorities, in turn, will face growing health and social needs among their residents. In partnership with a local housing authority, we conducted a qualitative study to better understand the health and social needs of older adult public housing residents. We conducted semi-structured qualitative interviews with 27 older adults at two public housing sites in Austin, Texas; we asked about their experience of aging in public housing, their health, healthcare, and community life. Interviews were audio-recorded and transcribed; interviews were systematically coded and verified by a second coder. Themes were identified using comparative analysis. We interviewed 16 females and 11 males (mean age = 71.7 years). We identified three themes. Residents characterized good healthcare as that which is provided by physicians who are consistent educators that listen to residents' primary concerns. They defined health as being mobile and lacking pain. Finally, they desire more, recurring opportunities to learn about health and connect interpersonally within their housing community; they perceive limited meaningful relationships as a significant contributor to poor health among residents. The older adult public housing residents in our study outlined what good health and healthcare looks like. These themes can be utilized to improve relationships between residents and their healthcare providers. Social isolation can be mitigated through public housing programming that promotes physical and mental acuity.

### THE EFFECT OF BOTTLEIUM, A BOTTLE-TYPE AQUARIUM, OWNERSHIP ON COMMUNITY-DWELLING OLDER ADULTS

Mai Takase,[1] Ryogo Ogino,[1] Keishiro Yoshida,[2] Hikari Kusunoki,[2] and Tetsuya Kenmochi,[2] *1. The University of Tokyo, Tokyo, Tokyo, Japan, 2. GEX International Corporation, Higashiosaka, Osaka, Japan*

The pet ownership of ornamental fish acts positively towards the well-being of older adults. A Bottleium is an aquarium that uses a glass bottle instead of a tank. Its small size allows older adults to own ornamental fish as a pet. In this study, the effect of Bottleium ownership on the daily life of community-dwelling older adults was explored. A three-hour workshop to build one's own Bottleium (size: 11cm×11cm×20cm) was hosted at Toyoshikidai housing complex (Kashiwa, Japan). An ornamental fish and a freshwater snail were added to each bottle. Semi-structured interviews were conducted with participants one month after taking the Bottleium home (N=25). The Bottleium ownership acted as a stimulus to older adults. The effects on an individual were "trigger of conversation" and "development of responsibility." Participants living alone treated the fish as their companions and had light conversations. They carefully looked after the fish as would a pet owner. Furthermore, the Bottleium facilitated "interpersonal interactions." Basic information was provided during the workshop, but participants assisted each other in complementing the information, and they even visited each other's residences to observe the fish. Older adults who do not own the Bottleium also visited for this purpose. Two cases were reported where participants looked after each other's fish in times of hospitalization. The interpersonal interactions might have resulted from the nature of the housing complex, as participants were already familiar with each other. Similar phenomena could be anticipated at places such as apartments and nursing homes.

### SESSION 2872 (POSTER)

### SERVICES AND INTERVENTION: EXPERIENCES AND OUTCOMES

### AGE AND SEX DIFFERENCES OF COMORBIDITIES IN MEDICARE OLDER ADULTS WITH CHRONIC OBSTRUCTIVE PULMONARY DISEASE

Tham Le, and Linda Simoni-Wastila, *University of Maryland, Baltimore, Maryland, United States*

OBJECTIVES: Although comorbidity varies by sex and age, comorbidity variation among individuals with specific primary conditions is less well-understood. We sought to quantify chronic comorbidities in older adults with chronic obstructive pulmonary disease (COPD) using representative Medicare claims data. METHODS: This retrospective cohort study consisted of individuals aged 65+ with a COPD

diagnosis identified in a 5% Medicare sample enrolled between 1/1/2012-1/1/2015. We quantified the prevalence of 40 comorbidities and sex and age variation (≥ or ≤ 85 years). RESULTS: Of 461,268 eligible beneficiaries, 60% were female, 86% were white, with mean (SD) age of 79 (8) years. The majority (89.2%) had at least 5 comorbidities; 50.4% had ≥ ten comorbidities. Most prevalent conditions included: hypertension (92.6%), hyperlipidemia (86.4%), anemia (74.9%), rheumatoid arthritis (RA) (68.0%), congestive heart failure (CHF) (49.6%), diabetes (46.7%), depression (43.7%), peripheral vascular disease (PVD) (42.9%), and chronic kidney disease (CKD) (38.6.) Male patients had higher prevalence of CHF, diabetes, CKD, atrial fibrillation (AFib), AMI, cancer, tobacco use disorder, and liver disease, while females had higher prevalence of hypertension, anemia, RA, depression, asthma, osteoporosis, pain, hypothyroid, obesity, dementia, and glaucoma. Compared to patients under 85, those aged ≥85 years had higher prevalence of cardiovascular disease, depression, musculoskeletal conditions, cancer, dementia, glaucoma, and CKD; but lower prevalence of asthma, anxiety, and metabolic disorders. CONCLUSIONS: Older adults with COPD encounter a high prevalence of comorbidities. Comorbidity patterns differs across age and sex spectrum, highlighting the significance of age and sex in developing individualized clinical care and epidemiological research.

### BECOMING AN AUTOLOGOUS STEM CELL TRANSPLANT PATIENT

Sean Halpin,[1] Michael Konomos,[2] and Ivey Jowers,[3]
*1. University of Georgia, Decatur, Georgia, United States,*
*2. Emory University, Atlanta, Georgia, United States,*
*3. Winship Cancer Institute, Atlanta, Georgia, United States*

In the current study, we sought to examine how older patients incorporate the identity of a patient receiving autologous stem cell transplant (ASCT) for multiple myeloma (MM) into their daily lives. In this ethnographic study using interpretative phenomenological analysis, we observed pre-transplant education visits with 30 MM patients, followed by semi-structured interviews in their hospital rooms during transplant. The experience of receiving ASCT for MM required effort by patients to not only maintain their past identity but also establish a new patient identity. Reconciling these two identities required deliberate and emotionally draining effort from the patient. Results were organized into two overarching themes of social relationships and aesthetics with subthemes for each. Patients experienced challenges reconceptualizing their social support network to meet their changing needs; often with a spouse or child taking on a caregiving role. In regard to aesthetics, patients contended with the physical reminders of their new diseased identity, adopting various aesthetic strategies to either embrace or conceal bodily changes. Understanding methods MM patients who are receiving ASCT use to negotiate normalcy during treatment may be helpful for developing interventions for alleviating distress during this difficult time.

### HOW DO SENIOR CENTER ATTENDEES RATE THE IMPORTANCE OF PROGRAMS AND SERVICES COMPARED TO NON-ATTENDEES?

Ceara Somerville,[1] Saralyn Collins,[2] Shayna Gleason,[3] and Caitlin Coyle,[2] *1. University of Massachusetts Boston, Everett, Massachusetts, United States, 2. University of Massachusetts Boston, Boston, Massachusetts, United States, 3. University of Massachusetts, Boston, Boston, Massachusetts, United States*

Senior centers provide a wide range of programs and services to meet the needs of the growing aging population. As senior centers aim to serve all older community-members, it is important to assess the value of these services through the lens of both attendees and non-attendees of senior centers. Using a sample of 4,750 community-dwelling adults age 60 or older from Massachusetts, this project aims to analyze perceptions of program importance by center attendees versus non-attendees. Almost 60% of the sample never attend a senior center. Nearly a third of center attendees ranked exercise classes and education opportunities as important, compared to about 20% of non-attendees. Almost half of attendees rated application assistance and nutrition programs as not important, compared to 38% and 40% of non-attendees, respectively. Conversely, for all programs listed, non-users more frequently checked "unsure" of program/service importance. This was especially true for exercise classes, professional services, social or recreational activities, educational opportunities, and trips, for which 18% of non-attendees selected unsure. Center attendees more frequently view the programs/services offered at the senior center as important or very important, likely because they have greater awareness. Results highlight that that non-attendees are unsure of the value of programs or have no interest the programs and services provided. Not only do these results provide an opportunity to senior centers to assess how valuable certain programs and services are, they also emphasize the need for continued information streams regarding programs and services offered at senior centers to those who are unfamiliar.

### SESSION 2875 (POSTER)

### BRAIN AGING, COGNITIVE AGING, AND ALZHEIMER'S

### AGE 20 COGNITIVE ABILITY MODERATES THE LONG-TERM INFLUENCE OF LIFESTYLE BEHAVIORS ON BRAIN AGING IN LATE MIDLIFE

Carol Franz,[1] Teresa Warren,[2] and William Kremen,[3]
*1. University of California San Diego, La Jolla, California, United States, 2. San Diego State University, La Jolla, California, United States, 3. University of California San Diego, San Diego, California, United States*

We examined whether the longitudinal association between lifestyle behaviors and brain age is moderated by early general cognitive ability (GCA). The sample comprises 356 participants from the Vietnam Era Twin Study of Aging (VETSA). At mean age 40 (SD 2.7; range 34-44) a positive lifestyle index was created comprising three self-reported behaviors: not smoking, zero to moderate alcohol consumption, and high social engagement. GCA at mean age 20 was assessed with the Armed Forces Qualification Test. At mean age 68 (SD 2.6; range 61-72), participants underwent structural magnetic resonance imaging which was used to create predicted brain age difference (PBAD) scores. Multivariate models included GCA, lifestyle and their interaction as IVs, adjusted for age, ethnicity, APOE genotype, height, and family membership. Age 20 GCA and age 40 lifestyle significantly predicted age 68 PBAD [$F=5.83$; $p=.02$ and $F=15.14$; $p<.001$, respectively]. Both positive behaviors and higher age 20 GCA were associated with less brain aging. The GCA-lifestyle interaction was also significant. Those with both lower age 20 GCA and fewer positive behaviors had older brains relative to chronological age [F=5.00; p=. 03]. When GCA was high, however, participants had younger brains, regardless of lifestyle behaviors, suggesting a protective effect of early high GCA or cognitive reserve on later brain health. However, for those with lower cognitive reserve, positive lifestyle behaviors appeared to be protective against brain aging nearly three decades later. Results highlight the important role of cognitive reserve and lifestyle factors for later life brain health.

### AGING RELATED TRANSCRIPTOMIC CHANGES IN THE MOUSE MODELS OF ALZHEIMER'S DISEASE

Asli Uyar,[1] Ravi Pandey,[1] Christoph Preuss,[2] Kevin Kotredes,[2] Gareth Howell,[2] Michael Sasner,[2] and Gregory Carter,[2] *1. The Jackson Laboratory, Farmington, Connecticut, United States, 2. The Jackson Laboratory, Bar Harbor, Maine, United States*

Alzheimer's Disease (AD) is characterized by multiple clinical phenotypes and molecular signatures at different stages of the disease and aging is the major risk factor for sporadic AD. Aging and AD are linked at molecular, cellular and systems level with commonalities in inflammation and associated immune response in the brain. Mouse models of AD were developed that mimic various aspects of aging-associated neurodegeneration and inflammation. Research in mouse models of AD showed that drugs and treatments designed for AD can decelerate aging phenotypes suggesting efficient utilization of these models in aging research. We analyzed RNA-Seq transcriptomic data from transgenic mouse models of familial AD (APP/PS1 and 5XFAD) and knock-in mouse models of late-onset AD (APOE and TREM2) at the ages between 4-months and 24-months. The number of differentially expressed genes between transgenic/knock-in and WT mice increased by age in all mouse models. Gene set enrichment analysis identified metabolic pathways, including oxidative phosphorylation, altered in an age and genotype related manner in the brain of APP/PS1 and 5XFAD mice that recapitulate major features of amyloid pathology. Immunity related pathways were enriched in APOE4 model carrying Trem2*R47H mutation at >12 months-old. We also mapped the transcriptional signatures to co-expression gene modules of human LOAD from the AMP-AD consortium and observed correlations specific to each mouse model. Our study provides a detailed view of how the aging interacts with AD-relevant pathologies at the transcriptome level and demonstrates potential translational relevance of the AD mouse models in the context of human aging.

### ALZHEIMER'S DISEASE-ASSOCIATED PATHOLOGY IN A TRANSGENIC MOUSE MODEL RESULTS IN ALTERED VOIDING FUNCTION

Cara Hardy,[1] Dawn Rosenberg,[1] Ramalakshmi Ramasamy,[2] Xiangyou Hu,[1] and Phillip Smith,[1] *1. UConn Health, Farmington, Connecticut, United States, 2. UConn Health, Farminton, Connecticut, United States*

Alzheimer's disease (AD) is a devastating disorder primarily affecting older adults and is the most common neurodegenerative disease in the US. More than one in three AD patients experience AD-associated urinary dysfunction (ADUD), which directly contributes to their institutionalization. While ADUD has been clinically regarded as a result of poor cognitive control over urinary function, the physiology underlying loss of urinary control remains unknown. We hypothesize that beta-amyloidosis in the CNS results in pathologic changes in urinary structure and function. Male and female Tg-APP/PS1DE9 mice were used before plaque deposition (4-6 months) and after plaque accumulation (8-10 months) and compared to their WT littermates. Pressure-flow cystometry was conducted under urethane anesthesia to assess urinary performance at the level of the autonomic nervous system in the absence of cortical control. Pharmacomyography was performed on bladder strips to determine tissue-level changes in the absence of CNS input. In Tg-APP/PS1DE9 mice, plaque accumulation resulted in diminished volume sensitivity and decreased voiding efficiency. Pharmacologic studies showed aberrant drug responses, altered cholinergic signaling, and decreased resilience of tissue longevity after plaque accumulation. Based on our findings, we conclude that the AD-related pathology of Aβ accumulation results in a distinct urinary phenotype in our model, analogous to the ADUD observed in AD patients. Establishing and expanding models of ADUD to other mouse models of AD-associated pathology may improve the efficacy of treating ADUD and increase quality of life for patients and their caregivers.

### CAROTID REVASCULARIZATION IMPROVES BALANCE AND MOBILITY, PARTICULARLY IN PATIENTS THAT ARE MOST IMPAIRED

Vicki Gray,[1] Sarasijhaa Desikan,[1] Amir Khan,[2] Dawn Barth,[3] Siddhartha Sikdar,[2] John Sorkin,[3] and Brajesh Lal,[4] *1. University of Maryland School of Medicine, Baltimore, Maryland, United States, 2. George Mason University, Fairfax, Virginia, United States, 3. University of Maryland School of Medicine, Balitmore, Maryland, United States, 4. University of Maryland, Baltimroe, Maryland, United States*

Balance and mobility function worsen with age, and more so for those with underlying diseases. Our research has demonstrated that asymptomatic carotid artery stenosis (ACAS) is associated with worse balance and mobility, and a higher fall risk, compared to older adults with similar comorbidities, but without ACAS. Thus, ACAS, with attendant blood flow-restriction to the brain is a potentially modifiable risk factor for balance and mobility dysfunction. The purpose of this study was to evaluate the impact of restoring blood flow to the brain by carotid revascularization, on balance and mobility in patients with high-grade ACAS (≥70% diameter-reducing stenosis). Twenty adults (67.0±9.4 years) undergoing carotid revascularization for high-grade stenosis were enrolled. A balance and mobility assessment was performed before- and six weeks- after carotid revascularization and included: Short Physical Performance Battery (SPPB), Berg Balance Scale (BBS), Four Square Step Test (FSST), Dynamic Gait Index (DGI) Timed Up and Go (TUG), gait speed, MiniBESTest, and Walk While Talk (WWT) test. Paired t-tests assessed changes in outcome measures between the two-time points. Significant improvements were observed in measures that combined walking with dynamic movements, DGI (P=0.003), and MiniBESTest

(P=0.021). Pearson's correlations examined the relationship between balance and mobility before surgery and change score after surgery. Patients with lower baseline DGI and MiniBest scores demonstrated the most improvement on follow-up testing (r=-0.70, p=0.001, and r=-0.59, p=0.006, respectively). In conclusion, revascularization of a carotid artery stenosis improves balance and mobility; the greatest improvements are observed in those patients that are the most impaired.

### COGNITIVELY IMPAIRED OLD MICE DISPLAY CORRELATED REDUCTION IN CORTICAL NMDA RECEPTOR AND COMPLEX IV

Ramkumar Thiyagarajan,[1] Yonas Redae,[1] Kenneth Seldeen,[2] and Bruce Troen,[1] *1. University at Buffalo, Buffalo, New York, United States, 2. University of Buffalo, Buffalo, New York, United States*

Cognitive decline in older adults represents a major challenge since cognitive impairment is found in 10% of those ≥ 65 and 50% ≥ 85. Thus it is increasingly important to understand the impact of aging on cognitive health. We performed a battery of tests to assess cognition in 6 month-old (n=12) and 24 month-old (n=8) C57BL/6J mice, equivalent to 30 and 70 year old humans, respectively, and also assessed protein markers in cortex for mitochondrial health and cognition. We found that aged mice displayed fewer spontaneous alternations in the T maze test (p=0.034) and lower recognition of novel objects (p=0.022). In addition, aged mice showed prolonged escape time in the Barnes maze (p=0.035), all of which taken together suggest reduced capacity for learning and recall. Aged mice also exhibited diminished nest building (p<0.001), revealing an impaired functional capacity analogous to the instrumental activities of daily living (IADL) geriatric assessment. We found reduced mitochondrial complex IV expression in the cortices of aged mice concomitant with less expression of N-Methyl D-Aspartate (NMDA) receptor subunits 1, 2A and 2B. The cortices from old mice also exhibited greater expression of immature brain derived neurotrophic factor (pro-BDNF). The alterations in NMDA receptors and pro BDNF are consistent with memory impairment and greater neuronal cell death. Therefore, aged mice exhibit significantly reduced recall and learning ability alongside marked alterations in mitochondrial complex, NMDA receptor, and pro-BDNF expression. Studies are underway to assess whether these molecular changes are responsible for the cognitive declines with aging.

### CO-OCCURRENCE OF PHYSICAL AND COGNITIVE DECLINE IN VERVET MONKEYS (CHLOROCEBUS AETHIOPS SABAEUS)

Brett Frye,[1] Carol Shively,[2] Suzanne Craft,[1] Thomas Register,[1] Matthew Jorgensen,[1] Caitlin Latimer,[3] Christie Scott,[1] and Hannah Register[1] *1. Wake Forest School of Medicine, Winston-Salem, North Carolina, United States, 2. Wake Forest University Alzheimer's Disease Research Center, Winston-Salem, North Carolina, United States, 3. University of Washington, Seattle, Washington, United States*

Age-related neurodegeneration associated with Alzheimer's (AD) disease begins in middle age, well before the onset of symptoms. Therefore, translational models to identify modifiable risk factors in middle-age are needed to understand etiology and identify therapeutic targets. Vervet monkeys (Chlorocebus aethiops sabaeus), like humans, naturally develop several risk factors for AD with age, including obesity, prediabetes, and hypertension. Furthermore, older vervets exhibit accumulation of amyloid and tauopathies, decreased brain volumes, and physical declines in gait speed, suggesting that these NHPs may be useful models of early AD-like neuropathology. Currently, we are investigating the extent to which cognitive and physical decline co-occur in 20 elder (mean age=23 years, ~equivalent to a human age of 80 years) and 10 middle-aged (mean age=11 years) females through assessments of physical performance, executive function, social cognition, and short-term memory. These measures are part of a larger study to integrate physical, social, and cognitive function with measures of body composition, metabolic profiles, CSF, blood, neuroimages, and neuropathology. While tests of social cognition and short-term memory are ongoing, assessments of executive function indicate that performance declines with age (N=26; p<0.05; R-squared=0.23). Furthermore, animals that exhibit slower gait speed also perform poorly on the executive function task (N=26, p<0.05; R-squared=0.25). These preliminary results suggest that accelerated aging co-occurs in multiple systems in vervets. This study will enable examination of temporal relationships between physical and cognitive declines. Ultimately, this comprehensive, integrative whole-body approach will help clarify the mechanisms underlying divergent aging trajectories and inspire interventions that promote multi-system healthy aging.

### DETERMINING THE ROLE OF APOE4 IN AGE-RELATED CEREBROVASCULAR DECLINE

Kate Foley,[1] Peter Borchian,[2] Dylan Garceau,[1] Kevin Kotredes,[1] Paul Territo,[2] Michael Sasner,[1] Gareth Howell,[1] and Cory Diemler,[1] *1. The Jackson Laboratory, Bar Harbor, Maine, United States, 2. Indiana University, Indianapolis, Indiana, United States*

Cerebrovascular decline occurs during aging and may be critical during prodromal phases of Alzheimer's disease (AD). The E4 allele of apolipoprotein E (APOE4) is the greatest genetic risk factor for AD and decreased longevity and studies suggest APOE4 increases risk for age-dependent cerebrovascular damage. To study the relationship between APOE4 and age-related cerebrovascular decline, male and female C57BL/6J (B6) mice carrying combinations of APOE alleles including APOE4 (risk) and APOE3 (neutral), as well as B6 controls were assessed at a variety of ages from 4 to 24 mos for cognitive ability, biometrics and cerebrovascular health including i) PET/MRI using 64Cu-PTSM (perfusion) and 18F-FDG (metabolism), ii) transcriptional profiling and iii) immunofluorescence. Despite no cognitive decline, male APOE4 mice showed hypo-perfusion and hypo-metabolism by 12 mos, while female APOE4 mice showed an uncoupled hyper-perfusion and hypo-metabolism phenotype. Transcriptional profiling showed differential expression of genes involved in regulation of cerebral perfusion, glucose transportation and metabolism in APOE4 mice. An age-dependent blood brain barrier compromise was also apparent in the brains of female APOE4 mice. Physical activity reduces risk for human AD and our data shows exercise improves cerebrovascular health in mice. However, the effects to cerebrovascular health in individuals carrying genetic risk factors such as APOE4 are not known. To determine whether exercise can overcome APOE4-dependent cerebrovascular damage, APOE mice are being exercised from 2-4 and to 2-12 mos. Transcriptional profiling and immunofluorescence will determine whether the benefits of exercise to the cerebrovasculature are modulated by genetic risk factors such as APOE4.

### DOES INFLAMMATION MEDIATE THE ASSOCIATION BETWEEN SLEEP DURATION AND INCIDENT DEMENTIA AMONG OLDER ADULTS?

Chenkai Wu,[1] and Weihao Xu,[2] *1. Duke Kunshan University, Kunshan, Jiangsu, China, 2. Chinese PLA General Hospital, Beijing, China*

Sleep duration is a risk factor for multiple health outcomes. Growing attention has been directed to the association between sleep duration and dementia; however, results were inconsistent and the mechanisms remained largely unknown. We hypothesized that elevated levels of inflammation markers— C reactive protein (CRP), interleukin-6 (IL-6), and tumor necrosis factor-α (TNF-α)—would mediate the association between sleep duration and dementia among older adults. Data were from the Health, Aging, and Body Composition Study; 3,010 participants free of dementia at baseline were included. Sleep duration was classified into: short (<6 hours), normal (6-8 hours), and long (>8 hours). Incident dementia was defined as (i) use of prescribed dementia medications, (ii) adjudicated dementia diagnosis, or (iii) a race-stratified cognitive decline >1.5 SDs from the baseline mean. We used Cox models to examine the associations among sleep duration, inflammation, and dementia. The average age was 73.6 years (SD=2.9); 49% were male and 41% were black. During 10 years of follow-up, 515 participants (17.1%) developed dementia. Long sleep duration was associated with higher hazard of dementia than normal sleep duration (HR=1.50, 95%CI=1.02-2.21). This association was attenuated by approximately 10% when CRP or IL-6 was added in the model. When all three inflammation markers were included in the model, the hazard ratio of long sleep duration was reduced by nearly 30% and no longer significant (HR=1.36, 95%CI=0.89-2.08). Long sleep duration was associated with high risk of incident dementia among older adults and the association was partly explained by elevated levels of inflammation markers.

### EFFECT OF OSTEOARTHRITIS ON PREFRONTAL CORTICAL ACTIVATION PATTERNS DURING DOWNWARD REACHING IN OLDER WOMEN

Manuel Hernandez, Yang Hu, and Alka Bishnoi, *University of Illinois at Urbana-Champaign, Urbana, Illinois, United States*

Downward reaching may lead to falls in older adults, but the underlying mechanisms are poorly understood, particularly in older women with osteoarthritis. Given the importance of attentional resources when maintaining balance in balance-demanding conditions, functional near-infrared spectroscopy may provide a lens to the attentional resource allocation needed to maintain balance while reaching down to the ground with or without full contact with the floor. We examined the changes in the executive control of downward reaching movements in older women with osteoarthritis. We hypothesized that prefrontal cortical activation would be higher in older women with osteoarthritis, compared to age-matched controls, particularly as the balance demands increased. Older women with osteoarthritis (n=7, mean±SD age: 66±3 years) and age-matched controls (n=10, mean±SD age: 67±6 years) were recruited from the local community. The effect of the base of support and target position (toe or maximal forward distance along ground) on attentional resources in older women with osteoarthritis were evaluated using the average oxygenated hemoglobin levels as a measure of prefrontal cortical (PFC) activation. Significant base of support by cohort and target by cohort interactions were observed on average PFC activation levels (P<0.005). As expected, PFC activation levels increased as the contact with the floor decreased and as the target distance increased, but older women with osteoarthritis were unable to increase their PFC activation levels as much as age-matched controls. In conclusion, these data suggest that older women with osteoarthritis may not be adequately modulating attentional resources to meet high-balance demanding tasks.

### EFFECTS OF CNS DEMYELINATION AND MYELIN RECOVERY ON URINARY PHYSIOLOGY

Ramalakshmi Ramasamy,[1] Dawn Rosenberg,[2] Cara Hardy,[2] Stephen Crocker,[2] and Phillip Smith,[2] *1. UConn Health, Farminton, Connecticut, United States, 2. UConn Health, Farmington, Connecticut, United States*

Multiple sclerosis (MS) is a chronic inflammatory demyelinating disease of the central nervous system (CNS). Of note, over 80% of MS patients have urinary symptoms as one of their earliest symptoms. Since MS patients often live into older age, urinary incontinence and retention are significant problems for which few if any effective preventive or therapeutic options are available. The mechanisms by which MS contributes to urinary dysfunction are not well understood. We propose to elucidate the impact of demyelination on urinary performance using the cuprizone model, a model used to study the effects of CNS demyelination and spontaneous remyelination. We hypothesize that CNS demyelination in the cuprizone model will result in aberrant changes in urinary function, and that after remyelination occurs this dysfunction will be alleviated. C57Bl/6 mice were treated with cuprizone (0.2% w/w) for four weeks to induce demyelination. One group was allowed four additional weeks to recover from demyelination, while the other continued cuprizone treatment. Following this eight-week treatment, pressure/flow cystometry, electromyography, and molecular studies were performed to assess demyelination-induced differences in urinary performance. Cuprizone-recovery mice displayed improvements in cystometric function compared to their demyelinated littermates, as seen through improved volume sensitivity and voiding efficiency. Pharmacologic studies showed no significant changes in contractile responsiveness. Thus, we conclude that CNS demyelination results in aging-like phenotype and urinary dysfunction consistent with that observed in clinical disease. Therapeutics aimed at increasing the remyelination potential of the CNS neurons offer the possibility of alleviating urinary dysfunction associated with MS.

### FUSOGENIC LIPOSOMES DELIVER RESVERATROL TO BRAIN MICROCIRCULATION AND IMPROVE NEUROVASCULAR COUPLING IN AGED MICE

Adam Nyul Toth,[1] Tabea Wiedenhoeft,[2] Stefano Tarantini,[1] Tamas Csipo,[3] Priya Balasubramanian,[3] Anna Csiszar,[1] Agnes Csiszar,[2] and Zoltan Ungvari,[4] *1. University of Oklahoma Health Sciences Center, Oklahoma City, Oklahoma, United States, 2. Institute of Complex Systems, Jülich, Germany, 3. OUHSC, Oklahoma City, Oklahoma, United States, 4. University of Oklahoma Health Sciences Center, Oklahoma City, United States*

Adjustment of cerebral blood flow (CBF) to the increased oxygen and nutrient demands of active brain regions via neurovascular coupling (NVC) has an essential role in maintenance of healthy cognitive function. In advanced age, cerebromicrovascular oxidative stress and endothelial dysfunction impair neurovascular coupling, contributing to age-related cognitive decline. Recently we developed a resveratrol (3,4′,5- trihydroxystilbene)-containing fusogenic liposome (FL-RSV)-based molecular delivery system that can effectively target cultured cerebromicrovascular endothelial cells, attenuating age-related oxidative stress. To assess the cerebromicrovascular protective effects of FL-RSV in vivo, aged (24-monthold) C57BL/6 mice were treated with FL-RSV for four days. To demonstrate effective cellular uptake of FL-RSV, accumulation of the lipophilic tracer dyes in cells of the neurovascular unit was confirmed using two-photon imaging (through a chronic cranial window). NVC was assessed by measuring CBF responses (laser speckle contrast imaging) evoked by contralateral whisker stimulation. We found that NVC responses were significantly impaired in aged mice. Treatment with FL-RSV significantly improved NVC responses by increasing NO-mediated vasodilation. These findings are paralleled by the protective effects of FL-RSV on endothelium-dependent relaxation in the aorta. Thus, treatment with FL-RSV rescues endothelial function and NVC responses in aged mice. We propose that resveratrol containing fusogenic liposomes could also be used for combined delivery of various anti-geronic factors, including proteins, small molecules, DNA vectors and mRNAs targeting key pathways involved in microvascular aging and neurovascular dysfunction for the prevention/treatment of age-related cerebromicrovascular pathologies and development of vascular cognitive impairment (VCI) in aging.

### IMPAIRED MITOCHONDRIAL AND GLYCOLYTIC FUNCTIONS IN PERIPHERAL BLOOD LEUKOCYTES OF ALZHEIMER'S DISEASE

Ravindra Bharadwaj,[1] Hayley Gibler,[2] Sanjay Srivastava,[3] and Hena Tewari,[4] *1. Texas Tech University Health Sciences Center, Amarillo, Texas, United States, 2. Tecas Tech HSC AMA, Amarillo, United States, 3. Texas Tech HSC, Amarillo, United States, 4. Texas Tech HSC - AMA, Amarillo, Texas, United States*

Recent failures of the trials targeting amyloid to treat Alzheimer's disease (AD) are prompting scientists to explore other pathological pathways. Brains of AD patients have been noted to have impaired mitochondrial function. It has not yet been determined if AD is caused by a systemic defect in cellular bioenergetics. To determine the cellular bioenergetics, we compared the Oxygen Consumption Rate (OCR – indicating oxygen dependent respiration) and Extra Cellular Acidification Rate (ECAR – indicating glycolytic function) in leukocytes of collected blood samples of Alzheimer's and non-dementia patients. Methods: After IRB approval and consents, blood samples from each clinically diagnosed Alzheimer's and age matched normal subjects were collected. Immediately after collection the blood samples were analyzed using Agilent Seahorse XFe/XF Analyzer as per protocol by manufacturer. Results: Impaired mitochondrial and glycolytic functions were noted in Alzheimer's patients as compared to normal subjects. OCR was significantly lower in Alzheimer's patients. A lower rate of respiration was noted both at basal as well as maximal respiration. Reduced spare respiration capacity was also noted in response to the stressors. Similarly reduced ECAR and reduced glycolytic reserve was also noted in Alzheimer's patients, indicating impaired oxygen independent mitochondrial respiration. Discussion: This pilot study demonstrates that there is an impaired mitochondrial and glycolytic function in the peripheral blood cells. This indicates towards a systemic nature of the disease and a potential future bio-marker. Further studies should be planned in this direction.

### INCREASED SERUM IL 6 ASSOCIATED WITH MORTALITY AND COGNITIVE IMPAIRMENT IN ELDERLY BRAZILIANS

Marcelo Caldeira,[1] Milena Brunialti,[2] Reinaldo Salomão,[2] Clineu Almada Filho,[3] and Maysa Cendoroglo,[3] *1. Universidade federal de São Paulo, SAO PAULO, Sao Paulo, Brazil, 2. UNIVERSIDADE FEDERAL DE SÃO PAULO, SAO PAULO, Brazil, 3. UNIVERSIDADE FEDERAL DE SÃO PAULO, SAO PAULO, Sao Paulo, Brazil*

Immunological and inflammatory changes are gaining importance in aging as they are associated with functional limitations and mortality. Chronic inflammation associated with aging (Inflammaging) is a systemic and subclinical condition, characterized by changes in the levels of interleukins such as IL1, IL4, IL6, IL8, IL10 and TNF alpha, associated with genetic, physiological and environmental factors, whose importance is to be directly associated with morbidity and mortality in the elderly. Objective: To evaluate, through a longitudinal study, the relationship between chronic inflammation associated with aging and possible outcomes, such as cognitive changes and mortality in independent oldest old adults. Methods: were evaluated 201 elderly, aged 80 years or older, community residents, with preserved cognition, without acute diseases and with controlled chronic diseases. In a 02 years of interval, laboratory collections of inflammatory markers (IL 1, IL 4, IL6, IL10, TNF alpha and CRP) were performed and outcomes such as cognitive impairment and deaths were evaluated. Results: There was a correlation between increased serum IL6 and cognitive impairment, in the group of women (p-value = 0.008) and in the group All (p-value = 0.022). In the group of men, there was a significant difference between the increase in IL6 values (p-value = 0.028) and CRP (p-value = 0.016) in relation to deaths. Conclusion: The results of this longitudinal study showed and confirmed the positive association between changes in inflammatory markers such as IL6 and outcomes such as cognitive impairment and mortality also in elderly Brazilians.

### LOW DOSE BRAIN RADIATION REDUCES ELEVATED PLASMA BRANCH CHAIN AMINO ACID LEVELS IN APP/PS1 MICE

Michael Maddens,[1] George Wilson,[2] Stewart Graham,[2] and Ali Yilmaz,[2] *1. William Beaumont Hospital, Royal Oak, Michigan, United States, 2. Beaumont Hospital - Royal Oak, Royal Oak, Michigan, United States*

Branch Chain Amino Acids (BCAA) have recently been implicated in Alzheimer's Disease (AD). We previously showed that low dose brain radiation (RT) [5 fractions of 2 Gy] reduces amyloid-beta plaque burden and results in improved cognition in the APP/PS1 model of AD. In this study we investigated whether this schedule of radiation altered the metabolomic profile of serum. 10 month old male (M) and female (F) APP/PS1 mice were either treated with whole brain radiotherapy (5 x 2 Gy) or received sham irradiation. Eight weeks later the animals were euthanized and blood, urine and brain tissue collected. 1H NMR spectra were acquired. 256 transients were acquired for each sample and chemical shifts (δ) are reported in parts per million (ppm). Analysis included: 3 F and 5 M with no transgene (as a background controls), 5 F who received no RT, 7 F who received RT, 12 M who received RT and 12 M who received no RT. A total of 46 metabolites were analyzed. The most significantly changed metabolites were the BCAAs leucine, isoleucine and valine.. The effect was most pronounced in female mice where levels were reduced to those found in non-transgenic mice. APP/PS1 mice spontaneously display increased plasma BCAA, suggesting that AD pathology potentiates defects in BCAA metabolism, putting patients with AD at a higher risk of BCAA-induced brain damage. Reduction of these levels by low dose radiation may be beneficial.

### NEUROFILAMENT LIGHT CHAIN AND COGNITIVE PERFORMANCE IN SELECTED SIBLINGS FROM THE CATSLIFE STUDY

Chandra Reynolds,[1] Andrew Smolen,[2] Christopher Link,[3] and Sally Wadsworth,[2] *1. University of California, Riverside, Riverside, California, United States, 2. University of Colorado Boulder, Boulder, Colorado, United States, 3. University of Colorado, Boulder, Colorado, United States*

Markers of neurodegeneration such as neurofilament light chain (NfL) may be elevated with neurological diseases such as multiple sclerosis (MS) as well as Alzheimer's disease. NfL is a marker of axonal integrity where higher values positively relate to the degree of damage. NfL shows variations in early adulthood among healthy individuals and may relate to executive function performance in otherwise healthy individuals aged 19-32 years. In the ongoing CATSLife (Colorado Adoption/Twin Study of Lifespan behavioral development and cognitive aging) Quanterix Simoa assays of NfL were measured in 34 individuals selected based on self-reported neuroinflammatory conditions (N = 5) or by APOE genotype (N_nonE4 = 18, N_E4 = 16). The distribution of NfL was consistent with other studies of early-mid adulthood (range = 1.3 - 22.3 pg/ml). Based on partial regression weights predicting log-transformed NfL, NfL was higher in cases (exp(b)=1.08 pg/ml), in males (exp(b)=1.25 pg/ml), by age (exp(b)=1.03 pg/ml per year) and in APOE E4 carriers (exp(b)=1.11 p/mg). Moreover, correlations partialed for age, sex, APOE e4 and case status suggest higher NfL may be associated with lower Full Scale IQ and general cognitive ability (r's = -.18 and -. 28) overall and may be more evident among APOE E4 carriers (r's = -.42 - .44, partialed for age, sex, case status). In this pilot study, the observed NfL associations with general cognitive ability, particularly among APOE E4 carriers, suggests NfL may be a salient biomarker of cognitive functioning by early- to mid-adulthood.

### NMN RESCUES ENDOTHELIAL FUNCTION AND NEUROVASCULAR COUPLING, IMPROVING COGNITIVE FUNCTION IN AGED MICE

Stefano Tarantini,[1] Andriy Yabluchanskiy,[2] Praveen Ballabh,[3] Eszter Farkas,[4] Joseph Baur,[5] David Sinclair,[6] Anna Csiszar,[1] and Zoltan Ungvari,[7] *1. University of Oklahoma Health Sciences Center, Oklahoma City, Oklahoma, United States, 2. Biochemistry and Molecular Biology, Oklahoma City, United States, 3. Albert Einstein College of Medicine, Bronx, NY, United States, 4. Faculty of Medicine, Szeged, Hungary, 5. Perelman School of Medicine at the University of Pennsylvania, Philadelphia, Pennsylvania, United States, 6. Harvard Medical School, Boston, United States, 7. University of Oklahoma Health Sciences Center, Oklahoma City, United States*

Adjustment of cerebral blood flow (CBF) to neuronal activity via neurovascular coupling (NVC) has an essential role in maintenance of healthy cognitive function. In aging increased oxidative stress and cerebromicrovascular endothelial dysfunction impair NVC, contributing to cognitive decline. There is increasing evidence showing that a decrease in NAD+ availability with age plays a critical role in a range of age-related cellular impairments but its role in impaired NVC responses remains unexplored. The present study was designed to test the hypothesis that restoring NAD+ concentration may exert beneficial effects on NVC responses in aging. To test this hypothesis 24-month-old C57BL/6 mice were treated with nicotinamide mononucleotide (NMN), a key NAD+ intermediate, for 2 weeks. NVC was assessed by measuring CBF responses (laser Doppler flowmetry) evoked by contralateral whisker stimulation. We found that NVC responses were significantly impaired in aged mice. NMN supplementation rescued NVC responses by increasing endothelial NO-mediated vasodilation, which was associated with significantly improved spatial working memory and gait coordination. These findings are paralleled by the sirtuin-dependent protective effects of NMN on mitochondrial production of reactive oxygen species and mitochondrial bioenergetics in cultured cerebromicrovascular endothelial cells derived from aged animals. Thus, a decrease in NAD+ availability contributes to age-related cerebromicrovascular dysfunction, exacerbating cognitive decline. The cerebromicrovascular protective effects of NMN highlight the preventive and therapeutic potential of NAD+ intermediates as effective interventions in patients at risk for vascular cognitive impairment (VCI).

### ODOR SENSITIVITY AS A BIOMARKER OF AGING

Marjana Sarker,[1] and Scott Leiser,[2] *1. University of Michigan Medical School, Ann Arbor, Michigan, United States, 2. University of Michigan, Ann Arbor, Michigan, United States*

Recent studies support the deterioration of the sense of smell as an important biomarker for cognitive impairment diseases, including Alzheimer's disease. The model organism C. elegans has a well-studied olfactory system, which provides an ideal platform to measure loss of smell with aging. The goal of our project is to use the short lifespan and olfactory changes observed in nematodes to identify mechanisms to slow aging and treat age-related diseases. Our approach is to utilize worms at various times of their healthy adult lifespan and to test for their sensitivity to known attractants such as benzaldehyde. These odorants are largely detected by the main AWC olfactory neurons. It is well documented that the responsiveness of AWC decreases with age. Our paradigm is to briefly fast worms to increase motivation before testing their ability to discriminate odors. Our results show that younger worms actively move toward the attractant and show preference for specific attractants. However, older worms frequently do not respond to attractive odors and remain near the point of origin, regardless of motility. These results indicate a decreased odor response with age. Our current work focuses on identifying genes and compounds that positively affect this odor response in older animals. The resulting data can then be tested for their efficacy to improve other aspects of healthspan and potentially longevity.

### QUANTITATIVE EEG FEATURES OF LEVEL OF CONSCIOUSNESS IN CRITICALLY ILL NONAGENARIANS

Shawniqua Williams Roberson, and Kevin Haas,
*Vanderbilt University Medical Center, Nashville, Tennessee, United States*

The standard for monitoring sedation levels in critically ill patients is intermittent bedside evaluation, and is prone to anchoring bias. Quantitative electroencephalography (qEEG) allows automated processing of recorded brain electrical activity and could be used to continuously monitor level of consciousness in critically ill patients. The majority of qEEG studies have included persons 80 years of age or less, and the qEEG profiles of nonagenarians have been incompletely characterized. Knowledge of the qEEG patterns of patients 90 years and older is essential for appropriate interpretation of such metrics in this population. This retrospective cohort study characterized qEEG profiles of acutely ill nonagenarians. We investigated whether the relationship between qEEG and level of consciousness differed between patients with and without a history of dementia. We included patients 90-100 years old admitted to Vanderbilt University Medical Center who underwent EEG and as part of their clinical care. We compared qEEG features to nursing-defined level of arousal as measured by the Richmond Agitation-Sedation Scale (RASS) in patients with and without history of dementia. Between January and December 2019, 26 nonagenarians underwent EEG for clinical purposes. One study was excluded due to excessive artifact. Of the remaining, 6 (24%) were male and 18 (72%) were Caucasian. Among all patients, RASS decreased with increases in EEG theta variability (coefficient -7.7, 95%CI -10.6 to -4.8). This relationship was not significantly modified by history of dementia (coefficient of interaction term -0.36, 95%CI -3.7 to 2.9). Dementia does not impact qEEG features of level of consciousness in nonagenarians.

### SESSION 2876 (POSTER)

### METABOLISM

### 4:10 CYCLES OF VERY LOW CALORIES PROTECT AGAINST TUMOR XENOGRAFTS, BUT NOT METASTASES IN THE ABSENCE OF CHEMOTHERAPY

Laura Corrales-Diaz Pomatto, Oye Bosompra, Sarah Wong, Monica Bodogai, Jonathan Kato, Melissa Carpenter, Arya Biragyn, and Rafael de Cabo,
*National Institute on Aging, Bethesda, Maryland, United States*

Cancer is a leading cause of mortality, with its incidence only expected to rise with an increasingly aging population. Dietary interventions, primarily caloric restriction (CR), lower cellular energy metabolism and have long been utilized to slow the aging process and protect against age-related diseases, including cancer. However, due to the stringency of CR, dietary alternatives that offer the same beneficial outcomes in cancer prevention and longevity have become increasingly attractive. Periodic cycles (4 days twice a month) of low caloric intake followed by a standard ad libitum (AL) diet was previously shown to promote health-span in mice and humans and protect against primary tumorigenesis and enhanced the effects of chemotherapy. The aim of our study was to compare the tumorigenic potential of 4T1 cells, a murine model of stage IV breast cancer, in young and aged female BALB/c mice fed either periodic cycles of low caloric diets versus chronic 20% CR. Compared to AL controls, we found a significant delay in primary tumor growth in mice regardless of diet composition by the 4:10 cycles of very low caloric intake. However, unlike in CR, CR-alternative diets were not protective against lung metastases in the absence of chemotherapy. Our study sheds light into the underlying differences of calorie-based interventions in the absence of chemotherapy.

### A 3-WEEK TRYPTOPHAN-DEFICIENT DIET RESULTED IN DECREASED BODY WEIGHT AND INCREASED TRABECULAR BONE MASS IN MICE

Carlos Isales,[1] Kehong Ding,[1] Meghan McGee-Lawrence,[1] Wendy Bollag,[1] William Hill,[2] Sadanand Fulzele,[1] Mohamed Awad,[1] and Mark Hamrick[1], *1. Augusta University, Augusta, Georgia, United States, 2. Medical University of South Carolina, Charleston, South Carolina, United States*

Tryptophan is an essential amino acid with a variety of bioactive metabolites including serotonin, melatonin and nicotinamide. Dietary Trp restriction results in increased lifespan but with detrimental side effects. The initial catabolite in the Trp breakdown pathway, kynurenine, increases with aging and induces bone loss. Thus, we hypothesized that eliminating Trp in the diet of older mice might be osteoprotective. In an IACUC-approved protocol, we fed either 0, 0.2 (standard), 0.7 or 1.25% Trp-containing diets to aged (23-month-old) C57BL/6 mice for a planned eight weeks. There was a rapid decrease in body weight in the mice fed the 0% tryptophan diet and the mice had to be sacrificed at three weeks (25.2±1.4 vs 35.4±3.6 vs 33.9±2.4 vs 33.4±3.1

gm, (Means+SD, No Trp: 0%; Standard Trp 0.2%; High Trp: 0.7%; High Trp: 1.25%, p=0.0004, 0 vs 0.2%, n=9-10/group). Removal of Trp from the diet had a differential effect on bone mineral density (BMD). Total BMD was increased in the low Trp group: (0.0548±0.0017 vs 0.518±0.0021 vs 0.0526±0.0021 vs 0.0524±0.0022, Means+SD, No Trp: 0%; Standard Trp 0.2%; High Trp: 0.7%; High Trp: 1.25%, p=0.016, 0 vs 0.2%). Femoral BMD did not change; however, spinal BMD rose (0.0603±0.005 vs 0.0477±0.06 vs 0.0505±0.005 vs 0.0535±006; Means+SD, Trp 0% vs 0.2%, p=0.00009). The mice on the 0% Trp diet also had lower body fat (22.4±2.3% vs 26.7±3.3%; Means+SD, Trp 0 vs 0.2%, p=0.005). Thus, dietary Trp restriction resulted in a beneficial increase in spinal BMD, though at least in part related to weight loss.

### A METABOLIC STRESS RESPONSE

Scott Leiser,[1] Christopher Choi,[2] Ajay Bhat,[3] and Charles Evans,[1] *1. University of Michigan, Ann Arbor, Michigan, United States, 2. University of Michigan Medical School, Ann Arbor, Michigan, United States, 3. University of Michigan, ANN aRBOR, Michigan, United States*

An organism's ability to respond to stress is crucial for long-term survival. These stress responses are coordinated by distinct but overlapping pathways, many of which have been found to also regulate longevity in multiple organisms across species. Despite extensive effort, our understanding of these pathways and how they affect aging remains incomplete and thus is a key area of study in Geroscience. Our previous work identified flavin-containing monooxygenase-2 (fmo-2) as a key longevity-promoting gene downstream of at least three longevity promoting pathways, including the hypoxic response, the pentose phosphate pathway, and the dietary restriction pathway. Based on the commonalities of these pathways, we hypothesized that fmo-2, a classically annotated xenobiotic enzyme, might play a key endogenous role in responding to metabolic stress. Our resulting data, using metabolic profiling and further epistatic analysis, both support this hypothesis and link fmo-2's mechanism to modifications to one-carbon metabolism (OCM), a key intermediate pathway between the nucleotide metabolism, methylation, and transsulfuration pathways. Using mathematical modeling and a novel metabolomics approach, we were able to further identify the likely mechanism of fmo-2-mediated metabolic effects, and connect them to both OCM and downstream components. We propose a model whereby nematode fmo-2 represents a class of enzymes that are able to modify large aspects of metabolism, similar to how transcription factors modify gene expression, and that fmo-2 is a key member of a conserved metabolic stress response.

### DEFINING THE RESPONSE OF CAENORHABDITIS ELEGANS TO MULTIPLE SIMULTANEOUS STRESSORS

George Sutphin, Hope Dang, Emily Turner, Destiny DeNicola, Sage Hamming, Amanda Furtmann, and Delaney Kelser, *University of Arizona, Tucson, Arizona, United States*

Cells are constantly subjected to a variety of intrinsic and extrinsic stresses—oxidative, protein misfolding, osmotic—and respond by activating a range of molecular pathways to mitigate and repair damage—oxidative stress response, unfolded protein response, osmotic stress response. While individual stress response pathways have been described in detail, and some interventions improve resistance to multiple forms of stress (e.g. dietary restriction, insulin signaling inhibition), surprisingly little is known about how these responses differ when cells are challenged with multiple types of stress simultaneously. The molecular architecture underlying multi-stress response has broad implications for aging and age-associated disease. One characteristic of aging is a progressive increase in multiple categories of cellular stress accompanied by a decline in cellular stress response capability. Human diseases rarely involve a single form of stress—Alzheimer's disease is characterized by neuroinflammation, oxidative stress, and misfolded proteins, while cancer exhibits oxidative stress, DNA damage, and localized hypoxia. Determining how cells respond differently to one form of stress in the presence of another is critical to building an accurate model of the aging cellular environment. We are using Caenorhabditis elegans to systematically evaluate the molecular network that cells employ when challenged by multiple simultaneous stressors and how different stress combinations impact organismal survival and health. Here we present our initial characterization of C. elegans response to multiple categories of cellular stress (oxidative, osmotic, ER, Golgi, heavy metal), which stressors elicit non-additive interactions when combined, and how these combinations impact survival, health, and established stress response pathways.

### DPP4 GENE POLYMORPHISM ASSOCIATES WITH TYPE 2 DIABETES MELLITUS IN OLDER ADULTS

Elisa Alves,[1] Audrey Tonet-Furioso,[2] Izabela D. Bastos,[2] Clayton Moraes,[2] and Otávio Nóbrega,[2] *1. Universidade de Brasília, Brasília, Brazil, 2. Universidade de Brasília, Brasília, Distrito Federal, Brazil*

Genetic variations of the dipeptidyl peptidase 4 enzyme (DPP-4) have been associated with glycemic disorders, especially among older adults. The objective of this study was to investigate the association of a single nucleotide polymorphism on the DPP4 gene with the occurrence of type 2 diabetes mellitus in a sample of older community-dwelling patients. Clinical, anthropometric and physiological characteristics as well as biochemical data related to lipidemia, glycemia and hormonal factors and variables related to the lifestyle were analyzed among 338 individuals aged 60 years and older from two general geriatric outpatient clinics in the Federal District, Brazil. Genotypes related to the polymorphism rs3788979 (A/G) of the DPP4 gene of these patients were determined by conventional polymerase chain reaction followed by enzymatic restriction, whereas the serum levels of DPP-4 were assessed by colorimetric immunoassay. Among the results obtained, there was clear variation in the glycemic levels both in terms of blood glucose and glycosylated hemoglobin according to DPP-4 genotypes, with increased levels between homozygotes for the G allele. However, no association was found between genotypes and occurrence of type 2 diabetes mellitus in patients. No other clinical, biochemical or anthropometric variables were influenced by the polymorphism. In our conditions, there was no association between genotypes and DPP-4 levels. Considering that DPP-4 is involved in the metabolism of incretins and directly interferes with glycemia in organisms, our result corroborates that genetic variations of this enzyme can affect glycemic homeostasis, despite not being determinant for the occurrence of type 2 diabetes mellitus.

### EFFECT OF NUTRITIONAL STRESS ON THE EXPRESSION OF SIRTUIN GENE FAMILY

Xiaomin Zhang,[1] Fathima Ameer,[2] Jasmine Crane,[3] Gohar Azhar,[3] and Jeanne Wei,[3] *1. University of Arkansas for Medical Sciences, Little Rock, Arkansas, United States, 2. University of Arkansas for Medical Sciences, University of Arkansas for Medical Sciences, Arkansas, United States, 3. UAMS, Little Rock, Arkansas, United States*

The sirtuin (SIRT) proteins are a highly conserved family of NAD+-dependent deacetylases that regulate histones as well as non-histone proteins. Seven sirtuin genes have been identified (SIRT1 to SIRT7) in mammals. SIRT1, SIRT6, and SIRT7 are primarily localized in the nucleus. SIRT2 is localized mainly in the cytoplasm. SIRT3, SIRT4, and SIRT5 are often located in the mitochondria. Therefore, the sirtuin family proteins exert their diverse functions at various cellular locations and regulate metabolism, stress responses, growth and aging processes. The sirtuin proteins are often considered as nutrient sensors. This study assessed the expression of sirtuin genes in C2C12 muscle cells under glucose stress conditions at different time points. Expression of all seven sirtuins was confirmed by Real-Time PCR analysis. SIRT1 (24 h) and SIRT3 (6 h and 15 h) are highly expressed under low glucose (2.7 mM) and high glucose (25 mM) conditions, whereas SIRT2 and SIRT4, SIRT5, SIRT6 and SIRT7 expressions were either relatively lower or there was no significant change under glucose stress conditions. Our results indicate that SIRT1 and SIRT3 demonstrated the greatest fluctuation in response to glucose stress, whether high or low glucose. These findings will help elucidate the role of sirtuins in the regulation of cellular processes, including metabolism. It will also help to enhance our understanding of the roles of sirtuin genes in regulation of blood sugar fluctuation in normal persons and diabetic patients, as well as in elderly individuals, many of whom are insulin resistant and "prediabetic" or diabetic.

### EFFECT OF PPARΓ INHIBITION ON BONE IN AGED ANIMALS

Xingming Shi,[1] Kehong Ding,[2] Yun Su,[2] and Carlos Isales,[1] *1. Augusta University, Augusta, Georgia, United States, 2. augusta university, Augusta, Georgia, United States*

Aging is accompanied by bone loss and marrowfat accumulation. PPARg is a key factor regulating adipocyte differentiation. Marrowfat secretes large quantities of factors including adipokines, cytokines and chemokines that contribute to bone loss. We hypothesized that inhibition of PPARg would reduce adipocytes and adipose-generated inflammatory factors, and thus decrease aging-induced bone loss. To test this hypothesis, we treated young (6mo) and old (23mo) female C57BL/6 mice with a PPARg antagonist GW9662 (1mg/kg body weight, IP injection for 6wks) and examined the effects on bone, immune cell lineage skewing, and the expression of inflammation related genes. DXA analysis data showed that GW treatment had no effect on vertebral and femoral BMD and BMC in young or old mice. u-CT analysis showed that GW treatment reduced BV/TV in old mice but had no effect in young mice. FACS analysis data showed that GW treatment significantly increased lymphoid (CD3) and decreased myeloid (CD11b) lineage cells only in young mice. GW treatment significantly decreased CD4 and CD8 T cell subpopulations in young mice, but had no effect in old mice. Interestingly, Nanostring analysis of inflammation genes revealed an opposite effects of GW treatment in young vs. old mice, i.e., genes whose expression were downregulated by GW in bone cells of the young mice were upregulated in the old mice. These results suggested that systemic inhibition of PPARg, while enhancing CD3 and reducing CD11b cell populations in the bone marrow of young mice, may have a negative effect on bone architecture in old mice.

### GREATER SKELETAL MUSCLE OXIDATIVE CAPACITY IS ASSOCIATED WITH HIGHER RESTING METABOLIC RATE: RESULTS FROM THE BLSA

Marta Zampino,[1] Richard Semba,[2] Fatemeh Adelnia,[3] Jennifer Schrack,[4] Richard Spencer,[1] Kenneth Fishbein,[1] Eleanor Simonsick,[1] and Luigi Ferrucci,[1] *1. National Institute on Aging, Bethesda, Maryland, United States, 2. Johns Hopkins University, Baltimore, Maryland, United States, 3. Vanderbilt University Medical Center, Nashville, Tennessee, United States, 4. Johns Hopkins Bloomberg School of Public Health, Baltimore, Maryland, United States*

Resting metabolic rate (RMR) tends to decline with aging. The age-trajectory of decline in RMR is similar to changes that occur in muscle mass, muscle strength and fitness. However, while the decline in these phenotypes have been related to changes of mitochondrial function and oxidative capacity, whether lower RMR is associated with poorer mitochondrial oxidative capacity is unknown. In 619 participants of the Baltimore Longitudinal Study of Aging, we analyzed the cross-sectional association between RMR (kcal/day), assessed by indirect calorimetry, and skeletal muscle maximal oxidative phosphorylation capacity, assessed as post-exercise phosphocreatine recovery time constant (tau-PCr), by phosphorous magnetic resonance spectroscopy. Linear regression models were used to evaluate the relationship between tau-PCr and RMR, adjusting for potential confounders. We found that independent of age, sex, lean body mass, muscle density and fat mass, higher RMR was significantly associated with shorter tau-PCr, indicating greater mitochondrial oxidative capacity. In conclusion, higher RMR appears to be associated with a higher mitochondrial oxidative capacity in skeletal muscle. This association may reflect a relationship between better muscle quality and greater mitochondrial health.

### INHIBITION OF KYNURENINE METABOLISM AND ITS EFFECT IN MITOCHONDRIAL FUNCTION IN HEPATOCELLULAR CARCINOMA

Raul Castro-Portuguez,[1] Samuel Freitas,[2] and George Sutphin,[1] *1. University of Arizona, Tucson, Arizona, United States, 2. University of Arizona, Tucson, United States*

Hepatocellular carcinoma (HCC) is the most prevalent cancer in the liver. The majority of ingested tryptophan is processed in the liver through the kynurenine pathway, the endpoint of which is de novo NAD+ biosynthesis.

Dysregulation of tryptophan-kynurenine metabolism and NAD+ synthesis may promote mitochondrial malfunction, tumor reprogramming, and carcinogenesis. Using a publicly available gene expression dataset from liver hepatocellular carcinoma (LIHC) samples available through The Cancer Genome Atlas (TCGA; n = 371), we employed Principal Component Analysis (PCA), hierarchical clustering, gene-pattern expression profiling, and survival analysis to cluster patients and determine overall survival. Our analysis of genes encoding kynurenine pathway enzymes determined that patients with high QPRT expression had a poor prognosis with decreased median survival, with no effect on the maximum survival. There is a significant difference in the survival between patients with high QPRT expression relative to patients with high HAAO/AFMID expression (HR = 1.2, [95% CI 0.5-1.8] P = 0.0181, Gehan-Breslow-Wilcoxon Test). Patients with high QPRT expression have higher survival rates compared with low QPRT expression (HR = 1.4, [95% CI 0.9-2.2] P = 0.0344, Gehan-Breslow-Wilcoxon Test). To test the consequences of kynurenine-pathway inhibition in mitochondrial function and morphology we use 4-Cl-3HAA, an irreversible HAAO inhibitor, and observed a small increase in mitochondrial fragmentation in HepG2 cells after 24 hours of treatment. We conclude that kynurenine metabolism may be useful as a biomarker to predict patient prognosis among HCC patients. In ongoing work, we are testing QPRT inhibitors in cell culture as a potential adjuvant for chemotherapies.

### LATE-ONSET INTERMITTENT FASTING DECREASES AGING-RELATED FRAILTY

Yoko Henderson,[1] Nazmin Bithi,[2] Christopher Link,[3] Jie Yang,[1] and Christopher Hine,[1] *1. Cleveland Clinic Lerner Research Institute, Cleveland, Ohio, United States, 2. Lerner Research Institute, Cleveland Clinic, Cleveland, Ohio, United States, 3. Cleveland Clinic, Cleveland, Ohio, United States*

Global average life expectancy continues to rise. As aging increases likelihoods of exhibiting geriatric syndromes (a.k.a. frailty), there is a need for effective anti-aging treatments. Multiple studies have shown the positive effects of dietary restriction (DR) on lifespan in various model organisms. However, DR is not widely implemented in older adults due to issues with patient compliance and the overall lack of understanding on the effects of DR initiated later in life. Thus, the present study tested whether late-life DR, specifically Every-Other-Day (EOD) fasting, attenuates aging-related frailty using a modified and simplified frailty index in mice. Briefly, 20-month old male and female C57BL/6 mice (human equivalent of 65 years) that had been on a control chow diet ad libitum during adulthood were placed on EOD fasting or ad libitum feeding for 2.5 months. Their frailty index was identified using an indirect calorimeter, glucose tolerance test, novel object place recognition test, forelimb grip strength meter, and rotarod. We found that late-life EOD fasting decreased overall caloric intake in males but not in females. In addition, EOD fasting significantly improved metabolic, musculoskeletal, and cognitive endpoints in male mice, but enhanced only some of these in female mice. Furthermore, EOD fasting improved hydrogen sulfide (H2S) production capacity and its associated sulfhydration signaling in tissues, which positively correlated with improvements in frailty measures. We conclude that EOD fasting implemented late in life can have therapeutic potential in the clinic. We are currently investigating the necessity of H2S production for DR mediated benefits and longevity.

### LATE-ONSET PHARMACOLOGICAL OR DIETARY INTERVENTIONS IMPROVE HEALTHSPAN AND LIFESPAN IN MALE AND FEMALE MICE

Sarah Mitchell,[1] Michael MacArthur,[1] Alice Kane,[2] Margaret Torrence,[3] Huseyin Mehmet,[4] James Vath,[5] Brendan Manning,[1] and James Mitchell,[6] *1. Harvard T.H. Chan School of Public Health, Boston, Massachusetts, United States, 2. Harvard Medical School Center for Animal Resources School of Public Health, Boston, Maine, United States, 3. Harvard TH Chan School of Public Health, Boston, Massachusetts, United States, 4. Zafgen, Inc., Boston, Massachusetts, United States, 5. Zafgen, Inc, Boston, Massachusetts, United States, 6. ETH Zurich, Zurich, Massachusetts, Switzerland*

While late-onset dietary or pharmacological interventions can extend longevity in rodents, whether or not they can be used to reverse or forestall onset of aging-related symptoms (i.e. frailty) remains untested. Here, we employed three interventions to test this hypothesis. Male and female C57BL/6 mice were randomized to one of four groups: control, 15% calorie restriction (15CR), 0.1% Methionine Restriction (MR)or ZGN1062 (1.5mg/kg, drug in feed) starting at 21mo. Healthspan measurements (mouse clinical frailty index (FI), blood collection, and hematology) were performed every three months, and survival was assessed for all mice. At baseline there were no significant differences in frailty index. After 6mo FI increased consistent with reduced healthspan in control males (0.23□0.01 to 0.34□0.01 A.U, p<0.0001) and females at this age (0.19□0.03 to 0.24□0.01, p<0.0001). Male 15CR, MR and ZGN1062 mice had significantly lower FI scores at 27mo age (15CR: 0.32□0.01, p=0.02; MR: 0.31□0.01, p=0.0009; ZGN1062: 0.30□0.01, p<0.0001). Female mice were less frail than males at 27mo, suggesting sexual dimorphism in the timing of frailty onset in mice. ZGN1062 significantly extended lifespan in males (HR=0.56, p=0.007) and females (HR=0.46, p=0.001). There was a sexual dimorphism in the ability of 15CR and MR to extend lifespan, and a trend towards increased lifespan in males (HR=0.69, p=0.057 and HR=0.71, p=0.09) but not in females. Histological analysis for cause-of-death is ongoing. Taken together these data suggest that a pharmacological intervention associated with weight loss, which may be a more practical therapeutic strategy towards mitigation of age-related healthspan decline than dietary restriction-based interventions.

### LONGEVITY-EXTENDING METAP2 INHIBITORS INDUCE CALORIC RESTRICTION THROUGH P53-DEPENDENT INDUCTION OF GDF-15

Michael MacArthur,[1] Sarah Mitchell,[1] Jon Jung,[2] Margaret Torrence,[3] Alice Kane,[4] Huseyin Mehmet,[5] Brendan Manning,[1] and James Mitchell,[6] *1. Harvard T.H. Chan School of Public Health, Boston, Massachusetts, United States, 2. University of Glasgow, Glasgow, Scotland, United Kingdom, 3. Harvard TH Chan School of Public Health, Boston, Massachusetts, United States, 4. Harvard Medical School Center for Animal Resources School of*

*Public Health, Boston, Maine, United States, 5. Zafgen, Inc., Boston, Massachusetts, United States, 6. ETH Zurich, Zurich, Massachusetts, Switzerland*

MetAP2 is a 67kDa protein which sits at the translation initiation complex and cleaves N-terminal methionine off of nascent peptides. Inhibitors of MetAP2 cause profound weight loss secondary to decreased food intake. These inhibitors also significantly extend longevity in mice in late-life intervention. However, the exact mechanism of action causing decreased food intake is not known. Here we investigated the molecular mechanism and target tissue of a MetAP2 inhibitor's (Zgn1062) anorectic effects. First we identified the target tissue by testing targeted Zgn1062 delivery to specific brain regions. Delivery to the medio-basal hypothalamus did not have a significant effect but delivery to the lateral ventricle resulted in significantly decrease food intake and body weight after 2 and 14 hours. When we delivered a neuron-targeted AAV encoding MetAP2 shRNA we saw decreased efficacy of MetAP2 confirming the required for neuronal MetAP2 for anorectic effects. To determine the molecular mechanisms we performed RNAseq of wildtype and MetAP2 KO HT1080 cells across a timecourse of Zgn1062 treatment. The main pathway activated across timepoints in MetAP2-dependent manner was P53 signaling. A main P53 target that was upregulated was the known anorectic peptide GDF15. We confirmed GDF15 increases in vivo at both mRNA (liver and intestines) and protein level (serum) in response to Zgn1062. We also found that Zgn1062 treatment reduces senescent cell burden in visceral adipose tissue in vivo and reduces SASP gene expression in fat explants ex vivo. We hypothesize that Zgn1062's potent P53 activation may play a role in clearance of senescent cells.

### METABOLIC REGULATION OF LONGEVITY BY ONE-CARBON METABOLISM AND FLAVIN-CONTAINING MONOOXYGENASE

Christopher Choi,[1] Scott Leiser,[2] Charles Evans,[2] and Daniel Beard,[2] *1. University of Michigan Medical School, Ann Arbor, Michigan, United States, 2. University of Michigan, Ann Arbor, Michigan, United States*

Nematode flavin-containing monooxygenase-2 (fmo-2) is induced by dietary restriction and hypoxia, and is required for the longevity and health benefits of these pathways. It is also sufficient to confer these benefits when overexpressed. As FMOs are well-conserved across taxa, the fmo-2 mechanism has high translational potential. To determine the changes that occur following fmo-2 induction, we performed RNA-seq and untargeted metabolomics analyses. Our data reveal that one-carbon metabolism (OCM) is significantly altered by fmo-2 overexpression. OCM is a nexus for essential metabolic pathways, including transmethylation, transsulfuration, nucleotide synthesis, and amino acid metabolism. We hypothesized that fmo-2 confers longevity benefits by altering key metabolic processes within or downstream of OCM. To test this, we asked whether fmo-2 and OCM interact to regulate longevity by knocking down expression of genes involved with OCM and measuring lifespan and oxidative stress resistance. To understand the biological implications of these interactions, we generated a computational model using qPCR data of key OCM-related genes to predict changes in OCM metabolic flux. Our model predicts significant changes in OCM flux that are regulated by fmo-2 expression levels and are consistent with our RNAi and multi-omics results. We are now testing this model by knocking down genes downstream of OCM to determine their role in fmo-2-mediated benefits. Preliminary results support our hypothesis that changes in metabolic flux through OCM are involved downstream of fmo-2 expression, and may also implicate the UPRER in this pathway. Our future work will elucidate this mechanism and link stress perception and fmo-2-mediated longevity.

### NICOTINAMIDE AND SUGAR METABOLISM ASSOCIATED WITH MUSCLE MASS LOSS DURING CALORIE RESTRICTION IN OLDER ADULTS

Ellen Quillen,[1] Jingyun Lee,[2] Cristina Furdui,[1] Daniel Beavers,[1] and Kristen Beavers,[3] *1. Wake Forest School of Medicine, Winston-Salem, North Carolina, United States, 2. Wake Forest Baptist Health, Winston-Salem, North Carolina, United States, 3. Wake Forest University, Winston-Salem, North Carolina, United States*

Weight loss among older adults remains controversial due to lean mass loss and potential exacerbation of disability risk. Using the Medifast for Seniors clinical trial (NCT02730988), which investigated high protein supplementation (≥1.0 g/kg/d) during caloric restriction to preserve lean mass among 96 older adults (>70 years, 74% women, 27% black) with obesity (BMI: 35 kg/m^2), we applied untargeted metabolomics to identify small molecules associated with the highly variable change in lean muscle mass during weight loss. Forty-seven participants were randomized to high protein weight loss, and 92% lost at least 5% body weight over 24 weeks. Across DXA-ascertained measures of lean body mass, gynoid lean mass exhibited the broadest range of change: +4% to -12%. For 38 participants, untargeted metabolomics data was generated from fasted serum samples collected before and after intervention. 121 serum metabolites were identified and change from baseline was tested for correlation with percent change in gynoid muscle mass. Increasing nicotinamide levels were associated with a greater loss of gynoid muscle mass (R^2=0.22, p=0.0027). Pathway analysis was applied to identify Kyoto Encyclopedia of Genes and Genomes (KEGG) biochemical pathways containing multiple nominally-associated metabolites. The amino sugar and nucleotide sugar metabolism pathway was significantly enriched (p=0.006), containing four sugar metabolites associated with shifts in lean muscle mass. This pathway is important in the glycosylation of polysaccharides, a ubiquitous and important regulator of energy metabolism, but has not previously been linked to muscle mass and should be further interrogated in preservation of lean muscle during weight loss.

### POOR MITOCHONDRIAL HEALTH AND SYSTEMIC INFLAMMATION? TESTING OF A CLASSICAL HYPOTHESIS

Marta Zampino, Luigi Ferrucci, Richard Spencer, Kenneth Fishbein, Eleanor Simonsick, and Pei-Lun Kuo, *National Institute on Aging, Bethesda, Maryland, United States*

Chronic low-grade inflammation often occurs with aging and has been associated with negative health outcomes. Despite extensive research on the origins of "inflammaging", the causative mechanisms remain unclear. However, a connection between poor mitochondrial health and chronic inflammation has been hypothesized, with decreasing mitochondrial function occurring with age and precipitating an increase in reactive oxygen species and other pro-inflammatory macromolecules such as mitochondrial DNA. We tested this hypothesis on a population of 619 subjects from the Baltimore Longitudinal Study of Aging, measuring muscle mitochondrial oxidative capacity in vivo by phosphorus magnetic resonance spectroscopy (P-MRS), and plasma interleukin (IL)-6, the most widely used biomarker of inflammaging. The P-MRS-derived post-exercise phosphocreatine recovery time constant tau-PCr, a measure of oxidative capacity, was expressed as a categorical variable through assignment to quintiles. Participants in the first quintile of tau-PCr (best mitochondrial function) were taken as reference and compared to the others using linear regression analysis adjusted for sex, age, lean and fat body mass, and physical activity. Those participants with the lowest oxidative capacity had significantly higher log(IL-6) levels as compared to the reference group. However, data from the other quintiles was not significantly different from the reference values. In conclusion, severe impairment of oxidative capacity is associated with increased inflammation. This study design does not provide conclusive evidence of whether increased inflammation and impaired bioenergetic recovery are both caused by underlying poor health status, or whether mitochondrial deficits lead directly to the observed inflammation; we anticipate addressing this important question with longitudinal studies.

### ROLE OF INTRAMUSCULAR FAT AND LEAN MUSCLE IN SURFACE ELECTROMYOGRAPHY AMPLITUDE OF THE GLUTEUS MEDIUS IN OLDER ADULTS

Marcel Lanza,[1] Vicki Gray,[2] Alice Ryan,[3] Will Perez,[3] and Odessa Addison,[2] *1. University of Maryland, Baltimore, Baltimore, Maryland, United States, 2. University of Maryland School of Medicine, Baltimore, Maryland, United States, 3. Baltimore VA Medical Center, Baltimore, Maryland, United States*

Surface electromyography (sEMG) is frequently used to assess muscle activation in older individuals. Subcutaneous fat is one well-known factor that influences sEMG amplitude. The amount of intramuscular fat (IMAT) may negatively impact the muscles ability to produce force with aging, while high density lean tissue (HDL; fat free muscle) has an opposite effect. However, influence of IMAT or HDL on sEMG amplitude remains unclear. Thus, the aim was to investigate the influence of IMAT and HDL on sEMG amplitude of the gluteus medius (GM) muscle during a maximal voluntary isometric contraction (MVIC) in older adults. Twelve older adults (7 females; age: 71±3 y; BMI= 29±4 Kg/m2; X ± SD) underwent a CT scan to determine IMAT and HDL cross-sectional area in the GM. IMAT and HDL were normalized as a percentage of the total muscle area. Maximal hip abduction MVIC was measured at 30□ hip abduction in standing, while sEMG was recorded from the GM muscle. Spearman correlations showed a positive association between GM HDL and sEMG amplitude (r = 0.692, P = 0.013) and negative between GM IMAT and sEMG amplitude (r = -0.683, P = 0.014). This is the first study to demonstrate the amount of IMAT may limit the ability to activate the hip abductor muscle. Given that muscle activation is a determinant of strength, interventions to lower levels of IMAT and increase levels of lean muscle may be important to slowing decreases in strength with aging.

### SERUM FACTORS MEDIATE THE BIOENERGETIC BENEFITS OF EXERCISE TRAINING AND CALORIC RESTRICTION

Jenny Gonzalez-Armenta,[1] Barbara Nicklas,[2] and Anthony Molina,[3] *1. Wake Forest School of Medicine, Winston-Salem, North Carolina, United States, 2. Wake Forest University, Winston-Salem, North Carolina, United States, 3. University of California San Diego, La Jolla, California, United States*

Diet and exercise interventions have been shown to improve age-related decline in mitochondrial function. While the systemic benefits of diet and exercise are apparent, the mechanisms underlying these changes are not known. Our lab and others have used blood-based bioenergetic profiling to demonstrate that systemic bioenergetic capacity is related to many aspects of healthy aging, including: gait speed, grip strength, and inflammation. This work suggests a potential role for circulating factors in mediating systemic mitochondrial function. In this study, we developed a high-throughput respirometry assay to examine the effects of circulating factors on mitochondrial function of myoblasts in vitro. We used serum from older, overweight and obese adults who participated in a clinical trial comparing resistance training (RT) and resistance training plus caloric restriction (RT+CR). When combined, both interventions significantly increased serum-mediated basal (42.08 to 50.14 pmol/min, p=0.004), ATP-linked (35.57 to 42.37 pmol/min, p=0.006), and maximal respiration (132.30 to 150.00 pmol/min, p=0.02). With RT, we found significantly increased basal (40.80 to 53.85 pmol/min, p=0.01) and ATP-linked respiration (34.36 to 46.39 pmol/min, p=0.007) and trends for increased maximal respiration (130.09 to 153.24 pmol/min, p=0.10) and spare respiratory capacity (89.30 to 101.38 pmol/min, p=0.07). With RT+CR, there were trends for increased maximal respiration (134.32 to 147.06 pmol/min, p=0.10) and spare respiratory capacity (91.06 to 100.41 pmol/min, p=0.11). Additionally, we found that post-intervention serum-mediated basal and ATP-linked respiration were significantly and positively correlated with physical ability, as reported by SPPB score. Future studies will focus on identifying circulating factors responsible for these changes.

### SEX-DEPENDENT EFFECTS OF QUADRICEPS FAT CONTENT ON SINGLE MUSCLE FIBER SIZE IN OLDER ADULTS

Chad Straight,[1] Joseph Gordon III,[1] Aurora Foster,[1] Nicholas Remillard,[1] Bruce Damon,[2] Stuart Chipkin,[3] Jane Kent,[1] and Mark Miller,[1] *1. University of Massachusetts Amherst, Amherst, Massachusetts, United States, 2. Vanderbilt University, Nashville, Tennessee, United States, 3. University of Massachusetts Amherst, Amherst, United States*

Evidence suggests that ectopic fat deposition interferes with skeletal muscle structure and function, but few studies have examined underlying morphological and contractile properties at the single fiber level. Healthy older (65-75 y) men (n=9) and women (n=9) underwent dynamometry for assessment of knee extensor maximal torque, water-fat magnetic resonance imaging to quantify quadriceps muscle cross-sectional area (CSA) and fat fraction (FF), and vastus lateralis biopsies to determine morphology and function of type I and II muscle fibers. Despite similar body mass indices (24.4±1.3 vs. 24.6±0.5 kg·m2, p=0.93) and daily moderate-to-vigorous physical activity (46±7 vs. 41±9 min·d-1, p=0.67), women had greater FF (9.0±0.3 [range: 7.6-10.6] vs. 7.9±0.4 [6.0-9.7] %, p=0.04) than men, indicating increased adipose tissue deposition in skeletal muscle. Women also had smaller quadriceps CSA (39.8±1.8 vs. 57.9±1.3 cm2, p=0.01), specific torque (1.5±0.1 vs. 1.9±0.1 Nm·cm-2, p=0.01) and type II fiber CSA (3,943±312 [2,350-5,140] vs. 5,352±384 [3,560-6,590] µm2, p=0.01) than men. Type I CSA did not differ by sex (4,918±228 [3,740-5,600] vs. 5,630±440 [3,640-7,670] µm2, p=0.19). In older women, FF was inversely associated with single fiber CSA in type I (r= -0.81, p=0.02) and II (r= -0.76, p=0.03) fibers, and tended to be associated with slower myosin-actin cross-bridge kinetics (longer myosin attachment time) in type I fibers (r=0.65, p=0.08). These relationships were not observed in men. Overall, healthy older women have greater intramuscular fat than men, which may contribute to sex-specific effects on knee extensor specific torque through differences in muscle fiber size and cross-bridge kinetics.

### TIME-RESTRICTED FEEDING AND CALORIC RESTRICTION IMPACT ON SPONTANEOUS NEOPLASMS IN FEMALE MICE

Annamaria Rudderow, Eleonora Duregon, Michel Bernier, and Rafael de Cabo, *1. National Institute on Aging, Bethesda, Maryland, United States*

In older humans, multiple chronic diseases and increased life expectancy impose a disproportionate socioeconomic burden. Dietary interventions are valuable strategies for promoting healthy aging. Caloric restriction (CR) without malnutrition is a robust intervention able to delay disease onset and increase survival in model organisms. However, the impracticability of chronic CR outweighs the potential long-term benefits in humans. Time-restricted feeding (TRF), i.e. the limitation in the timing of food intake without necessarily reducing caloric intake, can protect against metabolic disorders through the synchronization of the circadian rhythm. This study compares whether limiting access to ad libitum (AL) food for a few hours per day mimics the beneficial effects of a CR diet. A large cohort of C57BL/6J female mice (n=250) was distributed into five feeding paradigms at midlife: AL, TRF for 8 hours, TRF for 4 hours, 20% CR and 20% CR fed twice a day (CRx2). Pathological analyses at death reveal a shift in fatal neoplasms toward an older age in TRF8 mice. AL mice had the highest prevalence of tumors (93%) and TRF4 had the lowest (77%). The highest tumor burden was observed in AL mice while CRx2 animals had the lowest number of neoplasms. Histiocytic sarcoma and lymphoma were the most represented malignancies, with CR mice exhibiting the highest rate of histiocytic sarcoma (75%) and the lowest rate of lymphoma (10%). These results indicate that time- and calorie-restricted feeding regimens can slow down malignant neoplasm progression and extend health span in female mice, even when started in adulthood.

### UNCOVERING THE SPECIFIC FUNCTIONS OF MIR-33 IN REGULATION OF FEEDING AND CARDIOMETABOLIC DISEASES LINKED TO AGING.

Nathan Price,[1] Xinbo Zhang,[1] Pablo Fernandez-Tussy,[1] Rafael de Cabo,[2] and Carlos Fernandez-Hernando,[1]
*1. Yale University, New Haven, Connecticut, United States, 2. National Institute on Aging, Bethesda, Maryland, United States*

Heart disease and metabolic dysfunction are two of the most important age related health issues, and feeding behavior is a critical factor contributing to these conditions. miR-33 promotes the development of atherosclerosis, by impairing macrophage cholesterol efflux and reverse cholesterol transport. Specific disruption of the interaction between miR-33 and the cholesterol transporter ABCA1 protected mice from atherosclerosis in a manner similar to that observed with loss or inhibition of miR-33. However, miR-33 has also been shown to impact other cellular functions, including targeting numerous mRNAs related to bioenergetics and inflammatory response, that may also contribute to its effects on atherosclerosis. Moreover, characterization of miR-33 deficient animals has revealed a strong predisposition to the development of obesity and metabolic dysfunction. While this phenotype appears to be due to alterations in feeding behavior, it is not clear what organ or organs are primarily driving this effect or what functions of miR-33 may be responsible. To address these questions, we have generated conditional miR-33 knockout mice to selectively remove miR-33 from a number of key metabolic tissues. Using these unique mouse models, we have performed an extensive characterization of how miR-33 impacts the function of different metabolic tissues in both chow and high fat diet fed mice, and assessed what impact this has on regulation of metabolic homeostasis and atherosclerosis. This work will improve our understanding of the mechanisms regulating feeding behavior and provide critical information for the development and evaluation of novel approaches to combat cardiometabolic diseases associated with aging.

### SESSION 2877 (POSTER)

### SENESCENCE, EPIGENETICS, AND METFORMIN

### A GENOME-WIDE INTEGRATIVE STUDY OF DNA METHYLATION, GENE EXPRESSION, AND LATER LIFE HAND GRIP STRENGTH

Mette Soerensen,[1] Jonas Mengel-From,[1] Kaare Christensen,[2] Lene Christiansen,[3] and Qihua Tan,[1]
*1. University of Southern Denmark, Odense C, Denmark, 2. University of Southern Denmark, Odense C, Syddanmark, Denmark, 3. Copenhagen University Hospital, Copenhagen Ø, Denmark*

Hand grip strength (HS) measures muscular strength and associates with multiple health outcomes and mortality. Studies of epigenetic and transcriptomic markers could help elucidate the biology behind HS; markers for which monozygotic (MZ) twins are excellent study populations. We performed integrated enrichment analyses (IEA) of an epigenome-wide association analysis (EWAS) and a transcriptome-wide association analysis (TWAS) of HS

in blood samples of 452 MZ twins (56-80 years of age). Unsupervised IEA were conducted by the KeyPathwayMiner algorithm, while supervised IEA were performed by the KEGG and Reactome databases. No individual CpG site or probe passed correction for multiple testing. Investigating the overlap in genes with p-values<0.01, 0.005 or 0.001 in the EWAS and TWAS, revealed 67, 21 and 2 unique genes, respectively. The latter 2 were TESK2 and VWA1. By the supervised approach, the 67-gene overlap identified three pathways related to "antigen processing and presentation", driven by HLA-A, HLA-B, TAP2 and PSME2. With the unsupervised approach the 21-gene and 67-gene overlaps revealed networks containing 7 and 19 genes, respectively. Exception nodes (added by the algorithm for structure) were CREBBP and CSNK2A2 for the former, and APP and HSP90AB1 for the latter. The remaining IEA revealed no gene sets or networks. Several of these genes have previously been linked to HS relevant traits, e.g. arthritis (HLA-A, HLA-B and TAP2), smooth muscle and cardiovascular function (TESK2, HLA-B and APP) and sarcopenia (HSP90AB1). Hence, this study reports genes and pathways previously reported for physical functioning, yet also novel candidates for further verification.

### A PANEL OF DNA METHYLATION AND PROTEOMIC BIOMARKERS FOR SPECIFIC AGING PATHWAYS

Albert Higgins-Chen,[1] Luigi Ferrucci,[2] and Morgan Levine,[3] *1. Yale School of Medicine, New Haven, Connecticut, United States, 2. National Institute on Aging, Bethesda, Maryland, United States, 3. Yale University School of Medicine, New Haven, Connecticut, United States*

Most aging biomarkers such as DNA methylation and proteomic clocks have focused on measuring overall "biological age," a single number that predicts age-related morbidity and mortality better than absolute chronological age. While intuitive and interpretable, this single biological age number does not account for the possibility that different individuals may preferentially experience aging in different molecular and cellular pathways, and therefore does not suggest personalized aging interventions. We reasoned that a panel of biomarkers each capturing specific aging pathways, such as mitochondrial dysfunction or cellular senescence, may capture the heterogeneity of aging better than existing composite measures. To address this, we employed weighted gene co-expression network analysis to cluster tissue-specific transcriptomes and the serum proteome into specific modules with distinct biological functions and characterized how these modules change with age. We trained DNA methylation proxies of these functional modules that we then applied to independent validation data to identify associations with age-related morbidity and mortality. Clustering analysis using the DNA methylation biomarkers showed that different individuals show distinct patterns of aging. These pathway-specific biomarkers will elucidate how different aging mechanisms interact with each other to produce the larger phenomenon of aging, and for evaluating novel therapeutics targeting specific hallmarks of aging.

### AGE-DEPENDENT CHANGES IN NUCLEAR MECHANOTRANSDUCTION AS A DRIVER OF SARCOPENIA

Shama Iyer,[1] Christopher Ward,[1] Joseph Stains,[1] Alice Ryan,[2] Eric Folker,[3] and Richard Lovering,[1] *1. University of Maryland School of Medicine, Baltimore, Maryland, United States, 2. Baltimore VA Medical Center, Baltimore, Maryland, United States, 3. Boston College, Chestnut Hill, Massachusetts, United States*

Informed by evidence that dysregulated nuclear dynamics and nuclear transport may contribute to atrophy in diseased skeletal muscle, the purpose of this study was to assess nuclear deformability, permeability, transport, and mechano-signaling outputs (YAP/TAZ, a marker of mechanoresponsiveness, and their downstream genes) in aging skeletal muscle. We hypothesized that aging muscle would show changes in: proteins within LINC (linker of the nucleus to the cytoskeleton) complex, lamina and nuclear pore complex (NPC), and mechano-signaling outputs, with consequent decreased nuclear deformability and increased permeability. We further expected an increase in nuclear strain would increase nuclear YAP/TAZ and downstream indicators of YAP activity (Ankrd1, Cyr61). We used young, adult and aged C57BL6 mice (~4, 14, and 26 months, respectively). Nuclei were less deformable to passive mechanical stretch ex-vivo in adult muscle fibers compared to young muscle fibers. LINC protein gene expression, YAP/TAZ protein, and expression of their downstream genes were significantly increased in adult muscles compared to young muscles. YAP/TAZ protein and their downstream genes were further increased in aged muscles, indicating hyperactivation of YAP/TAZ in aging muscle. Changes with aging in the lamina and NPC included a loss of lamin β1, Nup107 and POM 121, which could underlie the increased nuclear permeability we found in nuclei of aged muscle. In summary, these data highlight a possible role for LINC, lamina and NPC in changes of aging-related nuclear dynamics and mechano-sensing, and may represent therapeutic targets for sarcopenia. Future studies will examine how altering these components affects muscle function during aging.

### AGE-RELATED INCREASED ONSET AND PROGRESSION OF PROSTATE CANCER IS REVEALED IN NOVEL PTEN-NULL MOUSE MODELS

Qiuyang Zhang,[1] Sen Liu,[2] Bing Zhang,[3] Asim Abdel-Mageed,[2] Chad Steele,[2] Alun Wang,[2] S. Jazwinski,[2] and Leann Myers,[4] *1. Tulane University Schoole of Medicine, New Orleans, Louisiana, United States, 2. Tulane University School of Medicine, New Orleans, Louisiana, United States, 3. Tulane University School of Medicien, New Orleans, Louisiana, United States, 4. Tulane University School of Public Health and Tropical Medicine, New Orleans, Louisiana, United States*

Prostate cancer (PCa) is associated with advanced age. To better understand how age impacts PCa, it is critical to use PCa animal models generated at different ages (aged vs. non-aged). The PB-Cre4 driven phosphatase and tensin homolog (Pten) conditional knockout mouse model, which closely imitates human PCa initiation and progression. However, the Pten deletion is triggered in a 2-week-old prostate, when comparing the extent of PCa between aged and non-aged mice, it is difficult to distinguish the extent to which the onset and progression of PCa are due to the acceleration of the normal aging process or due to the manifestation of PCa pathologies over time. We present here a protocol to inject Cre-expressing adenovirus with luciferin tag intraductally into the prostate anterior lobes of Pten floxed mice, thus, the Pten-loss will be triggered at different ages post-Cre expression. The in vivo imaging of luciferin signals following viral infection was conducted to confirm the Cre expression and activity. Immunohistochemical staining was performed to confirm the Cre expression, Pten loss, and p-Akt and p-S6 activation. Prostate weight and histopathology were compared between aged and non-aged mice. The results showed that the virus infection was limited in the prostate glands and aged mice had significantly increased PCa onset and progression compared to young mice. Although technical skill is required to carry out this procedure and the success rate of viral infection is about 80%, this model of PCa is of great use to all investigators in the aging and cancer research field.

### AN APPROACH TO IDENTIFY NEW PLEIOTROPIC GENETIC LOCI FROM PUBLICLY AVAILABLE UNIVARIATE GWAS RESULTS

Yury Loika, and Alexander Kulminski, *Duke University, Durham, North Carolina, United States*

The connections between genes and multifactorial polygenic age-related traits are not trivial due to complexity of metabolic networks in an organism, which were primarily adapted to maximize fitness at reproductive age in ancient environments. Given this complexity, pleiotropy in predisposition to complex traits appears to be common phenomenon. Identifying mechanisms of pleiotropic predisposition to multiple age-related traits can be a key factor in developing strategies for extending health-span and lifespan. Correlation between complex traits may be a factor shedding light on these mechanisms. Recently, we used an omnibus test leveraging correlation between multiple age-related traits to gain insights into pleiotropic predisposition to them. The analysis using individual-level data identified large number of new pleiotropic loci and highlighted a novel phenomenon of antagonistic genetic heterogeneity, which was characterized by antagonistic directions of genetic effects for directly correlated traits. Here, we demonstrate feasibility of our approach using summary statistics from univariate genome-wide (GW) association studies (GWAS). Our analysis focused on the results for high density lipoprotein cholesterol (HDL-C) and triglycerides (TG) from the Global Lipids Genetic Consortium, which reported 94 GW significant loci ($p \leq 5\times10\text{-}8$). The traits' correlation was estimated from the individual level data. Our approach identified 28 loci with pleiotropic predisposition to HDL-C and TG at $p \leq 5\times10\text{-}8$, which did not attain univariate GW significance with either of these traits. Fifteen of them (53%) demonstrated antagonistic heterogeneity. These results show that our approach can be efficiently used in the analysis of summary statistics from published studies to identify novel pleiotropic loci.

### ANTI-AGING EFFECTS OF HYDROPHOBIC AND HYDROPHILIC COMPONENTS FROM IMMATURE PEAR FRUITS EXTRACT

Akira Ogita,[1] Wakae Murata,[2] Ken Yamauchi,[3] Akiko Sakai,[4] Yoshihiro Yamaguchi,[4] Toshio Tanaka,[4] and Ken-ichi Fujita,[4] *1. Osaka City University, Osaka, Japan, 2. Yonago National College of Technology, Yonago, Tottori, Japan, 3. Keio University, Yokohama, Kanagawa, Japan, 4. Osaka City University, Osaka, Osaka, Japan*

Cellular senescence, the decline of cellular function due to aging, causes gradual loss of physiological functions and induces some chronic diseases, which negatively affect the quality of human life. Intervention in the cellular senescence process may reduce these incidences and delay the progression of age-related diseases, thereby contributing to the longevity of human lifespan. The budding yeast, Saccharomyces cerevisiae, is a model system that can provide significant insights into the genetics and molecular biology of senescence and is a suitable cellular model for research on mammalian cells. In the 2019 GSA meeting, we had revealed that the prolongation of yeast cell lifespan was induced by the addition of immature pear fruits extracts (iPE). In this study, we have focused on investigating the anti-senescence effects of hydrophilic (WiPE) and hydrophobic (OiPE) components of iPE on yeast cells and their genes and their possible application in extending human lifespan. The anti-aging effects of iPE were investigated using a chronological lifespan assay on S. cerevisiae cells. The chronological lifespan of the yeast was significantly extended in those treated with both WiPE and OiPE at 1% (v/v). The expression of sirtuin-related genes, which regulate cellular senescence, was examined by RT-PCR. Interestingly, gene expression was found to be significantly increased only in WiPE treated cells. The results suggested that the different polarity components from iPE exhibited anti-aging effects on the cells via different mechanisms. Research on the identification of useful components in iPE and the possibility of application to mammalian cells is ongoing.

### BASELINE CHARACTERISTICS OF PARTICIPANTS IN A RANDOMIZED CONTROLLED TRIAL OF METFORMIN FOR FRAILTY PREVENTION

Tiffany Cortes,[1] Nicolas Musi,[2] Chen-pin Wang,[3] Joel Michalek,[4] and Sara Espinoza,[5] *1. University of Texas HSC at San Antonio, San Antonio, Texas, United States, 2. UTHSCSA, San Antonio, Texas, United States, 3. UT Health San Antonio, san antonio, Texas, United States, 4. UT Health Science Center San Antonio, San Antonio, Texas, United States, 5. University of Texas Health Science Center at San Antonio, San Antonio, Texas, United States*

We are conducting a double-blind, randomized controlled trial of metformin for frailty prevention. Participants are adults aged 65+ years with pre-diabetes assessed by 2-hour oral glucose tolerance test (OGTT). Those who are frail (Fried criteria) are excluded. Participants are randomized to metformin (maximum dose of 2,000 mg/day) vs. placebo and followed for 2 years. The primary outcome is frailty (category and score); secondary outcomes are physical performance and function (short physical performance battery, 6-minute walk, lower extremity strength), systemic and skeletal muscle tissue inflammation, muscle insulin signaling, insulin sensitivity (insulin clamp), glucose tolerance (OGTT), and body composition (dual-energy x-ray absorptiometry). Safety assessments occur every 3 months; frailty, systemic inflammation, and OGTT are assessed at baseline and every 6 months, and insulin clamp with muscle biopsies are assessed at baseline and every 12 months. To date, 85 subjects have been randomized; 120 completers are planned. Mean age is 72.8 ± 5.7 years, 55.3% are male, and 43.5% were Hispanic. Mean BMI is 30.2±5.8 kg/m2, waist circumference is 104.4 ±15.5 cm, fasting glucose is 102.3 ± 10.0 mg/dL, Hemoglobin A1c is 5.8 ±0.3, and glucose at 2 hours during OGTT is 167.3 ± 17.8 mg/dL. Metformin is being examined in this study as a potential therapeutic agent to prevent frailty in older adults with pre-diabetes. Findings from this trial may have future implications for the screening and potential treatment of pre-diabetes in older patients with metformin for the prevention of frailty.

### CELL-BASED AND PHARMACOLOGIC HORMONE THERAPY MAINTAIN DIASTOLIC FUNCTION AFTER OVARIECTOMY IN HYPERTENSIVE RATS

Leanne Groban,[1] Xuming Sun,[1] Sittadjody Sivanandane,[1] Hunter Hodge,[1] Hao Wang,[1] marina Lin,[1] and Emmanuel Opara,[2] *1. Wake Forest School of Medicine, Winston-Salem, North Carolina, United States, 2. Wake Forest School of Medicine, Winston-Salem, North Carolina, United States*

The role for hormone therapy in the maintenance of diastolic function upon ovarian senescence has not been clinically tested due to concerns for off-target health risks. We developed a cell-based hormone therapy (cHT) approach that recapitulates native cell–cell interactions between ovarian granulosa and theca cells in a 3D bioengineered construct to mimic the dynamic release of sex hormones. Our first report in ovariectomized (OVX) rats shows that cHT ameliorates various adverse somatic effects of hormone deficiency (e.g. bone loss). To extend these findings to cardiac health, we sought to determine the efficacy of cHT in preserving diastolic function in OVX-spontaneously hypertensive rats (SHR). 14 SHRs underwent bilateral OVX while 5 SHRs received sham surgery at 12 weeks of age. Following an 8-week washout, OVX rats were randomized to cHT or pharmacologic hormone therapy (pHT: E2 (10 mcg/kg/day) and P4 (2 mg/kg/day, s.c.) for 10 weeks and compared to OVX-vehicle and Sham. While uterine atrophy by OVX was minimized by cHT and pHT, hormone levels across OVX groups were not overtly different. Systolic blood pressure increased progressively over time ($P<0.01$), without a treatment effect. Even so, cHT and pHT prevented OVX-related reductions in myocardial relaxation and increases in Doppler-derived filling ($P<0.05$); paralleling the diastolic profile of Sham. Alongside superior diastolic function, 25% increases in cardiac interferon regulatory factor-4 (Irf-4) gene expression levels occurred in both hormone-treated OVX groups and Sham when compared to OVX-vehicle, suggesting a link between sex hormones and local immune modulation in the regulation of female cardiac health.

### CPG METHYLATION IN AGING: TRAJECTORIES OF INDIVIDUAL SITES

Margarita Meer,[1] and Morgan Levine,[2] *1. Yale School of Medicine, New Haven, Connecticut, United States, 2. Yale University School of Medicine, New Haven, Connecticut, United States*

Age-related changes in methylation in a set of genomic CpGs have been shown to form a kind of molecular clocks of aging – DNA methylation (DNAm) clocks. These markers are usually based on a small set of CpGs in every case, but 1) they rarely overlap between different clocks and 2) they are interchangeable, meaning that one can remove all clock sites from a data set and make a new clock of similar precision selecting a new set from the remaining sites. Nonetheless, only a fraction of CpG sites would be suitable for DNAm clocks. We performed an extensive analysis of all CpG sites aging behavior. Previous studies were focused on identifying positions where changes in DNAm correlate with age, but in this case, some of CpGs where DNAm changes occur in a non-linear way can be overlooked. We assessed the aging trajectory of every CpG, clustered CpGs by the type of aging behavior and applied a machine learning approach to construct a new kind of DNAm clocks based on the DNAm of these clusters. Since every cluster is composed of multiple CpGs, it makes this marker resistant to a common problem of missing data. Using blood, brain, skin, colon and liver samples we were also able to investigate tissue specificity of CpGs trajectories.

### DETRUSOR UNDERACTIVITY AS AN HCN-MEDIATED FAILURE OF RESILIENCE IN AGING

Dawn Rosenberg,[1] Cara Hardy,[1] Ramalakshmi Ramasamy,[2] and Phillip Smith,[1] *1. UConn Health, Farmington, Connecticut, United States, 2. UConn Health, Farminton, Connecticut, United States*

Sympathetic relaxation of the bladder wall permits low pressure urine storage and allows central regulation of afferent sensitivity to volume. Impaired regulation of volume sensitivity has been linked to symptoms of underactive bladder and cystometric detrusor underactivity. Hyperpolarization-activated cyclic nucleotide-gated channels (HCN channels) are mediators of sympathomimetic-induced detrusor relaxation in young mouse bladder tissue, however in bladder strips from old female mice, HCN blockade enhanced age-diminished isoproterenol-induced relaxation. We therefore hypothesized that loss of HCN would compromise cystometric function and enhance sympathomimetic responses in old mice. Male HCN1 KO mice (20-22 mo) and their WT littermates underwent pressure-flow cystometry under urethane anesthesia to assess urinary performance at the level of the autonomic nervous system in the absence of cortical control. Following cystometry, bladders were harvested and pharmacomyography was performed on bladder strips to determine tissue-level changes in the absence of CNS input. All mice responded to continuous-fill cystometry by establishing regular filling/voiding cycles. HCN KO mice function showed discrete changes in volume sensitivity vs. WT. Bladder strip studies showed minimal response to isoproterenol regardless of HCN status, and no significant differences in response to carbachol based on HCN status. We conclude that HCN status impacts the brainstem-bladder reflexic control over urine storage/voiding, but not by regulating bladder wall tensions during filling. The absence of HCN influence on the loss of end-organ responsiveness to sympathetic control in old mice points to an increasing dependency on central control mechanisms with aging.

### EFFECT OF COMBINED DASATINIB AND FISETIN TREATMENT ON SENESCENT CELL CLEARANCE IN MONKEYS

Ricki Colman,[1] Tamara Tchkonia,[2] Tamar Pirtskhalava,[2] Nino Giorgadze,[2] Larissa Prata,[2] Kalli Schaefer,[2] and James Kirkland,[2] *1. University of Wisconsin, Madison,*

*Madison, Wisconsin, United States, 2. Mayo Clinic, Rochester, Minnesota, United States*

Aging is the biggest risk factor for the most serious chronic diseases and disabilities. Cellular senescence, a state in which cells stop dividing but release factors that damage other cells, may contribute to both age-related and chronic diseases. Removal of senescent cells from aged mice has been shown to delay aging and age–related disabilities. Our goal was to determine the ability of potential senolytic agents to remove senescent cells in a primate model. Several agents and combinations were tested including Fisetin, Navitoclax, combined Dasatinib and Quercetin, and combined Dasatinib and Fisetin. Here we describe the Dasatinib and Fisetin trial. Dasatinib is an FDA approved oral anticancer drug that has been used to treat chronic myelogenous leukemia in humans. Fisetin is a flavonoid that can be found in many plants, particularly strawberries, and acts as a coloring agent. After baseline measurements, six older (mean age=21 years) female rhesus monkeys (Macaca mulatta) were given a combined oral dose of Dasatinib (5 mg/kg) and Fisetin (100 mg/kg) on two consecutive days. Animals were additionally assessed at 1- and 7-weeks following dosing. At 7 weeks post dosing, there were fewer ($p<0.05$) p16+ cells in the epidermis compared to baseline. Similarly, there was a reduction ($p<0.05$) in p21+ cells in the epidermis at 1- and 7-weeks post dosing compared to baseline. There were no negative outcomes associated with treatment. This study provides preliminary evidence for the senolytic potential of combined Dasatinib and Fisetin treatment and indicates that pharmacological mitigation of age-related changes is possible.

### ELEVATED GROWTH DIFFERENTIATION FACTOR-15 IS A BIOMARKER OF SARCOPENIA IN OLDER ADULTS

Miji Kim,[1] and Chang Won Won,[2] *1. College of Medicine, Kyung Hee University, Seoul, Republic of Korea, 2. Kyung Hee University Medical Center, Seoul, Korea, Seoul, Republic of Korea*

Growth differentiation factor 15 (GDF-15) is associated with disease progression, mitochondrial dysfunction and mortality. Elevated GDF-15 level was recently reported to be associated with poorer physical performance in healthy community-dwelling adults. However, the relationship between serum GDF-15 concentration and sarcopenia in community-dwelling older adults has not been well characterized. We analyzed 929 participants (mean age 75.9±8.9 years, 48.0% men) from the Korean Frailty and Aging Cohort Study who underwent assessment of serum GDF-15 concentration and sarcopenia. Participants with an estimated glomerular filtration rate <60 ml/min/1.73 m2 were excluded from this analysis. Sarcopenia status was determined as per the Asian Working Group for Sarcopenia (AWGS) 2019 guidelines. As per the AWGS 2019 algorithm, 154 (16.6%) participants in the study population were classified as having sarcopenia. Median serum GDF-15 concentration was elevated in the sarcopenic group vs. the non-sarcopenic group (920 vs. 793 pg/ml, $p<0.001$). In the multivariate analysis adjusted for potential confounders, the highest GDF-15 tertile (≥1245 pg/ml) was associated with a higher risk of sarcopenia vs. the lowest tertile (<885 pg/ml) (odds ratio [OR] = 1.95, 95% confidence interval [CI] 1.15–3.31). This association remained unchanged (OR = 1.90, 95% CI 1.14–3.23) after further adjustment for potential biomarkers (myostatin, dehydroepiandrosterone, and insulin-like growth factor-1). The OR per unit increase in log-transformed GDF-15 concentration was 3.59 (95% CI 1.21–10.70). To conclude, our results suggest that higher circulating GDF-15 concentration was independently associated with a greater risk of sarcopenia in community-dwelling older adults. Serum GDF-15 concentration can be a promising biomarker for sarcopenia

### EPIGENETIC SIGNATURES OF CELL STATES IN AGING

Diana Leung,[1] and Morgan Levine,[2] *1. Yale University, Elmhurst, New York, United States, 2. Yale University School of Medicine, New Haven, Connecticut, United States*

Epigenetic clocks based on DNA methylation (DNAm) show striking age correlations and predict various outcomes. Patterns of DNAm also reflect critical mechanisms in differentiation and proliferation. As such, an outstanding question is whether part of the signal epigenetic clocks are capturing represent shifts in the proportions of somatic stem cells, senescence cells, and/or tumorigenic cells. Here, we assembled various methylation datasets that captured relevant phenomena, including pluripotent stem cells, differentiation, senescence, and cancer, and performed weighted network analysis to cluster and compare DNAm modules. We find overlapping clusters between in vitro samples and in vivo tissue samples, suggesting that cell-level phenomena like cell replication, senescence, and cancer intersect with age-related epigenetic signatures. While the effects of aging manifest at multiple systems levels, from the genome to clinical phenotypes, these analyses may help provide insight to the contribution of cell phenotype dynamics to the general aging phenomenon.

### EXOSOMES DERIVED FROM SENESCENT CELLS PROMOTE CELLULAR SENESCENCE

Genxiang Mao,[1] and Xiaogang Xu,[2] *1. Zhejiang Hospital, hangzhou, Zhejiang Province, China, 2. Zhejiang Hospital, Hangzhou, Zhejiang, China*

Exosomes are one type of small-cell extracellular vesicles (sEVs), which together with the senescence-associated secretory phenotype (SASP) mainly constitute the senescent microenvironment and perform remotely intercellular communication. However, the effects of senescence on exosomes biosynthesis and secretion and its role in the cell senescence are still obscure. Here, we used human fetal lung diploid fibroblasts (2BS) passaged to PD50 to construct the senescent cells model in vitro, which were confirmed by senescence-related β-galactosidase staining, cell cycle distribution, and intracellular ROS levels. PD30 2BS was used as young control. We evaluated the exosomes derived from senescence and young control group respectively and investigated their regulation of senescence. We found that exosomes released from 2BS had typical sizes and cup-shapes morphology and their surface presented typical exosome-associated proteins. The number of exosomes secreted by senescent cells was significantly higher than that of young cells. Moreover, exosomal markers Alix, TSG101, and CD63 were all more expressed than young cells. Furthermore, we treat young cells with exosomes secreted by senescent cells, which can induce senescence-like changes in young cells, including increased SA-β-Gal activity, up-regulated p16 protein expression, and activation of the Notch signaling pathway. The above results imply that exosomes derived from senescent cells can promote cell senescence. The findings expand the current knowledge on exosomes-mediated aging and provide a novel understanding of the relationship between SASP and senescence. This study is supported by National Natural Science Foundation of China (No. 81771520 and 31702144).

### INTRANASAL OXYTOCIN IMPROVES LEAN MUSCLE MASS IN OLDER ADULTS WITH SARCOPENIC OBESITY: A PILOT STUDY

Jessica Lee,[1] Sara Espinoza,[2] Adetutu Odejimi,[3] Chen-pin Wang,[2] Vinutha Ganapathy,[2] Chiara Pascucci,[4] Nicolas Musi,[2] and Elena Volpi[3] *1. The University of Texas Health Science Center at Houston, Houston, Texas, United States, 2. University of Texas Health Science Center at San Antonio, San Antonio, Texas, United States, 3. The University of Texas Medical Branch at Galveston, Galveston, Texas, United States, 4. Sapienza University of Rome, Rome, Italy*

Obese older adults often have sarcopenia with increased functional impairments. Unfortunately, conventional weight loss treatments can lead to further muscle mass loss. Increasing evidence from animal studies suggests that the pituitary hormone oxytocin has trophic effects on skeletal muscle cells and can induce weight loss. We piloted a clinical trial testing whether intranasal oxytocin would decrease adiposity without lowering muscle mass in older adults with sarcopenic obesity. Twenty-one older (≥60years), obese (30-43kg/m2), sedentary (<2 strenuous exercises/week) adults with slow gait speed (<1m/sec) were randomized to intra-nasal oxytocin (24IU four times/day) or placebo for 8 weeks. Pre and post body mass index (BMI), 2-hour oral glucose tolerance test (OGTT), hemoglobin A1c (HbA1c), short physical performance battery (SPPB), and whole body lean and fat mass (via dual-energy X-ray absorptiometry) were assessed. Generalized estimation equation method was used to evaluate effects of oxytocin on these continuous measures. At baseline, results were: age 67.5±5.4years, 71% female, BMI 36.0±3.6kg/m2, HbA1c 5.7±0.4%, 2-hr OGTT glucose 140.8±4.1mg/dL, SPPB 9.2±1.9, fat mass 45,429±7,037g, and lean mass was 49,892±10,470g. From baseline to follow-up, total lean mass increased significantly (2,250g) in the oxytocin group (pre- vs. post-treatment difference of -690g in placebo and +1,559g in oxytocin, p<0.01). Oxytocin did not lead to significant changes in other measures. This data suggests that oxytocin leads to significant improvement in whole body lean mass. Future studies in a larger study population will help determine whether older adults with sarcopenic obesity may benefit from intranasal oxytocin to improve lean muscle mass and physical function.

### METABOLIC REGULATION OF THE SENESCENCE PROGRAM

Manali Potnis,[1] Timothy Nacarelli,[1] Eishi Noguchi,[1] Ashley Azar,[1] and Christian Sell,[2] *1. Drexel College of Medicine, Philadelphia, Pennsylvania, United States, 2. Drexel University, Philadelphia, Pennsylvania, United States*

Cellular senescence is a cell fate defined by an irreversible cell-cycle arrest and a pro-inflammatory secretory profile. It is a consequence of a shift in metabolism and rearrangement of chromatin. Accumulation of senescent cells is a universal hallmark of age-related pathologies suggesting these cells contribute to age-related susceptibility to disease. Here, we examine the interplay between two metabolic inhibitors of senescence: Rapamycin treatment and Methionine restriction (metR). We report that a combination of methionine restriction and rapamycin induces a metabolic reprogramming that prevents activation of the senescence program in human fibroblasts. The treated cells continue to divide at a slow rate at a high passage and lack senescence-associated markers and inflammatory cytokines. Genome-wide chromatin accessibility analysis reflects chromatin remodeling with distinctly increased accessibility of heterochromatic regions in treated cells. Further, Transcriptome-wide analysis reveals increased expression of specific methyltransferases which alter the trimethylation of H3, one of the strongest hallmarks of open chromatin. This may represent a mechanistic link between a major hallmark of senescence and nuclear events required for senescence.

### METFORMIN IMPROVES COGNITION BY REDUCING LEAKY GUT AND BENEFITING GUT MICROBIOME–GOBLET CELL–MUCIN AXIS

Hariom Yadav,[1] Shokouh Ahmadi,[1] Bo Wang,[2] Jamie Justice,[1] Jingzhong Ding,[1] Dalane Kitzman,[1] Donald McClain,[1] and Stephen Kritchevsky,[1] *1. Wake Forest School of Medicine, Winston-Salem, North Carolina, United States, 2. North Carolina A&T State University, North Carolina, United States*

Older adults are suffering from several aging-related illnesses including cognitive decline and effective strategies to prevent and/or treat them are lacking, because of a poor understanding of therapeutic targets. Low-grade inflammation is a key risk factor of aging-related morbidities and mortalities, and it is often higher in older adults. Although, precise reasons for increased inflammation remain unknown, however, emerging evidence indicates that abnormal (dysbiotic) gut microbiome and dysfunctional gut permeability (leaky gut) are linked with increased inflammation in older adults. However, no drugs are available to treat aging-related microbiome dysbiosis and leaky gut, and little is known about the cellular and molecular processes that can be targeted to reduce leaky gut in older adults. Here, we demonstrated that metformin, a safe FDA approved antidiabetic drug, decreased leaky gut and inflammation in older obese mice, by beneficially modulating the gut microbiota. In addition, metformin increased goblet cell mass and mucin production in the older gut, thereby decreasing leaky gut and inflammation. Mechanistically, metformin increased the goblet cell differentiation markers by suppressing Wnt signaling. Our results suggest that metformin can prevent and treat aging-related leaky gut and inflammation, by beneficially modulating gut microbiome/goblet cell/mucin biology.

### MITOCHONDRIAL DYSFUNCTION AND CELLULAR SENESCENCE DUE TO AGING CONTRIBUTES TO PROSTATIC FIBROSIS

Teresa Liu,[1] Emily Ricke,[2] Donald DeFranco,[3] and William Ricke,[4] *1. University of Wisconsin--Madison, Madison, Wisconsin, United States, 2. University of*

*Wisconsin - Madison, Madison, Wisconsin, United States, 3. University of Pittsburgh, Pittsburgh, Pennsylvania, United States, 4. University of Wisconsin Madison, Madison, Wisconsin, United States*

Aging is the single largest risk factor for many common diseases that burden public health. This is especially true in the prostate; as men age, the prostate undergoes a prototypical aging change, fibrosis. The aged fibrotic prostate causes urinary symptoms which will afflict nearly every man if they live long enough. However, the molecular mechanisms responsible for the aging-dependent promotion of fibrosis are largely unknown and understudied. In this study, we sought to reveal the contribution of mitochondrial dysfunction to fibrosis in the aging prostate, ultimately leading to prostate dysfunction, urinary symptoms, and overall poor health. The demise of mitochondrial function is well established in other aging-associated diseases but has not been investigated in the prostate. Our analysis revealed an increase in cellular senescence and mitochondrial dysfunction in BPH patient tissue compared to normal prostates. Furthermore, selective inhibition of mitochondrial complex I by rotenone in cultured human prostate fibroblasts led to myofibroblast phenoconversion as characterized by increased expression of Col1a1, Col3a1, and ACTA2. To determine the contribution of mitochondrial dysfunction on fibrosis and lower urinary tract dysfunction (LUTD), we examined the prostate lobes in both aging and steroid-induced LUTD in mice. These models have been extensively characterized and exhibit an age-mediated increase in LUTD and fibrosis. We observed a decrease in mitochondrial function and an increase in cellular senescence corresponding to an increase in fibrosis and LUTD. This suggests that normal aging-dependent reductions in mitochondrial complex I function in the prostate may promote fibrosis and contribute to urinary dysfunction.

### PRO-INFLAMMATORY CD8 T CELLS CONTRIBUTE TO AGE-RELATED LARGE ARTERY STIFFENING

Daniel Trott,[1] and Sunita Sharma,[2] *1. University of Texas at Arlington, Arlington, Texas, United States, 2. University of Texas at Arlington, ARLINGTON, Texas, United States*

We have shown that T cells accumulate around the aorta with advanced age and that both pharmacologic and genetic deletion of total T cells result in improved large artery stiffness in old mice. The purpose of this study was to test the hypothesis that CD8 T cells specifically mediate age-related large artery stiffening. We randomized old (22-24 month) C57Bl6 mice (n=5/group) to treatment with an anti-CD8 or isotype control antibody (100µg every 5 days) for 28 days. We assessed large artery stiffness using pulse wave velocity (PWV) before and after antibody treatment. We found that PWV was similar between isotype (301±14 cm/s) and anti-CD8 (292±18 cm/s) before treatment. Following treatment, anti-CD8 treated mice exhibited lower PWV (272±11 cm/s) compared to the isotype (315±14 cm/s). Following euthanasia, we assessed aortic T cell infiltration by flow cytometry we found that anti-CD8 treated mice exhibited blunted aortic total CD3+ (262±42 vs. 1400±94 cells per aorta) and CD8+ (52±19 vs. 565±139 cells per aorta) cells compared to isotype controls. In a separate cohort of mice we compared interferon (IFN)-γ and tumor necrosis factor (TNF)-α production of aortic infiltrating CD8 cells from young (4-6 months, n=5) and old mice (n=8) using flow cytometry. A greater proportion of CD8 cells from old aortas produced IFN-γ (70±3% vs. 46±6%) compared to young. Similarly, a greater proportion of aortic infiltrating CD8 cells from old mice produced TNF-α (35±6% vs. 17±3%) compared to young. Collectively, these results suggest that pro-inflammatory CD8 cells contribute to cell non-autonomous arterial aging.

### VACCINATION IMPROVES CD4 T HELPER SUBSET BALANCE IN AGED MICE BY OVERCOMING THE EFFECTS OF THE SENESCENT ENVIRONMENT

Blake Torrance, Erica Lorenzo, Spencer Keilich, Andrew Harrison, Iman Al-Naggar, and Laura Haynes, *UConn Health, Farmington, Connecticut, United States*

Influenza (flu) infection is a leading cause of morbidity and mortality in older adults. Although vaccination is the best strategy to combat the effects of flu infection, there is a marked decline in vaccine efficacy in older adults. We hypothesized that an increased level of basal inflammation, a hallmark of the aging, plays a role in this decreased vaccine efficacy and that a vaccination strategy designed to augment the aged response to flu would be beneficial. Adjuvanted vaccination with a conserved influenza protein enhanced viral clearance, antibody production, and protected against weight loss in both young and aged mice. Vaccination also decreased levels of inflammation brought about by leukocyte infiltration and induced a strong type 2 cytokine environment in the lung. Additionally, vaccination reduced the proportion of flu-specific FoxP3+ T regulatory cells (Tregs) and decreased levels of TGF-β in aged lungs following infection. Accumulation of senescent cells, another hallmark of aging, can contribute to the inflammatory environment via their senescent associated secretory phenotype (SASP). Supporting a role for senescent cells in exacerbating age-related deficits in the response to vaccination, treatment of aged mice with the senolytic drugs Dasatinib and Quercetin also decreased levels of Tregs and TGF-β in the lungs following flu infection. These findings give insight to how vaccination responses can be ameliorated in older adults by targeting both inflammatory and regulatory signals and how accumulation of senescent cells could influence the dysregulation of the immune response with age.

### SESSION 2878 (POSTER)

### ACUTE CARE AND HOSPITALIZATION

### A PROSPECTIVE STUDY OF FRAILTY AND 30-DAY FUNCTIONAL OUTCOMES OF HOSPITALIZED OLDER ADULTS WITH PNEUMONIA

Chan Mi Park,[1] Wonsock Kim,[1] Jong Hun Kim,[1] and Dae Kim,[2] *1. Korea University Anam Hospital, Seoul, Republic of Korea, 2. Hebrew SeniorLife, Harvard Medical School, Roslindale, Massachusetts, United States*

Pneumonia is a major cause of morbidity and mortality in older adults. The role of frailty assessment in older adults with pneumonia is not well defined. A prospective cohort study was conducted to investigate the clinical and functional outcomes of pneumonia in older adults with different levels of frailty. Between November 2019 and March 2020, we enrolled 92 adults who were 65 years or older and hospitalized for pneumonia at an academic medical center in Seoul, Korea. A deficit-accumulation frailty index (FI) was calculated using 50 items from comprehensive geriatric assessment (range: 0-1; higher values indicate greater frailty). The ability to perform 21 daily activities and physical tasks prior to the illness and after 30 days was self-reported. Primary outcome was death or functional decline from prior level at 30 days. The study cohort had a mean age of 79.4 (standard deviation [SD], 7.11) years, 38 (41.3%) women, and 20 (21.7%) nursing home residents. The mean FI was 0.29 (SD, 0.19), with 27 (29.4%) robust (FI<0.15), 21 (22.8%) pre-frail (FI, 0.15-0.24), 19 (20.7%) mild-to-moderately frail (FI, 0.25-0.44), and 25 (27.2%) severely frail (FI≥0.45) categories. Among 71 patients without maximum disability at baseline, 8 (11.3%) died and 39 (54.9%) experienced functional decline. The 30-day risk of primary outcome for increasing frailty categories were 47.8%, 57.9%, 77.8%, and 81.8%, respectively (p-for-trend=0.022). Our results indicate that pneumonia is a major disabling illness in older adults with frailty. A proactive geriatric evaluation and multidisciplinary intervention are needed to improve functional recovery in these patients.

### DECREASING EMERGENCY ROOM UTILIZATION IN HIGH RISK GERIATRIC PATIENTS

Shamsi Fani,[1] Lizette Munoz,[2] Susana Lavayen,[2] Blair McKenzie,[3] Audrey Chun,[4] Jeff Cao,[3] and Stephanie Chow,[5] *1. Mount Sinai Medical Center, New York, New York, United States, 2. Mount Sinai Hospital, New York, New York, United States, 3. The Mount Sinai Hospital, New York, New York, United States, 4. Icahn School of Medicine at Mount Sinai, New york, New York, United States, 5. Icahn School of Medicine at Mount Sinai, New York, New York, United States*

Background: The Acute Life Interventions Goals & Needs Program (ALIGN) at the Mount Sinai Hospital in New York City aims to work closely with high risk geriatric patients for short term intensive management of acute medical and social issues. Quantitative measures for determining success of the program is comparing emergency room visits and hospitalizations prior to and after enrollment with ALIGN. The Community Paramedicine service allows a paramedic, the ALIGN provider, and an emergency room physician to assess and triage patients in their home via video conference thereby avoiding ED visits for non-urgent services. Method: We reviewed the utilization of the Community Paramedicine service (from July 2017-February 2020) and its impact on ALIGN's efforts to reduce unnecessary ED visits and hospitalizations. Results: 36 patients were evaluated with the Community Paramedicine service (from July 2017-February 2020). 19 or 52.8% avoided an ED visit and 17 or 47.2% were transported to the ED. 12 or 70.6% were admitted to the hospital of those that were transported to the ED initially. Top reasons for transport to ED included generalized weakness, acute mental status change (AMS), and shortness of breath (SOB). Conclusions: A Community Paramedicine program utilized by a high risk geriatrics team like ALIGN is effective in reducing ED visits and hospitalizations for the elderly population who incur greater expenses to the health care system and traditionally have poorer health outcomes.

### DELAYED HOSPITAL DISCHARGE–THE EFFECT OF DEMENTIA AND GP CONTACTS PRIOR TO HOSPITALIZATION

Mari Aaltonen,[1] Shiraz El Adam,[2] Mariko Sakamoto,[3] Anne Martin-Matthews,[2] and Kimberlyn McGrail,[2]
*1. Tampere University, Tampere, Finland, 2. The University of British Columbia, Vancouver, British Columbia, Canada, 3. University of British Columbia, Vancouver, British Columbia, Canada*

Delayed hospital discharge, also known as alternate level of care (ALC) in Canada, refers to a stay in hospital when acute services are no longer needed but the patient occupies a hospital bed while waiting to be discharged to an appropriate care setting. ALC has negative consequences for both the service system (high costs, inappropriate use of hospital resources) and individuals (feelings of uncertainty, functional loss). Extensive administrative health care data from British Columbia, Canada, were employed to study differences in ALC between people with and without dementia in 2001/02, 2005/06, 2010/11 and 2015/16, and whether continuity in physician–patient relationships prior to hospitalization is associated with ALC. We analyzed designation of ALC and length of stay in ALC, using generalized estimating equations logistic regression and negative binomial regression analysis. Of all individual, residential area and health care related factors, dementia was the single most important factor increasing the odds of designation of ALC (OR 4.76, 95%CI 4.59, 4.93). Dementia also added to the length of stay in ALC. Proportion of patients designated to ALC increased over the study years. Higher number of visits to the same general practitioner (GP) prior to hospitalization decreased the odds of ALC, especially in people with dementia. As populations age, the number of people with dementia is increasing. Efforts to control ALC have resulted in greater concentration of ALC among people with dementia. Higher continuity GP care may be a way to help understand and control these trends.

### DEMENTIA NURSING COMPETENCY SCALE AT ACUTE HOSPITALS VERSION 2: DEVELOPMENT AND PSYCHOMETRIC EVALUATION

Chieko Greiner,[1] Shoko Urashima,[2] Hirochika Ryuno,[3] Yuko Yamaguchi,[3] and Atsuko Fukuda,[4] *1. KOBE UNIVERSITY, Kobe, Japan, 2. Osaka Saiseikai Ibaraki Hospital, Osaka, Japan, 3. Kobe University, Kobe, Japan, 4. Kobe University, Kobe, United States*

Under an increasing number of people with dementia worldwide, the number of patients with dementia in acute settings is also growing. For patients with dementia to receive treatment with peace of mind, nurses should have competency in caring for patients with dementia. Thus, the aims of this study were to develop the Dementia Nursing Competency Scale in Acute Hospitals version 2 (DNCS-AH-v2) and to evaluate its psychometric properties. The draft was distributed to a convenience sample of 3000 nurses at 300 acute hospitals. After confirming the ceiling effect and the floor effect, reliability and validity were verified using Cronbach's alpha, I-T correlation, test-retest reliability, G-P analysis, and exploratory and confirmatory factor analyses. This study was approved by the Institutional Review Board of Kobe University. Of a total of 3000 nurses, 878 (29.3%) responded to the questionnaire and 773 (25.8%)

valid responses were analyzed. Mean age and mean working experience of the participants were 37.4 ± 9.3 and 14.0 ± 8.7 years, respectively. The 27-item DNCS-AH-v2 was developed with five factors scored on a six-point Likert scale. Cronbach's alpha was .925, I-T correlation was between .46 and .68 (p<.01), ICC coefficient (1, 1) was .76 (p<.001), and significant differences were confirmed for G-P analysis (p<.001). For the confirmatory factor analysis, fit indices of CFI =.868, GFI =.868, and RMSEA =.065 were obtained. High reliability and moderate validity were confirmed for the DNCS-AH-v2. The developed DNCS-AH-v2 could be used to evaluate dementia nursing competency in acute hospitals.

### EFFECTIVE AND EVIDENCE-BASED: HOW THE 4MS AGE-FRIENDLY FRAMEWORK CAN RESULT IN IMPROVED CARE IN A RURAL PRIMARY CARE CLINIC

Leah Tobey,[1] and Robin McAtee,[2] *1. University of Arkansas for Medical Sciences, Little Rock, Arkansas, United States, 2. UAMS, Little Rock, Arkansas, United States*

As healthcare providers struggle to reframe aging, framing Age-Friendly care is also occurring. The Arkansas Geriatric Education Collaborative (AGEC) is a HRSA Geriatric Workforce Enhancement Program with an objective to improve clinical health outcomes of older adults (OA) in primary care settings. As a member of the 2020 Institute for Healthcare Improvement (IHI) Age-Friendly cohort, the AGEC has partnered with ARcare, an AR federally qualified healthcare clinic network, to implement the 4Ms in 4 rural clinics over 3 years. AGEC's first goal of working with rural primary care clinics is to improve their knowledge of best practices of caring for OA. This was started by providing Geriatric Interdisciplinary Team Training to clinic staff, obtaining baseline data of common health related indicators for OA and starting regular geriatric focused training. Training on the 4Ms (Matters, Medication, Mentation Mobility) framework was next and completed followed by planning and implementation. The process was well received and results are promising. Year 1 data in one clinic show incremental improvements over baseline data in several areas including assessing Mobility with fall screens which has improved over 50% in one year and annual wellness visits (where all 4Ms are reviewed) have increased 30%. However, several areas of opportunities for improvement have also been noted and turned into quality improvement projects (QI). This includes an opportunity to improve depression screens for the clinic's Mentation measure, which dropped almost 30% in one year. QI projects are ongoing to improve each of the elements of becoming age-friendly.

### ELECTRONIC HEALTH RECORD DATA CAN BE USED AT THE BEDSIDE TO IDENTIFY OLDER HOSPITALIZED PATIENTS WITH DELIRIUM

Ariba Khan,[1] Kayla heslin,[2] Michelle Simpson,[3] and Michael Malone,[4] *1. Advocate Aurora Health Care, Milwaukee, Wisconsin, United States, 2. Aurora Research Institute, Milwaukee, United States, 3. Advocate Aurora Health Care, Milwaukee, United States, 4. Advocate Aurora Health, Milwaukee, Wisconsin, United States*

Delirium is a serious condition that is often underrecognized. Several delirium predictive rules can assist in early detection. The coupling of prediction rules with features of the EHR are in their infancy but hold potential. This study aimed to determine variables within the EHR that can be used to identify older hospitalized patients with delirium. This is a prospective study among patients >=65 years admitted to the hospital. Researchers screened daily for delirium using the 3-D CAM. Predictive variables were extracted from the EHR. Basic descriptive statistics were conducted. Chi-squared and Fischer's exact tests were used to compare differences among those diagnosed with or without delirium as appropriate; binary logistic regression was used for multivariate modeling. Among 408 participants, mean age was 75 years, 61% were female, and 83% were black. The overall rate of delirium was 16.7% (prevalent delirium 10.5%; incident delirium 6.1%). There was no statistical difference in 30-day mortality (2.9% vs. 2.7%) or 30-day readmission (13.2% vs. 14.7%) rates between those with and without delirium (both P>0.05). Even so, patients with delirium were older, more likely to have a diagnosis of infection and/or cognitive impairment, as well as increased severity of illness (all P's <0.05). Moreover, patients with delirium had a lower Braden score and higher Morse fall score (both P's <0.01). In multivariate analysis, cognitive impairment (OR 5.49; 95% CI 2.77-10.87) and lower Braden scores (OR 1.29; 95% CI 1.18-1.41) remained significant predictors of delirium. Further research is needed to develop an automated EHR prediction model.

### ENHANCING OUR UNDERSTANDING OF TRANSITIONAL CARE PROGRAMS

Katherine McGilton,[1] Shirin Vellani,[2] Alexandra Krassikova,[2] Alexia Cumal,[3] Sheryl Robertson,[3] Constance Irwin,[2] Jennifer Bethell,[2] and Souraya Sidani,[4] *1. Toronto Rehabilitation - UHN, Toronto, Ontario, Canada, 2. Toronto Rehabilitation Institute-University Health Network, Toronto, Ontario, Canada, 3. Toronto Rehabilitation Institute-University Health Network, Toronto, Canada, 4. Daphne Cockwell School of Nursing, Ryerson University, Toronto, Canada*

Many hospitalized older adults experience delayed discharge. Transitional care programs (TCPs) provide short-term care to these patients to prepare them for transfer to nursing homes or back to the community. There are knowledge gaps related to the processes and outcomes of TCPs. We conducted a scoping review following Arksey & O'Malley's framework to identify the: 1) characteristics of older patients served by TCPs, 2) services provided within TCPs, and 3) outcomes used to evaluate TCPs. We searched bibliographic databases and grey literature. We included papers and reports involving community-dwelling older adults aged ≥ 65 years and examined the processes and/or outcomes of TCPs. The search retrieved 4828 references; 38 studies and 2 reports met the inclusion criteria. Most studies were conducted in Europe (n=19) and America (n=13). Patients admitted to TCPs were 59-86 years old, had 2-10 chronic conditions, 26-74% lived alone, the majority were functionally dependent and had mild cognitive impairment. Most TCPs were staffed by nurses, physiotherapists, occupational therapists, social workers and physicians, and support staff. The TCPs provided 5 major types of services: assessment, care planning, treatment, evaluation/care monitoring and discharge planning. The outcomes most frequently assessed were discharge destination, mortality, hospital readmission, length of stay, cost and functional status. TCPs that reported significant improvement in older adults' functions (which was the main goal of the TCPs) included multiple services delivered by multidisciplinary teams. There is a wide variation in the operationalization of TCPs within and between countries.

### HOME HEALTH CARE PRACTICES TO REDUCE ACUTE-CARE HOSPITALIZATIONS

Bonnie Albright,[1] and Yael Pelenur,[2] *1. University of Massachusetts - Boston, Boston, Massachusetts, United States, 2. University of Vermont, Burlington, Vermont, United States*

Avoidable acute-care admissions are linked to negative health outcomes for patients and are costly to insurers. Home health care agencies (HHAs) are important players in preventing these admissions. Research on best practices to prevent acute-care admissions in the home health care context is limited. The purposes of this study were both to discover what practices HHAs are implementing in Medicare-certified home health care episodes to prevent acute-care admissions, and to learn what barriers HHAs face in implementing these practices. This study used mixed methods including qualitative and quantitative elements. Seven key informant interviews were used to develop a web survey that was emailed to all Medicare-certified HHAs in Massachusetts (response rate 12.43%, n=23). Using qualitative methods including thematic review, open-coding, and member-checking this study developed a four-categorization method. The categories are assessment, interventions, communication, and global practices. The study also developed a taxonomy for describing barriers to implementing practices. The distribution of responses for the new taxonomic categories were: patient-related (32.35%), staffing-related (29.41%), software-related (17.65%), physician/hospital-related (14.29%), and reimbursement/regulation-related (5.88%). This study fills a gap in research by describing the realities of home health care practice in the context of avoidable acute-care admissions. The categorization of best practices and the taxonomy of barriers developed in this study provide frameworks for understanding HHA practice. Further research is needed to effectively reduce avoidable acute-care admissions during and after Medicare-certified home health care episodes.

### HOSPITAL DISCHARGE INTERVENTIONS: A COST-SAVINGS COMPARISON

Bonnie Albright,[1] and Yael Pelenur,[2] *1. University of Massachusetts - Boston, Boston, Massachusetts, United States, 2. University of Vermont, Burlington, Vermont, United States*

For an older patient, transitioning back into the community after an acute health incident is a critical juncture. Avoidable acute-care readmissions are expensive for hospitals and federal programs. Negative health outcomes for patients and negative financial outcomes for hospitals and federal programs have focused attention on effective discharge interventions to improve care transitions and decrease avoidable acute-care readmissions. This study compares cost and readmissions outcomes from peer-reviewed publication data for three discharge interventions: Care Transitions Intervention (CTI), Project RED (ReEngineered Discharge), and the Transitional Care Model (TCM). This study adjusted costs to 2015 rates and compared cost savings per patient, return on investment (ROI) and percent reduction of readmissions. Cost savings per patient (2015-adjusted) were found for all interventions: CTI ($152.89); Project RED ($327.03); TCM ($1565.84). ROI was positive for all interventions: CTI (832%); Project RED (535%); TCM (232%). Compared to control groups, intervention group readmissions were 3.6% lower for CTI (n.s.), 5.5% lower for Project RED (p<.05), and 13.2% lower for TCM (p<.05). These three discharge interventions differ in scale and intensity, but they all show cost savings and reductions in readmissions. The lower-cost intervention shows cost savings and ROI (CTI), and the more resource-intensive interventions (Project RED and TCM) reduce costs and statistically significantly reduce rates of readmission. Even with small budget dollars, hospitals have options for finding an effective discharge intervention to reduce costs and readmission rates.

### HOSPITALIZATION AND SUCCESSFUL AGING OF COMMUNITY-DWELLING OLDER ADULTS: USING THE KOREAN NATIONAL SURVEY DATA

Ji Yeon Lee,[1] Bora Kim,[2] Kyung Hee Lee,[1] and Changgi Park[3], *1. Yonsei University, Seoul, Republic of Korea, 2. Yonsei University College of Nursing, Seoul, Republic of Korea, 3. University of Illinois, Chicago, Illinois, United States*

Hospitalization experience can be an obstructive factor to successful aging. Although older adults who had hospitalization experience has been considered to have poor health status and low participation in one's life, it is not obviously evident whether hospitalization itself affects successful aging. This study aimed to investigate whether three components of successful aging (i.e., diseases and disease-related complications, physical and mental functions, and engagement with life) were different in community-dwelling older adults who had hospitalization experience for the past one year compared to the counterpart older adults without hospitalization experience. A secondary data analysis was performed using a nationally representative survey data in Korea. A total of 1,812 who had hospitalization experience were matched to 1,812 control counterpart using propensity score matching. Sampling weight of the survey was considered for all statistical analysis. The community-dwelling older adults with hospitalization experience were less likely to be aging successfully than the older adults without hospitalization experience. The older adults with hospitalization experience had more chronic illnesses and malnourishment; they had more impairment in physical function and depressive symptoms; they were less active in working, social activities, and traveling. However, there were no differences in cognitive function and religious activities between the groups. In conclusion, the community-dwelling older adults who had hospitalization experience have poor health status and less engagement in one's life in general after matching covariates using propensity score matching analysis. Therefore, more attention and assist are needed to the community-dwelling older adults with hospitalization experience to facilitate successful aging.

### LOW SERUM ALBUMIN IN PATIENTS WITH COEXISTING COGNITIVE IMPAIRMENT PREDICTS SURGICAL COMPLICATIONS

Coco Victoria Tirambulo,[1] Caitlyn McFadden,[1] David Lieberman,[2] and Mindy Fain,[3] *1. The University of Arizona Center on Aging, Tucson, Arizona, United States, 2. University of Arizona College of Medicine - Geriatrics, General Internal Medicine and Palliative Medicine, Tucson, Arizona, United States, 3. University of Arizona Center on Aging, Tucson, Arizona, United States*

Cognitive impairment (CI, ~15-20%) and malnutrition (~38.7%) are common concerns among older adults ≥65 years. CI and malnutrition may be used as predictive risk factors for poor surgical outcomes. The 2012 ACS NSQIP/AGS Best Practice Guidelines for the preoperative assessment of geriatric surgical patients classify severe nutritional risk as either having a BMI < 18.5 kg/m2, serum albumin (SA) < 3.0 g/dL and/or unintentional weight loss > 10%-15% within 6 months. Using SA as a surrogate marker for malnutrition, we evaluated the relationship between CI, malnutrition, and risk for poor surgical outcomes in a geriatric population. Electronic medical record chart reviews of patients (≥65 years old) undergoing elective intermediate or high-risk surgery (IHRS), between 2016 and 2019 in Tucson, AZ, were conducted. Pre-and-post assessment factors such as cognitive status via mini-cog, laboratory markers (SA), and hospital complications were examined. Multivariate regression analyses were performed to determine the association between cognitive status, SA levels, and hospital complications. Of the 173 patients undergoing IHRS included in this assessment (mean age: 75.5±7.4 years, [60-93 years], 54.9% male), 42.8% experienced hospital complications. Multivariate regression analysis revealed cognitive impairment and low SA levels were significantly associated with this outcome (p<0.05), adjusted for age and gender. We demonstrated MCI and low SA levels are risk factors of postoperative hospital complications among older patients undergoing elective IHRS. Increased understanding of predictive factors can help enhance prevention efforts, aiding in improving patient experiences and reducing patient and hospital costs.

### NURSING STAFFING IS ASSOCIATED WITH MOBILITY AND FOOD INTAKE IN OLDER HOSPITALIZED PATIENTS

Orly Tonkikh,[1] Efrat Shadmi,[1] and Anna Zisberg,[2] *1. University of Haifa, Haifa, Israel, 2. Faculty of Social Welfare and Health Sciences, University of Haifa, Haifa, Israel, Haifa, Isreal, United States*

Hospitalization processes related to patient mobility and food-intake significantly affect outcomes of older adults. Nurses are the front-line personnel responsible for promoting performance of these functioning-preserving processes. The degree to which nursing skill-mix is related to their performance is unclear. We investigated the association between staffing and hospitalization processes in a cohort of 836 older adults aged 70+ admitted to internal units for non-disabling conditions. Mobility and food-intake were assessed within 2 days of admission using validated questionnaires. Nurse-patient ratios and nursing skill-mix (i.e. registered nurses, nurse aides, and advanced practice nurses) were assessed using administrative and payroll/roster data. Decision-trees were developed for mobility and food-intake applying classification and regression tree analysis. The mobility decision-tree identified four characteristics that subdivided the patients into eight segments (nodes) (pre-admission functioning, sex, malnutrition risk and percent of advanced practice nurses). The food-intake decision-tree identified five characteristics (pre-admission functioning, sex, chronic morbidity, age and percent of nurse aids) that subdivided the patients into ten nodes. Percent of advanced practice nurses and the percent of nurse aids classified low functioning patients: higher percent of advanced practice nurses (>30% vs. ≤30%) was associated with higher probability of walking in corridors (20.7%) versus inside the room (4.3%), and higher percent of nurse aids (>23% vs. ≤23%) was associated with higher probability of eating more than half of the served meals (83.9%) versus others (66.3%). This study shows that staffing levels are associated with better performance of functioning-preserving processes. Future studies should investigate staffing interventions improving functioning-preserving processes.

### OLDER SEPSIS SURVIVORS SUFFER PERSISTENT DISABILITY BURDEN AND POOR LONG-TERM SURVIVAL

Robert Mankowski,[1] Stephen Anton,[2] Gabriela Ghita,[3] Christiaan Leeuwenburgh,[2] Lyle Moldawer,[4] Philip Efron,[4] Scott Brakenridge,[4] and Frederick Moore,[4] *1. University of Florida, Gainesville, Florida, United States, 2. Aging and Geriatric Research, Gainesville, United States, 3. Biostatistics, Gainesville, United States, 4. Surgery, Gainesville, United States*

As in-hospital sepsis mortality has decreased, more "sepsis survivors" are progressing into poorly characterized long-term outcomes. The purpose of this study was to describe the current epidemiology of sepsis in older adults compared to middle-aged and young adults. Design: Prospective longitudinal study with patients categorized into young (≤ 45 years), middle-aged (46-64 years) and older (≥ 65 years) patient groups. 328 sepsis patients were characterized by a) baseline demographics and predisposition factors, b) septic event, c) hospital outcomes and discharge disposition, d) 12-month mortality and e) Zubrod Performance status, physical function and cognitive function at three, six and 12-month follow-up. Follow-up visits were not completed due to death (in 68) and withdrawal of consent (in 32). Compared to young and middle-aged patients, older patients had: 1) significantly more comorbidities at presentation (example chronic renal disease 6% vs 12 % vs 21%), intra-abdominal infections (14% vs 25% vs 37%), septic shock (12% vs 25% vs 36%) and organ dysfunctions, 2) higher 30 day mortality (6% vs 4% vs 17%) and fewer ICU free days (median 25 vs 23 vs 20), 3) more progression into CCI (22%, vs 34% vs 42%) with higher poor disposition discharge to non-home destinations (19% vs 40% vs 62%), 4) worse 12-month mortality (11% vs 14 % vs 33%) and, 5) poorer Zubrod Performance status and objectively-measured physical and cognitive functions with slight improvement over 12 month follow-up. Conclusion: Compared to younger patients, older sepsis survivors suffer with both a higher persistent disability burden and 12-month mortality.

### PERCEPTIONS OF CARE AMONG OLDER ADULTS IDENTIFIED AS FALL RISK DURING A HOSPITAL STAY

BARBARA KING, *University of Wisconsin-Madison, Madison, Wisconsin, United States*

In-hospital falls are a significant clinical, legal and regulatory problem. The Centers for Medicare and Medicaid no longer reimburse hospitals for falls that result in injury, adding increase pressure on acute care settings to prevent falls. Additionally, evidence-based practice recommendations for fall prevention in hospitals do not exist, thus leaving administrators to create their own programs. One common strategy used by hospital providers to prevent falls is to restrict patient mobility. Little information on how older adult patients experience fall prevention during a hospital stay has been published. The purpose of this study was to understand perceptions of care among older adults identified as fall risk during a hospital stay. This qualitative study utilized inductive content analysis. Older adults (N=20) from a large academic medical center in the Midwest were recruited to participate in one-to one in-depth interviews. Open coding, categorization and abstraction was used to analyze the data. Three main categories were identified that summarized the older adult patient perception of hospitalization: Act of Caring, something they received from staff, provide to staff or provided to self; Being Restricted in movement resulting in either accepting or rejecting the restriction and Being Freed at discharge, often being told "just be careful". Older adult identified as fall risk described being restricted in movement during a hospital stay. Many passively accepted this restriction even though they felt a lack of movement would be harmful to them. Additional research on the patient experience with fall prevention is needed.

### PREVALENCE AND PREDICTORS OF MORTALITY FOR OLDER ADULTS REFERRED TO A HOSPITAL AVOIDANCE PROGRAM

Shahzad Gul,[1] Megan Freund,[2] Robert Sanson-Fisher,[3] Matthew Clapham,[4] and penelope Webster,[5] *1. John Hunter Hospital, Hunter New England Area Health Services, Cameron Park , New South Wales, Australia, 2. Health Behaviour Research Collaborative, School of Medicine and Public Health, Faculty of Health and Medicine, University of Newcastle, Callaghan, New South Wales (NSW), Australia, New Lambton Heights, New South Wales, Australia, 3. Health Behaviour Research Collaborative, School of Medicine and Public Health, Faculty of Health and Medicine, University of Newcastle, Newcastle, New South Wales, Australia, 4. Hunter Medical Research Institute, Hunter Medical Research Institute, New South Wales, Australia, 55. . Community Acute Post-Acute Care, Hunter New England Local Health District, NSW, Australia, 5. . Community Acute Post-Acute Care, Hunter New England Local Health District, NSW, Australia, New South Wales, Australia*

A cross sectional retrospective data linkage study of older adults discharged from local hospital avoidance program between January 2017 and January 2018 was undertaken (N=286; mean age 80.5 years). The prevalence of death at 3 months, 6 months, 12 months, 18 months and 33 months was calculated. Patient demographic characteristics associated with participant's risk of mortality at 33 months after discharge was examined using Cox multivariable regression. Patient demographic and health characteristics associated with participant mortality within 12 months of discharge was examined using multivariable logistic regression for patients with complete health characteristic data (n=195). The mortality prevalence was 17% at six months and the cumulative prevalence at one year, 18 months and 33 months post discharge were 24%, 29% and 36% respectively. Patient demographic characteristics associated with participants' risk of mortality at 33 months after discharge were gender, age and household arrangements. Health and demographic characteristics associated with mortality within 12 months of discharge were lower cognition, increased burden of comorbidity, decreased physical function, a weight less than 55 kilograms, older age and male gender. These results indicate that a significant proportion of people attending a hospital avoidance program are likely to be entering into the final year of their life. This suggests that hospital avoidance programs should routinely identify patients who are likely nearing end of life, and support advance care planning for this patient group.

### QUALITATIVE EXPERIENCES OF MULTI-SENSORY ENGAGEMENT FOR ACUTELY ILL PERSONS WITH DEMENTIA

Sheila Molony,[1] and Christine Waszynski,[2] *1. Quinnipiac University, Hamden, Connecticut, United States, 2. Hartford Hospital, Hartford, Connecticut, United States*

Delirium occurs in up to 25% to 50% hospitalized patients and the risk is increased for older adults with dementia. Multi-sensory stimulation has been effective in community and nursing home settings to reduce delirium in persons with dementia. This paper presents qualitative findings from field notes, observations and participant/family interviews. A sample of 25 individuals with dementia and were recruited from 2 medical units and randomized to intervention with two sessions in the multi-sensory "Hub" on 2 hospital days or receive usual care. One investigator recorded field notes prior to and during the intervention (or control). An RN in the Hub documented participant engagement. Audio-taped interviews with participants and family were recorded immediately after Hub visits. All data was transcribed and analyzed by two other team members using NVivo 12.0. Both coders reached consensus after independently coding the first 12 transcripts using open coding and descriptive content analysis. Pre-intervention data revealed common symptoms of delirium in both groups and post-intervention fatigue as a prominent theme. Participants in both groups were able to engage with the researcher and respond to interviews. Hub participants demonstrated high levels of engagement with some surprising positive responses and improvement from pre-intervention behavior. Qualitative findings from this pilot study highlight the abundance of delirium risk factors encountered by hospitalized older adults with dementia and demonstrate positive, engaging MMSE experiences, despite these risks. Further study is needed to identify longer-term impact.

### SURVIVAL AFTER DISCHARGE FROM GERIATRICS VS. INTERNAL MEDICINE WARDS, BY RISK STATUS AND DIAGNOSIS

Antonella Giordano,[1] Giulia Carreras,[1] Mauro Di Bari,[1] Luca Degli Esposti,[2] Paola Michelozzi,[3]

Roberto Bernabei,[4] Niccolò Marchionni,[5] and Daniela Balzi,[6], *1. University of Florence, Florence, Italy, 2. Clicon – Health, Economics & Outcome Research, Ravenna, Italy, 3. Regione Lazio, Roma, Italy, 4. Fondazione Gemelli, Rome, Italy, 5. University of Florence, Firenze, Italy, 6. ASL Toscana Centro, Florence, Italy*

In randomized clinical trials, compared to Internal Medicine (IM), admission to Geriatrics (G) improved clinical outcomes of frail older patients accessing the Emergency Department (ED). Whether this advantage is maintained also in the "real world" is uncertain. We compared long-term survival of patients admitted to G or IM wards after stratification for background risk and across a variety of discharge diagnoses. Data were derived from the "Silver Code National Project (SCNP)", an observational study of 180,079 unselected 75+ years old persons, admitted via the ED to IM (n=169,717, 94.2%) or G (n=10,362) wards in Italy. The Dynamic Silver Code (DSC), based on administrative data, was applied to balance for background risk between participants admitted to G or IM. One-year mortality was 33.7%, it was lower in participants discharged from G than IM (32.1 and 33.8%, respectively; p<0.001), and increased progressively across four DSC risk classes (p<0.001). Admission to G was associated with survival benefit in DSC class II to IV participants, with HR (95% CI) of 0.88 (0.83-0.94), 0.86 (0.80-0.92) and 0.92 (0.86-0.97), respectively. Cerebrovascular diseases, cognitive disorders, and heart failure were the ICD-9 coded diagnoses with the widest survival benefit from admission to G, which was mostly observed in DSC class III. In conclusion, admission to G may provide long-term survival benefit in subjects who, based on the DSC, may be considered at an intermediate risk. Specific clinical conditions should be considered in the ED to improve selection of patients to be targeted for G admission.

### THE RELATIONSHIP BETWEEN RACE AND ETHNICITY IN POST-SURGICAL DISCHARGE DISPOSITION

Caitlyn McFadden,[1] Coco Victoria Tirambulo,[1] David Lieberman,[2] and Mindy Fain,[3] *1. The University of Arizona Center on Aging, Tucson, Arizona, United States, 2. University of Arizona College of Medicine - Geriatrics, General Internal Medicine and Palliative Medicine, Tucson, Arizona, United States, 3. University of Arizona Center on Aging, Tucson, Arizona, United States*

Racial and ethnic disparities have been reported regarding outcomes of intermediate or high-risk surgical (IHRS) procedures. This study aimed to assess whether or not these disparities exist with respect to post-procedural discharge disposition (DD). EMR chart reviews of patients (≥65 years old) undergoing IHRS were conducted, 2016-2019 in Tucson, AZ. Race and ethnicity were reported as American Indian/Alaskan Native; Black or African American; More-Than-One-Race; Native Hawaiian/Pacific Islander; White/Caucasian; Unknown/Not Reported, Hispanic/Latino; Non-Hispanic/Latino; or Unknown/Not Reported. DD was reported as to home, home with home health, rehabilitation center, skilled nursing facility (SNF), death, or other. Kruskal-Wallis tests were performed to determine the association with DD. Of the 161 patients (mean age: 74.7±6.9, [60-91], 53.4% male) assessed, 15.4% were discharged to a facility other than home or home with home health. Ethnicity was significantly associated with discharge disposition (p<0.005). Non-Hispanic/Latinos (66.1%) were more likely to be discharged home compared Hispanic/Latinos (14.8%), while those Unknown/Not Reported were more likely to be discharged to a SNF. Although race was not significantly associated with DD (p<0.062) due to sample size, it is clinically important to consider as 59.9% of White/Caucasians were more likely to be discharged home compared to 1.6% of American Indian/Alaskan Natives; 1.6% Black or African Americans; 5.7 of More-Than-One-Races; and 12.0% Unknown/Not Reported. We demonstrated ethnic disparities affect postoperative DD. Although racial disparities were not significantly associated with DD, anticipating the impact of race and ethnicity to facilitate discharge planning that meets the goals of the older patient is crucial to care.

### THE ROLE OF COMORBID DISEASES IN GEOGRAPHIC DISPARITIES OF MORTALITY FROM SEPTICEMIA AMONG OLDER ADULTS ACROSS THE UNITED STATES

Julia Kravchenko, and Igor Akushevich, *Duke University, Durham, North Carolina, United States*

Sepsis is a prominent cause of death in the US. However, few studies examined its regional variations. Using the Multiple-Cause-of-Death and 5%-Medicare data, we analyzed mortality among the US patients aged 65+ who had septicemia and various comorbid diseases in the states with the highest (HI,FL,AZ,CT,CO, and MN—a.k.a. leading states) and lowest (AR,TN, LA,OK,KY,AL,MS, and WV–a.k.a. lagging states) life expectancy. In 2010-2018, age-adjusted mortality from septicemia was almost twice as high in lagging (85.2±0.5/100,000) comparing to leading (45.2±0.3/100,000) states. Between-the-states difference was the most pronounced at age 65-74 (44.7±0.4 vs. 20.8±0.2) and gradually reduced in older age groups. Higher mortality from septicemia in the lagging US states could be, at least in part, because of poorer health of the beneficiaries who enter Medicare at age 65. Mortality rates were the highest among septicemia patients who had comorbid cancer, ischemic heart disease, influenza/pneumonia, or kidney failure. Mortality rates of patients with co-existing Alzheimer's disease or arterial hypertension were lower, but these comorbidities contributed most to geographic disparities. The role of complications due to surgical and other medical procedures in disparities across the states was minimal. Substantial contributions to geographic disparities in septicemia mortality were detected not only for well-known and highly-lethal comorbidities such as diabetes, heart failure, and kidney diseases, but also for less frequently discussed conditions with lower per se mortality such as disease of intestine, skin/subcutaneous tissue, and cystitis, urethritis, or unspecified infections of urinary tract.

### TRANSLATING RESEARCH FINDINGS INTO AN EVIDENCED-BASED APPROACH TO IMPROVE HOSPITAL PRACTICES FOR MALNUTRITION

Rachel Deer, Janna Lehe, Colleen James, and Erin Hommel, *The University of Texas Medical Branch at Galveston, Galveston, Texas, United States*

Malnutrition is a common problem that often goes unrecognized. In a recent UTMB-OAIC funded pilot, we found 49% of older adults were at risk of malnutrition at hospital admission. Malnutrition is associated with increased length of stay, poorer patient outcomes, and higher risk of mortality. Also, malnutrition severity alters hospital reimbursement rates. In 2018, the UTMB health system recognized the need for institutional guidelines to help identify, diagnose, document, and code mild/moderate/severe malnutrition. At baseline, compared to similar academic medical centers, UTMB ranked in the bottom quartile for malnutrition diagnosis. A multidisciplinary committee formed with physicians, nurses, researchers, dieticians, coding, and information technology. Preliminary data from the pilot study found the Nutritional Risk Screen (NRS) had the best sensitivity, specificity, positive and negative predictive values. The NRS was made more user friendly with scripting/prompts in the electronic medical record (EMR) to improve consistency/compliance among nurses. The Subjective Global Assessment was used in EMR by dieticians to document malnutrition diagnosis. A Best Practice Advisory was created to give physicians the option to easily add malnutrition diagnosis to the problem list. Since “go-live” in February 2019, NRS completion improved from 10.6% to 70.0%. Coding of malnutrition increased from 3.7% to 5.8%. In a 6 month follow-up, 113 patients were found to have direct benefits from the new process, resulting in an estimated financial impact of $945,605. Going forward, we have identified multiple areas of continued education needs to further improve the implementation and uptake of the new screening tool and diagnostic processes.

### SESSION 2880 (POSTER)

### BIOLOGY OF AGING

### ADIPOSITY IS POSITIVELY ASSOCIATED WITH AD-SIGNATURE CORTICAL THICKNESS IN OLDER ADULTS

Tina Brinkley, Samuel Lockhart, Bonnie Sachs, Maryjo Cleveland, Benjamin Williams, James Bateman, Samantha Rogers, and Suzanne Craft, *Wake Forest School of Medicine, Winston-Salem, North Carolina, United States*

Mid-life obesity is associated with a higher risk for Alzheimer’s disease (AD). However, this association is attenuated or even reversed in late-life, when weight loss may be a preclinical sign of AD. While neuropathological changes likely occur alongside aging-related changes in body composition, this has not been largely investigated. We aimed to determine the association between adiposity and a specific pattern of reduced cortical thickness associated with AD risk and progression. Global and regional adiposity (via dual-energy x-ray absorptiometry) and AD-signature cortical thickness (via surface-based cortical analysis of 3T brain MRI scans) were measured in 35 middle-aged and older adults from the Wake Forest Alzheimer’s Disease Clinical Core (mean age: 69.4±7.8 years, 80% female, 91% White, 29% cognitively impaired). Partial correlations adjusted for age, sex, and cognitive status were examined overall and stratified by age (0.59, $p \leq 0.05$). No significant associations were observed in middle-aged adults. These findings suggest that AD-related cortical thinning may be accompanied by a global reduction in body fat among older adults.

### AGE-RELATED CHANGES IN MOLECULAR AND WHOLE MUSCLE FUNCTION: ROLE OF FAT CONTENT?

Joseph Gordon III,[1] Nicholas Remillard,[1] Chad Straight,[1] Rajakumar Nagarajan,[1] Bruce Damon,[2] Stuart Chipkin,[1] Mark Miller,[1] Jane Kent,[1] and Joseph Gordon[1], *1. University of Massachusetts Amherst, Amherst, Massachusetts, United States, 2. Vanderbilt University, Nashville, Tennessee, United States*

Decreases in muscle size and function are a general consequence of old age; the precise mechanisms of these changes remain unclear. Recent studies suggest that fat deposition in muscle may also contribute to dysfunction in older adults. Fat content was quantified in the quadriceps, and its effects on function in healthy young (21-45 y) and older (65-75 y) men and women (n=44) of comparable physical activity were compared. A subset of the young matched with the older group for muscle fat content were also examined. Peak fat-free whole muscle cross-sectional area (mCSA; cm2), volume (MV; cm3), fat content (fat fraction, FF; %), specific torque (Nm/mCSA) and peak contraction velocity (Nm·s-1) were determined using fat-water magnetic resonance imaging and dynamometry (0-300□·s-1). To examine potential molecular mechanisms of muscle weakness, vastus lateralis biopsies were obtained (n=31) and cross-bridge kinetics of type I and II fibers were determined. FF was higher in older adults than young (8.4±1.2% (SE), 7.6±1.4; p=0.03), while mCSA (48.9±10.4 vs. 64.2±17.3), MV (1536±532 vs. 2112±708), specific torque (2.6±0.4 vs. 3.2±0.4), and peak voluntary contraction velocity (422±20 vs. 441±23) were lower in older than young ($p<0.01$). Type II fiber myosin attachment rate was slower and attachment time longer in older muscle ($p<0.017$), providing a potential mechanism for the slowing of peak contraction velocity with age. Notably, differences at the whole muscle and molecular levels remained for the subset of young and older groups matched for FF, suggesting that fat deposition in muscle does not exacerbate age-related changes in function.

### ASSOCIATIONS BETWEEN A NEW BIOMARKER OF ELEVATED CHRONIC INFLAMMATION AND ACCELERATED AGING

Line Rasmussen, Avshalom Caspi, and Terrie Moffitt, *Duke University, Durham, North Carolina, United States*

To further understand and measure the association between chronic inflammation, aging, and age-related diseases, broadly applicable standard biomarkers of systemic chronic inflammation are needed. We tested whether elevated blood levels of the emerging chronic inflammation marker soluble urokinase plasminogen activator receptor (suPAR) were associated with accelerated aging, lower functional capacity, and cognitive decline. We used data from the population-representative longitudinal Dunedin Study (N=875). Plasma suPAR levels were analyzed at ages 38 and 45 years. We performed regression analyses adjusted for sex, smoking, and C-reactive protein. suPAR levels increased from 2.39 ng/mL (SD 0.89) at age 38 to 3.01 (SD 1.03) at age 45 years. Elevated suPAR was associated with accelerated pace of biological aging across multiple organ systems (β 0.28, 95% CI 0.21–0.35), older facial appearance (β 0.16, 95% CI 0.10–0.22), and with structural signs of older brain age (β 0.06, 95% CI -0.00–0.13). Moreover, participants with higher suPAR levels had lower functional capacity (more physical limitations [β 0.24, 95% CI 0.18–0.30]; slower gait speed [β -0.14, 95% CI -0.20; -0.08]) and greater decline in cognitive function (β -0.07, 95% CI -0.13; -0.01) from childhood to adulthood compared to those with lower suPAR levels. Finally, improvements in health habits between age 38 and 45 (smoking cessation or increased physical activity) were associated with less steep increases in suPAR levels over those years. Our findings provide initial support for the utility of suPAR in studying the role of chronic inflammation in accelerated aging and functional decline.

### GENE EXPRESSION PROFILING SUGGESTS DOWNREGULATION OF WNT PATHWAY SIGNALING WITH AGING

Allison Kuipers,[1] Cara Nestlerode,[1] Victor Wheeler,[2] Iva Miljkovic,[1] and Joseph Zmuda,[1], *1. University of Pittsburgh, Pittsburgh, Pennsylvania, United States, 2. Tobago Health Studies Office, Upper Scarborough, Tobago, Trinidad and Tobago*

The wingless (Wnt) pathway is involved in many age-related diseases and conditions, including cancer and cardiovascular disease. However, the impact of aging on Wnt pathway signaling remains largely unknown. Therefore, we surveyed peripheral blood expression of 43 Wnt pathway genes and tested for association with age in 369 Afro-Caribbean men recruited from the population of Tobago (mean age 64 years, range 51-89 years). Gene expression was measured in counts per sample, then normalized and background subtracted. All expression counts were transformed to normality after excluding any extreme outliers. Fourteen Wnt genes showed detectable expression in our sample and were examined further for association with age using linear regression both individually and using stepwise selection models to identify independent signals. A Bonferroni-corrected alpha for the 14 tested genes (α=0.0036) was used. Six of the 14 tested genes showed significant univariate correlation with greater age [r (gene): 0.169 (APC), 0.179 (AXIN1), 0.222 (CTNNB1), 0.211 (GSK3b), -0.178 (LEF1), -0.166 (TCF1)]. When combined, expression of four genes was determined to be marginally (P<0.05) independently associated with age (AXIN1, CDH2, CTNNB1, TCF1). After correction for multiple testing, a 10-year greater age was independently associated with 4% greater CTNNB1 expression and 8% lower TCF1 expression respectively (both P<0.0001), and this association accelerated after age ≥60 years (p-interaction 0.01 and 0.07, respectively). Greater expression of CTNNB1 and lower expression of TCF1 may indicate decreased Wnt pathway signaling. These results are the first to suggest that aging may be associated with alterations in Wnt pathway signaling.

### GENOME-WIDE LINKAGE ANALYSIS IDENTIFIES A NOVEL LOCUS FOR GRIP STRENGTH: THE LONG LIFE FAMILY STUDY

Adam Santanasto,[1] Mary Wojczynski,[2] Ryan Cvejkus,[3] Bharat Thygarajan,[4] Kaare Christensen,[5] and Joseph Zmuda,[3] *1. University of Pittsburgh, PITTSBURGH, Pennsylvania, United States, 2. Washington University School of Medicine, St Louis, Missouri, United States, 3. University of Pittsburgh, Pittsburgh, Pennsylvania, United States, 4. University of Minnesota, Minneapolis, Minnesota, United States, 5. University of Southern Denmark, Odense C, Syddanmark, Denmark*

Grip strength declines with aging, is an indicator of overall health, and predicts mortality among older adults. Herein, we quantified the genetic contributions to grip strength among 4534 individuals, belonging to 574 families in the Long Life Family Study (age 70.3 ± 15.7, range 24–110 years; 56% women). Grip strength was measured using a handheld dynamometer, and the maximum value of two trials in the stronger hand was used. Quantitative trait linkage analysis was completed using pedigree-based maximum-likelihood methods with logarithm of the odds (LOD) scores >3.0 indicating genome-wide significance. Linkage analysis in the top 10% of families contributing to LOD scores was also performed to allow for heterogeneity among families (HLOD). All analyses were adjusted for age, sex, height and field center. Grip strength was lower per one year of older age (β: -0.34 ± 0.01kg, p <0.01), and overall: 24.3% of men and 19.3% of women had "low" grip strength according to European Working Group on Sarcopenia definitions. Grip strength was highly heritable (h2 = 0.37, p<0.05). We identified a potentially novel locus for grip strength on chromosome 18p (LOD 3.18) with 26 families contributing to this linkage peak (HLOD = 10.94). Deep sequencing of the chromosome 18 region may yield fundamental insight on the biology of muscle weakness with aging, and may help identify novel therapeutic targets for treatment and prevention of this common condition.

### INTERACTIONS BETWEEN GENES FROM AGING PATHWAYS SIGNIFICANTLY INFLUENCE RISK OF ALZHEIMER'S DISEASE

Svetlana Ukraintseva,[1] Konstantin Arbeev,[1] Hongzhe Duan,[1] Igor Akushevich,[1] Mary Feitosa,[2] Kaare Christensen,[3] Eric Stallard,[4] and Anatoliy Yashin,[1] *1. Duke University, Durham, North Carolina, United States, 2. Washington University School of Medicine, St Louis, Missouri, United States, 3. University of Southern Denmark, Odense C, Syddanmark, Denmark, 4. Duke University, Biodemography of Aging Research Unit, Durham, North Carolina, United States*

Age is major risk factor for AD; however, relationships between aging and AD are not well understood. Decline in physiological resilience is universal feature of human aging that may also play role in AD. Aging-related pathways (such as IGF-I/P53/mTOR-mediated) that are involved in tissue resilience work in concert to decide outcomes of cell responses to stress/damage, such as survival, apoptosis, autophagy, etc. We hypothesized that interplay among genes in these pathways may influence AD risk as result of epistasis (GxG). We estimated effects of pairwise epistasis between SNPs in 53 genes from respective pathways on AD risk in the LLFS compared with other data (HRS, CHS, LOADFS). We found significant (fdr<0.05) GxG effects on AD risk in older adults across datasets. The SNP rs11765954 in CDK6 gene was involved in top GxG effects on AD in all datasets, when paired with SNPs in BCL2 and PPARGC1A. The CDK6 role in AD could be pleiotropic, depending on its activity in neurons: CDK6 expression is needed for DNA repair and neuronal survival; however, CDK6 overexpression may lead to the cell cycle reentry in postmitotic neurons resulting in apoptosis, which may contribute to neurodegeneration. CDK6 was earlier found to interfere with BCL2 effects on apoptosis, and with PPARGC1A effects on energy metabolism, which might contribute to observed GxG between these genes. We conclude that interactions among genes from biologically connected aging pathways may significantly influence AD risk. Uncovering such GxG effects has a potential to yield new genetic targets for AD prevention/treatment.

### INTERGENERATIONAL TRAUMA TRANSMISSION? TEST OF CELLULAR AGING IN MOTHERS EXPOSED TO SEXUAL ABUSE AND THEIR CHILDREN

Laura Etzel,[1] Waylon Hastings,[2] Brooke Mattern,[2] Christine Heim,[3] Jennie Noll,[2] Frank Putnam,[4] Monica Oxford,[5] and Idan Shalev,[2] *1. Penn State University, University Park, Pennsylvania, United States, 2. Pennsylvania State University, University Park, Pennsylvania, United States, 3. Charite Universitatsmedizin Berlin, Berlin, Germany, 4. University of North Carolina, Chapel Hill, North Carolina, United States, 5. University of Washington, Seattle, Washington, United States*

Exposure to maltreatment during childhood can lead to increased risk for poor health outcomes in adulthood. Child maltreatment and later poor health may be linked by premature biological aging. We tested whether childhood sexual abuse (CSA) is associated with telomere length (TL) in adult females. We further tested the hypothesis of intergenerational transmission of trauma by measuring TL in both CSA-exposed and non-exposed mothers and their children. TL was measured in a subset of participants and their children from a prospective-longitudinal cohort study of sexually abused females and a demographically matched comparison group. Linear regression models were used to test for associations between CSA-exposure and age-adjusted TL in females (N=108, mean age 36.3 years). Multilevel linear models were used to test the intergenerational effect of maternal-CSA exposure on age-adjusted TL in their children (N=124 children mean age 10.5 years across 61 mothers). CSA-exposure was not associated with TL in females. Replicating previous work in this area, maternal TL and sex were significant predictors of child TL in all models tested. Longer maternal TL predicted longer TL in children, and female children had longer TL than male children. Maternal-CSA exposure did not predict TL in children. This finding is in line with some previous results on CSA and TL measured in adulthood. Previous significant results associating child maltreatment with shorter TL in adulthood may be capturing a population of individuals exposed to either multiple types of maltreatment or maltreatment in childhood with concurrent TL measurements.

### METRICS OF PHENOTYPIC AGING FROM THE ENERGETICS PERSPECTIVE

Pei-Lun Kuo,[1] Michelle Shardell,[2] Jennifer Schrack,[3] Morgan Levine,[4] Eleanor Simonsick,[1] and Luigi Ferrucci[1] *1. National Institute on Aging, Bethesda, Maryland, United States, 2. University of Maryland School of Medicine, Baltimore, Maryland, United States, 3. Johns Hopkins Bloomberg School of Public Health, Baltimore, Maryland, United States, 4. Yale University School of Medicine, New Haven, Connecticut, United States*

Identifying the most critical metrics of aging is an ongoing challenge due to a lack of comprehensive measurements and heterogeneity of the aging process. Using the Baltimore Longitudinal Study of Aging, we developed a conceptual framework to identify metrics of aging that capture the hierarchical and temporal relationships between functional aging, phenotypic aging, and biological aging based on four hypothesized domains: energy regulation, body composition, homeostatic mechanisms, and neurodegeneration. Focusing on the energetics domain, we examined trajectories of eight phenotypes using more than 10 years of longitudinal data. The standardized Cronbach's alpha for these variables was 0.80, providing construct validity of our concept. We further implemented item response theory to integrate these phenotypes into a summarized energy score. Linear mixed models were used to assess the cross-sectional and longitudinal associations between the summarized energy score and physical functioning as measured by gait speed and time to walk 400m as quickly as possible (number of participants ~ 811, number of observations ~ 1700). After adjusting for age, sex, weight, and height, a higher summarized energy score was independently associated with faster baseline gait speed (0.13 m/s, p<0.001 ) and faster 400m time (-35.3 seconds, p<0.001), and longitudinally associated with slower gait speed decline (0.08 m/s/decade, p<0.001) and slower 400m time increase (-37.8 secs/decade, p<0.001). This work demonstrates the utility of our energetics domain-based summarized score. Moving forward, it will be important to clarify relationships between this summarized score and other functional metrics and assess its generalizability to the other cohorts.

### NEUTROPHIL-LYMPHOCYTE RATIO AND MORTALITY IN THE LONG LIFE FAMILY STUDY

Sithara Vivek,[1] Bharat Thygarajan,[1] Joanne M. Murabito,[2] Nicole Schupf,[3] Joseph Zmuda,[4] Jonas Mengel-From,[5] and Mary Wojczynski,[6] *1. University of Minnesota, Minneapolis, Minnesota, United States, 2. Boston Medical*

*Center, Boston, Massachusetts, United States, 3. Columbia University, New York, New York, United States, 4. University of Pittsburgh, Pittsburgh, Pennsylvania, United States, 5. University of Southern Denmark, Odense C, Denmark, 6. Washington University School of Medicine, St Louis, Missouri, United States*

Neutrophil to Lymphocyte Ratio (NLR) reflects the balance between the innate (neutrophils) and adaptive (lymphocytes) immunity. Though NLR is a strong predictor of mortality in the general population, the distribution of NLR and its association with mortality has not been evaluated in families with exceptional longevity. Hence, we evaluated this question in the Long Life Family Study, a family based study of exceptional longevity. We used data from offspring of long lived (n=2065) family members and spousal controls (n=673). We used multivariate linear regression models adjusted for age, family relatedness, sex, field center, BMI and comorbidities (diabetes, CVD, cancer) to evaluate differences in NLR between long lived family members and spousal controls. Cox proportional hazard models were used to examine the association between NLR and mortality. 157 (7.6%) offspring in long lived families and 68 (10.1%) spousal controls were deceased during 12 years of follow up. NLR was similar among offspring in long lived families and spousal controls (1.96±1.06 vs.1.98±1.28; p=0.64). There was a significant positive association between NLR and overall mortality [HR: 1.3, 95% CI (1.01, 1.67)), p:0.04]. There was no statistically significant difference in this association among offspring in long lived families and spousal controls (p for interaction =0.16). The association between NLR and overall mortality was no longer significant [HR: 1.24; p:0.36] after adjustment for IL-6 and hsCRP. These results suggest that NLR may be a predictor of mortality in families with exceptional longevity though this association may not be independent of other inflammatory biomarkers.

### RACIAL DIFFERENCES IN GLUCOSE HANDLING AND FUNCTION WITH OBESITY REDUCTION: PRELIMINARY FINDINGS

Michael Borack,[1] Marshall Miller,[2] Jamie Rincker,[1] Shelley McDonald,[1] Kathyrn Starr,[3] and Connie Bales,[3]
*1. Duke University, Durham, North Carolina, United States, 2. Duke University, Efland, North Carolina, United States, 3. Duke University School of Medicine, Durham, North Carolina, United States*

Blacks have higher rates of obesity and are twice as likely to develop diabetes as non-Hispanic whites. Obesity reduction can improve metabolic health, but physical function and glucose handling may be threatened by concomitant loss of muscle mass. These preliminary findings from a 4-mo. randomized controlled trial assess the racial differences in glucose handling and physical function in obese, older adults with prediabetes (Fasting Plasma Glucose (FPG) ≥95<126 mg/dL or HbA1c 5.7-6.4%) following obesity reduction. At 4 mo. endpoint, participants (n = 31; age = 68.1±5.4 years, BMI =36.0±4.7 kg/m2) had reduced (p<0.05) body weight in both Blacks (5.1%) and Whites (4.1%); HbA1c levels were also reduced (Blacks = -0.3 ±0.3; Whites = -0.1±0.3) with no difference by race. However, FPG was reduced for Blacks compared to Whites (-7.9±9.5 vs. -2.8±6.2 mg/dL; p<0.05). Short Physical Performance Battery (SPPB) score was lower for Blacks than Whites at both baseline (9.8±1.5 vs 10.9±1.2; p<0.05) and 4 mo. (10.17±1.4 vs 11.21±1.3; p<0.05), respectively. A trend towards improvement (p=0.08) in meters walked in 6 minutes was present in both Blacks (13.3±60.8) and Whites (20.0±36.3) with no between-group difference. Interestingly, at baseline, 41% of participants said they modified their behaviors due to a fear of falling despite having a mean SPPB score of 10.3±1.5. Following the intervention, fear of falling was reduced, with 35% of the participants reporting this behavior. Our findings illustrate that modest weight loss improves glucose handling, physical function and perceived fall risk for both Black and White older adults with prediabetes.

### SARC-F CAPACITY TO TRACKING SARCOPENIA IN WOMEN FROM MANAUS-AM

MYRIAN FABER,[1] and Fatima Baptista,[2] *1. University of the State of Amazonas, MANAUS, Amazonas, Brazil, 2. Faculty of Human Motricity, University of Lisbon, Faculty of Human Motricity, University of Lisbon, Lisboa, Portugal*

This study aimed to analyze the performance of SARC-F for the identification of sarcopenia in women from Manaus. For the analysis of the performance of the SARC-F, the criteria defined by the EWGSOP (2018) and the SDOC (2019) were considered. The sample consisted of 236 women aged 66.27±5.76 years. In addition to the SARC-F, a dynamometer was used to assess the muscle strength and gait speed was determined on a 2.44 m course. To identify sarcopenia, the following cut-off valueswere used: SARC-F ≥ 4 points; grip strength: EWGSOP <27kg for men and 16kg for women, SDOC <35.5kg for men and <20.0 kg for women; grip over body mass index (BMI, kg/m2) <1.05 for men and 0.79 for women; grip over body weight (BW, kg) <0.45 for men and <0.34 for women; gait speed ≤ 0.8 m/s for both men and women. The results revealed a prevalence of sarcopenia in 54% of the sample. The Kappa statistic intended to analyze the agreement between the SARC-F and the grip strength, the grip strength corrected for BMI or for BM, and the gait speed. The cross-classification analysis showed linear weighted Kappa coefficients near 0 with exception of gait speed (0.264±0.054, agreement in 61.2% of the participants) and grip strength when the cut off is 20 kg (0.248±0.062 - agreement in 63% of the participants). Cross-classification analysis between SARC-F and objective measures of physical capacity (grip strength and gait speed) showed linear weighted Kappa coefficients with slight or fair agreement in women from Manaus.

### THE ASSOCIATION OF FREEZER STORAGE TIME WITH VITAMIN K AND VITAMIN D CONCENTRATIONS IN HUMAN BRAIN TISSUE

Kyla Shea,[1] Xueyan Fu,[1] Gregory Dolinkowski,[1] Bess Dawson-Hughes,[1] Thomas Holland,[2] Klodian Dhana,[2] and Sarah Booth,[1] *1. Tufts University, Boston, Massachusetts, United States, 2. Rush University, Chicago, Illinois, United States*

Vitamins K and D are present in the human brain and have been implicated in Alzheimer's disease and related dementias

(ADRD). Because the use of banked brain tissue in ADRD research is increasing, we evaluated the stability of vitamin K and vitamin D in human brain tissue over long-term freezer storage using samples obtained from the Rush Memory and Aging Project (n=500, mean age=91, 29% male). Specimens were stored at -80□C until analyzed. Vitamin K (menaquinone-4, MK4) and vitamin D (25(OH)D) were measured in four regions (mid-temporal and mid-frontal cortexes, cerebellum, anterior watershed white matter) and averaged across regions. Storage time was categorized into two-year increments. Differences in MK4 and 25(OH)D concentrations according to storage time were evaluated using general linear models. MK4 concentrations did not differ in brains stored ≤8 years (geometric mean±SEM MK4 pmol/g: storage ≤2.0 years=1.2±0.1, 2.1-4.0 years=1.2±0.1, 4.1-6.0 years=1.4±0.1, 6.1-8.0 years=1.4±0.2; p≥0.21). MK4 in brains stored >8.0 years (0.8±0.1 pmol/g) was 33% lower than the concentration in brains stored ≤2.0 years (p=0.005). The 25(OH)D concentrations did not differ in brains stored ≤6 years (geometric mean±SEM 25(OH)D pmol/g: storage ≤2.0 years=1.2±0.1, 2.1-4.0 years=1.1±0.1, 4.1-6.0 years=1.2±0.1; p≥0.37). The 25(OH)D concentration in brains stored >6.0 years was 31-37% lower than that in brains stored ≤2.0 years (6.1-8.0 years=0.8±0.06, >8.0 years=0.7±0.04; p<0.001). MK4 and 25(OH)D appeared to be stable in human brain tissue stored at -80oC for up to 8 and 6 years, respectively. Storage time merits consideration when designing and interpreting studies that relate brain nutrient concentrations to ADRD.

### THE FOXO3 LONGEVITY GENOTYPE IS ASSOCIATED WITH BETTER SURVIVAL AFTER ACUTE MYOCARDIAL INFARCTION IN OLDER PATIENTS

Randi Chen,[1] Kazuma Nakagawa,[1] Kamal Masaki,[1] Tim Donlon,[2] Ayako Elliott,[1] Richard Allsopp,[2] D. Craig Willcox,[3] and Bradley Willcox,[4] *1. Kuakini Medical Center, Honolulu, Hawaii, United States, 2. University of Hawaii, Honolulu, Hawaii, United States, 3. Okinawa International University,, Ginowan, Okinawa, Japan, 4. Kuakini Health Systems, Honolulu, Hawaii, United States*

Background: Although numerous studies have been published on prognostic factors after a first acute myocardial infarction (AMI) among middle-aged men, little is known about the prognostic factors and genetic determinants for post-AMI elderly patients. Methods: The Kuakini Honolulu Heart Program (KHHP) is a prospective population-based study of cardiovascular disease (CVD) (defined as coronary heart disease (CHD) and stroke) among men of Japanese ancestry living in Hawaii. We identified 141 first AMI patients from participants of the KHHP exam 4 (1991-93) by ECG and/or cardiac enzymes (onset age: 73-96 years). Men who survived more than one year after AMI were followed for mortality from onset of AMI to December 2018 (all were deceased by 28 years of follow-up). All had FOXO3 genotype information available. Quantile regression was used to compare the median survival times of FOXO3-G allele carriers (longevity genotype) with FOXO3-TT homozygotes (common genotype). Results: Adjusting for age at AMI onset, baseline body mass index (BMI) and glucose levels (at exam 4), median survival times differed between FOXO3-G allele carriers and FOXO3-TT homozygotes by 2.1 years (95% CI=0.24-3.95, p=0.027). Adding other known CHD risk factors (i.e. hypertension, etc.) to the model reduced the difference to 2.03 years (95% CI=0.17-3.89, p=0.033). Furthermore, chi-square testing showed the mortality rate from CVD among FOXO3-G allele carriers was significantly lower than that among the common FOXO3-TT genotype. Conclusions: The data suggest that the FOXO3 longevity genotype reduces the risk of dying from CVD and extends survival time of elderly patients with acute myocardial infarction.

### THE IMPACT OF APOE E4 ALLELE IN THE EFFECTS OF PHYSICAL ACTIVITY INTERVENTIONS ON MAJOR MOBILITY DISABILITY INCIDENCE

Erta Cenko,[1] and Todd Manini,[2] *1. University of Florida, Gainesville, Florida, United States, 2. The University of Florida, Gainesville, Florida, United States*

The apolipoprotein E (ApoE) e4 polymorphism is traditionally linked to increased rates of cognitive impairments and Alzheimer's Disease. Recently, however, studies have connected ApoE e4 to self-reported mobility disability in those without cognitive impairment. We aimed to estimate the extent to which ApoE status is associated with incident major mobility disability (MMD) and whether it modifies the known beneficial effects of physical activity on incident MMD. Data were from 1,372 participants 70+ years old at risk for mobility impairments, enrolled in The LIFE Study. Participants were randomized to a structured physical activity (PA) or a health education program. The primary outcome was MMD, defined as the inability to walk 400 meters and measured every 6 months for an average follow-up period of 2.6 years. We used proportional hazards regression to examine the main effect of ApoE allele status and interaction with intervention group on MMD incidence. Cumulative MMD incident rates were similar in ApoE-e2 (31.46%), ApoE e3 (33.41%), and ApoE-e4 (33.33%) carriers. Compared to the common ApoE-e3, MMD risk was similar in ApoE-e2 (hazard ratio [HR], 0.945 [95%CI, 0.70-1.27], P = 0.71) and ApoE-e4 carriers ([HR], 1.187 [95%CI, 0.95-1.48], P = 0.41). Additionally, ApoE carrier status did not modify the positive effect that a PA intervention had on MMD risk (interaction p-value > 0.20). These results suggest that ApoE carrier status is not associated with incident mobility disability. ApoE carrier status does not impact the effect a structured PA program has on reducing the risk of MMD in older adults.

### SESSION 2881 (POSTER)

### CANCER

### CAUSES OF TIME TRENDS IN PREVALENCE AND MORTALITY OF MAJOR CANCERS AMONG U.S. OLDER ADULTS

Igor Akushevich, Arseniy Yashkin, Julia Kravchenko, and Anatoliy Yashin, *Duke University, Durham, North Carolina, United States*

The time trend of prevalence and mortality of major cancers is the result of three competing processes: changes in the incidence rate, stage-specific survival, and ascertainment at early stages. Partitioning approach allows for evaluating the relative contribution of each of these competing processes to the overall trend. In this report we applied the partitioning methodology developed for the SEER registry data for prostate, colorectal, lung, female breast, bladder, ovarian, stomach, pancreas, kidney, liver cancers and melanoma. The analysis involves the design and estimation of four models for each cancer site: i) incidence rate using the Armitage-Doll model with individual predisposition modeled by the gamma distribution, ii) probability of relative survival after cancer diagnosis using the Weibull model for time after disease onset, iii) frequencies of stage at onset, and iv) mortality in the general population using the Gompertz model. B-splines are used to fit the time patterns of model parameters obtained for each year Relative contributions of the partitioning components were evaluated for individual cancers (e.g., increase of prevalence in prostate cancer in 2000 was due to increased incidence (59%), improved survival (29%), and improve stage ascertainment (12%)) and compared among all considered cancers. The results were discussed in the light of the effect of the accumulation of survivors occurring in early years (due to improving survival) and their higher mortality (because of higher prevalence of survivors) in later years (i.e., mortality is transferred to latter time periods due to overall improvements in survival).

### COGNITIVE RESERVE, INCIDENT CANCER, AND RATE OF MEMORY DECLINE IN LATER LIFE

Monica Ospina Romero,[1] Willa Brenowitz,[2] Eleanor Hayes-Larson,[3] Sarah F. Ackley,[1] Elizabeth R. Mayeda,[3] M. Maria Glymour,[1] and Lindsay Kobayashi,[4] *1. University of California San Francisco, San Francisco, California, United States, 2. University of California, San Francisco, San Francisco, California, United States, 3. University of California Los Angeles, Los Angeles, California, United States, 4. University of Michigan, Ann Arbor, Michigan, United States*

Cognitive reserve (cognitive skills and abilities acquired before onset of brain pathology) helps maintain cognitive function during aging. Cognitive decline after cancer treatment, known as "chemobrain," is a prevalent outcome among older cancer survivors. It is unknown whether cognitive reserve buffers against acute neuropathological events such as cancer-related cognitive decline. We examined acute and long-term rate of memory decline associated with incident cancer diagnosis by education levels as proxy for cognitive reserve (low: <12 years; intermediate: 12 to <16 years; high: ≥16 years) in 14,449 adults aged 50+ in the US Health and Retirement Study from 1998-2016. Memory (z-scored) was assessed biennially as immediate and delayed word recall combined with proxy assessments. We used adjusted linear mixed models to determine long-term rates of memory decline before and after cancer diagnosis, and acute memory decline immediately after diagnosis (3,248 incident cases), and compared them with corresponding memory trajectories in cancer-free participants. Acute memory decline immediately after diagnosis was larger in those with low (-0.098 SD units, 95% CI: -0.150, -0.045) versus high (-0.038 SD units, 95% CI: -0.084, -0.008) education. Long-term memory decline after cancer was faster in those with low (-1.16 SD units/decade, 95% CI: -1.25, -1.07) versus high (-0.89 SD units/decade, 95% CI: -0.96, -0.82) education. Consistent with previous research showing an inverse cancer-dementia relationship, individuals with cancer had more favorable memory trajectories than cancer-free individuals with similar age and education. Among those with cancer, lower cognitive reserve was associated with greater acute and long-term memory decline after diagnosis.

### COMPARE BREAST CANCER SCREENING, DIAGNOSIS, AND TREATMENT BETWEEN MEDICARE PATIENTS WITH AND WITHOUT ADRD

Xingran Weng,[1] Chan Shen,[1] Monali Vasekar,[1] Sachin Gupta,[2] and Li Wang,[1] *1. Penn State College of Medicine, Hershey, Pennsylvania, United States, 2. Tower Health Medical Group, Wyomissing, Pennsylvania, United States*

Background: Incidence of both breast cancer and Alzheimer's disease and related dementias (ADRD) increases with advancing age. Little research has delineated breast cancer screening, diagnosis, and treatment among women with ADRD. Method: Surveillance, Epidemiology, and End Results (SEER)-Medicare linked data were used. Female breast cancer patients diagnosed between 2005-2015 were identified. Chi-square tests were conducted to compare the characteristics of two groups with and without ADRD. Multiple logistic regression models were estimated to explain the diagnosis and treatment differences. Results: A total of 44,112 female Medicare beneficiaries age 65 or older were identified. Patients with ADRD (17.5%) were less likely to receive breast cancer screening (42.8% vs. 46.6% for all data years combined, p<0.0001), more likely to be diagnosed with breast cancer after death by autopsy or death certificate (8.1% vs 2.0%, p<0.0001). Among those who are diagnosed before death, patients with ADRD were more likely to be diagnosed with breast cancer at age 75 and older (84.8% vs. 15.2%, p<0.0001). After adjusting for age, race, poverty level, marital status, cancer stage at diagnosis, cancer screening history, wellness visit history, comorbidity, and rural/urban residence, logistic regressions suggest that patients with ADRD were less likely to receive surgery (AOR=0.48, 95%CI: 0.45-0.52), radiation (AOR=0.41, 95%CI: 0.39-0.44), or chemotherapy (AOR=0.38, 95%CI: 0.35-0.41). Conclusion: Breast cancer screening was less utilized and breast cancer was diagnosed at an older age in patients with ADRD than those without. Treatments (surgery, radiation, and chemotherapy) were given less frequently to patients with ADRD.

### DEPRESSION MODERATES THE EFFECT OF PHYSICAL FUNCTIONING OVER TIME IN CANCER SURVIVORS

Patricia Bamonti,[1] Aanand Naik,[2] Jonathan Bean,[3] and Jennifer Moye,[4] *1. VA Boston Healthcare System, Brockton, Massachusetts, United States, 2. Center for Innovations in Quality, Effectiveness, and Safety (iQuest), Houston, Texas, United States, 3. Harvard Medical School, Boston,*

*Massachusetts, United States, 4. VA Boston Healthcare, Boston, Massachusetts, United States*

Cancer survivors are at-risk for physical functioning (PF) decrements particularly among older adults. However, few studies have examined moderators of PF over time which may guide rehabilitation interventions. This study examined the moderating effect of depressive symptoms on the association between PF at 6 months following cancer diagnosis and PF 18 months after diagnosis controlling for age, education, and treatment status (whether still in treatment). We hypothesized that the association between PF (T1) and PF (T3) would be attenuated by higher depression scores 12 months after diagnosis (T2). Participants (N = 170; Mage 65.3 +/- 9.17, 98.2% male; 81.2% White) with head and neck, esophageal, gastric, or colorectal cancers were recruited from tumor registries at two VAMCs. Self-report measures included demographics, treatment status, depression symptoms (PHQ-9), and physical functioning (PROMIS) were collected. Performance-based measure of PF (SPPB) was administered. Depression symptoms at Time 2 moderated the relation between performance-based PF (SBBP: $\Delta R2 = .07$, $F(6, 103) = 10.1$, $p < .001$) but not self-reported PF, PROMIS: $\Delta R2 = .001$, $F(6, 110) = 15.3$, $p =.733$. The turning point of non-significance to significance of SBPP T1-T3 was a PHQ-9 score of 7.2. In the absence of depression, the best predictor of future functioning is prior functioning. For those with PHQ-9 scores > 7.2 (28% of the sample), prior functioning does not predict future functioning. Depression should be measured closely with performance-based measures of PF, including gait speed, to improve prediction of future functioning and guide personalized interventions.

### DIETARY ACID LOAD, PAST SMOKING INTENSITY AND MORTALITY AMONG BREAST CANCER SURVIVORS

Tianying Wu,[1] Fang-Chi Hsu,[2] and John Pierce,[3] *1. San Diego State University, SAN DIEGO, California, United States, 2. School of Medicine, wakeforest, North Carolina, United States, 3. University of California at San Diego, San Diego, California, United States*

Cancer survivors are at accelerated risk of aging and more susceptible to unhealthy diets and lifestyles than people without cancers. However, current dietary guidelines for cancer survivors not quite different from that for general healthy population. Further, these guidelines are not specific for cancer survivors who are past smokers. Acid-producing diet can accelerate aging and stimulate cancer development if acid-base balance is not regulated properly. Cancer survivors and past smokers have reduced capacities to adjust acid-base balance. Thus, we conducted prospective cohort analyses among 2950 early-stage breast cancer survivors who enrolled in the Women's Healthy Eating and Living study and provided dietary information through 24-hour recalls at baseline and during follow-up. We assessed dietary acid load using two common dietary acid load scores, potential renal acid load (PRAL) score, and net endogenous acid production (NEAP) score. We assessed past smoking intensity by pack-years of smoking. After an average of 7.3 years of follow-up, there were 295 total death, and 249 breast cancer-specific death. Increased PRAL and NEAP scores were positively associated with total mortality and breast cancer-specific mortality (p for trend <0.1 for PRAL and <0.01 for NEAP). Further, dietary acid load and pack-years of smoking had joint positive associations with mortalities (Comparing the highest to the lowest categories, risk increased by 2.5-3 times; P for trend <0.01 for both PRAL and NEAP). Our study provides valuable evidence for adding dietary acid load to dietary guidelines for breast cancer survivors and developing specific guidelines for past smokers.

### PILOT STUDY OF A SURGICAL-ONCOLOGY GERIATRIC CO-MANAGEMENT PROGRAM

Megha Patel,[1] Stephanie Chow,[2] and Lizette Munoz,[3]
*1. Icahn School of Medicine at Mount Sinai Hospital, New York, New York, United States, 2. Icahn School of Medicine at Mount Sinai, New York, New York, United States, 3. Mount Sinai Hospital, New York, New York, United States*

Purpose: This study aims to evaluate clinical outcomes of a pilot co-management model for patients 65 years and older that were referred by their surgical oncologist for a comprehensive geriatric assessment prior to surgery. Methods: A retrospective chart review was conducted for 9 patients. Patients' pre-operative Charlson Comorbidity Index (CCI) and frailty index were measured. Additional measures included advanced care planning (ACP) documentation and whether patients transferred primary care. Post-operative courses and complications were followed, including length of stay (LOS) and discharge outcomes. Results: A total of 9 patient charts were reviewed. The average age was 79 years. The average CCI and frailty indices were 9 and 4, respectively. Every patient had ACP during the initial assessment. Five patients had multiple outpatient geriatrician visits. Of the 9 referrals, 7 proceeded with surgery. LOS ranged from 6 – 22 days, with a median and average of 8 and 11 days, respectively. Of those undergoing surgery, 4 had an inpatient geriatrics consult. Complications included 1 mortality, 2 aborted cases and 4 with other complications. Four patients were discharged to previous living situations and 2 to SAR. Two patients had one ED/UC visit and 2 had multiple readmissions. No patients transferred their primary care. Conclusion: This is a small pilot showing a promising collaboration between geriatrics and surgical oncology. It outlines a supportive framework for initial and peri-operative geriatric assessments with favorable experiences for both providers. More studies are necessary to make clinical associations with this co-management model.

### RELIABILITY OF THE CAPACITY TO CONSENT TO TREATMENT INSTRUMENT IN METASTATIC CANCER

Mackenzie Fowler,[1] Dario Marotta,[2] Richard Kennedy,[1] and Kristen Triebel,[1], *1. University of Alabama at Birmingham, Birmingham, Alabama, United States, 2. Student, Birmingham, Alabama, United States*

Metastatic cancer patients undergo numerous treatment strategies with known cognitive side effects. It is unclear how medical decision-making capacity (MDC) is impacted by cognitive deficits in metastatic cancer. This study examines reliability of an objective measure of MDC compared with self-report. Participants with newly diagnosed metastasis to the brain and other sites were enrolled at the University of Alabama at Birmingham over seven years. At the study visit, participants completed a comprehensive neuropsychological battery including memory and executive functioning, and the Capacity to Consent to Treatment Instrument (CCTI) assessing medico-legal domains of MDC. The CCTI is a reliable and valid instrument containing two vignettes of hypothetical medical situations assessing four standards of consent. We examined reliability between the CCTI and self-reported MDC using Gwet's AC1 statistic. Participants with brain metastasis with impairment on the CCTI demonstrated significantly lower executive functioning and memory skills than those without impairment (Trails B Raw: 158.20±86.82 vs. 118.90±77.98 p: 0.0248; Digit-Span Raw Forward: 9.13±2.30 vs. 10.12±2.03 p: 0.0254). There were no significant differences between intact and impaired participants with other metastases. Low reliability was observed between self-report and all medico-legal standards on the CCTI across both metastatic groups [Gwet's AC1 Appreciation: 0.70 (0.58, 0.81); Reasoning: 0.34 (0.16, 0.52); Understanding: 0.44, (0.27, 0.60)]. Self-rating of MDC is unreliable in metastatic cancer patients. Patients with metastases (particularly brain metastases) may lack awareness of deficits in their MDC, so providers must affirm proper autonomy in their decisions.

### SPATIAL ANALYSIS OF PROSTATE CANCER IN TEXAS COUNTIES

Ganesh Baniya, and Joseph Oppong, *University of North Texas, Denton, Texas, United States*

Prostate cancer (PCa) is the most common cancer in American men, with estimated 191,930 new cases and about 33,330 deaths for 2020 (American Cancer Society (ACS), 2020). In Texas, there were 10,660 new cases and 1,900 deaths from prostate cancer in 2019 and only about 53.6% of men had ever talked to a healthcare professional about the advantages of the PSA test (Texas Department of State Health Services, 2018). The incidence of PCa is about 60% higher in blacks than in whites for unknown reasons (ACS, 2019), and once cancer has reached to its latent state, other comorbidities such as low sexual satisfaction, urinary incontinence and obstruction, and bowel dysfunction can decrease the quality of life even after surgery. A four-fold, worldwide increase in males above 65 years is expected by 2050 (Ferlay et al., 2010). Thus, with an increasing elderly population, the percentage of men needing new diagnoses and effective treatment will also rise. Previous studies have identified increasing age, African ancestry, a family history of the disease, and certain inherited genetic conditions as significant risk factors for prostate cancer (ACS, 2019). This study examined the geography and change at county-level PCa mortality rates in Texas from 1999-2009 using Texas vital web dataset in relation to smoking, race/ethnicity, obesity, alcohol consumption, and insurance coverage. Results showed that PCa is concentrated mostly in the western, eastern and central parts of TX and is positively correlated with smoking, obesity, PCP per 100,000 and negatively related to alcohol consumption and percent uninsured.

### THE MODERATING ROLE OF AGE ON COGNITION AMONG OLD CANCER SURVIVORS: COMPARISON WITH OLDER ADULTS WITHOUT CANCER

Kun Wang,[1] Hee Yun Lee,[1] and Jessica Neese,[2]
*1. University of Alabama, Tuscaloosa, Alabama, United States, 2. MSW, Tuscaloosa, Alabama, United States*

Considering that 64% of cancer survivors are older adults, it is plausible that the overlap of cancer and old age may worsen older cancer survivors' cognitive functioning. The current study aimed to test the effect of cancer history on cognition in comparison with older adults without cancer history and examine how age groups moderated the association between cancer and cognition. A subsample of 9197 participants drawn from the Health and Retirement Study (HRS) Wave13 were included in this cross-sectional study. Total word recall (score range: 0-20), mental status (0-15), and total cognition (0-35) were the three continuous dependent variables. Multilinear regressions were conducted with and without the interaction term (cancer history * age group). Findings showed cancer survivors tended to be older, Non-Hispanic white males, and higher educational attainment. Cancer history (B= .14, $p < .05$) was significant only for mental status. Cancer survivors had higher mental status scores than older adults without cancer history. The moderation effects of age groups on total word recall ($p < .05$), mental status ($p < .01$) and total cognition ($p < .01$) were significant only in the 85+ group. After adjusting for other variables, cancer survivors in the 85+ age group had significantly higher mental status and total cognition scores than their non-cancer counterparts. Posttraumatic growth can potentially explain the surprising result that older cancer survivors had higher cognition scores. Another possible explanation is that high cognitive functioning lengthened survival, and not cancer increased cognition. More experimental studies are needed to explore the current study's findings further.

### SESSION 2882 (POSTER)

### CAREGIVING

### A COMMUNITY RESOURCE REFERRAL INTERVENTION FOR INFORMAL CAREGIVERS OF PEOPLE WITH DEMENTIA

Kelly Boyd,[1] Kate Doyle,[2] Soo Borson,[3] Elbert Huang,[2] Stacy Lindau,[4] Aviva Nathan,[5] Katherine Thompson,[6] and Jennifer Makelarski,[2] *1. The University of Chicago, Chicago, Illinois, United States, 2. University of Chicago, Chicago, Illinois, United States, 3. University of Washing, Seattle, Washington, United States, 4. University of Chicago, Chicago, United States, 5. University of Chicago, Chicag, Illinois, United States, 6. University of Chicago Medicine, Chicago, Illinois, United States*

The National Alzheimer's Project Act (PL-111-375) calls for connecting dementia caregivers to community resources, but few caregiver interventions have focused on this component of support. CRx-D is a theory-driven, scalable intervention that addresses common caregiver needs through: (1) education to normalize needs, (2) coaching on resource activation and (3) tools to promote access, including a printed list of nearby resources ("HealtheRx-D"), an online resource finder, proactive and ongoing text message support. We pretested the intervention arm of CRx-D among 10 caregivers of PWD to assess appropriateness, feasibility and fidelity. Patients presenting for care at 3 outpatient clinics were screened for eligibility, enrolled and given the intervention (12/19-01/20). Of 1,038 patients approached, 12 were caregivers of PWD; 10/12 met all eligibility criteria. All 10 eligible caregivers were enrolled in the study. Caregivers were, on average, 49 years old (range, 29-65), primarily female (n=8), Black/African American (n=9), and half were children of PWD (n=5). At baseline, several caregivers indicated unmet caregiving or health needs (caregiving education [n=7], free/affordable meals [n=5] and financial assistance [n=5]) indicating the intervention's appropriateness. Intervention components were delivered as intended to all caregivers, although one caregiver provided a landline so could not receive texts. A scalable resource referral intervention for caregivers is appropriate. Delivery is feasible with high fidelity. Identifying caregivers at the point of their own healthcare requires high volume screening. These findings will inform a randomized controlled trial of 344 caregivers of PWD to evaluate the impact of CRx-D on caregiver self-efficacy and satisfaction with care.

### CHALLENGES OF CAREGIVERS OF PATIENTS WITH ALZHEIMER'S DISEASE

Zara Sajwani, *The University of Texas at Arlington, Allen, Texas, United States*

The purpose of this presentation is to identify burden and problems experienced by caregivers of patients with Alzheimer's Disease (AD). AD results in gradual deterioration of cognition, language, and memory that can impact an individual's ability to independently perform daily functional activities (CDC, 2019). The role of caregivers is significant in providing assistance to the patients with chronic AD which can be a source of strain for caregiver population. About 16.3 million informal AD caregivers have spent 18.5 billion hours, which is equal to value of $234 billion, to assist patients with other dementia types and AD in 2018 (Alzheimer's Association, 2019). In-depth literature synthesis was carried out using multiple databases. Recent and relevant articles were selected to be added in the review. Due to responsibility of constant vigilance of AD patients, the caregivers may overlook their self-care needs and detach themselves from social life. Literature analysis revealed common challenges and needs of AD care partners including limited social engagement, concerns of sexuality, and sleep problems. Understanding caregiver problems will help nurses and other health care professionals to support families by planning preventive measures. Resources can be invested to improve physical and mental well-being of caregivers. Researches can be planned to bridge the knowledge gap identified through literature review on this topic. References Alzheimer's Association. (2019). Alzheimer's disease caregivers. http://act.alz.org/site/DocServer/caregivers_fact_sheet.pdf?docID=3022 Centers for Disease Control and Prevention. (2019). Alzheimer's disease and healthy aging. https://www.cdc.gov/aging/aginginfo/alzheimers.htm

### DEMENTIA CAREGIVER SATISFACTION AND UTILIZATION OF A TELEHEALTH APP: RESULTS FROM THE FAMTECHCARE TRIAL

Kristine Williams,[1] Clarissa Shaw,[2] Yelena Perkhounkova,[2] Maria Hein,[2] and Carissa Coleman,[3] *1. University of Kansas, Lawrence, Kansas, United States, 2. University of Iowa, Iowa City, Iowa, United States, 3. University of Kansas, Kansas City, Kansas, United States*

Technology can enhance support for families caring for persons with dementia but must be acceptable to be adopted. In the FamTechCare trial, caregivers used an app to videorecord care encounters that were reviewed by an expert panel who provided tailored feedback. The intervention reduced caregiver depression and improved caregiver competence. This mixed methods study reports caregiver satisfaction and utilization of the intervention and evaluation of the intervention by the expert panel. A convergent parallel mixed methods design was used to evaluate the satisfaction, usability, and feasibility of the intervention. Caregiver-person with dementia dyads were randomized to the FamTechCare video support or telephone attention control support groups. Caregivers completed a satisfaction survey at the completion of the 3-month trial. The number and duration of videos submitted and calls received by caregivers were used to evaluate utilization. Relationships between participant characteristics and satisfaction and utilization were evaluated. Feasibility and future directions for the intervention were assessed through interviews with the expert panel. The majority of caregivers in both groups reported benefits from participation. More FamTechCare caregivers found the interventionist support to be helpful ($p=.001$) and effective ($p=.020$) compared to attention control caregivers. FamTechCare caregivers of persons with more severe dementia were more likely to report that video recording intruded on their privacy ($p=.050$). Age, gender, education, dyad relationship, rural status, and type and severity of dementia were not associated with ratings of acceptability, ease of use, or intervention utilization. The expert panel rated FamTechCare as useful and identified adaptations to enhance feasibility.

### EFFECT OF FAMILY CAREGIVER NEED FOR TRAINING ON MEDICARE HOME HEALTH CARE

Julia Burgdorf, Elizabeth Stuart, and Jennifer Wolff, *Johns Hopkins Bloomberg School of Public Health, Baltimore, Maryland, United States*

Medicare home health providers are required to offer family caregiver training; however, there is little information regarding the impact of family caregiver training on home health care intensity. A better understanding of this relationship is necessary to inform development and prioritization of caregiver training interventions in this setting. This research assesses whether and how family caregiver need for training affects care intensity during Medicare home health. We examine 1,217 (weighted n=5,870,905) fee-for-service Medicare beneficiaries who participated in the National Health and Aging Trends Study (NHATS) between 2011-2015 and received Medicare-funded home health care within one year of survey. Using propensity score adjusted, multivariable logistic and negative binomial regression, we model the relationship between family caregiver need for activity-specific training and the number/type of visits received during Medicare home health. We found that older adults whose family caregiver required training on self-care tasks had greater odds of receiving any therapy visits (aOR: 1.70; 95% CI: 1.01, 2.86), aide visits (aOR: 2.12; 95% CI: 1.11, 4.05), or training visits (aOR: 1.49; 95% CI:1.01, 2.21). Older adults whose family caregiver required training on medication management had greater odds of receiving any nursing visits (aOR: 3.03; 95% CI: 1.06, 8.68) and incurred 1.06 (95% CI: 0.11, 2.01) additional nursing visits. Findings support the importance of connecting family caregivers to training resources. Additionally, findings suggest that home health providers should consider prioritizing training interventions which focus on caregiving activities most closely tied to resource utilization: self-care and medication management.

### EXPLORING FORMAL CAREGIVER BURDEN WITHIN NURSING HOMES: AN INTEGRATIVE REVIEW

Rachel Kunkle, Claudia Chaperon, and Ann Berger,
*University of Nebraska Medical Center, Omaha, Nebraska, United States*

The purpose of this integrative review was to explore formal caregiver burden in nursing homes among direct care nursing staff. The aim was to gain an understanding of the state of the science of formal caregiver burden, it's measurement, and its effect on resident care. Based on PRISMA guidelines, a systematic search of CINAHL, PubMed, PsycINFO and Embase databases was conducted using terms from the definition of formal caregiver burden, three conceptual models, and the thesaurus feature of each database for years ranging from 1979-2019. Inclusion criteria consisted of peer-reviewed articles in English that focused on the key terms of formal caregiver burden among direct care nursing staff only in nursing home facilities. Out of 925 citations, 20 articles met criteria; 15 quantitative and 5 qualitative studies. Sample sizes ranged from 11-1283, number of facilities from 1-55, and bed size from 31-203. Psychometric measures used in the studies reported lower validity and reliability, a variety of conceptual definitions interchangeably, and primarily studied nursing assistants (n=19). Five studies included nurses as formal caregivers, and one studied only nurses. Variables used to identify burden were racial disparities (n=2), trauma experience (n=1), depression (n=4), distress (n=3), mental health (n=1), stress (n=2), health complaints (n=1), and alienation (n=1). Two studies evaluated the effect on resident care, one finding significant results of in-service training contributing to positive attitudes of formal caregivers toward confused residents. Current limitations in the understanding of formal caregiver burden limit the advancement of research.

### FAMILY CAREGIVERS' SELF-EFFICACY AND ITS RELATIONS TO WELL-BEING: GENDER AND AGE DIFFERENCES

Yu-Ping Chang, Young Seo, and Tania Von Visger,
*University at Buffalo, Buffalo, New York, United States*

Family caregivers of older adults perceive their roles as beneficial as well as burdensome. They also report physical and psychological symptoms affecting their well-being. Research indicates that caregiver self-efficacy leads to positive outcomes such as decreased caregiver burden and stress. However, it is unclear how self-efficacy is related to their well-being, and how this association might differ by age and gender. We aim to explore the relationships between family caregiver self-efficacy and well-being, and determine if age or gender moderates these relationships. We analyzed responses of 2,652 family caregivers (68% female and 32% male; mean age= 61.2) from the National Study of Caregiving (NSOC) III (2017) cross-sectional survey. We used three composite variables measuring self-efficacy: feeling confident, adjusting changes, and recovering quickly. We used five composite variables measuring well-being: pain, sleep problems, positive and negative affects, and depression/anxiety. We conducted a design-based weighted logistic regression analysis to examine the relationships among variables of interest. Results indicated that low self-efficacy was associated with increased pain, sleep problems, positive and negative affects, controlling for caregivers' age, sex, and race/ethnicity. Examination of interactions indicated that the relationship between self-efficacy and pain and the relationship between self-efficacy and depression/anxiety were stronger in female caregivers, whereas age did not serve as a moderator in the relationship between self-efficacy and well-being. Our findings provide insight that can guide intervention development to improve family caregiver self-efficacy and well-being. Furthermore, future research may involve interventions with the consideration of family caregivers' gender.

### GENDER DIFFERENCES IN CAREGIVING OF OLDER ADULTS: A SYSTEMATIC REVIEW OF THE LITERATURE

Michael Bueno,[1] and Jo-Ana Chase,[2] *1. University of Missouri - Columbia, Columbia, Missouri, United States, 2. University of Missouri, Columbia, Columbia, Missouri, United States*

Caregivers of older adults with chronic illness provide essential care that benefits individuals and society. Many factors influence health outcomes related to the caregiving role. The purpose of this review is to explore how caregiver health outcomes may vary by gender. Data sources include CINAHL, Google Scholar, Google and PsychINFO. Eligible studies focused on gender-based differences in psychological and emotional outcomes of primary informal or familial caregivers (ie, spouses, children, grandchildren, nieces, nephews, neighbors, and friends) of an older adult with chronic illness. The Caregiver Identity Theory (CIT) guided this study. Twelve studies were eligible for this review. Consistent with the CIT, negative outcomes are associated with changing role identity throughout the care recipient's disease progression, and these outcomes differ between genders. Women tended to experience higher overall burden, stress, anxiety, shame and role strain; however, some studies found that men may be affected more during the initial transition into the caregiver role. Methodological limitations of primary research in this area included the use of cross-sectional design, poor generalization to US populations since most studies were conducted outside of the US, and disproportionately fewer male participants across studies. Overall, gender differences in health outcomes among caregivers exist, and other variables relating to gender, such as kinship and age, may also be important factors. Findings suggest health professionals should create individualized engagement, communication, and training strategies with considerations for gender differences in design. Future studies including more male caregivers and more diverse participants is warranted.

### HOW HEALTHCARE INTERACTIONS CONTRIBUTE TO BURDEN FOR CARE PARTNERS OF OLDER ADULTS

Anne Mueller, and Beth Fields, *University of Wisconsin–Madison, Madison, Wisconsin, United States*

As the aging population in the U.S. continues to grow, care partners (i.e. family and friends) are assuming increasingly intense and complex caregiving responsibilities. Care partner burden is associated with poorer health outcomes for older adults and more frequent rehospitalizations. This secondary data analysis aims to examine the relationship between different types of health care interactions and care partner burden. A total of 2,588 care partners of Medicare beneficiaries age 65 and older were included. Secondary analyses were conducted using cross-sectional data from the 2017 National Study of Caregiving. Logistic regression analyses were used to determine the relationship between health care interactions and care partner burden while controlling for demographic characteristics. The average care partner was 62 years old (range 18-98), female (68.1%), and white (62.8%). More than half of the care partners (51.3%) reported financial, emotional, and/or physical difficulty as a result of helping the older adult. Logistic regression analyses show that care partners who made medical appointments (AOR=2.04), accessed online medical information (AOR=1.55), and coordinated care between medical providers (AOR=2.15) were significantly more likely to report burden. Care partners are important allies in supporting the health of older adults but may experience excess burden due to health care interactions. Practitioners and researchers may need to evaluate ways to improve the ease, efficiency, and accessibility of different types of health care interactions for care partners of older adults. A better understanding of factors that contribute to care partner burden may inform tailored interventions and future health and aging policies.

### I DON'T WANT TO SEE IT END: A FAITH COMMUNITY NURSE INTERVENTION FOR OLDER ADULTS AND CAREGIVERS

Loralee Sessanna,[1] Sherry Pomeroy,[2] and Yvonne Askew,[3] *1. University at Buffalo, Buffalo, New York, United States, 2. Orchard Park Presbyterian Church, Buffalo, New York, United States, 3. Catholic Health Buffalo, New York, Buffalo, New York, United States*

The Faith Community Nurse (FCN) provides population-based and other nonclinical services in community settings that are not commonly available to the older adult population. The purpose of the FCN Connections (FCNC) study was to test the impact of the FCNC intervention on the health and well-being of clients (C) age 60 and older with chronic diseases and caregiver (CG) dyads (CCGD) by embedding a FCN in a primary care practice (PCP). A mixed method design was used with two cohorts over 18 months (N = 13 CCGD). Experienced FCNs led the components of the intervention in the CCGD home, including client health assessment, education and support while identifying their caregiver's perceived needs, providing education and spiritual/emotional support, linking to resources, and connecting the CCGD with the PCP. The CCGD completed measurements at baseline, 6 and 12 weeks. CG knowledge, preparedness, self-efficacy with caregiving activities, and spirituality were measured with statistically significant CG improvement for handling emergencies, making caregiving activities pleasant, and taking care of emotional needs of the client. Spirituality, self-esteem, function, depression, and healthy living domains were measured for clients. At 12 weeks, semi-structured interviews with the CCGD were conducted and transcribed. An experiential/phenomenological framework and Reflexive Thematic Analysis were used to analyze data which generated one overarching theme, 'I don't want to see it [study] end' and key themes, Theme 1 [client]: 'She was always there to help' and Theme 2 [caregiver]: 'It's like you took the pressure off me for a while' describing the CCGD's FCNC experience.

### INTERVENTIONS TO ENHANCE RESILIENCE AMONG FAMILY CAREGIVERS: A SYSTEMATIC REVIEW

Nai-Ching Chi,[1] Soojeong Han,[2] Ying-Kai Fu,[1] Lynn Nakad,[1] and George Demiris,[2] *1. University of Iowa, Iowa City, Iowa, United States, 2. University of Washington, Seattle, Washington, United States, 3. University of Pennsylvania, Philadelphia, Pennsylvania, United States*

Long-term caregiving can negatively impact caregivers' physical and psychological well-being and are associated with increased morbidity and mortality among caregivers. A more positive caregiving experience is associated with increased coping skills and resilience. Caregiver-targeted interventions designed to promote caregivers' resilience may lead to improved caregivers' well-being, the quality of caregiving, and care recipients' outcome. However, a comprehensive evaluation of effective interventions that enhance resilience among caregivers is lacking. Therefore, the purpose of this study is to critically evaluate existing training programs to inform clinical practice and future program design. A literature review was conducted to search available articles published before June 2018 in databases including PubMed, CINAHL, PsycINFO, and Scopus. Search strategies used index and keyword methods. The inclusion criteria were peer-reviewed, research studies published in English that evaluated an intervention primarily aimed to improve family caregivers of adult patients' resilience or had a primary outcome of resilience. Seven studies fit the criteria identified. Six studies used psychosocial therapy and one used pharmacological therapy to promote caregivers' resilience.

Several effective resources were identified, such as counseling, self-help manuals, psychoeducation, cognitive behavioral therapies, and the use of an antidepressant. All studies reported positive improvements in caregivers' outcomes and six studies reported caregivers had improved resilience after receiving interventions. However, most interventions require health professionals to train patients and caregivers for multiple sessions. Future studies should develop feasible, effective, and less labor-intensive interventions, such as self-help manuals, telehealth interventions, or interventions delivered during routine visits.

### MEASURING CAREGIVER PERSON-CENTERED COMMUNICATION FOR PERSONS WITH DEMENTIA VIA VIDEO OBSERVATIONS

Ying-Ling Jao,[1] Carissa Coleman,[2] Kristine Williams,[3] Diane Berish,[1] WEN LIU,[4] Amy Berkley,[2] and Yo-Jen Liao,[1] *1. Pennsylvania State University, University Park, Pennsylvania, United States, 2. University of Kansas, Kansas City, Kansas, United States, 3. University of Kansas, Lawrence, Kansas, United States, 4. University of Iowa, Iowa City, Iowa, United States*

Communication is fundamental for daily care activities in nursing homes (NHs). Second-by-second behavioral coding of video observations is an ideal approach to examine the interactive nature of communication but requires a reliable coding scheme. Recent studies have adapted the Peron-Centered Behavioral Inventory (PCBI) and Task-Centered Behavioral Inventory (TCBI) to analyze caregiver communication during mealtime interactions, but their use for coding general daily caregiving activities has not been widely evaluated. This pilot study adapted the PCBI and TCBI of video observations and determined their inter-rater reliability for measuring caregiver verbal communication with persons with dementia (PwD). We analyzed videos from a randomized controlled trial of an intervention to improve caregiver communication in NHs. We selected one 1-minute segment from 12 videos that included interactions of caregiver-resident dyads. One research assistant transcribed caregivers' verbal communication and segmented the communication into utterances. Two other research assistants independently coded each utterance using the adapted PCBI and TCBI. The coding scheme was expanded by modifying the existing operational definitions, adding three new codes, and developing a coding decision guide. Residents were Caucasian (100%), mean age 86 years with dementia and resistive behaviors. The adapted PCBI and TCBI had an inter-rater reliability of Kappa=0.656 ($p<.001$) across the 12 videos. Overall, our adapted PCBI and TCBI showed substantial inter-rater reliability. The results support the use of our adapted PCBI and TCBI to distinguish between person-centered and task-centered communication in video observations, which, in turn, allows for sequential analysis to examine the impact of caregiver communication on PwD.

### QUALITY OF CARE PROVIDED BY FAMILY CAREGIVERS OF OLDER PEOPLE WITH DEMENTIA: MEASUREMENT AND INTERPRETATIONS

Afeez Hazzan, *State University of New York at Brockport, Hilton, New York, United States*

Family caregivers of older people living with dementia are relatives, friends, or neighbors who provide assistance related to this condition, but who are unpaid for the services they provide. Although caregiving could be personally rewarding, many caregivers report a high level of strain. Compared to caregivers of older adults who do not have dementia, family caregivers of older people living with dementia report lower quality-of-life (QoL). In a published systematic review examining the relationship between family caregiver QoL and the quality of care provided, only one study was found to be somewhat relevant. The study suggested that the primary reason for an absence of research into the link between family caregiver QoL and quality of care was the absence of a questionnaire for measuring quality of care in dementia. Therefore, any attempt to investigate the impact of caregiver QoL on the care provided to older people with dementia must first address the lack of an instrument to measure quality of care. To address this issue, we interviewed approximately 20 family caregivers in order to elicit feedback on measurements and interpretation of the quality of care provided by family caregivers of older people living with dementia. Content analysis of the interview transcripts revealed that the quality of relationships with family, caregiver availability to provide or supervise care, and availability of paid or volunteer help are important for the quality of care provided. These results have important implications, particularly for the development of an instrument to measure quality of care in dementia.

### SYMPTOM EXPERIENCE AMONG FAMILY CAREGIVERS: SYMPTOM CLUSTER ANALYSIS

Yu-Ping Chang, Young Seo, and Tania Von Visger, *University at Buffalo, Buffalo, New York, United States*

Family caregivers of older adults experience physical and psychological symptoms such as fatigue, insomnia, depression, or anxiety which may negatively impact their quality of life. Although those symptoms often co-occur, there is no prior research on symptom clusters identification among family caregivers. Symptom cluster analysis in chronic disease populations demonstrates usefulness in clinical management. An in-depth understanding of the pattern of symptom experience can guide intervention development to improve caregiving experience, coping skills, and well-being. This study aimed to identify symptom clusters as experienced by family caregivers in the US. We analyzed responses of 2,652 unpaid caregivers from the National Study of Caregiving (NSOC) III (2017) survey to identify underlying symptom clusters. The NSOC is a nationally representative survey of family caregivers to older adults with limited functions in daily activities. We used 17 binary items measuring the physiological and psychological symptoms of caregivers. We conducted a hierarchical cluster analysis with Ward linkage method to identify the symptom clusters. Three symptom clusters were identified based on the optimum silhouette width as follows: (1) Cluster 1 (lack of cheerfulness, peacefulness, and full of life, breathing problems, and sleep interrupted); Cluster 2 (feeling lonely, down, bored, nervous, worrisome, upset, and having little interest); and (3) Cluster 3 (limited strengths in arms and legs, having low energy, pain, and troubles falling back asleep). Our findings indicate that physical and psychological symptoms are highly preventable in family caregivers. Future research on symptoms management strategies can focus on targeting multiple symptoms based on their co-occurence.

### TAILORED STRATEGIES AND SHARED DECISION-MAKING FOR CAREGIVERS MANAGING PATIENTS' PAIN: A WEB APP

Nai-Ching Chi,[1] Lynn Nakad,[1] Ying-Kai Fu,[1] Ibrahim Demir,[1] Stephanie Gilbertson-White,[1] Keela Herr,[1] George Demiris,[2] and Lee Burnside[3], *1. University of Iowa, Iowa City, Iowa, United States, 2. University of Pennsylvania, Philadelphia, Pennsylvania, United States, 3. University of Washington, Seattle, Washington, United States*

Family caregivers (FCs) are essential in helping manage pain for patients who receive hospice care at home, but an accessible resource to prepare FCs in pain management is lacking. This study aims to develop an accessible resource that aids FCs in pain management. First, we conducted two literature reviews and two secondary data analyses to investigate the challenges that FCs face when managing pain for patients. Based on the results, we identified 20 common challenges that prevent FCs from providing effective pain management. Second, we developed evidence-based content to address each identified challenge, and a library to provide FCs with pain education. Third, experts and clinicians (n=10) in hospice care validated the content, and FCs (n=10) gave feedback on how to improve the understandability of the content. Currently, we are converting the content to a web app prototype. The web app will consist of: (1) an assessment tool with questions to assess the FCs' challenges when managing patients' pain, (2) computer-generated strategies tailored to address the identified challenges, (3) discussion questions for FCs to use with their care team to prioritize goals of pain management, and (4) a library to enhance access to educational materials. This study presented the process of developing an evidence-based and user-centered web app. This study will advance the field of pain management in hospice care by providing accessible and evidence-based tools to support and engage FCs in pain management. Also, this web app will allow best practices to be quickly translated from research into practice.

### THE BREAKING POINT: FAMILY DEMENTIA CAREGIVERS' RECOGNITION OF THE NEED TO INTERVENE

Candace Harrington,[1] Cheryl Dean-Witt,[2] and Frances Hardin-Fanning,[3] *1. University of Louisville, Louisville, Kentucky, United States, 2. University of Louisville, Louisville,, Kentucky, United States, 3. University of Louisville, Winchester, Kentucky, United States*

Family dementia caregivers are an under-recognized and valuable geriatric workforce whose services have broad implications for health care systems. Family dementia caregivers may experience uncertainty, loss of role identity, involuntary role assumption, or undesirable life transitions. Subsequent unintentional mistreatment or abuse of their family care recipient may occur. Approximately 50% of caregivers admit to some form of mistreatment of their loved one who lives with dementia. Using Selder's (1989) life transition theory, this qualitative study explored family members' life transition process toward their new role identities as family dementia caregivers to better understand the personal and historical contexts of caregiving. Semi-structured interviews were conducted with 10 participants to answer the questions: How does one acquire the role of primary family dementia caregiver?" and "How do personal and historical contexts inform the family dementia caregiver role?" Richness of data drove our sample size. Epistemological integrity ensured trustworthiness and rigor. A modification of Colaizzi's (1978) analytic method was used for interpretative phenomenological analysis. The emergent themes uncovered by participants' statements included: "It's my turn", "the breaking point", and "a fine line" with the subtheme "balancing dignity and safety". Participants described their introspective journeys toward a changed reality as family dementia caregivers. Our findings suggested the need for early recognition and vigilance to prevent the exploitation and mistreatment of those with dementia. Rural agriculture-based family caregivers in our study described unique and challenging characteristics. Further research is needed to explore the implications of these contextual nuances for rural agriculture-based family dementia caregivers.

### UNDERSTANDING THE LINK BETWEEN FAMILY CAREGIVER QUALITY OF LIFE AND THE CARE PROVIDED TO OLDER PEOPLE WITH DEMENTIA

Afeez Hazzan, *State University of New York at Brockport, Hilton, New York, United States*

Dementia is one of the most rapidly growing diseases in the United States. In 2018, the direct costs to American society of caring for older people with dementia was approximately $277 billion. Primary informal caregivers are mainly responsible for the care of older people with dementia including Alzheimer's disease. Caregivers perform a myriad of duties ranging from shopping for their loved ones' groceries, helping with medications, and managing finances. The caregiving role becomes more demanding as the disease progresses over time, and studies have shown that the quality-of-life (QoL) experienced by caregivers of older adults who have dementia is lower than the QoL of caregivers for older people who do not have dementia. To the best of our knowledge, there has been no research conducted to investigate whether lower caregiver QoL affects the level or quality of care that caregivers provide to persons with dementia. In the current study, we interviewed family caregivers living in Rochester, New York to inquire about their quality of life and the care provided to older people living with dementia. Further, caregivers completed the 36-item Short Form Health Survey (SF-36) as well as a draft questionnaire for measuring the quality of care provided to older people living with dementia. Both quantitative and qualitative findings from this study reveals important relationships between family caregiver QoL and the care provided, including the impact of social support and financial well-being. The study findings could have significant impact, particularly for the provision of much needed support for family caregivers.

### USE OF HOMECARE NURSING AND SENSE OF SECURITY AMONG FAMILY CAREGIVERS OF OLDER ADULTS

Ayumi Igarashi, Madoka Katayama, Mariko Sakka, Maiko Noguchi-Watanabe, Chie Fukui, Asa Inagaki, Satomi Kitamura, and Noriko Yamamoto-Mitani, *The University of Tokyo, Tokyo, Japan*

Having a sense of security for living at home is essential to continue living at home and it is important to develop a community care system that enables high sense of security. Because frail to dependent older adults often accompany various conditions that require medical attention, receiving homecare nursing might allow the family caregiver to feel safe in living at home. Therefore we examined whether receiving homecare nursing service contributes to a higher family caregiver sense of security. We conducted a questionnaire survey regarding older adults who were 75 years and older with at least some sort of community care services, and their family caregivers (n=776). Family sense of security was measured using 5 items (e.g., "I feel secure about my relative living at home with help of available services"), rated on 5-point Likert scales. Characteristics of older adults were obtained from their nurses or care managers. The mean age of the older adults was 85.8 years, 58% female, and 8 % living alone. The mean total family sense of security was 20.6 (standard deviation = 3.7) out of 25. In multiple regression analysis, the family sense of security was positively associated with the use of homecare nursing ($\beta$= 0.09, p=0.020), adjusting for participants' age, stability of their medical conditions, level of activities of daily living, use of medical procedures, living arrangements, and house call physician services. Homecare provided by nurses could contribute to longer staying at home among older adults by way of the higher family sense of security.

### SESSION 2883 (POSTER)

### COGNITIVE IMPAIRMENT AND COGNITION

### ALCOHOL CONSUMPTION AND COGNITIVE FUNCTION IN AN OLDER POPULATION IN CHINA

Anying Bai,[1] Bo Hou,[2] Junsong Chen,[1] and Boming Fu,[1] *1. Peking University, Beijing, China, 2. Bradford Teaching Hospitals NHS Foundation Trust, Bradford, England, United Kingdom*

The relationship between alcohol consumption and cognitive function has only been studied to a limited extent in China. This paper examines this relationship using the China Health and Retirement Longitudinal Study (CHARLS), a nationally representative dataset of the Chinese population aged over 45. Alcohol consumption was measured by drinking status (never, former, moderate, excessive drinkers) based on number of standard drinks per week. Mental status and episodic memory function were used as measures of cognitive function. Lagged dependent variable models were used to examine independent associations between alcohol consumption and cognitive function. Our models controlled for demographic factors, socioeconomic factors, baseline cognitive functioning and an indicator for lifestyle. We also tested for an inverted J shaped relationship between alcohol consumption and cognitive functioning. A total of 10404 nondrinkers (60.09%), 2450 former drinkers (14.15%), 1599 moderate drinkers (9.24%) and 1525 excessive drinkers (8.81%) were included. Compared to never drinkers, there were no statistically significant difference between this group and moderate drinking group. While, excessive drinkers were consistently associated with on average 0.13-point decrease in episodic memory scores (p =0.031). For mental intactness, there were no statistically significant differences between never drinkers and other groups. Furthermore, we did not find evidence to support an inverted J-shaped association between standard drinks per week and measures of cognitive functioning. Excessively drinking was associated with greater decline in episodic memory function, but not in mental intactness in elder Chinese population. There is no significant association between moderate drinking and cognitive functioning measures.

### AN ECOLOGICAL MODEL OF CARE FOR OLDER ADULTS WITH COGNITIVE IMPAIRMENT

Elizabeth Rhodus,[1] Graham Rowles,[2] Shani Bardach,[2] Elizabeth Hunter,[3] and Gregory Jicha,[2] *1. University of Kentucky, Richmond, Kentucky, United States, 2. University of Kentucky, Lexington, Kentucky, United States, 3. University of Kentucky, University of Kentucky, United States*

Living with dementia causes increasing dependence on the surrounding physical and social environment. There is limited information on environmental interactions of persons with cognitive impairment. Based on participant observation and repeated interviews with both members of nine dyads (primary care partner and person with cognitive impairment) in situ in their homes, a theoretical model depicting environmental interaction was developed. The model illustrates parallel and interwoven environmental experiences of each member of the dyad as they negotiate progressive cognitive impairment. Evolution of the dyad is situated within nested layers of the physical and contextual environment including physical structures, social norms, and political environments. Experiential elements for each member of the dyad are described. Elements include cognitive status, trial and error associated with care provision, adverse behavior linked with onset of caregiver burden, onset of a significant event leading to altered living situations, and maximum dependence on environmental factors prior to end of life. Evidence collected suggests that both persons of the dyad become increasingly susceptible to environmental influences with progression of the disorder. Implication of these findings offer a theoretical framework describing dyadic experiences of environmental interactions when living with dementia. This theoretical model provides a basis for clinical and social intervention to enhance the well-being of both members of the dyad. Interventions associated with environmental interactions may slow socially discordant behavioral manifestations associated with dementia and significantly improve quality of life for both members of the dyad.

### ASSOCIATION OF BALANCE SCORES WITH COGNITIVE FUNCTION IN OLDER ADULTS

Claire Meunier,[1] Ellen Smit,[1] Annette Fitzpatrick,[2] and Michelle Odden,[3] *1. Oregon State University, Corvallis, Oregon, United States, 2. University of Washington, Seattle, Washington, United States, 3. Stanford University, STANFORD, California, United States*

Previous research has reported an association between balance and cognitive function; however, there is a paucity of data on this relationship over time. This study examined the cross-sectional and longitudinal association between balance and cognitive function in 4,811 participants aged 65 years and older in the Cardiovascular Health Study (CHS). Cognitive function measures included the Modified Mini-Mental State Examination (3MSE) and Digit Symbol Substitution Test (DSST); measures were collected annually for six years, starting in 1992-1993. A tandem stance balance test was administered at baseline; this test was held for 5 seconds. Cross-sectional and longitudinal models were adjusted for demographics, behavioral and disease covariates. We found that participants with worse balance scores had lower cognitive function scores, and this effect was limited to participant who were above the median age (76 years) (p-value for interaction = 0.03 in a demographic-adjusted model). Participants 76 years and older who failed the balance test had an average adjusted decline of -0.97 (95% CI: 1.20, 2.29) 3MSE scores per year more than participants who completed the balance test. DSST showed similar results; participants with poor balance decreased -0.21 (95% CI: -0.37, -0.05) points per year more than participants who completed the balance test. The adjusted Cox proportional hazard model found participants with poorer balance had a higher risk of cognitive impairment over the six years (HR= 1.72 95% CI: 1.30, 2.29). A better understanding of the pathophysiological link between balance and cognition may inform strategies to prevent cognitive decline.

### ASSOCIATIONS BETWEEN SMOKING AND COGNITIVE FUNCTION AMONG COMMUNITY-DWELLING U.S. CHINESE OLDER ADULTS IN CHICAGO

Shengxi Sun,[1] Nannan Zhang,[2] Mengting Li,[3] and XinQi Dong,[3] *1. Rutgers, the State University of New Jersey, Chicago, Illinois, United States, 2. East China University of Science and Technology, Shanghai, China, 3. Rutgers University, New Brunswick, New Jersey, United States*

Previous studies on smoking and cognition reported mixed findings. The inconsistent results are partially explained by the fact that they were limited to specific populations and using different cognitive function measurements. This association between smoking and cognition has rarely been studied in the rapidly increasing U.S. Chinese older adults. This study aims to determine if smoking status and smoking amount are associated with global cognition and cognitive domains in U.S. Chinese older adults. Data was extracted PINE. Five cognitive function tests (East Boston Memory Test, East Boston Memory Delayed Recall, Digital Backward test, Symbol digit Modality Test, and MMSE) were used to measure cognitive domains including episodic memory, working memory, and processing speed. Five cognitive tests were converted to z scores and averaged to generate global cognition. Self-reported smoking status was used for generating smoking status and smoking amount (pack-years). Linear regression was used. The results showed that former smokers had lower global cognition (b=-0.111, SE=0.053, p<.05) and perceptual speed (b=-0.185, SE=0.066, p<.01) than never smokers; current smokers had lower global cognition (b=-0.240, SE=0.060, p<.001), working memory (b=-0.340, SE=0.083, p<.001) and perceptual speed (b=-0.370, SE=0.075, p<.001) compared with never smokers. Smoking pack-years is negatively associated with global cognition (b=-0.003, SE=0.001,p<.001), episodic memory (b=-0.005, SE=0.001, p<.001), and perceptual speed (b=-0.004, SE=0.001, p<.001). Findings revealed that among all smokers, current smokers had the worst cognition and heavier smoking was associated with worse cognition. Policymakers could take measures in lowering smoking amount among U.S. Chinese older adults to protect their cognition.

### BEHAVIORS CHARACTERISTIC OF AUTISM SPECTRUM DISORDER IN OLDER ADULTS WITH COGNITIVE IMPAIRMENT

Elizabeth Rhodus,[1] Justin Barber,[2] Erin Abner,[3] Shani Bardach,[2] Graham Rowles,[2] and Gregory Jicha,[2] *1. University of Kentucky, Richmond, Kentucky, United States, 2. University of Kentucky, Lexington, Kentucky, United States, 3. University of Kentucky, Lexington, United States*

Autism spectrum disorder (ASD) is commonly recognized by the time of adolescence, but is poorly understood in older adults. The possibility of late-life emergence of ASD has been poorly explored. In order to investigate late-life emergence of behaviors characteristic of ASD in MCI and AD, we surveyed caregivers of 142 older adults with neurodegenerative cognitive impairment using the Gilliam Autism Rating Scale-2. Participants with high autism index ratings (Autism 'Possible/Very Likely', n=23) reported significantly (statistically and clinically) younger age at onset of cognitive impairment than those who scored in the Autism 'Unlikely' range (n=119): 71.14±10.9 vs. 76.65±8.25 (p = 0.034). Additionally, those in Autism 'Possible/Very Likely' group demonstrated advanced severity of cognitive impairment, indicated by Clinical Dementia Rating Scale Sum of Boxes scores. Data demonstrate that ASD behaviors may appear de novo of degenerative dementia and such behaviors are more prevalent in those with early onset dementia. Further work elucidating a connection between ASD and dementia could shed light on subclinical forms of ASD, identify areas of shared neuroanatomic involvement between ASD and dementias, and provide valuable insights that might hasten the development of therapeutic strategies.

### COGNITIVE IMPAIRMENT AND SELF-CARE AMONG CONGESTIVE HEART FAILURE PATIENTS IN AN OUTPATIENT CLINIC

Chelsea Liu,[1] Nicole L. Williams,[2] Nisha Chandra,[3] Rosanne Rouf,[3] Andrew Gaddis,[2] Yessenia Gomez,[2] Tanya Simmons,[3] and Rebecca Gottesman,[2] *1. Johns Hopkins Bloomberg School of Public Health, Baltimore, Maryland, United States, 2. Johns Hopkins School of*

*Medicine, Baltimore, Maryland, United States, 3. Johns Hopkins Bayview Medical Center, Baltimore, Maryland, United States*

The burden of congestive heart failure (CHF) is the greatest among older adults. Cognition is important for carrying out self-care tasks such as monitoring sodium intake, but little is known about how cognition affects self-care in acutely ill CHF patients. We aimed to assess the association between cognition and self-care in CHF patients from an outpatient diuresis clinic. Cognitive function was measured using the Mini-Mental State Exam (MMSE) and other tests representing 5 cognitive domains. The Self-Care of Heart Failure Index (SCHFI), given to a subset of participants, consisted of 22 questions each scored on an ordinal scale of 1-4 with a total score ranging from 22-88; higher scores indicated better self-care. SCHFI questions were further categorized into maintenance, management and confidence sub-scores. Multiple linear regressions were used to analyze the association between neuropsychological test scores and SCHFI scores. A total of 68 CHF patients had complete SCHFI data, with a mean age of 65.6 years and a mean total SCHFI score of 70.9 points. Nine (13.2%) patients were cognitively impaired (MMSE<24). Older age, lower education and history of stroke were associated with cognitive impairment. After adjusting for age, education, diabetes, and depressive symptoms, no associations were observed between the other neuropsychological test scores and any of the SCHFI scores. Though findings suggest that cognition is not associated with self-care, the analysis may have been underpowered. Further evaluation of a greater number of CHF patients is needed to understand the implications of cognition on self-care and provide guidance for interventions.

### CONTEMPLATIVE PRACTICES AND LATER LIFE MEMORY AMONG WOMEN

Nirmala Lekhak,[1] and Tirth Bhatta,[2] *1. University of Nevada, Henderson, Nevada, United States, 2. University of Las Vegas Nevada, Las Vegas, Nevada, United States*

Contemplative practices such as meditation, yoga, and prayer had been used as coping resources to reduce the adverse impacts of stressful life experiences. Despite emerging scholarship on the benefits of contemplative practices for cognitive health, scant research has examined the influence of such practices on both episodic and working memory among women in later life. While the use of private prayer outside of church or temple has been shown to have statistically significant positive effect on episodic memory among older adults, previous studies have relied on measures that fail to capture various aspects of meditative practices (e.g., mental imagery, relaxation). Drawing from the Study of Women's Health Across the Nation (n=2245) conducted during 2006-2008, this study investigates the effect of contemplative practices (e.g. meditation / imagery / relaxation technique, yoga, and prayer) on episodic and working memory. Multivariate regression model estimates suggests women who either used meditation, imagery or relaxation techniques had significantly better episodic (b=0.61, p=0.001) and working memory (b=0.32, p<0.05) as compared to those who did not use those methods. Surprisingly, the influence of prayer on episodic memory was negative (b=-0.36, p<0.05), while the influence of yoga on memory was not statistically significant. Our findings underscore the need to incorporate meditative practices in non-pharmacological interventions that are designed to improve later life memory.

### DO MEMORY PROBLEMS IN OLDER DRIVERS IMPACT DRIVING FREQUENCY IN METRO VERSUS NON-METRO COMMUNITIES?

Tyler Orr,[1] and Randall Rupper,[2] *1. Brigham Young University--Provo, Provo, Utah, United States, 2. Salt Lake VA, Salt Lake City, Utah, United States*

Perceived memory problems may cause older adults to limit functional activities such as driving. For those individuals living in non-metropolitan communities, greater distances between activities, lack of public transportation, and fewer support systems may make reducing driving frequency less feasible. We hypothesized that older adults in non-metropolitan communities would be more likely to continue frequent driving even if they also perceived memory problems. We used the National Health and Aging Trends Study to examine the association between reported memory difficulty and the frequency of driving. These data were then stratified by metropolitan versus non-metropolitan classification using both ordinal logistic regression and Chi-Squared testing. In both metropolitan and non-metropolitan communities, respondents were more likely to report reductions in driving frequency if they also reported memory problems. However, in both metropolitan and non-metropolitan communities, the majority of respondents reporting fair or poor memory continued to report frequent driving; and, there were no statistical differences in frequency of reported driving between metropolitan and non-metropolitan respondents. These analyses suggest that strategies are necessary in both metropolitan and non-metropolitan areas to help older drivers with perceived memory difficulties to recognize when they need to limit driving. Further research is necessary to determine which strategies are likely to be effective in metropolitan and/or non-metropolitan communities.

### IMPACT OF COGNITIVE FUNCTIONING AND AGE ON PATIENT-REPORTED OUTCOMES IN PARKINSON'S DISEASE

Ann Gruber-Baldini,[1] Haesung Kim,[1] Jay Unick,[2] and Lisa Shulman,[1] *1. University of Maryland School of Medicine, Baltimore, Maryland, United States, 2. University of Maryland School of Social Work, Baltimore, Maryland, United States*

Cognitive impairment is prevalent in Parkinson Disease (PD) and increasing age is a PD risk factor. Age and cognition may impact patient-reported outcome measures (PROMs) level, reliability, or validity of responses. This study investigated the relative impact of cognitive function and age on PROMs in PD. Cross-sectional data (n=676) included assessments of age, cognition (Montreal Cognitive

Assessment; MoCA) and PROMIS-29 Profile (physical functioning, anxiety, depression, fatigue, sleep disturbance, social functioning, pain). Analyses examined differences by age and MoCA in: 1)Level—correlations, multivariable regressions controlling for disease severity (UPDRSmotor, PD duration), comorbidity (CIRS-G), demographics; 2) Reliability--Cronbach's alpha, and 3)Validity--correlations of PROMIS physical function with physician assessments. Sample was age M=68.0(SD=9.1); range=36-93 years, 64% male, 87% white, 37% college educated, PD duration M=8.2(SD=6.1) years, and MoCA M=24.3(SD=4.9; range 2-30). Greater cognitive impairment was consistently associated with greater physical/mental impairment (r=.14-.45; p<.05), except for sleep disturbance (r=-.07, p=.08) Multivariable regressions found cognition remained a significant predictor of physical functioning, anxiety, and depression older age predicted anxiety and social functioning. Comorbidity was the greatest predictor across all the PROMs (r=.22-.45). Reliability for PROMIS measures was excellent (alpha>.8) across cognitive and age groups, except for Fatigue at MoCA.36) across cognition and age groups. Cognitive impairment in PD is associated with lower physical function and mental health levels. Reliability and validity of most PROMs in PD are neither impacted by cognition nor age.

### INCIDENT COGNITIVE IMPAIRMENT DURING AGING IN RURAL SOUTH AFRICA: EVIDENCE FROM THE HAALSI COHORT, 2014 TO 2019

Lindsay Kobayashi,[1] Meagan Farrell,[2] Kenneth Langa,[1] Nomsa Mahlahela,[3] Ryan Wagner,[3] and Lisa Berkman,[2] *1. University of Michigan, Ann Arbor, Michigan, United States, 2. Harvard T. H. Chan School of Public Health, Cambridge, Massachusetts, United States, 3. University of the Witwatersrand, Acornhoek, Mpumalanga, South Africa*

We estimated the incidence of cognitive impairment and its key sociodemographic, social, and health-related predictors at the first longitudinal follow-up of the population-representative "Health and Aging in Africa: A Longitudinal Study of an INDEPTH Community in South Africa" (HAALSI) cohort of adults aged ≥40 in rural Agincourt, South Africa. Cognitive impairment was defined as scoring ≥1.5 SD below the baseline mean composite time orientation and episodic memory score, or requiring a proxy interview with "fair" or "poor" proxy-reported memory. Activity of daily living (ADL) limitations were compared according to incident cognitive impairment status. Incidence rates (IRs) and rate ratios (IRRs) for cognitive impairment according to sociodemographic, social, and health-related predictors were estimated using Poisson regression with robust standard errors, and weighted to account for mortality. Over a 3.7-year mean follow-up, 309/3,861 at-risk participants newly developed cognitive impairment (IR=24.0/1000 person-years (PY); 95% CI: 21.6-26.8). Incidence increased from IR=9.1/1000 PY (95% CI: 5.5-16.1) among those aged 40-44 at baseline to IR=76.5/1000 PY (95% CI: 63.2-93.4) among those aged 80+. At least one ADL limitation was prevalent in 39% of those with incident cognitive impairment, compared to 7% of non-impaired participants. Incident cognitive impairment did not vary by sex/gender, HIV status, or cardiovascular factors, but was strongly graded according to education, literacy, household assets, employment, marital status, and frequency of alcohol consumption. This study presents one of the first incidence rate estimates for cognitive impairment in sub-Saharan Africa. Social disparities in cognitive impairment were apparent in patterns similar to many high-income countries.

### LOW COGNITIVE FUNCTION IS ASSOCIATED WITH REDUCED FUNCTIONAL FITNESS AMONG COMMUNITY-DWELLING OLDER ADULTS

Michelle Gray,[1] Joshua Gills,[1] Jordan Glenn,[2] Erica Madero,[3] Aidan Hall,[2] nami Fuseya,[2] Jennifer Vincenzo,[4] and Nick Bott,[3] *1. University of Arkansas, Fayetteville, Arkansas, United States, 2. Neurotrack Technologies, Redwood City, United States, 3. Neurotrack Technologies, Redwood City, California, United States, 4. UAMS, Fayetteville, Arkansas, United States*

Among older adults over 70, 22-30% report difficulty performing at least one activity of daily living (ADL). While the precipitants of ADL decline are multifactorial, over 50% of cognitively impaired adults require assistance with ADLs. The exact relationship between cognitive and functional decline remains unknown, but it is important to understand their relationship. Eighty-three older adults (80.9 + 5.4 years) enrolled in this study and completed functional fitness and cognitive assessments. Functional fitness assessments included: Short Physical Performance Battery (SPPB), 10-meter walk, dual-task, and power chair stand (average and peak). Cognition was assessed using the Montreal Cognitive Assessment (MoCA) and Visual Paired Comparison task (VPC). Categories of low cognitive function (LCF) and high cognitive function (HCF) were determined by VPC scores. SPPB was 10.2% greater among the HCF group. The HCF group walked 12.6% (0.16 m/s) faster than the LCF group. Dual-task (fast) performance was 13.2% faster among the HCF group. Additionally, when rising from a seated position during the average and peak power chair stand task, the HCF group moved 16.7% and 16.1% faster than the LCF group, respectively. MoCA scores were 2.8 points greater among the HCF group. Based on the current results, significant differences exist between cognitive groups suggesting a relationship between functional fitness and cognition. What remains unknown is the ability to influence functional fitness by changing cognition or vice versa. Future research is warranted to determine the relationship of change in either domain over time.

### PROMOTING CONVERSATIONS ABOUT COGNITIVE DECLINE BETWEEN OLDER ADULTS AND PRIMARY CARE PROVIDERS

Benjamin Olivari,[1] Christopher Taylor,[2] Nia Reed,[2] and Lisa McGuire,[2] *1. CDC, Atlanta, Georgia, United States, 2. Centers for Disease Control and Prevention, Atlanta, Georgia, United States*

Alzheimer's disease and related dementias often begin with symptoms of mild memory loss, eventually leading to more severe cognitive impairment, functional impairment, and ultimately, death. Data from the Behavioral Risk Factor Surveillance System core questions related to chronic diseases and from the cognitive decline optional module on subjective cognitive decline (SCD) from the years 2015-2018 were aggregated across the participating 50 states, D.C., and Puerto Rico for this analysis. Among U.S. adults aged 65 years and older, only 39.8% (95%CI=37.6-42.1) of those experiencing SCD reported discussing their SCD symptoms with a healthcare provider. The prevalence of discussing SCD symptoms with a provider was higher among those with at least one chronic condition than among those with no chronic conditions. 30.7% (28.6-32.8) of those aged 65 years and older reported that their SCD led to functional limitations and 28.8% (26.5-31.2) needed assistance with day-to-day activities. For patients aged 65 years and older, Welcome to Medicare visits and Medicare Annual Wellness Visits are critically underutilized primary care access points. Primary care providers can manage chronic conditions, cognitive health, and initiate referrals for testing. Efforts to promote the use of toolkits and diagnostic codes that are available to primary care providers to initiate conversations about memory loss with patients may be utilized to improve detection, diagnosis, and planning for memory problems. Discussions may lead to earlier detection and diagnosis of cognitive impairment, such as Alzheimer's disease, or other treatable conditions such as delirium or pressure in the brain and avoid costly hospitalizations.

### RECIPROCAL ASSOCIATION BETWEEN COGNITIVE FUNCTION AND ORAL HEALTH: A SYSTEMATIC REVIEW AND META-ANALYSIS

Xiang Qi,[1] Zheng Zhu,[2] and Bei Wu,[3] *1. New York University, jersey city, New Jersey, United States, 2. Fudan University, Shanghai, China, 3. New York University, New York, New York, United States*

Increasing evidence suggests that there is a linkage between cognitive function and oral health. However, there are few systematic reviews with meta-analysis have been conducted to evaluate the strength of this association. Moreover, existing studies usually focused on unidirectional associations between cognitive function and oral health; no research has demonstrated this inter-relationship in a longitudinal study. This study aims to systematically assess the magnitude of the bidirectional association between oral health and cognitive decline for studies using longitudinal data. Six international databases were searched up until December 31, 2019. Random-effects pooled Risk Ratios (RRs) with 95% confidence intervals (CIs) were calculated. The grading of recommendations assessment, development, and evaluation (GRADE) system was used to assess the quality of evidence. From 13,251 potentially eligible articles, 54 longitudinal studies were included in the systematic review and 18 were in meta-analysis. Meta-analysis was performed for tooth loss and periodontitis disease. Random effects analysis showed, with statistically low heterogeneity, Risks of cognitive decline included suboptimal dentition (<20 teeth) (RR 1.44, 95% CI 1.03-3.65) and periodontitis diseases (RR 1.48, 95% CI 1.22-1.81). Cognitive decline was a risk factor for tooth loss (RR 1.54, 95% CI 1.23-9.69). The overall quality of evidence, however, was rated as very low. The result of this review highlights that cognitive decline is a risk factor for poor oral health, and older adults with suboptimal oral health appear to have an increased risk of cognitive impairment. More studies with rigorous designs are needed to further examine this association.

### SCREENING NEUROCOGNITIVE DISORDERS IN PRIMARY CARE SERVICES: THE QUICK MILD COGNITIVE IMPAIRMENT APPROACH

Constanca Paul,[1] Susana Sousa,[1] Pedro Santos,[2] Rónán O'Caoimh,[3] and William Molloy,[4] *1. University of Porto, Porto, Portugal, 2. Biomedical Sciences, Abel Salazar Biomedical Sciences Institute - University of Oporto, Porto, Portugal, 3. National University of Ireland, Galway City, Galway, Ireland, 4. St. Finbarr's Hospital, Cork, Ireland*

Neurocognitive Disorders (NCD) is an increasingly common condition in the community. The General Practitioner (GP) in Primary Care Services (PCS), have a crucial role in early detection of NCD and is usually the first professional to detect the signs of MCI. The objective of this study was to test the feasibility and utility of the cognitive screening instrument QMCI in Primary Care. A community sample of 436 people 65+ living in the community was randomly selected from a larger group of old people with mental health concerns (N=2734), referred by their GPs. The mean age of the sample was 75.2 years (sd 7.2), with 41.3% men and 58.7% women; 60.4% married followed by 28.7% widows. The education level was low with 21% illiterate and 69,8% people with 4 years education. The QMCI mean was 37.1/100 (sd 16.2). The amount of people screening positive for cognitive impairment QMCI (<62/100) was 94.2%. In the distribution of people with cognitive impairment by Global Deterioration Scale (GDS) three recoded categories, of the 286 people 76,1% where classified as having very mild or mild impairment, 19,4% moderate or moderately serious and 4,5% severe or very severe impairment. These results confirm the perception of GPs about their clients having mental health concerns and the ability of QMCI accurately discriminate MCI. The QMCI is very brief (3-5mins) fitting the short time of GPs to assess cognitive status and timely refer clients to nonpharmacological interventions that could postpone NCD symptoms.

### SUBJECTIVE COGNITIVE IMPAIRMENT IN LIVER TRANSPLANT RECIPIENTS

Dami Ko,[1] Mary Dietrich,[2] Katherine Gifford,[2] and Sheila Ridner,[2] *1. Northeastern University, Jamaica Plain, Massachusetts, United States, 2. Vanderbilt University, Nashville, Tennessee, United States*

Cognitive impairment is an emerging health concern in the liver transplant (LT) population. To successfully address this condition and improve patient-centered outcomes (e.g., quality of life), it is essential to involve LT recipients and caregivers in developing care plans for cognitive impairment and take into account their subjective ratings regarding recipients' cognitive function. Although a high degree of agreement between recipient-rated and caregiver-rated cognitive function is desirable to reduce conflicts in developing plans, disagreement between the two may exist. This cross-sectional study is the first to examine the correlation between recipient-rated and caregiver-rated cognitive function in the LT population. Sixty pairs of adult LT recipients (mean age 60.4±6.9) and their caregivers (mean relationship with recipients 35 years) participated in this study. Two versions of Modified Everyday Cognition (ECog), one for LT recipients and the other for caregivers, were used to assess recipient-rated and caregiver-rated cognitive function in six domains, including memory and planning. Significant intra-class correlations were found in the ECog total and domain scores. The correlation coefficient of the ECog total score was 0.48, indicating a fair correlation, and the coefficients of the ECog domains ranged between 0.35 and 0.56, indicating poor to fair correlations based on the guideline of Cicchetti (1994). These findings suggest that a tailored approach to addressing the poor agreement between recipients and caregivers should be adopted to develop successful treatment plans for cognitive impairment. Future studies should examine the degree of agreement between objective (e.g., cognitive tests) and subjective ratings of cognitive function.

### THE ASSOCIATION BETWEEN DIABETES AND COGNITION AMONG OLDER HISPANICS IN THE UNITED STATES AND MEXICANS IN MEXICO

Jaqueline Avila, Rebeca Wong, and Rafael Samper Ternent, *The University of Texas Medical Branch at Galveston, Galveston, Texas, United States*

The objective is to assess if the effect of diabetes on cognition differs by race/ethnicity in the U.S. and how this association differs between older Hispanics in the U.S. and older Mexicans in Mexico. Data comes from a sample of older adults 50 and older with direct interviews from the 2012 waves of the Health and Retirement Study (N=17,810) and the Mexican Health and Aging Study (N=13,270). Cognition was measured as a total cognition score. OLS regressions were used to test the association between diabetes and cognition by race/ethnicity in the U.S. and among older Mexicans in Mexico. Results showed that Non-Hispanic Whites (NHW) had the highest cognition scores in the U.S., followed by Hispanics and non-Hispanic blacks (NHB). Mean cognition score of older Mexicans was higher than for NHB and Hispanics in the U.S. but lower than NHWs. The prevalence of diabetes was highest among Hispanics (32.3%), followed by NHB (30.6%) and NHW (19.9%). The prevalence of diabetes in Mexico was like those NHW in the U.S. (19.9%). In the U.S., the effect of being NHB and Hispanic (compared to white) on cognition was equivalent to having 5.3 and 2.4 fewer years of education, respectively. However, the effect of diabetes on cognition did not differ by race/ethnicity. The final analysis will include a direct comparison between Hispanics in the U.S. and a matched sample of older adults in Mexico with similar sex and age to test differences in the effect of diabetes on cognition between these two samples.

### THE RELATIONSHIP BETWEEN DAILY RAPID-EYE MOVEMENT SLEEP AND COGNITIVE FUNCTIONING IN OLDER ADULTS

Tiana Broen,[1] Tomiko Yoneda,[1] Jonathan Rush,[1] Jamie Knight,[2] Nathan Lewis,[1] Rebecca Vendittelli,[1] Scott Hofer,[1] and Andrea Piccinin,[3] *1. University of Victoria, Victoria, British Columbia, Canada, 2. University of Victoria, University of Victoria, British Columbia, Canada, 3. Psychology, Victoria, British Columbia, Canada*

Previous cross-sectional research suggests that age-related decreases in Rapid-Eye Movement (REM) sleep may contribute to poorer cognitive functioning (CF); however, few studies have examined the relationship at the intraindividual level by measuring habitual sleep over multiple days. Applying a 14-day daily diary design, the current study examines the dynamic relationship between REM sleep and CF in 69 healthy older adults (M age=70.8 years, SD=3.37; 73.9% female; 66.6% completed at least an undergraduate degree). A Fitbit device provided actigraphy indices of REM sleep (minutes and percentage of total sleep time), while CF was measured four times daily on a smartphone via ambulatory cognitive tests that captured processing speed and working memory. This research addressed the following questions: At the within-person level, are fluctuations in quantity of REM sleep associated with fluctuations in next day cognitive measures across days? Do individuals who spend more time in REM sleep on average, perform better on cognitive tests than adults who spend less time in REM sleep? A series of multilevel models were fit to examine the extent to which each index of sleep accounted for daily fluctuations in performance on next day cognitive tests. Results indicated that during nights when individuals had more REM sleep minutes than was typical, they performed better on the working memory task the next morning (estimate = -.003, SE = .002, p = .02). These results highlight the impact of REM sleep on CF, and further research may allow for targeted interventions for earlier treatment of sleep-related cognitive impairment.

### SESSION 2884 (POSTER)

### DEMENTIA

### BEHAVIORAL PATTERNS OF CARE PROVIDERS AND RESISTIVENESS TO CARE OF PERSONS WITH DEMENTIA

Bo Ra Kim,[1] Kyung Hee Lee,[1] Eunhee Cho,[1] Heejung Kim,[1] and Bora Kim,[2] *1. Yonsei University, Seoul, Republic of Korea, 2. Yonsei University College of Nursing, Seoul, Republic of Korea*

Resistiveness to care in persons with dementia is a distinct obstacle for care providers to provide oral care. It is necessary to find out specific behaviors of care providers that affect resistiveness to care. This is a secondary data analysis to identify behaviors of care providers related to resistiveness to care using 70 videos that were taken when 23 persons with dementia received oral care in long-term care facilities at baseline, month 3 and month 6. The behavior patterns of care providers and resistiveness to care of persons with dementia were extracted in the form of the frequency of each behavior using a coding scheme for behavioral observation. The coding scheme for behavior patterns of care providers consists of 2 dimensions: person-centered behaviors including 18 items and task-centered behaviors including 4 items. In addition, the coding scheme for resistiveness to care of persons with dementia includes 13 items. The data were analyzed using a linear mixed model analysis considering the repeated measured data. Among person-centered behaviors, 'assessing the comfort of resident,' and 'cooperatively negotiating' were less likely to occur resistiveness to care. However, 'physically controlling' was the most significant to increase the occurrence of resistiveness to care of persons with dementia. Therefore, it is recommended that care providers try to understand the causes of discomfort for persons with dementia and meet his/her needs rather than to physically control when providing oral care.

### CHINESE AMERICAN CAREGIVERS OF OLDER ADULTS WITH ALZHEIMER'S DISEASE AND OTHER DEMENTIAS: A SCOPING REVIEW

Mengyao Hu, Laura Grunin, and Bei Wu, *New York University, New York, New York, United States*

With the rapid growth of aging populations, the number of older adults with dementia is increasing worldwide. While there is a significant amount of research on dementia caregivers, we know very little about Chinese American (the largest subgroup of the Asian American population in America) caregivers. Therefore, the aims of this study are to 1) conduct a scoping review by identifying existing studies on Chinese American dementia caregivers, 2) present the current state of the science on Chinese American dementia caregiving, and 3) provide direction for future research. Twenty-one studies were included in the final review with 3 main themes synthesized (care experience, utilization of programs/services, and recruitment for caregivers). Care experience included illness perception towards Alzheimer's Disease and related dementia (ADRD) such as stigma and normalization of the disease process. Filial piety was another important cultural belief underpinning care experience. An underutilization of supportive programs/services among this population was identified. Additionally, the few existing programs/services for Chinese American caregivers as well as the barriers encountered when seeking these programs/services were seen in the literature. The strategies and barriers of the included research articles for recruitment of Chinese American caregivers are also discussed in this study. These findings provide an overview of the current knowledge about Chinese American caregivers and serve as a stepping stone for future studies on similar populations in promoting caregiver's health and developing culturally sensitive caregiver support services.

### CONTENT ANALYSIS OF CHURCH LEADERS' PERCEPTIONS AND ATTITUDES TOWARD DEMENTIA

Kimberly Foster,[1] and Fayron Epps,[2] *1. Georgia State University, Atlanta, Georgia, United States, 2. Emory University, Georgia, United States*

Individuals with dementia face many challenges, including the reactions others have to their diagnoses and associated symptoms. Those affected by dementia often seek support from their faith communities and find many of their faith leaders are unable to respond to their needs due to a lack of awareness. In an effort to transform the perception of dementia within the faith community, dementia educational workshops for church leaders were held at three African American churches in Metropolitan Atlanta. As part of the dementia workshop education, an exercise was used to assess attendees' perceptions and attitudes toward dementia pre and post workshop. One hundred and eight participants took part in this exercise. At the beginning and end of the workshop, participants were asked to write a word or short phrase that came to mind when they thought of dementia. Qualitative content analysis was conducted and 15 codes were extracted and categorized into 3 groups: positive (e.g., supportive); negative (e.g., horrified); and neutral (e.g., information-seeking). Before the workshop, participant responses trended towards negative responses (e.g., fear, loss, damn). After the workshop, participants expressed in writing more neutral and positive phrases and words (e.g., support, hopefulness, caring). The findings indicate that with training, church leaders can change their perceptions and attitudes toward dementia to a more accepting and positive stance. Ultimately, continued education in the faith communities may allow for families affected by dementia to feel comfortable seeking help from their church, a very important resource in their community.

### DELIVERING AND EMBEDDING DEMENTIA-FRIENDLY TRAINING IN A HEALTHCARE SYSTEM

Ellen Schneider,[1] Maureen Dale,[2] Krista Wells,[3] John Gotelli,[3] Carol Julian,[2] Cristine Henage,[2] Jan Busby-Whitehead,[2] and Ellen Roberts[2], *1. University of North Carolina at Chapel Hill, Raleigh, North Carolina, United States, 2. University of North Carolina School of Medicine, Chapel Hill, North Carolina, United States, 3. UNC Health Care, Hillsborough, North Carolina, United States*

Alzheimer's disease is the 4th leading cause of death in North Carolina for people 65 and older. People with dementia are hospitalized more often and have prolonged stays, poorer outcomes, higher costs, and increased readmission rates. Hospital employees have expressed the desire to have specialized training to learn how to more effectively communicate with and provide better care to patients with dementia. To address identified patient and hospital employee needs, the University of North Carolina (UNC) Center for Aging and Health is disseminating hospital-specific dementia-friendly training at five hospitals within the UNC

Health System. The training is being delivered via online modules and follow-up didactic sessions over a three-year period to clinical and non-clinical staff who interact with patients. To date, 1,948 employees at three of the five hospitals have launched the online training; 1,102 have completed the training. The pilot training took place at the UNC Hospitals--Hillsborough Campus ("Hillsborough Hospital") in 2019. Hillsborough Hospital staff (n=195) who participated in the dementia friendly training completed a survey to assess their ability to recognize symptoms and provide appropriate care to dementia patients pre- and post-training. Clinical staff answered 23 Likert scale self-efficacy questions; non-clinical staff answered the first 12 of these questions. Positive change in self-efficacy ratings from pre- to post-training was significant for every question (p < .0001). Additional results will be included in the poster. The dementia-friendly hospital initiative is preparing employees to provide better care for people with dementia and is effective in increasing employee self-efficacy.

### DEMENTIA ADJUDICATION TRIGGERS ASSOCIATED WITH INCREASED MORTALITY FOR OLDER AUSTRALIANS: EVIDENCE FROM ASPREE

Jane Banaszak-Holl,[1] Xiaoping Lin,[1] Jing Xie,[2] Stephanie Ward,[3] Henry Brodaty,[4] Raj Shah,[5] Anne Murray,[6] and John McNeil,[1] *1. Monash University, Melbourne, Victoria, Australia, 2. Peter Mac Cancer Centre, Melbourne, Victoria, Australia, 3. UNSW - Sydney, Sydney, New South Wales, Australia, 4. UNSW Sydney, Sydney, New South Wales, Australia, 5. Rush University, Chicago, Illinois, United States, 6. University of Minnesota, Minneapolis, Minnesota, United States*

Research Aims: This study seeks to understand whether those with dementia experience higher risk of death, using data from the ASPREE (ASPirin in Reducing Events in the Elderly) clinical trial study. Methods: ASPREE was a primary intervention trial of low-dose aspirin among healthy older people. The Australian cohort included 16,703 dementia-free participants aged 70 years and over at enrolment. Participants were triggered for dementia adjudication if cognitive test results were poorer than expected, self-reporting dementia diagnosis or memory problems, or dementia medications were detected. Incidental dementia was adjudicated by an international adjudication committee using the Diagnostic and Statistical Manual for Mental Disorders (DSM-IV) criteria and results of a neuropsychological battery and functional measures with medical record substantiation. Statistical analyses used a cox proportional hazards model. Results: As previously reported, 1052 participants (5.5%) died during a median of 4.7 years of follow-up and 964 participants had a dementia trigger, of whom, 575 (60%) were adjucated as having dementia. Preliminary analyses has shown that the mortality rate was higher among participants with a dementia trigger, regardless of dementia adjudication outcome, than those without (15% vs 5%, X2 = 205, p <.001). Conclusion: This study will provide important analyses of differences in the hazard ratio for mortality and causes of death among people with and without cognitive impairment and has important implications on service planning.

### DEMENTIA CARE TRAINING REGULATIONS AND DEFICIENCIES OF CARE FOR INAPPROPRIATE PSYCHOTROPICS USE IN NURSING HOMES

Jung Min Yoon,[1] Alison Trinkoff,[1] Carla Storr,[1] and Elizabeth Galik,[2] *1. University of Maryland, Baltimore, Baltimore, Maryland, United States, 2. University of Maryland, Baltimore, Maryland, United States*

Psychotropics are often used to manage behavioral and psychological symptoms of dementia (BPSD) in nursing homes (NHs), despite their adverse effects and lack of efficacy. NHs can be flagged for inappropriate psychotropics use as a deficiency of care (F-tag 758). To improve dementia care, 15 states require dementia-specific in-service training for NH nursing staff with specific training content and hours. The study aimed to relate the occurrence of F-758 citations to the presence of dementia-specific in-service training regulations, stratified by nurse staffing levels (<75th vs ≥75th percentile of nurse hours per resident day, HPRD). Certification and Survey Provider Enhanced Reporting data (n=14,548 NHs) from 2017-18 were used, containing 1,872 NHs with F-758 tags related to care of residents with dementia. NHs in states specifying training content and hours had significantly lower odds of receiving F-758 tags (OR=0.75, 95% CI=0.60-0.94). Among NHs with lower registered nurse HPRD, those in states regulating training content and hours had significantly lower odds of receiving F-758 tags (OR=0.66, 95% CI=0.49-0.89), with similar findings among NHs with lower certified nurse assistant HPRD (OR=0.69, 95% CI=0.51 52-0.91 92). This study found that required dementia-specific in-service training may be helpful in facilities with lower staffing. It is recommended that states develop more comprehensive, robust dementia care training regulations for NH nursing staff.

### DEMENTIA DIAGNOSIS AND CHALLENGES IN MINORITY POPULATIONS

Halima Amjad,[1] Marcela Blinka,[2] Jennifer Aufill,[3] and Quincy Samus,[1] *1. Johns Hopkins University, Baltimore, Maryland, United States, 2. Johns Hopkins University, School of Medicine, Baltimore, Maryland, United States, 3. Johns Hopkins Bloomberg School of Public Health, Baltimore, Maryland, United States*

Alzheimer's disease and related dementias are underdiagnosed in the United States, with potentially higher rates of underdiagnosis among minority groups. Our objective was to examine perceptions of dementia, the utility and timeliness of diagnosis, and experiences obtaining diagnosis and care in minorities. We recruited 17 family caregivers of African American (n=11), Latino (n=3), and Asian (n=3) persons with dementia (PWD) to complete surveys and semi-structured interviews. Caregivers were mostly female (n=14), children of PWD (n=14), and had greater than high school education (n=16). Mean PWD age at diagnosis was 76 years (range 63-90) with mean 17 months from symptom observation to diagnosis (range 0.5-36 months). Interview themes were coded using a grounded theory approach. Emerging themes related to concerns prior to diagnosis, diagnosis experiences, timeliness of diagnosis, ways to improve diagnosis and care, familiarity with dementia, and stigma. Poor memory was the most common early concern; caregivers also noted behavioral symptoms, weight loss, family stress, and PWD vulnerability. Caregivers recalled key moments when they knew something was wrong. Primary care was the most frequent starting point in diagnosis; longstanding primary care relationships both facilitated and hindered diagnosis. Nine of the 17 caregivers felt diagnosis was delayed. Caregivers preferred clinicians who were forthcoming with the diagnosis and what to expect and noted the importance of family meetings or counseling. Prior experience or knowledge of dementia was common. Caregiver perspectives and experiences elicited in this study may be translated to interventions and clinical practices that proactively detect and address dementia in minorities.

### DISCUSSIONS BETWEEN PEOPLE WITH DEMENTIA AND CARE PARTNERS ABOUT END-OF-LIFE CARE PLANNING

Megan Shepherd-Banigan,[1] Cassie Ford,[2] Emmanuelle Belanger,[3] and Courtney Van Houtven,[1] *1. Duke University School of Medicine, Durham, North Carolina, United States, 2. Duke School of Medicine, Durham, North Carolina, United States, 3. Brown University School of Public Health, Providence, Rhode Island, United States*

Advanced care planning (ACP) leads to better end-of-life (EoL) care. Yet, some care-partners are unaware of the person with dementia's (PwD) preferences. Care-partners play an important role in urging PwD to consider their EoL care wishes early in their disease course and to document those wishes. However, it is unknown whether discussions between care-partners and PwD are associated with documenting EoL care plans. We apply generalized linear models to baseline data from the CARE-IDEAS study which includes a sample of patients who received an amyloid PET scan and their care-partners (n=1,672). We examine the association between PwD report of having discussed EoL care with their care-partner and PwD report of having documented their plans through an advanced directive, a living will, or designating a health care proxy. PwD who reported speaking with their care-partners about EoL care were 10% (marginal probability (MP) 0.10; 95% CI: 0.8, 0.13) more likely to have documented their EoL care wishes. Furthermore, if PwD and care-partners agreed that they had discussed EoL care, PwD were 7% (MP 0.7; 95% CI 0.04, 0.10) more likely to report that they documented their EoL care plans. The positive association between communicating with care-partners about EoL care and having formal EoL care plans suggests that the ACP process could be a systematic approach to increase the care-partner's knowledge of PwD EoL wishes. These results also suggest that increasing involvement of care partners in ACP may encourage patients to document their wishes at end of life.

### EFFECTS OF AMBIENT BRIGHT LIGHT ON NEUROBEHAVIORAL SYMPTOMS IN DEMENTIA: A SYSTEMATIC REVIEW

Ying-Ling Jao,[1] Julian Wang,[1] Yo-Jen Liao,[1] Diane Berish,[1] Kimberly Van Haitsma,[2] and Marie Boltz,[2] *1. Pennsylvania State University, University Park, Pennsylvania, United States, 2. Penn State University, University Park, Pennsylvania, United States*

Most persons living with dementia (PLwD) experience behavioral and psychological symptoms of distress in dementia (BPSDs). Despite increased utilization of bright light to improve BPSDs, the evidence of effectiveness and dosage using ambient light has not been comprehensively examined. This review synthesized research evidence on the effect of ambient light on BPSD in PLwD. A literature search conducted in Scopus, PubMed, CINAHL, and Web of Science included keywords: dementia, bright light, ambient light, indirect light, behavior symptoms, agitation, wandering, depression, aggression, and apathy. Original studies that examined the effect of ambient light on BPSDs were included. Six studies were identified. Sample size ranged from 6 to 189. Lighting delivery methods included a lighting table and ceiling-mounted fixtures in public areas and/or participant's bedroom. Lighting intensity ranged from 1,000-3,000 lux, color temperature ranged from bluish white to warm white, and exposure duration ranged from 4 to 24 hours a day. PLwD with higher light exposure showed more pleasure and alertness. Ambient bright lighting showed mixed results in reducing agitation with one study reporting increased agitation. Three out of four studies showed positive effects upon depressive symptoms. Ambient bright light positively impacted pleasure and alertness. Mixed results on agitation and depressive symptoms may be explained by differences in illuminance, color, duration, and targeted lighting positions. Further studies are needed to confirm the positive effects of ambient light on BPSD. Accurate lighting exposure measurements related to spectral compositions and dosage for individual PLwD would help explain the underlying relationships between lighting and BPSD.

### EVALUATION OF INTERVENTION ACCEPTABILITY IN COMMUNITY STUDIES WITH INDIVIDUALS AND DYADS AFFECTED BY DEMENTIA

Melissa Harris,[1] and Marita Titler,[2] *1. University of Michigan, Madison Heights, Michigan, United States, 2. University of Michigan, Ann Arbor, Michigan, United States*

Multiple non-pharmacologic interventions for symptom management in community dwelling individuals with dementia have demonstrated effectiveness, but have had limited uptake in practice. Prior reviews have evaluated acceptability of interventions for caregivers, but none have evaluated interventions for care recipients with dementia and dyads. This review synthesized the evidence about intervention acceptability for dyads (individuals with dementia and informal caregivers) and individuals with dementia residing at home. Four databases were searched (PubMed, CINAHL, AgeLine, PsycINFO) using inclusion criteria of: intervention studies, community dwelling individuals with dementia or dyads of care recipients and informal caregivers, non-pharmacologic intervention, evaluation of intervention acceptability. Gray literature and non-English articles were excluded. 173 citations were screened by title and abstract, 38 were reviewed by full text, and 19 studies were included. 18 studies focused on dyads, and 13 different non-pharmacologic intervention types were evaluated across studies. Qualitative (n=3), quantitative (n=8) and mixed methods (n=8) were used to evaluate acceptability. Approaches and measures of acceptability included field notes, behavioral checklists, focus groups, semi-structured interviews, questionnaires, and completion rates of intervention sessions and outcome measures. Although participants' benefit and satisfaction with the interventions were high across studies, variability in definitions of acceptability, the methods and measures used constrain the interpretation and generalizability of findings. Psychometric properties of quantitative questionnaires were not addressed even as the most basic level of face or content validity. To enhance the applicability of non-pharmacologic treatments for this population, future research should emphasize the evaluation of intervention acceptability, as well as effectiveness.

### FROM DEFEAT TO EMPOWERMENT: USER EXPERIENCES FROM A HEALTH PROMOTION INTERVENTION FOR PEOPLE WITH DEMENTIA

Martine Kajander,[1] Martha Therese Gjestsen,[2] and Ingelin Testad, *1. Centre for Age-related Medicine - SESAM, Stavanger University Hospital, Stavanger, Norway, Sandnes, Norway, 2. Centre for Age-related Medicine - SESAM, Stavanger University Hospital, Stavanger, Norway, Stavanger, Norway, 3. Centre for Age-Related Medicine - SESAM, Stavanger University Hospital, Stavanger, Norway*

Empowering people with early-stage dementia through the provision of information and support has gained an increasing focus as the number of people with dementia increases worldwide. Health Promotion is a mean to empower the person affected to take an active role in the situation, and taking steps themselves to adjust and cope with the condition. The aim of this study was to explore the experiences of people with early-stage dementia provided with support and information through a 12-week Health Promotion course. Data comprises separate individual semi-structured interviews with 32 people with dementia after attending the course. For each participant, a carer was also interviewed. Interviews were analysed using systematic text condensation. Four categories emerged from the analysis. These were: (I) bridging the post-diagnostic information gap, (II) promoting healthy behaviours, (III) meeting others with early-stage dementia, and (IV) coming to terms with the diagnosis. The results demonstrated that the intervention was well received by participants; learning about dementia, meeting others in the same situation and focussing on maintaining a healthy lifestyle empowered and motivated participants. The participants' carers found the course booklet especially useful and it improved family communication. In conclusion, a 12-week Health Promotion course has the potential to empower people with dementia to cope with their condition through the provision of information, peer-support, which in turn can improve family communication and ease the process of accepting the diagnosis.

### HARMONY STUDY: PIMAVANSERIN SIGNIFICANTLY PROLONGS TIME TO RELAPSE OF DEMENTIA-RELATED PSYCHOSIS

Pierre Tariot,[1] Erin P. Foff,[2] Jeffrey L. Cummings,[3] Maria-Eugenia Soto-Martin,[4] Bradley McEvoy,[5] Srdjan Stankovic,[2] and Amy Howard, *1. Banner Alzheimer's Institute, Pheonix, Arizona, United States, 2. ACADIA Pharmaceuticals Inc., Princeton, New Jersey, United States, 3. Cleveland Clinic Lou Ruvo Center for Brain Health, Las Vegas, Nevada, United States, 4. Gerontopole Alzheimer Clinical Research Center/University Hospital of Toulouse, Toulouse, France, 5. ACADIA Pharmaceuticals Inc., San Diego, California, United States*

Dementia-related psychosis (DRP) is common among patients with Alzheimer's disease (AD), Parkinson's disease (PD), dementia with Lewy bodies (DLB), frontotemporal dementia (FTD), and vascular dementia (VaD) and is associated with poor outcomes. HARMONY (NCT03325556) was a Phase 3, placebo-controlled, randomized, relapse-prevention study evaluating the efficacy and safety of pimavanserin for treating hallucinations and delusions associated with DRP. Patients with dementia and moderate-severe psychosis received open-label (OL) pimavanserin for 12 weeks. Patients with sustained response (≥30% reduction in Scale for the Assessment of Positive Symptoms hallucinations+delusions Total Score AND Clinical Global Impression-Improvement score of much/very much improved) at Weeks 8 and 12 were randomized 1:1 to continue pimavanserin or receive placebo for up to 26 weeks in the double-blind (DB) period. The primary endpoint was time from randomization to relapse of DRP. 392 patients enrolled. 217 (61.8%) eligible patients experienced sustained response and were randomized. OL response was similar regardless of dementia subtype (randomization rates: 59.8% AD, 71.2% PDD, 71.4% VaD, 45.5% DLB, 50.0% FTD), baseline disease characteristics, age, dementia severity, or previous drug therapy. The study stopped early for superior efficacy when a prespecified interim analysis revealed >2.8-fold reduction in risk of relapse with pimavanserin (hazard ratio: 0.353; 95% CI: 0.172, 0.727; 1-sided p=0.0023). Adverse event rates were low and balanced (OL: 36.2%; DB: 41.0% pimavanserin, 36.6%

placebo). No negative impact on cognition or motor function was observed. The Harmony study demonstrated a robust decrease in hallucinations and delusions and significant maintenance of efficacy with pimavanserin treatment in DRP.

### INITIAL DERIVATION OF POSITIVE DEMENTIA-RELATED OBSERVATIONAL MEASURES: A DESCRIPTIVE ETHNOGRAPHY

Maya Staehler,[1] Clark Benson,[1] Jordan Madden,[2] Laura Block,[1] and Andrea Gilmore-Bykovskyi,[3]
*1. University of Wisconsin-Madison School of Nursing, Madison, Wisconsin, United States, 2. Mayo Clinic, Rochester, Minnesota, United States, 3. University of Wisconsin-Madison, Madison, Wisconsin, United States*

Person-centered caregiving approaches emphasize efforts to protect and maintain the personhood of people living with dementia (PLWD). The influence on person-centered caregiving approaches on PLWD have predominantly focused on deficit-oriented outcomes, such as absence or reduction of behavioral symptoms. While important to quality of life, the absence of measurable "positive" responses to person-centered caregiving approaches limit opportunities to specify sensitive and meaningful outcome measures that more holistically represent PLWD's care experiences as more than the absence of a negative outcome. To address these gaps, we conducted a secondary analysis of video-observations of PLWD (N=9) surrounding mealtime cares using a descriptive ethnographic approach. Our objectives were to descriptively summarize specific responsive behaviors demonstrated by PLWD surrounding person-centered caregiving interactions, specifying observable features of these responses and consider their utility in future video-observational research. Findings indicate PLWD contribute both verbal and non-verbal communication surrounding person-centered approaches which can be characterized as conversational (starting conversation, answering or asking questions), expressing preferences (indicating needs and preferences, agreeing or disagreeing, complying with or refusing caregiver requests, permission granting), emotional responses (mirroring caregivers' emotions, demonstrating emotion e.g. smiling), and reflexive (mirroring of caregiver's actions), with overlap between categories. Findings suggest that PLWD not only contribute and respond in meaningful ways to person-centered interactions, but also initiate a significant number of these interactions. This study contributes to a growing body of research and advocacy that examines the personhood and abilities of PLWD and establishes the utility of observational data in studying PLWD contributions.

### LIFE COURSE SOCIOECONOMIC STATUS AND LATER LIFE ALZHEIMER'S DISEASE-RELATED NEUROPATHOLOGICAL LESIONS

Sarah Tom,[1] Amol Mehta,[1] Stepanie Izard,[1] Paul Crane,[2] David Bennett,[3] Philip De Jager,[1] and Julie Schneider,[4]
*1. Columbia University, New York, New York, United States, 2. University of Washington, Seattle, Washington, United States, 3. Rush University Medical Center, Chicago, Illinois, United States, 4. Rush Alzheimer's Disease Center, Chicago, Illinois, United States*

While higher life course socioeconomic status (SES) is associated with lower Alzheimer's Disease (AD) risk, relationships with AD-related neuropathological lesions are unclear. We hypothesize that high SES in early, mid and late life will be associated with lower frequency of AD-related pathological lesions. The Rush Memory and Aging Project is a cohort of 2025 people age ≥ 65 years from Northeastern Illinois recruited 1997 – 2018; 972 participants died. We created binary variables for Braak stage (0-II versus III-VI), NIA-Reagan score (low likelihood/no AD pathology versus high/intermediate likelihood), presence of microinfarcts and, separately, macroinfarcts, and life course SES based on median for late life (baseline income), midlife (income at age 40 years), and early life (composite of parental education and number of siblings). Logistic regression models adjusted for ages at baseline and death, sex, presence of APOE-Ɛ4 alleles, and separately, vascular factors and education. Of 761 participants with relevant data, 69% were women, and mean ages at baseline and death were 83 + 6 years and 90 + 6 years, respectively. High early life SES was related to lower frequency of AD pathology (OR= 0.69, 95% CI 0.50, 0.96) and macroinfarcts (OR= 0.69, 95% CI 0.51, 0.94). Results were similar when adjusting for vascular factors; adjustment for education modestly attenuated these associations. Midlife and late life SES were not associated with AD-related neuropathological lesions. High early life SES was related to lower frequency of AD pathology and macroinfarct presence. Environment during early development may influence later life brain aging.

### OUTCOMES OF PRIMARY CARE FOR PEOPLE WITH DEMENTIA: WHAT'S IMPORTANT?

Laura Wray,[1] Bonnie Vest,[2] Laura Brady,[3] Christina Vair,[4] Gregory Beehler,[5] and John McCarten,[6] *1. Veterans Health Administration, Buffalo, New York, United States, 2. University at Buffalo, Buffalo, New York, United States, 3. SUNY University at Buffalo, Buffalo, New York, United States, 4. Department of Veterans Affairs - Salisbury VA Health Care System, Salisbury, North Carolina, United States, 5. Buffalo VAMC, Buffalo, New York, United States, 6. Minneapolis VA Medical Center, Minneapolis, Minnesota, United States*

People with dementia (PWD) typically receive most of their healthcare in primary care (PC), but neurocognitive disorders can be challenging to recognize, assess, and manage in that setting. As a result, cognitive impairment in older adults is often missed or not addressed until later stages. The result is poor management of comorbid health conditions, increased healthcare utilization, and negative outcomes for the patient and family. Further, strategies for improvement and barriers to high quality PC for PWD have received limited attention. To improve PC for PWD, it is essential to understand what care outcomes should be targeted. To address this gap, we used a qualitative approach to examine potential outcomes of PC from the perspectives of older adults, family caregivers, primary care teams, and geriatrics specialists (n=79) from two Veterans Health Administration healthcare systems. Participants were interviewed individually or in focus groups. A directed content analysis based on the adapted Donabedian model was employed and expanded to fully capture transcript content. Three main categories of outcomes were identified: Personhood (i.e., independence), Physical Health and Safety, and Quality of Life. Regardless of participant type, respondents focused on similar desired outcomes and, notably, identified outcomes as important for both patients and their broader social context (i.e., caregivers, family). Discussion will: show how findings align with work conducted in specialty and residential care; describe how challenges to attaining these outcomes in PC can be overcome; and, challenge cognitive screening recommendations for PC that are based primarily on risk/benefit analysis of medication-focused outcomes.

### POLYSOMNOGRAPHIC SLEEP PARAMETERS: NOVEL DIGITAL BIOMARKERS FOR DEVELOPING DEMENTIA

Javad Razjouyan,[1] Sara Nowakowski,[2]
Amir Sharafkhaneh,[3] Mark Kunik,[1] and Aanand Naik,[1]
*1. Center for Innovations in Quality, Effectiveness, and Safety (iQuest), Houston, Texas, United States, 2. Baylor College of Medicine, Houston, Texas, United States, 3. Michael E. DeBakey VA Medical Center, houston, Texas, United States*

Neuroprotection, early diagnosis, and behavioral intervention are priorities for dementia research. Sleep is emerging as an important potential remediable risk factor. Polysomnography using various digital parameters, qualitatively and quantitively, evaluates sleep. In this study, we examined whether sleep parameters derived from attended overnight polysomnography (PSG) studies are associated with developing dementia. We retrieved 61,165 free-text PSG reports from the VA national electronic health records from 2000 to 2019. Patients with dementia diagnosis up to one-year after PSG were excluded. Patients who developed dementia >1 year after PSG (Dem) were classified using all-cause dementia ICD-9/10 codes documented on two separate visits starting a year after the PSG until the end of 2019 in a 1-year sliding period. Patients with no ICD-9/10 dementia codes (NDem) were propensity matched 1:1 for age, gender, race, ethnicity, BMI, and Charlston comorbidity index to the Dem group (n=1,534). We used natural language processing to identify sleep onset latency (SOL), total sleep time (TST), and apnea-hypopnea index (AHI). Univariate analysis was used to compare the groups. TST (254 v 266m, p=0.001) were significantly shorter and SOL (28 v 31m, p=0.047) were significantly prolonged in Dem compared to NDem. The odds ratio of individuals with an AHI ≥ 15 was significantly higher in Dem compared to NDem group (1.18, 95%CI: 1.04-1.35). Patients with incipient dementia exhibited longer SOL, shorter TST, and a greater proportion had moderate-to-severe apnea compared to patients without dementia. These objective sleep parameters may serve as potential biomarkers for patients at risk for developing dementia.

### RECOGNIZING HEALTHCARE NEEDS AMONG OLDER ADULTS WITH DEMENTIA AND WITH MIGRANT BACKGROUND

Mary Dioise Ramos, *Kennesaw State University, Marietta, Georgia, United States*

Dementia, a syndrome affecting millions of people worldwide, can be challenging, especially in patients with a migrant background. Language barriers and language-based diagnostic tools, cultural differences in the perception of the syndrome as well as restricted access to healthcare can influence medical care. Healthcare providers play a key role in diagnosing persons with dementia and they are in the best position to raise awareness about dementia among older adults. They examine a large number of patients and are generally the first point of contact for people with any health complaints. Therefore, they are able to identify treatable causes of the syndrome at an early stage to prevent irreversible health impairment. This study investigates whether health care providers feel prepared to meet the diagnostic needs of these patient groups and whether there are challenges and support needs. A cross-sectional study is used in this study among a random sample of healthcare providers. Healthcare providers experienced barriers especially the uncertainties in identifying dementia in patients with a migrant background. Language barriers that affected or prevented diagnostics and information deficits in patients with a migrant background were the most frequently reported barriers. Lack of acceptance of the syndrome was also common. Public health measures supporting healthcare providers in their interaction with a migrant background patient as well as information and services for patients with dementia are needed. Efforts to facilitate access to interpreting services and to focus on people with a migrant background in healthcare are necessary.

### SELF-PERCEPTIONS OF DRIVING ABILITY IN OLDER ADULTS WITH AND WITHOUT ALZHEIMER'S DISEASE

Rebecca Davis, and Megan Owens, *Grand Valley State University, Grand Rapids, Michigan, United States*

A concern for families and persons with dementia is determining when driving cessation is necessary. Older adults often modify driving behaviors as they age due to self-perception of a decline in their abilities. However, it is not known how persons with Alzheimer's disease modify driving behaviors. The purpose of this study was to determine difference in how similarly aged persons with and without Alzheimer's disease perceive their driving behaviors and modify them. Data were collected for 48 persons with early stage AD or mild cognitive impairment (MCI) due to AD; and 53 similarly aged older adults without cognitive diagnoses, using a survey on driving frequency, habits, and wayfinding ability. Chi-square analysis was used to determine any statistically significant differences in reported driving habits and difficulties between the study groups. Results from the analysis showed that the majority of the sample was still driving (n=81, 83%). Persons with AD/MCI (n=33, 73%) were less likely to be driving than the control group (n=48, 91%). Of those who were still driving, those with AD/MCI were more likely to limit driving frequency, avoid driving alone and in unfamiliar routes. Worries about getting lost were more frequently noted in those with AD/MCI. The AD group was more likely to make modifications to their driving such as limiting driving to daylight hours and not driving alone. These important results indicate persons with early stage AD/MCI may have self-awareness about driving impairments and make modifications to their driving habits that may increase their driving safety.

### SESSION 2885 (POSTER)

### DEPRESSION, ANXIETY, AND MENTAL HEALTH

### A PRACTICAL PROTOCOL FOR REPORTING DEPRESSIVE SYMPTOMS IN A CLINICAL TRIAL OF GRANDMOTHER CAREGIVERS

McKenzie Wallace,[1] Alexandra Jeanblanc,[2] and Carol Musil,[2] *1. Case Western Reserve University, Lorain, Ohio, United States, 2. Case Western Reserve University, Cleveland, Ohio, United States*

Intervention studies conducted in caregivers often focus on improving mental health. Consequently, researchers may discover incidental findings like elevated depressive symptoms. Researchers have an ethical obligation to report incidental findings to participants, but no protocols exist for reporting behavioral health symptoms. This presentation describes a protocol for reporting elevated depressive symptoms to participants, based on the protocol used in a national randomized clinical trial of stress-reduction methods for 348 grandmothers raising grandchildren. Each questionnaire included the CES-D scale and was scored immediately after completion. Based on our previous work showing higher CES-D scores in custodial grandmothers, and the desire to balance the principles of beneficence and nonmaleficence, we established a cut-off score of 30. A registered nurse on the research team called participants with scores over 30 and ascertained whether the participant 1) was aware of the problem and 2) had sought help, and then offered additional resources. Overall, 94 (27%) participants had a CES-D score > 30. The majority (91%) were aware of the problem. About a third of the participants were on medication for their symptoms, and a third were seeing a therapist. Nine participants were not aware they had depressive symptoms. While most of our participants were aware of their depressive symptoms, they were appreciative of our call and several noted that they would speak to their provider. Future studies should consider how to implement a similar protocol to deliver information critical to the mental health of participants.

### ASSOCIATION OF LONELINESS AND SOCIAL ISOLATION WITH HEALTHSPAN AND LIFESPAN IN THE U.S. HEALTH AND RETIREMENT STUDY

Christopher Crowe,[1] Dayoon Kwon,[2] and Daniel Belsky,[3] *1. Columbia University, New York, New York, United States, 2. Columbia University Mailman School of Public Health, New York, New York, United States, 3. Columbia Mailman School of Public Health, New York, New York, United States*

Loneliness and social isolation present emerging public health challenges for aging populations. To inform efforts against loneliness and social isolation, research is needed to evaluate impacts of these conditions on patterns of adverse aging outcomes. We used longitudinal, repeated measures data to isolate effects of loneliness and social isolation from effects of socioeconomic circumstances and psychological vulnerabilities, rule out reverse-causation, and test dose-response in associations with morbidity, disability, and mortality. We also explored the biological basis of relationships by testing associations with biological aging. We analyzed data from more than 12,000 adults aged 50-95 in the US Health and Retirement Study (HRS) who reported on symptoms of loneliness and social isolation at 2-3 time points during 2006-2014. We measured loneliness using the 3-item Revised UCLA Loneliness Scale and social isolation using a 6-item scale. We tested associations with chronic disease, disability, and biological aging in 2016 and with mortality through 2018. We found (1) associations of loneliness and social isolation with adverse aging outcomes are not fully explained by socioeconomic circumstances and psychological vulnerabilities; (2) the direction of relationships with disability indicates temporal precedence of loneliness and social isolation; (3) there is a dose-response in associations with loneliness; and (4) there is evidence of a biological mechanism explaining associations with loneliness and social isolation. Findings add to the emerging literature on health sequelae of loneliness and social isolation. Assessment of loneliness and social isolation in surveillance and intervention with older adults can enhance risk assessment for adverse aging outcomes.

### DEPRESSIVE SYMPTOMS AMONG OLDER, SEXUAL MINORITY ADULTS AND THE INFLUENCE OF MARITAL STATUS

Briana Mezuk,[1] Rachel Bergmans,[2] and Victoria Schoebel,[3], *1. University of Michigan School of Public Health, Ann Arbor, Michigan, United States, 2. University of Michigan Medical School, Ann Arbor, Michigan, United States, 3. University of Michigan, Charlotte, North Carolina, United States*

Adverse health outcomes, including poor mental health and depression, tend to be more common among those who are lesbian, gay, or bisexual (LGB). Minority stress theory posits that chronic minoritization contributes to disparities in depression. Factors like social support and income can improve mental health outcomes, and these resilience-promoting factors can be gained through marriage. However, whether marriage improves mental health outcomes in old age regardless of sexual orientation is not well established. This study aims to determine if depressive symptoms differ by sexual orientation in old age, and to test whether the association between depression and sexual orientation was moderated by marital status. The 2016 Health and Retirement Study, a nationally representative sample of U.S. adults older than 50 years (n=4,253), was the first wave to include respondent sexual orientation. Depressive symptoms were measured on the 8-item Center for Epidemiologic Studies Depression Scale (CES-D). Those with a score greater than or equal to 3 were considered to have elevated depressive symptoms. When testing main effects in the adjusted model, depression was not more common among LGB persons than heterosexual persons (OR=0.95; 95% CI=0.46-2.00). Yet, marital status significantly moderated the relationship between sexual orientation and depression (p=0.034). Among heterosexual adults, being married was protective against depression (OR=0.48; 95% CI=0.32-0.71) when compared to being never married, whereas marriage was not protective among LGB adults (OR=0.95; 95% CI=0.26-3.45). Findings indicate that LGB adults do not experience the same mental health benefits of marriage as heterosexual individuals.

### DEPRESSIVE SYMPTOMS LONGITUDINALLY MEDIATE THE NEGATIVE EFFECTS OF HBA1C ON MEMORY CHANGE OVER 6 YEARS

A. Zarina Kraal,[1] Vicki Ellingrod,[2] and Laura Zahodne,[1] *1. University of Michigan, Ann Arbor, Michigan, United States, 2. University of Michigan, Ann Arbor, United States*

Elevated glycated hemoglobin (HbA1c) has been associated with worse episodic memory in Type 2 diabetes. Prospective links between HbA1c and incident depressive symptoms suggest that behavioral mechanisms may underlie this association. Elevations in HbA1c may lead to depressive symptoms via biochemical changes directly caused by Type 2 diabetes or through distress associated with managing the treatment demands of the disease. This study aimed to determine whether depressive symptoms longitudinally mediate associations between HbA1c and episodic memory over 6 years. Participants (N=2,155) comprised adults aged 51+ with self-reported Type 2 diabetes and clinically elevated HbA1c (≥5.7%) at the time the Health and Retirement Study initiated blood collection. A longitudinal mediation model quantified associations between baseline HbA1c and 6-year change in episodic memory through 4-year change in depressive symptoms, controlling for baseline socio-demographics, other health conditions, and medication adherence. HbA1c was assayed from dried blood spots. Depressive symptoms were self-reported twice over four years. Episodic memory, assessed three times over six years, was a z-score composite of immediate and delayed recall of a word list. Increased depressive symptoms four years after baseline partially mediated the negative association between baseline HbA1c and 6-year memory decline. Depressive symptoms partially mediated the concurrent association between HbA1c and memory at baseline. This longitudinal study provides evidence that the deleterious effects of HbA1c on subsequent episodic memory may operate partly through behavioral mechanisms. Depressive symptoms may represent a critical target for interventions to reduce the enduring negative effects of hyperglycemia on memory aging in Type 2 diabetes.

### EVIDENCE OF MEDIATION BY DEPRESSIVE SYMPTOMS IN THE ASSOCIATION BETWEEN FRAILTY AND COGNITIVE DECLINE IN OLDER ADULTS

Nicholas Resciniti, Matthew Lohman, and Anwar Merchant, *University of South Carolina, Columbia, South Carolina, United States*

Frailty and pre-frailty have been shown to predict cognitive decline in older adults; however, knowledge about mediating pathways in this association is lacking. This study aims to assess if depressive symptoms mediate the relationship between frailty and cognitive function. Frailty status, cognitive function scores, and depressive symptoms were measured in 4,672 community-dwelling US adults ≥65 and older from the Health and Retirement Study from 2006-2010. Fried's frailty phenotype criteria (weakness, slowness, physical inactivity, low weight, and exhaustion) were used to categorize individuals as frail (3-5 criteria), pre-frail (1-2 criteria) and robust (0 criteria). Memory recall and global mental status (from 0-35) was used to determine cognitive function. The CES-D was used to assess depressive symptoms. A causal mediation analysis was performed to estimate the direct effect (DE), the direct effect (DE), and the indirect effect (IE). Both frailty (TE: β=-1.22; 95% CI: -1.75, -0.68) and pre-frailty (TE: β=-0.52; 95% CI: -0.86, -0.18) were found to be associated with lower cognitive scores, after adjusting for confounders. There was significant but small IE between frailty status and declining cognitive scores mediated by depressive symptoms [frailty: β=-0.09 (95% CI: -0.14, -0.03); pre-frailty: β = -0.03 (95% CI: -0.06, -0.01). Additionally, the DE was significant for frailty (β=-1.13; 95% CI: -1.67, -0.59) and pre-frailty (β=-0.49 95% CI: -0.82, -0.15). This study provides evidence that depressive symptoms may mediate the association between frailty and cognitive decline. The results suggest possible intervenable pathways for preventing or delaying cognitive decline.

### FACTORS ASSOCIATED WITH DEPRESSIVE SYMPTOMS AMONG U.S. OLDER CHINESE IMMIGRANTS

Ying-Yu Chao,[1] Jin Young Seo,[2] Mei-Lan Chen,[3] and Peijia Zha,[4] *1. Rutgers School of Nursing, Newark, New Jersey, United States, 2. Hunter College, CUNY, New York, New York, United States, 3. Georgia State University, Atlanta, Georgia, United States, 4. Rutgers, The State University of New Jersey, Newark, New Jersey, United States*

Purpose: Chinese Americans represent the largest Asian ethnic subgroup in the United States. Depression is the most common mental health problem among older adults. However, we have a limited understanding of depressive symptoms among older Chinese immigrants. The study aimed to examine the potential factors associated with depressive symptoms among older Chinese immigrants in U.S. Methods: We recruited participants from psychiatric clinics who sought professional help in New York City. Inclusion criteria were Chinese immigrants from Asian countries 50 years or older; able to speak and understand either Mandarin or Cantonese; and had a diagnosis with major depressive disorder. Depressive symptoms were measured with Quick Inventory of Depressive Symptomatology; cognitive function was measured with Montreal cognitive assessment; sleep quality was measured with Pittsburgh Sleep Quality Index, and physical activity was measured with International Physical Activity Questionnaire. Descriptive statistics and multiple regression were performed. Results: Participants were ninety-nine Chinese older immigrants (mean age: 60.69 ± 7.62 years). Participants who had more children ($p < .05$), poor health status ($p < .01$), poor quality of life ($p < .01$), less social support ($p < .01$), and need help with activities of daily living ($p < .05$) had more depressive symptoms. Cognitive function, sleep quality, and physical activity were significantly associated with depressive symptoms. Conclusions & Implications: Poor cognitive function, poor sleep quality, and less physical activity were associated with depressive symptoms. Our results provide knowledge for developing culturally tailored self-management interventions for older Chinese immigrants with depressive disorder in managed care settings.

### FACTORS ASSOCIATED WITH SOCIAL ISOLATION AMONG OLDER ADULTS: THE SINGAPORE CHINESE HEALTH STUDY

Jon Barrenetxea,[1] Yang Yi,[1] Woon Puay Koh,[1] and Feng Qiushi,[2] *1. Duke-Nus Medical School, Singapore, Singapore, 2. National University of Singapore, Singapore, Singapore*

Social isolation is a determinant of mortality and well-being among older people. Factors associated with isolation could be different in societies where older adults live mainly with family, as individuals might feel isolated despite living with others. We studied the factors associated with isolation among 16,948 older adults from follow-up 3 of the Singapore Chinese Health Study, a population-based cohort of older Singapore Chinese (mean age of 73, range: 61-96 years). We defined social isolation as having "zero hour per week" of participation in social activities involving 3 or more people and scoring the lowest decile on the Duke Social Support Scale of perceived social support. We used multivariable logistic regressions to compute odds ratio (OR) and 95% confidence interval (CI) for factors associated with likelihood of social isolation. Although only 14.4% of isolated participants lived alone, living alone remained a significant factor associated with isolation (OR 1.93, 95% CI 1.58-2.35), together with cognitive impairment (OR 1.73, 95% CI 1.46-2.04) and depression (OR 2.44, 95% CI 2.12-2.80). Higher education level was inversely associated with isolation (p for trend<0.001). In stratified analysis, among those living alone, compared to women, men had higher odds of social isolation (OR 2.18, 95% CI 1.43-3.32) than among those not living alone (OR 0.99, 95% CI 0.84-1.17) (p for interaction<0.001). Our results showed that living alone, cognitive impairment and depression were indicators of isolation among older Singaporeans. In addition, among those living alone, men were more likely to experience social isolation than women.

### FUTURE CONFIDENCE AND SELF-REPORTED HEALTH AMONG THE BEREAVED ELDERLY IN CHINA

Haimin Pan,[1] and Qian Liu,[2] *1. Xiamen University, Xiamen, China, 2. Hunan Normal University, Changsha, China*

Time perspective serves as an important determinant for the elderly's health-related outcomes, including those in bereavement. Confidence in the future (CF) seems a reflecting viewpoint a in this line, however, it is hardly investigated as a predictor for health among the elderly. This study centered on the casual relationship between CF and self-reported health (SRH) among the bereaved older Chinese adults. Data from a sample of 376 elders in bereavement were finally used for analysis. The sample was extracted from a national representative data, namely, China Family Panel Studies (CFPS), and contained 2012, 2014, 2016, and 2018 waves of information. A across-lagged model with four time points was used to explore the longitudinal relationship between CF and SRH among the bereaved elderly in China. This model achieved acceptable indices of goodness of fit, and the results revealed that SRH positively predicted future confidence in general among the senior participants, while CF failed to predict SRH at any point of time. The cross-sectional associations between CF and SRH were generally positively significant. The results implied the casual effect of SRH on CF, which was different from the cross-sectional relationship between CF and SRH. It seemed that enhancing the oral health of the bereaved elderly could be beneficial for increase in their CF. Tailored services are recommended for improving the perception of the bereaved elderly on their general health, and thus strengthening their positive perspectives for future.

### GEOGRAPHIC REGIONAL VARIATION IN PATTERNS OF RACIAL AND ETHNIC DISPARITIES IN LATE-LIFE DEPRESSION

Chirag Vyas,[1] Charles Reynolds III,[2] David Mischoulon,[1] Grace Chang,[3] and Olivia Okereke,[1] *1. Massachusetts General Hospital, Boston, Massachusetts, United States, 2. University of Pittsburgh Medical Center, Pittsburgh, United States, 3. VA Brockton Healthcare System, Brockton, United States*

There is evidence of racial/ethnic disparities in late-life depression (LLD) burden and treatment in the US. Geographic region may be a novel social determinant; yet, limited data exist regarding the interplay of geographic region with racial/ethnic differences in LLD severity, item-level symptom burden and treatment. We conducted a cross-sectional study among 25,503 men aged 50+ years and women aged 55+ years in VITAL-DEP (VITamin D and OmegA-3 TriaL-Depression Endpoint Prevention), an ancillary study to the VITAL trial. Racial/ethnic groups included Non-Hispanic White, Black, Hispanic, Asian, and other groups (Native American/Alaskan Native and other/multiple/unspecified-race/ethnicity). We assessed depression status using: the Patient Health Questionnaire-8 (PHQ-8); self-reported clinician/physician diagnosis of depression; medication and/or counseling treatment for depression. In the full sample, Midwest region was significantly associated with 12% lower severity of LLD, compared to Northeast region (rate ratio (RR) (95% confidence interval (CI)): 0.88 (0.83-0.93)). However, racial/ethnic differences in LLD varied by region. For example, in the Midwest, Blacks and Hispanics had significantly higher depression severity compared to non-Hispanic Whites (RR (95% CI): for Black, 1.16 (1.02-1.31); for Hispanic, 2.03 (1.38-3.00)). Furthermore, in multivariable-adjusted logistic regression models, minority vs. non-Hispanic White adults had 2- to 3-fold significantly higher odds of several item-level symptoms across all regions, especially in the Midwest and Southwest. Finally, among those endorsing PHQ-8≥10, Blacks had 60-80% significantly lower odds of depression treatment, compared to non-Hispanic Whites, in all regions. In summary, we observed significant geographic variation in patterns of racial/ethnic disparities in LLD outcomes. This requires further longitudinal investigation.

### GLOBAL COGNITION MODIFIED THE LONGITUDINAL RELATIONSHIP BETWEEN ANEMIA AND DEPRESSION IN OLD AGE: THE IMIAS STUDY

Tamer Ahmed,[1] and Helen-Maria Vasiliadis,[2] *1. University of Sherbrooke, Montreal, Quebec, Canada, 2. University of Sherbrooke, Sherbrooke, Quebec, Canada*

Background: We examined the longitudinal relationships between hemoglobin concentrations or the severity of anemia and depression and whether baseline cognitive function modifies these longitudinal relationships over 4 years of follow-up. Methods: A total of 1608 community-dwelling older adults from the International Mobility in Aging Study (IMIAS) aged 65 to 74 years were recruited in Natal (Brazil), Manizales (Colombia), Kingston (Ontario, Canada), and Saint-Hyacinthe (Quebec, Canada). The study outcome was depression, defined by a score of 16 or over in the Center for Epidemiologic Studies Depression Scale (CES-D). Longitudinal associations over four years follow-up were examined using generalized estimating equations. Models reported were either unadjusted and adjusted for research sites, alcohol drinking status, body mass index, chronic conditions, activities of daily life disabilities, and polypharmacy. Results: Longuitinal relationships suggested an evidence of multiplicative interaction by baseline global cognition in which 1g/dL increase in hemoglobin concentrations there was a significant reduction in the risk of depression with a stronger effect among participants with good cognitive function (Odds Ratio (OR)=0.85, 95% CI: 0.78-0.92) compared to those with poor cognition (OR=0.89, 95% CI: 0.80-0.97). Anemia and poor cognition at baseline were associated with an increased risk of depression over 4 years of follow-up (OR=5.80, 95% CI: 1.84-18.23). Global cognition was an effect modifier of the longitudinal association between the severity of anemia and depression. Conclusion: In international samples of older adults, hemoglobin concentrations, as well as the severity of anemia, were independent risk factors for depression and these associations differed by global cognitive function.

### JOB STRAIN AND MENTAL HEALTH AMONG OLDER ADULTS: RESULTS FROM THE NATIONAL HEALTH INTERVIEW SURVEY

Christina Hatten,[1] Melissa DuPont-Reyes,[2] Tailisha Gonzalez,[3] Rosenda Murillo,[4] Sandra Echeverria,[5] and Elizabeth Vasquez,[6] *1. University at Albany, State University of New York, Albany, New York, United States, 2. Texas A&M University, College Station, Texas, United States, 3. CUNY School of Public Health, New York, New York, United States, 4. University of Houston, Houston, Texas, United States, 5. University of North Carolina, Greensboro, Greensboro, North Carolina, United States, 6. University at Albany (SUNY), Rensselaer, New York, United States*

Increased life expectancy and retirement age have contributed to a gradual aging of the US workforce. Extant literature has identified job strain (e.g. job demands, work-related stressors etc.) as a risk factor for both physical and mental health outcomes in working populations. However, there is comparatively less evidence examining job strain and mental health among older working adults. We utilized National Health Interview Survey data (2015), which contained detailed information on work conditions, to investigate the relationship between fear of job loss and self-rated mental health among working adults at 25-54 and 55+ years of age. We fit multiple logistic regression models that accounted for the complex survey design. Mental health was assessed using the K6 screening instrument that measures nonspecific psychological distress. In the sample of 16,291 working adults, 11.6% worried about job loss and 13.3% reported moderate/severe mental distress. Overall, we found that the odds of poor self-rated mental health among those who reported fear of job loss were 2.35 times that of respondents with perceived job security after adjusting for age, sex, educational attainment, race and/or ethnicity, body mass index, and smoking behaviors (95% Confidence Interval (CI): 2.01-2.75). Age-stratified results showed that perceived job insecurity corresponds with a 2.33 odds of mental distress among workers aged 25-54, and a 2.44 odds of mental distress among older workers. Our findings highlight the importance of age dynamics in mental health for working adults and suggest that job strain presents a persistent challenge for the wellbeing of workers.

### OLDER ADULTS IN POOR HEALTH RECEIVED SOCIAL SUPPORT MAINLY FROM FAMILY: THE SINGAPORE CHINESE HEALTH STUDY

Jon Barrenetxea,[1] Yang Yi,[1] Kyriakos S. Markides,[2] Woon Puay Koh,[1] and Feng Qiushi,[3] *1. Duke-NUS Medical School, Singapore, Singapore, 2. The University of Texas Medical Branch at Galveston, Galveston, United States, 3. National University of Singapore, Singapore, Singapore*

While having social support can contribute to the health of older adults, those in poor health may be limited in their capacity to receive social support. We studied health factors associated with social support among 16,948 participants from follow-up 3 of the Singapore Chinese Health Study, a population-based cohort of older Singapore Chinese. Participants were interviewed at mean age of 73 years (range from 61 to 96 years) using the Duke Social Support Scale. Latent Class Analysis (LCA) was applied to derive groups based on the source and intensity of social support. We ran multivariate logistic regression models to study health factors associated with group membership. LCA revealed four groups in increasing social support scores: The “family restricted”, who had the lowest social support scores and only received support from family (50%); the “loners”, who had some support from extended family and non-family (5%); the “family oriented”, who had broad family support and some non-family support (28%); and the “overall supported”, who had the highest social support scores and received broad support from family, extended family and non-family (17%). Compared to the “overall supported” group, health factors associated with being “family restricted” were: having instrumental limitations [odds ratio (OR) 1.34, 95% confidence interval (CI) 1.19-1.50], having poor self-rated health (OR 1.40, 95% CI 1.28-1.54), being depressed (OR 2.49, 95% CI 2.21-2.81) and being cognitively impaired (OR 1.19, 95% CI 1.04-1.37). Our results showed that older adults in poor health received social support mainly from family.

### OLDER ADULTS’ SUBJECTIVE WELL-BEING FOLLOWING AN EXERGAME INTERVENTION “BANDIT THE DOLPHIN”: FOCUS GROUP FINDINGS

Brittany Drazich,[1] Breanna Crane,[2] Kyle Moored,[3] Karl Shieh,[4] Janiece Taylor,[1] and Michelle Carlson,[2] *1. Johns Hopkins School of Nursing, Baltimore, Maryland, United States, 2. Johns Hopkins Bloomberg School of Public Health, Baltimore, Maryland, United States, 3. Johns Hopkins School of Public Health, Baltimore, Maryland, United States, 4. Johns Hopkins University, Johns Hopkins University, Maryland, United States*

Due to generational mental illness stigma and under diagnosis of mental illness, older adults do not always receive the mental health help that they need. One unique technology that has the potential to improve mood in older adults is exergames, or exercise video games. The objective of this substudy (main study: Stimulation With Intricate Movements "SWIM" Study) was to explore older adults' mood following an exergame intervention called "Bandit the Dolphin," created by the Johns Hopkins KATA Studio. Researchers conducted three focus groups with 14 community-dwelling older adult participants who took part in the SWIM Study exergame intervention. The semi-structured focus groups were transcribed, coded, and analyzed using deductive and inductive techniques described by Ray Maietta's "sort and sift, think and shift" method. Three themes related to playing "Bandit the Dolphin" and mood emerged. First, participants described their perceived association between activity and mood. Participants felt that both active and passive activities, "Bandit the Dolphin" and otherwise, improved their mood through the "fun" factor, and through feelings of achievement. Second, the participants described that the competition and frustration of playing "Bandit the Dolphin" increased eventual feelings of achievement. Third, participants described how feelings of immersion, or being absorbed in the game, helped them forget their other life concerns. These findings provide a better understanding of older adults' perceived relationship between an exergame intervention, "Bandit the Dolphin," and short-term improved mood. Future health and engineering researchers should explore exergames as a potential tool to improve the mental health of older adults.

### THE ABSENCE OF CLOSE SOCIAL NETWORK CONNECTIONS: A CASE STUDY INVESTIGATION IN SOCIAL ISOLATION

Raeann LeBlanc,[1] and Rachel Walker,[2] *1. University of Massachusetts, Amherst, Amherst, Massachusetts, United States, 2. University of Massachusetts Amherst, Amherst, Massachusetts, United States*

Social isolation is an emerging phenomenon known to significantly influence health outcomes and carries specific risk in older age. As part of a larger study exploring the social network effects on health among persons age 65 and above, it was found that four cases from a larger sample (n=89) could not name any one from their social relationships supportive to them in managing their health while living with complex chronic health needs. In addressing these findings, these cases, bounded spatially by individual social networks and temporally by the time of the study interview, served the basis for intensive analysis using multiple data points from in-depth interviews and survey measures. Measures included descriptive data (social networks, demographics) and measures of health (SF-12), and social support (MOS-SSS). Each participant case identified as female gender and single, three lived alone, had a high burden of chronic conditions and poor health. All had recent hospitalizations (1 or more within the past year). The structures of their social networks varied in type of relationship roles and size. Social support was perceived overall as low. This study offers a specific contribution to research on social connection/isolation. This phenomenon is relative to specific contexts. Findings emphasize that social isolation can be specific to certain aspects of identity and poorer health in older age. Additional research on the functions and qualities of social networks, in addition to the structure, are important to specify in future research and knowledge development for practice assessment to determine social connection and isolation.

### THE MENTAL HEALTH OF OLDER VETERANS AGES 58-99 YEARS: 2016-2017 VE-HEROES FINDINGS

Yasmin Cypel,[1] Paula Schnurr,[2] Robert Bossarte,[3] William Culpepper,[1] Aaron Schneiderman,[1] Fatema Akhtar,[1] Sybil Morley,[4] and Victoria Davey,[1] *1. U.S. Department of Veterans Affairs, Washington, District of Columbia, United States, 2. U.S. Department of Veterans Affairs, White River Junction, Vermont, United States, 3. West Virginia University, Morgantown, West Virginia, United States, 4. U.S. Department of Veterans Affairs, Canandaigua, New York, United States*

Mental health and its correlates were examined in U.S. Vietnam War veterans approximately fifty years after the War. The 2016-2017 VE-HEROeS (Vietnam Era Health Retrospective Observational Study) was a mail survey of the health of U.S. Vietnam War veterans who served between February 28, 1961 and May 7, 1975 and matched US non-veteran controls. 'Veteran status' represented wartime experience for three cohorts: 'theater' veterans with service in Vietnam, Cambodia, or Laos, non-theater veterans with service elsewhere, and non-veterans with no military service. Veterans and non-veterans, aged 58-99 years, were randomly selected from a veteran sampling frame (n=9.87 million) derived from the Department of Veterans Affairs' USVETS dataset and a commercial address database, respectively. Questionnaires were mailed to 42,393 veterans and 6,885 non-veterans; the response rate for veterans was 45% (n=18,866) and 67% (n=4,530) for non-veterans. Weighted bivariate and multivariable analyses were conducted to examine poor overall mental health, via the SF-8TM Mental Health Component Summary score (MCS), and other mental health measures by veteran status and socioeconomic, health, and other military characteristics. Nearly 50% of all theater veterans reported poor overall mental health (MCS<50). Prevalence of mental health measures was greatest for theater veterans and successively decreased for non-theater veterans and non-veterans. Key correlates significantly (P< 0.02) associated with poor MCS included veteran status, race/ethnicity, income, physical health, health perception, trauma, distress, depression, posttraumatic stress disorder (Primary Care DSM-5 PTSD screen), and drug use. Results indicate a high burden of poor mental health among those who served in-theater.

### THE PREVALENCE OF PRIOR MENTAL HEALTH AND SUBSTANCE USE PROBLEMS IN OLDER PERSONS AND THEIR FAMILIES

Todd Becker,[1] John Cagle,[1] and Paul Sacco,[2] *1. University of Maryland, Baltimore, Baltimore, Maryland, United States, 2. University of Maryland, Baltimore, Maryland, United States*

Although research has shown mental health and substance use problems (MHSUPs) are fairly prevalent in older adults (OAs), less research has considered MHSUPs in hospice beneficiaries and their families. This secondary analysis filled this gap using the Health and Retirement Study's Core survey wave from 2014 and Exit wave data from 2016. These data are nationally-representative of OAs aged 50+. Each biennial wave introduces an experimental module to a random 10% of Core survey participants. One Core 2014 experimental module included self-report indicators assessing past MHSUPs, like depression and anxiety, using single items. Exit 2016 proxy-reported information about respondent deaths was used to create a decedent subsample. Descriptive statistics established MHSUP prevalence rates in OAs and their family. The self-report depression indicator was validated against the 8-item Center for Epidemiological Studies Depression Scale (CESD-8) at the ≥3, ≥4, and ≥5 cut points using $\chi 2$ analyses. The full sample's (N=1,461) average age was 68 years. Participants were mostly non-Hispanic (87.5%), White (72.8%), and female (59.7%). The decedent subsample (n=64) was bifurcated by hospice (54.7%) versus nonhospice (45.3%) utilization. Most participants in the full sample (63.9%), hospice decedent subsample (77.1%), and nonhospice decedent subsample (75.9%) endorsed at least one MHSUP. Depression and anxiety were the most common MHSUPs in each study sample. The CESD-8 was associated with the self-report depression indicator across all cut points (p<.001). Practitioners, policymakers, and researchers should consider the high prevalence rates of MHSUPs found in OAs and their families when designing programs, policies, and research.

### TRAJECTORIES OF DEPRESSIVE SYMPTOMS IN COMMUNITY-DWELLING KOREAN OLDER ADULTS

Jinhee Shin, and Eunhee Cho, *Yonsei University, Seoul, Republic of Korea*

Objectives This study aimed to identify trajectories of depressive symptoms and investigate predictive variables of latent class in Korean community-dwelling older adults. Methods Study participants comprised 2,016 community-dwelling Korean adults aged over 65 years, using data from the Korean Longitudinal Study of Aging (KLoSA) from 2006–2016. The KLoSA, a nationally representative panel survey, has been conducted biannually since 2006. We used latent class growth analysis to identify depressive symptom trajectories. Multinomial logistic regression analysis was conducted to identify predictors of each class of depressive symptoms. Results Five depressive symptom trajectory groups were identified: Class 1, no depressive symptom (13.8%); Class 2, low depressive symptom (32.8%); Class 3, decreasing depressive symptom (10.6%); Class 4, increasing depressive symptoms (24.0%); and Class 5, persistent depressive symptoms (18.8%). We found that older adults followed five distinct depressive symptom trajectories over 10 years. Mini-Mental State Examination scores, number of chronic diseases, educational level, gender, current employment, contact with children, and social activity were associated with a higher risk of these trajectories. Conclusions Depressive symptoms are associated with social networks as cognitive function scores increase and number of chronic diseases decrease. Interventions to strengthening existing social networks and developing relationships should be tailored to target specific needs for each trajectory, and chronic disease management, including cognitive function, may be beneficial in preventing depressive symptoms among older adults. KEYWORDS Older adults, Depressive symptom, Trajectory, Latent class growth analysis, Korean

### SESSION 2887 (POSTER)

### EPIDEMIOLOGY

### ASSOCIATION OF VAGINAL MICROBIOTA WITH THE GENITOURINARY SYNDROME OF MENOPAUSE ACROSS REPRODUCTIVE STAGES

Michelle Shardell,[1] Patti Gravitt,[1] Jacques Ravel,[1] Anne Burke,[2] and Rebecca Brotman,[1] *1. University of Maryland School of Medicine, Baltimore, Maryland, United States, 2. Johns Hopkins Medical Institutions, Baltimore, Maryland, United States*

The genitourinary syndrome of menopause (GSM) is a collection of signs and symptoms related to estrogen decline that involves physical changes to the vulva, vagina, and lower urinary tract. GSM signs and symptoms may occur during any reproductive stage but are most common during menopause. Vaginal microbiota, particularly Lactobacillus spp., protect the female genital tract from multiple conditions; however, Lactobacillus spp. abundance declines during menopause. We aimed to determine the longitudinal association of vaginal microbiota with GSM signs and symptoms across reproductive stages. In a two-year cohort study comprising 750 women aged 35-60 years who contributed 2111 semiannual person-visits, low-Lactobacillus spp. vaginal microbiota communities were observed at 21.2% (169/798), 22.9% (137/597), and 49.7% (356/716) of person-visits among pre-, peri-, and post-menopausal women, respectively (p<.001). After covariate adjustment, low-Lactobacillus spp. communities characterized by high Atopobium and Megasphaera relative abundance were associated with vulvovaginal atrophy relative to high-Lactobacillus spp. communities dominated by L. crispatus (OR[Odds Ratio]=3.04, 95% Confidence Interval[CI]=1.02-9.06) among post-menopausal, but not among peri- or pre-menopausal women. Also, post-menopausal women with low-Lactobacillus spp. communities reported decreased libido (OR=1.79, 95%CI=1.04-3.12) and vaginal dryness (OR=1.61, 95%CI=0.89-2.90) more frequently than their counterparts with high-Lactobacillus spp. communities, but not among peri- or pre-menopausal women (p for interaction<.05). Specifically, low-Lactobacillus spp. communities characterized by high Atopobium and Megasphaera relative abundance were related to both decreased libido (OR=2.82, 95%CI=1.11-7.14) and vaginal dryness (OR=3.50, 95%CI=1.18-10.44) compared with high-Lactobacillus spp. communities dominated by L. gasseri/L. jensenii. Vaginal microbiota, particularly Lactobacillus spp., and menopause may synergistically influence GSM.

### DETERMINANTS OF SATISFACTION WITH HEALTH SERVICES BY DIMENSIONS OF CARE: AN AGE-STRATIFIED ANALYSIS

Helen-Maria Vasiliadis,[1] Isabelle Pitrou,[2] and Djamal Berbiche,[3] *1. University of Sherbrooke, Sherbrooke, Quebec, Canada, 2. University of Sherbrooke, Longueuil,*

*Quebec, Canada, 3. universite de Sherbrooke, Sherbrooke, Quebec, Canada*

Studies that examined satisfaction with care in older adults are scarce. The aim of this research was to analyse satisfaction among older adults considering mental health, socio-clinical and health system factors and by age category. Data come from the Étude sur la Santé des Aînés Services study including 1,624 adults aged ≥65 years recruited between 2011-2013 in primary care in Quebec. Patient satisfaction was assessed during interviews with questions adapted from the Primary Care Assessment Survey. Mental health (anxiety, depression, suicidal ideation, psychological distress, cognition), social support, quality of life, the presence of pain and chronic conditions were self-reported. Health service use was extracted from administrative registries. Logistic regressions stratified by age were used to examine the associations of low satisfaction in three dimensions of care. For continuity of care, the determinants of low satisfaction were pain and attraction index for psychiatric services in adults 65 to 75 years versus anxiety, cognition and hospitalizations in adults 75 years and older. For patient-provider interactions, the determinants were psychological distress, attraction index for psychiatric services in adults 65 to 75 years versus quality of life and cognition in adults 75 years and older. For adequacy of care, anxiety, psychological distress, social support, pain, quality of life and attraction index for psychiatric services were significant in adults 65 to 75 years versus quality of life and cognition in adults 75 years and older. Results highlight different patterns of satisfaction by age category that should be used to improve care delivered in primary care.

### DEVELOPMENT OF A CLAIMS-BASED ALGORITHM TO IDENTIFY PATIENTS WITH AGITATION IN ALZHEIMER'S DEMENTIA

Steve Hwang,[1] Christie Teigland,[2] Zulkarnain Pulungan,[2] Alexis Parente,[2] and RoseAnn DePalma, *1. Avanir Pharmaceuticals Inc, Aliso Viejo, California, United States, 2. Avalere Health, Washington, District of Columbia, United States*

Agitation is common in patients with Alzheimer's dementia. Lack of a consensus definition has limited our understanding of the prevalence, patient profile, and added healthcare burden of agitation in Alzheimer's dementia (AAD). We developed an algorithm to identify AAD patients using 100% of Medicare Fee-For-Service administrative claims from 2011-2017. We adapted the International Psychogeriatric Association (IPA) 2015 definition, which had not been tested using real-world data. Patients were required to have 2+ claims ≥30 days apart for Alzheimer's disease and dementia, and continuous enrollment with medical/pharmacy coverage for 6-months pre- and 12-months post-index diagnosis. The AAD cohort included patients with 2+ claims ≥14 days apart with ICD-9-CM/ICD-10-CM codes selected based on the IPA definition (e.g., dementia with behavioral disturbance, irritability/anger, restlessness/agitation, violent behavior, impulsiveness, wandering). Patients with severe psychiatric disorders were excluded. The final population included 255,669 patients with (34.6%) and 482,710 patients without agitation (65.4%). The mean age in both populations was 82 years. Although the majority of patients in both groups was female, the proportion of males was slightly larger in the AAD group (31.2% vs 29.7%). Patients in the AAD group were also more likely to be low-income (dual-eligible: 44.0% vs 39.6%), disabled (10.4% vs 9.3%), and using antipsychotic and antidepressant medications. The 2 populations had similar comorbidity rates. AAD prevalence may be underestimated using claims data, given imprecise and under-coding. These findings suggest AAD patients can be identified using a claims-based algorithm to support early interventions that can potentially improve outcomes and reduce costs of care.

### EXCLUSION OF MEDICARE ADVANTAGE ENROLLEES FROM MEDICARE HEALTH OUTCOMES ANALYSES: POTENTIAL FOR BIAS

Arseniy Yashkin, Igor Akushevich, and Anatoliy Yashin, *Duke University, Durham, North Carolina, United States*

The aim of this paper is to assess the extent of the potential bias introduced by the exclusion of the Medicare Advantage (MA) population– an increasingly sizeable (31% of all beneficiaries in 2017) subset of the Medicare population which does not provide claims data to the Centers for Medicare and Medicaid Services– from Medicare-based health outcomes and epidemiologic analyses. Using self-reported data from the Health and Retirement Study together with monthly information on Medicare enrollment, we compared MA enrollees with individuals enrolled in traditional Medicare (TM) on 42 variables representing demographic, socioeconomic, adverse health behavior and health status-related characteristics over the 1991-2015 period. We used both univariate analysis (t-tests and standardized differences) and multivariate logistic regression to compare the two groups. We found that apart from differences in economic (higher in TM group) and education status (lower in MA group) – which have been increasing in magnitude over the 1991-2015 period– the MA subset was highly comparable with the traditional Medicare (TM) population. Even though the TM population was characterized by slightly higher levels of morbidity, the resulting crude prevalence rates for common age-related diseases in the TM/MA groups were within each other's 95% confidence intervals and did not represent a major source of bias. MA membership was not associated with increased mortality at any point over the 1991-2015 period. We conclude that exclusion of the MA population will not lead to notable bias in health outcome analyses apart from those for which income and education are important explanatory factors.

### FUNCTIONAL CORRELATES OF SELF-REPORTED ENERGY IN THE HEALTH, AGING, AND BODY COMPOSITION STUDY

Rebecca Ehrenkranz,[1] Andrea Rosso,[2] Briana Sprague,[1] Qu Tian,[3] Eleanor Simonsick,[3] Nancy Glynn,[1] Caterina Rosano,[1] and Theresa Gmelin,[1] *1. University of Pittsburgh, Pittsburgh, Pennsylvania, United States, 2. School of Public Health, University of Pittsburgh, Pittsburgh, Pennsylvania, United States, 3. National Institute on Aging, Bethesda, Maryland, United States*

While fatigue in older age is well studied, the clinical relevance of maintaining higher energy late in life is less understood. We explored associations of self-reported energy with cognitive performance, depressive symptoms, and physical function in the Health, Aging and Body Composition study (n=2,529, mean age =75.9, 63.5% white, 44.9% men). Self-reported energy over the past month was recorded from 0-10 (least to most energy) and dichotomized at the median (≥7=high energy). Cognitive performance was measured using Modified Mini-Mental State Examination and Digit Symbol Substitution Test. Depressive symptoms were measured using the Center for Epidemiologic Studies Depression scale. Physical function was assessed via fitness (timed 400-meter walk), self-reported physical activity, and usual and rapid gait speed. Variables bivariately associated with energy entered a logistic regression model with higher energy as the outcome, adjusted for demographics, chronic conditions, strength, and body mass index (BMI). Overall, 58% of the sample reported high energy, and self-reported energy was greater for males and those without chronic conditions ($p<0.05$). Lower odds of higher self-reported energy were found for participants with more depressive symptoms (aOR 95% CI= 0.55 [0.50, 0.62]) and longer time to walk 400m (aOR = 0.79 [0.70, 0.89]). Increased odds of higher self-reported energy were found for participants with faster usual and rapid gait speeds (aOR = 1.3 [1.2, 1.5]; aOR = 1.2 [1.1 – 1.4], respectively). Associations with cognitive performance were not significant. Higher self-reported energy reflects fewer depressive symptoms and greater physical function independent of demographics, chronic conditions, strength, and BMI.

### IDENTIFYING A POPULATION LIVING WITH ALZHEIMER'S: CONCORDANCE BETWEEN MEDICARE CLAIMS AND SURVEY REPORTS

Ayse Akincigil,[1] and Camille McKenzie,[2] *1. Rutgers, The State University of New Jersey, New Brunswick, New Jersey, United States, 2. Rutgers University--New Brunswick, New Brunswick, New Jersey, United States*

Community-level estimates of Alzheimer's disease and related dementias (ADRD) are necessary to assess health care needs and supports (to patients and family members), determine the burden of disease, conduct public health planning, improve access and care quality improvement, and to build a workforce with the necessary skills. Data from Medicare claims can provide efficient and timely estimates. However, earlier studies suggest that identifying ADRD populations solely from Medicare claims fails to capture many individuals that live with ADRD, with false-negative cases as high as 60%. We examined nationally representative data from the 2015-2017 Medicare Current Beneficiary Survey (MCBS) to assess the claims-based case ascertainment method, covering the transition to the International Classification of Diseases, Tenth Revision (ICD-10). The study population included community dwellers aged 65 or older, enrolled in traditional fee-for-service (n=12,409). Claims based method identified 1,325 cases (10.7% prevalence). However, there were 196 (1.6%) additional cases that self/proxy reported ADRD, but there was no ADRD diagnosis in any of their Medicare claims (hereafter referred to as self-report only). On average, the self-report only group reported higher numbers of limitations in activities, or instrumental activities of daily living, worse overall health, and more difficulty in concentrating or remembering, suggesting they are likely to be false negatives under claims-based case ascertainment method. In conclusion, claims based case ascertainment methods failed to capture some individuals with ADRD, but the magnitude of false-negative cases declined substantially in the era of ICD-10.

### OUTPATIENT PHYSICAL THERAPISTS SHOULD BE COMPETENT IN CARE OF OLDER ADULTS: A TOTAL POPULATION REGISTER-BASED STUDY

Solveig Arnadottir, *University of Iceland, Reykjavik, Iceland*

In Iceland, outpatient physical therapy (OPT) is traditionally not focused on older clients. Yet, the Icelandic population is aging as other populations in the world, and national policies endorse aging in place. The objective of this study was to explore 17 years of demographic information on OPT clients and to identify if this information reflects the total population aging. The research was built on 17 years (1999-2015) of complete data from: the Icelandic Health Insurances register with information on the total population of OPT clients (N=172071), and the Statistics Iceland register with demographic information on the total general population. The results revealed that in 1999, older adults comprised 18.3% of all OPT clients, and in 2015 it had increased to 23.5% Therefore, OPTs were 23% more likely to treat an older adult in 2015, compared to 1999 (Risk Ratio [RR] 1.23; 95% Confidence Interval [CI] 1.19-1.27). In the same time period older people became 15% more prevalent in the general population (RR 1.15; 95%CI 1.10-1.21). Linear modelling revealed a yearly 3.45% (95%CI 3.05-3.85) increase from 1999 to 2015 in the overall proportion of older OPT clients. This yearly trend, however, varied depending on age group and sex with the highest yearly increase in the ≥ 85 years old men (9.1%; 95%CI 7.90-10.35). This case of Iceland presents 17 years of continuous growth in older adults seeking OPT service. These findings reinforce an urgent need to enhance the geriatric competence of OPTs, who in their clinical practice frequently encounter older adults.

### PREDICTING LIFE EXPECTANCY USING VETERANS AFFAIRS ELECTRONIC HEALTH RECORD DATA

Alexandra Lee,[1] Bocheng Jing,[2] Sun Young Jeon,[1] John Boscardin,[1] and Sei Lee,[1] *1. University of California, San Francisco, San Francisco, California, United States, 2. San Francisco VA Medical Center, San Francisco, California, United States*

Electronic health records (EHRs) are a rich source of health data that could be used to create individualized life expectancy predictions to aid in clinical decision-making for long-term preventative treatments, such as cancer screening. Few previous studies have incorporated all possible predictors from the EHR. We aimed to screen and incorporate a large number of possible predictors from EHR data into a life expectancy (LE) prediction equation. Using the national Veteran's Affairs (VA) EHR databases, we identified all patients aged 50+ with a primary care visit during 2005 and assessed demographics, diseases, medications, laboratory results, healthcare utilization, and vital signs during the one year prior to the visit. Mortality follow-up was complete through 2017. We used an 80% random sample for model development and a 20% random sample for model validation. We used a Gompertz survival model with backwards selection to identify approximately 100 variables for the final LE prediction equation. In 1,263,595 VA patients, the mean age was 68 years and the majority were male (94%) and white (87%). During 12 years of follow-up, 602,576 (47.7%) died. Of 930 predictors from the EHR, 99 were included in the LE prediction equation. Harrell's C-statistic was 0.7705 (95%CI: 0.7693, 0.7718). The model estimated 10-year life expectancy with sensitivity of 81.6% (81.4%, 81.8%) and specificity of 68.8% (68.5%, 69.1%). In conclusion, we developed an LE prediction equation from hundreds of predictors in the VA EHR with good discrimination and calibration that may help clinicians weigh the potential benefit of long-term preventative treatments.

### SLEEP QUALITY AMONG OLDER WOMEN AND MEN IN THE UNITED STATES BY SEXUAL ORIENTATION

Adena Galinsky,[1] Karen Fredriksen Goldsen,[2] James Dahlhamer,[1] and Tina Norris,[1] *1. National Center for Health Statistics, Hyattsville, Maryland, United States, 2. University of Washington, Seattle, Washington, United States*

Sleep problems may increase the risk for, and result from, other health problems and negatively impact quality of life. Lesbian, gay, and bisexual older adults report more sleep problems compared to their straight counterparts when such problems are measured in the aggregate (e.g. "one or more of four specific sleep problems"). However, scant national research has examined if specific types of sleep problems vary by sexual orientation among older adults. Using 2015-2018 National Health Interview Survey (NHIS) data, we used logistic regression to separately model five sleep problems among women 50+ and men 50+ (lesbian/gay women: n=377, bisexual women: n=142, straight women: n=33,216; gay men: n=508, bisexual men: n=115, straight men: n=25,998) as functions of sexual orientation, controlling for age, race, education, and income. Sexual minority older adults were more likely than their straight counterparts to have taken sleep medication in the past week (women AOR=2.04, 95% CI:1.55, 2.67; men AOR=1.81, 95% CI:1.36, 2.40). The only other difference by sexual orientation found for men was bisexual older men's greater likelihood, compared to straight men, of having difficulty falling asleep (AOR=2.02, 95% CI: 1.08, 3.79). Older women did not differ by sexual orientation in difficulty falling asleep, difficulty staying asleep, or waking up not feeling rested for four or more days in the past week, or meeting National Sleep Foundation recommendations for hours of sleep per night, whether lesbian/gay and bisexual women were examined together or disaggregated. Future research may examine why sleep quality only sometimes varies by sexual orientation.

### USING BIG DATA FOR CLINICAL DECISION MAKING

Diana Woods,[1] and Maria Yefimova,[2] *1. Azusa Pacific University, Marina del Rey, California, United States, 2. VA Palo Alto Healthcare System, Redwood City, California, United States*

The current workforce is ill prepared for the rise in Americans 65 and older from 46.3 million in 2010 to 98.2 million by 2050, a national increase of 112.2 % accompanied by increasing chronic conditions. The increase in older Americans, the prevalence of those with dementia, accompanied by behavioral symptoms of dementia (BSD) is increasing. Innovative technology may alert health providers to early signs of decline in frail older adults with multiple chronic conditions. Remote monitoring in the home and community living spaces can address complex care needs for older adults. Monitoring may identify and predict deviations in a person's daily routine that herald a change in a chronic condition. We present two examples that can potentially assist in clinical decision making. The first exemplar used 24/7 sensor data to identify changes, potentially clinically significant, such that early intervention may prevent hospitalizations; the second exemplar presents the use of pattern recognition software (THEME TM) for temporal pattern analysis, to identify and quantify behavior patterns with regard to intensity, frequency and complexity, such that interventions may be individually tailored and timed. Clinical researchers and technology developers need to collaborate early in the process to consider the sources and frequency of clinical measures for meaningful predictions. One major challenge lies in the interpretation of the vast amounts of within individual data. Our insights strive to improve future interdisciplinary development of monitoring systems to support aging in place and support clinical decisions for timely and effective care for frail older adults.

### VALIDATING OLDER ADULT MORBIDITY TRAJECTORIES USING MULTIPLE COMORBIDITY INDICES

Michael Newman,[1] Heidi Hanson,[2] Karen Schliep,[3] Samir Abdelrahman,[2] Jim VanDerslice,[2] Ken Smith,[2] and Christy Porucznik,[2] *1. University of Utah, Holladay, Utah, United States, 2. University of Utah, Salt Lake City, Utah, United States, 3. University of Utah, Salt Lake City, United States*

Many older adults lead healthy lives while aging, with little or no morbidity. This group has been identified as "Escapers", for escaping the 10 most common lethal diseases in older adults. "Morbidity Trajectories" (MOTRs) are a metric based on the temporal patterning of comorbidity, which is used to characterize changes in disease status as a person ages. While these trajectories have been used to identify Escapers in various populations, they are sensitive to the choice of the disease metric. This study seeks to describe the differences in MOTR scale by alternative comorbidity indices. Understanding these differences is important because of the need to validate the potential end-point in health trajectory risk scores that may be used in a clinical setting. We found that 15-19 percent of a Medicare utilizing population (n=321722) aged >= 65 between 1992 and 2012 fall into the Escaper category, where there is a consistent Quan modification Charlson Comorbidity Index (CCI) score of 0 during the entire study period. Using the vanWalraven (vW) Elixhauser Comorbidity Index modification, we found that about a third (35.2%) of the study population have a vW Elixhauser score of 0 over the span, a significantly higher portion than the CCI estimate. We will discuss this difference and the resulting varying trajectories from each of these indices. Future work includes further validation of the MOTR scale using unsupervised machine learning clustering methods, and using supervised machine learning models to identify clinical factors and early life conditions that may influence MOTR membership.

### SESSION 2888 (POSTER)

### FRAILTY AND SARCOPENIA

### EFFECTIVENESS OF A 6-MONTH MULTICOMPONENT TRAINING ON PHYSICAL FRAILTY IN OLDER ADULTS WITH COGNITIVE IMPAIRMENT

Oscar Ribeiro,[1] Flavia Borges-Machado,[2] Duarte Barros,[2] and Joana Carvalho,[2] *1. Department of Education and Psychology, University of Aveiro, Aveiro, Portugal, 2. Faculty of Sports, University of Porto, Porto, Portugal*

Multicomponent training (MT) that combines aerobic, strength, postural, and balance exercises seems to be safe and effective to prevent and even reverse early stages of frailty, as well as counteract the physical consequences of cognitive impairment. This study analyzes the effectiveness of MT on physical frailty in older adults diagnosed with cognitive impairment. Forty-nine subjects (34 women) diagnosed with mild cognitive impairment (MCI) or dementia were allocated into exercise group (EG; 75.80±6.60 years; age range: 61-90) or a control group (CG; 82.56±5.45 years; age range: 73-90). EG was submitted to a 6-month MT intervention (2x/week, 50min). CG had a monthly recreational session. Sample mean of MMSE at baseline was 21.02 (±4.98). Participants were categorized as frail, pre-frail and robust according to a validated physical performance battery - Short Performance Physical Battery (SPPB). Overall, in baseline there were 12.5% frail and 37.5% pre-frail individuals in the EG group; in the CG there were 36% and 32%, respectively. Data from SPPB total score revealed that EG group increased the performance over time [t(23)=3,94; Δ=1.21 (±1.50); p<0.001], as opposite to CG individuals [t(24)=-2.26; Δ=-0.92 (±2.04); p=0.034]; also, there were 8.3% frail and 29.2% pre-frail in EG, and 40% and 32% in CG after intervention. By improving lower extremity functionality of patients diagnosed with cognitive impairment, MT intervention may be an important non-pharmacological strategy to improve physical frailty status and modify subjects' condition from frail to pre-frail or even robust. Supported by IPDJ & FCT: CIAFEL (FCT/UIDB/00617/2020), "Body and Brain" (POCI-01-0145-FEDER-031808), PhD Grant (SFRH/BD/136635/2018).

### FRAILTY CHANGE SCORES OVER LONG-TERM FOLLOW UP IN PATIENTS WITH CORONARY ARTERY DISEASE

Shahyar Gharacholou,[1] Carolyn Flock,[2] Ryan Lennon,[2] Joshua Slusser,[2] Leslie Cooper,[1] and Mandeep Singh,[2] *1. Mayo Clinic, Jacksonville, Florida, United States, 2. Mayo Clinic, Rochester, Minnesota, United States*

Background: Frailty assessment has often been performed only at baseline in cohort studies. Little is known regarding factors associated with changes in frailty indices over follow up in patients with coronary artery disease (CAD). Methods: Between 11/2008 - 8/2012, 142 community-dwelling adults ≥65 years of age with prior history of CAD (angina or revascularization) participated in a study of frailty assessment at Mayo Clinic Health System in La Crosse, WI. A sample of participants (n=45) were included for frailty re-assessment using the Fried frailty criteria approximately 5 years after their baseline measures. Frailty classification was based on absence of deficits (non-frail), 1-2 deficits (intermediately frail), or 3 or more deficits (frail). Factors associated with a change in frailty indices were studied. Results: There were 45 patients that had a second assessment of frailty indices. At baseline, 24 patients (60%) were not frail while 16 patients (40%) had at least 1 frail feature. At follow up, 20 patients (50%) were not frail while 20 patients (50%) had a frail feature. Those improving were more often being married, had prior revascularization, and were without angina. Interval development of slower gait speed (r = 0.46; p=0.004) and decreased grip strength (r = -0.39; p = 0.01) were associated with worsening frailty. Conclusions: Older adults with CAD are not often frail by standard criteria; however, incident deficits develop during long term follow-up. Spousal support, absence of angina, and change in functional indices are less often associated with frailty features.

### FRAILTY, WITH OR WITHOUT COGNITIVE IMPAIRMENT, IS A STRONG PREDICTOR OF FUTURE RECURRENT FALLS

Meiling Ge,[1] Qian-Li Xue,[2] Eleanor Simonsick,[3] Birong Dong,[4] and Judith Kasper,[5] *1. West China Hospital Sichuan University, Chengdu, Sichuan, China, 2. Johns Hopkins School of Medicine, Baltimore, Maryland, United States, 3. National Institute on Aging, Bethesda, Maryland, United States, 4. West China Hospital, Sichuan University, Chengdu, Sichuan, China, 5. Johns Hopkins Bloomberg School of Public Health, Baltimore, Maryland, United States*

The associations between physical frailty and cognitive impairment with falls history are well-established. However, their associations with prospectively ascertained recurrent falls are unknown. We used data from the National Health Aging Trends Study (NHATS) and marginal means/rates model to analyze the associations between frailty and cognitive impairment and recurrent falls over 6 years (2012-2017). Of the 6,000 older adults, 1,787 (29.8%) had cognitive impairment only, 334 (5.6%) had frailty only, 615 (10.3%) had both, and 3,264 (54.4%) had neither. At baseline, compared to the group without cognitive impairment or frailty, individuals with both frailty and cognitive impairment were more likely to have a history of falls (odds ratios (OR)=2.48, 95% confidence interval (CI)=1.98-3.11), followed by those with frailty only (OR=1.53, 95% CI=1.19-1.97), and those with cognitive impairment only (OR=1.22, 95% CI=1.05-1.41), after adjusting for age, sex, race, education, living alone, obesity, comorbidity, and mobility disability. In longitudinal analysis, those with frailty and cognitive impairment alone or together at baseline had higher rates of recurrent falls than those without cognitive impairment or frailty (cognitive impairment only: rate ratios (RR)=1.07, 95% CI=1.00-1.13; frailty only: RR=1.31, 95% CI=1.18-1.44; both: RR=1.28, 95% CI=1.17-1.40). The risk appeared to be comparable between those with frailty alone or those with frailty and cognitive impairment. These findings offered robust evidence for a strong predictive association between frailty and recurrent falls in non-institutionalized older adults, and the association remained strong with or without comorbid cognitive impairment.

### HIGH INTENSITY WALKING REDUCES FRAILTY AND IMPROVES PHYSICAL FUNCTION AMONG OLDER ADULTS LIVING WITH HIV

Margaret Danilovich,[1] Chad Achenbach,[2] Chen Yeh,[3] Lauren Balmert,[2] and Baiba Berzins,[3] *1. CJE SeniorLife, Evanston, Illinois, United States, 2. Northwestern University, Chicago, Illinois, United States, 3. Northwestern University, Chicago, United States*

Walking is a preferred mode of exercise for older adults, yet limited evidence exists on the optimal intensity to promote health gains, particularly among those with the frailty syndrome. Older adults aging with HIV have physical function impairments and higher prevalence of frailty, yet there is a paucity of evidence on therapeutic options to help these individuals maintain or improve their physical functioning. The purpose of this study was to investigate the feasibility and efficacy of a high intensity walking training (HIWT) intervention for pre-frail and frail older adults living with HIV. We enrolled n=11 older adults (>50 years of age and pre-frail or frail on the SHARE-FI). Participants underwent 16 walking sessions 2x/week consisting of 5 minute intervals of stair climbing, fast walking, weighted walking, balance tasks, and steps ups for a total of 30 minutes of high intensity (>70% of HRmax or >15 Rating of Perceived Exertion) activity. All participants were able to achieve the targeted high intensity levels throughout the sessions. We used a t-test to compare pre-post test means on a variety of physical performance measures. We found statistically significant improvements in the frailty score on the SHARE-FI, PROMIS fatigue, self-selected gait speed, Short Physical Performance Battery (SPPB), and Six Minute Walk Test. Importantly, all improvements were far above minimally clinically important differences suggesting this novel exercise approach may contribute to substantial improvements in physical function to reduce frailty in this population of older adults. Participants had no adverse events and were highly satisfied with training.

### IMPACT OF FRAILTY ON MEDICAL AND LONG-TERM CARE EXPENDITURES FOR THE ELDERLY AGE 75 OR OVER IN JAPAN

Hiroto Yoshida,[1] and Yuriko Kihara,[2] *1. Tohoku Bunka Gakuen University, Sendai, Japan, 2. Japan Health Care College, Eniwa, Hokkaido, Japan*

This study examined the impact of frailty on medical and long-term care expenditures in an older Japanese population. The subjects were those aged 75 years and over who responded to the survey (March 2018) in Bibai, Hokkaido, Japan (n=1,203) and have never received certification of long-term care insurance at the survey. We followed up 867 individuals (72.1%) until the end of December 2018 (10 month-period). We defined frailty as a state in performing 4 items and over of 15 items which were composed of un-intentional weight loss, history of falls, etc. Among 867 subjects, 233 subjects (26.9%) were judged to be frailty group, and 634 subjects (73.1%) non-frailty group. We compared period to the new certification of long-term care insurance (LTCI), accumulated medical and long-term care expenditures adjusted for age and gender between the two groups during the follow-up period. Cox proportional hazard models were used to examine the association between baseline frailty and the new certification of LTCI. The relative hazard ratio (HR) was higher in frailty group than non-frailty group (HR=3.51, 95% CI : 1.30-9.45, P=.013). The adjusted mean accumulated medical and long-term care expenditures per capita during the follow-up were significantly (P=.002) larger for those in the frailty group (629,699 yen), while those in the non-frailty group were 450,995 yen. We confirmed strong economic impact of frailty in the elderly aged 75 or over in Japan.

### INFLUENCE OF FRAILTY ON DISCHARGE SETTING FOR OLDER ADULTS WITH HIP FRACTURE AT INPATIENT REHABILITATION FACILITIES

Christine Tocchi, Shamatree Shakya, Sathya Amarasekara, and Michael Cary, *Duke University, Durham, North Carolina, United States*

Inpatient rehabilitation Facilities (IRFs) provide intensive rehabilitation therapy to patients to reduce functional impairment, enhance independence and return patients back to the community. Determination of eligibility for IRF is currently based on a preadmission screening. Frailty, a pervasive characteristic in older adults with hip fractures has not been examined as a clinical factor influencing function and discharge destination IRF outcomes. This study purpose was to determine the prevalence of frailty among older adult IRF patients with hip fractures and determine the association between frailty and function and discharge destination among IRF hip fracture patients. A retrospective cohort study design using CMS 2014 Inpatient Rehabilitation Facility-Patient Assessment Instrument file. Frailty was measured using a Frailty Index of 30 items. The final sample included 26,134 patients. Frailty, pre-frailty, and nonfrailty were present in 0.92% (n=24043), 3.3% (n=862), and .076% (n=199) of hip fracture patients, respectively. The majority (65%) of the patients were discharged home. There were significantly greater proportion of females than males discharged home and those of white race, 65 to 74 years of age, and those with higher functional status. Regression analysis showed significantly lower functional status at discharge (p < .0001) for males and those of non-white race, older age and frail. Study implications include the use of frailty status to identify hip fracture patients at high risk for adverse outcomes and need for future studies to explore the potential of frailty to provide value-added utility to IRF clinical settings and identify ongoing opportunities to guide person-centered care.

### MICROSTRUCTURAL NEUROIMAGING OF FRAILTY IN COGNITIVELY NORMAL OLDER ADULTS

Qu Tian,[1] Susan Resnick,[1] Bennett Landman,[2] and Luigi Ferrucci[1] *1. National Institute on Aging, Bethesda, Maryland, United States, 2. Vanderbilt University, Nashville, Tennessee, United States*

Physical frailty is an age-related clinical syndrome that is related to adverse health outcomes, including cognitive impairment and dementia. Recent studies have shown structural neuroimaging correlates with frailty. However, most existing evidence relies on brain volumetric measures. Whether brain microstructure is associated with frailty and its spatial distribution have not been explored. In the Baltimore Longitudinal Study of Aging, we identified 776 cognitively normal participants aged 50 and older who had concurrent data on frailty and brain microstructure by diffusion tensor imaging (DTI), including mean diffusivity (MD) of gray matter and fractional anisotropy (FA) of white matter. We first identified neuroimaging markers that were associated with frailty score (0-5) and further examined their relationships with frailty status (0: non-frail, 1-2: pre-frail, 3+: frail) using multivariate linear regression. Models were adjusted for age, sex, race, years of education, and Apolipoprotein E e4 carrier status. DTI-based neuroimaging markers that were associated with frailty status were localized in the supplementary motor area of the frontal lobe, several subcortical regions (putamen, caudate), and body and splenium of corpus callosum. This study demonstrates for the first time that microstructure of both gray and white matter differs by frailty levels in cognitively normal older adults. Brain areas were not widespread, but mostly localized in gray matter subcortical motor areas and white matter corpus callosum. Whether changes in brain microstructure precede future frailty development warrants further investigation.

### PERCEPTIONS AND UNDERSTANDINGS OF FRAILTY LANGUAGE: A SCOPING REVIEW

Mariko Sakamoto,[1] Pamela Durepos,[2] Kyla Alsbury,[3] Patricia Hewston,[2] Alyson Takaoka,[2] and Julia Borges[4], *1. University of British Columbia, Vancouver, British Columbia, Canada, 2. McMaster University, Hamilton, Ontario, Canada, 3. University of Toronto, Toronto, Ontario, Canada, 4. Dalhousie University, Halifax, Nova Scotia, Canada*

Diagnosing and responding to frailty in older adult populations is of growing interest for health care professionals, researchers and policymakers. Preventing frailty has the potential to improve health outcomes for older adults, which in turn has significant implications for health care systems. However, little is known about how older people understand and perceive the term "frailty", and what it means for them to be designated as frail. To address this concern, a scoping review was undertaken to map the breadth of primary research studies that focus on community-dwelling older adults' perceptions and understanding of frailty language, as well as explore the potential implications of being classified as frail. Searches were conducted in MEDLINE, Ageline, PsychInfo, CINAHL and EMBASE databases for articles published between January 1994 and February 2019. 4639 articles were screened and ten articles met the inclusion criteria, detailing eight primary research studies. Using content analysis, three core themes were identified across the included studies. These themes included: 1) understanding frailty as a multidimensional concept and inevitable consequence of aging, 2) perceiving frailty as a generalizing and harmful label; and 3) resisting and responding to frailty. Recommendations stemming from this review include the need for health care professionals to use person-centered language with older adults, discuss the term frailty with caution, and be aware of the potential consequences of labeling a person as frail. Importantly, this review demonstrates that for frailty interventions to be successful and meaningful for older adults, ongoing and critical examination of frailty language is necessary.

### PHASE ANGLE AS A BIOELECTRICAL MARKER TO IDENTIFY SARCOPENIA

Lisha Hou, and Jirong Yue, *West China Hospital, Chengdu, Sichuan, China*

Background: Phase angle (PhA) has been suggested as an indicator of cellular death and nutritional status. We aimed to evaluate the performance of phase angle as a sarcopenia marker among 50 years older and determine the optimal cut-off values. Materials and Methods: A cross section of 4500 with ≥50 years were assessed in terms of sarcopenia with bioelectrical indices. Phase angle can be determined through bioelectrical impedance analysis (In Body 770). Muscle strength and physical function were measured using hand grip and 4 m walking speed. Significant determinants of sarcopenia were further analyzed with multivariate logistic regression analysis. Results: 869 patients (19.31%) were diagnosed with sarcopenia. The average PhA was 5.03 ± 0.64° (Male: 5.31 ± 0.66°; Female: 4.87 ± 0.57°). After adjusting age, gender, race, occupation, BMI, marital status, smoking, drinking, exercise, chronic disease and ADL, phase angle was still independent associated factors with sarcopenia: phase angle (OR=0.25, 95% CI: 0.203-0.308, P < 0.001). Receiver operating characteristic analysis revealed that the optimal phase angle cutoff value to detect sarcopenia was ≤4.9º (AUC=0.768). Conclusions: Bioelectrical phase angle can be an useful bioelectrical marker to identify sarcopenia.

### PREVALENCE AND ASSOCIATED RISK FACTORS OF SARCOPENIA AMONG ELDERLY WITH DIABETES IN JAPAN AND TAIWAN

Yuko Yamaguchi,[1] Chieko Greiner,[2] Hirochika Ryuno,[1] Pi-Hsia Lee,[3] Hsin-Yen Yen,[3] Shu-Chun Lee,[3] Chiou-Fen Lin,[3] and Ting-I Lee,[3] *1. Kobe University, Kobe, Japan, 2. KOBE UNIVERSITY, Kobe, Japan, 3. Taipei Medical University, Taipei, Taiwan (Republic of China)*

The aim of this study was to investigate the prevalence of sarcopenia and its associated risk factors among elderly with diabetes in Japan and Taiwan. A cross-sectional study was conducted through convenience sampling. This study was approved by the institutional review boards of Kobe University Graduate School of Health Sciences (No. 543) and Taipei Medical University (No. N201905065). Of the 114 Japanese participants (24.9% females) and 226 Taiwanese participants (75.1% females), the mean age were 73.7±6.9 years and 74.2±6.6 years, respectively. Sarcopenia was found in 10.6% in Japan and 11.1% in Taiwan with no significant difference between both countries. Older age, poor relationships with neighbors, and poor consciousness for health management were significantly associated with sarcopenia in both countries. Additionally, Japanese participants with sarcopenia had significantly longer duration of diabetes, higher level of emotional distress assessed by the Problem Areas In Diabetes (PAID) scale, and lower level of total protein, but no significant differences were shown in Taiwanese participants. Binomial logistic regression analyses were performed to detect risk factors related to sarcopenia. After adjusting for age, sarcopenia was found to be significantly associated with poor consciousness for health management in Japanese participants (OR:0.12 P=0.02) and significantly associated with poor relationships with neighbors and poor consciousness for health management in Taiwanese participants (OR:0.18 P=0.008 and OR:0.45 P=0.04, respectively). These findings are expected to contribute to the development of effective strategies for preventing sarcopenia among elderly with diabetes in Japan and Taiwan.

### QIGONG EXERCISE IN FRAILTY, PSYCHOLOGICAL HEALTH, AND SPIRITUAL WELL-BEING OF MIDDLE-AGED AND OLDER BLACKS

Pei-Shiun Chang,[1] Yvonne Lu,[2] Susan Ofner,[2] and Chi Nguyen,[3] *1. Indiana University at Bloomington, Bloomington, Indiana, United States, 2. Indiana University, Indianapolis, Indiana, United States, 3. Indiana University, Bloomington, Indiana, United States*

Interventions are needed to address the frailty and psychological health in middle-aged and older African Americans. Qigong, a traditional medicine exercise, consists of gentle body movements, breathing exercise, and meditation. The benefits of Qigong exercise have been widely reported in Asian adults but there have been no known studies testing Qigong exercise in the African American population. The purpose of this pilot study was to evaluate the potential benefits of an 8-week Qigong exercise in physical and functional ability, balance, frailty, depression and anxiety, and spiritual well-being in community-dwelling middle-aged African Americans using a single group design. Fifteen African Americans aged 45 to 85 years were recruited to receive Qigong exercise over 16 bi-weekly, one-hour sessions. The Layers Module that unfolds the complex multidimensional benefits of Qigong exercise on physical, mental, and spiritual dimensions was used to guide the outcome measures. Data were collected at baseline and 14 days post intervention. Results showed positive trends in repeat chair stands, physical function, and spiritual well-being ($p<0.05$) with effect sizes ranging from 0.45 to 0.87. Despite no significance, over 52% of the participants showed improved depression scores, fast gait speed, and standing balance than baseline. Nearly 42% demonstrated some levels of frailty improvement than the baseline. No adverse events related to Qigong exercise intervention was reported. Qigong exercise is a potentially promising intervention which needs further testing in a randomized clinical trial.

### RELATIONSHIP BETWEEN PERCEIVED PHYSICAL AND MENTAL FATIGABILITY AND PHYSICAL PERFORMANCE IN VERY OLD ADULTS

Yixin Hu,[1] Zhuangzhuang Zhang,[2] Woei-Nan Bair,[3] Anying Bai,[4] and Li Fan,[5] *1. Chinese PLA General Hospital, Beijing, China, 2. Peking University Health Science Center, Beijing, China, 3. University of the Sciences in Philadelphia, Philadelphia, United States, 4. Peking University, Beijing, China, 5. Chinese PLA hospital, Beijing, China*

To investigate the relationship between perceived physical and mental fatigability and physical performance in community-dwelling very old adults (≥80 years). We examined the association in one retired community in Beijing including 404 very old adults. Pittsburgh Fatigability Scale (PFS), Chinese version, was used to assess perceived fatigability in physical domain (PFS-P) and mental domain (PFS-M). High fatigability is defined as PFS-P ≥ 15, and PFS-M ≥ 13. Physical performance measures include grip strength, usual gait speed, chair stand and Short Physical Performance Battery (SPPB) test. Women have higher PFS scores (both PFS-P & PFS-M) and higher prevalence of high fatigability than men. After adjusting for sex, usual gait speed and SPPB scores were significantly associated with PFS-P & PFS-M, while grip strength and chair stand performance were significantly associated with PFS-P only. After multivariable adjustment, usual gait speed (B=-3.745, P=0.021) and chair stand performance (B=0.335, P=0.005) were significantly associated with PFS-P, while usual gait speed (B=-2.656, P=0.006) and SPPB scores (B=-0.214, P=0.029) were significantly associated with PFS-M. Perceived physical and mental fatigability is highly prevalent in very older adults and they differ by sex. The significant associations between PFS scores and performance measures suggest that PFS is of potential clinical importance, especially when testing performance measures are not feasible. Utilization of PFS score can assist in identifying target populations who are at risk of reduced physical functions, such as older with depression, older women. Interventions to improve usual gait speed are likely to reduce both perceived physical and mental fatigability.

### SELF-REPORTED HEALTH AND SARCOPENIA PHENOTYPES: DATA FROM THE NATIONAL HEALTH AND AGING TRENDS SURVEY

John Batsis,[1] Cameron Dowd-Sivigny,[2] Christian Haudenschild,[3] Rebecca Crow,[4] Tyler Gooding,[5] Matthew Lohman,[6] and Elizabeth Vasquez,[7] *1. Dartmouth College, Lebanon, New Hampshire, United States, 2. University at Albany: School of Public Health, Rensselaer, New York, United States, 3. Dartmouth College Geisel School of Medicine, Lebanon, United States, 4. dartmouth hitchcock, lebanon, New Hampshire, United States, 5. Dartmouth Hitchcock Medical Center, Lebanon, New Hampshire, United States, 6. University of South Carolina, Columbia, South Carolina, United States, 7. University at Albany (SUNY), Rensselaer, New York, United States*

Background: Obesity in combination with sarcopenia (age-related loss of muscle mass, strength or function) is increasing in adults aged ≥65 years which places individuals at risk for functional decline and worse health. We ascertained the relationship between sarcopenic obesity and self-reported health in a representative US population. Methods: We identified participants ≥65 years with grip strength and body mass index (BMI) measures from the baseline wave of the National Health and Aging Trends Survey. Sarcopenia was defined using the Sarcopenia Definitions and Outcomes Consortium grip strength cut-points (males<35.5kg; females<20kg), and obesity was defined using standard World Health Organization BMI categories. We also assessed grip divided by BMI cut-points (males<1.05; females<0.79). Self-reported health was evaluated using a one-item question (Excellent/Very Good/Good vs. Fair/Poor). Logistic regression models adjusted for age, sex, smoking, education, comorbidities, and an ability to walk. Results: Of the 8,245 participants (59.7% female), median age category was 75-79, and mean grip strength and BMI were 26.0 kg and 27.0 kg/m2, respectively. Prevalence of sarcopenia and sarcopenic obesity was 12.7% and 41.1%. Compared to those without sarcopenia or obesity, the odds of impaired self-reported health using grip strength defined sarcopenia cut-points was higher in sarcopenic obesity (OR 1.87 [1.50,2.32]), sarcopenia (OR 1.79 [1.50,2.14]), and obesity (OR 1.24 [0.99,1.56]). Using the Grip divided by BMI cutpoints, we found the odds of low self-reported health in sarcopenia was OR 1.38 [1.12, 1.59]. Conclusions: Both sarcopenia and sarcopenic obesity are associated with an increased odds of decreased self-reported health.

### SOCIAL DETERMINANTS AND FRAILTY IN HIGH-NEED, HIGH-RISK VETERANS

Kiranmayee Muralidhar,[1] Willy Marcos Valencia,[2] Fei Tang,[2] and Stuti Dang,[2] *1. University of Miami, Miami, Florida, United States, 2. Miami VA Healthcare System, Miami, Florida, United States*

The VA Geriatrics and Extended Care Data Analysis Center uses national predictive modelling to identify High-Need High-Risk (HNHR) Veterans, to provide targeted services and reduce hospitalization and institutionalization risk. To learn the needs of Miami VA HNHR Veterans, we mailed a needs-assessment survey to 2124 Veterans, of whom 634 responded (29.8% response rate). The average respondent age was 70.5±9.2. Among them, 127(20%) were <65 years old, 326(51.4%) were 65-74, and 179(28.2%) were ≥75; 389(61.4%) White, 225(35.5%) Black/African Americans; 515(81.2%) were Non-Hispanic, 111(17.5%) Hispanic/Latino; 173(27.3%) were high school graduates, 350(55.2%) had at least some college credit, 39(6.2%) had a master's degree or more and 536(84.5%) were health literate. As per Morley's FRAIL scale, 266(42%) were frail, 242(38.2%) were pre-frail and 87(13.7%) were robust. Social risk factors possibly associated with frailty were analyzed using ordinal logistic regression. Univariate analysis showed significant association with poor health literacy, having a caregiver, social isolation, transportation trouble, delayed or missed doctors' appointments due to transportation, a negative perception of aging, likelihood of depression, being homebound, inability to use the internet, lack of technology for video conferencing and lack of email use (p≤0.01). Through multivariate ordinal logistic regression analysis, adjusting for patients' age and Jen Frailty Index, we found that the same social risk factors other than internet use showed significant association with frailty (p≤0.01). HNHR Veterans have complex social needs with a limited ability to manage their chronic conditions, necessitating interventions that address not only their medical issues but also their access barriers and social support.

### THE ASSOCIATION BETWEEN SELF-REPORTED AND OBJECTIVELY MEASURED ENERGY LEVEL IN OLDER ADULTS

Qu Tian,[1] Nancy Glynn,[2] Rebecca Ehrenkranz,[2] Briana Sprague,[2] Andrea Rosso,[3] and Caterina Rosano,[2] *1. National Institute on Aging, Bethesda, Maryland, United States, 2. University of Pittsburgh, Pittsburgh, Pennsylvania, United States, 3. School of Public Health, University of Pittsburgh, Pittsburgh, Pennsylvania, United States*

Energy is an important concept in human health and diseases. Self-reported energy has been described as "the individual's potential to perform physical and mental activity" and "the individual's energy availability". However, little empirical data exists on whether self-reported energy level is related to objectively measured energy level. Prior research suggests that more energy availability is associated with higher physical activity level. It remains unclear whether self-reported energy availability would be associated with objectively measured energy level, such as active energy expenditure and total energy expenditure. Using data from the Health, Aging and Body Composition Study, we identified 94 participants (mean age=86.2±2.4 y/o, 46%blacks, 52%women) with concurrent data on self-reported energy (scale 0-10) and objective energy level by the SenseWear Armband. We examined cross-sectional associations of self-reported energy with objectively measured energy and physical activity levels using Spearman correlation. Greater self-reported energy level was associated with higher daily active energy expenditure in kcal (r=0.30,p=0.004), higher METs (r=0.33,p<0.001), more minutes of physical activity (r=0.35,p<0.001), and more step counts (r=0.36,p<0.001). Self-reported energy was not associated with total energy expenditure (p=0.87) or estimated resting metabolic rate (p=0.53). Self-reported energy may reflect an individual's activity energy expenditure but not total energy expenditure. It further supports the hypothesis that energy availability even by self-report connects to physical activity behavior. Whether self-reported energy correlates with other health outcomes warrants further investigation.

### THE ELECTRONIC FRAILTY INDEX AND AREA DEPRIVATION INDEX: INDEPENDENT CONSTRUCTS IN RISK STRATIFICATION

Kathryn Callahan,[1] Kristin Lenoir,[2] and Nicholas Pajewski,[1] *1. Wake Forest School of Medicine, Winston-Salem, North Carolina, United States, 2. Wake Forest University Health Sciences, Winston-Salem, North Carolina, United States*

Frailty and social determinants of health (SDOH) have been associated with mortality for older adults. Given time limitations, passive electronic tools such as the eFI and publicly available data such as the Area Deprivation Index (ADI) hold appeal for targeting limited resources to support at-risk older adults. Literature is conflicting with regards to the relationship between frailty and SDOH. A retrospective, observational cohort of adults 65+ (n=44,548) identified as part of the Wake Forest Baptist Health (WFBH) accountable care organization was used to evaluate the association between ADI, eFI, and mortality between 1/1/2019 and 1/1/2020. A cox proportional hazard model was fit, adjusting for age, sex, race, and weighted Charlson Comorbidity Index. Sources of mortality data include claims data, the EHR at WFBH, and NC Vital Statistics. Block-level geographic identifiers (GEOID) were extracted and used to merge ADI national percentiles (Neighborhood Atlas), derived from U.S. Census 5-year American Community Survey estimates, which incorporates 17 SDOH measures (e.g., income, education, housing, employment.) Frailty was calculated by the WFBH eFI. 9216 (20.7%) were frail by eFI (eFI>0.21) and 235 (0.5%) died. The interaction between ADI tertile and eFI category was not significant (p=0.78). Being frail was associated with poorer survival when compared to the fit group; HR= 1.94 (95% CI =1.23, 3.08; p<0.01.) Survival did not differ between the maximum deprivation tertile and the minimum tertile, HR=1.21 (95% CI=0.88-1.68, p=0.25). Frailty and SDOH may represent independent constructs in risk stratification for older adults. Future work will explore associations within healthcare utilization.

### THE IMPACT OF THE RISK FOR SARCOPENIA ON FRAILTY AMONG OLDER ADULTS WITH PHYSICAL FUNCTIONAL DEPENDENCY

Hongting Ning,[1] Yinan Zhao,[2] Huijing Chen,[1] lulu Liao,[2] Hengyu Hu,[1] and Hui Feng,[2] *1. Central South University, Changsha, China, 2. central south university, Changsha, China*

Objective: 1) to assess the prevalence of the risk for sarcopenia, as well as frailty among older adults with physical functional dependency in a nationally representative sample; and 2) to identify the impact of the risk for sarcopenia on frailty in this vulnerable population in China. Methods: A total of 2,323 participants (age ≥ 60 years old) with physical functional dependency in five provinces in China were enrolled using a multistage cluster sampling scheme. Physical function was measured by the Barthel Index (BI), the risk for sarcopenia was defined as "calf circumference (< 34 cm in men, < 33 cm in women)" according to the 2019 consensus proposed by the Asian Working Group for Sarcopenia, and frailty was assessed by the FRAIL scale. Multivariate binary logistic regression was used to assess the impact of the risk for sarcopenia on frailty. Results: The prevalence of the risk for sarcopenia and frailty were 41.0% and 30.9%, respectively. Logistic regression analysis shows that the risk for sarcopenia was significantly associated with frailty (odds ratio 1.51, 95% confidence interval 1.19-1.90, p = 0.001) after adjustment for demographic and psychosocial factors, as well as health-related factors. Conclusion: This study shows that the risk of sarcopenia and frailty are prevalent, and the presence of the risk of sarcopenia increased the risk of frailty in older Chinese adults with physical functional dependency. Key Words: the risk for sarcopenia; frailty; older adults; physical functional dependency.

### THE QUANTILE FRAILTY INDEX: A CUTPOINT-FREE APPROACH TO BIOMARKER-BASED HEALTH ASSESSMENT

Garrett Stubbings, Spencer Farrell, Arnold Mitnitski, Kenneth Rockwood, and Andrew Rutenberg, *Dalhousie University, Halifax, Nova Scotia, Canada*

We develop a frailty index (FI) from continuous valued biomarker measurements that does not use thresholds to binarize deficits. In this work we construct a quantile frailty index (FI-Q) directly from risk quantiles, without binarizing the deficits. FI-Q is the average risk quantile for an individual in the population with respect to the set of measured biomarkers. We show that FI-Q predicts adverse health outcomes better than either a quantile-based cutpoint approach or an FI-Lab method used in previous studies. We also address practical questions such as how to use longitudinal data. We use data from the English Longitudinal Study of Ageing (ELSA) for longitudinal analysis and data from the National Health and Nutrition Examination Survey (NHANES) and the Canadian Study of Health and Aging (CSHA) to compare predictive value of FI-QM with previous FI-Lab studies.

### SESSION 2889 (POSTER)

### LONG-TERM CARE

### ASSESSING FUNCTIONAL NEED OF MINORITY OLDER ADULTS ACROSS TWO LONG-TERM SERVICES AND SUPPORTS SETTINGS

Jasmine Travers,[1] Andrew Cohen,[2] Norma Coe,[3] and Mary Naylor,[4] *1. Yale University, New York, New York, United States, 2. Yale University, New Haven, Connecticut, United States, 3. University of Pennsylvania, Philadelphia, Pennsylvania, United States, 4. NewCourtland Center for Transitions and Health, Philadelphia, Pennsylvania, United States*

Research has suggested that growth in Black and Hispanic older adults' nursing home (NH) use may be the result of disparities in options for long-term services and supports (LTSS). To investigate this issue, we aimed to determine whether there were no differences in the functional needs of racial and ethnic groups who received care in NHs versus the community. We identified respondents aged ≥65 years in the 2016 Health and Retirement Study who reported requiring caregiving help. We compared the site of care for Black and Hispanic older adults (minority group) to White older adults (comparison group). We performed unadjusted analyses to assess the association of functional need with community vs. NH care. Functional need was operationalized using a functional-limitations score and six individual activities of daily living (ADL). There were 186 minority older adults (community=78%, NH=22%) and 357 White older adults (community=50%, NH=50%). Across settings, minority older adults did not differ in age, marital status, and income, but a greater percentage of men were in NHs (48% versus 28%; p=0.01). The functional-limitations score was higher in NHs than in the community for both groups. Functional needs for the minority group were similar across the two settings in 2/6 ADLs (dressing p=0.11, toileting p=0.09), while White older adults in NHs were more impaired in all ADLs. Functional need for minority older adults primarily differed by setting while demographics did not. These are important factors to consider when implementing programs to keep older adults out of NHs and in the community.

### DIRECT CARE WORKFORCE: WHERE THE BOYS REALLY ARE

Christopher Kelly,[1] Jerome Deichert,[2] and Lyn Holley,[1] *1. University of Nebraska Omaha, Omaha, Nebraska, United States, 2. University of Nebraska at Omaha, Omaha, Nebraska, United States*

Purpose: This study describes the continued growth of male direct care workers (DCWs) and identifies the occupations with the greatest concentrations of male DCWs by utilizing the expanded information available in the 2018 American Community Survey (ACS). Design and Methods: Data were taken from the 1% Public Use Microdata Sample (PUMS) from the 2018 ACS. Beginning in 2018, the ACS separated the single occupation category nursing, psychiatric, and home health aides into three categories: home health aides, nursing assistants, and orderlies and psychiatric aides. Results: Between 2000 and 2018, the total number of male DCWs in the U.S. increased 118% to 474,925, with more than half (52.6%) in 2018 employed as nursing, psychiatric, and home health aides. Among these 250,139 aides, 62% (154,557) were employed as nursing assistants, 23% (57,126) worked as home health aides, and 15% (38,456) were employed as orderlies and psychiatric aides. However, 60% of all orderlies and psychiatric aides were male; this was the only occupation in the direct care workforce in which men were in the majority. Implications: The majority of male DCWs work as nursing, psychiatric, and home health aides and the new occupation classifications in the ACS reveal that while most work as nursing assistants and home health aides, the one occupation with a majority male workforce was orderlies and psychiatric aides. These findings suggest that the greatest need for male DCWs may be as orderlies and psychiatric aides, occupations in which size and physical strength are important factors.

### EMPTY BEDS IN NURSING HOMES FILLED BY YOUNGER INDIVIDUALS FOLLOWING ACA MEDICAID ELIGIBILITY EXPANSION

Ashley Ritter, Norma Coe, and Salama Freed, *University of Pennsylvania, Philadelphia, Pennsylvania, United States*

National decline in nursing home occupancy rates coupled with expansion of Medicaid insurance eligibility under the Affordable Care Act (ACA) potentially create an opportunity for younger, low-income individuals to enter nursing homes following a hospitalization. Changes in the population of individuals using nursing homes could result in downstream consequences on facility payor mix, case severity index, and ultimately patient outcomes. This study measures the effect of ACA Medicaid eligibility expansion on the patient population using nursing homes, accounting for the nursing homes' occupancy rate. Data were obtained from the publicly available national dataset, LTCfocus (2009-2016). Difference in differences estimation with time and state fixed effects was utilized to examine the effect of ACA Medicaid eligibility expansion on two outcomes, 1) average age in years of residents as of April 1 and 2) the proportion of individuals covered by Medicaid insurance at the facility level. Results show facilities with pre-ACA occupancy rates between 40% and 50% demonstrated the largest decrease in average age by year three, 1.32 years [95% CI: -2.257, -0.385]. Facilities with a pre-ACA occupancy rate of 60-70% demonstrated the largest increase in the proportion of individuals covered by Medicaid in year one, a 5.5 percentage point increase [95% CI: 0.009, 0.102]. In summary, Medicaid expansion under the ACA resulted in an increase in younger individuals and individuals covered by Medicaid using nursing homes, varying across pre-ACA occupancy rates. It remains to be studied if increased utilization of this high cost setting provides superior patient outcomes for these populations.

### EVIDENCE FOR PUBLICLY REPORTED QUALITY INDICATORS IN RESIDENTIAL LONG-TERM CARE: A SYSTEMATIC REVIEW

Franziska Zúñiga,[1] Magdalena Osinska,[2] and Franziska Zuniga, *1. Basel University, Basel, Basel-Stadt, Switzerland, 2. University of Basel, Basel, Switzerland*

Quality indicators (QIs) are used internationally to measure, compare and improve quality in residential long-term care. Public reporting of such indicators allows transparency and motivates local quality improvement initiatives. However, little is known about the quality of QIs. In a systematic literature review, we assessed which countries publicly report health-related QIs, whether stakeholders were involved in their development and the evidence concerning their validity and reliability. Most information was found in grey literature, with nine countries (USA, Canada, Australia, New Zealand and five countries in Europe) publicly reporting a total of 66 QIs in areas like mobility, falls, pressure ulcers, continence, pain, weight loss, and physical restraint. While USA, Canada and New Zealand work with QIs from the Resident Assessment Instrument – Minimal Data Set (RAI-MDS), the other countries developed their own QIs. All countries involved stakeholders in some phase of the QI development. However, we only found reports from Canada and Australia on both, the criteria judged (e.g. relevance, influenceability), and the results of structured stakeholder surveys. Interrater reliability was measured for some RAI QIs and for those used in Germany, showing overall good Kappa values (>0.6) except for QIs concerning mobility, falls and urinary tract infection. Validity measures were only found for RAI QIs and were mostly moderate. Although a number of QIs are publicly reported and used for comparison and policy decisions, available evidence is still limited. We need broader and accessible evidence for a responsible use of QIs in public reporting.

### EXPLORING LEADERSHIP, STRESS OF CONSCIENCE, AND PERSON-CENTERED CARE IN NURSING HOMES

Annica Backman,[1] karin sjögren,[2] Hugo Lovheim,[3] Marie Lindkvist,[4] and David Edvardsson,[5] *1. Umea University, Umea, Sweden, 2. Departement of Nursing, Umea, Sweden, 3. Umeå University, Umea, Sweden, 4. Department of Epidemiology and Global Health, Umea, Sweden, 5. La Trobe University, Melbourne, Victoria, Australia*

On a daily basis, many care situations contain difficult issues and challenges for care providers. Stress of conscience, such as feelings of guilt, can be experienced by staff when not fulfilling ethical obligations to the residents. Although leadership has been advocated as a key component for staff work perceptions as well as for person-centred care, the impact of nursing home managers' leadership on levels of stress of conscience among staff and the extent to which person-centred care (PCC) is provided is yet to be explored. Thus, the aim was to explore the relationship between leadership, stress of conscience and PCC as perceived by staff. The study was based on a cross-sectional national survey of 3084 staff and their managers in 189 nursing homes throughout Sweden. Descriptive statistics and regression modelling were used to explore associations. The preliminary results showed that leadership was negatively associated to stress of conscience and positively associated to PCC. PCC were negatively associated to stress of conscience. Additional findings will be presented. This indicates that nursing home managers' leadership seem to beneficially impact staff work situation in terms of stress of conscience and person-centred care provision.

### FACTORS ASSOCIATED WITH FOOD INTAKE, NUTRITIONAL STATUS, AND FUNCTION AMONG NURSING HOME RESIDENTS WITH DEMENTIA

Murad Taani,[1] and Adam Plotkin,[2] *1. University of Wisconsin-Milwaukee, Milwaukee, Wisconsin, United States, 2. Cornell College, Cornell College, United States*

Declined food intake is prevalent among long-term care (LTC) residents with dementia and associated with deleterious health outcomes. This study explores food intake, nutritional status, and function and its associated factors in LTC residents with dementia. Data from 82 LTC residents with dementia were used in this secondary analysis. In the primary study, appetite was assessed using the Short Nutritional Assessment Questionnaire (SNAQ). Dementia level, comorbidity, agitation, pain, mood, food intake, nutritional status, and function variables were extracted from the electronic medical record, including the most recent Minimum Data Set (version 3.0) assessment. The majority of residents were either malnourished or at risk of being malnourished and demonstrated a worse appetite than previously described in the literature. Comorbid illness, depressed mood, and appetite were associated with 37.1% of the variance in food intake over 30 days. Dementia level and appetite were associated with 22.2% of the variance in nutritional status. Food intake and nutritional status were associated with 29.1% of the variance in function. This study also highlights a new demographic that may require extra assistance in combating declined food intake: LTC residents with dementia who reside in a facility that follows restrictive food practices such as a kosher diet. The potential reversibility of factors associated with food intake and nutritional status provides opportunities for intervention.

### FACTORS RELATED TO THE WELL-BEING OF OLDER CHINESE LIVING IN INSTITUTIONAL CARE FACILITIES: A SYSTEMATIC REVIEW

Kun Wang, *University of Alabama, Tuscaloosa, Alabama, United States*

Due to the fast pace of population aging and the "4-2-1" family structure, institutional care was proposed as "support" for the elderly care service system in China. The purpose of this systematic review paper was to identify factors that are associated with the well-being of older residents living at institutional care facilities in China. Studies were included if participants (1)aged 60 years or older, (2) were living at an institutional care facility in mainland China. Studies were excluded if participants (1) were Chinese Immigrants, or residents in Hong Kong, Macao, and Taiwan, (2) were cognitively impaired, or (3) at the end of their lives and need palliative care in institutional facilities. A total of 12 articles were selected in this review study based on PRISMA guidelines: 10 quantitative studies and 2 qualitative studies. Anderson healthcare utilization model was used in this study to categorize related factors into three dynamics: predisposing factors, enabling factors, and need factors. Among predisposing factors, older, more educated, widowed adults with higher income were more likely to have higher levels of well-being in institutional care facilities. Social supports, such as family visit, activity engagement and peer support, were very important enabling factors. The actual need, such as ADL, health status and depression, was another important dimension for the well-being of older Chinese living in institutional care facilities. Aiming at increasing older residents' well-being, the present study suggested more tailored interventions should be designed and implemented to enhance their social support, activity engagement and peer support.

### MAKING QUALITY IMPROVEMENT DATA MEANINGFUL FOR LONG-TERM CARE ADMINISTRATORS

Lisa Cranley,[1] Lori Weeks,[2] TKT (Thomas) Lo,[3] Peter Norton,[4] and Carole Estabrooks,[3] *1. University of Toronto, Toronto, Ontario, Canada, 2. Dalhousie University, Halifax, Nova Scotia, Canada, 3. University of Alberta, Edmonton, Alberta, Canada, 4. University of Calgary, Calgary, Alberta, Canada*

Tailoring feedback data to engage end-user stakeholders when sharing organizational context data is a central component of quality improvement and integrated knowledge translation. For over a decade, our research team has collected survey data (using the validated Alberta Context Tool) on modifiable aspects of organizational context from long-term care (LTC) staff (e.g., nurses, unregulated providers) across a representative cohort of 94 LTC facilities in Western Canada. We have fed back data at the facility and care unit level with the goal of making research findings more useful for decision-making and aiding improvement efforts. We have used a binary method (more favourable / less favourable organizational context) to report multidimensional data. While useful to our stakeholders (e.g., administrators) we are continually seeking ways to increase the detail in our reporting, while maintaining usability for stakeholders. We have now developed a more detailed method – the context rank summary, which displays rankings of care units within and across LTC facilities. In this study, we used a qualitative descriptive design to explore perspectives of administrators and managers (leaders) from LTC facilities on the two different methods for reporting survey data. We conducted a total of three focus groups with 16 leaders in the Maritimes and Ontario, Canada. Transcripts were analysed using content analysis. Leaders preferred a feedback report that combines a binary method with the greater detail of the context rank summary. Providing organizational context data that is more meaningful, relevant and actionable could offer an additional path to identifying areas for improvement.

### MEALTIME CARE FOR PEOPLE WITH DEMENTIA: WHAT DO NURSING HOME STAFF THINK?

James Faraday,[1] Clare Abley,[2] Catherine Exley,[1] and Joanne Patterson,[3] *1. Newcastle University, UK, Newcastle upon Tyne, United Kingdom, 2. The Newcastle upon Tyne Hospitals NHS Foundation Trust, UK, Newcastle upon Tyne, United Kingdom, 3. The University of Liverpool, UK, Liverpool, United Kingdom*

More and more people with dementia are living in nursing homes (NH). Often, they depend on NH staff for help with eating and drinking. It is important that staff have the skills and support they need to provide good care at mealtimes. This qualitative study explores mealtime care for people with dementia, from the perspective of NH staff. Semi-structured interviews with NH staff (n=16) were carried out in two nursing homes. The homes were chosen to have diverse characteristics: one home had a large number of beds and was part of a small local organization; the other had a small number of beds and was part of a large national organization. Various staff members were interviewed, including direct care staff, senior carers, nurses, managers, and kitchen staff. Interviews were audio-recorded and transcribed verbatim. A constant comparison approach was taken, so that data from early interviews were explored in more depth subsequently. From the analysis, five themes emerged as important in mealtime care for people with dementia living in nursing homes: Setting the right tone; Working well as a team; Knowing the residents; Promoting autonomy and independence; Gently persevering. This work forms part of a larger ethnographic study on the topic, which includes data from residents with dementia, and family carers. Results will inform the development of a staff training intervention to optimize mealtime care for this population.

### MEASURING ADOPTION OF PATIENT PRIORITIES-ALIGNED CARE USING NATURAL LANGUAGE PROCESSING

Javad Razjouyan,[1] Jennifer Freytag,[1] Edward Odom,[2] Lilian Dindo,[3] and Aanand Naik,[1] *1. Center for Innovations in Quality, Effectiveness, and Safety (iQuest), Houston, Texas, United States, 2. Michael E. DeBakey VA Medical Center, houston, Texas, United States, 3. Michael E. DeBakey VA Medical Center, Houston, Texas, United States*

Patient Priorities Care (PPC) is a model of care that aligns health care recommendations with priorities of older adults with multiple chronic conditions. Social workers (SW), after online training, document PPC in the patient's electronic health record (EHR). Our goal is to identify free-text notes with PPC language using a natural language processing (NLP) model and to measure PPC adoption and effect on long term services and support (LTSS) use. Free-text notes from the EHR produced by trained SWs passed through a hybrid NLP model that utilized rule-based and statistical machine learning. NLP accuracy was validated against chart review. Patients who received PPC were propensity matched with patients not receiving PPC (control) on age, gender, BMI, Charlson comorbidity index, facility and SW. The change in LTSS utilization 6-month intervals were compared by groups with univariate analysis. Chart review indicated that 491 notes out of 689 had PPC language and the NLP model reached to precision of 0.85, a recall of 0.90, an F1 of 0.87, and an accuracy of 0.91. Within group analysis shows that intervention group used LTSS 1.8 times more in the 6 months after the encounter compared to 6 months prior. Between group analysis shows that intervention group has significant higher number of LTSS utilization (p=0.012). An automated NLP model can be used to reliably measure the adaptation of PPC by SW. PPC seems to encourage use of LTSS that may delay time to long term care placement.

### MEDICARE ANNUAL WELLNESS VISIT FOR NURSING HOME RESIDENTS

Theodore Malmstrom,[1] Milta Little,[2] Angela Sanford,[1] Marla Berg-Weger,[3] and John Morley,[1] *1. Saint Louis University School of Medicine, Saint Louis, Missouri, United States, 2. Duke University School of Medicine, Durham, New York, United States, 3. Saint Louis University, St. Louis, Missouri, United States*

The 2010 Patient Protection and Affordable Care Act includes the Annual Wellness Visit (AWV) for older adult (OA) patients. Medicare pays for an initial AWV per beneficiary and subsequent visits annually. Many Medicare beneficiaries have not taken advantage of the AWV preventive health benefit. The Saint Louis University Geriatrics Workforce Enhancement Program (GWEP) developed an AWV for OA, NH residents. This project describes the NH AWV and reports results. Data include age, gender, comorbidities, medications, hospitalizations, depression, frailty, pain, sarcopenia, sensory impairment, cognition, nutrition, smoking, falls, and advance directives. Two suburban academic for-profit NHs are included in this study (2016-17). OA NH residents (N=247) completed an AWV and 36.8% (n=91) had a 1-year follow-up AWV. OA NH residents were female (n = 177, 71.7%) and a majority ages 75+ (n = 172, 69.7%). Most (96.3%) had a documented advance directive. Comorbidities (7.8±2), polypharmacy (92.3%), vision impairment (52.8%), hearing impairment (52.8%), depression (65.2%), frailty (75.7%), sarcopenia (84.4%), risk of weight loss (53.9%), MCI (11.7%), and dementia (75.8%) were prevalent. Among OA NH residents (n=91) with an AWV follow-up, there was modest worsening in total comorbidities and medications as well as frailty, sarcopenia, and cognition scores (ps≤0.05). Pain, depression, and nutrition did not change. To our knowledge, no one has specifically analyzed the Medicare AWV in NHs. Data from the traditional AWV is an extension of the routine clinical care of OAs and therefore could also be useful for healthcare professionals focused on providing care to OA patients in the NH setting.

### PREDICTORS OF EFFORT-REWARD IMBALANCE AMONG EMPLOYEES PROVIDING THREE TYPES OF LONG-TERM CARE SERVICES IN JAPAN

Ayumi Honda,[1] Elizabeth Fauth,[2] Yin Liu,[2] and Sumihisa Honda,[1] *1. Nagasaki University Graduate School of Biomedical Sciences, Nagasaki, Japan, 2. Utah State University, Logan, Utah, United States*

Increasingly, employees are leaving their jobs as long-term care workers in Japan. The purpose of this study was to identify predictive factors of effort-reward imbalance (ERI) among employees in long-term care, to better understand factors associated with excessive effort and reduced reward. This cross-sectional study included 944 participants providing three types of long-term care: home-based (n=201), community-based (n=128), and institutional (n=615). Multiple logistic regression analysis was used to identify factors associated with self-reported ERI, where higher ERI scores indicated greater work-related efforts and lower rewards. Key independent variables included type of occupation, employment status, position, daily working hours, job satisfaction, and annual income. Our results showed that low job satisfaction was the sole common factor associated with ERI in employees across all three types of long-term care. Other predictive factors for ERI differed by type of long-term care services. Working longer hours predicted ERI in community-based and institutional care employees, but not home-based care employees. For institutional care employees, being a care manager, holding a position of department head, having family-related stress were risk factors for ERI, suggesting that in this setting, the rewards of higher income and more prestige in leadership positions are offset by greater work-related demands. In conclusion, factors associated with ERI were both common and distinct among employees providing different types of long-term care services. Adjusting work demands and working hours, and identifying unique contributors to ERI within specific long-term care settings may help with job retention in these occupations.

### RESEARCH ON THE RELATIONSHIP OF THE ORGANIZATION CAPACITY AND JOB SATISFACTION IN NURSING HOMES

lulu Liao,[1] Huijing Chen,[2] Yinan Zhao,[1] Hongting Ning,[2] and Hui Feng,[1] *1. central south university, Changsha, China, 2. Central South University, Changsha, China*

Objective: To explore the occurrence of organization capacity and to examine the relationships among the organization capacity and job satisfaction in nursing homes in China. Methods: A total of 577 participants in sixteen nursing homes in Hunan province of China were enrolled using a cluster randomized controlled trial. Staff job satisfaction was measured by Minnesota Satisfaction Questionnaire(MSQ) in short form, nursing homes' organization capacity was assessed by Shortell's Organization and Management Survey. One-way analysis of variance examined differences between variables. The relationship and correlation of organization capability, MSQ total score and scores in each dimension was analyzed. Results: Overall score of nursing homes' organization capacity was 99.5±8.6(level ranking, 21–105), and MSQ score was 78.2±8.2 (level ranking, 20–100). Staff job satisfaction levels were significantly different among nursing staffs with different levels of organization capability (F=9.40, P < 0.001). Correlation analysis showed that organization capability level was positively correlated with staff job satisfaction (r=0.52, P< 0.001). And Correlation analysis showed that Working conditions (r = 0.46, P < 0.001), Leaders (r = 0.46, P < 0.001), Responsibility (r = 0.51, P < 0.001) and External reward (r= 0.42, P < 0.001) were positively associated with organization capability. Conclusion: The organization capability can be improved by active staff job satisfaction. But the job satisfaction of nursing staffs was at a moderate level. Thus effective measures such as creating a supportive work conditions, appointing suitable leaders, enhancing responsibilities and providing external reward should be considered by nursing managers to promote organization capability in nursing homes.

### STRESS AND NURSING HOME ADJUSTMENT: MEDIATION BY RESOURCEFULNESS AND SELF-EFFICACY

Huimin Xiao, and Binbin Yong, *Fujian Medical University, Fuzhou, China*

Relocation to a nursing home is often assumed to be associated with stress for older adults. This study aimed to explore how stress affect psychological adjustment of nursing home residents. A cross-sectional survey was conducted. A sample of 386 nursing home residents was recruited from 11 nursing homes in Fujian Province, Southeast China. The Nursing Home Adjustment Scale, Perceived Stress Scale, Resourceful Scale, and General Self-Efficacy Scale were adopted to collect data. The path analysis was used to analyze the relationship of stress, nursing home adjustment, resourcefulness, and self-efficacy. The results indicated that stress directly caused poor nursing home adjustment. It also indirectly affected nursing home adjustment through the mediators of resourcefulness and self-efficacy, respectively. Furthermore, chained mediation was found from stress to nursing home adjustment through resourcefulness, and then through self-efficacy to cope with the negative affect. The current study contributes to the understanding of the mechanism of stress on nursing home adjustment in older adults. To improve their psychological adjustment, additional focus should be placed on enhancing resourcefulness and self-efficacy in nursing home residents.

### THE ACTION PROJECT METHOD APPLIED IN NURSING HOME SETTINGS

Charlotte Jensen,[1] Andrea Gruneir,[2] Matthias Hoben,[2] Jaclyn Tompalski,[3] Adam Easterbrook,[4] Janice Keefe,[5] Carole Estabrooks,[2] and Sheila Marshall,[6] *1. Staff, Edmonton, Alberta, Canada, 2. University of Alberta, Edmonton, Alberta, Canada, 3. Carlton University, Ottawa, Ontario, Canada, 4. Centre for Health Evaluation and Outcomes Sciences, Vancouver, British Columbia, Canada, 5. Mount Saint Vincent University, Halifax, Alberta, Canada, 6. University of British Columbia, Vancouver, British Columbia, Canada*

The action-project method (APM), developed in counselling psychology and used in various disciplines, has been shown to be useful for understanding major life transitions in different contexts. We argue that the APM is beneficial for studying the impact of nursing home (NH) home admission and daily life of residents and their families/friends. The APM enables researchers to explore how residents and their families/friends experience NH-life at individual and supraindividual levels of analysis. We applied the APM to solicit the views of residents and individuals close to them to understand their priorities for quality care. The APM data collection consisted of three stages. First, a resident and family member or other caregiver met with the interviewer who initiated a conversation about their experience in the NH. The interviewer then left the room but video-recorded the conversation. Second, the interviewer met with each participant to review the video with each participant offering reflection on the original conversation. These sessions were also recorded. Following transcription and analysis of the conversations, 3 lay-language narratives were created: 1 for each individual and 1 for the pair. Third, participants reviewed their own and the pair's narrative for additional comments. The APM offers a means to give a voice to NH residents and allows for people to talk about their experiences without the presence of a researcher. By using the APM, researchers can break down individual actions of participants and how these actions come together to form the project of navigating care in NHs.

### THE COLUMBIA BEHAVIOR SCALE FOR DEMENTIA: VALIDITY AND RELIABILITY IN LONG-TERM CARE SETTINGS

William Mansbach, and Ryan Mace, *Mansbach Health Tools, LLC, Simpsonville, Maryland, United States*

Numerous neuropsychiatric inventories have been created for behavioral and psychological symptoms of dementia (BPSD). These inventories are seldom used in long-term care (LTC) settings due to questionable psychometrics, lengthy administration, and reliance on knowledgeable informants. The Columbia Behavior Scale for Dementia (CBS) is a rapidly administered BPSD rating tool that was developed for LTC residents. The 11-item CBS can be completed in less <5 minutes independently, with nursing staff, or in conjunction with interdisciplinary care teams. LTC residents (N = 350) participated in a validation study in Maryland, USA (M age = 78.38, SD = 10.82). Internal consistency ($\alpha = 0.75$) and inter-rater reliability ($r = 0.99$) for the CBS were strong. CBS scores were not biased by informant type ($p > 0.05$): GNAs/CNSs (40.69%), nurses (36.10%), other facility staff (23.21%). Diagnostic validity was confirmed by significantly higher CBS scores ($p < 0.001$; large effect, d = .63) for LTC residents with dementia (n = 197, M = 4.63, SD = 4.58) versus those without dementia (n = 145, M = 2.17, SD = 2.87). Higher CBS scores were significantly associated with greater impairment on cognitive instruments (r range = -0.25, -.36) and increased mood dysfunction (r range = 0.20, 0.26), indicating convergent validity. Principal components analysis produced three CBS factors, psychosis, aggression, and non-aggressive motor disinhibition, which significantly identified LTC residents with greater odds for antipsychotic use. Results will be discussed in terms of right-sizing antipsychotic utilization, improving nonpharmacological behavior management, and enhancing the dementia literacy of nursing staff.

### THE INFLUENCE OF RACE AND GENDER ON STAFF-RESIDENT INTERACTIONS IN NURSING HOMES

Rachel McPherson,[1] Barbara Resnick,[2] and Elizabeth Galik,[3] *1. Univesity of Maryland, Baltimore, Maryland, United States, 2. University of Maryland School of Nursing, Baltimore, Maryland, United States, 3. University of Maryland, Baltimore, Maryland, United States*

Communication and interactions are an integral part of care in long-term care settings. Resident variables, such as race and gender, shape communication and interaction between staff and residents. The Quality of Interactions Schedule (QuIS) was developed to measure the quality of verbal and nonverbal interactions among nursing staff and older adults initially for those in acute care and later used as well in a variety of long term care settings. A quantified measurement of the quality of interactions between residents and staff was created to quantify the QuIS. The purpose of this study was to describe the gender and racial differences in scored quality of interactions. Data for the present study was based on baseline data from the Evidence Integration Triangle for Behavioral and Psychological Symptoms of Dementia (EIT-4-BPSD) implementation study. A total of 535 residents from 55 settings were included in the analyses. An analysis of covariance was conducted to determine a difference in QuIS scores between males and females while controlling for age. The second model tested for differences in QuIS scores between blacks and whites while controlling for age and gender. There was not a statistically significant difference in QuIS scores between male and female residents. There was a significant difference in QuIS scores between those who were black versus white, such that those who were black received more positive interactions from staff than those who were white. Future work should focus on a deeper examination of resident factors and staff factors that may influence these interactions.

### SESSION 2890 (POSTER)

### PHYSICAL ACTIVITY, EXERCISE, AND REHABILITATION CARE

### ASSOCIATION BETWEEN ACTIVITY DIVERSITY AND FRAILTY IN COMMUNITY-DWELLING OLDER JAPANESE: A CROSS-SECTIONAL STUDY

Junta Takahashi,[1] shuichi Obuchi,[1] Hisashi Kawai,[1] Kaori Ishii,[2] Koichiro Oka,[2] Yoshinori Fujiwara,[3] Hirohiko Hirano,[1] and Kazushige Ihara,[4] *1. Tokyo Metropolitan Institute of Gerontology, Itabashi-Ku, Tokyo, Japan, 2. Waseda University, Tokorozawa, Saitama, Japan, 3. Tokyo Metropolitan Institute of Gerontology, Tokyo, Japan, 4. Hirosaki University, Itabashi-Ku, Tokyo, Japan*

In addition to intensity and quality, diversity of activity will be an important factor to explain health outcomes among older adults. Few studies, though, examined an association between activity diversity and health outcomes. This study aimed to examine the association between activity diversity and frailty among community-dwelling older Japanese. Participants were community-dwelling older adults who participated in a cohort study, the "Otassya-Kenshin" in 2018 . The participants were asked frequency of 20 daily activities, inside/outside chores, leisure activities with/without physically, direct/indirect contact with friends and so on, in a week and activity diversity score were calculated using the formula of Shannon's entropy. Frailty was defined by the Japanese version of the Cardiovascular Health Study criteria. The difference in diversity score between frail and non frail were examined by t-test. Logistic regression analysis with covariates, age, sex, economic status, living alone, BMI, Mini-Mental State Examination, and IADL was adopted to find association between activity diversity score and presence of frailty. Of 652 participants (age: 72.8±6.3, women: 60.6%) analyzed, 27 (4.1%) were defined as frail. Frailty group revealed significantly lower activity diversity score than non-frailty group (0.66±0.11 vs 0.75±0.08, $P<0.01$). 0.2 point of decrease in diversity score increase 5 times chance of frailty after controlling covariates. We found significant relationship between activity diversity and health outcome among older subjects. The activity diversity may provide additional information to number or intensity of activity.

### ASSOCIATION OF PHYSICAL ACTIVITY, MUSCLE MASS, STRENGTH, AND FUNCTION TO QUALITY OF LIFE IN OLDER ADULTS

Murad Taani,[1] Chi Cho,[2] and Julie Ellis,[2] *1. University of Wisconsin-Milwaukee, Milwaukee, Wisconsin, United States, 2. University of Wisconsin Milwaukee, Milwaukee, Wisconsin, United States*

Physical inactivity and loss of muscle mass, strength, and function are associated with negative outcomes including disability and a decline in health-related quality of life (HRQoL) among older adults. Older adults living in continuing care retirement communities (CCRCs) are at greater risk for declining physical activity and muscle outcomes compared to community-dwelling older adults. Few researchers studying the association of muscle and physical activity have examined the distinction between physical and mental HRQoL. Understanding the differential association of physical and mental HRQoL to physical activity and muscle outcomes can inform the development of useful interventions. The aim of this study was to examine the relationships between physical activity, muscle mass, strength, function and physical and mental HRQoL. Using a descriptive, correlational design, 105 older adults living in CCRCs were recruited. Light physical activity (LPA), moderate physical activity (MPA), sedentary behavior, and steps per day were assessed using ActiGraph GT3X. Appendicular skeletal muscle mass (ASMM) was assessed with bioelectrical impedance spectroscopy, handgrip strength with JAMAR Smart Hand Dynamometer, muscle function with the Short Physical Performance Battery (SPPB) test, and physical and mental HRQoL with the SF-36 questionnaire. The mean age of participants was 83 (SD=7.4). Using multiple regression models adjusted for sex and age, steps per day and SPPB score explained 38.4 % of the variance in physical HRQoL. Handgrip strength explained 8 % of the variance in mental HRQoL. These findings suggest that QoL improvement programs should include components to improve physical activity, muscle strength and function.

### CONTRACTION TYPE INFLUENCES CRITICAL AGES FOR DECLINES IN LOWER BODY SPECIFIC FORCE IN WOMEN AGES 20 TO 89 YEARS

Ryan Miller, Eduardo Freitas, Aaron Heishman, Debra Bemben, and Michael Bemben, *University of Oklahoma, Norman, Oklahoma, United States*

The ability for a muscle to produce force relative to its size, specific force, is an important characteristic for healthy aging. Few studies have identified the influence that different muscle groups or contraction types may have on the onset of declines in specific force. Therefore, the aims of the current study were to identify critical ages for changes in upper (quadriceps and hamstrings) and lower leg (soleus and tibialis anterior) specific force and to determine if the onset of decline is influenced by contraction type (isometric or dynamic) in women aged 20 to 89 years. One-hundred and fifty-two women (47.1±17.7years, 164.2±7.0cm, 67.1±10.7kg) matched for physical activity with approximately 10 participants per five-year interval (20-24, 25-29years, etc.), were included in the present analysis. Specific force was calculated from peak torque values measured from isometric (ISOM) or isokinetic testing (60deg/s and 240deg/s) and made relative to muscle area measured by peripheral quantitative computed tomography. An iterative segmental modelling approach was performed to identify critical age periods for changes in specific force. For the upper leg, ISOM and 60deg/s specific force was maintained across the lifespan, whereas 240deg/s revealed a critical age of 35.2±5.3years. Critical ages were identified for all contraction types for the lower leg and occurred earlier with increasing velocity (ISOM: 63±6.6years, 60deg/s: 53.9±4.5years, and 240deg/s: 49±3.9years). These data suggest that muscle groups of the leg do not display uniform changes across different contraction types and that high velocity contractions display earlier declines across the lifespan.

### CUMULATIVE EFFECTS OF PHYSICAL ACTIVITY, DIETARY VARIETY, AND SOCIAL PARTICIPATION ON ACTIVE LIFE EXPECTANCY

Satoshi Seino,[1] Akihiko Kitamura,[1] Yui Tomine,[1] Mariko Nishi,[2] Yu Nofuji,[3] Yuri Yokoyama,[2] Hidenori Amano,[4] and Shoji Shinkai,[4] *1. Tokyo Metropolitan Institute of Gerontology, Itabashi-ku, Japan, 2. Tokyo Metropolitan Institute of Gerontology, Itabashi, Japan, 3. Tokyo Metropolitan Institute of Gerontology, Itabashi-ku, Tokyo, Japan, 4. Tokyo Metropolitan Institute of Gerontology, Tokyo, Japan*

Regular physical activity, dietary variety, and active social participation are modifiable and influential factors of adverse health outcomes. However, the cumulative effects of these behaviors are unknown. We examined these cumulative associations with active life loss in older adults. We analyzed 3-year longitudinal data from 7246 initially nondisabled residents aged 65-84 years from 18 districts of Ota City, Tokyo. Sufficiency of moderate- to vigorous-intensity physical activity (MVPA) of ≥150 minutes/week, dietary variety score (DVS) of ≥3, and social participation of ≥1 time/month were assessed using self-administered questionnaires. We operationally defined active life loss for individuals as being newly certified for long-term care insurance or death without prior certification. Multilevel survival analyses were applied to calculate hazard ratios (HRs) and 95% confidence intervals (CIs). During an average follow-up of 2.9 years, the cumulative incidence of active life loss was 11.3% (817 individuals: 650 new certifications and 167 deaths without prior certification). Multivariate-adjusted HRs (95% CIs) for active life loss were 0.73 (0.58-0.92) in only MVPA of ≥150 minutes/week, 0.88 (0.67-1.15) in only DVS of ≥3, 0.75 (0.51-1.09) in only social participation of ≥1 time/month, 0.56 (0.45-0.70) in the group satisfying any two, and 0.52 (0.40-0.67) in the group satisfying all three behaviors, compared with a reference group that did not satisfy any of the behaviors. Sensitivity analysis that excluded active life losses during the first year showed similar results. The combination of regular physical activity, dietary variety, and social participation further enhances the effects on active life expectancy than individual practices.

### EFFECT OF A TAI CHI-BASED EXERCISE PROGRAM ON COGNITIVE FUNCTION AMONG OLDER ADULTS WITH DEMENTIA

Rhayun Song,[1] Myonghwa Park,[2] Misook Jung,[3] Hyun Li Kim,[3] and Hanna Lee,[3] *1. Chungnam National University, Dae Jeon, Republic of Korea, 2. Chungnam National University, Daejeon, Taejon-jikhalsi, Republic of Korea, 3. Chungnam National University, Daejeon, Republic of Korea*

Cognitive function with dementia is strongly associated with physical function decline. A low intensity aerobic exercise, such as Tai Chi, may help either prevent or slow down cognitive impairment. This study aimed to examine the effect of Tai Chi-based exercise program on cognitive function among older adults with early dementia. Individuals registered at the Dementia Support Center were invited to participate in the Tai Chi-based exercise program twice a week for one-hour session for 20 weeks. The comparison group with the same inclusion criteria but not participated in any formal exercise program were recruited by matching with age, education, and pre-cognitive function. Cognitive function (MOCA-K) was measured at the pretest and at the completion of the study period. Fifty-two older adults with dementia (29 in Tai Chi group, 23 comparisons) with the mean age of 80.5 years completed all measurements. All participants had at least one chronic disease. About 50% of the participants received no formal education. At the completion of the study, Tai Chi group improved their cognitive function, while their counterpart remained similar in their MOCA-K score, specifically in attention (F=5.21, p=.027) and short term memory recall (F=6.66, p=.013). In conclusion, Tai Chi-based exercise program was safely and effectively applied to older adults with early dementia. The participants were able to follow the movements with the attendance rates of 80% during the study period. Further studies are warranted to explore the relationship between physical exercise and cognitive function in this population with various types of cognitive impairment.

### EFFECTS OF RESISTANCE EXERCISE ON MENTAL HEALTH IN OLDER CHINESE AMERICANS: A RANDOMIZED CONTROLLED TRIAL

Mei-Lan Chen,[1] Elisabeth Burgess,[1] Ying-Yu Chao,[2] Douglas Gardenhire,[1] and Ruiyan Luo,[1] *1. Georgia State University, Atlanta, Georgia, United States, 2. Rutgers School of Nursing, Newark, New Jersey, United States*

Regular exercise has shown to be potentially beneficial for improving mental health in older adults. However, few studies evaluated the effect of resistance exercise on psychological well-being in older Chinese Americans. The purpose of this two-arm randomized controlled trial (RCT) was to test the effects of resistance exercise training on stress, depression, and social engagement in community-dwelling older Chinese Americans. A total of 30 older adults (mean age 77.9 ± 5.0 years) were randomly assigned into the resistance exercise intervention group (n = 15) or the wait-list control group (n = 15). The resistance training intervention includes 50-min group exercise session twice weekly for 12 weeks. Participants' perceived stress, depressive symptoms, and social engagement were measured at baseline and 12 weeks follow-up. Descriptive statistics and t tests were performed for data analysis. The results revealed that the resistance exercise intervention group had significant improvements in perceived stress, depressive symptoms, and social engagement after receiving the 12-week intervention. At baseline, there were no significant differences between the intervention and the control groups on perceived stress, depressive symptoms, and social engagement. However, older adults received resistance exercise training had greater improvements in stress levels, depressive symptoms, and social engagement than their control counterparts at 12 weeks follow-up. The findings suggest resistance exercise has positive effects on psychosocial well-being for older adults. Further larger RCTs are needed to assess long-term effects of the resistance exercise intervention.

### FEASIBILITY OF A GROUP VERSION OF THE LIFESTYLE-INTEGRATED FUNCTIONAL EXERCISE PROGRAM

Carl-Philipp Jansen,[1] Corinna Nerz,[2] Sarah Labudek,[3] Jochen Klenk,[2] Lindy Clemson,[4] Clemens Becker,[2] and Michael Schwenk,[3] *1. Heidelberg University, Karlsruhe, Germany, 2. Robert-Bosch Hospital Stuttgart, Stuttgart, Germany, 3. Heidelberg University, Heidelberg, Germany, 4. Unversity of Sydney, Sydney, Australia*

In a randomised noninferiority trial, it is investigated whether a group version of the Lifestyle-integrated Functional Exercise program (gLiFE) is non-inferior to the individually delivered LiFE in terms of feasibility and effectiveness. While effectiveness evaluation is ongoing, feasibility results are already available. Participants (>70 years; confirmed fall risk) were randomized in either LiFE or gLiFE and participated in the same strength and balance exercises, however, based on different approaches of delivery. LiFE participants received seven home visits; gLiFE was delivered in seven group sessions. Feasibility was defined as willingness to participate, adherence to group/home visits, and drop-outs. Predictors for intention to participate were calculated using regression. N=310 participants were randomized to LiFE (n=156) or gLiFE (n=154). n=51 (16%) of the participants dropped out after baseline. Attendance analyses showed that when excluding drop-outs, 100% (iLiFE) and 88% (gLiFE) took part in at least 5 of the 7 meetings. Self-efficacy and outcome expectancies, but not risk perception, were predictors of the intention to participate (F(3,193)=24.84, p<.001). In this first study comparing a group-based LiFE format with the original LiFE, feasibility of both formats was shown in terms of high attendance and less drop-outs than expected in this target group. Compared to other studies involving group based training, compliance to intervention (defined as having absolved at least 5 sessions) was high in both formats. Lower attendance in gLiFE can be explained by inflexible scheduling as compared to making individual home visit appointments. Whether lower gLiFE adherence translates into lower effectiveness is currently analysed.

### INFLUENCE OF PHYSICAL ACTIVITY ENERGY EXPENDITURE ON FUNCTIONAL FITNESS AMONG OLDER ADULTS

Sally Paulson,[1] and Michelle Gray,[2] *1. Mount St. Joseph, Cincinnati, Ohio, United States, 2. University of Arkansas, Fayetteville, Arkansas, United States*

Remaining physically active as one ages plays a critical role in maintaining health and improving functional capacity. Further, older adults can see additional health-related benefits by increasing intensity, duration, frequency, and/or levels of physical activity. However, there is limited research examining physical activity energy expenditure (PAEE) and measures of functional fitness. Therefore, the purpose was to examine differences between older adults with varying levels of PAEE on selected measures of functional fitness. A sample of 25 adults (age: 74.0±7.1 years) were recruited from an urban area and divided into two groups. PAEE was calculated using the total caloric expenditure per week for all exercise-related activities from a self-reported PA questionnaire. Group one expended less than 3,000 calories per week and group two spent more than 3,000 calories per week performing PA. The selected measures of functional fitness were a 4-m gait speed (GS), 30-s chair stand test (CS-30), 2-min step test (ST), and the 8-foot up and go test (GUG). Data were analyzed using a one-way ANOVA. There was a statistically significant difference between the groups on GS ($F1, 24 = 9.29$, $p < .01$) and CS-30 ($F1, 24 = 4.37$, $p = .05$). The results yielded a trend for the GUG ($p = .06$). However, there was not a difference between the groups on the ST ($p = .11$). These results suggest older adults expending more than 3,000 calories per week performing PA walk faster and have greater lower-body strength.

### INTER-INDIVIDUAL DIFFERENCES IN EXERCISE RESPONSES IN ALZHEIMER'S DISEASE

Fang Yu,[1] Dereck Salisbury,[2] and Michelle Mathiason,[3]
*1. Arizona State University, Minneapolis, Minnesota, United States, 2. University of Minnesota, Minneapolis, Minnesota, United States, 3. University of Minnesota, Minneapolis, United States*

Aerobic exercise is widely supported as a disease-modifying treatment for Alzheimer's disease (AD) in animal models; however, its effects on cognition have been mixed in human studies, which may be attributable to inter-individual differences in aerobic fitness and cognitive responses to aerobic exercise. This study evaluated inter-individual differences in aerobic fitness and cognitive responses to 6-month aerobic exercise in participants with AD dementia by secondarily analyzing the FIT-AD Trial data. Aerobic fitness with the shuttle walk test (SWT), 6-minute walk test (6MWT), and maximal oxygen consumption (VO2max) from cycle-ergometer exercise test, and cognition with the AD Assessment Scale–Cognition (ADAS-Cog). Inter-individual differences were calculated as the differences in the standard deviation of 6-month change (SDR) in outcomes between the intervention and control groups. The sample size was 78 (77.4±6.3 years old, 15.7±2.8 years of education, 41% women). VO2max was available in 26 participants (77.7±7.1 years old, 14.8±2.6 years of education, 35% women). The results show that the SDR was 37.0, 121.1, 1.7, and 2.3 for SWT, 6MWT, VO2max, and ADAS-Cog, respectively, but there were no statistically significant differences between the intervention and control groups in these measures over six months. Our results indicate that inter-individual differences exist in aerobic fitness and cognitive responses to aerobic exercise in AD, which contributed to the favorable, but not statistically significant between-group differences in aerobic fitness and cognition. To conclude, our study is the first to demonstrate inter-individual differences in the responses to aerobic exercise in AD dementia using SDR.

### IS HANDGRIP STRENGTH A VALID AND RELIABLE MEASURE IN OLDER ADULTS?

Jefferson Spicher, Amy Silva-Smith, and Melissa Benton,
*University of Colorado Colorado Springs, Colorado Springs, Colorado, United States*

Handgrip strength is related to mortality, disability, functional independence, and quality of life in older adults, and cut points for diagnosis of sarcopenia have been proposed. However, there is no standardized procedure or device, so measurement may not be accurate. To assess validity and reliability we compared hydraulic (HD) versus digital (DD) handgrip dynamometers. Sixty-seven older (76.2 ± 0.9 years) men (n=34) and women (n=33) completed two measurements on sequential days (T1, T2) using both devices in random order. Participants sat in a chair with the device held in their dominant hand, their arm supported on a table or other stable surface, their wrist in a neutral position, and their elbow bent at a 90° angle. To avoid muscle fatigue that has been attributed to multiple attempts, participants squeezed the device one time as hard as possible for 3 seconds. Strong ($p<0.001$) intraclass correlations were observed for both devices (HD=0.98; SS=0.96) indicating good reliability. However, there were significant differences between devices and between measurements. Strength measured with HD was greater than DD at T1 (27.4 ± 1.4 vs. 23.4 ± 1.1 kg, $p<0.001$) and T2 (25.3 ± 1.4 vs. 21.8 ± 1.2 kg, $p<0.001$). Day-to-day measurements were also significantly different. Between T1 and T2 strength decreased 8% with HD ($p<0.001$) and decreased 7% with DD ($p=0.001$). In this group of older adults, significant differences in handgrip strength were observed between devices and timepoints indicating poor validity. As a diagnostic tool, standardization is needed for handgrip measurement procedures to improve accuracy.

### MINDFULNESS MEDITATION AND TAI CHI CHUAN ON SLEEP DISTURBANCE IN CHINESE OLDER PEOPLE: A RANDOMIZED CONTROLLED TRIAL

Sunny Chan,[1] Wai Chi Chan,[2] Siu Man Ng,[2] and Chong Ho Alex Yu,[3] *1. The Hong Kong Polytechnic University, Hong Kong, China, 2. The University of Hong Kong, Hong Kong, China, 3. Azusa Pacific University, Azusa, California, United States*

Sleep disturbances are common during the aging process and can result in a reduced quality of life. Many older people who experience sleep disturbances would consider turning to complementary and alternative medicine (CAM) due to the limitations of traditional pharmaceutical or psychological and behavioural treatments. Mindfulness Meditation (MM) and Tai Chi Chuan (TCC) are two common forms of mind-body based CAM. The former focuses more on mind-based practices whereas the latter emphasizes predominantly on body or movement-based practices. An etiological model of sleep disturbance (Shallcross et al., 2019) can lay the groundwork for a better understanding of the mechanisms of MM and TCC in relation to sleep disturbances. This study aims at comparing the effects of MM and TCC with Sleep Hygiene Education (SHE) control group. A three-armed randomized controlled pilot trial was conducted involving 45 community-dwelling older adults aged 65 to 82 with symptoms of sleep disturbance. Moderate effect sizes (Cohen's $d = 0.7$ and 0.56) were found for the primary outcome of insomnia severity at post-intervention as comparing MM and TCC groups with SHE control group, respectively. More specific, participants in the MM group showed more amelioration on mental health status, introspective awareness, and objective measure of EEG-based brain arousal level; whereas participants in the TCC group showed better improvement on physical health status and subjective measure of hyperarousal. Findings demonstrate the unique therapeutic effects of MM and TCC on improving sleep problem in older people. The application in a Chinese context will be discussed.

### POST-ACUTE REHABILITATION IN PERSONS WITH DEMENTIA: DOES IT MAKE A DIFFERENCE?

Jane Flanagan,[1] Marie Boltz,[2] and Ming Ji,[3] *1. Boston College, Chestnut Hill, Massachusetts, United States, 2. Penn State University, University Park, Pennsylvania, United States, 3. U. South Florida, Tampa, Florida, United States*

Persons with dementia are about two times more likely to be hospitalized than their peers who are cognitively healthy. These individuals are frequently discharged to skilled nursing facilities or nursing home settings, to receive short-term, post-acute, rehabilitative care. The rehabilitative care, Physical Therapy (PT) and Occupational Therapy (OT), provided in skilled nursing facilities (SNFs) aims to restore the person to their pre-hospitalization functional status and assist the person to return home. This study used MDS assessment data of 6396 people, age 65 years and older with dementia, admitted to SNFs in 2013 from acute care hospitals in Massachusetts to assess the effects of OT and PT on the change in physical function of nursing home residents admitted to the nursing home after hospitalization. Multiple linear regression analyses. The sample was mostly female (64.1%), non-Hispanic (98.86%), and white (93.71%), with a mean age of 85.3 (SD=6.85). After controlling for age, gender, race and comorbidities, and delirium, rehabilitation interventions (OT, PT or OT+PT) did not have any significant effect on changes in physical function among residents with dementia (p for OT = 0.14; p for PT=0.59; p for OT+PT:= 0.32). Additionally, non-white residents had poorer function at three months (β =1.86, 95% CI:-3.57- -0.16). The results indicate for persons with dementia admitted to SNFs, OT, PT or OT+PT did not lead to a significant improvement physical function. More innovative and effective interventions should be developed to improve physical function in persons with dementia post-hospitalization.

### PREFRONTAL CORTEX HEMODYNAMICS DURING EXERCISE IN OLDER ADULTS WITH MOTORIC COGNITIVE RISK SYNDROME

Kell Grandjean da Costa,[1] Eduardo Fontes,[2] Alekya Menta,[2] Sarah White,[3] Roger Fielding,[4] Christopher Kowaleski,[5] Nathan Ward,[1] and Kieran Reid,[4] *1. Tufts University, Medford, Massachusetts, United States, 2. Tufts University, Medford, United States, 3. Tufts University, Boston, United States, 4. Tufts University, Boston, Massachusetts, United States, 5. Somerville Council on Aging, Somerville, Massachusetts, United States*

The motoric cognitive risk syndrome (MCR) is a recently described pre-dementia syndrome in older adults characterized by slow gait coupled with subjective cognitive complaints. While previous studies have demonstrated the benefits of exercise on cerebral hemodynamics in healthy older adults, to date, no study has characterized the effects of exercise on these parameters among more vulnerable older persons with MCR. To address this knowledge gap, we investigated how the brain area responsible for high-order cognitive function (i.e., prefrontal cortex) is affected during acute cycling exercise in 19 older adults with MCR (Age (mean ± SD): 73.7 ± 7.1 years; BMI: 32.1 ± 5.5 kg/m2; gait speed: 0.55 ± 0.1 m/s; Modified Mini-Mental score: 91.8 ± 6.8; 74% female). Participants performed an incremental submaximal cycling test and we used functional near-infrared spectroscopy to assess changes in concentrations of Oxyhemoglobin (O2Hb), Deoxyhemoglobin (dHb) and total hemoglobin (Hbt) during exercise. Results showed that participants cycled for 4.9 ± 0.5 minutes, achieved a submaximal load of 54.7 ± 17.3 watts, a peak exercise heart rate of 95.7 ± 14.7 beats/min and a rate of perceived exertion (13.8 ± 2.0). Compared to baseline, there was an increase of 97.3 % in the O2Hb, 86 % on the Hbt and an 87.9 decrease of dHb while exercising. Our findings suggest that acute exercise at light through moderate intensity increases prefrontal cortex oxygenation in older adults with MCR. Additional studies are also warranted to characterize the effects of chronic exercise on cerebral hemodynamics in at-risk older adults.

### RELATIONSHIP BETWEEN PHYSICAL ACTIVITY AND FUNCTION WITH QUALITY OF LIFE IN COMMUNITY-LIVING OLDER ADULTS

Milan Chang,[1] Olof Geirsdottir,[1] Inga Thorsdottir,[1] Palmi Jonsson,[1] Alfons Ramel,[2] and Milan Chang Gudjonsson, *1. University of Iceland, Reykjavik, Iceland, 2. University of Iceland, Reykjavik, Capital area, Iceland*

Background: Quality of life (QOL) is a multidimensional concept which is often used as an evaluation of a person's health and psychological status. Increasing longevity can be associated with better QOL as long as older adults are independent in daily life. The aim of the study was to examine the associations of QOL with muscle strength and physical function among community-dwelling older adults. Methods: The current cross-sectional study had 225 participants (73.7±5.7yrs, 58.2% female) living in Reykjavik, Iceland. QOL measured using the 36-item short-form survey (SF-36). Covariates were anthropometrics, muscle strength, physical function including timed up and go test (TUG), and 6-minute walking distance (6MWD), physical activity per week (PA). Linear regression analysis was used to examine the association of QOL with physical function. Results: The mean QOL score for the study population was 54.9±6.13. The analysis was adjusted for age and gender, body mass index, height, and PA. We found that QOL was associated with better grip strength (B=1.4, P<0.0001), 6MWD (B=0.03, P<0.0001), slower TUG (B=-0.9, P<0.0001), and higher PA (B=0.03 m, P=0.039). However, QOL was not associated with quadriceps leg strength. Conclusion: The study suggests that QOL was associated with better physical function including grip strength, walking ability and the level of PA among community-dwelling older adults in Iceland.

### SATISFACTION WITH OUTDOOR ACTIVITIES OVER TIME AMONG LONG-TERM SERVICES AND SUPPORTS RECIPIENTS

Justine Sefcik,[1] Karen Hirschman,[2] Darina Petrovsky,[3] Liming Huang,[3] Nancy Hodgson,[4] Liza Behrens,[5] and Mary Naylor,[2] *1. Drexel University, Philadelphia, Pennsylvania, United States, 2. NewCourtland Center for Transitions and Health, Philadelphia, Pennsylvania,*

*United States, 3. University of Pennsylvania, Philadelphia, Pennsylvania, United States, 4. University of Pennsylvania, Cherry Hill, New Jersey, United States, 5. University of Pennsylvania School of Nursing, Philadelphia, Pennsylvania, United States*

Approximately 85% of older adults have at least one chronic health condition. The onset of chronic health conditions and mobility issues can constrain activities, including outdoor recreation. There is limited knowledge of older adults receiving long-term services and supports (LTSS) and their satisfaction with outdoor activities over time after enrolling in services. This study examined predictors of change in ratings of satisfaction with outdoor activities. A secondary analysis was conducted of data involving structured interviews with older adults (N=470) over the first two years of receiving LTSS (Health-Related Quality of Life: Elders in Long-Term Care; R01AG025524). Participants lived in assisted living communities, nursing homes, or their home. A single item on satisfaction with outdoor activities (assessed using a 5-point Likert scale: not at all to extremely satisfied) was the primary outcome. Mixed effects linear regression modeling using a backward elimination process was used for building a final multivariable model. In the final model, older age ($p<0.001$) and higher overall quality of life ratings ($p<0.001$) at baseline were associated with slower rates of increase in outdoor satisfaction over time. Higher education level ($p=0.035$) at baseline was associated with a faster rate of increase in outdoor satisfaction over time. Additionally, those who moved into an assisted living community ($p=0.024$) or nursing home ($p=0.016$) at baseline were associated with faster rates of increase in outdoor satisfaction over time compared to those in the home. Knowledge of factors influencing satisfaction with outdoor activities can assist interdisciplinary teams implement interventions for individual or organizational changes.

### SEX DIFFERENCES IN ENERGETIC COST OF WALKING AND LATER-LIFE FUNCTION AND PHYSICAL ACTIVITY PATTERNS

Matthew Peterson,[1] Kira Byrd,[1] and Andrew Frohle,[2]
*1. Campbell University, Buies Creek, North Carolina, United States, 2. Wright State University, Dayton, Ohio, United States*

Aerobic fitness is a strong predictor of functional independence with aging. Variability in aerobic fitness is due, in part, to the efficiency of the movement of body mass while ambulating [energetic cost of walking (ECoW)]. ECoW may serve as a low burden measure of fitness compared to volitional treadmill tests. We assessed the predictive ability of ECoW with later-life function and physical activity participation in healthy adults. N=75 (age = 54.7 +/- 9.2; 52% female) completed a treadmill test with indirect calorimetry, during which time a warm-up of 1.5 mph at 0% grade was uniform. ECoW was quantified as steady state volume of oxygen consumption (ml/kg/min) subtracted by the calculated metabolic cost of walking at given speed and grade (=7.5 ml/kg/min). Later-life (median=9 years follow-up) function was assessed using the SF-36 physical functioning subscale, and the Baecke questionnaire for physical activity. Mean EcoW was similar to calculated cost (difference = -0.14 ml/kg/min) but had large variation (SD = 2.7; range = -4.4 to +17 ml/kg/min). In females, higher ECoW was predictive of better later-life function (b=1.9; p=0.04) and higher physical activity levels (b=0.09; p=0.001). In males, lower ECoW was predictive of higher physical activity levels only (b=-0.04; p=0.01). All models controlled for % body fat, age, and comorbidities. Surprisingly, in females, higher ECoW was associated with better later-life health outcomes. We hypothesize that this phenomenon may be similar to the obesity paradox, in that relatively higher non-metabolic tissue in females may serve as a stimulus for muscle and functional preservation.

### THE ASSOCIATION OF LIGHT PHYSICAL ACTIVITY WITH HEALTH INDICATORS AMONG MIDDLE-AGED AND OLDER ADULTS

Kristen Moore,[1] Kaigang Li,[1] Manfred Diehl,[1] Katherine Thompson,[2] and Abigail Nehrkorn-Bailey,[1]
*1. Colorado State University, Fort Collins, Colorado, United States, 2. University of Chicago Medicine, Chicago, Illinois, United States*

Physical Activity (PA) Guidelines for Americans recommend engagement in moderate or moderate-to-vigorous physical activity (MVPA) for middle-aged and older adults. Although these guidelines encourage adults to "move more and sit less," there are no explicit recommendations for engaging in Light PA (LPA). The purpose of this study was to examine the association between LPA and health Indicators among middle-aged and older adults, in the context of sedentary time (ST) and MVPA. We used baseline data from 171 individuals (Mean age: 59.3±8.51 years), participating in Colorado State University's AgingPlus program. Selected health Indicators included Body Mass Index (BMI), waist circumference (WC in cm), mean arterial pressure (MAP in mmHg), grip strength (GS in lbs), and indirect VO2max (ml/kg/min). ST and PA were measured using Actigraph accelerometers worn for 7 days (excluding wear time <500 mins/day). Linear regression analyses, controlling for sex, age group, and race, indicated that more ST was associated with greater BMI (B=3.33), greater WC (B=3.35), and lower VO2max (B=-3.09). More LPA was associated with lower BMI (B=-4.47), lower WC (B=-5.0), lower MAP (B=-2.81), and higher VO2max (B=4.64). MVPA was associated with lower BMI (B=-3.04), lower WC (B=-4.21), higher VO2max (B=4.74), and higher grip strength (B=2.33). In conclusion, more ST was associated with indicators of poorer physical health. LPA, similar to MVPA, was associated with indicators of better physical health and performance. Future longitudinal and experimental studies examining the causal relationship between LPA and physical health and performance are warranted.

### THE INVENTORY OF PHYSICAL ACTIVITY BARRIERS: DEVELOPMENT AND PRELIMINARY VALIDATION

Mariana Wingood,[1] Nancy Gell,[2] Denise Peters,[3] and Tiffany Hutchins,[3] *1. University of Vermont, Waterbury, Vermont, United States, 2. The University of Vermont, Burlington, Vermont, United States, 3. University of Vermont, Burlington, Vermont, United States*

Inactivity levels among community-dwelling adults 50 years and older is a healthcare concern, particularly when examining the association between increasing age, inactivity, and risk of non-communicable diseases. To confront this concern, healthcare providers need to address the reasons for inactivity. Unfortunately, limited tools exist to address barriers of physical activity (PA). The purpose of our study was to develop and psychometrically evaluate a PA barrier scale for adults 50 years and older. The scale is called the Inventory of Physical Activity Barriers (IPAB) and it was developed, refined, and evaluated using a cross-sectional and a modified Delphi study. We had two groups of participants: 39 adults (50 years and older) provided survey pilot data for psychometric evaluation, and nine interprofessional PA experts assisted with finalizing the scale. Participants completed a demographic questionnaire, Physical Activity Vital Sign questionnaire, and IPAB. The IPAB's refinement was guided by item-scale correlations, descriptive statistics, and consensus among the PA experts. Construct validity was examined by comparing mean IPAB scores of inactive and active participants via independent t-test. Internal consistency was assessed via Cronbach Alpha. The IPAB was refined from 172 items to 40 items and found to be internally consistent ($\alpha$=.97) and able to differentiate individuals who do and do not meet the recommended 150 minutes of weekly PA (p=0.01). These preliminary results show the IPAB is a reliable assessment of PA barriers for adults 50 years and older, and are promising for the scale's construct validity and support further psychometric evaluation of the tool.

### THE RELATIONSHIP BETWEEN COGNITIVE DECLINE AND SEDENTARY TIME

Lorraine Phillips, and Mary Bowen, *University of Delaware, Newark, Delaware, United States*

Early identification of functional decline in older adults with mild cognitive impairment (MCI) provides the opportunity to initiate behavioral interventions to slow decline. More frequent breaks in sedentary time has been associated with greater lower extremity function. This longitudinal study examined the effect of 6-month change in cognitive function on monthly sedentary time, controlling for lower extremity function, among community-dwelling older adults with MCI. Twenty adults with Montreal Cognitive Assessment Score (MoCA) between 19-25, who were age $\geq$ 60 years old, and ambulatory, wore an actigraph for 6 months and participated in monthly in-person assessments. Measures included MoCA change (baseline to month 6), Short Physical Performance Battery (SPPB; baseline, months 3 and 6); sedentary time and physical activity intensity; and falls (monthly). The sample was 70% female, 60% non-Hispanic white, with a mean age of 77 years. Sixteen participants provided complete data for mixed-model analysis. Over 6 months, 11 falls occurred among 7 participants. The mean MoCA score declined from 22.7 to 21.9 while SPPB remained stable. Overall time spent in sedentary behavior was high (71%) and physical activity intensity was low (light and moderate combined= 26.1%). Results of multi-level analysis with sedentary time as a continuous Level-1 variable and MoCA change scores, SPPB scores, and age in Level-2 showed that negative change in MoCA ($\beta$=-0.11; p$\leq$0.05) was associated with increased sedentary time. Given sedentary time increases as cognitive function declines, older adults with MCI could benefit from interventions designed to interrupt sedentary time as well as increase physical activity.

### TIME-VARYING ASSOCIATIONS OF PHYSICAL ACTIVITY ON WEEKDAYS AND WEEKEND DAYS IN MINORITY OLDER ADULTS

Derek Hevel,[1] Laurie Kennedy-Malone,[2] Kourtney Sappenfield,[1] Heidi Scheer,[1] Christine Zecca,[1] and Jaclyn Maher,[1] *1. University of North Carolina at Greensboro, Greensboro, North Carolina, United States, 2. UNCG School of Nursing, Greensboro, North Carolina, United States*

Older adults are insufficiently physically active, and therefore, are at an increased health risk. However, less is known about the moment-to-moment physical activity behavior patterns that change across the day. The current study utilized accelerometry to better understand diurnal physical activity patterns. Minority older adults (N=91, age M=70.14, 96% Black/African American) participated in an 8-day study where they wore an ActivPAL accelerometer to measure physical activity. Physical activity was operationalized as stepping in the 60-minute window (±30-min) around a given moment. Time varying-effect modeling was used to determine how physical activity patterns change over the course of weekdays and weekend days. Results are rounded to the 5-min interval. On weekdays, time spent stepping increased from 8:00am (B=7.16, 95%CI: 5.04, 9.27) until peaking at 11:10am (B=8.46, 95%CI: 6.59, 10.33), slowly decreased to the lowest point at 6:10pm (B=6.72, 95%CI: 4.82, 8.62), and then increased slightly in the evening. On weekend days, time spent stepping was lowest at 8:00am (B=4.47, 95%CI: 2.14, 6.80), peaked at 12:20pm (B=5.50, 95%CI: 3.22, 7.78), gradually decreased until 5:50pm (B=5.21, 95%CI: 2.90, 7.52), and then increased slightly in the evening. Minority older adults engage in more stepping time around mid-day, but less stepping in the late afternoon to early evening on both weekdays and weekend days. Self-care activities (e.g., mealtime, bed-time routines) may influence older adults' physical activity. This work identifies vulnerable times during the day when older adults engage in relatively low levels of physical activity which may be of interest for interventions.

### SESSION 2891 (POSTER)

### TECHNOLOGY

### A RURAL TELE-VIDEO MODEL: RESULTS FROM EDUCATING PRACTITIONERS ABOUT OPIOIDS AND ALTERNATIVE PAIN MANAGEMENT

Leah Tobey,[1] Robin McAtee,[2] and Corey Hayes,[2] *1. University of Arkansas for Medical Sciences, Little Rock, Arkansas, United States, 2. UAMS, Little Rock, Arkansas, United States*

Providers in Arkansas wrote 105.4 opioid prescriptions for every 100 persons in 2017-nearly twofold greater than the average U.S. rate of 58.7 opioid prescriptions (CDC, 2017). This makes AR the second highest opioid prescribing state. AR-IMPACT (Arkansas Improving Multi-Disciplinary Pain Care and Treatment) is a tele-video case conference education model which was designed to improve AR providers' knowledge and behavior in pain management and improve awareness of opioid-sparing alternatives. Conference panelists include a geriatrician, psychiatrist, physical therapist, psychologist, pharmacist, a chronic pain specialist and various guest speakers such as the Arkansas Drug Director. During weekly tele-video conferences, relevant actionable cases were presented regarding treating diverse patients who have chronic pain. AR-IMPACT provided hundreds of practitioners with up-to-date data and education regarding opioid use and misuse, methods and resources regarding titrating and eliminating opioids, and viable alternative pain-management solutions such as motivational interviewing and dry-needling. This poster will present the background of the opioid crisis in rural Arkansas followed by the specifics of the development and application of the AR-IMPACT tele-video model. Specific older adult cases studies, quantitative and qualitative outcomes, feedback from presenters and participants, and lessons learned will be reviewed. Specifically, lessons learned center around the practitioners' culture of pain management and how that culture must evolve and change. New methods of pain management must be taught to practitioners and patients and adopted as the new norm. This will demonstrate that programs like AR-IMPACT are crucial in helping make that cultural change happen.

### A SCOPING REVIEW OF CURRENT TELEHEALTH INTERVENTIONS FOR CARE PARTNERS OF PERSONS WITH DEMENTIA

Leslie Tran,[1] Robin Tarter,[1] and Allison Lindauer,[2]
*1. Oregon Health and Science University, Portland, Oregon, United States, 2. Oregon Health & Sciences University, Portland, Oregon, United States*

Many burdens come with caring for someone with dementia, but telehealth interventions can provide information and support and reduce barriers to access. In this review we describe current telehealth interventions for family care partners of people diagnosed with Alzheimer's disease or related dementias (ADRDs). We conducted a systematic literature search using PubMed. Inclusion criteria were peer-reviewed, data-based research articles focusing on technological clinical interventions, published within the past two years, with participants providing unpaid care to persons with a diagnosis of ADRDs. We identified 53 relevant articles, of which 13 met our criteria. Included studies fell into three categories: peer support groups facilitated by gerontological social workers and psychologists, psychoeducation and behavior modification interventions delivered by nursing professionals, and symptom management advising overseen by physicians. Different technologies were used including computers, iPads, smartphones, and smartwatches. The duration of interventions also ranged from four weeks to three months, representing varying approached to participant engagement. The majority of caregivers were women, non-Hispanic white, spouses. Based on our findings of a lack of diversity in the samples of extant studies, and the need for interventions tailored to specific stages of ADRDs, future researchers can design studies to address these gaps. Overall, the interventions' effectiveness and participant satisfaction were high, resulting in improvements in burden, competence, and coping skills. Although every approach has its own strengths and weaknesses, we believe that the continued expansion of telehealth interventions will not only offer many benefits, but also transform the delivery of health care.

### ACCEPTABILITY AND FEASIBILITY OF ACTIVPAL ACTIVITY TRACKER FOR PRE-FRAIL AFRICAN AMERICAN OLDER ADULTS

Yi-Ling Hu,[1] Camila Rodriguez Valenzuela,[2] Ryan Jones,[2] Natalie Gamalski,[2] and Heather Fritz,[2] *1. Wayne State University, Detroit, Michigan, United States, 2. Wayne State University, Wayne State University, Michigan, United States*

Frailty is more prevalent among African American (AA) older adults compared to their White counterparts. Physical activity (PA) interventions are effective to attenuate frailty progression. The Activpal is adhered to the thigh and allows unobtrusive PA measurement for up to 14 days. However, few studies have tested the acceptability and feasibility of activity trackers among older pre-frail AA. In this study, pre-frail AA older adults were asked to wear the ActivPAL tracker on their thigh for 7 consecutive days. Acceptability was measured by compliance. Feasibility was evaluated by the valid number of days and programing errors. Intraclass correlation coefficients were used to assess the agreement of light to vigorous PA level between ActivPAL and the Community Healthy Activities Model Program for Seniors (CHAMPS). In a sample of 28 pre-frail AA older adults (mean age= 73.21,SD= 9.37, 100% female), 24 completed the 7-day data tracking. Reasons for non-compliance included skin irritation (n=1), and fear of causing other health issues (n=3). The number of valid days was= 6.9 0.33 with 1 incident of programming error. The agreement between ActivPAL and CHAMPS was fair (ICC =0.57, [95%CI]= -.08 -.85) with most participants (61.6%) over-reporting PA on CHAMPS. The ActivPAL accelerometer was acceptable and feasible to use among pre-frail AA older adults. Communication prior to applying the accelerometer is crucial to prevent non-compliance. The results confirm prior studies, which suggest that older adults may over-report PA levels. ActivPAL offers a feasible and accurate approach to assess PA among older AA.

### BARRIERS TO DEVELOPMENT OF AN APP FOR IMPROVING STAFF-FAMILY COMMUNICATION AT ISRAELI GERIATRIC FACILITIES

Rinat Cohen,[1] Gal Maydan,[2] Shai Brill,[3] and Jiska Cohen-Mansfield,[4] *1. School of Public Health, Sackler Faculty of Medicine, Tel Aviv University, Hadera, Israel, 2. Beit Rivka Medical Center, Petach Tikva, Israel, 3. Clalit Health Services, Tel Aviv, Israel, 4. Tel Aviv University, Tel Aviv, Israel*

Family caregivers (FCs) of institutionalized noncommunicative older persons reported multiple unmet communication needs focusing on the need to receive reliable and regular updates on the patient's condition. We have developed a mobile app for improving communication between FCs and healthcare professionals (HPs), based on 152 interviews with FCs and 13 discussion groups with HPs from four Israeli geriatric facilities. Both parties participated in app planning, tailoring it to their needs and abilities. App use implementation encountered major obstacles including the bureaucratic process concerning signing contracts between the university and software development firms, which hindered the process for a full year; data security department required disproportionate security levels that interfered with user experience and delayed the development process; the study's definition varied across different ethics/Helsinki committees (Institutional Review Boards; IRBs), which led to different demands, e.g., insurance for medical clinical trials although no drugs or medical device were involved; lack of cooperation by mid-level staff members despite the institutional adoption of the app project; low utilization by HPs resulted in FCs not receiving timely responses. Despite these and other obstacles, we tested app use for 15 months in one facility in a pre-post-design with intervention and control groups, and we have since begun testing it in another facility. FCs who had used the app had positive feedback and wished to continue using it. App use optimization requires implementation planning, assimilating changes in each facility's work procedures and HP's engagement and motivation and thus depends on institutional procedures and politics.

### CAN ROBOTS ENCOURAGE SOCIAL ENGAGEMENT AMONG OLDER ADULTS?

Yichun Lin,[1] Jing Fan,[2] Mary Dietrich,[2] Linda Beuscher,[2] Paul Newhouse,[2] Nilanjan Sarkar,[2] and Lorraine Mion,[3] *1. the Ohio State University, Columbus, Ohio, United States, 2. Vanderbilt University, Nashville, Tennessee, United States, 3. The Ohio State University, Columbus, Ohio, United States*

Apathy in older adults in long term care (LTC) settings is common, associated with morbidity and mortality, and requires extensive personnel time. Most LTC sites are limited in their ability to provide activities. We conducted a 3-month pilot study at two LTCs to determine whether a robot, with/without virtual reality (VR), was successful in encouraging social engagement between older LTC adults. Three robot activities were offered twice weekly for three weeks (6 sessions). Two activities with VR consisted of two book sorting games. One activity was "Simon Says" where older adults took turns as leaders. Demographics and cognitive data were collected. Videos were coded and analyzed using Noldus Observer: activity engagement as visual and verbal attention to the robot activity and social engagement as visual and verbal attention towards their partner. Participants were 2 men and 14 women, mean age 83. One dropped out because of hearing problems; one dropped out because of cognitive impairment. Fourteen, ie 7 pairs, attended all 6 sessions; ten had MCI and one had AD. Social and activity engagement varied by activity and by participant. Participants' perceptions (7-point Likert scale) remained positive over time (6.33 (SD 0.94) to 6.52 (SD 0.61)) but decreased slightly for the repeat activities (6.19 (SD 1.01) to 5.96 (SD 1.13)). Robots hold promise in LTC as ways to engage older adults who suffer from apathy. Further work is necessary to elucidate participant- and activity-level characteristics most conducive for success and mechanisms to increase the number and variety of activities.

### EFFECTIVENESS OF SERVO-ASSISTIVE ROBOTIC ROLLATOR (RT.2) AMONG OLDER ADULTS LIVING IN THE COMMUNITY

Takashi Yamanaka,[1] Kiwami Kidana,[1] Maiko Mizuki,[1] and Toshihumi Matsui,[2] *1. The University of Tokyo, Tokyo, Japan, 2. Oouchi Hospital, Tokyo, Japan*

Older adults tend to need assistance for ambulation with the progression of aging or when suffering from diseases. With technological advances, servo-assistive robotic rollators are available besides canes and walkers to assist disabled older adults. This study aimed to investigate the appropriate person and conditions for using a servo-assistive robotic rollator and its effects. Participants were 10 older adults living in the community (80.5±9.7 years; 4 males and 6 females) who used a servo-assistive robotic rollator (RT.2). After evaluating their physical (body composition, diseases, care need level, and SF-36), mental (MMSE, GDS-15, and WHO-5), and living conditions, they began using the device in daily life. We evaluated their ways of using it and the effects of its use through our observation and their self-report. Participants suffered from a stroke, spinal bone fracture, Parkinson's disease, osteoarthritis of the knees, or optic neuromyelitis. At the study's onset, cognitive impairment (MMSE<23/30), depressive states (GDS-15≧5), decreased grip strength, and decreased muscle mass (InBody S10) was found in one, three, six, and one participant, respectively. Most participants had a clear purpose for using it, such as going outside by foot or maintaining muscle strength. During the three-month observation period, no participant fell while using it. Some participants used it in a rehabilitation program at home, while others used it in daily life and went to several places with its assistance. Servo-assistive Robotic Rollators enabled older adults with difficulty in ambulation to walk outside safely and provided a greater opportunity to participate in society.

### ENGAGING OLDER MINORITY ADULTS IN A SMARTPHONE-BASED ECOLOGICAL MOMENTARY ASSESSMENT STUDY OF SEDENTARY BEHAVIOR

Laurie Kennedy-Malone,[1] Derek Hevel,[2] Kourtney Sappenfield,[2] Heidi Scheer,[2] Christine Zecca,[2] and Jaclyn Maher,[2] *1. UNCG School of Nursing, Greensboro, North Carolina, United States, 2. University of North Carolina at Greensboro, Greensboro, North Carolina, United States*

Minority older adults engage in excessive levels of sedentary behavior (i.e., sitting). Smartphone-based ecological momentary assessment (EMA) methods can provide novel insights into the modeling and prediction of activity-related behaviors. Yet, minority groups report barriers to participating in mobile health research (e.g., distrust, lack of interest, underrepresentation in research). This abstract reports on strategies used to recruit minority older adults and acceptability of an 8-day smartphone-based sedentary behavior EMA study in this population. Researchers partnered with existing community organizations servicing the target population (i.e., independent living communities, congregate meals sites, and churches) and trusted individuals within these organizations to facilitate introduction of the research team/study. In total, 123 older minority adults were recruited, 102 met inclusion criteria and 91 completed the study. During the study, participants answered 6 electronic EMA questionnaires/day and wore an ActivPAL activity monitor continuously. Participants received one-on-one training on these procedures and received check-in calls to monitor progress. Open-ended questions administered at the end of the study revealed the most enjoyable aspects of the study were the ability to learn more about themselves, contributing to science and/or their community, engaging in a new activity followed by receiving financial compensation and having someone checking on their progress. Least enjoyable aspects of the study included frequency of EMA questionnaires, apprehension of missing EMA questionnaires, carrying the smartphone, and difficulty wearing the activity monitor. Almost all participants (95%) expressed interest being contacted for future studies. Implications for future technology-based research regarding minority older adults' activity-related behaviors will be discussed.

### EXPLORING TECHNOLOGY PERCEPTIONS AND INTENTIONS TO USE IN OLDER ADULTS

Stephanie Williams,[1] Elizabeth Orsega-Smith,[2] and Laurie Ruggiero,[2] *1. Discover, New Castle, Delaware, United States, 2. University of Delaware, Newark, Delaware, United States*

By the year 2035, the older adult population is expected to expand to 78 million in the United States. Advancing technology has made aging in place a more accessible possibility; however, understanding what is preventing this population from adopting the advancing devices remains to be a challenge as the presence of a digital divide continues to exist. A 34-question survey adapted from the Technology Proficiency Self-Assessment Questionnaire, and the National Technology Readiness survey was administered to 101 participants over the age of 50 across five local senior centers. The average age range was 70-79 and most were female (79.2%), white (69%), and owned or had access to technology such as a computer or cell phone (93%). Examples of findings include 86% felt technology limited human interaction and 69% felt the use of technology could lead to security risk and a breach of privacy, while 79% felt technology could improve their quality of life. Results found 60-69-year-olds were significantly more likely ($p<.05$) to have or use technology versus 80-89-year-olds. Correlation between perception and intent to use technology among older adults was positive with a coefficient value of .59($p<.01$). Showing a relationship between perceptions and behavioral intentions to use technology, specifically in 60-69-year-olds. This study found access to technology (i.e. computers, cell phones, internet) was not a driving factor of usage among the older population attending a senior center. To increase understanding further exploration of perceptions and intentions to use technology is warranted in the older adult population.

### FEASIBILITY TO TRAIN OLDER ADULTS IN INDIA TO USE SMART-PHONE TO INCREASE PARTICIPATION IN DAILY ACTIVITIES

Asish Das,[1] and Amit Sethi,[2] *1. Manipal Academy of Higher Education, Manipal, Karnataka, India, 2. Occupational Therapy, Pittsburgh, Pennsylvania, United States*

Though 60% of 104 million older adults own a smart phone in India, up to 85% of these individuals find challenging to use smart-phone during daily activities. This is problematic because inability to use smart-phone limits their ability to participate in daily activities such as, managing their health, paying bills, shopping and communicating with their loved ones, resulting in social isolation and dependence on others. The purpose of this study was to examine the feasibility of training older adults to use smart-phone to increase participation in daily activities. This preliminary study was conducted over two phases. In phase-I, we used the principles of stakeholder engagement to interview 12 older adults to identify the barriers associated with the use of technology using a brief survey. Based on the results of Phase I, we implemented a single education and training session for 42 participant (> 60 years) in using smart-phone based applications in Phase II. Older adults were trained to use smart-phone based applications to manage money using banking apps, pay bills, shop, manage health to monitor vitals and medications and communicate with their loved ones. All participants rated their satisfaction with the program, 70% participants showed the ability to use the applications independently. Seventy five percent rated the program extremely useful, and 70% rated it extremely relevant to them. It is feasible to train older adults in using smart-phone based applications. Future studies need to focus on providing follow-up sessions to increase retention and carry-over to increase participation in daily activities.

### IT IS NOT FOR ME: WHY AGE MATTERS IN TECHNOLOGICAL INNOVATION

Irene Blackberry,[1] and Clare Wilding,[2] *1. La Trobe University, Wodonga, Australia, 2. La Trobe University, La Trobe University, Australia*

During 2017-2019, we conducted a randomized stepped-wedge cluster trial of an online program to increase ease of access to information and support for caregivers of a person living with dementia in rural Australia. The program, called Virtual Dementia Friendly Rural Communities (Verily) Connect, was innovative; it consisted of a custom-designed website and mobile application (app) in Android and iOS, use of videoconferencing for peer support, chat forum and use of volunteers to build technological skills and confidence for online users. The program overcame barriers experienced by rural Australian caregivers, particularly geographical distance from services and other caregivers, stigma, and lack of localized supports. Although participants identified that Verily Connect program afforded several benefits, including increased flexibility in accessing support, easier access to localized dementia-related information, and more opportunities for attaining support, uptake of the program was disappointingly low. A strong theme in the program evaluation, which used the Consolidated Framework of Implementation Research, was that although the program was perceived as advantageous, adaptable, of high quality, and easy to implement, engaging participants failed because the target users, people over 65 years, eschewed involvement with online technology. Our future research plans include finding ways to offer hybrid online/face-to-face support programs specifically designed for older adults, so that older adults have equitable opportunities to access technological innovations. Older adults ought not to be disadvantaged because it is difficult to keep pace in a rapidly changing world.

### NOVEL ANALYTIC APPROACHES TO INVESTIGATE MINUTE-LEVEL ACTIGRAPHY AND ASSOCIATIONS WITH PHYSICAL FUNCTION

Kaiyuan Hua,[1] Sheng Luo,[1] Katherine Hall,[2] Miriam Morey,[3] and Harvey Cohen,[4] *1. Duke University, Durham, North Carolina, United States, 2. Duke University, Veterans Health Administration, Durham, North Carolina, United States, 3. Veterans Health Administration, Durham,*

*North Carolina, United States, 4. Duke University, School of Medicine, Durham, North Carolina, United States*

Background. Functional decline in conjunction with low levels of physical activity has implications for health risks in older adults. Previous studies have examined the associations between accelerometry-derived activity and physical function, but most of these studies reduced these data into average means of total daily physical activity (e.g., daily step counts). A new method of analysis "functional data analysis" provides more in-depth capability using minute-level accelerometer data. Methods. A secondary analysis of community-dwelling adults ages 30 to 90+ residing in southwest region of North Carolina from the Physical Performance across the Lifespan (PALS) study. PALS assessments were completed in-person at baseline and one-week of accelerometry. Final analysis includes 669 observations at baseline with minute-level accelerometer data from 7:00 to 23:00, after removing non-wear time. A novel scalar-on-function regression analysis was used to explore the associations between baseline physical activity features (minute-by-minute vector magnitude generated from accelerometer) and baseline physical function (gait speed, single leg stance, chair stands, and 6-minute walk test) with control for baseline age, sex, race and body mass index. Results. The functional regressions were significant for specific times of day indicating increased physical activity associated with increased physical function around 8:00, 9:30 and 15:30-17:00 for rapid gait speed; 9:00-10:30 and 15:00-16:30 for normal gait speed; 9:00-10:30 for single leg stance; 9:30-11:30 and 15:00-18:00 for chair stands; 9:00-11:30 and 15:00-18:30 for 6-minute walk. Conclusion. This method of functional data analysis provides news insights into the relationship between minute-by-minute daily activity and health.

### PERCEIVED VALUE OF USING A DIGITAL TOOL TO SCREEN FOR ELDER MISTREATMENT IN THE EMERGENCY DEPARTMENT

Fuad Abujarad,[1] Thomas Gill,[1] Michael Pantalon,[1] Karen Jubanyik,[1] James Dziura,[1] Gail D'Onofrio,[1] and Esther Choo,[2] *1. Yale University, New Haven, Connecticut, United States, 2. Oregon Health & Science University, Portland, Oregon, United States*

A major barrier to reducing Elder Mistreatment (EM) is an inability to accurately identify victims. We conducted a qualitative study to evaluate stakeholders' perceived value and likelihood of adopting a tablet-based digital health tool to facilitate screening and prompt self-disclosure of EM in emergency departments (ED). The interactive tool utilizes virtual coaching, interactive multimedia libraries (graphics, animations, etc.), electronic screening, and brief motivational interviewing designed to enhance identifying EM among older adults. We conducted 3 focus groups with stakeholders, including 24 adults 60+ years, 2 social workers, 2 caregivers, and 2 ED clinicians. Two focus groups included only older adults, while one included representatives of all stakeholders. The main findings include: using a female voice for the tool narrator, larger font size, more multimedia, and headphones for privacy; and making a person available during screening if assistance is needed. Stakeholders indicated that it is difficult for victims to ask for help and any type of mistreatment screening would be helpful. On a 7-point Likert scale ranging from "1=Very Comfortable" to " 7=Very Uncomfortable", older adults scored 2.8 on average for whether they would feel comfortable using a tablet to screen for EM. Some said digital screening would maintain privacy and anonymity. Stakeholders highlighted the need to explain community resources available to older adults once EM is disclosed, especially resources offering help to the caregiver. In summary, this qualitative study supported using tablet-based screening for EM and highlighted the need to target stigma related to EM disclosure and fear of retaliation.

### PREVALENCE AND PERCEIVED USEFULNESS OF ASSISTIVE TECHNOLOGY IN MIND AT HOME DEMENTIA COHORT

Michael Morreale,[1] Deirdre Johnston,[2] Morgan Bunting,[3] Inga Antonsdottir,[4] and Quincy Samus,[4] *1. Johns Hopkins University, Swarthmore, Pennsylvania, United States, 2. Johns Hopkins Hospital, Baltimore, Maryland, United States, 3. Johns Hopkins University, Halethorpe, Maryland, United States, 4. Johns Hopkins University, Baltimore, Maryland, United States*

Dementia is generally characterized by both an increasing dependence in activities of daily living over the course of the illness, and a decreasing ability to self-manage everyday tasks. This places persons at risk for a number of undesirable outcomes including increased risk for injury in the home, increased risk for medical and behavioral complications, risk of premature institutionalization, and excessive burden on family caregivers (CG).3,4 Assistive Technology Devices (AT-Devices) could represent an efficient resource for supporting daily tasks while reducing both CG care burden and adverse risk to the person with dementia (PWD).3,4 In the context of a larger dementia care intervention clinical trial (The MIND at Home program) that involved persons living with dementia at home and their family caregivers, we conducted a supplemental baseline survey on 59 participants and their CGs to better understand the current prevalence of AT-Device use and which devices would be perceived as "most helpful". Our analysis showed that 51% of our study population used at least 1 of our listed AT-Devices. The most common AT-Device used at baseline were door guards (29%), tablets/smartphones (20%), and constant temperature shower nozzles (13%). Our survey demonstrated devices perceived as most useful included: shower nozzles, GPS locating devices, door guards, and Bluetooth tracking stickers. Individuals who endorsed African-American/Other race were significantly more likely to use at least one AT-Device than those who identified as Caucasian (OR: 4.80; 95% CI: 1.50-17.58). This significance was lost during adjustment for other demographic variables (sex, age, cohabitation status, and dementia severity).

### TELEHEALTH USE IN A HOME-BASED DEMENTIA CARE-COORDINATION PROGRAM

Deirdre Johnston,[1] Melissa Reuland,[2] Kelly Marshall,[3] Inga Antonsdottir,[4] Morgan Bunting,[5] and Quincy Samus,[4] *1. Johns Hopkins Hospital, Baltimore, Maryland, United States, 2. Johns Hopkins University School of Medicine, Baltimore, Maryland, United States, 3. JHHCG, Baltimore, Maryland, United States, 4. Johns Hopkins University, Baltimore, Maryland, United States, 5. Johns Hopkins University, Halethorpe, Maryland, United States*

In the coming decades, greater numbers of people will either have Alzheimer's Disease or a related dementia or will take care of a family member with dementia. The dementia syndromes are associated with increased risk of medical, social, and behavioral complications in both the person with dementia (PWD) and the caregiver (CG), many of which are preventable. These complications, and the dementia itself, can impede access to care and ultimately hasten residential care placement, which can be both undesirable and costly. A nearly universal unmet need in PWD/CG dyads is dementia-specific education. Therefore, it is vital we find ways to support and provide education to CG/PWD dyads to manage dementia in the community and home setting. MIND at Home is a dementia-care model developed and tested at Johns Hopkins University School of Medicine to minimize dementia complications and delay institutionalization by training non-clinical Memory Care Coordinators (MCCs) working under clinical supervision to support and guide PWD/CG dyads in the community. MCCs collaborate with CGs and PWDs in the community using an individualized care plan structured around the dyads' specific dementia-related needs. This presentation will describe how the MIND at Home team used handheld tablets to connect MCCs to clinicians from participants' homes, and will report on challenges encountered, strategies to address them, and participant and caregiver satisfaction with the telehealth experience.

### USABILITY AND FEATURE EVALUATION OF THE AMAZFIT BIPS SMART WATCH IN THE PRECISION START LAB

Jo-Ana Chase,[1] Chelsea Howland,[2] Malaika Gallimore,[3] and Blaine Reeder,[3] *1. University of Missouri, Columbia, Columbia, Missouri, United States, 2. University of Missouri - Columbia, Sinclair School of Nursing, Columbia, Missouri, United States, 3. University of Missouri, Columbia, Missouri, United States*

Interventions utilizing consumer-grade wearable and mobile devices may support older adult health and wellness. However, rapid technology change and short industry product release cycles limit timely incorporation of these devices. We developed a novel, multi-stage process to rapidly move from within-team evaluations to lab- and field-based participants studies based on small-sample technology testing methods from Human-Computer Interaction. We present findings from a first-stage evaluation of the Amazfit Bips smart watch for potential use in studies with older adults as part of the methodology validation. A four-person research team conducted evaluations using: 1) a wearables framework for user experience and feature availability; and 2) the System Usability Scale (SUS). Evaluators wore the watch seven days straight from the box. User experience checklists indicated high usability. However, corresponding comments identified challenges with downloading the mobile app, pairing the watch and phone, navigating watch and mobile interfaces, and privacy controls. Average SUS score was 65.6 indicating marginal usability (C grade). While meeting study goals, divergence in usability perceptions suggest the process could be improved by completing each set of instruments separately for the watch and mobile app rather than all at once. Given failures in pairing, app navigation challenges, and small screen size, the Amazfit Bips may be best suited for studies among older adults with a high degree of technical proficiency. For those with little technical experience or high disease burden, training materials and dedicated training with support may be required. Future steps are lab- and field-based tests with older adult participants.

### USABLE AND PRIVACY-ENHANCED TELEPRESENCE ROBOTS FOR OLDER ADULTS AGING IN PLACE

Xian Wu,[1] and Jenay Beer,[2] *1. University of Georgia, Athen, Georgia, United States, 2. Institute of Gerontology, Athens, Georgia, United States*

Aging population is growing rapidly in the United States and people are living longer. Maintaining health and wellness while aging-in-place is crucial for older adults. Telepresence technology is beneficial for this target population to stay socially connected, as well as utilize telehealth and telemedicine services. However, such technology was not specifically designed for older adults. For older users to adopt telepresence, it is important to ensure that they do not experience adoption barriers, such as issues with usability and privacy. This research used a user-centered evaluation to design, develop, and test telepresence user interfaces (UI). Thirty older adults (aged 60+) participated in a within-subjects evaluation of two telepresence UIs: 1) the controlled condition - a generic UI, called Presence, based on currently available telepresence systems; and 2) the experiment condition - an enhanced custom telepresence UI that was designed follow human factors and design principles for older adults, named InTouch. Participants tested both UIs in a virtual home environment developed in Unity. Qualitative and quantitative results suggest that older adults perceived the experiment condition – InTouch, to be more usable and private – and our older users provided insight on which usability and privacy features were critical for them. By investigating the design of telepresence for older users, and applying those findings to design recommendations, we aim to improve the ease of use and privacy level of telepresence– not only for our target users but for all users who wish to enhance social connectedness and utilize telehealth.

### USE OF GPS TRACKING TECHNOLOGY TO MEASURE MOBILITY IN OLDER ADULTS: A SYSTEMATIC REVIEW

Jane Chung,[1] Lana Sargent,[1] and Roy Brown,[2] *1. Virginia Commonwealth University School of Nursing, Richmond, Virginia, United States, 2. Virginia Commonwealth University, Richmond, Virginia, United States*

Global positioning system (GPS) tracking technology is increasingly used in aging research to objectively measure the spatial and temporal aspects of mobility in older adults. The review aims to systematically synthesize the literature to identify GPS-driven mobility measures and potential determinants of mobility limitation for community-dwelling older adults. A systematic search of six electronic databases was conducted. A total of 4897 articles were found with 2578 left to review after deduplication. Twenty-five studies met inclusion criteria: 24 cross-sectional studies and one follow-up study that measured mobility changes over time. Various types of GPS devices were used, including smartphones, GPS watches, or portable data logging kits. The GPS tracking period ranged from 1 to 30 days. The daily device wear time varied from 10 to 24 hours. Commonly reported GPS-based mobility measures included time out of home, distance moved, the number of out-of-home trips or walking tracts, the number of visited places, life-space area, and walking speed. Twenty-one studies reported some aspects of demographic, physical, psychosocial, or environmental factors related to the levels of GPS-based mobility. GPS tracking technology can continuously record individuals' activities and functional abilities within their life space. We found that there was heterogeneity in ways of applying GPS technology and defining and measuring mobility in community-dwelling older adults. Given the lack of consistency in GPS-based mobility assessment, a clear definition of mobility and standardization of GPS data collection and analysis are required for comparison across studies and better understanding determinants of mobility limitation in community-dwelling older adults.

### USE OF VIDEO-ENABLED TABLET FOR VIRTUAL CARE AMONG OLDER VETERANS

Maria Yefimova,[1] Jiaqi Hu,[2] Cindie Slightam,[2] Liberty Greene,[3] Camila Chaudhary,[3] Leonie Hayworth,[4] and Donna Zulman,[3] *1. VA Palo Alto Healthcare System, Redwood City, California, United States, 2. VA Palo Alto Healthcare System, Palo Alto, California, United States, 3. VA Palo Alto Healthcare System, Menlo Park, California, United States, 4. Veterans Health Administration, Palo Alto, California, United States*

With the proliferation of virtual care, healthcare systems are exploring ways to bridge the digital divide among vulnerable patients. Department of Veterans Affairs (VA) is distributing devices for qualifying Veterans to enable video visits with medical providers at home, yet their use among older patients is unknown. This retrospective cohort study used administrative data to characterize the use of VA-loaned iPads among older Veterans compared to younger Veterans and identify demographic predictors of utilization. Among 16,385 patients who were shipped a VA-loaned iPad in 2014-2019, 33.66% (n=5,516) were over 65 years old, and 3.1% (n=503) were over the age of 85. Two thirds (n=6799) of younger patients had a video visit (mean=3 visits) with provider using iPad in the 6 months since shipment, compared to 50% (n=253) of 85+ year-olds (mean=1.8 visits). Most common types of virtual visits for the oldest old patients were for geriatrics or home-based primary care, compared to mental health visits among younger patients. Logistic regression identified characteristics of older patients who were more likely to use iPads, such a marital status, urban location, and lower disease burden, which is similar to their younger counterparts. While older age groups used VA-loaned tablets less frequently, those who engaged with the devices were similar in demographics as their younger counterparts. Older patients used iPads differently, with higher engagement in geriatric and primary care services. Providing devices for virtual care may allow health systems to more easily reach older patients in the comfort of their home.

### USING WEARABLE SENSORS TO REMOTELY EVALUATE PROGRESS ON GOALS SET BY PATIENTS WITH DEMENTIA

Jennifer Freytag,[1] He Zhou,[2] Lilian Dindo,[3] and Aanand Naik,[1] *1. Center for Innovations in Quality, Effectiveness, and Safety (iQuest), Houston, Texas, United States, 2. iCamp, Baylor College of Medicine, Houston, Texas, United States, 3. Michael E. DeBakey VA Medical Center, Houston, Texas, United States*

Patient Priorities Care (PPC) is a communication framework designed to facilitate priorities-based conversations between clinicians and older adults with multiple chronic conditions. PPC focuses on collaboratively setting specific and measurable goals based on what matters most, and patients often set goals involving physical activity. Measuring goal achievement for patients with dementia is difficult because they often struggle to report activity accurately. In this pilot study, we assessed the feasibility of using a wearable sensor to evaluate patient-defined goal achievement. The wearable sensor measured daily mobility and sleep performance, including length of walking bouts, time sitting, postural transitions, and sleep duration. Patients wore pendant sensors for 48 hours at baseline (before PPC conversations) and 3-6 month follow-up. We present a case in which remote monitoring exhibited evidence of goal achievement and another case in which monitoring exhibited failure to achieve a goal. In the former, the patient set a goal of walking his dogs daily. At baseline, mobility performance suggested that he was not engaged in this activity. At follow-up, in contrast, all parameters showed accomplishment of the patient's goal. In the latter case, the patient set a goal of maintaining his habit of walking a mile per day. At follow-up, sensor measures showed that the patient was not making progress on this goal. Conclusion: Remote monitoring using wearable sensors can provide objective information about goal achievement for patients with dementia. Sensor measurement can assist clinicians in evaluating goal achievement for patients with dementia and adjusting care based on these measures.

### VIRTUAL REALITY BASED INTERVENTIONS AMONG PEOPLE WITH DEMENTIA: APPLICATIONS AND HEALTH-RELATED OUTCOMES

Yu-Ping Chang,[1] Yanjun ZHou,[2] and Hanbin Zhang,[2] *1. University at Buffalo, Buffalo, New York, United States, 2. University at Buffalo, Buffalo, United States*

The use of virtual reality (VR) technology to improve health among older adults has been receiving increased attention. VR technology has many applications and can produce benefits for people with various medical conditions such as symptom reduction or improving the diagnostic process. Despite the increase in the number of research studies of VR technology, little is known about how it has been used to improve health-related outcomes among people with dementia. This systematic review aimed to synthesize research evidence regarding the scope and impact of VR based interventions among people with dementia. Five databases, CINAHL, Embase, PsychINFO, PudMed, and Web of Science were searched to identify studies leveraging VR to facilitate interventions designed for people with dementia. Multiple keywords were used in combination including: dementia, Alzheimer's, VR, virtual reality, and Virtual Reality

Exposure Therapy. Articles were screened if they were published between 2000 through 2020. Fifteen studies met the criteria, with ten utilizing a randomized controlled trial. The VR based interventions in those studies included virtual physical exercises, virtual forests or natural landscapes, and virtual basic living activities. The results of these studies show that VR as a display medium can greatly improve decision-making, hearing, vision, motor ability, and memory in people with dementia residing either in the community or in long term care settings. Our review demonstrated that VR showed positive benefits through various applications for people with dementia. Future research is needed to make VR interventions more customized toward effectively meeting the needs of people with dementia.

### SESSION 2892 (POSTER)

### AGING IN PLACE (HS)

### AMBULATORY CARE PREFERENCES OF OLDER PEOPLE AGE 80 AND OVER: A SYSTEMATIC REVIEW OF QUALITATIVE STUDIES

Angélique Herrler,[1] Helena Kukla,[1] Vera Vennedey,[2] and Stephanie Stock,[2] *1. University of Cologne, Cologne, Germany, 2. Universital Hospital of Cologne, Cologne, Germany*

According to the United Nations, the number of people aged 80 and over is expected to treble by 2050 globally. But research on the preferences for care of this age group grows slowly. To achieve high-quality patient-centered care, we need to understand the oldest people's specific living circumstances, care preferences and goals. The aim of the study was to synthesize findings about ambulatory care preferences, experiences and expectations of people aged 80 and over. We systematically searched Medline, CINAHL, PsycInfo, Web of Science Core Collection and Google Scholar for qualitative studies published until October 2019 and additionally conducted forward and backward citation search for included studies. Two independent reviewers assessed studies for eligibility criteria and quality. We performed a thematic synthesis of study findings as developed by Thomas and Harden using MAXQDA-20 content analysis software. Twenty-three studies were included. They were mainly conducted in Europe, used face-to-face interviews, reported on ambulatory home care and used qualitative content or thematic analysis. The meta-synthesis revealed two fundamental themes from the perspective of older people: feeling safe and feeling valued in their relationships with caregivers and in their care environment. This was shown, for instance, in preferences for coordinated care, high continuity of caregivers, personal attention and interactions based on trust and respect. In practice, the older persons' preferences should be integrated into care planning and policies to ensure patient-centered care.

### CARE MANAGERS HAVE FEW OPTIONS FOR HOME MODIFICATION BECAUSE THEY ARE NOT SPECIFIED IN ARCHITECTURE

Kazunori Yoshida, Yoritaka Harazono, Toko Funaki, and Akiko Nishino, *The University of Tokyo, Tokyo, Japan*

In this paper, we aim to clarify the cause of the difficulty in home modification. The aging society becomes larger where older people have difficulty in living home because of weakened body functions. To maintain quality of life, it is important to modify houses. In Japan, home modification is conducted by care managers, who are originally from nurses, helpers, and so on. However, to modify houses, we hypothesized that it is needed to have knowledge about not only body function but also architecture. Because of this, home modification should be difficult for care managers. For this problem, we aim to clarify the difficulty in home modification. In November 2018, we took part in the teaching course for care managers about home modification and asked care managers the number of home modification they conducted and what they have difficulty in. As a result, we asked for 57 care managers, who have experience as care managers for 39 months in average. Home modification was mainly conducted for setting handrails (four for a care manager in average). It was also revealed that experience of modification for handrails and doors are larger when the experience of care manager becomes longer, but other modification is not the case. The care managers told us that they cannot understand architecture. This result indicates that care managers cannot think of many options for modification because of their little knowledge about architecture. Therefore, it should be needed to combine the architects and care managers for appropriate home modification.

### EFFECT OF PERCEIVED SOCIAL SUPPORT ON MORTALITY RATE AMONG OLDER CHINESE AMERICANS

Charu Verma,[1] Mengting Li,[2] and XinQi Dong,[2] *1. Rutgers University--New Brunswick, New Brunswick, New Jersey, United States, 2. Rutgers University, New Brunswick, New Jersey, United States*

Most existing studies have examined the relationship between social support and health in cross-sectional data. However, the changing dynamics of social support over time and its relationship with all-cause mortality have not been well explored. Using data from the Pine Study (N = 3,157), this study examined whether social support was associated with time of death at an 8 years follow-up among older Chinese Americans. Social support from a spouse, family members and friend were collected at the baseline using an HRS social support scale. Perceived social support and time of death were ascertained from the baseline through wave 4. Cox proportional hazard models were used to assess associations of perceived support with the risk of all-cause mortality using time-varying covariate analyses. Covariates included age, sex, education, income, and medical comorbidities. All study participants were followed up for 8 years, during which 492 deaths occurred. In multivariable analyses, the results showed that positive family support [HR 0.91; 95% CI (0.86, 0.98)] and overall social support [HR 0.95; 95% CI (0.92,0.98)] were significantly associated with a lower risk of 8-year mortality. Results demonstrate robust association in which perceived positive family and overall social support over time had a protective effect on all-cause mortality risk in older Chinese Americans. Interventions could focus on older adults with low social support and protect their health and well-being. Future studies could further explore why social support from family is different from social support from other sources regarding mortality risk in older Chinese Americans.

### FACTORS RELATED TO THE DIGNITY OF OLDER PEOPLE IN TEHRAN IN 2020

Ali Darvishpoor Kakhki, Fereshteh Moradoghli, Roghayeh Esmaeili, and Ali Kakhki, *Shahid Beheshti University of Medical Sciences, Tehran, Iran*

The elderly are the fastest growing segment of the population globally. This is also the case in developing countries such as Iran. The fear of aging and the refusal to accept older adults into the mainstream of society affects the dignity of older people. This study was conducted to describe factors and dignity of older people in Tehran, Iran. This descriptive, correlational study was conducted on a sample of 215 older people above age 60 in 10 public parks in five regions of Tehran in 2020. A socio-demographic questionnaire and patient dignity inventory were used for data collection. Content validity and Cronbach's alpha were used for evaluating the validity and reliability of questionnaires. Data were analyzed with SPSS. 60% of subjects were male and 40% female with a mean age of 68(± 5.05) year. The mean scores for dignity domains ranged from 67.30 (±17.56) for symptom distress, to 93.01(± 10.90) for dependency on a 100-point scale. The scores of dignity domains were significantly associated with job status, housing status, income source, health insurance, chronic diseases, annual physician referral rates, and hospitalization in last year. The best predictors of dignity were health insurance and annual physician referral rates. The findings of this study showed that the dignity of older people is related to a number of factors. Monitoring modifiable factors such as health insurance and annual physician referral rates and non-modifiable factors such as chronic diseases will help us to preserve or improve the dignity of older people.

### IF YOU DON'T USE IT, YOU LOSE IT: PERSPECTIVES OF OLDER ADULTS ON AGING IN PLACE IN SUBSIDIZED HOUSING

David Reyes-Farias,[1] Momana Jahan,[1] Sonia Pandit,[1] Carolina Reid,[2] Barbara Resnick,[3] and Rebecca Brown,[1]
*1. University of Pennsylvania, Philadelphia, Pennsylvania, United States, 2. University of California, Berkeley, Berkeley, California, United States, 3. University of Maryland School of Nursing, Baltimore, Maryland, United States*

Nearly 3 million older Americans with low incomes live in subsidized housing. This population has disproportionate rates of functional impairment, cognitive impairment, and nursing home admission. Patient-centered interventions are needed to improve aging in place for this population, but little is known about resident perspectives on this topic. We interviewed 58 residents aged 62 or older and 8 caregivers from 7 housing sites. We analyzed transcripts using qualitative thematic analysis. Participants reported that several factors impacted their ability to age in place. First, most participants noted the importance of physical environment, including the design and location of their apartment building. Physical accessibility and proximity to community resources facilitated aging in place, while features such as heavy doors and smoke in communal areas posed challenges. Second, most participants identified the importance of the building's social environment; support received from other residents and on-site staff facilitated aging in place, while gossip and unpleasant residents were barriers. Third, participants noted that health issues such as arthritis limited their ability to function independently, regardless of environment. Participants emphasized the importance of physical activity for preventing functional decline, stating, "if you don't use it, you lose it." Older adults living in subsidized housing view their environment as central to their ability to age in place. Our findings suggest that interventions to improve aging in place in these settings must focus not only on optimizing residents' physical function, but also on using the environment to promote resident independence and quality of life.

### OLDER ADULTS' EXPERIENCE OF HEALTH AND LIFE EVENTS AS REPORTED ONLINE OVER 150 CONSECUTIVE WEEKS

Jeffrey Kaye,[1] Nora Mattek,[2] Zachary Beattie,[2] Nicole Sharma,[2] Thomas Riley,[2] and Hiroko Dodge,[3]
*1. Layton Alzheimer's Disease Center, Portland, Oregon, United States, 2. Oregon Health & Science University, Portland, Oregon, United States, 3. Pregon Health & Science University, Portland, Oregon, United States*

Key activities and life events that impact quality of life and health may be gleaned from cross-sectional annual surveys or for medical data, derived intermittently from electronic health records. These methods cannot reflect the day-to-day and week-to-week changes that typically occur in domestic life. To assess the frequency and types of major life activities and events occurring at finer-grained temporal scale, we queried online, every week, three domains of activity and function (life events such as having an overnight visitor, travel away from home; health-related events such as medication changes, falls, ER/hospital visits, health limitations; and internal states such as pain, mood, or loneliness). Over a mean assessment period of 2.9 ± 1.2 years, 16,738 online surveys were completed by 129 community-residing volunteers with mean age 84 years. Overall the most frequent events reported were physical health limitations (14%), travel away from home (12%) and overnight visitors (9%). Needing new in-home assistance for medication management, bathing, dressing or grooming was rare (1%). Accidents were also rare (1%). Low mood, pain ratings (0-10 scale) and loneliness were infrequently reported; 4% low mood, 8% pain rated > 5, 3% reporting loneliness. Low mood and increased pain intensity were highly correlated with health changes (medication changes, ER/hospital visits, health limitations, falls; all $p<0.0001$). Continuous home-based online assessment of life activities and health can provide a more detailed and timely characterization of older adult function. These data may be used to guide more timely and effective health maintenance programs and interventions.

### REFRAMING AGING: PENN'S VILLAGE MEMBERS THRIVE WHILE AGING IN PLACE

Lois Evans,[1] Jane Eleey,[2] and Avalie Saperstein,[3]
*1. University of Pennsylvania School of Nursing, Philadelphia, Pennsylvania, United States, 2. Penn's Village, Philadelphia, Pennsylvania, United States, 3. Penn's Village, Meadowbrook, Pennsylvania, United States*

Villages help older neighbors age-in-place as they manage their environments, take advantage of opportunities for social and civic engagement, and improve or maintain health and well-being. National surveys repeatedly indicate that older adults prefer community living as long as possible. But communities change, and post-retirement living may require rebuilding social connections with old and new neighbors. Fortunately, today's retirees bring a wealth of knowledge and skills to later life which they are happy to share. Drawing on the talents and career experiences of older adults in Center City, Philadelphia, Penn's Village (PV) was created in 2007 to address the needs and wishes of neighbors wanting to stay in their own homes as they aged. A member of the Village-to-Village Network, PV (a 501c3) has itself matured through board development, strategic planning, and member engagement. In reframing aging in Center City, PV currently offers its over 300 members and volunteers an array of educational and recreational programs (e.g., talks, affinity groups, social events and outings); services (including transportation, home repairs, IT support, companionship, accompaniment to medical appointments), and opportunities to use their personal knowledge and skills to help their neighbors-- as drivers, companions, volunteer staff and co-chairs/members of committees (e.g., Board of Directors, Program, Welcoming, Marketing & Communications, Finance, Fundraising). In FY2019, volunteers provided nearly 1200 services to PV members. Our most recent survey responses indicate that 82% of volunteers found their work highly meaningful and 79% of those who received services believed their quality of life was greatly improved.

### THE FACTORS ASSOCIATED WITH DAILY LIFE SUPPORT FOR SINGLE OLDER ADULTS TO REMAIN AT THEIR HOME

Rika Sakurai, Asa Inagaki, Yukitsugu Komazawa, Mari Kimata, and Jun Goto, *The University of Tokyo, Tokyo, Japan*

Japan aims to enable older adults to remain at home in their familiar environment. However, the factors associated with daily life support for older adults who require medical and nursing care to remain at home are unclear. This study aimed to clarify the factors associated with daily life support for single older adults needing medical and nursing care to remain at home. Our participants were single older adults aged 65-94 years receiving medical and nursing care and their care providers. First, we analyzed records, which were written by care providers, regarding ten older adults who received medical and nursing care from 2014 to 2018. We categorized occurrences which exert single older adults' life on change into six factors, such as gradual frailty and loss of a loved one. Then, to consider how they experience these factors, we conducted semi-structured interviews with three additional older adults who were single and received home visiting nursing care service in 2020. During this process, four multidisciplinary researchers discussed the factors associated with daily life support for single older adults; finally, three factors were derived. The first one pertained to health conditions: receiving sufficient medical and nursing care maintain older adults' physical condition. The second related to the environment: maintaining social interactions (neighbors and friends). The third pertained to older adults' values and meanings to remain at home. This study suggests that care providers focus on these three factors to help older adults who received medical and nursing care to remain at their home.

### URBAN-RURAL DIFFERENCES IN GEODETIC DISTANCE BETWEEN HOME AND THE NEAREST PHARMACY AMONG OLDER PENNSYLVANIA ADULTS

Shivani Khan,[1] Debra Heller,[1] Leroy Latty,[1] Michelle LaSure,[1] and Theresa Brown,[2] *1. Magellan Rx Management/PACE, Harrisburg, Pennsylvania, United States, 2. Pennsylvania Department of Aging/The PACE Program, Harrisburg, Pennsylvania, United States*

Aim: We examined differences in geodetic or straight line distance between home and the nearest community pharmacy among rural and urban older adults in Pennsylvania. Method: The addresses of 241,398 older adults (≥65 years) and 2,880 community pharmacies enrolled in Pennsylvania's Pharmaceutical Assistance Contract for the Elderly (PACE) program in 2018 were geocoded. We identified pharmacies in the same or adjacent counties for each enrollee and measured the geodetic distance between home and those pharmacies. The pharmacy with the shortest distance from home was identified as the nearest pharmacy for each enrollee. Enrollees' home addresses were categorized as urban or rural at the county level, based on the Center for Rural Pennsylvania's definitions. T-tests and chi-squared tests were used for analyses. Results: Overall, 37% were rural older adults and the mean distance between home and the nearest pharmacy was 1.60 ± 2.21 miles. The mean distance between home and the nearest pharmacy was significantly greater in rural compared to urban older adults (2.78 ± 2.93 versus 0.91 ± 1.19; p<.0001). A higher proportion of rural older adults resided >5 miles away from the nearest pharmacy compared to urban older adults (19.19% versus 1.80%; p<.0001). Moreover, 2.96% of rural older adults resided >10 miles away from the nearest pharmacy compared to 0.08% of urban older adults (p<.0001). Conclusion: Older patients in rural counties need to travel longer distances for pharmacy access than in urban counties. Efforts to provide convenient access to medications and pharmacy services for rural older patients are necessary.

### SESSION 2893 (POSTER)

### ASSESSMENT

### A SYSTEMATIC REVIEW OF MISSOURI LYMPHEDEMA SYMPTOM ASSESSMENT TOOLS IN ORIGINAL RESEARCH AND REVIEW ARTICLES

Christiana Oyewusi, and Jane Armer, *University of Missouri-Columbia, Columbia, Missouri, United States*

Despite advances in cancer treatment, many survivors face a significant challenge of cancer-related lymphedema, together with aging. Aging results in structural changes in the lymphatic system. Beginning in 1998, tools have been developed at the University of Missouri to assess symptoms of lymphedema in cancer patients. The objective of this review was to synthesize evidence regarding use of

Missouri lymphedema symptom assessment tools in original research and review articles. A search of six electronic databases was conducted for articles published within 1998 and 2018 on review and use of the tools which are Lymphedema and Breast Cancer Questionnaire (LBCQ), Melanoma and Lymphedema Questionnaire (MELQ), and Gynecologic Cancer and Lymphedema Questionnaire (GCLQ). In all, 210 articles were retrieved, and 32 full-text articles meeting the inclusion criteria were reviewed. The studies reported a cumulative number of 5,872 study participants. Most manuscripts (70.97%) reported data on breast cancer lymphedema, 19.35% on gynecological cancer lymphedema, 3.23% on breast cancer and melanoma lymphedema, and 6.45% on melanoma. The use of LBCQ was reportedly more than the use of GCLQ and MELQ. Tool reliability ranged from r = 0.785 - 0.82 for LBCQ and 0.95 for internal reliability for GCLQ. The tools have been used in many countries including the United States of America. The importance of using valid and reliable quantitative measures in lymphedema symptom assessment across diverse populations and sites cannot be overstated. The tools have been modified and used over the past 20 years in various settings and across languages and cultures.

### DEVELOPING A QUESTIONNAIRE TO ASSESS OPINIONS REGARDING THE IMPORTANCE OF BATHING AND WASHING WITHOUT WATER

Fabian Groven, Jan Hamers, Gaby Odekerken-Schröder, and Sandra Zwakhalen, *Maastricht University, Maastricht, Limburg, Netherlands*

Bathing is one of the most performed activities among nurses. Although care recipients experience bathing as an important activity in daily living, nurses often undervalue this care task. We developed a questionnaire to measure nurses' opinions regarding 1) the importance of the bed bath, and 2) a bathing innovation known as Washing Without Water. Construction of the questionnaire items was based on literature and interviews with nursing home residents (n=8), their family (n=5) and nurses (n=6). After items construction, nurses and nursing students (n=124) completed the questionnaire to assess the questionnaire's internal consistency (IC) and construct validity. Cronbach's alpha coefficients were analyzed as an indicator for IC and items were deleted if this resulted in improved IC. To analyze the construct validity, a Principal Component Analyses (PCA) with Direct Oblimin Factor rotation was performed. The final scale consists of two subscales. The first subscale measures nurses' opinions about the importance of the bed bath and consists of 12 items. The second subscale consists of 17 items and aims to inventory nurses' opinions about the Washing Without Water innovation. The Cronbach's alpha coefficients are high (.81 for the first and .89 for the second subscale). The PCA results show a one factor loading for both subscales, explaining 33,20% and 37,08% of the variance for the first and second subscale respectively. Results indicate a reliable and valid questionnaire to measure nurses' opinions related to the bed bath, which can support health care institutions in evaluating the bed bathing process.

### EVALUATION OF AGILITY AND WALKING ABILITY IN ELDERLY PEOPLE

Ken Yamauchi,[1] Hiromichi Hasegawa,[2] Akira Ogita,[3] Tutomu Ichikawa,[4] Shota kagawa,[5] and Yamauchi Ken, *1. Keio University, Yokohama, Kanagawa, Japan, 2. NPFC, Nagakute, Aichi, Japan, 3. Osaka City University, Osaka, Japan, 4. Matsuyama Shinonome Junior College, Matsuyama, Ehime, Japan, 5. Tohto University, Chiba-shi, Chiba, Japan*

An important aspect of independent living in a super-aging society is the achievement of "successful aging" in mind and body. Recent large-scale epidemiological studies found that reduced walking ability significantly affects "extended healthy life expectancy" and "successful aging." In terms of anatomy and physiology, "usual aging" (i.e., the inevitable decrease in walking function due to aging) occurs unless one undertakes daily physical activity. Conversely, aggressive moderate and habitual physical activity may lead to "successful aging" (i.e., maintained and improved walking function). Previous studies revealed that agility and walking ability are related to healthy life expectancy. Thus, we measured agility with a stick response test to investigate its relationship to gait, and determined whether this test could be substituted for a battery test of walking ability. This test has the convenience of shortening measurement time and requiring a wider measurement place than gait evaluation performed by actually walking. Correlation was observed between the rod response test and stride length (r = 0.60), walking speed (r = 0.38), and walking ratio (r = 0.34). Therefore, we propose that the rod response measurement is simpler than a simple walking ability test and can be used as a physical function measurement item that predicts the health and longevity of the elderly.

### EXAMINATION OF ADVANCED FUNCTION AND ITS CORRELATES IN A COHORT OF COMMUNITY-DWELLING OLDER ADULTS

Daniel Liebzeit,[1] Wan-chin Kuo,[2] Beverly Carlson,[3] Kimberly Mueller,[2] and Lisa Bratzke,[2] *1. William S. Middleton Memorial Veterans Hospital, Verona, Wisconsin, United States, 2. University of Wisconsin-Madison, Madison, Wisconsin, United States, 3. San Diego State University, San Diego, California, United States*

Function in older adults includes multiple domains, from basic to "advanced," but we remain limited in detection of advanced function (engagement in social, leisure, and productive activities). The objective is to describe advanced function and examine relationships with basic function and health outcomes in community-dwelling older adults aged 55-65 years. This is an analysis of existing data from a large, ongoing cohort study, The Wisconsin Registry for Alzheimer's Prevention (WRAP R01 AG027161). We used a 1:1 prospective case–control design to examine whether older adults with lower advanced function (lower functioning group) at wave 3 showed lower IADLs and poorer health outcomes in wave 4, compared to those with higher advanced function (higher functioning group). The lower functioning group had a mean advanced function score of 74.4 (SD = 10.1), compared to 98.7 (SD = 11.1) in the higher functioning group. The mean IADL scores were similar in the two groups (p = 0.123). The lower functioning group had significantly lower self-rated health (mean = 3.52; SD = .79) than higher functioning group (3.70; 0.79) and a significantly lower proportion of individuals with unimpaired, stable cognition (77%) than the higher functioning group (85%). The lower functioning group had higher rates of comorbidities (4.25 vs. 3.96), mortality (4 vs. 1), and depressive symptoms (CES-D: 7.17 vs. 6.09), although results were not significant at $\alpha=0.05$. This study provides a foundation for examining advanced function, which may be an early indicator of poor health outcomes in older adults.

### MEASURING HAND FUNCTION IN OLDER ADULTS: THE NEED FOR BETTER ASSESSMENT TOOLS

Rachel Logue,[1] Elana Goldenkoff,[2] Michael Vesia,[2] and Susan Brown,[2] *1. University of Michigan, Ann Arbor, Michigan, United States, 2. University of Michiagn, Ann Arbor, Michigan, United States*

Aging is associated with a decline in hand muscle strength, dexterity, and tactile perception, leading to difficulties in activities of daily living and reduced independence (Millan-Calenti et al., 2010). However, current assessments do not adequately capture sensorimotor skills that underlie everyday activities such as dressing and food preparation. This study examined the ability of two novel assessment devices to detect age-related changes in hand force control and tactile pattern discrimination. Sensorimotor function was assessed in 13 healthy older adults (mean age 72.2 +/- 5.5y) and 13 young adults (mean age 20 +/- 1.4y). Maximum grip force (MVC), tactile sensation, and hand dexterity were measured using standard clinical techniques. Novel assessments consisted of submaximal (5-20% MVC) grip force tracking and computer-controlled tactile pattern recognition. Monofilament testing of tactile sensation was normal in the older group. In contrast, both the accuracy and speed associated with identifying tactile patterns was significantly worse in older ($p<0.001$) compared to young adults for both hands. While maximum grip force was similar in both groups, the ability to smoothly produce ($p<0.05$) and maintain ($p<0.02$) low grip force levels was compromised in older adults. Manual dexterity (Grooved Pegboard test) was significantly reduced in the older group ($p<0.001$) regardless of hand. These results indicate that the ability to extract meaningful information from tactile feedback and control low levels of force - aspects of fine hand control associated with activities of daily living – are impaired in older adults and underscore the need for more sensitive measures of hand function.

### PHYSICIAN ATTITUDES AND CONFIDENCE TOWARD DEMENTIA CAPABILITY: SCREENING, DIAGNOSES, AND REFERRALS

Tamara Herrick,[1] and Michelle Ward,[2] *1. Mainehealth, portland, Maine, United States, 2. UMAINE, Portland, Maine, United States*

The MaineHealth Alzheimer's Disease Partnership is working to improve integration between the healthcare system and community partners through training and a referral network. Primary care providers are often the first to assess cognitively impaired patients, so it is important to understand their attitudes and confidence in dealing with dementia. The objective of this study is to determine barriers to care and evaluate healthcare providers' attitudes towards their dementia capability, which includes screening for cognitive impairment, disclosing diagnoses, and making referrals to community-based organizations or specialists. A 27-item survey was developed and sent to 474 providers from MaineHealth practices via email. Fifty-three providers responded to the survey. Five healthcare professionals also took part in a focus group; looking more specifically at challenges encountered throughout the dementia care system. This poster will present the findings from the survey and focus group. There was strong agreement that much can be done to improve the quality of life for patients with dementia (86% agreed/strongly agreed) and that screening all patients over age 65 is important (85% agreed/strongly agreed). Confidence levels in ability to diagnose dementia, provide memory loss information, and refer patients to specialists were significantly associated with training ($p<.05$). The majority of providers identified barriers to cognitive screening and referring patients to community-based organizations, showing that improvements are needed at the system level to remove these barriers. Overall, the results suggest that dementia specific training can improve confidence in care and allow physicians to provide more information about memory loss to patients.

### SESSION 2894 (POSTER)

### PAIN: SYMPTOMS AND MANAGEMENT

### ATTITUDES OF OLDER ADULTS WITH CHRONIC MUSCULOSKELETAL PAIN TOWARD VIRTUAL REALITY

Lynn Nakad, and Barbara Rakel, *University of Iowa, Iowa City, Iowa, United States*

Older adults are especially susceptible to chronic musculoskeletal (MSK) pain. Pharmacological treatment often provides inadequate relief and overuse can lead to adverse events. Preliminary research has demonstrated the effectiveness of virtual reality (VR) distraction therapy for chronic pain. Understanding attitudes of older adults towards VR distraction therapy will help develop and optimize this therapy for this population. Therefore, the purpose of this study is to explore the attitudes and treatment acceptability towards the use of immersive VR distraction therapy of older adults suffering from chronic MSK pain. This descriptive, exploratory study used mixed methods. Data collection consisted of a consent process, eligibility screening, survey completion, 2 VR simulations (passive and active) lasting 10-minutes each, and either a focus group or interview. Survey data was used to measure: pain intensity and interference, pain catastrophizing, treatment acceptability, usability, and side effects. A total of 21 older adults completed the study. Treatment acceptability was high with an average score of 32.5 out of 40. However, average usability scores (62.9 out of 100) indicated a need for system improvements. Few participants (14%) experienced moderate to severe side effects. The following themes were identified: 1) VR is an enjoyable distraction; 2) Perceived effectiveness depends on chronic pain experience; 3) VR simulation experiences should be individualized; 4) Design considerations to improve usability in older adults; 5) Recommendations for future directions. The findings from this study can inform intervention design considerations and future directions for the use of VR technology for chronic pain management in older adults.

### BACK PAIN AND HEART FAILURE IN OLDER ADULTS: FINDINGS FROM THE HEALTH ABC STUDY

Jie Chen,[1] Yiming Zhang,[1] Eleanor Simonsick,[2] Angela Starkweather,[1] Ming-Hui Chen,[1] Paula McCauley,[3] Kendra Maas,[1] and Xiaomei Cong,[1] *1. University of Connecticut, Storrs, Connecticut, United States, 2. National Institute on Aging, Bethesda, Maryland, United States, 3. UConn Health, Farmington, Connecticut, United States*

Both back pain and heart failure (HF) have negative influence on all aspects of life. Little is known about the impact of back pain on older adults with HF. We include 1295 older adults who had data collected in the 11th year (2007-2008) of the Health, Aging and Body Composition (Health ABC) study to evaluate the effect of back pain on health status among older adults with and without HF. The participants aged 79-91, 54.8% were female and 34.8% were African American. Among 94 participants with HF, 63 (67.0%) had back pain; among 1201 participants without HF, 649 (54.0%) had back pain. Females reporting back pain had 4.76 (95% CI: 1.83, 12.37) times the odds of having HF compared to those without back pain. Male with back pain, compared to those without back pain, had 1.14 times (95% CI: 0.65, 2.02) the odds of having HF. Depressive symptoms were measured by the Center for Epidemiological Studies-Depression (CES-D) scale. Performance and functions were measured by the Established Populations for Epidemiologic Studies in the Elderly (EPESE) performance score, the Health ABC performance battery score and self-reported difficulty with functional tasks. These symptom and performance measures were significantly associated with both back pain and HF, but not the interaction terms of back pain and HF after adjusting demographic variables including gender, race, smoking status and BMI category. The high incidence and negative impact of back pain highlighted the needs of developing strategies in pain management among older adults with and without HF.

### COMPLEMENTARY AND INTEGRATIVE HEALTH APPROACHES ARE UNDERUSED AMONG OLDER VETERANS WITH MUSCULOSKELETAL PAIN

Ling Han,[1] Robert Kerns,[2] Melissa Skanderson,[3] Stephen Luther,[4] Samah Fodeh,[5] Joseph Goulet,[3] and Cynthia Brandt,[6] *1. Yale School of Medicine, New Haven, Connecticut, United States, 2. VA Connecticut Healthcare System, West Haven, Connecticut, United States, 3. VA Connecticut Healthcare System, West Haven, CT, West Haven, Connecticut, United States, 4. James A. Haley Veterans Hospital, Tampa, Florida, United States, 5. Yale School of Public Health, West Haven, Connecticut, United States, 6. Yale School of Public Health, west haven, Connecticut, United States*

Complementary and integrative health (CIH) approaches are recommended in national policy guidelines as viable options for managing chronic pain, yet their use among Veterans has been suboptimal, especially for older Veterans. We identified 64,444 Veterans with a diagnosis of musculoskeletal disorders (MSD) who reported a moderate to severe pain intensity during primary care visits in 2013 from the Veterans Health Administration (VHA) electronic records. Using natural language processing (NLP), CIH use (acupuncture, chiropractic care and massage) was documented for 8169 (6.5%) of 125408 primary care visits in providers' progress notes. Compared to their younger counterparts, older Veterans aged ≥ 65 years had 21% lower likelihood of using CIH during the year [Odds Ratio (OR): 0.79; 95% Confidence Intervals (CI): 0.73, 0.86] after accounting for demographic, clinical, temporal and spatial confounding using a generalized estimating equation logistic model. Non-white race/ethnicity, tobacco use, medical comorbidities and diagnosis of alcohol or substance use disorders were independently associated with less CIH use (ORs ranging 0.97-0.80, p<0.03-0.0001); whereas female gender, being married and number of MSD diagnoses were associated with greater CIH use (ORs ranging 1.13-1.30, p<0.0001). Redefining CIH use as chiropractic care alone [4.8% person-visits; OR: 0.78 (95% CI: 0.70, 0.86)] or incorporating structured data [9.0% person-visits; OR: 0.76 (95% CI: 0.70-0.82)] in the adjusted GEE model derived consistent results. Research to identify and address barriers to CIH use among older Veterans is encouraged.

### DEVELOPING AN RAI-MDS BEHAVIOR-BASED PAIN SCALE FOR LONG-TERM CARE RESIDENTS WITH ADVANCED DEMENTIA

Jennifer Knopp-Sihota,[1] Matthias Hoben,[2] Jeff Poss,[3] and Carole Estabrooks,[2] *1. Athabasca University, Edmonton, Alberta, Canada, 2. University of Alberta, Edmonton, Alberta, Canada, 3. University of Waterloo, Vancouver, British Columbia, Canada*

In Canadian and many international long-term care (LTC) facilities, pain assessment frequently relies on data from the Resident Assessment Instrument – Minimum Data Set 2.0 (RAI-MDS). The RAI-MDS produces a two-item scale, measuring both pain frequency and pain intensity. This scale correlates well with self-reported pain in cognitively intact LTC residents, but despite repeated testing, is less valid for use in residents with more advanced cognitive impairment who are unable to self-report their pain. In this study we aimed to develop and validate a behaviour-based pain assessment scale for long-term care residents using data available in the RAI-MDS. To construct our initial scale, we reviewed the literature and compiled a list of observable indicators of pain (e.g., grimacing) and linked these with 28 similar items available in the RAI-MDS. Using Delphi techniques, we further refined this to 20 items. We then evaluated the psychometric properties of our scale using two independent, representative samples, of urban LTC residents in Western Canada. Exploratory factor analyses were conducted in sample one (n=16,282) and confirmatory factor analyses (CFA) were then conducted in sample two (n=15,785) in order to test, and confirm, our model. A two-factor solution was identified grouping RAI-MDS items into subscales 1) change in status (e.g., new onset restlessness) and 2) behaviours (e.g., crying). Commonly recognized model fit indices were acceptable suggesting the adequacy of the two-factor solution. Results provide preliminary support for the use of behavioural-based pain assessment scale using RAI-MDS data. Further evaluation and validation of our scale is warranted.

### PAIN AND ITS MANAGEMENT: STRATEGIES AND OUTCOMES IN OLDER ADULTS WITH OR AT RISK FOR KNEE OSTEOARTHRITIS

Alisa Johnson,[1] Staja Booker,[2] Josue Cardoso,[3] Burel Goodin,[4] Kimberly Sibille,[5] and Roger Fillingim,[6] *1. University of Florida, Gainesville, Florida, United States, 2. The University of Florida, Gainesville, Florida, United States, 3. Pain Research and Intervention Center of Excellence, Gainesville, Florida, United States, 4. University of Alabama at Birmingham, Birmingham, Alabama, United States, 5. UNiversity of Florida, Gainesville, Florida, United States, 6. University of Florida, Pain Research and Intervention Center of Excellence, Gainesville, Florida, United States*

Knee osteoarthritis (KOA) is a leading cause of mobility disability that is characterized by chronic pain among older adults. Non-Hispanic Blacks (NHBs) suffer disproportionately from non-Hispanic Whites (NHWs), reporting higher pain intensity and disability. It is unclear how these differences in symptomatology translate into different patterns of utilization for self-management (SM) of pain, and if such patterns are associated with underlying biological pain mechanisms. This multisite observational study examined (1) use of self-management strategies among older NHB and NHW adults with/at risk for KOA and (2) associations among self-management strategies, clinical and experimental pain. After approval from institutional IRBs, NHB and NHW older adults (N= 202) with knee pain completed the McGill Pain Questionnaire-Short Form, questions on treatment strategies (e.g., massage, ice, heat, medications), and quantitative sensory testing. Covariates included study site and education. On average, participants reported using 2 ± 1.65 SM strategies, with 79% endorsing at least one SM strategy. Analysis of covariance revealed that clinical pain differed by race/ethnicity and use of SM and/or medical treatments (p's < 0.01). SM use did not differ by race/ethnicity, $p = 0.15$, but did differ significantly by gender, $p < 0.05$. Multiple linear regression demonstrated significant positive associations between SM and heat pain sensitivity for both NHBs and NHWs, ($p < 0.05$). SM is an important component of OA management for NHBs and NHWs. Our study is one of the first to show that SM use is significantly associated with pain mechanisms. Improved understanding will facilitate better mechanism-targeted pain management.

### PAIN-ASSOCIATED CLUSTERS AMONG NURSING HOME RESIDENTS

Andrea Budnick, Reinhold Kreutz, Adelheid Kuhlmey, and Dagmar Dräger, *Charité - Universitätsmedizin Berlin, Berlin, Berlin, Germany*

Chronic pain is common in older adults, particularly among nursing home residents (NHR). Internationally, the reported pain prevalence among NHR ranges from 3.7% to 79.5%. At least one in two German NHR are diagnosed with pain. Unrelieved chronic pain is associated with reduced physical functioning and psychological parameters. Given the high prevalence of pain among NHR, we hypothesized that there were likely pain-associated clusters in our target group. Clustering is an opportunity to identify differences in pain management and may enable better targeted health-service delivery for professionals. There are no available data regarding pain-associated clusters (sub-groups of NHR) based on different items measuring pain. This study was performed using baseline data, and was part of a cluster-randomized controlled trial conducted in 12 nursing homes in Berlin. We assessed pain using the German Brief Pain Inventory (BPI-NHR) among 137 NHR (mean age, 83.33 years) capable of self-report. We performed hierarchical agglomerative cluster analysis to generate three clusters (naming is based on the mean value of each BPI-NHR item in each cluster): pain-relieved (46.72 %), pain-restricted (22.63 %), and severe pain (30.66 %). Body-Mass-Index ($F(2,129) = 4.274$, $P = 0.016$), Barthel-Index ($F(2,133) = 3.246$, $P = 0.042$), and appropriateness of pain medication ($F(2,119) = 12.007$, $P = 0.000$) differed between clusters. Parameters associated with an increased or decreased risk of being in a pain-diagnosed cluster will be discussed. The observed need for clinical interventions aiming at shifting from pain-diagnosed clusters to pain-relieved status will be reflected.

### PATIENT-REPORTED FACTORS ALLEVIATING PAIN AMONG PERSONS WITH CANCER

Dottington Fullwood,[1] Roger Fillingim,[2] and Diana Wilkie,[3] *1. The University of Florida, Gainesville, Florida, United States, 2. University of Florida, Pain Research and Intervention Center of Excellence, Gainesville, Florida, United States, 3. University of Florida, Gainesville, Florida, United States*

Pain impacts wellbeing and is among the most common symptoms of cancer. Factors that decrease pain severity have been understudied despite their importance for high-quality cancer care. The study purpose was to describe pain alleviating factors and their association with type of cancer. This secondary comparative analysis included 579 participants from studies of inpatients and outpatients with cancer (mean age=58.7±12.3; 27.3% female; 85.5% White, 5.7% Black, 7.6% Other). They completed the McGill Pain Questionnaire on paper or a tablet computer. To determine factors that alleviated pain, we focused on the open-ended question: 1) What kinds of things relieve your pain? We coded text responses into six outcome categories: 1) Activity level, 2) Cognitive, 3) Environmental, 4) Medical, 5) Physical, and 6) Sedentary behavior. We counted the number of activities/factors in each category and conducted multivariable regression analysis adjusting for sociodemographic constructs. Adjusted models revealed that activity ($\rho=0.02$), cognition ($\rho<0.001$) and medication ($\rho<0.001$) were more often endorsed as alleviating factors among individuals living with lung cancer compared to head and neck cancer participants. Those diagnosed with lung cancer ($\rho=0.02$) and males ($\rho=0.02$) utilized significantly less physical alleviating factors than head and neck cancer individuals and females. This is the first study to examine pain-alleviating factors among individuals living with cancer. These findings contribute new information regarding activities that alleviate pain among cancer survivors. These findings could inform interventions to promote safe, personalized care designed to alleviate cancer-pain.

### THE PAIN SITUATION OF OLDER HOME-CARE RECIPIENTS AND INFLUENCING FACTORS

Dagmar Dräger, Reinhold Kreutz, Adelheid Kuhlmey, Andrea Budnick, and Dagmar Draeger, *Charité - Universitätsmedizin Berlin, Berlin, Berlin, Germany*

Chronic pain is a common symptom among older people. The international prevalence rate reaches 50% for older home-care recipients (aged ≥60). The most common causes of pain among older people are degenerative arthropathy and musculoskeletal diseases. Care recipients (81% aged ≥65) constitute a specific sub-group among pain patients, due to the restrictions they experience. In Germany, the prevalence rate in this group is 70%. Currently, no comprehensive information on the pain situation of older home-care recipients exists in Germany. The findings presented are based on a cross-sectional study of older (aged ≥65) home-care recipients (SGB XI) in Berlin, with chronic pain (n=225), capable of self-report (MMST≥18). Structured interviews comprised the primary data source. The pain situation was determined using the German Brief Pain Inventory (BPI-NH). Multiple regression analysis was applied to test how the most severe pain (dependent variable) was influenced by socio-demographic and medical parameters, mental and physical restrictions and pain medication. Analyses of the pain situation show a value of M=4.81 (SD±1.88) on the BPI intensity index, and a BPI pain interference index of M=5.47 (SD±2.15). The most intense pain averaged 6.96 (SD±2.15). On average, respondents reported 16.20 (SD±13.25) pain locations (range: 0-65). The number of pain locations, alongside other factors, had a significant influence, $R^2$=0.038 (corrected $R^2$=0.034), F (1.219) = 8.760, p<0.01), on pain intensity. The findings show severe pain intensity among older home-care recipients not reported in previous findings (e.g. in long-term in-patient care). Action in medical care, nursing care and educational aspects is urgently needed.

### TRANSCRANIAL DIRECT CURRENT STIMULATION COMBINED WITH MEDITATION FOR OLDER ADULTS WITH KNEE OSTEOARTHRITIS

Hyochol Ahn, Lindsey Park, and Hongyu Miao, *University of Texas Health Science Center at Houston, Houston, Texas, United States*

Osteoarthritis (OA) of the knee is one of the most common causes of pain in older adults. Recent evidence suggests that knee OA pain is characterized by alterations in central pain processing in the brain. Two nonpharmacological pain treatments, transcranial direct current stimulation (tDCS) and mindfulness-based meditation (MBM), have been shown to improve pain-related brain function in older adults with knee OA. Because tDCS promotes neuroplasticity, it may potentiate the effect of MBM that also stimulates adaptive changes in the brain. However, no studies have examined whether tDCS combined with MBM can reduce OA symptoms in older adults with knee OA. Thus, the purpose of this study was to examine the preliminary efficacy of tDCS combined with MBM in older adults with knee OA. Thirty participants with knee OA were randomly assigned to receive 10 daily sessions of home-based 2 mA tDCS combined with active MBM for 20 minutes (n=15) or sham tDCS combined with sham MBM (n=15). We measured OA-related clinical symptoms using the Western Ontario and McMaster Universities Osteoarthritis Index (WOMAC). Participants (60% female) had a mean age of 59 years. Active tDCS combined with active MBM significantly reduced scores on the WOAMC (Cohen's d = 0.83, P = 0.02). Participants tolerated tDCS combined with MBM well without serious adverse effects. Our findings demonstrate promising clinical efficacy of home-based tDCS combined with MBM for older adults with knee OA. Future studies with larger-scale randomized controlled trials with follow-up assessments are needed to validate our findings.

### YOU HAVE ONE REMINDER: SELF-MANAGE YOUR PAIN

Marcia Shade,[1] Kyle Rector,[2] Rasila Soumana,[1] and Kevin Kupzyk,[1] *1. University of Nebraska Medical Center, Omaha, Nebraska, United States, 2. University of Iowa, Iowa city, Iowa, United States*

Pain has a significant impact in the lives of aging adults. As the population of America grows older, they need to be encouraged to use pain self-management strategies. Technology is an option, but current pain management applications have usability limitations in older adults. This feasibility study describes the usability of voice assistant reminders for pain self-management. We enrolled 15 community-dwelling aging adults with chronic pain. Participants created two pre-determined voice assistant reminder tasks: 1) to take scheduled pain medication, and 2) to write in a pain diary. We collected data on demographics, pain, confidence of managing symptoms, and objective ease of use. After four weeks, we collected information about subjective ease of use and usefulness of the voice assistant. Participants were mostly female, average age 65 years; reporting moderate pain severity 4.58 (SD 2.29) and pain interference 3.94 (SD 2.62). The mean PROMIS self-efficacy for managing symptoms score was 50.8 (SD 8.2). Voice assistant usability was above average (78 out of 100). The median time to make a voice assistant profile was five minutes (SD 7.5), with a median of seeking help two times. No significant relationships were found between pain and usability. Three participants made physical activity and distraction reminders to self-manage pain. Voice assistant reminders were perceived as consistent, easy to set up and helpful for accountability. Older users may provide helpful feedback for development and testing of voice assistant software for pain self-management. Voice assistants may provide helpful reminders that encourage the completion of pain self-management strategies.

### SESSION 2895 (POSTER)

### MEDICATION ISSUES

### A WASTEFUL AND HARMFUL PRACTICE: SLIDING SCALE INSULIN USE AMONG NURSING HOME VETERANS

Kenneth Lam,[1] Siqi Gan,[2] Bocheng Jing,[3] Brian Nguyen,[3] and Sei Lee,[2] *1. VA Medical Center--San Francisco, San Francisco, California, United States, 2. University of California, San Francisco, San Francisco, California, United States, 3. San Francisco VA Medical Center, San Francisco, California, United States*

The American Medical Directors Association and the American Diabetes Association discourage the use of sliding scale insulin (SSI) in nursing home residents with diabetes due to its association with hypoglycemia, hyperglycemia, nursing burden, and patient discomfort. However, prevalence of SSI use is unclear. We used Veterans Affairs (VA) data from October 2013 to September 2016 to determine the weekly prevalence of SSI among 22,847 veterans with diabetes admitted to VA nursing homes (NHs). Average age was 75.3 (SD 8.3) years, mean A1c was 7.3% (SD 1.6%) and 57% were admitted from hospital. We first identified residents receiving any short-acting insulin. We then classified short-acting insulin use into three mutually exclusive regimens: (1) fixed scheduled doses, (2) SSI, defined as a variable dose of short-acting insulin without a concurrent fixed dose or (3) bolus with correction (BWC), defined as a variable dose given concurrently with a fixed dose that day. During the first week of NH admission, 64.7% of residents with diabetes received no short-acting insulin, 7.4% received fixed scheduled doses, 6.3% received BWC and 21.4% were on SSI. At week 12, the prevalence of fixed dose and BWC regimens was unchanged from baseline (fixed dose = 8.4%; BWC = 7.0%). In contrast, the prevalence of SSI decreased weekly to 15.8% (p for linear trend < 0.0001). Although SSI prevalence decreased from week 1 to week 12, 51% of residents on short-acting insulin were still using SSI in their 12th week of their NH stay.

### ASSOCIATION BETWEEN PRESCRIPTION OPIOID USE AND MORTALITY IN COMMUNITY-DWELLING OLDER ADULTS

Carina D'Aiuto,[1] and Helen-Maria Vasiliadis,[2] *1. Université de Sherbrooke, Longueuil, Quebec, Canada, 2. University of Sherbrooke, Sherbrooke, Quebec, Canada*

Prescription opioid use is concerning among older adults. Yet, few studies have examined the impact of opioid use on mortality by considering multimorbidity. Our sample includes 1586 older adults aged ≥65 recruited in primary care from 2011-2013 in a large health administrative region in Quebec and participating in the ESA-Services study, a longitudinal study on aging and health service use. An opioid prescription delivered in the 3 years prior to the baseline interview was identified using the provincial pharmaceutical drug registry. Mortality was ascertained from the vital statistics registry until 2015. The presence of chronic diseases was based on self-reported and physician diagnostic codes in health administrative databases. Physical multimorbidity was defined as ≥3 chronic physical conditions from either source. Physical/psychiatric multimorbidity was defined as ≥3 chronic physical conditions and ≥1 common mental disorder from either source. Logistic regression analyses were conducted to examine the association between opioid use and mortality, controlling for sociodemographic factors. Interactions were tested for opioid use and multimorbidity. Older adults with physical multimorbidity using opioids were 1.76 (95%CI: 1.02-3.03) times more likely to die than those not using opioids. Those with physical/psychiatric multimorbidity using opioids were 2.27 (95%CI: 1.26-4.09) times more likely to die than those not using opioids. Older age, male sex, and single marital status significantly increased the risk of mortality. Overall, opioid use increases the risk of death in older adults with multimorbidity. The presence of mental disorders further increases the risk of death in older adults with physical multimorbidity using opioids.

### CAREGIVERS' PERSPECTIVES OF MEDICATION MANAGEMENT ADVICE FOR PEOPLE WITH DEMENTIA AT HOSPITAL DISCHARGE

Mouna Sawan,[1] Yun-Hee Jeon,[1] Christine Bond,[2] Timothy Chen,[1] Sarah Hilmer,[3] and Danijela Gnjidic,[1]
*1. The University of Sydney, Camperdown, Sydney, New South Wales, Australia, 2. University of Aberdeen, Aberdeen, Scotland, United Kingdom, 3. Kolling Institute of Medical Research, St Leonards, New South Wales, Australia*

People with dementia admitted to hospitals are more likely to be exposed to inappropriate polypharmacy and experience worse outcomes than people without dementia. Family and informal caregivers play an important role in managing medications across transitions of care; however, studies describing the experiences of medication guidance provided to caregivers at hospital discharge are limited. We have explored caregivers' perceptions on the quality of and factors that influence caregiver participation in medication guidance at discharge. A qualitative approach using semi-structured interviews was conducted with 29 caregivers of people with dementia across Australia by telephone. Purposive sampling was used to ensure maximum variation of diverse perspectives. Content analysis was used to derive themes. Three themes were derived from analysis: inconsistent approaches to provision of medication information at discharge, caregiver awareness to advocate for the care recipient and managing competing priorities. Some caregivers reported inadequate information was provided because the information was communicated to the patient without the caregiver being present. Other caregivers stated a medication list, discharge summary and discussion with a healthcare profession provided useful information. Caregiver involvement in discussions on medication guidance at discharge was influenced by caregiver awareness to advocate for the care recipient to ensure medication safety and managing competing priorities at the time of discharge to manage stress. Caregivers flagged the need to establish structured caregiver education at discharge and community-based services to manage medications safely. Future studies are needed to explore development of resources to caregiver encourage participation during medication guidance at discharge.

### EVALUATION OF A COLLABORATIVE CARE MANAGEMENT PROGRAM FOR COMMUNITY-DWELLING OLDER ADULTS ON HIGH-DOSE OPIATES

Shahrzad Mavandadi,[1] Kristin Foust-Montague,[2] Elizabeth Grecco,[2] David Oslin,[3] and Joel Streim,[3]
*1. Corporal Michael J Crescenz VA Medical Center, Aliso Viejo, California, United States, 2. Perelman School of Medicine at the University of Pennsylvania, Philadelphia, Pennsylvania, United States, 3. Perelman School of Medicine at the University of Pennsylvania, Philadelphia, United States*

Treating pain in later life is complex, and there are significant safety risks associated with the use of analgesics, particularly opioids. This study examined preliminary results from a pilot study of a telephone-delivered collaborative care service designed for community-dwelling older adults with chronic pain receiving prescriptions for high doses of opioids

(i.e., >120 mg morphine-equivalent dose). Eighty-two older adults referred by the Pennsylvania Department of Aging's pharmaceutical assistance program for low-income seniors (PACE/PACENET) were eligible and enrolled in the program (i.e., the University of Pennsylvania/PACE Behavioral Health Laboratory). Patients were on average 73.5 (+/-6.1) years old, and the majority were white (91%) and female (70%). Patients completed a comprehensive baseline clinical assessment capturing their mental health, cognition, pain and functional status, as well as self-reported daily opioid dose and biopsychosocial needs. Patients were considered engaged in the program if they completed 2+ additional follow-up contacts with a care manager. During these contacts, care managers offered individualized treatment planning, with the goal of opioid dose reduction to safer levels. Of the 82 patients completing the baseline, 53 (65%) engaged in the program. At their last clinical contact, 91% of engaged patients achieved dose reductions (with 66% achieving dose reductions of >20% and 30% reporting doses <120 mg morphine-equivalent dose). Engaged patients also reported significant reductions in pain severity (p=0.05) and depressive symptoms (p=0.003) at the last contact relative to baseline. Findings support the feasibility of a community-based, collaborative care model for pain management and suggest the potential for positive treatment outcomes.

### FACTORS ASSOCIATED WITH PRESCRIPTION OPIOID USE AMONG COMMUNITY-DWELLING OLDER ADULTS

Carina D'Aiuto,[1] and Helen-Maria Vasiliadis,[2]
*1. Université de Sherbrooke, Longueuil, Quebec, Canada, 2. University of Sherbrooke, Sherbrooke, Quebec, Canada*

Opioid use is a growing concern in North America, particularly among older adults. Despite the opioid crisis and the aging population, few studies have evaluated the factors associated with opioid use among older adults. Our sample includes 1657 people aged ≥65 years recruited in primary care clinics from 2011 to 2013 in the Montérégie region of Québec and participating in the "Étude sur la Santé des Aînés" ESA-Services study, a longitudinal study on aging and health service use. The presence of chronic diseases was identified through self-reported health survey data linked to health administrative data. Opioid prescriptions were identified using the provincial pharmaceutical drug registry for those covered under the public drug insurance plan. Logistic regression analyses were conducted to examine the factors associated with opioid use over a 4-year period. 31.9% of participants used opioids. Factors associated with opioid use included: female sex (OR=1.24, 95%CI: 1.01-1.53), annual household income of <$25,000 (OR=1.25, 95%CI: 1.01-1.55), level of social support (OR=0.85, 95%CI: 0.73-0.99), and presence of pain/discomfort (OR=1.66, 95%CI: 1.34-2.04). Further, participants with ≥3 chronic physical conditions also reporting anxiety and/or depression were 3.63 (95%CI: 1.83-7.18) times more likely to use an opioid than those with 0-2 chronic physical conditions and no common mental disorder. Moreover, those with moderate, high, and very high psychological distress were more likely to use an opioid than those with a low psychological distress. Our findings suggest that, among other factors, physical and psychiatric multimorbidity is strongly associated with prescription opioid use in older adults.

### NURSE PRACTITIONERS' EXPERIENCES OF POLYPHARMACY IN COMMUNITY-DWELLING OLDER ADULTS

Christine Pariseault, Nancy Sharts-Hopko, and Elizabeth Blunt, *Villanova University, Villanova, Pennsylvania, United States*

Numerous studies exist that define polypharmacy and its impact on health. Additionally, the literature is rich in studies documenting the benefits of care provided by nurse practitioners. A gap in research exists at the intersection of the value of nurse practitioners in caring for older adults and their management of polypharmacy. Coinciding with a growth of America's older adult population and the need for adequate care, the purpose of this study was to explore the experiences of nurse practitioners caring for older adults experiencing polypharmacy. A qualitative descriptive study was conducted using a purposive sampling of nurse practitioners who care for older adults. Interviews were conducted and data was analyzed for themes. Four themes emerged: defining polypharmacy, communicating and collaborating, clinical judgement of nurse practitioners in relation to polypharmacy, and medication issues of older adults. Major themes emerged that depict the complexity of medication management in older adults as well as the important role of NPs in providing care to older adults. The significance of the study findings to future practice includes improving communication and collaboration of prescribing health care providers, better identification and management of polypharmacy, and improving the health care delivered to older adults. Safe and effective prescribing for older adults requires NPs consider the unique needs of each older adult while utilizing technology to support collaboration and decision making.

### NURSE STAFFING AND NURSING HOME DEFICIENCY OF CARE FOR INAPPROPRIATE PSYCHOTROPICS USE IN RESIDENTS WITH DEMENTIA

Jung Min Yoon,[1] Alison Trinkoff,[1] Carla Storr,[1] and Elizabeth Galik,[2], *1. University of Maryland, Baltimore, Baltimore, Maryland, United States, 2. University of Maryland, Baltimore, Maryland, United States*

Psychotropics use to manage behavioral and psychological symptoms of dementia (BPSD) in nursing homes (NHs) has been the focus of policy attention due to their adverse effects. We hypothesized that NHs with lower nursing staffing would have greater reliance on psychotropics use to control BPSD. A NH deficiency of care can be cited for inappropriate psychotropics use (F-tag 758). The association between the occurrence of F-758 tags and nurse staffing in residents with dementia was examined using the 2017-18 Certification and Survey Provider Enhanced Reporting data (n=14,548 NHs). Staffing measures included nursing hours per resident day (HPRD) and registered nurse (RN) skill-mix. Generalized linear mixed models that included covariates (NH location, bed size, ownership, proportion of residents with dementia/depression/psychiatric disorders and with Medicare/Medicaid) estimated the magnitude of the associations. There were 1,872 NHs with F-758 tags indicating inappropriate psychotropics use for NH residents with dementia. NHs with greater RN and certified nurse assistant (CNA) HPRD had significantly lower odds of F-758 tags (OR=0.59 54, 95% CI=0.47 44-0.73 66; OR=0.87, 95% CI=0.77-0.99, respectively) and similar findings were found in NHs with greater RN skill-mix (OR=0.14 10, 95% CI=0.05 04-0.37 25). There were no significant associations between the occurrence of F-758 tags and licensed practice nurse and unlicensed nurse aide HPRD. This study found that RN and CNA staffing had inverse associations with inappropriate psychotropic use citations among residents with dementia. NHs with higher RN staffing ratios may be better able to implement alternatives to pharmacological approaches for BPSD. It is suggested that NHs be equipped with adequate nurse staffing levels to reduce unnecessary psychotropics use.

### PHARMACIST-LED AUDITS FOR OLDER ADULTS WITH CANCER YIELD SIGNIFICANT INTERVENTIONS

Hilary Erdeljac,[1] Stephanie Yager,[2] Megan McClain,[2] Sarah Wall,[2] Carolyn Presley,[2] Edmund Folefac,[2] and Ashley Rosko,[3] *1. The Ohio State University, Columbus, Ohio, United States, 2. James Cancer Hospital and Solove Research Institute at The Ohio State University Wexner Medical Center, Columbus, Ohio, United States, 3. The James Cancer Hospital, The Ohio State University, Columbus, Ohio, United States*

Older adults with cancer have comorbidities that require medical management and confounders of chemotherapy and supportive medications exacerbate polypharmacy. A multidisciplinary team model was created to address these needs within the Cancer Aging and Resiliency (CARE) clinic. To reconcile medications for accuracy, compliance, side effects, and effectiveness, a pharmacist-led audit includes identification of potential therapeutic duplications, drug-drug interactions, or potential medication inappropriateness identified using Beers criteria. A pharmacist led review of patient's prescriptions can identify drug therapy problems (DTP) and result in safer medication management. METHODS: A retrospective review of pharmacy specific interventions was conducted using CARE Clinic patient data from February 2016 to October 2019 evaluating data from n=259 patients. RESULTS: A preliminary analysis of n=137 patients who had received medication reconciliation were included. The mean number of medications per patient was 13.1 ± 5.7 and 457 DTP were identified leading to 523 medication related interventions. There was an average of 3.3 DTP per patient. The most common types of DTP included medication reconciliation (n=137, 30.0%), potentially inappropriate medication (PIM) (n=74, 16.2%), administration/technique (n=35, 7.7%), and drug-drug interaction (n=28, 6.1%). The most frequent types of interventions involved education to the patient (n=166, 31.7%), medication reconciliation (n=137, 26.2%), medication discontinuation (n=84, 16.1%), patient to discuss further with physician (n=39, 7.5%), and medication initiated (n=35, 6.7%). Updated results involving approximately 259 patients will be presented. CONCLUSION: Comprehensive medication review within a multidisciplinary setting for the management of older adults with cancer can reduce polypharmacy and inappropriate medication use.

### POLYPHARMACY AND POTENTIALLY INAPPROPRIATE MEDICATIONS IN PATIENTS WITH DEMENTIA

Ryo nonaka, Atsuhiro Kanno, Takahiro Ohara, Kazuhiro Sumitomo, Shigeru Sato, Isabelle Miyazawa, Nobuo Koinuma, and Katsutoshi Furukawa, *Tohoku Medical and Pharmaceutical University, Sendai, Japan*

INTRODUCTION: The use of polypharmacy and potentially inappropriate medication (PIM) is a critical issue in geriatrics. Furthermore, the number of patients with dementia is dramatically increasing worldwide. In this study, we investigated: (1) if the states of polypharmacy and PIM differed in patients with and without dementia; (2) the types of medicine that were commonly prescribed; (3) the types of dementia that resulted in the prescription of multiple medicines; (4) if there was a correlation between the number of medicines and the number of medical institutions (hospitals and clinics) that the patients attended. METHODS: In total, 216 patients who were 65 years of age and older were analyzed. The number of medicines prescribed and the medical institutions they attended were counted through the electronic medical charts of Tohoku Medical and Pharmaceutical University Hospital. We employed the Beers 2019 criteria for the definition of PIM. RESULTS: (1) The number of prescribed medicines was not significantly different between patients with dementia and those without dementia. (2) Antihypertensives and gastro-intestinal medicines were the most commonly prescribed medicines in patients with and without dementia. (3) Patients with "dementia with Lewy bodies" and "mixed dementia" were prescribed the highest number of medicines. (4) The number of medicines and PIM use were significantly and positively correlated with the number of medical institutions. CONCLUSIONS: The number of medical institutions strongly affected the number of medicines prescribed and PIM use. Efforts should be made to organize and reduce the number of medical institutions that a patient attends.

### POLYPHARMACY IN THE OLDER ADULT: MEDICATION REVIEW IN THE COMMUNITY

Staci Pacetti,[1] Margaret Avallone,[1] and Elyse Perweiler,[2] *1. Rutgers University-Camden, Camden, New Jersey, United States, 2. Rowan University School of Osteopathic Medicine, Stratford, New Jersey, United States*

Polypharmacy remains a significant healthcare issue in the United States, resulting in drug interactions, adverse drug reactions, and potentially dangerous complications. Polypharmacy, often defined as the simultaneous use of five or more medications, may lead to adherence problems and an increased risk of hospitalizations and death, particularly in the older population. Medication management was incorporated into undergraduate nursing clinical experience in an affordable housing urban community as part of the New Jersey Geriatric Workforce Enhancement Program (NJGWEP), a 5-year grant supported by DHHS-HRSA. This paper will describe the first phase of this project, which involved the determination of the prevalence of polypharmacy and high-risk medications in this setting. The charts of sixty residents were reviewed and along with demographic information, the following data was collected: total number of medications (prescription, non-prescription and herbal supplements), high-risk medications using Beers criteria and identification of common themes or issues. The average age of the residents was 72 years, with cardiovascular diseases and diabetes mellitus being the most common comorbidities. Fifty-two of the sixty residents (86%) received five or more medications daily, meeting the definition of polypharmacy. The average number of medications taken on a daily basis was 8.9. Among the residents, centrally-active agents such as gabapentin, tramadol and lorazepam were the most commonly prescribed medications. The second phase of this project will include implementation of a medication reconciliation process to identify potential issues using the Beers criteria and implement appropriate interventions to ensure safe medication practices in this high-risk population.

### PRESCRIBING TRENDS OF ANTICHOLINERGIC DRUGS BETWEEN 1989 AND 2017: A UK BIOBANK STUDY

Jure Mur,[1] Simon Cox,[1] Riccardo Marioni,[1] Tom Russ,[2] and Graciela Muniz Terrera,[3] *1. University of Edinburgh, Edinburgh, United Kingdom, 2. University of Edinburgh, Edinburgh, Scotland, United Kingdom, 3. University of Edinburgh, University of Edinburgh, Scotland, United Kingdom*

Prescription drugs with anticholinergic properties are commonly prescribed and negatively impact physical performance, cognitive function, and increase the risk of falls and dementia. The prevalence of anticholinergic drugs is high in later life, when there is an increased risk of adverse drug effects. Recent, in-depth longitudinal analyses of specifically anticholinergic prescribing in Europe is lacking. Prescriptions for the UK-Biobank participants (n=222,122) were ascertained from primary care electronic patient records. We assigned anticholinergic activity to each drug by using a composite score. We used linear regression to study the association between current anticholinergic burden and time period, explore secular trends in anticholinergic use, and various demographic factors. We further explored the results in the context of different classes of prescriptions drugs. 74 distinct drugs in the sample (1.1%) had anticholinergic effects. An individual's overall anticholinergic burden increased nonlinearly (linear estimate=0.474, quadratic estimate = 0.094, both p<2.2x10-16) between 1989 (mean=0.09, σ=0.009) and 2000 (mean=0.22, σ=0.006) and increased nonlinearly (linear estimate=0.282, quadratic estimate=0.074, both p<2.2x10-16) from 2000 to 2016 (mean=0.27, σ=0.009). The proportion of patients prescribed at least one anticholinergic drug per month increased from 6.1% to 16.7% from 1989 to 2000 and increased to 18.6% by 2016. When adjusted for sex and polypharmacy, age was negatively associated with recent cross-sectional anticholinergic burden (estimate=-0.042, p<2.2x10-16). Our results demonstrate an increase in prescribing of anticholinergic drugs over the past 30 years and indicate contemporary deprescribing of anticholinergic drugs in the later decades of life.

### SELF-MEDICATION PRACTICE AMONG OLDER ADULTS: PREVALENCE AND ASSOCIATED FACTORS

Medea Locquet,[1] Charlotte Beaudart,[1] Jean-Yves Reginster,[1] and Olivier Bruyère,[2] *1. University of Liège, Liège, Belgium, 2. University of Liège, liege, Belgium*

The self-medication practice, or the self-administration of medicine or dietary supplements without any physician's advice, represents a public health issue due to its potentially related adverse health events. In older adults, few data are available on self-medication behaviors. The main objective of our study was thus to assess the prevalence of self-medication in a population of older adults. As a secondary exploratory analysis, we seek to determine the determinants of self-medication practice and its association with adverse health outcomes. Our sample was comprised of men and women aged 65 and over from the SarcoPhAge study. Data regarding self-administration of substances were collected through self-administered questionnaire. Clinical and socio-demographic characteristics of the participants as well as medical history were recorded by the investigator. A total of 272 older adults were recruited with a mean age of 77.3±5.2 years. Among them, 119 (43.8%) reported resorting to self-medication (38.6% self-administration of vitamins and dietary supplements, 28.5% self-administration of drugs). Participants which resort to self-medication were older (78.9±4.7 years versus 77.1±3.9 years, p=0.04) and had a lower body mass index (25.7±3.9 versus 27.3±5.2, p=0.006). After adjustments for confounding factors, significant associations were noted between self-medication practice and fractures (OR=3.36[1.02-11.28]) and hospitalizations (OR=1.77 [1.03-3.05]) occurred during the previous year. The practice of self-medication seems widespread among the older age. It seems more frequent in more vulnerable subjects and having experienced adverse health outcomes. This type of health practice must therefore attract the attention of health actors.

### TEACHING NURSE PRACTITIONER STUDENTS ABOUT POLYPHARMACY THROUGH A LIVED EXPERIENCE

Jennifer Kim, Kanah Lewallen, and Taylor Boll, *Vanderbilt University, NASHVILLE, Tennessee, United States*

Polypharmacy (typically defined as the concomitant use of 5 or more medications) affects 40-50% of older adults in the U.S., and is associated with geriatric syndromes, a higher risk of medication non-adherence, and adverse drug events. Medication non-adherence is a common frustrating clinical issue for clinicians who provide care for older adult patients. Simultaneously, patients often find medication regimens to be complicated and confusing. This may contribute to medication non-adherence, which may further lead to adverse drug events and/or negative health outcomes. The more medications a patient is taking, the higher the risk for non-adherence. Thirty-eight students enrolled in an adult-gerontology primary care nurse practitioner program were given a bag of five mock medications that are commonly prescribed for older adults. Students were instructed to follow the directions on each of the bottles for approximately one month. A private messaging system was available for students if refills were needed or if they had questions about their medications. A debriefing session for this month-long, ungraded simulation was held, at which time students returned medication bottles. Pill counts were not analyzed, but all returned bottles contained mock medications. Approximately 52.6% of students estimated adhering to the medication regimen 0-24% of the time, whereas 26.3% reported an adherence rate of 25-50%. The most commonly cited barrier to adherence (55.3%) was “forgetfulness”. Nearly all students (89.5%) reported that the exercise “very much” increased their awareness of challenges patients face when managing medications, and 97% cited an increased awareness of ways to improve medication adherence.

### THE INVISIBLE EPIDEMIC: A SPOTLIGHT ON THE OPIOID CRISIS AMONG OLDER ADULTS

Jessica Hsieh,[1] Lynn McDonald,[1] Adriana Shnall,[2] Raza Mirza,[3] Samir Sinha,[4] Andrea Austen,[5] Laura Tamblyn Watts,[6] and Christopher Klinger,[1]
*1. University of Toronto, Toronto, Ontario, Canada,*
*2. Baycrest Health Sciences, North York, Canada,*
*3. National Initiative for the Care of the Elderly, Toronto, Ontario, Canada, 4. Sinai Health, Toronto, Canada, 5. City of Toronto, Toronto, Ontario, Canada, 6. CanAge, Toronto, Canada*

Canada is facing an opioid crisis, with more than 3,900 related deaths occurring in 2017. Almost 30% of those deaths were among seniors; older adults also have the highest rates of opioid hospitalization and poisoning. Despite these statistics, little is known about the specifics. A comprehensive scoping review following Arksey and O’Malley’s (2005) framework was conducted to establish the magnitude of the problem, describe treatment approaches alongside prevention, and identify implications for practice, policy and research. Eight major electronic databases in medicine and the social sciences were searched alongside the grey literature, and a stakeholder consultation convened to validate results. A total of 6,814 articles, reports and thesis were identified. Forty-five sources met inclusion criteria, the majority stemming from the United States. Most were literature reviews, cohort and cross-sectional studies, with almost half also taking a gendered approach. Four predominant themes emerged from the thematic content analysis: 1) Medical Applications of Opioids; 2) Problematic Opioid Use; 3) Treatment and Prevention Strategies; and 4) Recommendations. Data highlighted ‘the invisible epidemic’, with treatment strategies to be specifically tailored to this population in light of metabolic differences and drug interactions as part of aging. Seniors are part of the current epidemic, with tailored approaches needed to ensure adequate, evidence-based counteraction – including seniors’ voices alongside further research in this regard. Education and training for prescribers needs to be enhanced alongside cross-jurisdictional drug monitoring programs to avoid drug interactions and misuse/fraud.

### SESSION 2899 (POSTER)

### CARDIOVASCULAR HEALTH AND DISEASE

### A SAS MACRO FOR COSINOR ANALYSIS OF AUTOMATED HEART RATE DATA IN THE ICU

Margaret Doyle, Terrence Murphy, and Melissa Knauert, *Yale University School of Medicine, New Haven, Connecticut, United States*

Cosinor analysis, developed by Franz Hallberg and colleagues in the 1960s, allows for the fitting of a cosine curve to data of known period and is frequently used in the analysis of circadian data. While software packages exist which will perform cosinor analyses, SAS has historically steered users to a reference (Ferrarra, 1993) that contains a flaw that results in miscalculation of the acrophase (peak) and nadir (minimum) values about half the time. The R package ‘cosinor’ includes the same error as the SAS reference. The macro we have written 1.) outputs a dataset with the correct values of acrophase, mesor, amplitude, nadir p-value for rhythmicity and R-squared for a curve fit to an individual’s data and 2.) plots the resulting curve and underlying data. While the macro does not allow for the simultaneous analysis of data from multiple individuals it will output the data from multiple curves so that these parameters can be used in downstream data analysis. We demonstrate the use of this macro on a dataset of automated heart rate data collected on patients in the intensive care unit. Given the growing use of automated data collection, it is vitally important that software that provides accurate statistical analysis of circadian phenomena be made widely available to clinical investigators.

### ASSOCIATION BETWEEN FRAILTY AND GUT MICROBIOTA METABOLITE TMAO IN OLDER PEOPLE WITH CARDIOVASCULAR DISEASE

Wei He, Hua Wang, and Jiefu Yang, *National Center of Gerontology, Beijing, China*

The potential for the gut microbiota to affect health has particular relevance for older adults. Recent evidence suggests that microbiota-derived metabolites may modulate aging-related changes in immunity, sarcopenia, and cognitive function, all of which are elements of frailty. Trimethylamine N-oxide (TMAO) produced by the metaorganismal metabolism of choline, has been implicated in disease pathogenesis. However, relatively little geroscience research has been carried out on TMAO , and even less on other gut microbiota metabolites. The purpose of this study was to explore the relationship between frailty and circulating TMAO concentration. Data and fasting blood samples came from a prospective comprehensive geriatric assessment cohort of older adults ($age \geq 65$, n=451) with cardiovascular diseases. The frailty index based on the accumulated deficits model (48 variables) was used for evaluating the status of frailty. TMAO levels differed between groups with a significant increase for people with frailty ($p < 0.001$). Compared with the lowest quartile of TMAO levels, patients in the highest quartile had increased 3.07-fold risk of frailty (OR=3.07, 95%CI,

1.69-2.97). After adjusting for age, gender, BMI, history of diseases, hsCRP, LDLc, TMAO levels remained associated with frailty (OR=2.11, 95%CI, 1.01-4.38). Similarly, a cubic spline curve showed a dose-dependent relationship between the odds ratio for the risk of frailty and circulating TMAO in a linear trend (p = 0.006). This study suggests that circulating TMAO are independently associated with frailty in older adult with cardiovascular diseases. Efforts to further characterize the relationship between gut microbiota metabolite and frailty should be further pursued.

### ASSOCIATION OF BRAIN NATRIURETIC PEPTIDE WITH MORTALITY IN EXCEPTIONALLY LONG-LIVED FAMILIES

Allison Kuipers,[1] Robert Boudreau,[1] Mary Feitosa,[2] Angeline Galvin,[3] Bharat Thygarajan,[4] and Joseph Zmuda,[1] *1. University of Pittsburgh, Pittsburgh, Pennsylvania, United States, 2. Washington University School of Medicine, St Louis, Missouri, United States, 3. University of Southern Denmark, Odense C, Midtjylland, Denmark, 4. University of Minnesota, Minneapolis, Minnesota, United States*

Natriuretic peptides are produced within the heart and released in response to increased chamber wall tension and heart failure (HF). N-Terminal prohormone Brain Natriuretic Peptide (NT-proBNP) is a specific natriuretic peptide commonly assayed in persons at risk for HF. In these individuals, NT-proBNP is associated with future disease prognosis and mortality. However, its association with mortality among healthy older adults remains unknown. Therefore, we determined the association of NT-proBNP with all-cause mortality over a median follow-up of 10 years in 3253 individuals free from HF at baseline in the Long Life Family Study, a study of families recruited for exceptional longevity. We performed cox proportional hazards analysis (coxme in R) for time-to event (mortality), adjusted for field center, familial relatedness, age, sex, education, smoking, alcohol, physical activity, BMI, diabetes, hypertension, and cancer. In addition, we performed secondary analyses among individuals (N=2457) within the normal NT-proBNP limits at baseline (<125pg/ml aged <75 years; <450pg/ml aged ≥75 years). Overall, individuals were aged 32-110 years (median 67 years; 44% male), had mean NT-proBNP of 318.5 pg/ml (median 91.0 pg/ml) and 1066 individuals (33%) died over the follow-up period. After adjustment, each 1 SD greater baseline NT-proBNP was associated with a 1.30-times increased hazard of mortality (95% CI: 1.24-1.36; P<0.0001). Results were similar in individuals with normal baseline NT-proBNP (HR: 1.21; 95% CI: 1.11-1.32; P<0.0001). These results suggest that NT-proBNP is a strong and specific biomarker for mortality in older adults independent of current health status, even in those with clinically-defined normal NT-proBNP.

### EXAMINING ANKLE FOOT ORTHOSIS WEAR TIME IN PATIENTS WITH PERIPHERAL ARTERY DISEASE

Sara Myers,[1] Danae Dinkel,[1] Mahdi Hassan,[2] Holly Despiegelaere,[3] Jason Johanning,[4] and Iraklis Pipinos,[4] *1. University of Nebraska at Omaha, Omaha, Nebraska, United States, 2. University of Nebraska Omaha, Omaha, Nebraska, United States, 3. VA Medical Center, Omaha, United States, 4. University of Nebraska Medical Center, Omaha, Nebraska, United States*

Peripheral artery disease (PAD) impacts over 8.5 million Americans and the prevalence of PAD increases with age. PAD restricts blood flow to the leg and its most common manifestation is claudication, a severe impairment of walking produced by ischemia-related, leg pain during exercise. An ankle foot orthosis (AFO) could improve these symptoms. To understand the potential impact of AFO usage, it is critical to determine wearability of the device in patients with PAD. The purpose of this study was to monitor wear time of an AFO and explore perceptions of the device. Participants (n=14) with PAD and claudication wore an AFO for three months. An accelerometer was placed directly on the AFO for 7 days and participants completed semi-structured interviews at midpoint (1.5 months) and post intervention (3 months). Based on accelerometer data at midpoint participants wore the AFO for an average of 4.9±2.3 out of 7 days and for an average of 7.5±4.2 hours each day. At post, participants wore the AFO for an average of 4.8±2.2 days for an average of 7.4±4.6 hours per day. In the interviews, almost all participants noted multiple barriers to wearing the AFO such as difficulty putting the AFO on and off, using stairs, walking on uneven ground, and driving. Our study found that participants wore the AFO ~7 hours/day but experienced barriers which may have limited their wear outside of these monitoring periods suggesting patients would wear an assistive device if design could be improved to address barriers.

### IDEAL CARDIOVASCULAR HEALTH IS ASSOCIATED WITH SLOWNESS AMONG COMMUNITY-DWELLING OLDER ADULTS

Heeeun Jung,[1] Miji Kim,[2] Hayoung Shim,[3] Yuri Seo,[1] Seoyoon Jane Lee,[4] and Chang Won Won,[5] *1. Graduate School, Kyung Hee University, Seoul, Republic of Korea, 2. College of Medicine, Kyung Hee University, Seoul, Republic of Korea, 3. Kyung Hee University, Seoul, Korea, Seoul, Republic of Korea, 4. Elderly Frailty Research Center, Kyung Hee University, Seoul, Republic of Korea, 5. Kyung Hee University Medical Center, Seoul, Korea, Seoul, Republic of Korea*

Slowness is associated with increased disability and mortality in older people. However, the relation between ideal cardiovascular health (CVH) and slowness in community-dwelling older adults is uncertain. We examined the prevalence of ideal CVH in Korean older adults and its association with slowness in community-dwelling older adults. We analyzed 2,597 participants (mean age 76.0±3.9 years, 54.4% women) without cardiovascular disease from the Korean Frailty and Aging Cohort Study. The usual gait speed over a distance of 4 m was measured using an automatic timer, and slowness was defined as a speed <1.0 m/s. Ideal CVH was described as attainment of ideal health behaviors (no smoking, regular physical activity, ideal body mass index, and healthy diet) and optimal health factors (blood pressure, HDL-cholesterol, and glycated hemoglobin). Multiple logistic regression analysis was used to examine the association between the CVH score and slowness. Ideal CVH was present in 785(30.2%) subjects. Considering those with poor level of CVH were as the reference group, the odds ratios

[OR] for slowness were 0.55 (95% confidence interval [CI], 0.39-0.77) for those with intermediate level of CVH, and 0.28 (95% CI, 0.17-0.45) for those with ideal level of CVH after adjustment for potential confounders. Among ideal CVH components, behavioral CVH score (OR 0.65, 95% CI 0.58-0.74) was significantly associated with slowness vs. the biological CVH score (OR 0.95, 95% CI 0.84-1.07). This study indicates that ideal CVH is significantly associated with a lower risk of slowness in community-dwelling older adults. A better CVH may help prevent slowness.

### INVESTIGATION OF ROLL-OVER CHARACTERISTICS IN HEALTHY OLD INDIVIDUALS AND PATIENTS WITH PERIPHERAL ARTERY DISEASE

Sara Myers,[1] Ganesh Bapat,[1] Jason Johanning,[2] and Iraklis Pipinos,[2] *1. University of Nebraska at Omaha, Omaha, Nebraska, United States, 2. University of Nebraska Medical Center, Omaha, Nebraska, United States*

Atherosclerotic blockages in the leg arteries, cause leg pain with ambulation (called claudication), impair gait and substantially reduce the walking ability of patients with peripheral artery disease (PAD). Ankle-foot orthoses are being developed and applied so patients can walk more and with less pain. Roll-over shape (ROS) is a potential design objective for such assistive devices. In the proposed work, we study roll-over characteristics in patients with PAD and healthy older subjects. Gait data of ten healthy older individuals (Age: 72.8 ± 5.5 years) and twenty patients with PAD (Age: 64.1 ± 6.6 years) were collected at self-selected walking speed. In patients with PAD, gait data were collected before the onset of pain and after claudication pain was induced. To generate ROS, the center of pressure data was transformed to the shank-based coordinate system and circular arcs were fit using an optimization program in MATLAB. Independent t-tests with Bonferroni corrections were used to separately compare roll-over radius differences (p<0.05) between healthy older to both walking conditions in patients with PAD. The mean roll-over radius was not significantly different between healthy older vs PAD pain-free (p=0.468) or PAD pain-induced (p=0.289) walking conditions. Our results indicate invariance of ROS radius in patients with PAD, which is consistent with previous literature showing general invariance of ROS in healthy young individuals. Previous biomechanical studies show gait kinematics and kinetics are more affected by PAD than by age. Future studies should focus on the potential adaptive mechanisms in patients with PAD achieving invariant ROS.

### LONG LIFE FAMILY STUDY SHOWS REDUCED CORONARY ARTERY DISEASE DESPITE HIGH POLYGENIC HAZARD SCORES

Mary Feitosa,[1] Allison Kuipers,[2] Mary Wojczynski,[1] Lihua Wang,[1] Thomas Perls,[3] Kaare Christensen,[4] Joseph Zmuda,[2] and Michael Province,[5] *1. Washington University School of Medicine, St Louis, Missouri, United States, 2. University of Pittsburgh, Pittsburgh, Pennsylvania, United States, 3. Boston University School of Medicine, Boston, Massachusetts, United States, 4. University of Southern Denmark, Odense C, Syddanmark, Denmark, 5. Washington University School of Medicine, SAINT LOUIS, Missouri, United States*

Polygenic hazard scores (PHS) for coronary artery disease (CAD) quantify individuals with age-specific genetic risk for CAD. We evaluated how well the PHS predict age at onset of CAD in the Long Life Family Study (LLFS; families selected for exceptional longevity), compared to the Family Heart Study (FamHS; random families and high CAD-risk families). LLFS contains 4572 European ancestry (EA) individuals from 581 families (age: 74.0 ± 14.3, range: 22-110 years). FamHS Random has 1806 EA individuals from 454 families (age: 56.2 ± 13.5, range: 22-91 years), while FamHS High CAD-risk has 2301 EA individuals from 553 families (age: 53.2 ± 12.8, range: 21-93 years). We generated the PHS from 176 published SNPs from GWAS for CAD (p< 5.0 x 10-8, r2< 0.2). In each of the extremes of the CAD PHS distributions (75%), Kaplan-Meier method showed that the LLFS presented significant delayed age at onset of CAD compared to FamHS (random and High CAD-risk: P<0.0001). A Cox proportional hazards regression model accounting for CAD age at onset, using family bootstrap (N= 1000) to correct for family relatedness, replicated these results. For example, in the top-25% CAD-PHS when comparing to FamHS high-risk, the LLFS CAD hazards ratio was 0.127 (95% CI: 0.099, 0.164). Our findings suggest that, while PHS captured some of the risk of CAD in LLFS, part of the predisposition remains to be determined. Other relevant factors, including additional genetic discoveries and lifestyle-environment influences are needed to fully determine CAD risk in extreme samples.

### SYSTEMATIC REVIEW OF GUIDELINES ON ANTIHYPERTENSIVE TREATMENT IN OLDER ADULTS: SPRINTING TO MORE HETEROGENEITY?

Jonathan Bogaerts,[1] Leonie von Ballmoos,[2] Wilco Achterberg,[1] Jacobijn Gussekloo,[1] Sven Streit,[2] Milly van der Ploeg,[3] Yvonne Drewes,[1] and Rosalinde Poortvliet,[1] *1. Leiden University Medical Center, Leiden, Zuid-Holland, Netherlands, 2. University of Bern, Bern, Bern, Switzerland, 3. Leiden University, Leiden, The Hague, Netherlands*

Clinical trials have demonstrated that antihypertensive treatment (AHT) in older adults is beneficial. Longitudinal studies, in contrast, have shown that low blood pressure is associated with higher all-cause mortality, especially in frail older adults. Despite the high quality of the available evidence, its translation into clinical guidance for the heterogeneous older population is challenging. To give a systematic overview of blood pressure targets for older adults recommended in clinical guidelines, we searched PubMed, Embase, Emcare, and five guideline databases. We selected guidelines with numerical thresholds for the initiation or the goal of non-disease-specific AHT (January 2008-October 2019). Guidelines with advices concerning AHT in older adults were analyzed. We appraised the guideline quality with the AGREEII-instrument. Of the 44 guidelines containing a numerical threshold for the initiation or the goal of AHT, 33 (75%) provided recommendations concerning AHT for older adults. Nineteen advised a higher target of systolic blood pressure (SBP) for older adults in comparison with the middle-aged population and 3 more recent advised a lower target. Over half (19/33) recommended to treat hypertension in the oldest old to a SBP <150 mmHg, while others advised intensive treatment to targets <120 mmHg. Although many guidelines mentioned frailty, only three gave specific thresholds and targets for frail older adults. The quality of the guidelines was not related with the recommended targets. Targets of AHT in older adults in international guidelines range from less strict to more intensive in comparison with the middle-aged and are set on chronological rather than biological age.

### THE DECREASED COMPLEXITY OF BLOOD PRESSURE DYNAMICS IS ASSOCIATED WITH HIGHER WHITE MATTER LESIONS IN OLDER ADULTS

Xin Jiang,[1] Xia Gao,[2] Hui zhang,[1] Wuhong Deng,[1] Wen Fu,[1] Brad Manor,[3] Lewis Lipsitz,[4] and Junhong Zhou,[5] *1. Shenzhen People's Hospital, Shenzhen, Guangdong, China, 2. Shenzhen People's Hospital, Shenzhen, China, 3. Hebrew SeniorLife/Harvard Medical School, Boston, Massachusetts, United States, 4. Hebrew SeniorLife and Harvard Medical School, Boston, Massachusetts, United States, 5. Harvard Medical School/ Hebrew SeniorLife, Roslindale, Massachusetts, United States*

White matter lesions (WML) are highly prevalent in older adults and thought to represent cerebral microvascular disease, contributing to slow gait and dementia. Hypertension is associated with WML. However, the underlying mechanism of this association is unclear. The complex beat-to-beat BP fluctuations represent the influence of BP regulatory mechanisms over multiple time scales. The association between WML and abnormalities in BP regulation may be manifest as a loss of complexity in BP dynamics. The aim of this study is thus to explore the relationships between hypertension, BP complexity, and WML in older adults. Twenty-two older adults with hypertension (SBP>140 mmHg) and 19 age-matched older adults without hypertension (i.e., control) completed this study. Their whole-brain WML were assessed by two neurologists using the Fazekas Scale. Greater score reflects higher WML grade. Each participant completed a 10-minute BP assessment when sitting quietly following the MRI. The continuous SBP and DBP series were recorded, and the complexity of them was quantified using multiscale entropy (MSE). Lower MSE reflects lower complexity. Compared to the controls, hypertensives had significantly greater Fazekas scores (i.e., higher WML grade) (F=4.8, p=0.02) and lower complexity of SBP and DBP (F>3.7, p<0.01), after adjusting for age. Across two cohorts, those with lower SBP and DBP complexity had higher Fazekas score (r<-0.51, p<0.01), and this association was independent of age and group. These results suggest that WML are associated with a loss of complexity in BP dynamics. Future longitudinal studies are needed to examine the causal relationship between WML and BP.

### VARIATION IN THE ASSOCIATION OF BODY TYPES TO DECREASED HIGH-DENSITY LIPOPROTEIN IN OLDER ADULTS (NHANES 2005-2014)

Queendaleen Chukwurah, *Tulane University, New Orleans, Louisiana, United States*

General obesity and central obesity represent cardiovascular disease risk factors and are known to be related to dyslipidemia. I examine the variation in the association of combined body mass index/waist circumference classification to decreased high-density lipoprotein cholesterol (HDL-C). Body mass index /waist circumference (WC) cut off values were used to create six body types: normal weight with normal WC (NWT-NWC), overweight with normal WC (OWT-NWC), obese with normal WC (O-NWC), normal weight with high WC (NWT-HWC), overweight with high WC (OWT-HWC), and obese with high WC (O-HWC). HDL-C was defined as decreased if < 40 mg/dl for men or < 50 mg/dl for women and normal if ≥ 40 mg/dL for men or ≥ 50 mg/dL for women. Sample population included 5,772 participants of the National Health and Nutrition Examination Survey (NHANES 2005-2014) aged 50 years and older. The mean (SD) age was 61.8 (0.2), and 50.5% were females, while 10% were minority. The prevalence of decreased HDL-C was 29.1%. Analysis involved weighted multivariable logistic regression adjusted for age, race-ethnicity, gender, education, poverty-income-ratio, smoking, and alcohol intake. Regression reveals a higher likelihood of decreased HDL-C for OWT-NWC (aOR 2.12 95% CI 1.43,3.15 ), NWT-HWC (aOR 2.57 95% CI 1.59,4.16 ), OWT-HWC(aOR 3.09 95% CI 2.29,4.15 ), and O-HWC (aOR 5.30 95% CI 4.01,6.86 ) when compared to NWT-NWC. These associations are important to public health practice and policies as it demonstrates the implications of the parallel use of anthropometric measures for all body weights in health-risk assessments of older adults.

### SESSION 2900 (POSTER)

### SENSORY HEALTH AND IMPAIRMENT

### A CONCEPTUAL FRAMEWORK FOR ENGAGED COMMUNICATION AT ADULT DAY SERVICES: A MIXED-METHODS STUDY

Sara Mamo,[1] Kara Wheeler,[1] and Olivia Perry,[2] *1. University of Massachusetts Amherst, AMHERST, Massachusetts, United States, 2. University of Massachusetts Amherst, Amherst, Massachusetts, United States*

Adult Day Services provide an opportunity for social engagement for older adults who might otherwise become isolated. Communication environments at many Adult Day Centers can be difficult for participants due to the high prevalence of hearing loss and poor acoustics in large activity rooms. The purpose of this study is to understand the hearing and social health status of participants across multiple group care settings as well as participants' challenges and motivations to engage in social communication. A mixed methods approach was undertaken. Seventy-two participants from two Programs for All-inclusive Care for the Elderly (PACE®) completed quantitative measures: hearing test, cognitive screener (MOST™), IOM Social and Behavioral Determinants of Health, UCLA Loneliness Scale, and Instrumental Activities of Daily Living. Using maximum variation sampling based on hearing status and UCLA loneliness scores, ten participants were invited to complete one-on-one semi-structure interviews. Interviews aimed to learn more about how and why participants did and/or did not engage in social communication with other PACE

participants. Transcripts were coded and thematic analysis was used to identify common barriers and motivations to communication. A conceptual framework was developed by integrating quantitative and qualitative findings to recognize what contributes to meaningful interactions or engaged communication. The findings from this study will contribute to the development of an intervention to address hearing loss and support communication for older adults in group care settings. In order to maximize the potential benefit of attending group-based day services, the communication barriers and motivations of older adults need to be addressed.

### DUAL EFFECTS OF HEARING LOSS AND DEMENTIA ON FUNCTIONAL LIMITATIONS IN OLDER ADULTS

Michael McKee,[1] Yunshu Zhou,[1] Joshua Ehrlich,[1] Elham Mahmoudi,[1] Jennifer Deal,[2] and Nicholas Reed,[3]
*1. University of Michigan, Ann Arbor, Michigan, United States, 2. Johns Hopkins Bloomberg School of Public Health, Baltimore, Maryland, United States, 3. Johns Hopkins University, Baltimore, Maryland, United States*

Age-related hearing loss (HL) is both common and associated with elevated risk for cognitive decline and poorer health. To care for an aging population, it is critical to understand the effect of coexisting HL and dementia on functional activities. The effect of co-existing dementia and self-reported HL on daily functioning were assessed. A cross-sectional analysis was performed using nationally-representative data from the 2015 National Health and Aging Trends Study consisting of U.S. adults 65+. The sample included 1,829 adults with HL (22.8%) and 5,338 adults without HL. Multivariable Poisson regression was used to model the independent effects and interaction of self-reported HL and dementia status on three validated functional activity scales (self-care, mobility, and household). All analyses adjusted for sociodemographic and medical factors. HL participants were more likely to be white, older, male, less educated ($p <0.01$). 8.4% had possible dementia and 6.5% had probable dementia. Respondents with HL or possible or probable dementia had significantly lower mobility, self-care, and household activity scores ($p<.001$ for all comparisons) compared to their peers. A small yet significant interaction was present in all models, suggesting that HL respondents with co-occurring dementia had lower mobility, self-care, and household activity scores than predicted by the independent effects of dementia and self-reported HL ($p<.001$ for all comparisons). Older adults with co-occurring dementia and HL are at increased risk for poor functioning and should be screened by healthcare providers. Future work should consider the impact of intervention in this vulnerable/at-risk population.

### HEARING LOSS AND HEALTHCARE SATISFACTION

Anna Jilla, and Nicholas Reed, *Johns Hopkins University, Baltimore, Maryland, United States*

Patient satisfaction with care has become increasingly important since Medicare's shift to value-based reimbursement models using the Hospital Care Quality Information from the Consumer Perspective survey. Effective communication plays an underappreciated role in satisfaction with care. Hearing loss impacts two-thirds of adults over 70 years and is a barrier to communication. Previous research has found that adults with hearing loss have poorer health outcomes and incur higher medical expenditures. The present study aims to explore the association of hearing loss on satisfaction with care. Nationally representative data from the 2015 Medicare Current Beneficiary Survey (MCBS) collected information on self-reported hearing with a hearing aid (no trouble, a little trouble, or a lot of trouble) and satisfaction with quality of health care (very satisfied/satisfied and dissatisfied/very dissatisfied) for all adult Medicare beneficiaries. A weighted sample of 48.6 million Medicare Beneficiaries was analyzed using logistic regression, adjusted for sex, race, educational attainment, income, general health, and functional limitations in instrumental activities of daily living. The adjusted model found that compared to adults with no hearing trouble, those with a little trouble hearing and a lot of trouble hearing had 1.47 times (95% CI: 1.06, 2.03) and 1.74 times higher odds (95% CI: 1.15, 2.62) higher odds of reporting dissatisfaction with care, respectively. Hearing loss, possibly mediated by its impact on communication, is associated with satisfaction with care. Given the emphasis placed on patient-reported satisfaction value-based reimbursement programs, hearing loss may represent a potential, low-risk/high-reward area of intervention to improve satisfaction.

### HEARING LOSS HEALTH LITERACY DISCREPANCY BETWEEN YOUNGER AND OLDER ADULTS

Erika Squires, and Hua Ou, *Wayne State University, Detroit, Michigan, United States*

Increasing the accessibility and affordability of hearing healthcare is a public health concern. Because low health literacy is a significant barrier to the use of existing effective healthcare services, it is critical to assess and understand health literacy deficits specific to hearing loss before implementing interventions. The purpose of this cross-sectional study was to identify differences in hearing loss health literacy among older and young adults, which is warranted because older adults are at-risk for lower levels of health literacy compared to their younger counterparts. Adults across the lifespan ($n = 170$) completed the Hearing Loss Health Literacy Assessment Tool, which includes self-rated ability to access/obtain, understand, and appraise hearing health information, as well as apply information to manage life with hearing loss. Results from an independent samples t-test indicated that older adults ($M = 6.3$, $SD = 1.45$, $n = 54$) self-reported significantly higher overall hearing health literacy than younger adults ($M = 5.37$, $SD = 1.27$, $n = 116$), $t(168) = 4.22$, $p < 0.0001$. Participants rated their ability to access/obtain information significantly lower than the other subscales. Age-differences in self-rated hearing health literacy exist. Findings from this study receive support from evidence indicating that the readability and suitability of the majority of patient education materials on hearing loss are not appropriate for the average U.S. adult. This investigation provides further evidence that the availability and accessibility of patient education materials on hearing loss is an important barrier that contributes to the limited use of hearing health care.

### MODELING HEARING LOSS PROGRESSION AND ASYMMETRY IN THE OLDER OLD: A NATIONAL POPULATION-BASED SURVEY

Rahul Sharma,[1] Anil Lalwani,[1] and Justin Golub,[2]
*1. Columbia University Irving Medical Center,*

*New York, New York, United States, 2. NewYork-Presbyterian Hospital/Columbia University Irving Medical Center, New York City, New York, United States*

The progression and asymmetry of age-related hearing loss has not been well characterized in those 80 years of age and older because public datasets mask upper extremes of age to protect anonymity. We aimed to model the progression and asymmetry of hearing loss in the older old using a representative, national database. This was a cross-sectional, multicentered US epidemiologic analysis using the National Health and Nutrition Examination Study (NHANES) 2005-2006, 2009-2010, and 2011-2012 cycles. Subjects included non-institutionalized, civilian adults 80 years and older (n=621). Federal security clearance was granted to access publicly-restricted age data. Outcome measures included pure-tone average air conduction thresholds and the 4-frequency pure tone average (PTA). 621 subjects were 80 years old or older (mean=84.2 years, range=80-104 years), representing 10,600,197 Americans. Hearing loss exhibited constant acceleration across the adult lifespan at a rate of 0.0052 dB/year2 (95% CI = 0.0049, 0.0055). Compounded over a lifetime, the velocity of hearing loss would increase five-fold, from 0.2 dB loss/year at age 20 to 1 dB loss/year at age 100. This model predicted mean PTA within 2 dB of accuracy for most ages between 20 and 100 years. There was no change in the asymmetry of hearing loss with increasing age over 80 years (linear regression coefficient of asymmetry over age=0.07 (95% CI=-0.01, 0.24). In conclusion, hearing loss steadily and predictably accelerates across the adult lifespan to at least age 100, becoming near-universal. These population-level statistics will guide treatment and policy recommendations for hearing health in the older old.

### RANDOMIZED CONTROLLED TRIAL OF TAILORED MUSIC LISTENING INTERVENTION FOR SLEEP IN DEMENTIA

Darina Petrovsky,[1] Shana Roan,[1] Nalaka Gooneratne,[1] Joke Bradt,[2] Laura Gitlin,[2] and Nancy Hodgson,[3]
*1. University of Pennsylvania, Philadelphia, Pennsylvania, United States, 2. Drexel University, Philadelphia, Pennsylvania, United States, 3. University of Pennsylvania, Cherry Hill, New Jersey, United States*

Sleep disruption in older adults living with Alzheimer's disease and related dementias (ADRD) is debilitating and contributes to increased institutionalization, reduced cognitive function, and accelerated disease progression. Given the potential harmful effects of pharmacologic treatment, non-pharmacologic approaches, such as music, may provide a safer alternative to improve sleep quality in this vulnerable population. No empirically validated music protocol exists to address sleep disruption in older adults with ADRD living at home. Therefore, the specific aims of this wait-list randomized controlled trial were to examine the 1) feasibility; 2) acceptability; and 3) preliminary efficacy of a tailored music intervention in home-dwelling older adults with ADRD with sleep disruption and their caregivers. This presentation focuses on baseline characteristics of dyads, which included persons with ADRD and their caregivers who have completed the clinical trial so far (N=28). The mean age of persons with ADRD was 71.6 (SD: 7.6). The mean age of caregivers was 58.7 (SD: 16.7). Sixty-eight percent (n=19) of persons with ADRD were female. Similarly, the majority of caregivers were female (n=20, 71.4%). Seventy-four percent of persons with ADRD scored 0.5 on the Clinical Dementia Rating instrument, indicative of very mild dementia. The majority of dyads identified themselves as non-Hispanic (>92%). Seventy-nine percent of persons with ADRD identified themselves as Black or African-American (n=22, 79%), while 82.1% of caregivers identified themselves as Black or African American. Preliminary analysis of qualitative data indicates high acceptability of the intervention. Results from this research study will inform a future efficacy trial.

### SELF-REPORTED UPTAKE OF CLINICAL PREVENTIVE SERVICES BY VISION IMPAIRMENT STATUS

Lama Assi,[1] Ahmed Shakarchi,[1] Orla Sheehan,[2] Nicholas Reed,[3] and Bonnielin Swenor,[3] *1. Johns Hopkins Wilmer Eye Institute, Baltimore, Maryland, United States, 2. Johns Hopkins University School of Medicine, Baltimore, Maryland, United States, 3. Johns Hopkins University, Baltimore, Maryland, United States*

Disease prevention is central to healthy aging. People with vision impairment are more likely than those without to report barriers to accessing health care and have unmet health care needs. We examined the association between functional vision impairment and preventive care uptake among adults aged 65 years and older in the 2016 and 2018 Behavioral Risk Factor Surveillance System (BRFSS) survey. The outcome of interest was being up-to-date with the recommended core clinical preventive services, as defined by Healthy People 2020: influenza and pneumococcal vaccination, and colorectal cancer screening for men, with the addition of breast cancer screening for women. Self-reported vision impairment was defined as blindness or serious difficulty seeing, even when wearing glasses. In models adjusted for sociodemographic characteristics (including age), access to care, and health/functional status, there was no difference in the odds of reporting being up-to-date with the recommended core preventive services among men with vision impairment compared to those without (odds ratio [OR]=0.90, 95% confidence interval [CI]=0.8-1.01); however, men with vision impairment were 0.82 times (95% CI=0.71-0.94) less likely than those without to report being up-to-date with colorectal cancer screening. Women with vision impairment were less likely than those without to report being up-to-date with the recommended core preventive services (OR=0.77, 95% CI=0.69-0.87); among the different services, the odds were lowest for reporting breast and colorectal cancer screening. These findings suggest that to achieve higher rates of preventive care uptake, especially cancer screening, older adults with vision impairment may be a special group to target.

### SUPPORTIVE HOME ENVIRONMENTS AS A MEDIATOR BETWEEN SENSORY IMPAIRMENT AND DISABILITY

Bernard Steinman,[1] Jon Pynoos,[2] Casandra Mittlieder,[1] Julie Overton,[2] and Treva Sprout Ahrenholtz,[1]
*1. University of Wyoming, Laramie, Wyoming, United States, 2. University of Southern California, Los Angeles, California, United States*

Home environments are important to older adults and can help to preserve independence in every day functioning when they are supportive. Nevertheless, over time, the quality of "fit" between individuals and their homes can decrease because of age-related physical changes. A significant proportion of older Americans experience sensory impairments that impact their capacity to perform daily living activities necessary to remain independent at home. Home environments designed to support access and safety have potential to ameliorate disability associated with declines in sensory status. Data from the National Health and Aging Trends Study (NHATS) were analyzed to assess the role of supportive home environments in mediating the relationship between self-reported measures of vision impairment, hearing impairment, and dual vision-hearing impairment and related ADL/IADL outcomes in community-dwelling older adults. Guided by the International Classification of Functioning, Disability and Health (ICF), regression models included covariates for sociodemographics, chronic conditions, mobility functioning, and participation. Supportive home environments were operationalized using indicators of whether participants had access to homes from the outside without having to use stairs; presence of a bedroom, kitchen, and full bathroom with a shower or tub on the same floor; and whether bathroom fixtures had been modified with features such as grab bars. Results suggest a statistical relationship between sensory function and disability that is explained in part by the lack of supportive home features. Implications are that older adults with sensory impairments can benefit greatly by improving environments in areas of the home that are known to cause difficulty.

### THE ASSOCIATION OF SENSORY IMPAIRMENT WITH INCIDENT DISABILITY-RELATED CESSATION OF EMPLOYMENT IN OLDER ADULTS

Ahmed Shakarchi,[1] Emmanuel Garcia Morales,[2] Nicholas Reed,[3] and Bonnielin Swenor,[3] *1. Johns Hopkins Wilmer Eye Institute, Baltimore, Maryland, United States, 2. Johns Hopkins Bloomberg School of Public Health, Baltimore, Maryland, United States, 3. Johns Hopkins University, Baltimore, Maryland, United States*

Sensory impairment (SI) is common among older adults, and it is an increasingly important public health challenge as the population ages. We evaluated the association between SI and incident disability-related cessation of employment in older adults using the population-based Health and Retirement Study. Participants employed in 2006 completed biennial interviews until self-reported incident disability-related cessation of employment. Participants were censored at loss to follow-up, retirement, or 2018. Participants rated their vision and hearing, using eyeglasses or hearing aids if applicable, on a Likert scale (poor, fair, good, very good, excellent). SI was defined as poor or fair ability, and SI was categorized as neither SI (NSI), vision impairment alone (VI), hearing impairment alone (HI), and dual SI (DSI). Cox proportional hazard regression assessed the association between SI and incident disability-related cessation of employment, adjusting for demographic and health covariates. Overall, 4726 participants were included: 421 (8.9%) were with VI, 487 (10.3) with HI, and 203 (4.3%) with DSI. Mean age was 61.0 ± 6.8 years, 2488 (52.6%) were women, and 918 (19.4) were non-White. In the fully adjusted model, incident disability-related cessation of employment over the 12-year follow-up period was higher in VI (Hazard Ratio (HR)=1.30, 95% confidence interval (CI)=0.92, 1.85), HI (HR=1.60, CI=1.16, 2.22), and DSI (HR=2.02, CI=1.38, 2.96). These findings indicate that employed older adults with SI are at increased risk of incident disability-related cessation of employment, and that older adults with DSI are particularly vulnerable. Addressing SI in older adults may lengthen their contribution to the workforce.

### THE ASSOCIATION OF VISION, HEARING, AND DUAL SENSORY IMPAIRMENTS WITH WALKING SPEED AND INCIDENT SLOW WALKING

Ahmed Shakarchi,[1] Varshini Varadaraj,[2] Lama Assi,[1] Nicholas Reed,[2] and Bonnielin Swenor,[2] *1. Johns Hopkins Wilmer Eye Institute, Baltimore, Maryland, United States, 2. Johns Hopkins University, Baltimore, Maryland, United States*

Vision (VI), hearing (HI) and dual sensory (DSI, concurrent VI and HI) impairments are increasing in prevalence as populations age. Walking speed is an established health indicator associated with adverse outcomes, including mortality. Using the population-based Health and Retirement Study, we analyzed the longitudinal relationship between sensory impairment and walking speed. In multivariable mixed-effects linear models, we found differences in baseline walking speed (m/s) by sensory impairment: Beta=-0.05 (95%CI=-0.07, -0.04), Beta=-0.02 (95%CI=--0.03, -0.003), and Beta=-0.07 (95%CI=--0.08, -0.05) for VI, HI and DSI, respectively, as compared to those without sensory impairment. However, similar annual declines (0.014 m/s) in walking speeds occurred in all groups. In time-to-event analyses, events were defined as "slow walking" (speed <0.60m/s) and "very slow walking" (<0.40m/s). Incident "slow walking" was 43% (95%CI=25%, 65%), 29% (95%CI=13%, 48%) and 35% (95%CI=13%, 61%) greater in VI, HI and DSI, respectively, than the no sensory impairment group, while incident "very slow walking" was 21% (95%CI=-4%, 54%), 30% (95%CI=3%, 63%) and 89% (95%CI=47%, 143%) greater; the increase was significantly greater in DSI than VI and HI. These results suggest that older adults with vision and hearing impairments walk slower and are at increased risk of slow walking than older adults without these sensory impairments. Additionally, older adults with DSI are at greatest risk of very slow walking.

### VISUAL IMPAIRMENT AT AGE 85 PREDICTS SUBSEQUENT COGNITIVE DECLINE AT AGE 90

Jeremy Jacobs,[1] and Jochanan Stessman,[2] *1. Hadassah Hebrew University Hospital, Jerusalem, Yerushalayim, Israel, 2. Hadassah Hebrew University Hospital, Mt Scopus Jerusalem, Israel, Jerusalem, Israel*

Although the impact of visual impairment (VI) upon functional status and mortality among older people is recognized, its relationship to cognitive function is unclear. We examined the association between VI and subsequent cognitive decline from age 85-90 among subjects from the Jerusalem Longitudinal Study (1990-2020), which follows a representative study sample born 1920-21. Assessment at age 85 (2005) and age 90 (2010) included Snellen visual testing and Mini Mental State Examination (MMSE) for 488 subjects. VI was defined as corrected best eye vision ≤ 40/60. Dementia was defined as MMSE ≤24/30, after visually-dependent items (drawing, writing, reading) were excluded from the MMSE, and the score (maximum=27) was corrected to a maximum of 30 by multiplying by a factor of 1.111. At age 85 frequency of VI was 40.1% (198/488) and 86.9% (424/488) of subjects were non-demented (MMSE ≥24/30). Between ages 85-90 the mean decline in MMSE among all subjects with VI vs. non-VI was 5.2±7.7 vs. 3.9±6.7 (p=0.053), among non-demented subjects was 5.2±7.8 vs. 3.5±6.3 (p=0.002), and the transition to dementia was 34% vs. 20% (p=0.004) respectively. In logistic regression analysis among non-demented subjects at age 85, the unadjusted odds ratio (OR) for transition to dementia by age 90 associated with VI at age 85 was OR 1.95, 95%CI 1.24-3.06, p<0.01, and after adjusting for gender, years of education, depression and diabetes, the adjusted OR was 1.74, 95%CI 1.09-2.76, p<0.05. In conclusion, visual impairment at age 85 is independently associated with subsequent cognitive decline and the development of dementia.

### SESSION 2901 (POSTER)

### GERONTOLOGY AND GERIATRICS EDUCATION

### ATTITUDES AND INTENTION TO WORK WITH OLDER ADULTS BY USA AND ISRAELI NURSING STUDENTS: MEASURE ADAPTATION AND TESTING

Catherine Van Son,[1] Anna Zisberg,[2] and Ksenya Shulyaev,[3] *1. Washington State University, Vancouver, Washington, United States, 2. Faculty of Social Welfare and Health Sciences, University of Haifa, Haifa, Israel, Haifa, Isreal, United States, 3. University of Haifa, Mount Carmel, Hefa, Israel*

More nurses are needed to meet the healthcare needs of older adults. Valid and reliable measures are lacking that assess attitudes and intentions of nursing students to select a career in geriatrics. This paper discusses the applicability of the Carolina Opinions on Care of Older Adults (COCOA) instrument on attitudes towards and intention to work with older adults among nursing students. A cross-sectional survey design was used with samples of nursing students from the United States (N=122) and Israel (N=109). Students completed the COCOA instrument and the measure's dimensionality, construct validity, reliability and equivalence among the nursing students was tested. Exploratory Factor Analysis (EFA) produced 5 factors explaining 59% of the variation with most of the items (17, 71%) loading above 0.40 on subscales according to the instrument's original structure. Four items loaded on different subscales than originally described. One original subscale was eliminated (Value of Older Adults), and one new subscale was created (Older versus Younger Adults). The reliability scores were good to acceptable for all subscales (.60 - .80). Confirmatory FA supported the data's fit to the modified COCOA instrument (CMIN/DF=1.55, CFI=.93, IFI=.93, RMSEA=.05). The analysis of model equivalence for USA and Israeli samples revealed significant differences attributed to Experience in Caring for Older Adults subscale. The modified COCOA instrument demonstrated good construct validity and reliability and can serve as estimation of nursing students' attitudes towards older adults and intention to choose geriatrics as a career. Further studies are needed to test its predictive validity.

### ATTITUDES OF GRADUATING MEDICAL AND NURSING STUDENTS TOWARD OLDER PERSONS IN GHANA

Grace Karikari,[1] Huber Lesa,[2] David Lohrmann,[2] Margaret Adamek,[3] and Karo Omodior,[2] *1. Indiana University School of Public Health, Grand Forks, North Dakota, United States, 2. Indiana University Bloomington, Bloomington, Indiana, United States, 3. Indiana University Purdue University Indianapolis, Indianapolis, Indiana, United States*

Purpose: Differences in attitudes between graduating medical and nursing students toward older persons in Ghana were compared. Additionally, the association between the overall quality of students' experiences with older persons and their attitudes was examined. Materials and Methods: A sample of 135 final year medical and nursing students from a public institution in Ghana participated in a cross-sectional study by completing a web-based self-administered questionnaire consisting of the 14-item University of California at Los Angeles Geriatric Attitudes (UCLA-GA) scale, and demographic questions. Data analysis involved a two-sample t-test and a one-way ANOVA. Results: Most participants (82.2%) held positive attitudes towards older persons. The mean score for the UCLA-GA scale that assessed attitudes of students towards older persons was 3.41 ± 0.41 (min: 2.29, max: 4.64); differences in attitudes between the two groups was significant (p = 0.001). Medical students had more positive attitudes toward older persons than nursing students. The association between students' attitudes and the overall quality of their experiences with older persons was significant (p = .001). Students whose experiences with older persons were negative had the least positive attitudes. Conclusion: Considering the impact negative experiences with older persons may have on students' attitudes, attention should be given to creating positive experiences through clinical and community-based exposures. Direct interactions with older persons who are active and living independently may challenge some common stereotypes such as older persons are infirm and senile, and foster positive attitude development.

### DEVELOPMENT OF AN EDUCATIONAL INTERVENTION AND PATIENT-FAMILY FACING MATERIALS FOR DEPRESCRIBING IN HOME HOSPICE

Jennifer Tjia,[1] Mardie Clayton,[2] Vennesa Duodu,[3] Geraldine Puerto,[3] Lynley Rappaport,[3] and Susan DeSanto-Madeya,[4] *1. UMass Medical School, Worcester, Massachusetts, United States, 2. University of Utah, Salt Lake City, Utah, United States, 3. Epidemiology, Worcester, Massachusetts, United States, 4. Boston College, West Roxbury, Massachusetts, United States*

Deprescribing is emerging as a clinical intervention to optimize quality of life, improve patient safety, and reduce burden for older adults with serious illness and their caregivers. Few resources are available to educate and engage clinicians, older adults and their families in discussions about deprescribing. This presentation describes the development of educational intervention materials, including clinician, patient and family-facing materials, about medication management and deprescribing for seriously ill older adults in home hospice. An environmental scan of the existing deprescribing resources was conducted; a state-of-the-art educational program for hospice deprescribing was located and used as the basis for an innovative clinician-facing educational intervention. A stakeholder panel of 2 hospice administrators, 3 nurses, 2 physicians, 2 pharmacists, and 2 former family caregivers, drawn from 2 geographically diverse hospice agencies, reviewed the content of the educational program and made recommendations for additional content. Iterative rounds of development and feedback resulted in: (1) a 3-part, 1 hour, clinician educational program with CE credits for nurses that presents a standardized deprescribing approach that aligns medication prescribing with the goals of patients and caregivers, and (2) a patient/family caregiver medication management notebook for intervention group participants (addressing management of common symptoms, medications in hospice, and deprescribing) and an adapted version for attention control participants. A professional designer created thematic coherence for all materials that were well received by stakeholder panelists and hospice staff. Ultimately, educational materials will support efforts to standardize deprescribing approaches that aim to optimize end-of-life care outcomes for patients and family caregivers.

### GERIATRIC FAST FACTS: FOUR YEARS OF GROWTH AND REACH

Edmund Duthie,[1] Kathryn Denson,[2] Deborah Simpson,[3] Steven Denson,[2] and Michael Malone,[3] *1. Medical College of Wisconsin, Milwaukee, Wisconsin, United States, 2. Medical College of Wisconsin, Wauwatosa, Wisconsin, United States, 3. Advocate Aurora Health, Milwaukee, Wisconsin, United States*

Clinical teachers are increasingly challenged to find the time to provide point of care education for learners. To meet this challenge, an interprofessional team from competing health care systems created and sustained Geriatric Fast Facts (GFF): easily accessible, concise 1-2 page topic summaries for clinical teachers to use (in lieu of the mini-lecture) with learners at the point of care. Designed by geriatrics educators in consultation with IT experts, we launched a mobile enabled website that is indexed and searchable by free text or topic, organ system, ACGME competency, disease, and the “underlying science” for the disease/illness. GFF topics are authored by subject matter experts with peer review by senior geriatricians. Brief quizzes test learners’ knowledge with score reports. Our 4-year results reveal that: 1) “Geriatric Fast Facts” appear in the top Google 10 listing; 2) Search engines account for 39% of site traffic; 3) 60% of unique users (N=29,000) are via direct link; 4) 19% via referral from another site. Our “bounce rate” (55%) is ideal as users quickly gain the information sought - affirmed by our session duration which averages < 2 minutes. Our Google Analytics results reflect steady growth and international reach: 73% of sessions are from the U.S., 5% from Canada, 22% international and growing! In summary with our population continuing to age and time for teaching being limited, GFFs are quick, accessible, evidence-based resources for point of care teaching.

### LEARNER ATTITUDES ON INTERPROFESSIONAL EDUCATION THROUGH FORMAL INTERPROFESSIONAL CASE DISCUSSIONS.

Anne Halli-Tierney,[1] Dana Carroll,[2] Robert McKinney,[1] and Rebecca Allen,[3] *1. The University of Alabama, Tuscaloosa, Alabama, United States, 2. Auburn University Harrison School of Pharmacy, Tuscaloosa, Alabama, United States, 3. Alabama Research Institute on Aging, Tuscaloosa, Alabama, United States*

Interprofessional education case sessions allow learners to apply discipline-specific knowledge to real-life scenarios through facilitated discussion of a patient case. Our interprofessional case discussion was implemented for learners to develop care plans for complex geriatric patients; learners have intentional time to learn with, from and about each other’s roles in geriatric patient care. Cases were de-identified from actual complex patients seen in geriatrics clinic. All learners receive the case and work through it from their discipline’s perspective, then join a facilitated group discussion to develop collaborative care plans. At session end participants are surveyed using the ICCAS and qualitative comments about perceptions on interprofessional case learning. Thirty-five learners (47%) completed the feedback survey. Disciplines represented were medicine, pharmacy, psychology and social work. Thirty learners (85%) indicated the case discussion session was very educational (n=22, 62%) to educational (n=8, 23%). Themes used most frequently regarding what was most educational were: “different professional approaches”, “professional roles”, “collaboration” and “problem solving”. Typically, learners were unable to identify “least educational” components to the activity, but some learners found the pace of information presentation too rapid or felt other professionals did not provide enough context for the suggestions they made for the case. Suggestions to improve the interprofessional case discussion activity included increased “time for discussion and consensus building”, more session structure, and addition of other professionals (e.g., nutrition, law). The majority of interprofessional learners participating in interprofessional case sessions have found them to be educational. Feedback from learners aligns with goals of interprofessional education.

### LEVERAGING PREHOSPITAL CARE TO ADDRESS ELDER MISTREATMENT: DEVELOPMENT OF AN ONLINE TRAINING FOR EMS

Kristin Lees Haggerty,[1] Melanie Miller,[1] Dana Wardlaw,[2] Athi Myint-U,[1] Randi Campetti,[1] Brad Cannell,[3] and Kristin Lees, *1. Education Development Center, Waltham, Massachusetts, United States, 2. Education Development Center, Waltham, United States, 3. University of Texas Health Science Center at Houston School of Public Health, Dallas, Texas, United States*

Elder abuse affects an estimated 1 in 10 older adults in the US and has devastating consequences for their health and well-being yet is widely under-recognized. Prehospital emergency medical service (EMS) providers are particularly well-positioned to identify older adults who are at risk of or experiencing mistreatment, and to report and intervene as appropriate. However, many EMS providers across the country lack the training and tools required to facilitate consistent identification and intervention. Recognizing the critical need for easily accessible, comprehensive, and relevant training, Education Development Center received funding from RRF Foundation for Aging to develop and pilot test the Elder Mistreatment EMS Training Curriculum (EM-ETC). The EM-ETC aims to improve identification, referral, and linkage to coordinated care and support services for older adults who are at risk of mistreatment. In this presentation, we will describe our iterative and collaborative process for developing an interactive online training program for EMS providers. The training leverages the unique circumstances of prehospital care to prepare EMS providers to recognize and respond effectively to elder abuse and help them to fulfill state licensing requirements. We will present findings from our formative research comprising consultation with subject matter experts and state offices of EMS education, focus groups with EMS and Adult Protective Services providers, and user testing with EMS providers.

### UNDERSTANDING MEDICAL STUDENTS' KNOWLEDGE OF OPIOID USE DISORDER: A PRELIMINARY STUDY

Sweta Tewary,[1] Ariel Kidron,[2] Naushira Pandya,[2] Jim Howell,[2] Marie Florent-Caree,[2] Annisah Ishmael,[2] and Rebecca Cherner,[2] *1. Nova Southeastern University, Fort Lauderdale, Florida, United States, 2. NSU, Davie, Florida, United States*

The rise of the opioid epidemic over the last two decades has increased the mortality rate, healthcare cost, and drug overdose deaths across the country. Practicing physicians are lacking in education regarding non-opioid alternatives to pain management, prevention, diagnosis, and treatment of opioid use disorder (OUD). Existing literature suggest a link between knowledge discrepancy and opioid use among clinicians resulting in patient's abuse of opioids. Therefore, it is important to educate medical students at the start of their career. This preliminary study assesses the current knowledge and perceived skills of medical students regarding (OUD)/opioid misuse and related content in order to identify gaps and provide necessary education. The study used a pre-post survey method to understand the demographics, medical, and clinical knowledge about opioid use, abuse, and clinical knowledge regarding patient opioid overdose. The self-administered survey was administered to all students 18 years or older, M1- M4 enrolled in NSU-KPCOM. A total of 1164 students met these criteria. However, only 137 students participated in the Pre-survey collected from August 2019 to September 2019. Approximately 12% of the eligible students participated in the pre-survey. Data was analyzed using frequencies and percentages. Results of the pre-survey suggest a progressive increase in opioid knowledge from M1 to M4 years. Results of the study suggest investigating a relationship between medical education and knowledge of opioid usage, with a specific lens aimed at assessing the efficacy of opioid education during second and third years of medical school

### USING QUALITY MEASUREMENT TO SUPPORT HEALTHY AGING: THE ADULT IMMUNIZATION STATUS COMPOSITE MEASURE

Courtney Barbera, *Discern Health, Baltimore, Maryland, United States*

Vaccine-preventable diseases remain a significant public health threat, especially for older adults and those with compromised immunity. Efforts to drive vaccine uptake can include quality measures, which help providers, health care organizations, payers, and others to benchmark and track immunization activities. To help address low immunization rates in adults, the National Committee for Quality Assurance (NCQA) released the Adult Immunization Status (AIS) composite measure, which calculates a single rate for health plan performance on influenza, Td/Tdap, shingles, and pneumococcal immunizations. Discern Health and the American Medical Group Association (AMGA) performed a mixed-methods study using de-identified patient data from three healthcare organizations to analyze feasibility of the AIS measure in quality programs at the medical group-level. The team also team conducted qualitative interviews with the organizations to understand best practices for improving immunization rates in adults. The study showed that the AIS measure is feasible for use at the medical-group level. It also revealed that adults 50 years of age and older were less likely to receive all age-appropriate vaccinations than those younger than 50. These findings indicate that there is a significant gap in rates of immunizations among older adults and point to potential solutions that can be supported through use of the AIS measure.

### SESSION 2902 (POSTER)

### CHRONIC DISEASE AND MULTIMORBIDITY

### ABILITY AND PERFORMANCE OF DAILY ACTIVITY FOR OLDER ADULTS WITH MULTIPLE CHRONIC CONDITIONS

Tara Klinedinst, and Juleen Rodakowski, *University of Pittsburgh, Pittsburgh, Pennsylvania, United States*

Older adults with multiple chronic conditions (MCC), and especially with added depression, have lower levels of daily activity than those without MCC. Engagement in daily activities can be measured by ability (what individuals can do) and performance (what individuals actually do). Understanding this difference is critical to developing interventions that align with the daily activity needs of older adults with MCC. The aim of this study was to understand the relationships between ability and performance of instrumental activities of daily living (IADL) among older adults with varied MCC and depression status. We conducted a cross-sectional study using data from the National Health and Aging Trends Study. We used MANOVA to detect differences in ability and performance of IADL by MCC status and t-tests to test the difference between ability and performance for our sample. There was an effect of MCC status on ability and performance for older adults in this sample, $F$ (10,13386) = 67.12, p < .001, ηp2 = .05; performance and ability were lowest for MM-D, and highest for no MCC. Post-hoc t-tests revealed a difference between the mean scores for ability (M = .79, SD = .29) and performance (M = .92, SD = .23) across all groups of MCC status, t (6693) = -50.174, p < .001. Older adults with MCC, particularly with depression, displayed diminished performance of IADL, although they have the ability. Our results suggest interventions should not only address what individuals can do, but also what they actually do in their context.

### ACTIVITY AND COGNITION IN OLDER ADULTS WITH AND WITHOUT SLEEP APNEA

Nora Mattek,[1] Jeffrey Kaye,[2] and Zachary Beattie,[1]
*1. Oregon Health & Science University, Portland, Oregon, United States, 2. Layton Alzheimer's Disease Center, Portland, Oregon, United States*

Many older adults have sleep apnea. It often goes undiagnosed. Sleep apnea is associated with decreased oxygen to the brain, fragmented sleep, shorter sleep duration, impaired cognition, increased risk for dementia, and less clearance of amyloid and tau. Little longitudinal data are available identifying objective measures of sleep apnea over time that may facilitate improved identification of affected persons. One hundred ninety-five participants age 57 or older were enrolled as part of Collaborative Aging Research using Technology (CART) initiative, a national study examining the feasibility of unobtrusive remote sensing and monitoring of cognitive, behavioral, physiological, and health-related activities. Nightly hours of sleep and total daily step count were acquired using a wrist worn device. Sixty (31%) reported having sleep apnea. Those with sleep apnea had a significantly lower MoCA total score than those without (22.9 vs. 24.2, p<0.01) and were more likely to be classified as MCI by MoCA cutoff <23 (49% vs. 24%, p<0.001). Volunteers who live alone were less likely to be diagnosed with sleep apnea (p<0.01). Males were much more likely to report having sleep apnea than females (p<0.0001). Of those with watch-derived activity data, sleep apnea cases had shorter mean sleep duration (7.2 vs. 7.8 hours, p=0.05) and fewer total daily steps than those without sleep apnea (2384 vs 3327, p=0.04). Sleep apnea may be identified by its association with mildly impaired cognition, with shorter sleep duration, and less total daily activity as measured at home via remote continuous monitoring techniques.

### BIDIRECTIONAL ASSOCIATION BETWEEN KNEE OSTEOARTHRITIS AND DEPRESSIVE SYMPTOMS: A NATIONWIDE COHORT STUDY

Han Lu, Shaomei Shang, Limin Wang, and Hongbo Chen,
*Peking University, Beijing, China*

Both knee osteoarthritis (KOA) and depressive symptoms are common health issues affecting the quality of life of old adults. Although it is presumed that KOA has a bidirectional relationship with the depressive symptoms, no cohort study has proven it. This is the first study to determine the strength of association for the bidirectional relationship between KOA and depressive symptoms. Data were gathered from the nationally survey of China Health and Retirement Longitudinal Study in 2011-2015. The presence of depressive symptoms was defined by the 10-item Center for Epidemiologic Studies Depression Scale score of 10 or higher. The adjusted Cox proportional hazards regression model was conducted to estimate hazards ratios (HRs). Controlled covariates include gender, age, education, marital status, residence, number of chronic diseases, and disability. The analysis of KOA predicting the depressive symptoms onset consisted of 4,377 participants free from depressive symptoms at baseline. During 4 years follow-up, diagnosed KOA participants were more likely to have depressive symptoms than their peers without KOA (HR = 1.50, 95% CI: 1.23-1.83). The parallel analysis of depressive symptoms predicting KOA onset included 6,848 participants without KOA at baseline, those with depressive symptoms had a higher relative risk of developing KOA (HR = 1.64, 95% CI: 1.41-1.92). Our results provide compelling evidence that the KOA-depressive symptoms association is bidirectional, highlighting the importance of evaluating the relationship between physical and mental health among older people. Particularly, taking this association into consideration in the risk assessment and primary prevention of KOA and depression symptoms.

### DISTINCT TRAJECTORIES OF RECOVERY WITHIN THE FIRST YEAR AFTER HIP FRACTURE

Heather Mutchie,[1] Denise Orwig,[2] and Ann Gruber-Baldini,[3] *1. University of Maryland Baltimore, Baltimore, Maryland, United States, 2. Division of Gerontology, Baltimore, Maryland, United States, 3. University of Maryland School of Medicine, Baltimore, Maryland, United States*

Hip fracture patients experience heterogenous pathways of recovery. Knowing about these different groups can guide rehabilitation efforts. The sample was 339 hip fracture patients age 65+ recruited to the Baltimore Hip Studies 7th cohort [2006-2011; men(n=168) women(n=171)] assessed at baseline, 2.6, and 12 months. Group based trajectory modeling assessed participants' cognitive and functional recovery trajectories as measured by the Modified Mini-Mental State Examination(3MS;0-100), Hooper Visual Organization Task(HVOT,0-30), Trail-Making Task(TrailsA,B:0-301s), Short Physical Performance Battery(SPPB,0-12), Lower Extremity Physical Activities of Daily Living(LPADL,0-12), and physical activity (YALE, hours/week). Participants were on average 80.91(7.88) years old with 13(3.41) years of education. Two groups were identified for Trails A [Consistent high performance (85.3%), declined performance (14.6%)] and YALE [low exercise(94.9%), small decline(5.0%)]. Three groups were identified for 3MS [consistently high(76.3%), consistently borderline(17.1%), consistently low-performing(6.4%)] and SPPB [failure(19.3%), low-performing with small improvement(55.7%), moderate improvement(24.9%)]. Four groups were identified for HVOT[low scoring(14.2%), mid-range(31.9%), borderline(32.4%), well-performing(21.3%)], Trails B [consistent high-performance(40.2%), improved(18.6%), declined(12.0%), consistent failure(29.1%)], and LPADL[low-performing(27.0%), small improvement(50.0%), dramatic improvement(17.0%), high-performing(5.7%)]. The polynomial functions of the trajectory groups were primarily quadratic with some cubic functions best representing the recovery trajectories. Many of the groups have similar trajectories but at different magnitudes. For most tests performance at baseline was able to differentiate performance at 1 year. These data show that there are very few changes in high or low performing groups, but mid-range participants can experience volatility.

### DOES THE COEXISTENCE OF FOOD INSECURITY AND OBESITY IN OLDER ADULTS EXPLAIN VARIATIONS IN GLYCATED HEMOGLOBIN?

Queendaleen Chukwurah, *Tulane University, New Orleans, Louisiana, United States*

Obesity and food insecurity are known public health concerns for older adults, and both are independent predictors of glycated hemoglobin (HbA1c). I examined the impact of the co-existence of food insecurity and obesity on HbA1c using the National Health and Nutrition Examination Survey (NHANES), 2005-2014. Body mass index /waist circumference (WC) cut- off values were used to create six body types: normal weight with normal WC, overweight with normal WC, obese with normal WC, normal weight with high WC, overweight with high WC, and obese with high WC. HbA1c was defined as normal < 5.7% and abnormal >5.7%. Food security status (FSS) was defined following USDA protocols (food secure-FS, food insecure-FI). The sample population included 5,772 participants 50 years and older with mean (SD) age of 61.8 (0.2). A weighted multivariable logistic regression controlling for age, gender, race/ethnicity, education, and poverty-to-income ratio was run for this analysis. The proportion of older adults with both FI and obesity with high WC (51.1%, p<0.0001) was significantly higher than those FS (37.5%). Logistic regression model with body types and FSS had a maximum-rescaled R-square (MRRS) of 0.147 vs. 0.093 and 0.144 for FSS and body types alone. An increase in MRRS in the model with both body types and FSS compared to the models containing only body types or FSS demonstrates an improved model for fitting abnormal HbA1c levels. The knowledge of this effect may benefit health risk assessment and management in this population.

### METABOLIC AND INFLAMMATORY BIOMARKERS, MULTIMORBIDITY COMBINATIONS AND DISABILITY BURDEN AMONG OLDER AMERICANS

Anda Botoseneanu,[1] Sheila Markwardt,[2] and Ana Quinones,[3] *1. University of Michigan, Dearborn, Michigan, United States, 2. School of Public Health - Portland State University, Portland, Oregon, United States, 3. Oregon Health & Science University, Portland, Oregon, United States*

Specific multimorbidity combinations, in particular those including arthritis, stroke, or cognitive impairment have been associated with high burden of ADL/IADL disability. However, the biologic underpinnings of these associations are still unclear. We used data from the Health & Retirement Study (N=5,359, age 65 years or older at baseline) and negative binomial regression models to evaluate if metabolic and inflammatory biomarkers [HbA1c, HDL-cholesterol, C-Reactive Protein (CRP)] mediate the association between specific multimorbidity combinations (at baseline in 2010-2012; grouped around one of eight index diseases: arthritis, cancer, cognitive impairment, diabetes, heart disease, hypertension, lung disease, and stroke) and ADL/IADL disability (at subsequent wave in 2012-2014). Results were adjusted for sociodemographic characteristics, body-mass index, number of coexisting chronic diseases, and baseline ADL/IADL score. HbA1c (IRR=1.01, p=0.004) and CRP (IRR=1.01, p=0.003), but not HDL, were positively associated with the number of coexisting diseases. After adjustment for coexisting diseases, higher HbA1c was associated with greater ADL/IADL limitations for multimorbidity combinations including arthritis (IRR=1.11, p=0.047) and stroke (IRR=1.11, p=0.047), but not for combinations centered around other diseases, while CRP was no longer significantly associated with ADL/IADL limitations for any of the multimorbidity combinations. Models accounting for HbA1c and CRP, respectively, showed that only combinations including cognitive impairment had greater ADL/IADL limitations (IRR=1.45, p=0.015) compared to combinations without cognitive impairment. Insulin resistance and inflammation are strongly associated with the burden of multimorbidity; this strong association, rather than the biomarkers per se, appears to explain the greater ADL/IADL burden observed with all disease-specific multimorbidity combinations, except cognitive impairment.

### MODERATING EFFECT OF AGE ON THE RELATIONSHIP BETWEEN PATIENT ACTIVATION AND DIABETES SELF-CARE ACTIVITIES

suhyun kim, *Kyungpook National University, Daegu, Republic of Korea*

Purpose: The purpose of the study was to identify the moderating effect of age on the relationship between patient activation and diabetes self-care activities among Korean patients with type 2 diabetes mellitus. Methods: For the cross-sectional correlational study utilizing secondary data analysis, 155 patients diagnosed with type 2 diabetes were divided into two age groups: < 65 and ≥ 65 years. Original data were collected in ambulatory endocrinology units of two tertiary hospitals in South Korea from September 2016 to July 2017. Multivariate regression analyses were used, including interaction variable to detect a moderating effect of age group. Results: The level of diabetes self-care activities was similar between patients aged 65 or over and those aged less than 65 (with the possible range from 0 to 7) (3.71±1.22 vs. 3.53±1.20, t = -0.58, p = .561). Patient activation level was lower for patients aged 65 or over than those aged less than 65 (with the possible range from 0 to 100), but there was no statistical difference (61.54±4.01 vs. 68.66±16.45, t =1.68, p = .095). In multiple linear regression analysis, there was a significant interaction effect of age group (≥ 65 vs. < 65 years) and patient activation on diabetes self-care activities, controlling for the demographic and clinical variables (standardized beta = -.69, p = .021, 95% CI [-.08, -.01]). Conclusion: To encourage diabetes self-care activities, it is necessary to consider the limited effect of patient activation level for patients with type 2 diabetes aged 65 or over.

### OLDER ADULTS AGING WITH HIV: A GROWING POPULATION EXPERIENCING COMORBIDITIES AND SOCIAL ISOLATION

Carole Treston, *Association of Nurses in AIDS Care, Phila, Pennsylvania, United States*

Significantly more than half of people living with HIV in the United States are over age 50 and at least half of that number are over 70 years old. Advances in antiretroviral treatments continue to extend the lifespan of people with HIV. However, people aging with HIV, particularly those diagnosed earlier in the epidemic, known as "long term survivors" are likely to face a myriad of challenges: clinical, psychosocial, financial, and logistical. Aging with HIV is a complex mix of long-term treatment effects, early onset of general aging, comorbidities and other confounding factors including mental health and psycho-social factors that affect quality of life. Older persons living with HIV have experienced tremendous loss, stigma and discrimination, including within the healthcare system. Now, renewed losses amplified by the emergence of multiple comorbidities including cardiovascular and metabolic disease, HIV associated neurocognitive disease, other neurological disability, diminished bone health and frailty and other conditions can impair quality of life significantly. A review of the common comorbidities experienced by people aging with HIV and the intersection with social isolation, stigma and loss will be presented. Strength based, holistic care that focuses on resilience, and includes advocacy, social networks and care coordination delivered by nurses and nurse practitioners as part of a collaborative inter-professional education program at the Association of Nurses in AIDS Care to address the unique challenges experienced by PLWH will be described.

### PREVALENCE AND RISK FACTORS OF POSTPRANDIAL HYPOTENSION AMONG JAPANESE OLDER ADULTS IN A FACILITY

Noriko Suzuki,[1] Masahiko Hashizume,[2] and Hideyuki Shiotani,[3] *1. Seisen Jogakuin College, Nagano-shi, Nagano-ken, Japan, 2. Arima Onsen Hospital, Kobe, Japan, 3. Kobe University, Kobe city, Hyogo, Japan*

Postprandial hypotension (PPH) is an unrecognized sudden drop of blood pressure (BP) after meals and a hidden problem among older people including those living in long-term care facilities (LTCFs). Though PPH causes dizziness, falls, and syncope, it has received little attention from¬¬¬ healthcare workers (HCW) including caregivers, nurses and physicians, and risk factors of PPH should be carefully assessed to improve quality of life. Therefore, we aimed to examine the prevalence and risk factors of PPH in a LTCF in Japan. Participants were 114 older adults living in a LTCF in Japan (mean age 85.9 years old; 85 female (74%)). To examine PPH, blood pressure (BP) was measured before and after lunch. BP after meal was measured four times every 30 minutes. PPH is defined as a BP drop of 20 mmHg or more and we also defined a BP drop within a range of 19 to 15 mmHg as potential-PPH. As risk factors, we compared systolic and diastolic BP at baseline, body mass index, pulse rate, disease and complications between groups with/without PPH. The prevalence of PPH was 41% (47/114) and 52% with potential-PPH; 11% (13/114) added. Among risk factors, systolic BP was significantly higher in those with PPH (142.6 vs 123.5 mmHg, p <0.001). This study revealed that PPH & potential-PPH occurred in half of the subjects in a LTCF in Japan. HCW need to focus on high systolic BP to predict PPH and future research is necessary to prevent and cope with PPH for older people.

### RACIAL, ETHNIC, AND PLACE-OF-BIRTH DISPARITIES IN BODY TYPES: ASSOCIATION TO GLYCATED HEMOGLOBIN IN OLDER ADULTS

Queendaleen Chukwurah, *Tulane University, New Orleans, Louisiana, United States*

Obesity, a public health concern for older adults, contributes to abnormal glycated hemoglobin (HbA1c). We examine place of birth disparities in the prevalence rate of body types and their association to HbA1c using the National Health Nutrition and Examination Survey (NHANES) III. Body mass index /waist circumference (WC) cut off values were used to create six body types: normal weight with normal WC, overweight with normal WC, obese with normal WC, normal weight with high WC, overweight with high WC, and obese with high WC. Abnormal HbA1c was defined as HbA1c >5.7%. Weighted multivariable logistic regression adjusted for age, gender, education, and poverty-income-ratio was run. The sample population included 4,584 participants aged 50 years and older identifying as non-Hispanic whites (NHW), US-born Hispanics (USB-H), and foreign-born Hispanics (FB-H). The mean (SD) sample age was 63.9 (0.3). USB-H had the highest proportion of obese with high WC (35.6%,p<0.0001) compared to NHW (26.6%) and FB-H (22.2%). USB-H (aOR 1.97 95% CI 1.45,2.68) and FB-H (aOR 1.51 95% CI 1.10,2.06) had higher odds of abnormal HbA1c compared to NHW. Overweight with high WC (aOR 1.47 95% CI 1.11-1.93) and obese with high WC (aOR 2.11, 95% CI 1.60-2.79) had a high likelihood of abnormal HbA1c compared to normal weight with normal WC. Further adjustment for co-morbid conditions yielded a significantly improved fitting model (Maximum-rescaled R-square (MRRS) =0.1997,p<0.0001) compared to that further adjusted for health-related behaviors (MRRS=0.082,p<0.0001). The knowledge of these associations in an at-risk sub-population is insightful for clinical assessments and preventive interventions.

### SEQUENTIAL PATTERNS OF MULTIMORBIDITY IN A NATIONALLY REPRESENTATIVE COHORT STUDY OF AGING

Nicholas Schiltz,[1] Yicheng Hu,[1] Megan Foradori,[1] and Jessica Cooke Bailey,[2] *1. Case Western Reserve University, Cleveland, Ohio, United States, 2. Case Western Reserve University School of Medicine, Cleveland, Ohio, United States*

Multimorbidity – the presence of two or more chronic health conditions – is common among older adults. Despite this, relatively little is known about the epidemiology of specific sequences of disease onset that occurs in mid-life and older adults over time. This may be attributed to the sheer number of possible permutations, which is difficult to handle with traditional methods. This is a retrospective cohort study using the Health & Retirement Study (HRS), a nationally-representative panel survey of aging. The study population included all adults age 50 and older that had no reported chronic disease at baseline (n=5567). We use a data mining algorithm, Sequential Pattern Discovery using Equivalence classes (SPADE), to identify all possible sequences of eight self-reported age-related chronic diseases: hypertension, arthritis, diabetes, cancer, stroke, heart disease, chronic lung disease, and psychiatric disorders. There were 67 unique sequences of disease identified that occurred in at least 1% of the study population. The most common two event sequence was Arthritis=>Hypertension (15.5% of all subjects), and the second most common was Hypertension=>Arthritis (9.6%). The most common three-way sequence was Arthritis=>Hypertension=>Heart Disease (1.8%). Arthritis=>Stroke occurred in 1.5% of subjects and was associated with the highest mortality rate (71.3% of subjects died). Sequential pattern mining allows for the discovery of longitudinal patterns of disease that frequently occur in older adults and advancements in our understanding of the epidemiology of multimorbidity. Future applications may include predicting a given patient's disease trajectory based on their life course and disease history.

### SEX DIFFERENCES IN BONE-ACTIVE MEDICATION UTILIZATION BEFORE AND DURING THE YEAR AFTER HIP FRACTURE

Jennifer Kirk,[1] Denise Orwig,[2] Ann Gruber-Baldini,[3] Marc Hochberg,[3] Jay Magaziner,[4] and Alan Rathbun,[4] *1. University of Maryland Baltimore, Laurel, Maryland, United States, 2. Division of Gerontology, Baltimore, Maryland, United States, 3. University of Maryland School of Medicine, Baltimore, Maryland, United States, 4. University of Maryland, Baltimore, Baltimore, Maryland, United States*

Bone-active medications (BAM) [prescriptions (RxBAM) and supplements(calcium/vitamin D] increase bone mineral density and reduce osteoporotic fracture risk. However, RxBAM utilization rates are low, and it is unclear who is treated with BAMs before/after a hip fracture. This study examined sex differences in BAM use at baseline and predicted the probability of RxBAM use during follow-up(2, 6, and 12-months). The sample included frequency-matched males and females 65 years or older from the Baltimore Hip Studies' seventh cohort. Differences in baseline characteristics between males and females with complete data(n=313) were assessed using t-tests and chi-square tests. Generalized estimating equations(GEE) predicted the probability of RxBAM use by sex among participants(n=270) with outcome data during follow-up adjusted for baseline characteristics. Prior to fracture, there were sex-differences in BAM use, with fewer men than women taking RxBAMs(9% versus 26%), calcium(18% vs. 57%) and vitamin D (55% vs. 68%). These differences remained over the year post-hip fracture. Only 12(3.5%) participants took RxBAM the entire study period. Of RxBAM users n=70(26%), there were few new-users (n=35), and many participants stopped or never started treatment. Unadjusted GEEs showed that men were less likely to use RxBAM (OR= 0.42; 95% CI:0.22,0.78, p=.007), during the hip fracture recovery period compared to females. However, after controlling for differences in baseline characteristics between males and females, particularly pre-fracture BAM medication use, the observed association (OR=0.62; 95% CI:0.29, 1.31; p= 0.23). RxBAM use was low, especially in men and may contribute to the high rates of preventable subsequent osteoporotic fractures and post-fracture mortality.

### THE ACCEPTABILITY OF TECHNOLOGY IN HEALTH CARE AMONG OLDER KOREAN ADULTS WITH MULTIPLE CHRONIC CONDITIONS

Ji Yeon Ha,[1] and Hyeyoung Park,[2] *1. KONYANG UNIVERSITY, Daejeon, Republic of Korea, 2. University of Massachusetts Amherst, Amherst, Massachusetts, United States*

Background: Although there are benefits in utilizing ICT in health care, older adults have challenges in employ technologies in their health care management due to the changes in cognitive and physical functions, low motivation to use technology, and low computer/internet literacy (Adebayo et al, 2017; Wildenbos et al, 2018). The purpose of this study is to investigate the acceptance of technology among older Korean adults with multiple chronic conditions and examine factors associating with the acceptance of the technology. Method: The participants were 226 community-dwelling older adults who have more than two chronic conditions. Directed by the senior technology acceptance model (Chen & Chan, 2014), demographics, gerontechnology self-efficacy, gerontechnology anxiety, facilitating conditions, self-reported health conditions, cognitive ability, social relationship, attitude to life and satisfaction, physical functioning, and acceptance of technology were surveyed using a self-reported questionnaire. Findings: Older Korean adults with multiple chronic conditions showed a moderately high technology acceptance score (M = 25.35, SD = 5.28). There were significant differences in the acceptance of technology depending on age (r=-0.241, p<.01), cognitive ability (r=0.225, p<.01), gerontechnology self-efficacy (r=0.323, p<.0001), and facilitating conditions (r=0.288, p<.0001). Conclusion: While older age were associated to the acceptance of technology, gerontechnology self-efficacy which is one's judgment of their ability to perform a task successfully using gerontechnology and facilitating conditions which are environmental factors that help older adults use gerontechnology easier were positively associated with the acceptance of technology among older Korean adults with multiple chronic conditions.

### USE OF THE NEW ICD-10 VISION CODES AMONG MEDICARE BENEFICIARIES WITH STROKE

Kimberly Hreha,[1] Kenneth Ottenbacher,[1] Joshua Ehrlich,[2] and Heather Whitson,[3] *1. The University of Texas Medical Branch at Galveston, Galveston, Texas, United States, 2. University of Michigan, Ann Arbor, Michigan, United States, 3. Duke University, Durham, North Carolina, United States*

Older adults can experience vision impairment following stroke in combination with pre-existing ophthalmologic disease. The new ICD-10 coding system for identifying vision related health conditions provides a much higher level of detail for coding these complex scenarios than the previous ICD-9 system. While this new coding system has advantages for clinical care and billing, the degree to which providers are utilizing the expanded code structure is unknown. The study objective was to describe the use of ICD-10 vision codes in a large cohort of stroke survivors. We used a retrospective cohort design to study national 100% Medicare claims files from 2015 through 2017. Data were analyzed using all available ICD-10 vision codes for beneficiaries who had an acute care stay because of a stroke and who also had an ICD-10 visual code recorded at least once in their claims chart. The cohort (n= 269,314) was mostly female (57.1%) with ischemic stroke (87.8%). Approximately 15% were coded as having one or more vision impairments. Unspecified glaucoma was the most frequently used code among men (2.83%), beneficiaries over 85+ (4.80%) and non-Hispanic blacks (4.12%). But multiple vision codes were used in few patients, overall (0.6%). Less than 3% of those in the oldest group (85+ years) had two vision codes noted in their claims. Despite more available codes, the coding used to describe the vision impairments in this population of stroke survivors was not specific or diverse. Hospital providers should pay attention to specificity in order to improve coding practices.

### SESSION 2906 (POSTER)

### HEALTH AND HEALTH PROMOTION

### ALTERED FEBRILE RESPONSES IN OLDER ADULTS: A SYSTEMATIC REVIEW

Barbara Holtzclaw, *University of Oklahoma Health Sciences Center, Norman, Oklahoma, United States*

Human and animal studies support generalizations that older adults are less able than younger adults to mount an effective febrile response. Beyond difficulties this presents for assessing signs and symptoms of infection, concern exists that older adults may lack fever's protective immuno-stimulant benefits. Fever is a systemic physiological host response to a pyrogen resulting in release of proinflammatory cytokines that produce a regulated elevation of thermoregulatory set-point. Heat is generated, by shivering and molecular activity, and conserved, by vasomotor activity, elevating and maintaining body temperature at the higher set-point level. Because immunological, vasomotor, and kinetic activities raise body temperature, age-associated alterations have been hypothesized to explain blunted febrile responses in older adults. Purpose: A systematic review was done to 1) determine factors underlying presumed origins and alterations in older adults' febrile responses. 2) assess for gaps and controversies in emerging research that could inform care decisions. Comparisons of disciplinary assumptions, perspectives, and cross-disciplinary interpretations sought relevance to interdisciplinary care. Methods: Search of literature databases: Medline (OVID), and CINAHL (EBSCO). PubMed, and included relevant animal and human research findings since 2000 from physiology, gerontology, immunology, infectious disease, clinical medicine, and nursing. Findings: Altered innate immunity in sepsis shows early hyper-reactive response, prolonged inflammatory activity, and fever response contributing to cardiovascular and neurological morbidity, not temperature elevation. Morbidly was attributed to disease not age. Conclusions: Hazards of blunted febrile temperatures include undetected infections and possible loss of immune benefits. Significant evidence of age-related diminished febrile temperature's immune consequences shown with animal models.

### ANNUAL FOOT EXAMS AND INCIDENT AMPUTATION

Latricia Allen,[1] Anjali Khakharia,[2] Lawrence Phillips,[2] Theodore Johnson,[3] Constance Uphold,[4] Molly Perkins,[5] and Camille Vaughan,[5] *1. Emory University School of Medicine, Decatur, Georgia, United States, 2. Emory University, Decatur, United States, 3. Emory University School of Medicine, Atlanta, Georgia, United States, 4. eVA Geriatric Research Education and Clinical Center, Decatur, Florida, United States, 5. Emory University, Atlanta, Georgia, United States*

Diabetes-related lower extremity amputations (LEA) are high cost and high prevalence. Individuals with complications such as neuropathy, foot deformity, history of diabetic foot ulcer or LEA increased morbidity and mortality.1 Current national recommend a foot exam for individuals with diabetes annually or more often depending on risk for LEA.2, The purpose of this pilot study was to examine the relationship between annual foot exams and incident lower extremity amputation in a large Veteran cohort. We conducted a secondary analysis of a national VA Diabetes administrative dataset registry for Veterans with diabetes aged 65 and older during the period of fiscal year 2002-2014 (n=1,544,654; mean age 77.6 years; 97.9 % male). Using logistic regression, we examined the association between annual foot exams and incident LEA. Our analysis was adjusted for demographics, comorbidities, and LEA foot risk. The study included 18,759 (1.21%) Veterans with incident LEA and foot exams, 2,234 (0.14%) Veterans with incident LEA without foot exams. Median age range was 65-75 years old. Gangrene, osteomyelitis, foot ulcers, and neuropathy were the covariates with the highest risk of incident LEA with foot exam. Foot exams did not reduce the risk of LEA when examining Veterans with incident LEA (unadjusted OR of 1.62 (CI 1.56 - 1.69), p<.0001 and adjusted OR was 1.77 (CI 1.69 -1.86), p<.0001. Annual foot exams were not protective for LEA in Veterans with foot exams and incident LEA. Additional research is warranted to examine this relationship considering the effect of early intervention on LEA risk.

### DESIGN AND PRELIMINARY EVALUATION OF A COMMUNITY-BASED BRAIN HEALTH PROMOTION AND WELLNESS PROGRAM

Laura Block,[1] Megan Peck,[1] and Andrea Gilmore-Bykovskyi,[2] *1. University of Wisconsin-Madison School of Nursing, Madison, Wisconsin, United States, 2. University of Wisconsin-Madison, Madison, Wisconsin, United States*

Recommendations for risk reduction of dementia and cognitive decline emphasize addressing modifiable risk factors including physical activity, cognitive stimulation, and socialization. However, existing resources and programs to promote brain health through multifaceted risk factor reduction are often costly, challenging to individualize, and limited in their delivery format, narrowing their accessibility among older adults. To address community identified needs for brain health promotion resources, we applied a user-centered design approach to develop, implement, and evaluate feasibility/acceptability of a community-based brain health promotion program: Strength & Resilience Brain Health and Wellness Program. Design requirements were specified through ongoing end-user feedback via structured and open-ended surveys, group dialogue, and facilitator memoing which informed needed iterative refinements of program components. The resultant program incorporates information about brain health/dementia prevention, physical exercise, and cognitively-stimulating activities targeting attention, focus, problem solving, and communication. Integrated across components are deliberate adaptations for physical/cognitive abilities and activities to facilitate group cohesion and reduce stigma. Across two 10-week offerings, we found the program to be feasible as demonstrated through high enrollment (N=44) and retention (75%). A quarter of participants self-disclosed a dementia diagnosis in baseline surveys. Participants rated program components as acceptable, and perceived positive influences on target risk factors particularly social isolation, sense of self-worth, and cognitive stimulation. The current study provides a platform for a feasible and scalable group, community-based brain health promotion program, and suggests participant-centered outcomes extend beyond cognitive domains. More rigorous evaluation of the program is needed to evaluate fidelity across components and standardized outcomes.

### DETERMINANTS OF ADHERENCE TO ADA TYPE II DIABETES MELLITUS GUIDELINES: IMPLICATIONS FOR LONGEVITY

Arseniy Yashkin, Igor Akushevich, and Anatoliy Yashin, *Duke University, Durham, North Carolina, United States*

The aim of this study was to identify the differences in terms of demographics, socioeconomic status and overall levels of morbidity-related health burden between population strata characterized by high levels of adherence to American Diabetes Association screening guidelines and their low-adherence counterparts. Factor analysis was used to create a single continuous measure of adherence which was stratified and analyzed using the Cox proportional hazards model to identify adherence levels associated with protective effects for mortality. Based on the results, the entire population of Health and Retirement Study respondents newly diagnosed with diabetes mellitus, type II was then stratified into four levels of adherence – excellent, sufficient, insufficient, poor – based on the strength of the protective effect associated with that level of the adherence factor and compared. Mortality in the group associated with excellent adherence was 41 to 57 percentage points lower than among their counterparts. High levels of adherence were associated with White and Hispanic race, low morbidity burden, high education and economic status, and low levels of functioning limitations. Based on race-specific survival function estimates we found that the life expectancy at age 65 of an individual newly diagnosed with type II diabetes mellitus could be improved from 14.97 to 19.64 years for whites, 13.36 to 19.58 years for African Americans and 14.92 to 21.28 for Hispanics if average adherence levels are increased to the highest levels observed in our study. Finally, we found that adherence levels were improving over the 1991-2015 period suggesting successful diabetes awareness efforts.

### DO CENTENARIANS WITH HIGHER LIFE SATISFACTION LIVE LONGER?

Oscar Ribeiro,[1] Lia Araújo,[2] Rosa Marina Afonso,[3] and Laetitia Teixeira,[4] *1. Department of Education and Psychology, University of Aveiro, Aveiro, Portugal, 2. Instituto Politécnico de Viseu, Viseu, Portugal, 3. Universidade da Beira Interior, Covilhã, Portugal, 4. ICBAS-UP, Porto, Portugal*

Subjective well-being (SWB) is defined as a person's cognitive and affective evaluation of life and has been recognized as one of the main psychological factors associated with better health and longevity in different age groups. Several studies evidenced its influence on all-cause mortality, but such a relation has been scarcely explored in individuals aged 100 years and over. The aim of this study is to evaluate the role of SWB in the survival of a sample of centenarians. Two studies conducted in Portugal (PT100 Oporto and PT100 Beira Interior) followed individuals from the age of 100+ years, checking their survival every six months over the period of December 2013 until June 2019. The Satisfaction With Life Scale (SWLS; Diener, Emmons, Larsen, & Griffin, 1985) was used at baseline as a measure of subjective well-being. Given that this is a self-reported scale, only a subsample of centenarians with cognitive capacity were included in this study (n=82; 67 (80.7%) women; mean age at baseline 101 years (sd=1.3 years)). Results obtained through a univariate Cox proportional hazards model suggest that longer survival was associated with higher levels of life satisfaction, highlighting the importance of this psychological dimension for longevity even at very advanced ages.

### EVIDENCE FOR THE EFFECTIVENESS OF HEALTH AND SOCIAL SERVICES PARTNERSHIPS

Traci Wilson,[1] Suzanne Kunkel,[1] Amanda Brewster,[2] Jane Straker,[1] Elizabeth Blair,[3] and Marisa Scala-Foley,[3] *1. Miami University, Oxford, Ohio, United States, 2. University of California, Berkeley, Berkeley, California, United States, 3. National Association of Area Agencies on Aging, Washington, District of Columbia, United States*

Integration of health and social services is touted as a key method to address social needs and improve population health. We will share the latest evidence on how Area Agency on Aging (AAA) partnerships with health care entities and other organizations improve health outcomes for older adults, while reducing health care costs. AAAs are community leaders in cross-sectoral partnerships that effectively address social determinants of health for older adults, who account for a substantial share of overall health care spending. Results of a longitudinal study (2008 – 2016) which links data from four waves of the National Surveys of AAAs to data on county-level health outcomes show that AAA–health care partnerships and programs reduced health care utilization and costs. AAA partnerships with hospitals reduced Medicare spending by $136 per beneficiary. AAA involvement in evidence-based health promotion programs decreased potentially avoidable nursing home use by nearly one percentage point (representing a change of 6.5%). Finally, we will describe the prevalence and nature of contracting relationships between community-based organizations and health care entities, based on data from the 2020 CBOs and

Health Care Contracting Request for Information, the third national RFI of AAAs, Centers for Independent Living, and other aging and disability community-based organizations.

### FACILITATORS AND BARRIERS TO ENGAGING STAKEHOLDERS IN USE-INSPIRED AGING RESEARCH

Rose Ann DiMaria-Ghalili,[1] Justine Sefcik,[2] Katherine Clark,[2] Kathleen Fisher,[3] Jina Huh-Yoo,[2] Jana Hirsch,[4] and Laura Gitlin,[2] *1. Drexel University, Jenkintown, Pennsylvania, United States, 2. Drexel University, Philadelphia, Pennsylvania, United States, 3. Drexel University College of Nursing and Health Professions, Philadelphia, Pennsylvania, United States, 4. Drexel University School of Public Health, Philadelphia, Pennsylvania, United States*

The Cell2Society Aging Research Network at Drexel University is a university-wide, novel ecosystem for the pursuit of use-inspired aging research focused on topics from cellular to societal level. Our goals include: 1) Engaging stakeholders to jointly participate in age-related research; 2) Collaborating to build an infrastructure for age-related research through team science; 3) Develop and implement person-centered research that matters to older adults. We apply interdisciplinary, use-inspired approaches in three areas of relevance to older individuals, their families, healthcare and payment systems, communities and policy makers: (a) preventing and managing chronic conditions, (b) enhancing active and purposeful living, and (c) enabling aging in place in home and communities. Our initial qualitative study described facilitators and barriers experienced by community-based service providers (N=9) and payers/providers (N=5) in the greater Philadelphia area when engaging with academic investigators. We conducted three focus groups. Participants were mostly female (64%), white (64%) and were at their organization between 1-10 years (79%).Conventional content analysis revealed that successful research partnerships were facilitated by: 1) trusted investigators and academic institutions and 2) demonstrated collaborative qualities of investigators (e.g., good communication; provides staff education). Negative experiences engaging with academic-led research were related to: 1) research leaving the community (e.g., results never being shared) and 2) organizational limitations (e.g., lack of internal resources to complete projects). These findings will inform the development of stakeholder-academic partnerships from 2020 onward to design use-inspired aging research initiatives.

### GENDERED ASSOCIATION BETWEEN EMPLOYMENT STATUS AND UNMET HEALTH CARE NEEDS AMONG MIDDLE-AGED KOREANS

Si Young Song, Hey Jung Jun, Susanna Joo, and Sun Ah Lee, *Yonsei University, Seoul, Republic of Korea*

Previous studies show that working people are less likely to experience unmet health care needs than non-workers. Also, employment and health are located in gendered social contexts. The present study aims to examine the moderating effect of gender on the association between employment status and unmet health care needs among middle-aged Koreans. We conducted logistic regression using the Korean Health Panel data (in 2016 and 2017; N=2,573, age range=45-64). Having experiences unmet health care needs in 2017 was the binary dependent variable. Employment status in 2016 was the binary independent variable and gender was the moderating variable. Age, education level, marital status, annual income, household type, type of medical security, disability, self-rated health, the number of chronic diseases, and stress level in 2016 were also in the analytic model based on the Andersen's health behavioral model. The percentages of middle-aged people experiencing unmet health care needs were 18% for working men, 11% for non-working men, 13% for working women, and 16% for non-working women. The result showed there was significant moderating effect of gender (B= .72, $p< .05$). Specifically, working men were less likely to experience unmet health care needs than non-working men. On the contrary, there was not the significant difference in experiencing unmet health care needs between working and non-working women. It indicates that it is necessary to supplement medical services for especially for middle-aged men who are not employed because they might experience considerable amounts of unmet health care needs.

### HEALTH BEHAVIORS AND EHEALTH LITERACY AMONG OLDER ADULTS, HINTS 2019

Ruth Sanchez, Hannah Kay, Pooja Srikanth, Lyndsey Sandow, and Michelle Zhang, *University of Texas at Austin Dell Medical School, Austin, Texas, United States*

With rapid shifts in how health information is reported and consumed, providers and patients must consider their electronic or "eHealth" literacy. The purpose of this study was to analyze how older adults (age 60+) seek health information in the context of online and offline resources and how eHealth literacy correlates with health behaviors. We performed a cross-sectional analysis of a nationally representative sample of 2,587 U.S. older adults drawn from the Health Information National Trends Survey (HINTS) Iteration 5 Cycle 3. Weighted descriptive analyses were conducted to examine the association between CDC-recommended health behavior guidelines on produce consumption and exercise, eHealth literacy, and sociodemographics. Weighted logistic regression analyses were conducted with STATA 16.0 to assess the relationship between healthy behaviors and eHealth literacy controlling for sociodemographics. The weighted sample reported the following demographic characteristics: average age 71 years (range 60-98), 53.6% female, 73.8% White, 9.7% Black and 8.6% Hispanic. Of older adults, 26.7% performed 2 or more health behaviors regularly. Among older adults, those who have looked up medical information using electronics are 1.79 (95% confidence interval: 1.24, 2.58) times more likely to meet 2 or more CDC-recommended health behavior guidelines as compared to those that have not, after controlling for survey group, education, race/ethnicity and gender. Access and utilization of online resources among older adults may influence their health behaviors and health outcomes. Providers should consider the eHealth literacy of their older adult patients and direct them to appropriate and reliable online resources.

### IMPACT OF ORAL HEALTH ON QUALITY OF LIFE AND SUBJECTIVE WELL-BEING IN COMMUNITY-DWELLING OLDER ADULTS IN MEXICO

Irma Díaz,[1] Neyda Ma. Mendoza- Ruvalcaba,[2] Elva Dolores Arias,[3] and Julio Diaz,[4] *1. University of*

*Guadalajara, Guadalajara, Mexico, 2. University of Guadalajara CUTONALA, Guadalajara, Jalisco, Mexico, 3. University of Guadalajara, Guadalajara, Mexico, 4. Hospital Civil Fray Antonio Alcalde, Guadalajara, Mexico*

Objective: Associate the impact of oral health with quality of life and subjective well-being in the community-dwelling older adults in Mexico. Methods: Non-random sample; 326 subjects: age collected (60-69 / ≥ 70); gender (male / female); marital status (couple / no partner); schooling (0-6 years / ≥7); income for basic needs (yes / no); no depression (GDS-15), no cognitive impairment (MMSE) and comorbidity (no disease / ≥ 1 disease) to control biases. Oral conditions; Caries index (ICPOD) WHO criteria: Very low-Low; Moderate and High. Need for dental prostheses (WHO Manual): No prostheses needed (27-28 natural teeth or fixed / removable / total combination; Need prosthesis: 2-28 tooth without replacement. Xerostomia (Thomson Inventory); moderate to severe xerostomia > 17 points. Dependent variables: Quality of Life Related to Oral Health (GOHAI); 57-60 points: High perception. Subjective well-being: Moral Scale of the Geriatric Center of Philadelphia (PGCMS): Low score (0-11). Results: Age: 71.84 ± 7,278; female / male (70.9 / 29.1%). Controlling confounding factors, multiple logistic regression showed that the need for multi-unit or total prostheses; high CPOD index; severe xerostomia; and low perception of well-being subjective, were associated with low GOHAI scores: P = 0.000; P = 0.004; P = 0.003; P = 0.02 respectively. Subjective well-being only was associated with severe xerostomia and low CVRSO perception: P = 0.0 1; P = 0.02 respectively. Conclusion: Taking into account various confounding factors, the Quality of Life related to Oral Health was the most affected by the deterioration of oral health.

### MENOPAUSE SYMPTOM EXPERIENCE AND PERCEIVED WELL-BEING AMONG HIV POSITIVE AND NEGATIVE OLDER WOMEN IN IBADAN, NIGERIA

Olubukola Omobowale,[1] and Olubukola Adesina,[2]
*1. University of Ibadan, Nigeria, Ibadan, Oyo, Nigeria, 2. University of Ibadan, Ibadan, Oyo, Nigeria*

Globally, people are living longer with the Human Immunodeficiency Virus (HIV) and older individuals are becoming infected. Menopause symptoms affect women's health and are associated with perceived declines in wellbeing. This study assessed and compared the menopause symptom experience and perceived wellbeing among HIV positive and negative older women in Ibadan Nigeria Focus group discussions were conducted among menopausal women attending the ARV and GOP clinics at the University College Hospital Ibadan. Opinions of discussants on knowledge and experience of menopausal symptoms, perceptions about the menopause and perceived health status were explored. A total of 90 HIV positive and 92 HIV negative women aged between 40 to 60 years were sampled. Knowledge of the cause of menopause was poor, with more HIV positive women opining that sexual promiscuity causes menopause. The majority of the discussants had adequate knowledge of menopausal symptoms with most of them reporting vasomotor and musculoskeletal symptoms. In both groups, perceptions about the menopause were generally positive as most of them opined that the menopause means freedom from sexual activity and child birth. More HIV negative women perceived themselves to be in good health compared to HIV positive women. Menopause induces many of the same metabolic changes that are being observed with HIV infection, and this may affect the health and quality of life of aging women with HIV infection. There's a need for health education and health promoting interventions that will help these women in coping with the double burden of HIV infection and menopause.

### POSTMENOPAUSAL BILATERAL OOPHORECTOMY IS ASSOCIATED WITH AN INCREASED RISK OF RESTLESS LEG SYNDROME

Nan Huo, Carin Smith, Liliana Gazzuola Rocca, Michelle Mielke, and Walter A Rocca, *Mayo Clinic, Rochester, Minnesota, United States*

Restless legs syndrome (RLS), a common neurologic disorder, is more prevalent in women than in men. Evidence indicates the potential role of female hormones in the pathophysiology of RLS. However, few studies have examined the relationship between premenopausal bilateral oophorectomy (BSO) and the risk of RLS. The Mayo Clinic Cohort Study of Oophorectomy and Aging-2 (MOA-2) includes all women residents of Olmsted County, Minnesota, who underwent premenopausal BSO before the age of 50 years for a non-cancer reason between 1988 and 2007 (n=1,653). In addition, 1,653 age-matched (± 1 year) referent women who did not undergo BSO prior to index-date were included. Survival analyses were used to examine the association between BSO and risk of RLS (defined by DSM-IV criteria) after adjustments using inverse probability weighting. The median (IQR) age at index-date was 40.0 (40.0-47.0) years for the BSO and 40.0 (40.0-47.0) for the non-BSO cohorts. Women who underwent BSO before menopause had a higher risk of RLS (HR, 1.47; 95% CI, 1.08-2.01). In addition, women who underwent BSO before 46 years (HR: 1.53, 95% CI, 1.03-2.28) or received BSO without benign ovarian indications (e.g., cysts, endometriosis, HR: 1.69, 95% CI, 1.11-2.56) were at greater risk of RLS. Estrogen use after BSO did not affect the risk of RLS. Premenopausal women who underwent BSO had an increased risk of RLS, especially those younger than 46 years and without benign medical indication. Women considering BSO for ovarian cancer prevention should be informed of the increased risk of neurological disorder including RLS.

### PREDICTING PERCEIVED HEALTH OF OLDER ADULTS: THE ROLE OF HEALTH, PERSONALITY, AND RESILIENCE

Rotem Arieli, Joseph Kim, and Peter Martin, *Iowa State University, Ames, Iowa, United States*

Past research has not addressed how domain-specific "health" personality traits are associated with resilience and well-being. The purpose of this study was to determine pathways from health personality to perceived health, mediated by resilience. Data included 3,907 participants, 65 and older, collected by a large provider of Medicare Supplemental Health Insurance. The Health Personality Assessment (health neuroticism, health extraversion, health openness, health agreeableness, and health conscientiousness), Brief Resilience Scale, and perceived health were measured. Structural equation modeling and bootstrap mediation were conducted in Mplus. The hypothesized model resulted in a marginal fit, so direct paths from health openness and health conscientiousness to perceived health were added, resulting in an improved fit, $\chi 2(192)=1660.96$, RMSEA=.04, CFI=.95; $\chi 2\Delta(2)=403.99$, p<.001. Health neuroticism and health extraversion negatively predicted perceived health, fully mediated by resilience, $\beta$=-.11, p<.001, and $\beta$=-.01, p<.05, suggesting that people anxious about their health or that talk about their health had significantly lower levels of resilience. Resilience positively predicted perceived health, indicating that more resilient people reported better health. Higher levels of health openness predicted significantly lower levels of perceived health, $\beta$=-.19, p<.001. Greater levels of health conscientiousness predicted better perceived health, $\beta$=.20, p<.001, and resilience in-turn positively related to perceived health, $\beta$=.08, p<.001. Health personality and resilience explained 25.3% of variance in perceived health. This study exemplifies the importance of health personality and resilience in predicting perceived health for older adults. Future research should examine interventions focused on health personality increasing resilience, as older adults with higher resilience reported significantly better health.

### PRESERVING THE ACTIVITIES OF DAILY LIVING INDEPENDENCE IN LATE LIFE

Pao-feng Tsai,[1] Thomas Jakobs,[2] and Reid Landes,[3]
*1. Auburn University, Auburn, Alabama, United States,*
*2. InvoTek Inc., Alma, Arkansas, United States,*
*3. University of Arkansas for Medical Sciences, Little Rock, Arkansas, United States*

Levels of Assistance (LoA) is an effective caregiving intervention for maintaining activity of daily living (ADL) independence. It is a structured, almost prescriptive, approach to encourage completing ADLs as independently as an elder's capabilities permit. With appropriate prompts and assistance during dressing, elders can overcome disability, express retained competencies, and experience success. Simultaneously, caregivers learn to view their functions as maintaining the quality of life of able elders, and they receive reinforcement from elders who are more confident and happier. This study is a continuation of a previous project that created and tested a computer application training program for LoA in nursing homes. We refined the app to include grooming LoA and tested on 10 certified nursing assistant (CNA)/resident dyads at a local nursing home. The pilot results showed, although we did not see consistent improvement in CNA's dressing LoA, we achieved 10% to 30% improvement in grooming LoA. This indicates that the dressing assistance training is able to transfer to grooming LoA. With only an average of one-hour app training, this improvement is cost effective as compared to training provided by care professionals. Future studies should consider incorporating a culture change strategy to improve CNAs' intention for assisting elders. In addition, the training program should be offered in the initial hire to achieve maximum effect.

### PRODUCTIVE AGING AND WORK: OVERCOMING BARRIERS TO CREATING AGE-FRIENDLY WORKPLACES AMONG OLDER WORKERS IN NIGERIA

Olubukola Omobowale, *University of Ibadan, Nigeria, Ibadan, Oyo, Nigeria*

Due to global demographic shifts, the issue of older workers' health and productive aging is becoming much more pressing and Nigeria, the most populous country in Africa is not left behind. Productive aging involves providing a safe and healthy work environment for everyone using strategies that allow workers to function optimally at all ages, hence this study assessed the barriers to creating age friendly workplaces among older workers in Nigeria. A community based study was conducted among workers in Federal Government establishments aged 60 years and above. Using a Focus Group Discussion guide and an adapted checklist on age friendly workplaces, 16 strategic domains affecting older workers' health and age friendly workplaces were explored. Data were analyzed thematically. A total of 150 older workers aged between 60-70 years were sampled. There is no formal older worker's health initiative in all the establishments. Only two, of the sixteen domains checked were present. Other domains remain largely neglected by the management. Barriers to age-friendly workplaces include poor knowledge of the strategies, lack of political will and ageism. The concept of older workers' health is a strange phenomenon among the respondents. Organizations need to invest in developing a robust package to help older workers use their skills and create age friendly workplaces that ensure safety, health and well-being of older workers, from their first day on the job to their last. Government policies targeted at productive aging and work should be put in place in order to create age-friendly workplaces for older workers.

### RECIPROCAL EFFECTS BETWEEN LIFE SATISFACTION AND SLEEP DURATION IN CHINESE OLDER ADULTS

Qian Liu,[1] Haimin Pan,[2] and Yaolin Pei,[3] *1. Hunan Normal University, Changsha, China, 2. Xiamen University, Xiamen, China, 3. New York University, New York, New York, United States*

Sleep problems are prevalent among older adults. It is evident that sleep duration, as an important indicator of sleep quality, is closely associated with life satisfaction. However, it remains unclear as to whether sleep duration influences life satisfaction, or whether lower levels of life satisfaction increase the likelihood of sleep duration decline. This study examined the directional relationship between life satisfaction and sleep duration among Chinese older adults. Data were derived from the China Health and Retirement Longitudinal Study (2011, 2013, 2015 waves; age≥60 year; n = 5689). A cross-lagged panel model (path analysis) with three time points was used to jointly examine the longitudinal reciprocal effects between life satisfaction and sleep duration. This model achieved acceptable indices of goodness of fit. The results revealed that sleep duration were positively associated with life satisfaction at all time points (2011 to 2013: B=.06,SE=.01,p<.0001; 2013 to 2015: B=.05,SE=.01,p<.0001), and life satisfaction also positively predicted sleep duration across timepoints (2011 to 2013: B=.07,SE=.01,p<.0001; 2013 to 2015: B=.03,SE=.01,p<.05). These associations remained unchanged when taking demographics, and noontime napping into account. The findings indicate that the relationship between life satisfaction and sleep duration is bidirectional. Sleep duration may present as a mechanism for the relationship between life satisfaction and health, and suggests that effective treatment of sleep duration may improve life satisfaction.

### T3:T4 RATIO CAN DISTINGUISH BETWEEN ADAPTIVE CHANGES AND TRUE SUBCLINICAL HYPOTHYROIDISM IN OLDER ADULTS

enoch abbey,[1] John McGready,[2] Eleanor Simonsick,[3] and Jennifer Mammen[4], *1. Johns Hopkins University, Baltimore, Maryland, United States, 2. Johns Hopkins School of Public Health, Baltimore, Maryland, United States, 3. National Institute on Aging, Bethesda, Maryland, United States, 4. Johns Hopkins School of Medicine, Baltimore, Maryland, United States*

Because of heterogeneity in hormonal aging,1 we believe isolated elevated TSH is insufficient to drive clinical decision making for thyroid hormone replacement in older adults. We performed a cross-sectional study involving 63 older adult participants of the BLSA in order to assess the diagnostic value of individual hormone levels or free T3: free T4 ratio for differentiating thyroid-aging phenotypes. We defined two phenotypic groups, central adaptation and primary hypothyroidism, both with a rising TSH and with a rising or falling FT4 respectively. Fifty-four percent of study participants were male, the average age was 78.8 years, and 66.7% had the primary hypothyroidism phenotype. The unadjusted odds ratio of having the central adaptation phenotype is 23.40 (95% CI 3.66-149.73) for every unit increase in the FT3:FT4 ratio. The ROC curve had a C-statistic of 0.815. Similarly, FT4 alone distinguished the phenotypes with a C-statistic of 0.864. In contrast, TSH and FT3 were not predictive (C-statistic of 0.617, and 0.479 respectively). When the analysis is limited to the 24 individuals with elevated TSH, the ratio remains predictive (0.839). Both the higher FT4 and the lower ratio found in individuals with adaptive changes are consistent with a physiology similar to the adaptations seen in acute illness. This supports the hypothesis that elevated TSH can represent a response to stressors with aging and doesn't always warrant treatment with thyroid hormone. Our findings suggest that full thyroid function panel can be used to make better diagnostic decisions in older adults.

### WEIGHT LOSS MOTIVATION IN OLDER PRE-DIABETICS: PRELIMINARY EVIDENCE BY RACE IN THE EGGSPDITE STUDY

Marshall Miller,[1] Michael Borack,[2] Jamie Rincker,[2] Shelley McDonald,[2] Kathyrn Starr,[3] and Connie Bales,[3] *1. Duke University, Efland, North Carolina, United States, 2. Duke University, Durham, North Carolina, United States, 3. Duke University School of Medicine, Durham, North Carolina, United States*

Obesity hastens functional decline and intensifies chronic health conditions among older adults. Late-life obesity is of particular concern for older African Americans, who are at increased risk for obesity and type 2 diabetes and for whom weight loss interventions can be less effective. However, obesity interventions have been under-studied in this population; little is known about potential differences in motivation for change by race. The ongoing Eggs PreDiabetes Intervention Trial (EGGSPDITe) is a randomized controlled trial of expedited weight loss in older (60+ years) Black and White adults with prediabetes. Participants completed both the Stages- and Processes of Change questionnaires in Weight Management (S-Weight and P-Weight) at baseline and end of 4-month intervention. Preliminary combined-group analysis indicates that, while White participants reported a higher average stage of change at baseline, there was no difference by race ($ps < 0.05$) in changes for body weight, fat mass, and hemoglobin A1c at endpoint. Reductions in weight consequences evaluation (WCE) and increases in weight management actions (WMA) subscales were observed in both races ($ps < 0.05$), with a trend toward decreased emotional re-evaluation (EmR; $p = 0.06$). Of the processes of change subscales, only WCE differed by race ($p < 0.05$), with White participants showing ≈13.5% higher utilization, relative to Black participants, at both time points. These preliminary findings suggest that weight loss interventions can be equally effective among black and white older adults, although motivations for weight loss may differ by race.

### WHAT ACCOUNTS FOR PHYSICAL AND EMOTIONAL HEALTH? INFLUENCE OF HEALTH, PERSONALITY, AND HEALTH ACTIVATION

Joseph Kim, Nicholas Cone, Rotem Arieli, and Peter Martin, *Iowa State University, Ames, Iowa, United States*

The purpose of this study was to identify associations among health personality, health activation, and emotional and physical health, and to identify direct and indirect effects. Participants in the study consisted of 3907 older adults, 65 years of age and older. Measures used in the analyses were the Health Personality Assessment, the Consumer Health Activation Index, and The Veterans RAND 12-Item Health Survey. Structural equation modeling with bootstrap sampling estimation was conducted to examine direct and indirect effects. The measurement model, X2(307)=2142.34, CFI=0.96, RMSEA=0.04, and structural model, X2(313)=2167.36, CFI=0.96, RMSEA=0.04 yielded an acceptable fit. Significant direct paths were observed between health personality factors and health activation, and in turn health activation to emotional and physical health. The results indicate that older adults with lower levels of Health Neuroticism, lower Health Openness, higher Health Agreeableness, and higher Health Conscientiousness had higher levels of health activation. In addition, older adults with higher levels of health activation had higher emotional and physical health. Also, direct paths from health personality to emotional and physical health were observed. Lastly, significant indirect effects were health activation had a significant positive indirect effect on physical health through Health Agreeableness. Health activation had a significant negative indirect effect on emotional health through Health Neuroticism and Health Openness. The implication of this study is that health activation has a significant role in the emotional and physical health of older adults through health personality dispositions. In addition, health personality factors directly influence the emotional and physical health of older adults.

### SESSION 2907 (POSTER)

### MOBILITY DISABILITY

### ASSOCIATIONS BETWEEN COGNITIVE FUNCTION, BALANCE, AND MOBILITY IN COMMUNITY-DWELLING OLDER ADULTS WITH COPD

Shweta Gore,[1] Jennifer Blackwood,[2] and Tyler Ziccardi,[2] *1. MGH Institute of Health Professions, Charlestown, Massachusetts, United States, 2. University of Michigan-Flint, Flint, Michigan, United States*

Older adults with chronic obstructive pulmonary disease (COPD) are at risk for physical and cognitive impairment. Cognitive function is associated with falls in older adults however it is unknown if a relationship exists between cognitive function and falls in COPD. The aim of this study was to examine the relationships between cognitive function and balance and mobility in older adults with COPD. A secondary analysis was performed using data from the 2010 wave of the Health and Retirement Study (HRS) (N=4051). Cognitive (immediate and delayed recall, executive function) and physical (gait speed, tandem balance time) measure data was extracted from older adults with COPD (N=382) and an age matched control group without COPD (N=382) who met inclusion/exclusion criteria. Multivariate linear regression modeling was performed to examine associations between cognitive function and mobility or balance while controlling for age, gender, BMI, grip strength, and education. We found that in COPD, immediate word recall, delayed word recall, orientation, and executive function ($\beta$ ranging from 0.004-0.02) were significantly associated with gait speed while only delayed word recall ($\beta = 0.122$, $p < .05$) was associated with tandem balance. These same associations did not exist in those without COPD. In older adults with COPD, cognitive function is associated with balance and mobility. Screening for cognitive function, specifically delayed recall, should be a part of the management of falls in this population.

### CLINICAL SCREENING FOR LOWER LIMB MUSCLE WEAKNESS IN COMMUNITY-DWELLING OLDER WOMEN

Daniela Abreu,[1] and Jaqueline Porto,[2] *1. University of Sao Paulo, Ribeirao Preto, Sao Paulo, Brazil, 2. Ribeirão Preto School of Medicine of the University of São Paulo, Ribeirão Preto, Sao Paulo, Brazil*

The objective of the present study was to evaluate the ability of the five times sit to stand test (5TSST), grip strength (GS) and step test (ST) to detect older women with reduced lower-limb muscle strength (LLMS), and to investigate the clinical usefulness of the combination of such tests. One hundred and nineteen older women were submitted to the 5TSST, GS, ST and lower limb peak torque by an isokinetic dynamometer. The capacity of the clinical tests to detect older women with reduced LLMS was measured using the ROC curve, followed by calculation of posttest probability (PoTP). The results show that a ST score of 0.24 cm per cm of participant's height shows the best PoTP for a positive test (72%). However, the combination of the ST and 5TSST enhances the accuracy from 48% (prevalence of weakness in the population) to 82.6% if both tests are positive, and decreases the PoTP from 48% to 11.4% if both tests are negative. The inclusion of GS provided additional benefits of small magnitude. In conclusion, the ST performed alone or in combination with 5TSST could be an alternative for clinical screening of LLMS reduction in older women. The early identification of impairment of lower-limb muscle strength in independent older adults may favor early intervention and prevention of negative outcomes such as falls and functional limitations.

### DIETARY QUALITY AMONG OLDER OVERWEIGHT OR OBESE VETERANS WITH DYSMOBILITY

Elizabeth Parker,[1] Lauren Bergamo,[2] Will Perez,[2] Leslie Katzel,[2] Alice Ryan,[2] Steven Prior,[3] Monica Serra,[4] and Odessa Addison,[5] *1. University of Maryland School of Medicine, Baltimore, Maryland, United States, 2. Baltimore VA Medical Center, Baltimore, Maryland, United States, 3. University of Maryland School of Public Health, College Park, Maryland, United States, 4. UTHSCSA, San Antonio, Texas, United States, 5. University of Maryland School of Medicine, Glen burnie, Maryland, United States*

Older adults have unique dietary challenges due to a myriad of factors including age-related taste and smell changes and lack of nutrition knowledge that increase the risk for poor dietary quality. Healthier dietary quality is associated with higher muscle mass, strength and physical performance which may reduce the development of frailty and disability later in life; however, few studies have examined dietary quality among older Veterans with limited physical functioning. The purpose of this study was to examine overall dietary quality among older, overweight/obese veterans with dysmobility. Habitual dietary intake was assessed at baseline using three, nonconsecutive 24-hour recalls and used to calculate healthy eating index (HEI-2015; higher scores indicate higher diet quality). Twenty-eight participants were included in analysis: 93% male; 54% black; aged=69.5±7.0 years; BMI=35.5±5.4 kg/m2. Means and standard deviations were calculated for average intake of total daily energy (2184±645 kcals) and protein (0.89±0.3g/kg), daily servings of fruits (0.84±0.94) and vegetables (1.3±0.87), and HEI-2015 (52.8±13.4). Overall, 96% consumed fewer than the recommended 5 daily servings of fruits and vegetables and 68% consumed <1.0g/kg/d of protein (1.0-1.3g/kg/d recommended for older adults). Mean HEI-2015 was below the US national average for adults >65 years (2015-2016 NHANES 65+ years: 64.0), suggesting poor dietary quality among our sample. This pilot study suggests that dietary intake quality is suboptimal in older, obese Veterans with disability and highlights the need to identify strategies that improve dietary intake quality of older Veterans who may benefit from obesity and disability management.

### DISABILITY AND SYMPTOM BURDEN AMONG THE VERY OLD: COMPARISON OF SURVIVORS AND DECEDENTS

Xinran Liu, and Steven Albert, *University of Pittsburgh, Pittsburgh, Pennsylvania, United States*

How does disability and symptom burden among the very old differ between those who die and those who do not die over 12 months? We explored patterns of disability and symptom burden in the Health ABC cohort study, which involved quarterly phone interviews in 2011-14 (years 15-17). A proxy completed the interview when the proband was unable to participate. We identified a sample of 291 decedents with at least 1-year of follow-up before death and matched a 1:1 sample of survivors at the time of death by race, sex, and age (within ±3 years). 252 decedents (age 90.0±3.03, 65.1% Black, 52.4% female) and 288 survivors (age 90.1±3.03, 64.9% Black, 52.4% female) with at least 3 quarterly interviews were included for analysis. Decedents had a higher proportion of proxy-reported interviews compared to survivors (40.9%vs16.0%, P<0.01). Disability prevalence among decedents was significantly higher (P<0.01) compared to survivors (using an assisted walking device, 62.3%vs37.4%; difficulty getting in/out of bed, 32.0%vs19.4%; difficulty bath/shower, 28.9%vs10.0%; difficulty dressing, 19.0%vs8.7%). Decedents and survivors differed significantly (P<0.05) in self-reported number of symptoms (2.35vs1.78), severity of disability due to shortness of breath (4.09vs2.04), constipation (3.97vs1.74), and difficulty concentrating (1.98vs1.25). Decedents also had a significant higher score (P<0.01) on self-reported loss of appetite (2.24vs1.91) and worse global quality of life rating (3.04vs2.64), compared to survivors. The patterns were similar in proxy-reported group and in the group with a combination of self-and proxy-reported interviews. Even in very late old age, disability and symptom burden increase with the approach of death.

### DISABILITY DURING THE LAST YEARS OF LIFE AMONG NONAGENARIANS: THE VITALITY 90+ STUDY 2001-2014

Linda Enroth,[1] Pheck Hwa Soo,[2] Lily Nosraty,[1] Kristina Tiainen,[1] Jani Raitanen,[1] Marja Jylhä,[1] and Mari Aaltonen,[1] *1. Tampere University, Tampere, Finland, 2. JGZ Kennemerland, Velserbroek, Netherlands*

Increasing life expectancy has postponed the last years of life to older ages. Previous studies have demonstrated that disability is determined by age, age at death and closeness to death but only few have focused on oldest old population. We examined disability during the last years of life among people aged 90 years and older between 2001 and 2014 and assessed whether it varied by age at death, sex and study year. We used population-based survey data from the Vitality 90+ Study years 2001, 2003, 2007, 2010 and 2014 (N=5711, response rate 77-86%) linked with dates of death from Statistics Finland. Disability was defined as dependency in daily activities (dressing, getting in and out of bed) and mobility (moving indoors, walking 400m, using stairs). We analyzed disability stratified by closeness to death and age at death for men and women in each study year with logistic regression method. Disability in daily activities and mobility increased systematically with closeness to death (>4, 3-3.99, 2-2.99, 1-1.99 and 1> years to death) for both sexes in each study year. Also higher age at death (90-91 vs. 94+ years) was associated with disability. These associations remained consistent throughout the study period. This study shows that in the oldest old population both closeness to death and age at death determine the level of disability. We suggest that the complex and resource-draining care needs at the end of life will increase with growing number of people living their last years of life in very old age.

### EFFECTS OF A MULTIDISCIPLINARY INTERVENTION ON DAILY-LIVING GAIT AMONG OLDER ADULTS WITH PARKINSON'S DISEASE

Jeffrey Hausdorff,[1] Moriya Cohen,[2] Natalie Ganz,[3] Yitchak Green,[4] Inbal Badichi,[5] and Tanya Curevich,[3] *1. Tel Aviv Sourasky Medical Center, Tel Aviv, Tel Aviv, Israel, 2. EZRA–LEMARPE, Bnei Bar, Israel, 3. Tel Aviv Sourasky Medical Center, Tel Aviv, Israel, 4. EZRA–LEMARPE Organization, Bnei Brak, Israel, 5. EZRA–LEMARPE Orginization, Bnei Brak, Israel*

Multidisciplinary interventions can improve gait and balance in patients with Parkinson's disease (PD). However, it is not yet known if these interventions also positively impact the quality of daily-living walking. We, therefore, examined the effects of a multidisciplinary, intensive out-patient rehabilitation program (MIOR) as delivered by the rehabilitation center of EZRA–LEMARPE organization on gait and balance as measured in the clinic and on every-day walking, as measured during 1-week of continuous measurement. 46 PD patients (age: 70.05±7.71; gender: 31.3% women; disease duration: 8.85±6.27 yrs) were evaluated before and after participating in 8-weeks of physical, occupational, and hydro-therapy, boxing, and dance (3 days/week; 5 hrs/day). After the intervention, clinical measures of balance (MiniBest Test of Balance delta: 1.82±3.30 points, p=0.001), mobility (TUG delta: -1.78±6.15sec; p=0.001), and usual-walking speed (delta 19±16cm/s; p<0.001) improved. Daily-living step counts and daily-living gait quality did not change (p>0.5). In exploratory analyses, subjects were categorized as responders (Rs) and non-responders (NRs) based on changes in their daily-living walking gait speed. Rs increased their daily-living gait speed (delta: 10±14cm/s; p<0.001); NRs did not. Rs (n=21) also improved their daily-living gait quality measures (e.g. stride regularity, step length, stride time variability). At baseline, disease severity (MDS-UPDRSIII) was lower (p=0.02) in Rs (25.33±11.47), compared to the NRs (34.38±14.27). These results demonstrate that improvements in the clinic do not necessarily transfer to improvements in daily-living gait. Further, in select patients, MIOR can ameliorate daily-living walking quality, potentially reducing the risk of falls and other adverse outcomes associated with impaired mobility.

### MOBILITY MATTERS: A PILOT STUDY ON INCREASING HEALTH LITERACY AMONG FALL-RISK ELDERS

Mary Milidonis,[1] Jane Keehan,[2] Rebecca Deuley,[2] Sara Formoso,[2] Katherine Montgomery,[2] and Karen Kopera-Frye,[3] *1. Cleveland State University, Cleveland, Ohio, United States, 2. Cleveland State University, Cleveland, United States, 3. New Mexico State University, Las Cruces, New Mexico, United States*

Mobility is important to sustain for older adults to live independently. The purpose of this project was to evaluate teach back and ask me 3 interventions with a health education program that included Otago strength and balance exercise and a walking program The pilot program, Mobility

Matters, was completed with 16 older adults (mean age =76, range 63-87, SD = 8.6), 69% African American, 94% female. Older adults with moderate fall risk were recruited from community centers and participated in a 3-month program where they were paired with physical therapy students for pre- and post-intervention assessment. Participants were randomly assigned to a health literacy intervention group (HLG) (n=9) and received teach back and ask me 3 intervention twice a month for three months. The control group (n=7) received the same program of balance exercises/ walking program and after 3 months was given the health literacy intervention. Groups were not significantly different on age, gender and REALM scores. Assessment measures included: timed up and go, 30 second chair rise, 4 stage step test, 6 minute walk test, and activity balance confidence scale (ABC). Paired t-test analysis revealed mean significant differences on the measures of four stage balance test (p =.008), six-minute walk test (p=.026) and approached significance on ABC (p=.054). No significant differences were found for the non-health literacy group on all measures. The results suggest that health literacy intervention may improve outcomes for health education interventions with balance and aerobic exercise.

### MOTOR AND PHYSICAL FUNCTION IMPAIRMENTS IN MIDDLE-AGED AND OLDER ADULTS IN THE BALTIMORE LONGITUDINAL STUDY OF AGING

Yurun Cai,[1] Qu Tian,[2] Yuri Agrawal,[3] Eleanor Simonsick,[2] and Jennifer Schrack,[1] *1. Johns Hopkins Bloomberg School of Public Health, Baltimore, Maryland, United States, 2. National Institute on Aging, Bethesda, Maryland, United States, 3. Johns Hopkins University School of Medicine, Baltimore, Maryland, United States*

Older adults experience motor function decline early in the disablement process, impacting daily activities and contributing to adverse health outcomes. Few studies have comprehensively examined the interrelationships among motor and functional impairments and investigated whether their contributions to mobility difficulty vary in well-functioning older adults. We examined direct and indirect associations of motor and physical function impairments with slow gait speed (<1.0m/s) and mobility difficulty using structural equation modeling (SEM) among 858 participants aged ≥50 years in the BLSA (mean age=74.1±10.6, 55% women). Motor and physical function tests included grip strength, knee extension strength, proprioception, finger tapping, standing balance (semi-, full-tandem, single-leg), repeated chair stands, and usual gait speed. Mobility difficulty was defined as self-reported difficulty in walking ¼ mile or climbing stairs. Motor and physical function impairments increased linearly with age, with 27.6% of participants having slow gait speed and 10.4% having mobility difficulty. Age-adjusted SEMs identified chair stands pace as the strongest predictor of slow gait speed, followed by latent factors of upper and lower extremity muscle strength and standing balance. Chair stands pace was the strongest predictor of mobility difficulty, followed by gait speed. Latent factors of muscle strength, proprioception, finger tapping, and standing balance were indirectly associated with mobility difficulty via gait speed. All models showed good model fit (RMSEA<0.05, CFI>0.95). These findings suggest components of strength and balance are among the most important contributors to poorer functional performance in mid-to-late life. Future longitudinal studies gauging the effect of change in these factors are warranted.

### MOTOR AND PULMONARY FUNCTION AND MOBILITY DISABILITY AMONG BLACK AND WHITE OLDER ADULTS WITH AND WITHOUT HIV

Brittney Lange-Maia,[1] Aron Buchman,[1] Sue Leurgans,[2] Elizabeth Lynch,[1] Melissa Lamar,[1] Kristine Erlandson,[3] Lisa Barnes,[1] and Brittney Lange Maia,[1] *1. Rush University Medical Center, Chicago, Illinois, United States, 2. Rush Alzheimer's Disease Center, Chicago, Illinois, United States, 3. University of Colorado Denver-Anschutz Medical Campus, Aurora, Colorado, United States*

Black-White disparities in gait speed have been observed in studies of adults reporting HIV, consistent with work among older adults without HIV. However, it is unknown if racial differences exist among adults with HIV for other mobility-related factors. We aimed to determine if racial differences exist in mobility disability among older adults with and without HIV and assess if pulmonary and motor function contribute to mobility disability. We examined older adults age 50+ with HIV (N=177; 72% Black) and without HIV (N=191; 68% Black). Motor function summarized 10 motor performances including gait speed; pulmonary function summarized 3 measures assessed using hand-held spirometry. Mobility disability was based on self-report. In regression models adjusted for age, sex, medical conditions, and smoking, neither race nor HIV status were associated with mobility-related factors. However, in models stratified by HIV status, Blacks with HIV had worse motor (β=-4.3, p=0.04) and pulmonary function (β=-50.5, <0.001) and higher odds of mobility disability (odds ratio [OR]=2.9, 95% confidence interval [CI]=1.01-8.2) compared to Whites with HIV. Racial differences were not apparent among uninfected participants in motor function, pulmonary function, or mobility disability. In subsequent models, racial differences in mobility disability were attenuated and no longer significant in HIV when adjusting for motor function (OR=0.88 per/% higher motor composite, 95% CI=0.84-0.93). Racial differences in mobility disability in HIV were unaffected when controlling for pulmonary function. Results suggest that Blacks with HIV have greater mobility disability compared to Whites with HIV, and these differences are due to differences in motor function.

### REGIONAL ASSOCIATIONS OF CORTICAL THICKNESS WITH GAIT VARIABILITY: THE TASMANIAN STUDY OF COGNITION AND GAIT

Oshadi Jayakody,[1] Monique Breslin,[2] Richard Beare,[3] Velandai Srikanth,[3] Helena Blumen,[4] and Michele Callisaya,[5] *1. University Of Tasmania, Hobart, Australia, 2. University Of Tasmania, Hobart, Tasmania, Australia, 3. Monash University, Melbourne, Australia, 4. Albert Einstein College of Medicine, New York, New York, United States, 5. Monash University, Frankston, Australia*

Gait variability is a marker of cognitive decline. However, there is limited understanding of the cortical regions associated with gait variability. We examined associations between regional cortical thickness and gait variability in a population-based sample of older people without dementia. Participants (n=350, mean age 71.9±7.1) were randomly selected from the electoral roll. Variability in step time, step length, step width and double support time (DST) were calculated as the standard deviation of each measure, obtained from the GAITRite walkway. MRI scans were processed through FreeSurfer to obtain cortical thickness of 68 regions. Bayesian regression was used to determine regional associations of mean cortical thickness and thickness ratio (regional thickness/overall mean thickness) with gait variability. Smaller overall cortical thickness was only associated with greater step width and step time variability. Smaller mean thickness in widespread regions important for sensory, cognitive and motor functions were associated with greater step width and step time variability. In contrast, smaller thickness in a few frontal and temporal regions were associated with DST variability and the right cuneus was associated with step length variability. Smaller thickness ratio in frontal and temporal regions important for motor planning, execution and sensory function and, greater thickness ratio in the anterior cingulate was associated with greater variability in all measures. Examining individual cortical regions is important in understanding the relationship between gray matter and gait variability. Cortical thickness ratio highlights that smaller regional thickness relative to global thickness may be important for the consistency of gait.

### THE ASSOCIATION BETWEEN PERSONALITY AND HOMEBOUND STATUS IN OLDER ADULTS: RESULTS FROM THE NHATS

Xiaocao Sun,[1] Minhui Liu,[2] Christina E. Miyawaki,[3] Yuxiao Li,[4] Tianxue Hou,[4] Siyuan Tang,[1] and Sarah Szanton,[5] *1. Central South University, Changsha City, Hunan, China, 2. Central South University, Baltimore, Maryland, United States, 3. University of Houston, Seattle, Washington, United States, 4. Central South University, Changsha, Hunan, China, 5. Johns Hopkins University, Baltimore, Maryland, United States*

Personality is associated with predictors of homebound status like frailty, incident falls, and depression. It has been rarely investigated whether personality predicts homebound status among older adults. Using the combining cross-sectional data of the Year 2013 and Year 2014 data from the National Health and Aging Trends Study (NHATS), this study examined the association between personality traits and homebound status in a sample of community-dwelling older adults aged 65 years and older (N=2,788). Homebound status (non-homebound, semi-homebound, and homebound) was determined by the frequency, difficulty, and help of outdoor mobility. Personality traits, including conscientiousness, agreeableness, openness, extraversion, and neuroticism were assessed using the 10-item Midlife Development Inventory on a rating scale from 1 (not at all) to 4 (a lot). Each personality trait was included as a predictor in an ordinal logistic regression model to examine its association with homebound status after adjusting demographic and health-related covariates. The sample was on average 79±7.53 years old, non-Hispanic White (72.0%), female (58.6%), living alone (35.4%) or with spouse/partner only (37.4%). Seventy-four percent, 18%, and 8% of participants were non-homebound, semi-homebound, and homebound, respectively. Homebound participants tended to be less-educated older females. The average scores of conscientiousness, agreeableness, openness, extraversion, and neuroticism were 3.19±0.75, 3.57±0.56, 2.81±0.83, 3.13±0.75, and 2.22±0.86, respectively. Among these five personality traits, high conscientiousness (OR=1.34, p<0.001) and extraversion (OR=1.16, p=.03) were associated with a reduced likelihood of being homebound. These findings provided a basis for potential personality assessment to identify and protect individuals with high homebound risk.

### SESSION 2908 (POSTER)

### FALL RISK AND FALL PREVENTION

### A PRACTICAL AND VALIDATED FALL RISK SCREENING INSTRUMENT: A SYSTEMATIC REVIEW

Wytske Meekes,[1] J.C. Korevaar,[2] C.J. Leemrijse,[2] and L.A.M. van de Goor[2], *1. Tilburg University, Tilburg, Noord-Brabant, Netherlands, 2. Nivel (Netherlands Institute for Health Services Research), Utrecht, Utrecht, Netherlands*

Early detection of a high fall risk is important to start fall preventive interventions in time and to reduce fall risk among older people. Several fall risk screening instruments are available, however it is unclear which instrument is validated and most suitable for the primary care setting. This systematic review aims to identify the most suitable fall risk screening instrument(s) for the primary care setting (i.e. requires limited time, no expensive equipment and no additional space) with good prognostic ability to assess high fall risk among independently living older people. An extensive search was conducted in the databases PubMed, EMBASE CINAHL, Cochrane and PsycINFO. Twenty-six out of 2277 articles published between January 2000 and February 2019 were included. Six fall risk screening instruments were identified; TUG test, Gait Speed test, BBS, POMA, FR test, Fall History. Most articles reported AUCs ranging from 0.5-0.7 for all instruments. Sensitivity and specificity varied substantially across studies (e.g. TUG, sens.: 10-83.3%, spec.:37-96.6%). The results showed that none of the included screening instruments had sufficient (AUC>0.7) predictive performance (Šimundić, 2009). As suitability for the primary care setting prevails for now, Fall History appears to be the most suitable screening instrument. Compared to the other instruments, Fall History requires the least amount of time, no expensive equipment, no training, and no space (adjustments). Patient's fall history together with a health care professional's clinical judgment, might be a promising screening strategy for the primary care setting to identify high fall risk among older people.

### ACCURACY OF NURSES' FALL PREVENTION INTERVENTIONS IN PATIENTS WITH COGNITIVE IMPAIRMENT AND BEHAVIORAL SYMPTOMS

Deanna Gray-Miceli,[1] and Alison Kris,[2] *1. Thomas Jefferson University, Philadelphia, Pennsylvania, United States, 2. Nursing, Fairfield, Connecticut, United States*

Over 50 percent of older residents in nursing homes (NHs) fall each year. Many falls occur among NH patients with cognitive impairment and behavioral symptoms. Although NH nurses caring for patients who fall intervene to prevent fall recurrence, we know little about nurse's perceptions of the most effective interventions for management of falls related to behavioral symptoms. The purpose of this qualitative study is to describe and analyze nurse's perceptions of fall prevention interventions believed to be due to behavioral symptoms. This secondary analysis of existing qualitative data was conducted from a multi-site parent study in three NHs in the northeastern U.S. designed to test nurse's knowledge of falls prevention and interventions. Forty-seven registered or licensed practical nurses, English speaking who were full or part time employees were recruited to participate. Most were female (n=46; 98.7%) with a median age of 49.5 years and ten years experience. A grounded theory approach was used to explore qualitative data (Glaser & Strauss, 1967) about nurse's primary and secondary interventions. Qualitative data were collected in the form of responses to open ended questions from 47 nurses. The correctness of nurse interventions were independently evaluated, then validated among two independent experts. Cohen's κ was used to determine if there was agreement between the experts' judgement on correctness of the nurses' intervention. Results indicated a high level agreement between expert evaluations (κ =.727-.760, p<.001). Emergent themes in the nurse interventions included: seeking outside help, confronting behavior, completing additional assessments, and patient reassurance.

### BIDIRECTIONAL RELATIONSHIP BETWEEN SUBJECTIVE AGE AND FEAR OF FALLING: FINDINGS FROM THE NHATS

Yuxiao Li,[1] Minhui Liu,[2] Christina E. Miyawaki,[3] Tianxue Hou,[1] Xiaocao Sun,[4] Siyuan Tang,[4] and Sarah Szanton,[5] *1. Central South University, Changsha, Hunan, China, 2. Central South University, Baltimore, Maryland, United States, 3. University of Houston, Seattle, Washington, United States, 4. Central South University, Changsha City, Hunan, China, 5. Johns Hopkins University, Baltimore, Maryland, United States*

Subjective age refers to how young or old people experience themselves to be. It is associated with health status, physical function, and mental processes that influence the occurrence of fear of falling, which is a severe problem in older adults. The present study aims to contribute to existing knowledge by examining the relationships between subjective age and fear of falling. Participants were community-dwelling adults aged 65 years and older drawn from the 2011 (T1) - 2015 (T5) waves of the National Health and Aging Trends Study. Subjective age was measured by asking, "What age do you feel most of the time?" Fear of falling was measured with the question: "In the last month, did you worry about falling down?" The analyses included 1,984 participants without fear of falling at baseline, on average, 74.9±6.6 years old, female (53%), and non-Hispanic whites (76%). Eighty percent of the participants felt younger, and 4% felt older than their chronological age, but 21% of them had a fear of falling. Participants who experienced fear of falling at T5 tended to be female, non-Hispanic whites, and live alone. Generalized estimating equations revealed that an "older" subjective age independently predicted fear of falling (OR, 95%CI= 1.02, 1.01-1.02) controlling for demographics and health conditions. Fear of falling predicted an "older" subjective age (OR, 95%CI= 3.38, 1.72-6.66) after adjustment. These findings indicated bidirectional relationships between subjective age and fear of falling. Subjective age may help identify individuals who are at risk for fear of falling in older adults.

### EFFECTS OF A FALL PREVENTION PROGRAM ON FALL RISKS, FEAR OF FALLING, AND DEPRESSION IN OLDER ACTIVE ADULTS

James Karnes, Victoria Bates, Ashley Chafin, Sarah Fisher, Kelcie Gilmore, and Kelsey Pack, *georgia southern university, savannah, Georgia, United States*

Falls are the leading cause of injury in older adults; one in four older adults fall each year. The Otago Exercise Program (OEP) is an evidence-based fall prevention program that has been shown to reduce fall risk factors. However, exercise dosage is not known. The purpose of this study was to investigate the effectiveness of different frequencies of an OEP-based program on fall risk factors, fear of falling, and depression in older adults. Of 62 subjects initially recruited at community centers, 28 subjects met inclusion criteria and were assigned to control (Con), once-weekly (Grp 1), or twice-weekly (Grp 2) intervention groups based on subject attendance. Intervention consisted of a 12-week OEP-based program. Pre-intervention dependent variables included: 4-Stage Balance Test, 30-Second Chair Stand, Timed Up and Go (TUG), Geriatric Depression Scale (GDS), and Modified Fall Efficacy Scale (MFES). After 12 weeks, post-intervention testing assessed changes in these variables. Preliminary analysis of data using mixed design ANOVA (p.05) indicated significant changes between and within all groups for TUG and 30-Second Chair Stand. Results also suggested all groups improved on all variables with a direct relationship to exercise frequency. Furthermore, Grp2 improved more than Grp1 and Con in the 4-Stage Balance Test. These findings suggest an OEP-based falls prevention program performed 2x/week is more beneficial in decreasing fall risks and increasing lower extremity muscle strength than 1x/week. Moreover, results imply exercise frequency may be important in improving the magnitude of select falls risks variables.

### EFFECTS OF DANCE THERAPY ON BALANCE AND RISK OF FALLS IN OLDER PERSONS

Esha Chakravarty,[1] Indrani Chakravarty,[2] Ipsito Chakravarty,[3] and Prasenjit Bhattacharjee,[1] *1. Calcutta Metropolitan Institute of Gerontology, Mitcham, England, United Kingdom, 2. Calcutta Metropolitan Institute of Gerontology, Kolkata, United States, 3. Calcutta Metropolitan Institute of Gerontology, Kolkata, West Bengal, India*

Loss of balance and risk of falls is a major problem in older persons. Literature shows increasing use of yoga practices and dance therapy across Indian oldage homes and day care centres to improve balance and reduce risk of falls in older persons. Aim of this study is to evaluate the effects of dance therapy with focus on therapeutic movements derived from Indian classical dances on balance and risk of falls in older adults of Day Care Centres in Calcutta Metropolitan Institute of Gerontology, under Ministry of Social Justice and Empowerment, Govt. of India. Total of 24 older adults across 2 day care centres participated in the study attending dance therapy sessions for 3 months. All of them self reported problems of balance and repeated falls alongwith difficulties in performing Activities of Daily Living. Twenty one of them were females and 3 males. The mean age of the participants was 75.5 years. Limits of Stabililty (LOS) was used to measure balance and pre tests and post tests were performed. Results showed that the Limits of Stability were significantly higher (17.5%) in older persons after participating in the dance therapy sessions. This study supports that dance therapy using movements derived from Indian classical dance forms can support older persons to function with reduced risk of falls, improved balance, safely carry out mobility tasks and perform better Activities of Daily Living . Further studies can show how dance therapy can facilitate healthy ageing and influence State policies on healthy ageing.

### EVALUATION OF RACE AS A PREDICTOR OF FEAR OF FALLING IN BLACK OLDER ADULTS

Selena Washington,[1] Susan Stark,[2] Makenna Synder,[3] Yi-Ling Hu,[4] and Brittany Minor,[3], *1. Saint Louis University, Saint Louis, Missouri, United States, 2. Washington University in St. Louis, St. Louis, Missouri, United States, 3. Washington University, St. Louis, Missouri, United States, 4. Wayne State University, Detroit, Michigan, United States*

Fear of falling (FOF) is a common issue and health concern reported by adults 65 years of age and older.There is mixed evidence about potential disparity in fall incident rates due to race and FOF, and it is unclear if and under what circumstance falls rates and risks differ by racial ethnicity. The purpose of this study is to determine if race predicts fear of falling in older adults at greater risk for falls; and to examine the relationship between activities of daily living (ADL) and mobility performance measures with FOF. A cross-sectional observational study was used to examine predictors of FOF among community dwelling-older adults using data from two longitudinal randomized clinical trials (RCT). Participants (N=259) had a mean age of 75.7 ±7.4, 78.8% female, 80.64% Black, and 77.03% White or Other, with ≥ 1 fall in the last 12 months. Subjects completed a demographic profile; the Tinetti and Short Falls Efficacy Scale to assess FOF; and activities of daily living (ADL) and mobility performance scales to assess function. The chi square analysis revealed Black older adults were two times more likely to report FOF (OR = 2.17, 95% CI = 1.14, 4.15; p=.05) in comparison to White older adults. The regression analysis demonstrated that race is a significant factor to predict FOF (OR = .67, 95% CI = 1.21, 3.24; p=.05); and the descriptive analysis revealed significantly worse ADL and mobility function scores within the high FOF group.

### FALL PREVENTION IN DUTCH PHYSICIAN PRACTICES: A DESCRIPTIVE STUDY

Wytske Meekes,[1] C.J. Leemrijse,[2] J.C. Korevaar,[2] and L.A.M. van de Goor[2], *1. Tilburg University, Tilburg, Noord-Brabant, Netherlands, 2. Nivel (Netherlands institute for health services research), Utrecht, Utrecht, Netherlands*

Falls are an important health threat among frail older people. Physicians are often the first to contact for health issues and can be seen as designated professionals to provide fall prevention. However, it is unknown what they exactly do and why regarding fall prevention. This study aims to describe what physicians in the Netherlands do during daily practice in regards to fall prevention. About 65 physicians (34 practices) located throughout the Netherlands were followed up for 12 months. When a physician entered specific ICPC-codes related to frailty and falls in the Hospital Information System, the physician received a pop-up asking if the patient is frail. If so, the physician subsequently completed a questionnaire. The physicians completed 1396 questionnaires. More than half (n=726) of the patients had experienced a fall in the previous year and/or had a fear of falling (FOF) and 37% of these patients received fall prevention. Physicians did not know of 20% of the patients if they had experienced a fall and of 29% of the patients if they had a FOF. The three most often treated underlying causes were mobility problems, FOF and cardiovascular risk factors. The results show that physicians are not always aware of a patient's fall history and/or FOF and that only part of these patients receives fall prevention. Hence, it might be important to develop and implement strategies for systematic fall risk screening and fall prevention provision in the primary care setting to reduce falls among frail older people.

### IMPROVED PROCESSING SPEED IS A POTENTIAL MECHANISM BY WHICH EXERCISE REDUCES FALLS

Teresa Liu-Ambrose,[1] Jennifer Davis,[2] Ryan Falck,[1] and Karim Khan,[1] *1. University of British Columbia, Vancouver, British Columbia, Canada, 2. University of British Columbia, Kelowna, British Columbia, Canada*

A 12-month trial demonstrated the Otago Exercise Program (OEP), a home-based exercise program of strength and balance retraining exercises, significantly reduced the rate of subsequent falls among 344 older adults receiving care after a fall (JAMA, 2019). A significant improvement in processing speed, as measured by the Digit Symbol Substitute Test (DSST), was also observed. Given the DSST is a predictor of falls, we conducted mediation analyses to determine whether improved DSST mediated the effects of OEP on rate of: 1) total falls; 2) non-injurious falls; 3) mild injurious falls; and 4) severe injurious falls over the 12-month trial. Our causal mediation analyses were conducted using the mediation package in R, using quasi-Bayesian estimates and 95% confidence intervals. Compared with usual care, OEP significantly reduced the rate of total falls (IRR= 0.64; 95% CI: 0.44, 0.91; p= 0.013) and mild injurious falls (IRR= 0.49; 95% CI: 0.31, 0.77; p= 0.002). Improved DSST score was also associated with lower mild injurious fall rates (IRR= 0.95; 95% CI: [0.91, 0.99]; p= 0.014). Formal mediation analyses showed that improved DSST was a significant mediator of the effect of OEP on the rate of mild injurious falls (95% CI: -0.15, 0.00; p= 0.036). Improved processing speed may be a mechanism by which exercise reduces mild injurious falls.

### MEASURING FEAR OF FALLING AND APPRAISALS OF HARM

Helen Lach,[1] and Wanida Noimontree,[2] *1. Saint Louis University, St. Louis, Missouri, United States, 2. Faculty of Nursing Burapha University, Mueang Chon Buri, Chon Buri, Thailand*

Fear of falling (FOF) causes premature disability in older adults who limit their physical activities, making FOF an important area for aging research. Measures of falls self-efficacy have been widely used, but less attention has been paid to measuring the construct of fear. We re-assessed an older measure of fear and appraisals of harm to determine the factor structure and potential for a shorter version. Data was collected from 329 older adults from a variety of community setting (mean age 76.3, 84% female, 81% White); 63.8% reported FOF. Factor analysis resulted in four factors with eigenvalues greater than 1.0. Two factors achieved adequate Cronbach's alpha: appraisals of harm outcomes (0.924) and social stigma (0.815). Further analysis was used to identify a short form, resulting in a 6 item measure of appraisal of harm outcomes (Cronbach's alpha 0.86). The original and short versions were moderately correlated with falls self-efficacy as measured by the short form, and level of fear of falling using a 10-point rating scale (.327 - .492). Research is needed to further clarify psychological targets to help older adults remain physically active to improve quality of life.

### MEDICATION PRESCRIBED WITHIN 1 YEAR PRECEDING FALL-RELATED INJURIES IN OLDER ADULTS IN ONTARIO, CANADA

Yu Ming,[1] Aleksandra Zecevic,[2] Richard Booth,[2] Susan Hunter,[2] Andrew Johnson,[2] and Rommel Tirona,[2] *1. Western University, London, Ontario, Canada, 2. Western University, London, ON, London, Ontario, Canada*

Background: The consequences of fall-related injuries are becoming more significant due to ageing societies worldwide. This study aims to provide information on medications prescribed to older adults within one year before they experienced fall-related injury in Ontario, Canada. Methods: A population-based descriptive study of older adults (66 years and older) who experienced fall-related injury was conducted using administrative secondary health care data of Ontario. The percentages of patients prescribed each Anatomical Therapeutic Chemical 4th level medication class and fall-risk increasing drugs one year before their fall-related injuries was summarized. Results: From 2010 to 2014, 288,251 older adults (63.2% females) were admitted to Emergency Department due to fall-related injury, 39.9% were fall-related fractures, 12.6% were head injuries. One year prior to their injury, 48.46% of older adults were prescribed with statins; 35.23% were prescribed with diuretics; 26.84% were prescribed with antidepressants; 25.90% were prescribed with opioids and 16.61% were prescribed with anxiolytics. A higher percentage of females were prescribed with diuretics, antidepressants, and anxiolytics than males. 85 years and older people had higher percentage of prescription of diuretics, antidepressants and antipsychotics than other age group. Discussion: In general, older adults diagnosed with fall-related injuries were prescribed with more opioids, benzodiazepines and antidepressants than other general older adults. There were distinct patterns of prescription medication within each sex and age group (66-74 group, 75-84 group and 85 years and older group). Further association between medications and fall-related injuries need to be established using well-defined cohort studies.

### MOTORIC COGNITIVE RISK SYNDROME USING 3-ITEMS RECALL TEST AND ITS ASSOCIATION WITH FALLS

Hayoung Shim,[1] Miji Kim,[2] Heeeun Jung,[3] Yuri Seo,[3] Seoyoon Jane Lee,[4] and Chang Won Won,[5] *1. Kyung Hee University, Seoul, Korea, Seoul, Republic of Korea, 2. College of Medicine, Kyung Hee University, Seoul, Republic of Korea, 3. Graduate School, Kyung Hee University, Seoul, Republic of Korea, 4. Elderly Frailty Research Center, Kyung Hee University, Seoul, Republic of Korea, 5. Kyung Hee University Medical Center, Seoul, Korea, Seoul, Republic of Korea*

Motoric cognitive risk (MCR) syndrome is defined by the presence of subjective cognitive complaints (SCCs) and slow gait. Its components have synergistic effects to predict various adverse health outcomes. However, some studies reported SCCs might be influenced by cultural differences. Therefore, we devised another criterion to assess the cognitive aspect of MCR to enhance its utility irrespective of the cultural background of the subject by examining the association of MCR and its components with fall-related outcomes. This cross-sectional analysis included 2,641 community-dwelling older adults aged 70-84 years from the Korean Frailty and Aging Cohort Study. These participants did not have dementia and had no difficulties in performing activities of daily living. The newly devised criterion for cognitive aspects of MCR is based on three items recall test on Mini-Mental State Examination. One-hundred-ninety participants (7.2%) met the criteria of MCR using three items recall test. Unlike MCR using SCCs, newly defined MCR showed synergistic effects with fall-related outcomes. Participants with MCR using three items recall test showed a higher risk for falls in the past 1-year (odds ratio [OR] 1.59, 95% confidence interval [CI] 1.08-2.34), recurrent falls (OR 1.86, 95% CI 1.10-3.13), fall with injury (OR 1.60, 95% CI 1.10-2.34), fear of falling (OR 2.77, 95% CI 1.90-4.03), and low activities-specific balance confidence (OR 2.86, 95% CI 1.78-4.60) compared to each component, except for fall with fracture. We found MCR with three items recall test helped predict fall-related outcomes in clinical settings regardless of the cultural context.

### NURSES' FALL PREVENTION INTERVENTIONS IN NURSING HOME PATIENTS WITH ACUTE VERSUS CHRONIC UNDERLYING CONDITIONS

Deanna Gray-Miceli,[1] and Alison Kris,[2] *1. Thomas Jefferson University, Philadelphia, Pennsylvania, United States, 2. Nursing, Fairfield, Connecticut, United States*

Nursing Home (NH) nurses care for over 1.6 million older residents each year. Among this vulnerable population, an estimated 50 percent of older residents fall each year. Although licensed nurses caring for NH residents who fall intervene to prevent fall recurrence, we know little about nurse's perceptions of the most effective interventions for various types of falls they manage. The purpose of this qualitative study is to describe and compare licensed nurse's perceptions of fall prevention interventions believed to be due to acute underlying causes of a fall versus those believed to be due to chronic underlying conditions. This study is a secondary analysis of existing qualitative data from a multi-site parent study conducted in three NH sites in the northeastern U.S. designed to test nurse's knowledge of falls prevention and interventions. Forty seven registered or licensed practical nurses, English speaking who were full or part time employees were recruited to participate. Most were female (n=46; 98.7%) with a median age of 49.5 years and ten years' experience. Using Colazzi's (1978) method, 47 responses of nurse's were read from typed transcripts and analyzed independently by 2 judges. Significant statement were extracted to derive meanings and form themes. For falls related to acute causes, nurses most often stated they would collaborate with the physician, propose a blood pressure intervention and promote safety. For falls due to chronic causes, nurses promoted ambulation safety, pain interventions and collaborated with specialists. Since nurses intervened differently, identifying fall type is critical in selecting appropriate interventions.

### RURAL RESIDENCE, NUTRITION RISK, AND FALLS IN COMMUNITY-DWELLING OLDER ADULTS

Nancy Gell,[1] Caitlin Eckert,[2] Jennifer Schollmeyer,[3] Mariana Wingood,[4] and Emily Tarleton,[5] *1. The University of Vermont, Burlington, Vermont, United States, 2. University of Vermont, Burlington, Vermont, United States, 3. Support and Services at Home, South Burlington, Vermont, United States, 4. University of Vermont, Waterbury, Vermont, United States, 5. Northern Vermont University, Johnson, Vermont, United States*

Decreasing fall risk and maintaining independence is vital for community dwelling older adults. Nutritional status and rural residence may be independent predictors of falls. The aim of this study was to evaluate if nutritional status and rurality are positively associated with fall risk and predictive of future falls in community-dwelling older adults. We used data from a health risk assessment conducted by the Support and Services at Home organization serving Medicare beneficiaries in Vermont in 2017-2019 (N=3109; 79.6 years ±8.4, 75% female). Measures included the Fall Risk Questionnaire, Determine Nutrition Risk questionnaire, and fall history. Descriptive statistics from baseline measures and logistic regression analyses were used to identify predictors of a new fall with respect to rurality, fall risk, and nutritional status. At baseline, 67% of participants lived in rural communities, 37% had high nutrition risk, and 60% had elevated fall risk. Independently, rurality and high nutrition risk were significantly associated with fall risk ($p<0.001$) and high nutrition risk was associated with rurality ($p<0.001$). In the adjusted model, rural residence was not significantly associated with a fall within one year from baseline, whereas participants at high nutrition risk had a 50% higher odds of falling ($p= 0.001$). These findings suggest that falls may be associated with nutrition risk, but not living in a rural setting. Community-based initiatives should consider including nutrition screens as part of fall risk assessments. Further research is needed to understand the relationship between nutrition status and falls risk.

### SHOULD CAREGIVERS ATTEMPT TO ASSIST FALLING PATIENTS?

Tatjana Bulat,[1] Yvonne Friedman,[2] Blake Barrett,[2] Jason Lind,[2] Margeaux Chavez,[2] Linda Cowan,[2] Marie Martin,[3] and Alicia Shaw,[4] *1. JAH VA Hospital, Patient Safety Center of Inquiry, Tampa, Florida, United States, 2. Tampa VA Hospital, Tampa, Florida, United States, 3. VA North Texas, Dallas, Texas, United States, 4. Milwaukee VA, Milwaukee, Wisconsin, United States*

This quality improvement project seeks to provide guidance on whether caregivers should attempt to "assist" fallers, and if so, the safest way to minimize injury to themselves and the faller. Primary aims were to: 1) Identify common characteristics of documented assisted falls, 2) Identify cases where injuries to patients and/or staff occur, and 3) Provide guidance to the clinical field. Data sources for this project includes secondary databases of assisted falls events as well as primary data collection using computer simulation. Initial results for 2 VA quality tracking databases of assisted falls over a 9-year period are presented. Qualitative matrix analyses were conducted for both assisted falls datasets, which separately examined patient and employee injuries related to assisted falls. Two trained qualitative experts analyzed 195 fall narratives from the datasets to develop insights about the most common fall scenarios that result in injury. The most commonly reported assisted falls scenarios included 1) related to toileting, 2) while ambulating, and 3) while transferring from wheelchair. Findings of these analyses indicate current documentation does not capture the nuance of assisted falls. Additional variables such as 1) the direction of the fall; 2) the fall scenario; 3) how staff sought to assist; 4) staff injury description and 5) and other key variables (patient symptoms, environmental factors) that could improve fall documentation and understanding of assisted falls. Preliminary efforts are providing information for development of a computer simulation using a virtual environment to repeatedly test common fall scenarios and influences of caregiver assistance.

### STAFF AND ADMINISTRATOR PERCEPTIONS OF FALL RISK MANAGEMENT IN HOME- AND COMMUNITY-BASED SERVICE SETTINGS

Lisa Juckett, and Rachael Poling, *The Ohio State University, Columbus, Ohio, United States*

Older adults who receive home- and community-based services (HCBS), such as home-delivered meals and personal care assistance, are at particular risk for falls due to their extremely high prevalence of fall risk factors. HCBS organizations and their staff are well-positioned to implement fall risk screens with HCBS clients and then refer older adults to fall prevention services as needed; however, the extent to which HCBS organizations manage fall risk has yet to be systematically examined. The purpose of this qualitative study was to explore the barriers to and facilitators of implementing fall risk screens and fall prevention service referrals in HCBS organizations. A total of 26 HCBS staff members and administrators participated in semi-structured interviews and focus groups. Qualitative data were examined using directed content analysis guided by the Consolidated Framework for Implementation Research. HCBS staff expressed that strong rapport with clients allowed for them to address fall risk proactively but were concerned that their lack of fall prevention training precluded them from effective fall risk management. HCBS administrators perceived their organization to have reliable internal communication procedures which enhanced fall prevention care coordination but believed their limited connections with fall prevention service providers served as a barrier to referring at-risk clients to appropriate care. Accordingly, HCBS stakeholders are encouraged to develop strategies, such as providing fall prevention coaching and building a network of fall prevention service providers, that account for these barriers and facilitators in future efforts to support effective fall risk management with HCBS clients.

### STUDENT-LED FALL PREVENTION CARE MANAGEMENT REDUCED FALL RISKS AT ASSISTED LIVING FACILITIES

Hiroko Kiyoshi-Teo,[1] Claire McKinley-Yoder,[2] Erin Lemon,[3] and Olivia Ochoa,[3] *1. Oregon Health & Science University, Portland, Oregon, United States, 2. University of Portland, Portland, Oregon, United States, 3. Oregon Health Sciences University, Portland, Oregon, United States*

Older adults in residential care settings are four times more likely than those not living in care facilities to experience falls. Yet, fall prevention efforts at long-term care settings are under-resourced, under-regulated, and under-studied. To address this gap, we developed and studied the impact of a specialty clinical, Fall Prevention Care Management (FPCM), for nursing students to decrease older adults' fall risks. We enrolled assisted living residents that facility liaison identified as being high fall risk (fall rates or fall risk were not tracked at the study sites) and MOCA ≥15, in 2 assisted living facilities in Northwest USA. Participants received weekly, 1-hour, individual, semi-structured, Motivational Interviewing-based care management visits by same students over 6 visits. Changes in fall risks were measured by the CDC STEADI assessment (unsteadiness & worry), Falls Self-Efficacy Scale International-Short (FESI-S), and Falls Behavioral Scale (FAB). Twenty-five residents completed the study. Students addressed the following (multiple responses possible): emotional needs (n=23), improved motivation to prevent falls (n=21), and individualized education/coaching (i.e., exercise, mobility aids) (n=10-17). FESI-S score improved from 16.0 to 14.4 (p=.001; decreased fear. FAB score improved from 2.94 to 3.10 (p=.05; more frequent fall prevention behaviors). Frequency of those who felt steady while standing or walking increased (24% to 40%, p=.07) and those who did not worry about falling increased (20% to 36%, p=.08). FPCM clinical offered valuable opportunity to address unmet care needs of older adults to reduce fall risks.

### SESSION 2909 (POSTER)

### DIET AND NUTRITION

### ANTHROPOMETRIC MEASURES OF OBESITY AND FOOD CONSUMPTION AMONG U.S. CHINESE OLDER ADULTS

Shenglin Zheng, Qun Le, and XinQi Dong, *Rutgers University, New Brunswick, New Jersey, United States*

Most research uses body mass index (BMI) alone to measure obesity. Combined with waist circumference (WC), BMI may better identify obesity-related health risk. And diet is a key component of obesity management. To better understand the relationship between obesity and diet, this study aims to examine two anthropometric measures of obesity and the food consumption among U.S. Chinese older adults. Data were drawn from the PINE study wave III (2015-2017), a prospective cohort study of community-dwelling Chinese older adults (N=3053). We categorized participants into 6 groups: normal BMI (18.5-24.9) with normal WC (women ≤ 88cm and men ≤102cm), normal BMI with high WC (women WC >88cm and men WC >102cm), overweight (BMI=25.0-29.9) with normal WC, overweight with high WC, obese (BMI >30) with normal WC, and obese with high WC. A forty-eight-item food frequency questionnaire was used to measure frequencies of vegetables, fruits, grains, protein foods, dairy, sweets, and alcohol intake. Almost 12% participants had normal BMI but high WC and 10% were overweight with high WC. Participants who were overweight with high WC reported the highest intake of vegetables among groups. Participants with higher WC had significantly higher fruit consumption, compared to those with normal WC, regardless of their BMI. Spearman correlation analysis showed that being overweight with a high WC was correlated with higher frequencies of vegetables and fruits intake and having normal BMI with normal WC was correlated with higher alcohol intake. The findings provide new insights for future research and interventions on obesity/chronic disease management.

### ASSOCIATION OF SLEEP QUALITY AND NUTRITIONAL STATUS IN MIDDLE-AGED AND OLDER ADULTS: FINDINGS FROM THE WCHAT STUDY

Wanyu Zhao, Yan Zhang, and Birong Dong, *West China Hospital, Sichuan University, Chengdu, Sichuan, China*

Poor sleep quality and malnutrition are two common symptoms which are closely related to the health of middle-aged and older people, but few studies focus on the association between them. In this study, we aimed to identify associations between sleep quality and nutritional status in middle-aged and older adults. A total of 6792 community-dwelling adults aged 50 and older from the baseline of the West China Health and Aging Trend (WCHAT) study were analyzed in the present study. Sleep quality was assessed using the Pittsburgh sleep quality index (PSQI). Scores <=5, 6-10,11-15 and >=16 were categorized as good/mild impaired/moderate impaired/severe impaired sleep quality, respectively. Mini Nutritional Assessment Short Form (MNA-SF) was used to assess the nutritional status and a score<12 was identified as at risk of malnutrition. Logistic regression models were conducted to explore the associations. Of 6792 participants (mean age 62.41 ± 8.26 years, 62.49% women), 1831 (26.96%) had risk of malnutrition. The prevalence of participants with good/mild impaired/moderate impaired/severe impaired sleep quality were 53.72%, 35.54%, 9.61%, and 1.12%, respectively. In the logistic regression model, there were significant association between mild/moderate/severe impaired sleep quality and the presence of risk of malnutrition (OR=1.49, 95% CI=1.32, 1.68; OR=2.15, 95% CI=1.79, 2.59; OR=2.52, 95% CI=1.56, 4.06; all p<0.05) after adjusting for potential confounders. Sleep quality was significantly associated with malnutrition risk with a dosage effect among middle-aged and older adults. Our results highlight the importance of maintaining good sleep quality and nutritional status in middle-aged and older adults.

### BODY COMPOSITION BY CT VS. DXA: LONG-TERM PREDICTION OF MORTALITY IN THE HEALTH HEALTH ABC COHORT

Samaneh Farsijani,[1] Lingshu Xue,[1] Robert Boudreau,[1] Adam Santanasto,[1] Stephen Kritchevsky,[2] and Anne Newman[1] *1. University of Pittsburgh, Pittsburgh, Pennsylvania, United States, 2. Wake Forest School of Medicine, Winston-Salem, North Carolina, United States*

Background: Early work in the Health ABC cohort found that strength, but not muscle size predicted mortality. Recent literature suggests that body composition by computerized tomography (CT) and magnetic resonance imaging (MRI) predicts adverse health outcomes in diverse populations, but has not been directly compared to dual-energy X-ray absorptiometry (DXA) for predicting mortality. Objective: With long term follow-up, we reexamined body composition and mortality in Health ABC, comparing DXA and CT measures of muscle and fat. Methods: The Health ABC study assessed body composition in 2911 older adults (age 73.6±2.9 years) in 1996-97. Mid-thigh CTs were read for muscle area, intermuscular, subcutaneous-fat areas and muscle density (HU). DXAs were read for whole body fat mass and appendicular lean mass (ALM). Mortality was assessed every 6-months through 2014 (maximum 17.4 years). Cox proportional hazards models, adjusting for age, sex, race, height, weight, physical activity, smoking and comorbidities were used to assess mortality risk. Results: Strong correlations were observed between mid-thigh muscle and subcutaneous fat areas by CT and leg lean and fat mass by DXA (P<0.05). Lower mortality rates, per SD, were associated with higher CT muscle area (HR-men=0.76 [95%CI: 0.68-0.86]; HR-women=0.84 [0.75-0.94]), muscle density (HR-men=0.86 [0.79-0.93]; HR-women=0.89 [0.81-0.97]) and higher subcutaneous-fat (HR-men=0.90 [0.81-0.99]; HR-women=0.87 [0.77-0.98]), adjusting for covariates. Similarly for DXA, greater ALM (HR-men=0.56 [0.44-0.71]; HR-women=0.77 [0.59-1.01]) and higher total fat mass (HR-men=0.53 [0.40-0.72]; HR-women=0.58 [0.37-0.90]) were associated with lower risk of death. Conclusion: With long term follow-up, both CT and DXA assessments of body composition predicted all-cause mortality risk.

### DIETARY DIVERSITY AND DIETARY QUALITY: ASSOCIATIONS WITH BODY MASS INDEX CHANGE IN DIVERSE ADULTS WITH AGE

Marie Kuczmarski,[1] Rita Rawal,[1] May Beydoun,[2] Nancy Cotugna,[1] Benjamin Brewer,[1] Virginia Hughes,[1] Alan Zonderman,[2] and Michele Evans,[2] *1. University of Delaware, Newark, Delaware, United States, 2. National Institute on Aging, Bethesda, Maryland, United States*

Dietary Diversity (DD), a variety element of a healthful diet, can be measured by count, evenness and dissimilarity scores. This study explored the associations of DD, and dietary quality measured by Dietary Approaches to Stop Hypertension (DASH) score, with annualized Body Mass Index (BMI) change in a diverse sample. Participants, 1,104 African American (57.9%) and White (42.1%) adults, were from the longitudinal Healthy Aging in Neighborhoods of Diversity across the Life Span study. Mean±SE age at baseline was 48.3±0.20 years. The DD and DASH scores were calculated using four 24-hr recalls from baseline (2004-2009) and 1st follow-up wave (2009-2013). Count was based on consumption of ≥50% of an equivalent from 21 food groups. Evenness was derived using the Berry-Index adjusted by the food's health value; dissimilarity, by Mahalanobis Distance. The DASH score was computed using the Mellen formula. BMI was calculated from measured weight and height; change in BMI from 1st to 2nd follow-up waves (2009-2017). Linear regression results are expressed as β-coefficients □ standard error of means (β□SE). After adjusting for energy, age, sex, race, poverty status, education, and smoking, of the three DD measures, only mean count was associated with annualized change in BMI (8.166±3.575, p=0.023). Mean DASH score was inversely associated with BMI change (-6.599±2.690, p=0.014). Age and smoking were the only other significant predictors (-1.137±2.938, p<0.001), (-1.169±5.472, p=0.033), respectively. These findings provide evidence that high quality is associated with a decrease in BMI with age while high count scores are associated with a rise in BMI.

### DIETARY PROTEIN AND TRANSITIONS BETWEEN FRAILTY STATES AND TO DEATH IN ADVANCED AGE: LILACS NZ

Ruth Teh,[1] Ngaire Kerse,[1] Nuno Mendonca,[2] Oliver Menzies,[3] Tom Hill,[4] and Carol Jagger,[4] *1. University of Auckland, Auckland, Auckland, New Zealand, 2. Newcastle University Institute for Ageing, Newcastle upon Tyne, England, United Kingdom, 3. Auckland District Health Board, Auckland, Auckland, New Zealand, 4. Newcastle University, Newcastle upon Tyne, England, United Kingdom*

Modifiable factors delaying frailty progression are important in demographic ageing; health disparities for indigenous people require specific strategies. Does dietary protein intake impact transitions in frailty in Maori (indigenous) and non-Maori aged 80+ in New Zealand? LiLACS NZ is population based longitudinal cohorts of Maori aged 80-90 years and non-Maori aged 85 years, followed yearly to 5 years follow up. At 12 months follow up 459 participants contributed nutrient intakes from 24hr multiple pass recall dietary intake. Frailty states were derived from Fried frailty criteria. Mortality was established through National Health Index matching. Multistate modelling investigated the contribution of protein intake to transitions in frailty states and death using models of increasing complexity: 1)age, ethnicity; 2)sex; 3)disease counts; and 4)energy intake. Over 60% of the sample were prefrail throughout the study; disease burden differentiated frailty state. Of a total of 1269 transitions, 692 remained the same; the models used 549 transitions; 44% from robust to pre-frail or pre-frail to frail. Those recovering from pre-frail to robust had lower disease burden and higher nutritional intake. Those with higher protein intake were less likely to transition from robust to prefrail (model 4: per 1g/kg bodyweight/d: HR: 0.28, 95%CI: 0.08-0.91) and from pre-frail to robust (0.24 0.06-0.93). Increased protein intake was associated with increased transition from frailty to mortality but was moderated by energy intake. Greater protein intake in octogenarians in NZ was associated with both better and worse outcomes. Total energy intake tended to moderate negative outcomes.

### DIETARY QUALITY PREDICTS SHORT-TERM CHANGE IN MOBILITY LIMITATIONS IN OLDER U.S. ADULTS

Nicholas Bishop,[1] Sarah Ullevig,[2] Krystle Zuniga,[3] Kaipeng Wang,[4] and Julia Tucker,[5] *1. Texas State University San Marcos, San Marcos, Texas, United States, 2. The University of Texas at San Antonio, San Antonio, Texas, United States, 3. The University of Texas at Austin, Dell Medical School, Austin, Texas, United States, 4. University of Denver, Denver, Colorado, United States, 5. Texas State University, New Braunfels, Texas, United States*

Using a representative sample of older Americans, this project examines the association between dietary quality and short-term change in mobility limitations and whether this association differs for the food insecure or those receiving nutritional assistance. The sample was drawn from the 2012 Health and Retirement Study and 2013 Health Care and Nutrition Study and included 3,779 respondents representing a population of 37,217,566 adults aged 65 and older. Mobility limitations were operationalized as a log-transformed count of 11 indicators of limitation in physical mobility. Dietary quality was measured using the Alternative Healthy Eating Index-2010 (AHEI-2010) based on responses to a food frequency questionnaire. Food insecurity was a binary measure based on the USDA six-item short form. Nutritional assistance included receipt of supplemental food from sources such as food banks and/or reporting receipt of SNAP benefits (1=yes, 0=no). Autoregressive multiple regression was used to test whether AHEI-2010 predicted change in mobility limitations from 2012-2014 and whether food insecurity or receipt of supplemental food moderated this relationship. Around 10.7% of older adults were food insecure, and around 17.5% reported receipt of nutritional assistance. AHEI-2010 was associated with a slower decline in mobility limitations over the 2-year observational window (b=-0.014, SE=.003, p<.001), but food insecurity nor nutritional assistance were associated with changing mobility limitation, either directly or as moderators of the association between progressing limitations and AHEI-2010. These preliminary findings suggest dietary quality may be associated with disablement among older adults regardless of food environment.

### FOOD INSECURITY AND DENTAL CARIES IN U.S. AND SOUTH KOREAN OLDER ADULTS AGES 60+

Donald Chi,[1] Yujin Kim,[2] and JoAnna Scott,[3] *1. University of Washington, Seattle, Washington, United States, 2. Kangwon National University, Chuncheon, Kangwon-do, Republic of Korea, 3. UMKC, Kansas City, Missouri, United States*

There are no studies on the association between food insecurity and dental caries (tooth decay) in older adults. We analyzed nationally representative data from two countries – the U.S. and South Korea – to test the hypothesis that food insecurity would be positively associated with tooth decay in older adults ages 60+. The U.S. analyses focused on National Health and Nutrition Examination Survey data from three cycles (2011-12, 2013-14, and 2015-16) and the South Korean analyses focused on Korean National Health and Nutrition Examination Survey data from one cycle (2013-2015). Food insecurity was defined using the 18-item USDA Household Food Security Survey and a validated Korean version of the same survey. Tooth decay was defined as having at least one decayed tooth (no/yes). We ran logistic regression models to generate odds ratios and 95% confidence intervals (CI). The percentage of fully food secure older adults was 74.0% in the U.S. and 82.2% in South Korea. Twenty-three percent of U.S. older adults and 28% of South Korean older adults had at least one decayed tooth. Older adults in the U.S. with food insecurity were 2.03 times as likely to have untreated tooth decay (95% CI: 1.43, 2.87; P<0.0001). Findings were similar for South Korean older adults. Food insecurity is significantly associated with untreated tooth decay in both U.S. and South Korean older adults. Future research should identify the aspects of food insecurity that increase older adults' risk for tooth decay, with an emphasis on those that are amenable to population-based interventions.

### FOOD INSECURITY IS ASSOCIATED WITH DEPRESSIVE SYMPTOMS AMONG BRAZILIAN COMMUNITY OLDER ADULTS

Carolina Freiria,[1] Graziele Silva,[2] Larissa Hara,[2] André Fattori,[3] Flavia Borim,[1] Tábatta Brito,[4] and Ligiana Corona,[2] *1. University of Campinas, Campinas, Sao Paulo, Brazil, 2. University of Campinas, Limeira, Sao Paulo, Brazil, 3. University of Campinas, Campinas, Brazil, 4. Federal University of Alfenas, Alfenas, Minas Gerais, Brazil*

Food security can be defined as when the individual has access to food consumption in adequate quality and quantity, respecting aspects such as age, physiological condition and cultural habits. While international studies showed the association of Food Insecurity (FI) and many negative health outcomes, like depressive symptoms, less is known about food insecurity among older people in Brazil, especially about its association with health. The aim of this study is to analyze the relationship between FI and Depressive Symptoms (DS) among community older Brazilian adults. Were included in this study 493 community older people with 60+. Geriatric Depression Scale were used to measure DS and for assessment of FI was used the short version of the Brazilian Food Insecurity Scale, added with one question involving functional limitations to buy food. Logistic regression was used to estimate the odds ratio (OR) adjusted for covariates (e.g., sex, education, age and familiar income). The prevalence of FI were 42.4% and the prevalence of DS were 71.5% of population. The prevalence of DS was higher in the group with FI than among those without F (78.9% vs 65.8% respectively; p=0.001). In the adjusted regression analysis, the chance of presenting positive symptomatology for depression was 1.87 times higher among the older people with FI (CI 1.18 –2.91; p=0.007). The findings demonstrate high prevalence of FI and DS indicating the importance of FI screening among community-based older people in order to avoid possible negative health outcomes in this population, such as the development of depressive symptoms.

### FOUR EATING BEHAVIORS THAT MIGHT PREVENT METABOLIC SYNDROME ONSET IN OLDER ADULTS: COHORT STUDY

Sayori Wada,[1] Masahide Hamaguchi,[2] Nami Nakabe,[3] and Miho Ueda,[3] *1. Kyoto Prefectural University, Kyoto, Japan, 2. Kyoto Prefectural University of Medicine, Kyoto, Japan, 3. Japanese Red Cross Kyoto Daiichi Hospital, Kyoto, Japan*

Metabolic syndrome (MetS) is associated with cardiovascular disease and cancer, the leading causes of mortality and morbidity. Although eating behaviors may have an impact on the risk of MetS, exactly which behaviors can prevent MetS is not fully elucidated. We evaluated the onset of MetS in relation to eating behaviors among Japanese older adults aged 65 to 93. We enrolled individuals who underwent health checkups between April 2008 and March 2019, and performed a nine-year follow-up in this cohort study. Cox regression models were used to compare hazard ratios for MetS onset. Among the 2,661 older adults included, the mean age was 70.21 ± 0.089 years and 46% were women. During a mean follow-up of 1567.3 ± 19.3 months, 499 candidates (18%) developed MetS. The risk of MetS was significantly low in subjects in the "often eat vegetables" and "eat more than 30 items daily" groups (hazard ratio (HR) (95% confidence interval (CI)): 0.721 (0.595–0.872), p = 0.001; and 0.690 (0.545–0.874), p = 0.002, vs without the behavior, respectively). On the contrary, the risk of MetS was significantly higher in subjects in the "eat quickly" and "eat out more than twice a day" groups (HR (95% CI): 1.442 (1.208–1.721), $p < 0.001$; and 1.534 (1.245–1.890), $p < 0.001$, vs without the behavior, respectively). Four eating behaviors—regular vegetable consumption, eating more than 30 items daily, eating slowly, and refraining from eating out too often—might be beneficial with regard to preventing the onset of MetS.

### ORAL DYSPHAGIA AND ITS ASSOCIATIONS WITH NUTRITION, PHYSICAL FUNCTIONS, AND DEPRESSION FOR THE ELDERLY IN THE COMMUNITY

Yinan Zhao,[1] Hongting Ning,[2] lulu Liao,[1] Hengyu Hu,[2] Huijing Chen,[2] and Hui Feng,[1] *1. central south university, Changsha, China, 2. Central South University, Changsha, China*

Objectives: To explore the occurrence of oral dysphagia problems among elderly in community and to investigate whether the severity of oral dysphagia problems is associated with nutrition, physical functions and mental health. Methods: Trained nurses conducted a rigorous assessment of 679 elderly people living in communities in Zhuzhou. Basic personal information, The Short Form Mini Nutritional Assessment (MNASF), Activities of daily living (ADL), Patient Health Questionnaire-9 (PHQ-9) were used to assess each elderly. Oral dysphagia was assessed by means of a standardized questionnaire including clinically symptoms of oral dysphagia such as coughing and choking. Results: A total of 654 old people was enrolled in this study, including 250 (38.2%) developing the one or more symptoms of oral dysphagia, the most common symptoms were: drinking water choking (30.7%), followed by saliva (9.6%), dysphagia or pain (6.4%),food loss from the corner of the mouth (2.3%), foreign body sensation of the esophagus (1.8%),food residue (1.2%).The severity of oral dysphagia problems was linearly associated with nutrition, physical function, depression, education level and current care status. The higher the burden of oral symptoms, the lower the self-rated health. Conclusions: The occurrence of oral dysphagia symptom was associated in a stepwise fashion with nutrition, physical function, depression, education level and current care status. In the community, the early recognition, diagnosis and treatment of oral dysphagia is an important link of reducing the mortality and improving the rehabilitation outcome. Additionally, the psychological construction in the elderly should be taken seriously by their families and community workers.

### QUALITATIVE ASSESSMENT OF RESIDENT OBESITY IN NURSING HOMES BY MEDICAL PROVIDERS

John Harris,[1] Steven Handler,[1] Alison Trinkoff,[2] David Wolf,[3] and Nicholas Castle,[4] *1. University of Pittsburgh, Pittsburgh, Pennsylvania, United States, 2. University of Maryland, Baltimore, Baltimore, Maryland, United States, 3. Bellarmine University, Louisville, Kentucky, United States, 4. University of West Virginia, Morgantown, West Virginia, United States*

We present qualitative themes from an ongoing five-year AHRQ-funded project (R01HS026943) examining the various ways nursing homes provide care for residents with obesity to determine the most effective way to prevent adverse safety events for residents with obesity. Obesity is a common diagnosis among short- and long-stay residents, and in the past, nursing home administrators have reported concerns from admissions issues to negative resident outcomes. No studies have examined the medical provider's perspective on health of residents with obesity. In this abstract, we present three emergent themes from semi-structured interviews of medical providers (n=6) (nursing home medical directors, staff physicians, nurse practitioners) across the U.S. First, residents with obesity often have several complex and challenging medical conditions that require more services and health monitoring than most residents. Significant medical issues include diabetes, hypertension, cardiovascular disease, arthritis, and sleep apnea. Second, medical providers observe that it is difficult to provide daily custodial and nursing care, but the actual medical harm from substandard care is hard to quantify. Third, medical providers would like to help residents with obesity to lose weight and live healthier lives. There is, however, not an easy way to facilitate weight loss, due to limited resident physical activity, concerns about unhealthy weight loss, and difficulty changing established dietary habits of residents. These findings are limited by sample size, though themes have been consistent within the current participants. Comparing and contrasting these themes with other stakeholder groups (residents, nurse aides, administrators) interviews in the future will strengthen these findings.

### THE ASSOCIATION OF EARLY-MORNING EATING HABITS WITH HIGH NUTRIENT INTAKE BY OLDER JAPANESE ADULTS LIVING ALONE

Yoko Matsuoka, Junhua Zhang, Weida Lyu, Kei Aoyagi, Mai Takase, Ryogo Ogino, and Jun Goto, *The University of Tokyo, Tokyo, Japan*

The consumption of essential nutrition is fundamental to maintain the health of older adults. Conventional studies report that community dwelling older adults who live alone have the tendency to skip meals and result in low nutrition intake. However, the details regarding the dietary behavior and its association with the status of nutrition intake remain unclear. In this study, a cross-sectional analysis was conducted to explore the association between the time of meals and nutrition balance. In October 2019, questionnaire surveys were distributed to 184 participants of a lunch event dedicated to older adults living alone (Kashiwa, Japan). The time of meals, number of meals per day, consumption status of 10 food groups, and self-rated health were used for the analysis (N=165). As a result, older adults who usually ate their first meal between 5 to 8 A.M. ate three meals/day, while those who ate their first meal after 8 A.M. ate two meals/day. Those who ate their meal between 5 to 8 A.M., frequently consumed meat, fish and seafood, milk, and green and yellow vegetables compared to those who ate after 8 A.M. Self-rated health score was also high. The consumption of early-morning meals was associated with good dietary behaviors. The importance of eating breakfast has been emphasized, however, the time of the breakfast could vary among individuals. This study proposed the importance of considering the time of the meal. Development of intervention programs which encourage early-morning meal consumption might be helpful to form healthy dietary behaviors of older adults.

### UNDERSTANDING FOOD CONSUMPTION FREQUENCIES AMONG U.S. CHINESE OLDER ADULTS

Shenglin Zheng,[1] Dexia Kong,[2] Qun Le,[3] and XinQi Dong,[2] *1. Rutgers University, Chicago, Illinois, United States, 2. Rutgers University, New Brunswick, New Jersey, United States, 3. Rutgers University, New Brunswick,, New Jersey, United States*

A healthy diet is essential to various health outcomes that are common among minority aging populations. To explore frequencies and correlates of food consumption among U.S. Chinese older adults, this study used data from the Population-based Study of Chinese Elderly (PINE) collected in Chicago during 2015-2017 (N=3053). Food consumption frequencies of five food groups (vegetables, fruits, grains, protein foods, and dairy) were assessed by a validated 48-item food frequency questionnaire. All responses of consumption frequency were transformed to “times per day” and weighted by reported portion size. The average frequencies of vegetables, grains, and protein foods intake among the U.S. Chinese older adults were 2.02 (SD±1.32), 1.32 (SD±0.70), and 1.58 (SD±0.90) times/day, respectively. Fruits and dairy consumption frequencies were much lower: 0.76 (SD±0.70) and 0.48 (SD±0.53) times/day. In addition, higher levels of education were correlated with higher consumption of all five food groups. Being female was positively correlated with frequencies of fruits and dairy intake. In contrast, poorer life quality and having more children were correlated with less intake of all five food groups. Older age, preference to speak Cantonese/Taishanese compared to Mandarin/English, and poorer health status were also correlated with lower consumption frequencies of fruit and dairy. The study provides important dietary data of U.S. Chinese older adults and sheds light on significant socioeconomic correlates of food consumption. More in-depth investigations are needed to clarify the sociocultural determinants of dietary behavior and how they relate to different health outcomes among the U.S. Chinese population.

### VITAMIN D SUPPLEMENTATION AND MORTALITY IN OLDER ADULTS: A SYSTEMATIC REVIEW AND META-ANALYSIS

Yunli Zhao,[1] Shuli Jia,[2] Meiling Ge,[3] and Birong Dong,[1]
*1. West China Hospital, Sichuan University, Chengdu, Sichuan, China, 2. West China Hospital, Sichuan University, Chengdu, Shanxi, China, 3. West China Hospital Sichuan University, Chengdu, Sichuan, China*

Web of Science, Embase, Medline and Cochrane Central Register of Controlled Trails from their inception to 14 January 2020 were searched in this review. We included randomized controlled trials (RCTs), which compared vitamin D supplementation versus placebo or no intervention and reported mortality as one of outcomes in older adults. Two review authors extracted data independently. Fifty-one articles were included in the analysis, which generated a total pooled sample of 89,977 people and 9813 deaths. These trials were pooled in a meta-analysis, and the outcomes were expressed as risk ratios (RRs) and 95% confidence Intervals (CIs). Across all studies, vitamin D supplementation was not associated with all-cause mortality (RR 0.98, 95%CI 0.92 to 1.04, P=0.48, I2=14%; 89,977 participants; data from 51 trails). Vitamin D supplementation was significantly decreased cancer mortality ( RR 0.85, 95% CI 0.74 to 0.97, P= 0.02, I2=0%; 34364 participants; data from 6 trails). A subgroup analysis showed the associations between the length of vitamin D supplementation more than 3 years and all-cause mortality were statistically significance (RR 0.93, 95%CI 0.88 to 0.98, p = 0.01, I2=0%; 49336 participants; data from 17 trails). Subgroup analyses by vitamin D status, forms of vitamin D (vitamin D3, vitamin D2, alfacalcidol or calcitriol), dose showed no association with all-cause mortality. The evidence from pooled analysis of 51 RCTs undertaken in older adults shows vitamin D supplementation was not associated with all-cause mortality. More long-term trails are need to know weather vitamin D supplementation can decrease all-cause mortality in older adults.

### SESSION 2910 (POSTER)

### ADVANCE CARE PLANNING AND END-OF-LIFE ISSUES

### AN ADVANCE CARE PLANNING GROUP VISIT INTERVENTION FOR INDIVIDUALS WITH MILD COGNITIVE IMPAIRMENT

Andrea Daddato,[1] Prajakta Shanbhag,[2] Brianne Bettcher,[2] and Hillary Lum,[2] *1. University of Colorado School of Medicine, Parker, Colorado, United States, 2. University of Colorado School of Medicine, Aurora, Colorado, United States*

Among older adults without cognitive impairment, a novel advance care planning group visit (ACP-GV) intervention increased ACP documentation and readiness to engage in ACP. A key question is whether an intervention can be adapted to support people with mild cognitive impairment (MCI) and a family care partner. We used a human-centered design process, rapid-cycle prototyping, and qualitative methods to adapt an ACP-GV intervention to individuals with MCI and a study partner. In 2019, we convened a longitudinal cohort of six patient-study partner stakeholders in three focus groups to suggest intervention adaptations. We also conducted a single arm study of four ACP-GV interventions (n=13 dyads total) that were iteratively refined with input from the longitudinal focus groups and intervention participant feedback. Decision tools, resources and videos were used to describe the concept of ACP and flexibility in selecting a medical decision maker. Many ACP-GV participants strongly agreed that the group discussion gave them useful information (81%) and would recommend the ACP-GV to a friend (85%). Pre- and post-ACP readiness surveys indicated that participants were significantly more ready to talk to their medical decision maker about ACP (p=0.028), while study partners perceived their loved ones less ready to speak to their doctor about ACP following the intervention (p=0.031). Use of rapid prototyping allowed testing of different resources and tools aimed at helping individuals with MCI and their study partners discuss ACP. Future work is needed to understand the feasibility of implementing an ACP-GV intervention for individuals with MCI into clinical settings.

### CLINICIAN DISTRESS: A CONCEPT CLARIFICATION

Anessa Foxwell, *University of Pennsylvania, Philadelphia, Pennsylvania, United States*

In 2017, The National Academy of Medicine created the Action Collaborative on Clinician Well-Being and Resilience given the staggering statistics around distress and burnout, which is particularly high while caring for seriously ill older adults. This distress may be physical, emotional, spiritual, moral, ethical, existential or in degrees and combinations of multiple forms. Many crossover concepts are present in the literature, including moral distress, role stress, compassion fatigue, empathy, emotional labor, post-traumatic stress disorder, vicarious traumatization, and second victim. Because of the number of crossover concepts, we employed the Norris concept clarification method examining similar concepts to create an operational definition, construct a model, and develop possible research hypotheses of clinician distress. Articles were identified through PubMed, CINAHL, PsyhINFO, and SCOPUS with the expert assistance of a nurse librarian. Similar concepts in the literature were compared and contrasted with an emphasis on what encompasses clinician distress. Based on synthesis of the literature and existing concepts, clinician distress is defined as experiencing one or more negative emotions before, during, or after a clinical encounter that impacts the quality of care the clinician is able to provide to the patient. The result of this analysis provides some clarity around clinician distress and its surrogate terms, which presents opportunities for further investigation.

### EVALUATING AN ADVANCE CARE PLANNING SKILLS TRAINING FOR MEDICAL, NURSING, AND SOCIAL WORK STUDENTS

Todd Becker, and John Cagle, *University of Maryland, Baltimore, Baltimore, Maryland, United States*

Advance care planning (ACP) helps to ensure future healthcare is congruent with individual preferences. Curricula for health-focused professions rarely include ACP content. This is a repeated measures evaluation of an innovative, interprofessional student training to enhance the ability to lead ACP discussions. Outcomes were measured via self-report surveys at two timepoints (baseline = T1 [n = 35]; posttraining follow-up = T2 [n = 20]) and included validated measures of communication self-efficacy, ACP self-efficacy, and interprofessional teamwork. Data collection from a third timepoint (T3), following randomized group assignment is currently underway. The matched T1–T2 sample (N = 17) included students from medicine (64.7%), nursing (17.6%), and social work (17.6%). Participants were largely young (M=26 years), non-Hispanic (94.0%), White (59.0%), and female (71.0%). Paired t tests examined change from T1 to T2 for all outcomes. Despite worse communication self-efficacy (Mdiff=1.6, p<.001) and ACP self-efficacy (Mdiff=.92, p<.001), perceptions of interprofessional teamwork improved (Mdiff=3.0, p=.008). These T1–T2 findings mirror results from similar, prior research, which subsequently discovered that both self-efficacy outcomes and their effect sizes for change worsened at T2, but ultimately and substantially improved at T3. These fluctuations suggest participants initially overestimated their self-efficacy related to ACP at T1 and corrected their appraisals at T2. Other studies should account for this self-correction. Further replication is needed to understand the dip that appears to occur before anticipated improvements occur.

### HOSPICE FAMILY CAREGIVERS' USE OF AUDIO DIARIES

Kristin Cloyes,[1] Eliabeth Porter,[2] Maija Reblin,[3] Kathi Mooney,[2] Djin Tay,[2] and Lee Ellington,[2]
*1. University of Utah, Portland, Oregon, United States,*
*2. University of Utah, Salt Lake City, Utah, United States,*
*3. Moffitt Cancer Center, Tampa, Florida, United States*

Previous work has shown that emotional processing as part of diary writing improves well-being during and after stress. The purpose of this study was to determine the feasibility of verbal/audio diaries for home hospice family caregivers (HFCGs). We also describe diary content. As part of an ongoing multi-site, prospective longitudinal study, HFCGs of cancer patients report daily fluctuations in patients' and their own symptoms via an automated telephone system, including a recorded diary entry. HFCGs are randomly assigned instructions to either discuss additional symptoms or discuss their thoughts and feelings. Thirty-six (85.7%) participants to date have completed at least one audio diary. For this preliminary analysis, we selected the 14 longest diary recordings from each condition (n=28) to content analyze using Linguistic Inquiry and Word Count (LIWC) and NVivo 12. Participants are 78.6% female, 53 years of age on average, and most are spouse/partner (46.4%) or adult child (35.7%) caregivers. There was no difference in the overall positivity (23%) or negativity (77%) of words in either condition, but participants asked to express thoughts/feelings used significantly more anger-related terms (p=0.04) while those asked to describe symptoms used significantly more anxiety-related terms (p = 0.003). Time was the most common theme in both conditions but arose more frequently in the symptom condition (p=.08). Our findings suggest that most audio diaries are feasible for HFCGs, but varied prompts may facilitate different types of emotional expression. Future research should assess potential impact on emotional well-being and bereavement adjustment.

### HOW MANY PEOPLE NEED PALLIATIVE CARE AND HOW MANY MISS OUT?

Esther Davis,[1] Victoria Westley-Wise,[2] Stephen Moules,[1] Malcolm Masso,[1] Greg Barclay,[2] Zivai Nangati,[2] Joanne Davis,[2] and Kathy Eagar,[1] *1. University of Wollongong, Wollongong, New South Wales, Australia, 2. Illawarra Shoalhaven Local Health District, Wollongong, New South Wales, Australia*

With an ageing population and growing burden of chronic disease, the number of people requiring palliative or end-of-life care (P&EOLC) is set to rise. It is imperative to develop a comprehensive understanding of the quality and equity of P&EOLC provision to promote sustainable health systems. The aim of this presentation is to report an observational study of N=3,171 decedents across a Local Health Network to: 1) estimate the proportion who need, access and miss out on P&EOLC in the last 12 months of life, and 2) identify differences by clinical and sociodemographic characteristics. Analysis was performed on multiple integrated datasets containing routinely collected health and mortality data. Estimation methods based on underlying cause of death were applied to determine those decedents who could potentially benefit from P&EOLC. Results identified potential benefit to 75% of decedents, of which 62% received P&EOLC and 13% missed out. Decedents aged 85 years or more and from a residential aged care facility showed the lowest proportion of access. Decedents with diagnosis of liver or kidney failure and dementia received more P&EOLC than were expected to benefit. Multivariate logistic regression identified that diagnosis and no other clinical or sociodemographic factor was significantly associated with likelihood of accessing specialist palliative care, with cancer showing highest likelihood and heart failure lowest likelihood. This research highlights the value of population-based estimates to provide a 'whole of system view' of quality and equity of P&EOLC, with ready translation for service planners around resource allocation for need and likely benefit.

### IMPACT OF CULTURE AND RELIGION ON END-OF-LIFE DECISIONS AMONG ADVANCED CANCER PATIENTS IN DEVELOPING COUNTRIES

Justina Yevu Johnson,[1] and Lori Popejoy,[2] *1. University of Missouri, Columbia, Columbia, Missouri, United States, 2. University of Missouri, Columbia MO, Columbia, Missouri, United States*

Palliative care and end of life decisions are important components of quality care at the end-of-life. Individual's perception of cancer diagnosis is affected by their customs and traditions, religious orientations and stigma. Culture and religion as a social determinant of health affects people's interpretation of health and illness and is a major factor in deciding the type of care at end of life and death. The purpose of the review was to identify factors related to culture and/or religion that impact decision making at end of life among advanced cancer patients their primary family caregivers and healthcare providers. An extensive literature search was conducted in Psych Info, PubMed, Philosophy Index, Atlas Religion, and Academic Search Premier databases for primary studies on the topic. Primary studies conducted only in developing countries and among healthcare providers, advanced cancer patients and their primary family caregivers were included. Five studies met the inclusion criteria: two primary studies, one methodological paper, and two on perspectives. The studies reported economic status of the patient, family, culture, and religious beliefs as factors that affected decision making at the end of life. Improving cancer care in developing countries requires the accommodation of the culture, traditions, and religious beliefs of both healthcare providers, patients and family. Culturally appropriate care model is therefore needed to enhance palliative and end of life care in developing countries. Leininger's Cultural Care Theory seem an appropriate path to take.

### INTEGRATED MULTIDISCIPLINARY APPROACH TO ADVANCE CARE PLANNING FOR VULNERABLE OLDER ADULTS

Jennifer Gabbard, Nicholas Pajewski, Kathryn Callahan, Ajay Dharod, Kristie Foley, and Jeff Williamson, *Wake Forest School of Medicine, Winston-Salem, North Carolina, United States*

Advance Care Planning (ACP) is increasingly recognized as a crucial step to ensure patients receive medical care that consistent with their overall goals and values; however it remains suboptimal among vulnerable older adults. The objective was to determine whether a nurse navigator led ACP pathway plus a provider-facing EMR documentation program called ACPSmart improves ACP documentation within the EMR within an Accountable Care Organization (ACO) for vulnerable older adults. This was a randomized, pragmatic, effectiveness clinical trial was conducted from November 1, 2018 to November 1, 2019, at 8 primary care practices. Patients 65 years or older within an affiliated ACO with multimorbidity plus physical impairments, cognitive impairments, and/or frailty were included. Participants were randomized to either a nurse navigator led ACP pathway (NN) or usual care (UC). The primary outcome was documentation of new ACP discussion within the EMR at 12 months along with the quality of ACP discussions. Among 759 randomized patients (379 NN / 380 UC, mean age 77.7 years), compared to usual care, the NN led ACP pathway resulted in a higher rate of ACP documentation (42.2% vs 3.7%, p<0.001). There was also higher completion rates of ACP legal forms (24.3% vs 10.0%, p<0.001), MOST forms (10.3% vs 1.1%, p<0.001), along with documentation of medical decision maker (64% vs 35%, p<0.001). The nurse navigator led ACP pathway plus ACPSmart documentation program increased documentation of ACP within the EMR. This may mitigate prior barriers to ACP and could substantially improve ACP documentation within the EMR.

### NURSE-DRIVEN TRANSITIONS PROGRAM PROVIDES HIGH VALUE END-OF-LIFE CARE TO VETERANS WITH SERIOUS ILLNESS

Jane Driver,[1] Barbara Hayes,[2] Li Chen,[3] Jeffrey Gosian,[3] Lara Skarf,[4] Julie Paik,[3] and Amy Kind,[5] *1. VA Boston Medical Center, Newton, Massachusetts, United States, 2. West Roxbury VA Hospital, West Roxbury, Massachusetts, United States, 3. VA Boston Healthcare System, Boston, Massachusetts, United States, 4. VA Boston Healthcare System, West Roxbury, Massachusetts, United States, 5. VA Hospital Madison, Madison, Wisconsin, United States*

We developed the Supportive Coordinated Transitions of Care (SC-TraC) pathway at VA Boston to improve the quality of end-of-life (EOL) care. A nurse case manager (NCM) with training and experience in geriatrics and palliative care enrolled hospitalized patients with advanced illness (life expectancy < 2 years) who were not enrolled in hospice, and provided phone-based care coordination after discharge for up to 1 year. Our prior work found that SC-TraC patients were more likely to receive goal-concordant care, 60% more likely to enroll in hospice, twice as likely to die at home with hospice, and half as likely to die in an ICU, with no difference in survival. We worked with VA Geriatrics and Extended Care Data Analytics Center to calculate VA and Medicare/Medicaid cost data for a cohort of 104 SC-TraC cases and 104 carefully matched controls enrolled January 2017-June 2018, with follow-up through December 2019. Total cost data (VA + non-VA) was available for all patients up to 6 months following initial discharge. Difference in total cost per-patient was higher in SC-TraC patients at 30 days post-discharge (+ $3,258), but lower at 90 days (-$1,686) and 6 months (-$1,267). SC-TraC cost was substantially less in the last 30 days of life (-$-4,057). Cost differences were due to more home-based and less inpatient/institutional care in the SC-TraC cohort. This data suggests that the SC-TraC program promotes high value EOL care and is a financially sustainable model.

### PALLIATIVE CARE CONSULT FOR CLINICIAN DISTRESS THROUGH THE PHILOSOPHICAL LENSES OF GENDER NORMS AND PHENOMENOLOGY

Anessa Foxwell,[1] Salimah Meghani,[2] and Connie Ulrich,[2] *1. University of Pennsylvania, Philadelphia, Pennsylvania, United States, 2. University of Pennsylvania School of Nursing, Philadelphia, Pennsylvania, United States*

The National Academy of Medicine has raised significant concerns on clinician health and well-being as many experiencing burnout, post-traumatic stress, and depression. Indeed, clinicians experience a range of human emotions when caring for older adults with severe, life-limiting illnesses. These emotions may manifest in multiple ways and from various sources. Uncertain of how to attend to such distress, clinicians may consult a trusted resource, including the palliative care team. Palliative care specialists are trained to support the complexities and needs of patients and families; increasingly, however, palliative care consults are rooted in clinician distress. This session uses clinical case examples to explore the palliative care consult for distressed clinicians from two different philosophical perspectives: (1) phenomenology and (2) the social construct of gender norms. A phenomenological lens respects the unique, subjective lived experience of each individual in their day-to-day interactions with patients, families, and health care systems. Therefore, when caring for seriously ill older adults, clinicians may bring their own subjective experiences to the patient encounter and react differently to ethical dilemmas and conflicts that arise. The social construct of gender norms asks us to examine clinician distress from a different perspective. Here, the postmodern rejection of gender binarism allows clinicians to experience a spectrum of emotions and distress regardless of gender. Exploration through clinical cases will highlight the unique, varied experience of clinician distress and offer opportunities for future research into the role of palliative care teams in supporting distressed clinicians who care for seriously ill older adults.

### PALLIATIVE CARE RESEARCH: SUCCESSFUL RECRUITMENT AND RETENTION STRATEGIES OF PATIENT-CAREGIVER DYADS

Lissi Hansen,[1] Shirin Hiatt,[1] and Karen Lyons,[2] *1. Oregon Health & Science University, Portland, Oregon, United States, 2. Boston College, Chestnut Hill, Massachusetts, United States*

Research shows that the well-being of patients with serious illness and their family caregivers is significantly associated. Thus, to build the scientific knowledge upon which to establish high quality palliative and end-of-life care practices for these patients and their caregivers, research studies should include successful recruitment and retention strategies that focus on the patient-caregiver dyad. Aims: To review the literature focusing on successful dyadic recruitment and retention strategies and to describe successful recruitment and retention strategies, and attrition in a longitudinal study of end-stage liver disease (ESLD) patient-caregiver dyads. Methods: A five-year prospective longitudinal study of dyads included quantitative and qualitative data collected at 5 time points over 1 year: at baseline, 3, 6, 9, and 12 months. Results: Over a 32-month period 336 dyads were approached and 241 were enrolled. The refusal rate was 27 dyads (8.0%). Over the course of the study, 31 patients or caregivers withdrew for various reasons (too sick, liver transplantation). The attrition due to death of patients was 53 dyads (20.2%). Successful strategies used for recruitment and retention included tailoring to provider preference for referral, accommodating patient preference for data collection method, and having predictable and ongoing contact between a specific study staff and dyads. Conclusions: Less than 10 studies address recruitment and retention strategies most effective in dyadic research in various serious illnesses and clinical settings. Recruitment of ESLD patient-caregiver dyads is challenging. Future longitudinal dyadic studies of serious illnesses and palliative care may benefit from strategies learned from the current ESLD study.

### PRIORITIES FOR END-OF-LIFE CARE REPORTING IN NURSING HOMES: RESULTS FROM A MIXED-METHODS STUDY

Andrea Gruneir,[1] Matthias Hoben,[1] Charlotte Jensen,[2] Monica Buencamino,[3] Adam Easterbrook,[4]

Sheila Marshall,[5] Janice Keefe,[6] and Carole Estabrooks,[1] *1. University of Alberta, Edmonton, Alberta, Canada, 2. Staff, Edmonton, Alberta, Canada, 3. University of Alberta, Calgary, Alberta, Canada, 4. Centre for Health Evaluation and Outcomes Sciences, Vancouver, British Columbia, Canada, 5. University of British Columbia, Vancouver, British Columbia, Canada, 6. Mount Saint Vincent University, Halifax, Alberta, Canada*

The aim of the "Trajectories" project is to compile measures of nursing home (NH) quality to better characterize the final year of life for residents. In the first phase, we worked with various stakeholder groups to identify their priorities to focus the selection of possible outcomes relevant to end-of-life needs. Policy- and decision-makers from 5 Canadian health regions participated in an on-line, modified Delphi process to reach consensus on 3-4 measures of each burdensome symptoms and potentially inappropriate care practices. NH residents and families or care aides participated in an interview process using the Action Project Method. To date, all participants identified pain, mental health care, polypharmacy, and dyspnea as priorities. Policy- and decision-makers additionally identified infections and acute care transfers as priorities, while residents and families additionally identified mobility, cognition, and pressure ulcer care as priorities. There was general consistency across groups in terms of priorities but additional measures seemed to reflect either a system-wide or more personal perspective, depending on the source. Data collection with frontline staff and managers is on-going. Moving forward, we will use this list of prioritized outcomes to quantitatively assess the trajectories of these outcomes and associated factors, and to create a profile that allows for monitoring of end-of-life care in NHs.

### QUALITY OF END-OF-LIFE CARE FOR VIETNAM VETERANS: IMPLICATIONS FOR PRACTICE AND POLICY

Joan Carpenter,[1] Dawn Smith,[2] Hilary Griffin,[2] Daniel Kinder,[2] Joshua Thorpe,[2] Mary Ersek,[2] and Ann Kutney-Lee,[2] *1. Department of Veterans Affairs, Berlin, Maryland, United States, 2. Department of Veterans Affairs, Philadelphia, Pennsylvania, United States*

In federal response to the aging population of Vietnam-era Veterans, Congress directed the Department of Veterans Affairs (VA) to create a pilot program to identify and develop best practices for improving hospice care for this population. A first step in VA's response was to identify whether the end-of-life (EOL) care needs and outcomes of Vietnam-era Veterans differed from previous generations. Using medical records and bereaved family surveys, we examined clinical characteristics, healthcare utilization, and EOL quality indicators for Vietnam-era Veterans who died in VA inpatient settings between 2013-17. Contemporaneous comparisons were made with World War II/Korean War-era Veterans. Compared to prior generations, higher percentages of Vietnam-era Veterans had mental health/substance use diagnoses and disability. Similar percentages of family members in both groups reported that overall EOL care was excellent; however, post-traumatic stress disorder management ratings by families of Vietnam-era Veterans were significantly lower. Although current VA EOL practices are largely meeting the needs of Vietnam-era Veterans, greater focus on mental health comorbidity, including post-traumatic stress disorder, Agent Orange-related conditions, and ensuring access to quality EOL care in the community is warranted. Policymakers and healthcare professionals should anticipate more physical and mental health comorbidities among Veterans at EOL as Vietnam-era Veterans continue to age. Findings are being used to inform the development of standardized EOL care protocols and training programs for non-VA healthcare providers that are tailored to the needs of this population.

### REVERSALS IN DECISIONS ABOUT LIFE-SUSTAINING TREATMENT AMONG TERMINALLY ILL OLDER KOREAN PATIENTS

suhyun kim,[1] and Jung-Ja Choi,[2] *1. Kyungpook National University, Daegu, Republic of Korea, 2. Kyungpook national university, Daegu, United States*

Purpose: The purpose of this study was to investigate the patterns and factors of reversals in decisions about life –sustaining treatment (LST) among older patients with terminal stage of chronic cardiopulmonary diseases. Methods: In a retrospective correlational descriptive study, data were collected using medical chart review from 124 deceased older patients with terminal stage of cardiopulmonary disease who had made reversals of LST decisions in an academic tertiary hospital in 2015. Multivariate logistic regression analysis was used to identify the factors associated with the reversal to higher intensity of LST treatment. Results: Primary decision makers were offspring (72.6%), spouse (13.7%), acquaintance (8.9%), and patients (4.8%), in order. While 31.5% of the reversed decisions were made toward higher intensity of LST, 21.9% were made toward lower intensity of LST, and 46.6% were made for each treatment without change of overall code status. The use of inotropic was the most frequently reversed LST treatment (47.5%), followed by CPR (30.6%), intubation (27.4%), ventilator therapy (24.2%), and hemodialysis (17.8%). Patients who had lung diseases (vs. heart diseases), were single, divorced or bereaved (vs. married), and had acquaintance as a primary decision maker (vs. patients themselves) were significantly more likely to reverse the LST decisions to higher intensity of LST treatment. Conclusion: This study demonstrate the complex and turmoil situation of the LST decision making process among older patients with terminal stage of cardiopulmonary disease and suggests the importance of support for patients and families in their LST decision making process.

### SELF-REPORTED FEARS BY HOSPICE PATIENTS AT THE END OF LIFE

Jeannette Kates, *Thomas Jefferson University, Philadelphia, Pennsylvania, United States*

Fear is a common emotion that involves the intense anticipation of threat to a person. At end of life, this fear is often conceptualized as existential distress, which suggests a connection to spirituality. Processing impending death is essential to end-of-life closure and acceptance. Existing evidence suggests that spirituality is associated with greater coping, better psychosocial well-being, and dignified dying; however, the relationship between fear and spirituality at end of life, as well as the specific fears experienced, are not known. The purpose of this study was to explore the relationship between fear and spirituality in patients upon hospice admission. In this retrospective study, admission records from 154 hospice patients were reviewed. Hospice admission data from the psychosocial and spiritual assessments were analyzed using descriptive statistics, inferential statistics, and logistic regression. The average patient age was 81 years of age. A slight majority (51.3%) of patients admitted to fears upon hospice admission. Patients reported a range of one to six fears, with the most common fear being "pain and/or suffering." Forty-seven percent of the patients identified as being "spiritually active." Correlation analysis revealed no statistically significant relationship between fear and spiritually. Logistic regression analysis revealed some significant relationships between age and certain fears. Fear is a common symptom at the end of life, and appropriate emotional and psychological support should be provided to mitigate the fears. This study suggests that fears may be different for older adults.

### USING THE CONVERSATION STARTER KIT IN CANADA TO PROMOTE RESIDENT CARE PLANNING DISCUSSIONS IN LONG-TERM CARE

Sharon Kaasalainen,[1] and Tamara Sussman,[2] *1. McMaster University, Hamilton, Ontario, Canada, 2. McGill University, Montreal, Ontario, Canada*

Advance care planning (ACP) is still rare in Canadian long-term care (LTC) homes. Residents and their families view ACP as uncomfortable and difficult to implement, leading them to avoid these discussions. The purpose of this study was to explore the perceptions of LTC residents and their families about using an ACP tool called The Conversation Starter Kit. This study utilized a mixed methods approach. Data was collected in four LTC homes in Ontario, Canada from 78 residents and family members. Data was analyzed using thematic analysis and descriptive statistics. All participants read all sections but only 73% completed all sections of the toolkit. Participants spent an average of 52.3 minutes completing the toolkit and 36.4 minutes discussing it with their family members and/or LTC staff. Participants reported: a better understanding of ACP after using the tool (80%), that the tool helped clarify the available resources and/or choices (53%), and that they felt less apprehensive about ACP after using the tool (60%). Qualitative findings revealed many strengths (e.g., usefulness, ability to start difficult conversations, content and clarification), and weaknesses of the tool (e.g., redundant information, difficulty understanding the content and lack of information regarding medically assisted dying). Family members noted that the toolkit would have been helpful to receive earlier on in their family members' disease trajectory, perhaps before being admitted into LTC. These study findings support the feasibility and acceptability of the tool to engage residents and family members in/; ACP discussions in LTC.

### SESSION 2911 (PAPER)

### HOME-BASED CARE

### AGING IN PLACE IN SUBSIDIZED HOUSING: ROLES AND PERSPECTIVES OF STAFF MEMBERS

Sonia Pandit,[1] Momana Jahan,[1] David Reyes-Farias,[1] Barbara Resnick,[2] Carolina Reid,[3] and Rebecca Brown,[1] *1. University of Pennsylvania, Philadelphia, Pennsylvania, United States, 2. University of Maryland School of Nursing, Baltimore, Maryland, United States, 3. University of California, Berkeley, Berkeley, California, United States*

Nearly 3 million older Americans with low incomes live in subsidized housing. This population has disproportionate rates of functional impairment, cognitive impairment, and nursing home admission. Staff members who work in subsidized housing may have unique insight into how to improve aging in place for this vulnerable population, but little is known about their perspectives. We conducted 12 focus groups with 69 staff members from 7 subsidized housing sites. Staff included property managers, service coordinators, maintenance workers, administrative assistants, and security guards. Transcripts were analyzed using qualitative thematic analysis. The majority of participants noted that they "wear multiple hats" and their roles are not limited to professional responsibilities. Instead, their role is to maximize residents' quality of life. As a result, staff members often adopt personal roles akin to serving as residents' "surrogate family" members. This relationship gives staff a unique window into residents' lives which enables them to detect early warning signs among residents. For example, staff often observed changes in residents' physical appearance and hygiene, mood, behavior, and function which could indicate physical illness, mental illness, or cognitive impairment. Staff reported having some resources to address these warning signs, such as involving families and connecting residents to community resources. However, they also highlighted unmet needs to address these issues, such as on-site clinical staff and affordable mental health services. Our findings suggest that building staff are a valuable and underutilized resource for identifying at-risk residents in subsidized housing and helping to deliver interventions to improve aging in place.

### ARE URBAN HOME HEALTH AGENCIES MORE LIKELY TO ACHIEVE IMPROVEMENT IN CARE QUALITY?: A COHORT STUDY

Chenjuan Ma, *New York University, New York, New York, United States*

Home health care (HHC) is a core source of home- and community-based services to older adults "aging in place.' HHC quality is under increasing scrutiny. This study aimed to examine urban vs. rural disparities in HHC quality in the US. This is a cohort study using 2014-2019 national Home Health Compare data linked to Providers of Services (POS) files. Quality of HHC was measured by agency rates of 1) timely initiation of care and 2) hospitalization. We examined 6,448 home health agencies or 38,688 agency-years. At baseline, the mean rate of timely initiation of care was 91.3% (SD: 7.6; range: 28.0-100.0), with urban agencies performing worse (urban vs. rural: 91.0% vs. 92.3%); and the mean rate of hospitalization was 15.6% (SD: 3.7; range: 0.0-37.0), with urban agencies performing better (urban vs. rural: 15.3% vs. 16.4%). Estimates from multivariate hierarchical linear regressions showed that while the rates of timely initiation of care increased annually over time ($\beta$= 0.62, p=0.000), this improvement trends did not differentiate between urban and rural agencies (urban: $\beta$= 0.087, p=0.086). There was slight annual improvement in hospitalization rates over time ($\beta$= -0.07, p=0.0.003) and the trends in annual reduction in hospitalization rates were more significant in rural agencies than urban agencies (urban: β= 0.10, p=0.000). Our findings indicate urban (vs. rural) disparities in HHC quality and the trends of quality improvement.

### HELPING FILL THAT GAP: AGING IN PLACE AFTER DISASTER THROUGH THE LENS OF HOME-BASED CARE

Sue Anne Bell,[1] Lydia Krienke,[1] Raymond DeVries,[2] and Theodore J. Iwashyna,[2] *1. University of Michigan, Ann Arbor, Michigan, United States, 2. University of Michigan, Ann Arbor, Michigan, United States*

During a disaster, home-based care is intended to continue to function using existing care delivery models. Home-based care providers (HBCP) are often the closest contact with their clients—even during a disaster, seeing them in non-traditional care settings including shelters and hotels. This closeness and commitment to clients, gives HBCP unique insights into strategies to promote aging in place. The purpose of this study was to identify the individual and community-level support needs of older adults after a disaster through the lens of home-based care. Five focus groups were conducted with HBCP (n=27) in two disaster-affected settings: 2017's Hurricane Irma in Florida and Hurricane Harvey in Texas. Participants were identified by contacting home health agencies listed in an open-source database of agencies receiving Centers for Medicare and Medicaid Services funding. Data was manually coded using an inductive approach and themes were iteratively identified. Forty-nine codes were identified in the preliminary analysis, which were distilled into ten themes describing factors that influence care provision during and after disasters: patient autonomy/dependence, disaster-induced trauma, reluctance to evacuate, chronic disease exacerbation, unpreparedness, systemic inequality, provider preparedness actions, strong sense of community, mistrust of governmental authority, and the uniqueness of the patient and home-based care provider relationship. The perspective offered by HBCP illustrates the complexities of community-level preparedness and informal community support for chronically ill older adults surrounding disasters. Diverse groups involved in aging and disaster response can learn from strategies employed by HBCP during disasters to improve aging in place.

### RELATIONSHIPS OF SEVERE PAIN AND COGNITIVE IMPAIRMENT WITH ACUTE CARE USE IN HOME HEALTH PATIENTS

Jinjiao Wang,[1] Todd Monroe,[2] Adam Simning,[3] Xueya Cai,[1] Helena Temkin-Greener,[1] Fang Yu,[4] Thomas Caprio,[1] and Yue Li,[1] *1. University of Rochester, Rochester, New York, United States, 2. The Ohio State University, Columbus, Ohio, United States, 3. University of Rochester Medical Center, Rochester, New York, United States, 4. Arizona State University, Minneapolis, Minnesota, United States*

Pain assessment is challenging in patients with cognitive impairment that can lead to inappropriate pain management and unfavorable health outcomes. Using a 10% random sample of Medicare home health (HH) patients ≥ 65 years old from the 2017 Outcome and Assessment Information Set (OASIS) national dataset (N=646,109), we tested the relationships of cognitive impairment and constant, severe pain that interfered with daily living activities with acute care utilization (i.e., hospitalization and emergency department [ED] admission without hospitalization). Patients who had constant, severe, interfering pain (32.57%, N=210,444) were younger, more likely to be female, white, Medicare-Medicaid dually eligible, living alone, and having functional limitations and depressive symptoms, but less likely to have moderate-to-severe cognitive impairment (25.0% versus 31.5%, p<0.001) than others. In multivariable logistic regression models adjusting for the above covariates, when compared with patients with neither cognitive impairment nor severe, constant, interfering pain, those with both conditions were 17% more likely to have hospitalization (Odds Ratio [OR]=1.17, p<0.001) and 13% more likely to have an ED admission without hospitalization (OR=1.13, p<0.001). This was the first study that examined co-occurring pain and cognitive impairment in HH recipients using national OASIS data. Findings suggest that: 1) older HH patients with moderate-to-severe cognitive impairment have lower rates of reported pain that suggests under-recognition; and 2) having severe, interfering pain among cognitively impaired patients significantly increased their risk of acute care utilization. Therefore, systematic protocols and guidelines should be in place to facilitate pain assessment for improved outcomes among HH patients with cognitive impairment.

### SKILLED HOME HEALTH CARE AGENCY PERSPECTIVES ON COMMUNICATION WITH PHYSICIANS: A NATIONAL SURVEY

Jonathan Norton,[1] Amelie Nkodo,[1] Bhavana Nangunuri,[2] Danielle Pierotti,[3] Kimberly Carl,[4] Orla Sheehan,[1] Cynthia Boyd,[1] and Bruce Leff,[1] *1. Johns Hopkins University School of Medicine, Baltimore, Maryland, United States, 2. Eastern Virginia Medical School, Norfolk, Virginia, United States, 3. Visiting Nurse and Hospice for Vermont and New Hampshire, White River Junction, Vermont, United States, 4. Johns Hopkins Medicine, Baltimore, Maryland, United States*

BACKGROUND: Communication is important in the care of older adults receiving skilled home health care (SHHC). In a prior national survey, physicians viewed communication and care coordination with SHHC agencies as dismal. The views of SHHC personnel (Registered Nurses, Licensed Practical Nurses, Physical Therapists, Occupational Therapists, and Speech-Language Pathologists) on this issue have not been well studied. OBJECTIVE: To determine the effectiveness of communication between SHHC personnel and physicians who order SHHC services. METHODS: A nationally representative mailed survey of personnel from SHHC agencies identified through the 2016 Home Health Compare data set from the Centers of Medicare and Medicaid Services. RESULTS: 263 of 2000 surveys returned (13.2% response rate). Responding agencies were mainly proprietary (75.3%) and urban-based (83.7%). Most agencies were in the South (38.8%); 28.3% Midwest, 22.9% West, 12.1% Northeast. Only 62.2% of SHHC personnel completing start of care visits (n=202) reported being able to contact a physician when needed. The most common strategies used to contact physicians are phone (76.0%) and fax (11.2%). The greatest barriers to communication are having to communicate through a third party (64.9%) and a perception by SHHC personnel that "Physicians [are] not interested in communicating with SHHC Personnel" (45.1%). Failed communication resulted in delayed orders (70.8%) and sending a patient to the emergency room (37.1%). IMPLICATIONS: SHHC agency personnel experience significant barriers in communicating with physicians. Modes of communication remain rudimentary, and there are serious consequences of failed communication.

### SESSION 2912 (PAPER)

### BIOLOGY OF AGING I

### A VIRTUAL HEALTH LIBRARY FOR DEMENTIA PATIENTS AND CAREGIVERS TO IMPROVE QUALITY OF INFORMATION AND COMMUNICATION

Kirsten Mengell,[1] Alyssa Indelicato,[2] Sarah Lawler,[2] Nancy Smith,[3] Sharon Brangman,[2] and Telisa Stewart,[4]
*1. SUNY Upstate Medical University, Brewerton, New York, United States, 2. SUNY Upstate Medical University, Syracuse, New York, United States, 3. Nancy Smith Consulting, Syracuse, United States, 4. SUNY Upstate Medical University, Syracuse, United States*

Access to accurate health information is critical for patient decision making and health communication. Unfortunately, there is limited quality health information for dementia patients and their caregivers. A Virtual Health Library (VHL) provided access to credible health information through the electronic medical record (EMR) to reach the dementia patients and their caregivers. A VHL was created and brought together clinicians, caregivers, technology support personnel, public health professionals, and the health sciences library. The team identified areas of interest and met monthly to create VHL materials. Materials included voice-over slides and 1-page educational content that was uploaded to the EMR for patient and caregiver access. A baseline and final questionnaire assessed demographics, empowerment, and shared decision making for both the patient and the caregivers and a pre/post was created for each module to asses knowledge and stratification. Initially, 1331 patients with dementia were recruited for the project from a university geriatrics department. The population had a 28.3% enrollment in the EMR and only 3.8% used the EMR in the past six months. Of this pool, during the initial launch 32 patients and or caregivers completed the baseline within the first week. 98% of respondents were caregivers with an average age of 58.7. With the youngest caregiver 42 and the oldest 88. Of the caregiver's relationships to the patients is 43.3% their child, 40% their spouse, 10% other family, and 6.7% friends. During the project, participants improved access to health information and became empowered to engage with their healthcare provider.

### APOLIPOPROTEIN E GENOTYPE AND THE RELATIONSHIP BETWEEN CHITINASE 3–LIKE PROTEIN 1 AND POSTOPERATIVE DELIRIUM

Sarinnapha Vasunilashorn,[1] Long Ngo,[1] Sharon Inouye,[2] Tamara Fong,[3] Richard Jones,[4] Simon Dillon,[1] Towia Libermann,[1] and Edward Marcantonio,[1]
*1. Beth Israel Deaconess Medical Center, Boston, Massachusetts, United States, 2. Harvard University, Boston, Massachusetts, United States, 3. Institute for Aging Research / Hebrew SeniorLife, Boston, Massachusetts, United States, 4. Brown University, Providence, Rhode Island, United States*

Apolipoprotein E (APOE) ε4 does not confer increased risk of delirium in older surgical patients; however, ε4 status modifies the relationship of C-reactive protein (CRP) with delirium: increased risk for delirium in ε4 carriers with high CRP. We examine whether APOE genotype modifies the established association between inflammatory marker chitinase-3-like protein-1 (CHI3LI/YKL-40) and delirium in patients without dementia age≥70 undergoing major noncardiac surgery. We performed APOE genotyping using PCR, considering APOE ε4 vs. non-ε4 carriers. Plasma YKL-40, measured on postoperative day 2 by ELISA, was examined using sample-based quartiles (Q1-Q4). Delirium status was determined with daily interviews rating the Confusion Assessment Method, augmented by a validated chart review. We used generalized linear models adjusted for age, sex, surgery type, and stratified by APOE ε4 status. Among the 557 patients, 19% were APOE ε4 carriers, and 24% developed postoperative delirium. The YKL-40-delirium relationship differed by APOE status. Among APOE non-ε4 carriers, we found a significant relationship between YKL-40 and delirium (relative risk [RR](95% confidence interval [CI] for YKL-40 Q4 vs. Q1: 2.6(1.4-4.9) and Q3 vs. Q1: 2.3(1.2-4.5); p-trend<.01). Among APOE ε4 carriers, YKL-40 was not significantly associated with delirium (RR(95% CI) for YKL-40 Q4 vs. Q1: 2.0(0.6-6.6) and Q3 vs. Q1:1.1(0.3-3.5); p-trend=0.37). APOE non-ε4 carriers may have increased risk of delirium conferred by post-surgical inflammation specific to the type 2 immune response (high YKL-40). These results differ from prior results with CRP, and raise the possibility that APOE genotype may interact at different points in the inflammatory pathway leading to delirium.

### CIRCULATING PROCOLLAGEN TYPE III N-TERMINAL PEPTIDE AND PHYSICAL FUNCTION IN THE LONG LIFE FAMILY STUDY

Adam Santanasto,[1] Ryan Cvejkus,[2] Mary Wojczynski,[3] Nicole Schupf,[4] Megan Marron,[2] Kaare Christensen,[5] Bharat Thyagarajan,[6] and Joseph Zmuda,[2] *1. University of Pittsburgh, PITTSBURGH, Pennsylvania, United States, 2. University of Pittsburgh, Pittsburgh, Pennsylvania, United States, 3. Washington University School of Medicine, St Louis, Missouri, United States, 4. Columbia University Medical Center, New York, New York, United States, 5. University of Southern Denmark, Odense C, Syddanmark, Denmark, 6. University of Minnesota, Minneapolis, Minnesota, United States*

Circulating levels of procollagen type III N-terminal peptide (P3NP) may reflect increased fibrosis of skeletal muscle and other tissues with aging. In the current study, we tested if P3NP levels were associated with baseline and 7-year change in physical function among adults aged 39-104. Participants (n=400) were from the Long Life Family Study, a study of exceptional familial longevity. Plasma P3NP concentration was measured using a sandwich enzyme-linked immunosorbent assay (inter-assay

CVs <3%). At baseline and 7-year follow-up visits, physical function was measured using the Short Physical Performance Battery (score 0-12), which consists of gait speed, balance, and chair-rise tests. Grip strength was measured using a handheld dynamometer. The association between log-transformed P3NP and physical function was examined using Generalized Estimating Equations adjusted for familial relatedness, age, sex, height, weight, lifestyle characteristics, chronic disease prevalence and inflammatory cytokines. Participants were aged 73.1 ± 15.2 years, 54% female, had a BMI of 26.6 ± 4.3 kg/m2, and a gait speed of 1.0 ± 0.3 m/s. One standard deviation higher P3NP concentration was related to worse baseline SPPB score (β=-0.9points), gait speed (β=-0.05m/s), chair-rise time (β=8.34seconds), and grip strength (β=-2.0kg; all p<0.001). Higher P3NP concentration was also associated with greater declines in gait speed (β=-1.41, p<.001) and chair-rise performance (β=0.41, p<.001). Plasma P3NP concentration may be a strong, novel biomarker of current and physical function changes with aging. Future research is needed to extend our findings to a larger population, and determine the mechanisms underlying these associations.

### STRESSFUL LIFE EVENTS AND INFLAMMATION IN MIDLIFE: COMPARING ASSOCIATIONS WITH SUPAR, CRP, AND IL-6

Kyle Bourassa,[1] Line Rasmussen,[2] Terrie Moffitt,[2] and Avshalom Caspi,[2] *1. Duke University Medical Center, Durham, North Carolina, United States, 2. Duke University, Durham, North Carolina, United States*

Stressful life events are associated with poorer health, but the physiological mechanisms of this association are unclear. One mechanism that might play a role in this association is systemic inflammation. Using participants (n=828) from the Dunedin Longitudinal Study, we studied the association of stressful life events and inflammation from age 32 to 45. Inflammation was assessed using C-reactive protein (CRP), interleukin-6 (IL-6), and suPAR. We examined associations between stressful life events and systemic inflammation, as well as whether adverse childhood experiences (ACEs) moderated these associations. More adult stressful life events were associated with greater suPAR at age 38 (r = 0.12, p < .001) and age 45 (r = 0.19, p < .001). CRP and IL-6 did not evidence consistent associations at age 38 and 45. An increase in suPAR from age 38 to 45 was associated with more stressful life events in the interim, β = 0.10, p = .001. SuPAR at age 45 was independently associated with both childhood ACEs, β = 0.20, p < .001, and adult stressful life events, β = 0.18, p < .001. ACEs significantly moderated the association of stressful life events and suPAR at age 45, β = 0.12, p = .001, such that people with more childhood ACEs evidenced a stronger association between stressful life events and inflammation in midlife. These results were robust to controlling for clinical (sex, body mass, smoking) and childhood covariates (childhood IQ, SES, self-control). Systemic inflammation is one mechanism through which stressful life events could impact health.

### UNINTERRUPTIBLE POWER SUPPLY IMPROVES PRECISION OF TELOMERE LENGTH MEASUREMENT VIA QPCR

Waylon Hastings, and Idan Shalev, *Pennsylvania State University, University Park, Pennsylvania, United States*

Epidemiological literature has produced robust associations between telomere length (TL) and health, wherein individuals with shorter TL are at increased risk for chronic disease and death. Even so, technical challenges associated with TL measurement have led some to question their utility as a biomarker of aging. Several pre-analytical factors influence TL assessment via qPCR including tissue source, sample storage, and DNA extraction method. Additional work has investigated how conditions within the PCR run (e.g. mastermix reagents) influences precision and reproducibility of TL measurements. However, the impact of power supply remains unclear. Momentary fluctuations in power supply can affect the functioning of high-performance electronics, including real-time thermocylers. These fluctuations can be mitigated by using an uninterruptible power source (UPS), an electronic apparatus capable of supplying constant, uninterrupted voltage to high-performance electronics. The current study investigated how using a UPS influenced TL assessment via qPCR. Standard deviation and coefficient of variation across replicates were compared for samples assessed with or without the use of a UPS. Samples run with a UPS had significantly lower standard deviation (p<0.001) and coefficient of variation (p=0.002) than those run without a UPS. Notably, neither the efficiency of exponential amplification (p=0.674) nor the standard curve R2 (p=0.638) varied as a function of UPS status. Thus, UPS status decreases variability within sample replicates while maintaining the overall quality of the qPCR assay. *The work presented was supported by the Telomere Research Network, an NIH-sponsored working-group recently established to coordinate best practices for measuring telomere length in population-based research.

### SESSION 2913 (PAPER)

### BIOLOGY OF AGING II

### COGNITION, DEPRESSION, AND GENETICS: EXAMINING SEX DIFFERENCES USING POLYGENIC AND GENETIC INFERENCE TECHNIQUES

Erin Ware,[1] Arianna Gard,[1] Mingzhou Fu,[1] Lauren Schmitz,[2] Kelly Bakulski,[1] and Erin Bakshis Ware, *1. University of Michigan, Ann Arbor, Michigan, United States, 2. University of Wisconsin, Madison, Wisconsin, United States*

Alzheimer's disease and its related dementias (ADRD) are debilitating neurodegenerative diseases. As nearly two-thirds of persons diagnosed with Alzheimer's disease are women, more research is needed to understand sex differences in the biological mechanisms that underlie ADRD. Depression is a risk factor for Alzheimer's disease and higher rates of depression among women, compared to men, suggest that depression-related phenotypes and underlying biological factors may contribute to sex differences in ADRD. Using the Health and Retirement Study (N = 9908, European ancestry), a US panel-cohort study, the current analysis leverages Mendelian randomization techniques to assess sex-specific inferred causality of depressive symptoms on odds of dementia. All analyses assess most recent cognition and account for sex, education, study cohort, age and year of most recent cognition visit, and genetic ancestry principal components. A one standard deviation increase in depressive polygenic score was associated with 1.11 times higher odds of dementia (95% confidence interval: 1.02-1.21) relative to normal cognition. Each additional endorsed depressive symptom was associated with 1.13 times higher odds of dementia (95% confidence interval: 1.09-1.18) relative to normal cognition. Using the depression genetic instrument, a significant inferred causal relationship was observed between depressive symptoms and dementia (P=0.01, 1.73 odds ratio, 95% confidence interval: 1.12-2.67). When stratified by sex, this relationship was only significant in females (P=0.02, 1.76 odds ratio, 95% confidence interval: 1.08-2.87). These findings demonstrate that depressive symptoms are likely causally related to dementia, and this relationship is most pronounced in females.

### DEPRESSION AND PSYCHOLOGICAL WELL-BEING AS DISTINCT CONSTRUCTS: MUTUALLY EXCLUSIVE ASSOCIATIONS WITH BIOMARKER

Kheng Siang Ted Ng,[1] Shu Cheng Wong,[2] Glenn Wong,[3] Ee Heok Kua,[4] Anis Larbi,[3] and Roger C. M. Ho,[5]
*1. Yong Loo Lin School of Medicine, National University of Singapore, Singapore, 2. Duke-National University of Singapore, Singapore, Singapore, 3. Agency for Science, Technology and Research, Singapore, Singapore, United States, 4. Yong Loo Lin School of Medicine, National University of Singapore, Singapore, Singapore, 5. National University of Singapore, Yong Loo Lin School of Medicine, Singapore, Singapore*

Despite increasing emphasis on assessing the mental health of older adults, there has been inconclusive evidence on whether depression and psychological well-being (PWB) are fundamentally distinct constructs or representations of the opposite ends of the mental health spectrum. To instantiate either hypothesis, investigation of the associations between mental health scales and biomarkers have been proposed. First, we assessed depressive symptoms and PWB in community-dwelling older adults (N=59, mean age=67) using the Self-Rating Depression Scale (SDS) and Ryff's Scale of PWB (comprising six sub-scales). We measured a wide range of immune markers employing ELISA and flow cytometry. Subsequently, we used principal component analysis (PCA) to aggregate and derived biomarker factor scores. Lastly, multiple linear regressions were performed to examine the associations between the scales and the derived biomarker factor scores, controlling for covariates. PCA extracted six biomarker factors. Biomarker factor score 1 was significantly associated with PWB (β=-0.029, p=0.035) and the PWB sub-scale, self-acceptance (β=-0.089, p=0.047), while biomarker factor score 4 was significantly associated with the PWB sub-scale, purpose in life (β=-0.087, p=0.025). On the other hand, biomarker factor 6 was significantly associated with SDS (β=-0.070, p=0.008). There were mutually- exclusive associations between the scales with biomarker factor scores, supporting the hypothesis of distinct constructs. Our findings expanded the biomarkers of depression and PWB, deepening understanding of the biological underpinnings of depressive symptoms and PWB. These findings have implications in field work, since researchers could not infer one construct from the other, the examination of both constructs are essential.

### LIFESTYLE COMPLEXITY AND DEMENTIA RISK: EXAMINING MODERATION BY APOE GENOTYPE AND MILD COGNITIVE IMPAIRMENT

Kyle Moored,[1] Beth Snitz,[2] Jeanine Parisi,[1] Jeff Williamson,[3] Annette Fitzpatrick,[4] Steven DeKosky,[5] and Michelle Carlson,[6] *1. Johns Hopkins School of Public Health, Baltimore, Maryland, United States, 2. University of Pittsburgh School of Medicine, Pittsburgh, Pennsylvania, United States, 3. Wake Forest School of Medicine, Winston-Salem, North Carolina, United States, 4. University of Washington, Seattle, Washington, United States, 5. University of Florida College of Medicine, Gainesville, Florida, United States, 6. Johns Hopkins Bloomberg School of Public Health, Baltimore, Maryland, United States*

Prior studies suggest that the neuroprotective effect of physical exercise is moderated by APOE genotype and MCI status, but it remains unclear whether this extends to lifestyle complexity defined by a broader variety of physical, intellectual, and social activities. Participants were from the Ginkgo Evaluation of Memory (GEM) Study. We used 18 physical, intellectual, or social activities from the Lifestyle Activity Questionnaire. We performed latent class analysis to characterize subgroups with distinct activity response patterns and examined whether they have differential risk of incident dementia over time. A three-class model was chosen based on fit statistics and interpretability. Cox proportional hazards models, adjusted for potential demographic and health confounders, revealed that Class 1 (Highly intellectually/socially active) had a reduced risk of dementia compared to Class 3 (Less socially/less intellectually active; HR=.71, 95% CI: [.56,.88], p=.002). Class 2 (Socially/less intellectually active) did not differ in risk from Class 3 (HR=.90, 95% CI: [.73,1.1], p=.288). There was no evidence for effect modification for APOE e4 allele carriers (p's>.05), but the protective association for Class 1 only held for those without prevalent MCI at baseline (HR=.74, 95% CI: [.56,.98], p=.033). Results showed that subgroups characterized by a greater variety of social and intellectual activities had reduced risk for dementia, but only for those without MCI. This implies that late-life lifestyle complexity may be most neuroprotective for those in the preclinical stages of decline. Results also suggested that lifestyle complexity may act through a cognitive reserve pathway unrelated to amyloid pathology.

### MIDLIFE PLASMA Aβ AND LATE-LIFE RISK OF COGNITIVE IMPAIRMENT: THE ATHEROSCLEROSIS RISK IN COMMUNITIES STUDY

Kevin Sullivan,[1] Chad Blackshear,[1] A. Richey Sharrett,[2] Rebecca Gottesman,[3] David Knopman,[4] B. Gwen Windham,[1] Michael Griswold,[5] and Thomas Mosley,[1] *1. University of Mississippi Medical Center, Jackson, Mississippi, United States, 2. Johns Hopkins University, Baltimore, Maryland, United States,*

*3. Johns Hopkins School of Medicine, Baltimore, Maryland, United States, 4. Mayo Clinic, Rochester, Minnesota, United States, 5. The University of Mississippi Medical Center, Jackson, Mississippi, United States*

Plasma-based biomarkers of amyloid beta (Aβ), a neuropathological hallmark of Alzheimer's disease, show promise in predicting cognitive impairment and mapping onto cerebral amyloidosis, but little is known about how midlife plasma Aβ associates with late-life cognitive outcomes. Midlife plasma variants Aβ42 and Aβ40 were measured using a fluorimetric bead-based immunoassay in a subsample of visit 3 ARIC participants (1993-95; n=2585, mean age=59.4±5.2, 57% female, 23% African American). We investigated the relationship between midlife plasma Aβ and late-life mild cognitive impairment (MCI; n=923) and dementia (n=628) diagnosed from 2011-19. Multinomial logistic regressions estimated relative risk ratios (RRR) of MCI, dementia, and death vs normal cognitive status as a function of:(1) Aβ42:Aβ40 ratio, (2) Aβ42 and Aβ40 included as separate terms, and (3) Protected Aβ group (participants with Aβ42≥46 pg/ml and Aβ40 <233 pg/ml). Adjusters included age, sex, education, site-race, and APOE4. Every doubling of midlife plasma Aβ42:Aβ40 up to a threshold of 0.20 was associated with 41% lower risk of developing MCI/dementia in comparison to cognitively normal (RRR=0.59 [95% CI:0.42, 0.82]), with no association for ratio values ≥0.20. Every standard deviation increase in plasma Aβ42 was associated with 17% lower risk of dementia (RRR=0.83 [0.70, 0.99]), whereas every standard deviation increase in plasma Aβ40 was associated with 16% higher risk of MCI (RRR=1.16 [1.02, 1.31]). The protected midlife plasma Aβ group had 86% lower risk of late-life dementia vs all others (RRR=0.14 [0.04, 0.47]). Early measurement of plasma Aβ may prove an accessible and effective population screener for future cognitive impairment.

### SESSION 2914 (PAPER)

### CAREGIVING I (HS)

### DOING THE RIGHT THING: CAREGIVERS AND MEDICAL OR NURSING TASKS IN THE POST-ACUTE HOME CARE SETTING

Jo-Ana Chase,[1] David Russell,[2] Daniel Kaplan,[3] Michael Bueno,[1] Rungnapha Khiewchaum,[1] and Penny Feldman,[4] *1. University of Missouri, Columbia, Columbia, Missouri, United States, 2. Appalachian State University, Boone, North Carolina, United States, 3. Adelphi University, Cortlandt Manor, New York, United States, 4. Visiting Nurse Service of New York, New York, New York, United States*

Family caregivers often manage complex medical/nursing tasks (MNTs) for older adults returning home after a hospitalization. The purpose of this qualitative study was to describe caregivers' experiences leveraging diverse resources to manage MNTs for older adults receiving post-acute home health care services (HHC). In-depth telephone interviews were conducted with 20 caregivers of older adults who received HHC following hospitalization. Interviews were digitally audio-recorded, transcribed, and analyzed using directed content analysis. The Theory of Dependent-Care informed the analytical framework. We organized codes using three theoretical constructs related to managing older adults' MNT care needs ("tasks related to the other"), accessing existing social/environmental resources ("tasks related to the situation of care"), and working with the healthcare system ("tasks related to the system of care"). Caregivers' descriptions of MNTs included the complexity and socioemotional impact of assisting in these tasks (e.g., dependency, trust). When needed, caregivers' accessed social (e.g., family, friends) and environmental (e.g., neighborhood, housing) resources to help address the older adults' care needs. Caregivers also identified challenges and strategies for navigating and coordinating care and services within HHC and the larger healthcare system. Caregivers assisting with complex MNTs in the post-acute HHC setting need additional training and support. HHC providers can actively engage caregivers by tailoring training and support strategies, assessing social and environmental contexts and resources, and facilitating caregivers' navigation of the healthcare system. Future research could elucidate social and environmental factors associated with successful collaborative relationships among providers, older adults and their caregivers in the post-acute HHC setting.

### IMPACT OF FAMILY CAREGIVER TRAINING ON HOSPITALIZATION DURING MEDICARE HOME HEALTH CARE

Julia Burgdorf, and Jennifer Wolff, *Johns Hopkins Bloomberg School of Public Health, Baltimore, Maryland, United States*

Medicare home health providers are required to offer family caregiver training; however, there is little information regarding the impact of family caregiver training on patient outcomes in home health or other care delivery settings. A better understanding of this relationship is necessary to guide development of caregiver training interventions and inform policy discussions surrounding family caregiver training access. This research assesses whether and how unmet need for family caregiver training is associated with acute care utilization during Medicare home health. We examine 1,217 (weighted n=5,870,905) fee-for-service Medicare beneficiaries who participated in the National Health and Aging Trends Study (NHATS) and received Medicare-funded home health care between 2011-2016. We link NHATS data with home health patient assessments and Medicare claims, drawing measures of family caregivers' need for training from home health clinician reports and determining provision of training from Medicare claims. Using weighted, multivariable logistic regressions, we model the marginal change in probability of acute care utilization during home health as a function of family caregivers' unmet need for training. We found that older adults whose family caregivers had an unmet need for training had a probability of acute care utilization during home health that was 18 percentage points (p=0.001) greater than those whose family caregivers both needed and received training, holding all covariates at their means. Findings support the importance of connecting family caregivers to training resources and suggest one avenue by which investing in caregiver training may be cost-effective for integrated payers and providers.

### PERSPECTIVES OF FAMILY CAREGIVERS CONFRONTED WITH CARE-RESISTANT BEHAVIOR FROM PERSONS WITH DEMENTIA

Rita Jablonski,[1] Vicki Winstead,[2] and David Geldmacher,[3] *1. UAB School of Nursing, Birmingham, Alabama, United States, 2. University of Alabama at Birmingham, Alabaster, Alabama, United States, 3. UAB School of Medicine, Birmingham, Alabama, United States*

Problem: Care-resistant behavior is often bundled with other behavioral symptoms of dementia, but it is a unique behavior requiring targeted interventions. Purpose: To describe the experiences of caregivers receiving online coaching to manage care-resistant behaviors exhibited by persons with dementia. Design: Qualitative. Sample & Procedure: 20 caregivers (12 female, 8 male) were recruited from Memory Disorders and Geriatrics clinics to participate in 6 weeks of online coaching sessions delivered by a doctorally prepared nurse practitioner. Coaching sessions were recorded and transcribed. NVivo12 software was used to manage the thematic analyses. Results: Caregivers followed a general trajectory. They initially reported feelings of anger, frustration, and guilt. They believed that the person with dementia was purposefully "being stubborn and mean." As the coaching sessions progressed, these negative emotions and the attributions of intent altered. By the conclusion of the six weeks, caregivers expressed feelings of success and ingenuity in applying coaching strategies. Conclusion: Online coaching is an effective way to individualize strategies that enable the caregiver to manage and reduce care-resistant behavior. Implications: The use of a doctorally-prepared nurse practitioner to deliver coaching, while effective, is not sustainable. Next steps include developing a coaching training program that could be embedded into existing community resources for community-dwelling caregivers. Limitations: Participants were limited to referrals from two clinics in the same institution.

### RURAL-URBAN DIFFERENCES IN INFORMAL CAREGIVING, CAREGIVING INTENSITY, AND HEALTH-RELATED QUALITY OF LIFE

Steven Cohen, Julia McIlmail, and Mary Greaney, *University of Rhode Island, Kingston, Rhode Island, United States*

Introduction Rural areas in the US have a disproportionately high population of older adults and have reduced access to services. Older adults in rural areas are more reliant on family and friends for care. However, little is known about rural-urban disparities among the 40+ million caregivers nationwide. As rural-urban health disparities are pervasive among older adults, there is a need to understand how rural-urban disparities impact caregiving experiences and health-related quality of life (HRQoL). The objectives of this study were to examine rural-urban differences caregiving, caregiving intensity (caregiving hours/week and types of care provided), and caregiver HRQoL. Methods Data were abstracted from the 2009 Behavioral Risk Factor Surveillance System (latest dataset to include county of residence and caregiver module). The primary measure of rural-urban status was Index of Relative Rurality (IRR) decile. Associations between rural-urban status and caregiving and rural-urban differences in caregiving intensity and HRQoL were examined using generalized linear models, controlling for confounding and accounting for complex sampling. Results Rural respondents were more likely to be caregivers than urban respondents (IRR decile OR=1.015, 95%CI 1.014-1.016). Rural caregivers, on average, provided 2.43 hours/week more caregiving for each one-decile increase in IRR decile (95%CI 2.34-2.52) and had worse overall HRQoL (OR=1.34, 95%CI 1.33-1.35). Conclusion Rural informal caregivers offer higher levels of care than urban counterparts, and increased caregiving in rural areas is associated with reduced HRQoL. These results can inform policies designed to improve caregiver health, and facilitate the translation of existing programs and interventions to address rural caregivers' needs.

### THE HALF LIFE OF STRESS: CAREGIVER STRESS INCREASES NEXT-DAY SEVERITY OF BEHAVIORAL SYMPTOMS OF DEMENTIA

Carolyn Pickering,[1] Frank Puga,[2] Danny Wang,[1] and Maria Yefimova,[3] *1. University of Alabama at Birmingham, Birmingham, Alabama, United States, 2. The University of Alabama at Birmingham, Birmingham, Alabama, United States, 3. VA Palo Alto Healthcare System, Redwood City, California, United States*

The progressively lowered stress threshold theory posits that behavioral symptoms of dementia (BSD) are reactions to environmental and care related stressors. In line with this theory, this study tests whether the stress experienced the caregiver impacts BSD expression. Caregivers to persons with dementia recruited online completed daily diary surveys for 21 days reporting on daily contextual and environmental factors of caregiving life. Using multi-level modeling of diaries (n=911) nested within participants (N=51), two daily caregiver stressors (conflict with someone other than the person with dementia, stress about own personal health) were examined as predictors of BSD. The outcome of BSD was measured as severity (a rating on a Likert scale of 1-5 that was then person-centered to represent deviations from average severity). Covariates in the model included relationship type, caregiver age, as well as stressors to the care recipient known to increase BSD. On days when a caregiver had a conflict with others the severity of the care recipient's BSD increased by 1.44 points from average (p<.001, CI 1.05-1.75). In a time-lagged model, we observe that a caregiver having a conflict yesterday impacts today's BSD severity by an increase of 0.43 points from average (p = 0.027, CI 0.05-0.81). Caregivers' stress about their own health did not have a significant impact on the same day or next day BSD severity rating. These findings demonstrate that the well-being of the caregiver has measurable immediate impacts on the well-being of the person living with dementia, and suggests not all stress is equal.

### SESSION 2915 (PAPER)

### CAREGIVING II (HS)

### EMORY ROYBAL CENTER FOR DEMENTIA CAREGIVING MASTERY: DIVERSE RESPONSES TO LETTER OF INTENT CALL

Kenneth Hepburn,[1] Molly Perkins,[1] drenna Waldrop,[1] Leila Aflatoony,[2] Mi-Kyung Song,[3] and Carolyn Clevenger,[1]
*1. Emory University, Atlanta, Georgia, United States, 2. Georgia Tech, Atlanta, Georgia, United States, 3. Emory University, ATLANTA, Georgia, United States*

This new NIA-supported Roybal Center seeks to support Stage 1 pilot clinical trials of programs aimed at promoting caregiving competence and confidence in the great heterogeneity of dementia caregiving contexts. During our first cycle, we received 26 letters of intent (LOI) for full applications. Responses reaffirmed the Center's premise that dementia caregiving is remarkably varied in nature. While most proposed programs focused on generic caregiving, a number addressed caregiving issues facing specific ethnic/racial groups (African American; Korean American; Native Alaskan/American Indian; Latino), and several focused on specific dementing conditions (MCI, Lewy Body Dementia,TBI-based dementia). Most described programs centered on knowledge development and daily management skill issues (e.g., management of behaviors); others specified development of physical care skills. Decision-making and communication constituted the second most common topic. Over 40% proposed adaptation of existing programs; more than 25% proposed apps or technology interventions. Investigators represented a wide range of disciplines: 45% each from Health sciences (nursing, medicine, and social work) and Social/Behavioral sciences (principally psychology) and the rest from engineering and communications. LOIs varied most in their readiness to complete a clinical trial within a year. About 40% were in very preliminary stages; 25% were clearly poised for a Stage 1 trial; 15% did not sufficiently address the Center's aims. Key criteria for invitations to submit full applications (n=4) included: specificity of context; clinical trial readiness; reasonableness of proposed adaptation. These criteria should guide future LOIs addressing the diversity of important new research and intervention perspectives on the multifaceted work of caregiving.

### IDENTIFYING THE ROLE OF SPATIAL TRANSITIONS IN THE PALLIATIVE CARE EXPERIENCES OF UNHOUSED OLDER ADULTS

Ian Johnson,[1] and Michael Light,[2] *1. University of Washington, Seattle, Washington, United States, 2. Harborview Medicine, Seattle, Washington, United States*

The national population of unhoused older adults is predicted to nearly triple by 2030. Unhoused older adults have a mortality rate four to nine times higher than housed populations, and face structural barriers during illness trajectory that likely influence both where care takes place and processes around attaining psychosocial later-life goals. This study aimed to (1) document patterns in the healthcare trajectories of unhoused older adults and (2) examine the role of care transitions in psychosocial goal attainment. Retrospective chart review was completed in partnership with a mobile homeless palliative care team; additional data was gathered from provider focus group. Through a content analysis of this data, it was discovered that older unhoused palliative care patients experienced more transitions, and that numerous care transitions were associated with disruptions to goals. The type of care transitions patients experienced also did not reflect their later-life goals. Patients' movements impacted the role of formal and informal care networks in care and highlighted the implications of place in common psychosocial goals, such as family reconciliation. Unconventional neighborhood supports were found to help facilitate treatment adherence. These findings offer translational opportunities for further research, including "rapid respite" models and other innovations in mobile palliative care for unhoused and precariously-housed people with life-limiting illness.

### PREDICTORS OF CAREGIVER BURDEN IN DELIRIUM: PATIENT AND CAREGIVER FACTORS

Patricia Tabloski,[1] Franchesca Arias,[2] Nina Flanagan,[3] Tamara Fong,[4] Eva Schmitt,[5] Richard Jones,[6] Thomas Travison,[5] and Sharon Inouye,[7] *1. Boston College, Sudbury, Massachusetts, United States, 2. Marcus Institue for Aging Rsearch, Hebrew Senior Life, Boston, Massachusetts, United States, 3. Binghamton University, Vestal, New York, United States, 4. Institute for Aging Research / Hebrew SeniorLife, Boston, Massachusetts, United States, 5. Harvard Medical School, Boston, Massachusetts, United States, 6. Brown University, Providence, Rhode Island, United States, 7. Harvard University, Boston, Massachusetts, United States*

Delirium — an acute disorder of attention and cognition — is a common, life-threatening and costly syndrome occurring frequently in older hospitalized persons. The unexpected, rapid, and volatile nature of delirium can be difficult for family caregivers to experience and may contribute to subjective feelings of distress (i.e. "delirium burden"). The aim of this study was to examine whether pre-admission patient characteristics or patient-caregiver relationship and living arrangements were associated with caregiver burden as measured by the delirium burden scale for caregivers (DEL-B-C; score 0-40, higher score is more burden). Our sample consisted of 208 older adults and their caregivers from the Better Assessment of Illness (BASIL) study, an ongoing prospective, observational study of surgical and medical patients ≥70 years old; 22% of patients experienced delirium by the Confusion Assessment Method (CAM) and the average DEL-B-C score was 7.9, 95% CI(6.95-8.88). Results indicated that neither patient-caregiver relationship and living arrangement or patient factors including pre-admission pain, sleep disturbance, or new onset incontinence were significantly correlated with delirium-related caregiver burden. However, DEL-B-C scores were significantly higher in caregivers of patients with any ADL impairment (mean 8.5 vs. 5.2, p = .016) during hospitalization although none of the individual functional deficits alone were statistically significant. This finding suggests that the association of ADL impairment and DEL-B-C scores is not driven by a single functional domain. Future studies are needed to further understand how caregiver characteristics and patient factors occurring before and during hospitalization contribute to caregiver burden after the occurrence of delirium.

### TRAVELING THE PATH TO STROKE CAREGIVER READINESS

Barbara Lutz,[1] and Michelle Camicia,[2] *1. University of North Carolina-Wilmington, Wilmington, North Carolina, United States, 2. Kaiser Foundation Rehabilitation Center, Vallejo, California, United States*

Family members are often poorly prepared to assume the caregiving role post-stroke leaving them feeling overwhelmed, frustrated, and abandoned by the healthcare system leading to physical, mental, and emotional strain. To address this, we developed and tested the Preparedness Assessment for the Transition Home after stroke (PATH-s) instrument based on a theoretical framework for improving stroke caregiver readiness. Consecutive studies were conducted over the past 10 years to 1) develop the caregiver readiness theoretical model identifying gaps in caregiver preparation in 80 interviews with caregivers and stroke survivors as they transitioned home from inpatient rehabilitation care; 2) develop and validate the PATH-s instrument with 183 caregiver-stroke survivor dyads, and 3) develop and implement a corresponding catalogue of interventions developed in consultation with 5 expert rehabilitation nurse case managers to improve stroke caregiver readiness. The Improving Caregiver Readiness Model has 2 preparedness domains; commitment and capacity and six sub-domains. In a factor analysis each domain/sub-domain subscale in the PATH-s demonstrated satisfactory internal consistency (a=0.69-0.86). The overall mean score was 3.11 (range 1.68 to 4.00) with high internal consistency reliability (a=0.90). The PATH-s is highly correlated with the Preparedness for Caregiving Scale. The stroke survivor's total FIM score at discharge had a small but significant correlation with the PATH-s. Case managers find the PATH-s results and corresponding interventions helpful in tailoring transitional care plans. Caregivers worldwide describe the negative impacts of providing stroke care post-discharge. The Path to Stroke Caregiver Readiness Program shows promise for improving stroke caregiver preparation for discharge home.

### UNMET CAREGIVING NEEDS ARE ASSOCIATED WITH COGNITIVE FUNCTIONING AMONG OLDER SEPSIS SURVIVORS

Jo-Ana Chase,[1] Lizyeka Jordan,[2] Christina Whitehouse,[3] and Kathryn Bowles,[4] *1. University of Missouri, Columbia, Columbia, Missouri, United States, 2. Visiting Nurse Service of New York, New York, New York, United States, 3. Villanova University, Villanova, Pennsylvania, United States, 4. University of Pennsylvania School of Nursing, Philadelphia, Pennsylvania, United States*

Sepsis survivorship is associated with cognitive decline and complex post-acute care needs. Family caregivers may be unprepared to manage these needs, resulting in decline or no improvement in patient outcomes. Using a national dataset of Medicare beneficiaries who were discharged from the hospital for sepsis and received post-acute HHC between 2013 and 2014 (n=165,228), we examined the relationship between unmet caregiving needs and improvement or decline in cognitive functioning. Multivariate logistic regression was used to determine associations between unmet caregiving needs at the start of HHC and changes in cognitive functioning. Unmet caregiving needs included seven items from the start of care Outcome and Assessment Information Set (OASIS). Changes in cognitive functioning were measured using the start of care and discharge OASIS assessments. Twenty-four percent of patients either declined or did not improve in cognitive functioning from HHC admission to discharge, with variation seen by unmet need type. Sepsis survivors with unmet caregiving needs for activities of daily living assistance (OR 1.05, 95% CI 1.01, 1.09), medication assistance (OR 1.06, 95% CI 1.02,1.10), and supervision and safety assistance (OR 1.110, 95% CI 1.06,1.16) were more likely to decline or not improve in cognitive functioning, even after accounting for clinical and demographic characteristics. Older sepsis survivors with both cognitive impairment and unmet caregiving needs in the post-acute HHC setting are at high-risk for worsening cognition. Alerting the care team of cognitively impaired sepsis survivors with unmet caregiving needs may trigger evidence-based strategies to enhance caregiver training and reduce unmet caregiving needs.

### SESSION 2916 (PAPER)

### CHRONIC DISEASE AND MULTIMORBIDITY I

### CHALLENGES TO COMORBIDITY MANAGEMENT FOR OLDER HIV-POSITIVE INDIVIDUALS

Abigail Baim-Lance,[1] Christine Kerr,[2] and Diane Addison,[3] *1. Veterans Health Administration, Bronx, New York, United States, 2. Hudson River HealthCare, New Paltz, New York, United States, 3. CUNY Institute for Implementation Science in Population Health, New York, New York, United States*

70% of individuals with HIV in the United States are 50 years or older. These individuals' HIV is often well-managed, but they have acquired several comorbidities, including cardiovascular disease, non-AIDS defining cancers, renal disease, osteoporosis, liver disease, and neurocognitive disorders. Existing literature provides little guidance on structuring services to meet this burgeoning population's complex clinical and aging-related needs. To inform an approach, between April 2015 and June 2018, we conducted 13 exploratory qualitative group discussions with patients, providers and administrators receiving or providing HIV services in New York's Hudson Valley. We also conducted a retrospective electronic medical record chart review (n=50) of individuals >50 years receiving HIV care in 2017 at a Hudson Valley federally qualified health center (FQHC) to describe subspecialty referrals. Analysis of discussion groups highlighted challenges with initial access, the quality of encounters, and consistent follow-up. Patients thought 'ageism' contributed to poorer care quality, though extensive experience navigating the system was an advantage. The EMR review revealed patients receiving two referrals on average to (most commonly) ophthalmology/optometry (14%), gastroenterology (12%), dental (9%), cardiology (9%), and orthopedics (8%). Only half (54%) of scheduled referral appointments were attended. Documented barriers included insurance/costs, transportation, patient refusal, and fear of HIV disclosure. Findings suggest there are HIV-specific and non-specific barriers for older individuals with HIV, including perceived perceptions of aging. We have initiated a pilot study to investigate multilevel barriers and enablers to optimal comorbidity management. Gerontological insights brought to this analysis will support developing aging-appropriate clinical practices for this population.

### MULTIMORBIDITY ACCUMULATION BY RACE OR ETHNICITY AND BODY-WEIGHT STATUS AMONG MIDDLE-AGED AND OLDER AMERICANS

Anda Botoseneanu,[1] Sheila Markwardt,[2] Heather Allore,[3] Corey Nagel,[4] Jason Newsom,[5] David Dorr,[6] and Ana Quinones,[6] *1. University of Michigan, Dearborn, Michigan, United States, 2. School of Public Health - Portland State University, Portland, Oregon, United States, 3. Yale University, New Haven, Connecticut, United States, 4. University of Arkansas for Medical Sciences, Little Rock, Arkansas, United States, 5. Portland State University, Portland, Oregon, United States, 6. Oregon Health & Science University, Portland, Oregon, United States*

Obesity and multimorbidity are more prevalent among U.S. racial/ethnic minority groups. Evaluating racial/ethnic disparities in multimorbidity accumulation according to body-mass index (BMI) may guide interventions to reduce multimorbidity burden in vulnerable racial/ethnic groups. Data from the 1998-2016 Health & Retirement Study (N=8,106, 51-55 years at baseline) and generalized estimating equations models with inverse probability weights estimated the accumulation of seven chronic diseases (arthritis, cancer, diabetes, heart disease, hypertension, lung disease, and stroke) between racial/ethnic groups [Non-Hispanic White (reference; 64.2%), Non-Hispanic Black (21.6%), Hispanic (14.2%)]. Overweight and obesity were more prevalent in Black (82.3%) and Hispanic (78.9%) than White (70.9 %) participants at baseline. Initial burden of morbidity was higher among Black participants [risk ratio (RR) =1.3, $p<0.001$] but similar among Hispanic compared with White participants; and higher in overweight or greater BMI categories compared with normal BMI (RR=1.07, 1.15, 1.22, $p<0.001$, for overweight, obese 1, and obese 2/3 BMI, respectively). Disease accumulation did not differ among racial/ethnic groups. Higher BMI was associated with less disease accumulation compared with the normal BMI category (RR=0.99, 0.98, 0.97, all $p<0.001$, for overweight, obese 1, and obese 2/3 BMI, respectively, per two-year interval). Black participants crossed the threshold of multimorbidity (≥2 diseases) 4-6 years earlier than White and Hispanic participants. There are substantial differences in initial disease burden between Black and White middle-aged/older adults, but not in the accumulation of disease, suggesting the need to intervene prior to entering middle age to reduce disparities in the burden of multimorbidity among vulnerable racial minorities.

### MULTIMORBIDITY AND DEPRESSION INCIDENCE AMONG MIDDLE-AGED AND OLDER ADULTS IN CHINA

Fei Tang,[1] and Elizabeth Vasquez,[2] *1. University at Albany (SUNY), Albany, New York, United States, 2. University at Albany (SUNY), Rensselaer, New York, United States*

Previous studies suggested that multimorbidity (co-occurrence of two or more chronic conditions) is associated with increased risk of depression. However, limited studies have examined the relationships between multimorbidity and depression among Chinese middle-aged and older adults. The current study aimed to evaluate the associations between multimorbidity and incidence of depression and the potential mediation effect of functional limitation among Chinese middle–aged and older adults. Data of 8,093 individuals who participated in both wave 1 (2011) and wave 4 (2015) of China Health and Retirement Longitudinal Study (CHARLS) and were free of depression in wave 1 were included in the study. Multiple log-binomial regression models were used to evaluate the associations between multimorbidity and incident depression. Mediation analysis was conducted to examine the effect of functional limitation. A third of participants in our study were identified as having multimorbidity in wave 1 (N=2,479) and 23% participants were free of depression in wave 1 but had depression in wave 4 (N=1,827). After adjusting for potential confounders, multimorbidity was observed to be associated with depression incidence (RR: 1.31; 95% CI: 1.21 – 1.42). In addition, functional limitation mediated the relationship between multimorbidity and depression incidence. Our findings add to the literature on the potential associations between multimorbidity and depression incidence among Chinese middle-aged and older adults. Furthermore, the relationship between multimorbidity and depression incidence was observed to be mediated by functional limitation. Interventions that improve functional ability among Chinese middle-aged and older adults could potentially attenuate the effect of multimorbidity on depression incidence.

### PROPOSED FEATURES FOR A DIGITAL NUTRITION INTERVENTION FOR MANAGING PARKINSON'S DISEASE

Dara LoBuono,[1] Sarah Dobiszewski,[2] Alison Tovar,[2] Skye Leedahl,[2] Furong Xu,[2] Matthew Delmonico,[2] Leslie Mahler,[2] and Ingrid Lofgren,[2] *1. University of Rhode Island, North Haledon, New Jersey, United States, 2. University of Rhode Island, Kingston, Rhode Island, United States*

Digital health technologies can enhance quality of care for people with Parkinson's disease (PwPD) and their informal caregivers (ICG), but research in this area is lacking. Developing digital health nutrition services incorporating end-user preferences is essential. This cross-sectional, mixed-method study assessed 20 PwPD and their ICG. Participants reported sources they first search for when seeking health information, the amount of effort it takes to find the information, their confidence level in finding reliable health information, and their level of trust of information obtained. Semi-structured dyadic interviews obtained information about technology, digital health, and nutrition. Transcripts were double coded by two researchers to identify nutrition service features and a >80% agreement was achieved. The mean age was 68.1±11.2 years and 65% of PwPD were male, while 80% of ICG were female. Most PwPD and ICG (82.5%) went to the internet the last time they looked up health information, and about 1/3 reported it took a lot of effort to get this information. Nearly half were concerned about the quality of the information, and approximately 70% had issues trusting health information from government agencies or diet programs. Six themes around key features for a digital nutrition intervention emerged: tailored and specific, inclusion of caregivers, promote self-efficacy, from a nutrition expert, contain a social element, and include a follow-up session. The results suggest that digital health interventions will need to be tailored to meet the needs of PwPD and their ICG.

### THE INFLUENCE OF MULTIPLE FRAILTIES ON INSTITUTIONALIZATION AND MORTALITY IN COMMUNITY-LIVING OLDER ADULTS

Yunhwan Lee, *Ajou University School of Medicine, Suwon, Republic of Korea*

Frailty is a hallmark of accelerated aging, predisposing the older person to increased vulnerability to adverse health outcomes. Physical frailty is closely linked to other health dimensions, such as cognitive, psychological, and social functions. This study aims to examine the influence of multiple dimensions of frailty in predicting institutionalization and mortality. A nationally representative sample of Koreans 65 years or older from the Living Profiles of Older People Survey in 2008 was followed up for three years (n = 11,265). Physical frailty was defined as being prefrail or frail using the Fried phenotype model. Those with cognitive impairment, depressive symptoms, and social vulnerabilities, in addition to physical frailty, were considered to have cognitive frailty, psychological frailty, and social frailty, respectively. The proportional hazards model was used to analyze the risk of institutionalization and mortality by the total number and different combinations of frailties, adjusting for covariates. More than half (50.1%) of the participants exhibited multiple frailties, with 8.1% concurrently displaying frailty in all four domains (mixed frailty). The risk of adverse outcomes was elevated with a higher number of frailties, with hazard ratios of 2.59 (95% confidence interval [CI]: 1.52, 4.42) for institutionalization and 3.40 (95% CI: 2.50, 4.63) for mortality among those presenting mixed frailty. Whereas psychological frailty demonstrated a stronger predictive ability of mortality than institutionalization, the reverse was observed for social frailty. Multiple frailties are prevalent in late life. Acquiring more frailties raises the risk of adverse outcomes, with varying effects according to multidimensional frailty profiles.

### SESSION 2917 (PAPER)

### CHRONIC DISEASE AND MULTIMORBIDITY II

### DUAL-TASK GAIT COST AND FRONTAL LOBE INTEGRITY IN POSTSTROKE: RESULTS FROM ONDRI

Frederico Pieruccini-Faria,[1] Paula McLaughlin,[2] Benjamin Cornish,[3] Malcolm Binns,[4] Robert Bartha,[5] Bill McIlroy,[3] ONDRI collaborators,[6] Manuel Montero Odasso,[7] and Frederico Faria, *1. University of Western Ontario, London, Ontario, Canada, 2. Queens University, Kingston, Ontario, Canada, 3. University of Waterloo, Waterloo, Ontario, Canada, 4. Baycrest Health Sciences, Toronto, Ontario, Canada, 5. Robarts-Western University, London, Ontario, Canada, 6. Ontario Brain Institute, Toronto, Ontario, Canada, 7. The University of Western Ontario, London, Ontario, Canada*

Dual-task gait performance is a marker of motor-cognitive interactions modulated by the frontal lobes. After a stroke, gait disturbances are more evident, particularly when concurrently completing a mental task and walking, an effect called high dual-task cost (DTC). Following a stroke, the potential association of high-DTC, integrity of the frontal lobes and cognitive functioning is unclear. This study screened 161 participants with stroke history from the Ontario Neurodegenerative Disease Research Initiative (ONDRI)-cerebrovascular disease cohort (69.2±7.41 years of age; 31.7% women). Individuals scoring zero in the National Institute of Health Stroke Scale were analyzed (n=102). DTC was the percentage change in gait speed from the single to dual-task condition. Standardized normal-appearing white matter (NAWM) and grey matter (NAGM) volumes from superior, middle and inferior frontal lobe were compared between DTC quartiles (adjusted for age, sex, and education) using a multivariate model (MANOVA), with total frontal lobe volume as a covariate. Another model compared group performance across 5 adjusted cognitive domains (attention, memory, language, visuospatial performance, and executive functioning). Univariate tests revealed that NAWM volume in the superior frontal lobe (F=4.50; p=0.005; partial eta-squared=0.122) was significantly different across DTC quartiles. Contrast tests suggested that the first quartile had larger NAWM than the second and fourth. DTC quartiles also showed differences in attention (F=2.93; p=0.03; partial eta-squared=0.083) and contrast tests indicated that the first quartile performed significantly better than second and fourth. DTC poststroke may be a proxy for structural integrity of superior frontal lobe regions and attention.

### OLDER U.S. ADULTS WITH NOCTURIA OFTEN CANNOT USE THE ONLY FDA-APPROVED DRUGS FOR THE CONDITION

Theodore Johnson,[1] Kara Suvada,[2] Laura Plantinga,[2] Anna Mirk,[2] Alayne Markland,[3] Mohammed Ali,[2] Camille Vaughan,[2] and Kathryn Burgio,[4] *1. Emory University School of Medicine, Atlanta, Georgia, United States, 2. Emory University, Atlanta, Georgia, United States, 3. University of Alabama at Birmingham, BIRMINGHAM, Alabama, United States, 4. University of Alabama at Birmingham, Birmingham, Alabama, United States*

Nocturia, waking from sleep at night to void, is a common, bothersome symptom for which many older adults seek treatment. In 2017, the US FDA approved the first of two desmopressin analogues indicated for nocturia. While efficacious, these drugs can potentially cause severe hyponatremia that can be fatal, particularly if older adults have comorbid conditions (e.g., congestive heart failure, uncontrolled hypertension), laboratory abnormalities (e.g., low sodium, reduced renal function) and concomitant medications (e.g., diuretics, analgesics). Using secondary data from the National Health and Nutrition Examination Survey (NHANES) to identify a sample of U.S. adults 50 years and older with 2 or more nightly nocturia episodes, we determined the prevalence of contraindications, warnings, and need for more frequent monitoring indicated by the product label. Among the sample of 1,521 older respondents, 70.0% of those with nocturia had contraindications or a need for frequent sodium monitoring. Contraindications to desmopressin analogues were more prevalent with older age (27.6%, 34.5%, and 38.8%, for ages 50-64, 65-79, and 80+, respectively, $p<0.001$) as was the use of potentially interacting drugs (30.0%, 42.2%, 47.7%, respectively, $p < 0.001$). Most older adults with nocturia have medical conditions and concomitant medication use that makes very difficult the routine and safe use of the only FDA approved medications for nocturia. Providers prescribing desmopressin analogues in older adults need to carefully screen patients, employ frequent monitoring, and use shared decision-making to prevent undue harm.

### PERCEPTIONS OF A TRANSITIONAL CARE MODEL FOR OLDER ADULTS WITH MULTIMORBIDITY AND DEPRESSIVE SYMPTOMS

Maureen Markle-Reid,[1] Carrie McAiney,[2] Rebecca Ganann,[1] and Carly Whitmore,[1] *1. McMaster University, Hamilton, Ontario, Canada, 2. University of Waterloo, Waterloo, Ontario, Canada*

Transitioning from hospital to home is an important healthcare system priority. This paper reports on the qualitative findings from a larger mixed methods study designed to examine the implementation and effectiveness of a new transitional care intervention (Community Assets Supporting Transitions [CAST]). The goal of the CAST intervention is to improve the quality and experience of hospital-to-home transitions for older adults (≥ 65 years) with depressive symptoms and multimorbidity. Semi-structured interviews were completed with a sub-set of intervention group trial participants including 11 older adult participants and 1 caregiver, as well as 4 intervention nurses. A qualitative descriptive design was used to explore the perceived impacts of the CAST intervention on participants and their caregivers. Audio-recorded interviews were transcribed verbatim, with descriptive codes and themes generated using conventional content analysis. Patient participants indicated that the intervention resulted in improved access to information (e.g., medication review) and services (e.g., care coordination) that enhanced their self-management. Participants felt that the home visits and phone visits were valuable and helped to improve their mental health. Intervention nurses described advocating for patients to help achieve their needs. For example, nurses advocated for physiotherapy services to provide additional education to support patient mobility. Understanding patient, caregiver, and provider perceptions of the impact of the CAST intervention will help to identify how to improve the delivery of this transitional care intervention, to bridge the gap between hospital and community care, and to positively impact patient health outcomes.

### RURAL AND INDIGENOUS HEALTH DISPARITY IN MEDICAL SERVICE USE FOR DEMENTIA AND DIABETES MELLITUS IN MINNESOTA

Sung Han Rhew,[1] Patrick Bright,[1] Andrine Lemieux,[2] Wayne Warry,[2] and Kristen Jacklin,[3] *1. University of Minnesota, Duluth, Duluth, Minnesota, United States, 2. Memory Keepers Medical Discovery Team, University of Minnesota, Duluth, Duluth, Minnesota, United States, 3. Memory Keepers Medical Discovery Team, University of Minnesota, Duluth, Minnesota, United States*

Minnesota has shown relatively high growth of mortality from diabetes mellitus (DM) and dementia in recent years, especially in rural areas. Analysis of medical care utilization patterns may reveal the reasons for this trend. The goal of the present study was to characterize the Minnesota dementia and diabetes care landscape by rurality and geographic region. Specifically, we compared the Metro region to five other rural-urban regions. Disease-specific 2017 hospital admission and emergency department (ED) visit data was obtained from the State Center for Health Statistics and the Healthcare Cost and Utilization Project. We used the logistic regression analysis adjusted by multiple covariates to evaluate rural-urban differences in hospital admissions and ED visits. Age-adjusted rates of ED visits for both DM and dementia were significantly higher in rural zip code areas, especially in northeast regions. Rural areas had elevated odds for dementia hospital admissions (OR=1.05, $p<0.0001$) and ED visits (OR=1.24, $p<0.0001$), but decreased odds for DM hospital admission (OR=0.96, $p<0.0001$) and ED visits (OR=0.96, $p<0.0001$). This was particularly true in the northeast region (relative to Metro regions) where ED visits were less likely due to DM (OR=0.89, $p<0.0001$) but more likely related to dementia (ORs=1.42, $p<0.0001$). Geographic differences for ED visits due to DM were greater than those for dementia, with higher rates for rural as compared to urban regions (northeast MN compared to a large metropolitan region). This geographical mismatch between mortality rates and ED visit rates may illustrate the relative lack of access to health services in rural MN.

### TRAINING MEDICAL RESIDENTS TO ADDRESS SOCIAL DETERMINANTS OF HEALTH FOR BETTER HEALTH OUTCOMES

Angela Zell, Joan Ilardo, Adesuwa Olomu, Cristian Meghea, and Supratik Rayamajhi, *Michigan State University, East Lansing, Michigan, United States*

Gaps exist in training medical residents to assess social determinants of health (SDOH) related to chronic conditions. To address the need for better screening, we partnered with two Internal Medicine (IM) residency programs based in Lansing and Flint (Michigan) to pilot the Caring for Patients with Chronic Conditions (CPCC) project. IM residencies train internists with expertise in diagnosis, treating chronic conditions, promoting health through wellness education, and preventing and managing diseases. CPCC incorporated information during didactic sessions that residents could apply during their clinical activities that can influence their current and future clinical practice patterns. Presentations and panels from local community organizations on specific topics were incorporated into the curriculum that address needs of patients age 50 and older. To build on this education, the residents adapted the Office- Guidelines Applied in Practice (Office-GAP) checklist to identify SDOH affecting a patient's ability to managed chronic conditions. Using this tool: 1) involves resident training; 2) provides a decision support checklist; 3) influences patient activation; and 4) increases provider and patient communication through shared decision making. The checklist includes questions for patient response pertaining to SDOH that prevents them from managing their chronic conditions in addition to the level of action the patient is willing to do. Areas identified are discussed between patient and resident increasing patient activation. Referrals to community-based resources to identified SDOH needs are guided by the clinic's care manager. The Office-GAP tool is administered during three subsequent visits to ensure that patients actually accessed the community resources.

### SESSION 2918 (PAPER)

### COGNITION AND COGNITIVE IMPAIRMENT I

### LIFE COURSE SOCIOECONOMIC STATUS AND LATE-LIFE COGNITION AND COGNITIVE DECLINE IN THE RUSH MEMORY AND AGING PROJECT

Anna Krasnova,[1] Sarah Tom,[2] Linda Valeri,[1] Maria Glymour,[3] Paul Crane,[4] and David Bennett,[5]
*1. Columbia University Mailman School of Public Health, New York, New York, United States, 2. Columbia University, New York, New York, United States, 3. University of California, San Francisco, California, United States, 4. University of Washington, Seattle, Washington, United States, 5. Rush University Medical Center, Chicago, Illinois, United States*

The relationships among life course socioeconomic status (SES) measures with later life cognition and cognitive decline are unclear. We test the hypothesis that life-course SES is associated with late life level of cognition and rate of cognitive decline. The Rush Memory and Aging Project enrolled 1,864 dementia-free people aged ≥65 years between 1994 – 2018. Participants reported early life (parental education, number of siblings, and childhood financial need), mid-life (income at 40 years), and late life (baseline income) SES. Global cognitive function is a composite of 19 neuropsychological tests, administered annually. We utilized marginal structural models to assess the effect of SES (dichotomized at the median) at three life-course stages on late life global cognitive function and decline. We calculated inverse probability weights to adjust for socio-demographic confounders at each life-course stage. A total 1,063 participants had all relevant variables. Average follow-up was 4.4 years, and mean baseline age was 80.4 years. Most respondents were non-Hispanic white (89.7%) and female (74.1%). In the fully adjusted model, high childhood SES (coefficient 0.10; 95% CI 0.01, 0.20) and high late-life SES were associated with higher cognition intercept (coefficient 0.21; 95% CI 0.09, 0.32). High mid-life SES was associated with slower rate of cognitive decline (coefficient 0.02; 95% CI 0.001, 0.05). Childhood and late-life SES measures were not related to cognitive decline. Childhood and adult SES may reflect processes in building cognitive capacity, while midlife SES may reflect cognition maintenance. Interventions relating to SES across the life-course may benefit later life cognition.

### MACULAR GANGLION CELL–INNER PLEXIFORM LAYER AS A MARKER OF COGNITIVE AND SENSORY FUNCTION IN MIDLIFE

Natascha Merten, Adam Paulsen, A Alex Pinto, Yanjun Chen, Lauren Dillard, Mary Fischer, Carla Schubert, and Karen Cruickshanks, *University of Wisconsin-Madison, Madison, Wisconsin, United States*

Neurodegenerative diseases are public health challenges in aging populations. Early identification of people at risk for neurodegeneration might improve future treatment. Noninvasive, inexpensive screening tools are lacking but of great potential. Optical coherence tomography (OCT) measures nerve cell layer thicknesses in the retina, which is an anatomical extension of the brain and might be reflective of generalized neurodegeneration. We aimed to determine associations of macular ganglion cell-inner plexiform layer (mGCIPL) thickness with cognitive and sensorineural function in midlife. This study included 1880 Beaver Dam Offspring Study participants from the 10-year follow-up examination. We assessed cognition (principal component analysis of multiple cognitive test scores), cognitive impairment, hearing sensitivity thresholds and impairment, central auditory processing (% correct on a dichotic digits test), and visual and olfactory impairment. We measured mGCIPL using the Cirrus 5000 HD-OCT Macular Cube Scan. Multivariable linear and logistic regression models were used to determine associations of mGCIPL thickness and thin mGCIPL, defined as 1 standard deviation below average, with cognitive and sensorineural functions. Thinner mGCIPL was associated with worse cognition (0.01 standard deviation increase per µm thickness;95% confidence interval (CI) 0.01,0.02;p<.0001), worse central auditory function (0.07% increase per µm thickness;CI 0.01,0.13;p=.03) and visual impairment (Odds Ratio=0.95;CI 0.94,0.97;p<.0001). MGCIPL thickness was associated with hearing sensitivity in women only. There were no associations with impairments in hearing, olfaction and cognition. Results for thin group comparisons were consistent. MGCIPL thickness is associated with cognitive and sensorineural function and has potential as a marker for neurodegeneration in middle-aged adults.

### PRELIMINARY RESULTS OF MYCOG, A BRIEF ASSESSMENT FOR THE DETECTION OF COGNITIVE IMPAIRMENT IN PRIMARY CARE

Laura Curtis, Lauren Opsasnick, Julia Yoshino Benavente, Cindy Nowinski, Rachel O'Conor, Jordan Stoeger, Michael Wolf, and Richard Gershon, *Northwestern University, Chicago, Illinois, United States*

Early detection of Cognitive impairment (CI) is imperative to identify potentially treatable underlying conditions or provide supportive services when due to progressive conditions such as Alzheimer's Disease. While primary care settings are ideal for identifying CI, it frequently goes undetected. We developed 'MyCog', a brief technology-enabled, 2-step assessment to detect CI and dementia in primary care settings. We piloted MyCog in 80 participants 65 and older recruited from an ongoing cognitive aging study. Cases were identified either by a documented diagnosis of dementia or mild cognitive impairment (MCI) or based on a comprehensive cognitive battery. Administered via an iPad, Step 1 consists of a single self-report item indicating concern about memory or other thinking problems and Step 2 includes two cognitive assessments from the NIH Toolbox: Picture Sequence Memory (PSM) and Dimensional Change Card Sorting (DCCS). 39%(31/80) participants were considered cognitively impaired. Those who expressed concern in Step 1 (n=52, 66%) resulted in a 37% false positive and 3% false negative rate. With the addition of the PSM and DCCS assessments in Step 2, the paradigm demonstrated 91% sensitivity, 75% specificity and an area under the ROC curve (AUC)=0.82. Steps 1 and 2 had an average administration time of <7 minutes. We continue to optimize MyCog by 1) examining additional items for Step 1 to reduce the false positive rate and 2) creating a self-administered version to optimize use in clinical settings. With further validation, MyCog offers a practical, scalable paradigm for the routine detection of cognitive impairment and dementia.

### RELATIONSHIP BETWEEN PATIENT AND INFORMANT ASSESSMENT OF PERSONALITY AND COGNITIVE STATUS

Magdalena Tolea,[1] and James Galvin,[2] *1. University of Miami Miller School of Medicine, Boca Raton, Florida, United States, 2. University of Miami Miller School of Medicine, Palm Beach Gardens, Florida, United States*

Personality has been linked to risk of dementia. Most studies ask individuals to rate their own personality traits or for a knowledgeable informant to perform the rating; few collect data from both. When informants are asked to give an estimate of the patient's lifelong personality traits, they often describe personality before disease onset. When asked to self-rate, patients may instead assess their personality as they see themselves, providing a personality-state measure. The goal of this study was to assess agreement between two independent measures of personality and evaluate whether stage of cognitive impairment and characteristics of patients or caregivers impact concordance. In 79 consecutive patient-caregiver dyads presenting to our center (mean age:76.8±8.4; 44.1% female; 6% cognitively normal, 41% MCI; and 53% dementia) with in-depth psychosocial and neuropsychological evaluations, we found informants rated patients lower on openness (O) (ICC=0.434; 95%CI: 0.235-0.598) and agreeableness (A) (ICC=0.491; 95%CI: 0.302-0.643) and higher on extraversion (O) (ICC=0.396; 95%CI: 0.191-0.568) and neuroticism (N) (ICC=0.444; 95%CI: 0.247-0.607). Greater discordance was observed in established dementia (ICCE=0.497; 95%CI: 0.222-0.700; ICCA=0.337; 95%CI:0.031-0.586; ICCN=0.422; 95%CI: 0.191-0.683), compared with MCI (ICCO=0.568; 95%CI: 0.282-0.762). We explored the effect of patient and caregiver mood and caregiver burden on personality ratings. Although personality is typically described as a trait, we present evidence that in the eyes of patients, personality ratings may represent a state that changes across the spectrum of cognitive impairment. Understanding how patients and caregivers differentially perceive personality may assist in developing novel psychotherapeutic interventions and approaches dealing with behavioral manifestations of dementia.

### THE IMPACT OF COGNITIVE IMPAIRMENT ON RESOURCE UTILIZATION DURING MEDICARE HOME HEALTH CARE

Julia Burgdorf, and Jennifer Wolff, *Johns Hopkins Bloomberg School of Public Health, Baltimore, Maryland, United States*

Older adults with cognitive impairment have unique care needs that often lead to greater levels of health care utilization. Prior work suggests that older adults with cognitive impairment access home health care at higher rates; yet, recent Medicare home health payment system revisions exclude patient cognitive status when determining risk adjustment. This research examines the relationship between patient cognitive status and resource utilization during Medicare home health care. We examine 1,217 (weighted n=2,134,620) community-dwelling older adults who received Medicare-funded home health between 2011-2016, using linked nationally representative survey data from the National Health and Aging Trends Study (NHATS), home health patient assessment data, Medicare claims data, and Medicare Provider of Services files. We use weighted, multivariable negative binomial regressions to model the relationship between patient dementia status and the expected number of total visits and number of each visit type (nursing, therapy, and aide) during home health. Models adjusted for patient sociodemographic characteristics and health and functional status during home health, as well as home health provider characteristics. Among Medicare home health patients, the presence of cognitive impairment during home health is associated with 2.87 additional total visits (p<0.001), 1.27 additional nursing visits (p<0.01), and 1.23 additional therapy visits (p=0.04) during the home health episode. Findings suggest that recent revisions to the Medicare home health payment system may disincentivize home health care for older adults with dementia and/or financially penalize home health providers whose patient populations have a greater dementia burden.

### SESSION 2919 (PAPER)

### COGNITION AND COGNITIVE IMPAIRMENT II

### CERTIFIED NURSING ASSISTANTS' EXPERIENCES OF WORKPLACE VIOLENCE CARING FOR PERSONS WITH DEMENTIA

Chunhong Xiao,[1] Vicki Winstead,[2] Corteza Townsend,[3] and Rita Jablonski,[3] *1. The University of Alabama at Birmingham, Birmingham, Alabama, United States, 2. University of Alabama at Birmingham, Alabaster, Alabama, United States, 3. UAB School of Nursing, Birmingham, Alabama, United States*

Problem: Certified nursing assistants (CNAs) are the primary providers of direct care to persons residing in long term care facilities (LTCFs), many of whom have dementia. The need to deliver direct and intimate care increases CNAs' exposure to verbal and physical workplace violence. Purpose: To describe CNAs' experiences of physical and verbal workplace violence experienced during direct care activities in LTCFs. Design: Qualitative. Sample & Procedure: Ten African-American CNAs (9 female, 1 male) were recruited using snowball sampling from multiple LTCFs. Interviews were recorded and transcribed. NVivo12 software was used to manage the thematic analyses. Results: The identified themes were: 1) CNAs' perception that verbal and physical abuse was "part of the job" and unavoidable; 2) CNAs' feelings of minimization of the abuse by administration; and 3) inadequate CNA training to recognize and de-escalate triggers of verbal and physical violence, notably care-resistant behavior. Conclusion: The combination of institutional tolerance of workplace violence, coupled with CNAs' insufficient training in de-escalating volatile interactions with cognitively-impaired residents, is creating an unfavorable, possibly dangerous, workplace environment for CNAs. Implications: As more states elevate assaults on healthcare workers to felony crimes, there is an emerging risk of criminalizing dementia-related behavior in an attempt to address workplace violence. Interventions focused on helping CNAs recognize and de-escalate care-resistant behavior are necessary for violence prevention programs in LTCFs. Limitations: CNAs may have self-censored and under-described the severity of their experiences during face-to-face interviews, even with confidentiality protocols and the practice of off-site interviews.

### IDENTIFYING MEDICATION THERAPY PROBLEMS RELATED TO COGNITION AMONG OLDER ADULTS FOLLOWED BY A HOME-BASED CARE TEAM

Allison Levine,[1] Erin Emonds,[2] Marie Smith,[3] Nathaniel Rickles,[4] Richard Fortinsky,[5] and Alis Ohlheiser,[1] *1. UConn Health, Farmington, Connecticut, United States, 2. VA Boston Healthcare System, Boston, Massachusetts, United States, 3. University of Connecticut School of Pharmacy, Mansfield, Connecticut, United States, 4. University of Connecticut School of Pharmacy, Storrs, Connecticut, United States, 5. University of Connecticut, Farmington, Connecticut, United States*

Complications from dementia, depression, delirium (3Ds) and polypharmacy may accelerate patient decline. Cognitive vulnerabilities may be under-recognized and medication therapy problems (MTPs) overlooked, hindering optimal care. Clinical pharmacists on a multidisciplinary home-based care team (HBCT) being tested in a clinical trial were essential in identifying MTPs related to cognition. Medicare Advantage members >65 years old, living at home in Connecticut, with ICD-10 codes related to 3Ds were eligible. APRNs conducted in-home medication reconciliation along with medical and cognitive assessments. HBCT pharmacists assessed medication lists for MTPs related to indication, effectiveness, and safety (adverse events, interactions). After review by the HBCT APRN, geriatrician, and psychiatrist, salient pharmacist recommendations were forwarded to PCPs for consideration. Using retrospective analysis, MTPs and recommendations were classified based upon the Pharmacy Quality Alliance framework. MTP analysis included 105 patients enrolled from 2017-2019. We found 166 MTPs related to cognition, with a mean (SD) of 1.58 (1.35) (range 0-6) MTPs per patient. MTPs related to indication accounted for 34% (57/166) of total MTPs, of which 79% (45/57) were underuse and 21% (12/57) overuse; effectiveness represented 13% (22/166) of MTPs; safety represented over half (52%; 87/166) of total MTPs with benzodiazepines and anticholinergics commonly implicated. Common HBCT pharmacists' recommendations included discontinuation (23%; 38/166) and dose reduction (19%; 32/166). MTPs related to cognition were found among the overwhelming majority (79%) of patients. This work is significant because it supports the value of pharmacists on multidisciplinary teams to address cognitively harmful medications, dementia treatment side effects, and untreated cognitive conditions.

### INTENTION TO RECEIVE COGNITIVE SCREENING FOR ALZHEIMER'S DISEASE IN NONDEMENTED OLDER ADULTS

Juyoung Park,[1] Magdalena Tolea,[2] Lilah Besser,[1] and James Galvin,[3] *1. Florida Atlantic University, Boca Raton, Florida, United States, 2. University of Miami Miller School of Medicine, Boca Raton, Florida, United States, 3. University of Miami Miller School of Medicine, Palm Beach Gardens, Florida, United States*

The study explored factors associated with intention to receive cognitive screening for Alzheimer's disease (AD). It also examined whether self-efficacy mediates the relationship between knowledge about screening and the intention to be screened. A population-based, random-digit dialing survey was performed; 1,043 responses were collected from a sample of nondemented older adults living in urban, suburban, and rural areas. A majority were female (66.8%, n = 697) and White (82.7%, n = 863) with a mean age 62.6 years (SD = 10.2). Findings from regression analysis identified that being female ($\beta = .080$), being depressed ($\beta = .149$), and having a positive life orientation ($\beta = .120$) were significantly associated with the intention to receive cognitive screening, $p < .05$. Results indicated that older adults with a positive life orientation reported greater intention to be screened for AD, whereas depressed participants were more likely to plan to be screened for AD. Bootstrapping results identified a mediating effect of self-efficacy ($\beta = .2668$, $t = 7.3137$, $p < .0005$). Self-efficacy mediated the relationship between knowledge about screening and intention to be screened. Using self-efficacy as a mediation effect indicated that older adults with knowledge about screening understand the benefits of early screening and diagnosis and are more likely to have self-efficacy (i.e., confidence to consult with a physician), and thus are more likely to show intention to be screened. Intention to be screened for AD could increase public awareness by defining effective ways to assist older adults to seek a cognitive screen.

### SESSION 2920 (PAPER)

### DEMENTIA I

### A PATH TO EARLY DIAGNOSIS OF MCI AND DEMENTIA: INTEGRATING MYMEMCHECK INTO THE PRIMARY CARE WORKFLOW

William Mansbach,[1] Ryan Mace,[1] Theresa Frangiosa,[2] Virginia Biggar,[3] Meryl Comer,[4] and Steven Simmons,[5] *1. Mansbach Health Tools, LLC, Simpsonville, Maryland, United States, 2. UsAgainstAlzheimer's, Royersford, Pennsylvania, United States, 3. UsAgainstAlzheimer's, Washington, District of Columbia, United States, 4. UsAgainstAlzheimer's, Kensington, Maryland, United States, 5. Allegiance Housecalls LLC, North Potomac, Maryland, United States*

Barriers to the early detection of mild cognitive impairment (MCI) and dementia can delay diagnosis and treatment. myMemCheck® was developed as a rapid free cognitive self-assessment tool that can be completed in the practice setting or at home to identify older adults that would benefit from a more comprehensive cognitive evaluation for MCI and dementia. Two prospective cross-sectional studies (N = 59; N = 357) were conducted to examine the psychometric properties and clinical utility of myMemCheck®. myMemCheck® evidenced adequate reliability (test-retest, r = 0.67) and strong construct validity (η2 = 0.29, discriminating normal, MCI, dementia). Receiver operating characteristic analysis evidenced an optional myMemCheck® cut score for identifying older adults with MCI or dementia (sensitivity = 0.80, specificity = 0.67, positive predictive value = 0.91, negative predictive value = 0.43). myMemCheck® explained 25% of cognitive status beyond basic patient information. We provide specific suggestions for integrating myMemCheck® into practice to optimize workflow. Study results are further interpreted in the context of two national online surveys (healthcare professionals, N = 181; consumers, N = 1740). Healthcare professionals widely agreed on the need (94%) and importance (86%) of cognitive self-assessments. Public demand for cognitive self-assessment was confirmed by consumers who trialed myMemCheck® as part of their survey participation—86% agreed on the need for a tool like myMemCheck®. Mixed methods findings suggest that myMemCheck® could fast- track the diagnostic process, facilitate appropriate referrals for cognitive and neuropsychological evaluation, reduce assessment burden in healthcare, and prevent negative outcomes associated with undetected cognitive impairment.

### AGREEMENT BETWEEN PROXY-RATED AND SELF-REPORTED QUALITY OF LIFE FOR PERSONS WITH DEMENTIA

Mariko Sakka, Ayumi Igarashi, Chie Fukui, Maiko Noguchi-Watanabe, Asa Inagaki, Manami Takaoka, Hiromi Kobayashi, and Noriko Yamamoto-Mitani, *The University of Tokyo, Tokyo, Japan*

While quality of life (QOL) is an important endpoint of homecare for persons with dementia (PWD), PWDs often have difficulty in articulating their QOL by themselves. Instead proxy-rating is often used. However, evidence is still scarce regarding to what extent proxy-ratings reflect actual QOL of PWDs. We examined the association between self-report QOL by PWDs and proxy-rated QOL. We conducted a questionnaire survey to PWDs who were 75 years and older, their family, and homecare nurse in charge of the PWD. Two measures were used: 1) a newly developed, 4-item self-report for QOL of PWDs, and 2) a standardized, 6-item proxy-rating dementia QOL scale. In the self-report, the PWD were asked about their daily mood or satisfaction in life in brief, easy-to-understand sentences. The self-reports and proxy-ratings were compared using intraclass correlation coefficients (ICC). Data from 382 PWDs, 248 family caregivers and 124 nurses were used. The mean age of PWD was 85.9 years and 60.5% were female. The proxy-rating by nurses were more strongly associated with self-reports, compared to the association between family proxy rating and self-reports (r = 0.351, p < .001; r = 0.236, p < .001, respectively). Proxy ratings by spouses and biological children were significantly associated with self-report (r = 0.257, p =.004; r =. 204, p = .006, respectively), while rating by children-in-law were not (r = 0.217, p = .160). Proxy-ratings may not be an appropriate substitute for self-report. Homecare nurses may evaluate the QOL of PWD better than their family caregiver.

### CARING FOR OLDER ADULTS WITH VISION IMPAIRMENT AND DEMENTIA: DATA FROM THE NATIONAL STUDY OF CAREGIVING

Varshini Varadaraj,[1] Bonnielin Swenor,[1] Shang-En Chyng,[1] Orla Sheehan,[2] Ashley Deemer,[1] Joshua Ehrlich,[3] Jennifer Wolff,[4] and David Roth,[1] *1. Johns Hopkins University, Baltimore, Maryland, United States, 2. Johns Hopkins University School of Medicine, Baltimore, Maryland, United States, 3. University of Michigan, Ann Arbor, Michigan, United States, 4. Johns Hopkins Bloomberg School of Public Health, Baltimore, Maryland, United States*

We examined caregiving relationships for individuals with vision impairment (VI) and dementia, using 2011 National Health and Aging Trends Study (NHATS) data, a survey of Medicare beneficiaries, linked to the National Study of Caregiving, a survey of family/unpaid helpers to NHATS participants. VI was defined as self-reported blindness or difficulty recognizing someone across the street, watching television or reading newspaper print. Dementia was defined as probable dementia based on survey-report or AD8 criteria. Caregiving outcomes included: (1) hours of care provided in the last month and (2) number of valued activities affected by caregiving. Among 1,196 caregivers, 617 assisted older adults without dementia or VI (D-/VI-), 298 with dementia but without VI (D+/VI-), 143 without dementia but with VI (D-/VI+), and 138 with dementia and VI (D+/VI+). In fully-adjusted regression models, caregivers of older adults D+/VI+ spent twice as many hours (IRR=2.0; 95%CI: 1.5-2.7) providing care than caregivers of older adults D-/VI-; however, caregivers of adults D+/VI- and those providing to older adults D-/VI+ spent 1.5-times more hours (95% CI=1.2-1.7; 95% CI=1.1-2.0, respectively). Additionally, caregivers of older adults D+/VI+ reported 4 times as many valued activities were affected (95%CI=2.8-5.6) then caregivers of those D-/VI-, while caregivers of those D+/VI- reported 1.9-times (95% CI=1.3-2.8) and D-/VI+ 1.6-times (95% CI=1.1-2.3) more activities were affected. Our results suggest that caring for older adults with VI has similar demands as caring for older adults with dementia, but that these implications may be magnified when caring for older adults with both dementia and VI.

### CONCOMITANT CHOLINESTERASE INHIBITOR AND ANTICHOLINERGIC DRUG USE AMONG OLDER ADULTS WITH DEMENTIA

Elizabeth Carter, and Erwin Tan, *AARP, Washington, District of Columbia, United States*

Anticholinergic medications (ACh) are frequently prescribed to older adults despite being associated with impaired physical functioning. Moreover, the concomitant use of ACh and cholinesterase inhibitors (ChEI) reduces the body's response to both drugs, thereby diminishing the modest effectiveness of ChEI at slowing the progression of dementia symptoms. The objective of this study was to assess the risk of concomitant ACH/ChEI use on functional outcomes, including fall, fracture, and traumatic brain injury

(TBI). We conducted a retrospective review of data from the OptumLabs® Data Warehouse, a de-identified administrative claims database for commercially insured and Medicare Advantage (MA) enrollees, representing a diverse mixture of ages, ethnicities, and geographical regions across the U.S. Our cohort included adults 65 and older enrolled in MA between 1998 and 2017. Subjects were required to have dementia (by a diagnosis and/or prescription for a dementia drug (memantine or ChEI)) and at least one claim for ChEI during 12 months of follow-up. Subjects had to be enrolled in MA 6 months prior to the dementia index date. We defined concomitant ACh/ChEI use as an overlap of 30 days or more. Nearly one-third (29%) were concomitantly prescribed ACh and ChEI. Half (51%) of concomitant users were prescribed ChEI first, 46% were prescribed ACh first, and 3% received prescriptions on the same day. Results from multiple logistic regression analyses show that older adults with dementia who had concomitant ACh/ChEI use were 18%, 16%, and 25% more likely to experience a fall, fracture, or TBI, respectively, than those taking ChEI alone.

### HIGH OCCURRENCE OF DEMENTIA IN OLDER ADULTS RETURNING TO COMMUNITY FROM PRISON

Amy Byers,[1] Yixia Li,[2] and Lisa Barry,[3] *1. University of California, San Francisco / San Francisco VA Health Care System, San Francisco, California, United States, 2. Northern California Institute for Research and Education, San Francisco, California, United States, 3. UConn Center on Aging, Farmington, Connecticut, United States*

Very little is known about Alzheimer's Disease and related dementias (AD/ADRD) and diseases (e.g., Parkinson's/PD) in older adults returning to community from prison ("older reentry adults"). We utilized a national sample of older veterans to conduct the first study documenting the occurrence (prevalence/incidence) of AD/ADRD and related diseases in older reentry adults. We examined 28,235 reentry veterans who were aged 50 years and older at their most recent release date and Medicare beneficiaries, 2008 through December 31, 2017. AD/ADRD and related diseases were identified by ICD-9/10 codes in the electronic health record from Medicare and inpatient/outpatient Veterans Health Administration data. We examined distributions of AD/ADRD and related diseases across 4 age categories (sample %): 50-64 (55%), 65-74 (37%), 75-84 (7%), and 85+ (0.7%). Of the 28,235 veterans 50 years and older, 17% (n=4,725) had dementia (defined by AD/ADRD based on NIA criteria) or mild cognitive impairment (MCI) and 3% (n=794) had PD. Of those with dementia/MCI, 18% had AD and 26% MCI. Nearly 40% of dementia diagnoses occurred prior to/on most recent release date, and 40% of PD diagnoses occurred prior to/on most recent release date. Differences were significant across age groups (P<.001), with very high rates of diagnoses across all age groups, as well as indicative of high occurrence of early onset dementia and "accelerated aging" [50-64, 14%; 65-74, 18%; 75-84, 33%; and 85+, 53%]. This study is a first step in filling a major research gap by describing AD/ADRD, MCI and related diseases in reentry adults.

### SESSION 2921 (PAPER)

### DEMENTIA II

### APPLICATIONS OF STOCHASTIC PROCESS MODELS TO CONSTRUCTING PREDICTIVE MODELS OF ALZHEIMER'S DISEASE

Konstantin Arbeev, Olivia Bagley, Arseniy Yashkin, Hongzhe Duan, Igor Akushevich, Svetlana Ukraintseva, and Anatoliy Yashin, *Duke University, Durham, North Carolina, United States*

Large-scale population-based data collecting repeated measures of biomarkers, follow-up data on events (incidence of diseases and mortality), and extensive genetic data provide excellent opportunities for applying statistical models for joint analyses of longitudinal dynamics of biomarkers and time-to-event outcomes that allow investigating dynamics of biomarkers and other relevant factors (including genetic) in relation to risks of diseases and death and how this may propagate to the future. Here we applied one such model, the stochastic process model (SPM), to data on longitudinal trajectories of different variables (comorbidity index, body mass index, cognitive scores), other relevant covariates (including genetic factors such as APOE polymorphisms and polygenic scores, PGS), and data on onset of Alzheimer's disease (AD) in the Health and Retirement Study. We observed that different aging-related characteristics estimated from trajectories of respective variables in SPM are strongly associated with risks of onset of AD and found that these associations differ by sex, APOE status (carriers vs. non-carriers of APOE e4) and by PGS groups. The approach allows modeling and estimating time trends (e.g., by birth cohorts) in relevant dynamic characteristics in relation to the disease onset. These results provide building blocks for constructing the models for forecasting future trends and burden of AD that take into account dynamic relationships between individual trajectories of relevant repeatedly measured characteristics and the risk of the disease. Such models also provide the analytic framework for understanding AD in the context of aging and for finding genetic underpinnings of such links between AD and aging.

### CHEMOTHERAPY AND ANESTHESIA IN COLORECTAL CANCER SURVIVORS AND THE RISK OF ALZHEIMER'S DISEASE

Igor Akushevich,[1] Arseniy Yashkin,[1] Julia Kravchenko,[1] and Miklos Kertai,[2] *1. Duke University, Durham, North Carolina, United States, 2. Vanderbilt University Medical Center, Nashville, Tennessee, United States*

Exposures common in cancer patients—chemotherapy, surgical injury and/or anesthesia, alone or in combination with predisposing factors—have been suggested as potential risk factors for Alzheimer's disease (AD). We explored the relationship between chemotherapy and cumulative anesthesia exposure, and development of AD in colorectal cancer survivors. We conducted a retrospective cohort study of individuals age 65 and older diagnosed with colorectal cancer between 1998 and 2013, drawing on SEER-Medicare data and employing a proportional hazards model. We found that exposure to chemotherapy in colorectal cancer survivors demonstrated a protective effect for AD HR=0.821 (0.784-0.860). The beneficial effect held in race-, sex-, cancer-stage-specific subgroups, across chemotherapy agents (e.g., Fluorouracil, Oxaliplatin, or Fluorouracil+Leucovorin), in multivariable analyses, and in propensity score-based pseudorandomization based on 70 demographic, socioeconomic, cancer-diagnosis-related, and comorbidity variables. The effect was diminished or absent when non-AD dementias were analyzed. Findings further demonstrated that the association between chemotherapy exposure and AD was not affected by competing risk of long-term mortality or possible correlation between choosing chemotherapy and higher cognitive score or use of alternative health insurance. The effect of anesthesia on AD was not significant (0.998 per hour, 0.992-1.005) and this effect held in all subgroups, multivariable analyses, and for pseudorandomized subpopulations. Harmful effect was detected for cerebral degeneration, excluding AD, cognitive deficits following cerebral hemorrhage, cognitive disorder due to injury, hepatic encephalopathy, and hepatolenticular degeneration. Sensitivity analyses focused on SEER-Registry-specific effects and possible misspecifications in anesthesia records with alternative models demonstrated stability of estimates.

### DUAL DECLINE IN GAIT AND COGNITION IS ASSOCIATED WITH FUTURE DEMENTIA: EVIDENCE FOR A PHENOTYPE

Manuel Montero Odasso,[1] Mark Speechley,[1] Richard Camicioli,[2] Nellie Kamkar,[3] Qu Tian,[4] Luigi Ferrucci,[4] Nick Bray,[3] and Frederico Pieruccini-Faria[5] *1. The University of Western Ontario, London, Ontario, Canada, 2. The University of Alberta, Edmonton, Alberta, Canada, 3. Parkwood Research Institute, London, Ontario, Canada, 4. National Institute on Aging, Bethesda, Maryland, United States, 5. University of Western Ontario, London, Ontario, Canada*

BACKGROUND: The concurrent decline in gait speed and cognition are associated with future dementia. However, the clinical profile of those who present with dual-decline has not yet been described. We aimed to describe the phenotype and risk for incident dementia of individuals who present a dual-decline in comparison with non dual-decliners. METHODS: Prospective cohort of community-dwelling older adults free of dementia at baseline. We evaluated participants' gait speed, cognition, medical status, functionality, incidence of adverse events, and dementia biannually over 7 years. Gait speed was assessed with a 6-meter electronic-walkway, and global cognition was assessed using the MoCA test. We compared characteristics between dual-decliners and non dual-decliners using t-test, Chi-square, and hierarchical regression models. We estimated incident dementia using Cox models. RESULTS: Among 144 participants (mean age 74.23 ± 6.72 years, 54% women), 17% progressed to dementia. Dual-decliners had a three-fold risk (HR: 3.12, 95%CI:1.23-7.93, p=0.017) of progression to dementia compared with non dual-decliners. Dual-decliners were significantly older with a higher prevalence of hypertension and dyslipidemia (p=0.002). Hierarchical regression models show that age and sex alone explained 3% of the variation in the dual-decliners group, while adding hypertension and dyslipidemia increased the explained variation to 8% and 10 %, respectively. The risk of becoming a dual-decliner was 4-fold if hypertension was present. CONCLUSION: Older adults with concurrent decline in gait speed and cognition represent a group at the highest risk of progression to dementia. These dual-decliners have a distinct phenotype with a higher prevalence of hypertension, a potentially treatable condition.

### RECRUITMENT OF A DIVERSE RESEARCH COHORT IN A LARGE METROPOLITAN AREA FOR DEMENTIA INTERVENTION STUDIES

Melissa Reuland,[1] Deirdre Johnston,[2] Inga Antonsdottir,[3] Morgan Bunting,[4] and Quincy Samus,[5] *1. Johns Hopkins University School of Medicine, Baltimore, Maryland, United States, 2. Johns Hopkins Hospital, Baltimore, Maryland, United States, 3. Johns Hopkins School of Nursing, Baltimore, Maryland, United States, 4. Johns Hopkins University, Halethorpe, Maryland, United States, 5. Johns Hopkins University, Baltimore, Maryland, United States*

In the near future, the costs, both human and financial, of dementia care will grow exponentially. Over five and a half million older Americans are estimated to be living with Alzheimer's disease and related dementia (ADRD). By 2050, this is expected to increase to over 13 million, with persons of color being at the highest risk for developing dementia. Considerable federal, state and private funds have been committed to research to prevent, treat, and care for persons at risk for ADRD. However, enrollment of research participants, particularly those coming from diverse backgrounds into studies, is a perennial challenge and has serious implications. Between 2014 and 2019, a Johns Hopkins study team implemented a wide ranging research recruitment effort in the Baltimore-Washington DC area to enroll participants into two large federally funded dementia care coordination trials. A total of 2,063 study participants were self- or caregiver referred to these projects via referrals from organizations (e.g. religious, health, social service, aging, Medicaid clams) and targeted community outreach (e.g. events, media). Ultimately, 647 ADRD/study partner dyads were enrolled (31%). Outreach and recruitment challenges included stigma, lack of confirmed diagnosis, mistrust of research, and situational crises. The study team adapted enrollment criteria as challenges emerged, and ultimately spent $101,058 on outreach and recruitment to enroll 647 participant dyads. This represents a cost of $156.19 per dyad. This poster will provide background on the research program, detail the comprehensive outreach and recruitment strategies employed and their costs, and discuss best practices for recruiting this population.

### SESSION 2922 (PAPER)

### FALL PREVENTION I

### DISABILITY PREVENTION PROGRAM IMPROVES LIFE-SPACE AND FALLS EFFICACY: A RANDOMIZED CONTROLLED TRIAL

Minhui Liu,[1] Qian-Li Xue,[2] Laura Gitlin,[3] Jennifer Wolff,[4] Jack Guralnik,[5] Bruce Leff,[6] and Sarah Szanton,[7]

*1. Central South University, Baltimore, Maryland, United States, 2. Johns Hopkins School of Medicine, Baltimore, Maryland, United States, 3. Drexel University, Philadelphia, Pennsylvania, United States, 4. Johns Hopkins Bloomberg School of Public Health, Baltimore, Maryland, United States, 5. University of Maryland School of Medicine, Baltimore, Maryland, United States, 6. Johns Hopkins University School of Medicine, Baltimore, Maryland, United States, 7. Johns Hopkins University, Baltimore, Maryland, United States*

Life-space and falls efficacy are essential to physical independence and consistently predict disabilities. However, it remains unknown whether the CAPABLE, a disability prevention program, improves life-space and falls efficacy in older adults. We evaluated the effects of a home-based disability prevention program on life-space (N=194) and falls efficacy (N=233) among low-income older adults with restricted daily activities. The CAPABLE intervention consists of up to 6 one-hour home visits with an Occupational Therapist (OT), up to 4 one-hour home visits with a Register Nurse (RN), and up to $1300 worth of home repairs, modifications, and assistive devices with a handyman, during four months. Life-space and falls efficacy were measured by the Telephone Assessment of Mobility for homebound older adults and the Tinetti Falls Efficacy Scale at baseline and five months, respectively. Multinomial logistic regression and generalized linear models were used for data analyses. Participants in both samples were, on average, 75 years, predominantly black (86%) and female (85%-86%). Compared to participants in the control group, participants receiving the intervention were more likely to have improved (Odds Ratio [OR]: 2.10, 95% CI: 1.06-4.20) or unchanged overall life-space (OR: 2.62, 95% CI: 1.10-6.27) vs. decreased overall life-space. Participants who received the intervention also had significantly higher falls efficacy in performing daily activities (exponentiated coefficient: 1.11, 95% CI: 1.04-1.19). Life-space and falls efficacy can be significantly improved by CAPABLE program. These findings provide more evidence for the reasons for the increased physical independence.

### FEAR OF FALLING AND COGNITIVE DECLINE IN HEALTHY AGING ADULTS

Rebecca Kraut,[1] and Roee Holtzer,[2] *1. Yeshiva University, New York, New York, United States, 2. Yeshiva University, Bronx, New York, United States*

Fear of Falling (FOF) is common and associated with poor mobility in aging but whether persistence of FOF endorsement influences cognitive decline has not been reported. Here we determined the effect of FOF, measured dichotomously and after accounting for persistence, on decline in global cognitive function (GCF), memory, and attention/executive functions. Older adults with persistent FOF (n=81; mean age=77.63±6.67 yrs; %female=74.1), transient FOF (n=60; mean age=76.93±6.01 yrs; %female=61.7), and no FOF (n=286; mean age=75.77±6.42 yrs; %female=49.3) were included. FOF was assessed through yes/no responses to “do you have a fear of falling?” at baseline. GCF was assessed using RBANS; memory was assessed using a composite score comprising the immediate and delayed recall index scores from RBANS; attention/executive functions were assessed via a composite score comprising TMT A & B, letter and category fluency tasks, and digit symbol modalities. Cognitive measures were administered annually for up to six years. Linear mixed effects models revealed that persistent FOF was associated with a worse decline in GCF compared to both transient FOF (estimate=0.78, p=.022) and no FOF (estimate=0.75, p=.004). Persistent FOF was also associated with a worse decline in memory compared to those with transient FOF (estimate=0.08, p=.004) and those with no FOF (estimate=0.06, p=.006). Associations between FOF status and decline in attention/executive functions were not significant. These findings demonstrate that persistent FOF is a risk factor for cognitive decline in community-residing older adults.

### HOME SAFETY EVALUATION MODEL FOR OLDER ADULTS WITH RECURRENT FALLS

Willy Marcos Valencia,[1] Kimberly Cabrera,[2] and Vincent Hsu,[3] *1. Miami VA Healthcare System, Miami, Florida, United States, 2. Miami VAMC, Miami, Florida, United States, 3. University of Miami Miller School of Medicine, Miami, Florida, United States*

Recurrent falls are a major threat in older adults. Home environment can be a hazard, but it is potentially modifiable/reversible. In Miami VA, occupational therapists conduct home safety evaluations (HSE) to ascertain the need for modifications to reduce falls risk. We reviewed the cohort of high-risk, recurrent falls patients evaluated at our Falls Prevention Clinic (FPC) between August 2017 to November 2019, to evaluate the impact of HSE. We identified 48 Veterans, age 76.5±6.9 years, of whom 15 (31.3%) reported 1-2 falls/year, 18 (37.5%) reported 3-4 falls/year, and 15 (31.3%) reported ≥5 falls/year. Twenty-eight (58.3%) were offered a HSE. Within these subjects, 74.2% reported falling at least once within their home, 43.8% had fear of falling, 5 (17.9%) had a history of substance or alcohol abuse. We observed that 29 (60.4%) would benefit from the addition of grab bars and 26 (54.2%) could benefit from toilet adjustments. Twelve (25.0%) were recommended to install bed rails. Only 15 (31.3%) Veterans agreed to all recommendations, 25 (52.1%) declined due to preference, and 8 (16.7%) declined for other reasons. Only 8 (16.7%) of these Veterans lived alone. Another factor is that 11 (22.9%) Veterans were renting and 32 (66.7%) owned their homes. Addressing and improving environmental hazards may ameliorate the risk for recurrent falls. Our next steps are to evaluate the extent of home modifications, and the long-term changes in falls/year. Further research needs to determine the long-term efficacy and cost-effectiveness of HSE, and how it can be more accessible to the community.

### SESSION 2923 (PAPER)

### FALLS AND FALL PREVENTION

### BEYOND HIP FRACTURE: ORTHOPAEDIC TRAUMA IN AN AGING AMERICA

Lisa Reider,[1] Joseph Levy,[1] and Andrew Pollak,[2]
*1. Health Policy and Management, Baltimore, Maryland, United States, 2. University of Maryland, Baltimore, Maryland, United States*

Trauma related death and disability is common among working-age Americans, however the impact on older adults is consequential and increasing. Fractures are the most common traumatic injury diagnosis among Medicare beneficiaries, and though fragility fractures continue to be an important health problem, recent data indicate an increase in high-energy fractures. The purpose of this study was to produce national incidence estimates among US men and women ≥ 65 years using data from the 2003-2014 National Inpatient Sample (NIS). The study cohort included hospitalizations involving upper and/or lower extremity fractures which were further classified by mechanism as high or low energy using external cause of injury codes. Incidence was computed using survey weights provided by NIS, and population estimates from the Census Bureau. The incidence of high-energy fractures increased from 744.1/100,000 persons (95%CI: 681.1–807.1) in 2003 to 821.4/100,000 (95%CI: 795.0 – 874.8) in 2014 in women, and from 359.1/100,000 (95%CI: 331.4–386.8) to 408.2/100,000 (95%CI: 394.–809.2) in men. Over 80% were motor vehicle related. The greatest increase was among those ≥ 85 (1,856.4/100,000 to 2,126.3/100,000 in women; 1,069.1/100,000 to 1,215.1/100,000 in men). Simultaneously, the incidence of low-energy fractures declined: 748.4/100,000 (95%CI: 687.5–809.2) to 443.8/100,000 (95%CI: 423.5 -464.1) in women, and 310.6/100,000 (95%CI: 285 – 336.2) to 206.3/100,000 (95%CI: 196.5 - 216) in men. Results suggest that fractures commonly seen in younger adults will be seen more frequently in older age. It is therefore essential to establish treatment pathways to optimize outcomes for the growing number of injured older adults.

### DISENTANGLING AND QUANTIFYING MECHANISMS OF INCREASED FALL RISK AMONG OLDER ADULTS WITH DEPRESSION

Matthew Lohman, Nicholas Resciniti, and Anwar Merchant, *University of South Carolina, Columbia, South Carolina, United States*

Background: Older adults with depression are up to four times more likely to fall, yet the mechanisms by which depression increases fall risk are unclear. This study sought to quantify and compare the relative strength of selected mechanisms mediating the association between depression and falls. Methods: We used longitudinal linked data (2006 – 2010) from the Health and Retirement Study and Prescription Drug Study. The analytic sample included non-institutionalized adults age > 65 with data on physical functioning and medication use (n=3,565). Falls and injurious falls over the past two years were self-reported outcomes. Depression was measured using the Composite International Diagnostic Interview (CIDI). We used causal mediation analysis to estimate the total and direct associations between depression and falls and compared strength of three potential mediating mechanisms – frailty, cognition, and antidepressant (AD) use. Results: Of 190 participants reporting depression in 2006, 85 (44.7%) fell and 30 (15.8%) were injured from a fall between 2008 and 2010. Depressed individuals were 92% more likely to fall compared to non-depressed (OR=1.92, p<.01). We found significant indirect effects of AD use (indirect OR=1.17, p<.01) and frailty (OR=1.12, p=.013) representing 19% and 13% of the total effect of depression on falls, respectively. Cognition was not a significant mediator. Results were similar for falls leading to injury. Discussion: Results suggest that AD use and frailty explain a significant portion of the elevated risk for falls among depressed individuals. Identification of these and other mechanisms may inform clinical treatment decisions for older adults with depression.

### EXPERIENCE-BASED CO-DESIGN TO DEVELOP AN INJURY PREVENTION INTERVENTION IN SKILLED NURSING FACILITIES

Eleanor McConnell,[1] Sarah Berry,[2] Emily Hecker,[3] laurie herndon,[4] and Cathleen Colon-Emeric,[5] *1. Duke University Medical Center, Durham, North Carolina, United States, 2. Hebrew SeniorLife, Marcus Institute for Aging Research, Boston, Massachusetts, United States, 3. Duke University School of Medicine, Durham, North Carolina, United States, 4. Hebrew Seniorlife, Boston, Massachusetts, United States, 5. Duke University, Durham, North Carolina, United States*

Experience-based co-design (EBCD) improves clinical effectiveness and safety by incorporating end-user perspectives in the design of clinical interventions. To refine a centralized, multi-component fall-related injury prevention service (IPS) to be tested in skilled nursing facilities (SNFs) in a pragmatic trial, we employed a modified EBCD process. We first conducted in-depth interviews with SNF residents, family members, and staff (n = 28; three facilities in two states) regarding their experiences in falls prevention. We then engaged these and other stakeholders from multiple institutions (n=4) in a day-long co-design workshop with our interdisciplinary research team. Building upon themes drawn from the analysis of interviews, we targeted three intervention components that were refined during the workshop: de-prescribing process, osteoporosis treatment, and educational videoconferences. Key outcomes from the ECBD process included development of strategies to ensure that: (1) residents, families, and SNF staff are involved in communication about residents identified as high risk for fall-related injury, and in related treatment decisions; (2) approaches to monitoring for unintended consequences from the injury prevention plan are clearly understood by direct care staff and are compatible with existing workflow; (3) treatment plan risks and benefits are presented in a manner easily understood by stakeholders; and (4) staff education conferences build trust with the IPS nurse and provide direct care staff with support and advice about challenging cases. EBCD is a feasible approach to strengthen intervention development in SNFs and can lead to testable new ideas for protocol refinement to address diverse stakeholder perspectives.

### SYSTEMS ADDRESSING FRAIL ELDER CARE: AN IMPLEMENTATION STUDY

Harriet Aronow,[1] Linda Burnes Bolton,[2] Marcio Diniz,[1] Linda Kim,[3] and Bernice Coleman,[3] *1. Cedars Sinai, Los Angeles, California, United States, 2. Cedars Sinai Health System, Los Angeles, California, United States, 3. Cedars-Sinai, Los Angeles, California, United States*

SAFE CareTM was developed at one hospital and found to be an effective care model for frail older adults. SAFE CareTM includes nurse screening for frailty risks, multidisciplinary assessments, team huddles and care recommendations. Underlying implementation is an organizational change process. Study aim was to evaluate the implementation and outcomes of SAFE CareTM in three additional hospitals. Two units from each hospital were randomized to SAFE CareTM or usual care. Process evaluation employed semi-structured interviews. Inpatients were aged 65+ years with positive frailty risks (N = 1,151). Outcomes evaluated ICU admission, length of stay (LOS), and discharge destination. All outcome analyses were conducted with intention to treat models. Patients were on average 80 years old, 54% female, 58% Caucasian, 83% English speaking, with 3.4 positive frailty risks. Median LOS was 4.2 days, 6.5% ICU admissions, 32% discharge institutional care. Hospitals differed in patient demographics and outcomes. While no differences between treatment groups in patient demographics, intervention patients had more frailty risks and longer expected LOS. 62% of intervention unit patients received intervention. There were no univariate treatment effects on outcomes. In multivariate analysis, intervention unit patients had shorter LOS. While hospitals reported different experiences, all reported challenges in preparing the electronic health record to support SAFE CareTM. Staff reported increased interprofessional team communications. Differences among the hospitals in patients and organizational attributes argue strongly that implementation should be tailored to meet varying institutional needs while common measures and processes underlying implementation should be followed closely.

### WHOLE-PERSON, WHOLE-TEAM APPROACH TO QUALITY IMPROVEMENT: WHY PERSON-CENTERED CARE MATTERS

Ryann Engle,[1] A. Lynn Snow,[2] Valerie Clark,[3] Shibei Zhao,[3] Christopher Gillespie,[3] and Christine Hartmann,[3] *1. VA Boston Healthcare System, Boston, Massachusetts, United States, 2. Tuscaloosa VA Medical Center, Tuscaloosa, Alabama, United States, 3. Edith Nourse Rogers Memorial Veterans Hospital, Bedford, Massachusetts, United States*

The Department of Veterans Affairs (VA) began its culture transformation journey in 2006, supporting its nursing homes in providing high-quality, person-centered care in person-centered environments. We implemented a quality improvement intervention to support frontline staff from low-performing VA nursing homes in providing high-quality care using a whole-person, whole-team approach. The intervention consisted of a bundle with four components: 1) specialized frontline staff huddles that encouraged high-quality frontline staff communication and collaboration, 2) micro-root cause analyses and targeted interventions to promote resident sleep and reduce resident falls through individualized care, 3) in-depth frontline conversations regarding residents' distress behaviors and mobility, and 4) targeted, team-based, person-centered performance improvement projects. The intervention was implemented at 8 low-performing VA nursing homes (August 2018 - April 2019) via in-person and virtual sessions and facilitated through CLC-based champions and intervention team-based coaches. We monitored the intervention's impact using pre-post Centers for Medicare and Medicaid Services quality star ratings. We also conducted 17 post-intervention interviews with key informants at 7 participating nursing homes and conducted a content analysis of the data. Pre intervention, all 8 nursing homes had a history of being 1 or 2 stars in overall quality. Post intervention, 3 homes increased 1 star; 1 home increased 2 stars; 2 homes increased 3 stars; 2 homes increased 4 stars. Post intervention, participants perceived improved delivery of person-centered care (e.g., providing individualized sleep hygiene, de-implementing alarms). Our findings suggest a whole-person, whole-team intervention can effectively and efficiently improve both person-centered care and care quality.

### SESSION 2924 (PAPER)

### FALL PREVENTION II

### BIDIRECTIONAL LONGITUDINAL RELATIONSHIPS BETWEEN HOMEBOUND STATUS AND FALLS AMONG OLDER ADULTS

Minhui Liu,[1] Yuxiao Li,[2] Xiaocao Sun,[3] Christina Miyawaki,[4] Tianxue Hou,[2] Siyuan Tang,[3] and Sarah Szanton,[5] *1. Central South University, Baltimore, Maryland, United States, 2. Central South University, Changsha, Hunan, China, 3. Central South University, Changsha City, Hunan, China, 4. University of Houston, Houston, Texas, United States, 5. Johns Hopkins University, Baltimore, Maryland, United States*

Research has shown an association between homebound status and falls among older adults. However, this association was primarily drawn from cross-sectional studies. Using the National Health and Aging Trends Study, we examined 1) whether prior-wave falls predicted homebound status in a later wave in 2,916 non-homebound participants in Wave 1 and 2) whether prior-wave homebound status predicted falls in 2,512 participants with no falls in Wave 1. Homebound status (non-homebound and homebound) was determined by the frequency, difficulty, and needing help of outdoor mobility. Falls were ascertained by asking participants whether they had a fall in the last year. Generalized estimation equation models were used to examine their bidirectional association, adjusting for demographics, health-related, and behavioral factors. Participants who had fallen in later waves were more likely to be older non-Hispanic black, comorbid, and have more pain, depression, disabilities, worse health status vision impairment, and low physical activities. Participants who were homebound in later waves tended to older, female, non-Hispanic black, less-educated, living alone or with others only, comorbid, obese, and have more pain, depression, disabilities, worse health status, more hospitalizations, vision and hearing problems, and low physical activities. Previous falls significantly predicted later homebound status (adjusted odds ratio [OR]: 1.28, 95% CI: 1.09-1.50). Prior wave homebound status also significantly contributed to falls in the next year (adjusted OR: 1.28, 95% CI: 1.12-1.46). The bidirectional longitudinal association between homebound status and falls suggests a vicious circle between them. Fall prevention programs should particularly target homebound older adults for falls reduction.

### CAN FUNCTIONAL PERFORMANCE PREDICT LONG-TERM MORTALITY POST HIP FRACTURE IN OLDER ADULTS (65–100 YEARS OF AGE)?

Rashmita Bajracharya,[1] Denise Orwig,[2] Jay Magaziner,[2] and Jack M. Guralnik,[2] *1. University of Maryland, School of Medicine, Catonsville, Maryland, United States, 2. University of Maryland School of Medicine, Baltimore, Maryland, United States*

Functional performance measures (grip strength, Short Physical Performance Battery (SPPB), and 3-meter gait speed) represent underlying disease progression and predict mortality. However, there is little information regarding whether these measures assessed at 2-months post-hip fracture predict long-term mortality (10-year follow-up). To address this gap, a longitudinal analysis of Baltimore Hip Studies-7 cohort, with mortality verified by National Death Index, was conducted. Mean difference in 2-month functional performance measures (n=242, men n=121, female n=121) among those who survived and did not survive over 10 years was determined using t-test. Prediction of mortality by these measures, overall and by sex, was estimated using cox proportional hazard models, for which Hazard ratios (HR) with 95% confidence intervals (CI) were estimated. We found that, gait speed [0.47(standard deviation,SD=0.39) versus 0.31(SD=0.27)] and SPPB score [4.89(SD=3.31) versus 2.83(SD=2.24)] were significantly higher at 2 months among those surviving compared to those who did not. Adjusting for covariates, functional performance predicted long-term mortality in men and women. Increase in gait speed by 0.1m/s predicted 15% decrease in mortality for men [HR=0.85(0.55-0.96)] and 17% for women [HR=0.83 (0.74-0.93)]. Increase in SPPB by 1 unit predicted decrease in mortality by 14% for men [HR=0.86(0.77-0.95)] and 17% for women [HR=0.83(0.74-0.93). Increase in grip strength by 1 kg predicted 5% decrease in mortality for men [HR=0.94(0.92-0.97)] and 9% for women [HR=0.90(0.86-0.95)]. Functional performance measured at 2-months post-hip fracture predicted long-term mortality. Those with poor functional performance at 2-months can be referred for further assessment to optimize their care to promote survival.

### HIGHER-LEVEL COGNITIVE FUNCTION AND OBSTACLE ATTRIBUTES: AN FNIRS STUDY IN OLDER ADULTS WITH PARKINSON'S DISEASE

Jeffrey Hausdorff,[1] Topaz Sharon,[2] Ilan Kurz,[3] Hagar Bernad-Elazari,[1] Ira Galperin,[4] Nir Giladi,[4] Anat Mirelman,[1] and Inbal Maidan,[4] *1. Tel Aviv Sourasky Medical Center, Tel Aviv, Tel Aviv, Israel, 2. Tel Aviv University, Tel Aviv, Israel, 3. Ben Gurion University, Beer Sheva, Israel, 4. Tel Aviv Sourasky Medical Center, Tel Aviv, Israel*

Older adults with Parkinson's disease (PD) rely on prefrontal activation to compensate for impaired motor function during the performance of complex mobility-related activities such as obstacle negotiation. However, the influence of the properties of the obstacles on prefrontal activation has not been systematically evaluated. Here, we examined the effects of obstacle height and anticipation time on prefrontal activation in patients with PD and older adults. 34 patients with PD (age: 67.4±5.7 years; 14 women) and 26 older adult controls (age: 71.3±8.9 years; 11 women) walked in an obstacle course while negotiating anticipated and unanticipated obstacles at heights of 50 mm and 100 mm. Prefrontal activation was measured using functional Near-Infrared Spectroscopy (fNIRS); obstacle negotiation performance was measured using Kinect cameras. PD patients showed greater increases in prefrontal activation during and after obstacle crossing compared to the older adults (p<0.001). Obstacle height affected prefrontal activity only when crossing anticipated obstacles (time x height interaction, p=0.011); in that case, higher obstacles were accompanied by higher prefrontal activity. PD patients showed higher levels of activation during unanticipated obstacles, compared to anticipated obstacles (p=0.015). Different correlations between prefrontal activation and obstacle negotiation strategies were observed in the patients and the controls. These results point to the use of prefrontal activation as a compensatory mechanism in PD. Moreover, the higher activation of prefrontal regions during more challenging obstacles suggests that there is a greater reliance on cognitive resources in these demanding situations that may contribute to the higher risk of falls in patients with PD.

### MACHINE LEARNING IN AGING: AN EXAMPLE OF DEVELOPING PREDICTION MODELS FOR SERIOUS FALL INJURY IN OLDER ADULTS

Jaime Speiser,[1] Kathryn Callahan,[1] Jason Fanning,[2] Thomas Gill,[3] Anne Newman,[4] Marco Pahor,[5] Jack Rejeski,[2] and Denise Houston,[1] *1. Wake Forest School of Medicine, Winston-Salem, North Carolina, United States, 2. Wake Forest University, Winston-Salem, North Carolina, United States, 3. Yale University, New Haven, Connecticut, United States, 4. University of Pittsburgh, Pittsburgh, Pennsylvania, United States, 5. University of Florida, Gainesville, Florida, United States*

Advances in computational algorithms and the availability of large datasets with clinically relevant characteristics provide an opportunity to develop machine learning prediction models to aid in diagnosis, prognosis, and treatment of older adults. Some studies have employed machine learning methods for prediction modeling, but skepticism of these methods remains due to lack of reproducibility and difficulty understanding the complex algorithms behind models. We aim to provide an overview of two common machine learning methods: decision tree and random forest. We focus on these methods because they provide a high degree of interpretability. We discuss the underlying algorithms of decision tree and random forest methods and present a tutorial for developing prediction models for serious fall injury using data from the Lifestyle Interventions and Independence for Elders (LIFE) study. Decision tree is a machine learning method that produces a model resembling a flow chart. Random forest consists of a collection of many decision trees whose results are aggregated. In the tutorial example, we discuss evaluation metrics and interpretation for these models. Illustrated in data from the LIFE study, prediction models for serious fall injury were moderate at best (area under the receiver operating curve of 0.54 for decision tree and 0.66 for random forest). Machine learning methods may offer improved performance compared to traditional models for modeling outcomes in aging, but their use should be justified and output should be carefully described. Models should be assessed by clinical experts to ensure compatibility with clinical practice.

### MOBILITY SCREENING FOR FALL RISK ASSESSMENT IN A POPULATION-BASED SAMPLE OF OLDER ADULTS: AN ANALYSIS FROM THE CLSA

Marla Beauchamp, Ayse Kuspinar, Nazmul Sohel, Alexandra Mayhew, Lauren Griffith, and Parminder Raina, *McMaster University, Hamilton, Ontario, Canada*

Existing guidelines for fall prevention in older adults recommend mobility screening for fall risk assessment; however, there is no consensus on which test to use and at what cut-off. This study aimed to determine the accuracy and optimal cut-off values of commonly used mobility tests for predicting falls in the Canadian Longitudinal Study on Aging (CLSA). Mobility tests at baseline included the Timed Up and Go (TUG), Single Leg Stance (SLS), chair-rise, and gait speed test. Inclusion criteria were: age ≥ 65 years and history of a fall or mobility problem at baseline. Accuracy of fall prediction at 18-months for each mobility test was measured by the area under the receiver operating curve (AUC). Of 1,121 participants that met inclusion criteria (mean age 75.2 ± 5.9 years; 66.6% women), 218 (19.4%) participants reported ≥1 fall at 18-months. None of the mobility tests achieved acceptable accuracy for identifying individuals with ≥1 fall at follow-up. Among women 65-74 and 75-85 years, the TUG identified recurrent fallers (≥2 falls) with optimal cut-off scores of 14.1 and 12.9 seconds (both AUCs 0.70), respectively. Among men 65-74 years, only the SLS showed acceptable accuracy (AUC 0.85) for identifying recurrent fallers with an optimal cut-off of 3.6 seconds. Our findings indicate that for a population-based sample of community-dwelling older adults, commonly used mobility tests do not have sufficient accuracy for identifying fallers. The TUG and SLS can identify older adults at risk for recurrent falls, however their accuracy and cut-off values vary by age and sex.

### SESSION 2925 (PAPER)

### FRAILTY

### BEDSIDE ULTRASOUND MEASURES OF MUSCLE MASS AND FRAILTY MEASURES IN COMMUNITY-DWELLING OLDER ADULTS

Kenneth Madden, Boris Feldman, Shane Arishenkoff, and Graydon Meneilly, *University of British Columbia, Vancouver, British Columbia, Canada*

The age-associated loss of muscle mass and strength in older adults is called sarcopenia, and it is associated with increased rates of falls, fractures, hospitalizations and death. Sarcopenia is one of the most common physical etiologies for increased frailty in older adults, and some recent work has suggested the use of Point-of care ultrasound (PoCUS) measures as a potential measure of muscle mass. The objective of this study was to examine the association of PoCUS measures of muscle thickness (MT) with measures of frailty in community-dwelling older adults. We recruited 150 older adults (age >= 65; mean age 80.0±0.5 years, 66 women, 84 men) sequentially from 5 geriatric medicine clinics (Vancouver General Hospital). We measured lean muscle mass (LMM, by bioimpedance assay) and an ultrasonic measure of muscle quantity (MT, vastus medialis muscle thickness) in all subjects, as well as two outcome measures of frailty (FFI, Fried Frailty Index; RCFS, Rockwood Clinical Frailty Scale). In our models, MT showed an inverse correlation with the FFI (Standardized β=-0.2320±0.107, p=0.032) but no significant correlation with the RCFS (Standardized β = -0.025±0.086, p=0.776). LMM showed no significant association with either FFI (Standardized β=-0.232±0.120, p=0.055) or RCFS (Standardized β = -0.043±0.119, p=0.719). Our findings indicate that PoCUS measures show potential as a way to screen for physical manifestations of frailty and might be superior to other bedside methods such as bioimpedance assay. However, PoCUS measures of muscle thickness will likely miss patients showing frailty in the much broader context captured by the RCFS.

### FRAILTY AND TRAJECTORIES OF BLOOD PRESSURE AMONG OLDER MEXICAN AMERICANS OVER TIME.

Soham Al Snih, Martin Rodriguez, Lin-Na Chou, Kyriakos S. Markides, and Kenneth Ottenbacher, *The University of Texas Medical Branch at Galveston, Galveston, Texas, United States*

The objective of this study was to examine whether blood pressure (BP) trajectories differ by frailty status among older Mexican Americans. Data are from an 18-year prospective cohort study of 1,781 non-institutionalized Mexican American aged ≥ 67 years from the Hispanic Established Population for the Epidemiological Study of the Elderly (1995/96-2012/13). Frailty was defined as meeting three or more of the following: unintentional weight loss of >10 pounds, weakness, self-reported exhaustion, low physical activity, and slow walking speed. General linear mixed models were used to estimate trajectories of systolic and diastolic BP over an 18-year period as a function of frailty status. All variables were analyzed as time-dependent covariates except for gender and education. At baseline, 46.3% participants were non-frail, 44.8% were pre-frail, and 9.0% were frail; and the mean of systolic and diastolic BP was 136.9 mmHg (SD=18.6) and 77.3 mmHg (SD=12.2), respectively. Frail participants had greater systolic and diastolic BP decline over time than non-frail (estimate=-3.94, SE=0.88, p-value=<0.0001 and estimate=-1.32, SE=0.54, p-value=0.0138, respectively); and pre-frail participants had greater systolic BP decline than non-frail (estimate=-1.51, SE=0.54, p-value=0.0049), after controlling for all covariates. Those with high body mass index and hypertension with and without treatment had increased levels of systolic and diastolic BP over time. Older age, female gender, arthritis, diabetes, and stroke had decreased levels of diastolic BP over time. This study showed progressive decline in systolic and diastolic BP in frail compared to non-frail older Mexican Americans, which might have implications when treating frail older adults with hypertension.

### FRAILTY AS A MODERATOR OF THE RELATIONSHIP BETWEEN SOCIAL ISOLATION AND HEALTH OUTCOMES

Fereshteh Mehrabi, and François Béland, *University of Montreal, Montreal, Quebec, Canada*

Previous studies have reported that social isolation is a predictor of adverse outcomes, which is also closely associated with frailty. Very little is known about the moderating role of frailty on the impact of social isolation on health. We performed a cross-sectional analysis of the first wave of the FRéLE longitudinal study, consisting of 1643 Canadian community-dwelling older adults aged 65 years and over. Multivariate regression analysis was performed to examine the interaction between social isolation and frailty on health, controlling for socioeconomic characteristics and life habits. Social isolation was measured through social participation, social networks and support for different social ties namely, friends, children, extended family, and partner. In contrast to Berkman's theory on the impact of social isolation on health, we found that frailty had no modifying role on the effects of social isolation on health. Frailty was significantly associated with all adverse outcomes. Less social participation was associated with ADLs, IADLs, depression and cognitive decline. The absence of friends was associated with depression and cognitive decline. Less support from children and having no children were associated with ADLs, comorbidity and depression. Fewer contact with extended family and having no family members were notably associated with ADLs and IADLs. Those who received less support from a partner or had no partner were more depressed and had more difficulties in performing IADLs. This study suggests that older adults who participate in social activities and have social ties, feel better with respect to physical health than those who feel isolated.

### FRAILTY COMPONENT TRAJECTORIES AFTER RESTORATION OF KIDNEY FUNCTION AMONG KIDNEY TRANSPLANT RECIPIENTS

Nadia Chu,[1] Xiaomeng Chen,[1] Dorry Segev,[1] and Mara McAdams-DeMarco[2], *1. Johns Hopkins School of Medicine, Baltimore, Maryland, United States, 2. Johns Hopkins Bloomberg School of Public Health, Baltimore, Maryland, United States*

Frailty is associated with decreased access to kidney transplantation (KT) and poor post-KT outcomes. Little is known about how an acute stressor, like KT, can impact the five physical frailty phenotype (PFP) criteria. We conducted a two-center prospective cohort study (2009-2019) of adult patients undergoing KT. PFP criteria were measured at KT admission, 1 month, 3 months, 6 months, 1 year, and annually thereafter post-KT. We used adjusted mixed effects models with fixed and random effects for person and time to describe repeated measures of continuous criteria components (weight, gait speed, grip strength, activity). We used an adjusted generalized estimating equation to quantify longitudinal, binomial response patterns of exhaustion. Among 1,410 KT recipients (mean age=53) followed for a mean of 1.9 years (IQR=0.1-3.2), 46.8% had low activity, 46.7% weakness, 29.0% exhaustion, 16.1% slowness, and 14.3% unintentional weight loss at KT admission. Among continuous components, weight worsened (0.3lb/month, 95%CI:0.2,0.4), while grip strength (0.09kg/month, 95%CI: 0.07,0.11) and activity (5.8Kcal/month, 95%CI: 3.3, 8.2) improved post-KT; gait speed remained stable (-0.0004s/month, 95%CI: -0.01, 0.0005). Additionally, likelihood of transitioning from being exhausted did not change (OR=1.0, 95%CI: -0.01, 0.0005). Trajectories differed by age, such that improvements were observed among younger recipients (<65 years), but not among older recipients (≥65 years) (p-interactions<0.05). After undergoing a common surgical stressor, KT recipients demonstrated weight gain as well as improvements in strength and activity. Despite benefits of restoration of kidney function, clinicians should consider monitoring KT recipients for persistent weight gain, exhaustion, and slowness post-KT, particularly among older adults.

### FRAILTY MODIFIES EFFECTS OF CHAIR YOGA ON CHRONIC PAIN IN OLDER ADULTS WITH OSTEOARTHRITIS

Juyoung Park,[1] and Zuyun Liu,[2] *1. Florida Atlantic University, Boca Raton, Florida, United States, 2. Zhejiang University, Zhejiang, China*

As a secondary analysis, this study used data from our previous 8-week chair yoga (CY) intervention trial with two-arm, access-blinded randomized controlled trial to examine modifying effect of baseline frailty on intervention effects of CY on pain and pain interference (i.e., consequences of pain on relevant aspects of life). Using the cumulative frailty index (FI) approach, we constructed the FI using 82 comprehensive deficits, including physical function, balance, fatigue, emotional well-being, and social activity. We calculated FI at baseline, 4 weeks, and 8 weeks. A linear mixed-effects model with random intercept was used, adjusting for research sites, cohort effect, and time. To test for potential modifying effects of baseline FI on the intervention effect by CY, we added a three-way interaction term: intervention (CY vs. Health Education Program), time, and baseline FI. A total of 112 participants (M = 75.3[7.5] years; 76% female, 40% White, 46% Hispanic) completed the study. Each 0.01 increment in baseline FI was associated with higher pain ($\beta = 0.28$, $p < .001$) and pain interference ($\beta = 0.51$, $p < .001$). There was a significant interaction effect among intervention, time, and baseline FI ($p = .02$ for pain, $p = .01$ for pain interference), indicating that participants with higher levels of baseline FI had greater declines in pain and pain interference. Frailty modified the intervention effect of CY on pain in older adults with lower extremity osteoarthritis, underscoring the importance of assessing frailty to improve management of pain in the population.

### SESSION 2926 (PAPER)

### FRAILTY AND SARCOPENIA II

### AIR POLLUTION AND GERONTOLOGICAL CONSTRUCTS AMONG PATIENTS WITH END-STAGE KIDNEY DISEASE

Mara McAdams-DeMarco,[1] Miranda Jones,[1] Yijing Feng,[2] Jeremy Walston,[2] Nadia Chu,[2] and Dorry Segev[2] *1. Johns Hopkins Bloomberg School of Public Health, Baltimore, Maryland, United States, 2. Johns Hopkins School of Medicine, Baltimore, Maryland, United States*

Frailty is triggered by inflammatory pathways among patients with end-stage kidney disease (ESKD). Exposure to air pollution is associated with increased inflammation and as such may be a determinant of frailty in patients with ESKD. Therefore, we sought to estimate the impact of household-level exposure to fine particulate matter (particles <2.5μm in diameter [PM2.5]) on frailty and other gerontological constructs among patients with ESKD. We leveraged a prospective, two-center cohort study of 1,482 adults with ESKD

(2014-2019) from 40 US states. The physical frailty phenotype (PFP), SPPB, ADL/IADL dependence and 3MS global cognitive impairment were assessed at transplant evaluation. Household-level air pollution was estimated as annual average PM2.5 concentrations at each participant's address using SEDAC national air pollution data. We estimated the odds of these gerontologic constructs using adjusted logistic regression by quartiles of PM2.5 concentrations accounting for confounders including socioeconomic status. Compared to patients with PM2.5 concentrations in the lowest quartile (<9.3μg/m3), those with exposure to the 3rd quartile (10.0-11.1μg/m3) had 1.50-fold (95%CI:1.04-2.17) increased odds of frailty. However, exposure to PM2.5 concentrations in the second (9.3-10.0μg/m3) and fourth quartiles (>11.1μg/m3) were not significant. Those with PM2.5 in the 3rd (OR=1.60, 95%CI:1.19-2.16) or 4th (OR=1.61, 95%CI:1.20-2.16) quartile had an increased risk of having dependence in ADLs or IADLs. PM2.5 was not associated with SPPB or cognitive impairment. Among ESKD patients, fine particulate matter was associated with greater frailty and dependence burden, although these association may not be linear. Further study of the role of inflammation on these associations are needed.

### BIDIRECTIONAL RELATIONSHIP BETWEEN SUBJECTIVE AGE AND FRAILTY: FINDINGS FROM THE NHATS

Yuxiao Li,[1] Minhui Liu,[1] Christina Miyawaki,[2] Xiaocao Sun,[1] Tianxue Hou,[1] Siyuan Tang,[1] and Sarah Szanton[3], *1. Central South University, Changsha, Hunan, China, 2. University of Houston, Houston, Texas, United States, 3. Johns Hopkins University, Baltimore, Maryland, United States*

Frailty is a clinical syndrome that becomes increasingly common as people age. Subjective age refers to how young or old individuals experience themselves to be. It is associated with many risk factors of frailty, such as increased depression, worse cognitive function, and poorer psychological wellbeing. In this study, we examined the relationship between subjective age and frailty using the 2011-2015 waves of the National Health and Aging Trends Study. Participants were community-dwelling older adults without frailty in the initial wave (N=1,165). Subjective age was measured by asking participants, "What age do you feel most of the time?" Based on the Fried five phenotypic criteria: exhaustion, unintentional weight loss, low physical activity, slow gait, and weak grip strength, frailty was categorized into robust=0, pre-frail=1 or 2; frail=3 or more criteria met. Participants were, on average, 74.1±6.5 years old, female (52%), and non-Hispanic White (81%). Eighty-five percent of the participants felt younger, and 3% felt older than their chronological age, but 41% of them were pre-frail/frail. Generalized estimating equations revealed that an "older" subjective age predicted a higher likelihood of pre-frailty and frailty (OR, 95%CI= 1.01, 1.01-1.02). In contrast, frailty predicted an "older" subjective age (OR, 95%CI= 2.97, 1.65-5.35) adjusting for demographics and health conditions. These findings suggest a bidirectional relationship between subjective age and frailty. Older people who feel younger than their chronological age are at reduced risk of becoming pre-frail/frail. Intervention programs to delay frailty progression should include strategies that may help older adults perceive a younger subjective age.

### DIFFERENCES IN CAUSE-SPECIFIC MORTALITY BETWEEN FRAIL MEN AND WOMEN IN THE UNITED STATES

Matthew Lohman,[1] Amanda Sonnega,[2] Amanda Leggett,[2] and Nicholas Resciniti,[1] *1. University of South Carolina, Columbia, South Carolina, United States, 2. University of Michigan, Ann Arbor, Michigan, United States*

While frailty is associated with risk of numerous adverse health outcomes including mortality, little is known about the most common specific causes of death among frail older adults or how these causes might differ by gender. This information may be important to understanding the frailty syndrome and to informing screening and treatment. We used linked data from the Health and Retirement Study (2004 – 2012) and the National Death Index (NDI). We analyzed data from HRS participants age 65 and older who completed a general health interview and physiological measures (n=10,490). Frailty was operationalized using the phenotype criteria – low weight, low energy expenditure, exhaustion, slow gait, and weakness. Causes of death were determined using International Classification of Diseases (v10) codes from death certificates. We used Cox proportional hazards to compare incidence of cause-specific mortality by frailty status and gender. The attributable risk of mortality due to frailty in the sample was 16.6% among women and 17.3% among men. Overall, frail older adults had greater risk of death from heart disease (hazard ratio (HR): 2.97; 95% CI: 2.18, 4.04), cancer (HR: 2.81; 95% CI: 2.01, 3.93), and dementia 2.86 (95% CI: 1.46, 5.58) but not cerebrovascular disease or accidents. Frail women were more approximately 29% more likely to die from heart disease than frail men. Findings suggest that frailty is a significant risk factor for mortality from several different causes, especially among women. Findings may help inform screening and treatment decisions for older adults at risk for frailty.

### INTERSECTION OF SEX AND FRAILTY IN HUMORAL IMMUNE RESPONSES TO INFLUENZA VACCINE IN COMMUNITY-DWELLING OLDER ADULTS

Janna Shapiro,[1] Helen Kuo,[1] Rosemary Morgan,[1] Huifen Li,[2] Sabra Klein,[1] and Sean Leng,[2] *1. Johns Hopkins Bloomberg School of Public Health, Baltimore, Maryland, United States, 2. Johns Hopkins School of Medicine, Baltimore, Maryland, United States*

Older adults bear the highest burden of severe disease and complications associated with seasonal influenza, with annual vaccination serving as the best option for protection. Variability in vaccine efficacy exists, yet the host factors that affect immune responses to inactivated influenza vaccines (IIV) are incompletely understood. We hypothesized that sex and frailty interact to affect vaccine-induced humoral responses among older adults. To test this hypothesis, community-dwelling adults above 75 years of age were recruited yearly, assessed for frailty (as defined by the Cardiovascular Health Study criteria), and vaccinated with the high-dose trivalent IIV. Humoral immune responses were evaluated via hemagglutination inhibition titers. The study began during the 2014-2015 influenza season, with yearly cohorts ranging from 76-163 individuals. A total of 617 vaccinations were delivered from 2014-2019. In preliminary analyses, the outcome of interest was seroconversion, defined as ≥ 4-fold rise in titers. Crude odds ratios suggest that females are more likely to seroconvert to influenza A strains (H1N1: OR = 1.39, (0.98-1.96) ; H3N2: 1.17 (0.85 – 1.62)), while males are more likely to seroconvert to the B strain (OR = 0.85 (0.60 – 1.22)). Furthermore, this sex difference was modified by frailty – for example, the odds of seroconversion to H1N1 were 65% higher for females than males among those who were nonfrail, and only 30% higher among females who were frail. Together, these results suggest that sex and frailty interact to impact immune responses to influenza vaccines. These findings may be leveraged to better protect vulnerable populations.

### URBAN-RURAL DIFFERENCES IN SARCOPENIA PREVALENCE AND NUTRITIONAL RISK FACTORS: THE NHANES (2001–2002 AND 2011–2014)

Jason Aziz,[1] Kieran Reid,[1] John Batsis,[2] and Roger Fielding,[1] *1. Tufts University, Boston, Massachusetts, United States, 2. Dartmouth College, Lebanon, New Hampshire, United States*

Background: Older adults living in rural areas experience health inequities compared to their urban counterparts. These include comorbidities, poor diet and physical inactivity; known risk factors for sarcopenia. No studies examining urban-rural differences in the prevalence of sarcopenia and slow gait speed among older adults in the United States exist. Objective: To compare the prevalence of sarcopenia and slow gait speed between urban and rural older adults living in the United States. As a secondary aim, we examined relationships between rural residency, total energy and total protein on gait speed and grip strength. Methods: We performed a secondary data analysis of two cohorts in the continuous NHANES (2001-2002 and 2011-2014), using gait speed or grip strength data, along with urban-rural status, dietary, examination, questionnaire and demographic data in older (≥ 60 yrs.) adults. Results: The prevalence of GripBMI weakness was higher in urban vs. rural participants (27.4% vs. 19.2%), whereas their absolute grip strength was lower (31.75(±0.45) vs. 33.73(±0.48)). Total energy, total protein and relative protein intakes were similar between urban and rural participants. Total energy intake was associated with gait speed and grip strength. Conclusions: Older adults living in urban areas of the United States, were weaker compared to their rural counterparts. Rural residency was not associated with gait speed or grip strength. Total energy intake was associated with slower gait speed but higher grip strength. This report is the first to examine urban-rural differences in sarcopenia and slow gait speed in older adults living in the United States.

### SESSION 2927 (POSTER)

### DEMENTIA AND COGNITIVE IMPAIRMENT I

### A SHORT, VALID, AND FLEXIBLE WEB-BASED SCREENER FOR MILD COGNITIVE IMPAIRMENT

Nicole Schmidt,[1] Laura Germine,[2] Eric Grodsky,[3] Chandra Muller,[4] John Robert Warren,[1] and Jennifer Manly,[5] *1. University of Minnesota, Minneapolis, Minnesota, United States, 2. Harvard Medical School, Belmont, Massachusetts, United States, 3. University of Wisconsin-Madison, Madison, Wisconsin, United States, 4. University of Texas at Austin, Austin, Texas, United States, 5. Columbia University, New York, New York, United States*

Clinical assessments for identifying mild cognitive impairment (MCI) can be costly and time-consuming. Few screeners for MCI exist that can be implemented quickly and outside of the clinic using flexible, cost-effective methods, such as web-based, mobile device-friendly assessments. Using data from middle-aged, racially and ethnically diverse Offspring Study participants (N=34 with MCI, N=54 without MCI; mean age: 53.9±3.4), we analyzed the sensitivity and specificity of several web- and telephone-based measures to identify MCI, after accounting for age and education. Web assessments included the Verbal Paired Associates (PA) and Visual PA tests. Phone assessments included the Number Series, Letter and Category Fluency, Number Span Forward & Backward, the AD8, and self-reported memory complaints. The discriminant ability of the web-based Visual PA test for MCI (ROC Area = .69) was comparable to phone-based measures, including the Category Fluency (ROC Area = .69), Number Span Forward (ROC Area = .61) and Backward (ROC Area = .67), and Letter Fluency (ROC Area = .68). Visual PA strongly predicted MCI, with a 98% reduction in the odds of MCI for every additional correct answer (OR=0.02), but our results are imprecise (95%CI: .000 to .76). A web-based Visual PA measure appears comparable to phone assessments in detection of MCI, although substantial uncertainty in its diagnostic precision remains. However, it is short, easily administered on a large-scale, and our evidence suggests that it can provide a sensitive and specific test to refer racially and ethnically diverse individuals for more thorough clinical assessment.

### AN INNOVATIVE MODEL OF DEMENTIA PROGRAMMING FOR COMMUNITY-DWELLING OLDER ADULTS WITH FAMILY CAREGIVERS

Gregg Gorzelle,[1] Michael Skrajner,[2] Cassie Best,[3] and Drew Walker,[4] *1. Hearthstone Alzheimer Care, Hiram, Ohio, United States, 2. Hearthstone Alzheimer Care, Wickliffe, Ohio, United States, 3. Hearthstone, Cleveland, Ohio, United States, 4. Hearthstone, Woburn, Massachusetts, United States*

Decide, Discover, and Do!TM (D3) is an alpha-version iPad application developed and evaluated in a National Institute on Aging-funded Phase 1 SBIR project. The goal of D3 is to enhance the quality of life and care for community-dwelling persons living with dementia whose primary care partners are family members. D3 consists of (1) evidence-based activities for care partners to facilitate with their loved ones and (2) video-based interactive training on best practices in dementia care, for care partners. The activities are unique in that they create an overarching narrative for daily activities that creates a consistent routine capitalizing on procedural memory. The activities build upon one another, starting with the persons living with dementia choosing a topic (e.g., nature) early in the day, followed by the dyad engaging in a tablet-based activity related to the topic (e.g., reading an article about forests), and culminating in an experiential activity (e.g., tasting various foods found in nature, e.g. wild raspberries). A total of 18 participants took part in the this feasibility study. The study examined the impact of D3 training modules on knowledge transfer (16% mean increase in care partner knowledge across three courses); satisfaction with the training modules (96% satisfaction across three courses); and satisfaction with the activities programming (91% satisfaction across all items for persons living with dementia and 99% for care partners). No distal changes related to care partner stigma nor caregiver burden were seen in this short study.

### EMERGENCY DEPARTMENT NURSES' EXPERIENCES IN CARING FOR OLDER ADULTS WITH DEMENTIA

YuJin Park,[1] Eun-Hi Kong,[2] and Hyejin Kim,[3] *1. Gachon Gil Hospital, Incheon, Republic of Korea, 2. Gachon University, Incheon, Republic of Korea, 3. Chung-Ang University, Seoul, Republic of Korea*

The aim of this study was to describe emergency-department nurses' experiences in caring for older adults with dementia. A qualitative descriptive method was employed. A convenience sampling method was used to recruit nurses from seven regional emergency medical centers. Data were collected through to in-depth interviews and field notes until data were saturated. A total of 23 emergency-department nurses participated in the first interview and 18 of them participated in the second interview. Data were analyzed using qualitative content analysis with the ATLAS. ti 8.2 software program. Through data analysis of interview data, 36 codes and 10 categories were identified. Finally, four themes emerged from data analysis: lack of preparation for dementia care, tired of caring for older adults with dementia, an inappropriate emergency-department environment for older adults with dementia, and needs for improvements in dementia care. Emergency-department nurses felt completely unprepared for dementia care and felt exhausted after caring for older adults with dementia. In addition, the emergency department is considered to be an environment that does not consider older adults with dementia and thus is unsuitable for older adults with dementia. Also, nurses require systematic support when caring for older adults with dementia and improvement in awareness in the emergency department. Study results provide important data to develop educational material for emergency care including criteria for emergency medical assessments and policies of emergency medical institutions. In addition, the results provide evidence for clinical practice guideline for nurses who care for older adults with dementia in the emergency departments.

### EXPLORING THE EXPERIENCES OF PERSONS LIVING WITH DEMENTIA AND THEIR CARE PARTNERS IN A MEMORY CAFÉ-STYLE PROGRAM

Morgan Bunting,[1] Inga Antonsdottir,[2] Marti Bailey,[3] and Quincy Samus,[2] *1. Johns Hopkins University, Halethorpe, Maryland, United States, 2. Johns Hopkins University, Baltimore, Maryland, United States, 3. Club Memory, Washington, D.C., United States*

Community-based supportive care interventions are needed to address unmet needs and maximize quality of life for persons living with dementias and their care partners (care dyad). Memory cafés and similar programs serve as a potentially important vehicle to reduce dementia stigmatization, improve inclusiveness, promote well-being, socialization and meaningful activity engagement, and disseminate dementia-related education and knowledge. Despite gaining popularity, little is known about how these programs impact participants or how they work best. The Club Memory® program in Washington, D.C. is an enhanced memory café-style program that delivers a normalizing environment, decreased negative stigma and a chance to experience a catalyst for change despite disease progression. Using qualitative methods, we investigated the perceived impact, benefits, and weaknesses of Club Memory from multi-stakeholder perspective, to explore how this program might be improved and standardized for broader implementation. We conducted two focus groups, one of care partners and one of persons living with dementia (N=12). Upon analyzing transcriptions, five common themes emerged among the focus groups: Atmosphere, Sense of Community, Skill-Building, Tools and Strength in Staff. Integrating perspectives from the care dyad revealed congruent thoughts on program perception, benefits and strengths, and opportunities for improvement. Club Memory® appears to be a valuable model that creates a stigma-free supportive environment, educates and engages the care dyad to foster understanding, confidence, curiosity and empowerment. Our findings contribute to a deeper understanding of the impact community-based strategies can positively impact the care dyad's life.

### HOW WELL DO VIDEOS ON YOUTUBE MEET INFORMATION NEEDS OF ADRD FAMILY CAREGIVERS? A QUALITATIVE ANALYSIS

Shinyi Wu,[1] Katherine Foster,[2] Magaly Ramirez,[3] Haomiao Jin,[1] and Elizabeth Joe,[4] *1. University of Southern California, Los Angeles, California, United States, 2. University of Southern California, Fountain Valley, California, United States, 3. University of Washington, Seattle, Washington, United States, 4. Keck School of Medicine, USC, Los Angeles, California, United States*

Family caregivers need access to information, education, and support for taking care of individuals with Alzheimer's disease and other dementia (ADRD). As YouTube is increasingly being used for sharing health information, videos regarding the disease and caregiving are becoming an important source of information to support family ADRD caregivers. This study aimed to assess the types and quality of information available on YouTube videos related to ADRD caregiving. A framework categorizing information needs of family ADRD caregivers was developed through a qualitative study with caregivers (N=21) and with healthcare and community stakeholders (N=6). The framework was used to conduct content analysis of YouTube videos. Nineteen categories of information needs were identified, including information about ADRD, healthcare services and treatment, available community resources, caregiving skills, and short- and long-term care. YouTube videos that met the keywords, language, and view selection criteria were evaluated by two coders on a developed rating scale to measure their relevance and helpfulness. A neurologist verified the ratings in 10% of the coded videos for quality assurance. There were 48 English and 23 Spanish videos met the selection criteria. More English (89.6%) than Spanish (56.5%) videos provided tips on handling specific ADRD symptoms. The majority categories of information needs (15 of the 19) were absent in most videos (87.0%, 89.6%). Many of the most watched videos were not rated as helpful. Community-based providers and healthcare organizations are encouraged to make high quality needed information in commonly accessed videos sharing service to support ADRD family caregivers.

### INFORMATION SEEKING BEHAVIOR OF LATINO FAMILY CAREGIVERS OF PEOPLE WITH ALZHEIMER'S DISEASE AND RELATED DEMENTIAS

Magaly Ramirez,[1] Sofia De Anda,[1] Haomiao Jin,[2] Joseph Herrera,[3] and Shinyi Wu,[2] *1. University of Washington, Seattle, Washington, United States, 2. University of Southern California, Los Angeles, California, United States, 3. Rancho Los Amigos, Downey, California, United States*

Latinos are disproportionately affected by Alzheimer's disease and related dementias (ADRD) compared to non-Latino Whites. Family caregivers need access to high-quality information, education, and support. The study objective was to understand the information seeking behavior of Latino caregivers of individuals with ADRD. We conducted qualitative interviews in Los Angeles County with Latino caregivers of individuals with ADRD (N=21) and with healthcare and community stakeholders serving Latinos with ADRD and their caregivers (N=6). Caregivers sought information about ADRD, healthcare services and treatment, available community resources, caregiving skills, and short- and long-term care. Having limited time to seek information, lacking skills to navigate the Internet, and insufficient availability of information created barriers to accessing information. With access to information, caregivers increased their knowledge about ADRD, enrolled the person with ADRD in a memory/dementia clinic, participated in treatment decisions, attended community events, learned how to respond to challenging behaviors, and enrolled in daycare/respite programs. Caregivers experienced barriers to using information due to the insurance and immigration status of the person with ADRD and due to services only being available in English. Our study contributes new knowledge on the accessibility and use of information by Latino caregivers and their unique barriers in transforming information access into meaningful results. Healthcare and community-based providers can use our study findings to tailor informational interventions to the needs and preferences of Latinos and to the unique barriers they face in using information to support their caregiving role.

### INITIAL TRANSLATION OF A DEMENTIA CAREGIVER INTERVENTION INTO A MOBILE HEALTH APPLICATION

Taylor Maynard,[1] Travis Frink,[2] Kunal Mankodiya,[2] Jennifer Davis,[3] Brian Ott,[1] Lisa Uebelacker,[4] and Geoffrey Tremont,[1] *1. Rhode Island Hospital, Providence, Rhode Island, United States, 2. University of Rhode Island, Kingston, Rhode Island, United States, 3. University of British Columbia, Kelowna, British Columbia, Canada, 4. Butler Hospital, Providence, Rhode Island, United States*

Caring for a person with dementia is associated with negative outcomes. Few caregiver interventions have been implemented in community settings. Mobile technology is one method for reaching many caregivers. This project translated two empirically-supported interventions for dementia caregivers into a mobile health application. A team of clinical researchers and computer engineers developed an App called CARE-Well (Caregiver Assessment, Resources, and Education) over 6 months. The group worked closely to do the following: 1). translate interventional content to be compatible with a mobile platform; 2). create new materials; 3). determine App components that captured key intervention areas; 4). troubleshoot formatting, technology, and data security; and 5). educate each other about respective areas of expertise. We developed a beta version of the App that included: 1). assessment of caregiver stress and care recipient behavioral problems; 2). psychoeducation; 3). goal diary; 4). managing behavior problems; 5). online message forum; and 6). video library. Several challenges arose during the App development process, such as how to create navigation paths and goal lists based off users' assessment responses, data storage and usage tracking, enlarging text, and how to ensure privacy and confidentiality in the online message forum. Our experience developing the CARE-Well App showed that translating behavioral interventions into mobile health applications is feasible and dependent upon regular communication among multidisciplinary team members. Next steps for the App include beta testing with dementia caregivers and conducting a pilot randomized trial to determine feasibility for a future trial and its effects on caregiver stress.

### LEAD IT!: AN APP TO ENABLE PERSONS WITH DEMENTIA TO LEAD GROUP ACTIVITIES FOR THEIR PEERS

Michael Skrajner,[1] and Gregg Gorzelle,[2] *1. Hearthstone Alzheimer Care, Wickliffe, Ohio, United States, 2. Hearthstone Alzheimer Care, Hiram, Ohio, United States*

LEAD IT! is an app that enables persons with early and middle stage dementia to lead activities for their peers—i.e., other persons with dementia (PWD). An alpha version of the app was tested in a Phase 1 SBIR project. The alpha version included three Montessori-inspired activities. While PWD ostensibly view LEAD IT! as a set of enjoyable activities, it is actually an evidenced-based intervention aimed at reducing responsive behaviors and enabling PWD to fill meaningful social roles. A total of 24 PWD participated in the Phase 1 study: five leaders and 19 players. LEAD IT! Programming was implemented for six weeks, twice per week. LEAD IT! produced higher levels of positive engagement and affect, and lower levels of negative engagement, as compared to standard, baseline activities—i.e., non-digital activities led by staff. More specifically, when compared to baseline programming, players exhibited an 82% increase in Constructive Engagement (P=0.000), 80% increase in Passive Engagement (P=0.000), 60% reduction in Other Engagement (P=0.035), and 171% increase in Pleasure (P=0.000). One limitation of the Phase 1 study is that, at least insofar as the intervention is only implemented twice per week for six weeks, the positive outcomes seem to be limited to the period of time during which PWD are participating in the activity—i.e., changes on global measures, such as quality of life and depression were not detected. Still, the promising results of this study suggest that LEAD IT! is worthy of further development and evaluation in a planned Phase 2 study.

### LIFE REVIEW IN DEMENTIA: THE VALUE OF IDENTITY

Diane Walsh,[1] Hanna Ulatowska,[2] Tricia Santos,[2] Sara Aguilar,[2] and Rebecca Patterson,[2] *1. The University of Texas at Dallas, Richardson, Texas, United States, 2. The University of Texas at Dallas, Dallas, Texas, United States*

The stories told by veterans with dementia provide new insights into person-centered care for healthcare professionals. In this qualitative exploratory study, life review in seven WW II veterans with mild-moderate dementia was examined. Participants with mild-moderate dementia were selected from a larger study of seventy oldest-old veterans. Semi-structured interviews were conducted to elicit testimonial language about memorable war experiences, including their perception and evaluations of the war. The interviewer provided questions and prompts to facilitate responses when necessary. Despite decreased declarative memory manifested via reduction in details and specificity of information within narratives, participants demonstrated a desire to share their war time experiences. Veterans selected appropriate memories to share, indicating their preserved sense of self. They provided a general evaluation of their wartime experiences when prompted. Veterans expressed various types of identity related to both their social origin and their experience of participating in the war. Identities of social origin revealed cultural identities which were expressed via their sense of humor. Wartime identities include being a survivor, a patriot, and a tolerant person. Veterans also described how the GI Bill helped them become college educated. This study suggests that identity is relatively preserved more so in individuals with mild versus moderate dementia when producing autobiographical stories. The process of life review brings identity to the surface. Acknowledging the identity of individuals with dementia is essential to care as it recognizes the value of the person. Additionally, eliciting significant autobiographical memories serves as a valuable means of social engagement and connection.

### MOVING TOGETHER: LIVESTREAM VIRTUAL GROUP MOVEMENT CLASSES FOR PERSONS WITH DEMENTIA AND CAREGIVERS

Deborah Barnes,[1] Cynthia Benjamin,[2] Amanda Lee,[3] Jennifer Lee,[4] Wolf Mehling,[4] Margaret Chesney,[3] and Rebecca Sudore,[3] *1. San Francisco VA Health Care System, San Francisco, California, United States, 2. Together Senior Health, San Francisco, California, United States, 3. University of California,San Francisco, San Francisco, California, United States, 4. University of California, San Francisco, San Francisco, California, United States*

MOVING TOGETHER classes deliver an online livestreaming version of the evidence-based Paired PLIÉ (Preventing Loss of Independence through Exercise) group movement program for people living with dementia (PLWD) and caregivers (CGs) that targets physical movements, mindful body awareness, and social engagement. Paired PLIÉ has been found to be associated with improvements in physical, cognitive, social/emotional function, and relationship quality. However, CGs also reported logistical challenges related to attending in-person classes. Our goal was to assess feasibility and satisfaction with an online version of the program developed by Together Senior Health, Inc. Fifteen of twenty-one participants (70%; 8 PLWD, 7 CGs) successfully completed the 12-week program, of whom all (100%) reported being highly satisfied with online delivery. Participants described physical, social and emotional benefits of participating: "I liked the activities with partner and that I could feel various parts of my body getting massage and movement (PLWD);" and "Really enjoyed this class, so did my body, brain and my spirit (CG)." Participants strongly supported the online format: "[I liked] BEING AT HOME! … Getting ready to go out, going out and coming home takes a lot of energy and we are not sure we would participate if it were not online (PLWD)." In conclusion, we found that online delivery of Moving Together is feasible and acceptable to PLWD and CGs. Offering livestream virtual group movement classes such as Moving Together could enhance accessibility to evidenced-based programs for PLWD and CGs.

### NON-PHARMACOLOGIC INTERVENTIONS FOR CAREGIVERS OF PEOPLE WITH DEMENTIA IN LATIN AMERICA: A REVIEW

Jose Aravena,[1] Jean Gajardo,[2] and Laura Gitlin,[3] *1. School of Public Health, Yale University, New Haven, Connecticut, United States, 2. Universidad San Sebastián, Santiago, Chile, 3. Drexel University, Philadelphia, Pennsylvania, United States*

In a scenario of increasing longevity and social inequalities, Latin-America is an important contributor to the worldwide dementia burden. Caregivers' health is fundamental to maintain the person with dementia quality of life. However, caregiving is a culturally sensible role that requires tailored solutions. The aim is to synthesize the evidence about non-pharmacologic interventions targeted to caregivers of people with dementia in Latin-American contexts. A comprehensive review of interventions in caregivers and persons with dementia in Latin-American countries was conducted using MEDLINE, Embase, PsycINFO, and Scopus with studies published until January 27th, 2020. Randomized clinical trials of non-pharmacologic interventions targeted to caregivers of people with dementia or dyads where included. Qualitative synthesis of the evidence was presented and analyzed. Overall, 9 pilot RCT were included for the final analysis (6 Brazil, 1 Colombia, 1 Mexico, 1 Perú). The biggest study recruited 69 caregivers and the smallest 13 dyads, with follow-up range of 3-6 months. 5 control groups received at least some other non-standard care type of intervention. 8 were targeted exclusively to caregivers (4 group intervention, 3 individual, and 1 combined) and 1 multicomponent intervention. Most frequent measured outcomes were caregiver's burden, anxiety, depressive symptoms, and quality of life, and person with dementia neuropsychiatric symptoms. Individual interventions report better results in caregiver parameters such as burden and depressive symptoms and person with dementia neuropsychiatric symptoms. Group interventions presented mixed results. Nevertheless, the quality of evidence was low. There is a critical need to study interventions for caregivers in Latin-American contexts.

### PERCEPTIONS OF TREATMENT BURDEN AMONG ELDERS WITH DIABETES AND COMORBID ALZHEIMER'S DISEASE AND RELATED DEMENTIAS

Victoria Vaughan Dickson,[1] Halia Melnyk,[2] Rosie Ferris,[3] Joshua Chodosh,[4] and Caroline Blaum,[3] *1. New York University, New York, New York, United States, 2. Nursing, New York, New York, United States, 3. NYU Langone Health, New York, New York, United States, 4. New York University Grossman School of Medicine, New York, New York, United States*

Background: An estimated 25% of older adults with diabetes (DM) may have co-occurring Alzheimer's Disease and Related Dementias (ADRD), complicated by multiple treatment plans and providers. Assessing treatment burden has been limited to patients' perspectives; little is known about caregiver perceptions of treatment burden despite their important role in personal care and treatment adherence. The purpose of this qualitative study was to describe caregiver perceptions of treatment burden for older adults with DM-ADRD. Methods: This qualitative study was conducted in the formative phase of "Enhanced Quality in Primary care for Elders with DM-ADRD (EQUIPED-ADRD) a pragmatic randomized controlled trial in a large, diverse healthcare system. A diverse sample of caregivers (n=15) of patients enrolled in the RCT participated in interviews about their caregiver role and perceptions of treatment burden of DM-ADRD clinical management. Qualitative data were analyzed using content analysis and themes about treatment burden were compared to domains on the Treatment Burden Questionnaire (TBQ). Results: Caregivers reported high levels of burden related to treatment plans for patients with DM-ADRD. Themes related to complexity and burden of medication management, monitoring (e.g., blood pressure, glucose monitoring), dietary and physical activity regimens, navigating healthcare providers and financial burden were reported. Caregivers also described high levels of emotional burden that was associated with patient's cognitive decline and family functioning stress. Conclusions: Interventions to reduce treatment burden for patients and caregiver should include activating social/nursing services, respite care and care coordination that may support caregivers especially as patient treatment increases in complexity over time.

### POSITIVE BEHAVIORS AND STRENGTHS OF PEOPLE WITH DEMENTIA

Diana DiGasbarro,[1] Courtney Whitaker,[2] and Benjamin Mast,[1] *1. University of Louisville, Louisville, Kentucky, United States, 2. Masonic Homes Kentucky, Louisville, Kentucky, United States*

Assessment of and care for people with dementia is shifting away from a purely medical disease model toward a more holistic, biopsychosocial approach. Within this movement is growing interest in balancing the negative, symptom-based view of people with dementia with better understanding of strengths and positive behaviors exhibited by people with dementia. The aim of this qualitative study was to gather perspectives of professional caregivers regarding positive behaviors and strengths observed in people with dementia. Data were obtained from three focus groups conducted at a memory care unit within a large long-term care facility. Focus group participants (N=14) worked in nursing and the activities department. Inductive methods were used to code and analyze focus group transcripts and recordings. Five major themes were identified: enduring abilities and values, enduring traits and strengths, sense of purpose and meaning, desire to be helpful, and prosocial behavior. These themes illustrate many domains in which professional caregivers have observed positive behaviors and strengths in people with dementia living in a long-term care facility. The results of this study contribute to the growing literature pertaining to the intersection of positive psychology and dementia research and practice. Future directions include development of an assessment tool to measure positive behavior and strengths in people living with dementia.

### PSYCHOMETRIC PROPERTIES OF THE CHINESE WAYFINDING EFFECTIVENESS SCALE

Yi-Chen Chiu,[1] and Donna Algase,[2] *1. Chang Gung University, Taoyuan County, Taiwan (Republic of China), 2. University of Michigan, Ann Arbor, United States*

Being unable to find one's way is a terrifying experience accompanied by feelings of fear or frustration. Persons with cognitive impairment (PWCIs) are apt to get lost and show difficulty in wayfinding effectiveness. Therefore, it is important to develop an instrument to measure the wayfinding effectiveness in PWCIs in northern Taiwan. This was a cross-sectional design with 180 PWCIs to examine the psychometric properties of the Chinese Wayfinding Efficient Scale (CWE) developed by Algase. An exploratory Factor Analysis with varimax rotation was computed to explore the underline conceptual structure of the CWE. The results derived 6 factors: FDPFE = Finding a destination and a path in familiar environments; FDPUFE=Finding a destination and a path in unfamiliar environments; SDAS = Sense of direction and analytic strategies; SDGS = Sense of direction and global strategies; LMD = Landmark and distance in familiar/unfamiliar environments; MAP = Using a map in familiar/unfamiliar environments. This solution explained 66.50% of variance. The internal consistency values for the total and subscales were excellent. Item 25 was single loaded, therefore, deleted. One-week test-retest reliability of the CWE total scale and subscales were estimated using a subset of data (n = 15) by calculating ICC. One subscale, Finding a distance and a path in familiar environments (FDPFE), did not have significant test-retest reliability but the rest of the subscales and total scale had excellent test-retest reliability (.81-1.00). The initial psychometric properties of the CWE were acceptable. Further research should explore the possible associate factors.

### RACIAL AND ETHNIC DISPARITIES AT END OF LIFE AMONG PERSONS WITH ALZHEIMER'S DISEASE AND RELATED DEMENTIAS

Morgan Rising,[1] and Nicole Hair,[2] *1. University of South Carolina, Columbia, United States, 2. University of South Carolina, Columbia, South Carolina, United States*

End-of-life (EOL) care for persons with Alzheimer's disease and related dementias (ADRD) is heterogeneous and, often, inconsistent with patients' priorities. While differences in physician-patient communication or the health care decision-making process likely contribute to disparities in EOL care, little is known about the relationships between race, advance care planning (ACP), and quality of care among persons with ADRD. The aim of this study was to (1) characterize trends in both ACP and EOL treatment intensity in persons with ADRD and (2) test whether racial differences in ACP mediate disparities in EOL care. We analyzed a population-based cohort of older adults with cognitive impairment or dementia who participated in the Health and Retirement Study (HRS) and died between 2000 and 2014 (n = 5,316). While participation in ACP among persons with ADRD increased from 2000 to 2014 (66% to 83%, P < 0.05), models stratified by race showed that differences in participation rates across white and nonwhite persons with ADRD persisted over the sample period. Racial disparities in the location of death, a proxy for the intensity of EOL care, narrowed from 2000 to 2014. However, next-of-kin surrogates of nonwhite persons with ADRD were much more likely to report the decedent received "all care possible... in order to prolong life". Assignment of a durable power of attorney was found to influence location of death, while both creation of a living will and participation in discussions about EOL care preferences were found to influence the likelihood that decedents received all possible life-prolonging treatments.

### REASONS FOR NON-PARTICIPATION IN AN ACTIGRAPHY STUDY IN AN ALZHEIMER'S DISEASE CENTER REGISTRY

Alex Laffer,[1] Hilary Hicks,[2] Genna Losinski,[1] and Amber Watts,[1] *1. University of Kansas, Lawrence, Kansas, United States, 2. The University of Kansas, Lawrence, Kansas, United States*

When recruited individuals decline to participate in research, it can lead to sampling bias, increased costs, and extended duration of data collection. Understanding reasons why eligible participants decline participation may improve study enrollment rates. We aimed to understand barriers to recruitment and data collection in older adults with and without Alzheimer's disease in the University of Kansas Alzheimer's Disease Center Registry annual visit. We recruited Registry participants to join an observational sub-study using wrist-worn actigraphy to measure physical activity and sleep. We analyzed reasons for non-enrollment from encounters with non-participating individuals. Of 104 encounters, 37 were never recruited due to appointment cancellation, rescheduling, or no-show. Of the remaining encounters, the most common reasons for non-participation were physical limitations (N = 13), study logistics (e.g., limited supplies; N = 12), participant travel plans (N = 10), and unknown (N = 8). Other categories (N = 6) included disinterest, study partner concerns about pragmatics (e.g., fear that an individual with AD would lose the ActiGraph), problems with the study design (e.g., lack of feedback to participants), and participants' limited availability or deferment to a later date. These findings offer insight into potential avenues to overcome barriers to participation in older adults already engaged in ongoing research through an Alzheimer's Disease Center Registry. Researchers could benefit from adapting study procedures to correct for reasons of non-participation. For example, giving more education and reassurance to potential participants about observation and giving feedback regarding activity patterns.

### STIGMATIZATION AND THE EXPERIENCE OF INFORMAL DEMENTIA CAREGIVERS IN NIGERIA

Candidus Nwakasi,[1] Kate de Medeiros,[2] and Darlingtina Esiaka,[3] *1. University of Southern Indiana, Evansville, Indiana, United States, 2. Miami University, Oxford, Ohio, United States, 3. Union College, Union College, New York, United States*

There is no formal word for dementia in Nigeria. Instead, some Nigerians, in their effort to make sense of dementia symptoms, use descriptions that may result in stigmatization of people living with dementia and their families. With Nigeria's rapid aging, increased risk of dementia, and lack of formal long-term care, this study focused on the impact of stigma on the caregiving experiences of Nigerian women. This exploration is significant as adult females in Nigeria are the pillar of informal caregiving in the country. The study employed a qualitative descriptive method. Semi-structured interviews were conducted with a purposive sample of 12 adult informal female caregivers in Anambra, Nigeria. Data were then transcribed, coded and analyzed for themes. Afterwards, focus groups of 21 adult Nigerians residing in Ohio, US, were conducted to offer more contextual insight on the findings. The three major themes identified were: 1) negative views of dementia symptoms (e.g., witchcraft, madness), 2) caregiving protects against stigmatization (e.g., by keeping family members out of sight), and 3) stigma and caregiving support such as adult children abandoning parents with dementia because of the stigma associated with dementia. Given the overwhelming presence of stigma in all aspects of dementia to include dementia caregiving, results point to the critical need for better strategies to help strengthen informal caregiving in Nigeria. This includes culturally appropriate dementia education for families and caregivers, and formal long-term care policies that include care support in a rapidly aging Nigeria.

### STIGMATIZING BELIEFS ABOUT ALZHEIMER'S DISEASE IN DIVERSE ETHNIC GROUPS OF ASIAN AMERICANS

Jiaming Liang, and Yuri Jang, *University of Southern California, Los Angeles, California, United States*

Cumulative studies have investigated Alzheimer's disease (AD)-related issues among Asian Americans, but few have considered ethnic diversities within the Asian group. Using an ethnic-diverse Asian American sample, the present study explored the prevalence, ethnic variations, and predictors of stigmatizing beliefs about AD: (1) AD is a normal process of aging, (2) it is embarrassing to have a family member with AD, and (3) social interactions with an AD patient should be avoided. Inspired by the sociocultural health beliefs model, a focus was given on the role of immigration and culture-related variables. Using data from the 2015 Asian American Quality of Life survey (N = 2609, age range = 18-98) that includes Chinese, Asian Indian, Korean, Vietnamese, Filipino, and other Asians, logistic regression was conducted to examine how each of the three stigmatizing beliefs would be predicted by (1) demographic variables and (2) immigration and culture-related variables. Results indicate that the prevalence of the stigmatizing beliefs about AD varied across ethnicities. More than 63% of Vietnamese associated AD with a normal process of aging, and about 10% of Chinese reported that they would feel embarrassed if their family member had AD. Logistic regression models demonstrated that advanced age, male gender, low education, and limited English proficiency increased the odds of reporting one or multiple stigmatizing beliefs about AD. The findings suggest a varying degree of AD-related misconceptions and stigmatization and call attention to the need for culturally sensitive community education on AD in Asian communities.

### STUDY PROTOCOL: PARTNERS AT MEALS TELEHEALTH INTERVENTION FOR CAREGIVERS OF PERSONS WITH DEMENTIA

Suparna Qanungo, Elaine Amella Krug, Kelly Martin, Martina Mueller, Mohan Madisetti, and Teresa Kelechi, *Medical University of South Carolina, Charleston, South Carolina, United States*

Background: As the aging population continues to increase, it is estimated that persons with dementias (PWDs) will reach 13 million by 2050. Lack of caregiver skills related to mealtime planning and the ability to cope with dysfunctional behaviors are well-documented factors that influence nutritional outcomes for PWDs, leading to social isolation, and negatively impacting their home stay. The aim of this study protocol is to test the effectiveness of a train-the-trainer program in which non-paid volunteers in respite care centers deliver a telehealth mealtime intervention for caregivers of PWDs, Partners at Meals. The program is based on the C3P Model of Changing the Place, People and Person. Methods: A cluster-randomized controlled trial with parallel mixed methods evaluation processes is being conducted. Caregivers and PWD dyads, receiving respite services are randomized to receive Partners at Meals or enhanced-usual-care for six months. Within the intervention group, dyads are partnered with a C3P-trained volunteer who works with caregivers to devise monthly mealtime plans. Under enhanced-usual-care, dyads receive standardized educational materials modified from The Savvy Caregiver Program for Alzheimer's caregiving. Primary outcomes include weight, calorie, protein and fluid intake of the PWDs and quality of life of the caregiver. Respite center administrators, program directors, volunteers and caregivers are evaluated for intervention fidelity, acceptability and sustainability. Implications: In this trial, we lay the groundwork to examine effectiveness and sustainability of a train-the-trainer telehealth program that could be widely disseminated for managing mealtimes in-the-home, while promoting quality of life of both the caregiver and PWDs.

### TELEPHONE-BASED HEALTH COACHING: REDUCTION IN ALZHEIMER'S RISK BEHAVIORS

Annie Rhodes,[1] Taylor Wilkerson,[2] Jennifer Inker,[1] Joann Richardson,[3] and Faika Zanjani,[1] *1. Virginia Commonwealth University, Richmond, Virginia, United States, 2. VCU, School of Social Work, Richmond, Virginia, United States, 3. VCU, Richmond, Virginia, United States*

Objective: Explore the feasibility of integrating intensive, telephone-based health coaching programs in low-income senior housing communities to reduce Alzheimer's risk behaviors. Design: Participants meeting study criteria: 60 years or older, a working telephone, no cognitive diagnoses, income below 1,000 USD monthly, and active cardiovascular or diabetic health symptoms were recruited from low-income housing units. Engagement in Alzheimer's risk behaviors: Cigarette use, alcohol overuse, polypharmacy, inactivity, depression, and cognition status, were measured at enrollment, and 12 weeks post. Weekly coaching sessions focused on reducing behavioral risk for Alzheimer's disease. Setting: Low-income senior apartments in Richmond, Virginia Participants: Twenty older adults, living in low income senior high rises. Participants were majority (95%) African-American (Mean= 69 years, SD=4.17, Range=61-77). Intervention: Participants engaged in a call with a coach for 12 weeks, focused on Alzheimer's risk reduction. Participants identified with coach specific behaviors to target. Primary Outcome Measure: Feasibility of telephone-based health coaching to reduce Alzheimer's Risk Behaviors. Feasibility is defined as participant engagement in health coaching and self-rated health outcomes. Results: Of the original 20 enrollees, 19 (95%) participated in coaching sessions. On average, 8.75 sessions were completed. All participants rated their experience as positive, and self-reported an improvement in health and healthy behaviors, in exit interviews. The coaching experience was rated 94.11 on a scale from (0-100). Participants rated their health coach, on average, 90.44 on a scale from (0-100). Participants rated their health improved as 92.37 on a scale from (0-100). Conclusion: Telephone-based health coaching was feasible based on participant engagement.

### SESSION 2928 (PAPER)

### HEALTH AND HEALTH PROMOTION I

### COPING CAPACITY AND EPISODIC MEMORY IN OLDER ADULTS WITH SMCS: THE MEDIATING ROLE OF HEALTHY LIFESTYLE

Feilong Wang,[1] Shijie Li,[1] Kaifa Wang,[2] and Yanni Yang,[1] *1. Third Military Medical University, Chongqing, China, 2. Southwest University, Chongqing, China*

Older adults with subjective memory complaints (SMCs) are at increased risk for episodic memory decline. Episodic memory decline is an important predictor of objective memory impairment (one of the earliest symptoms of Alzheimer's disease) and an often-suggested criterion of successful memory aging. Therefore, it is important to explore the determinant factors that influence episodic memory in older adults with SMCs. Roy adaptation model and preliminary evidence suggest that older adults with SMCs undergo a coping and adaptation process, a process influenced by many health-related risks and protective factors. This study aimed to explore the relationship between coping capacity and episodic memory, and the mediating role of healthy lifestyle between coping capacity and episodic memory in a sample of 309 community-dwelling older adults with SMCs. Results from the structural equation modeling showed that coping capacity directly affects episodic memory ($r=0.629$, $p < 0.001$), and there is a partial mediating effect (60.5%) of healthy lifestyle among this sample of older adults with SMCs. This study demonstrates that coping capacity and adaptation positively correlate with episodic memory in older adults with SMCs, and that these correlations are mediated by healthy lifestyle. The results suggest that older adults with poor coping capacity should be assessed and monitored regularly, and clear lifestyle-related interventions initiated by healthcare providers that promote healthy lifestyles may effectively improve coping capacity and episodic memory in this population group. Note: First author: Feilong Wang, Co-first author: Shijie li, Corresponding author: Yanni Yang

### DETERMINANTS OF MORTALITY AMONG OLDER ADULTS BY AREA-LEVEL MATERIAL AND SOCIAL DEPRIVATION.

Helen-Maria Vasiliadis,[1] Samantha Gontijo Guerra,[2] and Isabelle Pitrou,[2] *1. University of Sherbrooke, Sherbrooke, Quebec, Canada, 2. University of Sherbrooke, Longueuil, Quebec, Canada*

Although social inequalities are increasing worldwide, few studies have examined their consequences on mortality among older adults. The aim of this research was to examine determinants of mortality among older adults at the individual, health system and area level. Data come from the ESA-Services study conducted in 2011-2013 in Quebec including 1,765 adults aged ≥65 years. Mortality 3 years after the survey interview was obtained from vital statistics data. Material and social deprivation of area of residence was determined using the Pampalon index categorized into quintiles as follows: least deprived (1st quintile), middle quintiles (2nd, 3rd, 4th quintiles), most deprived (5th quintile). Other variables included clinical, psycho-behavioural, socio-economic and demographic factors. Cox regressions were used to examine the determinants of mortality while stratifying by level of area deprivation. Compared to most deprived areas, mortality was higher for those living in middle quintiles of deprivation. In the overall analyses, age, chronic conditions, social support and continuity of care were associated with mortality. When examining mortality by area of deprivation, results showed higher mortality ratios with cognitive impairment in middle and least deprived areas. In least deprived areas, female sex, the presence of bipolar disorder and dementia were associated with mortality. The strong associations between mortality, cognition, dementia, bipolar disorder and female sex in least deprived areas might be partially explained by a closer follow-up and earlier detection of this population. Continuity of care was also associated with significantly lower mortality ratios for those living in middle and most deprived areas.

### EFFECTIVENESS OF A CARE DELIVERY MODEL FOR HIGH-NEED COMMUNITY-DWELLING OLDER ADULTS

Kuei-Min Chen,[1] and Hui-Fen Hsu,[2] *1. Kaohsiung Medical University, Kaohsiung City, Taiwan (Republic of China), 2. Kaohsiung Medical University, Kaohsiung, Taiwan (Republic of China)*

The effectiveness of sufficient care coordination for high-need community-dwelling older adults has not been discussed. This study aimed to examine the effectiveness of a newly-developed care delivery model for high-need community-dwelling older adults. A cluster randomized controlled trial with repeated measures design was employed. A total of 145 high-need older adults participated in the study and were randomly assigned to either the intervention group or comparison group. A categorized list of care services based on the types of high-need older adults as the intervention allowed care coordinators to make adequate care service linkages. The intervention period ranged over 6 months with regulated home visits and assesssments. Functional ability, quality of life, depressive symptoms, and healthcare and social service utilizations were measured at baseline, and at 3 and 6 months into the intervention. The participants' satisfaction with care delivery was measured at the end of 6-month intervention. Results showed that the intervention group had a better functional ability, a higher quality of life, reduced depressive symptoms, and more efficient healthcare and social service utilizations than the comparison group at both the 3-month and 6-month intervals (all $p < .05$). By the end of the 6-month study, the intervention group were more satisfied with the care service linkages than the comparison group ($p < .05$). The positive effects of providing a categorized list of care services for care coordinators to make service linkages have been evidenced by the outcomes. The promising findings supported a further longer-term implementation of the care delivery model.

### INTERVENTIONS FOR IMPROVING PSYCHOSOCIAL ADJUSTMENT IN NURSING HOME RESIDENTS: A NETWORK META-ANALYSIS

Yanyan Zhang,[1] Huimin Xiao,[2] and Binbin Yong,[1]
*1. Fujian Medical University, Fuzhou, Fujian, China,*
*2. Fujian Medical University, Fuzhou, China*

This study aimed to identify the evidence of interventions for improving psychosocial adjustment in older adults relocating to nursing homes. We followed PRISMA-NMA guidelines to conduct a network meta-analysis. 12 electric databases were used to search for eligible randomized controlled trials (RCTs) and clinical trials (CCTs) from inception to August 27th, 2019. Two reviewers independently conducted article screening, data extraction, and risk of bias appraisal with the Revised Cochrane risk-of-bias tool for RCT and Risk Of Bias for CCT. The network plots were plotted to provide a visual representation of the evidence base. Bayesian fixed-effects pairwise and network meta-analysis were exhibited in the forest plot. A Bayesian network meta-analysis was performed on 30 eligible RCTs with 2119 participants, and 12 eligible CCTs with 491 participants. The quality of the most included studies was rated as moderate in RCTs and low in CCTs. Treatment effects showed that compared to conventional treatment, group reminiscence and group counseling resulted in significant improvements in loneliness (MD: 11, 95%, CI: 2.7–17, SUCRA: 99.5%; MD: 7.7, 95%, CI: 0.53–15, SUCRA: 53.9%, respectively). Similar results were obtained for art therapy (MD: 5.5, 95%CI: 0.8–10, SUCRA: 97.3%) in self-esteem. The model fit was good, and the inconsistency was low. Group reminiscence, group counseling, and art therapy are recommended for reducing loneliness and enhance self-esteem in nursing practice.

### SESSION 2929 (PAPER)

### HEALTH AND HEALTH PROMOTION II

### BODY MEASUREMENT AND HEALTH RISK EDUCATION EFFECTS ON OLDER ADULTS' HEALTH BELIEFS AND BEHAVIORS

Beatrice Gaynor, *University of Delaware School of Nursing, Newark, Delaware, United States*

Waist circumference (WC) measurement is an indicator of central obesity related disease risk that is rarely used in primary care (PC). The current PC practice of body mass index (BMI) calculation to screen for disease risk lacks specificity to the older adult habitus. Guided by the Health Belief Model, this study utilized a one-way analysis of covariance to examine the effect of experimental cues, WC measurement and central obesity disease risk education, compared to control cues, BMI and obesity classification, on older adults' health beliefs (perceived susceptibility and health benefits) and behaviors (diet and exercise) 6 weeks post cues/intervention. Of the 99 participants (control group [N=49]; experimental group [N=50]) 92% reported 'never' having WC measurement and 76% reported 'never' having BMI calculation in PC. Both groups reported high levels of perceived susceptibility and exercise at baseline. Changes in perceived susceptibility, diet, and exercise were non-significant in either group. There was a significant increase in perceived health benefits of WC measurement (p=0.01) and BMI calculation (p=0.01) in the experimental group compared to the control group. Willingness to exercise (p=0.007) significantly increased in the experimental group compared to the control group. The lack of BMI experience in both groups may have caused control cues to function as experimental cues in both groups. Thus, this study provides evidence that combined use of WC measurement, central obesity health risk education, BMI calculation, and obesity classification increase perceived benefits of body measurements and motivate physical activity in older adults over BMI and obesity classification alone.

### EFFECTS OF MEDICARE DRUG SUBSIDIES ON ADHERENCE FOR DIABETICS: EVIDENCE FROM A REGRESSION DISCONTINUITY DESIGN

Alexandra Glynn, Inmaculada Hernandez, and Eric Roberts, *University of Pittsburgh, Pittsburgh, Pennsylvania, United States*

Out-of-pocket prescription drug costs are rapidly rising, particularly for insulin, which is a life-saving drug used by 3.1 million diabetics on Medicare. High out-of-pocket costs place an accentuated financial strain on older adults with diabetes, many of whom have low incomes, and may impede medication adherence, leading to poor health outcomes. The Medicare Part D Low-Income Subsidy (LIS) program limits drug co-pays to under $8.50 per prescription and caps out-of-pocket drug costs for lowest-income recipients (<135% Federal Poverty Level, FPL), resulting in pronounced differences in out-of-pocket costs for those with marginally different incomes. Using detailed income data from the Health and Retirement Study linked to Medicare claims (2008-2016), we employed a regression discontinuity (RD) design to isolate the effects of differences in out-of-pocket costs at eligibility thresholds for the LIS. Diabetic beneficiaries whose income exceeded the LIS eligibility threshold had lower Part D spending (-$945/year, p=0.03, n=2,367) and adherence to oral antidiabetic drugs (-8%, p=0.02). We conducted secondary analyses at the eligibility threshold for Medicaid, as individuals whose income exceeds the eligibility limit for Medicaid (100% of FPL in most states) are significantly less likely to receive the LIS. Above the Medicaid eligibility threshold (n=2,295), annual spending on insulin was $395 lower (p=0.002) and proportion of insulin use was 6% lower (p=0.04). These results suggest low-income Medicare beneficiaries who are not shielded from out-of-pocket costs via the LIS are particularly sensitive to drug costs. Policy proposals to limit out-of-pocket costs could improve medication adherence to high-cost drugs for vulnerable beneficiaries.

### ENVIRONMENTAL INFLUENCES OF EXTREME HEAT ON THE HEALTH OF OLDER ADULTS: A RETROSPECTIVE STUDY

Mei Liu,[1] Carol Buller,[1] Barbara Polivka,[1] Terri Woodburn,[2] Mark Jakubauskas,[3] and Benjamin Wolfe,[3] *1. University of Kansas, Kansas City, Kansas, United States, 2. University of Kansas Edwards Campus, Overland Park, Kansas, United States, 3. University of Kansas, Overland Park, Kansas, United States*

Studies have suggested that extreme weather events have differential effects by age. By leveraging electronic medical records, we aim to analyze the environmental influence of extreme heat on the health of older adults. From our healthcare system's de-identified data warehouse, we extracted a retrospective cohort of 108,192 patients who were ≥65 years of age as of 1/1/2018 with pre-existing chronic conditions including diabetes, COPD, cardiovascular disease, or kidney disease. Extreme heat event period was defined as 5/1/2018 to 9/1/2018 (79 days with temperature ≥90o; 15 days of moderately poor/poor air quality index (AQI) [≥75] values) and the comparison period was defined as 5/1/2019 to 9/1/2019 (51 days with temperature ≥90o; 0 days with moderately poor/poor AQI values) in the Kansas City area. We randomly partitioned the study cohort into two sets and demonstrated the two patient sets were statistically similar (p>0.05) with respect to their demographic and underlying health conditions. Finally, we compared the respiratory, cardiovascular, and renal health outcomes between the 2018 and the 2019 cohorts. Most patients were Caucasians, female and had comorbid conditions. Results showed significantly higher number of all-cause emergency department visits (p=0.04) and outpatient visits (p=<.001) during the extreme heat event period in 2018. Analyses also showed significantly higher number of outpatient visits due to upper respiratory diseases (p=0.008) and acute renal failure (p=0.01) in 2018. In conclusion, extreme heat increased use of healthcare services in older adults with chronic conditions.

### SMOKING, BUT NOT OTHER ENVIRONMENTAL EXPOSURES, INCREASES RISK FOR ADVANCED BRAIN AGING

Nathan Whitsel,[1] Carol Franz,[2] and William Kremen,[3] *1. UCSD, San Diego, California, United States, 2. University of California San Diego, La Jolla, California, United States, 3. University of California San Diego, San Diego, California, United States*

Exposure to harmful substances and chemicals such as tobacco smoke, chemicals (e.g., herbicides, pesticides, Agent Orange) and metal dust has been associated with increased risk of developing cancer, cardiovascular disease, and other diseases that contribute to shorter life expectancy. Associations with brain health in relation to these exposures are less well studied. We examined the relationship between brain health and prolonged exposure to different harmful substances in 498 male participants average age 68 (range 61 to 73) from the Vietnam Era Twin Study of Aging

(VETSA). For self-reported tobacco smoke, herbicides/pesticides, and metal dust we created three groups reflecting recency of exposure (current/former/never). For Agent Orange we examined two exposure groups (ever/never). Brain health, defined as predicted brain age (PBAD), was evaluated by applying Brain-Age Regression Analysis and Computation Utility software (BARACUS) to magnetic resonance images collected at age 68. Tobacco smoking (r=-0.15, p=0.0004 ) was significantly correlated with PBAD and remained significant (F=5.56, p=0.005) in multivariate analyses adjusted for age, socioeconomic status (SES), age 20 general cognitive ability, and non-independence of twins within pairs. Never smokers had significantly younger brains than current or former smokers. PBAD did not differ for current versus former smokers. In other analyses, more advanced PBAD was associated with non-amnestic MCI. In this sample, tobacco smoking had the strongest relationship with overall brain health in late midlife compared with other types of environmental exposures, reinforcing its role in pathological aging and its importance as a public health priority.

### SESSION 2930 (POSTER)

### DEMENTIA AND COGNITIVE IMPAIRMENT II

### A QUALITATIVE STUDY ON MAKING RURAL COMMUNITIES MORE DEMENTIA-FRIENDLY

Melissa OConnor, Megan Pedersen, and Rachel Grace,
*North Dakota State University, Fargo, North Dakota, United States*

Recent studies on attitudes toward dementia in the United States, such as the World Alzheimer Report 2019, have found that fear and stigma are still widespread among the general public. This may be particularly true in rural communities. In the current study, community-dwelling adults in small Midwestern communities responded to the open-ended survey question, “What do you think could be done to make your community more welcoming for people with Alzheimer’s disease and other forms of dementia?” Participants (N=242) ranged in age from 18-88 (M=40, SD=21). The sample was 68% female, and 61% lived in communities of 50,000-150,000 people, while 39% lived in smaller towns. Most participants (61.2%) did not personally know someone with dementia. Data were collected via paper and telephone surveys. Responses to the open-ended question were analyzed using open, axial, and selective coding. The following themes emerged: greater exposure to individuals with dementia; educational workshops about dementia; more intergenerational programs; greater accessibility of respite care and other services; more fundraising efforts; and community leaders talking about dementia. Responses included, “Have more intergenerational programs that bring together Alzheimer’s patients and children in a positive environment.” “When I was in school, we visited an Alzheimer’s unit. That was a great experience.” “I know what it is, but I don’t know anything else. I wish I was more informed. I don’t know how to help.” These findings indicate that residents of rural communities are motivated to help individuals with dementia, but need more guidance, education, and personal connections/exposure.

### A SYSTEMATIC REVIEW OF OBSERVATIONAL DYADIC PERSONS LIVING WITH DEMENTIA: CAREGIVER COMMUNICATION INSTRUMENTS

Sohyun Kim, *University of Iowa, Iowa City, Iowa, United States*

To facilitate social interaction and providing quality care in persons living with dementia (PLWD), an effective means of evaluating communication quality between PLWD and their caregiver is needed. However, there is no systematic review of current instruments to assess dyadic PLWD-caregiver interactions in various care settings. The purpose of this review was to critically evaluate existing dyadic observational communication instruments used to provide recommendations. A systematic review using the Preferred Reporting Items for Systematic Reviews and Meta-Analyses Guideline was conducted. Literature that were published by May 2019 in English were searched from CINAHL, AgeLine, PsychINFO, Communication and Mass Media Complete, ProQuest Dissertations and Theses Global, and Scopus. Keywords were “communication strategy,” “communication,” “caregivers,” “dementia,” and any combination of these terms or MeSH terms. Data were extracted including development process, operational concept, target population and setting, items/scoring format, psychometric properties, and research/clinical use. A total of 3042 articles were identified and 15 instruments from 29 studies were evaluated by the scoring of 12 psychometrics: participants/items ratio, reliability (internal consistency, intra-rater, inter-rater), and validity (content, concurrent, predictive, known group difference, divergent/discriminant, convergent, structural). The total score was ranged from 0 to 22 (high quality: 16-22, moderate: 8-15, low: 0-7). There was no instrument with high quality assessing dyadic interaction. Only one instrument was evaluated as moderate quality (modified Behavioral Observation Scoring System, BOSS). While existing instruments are still in the early stages of development and testing, they demonstrate potential evidence that may require further testing before application in research and clinical practice.

### ACCELERATED DECLINE IN SENSE OF PURPOSE IN LIFE YEARS PRIOR TO DEMENTIA DIAGNOSIS

Nathan Lewis,[1] Jonathan Rush,[1] and Patrick Hill,[2]
*1. University of Victoria, Victoria, British Columbia, Canada, 2. Washington University in St. Louis, Saint Louis, Missouri, United States*

Research has pointed to sense of purpose in life as an important individual difference that promotes successful aging, predicting greater psychological wellbeing, physical functioning, and cognitive health in older adulthood. Despite such benefits, it is unclear how major life challenges such as the onset of cognitive impairment may impact the sense of purpose of older adults. Building on this, the present study examined long-term change in sense of purpose in a longitudinal sample of older adults who would later develop dementia. Data were from 341 participants without dementia at study intake who were subsequently diagnosed with dementia during the follow-up period (Mage = 81.92 years at intake, Mage = 89.19 years at diagnosis, 72.72% female). Participants completed annual assessments of purpose in life up to 17 years prior to dementia diagnosis (M = 6.99 assessments prior to diagnosis). Piecewise growth modeling examined change in sense of purpose in life with time structured as years to diagnosis. Results suggested a period of accelerated decline distinct from normative age-related change beginning around 5.60 years prior to dementia onset. Discussion will focus on two potential explanations: that perceiving declines in cognition may lead to diminished sense of purpose in life or that underlying changes in the brain may impact both cognition and sense of purpose resulting in concurrent decline over time.

### ADDRESSING ALZHEIMER'S RISK IN RACIALLY DIVERSE, LOW-INCOME OLDER ADULTS THROUGH TELEPHONE HEALTH COACHING

Faika Zanjani,[1] Annie Rhodes,[1] Taylor Wilkerson,[2] Jennifer Inker,[1] and Joann Richardson,[3] *1. Virginia Commonwealth University, Richmond, Virginia, United States, 2. VCU, School of Social Work, Richmond, Virginia, United States, 3. VCU, Richmond, Virginia, United States*

Preclinical Alzheimer's disease (AD) behavioral risk reduction needs to be more fully explored at the community-level. Connecting behavior change to AD can reduce individual-level helplessness for the disease. However, behavior change targeting AD prevention factors (e.g., alcohol, depression, physical inactivity, smoking, isolation, medication management) is extremely challenging for multiple reasons, including failures in connecting AD and health behavior risk, and due to individual-level motivational, self-efficacy, and knowledge barriers. Methods: As part of the Virginia Commonwealth University iCubed Health and Wellness in Aging Population Core, 20 diverse older adults (aged 60+) living in Richmond, VA, with incomes below $12,000/year and managing either diabetes/cardiovascular symptoms, were offered weekly telephone-based health coaching for 12-weeks, providing education, motivations, self-efficacy skills, and referral services, for AD behavioral risk factors. A patient preference health coaching behavioral change strategy was implemented, where the person decides which behavioral practices to target. All study subjects completed a behavioral-psychosocial baseline and 3-month follow-up assessment. Findings: The study demonstrated feasibility for implementing health coaching within low-income racially-diverse older adults. The study sample (n=20, mean age 69 years (range: 61-77 years) was 90% African American (n=18), and 55% males (n=11). Improvement in AD knowledge (F=4.19;p=.0565); cognitive functioning (memory (F=4.19;p=.0556); delayed memory (F=2.85;p=.1086); TrailsA (F=5.60;p=.0294)), alcohol-risk (F=3.33;p=.1108) and social isolation (F=4.11;p=.0569) trends were found at 3-month follow-up. Conclusions: The findings from this study exhibit positive trends in reducing AD risk. This study creates the impetus for future large-scale investigations and dissemination of findings to improve the lives of at-risk low-income aging adults.

### ADDRESSING GUN SAFETY AND DEMENTIA: TRAINING PROFESSIONALS IN THE COMMUNITY

Silvia Orsulic-Jeras,[1] Sara Powers,[1] Jessica Empeno,[2] Regina Lagasca,[3] Amy Abrams,[3] Sarina Barker,[3] Rebecca McDaniel,[3] and Marcie Hanna,[3] *1. Benjamin Rose Institute on Aging, Cleveland, Ohio, United States, 2. Lightbridge Hospice & Palliative Care, San Diego, California, United States, 3. Alzheimer's San Diego, San Diego, California, United States*

As the number of Alzheimer's disease and related dementia (ADRD) cases grow, first responders will have more frequent interactions with individuals living with ADRD within the community. Recognizing the lack of dementia training among first responders and through the support of an Administration for Community Living grant, Alzheimer's San Diego partnered with local law enforcement to support and educate first responders on local ADRD resources, referral systems for families and caregivers, and disease-related information, in hopes of building stronger dementia capable services within San Diego County. To date, 249 first responders have participated in dementia education trainings and completed the related evaluations that aimed to understand their experiences with and knowledge of dementia. On average, participants were 44.74 years old (SD=18.45) and served 8.72 years (SD=7.99) on their job. Majority were male (n=189), had some college education (n=94), and identified as White/Caucasian (n=105) or Hispanic/Latino (n=92). Although objective knowledge (as measured by a shortened version of the Alzheimer's Disease Knowledge Scale [ADKS]) did not significantly differ from pre- to post-test, participants' perceived subjective knowledge about dementia significantly increased from pre-test (M=1.17, SD=0.77) to post-test (M=1.89, SD=0.66); t(141)=10.56, p<.001. Encouragingly, participants who reported receiving prior training on dementia had significantly higher ADKS scores at both pre- and post-test compared to those who did not have prior training. Results highlight the need for ongoing dementia-related training for law enforcement and the importance of evaluation requirements. Discussion will focus on policy implications and how communities can support dementia capability among first responders.

### ASSESSING ALZHEIMER'S DISEASE SPECIFIC BARRIERS TO ENROLLMENT IN AN OBSERVATIONAL ACTIGRAPHY STUDY

Genna Losinski,[1] Alex Laffer,[1] Hilary Hicks,[2] and Amber Watts,[1] *1. University of Kansas, Lawrence, Kansas, United States, 2. The University of Kansas, Lawrence, Kansas, United States*

There are unique challenges to recruit and enroll individuals with Alzheimer's disease (AD) into research studies, and typical barriers to participation include the need for study partner involvement, use of invasive procedures (e.g., lumbar punctures), and lack of awareness of ongoing research. Failure to enroll this population impacts generalizability and external validity of results. The current study sought to explore reasons for non-participation in individuals with AD enrolled in the University of Kansas Alzheimer's Disease Center (ADC) Registry. Participants were approached at their annual registry visit and asked to participate in an observational sub-study that utilized wrist-worn actigraphy to measure physical activity and sleep over a one-week period. Of the thirty-six non-participation encounters that were recorded over a 2.5 year data collection period, 28% were never recruited due to appointment cancellation, rescheduling, or no-show. Of the remaining encounters, the three most common reasons for non-participation included: physical limitations of individuals with AD (15%), unknown

(28%), and study partner concerns regarding use of technology in individuals with impaired cognition due to AD (25%). Multiple study partners were concerned that the individual with AD would lose the watch, remove the watch from the wrist, or become irritated while wearing it. Findings suggest that the use of technology such as actigraphy presents an additional barrier to enrollment that is unique to individuals with AD. Future studies should consider potential interventions to address study partner concerns regarding use of technology in individuals with AD.

### ASSOCIATIONS BETWEEN SOCIAL ENGAGEMENT AND PHYSICAL ACTIVITY AMONG COMMUNITY-DWELLING OLDER ADULTS WITH DEMENTIA

Aya Yoshikawa, Matthew Smith, and Marcia Ory, *Texas A&M University, College Station, Texas, United States*

People with dementia (PWD) tend to experience loneliness and physical inactivity, which increases their risk of negative health outcomes such as depression and declined physical functioning. Research indicates the link between supportive social environments and physical activity (PA), yet this association is not fully studied among PWD. This cross-sectional study investigated associations between social engagement and moderate (i.e. walking for exercise) and vigorous PA among PWD. A subset sample of community-dwelling Medicare beneficiaries in the 2015 National Health and Aging Trends Study, representing PWD including possible (n = 779) and probable (n = 902) dementia, were analyzed using survey-weighted logistic regression analysis. All analyses were controlled for sociodemographic characteristics, living arrangement, physical functioning, and the number of chronic conditions. The majority of participants was 80 years or older (52.5%), female (53.1%), White (67.4%), had a high school or higher education (64.0%), and two or more chronic conditions (74.1%). Living alone (OR = 1.60, 95%CI 1.22, 2.10), knowing their community well (OR = 1.56, 95%CI 1.12, 2.16), and going out for enjoyment (OR = 1.93, 95%CI 1.37, 2.73) were associated with moderate PA. In addition to going out for enjoyment (OR = 2.40, 95%CI 1.65, 3.53), attending organized activities (OR = 1.97, 95%CI 1.15, 3.39) and working as a volunteer (OR = 1.63, 95%CI 1.04, 2.57) were associated with vigorous PA. Findings suggest that enjoyable activities and community familiarity may facilitate walking behavior among PWD. Integrating events and volunteer activities within communities is encouraged for supporting active living among PWD.

### BEST PRACTICES FOR INCLUDING INDIVIDUALS WITH DEMENTIA AS RESEARCH PARTICIPANTS

Katherine Judge, Morgan Minyo, Amanda MacNeil, Claire Grant, and Kaitlyn Lucas, *Cleveland State University, Cleveland, Ohio, United States*

Including individuals with dementia as participants in research studies can be a challenge given the cognitive symptoms experienced, including difficulties with attention, memory, language, and executive functioning and behavioral symptoms, such as agitation and frustration. Researchers, however, have begun to include individuals with dementia as more active participants in the research process. Specifically, individuals with dementia have provided self-report information about their illness experience, including how they cope and manage with their illness and how illness-related strains are related to psychosocial outcomes. Researchers also have starting using self-report data collection protocols instead of proxy-report measures to assess the impact of non-pharmacological interventions developed for individuals with dementia on well-being outcomes such as depressive symptoms, unmet needs, and relationship strain. To date, however, there are no clear guidelines or 'best practices' to assist researchers in developing study protocols that facilitate the inclusion and participation of individuals with dementia. This poster will present findings from a systematic literature review of studies that have successfully included individuals with dementia as research participants. Key areas examined include: 1) the focus and type of research question studied; 2) the use and type of cognitive screening tools for determining study eligibility; 3) implementation of qualitative versus quantitative research designs; 4) objective versus subjective measures; 5) data collection tools; 6) psychometric evidence of data obtained; and 7) how data have been analyzed. Findings highlight the importance of collecting data directly from individuals with dementia to further understand the illness experience and assess the impact of non-pharmacological interventions.

### DEMENTIA-RELATED ANXIETY AND GENERAL ILLNESS ANXIETY DIFFER BASED ON FAMILIAL EXPOSURE TO PERSONS WITH DEMENTIA

Erika Fenstermacher,[1] Alexandria Ebert,[2] Natalie Shook,[3] Jerin Lee,[3] Jenna Wilson,[2] and Ilana Haliwa,[2]
*1. West Virginia University, Pursglove, West Virginia, United States, 2. West Virginia University, Morgantown, West Virginia, United States, 3. University of Connecticut, Storrs, Connecticut, United States*

Dementia-related anxiety is a specific form of illness anxiety that has been associated with familial exposure to persons with dementia (FMwDs). However, it is unknown whether FMwDs is specifically associated with dementia-related anxiety or whether it is also related to general illness anxiety, which has broader health implications. Furthermore, the level of exposure to family members with dementia may matter. Thus, we examined whether level of familial exposure to dementia was related to general illness anxiety and dementia-related anxiety. Participants (N = 401) aged 18-76 years (M = 39) recruited through Amazon's Mechanical Turk completed an online survey. Dementia exposure was split into three levels: (1) not knowing a friend/family member with dementia (55.2%); (2) knowing a family member with dementia (33.9%); and (3) providing care for a family member with dementia (10.9%). Familial exposure to dementia was related to both general illness anxiety and dementia-related anxiety. Participants who provided care for FMwDs had significantly higher levels of illness anxiety than both people who had a FMwD and people who did not ($ps < .001$). Similarly, participants who provided care for FMwDs had significantly higher levels of dementia-related anxiety than participants who did not have a FMwD ($p < .01$). Caregivers play a critical role in the quality of life of those with dementia, however it is clear that the potential psychological impact of such work is pervasive. This study provides a foundation to explore differences between illness anxiety and dementia worry, and examine interventions to reduce anxiety among caregivers.

### DEVELOPMENT AND PSYCHOMETRIC EXAMINATION OF A SHORT-FORM DEMENTIA ATTITUDES SCALE

Alexandria Ebert,[1] Jenessa Steele,[2] and Julie Hicks Patrick,[1]
*1. West Virginia University, Morgantown, West Virginia, United States, 2. Radford University, Radford, Virginia, United States*

With the increase in research related to people directly and indirectly affected by Alzheimer's Dementia and related disorders (ADRD), there is a need for short, psychometrically-sound instruments to assess the variety of influences on the quality of life for the person with dementia (pwd) and their care partners. We sought to develop and assess a short version of the Dementia Attitudes Scale (DAS; O'Connor & McFadden, 2010) for use in such endeavors. Using a sample of 321 adults (Mage = 39.7, range 20 to 70 yrs; 47% female), we surveyed a host of individual characteristics (e.g., age, personality, experience with pwd) and several current measures of attitudes about pwd. Ten days later, 162 of these adults completed a retest of the DAS. Results of a confirmatory factor analysis supported the 2-factor solution reported by O'Connor and McFadden. Internal consistency was acceptable for the Comfort factor (Time 1alpha = .83; T2 alpha = .82) and for the Knowledge factor (Time 1 alpha = .86; Time 2 alpha = .87). These estimates are similar to those in the original report. Due to limitations of coefficient alpha we also used structural equations to examine the regression weights for each item as a function of chronological age and experience. These analyses showed that the factor structure was robust when considering these individual characteristics. Finally, we compared different scale lengths using a series of ROC curves. We discuss our results in the context of providing brief and psychometrically-sound measures for use in large survey studies.

### DISCORDANCE BETWEEN PATIENT AND CAREGIVER ON RATINGS OF PURPOSE IN LIFE FOLLOWING A DEMENTIA DIAGNOSTIC APPOINTMENT

Matthew Wynn,[1] Catherine Ju,[1] and Patrick Hill,[2]
*1. Washington University in St. Louis, St. Louis, Missouri, United States, 2. Washington University in St. Louis, Saint Louis, Missouri, United States*

Purpose in life has been linked with better well-being and reduced risk for major illness. As such, work has focused on understanding what leads to changes in sense of purpose during adulthood, with a focus on major life events. Receiving a dementia diagnosis is a major life event that could affect purpose in life both for older adults with dementia and their potential caregivers. To examine this issue participants answered questions at two timepoints, before their diagnostic appointment at a specialized memory clinic, and between two days and two weeks after the appointment. Participants provided self-report ratings of sense of purpose and as well as open-ended answers regarding their purpose in life. Data is available from both caregivers and patients and qualitative coding was performed on participants' open-ended responses. Our analysis revealed discordance between patients and caregivers, such that caregivers' ratings of patients were lower in terms of purpose in life ($t = -5.63$, $p < .001$) and being in worse health ($t = -3.41$, $p < .001$) than patients' ratings of themselves on the same measures. Discordance between caregiver and patient in the context of a dementia diagnostic appointment and outcomes associated with this discordance are discussed.

### DOES ANXIETY AFFECT THE CLOCK-DRAWING TASK IN PATIENTS IN A MEMORY CLINIC? A CLINICAL CORRELATION STUDY

Hamed Khachan, Mahak Kanjolia, and Anil Nair,
*Alzheimer's Disease Center, Quincy, Massachusetts, United States*

Background: It is unknown if anxiety levels affect performance on clock drawing test (CDT) in memory clinic patients. Method: We performed a retrospective analysis of memory clinic patients in the south shore of Boston from 2010 to 2019. We correlated anxiety screen data (GAD7) to CDT scores, based on contour, numbers, and hands placement. Univariate analyses used Spearman correlation. A multivariate regression model analzed GAD7 to covariates of CDT, age, sex, and race. Hypothesis : We hypothesized a positive correlation between anxiety levels scored by the GAD7 and CDT. Results: 994 patients in the memory clinic between 2010-2020 had analyzable data. Patients were 58.6% female, 84.6 % White. Mean age was 70.1±14.4, CDT 1.84±1.04. CDT score correlated significantly to race ($\rho$=-0.16, p< 0.001), age ($\rho$=-0.28, p<0.001), gender ($\rho$=0.05, p=0.16), but not GAD7 ($\rho$=0.05, p=0.27). Multivariate model confirmed the lack of association of anxiety scores to CDT (□= 0.08, p=0.78). GAD7 scores correlated to female gender (□= -1.16, p=0.04). Conclusions: CDT scores were not affected by anxiety as measured on GAD7 scores. However, a positive correlation was shown on anxiety scores in females to CDT completion.

### IS IMMEDIATE RECALL TEST SCORES AFFECTED BY ANXIETY IN A MEMORY CLINIC POPULATION? A CLINICAL CORRELATION STUDY.

Hamed Khachan, Anil Nair, Fioralba Andrea, and Mahak Kanjolia, *Alzheimer's Disease Center, Quincy, Massachusetts, United States*

Background: It is unknown if anxiety affects performance on immediate recall testing (IR) in memory clinic patients. Method: We performed a retrospective analysis of memory clinic patients in the south shore of Boston from 2010 to 2019. We correlated anxiety screen data (GAD7) to IR scores. Univariate analyses used Spearman correlation. A multivariate regression model analyzed GAD7 to covariates of IR, age, sex, and race. Hypothesis: We hypothesized a positive correlation between anxiety levels scored by GAD7 and IR. Results: 994 patients in the memory clinic between 2010-2020 had analyzable data. Patients were 58.6% female, 84.6 % White. The mean age was 70.1±14.4, IR 6.62 ± 5.4, GAD7 5.5±5.71. On univariate analysis, IR correlated significantly to age ($\rho$ = 0.08, p = 0.01), gender ($\rho$ = 0.06, p = 0.046), and race ($\rho$= - 0.25, p <0.001), but not to GAD7 ($\rho$=-0.07, p=0.14). The multivariate model confirmed the lack of association of anxiety scores to (□=-0.05, p=0.41) to GAD7 scores. IR task performance was significantly associated only to age (□= -0.04, p=0.03) and gender (□= -1.18, p=0.04) in the regression model. Conclusions: Immediate recall task performance was not significantly affected by anxiety measured by GAD7 scores in a memory clinic population. However, a negative correlation was shown on immediate recall scores in males and older subjects.

### MODERATING EFFECT OF COGNITIVE RESERVE ON BRAIN INTEGRITY AND COGNITIVE PERFORMANCE

Monica Nelson,[1] Ross Andel,[1] Julie Martinkova,[2] Kateřina Čechová,[2] Hana Marková,[2] and Jakub Hort,[2]
*1. University of South Florida, Tampa, Florida, United States, 2. Motol University Hospital, Prague, Czech Republic*

Dementia is arguably the most devastating condition of older adulthood with treatment options still elusive. Alzheimer's is the most prevalent form of dementia where cognitive deficits relate strongly to underlying brain pathology. However, there exist cases in which cognitive performance does not match the corresponding level of neuropathology. Attempts to explain this phenomenon often include the concept of cognitive reserve (CR), whereby greater CR (e.g., more education or higher occupational position) presumably results in less impairment relative to the extent of pathology early in disease progression but also greater impairment once cognitive symptoms manifest. We examined the influence of CR proxy variables (education and occupation) on the relationship between hippocampal volume and cognitive performance on tests of executive control and memory using data from the Czech Brain Aging Study (CBAS). Participants were cognitively normal/with subjective cognitive decline but without actual impairment (CN; n=115; M(age)=66.43; M(education)=15.90; 37 men) or had amnestic mild cognitive impairment (aMCI; n=165; M(age)=71.37; M(education)=14.92; 85 men). We found that hippocampal volume was significantly related to executive control (b=-.0001, p=.03) and memory (b=.0002, p<.001) for participants with aMCI, but only memory (b=.0002, p=.03) for CN participants. Occupational position moderated the association between memory and hippocampal volume in aMCI, with the result approaching significance (p=.07), whereby a greater link between memory problems and hippocampal atrophy was present in those previously in high occupational positions. No other moderations for occupational position or education emerged (ps>.25). We found evidence for the concept of CR using occupational position as proxy.

### MUSIC AND MEMORY IN DEMENTIA CARE: COMFORT WITH HARMONY

Deepa Vinoo, *NYC Health+Hospitals/Coler, Roosevelt Island, New York, United States*

Data show a substantial increase in the number of people with diagnosis of dementia nationally and globally. Behavioral disturbances among persons with dementia including agitation and psychosis form a constellation of symptoms referred to as behavioral and psychological symptoms of dementia (BPSD).BPSD impacts heavily on resident's quality of life, caregivers stress and management options for the team. FDA do not approve usage of psychotropic for Dementia related Behaviors.This study was conducted in six memory care units ,N-150.Prior to the project Psychotropic usage was 64% ,physical altercations, fallsand staff been out due to work related injury were high in those units. In 2014 a memory care team came together , identified gaps and created structured memory care units with memory care programs which includes , consistent trained staffing, meaningful engagement of residents, psychotropic stewardship program , shift from medical model to palliative model of care,person centered approach, music& memory program, shared governance, staff empowerment. All residents in those units were assessed for Psychotropic usage, falls, pain management. transfer to acute hospitals and 1;1 from 2014 to 2019. Results, Usage of Music& Memory increased from 4% 75%, Anti psychotic usage reduced by 18%, Falls reduced by 12%, Physical altercation decreased from 12 to 0, Improved pain Management .Significant reduction in transfer to acute hospitals. Reduced 1;1 from 6 to 1.Staff verbalized increased satisfaction and decreased stress. Improved family involvement. Increased bonding between residents, staff and families.Decresed work related injury while giving care.

### SOCIAL ENGAGEMENT AND COGNITIVE HEALTH: THE MEDIATING ROLE OF COGNITIVE AND PHYSICAL ACTIVITY

Takashi Amano,[1] Nancy Morrow-Howell,[2] Sojung Park,[2] and Brian Carpenter,[2] *1. Rutgers University-Newark, Newark, New Jersey, United States, 2. Washington University in St. Louis, St. Louis, Missouri, United States*

This study aimed to assess the association between social engagement and conversion from Cognitive Impairment No Dementia (CIND) to dementia and to investigate the mediating role of cognitive and physical engagements on that relationship. Data from two waves (2010 and 2014) of the psychosocial and core modules of the Health and Retirement Study (HRS) were used. The sample consisted of 929 people who had CIND in 2010 and participated in the survey in 2014. Latent Class Analysis (LCA) with eight indicators of social engagement (activities with children, volunteering with youth/others, attending educational course/organization, meeting up, speaking on the phone, writing or emailing) found three groups: formal and informal social engagement (20.7%), informal social engagement only (48.9%), and low social engagement (30.5%). Binary logistic regression analysis showed sub groups with higher levels and greater variety of social engagements were associated with lower probability of conversion to dementia in four years. Path analysis with structural equation modeling (SEM) framework showed the relationship between patterns of social engagement and lower conversion to dementia was mediated by having higher engagement in cognitive activities (e.g. home maintenance, playing sports), but not by engagement in physical activities (e.g. playing games, using computer). Results from this study implied (1) promoting active social engagement may be a promising intervention to prevent or delay conversion from CIND to dementia, and (2) promoting social engagement may be a particularly effective and efficient strategy since it promotes other activity engagements that may itself prevent or delay conversion from CIND to dementia.

### STRESS MEDIATING GENES IN AGING, HEALTH, AND LONGEVITY TRAITS: EFFECTS OF MULTIPLE INTERACTIONS

Anatoliy Yashin,[1] Dequing Wu,[2] Konstantin Arbeev,[1] Arseniy Yashkin,[1] Galina Gorbunova,[1] Igor Akushevich,[1] Matt Duan,[3] and Svetlana Ukraintseva,[1] *1. Duke University, Durham, North Carolina, United States, 2. Duke University, Durham, United States, 3. Duke.University, Durham, North Carolina, United States*

Persistent stress of external or internal origin accelerates aging, increases risk of aging related health disorders, and shortens lifespan. Stressors activate stress response genes, and their products collectively influence traits. The variability of stressors and responses to them contribute to trait heterogeneity, which may cause the failure of clinical trials for drug candidates. The objectives of this paper are: to address the heterogeneity issue; to evaluate collective interaction effects of genetic factors on Alzheimer's disease (AD) and longevity using HRS data; to identify differences and similarities in patterns of genetic interactions within two genders; and to compare AD related genetic interaction patterns in HRS and LOADFS data. To reach these objectives we: selected candidate genes from stress related pathways affecting AD/longevity; implemented logistic regression model with interaction term to evaluate effects of SNP-pairs on these traits for males and females; constructed the novel interaction polygenic risk scores for SNPs, which showed strong interaction potential, and evaluated effects of these scores on AD/longevity; and compared patterns of genetic interactions within the two genders and within two datasets. We found there were many genes involved in highly significant interactions that were the same and that were different within the two genders. The effects of interaction polygenic risk scores on AD were strong and highly statistically significant. These conclusions were confirmed in analyses of interaction effects on longevity trait using HRS data. Comparison of HRS to LOADFS data showed that many genes had strong interaction effects on AD in both data sets.

### THE EFFECTS OF DEMENTIA KNOWLEDGE ON DEMENTIA WORRY, ATTITUDES, SOCIAL COMFORT, AND AFFECT

Alexandria Ebert, Maya Huggins, Hannah Wagner, Zachary Lightcap, Tessla Muir, Mariah Faulkenberry, and Amy Fiske, *West Virginia University, Morgantown, West Virginia, United States*

Previous work has demonstrated that personhood-based knowledge of dementia is associated with less fear of dementia and greater social comfort with persons with dementia. Nonetheless, a causal link has not been tested. We examined the effects of types of dementia knowledge on dementia worry, fear, and social comfort, as well as affect more broadly and attitudes toward dementia, which have not previously been examined. Participants (N = 338) aged 19-78 (M = 44.82, SD = 16.66) were randomized into one of five experimental conditions: biomedical-knowledge (BK; read biological and medical facts about dementia); personhood-based knowledge (PBK; read accounts written by persons living with dementia); both BK and PBK; baseline control; and active control. Participants then completed outcome measures. Significant effects of knowledge on dementia worry ($p < .05$) as well as personal distress, empathic concern, and negative affect ($ps < .001$) emerged; groups did not significantly differ in attitudes, comfort, or dementia fear. Specifically, participants in the BK and PBK conditions exhibited significantly higher levels of personal distress than those in the control conditions. Similarly, participants in the PBK condition had significantly higher levels of negative affect than those in the control conditions and significantly higher levels of dementia worry than those in the baseline control condition. Participants in the PBK condition also had significantly higher levels of empathic concern than those in the biomedical knowledge and control conditions. Results suggest that although reading about dementia induces negative affect, it also induces empathic concern.

### TRAJECTORIES OF PURPOSE IN LIFE FOLLOWING A DEMENTIA DIAGNOSTIC APPOINTMENT

Matthew Wynn, Catherine Ju, and Patrick Hill, *Washington University in St. Louis, St. Louis, Missouri, United States*

Purpose in life has been linked with better well-being and reduced risk for major illness. As such, work has focused on understanding what leads to changes in sense of purpose during adulthood, with a focus on major life events. Receiving a dementia diagnosis is a major life event that could affect purpose in life both for older adults with dementia and their potential caregivers. To examine this issue participants answered questions at two timepoints, before their diagnostic appointment at a specialized memory clinic, and between two days and two weeks after the appointment. Participants provided self-report ratings of sense of purpose and as well as open-ended answers regarding their purpose in life. Data is available from both caregivers and patients and qualitative coding was performed on participants' open-ended responses. While there was no significant, mean-level change in purpose in life for patients ($t = -.14$, $p = .88$) or caregivers ($t = .73$, $p = .46$), some participants exhibited reliable change in sense of purpose. Factors underlying individual increases and decreases in sense of purpose following a dementia diagnostic appointment are explored and discussed.

### UNDERSTANDING ALZHEIMER'S DISEASE KNOWLEDGE IN LOW-INCOME, RACIALLY DIVERSE OLDER ADULTS

Faika Zanjani, Taylor Wilkerson, Annie Rhodes, Jennifer Inker, and Joann Richardson, *Virginia Commonwealth University, Richmond, Virginia, United States*

Individuals demonstrate varying levels of Alzheimer's disease (AD) knowledge, as well as commonly held misconceptions about the risk factors and nature of the disease. Older adults often demonstrate low scores on AD knowledge scales and African Americans are often specifically not aware of their higher AD risk status compared to other racial groups. We measured the Alzheimer's knowledge in 60+ community-dwelling adults, as part of a larger study on AD health coaching. Participants (n=20) were recruited from low-income communities within the Richmond, Virginia area. The study sample was 85% African American (n=17) and 55% male (n=11). Participants completed a behavioral psychosocial test battery, including the Alzheimer's Disease

Knowledge Scale. Similar to previous research, this sample of older adults held common misconceptions about AD, including the ideas that mental exercise can prevent AD (80% answered incorrectly) and individuals with AD are incapable of making decisions about their care (70% answered incorrectly). In this sample, the majority of African American older adults were aware of the fact that they have the highest risk for developing AD (20% answered incorrectly) compared to other racial groups. Analyses found no significant relationship between AD knowledge and health outcomes, alcohol consumption, or education. In conclusion to reduce AD risk, addressing AD knowledge in minority low-income population is important and needed. This is especially relevant since African American older adults are more likely to live in communities rather than nursing or assisted living facilities, receiving less access to interventions and research innovation.

### USES OF REMINISCENCE IN DEMENTIA CARE

Abdallah Abu Khait and Juliette Shellman, *University of Connecticut, Storrs, Connecticut, United States*

Improving the quality of life for older adults diagnosed with dementia is a public health research priority. Reminiscence is one non-pharmacological technique used to address the behavioral and psychosocial problems associated with dementia. The uses of reminiscence in dementia care have not yet been integrated, synthesized, critically analyzed, or delineated in the literature. Whittemore and Knafl's five-step method provided the framework for this integrative review. A comprehensive search of PubMed, CINAHL Plus, SCOPUS, and PsycINFO was undertaken. Articles published in English, focused on participants with a diagnosis of mild to moderate dementia, and with evidence of using reminiscence in dementia care were included. Twenty-six studies published between 2009 and 2019 that met the inclusion criteria were analyzed. Four themes emerged from the integrated findings: (a) recovery from the darkness of depressive symptoms; (b) enhancement of cognitive functions and filling the memory gap; (c) living a fulfilling life in late adulthood, and (d) fulfilling reminiscence functions. The critical appraisal process revealed mixed effectiveness of the use reminiscence on health outcomes in dementia care due to diverse types of reminiscence, different outcomes measures, different data collection toolkits, and a lack of a standardized reminiscence protocol among research studies. Results from this review provide a better understanding of the potential benefits of using reminiscence in dementia care. However, improving the methodological rigor of future studies is necessary to attain conclusive evidence of the effectiveness of using reminiscence in dementia care. Implications of these findings for gerontological education, practice, and research will be presented.

### SESSION 2931 (PAPER)

### MOBILITY DISABILITY I

### ASSOCIATION OF AFFECT AND GAIT PERFORMANCE IN DUAL-TASK WALKING IN NON-DEMENTED OLDER ADULTS

Deepan Guharajan, and Roee Holtzer, *Yeshiva University, Bronx, New York, United States*

Aging populations are at increased risk to experience mobility disability, which is associated with falls, frailty, and mortality. Previous studies have not examined the concurrent associations of both positive and negative affect with gait velocity. We examined whether individual differences in positive and negative affect predicted dual-task performance decrements in velocity in a dual-task (DT) paradigm in non-demented older adults. We hypothesize that positive affect would be associated with lower DT costs, and negative affect would be associated with higher DT costs. Participants (N = 403; mean age, = 76.22 (6.55); females = 56%) completed the Positive and Negative Affect Schedule (PANAS) and a DT paradigm that involved three task conditions: Single-Task-Walk (STW), Alpha (cognitive interference requiring participants to recite alternate letters of the alphabet), and Dual-Task-Walk (DTW) requiring participant to perform the two single tasks concurrently. Gait velocity was assessed via an instrumented walkway. As expected, results of a linear mixed effects model (LME) showed a significant decline in gait velocity (cm/s) from STW to DTW (estimate = -11.79; 95%CI = -12.82 to -10.77). LME results further revealed that negative affect was associated with greater decline in gait velocity from STW to DTW (ie., worse DT cost) (estimate = -0.38; 95%CI = -0.73 to -0.03). Positive affect did not, however, predict DT costs in gait velocity (estimate = -0.09; 95%CI = -0.23 to 0.05). These findings suggest that increased negative affect interferes with the allocation of attentional resources to competing task demands inherent in the DT paradigm.

### COMBINING TDCS WITH A MOTOR-COGNITIVE TASK TO REDUCE THE NEGATIVE IMPACT OF DUAL-TASKING ON THE GAIT OF OLDER ADULTS

Jeffrey Hausdorff,[1] Nofar Schneider,[2] Marina Brozgol,[1] Pablo Cornejo Thumm,[3] Nir Giladi,[1] Rachel Katz,[1] Anat Mirelman,[1] and Brad Manor[3] *1. Tel Aviv Sourasky Medical Center, Tel Aviv, Tel Aviv, Israel, 2. Tel Aviv University, Tel Aviv, Israel, 3. Hebrew SeniorLife/Harvard Medical School, Boston, Massachusetts, United States*

The simultaneous performance of a secondary task while walking (i.e., dual tasking) increases motor-cognitive interference and fall risk in older adults. Combining transcranial direct current stimulation (tDCS) with the concurrent performance of a task that putatively involves the same brain networks targeted by the tDCS may reduce the negative impact of dual-tasking on walking. We examined whether tDCS applied while walking reduces the dual-task costs to gait and whether this combination is better than tDCS alone or walking alone (with sham stimulation). In 25 healthy older adults (aged 75.7±10.5yrs), a double-blind, within-subject, cross-over pilot study evaluated the acute after-effects of 20 minutes of tDCS targeting the primary motor cortex and the dorsal lateral pre frontal cortex during three separate sessions:1) tDCS while walking on a treadmill in a virtual-reality environment (tDCS+walking), 2) tDCS while seated (tDCS+seated), and 3) walking in the virtual-reality environment with sham tDCS (sham+walking). The complex walking condition taxed motor and cognitive abilities. During each session, single- and dual-task walking and cognitive function were assessed before and immediately after stimulation. Compared to pre-tDCS performance, tDCS+walking reduced the dual-task cost to gait speed (p=0.004) and other gait features (e.g., variability p=0.02), and improved (p<0.001) executive function (Stroop interference score). tDCS+seated and sham+walking did not affect the dual-task cost to gait speed (p>0.17). These initial findings demonstrate that tDCS delivered during challenging walking ameliorates dual-task gait and executive function in older adults, suggesting that the concurrent performance of related tasks enhances the efficacy of the neural stimulation and mobility.

### EFFECTS OF FNIRS PROCESSING METHODS ON PREFRONTAL HEMODYNAMICS DURING SINGLE AND DUAL TASK WALKING IN OLDER ADULTS

Meltem Izzetoglu,[1] and Roee Holtzer,[2] *1. Villanova University, Villanova, Pennsylvania, United States, 2. Yeshiva University, Bronx, New York, United States*

functional near infrared spectroscopy (fNIRS) has been increasingly used to assess changes in the hemodynamic response during active walking in aging and disease populations. Key findings revealed that HbO2 in the prefrontal cortex (PFC) increased from single-task-walk (STW) to dual-task-walk (DTW) due to the greater cognitive demands inherent in the latter condition. However, previous studies utilized a limited and inconsistent number of algorithms and filters to remove artifacts from fNIRS-derived brain activation data. Critically, there is no gold standard for artifact removal at the present time, which reduces replicability and generalizability. To address this critical limitation, we have reanalyzed a large dataset of older adults (n=83) who underwent our walking protocol by using different hemodynamic conversion parameters (molar extinction coefficients and age and wavelength dependent differential pathlength factors) and applying different filters having various cut-off frequencies for artifact removal. On the extracted hemodynamic responses, namely oxygenated-hemoglobin (HbO2) and deoxygenated-hemoglobin (Hb), linear mixed effect model results indicated that task effects showed similar significant increases in HbO2 from STW to DTW (range of effect sizes was 0.59 to 0.64) and as well as the expected decline in Hb from STW to DTW (range of effect sizes was 0.18 to 0.32) irrespective of the methods used. In addition, the intraclass correlations suggested excellent reliability across methods (HbO2 range = 0.982 to 0.996; Hb range = 0.883 to 0.984). In conclusion, these findings provide strong support to previously published articles but also highlight the need to establish a gold standard for fNIRS processing.

### NEURAL INEFFICIENCY DURING DUAL-TASK WALKING IN OLDER ADULTS: THE MODERATING EFFECT OF BRAIN MORPHOLOGY

Daliah Ross,[1] Mark Wagshul,[2] Meltem Izzetoglu,[3] and Roee Holtzer,[1] *1. Yeshiva University, Bronx, New York, United States, 2. Albert Einstein College of Medicine, Bronx, New York, United States, 3. Villanova University, Villanova, Pennsylvania, United States*

Aging is accompanied by changes in cognitive, physical, and brain function. Research has found increased activation in the prefrontal cortex (PFC) in dual-task-walk (DTW) as compared to single-task walk (STW) due to increased cognitive demands in the former condition. Older adults have more difficulty with DTW than younger adults, and difficulty with DTW has been found to predict functional decline in aging. As the population gets older, it is important to identify biomarkers predictive of functional decline. The neural inefficiency hypothesis posits that some people, including older adults, utilize more neural resources than others without showing benefits to behavioral performance. We examined the moderating effect of cortical thickness on PFC activation from STW to DTW. We predicted that less cortical thickness would be associated with increased PFC activation but without benefit to behavioral performance, to compensate for limited neural resources. Participants were 55 right-handed community-residing healthy older adults (mean age = 74.84 + 4.97; %female = 49.1). Cortical thickness was measured via MRI. Activation in the PFC was measured via functional near-infrared spectroscopy-derived (fNIRS) oxygenated hemoglobin (HbO2) levels. Analyses controlled for behavioral performance. Linear-mixed-effects models revealed that cortical thickness in several brain regions significantly moderated the increase in HbO2 from STW to DTW, p < 0.01. Thinner cortex, in regions implicated in walking and cognition, was associated with a greater increase in HbO2 levels. Supporting the neural inefficiency hypothesis, results suggest that older adults with greater cortical thinning over-activate the PFC during attention-demanding walking.

### THE EFFECTS OF PAIN ON BRAIN AND BEHAVIOR DURING COGNITIVE AND MOTOR PERFORMANCES IN OLDER ADULTS

Hannah Pakray,[1] Elizabeth Seng,[1] Meltem Izzetoglu,[2] and Roee Holtzer[1], *1. Yeshiva University, Bronx, New York, United States, 2. Villanova University, Villanova, Pennsylvania, United States*

Pain is prevalent and associated with adverse outcomes in older adults. The moderating effect of pain on cortical control of locomotion has not been assessed. This study examined the effects of subjective pain on changes in fNIRS-derived oxygenated hemoglobin (HbO2), gait velocity, and cognitive accuracy from single to dual-task walking conditions among older adults. Participants (n=383; 55% female; mean age=76) were community-residing older adults. Participants completed two single tasks [Single-Task-Walk (STW) and Cognitive Interference (Alpha)] and the Dual-Task-Walk (DTW), during which participants performed the two single tasks simultaneously. The MOS- PSS and PES were used to assess pain severity and interference. Gait velocity was assessed with an electronic gait mat, cognitive accuracy was measured using the rate of correct letter generation. Linear mixed effects models revealed that HbO2 increased in DTW compared to STW (p < .001) and Alpha (p < .05). The presence of perceived pain was associated with an attenuated increase in HbO2 from Alpha to DTW (p < .05). Amongst those with pain, worse pain severity was associated with an attenuated increase in HbO2 from STW to DTW (p < .001). Pain interference did not moderate the increase in HbO2 from single to dual tasks. Pain did not have a moderating effect on behavioral outcomes. These results indicate that task-related changes in the hemodynamic response in the prefrontal cortex during walking may be a sensitive marker to the effects of subjective pain on brain function in older adults.

### SESSION 2932 (PAPER)

### MOBILITY DISABILITY II

### FUNCTIONAL MEASURES ARE SEVERELY UNDER-CAPTURED IN ELECTRONIC HEALTH RECORDS

Nicholas Schiltz, Megan Foradori, Andrew Reimer, and Mary Dolansky, *Case Western Reserve University, Cleveland, Ohio, United States*

Electronic health records (EHR) data are increasingly used to inform clinical care decisions, assess quality of care, and identify patients at high-risk of poor outcomes (e.g. re-admission). Functional measures—including mobility and the ability to perform activities of daily living (ADLs)—are key indicators associated with health-related quality of life and chronic disease management in older adults. The goal of this analysis was to quantify the extent that measures of function are used in a national pool of structured EHR data. We used 2017-2019 data from IBM Watson Health Explorys, representing EHR data from 27 health systems and 360 hospitals nationwide (n=5,224,530 adults age 65 and older). Structured EHR data were mapped to SNOMED-CT codes that identifed six categories of function: mobility, fine motor, gross motor, large muscle, ADLs, and instrumental ADLs. Results indicated that only 3 of the 6 categories were used: ADLs (4.2% of study population), mobility (3.2%), and gross motor skills (2.4%). Fine motor, IADLs, and large muscle function were not recorded in any patients. These results indicate that functional measures appear to be under-reported in structured EHR data when compared to published estimates of the population prevalence. In conclusion, measures of function and mobility remain largely unused in structured EHR data, likely because this information is either not assessed, unavailable for inclusion, or is captured in a non-structured format (e.g. clinical notes). Comprehensive functional measures need to be added to EHRs to assess quality and improve delivery and outcomes in older adult patients.

### LIFE-SPACE IN A NATIONAL COHORT OF U.S. OLDER ADULTS: NORMATIVE DATA FOR THE UAB LIFE-SPACE ASSESSMENT

Alexander Lo,[1] Virginia Wadley,[2] Michael Crowe,[2] Cynthia Brown,[2] and Richard Kennedy,[2] *1. Northwestern University, Chicago, Illinois, United States, 2. University of Alabama at Birmingham, Birmingham, Alabama, United States*

The University of Alabama at Birmingham Life-Space Assessment (LSA) is a self-reported measure for assessing community mobility. Restricted mobility is correlated with a number of adverse health outcomes, including mortality, frailty, cognitive decline, and nursing home admissions. Thus, it is important for providers to understand how the LSA score of a patient compares to the general population. To facilitate such comparisons, we developed demographically adjusted norms for the LSA and its correlation with other functional measures. Norms were based on 15,390 participants age 45 and older in the National Institutes of Health-funded REasons for Geographic and Racial Differences in Stroke (REGARDS) study, a national, population-based, longitudinal study investigating the causes of excess stroke mortality among African Americans and individuals living in the Southeastern US stroke belt region. LSA scores declined from a median of 100 in the 45-54 age range to a median of 59.7 in the 85 and older age range, with higher median scores in males. LSA scores showed modest but significant positive correlations with SF-12 Physical Component and Mental Component, Center for Epidemiologic Studies Depression Scale, and Six Item Screener cognitive scores, as well as modest but significant negative correlations with AD8 Dementia Screening, Katz Activities of Daily Living, and Timed Walk scores. The LSA is a brief, easily administered measure that offers a valid method of assessing community mobility in the older adult population.

### LIFESTYLE ADJUSTMENT AND MOBILITY-RELATED GOAL SETTING AFTER DRIVING CESSATION WITH PEOPLE LIVING WITH DEMENTIA

Theresa Scott,[1] Jacki Liddle,[1] Elizabeth Beattie,[2] Louise Gustafsson,[3] Geoffrey Mitchell,[1] Donna Rooney,[4] and Nancy Pachana,[1] *1. The University of Queensland, St Lucia, Queensland, Australia, 2. Queensland University of Technology, Brisbane, Australia, 3. Griffith University, Brisbane, Queensland, Australia, 4. The University of Queensland, Brisbane, Queensland, Australia*

Community mobility is an important social determinant of health. For people living with dementia, the forfeiture of a driving licence can signal a loss of independence, limiting access to activities outside of the home. Loss of community connectivity and social participation has a substantial impact on quality of life and may lead to depression and more rapid cognitive decline. This study is focused on a driving cessation intervention that helps people with dementia identify personal goals that are framed around community mobility and adjusting to life without driving. Health professionals work with participants to translate these into specific, practical and achievable outcomes by program end. Participants may nominate more than one goal. This study reports on goal setting and achievement. Using a modified version of the Canadian Occupational Performance Measure it examines pre- to post-intervention achievement of, and satisfaction with, identified goals for 17 participants living with dementia aged 63-93 (M=75.24, 76% male) from regional and metropolitan Australia. Thematic analysis of clinical interviews and field notes highlighted the range of desired goals, and the challenges posed and problem-solving strategies used in setting realistic, non-driving goals. Significant positive improvements were found across a total of 29 goals for (i) performance $t(28) = -10.01$, $p < .000$, and (ii) satisfaction, $t(28) = -10.32$, $p < .000$. The implications for practice are that supportive goal-setting of personally relevant objectives and valued activities following driving cessation may be effective in lessening some of the negative effects of giving up driving for people with dementia.

### PREDICTORS OF PHYSICAL RESILIENCE IN OLDER ADULTS

Catherine O'Brien, and Roee Holtzer, *Yeshiva University, Bronx, New York, United States*

Physical resilience (PR), which denotes one's ability to resist functional physical decline, can be operationalized through longitudinal assessments on the Short Physical Performance Battery (SPPB). Dual-task walking (DTW) is predictive of adverse outcomes but its role in predicting incident PR has not been assessed. Herein, we determined whether velocity during Single-Task-Walk (STW) and Dual-Task-Walk (DTW) conditions predicted incident loss of PR and identified moderators of this relationship. Participants were 163 (mean age=75.5; %female=52) non-demented, community-dwelling older adults with baseline SPPB scores of 10-12. At baseline, individuals completed neuropsychological testing, the SPPB and DTW paradigm. Cognitive reserve was evaluated using the Wide Range Achievement Test (WRAT-3) and speed of processing was assessed using the Symbol Digit Modalities Test (SDMT). Individuals with SPPB scores < 10 were categorized as not physically resilient. Those with scores of 10 or higher were categorized as physically resilient. At three-year follow up 75.4% (n=123) of participants remained physically resilient while 24.5% (n=40) lost PR. Binary logistic regression revealed that slower DTW (OR= 0.96, p= 0.033, 95%CI [.926, .997]), but not STW velocity (OR= 1.00, p= 0.861, 95%CI [0.962, 1.048]), was a significant predictor of PR loss. Moreover, moderation analyses revealed that DTW velocity predicted PR loss only among individuals who had lower baseline scores on the WRAT-3 (OR=0.937, p=0.004, 95%CI [.896, .979]) and SDMT (OR=0.949, p=0.018, 95%CI [.909, .991]). We propose that cognitive reserve and speed of processing influenced the utility of DTW velocity in predicting PR loss among community-residing older adults.

### TRAJECTORIES OF WALKING SPEED AND CAUSE-SPECIFIC MORTALITY: EVIDENCE FROM ENGLAND

Giorgio Di Gessa,[1] and Paola Zaninotto,[2] *1. University College London, London, England, United Kingdom, 2. University College London, London, United Kingdom*

Decreased walking speed can predict adverse health-related outcomes such as falls and admissions to hospital. Experiencing fast decline in walking speed has also been associated with increased risk of ‘all-cause’ mortality. In this study, we investigate the links between walking speed trajectories and specific causes of death. We used data from the English Longitudinal Study of Ageing, a large nationally representative survey which collects information biennially on people aged 50 and over in England since 2002. The sample consisted of 4,112 respondents eligible for a walking speed test at baseline who had not died before 2006. Rate of change in walking speed was derived from growth curve models and categorised in three trajectories (slow, moderate, and fast decline). We used competing risk analysis to explore the relationships between these trajectories and mortality, and their interactions with baseline wealth. During a mean of 9.5 years of follow-up, 1543 participants (37%) died (639 from cardiovascula disease -CVD, 311 from respiratory disease -RD, and 593 from cancer). Results suggest a significant difference in mortality across walking speed trajectories (with increased risk of death among those with fast declines) for CVD and RD deaths (P<0.001), even after controlling for baseline characteristics. There was no significant difference for cancer deaths (p=0.44). Further stratified analyses suggested that fast decline was associated with higher CVD and RD mortality even among those with an initial fast walking speed (>1.22 m/s). Strategies to maintain motor performances in later life have the potential to preserve life.

### SESSION 2933 (POSTER)

### DEMENTIA AND COGNITIVE IMPAIRMENT III

### CAN SOCIAL CONNECTIONS IMPROVE DEMENTIA KNOWLEDGE AMONG HOMEBOUND COMMUNITY-DWELLING OLDER AFRICAN AMERICANS?

Maritza Dowling,[1] Hiroko Dodge,[2] Antonio Puente,[3] and Beverly Lunsford,[4] *1. George Washington University - School of Nursing, Washington, District of Columbia, United States, 2. Pregon Health & Science University, Portland, Oregon, United States, 3. School of Medicine & Health Sciences, Washington, District of Columbia, United States, 4. George Washington University-School of Nursing, Washington, District of Columbia, United States*

Despite the clear and compelling association between social connections and well-being, the underlying mechanisms that help stave off adverse health impacts are not well understood, particularly among older adults in disadvantaged groups. Social relationships in older age may be instrumental for exchanging and gaining knowledge that further influence health and help increase awareness about misconceptions and lifestyle behaviors known to delay or reduce cognitive decline. This study used cross-sectional survey data from 147(aged 58-90 years; 75% female) low-income African American homebound community dwellers to investigate heterogeneity in dementia literacy profiles and its association with social connectedness. Eleven items (false=0, true=1, don’t know=2) from a validated instrument were used to measure dementia literacy (DL). The Lubben’s social network scale was used for a social connectedness construct. We employed a 2-stage latent class modeling approach to examine heterogeneity in DL and estimate the regressions among the derived classes and the predictors (social connectedness, education level and age). A 3-class model produced a reasonable fit and classification (entropy=0.852) of “dementia literacy patterns” labeled as (high-literacy:37%), moderate-literacy:45.2%), low-literacy:17.8%)). Social connectedness was highly predictive of class membership. A high level of social relationships increased the probability of being in the “high-dementia-literacy” class compared to the “low-dementia-literacy” class (OR=2.189, p=0.016). For a unit increase in social connectedness, the odds of being in the “high-dementia-literacy” class compared to the “low-dementia-literacy” class increased by a factor of 2.2. Tailored and focused interventions to reduce social disconnectedness may also help increase dementia awareness and reduce barriers to early diagnosis.

### COGNITIVE ACTIVITY AS A MODERATOR OF EDUCATIONAL ATTAINMENT AND WORK STATUS IN COGNITIVE AGING

Mirjam Stieger, and Margie Lachman, *Brandeis University, Waltham, Massachusetts, United States*

Cross-sectional findings showed that education differences in memory performance were moderated by frequent cognitive activity (Lachman et al., 2010). The present study examined whether frequent cognitive activity could compensate for lower education when focusing on change in cognitive performance across nine years. The study also explored whether cognitive activity can slow down declines in retired adults as previous research suggested that retiring is associated with an increased risk of cognitive decline (e.g., Wickrama et al., 2013). Longitudinal data from the MIDUS study included N = 3,325 middle-aged and older adults. Outcome variables were two factors of cognitive performance: Episodic Memory (EM) and Executive Functioning (EF). Independent variables were years of education, work status (working vs. retired), and frequency of cognitive activity. The results suggest that cognitive activity moderated the effect of educational attainment on change in EM. Individuals with both higher education and cognitive activity showed the smallest declines in EM. Individuals with lower educational attainment but high cognitive activity had less decline in EM compared to their low education counterparts. Those who increased their cognitive activity over time showed less decline in EF. In terms of work status, working adults had less decline in EM and EF compared to retired adults and retired adults who did not maintain their cognitive activity declined more in EF. The results emphasize the importance of frequent engagement in cognitive activity across the lifespan, which can attenuate cognitive declines especially among those who have lower education or have retired.

### COGNITIVE PERFORMANCE IN OLDER ADULTS WITH SUBJECTIVE COGNITIVE DECLINE

Vanessa Taler,[1] Cassandra Morrison,[1] and Christine Sheppard,[2] *1. University of Ottawa, Ottawa, Ontario, Canada, 2. Sunnybrook Research Institute, Toronto, Ontario, Canada*

Subjective cognitive decline (SCD) refers to a perceived decline in cognitive function in the absence of neuropsychological deficits. Older adults with SCD are at increased of subsequent development of mild cognitive impairment or dementia. We had 224 adults aged 65+ complete questionnaires assessing their subjective appraisal of their cognitive function, including questions about word-finding difficulty, memory, and attention/concentration. Participants also completed the Montreal Cognitive Assessment (MoCA). All participants exhibited cognitive performance that was within normal limits for age and education. In total, 29.5% of participants reported word-finding difficulties, 16.5% reported difficulties with remembering things, and 8.5% reported difficulties with attention/concentration. We found that (1) self-reported word-finding difficulties were associated with lower performance on delayed word recall, and (2) self-reported difficulties in concentration/attention or memory were associated with lower performance on the abstraction subtask in the MoCA. No other MoCA subtasks were associated with self-reported cognitive function. A subset of the participants (n=69) also completed a battery of tasks assessing semantic function, including picture naming, associative matching tasks, identification of semantic features, and semantic questions. Again, self-reported word-finding difficulty predicted lower performance on semantic tasks. These results suggest that older adults may be aware of changes in their cognitive performance prior to objective neuropsychological impairment. Moreover, their awareness appears to be domain-specific: self-reported language difficulty is associated with lower performance on language-based tasks, while self-reported difficulty in memory, attention, or concentration is associated with lower performance on an abstraction task.

### DEPRESSION AND COGNITIVE FUNCTIONING AMONG ADULTS AGE 60 AND OVER: UNITED STATES, 2011-2014

Ellen Kramarow, and Debra Brody, *National Center for Health Statistics, Hyattsville, Maryland, United States*

Previous studies have noted an inverse association between depression and cognitive functioning. The objective of this research is to explore this relationship with data from a nationally representative survey containing validated measures of cognition, depression, and other health conditions. The study population was respondents aged 60 and over who completed the examination component of the 2011-2014 National Health and Nutrition Examination Survey (NHANES) (N=3,472). Cognitive tests included the CERAD word list learning trials, measuring immediate and delayed memory, and the Digit Symbol Substitution test (DSST), measuring attention and processing speed. The presence of depressive symptoms was based on a score of 10 or higher out of 27 from the Patient Health Questionnaire (PHQ-9). Statistical analyses included regression models with low cognitive performance (scoring in the lowest 25th percentile) as the dependent variable. Results from regression models showed that having depressive symptoms significantly increased the odds of scoring in the lowest 25th percentile of both the DSST (OR = 3.4) and the CERAD test (OR = 1.7), controlling for age, sex, and race and Hispanic origin. Adding in a measure of heart disease showed an independent effect of heart disease on low cognitive performance (OR = 1.7 for DSST and OR = 1.3 for CERAD test), while the effect of depression was lessened but still statistically significant. In this study, depression is associated with cognitive functioning, but its effect may be attenuated by the presence of other chronic health conditions.

### DEVELOPING A SPATIAL-SKILLS-FOCUSED MUSIC PROGRAM FOR OLDER ADULTS WITH CHANGES IN COGNITION

Jennie Dorris, and Juleen Rodakowski, *University of Pittsburgh, Pittsburgh, Pennsylvania, United States*

Older adults with cognitive changes need stimulating programming to maximize their cognitive abilities. One area to maximize includes spatial skills, its decline can lead to disorientation and wandering. Music has potential to maximize spatial skills: reading music's notation is associated with enhanced spatial skills in children and professional musicians. It's critical to understand the potential impact of a spatially focused music program for older adults with changing cognition; if successful, future music programs could support people staying orientated in their environments and living independently longer. We developed and assessed a six-week marimba program focused on reading music with 15 older adults ages 65-89 with changes in cognition. We compared their scores on the Orientation Test from the Test of Visual Perceptual Skills pre- and post-intervention and assessed if participants self-selected to read music notation. Participants scored an average Modified Mini Mental State Examination (3MSE) score of 81.3 (SD = 11.0). On average, participants' scores on the Orientation Test moved from 13.4 (SD =1.9) to 14.1 (SD= 2.7), providing a cohen's d effect size of 0.3. Over the six weeks, 11 out of the 15 participants selected to read music for at least one class, indicating a statistically significant change using the Wilcoxon signed-rank test (Z = -3.16, p < 0.01), suggesting that older adults with cognitive changes may be able to learn to read music. This is important, as a spatially focused music program may maximize spatial skills that older adults need to successfully navigate their world safely and independently.

### DIVERSE SOURCES OF SOCIAL SUPPORT AND COGNITIVE FUNCTIONING BY RACE, ETHNICITY, AND NATIVITY

G. Robin Gauthier,[1] Marc Garcia,[2] and Catherine García,[3] *1. University of Nebraska-Lincoln, Lincoln, Nebraska, United States, 2. University of Nebraska, Lincoln, Nebraska, United States, 3. University of Southern California, Los Angeles, California, United States*

This study examines the relationship between social support profiles and cognitive functioning by race, ethnicity and nativity in older adults using cross-sectional data drawn from the Health and Retirement Study (2010 and 2012). We employed a hierarchical clustering routine to generate nine support profiles that differentiated three sources of support: children, wider family relationships and friendships. Cognitive functioning was measured as the score on the Telephone Interview for Cognitive Status (TICS-m), a 27 point scale of cognitive function. Our approach explicitly acknowledges the ambivalence and multidimensionality of close relationships and the resources embedded within them. Descriptive analyses revealed significant differences in access to support across demographic groups. White respondents are over-represented in profiles that are characterized by support from friends, and under-represented in family support profiles. The reverse is found among Foreign-born Hispanic respondents who are over-represented in the profiles characterized by high family support and under-represented in those with high friend support. Native-born Hispanic respondents and Black respondents have less clear support patterns, although both are more likely to receive support from family and children compared to friends. Findings from the poisson regression suggest that the relationship between familial support and cognitive decline is stronger among Hispanic respondents, particularly those who are foreign born. These findings are supported even with the inclusion of other relationship quality indicators including negative support and frequency of contact.

### DOES COGNITIVE SELF-REPORT MEASURE TYPE DIFFERENTIALLY PREDICT COGNITIVE DECLINE? A SYSTEMATIC REVIEW

Rachel Wion,[1] Nikki Hill,[2] Tyler Bell,[2] Jacqueline Mogle,[2] Jennifer Yates,[3] and Iris Bhang,[2] *1. Indiana University, Indianapolis, Indiana, United States, 2. Pennsylvania State University, University Park, Pennsylvania, United States, 3. University of Nottingham, Nottingham, United Arab Emirates*

Up to 47% of older adults without measurable cognitive impairment report difficulties with memory and thinking which potentially increases their risk for developing cognitive decline. Many measures are used for assessing self-reported cognition; however, certain types of these measures may be more predictive of cognitive decline. The purpose of this systematic review was to compare the role of cognitive self-report measure types in predicting risk for cognitive decline. PubMed, CINAHL, and PsycINFO databases were searched using the following inclusion criteria: longitudinal studies, outcome of cognitive decline, and two or more cognitive self-report measures. A total of 4,319 articles were identified during the initial search and narrowed to 19 final articles. The Quality in Prognosis Studies tool was used to determine study quality. Six comparison themes emerged during synthesis: self-reported cognition or memory with or without worry; self-reported global cognition or self-reported memory; self-reported memory decline and self-reported executive function decline; self-reported cognition and self-reported memory by others; self-reported memory and self-reported memory problems in comparison with peers; and self-reported memory and self-reported memory affecting daily function. Self-reported memory decline with worry and self-reported memory problems by others were most predictive of future impairment. It was difficult to definitively determine whether certain cognitive self-report measure types where more predictive of risk for cognitive decline because there were very few articles in some of the comparison groups. Future investigations of self-reported cognition should focus on using measures that have been shown to be the most efficacious at predicting risk for cognitive decline.

### EEG MEASURES OF VALUE-DIRECTED STRATEGIC PROCESSING IN OLDER ADULTS WITH AND WITHOUT MILD COGNITIVE IMPAIRMENT

Lydia Nguyen,[1] Shraddha Shende,[1] Daniel Llano,[2] and Raksha Mudar,[1] *1. University of Illinois at Urbana-Champaign, Champaign, Illinois, United States, 2. University of Illinois at Urbana-Champaign, Urbana, Illinois, United States*

Value-directed strategic processing is important for daily functioning. It allows selective processing of important information and inhibition of irrelevant information. This ability is relatively preserved in normal cognitive aging, but it is unclear if mild cognitive impairment (MCI) affects strategic processing and its underlying neurophysiological mechanisms. The current study examined behavioral and EEG spectral power differences between 16 cognitively normal older adults (CNOA; mean age: 74.5 ± 4.0 years) and 16 individuals with MCI (mean age: 77.1 ± 4.3 years) linked to a value-directed strategic processing task. The task used five unique word lists where words were assigned high- or low-value based on letter case and were presented sequentially while EEG was recorded. Participants were instructed to recall as many words as possible after each list to maximize their score. Results revealed no group differences in recall of low-value words, but individuals with MCI recalled significantly fewer high-value words and total number of words relative to CNOA. Group differences were observed in theta and alpha bands for low-value words, with greater synchronized theta power for CNOA than MCI and greater desynchronized alpha power for MCI than CNOA. Collectively, these findings demonstrate that more effortful neural processing of low-value words in the MCI group, relative to the CNOA group, allowed them to match their behavioral performance to the CNOA group. Individuals with MCI appear to utilize more cognitive resources to inhibit low-value information and might show memory-related benefits if taught strategies to focus on high-value information processing.

### EFFICACY OF DIFFERENT TYPES OF EXERCISE ON COGNITIVE FUNCTION IN PEOPLE WITH MCI OR DEMENTIA: A NETWORK META-ANALYSIS

Qiaoqin Wan,[1] Xiuxiu Huang,[2] Xiaoyan Zhao,[1] Bei Li,[1] Ying Cai,[1] and Shifang Zhang,[1] *1. Peking University, Beijing, China, 2. Peking university, Beijing, China*

With the accelerating progress of population aging, cognitive dysfunction is becoming increasingly prevalent. Exercise, as a promising non-pharmaceutical therapy, showed favorable effects on cognitive function. But which type is the most effective exercise treatment is still unclear. This study compared the efficacy of different types of exercise interventions based on network meta-analysis and aimed to explore the optimal exercise treatment for cognitive decline. The electronic databases of PubMed, Web of Science, Embase, Cochrane Central Register of Controlled Trials, SPORTDiscus, PsycInfoy, and OpenGrey were searched from inception to September 2019. We only included randomized controlled trials that examined the effectiveness of exercise interventions in people with MCI or dementia. Primary outcomes were global cognition, executive function and memory function. Standard mean difference (SMD) and its 95% confidence interval (CI) were calculated to estimate the effect sizes. Finally, 73 articles with 5748 participants were included. The results showed all kinds of exercise interventions were effective on global cognition and resistance exercise was probably the most effective exercise treatment to prevent the decrease of global cognition (SMD=1.05, 95%CI 0.56-1.54), executive function (SMD=0.85, 95%CI 0.21-1.49) and memory function (SMD=0.32, 95%CI 0.01-0.63) for people with cognitive dysfunction. Subgroup analysis revealed multi-component exercise showed more favorable effects on global cognition (SMD=0.99, 95%CI 0.44-1.54) and executive function (SMD=0.72, 95%CI 0.06-1.38) in people with MCI. In conclusion, resistance exercise tended to be the optimal exercise type for people with cognitive dysfunction, especially for people with dementia. And multi-component exercise also should be recommended for people with MCI.

### FAVORITE ACTIVITY PATTERNS AMONG OLDER ADULTS WITH AND WITHOUT DEMENTIA: FINDINGS FROM THE NHATS

Tianxue Hou,[1] Minhui Liu,[2] Christina E. Miyawaki,[3] Yuxiao Li,[1] Xiaocao Sun,[4] Siyuan Tang,[4] and Sarah Szanton,[5] *1. Central South University, Changsha, Hunan, China, 2. Central South University, Baltimore, Maryland, United States, 3. University of Houston, Seattle, Washington, United States, 4. Central South University, Changsha City, Hunan, China, 5. Johns Hopkins University, Baltimore, Maryland, United States*

Favorite activities are usually meaningful and valuable to older adults. However, information on favorite activity patterns and their relationship with cognitive function from large samples is still limited. Using Round 1 data from the National Health and Aging Trends Study, we examined favorite activity patterns among community-dwelling older adults with and without dementia (N=6,565). Based on the 8-item Ascertain Dementia (AD8) dementia screening interview, participants were classified into no dementia, possible dementia, and probable dementia. Favorite activity was assessed by asking participants, “What their favorite activity they are currently able to do?” Multinomial logistic regression models were used to examine the association between each of the top three favorite activities and the cognitive impairment categories, controlling for demographics and general health. The sample was on average, 77±7.45 years old, non-Hispanic White (69.8%), female (57.3%), and 35.0% had high school education. The three most popular favorite activities among probable dementia participants were watching TV, walking, and outdoor maintenance. Participants who liked watching TV most were more likely associated with possible dementia (Relative Risk Ratio [RRR] = 1.49, p=0.044) compared to participants without favorite activities. Participants who liked walking most were less associated with possible dementia (RRR=0.58, p=0.003) and probable dementia (RRR=0.39, p<0.001) compared to those without favorite activities. Similarly, participants who liked outdoor maintenance most were less likely to develop possible dementia (RRR=0.48, p<0.001) and probable dementia (RRR=0.27, p<0.001) than participants without favorite activities. Researchers may use older adults’ “active” favorite activities to create tailored interventions to slow dementia progression.

### LIKELIHOOD OF PARTICIPATION IN HOME-BASED COGNITIVE ASSESSMENT: THE ROLE OF SUBJECTIVE COGNITIVE DECLINE AND AGE

Moriah Splonskowski,[1] Holly Cooke,[2] and Claudia Jacova,[2] *1. Pacific University, Forest Grove, Oregon, United States, 2. Pacific University, Hillsboro, Oregon, United States*

Home-based cognitive assessment (HBCA) services are emerging as a convenient alternative to in-clinic cognitive assessment and may aid in mitigating barriers to detecting cognitive impairment (CI). It is unknown which older adults would be likely to participate in HBCA. Here we investigated the role of age and Subjective Cognitive Decline (SCD). SCD has demonstrated an increased risk for progression to CI/dementia. A nation-wide community-dwelling sample of 494 adults age 50+ were recruited via Amazon Mechanical Turk to complete an online survey assessing perceptions around HBCA and SCD. Our sample was 91.9% White and 66.8% female. It consisted of 174 respondents aged 50-60, 265 aged 61- 70, and 55 aged 71-79. Age groups were comparable with respect to their acceptance of cognitive assessment (Range 4-20, higher score=higher acceptance, 7.9±3.3, 8.15±3.2, 8.05±3.43) and SCD-Q total (43.1±5.8, 43.2±5.7, 43.3±5.7). Correlation analysis revealed a relationship between SCD-QSCD total and perceived likelihood of participation in HBCA for those ages 61-70 (r(263) = .222

p = .000), but not for ages 50-60 or 71-79 (r(172) = .102 p = .152; r(53) = -.102 p = .458). Our findings suggest that SCD influences the likelihood of participation in HBCA for older adults' transitioning to old age (61-70). Findings show that for adults transitioning into old age (61-70), perceived cognitive state influences their likelihood of participation in HBCA. Importantly, concerns about CI/dementia may generate more favorable perceptions of HBCA for this group.

### MEDIATORS OF THE RELATIONSHIP BETWEEN FUNCTIONAL LIMITATIONS AND LONELINESS AMONG HOMEBOUND AFRICAN AMERICANS

Maritza Dowling,[1] Hiroko Dodge,[2] Antonio Puente,[3] and Beverly Lunsford,[4] *1. George Washington University - School of Nursing, Washington, District of Columbia, United States, 2. Pregon Health & Science University, Portland, Oregon, United States, 3. School of Medicine & Health Sciences, Washington, District of Columbia, United States, 4. George Washington University-School of Nursing, Washington, District of Columbia, United States*

Physical/functional limitations are known to lead to loneliness, but there is little research on protective factors that may mediate their adverse association, particularly in vulnerable populations. This study used cross-sectional survey data from 147 (aged 58-90 years; 75% females) predominantly low-income African American homebound community dwellers to investigate the role of resilience and social connectedness in mediating the effect of physical/functional limitations on loneliness. Items from validated instruments were used to measure four latent variables (physical/functional limitations, resilience, loneliness and social connectedness). Structural parallel mediator models estimated each path in the mediation analysis controlling for gender, education, and age. Confidence intervals for mediation effects were generated via bias-corrected bootstrapping with 10,000 replications. The total effect of physical/functional limitations on perceived loneliness was significant. Social connectedness and resilience fully and significantly mediated the relationship between physical/functional limitations and loneliness. The indirect effect (B =.143; 95% CI = .047, .280) for the physical/functional limitations-resilience-loneliness pathway indicated that the positive effect of physical/functional limitations on loneliness was approximately 0.143 points lower as mediated by higher resilience. The indirect path of physical/functional limitations on loneliness through the mediation of social connectedness (B =.077; 95% CI = .010, .168) yielded a reduction of .077 points. The total amount of variance in feelings of loneliness accounted for by the overall model, which included the proposed mediators and control variables, was 48.6%. Interventions to build resilience and social interactions may attenuate the effects of physical/functional disability and loneliness on health outcomes in individuals at risk.

### MEMORY MATTERS: EDUCATION ABOUT COGNITION IN TWO NATURALLY OCCURRING RETIREMENT COMMUNITIES

Gregory Hinrichsen,[1] Joyce Fogel,[1] Anne Foerg,[2] and Rosanne Leipzig,[1] *1. Icahn School of Medicine at Mount Sinai, New York, New York, United States, 2. JASA, New York, New York, United States*

"Memory Matters" is a program for older residents and staff of two New York City naturally occurring retirement communities (NORC). The program is in its third year of implementation with the broader goals of enhancing resident knowledge of normal cognitive changes and cognitive impairment including risk factors for dementia. The emphasis is on engaging residents to improve brain health. With staff, goals are to enhance their capacity to understand, identify, refer, and support residents with cognitive impairment. This is done with case conferences, consultation, lectures, and workshops. For residents, the program provides lectures, interactive workshops, films, as well as periodic "Ask the Doc" individual meetings where residents can discuss cognitive and related concerns. A geriatrician and geropsychologist conduct these activities with invited guest speakers including geriatric fellows. Yearly, approximately 180 unique individuals are served in this program. Resident program satisfaction surveys were favorable. For example, 80% of residents replied "a lot" to the question, "Did you find this presentation interesting?", in the past six months of programming. Building on these efforts, we conducted a formal survey with 249 resident participants of one NORC that found: 15% have dementia/experience memory loss; 12% live with someone with dementia/ memory issues; 16% identify as a dementia caregiver; and 75% are concerned about developing dementia. We then hosted a community-wide forum to discuss survey results. Our experience has underscored the need for expansion of our efforts. Future plans include creating a "dementia friendly" NORC. Funded by the UJA-Federation of New York.

### PATTERNS OF COGNITIVE FUNCTION TRAJECTORY AND THEIR ASSOCIATED FACTORS IN KOREAN OLDER WOMEN IN SINGLE HOUSEHOLDS

DoKyung Yoon, Hey Jung Jun, and Sun Ah Lee, *Yonsei University, Seoul, Republic of Korea*

The aim of this study was to identify distinct patterns of cognitive function trajectory among Korean older women in single households and to investigate their associated factors. We used data from the five waves of the Korean Longitudinal Study of Ageing (KLoSA), a nationally representative longitudinal survey collected biennially from 2006 to 2014. The sample was 244 women aged 65 or above who had maintained the single household status for the five waves. Using Growth Mixture Modeling, distinct patterns of cognitive function trajectory were identified. Then, multinomial regression analysis was employed to examine the associations between identified trajectories and various factors (socio-demographic, health-related, psychological, and interpersonal factors at wave 1). Results showed that there were three distinct patterns of cognitive function trajectory: 'maintaining-low' (20.9%), 'moderate-decreasing' (33.6%), and 'maintaining-high' (45.5%). Specifically, compared to the 'maintaining-low' group, younger women were more likely to belong to the 'moderate-decreasing' group and the 'maintaining-high' group. Furthermore, those with higher household income, and who participated in more various types of social activities (e.g., participation in leisure/culture/sports clubs, volunteering work) were more likely to belong to the 'maintaining-high' group. Compared to the 'moderate-decreasing' group, those who were younger and participated in more various types of social activities were more likely to belong to the 'maintaining-high' group. Findings suggest that socioeconomic status and social engagements may be critical for cognitive function among older women in single households, and encouraging them to remain socially active may help prevent poorer cognitive outcomes.

### PSYCHIATRIC DISORDERS AND MILD COGNITIVE IMPAIRMENT IN OLDER VETERANS WITH SUBJECTIVE MEMORY COMPLAINTS

Mia Delgadillo,[1] Megan Frank,[1] Aidan Boese,[1] Tilman Schulte,[1] and J. Kaci Fairchild,[2] *1. Palo Alto University, Palo Alto, California, United States, 2. VA Palo Alto Healthcare System, Palo Alto, California, United States*

Psychiatric disorders pose a unique risk for Alzheimer's disease (AD). Prior research indicates psychiatric disorders in MCI increase AD vulnerability. Less research has been done to understand how psychiatric disorders may affect the development of MCI. Understanding these potentially modifiable risk factors is important as they may represent a potential target of intervention for secondary prevention of AD. The present study examines the relationship between psychiatric disorders and amnestic MCI (aMCI) in a sample of Veterans with subjective memory complaints. The sample included 150 older adults with subjective memory complaints (90% male, age = 70.6±8.2). aMCI diagnosis was based upon performance on the delayed recall trials of the Rey Auditory Verbal Learning Test and Logical Memory II of the Wechsler Memory Scale-4th edition. Psychiatric disorders (e.g., Mood Disorders, Anxiety Disorders, and Substance Use Disorders) were assessed using the Mini Neuropsychiatric Interview for DSM-IV. Logistic regression modeling demonstrated that diagnosis of anxiety disorders, but not mood or substance use disorders, was significantly associated with aMCI status. Specifically, older adults with an anxiety disorder were less likely to have aMCI than those older adults without an anxiety disorder. Additional analyses revealed that within those with aMCI (n=107), persons with a psychiatric disorder were significantly younger than those without a psychiatric disorder by an average of 6 years. These findings support prior research on the complex relationship of anxiety and cognitive impairment as well as suggest that those with psychiatric disorders may be at risk for developing aMCI at younger ages.

### RACIAL AND ETHNIC HEALTH INEQUALITIES IN ACCELERATED TRANSITIONS IN MILD COGNITIVE IMPAIRMENT AND DEMENTIA

Ginny Natale,[1] Yun Zhang,[2] and Sean Clouston,[2] *1. Stony Brook University, Stony Brook, New York, United States, 2. SUNY Stony Brook University, Stony Brook, New York, United States*

Background: It is unclear how the expected risk of Mild Cognitive Impairment (MCI) and conversion to dementia in the United States differs by Race/ethnicity and gender in older adults. It is unclear whether expectations about racial disparities have changed in light of changing trends in obesity and diabetes. Method: We analyzed six waves of the National Health and Aging Trends Study (NHATS), a nationally representative prospective cohort study of U.S. residents aged 65 and older. Participants were classified into four clinically meaningful mutually-exclusive groups: unimpaired, MCI, probable dementia and death. We used multistate survival models to examine individuals' cognitive transitions and reported predictors using hazards ratios. Results: Participants (n=6,078) were 77 years old, on average, and the majority were white, females and high school graduates. Increased age was associated with risk of MCI and death. Adjusted for obesity status and diabetes, Hispanics were had a net reduced risk of death from dementia (HR=0.60; 95% CI [0.38-0.96]) but lived longer with MCI and dementia before death. Cognitively normal Blacks faced significantly increased risk of developing MCI (HR=2.54 95% CI [1.81-3.53]) and dementia (HR=1.77 95% CI [1.34-2.35]). Obese and overweight elderly had reduced risk across transitions to MCI then to dementia, but similar risks of death compared to lower body mass indices (BMI). BMI and diabetes reveal a suppression effect and racial inequalities became more apparent. Discussion: Results showed persistent racial inequalities in MCI, probable dementia, and death, supporting the theory that racial/ethnic factors are important to expectations of aging Americans.

### SEVERITY OF COGNITIVE IMPAIRMENT MODERATES THE EFFECT OF DEPRESSION ON MOTIVATION

Claire Growney, Xianghe Zhu, Julia Sorensen, Emily Smith, and Shevaun Neupert, *North Carolina State University, Raleigh, North Carolina, United States*

Motivation to engage in cognitive activities is a critical feature of cognitive health, especially for older adults. A growing body of literature demonstrates that personal resources such as mental health contribute to older adults' motivation. However, little is known about factors that contribute to motivation for cognitive engagement in older adults already experiencing significant decline, a population for whom engagement may be uniquely consequential. In the present study, older adults (age 61-85) with cognitive impairment (Short Blessed scores of 7+) and/or dementia diagnoses completed a series of assessments of cognitive ability, well-being, and motivation to engage in cognitively demanding activities as a part of a larger project. Linear regression analyses revealed that cognitive ability moderated the effect of depression on motivation to engage in cognitively demanding activities. For those with relatively mild levels of cognitive impairment, depression was associated with lower levels motivation, whereas depression was unrelated to motivation for those with more severe cognitive impairment. Notably, results were unchanged when controlling for physical health, suggesting that depression has unique effects on motivation for these individuals. Results contribute to a growing body of literature demonstrating that mental health is particularly consequential for individuals in earlier stages of cognitive decline. We suggest that interventions targeting mental health in individuals beginning to experience cognitive decline may be effective in increasing motivation to engage in activities that are beneficial for cognitive health and that such interventions may be most effective when implemented at the earliest stages of dementia.

### THE IMPACT OF BILINGUALISM ON COGNITIVE FUNCTIONING IN OLDER ADULTS

Hillary Rouse,[1] Brent Small,[1] and John Schinka,[2] *1. University of South Florida, Tampa, Florida, United States, 2. University of South Florida, tampa, Florida, United States*

Research on bilingualism has found inconsistent results regarding its potential benefit on the cognitive abilities of older adults. The goal of the current study was to evaluate differences in cognition on a wide array of neuropsychological assessments between monolingual and bilingual cognitively healthy older adults who specifically speak only English and/or Spanish. The sample included cognitively intact older adults who were either monolingual (n=247) English speakers or bilingual (n=42) in English and Spanish. Performance was compared between groups from a battery of neuropsychological assessments that measured executive function, attention, short-term memory, and episodic memory. Compared to English and Spanish bilinguals, monolingual English speakers performed significantly better on a variety of tasks within the domains of executive function, attention, and short-term memory. No significant differences were found in favor of the bilinguals on any domain of cognitive performance. In the present study, we failed to observe a significant advantage for English and Spanish bilingual speakers on the cognitive performance of older adults when compared to monolingual English speakers. This study suggests that the bilingual advantage may not be as robust as originally reported, and the effects of bilingualism on cognition could be significantly impacted by the languages included in the study.

### THE ROLE OF STRESSFUL CHILDHOOD EXPERIENCES IN SHAPING LATER-LIFE MEMORY LOSS AMONG BLACK AND WHITE U.S. ADULTS

Caroline Hartnett, *University of South Carolina, Columbia, South Carolina, United States*

Cognitive decline common in the U.S. and greatly impacts quality of life, both for those who experience it and for those who care for them. Black Americans experience higher burdens of cognitive decline but the mechanisms underlying this disparity have not been fully elucidated. Stress experienced in early life is a promising explanatory factor, since stress and cognition are linked, childhood stressors been shown to have a range of negative implications later in life, and Black children experience more childhood stressors than White children, on average. In this paper, we use data from the Behavioral Risk Factor Surveillance System (BRFSS) to examine whether stressful experiences in childhood help explain Black-White disparities in memory loss. These data were available for 5 state-years between 2011 and 2017 (n=11,708). Preliminary results indicate that, while stressful childhood experiences are strongly associated with memory loss, stressful experiences do not mediate the association between race and memory loss. However, race does appear to moderate the association between stressful childhood experiences and memory loss. Specifically, stressful experiences are associated with a higher likelihood of memory loss for Black adults compared to White adults.In addition, there seem to be some noteworthy patterns across different types of experiences (i.e. parental drinking may predict later memory loss more strongly for Black adults than White adults, but parental hitting may predict memory loss more strongly for White adults than Black adults).

### TYPE OF TEA CONSUMPTION AND MILD COGNITION IMPAIRMENT IN OLDER ADULTS

Yao Yao,[1] Huashuai Chen,[2] Danan Gu,[3] and Yi Zeng,[1] *1. Peking University, Durham, North Carolina, United States, 2. Duke University, Durham, North Carolina, United States, 3. United Nations, New York, New York, United States*

Existing studies have testified the neuroprotective qualities of tea. As there are several types of tea, question on which type of tea may exert substantial influence on cognitive health is intriguing and remains unknow. We aim to estimate the association between type of tea consumption and mild cognition impairment (MCI) using a nationally representative dataset of older population in China. Type of tea consumption was classified as three groups: Green, fermented (White, Oolong, Black, and Pu'eh), and flower tea. The Mini-Mental State Examination (MMSE) was adopted to assess cognitive function. We conducted multivariate logistic regressions to evaluate the association between type of tea drinking and cognition outcomes (MMSE score and MCI). Potential confounders including sociodemographic factors, health conditions, dietary patterns, lifestyles, activities of daily living, mental health, and living environments. A total of 10,923 participants (mean age: 85.4 yr; female: 53.5%) included in the study. The type of current tea consumption among the participants were: 2143 for green tea, 1302 for fermented tea, and 844 for flower tea. Compared to those who had no habit of tea consumption, the odds ratio of MCI in green tea drinkers was 0.80 (0.68-0.95), in fermented tea drinkers was 1.07 (0.89-1.30), and in flower tea drinkers were 0.85 (0.67-1.09). Our study showed green tea and flower tea consumption associated with lower odds of MCI, while the association was not found among fermented tea drinkers. Future experimental and longitudinal studies are warranted to illustrate the association between varied type of tea and cognitive health.

### SESSION 2934 (POSTER)

### FALLS, FRAILTY, AND PHYSICAL ACTIVITY

### A CONCEPTUAL FRAMEWORK OF PERSON-ENVIRONMENT RELATIONS IN THE OUT-OF-HOME MOBILITY OF PEOPLE LIVING WITH DEMENTIA

Kishore Seetharaman, Habib Chaudhury, and Atiya Mahmood, *Simon Fraser University, Vancouver, British Columbia, Canada*

This paper proposes a conceptual framework on the relationship between neighbourhood mobility and developmental outcomes of persons living with dementia. While there is growing evidence on the importance of out-of-home mobility for community-dwelling people with dementia, there is a lack of theoretical understanding of the person-environment (P-E) interactions involved in out-of-home mobility in the context of dementia and their influence on psychosocial outcomes. The proposed framework adapts Chaudhury and Oswald's Integrative Conceptual Framework of Person-Environment Exchange to address the influence of out-of-home mobility-related P-E interactions on processes of agency and belonging for people living with dementia and the cumulative effect on developmental outcomes of autonomy and identity. The framework describes a linear sequence of four components: (i) out-of-home mobility-related P-E interactions determined by individual, social, physical environmental, and technological factors; (ii) mobility-related environmental barriers and facilitators and perception of out-of-home mobility-related risk and benefit; (iii) interrelated positive and negative processes of behaviour-driven agency and experience-driven belonging; and (iv) interrelated developmental outcomes of autonomy and identity. This framework advances our conceptual understanding of the relations between the experience of living with dementia and different aspects of the neighbourhood environment by bridging objective and subjective dimensions of P-E interactions and framing out-of-home mobility as an important contributor in maintaining identity and autonomy. The framework could inform empirical research in this area, evaluation of dementia-friendly community initiatives, and policy decisions by drawing attention to both the functional aspects and meanings associated with out-of-home mobility for persons living with dementia.

### ADAPTIVE CAUSAL THINKING ABOUT MOBILITY CHALLENGES: IMPLICATIONS FOR QUALITY OF LIFE

Judith Chipperfield,[1] Jeremy Hamm,[2] Patricia Parker,[1] Maria Krylova,[1] Loring Chuchmach,[1] Raymond Perry,[1] Cheliza Krause,[1] and Christiane Hoppmann,[3] *1. University of Manitoba, Winnipeg, Manitoba, Canada, 2. North Dakota State University, Fargo, North Dakota, United States, 3. University of British Columbia, Vancouver, British Columbia, Canada*

Weiner's attribution theory posits that it is adaptive to ascribe challenges to controllable causes (e.g., insufficient effort, bad strategies) and maladaptive to ascribe them to uncontrollable causes (e.g., old age). This is supported by our prior research that showed a heightened risk of mortality when mobility challenges were attributed to old age. The present pilot study randomly assigned older adults (N=36) in a day hospital to either an attributional retraining (AR) intervention group that viewed a video intended to shift causal thinking regarding mobility challenges (uncontrollable→controllable causes), or to a comparison group (No-AR). Participants completed a Time1 survey, the AR intervention (one week later), and a Time2 follow-up survey two weeks later. A manipulation-check revealed that AR was effective in shifting causal thinking away from maladaptive causes; a decline in the endorsement of the old age attribution was observed in the AR group (Ms=2.61 vs. 2.06; p=.02), but not in the No-AR group (Ms=2.45 vs. 2.35, p=.30). The AR and No-AR groups were equivalent at Time 1 on two quality-of-life outcomes: helplessness and perceived control (PC) over health. However, helplessness declined (Time1-Time2) in the AR group (Ms=1.13 vs. 0.73, p=.03), whereas it was relatively stable in the No-AR group (Ms=1.42 vs. 1.26, p>.20). Moreover, PC increased marginally in the AR group (Ms=6.50 vs. 6.69; p=.06), but declined in the No-AR group (Ms=6.20 vs 5.45, p=.05). Together, these findings suggest that attributions can be shifted away from uncontrollable causes and that this shift can have a protective effect that benefits quality-of-life.

### AWARENESS OF LOSSES AND NOT GAINS PREDICTS MODERATE-TO-VIGOROUS PHYSICAL ACTIVITY

Erica O'Brien,[1] Manfred Diehl,[2] Eric Cerino,[1] and David Almeida,[1] *1. Pennsylvania State University, University Park, Pennsylvania, United States, 2. Colorado State University, Fort Collins, Colorado, United States*

The current study used data from a nationally representative sample of middle-aged and older adults to examine the synergistic relationship between negative and positive self-perceptions of aging on two types of physical activity (PA) and sedentary behavior. Participants from Wave 3 of the National Study of Daily Experiences reported on their awareness of age-related losses and gains, the frequency, duration, and intensity of their PA, and their sedentary behavior across seven days. We performed separate hierarchical models that regressed moderate-to-vigorous exercise, walking, and sitting on perceived losses and gains. Results revealed a significant main effect of perceived losses on moderate-to-vigorous exercise (p=.005, 95% CI=[-121.79,-22.267]). People who perceived more losses than average also reported engaging in fewer minutes of effortful PA. Results also suggested a marginal trend for more perceived losses to predict less walking (p=.055, 95% CI=[-42.85,0.42]) but to have no impact on sitting time (p=.58). Neither perceived gains nor the interaction between perceived gains and perceived losses emerged as significant predictors (ps>.17). Previous work demonstrates a facilitative role played by positive (self-)perceptions of aging on engagement in preventive health behaviors that have important impacts on long-term developmental outcomes. We explored additive and multiplicative effects associated with gain- and loss-related perceptions on PA and sedentary behavior to improve our understanding of the psychological context that surrounds the views of aging and health link. Initial findings suggest differential consequences of positive and negative self-perceptions of aging on distinct health-related behaviors, with the latter being stronger and contributing to activity inhibition.

### CHANGES IN LEISURE SELF-EFFICACY AND FALL RISK: ONE-YEAR RESULTS OF N'BALANCE, A FALL PREVENTION INTERVENTION PROGRAM

Laura Payne,[1] Cathy Headley,[2] Christine Katzenmeyer,[3] and Chungsup Lee,[4] *1. University of Illinois @ Urbana-Champaign, Urbana, Illinois, United States, 2. Rockford University, Rockford, Illinois, United States, 3. Consortium for Older Adult Wellness, Lakewood, Colorado, United States, 4. California State University, Long Beach, Long Beach, California, United States*

Fear of falling can prevent people from engaging in valued leisure activities. Yet few studies have examined the role of leisure self-efficacy in fall prevention (Datillo, Martire, Proctor, 2012). The purpose of this study was to assess how participation in a fall prevention program affected worrying about falls, self-reported falls, and leisure self-efficacy in older adults over a 1-year fall prevention intervention. N 'Balance is an 8-week community-based multi-modal fall prevention program. This community intervention study included a treatment (N=50) and control group (N=42). Data were collected in four waves: 1) pre-program physical assessment and survey, 2) post-program physical assessment and survey, 3) six month follow-up survey, and 4) 12 month follow-up survey. Measures included the Activities-Specific Balance Confidence Scale (Powell, Myers 1995), self-reported worry about falling and the leisure self-efficacy scale. Analysis of covariance was used to assess the group x time effects of N

'Balance on worry about falling, leisure self-efficacy and the number of falls in the last 12 months, while controlling for age and subjective health. From baseline to six months post intervention, fall worry decreased significantly for the treatment group and increased for the control group (p< .05). However, there was no significant change over time in the number of self-reported falls by either group. Leisure self-efficacy was higher at baseline for the treatment group and decreased significantly from 6 to 12-months post N Balance participation, whereas the control group had lower leisure self-efficacy at baseline and increased significantly over the 1-year study period (p<.05).

### DIFFERENCES IN CHARACTERISTICS ASSOCIATED WITH MOBILITY IN OLDER DWELLERS OF A HILLSIDE RESIDENTIAL COMMUNITY

Ryoichi Nitanai, Ryogo Ogino, Daisuke Umemoto, Yu Matsumura, Rika Sakurai, Kazutate Hosogaya, Takaaki Endo, and Jun Goto, *The University of Tokyo, Tokyo, Japan*

In a super-ageing society, sustaining outings as an elderly person is necessary for maintaining good health and preventing frailty and social isolation. Avoiding driving cars and walking due to ageing are among factors that deter the elderly from going out. However, the characteristics associated with mobility on foot and by car in actual outings of the elderly have not been examined. To explore these characteristics, we conducted interviews with 23 elderly participants living in a suburban hillside residential community in Japan, and investigated the destinations, routes, and means of all their outings for a certain week. Then, spatial analysis was conducted to identify differences in behavioral characteristics associated with mobility on foot and by car/taxi among three age groups: 70–79, 80–84, and 85+. Consequently, two inclinations were identified. First, the older the group, the smaller the area of the outing on foot due to difficulties in walking on slopes for a long duration with luggage. Second, the use of a car/taxi varies among the three groups. While the 70–79 age group used cars/taxis for district-to-district trips, the 80–84 age group rarely used them, and further, the 85+ age group mainly travelled by them one-way or made back-and-forth trips. To summarize, for older suburban dwellers in a hillside residential community, mobility on foot is lowered by physical and mental weakness and the landscape. Therefore, even though mobility increases by using cars/taxis, willingness for outing has changed with ageing, and thus, they make limited use of opportunities.

### EXPLORING THE BENEFITS OF ASSISTIVE DEVICES USE IN OLDER ADULTS WITH IMPAIRED LOWER LIMB FUNCTION

Tai-Te Su,[1] and Shannon Mejia,[2] *1. University of Illinois at Urbana-Champaign, Urbana, Illinois, United States, 2. University of Illinois at Urbana-Champaign, Champaign, Illinois, United States*

Assistive technologies are an essential component of meeting the needs of an aging population. With advancing age, chronic conditions and physiological changes can result in impaired lower limb function among older adults, which may in turn limit their ability to perform daily activities or even walking. Informed by the continuity theory, we conceptualize assistive devices (ADs) as a resource that older adults with mobility limitations can leverage to remain active and mobile in late life. However, evidence remains scarce to determine the extent to which using ADs could create a measurable change in older people's experienced well-being. Using data from the 2012 wave of the Health and Retirement Study, our study aimed to examine the potential psychological benefits that accompany ADs use in a sample of older adults with at least some limited lower limb mobility (n = 505, 59% female, 85.6% white, age = 75.8 ± 6.7). Results from multiple linear regression showed that although AD use was not directly associated with global well-being, those who used ADs reported more positive experience while walking (b = 0.65, SE = 0.23, p<.01) and traveling (b = 0.92, SE = 0.43, p<.05). Additionally, our results indicated that AD users had higher self-efficacy compared with nonusers (b = 0.40, SE = 0.20, p<.05) after controlling for age, gender, socioeconomic status, as well as physical function level. We suggest that AD provision should be considered in intervention strategies to increase well-being and quality of life in older adults with impaired lower limb function.

### GENDER DIFFERENCES IN MORTALITY RISK AFTER DRIVING CESSATION AMONG OLDER MEN AND WOMEN: A MEDIATION ANALYSIS

Jiao Yu,[1] Eva Kahana,[1] Boaz Kahana,[2] and Yuhan Zhang,[1] *1. Case Western Reserve University, Cleveland, Ohio, United States, 2. Cleveland State University, Cleveland, Ohio, United States*

Driving is the most important personal transportation mode in the US for maintaining mobility. Previous studies of older adults who stop driving have identified several health risks associated with driving cessation, including less access to health care, increased dependency, social isolation, and elevated risk of mortality. The purpose of this analysis was to examine driving status as a predictor of mortality among community-dwelling older men and older women. Data were drawn from a prospective panel study of successful aging project of 1000 older adults (mean age = 80). Participants' driving status was measured at baseline and mortality rates were observed across the subsequent 10 years. Extended Cox proportional hazard model indicated a 76% (p<0.001) significantly higher mortality risk for non-drivers versus drivers. This relationship was mediated by health conditions and functional status for both older men and older women. Among older men, health status fully mediates the association between driving cessation and mortality risk. A partial mediation effect of health status on the association between driving cessation and mortality risk was found among older women. Older women who stopped driving faced 56% (p>0.01) higher relative mortality risk than their driver counterparts. Social and cultural issues such as gender stereotype, autonomy, and social connection with their implication for driving may explain existing gender differences among older adults.

### GENDER DIFFERENCES IN PREDICTIVE FACTORS OF OLDER ADULTS' IADL RECOVERY FOLLOWING HIP FRACTURE

Elise Colancecco,[1] Ann Kolanowski,[2] and Vernon Chinchilli,[3] *1. Moravian College, Summit Hill, Pennsylvania, United States, 2. Penn State University, University Park, Pennsylvania, United States, 3. Penn State College of Medicine, Hershey, Pennsylvania, United States*

After hip fracture, older adults experience poor functional outcomes including a lack of IADL recovery. Gender differences exist in risk, incidence, mortality, and complication rates; yet, analyses of predictive factors of IADL especially by gender are often not conducted. The purpose of this study was to investigate gender differences in predictive factors of IADL recovery for older adults at two and six months following hip fracture. This secondary analysis used data (n=326 with IADL of n=399) the Baltimore Hip Studies (BHS-7 cohort). Participants were >65 years of age and community-dwelling. Men were sequentially enrolled; women were frequency-matched. Data analysis required building a shared parameter model was built that incorporated an ordinal logistic regression within a generalized linear mixed-effects model, in conjunction with a time-to-event hazards regression model for the time to death or withdrawal. Predictive factors included: age, race, marital status, and comorbidities; physical function; cognitive status (3MS); and psychosocial function (depression [CES-D], resilience, fear of falling, social participation, and perceived health status. Results indicated that higher age (OR 1.1 95% CI 1.05, 1.15, p< .01), greater comorbidity burden (OR 1.31 95% CI 1.08, 1.6, p < .01), poorer baseline Lower Physical ADL ( OR 1.8 95% CI 1.54, 2.15, p< .01), better cognitive function (OR 0.95 95% CI 0.9, 1; p= 0.047) and poorer LPADL recovery (OR 1.27 95% CI 1.07, 1.5, p< .05) significantly impacted IADL recovery. The stratified (by gender) model was not as strong as the full model, but did indicate some gender differences may exist.

### HIGH COGNITIVE LOAD SITUATIONS DECREASE BOTH GAIT AND COGNITIVE PERFORMANCE

Hyeon Jung Kim, Farahnaz Fallahtafti, Jennifer Yentes, Dawn Venema, and Julie Blaskewicz Boron, *University of Nebraska Omaha, Omaha, Nebraska, United States*

A high cognitive load situation (HCLS) is completing two or more tasks simultaneously (i.e. walking while talking). Differential allocation of attentional demands creates HCLS, potentially deteriorating cognitive and/or gait performance, impacting fall risk. This study investigated whether different load types [(Single-task (ST): talking/walking only, and HCLS: walking while talking on a phone)] impacted gait and cognitive performance among young (n=8; age=23.16±1.96yrs), middle-aged (n=14; age=44.79±7.42yrs), and older (n=15; age=74.47±3.91yrs) adults. In 3-minute trials, participants completed single-task walking (ST-W) and phone conversations with easy (e.g., favorite food, ST-E) and difficult (e.g., personal relationships, ST-D) topics, and also combined walking and talking (easy: HCLS-E and difficult: HCLS-D). For gait, speed, step length (SL) and stride width (SW) were analyzed with 3(ST-W, HCLS-E, HCLS-D) x 3(Age) repeated-measures ANOVAs. HCLS resulted in slower speed (p <.001, shorter SL (p <.001), and wider SW (p=.008) across groups. Older adults exhibited shorter SL across walking conditions (p=.002) compared to young and middle-aged. For cognition, Word Count (WC) and Authenticity (i.e. honesty) were analyzed with 2(Evs.D) x 2(STvs.HCLS) x 3(Age) repeated-measures ANOVAs. Main effects emerged for conversation topic in WC (p=.04) and Authenticity (p<.001); difficult topics negatively impacted participants' cognitive performance, likely resulting from higher attention to maintain conversations without personal interactions (i.e. visual cues). Marginal age-group differences (p=.056) revealed older age resulted in less authentic conversations. The HCLS in this study negatively impacted gait and cognitive performance. Understanding this relationship may ultimately inform development of interventions to improve allocation of attentional demands, potentially mitigating fall risk.

### INFLUENCE OF DRIVING AND TRANSPORTATION ACCESS ON SOCIAL ISOLATION RISK AMONG OLDER ADULTS

Matthew Smith,[1] Caroline Bergeron,[2] Matthew Barrett,[1] Leigh Ann Eagle,[3] and Sue Lachenmayr,[3] *1. Texas A&M University, College Station, Texas, United States, 2. Institut national de santé publique du Québec, Québec City, Quebec, Canada, 3. MAC, Salisbury, Maryland, United States*

Background. Transportation is essential to accessing healthcare and community services, but the inability to find transportation may hinder social interactions and connectivity. This study examined driving and transportation access associated with self-reported social isolation risk among adults age 60 years and older. Methods. The Upstream Social Isolation Risk Screener (U-SIRS) was developed to assess social isolation risk among older adults within clinical and community settings. Comprised of 13 items (Cronbach's alpha=0.80), the U-SIRS assesses physical, emotional, and social support aspects of social isolation. Using an internet-delivered survey, data were analyzed from a national sample of 4,082 adults age 60 years and older. Theta scores for the U-SIRS served as the dependent variable, which were generated using Item Response Theory. An ordinary least squares regression model was fitted to identify transportation-related indicators associated with social isolation risk. Results. Approximately 13% of participants did not drive and 18.2% reported not being able to identify a ride or transportation when needed. Higher U-SIRS scores were reported among participants who did not drive (B=0.034, P=0.020). Lower U-SIRS scores were reported among those who live with a spouse/partner (B=-0.153, P<0.001) and those who reported the ability to get a ride from a family member (B=-0.160, P<0.001), friend (B=-0.256, P<0.001), or taxi (B=-0.032, P=0.044). Every additional source of transportation available significantly reduced participants' U-SIRS score (B=-0.239, P<0.001). Conclusion. Given transportation options may reflect physical functioning, social networks, and socioeconomic status, study findings suggest transportation access is an important contextual factor associated with social isolation risk.

### NEIGHBORHOOD WALKABILITY AMONG OLDER ADULTS WITH AND WITHOUT PHYSICAL DISABILITIES

Rie Suzuki,[1] Jennifer Blackwood,[2] and Noah Webster,[3]
*1. University of Michigan--Flint, Flint, Michigan, United States, 2. University of Michigan-Flint, Flint, Michigan, United States, 3. University of Michigan, Ann Arbor, Michigan, United States*

Older adults with physical disabilities (PDs) often experience obstacles to walking locally. Although health promotion programs targeting physical activity are available in lower income, few studies have compared the walking experiences of older adults in these communities who have PDs with those who do not. The purpose of this study was to compare perceptions of neighborhood walkability among adults living in lower income communities with and without PDs. Participants (N=132) were recruited in 2018 at a regional health clinic in Flint, MI. To be eligible, participants had to be over 65 years old and Flint residents. A subsample (N=12) were then followed up with in 2019/2020. We defined PDs as having difficulty performing one or more activities of daily living. Descriptive statistics and analysis of covariance (ANCOVA) were performed. Of the 132 participants, the mean age in 2018 was 69.75 (SD=5.00). The majority were female (68%); African American (80%); single, divorced, or widowed (80%); and educated below GED level (84%). Older adults with PDs were less likely than those without to visit stores within walking distance and walk in their neighborhoods, and more likely to complain about a lot of traffic along the street. Analysis of the longitudinal data show that older adults who had PDs at time 1 were more likely at time 2 to 1) state that their neighborhoods were unsafe; and 2) perceive their neighborhoods more negatively. Findings suggest it is essential to develop disability-friendly support systems and accommodations to encourage walking in lower income communities.

### OLDER ADULTS' PERCEPTIONS OF FALLS AND FALLS PREVENTION: AN INTERVIEW-BASED STUDY

Danielle Catona, *George Mason University, Laurel, Maryland, United States*

The aim of this study was to gain an understanding of older Americans' perceptions of falls and strength and balance exercise (SBE) as a means of falls prevention. Face-to-face, semi-structured interviews were conducted with 72 community-dwelling adults aged 65 to 89 years recruited from a variety of settings. Data were coded inductively to identify themes present within participants' responses. This process included open coding and creating categories. Data revealed four themes related to falls: (1) others are at risk of falling, but not me, (2) people who fall experience bodily harm, (3) people who fall are a burden to others, and (4) people who fall end up in nursing homes. Four themes emerged related to benefits/facilitators of SBE: (1) SBE enables older adults to remain active and independent, (2) SBE provides an opportunity for older adults to socialize, (3) SBE has positive physical and mental health effects for older adults, and (4) healthcare providers advise older adults to perform SBE. There were three barriers associated with SBE: (1) having limited/no prior SBE experience, (2) having a pre-existing condition, and (3) disliking group-based, SBE classes. Study findings suggest older adults underestimate their risk of falling compared to their peers. As a result, SBE interventions may be promoted more effectively by highlighting personal and social benefits associated with SBE rather than physical risks associated with falls. Additionally, personal recommendations from healthcare providers as well as identification of modified and home-based programs may increase participation in SBE interventions.

### PHYSICAL ACTIVITY PROGRAMMING AND PHYSICAL FUNCTION OF OLDER ADULTS IN ADULT DAY CENTERS: A MIXED-METHODS APPROACH

Yuliana Soto,[1] Susan Aguinaga,[2] and Jacqueline Guzman,[2]
*1. University of Illinois--Urbana-Champaign, champaign, Illinois, United States, 2. University of Illinois at Urbana-Champaign, Urbana, Illinois, United States*

With increased prevalence of Alzheimer's disease, there is a need for long-term care services (e.g., Adult Day Centers (ADCs)) to provide physical activity (PA) programs to maintain physical function of older adults. ADCs report offering PA programs; however, information on PA programs and physical function of participants attending ADCs is limited. The study aims to a) explore perspectives of ADC directors on PA programming; b) examine physical function in older adults attending ADCs. A cross-sectional mixed-methods study was conducted among ADC directors and attending participants. Interviews were conducted with ADC directors to assess barriers and facilitators of PA programming. Physical function was assessed among ADC participants via the Short Physical Performance Battery (SPPB) and Timed Up and Go (TUG). Five director interviews were conducted and three major themes emerged; 1) current PA programming limited by fear of falls, 2) staff training and retention, and 3) diversifying PA programming. Twenty-nine ADC participants enrolled in the study, Mage= 74.5±8.2 years; BMI= 29.2 ±7.4 kg/m2; MMSE= 25.6 ±3.3; 51.7% (n=15) African American; 79.3% (n=23) males. ADC participants scored 6.7±3.1 on the SPPB and 15.4±5.3 seconds on the TUG. Directors expressed the importance of PA; however, mentioned current programming was limited due to risk of falls and untrained staff in PA. Findings indicate that older adults attending ADCs have physical function scores indicative of high fall risk. Future PA programming may consider including alternative forms of PA while embedding falls prevention strategies to reduce risk of falls and improve physical function among ADC participants.

### PREDICTING HOSPITAL OUTCOMES USING THE REPORTED EDMONTON FRAIL SCALE-THAI VERSION

Inthira Roopsawang,[1] Hilaire Thompson,[2] Oleg Zaslavsky,[2] Basia Belza,[3] and Suparb Aree-Ue,[4]
*1. Mahidol University, Bangkok, Thailand, 2. University of Washington, Seattle, Seattle, Washington, United States, 3. University of Washington, Seattle, Washington, United States, 4. Mahidol, Bangkok, Thailand*

Backgrounds Frailty is a common geriatric condition leading to poor surgical outcomes. Having a valid frailty measure has the potential to improve surgical care quality. Objectives To test the ability of the Reported Edmonton Frailty Scale-Thai version (REFS-Thai) in predicting hospital outcomes compared with the American Society of Anesthesiologists physical status classification (ASA) and the Elixhauser Comorbidity Measure (EMC) in older Thai orthopedic patients. Methods A prospective study was conducted on hospitalized older adults scheduled for elective orthopedic surgery. Multiple Firth logistic regression modeled the effect of frailty on postoperative complications, postoperative delirium (POD), and discharge disposition, while the length of stay (LOS) was examined by Poisson regression. The area under the receiver operating characteristic curve (AUC) and mean squared errors (MSE) were used to compare the predictive ability of the instruments. Results Two hundred participants with mean age of 72 (range 60-94 years) were mostly female, 23% were frail. Adjusting for other variables, frailty was significantly associated with postoperative complications (OR = 2.38, p = 0.049), POD (OR = 3.52, p = 0.034), and prolonged LOS (relative risk [RR] = 1.42, p = 0.043). The REFS-Thai alone shows good performance in predicting postoperative complications (AUC = 0.81, 95% CI = 0.74-0.88) and POD (AUC = 0.81, 95% CI = 0.72-0.90). The combination of REFS-Thai with ASA and EMC demonstrates an improved predictability. Conclusion The REFS-Thai was useful in predicting adverse outcomes in surgical orthopaedic older adults. Integrating the REFS-Thai for preoperative assessment may be useful for enhancing care quality.

### PREDICTORS FOR CONFIDENCE IN SELF-MANAGEMENT OF FALLS AND THE ROLE OF FEAR OF FALLS

Qiwei Li, and Becky Knight, *University of North Texas, Denton, Texas, United States*

Falls have been a crucial threat for older adults to stay independent. Once they have fallen, older adults are more likely to receive injuries and become people with disabilities. Conventionally, the measurement of fall efficacy focused on the capacity of performing certain activities such as walking or bathing without a fall. However, given the fact that one out of five older adults fall every year, self-efficacy in self-protection when falls do happen calls for a better understanding of confidence in self-management of a fall. Among predictors for fall prevention outcomes, “fear of falls” has received attention. However, “fear of falls” was largely missing in studies exploring self-management of falls in scenarios where falls do happen. This study explores the predictors for CSMoF including “fear of falls”. A series of simultaneous and hierarchical regression analyses with related interaction analyses and a path model were applied to determine the contribution of each predictor variable and the mediating role of “fear of falls”. The findings of the study reported that demographic characteristics, chronic conditions, and perceptions of falls were associated with CSMoF. The path analysis confirmed the mediating role of “fear of falls” as the indirect effects were occupying substantial percentages in the total identified effects. “Fear of falls” should continue to be a core of fall prevention programs and is particularly important for programs that aim to teach older adults what to do when they fall, whom to call for help, and how to avoid injuries upon falling.

### THE INTERRELATIONSHIP BETWEEN SUBJECTIVE HEALTH, COGNITIVE DECLINE, AND LATE LIFE DISABILITY: A PATH ANALYSIS

Sarah Hubner, Hyeon Jung Kim, Brenda Nguyen, Brooke Hansen, and Julie Blaskewicz Boron, *University of Nebraska Omaha, Omaha, Nebraska, United States*

Relationships between mental, physical and cognitive health can differentially impact individuals’ ability to function in everyday life. As people age, this can further influence independence and quality of life. To better understand these relationships, the current study implemented path analysis to investigate the impact of subjective mental, physical, and cognitive health on disability. Analyses explored relationships between demographic variables, subjective mental and physical health, cognitive decline, and self-reported disability (difficulty Walking/Climbing stairs [WC], Dressing/Bathing [DB], and Doing Errands [EA]). Data from the Behavioral Risk Factor Surveillance System were examined. The most recent four waves (2015-2018) of available data from states utilizing the Cognitive Decline Module were included (50 states, two territories). Path analyses were conducted and modeled in AMOS. Measures of CFI (0.986), TLI (0.938), and RMSEA (0.046) indicate good model fit. Listwise deletion was utilized (n=212117) . Respondents were aged 45+ and were generally white (82.8%), female (58.7%), and of “good/very good” subjective general health (64.0%). Results revealed being non-white (WCΣβ=0.028, DBΣβ=0.021, EAΣβ=0.025, all p’s<.001), of older age (WCΣβ=0.124, DBΣβ=0.004, EAΣβ=0.001, all p’s<.001), female (WCΣβ=0.016, DBΣβ=0.013, EAΣβ=0.016, all p’s<.001), poorer mental health (WCΣβ=0.080, DBΣβ=0.082, EAΣβ=0.116, all p’s<.001), poorer physical health (WCΣβ=0.410, DBΣβ=0.294, EAΣβ=0.314, all p’s<.001), and presence of subjective cognitive decline (WCΣβ=0.107, DBΣβ=0.107, EAΣβ=0.138, all p’s<.001) all had a positive total effect on disability. Ultimately, these results indicate the interrelationship between subjective health and self-reported ability/disability. These findings may help to improve care considerations for an aging population by serving as indicators for needs for assistance and support.

### THE LINK OF THREE-DIMENSIONAL FRAILTY INDEX WITH QUALITY OF LIFE AND FEAR OF FALLING AMONG TAIWANESE OLDER ADULTS

Duan-Rung Chen,[1] Chun-Tung Kuo,[2] and Peng-Yu Chen,[2] *1. National Taiwan University, Taipei, Taiwan (Republic of China), 2. institute of Health Behaviors and Community Sciences, Taipei, Taiwan (Republic of China)*

Objective. Frailty has received increasing attention as a way of understanding gradual losses in one or more domains of human functioning (physical, psychological, and social) in older adults. Studies suggested that frailty is related to lower quality of life (QoL) and the fear of falling (FoF). The most commonly used frailty criteria is the Fried Phenotype, which solely focuses on physical dimension of frailty. This study aims to evaluate the three-dimensional frailty index (namely, physical, psychological and social), and its association with QoL and FoF in a sample of community-dwelling Taiwanese older people. Methods. A total of 751 older adults aged 65 years and older (mean age 73.69 yrs ; SD=6.6) were included from May 2019 to Jan 2020 in Taipei City. The 8-Item Short-Form Health and the Falls Efficacy Scare International (FES-I) were used. Structural equation models (SEM) were employed to examine the association of the three-dimensional frailty index with QoL and FoF. Results. The SEM results confirmed a three-dimensional frailty index (physical, psychological and social frailty), and it is significantly associated with OoL and FoF. Physical frailty had the strongest association with PCS and FES-I, yet social frailty with MCS. Conclusion. Public health efforts to prevent elderly frailty should not solely focusing on physical aspect of frailty.

### THE OKLAHOMA FALLS PREVENTION PROGRAM TARGETING RURAL COMMUNITY-DWELLING OLDER ADULTS

Keith Kleszynski,[1] Janis Campbell,[2] Omolara Henley,[3] and Lee Jennings,[1] *1. University of Oklahoma Health Sciences Center, Oklahoma City, Oklahoma, United States, 2. University of Oklahoma, Oklahoma City, Oklahoma, United States, 3. University of Oklahoma Health Science Center/OHAI, Oklahoma City, Oklahoma, United States*

The Oklahoma Healthy Aging Initiative is a statewide health promotion program for older adults based at the University of Oklahoma. Seven staff educators and 32 volunteers delivered 2 community-based fall prevention programs, Staying Active and Independent for Life (SAIL) and Tai Chi Quan: Moving for Better Balance (TCBB) to 763 older Oklahomans in 71 sites across the state over 9 months. For both programs, twenty-four 60-90 minute classes were delivered over 12 weeks with pre and post assessments completed at the first and last class, respectively. Two hundred ninety eight participants (39%) completed at least 75% of class sessions and to date 140 completed a posttest evaluation and were included in the evaluation. Participants were mostly older (87% ≥60 years), female (86%), college educated (45%), white (87%), and most participated in TCBB (89%). Participants improved in 2 physical performance measures: mean 30-second chair stands increased from 11.5 (SD3.8) to 13.1 (SD3.4) stands (p<0.0001); and mean timed up and go time decreased from 10.0 (SD2.9) to 9.4 (SD2.9) seconds (p=0.004 ). More participants reported vigorous or moderate activity at least 3 times per week after program completion, 134 (96%) vs. 114 (81%), p=0.0001. There was no difference in measures of global health, satisfaction with social roles and activities, or companionship with participant mean scores near the upper range of these scales at baseline. Older Oklahomans participating in community-based exercise report good overall health and report high social connection. Future efforts will focus on more socially isolated older adults and diverse communities.

### THE ROLE OF FUNCTIONAL RISK AND FEAR OF FALLING IN OLDER ADULTS' EVERYDAY WALKING ACTIVITY

Carl-Philipp Jansen,[1] Jochen Klenk,[2] Corinna Nerz,[2] Sarah Labudek,[3] Franziska Kramer,[3] Clemens Becker,[2] and Michael Schwenk,[3], *1. Heidelberg University, Karlsruhe, Germany, 2. Robert-Bosch Hospital Stuttgart, Stuttgart, Germany, 3. Heidelberg University, Heidelberg, Germany*

Some persons have low functional risk (FR) but also high levels of fear of falling (FOF), in some it may be the exact opposite; in others, FOF matches actual functional risk. In order to characterise older persons in this respect, Delbaere et al. (2010) defined four groups: 'vigorous' (low FR/FOF), 'anxious' (low FR/high FOF), 'stoic' (high FR/low FOF), and 'aware' (high FR/FOF). We examined how the proposed group model translates into actual walking behaviour and explored whether group differences in walking occur due to FR level rather than the amount of FOF. Group allocation of N=294 participants was determined based on previously published cut-offs for FR (high vs. low Timed Up-and-Go) and FOF (high vs. low Short Falls-Efficacy Scale International). Walking activity was operationalised as mean number of steps per day over one week, assessed using 'activPAL4™ micro' accelerometers. Number of steps in the four groups were 6,335 ('vigorous'), 5,782 ('anxious'), 4,851 ('stoic'), and 4,627 ('aware'). Linear regression results showed that in the two low FR groups, those with high FOF did not differ significantly from the reference group with low FOF (anxious - vigorous: B=-645.3 steps, p=.157); however, the two groups with high FR showed a significantly different number of steps than the 'vigorous' group, irrespective of their FOF (aware-vigorous: B=-1536.1 steps, p=.002; stoic-vigorous: B=-1314.8 steps, p=.005). This means that FR outperformed FOF in their association with walking behaviour, i.e., participants can be better separated in their daily walking behaviour by FR than by FOF.

### THE ROLE OF GENDER IN THE TRANSITION TO DRIVING CESSATION IN PERSONS WITH DEMENTIA

Stephanie Yamin,[1] Roxana Manoiu,[2] Gary Naglie,[3] Sarah Sanford,[4] Elaine Stasiulis,[5] Brenda Vrkljan,[6] and Mark Rapoport,[7] *1. Saint Paul University, Ottawa, Ontario, Canada, 2. Saint-Paul University, Saint-Paul University, Ontario, Canada, 3. University of Toronto, Toronto, Ontario, Canada, 4. Baycrest Health Sciences, Toronto, Ontario, Canada, 5. Baycrest Rotman Research Institute, Toronto, Canada, 6. McMaster University, Hamilton, Ontario, Canada, 7. Sunnybrook Health Sciences Centre, Sunnybrook Health Sciences Centre, Ontario, Canada*

Driving often provides a sense of independence, quality of life and emotional wellbeing. For older adults living with dementia, driving cessation eventually becomes inevitable. Driving cessation has been shown to negatively impact older adults' mobility and, consequently, quality of life. Caregivers of persons with dementia (PWD) who have ceased driving are also impacted as they often become responsible for meeting the mobility needs of PWD and they provide emotional support in respect to this significant life transition.

To date, there is little information on the role of gender in the transition to driving cessation in PWD. The purpose of this study was to examine the role that gender plays among drivers and ex-drivers with dementia from the perspectives of PWD, their caregivers, and healthcare practitioners. Secondary thematic analyses were conducted from a pre-existing sample of persons with dementia (N=10), family caregivers (N=13), and healthcare practitioners (N=6) who participated in interviews and focus groups about their experiences around driving cessation in the context of dementia. Data analyses involved an inductive thematic technique that allowed for generating themes. The main themes identified gender differences as a significant factor in: (1) difficulty accepting driving cessation (2) driving as it is tied to identity, (3) emotional responses to driving cessation, (4) driving as part of the caregiving role. The findings suggest that there is a need for tailored interventions for men and women who lose their ability to drive, in addressing their unique emotional responses and in supporting them through this important life transition.

### TRENDS IN DISABILITY AMONG ADULTS 55-64 IN THE UNITED STATES AND ENGLAND FROM 2002 TO 2016

HwaJung Choi,[1] Robert Schoeni,[2] Tsai-Chin Cho,[2] and Kenneth Langa,[1] *1. University of Michigan, Ann Arbor, Michigan, United States, 2. University of Michigan, Ann Arbor, United States*

The paper's goal is to assess whether and, if so, the extent to which prevalence in disability of adults near retirement ages in the US increased over time compared to their peers in England and examine income group differences in the relative trends. This study uses 2002-2016 Health and Retirement Study (HRS) and English Longitudinal Study of Ageing (ELSA) focusing on adults aged 55-64. Annual percent changes over the period of 2002-2016 for limitations in instrumental activities of daily living (IADL) and activities of daily living (ADL) are estimated for each survey (HRS and ELSA) using multivariable logistic regressions to adjust for individual-level characteristics While disability prevalence of adults ages 55-64 in England improved over the years of 2002-2016 (annual % change= -2.01 for IADL; - 2.53 for ADL), disability prevalence of US adults has not improved and in fact even worsened in terms of IADL (annual % change= +1.35). There are substantial variations in the IADL/ADL trends by income groups. In the US, the adverse trends in disability were more pronounced among the lowest income groups (annual % change in IADL=1.76 for bottom 20% vs. -2.08 for top 20%; annual % change in ADL=1.08 for bottom 20% vs. -2.08 for top 20%). In England, the disability status improved over time for all but the lowest income group. We will examine further to identify specific factors contributing to divergent/convergent trends in disability between the US and England.

### USE OF SITTERS FOR PATIENT SAFETY IN A VETERAN POPULATION

Denise Kresevic,[1] and Christopher Burant,[2] *1. Louis Stokes Cleveland VA Medical Center, Sagamore Hills, Ohio, United States, 2. Case Western Reserve University, Parma, Ohio, United States*

Patient safety including falls risk, is a high priority and an increasing challenge for all health care facilities. Safety risk factors include both physical factors and psychological factors. One common strategy to increase safety has been the use of "sitters.". Studies on functions and outcomes are conflicting some have reported no differences in falls, decreases in falls and restraints, and increases in falls. A total survey sample of 22 "sitters" and 56 registered nurses conducted at a large midwestern VA facility to assess perceptions of sitters. Groups were similar in ages (41-50 years) with sitters having slightly more experience (11-20 years) versus nurses (6-10 years). Safety conditions most likely to be identified with sitter usage were delirium, elopement, and being a hospice patient. Sitters were more likely to identify falls risk, sitters 63% of time versus RN perception 30.9% (Chi Square=7.0, df=l, p=.008); dementia 59% vs 13% (Chi square=17.15, df=1, p=.001); and weakness 66.7% vs 18.2% (Chi square=16.54, df=1, p=.001). Sitters were more likely to have training in delirium 55% vs nurses, 34% (Chi sq=2.557, df=1 p=.11). Nurses identified that the use of sitters were very likely to prevent falls 29.8%, calm patients 25.2%, maintain lines 25.2%, prevent elopement 30.5% and redirect patients 29.7%. Nurses identified the following available safety strategies: alarms (67.2%), adjusting assignment (47.2%), music therapy (5.4%), use of restraints (<2%), pet therapy (<2%), and video monitoring (<1%). Implementation of safety programs must address availability of multiple strategies including: matching sitter competencies with patient populations served.

### SESSION 2935 (POSTER)

### MENTAL AND PSYCHOLOGICAL HEALTH

### AFFECTIVE RESPONSE DURING A SORTING TASK FOR GERIATRIC HOARDING

Mary Dozier,[1] Amy Young,[2] and Scott Camilleri,[2] *1. Mississippi State University, Mississippi State, Mississippi, United States, 2. Mississippi State University, Mississippi State, United States*

Previous research suggests older adults with hoarding disorder are more likely to express non-fear-based emotions when sorting. The purpose of this study was to examine the emotions expressed by eight rural-dwelling older adults with hoarding disorder when sorting and discarding possessions. The study took place in participants' homes and involved a 15-minute behavioral sorting task where participants were asked to sort through personal items and make a decision to either keep or discard each item. Participants were asked to rate their Subjective Units of Distress (SUDs) and to state their emotional state prior to the sorting task, every 5 minutes throughout the test, and after completing the task. Four participants did not complete the sorting task due to a lack of a desire to discard objects (e.g., "I want to keep it all"). For the four participants who did complete the task, an average of 53 items were sorted and an average of 24 items were discarded. The average decrease in SUDs from pre-task to post-task was 40. Overall, most participants reported feeling positive emotions before, during, and following the task, with the most commonly reported emotion being joy. This study supports prior research suggesting that not all hoarding is fear based and that older adults may be more motivated by increasing positive associations with sorting and discarding. A focus on increasing patients' insight and sense of self-efficacy may lead to increased treatment gains for older adults with hoarding disorder.

### ASSOCIATION BETWEEN DISCRIMINATION AND DEPRESSIVE SYMPTOMS AMONG OLDER BLACKS: A PILOT STUDY

Tomorrow Arnold,[1] Courtney Polenick,[1] and Frederic Blow,[2] *1. University of Michigan, Ann Arbor, Michigan, United States, 2. University of Michigan Medical School, Ann Arbor, Michigan, United States*

Racial minority status may be a major source of both acute and chronic stress (e.g., racial discrimination) that contributes to depression among older adults. Indeed, older African Americans experience more psychological distress than their White counterparts largely due to chronic stressors including racism. Yet, little is known about particular aspects of racial discrimination that are most strongly associated with depressive symptoms in middle and later life. The present study is a part of an ongoing pilot project and sought to examine the associations between discrimination experienced in the past month [i.e., perceived ethnic/racial discrimination (PED), everyday discrimination (ED), and heightened vigilance to discrimination (HVD)] and past-month depressive symptoms among Black/African American adults aged 50 to 80 (N = 106). More frequent PED was associated with greater severity in overall depressive symptoms as well as affective and somatic symptoms individually. More frequent ED was associated with greater affect symptom severity only. Increased HVD was associated with greater overall depressive symptom severity, particularly affective symptoms. Discrimination was also differently correlated with specific depressive symptoms. PED was associated with back pain, anhedonia, loss of interest in sex, difficulty breathing, anxiousness, inability to concentrate, and suicidal ideation. Greater ED was associated with anhedonia, chest pain, and feelings of guilt/self-reproach. Higher HVD was associated with chest pain and feelings of guilt/self-reproach. These results suggest that experienced discrimination and attempts to prepare for these experiences may play a role in the presentation of depressive symptomatology and vary by type of symptoms experienced among older Blacks.

### ASSOCIATIONS BETWEEN SELF-ESTEEM WITH DIVERSE DELETERIOUS MENTAL HEALTH OUTCOMES AMONG OLDER ADULTS

Marissa Pifer, Daniel Segal, and Meghan Marty, *University of Colorado at Colorado Springs, Colorado Springs, Colorado, United States*

Research suggests meaningful relationships between self-esteem and various deleterious mental health outcomes. However, these relationships are less well understood in older adults, where age-specific models of self-esteem are lacking. In this study, older adult participants (N = 284, M age = 73.3 years) anonymously completed a series of questionnaires as part of a larger battery. Measures examined for this study included the Single-Item Self-Esteem Scale (SISE), Patient Health Questionnaire (PHQ-9), Beck Hopelessness Scale (BHS), Three-Item Loneliness Scale (3LS), Sense of Belonging Inventory – Psychological Subscale (SOBI-P), and the Geriatric Anxiety Scale (GAS). A multiple regression was conducted to examine how these mental health variables predict self-esteem. The model explained a significant 39% of the variance in self-esteem, F (5,234) = 29.68, p < .001, with two significant predictors. Specifically, lower scores on the PHQ-9 (β = -3.06, p < .01) predicted higher scores on the SISE, whereas higher scores SOBI-P (β = .27, p < .01) predicted higher scores on the SISE. Scores on the 3LS (β = -.09), BHS (β = -.03), and GAS (β = -.03) did not emerge as significant predictors in the model. Results from this study indicate that lower depression and higher sense of belonging have unique contributions to self-esteem for older adults, controlling for the shared variance from other mental health variables, such as loneliness, hopelessness, and anxiety. Although causality was not assessed, future prospective studies should examine whether interventions focusing on reducing depression or increasing feelings of belongingness enhance self-esteem for older individuals.

### CONFIRMATORY FACTOR ANALYSIS OF THE PERCEIVED STRESS SCALE-10 ACROSS AGE GROUPS

Robert Intrieri, and PAIGE GOODWIN, *Western Illinois University, MACOMB, Illinois, United States*

The Perceived Stress Scale-10 (PSS; Cohen et al, 1983) was developed to measure subjective elements of stress. Most measures focus on objective characteristics of stress (e. g., frequency of occurrence) and specific situations that produce stress (e. g., divorce) but ignore the cognitive appraisal associated with stressful stimuli. The PSS-10 assesses the interplay between stressor and appraisal-mediated coping ability. Factor analytic studies provide support for two factors: perceived helplessness and perceived self-efficacy (see Roberti et al. 2006). The current study presents data from 591 people across three groups: 221 young adults (mean age 19.31, SD = 1.21), 283 middle-age adults (mean age 48.27, SD = 5.14), and 109 older adults (mean age 72.95, SD = 7.22). An ordinal confirmatory factor analysis (CFA) using robust weighted least squares (WLSMV) evaluated invariance across age groups. Results showed CFI/TLI values of .964/.953, 965/.960, and .964/.969 for Configural (CI), Metric (MI), and Scalar (SI) models. The RMSEA for CI, MI, and SI models was .086, .081, and .071. Based upon recommendations of Cheung and Rensvold (2002), Sass (2011), and Chen (2007), a cutoff of ΔCFI ≥ 0.01 was established as evidence of invariance. The ΔCFI between CI and MI models was < .01 so analysis continued with the SI test. Once again, ΔCFI between MI and SI models was < 0.01 which did not justify rejection of the null hypothesis. Based on these analyses, PSS-10 scores are valid across multiple age groups. Further, results support the multidimensional nature of the PSS-10.

### DOES THE HOME-VISIT COMMUNICATING SERVICE BY POSTAL WORKERS IMPROVE THE MENTAL HEALTH OF OLDER PERSONS LIVING ALONE?

Hiroki Inagaki,[1] and Shuichi Awata,[2] *1. Tokyo Metropolitan Institute of Gerontology, Itabashi-ku, Tokyo, Japan, 2. Tokyo Metropolitan Institute of Gerontology, itabasi-ku, Tokyo, Japan*

In Japan, the number of older households living alone or married couples is increasing as society ages. For such households, Japan Post offers "Mimamori Home-visit service" a fee-based service where postal workers visit once a month to check their living and health conditions. We examined whether the use of this service improves the mental health of users. There were 10,592 service users as February 2019. The survey targeted 524 people (356 women) who started using the system in January or February (wave1) and continued using until August 2019 (wave2). The mean age was 79.5 years. Visiting postal workers conducted tablet-based interviews to assess social support networks (LSNS-6) and mental health (WHO-5). Information on gender, age, and family form (living alone or not) was provided by Japan Post. The WHO-5 average score was 16.4 for wave1 and 16.3 for wave2. Changes in mental health (WHO-5 scores) at 2 time points were compared by ANCOVA using family form (living alone or not) and social isolation (12 points or less/13 or more for LSNS-6) as explanatory variables and gender and age as covariates. The results showed a significant interaction between the 3 factors of time, family form, and social isolation. WHO-5 scores increased (14.2 to 15.3) in the group that lived alone and had 12 or less of LSNS-6. In the other group, the score was no change or lowered. It has been shown that mental health improves with the use of monitoring services in elderly people living alone and in social isolation.

### EFFECTS OF THE JC JOYAGE STEPPED-CARE MODEL ARE DIFFERENTIALLY ASSOCIATED WITH OLDER ADULTS' BASELINE PROFILES

Tianyin Liu,[1] Gloria Hoi Yan Wong,[1] Shiyu Lu,[1] and Terry Lum,[2] *1. The University of Hong Kong, Hong Kong, China, 2. hku, Pokfulam, China*

Depression is a multidimensional construct consists of different symptom clusters. This paper aims to investigate if the impact of a stepped-care model, JC JoyAge, differs for older adults with different baseline depressive symptom clusters. Data came from 702 older people aged 65 and over who completed the JoyAge program. Their depression (measured by Patient Health Questionnaire-9 [PHQ-9]), anxiety, loneliness, and cognition were assessed by social workers at baseline and 12-months follow-up. Among them 609 were at risk or with mild symptoms and received group-based preventive care (prevention group), and 93 had moderate or above symptoms and received intensive intervention (intervention group). Their responses to PHQ-9 were coded to indicate affective, cognitive, behavioral, and somatic symptom clusters. It was found that somatic complaints had the highest prevalence (91%), followed by affective (83%), behavioral (60%), and cognitive symptoms (41%). Logistic regressions were used to estimate the effects of the program. For the prevention group, the JoyAge preventive care is more effective among those who reported behavioral symptoms (b=0.44, p<0.05, OR=1.55, 95% CI: 1.01, 2.40), but less so in those who had cognitive appraisal issues (b=-0.42, p<0.05, OR=0.66, 95% CI: 0.46, 0.96). For the intervention group, the JoyAge intervention was more effective in treatment among those who reported more affective symptoms (b=0.46, p<0.05, OR=1.59, 95% CI: 1.05, 2.42). The benefits of JC JoyAge stepped-care are differentially associated with participants' baseline profile. Participants' overall depressive symptom severity and the presentation of symptom clusters need to be taken into consideration when delivering the services.

### EMENTAL HEALTH LITERACY AND THE RELATIONSHIP TO BARRIERS TO MENTAL HEALTH CARE

Eve Root, and Grace Caskie, *Lehigh University, Bethlehem, Pennsylvania, United States*

According to the American Psychological Association (2017), one in four individuals who are 65 years and older experience a mental health problem; however, many older adults do not receive the services they need and deserve (Karlin, 2008). The current study utilizes a new concept similar to eHealth Literacy called eMental Health Literacy, defined as the degree to which individuals obtain, process, and understand basic mental health information and services needed to aid their recognition, management, or prevention of mental health issues. The relationship of eMental Health Literacy to perceived barriers to receiving mental health services was examined in a sample of middle-aged and older adults. We hypothesized that higher eMental Health Literacy would predict fewer reported barriers to mental health services. A sample of 243 participants (M=63.33, range=55-80 years) were recruited online through Amazon Mechanical Turk to complete measures assessing eMental Health Literacy (eMHEALS) and mental health barriers (BMHSSS-R). After adding two correlated errors, a structural equation model specifying eMHEALS as a predictor of extrinsic and intrinsic barriers to mental health services achieved good fit ($\chi 2(60)=170.014$, p<.001, SRMR=.068, CFI=.944, GFI=.901, TLI=.927, RMSEA=.087). All indicators were significantly related to their latent construct (p<.001). The results indicate higher eMental Health Literacy significantly predicted fewer reported intrinsic and fewer extrinsic barriers to mental health services. These relationships were statistically significant even when examined across differing socioeconomic status and age. These findings indicate eMental Health Literacy may have significant impact on the way individuals in later life navigate through the mental healthcare system.

### HEALTH CHARACTERISTICS AND TREATMENT PATTERNS OF VHA VETERANS DIAGNOSED WITH PTSD IN LATER LIFE

Linh Dang, and Jenefer Jedele, *Veterans Health Administration, Ann Arbor, Michigan, United States*

Older adults may exhibit symptoms of Post-Traumatic Stress Disorder (PTSD) after a traumatic event, even if they do not meet the criteria for a full diagnosis. This sub-clinical

PTSD affects 7-15% of older adults and is associated with elevated depressive symptoms, suicidal ideation, and poorer physical health. Individuals with sub-clinical PTSD often experience worsening symptoms, resulting in a full diagnosis of PTSD, albeit late onset. This evaluation informs the Veterans Health Administration (VHA)'s understanding of the developmental course of PTSD in older Veterans. VHA administrative data were used to examine health characteristics and service utilization in the five years before and five years after the initial VHA documentation of PTSD. We identified a cohort of Veterans (n=27,610), alive and with at least 1 encounter in all evaluation years, with a first PTSD diagnosis documented between ages 50-59, a cohort outside of the average age of diagnosis, but before Medicare eligibility. We compared periods before and after diagnosis across different ages of first diagnosis (50-54, 55-59). Veterans diagnosed at later ages (55-59) had a greater number of mental and physical health conditions. Increasing VHA use preceded a PTSD diagnosis in both groups, but the increase was steeper among those diagnosed at 55-59. Future analyses will compare these patterns to those of Veterans diagnosed at younger ages. Findings from this work may provide a profile of persons at risk for late-life PTSD for use in targeting interventions earlier to reduce the risk of developing worsening PTSD symptoms in late life.

### IMPLEMENTING AN INTERDISCIPLINARY COMMITTEE TO REDUCE PSYCHOTROPIC DRUG AND INCREASE BEHAVIORAL INTERVENTION USE

Lauren Hess Conrad,[1] and David Portman,[2] *1. Butler VA Health Care System, Pittsburgh, Pennsylvania, United States, 2. VA Butler Healthcare System, Butler, Pennsylvania, United States*

In Fiscal Year (FY) 2018, the Butler VA Health Care System's Psychotropic Medication and Behavior Management Committee was identified as a Veterans Integrated Service Network 4 Best Practice. The goal of this committee is to reduce unnecessary psychotropic medication use and polypharmacy and to increase behavioral intervention implementation among Community Living Center (CLC) Veterans. This committee meets quarterly to review Psychotropic Drug Safety Initiative data, behaviors and behavior care plans, and all psychotropic medications prescribed to Veterans. Psychiatric diagnoses, changes to psychotropic medications, and appropriate behavioral interventions are discussed. Committee members take responsibility for action items in accordance with their discipline; documentation of recommendations are made in quarterly behavioral health assessments in CPRS; and follow-up on action items is completed at twice weekly interdisciplinary treatment team meetings, weekly behavior rounds, and/or as needed. From the first quarter (Q1) of FY16 to Q1 FY20, the Butler VA CLC has seen decreased prescriptions of 2 or more anticholinergics (6.6% to 0.80%), antihistamines (12.5% to 5.9%), benzodiazepines (24.7% to 11.0%), and benzodiazepines or sedative hypnotics (23.2% to 9.0%). While prescription of antipsychotic use has increased (Q1 FY20 = 23.8%), the committee will follow Long Term Care Institute guidelines for gradual dose reductions, behavioral interventions, and as needed psychotropic medication PRN use. This committee provides an interdisciplinary forum to discuss and implement beneficial changes to pharmacological and non-pharmacological interventions among all CLC Veterans. The committee is a valuable process for monitoring and reinforcing best practices that may be easily replicated across VA CLCs nationwide.

### MARITAL RELIGIOUS HOMOGAMY AND DIMENSIONS OF WELL-BEING IN LATER LIFE: EVIDENCE FROM THE UNITED STATES

Laura Upenieks,[1] Markus Schafer,[2] and Jeremy Uecker,[3]
*1. University of Texas at San Antonio, San Antonio, Texas, United States, 2. University of Toronto, Toronto, Ontario, Canada, 3. Baylor University, Waco, Texas, United States*

Past research points to the importance of couple-level religious similarity for multiple dimensions of older adults' partnership quality and stability, but we have a limited understanding of whether religious homogamy matters for the well-being of seniors. This study uses dyadic data from the National Social Life, Health, and Aging Project (NSHAP), a representative sample of 953 individuals ages 62–91 plus their marital or cohabiting partners. Using actor-partner interdependence models in the general structural equation model framework (GSEM), we find that religious attendance homogamy is beneficial for the physical health of men and the mental health and self-reported happiness of women. There were no associations between religious homogamy for religious importance detected. Taken together, our results attest to the ongoing importance of religious similarity—service attendance, in particular—for mental and physical well-being in later life. Future research is needed to more fully examine which mechanisms account for these patterns.

### OBJECTIVE AND SUBJECTIVE COGNITIVE FUNCTION, AND RELATIONS WITH QUALITY OF LIFE AND PSYCHOLOGICAL DISTRESS

Hallie Nuzum,[1] Katherine Dorociak,[1]
Shirit Kamil-Rosenberg,[1] Peter Louras,[2]
Mandana Mostofi,[2] and J. Kaci Fairchild,[1] *1. VA Palo Alto Healthcare System, Palo Alto, California, United States, 2. Palo Alto University, Palo Alto, California, United States*

Objective and subjective cognitive function have been associated with decreased quality of life and increased psychological distress in older adults. The present study examined relations of objective and subjective cognition with quality-of-life and mental-health outcomes in individuals with amnestic mild cognitive impairment (aMCI). The sample included 98 older adults with aMCI (92.5% male, age = 70.9±9.2 years). Measures included objective cognition (i.e., attention, memory, language, visuospatial abilities, processing speed, executive function, and overall), subjective memory (Multifactorial Memory Questionnaire [MMQ]), quality of life (Dementia Quality of Life [DQoL]), and mental health (Geriatric Depression Scale, Geriatric Anxiety Inventory, and Penn State Worry Questionnaire). Objective and subjective cognition were weakly correlated (range $|r| = .00–.23$). Objective cognitive measures were largely uncorrelated with quality of life or mental health, with only two significant ($p < .05$) correlations between Processing Speed and Worry (r = -.24), and Overall Cognition and DQoL Aesthetics (r = .20). Subjective cognition was more strongly correlated with quality of life, including significant (p < .01) correlations between MMQ Abilities and DQoL Negative Affect (r = -.38), and MMQ Contentment and DQoL Positive Affect (r = .28). Additionally, MMQ Contentment and Abilities were significantly (p < .01) negatively correlated with all three mental-health outcomes (range |r| = .28–.43). This study demonstrated that subjective memory, particularly affect and self-appraisal regarding one's memory capabilities, is more closely related to quality-of-life and mental-health outcomes than objective cognitive performance in an aMCI sample, and, therefore, may represent important targets for intervention.

### PARALLEL PROCESS LATENT GROWTH MODELING OF THE RELATIONSHIPS BETWEEN SOCIAL COHESION AND MENTAL HEALTH IN LATER LIFE

Sok An,[1] Kyeongmo Kim,[2] and Minhong Lee,[3] *1. Korea Rural Economic Institute, Austin, Texas, United States, 2. Virginia Commonwealth University, Richmond, Virginia, United States, 3. Dong-eui University, Busan, Pusan-jikhalsi, Republic of Korea*

Previous literature suggests that social factors (e.g., social cohesion, social support) are protective predictors of mental health problems. However, there might be a reciprocal relationship between social factors and mental health and the relationship changes over time. Therefore, this study examined the longitudinal relationship between community social cohesion and mental health using a latent growth curve model with 8 waves of the National Health and Aging Trends Study (NHATS; 2011-2018), a nationally representative panel study of Medicare beneficiaries in the United States. Social cohesion measured the perceived level of mutual trust by three items (score range: 0-6) and mental health was measured by PHQ-4 (score range: 0-12). The final model including covariates (age, gender, functional disabilities) fit the data well: $\chi 2$=1036.383, p<.001; RMSEA=.037; CFI=.960; and SRMR=.070. Initial level of social cohesion was negatively associated with initial level of mental health problem ($\beta$=–.23, p< .001), suggesting that higher levels of social cohesion was associated with lower levels of mental health problems. The covariance between social cohesion slope and mental health slope was significant ($\beta$=–.16, p< .01), suggesting an increase in social cohesion was associated with a decrease in mental health problems over time. Functional disabilities significantly influenced mental health over time, while functional disabilities did not influence social cohesion consistently. This study adds to the growing literature on the ways mental health status and social connection have reciprocal relationships over time. Therefore, mental health status in later life could be decreased by improving social cohesion and connectedness with the community.

### PATIENT-REPORTED OUTCOME MEASURES IN OLDER VETERANS INITIATING A NEW EPISODE OF MENTAL HEALTH CARE

Jenefer Jedele,[1] Karen Austin,[1] and Sandra Resnick,[2] *1. Veterans Health Administration, Ann Arbor, Michigan, United States, 2. Veterans Health Administration, West Haven, Connecticut, United States*

The VA Measurement Based Care (MBC) in Mental Health (MH) Initiative supports implementing patient reported outcome measures (PROMs) for MH treatment planning and shared decision-making as a routine aspect of care. Using VHA administrative data, we identified Veterans initiating a new MH treatment episode (index encounter), i.e. prior 6-months without VHA MH encounters. We compare MH diagnoses, medications, and encounters during the 6-months from and including the index encounter by age (50-64; 65-79; 80+) between Veterans receiving 1 or more measures (PROM) to those receiving none (noPROM). The percentage of PROM Veterans decreased with age: 26.7% (50-64); 18.5% (65-79); 12.5% (80+). Consistent across age, PROM Veterans had more encounters than noPROM Veterans. In the year before treatment initiation, a smaller percentage of PROM Veterans had multiple MH diagnoses (21.0% v. 29.1%). At treatment initiation, both groups were equally likely to have multiple diagnoses (20.7% v. 20.1%); a higher percentage of the noPROM group were diagnosed with schizophrenia (3.8% v. 1.0%), bipolar (4.5% v. 2.2%), or PTSD (29.2% v. 21.8%). Substance use disorder and major depression were more prevalent in the PROM group. These patterns held across age categories. A smaller percentage of PROM Veterans had been prescribed psychotropic medication during the index encounter (32.8% v. 42.8%). For PROM Veterans, an average of 3 measures were received 1.5 months apart. The number of measures declined and the interval between measures increased with age. Potential barriers and possible efforts to target the use of PROMs with older Veteran patients are discussed.

### PERCEIVED PARTNER RESPONSIVENESS ATTENUATE THE LINK BETWEEN MENOPAUSAL SYMPTOMS AND DEPRESSED AFFECT

Kayeon Lee, Hey Jung Jun, Susanna Joo, and Sun Ah Lee, *Yonsei University, Seoul, Republic of Korea*

The years around menopause are the time that associates not only with hormonal changes but with psychological and social transitions, and previous studies have consistently revealed the relationship between menopause and depression. The present study examined the moderating effect of perceived partner responsiveness (PPR) on the association between menopausal symptoms (MS) and depressed affect (DA). The sample was middle-aged climacteric women (N = 754, Age=49-60) from the second wave of Midlife in the United States (MIDUSII). Measurement for MS consisted of the frequency of five symptoms in the past 30 days (insomnia, heavy sweating, painful intercourse, hot flashes, and irritability). PPR was assessed using three items matched the core components of responsiveness (understanding, validating, and caring). Results revealed that there were significant interactions between menopausal symptoms and PPR (b = 0.05, p < 0.039). Specifically, the level of elevation of DA in response to MS was smaller in women with higher levels of PPR (b = 2.93, p < 0.001) than in those with lower levels of PPR (b =3.05, p < 0.001). According to the region of significance analysis, the coefficients of MS on DA were significant within the -2SD to +2SD range of PPR, but it decreased as the PPR increased. Findings suggest that partners' careful responsiveness may mitigate the detrimental effects of MS on DA among climacteric women.

### PRESENCE OF MEANING MEDIATES THE RELATIONSHIP BETWEEN MEANING SEARCH AND OUTCOME: A CROSS-CULTURAL STUDY

Nicole Long Ki Fung, and Helene Fung, *The Chinese University of Hong Kong, Hong Kong, China*

Search for meaning (SFM) is associated with many well-being measures. The mechanism behind remains unclear. This study explores presence of meaning (POM) as a mediator to explain the association. While dialectical thinking in Eastern cultures values both process and outcome, oppositional thinking in Western cultures makes the two opposing. Since dialectical thinking increases with age, we hypothesize that with increased age, SFM is associated with POM more positively (less negatively). This heightened POM results in better well-being. We surveyed 2014 participants (aged 18-96, Mage= 55.6) in Eastern cultures: Hong Kong and Taiwan; Western cultures: Germany, United States and the Czech Republic. In Eastern cultures, SFM was positively associated with POM and life satisfaction. POM partially mediated the relationship between SFM and life satisfaction (b=0.328, p<.001). With age, SFM was associated more positively with POM and life satisfaction (b=0.009, p<.001). While POM partially mediated the relationship in younger adults (b=0.162, p<.001), full mediation was found in older adults (b=0.451, p<.001). In Western cultures, SFM was negatively associated with POM and life satisfaction. POM partially mediated the relationship between SFM and life satisfaction (b=-0.120, p<.001). With age, the negative association of SFM with POM and life satisfaction was attenuated (b=0.002, p<.001). These finding suggested that SFM becomes more beneficial to older adults across culture via establishing POM. Identifying factors that facilitate the process of achieving meaning through searching is therefore important.

### RURAL HOARDING IN OLDER ADULTS: A PILOT CHARACTERIZATION STUDY

Mary Dozier, *Mississippi State University, Mississippi State, Mississippi, United States*

There has been limited research on geriatric hoarding disorder in rural areas. Older adults living in rural areas are more likely to feel stigmatized due to mental health difficulties and to have multiple barriers to healthcare. The purpose of this study is to present the clinical picture of eight older adults (mean age 68, range 57-92) with hoarding disorder who live in the rural southeastern U.S. Participants completed a semi-structured interview and the NIH Toolbox Emotion and Cognition Batteries in their homes. Participants were mostly female (n = 6) and identified as White (n = 5) or African American (n = 3). All participants reported being Christian. Most participants were divorced or had never married (n = 6). All participants reported having at least one current medical condition, with the most commonly reported diagnosis being high blood pressure (n = 4). Half of participants reported that they had experienced at least one intervention from their family; however, only one participant reported ever experiencing an intervention from another source (i.e., property manager). On average, participants reported having a low level of emotional support and life satisfaction and a high level of loneliness and somatic symptoms of fear. Participants' performance on tests of cognitive functioning was worst for processing speed, with three out of the eight participants performing at the level of borderline impairment or worse. Understanding the clinical presentation of hoarding disorder in rural-dwelling older adults is the first step to the development and implementation of evidenced-based treatments in this population.

### SUICIDE RISK IN OLDER ADULTHOOD: DIFFERENTIAL PATHWAYS BASED ON RACE

Matthew Fullen,[1] Mary Mize,[2] Jihee Hong,[2] Laura Shannonhouse,[2] and Jordan Westcott,[1]
*1. Virginia Tech, Blacksburg, Virginia, United States,*
*2. Georgia State University, Atlanta, Georgia, United States*

Late-life suicide is a complex public health issue, and older adults have a higher risk threshold than the national average (Drapeau & McIntosh, 2020). Most late-life suicide research focuses on elevated risk of older white males, and less is known about risk factors among Black older adults (Joe et al., 2014). Although fewer Black older adults die by suicide than White older adults, forms of suicidality do not differ between Black and White older adults (Cohen et al., 2008). Suicide risk factors, such as psychological distress (Watkins & Johnson, 2018) and chronic pain (Bazargan et al., 2016), are prevalent among Black older adults. According to the Interpersonal Theory of Suicide (IPTS; Van Orden et al., 2016), thwarted belongingness and perceived burdensomeness inform the development of suicidal desire. These findings have been corroborated among older adult samples, though lacking racial diversity. To better understand how the IPTS functions for older adults, and probe whether suicide risk pathways operate differently depending on race, we used data from over 400 homebound older adults residing in a U.S. metropolitan area to clarify if this suicide risk pathway is similar for Black and White older adults. Race moderated the relationship between physical and psychological pain and thwarted belongingness and perceived burdensomeness, with pain among Black older adults having a greater impact on their sense of belonging and burdensomeness. Findings illuminate the need for culturally nuanced understandings of suicidality in older adulthood. The presenters will demonstrate these results and discuss implications for cross-cultural suicide prevention frameworks.

### THE IMPACT OF FINANCIAL COACHING ON HEALTH AND FINANCES IN OLDER SCAM AND ID THEFT VICTIMS

Peter Lichtenberg, LaToya Hall, Rebecca Campbell, and Evan Gross, *Wayne State University, Detroit, Michigan, United States*

Population based and clinical samples consistently demonstrate that older financial exploitation (FE) victims are vulnerable across multiple domains. Our earlier research indicated that older scam and identity theft victims were vulnerable across multiple health and mental health dimensions as compared to a control group. The current work is a longitudinal study of 20 financial coaching participants and 20 controls. Program participants received individual financial coaching to help with credit problems, reporting and collections after they were scam or identity theft victims. Both groups received baseline assessment and 6 month followups (from the time of the end of the coaching). The mean age of the sample was 69 years, with a mean of 15 years of education. Seventy percent of the sample were women and African Americans. Control participants had fewer health conditions and better memory and executive functioning at baseline as compared to coaching participants. While there were no significant health measure changes in the control group, the coaching group demonstrated significantly decreased anxiety at follow up and trends for improved executive functioning and IADLs. Further, while financial stress and social support were significantly lower for the coaching group at baseline, at followup there were no group differences in these measures. The study results provide evidence that the financial coaching program has served as a protective factor in negating many of the adverse outcomes associated with FE and supports financial coaching programs as an appropriate intervention. In addition the coaching services saved or returned monies to nearly 65% of participants.

### THE IMPORTANCE OF POSITIVE EVENTS WHEN LIVING WITH CHRONIC PAIN

Julie Kircher,[1] Susan Charles,[2] Nancy Sin,[3] and David Almeida,[4] *1. University of California, Irvine, Irvine, California, United States, 2. BSS, Irvine, California, United States, 3. University of British Columbia, Vancouver, British Columbia, Canada, 4. Pennsylvania State University, University Park, Pennsylvania, United States*

Chronic pain is a common condition in later life that is related to high levels of anxiety and depression. One reason why chronic pain is related to affective distress is that this condition may prevent people from deriving the same positive emotions from enjoyable activities. Few studies, however, have examined how exposure and reactivity to daily events differ by chronic pain status. We hypothesized that those with chronic pain will have less exposure and less positive affect reactivity to positive daily events compared to those without chronic pain. Participants from the diary substudy of MIDUS (N = 1,733; nChronicPain = 658, nNoPain = 1,075; M = 56 years-old) completed eight interview days. Chronic pain status was unrelated to the frequency of positive events. Multi-level models revealed that although people with chronic pain had lower levels of daily positive affect, they reacted more positively to daily events ($\gamma = -.033$, SE = .010, $p < .0001$). As a result, levels of daily positive affect on days when people experienced a positive event did not vary by pain status (MChronicPain = 2.73, MNoPain = 2.75). People with chronic pain averaged higher levels of daily negative affect compared to people without chronic pain (MChronicPain = .21, M NoPain =.20), but, on days when they experience a positive event, those with chronic pain had a greater decrease in their negative affect. Findings suggest that positive events impact those with chronic pain more than they do individuals without chronic pain.

### SESSION 2936 (POSTER)

### GENDER AND SEXUALITY

### AGING AND OLD AGE IN COLOMBIAN TRANS WOMEN: A GROUNDED THEORY APPROACH

Maria Reyes,[1] Maldonado Daniela,[2] Méndez Carlos,[3] Maria Ariza,[4] Vannesa Arias,[3] and Isabella Pachon,[3] *1. Universidad El Bosque, Bogota, Colombia, 2. Red Comunitaria Trans, Bogota, Distrito Capital de Bogota, Colombia, 3. Universidad El Bosque, Bogota, Distrito Capital de Bogota, Colombia, 4. Universidad El Bosque, BOGOTA, Distrito Capital de Bogota, Colombia*

Trans people around the world represent one of the most marginalized and stigmatized groups in society who are at high risk of discrimination, violence and abuse (White Hughto, Reisner, & Pachankis, 2015). In Colombia, older adults face a situation of vulnerability and poverty, and this situation is even more dramatic for older people with diverse gender identities. The research focused on understanding the challenges that a group of Colombian trans women experience in the process of aging and old age. An exploratory qualitative research project was carried out using constructionist Grounded Theory. Twenty five trans-women from 50 to 67 years old who live in Bogotá, Colombia participated. The data were collected using semi-structured interviews. The results address three main research questions: (a) How the participants overcome the life expectancy and achieve middle and older adulthood? (b) What are the barriers the participants faced in the aging process? (c) How do the group of Colombian trans women imagine and considered a successful aging? The results evidenced that the process of aging of the participants was influenced by six psycho-socio-cultural categories. A central category that was identified as opportunity, which was influenced by five categories: a) Violence, discrimination and transphobia, b) Transit process, c) Personal strengths d) Mobilization and activism and e) experience and perception the old age. Discussion. The challenges that the participants experienced were those associated with the process of aging and to their gender identity. Trans women achieve middle and old adulthood for their personal strengths, activism and mobilization.

### ARE OLDER ADULTS SEXUALLY WELL: A COMPARATIVE QUALITATIVE STUDY IN PORTUGAL AND ROMANIA

Sofia von Humboldt,[1] Georgeta Niculescu,[2] Gail Low,[3] and Isabel Leal,[1] *1. Instituto Superior de Psicologia Aplicada (ISPA), Lisbon, Portugal, 2. Romanian Association for Person Centred Psychotherapy (ARPCP), Bucharest, Romania, 3. Faculty of Nursing, University of Alberta, AB, Edmonton, Canada, Alberta, Canada*

Objective: This study aims the perspectives of older adults on their sexual well-being. For this purpose, a qualitative research was carried out, which analyzes older adults' perspectives on indicators of sexual well-being in Portugal and Romania. Methods: Forty seven older participants aged 65 to 91 years, were interviewed. Participants lived in the community. All the interviews went through content analysis. Results: Preliminary results of content analysis generated 5 themes for the Romanian sample: Supportive relationship ($k = .92$, $p < .01$); positive financial situation ($k = .91$, $p < .01$); good health ($k = 94$, $p < .01$); education ($k = .88$, $p < .01$); and family support ($k = .89$, $p < .01$); and five themes for the Portuguese sample: Supportive relationship ($k = .91$, $p < .01$); demonstration of love ($k = .91$, $p < .01$); sharing joint activities ($k = 92$, $p < .01$); positive attitude and good humor ($k = .91$, $p < .01$); and open communication ($k = .99$, $p < .01$); Conclusions: This study highlighted the perspectives of Portuguese and Romanian older adults concerning sexual well-being. For both samples, showing a supportive relationship with a partner was the more frequent theme. Keywords: Content analysis; cross-national; older adults; qualitative study; sexual well-being.

### AS WE AGE: LISTENING TO THE VOICES OF LGBTQ OLDER ADULTS

Cory Cummings,[1] Jennifer Dunkle,[2] Bianca Mayes,[3] Carolyn Bradley,[4] and Porsha Hall,[5] *1. Monmouth University, West Long Branch, New Jersey, United States, 2. Stockton University, Atlantic City, New Jersey, United States, 3. Garden State Equality, Asbury Park, New Jersey, United States, 4. Monmouth University School of Social Work, West Long Branch, New Jersey, United States, 5. SAGE (Advocacy and Services for LGBT Elders), New York, New York, United States*

LGBTQ older adults present a range of biopsychosocial needs and life experiences that may differ from the general population of older adults. Researchers have broken LGBTQ older adults into three age brackets: the Invisible Generation born before the 1920s; the Silent Generation born in the 1930s and 1940s; and the Pride Generation born in the 1950s and 1960s (Fredriksen-Goldsen, 2016). Research is emerging on health disparities and is fueling calls for inclusive services for this population. This paper session reports on the work of a research collaborative between social work and public health (two universities and a statewide advocacy organization). A qualitative study, designed as phase one a statewide need assessment, engaged ten focus groups (N=48 participants) throughout a mid-Atlantic state. Study aims were to better understand the experiences and perceptions of LGBTQ older adults now and expectations and plans for care as they age. Findings included (1) emphasis on the nuance of connection as an ageing LGBTQ adult; (2) expectations for quality of services; (3) realities of planning for future living arrangements; and (4) two sides of advocacy, as both a personal responsibility and a call for allyship. Recommendations will be made on how attendees can: evaluate agency policies and procedures to create safe spaces and inclusive services, engage in needs assessments of older LGBTQ+ adults in their own communities, and advocate at the State and Federal levels to strengthen services in the aging network to better serve this group, with specific focus on the Older Americans Act.

### ASSOCIATION BETWEEN CHRONIC ILLNESS, MASCULINITY, AND WELL-BEING IN OLDER BLACK MEN

Hana Young,[1] Jessalyn Li,[1] and Darlingtina Esiaka,[2]
*1. Union College, Schenectady, New York, United States,
2. Union College, Union College, New York, United States*

Research suggests that living with a chronic illness has deleterious impacts on the well-being and quality of life of aging adults. Specifically, it opposes traditional masculine constructs in men and impacts their physical and mental health outcomes. In this study, we examined the lived experiences of Black men, aged 55 years and over, and diagnosed with chronic illness using life history narratives. Participants responded to open-ended questions such as "what aspects of living with illness do you find relatively difficult or easy?" and "what situations make you particularly aware of your illness?" Common themes that emerged from the participants' responses were the performance of masculinity, fulfilling family duty and obligation, limited sexual encounters, and feelings of exclusion in one's community. Additionally, participants stated that chronic illness impacts their mental well-being and triggers behavioral responses that exacerbate their ability to cope with the illness. Their responses highlight the conflict between traditional masculine expectations and the presence of chronic illness and illustrate the extent to which 'manhood' is a determinant of health even in older men. Our findings can inform the development of tools and interventions designed to improve the experience of well-being among Black men aging with chronic illness.

### FINANCIAL DECISION MAKING AND SURVEY RESPONSES OF OLDER COUPLES

Angela Curl, and Estevan Tobias Molina, *Miami University, Oxford, Ohio, United States*

The Health and Retirement Study (HRS) asks mid-life and older couples to identify the person most knowledgeable about household finances to provide responses to financial questions ("financial respondent" [FR]). The purpose of this study is to analyze predictors of a female FR, with a focus on gendered financial decision making. This study analyzed HRS data from 2012 to 2018. Our sample consisted of 5,038 married/partnered couples over the age of 50 (at baseline), after eliminating cases with missing data on study variables. Using HLM 6.08 software, we computed the odds of couple households having a female FR. Our main predictor was the amount of female financial decision making power, as measured using eight Likert scale items (0=male always makes this decision, 4=female always makes this decision), for purchases (e.g., cars, major appliances) and financial activities (e.g., paying bills, buying groceries); summed into a scale (range: 0-32). Our models controlled for race, ethnicity, education, income, age, health, cognitive ability, and depressive symptoms. Results indicate that men were more likely to be the FR, but when the female makes more household financial decisions then she is more likely to be the FR (OR = 1.16, p<.01). Black race, lower income, and time also increased the odds of a female FR. This study highlights women's historical and ongoing limited power in financial matters of married couples. Financial knowledge and decision making skills should be encouraged for both partners in couple households.

### GENDER AND SEXUALITY DIFFERENCES IN THE LINK BETWEEN MARITAL QUALITY AND HEALTH AMONG OLDER ADULTS

Yingling Liu, *Baylor University, Waco, Texas, United States*

Research shows that married people tend to have better health than those who are not married, but not all marriages are equally protective. Aversive marriage has been proved to be negatively related to health. Although many studies have found gender differences in the relationship between marital quality and health, little research has done to explore the potential mechanism behind. The aim of this longitudinal study was to investigate the relationship between marital quality and health, as well as to determine how gender and sexuality play a role in this relationship. Using a sample of older American adults aged 57-85 from the Wave 1 and 2 National Social Life, Health, and Aging Project (N=1250 married individuals), results from the statistical model showed that compare to supportive marriage, men who are in aversive and indifferent marriages have significant worse self-rated physical health, feel less happy, and more depressed over five years period of time. For women, however, indifferent and aversive marriage is only associated with less happiness over time. Ambivalent marriage has no significant relationship with any health outcome either for men or for women. In addition, results from the mediation analysis suggested that the frequency of sex explains the relationship between marital quality and health only for men but not for women. These important findings shed light on the different mechanisms behind marital quality and health between men and women. The potential benefit of this study provides a basis for developing a marriage counseling strategy for older men and women.

### PERCEIVED COGNITIVE ABILITY IN LESBIAN, GAY, BISEXUAL, AND HETEROSEXUAL VIETNAM ERA VETERANS

Michelle Hilgeman,[1] John Blosnich,[2] Yasmin Cypel,[3] Fatema Akhtar,[4] Aaron Schneiderman,[5] Erick Ishii,[6] Dennis Fried,[7] and Victoria Davey,[4] *1. VA Medical Center--Tuscaloosa, Tuscaloosa, Alabama, United States, 2. University of Southern California, Los Angeles, California, United States, 3. U.S. Department of Veterans Affairs, Washington DC, District of Columbia, United States, 4. U.S. Department of Veterans Affairs, Washington, District of Columbia, United States, 5. U.S. Department of Veterans Affaris, Washington, District of Columbia, United States, 6. Department of Veterans Affairs (10. P4. Q), Washington DC, United States, 7. VA New Jersey HCS, EAST ORANGE, New Jersey, United States*

Lesbian, gay, and bisexual (LGB) Veterans report stress (e.g., discrimination under Don't Ask Don't Tell policies) and mental health conditions (e.g., depression) that may increase risk for neurocognitive changes like dementia. Subjective cognitive decline (SCD) can be an early indicator of neurocognitive change – yet no known studies have examined SCD in LGB Veterans. Cross-sectional data from the Vietnam Era Health Retrospective Observational Study (VE-HEROeS) were examined for 260 LGB and 17,796 heterosexual Veterans. VE-HEROeS is the latest probability-based survey of Vietnam Era Veterans (1961–1975) as older adults (2016-2017). SCD was assessed using two subscales of the Functional Assessment of Cancer Therapy-Cognitive Instrument Version-3 (FACT-Cog). Good reliability was observed in this sample: Cronbach's alpha =.94 for the 7-item Perceived Cognitive Abilities subscale and .88 for the 4-item Comments from Others. Analyses were weighted to account for the complex survey design. LGB Veterans were slightly younger (M=68.3, range 59-84) than heterosexual Veterans (M=69.1, range 58-99, p=.03); were more likely to be female (13% vs 3%, p<.01); and had fewer people living in the household (M=1.7 vs. M=2.1, p<.01). LGB Veterans were also more likely than heterosexual Veterans to report feeling depressed most or all of the time over the past 30 days (5.7% vs. 3.6%, respectively, p<0.01) on a single 5-point Likert-scale. SCD indicators did not vary by Veteran sexual orientation (M=19.69 and M=19.69; M=14.2 and M=14.1) and were elevated compared to published studies in healthy adult samples. More work is needed to examine neurocognitive risk factors in aging LGB Veterans.

### PORNOGRAPHY: HOW DOES THE BOOMER GENERATION AND OLDER PREDICT OTHERS' VIEWING TIME?

Katie Sakel,[1] and Joshua Grubbs,[2] *1. Bowling Green State University, Gibsonburg, Ohio, United States, 2. Bowling Green State University, Bowling Green, Ohio, United States*

The increase of exposure to online pornography has decreased the age of initial exposure to pornography. However, very little is known about the outcomes resulting from increased pornography exposure in the Baby Boomer generation and beyond. The current study asked what predictors were significant in individuals born in 1965 and earlier when predicting the perceived pornography viewing time for the average man and woman. To answer this question, a nationally representative population (N = 1073, 510 males) completed a web-based survey measuring the age of the participant, gender of the participant, self-directed sexual behaviors ("How frequently have you masturbated while viewing pornography alone?"), partner-directed sexual behaviors ("How frequently have you viewed pornography with a partner?"), a religiosity index ("How important is your religion?"), and the predicted perceived time that a woman and man watches pornography, Results showed that perceived time that the average man spent viewing pornography was significantly predicted by age of the participant, gender of the participant, self-motivated sexual behaviors and partner-motivated sexual behaviors. Religiosity was not a significant predictor. In the regression predicting perceived time that the average woman viewed pornography were age of the participant, self-motivated sexual behaviors, and partner-motivated sexual behaviors. Gender of the participant and religiosity of the participant were not significant predictors. Further research should expand this work to a lifespan perspective and longitudinal studies.

### PRELIMINARY FINDINGS OF NEEDS OF OLDER CAMBODIANS IN THREE PROVINCES

Kakada Kuy, *Washington University St Louis, University City, Missouri, United States*

The most recent national survey of older Cambodians was conducted in 2004. To further our understanding of Cambodia's older adults, new data are required. This primary data collection study aimed to assess physical, psychological and social needs of older Cambodians and examine gender differences in those needs. We collected data in 2019 in three provinces in the northwestern region of Cambodia (N=210). Due to older Cambodians' higher level of illiteracy, research assistants filled out paper surveys for them during individual interviews. Then responses were transferred into an e-survey database for data management. Physical needs were examined with self-rated health and health complaints. Psychological wellbeing and depression scores were used to measure psychological needs. For social needs, we examined social support using Social Network and Social Support scale. More than half of older Cambodians had less than good physical health and reported numerous health symptoms. Yet, three quarter reported higher than average psychological wellbeing and a low level of depression. Some of the samples showed modest level of social support in their family and community. Furthermore, independent two-sample T-tests showed that older men and women did not differ in physical and social needs ($p<.001$). Older women, however, did poorly in psychological needs compared to men ($p<.001$). Men also had lower level of depression and better psychological wellbeing ($p<.001$). Future studies shall further investigate other aspects of Cambodia's aging population in which gender differentials may also exist.

### RELATIONSHIP BETWEEN HEALTH AND WELL-BEING AND ADHERENCE TO SOCIAL NORMS ACROSS ADULTHOOD

Giselle Ferguson,[1] Martin Sliwinski,[2] and Stacey Scott,[1]
*1. Stony Brook University, Stony Brook, New York, United States, 2. Penn State University, University Park, Pennsylvania, United States*

When an individual deviates from social norms, others implement stressful social consequences to discourage deviance. Over a lifetime, this stress may accumulate, causing physiological wear-and-tear and poorer health and well-being. However, older adults may choose to surround themselves primarily with close, supportive others, so they may no longer be exposed to consequences for deviance, and would then be shielded from downstream effects. Alternatively, adults from older cohorts may value traditional norms more strongly, and would therefore experience greater effects of norm deviance. We expected that individuals who adhered to norms would report better health and well-being than individuals who deviated from norms, and that this effect would be either absent or intensified for older adults. We tested this prediction with a secondary data analysis of n=220 adults ages 25-65 (63.2% women). We operationalized individuals' adherence to their respective gender norms and to general, non-gendered norms using demographic and questionnaire responses. Greater general norm adherence significantly predicted better health and well-being, independent of age. For masculine norms, work-primacy norm adherence predicted better well-being, but anger and self-reliance norm adherence predicted worse health and well-being in men. Age moderated this effect: with older age, the effect of anger was reversed such that greater anger predicted better health among men. For feminine norms, adherence did not predict better outcomes in women at any age. Although the relationship between gendered norm adherence and health and well-being may depend on cohort, the results suggest that general norm adherence does influence health and well-being.

### SELF-RATED HEALTH IN OLDER ADULTS (60+) WHO ARE SEXUALLY ACTIVE: ASSOCIATION WITH PLEASURE

Nicole Viviano, *University of Maryland, Baltimore, Gaithersburg, Maryland, United States*

Older adults who engage in sexual activity (SA) tend to have greater enjoyment in life compared to their less sexually active counterparts. Further, older adults tend to report more satisfying or pleasurable SA when less health conditions are reported. Although self-rated health (SRH) is often incorporated in studies concerning SA, it is not always included when examining sexual intimacy, pleasure, and pain/discomfort. It is important to understand how SRH is associated with both SA and sexual pleasure as it could indicate when providers need to initiate conversations with their patients regarding safe sex practices and/or diagnose related health conditions (i.e. erectile dysfunction, vaginal dryness, etc.). The current study examined the association between SRH and experiencing pleasurable sexual interactions in older adults (60+). The Midlife in the United States (MIDUS) Refresher dataset (2011-2014) was used for this secondary data analysis. The dependent variable is pleasure in sexual interaction; the independent variable is self-rated health. Covariates include marital status, race, sex, sexual intimacy and sexual pain/discomfort. An ordinal logistic regression shows that for every one-unit increase in SRH, there is 1.341 times odds of experiencing pleasure in sexual interactions in older adults 60+ after adjusting for covariates. Individuals who had higher SRH had increased odds of having pleasurable sexual encounters. It is already clear that older adults are engaging in sexual activity, despite stereotypical myths. Further understanding the relationships between factors of SA like pleasure, pain/discomfort, and intimacy and health factors might allow for the extension of a healthy sexual life.

### SEX DIFFERENCE IN SELF-REPORTED SEXUAL FUNCTIONING AMONG FRAIL OLDER ADULTS

Brianne Olivieri-Mui,[1] Sandra Shi,[2] Ellen McCarthy,[3] and Dae Kim,[1] *1. Hebrew SeniorLife, Harvard Medical School, Roslindale, Massachusetts, United States, 2. Beth Israel Deaconess Medical Center, Boston, Massachusetts, United States, 3. Hebrew SeniorLife, Harvard Medical School, roslindale, Massachusetts, United States*

Frailty may differentially impact how older adult males and females perceive sexual functioning, an important part of well-being. We assessed the level of frailty (robust, pre-frail, frail) for anyone with data on 11 sexual functioning questions asked in wave 2 of the National Social Life, Health, and Aging Project, 2010-2011 (n=2060). Questions covered five domains: overall sexual function (OSF), sexual function anxiety (SFA), changes in sexual function (CSF), erectile/vaginal dysfunction (EVD), and masturbation. Logistic regression identified sex differences in frailty and reporting worse sexual functioning. Linear regression predicted the number of domains reported as worse. Among males (n=1057), pre-frailty meant higher odds of reporting SFA (OR 1.8 95%CI 1.2-6.6), CSF (OR 1.7 95%CI 1.1-2.7), and EVD (OR 1.5 95%CI 1.0-2.2). Among females (n=1003), there was no difference in reporting by frailty. Females were more likely to report worse OSF (Robust: OR 7.4, 95%CI 4.8-11.4; Pre-frail: OR 6.2, 95%CI 3.9-9.9; Frail: OR 3.4 95%CI 1.7-6.6), but less likely to report SFA (Robust OR .3, 95%CI .2-.5; Pre-frail OR .2, 95%CI .1-.3; Frail OR .2 95%CI .1-.3). Pre-frail and frail females reported fewer domains as worse (Pre-frail coefficient -0.21 SE 0.09, Frail -0.43 SE 0.14). As frailty worsened, males reported more domains as worse (Pre-frail 0.24 SE 0.07, Frail 0.29 SE 0.08). Self-reported sexual functioning differs by sex at all levels of frailty, and reporting by males, but not females, changes with frailty. Providers should be aware that sexual functioning is of importance to both sexes despite varying degrees of frailty.

### SEX IN LATE LIFE: ASSESSING ADULTS' EXPECTATIONS AND THE ROLE OF HEALTHCARE PROFESSIONALS

Jill Naar,[1] Raven Weaver,[2] and Shelbie Turner,[3]
*1. Appalachian State University, Boone, North Carolina, United States, 2. Washington State University, Pullman, Washington, United States, 3. Oregon State University, Corvallis, Oregon, United States*

Sexual activity contributes to quality of life throughout the lifespan. However, stigma about sex in late life influences older adults' perceptions and healthcare professionals' perceptions of older adults' sexual health/behaviors. Using a multi-methods approach, we examined attitudes and knowledge about sexual health/behaviors in late life. Using longitudinal data from the Midlife in the US Study (Wave 1-3; N=7049), we ran age-based growth curve models to analyze changes in levels of optimism about sex in their future. We also piloted a survey with healthcare professionals assessing attitudes, knowledge, and awareness of policy about sexual health/behaviors among older adults. Adults' expectations became less optimistic with increased age ($\beta = -0.1$, SE = 0.003, $p < .0001$). Men were more optimistic than women at age 20 ($p = 0.016$), but men's optimism decreased over the life course at a faster rate than did women's ($p < .0001$), so that from ages 40-93, men were less optimistic than women. Among healthcare professionals (N=21), the majority indicated never or rarely asking their clients about sexual history or health/behaviors; however, they indicated some knowledge about issues relevant to older adults (e.g., safe-sex practices, sexual dysfunction). Few indicated awareness about policies related to sexual behavior among residents (i.e., issues of consent, STIs). Among adults, there is a need to address declining optimism for expectations about sex in late life. Health professionals are well-situated to raise awareness and normalize discussions about sexual health, thus countering negative stigma and contributing to increasing optimism for expectations to remain sexually active.

### SEXUAL EXPRESSION IN OLD AGE: A QUALITATIVE STUDY

Sofia von Humboldt, José Alberto Ribeiro-Gonçalves, and Andrea Costa, *Instituto Superior de Psicologia Aplicada (ISPA), Lisbon, Portugal*

Objective: This study aims to analyze how older adults express themselves sexually. For this purpose, a qualitative research was carried out, which analyzes their perspectives at a cross-national level. Methods: Four hundred and ninety five older participants aged 65 to 98 years, were interviewed. Participants were of three different nationalities and lived in the community. All the interviews went through the process of verbatim transcription and subsequent content analysis. Results: Results of content analysis produced nine themes: Tender and care (k = .91, $p < .01$); altruism and gratitude (k = .81, $p < .01$); attractiveness (k = 94, $p < .01$); positive communication (k = .89, $p < .01$); sexual activity (k = .88, $p < .01$); good health and physical condition (k = .96, $p < .01$); supportive relationship (k = .84, $p < .01$); eroticism (k = .94, $p < .01$); and feeling active and alive (k = .92, $p < .01$). Conclusions: This study stressed different ways on expressing sexuality in old age and underlined the importance of tender and care and eroticism for older adults who are sexually active. Keywords: Content analysis; older adults; qualitative study; sexual expression.

### SEXUALITY IN LATER LIFE AND MENTAL HEALTH AMONG OLDER BLACK GAY MEN

Darlingtina Esiaka,[1] Alice Cheng,[2] and Candidus Nwakasi,[3]
*1. Union College, Union College, New York, United States, 2. Union College, Schenectady, New York, United States, 3. University of Southern Indiana, Evansville, Indiana, United States*

Self-acknowledgement and integration of racial and sexual identities are significant to one's overall sense of identity because of their implications for mental health and wellbeing. These issues are important as one ages because older people experience a wide range of factors that add layers to their ability to (re)integrate subsets of their identity into their overall self-identity such as age and age-related disabilities. This study examined the intersection of race and sexual identities on overall health status in older Black gay men, a demographic group that has historically received less attention. Data from the Social Justice Sexuality (SJS) survey of LGBTQ+ people of color which occurred over a 12-month period in the United States were analyzed. Participants (N=160), 50 years and over, responded to questions about their sexuality, social identity, family dynamics, community connection and engagement, and mental and physical health. Results show an association of mental wellbeing with racial and sexual identities. Further, results show that a strong sense of connection to other sexual minorities is positively associated with mental health in older Black gay men. We discuss the implication of findings for mental health interventions targeting this gendered population.

### WHO MASTURBATES? IT DEPENDS: PREDICTORS OF MASTURBATION BY GENDER AND PARTNERSHIP OF OLDER ADULTS

Patricia Barrett,[1] and Elisabeth Burgess,[2] *1. Georgia State University, Marietta, Georgia, United States, 2. Georgia State University, Atlanta, Georgia, United States*

Studies on masturbation often exclude older adults. While the frequency of masturbation is known to decline over the life course, research has neglected masturbation in older adulthood. In the adult population, the connection between partnered sex and masturbation has been well established; this relationship is mediated by individual's contentment with their sex life. Furthermore, the sex/masturbation relationship is distinct by gender. This study seeks to understand how these relationships exist and influence masturbation among older adults. Using data from the third wave of the National Social Life, Health and Aging Project (NSHAP) (N=2,006), we completed logistic regressions to examine how sexual thoughts, frequency and contentment with partnered sex predict older adult's likelihood of masturbating. Preliminary analyses, controlling for partnership status, gender, age, race, mental and physical health, indicate that frequent sexual thoughts (OR 4.52) and discontentment with sex frequency (OR 1.46), rather than frequent partnered sex, are significantly associated with an increased likelihood of masturbating in the year prior. Employing separate regressions for gender, bothersome sexual dysfunctions and higher depression scores were significantly associated with increased odds of masturbating for women, but not men. For partnered older adults, only those who reported relationships that were both physically and emotionally satisfying were less likely to masturbate (OR 0.54). To our knowledge, this is the first study that considers masturbation as a sexual behavior with distinct predictors by gender and partnership status. We conclude by discussing the implications of nuanced and varying sexual desires and behaviors among the older adult population.

### SESSION 2937 (POSTER)

### TECHNOLOGY I

### DOES AGEISM WIDEN THE DIGITAL DIVIDE? AND DOES IT VARY BY GENDER?

Eun Young Choi,[1] Youngsun Kim,[2] Edson Chipalo,[3] and Hee Yun Lee,[4] *1. Leonard Davis School of Gerontology, Los Angeles, California, United States, 2. Kyunghee University, Yongin-si, Republic of Korea, 3. University of Alabama, Tuscaloosa, United States, 4. University of Alabama, Tuscaloosa, Alabama, United States*

Existing literature informed that ageism might affect Internet use among older adults, further widening the digital divide among age groups. However, little empirical studies have been conducted on this topic. Our study aims to investigate (1) the current use of the Internet by gender, (2) the association between ageism and Internet use, and (3) potential gender differences. A cross-sectional data drawn from the 2016 Health and Retirement Study (HRS) was analyzed. Separate multiple regression analyses were conducted by gender to determine the varying impact of ageism on Internet use. We used two types of ageism (1) internal ageism (ingroup discrimination) and (2) external ageism (discrimination from external entities) to observe each type's contribution to Internet use. About half of the sample (52% male and 54% female) reported using the Internet "daily," while a quarter (26% male and 25% female) responded, "never/ not relevant." No significant differences between gender were found in levels of Internet use, the rates of external ageism, or the degree of internal ageism. A higher level of ageism was associated with a lower level of Internet use. Interaction effects between age groups and ageism varied across gender: external ageism had interaction effects on men's Internet use whereas internal ageism showed significant results for women. Our findings suggest that ageism may influence Internet use and its impact differs by gender. Gender-tailored intervention strategies should be developed to help older individuals to diminish the adverse effects of ageism on Internet use.

### EDUCATION AND LITERACY IN THE DIGITAL DIVIDE: CROSS-NATIONAL COMPARISON OF EAST ASIA AND THE USA

Taka Yamashita,[1] Giyeon Kim,[2] Chih-ling Liou,[3] Takatoshi Ando,[4] Darren Liu,[5] and Anthony Bardo,[6] *1. University of Maryland, Baltimore, Maryland, United States, 2. Chung-Ang University, Seoul, Republic of Korea, 3. Kent State University at Stark, North Canton, Ohio, United States, 4. Yokohama National University, Yokohama, Japan, 5. Des Moines University, West Des Moines, Iowa, United States, 6. University of Kentucky, Lexington, Kentucky, United States*

The disproportionate access to, and utilization of, modern information, communication and computer technologies in later life (or Digital divide) is widely recognized as a major problem in economically developed nations. Extant research has established that the digital divide is largely contingent on advanced age and socioeconomic status (e.g., educational attainment). Yet, most studies have exclusively focused on Western populations. Thus, the objective of this study was to examine patterns in the digital divide by educational attainment, and identify the role that basic skills (i.e., literacy) play in shaping cross-national differences. Internationally representative data of adults aged 45 years and older from Japan, South Korea, and the US were obtained from the Program for International Assessment of Adult Competencies (PIAAC). The PIAAC data include information on everyday digital technology use (e.g., computer device, email, online information and transactions), sociodemographic characteristics, and systematic literacy assessments (score 1-500). Based on results from weighted binary logistic regression models that used the moderator function, the effects of education on email use were significantly weaker (odds-ratio = 0.78, $p < 0.05$) in Japan vs. US, but equivalent in Japan vs. South Korea ($p > 0.05$). Additionally, education (odds-ratio = 1.22, $p < 0.05$) and literacy (odds-ratio = 1.01, $p < 0.05$) were consistent predictors a digital divide, and the effects of literacy were similar across all three nations ($p > 0.05$). Theoretical explanations of findings and other results are discussed in cultural contexts.

### EXPLORING APPLICATION OF SMART COMPANION ROBOTS TO FACILITATE FAITH-BASED MINISTRIES FOR OLDER HOMEBOUND ADULTS

Christianah Adebayo, and Alex Bishop, *Oklahoma State University, Stillwater, Oklahoma, United States*

This study examined perceptions regarding application of smart robot companions to facilitate homebound religious ministry programming to older adults. A total of N= 7 participants consisting of ministers, pastoral volunteers, and older adult members from Methodist, Presbyterian, and Roman Catholic traditions engaged in a 60-minute focus group. One key goal was to address the question: If you could design a robot for ministry to homebound older adults what would you want it to do? All responses were recorded, transcribed and coded for thematic content. Three core themes emerged relative to spiritual privacy, prospective intervention, and inclusive monitoring. Relative to spiritual privacy, participants expressed concern regarding robotic ability to maintain the spiritual privacy of the older homebound adult, especially when engaged in faith-based behaviors such as private prayer, confession, and pastoral counseling. Second, participants suggested that a robot companion serve as a memory aide for older homebound members. In particular, participants acknowledged a need for robot intervention relative to sending alerts concerning prayer requests, reminders of upcoming church-related events, and notices concerning future homebound ministry visitations. Finally, participants indicated an interest for the robot to actively monitor community inclusion, noting that many older homebound adults are left out of weekly church-related services or events and experience seclusion, yet most "retain a desire be included and remain an integral part of their faith community." Results will be used to present a conceptual model for a smart robotic faith assistant designed for delivery of homebound ministries to older adults.

### FEASIBILITY AND ACCEPTABILITY OF AN MHEALTH DELIVERED MINDFULNESS THERAPY FOR CAREGIVERS

Elissa Kozlov, and XinQi Dong, *Rutgers University, New Brunswick, New Jersey, United States*

Decades of research have documented the profound, negative effects of caregiving on unpaid caregivers. Mindfulness Therapy (MT) is a promising, non-pharmacological technique with proven efficacy and effectiveness in managing stress, depression and anxiety in diverse populations. While the evidence-base for MT in caregiving is growing, traditional MT (8+ hours of face-to-face treatment with trained providers) is likely not a realistic treatment model for most caregivers due to lack of trained personnel, time constraints of the caregiver, and reimbursement issues. Therefore, in order to meet the unique needs of caregivers of older adults with cognitive impairment, an innovative delivery model is required. MHealth can be a useful tool to deliver behavioral interventions, as it overcomes barriers of traditional psychotherapy such as provider availability, scheduling conflicts, and cost. The objective of this paper is to report the feasibility, acceptability and preliminary efficacy of a pilot trial of mHealth delivered MT for stress and caregiver burden in caregivers of persons with dementia. The average age of participant was 63.2 years old. After two weeks, 93% of participants reported using the mindfulness app for an average of 48.38 minutes per week. At eight weeks, 88% of users reported using the mindfulness app for an average of 35 minutes per week. At 8 weeks, 100% of users reported practicing mindfulness without using the app for an average of 45.6 minutes per week. MHealth mindfulness therapy appears to be a feasible method of delivering mindfulness to caregivers of older adults with memory impairment.

### PATTERNS OF INTERNET USE, LONELINESS, AND MENTAL AND PHYSICAL HEALTH AMONG KOREAN BABY BOOMERS

Jeehoon Kim,[1] Jwakyum Kim,[2] Ji Hyun Lee,[3] and Gyounghae Han,[4] *1. Idaho State University, Pocatello, Idaho, United States, 2. Kkottongnae University, Cheongju, Republic of Korea, 3. Florida State University College of Medicine, Tallahassee, Florida, United States, 4. Seoul National University, Seoul, Republic of Korea*

Introduction. Korean Baby Boomers are more affluent and gained higher education than past generations. Their relatively younger age (born between 1955 and 1963), coupled with more personal resources can help the Boomers have easier access to digital devices, but little is known about their patterns of online activities and how diversity in online activities is associated with health status among Korean Boomers. Methods. Using data from the 2012 Korean Baby Boomers Panel Study (n=3,272), we conducted latent class analysis based on ten online activities. Three classes were identified with different patterns of online activities; namely, maximizers (n=315), selective users (n=1,435), and rare users (n=1,522). Multinomial logistic regression analysis was performed to examine factors associated with different groups. Ordered probit regression and multiple regression analysis were employed to predict depressive symptoms and self-reported health of Korean Boomers, respectively. Results. Selective users used the internet for information search, e-mail, SNS, streaming music, or gaming, whereas maximizers engaged actively on all activities. Rare users were minimally engaged in information search. Both selective users and maximizers were more actively engaged with social relationships and social group activities compared to rare users. Maximizers were more likely to experience loneliness than rare users. Selective users were less likely to report depressive symptoms and had better health than rare users. Discussion. Moderate engagement with digital technology supported Korean Boomers in maintaining social relationships and better health. Intervention strategies for alleviating loneliness and preventing social isolation for maximizers, thus helping them maintain better health, will be discussed.

### TECHNOLOGY IMPLEMENTATION IN PERSONS WITH DEMENTIA: A SYSTEMATIC REVIEW AND META-ANALYSIS

Sarah Hubner, Julie Blaskewicz Boron, and Brenda Nguyen, *University of Nebraska Omaha, Omaha, Nebraska, United States*

Maintaining independence and quality of life (QOL) is a primary goal for adults. To support this goal, assistive and interactive technology (AIT) has been implemented to improve function and mitigate disease. To assess AIT effect on QOL in community-dwelling persons with dementia and mild cognitive impairment (MCI), a systematic review was performed, and articles were prepared for meta-analysis. Electronic database searches were carried out in PubMed, Cumulative Index for Nursing and Allied Health Literature, PsychINFO, and Web of Knowledge/Web of Science. Peer-reviewed journal articles published in English between January 2010 and February 2020 were included in the search. Studies investigated personal AIT use aimed at improving QOL (i.e. satisfaction/mood, functional ability, psychological/social function, independence). Technology was implemented in the home in everyday life. Studies were limited to those including community-dwelling participants aged 65+ with a diagnosis or report of MCI or dementia. Initial search resulted in 2624 total titles. After duplicate deletion, 1546 unique articles were identified. After title and abstract deletion, 60 articles were screened at full-text. After full-text screening, five usable articles remained. Usable studies presented: 1) a digital tablet companion, 2) a digital reminder calendar, 3) a medication-adherence bottle, 4) an automatic medication dispenser, and 5) a comprehensive tele-care computer system. These studies provide outcome measures focused on functional improvement and/or subjective QOL, informing future research in AIT implementation. Additionally, the severe paucity of applicable studies indicates a need for increased primary research on intersections between dementia and technology in the home environment.

### THE DISPARITIES IN ACCESSING ONLINE HEALTH INFORMATION AND HEALTH CARE UTILIZATION AMONG OLDER AMERICANS

Darren Liu,[1] Taka Yamashita,[2] Betty Burston,[3] and Jennifer Keene,[3] *1. Des Moines University, West Des Moines, Iowa, United States, 2. University of Maryland, Baltimore, Maryland, United States, 3. University of Nevada Las Vegas, Las Vegas, Nevada, United States*

Digital divide or unequal access to internet and other technology result in health disparities among older adults. This study examined possible disparities in accessing online health-related technology, and the associations between the online health-related technology use and health care utilization among older adults in the U.S. The data comprised a sample of 1,497 older adults aged 51 and older which were obtained from the 2014 Health and Retirement Study (HRS)'s supplemental module (Health behaviors). We used the survey-weighted negative binomial regression and binary logistic regression for the analyses. The results showed that older age, racial/ethnic minorities (e.g., Black and Hispanic vs. Whites & Other), being married, lower educational attainment, lower-income, being uninsured and reporting poorer health were associated with lower utilization of online health-management tools. In Addition, the use of online health-management tools was associated with 34% greater mean number of doctor visits (Incidence-Rate-Ratio = 1.34, S.E. = 0.10, $p < 0.05$) than non-use. However, the use of online health-management tools was not associated with hospitalization. Indeed, only health care needs - the self-rated health (Odds-Ratio = 0.58, S.E. = 0.18, $p < 0.05$) and the number of chronic conditions were associated hospitalizations (Odds-Ratio = 1.68, S.E. = 0.07, $p < 0.05$). More research is needed to clarify the purposes (e.g., prevention vs. treatment) and outcomes of health care service utilization within the context of health care technology use. Yet, it is important to proactively address digital divide as one of upstream strategies for reducing health and health care disparities.

### THE RELATIONSHIP BETWEEN TECHNOLOGY USE AND COLON CANCER SCREENING

Na Sun, *Miami University, Oxford, Ohio, United States*

Population aging is accompanied by an increase in chronic diseases such as cancer. Colon cancer is the third most common cancer and a leading cause of cancer death. Screening tests can aid early detection and treatment. It is unclear how information and communication technology (ICT), especially media through mobile devices, influences cancer screening. This study analyzes the relationship between ICT usage and colon cancer screening among U.S. adults. Data are from the second cycle of the Health Information National Trend Survey 5. Cancer screening included having one of the following: colonoscopy, sigmoidoscopy and/or stool blood test to check for colon cancer. Approximately 70% of respondents had at least some college education, 51% were female, and the mean age was 48 years. More than half of respondents report using apps on a tablet or smartphone for health and wellness purposes, and around 70% of them used apps for health communication and decision-making. Based on results of a binary logistic regression model, people who use mobile apps for discussions with health care providers ($p<0.01$), who use the internet to look for information about cancer ($p <0.01$), and who do not use mobile apps to make decisions about how to treat an illness or condition ($p<0.01$) are more likely to conduct cancer screening. ICT usage may enable people to gather information about cancer screening and improve patient and physician communication. Future studies should explore longitudinal associations between ICT usage, cancer screening, and cancer outcomes.

### UNDER THE DIGITAL BRIDGE: INVESTIGATING TROLLING BEHAVIORS IN BABY BOOMERS

Kristen Hardin-Sigler, Rebecca Deason, Stephanie Dailey, Natalie Ceballos, and Krista Howard, *Texas State University, San Marcos, Texas, United States*

Internet trolling, or the intentional disturbance or upsetting of others on social media for personal amusement, has become increasingly prevalent in recent years (Howard et al., 2019). Current research focuses on these destructive social media behaviors in younger populations, therefore this study set out to investigate the gender differences of trolling behaviors in Baby Boomers. Participants (N = 140), ages 54 and older, were recruited from the Amazon Mechanical Turk and were compensated for their participation. Participants completed a survey investigating their likelihood to engage in trolling behaviors, the extent to which they enjoy trolling, and their feelings while trolling. Results indicated that while there were no significant differences between men and women in their need, intensity of use, or addiction to social media, men were significantly more likely to engage in trolling behaviors than women. Men reported posting to upset others ($p = .018$), as well as commenting to upset others ($p = .053$), more often than women. Furthermore, when engaging in these behaviors, men reported feeling intelligent ($p = .013$), confident ($p = .024$), superior ($p = .053$), and happy ($p = .012$), more often than women. However, these results could be indicative of a more sinister issue. Men also reported more often that their reasons for engaging in trolling behaviors were feelings of loneliness ($p = .005$) and anxiety ($p = .010$). This indicates that these trolling behaviors may then be a way for men to seek out some form of "social support" in the online community.

### VOICE TECHNOLOGIES TO SUPPORT AGING IN PLACE: OPPORTUNITIES AND CHALLENGES

Alisha Pradhan,[1] and Amanda Lazar,[2] *1. University of Maryland, College Park, College Park, Maryland, United States, 2. University of Maryland, College Park, Maryland, United States*

Technology to support aging in place has been a topic of interest. Research indicates that older adults are increasingly using commercially available voice assistants in smart speakers. These devices enable non-visual interaction that does not require extensive expertise with traditional mobile or desktop computers, thus offering new possibilities of access to digital technology. We conducted two different studies with individuals aged 65 years old or above—a three week smart speaker deployment study with individuals who did not use computing devices regularly and a workshop on customizing internet of things technology with tech savvy individuals. Our findings indicate specific ways that these voice technologies might support aging in place, including ease of use and due to their not being identified with aging-specific technologies. We observed that participants consistently used their voice agent for finding online information, particularly health-related, emphasizing the need to revisit concerns about credibility of information with this new interaction medium. And, although features to support memory (e.g., setting timers, reminders) were initially perceived as useful, the actual usage was unexpectedly low due to reliability concerns. Our work provides a basis to understand older adults' perceptions and usage of current voice technologies. We also identify opportunities for customizing voice technologies to better support aging in place.

### SESSION 2938 (POSTER)

### ISOLATION AND LONELINESS

### A LONGITUDINAL STUDY OF THE IMPACT OF LONELINESS ON PERSONAL MASTERY AMONG OLDER ADULTS IN SINGAPORE

Emiko Takagi,[1] Yasuhiko Saito,[2] and Angelique WM Chan,[3] *1. San Francisco State University, San Francisco, California, United States, 2. Nihon University, Tokyo, Japan, 3. Duke-NUS Medical School, Singapore, Singapore*

This study uses longitudinal data to examine the association between older adults' sense of mastery and loneliness. We examined the data of a nationally representative sample of adults 60 years and older in Singapore (Wave1, n=4,990) from the Panel of Health and Aging among Older Singaporeans Survey. The initial participants were followed up in 2011 (Wave2, n=3,103) and in 2015 (Wave3, n=1,572). At each wave, emotional loneliness was assessed using the UCLA three-item loneliness scale and sense of mastery was measured with the five items from the Pearlin Mastery Scale. We conducted cross-lagged regression analyses where loneliness and personal mastery scores in each wave were treated as endogenous variables along with covariates including demographic characteristics, health conditions, and the overall strength of social network measured by Lubben Social Network Scale. The results showed that loneliness in wave 1 and wave 2 respectively predicted a lower level of personal sense of mastery in subsequent waves. However, the other direction, the influence of personal mastery in wave 1 and wave 2 on loneliness at subsequent waves, was not significant. Furthermore, the analysis showed that older adults' relatively strong social network was related to a lower level of loneliness and a higher sense of mastery at Wave 3. The finding suggests that loneliness plays a critical role in influencing older adults' personal sense of mastery and that the strength of social network is an important mediator of loneliness and personal sense of mastery amongst older adults and a potential area for intervention.

### ASSOCIATIONS BETWEEN HEARING LOSS, LONELINESS, AND SOCIAL ISOLATION: A SYSTEMATIC REVIEW

Jonathan Suen,[1] Aishwarya Shukla,[1] Adele Goman,[1] Carrie Price,[1] Frank Lin,[2] and Nicholas Reed,[1] *1. Johns Hopkins University, Baltimore, Maryland, United States, 2. Johns Hopkins University School of Medicine, Baltimore, Maryland, United States*

Hearing loss is highly prevalent among older adults, as is occurrences of loneliness and social isolation. Both loneliness and social isolation are also associated with insidious outcomes such as earlier mortality from all-causes and higher prevalence of chronic comorbidities. The purpose of this review is to synthesize published investigations that report on the associations between hearing loss with loneliness and social isolation. A systematic search through PubMed, Embase, CINAHL Plus, PsycINFO, and the Cochrane Library identified an initial total of 2495 references. Two independent reviewers screened articles for inclusion, with a third reviewer adjudicating. Studies published in English of older adults with hearing loss that also assessed loneliness and/or social isolation using a validated measure were included. Investigators used a modified Newcastle-Ottawa Scale (NOS) to appraise study quality. A final total of 14 articles were included in the review. The majority (12/14) were cross-sectional in design. Assessment methods were varied across hearing status, loneliness, and social isolation. Despite this heterogeneity, most multivariable adjusted investigations revealed that hearing loss was significantly associated with higher risks for both phenomena. Several studies also revealed this association to vary across gender, with women showing a stronger association than men. Our findings indicate that hearing loss is associated with both loneliness and social isolation, which have important implications for the cognitive and psychosocial health of older adults. Future investigations should examine possible underlying mechanisms of these relationships, as well as the efficacy of interventions through aural rehabilitation programs in addressing loneliness and social isolation.

### ASSOCIATIONS OF DEPRESSION AND SOCIAL ISOLATION RISK AMONG ADULTS AGE 60 AND OLDER

Matthew Smith,[1] Lesley Steinman,[2] Matthew Barrett,[1] Leigh Ann Eagle,[3] Sue Lachenmayr,[3] and Basia Belza,[2] *1. Texas A&M University, College Station, Texas, United States, 2. University of Washington, Seattle, Washington, United States, 3. MAC, Salisbury, Maryland, United States*

Background. Depression and social isolation are believed to be strongly interrelated. Social isolation can lead to depression because of reduced human contact and connectivity. Depression can cause withdrawal from interpersonal encounters and fuel feelings of social isolation. Despite causality, this study aimed to examine the relationship between depression and social isolation risk among older adults. Methods. Using an internet-delivered survey, data were analyzed from a national sample of 4,082 adults age 60 years and older. The survey intended to validate the Upstream Social Isolation Risk Screener (U-SIRS), a 13-item screener (Cronbach's alpha=0.80) to assesses physical, emotional, and social support aspects of social isolation. Theta scores for the U-SIRS served as the primary independent variable, which were generated using Item Response Theory. Depression was the dependent variable for this study, which was identified using the PHQ-2 (scores of 3+ indicated risk for depression). Binary logistic regression was used to identify factors associated with depression. Results. Participants' average age was 69.6(±5.2) years, 59% of participants were female, and 9% met depression criterion. Depressive symptomology and U-SIRS theta scores were positively significantly correlated (r=0.56, P<0.001). Participants with higher U-SIRS theta scores (OR=3.52, P<0.001), with more chronic conditions (OR=1.16, P<0.001), and without people they felt close to and could call for help (OR=1.76, P=0.004) were more likely to report depression. Conclusion. Given the strong interrelation of depression and social isolation risk, coordinated efforts are needed to both treat depressive symptomology and link older adults to resources and services that facilitate meaningful interactions with others.

### BEST PRACTICES AND POLICIES FOR ADDRESSING SOCIAL ISOLATION AMONG OLDER ADULTS

Raza Mirza,[1] Samir Sinha,[2] Andrea Austen,[3] Anna Liu,[4] Jaemar Ivey,[5] Michelle Kuah,[6] Lynn McDonald,[7] and Jessica Hsieh,[7] *1. National Initiative for the Care of the Elderly, Toronto, Ontario, Canada, 2. Sinai Health, Toronto, Canada, 3. City of Toronto, Toronto, Ontario, Canada, 4. Ontario Ministry of the Environment, Toronto, Canada, 5. University of Toronto, Toronto, Canada, 6. National Initiative for the Care of the Elderly, Toronto, Canada, 7. University of Toronto, Toronto, Ontario, Canada*

Isolation has been flagged as a major health and social problem for seniors. Yet, many seniors themselves, their friends/family, and carers for seniors may not recognize risk factors for isolation or know what to do if a senior is isolated. Results from the 2016 General Social Survey noted that 27% of seniors reported they were not socially connected with others, with 20% reporting that they lacked support to carry out chores, and 17% reported feeling isolated. However, there has yet to be a comprehensive review of the evidence to suggest what has emerged as best practices and key policy enablers. To address this, seniors and other key stakeholders (n=200) in the community were interviewed on their perspectives and experiences of social isolation. Additionally, three focus groups (n=24) were conducted, along with a consensus meeting, to identify top priorities, best practices and develop implementation strategies. The priority areas identified were: 1) opportunities for seniors to network and be part of the social fabric; 2) initiatives promoting inclusive community development; 3) programs that promote education related to social isolation; 4) develop services that place an emphasis on partnerships/collaborations; 5) services that are sustainable over the longer term. By mapping the best, emerging practices and policies for social isolation, the ability to synthesize the evidence on social isolation and co-create knowledge translation tools with seniors and other stakeholders will be possible. This will help identify solutions and policies that can be used by governments, health systems, and individuals to comprehensively target social isolation.

### DETERMINANTS OF SOCIAL INCLUSION AND THEIR EFFECT ON THE WELLBEING OF OLDER ADULTS

Keya Sen,[1] Victor Prybutok,[2] and Gayle Prybutok,[3] *1. University of North Texas, Denton, Texas, United States, 2. UNT, Denton, Texas, United States, 3. UNT, Denton, United States*

Social inclusion fosters interpersonal relationships that reduce social isolation and enhance wellbeing in older adults. This study finds that socially engaged older adults are less likely to decline in health and have more wellbeing than those less engaged. The connection between wellbeing and social engagement is examined with hypotheses that there is a significant linear relationship between wellbeing and age, ethnicity, gender, the involvement and perception of participatory activities, community dwelling and the use of technology among older adults. A multiple linear regression on 4621samples obtained from National Health and Aging Trend Study, Round 8 shows that social engagement explained a unique variance in wellbeing (34.5%) suggesting that more social connections, via social activities, community-dwelling, mobility, and use of technology, there is enhanced health and fewer chances of cognitive decline in older adults. The use of text messaging and emails had a moderating effect on cognition and wellbeing of older adults. It is suggested that existing low-cost community programs targeting the so-called social determinants of health can be reworked to address social isolation and foster knowledge and technology skills in the older population. Directions for future research include examining human behaviors and perceptions to stay connected.

### DUAL TRAJECTORIES OF SOCIAL ISOLATION AND DEMENTIA IN OLDER ADULTS: A POPULATION-BASED LONGITUDINAL STUDY

Xiaoling Xiang,[1] Patrick Ho Lam Lai,[2] Yihang Sun,[1] and Ruth Dunkle,[1] *1. University of Michigan, Ann Arbor, Michigan, United States, 2. University of Michigan-Ann Arbor, Ypsilanti, Michigan, United States*

The purpose of this study was to identify patterns of changes in social isolation and dementia and the interrelations between these developmental trajectories. The study sample included 7,609 Medicare beneficiaries age 65 and older from the National Health and Aging Trends Study 2011 through 2018 surveys. A group-based dual trajectory modeling approach was used to identify distinct groups of developmental trajectories for social isolation and dementia status over the 8-year period. The dual model provided estimates of conditional and joint probabilities linking the two sets of trajectory groups. Changes in social isolation over an 8-year period followed four trajectories: rarely isolated (62.2%), steady increase (13.5%), steady decrease (7.4%), and persistently isolated (16.9%). Changes in dementia risk also followed four trajectories: persistently low risk (80.4%), increasing with early onset (3.9%), increasing with late onset (4.5%), and persistently high risk (11.2%). Over two-third (68%) of the persistently low dementia group were also in the rarely isolated group. Both increasing dementia groups were composed mainly of individuals from the increasing social isolation group (40-43%) and persistently isolated group (24-29%). The persistently high dementia group had the most overlap with the decreasing social isolation group (47%), followed by the persistently isolated group (28%). For the most part, social isolation and dementia evolve in the same direction for older adults over an 8-year period. However, the pattern of associations between these developmental trajectories is complex and may be reversed among long-term dementia survivors.

### FAMILY SOLIDARITY, SOCIAL SUPPORT, LONELINESS, AND WELL-BEING AMONG OLDER ADULTS IN RURAL CHINA

Xiaoyan Zhang, and Merril Silverstein, *Syracuse University, Syracuse, New York, United States*

China is experiencing a large increase in elderly population. In 2019, China's population aged 60 and above had reached 253 million, accounting for 18.1% of the total population (National Bureau of Statistics of China, 2020). By 2050, the number of adults aged 60+ would be up to 430 million, reaching one third of the total population (Du, Zhai & Chen, 2005). Considering such a rapid aging process and the existing large number of older adults in China, it becomes imperative to investigate how psychosocial factors affect this group's subjective well-being. This study proposed that, among older adults, higher support received from each of the three relational sources (adult children, family and friends) were associated with reduced loneliness and improved well-being. Structural equation modeling was conducted using a sample of rural adults aged 60 and older (N= 1142) from the 2018 wave of data from the Longitudinal Study of Older Adults in Anhui Province, China. Findings indicated that support from adult children directly and indirectly decreased older adults' depression and improved their life satisfaction through loneliness; while support from family members directly decreased depression but did not directly improve life satisfaction or indirectly improve well-being through loneliness. Although support from friends did not have a significant impact on older adults' well-being, it indirectly improved well-being through reduced loneliness. Findings have implications for programs or interventions targeting both parent -adult-child support and friends support and reducing rural older adults' loneliness.

### LONELINESS AND PURPOSE IN LIFE ARE IMPORTANT PREDICTORS FOR FUTURE CARE PLANNING

Wingyun Mak,[1] and Silvia Sörensen,[2] *1. The New Jewish Home, New York, New York, United States, 2. University of Rochester, Rochester, New York, United States*

Experiencing purpose and social connection in later life is associated with better quality of life, better cognition, less morbidity, and lower risk of mortality. People who experience less purpose in life are more likely to report loneliness (Neville et al., 2018), and those with vision impairment are at greater risk for loneliness than the general population (Brunes et al., 2019). Planning for future care may be one way to enhance late life outcomes, but it is unclear how loneliness and purpose in life are related to planning behaviors in older people with vision loss. Using a sample of 200 older adults who were diagnosed with macular degeneration, this study explored the association of loneliness and purpose in life on future care planning variables after controlling for basic health and demographic variables. Hierarchical regressions showed that 1) people who are lonelier ($\beta$=.26, p<.05) but report greater purpose in life ($\beta$=.19, p<.05) are more aware of future care needs ($\Delta$R2= .14, p<.001); 2) people who are lonelier ($\beta$=-.16, p<.05) but report greater purpose in life ($\beta$=-.46, p<.001) are less avoidant of planning ($\Delta$R2= .14, p<.001); and 3) people who report greater purpose in life are more likely to gather information ($\beta$=.24, p<.05; $\Delta$R2= .04, p<.05) and establish concrete plans related to planning for future care ($\beta$=.25, p<.05; $\Delta$R2= .06, p<.001). These results suggest that having purpose in later life may boost planning behaviors, while those who are lonely may need help translating their awareness of future care needs into planning behaviors.

### MORE EXERCISE IS LINKED TO LESS LONELINESS IN ADULT DAY HEALTH ATTENDEES

Wingyun Mak, *The New Jewish Home, New York, New York, United States*

There is a growing evidence that more physical activity is linked to less loneliness (Cigna, 2018; Pels & Kleinert; 2016). It is less clear whether this relationship persists in older adults who are attending adult day health services and often have multiple comorbidities. In this study, we examined whether spending more time exercising would significantly predict whether adult day health attendees reported loneliness. We used UAS-NY data from a sample of 221 adult day health attendees from 2019 to early 2020 who scored five or greater on the Nursing Facility Level of Care Index, which is a score derived from assessments of cognition, communication and vision, mood and behavior, functional status, continence, and nutritional status. A logistic regression showed that after controlling for demographic variables, cognition, health, and quality of family relationships, participants were less likely to report loneliness if they had been attending adult day health services for at least six months or more (B=.86, p<.05), spent less time alone (B=-.45, p<.05), and exercised more (B=.47, p<.05; $\chi$2(3)=15.6, p=.001). These results suggest that in addition to participating in adult day services and spending more time with others, exercising may have an impact on the experience of loneliness.

### RESILIENCE OF HMONG OLDER ADULTS: COPING WITH LONELINESS

Cindy Vang,[1] Michael Sieng,[2] and Mingyang Zheng,[3] *1. University of Minnesota, Twin Cities, Saint Paul, Minnesota, United States, 2. Arizona State University, Tempe, Arizona, United States, 3. University of Minnesota-Twin Cities, Saint Paul, Minnesota, United States*

Older refugees are especially susceptible to loneliness with their history of trauma, forced migration, and social isolation in host countries. Rates of loneliness among older immigrants range from 24% to 50%. Despite their heightened vulnerability to loneliness, coping mechanisms among this population remain understudied. The purpose of this study was to examine how community-dwelling Hmong older adults, an aging refugee group, cope with loneliness. The data was drawn from a larger constructivist grounded theory study aimed at understanding the loneliness experiences of community-dwelling Hmong older adults. Semi-structured individual interviews were conducted in the Hmong language with 17 Hmong age 65 and older residing in Northern California. Data was collected and analyzed in an iterative and comparative process using initial coding, focused coding, and connecting the focused codes to form categories and subcategories. Five coping mechanisms emerged from the data: (a) religious and spiritual beliefs; (b) social support; (c) wandering; (d) activity engagement; and (e) avoidance and control. Coping mechanisms utilized by Hmong older adults in this study highlighted the resilience of this aging population and the lack of culturally-relevant programs to prevent and address their persistent loneliness and emotional distress. Implications for research, practice, and policy suggests the need for greater culturally- and linguistically-competent services informed by Hmong older adults.

### SOCIAL ISOLATION AND ITS RELATED FACTORS IN PARTICIPANTS OF A SENIOR CITIZENS' COLLEGE IN JAPAN

Hayato Uchida,[1] Yoshinori Fujiwara,[2] and Shinro Matsuura,[3] *1. University of Hyogo, Himeji, Hyogo, Japan, 2. Tokyo Metropolitan Institute of Gerontology, Tokyo, Japan, 3. Matsuura Clinic, Himeji, Hyogo, Japan*

The aim of this study was to clarify the social isolation and its related factors of participants in senior citizen's college in Japan. The participants were 104 persons (age 73.3+/-4.3) aged sixty-five years or over living in Hyogo Prefecture, Japan. They were the students of a senior citizens' college established by Himeji City, Hyogo Prefecture in 1981. We conducted a cross- sectional study that included age, family structure, employment status, financial status, self-related health, presence of chronic disorders, instrumental activities of daily living, dietary variety score (1-10). The mental health well-being was assessed using the Japanese version of the World Health Organization Mental Health Well-being Index-five items (WHO-5). We defined social isolation as seeing friends or relatives less than two or three times a month. We carried out the survey in November in 2019. Selected variables were compared after dividing the participants into "socially isolated group" and "not socially isolated group". A t-test, a chi-square test, and logistic regression analysis were conducted to examine the related factors of social isolation in this study. Of the 104 participants who were analyzed for social isolation, 21 (20.2%) were found to be socially isolated. From the results of multivariate logistic regression analysis, "lower mental health" (OR=4.013, 95%CI=1.745-10.435) and "lower self-related health" (OR=2.583, 95%CI=1.103-9.564) were independently associated with social isolation. These results suggest that, to prevent the social isolation of the participants in senior citizen's college in Japan, it is necessary to develop and implement the approach which promote mental health and self-related health.

### SOCIAL MEDIA USE AND LONELINESS AMONG OLDER ADULTS: THE MEDIATING ROLE OF SOCIAL CONTACT AND PERCEIVED SOCIAL SUPPORT

Kunyu Zhang,[1] Jeffrey Burr,[2] Kyungmin Kim,[2] Nina Silverstein,[3] and Qian Song,[2] *1. University of Massachusetts Boston, Woburn, Massachusetts, United States, 2. University of Massachusetts Boston, Boston, Massachusetts, United States, 3. University of Massachusetts Boston, Needham, Massachusetts, United States*

Loneliness is a risk factor for poor quality of life among older adults. Social media use provides a new dimension of communication for older adults to connect with people and to maintain social relationships. However, research has been inconclusive about whether social media use reduces loneliness among older adults, which is due in part to a lack of appropriate measures for capturing different types of social media use. Furthermore, little is known about the underlying mechanisms through which social media use is associated with loneliness. This study investigates the association between social media communication with close social ties and loneliness among community-dwelling older adults (65+), and further examines the mediating role of social contact and social support in the association. Data from the 2014 wave of the Health and Retirement Study (HRS) are analyzed to address our research questions (N = 4,184). Path analyses are employed to examine the relationships among social media communication with close social ties (i.e., children, family, and friends), frequency of contact with social ties (i.e., phone, in-person contact, write/email), perceived social support from social ties, and loneliness (R-UCLA loneliness scale). The results show that a higher level of social media communication is associated with lower levels of loneliness through social contact and perceived social support. Moreover, the relationship between social media communication and perceived social support is partially mediated by social contact. These findings suggest that social media communication may be considered an intervention that may reduce loneliness among older people.

### SOCIAL PROBLEM-SOLVING AND SUICIDE RISK ACROSS THE LIFESPAN: A QUALITATIVE INVESTIGATION

Ali Molaie, Adrienne Chong, and Jane Fisher, *University of Nevada, Reno, Reno, Nevada, United States*

Social disconnectedness, or thwarted belongingness (TB) is a risk factor for suicide across the lifespan. Perceived time left (PTL) to live may impact perceptions of hopefulness for future social connectedness, and thus the generation of problem-solving strategies for decreasing TB and the risk for suicide. We conducted an exploratory analysis of the quantity and quality of older and younger adults' recommendations for decreasing TB of characters in vignettes who were depicted as having high or low TB (i.e., described as lonely or not lonely) and high or low PTL (i.e., described as being 35-years-old or 85-years-old). We also examined the relation between participants' social recommendations and suicide risk. Participants with higher suicide risk endorsed higher hopelessness on the High TB/Low PTL vignette and were more likely to recommend contact with family as a means of reducing TB ($\chi^2(1) = 4.25$, $p = 0.04$) than were participants with lower suicide risk. Participants with higher suicide risk were also more likely to recommend social activity with friends than were participants with lower suicide risk on the High TB/High PTL vignette, ($\chi^2(1) = 6.66$, $p = 0.01$) and on the High TB/Low PTL vignette ($\chi^2(1) = 6.14$, $p = 0.02$). Additionally, age differences were observed such that older participants made fewer recommendations involving socializing with family and friends than did younger adults, and provided more nonsocial or made no recommendations across vignettes. The results suggest that social problem-solving may be an important differentiating variable among older and younger adults at risk for suicide.

### THE DEVELOPMENTAL CONTEXT OF SOCIAL HOPEFULNESS AND SUICIDE RISK

Ali Molaie, Adrienne Chong, and Jane Fisher, *University of Nevada, Reno, Reno, Nevada, United States*

Social disconnectedness, or thwarted belongingness (TB) has been documented as an important risk factor for suicide across the lifespan. Perceived time left (PTL) may impact perceptions of hopelessness for social connectedness and therefore inform developmentally sensitive trajectories of suicide risk. This study examined interpersonal conditions associated with social hopelessness in younger and older adults in order to identify variables important for conceptualizing suicidality across the lifespan. We compared younger and older adults' perceptions of social hopelessness for characters in vignettes that were depicted as having high or low TB (i.e., described as lonely or not lonely) and high or low PTL (i.e., described as being 35-years-old or 85-years-old). Additionally, we examined the relation between social hopelessness and suicide risk (using the Suicide Behaviors Questionnaire—Revised). Participants included 135 younger (M = 19.32) and 69 older (M = 74.91) adults. Older adults endorsed less social hopelessness than did younger adults on the High TB/Low PTL vignette, $t(102.15) = -4.88$, $p < 0.001$, as well as the Low TB/Low PTL vignette, $t(194) = -2.04$, $p = 0.04$. Participants with higher suicide risk also endorsed higher social hopelessness on the High TB/Low PTL vignette than did participants with lower suicide risk, $t(194) = -2.10$, $p = 0.04$. Younger adults and participants with higher suicide risk across both age groups reported less optimism for characters' future social connectedness, particularly for those portrayed as older. This study provides support for the importance of developmentally informed conceptualizations of risk factors for suicide, including social hopelessness.

### THE EFFECT OF COGNITIVE IMPAIRMENT ON LONELINESS IN OLDER ADULTHOOD: EVIDENCE FROM HRS 2008-2018

Ji Hyun Lee, Martina Luchetti, Angelina Sutin, and Antonio Terracciano, *Florida State University College of Medicine, Tallahassee, Florida, United States*

Background: People experience loneliness when there is a mismatch between desired and actual social interaction. Demographic and health factors have been implicated in loneliness; less is known about the unique association of cognitive impairment on loneliness in older adulthood. Purpose: This study examined the link between cognitive impairment status and level and change in loneliness over a 9-year period and whether it is independent of physical health, depression, and social isolation. We examine the associations for overall and the emotional and social loneliness sub-domains of loneliness. Methods: Data were from the Health and Retirement Study 2008-2018 waves (N = 8,269, age 50+). Cognitive impairment status was categorized using mTICS. Loneliness was measured with 11-item UCLA Loneliness scale. Multilevel modeling was used to analyze the effects of cognitive status on loneliness, controlling for time-varying functional limitation, disease burden, social contact, and depression. Results: Cognitive impairment not dementia (CIND) was associated with higher loneliness ($b = .04$, $p < .001$). CIND ($b = .03$, $p = .036$) and dementia ($b = .09$, $p = .017$) were linked to higher emotional loneliness but were not independent of social isolation and depression. Those with CIND had higher social loneliness ($b = .04$, $p = .016$), even after adjusting for covariates. The trajectory of loneliness did not vary by cognitive status. Conclusions: Cognitive impairment is a risk factor for loneliness among older adults. Those with mild cognitive impairment experienced heightened loneliness, especially for social belongingness. Cognitive function should be considered in designing interventions for loneliness.

### THE EFFECT OF LONELINESS ON COGNITION: A LONGITUDINAL STUDY

Robert Maiden,[1] Larry Greil,[2] and Bert Hayslip Jr,[3]
*1. Alfred University, Alfred, New York, United States, 2. Sociaology, Alfred, United States, 3. University of North Texas, Denton, Texas, United States*

A panel study was conducted among 244 older adults (52-years-old to 92) to explore whether social engagement and loneliness are associated with cognitive ability. Measures of crystallized (Gc) and fluid (Gf) ability were collected at two points in time. Using latent variable SEM with separate models for men and women, Gc and Gf at W2 were regressed on perceived general health, social support, sociability, loneliness and involvement in organizational activities, controlling for Gc and Gf at W1. Fit statistics were adequate. Among women, Gc at W1 was associated with perceived health (B=1.03, p=.000), while Gf at W1 was associated with perceived general health (B=1.28, p=.010) and organizational involvement (B=1.8, p=.019). Gc at W2 was associated with Gc at W1 (B=.61, p=.000), and age (B=-.12, p=.007), while Gf at W2 was associated with Gf at W1 (B=.74, p=.000), age (B=-.08, p=.008), and loneliness (B=-.78, p=.038). Among men, there were no significant associations between either Gc at W1 or Gf at W1 and other variables. Gc at W2 was associated with Gc at W1 (B=.29, p=.031), while Gf at W1 was associated with Gf at W2 (B=7.9, p=.000) and perceived general health (B=2.46, p=.006). These findings suggest that loneliness and organizational involvement are associated with lower Gf scores among women but not among men. Gc was not associated with loneliness or organizational involvement for either women or men. This suggests that interventions targeting the prevention of loneliness and the promotion of organizational involvement may enhance cognitive functioning in later life among women.

### THE EFFECTS OF PET OWNERSHIP ON PSYCHOLOGICAL WELL-BEING AMONG SOCIALLY ISOLATED OLDER ADULTS

Tomoko Ikeuchi,[1] Takumi Abe,[2] Yu Taniguchi,[3] Satoshi Seino,[4] Yui Tomine,[4] Chiho Shimada,[4] Akihiko Kitamura,[4] and Shoji Shinkai,[1] *1. Tokyo Metropolitan Institute of Gerontology, Tokyo, Japan, 2. Tokyo Metropolitan Institute of Gerontology, Tokyo, United States, 3. National Institute for Environmental Studies, Tsukuba, Ibaraki, Japan, 4. Tokyo Metropolitan Institute of Gerontology, Itabashi-ku, Japan*

Accumulating evidence suggests that pet ownership associates with positive psychological outcomes (i.e., less loneliness, lower depression, etc.) in older adults. Yet, the role of pet ownership in psychological well-being (PWB) of socially isolated older adults is not fully explored. In this study, we hypothesized that pet (i.e., dog or cat) ownership would have positive effects on PWB among socially isolated older adults. The study used cross-sectional data of 9875 community-dwelling older adults collected in 2016 in a metropolitan area of Japan. Overall, 2841 (28.8%) participants were socially isolated (i.e., having social interactions with others less than once a week). Stratified by dog and cat ownership, 3143 (31.8%) were current or previous dog owners, and 1724 (17.5%) were current or previous cat owners. PWB was dichotomized using a score of the WHO-5 Well-Being Index, and 2730 (27.6%) were identified as low levels of PWB. Logistic regression models, adjusted for demographic confounders, showed that social isolation was associated with lower PWB (OR: 2.39; 95% CI: 2.17, 2.64) and lower odds of having a dog (OR: 0.70; 0.63, 0.77). When social isolation and dog ownership were entered into a model simultaneously as independent variables, dog ownership was associated with greater PWB (OR: 0.90; 0.81, 0.99). There was a significant partial mediating effect of dog ownership found on the association between social isolation and PWB (Sobel test p=.034). No such associations were observed in cat ownership. Our results suggest that having a dog may be effective for increasing PWB for socially isolated older adults.

### THE JOINT EFFECTS OF LONELINESS AND DEPRESSION ON MORTALITY: DOES GENDER MATTER?

Ted Kheng Siang Ng,[1] Abhijit Visaria,[2]
Angelique WM Chan,[2] and Kheng Siang Ted Ng,[3]
*1. DUKE-National University of Singapore, Singapore, Singapore, 2. Duke-NUS Medical School, Singapore, Singapore, 3. Yong Loo Lin School of Medicine, National University of Singapore, Singapore*

Loneliness and depression are both associated with an increased risk of all-cause mortality among older adults. However, the evidence on the joint effect of loneliness and depression is scarce. Furthermore, previous research has rarely examined the modifying effects of gender. We investigated these questions using the Panel on Health and Aging of Singaporean Elderly, a nationally-representative cohort study of community-dwelling older Singaporean adults aged 60 and above, conducted in 2009 with two follow-up waves in 2011 and 2015 (N=4536). We operationalized six groups based on three categories of loneliness measured using the 3-item University of California, Los Angeles (UCLA) loneliness scale: always lonely, sometimes lonely, and never lonely; Two categories of depressive symptom scores were measured using the 11-item Center for Epidemiologic Studies Depression Scale (CES-D) scale: depressed and not depressed. Cox proportional hazards models were employed to estimate the mortality risks for each group, with an extensive set of covariates. Due to significant differences in the prevalence of loneliness and depression in different genders, we conducted gender-stratified analyses. Compared to being not depressed and never lonely, women who were depressed and sometimes lonely and who were not depressed but always lonely had a higher mortality risk. Men who were not depressed but sometimes lonely had a higher mortality risk. We conclude that loneliness appears to be the predominant construct in conferring excess mortality risk. Health policies and interventions addressing the factors common and unique to each gender may improve psychological well-being at older ages, thereby extending the lifespan.

### THE MODERATING IMPACT OF ATTENDING SERVICES ON THE EFFECT OF OLDER ADULTS' DISCRIMINATION EXPERIENCES ON LONELINESS

Dahee Kim,[1] and Kyuho Lee,[2] *1. Iowa State University, Ames, Iowa, United States, 2. Daegu University, Gyeongsan, Kyongsang-bukto, Republic of Korea*

Research has shown that perceived discriminations impact physical and mental health in later life. Discrimination experiences could make older adults consider themselves as a social misfit and decrease their social interactions, which finally increases their loneliness. Religious behaviors has been reported as a key factor of a lower sense of isolation. Considering that religious behaviors provide opportunities to engage in more extensive social networks and have supportive social ties with community members, attending religious services might decrease the impact of older adults' perceived discrimination on loneliness. The current research aims to examine the moderating role of religious services attendance in the association between older adults' perceived discrimination and loneliness. We used data of 4,488 adults aged 50 to 80 (M=66.27, SD=10.15) from the Health and Retirement Study (HRS) collected in 2012 and 2014. Linear regression analysis was performed to investigate whether older adults' religious service attendance might decrease the impact of their perceived discriminations in daily life on the level of loneliness. The results indicated that more perceived discriminations older adults face on a daily basis were significantly associated with higher levels of loneliness. However, participants who frequently attended religious services showed a lower impact of perceived discriminations on their loneliness. These findings highlight the positive effects of engaging in religious activities on discriminated older adults' social well-being. These findings also emphasize the role of the religious community as a social resource for socially marginalized older adults.

### SESSION 2939 (POSTER)

### ATTITUDES ABOUT AGING I

### ARE OLDER ADULTS A BURDEN ON SOCIETY? ETHNIC DIFFERENCES IN WHAT YOUNGER NIGERIANS THINK

Abolade Oladimeji, *case western reserve university, cleveland, Ohio, United States*

Ageism is on the rise in Nigeria. Recently congress passed a bill on "not too young to rule" a campaign to advocate for younger Nigerians to take over leadership positions from older political officeholders. This paper focuses on ethnic differences among Nigerians who think older adults are a burden on society. Our sample included 1,693 randomly selected Nigerians (18 yrs-59yrs) who were in wave six of the world values survey conducted in 2012 comprising of the three major ethnic groups in Nigeria; Hausa (33%), Igbo (24%), and Yoruba (25%). Using multivariate logistic regression, results show that among the Hausas; life satisfaction, education, employment, gender, income, and geography predict bias towards older adults (OR=1.12*,.89*,.61*,.63*,1.12*,.74***) respectively. Among the Igbos; only education predicts bias towards older adults (OR=.75***). Among the Yorubas; income and geography predict bias towards older adults (OR=.86*, 1.20*) respectively. These findings underscore that several individual resources, as well as relationship status, are associated with a bias towards the elderly in Nigeria. The results reinforce the idea that the underlying causes of ageism might be structural such as political failure, poverty, illiteracy, and rurality. To change how younger generations, think of older adults, macro-level interventions and policies need to be implemented.

### ASSOCIATIONS BETWEEN SUBJECTIVE AGE, CONTACT WITH OLDER ADULTS, AND AGE STEREOTYPES AMONG YOUNGER ADULTS

Maria Kurth,[1] and Robert Intrieri,[2] *1. Oregon State University, Corvallis, Oregon, United States, 2. Western Illinois University, MACOMB, Illinois, United States*

Subjective aging (SA) has generally focused on middle-age and older adults in relation to physical and psychological health for the last 70 years (see Barak & Stern, 1986). Kornadt et al. (2019) recently called for more research examining: co-development of age stereotypes and SA, and this association across the lifespan. Literature examining SA and age stereotypes among younger adults is limited and suggests that age stereotypes are not directly associated with SA (Packer & Chasteen, 2006). Increased contact with older adults, however, is associated with less ageist attitudes (Bousfield & Hutchinson, 2010). This study examined SA and the associations between contact frequency and ageism. The sample consisted of 467 undergraduate students (Mage = 21.48, SDage = 2.63). Subjective age was assessed by asking How old do you feel compared with others your age?, and was scored on a 5-point scale from younger all the time (5) to older all the time (1). Ageism was assessed with the Aging Semantic Differential (ASD), which contains four factors. Results showed significant effects across felt age for contact frequency ($F(4, 406) = 3.841$, $p = .004$). Results for the ASD factors were mixed with Autonomy and Integrity showing significant effects for SA ($F(4, 405) = 2.763$, $p = .027$; $F(4, 405) = 2.773$, $p = .027$ respectively). Instrumentality and Acceptance were nonsignificant. Results suggested feeling older all the time is related to more contact, but more negative attitudes- this increased contact might providing priming for more ageist attitudes (Eibach et al., 2010).

### CROSS-VALIDATION OF A SCALE TO MEASURE CONTACT WITH OLDER ADULTS

Robert Intrieri,[1] and Maria Kurth,[2] *1. Western Illinois University, MACOMB, Illinois, United States, 2. Oregon State University, Corvallis, Oregon, United States*

According to Allport (1954) intergroup contact would reduce prejudice that in-group members would experience toward out-group members. Allport also theorized that positive intergroup contact would follow after four conditions were met: (a) equal group status within the situation, (b) common goals, (c) intergroup cooperation, and (d) the support of authorities, law, or custom. While contact with older adults is a principal influence on attitudes toward older people, there is a paucity of adequate contact measures. This study assessed the cross-validity of the factor structure of an instrument to measure contact with older adults. The convenience sample consisted of 470 participants (61% male) from an undergraduate student subject pool (M = 20.67, SDtotal = 3.37). Participants were predominantly Caucasian (n=176, 48.9%), African American (n=103, 28.6%), and Hispanic/Latino/a (n=51, 14.2%). Results of a confirmatory factor analysis showed the three-factor model exhibited a reasonable fit to the data □□ (41, n = 360) = 191.797; p<.0001, CFI =.971; TLI =.961; RMSEA = .101 (90% CI, 0.087-0.116) SRMS = .042. An additional model examining the relationships between a single indicator of contact frequency and the three-factor COA scale revealed similar fit statistics □□ (41, n = 360) = 191.797; p<.0001, CFI =.967; TLI =.955; RMSEA = .092 (90% CI, 0.079-0.105) SRMS = .044. The findings provide clear and consistent evidence across independent samples that the covariances among the items are best explained through a latent structure that consists of three meaningful factors ("General Contact", "Positive Experience, and "Negative Experience").

### GENDER CODES AND AGING: COMPARISON OF FEATURES IN TWO WOMEN'S MAGAZINES

Ann O'Hanlon,[1] Therese Mendez,[2] and Melissa Morrissette,[1] *1. University of New Orleans, New Orleans, Louisiana, United States, 2. University of New Orleans, New Orleans, LA, Louisiana, United States*

Magazines and other media promote beauty standards and gender roles in feature articles and advertising. Publications present idealized images of women often in contrast to the average reader's appearance. Analyses of such images suggest gender roles are reinforced through subtle cues embedded in hand gestures, eye gaze, head posture, and body position (Goffman, 1976). This study analyzed a recurrent feature in two different magazine presenting an idealized standard of aging to mature women. The first magazine, MORE, featured mature women, typically between the ages of 40 and 60, with the banner of "This is what (woman's age) looks like." MORE magazine is no longer in press, but another magazine, Women's Day, began a similar recurrent column featuring a women between 40 and 60 with the title "Own Your Age—Yes, I am (women's age)." Both features included copy describing the woman's perspective on life and aging and a listing of specific beauty products that she uses. These features were analyzed as advertisements, because they promote a message about being a woman of a certain age and the specific products used to achieve that look. Three researchers coded 43 images from MORE magazine and 30 images from Woman's Day for physical characteristics of aging and evidence of Goffman's gender codes. Most photos presented women who appeared younger than their stated age. Images showed the presence of Goffman's gender codes including feminine touch, ritualization of subordination, licensed withdrawal, and infantilization and were more prevalent in the MORE feature than Woman's Day column.

### GENDER DIFFERENCES IN ATTITUDES TOWARD AGING AND ITS LONGITUDINAL IMPACT ON PSYCHOLOGICAL HEALTH

Eun Young Choi,[1] Yujin Franco,[2] and Elizabeth Zelinski,[2] *1. Leonard Davis School of Gerontology, Los Angeles, California, United States, 2. University of Southern California, Los Angeles, California, United States*

Individuals with negative attitudes towards own aging (ATOA) experience worse psychological health in later life. At the intersection of sexism and ageism, women are likely to have greater concerns about growing older and hold more negative views of aging than their men counterparts. However, the impact of gender on the relationship between ATOA and psychological health is unclear. Moving forward, the current study aims to examine 1) gender differences in longitudinal changes in ATOA and 2) whether gender moderates the association of ATOA with cognitive function and depressive symptoms. Using three waves (2008, 2012, and 2016) from the Health and Retirement Study, a total of 6,675 adults aged 50+ (60% female) were analyzed. A series of multilevel growth curve analyses were performed to investigate the 8-year changes in ATOA and within- and between-person effects of ATOA on cognitive function and depressive symptoms. The models controlled for demographic, socio-economic, and physical health characteristics. Women had more negative ATOA at baseline compared to men, but not in rates of change. When levels of ATOA were more negative, both cognitive performance and depressive symptoms were poorer over time between individuals as well as within-person. We found that the detrimental effects of negative ATOA on depressive symptoms were stronger for women, but there were no significant gender differences in relation to cognitive functioning. Our findings demonstrated that women view aging more unfavorably than men, and the effects of endorsing negative ATOA are more pronounced on women's mental health.

### HOW GROWING OLDER WITH THEIR BEST FRIEND CHANGES OLDER ADULT MEN'S PERSPECTIVES ON AGING

Ranell Mueller, *St. Francis University, DUNCANSVILLE, Pennsylvania, United States*

Knowledge of the deeper meaning of how growing older alongside companion animal dogs affects men's perspectives of aging is limited. This study employed qualitative research methods utilizing individual interviews and panel discussions with older adult men in order to investigate the dynamic phenomenon of their personal experience of growing older alongside their aging companion animal dogs. Individual audio-recorded and in-depth interviews and repeated panel discussions with a sub-group of participants, convened as a panel over a three-month period, explored behavioral and emotional manifestations of aging along with a companion animal. Analysis involved open, axial, and selective coding of transcripts to reveal underlying patterns within the data. Outcomes included insight into the role of dogs in the men's perspectives of attitudes toward aging, toward not only themselves as older adult men, but also toward their aging dogs. Findings reveal that the older adult men felt empathy for their aging dogs, which translated to feeling empathy for themselves as well as other older adults. The men revealed attitudes of being sympathetic and understanding of the aging process simply as a result of aging alongside their aging dogs. A prominent idea that emerged was a feeling of grace toward oneself, others and their aging dogs. This study offers insight into the deeper understanding of one effect caring for older companion animals has on older adult men's attitudes toward aging. This study provides movement toward a theory of the role of dogs in the development of older adult men's attitudes toward aging.

### I'D RATHER BE . . . : ACTIVITIES OLDER ADULTS REPORTED LEADING TO DECREASED ENGAGEMENT WITH COGNITIVE TRAINING

Erin Harrell,[1] and Nelson Roque,[2] *1. University of Alabama, Tuscaloosa, Tuscaloosa, Alabama, United States, 2. Pennsylvania State University, University Park, Pennsylvania, United States*

One modifiable risk factor of dementia is cognitive inactivity. Given cognitive ability is closely tied to continual performance of instrumental activities of daily living, cognitive training programs continue to be explored as a way to boost cognition and allow older adults to remain independent longer. While the efficacy of cognitive training is controversial, identifying activities older adults are willing to limit in exchange for cognitive training provides valuable information in relation to designing cognitive training programs that appeal to older adults. Using a qualitative approach, this study highlights activities older adults (ages 64+) noted as contributing to decreased gameplay of a cognitive training program on a tablet device. We found that respondents (61%) noted playing less as a result of entertainment activities (i.e., reading and playing games), social activities (31%) and travel (27%). Findings have implications for device form factor in administering cognitive training and other programs.

### INDIVIDUALISM INCREASES THE INFLUENCE OF PERCEIVED COMPETENCE OF OLDER ADULTS ON ATTITUDES TOWARD THEM

Amber Xuqian Chen,[1] and Helene Fung,[2] *1. The Chinese University of Hong Kong, Hong Kong, China, 2. The Chinese University of Hong Kong, Sha Tin, China*

Negative views of ageing can lower respect for older adults. Yet, negative views of ageing vary across cultures. Asian collectivistic cultures are assumed to respect older adults more than Western individualistic cultures do. However, recent empirical findings on this cross-cultural comparison have suggested that negative attitudes toward older people are also prevalent, or even more evident in collectivistic cultures than individualistic cultures. Using data from the sixth wave of the World Values Survey, a dataset consisting of 75,650 individuals from 56 societies, we employed Linear Mixed Modeling to test the association between perceived competence of older adults and respect towards them. We also explored and the moderating role of culture on this association. In the present study, perceived competence of older adults was indexed as a proportional score representing the relative perception of competence (i.e. relative competence perception = competence / (competence + friendliness). Results showed that individuals tended to respect older adults who were more competent or friendly. Furthermore, individuals who were more individualistic respected older adults more when older adults were perceived to be more competent relative to friendly. This pattern was reversed in individuals who were less individualistic. These findings suggest that whether people who differ on personal individualistic values respect older adults depends on whether older adults are perceived to be competent versus friendly. Findings from this study highlight the importance of changing cultural values on ageism attitudes, especially the potential effects of rising individualism on negative attitudes of ageing in Asia.

### NORTH AMERICAN AND INTERNATIONAL STUDENTS' PERSPECTIVES ON OLDER ADULTS

ADAM SHEA,[1] Jessica Strong,[2] and Kirsten Graham,[3] *1. university of prince edward island, Charlottetown, Prince*

*Edward Island, Canada, 2. University of Prince Edward Island, Charlottetown, Prince Edward Island, Canada, 3. Southern Utah University, Cedar City, Utah, United States*

Ageism is recognized around the world as detrimental to older adults' health and well-being, and there are differences in how cultures view older adults. Infrequently are ageist attitudes among cultures compared within one study. Here, we sought to examine views on older adults across cultures in a sample of university students attending school in North America (n=31). As part of a larger survey of ageist attitudes, we conducted a thematic analysis on open-ended responses to the question "How are older adults viewed in your culture?" Half of the respondents were international students. Results found similarities and differences between groups. First, both groups saw older adults as individuals who are and should be respected. Second, however, North American students viewed older adults as "important" and "role models", whereas international students viewed older adults as "leaders" of and at the "head of the family". Third, North American students saw older adults as "needing extra help." In contrast, international students reported that families "should provide care" as a duty or responsibility. Fourth, North American students believed older adults provide "wisdom," "love," and "support" but the international students felt older adults provided knowledge, experience, and opinions that are valued by the family and society that are important to decision making. Finally, North American students describe negative perceptions and experiences with older adults, which was completely absent from the international students responses. Results are discussed in a cultural context of personal and formal relationships with elders.

### SELF-RELATED VIEWS OF AGING AFTER AGE 50: THE LONG-TERM EFFECTS OF POOR HEALTH IN CHILDHOOD

Jacqui Smith, and Marina Larkina, *University of Michigan, Ann Arbor, Michigan, United States*

Age stereotypes and expectations about one's own aging commence in childhood but most research focuses on predictive associations with midlife health behaviors, later-life chronic conditions, and longevity. Surprisingly little is known about the role of poor childhood health in these associations. This study aims to fill this gap. Using data from the Health and Retirement Study (HRS: N = 5807; aged 50-98), we investigated whether diagnosed chronic illness before age 16 and self-rated childhood health predict late-life self-perceptions of aging (SPA) and subjective age discrepancy (AD). We conducted multivariate multiple regressions to determine the joint and unique effects of childhood health. Models included controls for current health (functional limitations), memory status, and demographic covariates (age, gender, race/ethnicity, marital status, and education). Multivariate tests (Pillai's trace) revealed that both childhood health indicators were significant predictors. Over and above all covariates and the covariation of the two views of one's own aging, univariate models showed that the number of childhood diagnoses was significant predictor of AD ($p < .007$) but not for SPA. In contrast, self-rated childhood health was a significant predictor of SPA ($p < .001$) but not for AD. This study provides new insight into precursors of self-evaluations of aging. Whereas childhood diagnoses of chronic illness attenuated the extent that individuals felt younger than their actual age, ratings of poor childhood health enhanced negative SPA. The non-normative experience of poor health in early life is a lifelong foundation for both late life beliefs and health.

### THE EFFECT OF NONESSENTIALIST BELIEFS ABOUT AGING ON HEALTH BEHAVIOR INTENTIONS

Ga-Eun (Grace) Oh, *Open University of Hong Kong, Kowloon, China*

Globally, as people expect the longer life expectancy than ever before, people have increasing concerns about their health and aging. Although what people believe regarding aging can affect their health behaviors, limited research has investigated which beliefs regarding aging influence health behaviors. Previous research has shown that essentialist beliefs about aging reflect beliefs that the aging process is fixed, while nonessentialist beliefs about aging reflect the beliefs that the aging process is rather malleable. Since beliefs in nonessentialism regarding aging imply the benefits of health-promoting behaviors, we examine if manipulating nonessentialist beliefs about aging could contribute to intentions to engage in health behaviors. We also investigate if age and income might moderate the effect of nonessentialist beliefs. We conducted an experiment with a sample of American participants of varied ages (n = 599). The results showed that compared to essentialist beliefs, nonessentialist beliefs regarding aging significantly increased an intention to eat healthy food but they did not improve intentions of other health-promoting behaviors in terms of regular exercise and consumption of fruits and vegetables. Income moderated the effect of essentialist beliefs on an intention to eat fruits and vegetables. Specifically, nonessentialist beliefs had a positive effect among high-income people but rendered a negative effect among low-income people. Together, the present findings provide initial evidence that nonessentialist beliefs have a potential to promote health behaviors and call for the further investigation of the effects of educating nonessentialist beliefs on actual health behaviors and the boundary conditions of the effects.

### THE IMPACT OF MEMORY STEREOTYPE THREAT ON MEMORY AND MEMORY SELF-EFFICACY IN OLDER ADULTS

Lauren Fredriksen, Renee' Zucchero, Brock Partlow, Ruth Infante, Janie Taylor, and Haley Washburn, *Xavier University, Cincinnati, Ohio, United States*

This study examined the impact of memory stereotype threat on memory duration (e.g., short-term and long-term) and modality (e.g., verbal and non-verbal), and memory self-efficacy in older adults who live independently (Mage = 77 years). Participants (N= 66) were randomly assigned to a group that received either neutral instructions or memory stereotype threat inducing instructions. All participants completed the California Verbal Memory Test-Second Edition (CVLT-2), the Rey Complex Figure Test (RCFT), a memory self-efficacy measure, and a demographics survey. An independent samples t-test indicated participants in the stereotype threat group reported significantly lower memory self-efficacy than participants in the neutral group. The main effect of the within-subjects factor of a 2x2 mixed analysis of variance (ANOVA) indicated that participants performed significantly better on short-term non-verbal memory than long-term non-verbal memory. There was no significant difference between the neutral and stereotype threat groups in memory modality or duration. These results may indicate that the instructions used to induce memory stereotype threat were not phrased strongly enough to elicit poorer performance on the CVLT-2 and RCFT in the memory stereotype threat group. Additionally, participants reported having a high level of education (i.e., a master's degree was the modal educational level), which may have served as a buffer for memory stereotype threat. The findings call for future research to explore the impact of level of education on memory self-efficacy in older adults. Also, future research may focus on the impact of the strength of memory stereotype threat on memory performance.

### THE INFLUENCE OF AGING ATTITUDES ON THE RELATIONSHIP BETWEEN LONELINESS AND PERCEIVED HEALTH

Kate Kondolf, Zoë Shelton, and Silvia Sörensen,
*University of Rochester, Rochester, New York, United States*

Loneliness has negative implications for both psychological and physical wellbeing. Age-related impairments further limit social ties, making older adults with vision loss more susceptible to loneliness. Negative age stereotypes directed at the self over the course of one's personal aging process (Levy, 2009) have harmful effects on cardiovascular health (Levy et al., 2009), engagement in health behaviors (Stewart et al., 2012), longevity (Sun et al., 2017), and psychological well-being. Feelings of loneliness are strongest among individuals who believe that loneliness is a part of aging (Pikhartova et al., 2015). Although loneliness and aging attitudes are both closely linked to health, their interplay has not been investigated within older adult populations. We hypothesized that attitudes about aging would increase as a result of loneliness and thus help explain the relationship between loneliness and perceived health. This study used baseline data from an intervention study of older adults with Macular Degeneration (N=224, aged 60-96, 63.4% female, 20% low-income). Measures: 8-item UCLA Loneliness Scale, Attitudes toward Own Aging scale (ATOA for self-stereotypes, Lawton, 1975), One-item self-reported health. Results: Linear regression showed significant relationships between loneliness and health ($\beta$=-.145, p<.05), loneliness and ATOA ($\beta$=.32, p<.001), and self-stereotypes and health ($\beta$=-.45, p<.001). Adding ATOA to the model regressing health on loneliness rendered the direct effect of loneliness on health non-significant ($\beta$=-.014, p=.833), suggesting a mediator effect. Analyses controlling for age, gender, and education yielded comparable results, with the explained variance increasing from 5% (demographics and loneliness) to almost 24% (adding ATOA as mediator).

### THE SOCIAL PATTERNS OF AGE DISCRIMINATION: AN ANALYSIS OF THE MACRO-PERSPECTIVE-INTERPRETATIONS

Stefan Hopf, *National University of Ireland Galway, Galway, Ireland*

Modern societies can be regarded as service economies, consequently accessing services is an essential part of social and economic participation. Direct and indirect indiscrimination act as barriers to accessing and using services and one way to address these barriers is to implement anti-discrimination legislation and policy. From a sociological point of view, such policies and legal frameworks can be described as elements of the social discourse in these areas. These texts, along with their implicit and explicit interpretations of the problem, represent the official and legitimised stake of the socially available stock of knowledge of what constitutes age discrimination. Hence the shape and contribute to the general understanding of age discrimination. The study aims to investigate the interpretation patterns offered by the "supply" side, that is by those actors who in their work refer to but also (re-) shape and disseminate the problem interpretation contained in the official texts. To address this aim, focus groups with stakeholders and semi-structured interviews with legal and policy experts were conducted in Austria and Ireland. The findings highlight that experts and stakeholders' definitions of age discrimination usually extend past legal and policy concepts. The expert and stakeholder approaches differ in their starting points for describing the problem, ranging from vulnerability considerations to human rights-based concepts and more structurally orientated needs-based criteria. Finally, the analysis also reveals a central distinguishing feature of age discrimination, namely the "de-temporalization" and "de-historicization" of the person, which is of equal importance as the de-individualization as a consequence of stereotyping

### WHAT ARE THE MOST DISTRESSING ASPECTS OF EXPERIENCING ELDER ABUSE? FINDINGS FROM A QUALITATIVE STUDY WITH VICTIMS

Jessica Hsieh,[1] David Burnes,[1] Clara Scher,[2] Paula Zanotti,[2] Chelsie Burchett,[3] Jo Anne Sirey,[2] and Mark Lachs,[4] *1. University of Toronto, Toronto, Ontario, Canada, 2. Weill Cornell Medicine, White Plains, New York, United States, 3. Weill Cornell Medicine, New York, New York, United States, 4. Weill Cornell Medicine, New York City, New York, United States*

Adult protective services and other community-based agencies respond to hundreds of thousands of elder abuse cases annually in the United States; however, few studies include elder abuse victims' voices. This study explored the most distressing aspects of elder abuse, as identified by victims themselves; to date, this is the first known study on this topic. Guided by a phenomenological qualitative methodology, this study conducted in-person, semi-structured interviews with a sample of elder abuse victims (n = 30) recruited from a community-based elder abuse social service program in New York City. To enhance trustworthiness, two researchers independently analyzed transcript data to identify key transcript codes/themes. Distressing aspects of elder abuse were identified across three key domains, related to feelings of loss (50% of codes), threats/negative consequences (55%), and client-needs/system incongruity (14%). Specifically, the first theme represented outcomes related to loss of relationships (19% of 'loss' codes), personhood (16%), credibility (19%), faith/trust in others (38%), and finances (8%). The second theme looked at threats to physical self (34% of 'threat' codes), psyche (39%), and others, including the perpetrator (27%). The third theme focused on mismatches in client/

system goals (50% of 'incongruity' codes) and legal system involvement (50%). The findings in this study provide a comprehensive and conceptually organized range of aspects to serve as infrastructure for the development of meaningful interventions to address the needs of victims. This study represents one of the largest efforts to understand and integrate the perspectives and needs of victims into elder abuse intervention practice/research to date.

### SESSION 2940 (POSTER)

### DIVERSE RACE AND ETHNIC POPULATIONS I

### ASIAN AMERICAN OLDER ADULTS AND SOCIAL ISOLATION: A SYSTEMATIC LITERATURE REVIEW

Simona Kwon, Deborah Min, and Stella Chong, *NYU School of Medicine, New York, New York, United States*

Asian Americans are the fastest growing racial and ethnic minority group in the United States, whose population is aging considerably. Previous studies indicate that social isolation and loneliness disproportionately affects older adults and predicts greater physical, mental, and cognitive decline. A systematic literature review using PRISMA guidelines was conducted to address this emerging need to understand the scope of research focused on social isolation and loneliness among the disparity population of older Asian Americans. Four interdisciplinary databases were searched: PubMed, CINAHL, PsycINFO, and AgeLine; search terms included variations on social isolation, loneliness, Asian Americans, and older adults. Articles were reviewed based on six eligibility criteria: (1) research topic relevance, (2) study participants aged >60 years, (3) Asian immigrants as main participants, (4) conducted in the United States, (5) published between 1995-2019, and (6) printed in the English language. The search yielded 799 articles across the four databases and 61 duplicate articles were removed. Abstracts were screened for the 738 remaining studies, 107 of which underwent full-text review. A total of 56 articles met the eligibility criteria. Synthesis of our review indicates that existing research focuses heavily on Chinese and Korean American immigrant communities, despite the heterogeneity of the diverse Asian American population. Studies were largely observational and employed community-based sampling. Critical literature gaps exist surrounding social isolation and loneliness in Asian American older adults, including the lack of studies on South Asian populations. Future studies should prioritize health promotion intervention research and focus on diverse understudied Asian subgroups.

### ATTACHMENT TO HOME AND COMMUNITY IN OLDER RURAL AFRICAN AMERICANS IN MISSISSIPPI

Carolyn Adams-Price, Muhammed Riaz, Margaret Ralston, and Antonio Gardner, *Mississippi State University, Mississippi State, Mississippi, United States*

The purpose of this study was to examine qualitatively attachment to home and community in older rural African Americans in the Deep South. Sixty adults aged 52-79 (mean age 64.7, 24 males and 36 females) were interviewed using semi-structured interviews. Participants lived in two micropolitan counties in Mississippi, with most living in one of two mostly African American communities with fewer than 1000 residents. Interviewees were asked about their attachment to their house, the land the house is on, and the community in which they live. Interviews were recorded and transcribed, and transcriptions were analyzed for themes by two qualitative researchers using phenomenological analysis. The two researchers uncovered similar themes; discrepancies were discussed and integrated, and checked for consistency with the original text. The most prominent themes for attachment to home were sense of ownership/having built or made home their own, legacy/generational/historical attachment, sense of peace and safety, solitude and privacy, and attachment to specific features. Interviewees felt that their homes were a part of who they are and a part of their personal and family history; they also reported that their homes were safe and comfortable havens. When participants were asked about their attachment to their community, four themes emerged: socialization/friendly visiting, family and close ties, religious/spiritual, and solitude/quiet community. These results will be discussed in the context of Wahl's 2012 model, which asserts that older adults' attachment to place is a function of agency and belonging; belonging was a more prominent theme in this group.

### BLACK-WHITE DIFFERENCES IN HEARING PROBLEMS AMONG OLDER AMERICANS: FINDINGS FROM TWO LARGE REPRESENTATIVE SURVEYS

Esme Fuller-Thomson, ZhiDi (Judy) Deng, and Senyo Agbeyaka, *University of Toronto, Toronto, Ontario, Canada*

The purpose of this study is to investigate Black-White differences in hearing problems among older adults living in the United States. Secondary data analyses were conducted using the 2017 American Community Survey (ACS) with a replication analysis in the 2016 ACS. The ACS is an annual nationally representative survey of Americans living in community settings and institutions. The sample size of older Americans (age 65+) in 2017 was 467,789 Non-Hispanic Whites (NHW) and 45,105 Non-Hispanic Blacks (NHB). In the 2016 ACS, there were 459,692 NHW and 45,990 NHB respondents aged 65+. Measures of hearing problems, age, race/ethnicity, education level and household income were based on self-report. Data were weighted to adjust for non-response and differential selection probabilities. The prevalence of hearing problems was markedly higher among older NHW (15.4% in both waves) in comparison to NHB (9.0% in 2017; 9.4% in 2016; both $p<.001$). In the 2017 ACS, the age-sex adjusted odds of hearing loss were 69% higher for NHW compared to NHB, which increased to 91% higher odds when household income and education level were taken into account (OR=1.91; 95% CI=1.85, 1.97). Further analyses by 10 year age cohorts indicated comparable findings (fully adjusted ORs range from 1.89 to 1.98). Findings from the 2016 ACS were very similar (e.g., 65+ fully adjusted OR=1.81). NHW have a much higher prevalence and almost double the odds of hearing loss compared NHB. Future research should investigate if melanin plays an otoprotective role through enhancing the antioxidant capability of cochlea.

### CHANGE IN COGNITIVE PERFORMANCE BY RACE OR ETHNICITY AND MULTIMORBIDITY AMONG OLDER AMERICANS

Ana Quinones,[1] Siting Chen,[2] Anda Botoseneanu,[3] Heather Allore,[4] Jason Newsom,[5] Stephen Thielke,[6] and Jeffrey Kaye,[7] *1. Oregon Health & Science University, Portland, Oregon, United States, 2. OHSU-PSU School of Public Health, Portland, Oregon, United States, 3. University of Michigan, Dearborn, Michigan, United States, 4. Yale University, New Haven, Connecticut, United States, 5. Portland State University, Portland, Oregon, United States, 6. University of Washington, Seattle, Washington, United States, 7. Layton Alzheimer's Disease Center, Portland, Oregon, United States*

Understanding factors that influence cognitive performance remain critical priorities, particularly among racial/ethnic groups that have higher prevalence of dementia. This study assesses race/ethnic (non-Hispanic white, non-Hispanic black, Hispanic) differences in cognitive performance in adjusted models accounting for co-existing self-reported chronic conditions (arthritis, diabetes, cancer, depressive symptoms, cardiovascular disease, hypertension, lung disease, osteoporosis, stroke), age, sex, education, and income. Data from the 2011-2017 National Health and Aging Trends Study (NHATS), a nationally-representative sample of Medicare beneficiaries (N=7,041, mean age=77.5), were used to estimate a series of cross-sectional multivariable linear regressions to evaluate race/ethnic differences in cognitive performance scores on the NHATS cognitive composite test of memory, orientation, and executive function domains (range 0-33) over seven years. In adjusted models, black participants had lower cognitive scores relative to white participants in 2011 (b=-2.25, 95% CI[-2.52, -1.98]) and by the end of the observation period in 2017 (b=-3.24, 95% CI[-3.72, -2.76]). Similarly, Hispanic participants experienced lower cognitive scores relative to white participants in 2011 (b=-2.31, 95% CI[-2.77, -1.84]) which persisted to the end of the observation window (b=-2.77, 95% CI[-3.66, -1.89]). Racial/ethnic groups had significantly lower cognitive scores relative to white Medicare beneficiaries over seven years of assessment. These analyses build toward longitudinal analyses of repeated observations of cognitive performance. Given the broad clinical and policy implications involved in caring for persons with dementia, it will be important to intervene earlier on modifiable risk factors to postpone cognitive declines among older minority ethnic adults.

### ETHNICITY MODERATING THE RELATIONSHIP OF COGNITION FUNCTION OF PATIENTS WITH DEMENTIA ON CAREGIVER DEPRESSION

Alan Palmer,[1] Rowena Gomez,[2] Eric Taylor,[2] Eliot Steer,[2] and Megan Frank,[2] *1. Palo Alto University, Sunnyvale, California, United States, 2. Palo Alto University, Palo Alto, California, United States*

The purpose of this study was to see if ethnicity (African-American and Caucasian) moderates the predictive effects of cognition functioning in patients with dementia on caregivers' severity of depressive symptoms. Secondary data analyses were conducted from Resources for Enhancing Alzheimer's Caregiver Health (REACH II; 2001-2004). The participants consisted of 214 African American and 321 Caucasian participants (N = 535). The assessment battery included the Center for Epidemiologic Depression Scale (CES-D) to measure depression severity, Mini-Mental State Exam (MMSE) to measure level of cognitive function, and demographic questionnaire to gain information about caregivers and care-recipients. ANOVAs and ANCOVAs were used to examine ethnic group differences in care-recipient cognitive functioning in predicting caregiver depression. Caucasian caregivers reported significantly higher levels of depression and care-recipients' cognitive function compared to African American caregivers, ps<.05. A custom ANCOVA indicated a significant interaction between ethnicity and care-recipient cognitive functioning on caregiver depression with greater effects of care-recipient cognitive function on caregiver depression for the African American caregivers than for the Caucasian caregivers, p=.02. Descriptively, the depression severity for the Caucasian caregivers remained relatively high across levels of care-recipients' cognition. The findings indicated that ethnicity moderated the effects of care-recipient cognitive functioning on caregiver self-report of depressive symptoms. These findings suggest greater resiliency in African -American caregivers supporting their dementia or dementia-related condition care-recipients (Dias et al., 2015). These findings support the need to develop cultural specific interventions to better support the wellbeing of caregivers of care-recipients with dementia or dementia-related conditions.

### HISPANICS AND HOSPICE CARE: A SYSTEMATIC REVIEW

Valeria Cardenas, Susan Enguidanos, and Gillian Fennell, *University of Southern California, Los Angeles, California, United States*

Hospice care has demonstrated improved pain and symptom relief for patients at end-of-life, however, Hispanics have significantly lower rates of hospice use compared to Whites. Moreover, few studies have examined factors associated with these lower enrollment rates and barriers to hospice care experienced by Hispanics. This systematic literature review aims to provide a comprehensive overview of studies examining Hispanic hospice use. We conducted a comprehensive search using three electronic databases (Ovid Medline, EMBASE, and CINAHL) from January 1946 to March 2019 using MESH terms for Hispanics, hospice, and end-of-life care. Our review was guided by the Preferred Reporting Items for Systematic Reviews and Meta-Analysis Protocols (PRISMA-P). Studies of Hispanic adults living in the United States that examined hospice use, outcomes of care, or knowledge and attitudes towards hospice were included. Commentaries, case studies, editorials, literature reviews were excluded. Of the 4,230 abstracts reviewed, 43 peer-reviewed articles met the inclusion criteria. Among these studies, barriers to hospice among Hispanics included lack of hospice knowledge and awareness, language barriers, and cultural barriers. Among most studies, Hispanics were less likely to receive hospice care than Whites, although some studies found that among those that enrolled in hospice, Hispanics had longer lengths of stay than whites. Overall few studies examined Hispanics use of hospice, and among those we found most were of moderate and low quality. More research is needed to understand the full range of Hispanics experiences of hospice care. Such research could guide efforts to develop culturally tailored care for this community.

### INFORMING THE FUTURE: CANCER PREVENTION AND DIAGNOSIS BELIEFS AMONG OLDER LATINO IMMIGRANTS

Iraida Carrion,[1] Malinee Neelamegam,[2] and Jane Roberts,[3] *1. University of South Florida, Tampa, Florida, United States, 2. Yale School of Public Health, New Haven, Connecticut, United States, 3. University of South Florida, Sarasota, Florida, United States*

Despite emerging research on Latinos and cancer (Carrion et al., 2018), there are no data regarding beliefs about cancer prevention and diagnosis in older Latinos residing in Central Florida. Similarly, to the US in general, Central Florida's older Latino population is growing. Compared with other national samples, Latino immigrants in the southern U.S. report poorer health than in other regions (Siegel et al., 2015). Using convenience sampling (N = 168), univariate analysis was done to recognize the study population's characteristics. Frequencies were assessed to understand participants' responses to questions on cancer-related attitudes. The effects of age, country of origin, length of stay in the U.S., and marital status were assessed using logistic regression. Of the 168 individuals in the study, 34.5% were male with a mean age of 67.9, and a majority had at least a high school education and 25.8 years residing in the U.S. The participants were aware that tobacco use can cause cancer (93.5%) and that smoking affects the smoker as well as their family members (84.5%). They were also aware that mammograms facilitate early diagnosis of breast cancer (81.5%) and of the association between prolonged sun exposure and skin cancer (86.9%). However, specific knowledge about early diagnosis was low. Only 29.2% of participants knew that breast cancer can be diagnosed early, which was similar to the response toward early diagnosis of prostate cancer (24.4%). Among the participants, 26.2% were categorized as having poor knowledge of cancer prevention.

### LIFELONG BILINGUALISM AND LITERACY SKILLS USE AMONG HISPANIC ADULTS

Roberto Millar,[1] Shalini Sahoo,[2] Taka Yamashita,[3] and Phyllis Cummins,[4] *1. Graduate Student, Baltimore, Maryland, United States, 2. University of Maryland, Baltimore, Elkridge, Maryland, United States, 3. University of Maryland, Baltimore, Maryland, United States, 4. Miami University, Oxford, Ohio, United States*

Literacy skills use in everyday life is associated with social (e.g., civic participation) and economic (e.g., higher wages) benefits throughout the life course. Yet, the use of literacy skills may be lower among immigrants and those with limited language proficiency. No prior research examined associations between bilingualism in early life — a critical period for language acquisition — English literacy skills, and the use of literacy skills in later life of Hispanic adults. The objective of this study is to determine whether lifelong bilingualism (i.e., learning a second language in childhood and still understanding today) is associated with Hispanics' everyday use of literacy skills later in life. Nationally representative data (n = 412) of Hispanics 35 years and older were obtained from the 2012/2014 Program for International Assessment of Adult Competencies (PIAAC). A series of logistic regression models were used to examine the associations between lifelong bilingualism and several measures of literacy skills use in everyday life. Results showed that lifelong bilingualism (vs. being monolingual) was negatively associated with the daily use of letters, notes, and e-mails (Odds Ratio = 0.426, $p< 0.05$), reading financial statements (Odds Ratio = 0.474, $p< 0.05$), and reading diagrams (Odds Ratio = 0.391, $p< 0.05$). Additionally, literacy was a consistent predictor of skill use. While bilingualism is generally beneficial for aging adults, our findings suggest that, among Hispanics, lifelong bilingualism is associated with the less frequent use of literacy skills later in life. We discuss the possible theoretical and practice implications of these findings.

### LITERACY SKILLS, IMMIGRATION, AND MOTIVATION TO LEARN AMONG MIDDLE-AGED AND OLDER ADULTS IN THE UNITED STATES

Shalini Sahoo,[1] Roberto Millar,[2] Taka Yamashita,[3] and Phyllis Cummins,[4] *1. University of Maryland, Baltimore, Elkridge, Maryland, United States, 2. Graduate Student, Baltimore, Maryland, United States, 3. University of Maryland, Baltimore, Maryland, United States, 4. Miami University, Oxford, Ohio, United States*

Education and training over the life course or lifelong learning has become critical in the fast-changing U.S. society. Foundation skills (e.g., literacy), motivation, curiosity, as well as access to learning opportunities are essential to promote lifelong learning. Despite the importance of these promoting factors, empirical research focusing on complex relationships between literacy skills, immigration and motivation to learn (MtL) among middle-aged and older adults is scarce. The objective of this study is to examine how literacy skills and immigration (vs. U.S. born) are associated with MtL among middle-aged and older adults in the U.S. Nationally representative data (n = 8,670) of adults aged 45 years and older were obtained from the 2012/2014 Program for International Assessment of Adult Competencies (PIAAC). Structural equation models were constructed to examine the formerly tested and validated latent MtL construct based on four 5-point Likert-type scale items among the sub-population of interest. Results showed that higher literacy skills (0-500 points; $b = 0.002$, $p < 0.05$) was associated with greater MtL. Additionally, immigrants were less likely ($b = -0.114$, $p < 0.05$) to have greater MtL than those who are non-immigrants (i.e. U.S. born). Higher literacy skills may indicate positive experiences in previous adult education and training and greater readiness for further learning. Findings from this study provide new empirical evidence of lifelong learning determinants. Educators and researchers should be aware of limited literacy and being an immigrant as potential barriers to knowledge-seeking in later life.

### PREDICTORS OF CANCER DETECTION BEHAVIORS AMONG OLDER NIGERIAN MEN

Kelsey Brodie,[1] Darlingtina Esiaka,[2] and Candidus Nwakasi,[3] *1. Union College, Schenectady, New York, United States, 2. Union College, Union College, New York, United States, 3. University of Southern Indiana, Evansville, Indiana, United States*

Recent research emerging from Nigeria suggest an increasing mortality due to cancer, especially among older

Nigerian men who are at a higher risk of being diagnosed at fatal or advanced stage of cancer. With older age as a significant risk factor for cancer development such as prostate cancer in men, this study explored factors that influence cancer detection behavior among aging Nigerian men. Specifically, we examined possible predictors of current and future intentions to engage in early cancer detection behaviors among Nigerian men. Participants (N=143), with a mean age of 44.73 (SD = 6.15), responded to measures assessing health (cancer detection behaviors), social (masculinity, self-esteem, attachment), and psychological (active coping) factors. Demographic and ecological questions were also included in the survey. Results revealed that education, masculinity, and anxious attachment were significant predictors of current cancer detection behavior. Education, masculinity, and anxious attachment also predicted future cancer screening intentions. We discuss the implication of result for health policy, health education and cancer prevention interventions for Nigerian men and for the global campaign for early cancer detection.

### RACE AND EDUCATIONAL DISPARITIES IN ADVANCE DIRECTIVE COMPLETION: ENCOURAGING TRENDS IN THE UNITED STATES

Catheryn Koss, and Rebecca Hensley, *California State University, Sacramento, Sacramento, California, United States*

Advance directives (AD) help to ensure patients' wishes are honored and contribute to improved end-of- life care. Race and education disparities in advance directive completion have been extensively documented. This study examined five waves of U.S. Health and Retirement Study exit survey data (N = 7,067) to examine to what extent these disparities have expanded or diminished over the past decade. Overall, advance directive completion increased from about 63% among participants who died in 2005-06 to about 73% among those whose deaths occurred between 2015 and 2016. Non-Hispanic whites were almost four times as likely to have advance directives compared to Hispanics or African Americans across this time period (OR=3.90. p<.0001). However, the growth rate in advance directive completion among non-Hispanic whites was significantly slower than for non-whites (OR=.90, p<.01). Compared to those with a high school education or less, those with some college (OR=1.67, p<.0001) and those with at least a college degree (OR=2.02, p<.0001) were significantly more likely to have advance directives across the time period. There were no significant differences in growth rates of advance directive completion for the different educational categories. These results suggest that educational disparities in advance directive completion are fairly stable, but that race disparities may be diminishing.

### RACIAL OR ETHNIC AND MULTIMORBIDITY DIFFERENCES IN FUNCTIONAL LIMITATION TRAJECTORIES AMONG OLDER AMERICANS

Ana Quinones,[1] Miriam Elman,[2] Anda Botoseneanu,[3] Jason Newsom,[4] David Dorr,[1] Corey Nagel,[5] and Heather Allore,[6] *1. Oregon Health & Science University, Portland, Oregon, United States, 2. OHSU-PSU School of Public Health, Portland, Oregon, United States, 3. University of Michigan, Dearborn, Michigan, United States, 4. Portland State University, Portland, Oregon, United States, 5. University of Arkansas for Medical Sciences, Little Rock, Arkansas, United States, 6. Yale University, New Haven, Connecticut, United States*

Racial/ethnic minority groups in the U.S. are at risk for greater co-existing chronic disease (multimorbidity) burden and experience greater functional limitations relative to non-Hispanic white peers. To target programs designed to preserve functional independence, this study aims to identify temporal trends of functional limitation among race/ethnic groups and within the context of multimorbidity. Data from the Health & Retirement Study (2000-2014, N=16,959, 65 years of age and older, community-dwelling adults) were used in generalized estimating equation (GEE) models to assess changes in functional limitations over time (combined activities of daily living [ADL] and instrumental activities of daily living [IADL], range 0-11). Models were adjusted for race/ethnicity (non-Hispanic black, Hispanic, non-Hispanic white), self-reported chronic disease categories (no/one, ≥2 somatic, somatic-depression; of arthritis, cancer, diabetes, heart disease, high depressive symptoms [CES-D8≥4], hypertension, lung disease, stroke), age at baseline, sex, body-mass index, education, partnered, net worth, and time. In adjusted GEE models, Hispanic and black respondents experience 1.4 times greater counts of functional limitations, respectively, relative to white respondents (incidence rate ratio [IRR]= 1.4, 95% CI[1.17, 1.66], IRR=1.4, CI[1.26, 1.61]); however, temporal trends were similar. With regard to multimorbidity categories, somatic or somatic-depression multimorbidity were each associated with 2.2 or 3.5 times greater functional limitations, respectively, relative to having no/one condition (IRR=2.2, CI[2.06, 2.39], IRR=3.5, CI[3.18, 3.74]). There are marked differences in functional limitation levels between minority ethnic and white groups, as well as among chronic disease combination groups, suggesting the need to intervene in middle-age to reduce disparities.

### SELF-RATED HEALTH STATUS AS A PREDICTOR OF EXECUTIVE FUNCTION IN OLDER LATINOS

Jacqueline Guzman,[1] Yuliana Soto,[2] David Marquez,[3] and Susan Aguinaga,[1] *1. University of Illinois at Urbana-Champaign, Urbana, Illinois, United States, 2. University of Illinois–Urbana-Champaign, champaign, Illinois, United States, 3. University of Illinois at Chicago, Chicago, Illinois, United States*

Latinos have high risk of Alzheimer's disease and related dementias (ADRD). Self-rated health (SRH) has been used to predict cognitive decline. Early detection of executive function changes may help identify those at higher risk of cognitive decline. The purpose of this study was to examine the relationship between SRH and executive function in Latinos. Latinos (N=333, 84.4% female, Mage= 64.9 ± 7.08) from the BAILA randomized controlled trial self-rated their health as 1) poor/fair, 2) good, and 3) very good/excellent. Executive function was assessed by the Trail-making B, Verbal Fluency, Stroop C & CW, and the Digit Modality tests and stratified by SRH. One-way analysis of variance showed that the effect of SRH was significant for Trails B, F(2,298)=4.01, p=.019 and Stroop CW, F(2,298)=3.07, p=.048. Tukey's test indicated that participants who rated their health as fair/poor took longer to complete Trails B (M=196.78±83.0 seconds) compared to those who rated their health as good (M=185.25 ± 85.1 seconds) and very good/excellent (M=149.25±95.3 seconds). Stroop CW results demonstrated that those in the fair/poor health category scored lower (M=17.22±6.6) than those in good (M=19.70±8.5 words/minutes) and very good/excellent health categories (M=18.73±8.2 words/minute). In sum, the results suggest SRH is related to executive function such that lower categories of SRH are indicative of poorer executive function. SRH might be used as a proxy for executive function and as a tool that community leaders can use to identify individuals at high risk of ADRD in need of behavioral interventions.

### THE AFRICAN IMMIGRANT MEMORY LOSS PROJECT: A UNIVERSITY-COMMUNITY PARTNERSHIP

Manka Nkimbeng,[1] Hayley McCarron,[2] Gabriela Bustamante,[2] Wynfred Russell,[3] Tetyana Shippee,[2] and Joseph Gaugler,[2] *1. Johns Hopkins University school of Nursing, Minneapolis, Minnesota, United States, 2. University of Minnesota, Minneapolis, Minnesota, United States, 3. African Career, Education & Resource, Inc., Brooklyn Park, Minnesota, United States*

The few studies on dementia prevalence in immigrant communities show that immigrants from diverse racial/ethnic backgrounds have a higher prevalence of dementia compared with their U.S.-born counterparts. However, this body of work is small, resulting in a lack of reliable estimates of dementia prevalence among African immigrants. The AIMLP is a partnership between the African Career, Education, and Resources, Inc. (ACER) and the Families and Long-Term Care Projects (FLTC) of the University of Minnesota School of Public Health. Guided by an advisory board, the goal of this project is to develop culturally informed instruments, and use these to collect data to identify dementia care needs, knowledge, and resources in the African immigrant community. Study implementation started in August 2019, five advisory board meetings have been convened and 2 pilot focus groups have occurred. Twelve individuals participated in the focus groups. The majority (90%) were from Liberia and 60% were over the age of 55. Two participants currently care for a family member with dementia. Preliminary findings reveal a great need for education on dementia, and general lack of awareness on management, and limited access to services/supports. Focus groups will be finalized in March and the study survey will be developed and administered in the summer. These survey findings will be available and presented at the conference in November 2020. This is the first project to identify the extent of dementia care needs and resources among African immigrants; which will inform interventions for this population.

### THE ASSOCIATION BETWEEN HEARING LOSS AND COMPLETION OF ADVANCE CARE PLANNING AMONG OLDER ADULTS

Junghee Han,[1] and Junghyun Park,[2] *1. Calvin University, Grand Rapids, Michigan, United States, 2. New York University, New York City, New York, United States*

Background: Ensuring access to quality end-of-life (EOL) care for all older adults is emerging public health concern. Hearing loss (HL) is the third most common chronic disease affecting older adults and a major impediment to access healthcare services. However, little is known about the impact of HL on advance care planning for older adults. Method: A sample of 1,862 older adults (≥65 years) was drawn from the National Health and Aging Trends Study (NHATS). HL was determined by self-report and advance care planning was measured by asking if an individual completed living wills or the Durable Power of Attorney for Health Care (DPAHC). Covariates included age, gender, race, marital status, education, religion, nativity, depression, region, facility status, regular doctor availability, Medicaid, hospitalization, cognition, perceived health status and a presence of chronic disease. Results: Descriptive statistics revealed that nearly 67% of older adults with HL completed the DPAHC, and the majority of them (71%) also had living wills. Multivariable logistic regression analyses showed that HL was significantly associated with completion of DPAHC and living wills, after controlling for a list of covariates (OR=0.50, $p<0.05$). Conclusions: The findings show HL is a significant predictor of completion of any type of advance directives. Facilitating effective communication in advance care planning for older adults with HL is needed. Healthcare provider should make health information accessible to them to get quality EOL care.

### THE GENDERED EFFECTS OF EDUCATION AND ACCULTURATION ON OLDER KOREAN IMMIGRANTS' COGNITIVE FUNCTION

Eun Young Choi,[1] Yuri Jang,[2] and David Chiriboga,[3] *1. Leonard Davis School of Gerontology, Los Angeles, California, United States, 2. University of Southern California, Los Angeles, California, United States, 3. University of South Florida, Tampa, Florida, United States*

This study advances knowledge concerning older immigrants' cognitive health by examining the role of gender in the context of education, acculturation, and cognitive function. Guided by an emerging Gendered Process of Acculturation framework, we hypothesized that the cognitive health of men and women would benefit differently from a high level of education and acculturation. Cognitive health was measured with the Mini-Mental State Examination. Data were drawn from the Study of Older Korean Americans (SOKA), a multi-site survey of Korean Americans aged 60 and over (N = 2,061). Multivariate linear regression analyses were conducted to investigate the moderating role of gender in the effects of (1) education, (2) acculturation, and (3) the association of education and acculturation on cognitive function. Gender was found to be a significant moderator in the relationship of education and acculturation with cognitive health: the positive effect of both education and acculturation was greater among women than men. Furthermore, the three interaction among education, acculturation, and gender was significant: the positive impact of education on cognition was particularly pronounced among women with low acculturation and eliminated gender differences in cognitive status. Our findings suggest that gender plays a critical role in determining the cognitive health benefit arising from education and acculturation singularly and in concert, highlighting the vulnerability of women with low education and acculturation. These findings have implications for social interventions targeting immigrant populations.

### THE INFLUENCE OF ORGANIZATIONAL CLIMATE ON THE ENROLLMENT OF PEOPLE OF COLOR INTO CLINICAL TRIALS

Marcela Blinka,[1] Karlynn BrintzenhofeSzoc,[2] and James Zabora,[3] *1. Johns Hopkins University, School of Medicine, Baltimore, Maryland, United States, 2. University of Cincinnati, Cincinnati, Ohio, United States, 3. Sidney Kimmel Comprehensive Cancer Center at JH, Baltimore, Maryland, United States*

A significant need exists to improve enrollment of people-of-color (POC) in randomized clinical trials as the gold standard for evaluating new therapies so that health disparities can be reduced. Improving representation in clinical trials is expected to improve the applicability of clinical findings more broadly, especially for minority subgroups. This cross-sectional study assessed the perception of organizational climate (OC) among staff in five cancer research groups (CRGs) of a mid-Atlantic NCI-designated comprehensive cancer center. The Organizational Climate Measure (OCM), a measure of four OC models of the Competing Values Framework, was used. Enrollment data were obtained from the 2016 Cancer Research Management System data and 2017 enrollment rates. A statistically significant difference in mean scores was found in the Pressure to Produce Scale ($F = 4.21$, $p < .05$). One CRG's staff reported lower pressure to meet targets than three other CRGs ($p < .05$) and trending towards significance for the fourth CRG ($p < .08$). This suggests that organizational priorities considering all patients as potential clinical trial participants increases accrual. A statistically significant negative relationship between the Outward Focus sub-scale and POC enrollment ($r = -.228$, $p < .05$) was also found, suggesting that an external organizational focus, alone, is insufficient to increase POC accrual. Implications for future research include 1) does the organizational priority of decreasing the pressure to produce influence accrual of POC? and 2) does an OC that is flexible and externally focused lead to a more effective means of accruing POC as clinical trial participants?

### THE MEDIATION ROLE OF SOCIOECONOMIC STATUS ON RACIAL AND ETHNIC DIFFERENCES IN ADVANCE CARE PLANNING

Junghee Han,[1] and Taekbeen Nam,[2] *1. Calvin University, Grand Rapids, Michigan, United States, 2. Grand Rapids, Michigan, United States*

Background. Most research on EOL care planning has focused on racial/ethnic differences in completing advanced directives (AD) rather than the pathways of the disparities. Therefore, this study aims to examine the mediating role of education and income in racial/ethnic differences in EOL care planning. Methods. A secondary data analysis of Health and Retirements Study (HRS) 2004-2014 wave was used. The sample included 6,518 participants (($\geq$ 65 years old). The independent variable measured the respondents' race and ethnicity and the dependent variable measured the completion rate of living wills or the Durable Power of Attorney of Health Care (DPAHC). Covariates included gender, age, marital status, religion, place f birth, educational attainment, income, cognitive function, limitations in physical functioning, geriatric syndromes, and the number of progressive chronic disease. Results. The hierarchical logistic regression analysis showed that race/ethnicity was a significant predictor of completing AD ($p \leq 0.001$). Mediation analysis, Karlson, Home, and Breen (KHB), revealed that both education and income explained 14.4% of racial/ethnic differences in completion of living wills for non-Hispanic Blacks and 17.6% for Hispanics. Similarly, education and income accounted for 17.6% of racial/ethnic disparities in completion of DPAHC for non-Hispanic Blacks and 20.9% for Hispanics. In particular, education had stronger mediating effect on the outcome variables than income. Discussion. The findings suggest the importance of targeted educational interventions for people of color with lower SES to raise their awareness of benefits of advance care planning and increase their access to higher quality of EOL care.

### TOWARD A COMPARATIVE STUDY OF COLLECTIVE MEMORY AND CITIZENSHIP AMONG OLDER ADULTS WITH DEMENTIA

Jessica Robbins, *Wayne State University, Detroit, Michigan, United States*

This poster explores new directions for understanding possibilities for citizenship among older adults with dementia, who often experience loss of citizenship and diminishment of personhood due to their diagnosis. Drawing on data from two distinct ethnographic studies—one on memory and personhood in a day center for people with Alzheimer's disease in Poland, and the other on gardening and wellbeing among older African Americans without cognitive impairment in Detroit—this poster asks how the collective past may shape experiences of dementia and possibilities for citizenship in the present. In Poland, practices of remembering involving collective memory can sustain personhood and foster ties of relatedness among people with dementia. This apparent paradox between people with dementia's loss of memory and their capacity to build social relations based on remembering can be resolved through expanding understandings of personhood to include practices of remembering involving collective pasts (e.g., shared national frameworks, embodied practices of sociality). In Detroit, gardening fosters connections with the past, as older African Americans are reminded of deceased loved ones through practices and the plants themselves. These intimate connections and everyday activities are situated in racialized histories of migration, disinvestment, and "revitalization," even as they provide the means to cultivate life in the present. This poster concludes that the potential for collective pasts to generate life in the present can become evident through ethnographic research among people with dementia, and in particular, through studying gardening among people with dementia.

### UNDERSTANDING TRANSITIONS IN IADL AND THEIR CONSEQUENCES FOR COGNITIVE IMPAIRMENT AMONG OLDER MEXICAN AMERICANS

Jiwon Kim,[1] Jacqueline Angel,[2] and Sunshine Rote,[3] *1. University of Texas, AUSTIN, Texas, United States, 2. The University of Texas at Austin, Austin, Texas, United States, 3. University of Louisville, Louisville, Kentucky, United States*

Mexican Americans tend to live longer lives than other ethnic groups, but it remains unclear how this trend influences the trajectory of disability and its consequences for cognitive frailty. Building on previous research, we assess transitions in IADL among the oldest old. We use data from three waves of the Hispanic Established Population of the Epidemiologic Study of the Elderly (H-EPESE) to investigate trajectories of IADL disability as individuals' age into their 80s and 90s, a period of the life course with much higher rates of morbidity and decreasing socioeconomic resources. The H-EPESE is a benchmark longitudinal cohort study based on an original sample of 3,050 Mexican-Americans aged 65 and older in the Southwestern United States. Our modeling approach estimates transitions in patterns of IADL employing the Latent Transition Analysis (LTA). Results revealed three heterogeneous latent classes: high IADLs, difficulty in transportation and mobility, and low IADLs. Those with high IADLs tended to remain in the same class over time. Individuals having difficulty in transportation and mobility tended to stay in the same class or transfer to high IADLs, whereas those with overall low IADLs transferred to either the same class or to difficulty in transportation and mobility. Additional analysis revealed that cognitive impairment was a significant predictor of instrumental disability over time. Furthermore, females were more likely than males to belong to difficulty in transportation or mobility class than to the low IADL class. Our results highlight the long term consequences of cognitive decline on IADL limitations.

### WORK AND SELF-RATED MEMORY AMONG NATIVE HAWAIIAN AND OTHER PACIFIC ISLANDER OLDER ADULTS

Yeonjung (Jane) Lee,[1] and Tyran Terada,[2] *1. University of Hawai'i at Mānoa, Honolulu, Hawaii, United States, 2. University of Hawai'i, Pearl City, Hawaii, United States*

According to the productive aging framework, productive activities can function as protective factors of cognitive health. Productive activities, such as work, have been linked to positive cognitive health outcomes in older adults. Yet, less is known about if the beneficial effects of work on cognition extend to the Native Hawaiian and Other Pacific Islander (NHOPI) older adult population. Thus, the purpose of this research is to investigate how work is associated with self-reported memory/concentration among NHOPI older adults. Moreover, the moderating role of education was explored. Using data from the 2014 Native Hawaiian and Pacific Islander National Health Interview Survey (NHPI NHIS), the study explores the associations between work and self-rated levels of difficulty remembering or concentrating. A total of 1,045 older adults ages 50 years and older were included for analyses. Weighted multivariate analyses with multiple imputation techniques were used. The NHPI NHIS is the first federal survey focusing on the NHOPI population in the United States. Those who were engaged in work had lower odds of having severe difficulty memorizing or concentrating while controlling for other sociodemographic and behavioral factors. Interestingly, there was a significant interactive effect of work and education on self-rated memory. Those with lower education levels have lower self-rated memory, but the odds of having memory difficulty decreased when they worked. Findings highlight the importance of productive aging in promoting healthy cognitive aging. Research and practice addressing productive aging and cognition should provide culturally relevant resources to NHOPI older adults.

### SESSION 2941 (POSTER)

### DIVERSE RACE AND ETHNIC POPULATIONS II

### AGING WITH HIV: INFLAMMATION IS ASSOCIATED WITH PAIN, POORER PHYSICAL FUNCTION, AND FRAILTY

Heather Derry, Carrie Johnston, Chelsie Burchett, Eugenia Siegler, and Marshall Glesby, *Weill Cornell Medicine, New York, New York, United States*

With advances in antiretroviral therapies, people living with HIV have life expectancies similar to their HIV-negative peers. Yet, they experience elevated multi-morbidity that can compromise quality of life as they age. Links between inflammation and accelerated aging may inform interventions, but these links are understudied in older adults with HIV. We investigated cross-sectional relationships between inflammation, well-being, and geriatric syndromes among 161 HIV-positive older adults. Participants provided fasting blood samples (for serum cytokines and CRP) and completed surveys (MOS-HIV; falls) and cognitive (MoCA) and frailty assessments (using Fried criteria). Adjusted linear and logistic regression models tested relationships between inflammatory markers and age-related health outcomes, controlling for age, gender, BMI, race, comorbidity burden, statin use, and smoking status. 93% had suppressed viral load. 11% had CRP levels suggesting possible acute illness (>10 mg/L) and were excluded from analyses. Participants with higher IFN-$\gamma$ reported greater pain ($p=0.003$), greater cognitive complaints ($p=.02$), and worse physical function ($p=0.04$), than those with lower IFN-$\gamma$. Similarly, higher IL-6 levels were related to worse physical function ($p=0.01$) and slightly greater cognitive complaints ($p=0.06$), but were not significantly related to pain in adjusted models. Compared to those with lower IL-6, those with higher IL-6 levels were more likely to be frail ($p=0.04$). CRP was not significantly related to these outcomes. Six-month fall history and objective cognitive scores were not significantly related to the assessed inflammatory markers. Our results illustrate key, expected links between inflammatory processes, frailty, physical function, and pain among older adults with HIV.

### CHANGE OVER TIME IN LINGUISTIC ACCULTURATION: A COMPARISON BETWEEN CUBAN AND NON-CUBAN HISPANIC IMMIGRANTS

David Chiriboga,[1] Nan Sook Park,[1] Yuri Jang,[2] and Victor Molinari,[1] *1. University of South Florida, Tampa, Florida, United States, 2. University of Southern California, Los Angeles, California, United States*

While acculturation and its implications for mental health have received extensive attention in studies with older immigrant populations, change over time in acculturation less so. This paper examines change over a two-year period in levels of linguistic acculturation among Cuban (n = 186) and non-Cuban Hispanic (n = 116) immigrants living in Florida. Data came from the waves of the Survey of Older Floridian (SOF), conducted using a statewide sampling frame based on population densities, with a computer-assisted telephone interview strategy. Calls were initiated through random digit dialing, and a follow-up interview was added approximately two years later. Measures included six acculturation items, one dealing with the participant's language preference for the interview itself and five items dealing with language ability and usage. Results indicated that non-Cuban Hispanics were significantly more acculturated than Cuban Hispanics, and that at least 75% of Wave 2 acculturation was accounted for by Wave 1 status. After controlling for initial levels of acculturation, those who at first interview reported more economic problems and those reporting fewer depressive symptoms were more acculturated at follow-up. It was concluded that while linguistic acculturation is relatively stable, interventions to increase acculturation have implications for mental health service utilization.

### CONSCIOUS AND NON-CONSCIOUS PROCESSES REGULATE MINORITY OLDER ADULTS' SEDENTARY BEHAVIOR

Jaclyn Maher,[1] Derek Hevel,[1] Kourtney Sappenfield,[1] Heidi Scheer,[1] Christine Zecca,[1] and Laurie Kennedy-Malone,[2] *1. University of North Carolina at Greensboro, Greensboro, North Carolina, United States, 2. UNCG School of Nursing, Greensboro, North Carolina, United States*

Accumulating evidence suggests that sedentary behavior (SB), or time spent sitting, is regulated by both conscious (e.g., intentions) and non-conscious (e.g., habits) motivational processes. Much of the work investigating these processes has employed summary-based measures of typical motivation and behavior. This study employed ecological momentary assessment (EMA) methods and accelerometry to determine the extent to which conscious and non-conscious processes regulate minority older adults' momentary decisions to engage in SB. Over the course of the 8-day study, minority older adults (N=91; age range: 60-89 years, 96% Black/African American) answered 6 EMA questionnaires/day on a mobile phone and wore an ActivPAL activity monitor to measure SB. EMA questionnaires assessed momentary intentions to limit SB over the next two hours. SB habit strength was self-reported at an introductory session. Results from a multilevel linear regression model indicated that on occasions when individuals had stronger intentions than usual to limit SB, they subsequently engaged in less SB (b=-3.72, p<0.01). Individuals who had stronger SB habits, tended to engage in more SB (b=3.00, p<0.01). An additional multilevel model revealed that habits did not significantly moderate the association between momentary intentions and subsequent SB (b=-1.06, p=0.09). In conclusion, minority older adults' momentary SB appears to be directly influenced by both conscious and non-conscious motivational processes, though the interactive effects are unclear. Interventions to reduce minority older adults' SB should include content to increase intentions to limit SB (e.g., information on instrumental and affective consequences) and disrupt habitual SB (e.g., action planning).

### DEPRESSIVE SYMPTOMS IN OLDER AFRICAN IMMIGRANTS: A DESCRIPTIVE STUDY

Manka Nkimbeng,[1] Zachary Baker,[2] Janiece Taylor,[3] Sarah Szanton,[4] Tetyana Shippee,[2] and Joseph Gaugler,[2] *1. Johns Hopkins University school of Nursing, Minneapolis, Minnesota, United States, 2. University of Minnesota, Minneapolis, Minnesota, United States, 3. Johns Hopkins School of Nursing, Baltimore, Maryland, United States, 4. Johns Hopkins University, Baltimore, Maryland, United States*

In FY 2018-2019, the National Institutes of Health devoted $2,387,505,711 to projects studying depression. Before and following their arrival into the United States stressful life circumstances may render African immigrants particularly at risk for depression. The objective of this study is to provide an estimate and identify correlates of depressive symptoms in older (≥50 years) African immigrants. We performed secondary data analyses of the Older African Immigrant Health study (n = 148). Bivariate analyses evaluated associations between depressive symptoms and sociodemographic and immigration-related factors. Depressive symptoms were measured with the PHQ-8 scale and scores of ≥ 5 were considered indicative of depressive symptoms. The mean age of participants was 62 years (SD:8.2), 61% were female, 30% had less than high school education, and 58% reported having health insurance coverage. Thirty percent of the sample had depressive symptoms (PHQ-8 score of ≥ 5) but only one individual would be classified as having moderately severe or severe depression (PHQ-8 ≥15). Depressive symptoms did not differ by age, marital status, education, or income. There was a statistically significant difference in depressive symptoms by reason for migration, recruitment location, and employment status. Although only one participant would be classified as severely depressive, a large proportion of this sample had depressive symptoms. Mental health concerns were reported as a significant health problem for African immigrants visiting a community service organization in New York. More research is needed to examine the prevalence, immigration-related correlates, predictors, and health ramifications of depression in older African immigrants.

### HOUSING INSECURITY AND THE EMOTIONAL AND PHYSICAL HEALTH OF AFRICAN AMERICANS

Aarti Bhat,[1] August Jenkins,[2] and David Almeida,[3] *1. Pennsylvania State University, Pennsylvania State University, Pennsylvania, United States, 2. Penn State, University Park, Pennsylvania, United States, 3. Pennsylvania State University, University Park, Pennsylvania, United States*

Housing insecurity—or limited and/or unreliable access to quality housing— is a potent on-going stressor that can adversely impact individual well-being. This study extends previous research by investigating the impact of housing insecurity on both the emotional and physical health of aging African American adults using the Midlife in the United States (MIDUS) Refresher oversample of African Americans collected from 2012-2013 (N = 508; M age = 43.02; 57% women). Participants reported on their negative affect, number of chronic health conditions experienced in the last year, and experiences of housing insecurity since the 2008 recession (e.g., homelessness, threatened with foreclosure or eviction, lost home). Negative affect and chronic conditions, respectively, were regressed on housing insecurity, and the potential moderating effect of age was tested. Results showed that housing insecurity was associated with more negative affect (B = 0.05, SE = 0.03, p = .002) and chronic health conditions (B = 0.26, SE = 0.03, p < .001). Additionally, the association between housing insecurity and negative affect was moderated by age (B = -0.11, SE = 0.00, p = .019), such that the effect of housing insecurity on negative affect was stronger for younger adults than for older adults. These results suggest that experiences of insecure housing leave African American adults vulnerable to compromised emotional and physical health, however, the negative effects of housing insecurity may attenuate with age.

### INTERGENERATIONAL MENTORING ON HEALTH PROMOTION TO IMPROVE HEALTHY LIFESTYLES AMONG LATINO FAMILIES

Karen Kopera-Frye, Ashley Graboski-Bauer, and Ana Luisa Gonzalez-Marquez, *New Mexico State University, Las Cruces, New Mexico, United States*

New Mexico now has the 32nd highest adult obesity rate; approximately one-third are Latinos. Obesity rates in NM for children aged 2-11 years range from 11 – 14%. Mentoring as a health promotion or intervention strategy has become widespread. However, few programs have focused on several generations reciprocally influencing each other in healthy behaviors. Project I'M HIP (Intergenerational Mentoring Health Information Pathways)'s goals included: Providing an innovative, multigenerational educational program to promote greater maternal, child, and grandparent well-being, healthier lifestyle behaviors, and support continued healthy home environments by empowering the families with knowledge. Three cohorts of 30 families (1 parent, 1 child, 1 grandparent/other relative) were recruited for Program I'M HIP. This Program utilized culturally sensitive Evidence-Based Programs (EBPs). Monthly educational sessions focused on physical activity and adapting meals to be healthy. Project outcomes included exercise frequency, Body Mass Index (BMI), and a knowledge quiz assessing healthy meal facts, exercise knowledge via a 10-item quiz; all assessments pre- and post-program. Program outcomes included: 100% of the parents shared at least 1 fact on nutrition or exercise with other relatives, thus affecting another household; paired t-test analyses revealed significant changes in knowledge quiz total scores ($t_{70} = 5.03$, $p < .0001$), increased exercise frequency ($t_{72} = 2.106$, $p < .05$); no significant change in BMI from pre- to post-assessments; and children corrected their parents on proper diet; all demonstrating the reciprocal mentoring effects of parent, child, and other relative on health behaviors.

### IS CHILDHOOD DISADVANTAGE TEMPORARY OR PERMANENT? EVIDENCE FROM THE STUDY ON GLOBAL AGEING AND ADULT HEALTH IN GHANA

Paul Ayernor, *City University of New York-CUNY, Staten Island, New York, United States*

The paper assesses whether childhood socioeconomic status have a temporary or permanent effect on adult health status and well-being. The study uses cumulative inequality theory to explain disparity in health status and well-being at older ages in Ghana. Data comes from the 2007-2008 World Health Organization global study of ageing in Ghana (SAGE). The study utilizes wave 1 of the data, with retrospective questions about early childhood socioeconomic status. The study uses ordinal logistic regression models to assess the relationship between childhood socioeconomic status and self-report health on one hand and wellbeing on the another. The results show that father's education is a significant predictor of health status and wellbeing at older ages. Specifically, the odds of reporting good and moderate health status and wellbeing are 1.29 and 2.22 times higher among older adults whose fathers have primary education or higher. As expected, the odds of reporting moderate or good health status and wellbeing decrease with increasing age and also for women. In terms of interaction effects, those aged 60-69 years whose fathers have primary education are less likely to report good and moderate health. In contrast, those who are aged 70-79 years old and have fathers with secondary or higher education are 2.51 times more likely to report good and moderate wellbeing. There is strong evidence of compensation among those who keep once or twice contact with social ties.

### ORAL HEALTH AND DENTAL CARE IN OLDER KOREAN IMMIGRANTS: A QUALITATIVE STUDY

Yuri Jang,[1] Min-Kyoung Rhee,[1] Jeong Chung Hyeon,[1] Eun Young Choi,[2] Juyoung Park,[1] and Nan Sook Park,[3], *1. University of Southern California, Los Angeles, California, United States, 2. Leonard Davis School of Gerontology, Los Angeles, California, United States, 3. University of South Florida, Tampa, Florida, United States*

Many segments of the U.S. population continue to experience a disproportionate burden of oral disease and inequities in dental care, and older Asian immigrant populations are among those at high risk. Responding to the needs to attend to ethnic and geographic variations among older Asian Americans and to better understand contextual factors that shape their experiences of oral health and dental care, the present study conducted in-depth interviews with eighteen older Korean immigrants in the Los Angeles Greater area. The qualitative inquiries were theoretically guided by the three core categories of the Andersen's (1968, 1997) health service model: oral health needs, service barriers, and service outcomes. Using the constant comparison method, themes and sub-themes within each category were derived. The eight themes emerged from the qualitative data were: (1) oral health problems, (2) perceived need, (3) insurance and finance, (4) language barriers, (5) social support, (6) knowledge and belief, (7) satisfaction with service, and (8) areas of improvement. The findings demonstrated varied experiences associated with oral health and dental care of older Korean immigrants and informed the development of services and programs responsive to the identified needs and barriers.

### PHYSICAL HEALTH CONSTRAINTS AND PSYCHOLOGICAL DISTRESS IN OLDER ASIAN AMERICANS: THE ROLE OF PERCEIVED HEALTH

Hyunwoo Yoon,[1] Yuri Jang,[2] and Seoyoun Kim,[3]
*1. Portland State University, Portland, Oregon, United States, 2. University of Southern California, Los Angeles, California, United States, 3. Texas State University, San Marcos, Texas, United States*

Given the importance of understanding the underlying dynamics of physical and mental health in old age, the present study explored the roles of physical health constraints in predicting subjective health perception and psychological distress among older Asian Americans. Guided by the Health Belief Model, we also examined whether subjective health perception would function as a mediator in the link between physical health constraints and psychological distress. Using data from 533 Asian Americans aged 60 and over (mean age=69.4, SD=6.88) in the 2016 Asian American Quality of Life Study, the direct and indirect effect models were tested with multivariate linear regressions and the PROCESS macro. Advanced age, unmarried status, lower levels of acculturation, and more chronic physical conditions were significant predictors of psychological distress. When subjective health perception was added to the model, an additional 5% of the variance was accounted for, resulting in 25% of the total variance explained by the estimated model. Negative health perception was a significant predictor of increased level of psychological distress. Supporting the mediation hypothesis, all direct paths among physical health constraints, subjective health perception, and psychological distress were significant. The indirect effect of physical health constraints on psychological distress through subjective health perception status was significant, as evidenced by the 95% bootstrap confidence interval for the indirect effect not containing zero (.07, .28). The findings not only help better understand the psychological mechanisms that underlie physical health constraints and psychological distress but also suggest avenues for interventions.

### PNEUMOCOCCAL PNEUMONIA VACCINATION AMONG OLDER AFRICAN AMERICAN AND HISPANIC MEN WITH CHRONIC CONDITIONS

Matthew Smith, Ledric Sherman, Daunte Cauley, Brittany Badillo, and R. Kirby Goidel, *Texas A&M University, College Station, Texas, United States*

The CDC estimates that 70% of adults ages 65 years and older received the pneumococcal pneumonia vaccination (PPV). However, documented PPV rates are substantially lower for men, especially non-white men. This study examined common and unique factors associated with PPV among racial/ethnic minority men age 65 and older with one or more chronic conditions. Data were analyzed from a national sample of 470 African American (n=267) and Hispanic (n=203) males using an internet-delivered questionnaire. Two binary logistic regression models were fitted to compare factors associated with PPV. On average, participants were age 70.1(±4.5) years and reported 3.9(±2.6) chronic conditions. PPV rates were 56.8% and 43.2% among African American and Hispanic males, respectively. Across models, men who received vaccines for influenza ($P<0.001$) and shingles ($P<0.01$) were more likely to receive the PPV; whereas, those who reported more disease self-care barriers were less likely to receive the PPV ($P<0.05$). Among African American males, those who were widowed (OR=3.80, P=0.022) and had an annual eye examination (OR=3.10, P=0.001) were more likely to receive the PPV; whereas, divorced/separated men were less likely to receive the PPV (OR=0.33, P=0.003). Among Hispanic males, those who took more medications daily (OR=1.36, P=0.005), reported higher disease self-management efficacy (OR=1.15, P=0.011), and had a colon cancer test in the past 12 months (OR=3.55, P=0.007) were more likely to receive the PPV. Findings suggest the need for culturally tailored education and self-management interventions to increase PPV and preventive healthcare service utilization among older racial/ethnic minority men.

### PREDICTORS OF SEEKING HEALTH INFORMATION AND MENTAL HEALTH SUPPORT IN U.S. PRISONS: A STUDY USING 2014 PIAAC DATA

Candidus Nwakasi,[1] Darlingtina Esiaka,[2] and Janardan Subedi,[3] *1. University of Southern Indiana, Evansville, Indiana, United States, 2. Union College, Union College, New York, United States, 3. Miami University, Miami University, Ohio, United States*

Being in prison increases the vulnerability to poor health, especially mental illnesses. This is evident in the documented health disparities between prison inmates and the general population. For example, suicide rates among inmates are higher than in the general population. There is an urgent need to understand how inmates experience mental well-being. This is important as some inmates serve long/life sentences and some will need to successfully re-integrate into the society. Although they have a constitutional right to health care access through the Eight Amendment, little is known of the health information and mental health support seeking patterns among inmates. The current study examined factors associated with the amount of health information accessed, and participation in mental health support groups in US prisons. Data (N= 645) from the Program for the International Assessment of Adult Competencies (2014) were analyzed using linear and logistic regressions. Sample weights were applied in the analyses. Results show statistically significant relationships between amount of health information acquired and age (66 years and above), race, health-status, readiness to learn, literacy skill, and numeracy skill. Social trust moderated the effect of education on the odds of participating in mental health support groups. Also, gender, work duration, attending substance abuse support and life skills groups were significant predictors. Our study may provide insight for stakeholders (e.g., policymakers, clinicians, social workers, and wardens, etc.) working in partnership to deliver a more tailored health interventions for inmates, by highlighting key contextual issues predicting mental health and well-being within prison settings.

### SOCIODEMOGRAPHIC DISADVANTAGE, LIVING WITH A SMOKER, AND HEALTH RISK BEHAVIORS IN MIDDLE-AGED AND OLDER WOMEN

Carole Holahan,[1] Charles Holahan,[1] Sangdon Lim,[2] Yen Chen,[2] and Daniel Powers,[2] *1. University of Texas at Austin, Austin, Texas, United States, 2. The University of Texas at Austin, Austin, Texas, United States*

Sociodemographic disadvantage places individuals at risk for an unhealthy lifestyle (Kushi et al., 2012; Shanker et al., 2010), as well as for exposure to second-hand household smoke (Gan et al., 2015; Zhang et al., 2012). However, the role of living with a smoker in the association between sociodemographic status and health behavior is unstudied. This study investigated the role of living with a smoker in partially explaining the link between sociodemographic disadvantage and physical inactivity and poor dietary behaviors. The study used limited access data from the Women's Health Initiative Observational Study obtained from NHLBI. Participants were 83,597 women ranging in age from 49 to

81; 6038 participants lived with a smoker. Cross-sectional logistic regression analyses examined paths in the models; bias-corrected bootstrapped confidence intervals tested indirect effects in probit analyses. Analyses controlled for age, ethnicity, marital status, and participants' current smoking status. Results demonstrated a significant association ($p < .001$) between sociodemographic disadvantage (composite of low education and low income) and living with a smoker (OR = 1.74). The unstandardized indirect effects (CIs are in brackets) from sociodemographic disadvantage through living with a smoker to no exercise, no walking, high percent dietary fat, and low servings of fruits and vegetables through living with a smoker were statistically significant (.023 [.019, .028], .026 [.023, .033], .041 [.037, .047], and .032 [.027, .036], respectively). These findings illustrate the need to address multiple non-smoking health risk behaviors in household smoking interventions for disadvantaged families. This project was supported by the NIH/NCI (R03CA215947).

### STRESS, HOPE, AND DEPRESSION AMONG OLDER ADULTS LIVING IN PUBLIC HOUSING

Mignon Montpetit,[1] LaKeesha James-Smith,[2] and Amy Gourley,[3] *1. Illinois Wesleyan University, Bloomington, Illinois, United States, 2. Bloomington Housing Authority, Bloomington, Illinois, United States, 3. Illinois Wesleyan University, Carlock, Illinois, United States*

Individuals living in public housing often experience myriad stressors related to poverty and mental illness. The current study explores how hope impacts the relationship between stress and depression in a sample of adults (aged 51-90 years; Mage= 63.3 years; SDage= 8.6 years) living in public housing. Questionnaire data were collected before and after running an intervention geared toward improving residents' well-being. Results of the initial questionnaire study suggest that hope moderates the stress -> depression relationship ($p = .001$), with effects in the expected directions: individuals exhibiting higher-than-average levels of stress and below-average hope reported the highest levels of depression. Data further suggest modest increases in hope post-intervention ($p = .06$). Overall, results suggest that hope may be important in helping mitigate the impact of life stress on vulnerable individuals, and that it can be augmented in the context of a short-term, cost-effective intervention.

### THE ASSOCIATION BETWEEN DISCRIMINATION AND SELF-MASTERY IN U.S. CHINESE OLDER ADULTS

Mailun Zhang,[1] Mengting Li,[2] and XinQi Dong,[2] *1. staff, New Brunswick, New Jersey, United States, 2. Rutgers University, New Brunswick, New Jersey, United States*

Perceived discrimination related to one's racial/ethnic membership has been linked to negative impact on the health and wellbeing of minority populations. While the anti-Chinese sentiment in the US dates to the 19th century, discrimination experienced by this population and its impact has been poorly understood. Self-mastery is a protective psychological resource reflecting one's ability to cope with stressors. This study examines the interaction between discrimination and self-mastery among 3,157 US Chinese older adults. Data were obtained from the Population Study of Chinese Elderly in Chicago (PINE) collected between 2011 and 2013. Self-mastery was measured using the Pearlin Mastery Scale. Discrimination was measured using the Experiences of Discrimination instrument. Linear regression was used. Discrimination experiences were found common (21.3%) among the US Chinese older adults. Younger age, male gender, higher levels of education, higher income, being married, more children, and fewer medical comorbidities were associated with a higher sense of self-mastery. After controlling for these potential confounders, discrimination experiences appeared to be significantly associated with lower self-mastery. Specifically, people who have experienced discrimination when getting hired (Beta [B]=-4.47,Standard Error [SE]=1.04, $p<0.01$), in working environment (B=-1.13,SE=0.52, $p<0.05$), getting health care (B=-3.45,SE=0.85, $p<0.01$), getting services in a store or restaurant (B=-2.12,SE=0.78, $p<0.01$), getting credit, bank loans, or a mortgage (B=-6.86,SE=2.83, $p<0.05$) and interacting with police or in the courts (B=-4.15,SE=1.48, $p<0.01$) were associated with lower levels of self-mastery. The findings suggested that discrimination experiences might be harmful by diminishing one's protective coping mechanism, which warrants longitudinal studies among minority aging populations to clarify.

### THE HAPPY CAMPAIGN: ASSESSING THE EFFECTS OF A COMMUNITY-WIDE INTERVENTION ON OLDER ADULTS LIVING IN PUBLIC HOUSING

Amy Gourley,[1] Mignon Montpetit,[2] and LaKeesha James-Smith,[3] *1. Illinois Wesleyan University, Carlock, Illinois, United States, 2. Illinois Wesleyan University, Bloomington, Illinois, United States, 3. Bloomington Housing Authority, Bloomington, Illinois, United States*

In the field of developmental psychology, the Stress-and-Coping model (Lazarus & Folkman, 1984) posits that individual differences in biological, psychological, and social risk and protective factors serve to increase or buffer the impact of stressful experiences on psychological well-being later in life. Importantly, research suggests that residents of public housing generally experience more risk factors than elders at large (Rabins, et al. 1996). The present study examines the impact of a programmatic intervention, The Happy Campaign, on individuals living in public housing in a small Midwestern city (aged 51-90 years; Mage= 63.3 years; SDage= 8.6 years). Goals of the Happy Campaign were to improve residents' coping skills and increase perceived support. Results demonstrated significant improvement in key aspects of well-being post-intervention; these included significant increases in exercise ($p = .04$), self-reported health ($p = .01$), as well as decreases in negative affect ($p= .008$). Data also show a moderate increase in residents' hope post-intervention ($p = .06$). Although future research is needed to account for confounding variables that arose in conducting research in this community setting, these data provide preliminary evidence that a broad-based, environmental intervention may offset the myriad risks faced by particularly vulnerable elders, and even augment well-being.

### TRADITIONAL CULTURAL PRACTICES PROVIDE MORE FREQUENT FEELINGS OF HAPPINESS AND CALM FOR NATIVE AMERICAN ELDERS

Collette Adamsen,[1] and Ramona Danielson,[2] *1. University of North Dakota, Grand Forks, North Dakota, United States, 2. North Dakota State University, Fargo, North Dakota, United States*

Though American Indian, Alaska Native, and Native Hawaiian (AI/AN/NH) elders are an understudied population, available data demonstrates higher rates of depressive symptoms among these elders. In addition, AI/AN/NH elders are a medically underserved population, with geographic isolation a common barrier to accessing emotional/mental health services. However, cultural practices are important sources of resilience for AI/AN/NH elders. Survey data from "Identifying our Needs: A Survey of Elders" Cycle VI (2014-2017), conducted by the National Resource Center on Native American Aging, were analyzed using ordinal logistic regression; N=18,134 adults age 55+ from 164 tribal survey sites. Respondents indicated how often (from none to all of the time) they participate in cultural practices (e.g., traditional food, music, customs). Frequency of participation varied; 27% of elders reported participating in traditional cultural practices a good bit of the time or more, 28% some of the time, 18% a little of the time, and 27% did not ever participate. We explored the relationship between frequency of cultural practices and frequency of feelings of happiness, calm/peacefulness, nervousness, and being downhearted/blue. A significant positive association was found between higher frequency of cultural participation and feelings of happiness and calm/peacefulness; no association was found with nervousness or depressive symptoms. While the frequency of participation by elders in cultural practices is directly related to better self-reported levels of happiness and peacefulness, nearly half (45%) never/almost never participated in these types of practices, which underscores the need to support availability of and elders' participation in cultural practices.

### VIEWS ON HIV DISCLOSURE AMONG OLDER ADULTS LIVING WITH HIV WHO ARE CHILDHOOD SEXUAL ABUSE SURVIVORS

Monique Brown, Titilayo James, Chigozie Nkwonta, and Amandeep Kaur, *University of South Carolina, Columbia, South Carolina, United States*

Rates of childhood sexual abuse (CSA) among people living with HIV are twice the CSA estimates among the general population. These statistics suggest that CSA prevalence may range from 16-22% among older adults living HIV (OALH). HIV disclosure continues to be a key consideration among people living with HIV. However, studies examining the views on HIV disclosure among OALH who are CSA survivors are lacking. Therefore, the aim of this study was to explore the views on HIV disclosure among OALH who are CSA survivors using a qualitative approach. Twenty-four adults aged 50-67 years, living with HIV and with a CSA history participated in the study. In-depth semi-structured interviews were conducted, audio-recorded and were analyzed using thematic analysis. The iterative analytic process included discussion of initial thoughts and key concepts, identification and reconciliation of codes, and naming of emergent themes. Three themes emerged: "You don't have to tell the person if it's just casual sex", "Nothing ought to be hidden especially when you get ready to engage in sex", and "As for me, I don't disclose". Views on HIV disclosure among OALH varied. Some participants stated that disclosure of HIV status should be dependent on the type of sexual relationship, while some OALH stated that participants should disclose regardless of type of relationship. Some participants were hesitant to disclose their HIV status due to anticipated HIV-related stigma. Understanding the perspectives on HIV disclosure among OALH with a CSA history may help to inform disclosure intervention programs for this vulnerable population.

### DIVERSE RACE AND ETHNIC POPULATIONS II

### A MODERATING MODEL OF SELF-EFFICACY IN CAREGIVERS OF LATINA BREAST CANCER SURVIVORS

Megan Thomas Hebdon,[1] Tracy Crane,[2] Pamela Reed,[2] and Terry Badger,[2] *1. University of Utah, Blacksburg, Virginia, United States, 2. University of Arizona, Tucson, United States*

In caregivers of Latina breast cancer survivors, contextual factors such as informational support, Anglo-orientation, and spiritual well-being may affect physical and mental health. The purpose of this study was to test if caregiver self-efficacy moderated relationships between contextual factors and health outcomes. A model, derived from Bandura's Social Cognitive Theory, included self-efficacy cancer knowledge (survivor) and self-efficacy symptom management (caregiver) as moderators of relationships between contextual factors and global health and depression. Secondary analysis of baseline caregiver data from an experimental study testing two psychoeducational interventions with Latina breast cancer survivors and their caregivers was conducted. Both self-efficacy measures were tested as moderators for relationships between contextual factors and health outcomes with fixed cutoffs (medium: mean, low/high: ±1 SD). Caregiver participants (N=233) were 43 years on average (SD=13), primarily women (70%), low-income (78%), and of Mexican-American ethnicity (55%). Anglo-orientation was significantly associated with global health ($r(233)=.27$, $p<.001$) and depression ($r(233)=-.13$, $p=.05$). High levels of self-efficacy cancer knowledge strengthened the negative relationship between depression and Anglo-orientation, while a slightly positive relationship was noted at low self-efficacy levels. Informational support was significantly related to global health ($r(233)=.39$, $p<.001$) and depression ($r(233)=-.43$, $p<.001$). Self-efficacy symptom management strengthened the negative relationship between informational support and depression. Correlational and moderation relationships were not significant for spiritual well-being. Both caregiver- and survivor-focused self-efficacy affected relationships between contextual factors and depression in caregivers of Latina breast cancer survivors. Further research should address both types of self-efficacy in caregiver health.

### AGE DIFFERENCES IN ENGAGEMENT IN HONG KONG ANTI-EXTRADITION BILL PROTEST: THE ROLE OF SELF-RELEVANCE

Tze Kiu Wong,[1] Dwight C.K. Tse,[1] Nicole Long Ki Fung,[2] and Helene Fung,[3] *1. The Chinese University of Hong Kong, Hong Kong, China, 2. the Chinese University of Hong Kong, Hong Kong, China, 3. The Chinese University of Hong Kong, Sha Tin, China*

Older adults were found to be less involved in non-institutional political actions than younger people did, and our previous work found that self-relevance mediated this age difference. In this study, we attempted to replicate the finding in a real-life social movement. We recruited 1037 participants (aged 18-84) during the anti-extradition bill movement in Hong Kong in September 2019. They responded to questions of how relevant and important the movement was to them, and whether they had taken part in a list of 8 political actions (e.g. signing petitions, joining rallies). Older adults indeed participated less in the movement compared with younger adults, and the age difference could partly be attributed to a lower perceived relevance of the movement. The finding suggested emphasizing on self-relevance as a potential way to promote political participation in older adults.

### AN EMPIRICAL ANALYSIS OF THE DETERMINANTS OF MORTALITY RISKS OF CHINESE CENTENARIANS IN THE ERA OF LONGEVITY

Jiehua Lu,[1] and Hui Zhu,[2] *1. Peking University, Beijing, China, 2. Nankai University, Tianjin, China*

With the rapid socioeconomic development and the increasing average life expectancy, China has shifted into an era of longevity. One significant feature of this era is the increasing size of Chinese centenarians and their health status are attracting increasing attention from academic communities. By using Chinese Longitudinal Healthy Longevity Survey (CLHLS), this paper employs Cox model to pinpoint the key influencing factors of mortality risk of Chinese centenarians. Our findings turn out that compared with the other elderly population, the mortality-risk determinants among centenarians are unique. The most important risk of the latter comes from their objective health status. The worse the health situation, the higher mortality risk is. Meanwhile, this rate is less affected by social and economic conditions. However, gender and smoking habit all play parts in the mortality risk of centenarians. This study comprehensively understands the key factors of the mortality risk of Chinese centenarians, which is of great significance for reducing centenarians' mortality risks and enhancing their health and well-beings.

### CAREGIVERS' BELIEFS AND STRESS AMONG RURAL AND URBAN AFRICAN AMERICAN CHRONICALLY ILL CAREGIVERS

Idethia Shevon Harvey, *Texas A & M University, Bryan, Texas, United States*

Introduction: African American women bear a disproportionate burden of chronic disease and disability compared to non-Hispanic whites, particularly rural African American women. The number of African American women providing informal care is increasing, and constant stress among caregivers produces long-term effects among middle-aged and older individuals. The objective of this study was to examine the relationship between stressors and attitudes of caregiving among chronically-ill African American rural and urban women. Methods: The sample included 519 rural and urban African American female caregivers (M = 53.8 years; SD = 15.05), with at least one chronic condition, participated in the Black Rural and Urban Caregivers Mental Health and Functioning Study. Descriptive statistics, Pearson correlation, and hierarchical linear regression analyses were performed among rural women with chronic conditions (N=265) and urban women with chronic conditions (N=254). Results: The number of chronic diseases was the most significant predictors, explaining 16% of stress, followed by caregiver beliefs, socio-demographic factors, and caregiving related factors ($F = 21.50$, $p < 0.01$). Discussion: The findings from this study can provide rural health care providers and rural health educators with a basis to assess potential support and disease management programs among chronically-ill caregivers.

### FACTORS AFFECTING SUBJECTIVE WELL-BEING AMONG OLDER ADULTS IN INDIA

Kasturi Banerjee, and Tamara Baker, *University of Kansas, Lawrence, Kansas, United States*

India has a rapidly expanding aging population whose unique mental health needs remain largely unexplored. Existent preliminary data however, show a significant association between life satisfaction and depressive symptoms within this population. Yet, little is known regarding the specific social and behavioral factors that may influence this relationship. Using data from the Longitudinal Aging Study in India (LASI) Pilot survey, the current study aimed to examine demographic and psychosocial factors associated with life satisfaction and subjective well-being among older adults 45+ years of age in the Indian states of Punjab, Rajasthan, Kerala and Karnataka. Results from the multivariate analyses indicated that age, household resources, neighborhood safety, religion, literacy status and participation in social activities are significantly associated with life satisfaction. Belonging to a southern state ($\beta=.156$; $p<0.001$), being financial provider status ($\beta=-.073$; $p<0.001$) and not being a care provider ($\beta=.105$; $p<0.01$) were significant predictors of greater life satisfaction. These findings are consistent with previous exploration of state level disparities regarding accessible resources and quality of life, and similarly the need to better understand the role of financial difficulties and caregiving burden among this population. These findings suggest the need to use qualitative assessment that explores the role additional factors such as social engagement and perceived neighborhood support have on this population's subjective well-being; thereby shaping public policy, focus resources, and form the foundation of intervention programs.

### GENDER, RACE, AND IMMIGRANT STATUS: INTERSECTING IMPLICATIONS FOR HEALTH IN MIDDLE AND LATER LIFE

Margaret Penning, Neena Chappell, Sean Browning, and Helena Kadlec, *University of Victoria, Victoria, British Columbia, Canada*

Although the negative implications of gender, race and immigrant inequalities for health and well-being in the middle and later years of life are well-documented, there is a lack of research addressing their combined implications as well as the mechanisms linking them to various health-related outcomes. Yet, as intersectionality theory reminds us, the consequences of gender, race, immigrant and other inequalities for physical and mental health outcomes must be understood in terms of these overlapping social identities. Moreover, linking intersectionality to stress process theory provides us with an explanation of the mechanisms potentially linking intersecting structural inequalities to health outcomes. This paper draws on data from the Canadian Longitudinal Study on Aging (CLSA - N=51,338) to assess the additive and interactive implications of gender, race and immigrant status for physical and mental health outcomes, together with the mediating effects of primary and secondary stressors on these outcomes. The results of a series of weighted least squares regression analyses suggest that immigrant status interacts with race and/or gender to influence health outcomes. Socioeconomic and other stressors also play a role in linking these intersecting structural inequalities to health outcomes. Overall, our findings provide initial support for the value of linking intersectionality and stress process frameworks for an understanding of the health implications of structural inequalities in middle and later life.

### GENTRIFICATION AND CHRONIC CONDITIONS IN OLDER ADULTS: SERVICE PROVIDERS' PERSPECTIVES

Ronica Rooks, *University of Colorado Denver, Denver, Colorado, United States*

Where we live impacts our health, but this is more apt for older adults (aged 55+) aging-in-place in their neighborhoods. Gentrification, i.e. the transformation of neighborhoods from low to high value, can put community-dwelling older adults at risk for residential displacement with limited retirement incomes and financial stressors like increased housing costs and property taxes, residential turnover and changing access to resources. As a place-based stressor, gentrification may exacerbate social vulnerabilities (e.g., lower socioeconomic status and racial/ethnic minority status) related to chronic condition (CC) disparities. But, little gentrification research focuses on these issues. This research examines associations between gentrification and older adults' CC management related to broader social determinants in Hamilton, Ontario, Canada from health and social service providers' perspectives. Hamilton, a recovering steel industry city with in-migration from Toronto, is experiencing higher costs of living, income inequality and tension with recent gentrifiers. I conducted key informant interviews with service providers in city government and community-based organizations using thematic analysis. Across providers, food insecurity, social isolation and displacement were the biggest issues associated with gentrification and CC, particularly for older adults with lower incomes and government disability support. Results thus far reveal Hamilton has numerous older adult-focused providers, but older adults often have difficulties accessing services due to a lack of knowledge, not always asking or realizing when they need help and coordinated referral difficulties across providers. To address these challenges, providers consider environmental scans, mapping resources and advertisement in an online community information database from the city's public library.

### IMMIGRANT STATUS PREDICTS WORSE SUBJECTIVE MEMORY COMPLAINTS

Yujin Franco, and Yujin Jeong, *University of Southern California, Los Angeles, California, United States*

The "Immigrant Health Paradox" suggests that immigrants experience better health and lower mortality than the U.S.-born population despite their lower average socioeconomic status. However, it is unclear whether this health advantage extends to all areas of cognitive functioning. This study investigates cognitive functioning and Subjective Memory Complaints (SMCs) as a function of immigrant status and identifies predictors of cognitive decline as well as SMCs among the immigrant population. Data were drawn from the 2010 wave of the Health and Retirement Study. The sample consisted of 9,812 older adults aged 65 and older (8,873 U.S.-born and 939 foreign-born). Logistic regression was used to examine whether immigrant status was associated with cognitive functioning and SMCs, controlling for socio-demographic (age, gender, education, and marital status), health conditions (diabetes, depression, hypertension, and stroke), and functional limitations. Being foreign-born was not a significant predictor of dementia (OR:1.18, 95% CI: 0.83-1.67). However, immigrants were 41% more likely to report SMCs compared to U.S.-born respondents (OR:1.41, 95% CI: 1.17-1.69). Among the immigrant population, immigrants with less than a high school education showed four- and two-times higher odds of having dementia and reporting SMCs than those with more than high school. It is necessary to provide dementia education and screening to immigrants, especially those with low education, as this may contribute to reducing disparities in cognitive functioning within the older population.

### MORE THAN MEETS THE EYE: INCOME AND RACIAL DISPARITIES IN VISION HEALTH OF OLDER ADULTS

Asif Iqbal,[1] and Ananya Sarker,[2] *1. Case Western Reserve University, Cleveland, Ohio, United States, 2. Case Western Reserve University, Cleveland, Ohio, United States*

By the end of this decade, the USA is projected to experience an increase of more than 50% in the number of people with poorer vision health as the population grows older. Using data from the NHANES III (1988–1994) for 7,186 White, Black, and Hispanic adults of ages 50 to 90 (Mean=68.23, SD=10), this paper examines the racial/ethnic, and socioeconomic disparities in vision health in the older adult population. The focus of this paper is on Visual Impairment indicators: full/partial blindness and trouble seeing with glasses to demonstrate vision health disparities in these race/ethnic groups in terms of family income using logistic regression analysis. Controls include demographic characteristics like age, gender, marital status, region, education and behavioral features like alcohol consumption and smoking. We explore another component of vision health: days since last visit to healthcare provider to evaluate the inequalities in access to health care using OLS regression analysis. In Whites (OR=.85) and Blacks (OR=.63), people with less family income are more likely to experience blindness, however, there exists no significant variability in blindness in terms of family income among Hispanics. In Black (OR=.82) and Hispanics (OR=.85), people with less family income are more likely to have trouble seeing even with glasses, however, this relationship does not exist among Whites. Days since last visit can be explained by income for Whites (Beta=-92), not for Blacks and Hispanics. This compounded disparity puts a disproportionate economic burden on minority groups, but the current Medicare policy fails to address that.

### PHYSICAL ACTIVITY AS A MEDIATOR IN THE RELATIONSHIP BETWEEN RACE OR ETHNICITY AND CHANGES IN MULTIMORBIDITY

Jason Newsom,[1] Emily Denning,[1] Ana Quinones,[2] Miriam Elman,[3] Anda Botoseneanu,[4] Heather Allore,[5] Corey Nagel,[6] and David Dorr,[2] *1. Portland State University, Portland, Oregon, United States, 2. Oregon Health & Science University, Portland, Oregon, United States, 3. OHSU-PSU School of Public Health, Portland, Oregon, United States, 4. University of Michigan, Dearborn, Michigan, United States, 5. Yale University, New Haven, Connecticut, United States, 6. University of Arkansas for Medical Sciences, Little Rock, Arkansas, United States*

Racial/ethnic disparities in multimorbidity (≥2 chronic conditions) and their rate of accumulation over time have been established. Studies report differences in physical activity across racial/ethnic groups. We investigated whether racial/ethnic differences in accumulation of multimorbidity over a 10-year period (2004-2014) were mediated by physical activity using data from the Health and Retirement Study (N = 10,724, mean age = 63.5 years). Structural equation modeling was used to estimate a latent growth curve model of changes in the number of self-reported chronic conditions (of nine) and investigate whether the relationship of race/ethnicity (non-Hispanic Black, Hispanic, non-Hispanic White) to change in the number of chronic conditions was mediated by physical activity after controlling for age, sex, education, marital status, personal wealth, and insurance coverage. Results indicated that Blacks engaged in significantly lower levels of physical activity than Whites (b = -.171, □ = -.153, p < .001), but there were no differences between Hispanics and Whites (b = -.010, □ = -.008, ns). Physical activity also significantly predicted both lower initial levels of multimorbidity (b = -1.437, □ = -.420, p < .001) and greater decline in multimorbidity (b = -.039, □ = -.075, p < .001). The indirect (mediational) effect for the Black vs. White comparison was significant (b = .007, □ = .011, 95% CI [.004,.010]). These results provide important new information for understanding how modifiable lifestyle factors may help explain disparities in multimorbidity in middle and later life, suggesting greater need to reduce sedentary behavior and increase activity.

### RACIAL DISPARITIES IN COGNITIVE FUNCTIONING: THE MEDIATING ROLE OF SOCIAL RESOURCES

Kazi Sabrina Haq, and Margaret Penning, *University of Victoria, Victoria, British Columbia, Canada*

Despite frequent recognition of disparities in cognitive functioning between White and non-White older adults, the pathways or mechanisms through which race affects cognitive functioning have yet to be elucidated. The research questions addressed in this paper are: 1) Is there a relationship between racial minority status and cognitive functioning in middle and later life? 2) To what extent do social resources (i.e., social support, social networks, and social participation) mediate the relationship between racial minority status and cognitive functioning? 3) Finally, drawing on intersectionality theory, if social resources do mediate the relationship between racial minority status and cognitive functioning, to what extent is this mediation effect moderated by the interaction of gender and Socioeconomic Status (SES)? Using cross-sectional data drawn from the Canadian Longitudinal Study on Aging (CLSA) with a sample of over 50,000 Canadians (2010-15) aged 45 to 85 years, multivariate regression analyses (OLS, logistic, multinomial logistic) assess the mediating effect of social resources on the relationship between racial minority status and cognitive functioning. Controlling for age, gender and other relevant determinants, preliminary results reveal that racial disparities in cognitive functioning (i.e., lower cognitive test scores) exist in Canada and that this relationship is partially mediated by some indicators of social resources (e.g., functional social support, emotional social support). Our findings suggest the need for interventions targeted at increasing social resources for racial minority groups to cope with the risk of developing cognitive impairment in later life.

### RELATIVE IMPORTANCE OF POSITIVE AGING DIMENSIONS AMONG LATINO OLDER ADULTS AND SERVICE PROVIDERS

Jennifer Smith, Cate O'Brien, and Joseph Bihary, *Mather Institute, Evanston, Illinois, United States*

The variation in Latino older adults' conceptualizations of positive aging across studies suggests greater attention should be paid to within-group factors. The purpose of the current study was to identify which factors are important to positive aging from the perspective of Latino older adults, and whether the importance of these factors varied based on participant characteristics. A second aim of this study was to examine whether there are differences in views of successful aging between Latino older adults and service providers who support aging Latinos. The current study was conducted as part of a broader research project investigating Latino older adults' perceptions of positive aging. Latino older adults (n = 93) and aging services providers (n = 45) rated the importance of a series of statements related to positive aging. Mixed-methods analysis of the statements identified nine distinct dimensions (Positive Outlook, Spirituality/Religion, Healthy Behaviors, Independence, Self-Care, Support for Others, Social Support, Leisure Activities, and Adaptability). Latino older adults rated Positive Outlook and Spirituality highest on importance, and ratings differed based on gender and other individual difference characteristics. For example, men placed greater relative importance on Independence and Support for Others compared to women, and younger participants rated Independence higher on importance compared to older participants. In addition, Latino older adults (vs. providers) placed greater importance on all aspects of positive aging, with greatest mean differences related to providing Support for Others and Spirituality. These findings have implications for wellness programs for Latino older adults and training for service providers.

### SOCIAL ENGAGEMENT AND EPISODIC MEMORY IN NON-HISPANIC BLACK AND WHITE OLDER ADULTS

Abbey Hamlin,[1] A. Zarina Kraal,[2] and Laura Zahodne,[2]
*1. University of Michigan, Highland, Michigan, United States, 2. University of Michigan, Ann Arbor, Michigan, United States*

Social engagement may confer cognitive benefits in older adulthood, but studies have typically been restricted to largely non-Hispanic White (NHW) samples. Levels of social engagement vary across race such that NHW report larger social networks, more frequent participation in social activities, and greater social support than non-Hispanic Blacks (NHB). Associations between social engagement and cognition may also vary by race, but research is sparse. The current cross-sectional study examined associations between different aspects of social engagement and episodic memory performance, as well as interactions between social engagement and race among NHB and NHW participants in the Michigan Cognitive Aging Project ($N = 247$; 48.4% NHB; age = $64.19 \pm 2.92$). Social engagement (network size, activities, support) was self-reported. Episodic memory was a z-score composite of immediate, delayed, and recognition trials of a list-learning task. Separate hierarchical linear regression models quantified interactions between race and each of the three social engagement variables on episodic memory, controlling for sociodemographics, depressive symptoms, and health conditions. Results showed a main effect of more frequent social activity on better episodic memory, as well as an interaction between race and social support indicating a significant positive association in NHB but not NHW. These preliminary findings suggest that participating in social activities may be equally beneficial for episodic memory across NHB and NHW older adults and that social support may be particularly beneficial for NHB. Future research is needed to determine the potential applications of these results in reducing cognitive inequalities through the development of culturally-relevant interventions.

### THE TRANSLATION AND CROSS-CULTURAL ADAPTATION OF AN ARABIC VERSION OF THE REMINISCENCE FUNCTIONS SCALE

Abdallah Abu Khait,[1] and Juliette Shellman,[2] *1. University of Connecticut, Storrs, Connecticut, United States, 2. University of Connecticut, Storrs Mansfield, Connecticut, United States*

The Reminiscence Functions Scale (RFS), a 43 item reliable and valid scale, measures eight specific reasons as to why individuals reminisce: (a) identity (b) death preparation; (c) problem-solving; (d) bitterness revival; (e) boredom reduction; (f) intimacy maintenance; (g) conversation; and (h) teach/Inform others. Research indicates that certain reminiscence functions have a positive impact on the mental-health and well-being of older adults. However, no known studies have been conducted in Arab countries examining the relationship between reminiscence functions and mental health outcomes due to the lack of an Arabic version of the RFS. The purpose of this study was to translate the RFS from English to Arabic (Modern Standard Arabic), back-translate from Arabic to English, and compare the two English versions for equivalence and accuracy through a multi-step translation method. A team of bilingual, bicultural, Arabic speaking experts assembled to conduct the forward, back translation and harmonization process. In the next step, professionals with expertise in linguistics communication sciences and disorders, Arabic literature, geriatric nursing, and medicine reviewed the translated documents to assess the content (relevant to the target culture) and semantic equivalencies (similarity of meaning in the target culture). Challenges that occurred during the study included finding nuanced translation equivalences for Likert scale responses, translation of idioms such as "when time is heavy on my hands", and logistical issues such as coordinating virtual meetings for the team of experts. Lessons learned during the translation process and implications for use of the RFS-Arabic version with Jordanian older adults will be presented.

### UNDERSTANDING THE IMPACT OF PAIN AMONG LATINX OLDER ADULTS

Calia Morais,[1] Michael Robinson,[2] Roger Fillingim,[1] and Emily Bartley,[3] *1. University of Florida, Pain Research and Intervention Center of Excellence, Gainesville, Florida, United States, 2. University of Florida, Center for Pain Researach and Behavioral Sciences, Gainesville, Florida, United States, 3. University of Florida, Pain Research and Intervention Center of Excellence, Gainesville, United States*

Pain is the number one reason for seeking medical attention, and a top contributor to healthcare costs in the United States. Considerable evidence highlights racial/ethnic disparities in pain with Latinx communities being disproportionally affected at higher rates. Compared to other ethnic minority groups, Latinx older adults are more socioeconomically disadvantaged and experience higher rates of disability and frailty (Garcia, Downer, Crowe, & Markides, 2017). Given the rapidly growing population of Latinx, it is anticipated that the incidence of pain and its associated impact will adversely affect the quality of life of many older adults. The public health focus on improving pain management with non-opioid treatment options also highlights the need to provide culturally responsive care for Latinx older adults. However, limited efforts have addressed this important target for intervention. Following the biopsychosocial model of pain, this presentation will provide an overview of biological, psychosocial and cultural factors influencing pain disparities among older adults. Findings from experimental and clinical settings will be discussed. The presenter will also review current evidence highlighting the preference for self-management strategies for pain management among Latinxs, as well as the need to increase accessibility to psychosocial treatments for chronic pain, such as cognitive behavioral therapy for pain (CBT-pain). Furthermore, preliminary findings from a qualitative study will be presented to illustrate the need to offer culturally adapted treatments for pain management (Torres, Thorn, Kapoor, & DeMonte, 2017). Future research is needed to study sociocultural factors influencing pain disparities to help identify modifiable targets for intervention.

### SESSION 2943 (POSTER)

### FAMILY AND CROSS-GENERATIONAL RELATIONSHIPS

### ADULT CHILDREN'S EDUCATION ATTAINMENT AND PARENTS' SUBJECTIVE WELL-BEING IN CHINA

Dan Chen, and Yuying Tong, *The Chinese University of Hong Kong, Hong Kong, China*

Parent-child tie is important for parental later life due to insufficient old-age support in developing contexts. Parents often anticipate they would share the returns of children's education for their early period investment. Previous studies show that adult children's education is positively associated with parents' survival and physical health in both low- and middle-income countries. What's less discussed in literatures is the effect of adult children's education on parental subjective wellbeing. Drawing the China Health and Retirement Longitudinal Study (CHARLS), this study intends to explore the effect of adult children's education attainment on parents' life satisfaction. This study uses information from baseline wave in 2011 and latest wave in 2015 of CHARLS. The analytic sample restricts to adult children aged between 25 and 49 with the highest education among all children of a parent who are aged between 50 and 84. To handle the reversed causality, this study adopts lagged effect model and controls for baseline subjective wellbeing. Instrumental variables (IV) are also used to handle the endogeneity issue existing between children's education and parental wellbeing to conclude a causal effect. The preliminary results without IV reveal that association between children's schooling years and parents' life satisfaction is non-linear. However, results with IV show that adult children's schooling years are negative associated with parents' life satisfaction. This study firstly draws attention on negative sides of children's education attainment on parental subjective wellbeing which implies more studies to unfold the mechanisms underlying the association.

### CAREGIVER RELATIONSHIPS AND DISTRESS MODERATED BY COLLECTIVISM

Eunbea Kim,[1] Mary Rogers,[2] Erica Szkody,[2] and Cliff McKinney,[2] *1. Mississippi State University, Gyeongsan-si, Kyongsang-bukto, Republic of Korea, 2. Mississippi State University, Mississippi State, Mississippi, United States*

The number of older populations raising their grandchildren has increased. Past research has indicated the distress custodial grandparents' experience is related to their family relationships (Hayslip, Shore, & Emick, 2006). Family relationships are also influenced by a variety of factors such as social history, culture, family structure, and individual differences (Uhlenberg & Kirby, 1998). The current study evaluated the influence of culture on the relationship between caregiver relationship quality and mental health by examining 885 children (18-25 years; M=18.93). This study also compared the difference in cultural impact between custodial grandparents-grandchildren and biological parents-children. Measures included the Network of Relationships Inventory, Hofstede Cultural Questionnaire, and Adult Behavior Checklist. Path analysis was conducted using AMOS 26.0 which resulted in an interaction between relationship closeness and collectivism to predict custodial grandparent depressive symptoms. Custodial grandparents who reported a lower level of closeness with their grandchildren in a higher collectivistic culture reported a significantly higher level of depression symptoms than those in a more individualistic culture, particularly for custodial grandmothers. However, custodial grandparents who reported a higher level of closeness with their grandchildren in a higher collectivistic culture reported significantly lower levels of depressive symptoms than those in a more individualistic culture. Furthermore, compared to biological parents, custodial grandparents reported significantly lower levels of depressive symptoms when reporting higher collectivistic culture. These findings will inform the need for more research to assess factors of cultural features that reduce psychological problems and support family relationships to adapt psychological therapies in older adults.

### DYADIC PATTERNS OF MARITAL QUALITY DURING LATER LIFE

Jennifer Bulanda,[1] Taka Yamashita,[2] and J. Scott Brown,[1]
*1. Miami University, Oxford, Ohio, United States,*
*2. University of Maryland, Baltimore, Maryland, United States*

Although earlier cross-sectional studies suggested a U-shaped curve in marital quality over the life course, recent longitudinal studies find stability or continual decline (Proulx, Ermer, & Kanter, 2017). It is important to better understand patterns of marital quality during later life, as marital quality is associated with older adults' marital stability, health, and longevity. However, few studies have utilized couple-level data to examine marital quality trajectories, and only one has examined dyadic patterns during later life (Wickrama et al., 2020). We use nationally-representative data from the 2006-2016 waves of the Health and Retirement Study (HRS) to examine positive and negative dimensions of marital quality among a sample of continuously-married couples over age 50 in which both partners provided data on marital quality at three time points ($n = 1,389$ couples). A survey-weighted latent growth curve model simultaneously examines two marital quality trajectories: own and spouse's. Preliminary results show that mean baseline positive and negative marital quality are similar for own and spouse's trajectories. Although growth rates are statistically non-significant for positive marital quality, the variance of growth rate is statistically significant for spouse's trajectory (0.001, $p < 0.05$), and greater baseline own positive marital quality is associated with negative growth of spouse's positive marital quality. Growth rates are similar for own and spouse's trajectories of negative marital quality, and variance of growth rate is statistically significant for spouse's negative marital quality trajectory. Results point to stability in marital quality over time, and suggest the importance of using couple-level data.

### EXPANSION OF THAILAND'S SOCIAL PENSION POLICY AND ITS IMPLICATIONS FOR FAMILY SUPPORT FOR OLDER PERSONS

Bussarawan Teerawichitchainan, *National University of Singapore, SINGAPORE, Singapore*

Thailand is among few developing countries that have provided universal social pension for its older adults since 2009. Analyzing nationally-representative data from the Surveys of Older Persons in Thailand, we address the extent to which older Thais have benefited from the policy and describe the socio-demographic correlates of older persons who primarily rely on OAA as their main income source. Importantly, we examine how reliance on OAA may have implications for intergenerational exchanges between older parents and adult children as well as the well-being of older Thais. Results show significant changes over the last decade in the patterns of old-age income sources and filial economic support for older parents, particularly after the universalization of the OAA policy. We find the declining importance of children and rising significance of OAA as the primary income source. Older persons whose main income source was OAA were considered socially and economically vulnerable, although it was men rather than women who were more likely to depend on OAA as the main income source. Results further indicate that reliance of OAA as the main income source is not associated with a reduction in non-monetary familial support (intergenerational coresidence and social support) for aged parents. Nevertheless, we find that older parents whose main income was OAA were consistently less likely to report income adequacy and psychological well-being compared to others. This evidence suggests that while the OAA scheme may have tackled old-age poverty issues, it has little impact in dampening economic and social inequality among older Thais.

### EXPERIENCES AND IMPLICATIONS ON FAMILY RELATIONSHIPS FOR GRANDMOTHERS RAISING THEIR GRANDCHILDREN

Carol Musil, Elizabeth Tracy, Rashon Braxton, McKenzie Wallace, and Alexandra Jeanblanc, *Case Western Reserve University, Cleveland, Ohio, United States*

In the U.S., over 2.7 million grandparents are primary caregivers to grandchildren. It is critical to understand the experiences of grandparent caregivers to design tailored, supportive programs. Our aim was to analyze written journals of 129 grandmothers with respect to the impact of raising grandchildren on relationships with family members. As part of a national RCT study of grandmother caregivers, participants completed daily journals for 4 weeks. Employing thematic data analysis, three members of the research team coded using NVIVO 12 Plus. The research team met regularly to compare and resolve discrepancies in coding. Percent agreement was > 80%. Relationships with the grandchild's mother were characterized by anger/tension, resentment, and the realization that the mother was incapable of parenting while at the same time expressing worry/concern for her. Relationships with the grandchild's father mirrored these dynamics while also depicting the father as a distant figure, inconsistent, and financially absent. Spousal relationships were marked by challenges faced by the spouse, their shared role/influence as a grandparent, and the quality of their time spent together. The other relationships described were often focused on their other adult children, as well as their own adult siblings. Grandmother caregivers expressed stress or strain and frustration within these relationships. Caregivers also verbalized gratitude for support from family members within the context of familial and financial stress. Grandmother caregivers may need support in managing familial relationships and stress within these relationships, which may be a target for future interventions.

### FAMILY VALUE MATTERS: INTERGENERATIONAL SOLIDARITY AND LIFE SATISFACTION OF CHINESE OLDER MIGRANTS

JUN YAO,[1] Li zhang,[2] and Peiyi Lu,[3] *1. NANJING MEDICAL UNIVERSITY, NANJING, Jiangsu, China, 2. Nanjing University of Chinese Medicine, NANJING, Jiangsu, China, 3. Iowa State University, Ames, Iowa, United States*

The number of older adults who migrate due to family reasons has been increasing rapidly in China in the past decade. However, few empirical studies have focused on this group. This study focused on them and explored the association of intergenerational solidarity with older migrants' life satisfaction when they were adapting to the new environment. We surveyed 340 older adults who migrated to Nanjing with their children either to help care for the grandchildren or enjoy retirement life. Respondents were recruited from the community. Structural equation modeling was adopted to analyze the associations among intergenerational solidarity, loneliness, aging perception, and life satisfaction. Results showed intergenerational solidarity was negatively correlated with loneliness ($r=-0.304$) but positively correlated with life satisfaction ($r=0.386$). Loneliness was linked to lower life satisfaction($r=-0.517$). Path analyses showed that loneliness played a partial mediation role on the relationship of intergenerational solidarity and life satisfaction. Aging perception negatively moderated the association between intergenerational solidarity and loneliness, and also negatively moderated the mediating effect of loneliness on intergenerational solidarity and life satisfaction. It is concluded family values played important roles in Chinese older migrants' mental health. When they migrate to a new city, intergenerational solidarity can help ease their loneliness and subsequently improve their life satisfaction, which finally help them adapt to the new environment. Positive perception towards aging also helps improve their well-being after migration. Based on these findings, we suggest practitioners design education program to promote family values among the family with older migrants.

### GRANDPARENTING AND THE RECEIPT OF FILIAL PIETY FROM ADULT CHILDREN IN CHINESE AMERICAN FAMILIES

Jeung Hyun Kim, *Syracuse University, Syracuse, New York, United States*

The current study explores the association between grandparent caregiving by Chinese American elders and their perceived receipt of filial support from their adult children, called filial piety (xiao). Many studies find a correlation between grandparent caregiving and filial behaviors from their adult children, which is notably higher among minority families, especially among Asians than among white families, stimulated by the norm of reciprocity, familism, and extended kinship. Drawing from the theory of intergenerational relationships, social exchange theory, and the role theory, this study questions whether a more active engagement in grandparenting renders higher levels of filial piety returns from adult children. It uses the PINE data, a survey on the wellbeing of Chinese American elders in Chicago. The results show that more hours of grandparent caregiving relate to higher returns of filial piety perceived by older parents. Correspondingly, though with a marginal significance, more pressures to take care of a grandchild from adult children reduce the elders' perception of filial piety receipt. No interaction effect is found between the grandparenting hours and the pressure from adult children. Additionally, Chinese American elders possessing higher levels of education, mastery, and longer stays in the US perceive lower levels of filial piety receipt from adult children. Discussion will focus on how grandparent caregiving can be mutually beneficial and strengthen intergenerational relationships among Chinese American families.

### HEARING THE UNHEARD: PROMOTING ACTIVE AND HEALTHY AGING WITH FAMILY LEISURE

Tia Rogers-Jarrell,[1] and Brad Meisner,[2] *1. York University, Mississauga, Ontario, Canada, 2. York University, North York, Ontario, Canada*

Grandparents are playing an increasing role in the lives of their grandchildren. Participating in this role enriches grandparent's well-being through feelings of enjoyment and satisfaction. Research also indicates participating in family leisure positively improves family communication, creates higher quality relationships, and enhances family cohesiveness and connectedness. However, the grandparent perspective, and their inclusion in family leisure, is often overlooked, rendering their voices invisible. Given the growing aging population, and the role grandparents play, their involvement in, and experience with, family leisure is becoming increasingly relevant. Therefore, the purpose of this paper was to critically review and explore the available literature on grandparents (ages 65+) experiences with family leisure. This paper critically reflected on benefits of family leisure participation according to the World Health Organizations active aging and healthy aging ideologies. Participation in family leisure provides grandparents with opportunities for social participation, remaining active and engaged through generativity and contribution to family, as well as building, maintaining and enhancing relationships. More research that is inclusive of grandparent's perspective of, and experience with, family leisure is needed. By taking a socioecological approach to understanding the experiences of grandparents' participation in family leisure, clear suggestions can be made on how to support grandparent's involvement in family leisure at the interpersonal, organizational, institutional, community, and policy levels. In particular, future actions and initiatives should simultaneously support family leisure across and within these domains. Promoting family leisure is one way to support active and healthy aging not only among older adults, but among all individuals.

### INTERGENERATIONAL RELATIONSHIPS IN AN ECONOMICALLY VULNERABLE COMMUNITY: FINDINGS FROM THE FLINT WOMEN'S STUDY

Rodlescia Sneed, *Michigan State University, Flint, Michigan, United States*

Older women in economically disadvantaged communities often balance a range of relationships that present both benefits and challenges. The current study describes older women's perceptions of the benefits and challenges of maintaining intergenerational relationships with younger women and children in their community. We used secondary data from the Flint Women's Study, a qualitative interview project that included 60-90 minute structured interviews with 100 women who either lived or worked in the Flint, Michigan metropolitan area. Interviews were recorded, transcribed, and coded using the belongingness theory framework. Special populations (including older women) were coded in the dataset. The main benefits of intergenerational relationships included feeling valued, social connection, and giving back to future generations. In particular, intergenerational relationships allowed older women in this economically disadvantaged community to leave behind a meaningful social legacy in the absence of a meaningful economic legacy. Despite the perceived benefits, older women had difficulty effectively forming intergenerational relationships. Many reported being naturally isolated from those of younger generations. For those who were not isolated, many cited challenges related to grandparent caregiving, which was often connected to workforce conflicts, financial burden, caring for multiple generations well into adulthood, managing grandchildren's traumas, and a lack of technological proficiency needed to meet grandchildren's educational needs. Participants described the impact of these challenges on older women who lived in disadvantaged communities. Future programming should consider the needs of older women in disadvantaged communities and provide resources to maximize the benefits and minimize the challenges of intergenerational relationships in this population.

### KEY COMPONENTS OF INTERVENTIONS FOR GRANDPARENTS RAISING GRANDCHILDREN: A SYSTEMATIC REVIEW

Athena Chung Yin Chan,[1] and Timothy Piehler,[2] *1. University of Minnesota–Twin Cities, Saint Paul, Minnesota, United States, 2. University of Minnesota – Twin Cities, St. Paul, Minnesota, United States*

Increasingly grandparents provide primary or supplementary care for their grandchildren globally. In view of the challenges faced by grandparent caregivers, a number of intervention programs were designed to act as a buffer to the potential negative impacts on their well-beings. A recent meta-analysis (Chan et al., 2019) shows that interventions for grandparent caregivers have small to moderate effect sizes for increases in social support and parenting skills. However, little is known how the multi-component interventions work. The aim of this review is to categorize the key components of intervention programs and determine the direct effect of components on grandparent caregivers' outcomes (i.e., physical, mental health and well-being). A component-level approach is adopted to disentangle the 'active ingredients' of the interventions. Peer-reviewed articles were searched via five electronic database (published before January 2020). A keyword search was performed based on combinations of two groups of terms: (1) grandparent caregivers and (2) intervention, training, and support group. Studies adopting an experimental or pre/post design and reporting quantitative changes in grandparents' outcomes were included. Studies focusing on the special needs of grandchildren were excluded. Fourteen interventions were identified from over 20 included studies. Preliminary analyses show that different physical and mental outcomes of grandparent caregivers were predicted from key components of education, skills training, relaxation and psychosocial support. With the goal of developing evidence-based interventions for grandparents raising grandchildren, this synthesis offers insight into the mechanisms of an intervention, which informs its generalizability or applicability to a new context.

### MARRIAGE AND MORTALITY: THE IMPORTANCE OF NON-SPOUSAL SUPPORT FOR NEVER-MARRIED OLDER ADULTS

Adam Roth, and Siyun Peng, *Indiana University, Bloomington, Indiana, United States*

Married individuals have been shown to consistently outlive their unmarried peers. Although numerous factors contribute to such mortality disparities, spousal support serves as one of the most central marital resources that reduces mortality risk for married older adults. Unmarried older adults, who lack access to such support, tend to rely more heavily on extended family and friends for their social needs. Yet it is unclear whether support from these non-spousal sources can be successfully substituted for spousal support to provide unmarried older adults with similar protection against mortality. In the present study, we use nationally representative data from the National Social Life, Health, and Aging Project to assess whether support from family and friends reduces the mortality differences between married and unmarried older adults. Although we examine all forms of singlehood (i.e., divorce, widowhood, and never married), we pay primary attention to never married older adults in relatively to their married peers as they have been exposed to a lifetime without a spouse. We find that never married older adults are especially sensitive to non-spousal support. More specifically, never married respondents who reported low levels of support were far more likely to die than married respondents who had similar levels of non-spousal support. Yet when support was high, never married respondents were neither more nor less likely to die than their married peers.

### MEN'S HEALING WORK: RECONCILING INTERGENERATIONAL AMBIVALENCE THROUGH FATHERHOOD, LETTER WRITING, AND COMMUNITY

Allen Kim, *International Christian University, Tokyo, Japan*

Due to globalization, new ideals of fatherhood are challenging traditional paternal roles in South Korea. Contemporary fathers striving to emulate more engaged parenting sometimes wrestle with painful recollections of their own fathers' stern, distant, and patriarchal approach. How do men reconcile their aspirations for their own development as parents when conflicted relationships with their fathers? Motivated by the concept of intergenerational ambivalence, this study analyzes letters South Korean men write to their fathers as assigned homework for Father School, an international men's movement that aims to make men more nurturing. Under Father School direction, men adopt a life course frame that allows them to reconcile their mixed feelings toward their aging fathers. Analysis points to three life course discursive strategies that permit men to balance negative judgments with positive ones: 1) sharing with their parent the life stage as worker and father; 2) appreciating historically-situated differences between twentieth and twenty-first century lives and parenting imperatives; and, 3) drawing on deeply rooted filial norms to take responsibility for their own role in intergenerational conflicts. In addressing how these men manage intergenerational ambivalence, the article moves beyond prior research to extend the concept to father-son dyads, the Asian context, and the neglected meso-level where organizations may actively structure reconciliation.

### MULTIDIMENSIONAL PROFILES OF RELIGIOSITY: DO THEY MATTER FOR GEN-XERS' PSYCHOLOGICAL AND FAMILIAL WELL-BEING?

Kent Jason Cheng, Woosang Hwang, Jeung Hyun Kim, Merril Silverstein, and Maria Brown, *Syracuse University, Syracuse, New York, United States*

Although researchers have suggested that religiosity is a multidimensional construct, less is known about the long-term effects of religiosity profiles on Generation X (born between 1965 and 1980) adults' psychological and familial wellbeing over the life courses. Thus, the goal of this study is (1) to identify unobserved profiles of young-adult Gen-Xers' religiosity based on religious attendance, religious intensity, spirituality, and religious ideology, (2) to investigate demographic factors that predict membership in these religiosity latent classes, and (3) to examine how these profiles of religiosity predict Gen-Xers' psychological wellbeing (self-esteem, life satisfaction, and depression) and familial wellbeing (martial satisfaction, and affectual and associational solidarity toward their aging parents) in early and middle adulthood. We selected 462 Gen-Xers from the Longitudinal Study of Generation in the 2005 (mean age = 30.25) and the 2016 waves (mean age = 41.25). In terms of data analysis, the three-step latent class analysis was conducted. We identified four religiosity profiles among young-adult Gen-Xers: strongly religious, weakly religious, literalists but not religious, religious but not literalists. Less educated single Gen-Xers were more likely to be in the strongly religious class, and less likely to be in other classes. Gen-Xers in the strongly religious class reported high scores of psychological and familial wellbeing than those in other religiosity classes. Given that the religiosity of the U.S. population has declined substantially over the past few decades, our findings indicate that religiosity is an important resource for Gen-X adults' psychological and familial wellbeing.

### OLDER ADULT VOLUNTEERS IN INTERGENERATIONAL PROGRAMS IN EDUCATIONAL SETTINGS ACROSS THE GLOBE

Youjung Lee,[1] Young-Mi Kim,[2] Laura Bronstein,[3] and Vince Fox,[4] *1. Binghamton University, State University of New York, Binghamton, New York, United States, 2. Dong-Eui University, Busan, Republic of Korea, 3. Binghamton University, Binghamton, New York, United States, 4. Broome County Office for Aging, Binghamton, New York, United States*

Volunteerism is a global phenomenon that aids multiple generations. Considering the positive evidence of volunteering among older adults and their desire for intergenerational engagement, it is important to explore older adult volunteers'

experiences in intergenerational programs with a specific focus on the cultural and social impacts of volunteering in educational settings in later years. Using a phenomenological qualitative approach, 43 interviews with older adult volunteers (23 in Korea and 20 in the USA) in intergenerational programs were conducted. Participants were recruited from the Beautiful Story Grandma (BSG) in Korea and the Foster Grandparent Program (FGP) in the USA in 2019. Due to the prescribed nature of the BSG, all of the Korean volunteers were female. The USA volunteers from the FGP included three African Americans, one Asian, and 16 White older adults. Two FGP volunteers were male. Korean participants lived primarily in Busan Metropolitan city (mean age: 63, range: 61-73). The USA volunteers were from urban/suburban areas New York State (mean age: 74, range: 60-84). Two major themes emerged from the interviews revolving around the role of culture and other demographics in the experience of volunteering: (1) Older adults experience benefits from volunteering to support the younger generation that transcend demographic and geographic differences; and, (2) distinctive cultural views of education lead to different experiences of volunteering in the two countries. The comparative research highlights the need for development of a model for culturally responsive practice with older adult volunteers in a global context.

### OLDER COUPLES' MARITAL QUALITY AND HEALTH BEHAVIORS

Angela Curl, Jennifer Bulanda, and
Amy Restorick Roberts, *Miami University, Oxford, Ohio, United States*

Health benefits of marriage may stem in part from spouses discouraging unhealthy behavior and encouraging healthy practices. Although studies show spousal effects on health behaviors, few have assessed whether spousal effects vary by the quality of the marital relationship. Spouses in low-quality marriages may be less likely to engage in joint activities that promote health (e.g., shared exercise), make fewer attempts at monitoring their spouse's health behaviors, and be less successful in their attempts to intervene. Those in unhappy relationships may also use unhealthy behaviors as maladaptive coping strategies to deal with marital stress. We use dyadic data from couples over age 50 in the 2006 and 2008 waves of the Health and Retirement Study (HRS) to examine how both spouses' ratings of positive and negative dimensions of marital quality are associated with their own and their spouses' exercise and smoking (n=3,498 couples). Using HLM software, we estimated actor-partner interdependence models (APIM). Results indicate that both own and husbands' ratings of positive marital quality are significantly associated with wives' odds of smoking. Own perceptions of negative marital quality and wives' perceptions of both positive and negative marital quality are associated with husbands' odds of smoking. For wives, neither own nor spousal marital quality is significantly related to exercise. For husbands, however, wives' higher positive marital quality and lower negative marital quality are associated with increased exercise. Strategies to improve marital quality may promote healthy behaviors among older adults, particularly for husbands.

### RELATIONSHIP QUALITY AND DEPRESSIVE SYMPTOMS AMONG VERY OLD PARENTS AND THEIR OLDER CHILDREN IN SOUTH KOREA

Gyounghae Han,[1] Joohong Min,[2] Joohyun Kim,[1]
Kyungmin Kim,[3] and Kathrin Boerner,[3] *1. Seoul National University, Seoul, Republic of Korea, 2. Jeju National University, Jeju, Republic of Korea, 3. University of Massachusetts Boston, Boston, Massachusetts, United States*

Research has consistently reported the association between intergenerational relationship quality and mental health outcomes in later life. However, few studies have examined the link among very old parents and their older children, and even fewer studies investigated whether the relationship quality matters similarly to parents and children. Employing a dyadic approach, this study examined how one's own and partner's perceptions of relationship quality (i.e., support and conflict) are associated with depressive symptoms among very old parents and their children. Data from 105 dyads of parents (age 81-97; $M = 87.92$, $SD = 2.80$) and their children (age 65-72; $M = 65.87$, $SD = 1.23$) in South Korea were used. Results showed that parents tended to report significantly higher levels of intergenerational support and lower levels of intergenerational conflict, compared to their children. Regarding the actor effects of relationship quality, one's own perceptions of intergenerational conflict were positively associated with depressive symptoms for both parents ($\beta = 0.26$, $p < .01$) and children ($\beta = 0.37$, $p < .001$), whereas intergenerational support was not significant. In terms of the partner effect, intergenerational support (reported by parents) was negatively related to depressive symptoms only for children ($\beta = -0.21$, $p < .01$), but the partner effect of conflict was not significant. The findings highlight the centrality of perceptions of intergenerational relationship in understanding well-being in later life. Further, children's depressive symptoms were susceptible to how their parents view the relationship. Findings were discussed in the context of Intergenerational Stake Theory.

### RESPECT YOUR ELDERS: THE RELATION BETWEEN GENERATIVITY AND LIFE SATISFACTION IN CUSTODIAL GRANDPARENTS

Rachel Scott,[1] Danielle Nadorff,[2] Loriena Yancura,[3] and
Melissa Barnett,[4] *1. Mississippi State University, Starkville, Mississippi, United States, 2. Mississippi State University, Mississippi State, Mississippi, United States, 3. University of Hawai'i, Honolulu, Hawaii, United States, 4. The University of Arizona, Tucson, Arizona, United States*

In Erikson's (1950) theory of psychosocial development, generativity is defined as the drive to benefit future generations and leave a legacy. The prototypical individual satisfies this generative desire through parenthood in midlife. This stage has been shown to expand into later life due to grandparenthood (Erikson, 1982). Generativity has been shown to predict life satisfaction, but the amount of generative concern and action can be impacted by factors such as perceived respect from younger generations (Cheng, 2009). The current study examined adults aged 40 and older (M age = 61 yr) using a nationwide sample from Qualtrics Panel Service to assess the extent to which perceived respect moderates the relation between generativity and life satisfaction in custodial grandparents. Results indicated that perceived respect from the grandchild was found to have a moderating effect on the relation between generativity of custodial grandparents and life satisfaction. Results suggest that for those who perceive low levels of respect from their grandchildren, the more generativity they express, the lower their life satisfaction. For those who perceive higher levels of respect from their grandchildren, the more generativity they express, the higher their life satisfaction. These findings suggest that as attempts to be generative increase, life satisfaction fluctuates. This may in turn impact the likelihood of generative actions from the custodial grandparent.

### ROLE CENTRALITY AND SHARED ACTIVITIES WITH GRANDCHILDREN: EFFECTS ON GRANDPARENT DEPRESSION

Madeline Marello, and Julie Patrick, *West Virginia University, Morgantown, West Virginia, United States*

Research shows that physical and mental health are closely linked (Ohrnberger, Fichera, & Sutton, 2017). Further, social role theory states that holding and enacting valued roles, such as grandparenting, can buffer the negative effects of health on depression (Reitzes & Mutran, 2004). Using data from 247 grandparents (Mean age = 66.5; range 42 to 90 years; 46.2% grandfathers), we examined the differences between 164 custodial and 83 traditional grandparents on whether grandparent role centrality and engagement with grandchildren altered the effects of physical health on depression. The multigroup moderated moderation model was significant (X2(DF=30, N = 247) = 1610.78, $p < .001$; R2 = .797). We examined whether the paths were moderated by custodial status. Among custodial grandparents, role centrality ($\beta$ = -.482**) and shared activities ($\beta$ = -.493***) were significant predictors of depressive symptomatology. Moreover, the interaction between physical health and activities ($\beta$ = .488***) and between physical health and role centrality ($\beta$ = .522**) also accounted for significant variance among custodial grandparents. Custodial grandparents in poorer health who valued the grandparent role and those in poorer health who engaged with their grandchildren experienced fewer depressive symptoms. No such patterns were observed for traditional grandparents. Although we had anticipated that the interaction between role centrality and engagement with grandchildren would predict depressive symptoms, the interaction did not reach significance. Results are discussed in terms of the need to examine the differences of family/social contexts in grandparent populations.

### THE EFFECTS OF GRANDCHILD CARE ON MENTAL HEALTH AMONG CHINESE ELDERLY: THE MEDIATING EFFECTS OF SOCIAL NETWORKS

Dan Tang,[1] Jie Qiu,[2] and Kun Zhang,[1] *1. Renmin University of China, Beijing, China, 2. Qingyuan Polytechnic, Qingyuan, China*

Using the data of 2014 baseline survey of the China Longitudinal Aging Social Survey (CLASS), which provides a sample of older Chinese who had grandchild younger than 18 years old, this study examines the associations among grandchild care, social networks, and depressive symptoms among Chinese older adults. The older adults are divided into three groups basing on the frequency of their behaviors of taking care of grandchildren. The three groups are 'no care, providing care occasionally, providing care frequently'. The mediating and moderating effects of social networks between grandchild care and depressive symptoms are tested. Results show that older adults who provide grandchild care report superior social networks and better mental health than those who don't provide grandchild (reference group). After controlling the related variables, the older adults who provide grandchild occasionally benefit more than those who take care of grandchild frequently. Grandchild care is related to larger social networks, and the social networks are fully mediating the association between grandchild care and depressive symptoms.

### THE IMPACT OF TAKING CARE OF GRANDCHILDREN ON HEALTH OUTCOMES IN JAPANESE COMMUNITY-DWELLING ELDERLY

Yoshinori Fujiwara,[1] Kumiko Nonaka,[2] Masataka Kuraoka,[1] Sachiko Murayama,[1] Yuta Nemoto,[3] Hiroshi Murayama,[1] Yoh Murayama,[1] and Erika Kobayashi,[1] *1. Tokyo Metropolitan Institute of Gerontology, Tokyo, Japan, 2. Tokyo Metropolitan Institute of Gerontology, Tokyo, Japan, Tokyo, Japan, 3. Tokyo Metropolitan Institute of Gerontology, Tokyo, Tokyo, Japan*

Taking care of grandchildren may provide health benefits to older adults due to keeping their social roles and feeling more generative; however, we have scarce knowledge of the relationships in Asian countries. This study addressed this question in older Japanese. The data was obtained from a two-year follow-up mail survey conducted in 2016 on 3,116 randomly selected older Japanese, aged 65-84 years, living in a metropolitan area. The main outcome was deterioration of health assessed by the Self-Rated Health (SRH), WHO-5, and Instrumental Activities of Daily Living (IADLs), defined as decline in 1 or more points obtained after 2 years of follow-up. The frequency of taking care of grandchildren was assessed as every day, 4-6 days per week, 1-3 days per week, 1-3 days per month, several days per year, and none. A multiple linear regression examined the impact of taking care of grandchildren as a predictor of protection of decline in SRH, WHO-5 and IADLs. The models were adjusted for confounding factors. Of 1,561 who responded to the follow-up survey, 959 people had grandchildren at baseline. The subjects had a mean age of 73.2±5.3 years, and mean scores of SRH:2.1±0.6; WHO-5;16.1±5.3, IADLs; 4.9±0.6 (higher scores represent higher evaluation). The higher frequency of taking care of grandchildren were longitudinally associated with less decline in SRH, WHO-5, and IADLs (standardized partial regression coefficient, $\beta$=-0.090, p=0.013; $\beta$ =-0.023, p=0.547; $\beta$ =-0.107, p=0.008, respectively). In conclusion, taking care of grandchildren might be a protective factor of comprehensive and functional health deterioration.

### THE ROLE OF INTERGENERATIONAL RELATIONSHIPS: APPLYING THE FAMILY STRESS MODEL TO GRANDFAMILIES

Rachel Scott,[1] Danielle Nadorff,[2] Loriena Yancura,[3] and Melissa Barnett,[4] *1. Mississippi State University, Starkville, Mississippi, United States, 2. Mississippi State University, Mississippi State, Mississippi, United States, 3. University of*

*Hawai'i, Honolulu, Hawaii, United States, 4. The University of Arizona, Tucson, Arizona, United States*

The Family Stress Model (FSM) of Economic Hardship (Conger, Rueter, & Conger, 2000) was developed to explain the impact of financial stress on families through links between economic difficulties, parental emotional distress, marital conflict, disrupted parenting behaviors, and child maladjustment. The FSM has been cross validated in samples of custodial grandparents (i.e., grandparents who provide substantial care for their grandchildren; Smith et al., 2017). The current study modified the FSM by replacing inter-parent relationship difficulties with inter-generational relationship problems between the custodial grandparents and their children to ultimately examine the adjustment of the grandchildren. This change to the model is supported by prior research conducted on intergenerational stress impacting the parenting and subsequent development of children in grandfamilies (Barnett, Mills-Koonce, Gustafsson, & Cox, 2012). Using a nationwide sample of 317 custodial grandparents aged 40 and older (M = 61 yr) the fit of the modified model was tested using AMOS 26. Latent variables in the model included Economic Pressure, Caregiver Distress, Disrupted Parenting, Intergenerational Relationship, and Child Adjustment. Moderate fit was achieved ($\chi 2(308) = 574.88$; CFI = .896; RMSEA = .052). All pathways were significant with the exception of Disrupted Parenting to Child Adjustment. These results indicate that intergenerational relationships are an important predictor of child adjustment, and an applicable substitute for inter-partner relationships when modeling family stress in custodial grandfamilies. Details and clinical implications will be discussed.

### TOO MANY TREATS OR NOT ENOUGH TO EAT? THE IMPACT OF CUSTODIAL GRANDPARENTS ON FOOD SECURITY AND NUTRITION

Danielle Nadorff, and Rahel Mathews, *Mississippi State University, Mississippi State, Mississippi, United States*

In the US, 28.5% of seniors are obese, with a BMI of 30 plus. The prevalence of obesity for children is also an alarming 17%, making it one of the primary public health burdens. According to the socio-ecological model, a child's weight status can be influenced by factors related to parenting style, family, and the community. The literature reflects a significant emphasis focusing on children and their parents. However, according to the US Census, 7.5 million grandchildren are living with their grandparents, with about 1/3 of these residing in skipped-generation households. There are essential age-related differences in food preparation and eating behaviors between middle-aged and older grandparents and younger adult parents that may influence their children's eating behaviors. Grandparents may provide a positive feeding environment, including role-modeling healthy food intake, teaching children about nutrition, and involving them in mealtimes and cooking, monitoring and encouraging children to eat nutritious foods, especially vegetables and regularly serving vegetables. However, grandparents have also reported providing energy-dense and nutrient-poor food and drinks and used food as a reward or gift. The current study aims to investigate the influence of caregiver type (grandparents only, parents only, or multigenerational households) on children's nutrition, food security, and BMI. One-way ANCOVAs controlling for SES found that grandparent-headed households had children with more deficient diet and higher BMIs, but also less food insecurity. These results indicate that age-related changes in caregiver type are an important predictor of children's nutritional health. Details and clinical implications will be discussed.

### SESSION 2944 (POSTER)

### CAREGIVING I (BSS)

### A SYSTEMATIC REVIEW OF INTERVENTIONS THAT REDUCE FAMILY AND FRIEND CAREGIVING TIME

Zachary Baker,[1] Eric Jutkowitz,[2] and Joseph Gaugler,[1]
*1. University of Minnesota, Minneapolis, Minnesota, United States, 2. Brown University, Providence, Rhode Island, United States*

The decreasing number of family/friend caregivers available to help the rising number of older adults is creating a critical family care gap. For this reason, there is a growing need for interventions that reduce family/friend caregiving time. We systematically reviewed five electronic databases to identify randomized trials, case control, quasi-experimental, and cross-sectional studies that evaluated a modifiable element that could be targeted for interventions with care recipients 65+ and/or their family/friend caregivers and reported on an outcome of time spent caregiving. We excluded studies without a comparison, broadly defined. The initial search included 1,812 unique records. Following abstract and title screening 311 full-texts were reviewed. Fifty-five studies published between the years of 1990 and 2019 met inclusion criteria. Studies predominantly focused on care recipients with dementia (58%) and were largely conducted in western countries (91%). The categories of interventions reviewed included pharmaceuticals (25%), public long-term care financing (7%), case management (7%), care setting (16%), technology (7%), multi-component interventions (9%), skills building (15%), additional formal expertise/care (9%), and other (5%), with one study falling into multiple categories. Pharmaceuticals, case management, care setting, and multi-component interventions demonstrated promising evidence to reduce family/friend caregiving time. Methodologically, studies were inconsistent in measurement and ascertainment of caregiving time. Given the public health concerns of reduced availability of family/friend caregivers for older persons in the upcoming decades, caregiving interventions should consider measurements of caregiving time as key outcomes.

### CAREGIVER DEPRESSION, CHANGE IN THE SEVERITY OF NEUROPSYCHIATRIC SYMPTOMS, AND SUBSEQUENT CAREGIVER ABUSE

Boye FANG,[1] Elsie Yan,[2] Yaolin Pei,[3] and Gengzhen Chen,[4]
*1. Sun Yat-Sen University, Guangzhou, China, 2. The Hong Kong Polytechnic University, Hong Kong, China, 3. New York University, New York, New York, United States, 4. Shantou University Medical College, Shantou, China*

Objectives: To examine whether caregiver (CG) depression predicts subsequent occurrence of elder abuse and whether change in the severity how care-recipient change in the severity of care recipient (CR) neuropsychiatric symptoms influence this association. Methods: Using two-year longitudinal data, we analyzed a consecutive sample of 800 Chinese family CGs and their CRs with dementia recruited from the geriatric and neurological departments of three Grade-A hospitals in People's Republic of China (PRC). All the participatory dyads were assessed between September 2015 and February 2016 and followed for two years. Results: CG depression at baseline predicted the subsequent occurrence of physical and psychological abuse and CG neglect. However, change in the severity of CR neuropsychiatric symptoms altered these relationships. Specifically, while unchanged and increased CR neuropsychiatric symptoms heightened the positive effect of CG depression on subsequent abuse, decreased CR neuropsychiatric symptoms protected older adults from abuse by a depressed CG. Conclusion: This study showed the differential impact of CG depression on subsequent occurrence of elder abuse depending on the change in the severity of neuropsychiatric symptoms related to the CRs. The present findings provide important insights to the design of a systematic and integrative intervention protocol for elder abuse that simultaneously focuses on treating CG depression and CR neuropsychiatric symptoms.

### CYCLE OF DISADVANTAGES: AN EXPLORATION OF YOUNG AND OLDER FEMALE DEMENTIA CAREGIVERS' EXPERIENCES

Kafayat Mahmoud,[1] and Candidus Nwakasi,[2] *1. University of Kansas, Lawrence, Kansas, United States, 2. University of Southern Indiana, Evansville, Indiana, United States*

Families play an essential role in providing care for older persons in Nigeria, a responsibility that is culturally assigned to women. Caregiving is made even more difficult given the increasing rates of older persons with dementia and the limited capacity of caregivers to provide adequate care. We explored the experiences of two female dementia caregivers and how they cope with other competing responsibilities. The research method employed a case study approach, which followed the lived experiences of two Nigerian women – a 75-year-old woman (Ada) who cares for her husband, and a 35-year old woman (Chika) who cared for her father. The research adopted the life course concept of 'linked lives' and the Family Stress Process model to explore and identify the continued embeddedness of persons within their family networks and relationships, which may have deleterious effects on the wellbeing of caregivers. This study revealed that married women providing care to older parents are exposed to domestic violence from their spouses who deem them uncommitted to their (husbands') families. These women continue to remain in such abusive relationships due to financial dependence and unwillingness to leave their children. Older women caregivers, on the other hand, over time, begin to develop physical limitations, which limit their ability to provide care for their spouses and subsequently must depend on unemployed children who live close by. This study highlights the complicated cycle of exposure to socio-economic and health disadvantages which women experience as caregivers across two generations.

### FAMILY AVAILABILITY AND ITS INFLUENCE ON INFORMAL AND FORMAL CARE USED BY ADULTS WITH DEMENTIA IN THE UNITED STATES

HwaJung Choi,[1] Michele Heisler,[2] Tsai-Chin Cho,[2] and Cathleen Connell,[1] *1. University of Michigan, Ann Arbor, Michigan, United States, 2. University of Michigan, Ann Arbor, United States*

This research is to provide national estimates of spouse and adult child availability to care for adults 55+ with dementia and to examine associations between availability and formal and informal care utilization. Only 23% of adults with dementia had a non-disabled spouse; 66% had an adult child living less than 10 miles away. Substantial variations in family availability were discovered across demographic and socioeconomic groups. For example, 29% of non-Hispanic blacks vs. about 40% of other racial/ethnic groups (OR=0.63; p<0.001) had a spouse. Only 16% of the bottom wealth quartile had a spouse compared to 61% of the top quartile (OR=0.13; p<0.001). In contrast, the greater share of non-Hispanic blacks than non-Hispanic whites had a coresident adult child (OR=2.07; p<0.001) and a non-employed adult child (OR=1.45; p<0.001). Hispanics had the most family availability from both spouse and child. Having a spouse was significantly associated with a lower probability of receiving formal care; AOR=0.54 (95% CI 0.46-0.64) for any formal care; AOR=0.50 (95% CI 0.39-0.64) for institutional care. Having a coresident adult child in year T-2 also substantially reduced the probability of receiving formal care in year T (AOR=0.37; 95% CI 0.29-0.48). The presence of a spouse and co-resident adult child significantly reduce the use of formal care by adults with dementia in the US. Policies and interventions that rely on family members to provide dementia should reflect the substantial heterogeneity in potential family availability across racial/ethnic and socioeconomic groups.

### MALE FAMILY CAREGIVERS' WELL-BEING: BALANCING WORK AND CARE FOR PARENTS IN JAPAN

Hiroko Umegaki-Costantini, *SciencesPo, Paris, France*

Despite substantial discussions on caregivers in policy making and academic research, a rapidly emerging new group of middle-aged male family caregivers who often also work are largely understudied. Inadequate understanding of male family caregivers jeopardises not only themselves but also care recipients, other family members and society: better care for older people depends on caregivers' well-being. I address this gap by understanding how and why gender affects male family carers' choices of care practices, and how male caregivers as 'gendered beings' sustain their well-being while reconciling work and care for their parents. Through in-depth-qualitative narrative interviews based on social anthropology of everyday life with 23 male working family carers in Tokyo in 2019, I find that men select care practices with priority given to familiar tasks deemed appropriately masculine. To perform 'appropriate' care for their parents, such men tend to approach care as they do their 'work': they 'work-nise' care. Such a gendered approach to care practices has important implications for addressing gender in public interventions. Also, I find that for male family working carers, achieving well-being does not mean balance in work, care and family. Rather, for them a source of well-being is from fulfilling filial duty as sons, which, however, requires making an appropriate effort and devoting themselves to some extent to support their parent(s). Thus, such men need to be embedded in a care and employment system that enables them to achieve such well being that, in turn, significantly impacts their care provision.

### MILLENNIALS AS CAREGIVERS: RESULTS FROM THE BRFSS, 44 STATES, DC, AND PUERTO RICO, 2015-2018

Nia Reed,[1] Erin Bouldin,[2] Christopher Taylor,[1] and Lisa McGuire,[1] *1. Centers for Disease Control and Prevention, Atlanta, Georgia, United States, 2. Appalachian State University, Boone, North Carolina, United States*

Millennials (born 1981-1996), now outnumber Baby Boomers. Millennials have the unique task of potentially providing care for their own children, their aging parents, and grandparents, making them the first "club sandwich" generation. We utilized data from 44 states, the District of Columbia, and Puerto Rico from the 2015-2018 Behavioral Risk Factor Surveillance System to estimate the prevalence of informal caregiving among Millennials (based on age at the time of survey completion: 18-34 in 2015, 19-35 in 2016, 20-36 in 2017, and 21-37 in 2018). We conducted log-binomial regression analyses to compare mental and physical health among Millennials, adjusting for age, sex, education, and race/ethnicity. Among 37,780 Millennials included, 18.7% provided regular care/assistance to a friend/family member with a health problem, long-term illness, or disability in the past month. Of these caregivers, 36.3% provided the most care to a parent/parent-in-law and 21.8% to a grandparent. Half of Millennials had ≥1 children under age 18 in their home, regardless of caregiving status. In adjusted models, Millennial caregivers had higher prevalence of frequent mental distress [≥14 days in the past 30 days when mental health was not good] (prevalence ratio [PR]=1.72, 95% confidence interval [CI]: 1.50-2.00, $p<0.001$), higher prevalence of fair/poor health (PR=1.42, 95%CI: 1.20-1.68, $p<0.001$), and higher prevalence of ≥1 chronic health conditions [current asthma, arthritis, cancer, cardiovascular disease, diabetes, or COPD] (PR=1.69, 95%CI: 1.51-1.90, $p<0.001$) than non-caregivers. Nearly one in five Millennials was providing informal care, and Millennial caregivers reported significantly worse mental and physical health than their non-caregiving peers.

### PARTNER EFFECTS ON DEPRESSED MOOD IN CAREGIVING DYADS ARE MOST PRONOUNCED IN CANCER CAREGIVING, PARTNER CAREGIVING

Nadia Al Nassar and Kristin Litzelman, *University of Wisconsin-Madison, Madison, Wisconsin, United States*

Theory and empirical evidence indicate that the well-being of caregivers and their care recipients is interrelated, although conflicting evidence has emerged across different caregiving populations. To establish a more nuanced understanding of this phenomenon, we used data from the National Health and Aging Trends Survey and the National Survey on Caregivers (2015 and 2017, n=759 dyads with complete longitudinal data) to construct actor-partner interdependence models assessing how spillover of depression varies by care recipient health condition (cancer, dementia, stroke, diabetes, or other conditions) and relationship type (spouse/partner, child, or other). Across condition types, the largest magnitude partner effects were observed in dyads with cancer, in which a one-point increase in caregiver depressed mood was associated with a 0.23-point increase in subsequent care recipient depressed mood ($p=0.02$) and a one-point increase in care recipient depressed mood was associated with a 0.33-point increase in subsequent caregiver depressed mood ($p<0.01$). Moderation by cancer status was statistically significant (pinteraction=0.03). Among spouse/partner caregivers, caregivers' depressed mood was associated with subsequent depressed mood in the care recipient ($p<0.05$) but there was no evidence of spillover from the care recipient to the caregiver. Conversely, in both adult child caregivers and other caregivers, there was evidence for spillover from the care recipient to the caregiver ($p<0.05$) but not the reverse. The findings show that the interrelationship in the well-being of caregivers and care recipients varies by key caregiving characteristics, with implications for the development, dissemination, and implementation of interventions targeting caregiver, care recipient, and dyadic well-being.

### PERCEPTIONS OF CAREGIVING: RACIAL EXAMINATIONS OF RESILIENCY AND STRESS

Athena Koumoutzis,[1] and Kelly Cichy,[2] *1. Miami University, Oxford, Ohio, United States, 2. Kent State University, Kent, Ohio, United States*

Adult children are at risk of emotional strain when parental caregiving needs emerge. Pearlin's Stress Process Model (1990) and caregiver studies suggest minority caregivers report lower subjective caregiving burden, however, few studies simultaneously consider both the stresses and rewards of caregiving. Using data from Wave II of the Family Exchange Study (N = 243), we examine racial differences in midlife adults' perceptions (i.e., stress and rewards) of assisting their parents with activities of daily living (ADLs) and the associations between perceptions of ADL assistance and emotional well-being among adults who help their parents with ADLs. Compared to non-minority caregivers (M = 4.18, SD = 0.91), minority caregivers (M = 4.45, SD = 0.84) found it more rewarding to help their mother ($t(314) = -2.54$, $p < .05$), whereas non-minority caregivers (M = 2.25, SD = .1.27) found it more stressful to help their father than did minority caregivers (M = 1.64, SD = 0.99), $t(162) = 3.01$, $p < .01$). After controlling for demographics and ADL needs, linear regression analyses revealed that the stress of helping parents predicted depression ($F(6, 189) = 5.30$, $p < .001$) and race moderated the association ($p < .01$); the association was only significant for minority caregivers ($p < .05$). Implications will be discussed.

### PSYCHOLOGICAL WELL-BEING AND POSITIVE ASPECTS OF CAREGIVING: WHAT'S THE RELATIONSHIP?

Kelly O Malley,[1] and Sara Qualls,[2] *1. New England GRECC, VA Boston, Boston, Massachusetts, United States, 2. University of Colorado Colorado Springs, Colorado Springs, Colorado, United States*

Many family caregivers derive meaning and purpose from caring for a loved one. Research on the mechanisms underlying positive aspects of caregiving have explored appraisal, reciprocity, resilience, gratitude, mastery, and appreciation. This study aimed to explore how individual personality factors, using the framework of psychological well-being (i.e., autonomy, positive relationships, purpose, personal growth, self-acceptance, and environmental mastery) proposed by Ryff (1989), may influence positive aspects of caregiving. N = 452 family caregivers completed informed consent, provided demographic information, and completed study measures using an online research platform. Multiple regression equations were calculated to assess which aspects of psychological well-being predicted two positive aspects of caregiving: competence and personal gain. The model explained 22% of the variance in caregiving competence, $F(6, 445) = 21.01$, $p < .001$. Autonomy ($\beta = .26$, $p < .001$) and self-acceptance ($\beta = .18$, $p = .02$) contributed most significantly to feelings of competence. The model explained 14% of the variance in personal gain, $F(6, 445) = 12.11$, $p < .001$. Self-acceptance ($\beta = .19$, $p = .02$), personal growth ($\beta = .18$, $p = .01$) and autonomy ($\beta = .13$; $p = .03$) contributed most significantly feelings of personal gain. Psychological well-being, specifically feelings of autonomy and self-acceptance, are modest predictors of feelings of competence and gain, and psychological well-being appears to have an effect on positive aspects of caregiving. Interventions for family caregivers should consider ways of building autonomy, self-acceptance, and growth to increase feelings of competence and gain.

### RECRUITING RACIALLY AND ETHNICALLY DIVERSE DEMENTIA FAMILY CAREGIVERS FOR SURVEY RESEARCH

Kyra Mendez, and Hae-Ra Han, *Johns Hopkins University School of Nursing, Baltimore, Maryland, United States*

Recruiting racially and ethnically diverse dementia family caregivers (FCGs) for research can be challenging. The purposes of this presentation are to describe methods of successfully recruiting racially and ethnically diverse dementia FCGs for survey research and to share lessons learned. This study aimed to recruit dementia FCGs who have a chronic health condition and access to a mobile device. FCGs were primarily recruited from the Baltimore-Washington Metropolitan area to complete an online or phone survey about their technology use. A variety of recruitment methods were used including: posting ads in a local newspaper targeting older adults, partnering with a local Alzheimer's research center and memory treatment center, attending community events, using online research registries, and posting online advertisements. The most successful method of recruiting minorities was by attending community events for caregivers and talking directly with community members about the research study. Online recruitment methods were less successful, but yielded the greatest diversity of participants, including Asian, Native American, mixed race, and African American FCGs. Some challenges associated with recruiting minority FCGs were working with primarily immigrant communities; recruiting FCGs who do not speak English; and building trust among communities that have been negatively impacted by research. Suggestions for future research include using recruitment strategies that enable researchers to build rapport with FCGs, engaging community stakeholders to understand your source population, and using a variety of recruitment methods. Also, online recruiting through credible sources appears to be a somewhat feasible method of recruiting diverse FCGs for survey research.

### RESILIENCE AND SERVICE NEEDS AMONG AFRICAN AMERICAN CAREGIVERS OF PEOPLE WITH DEMENTIA: A PILOT MIXED-METHODS STUDY

Heehyul Moon,[1] Sunshine Rote,[1] Hallie decker,[2] Burton Kelsey,[3] Chelsea Burton,[2] Sharon Moore,[4] and tewsdaay babicka,[2] *1. University of Louisville, Louisville, Kentucky, United States, 2. u of l, Louisville, United States, 3. U OF L, LOUISVILLE, United States, 4. u of l, louisville, United States*

African American caregivers face unique challenges and strengths in addressing the needs of dementia care recipients. The purpose of the current study was to explore the roles of the familism and faith and service preferences and needs for African American dementia caregivers. Through collaborative partnerships among the research team, Alzheimer's' Association, Area Agency on Aging, and local churches, we obtained focus group and survey data (N=30) from African American dementia caregivers. Most caregivers were female (90%) and older than 51 years and had provided care more than 3 years. CGs showed strong cultural reasons (e.g., faith, duty, paying back) for providing care (range 0-40;M= 34.5; SD= 5.2). Focus groups data, which was audio taped, transcribed, and analyzed by three researchers using content analysis, revealed three major themes related to dementia care experiences and needs. First, caregivers explained positive aspects of caregiving for both the individual caregiver and family (growth in faith, sense of self -efficacy, feeling of gratefulness). Second, caregivers also described negative aspects that pose substantial challenges such as lack of services, lack of balance in life, family conflicts over care, and mistrust based on their previous experiences with existing health care systems. Third, caregivers described their knowledge of dementia and shared their self-care and coping strategies and a need for support group services. The findings show an urgent need to implement culturally responsive services, especially in undeserved areas and populations, for caregivers to maintain or improve their emotional well-being and quality of care as well as family relationships.

### ROLE OVERLOAD, PERCEIVED GRATITUDE, AND HEALTH AMONG SPOUSAL CAREGIVERS: A LONGITUDINAL STUDY

Suyoung Nah,[1] and Lynn Martire,[2] *1. The Pennsylvania State University, State College, Pennsylvania, United States, 2. The Pennsylvania State University, University Park, Pennsylvania, United States*

Spouses are likely the most vulnerable caregivers due to their advanced age and more intense caregiving than other family members. Thus, it is crucial to identify risk and protective factors for spousal caregivers' health. According to Pearlin's stress process model, caregivers' subjective stressors and resources may impact their health. We focused on spousal caregivers' role overload, a critical indicator of subjective stressors, and their perception that the partner is grateful for their help, which may be beneficial for their health but has rarely received attention. This study tested the hypotheses that spousal caregivers' higher role overload and lower perceived gratitude are associated with poorer physical and mental health over time. We also examined whether greater perceived gratitude buffers the negative relationships between role overload and health. We focused on 223 spousal caregivers of older adults without dementia from the 2015 and 2017 National Study of Caregiving. Autoregressive models revealed that spousal caregivers' higher role overload at baseline was associated with poorer self-rated health at follow-up (b = -0.23, p < .05), but not with depressive symptoms or anxiety. Spousal caregivers' greater perceived gratitude at baseline was associated with lower anxiety at follow-up (b = -0.32, p < .01). There were no moderating effects of perceived gratitude on the relationships between role overload and health. These findings suggest that spousal caregivers' role overload is a risk factor for their physical health, while their perception that the partner is grateful for their help serves as a protective factor for their mental health.

### SPOUSAL ACTIVITY LIMITATIONS AND DEPRESSIVE SYMPTOMS: BENEFITS OF SPOUSAL CAREGIVING AND COSTS OF SPOUSAL PAIN

Sae Hwang Han,[1] Kyungmin Kim,[2] and Jeffrey Burr,[2]
*1. University of Texas at Austin, Austin, Texas, United States, 2. University of Massachusetts Boston, Boston, Massachusetts, United States*

Experiencing difficulties in performing basic activities of daily living poses significant challenges for older adults living with such limitations and also for their spouses. A growing body of evidence demonstrates cross-spousal linkages between activity limitations and depressive symptoms. However, under what conditions these linkages may be strengthened or weakened has received little attention in the literature. We addressed this gap by examining whether a) providing spousal caregiving and b) spousal pain moderated the link between spousal activity limitations and one's own depressive symptoms. We used seven waves of longitudinal household data from the Health and Retirement Study (2004-2016; N=12,369) to estimate within-person associations between spousal activity limitations and depressive symptoms, focusing on the moderating roles of caregiving behavior and spousal pain. In particular, asymmetric fixed effects models were used to estimate the unique effects of transitioning into a spousal caregiver role in the context of spousal activity limitations. Results from multilevel models were gendered. For wives (but not for husbands), transitioning into a caregiver role to provide spousal care alleviated depressive symptoms associated with spousal activity limitations, whereas depressive symptoms were increased when husbands with activity limitations also reported frequent, moderate to severe pain. Our findings indicate that the link between spousal activity limitations and depressive symptoms is not uniform, and that the cross-spousal association may best be understood when relevant contextual factors are considered. The findings are also in line with recent studies showing that caregiving may also lead to enhanced well-being and reduced mortality risk under some circumstances.

### THE DYADIC EFFECTS OF PERCEIVED SUPPORT ON DEPRESSION IN SPOUSAL CARE PARTNERS

Kylie Meyer,[1] Neela Patel,[1] and Carole White,[2]
*1. UT Health Sciences at San Antonio, San Antonio, Texas, United States, 2. UT Health San Antonio, San Antonio, Texas, United States*

Relationship quality is an important factor affecting care partners' health and wellbeing. Supportive marital relationships are associated with better physical and subjective health, whereas strain is associated with poorer health. Recent studies now indicate a dyadic effect of relationship quality on health outcomes, such that an individual's perceptions of their relationship also affects their partner's outcomes. Few studies have examined the dyadic effects of relationship quality on mental health among older cognitively intact caregiving couples. To address the lack of dyadic research about how perceived support from one's spouse related to experiences of depression for individuals and their care partners, we apply cross-sectional actor partner interdependence models (APIMs) to data from the Health and Retirement Study (HRS) (N=490 dyads). APIM regression models controlled for participant demographic characteristics, relationship length, and care recipient functional ability. Findings showed that positive perceived support from a spouse had a stronger negative association with one's own depression for care recipients than for caregivers. Similarly, greater negative perceived support from a partner was associated with higher levels of depression; whether the partner was the caregiver or care recipient did not make a difference in this model. Although there are hundreds of caregiver interventions to address caregivers' mental health, few have demonstrated improvement in care recipient outcomes. Observation of both actor and partner effects in this study suggests there may be opportunities to improve care recipient and caregiver mental health by targeting interventions to promote high quality relationships with caregivers or both members of the care dyad.

### THE EFFECTS OF TRANSITIONS IN CAREGIVING AND CHANGES IN SOCIAL PARTICIPATION ON OLD ADULTS' DEPRESSIVE SYMPTOMS

Xinyi Zhao,[1] Huiying Liu,[2] and Quan Zhang,[3],*1. Peking University, Beijing, China, 2. Central South University, Changsha, Hunan, China, 3. Peking University, Beijing, China, China*

Objectives: This study aimed to investigate the effects of transitioning into spousal caregiving, changes in social participation, and their interactions on depressive symptoms among community dwelling old adults over time. Methods: The samples included old adults who were non-caregivers at 2011 baseline of China Health and Retirement Longitudinal Study (CHARLS) and joined the follow-up surveys in 2013 and 2015. Generalized estimating equations (GEE) was used to analyze the effects of caregiving transitions (transitioning into low-intensity or high-intensity caregivers versus the non-caregivers) and changes in social engagement on the depressive symptoms over time. Results: The results showed that old adults who transitioned into spousal caregiving over a 4-year period reported more depressive symptoms than those remained non-caregivers. Old adults who continued or increased social participation reported fewer depressive symptoms than those without social participation. Individuals who continued social participation during the transitions into high-intensity caregiving showed less severe elevated depressive symptoms than their counterparts who did not engage in social participation. Conclusion: The results highlighted that continuous social participation might be a protective factor for old adults against negative psychological outcomes during the transition to high-intensive caregiving.

### THE PATTERNS OF CAREGIVING ACTIVITIES FOR FAMILY CAREGIVERS OF OLDER ADULTS: A LATENT CLASS ANALYSIS

Jing Huang,[1] Pui Hing Chau,[1] Edmond Pui Hang Choi,[1] Bei Wu,[2] and Vivian Lou,[1] *1. The University of Hong Kong, Hong Kong, China, 2. New York University, New York, New York, United States*

The purposes of this study were to identify the patterns of caregiving activities among family caregivers in Hong Kong and to examine their associations with characteristic factors and caregiver burden. The data was from the cross-sectional survey on the profiles of family caregivers of older adults in Hong Kong. 932 family caregivers were classified into different classes by using the Latent class analysis (LCA) according to their engagements in the 17 daily caregiving activities: 6 activities of daily living (ADLs), 8 instrumental activities of daily living activities (IADLs), emotional support, decision-making, and financial support. Five classes were revealed and labeled "Total All-round Caregiving" (Class I: 19.5%), "Partial All-round Caregiving" (Class II: 8.2%), "ADLs Free Caregiving" (Class III: 23.8%), "ADLs & Partial IADLs Free Caregiving" (Class IV: 32.5%), "Financial Caregiving" (Class V: 16.0%), respectively. Results from multinomial logistic regression found that the following factors were associated with the class membership: care recipients' age, medical diagnoses, and caregivers' gender, job status, marital status, self-rated economic status, living with care recipients, and caring for ≥40 hours per week. Findings from multiple linear regression showed caregivers with different patterns of caregiving activities reported different levels of caregiver burden. Caregivers in Class I have been found with the highest caregiver burden. This is the first study that has applied LCA to capture the patterns of caregiving activities among family caregivers. Identification of caregiving activity patterns and examination of their characteristics and caregiver burden can help healthcare providers to shift to prioritized and targeted caregiver support.

### THE SPIRITUAL DIMENSIONS OF CARING FOR A FAMILY MEMBER WITH EARLY-STAGE DEMENTIA

Jocelyn McGee, Dennis Myers, Rebecca Meraz, Janet Galarza, Morgan Davie, and Jensen Smith, *Baylor University, Waco, Texas, United States*

There are approximately 16.1 million family members caring for persons with Alzheimer's Disease and other dementias in the USA. However, few studies have examined the role of spirituality/religiosity as it relates to the caregiving experience, particularly in family members of persons in the early-stages of dementia. In this cross-sectional qualitative study, one-on-one, in-depth, 60-90 minute interviews were conducted with family members of persons assessed to be in the early-stages of dementia (n = 26). A structured interview, "The Spiritual and Religious Dimensions of Living with Dementia" developed by McGee, J. & Carlson-Zhao, H. (2012), was utilized. Interviews were recorded and transcribed by members of an interdisciplinary team of gerontology researchers (nursing, psychology, and social work). Narrative data were examined through thematic analysis approach, supported by NVivo (version 12.0) software, and a modified constant comparison analysis approach (Glaser & Strauss, 1967). Participants reported a diverse range of spiritual/religious beliefs, practices, and experiences including how these intersected with their adaptation to the opportunities and challenges of the caregiver role. The importance of intrapersonal processing, family connection, and community support for managing the ambiguity of this role was expressed. The interview questions were reportedly therapeutic for some participants suggesting the need to include similar questions as part of the assessment in clinical care settings. Indeed, interviewing caregivers of people with early-stage dementia may serve to improve clinical outcomes by identifying important aspects of spiritual/religious coping that can be encouraged as well as spiritual struggles that need to be addressed. Additional research is needed.

### UNRAVELING THE HEALTH-PROTECTIVE EFFECTS OF SENSE OF COHERENCE IN DEMENTIA CAREGIVING

Doris Yu, *The University of Hong Kong, Hong Kong, China*

The health protective effects of sense of coherence in a stressful encounter is well recognized. Representing the extent an individual can comprehend, manage and make meaning out of a caregiving situation, little is known about how SOC shapes the dementia caregiving process in a way to benefit the carers' health outcomes. This exploratory study recruited 401 family cares of dementia in Hong Kong. Over 30% of them reported moderate to severe depression, and those with low sense of coherence were most affected. Further analysis indicated that the health protective effects of SOC was mainly mediated through a higher level of perceived gain and more effective coping. In particular, those who can make meaning out of the caregiving situation were most benefited by such a mechanism. The study provides direction on ways to strengthen the health risk identification for family carers and enhance their role adaptation. After this presentation, the participants are able to: 1) Understand the role of sense of coherence in shaping the health of dementia family caregivers. 2) Understand the mediating pathways which explain the health benefit of sense of coherence for dementia family caregivers. □ Gain critical insights for developing health risk identification strategies and tailored interventions to protect the dementia family caregivers from depression.

### SESSION 2945 (POSTER)

### CAREGIVING II (BSS)

### A PILOT STUDY OF THE EFFECTS OF FORMAL SERVICE ON JAPANESE FAMILY CAREGIVERS' DAILY FLUCTUATION OF STRESS

Koji Abe, *Kagoshima University, Kagoshima-shi, Kagoshima, Japan*

Purpose: Recent studies report daily fluctuations in stress among family caregivers of older individuals with dementia. Several studies focused on daily stressors or behavioral and psychological symptoms of dementia and use of adult day services. Most previous studies on daily fluctuations of caregivers' stress have used a daily diary approach. This approach involves creating multiple daily reports, making it possible to examine between-person differences and within-person processes of change. However, only few studies used this approach for family caregivers in Asian countries. Therefore, this study examines the applicability of a daily diary approach for Japanese family caregivers and the effects of formal care services on their stress and depression. Methods: Participants were 13 family caregivers of individuals with dementia using formal care services in a rural area in Japan. They were assessed through self-administered questionnaires including use or nonuse of formal care services, caregiving stressors (DASC-8), depressive symptoms (K-6), and caregiving stress for 7 days. Generalized linear mixed models (GLMM) with data nested within persons were used to examine the effects of formal services on stress and depression. Results: For the GLMM procedure, this study used caregiving stressors and stress variables as fixed effects and participants as random effects. Results indicated that use of formal services significantly lowered caregivers' stress and depression. Conclusion: The findings demonstrate the applicability of a daily diary approach and the effectiveness of formal services on the stress of Japanese family caregivers.

### ASSESSING THE IMPACT OF OLDER AMERICANS ACT–FUNDED SERVICES ON CAREGIVER STRESS

Racheal Chubb,[1] and Janet Wilmoth,[2] *1. Syracuse University, Felts Mills, New York, United States, 2. Syracuse University, Syracuse, New York, United States*

Funding for OAA programs that support caregivers and care recipients has steeply declined since 2010. This is potentially problematic given these programs provide services that may reduce caregiver stress. To better understand what and for whom caregiving services reduces stress, we used data from the National Survey of Older Americans Act Participants (NSOAAP) to estimate logistic regression models predicting caregiver stress. These models include the following measures: services provided to the care recipient, services received by the caregiver, and caregiver satisfaction with services received; caregiver health, age, race, and gender; and care recipient health, age, gender, relationship to the caregiver, and coresidence with the caregiver. The results indicate that caregivers who were satisfied with services were less likely to be stressed compared to those who are not satisfied with services. Those who received respite care and counseling services were less likely to be stressed than those who attended classes and training. Consistent with the literature, caregivers who reported better health or cared for someone in better health were less likely to be stressed. Caregivers were also more likely to be stressed if they provided help with medical care compared to ADLs or if they lived with the care recipient. Overall, the results underscore the importance of continued, and possibly expanded, OAA funding for caregiver support services, especially those that provide respite and counseling to individuals who are providing care to frail and co-residential older adults.

### CAREGIVER KNOWLEDGE OF LONG-TERM SERVICES AND SUPPORTS: EFFECTS OF RURALITY AND SUPPORT

Lauren Stratton, Nichole Richter, Mack Shelley, and Jennifer Margrett, *Iowa State University, Ames, Iowa, United States*

Caregivers often lack knowledge regarding available long-term services and supports (LTSS). Certain barriers, such as rurality and levels of social support, may contribute to a lack of knowledge and accessibility of LTSS. The Caregiver Beginnings Workshop, held in 12 communities throughout 11 counties in Iowa, was a one-time educational session created to increase knowledge and awareness of LTSS. Data were collected from pre- and post-tests completed during the workshop (N = 98). To assess caregivers' initial knowledge of LTSS, a hierarchical regression model was estimated to examine knowledge of LTSS in caregivers as predicted by caregiver education, number of health problems in care recipient, relationship type, feelings of social support, and rurality. Results showed that rurality ($\beta = 0.33$, $p = 0.047$) and infrequent or no support ($\beta = -0.30$, $p = 0.02$) were significant predictors (R2 = 0.21), indicating that caregivers living in rural areas reported higher knowledge of LTSS and those who reported infrequent or no support reported less knowledge. Additional analyses examined county-level data to better understand the availability of community resources in rural areas. County-level variables (e.g., number of home healthcare services, education level, income, health status) were included in a regression model to predict knowledge of LTSS. The results indicated that median income ($\beta = -0.32$, $p = 0.002$) and an educational attainment of an associate's level degree or higher ($\beta = -0.30$, $p = 0.004$) were significant predictors. Discussion focuses on the importance of support and accessible resources for caregivers in all geographic areas.

### CHALLENGES OF RECRUITING CAREGIVER AND CARE RECEIVER DYADS FOR A RANDOMIZED CLINICAL TRIAL

Christine Fruhauf,[1] Arlene Schmid,[2] Neha Prabhu,[1] Laura Swink,[3] Jennifer Portz,[4] Heather Leach,[1] Mary Hidde,[1] and Marieke Van Puymbroeck,[5] *1. Colorado State University, Fort Collins, Colorado, United States, 2. Colorado State University, Fort Collinc, Colorado, United States, 3. UCHealth Anschutz, Denver, Colorado, United States, 4. University of Colorado Anschutz, Aurora, Colorado, United States, 5. Clemson University, Clemson, South Carolina, United States*

The recruitment of participants for chronic disease clinical trial research is often challenging. Further complicating participant recruitment occurs when the intervention is dyadic (i.e., simultaneously includes both care receivers and caregivers or recruiting pairs of people). Despite the strong support in favor of dyadic interventions for certain chronic diseases (e.g., among cancer and stroke survivors and their caregivers), researchers have not systematically shared challenges and opportunities for dyadic recruitment. During the recruitment for a yoga and self-management education intervention for people with chronic pain and their caregivers, several steps were taken to recruit and screen potential participants for the study. In this presentation, we will provide an overview of common recruitment challenges for physical activity and chronic disease self-management studies as well as the actual challenges encountered and our procedures for overcoming these obstacles. We will present our consort figure with attention toward inclusion and exclusion criteria of both care receivers and caregivers. Additional discussion will include specific challenges encountered when recruiting and screening caregivers (i.e., after the care receiver has been screened). The need for innovative clinical trial research with caregivers and care recipient dyads is essential as new care practices continue to evolve and demands on health care utilization increase. Lessons learned from this study may prove useful for future researchers as they embark on developing and testing dyadic interventions among adults with chronic disease and their caregivers.

### DEVELOPMENT OF AN INNOVATIVE FINANCIAL LITERACY AND PREPAREDNESS PROGRAM FOR FAMILY CAREGIVERS

Katherine Judge,[1] and Sam Fazio,[2] *1. Cleveland State University, Cleveland, Ohio, United States, 2. Alzheimer's Association, Chicago, Illinois, United States*

Informal caregivers provide the bulk of daily care and assistance to older adults needing help. Tasks range from assisting with transportation, coordinating care and appointments, household tasks, emotional and social support, and personal care. Caregivers also assist with financial care-related issues. This assistance ranges from helping pay bills, making health-care decisions, to paying out-of-pocket care expenses. Research on financial care-related issues greatly lags behind other areas of caregiving. Additionally, few programs have been developed that specifically address these financial issues and how best to provide timely and personalized information for families. The following poster will present an innovative program that addresses these gaps within the literature and fills the void facing families in navigating key financial care-related decisions. Funded by the Administration on Community Living, the evidence-informed program was developed based on findings from a comprehensive literature review, an environmental scan, market analysis, and feedback from focus groups. The program includes educational information, skills-training, and resources for caregivers across the following content areas: Introduction to Costs of Caregiving, Benefits of Early Planning, Avoiding Financial Abuse and Fraud, Conversations about Finances, Assessing Financial Needs, Creating Action Plans, and Finding Financial Support. The program also addresses specific financial needs facing caregivers of individuals with Alzheimer's disease and related dementias. Discussion will highlight key aspects of the program, including the standardized yet flexible and tailored approach for addressing families' specific financial care-related needs, along with next steps in program implementation and evaluation.

### DO THE REASONS EMERGING ADULTS BECOME INFORMAL CAREGIVERS RELATE TO FUTURE WILLINGNESS TO CARE? A MIXED-METHODS STUDY

Anastasia Canell, Hannah Bashian, and Grace Caskie, *Lehigh University, Bethlehem, Pennsylvania, United States*

Approximately 12-18% of family caregivers to older adults in the U.S. are emerging adults (aged 18-25), yet minimal research focuses on this subgroup of caregivers (Levine, 2005). Although several theories have developed to explain the growing number of emerging adults assuming informal caregiving roles (e.g., alleviating burden on middle-aged caregivers, family obligation; Dellmann-Jenkins & Brittain, 2003), the reasons why emerging adults become caregivers have not been studied. In the current study, a sample of 248 emerging adult caregivers were asked to describe the circumstances that led to providing unpaid care to an older adult. Using discovery-oriented coding methodology, 11 themes emerged within participants' responses: care-recipient illness (35.5%), familial relationship (35.5%), care-recipient became dependent (23.8%), proximity (13.7%), only option (10.1%), reciprocal care (8.9%), availability (8.5%), age-related decline (6.9%), monetary restrictions (6.9%), care-recipient desire (6.0%), and community service (4%). Follow-up analyses found that participants who identified familial relationships (e.g., "They are my parents so I felt obligated...") were less likely to endorse willingness to provide nursing care in the future compared to those who did not identify familial relationships (p=.032). Participants who identified care-recipient dependency as a circumstance leading to caregiving (e.g., "My grandmother fell and was in rehab...") were more likely to endorse willingness to provide instrumental (p=.034) and emotional (p=.047) care in the future than those who did not identify care-recipient dependency. These results demonstrate the unique reasons that may lead emerging adults to begin caregiving and how these reasons relate to future willingness to care for an older adult.

### ENHANCING DEMENTIA CAREGIVERS' EMOTIONAL COPING IN AN INTERGENERATIONAL EXPRESSIVE ARTS BASED THERAPY PROGRAM

Keisha Carden,[1] Rebecca Allen,[2] Katherine Ramos,[3] Daniel Potts,[4] and Keisha Ivey *1. University of Alabama, Tuscaloosa, Alabama, United States, 2. Alabama Research Institute on Aging, Tuscaloosa, Alabama, United States, 3. Duke University School of Medicine, Raleigh, North Carolina, United States, 4. Tuscaloosa VA Medical Center, Tuscaloosa, Alabama, United States*

Dementia caregivers often experience elevated levels of stress and are at increased risk for psychological disorders. Recent research has implicated emotional avoidance, psychological inflexibility, and related processes in the development and maintenance of psychopathology. Addressing these processes is essential for improving caregiver mental health. This pilot study examined changes in emotional avoidance, psychological inflexibility, and anticipatory grief among familial caregivers of individuals with dementia enrolled in an intergenerational expressive arts program (Bringing Art to Life). Caregivers were an average age of 63.32 (11 NHW, 8 AA; 13 Women, 6 Men). On average, caregivers' emotional avoidance and psychological inflexibility (M = 19.63, SE = 2.16) decreased significantly after participating in the program (M = 15.06, SE = 1.80). This difference, -4.57, BCa 95% CI [1.82, 7.13], was significant, t(15) = 3.81, p = .002, and represented a medium-sized effect, d = 0.70. Overall experience of anticipatory grief (M = 51.88, SE = 3.26) also decreased (M = 48.00, SE = 2.79). More specifically, caregivers'

reported sadness and loneliness (M = 17.63, SE = 1.37) decreased significantly (M = 14.5, SE = 0.95), BCa 95% CI [1.25, 5.00], t (15) = 3.21, p = 006, and represented a medium-sized effect, d = 0.64. These findings illuminate novel ways in which interventions might both target underpinnings of the caregiver stress process and improve the dyadic relationship. Specific intervention elements are explored and recommendations are made for future intervention development and implementation as well as measurement based outcome tracking.

### PATHWAYS TO MENTAL HEALTH–RELATED TREATMENT AMONG CAREGIVING SPOUSES

Hyojin Choi,[1] Kristin Litzelman,[1] Molly Maher,[2] and Autumn Harnish,[3] *1. University of Wisconsin-Madison, Madison, Wisconsin, United States, 2. University of Wisconsin - Madison, Madison, Wisconsin, United States, 3. University of Wisconsin-Madison, Fitchburg, Wisconsin, United States*

Spouses of cancer survivors are 33% less likely to receive guideline-concordant depression treatment than other married adults. However, depression is only one of many manifestations of psychological distress for caregivers. This exploratory study sought to assess the paths by which caregivers access mental health-related treatment. Using nationally representative data from the Medical Expenditures Panel Survey, we assessed the proportion of caregivers who received a mental health-related prescription or psychotherapy visit across care settings (office based versus outpatient hospital, emergency room, or inpatient visit), provider type (psychiatric, primary care, other specialty, or other), and visit purpose (regular checkup, diagnosis and treatment, follow-up, psychotherapy, other). In addition, we assessed the health condition(s) associated with the treatment. The findings indicate that a plurality of caregivers accessed mental health-related treatment through an office-based visit (90%) with a primary care provider (47%). A minority accessed this care through a psychologist or psychiatrist (11%) or a physician with another specialty (12%) or other provider types. Nearly a third accessed treatment as part of a regular check-up (32%). These patterns did not differ from the general population after controlling for sociodemographic characteristics. Interestingly, mental health-related treatments were associated with a mental health diagnosis in only a minority of caregivers. The findings confirm the importance of regular primary care as a door way to mental healthcare, and highlight the range of potential paths to care. Future research will examine the correlates of accessing care across path types.

### PATTERNS OF OBLIGATION OF CARE AMONG URBAN AND RURAL AMERICANS

Johanna Sosa,[1] Darlingtina Esiaka,[2] and Candidus Nwakasi,[3] *1. Union College, Schenectady, New York, United States, 2. Union College, Union College, New York, United States, 3. University of Southern Indiana, Evansville, Indiana, United States*

In recent years, there is concern about the shortage of adult children who are active participants in the care of their older parents (those 65 years and above). Reviewed studies suggest that attitudes towards the provision of care for older parents differ and may depend on ecological affordances. Particularly, demographic changes due to urbanization shape constructions of obligation to provide eldercare. This study examined patterns of filial responsibility and felt obligation of adult children toward their older parents among rural and urban dwellers in the US. Participants (N= 187) responded to questionnaires assessing filial responsibility, felt obligation to parents, and additional social and demographic characteristics. Results show that rural Americans reported higher levels of filial responsibility and felt obligation to older parents. Also, filial responsibility is associated with the felt obligation for contact with parents, avoidance of conflict with parents, provision of assistance and care, self-sufficiency and independence from parents, and sharing of personal experiences with parents. Our findings indicate the existence of cultural ecological contexts that afford a sense of duty, care, and assistance to older parents. We discuss strategies that acknowledge the relevance of these factors in promoting participation in eldercare.

### POSITIVE ASPECTS OF CAREGIVING AMONG FAMILY CAREGIVERS OF INDIVIDUALS WITH DEMENTIA

Mary Grace Asirot,[1] Anna Papazyan,[2] and Yeonsu Song,[1] *1. UCLA, Los Angeles, California, United States, 2. VA Greater Los Angeles Healthcare System, North Hills, California, United States*

Traditionally, caregiving for individuals with dementia has been viewed as a negative experience. Understanding positive aspect of caregiving and related factors is important to improve health among family caregivers. We analyzed baseline data from an ongoing dyadic sleep education trial for individuals with dementia and their caregivers (N=21 dyads; mean age 70.8± 11.1 for caregivers, 80.5± 8.3 for care-recipients). The Positive Aspects of Caregiving (PAC 9-item) was used to assess subjective satisfaction with caregiving. Other measures included Zarit Burden Interview (ZBI), SF-12 Health Survey (SF-12v2), Revised Memory and Behavior Problems Checklist (RMBPC), and Pittsburgh Sleep Quality Index (PSQI). Pearson correlations and t-tests were calculated for analyses. Caregivers most frequently endorsed that caregiving enabled them to appreciate life more (n=16 agreed a lot). Caregivers who began providing care within the first few months of the care-recipient needing care (n=16) had greater positive aspects of caregiving than those who started providing care sometime later (n=5) (36.37±7.33 versus 25.8±8.29, p=0.01). Caregivers with higher PAC scores had lower ZBI score (r=-0.49, p=0.02), better mental health on the SF-12v2 (r=0.53, p=0.01), less distress related to care-recipient behaviors on the RMBPC (r=-0.50, p=0.02), and lower PSQI subscale (perceived sleep quality) score (r= -0.46, p=0.04). Findings suggest that higher positive caregiving experience was associated with better mental health and sleep quality, and less burden and distress from the care-recipients behaviors. More research is needed to better understand this relationship and to determine possible interventions to increase positive aspects of caregiving.

### PREVALENCE OF HEAVY ALCOHOL USE BY CAREGIVERS OF PERSONS WITH ALZHEIMER'S DISEASE OR RELATED DEMENTIA

Zachary Kunicki, and Richard Jones, *Brown University, Providence, Rhode Island, United States*

Some caregivers of persons with Alzheimer's Disease and related dementias (ADRD) are known to be under high levels of burden, which is associated with higher levels of anxiety, depression, and stress. Previous research has established anxiety, depression, and stress are associated with heavy alcohol use, but little research has examined heavy alcohol use among ADRD caregivers. Heavy alcohol use could influence the ability of ADRD caregivers to provide care. The purpose of this study was to explore the prevalence and prevalence correlates of heavy alcohol use among ADRD caregivers using the 2016 Behavior Risk Factor Surveillance Survey (BRFSS). We identified 2,028 persons among the 486,303 BRFSS respondents who were the primary informal caregivers of a person with ADRD. Among them, the prevalence of heavy alcohol use was 6.3 per 100 persons. Adult child caregiver relationship, positive smoking status, and fewer hours of providing care per day were all positively and significantly associated with heavy alcohol use. Notably, sex was not. Future research should examine if heavy alcohol use by ADRD caregivers is related to personally and clinically relevant outcomes of care provided to the persons with ADRD.

### SELF-EFFICACY AMONG CAREGIVERS OF PEOPLE WITH DEMENTIA AND ITS IMPACT ON CAREGIVERS' HEALTH

Soyeon Cho, *City University of New York/CityTech, Brooklyn, New York, United States*

Self-efficacy is construct which is associated with positive thinking. It has been examined in caregiving studies to alleviate caregivers' negative health outcomes. However, little is known about Asian American caregivers' self-efficacy on their psychological and physical outcomes, especially caregivers with people with dementia. Thus, the present study examined self-efficacy of caregivers as a potential mediator in the association between caregiving role captivity and depressive symptoms among older Korean Americans. Data were driven from a cross-sectional study of 175 community-dwelling Korean American older adults (aged 60 and older) in 2019. The direct significant relation between caregiving role captivity and depressive symptoms became insignificant after self-efficacy was introduced, which demonstrates a full mediation effect of self-efficacy. Results suggest that even in the presence of caregiving role captivity, mental well-being such as depression of caregivers can be maintained by having competence in self-management of their own health.

### SHARED DECISION MAKING BETWEEN OLDER ADULT HOME HEALTH PATIENTS AND THEIR CAREGIVERS: A DYADIC COPING PERSPECTIVE

Djin Tay, Lee Ellington, Gail Towsley, Katherine Supiano, and Cynthia Berg, *University of Utah, Salt Lake City, Utah, United States*

Older adult Home health (HH) patients comprise a medically frail population with increased inpatient and emergency department utilization. Despite the need for advance care planning among this population, rates are suboptimal. Patients rely increasingly on caregivers to advocate and coordinate their care particularly at the end of life; however surrogate decision makers are often underprepared for their roles in end-of-life decision making. This study examined shared decision making processes among older adult HH patients and caregivers during a shared decision making intervention guided by the Developmental-Contextual Model of dyadic coping (DCM). Purposive recruitment of N=18 HH patient-caregiver dyads was conducted. Patients were 55 years and above and participated with a family or non-family caregiver they nominated to the study. A 10-41 minute long video-recorded advance care planning intervention was conducted in patients' homes and analyzed for non-verbal and verbal interactions using Noldus Observer XT 14.0. Theoretically-derived codes were applied deductively in a content analysis to examine dyadic processes associated with interactions suggesting agreement (convergent interactions) and disagreement (divergent interactions). Convergent interactions demonstrated greater alignment in illness representations and shared appraisals, and processes involving support, negotiation, and confirmation of preferences were noted. Convergent interactions also facilitated joint planning for future decisions. Disagreement on illness representations and/or shared appraisals, and overriding another's preference was observed with divergent interactions. This study builds the groundwork for intervention refinement to promote constructive decision making and address non-constructive decision making among patient and caregivers for advance care planning.

### SPOUSAL CAREGIVING AND COGNITIVE TRAJECTORIES: DOES CARE RECIPIENT DEMENTIA STATUS MATTER?

Taylor Atkinson, Dylan Jester, and William Haley, *University of South Florida, Tampa, Florida, United States*

Caregiving is often considered stressful, even more so if the care recipient has been diagnosed with dementia. The current study examines the rate of cognitive decline of spousal caregivers of persons with dementia (CG-D) when compared to spousal caregivers of persons without dementia (CG) before and after the death of the care recipient. Health and Retirement Study (HRS) data from 1998-2016 were used to examine cognitive trajectories of CG-D (n=364) and CG (n=1,649) before and after the care recipient death. Cognition was measured through the HRS's shortened Telephone Interview of Cognitive Status and separated into measures of total cognition and memory. Covariates included age, education, sex, race, ethnicity, care hours, frailty, socioeconomic status, nursing home placement of the recipient, and whether the death was expected. Piecewise mixed models were constructed to examine two two-year periods of decline leading up to the death of the care recipient, and two two-year periods of decline after the death of the care recipient. CG-D and CG declined at equivalent rates on measures of total cognition and memory ($ps > .05$). In all caregivers, total cognition and memory declined at a stable rate before the death of the care recipient. However, an accelerated decline was evident after the death of the care recipient ($ps < .001$). Our results suggest that cognitive decline is not differentially affected by care recipient dementia diagnosis. We find evidence that the death of a spousal care recipient is accompanied by hastened cognitive decline in our population-based sample.

### THE ASSOCIATION BETWEEN CAREGIVING BURDEN AND DEPRESSIVE SYMPTOMS AMONG U.S. CHINESE ADULT CHILDREN

Shuting Liang,[1] and XinQi Dong,[2] *1. Rutgers University–New Brunswick, Chicago, Illinois, United States, 2. Rutgers University, New Brunswick, New Jersey, United States*

Existing research has showed the impact of caregiving burden on physical and psychological outcomes among adult children, but less have examined its association among Chinese immigrants in the US. This research will present the association between caregiving burden and depressive symptoms among U.S. Chinese adult children. Cross-sectional data were drawn from the PIETY study with 547 Chinese adult children aged over 21 years old in the greater Chicago area between 2012-2014. Caregiving burden was assessed by 24-item caregiver burden developed by Novak and Guest and is composed of five factors: time-dependence, developmental, physical, social, and emotional burden. Depressive symptoms were assessed by the nine-item Patient Health Questionnaire. Logistic regression analysis was conducted. In our sample, 241 (44%) adult children had depressive symptoms and 174 (72.2%) were female. In the result of multivariate analysis, after adjusting for covariates, developmental burden (Odds ratio [OR] 1.13 [1.05-1.21]), physical burden (OR 1.17 [1.06-1.28]), social burden (OR 1.20 [1.08-1.32]), and emotional burden (OR 1.22 [1.11-1.35]) were positively associated with reporting any depressive symptoms. However, the time-dependence burden was not associated with depressive symptoms. The findings highlight the potential impact of caregiving burden on depressive symptoms and how different domains of caregiving burden are associated with depressive symptoms among Chinese caregivers in the U.S. Future research should include multidimensional social supports or acculturation as underlying factors which might affect the relationship between caregiver burden and depressive symptoms across Chinese community in the US.

### THE IMPACTS OF DISCRIMINATION AND FILIAL CAREGIVERS' AGE ON ASPECTS OF PHYSICAL HEALTH

Barbara Hodgdon, and Jen Wong, *The Ohio State University, Columbus, Ohio, United States*

Filial caregivers (e.g., individuals caring for a parent or parent-in-law) are a part of the growing number of family caregivers in midlife and late adulthood. The responsibilities that filial caregivers navigate in midlife and late adulthood may expose them to multiple types of discrimination that may decrease their physical health, though this relationship has been understudied. As numbers of family caregivers grow, it is important to examine the potential vulnerability of younger and older filial caregivers' physical health in the context of discrimination. Informed by the life course perspective, this study compares the physical health of younger (aged 34-64) and older (aged 64-74) filial caregivers who experience discrimination. Filial caregivers (N=270; Mage=53; SD=9.37) from the Midlife in the United States (MIDUS-II) Survey reported on demographics, family caregiving, daily discrimination, self-rated physical health, and chronic conditions via questionnaires and phone interviews. Regression analyses showed no differences between younger and older adults' self-rated physical health or average chronic conditions. However, moderation analyses revealed that younger filial caregivers who experienced greater discrimination reported poorer self-rated physical health than their older counter parts as well as younger and older filial caregivers who experienced less discrimination. Additionally, younger caregivers with greater discrimination exposure exhibited more number of chronic conditions as compared to other caregivers. The study results highlight the impact of the intersection between filial caregivers' age and discrimination on physical health. Findings have the potential to inform programs that could promote the health of filial caregivers in the face of discrimination.

### THE INFLUENCE OF DEMENTIA CAREGIVING STYLES ON CAREGIVER DISTRESS AND THE PERSON WITH DEMENTIA'S QUALITY OF LIFE

Amanda Leggett,[1] Cathleen Connell,[2] Laura Gitlin,[3] and Helen Kales,[4] *1. The University of Michigan, Ypsilanti, Michigan, United States, 2. University of Michigan, Ann Arbor, Michigan, United States, 3. Drexel University, Philadelphia, Pennsylvania, United States, 4. UC Davis, Sacramento, California, United States*

Building vertically upon the Stress Process Model, dementia caregivers' cognitive-behavioral management styles are an understudied area with implications for dyadic care outcomes and tailoring of care interventions. We consider whether membership in five previously classified caregiving styles (Externalizers, Individualists, Learners, Adapters, Nurturers- which vary in their adaptability, dementia understanding, and behavioral management practices) impacts caregivers' experiences of care-related stress and the quality of life of the person with dementia (PWD). Participants included 100 primary family caregivers for PWDs who were 74% female, 18% non-White, and on average 64 years old. Utilizing linear regressions, each caregiving style was considered as a key predictor (reference: Externalizers- poor understanding, non-adaptable approach, and punitive behavioral strategies) of the Caregiver Assessment of Function and Upset (CAFU) upset score, Neuropsychiatric Inventory (NPI-C) distress scale, Zarit Burden Interview (ZBI), and PWD quality of life (QOL-AD) scale controlling for demographics, care duration, co-residency, and dementia severity. Relative to Externalizers, Nurturers (understanding, adaptability, positive engagements) had less CAFU upset ($\beta$=-0.4, p<.01), less NPI-C distress ($\beta$=-0.3, p<.05), and greater QOL-AD for the PWD ($\beta$=0.4, p<.01). Learners (recognize need to change care approach, attempting adaptability, trial-and-error behavioral care) also showed significantly lower NPI-C distress than Externalizers ($\beta$=-0.5, p<.01). Thus caregiving styles with more dementia understanding, adaptability and positive behavioral strategies showed less distress and better PWD QOL. Corresponding with recent dementia care summits calling for identification of caregivers at greatest risk for poor outcomes, targeting and tailoring interventions based on caregiving styles may lead to great public health impact.

### THE RELATIONSHIP BETWEEN FUNCTIONAL DECLINE OF CARE RECIPIENTS AND HEALTH OF CAREGIVERS

Jinmyoung Cho, and Alan Stevens, *Baylor Scott & White Health, Temple, Texas, United States*

Pearlin's stress-process model (2010) depicts that functional decline in care-recipient would shape caregiving burden and impact on caregiver's health. With this background, we explored how the changes in care-recipients' physical and cognitive functioning are related to the caregivers' physical health. A total of 853 care-recipients from the Rounds 1 and 5 of the National Health and Aging Trend Study (NHATS) and their 1,303 caregivers from Round 5 of National Study of Caregiving (NSOC) were included. Multiple regression analyses were conducted to identify correlates of self-rated health and the number of chronic conditions with the change in physical and cognitive functioning from Round 1 to 5 and multidimensional caregiver burden. Physical functioning measured by the NHATS short physical performance battery included balance stands, walking chair stands, grip strength, and peak airflow. Memory, orientation, and executive functioning measured cognitive functioning. Multidimensional caregiver burden includes four domains (emotional, psychological, relationship, and resilience) identified with factor analysis. Background factors (recipient's age, assisting recipients for 5 years, race/ethnicity, and number of chronic conditions of recipient) were included as covariates. After controlling covariates, the data showed that 5-year change of physical functioning and caregiver's emotional burden were negatively significant for self-rated health; and assisting care-recipients for 5 years or more was significant for more numbers of chronic conditions among caregivers. Findings highlight that caregivers' physical health is closely associated with care-recipient's functional decline for long-term caregiving experiences. Further investigation on caregiver's physical health using multiple health outcomes is needed to promote physical health in long-term caregivers.

### SESSION 2946 (POSTER)

### COGNITION AND COGNITIVE FUNCTIONING

### 5-COG STUDY: CROSS-CULTURAL COMPARISON OF SUBJECTIVE COGNITIVE COMPLAINTS IN A DIVERSE PRIMARY CARE POPULATION

Emmeline Ayers,[1] Erica F. Weiss,[2] and Joe Verghese,[1]
*1. Albert Einstein College of Medicine, Bronx, New York, United States, 2. Montefiore Medical Center, Bronx, New York, United States*

Subjective cognitive complaints (SCC) are risk factors for cognitive decline in older adults. A link between SCC and depressive symptoms has also been reported. These associations have not been much studied in non-White populations. We examined the relationship of SCC with cognitive function and depressive symptoms in adults aged 65 and older attending a primary care clinic in the Bronx. Five common SCC questions (four memory-related and one non-memory-related) were identified by literature review. Linear regressions, adjusted for age, sex and education years, were used to examine associations between individual SCC and cognitive function (Montreal Cognitive Assessment (MoCA) score and Hopkins Verbal Learning Test (HVLT) recall score) and depressive symptoms (Geriatric Depression Scale (GDS) score) for Hispanic (n=53) and non-Hispanic Black (n=47) adults. Mean number of SCC was similar for Blacks and Hispanics (2.3 vs. 2.4, p=0.752). Hispanics performed worse on the MoCA than Blacks (16.4 vs. 18.5, p=0.012), but education explained this difference. GDS and HVLT were similar across groups. For Hispanics only, a response of fair or poor to the question "how is your memory for a person your age?" was associated with worse MoCA scores (β -2.6; p=0.008). SCC were not associated with HVLT scores for either group. Four SCC for Blacks and two for Hispanics were associated with worse GDS scores. In an urban clinic population, SCC for Blacks and Hispanics were associated more with depressive symptoms than cognition. Further research is needed to identify SCC that better correlate with cognitive function in diverse populations.

### AGE-RELATED PATTERNS IN THE SUBJECTIVE APPRAISAL OF COGNITION

Claudia Jacova,[1] Samantha Smith,[2] and Frank Robertson,[1]
*1. Pacific University, Hillsboro, Oregon, United States,*
*2. Student, Hillsboro, Oregon, United States*

Subjective cognitive decline (SCD) is a construct of high interest in aging and dementia because individuals endorsing it are at higher risk of developing cognitive problems. It is unclear how individuals arrive at the judgement that they have SCD. Here we aimed to understand which SCD symptoms give rise to the perception of decline as older adults age. Community-dwelling adults (N=494, mean age=63.6, SD=5.44), completed the Subjective Cognitive Decline Questionnaire (SCD-Q) online, using an online crowdsourcing site. The SCD-Q consists of one global question regarding self-perceived decline (yes/no) and 24 questions about everyday functioning which we utilized to form a memory, language, and executive functioning domain score, higher for greater perceived decline. Logistic regression revealed that memory and language domains predicted the likelihood of endorsing SCD for adults aged >64 (Memory: OR=1.76, CI=1.47-2.05; Language: OR=1.66, CI=1.30-2.02). Only the memory domain predicted the likelihood of endorsing SCD for adults <63 (OR=2.69, CI=2.35-3.02). Executive functioning domain scores did not play a role in the relationship between SCD likelihood in either age group. The higher the self-perceived memory or language decline, the more likely older adults are to conclude they have SCD. Our results suggest there is an age-related trajectory in how people evaluate their cognition, with younger people only considering memory and older people considering both memory and language. Clinicians should be aware of this trajectory when examining patients with SCD. Executive functions should be specifically queried because they may not emerge from older adults' self-reported cognitive problems.

### ASSOCIATIVE IMAGERY AS A STRATEGY TO IMPROVE DESTINATION MEMORY IN YOUNGER AND OLDER ADULTS

Tara Johnson,[1] Katie Stanko,[2] and Susan Jefferson,[3]
*1. Indiana University of Pennsylvania, Indiana,*

*Pennsylvania, United States, 2. University of Pittsburgh, Pittsburgh, Pennsylvania, United States, 3. Pittsburgh VA Health Care System, Pittsburgh, Pennsylvania, United States*

Destination memory errors (inability to remember to whom information was shared) affects all ages, but older adults are particularly vulnerable due to poor source monitoring. Individuals may assume information was already shared when it was not or repeat previously shared information. The current study explored two mental imagery strategies (vivid imagery, visualizing context) to improve destination memory. Using a software program, younger and older adults told randomly generated facts to random celebrity faces. Participants were unaware of the upcoming memory tests. The control group did not use a strategy. The imagery group used vivid imagery to connect the fact and face (e.g., visualize Oprah on a dime to remember Oprah was told that dimes have 118 ridges). The context group visualized a provided context (e.g., grocery store) when telling a fact to a face. Assessments of performance on item memory (facts, faces) as well as destination memory (face-fact pairings) were counterbalanced. Results indicated an associative memory deficit among older adults, which was driven by a higher rate of false alarms. However, across all adults, the vivid imagery condition was more accurate than the control condition, and they demonstrated fewer false alarms. These findings suggest that older adults can use mental imagery to reduce false alarms and improve destination memory performance. Implications include reducing age stereotypes, improving conversations, and decreasing potentially dangerous situations (e.g., withholding important health information thinking it already was shared with a doctor).

### CHRONIC STRESS, C-REACTIVE PROTEIN, AND COGNITION AMONG RACIALLY AND ETHNICALLY DIVERSE OLDER ADULTS

Emily Morris, and Laura Zahodne, *University of Michigan, Ann Arbor, Michigan, United States*

Objective: Previous research suggests that chronic stress is associated with worse cognitive aging, but minimal research has examined potential mechanisms and moderators of these associations. Chronic stress is known to increase inflammation (e.g., C-reactive protein [CRP]), which has in turn been associated with worse cognition among older adults. The present study examined whether (1) CRP mediates associations between chronic stress and episodic memory and verbal fluency; and (2) these relationships differ by race/ethnicity. Methods: Participants included 18,968 adults (64% non-Hispanic White; 19% non-Hispanic Black; 14% Hispanic; 3% non-Hispanic other race/ethnicity; Mage=71.8; SDage=6.0) from the Health and Retirement Study. Chronic stress was operationalized as the occurrence and impact of eight ongoing stressors. Cross-sectional, stratified mediation models were conducted for three cognitive outcomes: immediate recall, delayed recall, and verbal fluency. Covariates included sociodemographics and vascular disease burden. Results: Chronic stress was associated worse immediate recall (beta=-.028). Higher CRP was not associated with any cognitive domains. Non-Hispanic Black participants reported more chronic stress than non-Hispanic White and Hispanic participants. Chronic stress was less strongly associated with higher CRP in non-Hispanic Black (beta=-.035) participants than non-Hispanic White (beta=.046) or Hispanic (beta=.059) participants. Discussion: Chronic stress may negatively influence episodic memory, but findings do not suggest that CRP mediates links between chronic stress and cognition. CRP may not track as closely with chronic stress among non-Hispanic Black older adults who may experience additional risk factors for inflammation and/or adapt to increased chronic stress.

### CORTISOL RESPONSES TO A LABORATORY CHALLENGE: THE MODERATING ROLE OF COGNITIVE PERFORMANCE

Elise Grimm,[1] and Stefan Agrigoroaei,[2] *1. Universite Catholique de Louvain, Louvain-La-Neuve, Belgium, 2. UCLouvain, Belgium, Louvain-La-Neuve, Belgium*

Recent theoretical and empirical studies have considered higher cognitive performance as a protective factor with respect to reactivity, recovery and habituation to acute stressors. The goal of our study was to examine the individual role of inhibition, working memory, processing speed, reasoning, and category fluency in the regulation of the cortisol response to a laboratory challenge. Younger, middle-aged, and older participants (N =109, aged 22-84, M=55.90, SD=16.35) were invited to a laboratory session comprising a driving simulation and a set of cognitive tasks. At least one week in advance, baseline cognitive performance was measured using the Brief Test of Adult Cognition by Telephone (BTACT). Throughout the lab session, five saliva samples were taken, which allowed for the computation of a global measure of cortisol release (area under the curve (AUC)). Cortisol AUC was regressed on the individual BTACT cognitive tests, while controlling for age, sex, education, body mass index, physical activity, and time since awakening. The results revealed that inhibition and working memory significantly accounted for the cortisol response. These associations remained significant when other factors such as smoking, caffeine consumption, and medication use were included as covariates. The contributions of reasoning and speed of processing approached significance. Our findings contribute to the emerging evidence that cognitive functioning modulates stress responses to acute stressors. The findings are discussed in the context of cognitive interventions with transfers and implications for stress processes and healthy aging.

### DEVELOPMENTAL TRAJECTORY OF THEORY-OF-MIND DECLINE IN OLDER ADULTS

W. Quin Yow, Xiaoqian Li, and Jiawen Lee, *Singapore University of Technology & Design, Singapore, Singapore*

Theory-of-Mind (ToM) is critical to individual social competence and mental health across the lifespan (Frith, 2008). Though it is often discussed as one broad construct, ToM abilities can be viewed as following a developmental trajectory: from early emotion recognition and gaze following to more advanced inferences about others' beliefs, perspectives, and intentions (Hutchins et al., 2012). Despite current literature suggesting that ToM abilities may be impaired in late adulthood, there is no consensus regarding whether ToM abilities are differentially affected by age. In this study, we examined younger adults (N=18, aged 19-30) and older adults (N=13, aged 58-76) on their ToM competence across three levels of ToM abilities: Early-ToM (e.g., recognizing a happy face), Basic-ToM (e.g., perspective-taking and false-belief reasoning), and Advanced-ToM (e.g., inferring second-order emotion and false belief). All participants completed a Theory-of-Mind Task Battery consisting of three subscales that assessed the three levels of ToM, where participants viewed vignettes and answered questions about the protagonists' feelings and beliefs. Overall, younger adults outperformed older adults on the battery, $F(1,29)=7.34$, $p=.011$. However, a significant interaction between age and ToM levels ($p=.010$) revealed that Early and Advanced ToM ($ps>.25$) were not as affected by age as Basic ToM ($p=.007$). Older adults have difficulty in inferring others' perspectives/beliefs while their attributions of emotion and higher-order false beliefs are relatively preserved compared to the younger adults. These findings provide important insights into the impact of age on various levels of ToM and could help inform early detection of ToM decline in normal aging.

### DOES INSTRUCTION IMPROVE OLDER ADULTS' KNOWLEDGE OF MEMORY AGING?

Rachael Turner,[1] Celinda Reese,[1] Susan Brigman,[2] and Katie E Cherry,[3] *1. Oklahoma State University, Stillwater, Oklahoma, United States, 2. Franciscan Missionaries of Our Lady University, Baton Rouge, Louisiana, United States, 3. Louisiana State University, Baton Rouge, Louisiana, United States*

Memory loss happens in later life. For cognitively healthy older adults, deficits in memory in everyday life may be frustrating, but are less severe compared to the memory dysfunction observed in persons with progressive dementia syndromes, such as Alzheimer's disease (AD). Normal memory aging has been defined as benign memory deficits due to genuine maturational processes in otherwise healthy older adults. Pathological memory aging has been defined as memory dysfunction due to non-normative factors such as disease or trauma to the brain (Cherry & Smith, 1998). In the present research, we examined the effects of instruction on knowledge of memory aging issues among community-dwelling older adults. Participants ranged in age from 59 to 94 years. All were enrolled in a six-week lecture series on cognition in later life. They completed the Knowledge of Memory Aging Questionnaire (KMAQ: Cherry, Brigman, Hawley, & Reese, 2003) before and after the series. Results indicated that their knowledge of pathological memory aging was greater than their knowledge of normal memory aging, as expected. Importantly, both normal and pathological types of knowledge were impacted by instruction, as post-test scores were higher than pre-test scores for both scales. In addition, a select set of items reflecting ageist views were also impacted by instruction; scores on this subset were significantly improved (less reflective of ageist stereotypes) at the end of the lecture series. Implications for the design of educational programs on adult cognition for community-dwelling older adults are considered.

### EARLY- TO LATE-LIFE ENVIRONMENTAL FACTORS AND LATE-LIFE GLOBAL COGNITION: THE SONIC STUDY

Yoshiko Ishioka,[1] Yasuyuki Gondo,[2] Yukie Masui,[3] Takeshi Nakagawa,[4] Madoka Ogawa,[3] Hiroki Inagaki,[3] Saori Yasumoto,[2] and Tatsuro Ishizaki,[3] *1. Keio University, Minato-ku, Kanagawa, Japan, 2. Osaka University, Suita, Osaka, Japan, 3. Tokyo Metropolitan Institute of Gerontology, Itabashi-ku, Tokyo, Japan, 4. National Center for Geriatrics and Gerontology, Ōbu, Aichi, Japan*

Life environment across the life course—such as engagement in late-life leisure activity (LA), primary occupation, and early-life education—have been reported to be associated with better late-life cognitive outcomes. However, few studies have included all these factors from the past to the present due to the time-consuming procedure to measure all factors. This study examined (1) whether late-life LA is associated with better late-life cognition, after considering other life environments and (2) whether occupation, education, and childhood intelligence quotient have indirect effects on the late-life cognition through late-life LA. We used baseline data from the groups of 70- and 80-year-olds in the SONIC study ($N = 1721$ ). Global cognition was measured using the Montreal Cognitive Assessment. As for LA, participants were asked for yes/no answers to questions regarding their engagement in 158 activities. A latent factor representing LA was used in the analyses. We retrospectively evaluated the complexity of work with data, people, and things. As early-life environments, education and language and arithmetic abilities during elementary school were included in the analyses. Age and gender were controlled. A structural equation model showed that late-life LA was significantly associated with higher global cognition, even after controlling for all past factors (RMSEA = .050, GFI = .973, AGFI = .947). Sobel tests showed significant indirect effects of occupation, education, and childhood abilities on cognitive function. Results were robust across age and gender. It is suggested that engagement in LA explains individual differences in late-life cognitive function.

### EVIDENCE FOR AN AGE-RELATED POSITIVITY EFFECT IN METACOGNITIVE CONFIDENCE JUDGMENTS

Edie Sanders,[1] and Jane Berry,[2] *1. Florida State University, Tallahassee, Florida, United States, 2. University of Richmond, Richmond, Virginia, United States*

We examined age differences in metacognitive monitoring of emotionally-valenced stimuli. If older adults (OAs) are more focused on emotionally meaningful goals in late life (Carstensen, 2006), then they should demonstrate attentional and memory biases for positive stimuli over neutral and negative stimuli and, arguably, these cognitive biases should be reflected in their metacognitive judgments of learning. Judgments of learning (JOLs) for memory of positive, negative, and neutral words were collected. Younger adults (YAs) aged 18-23 years and OAs aged 65-90 years ($N = 85$) studied words in each valence category and made immediate JOLs, followed by a two-alternative forced choice (2AFC) recognition memory task. Analyses of JOLs revealed evidence for a positivity effect (Mather & Carstensen, 2005) in metacognitive confidence for OAs and an emotional salience effect in YAs (Tauber & Dunlosky, 2012; Zimmerman & Kelley, 2010). Predictably, YAs recognized more words than OAs, but valence did not affect number of words recognized and valence did not moderate age differences in recognition memory ($p = .055$). Memory monitoring as measured by resolution accuracy was equivalent in YAs and OAs (Hertzog

& Dunlosky, 2011). Positive affect was higher and negative affect was lower in OAs relative to YAs (Gallant, Spaniol, & Yang, 2019), lending additional evidence to an orientation toward the positive in older adulthood. These results are novel in that they demonstrate an age-related positivity effect that extends beyond the domains of memory and emotion to the domain of metacognitive aging. Discussion will focus on theoretical, methodological, and applied implications.

### HEART AND MIND: ESSENTIAL COMPONENTS IN SOUND DECISION-MAKING

Michaela Clark, Julie Hicks Patrick, and Michaela Reardon, *West Virginia University, Morgantown, West Virginia, United States*

Consumer tasks permit an ecologically-valid context in which to examine the contributions of affective and cognitive resources to decision-making processes and outcomes. Although previous work shows that cognitive factors are important when individuals make decisions (Patrick et al., 2013; Queen et al.), the role of affective components is less clear. We examine these issues in two studies. Study 1 used data from 1000+ adults to inform a cluster analysis examining affective aspects (importance, meaningfulness) of making different types of decisions. A 4-cluster solution resulted. In Study 2, we used affective cluster membership and cognitive performance as predictors of experimental decision-making outcomes among a subset of participants (N = 60). Results of the regression (F(2, 40) = 6.51, p < .01, R2 = .25.) revealed that both the affective clusters (b = .37, p = .01) and cognitive ability (b = -.30, p = .04) uniquely contributed to the variance explained in decision quality. Age did not uniquely contribute. Results are discussed in the context of developing measures that enable us to move the field forward.

### INFORMATION AVOIDANCE IN DECISION MAKING: DO THE REASONS VARY BY AGE?

Stephanie Deng,[1] Julia Nolte,[2] and Corinna Loeckenhoff,[2] *1. Cornell University, Rochester, Minnesota, United States, 2. Cornell University, Ithaca, New York, United States*

Older adults make up the majority of the U.S. patient population and age differences in information avoidance have potential implications for their ability to participate in informed medical decision making. Meta-analytic evidence suggests that older adults seek less information before making a decision than younger adults do (Mata & Nunes, 2010). However, age differences in explicit information avoidance have yet to be quantified. We hypothesized that older adults would avoid decision-relevant information more strongly than younger adults do. We also examined the self-reported reasons for information avoidance and hypothesized that older adults would express more concern about unwanted information influencing their affect (Reed & Carstensen, 2012) and decision preferences (Mather, 2006), both of which are known predictors of information avoidance (Woolley & Risen, 2018). To test these assumptions, we conducted a pre-registered online study involving three different health-related decision scenarios. For each scenario, an adult lifespan sample (N=195, Mage=52.95, 50% female, 71% non-Hispanic White) chose to either receive or avoid information. Responses were highly correlated across scenarios and results were pooled into a single avoidance measure. Analyses indicated that concerns about consequences for decision preferences positively predicted decision avoidance (p<.001), whereas concerns about consequences for affect did not (p=.079). Contrary to predictions, older age was not significantly associated with information avoidance (p=.827). Further, self-reported concerns about the influence of unwanted information on affect and decision preferences were negatively associated with age (ps<.001). This suggests that interventions to foster pre-decisional information seeking should be tailored to the target age group.

### JOB STRAIN AND LATE-LIFE COGNITION: FINDINGS FROM THE PUERTO RICAN ELDERLY HEALTH CONDITIONS STUDY

Taylor Vigoureux,[1] Monica Nelson,[1] Ross Andel,[1] Brent Small,[1] Michael Crowe,[2] Ana Luisa Davila,[3] and Taylor Drury,[1] *1. University of South Florida, Tampa, Florida, United States, 2. University of Alabama at Birmingham, Birmingham, Alabama, United States, 3. University of Puerto Rico, San Juan, Puerto Rico*

Chronic stress at work, represented by job strain, has been associated with adverse late-life cognitive outcomes in the United States and Western Europe. We examined the relationship between job strain and change in cognition in a less affluent, Hispanic sample of adults aged 60-100 from mainland Puerto Rico. Job strain indicators (i.e., job demands/job control/job strain) were quantified from (a) standardized occupation-based job strain scores from Karasek's Job Content Questionnaire (JCQ; n=1102), and (b) O*Net variables forming factors of job demands and job control (n=1639). Occupation information, covariates, and cognition came from the Puerto Rican Elderly Health Conditions (PREHCO) Study conducted in 2002-2003, with cognition follow-up in 2006-2007. All analyses controlled for age, sex, baseline depressive symptoms, baseline financial problems, and childhood economic hardship. Across both operationalizations of job strain indicators, higher job control was associated with less decline in late-life cognition (JCQ: b=.18, p<.05; O*Net: b=.31, p<.001) until controlling for education (JCQ: b=.09, p=.248; O*Net: b=.12, p=.097). Job strain was associated with more decline in cognition (JCQ: b=-.75, p<.05; O*Net: b=-.87, p<.05) until controlling for education (JCQ: b=-.49, p=.098; O*Net: b=-.46, p=.262). For Karasek's measure, the relationships were driven by more educated participants. Job control was related to less cognitive decline whereas strain related to more decline among older Puerto Ricans over four years, whether assessed with JCQ-based or O*Net-based scores. However, education emerged as more important for change in late-life cognition than job strain indicators overall, suggesting results that diverge from countries with higher average socioeconomic status.

### MOVEMENT THERAPY AND COGNITIVE FUNCTION IN MIDDLE-AGED AND OLDER ADULTS: A 10-YEAR STUDY

Kallol Kumar Bhattacharyya, Hongdao Meng, Gizem Hueluer, and Kathryn Hyer, *University of South Florida, Tampa, Florida, United States*

Cognitive function is an important component of healthy aging and physical activities have been shown to support late life cognitive function. However, it is unclear whether non-traditional physical activities provide additional benefits for cognitive function above and beyond traditional leisure physical activities. This study examines the associations between movement therapy and cognitive function in the US population. We used data from the waves 1, 2 and 3 (1995-2014) of the Midlife in the United States (MIDUS) study. MIDUS included a national probability sample of community-living adults aged 25-75 years old in 1995 (wave 1) and added the wave 2 cognitive functioning tests of executive function and episodic memory. We applied multivariate linear regression models to estimate the effect of movement therapy (wave 2) on the cognitive episodic memory and executive function (wave 3) while controlling the covariates (wave 2 sociodemographic factors, health, and cognitive function). A total of 2097 individuals aged 42-92 years (mean 64.4, sd 10.9, 55.6% women) were included in the analysis. Movement therapy was independently associated with better episodic memory (beta=0.117, p=0.02), but not with executive function (beta=0.039, p=0.14), after including control variables. The results suggest that movement therapy may be an effective non-pharmacological intervention to attenuate age-related cognitive decline in middle-aged and older adults. Future research should test whether these findings can be replicated in similar populations and if confirmed, interventions should incorporate a wider range of physical activities in community-living older adults with the goal of maintaining and improving physical and cognitive health.

### NEURAL INHIBITION TASK ELICITS AGE-ASSOCIATED CHANGES IN PREFRONTAL CEREBRAL OXYGENATION

Talia Salzman,[1] Tabassum Rahman,[2] Nadia Polskaia,[1] Gabrielle St-Amant,[1] Diana Tobón Vallejo,[3] Yves Lajoie,[1] and Sarah Fraser,[1] *1. University of Ottawa, Ottawa, Ontario, Canada, 2. University of Ottawa, Ottawa, Canada, 3. Universidad de Medellin, Medellin, Antioquia, Colombia*

Certain cognitive tasks, such as those involving inhibition, can influence an older adult's dual-tasking ability more than others. This study aimed to manipulate cognitive task difficulty to evaluate age-associated differences in brain activity and behaviour during walking. Nineteen younger (M=21.3, SD=3.9) and 20 older (M=71.8, SD=6.4) adults completed four cognitive-auditory tasks: simple reaction time (SRT; processing speed), Go-no-Go (GNG; neural inhibition), N-back (NBK; working memory) and Double number sequence (DNS; working memory) with or without self-paced walking. Trials took place under single cognitive (SC), single motor (SM) and dual-task (walking with a cognitive task; DT) conditions. Throughout each condition, cerebral oxygenation changes ($\Delta HbO2$) in the prefrontal cortex were acquired using functional near-infrared spectroscopy (fNIRS). Behavioural measures including response time (ms), accuracy (%) and gait speed (m/s) were also calculated. Repeated measures ANOVAs revealed that OAs exhibited greater $\Delta HbO2$ than YAs in the left hemisphere during the GNG inhibition task (p = 0.04). Activation in the right hemisphere also increased compared to the left during DNS DT (p = 0.05). Response times increased with increasing task difficulty and YAs were faster than OAs during NBK SC (p = 0.09). Neural findings revealed age-associated changes in prefrontal activation at the GNG inhibition difficulty level. Behavioural results indicated poorer performance with increasing task difficulty including slower response times in OAs. Moreover, gait speed and accuracy only decreased within task and difficulty. Therefore, understanding the neural and behavioural changes across task difficulty may help monitor cognitive decline and distinguish normal aging from disease states.

### NOW YOU SEE THEM, NOW YOU DON'T: AGE DIFFERENCES IN RISK AVERSION

Barış Sevi,[1] Jenna Wilson,[2] JoNell Strough,[2] and Natalie Shook,[1] *1. University of Connecticut, Storrs, Connecticut, United States, 2. West Virginia University, Morgantown, West Virginia, United States*

Older age has often, but not always, been associated with greater risk aversion. Some have suggested that age differences in risk may reflect age-related declines in cognitive abilities. This study investigated the robustness of age differences in risk aversion across three different risk-taking measures, after controlling for cognitive abilities. Community-dwelling younger (n = 75; 25-36 years, M age = 29.01) and older (n = 74; 60-90 years, M age = 69.11) adults completed self-report and behavioral measures of risk aversion and several measures of cognitive abilities. Results showed that older adults reported significantly greater risk aversion than young adults on the behavioral measure of risk (Balloon Analogue Risk Task, BART), but not on the self-report measures (Framing Task and Choice Dilemmas Questionnaire). Greater risk aversion on BART was significantly associated with lower analytic thinking, slower processing speed, and worse shifting of attention. Therefore, we tested the relation between age and risk aversion on the BART while controlling for these three cognitive abilities. Age differences in risk aversion remained significant even after accounting for cognitive abilities. Our results suggest that the lack of consistent age differences in risk aversion in the literature may at least partly be due to measurement differences, which raises concerns about the construct validity of these measures of risk aversion. Moreover, cognitive decline may not explain age differences in risk. Further research is needed to understand factors that dampen and heighten risk aversion in people of diverse ages.

### PREDICTING AGE FROM LARGE-SCALE BRAIN NETWORKS: EVIDENCE FROM THE CAM-CAN DATASET ACROSS THE LIFESPAN

Meghan Caulfield,[1] Irene Kan,[1] and Evangelia Chrysikou,[2] *1. Villanova University, Villanova, Pennsylvania, United States, 2. Drexel University, Philadelphia, Pennsylvania, United States*

Changes in cognition observed in aging (e.g. a shift from prioritization of fluid cognition in young adulthood toward an emphasis on crystalized knowledge and semantic cognition in older adulthood) are believed to reflect alterations in neural connectivity in aging. Recent work specifically highlights how increased connectivity between executive control (EC) regions and default mode network (DMN) may underlie characteristic shifts in cognitive abilities between younger and older adults. However, the contribution of the salience network, which plays a crucial role in mediating the dynamic interplay between EC and DMN, is relatively overlooked. To extend previous work, we used a large cohort (N = 547) of participants from the Cam-CAN database (18-88 years old) to examine whether resting-state functional connectivity between EC and DMN can reliably predict participant age. We further examined how addition of the salience network impacts the hypothesized increased connectivity between EC and DMN as a result of aging. A series of multiple regression analyses using functional connectivity and age as variables revealed that connectivity between EC and DMN regions (specifically between dorsolateral and ventromedial prefrontal cortex and parietal regions, including the precuneus) accounted for a significant portion of age variability and that the inclusion of the salience network improved the models' explanatory power. Follow-up analyses by age cohort further highlighted that these relationships dynamically change across the lifespan. We will discuss these findings in the context of default-executive coupling hypothesis for aging and propose avenues for future research in refinement of this model.

### SPOUSAL LOSS AND COGNITIVE FUNCTIONING: DO PRE-LOSS MARITAL QUALITY AND GENDER MATTER?

Joohong Min,[1] and Jieun Song,[2] *1. Jeju National University, Jeju, Cheju-do, Republic of Korea, 2. Waisman center, Madison, Wisconsin, United States*

Prior research has found that the risk of cognitive decline increases after the death of a spouse. In general, the impact of life transitions is contingent on contextual factors such as socio-demographic characteristics or relationship quality. However, there is limited research on how marital quality before spousal loss and gender influence the association between spousal loss and cognitive change. The current study examines the effects of spousal loss on change in cognitive functioning as well as the moderating effects of pre-loss marital quality and gender. Data from two waves of the Midlife in the United States (MIDUS) study were analyzed (MIDUS2: 2004-05, MIDUS3: 2013-14). The analytic sample consists of two groups: (1) 179 bereaved adults who were age 54 or older at MIDUS2 (M = 65.2, SD = 9.5) and whose spouses died between MIDUS2 and MIDUS3, and (2) 179 non-bereaved adults, matched with the bereaved group on age and gender, who did not experience spousal loss between the two waves. Cognitive function was assessed via BTACT (Brief Telephone Adult Cognition Test) at both waves. Regression results show that both pre-loss marital quality and gender significantly moderate the association between spousal loss and change in cognitive functioning. Specifically, relative to their counterparts, men and those who reported better marital relationships prior to spousal death had a greater risk of cognitive decline after a spouse's death. The findings suggest the significance of pre-loss marital quality and gender for cognitive changes in widowhood and have implications for the development of efficient interventions

### TAKE ACTION OR WORRY? OLDER ADULTS' ANTICIPATED RESPONSES TO CONCERNING COGNITIVE ASSESSMENT RESULTS

Claudia Jacova,[1] Sara Wong,[1] and Samantha Smith,[2]
*1. Pacific University, Hillsboro, Oregon, United States,*
*2. Student, Hillsboro, Oregon, United States*

Under a third of older adults (28%) report having ever received an assessment for cognitive problems in US primary care settings. Patient resistance is cited as a major reason cognitive assessments are not performed. Theoretical models emphasize the role of anticipated benefits and harms in shaping health behaviors. Accordingly, here we investigated older adults' anticipated actions and worries regarding their cognitive assessment results. A total of 393 community-dwelling respondents between ages 50 and 91, 65% female, 89% college/university-educated, with no diagnosed cognitive disorder, completed Attitudes Around Cognitive Testing (AACT) at primary care sites (n=98) and through an online platform (www.mturk.com) (n=298). AACT examines older adults' preferences and concerns about cognitive assessment. It includes questions about actions participants would take and worries they would have if assessment results indicated cognitive problems. Willingness to take part in testing (yes or unsure/no) was also assessed. We found that seeking a formal diagnosis (84%), talking to family about healthcare (77%), and planning one's own future (70%) were highly endorsed actions, and becoming depressed (48%), becoming anxious (47%), and losing driving privileges (41%) highly endorsed worries. Logistic regression showed that total worries and worry-action difference scores predicted reduced willingness (OR=0.84, CI=0.75-0.93 and OR=0.82, CI=0.74-0.82, respectively), whereas total actions did not. Our results suggest that older adults view concerning cognitive assessment outcomes as an opportunity for taking action as well as a reason for worrying. Both worries and actions appear to play a role in deciding whether to take part in a cognitive assessment.

### THE ASSOCIATION BETWEEN COGNITIVE FUNCTION AND PREVENTIVE CARE SERVICE UTILIZATION AMONG U.S. CHINESE OLDER ADULTS

Zhing Loong Poh,[1] Dexia Kong,[2] Mengting Li,[2] and XinQi Dong,[2] *1. Rutgers University, The State University of New Jersey, Chicago, Illinois, United States, 2. Rutgers University, New Brunswick, New Jersey, United States*

Preventive healthcare utilization is an important aspect of medical practice that facilitates the identification of chronic diseases at an early stage and increases options for treatment. Cognitive function plays an important role in individuals' utilization of preventive care services. However, our understanding of the relationship between cognitive function and preventive care utilization is limited, particularly in older minority aging populations. The study aims to assess the association between cognitive function and preventive healthcare utilization among U.S. Chinese older adults. Data were obtained from the Population Study of Chinese Elderly in Chicago (PINE). Five instruments were used to measure global cognition, including the Mini-Mental State Examination, East Boston Memory Test Immediate Recall and Delayed Recall, Digit Span Backwards, and Symbol Digit Modalities Test. Preventive care services included immunization (i.e. flu, pneumonia, and hepatitis B vaccines) and cancer screenings (i.e. colorectal, breast, cervical, and prostate). Multivariable regression analyses were used. The findings showed that higher level of global cognition was associated with higher utilization of pneumonia vaccination (OR=1.32, 95% CI= 1.14-1.52), hepatitis B vaccination (OR=1.24, 95% CI= 1.05-1.47), colon exam (OR=1.23, 95% CI= 1.07-1.41), mammogram (OR=1.46, 95% CI= 1.22-1.73), breast exam (OR=1.23, 95% CI= 1.04-1.46), and cervical exam (OR=1.38, 95% CI= 1.15-1.65). Future longitudinal studies are needed to elucidate potential mechanisms underlying the relationship between cognitive function and preventive care utilization among U.S. Chinese older adults. Study findings underscore the need to understand preventive care utilization patterns among U.S. Chinese older adults with low cognitive function.

### THE EFFECTS OF READING ENGAGEMENT ON OLDER ADULTS' RESOURCE ALLOCATION IN READING AND COGNITION

Giavanna McCall,[1] Sueyoun Hwang,[2] Takudzwa George,[2] Xiaomei Liu,[2] and Elizabeth A L Stine-Morrow,[3]
*1. University of Illinois at Urbana-Champaign, Urbana, Illinois, United States, 2. Beckman Institute, Urbana, Illinois, United States, 3. University of Illinois, Urbana, Illinois, United States*

With aging, print exposure has been shown to predict effective resource allocation during reading, as well as to account for growth in certain abilities. To test the hypothesis that literacy engagement has causal effects on reading processes and the cognitive abilities on which they depend, we developed a home-based literacy intervention administered via iPads. Participants (64% female; 60-79 years of age; < 10hrs/week of reading at baseline) were randomly assigned to an 8-week reading intervention group (developed in collaboration with a Champaign Library adult literacy specialist) or to a puzzle control, both of which involved ~8hrs/week of activity engagement. At pretest and posttest, participants performed a self-paced reading task to assess resource allocation to word and conceptual processing, and completed a battery of cognitive measures assessing episodic memory, working memory, and verbal fluency (alphas $\geq$ .79). Based on one-tailed tests, results showed greater positive change in working memory, $t(57)=1.69$, $p<.05$, for the literacy group relative to the control, and a marginally significant difference in change in episodic memory, $t(57)=1.62$, $p<.06$, but not in verbal fluency, $t< 1$. In addition, there was a significant difference between conditions in change in resource allocation to conceptual processing, $t(53)=1.75$, $p<.05$. Changes in working memory and conceptual processing were positively correlated ($r=.27$, $p=.02$). These findings suggest that reading engagement may be beneficial for older adults' growth in fluid ability, which impacts reading processes, so as to create a "virtuous spiral" of resilience between literacy engagement and ability.

### THE REMINISCENCE BUMP EFFECT IN AUTOBIOGRAPHIES

Hannah Benz,[1] Thomas Pierce,[2] and Grace Flood,[2]
*1. Radford University, Blacksburg, Virginia, United States, 2. Radford University, Radford, Virginia, United States*

The reminiscence bump effect refers to the tendency for older adults to recall more life events from their late teens through early thirties than from other periods of life. Participants in laboratory studies are often encouraged to recall events quickly and spontaneously, with the intention of eliciting those life events that come most easily to mind. The purpose of this study was to determine if a reminiscence bump effect is observed in accounts of life events that are carefully organized and narrated over prolonged periods of time; specifically, those presented in published autobiographies. 1911 life events were collected from the autobiographies of 16 authors. Four authors were female and 12 were male. The mean age of authors at the time of publication was 65.88 (SD = 13.94). 45.52% of life events reported in autobiographies occurred when authors were between 18 and 32 years of age, a value significantly higher than expected by chance [Chi-square (1, N = 1911) = 457.70, p < .001] and consistent with those found in laboratory studies supporting the reminiscence bump effect (e.g., Rubin, Wetzler, & Nebes, 1986). In addition, the 15-year reminiscence bump period represented an average of 22.77% of the authors' lives at the time they published their autobiography; however, events from the reminiscence bump period took up an average of 42.52% of the pages in their autobiographies. These data provide evidence for a reminiscence bump effect in carefully considered life story narratives which required months or years to complete.

### WEIGHT STATUS INFLUENCES CAROTID INTIMA-MEDIA THICKNESS TO EXECUTIVE FUNCTION AMONG OLDER ADULTS

Desiree Bygrave, and Regina Wright, *University of Delaware, Newark, Delaware, United States*

Carotid atherosclerosis has emerged as an early predictor of reduced cognitive function. Underlying this association are risk factors, such as overweight and obesity, that promote carotid atherosclerosis and poor cognitive outcomes. Given the prevalence of overweight and obesity among older adults, there is a critical need to better understand how atherosclerosis influences cognitive function in the context of elevated weight. To address this gap, the current study examined relations between carotid atherosclerosis (carotid intima-media thickness [IMT]), and attention (Trailmaking Test) and executive function (Verbal Fluency Test) performance, and whether they varied as a function of weight status (body mass index [BMI] classification). Data were analyzed from 162 older adults (mean age = 68.43y, 34% male, 41% African American), free of major disease. Mutliple regression and analysis of variance analyses, adjusted for age, sex, education and mean arterial pressure, showed a statistically significant IMT x BMI interaction for Verbal Fluency performance (p=.04) and a trending IMT x BMI interaction for Trailmaking A performance (p=.05). Simple effects analysis of IMT and Verbal Fluency performance showed that this association was most pronounced among those who are obese. Findings suggest atherosclerosis may influence executive function in the context of obesity among older adults. As the development of carotid atherosclerosis is strongly related to aging, our findings suggest that maintaining a healthy weight may reduce its impact on executive function in older adulthood.

### WHY OLDER ADULTS RECALL AUTOBIOGRAPHICAL MEMORIES FROM THEIR YOUTH

Tabea Wolf, and Daniel Zimprich, *Ulm University, Ulm, Baden-Wurttemberg, Germany*

When older adults are asked to remember their lives, they recall disproportionally more events from their youth (e.g., Rubin, Rahhal, & Poon, 1998). This phenomenon, called the reminiscence bump, is one of the most robust findings in autobiographical memory research. Whereas most explanatory accounts have focused on differential encoding and retention of memories experienced during one's youth (e.g., Rubin et al., 1998), recent research also puts emphasis on the retrieval of memories (e.g., Glück & Bluck, 2009; Rubin & Berntsen, 2003). In the present study, we take a functional perspective on the reminiscence bump and examine why older adults recall memories from their past. Participants (age 57-89; N = 112) reported memories in response to 30 emotionally neutral cue-words and self-rated each memory for serving directive, social-bonding, self-continuity, and mood-enhancing functions (Wolf & Demiray, 2019). The age distribution shows an early reminiscence bump located between the ages of 6 and 20 years. Compared to memories from later life periods, memories from the reminiscence bump more frequently serve self-continuity and less frequently directive and mood-enhancing functions. No differences were found regarding the use of memories for social-bonding. The results strengthen the assumption that experiences from one's youth serve to maintain a sense of self-continuity throughout the lifespan (e.g., Rathbone et al., 2008). To cope with current problems or emotions, however, older adults are more likely to draw on experiences from their adult life – probably because these experiences are more similar to what they are experiencing now.

### SESSION 2947 (POSTER)

### DEPRESSION AND ANXIETY

### A SYSTEMATIC REVIEW OF THE PSYCHOMETRIC PROPERTIES OF THE GERIATRIC ANXIETY INVENTORY

Philippe Landreville,[1] Alexandra Champagne,[1] and Patrick Gosselin,[2] *1. Université Laval, Québec, Quebec, Canada, 2. Université de Sherbrooke, Sherbrooke, Quebec, Canada*

The Geriatric Anxiety Inventory (GAI) is a widely used self-report measure of anxiety symptoms in older adults. Although much research has been conducted on the psychometric properties of the GAI, previous reviews have examined only a small proportion of studies and have not evaluated the methodological quality of this work. In view of this, we conducted a systematic review of the psychometric properties of the GAI and it's short form (GAI-SF). Relevant studies (N = 31) were retrieved through a search of electronic databases (Pubmed, PsycINFO, CINAHL, EMBASE and Google Scholar) and a hand search. The methodological quality of the included studies was assessed by two independent reviewers using the "COnsensus-based Standards for the selection of health status Measurement INstruments" (COSMIN) checklist. Based on the COSMIN checklist, internal consistency and test reliability were mostly rated as poorly assessed (63% and 72.7% of studies, respectively) and quality of studies examining structural validity was mostly fair (60% of studies). Both the GAI and GAI-SF showed adequate internal consistency and test-retest reliability. Convergent validity indices were highest with measures of generalized anxiety and lowest with instruments that include somatic symptoms. Substantial overlap with measures of depression was reported. While there is no consensus on the factorial structure of the GAI, the short version was found to be unidimensional. Our review therefore suggests that the GAI and GAI-SF have satisfactory psychometric properties while indicating that future efforts should aim to achieve a higher degree of methodological quality.

### CLINICAL DEPRESSION, DEPRESSIVE SYMPTOMS, AND DEPRESSED COGNITION IN CAREGIVING GRANDMOTHERS

Christopher Burant,[1] Alexandra Jeanblanc,[2] Carol Musil,[2] McKenzie Wallace,[3] and Zauszniewski Jaclene,[2] *1. Case Western Reserve University, Parma, Ohio, United States, 2. Case Western Reserve University, Cleveland, Ohio, United States, 3. Case Western Reserve University, Lorain, Ohio, United States*

Grandmothers living with or raising grandchildren have elevated levels of depressive symptoms compared to grandmothers who do not provide care. While the CES-D measures the somatic, positive and negative affect, and interpersonal strain symptoms experienced with depression, the Depressive Cognitions Scale captures the change in cognitive thinking that often precedes clinical depression. Our aim was to compare depressive symptoms and depressed cognitions between grandmother caregivers with a diagnosis of depression and those without in a nationwide sample of 342 grandmother caregivers. In the questionnaire, participants were asked whether they had a diagnosis of depression amongst other health conditions and also completed the CES-D and the Depressed Cognitions Scale. A score of 16 or greater on the CESD or a score above 7 on the Depressed Cognitions Scale can be used to identify individuals who may be at risk for depression. Grandmothers who had a diagnosis of depression were more likely to have CES-D scores 16 and above (79.7 %) as compared to those who were not (39.2%) (Chi Square=54.55, p<.001); and more likely to have higher depressed cognition scores 7 and above (71.3 %) as compared to those who did not (42.9%) (Chi Square=26.68, p<.001). Additionally, grandmothers who had depressed cognitions were more likely to have CES-D scores 16 and above (74.1 %) as compared to those who were not (33.1%) (Chi Square=57.56, p<.001). The elevated scores in participants who already have a diagnosis of depression indicates the need for potential interventions to further address depressive symptoms in grandmother caregivers.

### CONFIRMATORY FACTOR ANALYSIS OF THE GERIATRIC DEPRESSION SCALE TO MEASURE APATHY IN OLDER ADULTS

Mirnova Ceide, Aviva Cantor, Joe Verghese, and David Lounsbury, *Albert Einstein College of Medicine, Bronx, New York, United States*

Apathy is a psychological syndrome characterized by lack of motivation, interest, flattening of affect and social withdrawal; which has been associated with cognitive and functional decline in community dwelling older adults. Many of these associations have been investigated using subscales of depression tools, with varying sensitivity and specificity. The objective of this study was to develop an apathy measurement model, which distinguishes depression and apathy, using the Geriatric Depression Scale Long Form (GDS). We conducted an exploratory factor analysis (EFA) in a memory clinic cohort (N=81) who completed the GDS. Based on the EFA results, we ran a confirmatory factor analysis (CFA) in a community dwelling cohort of older adults (≥65 years old) enrolled in the Central Control of Mobility in Aging study (CCMA) and computed factor scores in both cohorts. CFA of the GDS in the CCMA cohort (N=538) yielded 3 factors: Depression (14 items; Cronbach's $\alpha$=0.77), Apathy (10 items; Cronbach's $\alpha$=0.63), and Cognitive concern (4 items; Cronbach's $\alpha$=0.546). In linear regression models, adjusted for age and gender, the memory clinic cohort had significantly higher Apathy factor scores ($\beta$ = .18, t(618) = 4.49, p < .001) factor scores compared to the CCMA cohort.Our findings suggest that the CFA derived apathy score is a reliable and valid tool to distinguish apathy from depression in older adults. This novel measurement model can be employed in both clinic and research populations to investigate the role of apathy in cognitive decline.

### DEPRESSION SCREENING AND RATES OF MOOD DISORDER DIAGNOSIS AND SUICIDE IN YOUNGER AND OLDER ADULTS

Julie Lutz,[1] Kenneth Conner,[1] Michael Hasselberg,[1] Kathleen Fear,[1] and Kim Van Orden,[2] *1. University of Rochester Medical Center, Rochester, New York, United States, 2. University of Rochester School of Medicine and Dentistry, Rochester, New York, United States*

Rates of suicide are elevated among older adults in the U.S. and around the world, with the highest rates in older men (CDC, 2019; WHO, 2014). More than half of older adults who die by suicide were in contact with a primary care physician within the month prior to death, and almost one-third within the prior week (Luoma et al., 2002; Stene-Larsen & Reneflot, 2019), demonstrating the importance of better identification and treatment of mental health issues among older adults across healthcare systems. Depression, a well-established risk factor for suicide, goes under-diagnosed and under-treated, especially among older adults (Bryant, 2010). The University of Rochester Medical Center has led integration of patient-reported outcomes assessment via large-scale implementation of PROMIS measures across multiple departments. We compared results of PROMIS depression screening from 1/1/2015 to 8/31/2019 with mood disorder diagnoses within the year prior and year following screening. Twenty-six percent (39491/154669) of adults under age 65 and 23% (11694/51702) of those age 65 and older screened positive for mild, moderate, or severe depression. Whereas 29.0% of younger adults who screened positive received mood disorder diagnoses, 22.1% of older adults received a diagnosis ($\chi 2(1)$=214.69, p<.001). Suicide outcomes using National Death Index data will also be reported. Results confirm depression is underdiagnosed (and, by extension, likely undertreated) at all ages, but show a distinct disparity for older adults, which may put them at greater risk for negative outcomes such as suicide.

### DEPRESSIVE SYMPTOM PROFILE TRANSITIONS FROM MIDLIFE TO LATE LIFE IN THE WISCONSIN LONGITUDINAL STUDY

Kyle Carr, *Boston College, Chestnut Hill, Massachusetts, United States*

Late-life depression is a significant public health problem among the growing elderly population in the United States. Structural social capital has previously been associated with lowering depressive symptoms in later life, but these results have been inconsistent. However, few studies have investigated this association when investigating different subtypes of depression. The current study used data from the Wisconsin Longitudinal Study (WLS) of 3,197 respondents to examine how structural social capital influences baseline depression statuses and transitions in these depression statuses. Latent class and latent transition analysis (LCA/LTA) were used to identify latent statuses at two time points in the WLS – 1992 and 2011 – as well as transitions between those statuses. Four depression statuses were identified at both time points: Very Depressed, Depressed and Lonely, Agitated and Restless, and Not Depressed. Gender, self-rated health, total assets, structural social capital measures, and polygenic score for depression were all predictors of baseline depression statuses. Transitions between depression statuses were associated with two forms of structural social capital – social support and social involvement. These findings add to the increasing number of studies investigating subtypes of depression in older adults as well as to scholars examining the association between structural social capital and depressive symptoms. Results suggest possible social and behavioral factors policymakers can use to identify risk of depression in mid-life and areas of intervention to improve depressive symptoms for aging adults.

### DIETARY BEHAVIOR AND DEPRESSIVE SYMPTOMS IN LATE-LIFE MARRIAGE

Talha Ali,[1] Gail McAvay,[2] and Joan Monin,[1]
*1. Yale University, New Haven, Connecticut, United States, 2. Yale School of Medicine, New Haven, Connecticut, United States*

Epidemiologic studies have linked dietary patterns to psychological health including depression, anxiety, and stress. However, no research has examined dyadic associations between dietary behavior and depressive symptoms in older married couples. In this study, one hundred and one couples 51 to 90 years of age who were married or in a marriage-like relationship and living together for at least 6 months were recruited. Participants completed questionnaires and self-reported their dietary behavior (i.e., the total number of meals, number of snacks, and number of fast-food meals eaten in a typical day and the number of meals they eat alone and eat sitting down). They also completed the 20-item Center for Epidemiologic Studies Depression Scale. Results of the Actor Partner Interdependence Models controlling for income, education, chronic conditions, and marital satisfaction, showed that for wives only, more meals eaten in a day were associated with lower depressive symptoms (actor effect). Additionally, more snacks eaten by the wife and more meals eaten alone by the wife were associated with higher depressive symptoms for the husband (partner effects). Findings suggest that wives' dietary behavior is particularly important, not only for their own but also their husbands' mental health in late-life marriage.

### EFFECTS OF PAIN AND DEPRESSION ON ADL DISABILITY OVER 6 YEARS OF FOLLOW-UP AMONG OLDER ADULT AMERICANS

Jaspreet Sodhi, and Soham Al Snih, *The University of Texas Medical Branch at Galveston, Galveston, Texas, United States*

The objective of this study was to examine the effect of co-occurring pain and depression on ADL disability over 6-years of follow-up among older adult Americans. We studied 5,236 participants aged 65 years and older from the National Health and Aging Trends Study (2011-2017) The primary outcome was ADL disability defined as any limitation in ADLs (eating, bathing, transferring, dressing, moving inside, and out of bed). The independent predictors were self-reported pain and depression. Covariates included socio-demographics (age, gender, marital status, race/ethnicity and years of formal education), body mass index, and comorbidities. Participants were categorized into four groups according to pain and depression: no pain and no depression, pain only, depression only, and depression and pain. Generalized Estimation Equation model was used to estimate the odds of ADL disability as a function of pain and depression. All variables were analyzed as time-varying except for age, race/ethnicity, and education. The odds of ADL disability as a function of pain only and depression only was 1.62 (95% CI 1.38-1.91) and 2.13 (95% CI 1.54-2.95), respectively. The odds of ADL disability as a function of pain and depression were 3.92 (95% CI 3.13-4.92). Older age, being married, Hispanics, and comorbid conditions were also predictive factors of ADL disability over time. Female participants and those with higher levels of education were less likely to report ADL disability over time. The findings suggest that both pain and depression significantly increased the risk of ADL disability in this population over 6-years.

### EFFECTS OF TREATMENT PREFERENCE ON ADHERENCE, ATTRITION, AND PROCESS MEASURES AMONG OLDER ADULT WORRIERS

Gretchen Brenes,[1] Heidi Munger Clary,[1] Michael Miller,[1] Jasmin Divers,[2] Andrea Anderson,[1] Gena Hargis,[1] and Suzanne Danhauer[1] *1. Wake Forest School of Medicine, Winston-Salem, North Carolina, United States, 2. NYU Winthrop Hospital, New York, United States*

Patient preference may be related to treatment outcomes through decreased rates of attrition and higher rates of adherence and patient satisfaction. We present findings from a 2-stage randomized preference trial of cognitive-behavioral therapy (CBT) and yoga for the treatment of late-life worry. We examine rates of preference for CBT and yoga, as well as the stability of these preferences over time. We also examine the impact of preference on adherence, attrition, and process measures (satisfaction, treatment expectancies, and working alliance). Five hundred participants were randomized to either the randomized controlled trial (RCT; N=250) or the preference trial (participants chose the treatment; N=250). All participants received 10 weeks of an intervention. Among those in the preference trial, 48% chose CBT and 52% chose yoga (p>.05). Strength of preference was similar between the groups; 73.3% and 76.2% reported a strong preference for CBT and yoga, respectively (p>.05). Fourteen percent of those who preferred CBT at baseline preferred yoga upon completion of the intervention, while 12.2% of those who preferred yoga at baseline preferred CBT upon completion of the intervention (p>.05). There were no significant differences between participants in the RCT and preference trial on intervention adherence, attrition, satisfaction, or working alliance (p's>.05). Treatment expectancies were higher for the preferred intervention (p's<.0001). Results suggest that older adults prefer CBT and yoga at similar rates, and these preferences are stable. Receiving a preferred treatment had no effect on adherence, attrition, satisfaction, or working alliance.

### GRANDPARENTING AND WELL-BEING OF HONG KONG OLDER ADULTS: THE MEDIATING ROLE OF INTERGENERATIONAL RELATIONSHIPS

Daniel W. L. Lai, Jessica J. Li, and Vincent Lee, *The Hong Kong Polytechnic University, Kowloon, China*

Grandparenting and intergenerational relationship play important roles in some older adults' later life, especially older people of Chinese culture. This study investigated the relationship between grandparenting activities, intergenerational relationship, and psychosocial well-being of Hong Kong Chinese older adults. A representative sample of 507 grandparents (aged 55+) were telephone surveyed in June to July 2019. Level of involvement in grandparenting activities was measured. Resilience and happiness were measured by Connor-Davidson Resilience Scale and Subjective Happiness Scale. Two single-item instruments were adapted to capture the relationships between older adults and adult children, and between grandparents and grandchildren, respectively. A series of linear regressions and mediation tests with bootstrap approach were performed to examine the relationships between grandparenting activities, intergenerational relationship, and resilience and happiness. After controlling for socio-demographics, the frequency of grandparenting activities correlated positively with resilience and happiness. The relationship was partially mediated by inter-generational relationships including the relationships with adult children and grandchildren. The findings have concluded that grandparenting involvement and satisfactory intergenerational relationship are protective factors of health and wellbeing. Future healthy aging policy-making or programming should expand the scope from focusing on individual older adults to strategies of achieving the family-friendly goal so that intergenerational relationships could be better nurtured, benefiting not just the family as a functional unit but also the older adults' healthy aging.

### INTERNET-BASED COGNITIVE-BEHAVIORAL THERAPY FOR DEPRESSION: A FEASIBILITY STUDY FOR HOMECARE OLDER ADULTS

Xiaoling Xiang,[1] Yihang Sun,[1] Shawna Smith,[1] Patrick Ho Lam Lai,[2] and Joseph Himle,[1] *1. University of Michigan, Ann Arbor, Michigan, United States, 2. University of Michigan-Ann Arbor, Ypsilanti, Michigan, United States*

This pilot study examined the feasibility of delivering internet-based cognitive behavioral therapy (iCBT) to homebound older adults with symptoms of depression who are recipients of non-medical home care. A feasibility open trial was conducted in the homes of homecare older adults (n=26). When possible, home care workers (HCWs) of older adults (n=13) were recruited to provide external support for iCBT usage. In cases where consistent assistance from the same HCW was not feasible, participants were given the choice of working on the program on their own (n=7) or receiving assistance from a research assistant (RA) (n=6). The mean therapy sessions completed was 4.7 out of 8 total sessions. The mean satisfaction rating was 7.7 (SD=2.9) and 86% would recommend the program to others with depressed mood. Significant reductions in depressive symptoms and anxiety symptoms and improvement on a quality of life measure were observed at post-test. The RA-supported group tended to have the best adherence, satisfaction, and reduction in depressive symptoms, followed closely by the HCW-supported group. The self-guided group had the lowest adherence, satisfaction, and symptom reduction. iCBT is a feasible and acceptable treatment modality for homebound older adults with depressive symptoms and potentially effective. Data from the participant exit interviews suggest a need for refining the existing treatment platform to better meet the needs and capabilities of homebound older adults. Future studies are warranted to examine treatment effectiveness as a function of HCW support.

### MEDIATING ROLE OF TRAIT MINDFULNESS ON THE RELATIONSHIPS BETWEEN AGE AND BOTH DEPRESSIVE AND ANXIETY SYMPTOMOLOGY

Scotti Howard, and Eric Allard, *Cleveland State University, Cleveland, Ohio, United States*

Previous research has shown that despite experiencing more negative life events, older adults maintain relatively high levels of well-being compared to their younger counterparts. This effect appears to be at least partially mediated by trait mindfulness in older adults (Raes et al., 2013). The current study expanded into an investigation as to how trait mindfulness might intervene on the relationship between age and other well-being indicators: anxiety and depressive symptomology. Participants included 30 older adults (aged 60-83) and 41 young adults (aged 18-35). Trait mindfulness was examined using the Mindful Attention Awareness Scale (MAAS), while depressive symptoms and trait anxiety were measured using the Center for Epidemiological Studies Depression Scale (CES-D) and the State-Trait Anxiety Inventory (STAI), respectively. Two separate mediated multiple regression models were conducted using Hayes' PROCESS Macro in SPSS. Trait mindfulness exhibited a significant indirect effect on the relationship between age and depressive symptoms ($\beta = -2.27$, $p < .005$), which was also seen for the relationship between age and trait anxiety ($\beta = -4.17$, $p < .001$). Older age predicted higher trait mindfulness, which in turn predicted diminished self-reported anxiety and depressive symptomology. Controlling for mindfulness in these models reduced the direct effect of age on depression and anxiety to non-significance. These findings imply that the relationship between age and trait mindfulness can be extended to alternative markers of well-being.

### REGIONAL BRAIN VOLUMES ASSOCIATED WITH DEPRESSION IN THE NATIONAL ALZHEIMER'S COORDINATING CENTER UNIFORM DATA SET

Shanna Burke,[1] Tan Li,[1] Adrienne Grudzien,[1] Christopher Barnes,[2] Kevin Hanson,[2] and Steven DeKosky,[3] *1. Florida International University, Miami, Florida, United States, 2. University of Florida, Gainesville, Florida, United States, 3. University of Florida College of Medicine, Gainesville, Florida, United States*

Depression has been associated with greater risk of Alzheimer's disease (AD), and existing research has identified structural differences in brain regions in depressed subjects compared to healthy samples, but results have been heterogeneous. We sought to determine the effect of depression on regional brain volumes by cognitive and APOE e4 status. Secondary analysis of the National Alzheimer's Coordinating Center (NACC) Uniform Data Set was conducted using complete MRI data from 1,371 participants (mean age: 70.5; SD: 11.7). Multiple linear regression was used to estimate the adjusted effect of depression (via the Neuropsychiatric Inventory Questionnaire) on regional brain volumes through measurement of 30 structural MRIs. Depression in the prior two years was associated with lower total brain, cerebrum,, and gray matter volumes and greater total brain white matter hyperintensities ($p<.05$). Greater volumes were also observed in all ventricular volume measures. Lower mean volumes were observed in six additional frontal lobe and parietal lobe cortical regions. Alternately, depression antecedent to the past 2 years correlated only with occipital lobe gray matter volumes (right, left, total). Our findings suggest that depression in the prior two years is associated with atrophy across multiple brain regions and related ventricular enlargement, even after controlling for intracranial volume and demographic covariates. The duration of depression influences results, however, as depression prior to 2 years before assessment was correlated with significantly fewer and different regional brain volume changes.

### THE EFFECT OF ANXIETY ON REGIONAL BRAIN VOLUMES IN THE NATIONAL ALZHEIMER'S COORDINATING CENTER UNIFORM DATA SET

Shanna Burke,[1] Tan Li,[1] Adrienne Grudzien,[1] Christopher Barnes,[2] Kevin Hanson,[2] and Steven DeKosky,[3] *1. Florida International University, Miami, Florida, United States, 2. University of Florida, Gainesville, Florida, United States, 3. University of Florida College of Medicine, Gainesville, Florida, United States*

Anxiety has been associated with greater risk of Alzheimer's disease (AD) and existing research has identified structural differences in regional brain tissue in anxious compared to healthy samples, but results have been variable and somewhat inconsistent. We sought to determine the effect of anxiety on regional brain volumes by cognitive and apolipoprotein e (APOE) e4 status using data from a large, national dataset. A secondary analysis of the National Alzheimer's Coordinating Center Uniform (NACC) Data Set was conducted using complete MRI data from 1,371 participants (mean age: 70.5; SD: 11.7). Multiple linear regression was used to estimate the adjusted effect of anxiety (via the Neuropsychiatric Inventory Questionnaire) on regional brain volumes through measurement of 30 structural MRI biomarkers. Anxiety was associated with lower total brain and total cortical gray matter volumes and increased lateral ventricular volume (p<.05). Lower mean volumes were also observed in all hippocampal, frontal lobe, parietal lobe, temporal lobe, and right occipital lobe volumes among participants who reported anxiety. Conversely, greater ventricular volumes were also correlated with anxiety. Findings suggest that anxiety is associated with significant atrophy in multiple brain regions and ventricular enlargement, even after controlling for intracranial volume and demographic covariates. Anxiety-related changes to brain morphology may contribute to greater AD risk.

### THE PENN STATE WORRY QUESTIONNAIRE IS INVARIANT ACROSS AGE GROUPS

Robert Intrieri, and PAIGE GOODWIN, *Western Illinois University, MACOMB, Illinois, United States*

Prevalence of GAD is between 3 to 5% with onset in the early to mid-twenties (Kessler et al. 2009). The Penn State Worry Questionnaire (PSWQ) is a 16-item self-report instrument assessing generalized anxiety symptoms (Meyers, et al., 1990; Molina & Borkovec, 1994). Brown (2003) and Olatunji et al. (2007) conducted Confirmatory Factor Analyses identifying a two factor model of Worry Engagement and Absence of Worry. No published studies have examined the factor structure of the PSWQ across age groups. The current study presents data from 612 people across three groups: 221 young adults (Mage = 19.31, SD = 1.21), 283 middle-age adults (Mage = 48.27, SD = 5.13), and 108 older adults (Mage = 72.95, SD = 7.19). An ordinal confirmatory factor analysis (CFA) using robust weighted least squares (WLSMV) tested for invariance across groups. Results showed CFI/TLI values of .983/.981, 984/.983, and .981/.984 for Configural (CI), Metric (MI), and Scalar (SI) models. The RMSEA for CI, MI, and SI models was .064, .061, and .059. Based upon Cheung and Rensvold (2002), Sass (2011), and Chen (2007), a cutoff of $\Delta CFI \geq 0.01$ was established as evidence of invariance. The $\Delta CFI$ between CI and MI models was < .01 so analysis continued with the SI test. The $\Delta CFI$ between MI and SI models was < 0.01 and did not justify rejection of the null hypothesis. These analyses suggest PSWQ scores are valid across age groups and provide additional support for the multidimensional nature of the PSWQ.

### THE WHO AND WHEN OF WORRY: THE FADING IMPACT OF GENDER ON WORRY AND ANXIETY SEVERITY AMONG OLDER ADULTS

Katie Granier,[1] Rebecca Ingram,[2] and Daniel Segal,[3]
*1. University of Colorado Colorado Springs, Colorado Springs, Colorado, United States, 2. UCCS, Colorado Springs, Colorado, United States, 3. University of Colorado at Colorado Springs, Colorado Springs, Colorado, United States*

Introduction. This study examined the influence of age and gender on diverse worry constructs and overall anxiety among younger (age 18-30) and older (age 65+) adults. Methods. 411 participants (311 younger, 100 older adults; 77.1% female) completed the PSWQ, BMWS, WDQ, and GAS online. Results. Among a series of 2x2 between-subjects ANOVAs, significant interaction effects between age and gender were found among all worry and anxiety measures. Specifically, there was a significant interaction effect on worry severity as measured by the PSWQ (F[1, 393]=4.28, p<.05), the WDQ (F[1, 397]=8.42, p<.01) and the BMWS (F[1, 396]=10.41, p<.01). Gender had a larger impact on worry among younger adults than older, though both age groups showed similar patterns of women reporting greater worry than men. Though both younger and older adults showed a gender difference in worry severity, this difference was mitigated by late life. There was also an interaction effect on anxiety (GAS total) in that younger women reported greater anxiety than younger men but older adults reported similar anxiety across genders, F(1, 384)=9.78, p<.01. Simple main effects analysis showed that younger women scored higher than older women on all measures of worry and anxiety, whereas younger men scored higher than older men on the PSWQ and WDQ but not the BMWS or GAS. Discussion. Consistent with previous literature, women reported greater worry and anxiety than men. However, this difference was mitigated and even extinguished among some measures in older adults. Possible explanations are discussed.

### SESSION 2948 (POSTER)

### HEALTH PROMOTION AND WELL-BEING I

### AN EXAMINATION OF THE MEIKIRCH MODEL'S MULTIDIMENSIONAL DEFINITION OF HEALTH AND AGING

Hillary Dorman,[1] Rebecca Allen,[2] Patricia Parmelee,[1] and Lisa McGuire,[3] *1. University of Alabama, Tuscaloosa, Alabama, United States, 2. Alabama Research Institute on Aging, Tuscaloosa, Alabama, United States, 3. Centers for Disease Control and Prevention, Atlanta, Georgia, United States*

The present study used the CDC's 2017 Behavioral Risk Factor Surveillance System and Structural Equation Modeling (SEM) to test the Meikirch model's theoretical definition of health and aging. This biopsychosocial and ecological framework considers the dynamic interplay between an individual's biology, health behavior and health potential, social surroundings, physical environment, and demands of living on both mental and physical health outcomes. A total sample of 96,568 adults were included with a mean age of 66.05 years (SD = 9.91). Individuals were classified in the following age groups: 43% middle-aged (45-64 years), 33% young-old (65-74 years), and 25% old-old adults (75+ years). The sample was largely female (61%), Non-Hispanic White (86%), and urban (67%). A CFA and WLSMV estimation was used to assess each latent construct. The overall SEM model was found to be a good fit (RMSEA=0.05, CFI=0.94, TLI=0.90), explained a significant portion of the variance in the health outcome (65%), and appeared to mimic prior literature on health variable relationships. An important finding within this study suggests the significant relationship between education, personally acquired potential, and health outcome variables. Underscoring health education and health literacy interventions may positively promote a person's health behavior, resource access, and health outcomes across the lifespan. The Meikirch model can be used as a framework in public health interventions to better understand health adaptation, as well as behavioral risks and systematic hurdles. Overall, the study emphasizes how understanding health is not exclusively an individual hurdle to tackle, but a communal goal.

### ARE WE MISSING THE TARGET WHEN MEASURING QUALITY OF LIFE?

Leah Janssen,[1] and Kate de Medeiros,[2] *1. Miami University, Hamilton, Ohio, United States, 2. Miami University, Oxford, Ohio, United States*

Over the last several decades, many instruments have been created to measure quality of life (QoL) in older adults, particularly for intervention research on individuals living with dementia. However, since definitions of QoL lack standardization across the research literature, the question of how to holistically capture an elusive and expansive concept such as QoL remain. This research uses qualitative content analysis to explore definitions and domains of QoL with an eye toward overlap and gaps. Definitions of QoL were extracted from gerontology encyclopedia entries and other peer-reviewed supplemental resources and analyzed for themes using Dedoose qualitative software. Results revealed three over-arching themes: no standardized or universal definition of QoL, use of subjective and objective factors for measurement, and varying domains of QoL. Additionally, we further distilled theme three to identify eight unique QoL domains: 1) economic/financial, 2) environment, 3) ADL/IADL function, 4) participation in activities, 5) personal resources, 6) physical health, 7) psychological well-being, and 8) social/relational, the total of which were only found in one of 15 definitions of QoL. Overall, findings led to an overarching definition of QoL that cuts across multiple dimensions and factors. We argue that by having all eight domains our understanding and measurement of QoL is enhanced, thereby improving our assessment of existing definitions of QoL, as well as the instruments used to measure QoL.

### ASSOCIATION BETWEEN MENTAL HEALTH CONDITIONS AND CARE FRAGMENTATION: A NATIONAL STUDY OF HIGH-RISK OLDER VETERANS

Ranak Trivedi,[1] Fernanda Rossi,[2] Sarah Javier,[2] Liberty Greene,[3] Sara Singer,[4] Megan Vanneman,[5] Mary Goldstein,[6] and Donna Zulman,[3] *1. VA Palo Alto/ Stanford University, Menlo Park, California, United States, 2. VA Palo Alto Health Care System, Menlo Park, California, United States, 3. VA Palo Alto Healthcare System, Menlo Park, California, United States, 4. Stanford University, Stanford, California, United States, 5. University of Utah School of Medicine, Salt Lake City, Utah, United States, 6. Stanford University, Palo Alto, California, United States*

Fragmented healthcare causes information loss, duplicative tests, and unwieldy self-care regimens. These challenges may be amplified among older, high-risk patients with co-occurring mental health conditions (MHC). We compared healthcare fragmentation for chronic physical conditions among Veterans with and without MHC (depression, PTSD, schizophrenia, bipolar disorder, anxiety, personality disorder, or psychosis based on ICD-9 codes). Sample included Veterans who were □65y, at high risk for 1-year hospitalization, and had □4 non-MHC visits during FY14. Visits were covered by Veterans Affairs (VA), VA-purchased care (both from VA Corporate Data Warehouse), or Medicare Parts A/B (claims data from VA Information Resource Center). Outcomes were two fragmentation measures calculated in FY15: 1) non-mental health provider count, where a higher number indicates more fragmentation, and 2) Usual Provider of Care (UPC), the proportion of care with the most frequently seen provider, where a higher number indicates less fragmentation. We used Poisson regression and GLM with binomial distribution and logit link to test the association between MHC status and fragmentation, controlling for sociodemographic characteristics (e.g., age), medical comorbidity, and driving distance to VA. Of the 125,481 Veterans included, 47.3% had 1+ MHC. Compared to older, high-risk Veterans without MHC, those with MHC saw fewer providers (pseudo R2 = 0.02) and had a higher UPC (more concentrated care; OR = 1.07). Within the VA, older, high-risk Veterans with MHC do not experience greater healthcare fragmentation. Further research is needed to determine if this is due to different needs, underuse, or appropriate use of healthcare across the groups.

### COMPARING A CLINICIAN ASSISTED AND APP-SUPPORTED POSITIVE PSYCHIATRY BEHAVIORAL ACTIVATION INTERVENTION

Ariane Massie,[1] Keri-Leigh Cassidy,[2] Michael Vallis,[3] David Conn,[4] Daria Parsons,[5] Julie Spence mitchell,[5] Claire Checkland,[5] and Kiran Rabheru,[6] *1. York University, North York, Ontario, Canada, 2. Dalhousie University, Dalhousie University, Nova Scotia, Canada, 3. Dalhousie University, Halifax, Nova Scotia, Canada, 4. University of Toronto, Toronto, Ontario, Canada, 5. Canadian Coalition for Seniors Mental Health, Markham, Ontario, Canada, 6. University of Ottawa, Ottawa, Ontario, Canada*

Positive psychiatry offers a unique approach to promote brain health and well-being in aging populations. Health interventions are increasingly becoming available using self-guided apps, however, little is known about the effectiveness of app technology or the difference between in-person versus self-guided app methodology for behavioural activation. The objective of this study was to investigate the difference in users and outcomes between two formats of a positive psychiatry intervention to promote brain health and well-being in later-life: (1) clinician-assisted, and (2) independent app use for self-management. As part of a larger national knowledge translation intervention two methods of a behavioural activation intervention (Clinician-assisted vs. Independent app use) were retrospectively compared. Main outcomes were patient characteristics (age, sex, and completion rate), psychological outcomes (health and resilience, and well-being), and behavioural outcomes (goal attainment, and items of goal SMART-ness). Clinician-assisted patients (n=254) were more likely to be male, older, and had lower health and resilience scores at baseline than Independent app users (n=333). Clinician-assisted patients had notably higher completion rates (99.2% vs. 10.8%). Psychological outcomes were similar regardless of intervention method for those who completed the intervention. Clinician-assisted patients had higher rates of goal attainment and goal SMART-ness. A preliminary goal setting methodology for effective behavioural activation, to promote brain health and well-ness, is given. Clinician-patient relationships were found to be an important factor for intervention completion, caution is given for app use referral. Results indicate a need for further exploration to determine best practices for health app use in clinical practice.

### DOES CHANGE PREDICT CHANGE IN THE CHANGE INTERVENTION STUDY?

Scott Maitland,[1] Paula Brauer,[1] David Mutch,[1] Dawna Royall,[1] Doug Klein,[2] Angelo Tremblay,[3] Caroline Rhéaume,[3] and Khursheed Jeejeebhoy,[4]
*1. University of Guelph, Guelph, Ontario, Canada, 2. University of Alberta, Edmonton, Alberta, Canada, 3. Laval University, Québec, Quebec, Canada, 4. University of Toronto, Toronto, Ontario, Canada*

Does change in one lifestyle factor (e.g., exercise/fitness) help explain change in another factor or process (i.e., dietary behavior or cardiovascular risk)? The current modeling was a secondary analysis of a primary care feasibility study of individualized lifestyle (diet and exercise) treatment of metabolic syndrome (n=293; mean age = 59yrs) that achieved 19% reversal over one year. Diet quality was assessed by the Healthy Eating Index (HEI) (2005 Canada); while fitness was assessed by several measures (VO2max, flexibility, curl-ups, push-ups). Three occasions (i.e., baseline, 3-, and 12-month) were examined using latent change score and latent growth curve models (in AMOS) to assess whether changes in one domain predicted changes in the remaining domains: (1) diet (measured by HEI or latent construct); (2) fitness (measured by VO2max percentiles or latent construct); and, (3) 10-year risk of cardiovascular disease (by Procam Risk score). Results showed significant improvement in all three domains separately during the intervention, with greater change between baseline and 3-month assessment and continued change between 3- and 12-months. Initial status variables on observed constructs were moderately positively correlated and change in dietary behavior was significantly related to change in fitness levels, but neither were significantly related to change in the 10-year risk of cardiovascular disease. In addition, the associations between change in diet and changes in fitness were inconsistent baseline to 3 months, and 3-12 months. These results offered new insight on relationships among interventions in a behavioural counselling program which can inform future programming.

### FEASIBILITY TEST OF A CUSTOMIZABLE RELATIONSHIP INTERVENTION FOR STROKE SURVIVOR-FAMILY CAREGIVER DYADS

Michael McCarthy,[1] Yolanda Evie Garcia,[1] Karen Lyons,[2] Angelica Sanchez,[1] and Tamilyn Bakas,[3] *1. Northern Arizona University, Flagstaff, Arizona, United States, 2. Boston College, Chestnut Hill, Massachusetts, United States, 3. University of Cincinati, Cincinnati, Ohio, United States*

A strong interpersonal relationship after stroke is important for the well-being of survivors and family caregivers. However, few interventions are designed to strengthen the relationship between members of the care dyad in order to prevent depression and other poor outcomes. The aim of this study was to feasibility test a quality of relationship intervention for stroke dyads called Hand in Hand (HiH). Sixteen survivor-caregiver dyads were recruited and randomized into either the HiH intervention group (n=8) or the Information, Support, and Referral (ISR) control group (n=8). HiH dyads received up to 8 sessions with a social worker in-person, by telephone, or by Zoom web conference, prioritized according to a 17-item screening tool with 17 corresponding HiH content areas. ISR dyads received up to 8 sessions that included information, active listening, and referrals as needed. Process, satisfaction, and pre/post outcomes data were collected for both groups. Seventy-five percent of HiH dyads completed over half the sessions which lasted, on average, 55 minutes (range 26-76). Sixty-two percent of ISR dyads completed over half the sessions which lasted, on average, 21 minutes (range 15-33). Dyads in both groups reported being satisfied with the program materials and processes. Survivors and caregivers in both groups experienced improvements in outcomes, particularly caregivers in the HiH group who showed improvements in communication, coping, subjective relationship quality, and depressive symptoms. Findings suggest that HiH is feasible to implement with stroke dyads and has promise for improving outcomes for participants. Additional research is needed to determine program efficacy.

### INNOVATIVE STRATEGIES TO REACH DIVERSE ELDERS: USING AGE-TASTIC TO IMPROVE HEALTH AND WELL-BEING

Jacquelin Berman,[1] Manoj Pardasani,[2] Madison Gates,[3] and Mebane Powell,[3] *1. NYC Department for the Aging, NYC, New York, United States, 2. Hunter school of social work, New York city, New York, United States, 3. NYC Department for the Aging, New York city, New York, United States*

This session will present the findings of a randomized control trial evaluating the impact Age-Tastic has on behavioral change. Age-Tastic is an eight-week intervention that uses game play, group facilitated discussion, and at home activities to promote positive behavioral change. There are five behavioral change domains: emotional well-being, nutrition, financial exploitation, falls prevention, and health literacy. A randomized control trial was conducted in New York City senior centers and include a diverse group of older adults. There were 98 older adults assigned to an experimental or control group. Participants in both groups completed a baseline survey, which was repeated at the end of the intervention and at week sixteen. The experimental (n = 64) and control (n = 34) groups did not significantly differ at baseline in regard to behaviors; however, upon completion of the intervention, the experimental group had significantly changed health behaviors ($p < .05$) compared to the control group. The behavior change reported by the experimental group was maintained at week sixteen ($p < .001$) with a medium effect size ($\varepsilon 2 = .17$). In addition the experimental group also reported significant improvements at weeks eight ($p < .001$) and sixteen ($p < .001$) with large effect sizes ($r2 = .62$ and $.52$, respectively). This study found that Age-Tastic is an effective intervention for engaging older adults holistically about their health and wellness and for promoting positive behavioral change among diverse racial and ethnic populations.

### NATIONAL HOSPITALIZATION TRENDS AMONG CENTENARIANS IN THE UNITED STATES

Sylvia twersky,[1] and Adam Davey,[2] *1. The College of New Jersey, Ewing, New Jersey, United States, 2. University of Delaware, Newark, Delaware, United States*

Continued increases in life expectancy mean than an unprecedented number of individuals are reaching centenarian status, often with complex health concerns. Little research has addressed acute care experiences of centenarians. To address this knowledge gap, we analyzed 10 years of hospital admissions data from a total of 52618 centenarians (aged 100 to 115 years) in the nationally representative National Inpatient Study (NIS) with the aim of characterizing centenarian (mean age 101.4 years, 80% women, 24.2% non-White, 91.3% Medicare, 72.2% emergencies, 18.6% urgent) hospitalizations. Mean length of stay (LOS) decreased from 6.1 to 5.1 days (2000-2009). Over the same period, mean total charges rose from $13373 to $25026; they were $4478 lower for women than men and $4032 more for centenarians age 105 years and older than ages 100 to 104, despite comparable LOSs. Mean LOS was approximately 0.7 days longer for non-Whites than Whites, and mean total charges were approximately $6531 higher for non-Whites than Whites. The 10 most common admission diagnoses were consistent with frailty and end stage cognitive impairment including pneumonia, congestive heart failure, urinary tract infections, fracture of neck of femur, septicemia, fluid and electrolyte disorders, aspiration pneumonitis, acute cerebrovascular disease, acute myocardial infarction, and gastrointestinal hemorrhage, and 10.0% of centenarians died in hospital. We link individual-level data with hospital and state characteristics over the same time period in order to provide a more detailed analysis of individual and system-level characteristics that contribute toward health disparities in hospitalizations and health outcomes among centenarians.

### OLDER ADULT REFUGEES' ROLES IN CREATING PARTNERSHIPS FOR CULTURALLY RESPONSIVE HEALTHCARE

Denise Lewis,[1] Desiree Seponski,[2] and Amber Kelley,[1]
*1. University of Georgia, Athens, Georgia, United States, 2. University of Georgia, University of Georgia, United States*

The aim of this presentation is to reveal the importance of engaging older refugee community members in creating partnerships with local healthcare providers to implement culturally responsive interventions. Such engagement invites older refugees' participation and encourages continued connection to country of origin beliefs and culture, particularly within the sphere of healthcare and medical family therapy. Cambodian and Laotian refugee families in coastal Alabama were interviewed via qualitative community-based participatory research and ethnographic, in-depth interviews focused on familial and communal processes. Local healthcare providers engaged in focus group discussions regarding cultural processes associated with health beliefs and behaviors and in periodic outreach through culturally responsive health fairs. We found that older adults play important roles in refugee populations as community leaders, problem-solvers, and legacy-carriers upholding traditional values and cultural continuity. They also maintain and promote continuity by employing traditional, complementary, or alternative medicine (TCAM). Recursive processes also emerged as older individuals sought care from younger community members in times of sickness, including having younger generations provide language translation and transportation to local healthcare centers and hospitals and in navigating the United States healthcare system. In addition, community members joined with local healthcare providers to aide in health and healthcare literacy among refugees and to educate local physicians on culturally responsive interventions. Implications include the inclusion of older adults in health decisions and the promotion and maintenance of community partnerships with health agencies that both encourage TCAM utilization and also allow for access to ongoing, appropriate treatment within the US healthcare system.

### PROVIDER-IDENTIFIED DEPRESCRIBING FACILITATORS AND STRATEGIES FOR OLDER ADULTS IN PRIMARY CARE: A TEAM ALICE STUDY

Laura Brady,[1] Susan LaValley,[2] Molly Ranahan,[3] Collin Clark,[4] Scott Monte,[5] David Jacobs,[6] Robert Wahler,[2] and Ranjit Singh,[7] *1. SUNY University at Buffalo, Buffalo, New York, United States, 2. University at Buffalo, Buffalo, New York, United States, 3. Erie County Medical Center, Buffalo, New York, United States, 4. University at Buffalo School of Pharmacy and Pharmaceutical Sciences, Buffalo, New York, United States, 5. University at Buffalo School of Pharmacy, Orchard Park, New York, United States, 6. University at Buffalo, Buffalo, United States, 7. State Univ. of New York at Buffalo, Buffalo, New York, United States*

Potentially inappropriate medications (PIMs) may harm adults over the age of 65, yet PIMs are prescribed at high rates. The process of deprescribing PIMs is challenging in the primary care setting, particularly among older adult patients with multiple chronic conditions. While barriers to deprescribing are well known, less data are available on the facilitators and strategies that primary care providers consider key to successful deprescribing. This study examines providers' perceptions and attitudes of deprescribing to identify individual and systems-level facilitators and strategies for successful deprescribing. Data were collected through semi-structured interviews with 20 providers recruited from primary care practices located in Western New York. Rapid thematic analysis was used to identify the facilitators and strategies providers perceived as important to successful deprescribing. Facilitators included providers adapting their approach to deprescribing PIMs based on their knowledge of the patient. Providers' own characteristics were also important, as were those of their organization, including whether a clinical pharmacist was available to consult. Strategies for deprescribing were patient-focused (e.g., adapting to patient's lifestyle), process-focused (e.g., patient education on polypharmacy), and medication-focused (e.g., tapering). It is clear that many of the primary care providers who treat a larger number of older adults are aware of the importance of deprescribing PIMs. However, deprescribing in a busy primary-care setting is challenging. Findings detailing providers' perceptions of facilitators and strategies for deprescribing can guide future interventions and target support to reduce the risk of harm from PIMs in older adults.

### THE CONTRIBUTION OF EIGHT MODIFIABLE RISK FACTORS TO DEMENTIA PREVALENCE IN JAMAICA

Kimberly Ashby-Mitchell, and Denise Eldemire-Shearer, *University of the West Indies, Kingston, Jamaica*

The number and proportion of dementia cases in Jamaica attributable to eight modifiable lifestyle factors was calculated using factor analysis in Stata and Levin's Attributable Risk formula (hypertension, diabetes mellitus, depression, low educational attainment, smoking, overweight, obesity and vision problems). Four sources of data were used: (1) risk factor prevalence data were obtained from the Jamaica Health and Lifestyle Survey, 2008, (2) relative risk data were sourced from published meta-analyses, (3) factor analysis in Stata version 15 was performed to estimate shared variance among risk factors utilising data from the Health and Social Status of Older Persons in Jamaica Study and, (4) estimated prevalence of dementia in Jamaica in 2012 was obtained from a 2018 publication by Shearer et al. Obesity was found to be responsible for the largest proportion of dementia cases (13.94%; 2508 cases) followed by hypertension (10.64%; 1917 cases), low educational attainment (7.21%; 1299 cases), overweight (6.42%; 1156 cases), smoking (3.90%; 702 cases), diabetes (3.38%; 608 cases), depression (1.00%; 181 cases) and impaired vision (0.002%; 34 cases%). Using this largely theoretical model, the eight factors examined account for approximately 46.0% of dementia cases. Dementia risk reduction programs that target even one of these factors has the potential to result in significant reduction in future dementia prevalence as they are all interrelated. In future work, as these data become available, the contribution of mid-life obesity, mid-life hypertension and physical inactivity will also be taken into consideration.

### UNDIAGNOSED DIABETES: IDENTIFYING THE COMMUNITY PATHS TO TYPE 2 DIABETES DIAGNOSTIC TESTING

Sarah Chard,[1] Laura Girling,[2] Loren Henderson,[1] and J. Kevin Eckert,[1] *1. UMBC, Baltimore, Maryland, United States, 2. University of Maryland, Baltimore County, Baltimore, Maryland, United States*

Over seven million U.S. adults are estimated to have undiagnosed diabetes and are at heightened risk of diabetes complications and poorer long-term glycemic control. Key to addressing undiagnosed diabetes is identifying how persons encounter diabetes testing in everyday life and the contextual factors that lead to consulting a health care provider. As part of the NIA-funded Subjective Experience of Diabetes Study we examined the pathways through which community-living African-American and non-Hispanic White older adults with type 2 diabetes (T2D) (N=75) received their T2D diagnosis. Systematic, thematic analyses using ATLAS.ti reveals three primary routes to diabetes diagnosis: diagnosis through continuity of primary care, diagnosis through happenstance testing, and diagnosis following the exacerbation of symptoms. While diagnosis as part of routine care was the least reported (N=13), participants' accounts suggest diagnosis in primary care validates the patient-provider relationship and provides an occasion to calmly establish a treatment plan. More frequently, however, diagnosis occurs through fortuitous encounters with glucose tests, e.g., through work or community research projects (N=15) or after symptoms become alarming and disrupt daily life (N=47). Participants' experiences in these latter two categories reveal the critical role of insurance and social prompts in the decision to consult a clinical provider regarding symptoms. At the same time, the abundance of over-the-counter therapies treating conditions commonly found early in the emergence of diabetes can delay clinical follow up. These findings highlight the importance of social prompts and community-based testing in the fight to reduce undiagnosed diabetes.

### WEIGHT CHANGE AND ALL-CAUSE MORTALITY IN LATER LIFE: FINDINGS FROM THE NATIONAL SURVEY OF THE JAPANESE ELDERLY

Hiroshi Murayama,[1] Jersey Liang,[2] Benjamin Shaw,[3] Anda Botoseneanu,[4] Erika Kobayashi,[1] Taro Fukaya,[1] and Shoji Shinkai,[1] *1. Tokyo Metropolitan Institute of Gerontology, Tokyo, Japan, 2. University of Michigan, Ann Arbor, Michigan, United States, 3. State University of New York, Rensselaer, New York, United States, 4. University of Michigan, Dearborn, Michigan, United States*

Recent studies predominantly in Western populations suggest that both weight gain and weight loss are associated with increased mortality risk in old age. However, evidence on this topic in Asian populations remains sparse. We examined the association between weight change and all-cause mortality in a nationally-representative sample of community-dwelling older Japanese. Data came from the National Survey of the Japanese Elderly (N = 4,869, age ≥ 60 years). Participants were followed for up to 30 years. Short-term (3 years) and medium-term (6 years) weight changes were classified as "loss ≥ 5%," "loss 2.5%–4.9%," "stable," "gain 2.5%–4.9%," and "gain ≥ 5%." Cox proportional hazards models were used to assess the relative (stable weight as reference) mortality risk associated with weight change categories. Short-term weight loss ≥ 5% was associated with higher mortality compared to the stable category, after adjusting for sociodemographic factors, health behaviors, and health conditions (hazard ratio = 1.36; 95% confidence interval = 1.22–1.51). The other weight change categories had no significant association with mortality. This was observed both among males and females. Moreover, the same pattern of results was observed when we used the medium-term weight change indicator. In conclusion, we found that both short- and medium-term weight loss greater than 5% increased the risk of dying among older Japanese; however, other types of weight change did not. This finding could inform clinical and public health approaches to body-weight management aimed to improve the health and survival of older adults, particularly in Asian populations.

### WELLNESS COACHING: PRACTICES AND PREFERENCES AMONG LIFE PLAN COMMUNITY RESIDENTS AND STAFF

Matthew Fullen,[1] Jennifer Smith,[2] Janis Sayer,[2] Philip Clarke,[3] and Connie Tomlin,[1] *1. Virginia Tech, Blacksburg, Virginia, United States, 2. Mather Institute, Evanston, Illinois, United States, 3. Wake Forest University, Terre Haute, Indiana, United States*

Wellness coaching, a process in which a coach and client partner together to address the client's wellness goals, can increase motivation and develop skills to enhance wellness and lifestyle balance among older adults. To understand wellness preferences among Life Plan Community residents, we surveyed a total of 447 residents from 10 Life Plan Communities. Participants were asked about perceptions of wellness, wellness activity preferences, motivators and barriers to participation in wellness activities, and wellness coaching program preferences. Twenty employees in wellness-related or leadership roles also completed a survey. To enhance our understanding of these perspectives on wellness coaching, nine residents and four employees participated in follow-up interviews. Among the study findings, the majority of resident respondents expressed an interest in improving almost all domains of wellness. Forty percent (40%) of participants said they were extremely or moderately likely to try wellness coaching, and about one-half (51%) said they believed they would benefit from wellness coaching. Staff indicated interest in implementing a wellness coaching program in their community, with 74% reporting at least a moderate likelihood of implementing a program if led by a staff member. Results indicated that emotional and vocational wellness programs were offered and attended less frequently than other types of wellness programs, which suggests that wellness coaching could help to address the need for more programming in these areas. In addition, findings suggested that implementation requires resident input to ensure buy-in. The survey results informed the development of recommendations for a resident wellness coaching program.

### SESSION 2949 (POSTER)

### HEALTH PROMOTION AND WELL-BEING II

### FACTOR ANALYSIS OF THE SHORT FORM COHEN MANSFIELD AGITATION INVENTORY AND MEASUREMENT INVARIANCE BY GENDER

Anju Paudel,[1] Barbara Resnick,[2] and Elizabeth Galik,[3], *1. University of Maryland, Glen Burnie, Maryland, United States, 2. University of Maryland School of Nursing, Baltimore, Maryland, United States, 3. University of Maryland, Baltimore, Maryland, United States*

Background: The Cohen-Mansfield Agitation Inventory (CMAI), available in both long and short versions, is a widely used measure to assess and evaluate agitation among older adults. There has been less psychometric testing of the short-form CMAI particularly with regard to the factor structure of this shorter measure. Purpose: The purpose of this study was to test the internal consistency, reliability and validity of short-form CMAI in a sample of nursing home residents and examine if it is invariant across gender. Specifically, it was hypothesized that consistent with the long form CMAI, the short-form CMAI would have three factors with acceptable internal consistency and item reliability. In addition, it was hypothesized that there would be no difference in factor structure and factor means across gender. Methods: This study utilized baseline data from a randomized trial including 553 residents from 55 nursing homes. Data was analyzed using structural equation modeling. Results: Confirmatory factory analysis supported the three-factor structure of short-form CMAI including aggressive ($\alpha$= 0.794), physically non-aggressive ($\alpha$= 0.617), and verbally agitated ($\alpha$= 0.718) behaviors; three items loading on physically non-aggressive behaviors had R2 close to 0.3 suggesting low reliability. Invariance testing confirmed that the shortened measure is invariant across gender. Conclusions: Short-form CMAI is a valid and reliable scale to assess agitation and gender differences in agitation in nursing home population. However, it could benefit from rewording the items with low reliability or, merging them with other similar items. Future work could also consider a four-factor structure for this shortened measure.

### FUTURE CARE PLAN AT THE CONTEMPLATION STAGE AMONG OLDER ADULTS

Abolade Oladimeji,[1] Eva Kahana,[2] Boaz Kahana,[3] and Jeongeun Lee,[4] *1. case western reserve university, cleveland, Ohio, United States, 2. Case Western Reserve University, Cleveland, Ohio, United States, 3. Cleveland State University, cleveland, Ohio, United States, 4. Iowa State University, Iowa City, Iowa, United States*

Previous studies have shown that making future care planning often involves several steps. This is consistent with the transtheoretical model of behavior change which identified stages in which behavior changes over time and progresses through a series of six stages (Prochaska & Norcross, 2001). Although most studies on future care planning among older adults have examined the progression from the pre-contemplation stage to an action stage, little is known about the unique characteristics of older adults at the contemplation stage. Our sample included 409 randomly selected older adults in Cleveland, Ohio who were in the first wave of a longitudinal study of successful aging (μage = 78.8, SD=5.74). We used multivariate logistic regression to predict older adults who are more likely to have thought of their future care plans (contemplation stage). Results indicate several individual and relationship factors such as marital status, engaging in health behaviors (not smoking), being young old, and having personal transportation (OR=.42, .63, 2.81, and 1.73) account for contemplating future care. These findings underscore that several individual resources, as well as relationship status, are associated with contemplation stage of future care planning. The results reinforce the idea that future care planning is a stage-based behavior that people engage in, rather than a stand-alone activity.

### GOING VIRAL: UNDERSTANDING MEDICAL MISINFORMATION AND OLDER ADULTS' VACCINE HESITANCY

Raza Mirza,[1] James Hull,[2] Siyu Peng,[3] Yalda Mousavi,[3] Muhammad Ibrar Mustafa,[3] Peter Papageorgiou,[3] Eric Rotgaus,[3] and Jessica Hsieh,[4] *1. National Initiative for*

*the Care of the Elderly, Toronto, Ontario, Canada, 2. National Initiative for the Care of the Elderly, Toronto, Canada, 3. University of Toronto, Toronto, Canada, 4. University of Toronto, Toronto, Ontario, Canada*

Influenza persists as a common communicable disease and remains a significant cause of disease burden across the world. Despite preventative therapies, such as influenza vaccination to reduce its spread and transmission, influenza continues to be a source of morbidity and mortality, even in developed countries. For the population over the age of 65, the effects of influenza virus may be more severe when they are compounded by pre-existing conditions and reduced natural immune function. In light of plateauing vaccination rates, a scoping review was conducted to map the literature and determine why seniors aged 65 and above refuse or fail to receive seasonal influenza vaccination. Nine peer-reviewed academic databases covering both social sciences and medical research were searched, along with the grey literature. A total of 6,562 references were identified; after the screening process, 118 references were included in the final review. Thematic analysis focused on the broad areas that positively or negatively influence older adults' decision-making regarding influenza vaccination, and this resulted in five main themes: (1) barriers to obtaining vaccination; (2) social factors; (3) personal characteristics; (4) individual subjectivity; and (5) direct clinical interventions. This review aims to identify gaps in knowledge and synthesize currently available information to make recommendations for future research, policy development and clinical practice. Increasing the vaccination rate among Canadian older adults will contribute to ongoing efforts to reduce the spread of the influenza virus among the population, reducing influenza-associated hospital admissions and deaths.

### HUM ALONG WITH THE SILENT DISCO HEADPHONES: LESSONS LEARNED IN IMPLEMENTING THE HEADPHONE PROGRAM IN A HOSPITAL UNIT

Lillian Hung, *University of British Columbia, Vancouver, British Columbia, Canada*

Silent disco headphones have been used among young people in concerts and parties; such headphones have extended distance coverage for broadcasting from a transmitter, features of noise cancelation, and three channels of music. Rather than using a speaker system, music is delivered by wireless headphones and facilitated by a DJ via a built-in microphone. No study has yet tested whether it is feasible to use such headphones to support well-being among older people in hospital settings. This study examined the feasibility of using silent disco headphones with older adults with dementia staying in a geriatric hospital unit. We employed a video-ethnographic design, including conversational interviews and observations, with video recording among ten patient participants in a hospital unit. Two focus groups were conducted with ten hospital staff across disciplines. Thematic analysis yielded three themes: (a) "it just made me feel happy, "(b) "it brings him back alive," (c) "it unlocks dementia". Delivering music and meditation programs via the silent disco headphones in the hospital unit has the potential to be a beneficial intervention that can enhance mood and energy, support self-expression, and promote wellness. Our findings suggested that witnessing the positive effects of headphones on patients changed the staff's view of how music could be used in the clinical setting to support patients' well-being. We identified enablers and barriers to implementing the headphone program in the hospital setting. Future research should further investigate how headphones may help to reduce stress and promote wellness for patients in the clinical environment.

### OLDER ADULTS LIVING WITH FOOD INSECURITY: THE IMPACT ON PSYCHOLOGICAL HEALTH

Robina Sandhu,[1] Victoria Marshall,[2] Carolyn Becker,[3] Lisa Kilpela,[1] and Keesha Middlemass,[4] *1. UT Health San Antonio, San Antonio, Texas, United States, 2. UT Health San Antonio, San Antonio, United States, 3. Trinity University, San Antonio, Texas, United States, 4. Howard University, Washington D.C., District of Columbia, United States*

Food insecurity (FI) refers to inadequate access to nutritious foods, either in terms of quality or quantity. In older adults, FI is associated with functional impairment, isolation, financial vulnerability, lower quality of life, and poorer health (e.g., diabetes, cardiovascular disease; Fernandes, et al). The effects of FI on psychological health broadly, however, have not been well-documented among older adults. This study sought to examine the impact of FI severity on psychological health indices among older adults. Older adult clients of local food pantries completed self-report measures of FI severity, worry, internalized weight stigma, trauma history, and eating disorder (ED) symptoms/behaviors. Participants (N=124, aged 66+) included: 68.5% women, 67.7% Hispanic, 75.8% □high school education, 51.0% household income < $10,000/year. Controlling for gender in all analyses, results indicated that FI severity predicted increased worry (p < .001, 21.9% variance), greater internalized weight stigma (p = .04, 3.9% variance), and a trend for increased risk for lifetime traumatic event exposure (OR = 1.4, 95% CI [.98, 2.01]). Regarding ED symptoms, male gender (OR = 6.60, 95% CI [1.96, 22.23] and higher FI severity predicted risk for self-induced vomiting in the past month (OR = 2.5, 95% CI [1.15, 5.36], risk for laxative/diuretic use for weight control (OR = 2.16, 95% CI [1.03, 4.52], and greater dietary restraint (p < .001, 16.1% variance). Male gender was associated with higher risk for binge eating in the past month (OR = 3.19, 95% CI [1.10, 9.24], while FI severity was not. Implications will be discussed.

### PATIENT-REPORTED HEALTH OUTCOMES AMONG OLDER ADULTS UNDERGOING TOTAL KNEE ARTHROPLASTY

Suttiwan Chawengkiattikul,[1] Suparb Aree-Ue,[2] Phichpraorn Youngcharoen,[3] and Viroj Kawinwonggowit,[4] *1. Faculty of Medicine Ramathibodi Hospital, Mahidol University, BKK, Thailand, 2. Mahidol, Bangkok, Thailand, 3. Mahidol University, BKK, Thailand, 4. Mahidol University, Bangkok, Thailand*

Backgrounds Total knee arthroplasty (TKA) is a remedy treatment for severe knee osteoarthritis; yet, postoperative outcomes vary. Preoperative patients' expectations to functional abilities are important factors influencing postoperative outcomes and satisfaction. Objectives To investigate the association among preoperative patients' expectations, post-operative functional abilities, and satisfaction to functional abilities among older adults undergoing TKA at 6-week after surgery. Methods Participants were 97 older adults who purposely selected based on the inclusion criteria. The data were collected at preoperative and postoperative TKA by using the Hospital for Special Surgery Knee Replacement Expectations Survey and the Knee and Osteoarthritis Outcome Score - function in daily living. The data analysis was performed by using descriptive statistics, paired t-test, and Pearson product moment correlation coefficient. Results Before surgery, patients' expectations to postoperative functional abilities had a high level with the total mean score of 70.21(SD =13.86). At 6-week after surgery, the overall functional ability had a significant improvement ($t = -9.229$, $p = .000$). Satisfaction to functional ability also had a high level (Mean ± SD = 71.15 ± 14.73). Patients' expectations to functional abilities had a significantly low positive correlation to postoperative functional ability and satisfaction ($r = .273$, $p < .05$; $r = .292$, $p < .01$, respectively). A significant moderate positive correlation between functional abilities and satisfaction to functional abilities was observed ($r = .603$, $p < .01$). Conclusion Understanding of expectations may be beneficial in gaining knowledge, paving expectations on possible outcomes, and enhancing the quality of care for these populations.

### SOCIAL DETERMINANTS OF UNMET HEALTHCARE NEEDS: COMPARISON BETWEEN MIDDLE-AGED AND OLDER PEOPLE

Sungjae Hong, *Emory University, Decatur, Georgia, United States*

This study aims to 1) examine unmet healthcare needs by age groups and 2) compare the social determinants of unmet healthcare needs between older and middle-aged people in Korea. This study employed a nationally representative dataset of the 2017 Healthcare Service Experience Survey in Korea. Unmet healthcare needs consisted of three categories by healthcare type: 1) clinic visitation, 2) treatment, and 3) medication. Independent variables include demographic, socioeconomic, and health status. Logistic regression models were estimated to reveal the social determinants of unmet healthcare needs of older (age≥65; N=2,178) and middle-aged (age 40~64; N=5,062) people. There was a positive gradient of unmet healthcare needs prevalence by age group, having the highest prevalence among older people (10.8%). While older people living alone were 1.70 times more likely to report any of unmet healthcare needs, there was no significant relationship between the two among middle-aged people. In addition, the effect of chronic disease morbidity on the probability of unmet healthcare needs was stronger among older people than it is among middle-aged people (OR=3.50 and 2.90, respectively). In contrast, the effect of household income was weaker than it is among middle-aged people (OR=1.73 and 2.95, respectively). The gradient of unmet healthcare needs by age group asks gerontologists and public healthcare scholars to focus on older people regarding unmet healthcare needs. Also, the difference between middle-aged and older people on social determinants of unmet healthcare needs implies different psychosocial pathways of unmet healthcare needs between the two age groups.

### SUBJECTIVE AND FUNCTIONAL HEALTH LITERACY SCREENING IN GERIATRIC PRIMARY CARE

Amy Albright,[1] Deanna Dragan,[2] Anne Halli-Tierney,[2] Dana Carroll,[3] and Rebecca Allen,[4] *1. University of Alabama, Northport, Alabama, United States, 2. The University of Alabama, Tuscaloosa, Alabama, United States, 3. Auburn University Harrison School of Pharmacy, Tuscaloosa, Alabama, United States, 4. Alabama Research Institute on Aging, Tuscaloosa, Alabama, United States*

The aim of the current study is to provide comprehensive health care to older adults by assessing physical and mental health in a geriatric primary care setting, including evaluation of both subjective and functional health literacy. Health literacy is vital to understanding medical information and making subsequent decisions based on this information. Knowledge of patient health literacy may be particularly important for care providers, as it can provide guidance on how to best communicate with the patient (Nouri & Rudd, 2015). It may be particularly important to monitor health literacy within older adults, as several studies (e.g., Kobayashi et al., 2015) have shown that health literacy decreases with mild cognitive impairment. Approximately 250 patients (mean age = 76; 74% female; 16% African American) attending an interdisciplinary geriatrics clinic in West Alabama have been recruited to take part in a variety of behavioral health screenings. The current study assessed subjective health literacy using questions developed by Chew, Bradley, and Boyko (2004) and functional health literacy using the Newest Vital Sign (Weiss et al., 2005). While there was a significant correlation between subjective and functional health literacy ($r = .43$, $p < .001$), 81% of patients reported adequate subjective health literacy, while only 41% demonstrated adequate health literacy on a functional screening measure. Based on these findings, self-reported health literacy may not necessarily be reflective of performance on more functional measures. Given the potential consequences of overestimating health literacy, this represents a serious barrier to patient care.

### THE ASSOCIATION BETWEEN OBESITY AND LONG-TERM COGNITIVE PERFORMANCE IN MIDDLE-AGED AND OLDER ADULTS

Andrew Fiscella, and Ross Andel, *University of South Florida, Tampa, Florida, United States*

Obesity is a growing epidemic in the United States and has been associated with negative health outcomes such as cardiovascular disease and diabetes. However, an obesity paradox has emerged which suggests that the effects of obesity may vary by age, with older adults potentially seeing a protective effect of obesity. This study examined the effects of overweight and obese status on cognitive performance at baseline and follow-up. It was hypothesized that obese middle-aged adults would perform worse than normal weight peers, but that reverse would be observed in older adults. Data from 701 participants in the Midlife in the United States study were included. Body mass index (BMI) and waist circumference were employed as measures of obesity. Z-scores for executive function, memory, and global cognition were used to quantify cognitive performance. While obese participants tended to perform worse on average than normal weight individuals there were no significant differences in performance between obese and normal weight participants in global cognition (p=.134), executive function (p=.164), or episodic memory (p=.708). Additionally, age did not moderate this relationship. However, there was a significant effect of education on all three domains. When stratified by education, participants with some college or higher, had a significant time*obesity*age interaction (F[3,328]=3.016, p<.05). For the oldest-old participants, executive function scores were higher for obese participants at follow-up compared to normal weight participants, but not at baseline. These findings suggest that level of education may serve as a form of cognitive reserve which compensates for deficits due to obesity.

### UP NORTH AND DOWN SOUTH: REGIONAL DIFFERENCES IN PAIN, RELIGIOUS COPING, AND NEGATIVE AFFECT IN OSTEOARTHRITIS

Katherine Cheesman,[1] Patricia Parmelee,[2] and Dylan Smith,[3] *1. The University of Alabama, Tuscaloosa, Alabama, United States, 2. University of Alabama, Tuscaloosa, Alabama, United States, 3. Stony Brook University, Stony Brook, New York, New York, United States*

Objective: This research examines regional differences (Northern vs. Southern) in pain, religious coping, and negative affect among African Americans (AA) and non-Hispanic Whites (NHW) over the age of 50 with physician-confirmed knee osteoarthritis (OA). Methods: As part of a larger study of racial/ethnic differences in everyday quality of life with OA, 116 persons were recruited from sites in Alabama (n = 64) and New York (n = 52). Participants completed global measures of pain (PGC Pain Scale) and religious coping (Brief RCOPE); daily variability in pain, coping, and affect was assessed using a daily diary methodology consisting of 4 daily phone calls over 7 days. Site comparisons were conducted using one-way multivariate analysis of covariance (MANCOVA) with covariates of race, sex, education, and marital status. Results: There was a significant multivariate effect of site on pain, religious coping, and affect, $F(5, 104) = 3.846$, $p = .003$, Wilk's $\Lambda = .844$, partial $\eta 2 = .156$. Follow-up univariate tests and mean examinations revealed that Southerners reported statistically more daily pain (M = 2.023, SD = .89), religious coping (M = .618, SD = .427), and negative affect (M = 6.556, SD = 2.661) than Northerners (M = 1.810, SD = .719; M = .386, SD = .417; M = 5.865, SD = 1.446). Implications: Results contribute to a growing understanding of how individuals use their religious beliefs to cope with daily pain. (Supported by R01-AG041655 D. Smith and P. Parmelee, PIs.)

### SESSION 2950 (POSTER)

### NURSING HOME, ASSISTED LIVING, AND LONG-TERM CARE

### ASSESSMENT OF A POTENTIAL UTI–USING VIDEOS TO ANALYZE A SIMULATED NURSING HOME EXPERIENCE

Donna Owen,[1] Alyce Ashcraft,[2] Kyle Johnson,[3] and Huaxin Song,[3] *1. Texas Tech University Health Sciences Center, Houston, Texas, United States, 2. Texas Tech University Health Sciences Cener, Lubbock, Texas, United States, 3. Texas Tech University Health Sciences Center, Lubbock, Texas, United States*

In the past, urinary tract infections (UTIs) have been attributed to poor hygiene, the natural course of aging, an unfortunate corollary of nursing home (NH) residence, and a condition routinely treated empirically with antibiotics. Recent UTI management consensus statements foretell a very different future and support the need to consider all UTIs in the NH as complex infections. Improving assessment capabilities of the NH nurse workforce is essential for improving quality of care. This study aimed to determine how, using simulation, licensed vocational nurses (LVNs) integrated a mobile decision-support app (MDS-app) into assessment of a NH resident with a potential UTI. The MDS-app directed the LVN to examine or question the resident (mannequin) to identify signs and symptoms developed as part of a simulated clinical scenario. MDS-app items were based on UTI practice guidelines. A descriptive, participant observation design (video-taped) was used with ten practicing LVNs. An observation checklist was used to examine audiovisual recordings and included frequency of verbal interaction (17.9+/-7.2), and eye contact (10.6+/-4.1). Participants (47%) were "glued to" the MDS-app without making resident eye contact or touching residents during the assessment. 60% of participants deviated from the app to ascertain urine odor and color; irrelevant symptoms for UTI diagnosis. Assessments required 11.20 (+/-4.67) minutes to complete. The MDS-app provided LVNs with needed focus on data driven by guidelines and not individual LVN preferences. Training LVNs should focus on integration of communication, assessment skills, and MDS-app use for evidence-based data collection as a basis for UTI treatment decisions.

### CHARACTERIZING MEALTIME VERBAL INTERACTIONS AMONG NURSING HOME STAFF AND RESIDENTS WITH DEMENTIA

WEN LIU,[1] Kristine Williams,[2] Melissa Batchelor,[3] Yelena Perkhounkova,[1] and Maria Hein,[1] *1. University of Iowa, Iowa City, Iowa, United States, 2. University of Kansas, Lawrence, Kansas, United States, 3. The George Washington University, Washington, DC, United States*

Mealtime difficulties are common in residents with dementia, leading to negative outcomes. Interaction with staff are critical to engage residents in eating. This study characterized dyadic verbal interactions (descriptive statistics), and relationships among verbal behaviors and between verbal behaviors and individual characteristics (bivariate analyses). This secondary analysis of 110 videotaped mealtime observations involved 25 residents and 29 staff (42 unique dyads) in 9 nursing homes (NH). Verbal behaviors (utterances) were coded using the Cue Utilization and Engagement in Dementia mealtime video-coding scheme, addressing 8 positive behaviors and 4 negative behaviors. Staff spoke three times more frequently (76.5%) than residents (23.5%). Nearly all staff utterances were positive (99.2%). 85.1% of residents' utterances were positive and 14.9% negative. Staff positive utterances were associated with staff negative utterances (p=.02), and resident positive

(p<.001) and negative (p<.001) utterances. Staff negative utterances were associated with resident negative utterances (p=.02), but not with resident positive utterances (p=.39). Resident positive and negative utterances were associated (p<.001). Staff positive utterances were associated with staff race (p=.01), and resident age (p=.01), dementia stage (p<.001), and eating function (p<.001). Resident positive utterances were associated with years staff worked as caregivers (p=.02) and in the current NH (p=.01), resident age (p=.04), comorbidity (p=.04), dementia stage (p=.01), and eating function (p=.003). Resident negative utterances were associated with dementia stage (p=.01). Dyadic mealtime interactions were dynamic, interactive and complex. Multiple resident and staff characteristics were related to mealtime verbal interactions. Findings may inform development of individualized, person-centered mealtime care interventions.

### CNAS' RATINGS OF NURSING HOME RESIDENTS' PAIN: THE ROLE OF CNA AND RESIDENT TRAITS

Emily Behrens,[1] Hyunjin Noh,[1] A. Lynn Snow,[2] and Patricia Parmelee,[3] *1. The University of Alabama, Tuscaloosa, Alabama, United States, 2. Tuscaloosa VA Medical Center, Tuscaloosa, Alabama, United States, 3. University of Alabama, Tuscaloosa, Alabama, United States*

Long-term care residents with and without cognitive impairment may experience undertreatment of persistent pain (Fain et. al, 2017). Certified nursing assistants (CNAs) are important sources of information about resident pain as they provide the majority of residents' hands-on care. Therefore, assessing the accuracy of CNAs' pain assessments and potential influencing factors may provide insight regarding the undertreatment of pain. Informed by prior research, this study examined resident pain catastrophizing and cognitive status as predictors of CNAs' pain assessment accuracy. CNA empathy was examined as a moderating variable. Analyses confirmed a relationship between pain catastrophizing and CNA pain rating accuracy (R^2 = .205, p < .01), reflecting lower accuracy of ratings for residents higher in catastrophizing. Hypotheses predicting a relationship between resident cognitive status and CNA pain rating accuracy and moderating effects of empathy were disconfirmed. Challenges of conducting research in long-term care are discussed.

### DEVELOPING AN INTERVENTION TO REDUCE SEDENTARY BEHAVIOR: ASSISTED LIVING RESIDENT FEEDBACK

Katelyn Webster, and Janet Larson, *University of Michigan School of Nursing, Ann Arbor, Michigan, United States*

Older adults in assisted living (AL) tend to be highly sedentary, which increases their risk of functional decline and frailty. Reducing sedentary behavior (SB) and replacing it with light physical activity (LPA) could have important implications for maintenance of functional abilities. The purpose of this qualitative study was to gather feedback from AL residents on a proposed exercise-specific self-efficacy enhancing intervention designed to promote LPA and reduce SB. We conducted one-on-one semi-structured interviews at four AL facilities with 20 residents ages 65-99 (mean age 83.1; 60% women). They were presented with the proposed intervention and were asked questions to inform the development and modification of the intervention. Data were analyzed with a thematic analysis approach. Specific recommendations included short intervention sessions of one hour and scheduled in the morning. Many residents thought a 16 week intervention was too long. We identified broader themes, including concerns about how the intervention would work for residents with a wide range of abilities and concerns about safety. They suggested that exercises will need to be highly modifiable. Most residents recommended framing the intervention message as increasing LPA rather than decreasing SB, because it would be more positive. All except one said they would want to participate in the intervention, but they were unsure how many other residents would want to be more active. The resident feedback and suggestions will guide development of the intervention and are important for increasing the probability that a future feasibility and acceptability trial of the intervention will be successful.

### DO NURSING HOME CULTURE CHANGE PRACTICES IMPROVE RESIDENTS' QUALITY OF LIFE AND PROMOTE A PERSON-CENTERED CLIMATE?

Minhong Lee,[1] Kyeongmo Kim,[2] and Sok An,[3] *1. Dong-eui University, Busan, Pusan-jikhalsi, Republic of Korea, 2. Virginia Commonwealth University, Richmond, Virginia, United States, 3. Korea Rural Economic Institute, Austin, Texas, United States*

Background and Purpose: Although nursing home culture change models (e.g., Green House, The Pioneer Network) in the U.S. have been proven to improve the quality of life of the residents by providing person-centered care and home-like environment, limited efforts have been made to implement a model in Korea. The purpose of this study was to examine whether the adapted culture change model would improve the quality of life of the residents and strengthen the person-centered climate. Methods: Employing a quasi-experimental design, the PI developed and implemented a 12-session culture change enhancement program to 32 staff in four nursing homes. Data were collected from 109 nursing home residents before and after the intervention, and key measures included the quality of life scale and the person-centered climate. The paired t-test analyses were performed with SPSS 26.0. Results: Paired samples t-tests showed that the culture change intervention significantly improved the quality of life of the residents. There were significant differences in the scores for privacy at pretest (M=2.63) and posttest (M=3.21); autonomy at pretest (M= 3.20) and posttest (M= 3.43); relationships at pretest (M= 2.88) and posttest (M= 3.36); individuality at pretest (M=3.26) and posttest (M=3.61). An average score for person-centered climate (M=6.25) were significantly higher than that of pretest (M=5.96) (p <.01). Conclusions and Implications: This study suggests that providing support and education to nursing home staff about the culture change strategies is essential to provide personalized services to nursing home residents and encourage residents to participate in the decision-making process.

### DO VOLUNTEERS MAKE A DIFFERENCE ON NURSING HOME QUALITY MEASURES?

Paul Falkowski,[1] Christopher Kelly,[2] and Nancy Kelley,[3]
*1. University of Nebraska - Omaha, Philadelphia, Pennsylvania, United States, 2. University of Nebraska Omaha, Omaha, Nebraska, United States, 3. University of Nebraska - Omaha, Omaha, Nebraska, United States*

The purpose of this study was to explore the relationship between nursing home volunteer programming and quality measures and increase the knowledge base of nursing home volunteer programming in various settings. Fifty-two nursing homes were surveyed using electronic surveys and personal interviews. Questions focused on the organization of the nursing home, characteristics of the volunteer program and volunteer activities. Of the 52 facilities surveyed, 19 were not part of a chain, 37 were in urban settings, and 24 were for-profit entities. Volunteers were used in 46 nursing homes with a mean number of volunteers of 51.7 onsite an average of 4.9 days per week. Bivariate analysis revealed statistically significant correlations ($p<.05$) between organizational and volunteer programming characteristics and six quality measures (pressure sores, urinary tract infections, depression, use of restraints, falls, use of antipsychotic and hypnotic drugs). Statistically significant ($p<.05$) inverse relationships were found between volunteers providing individualized activities (e.g., feeding assistance, combing hair, doing nails, and letter writing) and the incidence of urinary tract infections and the use of psychotropic drugs. Multiple regression analysis revealed a statistically significant ($p<.05$) inverse relationship between personal volunteer services such as combing hair and doing nails and the use of hypnotic drugs and antipsychotic drugs. This study indicates a significant correlation between volunteer programming and quality measure scores. A larger study of these relationships is indicated.

### EFFICACY OF LIFE REVIEW THERAPY INTERVENTION ON LIFE SATISFACTION AMONG OLDER ADULTS LIVING IN TAIWAN NURSING HOMES

Ching-Teng Yao, and Chia-Ju Lin, *Kaohsiung Medical University, Kaohsiung, Taiwan (Republic of China)*

Life review therapy, used as part of a comprehensive therapy plan for increasing the quality of life of the elderly, helps them to resolve their past conflicts, and accept their present conditions. This study tested the effectiveness of a structured life review therapy protocol on the life satisfaction of institutionalized older adults. A quasi-experimental design was adopted in this study. Fifty older adults aged 65 or above were recruited from nursing homes in southern Taiwan through convenience sampling. The participants in the intervention group carried out life review therapy for eight weeks in addition to their daily activities. The participants in the comparison group maintained their daily activities. Both groups were evaluated using a life-satisfaction scale including two aspects of life worries and situations in weeks 1 and 8. Data were collected at baseline (T1), immediately post-intervention (T2). Generalized estimating equations were used to examine the effect of the intervention on the outcomes. The overall life satisfaction increased significantly over time for the intervention group compared to the comparison group from week 1 to week 8. The life review therapy programs showed promising effects in improving the life satisfaction of older adults living in nursing homes.

### ENVY AND JEALOUSY OF LIVING-APART-TOGETHER RELATIONSHIPS IN CONTINUING CARE RETIREMENT COMMUNITIES

Chaya Koren,[1] and Liat Ayalon,[2] *1. University of Haifa, Haifa, Israel, 2. Bar-Ilan University, Ramay Gan, HaMerkaz, Israel*

Moving to a continuing care retirement community (CCRC) and living apart together (LAT) as a repartnering form, represent new late-life beginnings. A larger qualitative study on LAT relationships constructed in the CCRC identified envy and jealousy yet they were not examined in-depth. Envy is wanting something we lack whereas jealousy is fear of losing something that is ours to another. These emotions are rarely explored in the context of older adults' relationships. Our aim is to examine experiences of envy and jealousy from perspectives of residents aged 79 to 96 and staff, heuristically using Goffman's framework on (semi)-totalitarian institutions. 30 semi structured qualitative interviews were conducted in three CCRCs in Israel with 10 LAT residents, 10 residents not LAT, and 10 CCRC staff members including social workers. Analysis was conducted based on principles of thematic analysis and triangulation. Findings refer to kinds of envy, ignoring envy, and the development and consequences of jealousy and/or envy related to LAT in the CCRC. Conclusions address how semi-totalitarian CCRC features influence envy and jealousy experiences including implications for assisting social workers, older adults and their family members to adjust to life in the CCRC and assist CCRC management and staff to address possible consequences of envy and jealousy.

### EXAMINING SATISFACTION WITH ASSISTED LIVING AMONG RESIDENTS WITH AND WITHOUT DEMENTIA

Sarah Holmes,[1] Elizabeth Galik,[2] and Barbara Resnick,[3]
*1. University of Maryland Baltimore Doctoral Program in Gerontology, Baltimore, Maryland, United States, 2. University of Maryland, Baltimore, Maryland, United States, 3. University of Maryland School of Nursing, Baltimore, Maryland, United States*

Understanding residents' satisfaction with assisted living (AL) is essential for creating supportive environments that are focused on residents' needs and preferences. Nearly half of AL residents experience some level of cognitive impairment, although limited research has examined residents' satisfaction with AL particularly among those with cognitive impairment. The purpose of this study was to compare satisfaction with AL between residents with and without dementia. Baseline data from the Dissemination and Implementation of Function Focused Care in AL study was used in this analysis. A total of 481 AL residents were included in the sample. Measures included demographic information, Saint Louis University Mental Status Exam

(SLUMS), and the Resident Satisfaction Index. Controlling for age, gender, and comorbidities, multivariate analyses of variance was performed to consider the impact of cognitive status on residents' satisfaction with health care services, physical environment, relationships with staff, and social activities. The majority of participants were female (71%), White (97%), mean age was 89 years old (SD=7.43), and mean SLUMS score was 15.90 (SD=4.74). On average, residents were highly satisfied with AL reporting a mean score of 19.17 (SD=3.15). There were no significant differences in residents' satisfaction scores (p>.05) between residents with dementia and without dementia across all subdomains of satisfaction: health care services, physical environment, relationships with staff, and social activities. There may have been some bias in results due to social desirability. Further research should explore additional aspects of residents' satisfaction with staff such as whether or not person-centered care is provided.

### EXPECTATIONS REGARDING AGING AFTER RELOCATION TO AN ASSISTED LIVING COMMUNITY

Evan Plys, *University of Colorado Denver - Anschutz Medical Campus, Denver, Colorado, United States*

This study adds to the growing literature on attitudes toward aging in older adulthood by using a multidimensional measure to assess heterogeneous profiles of expectations regarding aging (ERA) in a sample of assisted living (AL) residents. The author analyzed secondary data from a cross-sectional quantitative study consisting of 202 residents of 21 ALs. Participants were mostly female (72%), white (90%), and widowed (59%); ages ranged from 51 to 100 (M = 83.05, SD = 10.32). Hierarchical Cluster Analysis identified four subgroups: (1) "healthy agers", n = 54, 27%, characterized by high physical, emotional, and cognitive ERA; (2) "cognitively intact", n = 41, 20%, characterized by low physical, low emotional, and high cognitive ERA; (3) "coping with decline", n = 56, 28%, characterized by moderate physical, high emotional, and low cognitive ERA; and (4) "unhealthy agers", n = 51, 25%, characterized by low physical, emotional, and cognitive ERA. Subgroups varied by mental health (healthy agers > unhealthy agers), cognitive ability (cognitively intact > coping with decline), and activity participation (coping with decline > unhealthy agers). Surprisingly, groups did not differ based on social support from co-residents, staff, or family. Results demonstrate that distinct subgroups of ERA exist among AL residents, supporting the utility of assessing ERA as a multidimensional construct in this setting. In addition, findings suggest that expecting to retain health and ability in at least one domain may protect against behavioral consequences of negative ERA. The author also discusses implications for future research and clinical practice in AL.

### HOW TO ACHIEVE THE DESIRED OUTCOMES OF A COMPLEX INTERVENTION IN NURSING HOMES: A THEORY OF CHANGE

Martin Dichter,[1] Jonas Hylla,[2] Almuth Berg,[3] Daniela Eggers,[4] Ralph Möhler,[5] Gabriele Meyer,[6] Sascha Köpke,[1] and Margareta Halek,[7] *1. University of Cologne, Cologne, Germany, 2. German Center for Neurodegenerative Diseases (DZNE), German Center for Neurodegenerative Diseases (DZNE), Germany, 3. Medical Faculty, Martin Luther University Halle-Wittenberg, Halle, Germany, 4. University of Lübeck, Lübeck, Germany, 5. Bielefeld University, Bielefeld, Germany, 6. Medical Faculty, Halle, Germany, 7. Witten/Herdecke University, Witten, Germany*

Background Recent systematic reviews suggest the effectiveness of complex psychosocial interventions to reduce sleep disturbances in people with dementia (PwD) living in nursing homes. However, it is unclear how and under which circumstances these interventions work and which components and processes are crucial determinants for effectiveness. Objectives To develop a Theory of Change (ToC) that describes a causal chain for the reduction of sleep disturbances. Design and Methods The ToC approach is a participatory method in intervention development to generate knowledge about how, why, and under which circumstances interventions are effective. We conducted two expert workshops, a subsequent expert survey (n=12), a systematic literature review, and expert interviews (day and night nurses). Results Necessary preconditions for the reduction of sleep disturbances were identified on staff, management and cultural levels of nursing homes. Intermediate goals like "individual knowledge on PwD is available", "a specific institutional concept to promote sleep is implemented", "person-centred care is implemented" and "sleep preferences of PwD are fulfilled" were defined. The intermediate goals, interventions, promoting and inhibiting factors as well as rationales were sorted into a causal chain. All intermediate goals were rated as relevant or highly relevant based on the expert survey. Conclusions The ToC model displays how a complex psychosocial intervention is likely to be effective in reducing sleep disturbances and meeting sleep preferences of PwD in nursing homes. The model is the basis for the development and evaluation of a planned complex psychosocial intervention to prevent and reduce sleep disturbances in PwD.

### INTERPERSONAL RELATIONSHIPS IN ASSISTED LIVING: FINDINGS FROM A SURVEY OF FAMILY MEMBERS AND STAFF

Francesca Falzarano,[1] M. Cary Reid,[2] Leslie Schultz,[3] and Karl Pillemer,[3] *1. Weill Cornell Medicine, New York, New York, United States, 2. Weill Cornell Medical College, New York City, New York, United States, 3. Cornell University, Ithaca, New York, United States*

In recent decades, assisted living facilities (ALFs) have grown dramatically as an alternative to nursing homes. Research in nursing homes has shed light on the nature of the relationships that exist between family members and staff. However, little is known about family-staff relations within ALFs. We present data from the first study to describe the prevalence of conflict and positive and negative family-staff interactions in ALFs and to examine whether positive and negative aspects of the relationship contribute to salient staff and family outcomes. We use data collected from 252 family members and 472 staff members across 20 ALFs from the Partners in Care in Assisted Living (PICAL) study. Participants completed measures including interpersonal conflict, depression, perception of treatment, and stress-related to caregiving. Results showed that conflict among family and staff members is relatively low in ALFs. For staff, interpersonal conflict and treatment by family members significantly predicted burnout and depression. For families, only gender significantly predicted burden. Subgroup analyses, however, indicated that the effect of interpersonal conflict was significantly associated with perceived caregiver burden among family members whose relative has dementia. Despite the relatively harmonious relationships identified among family members and staff in ALFs, sources of conflict and negative interactions were identified, revealing the influence these relationships have on both family and staff outcomes. These findings can inform intervention efforts targeting family-staff interactions within ALFs.

### JOB SATISFACTION IN THE LONG-TERM CARE WORKFORCE: HOW AGEISM PLAYS A ROLE

Alex Hennessa,[1] Tracey Gendron,[2] Verena Cimarolli,[1] Jennifer Inker,[2] Annie Rhodes,[2] and Robyn Stone,[1] *1. LeadingAge, Washington, District of Columbia, United States, 2. Virginia Commonwealth University, Richmond, Virginia, United States*

Prior research has demonstrated that ageism, specifically negative attitudes and behaviors about growing old, can be barriers to delivering high-quality long-term care (LTC), but little is known about how ageism may be related to job satisfaction – an important driver of workforce retention in LTC. Hence, the purpose of this study was to examine the role of ageism in job satisfaction in LTC. Our cross-sectional study used data collected from 265 staff members of aging services organizations (e.g. nursing homes, assisted living) representing the continuum of job types in LTC. The study examined the relationship between ageist attitudes (i.e. internalized and relational aging anxiety; affinity for older persons) and ageist behaviors, and job satisfaction when controlling for socio-demographic (i.e. age; gender; ethnicity) and employment-related variables (i.e. years of employment; advanced training in gerontology; direct care vs. managerial position). Results of a regression analysis showed that lower internalized aging anxiety and higher affinity for older people were significantly associated with higher levels of job satisfaction. Findings suggest addressing ageism to improve job satisfaction in LTC and provide some evidence for incorporating ageism screening and training into recruitment and onboarding of staff to enhance job satisfaction and to mitigate turnover.

### NURSING HOME STAFF'S PERCEPTIONS OF HEALTHCARE DECISION MAKING FOR UNBEFRIENDED RESIDENTS

Hyejin Kim,[1] Molly Perkins,[2] Thaddeus Pope,[3] Patricia Comer,[2] and Mi-Kyung Song,[5] *1. Chung-Ang University, Seoul, Republic of Korea, 2. Emory University, Atlanta, Georgia, United States, 3. Mitchell Hamline School of Law, Saint Paul, Minnesota, United States, 4. A.G. Rhodes Health & Rehab, Atlanta, United States*

'Unbefriended' adults are those who lack decision-making capacity and have no surrogates or advance care plans. Little data exist on nursing homes (NHs)' healthcare decision-making practices for unbefriended residents. This study aimed to describe NH staff's perceptions of healthcare decision making on behalf of unbefriended residents. Sixty-six staff including administrators, physicians, nurses, and social workers from three NHs in one geographic area of Georgia, USA participated in a 31-item survey. Their responses were analyzed using descriptive statistics and conventional content analysis. Of 66 participants, eleven had been involved in healthcare decision-making for unbefriended residents. The most common decision was do-not-resuscitate orders. Decisions primarily were made by relying on the resident's primary care physician and/or discussing within a facility interdisciplinary team. Key considerations in the decision-making process included "evidence that the resident would not have wanted further treatment" and the perception that "further treatment would not be in the resident's best interest". Compared with decision making for residents with surrogates, participants perceived decision making for unbefriended residents to be equally-more difficult. Key barriers to making decisions included uncertainty regarding what the resident would have wanted in the given situation and concerns regarding the ethically and legally right course of action. Facilitators (reported by 52 participants) included some information/knowledge about the resident, an understanding regarding decision-making-related law/policy, and facility-level support. The findings highlight the complexity and difficulty of healthcare decision making for unbefriended residents and suggest more discussions among all key stakeholders to develop practical strategies to support decision-making practices in NHs.

### ORGANIZATIONAL FACTORS ASSOCIATED WITH RETENTION OF CERTIFIED NURSING ASSISTANTS AND DIRECT CARE WORKERS

Katherine Kennedy, Robert Applebaum, John Bowblis, and Jane Straker, *Miami University, Oxford, Ohio, United States*

Low retention of certified nursing assistants (CNAs) and direct care workers (DCWs) continues to be an unresolved problem for nursing homes (NH) and assisted living (AL) settings. While numerous studies have examined predictors of CNA retention in NHs, little attention has been paid to differences between settings of long-term care. To inform practice and policy related to growth in the AL industry, this study compares the predictors of CNA and DCW retention rates. The 2017 Ohio Biennial Survey of Long-Term Care Facilities provides facility-level information from 968 NHs (91% response rate) and 708 ALs (88% response rate). Using regression analysis, we compare the factors that predict retention rates among providers with complete data on retention and controls. The same covariates relating to structural and financial characteristics, as well as staffing, management, and a number of retention best practices are used. Average DCW and CNA retention rates were 66% and 61% in ALs and NHs, respectively, with some settings reporting very low (and even 0%) retention over a year. AL and NH providers rated the problem's severity highest (6 out of 10) compared to retaining other licensed nurses. Similar and different predictors were found across financial, environmental, and managerial practices supporting retention. CNA and DCW retention strategies may not be equivalently meaningful between settings, given differing working environments, resources, and regulations. Aging services managers should be attuned to practices supporting retention in their industry.

### PREDICTING CHANGE IN DEPRESSIVE SYMPTOMS IN THE NURSING HOME

Joann Reinhardt,[1] Orah Burack,[1] Verena Cimarolli,[2] and Audrey Weiner,[1] *1. The New Jewish Home, New York, New York, United States, 2. LeadingAge, Washington, District of Columbia, United States*

Depressive symptoms are common among nursing home (NH) residents. Important predictors of depression to tease apart include demographic characteristics, physical status, functional ability, and chronic pain. A challenge to addressing depression is that a majority of NH residents have some level of dementia. Nonpharmacological management of depression in the NH is a recommended first line of treatment including: personalized activities, music therapy, repositioning, and attention to personal care needs (toileting, resting, and hydration). A holistic approach to the well-being of NH residents is the adoption of person directed (PDC) care models. In this study, predictors of decreased depression over time was examined in residents (N=144) living in two communities featuring PDC models, and those living in traditional care communities within the same NH. Care in the two PDC communities focused on provision of comfort care for persons with advancing dementia at-risk of not having their care needs met largely due to their inability to clearly communicate these needs. Care practices focused on knowing each elder deeply, and anticipating their needs. Care practices also included an emphasis on staff empowerment and meaningful life activities for residents. Traditional communities are those where PDC practices had not yet been incorporated. Data on demographic characteristics, cognitive status, physical and functional status, behavioral symptoms, and pain were extracted from the MDS. Results showed that being in the PDC group, less time in the nursing home, having less pain, and fewer behavioral symptoms were significant predictors of decreased depressive symptoms over a six-month period.

### PSYCHOSOCIAL REACTIONS TO RELOCATION TO NURSING HOMES IN CHINESE OLDER ADULTS

Xiuyan Lan,[1] Huimin Xiao,[2] and Ying Chen,[2] *1. Fujian Provincial Hospital, Fuzhou, China, 2. Fujian Medical University, Fuzhou, China*

This study aimed to elicit psychosocial reactions to relocation to nursing homes from older adults' perspectives with a qualitative interview design. Narratives from 23 Chinese nursing home residents from Fuzhou, China in a life review program were recorded, transcribed into sentences, and analyzed with the qualitative content analysis. It revealed five stages of psychosocial reactions to relocation to nursing homes as fear, struggle, compromise, acceptance, and contribution. The first stage resulted from negative labels attached to nursing homes, disconnection to the society, difficulties in establishing new relationships, and being abandoned by their families. The second stage described the behaviors of struggle: complain about family members, think of going back home, pray to have a change, and take action to leave. The third stage described the keys to compromise: choices between maintaining the harmony in family relation and companionship of relatives, choices between professional care and family care, and choices between costs and effects of family care and nursing home care. The fourth stage described how they accept nursing home life: accept the life and yet with worries, affirm benefits of living in nursing homes, and embrace the nursing home life. The last stage resulted from sense of ownership and giving full play to self-worth. This study generated new insights into the knowledge on psychosocial reactions to relocation to nursing homes and provided both family members and nursing home staff with a direction for how to promote a smoother relocation process.

### SENSE OF BELONGING, RELIGIOUS ACTIVITY, AND WELL-BEING IN LONG-TERM CARE RESIDENTS

Kelly Shryock, and Suzanne Meeks, *University of Louisville, Louisville, Kentucky, United States*

Residence in a long-term care (LTC) facility poses numerous challenges to psychological well-being and rates of depression are high. Sense of belonging (SoB) has been linked with measures of well-being in all age groups and interventions focused on improving SoB have been successful with college-age adults. It is unclear if SoB improves in LTC residents as they adjust to living in this environment or what factors predict poor SoB in this population. As part of a larger study of care preferences in LTC residents, participants (n= 76) completed measures of SoB, well-being, religious activity, and demographic information. SoB did not vary significantly based on duration of stay, age, gender, ethnicity, marital status, number of children, education, facility, cognitive functioning, or physical health. SoB was found to be significantly and positively correlated with participation in religious activities (r= .388, N=76, p=.001), private religious practices (r= .275, N=71, p=.020), and spirituality (r= .263, N=70, p=.028). There was also a significant positive correlation between SoB and positive affect (r= .450, N=74, p<.001) and SoB and life satisfaction (r= .393, N=74, p=.001). These results suggest that connections formed before admission to a LTC facility, including religious networks, are important to SoB and well-being and that individuals without or with low religious involvement may benefit most from interventions focusing on improving SoB in LTC residents.

### SESSION 2951 (POSTER)

### PERSONALITY

### ASSOCIATIONS BETWEEN THE FIVE FACTOR MODEL OF PERSONALITY AND DEMENTIA-RELATED ANXIETY

Stacy Yun,[1] Lisa Stone,[1] Evan Furr,[1] and Molly Maxfield,[2] *1. University of Colorado Colorado Springs, Colorado Springs, Colorado, United States, 2. Arizona State University, Phoenix, Arizona, United States*

The Five Factor Model (FFM) of normative personality is predictive of long-term outcomes, including well-being and anxiety. For example, people with anxiety disorders often report high Neuroticism and low Conscientiousness (Kotov et al., 2010). Dementia-related anxiety (DRA) is concern about developing dementia that can occur in individuals of any age and cognitive status (Kessler et al., 2012). This study assessed associations between the FFM and DRA and the extent to which other factors, such as demographics and variables related to DRA (i.e., external locus of control and lacking knowledge of dementia), contributed to relationships. Participants (N = 664; aged 18 to 81; M = 30.24) completed measures of the FFM, DRA, locus of control, and dementia knowledge. Hierarchical regression (block 1: basic demographics, block 2: DRA-related variables, and block 3: FFM) was computed. The set of predictors explained 17.9% of the variance in DRA, $F(14, 623) = 9.69$, $p < 001$. Being older, partnered, low on Conscientiousness and Openness, and having greater external locus of control and less dementia knowledge predicted higher DRA (p-values < .05). Surprisingly, Neuroticism was not predictive of DRA after controlling for demographic and DRA-related factors, indicating that the trait-like tendency towards emotional instability does not explain DRA. Longitudinal research can explore the course of relationships among Conscientiousness, Openness, and DRA over time to further examine significant effects of age, as expressions of personality change across the lifespan. Research targeting potentially modifiable factors (i.e., dementia knowledge) could help identify methods of reducing DRA.

### CAREGIVER NEUROTIC PERSONALITY TRAIT AND ELDER ABUSE—THE MODERATING ROLE OF CHANGE IN THE LEVEL OF CAREGIVER BURDEN

Boye FANG,[1] Elsie Yan,[2] Yaolin Pei,[3] and Xiaozhao Yang,[4] *1. Sun Yat-Sen University, Guangzhou, China, 2. The Hong Kong Polytechnic University, Hong Kong, China, 3. New York University, New York, New York, United States, 4. Sun Yat-Sen University, Guangzhou, United States*

Objectives: To investigate whether caregiver neuroticism has an effect on subsequent occurrence of elder abuse and whether change in the level of caregiver perceived burden alters this relationship. Methods: Using two-year longitudinal data, we analyzed a consecutive sample of 800 Chinese family caregivers and their care recipients with dementia recruited from the geriatric and neurological departments of three Grade-A hospitals in People's Republic of China (PRC). All the participatory dyads were assessed between September 2015 and February 2016 and followed for two years. Results: Significant increase in the prevalence was found for physical and psychological abuse, caregiver neglect, and financial exploitation. Caregivers high in neuroticism were more likely to engage in subsequent physical and psychological abuse, however, change in the level of caregiver perceived burden altered this association. Specifically, absence and alleviation of care burden during the two-year observation prevented the subsequent occurrence of physical and psychological abuse. Although caregiver neuroticism was also associated with subsequent caregiver neglect, caregiver perceived burden did not appear to have an impact on this relationship. Discussion: This study provided evidence that caregiver neuroticism was associated with subsequent physical and psychological abuse, while change in the level of caregiver perceived burden may alter this trajectory. These findings suggest the importance of implementing caregiver-centered intervention and prevention programs for elder abuse by specifically targeting at caregivers' behaviors related to their neurotic personality trait and cognitive appraisal of caregiving stressors associated with such personality trait.

### CHILDHOOD SOCIOECONOMIC STATUS, ADULT PERSONALITY, AND COGNITION: EARLY INDICATORS OF COGNITIVE IMPAIRMENT IN OLD AGE

Amanda Sesker,[1] Jason Strickhouser,[1] Páraic Ó Súilleabháin,[2] Antonio Terracciano,[1] and Angelina Sutin,[1] *1. Florida State University College of Medicine, Tallahassee, Florida, United States, 2. University of Limerick, Limerick, Ireland*

This study investigated the association between childhood socioeconomic status (cSES) risk of cognitive impairment but not dementia (CIND), cognitive impairment (dementia or CIND), and dementia and whether adult personality mediated this association. A sample of 10,289 participants (aged 50 and older) from the Health and Retirement Study (HRS) were followed across 2-year periods between 2006 - 2018. Estimates of mediation effects in Cox Proportional Hazards regressions were conducted using Mplus software to approximate the total effects of cSES on the cognitive outcomes and the natural indirect effects and natural direct effects derived when personality causally mediated this outcome. cSES was associated with increased risk of all three cognitive outcomes. Conscientiousness partially mediated the relationship between cSES and dementia, CIND, and cognitive impairment risk while neuroticism partially mediated dementia and impairment, but not CIND. Personality improved the overall model fit between cSES and both CIND and impairment, and conscientiousness was specifically associated with significantly lowered cognitive impairment risk over time. Conscientiousness and neuroticism substantially mediated the relationship between cSES and risk of impairment in old age. This research adds to lifespan models and suggests that distinct personality traits attenuate early childhood factors that contribute to lifespan development and cognitive aging. Conscientiousness in particular may act as a protective buffer mediating risk factors associated with cognitive impairment in old age.

### DAILY STRESSORS AND PHYSIOLOGICAL REACTIVITY: THE ROLE OF PERCEIVED STRESS, STRESS REACTIVITY, NEUROTICISM, AND AGE

MacKenzie Hughes, and Christopher Hertzog, *Georgia Institute of Technology, Atlanta, Georgia, United States*

Exposure to stressful events is an inevitable aspect of everyday life, such as encountering work deadlines or interpersonal conflicts. The body's physiological stress systems can become activated when exposed to stressors, resulting in increases in cortisol from the hypothalamic-pituitary-adrenal axis and/or increases in the alpha-amylase enzyme via the sympathetic-adrenal medullary system. We predicted physiological reactivity measured by salivary assay in an ecological momentary assessment (EMA) design, using The Perceived Stress Scale (PSS; Cohen, 1988), Perceived Stress Reactivity Scale (PSRS; Schulz, Jansen, & Schlotz, 2005), neuroticism, (Big Five Inventory; John, Donahue, & Kentle, 1991), and age. Adult participants (N=163) ages 20–80 years old (M=51.8 years) completed the individual differences measures and then provided seven saliva samples per day for 10 consecutive days while also completing five stress-related surveys per day via random-prompted EMA (Scott, Sliwinski, & Blanchard-Fields, 2013). Mean aggregate alpha-amylase correlated with the PSRS ($r = -.20$, $p < .05$) but not with the PSS or with neuroticism, suggesting alpha-amylase may be a more sensitive measure of subjective stress reactivity. In contrast, mean cortisol levels were not correlated with any of those measures. Age correlated with subjective stress ($r = -.23$, $p < .05$) and stress reactivity ($r = -.20$, $p < .05$) but not with hormone levels. Multilevel modeling will be used to evaluate within-person and between-person variation in stress hormones and EMA measures of daily stress in relation to the PSS, the PSRS, neuroticism, and age.

### FEELING OLDER AND CONSTRAINED: SYNERGISTIC INFLUENCES OF CONTROL BELIEFS AND SUBJECTIVE AGE ON COGNITION

Eric Cerino, Erica O'Brien, and David Almeida,
*Pennsylvania State University, University Park, Pennsylvania, United States*

Control beliefs are important correlates of cognitive health and aging. In addition, how old or young one feels is a self-perception of aging that may play a role in understanding control-cognition associations. We explored whether subjective age moderates associations among control beliefs and cognitive performance using data from the third wave of the national Midlife in the United States study. The analytic sample comprised of 2,621 adults aged 39–93 (Mage=64.06, SD=11.15; 55.51% female) that completed measures of control (mastery, perceived constraints), subjective age (how old you feel most of the time), and cognition (executive function, episodic memory) via telephone administration. Hierarchical regression analyses were conducted to examine whether mastery, perceived constraints, and subjective age were associated with cognitive performance, adjusting for chronological age, gender, education, marital status, and self-rated health. For executive function, there was a significant perceived constraints by subjective age interaction. Higher levels of perceived constraints were associated with worse executive function (Est.=-0.05, SE=0.01, p<.001), and this association was amplified among those with relatively older subjective ages (Est.-0.10, SE=0.02, p<.001). For episodic memory, higher levels of perceived constraints were associated with worse performance (Est.=-0.07, SE=0.03, p<.001), while reporting a more youthful subjective age was associated with better performance (Est.-0.10, SE=0.02, p<.001). Mastery was not associated with either cognitive domain (ps>.05). Results suggest that perceiving constraints in life may confer greatest risk to cognitive performance among adults who feel older than their actual age, whereas perceiving a more youthful subjective age may be more facilitative.

### HEALTH PERSONALITY, CONSUMER HEALTH ACTIVATION, AND LONELINESS

Nicholas Cone, and Peter Martin, *Iowa State University, Ames, Iowa, United States*

The purpose of this study was to identify relationships between health personality traits, consumer health activation (CHAI) and loneliness. Data for these analyses were collected by a large provider of Medicare Supplemental Health Insurance. The study consisted of 3,907 participants, 65 years and older. Participants were surveyed on health personality (e.g., Health Neuroticism, Health Extraversion, Health Openness, Health Agreeableness, and Health Conscientiousness), Consumer Health Activation, and Loneliness. Structural equation modeling and mediation were conducted through Mplus. The hypothesized model fit without direct paths from health personality to loneliness was not optimal. Adding direct paths from health neuroticism, health openness, and health agreeableness to loneliness resulted in an excellent fit, □2 (5) = 0.86, RMSEA = 0.00, CFI = 1.00. Health neuroticism and health openness were negatively related to health activation, which suggests respondents were less likely to be active about their health. Alternatively, health agreeableness and health conscientiousness were positively related to health activation, indicating more health activation. Mediation was tested for pathways from health personality dispositions to loneliness through health activation. The results suggest individuals higher in health neuroticism or health openness were less activated, which in turn indicated higher loneliness. Moreover, those higher in health agreeableness or health conscientiousness were more activated and indicated less loneliness. This study provides an understanding about loneliness through health personality and health activation. Future research should explore interventions for older adults with specific health personalities, or health activation to reduce loneliness levels.

### INTER-INDIVIDUAL AND INTRA-INDIVIDUAL RELATIONSHIPS BETWEEN NEUROTICISM AND COGNITION: A COORDINATED ANALYSIS

Tomiko Yoneda,[1] Jonathan Rush,[1]
Graciela Muniz Terrera,[2] Almar Kok,[3] Boo Johansson,[4]
Scott Hofer,[1] Daniel Mroczek,[5] and Andrea Piccinin,[6]
*1. University of Victoria, Victoria, British Columbia, Canada, 2. University of Edinburgh, University of Edinburgh, Scotland, United Kingdom, 3. Amsterdam University Medical Centres, Amsterdam, Netherlands, 4. University of Gothenburg, Göteborg, Sweden, 5. Northwestern University, Chicago, Illinois, United States, 6. Psychology, Victoria, British Columbia, Canada*

Existing literature indicates a relatively consistent relationship between neuroticism and cognitive functioning (CF). Interindividually, high levels of neuroticism may predispose individuals to cognitive aging and dementia-related neuropathology. Intraindividually, increases in neuroticism may be intrinsic to the aging process or to dementia pathology. These hypotheses are not mutually exclusive, though the relationships are rarely examined using the same individuals, which may contribute to publication bias and confusion regarding the hypotheses as mutually exclusive. Data were drawn from the Origins of Variance in the Oldest-Old (Sweden, Mage=83.6, 67% female), Swedish Adoption/Twin Study of Aging (Sweden, Mage=60.4, 59% female), and Longitudinal Aging Study Amsterdam (Netherlands; Mage=68.1, 52% female). Controlling for age, sex, education, and depressive symptoms, parallel process latent growth models were fit independently in each sample (NT=3293) to simultaneously estimate growth parameters of neuroticism with three measures of CF (processing speed, learning/memory, and reasoning). Multilevel meta-analysis estimated the pooled covariation between neuroticism and CF at baseline and overtime, revealing a significantly negative intercept-intercept relationship across datasets (covariance= -0.46, 95% CIs [-0.90,-0.02], z=-2.02, p=0.04, τ2=0.06). The slope-slope covariances were consistently negative, but the meta-analytic pooled estimate was not significant despite some significant individual estimates across studies. Overall, results provide some evidence for intraindividual and interindividual relationships between neuroticism and CF, such that higher neuroticism is associated with lower CF, and neuroticism tends to increase as CF decreases. Identification of the early indicators and risk factors for cognitive decline may facilitate development of screening assessments and aid in treatment strategies for dementia care services.

### NARCISSISM, SOCIAL ENCOUNTERS, AND MOOD IN LATE LIFE

Shiyang Zhang, Yee To Ng, and Karen Fingerman, *The University of Texas at Austin, Austin, Texas, United States*

Social contacts have a strong impact on older adult's well-being. However, narcissism (i.e., feelings of self-importance) may undermine interpersonal connections. Research with young adults has found that being narcissistic may generate feelings of boredom, irritation, and pride because narcissistic young adults oftentimes have difficulty maintaining attention, have greater sensitivity to negative social events (e.g. social rejections), and have an exaggerated sense of self-worth. Yet, we know little about narcissism in late life, particularly in a daily context. This study examined the associations between narcissism, social encounters, and mood (i.e., bored, irritated, lonely, proud) throughout the day. Older adults aged 65 + (N = 307) from the Daily Experiences and Well-being Study completed a measure of narcissism in a baseline interview. Then, in ecological momentary assessments (EMA), they reported social encounters and mood every 3 hours for 5 to 6 days. We found no significant associations between narcissism and number of social encounters throughout the day. Multilevel models revealed that older adults who scored higher on narcissism felt more bored and prouder throughout the day. Interaction terms involving narcissism and social encounters showed that during assessment periods when they had social encounters, participants who scored higher on narcissism reported a similar level of loneliness as when they were alone, whereas their peers who scored lower on narcissism experienced decreases in loneliness. Findings suggest that narcissism does not predict social encounters. However, older adults who are higher in narcissism may be less likely to be influenced by their social encounters.

### PERSONALITY AND HIGH BLOOD PRESSURE AMONG OLDER ADULTS: STATE ANXIETY AS A MEDIATOR

Lisa Stone,[1] Deborah Koh,[2] and Kendall Weber,[2]
*1. University of Colorado Colorado Springs, Colorado Springs, Colorado, United States, 2. University of Colorado at Colorado Springs, Colorado Springs, Colorado, United States*

High blood pressure (BP) is a prevalent medical condition among older adults, and previous research has consistently found that the Five Factor Model (FFM) of personality relates to elevated BP. However, variables that affect this relationship have been unexamined. Given the strong association between personality and state anxiety in later life, this study examined state anxiety as a mediator between the FFM and elevated BP. Participants consisted of respondents in the 2018 wave of the Health and Retirement Study (N=5225) who completed FFM and state anxiety questionnaires and had their BP measured. First, correlations were computed between the FFM and systolic and diastolic BP. Relationships were insignificant between systolic BP and the FFM. Conscientiousness (r = -.04) and Neuroticism (r = .03) were significantly correlated with diastolic BP. Next, a series of mediation models were computed. Controlling for sex, the test of indirect effects found that the connection from Conscientiousness to diastolic BP was significantly mediated by anxiety, Sobel Z test = -2.44, p = .01. Additionally, controlling for sex, the connection from Neuroticism to diastolic BP was significantly and fully mediated by anxiety, Sobel Z test = 2.02, p = .04. Although small effect sizes, Conscientiousness and Neuroticism were related to diastolic BP but not systolic BP, indicating personality differentially affects types of BP. The mediating role of state anxiety suggests that it can be used as a point of intervention to lessen the negative impact of low Conscientiousness and high Neuroticism on high diastolic BP among older adults.

### PERSONALITY DISORDERS AND SYSTEM USE AMONG OLDER VETERANS USING THE VETERANS HEALTH ADMINISTRATION

Jenefer Jedele, Cameron Griffin, and Sharon Nelson, *Veterans Health Administration, Ann Arbor, Michigan, United States*

Personality disorders (PD) are defined by longstanding patterns of dysfunctional behavior and associated with higher risk for adverse outcomes, such as suicide and comorbid mental health (MH). Despite recognition that PDs manifest differently as individuals age, few studies have examined PDs in older adults. VHA serves a significant population of older Veterans, providing a valuable opportunity to explore PD in older ages. Using FY2019 administrative data, we compare documented PDs, MH, and VHA use in a cohort of recent Veteran VHA users 50 years and older, across six age groups. At least one PD was documented for 0.8% of the total cohort, decreasing with age from 1.8% (50-54) to 0.2% (75+).

Among those with PD, 98% had a comorbid MH disorder with the greatest percent having a major depressive disorder (69.7%). Except for dementia, comorbid mental health disorders generally decreased with age. Suicide risk flags were documented for 8.2% of cohort Veterans with PD compared to 0.7% with MH only. Behavior risk flags were documented for 4.3% of cohort Veterans with PD compared to 0.3% with MH only. The presence of both flags decreased with age. Compared to cohort Veterans with MH only, those with PD had more than twice the number of outpatient MH encounters and 1.5 times more psychiatric hospitalizations and ED visits for MH. Differences in care were consistent across age. However, differences in use were greatest for Veterans ages 55-64. The need to address crises resulting in the use of inpatient and ED is discussed.

### PERSONALITY PATHOLOGY AND COGNITIVE AGING

Patrick Cruitt,[1] Patrick Hill,[1] and Thomas Oltmanns,[2]
*1. Washington University in St. Louis, Saint Louis, Missouri, United States, 2. Washington University in St. Louis, St. Louis, Missouri, United States*

Research on the relationship between normal-range personality and cognitive aging has demonstrated consistent, but modest, effects. The current investigation seeks to increase our understanding of unhealthy cognitive aging by examining the maladaptive extremes of personality. Borderline and avoidant personality disorder (PD), but not obsessive-compulsive PD, were hypothesized to show prospective associations with cognitive aging. The current investigation tested this hypothesis in two longitudinal studies of older adulthood: the Alzheimer's Disease Research Center cohort (ADRC, N = 434, Mage = 69.95, 56% women) and the St. Louis Personality and Aging Network study (SPAN, N = 1,058, Mage = 65.92, 54% women). The ADRC study administered a battery of neuropsychological tests to assess cognitive ability/memory. Borderline PD was measured with a composite derived from the NEO Five Factor Inventory. The SPAN study administered self-, informant, and interview measures of the three PDs, a free recall memory task, and an informant report measure of cognitive problems. Borderline PD features exhibited cross-sectional correlations with memory (ADRC: r = -.11; SPAN: all rs = -.08), general cognitive ability (ADRC: r = -.11), and informant reported cognitive problems (rs ranged from .15 to .39). These features also prospectively predicted changes in cognitive problems (Std. bs = .13 and .15), but not in memory or cognitive ability. Avoidant and obsessive-compulsive PD exhibited little association with cognitive aging. These findings suggest that borderline PD features may interfere with cognitive maintenance interventions. Furthermore, they argue for the development of PD treatments adapted for the context of later life.

### PRESENCE AND LEVELS OF RESILIENCE AND ITS SOCIODEMOGRAPHIC CORRELATES IN OLDER CHINESE AMERICANS

Jieyang Zheng,[1] Dexia Kong,[2] Mengting Li,[2] and XinQi Dong,[2] *1. Rutgers University, Chicago, Illinois, United States, 2. Rutgers University, New Brunswick, New Jersey, United States*

Resilience is defined as a personal quality that enables growth in knowledge, understanding and self-actualization in the face of adversity and life disruptions. Low levels of resilience can dispose older adults to higher risks for negative health outcomes in the aftermath of traumatic events. However, we have limited knowledge of resilience among minority aging populations. This study aims to examine the presence and levels of resilience and its sociodemographic correlates among U.S. Chinese older adults. Data were drawn from the Population Study of Chinese Elderly, an epidemiology study of U.S. Chinese older adults in the Greater Chicago area. Guided by a community-based participatory research approach, a total of 3,036 Chinese older adults aged 60 and above participated in face-to-face interviews from 2015 to 2017. Spearman's rank-order coefficient was utilized to test correlation. A 10-item validated Chinese version of the Connor-Davidson resilience scale was used to assess resilience. In our sample, 59.7% were female, and the average age was 75. The mean resilience score was 26.9, ranging from 1 to 40. U.S. Chinese older adults who were younger, male, married, had higher education and income, fewer children, better health status and quality of life, and improved health and have lived fewer years in the U.S. reported higher levels of resilience. Future longitudinal research is needed to investigate the protective effects of resilience among older Chinese Americans against mental and physical distress.

### SELF-REPORTED MEMORY PROBLEMS ARE RELATED TO DEPRESSIVE SYMPTOMS IN OLDER ADULTS WITH CERTAIN PERSONALITY TRAITS

Sakshi Bhargava,[1] Nikki Hill,[2] and Jacqueline Mogle,[2]
*1. The Pennsylvania State University, University Park, Pennsylvania, United States, 2. Pennsylvania State University, University Park, Pennsylvania, United States*

Self-reported memory problems and depressive symptoms tend to co-occur in older adults; however, this relationship may depend on personality traits and the type of self-reported memory assessed. Using a coordinated analytic framework, this study examined whether neuroticism, extraversion, and conscientiousness moderated the associations of older adults' self-reported memory with depressive symptoms at between- and within-person levels across three large, longitudinal datasets (range=8-12 years of follow-up) of community-dwelling older adults with no evidence of cognitive impairment (n=427-6,960; Mage: 69.47- 75.94; 72-84% White; 60-64% Female). Assessments of depressive symptoms (GDS-15 or CES-D) and self-reported memory (perceived memory decline, frequency of forgetting, and current memory rating) were taken annually or biennially; personality was assessed via the IPIP or NEO Five-Factor Inventory. Results were largely consistent across datasets. Specifically, between persons, self-reported memory problems (including perceived memory decline, higher frequency of forgetting, and lower current memory rating) were related to higher depressive symptoms only among older adults higher in neuroticism. In one dataset, results supported a protective effect of conscientiousness such that higher frequency of forgetting was related to lower depressive symptoms among older adults higher in this trait. Within persons and across datasets, at times when perceived memory decline was reported, or current memory rating was lower, depressive symptoms tended to be higher only in older adults higher in neuroticism. Results demonstrate the importance of considering personality traits and the type of self-reported memory when examining associations among reports of memory problems and depressive symptoms in cognitively intact older adults.

### THE EFFECTS OF WORD USE AND PERSONALITY PATHOLOGY ON DEPRESSION AND SUICIDAL BEHAVIOR IN OLDER ADULT INPATIENTS

Hyunyoung Ellen Park,[1] Helene Geramian,[2] Jennifer Ho,[2] Avner Aronov,[3] Wing Jin Mak,[4] Ira Yenko,[5] Dimitry Francois,[6] Richard Zweig,[2] and Hyunyoung Park,[2] *1. Yeshiva University Ferkauf Graduate School of Psychology, Bronx, New York, United States, 2. Yeshiva University Ferkauf Graduate School of Psychology, The Bronx, New York, United States, 3. Yeshiva University, Ferkauf Graduate School of Psychology, Brooklyn, New York, United States, 4. Ferkauf Graduate School of Psychology, Yeshiva University, The Bronx, New York, United States, 5. Yeshiva University Ferkauf Graduate School of Psychology, Mountain View, California, United States, 6. Weill Cornell Medicine, White Plains, New York, United States*

Few prior studies have examined whether word use is associated with personality pathology or is linked to depression and suicidal behavior in older adult inpatients. In this study, older adult depressed inpatients (N = 51; age M= 67, SD = 8.9; mean years of education = 15.49, SD = 2.6) provided narratives about high and low points in life, health challenges, and interpersonal conflicts, and completed measures of personality pathology (NEO Five Factor Inventory, Inventory of Interpersonal Problems-Personality Disorders-25), depression (Geriatric Depression Scale), and suicidal ideation or behavior (SCID-5). The Linguistic Inquiry and Word Count (LIWC) text analysis tool was used to examine the frequency of self-referential, negative emotion, or absolutist word use. After adjusting for word count, age, and occupational and marital status, the use of self-referential words in narratives about high points of life predicted suicidal behavior (OR = .563, p = .002), but word use did not predict depression or personality pathology. In regression analyses, neuroticism and maladaptive interpersonal functioning predicted depression (ß = .417, p = .002; ß = .364, p = .009, respectively) but not suicidal behavior. Together, these preliminary findings suggest that in depressed older adult inpatients, depression and suicidal behavior may have qualitatively different predictors.

### THE RELATIONSHIP BETWEEN BIG FIVE PERSONALITY TRAITS AND DRIVING STYLES IN OLDER ADULTS

Junyan Tian,[1] Anabella Raika,[1] Despina Stavrinos,[2] and Lesley Ross,[1] *1. Penn State University, State College, Pennsylvania, United States, 2. University of Alabama Birmingham, Birmingham, Alabama, United States*

Older adults' psychosocial factors, including personality, are correlated with driving performance and driving cessation. However, the relationship between personality and driving styles has been examined only among young and middle-aged drivers. This study examined the relationships of personality factors and self-reported driving styles among 72 healthy older drivers aged 65-85 (M=72.29, SD=5.36) using the Multidimensional Driving Style Inventory (MDSI) scale to measure reckless and careless, anxious, angry and hostile, and patient and careful driving styles. Personality was accessed with the Big Five Personality questionnaire. Correlational results indicated that less conscientiousness was significantly correlated with increased reckless and careless and less patient and careful driving styles; and lower agreeableness was significantly correlated with greater angry and hostile and less patient and careful driving styles. Being a man was associated with greater reckless and careless and angry and hostile driving styles. Age was not associated with driving styles. Accordingly, three regressions were tested. After controlling for gender, only lower conscientiousness was associated with greater reckless and careless driving style (β=-.007, p=.03). Men had a higher risk of reckless and careless (β=.342, p<.01) and angry and hostile (β=.392, p<.01) driving styles. Our results highlight the relationship between personality traits and self-reported driving styles among older adults, and how gender may influence some of these relationships. Future research should further investigate the associations between gender and personality traits and older adults' driving mobility and safety.

### SESSION 2952 (POSTER)

### PERSONALITY II

### AGE DIFFERENCES IN SELF-CONTINUITY IN THE GERMAN SOCIOECONOMIC PANEL

Corinna Loeckenhoff,[1] and Denis Gerstorf,[2] *1. Cornell University, Ithaca, New York, United States, 2. Humboldt University Berlin, Berlin, Berlin, Germany*

Self-continuity, the sense that one's personal past, present, and future selves are meaningfully connected, is unique to human beings. Self-continuity varies across individuals with higher levels conveying benefits for mental health and well-being, physical health and health-related behaviors, as well as financial planning and moral choices (for a review see Hershfield, 2019). From a developmental point of view, self-continuity emerges over the course of childhood, but less is known about its development in adulthood. Recent evidence indicates that higher chronological age is associated with higher perceived self-continuity among healthy adults. Studies further suggest that age effects are more pronounced for more distant time intervals but fairly symmetrical for past and future (for a review see Loeckenhoff & Rutt, 2017). However, prior work has predominantly relied on U.S. convenience samples raising questions about generalizability to broader population samples as well as cross-national consistency of the findings. To address these concerns, the present study examined self-continuity in the German Socio-Economic Panel (SOEP, 2017 Innovation Sample, n = 1659, aged 18-92, M = 62.8, SD = 18.1, 53% female). In addition to replicating the previously reported positive association between age and self-continuity (r = .17, p < .001) and testing for curvilinear effects, we report on the role of temporal direction (past vs. future), temporal distance (1, 5, and 10 years), and demographic factors (i.e., gender, education, and wealth). The present findings add to the literature on adult age differences in self-continuity. Practical implications and directions for future research are discussed.

### CONVERGENCE BETWEEN DSM-5 SECTION II AND III MODELS OF PERSONALITY DISORDERS AMONG OLDER ADULTS

Lisa Stone,[1] and Daniel Segal,[2] *1. University of Colorado Colorado Springs, Colorado Springs, Colorado, United States, 2. University of Colorado at Colorado Springs, Colorado Springs, Colorado, United States*

Introduction: The Personality Inventory for DSM-5 (PID-5) is a measure of the alternative model of personality disorders (PDs), proposed in Section III of the DSM-5, but the PID-5 has limited evidence of validity for use among older adults. This study examined the validity of the alternate model through associations with the 10 traditional PDs in DSM-5. It was hypothesized that the PID-5 would relate to the traditional PDs in patterns predicted by the alternate model. Method: Older adults (N = 202) completed the PID-5 and the Coolidge Axis II Inventory (CATI), a measurement of the 10 PDs. Results: Correlations were computed between the PID-5's 25 facets and the CATI's 10 PD scales. All facets were found to significantly (p < .001) and positively correlate with all 10 PD scales, with large effect sizes (> .30). Next, regressions were conducted, with the PID-5 facets predicting each PD scale. Overall, across the 10 regression analyses, the PID-5 facets accounted for significant variance in the CATI PD scales, ranging from 64% (Avoidant) to 71% (Obsessive-Compulsive). Discussion: Although some DSM-5 hypothesized facets were significant predictors, many unexpected significant relationships were also detected. Of the 10 PD models, seven models included more unpredicted significant traits than predicted ones; two models included more significant predicted traits than unpredicted ones; one model included an equal number of predicted and unpredicted significant traits. We found substantially more overlap between the PID-5 and CATI than anticipated in unpredicted directions, suggesting that the PID-5 has good specificity but lacks sensitivity.

### DOES TRAIT AFFECT IMPACT ACTIVITY ENGAGEMENT?

Rebekah Knight, Allura Lothary, and Thomas Hess, *North Carolina State University, Raleigh, North Carolina, United States*

Research has shown the amount of effort we expend towards our goals depends on a sense of self-efficacy, perception of task difficulty, and likelihood of achieving our goal. All of these processes are susceptible to the influence of affect. For example, negative moods may impede goal achievement by increasing perceptions of difficulty (Silvestrini & Gendolla, 2019). Negative experiences (such as past failures) can encourage these negative moods and subsequently impact self-efficacy (Esposito, Gendolla, & Van der Linden, 2014). Findings from self-efficacy research (e.g. Esposito et al., 2014) suggest that older adults may be particularly susceptible to the impacts of negative affect on effort mobilization, especially when tasks already seem challenging, with little chance of success. Perception of task difficulty, then, impacts the amount of effort exerted in completing the task. The present study sought to examine the factors that impact perceptions of difficulty and subsequent effort expenditure, represented by systolic blood pressure responsivity (SBP-R). Younger (N = 41) and Older (N = 163) adults completed a difficult cognitive task as part of a larger, longitudinal study, as well as measures of trait affect before study sessions. Our findings indicate younger adults exert less effort overall than older adults; however, when negative trait affect is considered, we find that higher levels of negative affect in older adults reduced task engagement. These results provide support for an effect of negative affect on task appraisals and posited age-related differences in effort mobilization.

### PERSONALITY AND WELL-BEING IN OLD AGE

Constanca Paul, *University of Porto, Porto, Portugal*

Personality is a controversial issues in Geropsychology. It is generally accepted that personality is relatively stable through life and plays a relevant role in life style and wellbeing. We assessed Personality with NEO (Neuroticism, Extroversion and Openness) in a community sample of 1322 individuals 55+ years, mean age 70.4 (sd=8.6) years, 71% women. The means were: Neuroticism 34.06(sd. 5.1); Extroversion 41.25(sd. 4.05), Openness 37.37(sd. 3.82), around 10 points higher than the normative Portuguese data. Considering the highest values of the 3th tertil in each domain, women have higher Neuroticism differing from men (p=0.001), and values raise with age along 3 age groups (p=0.001); Neuroticism has significant positive correlations (p<0.01) with loneliness, sleep problems, psychological distress, difficulties in ADL and IADL and self-perception of health, and negative correlations with social network, happiness and cognitive capacity. Extroversion do not differ between gender and varies by age group (p=0,04) in U inverted form. Extroversion is positively correlated with happiness and negatively with loneliness, self-perception of health, psychological distress, difficulties in ADL and IADL. Openness does not differ by gender but differ between age groups (p=0.18) in U shape and correlates negatively with cognitive capacity, and social network. Personality traits appear very relevant for successful aging as facilitators or risk factors of physical and mental health, and should be addressed to foster adaptation in old age.

### PERSONALITY TRAITS AND EMOTIONAL SUPPORT EXCHANGES AMONG OLDEST-OLD PARENTS AND OLDER-ADULT CHILDREN IN KOREA

Kyuho Lee, *Daegu University, Gyeongsan, Kyongsang-bukto, Republic of Korea*

Previous studies show that personality traits are predictors of individuals' exchanges (i.e., giving, perceiving, and evoking) in social support in general relationships. Less attention has been paid, however, to the roles of personality traits in parent-child relation, especially in the very old parent-child dyads. Focusing on personality's effects on the perception of received support, this study examines 1) whether personality traits are associated with a perception of received emotional support, 2) which personality traits work as a predictor of support perception, and 3) whether the similarity between parent and children exist in the patterns of personality-support relationships among Korean very old parent-child dyads. A total of 105 dyads of very old parents, 81 to 97 years old (M = 87.9, SD = 2.8), and their older-adult children, 65 to 72 years old (M = 65.9, SD = 1.2), participated in the study. The results of the actor-partner interdependence model reveal that less neurotic and more agreeable parents perceived more emotional support from children(actor-effects); parents with more agreeable children perceived more support from children(partner-effect). There was no actor- and partner effects of personality traits on the emotional support children perceived. Our findings show that children, as compared to their parents, are maybe less affected by parents' personality traits reflected by parents' interaction behaviors. We further provide explanations of each path from the personality traits to emotional support.

### PERSONALITY, DEPRESSION, SOCIAL FUNCTIONING, AND SUICIDAL BEHAVIOR IN DEPRESSED OLDER ADULT INPATIENTS

Ira Yenko,[1] Jennifer Ho,[2] Helene Geramian,[2] Wing Jin Mak,[3] Hyunyoung Ellen Park,[4] Avner Aronov,[5] Dimitry Francois,[6] and Richard Zweig,[2] *1. Yeshiva University Ferkauf Graduate School of Psychology, Mountain View, California, United States, 2. Yeshiva University Ferkauf Graduate School of Psychology, The Bronx, New York, United States, 3. Ferkauf Graduate School of Psychology, Yeshiva University, The Bronx, New York, United States, 4. Yeshiva University Ferkauf Graduate School of Psychology, Bronx, New York, United States, 5. Yeshiva University, Ferkauf Graduate School of Psychology, Brooklyn, New York, United States, 6. Weill Cornell Medicine, White Plains, New York, United States*

Older adults are at higher risk for completed suicide. However, research in late-life suicide for high-risk populations remains a neglected topic, with some researchers suggesting that our knowledge of risk factors and risk conferral remains incomplete and insufficient in their predictive ability. Personality processes, in the context of interpersonal problems, have been associated with suicidal behavior, depression, and social functioning, but have rarely been evaluated in samples of older adults during periods of highest risk. This study examined factors underlying the relationship between personality processes, depression, social role functioning, and suicidal behavior in older adult inpatients. It also examined the examined the additive effect of personality processes, social adjustment, and depression on suicidal behavior. Depressed middle aged and older adult inpatients (N=52; Age M= 66.88, SD= 8.76) completed self-report measures of personality pathology (IIP-PD-25), depression (GDS-30), social functioning (SAS-SR), and recent suicidal behavior (SIB). Our research found that while interpersonal pathology was positively associated with depression (GDS-30, ß = .37, p = .006) and social functioning (SAS-SR, ß = .384, p = .003), it was not associated with suicidal behavior. The combined model of social functioning and depression displayed a trend toward significance, but neither variable was robust enough to emerge as an independent predictor of suicidal behavior. However, bivariate analyses found moderate effect sizes between depression or social functioning and suicidal behavior. Risk for suicidal behavior likely involves dynamic, complex, and interrelated relationships with clinical implications regarding assessment within this population.

### SELF-EFFICACY PROXY PREDICTS PHYSICAL FRAILTY INCIDENCE OVER 8 YEARS IN NON-INSTITUTIONALIZED OLDER ADULTS

Melissa Hladek,[1] Jiafeng Zhu,[2] Brian Buta,[1] Sarah Szanton,[1] Karen Bandeen-Roche,[3] Jeremy Walston,[1] and Qian-Li Xue,[4] *1. Johns Hopkins University, Baltimore, Maryland, United States, 2. School of Public Health, Johns Hopkins University, Baltimore, Maryland, United States, 3. Johns Hopkins Bloomberg School of Public Health, Baltimore, Maryland, United States, 4. Johns Hopkins School of Medicine, Baltimore, Maryland, United States*

Physical frailty is defined as a syndrome of decreased physiologic reserve conferring vulnerability to functional decline, mortality and other adverse outcomes in response to a stressor. One potential modifiable risk factor of frailty is self-efficacy, which is confidence in one's ability to perform well at a task or domain in life. Self-efficacy is associated with improved health behavior and decreased chronic disease burden but has not been studied extensively in frailty research. Therefore, the purpose of this study was to evaluate a general self-efficacy proxy measure's ability to predict frailty in a nationally representative sample of older adults using data from the National Health and Aging Trends Study (NHATS) collected from 2011-2018. 4,835 older adults (65+) were dichotomized into low and high self-efficacy groups using the one-item self-efficacy proxy measure in NHATS. The Physical Frailty Phenotype was used to assess frailty. A discrete time hazard model was used to obtain incident hazard ratios of frailty in two models. Model 1 was adjusted for age, race, sex, education and income. Model 2 contained Model 1 covariates and activities of daily living and co-morbidities. We found that low self-efficacy predicted a 41% increased risk of developing frailty over 8 years after adjustment for socio-demographics (P<0.0001) and a 27% risk of incident frailty after further adjustment for activities of daily living and co-morbidities (P=0.004). This study provides preliminary evidence that self-efficacy may be a key modifiable element to incorporate into multi-modal frailty interventions.

### THE ASSOCIATION BETWEEN PERSONALITY TRAITS AND DEPRESSIVE SYMPTOMS IN U.S. CHINESE OLDER ADULTS

Maggie Li,[1] XinQi Dong,[2] and Dexia Kong,[2] *1. Rutgers University, Chicago, Illinois, United States, 2. Rutgers University, New Brunswick, New Jersey, United States*

Depressive symptoms are prevalent in the aging population and can negatively impact the health and well-being of older adults. Personality traits may interact with depressive symptoms, but there is currently limited knowledge regarding this relationship in minority aging research. This study aims to explore the associations between two personality traits, neuroticism and conscientiousness, and depressive symptoms in 3,157 U.S. Chinese older adults. Data were obtained from the Population Study of Chinese Elderly in Chicago (PINE) collected between 2011 and 2013. Neuroticism and conscientiousness were measured by the NEO Five-factor Inventory. Depressive symptoms were measured by the nine-item Patient Health Questionnaire (PHQ-9). 45.3% of the participants reported at least one depressive symptom. Controlling for potential confounders, logistic regression analyses showed that both traits were significantly associated with depressive symptoms. One unit increase in neuroticism was associated with 19% increased odds of having any depressive symptoms (odds ratio [OR]=1.19, 95% confidence interval [CI]=1.17-1.22). One unit increase in conscientiousness was associated with 5% decreased odds of having any depressive symptoms (OR=0.95, 95% [CI]=0.94-0.96). Results validate the significant association between both traits and depressive symptoms among U.S. Chinese older adults, adding to the psychological and cultural profiles of those who have experienced mental distress. More in-depth examination using culturally-tailored measurements for personality traits is encouraged in minority aging studies. The NEO inventory was developed from Western populations and hence might not adequately represent personality traits valued by non-Western cultures.

### THE RELATIONSHIP BETWEEN LIFESPAN VOLUNTEERISM AND PERSONALITY AMONG OLDEST OLD ADULTS

Gina Lee, and Peter Martin, *Iowa State University, Ames, Iowa, United States*

There has been a lack of studies exploring volunteerism from the life span perspective. This study aims to examine the relationship between personality and volunteerism among the oldest old population, using three types of volunteerism: “ever volunteered,” “last volunteered,” and “currently volunteering.” “Ever volunteered” assesses whether individuals ever volunteered in their life. “Last volunteered” examines when an individual last volunteered. “Currently volunteering” explores whether an individual is currently volunteering. By comparing the three volunteerism measures, this study took a life span view of volunteerism. Data of 208 oldest old adults, octogenarians (34.1%) and centenarians (65.9%), from the Georgia Centenarian Study were included in this study. The majority of the sample had volunteered sometime during their lifetime (88.9%), many of them still volunteered when they were in their 80s and 90s (40.4%), and the majority of the sample indicated that they were not currently volunteering (78.8%). Multiple regression analyses indicated that competence (a facet of conscientiousness) significantly predicted “ever volunteered,” and extraversion significantly predicted “last volunteered.” In other words, oldest old adults with high competence levels were more likely to have volunteering experiences in their life. Also, those with high levels of extraversion were likely to have more recent volunteering experiences. None of five personality traits significantly predicted “currently volunteering.” This study sheds light on the importance of different types of volunteerism which enables us to better understand the relationship between volunteerism and personality. We recommend future research to test the link between different types of volunteerism and well-being outcomes.

### SESSION 2953 (POSTER)

### SOCIAL DETERMINANTS OF HEALTH

### A CITY TOO BUSY TO REMEMBER? AGING, STRUCTURAL VIOLENCE, AND THE POLITICS OF FORGETTING IN ATLANTA’S GENTRIFICATION

John Pothen, Keland Yip, and Ellen Idler, *Emory University, Atlanta, Georgia, United States*

Can forgotten stories from the past inform a city’s future? As older adults continue to live longer and comprise more of the population than ever before, the suitability of gentrifying spaces for older adults aging in place is increasingly important. Critical theories of gentrification argue that remembering the experiences of older adults in this context - experiences of suffering, resilience, and structural violence - is essential to promote changes in support aging in place. In this study, we tell a story of individual experiences, structural violence, and aging in the ongoing gentrification of one neighborhood in southwest Atlanta. We construct this narrative through a qualitative analysis of 1,500 local newspaper articles from 1950 to the present day and 10 in-depth interviews with ex-residents of the neighborhood aged 65-87. Drawing on the theory of planetary rent gaps, we frame gentrification as a class struggle between property-owners and working class residents. We highlight the city government’s role as a facilitator for property-owners through projects including the Model City initiative, preparation for the 1996 Olympics, and ongoing development surrounding the Atlanta BeltLine. We show how these projects have affected the prospects for aging in place in general and, specifically, by affecting access to healthcare services. We share this story in an effort to combat the politics of forgetting and to inform a richer, more inclusive, and more equitable future for gentrifying spaces.

### ACUTE LIFE INTERVENTIONS, GOALS, AND NEEDS PROGRAM: SOCIAL DETERMINANTS OF HEALTH AMONG THE MOST VULNERABLE

Lizette Munoz,[1] Blair MacKenzie,[1] Audrey Chun,[2] Shamsi Fani,[3] Susana Lavayen,[1] and Stephanie Chow,[4] *1. Mount Sinai Hospital, New York, New York, United States, 2. Icahn School of Medicine at Mount Sinai, New york, New York, United States, 3. Mount Sinai Medical Center, New York, New York, United States, 4. Icahn School of Medicine at Mount Sinai, New York, New York, United States*

The Acute Life interventions Goals and Needs program(ALIGN) at Mount Sinai Hospital in New York City, is an inter-professional team dedicated to offering temporary intensive ambulatory care services to the most complex older patient population. This allows us to care for the most vulnerable population which often incur multiple hospitalizations, emergency room visits. Mr.C is a 81 yo male with past medical history of Chronic COPD, Depression, Gait instability, Mild Neuro-cognitive disorder, Hearing Loss, Coronary artery disease. Most significantly he had 3 ED visits, 1 admission, where he was found on the floor of his apartment after two days, by a meals on wheels volunteer. Team conducted a comprehensive assessment of Mr.C's social determinants of health and compiled a care plan. We learned that Mr.C does not like to bother others therefore found it difficult to seek help. Team built intensive rapport and gained his trust to help simplify medications, increase engagement and explore barriers to home care. Mr.C was connected to several community agencies including, meals on wheels for more stable food access, psychiatry to discuss depression and isolation, adult protective services for deep cleaning,financial management, pharmacy for blister packing, home care services and case management to continue encouragement with care plan. Mr.C is now able to reach out to the team as needed and has a navigator to help with managing care. This is one of many cases ALIGN encounters, that often go undetected due to comprehensive inter-professional care needed and minimal time given in traditional primary care.

### AGE-RELATED SOCIAL SELECTION AND ITS ASSOCIATED EMOTIONAL AND COGNITIVE COSTS ACROSS ADULTHOOD

Hsiao-Wen Liao,[1] and Laura Carstensen,[2] *1. Stanford University, Palo Alto, California, United States, 2. Stanford University, Stanford, California, United States*

Socioemotional selectivity theory maintains that goal prioritization differs across adulthood as a function of future time horizons. To prepare for a long and nebulous future, young adults prioritize learning and exploration over emotional meaning. Relieved from the burden to prepare, older adults prioritize emotionally meaningful goals. In the context of social relationships, younger adults include proportionally fewer familiar social partners in their social networks, whereas older adults' social networks encompass proportionally fewer unfamiliar social partners. Although social selection is considered adaptive, it inevitably involves gains and losses. The current study examined whether age-related selectivity correlates with (1) greater concurrent negative emotions in younger people, and (2) poorer cognitive performance in old age. A life-span sample (N = 258) completed a social networks questionnaire and cognitive tests. Daily emotional experience was assessed using experience sampling. A subset (N = 119) completed the cognitive tests again five years later. Results of multiple regression analysis, controlling for physical health and trait neuroticism, indicate that smaller proportions of familiar social partners in one's social network correlated with more frequent experience of negative emotions. Age moderated this association with a stronger association in younger than older people. Results of separate multiple regression analysis, controlling for baseline cognition, physical health, age, SES, and trait openness, indicate that a smaller proportion of social partners in one's outer social circle negatively predicted older adults' Digit Span Backward performance assessed five years later. We discuss our findings within the framework of gains and losses in life-span development.

### APPLYING ADMINISTRATIVE LINKAGE TO LONGITUDINAL AGING STUDIES: BOSTON EARLY ADVERSITY AND MORTALITY STUDY

Mina Antic,[1] Ashley Dorame,[2] Joseph Ferrie,[3] Maria Lopes,[2] Robert Waldinger,[1] Avron Spiro,[4] Daniel Mroczek,[5] and Lewina Lee,[2] *1. Massachusetts General Hospital, Boston, Massachusetts, United States, 2. Boston University School of Medicine, Boston, Massachusetts, United States, 3. Northwestern University, Evanston, Illinois, United States, 4. VA Boston Healthcare System, Waban, Massachusetts, United States, 5. Northwestern University, Chicago, Illinois, United States*

Adverse childhood experiences have been linked to poor adult health, yet the underlying pathways remain unclear. While longitudinal aging studies provide rich data on health trajectories in adulthood, two intrinsic limitations hamper progress in studying causal pathways: (1) reliance on retrospective assessment of early-life conditions, and (2) inadequate data coverage on lifespan developmental processes, especially in childhood. The Boston Early Adversity and Mortality Study (BEAMS) was designed to overcome these limitations by applying high-quality administrative record linkage to three longitudinal studies on aging that are over 50-years-old. BEAMS uses administrative linkage to acquire contemporaneous, early-life information on health, family, and environmental hazards from multiple databases. Our sample includes male participants from the VA Normative Aging (n=2280), Grant (n=456), and Glueck (n=268) Studies. BEAMS extends linkage to siblings, thus including women, so that our combined sample is representative of the early 1900s Northeastern U.S. population. Key steps in administrative linkage include coding identifiers from existing data; linkage to 1900-40 Censes, vital, and military (WWI, WWII, Veterans benefits) records; linkage to public databases for early-life lead exposure data, and later-life health information (Medicare, NDI). By linking records of study participants (74%-94% deceased) to numerous administrative databases, BEAMS will create a cradle-to-grave dataset with prospective data on early socioeconomic, psychosocial, and environmental exposures, and lifespan health data. BEAMS uses human review to achieve high-quality record linkage. Our methodology can be adopted by other longitudinal aging studies to overcome barriers in advancing causal knowledge on pathways linking early-life conditions to lifespan health outcomes.

### CHRONIC STRESS AND RISKS FOR MYOCARDIAL INFARCTION IN U.S. ADULTS

Heather Farmer,[1] Hanzhang Xu,[2] Ann Marie Navar,[2] Michael Nanna,[1] Linda George,[1] and Matthew Dupre,[1] *1. Duke University, Durham, North Carolina, United States, 2. Duke University School of Medicine, Durham, North Carolina, United States*

Long-term exposure to stress has been linked to multiple behavioral and biological responses that are detrimental to cardiovascular health, but the association between chronic stress and risks for acute myocardial infarction (MI) remains unknown. We examined the association between exposure to chronic stress and MI incidence from 2006 to 2016 using data from a nationally-representative prospective cohort study of adults aged 45 and older (n=15,109). Chronic stressors included ongoing issues related to personal health, social relationships, financial strain, housing, and caregiving responsibilities. Cox proportional hazards models were used to examine the association between the number of chronic stressors and MI while adjusting for confounding risk factors. More than half of the respondents reported ≥2 chronic stressors at baseline. Risks for MI increased incrementally from 1 chronic stressor (HR=1.28; 95% CI, 1.20-1.37) to ≥4 chronic stressors (HR = 2.71; 95% CI, 2.08-3.53) compared with those who reported no stressors. These risks were partly attenuated after adjustments for socioeconomic, psychosocial, behavioral, and clinical risk factors. The impact of chronic stressors was especially pronounced among adults with a history of MI (P value for interaction=.032). In adults with a prior MI, risks for a recurrent MI increased substantially from 1 chronic stressor (HR=1.31; 95% CI, 1.10-1.55) to ≥4 chronic stressors (HR = 2.92; 95% CI, 1.47-5.82) compared to those with no stressors. Chronic stress is a significant risk factor for acute coronary events in U.S. adults. More research is required to further understand the psychosocial, behavioral, and biological mechanisms underlying this association.

### EQUAL PROTECTION? DIFFERENTIAL EFFECTS OF RELIGIOUS ATTENDANCE ON BLACK-WHITE OLDER ADULT MORTALITY

Ellen Idler, *Emory University, Atlanta, Georgia, United States*

Social determinants of later life population health are “the circumstances in which we are born, grow up, live, work, and age” usually identified as power and status determinants: income, wealth, and education. Although rarely considered a social determinant of health, religious social ties are a familiar “circumstance” for many older persons, and there is considerable evidence linking religious attendance to all-cause mortality. There are race differences in both religiosity and mortality patterns: Black Americans show higher levels of both religious attendance and mortality compared with white Americans. This raises the question of equal protection of religious attendance: Is the protective effect of religious attendance on mortality weaker, stronger, or the same for whites and African Americans? The analysis employs 10-year longitudinal data from the Health and Retirement Study, 2004-2014 (N=18,346). In stratified models, after adjustment for sociodemographic factors and health, African Americans have a hazard ratio (HR) for frequent attendance at services that is more protective than for whites: .48 (95%CI: .35, .67) compared with .61 (95%CI: .53, .70). Health behaviors mediate 19% of the effect for blacks and 26% for whites; other social ties mediate 12.5% of the effect for blacks and 7% for whites. An interaction test shows a more protective effect of frequent attendance for blacks compared with whites (p<.000). Religious attendance may be more beneficial for African Americans who are multiply disadvantaged with respect to other social determinants of health. The mediation patterns also suggest that the mechanisms of effect for blacks and whites may differ.

### FINANCIAL STRAIN, SELF-RATED HEALTH, AND THE MEDIATING ROLE OF HEALTH BEHAVIORS DURING THE GREAT RECESSION

Lindsay Wilkinson, *Baylor University, Waco, Texas, United States*

Previous research has revealed the physical and mental health consequences of the economic recession that began in 2007 and ended in 2009. Despite accumulating evidence of the harmful health effects of the economic downturn on older adults, relatively little research has examined the mechanisms involved in this relationship. Moreover, most research on the financial crisis has relied on objective indicators of the recession, despite evidence demonstrating the predictive utility of subjective measures such as financial strain. Drawing on a subsample of respondents age 50 or older from the Health and Retirement Study (N = 2040), this research (1) examines whether initial financial strain and that due to the recession independently contribute to worsening self-rated health over the period of the recession and (2) investigates the role of health behaviors as mechanisms in this relationship. Using longitudinal lagged dependent variable models that adjust for pre-recession self-rated health, the results reveal that both initial and increased financial strain due to the recession were associated with worsening self-rated health between 2006 and 2009. In addition, increased financial strain during the recession was found to be associated with skipped or postponed health care visits and change in prescription drug use, such as pill splitting and reduced or skipped doses, which, in turn, were associated with decreased health ratings. The results from this study suggest two important pathways in the recession-health relationship and have implications for policies aimed at supporting older adults during future financial crises.

### HIGHER ED AS A LEVELER: HOW EARLY LIFE AND HIGHER EDUCATION SHAPE RESILIENCE AND HEALTH IN ADULTHOOD

Tyler Bruefach, and Dawn Carr, *Florida State University, Tallahassee, Florida, United States*

Growing evidence shows that individuals who have high levels of psychological resilience maintain higher levels of physical and psychological health in later life. Individuals cultivate psychological resilience over the life course, yet little research has explored its mechanistic effects on health during midlife. One source of resilience may be formal education, which is a well-established determinant of health in adulthood. Resilience might be one reason for this robust association, as education helps individuals develop greater psychological resources in adulthood. On the other hand, having a college degree also increases access to other health-promoting resources that can be leveraged over the life course, such as better-paying and higher-quality jobs. Using data drawn from the National Longitudinal Study of Adolescent to Adult Health (Add Health), the current paper examines: 1) how early-life factors shape psychological resilience in early adulthood (24-32 years); and 2) the effects of early adulthood resilience on the association between education and health in mid-life (36-44 years). Results show that psychological resilience and college education have significant direct effects on health in midlife, net of health in early adulthood. However, first-generation college graduates cultivate more psychological resilience from their educational attainment than do those with college-educated parents. That is, higher education serves as a leveler for health gaps in midlife for those with fewer resources available in early life by bolstering resilience. These results provide important insights about how early life factors play an important role in shaping successful aging processes.

### LONGITUDINAL ASSOCIATION BETWEEN EDUCATION AND DISABILITY IN OLDER ADULTS LIVING IN ICELAND

Milan Chang,[1] Olof Geirsdottir,[1] Lenore Launer,[2] vilmundur Gudnasson,[1] Palmi Jonsson,[1] Alfons Ramel,[3] and Milan Chang Gudjonsson *1. University of Iceland, Reykjavik, Iceland, 2. National Institute on Aging, Bethesda, Maryland, United States, 3. University of Iceland, Reykjavik, Capital area, Iceland*

BACKGROUND: Disabilities among older adults are associated with cumulative adversities such as low socioeconomic status (SES), poor nutrition, and lack of access to medical care and education. However, there is little evidence on the long-term association between education and disability status among older adults in Iceland. The aim of the study was to examine the association between mid-life education and prevalence of disability in activities of daily living (ADL) and mobility disability in late-life using 25 years of longitudinal data. METHODS: A large community-based population residing in Reykjavik, Iceland participated in a longitudinal study with an average of 25 years of follow-up (N=5764, mean age 77±6 yrs, 57.7% of women) Mid-life education was categorized into 2 groups (primary and secondary versus college and university). Disability status in late life was defined with ADL and mobility disability with a binary outcome (no difficulty versus any difficulty). Logistic regression analysis was used to examine the association. RESULTS: After controlling for age and gender, and midlife health risk factors, those who had high education at mid-life were less likely to have ADL disability (Odds Ratio (OR) = 0.75, 95% Confidence Interval (CI): 0.64 ~ 0.88, P ≤ 0.001) and mobility disability (OR = 0.72, 95% CI: 0.61 ~ 0.86, P < 0.001) compared with those who had low education in mid-life. CONCLUSION: People with high mid-life education were less likely to have ADL and mobility disability after 25 years later.

### MATHEMATICS AND RELATEDNESS: PREDICTING OPTIMAL SOCIAL ENGAGEMENT IN LATE LIFE

Christine Williams, Emmanuelle Tognoli, and Christopher Beetle, *Florida Atlantic University, Boca Raton, Florida, United States*

Multiple causes converge for older adults to shed social relationships. Lost opportunities for social engagement are tied to weakened cognitive reserve and under-optimal aging in health and disease. For example, a woman, 75, regularly strolls with younger friends. At 80, her reduced motor fitness makes it hard to keep pace and she withdraws her participation. With same-age peers, she might continue this healthy physical and social activity a few more years by unobtrusively shortening the outing or by slowing her pace. A man, 85, loves to debate politics with family, but his turn at talks diminish: his hearing loss (sensory) prevents quick grasp of the discussion; his slower verbal fluency (cognitive) hamper quick-witted replies. Both examples illustrate that social aging is not only a ¬¬¬property of the aging individual. Social context plays an important role. Our recently formed interdisciplinary group (geropsychiatric nurse, mathematical physicist and complexity scientist) is studying the systemic complexities of social aging with experiments and mathematical models. Our aim is to present our model and aging-focused hypotheses, as well as empirical validation in younger adults. Four key variables are group size and heterogeneity, and the strength and adaptability of social coordination. Our current results show that people coordinate better with others like them in pace, but they lose the ability to coordinate with people whose pace is different. We anticipate that our program of research will deliver evidence-based recommendations on social-engineering of activities that maximize opportunities for sustained interactions among older adults.

### NEIGHBORHOOD SOCIOECONOMIC POSITION AND SOCIAL SUPPORT IN A CLINICAL POPULATION OF OLDER ADULTS

Kristen Berg,[1] Adam Perzynski,[1] Nikolas I. Krieger,[2] Douglas Einstadter,[1] and Jarrod Dalton,[3] *1. Case Western Reserve University at MetroHealth, Cleveland, Ohio, United States, 2. Lerner Research Institute - Cleveland Clinic, Cleveland, Ohio, United States, 3. Cleveland Clinic Lerner College of Medicine at Case Western Reserve University, Cleveland, Ohio, United States*

Models of successful aging underscore the critical role of external social resources in older adults' health and well-being. Neighborhood socioeconomic position is known to influence health, but little is known about the linkages between neighborhood conditions, social relationships and health among older adults. We identified a cohort of 12,434 adults (aged 65+) who attended a Medicare Annual Wellness Visit from the NEOCARE Learning Health Registry. NEOCARE includes electronic health record (EHR) and neighborhood data from 1999-2017 on over 3 million unique Northeast Ohio individuals. The study population was 60% White, 32% Black or African American, 64% female, and 90% non-Hispanic. Over 60% were ages 65-74, 29% 75-84, and 10% 85 years or older (range from 65 to 101). We used ANOVA and chi square tests to examine variation in social support by quintile of the census tract area deprivation index. Compared to those in less disadvantaged neighborhoods, older adults in more disadvantaged neighborhoods were more likely to report needing help with care needs (Bonferroni-corrected x2=95.21, df=4, n=8,967, p<.001) but were less likely to report having help at home (x2=85.72, 4, n=12,354, p<.001). Similarly, adults in more disadvantaged neighborhoods reported less help available to them compared to those in more advantaged communities (F=39.31, df=4, n=12,099, p<.001). Furthermore, older adults living in more disadvantaged neighborhoods experienced significantly less functional independence (F=3.68, df=4 , n=8,571, p<.01). Our results suggest that the pathway from neighborhood socioeconomic conditions to successful aging includes perceived social needs and social support.

### OPIOID LITERACY IN AN IMMIGRANT GROUP IN RURAL AREA: USE OF SOCIAL DETERMINANT OF HEALTH FRAMEWORK

Hee Yun Lee,[1] Eun Young Choi,[2] Jieun Song,[3] Jamie Gajos,[4] and Yan Luo,[1] *1. University of Alabama, Tuscaloosa, Alabama, United States, 2. Leonard Davis School of Gerontology, Los Angeles, California, United States, 3. Waisman center, Madison, Wisconsin, United States, 4. The University of Alabama, Tuscaloosa, Alabama, United States*

Opioid overdose risk is particularly high in immigrant communities partly due to limited English proficiency (Guarino et al., 2015). Previous studies reported that social determinants of health (SDH) have been associated with risk for opioid overdose (Dasgupta et al., 2018). The current study examines the association between SDH and literacy of opioid overdose risk among the immigrant population living in a rural area. Specifically, we examine the association in various age groups including young adults (aged 20 to 34), middle-aged (aged 35 to 49), and older adults (ages 50 to 75). Data were drawn from a sample of Korean American immigrants residing in rural Alabama (N=225). The participants administered the Brief Opioid Knowledge (BOOK) Questionnaire (Dunn et al., 2016). Multiple regression analyses were conducted for three age groups to identify predictors of opioid literacy. Overall, older adults had lower levels of opioid literacy relative to their younger counterparts. Among young adults, low English proficiency, more chronic conditions, and greater depressive symptoms were significant predictors of limited opioid literacy. For the middle-aged adults, lower levels of health literacy and more pain symptoms were associated with limited opioid literacy. Among older adults, women, those with higher English proficiency, and lower health literacy had lower levels of opioid literacy. The findings demonstrated a greater vulnerability of older immigrants to limited opioid literacy. Different predictors based on SDH of limited opioid literacy across age groups have implications for tailored health promotion strategies to reduce opioid overdose risk.

### PERCEIVED SOCIAL INTEGRATION PREDICTS ENGAGEMENT AND RESPONSIVENESS TO POSITIVE EVENTS: TEST OF AGE MODERATION

Patrick Klaiber,[1] David Almeida,[2] and Nancy Sin,[1] *1. University of British Columbia, Vancouver, British Columbia, Canada, 2. Pennsylvania State University, University Park, Pennsylvania, United States*

Social Integration has important implications for health and well-being during adulthood. Being socially integrated might be an important resource that helps people to regularly engage in daily positive events. With older age, this resource might become increasingly important. However, being well socially integrated might also mean that people are certain that they experience more positive events in the future and thus, respond with less positive affect to any given positive event. We examined perceived social integration as a predictor of engagement and responsiveness to positive events using data from the National Study of Daily Experiences 2. 1904 adults (Mean age = 56.25, min = 33, max = 84) reported their daily positive affect and daily positive events during 8 consecutive days of telephone interviews. Perceived social integration was assessed at baseline. Adults higher in social integration experienced daily positive events more frequently (b = 0.01, SE = 0.003, p < .001), but showed less of an increase in positive affect on days with more-than-usual positive events (b = -0.003, SE = 0.001, p = .030). These models controlled for the Big Five personality traits, purpose in life, demographic variables and same-day occurrence of stressors. Age did not moderate the present associations. The present findings imply that social integration might be an important contributor to experiencing more positive events across adulthood. Being better socially integrated might also lead to responding with less positive emotions to any given positive event.

### PHYSICAL ACTIVITY AND NEIGHBORHOOD SOCIOECONOMIC DISADVANTAGE AMONG LOW-INCOME AFRICAN AMERICANS

Dextiny McCain,[1] Adrienne Aiken Morgan,[2] Karon Phillips,[3] and Keith Whitfield,[4] *1. North Carolina A&T State University, Kernersville, North Carolina,*

United States, 2. North Carolina A&T State University, Greensboro, North Carolina, United States, 3. UMBC, Baltimore, Maryland, United States, 4. Wayne State University, Detroit, Michigan, United States

Research shows regular physical activity (PA) is associated with better health and longevity; however, few studies consider contextual factors related to PA among African American (AA) older adults living in socioeconomically disadvantaged neighborhoods. The Physical and Cognitive Health Pilot Study (n=50) was used to examine associations between PA and level of neighborhood socioeconomic disadvantage among sedentary, AA older adults from four public housing communities in Durham, NC and Annapolis, MD (mean age=64.5; SD=10.42; 72% women). Participants were administered the Community Healthy Activities Model Program for Seniors (CHAMPS), a self-report questionnaire measuring weekly frequency and duration of PAs. Neighborhood socioeconomic disadvantage was defined by the Neighborhood Atlas Area Deprivation Index (ADI), which ranks neighborhoods according to Census block group/neighborhoods within each state and nationally. For the present sample, two of the Durham housing facilities were located in communities in the most disadvantaged block groups. Meanwhile, one Durham location and the Annapolis community were located in the least disadvantaged block groups. Bivariate correlations showed greater neighborhood socioeconomic disadvantage was associated with less participation in various PAs ($p<.05$). Next, ANOVA revealed the Annapolis group participated in statistically significantly more PAs, including visiting the senior center, church attendance, and light gardening ($p<.05$) compared to the most disadvantaged groups. The present findings suggest there are benefits to living in advantaged contexts despite lower-income status. These findings also suggest barriers within disadvantaged neighborhoods that limit access to recreational activities favorable to health status. Future research should address ways to overcome such barriers.

### RELIGION AND VOTING BEHAVIOR OF OLDER ADULTS WITH DISABILITIES IN GHANA

Kodwo-Nyameazea Yale, *University of North Carolina at Pembroke, Pembroke, North Carolina, United States*

Voting is a necessary and inherent right of citizens in democracies to select public office holders who decide how public goods and resources are distributed and maintained. It is therefore critical that all citizens who are eligible are able to participate in one of the key aspects of political participation – voting. This study focused on the factors that influence the ability of older adult Ghanaians with disabilities to vote in local and national elections. The study sample of 923 respondents was drawn from the second wave of WHO SAGE study on Global Aging and Adult Health. The results of the logistic regression analyses showed that religion influenced the voting behaviors of all the three people with disabilities groups included in the study. But certain groups are also influenced by interaction with community leaders and personal political interests and characteristics, including gender. Given these findings, it is suggested that an impact community be established around the meaning and ethics associated with the religious activities people with disabilities participate in, and engage them through civic engagement, and personal and community development activities that empower them to live meaningful lives.

### SOCIAL FRAILTY IS ASSOCIATED WITH THE RISK OF MALNUTRITION AMONG COMMUNITY-DWELLING OLDER ADULTS

Seoyoon Jane Lee,[1] Miji Kim,[2] Hayoung Shim,[3] Heeeun Jung,[4] Yuri Seo,[4] Chang Won Won,[5] and Jane Lee
*1. Elderly Frailty Research Center, Kyung Hee University, Seoul, Republic of Korea, 2. College of Medicine, Kyung Hee University, Seoul, Republic of Korea, 3. Kyung Hee University, Seoul, Korea, Seoul, Republic of Korea, 4. Graduate School, Kyung Hee University, Seoul, Republic of Korea, 5. Kyung Hee University Medical Center, Seoul, Korea, Seoul, Republic of Korea*

Social frailty does not merely affect the level of socialization, but also the means of obtaining resources for the daily lives of older adults. It is highly associated with the quality of life during advanced age. In this study, we analyzed the association between the Mini-Nutritional Assessment (MNA®) score (range 0-30) and 18 singular items and social frailty status of community-dwelling older adults. A total of 2,552 community-dwelling older adults aged 70-84 years (mean age 76.9±4.0, 51.8% female) from the Korean Frailty and Aging Cohort Study (KFACS) were assessed. The social frailty status was assessed in four categories- absence of economic resources, social resources, social activities, and social interactions. A higher MNA® score indicates better nutritional status. The prevalence of social frailty in older adults was 27.9% (mean age 78.1±4.0, 67.7% female). Approximately 40% of the participants were at risk of malnutrition or malnourished ($p<0.001$), while socially robust group accounted for 23% ($p<0.001$). They were at a higher risk of a lower MNA® score (odds ratio [OR] 0.90, 95% confidence interval [CI], 0.84-0.96). Socially-frail older adults have a higher possibility of not eating three full meals a day (OR 2.33, 95% CI 1.19-4.55) which increases the risk of malnutrition. In conclusion, social frailty, as a means of linking resources -including economic and social capitals- to the older adult population directly impacts the risk of malnutrition and requires an appropriate intervention.

### SOCIOECONOMIC CORRELATES OF HEALTHY AGING AMONG OLDER CAMBODIANS

Kakada Kuy, and Yuekang Li, *Washington University St Louis, University City, Missouri, United States*

Healthy aging is one of the most critical goals to attain on the World Health Organization's global aging agenda for developing countries. However, healthy aging has not been widely studied among the many older adults living in those nations. For example, most of the Cambodia's economically deprived older adults earn less than a dollar a day, while little scientific evidence is known about their healthy aging situation and their support system. Our study aimed to examine socioeconomic correlates of healthy aging among older Cambodians in three provinces. Data of a sample of older Cambodians ages 60 and above (N=210) from 12 districts were collected. Healthy aging was measured using the Healthy Ageing Index developed based on a Southeast Asian context. We measured social support using the Social Network and Social Support scale. Financial conditions were measured by an index derived from subjective and objective measurements. Multilevel mixed-effects models showed that better social supports from friends and family members, better financial conditions and education were associated with improved health aging among older Cambodians. Supports from friends had a stronger relationship than supports from family members. As one of the first studies examine the social determinants of health among older Cambodians, this study adds to the literature by substantiating the important roles of financial conditions and social support in determining their health and well-being. Findings point to the importance of improving living standard and maintaining social support of the older population in the country.

### STRESS IS A RISK FACTOR FOR SHINGLES AMONG OLDER ADULTS

Hyewon Kang, Eileen Crimmins, and Jennifer Ailshire, *University of Southern California, Los Angeles, California, United States*

Stress is a risk factor for shingles. Empirical evidence of how stress affects getting shingles is lacking for the older population. This paper examines how chronic stress and stressful events are associated with incident shingles in a nationally representative sample of the population over age 50, the Health and Retirement Study. Using data for 2010-2016, we tracked 12,628 persons aged 50 and older with no history of shingles at 2010 until the first shingles occurrence and linked shingles to chronic stress appraisal and stressful events in the prior period. Chronic stress appraisal was measured in eight life domains: health, spousal/children, finance, work, residence, relationship, alcohol/drug, and caregiving. Adverse life events including spousal loss, involuntary job loss, residential move, negative wealth shock, and spousal onset of disability were included in an index of stressful events. 3.3% of sample members developed new shingles cases. Regression results suggest that having a higher burden of stressful events significantly increased the risk for shingles (OR:1.13, 95% CI=1.05, 1.22), whereas ongoing chronic appraisal was not associated with shingles onset (OR:0.99, 95% CI=0.96, 1.03). Our findings highlight the importance of preventive efforts on stress management in reducing risks for zoster.

### THE EFFECT OF NEIGHBORHOOD COHESION ACROSS THE LIFESPAN ON COGNITIVE HEALTH

Elizabeth Munoz, Yee To Ng, and Sae Hwang Han, *The University of Texas at Austin, Austin, United States*

Neighborhood environments may serve as protective factors against cognitive impairment and decline. Recent evidence shows that neighborhood cohesion is associated with better cognitive health in adulthood. We extend the current literature by evaluating how neighborhood cohesion across the lifespan may influence cognitive function in adulthood. We used data from the Health and Retirement Study (HRS) and the HRS Life History Survey. Participants who were 50-89 years old at baseline, completed up to 10 longitudinal waves, and were not cognitively impaired in the last wave were included in the analyses (N=2,057). Early-life neighborhood cohesion was assessed with participants' retrospective ratings of sense of belonging in their local areas at age 10, when they first had a full-time job, and at age 40. Participants' assessment of neighborhood cohesion assessed at the final wave was treated as the current indicator of cohesion. Cognitive function was assessed with the full HRS cognitive battery. Preliminary findings from hierarchical mixed models showed an overall decline in cognitive function across time. Current and age 10 neighborhood cohesion were independently associated with better baseline cognitive function, but did not predict cognitive change. Interaction terms involving cohesion at age 10 and current neighborhood cohesion showed that current cohesion buffered the negative effect of low cohesion at age 10. No significant associations were observed for cohesion during the full-time job and age 40 periods. Although effect sizes were small, these results indicate that neighborhood cohesion in older adulthood may offset the detrimental effects of negative childhood environments on cognitive health.

### THE EFFECTS OF SEVERE AND FREQUENT BACK PAIN ON MENTAL HEALTH: DOES PERCEIVED SOCIOECONOMIC STATUS MATTER?

Christina Mu,[1] Dylan Jester,[1] Peggy Cawthon,[2] Katie Stone,[2] and Soomi Lee,[1] *1. University of South Florida, Tampa, Florida, United States, 2. California Pacific Medical Center, San Francisco, California, United States*

Back pain and mental health are related. The relationship may differ by socioeconomic status (SES); yet, research has not examined the moderating role of perceived SES. We examined if the association between back pain and poor mental health is more pronounced for older men with lower perceived SES. We used a sample of community-dwelling older men (>65yrs) with back pain from the Osteoporotic Fractures in Men Study (n=4,035). Participants reported their perceived SES in comparison to others in the community and in the nation (1=lowest—10=highest), back pain severity (mild—severe), and frequency (rarely—all of the time). Mental health was assessed with the 12-item Short Form Health Survey. Analyses were adjusted for sociodemographic and health covariates. Greater pain severity and higher pain frequency were associated with poorer mental health ($p<.001$). Only severe pain was associated with poorer mental health ($p<.001$). Pain 'some of the time' ($p=.02$), 'most of the time' ($p=.02$), and 'all of the time' ($p=.001$) were associated with poorer mental health. Adverse effects of pain were reduced with greater community SES ($p<.001$ for severe pain; $p=.02$ for 'all of the time' pain frequency) and greater national SES ($p=.01$ for severe pain; frequency n.s.). Reports of pain were worse for individuals with lower SES. Adverse associations of severe and high frequency back pain with poor mental health are more apparent in older men with lower perceived SES. Where one ranks oneself within their community or nation can influence the pain and mental health link.

### THE IMPACT OF CONSCIENTIOUSNESS ON PARTICIPANT DROP-OUT: A NOVEL METHOD FOR ESTIMATING MISSINGNESS

Tomiko Yoneda,[1] Nathan Lewis,[1] Jonathan Rush,[1] Andrea Piccinin,[2] Bryan James,[3] Scott Hofer,[1] and Graciela Muniz Terrera,[4] *1. University of Victoria, Victoria, British Columbia, Canada, 2. Psychology, Victoria, British Columbia, Canada, 3. Rush Alzheimer's Disease Center,*

*Chicago, Illinois, United States, 4. University of Edinburgh, University of Edinburgh, Scotland, United Kingdom*

Individuals low in conscientiousness are typically characterized by higher rates of dropout in longitudinal studies compared to individuals high in conscientiousness. Given that low conscientiousness is associated with increased risk of mortality and several adverse health behaviours and outcomes, attrition of individuals low in conscientiousness may result in systematic bias particularly relevant to developmental research focused on morbidity and mortality in older adulthood. Further, methods commonly used to estimate missing data require monotone coding patterns and untestable assumptions (e.g., MAR), and do not typically account for death as a competing risk factor. This project analyzed data drawn from the Memory and Aging Project (N=1156; Mage=79.2 years; 76.1% female) using multistate survival models to estimate the impact of conscientiousness on transitions between study wave participation over time (i.e., response, non-response), and death. With conscientiousness measured at baseline and death status determined by death records, complete state data are available for each study wave, unlike methods commonly used to model and estimate missingness. Adjusting for age, sex, and education, analyses revealed that higher levels of conscientiousness are associated with decreased likelihood of transitioning to non-response (HR= 0.97, CI's 0.95, 0.99) and death (HR=0.96, CI's 0.93, 0.99). These results suggest that over-sampling individuals low in conscientiousness during study recruitment may be important to better represent the general population, particularly when data are collected over several years or decades. Discussion will focus on how systematic bias introduced by higher response rates of individuals high in conscientiousness may impact health-related research based on longitudinal data.

### SESSION 2954 (POSTER)

### HEALTH BEHAVIORS

### A TIME-, GENDER-, AND DISEASE-STATE INVARIANT MODEL OF FITNESS ACROSS THE ADULT LIFESPAN

Scott Maitland,[1] Paula Brauer,[1] David Mutch,[1] Dawna Royall,[1] Doug Klein,[2] Angelo Tremblay,[3] Caroline Rhéaume,[3] and Khursheed Jeejeebhoy,[4] *1. University of Guelph, Guelph, Ontario, Canada, 2. University of Alberta, Edmonton, Alberta, Canada, 3. Laval University, Québec, Quebec, Canada, 4. University of Toronto, Toronto, Ontario, Canada*

In studies of community-based health behavior interventions (diet and physical activity) one goal in analysis is to show expected relationships between measures of intervention and clinically relevant outcomes. Many programs fail to show such clear links for many reasons beyond lack of intervention effectiveness. These secondary analyses were undertaken to assess if the measurement properties (stability and responsiveness) of intervention measures could have contributed to study findings. A feasibility study of lifestyle treatment of metabolic syndrome (n=293; mean age = 59yrs) had achieved 19% reversal over one year, yet neither diet quality nor fitness were associated with cardiovascular disease risk. Confirmatory factor analysis was used to examine fit of measurement models and factorial invariance was tested across three time points (baseline, 3-month, 12-month), gender (male/female), and disease status (diabetes) for the Healthy Eating Index (HEI) (Canada 2005) and several fitness measures (VO2max, flexibility, curl-ups, push-ups). The model fit for HEI was poor and could account for the lack of association seen in the original study. More development of diet quality measures is needed. The model for fitness, however, demonstrated excellent fit and displayed measurement equivalence across time, gender, and disease state. A higher degree of confidence exists when measurement equivalence/invariance is demonstrated, allowing for reliable tests of differences in comparison groups. The use of a multiple measure of fitness, including cardiorespiratory fitness, flexibility, and strength, helps eliminate limitations of using measures from a single domain or self-reported data is promising and should be considered in future work.

### AGING WITH AUTISM SPECTRUM DISORDER: AN EXPLORATION OF PHYSICAL ACTIVITY ENGAGEMENT

Danielle Waldron,[1] Beth Dugan,[1] Jeffrey Stokes,[1] Caitlin Coyle,[1] and John Kramer,[2] *1. University of Massachusetts Boston, Boston, Massachusetts, United States, 2. University of Florida, Gainesville, Florida, United States*

Adults with Autism Spectrum Disorder (ASD) participate in physical activity (PA) infrequently compared to adults in the general population. This is problematic as individuals with ASD suffer from disproportionate physical and mental health co-morbidities as well as diminished life expectancy, but do not reap the physical and mental health benefits of PA. This study used data from the National Core Indicators-In Person Consumer Survey (n=4,370; age: 18-78) to analyze factors associated with both aerobic PA and muscle strengthening (MS) activity in adults with ASD receiving state Developmental Disability Services. This research used multilevel logistic regression modeling, with mediation and moderation analyses to explore personal and environmental factors associated with PA/MS in this population. Findings indicated the following significant associations between community engagement and PA and MS: community contact (OR=1.17; $p<0.001$; OR=1.07; $p<0.001$), community group participation (OR=1.83; $p<0.001$; OR=1.91; $p<0.001$), and employment/day program participation (OR=1.32; $p<0.05$; OR=1.32; $p<0.001$). Additionally, at older ages, participants were less likely to engage in PA and MS three or more times a week (OR=0.99; $p<0.05$; OR=0.99; $p<0.05$). These findings indicate that increasing age is associated with decreased PA and MS activity in this group, while community engagement may facilitate their PA and MS activity. While much remains unknown about the population aging with ASD, it is evident that they suffer from poorer health than the general population and have experienced lifelong difficulties with socialization and communication. Greater access to community engagement opportunities may promote this population's healthy aging, as well as support their unique social needs.

### ASSESSING THE LONGITUDINAL RELATIONSHIP BETWEEN ALCOHOL USE AND DEPRESSION AMONG OLDER ADULT RETIREES

Antonia Diaz-Valdes Iriarte,[1] and Christina Sellers,[2]
*1. Universidad Mayor, La Reina, Santiago, Region Metropolitana, Chile, 2. Simmons University, Boston, Massachusetts, United States*

Alcohol use and depression are underrecognized problems affecting older adults, and its prevalence has increased for baby boomers compared to prior generations. Approximately 40% of people aged 65+, drink alcohol and approximately 19% experience. Additionally, the loss of important roles in life – such as being an employee, could negatively influence mental health at retirement. Although there is mixed evidence about the direction of the association between alcohol and depression in this population, and little is known about how retirement influence it. Generalized mixed models were performed to test the effect of retirement, alcohol use – drinks per day and binge drinking, and depressive symptoms among older adults. We draw data from the Health and Retirement study (n=11,164). Results suggest that being retired was associated with decreased depressive symptoms (b=-0.04, $p<0.05$) and increased drinks per day (b=0.01, $p<0.05$) and binge drinking (b=0.11, $p<0.05$). Each additional drink per day (b=-0.11, $p<0.05$) and binge drinking (b=-0.07, $p<0.05$) lead to decreased depressive symptoms. Similarly, increased drinking or binge drinking were associated to decreased depressive symptoms. Regular drinkers might increase their drinking to occupy the additional free time that comes with retirement, to alleviate anxiety and feelings of loneliness as they lost their working roles and they might have lost relatives, which might increase the craving for alcohol. Given the prevalence of depression and alcohol use, and its detrimental effects among older adults. Further research needs to be done to understands the particularities of this group and to generate interventions focused on prevention.

### DO CONTROL BELIEFS MODERATE THE RELATIONSHIP BETWEEN FUNCTION AND PHYSICAL ACTIVITY?

Elizabeth Teas, and Elliot Friedman, *Purdue University, West Lafayette, Indiana, United States*

Most older adults get far less than recommended levels of physical activity (PA), and interventions to improve PA have limited effectiveness. Barriers to PA include reduced physical function (PF) and diminished feelings of control, but their interactive influences on PA in older adults are unclear. Using two methodologies, the current study determined whether control beliefs modify the relationship between PF and PA. Data were from the second wave of the Midlife in the United States (MIDUS) study; the sample was constrained to participants with PA greater than 0 (n= 955, mean age= 54.27). PF (grip strength, gait speed, and chair stands) was measured during a clinic visit. Participants were asked about routine PA, from which a Metabolic Equivalent of Task score was calculated, and the extent to which they believed they have control over their life (0-7 scale). In linear regression models, including interactions between control beliefs and PF variables, gait speed and control were associated with PA; none of the interaction terms were significant. The second model used a person-centered approach to explore the potential of non-linear relationships and differences in groups of people by creating typologies. The group with low control and slow gait speed had significantly lower PA than the other three groups. Results suggest nuanced associations among PA, PF, and control beliefs where feelings of control may compensate for slow gait speed in particular. They also support the use of person-centered approaches to identify non-linear associations between modifiable protective factors and key outcomes in aging research.

### EFFECTIVENESS OF NUTRITIONAL EDUCATION FOR OBESE OLDER ADULTS WITH FRAILTY: A SYSTEMATIC REVIEW AND META-ANALYSIS

Yue-Heng Yin,[1] and Liu Yat Justina,[2] *1. The Hong Kong Polytechnic University, Hong Kong, China, 2. School of Nursing , The Hong Kong Polytechnic University, Hong Kong, China*

Obesity has been shown to intensify the decline of physical function and lead to frailty. Nutrition is an important method in managing obesity and frailty, while seldom reviews have ever explored the effects of nutritional education interventions. We conducted a systematic review (PROSPERO: CRD42019142403) to explore the effectiveness of nutritional education interventions in managing body composition and physio-psychosocial parameters related to frailty. Randomized controlled trials and quasi-experimental studies were searched in CINAHL, Cochrane Library, EMBASE, MEDLINE, PsycINFO, PubMed and Scopus from 2001 to 2019. Hand search for the reference lists of included papers was conducted as well. We assessed the quality of included studies by Cochrane risk of bias tool. Meta-analyses and narrative synthesis were used to analyse the data. Two studies with low risk of bias were screened from 180 articles, which involved 177 older people with an average age of 69.69±4.08 years old. The results showed that nutritional education was significantly effective in reducing body weight and fat mass than exercises, and it was beneficial to enhancing physical function and psychosocial well-being. But the effects of nutritional education in increasing muscle strength were not better than exercises. The combined effects of nutritional education and exercises were superior than either exercises or nutritional education interventions solely in preventing the loss of lean mass and bone marrow density, and in improving physical function. Due to limited numbers of relevant studies, the strong evidence of effectiveness of nutritional education interventions on reversing frailty is still lacking.

### EVALUATING A MOBILE-HEALTH-ENHANCED BEHAVIORAL LIFESTYLE INTERVENTION FOR FRAILTY IN DIABETES: A FEASIBILITY STUDY

Rozmin Jiwani, Jing Wang, Chengdong Li, and Sara Espinoza, *University of Texas Health Science Center at San Antonio, San Antonio, Texas, United States*

Type 2 diabetes (T2D) and overweight are significant predictors of frailty in older adults. There are no widely accepted intervention strategies for frailty. The Look AHEAD (Action for Health in Diabetes) trial demonstrated the efficacy of behavioral lifestyle intervention on weight loss and glycemic control in overweight older adults with T2D. It is unknown whether this intervention can prevent/delay frailty in older adults. We designed a feasibility study examining the effect of a behavioral lifestyle intervention enhanced with mobile technology (Fitbit) for self-monitoring of diet and physical activity on frailty and T2D outcomes over 6 months in overweight older adults diagnosed with T2D. Forty older adults were randomized to receive either 10 group sessions vs. one condensed session plus monthly phone calls for 6 months. In this analysis, we are reporting on Fitbit wear adherence and weight changes on the 20 participants in the group session for the first 6 group sessions. The study sample was aged 72.3±6.4 years; 62% female; 52% Hispanic; BMI 33.7±5.9 kg/m2; hemoglobin A1c 7.2%; frailty score 1.1±1.0 kg/m2. Thirteen (65%) are pre-frail, 6 (30%) are non-frail, and 1 (5%) is frail (using Fried criteria). Their weight (lbs.) changed from session 1 (210.2±42.5) to session 6 (196.8±44.2). Ten participants wore Fitbit every day between sessions, averaged at 92±12%. The preliminary evaluation showed the feasibility of using Fitbit to promote self-monitoring adherence in a behavioral lifestyle intervention and a positive trend for weight loss. Evaluating intervention effect on frailty at 6 months will provide us further insights.

### EXPLORING THE MOTIVATORS AND BARRIERS OF OLDER ADULTS PARTICIPATING IN AN INTERACTIVE EXERGAME INTERVENTION

Breanna Crane,[1] Brittany Drazich,[2] Kyle Moored,[1] and Michelle Carlson,[1] *1. Johns Hopkins Bloomberg School of Public Health, Baltimore, Maryland, United States, 2. Johns Hopkins School of Nursing, Baltimore, Maryland, United States*

Cognitive and physical activity are important to maintaining daily functioning in older adults. While bidirectional associations between cognitive and physical activity magnify with increasing age, elucidating shared benefits has been difficult as few interventions explicitly train on cognition and mobility simultaneously. We conducted focus groups among 14 older adults residing in an independent-living center who participated in an interactive video game study called Bandit the Dolphin, where participants simultaneously incorporated cognitive exercise and physical activity while navigating within a complex spatial environment to help Bandit jump, eat fish, and stun sharks. Using ‘sneaky exercise’ tactics, participants utilized upper extremities in conjunction with slight lower extremity movement to move Bandit within a 3-D oceanic environment. We conducted 3 semi-structured focus groups and analyzed the data using the “Sort and Sift, Think and Shift” method to assess general likes and dislikes as well as the primary motivators, barriers, and reasons for remaining in the study. Participants enjoyed the immersive nature, challenge, and “fun factor” of the game. Primary motivators for joining were generativity/helping others, self-improvement, from peer referrals, and because the study looked interesting. Key barriers reported in the study were exhaustion from standing, learning how to play in 3-D space, and frustration from lack of level advancement. Reasons for retention were due to the game being fun, a sense of duty, and fulfilling commitments. This information will guide ongoing research efforts to design interactive video game interventions that are enjoyable for older adults and maintain high retention rates.

### GENDER AND AGE MODERATE THE EFFECTS OF PARTNER SUBSTANCE USE ON PROBLEM DRINKING IN ADULTHOOD

Sara Miller, David Almeida, and Jennifer Maggs, *Pennsylvania State University, University Park, Pennsylvania, United States*

The current study examined whether partner substance use problems predict problem drinking and how gender and age moderate this relationship. Problem drinking refers to alcohol use resulting in alcohol dependence or health and social consequences. Participants were adults (n=2142, 53% female, mean age=54, range= 33-83) from Wave 2 of the Midlife Development in the United States (MIDUS) Study. Participants reported on both past 12-month problem drinking (e.g., emotional problems from drinking, urges to drink, month or “much time” drinking, drinking more to get effects, drinking more than intended, and alcohol-related role interference) and partner substance use problems. Results indicated that 22.2% of the sample reported at least one problem drinking behavior in the past year. Multiple linear regression analysis revealed a significant interaction between gender and partner substance use problems (b=0.05, p=0.01) such that for males having a partner with substance use problems was a risk factor for their own problem drinking. However, a three-way interaction with gender, age, and partner substance use problems (b=-0.41, p<0.01) indicated that partner substance use problems might have both gender and age-specific effects on problem drinking. Exploratory analyses of this interaction indicated that with age partner substance use problems might no longer promote risk for male problem drinking. Older adults are especially sensitive to the effects of alcohol for reasons such as lower tolerance, medication interaction, and health conditions. There is thus a need for identifying age-relevant factors associated with these drinking behaviors for intervention and prevention efforts.

### GROWTH CURVE OF DISABILITY OF OLDER ADULTS OVER A 12-YEAR PERIOD: CAN IT BE MODIFIED BY AGE OR ENGAGING IN ACTIVITIES?

Rongjun Sun, *Cleveland State University, Cleveland, Ohio, United States*

Adopting a growth curve model, this study aims to fit a growth trajectory of disability of older adults over a 12-year period, and to investigate whether such a trajectory is modified by initial age and level of engagement in activities. The data are from the Chinese Longitudinal Healthy Longevity Survey, which includes 16,064 individuals aged 60 or above in the first wave in 2002 who were followed-up in four more waves until 2014. Disability in this study is measured by having any difficulty in performing six activities of daily living. Activities include physical exercise and eight leisure activities. To rigorously test the causal effect of engaging in activities on disability, we adopted a time-lagged growth curve model. In addition, disability status in the initial wave was controlled at baseline and an array of health status measures, such as physical functioning and cognitive performance, were included as time-varying covariates. We introduced a random effect to control for unobserved heterogeneity between individuals. The results show a quadratic curve of disability over time with an accelerating pace in later waves. While initial age shows a moderate modifying effect, engaging in leisure activities substantially modified the trajectory: The probability of being disabled increased from 6.7% to 45.8% between the first and fourth follow-up for those inactive individuals. For those active older adults, it only increased from 3.1% to 18.0%. This study demonstrates that engaging in leisure activities can significantly reshape the trajectory of developing disability among older adults.

### HOME-BASED EXERCISE PROGRAM WITH TELEPHONE COACHING: A FEASIBILITY STUDY

Emma Gulley,[1] Joe Verghese,[1] Emmeline Ayers,[1] Tanya Verghese,[1] and Anne Felicia Ambrose[2]
*1. Albert Einstein College of Medicine, Bronx, New York, United States, 2. Montefiore, Bronx, New York, United States*

Exercise is crucial to maintain mobility, reduce falls and delay functional decline in older adults, but effective implementation strategies are lacking. Self-directed home-based exercise therapy is recommended by clinicians to overcome barriers such as cost, travel and availability. However, non-adherence is a major challenge due to lack of motivation, real time feedback or social support. To overcome these barriers, we conducted a feasibility study to evaluate a home-based exercise program with telephone coaching to improve mobility in frail older adults. Four non-demented, frail community-dwelling older adults were taught one of two exercise routines at our research center. The first involved complex exercises with internal and external cueing techniques that have been associated with neuroplasticity in previous studies (N=3). The second was a lesser cognitively demanding control program that included aerobic, balance and strengthening exercises (N=1). One week later, the participants were asked to repeat the exercises in their own home. The research assistant coached the patient over the telephone. A board-certified physiatrist was present during the home session to monitor adherence and fidelity to the protocol as well as address safety. The study produced qualitative findings regarding recruitment strategies, exercise feasibility, and other logistical issues relating to participant understanding, safety, and monitoring. Based on direct observation of participants at home, safety assessment protocols, instructions, and exercises were all refined. Building on this data, we plan to design a clinical trial to evaluate the impact of complex exercises designed to promote neuroplasticity and reduce cognitive and motoric decline in older adults.

### IMPROVING HEALTHY BEHAVIOR AND ADOPTING HEALTHY HABITS BY BODYWISE PROGRAM FOR OLDER ADULT DC RESIDENTS

Lillie Monroe-Lord,[1] Azam Ardekani,[2] James Lee,[1] and Elgloria Harrison,[1] *1. University of the District of Columbia, Washington, District of Columbia, United States, 2. Howard University, Beltsville, Maryland, United States*

An increase in physical activity (PA) level is associated with reduction in chronic diseases. However, the number of older adults who meet the PA guideline is low and decreases with age. Bodywise is a free program that aims to promote healthy behavior and to encourage older adults who live in Washington, DC to adopt a healthy habit by increasing their PA. The Bodywise program offers water aerobics, yoga, low-impact aerobics, and chair exercise classes, conducted by trained instructors at the University of the District of Columbia. This study reports an evaluation of the implementation of this program based on the surveys completed by participants after finishing the classes. Participants were DC residents aged 60 or older. According to the surveys, 68% of participants have adopted one or more healthy habits since joining the Bodywise program. In addition, more than 62% of participants have an increase in awareness of healthy behavior through this program. Overall satisfaction with the Bodywise program is 93%, satisfaction with the variety of classes offered is 72% and satisfaction with offered skill levels is 75%. Furthermore, 81% of participants reported overall satisfaction with their instructor, specifically 100% satisfaction related to their knowledge and skill level, and appropriateness of selected music, moves, and exercises. In conclusion, the Bodywise program leads to an overall change in both awareness of healthy behavior and development of healthy habits in participants. Further research can investigate the correlation of health awareness and healthy habits with overall satisfaction with the program and the instructor.

### PHYSICAL ACTIVITY, DIABETES, AND HEART CONDITIONS AMONG NATIVE HAWAIIAN AND OTHER PACIFIC ISLANDER OLDER ADULTS

Yeonjung (Jane) Lee and Tyran Terada *1. University of Hawaiʻi at Mānoa, Honolulu, Hawaii, United States*

The Native Hawaiian and Other Pacific Islander (NHOPI) older adult population remains understudied and are disproportionately affected by diabetes and heart conditions. Research has shown that participating in physical activity is a protective factor for many of the health conditions experienced by older adults. Yet, the link between physical activity, diabetes, and heart conditions among the NHOPI older adult population is limited. The purpose of this study is to identify the characteristics of NHOPI older adults and to explore the association between physical activity levels and diabetes/heart conditions. Methods and findings Using data from the 2014 Native Hawaiian and Pacific Islander National Health Interview Survey (NHPI NHIS), which is considered to have a representative sample of NHOPI, the study explores the associations between physical activity and diabetes/heart conditions. A total of 1,045 older adults ages 50 years and older were included for analyses. Weighted multivariate analyses with multiple imputation techniques were used. The NHPI NHIS is the first federal survey focusing on the NHOPI population of the United States with rich information on health. Results and Implications Those with who were engaged in a sufficient physical activity had lower odds of having diabetes or heart conditions than their counterparts without physical activity while controlling for other sociodemographic characteristics. Findings highlight the importance of physical activity promotion intervention in preventing cardiovascular disease. Research and practice addressing health disparities and cardiovascular conditions should leverage efforts to provide culturally relevant physical activity types and resources to NHOPI older adults.

### PHYSICAL ACTIVITY, OUTCOME EXPECTATIONS, AND SOCIAL SUPPORT IN DYADS OF FRAIL OLDER ADULTS AND THEIR HOME CARE AIDES

Lijuan Yin, and Naoko Muramatsu, *University of Illinois at Chicago, Chicago, Illinois, United States*

Frail community-dwelling older adults increasingly receive home care and continue to face barriers to participating in physical activity (PA) that could help maintain their function. Home care aides (HCAs) are well-positioned to promoting PA among older home care recipients because of their established relationship and regular interpersonal exchanges; yet, the role of HCAs in promoting and supporting PA in home care settings is seldomly studied. Using the quantitative and qualitative data from a 4-month home-based gentle PA intervention delivered by HCAs to their clients in a Medicaid-funded home care setting, the current study examined whether outcome expectations for exercise (OEE) held by HCAs led to client PA outcomes (i.e. functional limitations and physical performance) through social support for exercise (SSE) provided by HCAs. Longitudinal mediation analysis of 46 HCA-client dyads showed that higher baseline OEE held by HCAs were related to greater SSE reported by clients after the intervention ($p<.05$; bootstrapped standard errors), controlling for client-level covariates, including baseline OEE, age, gender, comorbidity, and whether HCA was client's family member. Unexpectedly, SSE did not have significant association with client PA outcomes nor mediated the relationship between OEE held by HCAs and client PA outcomes. Qualitative data suggested alternative factors may explain the results, such as clients' family beliefs in the intervention and clients' participation experiences (such as expectation fulfillment). Future research should consider older home care clients' family contexts to enhance our understanding of HCAs' roles in preserving the function of growing numbers of older home care recipients.

### PROBLEM DRINKING PREDICTS FUNCTIONAL HEALTH AND MORTALITY RISK 10 YEARS LATER IN THE MIDUS STUDY

Sara Miller, David Almeida, and Jennifer Maggs, *Pennsylvania State University, University Park, Pennsylvania, United States*

The current study examined whether problem drinking in older adulthood is associated with greater longitudinal risk of functional impairment and mortality through 2016. Problem drinking consists of patterns of alcohol use resulting in symptoms of alcohol dependence or health and social consequences. Participants were adults ($n=2654$, 56.1% female) from Wave 2 (mean age=55, range=30-84) and Wave 3 (mean age=64, range=39-93) of the Survey of Midlife Development in the United States (MIDUS) Study. Participants reported problem drinking behaviors (e.g., alcohol related role interference) and any disability in basic and instrumental activities of daily living (ADL, iADL). Mortality data was acquired from the 2016 MIDUS Mortality dataset. Results indicated that 20.7% of the sample reported at least one problem drinking behavior in the past year. Multiple linear regression analyses revealed that the sum of problematic drinking behaviors at Wave 2 predicted 10-year longitudinal change in impairments in ADL's ($b=0.05$, $p<0.01$) and iADL's ($b=0.05$, $p<0.01$) after controlling for age, education, gender, and previous ADL/iADL. Logistic regression results revealed that for every additional alcohol use problem reported at MIDUS 2, the odds of mortality increased by 1.74 ($b=0.55$, $p<0.01$), beyond controls for age and number of chronic conditions. The findings that problem drinking has a unique positive association with impaired functioning and mortality risk during older adulthood supports public health efforts to encourage reduced consumption, increased medical screening, and expanded treatment options.

### PROFILE GROUPS BASED ON LIFESTYLE AND DIFFERENCES IN MENTAL HEALTH AND COGNITION

Joseph Kim, and Kyuree Kim, *Iowa State University, Ames, Iowa, United States*

The purpose of this study was to identify the profiles of older adults according to lifestyle. Data for the study were from the 2017 Consumption and Activities Mail Survey (CAMS). CAMS 2017 is a questionnaire mailed to a subsample of respondents from the Health and Retirement Study. Participants were limited to older adults 65 and older, and the final sample consisted of 1136 older adults. The sample included 443 men and 693 women. Caucasians comprised 82.0% of the participants. Lifestyle was measured through items assessing the amount of time spent on activities. Due to high skewness, the items were dichotomized, 0=no time spent on activity and 1=time spent on the activity. Latent class analysis (LCA) was performed to identify groups based on lifestyle. LCA is a person-centered approach for identifying unobserved subgroups based on similarity in responses to items. Three lifestyle groups were identified. Group 1 was "Outgoing" with 471 individuals. Group 2 was "Adequate" with 229 individuals. Group 3 was "Inactive" with 436 individuals. An ANOVA was then conducted to assess mean differences in self-rated health, cognition, depressive symptoms, and loneliness for the three lifestyle groups. The "Outgoing" and "Adequate" groups had significantly higher scores on self-rated health and cognition, and in addition, significantly lower scores on depressive symptoms and loneliness compared to the "Inactive" group. No significant differences were observed between the "Outgoing" and "Adequate" groups. An implication from this study is the importance of maintaining an active lifestyle in later life for better mental health and cognition.

### SOCIAL RELATIONSHIPS AND ALCOHOL ABUSE IN OLDER AGES

Eric Vogelsang, *Cal State University-San Bernardino, San Bernardino, California, United States*

In an aging world, researchers and practitioners often extol the health benefits of social relationships (e.g., family ties, social participation) for older adults. Yet, they generally ignore how these same bonds and activities may contribute to negative health behaviors, such as alcohol use and abuse. Using data from the Wisconsin Longitudinal Study (16,065 observations from a cohort of 7,007 respondents), I examine how family structure, family history, and participating in certain social activities predict alcohol consumption and symptoms of alcoholism between ages 53 and 71. I find that having children and taking part in two particular social activities (meeting friends and group exercise) are associated with problematic drinking behavior. Moreover, religious participation and ever living with alcoholics were associated with reporting negative alcohol consequences, but not with alcohol consumption itself. These findings contextualize the increasing rates of alcohol abuse among older adults.

### SPORT INVOLVEMENT AMONG MIDDLE-AGED AND OLDER ADULTS WITH ARTHRITIS: MOTIVATIONS AND CONSTRAINTS TO PARTICIPATION

Megan Janke,[1] Julie Son,[2] Jill Naar,[3] Stephanie West,[3] Toni Liechty,[4] and Jen Wong,[5] *1. East Carolina University, Mount Berry, Georgia, United States, 2. University of Idaho, Moscow, Idaho, United States, 3. Appalachian State University, Boone, North Carolina, United States, 4. University of Illinois, Champaign, Illinois, United States, 5. The Ohio State University, Columbus, Ohio, United States*

Although participation in physical activity is recommended for adults with arthritis, research indicates individuals often stop participating in sports and physically active leisure due to the pain and symptoms associated with arthritis (Wilcox et al., 2006). Examining a group of older adults with arthritis, the present study examines motivations and constraints related to participating in sport and physically active leisure as well as how they negotiate constraints. Data (N=1203) were collected through an online questionnaire of adults aged 50 and older in the United States. This study includes individuals reporting a diagnosis of some form of arthritis (n=288; M age = 64.8, SD = 8.08). Approximately 32% self-reported participation in sport in the past 12 months. Descriptive statistics were conducted to explore motivations and constraints to sport involvement. Regressions were run to determine whether constraints and motivations explained adults' functional mobility and social wellbeing. The most commonly identified motivation for participation was for health purposes (80.2%). Constraints to participation included not being in good enough shape (51.9%) and not having others their age with whom to participate (47.4%). The most commonly identified constraint negotiation was to budget money (51.4%); this is not surprising since sport participation was perceived as expensive (41.3%). Motivations ($p<.01$) and constraints ($p<.001$) significantly predicted functional mobility; constraints significantly predicted some aspects of social wellbeing (i.e., coherence, contribution, actualization; $p<.05$) while constraint negotiation predicted social acceptance ($p<.05$) and integration ($p<.001$). Discussion will include implications and strategies for agencies and professionals who work with adults who have arthritis.

### THE STRESS-BUFFERING EFFECT OF DAILY PHYSICAL ACTIVITY ON NEGATIVE AND POSITIVE AFFECT

Sun Ah Lee,[1] Susanna Joo,[1] Kayeon Lee,[1] Hye Won Chai,[2] Hey Jung Jun,[1] and David Almeida,[2] *1. Yonsei University, Seoul, Republic of Korea, 2. The Pennsylvania State University, State College, Pennsylvania, United States*

Previous studies show that physical activity is beneficial for emotional well-being. This study extends prior research by examining whether engagement in physical activity moderates the association between daily stressor severity and daily emotional well-being. We used data from the second wave of the National Study of Daily Experiences, a subproject of the Midlife in the United States (MIDUS) study. Respondents (N = 1,851; ages 33 to84) reported their daily experiences across eight consecutive days. Multilevel models explored concurrent and lagged interaction effects between daily stressor severity and physical activity on negative and positive affect and whether these associations differed by age. Physical activity was measured by engagement in vigorous physical activity for at least 30 minutes. Results showed significant interactions between stressor severity and physical activity on same-day negative and positive affect. Specifically, stressor severity was associated with smaller elevation in daily negative affect on physically active days ($b = 0.08$, $p < 0.001$) compared to non-active days ($b = 0.11$, $p < 0.001$). Reductions in daily positive affect were greater on physically inactive days ($b = -0.11$, $p < 0.001$) compared to active days ($b = -0.08$, $p < 0.001$). These associations did not differ by age, but additional findings revealed that stressor severity was associated with greater elevation in negative affect among younger respondents ($b = 0.12$, $p < 0.001$) than older adults ($b = 0.10$, $p < 0.001$). These results highlight the importance of engagement in physical activity for emotional well-being under stressful situations in daily context.

### SESSION 2955 (POSTER)

### SOCIAL NETWORKS AND SUPPORT I

### BENEFITS OF VOLUNTEERING FOR MIDDLE-AGED AND OLDER ADULTS: COMPARISON BETWEEN TYPES OF VOLUNTEERING ACTIVITIES

Edwin K. H. Chung, Alfred H. K. Lam, Dannii Yeung, and Ka Hung Edwin CHUNG, *City University of Hong Kong, Hong Kong, China*

More middle-aged and older adults are often encouraged to volunteer (Gray et al., 2012). However, the effects of various volunteering activities on physical, psychological and cognitive health remain unknown. This study thereby aims to investigate such effects and the moderating effect of age in such associations. Data of 501 middle-aged and older Hong Kong Chinese adults (Mage = 53.06, SD = 4.55; and Mage = 70.46, SD = 7.34, respectively; range = 45 – 96) from a survey on Adult Development and Aging were analysed. Engagements in instrumental (e.g., food preparation, fundraising) and cognitively demanding volunteering (e.g., counselling, mentoring), hand-grip strength, life satisfaction, depressive symptoms, and cognitive functioning were measured. Factorial ANOVA revealed significant main effects of age group and volunteering type ($F = 29.71$, and $F = 3.96$, respectively, $ps < .001$), and an interaction effect of age and volunteering type ($F =1.80$, $p = .03$) on health outcomes. Comparisons among the four volunteering types (no volunteering, instrumental volunteering, cognitively demanding volunteering, and both types) revealed that individuals engaging in cognitively demanding volunteering had better hand-grip strength, life satisfaction, and cognitive functioning, and lower depressive symptoms than those who engaged in instrumental volunteering (all ps < .05). The health outcomes of instrumental volunteering were even worse than those who did not volunteer at all. These patterns were more prevalent in the middle-aged adults than in the older adults. Findings of this study indicated the beneficial effects of cognitively demanding volunteering, providing valuable directions for future programs on volunteering.

### COMPANIONSHIP WITH FAMILY, FRIENDS, AND NEIGHBORS IN LATER LIFE

Ikuko Sugawara,[1] Midori Takayama,[2] and Yoshiko Ishioka[2]
*1. University of Tokyo, Tokyo, Japan,*
*2. Keio University, Yokohama, Japan*

Companionship with close others are known to have a significant positive effect on our well-being in later years of life. At the same time, it is known that the frequency of meeting and chatting with others, an indicator of companionship, declines as we age. In this study we explore the situation of companionship among older-old and oldest-old people. The focus of this study on understanding how the aging process affects the experience of companionship and how people adapt to the loss of companionship. Semi-structured, in-depth interviews with 43 people aged 75 and older living in urban communities in Kawasaki, Japan. Participants were asked about everyday interactions with close others and feelings they experienced at the time. Interviews were transcribed and analyzed qualitatively. Chatting, going out for lunch or dinner, and going shopping were examples of activities older Japanese enjoyed with close others. Almost all respondents mentioned the loss of their old friends and siblings. They also mentioned that the decline in their physical and cognitive health, as well as that of their companions hindered shared activities they used to enjoy. They cherished positive interactions with others, although the frequency declined. Some respondents intentionally made new companions in the physical proximity, but it was hard to compensate for the loss of old companions with new one. These findings suggest that the value of companionship remains or even increase as we age. It would be important to identify environmental or social factors that may prevent the loss of companionship among older adults.

### INNOVATIVE EXPERIENCES AT WORK SUPPORT HIPPOCAMPAL MAINTENANCE IN LATE LIFE

Kaleena Odd,[1] Julie Blaskewicz Boron,[1] Aga Burzynska,[2] Jonathan Santo,[1] Paul Robinson,[2] Sherry Willis,[2] and K. Warner Schaie,[2] *1. University of Nebraska Omaha, Omaha, Nebraska, United States, 2. Omaha, Nebraska, United States*

Prior research has demonstrated the positive impact of occupational complexity on cognitive aging, however, neural underpinnings remain unclear. There is emerging evidence linking midlife managerial experience to slower hippocampal atrophy (Suo et al., 2012, 2017), supporting the brain maintenance model (i.e. preservation of young-like brain structure). However, occupational complexity, along with education, is known to be a proxy of cognitive reserve (i.e. mind's resistance to brain aging). The current study examined the influence of midlife work environment factors (i.e., autonomy, control, and innovation; Work Environment Scale, Moos, 1981) on change in hippocampal thickness, while controlling for education and age. We studied 150 participants (60-78 years, M = 66.27, SD = 5.20, 61% female) from the Seattle Longitudinal Study who had at least one MRI scan and remained cognitively normal between 2006 and 2014. Hypotheses were tested using multilevel modeling in Mplus; gender differences were examined. There was no substantial drop in model fit as a result of adding any of the significant effects. Innovation at work slowed the decrease in hippocampal thickness over time demonstrating the protective effect of more novelty, variety and change in work activities. There was a significant age by gender interaction, such that the decrease in hippocampal thickness was stronger for older women. Together, findings suggest that long-term impact of work environment on the hippocampus extends beyond the effects of education, particularly in men, supporting the brain maintenance hypothesis. Innovation at work should be considered in understanding protective/risk factors in hippocampal atrophy in older age.

### OCCUPATIONAL FUTURE TIME PERSPECTIVE MEDIATES AGE DIFFERENCES IN CONFLICT STRATEGIES

Dannii Yeung, and Alvin Ho, *City University of Hong Kong, Hong Kong, China*

Building on the theoretical framework of socioemotional selectivity theory (Carstensen, 2006), this presentation reports the findings of two studies conducted in Hong Kong Chinese workers to examine whether occupational future time perspective (OFTP) can account for the age differences in conflict strategies. Study 1 is a cross-sectional study with 416 working adults completed an online survey on conflict management (Mage=39.1 years, SD=12.1), and Study 2 is a laboratory study with 123 workers (Mage=40.1 years, SD=12.1) indicated their behavioural responses after watching hypothetical workplace conflict videos. In both studies, five conflict strategies (integrating, compromising, obliging, avoiding, and dominating) and OFTP (focus on opportunities and focus on limitations) were assessed. Parallel mediation analyses were performed. The results of Study 1 showed that both focus on opportunities and focus on limitations mediated the effects of age on obliging (b = -.006, SE=.002; and b = .006, SE=.002, respectively), avoiding (b = -.005, SE=.002; and b =.008, SE=.002, respectively), and dominating (b = -.014, SE=.003; and b = .009, SE=.002, respectively). Focus on opportunities could only account for the effects of age on integrating and compromising. The results of Study 2 showed that only focus on limitation could account for the age variations in the use of avoiding (b = .196, SE = .058) when facing intergenerational conflicts. The findings of this project reveal that the age-related focus on limitations increases older workers' likelihood to utilize maladaptive conflict strategies, such as dominating and avoiding, to deal with conflicts occurred in the workplace.

### RETHINKING SOCIAL RELATIONSHIPS: THE DIFFERENTIAL INVESTMENT OF RESOURCES MODEL OF SOCIAL DEVELOPMENT

Katherine Fiori,[1] Tim Windsor,[2] and Oliver Huxhold,[3]
*1. Adelphi University, Garden City, New York,*

*United States, 2. Flinders University, Adelaide, South Australia, Australia, 3. German Centre of Gerontology, Berlin, Berlin, Germany*

The empirical evidence concerned with the centrality of social relations to individual functioning across adulthood continues to accumulate. Theoretical development about age-related changes in social relationships, however, has lagged behind. In particular, existing theories either do not account for the influence of the developmental context, or are difficult to examine empirically because they do not posit specific testable mechanisms. We present a new conceptual model that we believe effectively incorporates much of the existing empirical work. The Differential Investment of Resources (DIRe) model has five distinct features. First, the model distinguishes between different types of 'social ties' by defining two crucial dimensions - closeness and kinship. Second, the investment of time and energy is defined as the core mechanism that explains the formation and maintenance of social ties. Third, individual characteristics, categorized as capacities, motivations, and skills, determine the amount, direction, and efficacy of the time and energy invested. Fourth, the model incorporates the developmental context: (a) in its effect on the social opportunity structure; (b) in its effect on time and energy; and (c) in its effect on the individual. Additionally, the social opportunity structure itself is determined by the individual's existing social network ties (i.e., social capital). Finally, the model describes how different types of ties, in turn, affect individual characteristics via social functions (social exchanges, social evaluations, and social influences). The proposed model will not only stimulate a healthy new debate in the field, but will also provide a theoretical basis for future research and hypothesis-testing.

### SOCIAL CONTACT AND WELLNESS OUTCOMES FOR OLDER ADULTS IN DIFFERENT RESIDENTIAL SETTINGS

Joseph Bihary, Jennifer Smith, Dugan O'Connor, Ajla Basic, Janis Sayer, and Cate O'Brien, *Mather Institute, Evanston, Illinois, United States*

More frequent social contact with others has been shown to be associated with positive well-being outcomes for older adults, who may be at increased risk of loneliness or isolation. The current study investigates whether the potential benefits of social contact might vary by social contact medium and by residence type (senior living (SL) settings vs. the broader community). 5,148 older adults residing in SL communities completed surveys on health and well-being. Data were combined with responses from 1,000 demographically similar older adults residing outside SL settings who participated in a similar study. Participants in both studies reported their frequency of social contact with friends (4 types: in-person meet-ups, speaking by phone, writing or emailing, and communicating via social media), subjective overall health, and life satisfaction. Results of multilevel regression analyses revealed that more frequent in-person social contact with friends was associated with more positive ratings of overall health only for participants in non-SL settings, but not those in SL. More frequent in-person social contact was also associated with higher life satisfaction, and this effect was stronger for non-SL participants. More frequent contact by phone and written letters/emails was associated with better ratings of overall health, regardless of residence type. In contrast, more frequent social media use was associated with lower ratings of overall health. Analyses controlled for age, gender, education, marital status, income, chronic health conditions, and depressive symptoms. The observed patterns of results speak to the possible protective benefits of social engagement for older adults in different residence settings.

### SOCIAL NETWORK TYPES AND HEALTH AMONG OLDER SINGAPOREANS

Rahul Malhotra, Pildoo Sung, and Angelique WM Chan, *Duke-NUS Medical School, Singapore, Singapore*

Little is known about the heterogeneity and dynamics in older adults' social networks and their bidirectional relationship with health in Asian societies. We investigate (1) social network types, (2) how network types predict health, and (3) whether health influences network types over time among older Singaporeans. We use data from Transitions in Health, Employment, Social engagement and Inter-Generational transfers in Singapore Study (THE SIGNS Study), a national longitudinal survey, collected in 2016-2017 (wave 1) and 2019 (wave 2). Latent class analysis is applied to identify distinct social network types and how they affect self-rated health after two years. Latent transition analysis is then employed to examine the pattern of change in network types between waves, and the relationship between baseline self-rated health and transition in network types. We identify six social network types: diverse, diverse but less socially engaged, immediate family, extended family, living alone yet diverse, and restricted (proportion at baseline: 7.2 %, 38.2 %, 14.1 %, 27.1 %, 7.0 %, and 6.4 %, respectively). Older adults in the 'living alone yet diverse' network type are less likely to report poor self-rated health after two years than those in the restricted and extended family network types. Additionally, we find that good health is related to more diversified network types—'diverse' and 'diverse but less socially engaged'—at baseline, and network types are relatively stable over two years. These findings contribute to the literature by capturing complexities in the reciprocal relationship between social network types and health in later life.

### SOCIAL NETWORKS AND DEPRESSION OVER TIME: THE MEDIATING ROLE OF SOCIAL SUPPORT AND SOCIAL ISOLATION

Abhijit Visaria, Pildoo Sung, and Angelique WM Chan, *Duke-NUS Medical School, Singapore, Singapore*

It is well established that a strong social network is an important factor for successful ageing, specifically for promoting and maintaining psychological wellbeing at older ages. Strong social networks are a source of social support especially at a time of need, and can also help older adults remain connected, active, and engaged in group activities. In this study, we seek to better understand the underlying pathways in the relationship between social networks and depressive symptoms, specifically comparing the extent to which the relationship is mediated by the receipt of material/monetary support, relative to perceived social isolation. We use data from the Panel on Health and Aging of Singaporean Elderly, a nationally-representative study of older Singapore citizens and permanent residents aged 60 years and older in 2009 (N=4990), with two follow-up surveys in 2011

(N=3103) and 2015 (N=1572). We conduct cross-lagged mediation analysis, and control for a number of potential confounders including cognitive function, chronic physical ailments, socioeconomic status, and demographic variables such as age, sex, marital status, and family size. Our preliminary analysis shows that a reciprocal relationship between social networks and depressive symptoms is mediated to a larger extent by social isolation compared to weaker social support.

### SOCIAL PARTICIPATION ACROSS MIDLIFE AND LATER LIFE: EVIDENCE FROM THE SILENT GENERATION

Eric Vogelsang, *Cal State University-San Bernardino, San Bernardino, California, United States*

Despite the well-established benefits of social participation for individuals and communities, little is known about how it varies throughout the life course. Drawing upon data collected between 1957 and 2011 by the Wisconsin Longitudinal Study (22,023 observations from a cohort of 6,627), this study provides four valuable results. One, I find evidence of five distinct social participation trajectories between the ages of 35 and 71; the majority of which demonstrate social disengagement over time. Two, these participation declines are primarily attributable to changes in meeting friends and group exercise activity. Three, the most pronounced activity differences separating those in more favorable and unfavorable participation trajectories are cultural event attendance and voluntary group membership. Lastly, I identify particular high school activities that are associated with social participation decades later. In total, these results highlight heterogeneity among different types of social activities, and underscore the possible consequences of membership decisions made in early adulthood.

### THE EFFECT OF OLDER ADULTS' VOLUNTEERING ON RELATIONAL SATISFACTION

Meeryoung Kim, *Daegu University, Gyeongsan, Republic of Korea*

Longevity is increasing in what is called the centenarian society. However, the average retirement age of Korea is the lowest among OECD countries. Because of increasing longevity, older adults need activities after retirement. Volunteering can be a substitute that allows Korean older adults to find a social identity. This study examined older adults' volunteering and how many kinds of volunteering affected relational satisfaction differently. This study used the 6th additional wave of the Korean Retirement and Income Study (2016). The target population of this study was ages over 60 and the sample size was 280. For data analysis, multiple regressions were used. Demographic variables were controlled. As for independent variables, reasons for volunteering whether they were motivated for self or for others were used. For dependent variables, relational satisfaction, such as family, human relation and overall life satisfaction was used. Volunteers' health is an important factor for relational satisfaction. If volunteering was self-motivated, satisfaction of both family and human relations were negatively affected. Reason for others also affected satisfaction of family and human relations negatively. Volunteering initiated by others increased satisfaction of family and human relations. Doing more than one kind of volunteering affected both satisfaction of family and human relations. For overall life satisfaction, the effect of volunteering for oneself was lower than other reasons. These findings implied that reasons for volunteering affected relational satisfaction differently. In addition, the activities of volunteering, such as taking part in one or more had different effects.

### THE EFFECTS OF EMPLOYMENT ON DEPRESSION AND LIFE SATISFACTION AMONG OLD-AGED USING THE DD METHOD COMBINED WITH PSM

Si Young Song, Hey Jung Jun, and Sun Ah Lee, *Yonsei University, Seoul, Republic of Korea*

The purpose of this study is to explore the effect of employment on depression and life satisfaction among old-aged. Using 12th (2017) wave and 13th (2018) wave of Korean Welfare Panel Study (KoWePS), three stages of analyses were conducted. First, through propensity score matching (PSM) method, sample with similar propensity scores was matched between the group that did not work in 12th wave but worked in 13th wave (experimental group, N=180), and the group that did not work in 12th and 13th wave (comparative group, N=180). Second, the matched sample was used to conduct multiple regression analysis with the group dummy variable (experimental group, comparative group) as an independent variable, and depression and life satisfaction as the dependent variables. Third, combined model of propensity score matching (PSM) and double difference (DD) method was conducted to more appropriately derive the net effect of employment. The results of multiple regression after propensity matching showed that employment had a positive effect on reducing depression ($B= -1.70$, $p< .01$) and increasing life satisfaction ($B= .12$, $p< .01$) in old-aged. Furthermore, in combined model of PSM and DD, life satisfaction was improved when employed compared to non-employed ($B= .15$, $p< .05$). The results of this study are meaningful in that the meaning of employment in old-aged is more clearly derived by solving selection bias and endogenous problems. Also, this study may provide reference for establishing welfare policies related to employment among old-aged.

### THE EFFECTS OF SITUATIONAL PEERS AND SUPPORT GROUPS ON THE WELL-BEING OF GRANDMOTHERS RAISING GRANDCHILDREN

Alexandra Jeanblanc,[1] Carol Musil,[1] Christopher Burant,[2] and McKenzie Wallace,[3] *1. Case Western Reserve University, Cleveland, Ohio, United States, 2. Case Western Reserve University, Parma, Ohio, United States, 3. Case Western Reserve University, Lorain, Ohio, United States*

Grandmothers raising their grandchildren face not only the demands of parenting, but the added burden of parenting a child they did not expect to raise. Similarly, grandmothers living in multigenerational households need to balance expectations and household/caregiving tasks across the generations. As part of a nationwide RCT designed to lessen the stress associated with the caregiving burden of raising grandchildren, we asked 342 grandmothers raising their grandchildren about their engagement with support groups and whether their social network included other grandfamilies. Here, we examine the effect of situational peers and support group engagement on grandmother's stress, reward, social support, mental health, mindfulness, and resourcefulness. Of our sample, 53.8% (N=184) reported belonging to a support group. The majority of participants (138) belonged to online support groups on Facebook, 41 reported participating in in-person support groups, 3 belonged to both in-person and online support groups, and 8 did not respond. When asked whether and how well our participants knew other families like theirs, 31% (106) said they did not know any, 40.6% (139) knew of at least one, but not well, and 28.4% (97) said they had at least one friend with a family like her own. Knowing other families like their own affected self-appraised stress, but no other outcomes. Grandmothers participating in support groups had lower mindfulness scores, higher stress, and worse mental health scores than grandmothers not participating in support groups, possibly reflecting the higher need for support among those participants.

### THE RELATIONSHIP BETWEEN SOCIAL SUPPORT, AGING EXPECTATIONS, AND HEALTH BEHAVIOR IN MIDDLE AGE

Hyun-E Yeom,[1] Misook Jung,[2] and Eunyoung Park,[1]
*1. Chungnam National University, Daejeon, Taejon-jikhalsi, Republic of Korea, 2. Chungnam National University, Daejeon, Republic of Korea*

Midlife is a critical period when individuals need to actively engage in healthy behaviors for healthy aging. Although both social relations and attitudes toward aging are factors related to health behavior, little is known about their relationships based on age-related differences. The purposes of this study were to investigate the influence of social support affecting health behavior through expectations regarding aging and to examine how age affects the relationship. A cross-sectional study was conducted with data from 245 midlife Koreans (mean age= 51.5) collected by a self-administered survey. Data were analyzed using the PROCESS macro in SPSS. Social support was significantly related to expectations regarding aging (r= -.135, p=.034) and health behavior (r= .223, p<.000). There was age-related difference in the relationship between social support and expectations regarding aging (β= .007, p=.038), indicating that the relationship was much stronger in the younger group. In addition, the influence of social support on health behavior through expectations regarding aging was significant in relatively young middle-aged individuals. Our findings emphasize the importance of supportive social relationships, which could affect expectations regarding aging linked to health behavior, especially for young middle-aged individuals. It is necessary to develop psycho-cognitive programs to activate social interaction and to improve positive attitudes toward aging for more active engagement in health behaviors in midlife individuals.

### SESSION 2956 (POSTER)

### TECHNOLOGY II

### A SYSTEMATIC REVIEW OF IN-HOME SMART TECHNOLOGY ADOPTION TO IMPROVE OLDER ADULT HEALTH AND FAMILY CAREGIVING

Geunhye Park, and Erin Robinson, *University of Missouri-Columbia, Columbia, Missouri, United States*

In-home and internet-based smart technologies to improve older adult health has been rapidly developing. Technologies such as in-home sensors and smart homes enable older adults to live independently and age in place. These technologies also assist informal caregivers in their roles, thus reducing caregiver burden. However, technology adoption among older adults and family caregivers has been relatively low and reasons for technology acceptance are complex. Therefore, the purpose of this study was to conduct a systematic review of the literature, examining acceptance and adoption of in-home, internet-based smart technologies that are designed to improve health outcomes of older adults and can assist family caregivers in providing supports. This study utilized the Rew method (2011) and included peer-reviewed research articles published between 1991 and 2019 and available in: ISI Web of Science; PubMed; Scopus; CINAHL; and PsycInfo. A total of 1,227 relevant articles were identified with the search strings used and a final sample of 48 articles were included after the title, abstract, and full article review processes. Findings highlight several facilitators and barriers to technology adoption. Some facilitators to adoption include: technology familiarity, safety/security, personally tailored, non-obtrusive design, easy access, and reduction of caregiver burden. A few barriers include: cost, difficulty to use, time, stigma, privacy, data accuracy, and confidence. Additional findings will also be presented. A more thorough understanding of these facilitators and barriers to acceptance/adoption is crucial for the successful dissemination of in-home, internet-based smart technologies. Increased adoption can improve older adult health and reduce caregiver burden.

### ADDRESSING THE ROLE OF SMART ROBOTIC HEALTH ASSISTANTS WITHIN THE HUMAN-MACHINE FRONTIER OF GERIATRIC HEALTHCARE

Nadia Firdausya,[1] Alex Bishop,[2] Barbara Carlson,[3] and Weihua Sheng,[2] *1. Oklahoma State University, stillwater, Oklahoma, United States, 2. Oklahoma State University, Stillwater, Oklahoma, United States, 3. University of Oklahoma Health Sciences Center, Oklahoma City, Oklahoma, United States*

Data for this study was acquired from three separate stakeholder focus group sessions involving nurse case managers (n = 5), social agency caseworkers (n = 5), and rural outreach providers (n = 5). Participants across all groups were asked to address the question: "When it comes to your work, what would you want a smart robot assistant to do for you?" Data from the three sessions were combined, transcribed verbatim, coded, and analyzed for thematic content. Three shared themes emerged, including health monitoring, behavioral intervention, and healthcare literacy. Relative to health monitoring, participants desired a robot that possessed functions in the form of "taking vital signs," and "tracking water and food intake." There was also a thematic agreement regarding behavioral intervention capabilities. Most notably, advisory stakeholders acknowledged a need for a smart robotic assistant to provide geriatric care recipients with "an alert or reminder to take medication." This was viewed as an essential intervention for improving medication adherence. Healthcare literacy emerged as a final theme among advisory groups. In particular, participants noted that a smart robot should assist with bi-directional communication and translation of health care information and instructions as a way to "minimize impediments of care due to language barriers." Findings will be further used to highlight how future integration of robotic health assistants represents a viable solution in helping geriatric healthcare workers work effectively alongside machines to meet the diverse care needs of older adults in both urban and rural settings.

### AGE DIFFERENCES IN THE RELATIONSHIP BETWEEN DAILY SOCIAL MEDIA USAGE AND AFFECT

Xin Yao Lin, and Margie Lachman, *Brandeis University, Waltham, Massachusetts, United States*

Social media platforms allow people to connect and share content online (e.g., Facebook, Twitter). Although older adults are becoming more frequent users of social media, there continue to be mixed views on whether social media positively or negatively impacts well-being. Past studies have mainly focused on cross-sectional analyses for individual differences. However, both the time spent on social media and one's affect can fluctuate on a daily basis. Thus, it is important to understand how the relationship between daily social media usage and affect varies within individuals from day to day. The current study adds to the literature by examining whether daily variations in time spent with social media are related to daily positive and negative affect and whether there are age differences in these relationships. The current study used an eight-day daily diary from the Midlife in the United States (MIDUS) Refresher dataset for 782 participants (ages 25-75). Multilevel modeling results revealed that age moderated the relationship between daily time spent on social media and negative affect: for younger adults, on days when they spent more time on social media, they had more negative affect. For older adults, on days when they spent more time on social media, they had less negative affect. Surprisingly, daily time spent on social media was not related to daily positive affect, nor did this relationship differ by age. Implications for future research are discussed with a focus on how social media usage can contribute to daily well-being for adults of different ages.

### AGE-COHORT DIFFERENCES IN LONGITUDINAL ASSOCIATIONS BETWEEN THE RATE OF INTERNET USE AND MEMORY FUNCTIONING

Vineet Raichur, Lindsay Ryan, RIchard Gonzalez, and Jacqui Smith, *University of Michigan, Ann Arbor, Michigan, United States*

Cross-sectional analyses of internet use patterns among older adults find that the rate of internet use is less with greater physical and memory difficulties. It is not clear, however, how age-cohorts differ in their internet use as physical and memory difficulties increase over time. In addition to factors such as increasing accessibility (cost) and social influences, the expansion and cognitive complexity of functions performed by the internet-enabled devices over time could influence internet use patterns. In this study, we investigate how the association between internet use and episodic memory difficulties over time varies between cohorts. We analyzed longitudinal data from the Health and Retirement Study (N = 15,703 in 2002; Aged 51 and older) between years 2002-2016 using mixed effects logistic regression models. Immediate and delayed word recall measures were used to assess episodic memory. Rate of internet use in the sample increased from 30% in 2002 to 53% in 2016. Rate of internet use among younger age groups was significantly higher in the baseline year. Younger age groups also showed a significantly higher rate of increase in internet use over time. In general, internet use decreased with episodic memory impairment. In addition to these effects, the effect of episodic memory on the rate of increase in internet use over time is lower in younger cohorts. These results indicate that younger cohorts of older adults are more likely to maintain internet use as they continue to age and therefore could better utilize technology for communication, social interactions and health interventions.

### DIGITAL HEALTH INFORMATION AS FRIEND OR FOE: THE QUESTION OF (MIS)TRUST AMONG OLDER INTERNET USERS

Gul Seckin,[1] Patricia Campbell,[2] and Megan Lawson,[2] *1. University of North Texas, Hickory Creek, Texas, United States, 2. University of North Texas, Denton, United States*

Gathering health information is among the major motivations for getting online among older adults who want to be better prepared with knowledge to manage their health and personal care. Prior research also showed significant gender differences in health-related use of the Internet. This research examined the effect of Internet use for health information on (a) mistrust of physician, (b) empowerment, (c) self-care, and (d) worry and/or anxiety. The sample (N=710; Mean= 48.82, SD=16.43) was randomly drawn from a national probability-based online panel. We performed gender-stratified sub-sample analyses of older respondents (age ≥60, N= 194). Hierarchical linear regression analyses showed that there is a negative association between older age and feeling empowered because of using the internet for health information ($\beta$ = -.23, p < .05) and a positive association between older age and mistrust of diagnosis and/or treatment of physician ($\beta$ = .19, p < .05). Study respondents did not report better self-care as a result of obtaining information from the Internet ($\beta$ = -.15, p > .05). Lastly, older adults reported less worry and/or anxiety because of information stumbled upon the Internet that may not be accurate ($\beta$ = -.25, p < .05). Sub-sample analyses showed that there are gender differences. Particularly, older men reported greater mistrust ($\beta$ = .32, p < .05), and less worry ($\beta$ = -.44, p ≤ .01) while these associations were not significant among older women. Results call for examination of the synergy of age and gender in perceived benefits of health-related Internet use.

### E-MAIL-RELATED PROBLEM-SOLVING BEHAVIORS ACROSS AGE GROUPS: AN ANALYSIS OF LOG FILE DATA

Taka Yamashita,[1] Leah Saal,[2] Roberto Millar,[3] Shalini Sahoo,[4] and Phyllis Cummins,[5] *1. University of Maryland, Baltimore, Maryland, United States, 2. Loyola University, Baltimore, Maryland, United States, 3. Graduate Student, Baltimore, Maryland, United States, 4. University of Maryland, Baltimore, Elkridge, Maryland, United States, 5. Miami University, Oxford, Ohio, United States*

Email is one of the most common and useful online communication tools. However, older adults tend to have difficulties fully taking advantage of email. Organizing the information in the email software environment and sending a message to selected recipients are examples of common email-related problem-solving. To date, little data are available to understand the email-related problem-solving behaviors of older adults. Nationally representative survey data and log-file data of the U.S. adults (n = 1,341) are derived from the 2012 Program for International Assessment of Adult Competencies (PIAAC), which provides computer-based assessment data on problem-solving skills. The PIAAC respondents used the computer device and solved the problems in the simulated email environment. Descriptive summary showed that those 55 years and older took longer (169 seconds), referred to the help menu (15%) and used the cancel button (26%) more often than younger age groups (e.g., age 25-34; 103 seconds, 3% and 17%, respectively) in one of the tasks. Additionally, binary logistic regression showed that taking longer time (odds-ratio = 0.99, $p < 0.05$) and using the help menu (odds-ratio = 0.85, $p < 0.05$) were associated with the incorrect answer to the email problem-solving, although the findings varied across different types of problems. These unique findings from the combination of survey and log-file data analyses suggested that some older adults may benefit from the training for common email-related problems rather than teaching themselves. Detailed descriptions of computer-based assessment log file data and other results are also evaluated in this study.

### FEASIBILITY AND ACCEPTABILITY OF APP-BASED MINDFULNESS-MEDITATION TRAINING FOR OLDER ADULTS

Leeann Mahlo,[1] and Tim Windsor,[2] *1. Flinders University, Woodside, South Australia, South Australia, Australia, 2. Flinders University, Adelaide, South Australia, Australia*

Few studies have focused on the utility of mindfulness-meditation for well-being in older adults. This study aimed to investigate the feasibility and acceptability of an app-based mindfulness-meditation program for community-based older adults. A convenience sample of 46 participants aged between 63 and 81 (M = 70.85, SD = 4.70) was recruited from the community. Participants were invited to engage with a 30-day app-based mindfulness-meditation program for 10-minutes daily on their smartphones. Each meditation session comprised focusing on the breath, mentally scanning the body, monitoring the mind's activity, and cultivating a nonjudgmental attitude toward experience. Participants completed psychosocial questionnaires at baseline, day 10, and day 30. On average, participants completed 25 sessions and almost 4 hours of application use across the 30-days. Results of linear mixed effects models showed significant improvements in positive affect, negative affect, and life satisfaction across the study interval, but no meaningful change in total or facet-level mindfulness or perceived stress. Furthermore, relative to high levels of smartphone efficacy, low smartphone efficacy was associated with higher perceived stress and negative affect, and less life satisfaction at baseline; and steeper improvements on these outcomes across the study interval. Results indicated that, on average, older adults found app-based mindfulness-meditation training interesting, enjoyable, valuable, and useful. The findings provide preliminary support for the feasibility and acceptability of an app-based mindfulness-meditation program with community-based older adults and demonstrate potential benefits for well-being. Furthermore, older adults' perceptions of smartphone competency may play an important role in the outcomes of app-based programs.

### GIVE PEOPLE WHAT THEY WANT, FEEDBACK: OLDER ADULT PERCEPTIONS ON COGNITIVE TRAINING FEATURES TO INCREASE ENGAGEMENT

Nelson Roque,[1] and Erin Harrell,[2] *1. Pennsylvania State University, University Park, Pennsylvania, United States, 2. University of Alabama, Tuscaloosa, Tuscaloosa, Alabama, United States*

According to Temporal Self-Regulation Theory (TST; Hall & Fong, 2007), adherence motivation can be driven by both positive and negative emotional reactions in which adherence is viewed in relation to gains versus losses. In a sample of 100 older adults (ages 64+), we explored participant-provided feedback related to intervention and game elements participants perceived would increase their adherence across five domains: (1) ability to adjust difficulty; (2) ability to change game design (from the preprogrammed American Western theme); (3) cognitive performance feedback; (4) ability to unlock extra game features (e.g., more levels); and (5) other elements. Ranked in order of frequency of endorsement, 53% endorsed: cognitive performance feedback; 47%: ability to adjust difficulty; 28%: ability to unlock new features; 27%: the 'Other' option; and 20% endorsed changing the game's theme. This work has implications for models of adherence, specifically, the role that expectations of later cognitive feedback might play.

### INCREASING ADOPTION AND UTILITY OF SMARTWATCHES IN OLDER ADULTS

Mitchell Roberts,[1] Erica Sappington,[1] Ali Yalcin,[1] Jeffrey Lowenkron,[2] David Amzallag,[3] and Carla VandeWeerd,[1] *1. University of South Florida, Tampa, Florida, United States, 2. The Villages Health, The Villages, Florida, United States, 3. Aviv Scientific, Bnei Brak, Israel*

Older adults (55+) represent one of the fastest growing groups adopting wearable devices (e.g. smartwatches). However, questions remain as to how older adults, healthcare providers and researchers can maximize the ability of these devices to maintain and improve health. The objective of this study was to understand the needs and preferences of older adults in wearable devices, as part of a Florida High Tech Corridor Matching Grant between The Villages Health, Aviv/Xtend Scientific, and The University of South Florida. Six focus groups were conducted with older adults (n=36) living in The Villages, Florida, and were stratified by gender. Topics such as adoption, benefits/concerns, usability, and potential gender differences were explored. Heart rate monitoring, calories burned, and step counting features were most often utilized by current wearable users (43%). Participants reported that the most important benefits of wearables were atrial fibrillation identification, fitness tracking and fall detection. Concerns included privacy, the "learning curve" and too frequent of notifications by the device. Across both genders, choice resonated strongly as a theme. Male and female participants desired personalized, easy to understand outcomes and control over data sharing preferences. Men were interested in continual access to wearable health data, whereas females were interested in daily or weekly summaries. Further, men reported higher on-going use and comfort with smartwatches as compared to females. Participants suggested "low, medium, and high-tech savvy" user profiles and supporting resources (e.g. 1-1 training, paper manuals) to enhance adoption. Recommendations for policy and practice are shared, in light of the findings.

### PHYSICAL INACTIVITY IN OLDER ADULT COUPLES: A DYADIC ANALYSIS USING CONTINUOUS IN-HOME MONITORING

Chao-Yi Wu,[1] Lyndsey Miller,[2] Rachel Wall,[3] Zachary Beattie,[1] Jeffrey Kaye,[4] and Lisa Silbert,[1]
*1. Oregon Health & Science University, Portland, Oregon, United States, 2. Oregon Health and Science University, Portland, Oregon, United States, 3. Portland Veterans Affairs Medical Center, Portland, Oregon, United States, 4. Layton Alzheimer's Disease Center, Portland, Oregon, United States*

Many older adults remain inactive despite the known positive health implications of physical activity (e.g. improved mood, reduced mortality risk). Physical inactivity is a known interdependent phenomenon in couples, but the majority of research identifies determinants of physical inactivity at the individual level. We estimated the average amount of physical inactivity for older adult couples and, using dyadic analysis, identified physical and mental health determinants thereof. Forty-eight heterosexual older adult couples (mean age=70.6, SD=6.63) from the Veterans Integrated Service Network 20 cohort of the Collaborative Aging Research using Technology (CART) initiative were included in this study. Both dyad members wore actigraph devices for a month. The average number per day of inactive periods (defined as no movement or sleep activity for ≥ 30 minutes) was estimated. Multilevel modeling revealed that, within couples, there was no difference between partners in the average number of inactive periods, but on average across couples, males had more inactive periods per day (13.4, SD=4.43) than females (12.3, SD=4.87). For males, older age was the only variable associated with more inactive periods ($\beta$=0.13, p=.013). For females, more depressive symptoms in men were associated with fewer inactive periods ($\beta$=-0.37, p=.002), and more dependence in completing their own IADLs predicted more inactive periods ($\beta$=2.80, p<.001). All models were adjusted for covariates. Viewing couples' activity as a unit, rather than as separate individuals, provides a novel approach to identifying pathways to reduce inactivity in older adults, especially when focusing on mental health issues and decreased independence within the couple.

### RECRUITMENT OF HARD-TO-REACH OLDER ADULTS IN TECHNOLOGY-BASED MENTAL HEALTH SERVICES STUDIES: LESSONS LEARNED

Patrick Ho Lam Lai,[1] Xiaoling Xiang,[2] Yihang Sun,[2] Joseph Himle,[2] and Ho Lam Lai *1. University of Michigan-Ann Arbor, Ypsilanti, Michigan, United States, 2. University of Michigan, Ann Arbor, Michigan, United States*

Homebound older adults are a hard-to-reach population with a high burden of depression and face substantial access barriers to mental health treatments. Internet-based psychotherapy is a promising strategy to address this persistent treatment gap, but older adults are severely underrepresented in internet-based psychotherapy trials. One challenge to advance this area of research and practice is the recruitment and retention of homebound older adults in clinical trials. Previous research has discussed the challenges of recruiting older adults in behavioral interventions and offered useful recommendations. However, recruiting homebound older adults, who face substantial mobility barriers, poses additional challenges not fully addressed in the literature. The expectation of using technology adds another layer of difficulty. In this presentation, we will discuss our group's experiences working with community partners to recruit and retain homebound older adults for a study on technology-based mental health treatment. We partnered with home care agencies, senior apartment buildings, and Meals-on-Wheels and experimented with a few different ways to recruit study participants. Issues related to accessibility, trust, and stigma emerged as important considerations when designing recruitment strategies and materials. The discussion will be an integration of our experiences and a review of previous literature on the challenges and recommendations for recruiting older adults in mental health services research.

### RURAL AND NON-RURAL DIGITAL DIVIDE PERSISTS IN OLDER ADULTS: INTERNET ACCESS, USAGE, AND PERCEPTION

Hee Yun Lee,[1] Eun Young Choi,[2] Youngsun Kim,[3] Jessica Neese,[4] and Yan Luo,[1] *1. University of Alabama, Tuscaloosa, Alabama, United States, 2. Leonard Davis School of Gerontology, Los Angeles, California, United States, 3. Kyunghee University, Yongin-si, Republic of Korea, 4. MSW, Tuscaloosa, Alabama, United States*

Despite the overall increase in Internet use among older adults, the digital divide within older Americans remains substantial. This trend is particularly true for older adults living in rural areas. Informed by the Social Determinants of Health Framework, our study aims to examine how one's residential area relates to (1) Internet Access, (2) subtypes of usage patterns, and (3) perceptions on technology use. Cross-sectional data were drawn from the 2012 Health and Retirement Study (HRS). The sample consisted of 18,196 older adults aged 50 and above (47.6% rural residents). A series of linear and logistic regression analyses were performed. Our models controlled for demographic characteristics, socioeconomic status, and health conditions. Compared to older adults living in urban areas, those residing in rural areas had 29% lower odds of internet access. Living in rural areas predicted lower levels of all sub-types of technology use (communication, financial, health, and media technology). In addition, non-users in rural areas showed more unfavorable perceptions of technology than urban residents. They were more likely to conceive technology as "too complicated", "too hard to learn", and "too difficult to keep up with all changes." Our findings suggest that substantial segments of older adults in rural areas are still behind in accessing and adopting digital technology. Targeted intervention efforts are urgently needed to reduce technology inequality including comprehensive plans to expand broadband access and building mobile technology infrastructure for rural communities.

### SAVVY SENIORS: A CONTENT ANALYSIS OF THE USABILITY OF OLDER ADULT RESOURCE WEBSITES

Kendall Weber,[1] Deborah Koh,[1] Lisa Stone,[2] and Andrew Lac,[2] *1. University of Colorado at Colorado Springs, Colorado Springs, Colorado, United States, 2. University of Colorado Colorado Springs, Colorado Springs, Colorado, United States*

Due to age-related declines, older adults often experience difficulties using the Internet and navigating websites, even among websites specifically designed for them. Subsequently, the National Institute on Aging (NIA) and National Library of Medicine (NLM; 2001) published guidelines to promote website usability among a later life population. Applying quantitative content analysis, this study examined the accessibility of senior resource websites based on NIA and NLM recommendations. Specifically, the relation between the type of organization sponsoring the websites (i.e., governmental, non-profit, or private) and overall accessibility and the relation between the number of resource categories (e.g., housing, financial, etc.) and overall accessibility were investigated. Font size, organization of presented information, and the color and contrast on the websites were coded to determine how they contribute to overall accessibility. Using a sample of the 100 most-searched senior resource websites, a one-way ANOVA indicated that the type of site on overall accessibility was not significant, $F(2, 97) = 2.29$, $p = .11$, $\eta2 = .04$. The multiple regression model showed that font size ($\beta = .30$, $p < .001$), organization of information ($\beta = .38$, $p < .001$), and color and contrast ($\beta = .45$, $p < .001$) were significant predictors of overall website accessibility and explained 62% of the variance, $F(3, 96) = 51.18$, $p < .001$. The findings suggest that various NIA guidelines differentially contribute to a website's accessibility, and further attention should be paid to cognitive aspects of resource websites.

### TECHNOLOGY AND SOCIAL ISOLATION AMONG OLDER ADULTS

Allison Dunatchik, and Jerry Jacobs, *University of Pennsylvania, Philadelphia, Pennsylvania, United States*

In this paper, we use time-use data from the US (2003-2018) and the UK (2001-2015) to address important questions about recent trends in social isolation and technology use among older adults aged 65 and over. Do new information, communication and robotic technologies contribute to growing social isolation among older adults or to growing opportunities for social connection? We believe that answers to these questions require a consideration of technologies that are already widely in use. We ask whether TV time is social time or whether people spend most of their time watching TV by themselves, whether this has changed over the past 15 years and what the potential implications are for wellbeing among older adults. The results show that TV time is a mixed experience among older adults. In the US and the UK, a substantial proportion of TV time – around half – is spent alone, with those who are not married, those in rented accommodations and those with poorer self-rated health spending more time watching TV alone. Controlling for demographic and household characteristics, TV time alone has increased in recent years, accompanying an increase in time spent alone in general in the UK and an increase in TV time in general in the US. Analysis of moment-to-moment wellbeing shows that individuals report lower positive affect while watching TV alone than while watching with others, suggesting the increase in time spent watching TV alone may have negative implications wellbeing among older adults.

### SESSION 2957 (POSTER)

### SOCIAL NETWORKS AND SUPPORT II

### AGE DIFFERENCE IN SOCIAL DISCOUNTING: GENEROUS LEVEL ALSO MAKES A DIFFERENCE

Hongmei Lin, and Xin Zhang, *Peking University, Beijing, China*

Social discounting refers to the phenomenon that individuals' generous behaviors decline as the social distance increases. But little is known how age could influence social discounting. The present study aimed at a more comprehensive understanding of age-related differences in social discounting. Moreover, as previous studies suggested that older adults are more loss aversion, we would also test whether framing (gain vs loss) could influence social discounting between two age groups. A mixed-model factorial design of 2 (age group: younger vs. older adults) × 2 (framing: gain vs. loss) × 2 (generous level: low vs. high) × 8 (social distance) was conducted, with a total of 78 younger adults and 82 older adults. A significant social distance × age interaction was found, which replicated previous studies suggesting that older adults are more generous toward socially distant others. Interestingly, a significant age × framing × generous interaction was also found, such that in low generous condition, older adults tend to be more generous than younger adults under both gain and loss framing, while such age difference disappeared in high generous condition. These findings indicate that generous level has a positive impact on people social discounting, inducing younger adults to get more generous. Contrary to our expectation, the framing of gain and loss seems not to wave individuals' social discounting. It seems that people think more seriously about the amount of allocation rather than framing.

### BEYOND RELIGIOSITY: A MODEL TO EXPLAIN SPIRITUALITY AMONG MIDDLE-AGED AND OLDER ADULTS

Carlyn Vogel, Debra Dobbs, and Brent Small, *University of South Florida, Tampa, Florida, United States*

Spirituality is difficult to define as researchers assign it different meanings and individuals' perceptions can vary. For example, spirituality may connect to religiosity, while others consider religiosity a less significant part of spirituality. This study investigates factors outside of religiosity that are significantly associated with spirituality to inform the characteristics of the concept. Webster's (2004) existential framework of spirituality was used to guide variable selection. The National Survey of Midlife in the United States wave three (MIDUS 3; 2013-2014; $n = 2,594$; $Mage = 63.5$, $SD = 11$, range = 39–92) was used to examine individuals' reported levels of spirituality. Multinomial logistic regression was conducted to examine factors related to low and high levels of spirituality compared to a moderate level. Participants with low spirituality were more likely to be male, less likely to be mindful, mediate/chant, feel a strong connection to all life, to indicate that they cannot make sense of the world, and to be religious. Participants with high spirituality were more likely to be female, have at least some college experience, be mindful, meditate/chant, feel deep inner peace, have a sense of deep appreciation, think that a sense of purpose is important for a good life, and have a high level of religiosity. Framed by Webster's conceptual model, the current study observed that religiosity is significantly associated with spirituality and that other mindfulness-based aspects are also present within this concept. Incorporating mindfulness with religious efforts will more accurately and holistically address spirituality.

### EVERYDAY GOAL PURSUIT IN OLDER COUPLES: LESSONS LEARNED FROM ELECTRONIC DAILY LIFE ASSESSMENTS

Elizabeth Zambrano Garza,[1] Christiane Hoppmann,[1] Maureen Ashe,[1] Denis Gerstorf,[2] Kenneth Madden,[1] and Victoria Michalowski,[3] *1. University of British Columbia, Vancouver, British Columbia, Canada, 2. Humboldt University Berlin, Berlin, Berlin, Germany, 3. University of British Columbia, Vancouver, Canada*

Being able to progress on and accomplish personal goals is an important source of satisfaction and meaning across the adult lifespan and into old age. This study focuses on the importance of close others, such as spouses, for facilitating goal progress when individual resources may no longer suffice. We used multilevel modelling to analyze data from 119 couples (M age = 70 years, SD= 5.9; 50% women). Participants reported their personal goals at baseline and subsequently provided simultaneous ratings of goal pursuit, effort, and partner involvement three times daily over 7 days. Our findings show that more effort and spousal involvement indeed go hand in hand with better goal progress. More in-depth follow up analyses will pinpoint the role of individual and partner effects as potential mechanisms underlying contributions to everyday goal pursuit as well as address stress related processes.

### GLOBAL SENSORY IMPAIRMENT INDEPENDENTLY PREDICTS DECREASED SOCIAL FUNCTION OVER TIME

Anna Huang,[1] Kristen Wroblewski,[2] Ashwin Kotwal,[3] Linda Waite,[2] Martha McClintock,[2] and Jayant Pinto,[2] *1. University of Chicago Medical Center, Chicago, Illinois, United States, 2. University of Chicago, Chicago, Illinois, United States, 3. University of California, San Francisco School of Medicine, San Francisco, California, United States*

The classical senses (vision, hearing, touch, taste, and smell) play a key role in social function by allowing interaction and communication. We assessed whether sensory impairment across all 5 modalities (global sensory impairment [GSI]) was associated with social function in older adults. Sensory function was measured in 3,005 home-dwelling older U.S. adults at baseline in the National Social Life, Health, and Aging Project and GSI, a validated measure, was calculated. Social network size and kin composition, number of close friends, and social engagement were assessed at baseline and 5- and 10-year follow-up. Ordinal logistic regression and mixed effects ordinal logistic regression analyzed cross-sectional and longitudinal relationships respectively, controlling for demographics, physical/mental health, disability, and cognitive function (at baseline). Adults with worse GSI had smaller networks ($\beta$=-0.159, p=0.021), fewer close friends ($\beta$=-0.262, p=0.003) and lower engagement ($\beta$=-0.252, p=0.006) at baseline, relationships that persisted at 5 and 10 year follow-up. Men, older people, African-Americans, and those with less education, fewer assets, poor mental health, worse cognitive function, and more disability had worse GSI. Men and those with fewer assets, worse cognitive function, and less education had smaller networks and lower engagement. African-American and Hispanic individuals had smaller networks and fewer close friends, but more engagement. Older respondents also had more engagement. In summary, GSI independently predicts smaller social networks, fewer close friends, and lower social engagement over time, suggesting that sensory decline results in decreased social function. Thus, rehabilitating multisensory impairment may be a strategy to enhance social function as people age.

### RELATIONSHIP STATUS, SOCIAL INTERACTIONS, AND CONVERSATIONS IN LATE LIFE

Yee To Ng,[1] Meng Huo,[2] Shiyang Zhang,[1] and Karen Fingerman,[1] *1. The University of Texas at Austin, Austin, Texas, United States, 2. University of California, Davis, Davis, California, United States*

Studies suggest spending more time interacting with and talking to others is associated with better well-being. Older adults with partners (e.g., married, cohabitated) may spend more time with their romantic partners and rely on them for support, whereas older adults without partners (e.g., widowed, divorced, never married) may have a greater reliance on other family members (e.g., grown children, siblings) and non-kin (e.g., friends). Yet, we know little about how older adults' relationship status affects their time spending alone or with other social partners, and the frequency of conversation throughout the day. Adults aged 65+ (N = 313) completed an interview about their relationship status and social partners. They then reported social encounters in ecological momentary assessments every 3 hours for 5 to 6 days. Participants also wore Electronically Activated Recorders which captured snippets of their conversation throughout the day. Older adults with partners reported 85% of time was with their romantic partners. Multilevel models revealed that compared to older adults with partners, older adults without partners were more likely to spend time alone and have encounters with friends throughout the day. Older adults without partners also engaged in fewer conversations throughout the day. Further, older adults without partners talked significantly more when they encountered friends than did older adults with partners. Findings suggest that friends are important in older adults' social networks particularly for those who do not have romantic partners. Daily contact with social partners facilitates conversations and that could have implications for emotional or cognitive health.

### SOCIAL SUPPORT FROM KIN AND MORTALITY RISK AMONG OLDER ADULTS IN A HISTORICAL AGING POPULATION

Julia Jennings, *University at Albany, SUNY, Albany, New York, United States*

Family are often sources of social, instrumental, and financial support for older adults. However, in many types of survey and archival data, details on the provision of support are lacking. This study examines the association between kin availability and cause-specific mortality among adults over age 60 using multiple longitudinal linked data sources from North Orkney, Scotland, 1851-1911. This study explores the relationships between cause of death and kin availability, as certain ailments may be amenable to interventions related to social support in this period while others may not. This approach will aid in interpreting the effects of social support that may be transmitted through kin networks. Reconstructed individual life courses (N=4,946) and genealogies, in combination with data on the proximity non-coresident kin, are used to examine kin availability and propinquity over the life course. Cause of death is available from death records and has been coded into the ICD. Orkney provides an interesting case study as longitudinal information is available on mortality and kin availability during a time of population aging. Kin availability is associated with longevity in this sample, while cause-specific analysis allows us to evaluate the role of social support in promoting longevity net of this association.

### THE 70- TO 85-YEAR-OLD SWEET SPOT: REGRESSION ASSOCIATIONS BETWEEN SPIRITUAL EXPERIENCE AND SUCCESSFUL AGING OUTCOMES

Stephen Fogle, *University of Nebraska Omaha, Omaha, Nebraska, United States*

Utilizing the MIDUS III dataset, this study conducted linear regression analysis for associations between daily spiritual experience and Successful Aging (N=2605). Age was measured in three groups: young-old (55-69), old (70-85), and old-old (86-100). Successful Aging was operationalized as Self-Reported Physical Health, Self-Reported Memory, Depressed Affect, and Life Satisfaction. Daily spiritual experience was measured with the Fetzer Institute five-item composite scale (Cronbach = .891). Analysis for each Successful Aging outcome was controlled for daily spiritual experience, physical and social neighborhood environment, age group, gender identification, co-habitation, income, education, cultural identification and disability. Regression analysis was undertaken for daily spiritual experience on the same control variables. Results found higher frequency of daily spiritual experience was significantly associated ($p = .000$) with better self-reported memory ($\beta$= .146***) and higher life satisfaction ($\beta$= .191***). Further, regression analysis revealed the 70-85 age group was significantly associated ($p = .000$) with better self-reported physical health ($\beta$= .123***), lower depressed affect ($\beta$= -.144***), and higher life satisfaction ($\beta$= .291***). Finally, the 70-85 age group was a stronger predictor of daily spiritual experience ($\beta$= .221***) than all other control variables except female gender identification ($\beta$= .244***). This study contributes evidence of associations between daily spiritual experience and Successful Aging outcomes, particularly memory and life satisfaction. This study demonstrates the advantage of measuring separate old age categories to reflect heterogeneity of the life course. Finally, this study underscores, “Why Age Matters”, through new evidence linking the 70-85 year old age group with daily spiritual experience and Successful Aging.

### TRAUMA AND RELIGIOUS COMPLEXITY IN LATER LIFE

Rennae Wigton,[1] Shannon Jones,[2] Austin Prusak,[2] and Andrew Futterman,[2] *1. Loyola University Maryland, Butler, Pennsylvania, United States, 2. Loyola University Maryland, Baltimore, Maryland, United States*

The present study examines the impact of traumatic life events on religious complexity in later life. We anticipated that those older adults experiencing stressors that produce significant personal vulnerability (e.g., life threatening illnesses) demonstrate reduced complexity of belief and behavior (e.g., less belief with doubt). From a sample of 278 semi-structured interviews of older adults (aged 55-101 years-old.) from six New England and New York states, we analyzed 166 interviews using grounded theory (Strauss & Corbin, 1990). Individuals who experienced trauma related to war, close familial loss, and/or severe physical illness tended to be “true believers,” (i.e., adhere to rigid belief orthodoxy; Hoffer, 1950). By contrast, those who experienced less severe trauma (e.g., minor illness, job loss) were less apt to describe rigid belief. Temporal proximity of trauma was not consistently associated with greater complexity of belief and behavior, in the sense that with great distance from trauma, individuals were able to “work through” their experiences of trauma, and thereby increase complexity of belief and behavior. This is consistent with findings by Harris and Leak (2015), Krause and Hayward (2012), and Wong (2013) that suggest that trauma leading to personal vulnerability leads to long-term physical, mental, behavioral, and spiritual deficits that rigid religious belief and behavior help to offset. These findings are discussed in terms of psychological theories of grief resolution, personal coping, and terror management.

### VOLUNTEERING AND COGNITIVE HEALTH AMONG MIDDLE-AGED AND OLDER ADULTS: THE MEDIATING ROLE OF FRIENDSHIP

Changmin Peng,[1] Jeffrey Burr,[1] Sae Hwang Han,[2] Kyungmin Kim,[1] and Jan Mutchler,[1] *1. University of Massachusetts Boston, Boston, Massachusetts, United States, 2. University of Texas at Austin, Austin, Texas, United States*

We lack knowledge about the underlying mechanisms that link formal volunteering to cognitive health. Friendships can be formed and improved through volunteering. Friendship is also beneficial to cognitive health as it often involves sharing information and promoting social interactions. This study investigated the potential mediating role of friendship (i.e., the number of close friends and the quality of friendships) for the association between volunteering (i.e., volunteering status and volunteering hours) and episodic memory among middle-aged and older adults in the United States, using data from the 2014 wave of the Health and Retirement Study (N = 6,029). Moderated mediation models were employed to test the mediation role of friendship for the association between volunteering and cognition for two age groups, middle-aged adults (age 50-64, n = 2,441) and older adults (age 65+, n = 3,588). The results showed that the quality of friendships, but not the number of close friends, mediated the relationship between volunteering (both status and hours) and episodic memory for middle-aged adults.

However, mediation effects of friendship were not discovered for older adults. Our findings emphasize the important role of the quality of friendship in understanding the cognitive benefit of volunteering among middle-aged adults. For older adults, the nonsignificant effects of friendship may be consistent with socioemotional selectivity theory, suggesting that older adults may not use volunteering to expand their social networks. Instead, they may participate in volunteer work for other reasons in the context of shrinking social networks.

### VOLUNTEERING AND FRIENDSHIP IN LATER LIFE: DOES GENDER MODERATE THE RELATIONSHIP?

Emily Lim, Changmin Peng, and Jeffrey Burr, *University of Massachusetts Boston, Boston, Massachusetts, United States*

Friendship, which is an essential part of social life and beneficial to individuals' well-being, plays a crucial role in maintaining social connectedness in late life. Volunteering is an avenue for older adults to stay socially engaged, and also provides older adults the opportunity to meet and make new friends. A limited literature suggests that volunteering may be associated with friendship, but many studies are limited by reliance on small, non-probability samples and overly simplistic analytic approaches. The literature is also unclear with respect to how volunteering behaviors relate to specific characteristics of friendships and whether there are gender differences underlying these relationships. Using the 2014 wave of the Health and Retirement Study (N=5,306), this study investigates the association between volunteering characteristics (i.e., volunteer status and hours) and friendship characteristics (i.e., the number of close friends, friendship quality, and contact frequency) among community-dwelling older adults. We also examine whether gender moderated these relationships. Results from linear regression analyses indicate that volunteer status and the number of volunteer hours were positively associated with each dimension of friendship. Also, the positive association between volunteering at 1–99 hours, 100–199 hours, and 200 hours or more and number of close friends, friendship quality, and contact frequency were stronger for older women than for older men. Findings demonstrated that volunteering is integral in shaping late-life friendships. The differential benefits of volunteering between older men and women also suggest that volunteering might be more critical for older women's friendships.

### SESSION 2958 (POSTER)

### END-OF-LIFE CARE AND DECISION MAKING

### A VIDEO DECISION AID FOR THE WEST VIRGINIA POLST: A RANDOMIZED, CONTROLLED TRIAL

Cherish Heard,[1] Emma Pino,[2] Jarred V. Gallegos,[2] Barry Edelstein,[3] and Alvin Moss,[2] *1. West Virginia University, Morgantown, West Virginia, United States, 2. WVU, Morgantown, United States, 3. WVU, Morgantown, West Virginia, United States*

Patients with serious medical conditions are faced with making decisions about treatments related to end-of-life care. The Physician Orders for Life Sustaining Treatment (POLST) is a document that allows patients to express preferences for four medical decisions including cardiopulmonary resuscitation, level of medical intervention, and medically administered fluids and nutrition. The purpose of the study was to develop and evaluate a video decision aid for the West Virginia POLST form. Sixty-four community-dwelling adults (50+) were recruited to participate. Participants were randomized to active control (exercise video) or intervention group (POLST video). Knowledge and decisional conflict were measured pre- and post-intervention. Participants were given a hypothetical vignette with medical information for the purpose of completing study measures. Separate MANCOVAs were conducted to explore the relation between treatment group and decisional outcomes at post-intervention for each medical decision, while controlling for numeracy and pre-intervention ratings of knowledge and decisional conflict. Results identified significant main effects of treatment group for each of the four medical decisions. Participants who viewed the video were more knowledgeable regarding CPR $F(1,59) = 42.844$, $p<.001$, medical interventions $F(1,59) = 20.475$, $p<.001$, and medically administered fluids $F(1,59) = 31.004$, $p<.001$, compared to participants assigned to the control group. Additionally, participants who viewed the video had less decisional conflict related to CPR $F(1,59) = 17.892$, $p<.001$, medical interventions $F(1,59) = 31.017$, $p<.001$, medically administered fluids $F(3,56) = 11.718$, $p=.001$, and nutrition $F(1,59) = 16.411$, $p<.001$, compared to participants in the control group. Conclusions and practical implications will be discussed.

### ASSOCIATION BETWEEN CAREGIVERS' AND CARE-RECIPIENTS' ADVANCE CARE PLANNING: AN EXPLORATORY STUDY

Hyunjin Noh, Lewis Lee, and Chorong Won, *The University of Alabama, Tuscaloosa, Alabama, United States*

Research on advance care planning (ACP) has highlighted major contributors to the completion of ACP documents. One of such contributors is knowledge about ACP, such as an advance directive or living will (LW). This study aims to 1) understand the initial exposure to ACP knowledge among informal caregivers' of chronically or seriously ill older adults and to 2) explore an association between caregivers' advance care planning and that of their care-recipients. Forty-four primary caregivers of cognitively impaired older adults were recruited at various community settings. A mixed-method design was used to qualitatively interview each participant face-to-face about his or her initial experience with ACP and to quantitatively ask if the participant completed a LW and if the care-recipient completed one as well. Qualitative content analysis of participant responses revealed that their initial experiences with ACP were mostly through their care-recipients, such as the care-recipient's ACP in previous hospitalizations or legal consultations. Chi-square test for independence was conducted to explore whether there is an association between caregivers' LW completion and that of care-recipients. The results show that there is a significant relationship between the two variables: $\chi 2$ (1, n = 44) = 8.84, $p < .001$, $\varphi = .49$. These findings suggest that secondary experiences with close one's ACP may serve as facilitator to one's ACP completion. Therefore, efforts to promote ACP should target a caregiver and care-recipient dyad so that caregivers as well as care-recipients may learn about and complete ACP documents.

### CHINESE AMERICANS' PRIORITIES AND PRACTICES FOR DESIGNATING A HEALTHCARE PROXY

Mandong Liu, and Iris Chi, *University of Southern California, Los Angeles, California, United States*

Many Americans need decision-making from someone else due to cognitive impairment. Chinese comprises the largest Asian group in the U.S.; few studies have investigated factors influencing Chinese Americans' health care proxy (HCP) designation. The aim of this study was to examine factors associated with Chinese Americans' HCP designation, using Andersen's Behavioral Model as a lens. Data were from the 2015 Asian American Quality of Life Survey. Hierarchical logistic regression analyses were conducted to test the incremental predictive power of predisposing, enabling, and needs factors. We found that age, marital status, religious affiliation, health insurance, acculturation, and self-rated health were predictors for HCP designation. The odds of having an HCP designation were 1.96 times higher for those aged 65 and above, compared to those aged 18-64 (p=0.045); The odds were 2.40 times higher for those who were married (p=0.006); The odds were 1.79 times higher for Protestants (p=0.042) and 2.25 times higher for Buddhists (p=0.025), compared to those with no religious affiliation; Having a health insurance increased the odds of having an HCP designation by 2.23 (p=0.022); For each additional unit in acculturation score, the odds of having an HCP designation increased by 0.65 (p=0.011); Compared to those who rated their health as excellent/very good/good, those rating health as fair/poor had a 2.97 times higher odds of HCP designation (p=0.001). We concluded that various factors influence Chinese Americans' HCP designation, and appropriate and innovative practices should be used to assist HCP discussion and designation among Chinese and other ethnic minority populations.

### CONVERSATIONS ABOUT DEATH AND DYING, END-OF-LIFE CARE PLANS AND PREFERENCES BETWEEN AGING PARENTS AND ADULT CHILDREN

Hyo Jung Lee, and Jacobbina Jin Wen Ng, *Nanyang Technological University, Singapore, Singapore*

This study aims to investigate whether attitude and perception on late-life death and dying, end-of-life care plans and preferences could be better understood from current values shared between aging parents and their adult children in the multi-cultural city-bound country, Singapore. We are in the process of interviewing 20 aging parent-adult child dyads. Up to date, six semi-structured interviews were completed and transcribed. We performed Content analysis to analyze the transcripts. Preliminary findings showed that both aging parents and adult children rarely discussed this issue, although parents had their own plans or preferences. The major barriers against open conversations about death and dying of aging parents include: the perception of not-yet time to talk about this issue (without knowing when the right time is) and tendency to have conversations about death in tandem with finances, but not death itself. Although specific end-of-life care plans or arrangements were not thought out thoroughly, aging parents expressed a high level of trust and reliance on close family members' decisions regarding their end-of-life care. They tended to agree on joint decision-making process within family, even though adult children had no or unmatched ideas about their aging parents' end-of-life wishes. This did not necessarily align with previous findings in Western countries, underscoring individuals' control over their own death and dying process. Open conversation within family, family-involved advance care planning, or joint decision-making processes may be warranted to promote quality of life and death in older Singaporeans and well-being of their family members of all ages.

### END-OF-LIFE DECISION MAKING IN CHRONIC LIFE-LIMITING DISEASE: A CONCEPT ANALYSIS AND CONCEPTUAL MODEL

Kristin Levoy,[1] Elise Tarbi,[1] and Joseph De Santis,[2] *1. University of Pennsylvania, Philadelphia, Pennsylvania, United States, 2. University of Miami, Coral Gables, Florida, United States*

Most deaths from chronic life-limiting diseases are preceded by end-of-life decisions, yet patients and caregivers are ill-equipped to make them. The lack of a common vocabulary surrounding end-of-life decision making and the paucity of conceptual models that explicate its components hamper improvements in clinical practice and research. Walker and Avant's method for concept analysis was used to investigate uses of "end-of-life decision making" in the literature in order to identify its components (antecedents, attributes, consequences), describe stakeholder roles (patients, family/caregivers, health care providers), and develop a conceptual model. An iterative search strategy resulted in 143 included sources. These encompassed 1,127,095 patients (primarily older adults), 3,384 family/caregivers, and 588 healthcare providers. Evidence revealed that end-of-life decision making is a process, not a discrete event. This begins with preparation (antecedents), which involves the designation of a decision maker and iterative patient, family/caregiver, and healthcare provider communication across the chronic illness. These preparatory processes inform end-of-life decisions, which possess three attributes: 1) serial choices in the terminal illness phase that are 2) weighed in terms of their potential outcomes 3) through patient and family/caregiver collaboration. The components impact patients' death experiences, caregivers' bereavement, and healthcare systems (outcomes). The resulting conceptual model highlights the larger context of preparation (beyond advance care planning) and the prominent role of caregivers in the end-of-life decision making process. Enhanced measurement must account for the dose, content, and quality of the preparation and decision components that collectively contribute to outcomes, which holds implications for practice improvements and research.

### ENGAGEMENT IN ADVANCE CARE PLANNING: THE ASSOCIATION WITH COGNITIVE IMPAIRMENT AND CARE PREFERENCES

Hyo Jung Lee,[1] and Giyeon Kim,[2] *1. Nanyang Technological University, Singapore, Singapore, 2. Chung-Ang University, Seoul, Republic of Korea*

Although there has been growing evidence that Advance care planning (ACP) benefits people with cognitive impairment nearing death, our understanding about this issue is still limited. This study examines whether cognitive impairment is associated with ACP engagement and end-of-life care preferences among older adults in the U.S. Using data from the 2012 National Health and Trends Study (n=1798, aged 65 to 101), we identified four levels of ACP engagement: None (28%), Informal ACP conversation only (12%), Formal ACP only (14%), and Both informal and formal ACP (46%). Older adults with None showed the highest prevalence of having cognitive impairment (17%), followed by those with Formal ACP only (15%) and the other two (6%, 6%). The results of Multinomial Logistic Regression showed that, compared to those without, respondents with cognitive impairment had 143% increased relative risk of having None (RR = 2.43, CI: 1.58-3.73) and 81% increased relative risk of completing Formal ACP only (RR = 1.81, CI: 1.11-2.95) relative to completing Both informal and formal ACP. In addition, respondents with None were more likely to prefer to receive all treatments available nearing death than those with any ACP engagement. Achieving high quality care at the end of life can be more challenging for older adults with cognitive impairment and their family caregivers due to the limited capacity. Although encouraged, informal ACP conversation with loved ones does not necessarily occur before the formal ACP, especially, for those with cognitive impairment. Therefore, they may merit more attention such as early ACP engagement.

### FACTORS PREDICTING SHORT LENGTH OF STAY IN HOSPICE PATIENTS WITH A PRIMARY DIAGNOSIS OF STROKE

Victoria Marino,[1] Maureen Templeman,[1] Ronald Schonwetter,[2] Debra Dobbs,[1] and William Haley,[1] *1. University of South Florida, Tampa, Florida, United States, 2. Chapters Health System, Tampa, Florida, United States*

Stroke is the second leading cause of death globally among people aged 60+, yet only 9% of hospice decedents have a primary diagnosis of stroke and little research has examined their end-of-life care experiences. Late referral and admission to hospice is an indicator of poor end of-life-care quality. This project identified factors predicting short stays in hospice, defined as a length of stay (LOS) of 14 days or less, via chart review of 100 hospice patients with a primary diagnosis of stroke. Of the 98 patients with complete data, 89% died in hospice; 11% were live discharges. Most patients were female, married, and referred to hospice from a hospital. Only 21% of patients entered hospice with a completed advance directive. Approximately 75% (n=73) of patients had a short LOS. Binary logistic regression indicated that gender, race, marital status, and having an advance directive at hospice admission were unassociated with LOS. Controlling for demographics, patients referred from home were 74% less likely to have a short LOS (OR=.26, CI=.08-.83) compared to those referred from a hospital, with a mean LOS of 44 and 13 days, respectively. Including Palliative Performance Scale (PPS) score in the model attenuated the effect of referral location. For every ten percent increase in PPS, participants were 85% less likely to have a short LOS (OR=.15, CI=.07-.32). Advance care planning should be more widely promoted among patients at high risk for stroke. There are opportunities for earlier referral to hospice for patients with a diagnosis of stroke.

### HOME HOSPICE VERSUS OTHER PLACES OF DEATH AND CAREGIVER STRESSES

Elizabeth Luth, and Holly Prigerson, *Weill Cornell Medical College, Larchmont, New York, United States*

As of 2017, more individuals in the U.S. die at home than in any other location. Hospice care was designed to provide support for people who are dying and their families. However, dying persons may have rapidly emerging needs that home hospice does not immediately meet, thereby, exposing family members to be “first responders.” Thus, home death may result in distress and burden for dying individuals’ family members, even when hospice is involved. This study uses multivariable regression analysis to explore the relationship between place of death and stressful end-of-life experiences in a sample of 185 patients with advanced cancer. We also analyze which end-of-life experiences are associated with death location. Compared to home hospice death, we found dying in a hospital was associated with fewer caregiver exposures to, and reports of fearfulness and helplessness in response to, stressful end-of-life events. Compared to home hospice death, hospital death was associated with decreased frequency of choking, falls, confusion/delirium, and feeling the patient has had enough. It was also associated with less fearfulness about choking and falls and less hopelessness about falls, the patient having enough, and thinking the patient was dead. Our results suggest home death with hospice care, often involves undesirable experiences that result in more caregiver fear and helplessness than dying in a hospital without hospice care. Research is needed to understand how to best support family members through stressful end-of-life experiences even when supported by home hospice services.

### HOW WILL I BE REMEMBERED? REFLECTION ON VIRTUES IN SELF-DEFINING MEMORIES ACROSS ADULTHOOD

Meghan McDarby,[1] Emily Mroz,[2] Susan Bluck,[2] and Brian Carpenter,[1] *1. Washington University in St. Louis, Saint Louis, Missouri, United States, 2. University of Florida, Gainesville, Florida, United States*

Reflection on memorialization may differentially influence nomination and narration of self-defining memories across the lifespan, including the extent to which positive character strengths (i.e., virtues) are represented. We investigated characteristics of self-defining memories across adulthood and in the context of being memorialized after death. Young, middle-age, and older adults were randomly assigned to narrate a memorialization-based self-defining memory (a memory to describe you after you are gone; n = 103) or current self-defining memory (a memory to describe your current self; n = 99). Participants rated qualities of their memory (e.g., personal significance) and the extent to which the memory represents them as virtuous (e.g., courageous, empathic, etc.). There were no age or condition differences in personal significance of the memory narrative (p = 0.43). However, there was an age-by-condition interaction for representations of virtue described in the self-defining memory, F(2, 199) = 3.94, p = 0.002. Young adults rated their self-defining memories as more virtuous in the memorialization condition than in the current self condition (p = 0.001).

Middle-age (p = 0.95) and older (p = 0.94) adults rated their self-defining memories as portraying similar levels of virtue across conditions. Unlike their middle-age and older counterparts, young adults report embodiment of virtue differently in unique contexts. Findings are discussed in the framework of how individuals' views of the self as virtuous change in relation to time lived and time left to live.

### LESSONS FROM WHAKAPAPA AND FILIAL PIETY: CAN SOCIAL WORK CAPITALIZE ON THE CONNECTION THAT SURVIVES DEATH?

Hong-Jae Park, *Western Sydney University, Penrith, Australia*

Ageing is part of life, and so is death. Although death will involve all of us over time, it is often regarded as a taboo topic, and bonds with the dead are seldom acknowledged in contemporary times. The paper presents selected insights on the connection that survives death, learned from a qualitative study on two indigenous knowledges—whakapapa (genealogical connections in Maori) and filial piety (respect/care for ancestors). Data were collected from interviews with 49 key informants (Maori=25; Korean=24) in 2018/19 in New Zealand and South Korea. The research findings indicate that the connectedness with ancestors or deceased loved ones is a significant part of the participants' mental and social lives. Māori (the first nation people of New Zealand) have established the unwritten convention of whakapapa as the core value that places whānau (family) at the centre of social relationships. In Korean culture, its filial piety/ancestor veneration tradition has emphasised the connection between deceased and living family members. Criticism about the traditions of whakapapa and filial piety was also raised by a few participants. The significance of this study is situated in the innovative perspective that the post-mortem relationship can be embodied, not only by the living who practise memorial respect for the dead, but also by those older people who establish after-life legacy before death. To help capitalise on this whakapapa connection, the so-called concept of "memorial social work" is presented as a potential area of social work practice, which has critical implications in the ageing/end-of-life related fields.

### MEANING MAKING AS A CENTRAL MECHANISM OF DIGNITY THERAPY FOR OLDER ADULTS WITH CANCER

Emily Mroz,[1] Carma Bylund-Lincoln,[1] Rachel Wisolmerski,[1] Diana Wilkie,[1] George Fitchett,[2] George Handzo,[3] Harvey Chochinov,[4] and Susan Bluck,[1]
*1. University of Florida, Gainesville, Florida, United States, 2. Rush University Medical Center, Chicago, Illinois, United States, 3. HealthCare Chaplaincy Network, New York, New York, United States, 4. University of Manitoba, Winnipeg, Manitoba, Canada*

Nearly 500,000 older Americans die a cancer-related death each year (National Vital Statistics Report, 2018). Following a diagnosis of a serious illness like cancer, maintaining a sense of dignity is central to a patient's wellbeing. Dignity Therapy (DT) was recently introduced as an intervention to enhance dignity for terminally ill patients (Chochinov et al., 2005). This therapy provides patients opportunities to foster a sense of dignity though making meaning of their lives (Hack et al., 2010). To date, whether meaning-making actually occurs as a central mechanism of effective DT has not been tested. The current study investigates (i) how often and in what forms meaning-making occurs during DT, and (ii) how patients' baseline feelings of dignity relate to meaning-making during DT. Participants were 25 male and female cancer outpatients (M age = 63.08; SD = 5.72). They completed the Patient Dignity Inventory (Chochinov et al., 2008) and then participated in Dignity Therapy with a trained provider. Sessions were audio recorded, transcribed, and reliably content-analyzed for meaning-making using an established coding scheme (Park & Folkman, 1997). Content-analysis revealed that all patients made meaning of past life events at least once (range: 1-12 occurrences). Multiple forms of meaning-making emerged, with Finding Benefit and Personal Growth most common. Patients reporting more dignity-related distress prior to DT showed greater meaning-making during the DT session ($r = .46$, $p < 0.05$). This study provides foundational evidence that meaning-making is a key mechanism of Dignity Therapy, helping older adults with cancer enhance dignity at end-of-life.

### RELIGIOSITY, COGNITION, AND ENGAGEMENT IN ADVANCE CARE PLANNING AMONG OLDER ADULTS

Richard Chunga, Jeffrey Stokes, and Beth Dugan,
*University of Massachusetts Boston, Boston, Massachusetts, United States*

Advance care planning (ACP) is an important process of discussion and documentation that may help older adults receive the end-of-life care they prefer. Although existing literature predominantly finds greater self-reported religiosity to decrease the rates of ACP, a clear consensus is not yet evident. Data from 3,182 adults aged 55 and older participating in the 2012 wave of the Health and Retirement Study were used to investigate this association and examine the moderating role of cognition. Participants reporting at least one of two ACP behaviors (written instructions and assigning a health care proxy) were categorized as formal planning only, engaging in only informal discussions was categorized as informal planning, and those who completed both or none were categorized accordingly. Cognition was measured using self-reported memory change over the last two years and with objective cognitive testing scores. Using multinomial logistic regression, three forms of ACP behaviors were regressed on a religiosity/spirituality scale, the two cognition measures, and demographic and psychosocial covariates. Greater religiosity was associated with a lower likelihood of engaging in both plans compared to none (OR=0.91, 95%CI=0.84-0.97), however this effect was no longer significant with the inclusion of race. Higher cognitive scores were associated with greater odds of engaging in informal-only (OR=1.07, 95%CI=1.04-1.10) and both plans (OR=1.04, 95%CI=1.01-1.06); subjective memory change was not associated with ACP. Neither cognitive measure significantly moderated the negative association of religiosity on ACP, suggesting that the awareness of worsening memory does not undermine the tendency to avoid planning among the highly religious.

### SYSTEMATIC REVIEW OF GRIEF INTERVENTIONS

Nicole Ricken, Alice Kim, Mona Khaled, Geoff Corner, and Christopher Beam, *University of Southern California, Los Angeles, California, United States*

Grief interventions address pain and suffering in response to the death of a significant other. Evidence-based grief psychotherapies treat normative grief to symptoms of persistent complex bereavement disorder, the latter of which is characterized by difficulty accepting the loss and persistent yearning for the decedent. We reviewed published randomized controlled trials (RCT) of grief-focused psychotherapies to test two hypotheses. First, participants receiving grief-focused psychotherapies should demonstrate decline of grief symptoms from pre- to post-intervention. Second, participants receiving grief-focused psychotherapies should demonstrate lower grief symptoms post-intervention than participants receiving control treatments. Published studies were identified using academic search engines (Web of Science, PsycInfo, and Google Scholar) and by reviewing reference sections of published RCTs. Twelve published RCTs were identified. Effect sizes (Hodges' g) and confidence intervals were calculated. Results support our first hypothesis and partially support our second hypothesis. Grief-related symptoms declined from pre- to post-intervention (g ranged from -0.39 to -2.51), with all studies reporting statistically significant effects. When comparing post-intervention differences in grief-focused psychotherapies versus control groups, effects were more variable (g ranged from -2.40 to 3.02), with seven studies demonstrating greater improvement among grief intervention recipients than control treatment recipients. Grief interventions appear to be effective for reducing grief-related symptoms pre- to post-intervention. However, they were only more effective than control treatments in just over half of published RCTs. While grief interventions were more effective, point estimate ranges are wide, suggesting that treatment effectiveness probably depends on factors other than the treatments themselves.

### THE PSYCHOSOCIAL AND SPIRITUAL COMPONENTS OF A GOOD DEATH

Quentin Maynard,[1] and Ellen Csikai,[2] *1. University of Southern Indiana, Newburgh, Indiana, United States, 2. University of Alabama, Tuscaloosa, Alabama, United States*

Advances in medical care led to more older adults living longer, with at least one chronic illness. Correspondingly, the disease trajectory and dying process may be prolonged, providing more time to plan for a 'good death.' A 'good death' is typically described in the literature according to medical and biological aspects, with limited explicit discussion of psychosocial and spiritual elements. The purpose of this study was to explore older adults' perceptions of psychosocial and spiritual factors that contribute to a death they would consider 'good'. Using descriptive qualitative research methods, 12 community-dwelling older adults in central Alabama participated in two in-depth interviews. The findings suggested a range of physical, psychological, social, and spiritual components contributed to the conception of a 'good death' and were often interrelated. For example, the medical treatment desired at end of life was influenced by the perceived effects on the participants' family/social network. Reflecting on previous experiences with death, along with motivations and values, guided what participants believed could help achieve their 'good death.' Most realized that preparation would allow for control over their experiences at end of life. The study findings emphasized the need to consider the holistic nature of advanced planning for the end of life according to what older adults and their families perceive as important to a 'good death'. Efforts by professionals to maximize quality of life throughout serious illness and near the end of life are essential and can be accomplished through understanding what a 'good death' means to older adults.

### THE RELATIONSHIP BETWEEN SUBJECTIVE AGE, HEALTH STATUS, AND ADVANCE CARE PLANNING

Ilana Engel, and Tamara Baker, *University of Kansas, Lawrence, Kansas, United States*

Current data show that how old a person feels, or subjective age (SA), may be associated with improved well-being and functioning, less cognitive impairment, and longer life. Yet, the relationship between SA and advance care planning (ACP) has yet to be examined. This is all the more important when determining the role SA has in end-of-life decision-making. Using data from the Health and Retirement Study (Wave 13, 2016), this study aimed to examine whether SA is associated with having a living will (LW), having a LW with life-limiting care, and having assigned a durable power of attorney. The sample included 3,165 participants 51+ years of age (mean age = 65.67; SD = 11.79). Analyses were conducted assessing the predictive value identified social, behavioral, and health factors have for ACP. Results from binomial logistic regression analyses indicated that participants who endorsed feeling older than their chronological age were similarly likely to have engaged in ACP as those who felt younger. As demonstrated previously, older chronological age was significantly associated with higher utilization of ACP (ps < .05). Those with cancer and women were significantly more likely to have a LW (ps <.05). The present study did not find evidence to support a significant relationship between SA and ACP. Lower overall engagement with ACP and treatment for specific conditions, such as cancer, may be more influential in determining who utilizes ACP. Future research should explore how SA may serve as a protective factor and/or a psychological mechanism that influences engagement in ACP.

### WHAT DO OLDER ADULTS CONSIDER A GOOD DEATH?

Ellen Csikai,[1] and Quentin Maynard,[2] *1. University of Alabama, Tuscaloosa, Alabama, United States, 2. University of Southern Indiana, Newburgh, Indiana, United States*

Without improved understanding and communication about what constitutes a 'good death', an extended dying process that does not attend to how serious illness affects older adults' quality of life near the end of life is likely (IOM, 2014). The primary aim of this study was to understand the concept of a 'good death' as defined by older adults. A scoping literature review of qualitative and quantitative research studies (published in peer-review journals since 2000) involving only community-dwelling older adults was conducted, using key search words: good death, dying well, older adults/elderly/seniors, gerontology/geriatrics and Boolean operators in

Abstracts in Social Gerontology, CINAHL Plus, PubMed, Academic Search Premier, PsychINFO. A total of 344 articles met the search criteria. Following initial systematic screening of titles/abstracts and full text review, five articles remained that met inclusionary criteria. Therefore, this analysis is based on a total of five articles. Each study was qualitative, with small samples and diverse populations of older adults. Among the factors perceived important, studies' shared results were dying in sleep/natural death/peaceful; family connection (e.g. time for closure, not being a burden) and faith/religiousness (e.g. getting 'right' with God, living with faith). Conceptions of a 'good death' often reflect primarily psychosocial/quality of life aspects, in addition to medical concerns. Health professionals need to know how a good death is defined by each individual in order to ensure holistic care and quality of life at the end of life according to older adults' expressed wishes.

### WHAT PREDICTS ADVANCE CARE PLANNING AMONG OLDER ADULTS: PERCEIVED HEALTH OR THE NUMBER OF CHRONIC CONDITIONS?

YuHsuan (Olivia) Wang, Susan Enguidanos, and Olivia Wang, *University of Southern California, Los Angeles, California, United States*

Advance care planning (ACP) is associated with improved quality of death and better end-of-life care. Studies have found that both health status and chronic illness influence rates of ACP. However, little is known about the relative association of each factor with engaging in ACP. This study aims to identify the extent to which the number of chronic conditions and self-rated health predict engaging in ACP. We used data from the Health and Retirement Study, a nationally-representative longitudinal survey of older adults. The sample consisted of 2016 core interview respondents. We conducted logistic regression models to examine the association between self-rated health and the number of self-reported chronic conditions with three dependent variables: (1) ACP engagement (n=687), (2) AD completion (n=1671), and (3) assignment of health care proxy (n=1668), while controlling for demographic characteristics,. Samples were weighted. Analysis revealed that reporting more chronic conditions was associated with higher odds of advance directive completion (OR:1.21, $p<.001$), ACP engagement (OR: 1.26, $p<.05$), and assigning medical proxies (OR: 1.32, $p<.001$). However, better self-reported health was associated with higher odds of having an AD (OR: 1.20, $p<.05$) and assigning medical proxies (OR: 1.27, $p<.01$). These findings suggest that greater number of chronic conditions increased the odds of having an AD, engaging in ACP and in assigning medical proxies; however, those reporting better health were more likely to have an AD and a medical proxy. Findings from this study point suggest that individuals with multimorbidities may be more open to engaging in ACP discussions and activities.

### WHAT WOULD PEOPLE THINK? SOCIAL NORMS, WILLINGNESS TO SERVE AS A SURROGATE, AND END-OF-LIFE TREATMENT DECISIONS

Rachael Spalding, JoNell Strough, and Barry Edelstein, *West Virginia University, Morgantown, West Virginia, United States*

Population aging has increased the prevalence of surrogate decision making in healthcare settings. However, little is known about factors contributing to the decision to become a surrogate and the surrogate medical decision-making process in general. We investigated how intrapersonal and social-contextual factors predicted two components of the surrogate decision-making process: individuals' willingness to serve as a surrogate and their tendency to select various end-of-life treatments, including mechanical ventilation and palliative care options. An online sample (N=172) of adults made hypothetical surrogate decisions about end-of-life treatments on behalf of an imagined individual of their choice, such as a parent or spouse. Using self-report measures, we investigated key correlates of willingness to serve as surrogate (e.g., decision-making confidence, willingness to collaborate with healthcare providers), and choice of end-of-life treatments. Viewing service as a surrogate as a more typical practice in healthcare was associated with greater willingness to serve. Greater decision-making confidence, greater willingness to collaborate with patients' physicians, and viewing intensive, life-sustaining end-of-life treatments (e.g., mechanical ventilation) as more widely accepted were associated with choosing more intensive end-of-life treatments. The current study's consideration of both intrapersonal and social-contextual factors advances knowledge of two key aspects of surrogate decision making—the initial decision to serve as surrogate, and the surrogate's selection of various end-of-life treatment interventions. Providers can use information about the role of these factors to engage with surrogates in a manner that better facilitates their decision making.

### WHO PREPARES MORE FOR END OF LIFE? THE FACTORS ASSOCIATED WITH END-OF-LIFE PLANNING AMONG OLDER ADULTS

Hayoung Park, Hey Jung Jun, Susanna Joo, and Sun Ah Lee, *Yonsei University, Seoul, Republic of Korea*

Being an "aged" society, only recently in Korea have there been growing interests in well-dying and end-of-life planning. Therefore, the purpose of this study was to investigate the factors associated with the level of end-of-life planning among Korean older adults. For the analyses, 2017 National Survey of Older Koreans was utilized and the sample was 10,073 older adults aged 65 and above. The independent variables were age, gender, employment, education level, marital status, household income, religion, number of children, and life satisfaction. The dependent variable, the level of end-of-life planning, was measured as the sum of five items of the question asking whether they have prepared the following items in preparation for end-of-life: shroud (8.3% prepared), cemetery/charnel house (25.1% prepared), mutual aid society (13.7% subscribed), will (0.5% prepared), lectures in preparation for end-of-life (0.4% participated). The results from multiple regressions using STATA 15.0 indicated that the level of end-of-life planning was significantly associated with age ($b=.02$, $p<.001$), gender ($b=-.07$, $p<.001$), education level ($b=.02$, $p<.01$), household income ($b=2.56$, $p<.001$), religion ($b=.06$, $p<.001$), number of children ($b=.04$, $p<.001$), and life satisfaction ($b=.17$, $p<.001$). That is, they have planned more for their end-of-life when older in age, female, more educated, wealthier, with religion, had more children, and more satisfied with life. The findings from this study suggest that political interventions are required to promote end-of-life planning education especially for less educated older adults with low income for their "well-dying". Further researches are needed to explore other personal/interpersonal factors associated with end-of-life planning.

### SESSION 2959 (POSTER)

### SUCCESSFUL AGING

### CONCEPT ANALYSIS OF AGING WITH DISABILITY IN ADULTS

Junha Park, *University of Wisconsin-Madison, Madison, United States*

People with long-standing physical disabilities are living longer than at any time in history, owing to advancements in medical science, rehabilitation, and social systems. Approximately 12–15 million people in the U.S. are aging with long-standing disabilities that were acquired before age 40 years. This population is forecast to grow, resulting in a subset within the aging community. More research is needed to increase understanding of successful aging with disabilities, the pathways of aging with disability, and unique characteristics of the aging process. The purpose was to define and clarify the conceptual meaning of aging with disability, identify its attributes, antecedents, and consequences, and explore the significance and implications of the concept in nursing. Rodgers' evolutionary method of concept analysis was used. Three databases, CINAHL, PubMed, and PsycINFO were used to retrieve literature. Inclusion criteria were publication year from 2001 to 2019, peer-reviewed academic journals, and English-language. Exclusion criteria included other than physical disability such as developmental, learning, or intellectual disability, focus on the aging process of people without disability, the main topic on the perspectives of caregivers or health care providers. Thirty-five articles met inclusion criteria. The analysis identified three antecedents (e.g., contributing barriers), three attributes (e.g., premature aging), and three consequences (e.g., accommodation needs). An understanding of the antecedents, attributes, and consequences of aging with disabilities will enhance quality of care including effective prevention and communication. Findings will guide researchers in developing a framework or theory to increase understanding of aging in individuals who acquired disability early in life.

### FROM THEIR PERSPECTIVE: BABY BOOMERS' HEALTH INFORMATION SEARCHING BEHAVIORS

Tammy Walkner, *University of Iowa, Solon, Iowa, United States*

People frequently turn to the Internet for health information, especially as they get older. But searches and results can be influenced by many factors including the comfort level for using digital technology, input from social connections, and personal knowledge. The Baby Boom generation is changing the age structure of the U.S. toward an older age demographic. Despite the significance of this cohort, little is known about how they apply health information to manage personal health. This study focused on Baby Boomers to increase knowledge about their health information searching behavior, the role social support plays in that behavior, and to better understand their perceptions of healthy aging. Social cognitive theory (Bandura, 2004) was the framework to investigate micro-level factors that may influence Baby Boomers' decisions about health information and whether they apply that information for healthy aging. Findings confirm Baby Boomers use online health information to advocate for specific treatments and more appropriate medications, to change their diet and exercise routines as well as to maintain their quality of life as they age. They also routinely rely on social connections when gathering information and implementing changes. Knowledge about Baby Boomers' health information seeking behaviors has broad implications for health care professionals, web page designers, policy makers, and others interested in promoting healthy aging. Insight into older adults' perceptions about using newer digital devices and their perseverance to learn new procedures like accessing electronic health records may spark changes in the communication between health care providers and patients of all abilities.

### GENERATIVITY AMONG JAPANESE ELDERLY: ITS ASSOCIATION WITH DEMOGRAPHIC CHARACTERISTICS AND HEALTH INDICATORS

Sachiko Murayama, Erika Kobayashi, Masataka Kuraoka, Kumiko Nonaka, Motoki Tanaka, Yuta Nemoto, Hiroshi Murayama, and Yoshinori Fujiwara, *Tokyo Metropolitan Institute of Gerontology, Tokyo, Japan*

Generativity is defined as concern and activity dedicated to contributing to the welfare of others, especially younger generations. Although generativity is postulated to be an important developmental task in old age, there are few reports of its related factors in Asian countries. The purpose of our study is to examine the gender difference of generativity and to explore the defining factors among Japanese elderly. During August to September 2016, we conducted a questionnaire survey for randomly selected 1,187 people aged 65–84 years in the Tokyo area (527 males, 660 females, mean age 72.6 years±5.5), and measured the following variables: generativity, gender, age, length of residence, parental status (having children or grandchildren), working status, commitment to child-rearing activities, mental health (WHO-5 scores), and Instrumental Activities of Daily Living (IADLs). As a result of Student's t-test, we found that males scored significantly higher on generativity than females ($t=2.678$, $df=1067.097$, $p<.01$). Moreover, we carried out multiple regression analysis, separated by gender. The results showed that, only among males, generativity was positively related to age ($\beta=.096$, $p<.05$) and having children ($\beta=.148$, $p<.001$). In addition, we found that generativity was positively associated with the following variables in both genders: having jobs, commitment to child-rearing activities, mental health, and IADLs ($\beta=.081–.318$, $p=.000–.032$). Among them, mental health and IADLs especially had strong effects on generativity ($\beta=.188–.318$, $p<.001$). We have concluded that the related factors of generativity differ between males and females, but regardless of gender, health indicators are strongly associated with generativity in old age.

### MEANING OF AGING, GEROTRANSCENDENCE, AND SUCCESSFUL AGING IN OLDER ADULTS WITH CHRONIC HEALTH CONDITIONS

Meredith Troutman-Jordan, *University of North Carolina, Charlotte, North Carolina, United States*

Aging presents change in the form of opportunities and challenges, from common physical alterations, to major life events. Perception of such events is greatly shaped by one's mental health, and is a major influence on gerotranscendence, a positive kind of aging involving redefinition of self, relationships, and proposed to be a precursor to successful aging. A mixed-methods cross-sectional descriptive design was used to study 50 older adults. Life Events Checklist, Gerotranscendence Scale, Herth Hope Index, and Successful Aging Inventory were administered. Mean participant age was 70.78 years; there were 9 males (18%), 41 females (82%), 13 were Black (26%), and 37 were White (74%). Participants reported a number of stressful events, most frequently transportation accidents, followed by other very stressful events or experiences, and sudden unexpected death of someone close. Gerotranscendence scores ranged from 0-10 (μ 6.88, a moderate score). Successful aging scores ranged from 40-79 (μ 62.33, a moderate score). A sub-sample of 6 participants engaged in semi-structured interviews, which were transcribed verbatim and subject to content analysis. Faith, displaced longing, temporal anticipation, proactive problem-solving/coping, and concern for future generations were emergent qualitative themes. Findings highlight opportunities for providers from multiple disciplines to target risks and possibilities for aging successfully and to promote hope, optimism, problem-solving skills, and gerotranscendence in all older adults, regardless of physical or functional health status.

### PERSPECTIVES OF PRODUCTIVE AGING: HOW OCCUPATIONS AFFECT HEALTH AND WELL-BEING OF OLDER ADULTS IN RETIREMENT

Marilyn Cole,[1] and Karen C Macdonald,[2] *1. Quinnipiac University, Stratford, Connecticut, United States, 2. Sacred Heart University, Bridgeport, Connecticut, United States*

A predicted surge in the aging population presents a major challenge for public health in the USA. As occupational therapy researchers, we wondered how continued engagement in productive occupations affected the health and well-being of older Americans. Today's retirees already understand the basics of successful aging, such as maintaining physical and mental fitness, and continuing an active lifestyle. Productive aging represents the next step: choosing roles and occupations that keep them engaged with others and their communities. As suggested by Clark (Jackson, Carlson, et al., 2012), occupations have the potential to provide a protective barrier to declining health, thereby lowering health care costs for the older population. Our Productive Aging qualitative studies (1 & 2) explore the lived experiences of life transitions, challenges, and adaptive skills implemented by participants (ages 65-80) in their own productive occupations. The findings of this follow-up study confirm and enhance themes of our first study (Cole & Macdonald, 2015): 1) prominence of the self-manager role, 2) intentionally maintaining social connections, and 3) engaging in self-fulfilling activities. Additional findings reflect current technology effects and elaborate how participants have met health challenges by adapting activities and environments (self-management), and using social resources (connections) to help them remain engaged in the occupations that give their lives meaning. Cole, M., & Macdonald, K. (2015). Productive aging: An occupational perspective. Thorofare, NJ: Slack, Inc. Clark, F., Jackson, Carlson, et al. (2012). Effectiveness of Lifestyle Intervention in promoting well-being of independently living older adults. Journal of Epidemiology and Community health.

### RESILIENCE IN YOUNG AND OLDER ADULTS: EUDAIMONIC WELL-BEING DESPITE LIFE CHALLENGES

Shubam Sharma,[1] Susan Bluck,[1] and Hsiao-Wen Liao,[2]
*1. University of Florida, Gainesville, Florida, United States,*
*2. Stanford University, Palo Alto, California, United States*

Although the types of events that occur across life phases may vary, experiencing multiple life challenges in a short timeframe can disrupt mental health (Zepinic, 2016). Maintaining self-continuity (i.e., sense of being the same person over time) when experiencing challenges may, however, foster resilience (Masten, 2001). This study investigated, in both young and older adults, whether: (1) experiencing multiple recent negative life challenges relates to lower current eudaimonic wellbeing, and (2) self-continuity acts as an internal resource for resilience. That is, whether having greater self-continuity ameliorates the negative association between frequent life challenges and well-being. Participants (N = 99 young, 87 older adults) reported all challenging events experienced in the last six years (Sarason et al., 1978). They also completed measures of eudaimonic Well-being (Ryff, 1989) and Sense of Self-continuity (past six years; e.g., Habermas & Köber, 2015). Older adults reported higher eudaimonic well-being than young adults ($p < .001$). Young adults reported experiencing more challenges ($p < .001$). Regardless, for both age groups, more frequently experiencing life challenges was associated with lower eudaimonic well-being ($p < .001$). Self-continuity mediated the association between more frequent experience of challenge and lower eudaimonic well-being: $\beta = .258$, SE = .061, 95% CI [.138, .378] ($p < .001$). Classic risk models focus on the negative effects of life's inevitable challenges. Our findings, grounded in a resilient aging framework, support the idea that individuals of any age can use internal resources (i.e., strong sense of self-continuity) to maintain or re-establish well-being.

### SELF-EFFICACY AS A MEDIATOR BETWEEN LIFESTYLE AND SELF-PERCEPTIONS OF AGING

Andrew Steward, and Leslie Hasche, *University of Denver, Denver, Colorado, United States*

A growing body of literature demonstrates the public health impacts of negative self-perceptions of aging (SPA). However, minimal research has explored mediating mechanisms as well as which lifestyle activities may influence SPA. Based on theory and prior research in successful aging, this study explored the impact of lifestyle activities on SPA and tested self-efficacy as a mediator between lifestyle activities and SPA. This study analyzed cross-sectional data from the psychosocial module in the 2018 wave of the Health and Retirement study (N = 2,675; mean age = 65.67/ SD = 9.95).

Eight SPA items (e.g., "Things keep getting worse as I get older") were derived from the Attitudes Toward Own Aging subscale of the Philadelphia Geriatric Center Morale Scale. 10 items measure self-efficacy on a 6-point Likert-type scale from strongly disagree to strongly agree. 21 lifestyle activities covering physical, intellectual, social, and spiritual domains were dichotomized into whether respondents participate at least monthly. Multiple regression analyses were utilized. Results indicate that self-efficacy explained 23% of the variance in SPA while holding constant age, gender, and functional ability. Sobel's test showed that self-efficacy mediated the relationship between lifestyle activities and SPA. Exercise explained the most variance in SPA, followed by computer use and volunteering. Building upon successful aging literature, this study demonstrates the impact of self-efficacy and helps distinguish which lifestyle activities may be most effective in improving SPA. In addition to individual-level lifestyle activities, the impact of structural interventions on SPA should be tested in future research.

### SUBJECTIVE SUCCESSFUL AGING: FACTORS RELATED TO A SELF-RATED PERCEPTION

Marlene Alvarado Rodriguez, and
Neyda Ma. Mendoza- Ruvalcaba, *University of Guadalajara, Guadalajara, Mexico*

Introduction Theoretical successful aging definitions may not consider the older adults perception´s on their own aging. The aim of this study is to analyze factors related to subjective perception of successful aging. Methods: Population based, random sample included n=401 community-dwelling older adults 60-years and older (mean age=72.51,SD=8.11 years,59.4% women). For measurement of subjective successful aging (S-SA), participants were asked to self-rate SA in a Likert scale: "do you believe/feel you are aging well?" Objective Successful aging (O-SA) was operationalized in accordance with Rowe & Kahn definition (no important disease, no disability, physical functioning, cognitive functioning, and being actively engaged). Sociodemographic and health data were also asked. Data were analyzed in SPSSv24. Results: In total 11% were successful agers according to objective measures, while 77.6% rated themselves as successful agers. In the Likert scale of S-SA, specifically 23.4% considered themselves as very much successful agers, 54.1% much, 20.2% somewhat, and only 2.2% said that not at all think they are aging successfully. Education was related to a better perception of S-SA, as well as life satisfaction (p=.000), better subjective health (p=.000), being a spiritual person (p=.000), and not feeling alone (p=.000). Age, marital status, sex, and life-long learning activities were not related to S-SA. Conclusion There is a disparity between subjective and objective successful aging rates Being a successful ager may have a different meaning for each person, and not necessarily involves established criteria. Criteria generated by older adults should be added in theoretical definitions and measures.

### SUCCESSFUL AGING IN THE NORTH: LESSONS LEARNED, LESSONS SHARED, AND ADVICE FROM THOSE AGING IN A GOOD WAY IN ALASKA

Jordan Lewis,[1] and Steffi Kim,[2] *1. University of Minnesota Medical School, Duluth, Minnesota, United States, 2. University of Alaska Anchorage, Anchorage, Alaska, United States*

This presentation presents and builds upon the Alaska Native Successful Aging model. Elders' conceptions of successful aging differ from the Western model, how rural and urban environments influence successful aging, and the lessons Elders pass down to ensure a healthy lifestyle are not common in the literature. 42 Alaska Native Elders from the Norton-Sound sub region of Alaska, 21 Alaska Native Elders from the Aleutian Pribilof Islands, 26 Elders from the Bristol Bay region, and 12 Elders residing in Anchorage, AK to provide an urban context of successful aging. A community-based, exploratory, qualitative research methodology was used to allow for co-learning between research team and tribal communities. 101 qualitative interviews were conducted, and team-based thematic analysis was used to establish codes and main themes to expand the Alaska Native Successful Aging model. We learned that emotional well-being, community engagement, spirituality, family and purposeful engagement, and physical health were instrumental in promoting successful aging within Norton Sound and Bristol Bay. These findings expanded Lewis's model to include gerotranscendence and generativity; Elders intentional relationships and stronger connections to traditional cultural and spiritual activities gave life deeper meaning and value. These findings argue the importance of including and emphasizing social components, historical perspective, and the importance of place, as well as generativity and gerotranscendence in program and policy development.

### SUCCESSFUL AGING: A COMPREHENSIVE OUTCOME USING LATENT PROFILE ANALYSIS

Kasey Longley, and Joseph Grzywacz, *Florida State University, Tallahassee, Florida, United States*

Understanding "successful aging" is a primary goal of gerontology and adult development researchers that has been motivated by rapid the increases in life expectancy over the last 100 years. Successful aging, as it is understood by Rowe and Kahn, is conceptualized multidimensionally in terms of limited disease and disability, high physical, mental and cognitive functioning, and active engagement with life. "Success" in all three domains reflects the idealized manifestation of "successful aging." Nevertheless, research on successful aging typically focuses on only one or two aspects of the model – most commonly physical disease or disability. The overall goal of this research is to advance understanding and subsequent attempts to promote holistic successful aging. Specifically, using key metrics from each domain of successful aging from the Midlife Development in the United States (MIDUS), this study characterizes distinct profiles of successful aging, and it describes the distribution of these profiles in the adult population. Results indicate 3 profiles. These are labeled as Successfully Aged, Somewhat Successfully Aged, and Least Successfully Aged. Approximately 82.1% of the population (mean age=50.5) is classified as Successfully Aged, whereas the remainder are classified in the Somewhat Successfully Aged (12.2%), and Least Successfully Aged (5.6%), respectively. As expected, those who were classified as Successfully Aged had the highest cognitive scores, sense of well-being, and self-rated health; and had the lowest number of age-related physical disabilities (i.e. cancer, stroke, osteoporosis, etc.) and mental health conditions (depression, anxiety disorder, and panic disorder). This outcome can be applied to multiple predictors.

### THE RELATIONSHIP BETWEEN ACTIVITY PARTICIPATION AND COGNITIVE FUNCTIONING

Sangha Jeon, and Susan Charles, *University of California-Irvine, Irvine, California, United States*

Researchers are growing increasingly interested in how the diversity of daily activities are related to well-being. The current study examined how both frequency and diversity in daily activities are associated with cognitive functioning. Participants from the third wave (2013-2016) of the Midlife Development in the U.S (MIDUS) survey (N=1281) completed both a telephone-based cognitive assessment and a mailed survey asking about participation in three different types of activities: cognitive (e.g., doing word games, attending educational lectures or courses), physical (e.g,. exercise, home chores), and social (volunteer work, attending sports or social groups). Frequency of activity participation was assessed with items asking how often they engaged in these activities, and diversity of activity participation was calculated by summing the number of activities they reported doing in each category. All analyses included sociodemographic variables, health status, and openness to experience as covariates. Findings from multiple regression indicated that greater frequency in all activities (cognitive, physical, and social) was related to higher levels of cognitive functioning. Greater diversity of social activity was also related to higher cognitive functioning. Education mediated the association between diversity in cognitive activities and cognitive functioning, suggesting that the link between higher levels of cognitive functioning and education may be partly attributed to people with higher levels of education engaging in greater diversity of cognitive activity.

### SESSION 2960 (POSTER)

### SLEEP AND AGING

### BETTER NIGHT'S SLEEP AND SUBJECTIVE COGNITION: THE ROLE OF DAY AND NIGHT WORK SHIFTS

Britney Veal, Christina Mu, and Soomi Lee, *University of South Florida, Tampa, Florida, United States*

Previous research indicates poor sleep and cognitive functioning are associated. Studies have yet to consider the role of work shift on this relationship. The current study examined the sleep and subjective cognition relationship in nurses, and if this relationship differed for day- and night- shift nurses. Sixty-one nurses (M=35.39, SD=11.73; 39 day-, 22 night-shift) reported their nightly sleep characteristics and next-day subjective cognition (i.e., processing speed, memory, and mental focus) using ecological momentary assessments for 2 weeks. Multilevel models controlled for sociodemographic characteristics and decomposed the variance attributed by between- and within-person levels. At the within-person level, better sleep the previous night was associated with better subjective cognition the following day. This relationship was more apparent in night-shift nurses than in day-shift nurses, such that (a) longer sleep duration predicted better mental focus (B=1.62, p<.05) and (b) higher sleep quality predicted better memory (B=8.67, p<.001). At the between-person level, better sleep overall was associated with better subjective cognition across days. This association was more apparent in day-shift nurses than in night-shift nurses, such that (a) better sleep quality and sufficiency predicted faster processing speed (B=34.33; B=26.28; p<.001) and (b) better sleep quality and greater sleep sufficiency predicted better memory (B=30.94; B=23.09; p<.001). Findings suggest that sleep characteristics are associated with subjective cognition in nurses day-to-day and on average. Specific sleep characteristics associated with subjective cognition differ between day- and night-shift nurses, presumably due to differences in their sleep issues and perceived cognitive abilities.

### BINGE DRINKING, DEPRESSIVE SYMPTOMS, AND SLEEP HEALTH IN MIDDLE-AGED AND OLDER ADULTS

Rebecca Lorenz, Samantha Auerbach, and Yu-Ping Chang, *University at Buffalo, Buffalo, New York, United States*

Unhealthy alcohol consumption such as binge drinking and depression are common problems among adults. The combined effect of binge drinking and depression might contribute to negative health outcomes, such as accidents, addiction, or sleep problems. Previous evidence has indicated that alcohol consumption differs by age. However, little is known about the association between binge drinking, depression, and sleep health, and how age might play a role in this association. This study aimed to examine the association between binge drinking, depressive symptoms, and sleep health in middle-aged and older adults and characterize any age differences. A total of 5191 middle-aged and older adults from the 2014 Core Survey of the Health and Retirement Survey (HRS) data aged 50 to 80 were included for this study. Binge drinking was defined as the consumption of 5 or more drinks (men) and 4 or more drinks (women) per drinking day. Depressive symptoms were measured using a validated 8-item Center for Epidemiologic Studies Depression Scale. Sleep health was assessed using a composite measure. Age was grouped into middle-aged (50-64.9 years) and older (65-79.9 years) adults. Multiple linear regression analysis was used to examine the associations between variables of interest. Our findings indicated that binge drinking and depressive symptoms negatively influenced sleep health among middle-aged adults, however this relationship was not found in older adults. Clinicians should simultaneously assess problematic alcohol consumption, depressive symptoms, and sleep health. Future research can develop and test age-specific interventions to reduce unhealthy drinking behaviors in middle-aged adults.

### DAILY ASSOCIATION BETWEEN SLEEP AND STRESSORS: ROLE OF CAREGIVING AT WORK AND HOME

Taylor Harris, Taylor Vigoureux, and Soomi Lee, *University of South Florida, Tampa, Florida, United States*

Previous research shows that adults with children experience poor sleep. We know that poor sleep is associated with experiencing more frequent and severe stressors (i.e, subjective feelings of believing his/her life is uncontrollable, unpredictable and overloading) the following day. This study examined whether the sleep—stressor relationship is stronger for individuals with children than those without. Participants were 61 oncology nurses (92% female). Participants completed a background survey that assessed sociodemographic and work characteristics. Using 14 days of ecological momentary assessments, participants reported their sleep characteristics daily upon waking. Three times daily, they also reported whether they experienced any stressors and how severe those stressors were. Multilevel modeling was used to assess whether the sleep—stressor relationship was stronger in nurses with children than those without. After controlling for sociodemographic covariates, poorer sleep quality was associated with more severe stressors. This daily association was moderated by the presence of children (B=-16.89, p<.01); the association was apparent for individuals with children (B=-5.74, p<.05), but not for those without. The daily association for sleep quality and stressor frequency also differed by the presence of children (B=-0.22, p<.01), although the slope for individuals without children did not reach the statistical significance. These findings suggest that individuals with children are at risk for experiencing a stronger linkage between poorer sleep and greater stressor severity. Improving sleep health among adults with children is critical for stress management. Future studies should examine whether age of children or number of children further influences the sleep—stressor relationship.

### DAILY ASSOCIATION BETWEEN SLEEP AND STRESSORS: ROLE OF PERSONALITY TRAITS

Angelina Venetto, Taylor Vigoureux, and Soomi Lee, *University of South Florida, Tampa, Florida, United States*

Poor sleep is associated with more stress across adult populations. The sleep—stress relationship is particularly important in nurses who are vulnerable to daily work-related stressors and poor sleep. Nurses with certain personality traits may be more vulnerable, however, the role that personality plays in the sleep-stress relationship has not previously been examined with lack of research in nurses. We examined how personality moderated the association between sleep characteristics and the perception of daily stressors in nurses. Participants were 61 oncology nurses who responded to a background survey that included a personality measure and completed 14 days of ecological momentary assessments. Each morning, participants reported sleep characteristics (i.e., perceived sleep sufficiency, sleep duration). Three times daily, participants reported their stressor experiences. We used multilevel models adjusting for sociodemographic characteristics, work shift, and work day. Results showed that on average across 2 weeks, participants with higher sleep sufficiency (β=-21.06, p<.05) and longer sleep duration (β=-11.80, p<.05) reported lower stressor severity. Agreeableness moderated the sleep duration—stressor severity association (β=25.07, p<.01), such that longer sleep duration was associated with lower stressor severity for participants with lower agreeableness (β =-17.39, p<.01), but not those with higher agreeableness (β=5.66, p>.05). These findings indicate that the protective nature of longer sleep duration on stressful experiences may not occur in nurses high in agreeableness. Nurses high in agreeableness may take on more responsibilities, exposing themselves to more daily stress. Thus, nurses who are high in agreeableness may be a good target population for stress-reduction interventions.

### DAILY RELATIONSHIPS BETWEEN PHYSICAL ACTIVITY AND SLEEP: DIFFERENCES BETWEEN SUBJECTIVE AND OBJECTIVE MEASURES

Alycia Bisson, Margie Lachman, and Alycia Sullivan Bisson, *Brandeis University, Waltham, Massachusetts, United States*

Although there is evidence that physical activity (PA) and sleep are related, it is unclear which aspects of these multidimensional constructs are involved. Many have examined differences in PA and sleep between persons, but few have tested daily associations within persons. The present study examined sleep (duration; hours spent asleep, WASO; wake after sleep onset, latency; time to fall asleep) and PA (total and intensity) over 7 days, using both a self-reported diary (subjective) and an ActiWatch (objective). Healthy adults between 34 and 83 came to University of Wisconsin, Madison to participate in the Midlife in the United States (MIDUS) Biomarker study (N=436, Mage: 56.92, SDage: 11.5). Subjective and objective measures showed differential relationships; subjective duration was higher, and latency was lower than objective measures. Some age differences were also found; older adults reported more WASO than middle-aged adults, but their WASO was similar according to actigraphy. Multilevel models revealed that total PA and intensity significantly predicted subjective and objective sleep measures, controlling for age, sex, and other demographic variables. More active participants had shorter sleep durations, WASO, and latency. Within-person analyses revealed that on days one is more active than average, sleep duration is shorter with less WASO across age. Although the negative relationship between PA and sleep duration was unexpected, it is possible that because more active individuals wake less during the night, they may need fewer hours of sleep because their sleep is more restful. Discussion will focus on possible mechanisms involved in linking PA and sleep.

### DAY-TO-DAY VARIABILITY IN LONELINESS: ASSOCIATIONS WITH AGE, SLEEP DURATION, AND HEALTH SYMPTOMS

Dakota Witzel, Shelbie Turner, and Robert Stawski, *Oregon State University, Corvallis, Oregon, United States*

Research has suggested that high day-to-day intraindividual variability (IIV) in positive emotions has negative impacts on well-being. Little research has explored the impact of IIV in negative emotions, particularly loneliness - a known risk-factor for poorer health in old age. With an estimated 25-29% of older adults reporting feelings of loneliness, it is imperative to examine age differences in the presence of IIV in loneliness and how this relates to sleep and physical health symptoms. Using data from the National Study of Daily Experiences (N=2022, Mage=56.24, Range=33-84), we examined whether (1) age was associated with IIV in loneliness, (2) greater IIV in loneliness was associated with lower average sleep duration and more physical health symptoms, and (3) age differences moderate the extent to which IIV in loneliness impacted the previous outcomes. Preliminary results indicated that age was associated with decreased IIV in loneliness (p<.001). Additionally, increased

IIV in loneliness and age was associated with significantly fewer physical health symptoms (ps<.05) but was not reliably associated with sleep duration. Finally, age and IIV in loneliness did not interact to predict either physical health symptoms or sleep duration. Older participants diminished IIV in loneliness – and lack of differential vulnerability to IIV in loneliness – is consistent with theoretical models of older adults' socioemotional regulation (i.e., socioemotional selectivity theory). Contrary to expectations, a higher variability in loneliness was related to lower average physical health symptoms. Discussion will focus on the meaning and importance of IIV in loneliness for older adults' health.

### DOES POOR SLEEP QUALITY PREDICT FUNCTIONAL LIMITATIONS?

Elliot Friedman, and Elizabeth Teas, *Purdue University, West Lafayette, Indiana, United States*

Previous work from our group (Friedman, 2016) linked sleep complaints to declines in mobility and risk of incident limitations over a 9-10 year follow-up among middle-aged and older adults. While these results suggest that poor sleep might undermine functional capacity, the self-report nature of the data leaves the robustness of this association unclear. The current study addressed this uncertainty by examining links between sleep and mobility limitations using subjective and objective assessments of both. Data were from the Midlife in the United States (MIDUS) study: the biomarker sub-sample (N = 664) from the original cohort (collected 2004-2006) and the Refresher cohort (collected 2011-2013). Sleep was assessed using the Pittsburgh Sleep Quality Index (PSQI; subjective) and 7 consecutive days of actigraphy (objective). Functional capacity was assessed by self-report of limitations and measured gait speed, grip strength, and chair stands. In linear regression models adjusting for demographic and health factors, lower PSQI scores (better sleep quality) predicted fewer reported limitations, stronger grip, quicker gait, and faster chair stands (all p<.01). Of the objective sleep metrics, time to fall asleep and time spent awake during the night predicted more self-report limitations, weaker grip (latency only), and slower gait speed and chair stands. These results extend our prior work by showing a) subjective sleep is linked to measured as well as self-reported physical function, and b) objective assessments of sleep predict reduced physical function, albeit to a lesser extent. They also brighten the spotlight on sleep as a key health determinant in older adults.

### EMOTIONAL TONE OF DREAMS AND DAILY AFFECT

Cassandra Richardson, Taylor Vigoureux, and Soomi Lee, *University of South Florida, Tampa, Florida, United States*

One theoretical function of dreams is emotion processing. However, few studies have examined how daily emotions in waking life (i.e., daytime affect) affect the emotional tone of dreams (i.e., dream affect) that night, and vice versa. This study examined daily bidirectional associations between dream affect and daytime positive and negative affect. Participants were 61 nurses who completed 2-weeks of ecological momentary assessments. If participants remembered the previous night's dreams (nparticipants=50; ndays=268), they reported the dream's emotional tone upon waking ('0'=very negative to '100'=very positive). Participants also responded to a short-version of the Positive and Negative Affect Scale three times/day. Multilevel modeling was used to evaluate two temporal directions (dream affect→ daytime affect or daytime affect→ dream affect) at the within- and between-person levels. After adjusting for sociodemographic covariates, at the within-person level, daily positive affect was higher and daily negative affect was lower than usual on days following more positive dream affect (B=0.19, p<.05; B=-0.26, p<.05, respectively). When we added the other temporal direction, today's positive or negative affect was not associated with dream affect that night. At the between-person level, nurses who reported more positive dream affect also reported more positive daytime affect (B=0.52, p<.01), but not less negative daytime affect (B=-0.34, p>.10). Findings suggest that dream affect is predictive of daily affect, but not the other way around. Future studies could further examine if emotions closer to sleep are more strongly associated with dream affect to motivate more precisely-timed affect interventions.

### FACTORS ASSOCIATED WITH PERCEIVED STRESS AMONG DEMENTIA CAREGIVERS WITH POOR SLEEP

Yeonsu Song,[1] Anna Papazyan,[2] Monica Kelly,[2] Sarah Choi,[1] Constance Fung,[2] Susan McCurry,[3] Cathy Alessi,[2] and Jennifer Martin,[2] *1. University of California Los Angeles, Los Angeles, California, United States, 2. VA Greater Los Angeles Healthcare System, North Hills, California, United States, 3. University of Washington, Seattle, Washington, United States*

Poor sleep among family caregivers of individuals with dementia is associated with higher levels of caregiver stress. Modifiable factors that increase risk of stress among caregivers with poor sleep are targets for intervention. This analysis aimed to identify factors associated with greater caregiver stress among caregivers with poor sleep. Baseline data from an ongoing trial of a dyadic sleep intervention program for individuals with dementia and their caregivers with poor sleep quality (defined by Pittsburgh Sleep Quality Index total score > 5; N=21 dyads; mean age 70.8± 11.1 for caregivers, 80.5± 8.3 for care-recipients) were analyzed. Caregiving factors included Zarit Burden Index (ZBI) and SF-12 Short Form Health Survey (SF-12v2). Care-recipient factors included Mini-Mental State Examination (MMSE) and Revised Memory and Behavior Problems Checklist (RMBPC). Stress was measured with the Perceived Stress Scale (PSS). Analyses included Pearson correlations and t-tests. Caring for multiple care-recipients (n=5: 24.8±2.7) was associated with higher (worse) PSS scores than caring for one care-recipient only (n=16: 19.6±3.7, p=0.011). Caregivers with higher PSS also had a significantly higher ZBI score (r=0.53, p=0.015), higher distress related to care-recipient behaviors on the RMBPC (r=0.57, p=0.009) and worse mental health on the SF-12v2 (r= -0.47, p=0.037). PSS was not associated with care-recipient MMSE. These findings suggest that caregivers with poor sleep who care for multiple care-recipients may be at higher risk of stress. This work also identified potential targets (e.g., caregiver burden, mental health, distress related to care-recipient behavior) for reducing stress in this population.

### LOW FREQUENCY EXTENSION FILTER AND ACTIGRAPH-CALCULATED SLEEP INTERVALS IN OLDER ADULTS

Hilary Hicks, Alex Laffer, Genna Losinski, and Amber Watts, *The University of Kansas, Lawrence, Kansas, United States*

Actigraphy has become a popular, non-invasive means of continuously monitoring physical activity and sleep. One optional setting, the low frequency extension (LFE) filter, reduces the movement threshold to capture low acceleration activity that is common in older adults. This filter significantly alters physical activity outcomes (e.g., step counts), but it is unclear if this has implications for sleep interval calculations that rely upon accurate differentiation between physical activity and sleep. We investigated the effects of the LFE filter on wrist-worn sleep estimates in older adults. Participants were 9 older adults who wore the ActiGraph GT9X on their non-dominant wrist for 7 days in a free-living environment. Raw data was processed with and without the LFE filter enabled, and sleep intervals were calculated by a proprietary ActiGraph algorithm. Paired samples t-tests demonstrated that the LFE filter generated significantly later bedtimes, fewer minutes spent in bed, shorter sleep duration, and fewer awakenings during the night compared to when the filter was disabled (all $p < .043$). Use of the LFE filter did not lead to differences in arise time, sleep latency, efficiency, or wake after sleep onset (all $p > .052$). While the LFE filter was designed to improve accuracy of physical activity estimates in more sedentary populations, these findings suggest that the LFE filter also has the potential to impact sleep estimates of older adults. Researchers using ActiGraph-calculated sleep would benefit from careful consideration of this software-dependent impact.

### MULTIDIMENSIONAL SLEEP HEALTH AND PHYSICAL FUNCTIONING IN OLDER ADULTS

Caitlan Tighe,[1] Ryan Brindle,[2] Sarah Stahl,[3] Meredith Wallace,[3] Adam Bramoweth,[1] Daniel Forman,[3] and Daniel Buysse,[3] *1. VA Pittsburgh Healthcare System, Pittsburgh, Pennsylvania, United States, 2. Washington and Lee University, Lexington, Virginia, United States, 3. University of Pittsburgh, Pittsburgh, United States*

Prior studies link specific sleep parameters to physical functioning in older adults. Recent work suggests the utility of examining sleep health from a multidimensional perspective, enabling consideration of an individual's experience across multiple different sleep parameters (e.g., quality, duration, timing). We examined the associations of multidimensional sleep health with objective, performance-based measures of physical functioning in older adults. We conducted a secondary analysis of 158 adults (Mage=71.8 years; 51.9% female) who participated in the Midlife in the United States (MIDUS) 2 and MIDUS Refresher studies. We used data from daily diaries, wrist actigraphy, and self-report measures to derive a composite multidimensional sleep health score ranging from 0-6, with higher scores indicating better sleep health. Physical function was assessed using gait speed during a 50-foot timed walk, lower extremity strength as measured by a chair stand test, and grip strength assessed with dynamometers. We used hierarchical regression to examine the associations between sleep health and gait speed, lower extremity strength, and grip strength. Age, sex, race, education, depression symptoms, medical comorbidity, and body mass index were covariates in each model. In adjusted analyses, better multidimensional sleep health was significantly associated with faster gait speed (B=.03, p=.01). Multidimensional sleep health was not significantly associated with lower limb strength (B=-.12, p=.89) or grip strength (B=.45, p=.40). Gait speed is a key indicator of functional capacity as well as morbidity and mortality in older adults. Multidimensional sleep health may be a therapeutic target for improving physical functioning and health in older adults.

### PERSONALITY AND SLEEP HEALTH: DO LIFESTYLE HABITS PLAY A ROLE?

Karley Deason, Christina Mu, and Soomi Lee, *University of South Florida, Tampa, Florida, United States*

The health behavior model proposes that healthy/unhealthy behaviors may play a role in the relationship between personality and health. Previous research shows that personality traits are linked to sleep, however, few studies have considered the moderating role of unhealthy behaviors in the personality—sleep relationship. The current study investigated the associations between specific personality traits and sleep and whether the associations were moderated by unhealthy behaviors. Participants were 61 oncology nurses (Mage=35.39, SDage=11.73). They responded to a background survey that assessed the big five personality traits and engagement in unhealthy behaviors (i.e., exercise, smoking, fast food and alcohol consumption). For two weeks, ecological momentary assessments captured daily variability in sleep (i.e., quality, sufficiency, onset latency, insomnia, duration). A series of multilevel models was used. After controlling for sociodemographics and work shift, higher conscientiousness was associated with greater sleep sufficiency (B=0.31, p<.05) and lower odds of having insomnia symptoms (OR=0.24, p<.05). Moreover, higher agreeableness was associated with longer sleep duration (B= 0.51, p<.05) and lower odds of insomnia symptoms (OR=0.29, p<.05). Other personality domains were not associated with sleep, however, extraversion interacted with unhealthy behaviors to be associated with sleep. Those who were more extraverted reported lower odds of insomnia and better sleep sufficiency; these associations were significant only for those with less unhealthy behaviors. Findings suggest that conscientiousness and agreeableness were associated with sleep health. The interaction between extraversion and unhealthy behaviors suggests that reducing unhealthy behaviors may be beneficial to improving sleep in individuals with certain personality traits.

### POOR SLEEP HEALTH AND NEXT DAY WORK IMPAIRMENT: THE MEDIATING ROLE OF FATIGUE

Breann LaRocque, Christina Mu, and Soomi Lee, *University of South Florida, Tampa, Florida, United States*

Nightly sleep impacts next-day alertness and cognitive functioning. For healthcare professions, work impairment can be life-threatening for patients. Thus, understanding how sleep affects work quality is imperative to promoting medical safety and overall health of workers. The current study investigated whether nightly sleep health is associated with next-day work impairment in nurses and whether this association is mediated by daily fatigue. Sixty nurses reported their sleep characteristics, fatigue, and work impairment using ecological momentary assessment for two weeks. We used a series of multilevel models (a path: sleep→fatigue, b path: fatigue→work impairment, c path: sleep→work impairment, c′ path: sleep and fatigue→work impairment), adjusting for sociodemographics and work shift. At the between-person level, poorer sleep quality was associated with greater work impairment (βc=-23.36, p<.001). This association was mediated by fatigue such that poorer sleep quality was associated with greater fatigue (βa=-19.54, p<.01), which was further associated with greater work impairment (βb=0.79, p<.001). After including fatigue, the association of sleep quality with work impairment was reduced (βc′ =-7.07, p=.08). Similarly, fatigue mediated the relationship between sleep sufficiency and work impairment (βa=-16.49; βb=0.79; βc=-19.36; p<.001; βc′ =-6.32, p=.05). At the within-person level, on days after long sleep duration (>8hrs), nurses reported greater work impairment (βc=10.08, p<.01), however, this was not mediated by fatigue. Our results suggest that poor sleep health may impair next-day work performance, mostly through increased fatigue. Future interventions for nurses can target daily fatigue to reduce the adverse effects of poor sleep on work impairment.

### RELATIONSHIPS AMONG TYPES OF ACTIVITY ENGAGEMENT AND SLEEP QUALITY AMONG OLDER ADULTS

Da Eun Kim, and Tonya Roberts, *University of Wisconsin–Madison, Madison, Wisconsin, United States*

There is increasing awareness that lack of activity engagement is associated with poor sleep quality. However, the majority of studies have focused on the effect of a single type of activity engagement on sleep quality. Little is known about the combined effect of multiple types of activity engagement on sleep quality. The aim of this study is to identify relationships among different types of activity engagement and sleep quality among older adults. This study is a secondary data analysis using the Health and Retirement Study data. The participants included 3,357 persons who were age 65 or older and who responded to survey modules on activity engagement and sleep quality in 2016. Before we conducted primary analysis, factor analyses and calculating coefficient omega were conducted to identify factor structure, construct validity and reliability of the activity engagement questionnaire. Then, regression was conducted to examine the relationships among multiple types of activity engagement and sleep quality after adjusting for covariates based on the senescent sleep model. Exploratory and confirmatory factor analysis showed the 14-item questionnaire was comprised of three factors; social, cognitive, and physical activity and the three-factor model showed adequate validity and reliability. In the regression model social (β=0.25, p=0.033) and cognitive (β=0.36, p=0.001) activity engagement were positively related to better sleep quality. Based on these results, future research is needed to identify the mechanisms in which social and cognitive activities influence sleep quality positively and to develop targeted activity interventions for older adults.

### SESSION 2961 (POSTER)

### CHRONIC DISEASE MANAGEMENT I

### CARDIOMETABOLIC RISK AND BIOMARKER TRAJECTORIES AMONG OLDER ADULTS: FINDINGS FROM THE HEALTH AND RETIREMENT STUDY

Qiao Wu, Eileen Crimmins, Jennifer Ailshire, Jung Ki Kim, and Erfei Zhao, *University of Southern California, Los Angeles, California, United States*

The deterioration of the cardiovascular system is a process associated with aging. Most of the prior works have examined changes in cardiometabolic risk (CMR) while aging at the population level using cross-sectional data, but we study within-person changes for total CMR and separate risk factors, including pulse pressure, resting heart rate, C-reactive protein, glycosylated hemoglobin (HbA1c), high-density lipoprotein cholesterol, total cholesterol, waist circumference, and obesity. We examine 8-year changes (from 2006 to 2014) among respondents from the Health and Retirement Study biomarker sample (n=19,776). We use growth curve models to identify differences at baseline and the changes while aging, by age, gender, race/ethnicity, and education. Blacks, the old-old, the less educated, and current smokers have higher baseline CMR. The total CMR increases while people age over 8 years. HbA1c, waist circumference, and pulse pressure increase significantly with age. A reduction in total cholesterol can be observed and is likely due to medication. The CMR increase is no longer significant after accounting for socioeconomic status. The next step of this study is to focus on the disparity of risk distribution, in order to identify the individuals that are most in need of specific care and support.

### EVALUATING THE ASSOCIATION BETWEEN SINGLE ITEM LITERACY SCREENER AND HEALTH OUTCOMES IN PATIENTS WITH LUNG CANCER

Julie Nguyen,[1] Caitlyn McNaughton,[2] and Jessica Sautter,[2] *1. University of the Sciences, Philadelphia, Pennsylvania, United States, 2. Lancaster General Health, Lancaster, Pennsylvania, United States*

Health literacy is becoming increasingly important in areas such as cancer care, where treatments are relatively difficult to navigate. This study aims to describe the how health literacy is associated with healthcare outcomes and health system usage among patients with lung cancer. Data include retrospective medical record data from 456 patients with lung cancer; half were age 70 and older. Patients were coded as having adequate or limited health literacy based on their response to their Single Item Literacy Screener (SILS). Data were collected from a 12 month period following diagnosis for each patient. One-third of patients had limited health literacy; this was significantly more common among adults age 70 and older. Patients with limited health literacy were more likely to have newly diagnosed lung cancers of stage 3B or higher (59.18% vs. 42.76%, p = 0.0011) compared to those with adequate health literacy. Patients with limited health literacy had higher median levels of depression based on the PHQ-9 questionnaire (4.0 vs. 3.0, p = 0.0082) and a higher median number of emergency department visits (1 vs. 0, p = 0.0156) and unplanned hospitalizations (1 vs. 0, p = 0.0044). Furthermore, patients with limited health literacy were more likely to have an emergency department visit or unplanned hospitalization sooner (p < 0.0001). These data illustrate a lower quality of life and a higher dependency on healthcare services for patients with limited health literacy. Assessment and interventions may be necessary to ensure access to quality healthcare.

### EXAMINING CARDIOVASCULAR DISEASE RISK PROFILES AMONG OLDER ADULTS WITH AND WITHOUT FIBROMYALGIA

Dylan Serpas, Barbara Cherry, and Laura Zettel-Watson, *California State University, Fullerton, Fullerton, California, United States*

Introduction: Cardiovascular diseases (CVDs) remain the leading cause of morbidity and mortality in the United States. Preexisting chronic health conditions may be confer increased CVD risk, specifically fibromyalgia (FM), a chronic condition characterized by widespread pain, fatigue, stiffness, and concentration problems. CVD risk increases with normal aging; however, characteristics of FM are suggested to exacerbate health profiles in normal aging processes that may contribute to increased CVD risk. Method: The sample included 221 older adults (M=63.40, SD=8.86; 82% female; 88% White/European American) and 55% reported an FM diagnosis. CVD risk factors were entered separately in a five-block hierarchical binary logistic regression model as predictors and included: cardiorespiratory fitness using the six-minute walk, BMI, standing and lying mean arterial pressure (MAP), and depression using the Beck Depression Inventory. Results: Logistic regression analyses revealed that poorer cardiorespiratory fitness (OR=.99, 95% CI=.99-1.00, p=.001), greater depressive symptoms (OR=1.35, 95% CI=1.19-1.53, p< .001) and lower standing MAP (OR=.98, 95% CI=.96-1.00, p=.036) were associated with higher odds of an FM diagnosis. However, no differences in lying MAP (OR=1.02, 95% CI=1.00-1.04, p=.137) or BMI (OR=1.02, 95% CI=.95-1.10, p=.644) for an FM diagnosis emerged. Discussion: These data support the importance of examining the health profiles of persons with FM in the context of CVD risk. Experiences of FM may produce distinct health profiles with characteristics that serve as both protective and risk factors in the context of CVD.

### FACTORS ASSOCIATED WITH PAIN INTERFERENCE AMONG BLACK AND WHITE OLDER ADULTS

Dottington Fullwood,[1] Roger Fillingim,[2] Alisa Johnson,[3] and Nancy Gell,[4] *1. The University of Florida, Gainesville, Florida, United States, 2. University of Florida, Pain Research and Intervention Center of Excellence, Gainesville, Florida, United States, 3. University of Florida, Gainesville, Florida, United States, 4. The University of Vermont, Burlington, Vermont, United States*

Pain interference (PI) is an indicator of pain impact and is associated with physical performance (PP). However, factors associated with PI among older adults are not well described, including associations with PP and racial differences. This study explored PI among older adults by race. Data were obtained from the 2013 Pain Supplement of the National Health and Aging Trends Study (N= 1,202; 59.9% female, 23.0% Black non-Hispanic). Interviews included questions on sociodemographics, multi-morbidities, pain intensity (0-10 scale), and PI overall. Participants were also asked "In the last month, how much did pain interfere with ADLs, household activities, going outside, shopping, social activities and walking, which was used to create a PI index (Range 0-18). Physical performance measures assessed balance, gait speed, and chair stands (Short physical performance battery; SPPB). Logistic and multivariable regression analyses were conducted to determine associations among PI with PP, pain intensity, and race. Older Black adults experienced higher pain intensity (3.90 vs. 3.03) and demonstrated greater PP limitations (5.4 vs. 7.1 SPPB score) compared to older White adults (p<0.001). Higher scores on the PI index were associated with worse PP, higher pain intensity, depression, multi-morbidity, and White race (p <0.001), independent of confounders. Similarly, more PI overall was associated with White race, higher pain intensity, worse PP, and multimorbidity (p<0.001). Despite higher pain intensity and worse physical performance, older Black adults reported lower PI than White older adults. Additional exploration is needed to discern the paradoxically lower PI among older Black adults, including potential resilience factors.

### GOOD, BAD, AND UGLY: PARTNER SUPPORT AND QUALITY OF LIFE AMONG COUPLES FACING SKIN CANCER

Laura Butner-Kozimor, and Jyoti Savla, *Virginia Tech, Blacksburg, Virginia, United States*

When older adults in partnered relationships face a skin cancer diagnosis of one partner, couples may rely on one another for support. Previous studies have found that perceived support can influence one's adjustment to the stressors associated with the skin cancer diagnosis, as well as influence the overall quality of life. Using dyadic data from 30 older couples (Mage = 70; SD = 7.25), this study examined positive and negative relationship-focused support strategies each partner provided and effects on the dyad's quality of life. Dyadic path analyses simultaneously examined the impact of support received by one's partner and its association with their own quality of life (actor effects) and their partner's quality of life (partner effects). Positive support received by either partner, in the form of active engagement, was not associated with quality of life. In contrast, negative support in the form of protective buffering received from supporting partners was associated with poorer quality of life for themselves (β = -.37, p = .05) as well as for partners with skin cancer (β = -.43, p = .01). Similarly, overprotection, also a negative support strategy, by supporting partners was associated with poorer quality of life for partners with skin cancer (β = -.63, p < .001). Findings illustrate that not all types of support are beneficial for the overall couple relationship and couple outcomes. Implications for practice and interventions for older couples facing a cancer diagnosis will be discussed.

### INVESTIGATING HEAD AND NECK CANCER SURVIVORS' EXPERIENCE OF SURVIVORSHIP CARE

Aaron Seaman,[1] Seyedehtanaz Saeidzadeh,[2] Nicholas Kendell,[1] Alan Christensen,[3] Timothy Thomsen,[1]

Heather Reisinger,[1] and Nitin Pagedar,[1] *1. Carver College of Medicine, University of Iowa, Iowa City, Iowa, United States, 2. College of Nursing, University of Iowa, Iowa City, Iowa, United States, 3. East Carolina University, Greenville, North Carolina, United States*

Head and neck cancer (HNC) accounts for 4% of all cancers diagnosed in the US, with 75% in adults over 55 years of age. HNC survivors must deal with the long-term consequences of the cancer and its treatments, which can have significant long-term physical, psychosocial, and financial consequences, dramatically impacting survivors' lives. While research has identified the unmet needs of HNC survivors, there has been little examination of HNC survivors' experiences living with a cancer history and engaging in survivorship care. To explore survivors' experiences and their attitudes toward their survivorship care, we conducted in-depth, semi-structured interviews with 22 HNC survivors whose survivorship care was managed within the HNC program of an academic tertiary care institution. Participants' mean age was 65 years old, ranging from 33 to 86. The majority of the participants were male (68%), white (96%), married (81%), and had some college education or a higher degree (81%). One third of participants (n=7) had rural residence, as defined by the Rural-Urban Continuum Codes. Participants reported a wide range of experiences based on multiple factors: cancer site, staging, and treatment; their expectations prior to treatment; and personal and social context. They varied in their approaches toward understanding and incorporating the impacts of their cancer experience, from physical side effects of treatment to social impacts. They described the importance of survivorship care both in physical and social terms. We discuss the implications of these results for future interventions to improve HNC survivorship care delivery.

### IS PAIN TIME PATTERN PREDICTIVE OF FUTURE TIME PERSPECTIVE IN OLDER CHRONIC PAIN PATIENTS?

Gillian Fennell,[1] Abby Pui Wang Yip,[2] M. Cary Reid,[3] Susan Enguidanos,[1] Elizabeth Zelinski,[1] and Corinna Loeckenhoff,[2] *1. University of Southern California, Los Angeles, California, United States, 2. Cornell University, Ithaca, New York, United States, 3. Weill Cornell Medical College, New York City, New York, United States*

Chronic pain patients constitute 65% of those ages 65 and older in the US. Many affected older adults are challenged to manage physical and psychological consequences tied to the intensity, interference, and temporal pattern of their pain. However, little quantitative research highlights the psychological impact of constant versus intermittent or ever-present-yet-variable pain, even though temporal pain pattern may have meaningful predictive power for wellbeing and future time perspective (FTP). A positive and expansive view of the future is particularly adaptive for this population because it is positively associated with treatment adherence. In this study, we analyzed secondary data to determine whether pain temporal pattern and pain duration are associated with differences in participant scores on Carstensen & Lang's Future Time Perspective scale. All participants (N=142) were 45 years old and older with non-cancer chronic pain lasting three months or more. There was no significant association between pain time pattern and FTP (p=.35). Additionally, controlling for pain duration, average FTP scores did not vary significantly as a function of time pattern (p=.07). Our analyses demonstrated no significant relationship between pain time pattern and FTP and no significant moderating effect of pain duration. However, in contrast to the previous literature, FTP was not significantly associated with age and negatively (rather than positively) associated with subjective health (r=-.08, p=.35; r=-.24, p<.01), thus raising concerns about the generalizability of these findings. Implications for understanding time perceptions in older pain patients are discussed.

### LONGITUDINAL CHANGES IN ARTHRITIS PAIN CONTRIBUTE TO SUBSEQUENT CHANGES IN BODY MASS

Deborah Finkel,[1] Charles Emery,[2] and Anna Dahl Aslan,[3] *1. Indiana University Southeast, New Albany, Indiana, United States, 2. Ohio State University, Columbus, Ohio, United States, 3. Jönköping University, Jönköping, Sweden*

Prior studies have documented that body mass index (BMI) is positively associated with bodily pain. However, data on the temporal sequence of BMI and pain suggest mixed results, with some studies indicating a bi-directional relationship, and other research among older adults supporting a uni-directional relationship from BMI to increased pain. Thus, it is critical to further examine temporal dynamics between changes in BMI and changes in bodily pain to help explicate possible mechanisms influencing the relationship. This study evaluated bivariate dynamic models of longitudinal change (McArdle & Hamagami, 2003) in the relationship between BMI and bodily pain with data from older adults participating in the Swedish Adoption/Twin Study of Aging (SATSA). The sample included 858 individuals aged 45-88 at intake, with up to eight waves of follow-up over 26 years. BMI {weight(kg)/[height(m)]2} was evaluated with objective measures of weight and height recorded by a study nurse. Pain symptoms were measured with six self-report pain questions, reflecting two pain factors: (1) pain in neck, back, or shoulder; and (2) hip pain, history of arthritis, or use of arthritis medications. Results indicated that the relationship between BMI and arthritis pain was uni-directional, with changes in pain symptoms contributing to subsequent changes in BMI, but no evidence that changes in BMI contributed to subsequent changes in pain symptoms. Model comparison indicated that the impact of pain on BMI was greatest before age 70, and then reduced somewhat after age 70, when the impact of other factors on BMI (e.g., ill health) likely increases.

### MEDICATION MANAGEMENT AMONG OLDER ADULTS WITH MULTIPLE CHRONIC CONDITIONS: WHAT ROLE DO CAREGIVERS PLAY?

Rachel O'Conor, Morgan Eifler, Andrea Russell, Lauren Opsasnick, Julia Yoshino Benavente, Laura Curtis, and Michael Wolf, *Northwestern University, Chicago, Illinois, United States*

Many older adults manage multiple chronic conditions (MCC) that require adherence to complex medication regimens. Few studies have investigated the degree to which caregivers support medication-related behaviors. We conducted semi-structured qualitative interviews with 25 caregivers of older adults with MCC to characterize caregiver medication assistance. Two coders used content and constant comparative analysis to analyze transcripts. The mean age of caregivers was 61 years; the majority were female (68%) and identified as non-white (Black, 52%; Hispanic, 8%). Caregivers were predominantly spouses (n=10), or children (n=11). Older adults were on average 73 years old, managing 5 chronic conditions and prescribed 7 medications. Caregivers acknowledged the importance of medications to the older adult's health, but their involvement in daily medication management was limited. Some caregivers preferred that the older adult continue these tasks to maintain autonomy, especially when caring for older adults who valued maintaining independence. Caregivers assumed medication responsibilities after older adults experienced sudden changes in health or upon observing non-adherence (e.g. full pill bottles). Older adults with higher medication burden (12+ medicines) adopted inefficient, cumbersome medication management practices; caregivers suggested simplified strategies, but the older adults refused to adopt recommended strategies. To combat resistance from the older adult, caregivers disguised assistance and deployed workaround strategies to monitor medication-taking behaviors. These findings suggest older adults and caregivers share a value of promoting independence of medication management, up until safety is seriously questioned. Additionally, there is a breakdown in communication at the time when older adults may benefit from increased caregiver involvement.

### PAIN ACCEPTANCE PREDICTS EXPANSIVE OUTLOOKS ON THE FUTURE IN OLDER CHRONIC PAIN PATIENTS

Gillian Fennell,[1] Abby Pui Wang Yip,[2] M. Cary Reid,[3] Susan Enguidanos,[1] Elizabeth Zelinski,[1] and Corinna Loeckenhoff,[2] *1. University of Southern California, Los Angeles, California, United States, 2. Cornell University, Ithaca, New York, United States, 3. Weill Cornell Medical College, New York City, New York, United States*

As chronic conditions continue to rise in the US, associated pain symptoms are rising as well, affecting 65% of those 65 and older. In an attempt to help patients lessen the burdensome physical/psychological effects of chronic pain, researchers have investigated the effectiveness of therapeutic interventions with pain acceptance-based models yielding the most promising effect sizes. However, these interventions do not explicitly account for how patients perceive their future. Qualitative work has shown that chronic pain patients with positive and expansive views of their futures report fewer pain-related anxiety and depression symptoms, and are more likely to engage in long-term (and often more effective) treatment regiments. This study aims to investigate whether pain acceptance scores predict future time perspective to enhance treatment effects of chronic pain interventions. Multivariate linear regression analyses were conducted with a sample of 148 non-cancer patients age 45 and older with chronic pain, i.e. pain lasting three or more months. Pain duration, neuroticism, sex, race, income, and age were included in the model to explore potential mediating or moderating effects. A significant positive association was found between pain acceptance and future time perspective (r=.42, p<.001, r2=.17). Additionally, with the inclusion of all covariates, our model significantly explained 24.1% of the variance in future time perspective in the sample, F(7,132)=5.99, p<.001. With an established association between these two psychological constructs, strategies to bolster future time perspective can easily be integrated into pain acceptance interventions for older chronic pain patients, hopefully pushing effect sizes past the 'moderate' level.

### PATIENT EXPERIENCE OF AN OSTEOPOROSIS TELEMEDICINE CLINIC

Melissa Steffen,[1] Jennifer Van Tiem,[1] Aaron Seaman,[2] Karla Miller,[3] Shylo Wardyn,[1] and Samantha Solimeo,[1] *1. VA Office of Rural Health Veterans Rural Health Resource Center-Iowa City (VRHRC-IC), Iowa City, Iowa, United States, 2. Carver College of Medicine, University of Iowa, Iowa City, Iowa, United States, 3. VA Office of Rural Health Veterans Rural Health Resource Center-Salt Lake City (VRHRC-SLC), Salt Lake City, Utah, United States*

Rural Veterans at risk of fracture due to osteoporosis remain underdiagnosed and undertreated, in part due to location-related barriers to accessing care. Despite lowered cost and travel barriers to osteoporosis care through implementation of a telehealth model directed at rural at-risk Veterans that took advantage of many strengths of the VA's healthcare system, only 30% of eligible Veterans accepted care. To understand low acceptance, we conducted 39 semi-structured telephone interviews with Veterans eligible for the clinic, including 19 who accepted screening and treatment, 12 who completed screening but declined treatment, and 8 who declined screening and treatment. Veterans who opted to be screened and/or treated for osteoporosis did so because: it was recommended by the VA; they were interested in learning more about their health; thought they may be at risk of osteoporosis; or believed screening would not cause them harm. Conversely, Veterans refused screening or treatment because of past negative experiences with medications, both bone and non-bone; a wish to not put anything else into their bodies; or the belief that their bone loss is not severe enough to warrant treatment. Outside medical professionals and peers influenced Veterans' decisions to not take or alter their treatment. Cost and travel distance remained a barrier for Veterans who did not live near a VA facility with the necessary screening and treatment infrastructure. Many barriers to osteoporosis care remain despite efforts to remove them. Delivery systems must account for both instrumental and social access to care to reduce fracture risk.

### THE EVALUATION OF A MODIFIED DECISION AID FOR OLDER WOMEN WITH LOW HEALTH LITERACY

Tamara Cadet,[1] and Mara Schonberg,[2] *1. Simmons University, Boston, Massachusetts, United States, 2. Beth Israel Deaconess Medical Center, Boston, Massachusetts, United States*

Given the lack of evidence recommending mammography for women >75 years, guidelines recommend that older women be informed of the uncertainty of benefits and of potential harms. The objective for this study was to evaluate the effect of a mammography decision aid (DA) designed for older women with low health literacy (LHL) on their decisional conflict and knowledge of mammography's benefits and harms in a pretest-posttest trial. Women, 75-89 years were eligible for this study if they had not had a screening mammogram in six months, a history of breast cancer/dementia and had LHL (defined as reporting difficulty completing medical forms on one's own or obtaining < college education). Forty-four women participated. their mean age was 78, 74% had a high school degree or less, and 53% were non-Hispanic White. Overall, women reported that the DA helped them prepare to talk with their clinician quite a bit (Mean =3.6/5.0 on preparation for decision making scale) and 97% found the DA helpful. Using McNemar's test, decisional conflict did not change and knowledge on a 10 item true/false test on mammography screening did not change; however, after receiving the DA women were correctly less likely to think that having a mammogram would prevent cancer. With the shift toward shared decision-making for women > 75 years, there is a need to engage women of all literacy levels to make these decisions and we have developed a DA on mammography screening for older women with LHL that these women perceive to be helpful.

### THE IMPORTANCE OF CANCER-RELATED AND OTHER HEALTH FACTORS ON COGNITION AMONG OLDER-ADULT LONG-TERM SURVIVORS.

Gabrielle Beck,[1] Gary Deimling,[1] Boaz Kahana,[2] Eva Kahana,[1] Erin Phelps,[1] and Spencier Ciaralli,[1]
*1. Case Western Reserve University, Cleveland, Ohio, United States, 2. Cleveland State University, Cleveland, Ohio, United States*

Previous research has identified cancer and cancer-treatment related effects on survivors' mental impairment including memory and concentration. However, research has not systematically examined the relative impact of cancer in the context of age and other age-related health challenges common in later life. This paper compares the effects of cancer-related factors with other health challenges faced by 471 older adult long-term survivors from an NCI-funded study of a randomly selected tumor registry sample from a major comprehensive cancer center. Having had chemotherapy is associated with several cognitive outcomes including memory and concentration. Survivors who reported more cancer-related symptoms during treatment reported a greater number of cognitive symptoms even decades after treatment. Importantly, other comorbid health problems as well as social factors were found to be important in explaining symptoms of cognitive impairment in this older adult sample. These findings suggest that health care and mental health providers consider the range of health challenges, including those related to cancer and its treatment, as they provide patient centered care.

### TRANSPORTATION BARRIER IN RURAL OLDER ADULTS' USE OF PAIN MANAGEMENT AND PALLIATIVE CARE: SYSTEMATIC REVIEW

Zainab Suntai, Chorong Won, and Hyunjin Noh,
*University of Alabama, Tuscaloosa, Alabama, United States*

Pain and symptom management is critical in ensuring quality of life for chronically or seriously ill older adults. However, while pain management and palliative care have steadily expanded in recent years, many underserved populations, such as rural older adults, experience barriers in accessing such specialty services, particularly due to transportation issues. The purpose of this systematic review is to examine the specific types of transportation-related barriers experienced by rural older adults in accessing pain and palliative care. Studies were searched through the following 10 databases: Abstracts in Social Gerontology, Academic Search Premier, CINAHL, MEDLINE, PsycINFO, SocINDEX with Full Text, Cochrane Database of Systematic Reviews, Nursing & Allied Health Database, Sociological Abstracts, and PubMED. Studies were chosen for initial review if they were written in English, full-text, included older adults in sample, and examined pain/palliative care/hospice, rural areas, and transportation. A total of 174 abstracts were initially screened, 15 articles received full-text reviews and eight met the inclusion criteria. Findings of the eight studies identified transportation-related issues as major access barrier to pain and palliative care among rural older adults: specifically, lack of public transportation; lack of special needs/wheelchair accessible vehicles; lack of reliable drivers; high cost of transportation services; poor road conditions; and remoteness to the closest pain and palliative care service providers. Results suggest that rural older adults have unique transportation needs due to the urban-centric location of pain and palliative care services. Implications for practice, policy and research with older adults are discussed.

### SESSION 2962 (POSTER)

### AGING IN PLACE (BSS)

### A COMMUNITY SPACE WITH DIVERSE ACTIVITIES SUPPORT OLDER ADULTS' SOCIAL PARTICIPATION AND SUSTAIN SOCIAL CONNECTION

Dongyool Kim, Taichi Masuda, Shuichiro Higuma, Yerim Yang, Sayaka Terazawa, Mai Takase, Ryogo Ogino, and Jun Goto, *The University of Tokyo, Tokyo, Japan*

Active older adults in Japan participate in multiple social activities to be socially involved. However, physical limitation and decline in enthusiasm due to ageing decrease their participation. Diverse activities should be available at one place, close to older adult's residence, to sustain social connections. A community space was launched at Toyoshikidai housing complex (Kashwia, Japan) in February 2018. The place offers about 25 activities per month. This research aimed to elucidate the relationship between activity type and motivation for participation, and study the effect of the community space on older adults' social connection. A cross-sectional questionnaire survey was conducted targeting the attendees of community space (February 2020). Of attendees, 68% lived within 10-minutes walking distance to the community space (N=101). The activities were classified into craft, exercise, and music. The motivation for attending craft events were information exchange and relaxation, as was health maintenance for exercise events. Participating in group performance was the motivation to attend active music event, and casual gathering and network expansion was for passive music event. The frequency of social participation outside the community space was low in the group aged over 75 years. This group attended the activities at the community space more frequently than did the younger group. Differences in the number of social connections were not found. This result implies that older adults maintained their social connection by attending activities held at the community space. The diverse programs and close location of the community space might have contributed to the motivation of participation.

### ASSOCIATION BETWEEN MEDICATION-RELATED PROBLEMS AND EMERGENCY ROOM VISITS AMONG COMMUNITY-DWELLING OLDER ADULTS

YuJun Zhu,[1] Marsha Meyer,[2] Denise Likar,[2] and Susan Enguidanos,[1] *1. University of Southern California, Los Angeles, California, United States, 2. SCAN Health Plan, Long Beach, California, United States*

Medication-related problems (MRPs) remain one of the largest health risks for older adults, yet there are few studies that identify the complete myriad of problems associated with medication use among community-dwelling older adults with chronic diseases. The aim of this study is to identify the range and quantity of MRPs among community-dwelling older adults and determine the relationship between number of MRPs and emergency room (ER) visits. Primary data were collected from a community medication program for diverse older adults (N=206). A comprehensive medication review was conducted to identify MRPs including adverse drug reaction, inappropriate storage, and non-adherence. We conducted multivariate logistic regression to examine the relationship between the number of MRPs and ER visits controlling for health conditions and demographic information including age, gender and race. The mean age of participants was 74.2 (SD=8.8) and 65% were female. Racial groups include Whites (39%), Blacks (21%), Hispanics (29%) and Asian/Pacific Islanders (11%). On average, participants had 5.3 (SD=2.3) health conditions and 18.8 (SD=5.9) MRPs, among which adherence (36%) and coordination of care (28%) were most commonly identified. About 30% had at least 1 ER visit in the previous 6 months. The number of MRPs was significantly associated with ER visits (OR=1.08, p=.03). Older adults experienced a variety of MRPs and for each problem experienced, the odds of having an ER visit increased by 8%. Interventions are needed to assess the myriad of MRPs among community-dwelling older adults and address those potential risks.

### EMERGENT BUSINESS MODELS FOR HOMECARE IN ENGLAND

Karla Zimpel-Leal, *University of Sheffield, Sheffield, United Kingdom*

The purpose of this study is to examine how emergent homecare business models are shaping the care market in England. Homecare providers for older people are facing a rise in demand for their services which is driven not only by an ageing population but also from a market demand for personalised care, choice, continuity of care, and real time availability. Combined with a turbulent political and policy environment, the current care landscape presented an opportunity for innovative and emergent homecare models to establish themselves and in some occasions disrupt the market by offering a more inducing service design and value propositions that better match customers' needs. Utilizing the Business Model Canvas, this study investigated various emergent models of homecare by using semi-ethnographic methods that included field observation and data collection, a narrative summary review and interviews. It has shown that homecare providers for an ageing customer base are becoming increasingly aware of emerging customer needs and expectations. Disruptive and emergent models such as uberisation, community-based, live-in and preventative models are becoming more pervasive in the current landscape. These models offer major shifts related to their value proposition, partnerships and customer segments. The value propositions are focused on several dimensions of wellbeing outcomes, choice and personalisation, whilst their care workforce is perceived as a major customer segments and their network of partners provides access to complementary services, investments and specialist knowledge. These changes are promoting more flexibility and responsiveness in the care market, enhancing service users' experience and encouraging workforce development.

### INVESTIGATING THE FACTORS THAT INFLUENCE SELF-EFFICACY AMONG HOME CARE WORKERS PROVIDING DEMENTIA CARE

Marie Savundranayagam, Anaya Ahmad, and Shalane Basque, *Western University, London, Ontario, Canada*

The self-efficacy beliefs of home care personal support workers (PSWs) play a crucial role in their professional competence and subsequent provision of quality care. Understanding the factors that influence self-efficacy of PSWs is critical to ensuring their job satisfaction and retention, and ultimately improving the quality of care provided to home care clients. Currently, there is a lack of literature exploring the factors influencing self-efficacy among home care PSWs who care for clients with dementia. Accordingly, the purpose of this study was to investigate the sources of self-efficacy for home care PSWs. Conventional content analysis of interviews with 15 home care PSWs yielded six categories of sources influencing self-efficacy: insufficient client information provided by employers, lack of supportive communication by employers, restriction of PSWs' discretion and autonomy by employers, insufficient practical dementia-specific training, sufficient work experience with clients with dementia, and feedback from family caregivers. These findings call for a multi-pronged approach to enhance the self-efficacy of PSWs. In particular, these findings offer specific areas of improvement for employers on how to best support their PSWs. They also highlight the significant role of dementia-specific education and training for PSWs regardless of their experience in the field. Finally, the findings emphasize the importance of family caregivers in the home care context. Taken together, the study's findings offer insights on how to best support PSWs and ensure stability in the dementia care workforce.

### LIVING WITHIN AND WITHOUT: LIFE-SPACE MAPPING TO VISUALIZE NEED AND RESOURCE ACCESS OF RURAL DEMENTIA DYADS

Julia Loup,[1] A. Lynn Snow,[2] and Michelle Hilgeman,[2] *1. University of Alabama, Tuscaloosa, Alabama, United States, 2. Tuscaloosa VA Medical Center, Tuscaloosa, Alabama, United States*

Rural-dwelling veterans with dementia (PWD) and their family caregivers (CG) have unique needs and resource access limitations. Life-Space assessment models suggest older adults' needs are reflected in their daily-life mobility and routines (Peel et al., 2005). Yet, medical treatment models seldom incorporate non-health related activities (e.g., transportation, groceries, distance to formal and informal support networks). This mixed-methods study proposes an exploratory life-space modeling visualization that integrates qualitative and quantitative daily-life data from rural dwelling dyads in Alabama. Two case studies are selected from a sample of 30 qualitative interviews to demonstrate this innovative analytic approach. One case depicts a married dyad (PWD and spousal CG) (CGage = 74; PWDage = 80, PWD MoCA score = 21) and the second visualization is of a PWD living alone (PWDage = 82, PWD MoCA Score = 20). Daily-life experiences and routines mentioned during interviews were categorized using a rapid analysis template approach and informed by unmet needs theories (Algase et al., 1996). Next, extracted data were placed into mapping visualization software. The maps include visual cues (colors, transportation routes, and icons) to designate met, unmet, and vulnerable needs and resources, allowing visual interaction with the two cases' dementia caregiving context and qualitative responses. Life-space maps may be useful tools to visualize resource access and assist integrated health care systems in better understanding daily interactions and intervention gaps for difficult to reach populations. Future developments include ecological momentary assessment and Global Positioning System (GPS) data to develop life-space maps using real-time data collection.

### LOW-INCOME COOPERATIVE LIVING ENVIRONMENTS: AN ANALYSIS OF UNIVERSAL DESIGN FEATURES THAT SUPPORT AGING IN PLACE

Migette Kaup,[1] Hannah Richardson,[1] Mikayla Adkins,[1] Brett LaFleur,[1] Sydney Tucker,[1] and Lauren Tines[2]
*1. Kansas State University, Manhattan, Kansas, United States, 2. StudioSIX5, Austin, Texas, United States*

Affordable housing for low-income older adults is in significant demand in the United States. Housing options available, however may not recognize how design can support social infrastructure in addition to basic physical accessibility. The aim of this research was to provide an analysis of the efficacy of the planning and design principles applied to a recently constructed cooperative living home. The goal was assess the universal design (UD) of features that supported aging in place, and, to inform the planning of future projects for the community. A post-occupancy evaluation process was conducted at the home, and all five residents consented to structured focus group discussions as well as individual interviews regarding their specific experiences living in the home. An environmental assessment was completed through detailed field notes, measurements, and photo documentation. An analysis of specific features of each room of the home was then assessed by the seven UD principles. Results revealed the specific features of each space that contributed to the completion (or the limitations) of basic activities of daily living as well as instrumental activities of daily living. These assessments were validated through feedback provided by the users. Many features of the home were found to be positive and supportive of equitable use by all residents. Other design attributes that could be improved were related to size and space for approach and use, and providing flexibility in use. Specific design changes based on resident input, such as kitchen function, storage safety, and lighting details, are highlighted and discussed.

### SAFETY OR AUTONOMY IN DEMENTIA CARE AT HOME, A QUALITATIVE STUDY ON FAMILY CAREGIVERS' EXPERIENCES

Vincent Moermans,[1] Michel Bleijlevens,[2] Hilde Verbeek,[2] Koen Milisen,[3] Bernadette Dierckx de Casterlé,[3] and Jan Hamers,[2] *1. Maastricht University, Riemst, Limburg, Belgium, 2. Maastricht University, Maastricht, Limburg, Netherlands, 3. KU Leuven, Leuven, Belgium*

Persons with Dementia (PwD) living in their own home become increasingly dependent on support from especially family caregivers who often are confronted with dilemmas. Family caregivers feel responsible for PwD and predominantly want to ensure their safety, whereas PwD wants to engage in meaningful activity and make decisions autonomously. This study aims to identify how family caregivers caring for PwD at home deal with these dilemmas. Using a grounded theory approach, this study conducted in-depth interviews with 8 family caregivers. Results show that dealing with the dilemma of safety versus autonomy is considered as a calculated risk. By timely entering into dialogue with their loved one, family caregivers try to create a safe environment taking into account the needs and preferences of PwD. However, when situations evolve to such an extent that family caregivers think unsafety will occur, they take over control and overrule the autonomy of the PwD. This often results in resistance to care in PwD. Dealing with the dilemma is a continuous and exhausting learning process due to the ever-changing behaviour of PwD. Family caregivers said that they are in need of support of professional caregivers, but that this need is met insufficiently. As a result, family caregivers have to rely mostly on their own intuition and earlier experiences. The conclusion is that family caregivers experience serious dilemmas in the care of PwD living at home. Professional support in making adequate decisions related to safety and autonomy is warranted.

### SOMEONE AT HOME: HOW KINSHIP SUPPORT INFLUENCES AGING-IN-PLACE DECISIONS AMONG CHILDLESS OLDER ADULTS IN RURAL CHINA

Chen Hongzhou, Vivian Lou, and Hongzhou Chen, *The University of Hong Kong, Hong Kong, China*

Background and aim: 80% of childless older adults in rural China choose to ageing-in-place (AIP) rather than moving to residential facilities, regardless the fact that they were financially constrained, scarily supported and some were physically disabled. This research explores the reason why AIP were prevailing preferred. Research design and Method: A qualitative method adopting constructive grounded theory approach was utilized in this research. 20 in-depth interviews were conducted among childless rural residents (aged 60 to 83 years old, 8 of them were over 75 years old) in Yunnan, one of the most economically disadvantaged provinces in China. Data was transcript and coded line-by-line, a in Vivo coding strategy was engaged to capture local language and meanings. Results: A phrase - 'there's someone at home' - was used by rural childless older adults to explain their AIP decision, which demonstrating the role of kinship relations. Three subthemes were emerged regarding to the phrase: 1) reciprocity, as the support were mutual and predictable; 2) justified conflicts, as older adults and 'someone' managing the support relation with subtle conflict; 3) unspoken agreement, as childless older adults being constrained by filial piety when negotiating for further support. All of sub-themes were related with sense of certainty. For participants who were over 75, growing old were "naturally" related with decreased social support. The daily-based kinship support and sense of certainty was particularly important among childless older adults who would like to choose AIP but still questioning the sustainability of self-reliance at an uncertain rural place.

### THE EFFECT OF NEIGHBORHOOD EXPERIENCES ON POSITIVE MENTAL HEALTH AMONG COMMUNITY-DWELLING OLDER ADULTS

Daniel R. Y. Gan,[1] Grand H.-L. Cheng,[2] Tze Pin Ng,[3] John Chye Fung,[3] and Im Sik Cho,[3] *1. Simon Fraser University, Vancouver, British Columbia, Canada, 2. The Open University of Hong Kong, Hong Kong, China, 3. National University of Singapore, Singapore, Singapore*

Given reduced life spaces, the neighborhood often functions as a social venue for older adults. Yet how these everyday social spaces affect older adults' psychosocial wellbeing remains largely unknown. Drawing on the GRP-CARE Survey data, this paper examined the relation between neighborhood experiences and positive mental health. Participants were 601 community-dwelling Singaporeans aged 50+ who lived in public housing neighborhoods. Neighborhood experiences were measured using the four-factorial, 16-item OpenX scale (Gan, Fung, Cho, 2019); positive mental health was measured using a six-factorial, 19-item scale (Vaingankar et al., 2011). Both scales have good psychometric properties and had been validated. Path analysis between relevant factors of both scales was conducted using Stata, within a theorized model of causation from neighborhood environment to social factors to psychosocial health. Age, education, ethnicity and sex were controlled for. Multiple linear regression analysis showed a strong, positive association between neighborhood experiences and mental health (p=0.000) even after controlling for personal traits (operationalized as depressive symptoms, GDS) in addition to sociodemographic variables. Path analysis showed that two distinct neighborhood health processes mediated this association. These were (1) the potential for a sense of community in the neighborhood improved emotional support, and (2) having better neighborly friendships improved interpersonal skills. These neighborhood health processes provide us with new lenses to understand older adults' everyday experiences of their neighborhoods. Community-based interventions to improve older adults' psychosocial wellbeing may be developed to facilitate these processes. Spatial and programmatic implications will be discussed in relation to age-friendly cities and communities (AFCC).

### THE INTERSECTIONALITY OF PERSON, SPACE, AND TIME FOR UNDERSTANDING AGING IN PLACE

Widya Ramadhani, Maurita Harris, and Wendy Rogers, *University of Illinois at Urbana-Champaign, Urbana, Illinois, United States*

Aging in place is interpreted differently across times and disciplines in the literature. Multiple interpretations of aging in place can lead to differences in expectations and goals when planning products, services, and technologies for older adults. We conducted a historical review across databases in the fields of anthropology, architecture, gerontol-ogy, medicine, psychology, and sociology to explore the evolution of 'aging in place' term across time and disciplines. We included articles that used the terminology "aging in place" or "ageing in place" in titles, abstracts, keywords, or subject. From the aging in place definition excerpts collected, we identified the preliminary themes and grouped them into three main themes: people, space, and time. Although the narrative of aging in place is highly related to living spaces, the cause and influencing factors are tied beyond the space. Person and time-related factors that are related to the aging experience im-pact the way aging in place is defined. When designing products, services, and technolo-gies to support successful aging in place, designers, researchers, policymakers, and care-givers should be aware that aging in place is at the intersection of personal, spatial, and temporal elements of older adults' lives. Based on the multiple perspectives of disciplines, we concluded that aging in place is beyond the matter of location, but also takes into account the person's capacity and the changes over the person's lifespan. Founda-tional understanding of the multiple factors that influence aging in place is critical to support older adults to have a healthy and optimal aging experience.

### TRAINING CLINICIANS TO SUPPORT AGING IN PLACE IN A MEDICAID HOME AND COMMUNITY-BASED WAIVER PROGRAM

Sandra Spoelstra,[1] Monica Schueller,[1] Alla Sikorskii,[2] and Katelyn Ware,[1] *1. Grand Valley State University, Grand Rapids, Michigan, United States, 2. Michigan State University, Grand Rapids, Michigan, United States*

A randomized trial in 18 home and community-based waiver sites implemented an aging-in-place model for 12,000 disabled older (mean age 78) adults. The trial was underpinned by the Knowledge-to-Action model and utilized 7 implementation strategies, including a 5.5-hour online social worker (SW) and registered nurse (RN) training. Baseline self-efficacy and attitudes of SWs and RNs were measured using validated scales, and knowledge uptake and satisfaction with a tool designed by the team. Characteristics, knowledge uptake, and satisfaction of SWs versus RNs were compared using t-, Wilcoxon, and chi-square tests. Two hundred forty-one RNs and 264 SWs participated. RNs were older (mean age 50; standard deviation [SD] 10.95) than SWs (41.35; SD=11.21) p<.01; and >90% overall were female. RNs had more professional experience, while SWs worked more years in the waiver (p<.01). SWs had greater self-efficacy (t(497)=2.99, p<.04), better attitudes (t(500)=2.59, p<.01), employability (t(500)=2.99, p<.04), and balance (t(491)=2.03, p<.05) than RNs. No differences were found on leadership, organizational culture, motivation, pressure to change, or attitude toward evidence-based practice. Knowledge uptake (range 1-16) was high and did not differ for RNs (Mean=15.2, SD=1.23) versus SWs (Mean=15.26, SD=0.89). Training content, format, role explanation, and information satisfaction (range 0-50) means also did not differ for RNs (Mean=35, SD=10.2) or SWs (Mean=34.9, SD=9.8). While many of the characteristics and outcomes were similar for RNs and SWs, SW's higher self-efficacy, better attitude, and employability despite less experience in the waiver indicate they may play a positive role in implementation of the intervention that is currently underway.

### SESSION 2963 (POSTER)

### ENVIRONMENT AND AGING (BSS)

### A STUDY ON COMMUNITY FORMATION PATTERNS BASED ON THE USAGE OF BENCHES BY SENIORS

Yunjeong Lee,[1] Akiko Nishino,[2] Yuji Matsuda,[2] Toshio Otsuki,[2] and Kazuhiko Nishide[2] *1. ChungNam Institute, Chungcheongnam-do, Republic of Korea, 2. The University of Tokyo, Tokyo, Japan*

In Japan, the rate of aging exceeds 27%, and the decline of communities has been an issue. Therefore, in this study we take the T housing complex as an example where the rate of aging is more than 40% and aim to understand the usage pattern of benches. An observational research was conducted for 19 days to examine the usage of a total of 23 benches, 17 existing ones and 6 newly installed ones, in the T housing complex. This investigation led to the findings. An average of 417 people used the benches in a day. Research determined that there are four different types of bench utilization, and they each present different ways of building relationships with other people. Bench use behavior was broken into four different categories. Sitting on a bench alone (without building relationships with others) was classified as "single use". Sitting on a bench with another person but not engaging in any relationship-building behavior was classified as "sharing". Sitting on a bench and exhibiting relationship-building behavior with others (such as through talking) was classified as "chatting". Sitting with companions in groups of 3 or more people and exhibiting relationship-building behavior was classified as "group use". Our findings provide evidence that a bench can be a device to foster the formation of diverse communities.

### ADULT AGE DIFFERENCES IN COPING STRATEGIES AFTER THE 2016 FLOOD

Katie E Cherry, Matthew Calamia, Emily M. Elliott, and Quyen P. Nguyen, *Louisiana State University, Baton Rouge, Louisiana, United States*

The 2005 Hurricanes Katrina and Rita had a devastating impact on south Louisiana, as did the more recent flooding in 2016. Multiple disaster exposures are associated with distress which may be lessened through adaptive coping behaviors, although prior disaster losses may affect current coping responses. In this study, we assessed self-reported coping strategies, resilience, and mental health outcomes after the 2016 flood. The sample was comprised of mostly middle-aged and older adults (N = 223, age range: 18-89 years). Three groups were compared: (1) non-flooded adults as controls, (2) once-flooded adults with structural damage to homes and property in 2016, and (3) twice-flooded adults who had relocated to Baton Rouge because of catastrophic losses in Hurricanes Katrina and Rita and they experienced damage in the 2016 flood. Analyses of variance confirmed that the three groups differed in coping responses, with non-flooded controls using significantly fewer strategies than their once and twice flooded counterparts. Correlation analyses demonstrated that age was positively associated with adaptive forms of coping (acceptance, religiosity) and negatively correlated with maladaptive coping (self-blame). These data suggest that awareness of prior severe weather experiences and catastrophic losses, which are likely for older adults living in hurricane prone areas, are an important consideration for disaster planning and the development of age-sensitive interventions to mitigate adversity.

### APPLYING THE PUSH-PULL FRAMEWORK TO DOWNSIZING IN LATE LIFE

Kyrsten Costlow,[1] Leona Yeager,[2] Shinae Choi,[1] Beverly Roskos,[3] and Patricia Parmelee,[2] *1. The University of Alabama, Tuscaloosa, Alabama, United States, 2. University of Alabama, Tuscaloosa, Alabama, United States, 3. University of Alabama, Tuscaloosa, Tuscaloosa, Alabama, United States*

One of the most frequently cited theoretical models of relocation decision-making is Wiseman's Behavioral Model of Elderly Migration. Based on this model, the present study used a push-pull framework to describe older adults' reasons for downsizing to a smaller home. A sample of 68 older adults who had downsized in the past year provided reasons for moving from their previous residence (push factors) and reasons for moving specifically to their new residence (pull factors). Participants rated the importance of each push/pull factor using a (1) not at all important to (4) extremely important scale. On average, participants rated pull factors (M = 3.63, SD = .31) as slightly more important than push factors (M = 3.54, SD = .40) in their decision to move. The most frequently reported push factors were declining health (n = 47, 22%), having too much space or maintenance (n = 40, 19%), and disliking the location of their previous residence (n = 30, 14%). The most commonly reported pull factors were the attractive location of the new residence (n = 60, 28%), social factors (e.g., being closer to family and friends; n = 36, 17%), and services or amenities available in the new residence (n = 32, 15%). Qualitative responses will be presented to illustrate the interaction between push and pull factors in participants' relocation decision-making. Findings are consistent with those identified in other studies on late-life relocation, establishing Wiseman's behavioral model as a useful framework for investigating downsizing in late life.

### BARRIERS TO DISASTER PREPARATION WHEN OLDER ADULTS ARE TAKING CARE OF OLDER ADULTS

Zhirui Chen, and Zhen Cong, *The University of Texas at Arlington, Arlington, Texas, United States*

This study examined the association between age and barriers for disaster preparation in the U.S., including the moderating effect of caring for an elderly person. Using a sample of 1147 individuals from the 2017 FEMA National Household Survey, we tested age as the key predictor, which had four groups: 18-44 (young age), 45-64 (middle age), 65-74 (young-old), and 75+ (old-old). Binomial logistic regression was conducted to investigate how age and the responsibility to take care of an older adult affected the likelihood of having preparation barriers, controlling for respondents' gender, education, race, home ownership, disability, and responsibility to take care of an older adult. The results showed that compared with other age groups, young-old were significantly less likely to have barriers for disaster preparation. In addition, taking care of an older adult increased the likelihood of having preparation barriers. Interestingly, relative to the young-old, caring for an older adult presented additional challenges for other age groups to have barriers for preparation. Our findings directed attention to age patterns in barriers for disaster preparation and heterogeneity among older adults, and also highlighted that caring for older adults could exacerbate barriers for disaster preparation. The life course perspective informed the discussion of results, which emphasized on life-span development and linked lives.

### COMPARISON OF THREE WAYFINDING ASSESSMENT TOOLS

Margaret Calkins,[1] and Rebecca Davis,[2] *1. IDEAS, Cleveland Hts., Ohio, United States, 2. Grand Valley State University, Grand Rapids, Michigan, United States*

An early, problematic symptom of aging, exacerbated by cognitive problems such as Alzheimer's disease, is difficulty finding one's way. Long term care (LTC) communities are especially challenging for wayfinding. This study compares three different tools to evaluate spatial complexity within eight LTC communities. The tools included computation of space syntax integration values using axial lines to determine how connected wayfinding routes are within the community. The Wayfinding Checklist tool (revised) rates the quality of signage, complexity of a building floor plan, and use of decorative elements. The Calkins Route Assessment tool evaluates route complexity using both plan and vertical visual field information (e.g. presence, location, clarity of signage and cues). The results of the analysis showed that the buildings varied with respect to integration, with some very well integrated floorplans and routes, and some that were very complex, with multiple jogs, turns, and limited integration. The Wayfinding Checklist echoed the variety of complexity of floorplans, but lowered scores for poor existence and design of signage and mostly substandard lighting for visibility. The Calkins Route Assessment tool added greater specificity to the space syntax analysis and Wayfinding Checklist, showing how visual elements (versus just visual access) can compensate for plans with low-integration. These study findings indicate that each tool was valuable for measuring wayfinding complexity, but measured different aspects of building and route complexity. Tools like these can be used to identify challenging wayfinding routes and buildings so that designers and clinicians can improve the overall design for wayfinding.

### CREATIVE CITIES CREATING CONNECTIONS: FOSTERING CROSS-AGE INTERACTION THROUGH LEISURE

Rachel Douglas, and Anne Barrett, *Florida State University, Tallahassee, Florida, United States*

Older and younger people interact infrequently in most social realms – a pattern stemming from institutional, cultural, and spatial age segregation. Increasing cross-age interaction offers promise as a strategy for not only enhancing social connections but also reducing ageist attitudes. We argue that a social realm with untapped potential for creating these connections is leisure, particularly within creative cities. To explore this possibility, our study examines cross-age interactions in Key West, Florida – a leisure-oriented city that promotes creativity through its social and built environment. Using participant-observation and interview data (n=126) from 2017 to 2019, we examined leisure experiences of tourists and residents, aged 23 to 83. Findings indicate that creative cultural contexts can counter ageist attitudes by promoting cross-age interaction. Data analysis revealed three processes encouraging these shifts – promoting diversity and acceptance, enhancing older adults' participation, and nurturing intergenerational bonds. Ongoing cross-age interactions were fostered through the island's welcoming philosophy and encouragement of out-group acceptance and diversity. They were further fostered by the island's wide array of leisure activities and proximity of shared spaces that enabled older adults to readily pursue leisure with younger people. Cross-age ties also were promoted by the island's focus on family-friendly leisure experiences. Our research highlights the potential of creative cities to promote age integration, and reduce ageism, through its cultural practices within leisure spaces.

### LIVING THROUGH DECLINE: RESIDENT PERCEPTIONS OF CHANGE WITHIN CLEVELAND'S URBAN NEIGHBORHOODS

Kaitlyn Langendoerfer, *Case Western Reserve University, Honesdale, Pennsylvania, United States*

Despite the vast amount of research focused on neighborhoods within the environmental gerontology, very little attention has been paid to learning how older residents make sense of and describe the changes that have occurred within their communities over the course of their lives. The purpose of this study was to provide a space for residents to tell their stories of what it was like to live through neighborhood decline within Cleveland. Older adults are an ideal group for examining perceptions of neighborhood decline as they have the perspective to address both the historical changes of their neighborhood and the biographical changes of their lives. This study utilized data from 4 years of ethnographic observations with over 30 older (age 60+), African-American adults who have aged in place within Cleveland since their childhood. Additionally, multiple in-depth life history interviews were conducted with 13 long-term residents. Data was analyzed using grounded theory techniques for emergent themes. While each resident had their own unique perspective of neighborhood change, common themes emerged related to 1) institutional decline, 2) changes in safety and crime and 3) changes to the people living in their communities. The findings suggest that resident perceptions can help us better understand how neighborhood dynamics work their way into the lives of long-term residents. The results are particularly important as they provide the foundation for exploring how residents responded to neighborhood decline and why residents remained within their neighborhoods when so many others left.

### PERCEIVED NEIGHBORHOOD ENVIRONMENTAL FACTORS RELATED TO SARCOPENIA IN URBAN-DWELLING OLDER ADULTS

Yuri Seo,[1] Miji Kim,[2] Hayoung Shim,[3] Heeeun Jung,[1] Seoyoon Jane Lee,[4] and Chang Won Won,[5] *1. Graduate School, Kyung Hee University, Seoul, Republic of Korea, 2. College of Medicine, Kyung Hee University, Seoul, Republic of Korea, 3. Kyung Hee University, Seoul, Korea, Seoul, Republic of Korea, 4. Elderly Frailty Research Center, Kyung Hee University, Seoul, Republic of Korea, 5. Kyung Hee University Medical Center, Seoul, Korea, Seoul, Republic of Korea*

Sarcopenia is common among older individuals and has adverse health outcomes. However, little is known about its association with neighborhood environmental factors. We explored the relationship between sarcopenia and neighborhood environmental factors among community-dwelling older adults aged 70–84 years in urban areas in the Korean Frailty and Aging Cohort Study. There were 1,776 participants in this cross-sectional study (mean age 75.9±3.8 years, 54.1% women). Sarcopenia was defined using the Asian Working Group for Sarcopenia guidelines. The neighborhood environmental factors were assessed using the 17-item Environmental Module of the International Physical Activity Questionnaire (IPAQ-E). The prevalence of sarcopenia was 22.5%. In the multivariate analysis adjusted for potential confounders, compared to the 5th quintile of the IPAQ-E score, the odds ratio (OR and 95% confidence interval [CI]) for sarcopenia in the 1st, 2nd, 3rd and 4th quintile were 2.14 (1.41-3.26), 1.70 (1.11-2.61), 1.76 (1.16-2.68) and 1.62 (1.07-2.47), respectively. Sarcopenia was associated with environmental factors including access to destinations ($\beta$ = -0.015) and neighborhood safety ($\beta$ = -0.008) (all $p<0.05$). Furthermore, no access to public transportation (OR 2.05, 95% CI 1.20-3.50), poor access to recreational facilities (OR 1.40, 95% CI 1.02-1.92), no presence of destination (OR 1.53, 95% CI 1.07-2.21), hill hazard (OR 1.34, 95% CI 1.02-1.77), and lack of safety from traffic (OR 1.35, 95% CI 1.02-1.79) was associated with an increased risk of sarcopenia. Our study suggests that neighborhood environmental characteristics are associated with sarcopenia and better neighborhood environmental strategies can help prevent sarcopenia among older adults.

### PERCEPTION OF NEIGHBORHOOD CHARACTERISTICS DURING CHILDHOOD AND COGNITIVE HEALTH AMONG OLDER ADULTS IN CHINA

Changmin Peng,[1] Sae Hwang Han,[2] and Jeffrey Burr,[1] *1. University of Massachusetts Boston, Boston, Massachusetts, United States, 2. University of Texas at Austin, Austin, Texas, United States*

Neighborhood environments shape the availability of resources for social engagement and social interaction, which are associated with better health outcomes. However, these contextual factors are also considered sources of potential social distress and tension, increasing the risk of subsequent health deficits, including cognitive decline. Our understanding of the linkage between childhood neighborhood environments and cognitive functioning in later life is limited. This study employed three waves of nationally representative data from the China Health and Retirement Longitudinal Study (2011-2015; N = 11,105) to investigate the relationship between self-reported neighborhood social cohesion during childhood (i.e., neighborhood safety, neighbors willing to help, and close-knit neighborhood) and cognitive functioning (Chinese version of TICS). We employed latent growth curve modeling to test hypotheses relating to life course models of childhood conditions and later life cognitive functioning (the long arm of childhood). The results showed that perceptions regarding the willingness of neighbors to help and close-knit neighborhood characteristics during childhood were positively associated with levels of later life cognitive function. Further, growing up in a neighborhood characterized by the willingness of neighbors to help others was negatively associated with the rate of cognitive decline, net of childhood and adulthood covariates. Self-report of neighborhood safety during childhood was unrelated to cognitive function (level and change). These findings underscored the long-term ramifications of childhood conditions as potential risk factors for later-life cognitive health. Social cohesion at the neighborhood level as experienced during childhood may be a protective factor for healthy cognitive aging among older Chinese adults.

### PERSONAL SUPPORT NETWORKS OF OLDER ADULTS IN RELATION TO THEIR PERCEPTIONS ABOUT DISASTER MANAGEMENT

Sato Ashida,[1] Lena Thompson,[1] Emily Hejna,[1] Sefonobong Obot,[2] and Haley Schneider,[1] *1. University of Iowa College of Public Health, Iowa City, Iowa, United States, 2. University of Iowa College of Public Health, Iowa City, United States*

Personal support networks are essential in ensuring the health and well-being of older adults especially in handling disaster situations. This study assessed the characteristics of personal support networks of older adults in relation to their perceptions about disaster management. Adults between ages 63 and 88 from Eastern Iowa participated in a survey prior to receiving a disaster preparedness education program. About half (47%) were living alone, 66% were female, and 53% had a high school diploma or fewer years of education. Forty-seven participants identified 308 support network members, with an average network size of 6.55 members ranging from 2 to 17. A greater number of network members with whom participants discussed disaster/emergency plans was associated with higher perceived response efficacy (preparation/planning "will help handle the situation better"; r=0.45, p=0.002), lower perceived barriers (preparation is "difficult to do"; r=-0.34, p=0.021), and higher self-efficacy to handle disaster situations (r=0.32, p=0.030). Having more members who would help "if something went wrong" was associated with higher self-efficacy to prepare

(r=0.44, p=0.002) and to handle disaster situations (r=0.34, p=0.021). Fatalistic perception that nothing can help handle disaster situations was associated with having fewer people who provide emotional support (r=-0.559, p=.003) and who participants trust (r=-0.46, p=.018). Older adults' social support network members may partly determine their perceptions regarding the importance of and their ability to prepare for emergency and disaster situations. Future studies may evaluate whether activating network member support and discussion would lead to increased motivation and preparedness among older adults.

### PHYSICAL AND SOCIAL NEIGHBORHOOD ENVIRONMENTS AND WELL-BEING

Midori Takayama,[1] Yoshiko Ishioka,[2] and Ikuko Sugawara,[3] *1. Keio University, Yokohama, Japan, 2. Keio University, Minato-ku, Kanagawa, Japan, 3. University of Tokyo, Tokyo, Tokyo, Japan*

Existing research has found effects of neighborhood environment on well-being. However, it is still not clear what features of neighborhood environments affect well-being for older adults and whether the impact of the environment varies depending on the health and economic conditions. In this study, we examined the relations between 4 physical and social neighborhood-context factors, that is, the availability of neighborhood physical resources (e.g. community centers and libraries), the walkability and accessibility, the availability of social resources (e.g. culture and recreation programs, and social care services), and the social inclusion (e.g. participation in decision making, and positive social attitude toward older adults), and individual-level well-being. Moreover, we examined the health and economic disparities of effect of neighborhood environments on well-being. We used data from locally representative longitudinal study of older Japanese aged 74 to 86 (N = 1388). Results from multi-level linear regression showed that after controlling individual variables having inhibitory/facilitatory effects of well-being, the availability of physical resources was associated with higher well-being score. Especially among older adults who had financial strains, the availability of physical environment had a positive effect on well-being more strongly. For older adults who had better physical functions, accessibility and walkability were associated with higher well-being score. The social inclusiveness was associated with higher well-being score among those who had no financial strain. These important findings demonstrate the need for more research exploring the underlying mechanisms. The potential benefits of this approach provide a basis for developing models of maintaining well-being for older adults.

### WHAT'S AGE HAVE TO DO WITH COMFORTABLENESS IN SEEKING AND PROVIDING SOCIAL SUPPORT AFTER A DISASTER?

Judith Robertson Phillips, and Edith Jimenez, *California State University San Marcos, San Marcos, California, United States*

Disasters are associated with loss of property and loss of psychological well-being. Receiving various types of social support, such as emotional or tangible support, from various sources, such as family or friends, have been found to reduce the adverse impact of a disaster on affected adult community residents. It is not well studied, though, why some adults will not seek or provide support after a disaster while other adults will. The purpose of this study was to explore how age might play a role in how comfortable adults were in seeking social support from others and how age might play a role in how comfortable adults were in providing social support to others after a disaster, the 2014 San Diego County, CA wildfires. One hundred and twenty-two community residents (18 to 80 years) were recruited with 33 adults identified as Secondary Disaster Survivors and 89 adults identified as Non-Victims. Analyses revealed that age did play a role in Secondary Survivors' comfortableness in seeking support from others; Older Adults (50-80) were significantly less likely than Middle-aged Adults (30-49) to feel comfortable in seeking support. There was no significant comfortableness difference between Middle-aged and Younger Adults (18-29) in seeking support. There were no age group differences in comfortableness in providing support to others for Secondary Survivors and Non-Victims. Implications from this data suggest that local disaster organizations should actively support affected older adults post-disaster while also including unaffected adults of all ages in the process of providing support to those in need.

### WHEN LESS IS MORE: DOWNSIZING, SENSE OF PLACE, AND WELL-BEING IN LATE LIFE

Kyrsten Costlow,[1] Shinae Choi,[1] Beverly Roskos,[2] and Patricia Parmelee,[3] *1. The University of Alabama, Tuscaloosa, Alabama, United States, 2. University of Alabama, Tuscaloosa, Tuscaloosa, Alabama, United States, 3. University of Alabama, Tuscaloosa, Alabama, United States*

The present study aimed to investigate the decision-making process and outcomes associated with downsizing to a smaller home in late life. Older adults who had downsized in the past year (n = 68) completed self-report measures of push-pull factors driving the decision to move, relocation controllability, sense of place (SOP), move satisfaction, and psychological well-being. It was hypothesized that the relation between push-pull factors and relocation outcomes (i.e., move satisfaction and psychological well-being) would be serially mediated by control and SOP. Haye's PROCESS macro was used to test serial multiple mediator models for each of the relocation outcomes. Placing greater importance on push relative to pull factors was associated with lower levels of well-being in three domains: environmental mastery (b = -5.52, p = .002), purpose in life (b = -3.94, p = .01), and self-acceptance (b = -3.61, p = .007). Results of serial mediation analyses suggested that older adults whose downsizing decisions were more strongly influenced by push factors felt less control over relocation, found it more difficult to develop SOP in the new home, and, in turn, experienced lower levels of psychological well-being and move satisfaction. These findings can be used to inform older adults' downsizing decisions and develop supports for relocating older adults.

### SESSION 2964 (POSTER)

### RETIREMENT (BSS)

### A LONGITUDINAL STUDY OF RETIREMENT TRANSITION AND ADVANCE DIRECTIVE COMPLETION

Catheryn Koss, *California State University, Sacramento, Sacramento, California, United States*

Advance directives (AD) help to ensure patients' wishes are honored and contribute to improved end-of-life care. According to normative life course theory, retirement is a significant role change that signals a transition into the third age and its socially prescribed activities. To the extent that ACP is viewed as something to do when one reaches a more advanced stage in life, retirement may spark recognition that planning for incapacity and the end of life is now personally relevant and appropriate. This study tested whether transitioning from work to retirement prompted AD completion. The sample included Health and Retirement Study participants 65 and older who, in 2012, had no ADs and were not completely retired (N = 919). Retirement was operationalized as both a categorical status and as a multistep process. Three waves of data were analyzed using multinomial logistic regression to test associations between retirement transition and advance directive completion. By 2014, 21% had completed ADs and another 17% completed them by 2016. Those who completely retired between 2012 and 2014 were almost twice as likely to complete ADs between 2014 and 2016. Graduated increase in level of retirement between 2012 and 2014 was associated with higher odds of new AD possession in 2016, but did not reach statistical significance at $p < .05$. These results suggest the period following retirement may be an optimal time to encourage patients and clients who have not already done so to complete advance directives.

### LINKING WORK-LIFE OCCUPATIONAL EXPOSURES WITH DISTRESS AND MORTALITY BEFORE AND AFTER RETIREMENT

Sarah Laditka, James Laditka, and Ahmed Arif, *University of North Carolina at Charlotte, Charlotte, North Carolina, United States*

Mental health problems have surpassed musculoskeletal injuries as causes of work disability. Workers in certain occupations may have high risks for mental health problems and premature death even after retirement. People in high risk occupations for many years may be particularly vulnerable, along with groups with higher health risks such as rural residents. Little research examines their occupation-related risks. No research has examined how occupational exposures affect mental health in retirement. We studied these life course risks using the nationally representative Panel Study of Income Dynamics, following participants 36 years beginning 1981, with annual measures of occupation and distress (n=16,994; 129,880 occupation measures; 415 deaths). We estimated hazards of developing distress in occupations hypothesized to have high and low distress risks, adjusted for factors associated with occupational choice and mental health including age, education, income, race/ethnicity, sex, childhood and midlife health, and family trauma. Compared to low risk occupations, working in high risk occupations was associated with 20% elevated odds of distress (adjusted odds ratio, OR 1.20, 95% confidence interval, CI 1.13 1.28) and 55% elevated risk of death (hazard ratio 1.55, CI 1.11-2.16). Each additional year in a high risk occupation increased the odds by 5%. Rural residents had the highest occupation-related distress risk (adjusted OR 3.05, CI 2.39-3.97). At ages 70+ each additional past exposure year was associated with 2% higher distress risk ($p<0.05$), and 4.6% higher mortality ($p<0.05$). Workers in certain occupations have high risks of psychological distress and death, even after retirement.

### RETIREMENT WITHOUT GOD: ACTIVISM AND COMMUNITY AMONG SECULAR PEOPLE OVER 60

Joseph Blankholm, *University of California, Santa Barbara, Santa Barbara, California, United States*

There are more than 1,400 nonbeliever communities in the United States and well over a dozen organizations that advocate for secular people on the national level. Together, these local and national groups comprise a social movement that includes atheists, agnostics, humanists, freethinkers, and other kinds of nonbelievers. Despite the fact that retired people over 60 dedicate most of the money and energy needed to run these groups, the increasingly vast literature on secular people and secularism has paid them almost no attention. Relying on more than one hundred interviews (including dozens with people over 60), several years of ethnographic research, and a survey of organized nonbelievers, this paper demonstrates the crucial role that people over 60 play in the American secular movement today. It also considers the reasons older adults are so important to these groups, the challenges they face in trying to recruit younger members and combat stereotypes about aging leadership, and generational differences that structure how various types of nonbeliever groups look and feel. This paper reframes scholarly understandings of very secular Americans by focusing on people over 60 and charts a new path in secular studies.

### SILVER FINANCE—ASSET MANAGEMENT OF ELDERLY PEOPLE IN THE MODERN ERA

Frances Yang,[1] Ka Hin, Edward CHEUNG,[1] and Vivian Lou,[2] *1. the University of Hong Kong, Hong Kong, China, 2. The University of Hong Kong, Hong Kong, China*

This study aims to explore the elderly people's knowledge, attitude, and understandings toward financial management, and how they behave in using IT-supported financial services. Mixed methodologies, including quantitative street survey and qualitative focus group interviews, are used. The result shows an active involvement with investment market of the mature segments despite claiming safe and stable returns as the paramount principle of asset management. They also have a substantial knowledge on some of the technological-enabled financial services, particularly the younger groups (age 50-59). Cybersecurity is their major concern and barrier on whether to use them or not. Besides, their value and behaviors of buying mature adult financial products should be understood within a context of traditional family culture value. Based on the above analysis, the elderly can be categorized into three major types in using IT-supported financial products and services, including proactive users, passive followers and hard opposition. On the one hand, this research breaks up the outdated stereotype of technology use among elderly people; on the other hand, aged people are facing the challenges of cognitive and physical decline led by ageing. As such, it is essential to conduct specific financial literacy intervention. This project brings new insights into the financial education which is appropriate to the elderly.

### SOCIAL PARTICIPATION AND DEPRESSIVE SYMPTOMS AMONG CHINESE RETIREES

Ziyao Xu, and Na Sun, *Miami University, Oxford, Ohio, United States*

With the rapid growth of the older population in China, the number of retirees is expanding. The retirement transition can lead to the loss of roles/challenge self-identity, increasing the risk of depression. Social participation may have a protective role in the context of retirement. The objective of this study was to examine the relationship between social participation and depressive symptoms among Chinese retirees. Using cross-sectional data from the 2015 China Health and Retirement Longitudinal Survey (CHARLS), we investigated 1949 retirees (Mean age = 66.11, SD=0.37). Social engagement was measured by summing participation in 10 social activities (e.g., interacting with friends, volunteering). Depressive symptoms were measured using the CESD-10; a score ≥ 10 was used to define depression. Participants, on average, engaged in 1.42 (SD=0.06) social activities and 16.8% of them had depression. Results of logistic regression indicate a significant relationship between social participation and depressive symptoms (p=0.014). For every additional social activity, the odds of depression decreased by 17%, holding age, gender, marital status, education, total income, and numbers of chronic diseases constant. Social activity is associated with depression among Chinese retirees. Future studies should explore the effects of specific types of activities on the mental health of Chinese retirees.

### SOCIAL SUPPORT CHANGES IN RESPONSE TO RETIREMENT

Brittany King, *Florida State University, Tallahassee, Florida, United States*

Retirement is an influential life course transition, that has potential to impact overall well-being as well as our social lives. The extent to which our social lives are changed is dependent upon the resources available both pre- and post-retirement. Research on retirement has focused on how well-being is changed, through measures such as depression, and conceptualize social support as a resource that can help offset some of the associated negative consequences. However, it is unclear how that resource of social support is itself being impacted. This study uses 2008-2016 Health and Retirement Study (HRS) data to assesses if how social support changes is dependent upon timing of retirement or whether the individual was forced to retire (N=1,933). Ordinary least squares regression (OLS) and marginal effects are used to assess the change in support and to test if the effects differentially impact certain groups. Preliminary results from this study show that men who have been retired for two waves report a significant negative change ($p<0.05$) in their in-person interactions with children, whereas women who have been retired for the same amount of time report a significant positive change ($p<0.01$) in their interactions with children. Additionally, women who are forced to retire report a significant ($p<0.05$) increase in their in-person interactions with children. These findings suggest that factors such as timing of retirement and forced retirement are important factors in understanding how received social support changes.

### WHAT IS THE PURPOSE IN RETIRING?

Rachel Best, and Patrick Hill, *Washington University in St. Louis, Saint Louis, Missouri, United States*

Sense of purpose is related to many positive physical and mental health outcomes (Boyle, Barnes, Buchman & Bennett, 2009). Unfortunately, sense of purpose declines as adults age and particularly with retirement (Pinquart, 2002). Retirement is often measured dichotomously, however, many people are retiring gradually and do not fit clearly as retired or not retired. Moreover, people retire for different reasons ranging from seemingly non-negative reasons (financial incentives or wanting to spend more time on non-work activities) to more negative reasons (physical health problems, mental health problems, cognitive decline, pressure to retire, or getting fired). The current study (N = 558; mean age = 60.17) used data from a Hawaiian sample in which participants completed self-report surveys on retirement status and activity engagement, as well as a measure of purpose (Scheier et al., 2006). We examined whether the role of retirement on sense of purpose differed by form of or motive for retirement. Results indicated the fully retired population reported significantly lower sense of purpose than both the still working and the partially retired populations, but these two groups did not differ. Participants who retired for negative reasons reported significantly lower sense of purpose than those who retired for non-negative reasons. Greater activity engagement was significantly related to sense of purpose for all retirement statuses. However, this relationship was stronger for the partially retired population. Future research should examine further the role of partial retirement for maintaining sense of purpose in older adulthood.

### SESSION 2965 (POSTER)

### ECONOMICS AND EDUCATION AND TRAINING

### A SHORT SCREENING MEASURE OF CONTEXTUAL VULNERABILITY FOR FINANCIAL EXPLOITATION

Rebecca Campbell, and Peter Lichtenberg, *Wayne State University, Detroit, Michigan, United States*

Financial exploitation (FE) among older adults is an expensive personal and societal problem. Many professionals who work with older adults need fast and reliable measures to detect FE, including: APS workers, financial professionals, and healthcare providers. Very few standardized measures exist to assess an older adult's risk for FE, and fewer still that do not require extensive training to administer. Lichtenberg et al. (in press) presented the Financial Exploitation Vulnerability Scale (FEVS), which assesses the context (i.e. financial, social, and psychological) in which an older adult is making a financial decision. This 17-item scale differentiates between older adults who've experienced FE and those who have not. It has excellent psychometric properties and clinical utility to detect FE beyond demographic and neuropsychological variables. We utilized exploratory factor analysis to examine the factor structure of the FEVS and reduce the number of items. Participant data (n=243) were drawn from two samples: a community-based sample and a group of individuals who were seeking financial coaching services following a financial scam or theft. Examination of the eigenvalues yielded a unidimensional scale. An iterative process was used to remove items that did not load on the single factor or with low corrected item total correlations. The final scale contained nine items and retained its ability to detect financial exploitation (AUC=0.778) and good internal consistency (a=0.845). This short form scale (FEVS-SF) is a brief, standardized screening measure to assess contextual vulnerability for FE that is accessible to professionals who work with older adults.

### ARE HOME CARE AIDES INTERESTED IN HELPING CLIENTS DO PHYSICAL ACTIVITY?

Naoko Muramatsu, Lijuan Yin, and Maria Caceres, *University of Illinois at Chicago, Chicago, Illinois, United States*

The current home care paradigm dictates home care aides (HCAs) provide prescribed help with activities of daily living, rather than stimulating older adults' reserves to maintain independence. Little is known about whether HCAs are interested in expanding their role to promote their clients' health. This study examined HCAs' interest in helping clients do physical activity among workers who care for their family members or clients assigned by home care agencies in a Medicaid-funded home care program. Data came from brief surveys completed by HCAs at state-mandated in-service training sessions (N=602; 42% caring for non-family clients only, 38% family clients only, 20% both family and non-family clients). Ordered logit analysis was conducted to examine whether HCAs caring for family clients differ from those caring for non-family clients in levels of interest in helping clients do safe physical activity as part of home care work, controlling for HCAs' age, gender, language (English/Spanish), and years of home care experience. HCAs' interest levels were high (62% very, 21% somewhat, 8% slightly, 9% not at all interested). Ordered logit analysis indicated that HCAs caring for family members had significantly higher interest levels than those caring for non-family clients only (83% higher among HCAs caring for both family and non-family clients, 30% higher among HCAs caring for family clients only). To reflect HCAs' interests and to maintain clients' independence, home care workforce training should direct its attention to empower HCAs to assume health-promoting roles.

### CULTIVATING GENERATIVE CONNECTION THROUGH AGRICULTURAL LEADERSHIP

Kyle Bower, *University of Georgia, Athens, Georgia, United States*

Adult agricultural leadership programs (ALP) address the needs of a diversifying society with pressing social, economic, environmental, and political challenges. Additionally, these programs offer transformative learning experiences that lead to a greater capacity of current and prospective leaders to become change agents in their communities. Our aging society generates a novel opportunity to reframe experiences of professional succession and retirement within the agricultural sector. In a profession where vitality, strength, and perseverance are fundamental, the agricultural industry needs leaders who remain aware of the foundational knowledge contributed by their predecessors. At the same time, it also necessitates innovation that may revolutionize the farming industry for decades to come. In this mixed-method study, we asked participants of an ALP in the Southeastern region of the U.S. to complete the Loyola Generativity Scale (N=45). Survey results (N=45; 60% response rate) indicated average overall generative (40.3; 40-41 scale average) concern. However, there was a considerable variation among participants, scores ranging from 25-57 (scale range 20-55). To understand the range of attitudes, we conducted interviews (N=10) to represent the distribution of scores by varying ages (M=38), gender, and educational background. Generativity Theory provided the foundation of our thematic qualitative analysis. We discuss the findings in terms of generative desire (motivational), beliefs (thoughts and plans), and actions (behaviors and applied meaning). Our quantitative and qualitative findings advance the conversation of the importance of maintaining social capital throughout one's career and identifying generative connections that assist with difficult transitions, such as retirement, in later life.

### DEVELOPING A DEMENTIA CARE TRAINING CURRICULUM FOR REGISTERED DIETITIANS

Joy Douglas,[1] Christine Ferguson,[1] and Beth Nolan,[2] *1. The University of Alabama, Tuscaloosa, Alabama, United States, 2. Positive Approach to Care, Ada, Michigan, United States*

Research supports the need for healthcare providers who are trained in providing care to older adults with dementia. However, few training options exist for Registered Dietitians (RDs) seeking dementia care training that is specific to nutrition. The purpose of this project was to adapt an existing dementia care training curriculum to meet the learning needs of RDs. The development team included two experts in dementia training and two RDs with expertise in gerontological nutrition. The new training module was based on the existing Positive Approach to Care™ (PAC) curriculum, which incorporates Kolb's Experiential Learning Theory and the Adult Experiential Learning Cycle. The development team first identified learning objectives for content that would be relevant to RDs who work with persons living with dementia, and modified components of the existing PAC curriculum to meet these objectives. After a preliminary pilot, the 2-hour program was presented to 20 RDs using a combination of lecture presentation, experiential learning, and skill-building techniques. Participants were provided written materials to reinforce the concepts presented. Participants answered five dementia-specific questions before and after the training, and overall, the average percentage of correct answers improved following the training. Two weeks following the training, participants completed an open-ended survey to provide feedback on the training. Participants responded favorably to the mixed learning formats in the training. When asked to rank their preferred learning methods, participants indicated lecture-based learning and experiential learning as their top preferred methods. These findings indicate that the adapted curriculum may improve dementia knowledge among RDs.

### ELDER FAMILY FINANCIAL EXPLOITATION: THE COMPLEXITY OF ROLES AND FAMILY CONTEXT

Athena Chung Yin Chan,[1] and Marlene Stum,[2]
*1. University of Minnesota–Twin Cities, Saint Paul, Minnesota, United States, 2. University of Minnesota, Twin Cities, Saint Paul, Minnesota, United States*

Despite elder family financial exploitation (EFFE) being recognized as the most prevalent type of elder abuse, little is known about the family context in which it occurs. To-date most EFFE research has focused on understanding the profiles of one victim and one perpetrator in reported cases. Informed by Family Systems Theory, this study offers new insight into the range and complexity of EFFE victim and perpetrator roles, family structures (relationship types across generations) and living arrangements. A mixed-methods dataset from a sample of non-perpetrator/non-victim concerned family members who experienced EFFE (most unreported to authorities) was utilized to map and analyze 23 family system genograms. The findings reflect four overall profiles when organized by the number of victim(s) and perpetrator(s) in each involved family system including: Single victim, single perpetrator (n=7), Single victim, multiple perpetrators (n=12), Two victims, single perpetrator (n=1), and Two victims, multiple perpetrators (n=3). Across the 4 profiles, most primary perpetrators moved in to live with the elder victims. For Single victim, single perpetrator cases, remarried spouses, as well as parent/adult child relationships in nuclear families with 2-3 adult children emerged. For Single victim, multiple perpetrators, up to five family perpetrators from 3 different nuclear families were involved, including adult children, their in-laws, and grandchildren as a common combination of perpetrators. The findings suggest EFFE is more complex than often assumed, involving multiple perpetrators and victims, and family relationship types beyond older parent/adult child. Implications for reframing risk profiles, assessment tools, and family-focused intervention strategies are discussed.

### FOSTERING AN UNDERSTANDING OF INTERPROFESSIONAL APPROACHES TO GERIATRICS

Anna Faul, Pamela Yankeelov, and Sam Cotton, *University of Louisville, Louisville, Kentucky, United States*

Serving older adults with multiple chronic conditions and variable social, emotional, or physical support effectively within the primary care setting requires an interdisciplinary approach to care, together with the integration of novel approaches to care coordination (Dorr et al, 2006). The purpose of this study is to examine the use of interprofessional learning models to educate a healthcare workforce that meets the needs of older adults by integrating geriatrics with primary care, maximizing patient engagement, and transforming the healthcare system. Specifically, the targeted learners for this curriculum were from a healthcare system in Belize that had no previous specialty training in interprofessional geriatrics care. The 4-day training took place in Belize with an interprofessional group of healthcare professionals that included social work, nursing and medicine. 100 learners participated in the trainings and including participants from social work, nursing and medicine. To evaluate the program, Kirkpatrick's Training Evaluation Model (Kirkpatrick & Kirkpatrick, 2005) was used to determine if learners were satisfied with the content (reaction), skilled (knowledge & skill) and confident in their abilities to utilize the curriculum (application of knowledge & skills). Analysis showed that learners, irrespective of discipline, were satisfied with the program. All disciplines experienced significant differences in their self-efficacy with working on interdisciplinary teams from pre to post assessments. Specifically, there was an increase in learner's confidence related to learning to work together cooperatively with other professions and how to communicate effectively with other members of an interprofessional team. Implications for future interprofessional curriculum will be discussed.

### LONGITUDINAL IMPACT OF AN INTERDISCIPLINARY COURSE ON AGING FOR FIRST-YEAR STUDENTS

Matthew Picchiello,[1] Nancy Morrow-Howell,[2]
Susan Stark,[2] and Brian Carpenter,[2] *1. Washington University in St. Louis, University City, Missouri, United States, 2. Washington University in St. Louis, St. Louis, Missouri, United States*

Undergraduate courses on aging have the potential to counteract negative stereotypes about older adults and to shift students' academic plans as they learn about aging-related opportunities. For six years we have taught an interdisciplinary course on aging for first-year undergraduate students. We present longitudinal data on students' attitudes and academic trajectories after taking the course. Students who took the course (n = 314) and comparable students who were not in the course (n = 353) were surveyed prior to and at the end of their first semester and at the end of each subsequent academic year. At each time point students rated the degree to which aging issues are relevant to their personal and professional lives. Students also reported aging-related curricular and extracurricular activities they pursued. Multivariate repeated-measures analyses revealed a significant interaction such that personal and professional relevance of aging issues were lower and remained stable for students not in the class, and were higher and increased for students in the class, $F(2,226) = 13.18$, $F(2,226) = 14.94$, $p$'s $< .01$. However, for course students, relevance returned to baseline levels by the end of their first year and remained constant in subsequent years. Results from chi-square analyses revealed that students in the class reported more engagement in aging-related courses, $\chi2(1) = 8.3$, research projects, $\chi2(1) = 90.1$, and extracurriculars, $\chi2(2) = 20.6$, $p$'s $< .01$. Results suggest that exposing students to information about aging early has the potential to alter academic trajectories, highlighting the importance of early education.

### LONG-TERM EFFECTS OF ABUSE IN LATER LIFE PERPETRATED BY FAMILY MEMBERS

Naomi Meinertz,[1] Pi-Ju Liu,[2] and Ron Acierno,[3] *1. Iowa State University, Ames, Iowa, United States, 2. Purdue University, West Lafayette, Indiana, United States, 3. UT Health Sciences Houston, Houston, Texas, United States*

Abuse in later life could potentially lead to lower levels of social support, especially when perpetrated by family members who are charged with protecting the older adult in their care. Using both waves of the National Elder Mistreatment longitudinal data (wave one collected in 2008 and wave two in 2015; N=774), long-term effects of abuse (i.e., physical, emotional, sexual, and financial) on levels of social support, physical health, and clinical depressive symptoms for respondents at or above the age of 60 years were analyzed. A multivariate analysis of variance showed that respondents abused at wave one (n=261) by a family member (B=-0.55, p≤0.001), a spouse or ex-partner (B=-0.349, p=0.02), or a non-relative or stranger (B=-0.301, p=0.026) had lower levels of social support eight years later at wave two. Those abused by a family member at wave one also experienced higher levels of depressive symptoms at wave two (B=-0.187, p=0.01). Perpetrator type did not predict general health at wave two. These results emphasize the long-term impact of abuse on the lives of older adults and highlight the importance trusted relationships, such as with family members, have on older adult health and wellbeing.

### PREDICTORS OF MOTIVATION TO LEARN AMONG MIDDLE-AGED AND OLDER MEN IN THE UNITED STATES

Taka Yamashita,[1] Roberto Millar,[2] Shalini Sahoo,[3] Thomas Smith,[4] and Phyllis Cummins,[5] *1. University of Maryland, Baltimore, Maryland, United States, 2. Graduate Student, Baltimore, Maryland, United States, 3. University of Maryland, Baltimore, Elkridge, Maryland, United States, 4. Northern Illinois University, DeKalb, Illinois, United States, 5. Miami University, Oxford, Ohio, United States*

Middle-aged and older men are less likely than women to participate in adult education and training (AET) outside of their work. AET is known to provide psychological, social and economic (e.g., job-related skills) benefits throughout the life course. Research has shown that motivation to learn (MtL) is the key to promoting AET. The objective of this study is to identify MtL predictors among middle-aged and older men in the U.S. Nationally representative data (n = 1,450) of men aged 45 years and older were obtained from the 2012/2014 Program for International Assessment of Adult Competencies (PIAAC). Structural equation models were constructed to examine how a latent MtL construct measured by four 5-point Likert-type itemsMtL might be predicted by participant characteristics. Results showed that having a postsecondary degree (vs. high school or less; $b = 0.19$, $p < 0.05$), higher literacy skills (0-500 points; $b = 0.01$, $p < 0.05$), at least one parent/guardian with a postsecondary degree (vs. those without; $b = 0.08$, $p < 0.05$) and better self-rated health ($b = 0.14$, $p < 0.05$) were associated with greater MtL. Additionally, Black ($b = 0.22$, $p < 0.05$) and Hispanic ($b = 0.19$, $p < 0.05$) men showed greater MtL than White men. Overall, socioeconomic status indicators and race/ethnicity were linked to MtL. Given the known challenges involved in middle-aged and older men's participationin AET (e.g., low and short-term participation), enhancing MtL may have long-term implications. Theoretical explanations and possible policy implications are evaluated.

### RETIREMENT PREPAREDNESS: HOW IMPORTANT IS BEING FINANCIALLY LITERATE?

Mengya Wang,[1] and Suzanne Bartholomae,[2]
*1. Iowa State University, ames, Iowa, United States, 2. Iowa State University, Ames, Iowa, United States*

Financial security in retirement is a major concern for many Americans. Numerous studies document that Americans are not prepared for retirement, with financial illiteracy cited as one reason Americans fail to plan. Employing data from the 2018 National Financial Capability Study (N=27,091), this study investigates actual financial literacy (AFL) and perceived financial literacy (PFL) and how combinations of this measure influences retirement planning, and varies based on years from retirement. This study found relatively low financial literacy and retirement preparedness levels among the US sample, even for those pre-retirees ages 55 to 64. Individually, PFL and AFL increased as one approached retirement. When combined, adults nearing retirement (55 to 64) comprised the greatest proportion of the high AFL and high PFL (29.9%) group compared to adults 20 years or more from retirement (18-44) who largely made up the low AFL and PFL (48%) group. Based on a logistic regression, adults closest to retirement (ages 55 to 64) are more likely to be planning compared to the other groups, as are adults who were financially confident, risk takers, highly educated, males, and white. Compared to adults with high AFL and high PFL, adults with low AFL and low PFL, or a combination (low PFL and high AFL, high PFL and low AFL) have lower odds of preparing for retirement. Both PFL and AFL influences retirement planning, and PFL may be as important as AFL. Our highlight the importance of policies and programs to support Americans with retirement planning.

### SEASON'S CHEATINGS: BEWARE OF HOLIDAY SCAMS

Lona Choi-Allum, and Alicia Williams, *AARP, Washington, District of Columbia, United States*

'Tis the season…to be on the lookout for possible scams and fraud. It's during this time of year that individuals are more focused on the spirit of the holidays and less focused on what may be happening with their pocketbook. AARP conducted a survey of 2,842 U.S. adults ages 18 and older to understand people's awareness of and experience with a variety of scams that are common around the holidays. The study explored experiences with purchasing gift cards, shipping/receiving packages, and charitable giving. In addition, the survey tested the knowledge of adults about several specific scams with a 'quiz' of five true or false statements. Results showed that one in six (17%) U.S. adults failed the quiz. When making gift card purchases, one in five U.S. adults have given and/or received a gift card that had no funds on it. And only about half of U.S. adults conduct research before making a monetary donation to charitable causes or organizations. Of those who do check out a charity first, over half (54%) did not make a donation based on what they found on charity rating sites. Also, U.S. adults say that packages are left outside of their home without requiring a signature. Half (50%) of U.S. adults say they never require a signature when shipping packages to home addresses while about one in seven (15%) always require a signature. More education is needed to raise awareness of scams that are common during the holidays.

### SUBSTANCE USE IS COMMON IN ELDER ABUSE CASES: QUALITATIVE FINDINGS FROM A MULTI-DISCIPLINARY TEAM

Seyed Parham Khalili,[1] Alyssa Elman,[2] Sunday Clark,[2] Yuhua Bao,[3] Yiye Zhang,[3] Philip Jeng,[3] Katherine Wen,[4] and Tony Rosen,[5] *1. Rutgers University, Philadelphia, Pennsylvania, United States, 2. Weill Cornell Medicine, New York, New York, United States, 3. Weill Cornell Medical College, New York, New York, United States, 4. Cornell University, Ithaca, New York, United States, 5. Weill Cornell Medicine / NewYork-Presbyterian Hospital, New York, New York, United States*

Elder abuse is mistreatment of an older adult by a caregiver or another person in a position with an expectation of trust. Adversely affecting as many as 10% of community-dwelling older adults in the US, it may include physical abuse, sexual abuse, psychological abuse, financial exploitation and neglect. Mental illness and substance use by caregivers, family members and victims themselves have been described as risk factors for multiple forms of elder abuse in prior cross-sectional analyses but the impact on these cases is poorly understood. To explore this association we conducted a focus group using a semi-structured format involving an inter-disciplinary group of elder abuse professionals that are part of the New York City Elder Abuse Center enhanced multi-disciplinary team (EMDT) in Staten Island, New York. Focus group participants reported that opioid, cocaine, cannabis and alcohol use is common among perpetrators of elder abuse, especially in cases of financial mistreatment, verbal and physical abuse. Other potential consequences included eviction of the older adult victim, co-dependency and involvement of the older adult in the procurement of illicit substances, and substance use by the older adult. Respondents specifically expressed concerns that the opioid epidemic, including rising heroin use, may be changing the frequency and nature of elder abuse, and that case investigations offer an opportunity to facilitate referrals for formal substance use disorder assessment and treatment. Future work includes additional focus groups and quantitative analysis to clarify the intersection between substance use and elder abuse and inform intervention and prevention strategies.

### THE EFFICACY OF PALLIATIVE CARE EDUCATION AND TRAINING IN PRIMARY CARE SETTINGS: A SCOPING REVIEW OF THE LITERATURE

Jessica Hsieh,[1] Raza Mirza,[2] Lynn McDonald,[1] and Christopher Klinger,[1] *1. University of Toronto, Toronto, Ontario, Canada, 2. National Initiative for the Care of the Elderly, Toronto, Ontario, Canada*

Primary care providers play an important role in palliative care service provision. A scoping review of the literature was undertaken in an attempt to gain insight into and awareness of palliative education and training needs in primary care settings. Four scientific databases covering the medical and social science fields were searched, alongside Grey literature. A total of 5,109 hits were generated, leading to 2,875 titles for individual review. Of these, 33 articles were included in the final review. Five major themes were identified: (1) communication skills; (2) knowledge of spiritual/psychosocial needs; (3) pain and symptom management; (4) cultural proficiency; and (5) experience working within interdisciplinary teams. Many primary care practitioners felt inadequately trained in palliative care and felt unprepared to provide the necessary care. Specifically, poor communication between healthcare professionals and patients were found to adversely affect the level of palliative care that is provided. Additionally, practical experience in palliative/end-of-life care was cited as one of the most beneficial methods in helping to improve knowledge about and ability to practice in the field. The majority of articles emphasized the need for education and training programs to enhance the quality of palliative/end-of-life care service provision. Palliative care education appeared to have numerous benefits, including enhancing providers' knowledge of and attitudes towards this subject, ability to provide palliative/end-of-life care, and self-perception of preparedness. As the landscape of education needs are constantly changing, this review serves as one of the steps in an ongoing evaluation of palliative care providers' training needs.

### THE FEASIBILITY AND ACCEPTABILITY OF TRAINING DIETITIANS TO HAND FEED PEOPLE LIVING WITH DEMENTIA

Christine Ferguson,[1] Joy Douglas,[1] and Beth Nolan,[2] *1. The University of Alabama, Tuscaloosa, Alabama, United States, 2. Positive Approach to Care, Ada, Michigan, United States*

The purpose of this study was to evaluate the feasibility and acceptability of a training program using the interactive Positive Approach to Care□ dementia care curriculum to Registered Dietitians (RDs). To recruit RDs, the Alabama Dietetic Association emailed its member twice about the training opportunity, and a maximum of 25 potential participants could register online. Of those who registered, 80% (20/25) attended the training, and all attendees agreed to the informed consent. The total cost for the two training sessions, including travel, supplies, and labor hours, was approximately $800. The primary challenge for the facilitator was accurately following the script due to time constraints. Two weeks after completing the training, participants answered open ended questions, and many shared how they enjoyed the hands-on activities and watching videos of people living with dementia being fed using the Hand-Under-Hand□ technique. Some participants would have preferred a longer workshop at a location that was more convenient for them. All participants shared how this training may impact their professional practice, such as how it changed the way they approach and communicate with people living with dementia. Since receiving the training, many shared how they have either already started or are interested in sharing the information with interdisciplinary healthcare professionals and/or caregivers. Overall, the training session is considered a feasible, acceptable, and low-cost approach to training RDs on providing hands-on care to people living with dementia. Lengthening the training may improve the replicability of the script in addition to providing opportunities for more hands-on activities.

### THE IMPACT OF EDUCATIONAL TRAINING FOR SUPPORTING CHINESE AMERICAN FAMILY CAREGIVERS

Man Wai Lun, *Borough of Manhattan Community College, new york city, New York, United States*

A pilot self-care educational training, focusing on common caregiving stress, self-care, conflicts, and communication within families for supporting caregiving for family members of old age, was offered to the Chinese American community. After the series of training, 16 participants were asked to evaluate their knowledge of family caregiving as well as the training. Results of the preliminary study revealed that most participants found the training informative but were not sure if they would continue to apply over time. Overall participants reported to be satisfied with the training and receptive to additional training in the future. Results encourage further implementing the training, and investigation of the longitudinal effect of the training to help with family caregiving issues.

### TURNING SENIORS' VOICES INTO ACTION ON ELDER ABUSE: FINDINGS FROM A PROJECT EMPOWERING OLDER ADULTS TO ASSIST PEERS

Jessica Hsieh,[1] Sharon Tan,[2] Raza Mirza,[3] and Lynn McDonald,[1] *1. University of Toronto, Toronto, Ontario, Canada, 2. University of Toronto, Toronto, Canada, 3. National Initiative for the Care of the Elderly, Toronto, Ontario, Canada*

Elder mistreatment, often understood in the context of abuse and neglect, is a growing concern for the health and wellbeing of seniors and their families. A 2015 Canadian prevalence study by the National Initiative for the Care of the Elderly (NICE) found that seniors who are mistreated are more likely to talk to peers, and not clinicians, police or family. However, a lack of knowledge, access to resources, and community stigma may limit seniors' abilities to address mistreatment. This study evaluated the impact on knowledge, attitudes, and behaviours of having seniors deliver workshops on mistreatment to other seniors. A seniors advisory committee developed content for sixteen workshops. Senior facilitators delivered sixteen workshops about mistreatment across Ontario. Participants completed pre/post-surveys assessing changes in knowledge, attitudes, and behaviours. Results indicate that workshops effectively increased awareness of mistreatment issues among participants, on average, by 37.32%. Participants experienced a 43.98% increase in their perceived preparation to provide information to an older adult asking about mistreatment. Barriers to help-seeking among seniors with knowledge of mistreatment include finding trustworthy sources and a lack of legal protection. Prior to the training, healthcare providers were the main sources of information for participants (57.75%); depending on the severity of the situation, 60.43% of participants indicated eventually reporting to police, who are not the preferred source of information. Senior-led workshops about mistreatment appear to be effective for increasing knowledge and encouraging disclosure and help-seeking behaviours. Results support prevention models that empower seniors to educate other seniors on issues around mistreatment.

### VULNERABILITY TO FINANCIAL SCAMS AMONG OLDER ADULTS: COGNITIVE AND PSYCHOSOCIAL FACTORS

Scott Beach,[1] Sara Czaja,[2] Richard Schulz,[1] David Loewenstein,[3] and Peter Lichtenberg,[4] *1. University of Pittsburgh, Pittsburgh, Pennsylvania, United States, 2. Weill Cornell Medicine, New York, New York, United States, 3. University of Miami, Miami, Florida, United States, 4. Wayne State University, Detroit, Michigan, United States*

This paper presents baseline results from a longitudinal study funded by National Institute on Aging examining financial exploitation (FE) among 720 White, African-American, and Hispanic adults age 60 and older. Our proposed conceptual model links socio-demographics, cognitive function, financial skills, and psychosocial factors to both scam exposure and vulnerability, which increases risk for experienced FE. This paper focuses on correlates of scam vulnerability, using a novel measure in which participants read six randomly ordered "scam" scenarios (investment opportunity; Medicare phishing and prescription drug fraud; health product; sweepstakes; telemarketing) and provide credibility ratings for each (1 = Not at all credible; 10 = Extremely credible). Ratings are coded "0" (credibility rating 1); "1" (ratings 2-4); "2" (ratings 5-10) and summed, with total "vulnerability" scores ranging from 0-12. Preliminary results from ~500 participants (Mean age = 73.6) show support for the proposed model. Older adults with poorer cognitive function in multiple domains (memory; reasoning ability; processing speed; other aspects of executive function; and crystallized intelligence); lower financial literacy; lower numeracy; those reporting difficulty with day-to-day financial management; and those performing worse on web-based banking skills tests were more vulnerable to scams. Scam vulnerability was a also higher among those with lower social integration; higher social isolation and loneliness; higher impulsivity; and higher ratings of depression. Use of "scam scenario" credibility ratings shows promise as a simple assessment approach for FE vulnerability. Understanding multiple pathways to FE is important to advance theory and for development of interventions to minimize risk among older adults.

### SESSION 2978 (POSTER)

### ATTITUDES ABOUT AGING II

### AGE-RELATED DIFFERENCE IN THE RELATIONSHIP BETWEEN AGE STEREOTYPES AND HEALTH BEHAVIOR IN MIDLIFE KOREANS

Hyun-E Yeom,[1] Eunyoung Park,[1] and Misook Jung,[2] *1. Chungnam National University, Daejeon, Taejon-jikhalsi, Republic of Korea, 2. Chungnam National University, Daejeon, Republic of Korea*

Aging stereotypes about health may affect the interpretation of health-related changes in midlife. The purposes of this study were to examine the influence of age-related stereotypic beliefs on health behavior linked to health status and stress and to investigate whether the relationship differs by age. This is a cross-sectional study using a convenience sample of 446 Korean middle-aged adults (mean age=50.7). Data were analyzed using the PROCESS macro approach of SPSS. Age-related stereotypic beliefs affected health behavior through the evaluation of health status (Index=-.006, 95% CI [-.002, -.001]) and related stress (Index=-.002, 95% CI [-.004, -.001]). The relationship between age-related stereotypic beliefs and health status significantly differed by age (β= -.02, p=.004), indicating that individuals with stronger age-related stereotypic beliefs tended to report poorer health status as they aged. With regard to the age difference, the influence of age-related stereotypic beliefs affecting health behavior through health status and related stress became relatively stronger when midlife individuals became older. The findings of this study highlight the importance of carefully assessing age-related beliefs based on consideration of age-related differences in middle-aged individuals. Developing psychoeducational strategies to modify the negative and erroneous aging-related beliefs and to improve health behavior is warranted.

### AWARENESS OF AGING MEDIATES THE RELATIONSHIP BETWEEN HEALTH AND CONTROL BELIEFS: A MICRO-LONGITUDINAL STUDY

Shenghao Zhang, and Shevaun Neupert, *North Carolina State University, Raleigh, North Carolina, United States*

Objective: Control beliefs are bidirectionally related to physical and cognitive health, but it is unclear how health influences control beliefs. Health-related experiences (physical symptoms and memory failures) on a particular day can make older adults more aware of their aging, and may subsequently lead to lower control beliefs. We propose that awareness of aging constructs (subjective age and awareness of age-related change [AARC]) could function as mediating mechanisms between health and control beliefs, and examine this relationship from both between- and within-person perspectives separately for domain-general and domain-specific control beliefs. Methods: Older adults (n=116) ranging in age from 60 to 90 (M=64.71) completed a nine-day daily diary study online, resulting in 743 total days. Participants reported their physical symptoms, memory failures, felt age, daily AARC gain and loss experiences, and control beliefs on Days 2-9. Results: Multilevel mediation results showed that between-person AARC losses mediated the relationship between physical symptoms and both domain-general and domain-specific control over physical symptoms. Between-person AARC losses also mediated the relationship between memory failures and both domain-general and domain-specific control over memory. AARC gains and subjective age did not mediate the relationship between health and control beliefs. Discussion: Our findings suggest that between-person differences in AARC losses function as underlying mechanisms linking health and control beliefs. Efforts to reduce AARC losses may lessen the negative impact of health problems on control beliefs for older adults.

### CULTURE, AGE, AND SELF-CONTINUITY: OLD CHINESE SHOWED LOWER CONTINUITY WITH THEIR PAST AND FUTURE SELF THAN AMERICANS

Yi Lu,[1] Lu Cong,[1] Corinna Loeckenhoff,[2] and Xin Zhang,[1] *1. Peking University, Beijing, China, 2. Cornell University, Ithaca, New York, United States*

As culture shapes the way people think and reason, it may also influence their perception of self-continuity, the psychological proximity to the past and the future, across the lifespan. Meanwhile, previous studies in America indicated that advancing age was associated with greater self-continuity. The present research is the first to simultaneously examine how age and culture interact with each other on individuals' continuity with past and future self. Using Ersner-Hershfield's visual scale, we assessed participants' temporal self-continuity at 3 past and 3 future time points (1 year vs. 5 years vs. 10 years) in a sample of 375 Chinese and 91 Americans. A 2(age: young vs. old) x 2(temporal direction: past vs. future) x 2(culture: Chinese vs. American) multilevel analysis was conducted. A significant interaction of age and culture was found, and such interaction revealed that younger Chinese and Americans shared a similar pattern on self-continuity at different temporal distances. However, older Chinese, compared with older Americans, presented a lower level of self-continuity and less variance across temporal distances, suggesting that older Chinese felt less connected with their recent self than both Americans and younger Chinese, and less connected with their remote self than older Americans. These findings fill the gaps in current research by revealing an opposite trend on self-continuity between older Chinese and Americans, and suggest more concern on country differences in this area.

### DEVELOPMENT AND VALIDATION OF THE AGE INTEGRATION SCALE: MEASURING OLDER ADULTS' INTERGENERATIONAL ATTITUDES

Carly Roman,[1] Elizabeth Zelinski,[1] and Christopher Beam,[2] *1. University of Southern California, Los Angeles, California, United States, 2. Dornsife College of Arts and Letters, Los Angeles, California, United States*

Opportunities for age integration (i.e., more intergenerational connections) are increasing among older adults, as a greater number live longer and are capable of interacting with younger individuals. Attitudes toward other generations can be considered a barrier that promotes or discourages intergenerational connections. While existing measures tend to focus on attitudes toward aging or older adults, they do not evaluate older adults' attitudes toward younger individuals. Item response theory (IRT) was used to create an attitude scale evaluating older adults' perceptions of their own age integration with younger individuals. Convenience sampling was used to recruit 100 older adults (55+ years old) who completed a 30-item age integration survey assessing their agreement (on a 5-point Likert scale) with statements about intergenerational beliefs and intentions to have intergenerational connections. IRT analyses supported a 10-item age integration scale indicating a unidimensional construct. The scale consists of items with moderate discrimination and difficulty levels on a 4-point Likert scale. Composite reliability of the 10-item scale was 0.83, which is considered substantial. Tests of convergent validity demonstrated that the total scale score correlated 0.68 with generativity. Discriminant validity tests suggest that the scale does not correlate strongly with satisfaction with life, purpose, depression, need for cognition, or considerations for future consequences, as the correlations ranged from -0.01 to 0.38. This novel measure provides an important, less-considered perspective of intergenerational relationships by assessing older adults' attitudes toward younger individuals. Future studies will validate this scale in a larger, more generalizable sample.

### PERCEPTION OF LIFE-CONTROL IS ASSOCIATED WITH MEDICAL CARE SATISFACTION IN CHRONICALLY ILL RURAL OLDER ADULTS

Anne Halli-Tierney, Hyunjin Noh, and Lewis Lee, *The University of Alabama, Tuscaloosa, Alabama, United States*

Prior studies show patient populations have varied experiences with healthcare systems, and this may influence satisfaction with medical care. Patients feeling control over life circumstances may have resilience and ability to adapt to adverse situations. Given socioeconomic and medical differences in older adults we examined factors influencing satisfaction with medical care in the setting of chronic conditions and pain. 100 older adults in rural West Alabama with chronic illnesses and pain were recruited from community senior centers and interviewed with a structured questionnaire. Participants were queried about medical interventions for chronic conditions and satisfaction with medical care. Overall assessment of life-control was measured by the West Haven Yale Multidimensional Pain Index (WHYMPI). Bivariate correlation and multivariate analysis were conducted. Correlations between satisfaction with medical intervention and life-control scores were significantly positive (r=.21, p<.05). Satisfaction with medical intervention and other covariates explained approximately 14.1% variance in life-control scores, R2=.141. Multivariate regression results confirmed those highly satisfied with medical intervention were marginally significantly likely to have increased life-control scores, b=.20, SE=.12, p=.088. Married persons were more likely to have higher life-control scores than those with other marital statuses, b=.84, SE=.34, p<.05. Income was positively associated with life-control scores, b=.18, SE=.08, p<.05. Older adults may perceive greater satisfaction from medical care if they feel greater control over life circumstances. Socioeconomic factors (marital status, income) are associated with life control. These findings can help predict satisfaction with healthcare and find ways to make healthcare more accessible to all.

### PHILIPPINE OLDER ADULTS' ATTITUDES TO LOVE AND REMARRIAGE

Ju Young Kim,[1] Hanzhang Xu,[2] Truls Ostbye,[3] and Grace Cruz,[4] *1. Duke University, Durham, North Carolina, United States, 2. Duke University School of Medicine, Durham, North Carolina, United States, 3. Duke, Durham, North Carolina, United States, 4. University of the Philippines, Diliman, Quezon City, Philippines*

Attitudes to love in older adults, often operationalized as acceptance of love and re-marriage in their 60s and 70s, is a key yet understudied component of aging in Southeast Asia. Using data from the 2007 Philippine Study on Aging that included 3105 older adults 60+, this study aimed to 1) describe the level of acceptance of love in older adults in the Philippines, 2)assess factors associated with acceptance of love, and 3)assess how acceptance of love is associated with social activity, life satisfaction, and health behaviors. Multivariate logistic regression was used to examine these associations while adjusting for age, gender, urban or rural residence, education, religion, marital status, self-reported health, comorbidity, and physical functioning). Only 1-in-5 older adults in the Philippines reported acceptance of love in older ages. Men and those with good health were more likely to report such acceptance, after adjustment for covariates (P<0.05). Although marital status alone had no association with acceptance of love, marital status interacting with gender showed significant associations with acceptance: unmarried men were more likely than married men to report acceptance. Individuals with lower acceptance of love were more likely to smoke (P<0.01). Attitude towards love was not significantly associated with social activity or life satisfaction after accounting for confounders. By evaluating the health and social outcomes associated with acceptance of love in older adults, this paper provided a better understanding of the utility of attitudes to love in older adults as a metric of elderly health in the Philippines.

### POSSIBLE SELVES ACROSS THE LIFESPAN

Victoria Chen, and Alysson Light, *University of the Sciences, Philadelphia, Pennsylvania, United States*

Possible selves are defined as "personalized representations of one's self in future states" (Cross & Markus, 1991). Research has also found that thinking frequently about possible selves predicts lower well-being, whereas thinking clearly about possible selves is associated with higher well-being (McElwee & Haugh, 2010). However, possible selves differ across the lifespan (Hooker & Kaus, 1994; Cross & Markus, 1991) and life stages can impact exploration of possible identities (Arnett, 2000). Thus we hypothesize that the relationship between thought about possible selves and well-being differs across the lifespan. In a cross-sectional survey, 240 participants (age range: 18-92) reported on frequency and clarity of possible selves, as well as general self-clarity (Campbell et al., 1996). Results indicate curvilinear moderation by age of the link between possible self-clarity and well-being, with the association being stronger among midlife adults than among younger adults and older adults. Also, as clarity of feared possible selves increases, self-concept clarity decreases. Similarly, frequency of thinking about feared possible selves was negatively correlated with self-concept clarity. However, clarity and frequency of thought about hoped-for positive possible selves had no correlation with self-concept clarity.

### PREDICTORS OF PSYCHOLOGIST TRAINEES' ATTITUDES TOWARD AND INTEREST IN FUTURE WORK WITH OLDER ADULT CLIENTS

Grace Caskie,[1] Abigail Voelkner,[2] and MaryAnn Sutton,[2] *1. Lehigh University, Bethlehem, Pennsylvania, United States, 2. Counseling Psychology, Bethlehem, Pennsylvania, United States*

By 2035, 25% of the growing older adult population may be in need of mental health services (Novotney, 2018; Vespa, 2018). However, only a small proportion of psychologists currently identify as geropsychologists; thus, the number of geropsychologists will be insufficient to meet these future demands. Identifying variables that explain the variability in current psychology trainees' expressed interest to engage in future clinical work with older adults is important so that training efforts can be targeted and the number of geropsychologists increased. Based on a multicultural framework and intergroup contact theory, this study examined contact with older adults, empathy, and multicultural competence as predictors of counseling and clinical psychology doctoral trainees' attitudes toward and interest in working with older adult clients. A sample of 311 doctoral trainees (234 clinical PhD/PsyD, 78 counseling PhD/PsyD) were surveyed online. Structural equation modeling tested the hypothesized interrelationships between study variables. The model showed good fit to the data ($\chi 2(82) = 179.803$, $p<.001$, TLI=.93, CFI=.94, RMSEA=.06, SRMR=.06). Greater contact with older adults was significantly related to more positive attitudes about older adults and greater interest in working with older adults. More positive attitudes was significantly related to greater interest in working with older adults. Empathy was significantly related to more positive attitudes, but to less interest in working with this age group. Increasing the amount of contact experiences with older adults as part of doctoral training programs in counseling/clinical psychology may help to enhance trainees' positive attitudes and interest in future clinical work with older adults.

### SOCIAL RELATIONSHIPS AND SELF-PERCEPTIONS OF AGING

Hanamori Skoblow, and Christine Proulx, *University of Missouri, Columbia, Missouri, United States*

Recent research has demonstrated that social relationships are positively associated with self-perceptions of aging (SPA; Santini et al., 2019), although to date, this evidence is cross-sectional. The current study builds on previous work and explores the longitudinal relation between social relationships and SPA, and whether these associations might be buffered by perceived mastery. Using repeated measures data from three waves (2008, 2012, and 2016) of the Health and Retirement Study (HRS), we examined how relationship quality (i.e., support and strain) influenced self-perceptions of aging among adults aged 65+ ($n = 1477$). Greater support from friends in 2008 was significantly associated with better SPA in 2016, and this effect was amplified by high levels of mastery in 2012. That is, older adults with high mastery and high friend support reported more positive SPA than individuals with less mastery or friend support, controlling for gender, age, race, education, income, depressive symptoms, self-rated health, and baseline SPA. Relationship quality with spouse, children, and other family members were not significant predictors of SPA, nor did mastery moderate the association between these relationships and SPA. These results provide evidence for the importance of interpersonal factors such as friendship quality and individual factors in understanding older adults' perceptions of the aging process.

### THE ASSOCIATION BETWEEN RECENT FAMILY DEATH AND SELF-PERCEPTIONS OF AGING AMONG MIDDLE-AGED AND OLDER ADULTS

Shu Xu, *University of Masschusetts Boston, Dorchester, Massachusetts, United States*

The loss of a family member may have a significant influence on one's aging experience in life. Self-perceptions of aging, which are an individual's beliefs or evaluation of their experiences of aging, have been described as an important factor for one's health and daily life. However, there is little research on the association between family death and self-perceptions of aging. This study examines the relationships between recent family death, self-perceptions of aging, and gender of the bereaved among middle-aged and older adults. Using nationally representative data from the Health and Retirement Study (HRS), we conducted cross-sectional analysis on adults age 50 years and older (n=1,839). Self-perceptions of aging were accessed by 8 items derived from the Attitudes Toward Own Aging subscale of the Philadelphia Geriatric Center Morale Scale and the Berlin Aging Study, and we considered recent family death (i.e., parental death, spousal death, sibling death and child death), as well as gender of the bereaved. Multiple linear regression analyses revealed that respondents who experienced recent family death report less positive self-perceptions of aging compared to those who did not experience recent family death ($t = 12.40$, $p < .01$). Recent parental death was more negatively related with self-perceptions of aging for bereaved women than for bereaved men ($\chi 2 = 4.28$, $p < .05$). Findings suggest that middle-aged and older adults experiencing recent family loss have less positive self-perceptions of aging, and gender of the bereaved plays an important role in the relationship between parental death and self-perceptions of aging.

### THE CREATIVE BENEFITS SCALE AS A PREDICTOR OF ATTITUDES ABOUT AGING IN MIDDLE-AGED AND OLDER WOMEN

Sarah Israel,[1] and Carolyn Adams-Price,[2] *1. Mississippi State University, Starkville, Mississippi, United States, 2. Mississippi State University, Mississippi State, Mississippi, United States*

Expectations about aging are connected with both positive traits (e.g., wisdom) and negative traits (e.g., physical frailty). Adams-Price et al. (2016) created the Personal Longevity Scale (PLS), which measures positive expectations (Hope) and negative expectations (Dread). The purpose of this study was to examine how psychological benefits gained from participating in creative hobbies may positively impact middle-aged and older women's attitudes about aging. In addition, we examined if participant age moderated this relationship. A total of 198 women, aged 50 to 82 years old, completed the Personal Longevity Scale (Hope and Dread subscales) and the Creative Benefits Scale (CBS; used to measure psychological benefits that people may experience as a result of long-term participation in a creative hobby). The CBS includes four subscales: gaining a sense of identity (Identity), getting a sense of relaxation (Calming), feeling closer to God or nature (Spirituality), and receiving recognition from others for one's hobby (Recognition). Single moderation models suggest that higher Identity scores were linked to more positive attitudes about aging but did not significantly predict negative attitudes. Age provided a marginally significant moderation effect to this relationship such that older women who received more of a sense of identity from their hobby reported the most positive attitudes about aging. Spirituality was linked to more positive attitudes about aging but did not significantly predict negative attitudes. Neither Calming nor Recognition were predictive of either positive or negative attitudes about aging. Implications for women's development will be discussed from an Eriksonian perspective.

### UNDERSTANDING ELDERSPEAK: AN EVOLUTIONARY CONCEPT ANALYSIS

Clarissa Shaw,[1] Jean Gordon,[1] and Kristine Williams,[2]
*1. University of Iowa, Iowa City, Iowa, United States,*
*2. University of Kansas, Lawrence, Kansas, United States*

Elderspeak is an inappropriate simplified speech register that sounds like baby talk and is commonly used with older adults, especially in health care settings. Understanding the concept of elderspeak is challenging due to varying views about which communicative components constitute elderspeak and whether elderspeak is beneficial or harmful for older adults. Rodger's evolutionary concept analysis method was used to evaluate the concept of elderspeak by identifying its attributes, antecedents, and consequences. A systematic search using the PubMed, CINAHL, PsychINFO, and Embase databases was completed, resulting in the review of 83 articles. Elderspeak characteristics were categorized by semantic, syntactic, pragmatic, paralinguistic, and nonverbal attributes. The primary antecedent to elderspeak is ageism in which old age cues and signs of functional or cognitive impairment lead to simplified communication from a younger caregiver. Research studies vary in reporting whether elderspeak facilitates or interferes with comprehension by older adults, in part depending on operational definitions and experimental manipulation. Overall, exaggerated prosody, a key feature of elderspeak, is found to reduce comprehension. Elderspeak is generally perceived as patronizing by older adults and speakers are perceived as less respectful. In persons living with dementia, elderspeak also increases the probability of resistiveness to care which is an important correlate of behavioral and psychological symptoms of dementia. Based on this concept analysis, recommendations for consistent operationalization of elderspeak in future research are made. A new definition of elderspeak is proposed, in which attributes that have been found to enhance comprehension are differentiated from from those that do not.

### WHAT FACTORS INFLUENCE THE PERCEPTION OF LONGEVITY?

Camela Barker,[1] Laura Lerebours,[1] Benjamin Gary,[1] Olivia Harper,[1] Devyn Dixon,[1] Tyson Brown,[1] Roland Thorpe, Jr.,[2] and Keith Whitfield,[3] *1. Duke University, Durham, North Carolina, United States, 2. Johns Hopkins University, Baltimore, Maryland, United States, 3. Wayne State University, Detroit, Michigan, United States*

Previous research has highlighted the link between health-related factors and longevity. In particular, indices such as poor cardiovascular reactivity, high BMI, stress, and respiratory functioning have been shown to increase risk of mortality. However, little is known about identifying specific health and psychosocial behaviors that compromise the perception of longevity in African Americans. The Study of Longevity and Stress in African Americans (SOLSAA) was designed to examine psychosocial and health factors that are characteristic of survivorship in African Americans families. Data from 570 participants in the SOLSAA were used to examine the relationship between psychosocial and health-related factors among individuals aged 50 to 99 years (M = 67.6, SD = 11.5). Perception of longevity was positively correlated with sex, r = .058, education, r = .021, diastolic blood pressure r = .067, respiratory functioning, r = .150 and body mass index, r = .027, but negatively correlated with age, r = -.205, systolic blood pressure, r = -.050, and perceived stress, r = -.106. Regression analyses revealed, not surprisingly, that as participants aged, perception of longevity decreased. Furthermore, women's perception of longevity was greater than that of men even after controlling for age. Given the longer life expectancy for women, this was also expected. Interestingly, high levels of perceived stress predicted reduced perception of longevity. These preliminary results suggest that the perception of stress is an important psychosocial factor for older African American's self-forecasting of their chances for longevity beyond just simply age or physical health status.

### SESSION 2979 (POSTER)

### CHRONIC DISEASE MANAGEMENT II

### A PRELIMINARY ANALYSIS OF HEARING LOSS AND ITS ASSOCIATION WITH MILD COGNITIVE IMPAIRMENT

Mary Caroline Yuk,[1] Rebecca Allen,[2] Marcia Hay-McCutcheon,[3] Dana Carroll,[4] and Anne Halli-Tierney,[1] *1. The University of Alabama, Tuscaloosa, Alabama, United States, 2. Alabama Research Institute on Aging, Tuscaloosa, Alabama, United States, 3. Audiology, Tuscaloosa, Alabama, United States, 4. Auburn University Harrison School of Pharmacy, Tuscaloosa, Alabama, United States*

Age related hearing loss, or presbycusis, is a global condition that is increasing in its prevalence. Despite being one of the most common chronic conditions among the older population, there is much more to understand about its association with other aspects of physical and emotional health and well-being. Current research is suggesting that hearing loss is more prevalent in those with cognitive impairment compared to those without cognitive impairment. This study analyzed the incidence of hearing loss and its linkage to mild cognitive impairment in a community-dwelling geriatric population. With the increasing prevalence of this condition in both rural and urban communities of Alabama, it becomes a more pressing matter to understand comorbidities and risk factors for future decline in functioning. This study was conducted in an interdisciplinary geriatrics primary care outpatient clinic in a Family, Internal, and Rural Medicine department affiliated with a university medical center in the Deep South. Ninety-one participants completed the Montreal Cognitive Assessment (MoCA) and a hearing screening. Hearing screenings were conducted in quiet rooms in the medical center using Phonak hearing screening cards. Detection of 500, 1000, 2000, and 4000 Hz tones was assessed. Pearson correlation analyses demonstrated an association between hearing loss mild cognitive impairment. Poorer hearing was significantly associated with lower scores on the MoCA. Conducting behavioral health screenings like this in other primary geriatrics clinics and community settings could improve care and identification of patient needs by integrating important data regarding comorbidities and independent living.

### AIN'T I SUPPOSED TO BE DOING SOMETHING?: IDENTIFYING AND DISCUSSING COGNITIVE ISSUES IN LUPUS

Ann Vandenberg,[1] Cristina Drenkard,[1] Felicia Goldstein,[1] Charmayne Dunlop-Thomas,[1] S. Sam Lim,[1] C. Barrett Bowling,[2] and Laura Plantinga,[3] *1. Emory University School of Medicine, Atlanta, Georgia, United States, 2. Duke University Medical Center, Durham, North Carolina, United States, 3. Emory University, Atlanta, Georgia, United States*

Lupus is a complex chronic disease associated with a high prevalence geriatric syndromes and reduced cognitive functioning, consistent with accelerated aging. Although patients with lupus commonly report cognitive symptoms or "lupus fog," cognitive assessment is not routine and little is known about day-to-day cognitive problems and their effect on chronic disease management. As part of a pilot exploring the use of a cognitive functioning report prototype for shared decision making in clinical encounters (Approaches to Positive Patient-Centered Experiences of Aging in Lupus study), we conducted four focus groups, two with lupus patients (n=18) and two with lupus providers (physicians and nurses; n=9) addressing cognition. We compared how the NIH Toolbox fluid cognition domains (episodic (retrospective) memory, working memory, processing speed, attention and inhibitory control, cognitive flexibility, and overall fluid cognition) matched with patient- and provider-identified cognitive problems and needs. In open questioning using a standard interview guide, patients identified all domains with rich experiential examples; providers identified fewer domains with less detail. An unanticipated additional domain was prospective memory, problems in remembering future actions, whether in the near future (e.g., forgetting what they needed to do on entering a room) or in the not-so-near future (e.g., forgetting about a medical appointment). Technological aids (e.g., smartphone alerts) were mentioned by some patients, but not providers, and represent a potential opportunity for medical care. Providers envisioned using cognitive assessment to distinguish cognitive issues (e.g., forgetting to take medication) from other problems (e.g., medication nonadherence) in order to tailor chronic disease management messaging.

### COPING STYLES AND STROKE KNOWLEDGE MEDIATE THE EFFECT OF SOCIAL SUPPORT AND UNCERTAINTY AMONG STROKE PATIENTS

Humin Zhang,[1] Jie Gao,[1] Yuyan Sun,[2] and Ziting Han,[3]
*1. Xinxiang medical university, Xinxiang, Henan, China,*
*2. Xinxiang Medical University, Xinxiang, Henan, China,*
*3. Xinxiang Medical University, ZhengZhou, China*

Objectives:This study aimed to investigate the mediating roles of coping styles and stroke knowledge between social support and uncertainty in illness among patients with primary stroke in China. Methods:The total of 204 Chinese primary stroke patients recruited using convenience sampling were asked to answer Mishel Uncertainty in Illness Scale for Adult(MUIS-A), Stroke Knowledge Questionnaire(SKQ), Social Support Rating Scale(SSRS), and Medical Coping Modes Questionnaires (MCMQ). Demographics characteristics of the patients were presented using descriptive statistics. We reported the relationship between the study variables using Pearson's Correlation Coefficients. We performed structural equation modeling to estimate the mediator effect of coping styles and stroke knowledge between social support and uncertainty in illness.Results: The results showed that 92% of patients with primary stroke had moderate above level of uncertainty in illness, with a mean score 75.04 (SD=9.61).Uncertainty was positively associated with coping styles ($r=0.232$, $P < 0.01$), and negatively associated with social support($r=-0.237$, $P < 0.01$) and stroke knowledge ($r=-0.386$, $P < 0.01$).The structural equation model indicated that the coping styles(19.8% of total effect) and stroke knowledge (38.5% of total effect)respectively acted as mediator role between social support and uncertainty in illness.Conclusions:Most patients with primary stroke present moderate above level of uncertainty in illness. stroke knowledge and coping styles were important mediating factors in the pathway between coping styles and uncertainty in illness. Our findings suggest the provision of stroke knowledge and training of coping styles for patients with primary stroke could alleviate their uncertainty in illness.

### FUNCTIONAL DISABILITY IN THE CONTEXT OF CANCER DIAGNOSIS: EXAMINING THE WITHIN- AND BETWEEN-PERSON ASSOCIATIONS

Rumei Yang,[1] Yin Liu,[2] and Elizabeth Fauth,[2] *1. Nanjing Medical University, Nanjing, China, 2. Utah State University, Logan, Utah, United States*

Physical functioning is essential for independent living and aging in place among older adults. Although older adults show a general trend of increasing functional disability, it was unclear how cancer diagnosis, which also has an increased incidence rate among older adults, may potentially aggravate the progress of functional decline at the within- and between-person levels. Using 3 waves of the China Health and Retirement Longitudinal Study (CHARLS, 2011-2015), the current study examined the trajectory of functional disability and associated factors over 5 years among older adults with (n = 139) and without cancer (n = 212), who were matched on key demographic characteristics. We considered four contributing factors to disability as specified in the Disablement Process model as covariates, in the context of cancer diagnosis timing at both the within- and between-person levels. These factors were psychological (i.e., depressive symptoms), physical limitations (i.e., pain, fall, and frailty), cognitive impairment (i.e., self-reported memory problems and word recall), and environmental constraints (i.e., social contact and availability of support). Consistent with the literature, there was a general trend of increasing functional disability among all participants over time. At the within-person level, although cancer diagnosis timing was associated with higher levels of functional difficulty, we found a slower increasing rate over time. At the between-person level, functional disability trajectory was not associated with cancer diagnosis timing. These findings suggest that maintenance of physical functioning should be considered at the within-person level, and personalized care is recommended for Chinese older adults with cancer.

### MARITAL STATUS AND GENDER DIFFERENCES IN END-OF-LIFE PLANNING

Lauren Stratton, Naomi Meinertz, and Suzanne Bartholomae, *Iowa State University, Ames, Iowa, United States*

Less than half of older adults make formal preparations to control difficult life transitions, yet planning can mitigate negative outcomes that accompany changes. To better understand multiple forms of end-of-life planning behaviors and the effect of demographic characteristics, we use data from the Understanding America Study (N=1812; aged 60+). We explored gender and marital differences in whether respondents created a will, living will, or declared a durable power of attorney for health care (DPOAHC), and who was chosen as DPOAHC (i.e., spouse, child, other relative). T-tests determined no significant gender differences in declaring a DPOAHC, creating a will, or living will. An analysis of variance (F=13.422, df=3, p<0.001) found married respondents more likely to declare a DPOAHC than separated/divorced (p<0.001) and never married respondents (p=0.002). Divorced respondents were less likely to declare a DPOAHC compared to widowed respondents (p<0.001) and widowed respondents were more likely compared to never married respondents (p=0.001). Additional analyses found the same pattern in who created a will or living will. A follow-up analysis of variance found no gender differences within marital status categories (i.e., married men versus married women). Overall, respondents who were never married were less likely to have prepared any forms of end-of-life planning. These results highlight the importance of having close relationships on the level of preparedness for end-of-life planning. Additional implications include increasing awareness about end-of-life planning for more vulnerable audiences. Future analyses will integrate findings regarding financial DPOAHC and examine other socio-demographic characteristics, such as education and race.

### PREDICTORS OF CHANGE IN OSTEOPOROSIS KNOWLEDGE, HEALTH BELIEFS, AND SELF-EFFICACY AFTER AN EDUCATION INTERVENTION

Philippe Ronel Labrias,[1] Richard Feinn,[2] Shanthi Johnson,[3] and Katherine McLeod,[1] *1. Frank H. Netter MD School of Medicine, Hamden, Connecticut, United States, 2. Frank H. Netter MD School of Medicine Quinnipiac University, Hamden, Connecticut, United States, 3. University of Alberta, Edmonton, Alberta, Canada*

Education interventions that increase osteoporosis knowledge and address health beliefs and self-efficacy help older adults make informed decisions to prevent and manage the disease. The aim of this study was to determine if clinical risk factors for osteoporosis moderate the effect of a multifaceted education intervention on osteoporosis knowledge, health beliefs, and self-efficacy. Patients 50 years and older with no prior diagnosis of or treatment for osteoporosis were referred by their primary care provider for bone mineral density testing by DXA and randomized to an osteoporosis education intervention group (n = 102) or usual care group (n = 101). Demographic and health history questionnaires, and validated tools to assess osteoporosis knowledge, health beliefs and self-efficacy were completed at baseline and 6-month follow-up. Results of the linear mixed-effects model showed a significant interaction with younger age (p=.024) on self-efficacy among patients in the intervention group compared to the usual care group. Patients with higher BMI had greater perceived health motivation (p=.026) in the intervention group. Compared to the usual care group, patients in the intervention group with higher vitamin D intake had greater perceived exercise (p=.020) and calcium benefits (p=.012) and those with a family history of osteoporosis had greater perceived susceptibility to osteoporosis (p=.045). By understanding the key factors that predict change in knowledge, health beliefs and self-efficacy after an education intervention compared to usual care, we can better tailor interventions to enhance prevention and management of osteoporosis.

### SOCIODEMOGRAPHIC AND MARITAL DIFFERENCES IN SPOUSAL INVOLVEMENT IN A PARTNER'S DIABETES DIET

Paulene Castro,[1] Kristin August,[2] and Dara Sorkin,[3] *1. Rutgers University-Camden, Oaklyn, New Jersey, United States, 2. Rutgers University, Camden, New Jersey, United States, 3. University of California, Irvine, Irvine, United States*

Spouses are commonly involved in their partners' diabetes management by supporting and regulating (i.e., controlling) their diet. Little is known, however, about what characteristics are associated with how and how often spouses are involved in this context. This study examined whether sociodemographic and marital characteristics helped explain some of the variability in diet-related spousal involvement in promoting a partner's adherence to a diabetes diet; specifically, whether gender, race/ethnicity, marital quality, and marital length were related to the frequency of spousal engagement in health-related social support and two types of health-related social control. Gender and race/ethnicity were examined as exploratory moderators of the associations between marital characteristics and spousal involvement. Data from two different data sets of older adults (55+ years) whose partners had type 2 diabetes were examined among four racial/ethnic groups (study 1 n = 205; study 2 n = 155). Regression analyses that controlled for patients' co-morbid health conditions revealed gender and racial/ethnic differences in the frequency of spousal involvement. In addition, marital quality was related to the frequency of support and positive forms of social control among most participants, particularly African American spouses. No associations between marital length and any type of spousal involvement were found, nor were there any gender differences in any of these associations. These findings provide insight into the importance of sociodemographic characteristics and marital quality in understanding spousal involvement in a partner's diabetes management.

### STEP UP: AN EVALUATION OF QUESTION-BEHAVIOR EFFECTS IN DIABETES FOOTCARE IN MIDDLE-AGED AND OLDER ADULTS

Anna Robertson,[1] Katherine Johanson,[1] Allison Walden,[2] Rebecca Ingram,[3] Lauren Schneider,[1] and Leilani Feliciano,[1] *1. University of Colorado, Colorado Springs, COLORADO SPRINGS, Colorado, United States, 2. University of Colorado, Colorado Springs, Colorado Springs, Colorado,*

*United States, 3. UCCS, Colorado Springs, Colorado, United States*

Type 2 diabetes mellitus (T2DM) is a major public health concern requiring engagement with specific self-care activities for improvement. Due to the increased risk for neuropathy, foot ulcers, and infections, engagement with certain footcare practices (e.g., inspecting feet and inside shoes, washing and drying between toes) is an important part of overall diabetes self-care. However, interventions targeting blood glucose monitoring, medication management, nutrition, and/or physical activity often precede footcare interventions. Question-Behavior Effects (QBEs), which describe the effects of behavior questioning on future engagement in that behavior, provide a unique framework for studying diabetes self-care. Although clinicians may not administer footcare interventions for people with T2DM, footcare behaviors are often still assessed (e.g., through questionnaires) in session and, consistent with QBEs, may improve through the repetition of questioning. The present study evaluated footcare QBEs (specifically, the number of days of foot inspection and cleaning in the past week) across 7 sessions of an at-home diabetes management program without intentional footcare intervention. Paired-samples t-tests (N = 78, ages 40-88 years) revealed significant improvements in the number of days of foot inspection and cleaning from intake to session 1 and intake to session 5. However, from intake to session 7, only significant improvements in foot inspection were detected. These findings reflect the potency of footcare QBEs in the absence of specific footcare interventions. They also reveal differential engagement in foot inspection and cleaning after an extended period of questioning, suggesting certain diabetes self-care behaviors may taper without the intentional introduction of intervention.

### STRATEGIES TO SUPPORT PHYSICAL ACTIVITY PARTICIPATION IN OLDER ADULTS WITH HEART FAILURE

Katherine Hall,[1] Amy Pastva,[2] Heather King,[2] Sean Lowers,[3] Julie Miller,[3] and Jennie Riley,[4] *1. Duke University, Veterans Health Administration, Durham, North Carolina, United States, 2. Duke University School of Medicine, Durham, North Carolina, United States, 3. Duke University, Durham, United States, 4. Duke Roybal Center, Durham, North Carolina, United States*

Physical activity (PA) is recommended for people living with heart failure (HF). Despite evidence of its benefits, participation in PA is low in this population, putting them at risk for loss of functional independence and additional health burdens. The aim of this pilot study was to ask older adults living with stable, chronic HF to identify strategies to support regular PA. Patients in an outpatient HF rehabilitation program were recruited to participate in focus groups about their PA knowledge, attitudes, and preferences as part of a stakeholder engagement project. At the beginning of the focus group, participants completed a questionnaire listing 8 potential strategies to optimize PA, and were asked to identify the top 4 strategies that they thought would be most beneficial to support regular PA participation. This was the focus of the current analysis. Thirteen adults with HF (M age=65; 46% female; 62% African American; M BMI=32.6 kg/m2) were enrolled. Top-rated strategies endorsed by participants to support long-term adherence to PA included provision of an exercise guide to support home-based exercise and supplement health provider-supervised exercise sessions (69%), group education classes (64%), completion of fitness assessments at regular intervals (62%), and provision of a transition pathway from an exercise rehabilitation program to a community-based exercise program (62%). The remaining strategies were endorsed by fewer than 50% of participants, and included remote delivery and support options. These results have important implications for future program development and implementation efforts to support PA among older adults with stable, chronic HF.

### SUPPORTING PERSONS WITH SEVERE DEMENTIA IN COMMUNICATING THEIR PREFERENCES

Vanessa Burshnic,[1] and Michelle Bourgeois,[2]
*1. Durham VA Medical Center, Durham, North Carolina, United States, 2. University of South Florida, Tampa, Florida, United States*

Government mandates require US nursing homes to provide preference-based, person-centered care. Persons with dementia (PWD) are less likely to have a role in preference assessments (PAs) used for care planning due to communication challenges associated with the disease. Thus, PWD are at risk of receiving de-personalized treatments. External supports (photograph and text cues) are known to improve communication in PWD. Yet these cues have never been studied with widely used PAs, such as the MDS 3.0 Section F and Preferences for Everyday Living Inventory (PELI). This study examined the effect of two PA conditions (externally supported; standard verbal) on preference consistency and response types (off-topic, clarification requests, elaboration) of residents with severe dementia (N=21) (BIMS < 7) when assessed twice, one-week apart. PA questions were derived from the MDS 3.0 Section F and PELI. As a social validity measure, naïve judges (N=10) listened to interviews and rated residents' communication clarity and their confidence understanding residents' preferences. Results showed that neither condition promoted significantly greater levels of consistency over time. Residents' 'clarification requests' were significantly fewer with use of external supports. Other response types were not significantly different across conditions. Judges' ratings were not significantly different across conditions; however, they rated residents' communication as clear and understandable overall. This study addresses a gap in current research and holds important implications for helping providers meet government mandates and enhance care plan participation by residents with severe dementia and other communication challenges.

### SESSION 2980 (POSTER)

### EMOTIONAL HEALTH AND REGULATION

### AGE AND COGNITIVE ABILITY PREDICT PERCEIVED DEMANDS OF SPECIFIC EMOTION REGULATION STRATEGIES

Claire Growney,[1] and Tammy English,[2] *1. North Carolina State University, Raleigh, North Carolina, United States, 2. Washington University in St. Louis, University City, Missouri, United States*

Certain emotion regulation (ER) strategies are often considered to be more or less demanding of cognitive resources. However, age-related differences in the perceptions of these demands are not yet understood. Older adults might perceive greater demands for certain strategies due to differences in cognitive ability and motivation to maintain emotional well-being. In the present study, we examined age and cognitive ability as predictors of perceived effort required to use ER strategies that span all families of the process model. A diverse sample of community participants (age 22-83) completed assessments of cognitive ability and perceived demands associated with ten ER strategies. Overall, response-focused strategies were rated as highest in demands whereas situation selection and savoring were perceived as least demanding. Older adults reported higher demands associated with situation selection, distraction, and detached reappraisal (but not positive reappraisal) compared with younger adults. Cognitive ability was not associated with perceived demands for ER strategies traditionally viewed as cognitively demanding (e.g., suppression). Rather, higher cognitive ability only predicted lower perceived demands for strategies often considered low in demand: situation selection and savoring. Perceived ER success was not consistently associated with age or cognitive demands. Results suggest that older adults view some, but not all, ER strategies as more demanding than younger adults do. The role of cognitive ability in age-related changes in ER may be more complex than previously expected. Notably, the lack of findings with perceived ER success suggest effort requirements associated with ER may not impede ability to successfully regulate across adulthood.

### AGE DIFFERENCES IN THE AFFECTIVE EXPERIENCE OF STATE CURIOSITY

Li Chu,[1] and Helene Fung,[2] *1. The Chinese University of Hong Kong, Shatin, New Territories, China, 2. The Chinese University of Hong Kong, Sha Tin, China*

Is feeling curious a pleasant, anxious or mixed feeling experience? Dual process theory posits that curiosity results from an optimal level of knowledge gap anxiety. Yet, personal growth facilitation model suggests that people are intrinsically curious, which is associated with positive affects. While curiosity may be pleasant or anxious, it may also be both. In fact, compared with younger adults, older adults were more likely to experience mixed emotions. However, very few studies investigated age differences in affective experience of curiosity, so the present study utilized a time-sampling dataset to address this question. This 14-day time-sampling study included 85 younger (43 females, age 18-30) and 83 older adults (40 females, age 60-85) who recorded momentary curiosity and affective experiences three times per day. Linear mixed-effects analysis revealed a significant 3-way interaction between age group, happiness and anxiousness on state curiosity (□=.20, SE=.05, p<.001). For younger adults, results suggested that curiosity was higher when they felt either happy or anxious but not when feeling both. Conversely, for older adults, curiosity was higher when they felt both happy and anxious concurrently. In other words, older adults were more likely to experience curiosity as a mixed emotional state, whereas younger adults were more likely to experience curiosity as a pure emotional state. This finding adds to the current mixed emotion and aging literature and has important implications for future interventions to enhance curiosity towards novelties for people from different age groups.

### AGE MODERATES THE EFFECT OF AWE ON COGNITIVE BUT NOT EMOTIONAL WELL-BEING

Laura Bernstein, and Julie Patrick, *West Virginia University, Morgantown, West Virginia, United States*

Evidence suggests that positive emotions may broaden and build our emotional and physical health, and cognitive resources (Fredrickson, 2001). A growing literature shows that happiness and joy can be powerful means for growth. In contrast to happiness, which pushes one to expand and accommodate, research suggests that awe, that feeling of being in the presence of something immense or transcendent, prompts the urge to assimilate. Although promising, few examinations have included older adults and a limited range of positive emotions have been examined. Thus, we sought to address this gap in the literature by assessing the influence of age and awe on emotional well-being. Data from 180 adults (M age ~ 38; range 18 – 89) were used to examine the main effects of age and dispositional awe (Shiota et al., 2006), and their interaction, on emotional well-being. Only 12% of the variance was explained [X2 (DF = 9) = 344.27, p < .001]. Awe was positively associated with emotional well-being (Beta = .280*), but neither age nor the interaction between age and awe contributed to the variance explained. We conducted a similar examination with perceived cognitive health [X2 (DF=9) = 337.09, p < .001; R^2 = .235]. A main effect for age and a significant age by awe interaction uniquely contributed to the variance explained in cognitive well-being. A similar model was tested with self-assessed health as the outcome. Neither main effects nor the interaction emerged as significant. Results are discussed within the context of age-invariant contributors to well-being.

### AGE SIMILARITY IN EMOTION PERCEPTION BASED ON EYE GAZE MANIPULATION.

Yosra Abualula,[1] and Eric Allard,[2] *1. Cleveland State University, Lakewood, Ohio, United States, 2. Cleveland State University, Cleveland, Ohio, United States*

The purpose of this study was to examine age differences in emotion perception as a function of emotion type and gaze direction. Old and young adult participants were presented with facial images showing happiness, sadness, fear, anger and disgust while having their eyes tracked. The image stimuli included a manipulation of eye gaze. Half of the facial expressions had a directed eye gaze while the other half showed an averted gaze. A 2 (age) x 2 (gaze) x 5 (emotion) repeated measures ANOVA was used to analyze emotion perception scores and fixation to eye and mouth regions of the face. The manipulation of eye gaze yielded more age similarities than differences in emotion perception. Overall, we did not detect age differences in recognition ability. However, we found that certain emotion categories differentially impacted emotion perception. Interestingly, we observed that an averted gaze led to beneficial performance for fear and disgust faces. Additionally, participants spent more time fixating on the eye regions of sad facial expressions. We discuss how naturalistic manipulations of various facial features could impact age-related differences (or similarities) in emotion perception.

### DOES DISCRIMINATION ATTENUATE AGE-BENEFITS IN EMOTIONAL HEALTH?

Daisy Zavala,[1] Elizabeth Munoz,[2] Martin Sliwinski,[3] and Stacey Scott,[4] *1. Stony Brook University, Nesconset, New York, United States, 2. The University of Texas at Austin, Austin, United States, 3. Penn State University, University Park, Pennsylvania, United States, 4. Stony Brook University, Stony Brook, New York, United States*

Perceived discrimination has been found to negatively impact emotional health (e.g., depression, psychological distress). Few studies, however, have examined this association across adulthood. Strength and Vulnerability Integration Theory (SAVI) proposes that there may be age-benefits in emotion regulation, however these may be reduced when faced with stressors. We examined whether two forms of stress related to discrimination (i.e., subtle and major) moderated expected age-benefits in emotional outcomes. We predicted that individuals who experienced more discrimination would display worse emotional outcomes, and that this effect would be stronger with older age. Participants were 334 diverse adults (25-65 years, Mage = 47, 63% Female) from Bronx, New York. They reported major and subtle discrimination experiences, depressive symptoms, positive affect (PA), and negative affect (NA). Subtle discrimination was associated with greater NA and depressive symptoms and lower PA (ps <.0001), but the associations with depressive symptoms and PA did not vary by age. Age, however, moderated the relationship between subtle discrimination and NA (b = .022, t (22) = 2.04, p<.05). Specifically, the slope between subtle discrimination and NA was stronger with older age. Greater major discrimination was associated with greater NA and depressive symptoms (p<.05), but these effects were age invariant. These results demonstrate that subtle and major discrimination are associated with poorer emotional health across individuals. Partially consistent with SAVI, stressful experiences like subtle discrimination may serve as a boundary condition for age-benefits in emotional outcomes.

### EFFECTS OF PERSONALITY AND EMOTION REGULATION IN DEPRESSED OLDER ADULT INPATIENTS

Wing Jin Mak,[1] Ira Yenko,[2] Avner Aronov,[3] Hyunyoung Park,[4] Helene Geramian,[4] Jennifer Ho,[4] Dimitry Francois,[5] and Richard Zweig,[4] *1. Ferkauf Graduate School of Psychology, Yeshiva University, The Bronx, New York, United States, 2. Yeshiva University Ferkauf Graduate School of Psychology, Mountain View, California, United States, 3. Yeshiva University, Ferkauf Graduate School of Psychology, Brooklyn, New York, United States, 4. Yeshiva University Ferkauf Graduate School of Psychology, The Bronx, New York, United States, 5. Weill Cornell Medicine, White Plains, New York, United States*

Personality pathology and emotion dysregulation are associated with impaired psychological and functional outcomes in adults. However, much less is known about their relationship with depression, social functioning, and suicidal behaviors among older clinical samples. The aim of this study was to examine how the two impact depression, social adjustment, and suicidal behavior in an inpatient sample of depressed adults 55 to 89 years of age (N=52). Personality (agreeableness and neuroticism) and suppression strategies (expressive and thought suppression) were first investigated individually and then jointly as a combined predictive model. Results found lower agreeableness predicted poorer social adjustment ($\beta$ = -2.871, p < .01), while higher neuroticism ($\beta$ = 0.426, p<.01) and greater use expressive suppression ($\beta$ =.253, p <.05) each predicted more severe depressive symptoms. Individuals high in neuroticism also evidenced greater use of both thought suppression and expressive suppression, while those who were more depressed endorsed poorer social functioning. Although not reaching the point of statistical significance, lower agreeableness, higher use of suppression strategies, and poor social functioning were found to be moderately associated with experience of recent suicidal ideation. Our findings demonstrated personality pathology and ineffective emotion regulation are promising potential pathways in the detection and intervention of psychological and functional impairments in depressed older adults. Our study also highlighted the need for more research examining the effects of personality pathology and emotional dysregulation from a functional perspective, which could enhance the focus of target problem areas in interventions for severely depressed older adults.

### EMOTIONAL AND SITUATIONAL ACCEPTANCE ACROSS THE LIFESPAN: A NOVEL SCALE

Hannah Wolfe, and Derek Isaacowitz, *Northeastern University, Boston, Massachusetts, United States*

Self-reported emotional well-being tends to increase with age (Charles & Carstensen, 2007), but evidence for age differences in emotion regulation strategies is mixed (Livingstone & Isaacowitz, 2019), and the strategy of acceptance, in particular, is relatively understudied. Acceptance involves the deliberate decision to not alter a situation or one's emotional response to it, and older adults report greater use of general acceptance (Shallcross, Ford, Floerke, & Mauss, 2013). Yet, no current scale distinguishes between situational and emotional acceptance; general acceptance is typically measured using a subscale of the Kentucky Inventory of Mindfulness Skills (KIMS; Baer, Smith, & Allen, 2004), which assesses judgments of emotions and thoughts. Therefore, a 6-item measure of situational acceptance was developed and administered to 24 younger adults (age 18-25) and 30 older adults (age 55+) on Amazon Mechanical Turk, along with the KIMS accepting subscale and Mindful Attention Awareness Scale (MAAS; Brown & Ryan, 2003). The situational acceptance scale achieved good reliability ($\alpha$=.721) and significantly correlated with the MAAS (r= .301, p=.027) and KIMS (r= .466, p<.001). Older adults tended to rate themselves as significantly higher on situational acceptance (M=29.83, SD=5.17) than younger adults (M=25.13, SD=5.72; t=-3.171, p=.003), and this pattern held for the MAAS and KIMS. These results confirm prior work suggesting older adults engage in acceptance more often than younger adults and expand this finding to situational, not just emotional, acceptance. Furthermore, skills related to mindfulness and acceptance appear to greatly overlap and may increase over the lifespan.

### EMOTIONAL HEALTH AND MORTALITY OF OLDER PARENTS OF CHILDREN WITH SERIOUS DEVELOPMENTAL DISABILITIES

Jessica Hoyle,[1] Sarah Laditka,[2] and James Laditka,[2]
*1. University of North Carolina - Charlotte, Charlotte, North Carolina, United States, 2. University of North Carolina at Charlotte, Charlotte, North Carolina, United States*

Caring for a child with serious developmental disability (SDD) involves stress as parents age and anticipate the child's future welfare. There are few studies of older parents of children with SDD. We followed parents for 18 years using the Panel Study of Income Dynamics (PSID). We defined SDD as: (1) autism, intellectual disability, learning disorder, epilepsy with seizures, attention deficit disorder/attention deficit hyperactivity disorder, or cerebral palsy; requiring (2) qualification for services and serious lasting impairment evidence. We used the PSID and its Child Development Supplement (1997-2007, 2014), linking children's and parents' data, with 5,780 parent-child dyads and 45,534 analytic observations. Parent outcomes included physician-diagnosed anxiety or depression, psychological distress (Kessler K6), and death. Discrete-time hazard analysis controlled for child and parent characteristics, and survey design. We identified parents of: Group 1, children without SDD or challenging behaviors; Group 2, SDD without challenging behaviors; Group 3, challenging behaviors without SDD; Group 4, SDD with challenging behaviors. Among parents ages 60+ and compared to Group 1, Group 2 through 4 odds ratios for anxiety/depression were, respectively, 1.28 (95% confidence interval, 0.63-1.92), 1.79 (1.35-2.24), and 2.62 (2.25-2.99), p-trend <.0001. Group 4 mental health risks were particularly high when children lived with the older parent: for anxiety/depression OR 6.75 (3.00-15.21), and distress OR 8.83 (3.58-21.77). At ages 60+ mortality was higher for parents of children with SDD (relative risk, RR 2.38, 1.21-4.67), especially Group 4 (RR 4.64, 1.39-15.47). Parents of children with developmental disabilities need emotional support, respite, and interventions addressing challenging behaviors.

### EMOTIONAL SALIENCE AND INTERPERSONAL PROBLEMS IN DEPRESSED OLDER ADULTS WITH PERSONALITY PATHOLOGY

Avner Aronov,[1] Helene Geramian,[2] Jennifer Ho,[2] Wing Jin Mak,[3] Ira Yenko,[4] Hyunyoung Ellen Park,[5] Dimitry Francois,[6] and Richard Zweig,[2] *1. Yeshiva University, Ferkauf Graduate School of Psychology, Brooklyn, New York, United States, 2. Yeshiva University Ferkauf Graduate School of Psychology, The Bronx, New York, United States, 3. Ferkauf Graduate School of Psychology, Yeshiva University, The Bronx, New York, United States, 4. Yeshiva University Ferkauf Graduate School of Psychology, Mountain View, California, United States, 5. Yeshiva University Ferkauf Graduate School of Psychology, Bronx, New York, United States, 6. Weill Cornell Medicine, White Plains, New York, United States*

Research demonstrates reciprocal relationships between personality and depression as well as the important role interpersonal conflicts play, but rarely explores these risk factors in older adults. This study aimed to examine relationships of personality traits, processes, and the impact of emotional involvement and distress during an interpersonal conflict on depression in older adults. The study also investigated whether emotional involvement or interpersonal distress moderate the relationship between personality pathology and depression. Depressed middle and older adult inpatients (N=37; mean age=65.73, SD=7.81; 56.8% female; 86.5% White/Non-Hispanic) completed self-reports and interview-based assessments regarding personality traits (NEO-FFI Neuroticism, Agreeableness), interpersonal problems (IIP-25), and depression (GDS). Narrative responses regarding an interpersonal conflict were obtained and rated for contamination themes as well as emotional involvement and distress. Overall, findings indicated that living with others predicted higher depression (p= .046) and was related to higher neuroticism and interpersonal problems. Personality traits (Neuroticism) (r= .485, p= .001) and processes (Interpersonal problems-trend) (β= .307, p= .058), as well as higher levels of emotional distress (r= .486, p= .001) and involvement (r= .475, p= .001) in an interpersonal conflict were also tied to depression in bivariate but not multivariate analyses. The moderating effects of emotional involvement or distress on the relationship between personality and depression were not supported. Depressed older inpatients who live with others appear at higher risk of depression. Personality traits and processes may be more distal risk factors for depression. Findings are discussed in relation to stress generation as well as clinical implications targeting emotional regulation.

### EXAMINING NOSTALGIA IN OLD LIFE: ANTECEDENCE AND OUTCOME

Linming Zhou,[1] Minjie Lu,[1] Chaobin Zhang,[2] and Helene Fung,[3] *1. Sun Yat-Sen University, Guangzhou, China, 2. Sun Yat-Sen University,Guangzhou, Guangzhou, China, 3. The Chinese University of Hong Kong, Sha Tin, China*

Nostalgia is a self-conscious, bittersweet but predominantly positive and fundamentally social emotion. The regulatory model of nostalgia suggests that experiencing nostalgia can buffer against social threat (e.g. social exclusion) by providing individuals with sense of social connectedness (Sedikides, et al., 2015). In the current research, we propose that this salutary effect of nostalgia may be stronger among older adults compared to younger adults because older adults value social meaningfulness to a greater extent. Fifty-nine younger adults (Mage = 20.15, SD = 0.215) and 56 older adults (Mage = 71.02, SD = 0.679) completed daily questionnaires three times a day for ten consecutive days, and reported their emotional experience and social activities. Results showed that perceiving social threat was positively correlated with nostalgia experience reported at the subsequent time point, and this association was stronger among older adults. In addition, nostalgia positively correlated with subsequent social activities among the older participants but not among the younger participants. These findings highlight that nostalgia brings beneficial psychological (sense of social connectedness) and behavioral (social engagement) outcomes to older adults.

### EXPLORING REACTIONS TO DISRESPECT AND SENSITIVITY TO SOCIAL REJECTION IN OLDER ADULTS

Amanda Chappell,[1] Kelly Smith,[1] Jenessa Steele,[2] and JoNell Strough,[1] *1. West Virginia University, Morgantown, West Virginia, United States, 2. Radford University, Radford, Virginia, United States*

Disrespect involves having low regard or low esteem for someone. Disrespect is a universal experience and has the potential to negatively impact relationships and fosters anger and aggression (Hawkins, 2015; Shwalb & Shwalb, 2006). In the current study, younger (ages 19-25) and older adults (ages 50-77) imagined a person they knew had disrespected them in six different hypothetical situations. For each situation, participants rated their emotional reactivity. Participants also indicated their sensitivity to social rejection (i.e. being left out/excluded). The primary research questions for this study included: do reactions to disrespect differ based on age? Also, does one's relationship status with a disrespect perpetrator matter? In the current study, participants had a stronger emotional reaction to disrespect when imagining the disrespect perpetrator was someone close to them rather than someone not close to them, regardless of age. An age by gender by relationship closeness ANOVA revealed three significant main effects: of age (older are less sensitive), gender (males are less sensitive), and relationship closeness (those more distant to perpetrator are less sensitive) on sensitivity to social rejection. Sensitivity to social rejection mediates the relationship between closeness to a disrespect perpetrator and emotional reaction, even after controlling for age as a covariate, $p<.001$. The findings of this study highlight the component of rejection that is typically involved within disrespect situations and may help to explain just why disrespect feels so hurtful. These findings also highlight that there may be some protection from disrespect based on age or gender.

### LOVE IN THE SHADOW OF OBLIVION: THE MEANING GIVEN TO LOVE IN COUPLES LIVING WITH ALZHEIMER'S DISEASE, FROM A FAMILY VIEW

Orit Shavit,[1] Aaron Ben-Ze'ev,[2] and Israel (Issi) Doron,[2] *1. Haifa University, zichron Yaacov, Hefa, Israel, 2. University of Haifa, Haifa, Israel*

Aims and objectives: To deeply understand the significance of love between spouses who live with Alzheimer's Disease (AD) in a familial aspect. Background: While there is extensive empirical knowledge on the subject of AD, as well as about love, little is known about love in old age, and even less is known about love between spouses who live with AD. This study is a pioneering study that describes love and relationship with AD. Design: A phenomenological qualitative research, which enables a close examination of the experience that accompanies the couple and their adult children from a family perspective that has not yet been examined. It belongs to the stream of Social Constructivism whereby the purpose of interviewing more than one family member was used to capture the process by which family members construct their identity as individuals and as a family unit. Methods: Forty-five in-depth interviews were conducted with n = 15 triads including the couple and their adult child, based on Interpretative Phenomenological Analysis (IPA). Results: Three central themes emerged: (1) The meaning of AD, (2) Continuity and discontinuity of love prior and with AD, (3) The meaning of love in coupled living with AD. Conclusions: Commitment with AD is a moral-ethical obligation, and not necessarily because of love, due to the increasing price of separation. Future research is discussed. Key words: Alzheimer's disease, Love, Relationship, Meaning, couples

### MODERATION OF PHYSICAL HEALTH ON EMOTIONAL HEALTH AMONG GRANDPARENTS RAISING GRANDCHILDREN

Yuqin Jiao,[1] Nathaniel Riggs,[1] Loriena Yancura,[2] Aimee Fox,[1] and Christine Fruhauf,[1] *1. Colorado State University, Fort Collins, Colorado, United States, 2. University of Hawai'i, Honolulu, Hawaii, United States*

An estimated 69.5 million older adults in the United States report being grandparents. It is also evident that the number of grandparents raising grandchildren among them is increasing. Although caring for their grandchildren often provides grandparents a sense of purpose and increased life satisfaction, unexpected responsibilities of parenting worsen the already challenging experience of aging (e.g., potential for poor physical and mental health). Thus, it is essential to study the well-being of grandparents raising grandchildren. Links between depressive symptoms and emotional health have been widely studied in previous research. However, limited research has focused on physical health as a potential moderator, despite indirect evidence shown that more depressive symptoms may be observed among those with worse physical health conditions. To address this, we used Optum® SF-36v2® Health Survey to collect physical- and mental-health data and Center for Epidemiological Studies Depression Scale (CES-D-10) to collect information on depressive symptoms in 137 grandparents raising grandchildren (age 40-83) before, immediately after, and six months after a six-week intervention focused on self-care practices. The presence of more depressive symptoms indicated worse emotional health. Physical health moderated these associations at all three time points, such that depressive symptoms were less strongly related to emotional health if grandparents self-reported better physical-health scores. These findings have important implications for future intervention studies. The importance of good physical health practices for grandparents raising grandchildren is evident, including increased physical engagement and better pain management.

### RESILIENCE FACTORS THAT MODIFY GENETIC RISK FOR ADHD MATTER FOR LATER LIFE EMOTIONAL AND COGNITIVE HEALTH OUTCOMES

Thalida Arpawong,[1] and Joel Milam,[2] *1. University of Southern California, Claremont, California, United States, 2. University of Southern California, Los Angeles, California, United States*

Having features of Attention Deficit Hyperactivity Disorder (ADHD) is associated with challenges with emotional regulation and cognitive function. Heritability for ADHD in adults is estimated to be 30%. The degree to which genetic risk for ADHD can be modified by protective factors, such as strong personal relationships and pursuing more education, to result in better emotional and cognitive outcomes at later ages is not well understood. We evaluated these relationships in a population-representative sample of older adults in the U.S. Health and Retirement Study, with 9,003 European Americans (EA; 57% women, age M=68.6, SD=10.4), and 1,622 African Americans (AA; 63% women, age M=64.4, SD=9.5). Outcomes included validated scales for psychological resilience, life satisfaction, depressive symptoms (DepSx), cognitive functioning, and impairment, assessed between 2008-2012. A genetic risk score for ADHD (GRS-ADHD) was calculated from a genomewide-scan, using a mixed ancestry sample. We used multivariable linear and logistic regression models, adjusted for age, gender, and genetic ancestry. We found a protective effect such that stronger personal relationships in adulthood reduced the inverse relationship between the GRS-ADHD and resilience and life satisfaction in later age (interaction p's<.004 in EAs), but not with DepSx. In contrast, strong parental relationships in childhood attenuated the association between the GRS-ADHD and later life DepSx (interaction p's<.007 in EAs and AAs) only. Education did not modify, but mediated the main effect of the GRS-ADHD on cognitive abilities and impairment in EAs and AAs. Findings have implications for later age health for those at greater genetic risk for ADHD.

### THE AGE AND WELL-BEING PARADOX REVISITED: A MULTIDIMENSIONAL PERSPECTIVE

Thomas Hansen, *Oslo Metropolitan University, Oslo, Norway*

Much gerontological research has focused on the paradoxical observation that older people, despite their lower objective quality of life, report higher well-being than younger people. High well-being in old age is believed to be caused by adaptation, emotional regulation, and accommodative strategies. We aim to add nuance by examining whether the "paradox" holds across dimensions of well-being; for men and women; in young old and old-old age; and before introducing statistical controls (e.g for health and social factors). Using fixed effects models and 15-years panel data from the Norwegian NorLAG study (n=2,700, age 40+), we explore age-related changes in cognitive, affective, and eudaimonic dimensions of wellbeing. Results indicate a general pattern of stability well into older age, but negative changes in advanced age, cross-sectionally and longitudinally, and for each well-being measure. Declines in well-being are less pronounced and with a later onset for the cognitive compared with the other measures. Results are similar for men and women. Loss of health and partner are the main causes of declining well-being in older age. Findings suggest qualifications to the "well-being paradox", e.g.: some dimensions of well-being remain more stable than others; across dimensions of well-being change is more negative in old-old than in young-old age; and patterns of increasing well-being in older age are more pronounced after controlling for age-related changes in health and social roles. We argue that the use of controls makes for false impressions of the psychological changes that actually occur when people grow older.

### THE LIFE ORIENTATION TEST: A CONFIRMATORY FACTOR ANALYSIS ACROSS THREE AGE GROUPS

Robert Intrieri, and PAIGE GOODWIN, *Western Illinois University, MACOMB, Illinois, United States*

The Life Orientation Test (LOT; Scheier & Carver, 1985) was developed as a measure of dispositional optimism. Optimism has been linked to positive life outcomes and is associated with psychological (Carver & Gaines, 1987; Scheier & Carver, 1985) and physical (Scheier & Carver, 1987; Scheier et al. 1989) well-being. The current study assessed 520 people placed into three age groups: young adult (n =149), middle-age adult (n = 252), and older adult (n = 119). The mean age for the young group was 19.24 (SD = 2.01), middle-aged (47.68 (SD = 4.75), and old was 71.99 (SD = 7.21). Data were submitted for a confirmatory factor analysis (CFA) which tested for invariance across the age groups. Previous factor analyses have identified two distinct factors representing optimism and pessimism. Results from the CFA showed that both Configural and Metric invariance models demonstrated acceptable fit for the two factor model (□2(df=57) = 61.92, p = 0.3047; □2(df=69) = 78.77, p = 0.1974). In contrast, Scalar invariance resulted in a poor fit across the three age groups (□2(df=81) = 139.288, p < 0.0001). Model comparisons revealed no significant differences between Configural and Metric models (□2(df=12) = 16.996, p = 0.1498). Model comparisons between Configural and Scalar and Metric and Scalar were (□2(df=24) = 78.947, p < 0.0001; □2(df=12) = 61.764, p < 0.0001). These results confirm previous research that shows a correlated two factor model consistent with the concept that optimism and pessimism are correlated elements rather than two ends of a continuum.

### THE MEDIATING EFFECT OF NEGATIVE EMOTION ON THE WELL-BEING CONSEQUENCE OF BELONGINGNESS DEFICIT ACROSS AGES

Vivian Hiu Ling Tsang,[1] and Helene Fung,[2] *1. The Chinese University of Hong Kong, Hong Kong, China, 2. The Chinese University of Hong Kong, Sha Tin, China*

Previous studies suggested that the negative influence of belongingness deficit on wellbeing may be driven by an increase in negative emotion, but the age difference of this mediating effect is still uncertain. This study tested the mediating effect of negative emotion in the influence of relationship quality on depressive symptoms across age. Based on the potential biological decline in emotional sensitivity and better management of emotion with age, we hypothesize that negative emotion may be a weaker mediator in driving the consequences of relationship quality among older adults than younger adults. A total of 494 participants (19 – 85 years old, 60% was female) participated in this study at three timepoints with a two-year interval across Hong Kong, USA and Germany. Results suggested a significant partial mediating effect of negative emotion (b = -.0920, 95%LLCI = -.1315 to 95%ULCI = -.0568). In particular, poorer relationship quality at timepoint 1 predicted more negative emotion at timepoint 2 and therefore predicted more depressive symptoms at timepoint 3. More importantly, the mediating effect was moderated by age significantly (b = .0025, 95%LLCI = .0009 to 95%ULCI = .0042). As expected, the mediating effect of negative emotion decreased with age and became not significant among individuals at older age (b = -.0344, BootLLCI = -.0805 to BootULCI = .0075). Future studies can further investigate on age-related mediators in driving the consequences of belonginess deficit.

### SESSION 2981 (PAPER)

### EMPLOYMENT INTERESTS OF OLDER ADULTS

### DISRUPTION AS ANTECEDENT TO NEW BUSINESS STARTUP: A STUDY OF ENTREPRENEURSHIP USING THE UNDERSTANDING AMERICA STUDY

Felichism Kabo,[1] Stewart Thornhill,[1] and Elizabeth Isele,[2] *1. University of Michigan, Ann Arbor, Michigan, United States, 2. GIEE (Global Institute for Experienced Entrepreneurship), North Yarmouth, Maine, United States*

We use data from a nationally representative study to examine antecedents of entrepreneurial activity in the US among younger (< age 50) and older (≥ age 50) adults using Shapero's and Sokol's Model of the "Entrepreneurial Event" (hereafter SEE). The "entrepreneurial event" here refers to the initiation of entrepreneurial behavior. The SEE approach assumes that individuals default to inertia until their lives are disrupted by exogenous factors such as life-changing events (Shapero & Sokol, 1982). The disruptor could be either positive or negative, and has the net effect of driving the individual to reconsider entrepreneurship as a viable opportunity (Krueger & Brazeal, 1994; Krueger & Day, 2010). We examine the correlation between negative disruptions and a person initiating entrepreneurial behavior (starting a new business), including whether the process is similar across younger and older adults. Using data collected in 2014 and 2019 from 3,586 individuals in the Understanding America Study panel, we run survey Cox proportional hazards models to analyze the effects of negative disruptions (getting separated or divorced) on starting a new business over a five-year period starting in 2014. We found that negative disruptions have a significant, positive effect but only among older adults. Further, the magnitude of that effect is about 3-7 times that of younger adults. Our findings support the validity of the SEE approach in advancing our understanding of the transition of individuals from potential to actual entrepreneurs. However, the findings suggest the SEE approach better explains this process in older rather than younger adults.

### HEALTH AND LABOR MARKET OUTCOMES AMONG OLDER ADULTS: CAUSAL EVIDENCE FROM KOREAN PANEL DATA AND DYNAMIC MODELING

Hoolda Kim,[1] and Sophie Mitra,[2] *1. Black Hills State University, Spearfish, South Dakota, United States, 2. Fordham University, Bronx, New York, United States*

Health and work are two key determinants of economic insecurity and wellbeing. Since economic insecurity may rise with age due to the deterioration of health and work ability, it is essential to study the causal relationship between health and labor market outcomes among older people. In Korea, the size of the older population has grown rapidly in recent decades but the pension system remains limited in terms of its reach and generosity, leaving a considerable number of older people suffering from economic insecurity. To investigate the two-way causal links between health and labor market outcomes, we use unique data on middle-aged and older Koreans from 12 waves of the Korea Welfare Panel Study (2006-2017) and two dynamic modeling approaches: the Arellano-Bond Generalized Method of Moment Model (AB-GMM) and the Maximum Likelihood Structural Equation Model (ML-SEM). Results point at gender differences regarding the direction of the causal link between health and labor market outcomes. Men are more likely to be in good health due to higher income or paid work. Women are more likely to earn a higher income and to work as paid employees because of good health. These effects particularly hold for Koreans aged 55-74 in urban areas.

### NEW INSIGHTS ON SELF-EMPLOYMENT OF OLDER ADULTS IN THE UNITED STATES

Joelle Abramowitz, *University of Michigan, Ann Arbor, Michigan, United States*

This work examines the nature of self-employment arrangements of older adults in the United States. Many people engage in self-employment - in the 2016 Health and Retirement Study (HRS), 20 percent of respondents working for pay reported being self-employed - yet there exists a dearth of data on these arrangements. This lack of data prevents consideration of important questions relevant to employment, inequality, and policy. Who works in different types of self-employment? What resources facilitate some individuals obtaining higher quality self-employment arrangements? To what extent does the income from different types of arrangements keep people out of poverty? Are different types of arrangements associated with individuals being happier and having more job satisfaction? This work leverages novel restricted-access data collected in the HRS in 2016 on the employer names and locations for individuals reporting self-employment along with respondent narratives on industry and type of work to classify self-employment reports into three entrepreneurial roles (own/run; manage; independent) across 14 different types of work. Using the breadth of information collected in the HRS and linkage to administrative records, this work then presents differences in characteristics, such as demographics, income, wealth, savings, health insurance coverage, home ownership, health status, and expectations of working longer, associated with different classifications of self-employment. Exploring these questions provides unique insights into the changing nature of work and the transition to retirement relevant to policy considerations across the health, insurance, and retirement income dimensions, among others.

### THE IMPACT OF THE FINANCIAL CRISIS ON ENTREPRENEURSHIP AMONG OLDER ADULTS

Julie Kim, *University of California, Irvine, Irvine, California, United States*

Older adults are assumed to be more averse to uncertainty than younger adults as economics and psychology studies based on prospect theory and related risk aversion theories show. Indeed, compared to younger adults, older adults engage in a smaller share of entrepreneurship. Yet adults 50 and over make up a steadily growing group of entrepreneurs in the US, and the 2008 financial crisis has not slowed them down. Sociological perspectives on older adults' entrepreneurship consider disruption in the structural conditions of the labor market as a result of the economic crisis and a culture of active aging to argue that older adults' entrepreneurial behaviors stem from structural and cultural roots. Analyses using 2003-2015 individual-level data from the Global Entrepreneurship

Monitor show that an economic downturn did not thwart older adults' entrepreneurial activities any more than it affected younger adults' activities. Further, older adults' odds of planning to start a business and actually starting a business were higher after the crisis than before the crisis. A closer examination reveals that older adults with less wealth generally have higher odds of pursuing entrepreneurship than other older adults. Meanwhile, wealthier older adults have higher odds of pursuing opportunity-driven entrepreneurship. Evidence suggests that the saliency of labor market conditions or the culture of active aging depends on older adults' social location.

### WORK AND FUNCTIONAL LIMITATIONS FOR OLDER WORKERS: DOES MEASUREMENT MAKE A DIFFERENCE?

Theresa Andrasfay,[1] Anne Pebley,[2] and Noreen Goldman,[1] *1. Princeton University, Princeton, New Jersey, United States, 2. University of California Los Angeles, Los Angeles, California, United States*

Social scientists have become increasingly interested in strenuous jobs as contributors to health inequality over the life course. Physically demanding work at later ages is of particular interest because it can have implications for retirement decisions, physical functioning, and disability, and strenuous jobs are prevalent among lower-income and minority older workers. Many studies have relied on occupational characteristics from the Occupational Information Network (O*NET), but few have assessed how these measures compare to self-reported occupational characteristics in terms of identifying social gradients in exposure and predicting future health outcomes. Using data from 16,683 respondents of the Health and Retirement Study, we obtained self-reported and O*NET measurements of general physical activity, frequency of lifting objects, and frequency of stooping/crouching required in the jobs they held at first interview. Pearson correlation coefficients revealed moderate correlations between the self-reported items and corresponding O*NET items. Though they are measured on different scales, both the self-reported and O*NET measures of physical demands revealed similar racial/ethnic and gender gradients in exposure to physically strenuous work. Lastly, we fit a series of random effects Poisson models to assess how these measures predict accumulation of functional limitations, a health outcome thought to result in part from strenuous working conditions. We found that while models using self-reported working conditions have the best fit with the data, models using the corresponding items in O*NET have comparable goodness-of-fit. These results suggest that, in the absence of self-reported physical occupational characteristics, O*NET characteristics provide a reasonable alternative.

### SESSION 2982 (PAPER)

### LIFE COURSE AND DEVELOPMENTAL CHANGE IN MIDDLE AND LATE LIFE

### A FAITH TO MOVE MOUNTAINS? CHILDHOOD ABUSE, RELIGIOUS CHANGE, AND MENTAL HEALTH AT MIDLIFE

Laura Upenieks, *University of Texas at San Antonio, San Antonio, Texas, United States*

Of all the various forms of adversity experienced during childhood, childhood maltreatment (emotional and physical abuse) is shown to have the largest impacts on mental health and well-being. Yet we still have a limited understanding of why some victims of early maltreatment suffer immense mental health consequences later on in the life course, while others are able to cushion the blow of these early insults. Using two waves of data from the National Survey of Midlife Development in the United States (MIDUS), this study considers change in religiosity as a buffer across three dimensions for victims of childhood abuse: religious importance, attendance, and the specific act of seeking comfort through religion. Results suggest that increases in religious comfort during adulthood are positively associated with adult mental health for victims of abuse, while decreases in religious comfort over time were associated with worse mental health. Changes in religious attendance and religious importance were not significant associated with mental health for victims of abuse. Taken together, my results show that the stress-moderating effects of religion for victims of childhood maltreatment are contingent on the stability or increases or decreases in religiosity over the life course, which has been overlooked in previous work.

### DO MEDIATORS LINKING CHILDHOOD CONDITIONS TO LATE-LIFE CHRONIC INFLAMMATION VARY BY RACE?

Kenneth Ferraro,[1] and Patricia Morton,[2] *1. Purdue University, West Lafayette, Indiana, United States, 2. Wayne State University, Detroit, Michigan, United States*

Recent findings suggest that childhood exposures can lead to chronic inflammation decades later, but the mechanisms underlying this relationship are relatively unknown. We investigate how childhood exposures influence adult chronic inflammation (measured by C-reactive protein) and examine five potential mediators comprising two midlife domains: socioeconomic status (SES) and health lifestyles. Using a sample of 8,891 adults aged 51 and older from the Health and Retirement Study (HRS), the analysis tests whether these life course mediators operate differently for Black, White, and Hispanic Americans. Among the six childhood domains examined, low SES and risky parental behaviors predict adult chronic inflammation, but adult health lifestyles mediate the effects of childhood SES and parental behavior. Adult SES also mediates the effect of childhood SES. Smoking and wealth exert stronger direct and indirect effects on adult inflammation for White Americans compared to Black Americans whereas BMI and exercise exert stronger direct and indirect effects for White Americans compared to Hispanic Americans. Although education mediated the effect of childhood SES on adult chronic inflammation, its effects did not vary by race. These results demonstrate that the physiological consequences of childhood exposures are carried into late-life via adult lifestyle factors and SES. In addition, the life course antecedents of chronic inflammation are distinct for Black, White, and Hispanic Americans. Future research investigating the early origins of adult health should consider not only multiple midlife mechanisms but also how resource mediation varies by race and ethnicity.

### GENE-ENVIRONMENT COVARIANCE AS A MECHANISM OF DECLINES IN OPENNESS IN LATE ADULTHOOD

Christopher Beam,[1] and Emily Sharp,[2] *1. Dornsife College of Arts and Letters, Los Angeles, California, United States, 2. Yale University, New Haven, Connecticut, United States*

We previously demonstrated that openness to experience declines with age and these declines correlate with mortality risk. We posited that decline in openness was related to change in behavior in relation to a change in future time perspective, defined here as dynamic changes in scope of time that influence persons' behavior. This idea, based on Baltes' selection, optimization, and compensation theory of lifespan development, suggests that with foreshortened time horizons individuals adapt their behavior leading to lesser engagement in novel experiences and relationships. The current study examined the genetically informed mechanisms underpinning the relationship between openness and mortality. Using identical and fraternal twins from the Swedish Adoption Twin Study of Aging (SATSA), we examined whether twins further from death nonrandomly select environments that maintain their openness scores while their co-twins nearer to death nonrandomly select environments that contribute to declines in openness. Using a sample of 822 twin pairs, we estimated a genetically-informed longitudinal model that quantified time-varying effects of twins' openness scores at time t-1 on latent nonshared environmental scores at time t. The model generates within-family gene-environment correlation, a statistical coefficient that quantifies the genetic basis for nonrandom exposure to environments. Results suggest significant time-varying correlations between twins' openness scores and their unique environments as well as increasing gene-environment correlation over time. Findings are consistent with the view that environments can support and reinforce maintenance of or declines in openness depending on the length of persons' time horizons.

### GENERATIVITY DEVELOPMENT ACROSS ADULTHOOD: A LONGITUDINAL INVESTIGATION

Niccole Nelson, and Cindy Bergeman, *University of Notre Dame, Notre Dame, Indiana, United States*

The developmental trajectory of Generativity, or investment in the next generation, has been theorized about for decades. Although Generativity is widely hypothesized to peak in midlife, and thus, follow a nonlinear change trajectory across adulthood, extant studies have been too limited in scope to formally test this hypothesis. Indeed, most existing studies on Generativity development have been cross-sectional, with the few longitudinal studies either only examining the first half of adulthood or using too few measurement points. The current study, therefore, aimed to address these limitations by investigating Generativity development in the context of an accelerated longitudinal design. Accelerated longitudinal designs capitalize on both cross-sectional and longitudinal data, combining age-heterogenous individuals' overlapping trajectories to estimate developmental change across the sample's age range. If cohort effects are not present in the estimated trajectory, this trajectory can be interpreted as developmental change. Participants included 876 age-heterogenous individuals from The Notre Dame Study of Health & Well-being (Mean age = 58.89; SD age = 9.42), a 10-year, longitudinal study of adult development and aging. Capitalizing on the age-heterogeneity of the sample at Time 1, two-level, multilevel modeling was employed to estimate Generativity development across ages 37 to 96. Results indicate that Generativity follows an age-graded cubic trend, with no apparent cohort effects. Specifically, Generativity peaks in early midlife, declines slightly before stabilizing across ages 47-77, and then declines sharply thereafter. Implications for lifespan developmental research, as well as health and well-being, will be discussed.

### IMPACT OF DOING GOOD ON THE AGING SELF: GROWTH AND CHANGE THEMES IN NARRATIVES OF PROSOCIAL BEHAVIOR

Laura Graham,[1] Jeanne Nakamura,[2] and Noah Ringler,[2] *1. University of California, Riverside, Santa Monica, California, United States, 2. Claremont Graduate University, Claremont, California, United States*

Contemporary theories consider development to be lifelong, suggesting a potential for personal growth in older adulthood. Narrative studies have found benefits of having growth themes in older adults' broad life stories, yet there is limited research focusing on the specific experiences in later life that elicit growth. One potential for personal growth during older age is through prosocial behavior, but studies have overlooked how older adults narrate such experiences and the perceived impact the narratives have on the self. We conducted interviews with a sample of 47 older adults engaged in prosocial commitments to examine the types of perceived self-change as well as patterns in high points and low points of the experience. Narrative analyses revealed the majority of participants reported a change in the self, and half of the sample used growth themes to characterize the self-change. Inductive coding of change narratives revealed an emergent category of virtuous change (e.g., transcendence, wisdom, humanity), as well as a variety of other change categories (e.g., competence, cognitive abilities, negative changes). Those who grew narrated high points and low points with more integration than those who did not, displaying a multifaceted, complex understanding of significant episodes through a blend of both positive and negative elements within one story. These findings suggest that an integrated understanding of prosocial experiences may provide an avenue toward personal growth. Implications for research and practice are discussed.

### SESSION 2983 (PAPER)

### RETIREMENT IN THE 21ST CENTURY

### COGNITIVE FUNCTIONING WHEN RETIRING: FINDINGS FROM A SWEDISH POPULATION-BASED LONGITUDINAL STUDY

Linn Elena Zulka, Isabelle Hansson, Valgeir Thorvaldsson, and Linda B. Hassing, *University of Gothenburg, Gothenburg, Sweden*

The effects of retirement on cognition are still unclear and empirical evidence is conflicting. Especially for retirement from cognitively demanding jobs, positive as well as negative effects have been reported. Leisure activity engagement has been hypothesized to play an important role in explaining the mixed evidence. In this study, we examine the interplay between job demands before retirement and changes in leisure activities before and after retirement and their relation to post-retirement cognitive functioning. Using data from the HEalth, Aging and Retirement in Sweden (HEARTS) study, cognitive trajectories before and after retirement were modeled in a multi-level piecewise model (N = 2688 observations). Post-retirement memory and reasoning ability were predicted by self-reported work demands and changes in leisure activity engagement. Results imply a stable increase in memory over the retirement transition and less steep increase in abstract reasoning after retirement. Work demands and leisure activity participation were not related to post-retirement cognitive change. Job demands and leisure activity engagement may not play an important role for short-term post-retirement cognitive functioning. These findings support the conclusion that retirement, independent of prior work demands, does not affect cognitive functioning negatively.

### HISTORICAL DIFFERENCES IN RETIREMENT ADJUSTMENT IN GERMANY

Georg Henning,[1] and Oliver Huxhold,[2] *1. German Centre of Gerontology, Berlin, Germany, 2. German Centre of Gerontology, Berlin, Berlin, Germany*

The nature of retirement has been constantly changing over the last decades. Retirement transitions of later-born cohorts differ from those of earlier-born cohorts in terms of sociocultural context and timing. In addition, today's retirees differ from those who retired earlier in historical time, for example with respect to gender composition, social resources and health status. Gerontological research has rarely addressed the question whether such developments translate into historical differences in retirement adjustment quality. In the current study, we investigated historical differences in perceived retirement adjustment. We distinguished developments for blue-collar and white-collar workers to detect potentially increasing social inequalities. Our pre-registered analyses were based on data from four waves of the German Ageing Survey. The sample included n = 990 participants interviewed either 1996, 2002, 2008 or 2014, who retired in a five-year period before the respective interview (1991-1996, 1997-2002, 2003-2008 and 2009-2014, respectively). Retirement adjustment was measured with three self-report items. Our preliminary results, based on multi-group structural equation modeling, do not provide evidence for a linear improvement or decline of retirement adjustment quality over historical time. White-collar worker reported better adjustment, but this effect was constant over historical time. Our results do not support ideas of fundamental historical differences or growing social inequalities in the individual experience of retirement.

### RETIREMENT FINANCIAL INSECURITY FOR MEN AND WOMEN IN CANADA: THE IMPACT OF LIFE COURSE WORK AND INCOME INSTABILITY

Miles Taylor,[1] Dawn Carr,[1] and Amelie Quesnel-Vallee,[2] *1. Florida State University, Tallahassee, Florida, United States, 2. McGill University, Montreal, Quebec, Canada*

Financial security is critical to overall well-being in retirement. Life course disruptions in work due to unemployment or disability may have lasting impacts on financial security in later life, and these effects may or may not be ameliorated by old age security programs and retirement benefits. Women are known to be particularly vulnerable for financial hardship in later life, making their life course experiences especially important in understanding financial well-being in retirement. We examine the Canadian Longitudinal and International Study of Adults (LISA) to assess later life financial insecurity in retirement, including 20 years of data on labor force experiences across adulthood drawn from linked tax records (N=2,353; N=1,079 men and N=1,274 women). The Canadian context is a useful complement to studies in the US due to its diverse and relatively generous public support programs, in addition to personal savings options for retirement. We find that among men, financial insecurity is tied to years reporting unemployment, where for women this outcome is more strongly linked to reports of disability and income assistance across adulthood. Although public and private retirement benefits do not offset these associations for either gender, receipt of income supplements reduce subjective financial insecurity among women. Among men, personal savings have the most robust effect in reducing financial insecurity. Our results suggest that in Canada, with its relatively generous provision of retirement support, life course work and health instability have lasting but differential effects for men and women in retirement.

### SUPPORT FOR A LOWER STATE PENSION AGE FOR DISADVANTAGED OLDER WORKERS

Jaap Oude Mulders,[1] Hendrik Van Dalen,[2] and Kène Henkens,[3] *1. Netherlands Interdisciplinary Demographic Institute, The Hague, Netherlands, 2. Netherlands Interdisciplinary Demographic Institute, Den Haag, Netherlands, 3. NIDI, Den Haag, Netherlands*

Due to policy reforms, early exit from the labor market has decreased substantially and people are participating in the labor market until much higher ages than before. As a result, there are increasingly many people that struggle to continue working until they can comfortably retire, for example due to chronic health conditions or having to provide informal care. A potential solution would be to grant earlier access to state pension benefits (such as Social Security) for disadvantaged older workers. While it is known that many people are supportive of such a policy, the question remains how much earlier access would be granted under which circumstances. Here, using a quasi-experimental vignette design (10,350 observations nested in 2,070 respondents), we study how much earlier Dutch people would like to grant access to disadvantaged older workers. Relevant characteristics of older workers that are judged are the age at which they started working, the level of physical strain in their job, whether they have chronic health conditions, and whether they provide informal care to a loved one. The result show that, on average, people would grant older workers with chronic muscoskeletal conditions or cardiovascular disease one year earlier access to the state pension than normal, while older workers that provide daily informal care would be granted 10 months earlier access. Cumulative disadvantage could lead to a maximum of three years earlier access to pension benefits. This study provides important insights into fairness considerations surrounding state pension provisions, and implications for practice will be discussed.

### WHO BENEFITS IN VITALITY AFTER RETIREMENT? FINDINGS FROM A 3-YEAR PANEL STUDY

Anushiya Vanajan,[1] Ute Bültmann,[2] and Kène Henkens,[3] 1. *Netherlands Interdisciplinary Demographic Institute, Den Haag, Netherlands,* 2. *University of Groningen, Groningen, Netherlands,* 3. *NIDI, Den Haag, Netherlands*

Background. Past studies have revealed the effect of retirement on various health measures. None, however, have studied retirement's effect on vitality, a holistic measure of physical and mental health. To boot, very few studies have addressed the heterogeneity in the health consequences of retirement. This study investigates the effect of retirement on vitality, and how this effect is influenced by 1) manual work and 2) baseline vitality. Methods. The analyses were based on two waves of the NIDI Pension Panel Survey, collected in the Netherlands in 2015 and 2018. Data from 4,156 older workers (N=4,156), of whom 1,934 (46.5%) retired between waves, were analysed. Vitality is assessed in three ways, as: 1) a composite measure of vitality, and its sub-components 2) energy and 3) fatigue. Results. Conditional Change OLS Regression Models demonstrate that retirement improves vitality and decreases fatigue. These effects were heterogeneous. Retirement was more advantageous for older workers who experienced poor vitality and increased fatigue before retirement. Likewise, older workers who were employed in manual work before retirement, experienced the largest gains in vitality and deepest declines in fatigue post-retirement. No such effects were found for energy. Conclusions. Older workers experiencing low baseline vitality and high baseline fatigue and those in manual labor may benefit from early retirement. Since early retirement is financially unfavorable, it is essential to provide these groups of workers with workplace vitality interventions that may not only improve their vitality and quality of working life, but also extend their participation in the labor market.

### SESSION 2984 (PAPER)

### FACTORS ASSOCIATED WITH DRIVING AND DRIVING CESSATION

### BALANCING MOBILITY AND DRIVING SAFETY: FACILITATORS AND BARRIERS FOR DRIVING FREQUENCY AMONG OLDER DRIVERS

Jiawei Cao, and Jonathon Vivoda, *Miami University, Oxford, Ohio, United States*

Being able to drive safely ensures older adults' transportation mobility and independence. It is also a key element for social participation and to achieve productive aging. However, when accounting for driving exposure, crash risk and crashed-related deaths increase for older drivers aged 70 years and plus. By analyzing data from NHATS, this study aimed to assess factors that might affect driving frequency (a five-category ordinal variable that measured how often people drove places). I examined whether productive aging activities (e.g., working, volunteering, caregiving, leisure, religious participation, civic engagement), perceived importance of social participation, mental wellbeing, physical capacity, cognition, sensory function, and community environment were either positively or negatively related to driving frequency. Age, gender, race, household size, marital status, and self-rated health were controlled for in this study. Results from ordinal regression showed that higher driving frequency was observed among older adults who reported that they worked for pay, provided care, visited friends and family, and went out for enjoyment. Higher driving frequency was also related to a higher perception of social participation importance. Inability to walk six blocks, lower scores on delayed word recall, and poorer vision were associated with lower driving frequency. Lower driving frequency was also found among women, married couples, and people with worth health and advanced age (80 years and older). These results suggest that higher driving frequency is associated with active social engagement and participation. New vehicle technologies or alternative transportation services and programs could be implemented to ensure safety, mobility, and social participation.

### DRIVING CESSATION AND SOCIAL PARTICIPATION IN LATE LIFE: A SYSTEMATIC REVIEW

Weidi Qin, *Case Western Reserve University, Cleveland, Ohio, United States*

Driving cessation is a major life transition in late life, and can affect the quality of social life in older adults. The present study aims to systematically review the literature on how driving cessation affects social participation among older adults in the US. The study selection followed the Preferred Reporting Items for Systematic Reviews and Meta-Analyses (PRISMA). Extant literature published from 1990 to 2019 that examined driving cessation and social participation or social engagement among older adults in the US was searched using eight search engines: PsycINFO, CINAHL, SocIndex, AgeLine, MedLine, Scopus, Transportation Research Board Publication Index, and Cochrane Library. Quantitative studies that met the inclusion criteria were reviewed. The assessment of methodological quality was also conducted for included studies. In total, seven studies met the inclusion criteria. Six of the included studies found significant relationships between driving cessation and at least one domain of social participation, such as volunteering, employment, leisure-time activities, and the frequency of contacts. However, the measures of social participation were inconsistent across studies, which might explain that no effects of driving cessation were found on the structure of social network, such as contacts with friends and extended family. There is a need to assist older adults in successfully transitioning to driving cessation and maintaining the social participation. The overall quality of included studies is moderate based on the assessment of risk of bias and confounding.

### DRIVING PRIVATE VEHICLES IN LATER LIFE: NECESSITY OR LUXURY?

Moon Choi, *KAIST, Daejeon, Taejon-jikhalsi, Republic of Korea*

The automobile industry in South Korea has rapidly expanded for the last half century. The current generation of older adults is the first generation that has experienced mass car ownership and also faced challenges related to driving cessation due to geriatric syndromes. This study aimed to explore diversity in reasons for driving private vehicles among Korean older adults. Data came from a nationally representative survey on adults aged 65 years and older conducted in August, 2019 (N=1,500; women 57.1%). One out of four respondents (24.5%) reported driving, and the current drivers were categorized into four groups by primary reason for driving: (a) for convenience, (b) for caregiving, (c) for survival (due to the lack of public transport) and (d) for living (i.e. taxi or bus driver). The results showed that current drivers were more likely to be men, younger and more educated compared to non-drivers. Conspicuously, those who reported driving for their family members with mobility needs were the second largest group after those who reported driving for convenience such as for grocery shopping (34.6% and 43.6%, respectively). The results of multinomial logistic regression model showed that older age, living in rural areas and poorer self-rated health were associated with driving for survival as compared to driving for convenience. About the odds of driving for caregiving compared to driving for convenience were higher for those married and living with their spouse than for those not-married. The results imply the importance of considering diversity in developing public policies for older drivers.

### ESTIMATING THE FREQUENCY OF SUICIDE RELATED TO DRIVING CESSATION AMONG OLDER ADULTS: A DATA SCIENCE APPROACH

Tomohiro Ko,[1] Viktoryia Kalesnikava,[2] and Briana Mezuk,[1] *1. University of Michigan School of Public Health, Ann Arbor, Michigan, United States, 2. University of Michigan, Ann Arbor, Michigan, United States*

Up to 35% of older adults in the United States engage in partial to complete driving cessation. Suicide risk is highest among older adults, and driving cessation can precipitate psychosocial changes linked with elevated risk of suicide, such as loss of independence, mobility, and socialization. Little is known about the association between suicide and driving cessation among older adults. We developed a supervised machine learning natural language processing algorithm to estimate the frequency of and characterize suicides associated with driving cessation from restricted-access case narratives in the National Violent Death Reporting System (NVDRS, 2003-2015) from suicides and undetermined deaths among adults 55 years and older in 27 states. Among 47,759 decedents, preliminary results identified 972 deaths associated with driving cessation, with a mean age of 71.4, 91.5% White, and 78.3% Male. 13.8% of the decedents of driving cessation-associated suicide had a crisis contributing to the suicide within 2 weeks of death. This analysis assesses the relationship between contextual geographic features such as population density and the distribution of driving cessation-associated suicides across the US. Government and clinical stakeholders should invest in public transportation infrastructure catered to older adults and implement policies to reduce the burden of driving cessation. Increased accessibility of technological innovations such as self-driving cars may also enhance quality of life for a growing older adult population.

### EXAMINING RISKY DRIVING BEHAVIOR AMONG OLDER DRIVERS USING SELF-REPORTED AND OBJECTIVE DATA

Jennifer Zakrajsek,[1] Lisa Molnar,[1] David Eby,[1] David LeBlanc,[1] Lidia Kostyniuk,[1] Nicole Zanier,[1] and Tina Sayer,[2] *1. University of Michigan, Ann Arbor, Michigan, United States, 2. Toyota Motor North America Research & Development, Ann Arbor, Michigan, United States*

Motor vehicle crashes represent a significant public health problem. Efforts to improve driving safety are multifaceted, focusing on vehicles, roadways, and drivers with risky driving behaviors playing integral roles in each area. As part of a study to create guidelines for developing risky driving countermeasures, 480 drivers (118 young/18-25, 183 middle-aged/35-55, 179 older/65 and older) completed online surveys measuring driving history, risky driving (frequency of engaging in distracted [using cell phone, texting, eating/drinking, grooming, reaching/interacting] and reckless/aggressive [speeding, tailgating, failing to yield right-of-way, maneuvering unsafely, rolling stops] driving behaviors), and psychosocial characteristics. A cluster analysis using frequency of the risky behaviors and seat belt use identified five risky behavior-clusters: 1) rarely/never distracted-rarely/never reckless/aggressive (n=392); 2) sometimes distracted-rarely/never reckless/aggressive (n=33); 3) sometimes distracted-sometimes reckless/aggressive (n=40); 4) often/always distracted-often/always reckless/aggressive (n=11); 5) no pattern (n=4). Older drivers were more likely in the first/lowest cluster (93.8% of older versus 84.2% of middle-aged and 59.3% of young drivers; p<.0001). Fifteen older drivers participated in a follow-up study in which their vehicles were equipped with a data acquisition system that collected objective driving and video data of all trips for three weeks. Analysis of video data from 145 older driver trips indicated that older drivers engaged in at least one distracted behavior in 115 (79.3%) trips. While preliminary, this suggests considerably more frequent engagement in distracted driving than self-reported and that older drivers should not be excluded from consideration when developing risky driving behavior countermeasures. Full study results and implications will be presented.

### SESSION 2985 (PAPER)

### MENTAL HEALTH AND OLDER ADULTS

### CHILDHOOD TRAUMA AND ADULT PSYCHOLOGICAL ADJUSTMENT: RESILIENCE IN OLDER ADULT SURVIVORS OF INSTITUTIONAL ABUSE

Shauna Mc Gee,[1] Andreas Maercker,[1] Alan Carr,[2] and Myriam Thoma,[1] *1. University of Zürich, Zürich, Switzerland, 2. University College Dublin, Dublin, Ireland*

Several international cohorts of older adults share past experiences of welfare-related adversity. In Ireland, reports of childhood maltreatment, neglect, and abuse within institutional welfare settings included a harsh regime, childhood labour, and physical and sexual assault. Preliminary research with these Irish survivors revealed a high prevalence of psychological disorders in adulthood. A pathological perspective of aging is often applied to such older adults, due to the long-term health consequences associated with childhood trauma. However, little is known about later life resilience or resilience mechanisms in this population.

Therefore, using conceptual models of resilience, this qualitative study aimed to investigate factors associated with resilience in older adult survivors of childhood institutional abuse. Participants were 17 Irish older adults, 50-77 years of age, with experiences of childhood abuse in institutional care settings. Semi-structured interviews were conducted, lasting 60-120 minutes. Transcribed interviews were analysed using the Framework Analysis method. Nine themes were derived from the data, including core, internal, and external resilience factors: Individual characteristics, personality characteristics, support systems, goal attainment, adaptive belief systems, processing, influential events and experiences, recognition and collective identity, and access to services. Results suggest that resilience can be understood not only as an inherent trait, but also as a learnable set of behaviours, thoughts, and attitudes, which can be supported by external resources in an older adults' environment. The identification of personal and contextual factors underpinning resilience in older adults with trauma experiences may help foster a more positive, strengths-based approach to aging in psychological research and practice.

### HEARING IMPAIRMENT AND RISK OF DEPRESSION IN OLDER ADULTS IN HEALTH ABC

Danielle Powell,[1] Joshua Betz,[2] Kristine Yaffe,[3] Stephen Kritchevsky,[4] Elsa Strotmeyer,[5] Eleanor Simonsick,[6] Frank Lin,[7] and Jennifer Deal,[2]
*1. Johns Hopkins University, Baltimore, Maryland, United States, 2. Johns Hopkins Bloomberg School of Public Health, Baltimore, Maryland, United States, 3. UCSF Weill Institute for Neurosciences, San Francisco, California, United States, 4. Wake Forest School of Medicine, Winston-Salem, North Carolina, United States, 5. University of Pittsburgh, Pittsburgh, Pennsylvania, United States, 6. National Institute on Aging, Bethesda, Maryland, United States, 7. Johns Hopkins University School of Medicine, Baltimore, Maryland, United States*

Whether hearing impairment (HI) is associated with depressive symptoms remains disputed for older adults, in part due to varying definition employed, use of subjective hearing measures, or cross-sectional analysis. We studied 1936 men and women (mean age 74.1 years, 41.7% black race) enrolled in the prospective Health, Aging and Body Composition study Hearing thresholds at 500-4000 Hz were averaged to create a pure tone average (PTA) and HI was defined using clinical cutpoints in the better-hearing ear. Depression was measured using the Center for Epidemiologic Studies Depression Scale (CES-D) or the CES-D 10, a revised 10 question scale depending on visit. Linear mixed effects models with random intercepts and slopes were used to estimate difference in rates of change in depressive symptomatology by hearing status over nine years. Cox proportional hazard models were used to examine the association between HI and incident depression defined as change in CES-D score >=10 points. In models adjusted for demographic and clinical covariates, participants with HI demonstrated a higher baseline prevalence of depressive symptoms compared to those with normal hearing (20.7% vs. 8.4%).Rates of change did not differ by HI status. Participants with moderate or greater HI had an increased risk of 9-year incident depression (HR=1.28, 95% CI: 1.00-1.62) compared to participants with normal hearing. HI is associated with increased risk of incident depression and a greater overall prevalence of depression compared to normal hearing, underscoring the importance of further research on whether rehabilitative therapies can mitigate this association.

### HIGHER RATES OF UNTREATED DISTRESS IN OLDER VERSUS YOUNGER ADULTS WITH INDOLENT LYMPHOMA

Kelly Trevino,[1] Peter Martin,[2] and John Leonard,[3]
*1. Memorial Sloan Kettering Cancer Center, New York, New York, United States, 2. Iowa State University, Ames, Iowa, United States, 3. Weill Cornell Medicine, New York, United States*

Indolent lymphomas are incurable but slow-growing cancers, resulting in a large number of older adults living with these diseases. Patients typically live with their illness for years with the knowledge that disease progression is likely. Yet, little is known about psychological distress in this population. This study examined rates of and the relationship between distress and mental health service use in older and younger adults with indolent lymphomas. Adult patients diagnosed with an indolent lymphoma (e.g., follicular lymphoma, marginal zone lymphoma) within the past six months completed self-report surveys of distress (Hospital Anxiety and Depression Scale; HADS) and mental health service use since the cancer diagnosis (yes/no). Descriptive statistics, t-tests, and chi-square analyses were used to examine study questions. The sample (n=84) included 35 patients 65 years or older. Across the entire sample, 21.4% screened positive for distress on the HADS; 58.8% of these patients did not receive mental health services. Older adults reported lower distress levels than younger adults (17.1% v. 24.5%; p=.038). Among younger adults, 50% of distressed patients received mental health services; only 20% of distressed older adults received mental health services. Distress was associated with mental health service use in younger adults (p=.004) but not in older adults (p=.17). Older adults with indolent lymphomas have higher levels of untreated distress than younger adults. Research on the mechanisms underlying these age differences (e.g., stigma toward mental health services, ageism) would inform interventions to increase rates of mental health service use and reduce care disparities due to age.

### PSYCHIATRIC HISTORY AND TRAJECTORIES OF COGNITIVE CHANGE PREDICT RISK OF NURSING HOME RESIDENCE

Maria Brown,[1] and Miriam Mutambudzi,[2] *1. Syracuse University, Syracuse, New York, United States, 2. University of Glasgow, Glasgow, Scotland, United Kingdom*

Previous research indicates that a history of psychiatric, emotional or nervous problems can affect cognitive function at age 65 and cognitive trajectories over time. To explore the potential impact of this relationship on nursing home use, we applied latent class growth curve models to five waves (1998-2008) of Health and Retirement Study data to identify four classes of cognition trajectories, defined by baseline cognitive function scores (low, medium, high) and rate of change (stable or declining). We then ran survival analyses using HRS years 2008 to 2016 to determine risk of nursing home residence based on psychiatric history and cognition trajectory class. We hypothesized that self-reported history of psychiatric, emotional or nervous problems will be associated with greater risk of nursing home residence, and that self-reported history of psychiatric, emotional and nervous problems will interact with cognition trajectories to predict level of risk of nursing home residence. Results indicate that psychiatric history is independently associated with greater risk of nursing home residence, net the effects of a variety of life course variables in the model. Further, psychiatric history interacts with "low cognition declining" and "medium cognition declining" trajectories to increase the risk of nursing home residence. Evidence of the relationship between psychiatric history, cognitive decline, and nursing home residence can be used to enhance public understanding of the impact psychiatric history has on the long-term care system, and to educate policy-makers and providers about the need for appropriate psychiatric training for staff in community-based and residential long term care programs.

### SOCIAL ENVIRONMENT AND MENTAL AND BEHAVIORAL HEALTH OUTCOMES IN OLDER ADULTS: A CRITICAL REVIEW

Atami Sagna De Main, and Bo Xie, *The University of Texas at Austin, Austin, Texas, United States*

Social environments are complex and critical to older adults' health. Older adults are vulnerable to lack of social connectedness, social engagement and social contribution. 10-43% of community-dwelling older adults suffer from social isolation and loneliness in the United States. Despite the growing evidence on the impact of social environment on older adults' health, it remains that the information about older adults' social environment and its relationships to mental/behavioral health is fragmented. The purpose of this study is to determine the state of the science on social environment components (social connectedness, social engagement, social contribution) and mental/behavioral health (depressive symptoms, anxiety, psychological well-being, substance use, mental healthcare utilization) among community-dwelling older adults (65+ years). Five databases - CINAHL, PubMed, PsycINFO, PsycARTICLES, SocINDEX - were systematically searched using PRISMA guidelines to identify relevant articles from 1990 to 2019. Eleven articles are included in this review, illustrating relationships between social environment and mental/behavioral health. The studies found that poor social connectedness, social engagement, and social contribution were significantly associated with older age, poor perceived health, depression and anxiety symptoms, poor psychological well-being, hopelessness, having multiple chronic conditions, and functional limitations. Low social connectedness was significantly related to poor utilization of mental health services. No significant association was found between social environment and substance use in older adults. The findings of this review add to the literature related to social environment being relevant to older adults' mental/behavioral health and highlight the need to further our understanding of their dynamic relationships and changes over time.

### SESSION 2986 (PAPER)

### TECHNOLOGY USE AND TRAINING

### A NOVEL METHOD FOR ASSESSING AND TRAINING EVERYDAY FUNCTIONAL SKILLS

Sara Czaja,[1] Philp Harvey,[2] and Peter Kallestrup,[3] *1. Weill Cornell Medicine, New York, New York, United States, 2. University of Miami Miller School of Medicine, Miami, Florida, United States, 3. i-Function, Miami, Florida, United States*

Older adults, especially those with cognitive impairments often experience difficulty performing everyday tasks such as medication management, which threatens independence. Thus, there is an interest in developing treatment approaches for those who are experiencing or at risk for cognitive problems. Cognitive remediation training (CRT) programs have shown to be effective in improving cognitive abilities, but there is limited evidence to suggest that CRT results in everyday task performance gains. This presentation will discuss findings from a trial evaluating an innovative computer -based functional skills assessment and skills training program (CFSAT), which includes ecologically valid simulations of everyday tasks (e.g., shopping, money and medication management). The sample includes non-cognitively impaired (NC) older adults (n=50) and those with mild cognitive impairment (MCI) (n = 40), ranging in age from 60 -86 years (M= 73.10; SD = 6.06), is primarily female (90%), and ethnically diverse (69% minority). Participants were randomized into the CFSAT condition or a CFSAT + CRT condition. Performance data includes real time measures of accuracy, response time and efficiency. The findings indicate that the assessment component of the CFSAT program differentiated between the NC and MCI groups at the baseline assessment. Both NC and MCI participants demonstrated improvements in performance following training across all tasks; though the MCI participants required more training. Participants who received CFSAT + CRT training demonstrated increased efficiency in skill acquisition. The results indicate that the CFSAT program is an efficacious tool for assessing and training functional performance in both non-cognitively and cognitively impaired older adults.

### AGING AND FINANCIAL CAPACITY: COGNITIVE CORRELATES OF AN EARLY INDICATOR OF FUNCTIONAL DECLINE

Katherine Wild,[1] Jennifer Marcoe,[1] Nicole Sharma,[1] Mary Siqueland,[1] Nora Mattek,[1] Jason Karlawish,[2] and Jeffrey Kaye,[3] *1. Oregon Health & Science University, Portland, Oregon, United States, 2. University of Pennsylvania, Philadelphia, Pennsylvania, United States, 3. Layton Alzheimer's Disease Center, Portland, Oregon, United States*

Financial capacity describes the ability to make and carry out sound financial decisions sufficient to meet an individual's needs for health and well-being. Impairments in financial capacity have been shown to be one of the earliest functional changes in patients with mild cognitive impairment (MCI), the precursor to Alzheimer's disease and related dementias. Recent developments using online automated monitoring of financial transactions promise a new way to identify the earliest signs of cognitive decline. We examine the feasibility of using secure online technology to link ongoing financial activity monitoring data with other objective measures of function and cognition in a cohort of independent living older adults. To date, 73 older adults (mean age = 76.8, MoCA = 25.9) have enrolled and are participating in a 12-month online financial monitoring program that tracks account activity and generates alerts for unusual or irregular transactions. At baseline participants are administered a battery of neuropsychological tests and the Financial Capacity Instrument (FCI), a measure of financial capacity using tasks of everyday financial activity. Financial monitoring data are collected continuously, and are summarized and reported monthly. Younger participants had more online transactions and higher FCI scores. FCI total score was positively correlated with animal fluency ($p < .02$), Trails A ($p < .03$) and B ($p < .0001$), and visual memory ($p < .0008$). Number of online transactions in one month was correlated with FCI score, and Trails B (faster time to completion). Lower MoCA scores were associated with higher number of alerts per month.

### CONSERVATION OF RESOURCES THEORY: TECHNOLOGY AND CAREGIVER STRAIN

Shinduk Lee,[1] Marcia Ory,[1] Deborah Vollmer Dahlke,[2] Tiffany Shubert,[3] Steve Popovich,[4] and Matthew Smith,[1]
*1. Texas A&M University, College Station, Texas, United States, 2. DVD Associates, LLC, Austin, Texas, United States, 3. Shubert Consulting, Chapel Hill, North Carolina, United States, 4. Clairvoyant Network, Austin, Texas, United States*

Using the Conservation of Resources Theory, this study examined how caregiver strain was influenced by care recipients' use of falls alert wearables. Online survey data from 486 unpaid caregivers for adults aged 50 and older were analyzed. Structural equation modeling was used to test the following hypotheses: (1) caregivers with fewer financial resources would engage in fewer resource conservation strategies (e.g., care recipients' use of falls alert wearables); (2) resource conservation strategy engagement would be associated less resource loss; and (3) the effect of resource conservation strategies on caregiver strain would be less salient than the effect of resources used on caregiving (e.g., time and social support). The hypothesized model had a good model fit (CFI=.910), with SRMR (.060) and RMSEA (.062) being close to .05. All hypothesized paths were statistically-significant, except for the direct effect of using falls alert wearables on social support ($p=.076$) and caregiver strain ($p=.135$). As hypothesized, higher income was associated with greater likelihood of using falls alert wearables ($b=.10$, $p>.022$). Technology use was associated with less time spent on caregiving ($b=-.16$, $p<.001$) and had statistically-significant indirect effects on caregiver strain ($b=-.03$, $p=.008$). The total effect of using falls alert wearables ($b=.04$, $p=.394$) on caregiver strain was less powerful than the effect of time ($b=.20$, $p<.001$) or social support ($b=-.28$, $p<.001$). Study findings suggest the benefits of using falls alert wearables to alleviate time-related burdens and downstream caregiver strain among unpaid caregivers. Future efforts should investigate the relative advantage of wearables for other caregiving purposes.

### I DO HELP: OLDER ADULTS AS DIGITAL MEDIA SUPPORT PROVIDERS FOR THEIR PEERS

Amanda Hunsaker, Minh Hao Nguyen, Jaelle Fuchs, Gökçe Karaoglu, Teodora Djukaric, and Eszter Hargittai,
*University of Zurich, Zurich, Switzerland*

Older adults include highly sophisticated digital media users among their numbers for whom diverse methods of using online technology are a daily occurrence. Given that some older adults are quite tech-savvy, it follows that they may also provide digital media support to others. This study examines technological support-giving abilities and experiences of older adults. We completed in-depth qualitative interviews with older adults (ages 59+) in Hungary, the Netherlands, Switzerland, and Turkey (N=63) exploring: (1) whether older adults give digital media support and reasons for (not) doing so; (2) how older adults provide support and to whom; and (3) experiences of providing support with IT security specifically. The research team conducted consensus meetings to identify themes and coding schemes, and memo writing to share findings and ensure reliability across and within coders. Overall, a large number of our participants reported giving digital media support, primarily within their social circles. The kinds of help provided highly varied from navigating the Internet to choosing devices and managing security settings. We also find mutual support – support given to each other – an important domain for how people in this age group share assistance for technical problems. While some participants did not provide help with digital technology because they lacked confidence to do so, others believed they could, but were never asked. That such a prominent amount of support providers exist in this age group implies that peer-led technical support approaches may be especially salient and effective in helping older adults use digital media.

### TECHNOLOGY USE AMONG CAREGIVERS FOR PERSONS WITH AND WITHOUT COGNITIVE IMPAIRMENT

Shinduk Lee,[1] Matthew Smith,[1] Deborah Vollmer Dahlke,[2] Tiffany Shubert,[3] Steve Popovich,[4] and Marcia Ory,[1]
*1. Texas A&M University, College Station, Texas, United States, 2. DVD Associates, LLC, Austin, Texas, United States, 3. Shubert Consulting, Chapel Hill, North Carolina, United States, 4. Clairvoyant Network, Austin, Texas, United States*

This study aims at describing technology use among caregivers for middle-aged or older adults with and without cognitive impairment (CI) and examining whether the associations between technology use, caregiver strain, and social support differ by care-recipient CI status. Online data from caregivers (n=561) for adults at aged 50 years and older were analyzed from a national caregiver and technology survey. Multiple binary items were used to indicate caregivers' use of various devices (smartphone, computer, e-reader, and wearable activity tracker) and applications (communication, online banking, navigation, online entertainment, medication alert/tracker, and physical activity tracker). Predictors were care-recipient CI status (having been diagnosed with cognitive problems versus no cognitive problems), caregiver strain, and social support. Multivariable logistic regression models were fitted for each technology and for testing effect modification by care- recipient CI status. All models were adjusted for total caregiving hours and caregiver age, sex, race/ethnicity, financial status, and residence. Almost half (47%) reported their care-recipient was diagnosed with CI. Caregivers for those with CI were more likely to use e-readers (adjusted odds ratio (AOR) = 1.55, p=.040), wearable activity trackers (AOR=1.77, p=.013), and medication alerts/trackers (AOR=2.59, p<.001). Generally, greater caregiving strain and social support were positively associated with use of multiple technologies (p<.05). No effect modification of caregiving strain and social support by CI status was observed (p>.05). Technology use differences among caregivers of persons with CI may be driven by care recipients' unique situations and demands. Future research should identify technology use benefits on caregiver health-related quality of life.

### SESSION 2987 (PAPER)

### SLEEP CONSIDERATIONS, DISTURBANCES, AND DISORDERS AMONG OLDER ADULTS

### ARE DIFFERENCES IN BEDTIMES BAD FOR RELATIONSHIPS? ASSOCIATIONS WITH ATTACHMENT AND CONFLICT IN MIDDLE-AGED COUPLES

Joshua Novak,[1] and Stephanie Wilson,[2] *1. Auburn University, Auburn, Alabama, United States, 2. Southern Methodist University, Dallas, Texas, United States*

A robust body of literature has found birdirectional associations between sleep quality and marital quality in couple relationships (Hasler & Troxel, 2010; Pearlin, 2010). Additionally, dyadic research shows that differences in couples' bed time routines and habits is associated with mental health outcomes (Chen, 2018), however the literature has not connected them with other marital processes that are mutable and clinically relevant. Attachment theory provides a clinically relevant framework that captures both interpersonal marital processes such as relationship conflict as well intrapersonal processes of individual emotional safety—essentially individuals' personal strategies to balance closeness and distance in a relationship (Feeney, 2002; Rhodes et al., 2001). The two main attachment styles related to sleep processes are attachment avoidance and attachment anxiety (Collins et al., 2002; Gun, 2015; Troxel, 2007). Utilizing data from 234 couple dyads, we investigated if differences in partners' bed times is associated with conflict frequency and attachment avoidance using a structural equation modeling approach. We controlled for a number of important factors and tested our hypothesized model against two plausible alternative models. Results revealed that greater difference in partners' bed times was associated with higher conflict frequency for both husbands and wives through higher men's attachment avoidance. Our findings highlight previous research on matched vs. unmatched couples on sleep routines, habits, and chronotypes (both morning or night vs. different; Larson et al., 1991) but highlight mutable and clinically relevant constructs for intervention. Implications for health promotion and marital therapy will be discussed.

### ASSISTIVE RELAXATION THERAPY FOR OLDER ADULTS WITH INSOMNIA AND MILD COGNITIVE IMPAIRMENT: A PILOT STUDY

Miranda McPhillips,[1] Junxin Li,[2] E. John Ward III,[3] and Nalaka Gooneratne,[1] *1. University of Pennsylvania, Philadelphia, Pennsylvania, United States, 2. Johns Hopkins School of Nursing, Baltimore, Maryland, United States, 3. University of Pennsylvania, School of Medicine, Philadelphia, United States*

Insomnia symptoms are prevalent in older adults with mild cognitive impairment (MCI) and can pose treatment challenges. Our objective was to test the preliminary efficacy of tablet-based assistive relaxation therapy (ART) to improve insomnia symptoms in community-dwelling older adults with MCI. ART involves breath-based relaxation techniques coupled with a physical anchoring task to redirect thoughts and disengage from pre-sleep anxiety-provoking cognitions. Using a pilot randomized controlled non-crossover design, 20 participants recruited from one urban adult day center were allocated in a 1:1 ratio to intervention or education only control group for a treatment period of two weeks. Our final sample (n=20) was balanced on all demographic and clinical variables and consisted of Black (100%), female (75%), older adults (mean age 68.85 ± 7.29) with mean Montreal Cognitive Assessment scores of 21.2 ± 2.48. All participants at baseline had insomnia symptoms (mean Insomnia Severity Index (ISI) score 15.8 ± 3.78) and poor sleep quality (mean Pittsburgh Sleep Quality Index (PSQI) score 12.95 ± 0.70); half had daytime sleepiness (Epworth Sleepiness Scale (ESS) score 10.15 ± 1.07). Compared to baseline, participants improved on ISI (9.83 ± 1.32; p=0.0002), PSQI (9.11 ± 1.02; p=0.0016) and ESS (8.17 ± 0.86; p=0.08). The intervention group had statistically significant mean change scores on ISI compared to the control (-7.5 ± 1.37 vs. -3.88 ± 1.48; p=.0461). There were no statistically significant between group differences on PSQI or ESS. Our preliminary results suggest ART therapy is an effective treatment for insomnia symptoms in older adults with MCI.

### POLYSOMNOGRAPHIC SLEEP IS ASSOCIATED WITH TIME TO DEVELOP DEMENTIA: A STUDY USING 19-YEAR VA NATIONAL EHR DATA

Sara Nowakowski,[1] Javad Razjouyan,[2] Amir Sharafkhaneh,[3] Mark Kunik,[2] and Aanand Naik,[2] *1. Baylor College of Medicine, Houston, Texas, United States, 2. Center for Innovations in Quality, Effectiveness, and Safety (iQuest), Houston, Texas, United States, 3. Michael E. DeBakey VA Medical Center, houston, Texas, United States*

Few studies have longitudinally investigated the association between objectively measured sleep and time to develop dementia. This study leverages polysomnography (PSG) sleep data extracted from the VA national electronic health records (VA-EHR) to assess the association between sleep and time to develop dementia. We identified 61,165 PSG reports from the VA-EHR from 2000 to 2019 using CPT codes. Patients who developed dementia were identified using all-cause dementia ICD-9/10 codes documented on two separate visits starting one year after the PSG study until the end of 2019 in a 1-year sliding period (n=1,534). Using the first appearance of ICD-9/10 code as dementia onset time, patients were clustered into 3 groups of early-, mid-, and late time to develop dementia (mean = 2.7, 7.5, 12.8 years, respectively). Natural language processing was used to extract sleep efficiency (SE) and sleep onset latency (SOL). Univariate analysis was used to compare the groups. After adjusting for age, SE was significantly higher in the late (76%) vs early (69%) group and SOL was significantly shorter in late (21m) versus early (33m) group. SE was higher and SOL was shorter in patients who developed dementia later compared to those who developed dementia earlier. Greater sleep continuity in late dementia onset group suggests that sleep may be a modifiable risk factor that could potentially delay the onset of dementia.

### THE IMPACT OF SLEEP DISTURBANCE ON REGIONAL BRAIN VOLUMES

Shanna Burke,[1] Tan Li,[1] Adrienne Grudzien,[1]
Christopher Barnes,[2] Kevin Hanson,[2] and Steven DeKosky,[3]
*1. Florida International University, Miami, Florida, United States, 2. University of Florida, Gainesville, Florida, United States, 3. University of Florida College of Medicine, Gainesville, Florida, United States*

Sleep disruption has been associated with increased beta-amyloid deposition and greater risk for later development of Alzheimer's disease. Studies indicate that sleep disturbance correlates with regional brain volumes, but data are limited. We sought to determine the effect of sleep disturbance on regional brain volumes by cognitive and apolipoprotein e (APOE) e4 status. We conducted a secondary analysis of the National Alzheimer's Coordinating Center (NACC) Uniform Data Set using complete structural imaging data from 1,371 participants (mean age: 70.5; SD: 11.7). Multiple linear regression was used to estimate the adjusted effect of sleep disturbance (via Neuropsychiatric Inventory Questionnaire) on regional brain volumes through measurement of 30 structural MRI biomarkers. Sleep disruption was associated with greater volumes in the right and left lateral ventricles and greater volume of total white matter hyperintensities ($p<.05$). Lower mean volumes in total brain, total gray matter, and total cerebrum grey matter volumes, and in 12 hippocampal, frontal, parietal, and temporal lobe volumes were observed among participants who reported sleep disturbance. Males, Hispanic participants, and those with less education were more likely to report sleep disruption. Cognitive status moderated the relationship between sleep disturbance and lateral ventricular volumes, while APOE e4 moderated the effect between sleep disturbance and parietal lobe volumes. These findings suggest that disrupted sleep is associated with atrophy across multiple brain regions and ventricular hydrocephalus ex vacuo, after controlling for intracranial volume and demographic covariates. The influence of cognition and APOE e4 status indicates that this relationship is affected by co-occurring physiological processes.

### SESSION 2988 (PAPER)

### HEALTH, WELLNESS, AND SUCCESSFUL AGING

### FRIENDSHIPS FORGED IN FITNESS: AN ETHNOGRAPHIC EXPLORATION OF OLDER WOMEN'S SOCIAL EXPERIENCES IN WATER AEROBICS

Britteny Howell, and Samantha Wanner, *University of Alaska Anchorage, Anchorage, Alaska, United States*

Research shows that participants, especially older women, are more likely to adhere to a fitness program when they have social supports. Gerontology research also demonstrates that the social relationships forged by older women at community and fitness centers can be long-lasting and provide a variety of supportive functions. Older adults respond well to pool- or water-based aerobic exercises that are safe on the joints and provide a comfortable environment away from the intimidating nature of the gym. Therefore, water-based classes provided at community fitness centers are well positioned to provide ample social opportunities to further reinforce continued physical activity for older women, resulting in health and quality-of-life improvements. This project is a 5-month ethnographic exploration of the social relationships created and maintained in the context of water-based fitness classes (water aerobics) at a local community center (YMCA) that is attended by a culturally diverse group of older adults. The friendships forged by women in the pool at the YMCA provide a variety of social supports that help to maintain healthy aging outcomes among participants. Drawing on components of Activity Theory and Social Support Theory, this presentation utilizes participant observation, semi-structured interviews, and questionnaires (N=35) to provide an anthropological "thick description" of the important role that fitness center friendships can form in the social lives of older women in the U.S.

### GEROTRANSCENDENCE AS A FACTOR OF ACTIVE AGING: PREDICTORS AND OUTCOMES

Olga Strizhitskaya, *Saint-Petersburg State University, Saint-Petersburg, Russia*

The image of modern aging had changed. While before ageing was associated with degenerative processes, today more older adults become active and meaningful members of the society. Still more knowledge is needed to help the majority of older adults to age in active and positive way. Solid body of research shows that in ageing the value of subjective factors dramatically increases. Gerotranscendence that suggests important positive personality changes to occur in aging, could be one of such mechanisms. The aim of the present study was to investigate which psychological characteristics were important for development of gerotranscendence and which positive outcomes it might cause. Participants were 200 older adults aged 60-89 (69% females). Methods: Gerotranscendence scale (Strizhitskaya), self-actualization test (Shostrom), Self-acceptance test (Pantileev), Psychological well-being scale (Ryff), Scale of social activity

(Strizhitskaya). Our results confirmed in consistence with literature that higher scores on self-acceptance and self-actualization favored development of gerotranscendence. We also found that developed gerotranscendence positively affected psychological well-being. New in our research was that we showed that people who demonstrated higher scores on gerotranscendence were more interested in maintaining social activities; they were interested in participation in social life of their community and were trying to continue active and meaningful social participation. The final model fitted the original data (Chi-square=12,168; df=11; p=0,103; CFI=0,944; GFI=0,973; RMSEA=0,051; PCLOSE=0,503). Thus gerotranscendence had positive effects on older adult's functioning and social participation.

### UNDERSTANDING WISDOM: AN INVESTIGATION OF GENDER DIFFERENCE IN OLD AGE

Kathryn Hartikka, *University of Florida, Gainesville, Florida, United States*

Understanding wisdom contributes to a larger dialogue on how best to serve older adults as they go through the aging process. Namely, by identifying gender differences in wisdom conceptualization, research can further gather information regarding the complex, implicit definitions. To investigate how older men and women differ in conceptualizing wisdom, semi-structured qualitative interviews with seven men and eight women (age range: 64-86 years) who scored above average on the Three-Dimensional Wisdom Scale (3D-WS), the Adult Self-Transcendence Scale (ASTI), and the Foundational Values Scale (FVS) were compared. In-depth analysis of semi-structured interviews uncovered spiritual and non-spiritual experiential-based knowledge, "good" or spiritual-based decision-making, and a selfless care for others as common themes among women. Men invoked similar themes, such as spiritual-based decision-making and experiential-based knowledge, yet also differed in conceptualizing wisdom by emphasizing themes such as growth through hard times and overcoming obstacles. Men also considered wisdom to be related to an open-mindedness about life and rarely noting selfless care for others as a characteristic of wisdom in contrast to female respondents. The findings confirm earlier quantitative research results on implicit wisdom theories that men are more likely than women to have a cognitive understanding of wisdom, whereas women are more likely than men to characterize wisdom as an integrative construct.

### WELLNESS FOR OLDER ADULTS LIVING WITH SERIOUS MENTAL ILLNESS: A PROPOSED PRACTICAL FRAMEWORK

Michelle Zechner,[1] Matthew Fullen,[2] Nora Barrett,[3] Margaret Swarbrick,[4] Carlos Pratt,[5] and Stephanie Santos,[6] *1. Rutgers, RBHS, Piscataway, New Jersey, United States, 2. Virginia Tech, Blacksburg, Virginia, United States, 3. Rutgers, Piscataway, New Jersey, United States, 4. Rutgers, PIscataway, New Jersey, United States, 5. Rutgers School of Health Related Professions, Piscataway, New Jersey, United States, 6. Rutgers University, Middlesex, New Jersey, United States*

Multi-dimensional wellness is a holistic, person-centered clinical approach, that offers promise as a framework for improving the health and well-being of older adults. This approach can be especially helpful for older adults who are experiencing mental health conditions as it focuses on strengths, resilience and recovery comprehensively. Few clinical interventions have addressed wellness in older adults with mental health conditions (Zechner et al., 2019).The authors reviewed two existing wellness frameworks: 1) grounded in extensive practical interventions for people living with mental health conditions (Swarbrick, 1997; 2006; 2012; 2017), and 2) a theoretical framework informed by a systematic literature review (Fullen, 2019). The two frameworks were compared for usefulness in older adults living with mental health conditions using the concept analysis strategies of analyzing the overlap and contrasting characteristics of concepts with related ideas, and then proposing a merged operationalized definition and practical implementation suggestions based on the characteristics of the original dimension (Mackeroff et al., 2016). The two wellness models described similar dimensions, but also represented distinct areas. We propose a synthesized framework combining the models to best serve older persons living with mental health conditions. The framework holds eight distinct areas 1) Developmental 2) Cognitive/ Intellectual, 3) Physical 4) Emotional 5) Social/ Relational, 6) Occupational/Vocational 7) Spiritual and 8) Contextual/Environmental/Financial. Inter-professional practical clinical strategies and policy suggestions will be presented for consideration. More work is underway to understand the salience of the proposed model for stakeholders and to empirically test how the proposed framework impacts outcomes.

### SESSION 2989 (PAPER)

### END-OF-LIFE AND ADVANCE CARE PLANNING

### DETERMINANTS OF ADVANCE DIRECTIVE COMPLETION AMONG OLDER CHINESE AMERICANS

Kaipeng Wang,[1] Bei Wu,[2] Fei Sun,[3] Dexia Kong,[4] and Yanqin Liu,[5] *1. University of Denver, Denver, Colorado, United States, 2. New York University, New York, New York, United States, 3. Michigan State University, East Lansing, Michigan, United States, 4. Rutgers University, New Brunswick, New Jersey, United States, 5. Arizona State University, Tempe, Arizona, United States*

Advance Directive (AD) allows older adults to communicate preferred care at the end of life. Numerous studies reported that ethnic minorities were less likely to complete AD then non-Hispanic Whites. However, determinants of AD completion among older Chinese Americans remain unknown. The present study aims to address this knowledge gap. Data came from a survey of 439 Chinese Americans aged from 51 to 103 living in two metropolitan areas in 2018. Participants' average year was 75 (SD=9.37). About 63% were women and 93% were born outside the US. Approximately 14% of participants completed an AD. Guided by the Andersen's Service Use Model, we used logistic regression to examine determinants of AD completion. Results show that older age ($OR = 1.06, p < 0.01$), being employed ($OR = 2.63, p < 0.05$), acculturation ($OR = 2.09, p < 0.001$), having US citizenship ($OR = 3.57, p < 0.01$), and expectation of intergenerational support ($OR = 1.84, p < 0.05$), were positively associated with AD completion. Physical and mental health needs were not significantly associated with AD completion. This is among the first studies focusing on AD completion among Chinese Americans, one of the fastest growing older minority populations in the US. Findings highlight the influence of socioeconomic and cultural factors on AD completion and illustrates the importance of developing culturally sensitive interventions to promote end-of-life care decision-making among older Chinese Americans.

### DISPARITIES IN EOL CARE BY DEMENTIA STATUS AND RACE

Joann Reinhardt, *The New Jewish Home, New York, New York, United States*

Prior research shows that minority and dementia status are associated with suboptimal end-of-life (EOL) care quality; care that is more aggressive, invasive, and futile. We conducted a retrospective study of EOL care for 300 decedents of varied race/ethnicity in a skilled nursing facility. The purpose of this secondary analysis was to test whether the EOL experience (medical orders in place, treatments, distressing symptoms, discussions with providers) differed by dementia status for different race/ethnic groups (Black, White, Hispanic). Chi-square tests were used to examine the relation between these four sets of EOL variables and dementia status (yes/no) separately for the three groups. Findings showed that for White decedents, PWD were less likely to have had a DNR or a DNI discussion with a provider in the nursing home. Also for White decedents, PWD were less likely to have had shortness of breath or pain. For Black decedents, PWD were more likely to have a DNR order. Also, for Black decedents, PWD were less likely to have been hospitalized. For Hispanic decedents, EOL variables and dementia status were not significantly associated. Overall, findings showed differences by race/ethnic groups in EOL experience based on dementia status. Black decedents with dementia were more likely to have escaped the acute care default. Findings for White decedents with dementia were mixed for aggressive versus comfort care. The EOL experience did not differ by dementia status for Hispanic decedents. Thus, efforts to promote positive EOL care for persons with dementia need to account for differences by race/ethnicity.

### UNRAVELING THE EFFECTS OF SOCIAL CLASS AND SYSTEMIC RACISM ON ADVANCE CARE PLANNING

Jenny McDonnell, *Emory University, Roswell, Georgia, United States*

While advance care planning (ACP) is recognized as a key facilitator of high-quality, goal-concordant end-of-life care, black Americans are less likely to participate in ACP than non-Hispanic whites (Carr 2011; Detering et al. 2010). There are divided explanations for why these disparities persist. Some scholars attribute racial disparities in end-of-life care to socioeconomic (SES) differences between black and white Americans citing blacks' and whites' differentiated access to, control over, and use of material resources (Wilson 1978; Yearby 2011). Others assert that health care preferences do not solely reflect lack of resources or health literacy, but that the larger social context frames care preferences differently across racial and ethnic groups in American society (Alegria et al. 2011; Sewell and Pingel forthcoming). By turning the analytical lens to class-privileged black Americans, I investigate whether racism overflows the margins of class disadvantage. Using data from the Health and Retirement Study, I ran logistic regression and moderation models. I found that class-privileged blacks are less likely to engage in ACP than both high-SES and low-SES whites. The interaction of race and SES was negatively and significantly associated with ACP (OR=0.91; $P<0.05$), indicating that SES has a stronger effect on the probability of ACP among whites than among blacks. Predicted probabilities show that 51% of low-SES whites are likely to engage in ACP compared to 32% of high-SES blacks. These findings indicate that racialized disparities in ACP exist independent of SES, and that the effects of SES and race are intersectional rather than simply additive.

### WHEN CANCER MEETS DEMENTIA: THE END-OF-LIFE CAREGIVING EXPERIENCE FOR OLDER ADULTS WITH COMORBID DEMENTIA AND CANCER

Gigi C.C. LING,[1] and Amy Y.M. CHOW,[2] *1. The Chinese University of Hong Kong, Hong Kong, China, 2. The University of Hong Kong, Hong Kong, China*

Older adults with comorbid dementia and cancer is an increasing phenomenon with the aging population worldwide. Caregivers of these older adults might have a totally different and unique end-of-life caregiving experience. This is because all physical and behavioral signs and symptoms of dementia and cancer may interact with each other and complicate the caregiving experience. The aims of this study was to understand and examine the end-of-life caregiving experiences for older adults with comorbid dementia and terminal cancer from the perspective of family caregivers. Twenty-one caregivers were invited to participate in a semi-structured interview that examined the end-of-life caregiving experiences, its impact and how they coped with the challenges they faced. The interviews were transcribed and analyzed using interpretative phenomenological analysis. The essential meaning of the phenomenon is understood as "grieving thrice, suffering dually and becoming one", characterized by how caregivers understood the meaning of togetherness after going through the time of recurring losses from dementia through cancer to death and experiencing ambiguous sufferings dually with their loved one. Ambiguous sufferings were not "there" before the diagnosis of cancer but emerge in the context of comorbid dementia and cancer and in the connection with the caregivers making interpretation and appraisal of their internal and external resources. These important findings fill in the knowledge gap in the literature related to end-of-life caregiving experience for older adults with comorbid dementia and cancer; and may guide the development of appropriate interventions to support the older adults and their caregivers in a holistic approach.

### SESSION 2990 (PAPER)

### RISK OF MILD COGNITIVE IMPAIRMENT AND DEMENTIA

### DECREASING SOCIAL SUPPORT ASSOCIATED WITH RISK OF MCI AND DEMENTIA IS PARTIALLY MEDIATED BY HIPPOCAMPAL VOLUME

Tara Gruenewald,[1] Andrew Petkus,[2] Xinhui Wang,[3] Diana Youan,[2] Margaret Gatz,[3] Mark Espeland,[4] and

Jiu-Chiuan Chen,[2] *1. Chapman University, Orange, California, United States, 2. USC Keck School of Medicine, Los Angeles, California, United States, 3. University of Southern California, Los Angeles, California, United States, 4. Wake Forest School of Medicine, Winston-Salem, North Carolina, United States*

Less supportive social relationships are linked to greater risk of cognitive decline in older adulthood. Few studies have examined if declines in social support predict risk of developing Mild Cognitive Impairment (MCI) or dementia and the neurobiological factors that may contribute to these associations. We analyzed data from 926 women in the Women's Health Initiative Memory Study-MRI (WHIMS-MRI) to examine whether low social support at baseline and declines over an 8-year period predicted subsequent risk of developing MCI/dementia. Social support (Medical Outcomes Study Social Support Scale) was self-reported at the baseline (1994-1998) and closeout (2004-2005) of the parent WHI hormone therapy clinical trial. Annual neuropsychological assessments were conducted in WHIMS (through 2018) to ascertain incident MCI/dementia; structural brain scans were performed in 2005-2006. Structural equation models assessed the association between level and change in social support and risk of incident MCI/dementia and putative mediation of these associations by structural brain variables in women free of MCI/dementia as of the trial closeout, adjusting for demographic, lifestyle, depression, and biomedical covariates. Both low baseline social support (HR=1.24 per 1-SD; p<.05) and declines in support (HR=1.18 per 1-SD; p<.05) predicted incident MCI/dementia risk. Women reporting decreasing social support had significantly lower hippocampal volumes (β=-.070; p<.05) which accounted for ~14% of the total effect of declining support on MCI/dementia risk. We will highlight the implications of these findings for understanding how changes in social support may be linked to risk of MCI/dementia, including potential bidirectional associations of changes in social support and neurobiological health.

### LANGUAGE AND MEMORY RESERVE MEDIATE PROTECTIVE EFFECTS OF SOCIAL SUPPORT ON MCI OR DEMENTIA RISK IN OLDER WOMEN

Andrew Petkus,[1] Tara Gruenewald,[2] Diana Youan,[1] Xinhui Wang,[3] Margaret Gatz,[3] Mark Espeland,[4] and Jiu-Chiuan Chen,[5] *1. USC Keck School of Medicine, Los Angeles, California, United States, 2. Chapman University, Orange, California, United States, 3. University of Southern California, Los Angeles, California, United States, 4. Wake Forest School of Medicine, Winston-Salem, North Carolina, United States, 5. Keck School of Medicine, Los Angeles, California, United States*

More supportive social relationships are protective of cognitive decline in older adulthood. Although supportive social relationships are hypothesized to promote cognitive reserve (CR; the cognitive adaption to neuropathology), it is unknown whether CR mediates associations between social support and risk of developing mild cognitive impairment or dementia (MCI/dementia). Data from 815 women (aged 73-87 years) participating in the Women's Health Initiative Memory Study-MRI cohort (WHIMS-MRI) and Women's Health Initiative Study of Cognitive Aging (WHISCA) were analyzed to examine whether domain-specific estimates of CR mediate associations between social support and incident MCI/dementia risk. Women completed the Medical Outcomes Study Social Support Scale (MOS-SS) in 2004-2005, a structural MRI (sMRI) of the brain in 2005-06, and annual extensive neuropsychological examinations till 2018. CR (6-months after completing the MOS-SS) was estimated across different domains (e.g. verbal memory, figural memory, language, visuospatial, and attention) as the residual variance after regressing out effects of sMRI variables, sociodemographic factors, and measurement error. Structural equation models were constructed to examine whether CR mediate associations between social support and MCI/dementia risk while adjusting for covariates. Higher social support was associated with lower MCI/dementia risk (hazard ratio=0.85 per 1-SD;p=0.037), higher language reserve (standardized β=0.09;p=0.008) and verbal memory reserve (standardized β=0.08;p=0.025). Language and verbal memory reserve each significantly explained approximately 14% of the protective effect of social support. Findings illustrate the heterogeneous effect of social support on CR, highlighting the importance of language and verbal memory reserve as mediators of the association between social support and MCI/dementia risk.

### SOCIAL INTEGRATION AND RISK OF DEMENTIA AMONG OLDER ADULTS

Yan Zhang,[1] Shannon Shen,[2] and Yulin Yang,[3] *1. Michigan State University, East Lansing, Michigan, United States, 2. Texas A&M University-San Antonio, San Antonio, Texas, United States, 3. University at Buffalo, Buffalo, New York, United States*

We examine the relationship between social integration and cognitive impairment and dementia among older adults using longitudinal data from Waves 1-8 of the National Health and Aging Trends Study (NHATS). The sample includes 7,492 respondents age 65 and older at baseline. We test multidimensional measures of social integration and cognitive well-being using discrete-time hazard models. The risk of dementia is calculated by a series of performance-based tests. Measures include levels of dementia: no dementia, cognitive impairment not dementia (CIND), and dementia, and three domains of cognition functioning: orientation, executive function, and memory. Social integration is an additive index measured by several questions, including marital status, living arrangement, social network, social contact, and social participation. Our results indicate that people with higher social integration have a lower risk of both cognitive impairment (not dementia) and dementia compared to those with lower social integration. This pattern continued across specific domains of cognitive functioning, including lower risk of orientation impairment, executive function impairment, and memory impairment for those with higher social integration. Tests of both gender and racial interactions did not yield any significant differences. Our findings demonstrate the strong association between social integration and lower risk of dementia among older adults. This study can speak to policy makers as the life expectancy of Americans increases and the aging population grows, highlighting the importance of giving support to older adults who are lack of social connectedness.

### SESSION 2991 (PAPER)

### LONELINESS AND ISOLATION IN LATER LIFE

### AGE-FRIENDLY ENVIRONMENT, SOCIAL SUPPORT, SENSE OF COMMUNITY, AND LONELINESS OF KOREAN ADULTS

Miri Kim, and Soondool Chung, *Ewha Womans University, Seoul, Republic of Korea*

Abrupt industrialization, urbanization, and the emergence of the nuclear family have contributed to the increase of single-person households, placing an emphasis on individualization in society. Because of such changes, more people are living alone and feeling lonely, alienated from society and family. This research examined the association between loneliness and age-friendly environment, a community equipped with policies, settings, services, and structures that promote active ageing and aging-in-place, with the mediating roles of social support and sense of community. The study analyzed a total of 590 respondents aged 45 and above using data from the age integration survey conducted by the OO Research Institute of South Korea. Structural equation modelling and the bootstrapping method were applied. Age-friendly environment was positively associated with social support (β=0.310,p=0.000) and sense of community (β=0.486,p=0.000). Social support was negatively associated with loneliness (β=-0.195,p=0.000). The full mediation effect of social support was observed in the pathway from age-friendly environment to loneliness (95% CI =[– 0.112, –0.028]). Social support was fundamental in lowering loneliness, and age-friendly environment could only reduce loneliness by following a pathway that enhances social support. In other words, despite how well an age-friendly environment is established, it would not decrease an individual's loneliness unless it also enhances social support level. This finding sheds importance in maintaining satisfactory relationships that enable sharing of feelings, and fulfilling emotional and social needs in lowering loneliness. Measures to increase social support level within community are suggested.

### SOCIAL ISOLATION AND LONELINESS: MODERATORS OF THE RELATIONSHIP BETWEEN SENSORY IMPAIRMENT AND COGNITION

Alison Huang,[1] George Rebok,[2] Bonnielin Swenor,[2] Jayant Pinto,[3] Linda Waite,[3] and Jennifer Deal,[1] *1. Johns Hopkins Bloomberg School of Public Health, Baltimore, Maryland, United States, 2. Johns Hopkins University, Baltimore, Maryland, United States, 3. University of Chicago, Chicago, Illinois, United States*

Hearing and vision impairment have been independently linked to accelerated cognitive decline in older adults, however there is limited evidence on the effect of dual sensory impairment (DSI) (both hearing and vision impairment) on cognition. Additionally, the impact of social isolation and loneliness, both correlates of DSI and independent risk factors for cognitive decline, on the DSI-cognition relationship has yet to be studied. Using data from the National Social Life, Health, and Aging Project (N=3,091), multivariable linear regression models were used to describe the cross-sectional relationship between self-reported functional sensory impairment (none, hearing only, vision only, DSI) and cognitive function, measured by the survey adapted Montreal Cognitive Assessment. We also included an interaction term in the model to investigate whether cognition is worse among older adults with sensory impairment who also are socially isolated or lonely. Participants in this sample are between 62-91 years with 15% reporting hearing impairment, 11% reporting vision impairment, and 7% reporting DSI. DSI was associated with significantly lower global cognitive function compared to no sensory impairment (-0.31 standard deviations (SD), 95% CI:-0.44 to-0.18), hearing impairment alone (-0.29 SD, 95% CI: -0.44 to -0.15), and vision impairment alone (-0.22 SD, 95% CI: -0.39 to -0.06). Furthermore, cognitive function was significantly worse among older adults with both DSI and smaller social networks (p-interaction <0.05). No differences in the DSI-cognition relationship were observed by level of loneliness. These findings add to the limited research on the relationship between DSI, social isolation and loneliness, and cognition.

### WHO WANTS TO BE ALONE? ANTECEDENTS OF MOTIVATION FOR SOLITUDE IN ADULTHOOD

Jing Yuan, and Daniel Grühn, *North Carolina State University, Raleigh, North Carolina, United States*

Objectives: As an inevitable part of daily life, solitude has both positive and negative consequences which are moderated by one's motivation for solitude. Self-determined motivation correlates with few psychological risks, whereas other-determined motivation correlates with higher risks (e.g., loneliness, depression, lower well-being). However, little is known about the antecedents of different motivations for solitude. The purpose of this study is to investigate the antecedents of motivation for solitude in a sample with younger, middle-aged, and older adults. Methods: We recruited 468 participants from Amazon Mechanical Turk and Introduction to Psychology class (age range: 17-70, M = 30.7, 50.4 % females). Preference and motivation for solitude were measured with the Preference for Solitude Scale and Motivation for Solitude Scale-Short Form. Age, sex, marital status, education level, living arrangement, instrumental activities of daily living (IADLs), civic engagement, social contact frequency, introversion, and empathy were measured as antecedents. Results: People with older age, higher empathy for fictional characters, lower personal distress, higher introversion, and females tended to have higher general preference for solitude. People with higher empathy for fictional characters tended to have higher self-determined motivation. People with higher empathy for fictional characters, lower empathic concern, higher personal distress, higher IADLs, and higher introversion were more likely to have higher other-determined motivation. Discussion: A person with an introverted personality, functional limitation, more negative empathic reactions towards others are likely to have maladaptive motivation for solitude and may need intervention. Future research should further investigate other antecedents for self-determined motivation.

### SESSION 2992 (PAPER)

### SOCIAL AND ECONOMIC CHALLENGES AND BARRIERS FOR OLDER ADULTS AND THEIR FAMILIES

### COGNITIVE DECLINE AND FINANCIAL WELL-BEING: EVIDENCE FROM THE COGNITIVE ECONOMICS STUDY

Yuci Zhou, *Massachusetts Institute of Technology, Cambridge, Massachusetts, United States*

Maintaining financial well-being is important for older adults, as they are generally decumulating wealth at this stage of life. We use survey data from the Cognitive Economics Study (CogEcon, 2008-2017) and Cognition and Aging in the USA Study (CogUSA, 2007-2014) to investigate the impact of cognitive decline on financial well-being of older adults in the United States. In our sample (N = 976), with an average starting age of 65 years old, we use linear regression, then add results including a control function approach to correct our estimates for sample selection and attrition. In particular, those who have poor health outcomes (including death) disproportionately attrit from the study and may bias the results of a naïve regression. The naïve regression results yield no relationship between cognition and financial well-being; however, after controlling for attrition, we find that a decline in crystallized intelligence is associated with a decrease in financial well-being, and changes in fluid intelligence are not associated with changes in financial well-being.

### DOES SPOUSE'S DEMENTIA DIAGNOSIS MAKE INDIVIDUALS SKIMP ON HEALTH CARE?

Yi Chen, *University of Southern California, Irvine, California, United States*

Dementia is a costly disease that places great burden on individuals, families, and health care systems. The substantial time and financial resources taken away by living with persons with dementia (PWDs) may make their spouses forgo needed health care, thus deteriorating long-term health. To quantify the effect of dementia on spouses' health investment, I employed a difference-in-difference approach, comparing use of preventive services and doctor visits before and after spouses' dementia onset. Using Health and Retirement Study (HRS) with linkage to Medicare claims, I identified 650 older adults whose spouses had incident dementia during 1993 to 2007, and matched them to 1,816 controls whose spouses were dementia-free. Primary analysis reveals that individuals whose spouse had dementia did not change their use of most health services, relative to dementia-free controls. In stratified analysis, middle-class individuals skimped on flu shot and diabetes screening. Providing help for activities of daily living (ADLs) was associated with 1.9 less doctor visits, the effect of which was stronger among females (2.5 less visits). Help with instrumental activities of daily living (IADLs) was not a predictor of any utilization outcome. In conclusion, externalities of dementia imposed on family members are more profound and complex than deprivation of time. Certain subgroups were worse off in health investment when facing the trade-off between caring for spouses with dementia and caring for themselves. When understanding dementia burden, the externality imposed on spouses and its heterogeneity should be considered.

### FINANCIAL LITERACY IN THE FAMILY CONTEXT: THE ROLE OF SPOUSAL EDUCATION AND GENDER AMONG OLDER COUPLES

Yang Li, *UMass Boston, Boston, Massachusetts, United States*

Despite recent advances in the literature on the association between one's own education and financial literacy, the role of the family context in financial literacy has received limited attention. I examine whether spousal education is associated with one's own financial literacy among older couples and whether this association differs by gender. Using data from the 2016 Health and Retirement Study (n=1,220), I employ a multilevel actor-partner interdependence model to examine the cross-partner effect of spousal education on own financial literacy among older couples. I analyze a set of regression models on pairwise data to estimate the moderating effect of gender. I find that having a college-educated spouse was associated with a higher likelihood of being financially literate and that wives' education attainment was associated with a higher likelihood of financial literacy for husbands. Understanding the role of spousal education in late-life financial literacy adds to our knowledge about the role of the family context as related to individual financial knowledge and skills. Older adults may acquire financial literacy within the family, such as learning from a spouse.

### OUT-OF-POCKET COSTS ATTRIBUTABLE TO DEMENTIA: A LONGITUDINAL ANALYSIS

Melissa Oney,[1] Lindsay White,[2] and Norma Coe,[3]
*1. University of Pennsylvania, PHILADELPHIA, Pennsylvania, United States, 2. RTI International, Seattle, Washington, United States, 3. University of Pennsylvania, Philadelphia, Pennsylvania, United States*

Alzheimer's disease and related dementias (ADRD) affects 5.5 million Americans, and is expensive despite the lack of a cure or even treatments effective in managing the disease. The literature thus far has tended to focus on the costs to Medicare, despite the fact that one of the main characteristics of ADRD (the loss of independence and ability to care for oneself) incurs costs not covered by Medicare. In this paper, we use survey data for 2002-2014 from the Health and Retirement Study to estimate the out-of-pocket costs of ADRD for the patient and their family through the first 8 years after onset of symptoms, as defined by a standardized 27-point scale of cognitive ability. A two-part model developed by Basu and Manning (2010) allows us to separate the costs attributable to ADRD into two components, one driven by differences in longevity and one driven by differences in utilization. We consider total out-of-pocket expenditures, as well as out-of-pocket expenditures by category (i.e. hospital, nursing home, doctor, prescription drug, and other). Our results suggest that the out-of-pocket costs of ADRD are quite substantial over the first 8 years after onset. We also find that out-of-pocket spending is decreasing over the first 8 years, similar to the trend seen in Medicare expenditures. The results of this study highlight the financial burden of ADRD, particularly for the population paying out-of-pocket for care.

### URBAN ENVIRONMENTAL BARRIERS AND FACILITIES TO MOBILITY AND PARTICIPATION FOR OLDER MOBILITY DEVICE USERS

W.Ben Mortenson,[1] Bill Miller,[2] Atiya Mahmood,[3] Francois Routhier,[4] and Delphine Labbe,[5] *1. University of British Columbia, Vancouver, British Columbia, Canada, 2. University of British Columbia, Vancouver, Canada, 3. Simon Fraser University, Vancouver, British Columbia, Canada, 4. University of Laval, Quebec City, Quebec, Canada, 5. University of Illinois at Chicago, Chicago, Illinois, United States*

Many people use mobility devices to get around. Unfortunately, these mobility device users frequently encounter environmental features and social practices that restrict mobility and social participation. For example, barriers in the built environment can exclude mobility devices users from certain spaces. They also report experiencing discrimination and stigma in the community. However, much of the research in this area has not examined the experiences of older mobility device users in a holistic manner. The purpose of our study was to explore the barriers and facilitators of mobility and participation among people who use wheeled mobility devices. This mixed-methods project used multiple participatory research methods including qualitative interviews, participant-led, community environmental audits, photovoice, mobility tracking using global positioning satellite data and building accessibility audits of participant nominated buildings. We used standardized tools to measure participants' perceived, physical functioning, anxiety and depression, mobility and mobility device confidence among device users living. The study included 104 participants (64 from the Metro Vancouver and 41 from Quebec City). The primary mobility devices used included manual and power wheelchairs, mobility scooters, canes, crutches and walkers. On average, participants were 58 years of age and 53% were female. Our analysis revealed four main themes: 1) wayfinding challenges; 2) barriers and facilitators in the built environment; 3) the influence of social practices; and 4) temporal and climatic fluctuations. Our findings identified policies and changeable features in the built and social environment that restrict accessibility, which could be remedied by working collaboratively with municipalities and service providers.

### SESSION 2993 (PAPER)

### FAMILY CAREGIVING FOR ADULTS WITH ALZHEIMER'S DISEASE AND OTHER RELATED DEMENTIAS

### CARE2CAREGIVER: A PEER SUPPORT HELPLINE FOR CAREGIVERS OF PERSONS LIVING WITH DEMENTIA

Michelle Zechner,[1] Margaret Swarbrick,[2] and Mary-Catherine Lundquist,[2] *1. Rutgers, RBHS, Piscataway, New Jersey, United States, 2. Rutgers, PIscataway, New Jersey, United States*

Caregivers of persons with Alzheimer's disease and Related Dementia (CADRD) provide a significant amount of support to their family members, however, experience many challenges and stress that impacts their quality of life, emotional and physical well-being. They also have difficulty accessing services. Peers supporters, or CADRD with specialized training, are an important resource to help alleviate stress related to caregiving and focus on wellbeing, yet few studies have examined peer support. Our retrospective cohort study examined four years of data collected from a peer support helpline for CADRD in a northeastern state. Data included: demographics, presenting issues, services provided to assess for changes across time and call frequency. The Peer Specialists were CADRD who received specialized training and supervision to provide telephonic peer support. Caregivers were primarily female, and the average age of caller increased significantly over time. Issues related to personal emotional wellbeing was the primary reason for the call. The primary type of support provided was peer support interactions, follow-up calls, and links to resources focused on caregiver well-being. Callers reported distress from offering care to multiple recipients. Electronic follow-up (e.g. email and chat) increased over time. Peer support helpline programs have the potential to address the practical, physical, social and emotional needs of CADRD in the location of the caregiver choice. This model has potential to reach the growing number CADRDs via an array of web-based services including chat, email and online support, which can help overcome transportation and other logistical barriers to support.

### DEMENTIA CAREGIVING AND COGNITION: AN EXTENSION OF THE STRESS PROCESS MODEL

Francesca Falzarano,[1] and Karen Siedlecki,[2] *1. Weill Cornell Medicine, New York, New York, United States, 2. Fordham University, New York, New York, United States*

As cases of Alzheimer's disease and related dementias (ADRD) continue to rise, informal caregivers are critical resources in providing dementia care, yet caregiving is associated with high levels of burden, stress, anxiety and depression. Caregiving can be a prolonged and stressful experience, and impaired cognitive functioning in caregivers could impact their own health and quality of life and compromise the quality of care provided to their care-recipient. Thus, the purpose of the current study is to use the Stress Process Model as a guiding theoretical framework to identify whether primary stressors (e.g., care recipient functional status, cognitive problems) or secondary stressors (e.g., loss of self, economic conflict) predict performance across seven domains of cognition in 50 primary ADRD caregivers. Hierarchical regression analyses were used to examine which primary and secondary stressors emerge as predictors of cognitive performance. Results indicated that primary stressors (e.g., problematic dementia behaviors and relational deprivation) significantly predicted working memory performance and secondary stressors (e.g., economic strain, loss of self) significantly predicted implicit memory performance. Additionally, higher levels of caregiver burden predicted worse performance on executive functioning and implicit memory measures. Overall, the findings of this study indicate that the stress associated with caregiving may have adverse effects beyond psychosocial outcomes, and findings can be used to inform policies and practices with regard to caregiver health and well-being.

### DEVELOPMENT OF A NOVEL MEASURE OF INFORMAL CAREGIVER BURNOUT

Nicholas James, and Daniel Paulson, *University of Central Florida, Orlando, Florida, United States*

Burnout is a concept which has permutated most settings over recent decades. However, due to its roots in occupational research there exists both theoretical and practical gaps to consider when measuring burnout within non-occupational settings, such as informal caregiving. This study developed and validated a measure of burnout for informal caregivers of individuals with Alzheimer's disease and dementia, the Informal Caregiver Burnout Inventory (ICBI). Theoretical and methodological implications are discussed. Development included a 10-step method for scale development proposed by Boateng and colleagues (2018). Expert feedback on item appropriateness and clarity was collected from 33 caregivers or related professional experts and used to modify the original item-bank. Following this, a national sample of 255 current caregivers was gathered. This survey included the ICBI, two gold-standard measures of burnout, and measures of depression and caregiver burden. Item reduction analysis was used to remove items with poor item-total and inter-domain correlations. The ICBI shows good item-agreement (Cronbach's alpha= .88) and principles of Item Response Theory were used to measure item- and scale-wide information captured. Convergent validity was then compared against other measures of burnout using Bland-Altman Plots. Divergent validity was similarly assessed by comparing the ICBI to a depression questionnaire. Finally, the predictive validity of each burnout measure was compared to their association with burden and depression. This study suggests that the ICBI may perform adequately as an index of caregiver burnout, and thus is address a methodological and clinical gap in current efforts to understand the dynamics of caregiving.

### IMPLEMENTATION OF THE FL-REACH CAREGIVER INTERVENTION: TRANSLATION IN AN OUTPATIENT MEMORY DISORDERS CLINIC

Tracy Wharton,[1] Daniel Paulson,[1] Nicholas James,[1] Rosemary Laird,[2] Barbara Mendez Campos,[2] and Gayle Shepherd,[2] *1. University of Central Florida, Orlando, Florida, United States, 2. Advent Health, Orlando, Florida, United States*

The REACH II intervention is the gold-standard in dementia caregiver interventions. The FL-REACH translation is a novel adaptation offered in both English and Spanish to an outpatient memory disorder clinic at an urban, Southeastern healthcare system. This pre-post feasibility trial involves 6 sessions (4 in person at the clinic and 2 by phone) with the identified caregiver and any other family who wish to attend, which may also include the person living with dementia. The program is focused on early stage post-diagnosis, and is structured around building rapport, empowering families to build support networks, and teaching skills and knowledge-based material. Twenty four of the 60-participant target sample have consented to participate in this ongoing study. Change on the Preparedness for Caregiving Scale is significant (t=3.03, p=.001, Cohen's d=2.49). Means for the Zarit Burden 12-item scale went from 24.5 to 13.17 (t=-6.65, p=.03, Cohen's d=3.53). Access by care recipients to dangerous objects decreased (67% to 14%). Confidence in ability to use behavioral strategies in caregiving increased from 8% at baseline to 72% at study completion. Satisfaction surveys indicate high satisfaction with all elements of the intervention. These outcomes are consistent with existing data regarding utility of the REACH framework and reflect feasibility of delivering an adapted program model in an outpatient clinic environment. A future randomized controlled trial should examine whether early intervention and training reduces utilization of emergency care over time and improves quality of life for families.

### SUICIDAL IDEATION IN DEMENTIA FAMILY CAREGIVERS

Frank Puga,[1] Danny Wang,[2] and Carolyn Pickering,[2] *1. The University of Alabama at Birmingham, Birmingham, Alabama, United States, 2. University of Alabama at Birmingham, Birmingham, Alabama, United States*

Family caregivers of individuals living with Alzheimer's disease or related dementias (ADRDs) are exposed to unique stressors that put them at risk for depression and suicidal ideation. To date, little is known about contextual factors surrounding suicidal ideation among ADRD family caregivers. We investigated individual caregiver characteristics (gender, age, relationship to care-recipient, history of depression and anxiety) and daily environmental stressors (behavioral symptoms of dementias; BSDs) associated with daily suicidal ideation using a micro-longitudinal design and ecological momentary assessment methods. Data were collected from a national sample of family caregivers (N=51) who completed daily diaries over 21 days (n=911). Suicidal ideation was endorsed on forty-seven days (5.16%) during the sampling period, with 11 participants (22%) endorsing suicidal ideation at least once. Suicidal ideation did not differ based on the caregiver's age and relationship to the care-recipient (spouse or child). Participants with a history of mild depression and anxiety endorsed more days with suicidal ideation. Finally, family caregivers were more likely to endorse suicidal ideation on a day when more than one type of BSD was reported (OR = 1.25, 95% CI: 1.04-1.50, p = 0.018) and when BSDs were perceived as more bothersome than average (OR = 1.12, 95% CI: 1.05-1.19, p < 0.001). In this investigation, we identified descriptive and predictive factors that will inform the development of targeted interventions for ADRD caregivers at high risk of suicidal ideation.

### SESSION 2994 (PAPER)

### CHRONIC DISEASE MANAGEMENT AND MODELS OF CARE

### CHARACTERISTICS OF OLDER VETERANS ENROLLED IN THE ILLINOIS MEDICAL CANNABIS PATIENT PROGRAM

Julie Bobitt,[1] and Hyojung Kang,[2] *1. University of Illinois at Urbana-Champaign, Champaign, Illinois, United States, 2. University of Illinois - Urbana/Champaign, Champaign, Illinois, United States*

Veterans often struggle with disabling conditions such as chronic pain and post-traumatic stress disorder (PTSD) that tend to worsen as they age. Common treatments for these conditions include the use of opioids and benzodiazepines, yet these medications tend to have unwanted side effects and can even result in addiction. While cannabis use in the US has increased significantly over the past decade, research regarding the risks and benefits is mixed and a growing number of research studies have highlighted the benefits of taking cannabis for medical purposes. While previous studies have looked at cannabis use in older adults and in Veterans over 18, no research has looked at cannabis use specifically in older Veterans. We surveyed adults age 60 and over who were enrolled in the Illinois Medical Cannabis Patient Program as of September 2019. We collected demographics, reason for enrolling in the medical cannabis program, history of cannabis, opioids, and/or benzodiazepines use, and health outcomes. Of 3,768 responses, 593 were Veterans. Older Veterans in our study were predominately male (92.1%), reported using cannabis primarily for pain (80.1%), PTSD or emotional health problems (50.1%) (i.e. anxiety and depression), and reported that cannabis use has positively impacted their quality of life (89.4%), health outcomes (81.9%), pain (86%), and sleep quality (77.1%). Understanding why older Veterans use cannabis and the outcomes they experience from cannabis use can inform state and federal policy makers and enhance clinical care practices.

### CHRONIC DISEASE SELF-MANAGEMENT: BEHAVIORAL INTERVENTION PREFERENCES INFORMED BY THE PEN-3 CULTURAL MODEL

Chivon Mingo, and Collins Airhihenbuwa, *Georgia State University, Atlanta, Georgia, United States*

Chronic Disease Self-Management Program (CDSMP) is an evidence-based program shown to improve health status, healthcare utilization, and health behaviors among individuals diagnosed with chronic conditions. Aging African Americans, a population at a greater risk of chronic disease diagnosis and burden, are underrepresented in the utilization of self-management behavioral programs. Previous research suggested that cultural distinctions (e.g., values, beliefs, preferences, experiences) impact the participation of racial/ethnic minorities in health-related research, health behavior outcomes, and healthcare utilization. Little is known about unique cultural influences on CDSMP utilization among this target group. To our knowledge, no research has applied a culture-specific theory to understand preferences or use of CDSMP among aging African Americans. Guided by the PEN-3 cultural model, this study examines preferences, barriers, and facilitators that may influence CDSMP utilization for the management of diagnosed chronic conditions. Using a qualitative research design, African Americans (N=50) from six Atlanta Metropolitan faith-based organizations participated in the 6-week CDSMP and one of six focus groups. Participants were largely female (70%) experiencing multiple chronic conditions (M=2) with an average age of 70. Focus groups were transcribed, and a thematic analysis was applied to identify emergent themes. Participants preferred programs that included family, community liaisons, and relevant advertisement material suggesting the importance of cultural identity. Accessing CDSMP at a familiar location with an endorsement of program benefit from trusted sources suggests the importance of relationship and expectations. Findings shed light on factors that may cause aging African Americans to embrace or avoid CDSMP as a healthcare option.

### LIFESTYLE CHALLENGES AMONG OLDER FEMALE CANCER SURVIVORS

Jessica Krok-Schoen,[1] Janell Pisegna,[2] Elizabeth Arthur,[2] Emily Ridgway,[3] and Ashley Rosko,[3] *1. Ohio State University, Columbus, Ohio, United States, 2. The Ohio State University, Columbus, Ohio, United States, 3. The James Cancer Hospital, The Ohio State University, Columbus, Ohio, United States*

The American Cancer Society recommends that survivors maintain a healthy lifestyle including a normal weight, being physically active, and maintaining a healthy diet to improve prognosis and health-related quality of life (HRQoL). Unfortunately, the majority of cancer survivors do not engage in a healthy lifestyle. The largest proportion of cancer survivors are older adults (≥65 years), yet they are often understudied, particularly regarding healthy lifestyles. This study sought to examine the lifestyle behaviors (maintaining healthy weight, dietary intake, physical activity) of older female cancer survivors and to identify associations with HRQoL. Older female cancer survivors (n=170) completed surveys to assess HRQoL (RAND-36), diet quality (HEI-2015), physical activity, malnutrition, and BMI. Descriptive analyses, correlations, and stepwise linear regressions were utilized. The majority of the sample (mean age=74.67±8.43 years) were white (90%), married (54.4%), college-educated (63.9%), and breast cancer survivors (67.4%). Self-reported health was very good (42.6%) and good (39.6%) and general HRQoL was 59.48±15.34 out of 100. Self-reported physical activity was low; 75.3%, 54.2%, and 68.1% reported no strenuous, moderate, and mild physical activity, respectively. Mean BMI was 27.71±6.24 with 64% of the participants being overweight or obese. Mean HEI-2015 scores were 66.39±10.00, below the "good" diet quality score of 80. Risk of malnutrition was present in 27.4% of participants. Regressions found that being White ($\beta$=-0.528, p=0.001) and lower BMI ($\beta$ =-0.405, p=0.024) were significant predictors of HRQoL. Results indicate the need for tailored health coaching for older cancer survivors regarding their lifestyle behaviors to improve prognosis and HRQoL.

### MULTIPLE CHRONIC CONDITION COMBINATIONS AND COGNITIVE TASK PERFORMANCE

Aaron Ogletree,[1] and Benjamin Katz,[2] *1. American Institutes for Research, Washington, District of Columbia, United States, 2. Virginia Tech, Blacksburg, Virginia, United States*

A growing body of literature describes important advances in the study of chronic conditions, most notably a paradigm shift from the study of individual chronic conditions to the study of multiple chronic conditions (MCCs). Despite these advances, little research has explored MCC combinations, and almost no published research has explored how MCC combinations are related to cognitive outcomes in older adult populations. Using data from the Health and Retirement Study, we categorized 17,349 older adults into one of 32 groups using self-reports of five of the most commonly diagnosed conditions. These included arthritis, diabetes, heart problems, hypertension, and respiratory problems. We utilized ANOVA to examine the associations between combinations of MCCs and performance on two cognitive tasks associated with executive function and fluid intelligence: verbal fluency and verbal analogies. Results demonstrated that older adults with a greater number of health conditions performed more poorly on both the verbal fluency (p<.0001) and analogies (p<.0001) tasks than those with fewer conditions. Some MCC combinations were associated with poorer cognitive task performance than other combinations: for example, older adults in the Heart-Hypertension-Respiratory group had an average score of 488.73 (SD=24.96) on the verbal analogies task and 14.06 (SD=7.06) on the verbal fluency task. Conversely, adults in the Arthritis-Heart-Respiratory group had average scores of 503.69 (SD=27.89) and 16.45 (SD=7.03), respectively, suggesting differential additive effects of MCCs. These findings demonstrate the complex associations of specific MCC combinations with cognitive performance and highlight the importance of better understanding the unique needs of older people with MCCs.

### UTILIZATION OF AN INTEGRATED MEDICARE ADVANTAGE MODEL IS POSITIVELY ASSOCIATED WITH MEDICATION ADHERENCE

Renae Smith-Ray,[1] Tanya Singh,[2] and Chester Robson,[3] *1. Walgreens Center for Research on Health and Wellbeing, Deerfield, Illinois, United States, 2. Walgreen Co, Riverside, Illinois, United States, 3. Walgreen Co, Deerfield, Illinois, United States*

An estimated 30% of U.S. healthcare costs are due to waste, inefficiencies, and excessive pricing. Research shows that integrated primary care models (IPC) improve health outcomes and reduce costs. Nearly all IPCs embed ancillary clinicians, including pharmacists, within the clinic. IPCs that embed a primary care clinic within a pharmacy are novel. This study describes the first known IPC for older adults that is based in a pharmacy and examines its impact on medication adherence. In January 2018, Walgreens launched an IPC focused on Medicare Advantage patients at select Kansas City Walgreens locations. Each morning the entire IPC team meets to review needs of patients who will be seen that day. Upon arrival, the patient is first seen by a pharmacist who completes medication and immunization reviews and fall risk screening. If a new medication is prescribed during the physician visit, the pharmacist returns to consult the patient. The IPC team works together to ensure that the Medicare Annual Wellness Exam is completed in entirety. We examined the impact of IPC utilization on adherence to the top seven chronic condition drug groups. IPC patients age 50+ with sub-optimal adherence (<80% proportion of days covered) during the year prior to the clinic opening were included (n=64). A Student's t-test revealed an 11% improvement in optimal adherence year-over-year between the pre- and post- periods (p<0.001). The pharmacy-based IPC is associated with improved medication adherence. Future research should examine the impact of this model on patient satisfaction and additional health outcomes.

### SESSION 2995 (PAPER)

### ATTITUDES, BELIEFS, AND PERCEPTIONS ABOUT AGING

### AWARENESS OF AGE-RELATED CHANGE, FUTURE TIME PERSPECTIVE, AND IMPLICATIONS FOR GOAL MANAGEMENT IN OLDER ADULTHOOD

Bethany Wilton-Harding,[1] and Tim Windsor,[2] *1. Flinders University, Adelaide, Australia, 2. Flinders University, Adelaide, South Australia, Australia*

How people manage their goals is central to adaptation across the lifespan. However, little is known about the individual difference characteristics that predict how and why people use different self-regulatory strategies. The present study aimed to investigate associations of perceived age-related gains and losses, and their interaction, as predictors of flexibility in goal management in older adulthood. We also examined whether future time perspective (FTP) mediated the relationship between AARC and goal flexibility, such that awareness of aging impacts perceptions of time remaining, impacting how individuals evaluate and manage their goals. A community-based sample of 408 adults aged between 60 and 88 years was recruited via an internet-based research platform. Participants completed questionnaire measures of AARC-gains, AARC-losses, FTP, goal disengagement, and goal re-engagement. A flexibility index reflecting tendencies toward use of both goal disengagement and re-engagement strategies was also analyzed. Although AARC-losses was associated with lower goal re-engagement and goal flexibility, this association was weaker among those with higher AARC-gains, indicating that AARC-gains may be protective in the relationship between AARC-losses and goal management. Furthermore, the association between AARC and goal management was mediated by FTP. Higher AARC-gains were associated with more open-ended FTP, which was associated with higher goal re-engagement and lower goal disengagement. On the other hand, higher AARC-losses was associated with more restricted FTP, which was associated with lower goal re-engagement, and higher goal disengagement. Results suggest that subjective awareness of aging (particularly awareness of gains), has important implications for goal management in older adulthood.

### CHALLENGING AND DISMANTLING AGEIST ATTITUDES, BELIEFS, AND BEHAVIORS THROUGH INTERGENERATIONAL PROGRAMS

Tia Rogers-Jarrell,[1] and Brad Meisner,[2] *1. York University, Mississauga, Ontario, Canada, 2. York University, North York, Ontario, Canada*

Age stereotypes are complex and multifaceted: individuals can demonstrate and embody numerous and varied positive and negative stereotypes. Therefore, solutions to combat age stereotypes must also be complex and multifaceted. Additionally, both social and physical forms of age segregation are common in our society. This causes fewer and fewer opportunities for younger and older people to interact. Intergroup Contact Theory suggests age stereotypes can be reduced through increased intergenerational contact. One way to encourage contact between younger and older populations is through intergenerational programming. However, there is a lack of literature investigating the effects of intergenerational programs on perceptions of aging. The purpose of this paper was to critically review and explore literature on intergenerational programs to understand how they influence age stereotypes and ageist attitudes. The available literature suggests that intergenerational programs involving young children (ages 4-8), adolescents (ages 11-18), or emerging adults (ages 19-26) interacting with older adults (ages 65+) can significantly reduce age stereotypes towards older adults. Additionally, older adults (ages 65+) negative beliefs and attitudes towards younger people (ages 4-26) can also be deconstructed after participation in intergenerational programs. Intergenerational programs act to break down age barriers and promote connections and understandings between generations. These programs challenge the belief that older and younger people should live and participate in spaces that are separate from one another. Providing opportunities for younger and older people to participate in intergenerational programs is one way to promote respectful relationships and enhance the quality of life and health of all generations.

### HEALTH DIFFERENCES AMONG PEOPLE WITH VARYING PROFILES OF AWARENESS OF POSITIVE AND NEGATIVE AGE-RELATED CHANGES

Serena Sabatini,[1] Obioha Ukoumunne,[1] Clive Ballard,[1] Manfred Diehl,[2] Hans-Werner Wahl,[3] Allyson Brothers,[2] and Linda Clare,[1], *1. University of Exeter, Exeter, England, United Kingdom, 2. Colorado State University, Fort Collins, Colorado, United States, 3. University of Heidelberg, Heidelberg, Baden-Wurttemberg, Germany*

Higher awareness of negative age-related changes (AARC-losses) is related to poorer mental and physical health whereas higher awareness of positive age-related changes (AARC-gains) is related to better mental health. Associations of health with AARC-gains and losses have been explored separately, but often people experience gains and losses concurrently. Using latent profile analysis, we identified at the cross-sectional level patterns of AARC-gains and losses and explored whether groups with distinct profiles of AARC-gains and losses differed in physical, mental, and cognitive health, and demographic characteristics. Analyses were based on the large-scale PROTECT study conducted in the UK (N= 6,192; mean age= 66.10(SD= 7.04); 76% women). A four-group solution revealed the best model fit (Akaike's information criterion= 156,061.93; Bayesian information criterion= 156,418.67); 45% of participants perceived many AARC-gains and few losses (Group 1); 24% of participants perceived moderate AARC-gains and few losses (Group 2); 24% of participants perceived many AARC-gains and moderate losses (Group 3); and 7% of participants perceived many AARC-gains and many losses (Group 4). The four groups differed meaningfully in health; Group 1 was the most healthy, followed by Groups 2, 3 and 4. Participants in Group 1 were most likely to perceive their health as excellent, reported the lowest levels of depression and anxiety, and showed the best cognitive performance. On average participants in Group 1 were younger, and more likely to be female, employed, and married, compared to other groups. Considering the co-existence of gains and losses is important when relating awareness of age-related changes to health.

### LOVING OTHERS: THE IMPACT OF COMPASSIONATE LOVE ON LATER-LIFE PSYCHOLOGICAL WELL-BEING

Eva Kahana,[1] Tirth Bhatta,[2] Boaz Kahana,[3] and Nirmala Lekhak,[4] *1. Case Western Reserve University, Cleveland, Ohio, United States, 2. University of Las Vegas Nevada, Las Vegas, Nevada, United States, 3. Cleveland State University, cleveland, Ohio, United States, 4. University of Nevada, Henderson, Nevada, United States*

Existing scholarship in social gerontology has surprisingly paid little attention to broader loving emotions, such as compassionate and altruistic love, as potentially meaningful mechanisms for improving later life psychological well-being. This study examined the influence of feeling love toward other persons and experiencing love from others on later life psychological well-being. We conducted a 3-wave longitudinal study of a representative sample of 340 ethnically heterogeneous community dwelling older residents of Miami, Florida. The increase in feeling of being loved (β=-1.53, p<0.001) and love for others (β=-1.43, p<0.001) led to decline in odds of reporting greater level of depressive symptoms over time. The odds of reporting higher level of positive affect were significantly greater for older adults who reported feeling loved by others (β=1.16, p<0.001) and expressed love for other people (β=1.18, p<0.01). Older adults who felt loved had 0.92-point lower ordered log odds of reporting higher negative affect than those who reported lower level of love. The impact of compassionate love on depressive symptoms and negative affect remained statistically significant even after adjustment for altruistic attitudes and emotional support. The influence of loving emotions on positive affect was, however, explained by altruistic attitudes and emotional support. Our findings underscore the powerful influence of both receiving and giving love for the maintenance of later life psychological well-being. We offer support for the expectation that love is a significant force in the lives of older adults that transcends intimate relationships.

### MEASURING SELF-PERCEPTIONS OF AGING: DIFFERENCES BETWEEN MEASURES WHEN PREDICTING HEALTH OUTCOMES

Jordan Boeder,[1] and Dwight Tse,[2] *1. Claremont Graduate University, Claremont, California, United States, 2. The Chinese University of Hong Kong, Hong Kong, China*

The majority of self-perceptions of aging (SPA) research uses either a combination of the Age-related Cognition (AgeCog) scales of Ongoing Development and Physical Loss, or the Attitudes Towards Own Aging (ATOA) subscale to assess views on aging. Although these scales are used interchangeably, the valence (positive/negative) and the specificity of the view on aging (domain-based/general) being assessed are not consistent. This study investigates how different measures of SPA relate to one another and whether they differentially predict various types of health outcomes (psychological/physiological; well-being/ill-being). Data from the 2008 and 2014 waves of the German Aging Survey (DEAS; n=3,745), a population-based representative survey of adults aged 40 to 95, was used to examine the relationship between the AgeCog scales and the ATOA subscale, as well as the differences in the types of health outcomes each predicts. The correlations between the AgeCog scales and the ATOA were higher than the correlation between the AgeCog scales (p < .001). The AgeCog scale of Ongoing Development significantly predicted psychological health outcomes across a six-year period, while the AgeCog scale of Physical Loss and the ATOA subscale predicted both physiological and psychological health outcomes. Evidence supports using the AgeCog scale of Ongoing Development to predict domain-relevant, psychological health outcomes. However, the multidimensionality of SPA is best measured by the ATOA subscale or a combination of the two AgeCog scales. Both forms of measurement were found to maximize the amount of explained variance for psychological and physiological indicators of well-being and ill-being.

### SESSION 2996 (PAPER)

### DISASTERS AND EMERGENCIES: A FOCUS ON THE COVID-19 CRISIS

### ENVIRONMENTAL INTERVENTIONS TO PROMOTE INFECTION CONTROL IN SENIOR-LIVING FACILITIES: CASES IN THE USA AND CHINA

ZHE WANG, and Bo li, *Henan University, Kaifeng, China*

In the evolving situation of COVID-19, an outbreak of the contagious disease in a nursing home near Seattle prompted urgent calls for precautionary tactics in senior-living facilities. Worldwide, the USA and China are the countries with the largest and second largest populations of people aged 80+. More than two million older adults live in senior-living facilities in the USA and the number of beds in senior-living facilities in China has increased to 6.5 million in recent five years. The risk of infections including COVID-19 is tripled in senior-living residents because of age, close living conditions, and underlying health conditions. Infectious diseases account for one third of all deaths in people age 65+ in the USA. COVID-19 has a case fatality rate of 2.3% overall and as high as 15% in patients age 80+ in China. Together with health and services interventions, environmental interventions should be considered to prevent the possible spread of infections in senior-living settings. Based on a literature review and using empirical data of 12 senior-living facility designs collected in both countries, this research analyzed the advantages and disadvantages of existing facility environments in terms of infection control. Environmental interventions to promote infection control are suggested in the context of individual facilities. Proposed interventions are analyzed at two levels: spatial design and design details. Five factors are included: visitors screening, ventilation, isolation rooms, hand washing, and daily temperature checks. From the perspective of infection control, the cross-country similarities and differences of senior-living facility designs are discussed.

### IMPLEMENTATION OF DISASTER PREPWISE, AN ONLINE DISASTER PREPARATION TOOL FOR OLDER ADULTS

Lena Thompson,[1] Emily Henja,[2] Travis Beckman,[3] and Sato Ashida,[1] *1. University of Iowa College of Public Health, Iowa City, Iowa, United States, 2. Rush University, Chicago, Illinois, United States, 3 Johnson County Homeland Security & Emergency Management Agency, Iowa City, Iowa, United States*

Older adults are less prepared for disaster situations than younger adults due to expenses, complicated preparation processes, and lack of support. We previously developed and tested a disaster preparedness program for older adults and showed its impact on improving preparedness and personal emergency support network. Using a 5-step community-engaged approach and in collaboration with our Stakeholder Advisory Board (SAB), we adapted this Disaster PrepWise program to an online platform and developed implementation plans within existing community-based service infrastructure. Community engagement processes allowed us to create an online tool that was considered "important" and "useful" by older adults and program deliverers. In terms of reach, 73 potential participants attended an introductory presentation and 47 participated. Those who participated were similar to those who did not in terms of gender and race. Twenty-seven participants completed surveys before and one-month after the program and reported engaging in an average of 2.9 additional recommended behaviors after intervention (t(26)=2.85, p=0.00). The major organizational barrier to adoption was limited staff time. Participating older adults reported adopting what they learned through the program in their daily life. Implementation process data showed multiple modifications made during delivery including delivery of the program in one instead of two sessions. The implementing organization reported their likelihood of maintaining this program within their practice as "extremely likely." Community-engaged approaches and process data led to an acceptable and useful program and implementation strategies that helped enhance preparedness behaviors among older adults. Future work will focus on program dissemination and larger scale evaluation.

### PANDEMIC PREPAREDNESS OF CARE HOMES SERVING OLDER ADULTS IN THE WAKE OF COVID-19: AN EXPLORATORY STUDY

Iftekhar Amin, *University of North Texas at Dallas, Dallas, Texas, United States*

Recent COVID-19 pandemic has disproportionately affected the older adult population worldwide. According to CDC, among older adults over 60 years the risk increases with age, with the highest risk of serious illness and death among those over 80 years. While public has been receiving messages about the risks and how to take preventive measures, it is not clear how the care homes serving older adults have been preparing. Data have been collected as part of an ongoing study from 30 independent living, assisted living, and memory care facilities across the United States. The centers were selected with a snowball sampling technique. Administrators of the centers were interviewed with a semi-structured questionnaire. It was apparent that although awareness of risks was high, preparation appears to be inadequate with little resources available at the time of the survey. Variation of preparedness based on the sociodemographic characteristics of the residents suggests that homes that serve predominantly minority and economically disadvantaged have greater likelihood of lacking preventive resources. It is critical that facilities serving older adults be prepared to ensure an effective healthcare response in the wake of novel viruses, such as COVID-19.

### SURVIVING TOGETHER BY STAYING APART: HOW OLDER ADULTS EXPERIENCED THE COVID-19 CRISIS

Julie Miller,[1] Lisa D'Ambrosio,[2] Alexa Balmuth,[1] Samantha Brady,[1] Martina Raue,[3] Taylor Patskanick,[2] John Rudnik,[2] and Joseph Coughlin,[2] *1. MIT AgeLab, Cambridge, Massachusetts, United States, 2. MIT, Cambridge, Massachusetts, United States, 3. Massachusetts Institute of Technology, Cambridge, Massachusetts, United States*

The repercussions of novel coronavirus (COVID-19) within the United States and across the globe are vast. Researchers at the MIT AgeLab conducted a national online survey (N=1200) related to COVID-19 in March 2020 with the goal of exploring participants' COVID-19-related attitudes, hygiene and consumer behaviors, and their use of technology to work, make purchases, stay informed, and stay socially connected. In this presentation, AgeLab researchers will describe findings among a sub-population of study participants deemed to be at highest health risks for the virus: adults ages 60 and over. Findings demonstrate that the largest group of participants stayed informed about COVID-19 primarily through online sources. As a result of the virus, most had made changes to their health and hygiene practices, including implementing social distancing measures, and the majority had changed at least one of their purchasing behaviors, most often buying more products than usual online. Second only to participants' physical health concerns related to COVID-19 were their financial health concerns, both related to near-term finances and longer-term financial goals. From this study, policy and practice implications emerge across public, private, and non-profit sectors that, taken together, can prepare older adults and their loved ones to survive and thrive during global emergencies.

### WHEN SOCIAL ISOLATION MIGHT SAVE YOUR LIFE: AN MIT AGELAB STUDY OF LIVING THROUGH THE COVID-19 CRISIS AT AGE 85 AND OVER

Julie Miller,[1] Taylor Patskanick,[2] Lisa D'Ambrosio,[2] Martina Raue,[3] Alexa Balmuth,[1] and Joseph Coughlin,[2] *1. MIT AgeLab, Cambridge, Massachusetts, United States, 2. MIT, Cambridge, Massachusetts, United States, 3. Massachusetts Institute of Technology, Cambridge, Massachusetts, United States*

Previous research shows that social isolation is associated with increased mortality rates among older adults. However, in the face of the novel coronavirus (COVID-19), social distancing was prescribed as a potentially lifesaving measure, particularly for older adults. In this session, MIT AgeLab researchers will present findings from a mixed methods study with the MIT AgeLab 85+ Lifestyle Leaders, a panel study of octogenarians and nonagenarians that began in September 2015. In March 2020, AgeLab researchers began conducting a series of telephone interviews (n=15) with Lifestyle Leaders and collected quantitative data via an online survey (n=25). Together, the interviews and survey inquired about Lifestyle Leaders' attitudes and behaviors related to COVID-19, as well as their uses of technology throughout the crisis. Findings suggest that fears about the impacts of coronavirus transcended multiple domains of the Lifestyle Leaders' lives, including their physical, socioemotional, and financial wellbeing. Lifestyle Leaders were asked about functional and emotional repercussions of social distancing and other precautions they had taken to reduce the spread of COVID-19 and to protect themselves from contracting the virus. Special attention was paid to differences in lived experiences of the COVID-19 crisis among Lifestyle Leaders who were living independently versus those who were living in assisted living or continuing care retirement communities. Findings from this study suggest that socially-connected emergency preparedness measures will become increasingly important for the growing number of octogenarians and nonagenarians.

### SESSION 2997 (PAPER)

### HOUSING, NEIGHBORHOODS, AND AGING IN PLACE

### CHANGES IN NEIGHBORHOOD-LEVEL SOCIOECONOMIC DISADVANTAGE AND OLDER AMERICANS' COGNITIVE FUNCTIONING

Jason Settels, and Anja Leist, *University of Luxembourg, Esch-sur-Alzette, Luxembourg*

Most studies of later life cognitive functioning have focused on individual-level variables. While some studies have examined neighborhood-level variables as influences upon older adults' cognitive functioning, this scholarship has neglected to consider neighborhoods in a dynamic context. The present study helps fill this research gap by considering how changing extents of neighborhood-level socioeconomic disadvantage cause changes in older residents' cognitive functioning. We employ waves 2 (2010-2011) and 3 (2015-2016) of the National Social Life, Health, and Aging Project (NSHAP) as our source of individual-level variables and the American Community Survey as our source of neighborhood-level variables. Our analytical sample includes 1,989 respondents who participated in both waves and were 50 to 90 years in age at wave 2 of the NSHAP. Through structural equation modelling, we find that rising neighborhood-level socioeconomic disadvantage significantly decreases older residents' cognitive functioning, both without and after controlling for baseline neighborhood-level socioeconomic disadvantage and cognitive functioning. Furthermore, approximately 7.2% of this effect is mediated through decreases in the sizes of networks of close confidants, and roughly 8.5% of this effect occurs through increased depressive symptoms. Our findings suggest that older adults' cognitive decline can be slowed down or prevented through improvements in their living environments. In particular, policies and programs that improve living spaces while also facilitating older residents' development of close and supportive confidant ties are likely to be particularly effective. Our study encourages further research on how neighborhood dynamics affect older persons' cognitive functioning.

### I NEVER THOUGHT ABOUT LEAVING: WHY RESIDENTS AGED IN PLACE WITHIN NEIGHBORHOODS EXPERIENCING URBAN DECLINE

Kaitlyn Langendoerfer, *Case Western Reserve University, Honesdale, Pennsylvania, United States*

Despite the rich literature on "aging in place", few studies have examined why older adults remain within neighborhoods that have experienced urban decline. This study defines "aging in place" as the process of aging within one's neighborhood over the life course. Further, much of the literature surrounding disadvantaged, urban communities have concentrated on those who left and portray those who remain as trapped. This is particularly true for older African Americans. The purpose of this study was to understand what influenced older, African American residents to stay within their declining neighborhoods. Data was utilized from 4 years of ethnographic observations with 30 older (age 60+), African-American adults who aged within Cleveland. Additionally, multiple in-depth life history interviews were conducted with 13 of these residents. Data was analyzed using grounded theory techniques. All residents expressed a desire to remain within their neighborhoods and many indicated that they never thought about leaving. While each resident had their own reasons for staying, common themes emerged related to: 1) autobiographical insideness, 2) sense of identity, 3) home ownership, and 4) interdependent lives. This study has important implications for research related to aging in place and place attachment. For one, it provides a counter narrative to the dominant notion that residents of poor neighborhoods would leave if they had the resources to relocate. Additionally, by using a narrative method, the older adults were able to explain for themselves why they stayed and describe the meaningful lives they created despite neighborhood decline.

### NEIGHBORHOOD DISADVANTAGE, COGNITION, AND HEALTH SELF-EFFICACY OF OLDER ADULTS IN A CLINICAL POPULATION

Kristen Berg,[1] Nikolas I. Krieger,[2] Douglas Einstadter,[1] Lorella Shamakian,[3] Jarrod Dalton,[4] and Adam Perzynski,[1] *1. Case Western Reserve University at MetroHealth, Cleveland, Ohio, United States, 2. Lerner Research Institute - Cleveland Clinic, Cleveland, Ohio, United States, 3. The MetroHealth System, Cleveland, Ohio, United States, 4. Cleveland Clinic Lerner College of Medicine at Case Western Reserve University, Cleveland, Ohio, United States*

The Medicare Annual Wellness Visit (MWV) includes an assessment of health risks for older adults in the United States. Research suggests that neighborhood-level social inequality influences multiple health outcomes. We sought to examine the association between neighborhood socioeconomic position and older adults' cognition, health management self-efficacy, and other health risks. We identified a cohort of 12,434 adults aged 65 and over from the NEOCARE Learning Health Registry who attended a routine MWV between 2011 and 2019. NEOCARE includes electronic health record and neighborhood data from 1999-2017 on over 3 million unique Northeast Ohio individuals. The study population was 60% White, 32% Black or African American, 64% female, and 90% non-Hispanic. Over 60% were ages 65-74, 29% 75-84, and 10% 85 years or older (range from 65 to 101). We used ANOVA and chi square tests to examine variation in health risks by quintile of census tract area deprivation index. Cognitive functioning differed across quintiles of area deprivation and Bonferroni-corrected tests indicated that adults in the most socioeconomically disadvantaged neighborhoods had lower average cognitive screening scores as compared to older adults in less disadvantaged areas ($F=53.50$, $df=4$, $n=12,204$, $p<.001$). The proportion of adults feeling efficacious in managing their health differed according to area deprivation, with adults in more disadvantaged neighborhoods having slightly lower self-efficacy, ($x2=11.01$, $df=8$, $n=11,937$, $p<.001$). Better understanding of the relationship between cognitive functioning and health self-efficacy and neighborhood environment is critical for designing programmatic and policy interventions aimed at supporting proactive aging in older adulthood.

### SOCIAL AND HEALTH DISPARITIES AMONG RELOCATED PUBLIC HOUSING RESIDENTS BY AGE: IMPLICATIONS FOR AGING IN PLACE

Nia Reed,[1] and Tiffany Young,[2] *1. Georgia State University, Atlanta, Georgia, United States, 2. Lenell and Lillie Consulting, LLC, New Bern, North Carolina, United States*

Research illustrates that neighborhood outcomes (including the built environment) influence the mental and physical health of vulnerable older adults (OA). Many OA aim to age-in-place but aging-in-place is less realistic for low-income OA because of gentrification and forced relocation. Examining neighborhood context is vital to understanding how the places we live contribute to well-being, yet, there is insufficient research on the biopsychosocial effects of forced relocation on low-income OA. To address this gap, our study uses aging-in-place theory to understand the association of neighborhood and health outcomes of relocated and nonrelocated low-income OA in public housing. This study includes three waves of data from Georgia State University's Urban Health Initiative Study. Participants (n=225) were categorized by age (young-old = 50-64; old-old = 65-74; and oldest-old = 75+). We conducted multivariate regression analyses to highlight relationships between neighborhood and health outcomes, and relocation. We used geocoding to provide within-group analysis of relocated residents to determine if geographic proximity to former public housing communities affected neighborhood and health outcomes. Results show that relocated OA have worse informal social control and neighborhood satisfaction outcomes, but better built environment conditions than those who aged-in-place. Mental health and physical functioning worsened for relocated OA. Relocation was associated with reduced social cohesion and worse built environment conditions for the oldest-old. Moderation analysis illustrated that OA who relocated farther away had worse neighborhood outcomes. Considering the importance of aging-in-place to OA well-being, policymakers may reconsider forced relocation and allocating funds to enhance neighborhoods.

### SESSION 2998 (PAPER)

### MOBILITY, DISABILITY, AND FUNCTIONAL IMPAIRMENT

### HEARING AND COGNITIVELY IMPAIRED LIFE EXPECTANCIES IN THE UNITED STATES

Jessica West, and Scott Lynch, *Duke University, Durham, North Carolina, United States*

As the population ages, increased prevalence of cognitive and sensory impairments may pose growing public health challenges. Among the nine modifiable risk factors for dementia, the highest percentage (9%) of dementia cases are attributed to hearing impairment. While much research has examined the relationship between hearing impairment and cognition, almost none has translated these relationships into a meaningful, life course metric: how many years of life individuals can expect to live with both impairments and how hearing impairment affects years lived with cognitive impairment. Our study fills this gap by using Bayesian multistate life table methods applied to nine waves of the Health and Retirement Study (1998-2014) to estimate years of life to be spent (1) with/without hearing and cognitive impairment, and (2) with/without cognitive impairment, conditional on having versus not having hearing impairment. Preliminary results for aim 1 reveal that at age 50, individuals will live 18.9 (18.7-19.2) years healthy, 4.3 (4.2-4.5) years hearing impaired but cognitively intact, 4.2 (4.0-4.3) years hearing unimpaired but cognitively impaired, and 2.3 (2.2-2.6) years with both impairments. Women will spend more years healthy, hearing unimpaired but cognitively impaired, or with both impairments; men will spend more years hearing impaired but cognitively intact. People with more education will spend more years hearing impaired but cognitively intact; people with less education will spend more years hearing unimpaired but cognitively impaired or with both impairments. Our study is one of the first to investigate the implications of hearing impairment for years of cognitively impaired life.

### MODERATING EFFECT OF PSYCHOLOGICAL RESILIENCE ON FUNCTIONAL IMPAIRMENT IN RELATION TO SUBSEQUENT DEPRESSION

Shinae Choi,[1] and Eun Ha Namkung,[2] *1. The University of Alabama, Tuscaloosa, Alabama, United States, 2. Brandeis University, Waltham, Massachusetts, United States*

The growing prevalence of functional impairment is a serious concern due to its relation to decreased quality of life in later life. Guided by the social convoy model and the stress process model, the present study investigated whether psychological resilience, particularly optimism and mastery moderated an association between functional impairment and subsequent depressive symptoms in later life. This study used data derived from two population-based national studies in the United States: 2012 and 2016 waves of the Health and Retirement Study (N = 5,035) and 2004 and 2013 waves of the Midlife in the United States (N = 2,476). Ordinary least squares regression was used to estimate the impact of optimism and mastery, respectively, on the associations between functional impairment (baseline measure at wave(t-1), changes over the study period from wave(t-1) to wave(t)) on subsequent changes in depressive symptoms. Across both studies, we found that having and developing functional impairment are related to increased number of depressive symptoms. Optimism independently predicted decreased depressive symptoms over the study periods and buffered the negative effects of functional impairment on depressive symptoms across the two studies. Specifically, the mitigating effects of optimism on depressive symptoms were greater for those with more numbers of functional limitations. The findings suggest that psychological resilience plays a key role in decreasing depressive symptoms, especially for midlife and older adults with functional impairment. The results also demonstrate the importance of examining both optimism and mastery when investigating psychological resilience and emotional well-being in older adults.

### OLDER ADULTS' ADOPTION OF MOBILITY DEVICES: INTERPLAY OF PREDISPOSING FACTORS AND PRECIPITATING HEALTH EVENTS

FARHANAZ SHARMIN, and Laura Sands, *Virginia Tech, Blacksburg, Virginia, United States*

Existing mobility limitations and chronic conditions increase likelihood of adopting mobility-related devices such as canes and walkers. Prior research has not considered how recent acute events such as falls and hospitalizations contribute to the adoption of mobility devices. We studied 4,592 older adults who responded to the 2015 and 2016 National Health and Aging Trends Study surveys, and classified adoption of mobility devices as: (i) Never users (did not use mobility devices either year) and (ii) New users (started using mobility devices in 2016). We determined through chi-square tests, that predisposing characteristics from 2015 that were significantly associated with being a New User in 2016 were: being female, aged 80+, minority race, having a high-school education or lower, living alone, being obese, and having a history of dementia, arthritis, stroke, mobility difficulties, falls, and hospitalization (all P's<0.05). We used logistic regression to determine the contribution of recent precipitating events on the adoption of mobility devices among older adults after controlling for 2015 characteristics that were significantly associated with being a New user. Precipitating events were significantly associated with being a New user of mobility equipment. Specifically, older adults who, between the 2015 and 2016 interviews, experienced a fall (OR=1.7; 95% CI=1.1-2.9), hospitalization (OR=3.7; 95% CI=2.3-5.9) or increase in mobility difficulties (OR=3.7; 95% CI=2.3-5.9) were more likely to be New users. Study findings reveal the importance precipitating events on the adoption of mobility devices, signaling the importance of assessing for need for mobility devices after these events.

### STEPPING OUT: A NOVEL PILOT FALLS PREVENTION PROGRAM FOR INDIVIDUALS WITH MILD COGNITIVE IMPAIRMENT

Barbara Fischer,[1] Allison Midden,[1] Aundrea Hoffmann,[1] Lynn DeWitt,[2] Kathryne Kohlman,[1] Lindy Clemson,[3] Katherine Sherman,[1] and Jane Mahoney,[4] *1. Clement J. Zablocki VA Medical Center, Milwaukee, Wisconsin, United States, 2. Clement J. Zablocki VA Medical Center, Green Bay, Wisconsin, United States, 3. Unversity of Sydney, Sydney, Australia, 4. University of Wisconsin - Madison, Madison, United States*

Objectives: Falls are the leading source of accidental injury and hospitalization among adults over the age of 65. Relative to people with intact cognition, individuals with cognitive impairment are at increased risk for falls; however, few falls prevention programs exist to specifically reduce and prevent falls in this population. To address this issue, we developed a novel, multifactorial, cognitively-based falls prevention program, Stepping Out. Based on the popular and effective evidenced-based program, Stepping On, Stepping Out was modified and tailored to the learning needs of individuals with Mild Cognitive Impairment (MCI). We hypothesized that older adults with MCI would find the program understandable, and that program participants would demonstrate reduced falls. Methods: Sixteen veterans, mean age of 77.5, diagnosed with MCI and at increased risk for falls participated in Stepping Out. Falls were collected for the six months prior to intervention and the six months during and after program participation. All participants completed post-program evaluations. Falls incidence was compared using a Wilcoxon paired signed rank test. Results: Stepping Out was found to be feasible and comprehensible by all participants. Program participants exhibited significantly reduced falls, with median reduction of two falls (p = 0.0020), and a range of zero to 12 falls. Discussion: With appropriate modifications, individuals with MCI were able to benefit from a cognitively-based falls prevention program and to reduce accidental falls incidence. Falls are an important and feasible target to address among individuals with cognitive impairment.

### THE MEDIATING ROLE OF OPTIMISM ON THE RELATIONSHIP OF ACTIVITIES OF DAILY LIVING AND WELL-BEING AMONG OLDER ADULTS

Kent Jason Cheng,[1] Darcy McMaughan,[2] and Matthew Smith,[2] *1. Syracuse University, Syracuse, New York, United States, 2. Texas A&M University, College Station, Texas, United States*

Limitations on activities of daily living (ADL) and instrumental activities of daily living (IADL) can be deleterious to an older person's life satisfaction and overall feelings of wellbeing. This study explored the possible mediating role of optimism on relationship between changes in ADL/IADL and life satisfaction over time. Using 2006-2016 data from the Health and Retirement Study (n=11,869), growth curve modelling was used to account for intra-individual and inter-individual changes in life satisfaction trajectories. All models controlled for age, sex, marital status, years of education, self-rated health, labor force status, log of household income, and attrition. In the baseline model without optimism and with all controls, coefficients for ADL (Beta=-0.13, P<0.01) and IADL (Beta=-0.12, P<0.01) were negatively significantly associated with life satisfaction. When optimism was introduced to the model, coefficients for both ADL and IADL increased by 0.01 and remained statistically significant, which suggests some mediating effects. When interaction terms between ADL/IADL and optimism were introduced, coefficients for ADL and IADL became statistically insignificant. However, the interaction between ADL and optimism (Beta=-0.02, P<0.05) was negatively significantly associated with life satisfaction. Findings suggest that optimism may protect against the negative impact of ADL/ IADL on life satisfaction. While changes in physical functioning and mobility may influence mental health status (e.g., depression, feelings of isolation), such consequences are not inevitable. Efforts are needed to highlight the positive aspects of aging and opportunities for life enrichment to increase morale and optimism among older adults.

### SESSION 2999 (PAPER)

### EXPERIENCES IN LONG-TERM CARE

### ADDRESSING UNDER-DETECTION IN MINIMUM DATA SET BEHAVIORAL MEASURES USING NIH STAGE III/IV EMBEDDED TRIAL DESIGN

Ellen McCreedy,[1] Roee Gutman,[1] James Rudolph,[2] Rosa Baier,[3] Kali Thomas,[1] Faye Dvorchak,[3] Jessica Ogarek,[4] and Vincent Mor,[3] *1. Brown University, Providence, Rhode Island, United States, 2. U.S. Department of Veterans Affairs Medical Center, Providence,*

*Rhode Island, United States, 3. Center for Long-Term Care Quality & Innovation, Providence, Rhode Island, United States, 4. Brown University, PROVIDENCE, Rhode Island, United States*

Using the National Institutes of Health (NIH) Stage Model framework, we are conducting a Stage III/IV embedded trial to evaluate the effects of personalized music on agitated behaviors in nursing home (NH) residents with dementia under two research conditions--less pragmatic, more researcher involvement (Stage III) and more pragmatic, less researcher involvement (Stage IV). We are conducting a three-year trial in 81 NHs, with 27 NHs receiving the intervention per year. Behavior frequency is assessed via resident MDS assessments, staff interviews, and direct observations of residents. During the first year, researchers interview NH staff and observe residents with dementia in 54 randomly selected NHs (27 treatment and 27 control, parallel design). MDS assessments are available for all 81 NHs throughout the three-year study (stepped-wedge design). In the 54 NHs in the parallel design, we compare staff interview and NH-conducted resident assessments and, using multiple imputation methods, we impute staff interview data for eligible residents of all 81 NHs in order to estimate the effect of the intervention under the step-wedge design. There are four key features of this trial: 1) combination of parallel and stepped-wedge designs; 2) equilibrating researcher-collected behavior data to NH-collected behavior data to impute research-collected behavior data for some residents; and 3) simulated resident selection process in control facilities to improve comparisons of effects across treatment groups. This design has the potential to shorten the research timeline by iteratively assessing real-world efficacy and large-scale effectiveness. Our design will inform pragmatic testing of other interventions with limited efficacy evidence.

### FAMILY CAREGIVER ROLE IN THE LONG-TERM SERVICES AND SUPPORTS OF INDIVIDUALS WITH DEMENTIA OVER TIME

Jennifer Reckrey, Evan Bollens-Lund, Mohammed Husain, Katherine Ornstein, and Amy Kelley, *Icahn School of Medicine at Mount Sinai, New York, New York, United States*

Family caregivers of persons living with dementia (PLWD) provide disproportionately high levels of care over a long and variable disease course, yet an understanding of the trajectory of care hours provided over time and the contributions of individual family members to overall care is lacking. This study used longitudinal data from the nationally representative Health and Retirement Study in order to compare the hours of care that spouses, children, and other family caregivers provide to those with and without dementia. During the last 10 years of life, family caregivers of PLWD provided nearly three times as many total care hours as compared to others (7,447 vs. 2,653 total hours). While care hours provided to PLWD increased steadily in each of the last 10 years of life (going from 4 hours/week 10 years before death to 33 hours/week the year before death, average annual increase 27%), care hours provided to others remained low and then nearly tripled in the last year of life to 22 hours/week on average. Adult children of PLWD provided 50% of total care hours, while adult children of others provided 41% of care hours. This study provides important insight into the high levels of year-over-year caregiving provided to PLWD by their family caregivers in general and by adult children in particular. Policies to support these caregivers must shift from short-term, episodic support to sustained assistance in acknowledgment of the key role family caregivers play in the long-term services and supports of PLWD.

### INTERGENERATIONAL COMMUNICATION AS NON-PHARMACOLOGICAL CARE IN A JAPANESE NURSING HOME

Toshiko Hamaguchi, *The University of the Sacred Heart, Tokyo, Japan*

Statistics shows that Japan now has 28.4% of population aged over 65, which marks the highest in the world. Moreover, one in four over 65 is said to have ADRD or MCI. Traditional family caregiving derived from filial piety is giving way to moving to care facilities in order to reduce a burden on children or get professional care. This suggests that everyday communication for older adults involves younger conversational partners with varying degrees of shared knowledge and experiences. Such intergenerational communication can be challenging and stressful on both sides. To date, empirical studies that observe interactional strategies of both younger caregivers and older adults is unknown. Using 21 recordings of weekly conversational activities (45-60 minutes) led by recreation workers (female, 40s) taken at a nursing home in Japan, this qualitative study demonstrates how (1) sharing of the past and present becomes a learning opportunity for both recreation workers and older participants with ADRD (aged 87-95) , (2) reference to nursing-home living establishes and reaffirms their interpersonal relationship, and (3) intergenerational conversation becomes a tool to express life satisfaction for the older participants. This evidence-based study proposes mundane conversations during recreational activities as part of non-pharmacological person-centered care which serves to improve the quality of life and life satisfaction of older adults at care facilities. It is therefore important to study communicative strategies of professional caregivers since they affect social engagement as well as emotional and psychological well-being of those at the end-of-life stage.

### MAYBE SHE'LL MAKE SOME FRIENDS?: FAMILY MEMBERS CULTIVATING INTIMACY IN ASSISTED LIVING

Andrea Fitzroy, Candace Kemp, and Elisabeth Burgess, *Georgia State University, Atlanta, Georgia, United States*

Intimacy continues to be important in later life, including for older adults in long-term care settings such as assisted living (AL). Our past work shows that intimacy is a multidimensional process and can involve a variety of partners. Drawing on data from the qualitative longitudinal "Convoys of Care" study (R01AG044368), we extend this research to examine the role family members play in cultivating intimacy and close relationships of AL residents. Using a grounded theory approach, we analyzed 2,224 hours of participant observation, and formal interviews with 28 assisted living residents (aged 58-96) and their formal and informal care partners (n=114) from four diverse AL communities. Findings show that family members can play integral roles in residents' experiences with intimacy, directly as relationship partners, and by facilitating or impeding residents' contacts with others. Family members cultivated residents' intimacy opportunities and experiences by direct engagement, resident advocacy, to non-involvement and disengagement. Family members' roles in cultivating intimacy fluctuated over time, increasing at times of health concerns and family change. Perceptive family members considered older adults' intimacy preferences when cultivating their intimate relationships. Family members concerned for the safety of their loved one sometimes acted as "gatekeepers" to intimacy by interfering in intimate relationships. We conclude with a discussion of implications for policy and practice aimed at improving the intimacy process and opportunities for older adults receiving long-term care.

### USING THE LIFE STORY BOOK WITH MENTALLY ALERT RESIDENTS OF NURSING HOMES

Theresa Chrisman, *University of Houston, Houston, Texas, United States*

Depression and lack of meaning in life (MIL) are common among residents of nursing homes (NHs) and contribute to a reduction in overall health and well-being. Life Story Book (LSB), a reminiscence intervention, is designed to provide a person with the opportunity to review their past and capture their life stories and photographs into a book. LSB has demonstrated positive outcomes for residents of NHs with dementia, yet little is known for residents without dementia. A switching replication design was used to examine the effects of LSB among 21 mentally alert residents from two NHs (NH-A and NH-B) in Houston, Texas. Participants in NH-A received three weeks of the LSB intervention, while NH-B received three weeks of care-as-usual; the intervention was then switched. The GDS-12R and the MIL questionnaire (MLQ) were used to measure depressive symptoms and MIL respectively. Participants from NH-A (n =11) and NH-B (n = 10) had a mean age of 75 years (SD =11.34); 81% female; 52% non-Hispanic white and 33% African American. Results from a one-way MANCOVA found no statistically significant difference on the GDS-12R and MLQ ($F(3, 14) = 2.50$, $p = .102$; Wilks' Lambda = .652; $\eta2 = .35$). Further analyses comparing the pre-intervention and post-intervention scores for the entire sample (N =21) found a significant reduction in depressive symptoms (M = 2.67; SD = 2.52) and (M =1.67, SD = 2.29); ($t(20) = 2.21$, $p = 0.039$). The potential benefits of LSB for mentally alert residents of NHs warrants further research.

### ALL-CAUSE DEMENTIA PREDICTION BY MACHINE LEARNING: THE HEALTH, AGING, AND BODY COMPOSITION STUDY

Chenkai Wu,[1] and Xurui Jin,[2] *1. Duke Kunshan University, Kunshan, Jiangsu, China, 2. Duke Kunshan University, Kunshan, China*

There are several shortcomings of the currently available risk prediction models for dementia. We developed a risk prediction model for dementia using machine-learning approach and compared its performance with traditional approaches. Data were from the Health, Aging, and Body Composition Study, comprising 3,075 older adults (at least 70 years). Dementia was defined as (1) use of a prescribed dementia medication, (2) adjudicated dementia diagnosis, or (3) a race-stratified cognitive decline>1.5 SDs from the baseline mean. We selected 275 predictors collected from questionnaires, imaging data, performance testing, and biospecimen. We used random survival forest (RSF) to build the full model and rank the importance of predictors. Subsequently, we built parsimonious models with top-20 predictors using RSF and Cox regression. A dementia risk score was developed using top-ranked variables. We used the C-statistic for performance evaluation. Over a median of 11.4 years of follow-up, 659 dementias (21.4%) occurred. The RSF model (both including all and top-20 variables) showed a higher C-statistic than the regression model. Digit symbol score, physical performance battery, finger tapping score, weight change since age 50, serum adiponectin, and APOE genotype were the top-6 variables. We created a dementia risk score (0-10) using the top-6 variables. A 1-unit increase in the risk score was associated with an 8% higher risk of dementia. The risk score demonstrated good discrimination (C-statistic=0.75). Machine learning methods offered improvement over traditional approaches in predicting dementia. The risk prediction score derived from a parsimonious model had good prediction performance.

### BUILDING, TESTING, AND LEARNING FROM NETWORK MODELS OF HUMAN AGING

Andrew Rutenberg, Spencer Farrell, Arnold Mitnitski, Kenneth Rockwood, and Garrett Stubbings, *Dalhousie University, Halifax, Nova Scotia, Canada*

We have developed computational models of human aging that are based on complex networks of interactions between health attributes of individuals. Our "generic network model" (GNM) captures the population level exponential increase of mortality with age in Gompertz's law together with the exponential decrease of health as measured by the frailty index (FI). Our GNM includes only random accumulation of damage, with no programmed aging. Our GNM allows large populations of model individuals to be quickly generated with detailed individual health trajectories. This allows us to explore individual damage propagation in detail. To facilitate comparison with observational data, we have also developed and tested new approaches to binarizing continuous-valued health data. To extract the most information out of available cross-sectional or longitudinal data, we have also reconstructed interactions from generalized network models that can predict individual health trajectories and mortality.

### COMBINING FRONTAL TDCS WITH WALKING REHABILITATION TO ENHANCE MOBILITY AND COGNITION: A PILOT CLINICAL TRIAL

David Clark,[1] Sudeshna Chatterjee,[1] Jared Skinner,[2] paige Lysne,[3] Samuel Wu,[4] Dorian Rose,[1] and Adam Woods,[5] *1. University of Florida, Gainesville, Florida, United States, 2. Appalachian State University, Boone, North Carolina, United States, 3. Aging and Geriatric Research, Gainesville, Florida, United States, 4. Department of Biostatistics, Gainesville, Florida, United States, 5. Clinical and Health Psychology, Gainesville, Florida, United States*

Walking function is compromised with older age, particularly for cognitively demanding complex walking tasks. Frontal lobe brain networks are important to both complex walking and cognitive function. There is a need for interventions that target this brain region. This pilot study assessed a novel intervention to enhance both walking and executive function in older adults. The primary hypothesis was that eighteen sessions of frontal lobe tDCS combined with complex walking rehabilitation would be feasible, safe, and show preliminary efficacy for improvements in walking and cognition. Eighteen participants were randomized to one of three intervention groups: active tDCS and rehabilitation with complex walking tasks (Active/Complex); sham tDCS and rehabilitation with complex walking tasks (Sham/Complex); or sham tDCS and rehabilitation with typical walking (Sham/Typical). Outcome measures included multiple tests of walking function, executive function, and prefrontal activity during walking measured by functional near infrared spectroscopy. For the walking tests, effect sizes for Active/Complex were generally higher than for Sham/Complex. The Sham/Typical group exhibited walking test effect sizes that were often larger than either of the complex walking groups, possibly due to higher intervention step count. For the executive function tests, effect sizes were largest for the Active/Complex group. Improvements in prefrontal activity during walking were observed, as conceptualized by the Compensation Related Utilization of Neural Circuits Hypothesis. These preliminary findings support that tDCS combined with complex walking rehabilitation in older adults is feasible and may enhance both walking function and executive function.

### CREATING A NOVEL PHYSICAL RESILIENCE MEASURE AMONG OLDER ADULTS: THE HEALTH, AGING, AND BODY COMPOSITION STUDY

Chenkai Wu,[1] Ya-Xi Li,[2] Michelle Odden,[3] and Megan Marron,[4] *1. Duke Kunshan University, Kunshan, Jiangsu, China, 2. Duke Kunshan University, Kunshan, China, 3. Stanford University, STANFORD, California, United States, 4. University of Pittsburgh, Pittsburgh, Pennsylvania, United States*

The concept of resilience has gained increasing attention in aging research; however, current literature lacks consensus on how to measure resilience. We constructed a novel resilience measure based on the degree of mismatch between persons' frailty level and disease burden and examined its predictive validity. We also sought to explore the physiological correlates of resilience. Participants were 2,457 older adults from the Health, Aging, and Body Composition Study. We constructed the resilience measure as the residual taken from the linear model regressing frailty on age, sex, race/ethnicity, 14 diseases, self-reported health, and number of medications. Participants were classified into three groups—adapters, expected agers, and premature frailers—based on residuals (less than, within, or above one standard deviation of the mean). Validation outcomes included years of able life (YAL), years of healthy life (YHL), years of healthy and able life (YHAL), disability, hospitalization, and survival. The average YHAL was 5.1, 7.7, and 9.1 years among premature frailers, expected agers, and adapters, respectively. Compared with premature frailers and expected agers, adapters had significantly lower rates of disability, hospitalization, and mortality and higher proportion surviving to 90 years. The likelihood of surviving to 90 years was 20.4%, 30.6%, and 39.7% among premature frailers, expected agers, and adapters. We developed and validated a novel approach for quantifying and classifying physical resilience in a cohort of well-functioning white and black older adults. Persons with high physical resilience level had longer healthy life span and lower rates of adverse outcomes.

### FACTORS ASSOCIATED WITH FRAILTY STATUS IN RELATIVELY HEALTHY COMMUNITY-DWELLING OLDER ADULTS

A R M Saifuddin Ekram,[1] Joanne Ryan,[2] Sara Espinoza,[3] Anne Murray,[4] Michael Ernst,[5] and Robyn Woods,[6] *1. ASPREE, Monash University, Prahran, Victoria, Australia, 2. School of Public Health and Preventive Medicine, Prahran, Victoria, Australia, 3. University of Texas Health Science Center at San Antonio, San Antonio, Texas, United States, 4. University of Minnesota, Minneapolis, Minnesota, United States, 5. College of Pharmacy, The University of Iowa, Iowa City, Iowa, United States, 6. Monash School of Public Health and Preventive Medicine, Prahran, Victoria, Australia*

Frailty is gaining importance as a predictor of disability and mortality in older people, and becoming frail poses a challenge for healthy aging. We investigated the prevalence and factors associated with pre-frail and frail status in a large study cohort of community-dwelling healthy older adults from Australia and the United States. A total of 19,114 individuals (87% Australian and 56% women) aged 65 years or older enrolled in a primary prevention clinical trial were evaluated. Frailty status was classified using the modified Fried phenotype criteria comprising of exhaustion, body mass index, grip strength, gait speed and physical activity. Prevalence and factors associated with frailty status (e.g. demographic characteristics and lifestyle factors) were reported using descriptive statistics along with a logistic regression model. At baseline, 2.3 % (95% CI, 2.1-2.5) of older trial participants were frail and 39.2% (95% CI, 38.5-39.9) were pre-frail, respectively. Women were more likely to be frail (65.1% vs 36.9%) and prefrail (58.0% vs 42.0%) than men. Lower levels of education (<12 years), living alone, ethnic minorities, current smoking and past alcohol use were some of the factors which were common among frail or prefrail. Despite being a relatively healthy cohort, more than one-third of the older trial participants were prefrail, which was more prevalent among specific subgroups of individuals. This study emphasizes the high burden of the prefrailty status even among an apparently healthy cohort of community-dwelling older people.

### FRAILTY STATUS AND ALL-CAUSE MORTALITY IN THE COMMUNITY-DWELLING OLDER PEOPLE: AN UMBRELLA REVIEW

A R M Saifuddin Ekram,[1] Joanne Ryan,[2] Carlene Britt,[3] Sara Espinoza,[4] and Robyn Woods,[3] *1. ASPREE, Monash University, Prahran, Victoria, Australia, 2. School of Public Health and Preventive Medicine, Prahran, Victoria, Australia, 3. Monash School of Public Health and Preventive Medicine, Prahran, Victoria, Australia,*

*4. University of Texas Health Science Center at San Antonio, San Antonio, Texas, United States*

Frailty is increasingly recognised for its association with adverse health outcomes including mortality. However, various measures are used to assess frailty, and the strength of association could vary depending on the specific definition used. This umbrella review aimed to map which frailty scale could best predict the relationship between frailty and all-cause mortality among community-dwelling older people. According to the PRISMA guidelines, Medline, Embase, EBSCOhost and Web of Science databases were searched to identify eligible systematic reviews and meta-analyses which examined the association between frailty and all-cause mortality in the community-dwelling older people. Relevant data were extracted and summarised qualitatively. Methodological quality was assessed by AMSTAR-2 checklist. Five moderate-quality systematic reviews with a total of 374,529 participants were identified. Of these, two examined the frailty phenotype and its derivatives, two examined the cumulative deficit models and the other predominantly included studies assessing frailty with the FRAIL scale. All of the reviews found a significant association between frailty status and all-cause mortality. The magnitude of association varied between individual studies, with no consistent pattern related to the frailty measures that were used. In conclusion, regardless of the measure used to assess frailty status, it is associated with an increased risk of all-cause mortality.

### FUNCTIONAL NEURAL UNDERPINNINGS OF INCREASED FALLS RISK

Meishan Ai,[1] Chun-Liang Hsu,[2] and Teresa Liu-Ambrose,[3] *1. Northeastern University, Boston, Massachusetts, United States, 2. Harvard Medical School, Roslindale, United States, 3. University of British Columbia, Vancouver, British Columbia, Canada*

Impaired cognition, especially in the domain of executive functions, is a major risk factor for falls in older adults. However, the underlying neural mechanism of the association between cognitive function and falls risk remains unclear. The current study compared the performance on mobility and cognitive assessments, and brain activation under Stroop task between fallers (>=2 falls in the past year) and non-fallers (0 or 1 fall in the past year). We found that during the incongruent condition of Stroop task, higher activation in precuneous, frontal and temporal areas was observed in fallers, while they showed comparable task performance as non-fallers. In addition, the contrast between congruent and incongruent conditions showed fallers exhibited increased activation in middle frontal region ($z>1.7$, $P<0.05$). Further, through mediation analysis, our data revealed that brain activation in temporal and frontal-paracingulate regions mediated the relationship between Montreal Cognitive Assessment and number of falls (confidence interval = 95%), after controlling for age and sex. Overall, our findings suggested that lower neural efficiency may be an underlying mechanism by which cognitive function is associated with falls risk.

### LOSS OF ARNT LIMITS IMPROVEMENT IN PHYSIOLOGICAL PERFORMANCE FOLLOWING AEROBIC EXERCISE IN AGING

Yori Endo,[1] Yuteng Zhang,[1] Bin Li,[1] Michael MacArthur,[2] and Indranil Sinha,[1] *1. Harvard Medical School, Boston, Massachusetts, United States, 2. Harvard T.H. Chan School of Public Health, Boston, Massachusetts, United States*

Hypoxia signaling is essential for angiogenesis and metabolic regulation during exercise. Our previous study has demonstrated an age-related loss of ARNT resulting in limited muscle regeneration. To explore the role of hypoxia signaling in physiological performance in relation to aging, we generated a mouse model with skeletal muscle-specific knockout of ARNT (ARNT mKO). ARNT mKO and ARNT WT mice were subjected to a sedentary activity or treadmill running exercise regime at an increasing speed of 8-12 m/min for 40 minutes, three times weekly over the course of 8 weeks. ARNT levels was 3-fold lower in old mice compared to young. The exercised WT mice exhibited 52% greater increase over the sedentary group in exercise endurance as measured by the maximum running distance (490.92±154.28 vs 237.76±135.19m, $p < 0.01$). In contrast, ARNT mKO mice did not benefit from exercise (231.85±198.61 vs 167.27±136.56m, p=0.41). The maximum running speed was severely restricted in the trained ARNT mKO mice versus WT (16±1.63 m/min vs 26.67±2.45 m/min, $p < 0.001$). Cross-sectional area of myofibers increased significantly following exercise in WT mice (2270 vs 2960 □m2, p=0,015) indicating muscle hypertrophic response, while no change was observed in the ARNT mKO group (2101 vs 2378□m2, p=0,21). Further, exercise increased femoral artery blood flow by 41% in ARNT WT mice, but not in ARNT mKO mice (898.96±52.33 vs 802.86±48.43, p=0.20). These data suggest that ARNT is essential for physiological response to exercise

### MEDITERRANEAN VERSUS WESTERN DIET EFFECTS ON CEREBRAL CORTICAL THICKNESS AND VOLUME IN CYNOMOLGUS MONKEYS

Brett Frye,[1] Suzanne Craft,[1] Thomas Register,[1] Jeongchul Kim,[1] Christopher Whitlow,[1] Samuel Lockhart,[1] and Carol Shively[2], *1. Wake Forest School of Medicine, Winston-Salem, North Carolina, United States, 2. Wake Forest University Alzheimer's Disease Research Center, Winston-Salem, North Carolina, United States*

Diet may influence the risk for cognitive decline and neurodegenerative disorders, including Alzheimer's disease (AD), but these relationships are difficult to study in humans. Cynomolgus macaques (Macaca fascicularis) are appropriate models for investigations of diet effects on the brain because, like humans, they are omnivorous, have complex central nervous systems, are susceptible to diet-induced diseases, and accumulate amyloid and tauopathies with age. Using structural magnetic resonance imaging, we examined diet effects on brain anatomy by measuring thickness and volume of several areas relevant to AD in 38 middle-aged females, at baseline and after Mediterranean or Western diet consumption for 36 months (equivalent to a 9-year follow-up in humans). Using repeated measures analysis, cortical thicknesses generally increased in the Western diet group. Western diets also resulted in increases in total brain volume and cortical gray matter and decreases in cerebrospinal fluid, white matter, and deep gray matter (striatum and thalamus) (all p's≤0.05). In contrast, thicknesses and volumes generally remained unchanged in animals consuming Mediterranean diets. Taken together, these findings demonstrate that Western diets induce widespread structural shifts which may increase risk of cognitive decline and neuropathology, whereas Mediterranean diets may exert a stabilizing influence on the brain. This study provides important insights about the significance of diet on brain structure and lays the groundwork for future investigations to uncover the molecular underpinnings of diet-induced changes in the brain. Mediterranean diet may protect against structural changes in brain that occur with age in those consuming a Western diet.

### OBSTACLE NEGOTIATION IN OLDER ADULTS: PREFRONTAL ACTIVATION INTERPRETED THROUGH CONCEPTUAL MODELS OF BRAIN AGING

David Clark,[1] Sudeshna Chatterjee,[1] Rachael Seidler,[2] Jared Skinner,[3] Dorian Rose,[1] paige Lysne,[4] and Adam Woods,[5] *1. University of Florida, Gainesville, Florida, United States, 2. Applied Physiology and Kinesiology, Gainesville, Florida, United States, 3. Appalachian State University, Boone, North Carolina, United States, 4. Aging and Geriatric Research, Gainesville, Florida, United States, 5. Clinical and Health Psychology, Gainesville, Florida, United States*

Age-related decline in executive function is associated with walking deficits in older adults. The main objective of this study was to better understand the cognitive control of obstacle negotiation in older adults by identifying predictors of prefrontal recruitment during the task. The study also examined the association between prefrontal recruitment and walking performance as well as interpretation of prefrontal activity relative to cognitive models of brain aging. Prefrontal oxygenated hemoglobin concentration (O2Hb) was measured by functional near-infrared spectroscopy during typical walking (Typical) and obstacle negotiation (Obstacles) tasks in older adults. The primary outcome was change in prefrontal recruitment (ΔPFR), measured as Obstacles ΔO2Hb minus Typical ΔO2Hb. Stepwise regression was used to identify potential predictors of ΔPFR. Additional analyses were conducted to further examine the relationship between ΔPFR and the identified predictors. Greater ΔPFR was predicted by lower age, worse executive function, and their interaction (R2=0.19 , p=0.02). Particularly, the effect of executive function on ΔPFR was more pronounced for "early aging" than for "late aging" older adults (p<0.001). Greater ΔPFR was significantly associated with a smaller reduction in walking speed during Obstacles compared to Typical. In conclusion, age, executive function, and their interaction predict prefrontal recruitment during obstacle negotiation in older adults. These findings are generally consistent with existing cognitive models of brain aging including neural inefficiency, compensatory overactivation, and capacity-limitation with a recruitment ceiling effect.

### P300 AMPLITUDE IN RELATION TO AGE, NEUROPSYCHOLOGICAL PERFORMANCE, AND GENETIC RISK FOR ALZHEIMER'S DISEASE

Julia Sheffler,[1] Greg Hajcak,[2] Cynthia Vied,[2] Melissa Meynadasy,[2] and Russell Mach,[2] *1. Florida State University College of Medicine, Tallahassee, Florida, United States, 2. Florida State University, Tallahassee, Florida, United States*

The P300 event-related potential (ERP) is associated with aging and risk for Alzhiemer's disease (AD) and mild cognitive impairment (MCI). Our study sought to replicate previous findings regarding P300 amplitude, age, and neuropsychological outcomes. We also sought to fill gaps in the literature by assessing associations in a primarily healthy sample of older adults (aged 60-75) and through use of comprehensive assessment procedures for ERPs, neuropsychological outcomes, and a genetic risk score (i.e., BDNF, APOE, and PSEN1 mutations). Approximately 25% of our total sample (N=72) met criteria for possible or probable mild cognitive impairment. We assessed whether the P300 elicited by auditory (oddball) and visual (go/nogo) paradigms were associated with performance across neuropsychological tests commonly used in clinical settings, which include cognitive domains of semantic, episodic, and visual memory, executive functioning, language (confrontation naming), abstract reasoning (visual and verbal), and attention. Further, we examined associations between P300 and multiple genetic risks for AD. Our findings demonstrated differences in outcomes between audio and visual tasks of P300, with visual tasks tending to show stronger relationships with neuropsychological and genetic factors. Neuropsychological measures of memory and executive functioning were most closely related to visual P300 amplitude. P300 amplitude was also significantly associated with a genetic risk score for AD, despite the sample generally performing in the normal range on most neuropsychological tasks. Overall, our study has implications for use of the P300 for early detection of risk for AD and for improving our understanding of the P300 as a cognitive biomarker.

### PAINTING BY LESIONS: FUNCTIONAL NETWORKS AFFECTED BY WHITE MATTER LESIONS ARE ASSOCIATED WITH POORER COGNITION

Rachel Crockett,[1] Chun Liang Hsu,[2] Roger Tam,[1] Todd Handy,[1] and Teresa Liu-Ambrose,[1] *1. University of British Columbia, Vancouver, British Columbia, Canada, 2. Harvard Medical School, Boston, Massachusetts, United States*

Cerebrovascular disease (CvD) is the second most common cause of dementia. Its associated pathology, such as white matter lesions (WML), is associated with reduced cognition. Due to the high variability, the relevance of WML location remains unknown. We hypothesised that although the location of WMLs may appear sporadic, they may actually lie within common functional networks. We used novel imaging methods to map the location of WMLs in a clinical sample with the functional connectivity associated with the same location in the human connectome. This identified the functional networks containing the largest WML load (>50%) in older adults with CvD. We then analyzed the association between level of disruption to these networks and measures of global cognition and executive functions. Included in this study were 164 older adults (>55 years old) with CvD. Cognition was assessed using the: 1) Montreal Cognitive Assessment (MoCA); 2) Stroop Colour Word Test; 3) Trail Making Tests; and 4) Digit Symbol Substitution Test. Our results found that the visual network and ventral attention network (VAN) surpassed the 50% overlap threshold with 85% and 66% overlap respectively. Additionally, after controlling for multiple comparisons and age, the level of disruption to the VAN was significantly associated with poorer global cognition, as measured by the MoCA (p=.001). These novel findings identify the functional networks most affected by the presence of WMLs in older adults with CvD and suggest that the disruption to the VAN caused by WML load may underlie the deficits seen in cognition in this population.

### PATTERNS OF WEIGHT CHANGE IN AGING AND DYING

Stephen Thielke, *University of Washington, Seattle, Washington, United States*

Little research has characterized the natural history of weight change in older adults. Different changes may occur during aging and dying. We analyzed 18 years of weight measures from a cohort of 736,361 Veterans, all of whom had died at age 70 or older. We produced summary measures that accounted for both chronological age and number of years before death. Several clear population-level trends appeared. (1) The average weight of the sample declined across all ages at a rate of about 0.18 BMI points per year. (2) Starting about seven years before death, the amount of loss began to accelerate, reaching a decline of 0.75 BMI points in the year before death. (3) Changes in weight relative to years of remaining life were independent of chronologic age. People who died at age 70 experienced, on average, the same type and duration of terminal decline as did those who died at age 95. (4) The dying process involved a cumulative loss of about 1.3 BMI points. (5) The distribution of weights during advancing age both declined and narrowed. (6) Disproportionate deaths occurred at the lower BMI ranges (below a BMI of 24), and especially below 18, regardless of age. (7) The finding in #5 is explained by the entire cohort losing weight, with death of the thinnest members. These findings argue for examining survival time in studies of weight change. They indicate that weight loss may be a natural part of dying, rather than a risk factor for it.

### RESTRICTED MEAN SURVIVAL TIME IN ACTION TO CONTROL CARDIOVASCULAR RISK IN DIABETES (ACCORD) BLOOD PRESSURE

Sandra Shi,[1] Lee-Jen Wei,[2] and Dae Kim,[3] *1. Beth Israel Deaconess Medical Center, Boston, Massachusetts, United States, 2. Harvard School of Public Health, Boston, United States, 3. Hebrew SeniorLife, Harvard Medical School, Roslindale, Massachusetts, United States*

Background: Restricted mean survival time (RMST) provides mean time lost or gained by an intervention. This may be a more intuitive way to understand treatment effect. Objective: To determine overall and subgroup treatment effects from blood pressure targets in older adults with diabetes. Methods: We analyzed the Action to Control Cardiovascular Risk in Diabetes (ACCORD) trial data. Our outcome was cardiovascular disease free survival. We measured 5-year RMST (days) between standard and intensive blood pressure control, and compared the difference by age (≥70 or <70 years old) and glycemic control (standard vs. intensive). Results: Over 5 years, those with intensive treatment lived, on average, 1716 days compared to 1714 days with standard treatment, with a RMST difference of 1.3 days (95% confidence interval, -22.1, 12.4). Among adults ≥70 years old, compared to standard treatment, intensive treatment resulted in an additional 13.6 (95% CI, -43.2, 70.3) days, where the difference was -0.5 (-20.8, 19.7) days for those aged <70 years old (p-for-interaction=0.673). Compared to standard treatment, intensive treatment resulted in 28.1 (0.4, 55.9) more days for those assigned to standard glycemic control, but it appeared to result in 25.2 fewer days (-52.3, 1.9) for those assigned to intensive glycemic control (p-for-interaction=0.007). Discussion: The benefit of intensive treatment over standard treatment varies by age and glycemic control. RMST difference may allow for more intuitive and personalized weighing of benefits and risks of intensive blood pressure control.

### ROLE OF GENETIC INTERACTIONS IN ALZHEIMER'S DISEASE: LESSONS FROM LONG LIFE FAMILY STUDY (LLFS)

Anatoliy Yashin,[1] Dequing Wu,[2] Konstantin Arbeev,[1] Eric Stallard,[3] Qihua Tan,[4] Alexander Kulminski,[1] Mary Feitosa,[5] and Svetlana Ukraintseva,[1] *1. Duke University, Durham, North Carolina, United States, 2. Duke University, Durham, United States, 3. Duke University, Biodemography of Aging Research Unit, Durham, North Carolina, United States, 4. University of Southern Denmark, Odense C, Denmark, 5. Washington University School of Medicine, St Louis, Missouri, United States*

Experimental and clinical studies of Alzheimer's disease (AD) provide plentiful evidence of AD heterogeneity and involvement of many interacting genes and pathways in regulation of AD-related traits. However, detailed mechanisms of genetic interactions (GxG) involved in AD remain largely unknown. Uncovering hidden patterns of such interactions from human data will help better understand the nature of AD heterogeneity and find new targets for AD prevention. In this paper, we applied a newly developed method of evaluating joint GxG effects on AD to analysis of the Long Life Family Study data. The analysis included several steps: (i) selecting candidate genes from stress response pathways that are thought to be involved in AD; (ii) estimating interaction effects of SNP-pairs on AD risk, and selecting the top interacting SNPs; (iii) running GWAS-like interaction analysis for SNP-pairs, with one SNP fixed; (iv) using characteristics of the detected SNP-pairs interactions to construct the SNP-specific Interaction Polygenic Risk Scores (IPRS); and (v) evaluating the effects of IPRSs on AD. We found that SNP-specific IPRS have highly significant effects on AD risk. For most SNPs involved in the significant interaction effects on AD, their individual effects were statistically not significant. Male and female analyses yielded different subsets of the top interacting SNPs. These results support major role of genetic interactions in heterogeneity of AD, and indicate that AD mechanisms can involve different combinations of the interacting genetic variants in males and females, which may point to different pathways of resistance/response to stressors in two genders.

### TORQUE AND VELOCITY DEPENDENCE OF MUSCLE FATIGUE IN AGING

Liam Fitzgerald,[1] Anthony Martin,[2] Rajakumar Nagarajan,[3] and Jane Kent,[3] *1. University of*

*Florida, 2. University of Massachusetts Amherst, Amherst, United States, 3. University of Massachusetts Amherst, Amherst, Massachusetts, United States*

Old age generally leads to smaller, weaker and slower skeletal muscles. To address the independent effects of weakness vs. slowing on fatigue in aging, we used a custom ergometer in a whole-body, 3 tesla magnetic resonance system to quantify knee extensor size, torque, velocity, power and intracellular energetics at baseline and during two 4-min fatiguing contraction protocols; one in which contraction velocity was constrained and torque varied (i.e., torque-dependent contractions; isokinetic, IsoK), and one in which torque was constrained and velocity varied (i.e., velocity-dependent contractions; isotonic, IsoT). On separate days, 10 young (27.5±1.2 yrs, 6 men) and 10 older (71.2±1.6 yrs, 5 men) healthy adults completed the IsoK (120°·s-1, 0.5 Hz) and IsoT (20% maximal torque, 0.5 Hz) protocols, with continuous measures of intracellular [Pi], pH, and [H2PO4-]. At baseline, contractile volume (803.5±72.3 vs. 1,125.6±109.9cm3), specific IsoK torque (0.035±0.004 vs. 0.058±0.007Nm.cm-3) and IsoT velocity (121.4±11.6 vs. 176.3±8.0deg.s-1) were greater in young than older (p≤0.023). Fatigue (%initial specific torque) was greater in young than older for IsoK (40.1±3.0 vs. 61.2±5.3%, p=0.0028), and accompanied by greater [Pi] and [H2PO4-] and lower pH in the young (p≤0.001). For IsoT, fatigue (%initial velocity) was not different between groups (young: 56.5±5.5 vs. older: 47.2±4.9%, p=0.661), despite lower pH and greater [H2PO4-] in young than old (p≤0.001). Collectively, these results reveal that normalizing dynamometer outputs to assess age-related differences in fatigue obscures baseline differences in muscle weakness. Further, our results suggest the contractile machinery may be less sensitive to changes in pH in older than young.

### WHOLE GENOME LINKAGE SCAN IDENTIFIES A NOVEL LOCUS ON 3Q28 FOR TG/HDL-C RATIO CHANGE OVER TIME

Ping An,[1] Allison Kuipers,[2] Iva Miljkovic,[2] Joseph Zmuda,[2] and Michael Province,[3] *1. Washington University School of Medicine, Chesterfield, Missouri, United States, 2. University of Pittsburgh, Pittsburgh, Pennsylvania, United States, 3. Washington University School of Medicine, SAINT LOUIS, Missouri, United States*

TG/HDL-C ratio (THR) represents a single inherited surrogate predictor of hyperinsulinemia or insulin resistance that is associated with premature aging processes, risk of diabetes and increased mortality. To identify genetic loci for THR change over time (ΔTHR), we conducted a whole genome linkage scan among subjects of European ancestry who had complete data from two exams collected about seven years apart from the Long Life Family Study (LLFS, n=3091), a study with familial clustering of exceptional longevity in the US and Denmark. Subjects with diabetes or using medications for dyslipidemia were excluded from this analysis. ΔTHR was derived using growth curve modeling, and adjusted for age, sex, PCs, familial membership, and then log-transformed to approximate normality. Our linkage scan was built on haplotype-based IBD estimation with 0.5 cM average spacing. Heritability of ΔTHR was moderate (46%), and evidence for significant linkage (LOD>3) was identified on 3q28 (LOD=4.1). This locus harbors ADIPOQ among several other promising candidate genes. Interestingly, several studies previously reported suggestive evidence of linkage at this locus for relevant traits including adiponectin, dementia, AD and SBP. This linkage signal was not explained by significant GWAS SNPs for LPL or those under the peak (LOD attenuated to 3.7). In conclusion, we found a novel genetic locus on 3q28 for ΔTHR in subjects without diabetes selected for exceptional survival and healthy aging. Further query of sequence elements including rare functional and regulatory variants at this locus is underway which may reveal novel insights on insulin resistance mechanisms for aging.

### SESSION 3000 (PAPER)

### IMPROVING DEMENTIA CARE

### CANADIAN CONSENSUS CONFERENCE ON PSYCHOLOGICAL AND NON-PHARMACOLOGICAL INTERVENTIONS FOR DEMENTIA

Debra Sheets,[1] Linda Clare,[2] Saskia Sivananthan,[3] Isabelle Vedel,[4] Teresa Liu-Ambrose,[5] Henry Brodaty,[6] Jim Mann,[7] and Carrie McAiney,[8] *1. University of Victoria, Victoria, British Columbia, Canada, 2. University of Exeter, Exeter, England, United Kingdom, 3. Alzheimer Society of Canada, Toronto, Ontario, Canada, 4. McGill University, Montréal, Quebec, Canada, 5. University of British Columbia, Vancouver, British Columbia, Canada, 6. UNSW Sydney, Sydney, New South Wales, Australia, 7. University of Victoria, Surrey, British Columbia, Canada, 8. University of Waterloo, Waterloo, Ontario, Canada*

Psychological and non-pharmacological interventions that could have a positive effect on outcomes important to persons living with dementia are essential to identify given the the limited efficacy of dementia medications and the diverse needs of persons living. In 2019, for the first time the Canadian Consensus Conference on the Diagnosis and Treatment of Dementia (CCCDTD) created a working group to develop recommendations related to a broad range of psychosocial and non-pharmacological interventions exist, typically aimed at improving cognition, symptoms, or well-being, as well as improving caregiver well-being and coping. The recommendations, primarily intended for primary care physicians, may also allow clinicians, organizations, and communities and help to better meet the needs of people living with dementia and their caregivers. A group of 11 experts, including persons living with dementia and informal caregivers, as well as clinicians and researchers from various organizations both nationally and internationally were invited to participate. A rapid review of meta-analyses and literature reviews on psychological and non-pharmacological interventions was conducted. The synthesized results were submitted for a consensus building approach using a Delphi method, involving a panel of more than 50 Canadian participants. Recommendations with a positive vote of 80% or more were considered to have reached consensus. All proposed recommendations reached consensus using the Delphi process. Details of the recommendations are presented. Five recommendations are made: group or individual physical exercise, group cognitive stimulation therapy, psychoeducational interventions for caregivers, dementia friendly organizations/communities, and case management.

### FEASIBILITY OF AUTOMATING FIDELITY MONITORING IN A DEMENTIA CARE INTERVENTION

Nancy Hodgson,[1] Ani Nencova,[2] Laura Gitlin,[3] and Emily Summerhayes,[2] *1. University of Pennsylvania, Cherry Hill, New Jersey, United States, 2. University of Pennsylvania, Philadelphia, Pennsylvania, United States, 3. Drexel University, Philadelphia, Pennsylvania, United States*

Careful fidelity monitoring is critical to implementing evidence-based interventions in dementia care settings to ensure that the intervention is delivered consistently and as intended. Most approaches to fidelity monitoring rely on human coding of content that has been covered during a session or of stylistic aspects of the intervention, including rapport, empathy, enthusiasm and are unrealistic to implement on a large scale in real world settings. Technological advances in automatic speech recognition and language and speech processing offers potential solutions to overcome these barriers. We compare three commercial automatic speech recognition tools on spoken content drawn from dementia care interactions to determine the accuracy of recognition and the guarantees for privacy offered by each provider. Data were obtained from recorded sessions of the Dementia Behavior Study intervention trial (NCT01892579). We find that despite their impressive performance in general applications, automatic speech recognition systems work less well for older adults and people of color. We outline a plan for automating fidelity in interaction style and content which would be integrated in an online program for training dementia care providers.

### HOSPITALIZATIONS IN ADULTS WITH ALZHEIMER'S DISEASE AND RELATED DEMENTIA—UNITED STATES, 2016–2017

Christopher Taylor,[1] Benjamin Olivari,[2] and Lisa McGuire,[1] *1. Centers for Disease Control and Prevention, Atlanta, Georgia, United States, 2. CDC, Atlanta, Georgia, United States*

Alzheimer's disease and related dementias (ADRD) are a significant public health burden. Collectively, ADRD have been called the most expensive chronic conditions in the United States due to outsized health care utilization. Data from the 2016 and 2017 Healthcare Cost Utilization Project National Inpatient Sample, an all-payer representative sample of US hospitalizations, were used to describe hospitalizations in adults ≥65 years with ADRD. Chronic conditions were defined using International Classification of Disease, Tenth Edition, Clinical Modification (ICD-10-CM) code definitions from the Centers for Medicare and Medicaid Services Chronic Conditions Warehouse. Code definitions from the Agency for Healthcare Research and Quality defined potentially preventable hospitalizations where admission might have been avoided by appropriate outpatient primary care management. One in six hospitalizations in adults ≥65 years were for persons with ADRD, including 1 in 3 adults ≥85 years. Among those with ADRD-related admissions, the most common reasons for admission, as defined by the principal diagnosis, were heart disease (18.1%), certain infections (14.5%), injuries (12.7%), respiratory illness (11.2%), and genitourinary conditions (10.4%). In hospitalized adults with ADRD, the prevalence of diagnosed urinary tract infection (37.0%)—a potentially preventable hospitalization—is more than double the prevalence in adults without ADRD (15.5%, prevalence ratio = 2.39, 95% confidence interval: 2.37-2.42). Common comorbidities, injuries, and potentially preventable hospitalizations all contribute to hospitalizations in adults with ADRD. Focusing on injury prevention and appropriate outpatient management of comorbidities in adults with ADRD might reduce the number of hospitalizations, including potentially preventable hospitalizations, among adults with ADRD.

### NURSE VISITS, SITE OF CARE, AND HOSPITALIZATION AMONG HOSPICE PATIENTS WITH DEMENTIA

Elizabeth Luth,[1] David Russell,[2] Holly Prigerson,[3] Kathryn Bowles,[4] and Miriam Ryvicker,[5] *1. Weill Cornell Medical College, Larchmont, New York, United States, 2. Appalachian State University, Boone, North Carolina, United States, 3. Weill Cornell Medicine, New York, New York, United States, 4. University of Pennsylvania School of Nursing, Philadelphia, Pennsylvania, United States, 5. Visiting Nurse Service of New York, New York, United States*

Persons with dementia comprise up to 50% of hospice patients and face an increased risk of burdensome, disruptive, and costly discharge from hospice due to hospitalization. The relationship between timing, dose and site of hospice care provided, all modifiable factors, and risk of hospitalization is poorly understood. We use a retrospective cohort analysis of 2,692 electronic health records of hospice patients with dementia who received care from a large hospice agency in New York City between 2013-2017 to determine the relationship between hospice service delivery (e.g., number and timing of nurse visits, home vs. facility-based) and risk of hospitalization (vs death). We control for demographic and clinical characteristics of patients. 9.36% of patients with dementia were hospitalized. Hospice service delivery factors were significantly associated with risk of hospitalization. Each additional nurse visit was associated with a 5% decrease in risk of hospitalization (AOR: 0.95, 95% CI: 0.92-0.98). Each additional day between last nurse visit and discharge was associated with a 7% increase in risk of hospitalization (AOR: 1.07, 95% CI: 1.04-1.11). Home hospice was associated with 97% higher odds of hospitalization (AOR: 1.97, 95% CI: 1.19-2.09). Hospice patients with dementia who receive services at home, receive fewer nursing visits, and have increased time between nursing visits are at increased risk for hospitalization. Research is needed to determine if increasing the number and timing of nursing visits can reduce risk of hospitalization in this population.

### THE NEED TO SEE THE WORLD: OUTINGS AND MEANINGFUL ENGAGEMENT AMONG ASSISTED LIVING RESIDENTS WITH DEMENTIA

Joy Ciofi,[1] Candace Kemp,[1] Stephen Duong,[1] Andrea Hill,[2] Anna Lisa Baidoo,[3] AprilSpring Wood,[1] Pamela Manley,[1] and Alexis Bender,[4] *1. Georgia State University, Atlanta, Georgia, United States, 2. Georgia*

*State University, Atlanta, United States, 3. Georgia State University, Macon, Georgia, United States, 4. Emory University, Atlanta, Georgia, United States*

Meaningful engagement is a promising avenue for enhancing the lives of persons with dementia. Yet, engagement can prove challenging across care settings. Here, we report on our ongoing 5-year NIA-funded study (R01AG062310) aimed at identifying best practices for promoting meaningful engagement opportunities among assisted living residents with dementia. Guided by grounded theory method, the study involves multiple modes of qualitative data collection, including: participant observation; resident record review; and in-depth interviews with residents (when possible), assisted living staff, residents' family and friends, volunteers, and others involved in residents' daily lives. We present analysis of data collected in 4 diverse care settings studied in-depth over a one-year period and focus on "outings" or instances when residents leave the assisted living setting. Findings show the variable nature, content, and frequency of outings and demonstrate the advantages, disadvantages, and challenges associated with taking persons living with dementia out of their living environments. Outings represent an opportunity "to get out," "see the world," and "not be stuck inside." Yet, outings are not universally successful and sometimes create frustration, distress or confusion. Multiple factors intersect to influence residents' opportunities to participate in and derive benefit from outings, including residents' cognitive and physical function, and personal preferences, and formal and informal care partners' knowledge, attitudes, behaviors, and resources. Care setting practices and policies, resources, size and location, and community connections also shape opportunities for and experiences with outings among residents living with dementia. We discuss the implications of our findings for research, policy, and practice.

### SESSION 3001 (PAPER)

### EDUCATION AND TRAINING FOR FRONTLINE WORKERS

### AN EXPERIMENTAL PROGRAM FOR EFFECTIVE COMMUNICATION OF MIDDLE-AGED AND OLDER ADULTS WITH PHARMACISTS IN TAIWAN

Yi-Wei Sun,[1] and Hui-Ching Weng,[2] *1. National Cheng Kung University, Taichung, Taiwan (Republic of China), 2. National Cheng Kung University, Tainan City, Tainan, Taiwan (Republic of China)*

Plenty of potential drug-related problems was noted in the middle-aged and older adults. Effective communication is essential for pharmacists to coordinate their opinions with patients to improve the effectiveness of drug treatment and reduce drug-related problems. However, few studies focused on patients' own abilities to communicate with pharmacists even though it is vital for the effectiveness of medical treatment. The purpose of this study was to design a communication training program for middle-age and older adults not only to enhance their understanding of pharmacists' responsibilities and the importance to communicate with pharmacists, but also to improve their self-efficacy about drug-related communication and the ability to list their latest medication. A non-randomized control-group pretest-posttest design was conducted in Tainan City. Through individual interviews of five pharmacists and a focus group interview, we ensured the appropriateness and expertise of the training program. 179 adults over 55 years old were distributed into experimental group or control group. The experimental group underwent the training program after the pretest, while no intervention was provided to the control group. Face-to-face and role-playing sessions were in process with a training booklet in the courses. Both groups completed the posttest four weeks after the pretest. This communication training program appears to be effective since the knowledge of pharmacists' responsibilities and self-efficacy of drug-related communication were significantly improved in the experimental group ($p<.05$) rather than control group. Completion of the recent medication list was also improved from 40.3% to 80.6% and was significantly higher than the control group.

### DEVELOPMENT OF AN INTERPROFESSIONAL TRAINING EDUCATION PROGRAM IN GERIATRICS

Sam Cotton,[1] Anna Faul,[2] Pamela Yankeelov,[1] and Joe D'Ambrosio,[1] *1. University of Louisville, Louisville, Kentucky, United States, 2. University of Louisville Trager Institute, Louisville, Kentucky, United States*

This study examines the development of an interprofessional training certificate program that prepares social work learners to infuse geriatrics and behavioral health into primary care settings. Since 2018, our program has trained 31 social work learners and 16 learners from counseling psychology and nursing. At the core of the certificate program is an emphasis on developing skills focused on the integration of geriatrics, behavioral health and primary care to address the lack of workforce trained at the intersection of these areas. Each series of workshop is aligned with core competencies that address the 4-M Model of Age-Friendly Health Care and SAMSHA's Core Competencies in Behavioral Health. Our professional certificate includes training in Motivational interviewing, as well as Cognitive behavioral therapy, Mindfulness based cognitive therapy and Problem-solving therapy, Narrative Therapy, Strategic Therapy, Systemic Therapy, Life Review and Reminiscence Therapy. Additionally, students receive professionalization trainings to help prepare them for the job market. To measure the efficacy of this curriculum program, we examined the outcomes related to student knowledge of geriatrics and behavioral health including knowledge attainment, fidelity to modalities, learner self-efficacy, and learner satisfaction. The results of this study showed that integrating interprofessional education into social work settings can lead to positive outcomes for student knowledge, self-efficacy and learning satisfaction. Additionally, we found that having a curriculum that focused on interprofessional teams contributed to higher self-efficacy in completing tasks compared to previous cohorts. This has implications for the way that we conceptualization the use of interprofessional education.

### IMPLEMENTING CHANGING TALK ONLINE TRAINING: A PRAGMATIC TRIAL TO IMPROVE DEMENTIA CARE COMMUNICATION IN NHS

Carissa Coleman,[1] Kristine Williams,[2] Yelena Perkhounkova,[3] Maria Hein,[3] Tim Beachy,[3] and Clarissa Shaw,[3] *1. University of Kansas, Kansas City, Kansas, United States, 2. University of Kansas, Lawrence, Kansas, United States, 3. University of Iowa, Iowa City, Iowa, United States*

The Changing Talk (CHAT) training effectively reduces elderspeak and subsequent behavioral challenges in residents with dementia in nursing homes. The Changing Talk: Online (CHATO) training was developed to increase staff access to education using a new online format. A pilot test was conducted to confirm the feasibility and effects of CHATO on training outcomes. In the initial nursing home, twenty-three direct care staff members in a small Midwestern nursing home enrolled in the course including 12 CNAs, 4 RNs, 2 LPNs, 2 CMAs, 1 Dietary Aide, 1 Social Worker, and 1 in Transportation. Two forms of a 13-item scenario-based test to measure knowledge gain were developed and tested. Of the 23 staff, 18 (78%) completed the post-test and 83% of completers achieved a post-test score of 70% or greater. Scores on the test improved from M=69% correct (SD=11.7) at pretest to 86% correct (SD=10.6) on posttest demonstrating knowledge gain (p=.024). Participants improved their recognition of elderspeak (21%) and person-centered communication (24%) in a video vignette and 86% self-reported improvement in their abilities to recognize ineffective communication and to apply more effective communication strategies in practice. A randomized control trial enrolled staff (N=187) in eight additional nursing homes. Preliminary results confirm improvements in test scores from M=70.6% correct (SD=15.8) at pretest to 77.2% correct (SD=14.1) on posttest and increased elderspeak recognition (p=.004). Relationships between nursing home characteristics, implementation strategies, and culture change measured by the Artifacts of Culture Change Tool and their relationship to communication outcomes will be presented.

### IMPROVING DEMENTIA CAPABILITY AMONG LAW ENFORCEMENT: EVALUATION OF DEMENTIA EDUCATION TRAINING IN SAN DIEGO COUNTY

Sara Powers,[1] Silvia Orsulic-Jeras,[1] Jessica Empeno,[2] Regina Lagasca,[3] Amy Abrams,[3] Sarina Barker,[3] Rebecca McDaniel,[3] and Marcie Hanna,[3] *1. Benjamin Rose Institute on Aging, Cleveland, Ohio, United States, 2. Lightbridge Hospice & Palliative Care, San Diego, California, United States, 3. Alzheimer's San Diego, San Diego, California, United States*

As the number of Alzheimer's disease and related dementia (ADRD) cases grow, first responders will have more frequent interactions with individuals living with ADRD within the community. Recognizing the lack of dementia training among first responders and through the support of an Administration for Community Living grant, Alzheimer's San Diego partnered with local law enforcement to support and educate first responders on local ADRD resources, referral systems for families and caregivers, and disease-related information, in hopes of building stronger dementia capable services within San Diego County. To date, 249 first responders have participated in dementia education trainings and completed the related evaluations that aimed to understand their experiences with and knowledge of dementia. On average, participants were 44.74 years old (SD=18.45) and served 8.72 years (SD=7.99) on their job. Majority were male (n=189), had some college education (n=94), and identified as White/Caucasian (n=105) or Hispanic/Latino (n=92). Although objective knowledge (as measured by a shortened version of the Alzheimer's Disease Knowledge Scale [ADKS]) did not significantly differ from pre- to post-test, participants' perceived subjective knowledge about dementia significantly increased from pre-test (M=1.17, SD=0.77) to post-test (M=1.89, SD=0.66); t(141)=10.56, p<.001. Encouragingly, participants who reported receiving prior training on dementia had significantly higher ADKS scores at both pre- and post-test compared to those who did not have prior training. Results highlight the need for ongoing dementia-related training for law enforcement and the importance of evaluation requirements. Discussion will focus on policy implications and how communities can support dementia capability among first responders.

### SESSION 3002 (PAPER)

### PHYSICAL ACTIVITY AND HEALTH

### ASSOCIATIONS BETWEEN PHYSICAL ACTIVITY AND LIVING ALONE: ARE THERE DIFFERENCES BY GENDER?

Jianjia Cheng,[1] and Emily Nicklett,[2] *1. University of Michigan, Ann Arbor, Ann Arbor, Michigan, United States, 2. University of Michigan, Ann Arbor, Michigan, United States*

Nearly one in three adults aged 65 and older live alone in the U.S. Despite this increasing trend, there remains little understanding regarding the relationship between living alone and physical activity (PA) in older adulthood. Is living alone a risk factor for lower levels of PA among older adults? The effects of living arrangements on health behaviors could differ by gender; however, findings from prior studies on this topic have been mixed or inconclusive. This is one of few studies to examine whether PA is associated with living alone (vs. living with others) and whether the association differs by gender using longitudinal data. Our data were drawn from 2006-2014 Psychosocial and Lifestyle Questionnaire of the Health and Retirement Study, a nationally representative sample of older adults in the U.S. (N=17371, mean age=75.4). PA was measured repeatedly using metabolic equivalents of task (MET) estimated values accounting for the vigor and frequency of self-reported PA (range 0-31). Using mixed-effects linear regression, we found that living alone was significantly associated with higher levels of PA (Coeff.: 0.41, p<0.001). When examined separately by gender, living alone was associated with significantly higher PA among women (Coeff.: 0.47, p<0.001) but not among men (Coeff.: 0.29, p=0.14) after controlling for marital status, other sociodemographic characteristics, and health-related indicators. Our study provides evidence of gendered differences in initiating and maintaining health behavior change in relation to living arrangements. Findings provide implications for the design of PA promotion programs and policies for older adults.

### DO AUTOMATIC HABITS PREDICT PHYSICAL ACTIVITY ENGAGEMENT IN OLDER PEOPLE?

Sophie Andrews,[1] Dinaz Parekh,[2] Brooke Brady,[2] Kim Delbaere,[1] Simon Killcross,[3] and Kaarin Anstey,[4]
*1. Neuroscience Research Australia, Randwick, New South Wales, Australia, 2. Neuroscience Research Australia, Randwick, Australia, 3. University of New South Wales, Kensington, Australia, 4. University of New South Wales, Sydney, Australia*

Sufficient physical activity is crucial to maintaining independence, health and wellbeing during ageing, yet physical inactivity is common in older people. Identifying factors associated with physical activity engagement is essential to develop novel approaches to increase activity in older people. Automatic, context-dependent habits may play an important role in physical activity behaviour. The current study aimed to investigate the relationship between physical activity behaviours and their automaticity in older people. 123 community dwelling Australians aged over 65 – 88 years (M=72.2; 81 women), recruited from participant registries, hospital noticeboards and community groups, completed an online questionnaire. Current physical activity levels were measured using the Incidental and Planned Exercise Questionnaire, and automaticity of those physical activity behaviours were measured using the Self-Report Habit Automaticity Index. Participants also reported demographic information, body mass index (BMI), medical history and current mood symptoms. Participants reported an average of 2.28 hours planned walking (SD=2.33), 5.81 hours planned moderate/vigorous exercise (SD=4.02), and 20.5 hours incidental activity (SD=15.52) per week. Multiple regression analyses revealed that after controlling for age, gender, BMI and depression symptoms, higher automaticity scores were associated with more hours per week of planned walking (p=.012), moderate/vigorous exercise (p=.038), and incidental activity (p=.017). Supporting older people to make their physical activity more habitual could therefore be an effective approach to increase levels of physical activity in this population.

### LONGITUDINAL RELATIONSHIPS BETWEEN FAVORITE ACTIVITIES AND HOMEBOUND STATUS IN OLDER ADULTS

Xiaocao Sun,[1] Minhui Liu,[2] Christina Miyawaki,[3] Yuxiao Li,[4] Tianxue Hou,[4] Siyuan Tang,[1] and Sarah Szanton,[5] *1. Central South University, Changsha City, Hunan, China, 2. Central South University, Baltimore, Maryland, United States, 3. University of Houston, Houston, Texas, United States, 4. Central South University, Changsha, Hunan, China, 5. Johns Hopkins University, Baltimore, Maryland, United States*

Favorite activities are usually meaningful to older adults and may influence their homebound status and vice versa. Using Round 1 (R1, in 2011) and Round 5 (R5, in 2015) data from the National Health and Aging Trends Study, we examined the patterns of favorite activity by homebound status and investigated their relationship among community-dwelling older adults (N=3,332). Homebound status (non-homebound, semi-homebound, and homebound) was determined by the frequency, difficulty, and needing help of outdoor mobility. Favorite activities were named by participants verbatim and then were classified into two categories (active and non-active) based on the estimated energy needed to perform the activity. Logistic regression models were used to determine whether homebound status at R1 predicted the types of favorite activity in R5, and ordinal logistic regression models for predictions from the types of favorite activity at R1 to homebound status at R5, adjusting for demographics and health conditions. The sample was, on average, 76±7.23 years old, non-Hispanic White (72.9%), and female (59.7%). Regardless of the homebound status, reading and walking are the top two favorite activities. Homebound older adults enjoyed "non-active" activities (e.g., watching TV), while non-homebound counterparts preferred "active" outdoor maintenance. Being homebound at R1 predicted non-active favorite activity in R5 (OR=.257, p<0.001), and R1 non-active favorite activity also predicted homebound status in R5 (OR=1.219, p =0.039). These findings provide new information on the activity preferences of older adults with different homebound status and how their preferences may influence their future homebound status.

### THE RELATIONSHIP BETWEEN PHYSICAL ACTIVITY LEVELS AND MANUAL DEXTERITY IN ADULTS OVER 60 YEARS OLD

Naomi Kim,[1] Rachael McGraw,[2] and Katie Thralls,[2]
*1. Seattle Pacific University, Spokane, Washington, United States, 2. Seattle Pacific University, Seattle, Washington, United States*

During aging there is a natural physiological decline that contributes to a loss of function needed for activities of daily living to maintain independence and high quality of life. Physical function needed for independence includes gross motor function (e.g., lower body strength for standing) and fine motor function (e.g., manual dexterity for dressing). Physical activity (PA) has shown to maintain fitness, such as muscular strength, to delay loss in gross motor function. However, there is limited research on the association between PA and fine motor function. The purpose of this study was to examine the relationship between meeting national Physical Activity Guidelines (PAG; >150 min./wk.) and manual dexterity in older adults (>60 years). Participants (N=45, Mean age = 80.2±8.2 years) completed an interview-assisted self-report of their PA level and an objectively measured manual dexterity assessment (i.e., Purdue Pegboard Test (PPT)). The PPT included four fine motor skill assessments. For all four PPT's, Analysis of Variance (ANOVA) tests showed a significant main effect for PA level, a main effect for age, and an interaction effect (PA*age) on manual dexterity for all PPTs (ps<0.05). Follow-up comparisons showed a significant main effect for PA level on manual dexterity for the older group (>80yrs; ps<0.05), and not for the younger group (ps>0.05). Pearson's r correlations showed significant moderate-positive correlations between activity level (min./wk.) and PPTs scores (r=0.45– 0.50; ps<0.005). These findings suggest that meeting PAG may be a preventative strategy to attenuate aging declines in manual dexterity to maintain hand function and independence.

### WEB-BASED PHYSICAL ACTIVITY INTERVENTION FOR COPD: DIFFERENCES BETWEEN RURAL AND URBAN VETERANS

Stephanie Robinson,[1] Stephanie Shimada,[1] Caroline Richardson,[2] and Marilyn Moy,[3] *1. Edith Nourse Rogers Memorial Veterans Hospital, Bedford, Massachusetts, United States, 2. University of Michigan, Ypsilanti, Michigan, United States, 3. West Roxburry VAMC, West Roxbury, Massachusetts, United States*

Physical activity is recommended for all patients with chronic obstructive pulmonary disease (COPD), a disease prevalent among older adults. Rural Veterans often lack access to in-person services to support behavior change. Technology can be used to overcome these barriers. This secondary analysis of a randomized controlled trial compared engagement (weekly website-logons) and efficacy (step-count change) measures of a 4-month technology-based physical activity intervention between rural and urban Veterans with COPD. Rural-urban commuting codes (RUCA) were used to classify participants (N=239; mean age=66.7±8.84 years) as 'rural' or 'urban'. Participants were randomized 2:1 to a pedometer and website with iterative, individualized step-count feedback and goals, motivational and informational messages, and an online community (intervention group, n=155, 45.8% rural) or to receive a pedometer-alone (waitlist-control group, n=84, 44% rural). General linear modeling estimated the adjusted effect of rurality on intervention response. There was no significant rural-urban difference in weekly website-logons (3.33 vs. 3.17; p=.364). There was a significant interaction between rurality and group on step-count change (p=.045). Among rural participants, both intervention and controls increased their step counts (567.32 vs. 732.30; p=.769). Among urban participants, the intervention group demonstrated a significantly higher change in step-counts compared to controls (1052.67 vs. -313.57; p=.008). Results suggest that rural participants increased their step-counts similarly whether they received a pedometer alone or used the intervention, whereas only urban participants who used the intervention increased their step counts. It is possible that simply receiving the pedometer alone was sufficient for behavior change in the rural participants.

### SESSION 3003 (PAPER)

### SOCIAL SUPPORT AND PSYCHOLOGICAL WELL-BEING

### DO SOCIAL SUPPORT AND STRAIN MEDIATE THE RELATIONSHIP BETWEEN CHILDHOOD EXPOSURES AND FRAILTY IN LATER LIFE?

Monica Williams-Farrelly, and Kenneth Ferraro, *Purdue University, West Lafayette, Indiana, United States*

Previous studies have identified the early origins of physical frailty, notably poor childhood health and socioeconomic status, but relatively few studies examine whether social support in later life mitigates the influence of early noxious exposures on frailty. Given the established relationship between health and social relationships in older adults, this research uses data from the Health and Retirement study (2004-2016) to examine whether social support and strain mediate the effect of childhood exposures on frailty in later life. A series of linear regression and pathway models were estimated to test whether childhood exposures, including socioeconomic status, infectious and chronic diseases, impairments, and risky adolescent parental behaviors, were associated with phenotypic frailty (Fried et al. 2001). After adjusting for demographic and adult factors, accumulated childhood misfortune was directly (b=0.015, p<.01) and indirectly (b=0.007, p<.001) associated with more frailty. Average social support, but not strain, from one's spouse, children, family and friends significantly mediated the relationship between accumulated misfortune and frailty (b= -.002, p<.01). Path analysis revealed that social support reduces later life frailty directly (b=-0.106 ,p<.001) and indirectly through a reduction in adult morbidity (b=-0.031, p<.001). However, counterintuitively we found that accumulated misfortune was associated with more social support. Supplemental analyses reveal that one or more infectious diseases in childhood were responsible for the positive relationship (b= 0.393, p<.001). These results have implications for how we may reduce the burden of frailty on those who have experienced misfortune early in life.

### INFLUENCE FACTORS AND LIFE SATISFACTION OF TYPES OF OLDER ADULTS' DAILY TIME USE USING LATENT PROFILE ANALYSIS

Joosuk Chae, Seok In Nam, Haesol Won, and Juyoung Lee, *Yonsei University, Seoul, Republic of Korea*

The purpose of this study is to divide the daily time use of older adults into types, examine the influence factors of each type, and verify the difference in life satisfaction between types. We used a total of six variables, three variables (essential, compulsory, and leisure time use) for weekdays and the same three variables for weekends. We used data from the sixth wave of the Korea Retirement and Income Study (n=3,993). Latent Profile Analysis was used to classify the older adults' (aged 65 and over) types of daily time use, resulting in a division into three types: essential activity-centered (16%), leisure-centered (26%), and balanced (58%). The weekend and weekday activities of each group did not differ. Compared with the balanced type, the significant factors of the essential activity-centered type were age (p<.01), work status (p<.001), and chronic disease (p<.001). In addition, the significant factors of the leisure-centered type were age (p<.05), gender (p<.05), subjective health (p<.001), work status (p<.001), and chronic disease (p<.001). Life satisfaction was lowest in the essential activity-centered type and highest in the balanced type; differences between the groups were identified (p<.000). Based on the results, we discuss practical interventions and the development of psychosocial programs for older adults. For the essential activity-centered type, living support is required because they spend more time sleeping and eating. In addition, we proposed the preparation of leisure programs applicable to the needs of older adults' of the leisure-centered type, since their life satisfaction was lower than the balanced type.

### INTERGENERATIONAL RELATIONSHIPS, SOCIAL SUPPORT, AND PSYCHOLOGICAL WELL-BEING AMONG KOREAN OLDER ADULTS

Yooumi Lee, and Janet Wilmoth, *Syracuse University, Syracuse, New York, United States*

This study investigates whether intergenerational relationships and social support improve the psychological well-being of Korean older adults. We examine whether intergenerational relationships and social support directly influence psychological well-being and the extent to which they mediate the distressing consequences of life events such as declining health and recent widowhood. Using longitudinal data from the 2006 to 2016 Korean Longitudinal Study of Aging, we explore depression trajectories among individuals who are 60 or older with at least one living adult child at baseline. Specifically, we converted data from 5,383 older adults into a person-period file with 24,726 observations over a ten-year period. Then we estimated linear growth curve models of depression trajectories separately for men and women using the Center for Epidemiologic Studies Depression Scale (CES-D). Results from the hierarchical linear models indicate that declining health and recent widowhood are positively related to depressive symptoms. Satisfactory intergenerational relationships and social support in the form of personal interactions and proximate living arrangements with adult children decrease depressive symptoms of older parents, especially among women. We conclude that the psychological benefits of intergenerational relationships and social support are contingent upon the vulnerability of older adults and discuss the implications for public policy.

### NEGATIVE MARITAL QUALITY, PURPOSE IN LIFE, AND DEPRESSIVE SYMPTOMS AMONG MIDDLE-AGED AND OLDER COUPLES

Elliane Irani,[1] Sumin Park,[2] and Ronald Hickman,[3]
*1. Case Western Reserve University, Ohio, United States, 2. Case Western Reserve University, Cleveland Heights, Ohio, United States, 3. Case Western Reserve University, Cleveland, Ohio, United States*

Negative marital quality is associated with poor health outcomes. Purpose in life can serve as a psychological resource to buffer the stressors experienced from a negative marital relationship. Yet, the associations among negative martial quality, a person's level of purpose in life, and depressive symptoms have not been fully explored in a dyadic context. We examined the actor (intra-individual) and partner (cross-spousal) effects of negative marital quality on depressive symptoms in couples and the potential mediating role of purpose in life. Structural equation modeling was used to analyze cross-sectional data on middle-aged and older married, heterosexual couples (N=1,235) who participated in the 2016 wave of the Health and Retirement Study. The final model had an acceptable fit to the data (TLI=.963, RMSEA=.040, SRMR=.038). At the actor level, negative relationship quality was positively associated with depressive symptom severity, and purpose in life mediated the relationship in wives and husbands. At the partner level, wives had more depressive symptoms when husbands reported higher negative marital quality. Comparatively, husbands had less depressive symptoms when their wives indicated a greater sense of purpose. Husbands also had a lower purpose in life when their wives had higher states of negative marital quality. This study highlights the psychological benefits of allaying negative perceptions of marital quality and enhancing the sense of purpose in middle-aged and older couples. The results support a focus on dyadic approaches to improve the psychological health, and potentially, the physical health status of middle-aged and older couples.

### THE EFFECT OF SOCIAL ACTIVITIES ON THE LIFE SATISFACTION OF KOREAN OLDER ADULTS

Meeryoung Kim, *Daegu University, Seoul, Republic of Korea*

As life expectancy increases, older adults need to find ways to occupy their time for 20-30 years. For Korean older adults, social activities such as having relationships with others as well as, involvement in organizations and volunteer work, are important for their social identity. Social activities are one of the categories of Rowe and Kahn's successful aging, this study examined the effect of having relationships, involvement in organizations and volunteering on the life satisfaction of older adults. This study used the 6th additional wave of the Korean Retirement and Income Study (2016). The target population was older adults (50~59, 60~74, 75+). The sample size was 1,921, 2,344 and 962 respectively. For data analysis, ANOVA and multiple regressions were used. The demographic variables were controlled. As for independent variables, having relationships, involvement in organizations, and volunteering were used. For the dependent variable, life satisfaction was used. Having relationships, involvement in organizations and volunteering were significantly different by age group. For each age group, the factors affecting life satisfaction differ. For the middle aged group, involvement in organizations and volunteering were significant factors affecting life satisfaction. For young-old adults, volunteering had the most significant effect on life satisfaction. Finally, for old-old adults, both having human relations and involvement in organizations were significant. These findings imply that social activities differ by age group. Also, the kind of activities affecting life satisfaction differ by age group. These findings imply that it is important for older adults to be involved in society, in various ways.

### SESSION 3004 (PAPER)

### SEXUAL HEALTH AND HIV/AIDS

### BARRIERS TO SUCCESSFUL AGING AMONG OLDER WOMEN LIVING WITH HIV: A QUALITATIVE STUDY

Anna Rubtsova,[1] Tonya Taylor,[2] Gina Wingood,[3] Igho Ofotokun,[4] Deborah Gustafson,[5] and Marcia Holstad,[1] *1. Emory University, Atlanta, Georgia, United States, 2. SUNY Downstate Health Sciences University, Brooklyn, New York, United States, 3. Columbia University Mailman School of Public Health, New York, New York, United States, 4. Emory University School of Medicine, Atlanta, Georgia, United States, 5. SUNY*

*Downstate Medical Center, Brooklyn, New York, United States*

Successful aging (SA) is the capacity of older people to thrive despite age-related changes and/or declines. Although our previous research found that older (age ≥50) women living with HIV (OWLH) can achieve SA, considerable barriers remain. The purpose of this qualitative study was to identify specific barriers to SA among OWLH. Our sample consisted of 29 OWLH recruited between October 2018 and March 2019 at two sites of Women's Interagency HIV Study (WIHS): Atlanta, GA and Brooklyn, NY. These participants were assigned to either semi-structured interviews (N=17: 8 interviews in Brooklyn and 9 in Atlanta) or focus group discussions (FGD: 1 FGD in Atlanta with 5 participants, and 1 FGD in Brooklyn with 7 participants). Our FGD and Interview Guides included questions focused on barriers to SA. Participants were, on average, 58 years old (range 50-73), 86% Black, 83% single, and 62% with annual income ≤ $12,000. All interviews and FGD were transcribed and coded using MAXQDA software. We used thematic coding within constructivist approach. Several themes emerged identifying the following SA barriers: multiple chronic conditions and pain (e.g., arthritis, neuropathy); polypharmacy and side effects of HIV medications ("it's wearing on me"); HIV-related stigma and loneliness ("I think my children would judge me if I would tell them I have it HIV"); substance use, giving up on yourself ("just sitting around, not doing anything"); and lack of access to resources and services (e.g., mental health providers, support groups). Our findings will help designing public health interventions promoting SA among OWLH.

### OLDER ADULT SEXUALITY, PARTNERSHIP, AND HEALTH: COHORT COMPARISONS OF BABY BOOMERS AND TRADITIONALISTS

Winnie Tong, and Linda Waite, *University of Chicago, Chicago, Illinois, United States*

This paper updates prior work on older adult sexuality, partnership, and health by examining the most current wave of the National Social Life, Health, and Aging Project (2015-16), a population-based study of health and social factors on a national scale. Comparing data from Wave I, Cohort 1 (2005-06) and Wave I, Cohort 2 (2015-16), we ask whether there are differences in partnership, sexual behaviors and health outcomes between two cohorts ('Traditionalists' vs. 'Baby Boomers'). Additionally, we examine whether sexual frequency is related to physical health, particularly the health conditions of arthritis, diabetes, cognitive impairment, and prior stroke, in both cohorts. We find significant differences between cohorts through a logistic model. For Traditionalists, age, gender, education level, partnership status and diabetes were all significantly related to sexual activity ($p < 0.001$). Older adults were less sexually active; men were more sexually active; the higher educated were more sexually active; diabetes patients were less sexually active; and partnered were more sexually active. For Baby Boomers, only age and partnership status were significantly related to sexual activity ($p < 0.001$); gender and diabetes diagnosis were also related ($p <0.005$). Significantly, partnership status for Boomers is negatively related to sexual activity; the other three relationships – age is related to less sexual activity, men have slightly higher sexual activity, and diabetes was related to less sexual activity – were as expected. Importantly, our findings may imply that partnership or marriage is not as significant to sexual activity, or to health outcomes, as previously believed.

### PSYCHOSOCIAL MECHANISMS OF SUCCESSFUL AGING AMONG OLDER WOMEN LIVING WITH HIV: A STRUCTURAL EQUATION MODEL

Anna Rubtsova,[1] Gina Wingood,[2] Igho Ofotokun,[3] Deborah Gustafson,[4] David Vance,[5] Anjali Sharma,[6] Adaora Adimora,[7] and Marcia Holstad,[1] *1. Emory University, Atlanta, Georgia, United States, 2. Columbia University Mailman School of Public Health, New York, New York, United States, 3. Emory University School of Medicine, Atlanta, Georgia, United States, 4. SUNY Downstate Medical Center, Brooklyn, New York, United States, 5. The University of Alabama at Birmingham, Birmingham, Alabama, United States, 6. Albert Einstein College of Medicine, Bronx, New York, United States, 7. University of North Carolina at Chapel Hill School of Medicine, Chapel Hill, North Carolina, United States*

Although older women living with HIV (OWLH) face challenges related to the intersection of HIV and aging, our published research found significant prevalence of self-rated successful aging (SRSA) in a sample of OWLH enrolled in the Women's Interagency HIV Study (WIHS). Studies in other populations link SRSA to positive psychosocial factors but little is known about SRSA mechanisms among OWLH. The purpose of this study is to test a conceptual psychosocial model of SRSA. Our sample (N=356) included OWLH enrolled in WIHS who participated in the "From Surviving to Thriving" (FROST) substudy and completed psychosocial and cognitive assessments: average age 56.5 years, 73% Black, 55% with annual income ≤ $12,000, 74% having 3 or more comorbidities, median CD4=673 cells/ml (Q1=486; Q3=880). SRSA was assessed using a research-based 10-point scale (higher scores=better outcomes). We conducted adjusted structural equation modeling. The global model included two latent variables -- protective attributes (composite of positive psychosocial factors: resilience, personal mastery, optimism, spirituality) and negative affect (composite of negative psychosocial factors: anxiety, depression, loneliness, internalized HIV-related stigma). The model showed good fit ($\chi2(65)=72.3$, $p=0.25$; RMSE=0.02; CFI=0.99) and explained 21% of variance in SRSA. Increased protective attributes were associated with improved SRSA both directly ($p<0.01$) and indirectly, via improved coping with stress ($p<0.001$). While negative affect did not have a direct effect on SRSA, it was indirectly associated with worsened SRSA via diminished protective attributes ($p<0.001$). Findings suggest the need for interventions enhancing positive and mitigating negative psychosocial factors to promote SRSA among OWLH.

### THE DEVELOPMENT OF A SCALE TO MEASURE HIV-PREVENTION FACTORS IN ADULTS AGE 50 AND OLDER

Rachel Wion,[1] Susan Loeb,[2] Jacqueline Mogle,[2] and Donna Fick,[3] *1. Indiana University, Indianapolis, Indiana, United States, 2. Pennsylvania State University, University Park, Pennsylvania, United States, 3. Penn State, University Park, Pennsylvania, United States*

Adults aged 50 and older are at risk for human immunodeficiency virus (HIV) infection. Currently, there are no measures specifically aimed at middle-aged and older adults to assess their HIV risk. Existing measures have been created for and mostly tested in adolescent and young adult populations. The purpose of this study was to modify and test existing instruments related to HIV prevention factors with an older adult population. Two rounds of an expert panel (N = 10) review were conducted to assess items from the Condom Use Self-Efficacy Scale and the Sexual Risks Scale for their applicability to older adults. Any items with content validity at the item level <0.78 were either discarded or modified. New items were also added. The final adapted HIV prevention scale had 31 items and was administered via an online survey. Single adults (N = 252) aged 50 to 85 who had been on at least one date over the past year participated in the study. The HIV prevention scale underwent confirmatory factor analysis. Model fit was estimated using maximum likelihood and standardized estimates were used for factor loadings. The items loaded on eight factors in three models: Model 1 (Mechanics, Advocacy, Intoxicants); Model 2 (Attitudes, Normative Beliefs, Perceived Susceptibility); and Model 3 (Intention, Expectations). There was adequate to excellent model fit. However, there were multiple correlations of error variances suggesting that while the items are appropriate for an older adult population, the scale will need adaptations prior to using for further data collection.

### THE SENIOR SEX EDUCATION EXPERIENCE STUDY: QUALITATIVE DATA FROM DEVELOPING AN ADULT SEX EDUCATION PROGRAM

Christina Pierpaoli-Parker, *University of Alabama at Birmingham, Vestavia Hills, Alabama, United States*

Older adults (OAs) account for an unprecedented proportion of STDs but receive limited sex education. We present preliminary data from the SEXEE study developing a sex education intervention for OAs 50+. The sample consisted of 17 OAs, ages 53 to 77, (M = 65, SD = 7.6; 64.7% female; 94.1% Caucasian) and seven primary care physicians, geriatricians, and other specialists (e.g. gynecologists), sampled purposively. Physicians completed a semi-structured interview to describe their experiences discussing sexual health with OAs, identify barriers to those discussions, and elicit recommendations for an educational curriculum. OAs participated in three separate focus groups to determine their interest in and suggestions for the intervention. Qualitative data underwent thematic coding separately by two researchers, with a third researcher resolving any discrepancy. One physician (14%) reported routinely assessing adults' sexual health; others only in the context of a specific presenting concern (e.g. ED). Though the physicians considered sexuality important component of QoL, many reported barriers to assessment and treatment, including insufficient time, training, and knowledge; concerns about personal and patient discomfort; and patient complexity. Of the OAs interviewed, 15 (88%) endorsed high interest in attending a sex education program. The most commonly recommended educational topics among physicians and OAs included: sexual changes with aging and management strategies; STDs and risk factors; tools for enlarging the sexual repertoire, myths about late life-sexuality; masturbation, and dating. Findings extend previous observations about clinical barriers to sexual health discussions and provide new insights for developing a sex ed intervention for this population.

### SESSION 3005 (PAPER)

### COGNITIVE FUNCTIONING

### FAMILY CAREGIVER MENTAL HEALTH: LINKING FAMILY CARE REGIME, INTERSECTIONALITY, AND STRESS PROCESS FRAMEWORKS

Sean Browning, and Margaret Penning, *University of Victoria, Victoria, British Columbia, Canada*

Although the implications of family care regime, social location, and stress process factors for the mental health of family caregivers have been well-documented individually, there is a lack of research that integrates these factors. Yet, linking family care regime and intersectionality approaches to stress process theorizing provides us with one possible explanation of the mechanisms potentially linking family care regime and intersecting structural inequalities to mental health outcomes. This paper draws on pooled data from the 2012 and 2016 European Quality of Life Surveys (EQLS - N=6,007) to assess direct and indirect associations between family care regime and the self-reported mental health (SRMH) of family caregivers, together with the additive and interactive associations involving social location (gender, age, socio-economic status, and marital status), and stress process factors (stressors and resources). The results of a series of weighted least squares regression analyses reveal that family care regime has a direct association with SRMH and that social location and stress process factors partially mediate this association. Additionally, the results suggest that additive and interactive social location factors have direct associations with SRMH and that stress process factors also partially mediate the association. Lastly, stress process factors are associated with SRMH as expected. Overall, our findings provide initial support for the value of linking family care regime, intersectionality and stress process frameworks for an understanding of the mental health implications of family caregiving.

### INITIAL RESULTS FROM AN EVERYDAY MEMORY AND METACOGNITIVE INTERVENTION FOR OLDER ADULTS

Ann Pearman, Christopher Hertzog, Emily Lustig, and MacKenzie Hughes, *Georgia Institute of Technology, Atlanta, Georgia, United States*

We report results from a new intervention study implementing an Everyday Memory and Metacognitive Intervention (EMMI). This intervention trains older adults on self-regulatory procedures for achieving everyday life goals by implementing a metacognitive perspective where participants learn mindful control over life tasks that place demands on planning and memory (e.g., learning new names and managing prospective memory demands). Fifty-three participants, age range 65 to 83, were assigned to either the EMMI treatment group (n = 32, mean age = 70.13, SD = 3.2) or a waitlist control group (n = 21, mean age = 71.76, SD = 4.7). Individuals with probable memory impairments, as indexed by low MOCA scores, were excluded from the study. Outcomes included daily diary reports of everyday memory errors and a prospective memory telephone task. EMMI participants had fewer reported memory errors per day (M = 0.42) than controls (M = 0.64), one-tailed p = .03. EMMI participants also performed better than controls on the telephone task outcome variables: total number of phone calls completed and mean absolute deviation of call times from scheduled times for successfully completed calls (ps<.001). Subjective outcomes, including personal memory beliefs, life satisfaction, and perceived stress, showed greater pretest-posttest improvement in the EMMI group compared to the control group. This study is a successful initial demonstration of the efficacy of our intervention for improving everyday cognition in older adults and highlights the possibility of improving success in memory-demanding everyday life contexts, thereby contributing to resilient aging in an older population.

### PATHWAY ANALYSIS OF LEISURE ACTIVITY AND COGNITIVE FUNCTION IN THE LONG LIFE FAMILY STUDY

Nicole Roth,[1] Paola Sebastiani,[2] Stephanie Cosentino,[3] Nicole Schupf,[3] Thomas Perls,[1] and Stacy Andersen,[1]
*1. Boston University School of Medicine, Boston, Massachusetts, United States, 2. Boston University, Boston, Massachusetts, United States, 3. Columbia University Medical Center, New York, New York, United States*

Familial longevity and greater involvement in activities purported to build cognitive reserve (e.g. education, cognitively stimulating leisure activity) have both been associated with better cognitive function in later life, yet little is known about how these protective factors relate with one another. In this work, we modeled the associations among familial longevity, proxies of cognitive reserve, and cognitive function in the Long Life Family Study (LLFS). We assessed cognitive function using a comprehensive battery of neuropsychological tests (i.e. Digit-Spans, California Verbal Learning Test, Rey-Osterrieth Complex Figure, phonemic fluency, category fluency, Word Generation, DKEFS Sorting Test, and logical memory) in a subset of LLFS family members and a referent cohort (N=314, mean age 75.7±14.6 years). To model these associations, we used a series of Bayesian hierarchical regression pathways that incorporate a random effect for family relatedness, adjusted by age and sex. All continuous variables were rescaled and bounded to be approximately between (0,1) in order to standardize regression coefficients and to allow for an asymmetrical beta-distribution. Controlling for education level, age, and sex, referents had greater engagement in late-life cognitive activities compared to LLFS family members, β=0.38 (95% CI: 0.18 to 0.57). In turn, those with higher markers of cognitive reserve exhibited better neuropsychological performance. Despite LLFS family members having lower participation in cognitively stimulating leisure activities, there were no differences between LLFS family members and referents on cognitive test performance. These results suggest long-lived family members may have more unique pathways (i.e. genetic/environmental) that preserve cognition later in life.

### THALAMIC VOLUME MEDIATES ASSOCIATIONS BETWEEN CARDIORESPIRATORY FITNESS AND MEMORY IN PEOPLE WITH PARKINSON'S

Andrew Petkus,[1] Megan Gomez,[2] Dawn Schiehser,[3] Vincent Filoteo,[3] Jennifer Hui,[4] Behnaz Jarrahi,[5] Sarah McEwen,[6] and Giselle Petzinger,[4] *1. USC Keck School of Medicine, Los Angeles, California, United States, 2. VA Long Beach Healthcare System, Long Beach, California, United States, 3. VA San Diego Healthcare System, San Diego, California, United States, 4. University of Southern California, LOS ANGELES, California, United States, 5. Stanford University, Stanford, California, United States, 6. Pacific Neuroscience Institute, Santa Monica, California, United States*

Cognitive deficits occur in patients with Parkinson's disease (PD), and cardiorespiratory fitness (CRF) is associated with both current and future cognitive decline in this disease. The underlying neurobiological factors explaining this relationship, however, are not well known. In this cross-sectional study we examined the associations between CRF and cognitive performance and whether such associations were mediated by grey matter volumes of basal ganglia structures. A total of 33 individuals with PD underwent structural magnetic resonance imaging (sMRI), CRF evaluation (VO2max), and neuropsychological assessment. Composite scores of episodic memory, executive functioning, attention, language, and visuospatial functioning were generated. Brain MRI morphological measurements was performed with the Freesurfer image analysis suite. Structural equation models were constructed to examine whether sMRI volume estimates of basal ganglia structures, specifically the thalamus and pallidum, mediated associations between VO2 max and cognitive performance while adjusting for age, education, PD disease duration, sex, and intracranial volume. Higher VO2max was associated with better episodic memory (Standardized β=0.390; p=0.009), executive functioning (Standardized β=0.263; p=0.021), and visuospatial performance (β=0.408; p=0.004). Higher VO2max was associated with larger thalamic (Standardized β=0.602; p<0.001) and pallidum (Standardized β=0.539; p<0.001) volumes. Thalamic volume significantly mediated the association between higher VO2max and better episodic memory (indirect effect=0.209) and visuospatial ability (indirect effect=0.178) performance (p<.05). The pallidum did not significantly mediate associations between VO2 max and cognitive outcomes. These results suggest the thalamus plays an important role in the association between CRF episodic memory and visuospatial functioning in individuals with PD.

### SESSION 3006 (PAPER)

### COGNITION AND EMOTIONS

### AGE AND DAILY POSITIVE EVENTS MODERATE THE LINK BETWEEN DEPRESSIVE SYMPTOMS AND INFLAMMATION

Jin Wen,[1] David Almeida,[2] and Nancy Sin,[1], *1. University of British Columbia, Vancouver, British Columbia, Canada, 2. Pennsylvania State University, University Park, Pennsylvania, United States*

Inflammation is a pathway underlying numerous aging-related conditions. Depression is related to elevated inflammation, whereas daily positive events have been linked to lower inflammation; these psychological experiences may interact with age to predict inflammation. The purpose of this study was to examine whether daily positive events moderate the association between depressive symptoms and inflammation in an adult lifespan sample. A sample of 343 adults ages 25-75 (55% women, 83% white) in the Midlife in the United States Refresher Study completed daily diary interviews for 8 evenings about their daily positive events. Depressive symptoms were assessed with the 20-item Center for Epidemiological Studies Depression scale, and blood samples were assayed for inflammatory markers interleukin-6 (IL-6) and C-reactive protein (CRP). On average, depression scores ranged from 0 to 44 (mean = 9.31, SD = 7.80), and participants reported 1.25 (SD = .70) positive events per day (range = 0–5). Depressive symptoms and daily positive events were separately associated with higher and lower log IL-6 and CRP, respectively. Depressive symptoms, daily positive events, and age interacted such that daily positive events predicted lower IL-6 (but not CRP) among midlife and older adults who reported lower depressive symptoms, whereas positive events were not related to inflammation among younger adults. Thus, these findings suggest that the protective association between daily positive events and inflammation was blunted when depressive symptoms were elevated and for younger adults. This work has implications for understanding age variations in the role of positive experiences in depression and inflammation.

### AGE ASSOCIATED WITH PREFERENCE FOR MORE AND EMOTIONALLY MEANINGFUL INFORMATION IN TIME-USE DECISIONS

Yochai Shavit, and Laura Carstensen, *Stanford University, Stanford, California, United States*

Socioemotional selectivity theory posits that goals change as a function of perceived time-horizons. As people age and time-horizons grow increasingly limited, people prioritize goals realized in the present over goals that will be realized in the distant future. Because goals that are emotionally meaningful are realized in their pursuit SST predicts that such goals will be prioritized by older people. Previous investigators have observed that age is negatively associated with search for information in decision making and attributed the finding to an effort to reduce cognitive load. In the present study, we hypothesized that because time becomes increasingly valued as it grows scarce, decisions about time use will show different patterns. We further hypothesized that age is associated with preference for information about present-oriented, namely emotional, benefits of activities over future-oriented benefits. 262 participants, aged 18-93 years, were presented with a hypothetical scenario inviting them to participate in one of four activities. On a grid containing 12 cells, participants were given the option to review information about activities' emotionally meaningful and future-oriented benefits. Participants could review as many cells as they wished before indicating their interest in the activity. Across all activities, age was associated with preference for reviewing emotionally meaningful information. Moreover, in this scenario age was associated with reviewing more not less information. Age-related preferences for more information was apparent despite a negative age association with recall-memory, suggesting that cognitive load does not deter information search when decisions align with socioemotional goals.

### AGE DIFFERENCES IN RESPONSE TO FRAMED SIDE EFFECTS INFORMATION ABOUT HYPOTHETICAL MEDICATIONS

Alyssa Minton, Madeline Nievera, Nathaniel Young, and Joseph Mikels, *DePaul University, Chicago, Illinois, United States*

Framing equivalent information as a gain (e.g., 90% survival rate) or a loss (e.g., 10% mortality rate) can differentially impact judgments and decision making, such that people make more favorable judgements when information is presented as a positive gain versus a negative loss. The current study investigated how framing and age influences evaluative judgments of hypothetical medications used to treat common health issues when the equivalent probability of experiencing a particular side effect was presented as a gain (e.g., "86% of people who took this medication did not experience rash outbreaks") or a loss (e.g., "14% of people who took this medication did experience rash outbreaks"). Younger and older adults were presented with health pamphlets for hypothetical medications with three unique side effects for each and indicated the medication's perceived riskiness, how positively and negatively they felt about the medication, and their likelihood to take the medication. Numeracy, risk-taking behavior, and current affective state were also measured. When information was presented in a loss frame, people reported more negative feelings about the medication, leading to greater perceived riskiness, IE = -.785, SE = .13, $p < .001$. Age indirectly influenced likelihood via the positive feelings about the medication, IE = .349, SE = .14, $p = .013$. Younger adults felt more positively about the medications than older adults, leading to an increased willingness to take the medication. These findings provide insight into how framing and age can differentially influence evaluative judgments, perceived risk, and willingness to take medication.

### IMPACT OF INSTRUCTIONS ON THE POSITIVITY EFFECT IN YOUNGER AND OLDER ADULTS' AUTOBIOGRAPHICAL MEMORIES

Karen Siedlecki, Jillian Minahan, and Neshat Yazdani, *Fordham University, Bronx, New York, United States*

The positivity effect (PE) refers to age-related enhancement in memory for positive information or decreased memory for negative information. The PE is thought to be the result of age-associated shifts in motivational goals towards enhancing well-being. Research has demonstrated the PE in laboratory settings, and a meta-analysis showed that the PE was most robust in conditions with fewer instructions/constraints (Reed et al., 2014). Findings related to the PE in autobiographical memory (AM) are mixed. Goals of the current study include examining 1) whether the PE was evident across four increasingly restrictive AM conditions (involuntary AMs, voluntary AM with no constraints, voluntary AM within a specific time frame, voluntary AM within a specific time frame and related to a specific cue word), and 2) whether available cognitive resources (assessed with the Stroop task and a trail-making test) influence the PE, since cognitive control is essential for achieving goals. Participants (N=126) completed the shortened Memory Experiences Questionnaire for each memory, which assesses several phenomenological characteristics, including memory valence. There were no significant differences in memory valence between the younger (n=69, ages 18-34) and older adult (n=57; ages 60-85) groups for involuntary AMs or the less constrained voluntary AMs, but there was a significant difference for the most constrained AM wherein older adults rated their AMs as more positive than younger adults. Performance on cognitive control measures did not influence the AM valence. These results highlight the importance of considering the effects of type of stimuli and instructions when studying the PE.

### THE ROLE OF ATTENTIONAL CONTROL IN ATTENTION TO EMOTIONAL STIMULI AMONG MIDDLE-AGED AND OLDER ADULTS

Yuen Wan Ho, and Derek Isaacowitz, *Northeastern University, Boston, Massachusetts, United States*

Prior studies have examined age differences in attention to emotional stimuli; in the current study, we considered how this might relate to dispositional measures of attentional control across age groups. Participants were 116 middle-aged (aged 35 – 64 years) and 39 older (aged 65-86 years) adults in the United States. In the study, participants filled in the Emotional Attentional Control Scale. Then participants watched fearful, happy, neutral, and disgusting videos. The gaze time for each video was measured by an eye tracker. Results did not show significant age difference in attention to happy and neutral videos. However, middle-aged adults gazed relatively more to the disgusting video and relatively less to the fearful video, t (115) =2.16, p =.03. The opposite pattern was found among older adults, t (38) =5.85, p<.001. Self-reported emotional attentional control was not significantly related to attention in either age group. These findings suggest that different stimuli may yield age differences in fixation that are a less consistent pattern with the age-related positivity effect reported in previous studies, and also that self-reported emotional attentional control may not relate to gaze patterns.

### SESSION 3007 (PAPER)

### COGNITIVE AND BRAIN FUNCTION AMONG DIVERSE RACIAL AND ETHNIC GROUPS

### CUMULATIVE ADVERSITY AND DEPRESSIVE SYMPTOMS: OPTIMISM AS A MODERATOR

Gabriella Dong, and Mengting Li, *Rutgers University, New Brunswick, New Jersey, United States*

Older immigrants encountered a wide range of traumatic events across the life span, before and after immigration, in the origin and host countries. Prior studies have shown that traumatic events were associated with depression, but less is known about what are the resilience factors against depression related to traumatic events. Optimism is one of resilience assets reflecting the extent to which people hold generalized favorable expectancies for their future. This study aims to examine whether optimism could moderate the negative impact of exposure to natural disasters, traumatic personal events, and historical events on depressive symptoms. The data were drawn from the Population Study of Chinese Elderly in Chicago (PINE) in 2017-2019, with a sample size of 3,125. Traumatic life events were evaluated by natural disasters (typhoon, earthquake, and tornado), personal events (e.g. death of a loved one, physical assault) and historical events (e.g. Japanese invasion of China). Depressive symptoms were measured by PHQ-9. Optimism was assessed by Revised Life Orientation test. Linear regression with interaction terms was used. Older adults with one additional exposure to natural disaster (b=0.34, SE=0.07, p <.001), personal event (b=0.32, SE=0.05, p <.001), and historical event (b=0.14, SE=0.04, p <.001) were associated with higher depressive symptoms. Optimism could buffer the negative impacts of natural disasters (b=-0.03, SE=0.01, p <.05), personal events (b=-0.05, SE=0.01, p <.001), and historical events (b=-0.02, SE=0.01, p <.01) on depressive symptoms. Psychological interventions are suggested to increase optimism of older adults with exposure to lifetime traumatic events to reduce their depressive symptoms.

### HOW ADOLESCENT SPORTS PARTICIPATION, FAMILY, AND SCHOOL RESOURCES IMPACT LATER-LIFE PHYSICAL ACTIVITY LEVELS

Thalida Arpawong,[1] Margaret Gatz,[1] Tara Gruenewald,[2] Catalina Zavala,[1] Dominika Seblova,[3] Jennifer Manly,[4] Susan Lapham,[5] and Carol Prescott[1] *1. University of Southern California, Los Angeles, California, United States, 2. Chapman University, Orange, California, United States, 3. Columbia University Medical Center, New York, New York, United States, 4. Columbia University, New York, New York, United States, 5. American Institutes for Research, Washington, District of Columbia, United States*

Engaging in physical activity (PA) in adulthood has multiple protective health effects in later ages. However, unknown are the extent to which PA habits are laid down earlier in life and persist into adulthood, and the extent to which greater opportunities for PA during adolescence stem from differences in socioeconomic status (SES) which then affect opportunities for PA. We investigated potential mechanisms underlying these relationships using the longitudinal Project Talent Twin and Sibling Study (assessments in 1960 and 2014). With this design, we are able to hold genetics constant by modeling relationships between monozygotic twins (who share 100% of their genes) and therefore isolate effects of contextual factors on later-life PA (mean age ~70). We found that higher family SES (ß=.39, p<.0001) and sports participation in adolescence (ß=.05, p<.0001) predicted PA 55 years later, adjusted for gender and physical limitations. This held true when partialling out genetic variation that could otherwise explain these relationships. More education also predicted later-life PA (ß=.12, p<.0001) separately from family SES and partially mediated the effect of family SES on PA. While school-level resources (e.g., availability of sports and recreation opportunities) did not predict later life PA, they did associate with adolescent sports participation (ß=.26, p=0.007). Overall, later-life physical activity was influenced by earlier life sports participation and education, with family rearing resources being more important than high school resources. As twin pair correlations suggest gender differences, future research will examine whether family or school resources differentially benefit males or females for later-life physical activity.

### RACIAL DISPARITIES IN COGNITIVE FUNCTION: THE ROLES OF CUMULATIVE STRESS EXPOSURES ACROSS THE LIFE COURSE

Ruijia Chen,[1] Jennifer Weuve,[2] Laura Kubzansky,[3] and David Williams,[3] *1. Harvard University, Cambridge, Massachusetts, United States, 2. Boston University, Boston, Massachusetts, United States, 3. Harvard T.H. Chan School of Public Health, Boston, Massachusetts, United States*

Introduction: Racial disparities in cognitive function have been well-documented in the literature, but factors driving the disparities remain under explored. This study aims to quantify the extent to which cumulative stress exposures across the life course explain Black–White disparities in executive function and episodic memory. Method: Data were drawn from the 2004–2006 wave of the Midlife Development in the United States (MIDUS) and the MIDUS refresher study (N=5,967, 5,277 White, 690 Black). Cumulative stress exposures were assessed by using 10 domains of stressors (e.g., financial stress, childhood adversity). Cognitive function was assessed using the Brief Test of Adult Cognition by Telephone. Marginal structural models were conducted to quantify the proportion of the effect of race/ethnicity status on cognitive function that can be explained by cumulative stress exposures. Result: Blacks reported higher levels of cumulative stress exposures and lower average levels of executive function and episodic memory than Whites. Cumulative stress exposures explained 8.43% of the disparities in executive function and 13.21 % of the disparities in episodic memory. Cumulative stress exposures had stronger effects on racial disparities in cognitive function in the older age group (age≥ 55 years old) than in the younger age group (age < 55 years old). Conclusion: Cumulative stress exposures explain modest proportions of racial disparities in levels of cognitive function. Interventions that focus on reducing stress exposures or improving coping resources among Blacks may help lessen racial disparities in cognitive function at the population level.

### SOCIAL ISOLATION AND COGNITIVE FUNCTION: EVIDENCE FROM OLDER CHINESE AMERICANS

Dexia Kong,[1] Ying-Yu Chao,[1] Fengyan Tang,[2] and XinQi Dong,[1] *1. Rutgers University, New Brunswick, New Jersey, United States, 2. University of Pittsburgh, Pittsburgh, Pennsylvania, United States*

Chinese older adults are particularly vulnerable to social isolation due to various barriers they face in developing/maintaining social networks (i.e. limited English proficiency and transportation barriers) in the U.S. However, the prevalence of social isolation and its potential health consequences in this rapidly growing minority aging population remain poorly understood. To address this knowledge gap, the current study examines the prevalence of social isolation, and the relationship between social isolation and cognitive function among U.S. Chinese older adults. Data were obtained from the Population-based Study of Chinese Elderly in Chicago collected between 2011 and 2013 (N=3,157). A four-item index (including living alone, not married, lack of confidant, and low participation in social activities) was constructed to assess social isolation (range: 0 to 4, a score of ≥2 was used to identify individuals who were most isolated). Cognitive function was measured by five validated instruments (range: -2.8 to 2.0). Nearly 22% of the sample were socially isolated. Multivariable linear regression analysis showed that social isolation accounted for 44% of variance in global cognitive functioning. Chinese older adults with greater levels of social isolation had poorer overall cognitive function (B= -0.05, SE=0.01, p=0.001). Study findings highlight the importance of addressing social isolation in cognitive aging among older Chinese Americans. Culturally tailored interventions facilitating the development of supportive social networks/support have the potential to mitigate cognitive decline in this population. Future longitudinal studies need to elucidate potential mechanisms underlying the relationship between social isolation and cognitive function. Practice implications will be discussed.

### THE EFFECTS OF THE BAILAMOS DANCE PROGRAM ON BRAIN FUNCTIONAL CONNECTIVITY OF OLDER LATINOS: AN EXPLORATORY STUDY

Guilherme Moraes Balbim,[1] Olusola Ajilore,[1] Melissa Lamar,[2] Kirk Erickson,[3] Susan Aguiñaga,[4] Eduardo Bustamante,[1] and David Marquez,[1] *1. University of Illinois at Chicago, Chicago, Illinois, United States, 2. Rush University Medical Center, Chicago, Illinois, United States, 3. University of Pittsburgh, Pittsburgh, Pennsylvania, United States, 4. University of Illinois at Urbana-Champaign, Urbana, Illinois, United States*

Compared to non-Latinos whites, older Latinos are at higher risk of cognitive impairment and engage in less leisure-time physical activity (PA). Resting-state brain functional connectivity (FC) is a putative biomarker for age-related cognitive decline. PA plays a role in FC of brain networks associated with cognitive decline. Objective: Investigate the effects of the BAILAMOS™ dance program on FC in three brain networks associated with age-related cognitive decline (Default Mode [DMN], Frontoparietal [FPN], and Salience [SAL] networks). Methods: Single-group pre-post design. Ten cognitively intact older Latinos participated in the four-month (2x/week for 60min) BAILAMOS™ dance program with four Latin dance styles. MRI was obtained pre- and post-intervention. FC was analyzed using the resting-state fMRI toolbox (CONN) via pairwise BOLD signal correlations and then converted into z-scores. We performed dependent t-tests, computed Cohen's d and 95%CI

for $p < 0.05$. Results: Within-FPN FC significantly increased ($t(9) = 2.35$, $p = 0.043$, $d = 0.70$) from pre (M=0.49±0.15) to post-intervention (M=0.59±0.13). In the DMN, we observed moderate effect size changes in the ratio of the FC between-networks by the FC within-networks (Mdiff = 0.10; 95%CI = -0.01; 0.21, $p = 0.08$, $d = 0.64$). Conclusions: The BAILAMOS™ program increased within-FPN FC, which is a cognitive-control network related to adaptive control and flexibility. Moderate changes between- vs. within-DMN FC suggest BAILAMOS™ also increased whole-brain DMN integration. Taken together, results might signal that Latin dance can combat the disruption of FC between the DMN and other networks, and within-FPN, which are associated with cognitive decline.

### SESSION 3008 (PAPER)

### RACE, SOCIETY, AND HEALTH OUTCOMES

### CHANGING EDUCATIONAL GRADIENTS IN LIFE EXPECTANCY WITH AND WITHOUT DISEASE AMONG U.S. OLDER ADULTS FROM 2000 TO 2010

Matthew Farina,[1] Phillip Cantu,[2] and Mark Hayward[1]
*1. University of Texas at Austin, Austin, Texas, United States, 2. The University of Texas Medical Branch at Galveston, Galveston, Texas, United States*

Recent research has documented increasing education inequality in life expectancy among U.S. adults; however, much is unknown about other health status changes. The objective of study is to assess how healthy and unhealthy life expectancies, as classified by common chronic diseases, has changed for older adults across education groups. Data come from the Health and Retirement Study and National Vital Statistics. We created prevalence-based life tables using the Sullivan method to assess sex-specific life expectancies for stroke, heart disease, cancer, and arthritis by education group. In general, unhealthy life expectancy increased with each condition across education groups. However, the increases in unhealthy life expectancy varied greatly. While stroke increased by half a year across education groups, life expectancy with diabetes increased by 3 to 4 years. In contrast, the evidence for healthy life expectancy provides mixed results. Across chronic diseases, healthy life expectancy decreased by 1 to 3 years for respondents without a 4-year degree. Conversely, healthy life expectancy increased for the college educated by .5 to 3 years. While previous research shows increases in life expectancy for the most educated, trends in life expectancy with chronic conditions is less positive: not all additional years are in lived in good health. In addition to documenting life expectancy changes across education groups, research assessing health of older adults should consider the changing inequality across a variety of health conditions, which will have broad implications for population aging and policy intervention.

### EXPLORING ACCULTURATION EXPERIENCES OF KOREAN IMMIGRANT OLDER ADULTS THROUGH EXPRESSIVE WRITING

Stephanie Rhee, *University of Wisconsin-Green Bay, Green Bay, Wisconsin, United States*

Korean immigrant older adults residing in areas without well-established Korean ethnic enclaves experience acculturative stress and depressive symptoms due to their lingual and cultural barriers. Expressive writing can be used as a culturally sensitive intervention to help those immigrants disclose their deepest thoughts and feelings related to their immigration and acculturation experiences. This study gathered qualitative data from the author's experimental study using expressive writing for first-generation Korean immigrant adults 60 to 88 years of age residing in Midwestern cities. Participants were instructed to write for 15-20 minutes per day in three consecutive days at their convenience in a comfortable and private setting and asked to return their writings by mail. A total of 22 participants returned their writings: 14 wrote about their past and current stressful experiences related to their immigration and acculturation, while eight wrote about their daily lives. Eight themes emerged from thematic coding processes guided by the grounded theory approach: (1) survival, resilience, hardiness, tenacity, pride; (2) lingual barriers; (3) religious faith; (4) gender difference in roles and values; (5) racial discrimination; (6) traditional strategies of acculturation; (7) family and intergenerational gap; and (8) aging. The themes illustrate the participants' lifelong efforts to shape their unique voices through heart-wrenching struggles, haunting wounds, and ethnic pride and resilience. The study findings suggest that culturally relevant programs and services are needed to facilitate social relationships and reduce lingual and cultural barriers for Korean immigrant older adults residing in non-ethnic enclaves.

### HIGH SCHOOL QUALITY AND 56-YEAR ALL-CAUSE MORTALITY RISK ACROSS RACE AND ETHNICITY

Dominika Seblova,[1] Kelly Peters,[2] Susan Lapham,[2] Laura Zahodne,[3] Tara Gruenewald,[4] Maria Glymour,[5] Benjamin Chapman,[6] and Jennifer Manly,[7] *1. Columbia University Medical Center, New York, New York, United States, 2. American Institutes for Research, Washington, District of Columbia, United States, 3. University of Michigan, Ann Arbor, Michigan, United States, 4. Chapman University, Orange, California, United States, 5. University of California, San Francisco, California, United States, 6. University of Rochester Medical Center, Rochester, New York, United States, 7. Columbia University, New York, New York, United States*

Having more years of education is independently associated with lower mortality, but it is unclear whether other attributes of schooling matter. We examined the association of high school quality and all-cause mortality across race/ethnicity. In 1960, about 5% of US high schools participated in Project Talent (PT), which collected information about students and their schools. Over 21,000 PT respondents were followed for mortality into their eighth decade of life using the National Death Index. A school quality factor, capturing term length, class size, and teacher qualifications, was used as the main predictor. First, we estimated overall and sex-stratified Cox proportional hazards models with standard errors clustered at the school level, adjusting for age, sex, composite measure of parental socioeconomic status, and 1960 cognitive ability. Second, we added an interaction between school quality and race/ethnicity.

Among this diverse cohort (60% non-Hispanic Whites, 23% non-Hispanic Blacks, 7% Hispanics, 10% classified as another race/s) there were 3,476 deaths (16.5%). School quality was highest for Hispanic respondents and lowest for non-Hispanic Blacks. Non-Hispanic Blacks also had the highest mortality risk. In the whole sample, school quality was not associated with mortality risk. However, higher school quality was associated with lower mortality among those classified as another race/s (HR 0.75, 95% CI: 0.56-0.99). For non-Hispanic Blacks and Whites, the HR point estimates were unreliable, but suggest that higher school quality is associated with increased mortality. Future work will disentangle these differences in association of school quality across race/ethnicity and examine cause-specific mortality.

### THE HEALTH AND WELL-BEING OF AFRICAN-AMERICAN OLDER ADULTS WITH A HISTORY OF INCARCERATION

Rodlescia Sneed, *Michigan State University, Flint, Michigan, United States*

African-Americans are overrepresented in the criminal justice system. Longer prison stays and release programs for older prisoners may result in an increased number of community-dwelling older adults with a history of incarceration. In recent years, there has been a substantial increase in research on health-related outcomes for currently incarcerated older adults; however, there has been little inquiry into outcomes for formerly incarcerated African-American older adults following community re-entry. In this study, we used secondary data from the Health and Retirement Study to describe employment, economic, and health-related outcomes in this population. Twelve percent of the 2238 African-Americans in our sample had been previously incarcerated. Those who had been previously incarcerated had higher rates of lung disease, arthritis, back problems, mobility problems, and mental health issues than their counterparts. They also had higher rates of hospitalization and lower use of dental health services. Further, while they did not experience lower employment rates than those with no criminal history, those who had been incarcerated had more physically demanding jobs and reported greater economic strain. Given the disproportionate incarceration rates among African-Americans, the aging of the prison population, and the increase in community re-entry for older prisoners, research that explores factors that impact the health and well-being of formerly incarcerated individuals has broad impact. Future work should focus on addressing the needs of this vulnerable population of African-American older adults.

### SESSION 3009 (PAPER)

### GENDER- AND SEX-CENTERED TOPICS IN AGING

### CAN WOMEN HAVE IT ALL: MARRIAGE, FAMILY, CAREER, AND GOOD HEALTH?

Julene Cooney, *Syracuse University, Manlius, New York, United States*

Women in the U.S. have been earning more education than their male peers since the mid-1990's. High power career positions that have historically been dominated by men are increasingly being filled by women. At the same time, since 2000, the U.S. workforce participation rate for women aged 25 – 55 has leveled off and begun to fall. Called the "mommy-wars" in the media, a heated debate has ensued between those women who feel that the role of mother should preempt seemingly selfish desires to engage in career work, while others firmly believe that it is possible to have it all; work, marriage and family. The purpose of this research project is to determine the health implications of each lifestyle for women later in life. Using the Health and Retirement Study, I compare self-rated health trajectories for the subset of women who are married, have children, and have attended college – those who are most likely to have the option to "opt-out" of the workforce for a stay-at-home mother lifestyle. Controlling for income and childhood health, and using growth curves and multivariate regression, preliminary results indicate that educated, married women who work full-time and raise children are the healthiest after age 50, while educated, married women who stay home with their children and do not work are the least healthy. In addition, these health trajectories have changed little since the 1950's, even as cultural expectations for women have shifted.

### CARDIOVASCULAR HEALTH AT THE INTERSECTION OF RACE AND GENDER: LIFE-COURSE PROCESSES TO REDUCE HEALTH DISPARITIES

Chioun Lee,[1] Soojin Park,[1] and Jennifer Boylan,[2]
*1. University of California, Riverside, Riverside, California, United States, 2. University of Colorado Denver, Denver, Colorado, United States*

Objective: Higher cardiovascular health (CVH) scores are significantly associated with reductions in aging-related disease and mortality but racial minorities exhibit poor CVH. We examine the degree to which (a) disparities in CVH exist at the intersection of race and gender and (b) CVH disparities would be reduced if marginalized groups had the same levels of resources and adversities as privileged groups. Methods: We used biomarker subsamples from the Midlife in the United States (MIDUS) core study and Refresher studies (N=1,948). Causal decomposition analysis was implemented to test hypothetical interventions to equalize the distribution of early-life adversities (ELAs), perceived discrimination, or adult SES between marginalized and privileged groups. We conducted sensitivity analyses to determine to what degree unmeasured confounders would invalidate our findings. Results: White women have the highest CVH score, followed by White men, Black men, and Black women. Intervening on ELAs reduces the disparities: White men vs. Black women (30% of reduction) and White women vs. Black women (15%). Intervening on adult SES provides large disparity reductions: White men vs. Black men (79%), White men vs. Black women (70%), White women vs. Black men (25%), and White women vs. Black women (32%). Among these combinations, interventions on ELAs and adult SES are robust to unmeasured confounders. However, intervening on discrimination makes little change in initial disparities. Discussion: Economic security in midlife for Blacks helps reduce racial disparities in cardiovascular health. Preventing exposure to ELAs among Black women may reduce their vulnerability to cardiovascular disease, compared to Whites.

### GENDER DIFFERENCES IN DISABILITY LIFE EXPECTANCY AMONG OLDER ADULTS IN COLOMBIA AGE 60 AND OLDER

Margarita Maria Osuna,[1] Matthew Farina,[2] and Jennifer Ailshire,[1] *1. University of Southern California, Los Angeles, California, United States, 2. University of Texas at Austin, Austin, Texas, United States,*

Life expectancy has increased rapidly in Colombia but it is unclear whether these added years of life are healthy years. The objective of this paper is to estimate gender differences in disability life expectancy for older adults. We use data from the first nationally representative survey of adults ages 60 and over in Colombia, the 2015 Survey of Aging, Health and Wellbeing (SABE) (N=23,694) to determine age- and gender-specific prevalence. Mortality data come from the Latin American Mortality Database. We created prevalence base life tables using the Sullivan´s method to calculate disability life expectancy. We define disability as having any reported difficulty with any of the following activities of daily living (ADL): eating, dressing, bathing, toileting, getting out of bed, and walking across a room. The age-adjusted prevalence of ADL disability was 8.7% for men and 12.4% for women. Life expectancy at age 60 for women was 20.6 years, of which 3.0 years (14%) were lived with disability. In contrast, men's life expectancy at age 60 was 18.2 years, of which 1.7 years (10%) were lived with disability. Women have lower mortality rates than men at older ages, but because they have higher disability prevalence, they live proportionately more years with disability relative to men. We also investigated patterns at different ages, and functional limitations, and found similar gender differences. This study provides new insights into how mortality and disability patterns at older ages contribute to the gender health disparity in older Colombians.

### SEX DIFFERENCES IN THE PREVALENCE OF PAIN TRAJECTORIES OVER A 14-YEAR PERIOD IN THE MEXICAN HEALTH AND AGING STUDY

Sadaf Milani, Martin Rodriguez, Rafael Samper Ternent, and Rebeca Wong, *The University of Texas Medical Branch at Galveston, Galveston, Texas, United States*

Pain increases with age, disproportionately affects females, and is a major contributor to decreased quality of life. Because pain is dynamic, trajectories are important to consider. However, few studies have looked at longitudinal trajectories of pain, by sex, using population data. We used data from the 2001, 2003, 2012, and 2015 waves of the Mexican Health and Aging Study, a nationally representative sample of Mexicans aged 50 and older. Individuals who had direct interviews at each time point and complete information on analytical variables were included (n=5,135). Pain reported at these time points was used to define pain trajectories as persistent pain, development of pain, diminishing pain, fluctuating pain, and never pain, by sex. Multinomial logistic regression was used to assess the association of sex with pain trajectories, adjusting for baseline demographic, behavioral, and health factors. Females had a higher prevalence of persistent pain (25.1% vs. 11.5%) while males had a higher prevalence of never pain (35.8% vs. 22.2%). Development of pain, diminishing pain, and fluctuating pain was similar between males and females (14.4% and 14.6%; 14.8% and 15.8%; 23.9% and 22.4%, respectively). Females had higher odds of persistent pain (OR: 2.13; 95%CI 1.67-2.70) and development of pain (OR: 1.40; 95%CI 1.11-1.75) compared to males. Overall, females had a much higher burden of persistent pain compared to males. Understanding trajectories of pain in later life and life course mechanisms for this large sex difference observed is crucial to improve quality of life.

### SESSION 3010 (PAPER)

### FAMILY CAREGIVING AMONG DIVERSE RACIAL AND ETHNIC POPULATIONS

### ATTITUDE TOWARD FAMILY INVOLVEMENT IN END-OF-LIFE CARE AMONG OLDER CHINESE AMERICANS

Kaipeng Wang,[1] Yanqin Liu,[2] Fei Sun,[3] Dexia Kong,[4] and Bei Wu,[5] *1. University of Denver, Denver, Colorado, United States, 2. Arizona State University, Tempe, Arizona, United States, 3. Michigan State University, East Lansing, Michigan, United States, 4. Rutgers University, New Brunswick, New Jersey, United States, 5. New York University, New York, New York, United States*

Family involvement is critical to end-of-life (EOL) care of older adults. Attitude toward family involvement in EOL care can be influenced by family relationship. Yet, mechanisms explaining such influence have not been examined among older Chinese Americans. This study aims to examine the association between family relationship and older Chinese Americans' attitude toward family involvement in EOL care and explore pathways of this association. Potential mediators include self-efficacy, perceived benefits, and perceived barriers of discussing EOL care with family members. Data were collected from 276 Chinese Americans aged 55+ in two metropolitan areas in 2018. Participants' average age was 74 years (SD=9.6). Approximately 64% of the sample were female. Most participants (57%) held positive attitudes toward family involvement in EOL care. Using the Structural Equation Modeling method, we found that family relationship had a significant positive total effect on positive attitude toward family involvement in EOL care (z=5.57, p<0.001). Indirect direct of family relationship on attitude toward family involvement in EOL care through both self-efficacy (z=3.13, p<0.01) and perceived barriers (z=2.30, p<0.05) of discussing EOL care with family members was significant. Results suggest that improving family relationship may increase elder's self-efficacy and reduce barriers of discussing EOL with family members, which is associated with more positive attitude toward family involvement in EOL care. Findings provide empirical evidence of how family relationship affects older Chinese Americans' attitude toward family involvement in EOL care and underline the need for family-centered interventions for older Chinese Americans.

### CO-DESIGNING AND EVALUATING A SELF-CARE CURRICULUM OF AN APP FOR CHINESE IMMIGRANT CAREGIVERS

Ying Wang,[1] Mandong Liu,[2] and Iris Chi,[2] *1. Lanzhou University, Lanzhou, Gansu, China, 2. University of Southern California, Los Angeles, California, United States*

Chinese immigrant caregivers face unique self-care difficulties in the United States due to language barriers, cultural isolation, and occupational stress. This study aimed to conduct a formative evaluation on a caregiver self-care curriculum of an app designed for Chinese immigrants in the United States. Using a co-design approach in 2019, 22 Chinese immigrant caregivers in Los Angeles county were recruited through purposive sampling method. The directed content analysis was adopted to analyze the qualitative data using NVivo 12.1.0 software. We organized the findings under two main contents: self-care and caregiving. Three categories were identified under the self-care content: physical health, emotional and mental health, and support resources. Sixteen subcategories under physical health (e.g., dietary supplements), five subcategories under emotional and mental health (e.g., depression) and eight subcategories under support resources (e.g., support and networking group, senior center) are suggested. Two categories were identified under the caregiving content: caregiving knowledge and skills, and community resources. Fourteen subcategories under caregiving knowledge and skills (e.g., care assessment) and six subcategories under community resources (e.g., medical emergency call) were mentioned. With this useful information, we could further refine the self-care curriculum to be more linguistically, culturally and occupationally sensitive for Chinese immigrant caregivers. Empowerment approach for enhancing the ability to caregiving and self-care should be emphasized in content design for immigrant caregivers. The co-design approach is crucial for planning of the program and intervention curriculum to improve understanding of the users' needs and better cater them.

### DATA FROM A MINDFULNESS PROGRAM FOR FAMILY CAREGIVERS TO PERSONS WITH DD, AND APPLICATION FOR OLDER INDIVIDUALS

Jeffrey Kahana, Lawrence Force, and Roger Monthie, *Mount Saint Mary College, Newburgh, New York, United States*

Parents who care for their children (young and adult) with developmental disabilities face many stressors and challenges. This paper reports on an intervention using mindfulness and cognitive reframing to improve psychological well-being of care-givers. We report on results based on 92 care-givers who participated in the program. An innovative component was utilizing parent care-givers along with trained peer facilitators. The program was conducted over six weeks, with three in person sessions, and three at home web-based sessions. The content emphasized mindfulness practice (meditation) along with cognitive reframing (aimed at boosting optimism) to address the stress family-caregivers experience in managing worry and the perceived lack of control that accompanies caring for children (young and adult) with developmental disabilities. Post-test data revealed increased awareness of stress coupled with greater competence in stress management. Given the life-long demands for care-giving of parents to the developmentally disabled, normative stressors of aging interact with stressors posed by care-giving demands. Implications for improving well-being of older parental care-givers will be discussed around the topics of (1) optimism and hope; (2) support of healthy behaviors; and (3) development of a mindset of gratitude.

### IT'S WHAT WE DO: MĀORI CARERS TALK ABOUT ROLES, CHALLENGES, AND COORDINATION IN CARING FOR OLDER FAMILY MEMBERS

Mary Simpson,[1] Kirstie McAllum,[2] Christine Unson,[3] Stéphanie Fox,[2] and Te Oha Hancock,[4] *1. University of Waikato, Hamilton, Waikato, New Zealand, 2. Universite de Montreal, Montreal, Canada, 3. Southern Connecticut State University, New Haven, Connecticut, United States, 4. University of Waikato, Hamilton, New Zealand*

As the population of Aotearoa/New Zealand heads towards one-in-four being aged over 65-years-and-over by 2040, it is anticipated that family members will play an increasingly important role in caring for older relatives with chronic and age-related health issues. Multi-generational, and in particular three-generational living arrangements, combined with family care of older relatives are a growing trend; a trend already even evident among Māori communities. This paper reports on a study that explored the care experiences and expectations of 14 past and current Māori carers (aged 23 to 72-years) of older relatives. Interviews were audio-recorded, transcribed verbatim, and coded independently. The initial thematic analysis revealed nine themes and participants were invited to feedback on the summary in person or in writing. The feedback resulted in the original themes being collapsed into four (with subthemes): "Care is Normal"; "Collective Coordination of Care"; "Insider-Carer—Outsider Perspectives on Caring"; and "Societal Influences on Family Care/Carers". Firstly, these themes highlight how Māori cultural norms infuse direct care, support, and coordination roles within family care of the older family member. Secondly, they reveal the challenges for family carers in talking about their work with others, especially (thirdly) in the face of negative attitudes towards care and carers of an older family member within wider society. These findings have implications for cultural and wider socio-political influences in socialising different groups to carer role expectations. If society is to better prepare future caregivers for their role, ongoing research is needed with the various cultural groups in Aotearoa/New Zealand.

### SESSION 3011 (PAPER)

### MODELS, RESEARCH METHODS, AND INNOVATION

### ACCESSIBILITY TO PARKS AND TRAILS AND PHYSICAL HEALTH MEASURES IN CATSLIFE: EVALUATING SELECTION

Chandra Reynolds,[1] Elijah Knaap,[2] Robin Corley,[3] Kyle Gebelin,[4] Elizabeth Munoz,[5] Soo Rhee,[3] and Sally Wadsworth,[3] *1. University of California, Riverside, Riverside, California, United States, 2. University of California-Riverside, Riverside, California, United States, 3. University of Colorado Boulder, Boulder, Colorado, United States, 4. University of California - Riverside, Riverside, California, United States, 5. The University of Texas at Austin, Austin, United States*

Associations of obesity and cardiovascular health with neighborhood walkability have been observed in the literature; however, limited research has evaluated self-selection that may underlie associations. We measured accessibility to parks and trails using geospatial measurements as well as self-reported activity-friendliness of neighborhoods (ease of walking, biking, and recreating) in 1140 participants (ages 28-49 years) in the ongoing Colorado Adoption/Twin Study of Lifespan behavioral development and cognitive aging (CATSLife). Physical health indicators included BMI, resting heart rate, and mean arterial blood pressure. The relative similarity of siblings' accessibility was evaluated to consider self-selection; all models were adjusted for sociodemographics including education. BMI was associated with accessibility to parks, with each increasing log(mile) distance associated with 1.2 BMI unit increase (se=.49, p<0.02). Self-reported neighborhood activity-friendliness was comparable in prediction of BMI (p<0.01). Greater trail accessibility was associated with lower resting heart rate (b=-.30, se=.14, p<0.04) and mean arterial pressure (b=-0.33, se=0.14, p<0.03), whereas self-reported neighborhood activity-friendliness was not associated (p>0.40). Measures of park accessibility tended to be more similar among identical twins (median ICC = 0.30) than fraternal twins or siblings (median ICC = 0.15) or siblings in adoptive families (median ICC = 0.12), excluding siblings who live together. Measures of trail accessibility were consistent across sibling types (median ICCs = 0.25-0.27). Sibling similarity for park accessibility modestly increased with genetic relatedness suggesting potential heritable contributions, whereas comparable similarity was apparent for trail accessibility. Altogether, small associations were observed for park and trail access with physical health, with indications of environmental selection.

### EVALUATING THE INTERNATIONAL CLASSIFICATION OF FUNCTIONING, HEALTH, AND DISABILITY MODEL AS AN AGING MODEL

Qiwei Li, and Becky Knight, *University of North Texas, Denton, Texas, United States*

The study provides a possible theoretical framework for future aging studies focusing on a comprehensive understanding of the relationship between physical functions, social participation, and context factors including environmental and personal variables. The International Classification of Functioning, Health, and Disability (ICF) model has received considerable studies in rehabilitation counseling fields as it bridges the gap between functional limitations and overall health status for social participation. The ICF model focuses beyond physical conditions and embraces social supports and personal coping styles. This study verifies the validity of the ICF model with a data set collected from a fall prevention program. For the methods, a structural equation modeling was estimated with latent variables including body structure, body functions, activities, and personal factors. The latent variables were suggested by the ICF framework. The results showed that the estimation outcome exhibited an acceptable goodness of fit, $\chi2(11) = 30.401$, p = .001 (due to the large sample size of 691), RMSEA= .051 [.030, .072], CFI = .968, TLI = .919, SRMR = .029. The equation level good of fit also was great with an overall R squared of .828. In conclusion, the ICF model was valid and has been tested in the aging studies with the data set collected from a fall prevention program for older adults. As the ICF model includes more variables than medical models such as personal attributions, a holistic understanding regarding aging experience among older adults from various backgrounds will become possible, which is an urgent need for diverse America.

### TESTING THE MISSING MECHANISM OF DEMOGRAPHIC AND HEALTH VARIABLES IN THE HEALTH AND RETIREMENT STUDY

Peiyi Lu, and Mack Shelley, *Iowa State University, Ames, Iowa, United States*

Studies using data from longitudinal health survey of older adults usually assumed the data were missing completely at random (MCAR) or missing at random (MAR). Thus subsequent analyses used multiple imputation or likelihood-based method to handle missing data. However, little existing research actually examines whether the data met the MCAR/MAR assumptions before performing data analyses. This study first summarized the commonly used statistical methods to test missing mechanism and discussed their application conditions. Then using two-wave longitudinal data from the Health and Retirement Study (HRS; wave 2014-2015 and wave 2016-2017; N=18,747), this study applied different approaches to test the missing mechanism of several demographic and health variables. These approaches included Little's test, logistic regression method, nonparametric tests, false discovery rate, and others. Results indicated the data did not meet the MCAR assumption even though they had a very low rate of missing values. Demographic variables provided good auxiliary information for health variables. Health measures (e.g., self-reported health, activity of daily life, depressive symptoms) met the MAR assumptions. Older respondents could drop out and die in the longitudinal survey, but attrition did not significantly affect the MAR assumption. Our findings supported the MAR assumptions for the demographic and health variables in HRS, and therefore provided statistical justification to HRS researchers about using imputation or likelihood-based methods to deal with missing data. However, researchers are strongly encouraged to test the missing mechanism of the specific variables/data they choose when using a new dataset.

### THE EFFECT OF LONG-TERM CHANGES IN DAILY STRESS PROCESSES ON PROSPECTIVE HEALTH: AN APPLICATION OF THREE-LEVEL SEM

Jonathan Rush,[1] Emily Willroth,[2] Eileen Graham,[3] Daniel Mroczek,[3] and David Almeida,[4] *1. University of Victoria, Victoria, British Columbia, Canada, 2. Northwestern University Feinberg School of Medicine, Evanston, Illinois, United States, 3. Northwestern University, Chicago, Illinois, United States, 4. Pennsylvania State University, University Park, Pennsylvania, United States*

The study of change over time, contexts, cohorts, and people is influenced by the sampling of observations within longitudinal studies. Intensive measurement designs, embedded within long-term longitudinal studies, provide new opportunities to understand changes in dynamic processes, as well as determinants and consequences of these changes over time. The present investigation examined whether short-term dynamic associations accounted for individual differences in prospective health functioning. We used measurement burst data from the National Study of Daily Experiences subsample (N = 2485) embedded within the Midlife in the United States longitudinal study. Two measurement bursts were separated by ten years, with each containing daily measures of stress and affect across eight consecutive days. Functional health was measured by basic and instrumental activities of daily living at three measurement waves spanning 20 years. Three-level structural equation models were fit to simultaneously model short-term within-person associations between stress and affect (i.e., stress reactivity) and long-term changes in these associations over the ten year period. Individual differences in long-term changes of the short-term dynamic association predicted both basic and instrumental activities of daily living at 20 year follow-up (estimate = 5.26, SE = 2.54, p < .01; and estimate = 5.48, SE = 2.81, p < .01, respectively). These effects were present after adjusting for mean levels of both stress and affect. We highlight how characterizing individuals based on the strength of their within-person associations across multiple time scales can be informative in predicting distal health outcomes.

### SESSION 3012 (PAPER)

### INTERGENERATIONAL CAREGIVING AND RELATIONSHIPS

### GENDER AND THE GRANDFATHER CAREGIVER EXPERIENCE

AviElle Raymore, *West Virginia University, Point Pleasant, West Virginia, United States*

In the United States, 2.7 million grandparents are responsible for a grandchild in their home. Grandfathers are present in the majority of grandparent caregiver households, but their contributions and voices are often overlooked. The aim of this study was to explore how grandfathers experience caregiving as men. Twelve grandfathers from the age of 50-76 years participated in the study. Two face-to-face, semi-structured interviews were conducted with eleven grandfather caregivers while a telephone interview was conducted with one grandfather. Interviews focused on their life story, experiences as grandfather caregivers, and views on male caregiving. Data were analyzed using coding and thematic analysis. Gender was important throughout grandfather's caregiving experiences. Grandfathers discussed their attitudes towards caregiving using language that reflected traditional gender norms. To them, women were nurturing caregivers while men were supposed to provide for their families as caregivers. Grandfathers appeared to stay connected to notions of traditional masculinities through participation in sports and physical play with their grandchildren and through their emphasis on men as responsible and providers. Grandfathers were aware that others may view them as incompetent caregivers, but they did not allow these stereotypes to affect how they viewed themselves as caregivers. These findings can improve the understanding of this population for service providers who work with grandparent caregivers. Providing better outreach for grandfather caregivers, strengthening programs and supports for them, and confronting attitudes or views towards male caregiving are important practice implications.

### GRANDPARENT RESILIENCE: IMPROVING SELF-EFFICACY IN GRANDPARENTS RAISING GRANDCHILDREN

Aimee Fox,[1] Nathaniel Riggs,[1] Loriena Yancura,[2] and Christine Fruhauf,[1] *1. Colorado State University, Fort Collins, Colorado, United States, 2. University of Hawai'i, Honolulu, Hawaii, United States*

Grandparents often protect against childhood trauma and promote resilience through their nurturance, love, and support when raising grandchildren. Despite the beneficial role grandparents have on their grandchildren, grandparents may experience challenges of their own, including physical, mental, and emotional health issues, lack of resources, and social isolation. Few interventions exist to help grandparents successfully adapt to the challenges they face as primary parenting figures. The purpose of this study was to test preliminary efficacy of a strengths-based intervention for grandparents raising grandchildren aimed at increasing self-care behaviors, managing emotions, and connecting to community resources. Grandparents (N = 137) providing primary care to grandchildren were recruited to participate in a single-group, pre- and post-test design, 6-week intervention. Self-efficacy was assessed at baseline, post-intervention, and at a 6-month follow-up. To evaluate who the intervention might be most beneficial for, grandparents' service knowledge, perceived support from others, and length of care provided, measured at baseline, were analyzed for moderating effects. Results of paired-samples t-tests reveal significant increases in self-efficacy (p = .013) from baseline to post-test, which were maintained at the 6-month follow-up (p = .010). Hierarchical multiple regression showed interaction effects of the hypothesized moderators were not significant, indicating improvements in self-efficacy regardless of individual variability at baseline. As demonstrated, interventions can be effective at increasing self-efficacy in grandparents raising grandchildren and strengths-based approaches have the potential to provide universal benefits to grandparents, thus improving functioning in grandfamilies and promoting the health and well-being of grandparents and their grandchildren.

### INTERGENERATIONAL CAREGIVING PATTERNS, BMI, AND GENDER GAPS AMONG THE SANDWICH GENERATION IN CHINA

Jingwen Liu,[1] and Feinian Chen,[2] *1. University of Maryland, college park, college park, Maryland, United States, 2. University of Maryland, College Park, College Park, Maryland, United States*

Existing literatures yield established evidence about the heightened stress brought by multiple roles and potential role overload across work-family context, but little is known about the BMI levels of the "sandwich" caregivers within families and the associated gender inequalities. Indeed, the Chinese pivotal generations are exposed to unshared stress and higher health risks considering that intergenerational support still predominates the caregiving patterns for the oldest old and dependent children under current socioeconomic backgrounds. Using 2011 and 2013 waves of China Health and Retirement Longitudinal Study (CHARLS, N = 12186), we examine associations of BMI and intergenerational caregiving patterns among the "sandwich generation" aged 45 to 69. We find that the sandwich generation with at least one parent alive and one grandchild under 16 have higher BMI (24.2, within obesity range) than their counterparts (23.7, within normal range). A higher proportion of females act as caregivers and especially high-intensity caregivers than males, and they also score one-unit higher in BMI than males (23.4). Fixed effect regression results indicate that simultaneous caregiving to both parents and young grandchildren significantly advances individuals' BMI levels, while no evidence shows similar negative effect of providing care to one generation. Moreover, high-intensity caregiving (1000 hours and above during the past year) is associated with elevated BMI for females but not for males. The gendered caregiving patterns and health implications inform the physical and psychological vulnerability of the pivotal generation and the necessities of gender-specific intervention in middle and later life.

### MULTIDIMENSIONAL PROFILES OF RELIGIOSITY AMONG GRANDPARENTS, PARENTS, AND GRANDCHILDREN

Joonsik Yoon, Woosang Hwang, Merril Silverstein, and Maria Brown, *Syracuse University, Syracuse, New York, United States*

Studies have frequently treated different measures of religiosity as cumulative components on a single scale of religiosity ranging in value from high to low, despite the broad consensus that religiosity is multidimensional in nature. Multidimensional typologies can be helpful not only in properly assessing and measuring religiosity in an increasingly diverse population, but could also help identify specific religious and spiritual differences within seemingly similar groups. The different dimensions of religiosity also allow us to classify populations according to their religious characteristics rather than according to social outcomes in relation to their religiosity. We apply latent class analysis and multiple group latent class analysis to multigenerational data from the Longitudinal Study of Generations (N= 1,726) to examine (1) what religiosity profiles exist across three generations (grandparents, parents, and grandchildren), and (2) how the patterns of religiosity profiles are the same or different across three generations. Results of the latent class analysis show that four religiosity subgroups are identified in the three generations: strongly religious, weakly religious, religious but not literalists, literalists but not religious. In addition, results of the multigroup latent class analysis show that the four religiosity subgroups are structurally the same across three generation. Our findings complement previous studies that show religiosity is a multidimensional construct, and demonstrate that religiosity class memberships are invariant across three generations. Furthermore, given that one in four U.S. adults consider themselves "spiritual but not religious" or "religious but not spiritual", our findings confirm similar patterns of religiosity subgroups in three generations.

### PROCESS-OUTCOME-RELATIONSHIPS AND SOLUTION-BASED INTERVENTIONS WITH GRANDPARENT CAREGIVERS

Julian Montoro Rodriguez,[1] Bert Hayslip Jr,[2] Jennifer Ramsey,[3] and Jane Jooste,[4] *1. University of North Carolina at Charlotte, Charlotte, North Carolina, United States, 2. University of North Texas, Denton, Texas, United States, 3. UNC Charlotte, Charlotte, North Carolina, United States, 4. Lewisville School District, Lewisville, Texas, United States*

Grandparents raising grandchildren face many challenges such as isolation, stigma, negative thoughts, and biases from age peers and service providers. In contrast, recent work suggests a need to re-frame grandparent caregiving in positive terms. In this light, the present study explored the processes key to the success of a solution-focused intervention with such persons. Fifty-two grandparents caregivers participated in a six-session solution focused intervention, using the theoretical framework of Selection, Optimization and Compensation (SOC) by Baltes & Baltes (1990). Throughout the program the emphases were on goal setting, solution focused thinking and communication skills. Participants were randomly assigned to intervention or waiting list control conditions. Within each session measures of hopefulness, positive thoughts about one's grandchild, solution focused thinking, self-rated stress and perceived goal attainment were taken. Results indicated significant decreases in stress and increases over six sessions in hopefulness, positive thoughts about the grandchild, being solution-oriented and success in overcoming barriers interfering with goal setting and implementation ($p<.05$). Measures of goal accomplishment, strategy usage, success and satisfaction with effort were uniformly high. Such findings speak to key processes underlying the efficacy of a solution-focused intervention with grandparent caregivers. Measures of such processes also predicted outcome measures, e.g., resilience, positive affect, caregiver strain, well-being, loneliness, depression, parental efficacy and adequacy of service needs met. These findings reinforce the importance of understanding change processes as predictors of solution-focused intervention outcomes with grandparent caregivers and are consistent with the need to emphasize such persons' strengths.

### SESSION 3013 (PAPER)

### CARING FOR OLDER PARENTS

### CARING FOR DYING PARENTS IN THE SHADOW OF CHILDHOOD MALTREATMENT: GENDERED AGENCY, CONSTRAINT, AND HEALTH

Robin Tarter, Dena Hassouneh, and Susan Rosenkranz, *Oregon Health and Science University, Portland, Oregon, United States*

Adult daughters represent the largest and fastest growing population of providers of unpaid care labor (UCL) to older adults with life limiting illness. Providing UCL to parents at the end of life is associated with significant and lasting risks of morbidity and mortality, especially for women with negative relationships with care recipients, and those who provide UCL based on constraining gendered expectations rather than agentic choice. While nearly one quarter of US women experience some form of maltreatment from parents during childhood, few studies have examined, or even acknowledged, the effect of trauma on the experience and health impact of family UCL. We used feminist poststructuralist informed dialogic narrative analysis to explore discursive constructions of agency and constraint in co-constructed life histories from 21 women who provided end of life UCL to older adult parents who maltreated them in childhood. For these women, parental childhood maltreatment influenced identity construction, social position, intersubjectivity, and vulnerability to victimization. For some, providing end-of-life UCL to the parents who maltreated them facilitated the mobilization of relational agency and identity validation. For others, providing UCL potentiated lifelong constraint, reinforcing their positions as non-agents and leading to significant psychical and emotional harm. End of life UCL for older adult parents represents a crucible out of which either healing or re-traumatization can arise. Our findings will be leveraged to inform clinical practice and policy to support the growing population women trauma survivors providing UCL to older adult parents, reducing negative outcomes for those at the greatest risk.

### RECIPROCAL ASSOCIATIONS BETWEEN NORMATIVE, AFFECTUAL, AND ASSOCIATIONAL SOLIDARITY WITH PARENTS IN YOUNG ADULTS

Jeung Hyun Kim, Woosang Hwang, Kent Jason Cheng, Maria Brown, and Merril Silverstein, *Syracuse University, Syracuse, New York, United States*

Intergenerational solidarity has become important as close family ties mobilize the provision of social support across generations and contribute to the family wellbeing. One popular approach to studying intergenerational cohesion in aging families is through the theoretical construct of intergenerational solidarity. However, less is known about the longitudinal and reciprocal associations between normative, affectual, and associational solidarity with mothers and fathers among young-adult children in the transition to adulthood. On the basis of the theoretical construct of intergenerational solidarity, we examined the reciprocal associations between three dimensions of intergenerational solidarity (normative, affectual, and associational) with parents in young-adult children from their early twenties to late thirties. Data were derived from 287 mother-son, 325 mother-daughter, 262 father-son, and 297 father-daughter groups who participated in the Longitudinal Study of Generations between 2000 and 2016. Autoregressive cross-lagged model with latent variables predicted the causal relations between three dimensions of solidarity across four parent-child groups. We found that young-adult sons' perceived associational solidarity with parents predicted normative solidarity over time, whereas young-adult daughters' perceived affectual solidarity with mothers predicted normative solidarity over time. In addition, young-adult daughters' perceived normative solidarity predicted affectual solidarity for fathers over time. The present study found that young-adult sons and daughters have different ways establishing normative solidarity in their early twenties to late thirties according to parents' gender. In addition, this study found that normative solidarity is beneficial for young-adult daughters developing emotional closeness with fathers over time.

### THERE AT ANY DISTANCE? GEOGRAPHIC PROXIMITY AND THE PRESENCE OF ADULT CHILDREN IN OLDER PEOPLE'S' CONFIDANT NETWORKS

Markus Schafer, and Haosen Sun, *University of Toronto, Toronto, Ontario, Canada*

Adult children are key members of their aging parents' close social network, often providing emotional and advisory supports. Still, adult children are not a guaranteed presence in older people's core discussion networks. Geographical distance is a leading explanation for why some children are excluded from the confidant network, but we hypothesize that certain parent- and dyadic-level factors make these intergenerational ties more resilient to distance. Using wave six of the Survey of Health, Ageing, and Retirement in Europe, we identified whether a living adult child was also a member of the parent's egocentric confidant network. We modeled the effect of the child (Level 1) and parent (Level 2) characteristics on the exclusion of a child from the core network using hierarchical logit models. We found that fifty-eight percent of children were excluded from a parent's network. Parents were more likely to exclude those who lived more than 25 km compared to children who lived within 5 km. The impact of distance was exacerbated among parents who were older, partnered, or had four or more children. Parents with higher education and good computer skills were less sensitive to longer distances when listing a child as a confidant. Finally, parents who had confidants outside of the nuclear family and who lived in Northern Europe were less likely to exclude a child over 100 km from their confidant network. Together, results indicate that a number of demographic factors and personal and social resources contribute to the elasticity of parent-child ties across long distances.

### WITHIN-FAMILY PATTERNS OF SHARING INSTRUMENTAL SUPPORT TO OLDER PARENTS OF MULTI-CHILD FAMILIES IN CHINA

Jia Chen,[1] and Xiaochen Zhou,[2] *1. Shanghai University, Shanghai, China, 2. University of Hong Kong, Hong Kong, China*

In different multi-child families, adult children may share their instrumental support to older parents in distinct ways regarding its family mean level and differentiation among multiple offspring within families. Based on the family systems theory and the collective ambivalence perspective, we aimed (1) to identify different within-family patterns in relation to multiple offspring's sharing instrumental support to an older parent in Chinese multi-child families; (2) to investigate potential individual and family predictors for different within-family patterns. Applying data from the China Family Panel Studies (2016, N=5791 older adults aged 60+), we employed latent profile analysis for classifying patterns and multinomial logistic regression for investigating predictors. Results showed three within-family patterns identified: independent (59.78%), highly-ambivalent (30.41%) and filial-cohesive (9.81%). Compared with the independent families, older parents in highly-ambivalent families were more likely to be older (OR=1.03), divorced/widowed (OR=0.61), to have lower educational levels(OR=0.84, ), poorer physical health (OR=0.92), to live in rural areas (OR=0.84), to have at least one adult daughter (OR=1.95)and one coresiding adult child (OR=3.22). Older parents in filial-cohesive families tended to be mothers (OR=0.82), divorced/widowed (OR=0.62), to have fewer adult children (OR=0.78) ,to have at least one adult daughter (OR=1.67) and one coresiding adult child (OR=2.16). The youngest adult children in filial-cohesive families tended to be older (OR=1.04). This study highlighted the importance of capturing different within-family dynamics regarding instrumental support to older parents among multiple adult children at the family level. It also uncovered the commons and differences between multi-child aging families in contemporary China.

### SESSION 3014 (PAPER)

### HEALTH AND SOCIAL EXPERIENCES OF FAMILY CAREGIVERS

### FAMILY DYNAMICS AND ELDER FINANCIAL EXPLOITATION BY FAMILY-MEMBER POWERS-OF-ATTORNEY AGENTS

Virginia Vincenti, and Bernard Steinman, *University of Wyoming, Laramie, Wyoming, United States*

Elder family financial exploitation (EFFE) is a growing problem likely to increase due to population aging. Older people, often considered vulnerable, are frequently targeted for financial exploitation. Relatives, identified as the largest group of perpetrators often misuse powers of attorney (POA); nevertheless, relationship complexity and dependencies, and family privacy result in underreporting and infrequent prosecutions. The aim of this research is to understand family risk factors that could be used for prevention and early detection. We hypothesized that family dynamics in EFFE families compared to non-EFFE families having family-member POA agents would be at greater risk when these risk factors were present. Our larger multi-state research team created a survey to collect demographic and situational data and to explore family-member and elder characteristics and specific family dynamics that could later place older relatives at risk for EFFE. Analyses consisted of testing whether poor family functioning, ineffective communication/problem-solving dynamics, resource exchange patterns, conflict before/during resource distribution, and entitlement attitudes were statistically associated with the occurrence of EFFE. Specifically, we tested a series of hierarchical logistic regressions to examine the association of family dynamic variables with EFFE. Results suggest that fairness conflict, exchange expectations, entitlement expectations, and communication patterns were statistically associated with EFFE. Current family communication patterns were not a statistical predictor of EFFE. These results could prompt older persons and relevant healthcare, legal, financial, law enforcement, social service, and counseling professionals to work proactively with families and mid-life and older adults to consider risk factors before making end-of-life decisions.

### HEALTH BEHAVIORS AMONG FAMILY CAREGIVERS PARTICIPATING IN THE PAID WORKFORCE

Abiola Keller, and Mauricio Garnier-Villarreal, *Marquette University, Milwaukee, Wisconsin, United States*

Up to 20% of family caregivers are also working full-time or part-time. The competing demands of caregiving and working may adversely affect the health of caregivers. In this study, we use data from the 2015 Behavioral Risk Factor Surveillance System Questionnaire-Caregiver module to examine the relationships between participation in the paid workforce and health behaviors among caregivers. Individuals with a positive response to the survey question, "During the past 30 days, did you provide regular care or assistance to a friend or family member who has a health problem or disability?" were identified as caregivers. Participation in the paid workforce was defined as being employed for wages or self-employed. We conducted separate survey-weighted multivariate regression analyses to examine the relationship between participation in the paid workforce and drinking, exercise, and smoking controlling for sociodemographic characteristics. For each outcome, all sociodemographic characteristic variables were entered into the model simultaneously. 55.6% of the 24,034 caregivers participated in the paid workforce. The survey-adjusted multivariate regression analyses showed that those in the paid workforce had an increased likelihood of binge drinking (OR = 1.39, 95% CI [1.14-1.69]) and decreased likelihood of engaging in leisure-time physical activity (OR = 0.83, 95% CI [0.73-0.95]) compared to those who were not in the paid workforce. The odds of smoking less frequently did not differ between the two groups (OR = 0.96, 95% CI [0.85-1.07]). Our findings underscore the need for workplace-based interventions aimed at promoting healthy behaviors among caregivers.

### THE CAREGIVER IDENTITY IN CONTEXT: THE CONSEQUENCES OF IDENTITY THREAT FROM SIBLINGS

Marissa Rurka,[1] J. Jill Suitor,[2] and Megan Gilligan,[3]
*1. Purdue University, Lafayette, Indiana, United States, 2. Purdue University, West Lafayette, Indiana, United States, 3. Iowa State University, Ames, Iowa, United States*

Although siblings represent central members of the networks of caregivers' and their parents, there has been limited attention to how siblings affect one another's well-being during caregiving. In this paper, we draw from theories of identity and stress to examine the impact that siblings have on caregivers' psychological well-being. Specifically, we employ a mixed-methods approach to explore whether caregivers' perceptions that their siblings are critical of the care they provide their mother are associated with higher depressive symptoms, as well as the mechanisms underlying this association. Using quantitative data collected from 406 caregivers nested within 230 families as part of the Within-Family Differences Study, we conduct mediation analyses to examine whether perceived sibling criticisms affect caregivers' depressive symptoms (a) directly, and/or (b) indirectly through sibling tension. We then analyze qualitative data collected from the same caregivers to gain insight into the processes underlying statistical associations. Quantitative analyses revealed that there was no direct relationship between perceived sibling criticisms and depressive symptoms; there was, however, an indirect relationship such that perceived sibling criticisms were associated with greater sibling tension, which in turn was associated with higher depressive symptoms. These quantitative findings were corroborated by qualitative analyses, which demonstrated that, in an effort to maintain their identity as a "good caregiver" despite sibling criticisms, caregivers often employed strategies that may have fueled tension in their sibling relationships. These findings highlight the importance of considering how the sibling networks in which caregivers are embedded shape their psychological well-being.

### THE IMPACT OF CAREGIVING INTENSITY AND RELIGIOSITY ON SPOUSE CAREGIVERS' HEALTH AND MORTALITY IN THE UNITED STATES (2004–2014)

Athena Koumoutzis,[1] and Nader Mehri,[2] *1. Miami University, Oxford, Ohio, United States, 2. Miami University, OXFORD, Ohio, United States*

Prior research has indicated that religiosity may buffer against the deleterious effects of caregiving. However, research is lacking in examining the role of religiosity and caregiving intensity in the context of caregiver wellbeing and mortality. Data come from the Health and Retirement Study (2004-2014 waves) and consisted of spousal caregivers and noncaregivers (n= 49,638 person-spells). Pearlin's Stress Process Model (1990) informed this study to analyze how religiosity impacts caregiver self-rated health and mortality by comparing the intensity of provided care among spousal caregivers and spousal noncaregivers. This study used two indicators to measure religiosity: 1) the importance of religion in life and 2) frequency of attending religious services. Bivariate probit model was used to model the impact of caregiving intensity and religiosity on self-rated health and all-cause mortality. After controlling for sociodemographic and health covariates, results showed that only the importance of religion in life predicted a better self-rated health among high intense spouse caregivers defined by providing >=14 hours of care per week. Findings suggest religiosity may buffer the adverse effect of caregiving stress on health for high intense spousal caregivers. Development and maintenance of religiosity may enhance positive aspects of caregiving and decrease caregiver burden.

### UNMET HOME- AND COMMUNITY-BASED SERVICE NEEDS AMONG INFORMAL CAREGIVERS IN RURAL AND URBAN AREAS

Erin Bouldin,[1] Jo-Ana Chase,[2] Christina Miyawaki,[3] David Russell,[1] Nancy Gell,[4] Christopher Taylor,[5] and Lisa McGuire,[5] *1. Appalachian State University, Boone, North Carolina, United States, 2. University of Missouri, Columbia, Columbia, Missouri, United States, 3. University of Houston, Houston, Texas, United States, 4. The University of Vermont, Burlington, Vermont, United States, 5. Centers for Disease Control and Prevention, Atlanta, Georgia, United States*

Home- and community-based services (HCBS) can reduce caregiver burden. We compared the prevalence of HCBS unmet needs among caregivers in rural and urban areas and identified factors associated with unmet HCBS needs. We used 2015-2018 Behavioral Risk Factor Surveillance System data, including the optional Caregiver Module, from 44 states, District of Columbia, and Puerto Rico. Caregivers were individuals providing care/assistance to a friend/family member with a long-term illness/disability during the past 30 days. Unmet needs were defined as needing, but not receiving, one or more of the following: caregiving classes, help accessing services, support groups, individual counseling, or respite care. "Rural" was defined as living outside Metropolitan Statistical Areas (available only for landline respondents). We calculated weighted estimates and used log-binomial regression to estimate adjusted prevalence ratios (PR). 19% of 25,180 caregivers lived in a rural area. Rural caregivers were less likely to report unmet HCBS needs (14.4% versus 20.6% urban, p<0.001), even after accounting for sociodemographic and caregiving characteristics (PR=0.81, 95% CI: 0.65-0.99, p=0.040). Unmet needs were more common among caregivers who provided more care, personal care, or care for someone with Alzheimer's disease/dementia, regardless of rural residence. Although rural individuals can experience more barriers to accessing health services, rural caregivers in our study reported fewer unmet HCBS needs than urban caregivers. Additional research is needed to determine if stronger systems of informal support in rural areas may explain this difference. Further investigation of factors contributing to differences in unmet service needs among rural and urban caregivers is needed.

### SESSION 3015 (PAPER)

### BIOBEHAVIORAL HEALTH

### BODY MASS INDEX INSTABILITY AND INCIDENT MILD COGNITIVE IMPAIRMENT AMONG BLACK OLDER ADULTS

Adrienne Aiken-Morgan,[1] Ana Capuano,[2] Zoe Arvanitakis,[2] and Lisa Barnes,[2] *1. North Carolina A&T State University, Greensboro, North Carolina, United States, 2. Rush University Medical Center, Chicago, Illinois, United States*

Despite general negative health effects, elevated body mass index (BMI) can be "protective" against poor health outcomes, including cognitive decline and mild cognitive impairment (MCI), in old age. However, few studies have examined the effects of BMI fluctuations (BMI instability) over time. The purpose of this study was to examine how BMI level at baseline and BMI instability is related to incident MCI in Black participants from the Minority Aging Research Study (MARS; N = 522, mean age = 73.5, mean education = 15.0; 76.5% women). Participants without cognitive impairment at baseline underwent annual clinical evaluations, including measurement of BMI and 19 neuropsychological tests for up to 15 years of follow-up to document MCI. 192 of 522 persons developed MCI. In Cox models adjusted for age, sex, and education, 1) higher baseline BMI, across the range of all values (mean=30.5; SD=6.5), was related to a decreased risk of MCI (Hazard Ratio = 0.97; 95% CI = 0.94-1.00); and 2) BMI instability (with a maximal range of 0-15.7; mean=3.2; SD=2.5) was related to an increased risk of MCI (Hazard Ratio = 1.09; 95% CI = 1.03-1.15). The present findings suggest that while late-life higher BMI level may protect against MCI, BMI instability over the years is detrimental to cognition in Black persons without dementia. Future research should investigate underlying mechanisms.

### CYSTATIN C TRAJECTORIES AMONG MIDDLE-AGED AND OLDER AMERICANS

Erfei Zhao,[1] Eileen Crimmins,[2] Jennifer Ailshire,[2] Jung Ki Kim,[3] and Qiao Wu,[2] *1. University of Southern California, Leonard Davis School of Gerontology, Los*

*Angeles, California, United States, 2. University of Southern California, Los Angeles, California, United States, 3. University of Southern California, Los Angeles, United States*

Deterioration in kidney functioning is associated with aging and is a major risk factor for mortality and other poor health outcomes. Medicare expenses for poor kidney functioning are about 100 billion dollars every year. High Cystatin-C is an indicator of poor kidney functioning. We do not know if cystatin-C increases gradually as an individual ages. We use the Health and Retirement Study 2006/2008 Biomarker sample with follow-up for 8 years to examine this. Demographic and socioeconomic differences in trajectories of Cystatin-C trajectories were examined for 22,984 participants aged 50 and older. Growth curve models reveal that, although Cystatin-C increases with age (beta=0.025, $p<0.001$), the annual increase varies by age (60-69 = 0.005, 70-79 = 0.013, 80+ = 0.017, $p<0.001$), controlling for other socioeconomic variables. Cystatin-C increases faster for males than females. Cystatin-C of non-Hispanic Whites is lower than non-Hispanic Blacks but higher than Hispanics; there is no racial/ethnic difference in change over time. People who spent fewer years in school have higher Cystatin-C, and college graduates have slower growth in Cystatin-C compared to people who did not graduate from high school. These novel findings highlight the disparities in the process of kidney aging among older Americans.

### INSULAR AND HIPPOCAMPAL CONNECTIVITY IS ASSOCIATED WITH PERCEIVED FINANCIAL EXPLOITATION IN OLDER ADULTS

Gali Weissberger,[1] Laura Mosqueda,[2] Annie Nguyen,[1] Jenna Axelrod,[1] Patricia Boyle,[3] Nathan Spreng,[4] and Duke Han,[1] *1. University of Southern California, Alhambra, California, United States, 2. Keck School of Medicine of the University of Southern California, Los Angeles, California, United States, 3. Rush University Medical College, Chicago, Illinois, United States, 4. McGill University, Montreal, Quebec, Canada*

Little is known about the neural correlates of financial exploitation (FE) in older adults. Cognitively-intact older adults who self-reported a history of FE (N=19; M age=69.84, SD=13.06) and demographically-matched non-FE older adults (N=16; M age=65.13, SD=8.48) underwent resting-state fMRI. Predefined regions of interest were prescribed using the Harvard-Oxford atlas for their involvement in tasks of economic decision making: insula, hippocampus, and the medial prefrontal cortex (mPFC). Analyses adjusted for age, education, sex, and MoCA scores; groups did not differ on these factors. Clusters were FDR-corrected with a threshold of $p<0.05$ (voxel threshold $p<0.005$), two-tailed. Compared to the non-FE group, the FE group exhibited greater functional connectivity (FC) between the right insula and left temporal lobe regions ($t(29)= -4.81$), and between the left insula and right temporal lobe regions ($t(29)= -5.78$). The FE group showed less FC between the left insula and two clusters in the right lateral occipital cortex ($t(29)= 5.18$) and left cerebellum ($t(29)= 4.68$). Additionally, FE was associated with greater FC between the right hippocampus and five clusters spanning the right temporal lobe, parietal lobe, and frontal pole ($ts(29)= -4.11$ to $-4.51$), and less FC between the right hippocampus and three clusters spanning the bilateral caudate and the left intracalcarine cortex ($ts(29)= 4.76$-$6.03$). Groups did not differ in FC patterns with the mPFC. Results suggest that FE is associated with whole-brain FC differences involving the insula and hippocampus among cognitively-intact older adults.

### SKIPPING MEALS IS ASSOCIATED WITH SYMPTOMS OF ANXIETY AND DEPRESSION IN U.S. OLDER ADULTS

Loretta Anderson, *University of Maryland School of Medicine, Reisterstown, Maryland, United States*

Previous studies have shown that higher levels of economic hardship in older adults is associated with increased odds of adverse health outcomes such as insomnia, anxiety, and depressive symptoms. The objective of this study was to determine if there was a differential association between individual measures of economic hardship and aforementioned adverse health outcomes. Cross-sectional analysis was conducted using data from the 2013 National Health and Aging Trends Study (NHATS). Logistic models were developed to assess the association between four measures of economic hardship which included not having enough money for food, utility bills, mortgage/rent, or medical bills/prescription drugs. Measures of adverse health outcomes were symptoms of depression, anxiety, and insomnia. There were 4467 community-dwelling older adults (65+) in the analyses. Results indicated those who skipped meals were more likely to have depression, anxiety, and insomnia symptoms than those who did not skip meals. After adjusting for race, age, gender, education, and total number of comorbid health conditions, skipping meals was associated with depression (OR=2.71, $p<.05$) and anxiety (OR=2.84, $p<.01$). Skipping meals did not have a statistically significant association with insomnia. The analysis for skipping meals showed a higher odds and more statistically significant results than the other measures of economic hardship listed above. These findings are relevant to population-based efforts to improve quality of life in aging populations and may be of interest to those researchers investigating the gut-brain axis. These findings may also inform future policy efforts to address health disparities and food insecurity in older adults.

### SESSION 3016 (PAPER)

### NATIONAL AND CROSS-CULTURAL STUDIES

### CHILDLESSNESS, INDIVIDUAL SOCIOECONOMIC RESOURCES, AND HEALTH: EXPLORING VARIATION IN 20 COUNTRIES

Nekehia Quashie,[1] Christine Mair,[2] Radoslaw Antczak,[3] and Bruno Arpino,[4] *1. TU Dortmund, Dortmund, Germany, 2. University of Maryland, Baltimore County, Baltimore, Maryland, United States, 3. Warsaw School of Economics, Warsaw, Poland, 4. University of Florence, Florence, Toscana, Italy*

Childless older adults may be at risk for poorer health cross-nationally, yet most studies on this topic analyze only a small number of countries and only 1 or 2 health outcomes. To our knowledge, two papers exist that explore associations between childlessness and multiple indicators of health using data from a large number of regionally diverse countries (e.g., 20 countries from North America, Asia, and Europe), but neither study includes an examination of socioeconomic resources. The level of health risk faced by childless older adults is likely to be distinctly shaped by older adults' socioeconomic resources (e.g., education, income, wealth). Associations between childlessness, socioeconomic resources, and health may also differ by country context. Using harmonized, cross-national data for adults aged 50 and older across 20 high- and middle-income countries (United States (HRS), European Union (SHARE), Mexico (MHAS), and China (CHARLS) from the Gateway to Global Aging data repository), we explore if and how individual-level socioeconomic resources (income, education, wealth) moderate associations between childlessness and five health indicators (self-rated health, ADL limitations, IADL limitations, chronic conditions, and depression). Results suggest that associations between childlessness and health outcomes vary by individual socioeconomic resources in some country contexts, but not in others. We discuss these findings in light of the impact of individual-level socioeconomic resources on older adults' support options and health outcomes cross-nationally.

### EAST AND WEST ETHNOGRAPHY IN ADULT DAY SERVICES: LESSONS FROM TAIWAN AND THE UNITED STATES

Chih-ling Liou,[1] and Sonia Salari,[2] *1. Kent State University at Stark, North Canton, Ohio, United States, 2. The University of Utah, Salt Lake City, Utah, United States*

Adult Day Services (ADS), an alternative to institutionalization, offer daytime programs for those with physical and/or cognitive impairments. ADS have grown rapidly in Taiwan and the U.S., to improve quality of life (QOL) and provide respite for caregivers. ADS researchers have focused primarily on caregiver outcomes, with less emphasis on consumers. Our study is among the first to examine these issues in a cross-cultural context. Surveys are insufficient for ADS attendees, since many have diminished capacity. We use ethnographic methods to more fully understand the life of clients in ADS in two countries. The focus is on clients and staff, while including the physical and social environments. Data were collected over 820 hours of observation and 77 interviews with key ADS stakeholders, including clients, for insider perspective. Comparisons between the two countries, found that infantilization (e.g., baby-talk, child-oriented activities and decor) occurred in all 10 settings to varying degrees and outcomes were shaped by culture. In Taiwan, infantilization embedded within a cultural tradition of respecting elders and exhibited as a parent-child role reversal, where staff played the adult-child role. Their over-helping and over-controlling were aimed to 'benefit' clients, who were seen as incompetent aging parents. In the U.S., infantilization resembled a primary school environment with elementary décor. Aides used baby-talk and 'taught' compulsory child-oriented songs and games. This effect was exacerbated when preschool-aged children were added, and all generations were treated similarly. Results can be translated to implement age-appropriate care environments across cultures to improve QOL in ADS.

### LATER-LIFE HEALTH DISPARITIES IN NEPAL: INTERSECTION OF GENDER AND SOCIOECONOMIC STATUS

Tirth Bhatta,[1] Neema Langa,[2] Nirmala Lekhak,[3] and Denise Burnette,[4] *1. University of Las Vegas Nevada, Las Vegas, Nevada, United States, 2. UNLV, Las Vegas, Nevada, United States, 3. University of Nevada, Henderson, Nevada, United States, 4. VCU, Richmond, Virginia, United States*

This study focuses on Nepal, a country still undergoing capitalist expansion, to examine intersectional effects of fundamental causes on later life health outcomes. Sandwiched between the republic of China and India, Nepal still has remnants of pre-capitalist social and economic formations. Despite growing focus on independent effects of SES and gender on health, the intersectional influences of such fundamental causes on later life health in Nepal has, however, been largely unexplored. Drawing from the World Health Survey (WHS) survey data (n=2,250 aged 50 years and older), we rely on negative binomial regression models to examine whether the effect of education and household wealth on chronic diseases and functional limitations differs between men and women. Findings indicate intersectional effects of gender, wealth, and education on health. Women do not incur health benefits from education and wealth. Statistically significant negative effect of education on functional limitations (OR=0.87, p<0.01) was documented only for men. Contrary to our theoretical expectations, we observed significantly higher count of chronic diseases among women (OR=1.13, p<0.01) with higher levels of education relative to lower educated women. Similarly, men in higher wealth quintiles reported significantly higher count of chronic diseases (OR=1.05, p<0.01) than their counterparts in lower wealth quintiles. Our study paves a way for future research on a range of structural mechanisms such as gendered labor market, patriarchal cultural expectations, and inequities in health care that could mediate intersectional effects of gender and education on later life health disparities in the Global South.

### SESSION 3017 (PAPER)

### RELATIONSHIPS, GENDER HEALTH, AND SEXUAL ORIENTATION

### DEALING WITH LONELINESS AMONG LGBT OLDER ADULTS: DIFFERENT COPING STRATEGIES

Natasha Peterson,[1] Jeongeun Lee,[2] and Daniel Russell,[1] *1. Iowa State University, Ames, Iowa, United States, 2. Iowa State University, Iowa City, Iowa, United States*

Older lesbian, gay, bisexual, transgender (LGBT) adults may be at risk for high levels of loneliness—a risk factor for worse health behaviors—as a result of historical and social discrimination. Some LGBT older adults may have estranged relationships with family members or have toxic relationships, consequently leaving them without adequate social support. The 2018 Loneliness and Social Connections survey by the AARP Foundation consists of a national sample of non-institutionalized individuals 45 and older, including 2905 individuals who identify as heterosexual and 318 who identify as LGBT. The study indicated individuals had similar levels of loneliness regardless of sexual orientation. However, significant differences between heterosexuals and LGBT participants were found in their communication and time usage when they are lonely. For example, heterosexual individuals socialize with friends in person more than homosexuals (t=-2.393, p<.05), whereas LGBT older adults use technology more to socialize with friends (t=3.749), p<.001. Further, findings revealed that older LGBT adults tend to engage in more risky or unhealthy behaviors when lonely than do heterosexual older adults (t=3.907, p<.001). Overall, the results indicate that while LGBT older adults may spend more time alone (t=7.350, p<.001), they are engaging in different types of activities, particularly involving technology to communicate with friends, suggesting compensation for their lack of in-person contact. By understanding how LGBT older adults cope with loneliness along with the risks and resources that have influences on their health disparities can be useful for developing interventions to improve the health and well-being in these communities.

### DOES SEXUAL ORIENTATION INFLUENCE TRAJECTORIES OF CHANGE IN HEALTH? A 20-YEAR FOLLOW-UP STUDY

Christi Nelson, and Ross Andel, *University of South Florida, Tampa, Florida, United States*

We examined the differences in physical health outcomes over a 20-year period between lesbian, gay, and bisexual (LGB) and heterosexual middle-aged and older adults. We also examined whether the associations were moderated by social support and affect. The analytical sample included 168 LGB adults and 336 propensity-matched heterosexual adults from the Midlife in the United States (MIDUS) study, ranging in age from 25 to 74 years (mean age=42.83) at baseline. Using negative binomial generalized estimating equations and mixed-effects analyses, data from three waves of MIDUS, spanning approximately 20 years from 1995 to 2014, were used to examine the associations between sexual orientation and the health outcomes (number of chronic conditions and functional limitations). Social support and affect were added to the models to test for moderation. The results found that LGB participants reported one more chronic condition at baseline and scored significantly higher for functional limitations. However, LGB participants increased less over time for number of chronic conditions than heterosexual participants, and there were no significant differences in terms of changes in functional limitation over time. Positive affect reduced the strength of the relationship between sexual orientation and functional limitations for LGB participants. No other moderating effects were significant. The results of this study suggest that LGB individuals may become resilient to the negative health effects of minority stressors over time. Interventions should focus on improving the health of LGB individuals when they are younger and more at risk of negative health outcomes.

### IT PROTECTS THEM FROM ME: REASONS OLDER ADULT BLACK WOMEN ENGAGE IN LIVING-APART-TOGETHER RELATIONSHIPS

Nytasia Hicks, *Miami University, Oxford, Ohio, United States*

The preference for living-apart-together (LAT) relationships, where individuals are committed to one another but reside in separate households, has increased among older adults. Despite the growing trend to LAT in later life, there is a dearth of literature on living-apart-together exploring the experiences of minority older adult sub-groups. Particularly, few studies have explored motivations for living-apart-together among minority older adult sub-groups. In this study, using a qualitative descriptive approach, reasons older adult Black women engage in living-apart-together relationships were explored. As part of a larger study, thirteen black women ages 59-74 (married and unmarried) completed two semi-structured phone interviews about their motivations for living-apart-together and how decisions, or lack thereof, to LAT were made. Inductive thematic analytic methods revealed three major motivations for LAT among this subgroup. Motivations included: (1) to protect freedom and self-governance; (2) to maintain current living arrangements with live-in family members; and (3) to maximize healthy relationship characteristics (e.g. individuality). Participants reflected that reasons to engage in LAT were influenced by the distance between living-apart-together partners, current caregiving roles, and level of commitment. As to how older adult black women decided to engage in living-apart-together relationships, Participants reported that LAT happened by coincidence or because of a partner's preference to maintain separate households. Boundary reinforcement regarding role strain was also identified as a core concept. Findings point to the need for applications to variations in relationship types among older adults regarding living arrangements, to professionals supporting aging families, and to further studies of family gerontology.

### MOTHERS' HEALTH AND RELATIONSHIPS WITH ADULT CHILDREN: THE MODERATING ROLE OF GENDER AND RACE

Catherine Stepniak,[1] J. Jill Suitor,[1] and Megan Gilligan,[2]
*1. Purdue University, West Lafayette, Indiana, United States, 2. Iowa State University, Ames, Iowa, United States*

Consistent with theories of the life course and intergenerational solidarity, families are generally closely tied groups in which one family member's event affects other members as well. Although the literature has documented that parents and adult children affect one another's well-being, less is known about how parents' health shapes relationship quality between family members. In this paper, we utilize data from the Within-Family Difference Study (WFDS) II to explore how mothers' functional limitations affect relationship quality between mothers and their adult children, as reported by both family members. We hypothesized that the association between mothers' health and intergenerational relationship quality would be moderated by gender and race. Using multi-level regression modeling, we found that mothers' reports of relationship quality were not predicted by the presence of mothers' functional limitations, nor were there any moderating effects of race or gender. In contrast, adult children who perceived that their mothers had limitations reported higher tension with them. Further, daughters were more likely than sons to report greater tension when they perceived that their mothers had limitations (differences between coefficients p < .10). White adult children reported lower levels of closeness and higher levels of tension when they perceived that their mothers had health limitations (differences between coefficients p < .05; p < .10 respectively). However, limitations did not predict Black children's reports of closeness or conflict with mothers. This study sheds new light on the complex ways in which race and gender moderate the role of mothers' limitations in intergenerational relationship quality.

### SESSION 3018 (SYMPOSIUM)

### ESPO/ BIOLOGICAL SCIENCES SECTION SYMPOSIUM: UNDERSTANDING THE COMPLEX AND INTERCONNECTED PATHWAYS OF AGING

### CIRCULATING CELL-FREE DNA IS ASSOCIATED WITH COGNITIVE OUTCOMES

Danielle Feger,[1] Lolita Nidadavolu,[1] Esther Oh,[2] Peter Abadir,[2] and Alden Gross,[3] *1. Johns Hopkins University, Baltimore, Maryland, United States, 2. Johns Hopkins University School of Medicine, Baltimore, Maryland, United States, 3. Johns Hopkins Bloomberg School of Public Health, Baltimore, Maryland, United States*

Cell death is a mechanism by which aging tissues are able to maintain homeostasis. DNA of nuclear and mitochondrial origin is released into circulation following apoptosis or necroptosis and can be quantified in the blood as circulating cell-free DNA (ccf-DNA). We hypothesized that higher levels of ccf-DNA would be associated with worse cognitive function. Ultra-sensitive digital PCR was used to measure ccf-DNA in participants from the Rush Alzheimer's Disease Center Religious Orders Study/Memory and Aging Project. Global cognitive function was derived from a composite of 19 tests on a neuropsychiatric battery. A total of 885 ccf-DNA samples were analyzed from N=624 participants. Generalized estimating equations were used to estimate the cross-sectional association between ccf-DNA and global cognition scores, while latent growth models were used to estimate the longitudinal association between ccf-DNA and global cognition scores. Multinomial logistic regression was used to estimate the odds of having mild cognitive impairment (MCI) or dementia at last study visit relative to normal cognition, based on levels of ccf-DNA. Higher ccf-DNA levels were associated with lower global cognition score (-0.10, [-0.18, -0.02]) cross-sectionally. Each 1-standard deviation increase in ccf-DNA was associated with more rapidly declining global cognitive function over time (-0.11, [-0.19, -0.03]). A dose-response relationship was observed between increasing levels of ccf-DNA and odds of MCI (odds ratio [OR] = 1.08, [0.83, 1.41]) and dementia (OR = 1.29, [1.06, 1.57]). Our results suggest that ccf-DNA may serve as a biomarker of global cognitive decline and dementia risk.

### DIFFERENCES IN LONGITUDINAL FASTING BLOOD GLUCOSE AND MORTALITY RISK ACROSS THE LIFESPAN BETWEEN MICE AND HUMANS

Dushani Palliyaguru, Eric Shiroma, John Nam, SLAM Investigators, Luigi Ferrucci, and Rafael de Cabo, *National Institute on Aging, Bethesda, Maryland, United States*

Aging profoundly affects metabolism where trajectories of metabolic indices serve as strong predictors of health, disease and mortality. Mice and non-human primates are widely used to model all aspects of human biology, including metabolism. However, there is limited knowledge on how different species metabolically age during their life course. Here, we compare longitudinal predictors of health and mortality of three major metabolic indices among mice, non-human primates and humans. Longitudinal fasting blood glucose, body weight and body composition over the lifespan were compared across species in mice (Study of Longitudinal Aging in Mice), Rhesus monkeys (NIA and Wisconsin colonies) and humans (Baltimore Longitudinal Study on Aging). Survival analysis was conducted to calculate the risk of death for subjects with highest and lowest quartiles of fasting blood glucose. We will present data highlighting species-specific mechanisms of glucose homeostasis over the lifespan and its association with mortality.

### SEX DIFFERENCES IN RESPONSE TO METFORMIN IN A LONGITUDINAL STUDY IN MICE

Camila Vieira Ligo Teixeira,[1] Irene Alfaras,[2] Simonetta Camadola,[1] Nathan Price,[3] Michel Bernier,[1] and Rafael de Cabo,[1] *1. National Institute on Aging,, Baltimore, Maryland, United States, 2. University of Pittsburgh, Pittsburgh, Pennsylvania, United States, 3. Yale University, New Haven, Connecticut, United States*

Sexual dimorphisms have been recognized in most aspects of human health and disease, and there is now clear evidence for more pronounced differences in response to pharmacological interventions of aging. Here, male and female C57BL/6J mice at six months of age were administered 0.5% metformin in their diet every-other-week (EOW) for the remainder of their lives. The intervention was well tolerated and did not result in lifespan extension. Male mice treated with metformin EOW lost weight and augmented lean-to-fat ratio. In contrast, EOW females were refractory to changes in body weight and body composition compared to controls. The intervention did not influence non-fasted plasma glucose levels, while causing an increase in lactate in mice of both sexes. Indirect calorimetry was performed to measure energy expenditure in EOW mice that were either ON or OFF metformin during testing. Focusing on the respiratory exchange ratio (RER), males, but not females, preferentially utilized carbohydrates (RER ~0.9-1.0) when OFF metformin, switching to lipids (RER ~0.8-0.9) when ON metformin. This resulted in significant differences compared to controls in both periods (OFF and ON). RER of EOW females was different from controls during OFF metformin (RER ~0.8-0.9), while exhibiting significantly lower RER when ON metformin (RER ~0.7-0.8). These results clearly point at a strong dimorphism in the action of metformin in mice, including its role in metabolic homeostasis and overall health span. A better understanding of how sex influences the health and prolongevity benefits of metformin will benefit our understanding of the longterm clinical implications.

### SESSION 3020 (PAPER)

### ACUTE CARE AND HOSPITALIZATION (PAPER)

### ASSOCIATION BETWEEN LIVING ARRANGEMENT AND ACUTE CARE USE IN OLDER MEDICARE HOME HEALTH PATIENTS

Jinjiao Wang, Meiling Ying, Xueya Cai, Thomas Caprio, Helena Temkin-Greener, and Yue Li, *University of Rochester, Rochester, New York, United States*

This secondary analysis used a 10% random sample from the national Outcome and Assessment Information Set (OASIS) of Medicare beneficiaries ≥ 65 years old who received home health (HH) care in 2017 (N=646,109). We examined the risk of hospital admission during a 60-day HH episode among Medicare home health patients in different living arrangements, including living alone at home (23.8%), living with other at home (64.8%), and residing in assisted facility (AL) facilities (11.4%). At the start of the HH episode, AL residents were older, more likely to have cognitive impairment, depressive symptoms, and limitations in activities of daily living (ADL) than those living at home at home (alone/with others). In the multivariable logistic regression model of hospital admission adjusting for demographic status (age, sex, race/ethnicity, Medicaid status), cognitive impairment, depressive symptoms, and ADL limitations, when compared to HH patients living with others at home (reference), AL residents were 15% less likely to have hospital admission (Odds Ratio [OR]=0.85, 95% Confidence Interval [CI]: 0.84, 0.88, p<0.001). HH patients living at home alone were not statistically significantly different from the reference (OR=0.99, 95% CI: 0.98, 1.01, p=0.47). HH patients in AL, despite having worse cognitive, mental, and physical function at baseline, had better outcomes than those living at home. This suggests 1) older adults living at home may have unmet health or personal care needs, and 2) synergies may exist between post-acute care through HH care and long-term care and support at AL that are critical to patient welfare.

### CARE TEAM MEMBERS' PERCEPTIONS OF AN INFORMATICS INTERVENTION TO IMPROVE GERIATRIC CARE ACROSS MULTIPLE SITES

Emily Franzosa,[1] Morgan Traylor,[2] Vivian Guerrero Aquino,[3] Kimberly Judon,[4] Ashley Schwartzkopf,[2] Brian Dixon,[5] and Kenneth Boockvar,[6] *1. James J. Peters VA Medical Center, Brooklyn, New York, United States, 2. Richard L. Roudebush VA Medical Center, Indianapolis, Indiana, United States, 3. VHA, New York, New York, United States, 4. US Department of Veterans Affairs - James J. Peters VA Medical Center, Bronx, New York, United States, 5. Center for Health Information and Communication, Health Services Research & Development Service, Indianapolis, Indiana, United States, 6. James J. Peters VA Medical Center, Bronx, New York, United States*

Electronic health information exchange (HIE) may improve care for geriatric patients receiving care across multiple sites by reducing test duplication, medication prescribing errors, and adverse events. This project evaluated the implementation of an HIE intervention at two VA medical centers offering VA providers real-time notification of non-VA inpatient or ED use, followed by post-hospital geriatric care coordination. We interviewed 23 providers (physicians, nurses, social workers and other care team staff) about their experiences with the program. Interviews were analyzed using the Consolidated Framework for Implementation Research (CFIR) to examine 1) goals and expectations for notifications and transitional care; 2) barriers to effective use of notifications and coordination; and 3) suggestions for improvement. Overall, care team members were positive about the intervention, noting it cut down on time searching for outside medical records and that care coordination visits were helpful in answering patients' questions and clarifying discharge instructions. However, some providers were not aware of the alerts, found the HIE interface challenging to use, or were concerned that expanding the program would create workflow issues. Suggestions for improvements included sharing information about newly prescribed medications, lab and radiological tests, and progress alerts during the episode of care; and including non-VA providers to facilitate care coordination. Social workers also asked to be included on alerts to improve follow-up. Our findings suggest HIE can be a useful tool for coordinating care across sites, provided information can be easily shared between all care team members and HIE interfaces are streamlined to reduce additional work.

### CLINICIANS' KNOWLEDGE AND ATTITUDES ABOUT DELIRIUM DETECTION AND MANAGEMENT

Priyanka Shrestha,[1] Erica Husser,[2] Diane Berish,[3] Long Ngo,[4] Marie Boltz,[5] Sharon Inouye,[6] Edward Marcantonio,[4] and Donna Fick,[7] *1. Penn State University, State College, Pennsylvania, United States, 2. Penn State, Spring Mills, Pennsylvania, United States, 3. Pennsylvania State University, University Park, Pennsylvania, United States, 4. Beth Israel Deaconess Medical Center, Boston, Massachusetts, United States, 5. Penn State University, University Park, Pennsylvania, United States, 6. Harvard University, Boston, Massachusetts, United States, 7. Penn State, University Park, Pennsylvania, United States*

Delirium is a serious and potentially life-threatening problem, but it remains clinically under-recognized. Various factors contribute to this under-recognition, including limited understanding of delirium, insufficient training and application of delirium assessments, potential stigma for the patient and increased workload for the clinician. As a part of an NIH funded study testing a rapid two-step delirium identification protocol at two hospitals in the U.S. (one urban and one rural), clinicians completed a 12-item survey to assess their knowledge and attitudes about delirium and their confidence in preventing and managing delirium. Survey response options followed a 5-point rating scale (strongly disagree, disagree, undecided, agree, strongly agree). The sample for this analysis included 399 clinicians (MDs=53; RNs=235; CNAs=111). Chi-square was used to test for group differences between clinician types. Less than half of the clinicians reported agreeing with the statement, "delirium is largely preventable" (MDs: 47%; RN: 44%; CNA: 41%, p-value=0.021). MDs and RNs indicated a high level of confidence in recognizing delirium while CNAs endorsed lower levels of confidence (MDs: 87%; RN: 81%; CNA: 65%, p-value=0.001). All types of clinicians reported lower confidence in managing delirium (MDs: 29%; RN: 36%; CNA: 44%, p-value=0.117). 47% of CNAs and 37% of RNs agreed there is a need for additional training in caring for persons with delirium while only 21% of MDs agreed (p = 0.031). Understanding how different types of clinicians think and feel about delirium will inform training and communication initiatives, clinical implementation, and research on best practices for delirium identification and management.

### POST-OPERATIVE DELIRIUM AND COGNITIVE DECLINE IN KIDNEY TRANSPLANT RECIPIENTS

Nadia Chu,[1] Xiaomeng Chen,[2] Dorry Segev,[2] and Mara McAdams-DeMarco,[3] *1. Johns Hopkins School of Medicine, Bethesda, Maryland, United States, 2. Johns Hopkins School of Medicine, Baltimore, Maryland, United States, 3. Johns Hopkins Bloomberg School of Public Health, Baltimore, Maryland, United States*

Post-operative delirium may be a marker for cognitive reserve, or a greater cognitive vulnerability to stressors. As a result, those with post-operative delirium may experience steeper decline in cognitive performance following stressors of surgery post-KT. We leveraged a single-center cohort study of 912 adult KT recipients with delirium assessments abstracted from medical records. Global cognitive function (3MS) and executive function (time to complete TMT-B minus TMT-A) were measured at time of KT, 1-month, 3-months, 6-months, 1-year, and annually thereafter post-KT. We used mixed effects models with fixed and random effects for person and time to describe repeated measures of cognitive performance and compared trajectories by post-operative delirium. Among 912 KT recipients, 44 (4.8%) had post-operative delirium. Post-operative delirium was associated with higher levels of cognitive impairment at KT (18.2% vs 8.0%), and was associated with lower 3MS component scores including in memory, identification/association, and orientation. After adjustment, those with delirium had 3MS scores that were on average 3.6 points lower than those without delirium (95%CI: -6.9, 0.3) at time of KT; delirium was not associated with differing global cognitive trajectories post-KT (difference=0.04 points/month, 95%CI:-0.1, 0.2). However, delirium was associated with lower executive function at KT (difference=44.0s, 95%CI: 17.4, 70.6) and steeper decline in executive function post-KT (difference=-1.1s/month, 95%CI:-2.1,-0.05). KT recipients with delirium experience greater decline in executive function, indicating greater cognitive vulnerability with potential vascular etiologies. Transplant centers should be aware of the cognitive risks associated with post-KT delirium and implement available preventative interventions to reduce risk of delirium.

### THE HARMONIZATION OF FOUR DELIRIUM INSTRUMENTS

Benjamin Helfand,[1] Elke Detroyer,[2] Koen Milisen,[3] Dimitrios Adamis,[4] and Richard Jones,[5] *1. University of Massachusetts Medical School, Newton, Massachusetts, United States, 2. KU Leuven, Leuven, Netherlands, 3. KU Leuven, LEUVEN, Vlaams-Brabant, Belgium, 4. Sligo Mental Health Services, Sligo, Ireland, 5. Brown University, Providence, Rhode Island, United States*

Delirium is a clinical syndrome characterized by acute cognitive dysfunction, which is pervasive in older persons. Delirium affects over 2.6 million Americans over age 65 annually. One major problem in detection of delirium is that over 40 different instruments have been created to identify delirium in different clinical settings. There is no single agreed upon reference standard instrument. In previous work, we performed a systematic review to identify the four most commonly cited and well-validated instruments for delirium identification. The aim of this study is to harmonize these four commonly used instruments: Confusion Assessment Method (CAM), Delirium Observation Screening Scale (DOSS), Delirium Rating Scale-Revised-98 (DRS-R-98), and Memorial Delirium Assessment Scale (MDAS). We used data from three separate sources (N = 1623). Participants were rated by multiple and overlapping instruments across studies, allowing us to apply item response theory linking procedures. We fit generalized structural equation models, and found unidimensional factor models fit well. We found the instruments were highly correlated (all r > 0.9) and kappa statistics for delirium case identification were high (range: 0.89 to 0.95). We generated crosswalks to map sum scores on one instrument to another. Our results suggest the same underlying construct, propensity to delirium, is measured across the four instruments. The crosswalks facilitate comparison and combination for immediate clinical use or for future meta-analyses. In future steps, we will use our results to find the optimal cut-points for use across all instruments to identify delirium.

### SESSION 3021 (PAPER)

### PALLIATIVE AND END-OF-LIFE CARE

### AGING AND ADVANCED DEMENTIA MATTERS: END-OF-LIFE CARE IN NURSING HOMES

Ruth Lopez,[1] Ellen McCarthy,[2] Meghan Hendricksen,[3] Susan McLennon,[4] Anita Rogers,[5] LaKeva Harris,[1] Ashley Roach,[6] and Susan Mitchell,[7] *1. University of Tennessee Knoxville, Knoxville, Tennessee, United States, 2. Hebrew SeniorLife, Harvard Medical School, roslindale, Massachusetts, United States, 3. Hebrew SeniorLife, Roslindale, Minnesota, United States, 4. University of Tennessee-Knoxville, KNOXVILLE, Tennessee, United States, 5. University of Tennessee at Martin, Parsons, Tennessee, United States, 6. University of Tennessee, Portland, Oregon, United States, 7. Marcus Institute for Aging Research, Roslindale, Massachusetts, United States*

Over 5 million Americans have dementia, and the majority will die in nursing home (NHs). While comfort is the main goal of care for most NH residents with advanced dementia, they commonly receive burdensome and costly interventions such as hospital transfers and feeding tubes that are of little clinical benefit. Despite 20 years of research and numerous experts and associations advocating a palliative approach to care, quantitative studies continue to demonstrate striking and persistent regional, facility, and racial differences, including: greater intensity care among African American versus White residents; greater intensity of care in the Southeastern US; and wide variation in care among NHs in the same region of the country. The reasons for these differences are poorly understood. Assessment of Disparities and Variation for Alzheimer's disease in Nursing home Care at End of life (ADVANCE) is a 3-year, NIA funded qualitative study of 16 NHs in 4 regions of the country which aims to explain regional and racial factors influencing feeding tube and hospital transfer rates. The purpose of this presentation is to present the methodology established in this study and to highlight factors challenging and enabling implementation of the study protocol. To date, data have been collected in 11 NHs, and include 135 staff interviews, 40 proxy interviews, and nearly 800 hours of observation. These findings demonstrate that although challenging, large qualitative research is possible and holds promise as an effective method to illuminate complex processes influencing end-of-life care for NH residents with advanced dementia.

### EVALUATION OF ONLINE PALLIATIVE CARE TRAINING FOR CERTIFIED NURSING ASSISTANTS

Jinsook Kim, and Jennifer Gray, *Northern Illinois University, DeKalb, Illinois, United States*

Palliative care for older adults is increasingly needed due to a burgeoning older adult population. Certified nursing assistants (CNAs) in skilled nursing facilities (SNFs) provide assistance with activities of daily living and comfort care. There, however, is a significant gap in evaluated palliative care trainings for CNAs. We used a waitlisted control group design to evaluate the effectiveness of an 8-module online palliative care training. CNAs (n=102) from 6 SNFs were randomly assigned to an experimental (n=51) and a control group (n=51) and completed a baseline evaluation. The experimental group took a posttest about palliative care knowledge upon training completion and a 1-month follow-up assessment about palliative care self-efficacy. The control group completed the assessments at the same time as the experimental group prior to receiving the training. The majority of the participants were female (92%). On average, participants were 31 years old, with 6.5 years tenure in the field. The retention rate was 90% at the posttest (n=92) and 82% at the 1-month follow-up (n=84). Palliative care knowledge (scored 0–100) significantly increased in the experimental group (mean 4.1, p < 05), with no significant change in the control group. Palliative care self-efficacy (scored 20-100) significantly improved from the baseline to follow-up in both groups (mean 4.3 and 5.8 respectively, p < 05) with no significant difference between study groups. The results indicate the effectiveness of an online palliative care training to improve CNA knowledge. Improvement in palliative care self-efficacy regardless of training participation warrants further exploration.

### GOALS-OF-CARE CONSULT AND FUTURE COSTS, ACUTE CARE AND HOSPICE USE IN MATCHED COHORTS OF AFRICAN AMERICANS AND WHITES

Lauren Starr,[1] Connie Ulrich,[1] Scott Appel,[2] Paul Junker,[3] Nina O'Connor,[3] and Salimah Meghani,[1] *1. University of Pennsylvania School of Nursing, Philadelphia, Pennsylvania, United States, 2. University of Pennsylvania Perelman School of Medicine, Philadelphia, Pennsylvania, United States, 3. University of Pennsylvania Health System, Philadelphia, Pennsylvania, United States*

African Americans receive less hospice care and more aggressive end-of-life care than Whites. Little is known about how palliative care consultation to discuss goals-of-care ("PCC") is associated with future acute care utilization and costs, or hospice use, by race. To compare future acute care costs and utilization and discharge to hospice between propensity-matched cohorts of African Americans with and without PCC, and Whites with and without PCC, we conducted a secondary analysis of 35,154 seriously-ill African American and White adults who had PCC at a high-acuity hospital and were discharged 2014-2016. We found no significant difference between African Americans with or without PCC in mean future acute care costs ($11,651 vs. $15,050, P=0.09), 30-day readmissions (P=0.58), future hospital days (P=0.34), future ICU admission (P=0.25), or future ICU days (P=0.30), but found greater discharge to hospice among African Americans with PCC (36.5% vs. 2.4%, P<0.0001). We found significant differences between Whites with PCC vs. without PCC in mean future acute care costs ($8,095 vs. $16,799, P<0.001), 30-day readmissions (10.2% vs. 16.7%, P<0.0001), future days hospitalized (3.7 vs. 6.3 days, P<0.0001), and discharge to hospice (42.7% vs. 3.0%, P<0.0001). Results suggest PCC decreases future acute care costs and utilization in Whites and, directionally but not significantly, in African Americans; and increases discharge to hospice in both races (15-fold in African Americans, 14-fold in Whites). Research is needed to understand how PCC supports end-of-life decision-making and hospice use across races and how systems and policies can enable effective goals-of-care consultations across settings.

### RECOGNIZING DECLINE AND SUPPORTING CARE TRANSITIONS IN OLDER ADULTS: HOMECARE NURSE PERSPECTIVES

Suzanne Sullivan, Catherine Mann, Samantha Mullen, and Yu-Ping Chang, *University at Buffalo, Buffalo, New York, United States*

Older adults with serious illness residing in the community are at risk for decline and death. Homecare Registered Nurses (RNs) are in an ideal position to recognize serious illness and engage older adults and their caregivers in discussions about goals for care, while guiding transitions to supportive care services such as palliative or hospice care. However, little is known about this process, or how homecare RNs act upon this information. Using a grounded theory approach, data were collected through focus group interviews with 35 RNs working in homecare. A social process rooted in relationship-based care over time was identified using the constant comparative method. RNs recognize serious illness and support care transitions by identifying changes in illness trajectories and assessing the impact of such changes on quality-of-life, adapting and accommodating care to support older adults in the home for as long as possible, communicating with the care team, engaging stakeholders, and maneuvering through complex systems of care; ultimately relinquishing care to other providers and settings. Our findings also reveal that RNs feel inadequately prepared and frustrated with a fragmented healthcare system and lack of collaboration among the team in supporting the best care transition for older adults and their caregivers. Our findings reinforce the importance of promoting care continuity in homecare settings whenever possible, suggesting a critical need to develop training and team processes that support and empower RNs, so that they may lead care transitions as changing needs emerge during serious illness management of older adults.

### SESSION 3022 (PAPER)

### ASSESSMENT (PAPER)

### BALANCE AND REACTIVE STEPS IN OLDER ADULTS WITH AND WITHOUT SELF-REPORTED MUSCULOSKELETAL CONDITIONS

Ferdinand Delgado, Cheryl Der Ananian, and Daniel Peterson, *Arizona State University, Phoenix, Arizona, United States*

Older adults with musculoskeletal conditions (MSC), including arthritis and osteoporosis, may have a higher risk of falls and falls-related injuries. Differences in balance between individuals with and without self-reported MSC are not well understood. Therefore, this study compared measures of balance (static and dynamic) and reactive stepping between older adults (N=99) with (75.79±5.38 years, n=38, 82% female) and without (75.93±6.36 years, n=61, 67% female) MSC. A cross-sectional design was used. Static balance was assessed via postural sway area (PSA) and PSA root mean square (PSARMS) during quiet stance. Dynamic balance was assessed with the Timed Up & Go (TUG), a dual-task cognitive TUG (TUG-COG), and the Four Square Step Test (FSST). Reactive stepping was measured as the first step latency, length, width, time, total number of recovery steps, and time until balance recovery after a backward lean and release. Linear regression was used to assess group differences. After adjusting for age, sex, body mass index (BMI), and grip strength, there were no significant differences between groups in static balance (PSA (p=0.884); PSARMS (p=0.246)) and reactive stepping outcomes (first step latency (p=0.184); total number of steps (p=0.423); step width (p=0.964)). The other reactive step outcomes are not reported since explained variance was not statistically significant (p>0.05). With dynamic balance, significant group differences showed individuals with MSC took more time to complete TUG (p=0.011) and TUG-COG (p=0.005), but not the FSST (p=0.493). Our findings suggest improving dynamic balance, especially with a walking component, in older adults with self-reported MSC is needed.

### BUILDING A FRAMEWORK FOR CARE OF OLDER PATIENTS IN AN ACADEMIC SETTING: HIGH RISK GERIATRICS AMBULATORY CARE PROGRAM

Stephanie Chow,[1] Audrey Chun,[2] Lizette Munoz,[3] Shamsi Fani,[4] Rolfe Sean Morrison,[1] Blair MacKenzie,[3] Susana Lavayen,[3] and William Hung,[5] *1. Icahn School of Medicine at Mount Sinai, New York, New York, United States, 2. Icahn School of Medicine at Mount Sinai, New york, New York, United States, 3. Mount Sinai Hospital, New York, New York, United States, 4. Mount Sinai Medical Center, New York, New York, United States, 5. Icahn School of Medicine at Mount Sinai; James J Peters VA Medical Center, Bronx, New York, United States*

BACKGROUND: Traditional models of geriatric medicine and health system reimbursement structure often force ambulatory care teams to function as high-volume delivery programs, thereby dis-servicing our most vulnerable and frail older patients. This “high cost and high needs” labeled demographic requires uniquely adapted plans from medical and social work providers. METHODS: To better examine opportunities for improved framework for geriatric ambulatory care, the Acute Life Interventions, Goals & Needs (ALIGN) Program has launched several inter-professional pilot programs, each with intention to explore components of health care service to older patients, and feasibility of implementation in other health care systems. Three current models include the ALIGN Program itself, a telemedicine community paramedicine program, and a geriatric surgery co-management program. RESULTS: Preliminary results are forthcoming, with initial promising findings. For the first 126 patients enrolled, mean emergency room (ED) visits 6 months prior to ALIGN enrollment were 1.7 visits per person, reduced to 0.7 ED visits/person 6 months post-graduation from the program, and 126 fewer ED visits. Mean hospitalization 6 months prior to enrollment was 0.32 per person, whereas 6 months post-graduation was 0.2 hospitalizations/person, totaling 40.32 hospitalizations saved. Mean length of stay in the hospital 6 months prior to ALIGN enrollment for the 22 patients admitted was 7.7 days, reduced to 7.3 days post-graduation, and 32 fewer hospital days in the small subset of patients requiring hospitalization despite program interventions. CONCLUSION: The ALIGN Program’s multi-professional and flexible modularity highlights promising innovative frameworks for ambulatory geriatrics care, warranting further exploration and collaboration.

### GRIP STRENGTH CHANGES IN BONE MARROW TRANSPLANT PATIENTS

Paulo Chaves,[1] Adrian Cristian,[2] and Marco Ruiz,[2]
*1. Florida International University, Miami, Florida, United States, 2. Baptist Health South Florida, Miami, Florida, United States*

Emerging evidence that cancers survivors may experience premature aging has prompted epidemiology research aimed at the characterization of key premature aging phenotypes. Physical functioning data relevant for premature aging phenotype classification in cancer survivors, however, remain scarce. We assessed the burden of dynapenia, a key sarcopenia indicator, and muscle strength loss in the understudied population of bone marrow transplant (BMT) patients. This study involved secondary analyses of data from patients who underwent bone marrow transplant (BMT) in a cancer Institute in South Florida (June 2018-April 2019) with traditional hematopoietic cell transplantation specific comorbidity index (HCT-CI) scores and other relevant information. T-tests for paired data assessed pre- vs. post-BMT changes in grip strength (GS) (n=9). Prevalence of dynapenia (GS<27Kg and <16Kg) in pre-BMT (n=20) and post-BMT (n=9) periods were calculated. Median age [25th-75th percentiles] age was 61 [55-68] years old. Fifty-percent were females. GS and HCT-CI scores were not correlated. Mean GS loss after BMT was 6.4 Kg (95% confidential interval: 2.5-10.4 Kg, p-value=.003) in post-BMT (median-time-after-BMT: 90 days) minus pre-BMT (median-time-before-BMT: 62 days) comparison. Proportion with dynapenia increased from 10% (1/10) to 20% (1/5) in women, and 10% (1/2) to 75% (3/4) in men. We observed substantial muscle strength loss and dynapenia burden in patients who underwent BMT. Considering that dynapenia is a major, potentially modifiable risk factor for frailty, which is not captured by HCT-TI, we speculated that dynapenia-related targeted assessments and interventions – preventive and/or rehabilitative – could offer complementary approaches for treatment enhancement in BMT patients.

### MAZE TEST SCORE ADJUSTMENTS WHEN USING NON-DOMINANT HAND IN FITNESS-TO-DRIVE ASSESSMENTS

Carolyn Unsworth,[1] Gemma Hext,[2] Anne Baker,[3] and Matthew Browne,[4] *1. Central Queensland University, Melbourne, Victoria, Australia, 2. Australian Catholic*

*University, melbourne, Victoria, Australia, 3. Australian Catholic University, Melbourne, Victoria, Australia, 4. CQUniversity, Bundaberg, Queensland, Australia*

Older drivers with health impairments may be required to undertake fitness-to-drive assessments. Scores on the Occupational Therapy-Drive Home Maze Test (OT-DHMT) can contribute to fitness-to-drive recommendations. The OT-DHMT is a short, timed maze test that has been shown to be valid and reliable, and norms are available for completion with a driver's dominant hand. However, the validity of a person's score when using their non-dominant hand to complete the test, for example following stroke, is unknown. This study aimed to determine if a person's OT-DHMT score time (in seconds) requires adjustment when completed with a non-dominant hand. The OT-DHMT was administered with a normative sample of 150 participants, aged 21-81 years (mean=48.6,SD=19.38). Overall, OT-DHMT score times were significantly faster when using a dominant (M=15.73) compared with non-dominant (M=17.64) hand, d=1.91 (CI 1.13, 2.69), t= 4.84,p< .01. Employing a generalised weighted least squares regression model indicated that multiplying a driver's non-dominant hand time by .833 seconds for drivers aged ≤60, and by .929 seconds for drivers aged 61+ can approximate dominant hand completion times. Adjusted scores can then be compared against normed scores to aid fitness-to-drive recommendations. The adjustment required for people aged ≤60 is larger than for older people, reinforcing previous findings that younger people have faster OT-DHMT completion times. These findings support the clinical utility and vaildity of using the OT-DHMT with older people undergoing fitness-to-drive assessment who may be required to use their non-dominant hand due to conditions such as stroke, arthritis or amputation.

### MOTOR ADAPTATION TO COGNITIVE CHALLENGES AND DESTABILIZING TREADMILL WALKING IN HEALTHY OLDER ADULTS

Pei-Chun Kao,[1] and Michaela Pierro,[2] *1. University of Massachusetts Lowell, Lowell, Massachusetts, United States, 2. Delsys Inc, Natick, Massachusetts, United States*

To develop effective fall prevention intervention, it is necessary to understand how older adults respond to challenges that demand cognitive-motor dual-tasking capability, an important capability in the daily lives. The purpose of this study is to investigate how older adults adjust their motor responses when encountering cognitive and walking perturbations simultaneously. We recorded kinematic data as subjects walked on a treadmill with or without 1) continuous random-amplitude treadmill platform sways (Perturbed vs. No-perturbed walking); and 2) each of the four cognitive tasks: Paced Auditory Serial Addition test (PASAT), clock test, visual color-word incongruent test (V-stroop), and auditory pitch-word incongruent test (A-stroop). We computed dynamic margins of stability (MOS), gait variability, and short-term local divergence exponent (LDE) of the trunk motion (local stability). Data of ten older subjects (age: 72.2±4.9) show that cognitive performance did not differ between standing, Perturbed or No-perturbed walking. Subjects demonstrated significantly greater local instability and variability in step measures, joint angle and MOS during Perturbed than No-perturbed walking (p<0.001). During dual-task conditions, subjects walked with significantly larger mediolateral MOS (MOSML) compared to walking only, especially during early phase of the trial. During Perturbed walking, subjects had significantly larger MOSML during PASAT and Vstroop than walking only. Our data showed that subjects tried to increase their dynamic MOS during Perturbed walking or a cognitive task more difficult or taxing visual attention. However, the adjustments do not sustain throughout the trial. These findings suggest older adults tend to prioritize cognitive over walking tasks even when encountering walking perturbations.

### SESSION 3023 (PAPER)

### CARDIOVASCULAR HEALTH AND DISEASE (PAPER)

### AGE-RELATED VARIATION IN CARDIOVASCULAR RISK ASSOCIATED WITH RED CELL DISTRIBUTION WIDTH

Jarrod Dalton,[1] David Zidar,[2] Sadeer Al-Kindi,[3] Nikolas I. Krieger,[4] and Adam Perzynski,[5] *1. Cleveland Clinic Lerner College of Medicine at Case Western Reserve University, Cleveland, Ohio, United States, 2. Louis Stokes Cleveland VA Medical Center, Cleveland, Ohio, United States, 3. University Hospitals Cleveland Medical Center, Cleveland, Ohio, United States, 4. Lerner Research Institute - Cleveland Clinic, Cleveland, Ohio, United States, 5. Case Western Reserve University at MetroHealth, Cleveland, Ohio, United States*

We previously showed that routine indicators of immune-hematologic function are strongly associated with all-cause and cardiovascular mortality, especially among older adults, in whom traditional atherosclerotic cardiovascular disease (ASCVD) risk factors predict poorly. The objective of this study was to quantify differences in the relationship between red cell distribution width (RDW) and 5-year risk of ASCVD events (stroke, myocardial infarction or cardiovascular death) as a function of age and the area deprivation index (ADI). We analyzed electronic health records of 76287 Cuyahoga County, Ohio residents who were over age 40 and who visited Cleveland Clinic Health System and/or MetroHealth System in two consecutive years between 2005 and 2015, the latter of which served as the index/baseline. Multivariable Cox regression was used, adjusting for sex, race/ethnicity, diabetes, systolic blood pressure and antihypertensive use. Generally, RDW levels in disadvantaged neighborhoods corresponded to people 15-20 years older from affluent neighborhoods. In a main-effects-only model, we found higher ASCVD event rates associated with age (hazard ratio [95% confidence interval] per 10 year increment: 1.34 [1.32–1.35]), ADI (top vs. bottom quintile: 1.30 [1.24–1.36]) and RDW (>16% vs ≤13%: 2.03 [1.94–2.12]). Age and RDW exhibited a synergistic interaction ($\chi 2$ = 17.7 on 2 df, p<0.001), with slightly larger RDW effects associated with increasing age, while evidence of differential RDW effects across ADI quintiles was weak ($\chi 2$ = 12.9 on 8 df, p=0.11). We conclude that RDW effects on ASCVD event risk are large, independent from traditional ASCVD risk factors and increase with advancing age.

### ASSESSING CARDIOMETABOLIC HEALTH RISK AMONG U.S. CHILDREN WHO LIVE IN GRANDPARENT-HEADED HOUSEHOLDS

MinKyoung Song,[1] Karen Lyons,[2] Laura Hayman,[3] Nathan Dieckmann,[1] and Carol Musil,[4] *1. Oregon Health & Science University, Portland, Oregon, United States, 2. Boston College, Chestnut Hill, Massachusetts, United States, 3. University of Massachusetts Boston, Boston, Massachusetts, United States, 4. Case Western Reserve University, Cleveland, Ohio, United States*

Many interventions have been designed to leverage parent-caregivers as change agents for improving children's cardiometabolic health (CMH), however very few have been designed to leverage grandparent-caregivers for that purpose. This is surprising since there has been a steady increase in children living in grandparent-headed households. As a first step in assessing the potential impact of interventions with grandparent-caregivers, we used data from the National Survey of Children's Health (2018) to compare CMH measures in children living in grandparent-headed households with CMH measures in children living in parent-headed households. Our hypothesis was that CMH risk might be higher in grandparent households – given that research shows that grandparents taking over caregiving from parents is associated with worse overall health outcomes for both grandparents and their grandchildren. Additionally, since research indicates that children who experience ≥ 4 adverse childhood experiences (ACEs) have significantly worse health outcomes, we assessed levels of ACEs. Our analytic sample included children aged 10-17 years (n=14,941). Adjusting for age, sex, race/ethnicity, and health insurance coverage status, children living in grandparent households were more likely to be obese (Adjusted Odds Ratio [95% confidence interval]= 2.04 [1.02, 4.09]), exposed to secondhand smoke (2.32 [1.49, 3.59]), and less likely to meet recommended age-appropriate standards for sleep (0.42 [0.27, 0.67]). The children living in grandparent households were more likely to experience ≥ 4 ACEs (8.59 [5.42, 13.62]). Our results provide indirect evidence that interventions with grandparent-caregivers may be particularly critical for improving CMH risk in families.

### ASSESSMENT OF APPROPRIATENESS OF CARDIOVASCULAR PREVENTIVE MEDICATION IN ADULTS ≥ 75 YEARS

Milly van der Ploeg,[1] Rosalinde Poortvliet,[2] Jacobijn Gussekloo,[2] and Yvonne Drewes,[2] *1. Leiden University, Leiden, The Hague, Netherlands, 2. Leiden University Medical Center, Leiden, Zuid-Holland, Netherlands*

Physicians are confronted with dilemma's on cardiovascular preventive medication for older people with complex health problems. With accumulation of diseases, limitations and shortening life expectancy, questions arise for whom cardiovascular preventive treatment is still appropriate (the expected benefits of treatment exceeds the negative consequences by a sufficiently wide margin resulting that treatment is worth doing). There is a need for more guidance in clinical situations. With the RAND/UCLA appropriateness method (RUAM), we investigated the appropriateness of cardiovascular preventive medication in adults ≥ 75 years. The RUAM is a systematic, formalized method, to combine available scientific evidence with the collective judgment of experts. Fourteen interdisciplinary panelists (9 physicians representing 6 medical disciplines, 1 medical ethics expert, 1 pharmacist and 3 older adults [lay man]), discussed and rated the appropriateness of starting and stopping of three medication groups (cholesterol lowering, blood pressure lowering and thrombocyte aggregation inhibitors) for different clinical scenarios (combinations of cardiovascular history, systolic blood pressure, complexity of health problems, age, life-expectancy, side-effects). Depending on the medication group, different patterns of appropriateness judgments across the clinical scenarios were found. In general, absence of cardiovascular disease, presence of complex health problems, a short life-expectancy or hindering side-effects were important factors to judge cardiovascular preventive medication as inappropriate. Results were summarized into colored reading maps. These findings can offer more guidance in clinical decision making about cardiovascular preventive treatment for adults ≥ 75 years.

### REDUCTION OF ELEVATED PROTON LEAK REJUVENATES MITOCHONDRIA IN THE AGED CARDIOMYOCYTE

Huiliang Zhang,[1] Nathan Alder,[2] Wang Wang,[1] Hazel Szeto,[3] David Marcinek,[1] and Peter Rabinovitch,[1] *1. University of Washington, Seattle, Washington, United States, 2. University of Connecticut, Mansfield, Connecticut, United States, 3. Alexandria LaunchLabs, New York, New York, United States*

Rational: Aging-associated diseases, including cardiac dysfunction, are increasingly common in the population. However, the mechanisms of physiologic aging in general, and cardiac aging in particular, remain poorly understood. While effective medical interventions are available for some kinds of heart failure, one age-related impairment, diastolic dysfunction in Heart Failure with Preserved Ejection Fraction (HFpEF) is lacking a clinically effective treatment. Methods and Results: Using the pH indicator cpYFP in the model of naturally aging mice and rats, we show direct evidence of increased mitochondrial proton leak in aged heart mitochondria following a pH gradient stress. Furthermore, we identified Adenine Nucleotide Translocator 1 (ANT1) as mediating the increased proton permeability of old cardiomyocytes. Most importantly, acute (2 hours) in vitro treatment with the tetrapeptide drug SS-31 (elamipretide) reverses age-related excess proton entry, decreases the mitochondrial flash activity and mitochondrial permeability transition pore (mPTP) opening and rejuvenates mitochondrial function. Moreover, we show that SS-31 benefits the old mitochondria by direct association with ANT1 and stabilization of the mitochondrial ATP synthasome, leading to substantial reversal of diastolic dysfunction. Conclusion: Our results uncover excessive mitochondrial proton leak as a novel mechanism of age-related cardiac dysfunction and elucidate how SS-31 is able to reverse this clinically important complication of cardiac aging.

### THE ASSOCIATION BETWEEN MYOCARDIAL STRAIN AND FRAILTY IN THE CARDIOVASCULAR HEALTH STUDY

Annabel Tan,[1] Sanjiv J. Shah,[2] Jason Sanders,[3] Bruce Psaty,[4] Anne Newman,[5] Chenkai Wu,[6]

Julius Gardin,[7] and Michelle Odden,[8] *1. Stanford University, Stanford, California, United States, 2. Northwestern University, Chicago, Illinois, United States, 3. Brigham And Women's Hospital, Wellesley, Massachusetts, United States, 4. University of Washington, Seattle, Washington, United States, 5. University of Pittsburgh, Pittsburgh, Pennsylvania, United States, 6. Duke Kunshan University, Kunshan, Jiangsu, China, 7. Rutgers New Jersey Medical School, Hackensack, New Jersey, United States, 8. Stanford University, STANFORD, California, United States*

Myocardial strain, measured by speckle tracking echocardiography (STE), is a novel measure of subclinical cardiovascular disease and may reflect myocardial aging. We aimed to explore the association between myocardial strain and frailty, a clinical syndrome of impaired resilience and lack of physiologic reserve. Frailty was defined in 4,042 participants of the Cardiovascular Health Study (CHS) as having 3 or more of the following clinical criteria: weakness, slowness, shrinking, exhaustion, and inactivity. We examined the cross-sectional and longitudinal associations of left ventricular (LV) longitudinal strain, LV early diastolic strain rate and left atrial reservoir strain with frailty in participants with no history of cardiovascular disease or heart failure at the time of echocardiography. In cross-sectional analyses, LV longitudinal strain, LV early diastolic strain, left atrial reservoir strain and LV ejection fraction (measured by conventional echocardiography) levels were lower (worse) among frail participants than among those who were not frail and pre-frail ($p<0.01$). This association of LV longitudinal strain and frailty was robust to adjustment by LV ejection fraction (adjusted OR: 1.34, 95% CI: 1.20, 2.09). Conversely, LV ejection fraction was not associated with frailty after adjustment for LV longitudinal strain. In longitudinal analyses, LV longitudinal strain and LV early diastolic strain were associated with incident frailty (adjusted OR: 1.49, 95% CI: 1.07, 2.08) and 1.65, 95% CI: 1.15, 2.25, respectively). In community-dwelling older adults without prevalent cardiovascular disease, worse LV longitudinal strain, reflective of subclinical myocardial dysfunction, was associated with frailty independent of LV ejection fraction and other risk factors.

### SESSION 3024 (PAPER)

### EPIDEMIOLOGY (PAPER)

### ACCELERATING TRANSLATION OF INTERVENTIONS: DOES IT NEED TO TAKE 17 YEARS FOR OLDER ADULTS TO BENEFIT FROM SCIENCE?

Janet Bettger, and Janet Prvu Bettger, *Duke University School of Medicine, Durham, North Carolina, United States*

Translation of evidence refers to widespread dissemination, adoption and implementation of interventions that can have a significant effect on population health. However, effective translation has been slow; significant lags and inconsistent uptake impede intended benefits for older adults. In response, interest and investments in implementation science as the study of methods to promote the adoption and integration of evidence into real-world settings have rapidly increased. By definition, the methodology applies to evidence-based practices, interventions, and policies. But the process of evidence generation can still be prolonged. This paper introduces a framework being tested at the Duke Roybal Center that integrates a model for behavioral intervention development and testing with principles of implementation science in order to accelerate translation across all phases of behavioral research. Attendees will first learn about the NIH Stage Model supported by NIA that guides researchers to identify, define, and clarify an array of activities across six stages of behavioral intervention development. These stages define components of intervention generation, pilot and then efficacy testing, effectiveness research and ultimately implementation of potent theory-driven interventions that improve health and well-being. With this foundation, the Duke framework will be presented to illustrate how concepts of several common implementation science frameworks and models can be integrated within the different stages. Interactive case studies will be used to illustrate application of this new integrated framework for evidence generation, accelerated implementation and scale-up, and pathways for translation. Integrating the Stage Model with principles from implementation science can accelerate translation.

### ALLOSTATIC LOAD AND BIOLOGICAL AGING INDICATORS IN THE MIDUS NATIONAL SURVEY

Waylon Hastings, Idan Shalev, and David Almeida, *Pennsylvania State University, University Park, Pennsylvania, United States*

Indices quantifying allostatic load (AL) and biological aging (BA) have received widespread use in epidemiological and health science literature. However, little attention has been paid to the conceptual and quantitative overlap between these indicators. By reviewing literature utilizing measures of AL and BA, we highlight differences with respect to biological markers employed and approach toward scale construction. Further, we outline opportunities where AL indices might be improved by adopting analytical features of BA measures. We demonstrate the utility of this approach using data from The MIDUS National Survey, constructing three indices of allostatic load: one standard approach modeled after Gruenewald et al, 2012, and two alternative formulations informed by BA procedures. The performance of AL indices are juxtaposed against two commonly employed indices of biological aging: Klemera-Doubal Method Biological Age and Homeostatic Dysregulation. All measures were significantly associated with chronological age. Alternative AL formulations were more strongly associated with biological aging measures than with the standard approach. MIDUS participants with increased allostatic load and older biological ages performed worse on tests of physical, cognitive, perceptual, and subjective functioning. Further, MIDUS participants with history of childhood-trauma and mental-health problems were measured as having increased AL and BA. Alternative AL formulations tended to have effect-sizes equivalent to or larger than those observed for BA measures. In conclusion, indices of allostatic load and biological age approximate similar processes when constructed with comparable biomarkers and rigor, in line with their conceptual overlap as proxies of cumulative wear and tear.

### PARENTAL LONGEVITY IS ASSOCIATED WITH BRAIN VOLUMES IN SELECTED AREAS

Qu Tian,[1] David Melzer,[2] Luke Pilling,[2] and Luigi Ferrucci[1]
*1. National Institute on Aging, Bethesda, Maryland, United States, 2. University of Exeter, Exeter, United Kingdom*

A few studies report that parental longevity is associated with faster gait speed, less memory decline, a lower risk of Alzheimer's disease, and lower white matter hyperintensities. Data on structural neuroimaging correlates of parental longevity and its spatial distribution are limited. This study aims to examine relationships of parental longevity with regional brain volumes. We identified 10,513 participants from UK Biobank (mean age=58±6, ranged40-70, 50%women) with data on parental longevity and information on MRI regional brain volumes that have been related to executive function (dorsolateral prefrontal cortex), memory (hippocampus, parahippocampal gyrus, inferior temporal lobe, middle temporal lobe) and motor function (precentral gyrus, putamen, caudate, corpus callosum). Participants were categorized based on whether at least one parent lived to age 85 or older or neither parent survived to age 85. Associations of parental longevity with each brain volume measure were examined using linear regression, adjusted for age, sex, education, ApoE e4 status, total gray matter atrophy, white matter hyperintensities, hypertension, and glucose. Compared to participants whose both parents died before 85, those with at least one parent surviving to 85 had greater brain volumes in hippocampus, parahippocampal gyrus, inferior temporal lobe, middle temporal lobe, and precentral gyrus in fully adjusted models (Bonferroni corrected p<0.01). There were no significant associations with volumes in dorsolateral prefrontal cortex, putamen or caudate. Parental longevity is associated with preserved brain structure localized in memory- and motor-related cortical regions. These findings support previous reports that parental longevity is associated with better memory and gait with aging.

### PHYSICAL ROBUSTNESS AND RESILIENCE AMONG LONG-LIVED FEMALE SIBLINGS: A COMPARISON WITH SPORADIC LONG-LIVERS

Angéline Galvin,[1] Svetlana Ukraintseva,[2] Konstantin Arbeev,[2] Mary Feitosa,[3] and Kaare Christensen,[4] *1. University of Southern Denmark, Odense C, Denmark, 2. Duke University, Durham, North Carolina, United States, 3. Washington University School of Medicine, St Louis, Missouri, United States, 4. University of Southern Denmark, Odense C, Syddanmark, Denmark*

Background: Long-lived individuals are central in studies of determinants of healthy longevity. However, few pro-longevity factors have been identified, presumably because of "phenocopies", i.e. individuals that live long by chance. Familial longevity cases may include less phenocopies than sporadic cases and provide better insights into longevity mechanisms. Here we examined whether long-lived female siblings have a better ability to avoid common diseases at ages 65+ (proxy for "robustness") and/or survive to extreme ages (proxy for "resilience") compared to sporadic long-livers. Methods: 1,156 long-lived female siblings were selected from three nationwide Danish studies (DOS, GeHA, LLFS) and age-matched with sporadic long-lived female control from the Danish population. Outcomes included cumulative incidence of common health disorders from age 65, and overall survival from 2006 onwards. Logistic and Cox models were used to evaluate incidence and survival respectively. Results: Long-lived female siblings had significantly lower risks of hypertensive (OR=0.84; 95%CI=0.71-0.99) and cerebrovascular (OR=0.73; 95%CI=0.55-0.96) diseases and depression (OR=0.74; 95%CI=0.62-0.88) at ages 65+, and better survival to extreme ages (HR=0.71; 95%CI= 0.63-0.81) compared to sporadic long-livers. After adjusting for diseases above, the association with mortality changed only marginally (HR=0.73 (0.64-0.83)). Conclusion: Familial longevity cases could be more informative for studying mechanisms of healthy longevity than sporadic cases. Long-lived female siblings demonstrate better physical robustness and resilience than their age-peers from general population, which might be attributed to a genetic component in familial longevity.

### SESSION 3025 (PAPER)

### PAIN AND PAIN MANAGEMENT

### PAIN INTERFERENCE: A BARRIER FOR DAILY LIVING ACTIVITIES IN OLDER ADULTS WITH MULTISITE MUSCULOSKELETAL PAIN

Yael Koren,[1] and Suzanne Leveille,[2] *1. University of Massachusetts Boston, Dedham, Massachusetts, United States, 2. University of Massachusetts Boston, Boston, Massachusetts, United States*

Almost half of older adults experience multisite musculoskeletal pain (MMP) contributing to difficulty in daily activities but little is known about specific domains by which pain interferes in daily living. This study aims to determine domains of pain interference (PI) related to MMP in a cohort of older adults living in the community. The MOBILIZE Boston Study (MBS) is a cohort study of 749 adults aged ≥70y. Musculoskeletal (MSK) pain was assessed using the joint pain questionnaire and grouped as: no pain, single site, and multisite pain. The Brief Pain Inventory PI sub-scale assessed level of interference (0-10 rating) in 7 categories in the previous week including general activity, mood, walking, work, relationships with people, sleep, and enjoyment of life. Interference items were grouped as: none (0 rating), mild (>0, ≤2), moderate ( ≥2, ≤5), and severe (≥5) PI. There was a strong gradient of PI according to pain groups with severe walking interference in 36.5% of those with MMP compared to 3.8% of those with no MSK pain. The least PI was in relationships with others (9.1% of MMP vs 1.1% of no MSK pain). Reports of interference in other domains were intermediate (20-26% of MMP vs 3-4% of no MSK pain). Women and those with Less education reported the most PI in every domain but no differences were observed by age. Greater attention to specific domains of pain interference such as walking could have substantial benefits for reducing the overall impact of MMP among older adults.

### PAIN MANAGEMENT AND COGNITIVE FUNCTION AMONG OLDER ADULTS IN CHINA

Xi Pan,[1] and Hongdao Meng,[2] *1. Texas State University, San Marcos, Texas, United States, 2. University of South Florida, Tampa, Florida, United States*

Chronic pain and cognitive decline are common age-related conditions affecting a large segment of older populations. Little is known about the pathway of cognitive functioning during the course of pain management in older adults. The current study aimed to examine the association between chronic body pain management and cognitive function over time among Chinese older adults. A total of 792 respondents aged 60 and above from urban and rural households in 28 provinces, 150 counties/districts, and 450 communities were selected from the China Health and Retirement Longitudinal Study (2013–2015). Cognitive function was measured in three domains: episodic memory, mental status, and global cognitive function. Difference-indifferences approach and mixed-effects linear regression models were employed to assess the association between chronic body pain management and cognitive function over time. Scores of mental status were found to decline slower by 0.49 unit (SE = 0.22, $p < 0.05$) in respondents who received pain management using analgesics, complementary and alternative medicine, or both from 2013 to 2015 after controlling for basic demographic and health confounders. Chronic pain management was associated with slower decline in domain-specific cognitive function, mental status over time. Findings of the study may contribute to understanding the mechanism of change in diverse cognitive abilities attributable to pain symptoms. More research is needed to elucidate the mediating effect of pain on cognitive decline, which could lead to testing of the impact of pain management on cognitive function among older population in both clinical and community settings.

### POOR SLEEP INCREASES NEXT DAY PAIN, PARTICULARLY FOR OLDER ADULTS WITH MILD COGNITIVE IMPAIRMENT

Xiaopeng Ji, Mary Bowen, and Mari Griffieon, *University of Delaware, Newark, Delaware, United States*

Sleep studies examine how pain is associated with poor sleep. However, emergent research suggests poor sleep increases pain and may interfere with activities of daily living (ADL) among older adults. This study will examine how poor sleep may affect next-day pain interference and how this relationship may vary by cognitive function. Ten community-dwelling older adults with lower extremity chronic pain wore an Actigraph GT9X Link for 7 days to measure poor sleep and next-day pain interference (Brief Pain Inventory; BPI). Multilevel mixed models accounted for intra-individual changes in sleep and pain interference and controlled for age, mild cognitive impairment (MCI) and depressive symptoms. Poor sleep among older adults with MCI (14 total observations) was also explored. Across 79 observations, increased number of awakenings ($\beta=0.03$; $p\leq 0.05$) and movement index scores ($\beta=0.08$; $p\leq 0.05$) were associated with increased next-day pain interference. In exploratory analyses, MCI intensified relationships between sleep efficiency ($\beta=-0.10$; $p\leq 0.05$), increased awakenings after sleep onset ($\beta=0.01$; $p\leq 0.05$) and increased length of sleep awakenings ($\beta=0.39$; $p\leq 0.01$) on next-day pain interference. This study's findings suggest poor sleep is associated with next-day pain interference and the ability to perform ADL. Older adults with MCI may be at an increased risk for poor sleep and pain-related interference in ADL. Interventions designed to moderate the association between poor sleep and pain in general and for adults with MCI in particular may be warranted.

### RECENT PAIN AMONG OLDER WOMEN AND MEN IN THE UNITED STATES BY SEXUAL ORIENTATION

Adena Galinsky,[1] Karen Fredriksen Goldsen,[2] James Dahlhamer,[1] and Tina Norris,[1] *1. National Center for Health Statistics, Hyattsville, Maryland, United States, 2. University of Washington, Seattle, Washington, United States*

Pain is not only a result of other health problems but an independent condition that can negatively impact quality of life. Lesbian, gay, and bisexual older adults report more pain compared to their straight counterparts when pain is measured in the aggregate (e.g. "one or more of the following types of pain"). However, scant national research has examined if specific types of pain vary by sexual orientation among older adults. Using 2015-2018 National Health Interview Survey (NHIS) data, we used logistic regression to separately model four types of pain among women 50+ and men 50+ (lesbian/gay women: n=377, bisexual women: n=142, straight women: n=33,216; gay men: n=508, bisexual men: n=115, straight men: n=25,998) as functions of sexual orientation, controlling for age, race, education, and income. Lesbian women and bisexual men were more likely (AOR=1.46, 95% CI:1.03, 2.08; AOR=2.95, 95% CI:1.08, 3.79, respectively), but bisexual women were less likely (AOR=0.6, 95% CI:0.33, 1.05), than their straight counterparts to have had a migraine or severe headache in the past three months. Bisexual men were also more likely than straight men to report lower back pain in the past three months (AOR=1.66, 95% CI: 1.02, 2.72). Sexual minority women were more likely than straight women to report joint pain in the past 30 days and lower back pain in the past three months. Future research may examine why the prevalence of specific types of pain vary by sexual orientation among older adults.

### THE MODEL OF EMPATHIC PAIN ASSESSMENT AND TREATMENT IN PERSONS WITH DEMENTIA

Lauren Starr,[1] and Kristin Corey Magan,[2] *1. University of Pennsylvania School of Nursing, Philadelphia, Pennsylvania, United States, 2. University of Rhode Island College of Nursing, Providence, Rhode Island, United States*

There are 5.8 million people with Alzheimer's dementia in the United States—81% are 75 years or older. Although half of persons with dementia regularly experience pain, their pain is underrecognized and undertreated, partly because clinicians experience challenges assessing pain in persons with dementia who cannot self-report. Evidence suggests clinician empathy is involved in pain assessment and treatment. Conceptual models guiding Alzheimer's research are lacking in the literature. To create an interdisciplinary, evidence-based model for understanding clinical empathy's relationship with the assessment and treatment of pain in persons with advanced dementia, we conducted a literature review of relevant manuscripts from 2000-2019 across disciplines and countries, emphasizing dementia studies and research conducted in the last decade. After performing quality appraisal using the Oxford Centre for Evidence-based Medicine's levels of evidence, we synthesized findings from 38 qualifying studies and developed a new conceptual model driven by observation of behaviors indicating pain in persons with dementia unable to self-report. The model represents the cognitive, affective, ethical, and behavioral components of clinical empathy involved in assessing and treating pain, relevant patient outcomes, and contextual factors influencing empathy and outcomes; and provides a framework for testing clinical empathy interventions to improve adverse outcomes in persons with advanced dementia. Understanding the relationship between clinician empathy and the assessment/treatment of pain in persons with dementia may improve care quality and help reduce pain behaviors in this population. This model may be used to inform pain research in persons with dementia and develop clinical interventions and clinician education programs.

### SESSION 3026 (PAPER)

### PHYSICAL ACTIVITY AND EXERCISE

### A LONGITUDINAL ANALYSIS OF SF-36 SCORES WITHIN THE CANDRIVE COHORT: AN EXAMPLE OF SURVIVOR BIAS

Michel Bedard,[1] Hillary Maxwell,[1] Isabelle Gelinas,[2] Shawn Marshall,[3] Gary Naglie,[4] Michelle Porter,[5] Holly Tuokko,[6] and Brenda Vrkljan,[7] *1. Lakehead University, Thunder Bay, Ontario, Canada, 2. McGill University, Montreal, Quebec, Canada, 3. University of Ottawa, Ottawa, Ontario, Canada, 4. University of Toronto, Toronto, Ontario, Canada, 5. University of Manitoba, Winnipeg, Manitoba, Canada, 6. University of Victoria, Victoria, British Columbia, Canada, 7. McMaster University, Hamilton, Ontario, Canada*

A bias inherent to prospective studies is focusing only on individuals who remain in the study; these individuals may differ from those who leave early. To examine this issue, we analyzed SF-36 scores by completion status for individuals enrolled in the seven-year Candrive cohort. The SF-36 provides a self-reported evaluation of health and well-being along two subscales, the Physical Component Summary (PCS) and the Mental Component Summary (MCS). Of 928 participants in the cohort, 887 had at least two consecutive years of data starting at baseline (age=76.17, SD=4.81; 61.9% male). A total of 142 participants had 7 years of data. Study discontinuation (due to withdrawal, driving cessation, or death) happened least in early years, and peaked after 6 years (n=235). When analyzed according to completion status, patterns of change in SF-36 scores varied. For example, participants with 7 years of data had mean PCS scores ranging from 51.41 (SD=7.92) at baseline to 46.93 (SD=9.46) at year 7, a change of 0.75 points per year. For those with only two years of data, scores were lower and dropped from 45.82 (SD=9.98) to 43.59 (SD=10.90), a change of 2.23 points over a single year (p<.001). Differences are also evident for other groups. While the results indicate relative stability of SF-36 scores among participants who remained in the study, participants who dropped out reported greater deterioration in scores. These results highlight important differences between participants based on completion status.

### EXERCISE STRATEGIES FOR OPTIMIZING AEROBIC CAPACITY AND SKELETAL MUSCLE PERFORMANCE IN OLDER ADULTS

Dallin Tavoian,[1] David Russ,[2] and Brian Clark,[1] *1. Ohio University, Athens, Ohio, United States, 2. University of South Florida Morsani College of Medicine, Tampa, Florida, United States*

Most older adults do not exercise regularly. Among those who do, the majority only perform one type of exercise, and— as such— are either not getting the benefits of endurance exercise or resistance exercise. The aim of this pilot study was to determine which standalone exercise strategy has the greatest effect on both cardiorespiratory and lower-extremity muscular function in insufficiently active older adults 60 to 75 years of age (N = 14). Participants were randomly assigned to either resistance training (RT, n=5), moderate intensity continuous training on a stationary bicycle (MICT, n=4), or high-intensity interval training on a stationary bicycle (HIIT, n=5) for supervised exercise sessions three times per week for 12 weeks. Maximal oxygen consumption increased a comparable amount in all groups (11.9±11.2% for HIIT vs. 8.0±14.8% for MICT vs 9.8±5.7% for RT). Leg extensor power did not change in the HIIT group (-0.34±5.2%), but increased by 5.2±9.7% in the MICT group and 14.5±26.1% in the RT group. Leg extensor strength decreased by 1.7±22.1% in the HIIT group and 0.6±6.4% in the MICT group, but increased by 27.3±21.2% in the RT group. These findings demonstrate that RT results in improved lower-extremity strength and power, as well as improvements in maximal aerobic capacity comparable to MICT and HIIT in older adults. Thus, RT should be promoted as an essential exercise strategy for older adults, particularly for individuals who are inactive or that are only performing one type of exercise regularly.

### PHYSICAL ACTIVITY PATTERNS AFTER RETIREMENT: THE REGARDS STUDY

Eric Shiroma,[1] J David Rhodes,[2] Aleena Bennet,[2] Monika M Safford,[3] Leslie MacDonald,[4] Steven P Hooker,[5] and Virginia Howard,[2] *1. National Institute on Aging, Bethesda, Maryland, United States, 2. University of Alabama at Birmingham, Birmingham, Alabama, United States, 3. Weill Cornell Medical College, New York, New York, United States, 4. National Institute for Occupational Safety and Health, Cincinnati, Ohio, United States, 5. San Diego State University, San Diego, California, United States*

Major life events, such as retirement, may lead to dramatic shifts in physical activity (PA) patterns. However, there are limited empirical data quantifying the magnitude of these changes. Our aims were to objectively measure PA before and after retirement and to describe changes in participation in various types of PA. Participants were employed black and white men and women enrolled in REGARDS (REasons for Geographic and Racial Differences in Stroke), a national prospective cohort study (n=581, mean age 64 years, 25% black, 51% women). Participants met inclusion criteria if they retired between their first and second accelerometer wearing

(2009-2013 and 2017-2018, respectively) and had valid accelerometer data (>4 days with >10 hours/day pre- and post-retirement). Accelerometer-based PA was categorized into average minutes per day spent in sedentary, light-intensity, and moderate-to-vigorous PA. Participants reported changes (less, same, more) in 12 types of PA. After retirement, participants decreased both sedentary time (by 36.3 minutes/day) and moderate-to-vigorous PA (by 5.6 minutes/day). Conversely, there was an increase in light-intensity PA (+18.1 minutes/day) after retirement. Participants reported changes in their participation level in various PA activities. For example, 41% reported an increased amount of TV viewing, 42% reported less walking, and 31% reported increased participation in volunteer activities. Findings indicate that retirement coincides with a change in the time spent in each intensity category and the time spent across a range of activity types. Further research is warranted to examine how these changes in physical activity patterns influence post-retirement health status.

### THE EFFECT OF THEORY-LED INTERVENTION FOR KNEE OSTEOARTHRITIS IN OLDER ADULTS: A CLUSTER RANDOMIZED TRIAL

Limin Wang, Hongbo Chen, Han Lu, Jieru Chen, and Shaomei Shang, *Peking University, Beijing, China*

Knee osteoarthritis (KOA) is a common joint disease in people over 60 years old. Exercise therapy is one of the most effective non-pharmacological treatments for KOA, but low exercise adherence needs to be improved. This two-arm cluster randomized trial study was to evaluate the effect of the transtheoretical model-lead home exercise intervention (TTM-HEI) program on exercise adherence, KOA symptom (pain intensity and joint stiffness) and knee function (lower limb muscle strength and balance) in Chinese older adults diagnosed with KOA. A total of 189 community-dwelling older adults with KOA (intervention group: n = 103, control group: n = 86) were enrolled from 14 community centers in Beijing, China in 2018. The intervention was a two-stage and 24-week transtheoretical model-based exercise program, and the control group underwent a same length but non-theory-based exercise program. Exercise adherence was measured at weeks 4, 12, 24, 36, and 48 after the program started, KOA symptoms and knee function were measured at baseline, week 24, and week 48. Results showed that the growth rate of exercise adherence in the intervention group increased 2.175 units compared with the control group (unstandardized coefficient of slope on group B2 = 2.175, $p < 0.001$), and the intervention program maintained participants' exercise adherence with 5.56 (SD = 1.00) compared with 3.16 (SD = 1.31) in the control group at week 48. In addition, TTM-HEI program showed significant effects on relieving KOA symptoms and improving knee function. This study provided an effective strategy for KOA intervention.

### TYPE 2 DIABETES REDUCES THE MUSCLE ANABOLIC EFFECT OF RESISTANCE EXERCISE TRAINING IN OLDER ADULTS

Amanda Randolph, Tatiana Moro, Adetutu Odejimi, Blake Rasmussen, and Elena Volpi, *The University of Texas Medical Branch at Galveston, Galveston, Texas, United States*

Type 2 Diabetes Mellitus (T2DM) accelerates the incidence and increases the prevalence of sarcopenia in older adults. This suggests an urgent need for identifying effective sarcopenia treatments for older adults with T2DM. It is unknown whether traditional approaches, such as progressive resistance exercise training (PRET), can effectively counteract sarcopenia in older patients with T2DM. To test the efficacy of PRET for the treatment of sarcopenia in older adults with T2DM, 30 subjects (15 T2DM and 15 age- and sex- matched controls) underwent metabolic testing with muscle biopsies before and after a 13-week full-body PRET program. Primary outcome measures included changes in appendicular lean mass, muscle strength, and mixed muscle fractional synthesis rate (FSR). Before PRET, BMI-adjusted appendicular lean mass was significantly lower in the T2DM group (0.7095±0.0381 versus 0.8151±0.0439, $p<0.0001$). As a result of PRET, appendicular lean mass adjusted for BMI and muscle strength increased significantly in both groups, but to a lesser extent for the T2DM group ($p=0.0009$) . Preliminary results for FSR (n=25) indicate that subjects with T2DM had lower basal FSR prior to PRET ($p=0.0197$) . Basal FSR increased significantly in the control group after PRET ($p=0.0196$), while it did not change in the T2DM group ($p=0.3537$). These results suggest that in older adults the positive effect of PRET on muscle anabolism and strength is reduced by T2DM . Thus, older adults with T2DM may require more intensive, multimodal and targeted sarcopenia treatment. Funded by NIH R01AG049611 and P30AG024832.

### SESSION 3027 (PAPER)

### SENSORY HEALTH AND IMPAIRMENT (PAPER)

### A SYSTEMATIC REVIEW OF VISUAL IMPAIRMENT AND COGNITIVE DECLINE AMONG OLDER ADULTS

Niranjani Nagarajan,[1] Bonnielin Swenor,[2] Lama Assi,[3] Joshua Ehrlich,[4] and Heather Whitson,[5] *1. Johns Hopkins University School of Medicine, Baltimore, Maryland, United States, 2. Johns Hopkins University, Baltimore, Maryland, United States, 3. Johns Hopkins Wilmer Eye Institute, Baltimore, Maryland, United States, 4. University of Michigan, Ann Arbor, Michigan, United States, 5. Duke University, Durham, North Carolina, United States*

Cognitive and visual impairments frequently coexist. With the aging of populations worldwide, the prevalence of these conditions are projected to increase substantially over time. A number of studies suggest that cognitive function and vision impairment are associated, and it is hypothesized to be due to a (1) common cause etiology, where both share common risk factors, and/or (2) causal association, where visual impairment causes cognitive decline. Sensory loss can lead to increased cognitive load, structural and functional changes in the brain, and/or decreased emotional, social, and physical well-being, all of which could potentially increase the risk of cognitive impairment. We conducted a systematic review of the existing literature, examining the association between cognitive and visual impairment among older adults. A total of 80 observational studies that reported a measure of association between visual and cognitive function and met the following criteria were included: (1) cross-sectional or longitudinal study design, (2) baseline mean age of participants ≥50 years, and (3) sample size of ≥100 participants. Of these 80 studies, 56 found a positive, significant association between visual function and cognitive decline. Forty-nine of the 56 studies used objective measures to test for visual acuity, contrast sensitivity, or visual fields. The sample included participants from 14 countries, including the US, UK, China, and Australia among others. Converging evidence of an association between vision impairment and subsequent cognitive decline suggests that visual impairment is a possible modifiable risk factor for cognitive decline and dementia. This hypothesis should be tested in prospective, controlled studies.

### COMPROMISED VISION IMPAIRS SUCCESSFUL AGING AMONG ASSISTED LIVING RESIDENTS

Judith Scott,[1] and Ann Mayo,[2] *1. University of Colorado Colorado Springs, Colorado Springs, Colorado, United States, 2. University of San Diego, San Diego, California, United States*

Community dwelling independent older adults' successful aging is known to be hampered by sensory and cognitive impairments. However, little is known about to what degree these impairments affect successful aging among assisted living (AL) residents. The purpose of this quantitative study, conducted in three (AL) facilities, was to describe factors affecting successful aging. A total of 88 older adults (M=89.0, SD=7.54), mostly women (n=68), completed hearing (CALFRAST-Strong procedure at 75cm, 35cm, and 2cm), vision (Jaeger reading [proximate], Snellen Acuity [visual acuity]), and cognitive screening (MiniCog, Borson et al), as well as the Lawton Instrumental Activities of Daily Living (IADL) and Successful Aging Inventory (SAI, Troutman et al, 2011). Most (68%) demonstrated hearing loss >25DB, with a significant difference demonstrated between age groups (age 65-89; n=38) (90-100; n=49) with the older group demonstrating worse hearing (F(1,80)=5.9, p=.017). Some vision compromise was noted for both reading (14.3%) and visual acuity (10.8%). Over one third of participants (34.1%) demonstrated compromised cognition. The SAI results indicated most participants were managing IADLs well (M=6.11, SD=1.42) and aging successfully (M=63.39, SD=9.04). Hearing, cognition and IADLs were not significantly related to successful aging. However, when compared to those without vision issues, participants with compromised vision, both reading and visual acuity, scored significantly lower on the SAI (reading F(1,75)= 24.9,p=.000; visual acuity F(1,28)=4.31, p=000). The infrastructure provided by AL settings may compensate for hearing, cognition, and IADL problems, but not as well for vision problems. Interventions supporting AL residents' vision should be a priority to improve successful aging.

### HYPOSMIA AND NEUROIMAGING SIGNATURE IN COMMUNITY-DWELLING OLDER ADULTS

Cynthia Felix,[1] Lana Chahine,[2] Honglei Chen,[3] Zichun Cao,[3] and Caterina Rosano,[1] *1. University of Pittsburgh, Pittsburgh, Pennsylvania, United States, 2. University of Pittsburgh School of Medicine, Pittsburgh, Pennsylvania, United States, 3. Michigan State University, East Lansing, Michigan, United States*

Olfaction declines with aging, and hyposmia, or impaired sense of smell, is associated with neurodegenerative disorders including Alzheimer's Disease (AD) and Parkinson's Disease (PD). Neuroimaging studies of hyposmia in AD/PD patients have often examined pathology-specific brain regions. Our knowledge of neural correlates in regions that mediate olfaction in community-dwelling older adults, is limited. We quantified mean diffusivity (MD) of the gray matter (GM) using diffusion tensor imaging in a community-dwelling sample of 308 older adults (mean age: 82.9 years, 58% women, 40% black). We focused on total brain and these regions involved in olfaction- olfactory bulb, amygdala, entorhinal cortex, orbitofrontal cortex, and hippocampus. Smell was tested with a scratch-and-sniff validated odor identification test, the Brief Smell Identification Test (BSIT). Hyposmia was defined as BSIT score of ≤8, assessed about 7 years prior to neuroimaging. In our sample, 23% had hyposmia, more in in men (30%) than in women (19%). Hyposmia was not significantly associated with cardiovascular risk factors such as hypertension; diseases such as stroke; age; race; cognitive or mobility functions (all p>0.1). In linear regression models adjusted for demographics and brain atrophy (total brain gray matter volume divided by intracranial volume), hyposmia was significantly associated with higher GM MD (lower microstructural integrity) of the left orbitofrontal cortex (standardized beta: 0.142, t=2.56, p=0.011). Understanding the neural substrates involved in hyposmia in aging is an important step towards advancing research on hyposmia in non-clinic-based, community-dwelling populations.

### OLFACTION AND PHYSICAL FUNCTION IN OLDER ADULTS: FINDINGS FROM HEALTH ABC

Yaqun Yuan,[1] Zhehui Luo,[1] Chenxi Li,[1] Eleanor Simonsick,[2] Eric Shiroma,[2] and Honglei Chen,[1] *1. Michigan State University, East Lansing, Michigan, United States, 2. National Institute on Aging, Bethesda, Maryland, United States*

The present study aims to investigate poor olfaction in relation to physical functioning in community-dwelling older adults and potential sex and race disparities. The analysis included 2511 participants aged 71-82 years (51.7% women and 38.4% blacks) from the Health Aging, and Body Composition (Health ABC) study. Olfaction was tested with the 12-item Brief Smell Identification Test (BSIT). Physical function measures included the Short Physical Performance Battery (SPPB), the Health ABC Physical Performance Battery (HABCPPB), gait speed of 20-meter walk, fast 400-meter walking time, grip strength, and knee extensor strength, repeatedly assessed annually or biennially for a follow-up of seven years. We analyzed each of these physical function measures using mixed models, adjusting for demographics, lifestyle, and comorbidities. For all measures except grip and knee extensor strength, poor olfaction was clearly associated with poorer physical performance at baseline and a faster decline over time. For example, at baseline, the multivariate adjusted SPPB was 8.23 ± 0.09 for participants with poor olfaction and 8.55 ± 0.09 for those with good olfaction (P = 0.02), after seven years of follow-up, the corresponding scores decreased to 6.46 ± 0.12 and 7.36 ± 0.10 respectively

(cross-sectional P<0.001, and P for olfaction-by-year interaction < 0.001). For grip and knee extensor strength, similar differences were suggested but didn't reach statistical significance. The overall results were similar by sex and race. In summary, poor olfaction is clearly associated with faster decline in physical functioning in older adults and future studies should investigate its potential health implications.

### THE ASSOCIATION BETWEEN HEARING AND PHYSICAL FUNCTIONING IN THE ATHEROSCLEROSIS RISK IN COMMUNITIES STUDY

Pablo Martinez-Amezcua,[1] Pei-Lun Kuo,[2] Kevin Sullivan,[3] Priya Palta,[4] A. Richey Sharrett,[5] Jennifer Schrack,[1] Frank Lin,[6] and Jennifer Deal,[1] *1. Johns Hopkins Bloomberg School of Public Health, Baltimore, Maryland, United States, 2. National Institute on Aging, Bethesda, Maryland, United States, 3. University of Mississippi Medical Center, Jackson, Mississippi, United States, 4. Columbia University Irving Medical Center, New York, New York, United States, 5. Johns Hopkins University, Baltimore, Maryland, United States, 6. Johns Hopkins University School of Medicine, Baltimore, Maryland, United States*

Hearing loss is highly prevalent among older adults and has deleterious effects on health. However, its association with physical functioning is not well defined. We investigated the cross-sectional association between hearing and physical function in 3,339 community-dwelling participants (mean age: 79 years, 59% women) of the Atherosclerosis Risk in Communities Study (ARIC). Hearing was measured by pure-tone average ([dB]) of 4 frequencies [0.5,1,2,4 kHz] and physical function was measured using the short physical performance battery (SPPB), which consisted of 3 performance-based tests (balance, gait speed, and chair stands) each scored ranging from 0-4, resulting in a total possible score of 0-12 (higher scores indicating better physical function). We estimated the association between hearing and physical function using continuous scores for each component of the battery, and the overall SPPB score categorized into high [10-12], intermediate [7-9], and low [≤6]) using ordinal logistic regression models. The SPPB scores were reversed for an easier interpretation of the odds ratios (OR). The category with better physical functions was the reference group for each model. After adjustment for demographics and comorbidities, poorer hearing (+10 dB in PTA) was associated with worse physical functioning: OR for lower balance score=1.17, 95% CI [1.08, 1.26]; OR for lower gait speed score=1.15, 95%CI [1.06, 1.25]; OR for lower chair stand score=1.07, 95% CI [1.04, 1.11]; and OR for lower overall SPPB category=1.15, 95%CI [1.07, 1.24]. Hearing loss is associated with poorer physical functioning, highlighting the potentially negative impact of hearing loss on mobility at older ages.

### SESSION 5000 (SYMPOSIUM)

### AGE AS AN OVERLOOKED ELEMENT OF DIVERSITY: APPROACHES TO ADDRESSING INTERGENERATIONAL PERSPECTIVES

Chair: Allyson Graf
Discussant: Amy Knepple Carney

Outside of gerontology, age is an often underappreciated element of diversity. At a time when all generations must work together to provide inclusive, multi-faceted solutions to today's societal problems, ageist and generational stereotypes are often barriers to meaningful intergenerational exchanges. Age derogation and negative stereotypes have been used to splinter communities, perpetuate misinformation, and trivialize intergenerational conversations. As researchers, educators, and practitioners, we understand why age matters, but our students, community leaders, and employers may not. It is our disciplinary obligation to convince those who ignore, dismiss, or misrepresent age of the importance of this aspect of diversity for navigating any multigenerational setting. In this talk, we provide three approaches to addressing age-related beliefs in the classroom. We begin by exploring the impact of generational stereotypes within minority communities. For the LGBT community, negative stereotypes coupled with rapid social change have lead to a growing generational gap. We then shift perspectives to examine the role that lifespan developmental psychology can play in preparing students to enter a diverse multigenerational workforce. Here, we discuss research on age identity and generational identity as distinct and self-enhancing life-span processes, and highlight the developmental barriers that must be navigated in order to foster intergenerational cohesion. Finally, we discuss the findings from Generation to Generation, an intergenerational discussion course for older and younger adults, designed to promote productive intergenerational contact. The results provide evidence that intergenerational discussion may facilitate improved connections between generations.

### UNPACKING "OK, BOOMER": USING LIFESPAN CONCEPTS TO EASE INTERGENERATIONAL CONFLICT

Allyson Graf, and Robin Bartlett, *Northern Kentucky University, Highland Heights, Kentucky, United States*

With the "OK, Boomer" media exchange in late 2019, intergenerational conflict is touted as existing at an all-time high. Although the age diversity of today's workforce is unprecedented, spanning nearly five generations of workers, generational stereotyping and its influence on the identities and experiences of those individuals is not new. In this talk, we will advocate for the role that lifespan developmental psychology can play in preparing students to enter a sometimes contentious, misrepresented multigenerational workforce. We will demonstrate the value of helping students distinguish normative age-graded, normative history-graded, and non-normative influences to better understand individual similarities and differences in developmental experiences. We will discuss research on age identity and generational identity as distinct and self-enhancing life-span processes. Finally, we will highlight the developmental barriers that must be navigated in order to foster intergenerational cohesion.

### DIVIDES WITHIN THE LGBT COMMUNITY: EXPLORING THE IMPACT OF GENERATIONAL STEREOTYPES

Kinsey Bryant-Lees,[1] and Mary Kite,[2] *1. Northern Kentucky University, Highland Heights, Kentucky, United States, 2. Ball State University, Muncie, Indiana, United States*

Age is a unique, often overlooked, aspect of identity, which is particularly problematic within the LGBT community. While sexuality, sexual orientation, and gender identity are central to the identities of LGBT people, they are verboten for older adults. Thus, older LGBT individuals' voices are inadvertently silenced. This talk will present data that demonstrates the reinforcing role that stereotypes play in maintaining the generational divide and address some of the unique intergenerational differences within the LGBT+ community that arise from differential experiences of cultural acceptance and historical events of LGBT youth coming of age across decades. For example, in the 1970's the DSM criteria would have classified LGBT people as mentally ill; stark contrast to the 2010's in which LGBT youth have grown up with legal protections against hate crimes, and marriage equality as a basic right. We will conclude with a discussion on cultivating community and productive conversations across generations.

### AGE AS AN ELEMENT OF DIVERSITY: INTERGENERATIONAL DISCUSSION TO IMPROVE AGE-RELATED ATTITUDES

Lisa Wagner,[1] and Tana Luger,[2] *1. University of San Francisco, San Francisco, California, United States, 2. Center for the Study of Healthcare Innovation, Implementation and Policy, Greater Los Angeles VA, Los Angeles, California, United States*

All generations must work together solving societal problems, yet age-related stereotypes are used to divide generations. Age derogation motivates younger people to vote by creating fear of an older White voting generation (Dear young people, don't vote; 2018), and to belittle older people ("Okay, Boomer…"). Demonizing older people creates prejudice within families asking that people target loved ones, for example, by pitting educational funding for young against health funding for older adults. Neither group wins when divisiveness occurs. Generation to Generation, an intergenerational course for older and younger adults, promotes intergenerational contact. Students discuss topical issues (e.g., racism) in multigenerational groups. Using pretest-posttest design, all students were invited to complete questionnaires at beginning and end of term. Younger adults reported significant increases in affection, comfort, kinship, engagement and enthusiasm for older adults, whereas older adults showed stability over time. Intergenerational discussion may facilitate improved connections between generations.

### SESSION 5001 (SYMPOSIUM)

### A GEROSCIENCE PERSPECTIVE ON COVID-19 – JOINT PROGRAM FROM THE AMERICAN AGING ASSOCIATION (AGE) AND THE GERONTOLOGICAL SOCIETY OF AMERICA

Chair: Rozalyn Anderson

Faculty will focus on the biology of aging as a contributor to the vulnerability in COVID-19. Faculty will present the latest concepts and insights that will advance our ability to confront this global outbreak. Our goal for this session is to connect with the concept of Geroscience and how ideas from aging biology research can be incorporated to improve outcomes and informed practice. Although the emphasis is on biology, the goal is to provide insight in a manner that is readily accessible to researchers across the aging spectrum that they might translate these ideas in the face of a very real-world challenge.

### DEVELOPING THERAPIES TO IMPROVE ANTIVIRAL IMMUNITY IN OLDER ADULTS

Joan Mannick, *resTORbio, Boston, Massachusetts, United States*

### COVID-19 IMMUNE RESPONSE, COMPLICATIONS, AND CURRENT STATUS

Albert Shaw, *Yale School of Medicine, New Haven, Connecticut*

### SESSION 5005 (SYMPOSIUM)

### AGE-FRIENDLY ONLINE TRAINING PROGRAMS FOR LONG-TERM SERVICES AND SUPPORTS STAFF TO IMPROVE CARE FOR OLDER ADULTS

Chair: Linda Edelman
Co-Chair: Kara Dassel

The purpose of our Geriatric Workforce Enhancement Program is to provide geriatric and primary care education and training to long-term care (LTC) providers and staff, health professions students and community members. Our LTC partners and the communities we serve are often very rural and travel to urban areas for training can be difficult. Therefore, we have developed four online training that are offered free to our partners and rural communities statewide. These programs are designed to integrate the aims of the Age-Friendly 4M's model (i.e., What Matters, Mobility, Medication, Mentation). The LTC nurse residency program provides gerontological nursing and inter-professional leadership training (all 4M's), in a synchronous online environment. The asynchronous Alzheimer's Disease and Related Dementias training modules educate LTC staff and family caregivers about types, diagnosis and care of older adults with dementia (Mentation and Medication). The asynchronous Opioid Use in LTC modules were developed with partners to deliver live at LTC staff trainings about opioid stewardship (Medication). The LTC Learning Communities are monthly tele-health sessions for inter-professional LTC teams to discuss current issues and propose solutions (all 4M's). We have successfully leveraged different synchronous and asynchronous online modalities to increase educational opportunities for formal and informal caregivers, including those in rural areas whose educational opportunities are geographically limited. To date our programs have reached over 500 individuals across our state, increasing knowledge about geriatric concepts, communication and team leadership. Moving forward, we will continue to develop and refine educational programs that promote the Age-Friendly geriatric-focused health care.

### DEVELOPMENT OF DEMENTIA BEST CARE PRACTICES IN LONG-TERM CARE SETTINGS ONLINE MODULES

Kara Dassel,[1] Larry Garrett,[2] Troy Andersen,[1] James Ballard,[1] Martin Freimer,[1] Jorie Butler,[1] and Linda Edelman,[1] *1. University of Utah, Salt Lake City, Utah, United States, 2. University of Utah - College of Nursing, Salt Lake City, Utah, United States*

One arm of the Utah Geriatrics Education Consortium focuses on providing Alzheimer's disease and related dementias (ADRD) education to formal and informal caregivers. An interdisciplinary group of faculty developed a 3-hour online training program focused on best care practices, recommended by the National Alzheimer's Association, for persons with ADRD residing in long-term care settings. Topic include: 1) Overview of Dementia (e.g., What are types of dementia and the associated symptoms?), 2) Understanding Behaviors and Your Approach (e.g., What are the best methods for recognizing and reporting behavior change?), 3) Effective Communication within Long-Term Care (e.g., What are the best strategies to enact effective team meetings?), and 4) Communication and Responding to Behaviors (e.g., What are methods for reducing patient agitation?). Throughout the four modules a case study follows "Mrs. Jones" to demonstrate the skills and techniques raised in each module. Pre- and post-evaluation questions are embedded in the training program.

### ONLINE OPIOID STEWARDSHIP TRAINING FOR LONG-TERM CARE STAFF

Linda Edelman,[1] Troy Andersen,[1] Cherie Brunker,[1] Nicholas Cox,[2] Jorie Butler,[1] Miguel Galindo,[3] Larry Garrett,[4] and Susan Butterwork,[5] *1. University of Utah, Salt Lake City, Utah, United States, 2. University of Utah - College of Pharmacy, Salt Lake City, Utah, United States, 3. University of Utah - School of Medicine, Salt Lake City, Utah, United States, 4. University of Utah - College of Nursing, Salt Lake City, Utah, United States, 5. Q-consult, LLC, St. Petersburg, Florida, United States*

Opioids are often the first-line chronic pain management strategy for long-term care (LTC) residents who are also at increased risk for opioid-related adverse events. Therefore, there is a need to train LTC providers and staff about appropriate opioid use and alternative treatment strategies. Our interdisciplinary team worked with LTC partners to identify staff educational needs around opioid stewardship. Based on this need's assessment, we developed eight modules about opioid use and risks for older adults, including those with dementia, recommendations for de-prescribing including other pharmacological and non-pharmacological alternatives, SBIRT, and motivational interviewing to determine "what matters". Each 20-minute module contains didactic and video content that is appropriate for group staff training or individuals and provides rural LTC facilities access to needed training in their home communities. Within the first month of launching online, the program received over 1100 hits and LTC partners are incorporating modules into clinical staff training schedules.

### SUCCESSES AND CHALLENGES OF FOSTERING AGE-FRIENDLY HEALTH SYSTEMS THROUGH A LONG-TERM SERVICES AND SUPPORTS ECHO

Cherie Brunker,[1] Jorie Butler,[1] Jacqueline Telonidis,[1] and Yin-Shun Chiu,[2] *1. University of Utah, Salt Lake City, Utah, United States, 2. University of Utah - College of Nursing, Salt Lake City, Utah, United States*

Launched in June 2016, the Utah Geriatric Education Consortium (UGEC) inter-professional Learning Community was developed by faculty in collaboration with primary care and community partners with a focus on Long Term Care. In 2019, UGEC expanded to include Assisted Living, Home Care and Hospice -5 primary care and 6 community long-term services and supports (LTSS) partners in rural and urban Utah with a primary objective of developing and training existing LTSS healthcare providers and direct care workers on the elements of age-friendly health systems ("4Ms": mentation, medication, mobility, and what matters) to enhance care and improve outcomes for older adults across the continuum of LTSS. Attendance in the monthly distance learning sessions averages 27.5. Sessions are rated highly and engage nurses, behavioral health workers, administrators, physicians, nursing assistants, occupational and physical therapists. The process, challenges, successes of transitioning to a LTSS ECHO (Extension for Community HealthCare Outcomes) will be discussed.

### THE UTAH EXPERIENCE: OUR SECRET SAUCE FOR LONG-TERM CARE NURSE RESIDENCY PROGRAMS

Nanci McLeskey,[1] Joan Gallegos,[2] and Jacqueline Telonidis,[1] *1. University of Utah, Salt Lake City, Utah, United States, 2. Comagine Health, Salt Lake City, Utah, United States*

Nurses working in long-term care (LTC) have a paucity of geriatric knowledge and leadership skills. One method of meeting this challenge is by offering an online geriatric-focused nurse residency program (NRP) to LTC nurses. The goal of the NRP is to provide geriatric nursing competencies to prepare LTC nurses to care for complex older residents and lead staff. Construct measures include geriatric knowledge and skills, self-rated efficacy, and attitudes towards QI. To date, 46 nurses have enrolled in the program. Completers' state they have increased knowledge and confidence; but turnover and workload issues often prevent nurses from completing the program. Thus, the current NRP with 6 nurse residents is streamlined to be completed in 4 months and with a focus on the 4 M's, leadership and geriatric competencies and resilience. Future plans are for the NRP to be available for nurses from all long-term services and supports settings.

### SESSION 5010 (SYMPOSIUM)

### AGE-FRIENDLY UNIVERSITIES: CONFRONTING AGEISM AND FOSTERING AGE INCLUSIVITY

Chair: Joann Montepare
Co-Chair: Kimberly Farah

The pioneering Age-Friendly University (AFU) initiative, endorsed in 2016 by GSA's Academy for Gerontology in Higher Education (AGHE), calls for institutions of higher education to respond to shifting demographics and the needs of aging populations through more age-friendly programs, practices, and partnerships. Over 65 institutions in the United States, Canada, European countries, and beyond have joined the network and adopted the 10 AFU principles. Despite the importance and appeal of the AFU initiative, individuals leading age-friendly efforts on their campuses have found that ageism in higher education is a persistent, yet overlooked, factor holding us back from embracing age diversity. This symposium will feature AFU partners who will discuss how ageism presents itself in higher education, along with offering recommendations for breaking it down and promoting greater age inclusivity. Montepare will open the session with an overview of systematic and implicit instances of ageism in higher education. Bowen and colleagues will then discuss results from an AFU Campus Climate Survey that examined the age attitudes of faculty, students, and staff along with their views about that nature of campus age-friendliness. Manoogian will discuss the value of approaching the teaching of age diversity from an intersectionality perspective. Reynolds and Kruger will provide theoretical framing and dissemination models for the GSA online course Ageism First Aid within various AFU and programmatic structures. Andreoletti and June will discuss how creating an age-inclusive AFU Learning Community can raise awareness about ageism across campus as well as in the community where a campus resides. Age-Friendly University (AFU) Interest Group Sponsored Symposium.

### AGEISM IN HIGHER EDUCATION: HISTORICAL REASONS AND CONTEMPORARY AGE-FRIENDLY SOLUTIONS

Joann Montepare, *Lasell University, Newton, Massachusetts, United States*

The need for greater attention to aging in higher education is indisputable. Changing age demographics are reshaping societies and challenging colleges and universities to consider how they can respond to aging populations through new approaches to teaching, research, and community engagement. However, ageism permeates academia in systematic as well as implicit ways, holding higher education back from expanding attention to aging. This presentation will describe how ageism manifests itself and how the pioneering Age-Friendly University (AFU) initiative with its ten guiding principles offers a framework to address the neglect of age in academia by advocating for greater age-diversity and inclusion. Special attention will be given to an AFU focus on breaking down age-segregation by bringing younger and older learners together around educational goals of mutual interest, engaging in collaborative teaching and learning experiences, and building intergenerational solidarity in line with social-psychological principles known to reduce prejudice and discrimination. Part of a symposium sponsored by Age-Friendly University (AFU) Interest Group.

### THE PERSONAL BELIEFS ABOUT AGING SCALE

Lauren Bowen,[1] Nina Silverstein,[2] Susan Whitbourne,[3] and Taylor Jansen,[4] *1. University of Massachusetts, Boston, Boston, Massachusetts, United States, 2. University of Massachusetts Boston, Needham, Massachusetts, United States, 3. University of Massachusetts Amherst, Boston, Massachusetts, United States, 4. University of Massachusetts Boston, Boston, Massachusetts, United States*

Most existing ageism scales (e.g., Fabroni et al., 1990; Laidlaw et al., 2007; Rupp et al., 2005) are designed to measure younger people's age attitudes, often asking respondents to affirm or reject stereotypes of older people. The Personal Beliefs about Aging (PBA) Scale gathers information about the degree to which respondents, regardless of age, value age diversity on their campus. Preliminary findings from a 2019-2020 University of Massachusetts system-wide electronic survey that yielded 2,563 responses across 3 (of 5) campuses indicate that most faculty (83%), staff (84%), and students (72%) perceived ageism as a serious problem in society; however far fewer considered ageism as a serious problem on their own campus, with students (20%) perceiving campus ageism to an even lesser extent than faculty (39%) and staff (36%). Part of a symposium sponsored by Age-Friendly University (AFU) Interest Group.

### ADOPTING AN INTERSECTIONALITY LENS WITHIN AN UNDERGRADUATE GERONTOLOGY CURRICULUM

Margaret Manoogian, *Western Oregon University, Monmouth, Oregon, United States*

Students who plan geriatric/gerontology careers typically learn the biopsychosocial domains of aging. Using intersectionality to understand older adults and family experiences (Calasanti & Kiecolt, 2012), however, offers students a deeper understanding of how aging adults may face interconnected oppressions and inequalities based on race/ethnicity, gender, age, sexual orientation, socioeconomic status, health, and other aspects of social location within micro and macro contexts. Through systematic assessment of student learning outcomes, a planned programmatic approach to integrating intersectionality was adopted within an undergraduate gerontology program. This multifaceted approach will be highlighted including new course development, course case studies, community member voices, practicum applied practices, and research activities.

**Calasanti, T. & Kiecolt, K. J. (2012) Intersectionality and aging families. In Blieszner, R., & Bedford, V. H. (Eds.), Handbook of families and aging (pp. 263-286). Santa Barbara, CA:** Praeger.
Part of a symposium sponsored by Age-Friendly University (AFU) Interest Group.

### THEORETICAL FRAMING AND DISSEMINATION OF AGEISM FIRST AID: A SIGNIFICANT TOOL FOR THE AFU MOVEMENT

Laurinda Reynolds,[1] and Tina Newsham,[2] *1. American River College, Sacramento, California, United States, 2. University of North Carolina Wilmington, Wilmington, North Carolina, United States*

By graduation most high school students have negative feelings about aging, the idea of working with older people is unappealing, and few choose to study aging. When non-traditional students of any age enroll after gaining experience in the workforce, they experience ageism and may not persist to degree completion. The value of Ageism First Aid (AFA) as an internal and external AFU tool is supported by Bronfenbrenner's Ecological Theory and Rogers' Diffusion of Innovation theory. AFA utilization during high school can increase traditionally-aged student enrollments. Its use for professional development on campuses can improve the campus climate for all constituents and ensure graduates will know how to avoid contributing to aging stigma. AFA use by providers of aging services and support can reduce the aging stigma older people experience. Attendees will receive handouts with a variety of dissemination models and information on how to gain institutional access to AFA. Part of a symposium sponsored by Age-Friendly University (AFU) Interest Group.

### CREATING AN AFU LEARNING COMMUNITY GROUP TO COMBAT AGEISM

Carrie Andreoletti, and Andrea June, *Central Connecticut State University, New Britain, Connecticut, United States*

Central Connecticut State University, a regional public university, joined the AFU global network in 2017. Through efforts to teach people what it means to be an AFU and infuse the AFU principles and practices into our campus culture, we recognized that our work must also include educating our faculty colleagues, staff, and administration about ageism and aging in general. Toward this end, we utilized our Center for Teaching and Faculty Development's small grant program to develop an AFU learning community group (LCG) that includes faculty, staff, and older learners interested in the AFU movement and how they can contribute to the AFU effort on our campus. This presentation will discuss how we have used the LCG as an opportunity to combat ageism and increase aging literacy by sharing resources such as Ageism First Aid, Reframing Aging, and AARP's Disrupting Aging classroom. Part of a symposium sponsored by Age-Friendly University (AFU) Interest Group.

### SESSION 5015 (SYMPOSIUM)

### AGE-FRIENDLY UNIVERSITY MODELS FOR ENGAGING WITH RETIRED COMMUNITY MEMBERS

Chair: Joann Montepare
Co-Chair: Kimberly Farah

The pioneering Age-Friendly University (AFU) initiative, endorsed in 2016 by GSA's Academy for Gerontology in Higher Education (AGHE), calls for institutions of higher education to respond to shifting demographics and the needs and interests of our aging populations through more age-friendly programs, practices, and partnerships. This symposium will focus on AFU Principle 9 that advocates for engaging actively with an institution's retired community. Appreciating the diversity of ways in which this principle can be put into practice, participants will discuss the various ways their AFU campuses have built connections with members of their local communities of retired adults. Montepare (Lasell University) will discuss the living and learning model of the institution's affiliated-retirement community (Lasell Village) that coordinates engagement in 450 credit hours of educational activity as a benefit of residency. Berman (Ithaca College) will discuss the Gerontology Institute's longstanding relationship with the Longview senior living community and how intergenerational educational and social experiences serve as enriching activities for students, faculty, and residents. Elfenbein (University of North Georgia) will discuss campus collaboration with The Wisdom Project, a community organization of adults 55+ interested in using their skills and talents to benefit their community through action and advocacy projects. Wenrick (Drexel University) will discuss Writers Room, a university-community literary arts program, and their intergenerational co-living initiative, creating a network of homeowners and student tenants whose shared interest in writing and storytelling forms the foundation for meaningful cohabitation.

### LIVING AND LEARNING PARTNERSHIPS ON AGE-FRIENDLY UNIVERSITY CAMPUSES

Joann Montepare, and Kimberly Farah, *Lasell University, Newton, Massachusetts, United States*

Campuses and communities have a long and varied history of partnerships around educational efforts. In response to current shifts in age demographics and the number of adults aged 65+ increasing dramatically, new partnerships around living and learning are emerging. In particular, university-based retirement communities (UBRCs) associated with institutions of higher education are growing as housing options for older adults in the United States. The establishment of the Age-friendly University (AFU) initiative offers a guiding framework for how UBRC partnerships may be effectively mounted. This presentation will use the case example of the Lasell University – Lasell Village partnership to demonstrate how AFU principles can be implemented through age-friendly curricular programming and intergenerational exchange. Logistical considerations such as individualized learning plans for UBRC residents, an on-campus location, shared resources, and governance policies will be also be discussed as components of successful partnerships.

### ENGAGING THE UNIVERSITY'S RETIRED COMMUNITY

Pamela Elfenbein, *University of North Georgia, Gainesville, Georgia, United States*

In support of our older adult community and in alignment with Age Friendly University principles, the University of North Georgia Center for Healthy Aging promotes wellness and disease prevention through active research and intervention, promotes leadership through mentorship and partnerships, fosters an environment of healthy aging through interdisciplinary service and research with community partners, and supports healthy aging through innovative interprofessional collaboration and research. This presentation offers insight into the ways we have engaged retired faculty and staff, with particular focus on a collaboration with The Wisdom Project, a community organization of adults 55+ interested in using their skills and talents to benefit their community through action and advocacy projects. While aligned with Principle 9, this presentation also offers insight regarding how the university supports additional AFU Principles.

### CULTIVATING INTERGENERATIONAL EXCHANGE: AN AGE-FRIENDLY UNIVERSITY MODEL FOR STRATEGIC PARTNERSHIP

Elizabeth Bergman, Mary Ann Erickson, and Jessica Valdez Taves, *Ithaca College, Ithaca, New York, United States*

Having recently celebrated its 20th anniversary, the Ithaca College/Longview Partnership is an enduring part of the Ithaca College experience. Longview is a senior living community located just across the street from campus on land donated by the college. A formal partnership has been in place since 1999, which provides a structure of support for various shared services and mutual access to educational and social opportunities for Ithaca College students, faculty, and staff, and Longview residents and staff. This presentation will include a brief overview of the history of the IC/Longview partnership, describe the infrastructure necessary for a successful partnership, and highlight some recent specific, high-impact programs that have made active engagement between Ithaca College and the Longview community possible.

### INTERGENERATIONAL HOMESHARING: COMMUNITY COHERED BY STORY

Rachel Wenrick, and Kirsten Kaschock, *Drexel University, Philadelphia, Pennsylvania, United States*

Drexel's Writers Room is a university-community literary arts program engaged in creative placemaking and art for social justice. Since 2014, our work has focused on two major themes: how we, as a community of writers ages 18-80, can create and connect through story; and how together we can address some of the changes we are seeing in the local neighborhood around Drexel's campus. This presentation will provide an overview of how the method of co-creation, core to our program since its inception, has allowed us to develop the concept for a writers house and intergenerational homesharing network as an aging-in-place and anti-displacement strategy; build the extensive cross-sector partner network needed to implement it, and earn crucial neighborhood and institutional support. The goal is for attendees to leave with ideas for how this concept might be adapted in their own communities.

### SESSION 5020 (SYMPOSIUM)

### AGHE PROGRAM OF MERIT: PRACTICAL AND TECHNICAL APPLICATION INFORMATION AND ASSISTANCE

Chair: Marilyn Gugliucci
Co-Chair: Donna Weinreich

At a time in education when it is important for programs at colleges and universities to be productive in teaching, student recruitment, and income generation, the AGHE Program of Merit review process can aid in advancing gerontology and health professions programs. The AGHE Program of Merit has expanded its review. Now Colleges of Osteopathic Medicine and all Health Professions program may apply for Program of Merit status.. The POM designation provides gerontology programs and those health professions program that include geriatrics/gerontology content in the curriculum with an AGHE "stamp of approval" which can be used to verify program quality to administrators, to lobby for additional resources, to maintain a quality program, to market the program, and to recruit prospective students into the program. This session will begin with the "Why," "What" and "How" of applying for Program of Merit and then provide time for small group consultation regarding the Program of Merit application. Preparation of the self-study document will be detailed by the experienced faculty members followed by the review process and timelines associated with application process. Free consultation available!

### AGHE PROGRAM OF MERIT FOR HEALTH PROFESSIONS EDUCATION

Donna Weinreich, *Western Michigan University, Kalamazoo, Michigan, United States*

In 2015, the Program of Merit was expanded and adapted to implement a voluntary evaluation process for health professions programs that are choosing to integrate gerontology/geriatrics competencies in order to prepare students for working with older adults as well as their informal care partners. These programs are now eligible to apply for the Program of Merit designation. The Program of Merit for Health Professions Programs is based on the AGHE Standards and Guidelines for Gerontology/ Geriatrics in Higher Education, Sixth Edition (2015), specifically Chapters 11 and 12. This session will provide an overview of the process, review the application content, and provide technical support for interested participants.

### AGHE PROGRAM OF MERIT FOR GERONTOLOGY PROGRAMS

Pamela Elfenbein, *University of North Georgia, Gainesville, Georgia, United States*

The POM designation provides gerontology programs with an AGHE "stamp of approval," which can be used to verify program quality to administrators, to lobby for additional resources to maintain a quality program, to market the program, and to recruit prospective students into the program. This worldwide process of evaluation for both Gerontology programs verifies for students that the program is consistent with globally vetted criteria in gerontology endorsed and recognized by AGHE; assures the public of the quality of programs and their graduates; clarifies for employers the knowledge and skills imparted to students who graduate from POM designated gerontology programs; informs campus administrators of global guidelines, expectations, and practice in gerontology education programs; and indicates to interested students that the program is of high quality. Those interested in the Gerontology Program of Merit will be able to ask questions, understand the application process, and ask for technical assistance.

### SESSION 5025 (SYMPOSIUM)

### ASSESSING AGE-FRIENDLINESS IN HIGHER EDUCATION: INTRODUCING THE INVENTORY AND CAMPUS CLIMATE SURVEYS

Chair: Nina Silverstein
Discussant: Nancy Morrow-Howell

The establishment of the Age-Friendly University (AFU) network and adoption of the 10 principles by institutions of higher education, was a major advance in the United Nations Sustainable Development Goal of promoting healthy and active aging through opportunities for intergenerational communities. AGHE endorsed the principles in 2016, since then over 60 institutions have joined the global network. Tools are needed to identify benchmarks that institutions can use to assess progress toward realizing the AFU principles on their own campuses. This symposium shares work done at the University of Massachusetts Boston, to develop and refine the AFU Inventory and Campus Climate Surveys (ICCS), a survey-based assessment instrument (developed from a prior pilot study in 2018) based on the premise that it is necessary to assess both the institution's actual age-friendly practices and its perceived age-friendliness or campus climate. In August, 2019, the University of Massachusetts President's office endorsed the 10 principles for the entire UMass system of 5 campuses, presenting an opportunity to assess a multi-campus system. To date, we have surveyed UMass Boston, UMass Lowell, UMass Dartmouth and UMass Medical (n=2,704). Testing and refinement of the AFU ICCS will contribute to both short- and long-term recommendations to assist in strategic planning by higher education institutions.

Whitbourne will present the Inventory reporting tool. Bowen will present the Climate Survey. Gautam and Revell will describe the AFU work at UMass Lowell and UMass Dartmouth respectively and the use of the assessment tools on their campuses; Morrow-Howell will serve as Discussant.

### THE AFU CAMPUS INVENTORY TOOL FOR ASSESSING AGE FRIENDLINESS IN THE OBJECTIVE ENVIRONMENT

Susan Whitbourne,[1] Nina Silverstein,[2] Lauren Bowen,[3] and Taylor Jansen,[4] *1. University of Massachusetts Amherst, Boston, Massachusetts, United States, 2. University of Massachusetts Boston, Needham, Massachusetts, United States, 3. University of Massachusetts, Boston, Boston, Massachusetts, United States, 4. University of Massachusetts Boston, Boston, Massachusetts, United States*

The AFU principles establish a framework that universities can use to set aspirational goals. As AFU principles become more widely adopted, there will be a need for institutions of higher education to evaluate their progress toward achieving these goals. In part, the AFU goals represent structural elements of the campus, or the "objective" environment. The AFU Campus Inventory provides an efficient, form-based, reporting tool for use by relevant campus departments to provide information on age-friendly practices, such as the prevalence of older people on campus as faculty, staff, and students; the provision of courses on aging, advising for older community members, and such amenities as reduced prices for various services. In this session, the Inventory will be demonstrated utilizing preliminary data from campuses in Massachusetts to illustrate its feasibility. Participants will be shown how to use the Inventory for adaptation at their own institutions.

### THE AFU CAMPUS CLIMATE SURVEY: ASSESSING PERCEPTIONS OF AGE FRIENDLINESS

Lauren Bowen,[1] Nina Silverstein,[2] and Susan Whitbourne,[3] *1. University of Massachusetts, Boston, Boston, Massachusetts, United States, 2. University of Massachusetts Boston, Needham, Massachusetts, United States, 3. University of Massachusetts Amherst, Boston, Massachusetts, United States*

While the AFU goals emphasize campus structures and practices (which can and should be evaluated), it is equally important to assess perceived age-friendliness by campus community members. Availability of academic and career services to students of all ages does not guarantee that older and younger students feel equally welcome and well-served. The Campus Climate Survey gathers the "perceived" data from faculty, staff, and students of all ages through a Likert-scale survey that asks perception questions about age-friendly services, facilities, and campus culture. Participants in this session will be shown how to use the CCS at their own institutions and learn its value through preliminary findings from 2,708 staff, student, and faculty responses from Massachusetts, such as: while the majority (68%) of campus staff members agreed that older adults feel welcome on campus, more than half (58%) anticipate facing age biases and challenges on campus as they age.

### AGE-FRIENDLY UNIVERSITY INVENTORY: PROCESS AND OUTCOME FROM UMASS LOWELL CAMPUS

Ramraj Gautam, Karen Devereaux Melillo, and andrew Hostetler, *University of Massachusetts Lowell, Lowell, Massachusetts, United States*

The UMass Lowell Center for Gerontology Research and Partnerships (CGRP) has been on the Age-Friendly University (AFU) trajectory since April 2018. In April 2019, at the CGRP-sponsored 5th Annual Healthy Aging Living Well Forum, qualitative analysis of table-top discussions identified themes related to the AFU Principles: Accessibility, care, communication community education, flexibility, finances/money, recreation, inclusive society, technology and transportation. In September 2019, in collaboration with the UMass President's Office and UMass Boston's Gerontology Institute, we launched the AFU Climate Survey to faculty, staff, and students on the UML campus. This was followed by the AFU Inventory assessments of 12 key campus leaders and offices. Three CGRP members solicited input via email, follow-up telephone communication and information from the University website. The results presented will focus on how other educational institutions might consider proceeding with this process and how to adapt the Inventory to meet other unique campus characteristics.

### AGE-FRIENDLY UNIVERSITY ASSESSMENTS: APPROACH BY THE UMASS DARTMOUTH CAMPUS

Andrew Revell, Brian Ayotte, and Jennifer Viveiros, *University of Massachusetts Dartmouth, North Dartmouth, Massachusetts, United States*

Representatives from The Ora M. DeJesus Gerontology Center at the University of Massachusetts Dartmouth began participating on the Age-Friendly University (AFU) panel at The Gerontological Society of America conference in November 2018. The recommendations and knowledge provided a foundation to begin informally surveying faculty and gerontology affiliates on age-friendly practices. In the fall of 2019, formal discussions began with our partners at other campuses, and in Spring 2020, the AFU Climate Survey was disseminated by email announcements to all students and employees on our campus, soon followed by the Inventory reporting tool to campus office administrators. Results of these assessments will be presented on the utility of these approaches and suggestions for refinement and broader dissemination.

### SESSION 5030 (SYMPOSIUM)

### ESPO/ ACADEMY FOR GERONTOLOGY IN HIGHER EDUCATION SECTION SYMPOSIUM: BRIDGING THE GAP: SHIFTING POPULATION DYNAMICS AND OPPORTUNITIES TO INFORM AND EDUCATE COMMUNITIES

Chair: Lauren Bouchard
Discussant: Marilyn Gugliucci

This symposium is intended to highlight population-shifts and their impact on the individual, family, institution, and community levels. Currently, increases in diversity among individuals under age 18 continue to outpace those of individuals age 65-and-older, creating a substantial racial/ethnic diversity gap between generations. As it relates to education-level, individuals age 65-and-older who have earned at least a bachelor's degree has increased from 5 percent in 1965 to 29 percent in 2018 (Population Reference Bureau, 2020). Population-shifts could be mitigated or better supported through enhancements to gerontology/geriatric education and training. This symposium will highlight the biopsychosocial aspects of these shifts and will link each aspect to the development of gerontology and geriatric curriculum. To start, presenter one will describe projects focused on community-based intergenerational programs to reduce social isolation and loneliness among rural older adults (Jill). Next, presenter two will describe findings from a university-based study, using surveys of aging and ageist attitudes to foster intergenerational connections between undergraduate students and older adult community members (Giselle). Presenter three will discuss a program to support the development of aging ministries to educate and support both caregivers and older adults in community settings (Lauren). Finally, presenter four will discuss findings from one-on-one interviews conducted with Chinese caregivers and Chinese geriatric social workers in efforts to develop and evaluate an end-of-life manual designed for Chinese immigrant caregivers (Mandong). The discussant will link the presenters' findings to implications for future gerontology and geriatric education and training while identifying key topics to inform and engage communities.

### REDUCING SOCIAL ISOLATION AND IMPROVING NUTRITION THROUGH INTERGENERATIONAL EXPERIENTIAL LEARNING

Jill Naar, *Appalachian State University, Boone, North Carolina, United States*

This presentation will focus on two projects that aim to inform and educate communities through community based intergenerational programs. An additional link is collaborating with community partner staff on the potential to improve health outcomes with the integration of generations in programming. Through inter-professional collaborations, these projects focus on two areas: reducing social isolation and loneliness among rural older adults through an intergenerational technology program with university students and modifying the implementation of an evidence-based preschool nutrition program for an intergenerational setting. This presentation will focus on the benefits of using community based intergenerational programs and experiential learning opportunities. Aims of this presentation are to highlight how issues related to social isolation and loneliness can be mitigated by incorporating experiential opportunities for university students while simultaneously supporting university students' preparation for engaging in an intergenerational workforce.

### STONY BROOK UNIVERSITY FORGING INTERGENERATIONAL LINKS THROUGH LIFE-STORY SHARING PROJECT

Giselle Ferguson, Caitlin Monahan, Sheri Levy, Suparna Rajaram, Lauren Richmond, and Stacey Scott, *Stony Brook University, Stony Brook, New York, United States*

According to the World Health Organization, the global population is aging, but ageism may now be the most socially "normalized" of any prejudice, more pervasive than sexism or racism. Ageism produces avoidant and disrespectful treatment of older adults and contributes to a shortage of college students seeking careers with older adults. To foster positive intergenerational contact and combat ageism, we organized life-story sharing by older adult community members in four undergraduate psychology courses with lifespan themes (Psychology of Aging, Memory, Death & Dying, Developmental Psychology; n≅500). A panel visited each class; instructors and graduate students facilitated discussion between students and panelists. Students completed pre- and post-surveys of ageism and attitudes toward aging. A majority of students recommended integrating the activity into future semesters. In free-responses, students also frequently expressed surprise that panelists reported not feeling different than they had at age 20, and that this information challenged previously held stereotypes.

### TAKING EDUCATION TO THE WORLD: IMPLEMENTING AGING CURRICULUM IN FAITH COMMUNITIES

Lauren Bouchard, *Concordia University Chicago, Chicago, Illinois, United States*

Religious communities are uniquely able to serve the needs of older adults, yet very little aging related training exists for lay people and clergy within religious congregations. This talk will discuss the needs of older adults in faith communities, intergenerational interventions, and formal educational training programs such as the Specialist in Aging Ministries program (SAM) developed by Concordia University Chicago as a way to educate and support caregivers and older adults in real world settings. The speaker will also discuss the ways gerontology curriculum has personally impacted her applied work with elders in faith communities including next steps for building and implementing curriculum and training for caregivers, older adults, and the community at large. Sociocultural implications and future directions will also be discussed.

### DEVELOPING AND REFINING AN END-OF-LIFE CARE MANUAL FOR CHINESE IMMIGRANT CAREGIVERS

Mandong Liu, and Iris Chi, *University of Southern California, Los Angeles, California, United States*

Planning for end-of-life (EOL) care in advance can enhance one's quality of life at EOL and decrease caregiver stress and anxiety. Culturally sensitive educational programs are needed to educate the public and encourage advance planning. This paper describes the team's efforts to develop and evaluate an EOL manual designed for Chinese immigrant caregivers. In 2019, one-on-one interviews were conducted with six Chinese caregivers and five Chinese geriatric social workers in Los Angeles County to obtain their feedback on manual improvement. Detailed suggestions included adding more content in the introduction to decrease fear for discussing death-related topics, such as using the concept of "life in four seasons"; having more case examples as how to initiate advance planning conversation with the older adult under different circumstances; adding content on how advance care planning and its documentation is legally protected, etc. Culturally sensitive advance planning community education is feasible among immigrant populations.

### SESSION 5035 (SYMPOSIUM)

### CAMPUS-WIDE BUY-IN FOR AN AGE-FRIENDLY UNIVERSITY: ONE GOAL AND MANY PATHS

Chair: Andrea Zakrajsek
Discussant: Carrie Andreoletti

Recognizing a growing aging population around the world as well as the many benefits of engaging learners at any age in higher education institutions (Kressley & Huebschmann, 2002; Morrow-Howell, et al., 2019; Silverstein, Choi, & Bulot, 2001), the Age-Friendly University (AFU) international initiative offers a medium to support diversity and inclusion efforts based upon age. Dublin City University (DCU), along with Arizona State University (ASU) and Strathclyde University, developed 10 Age-Friendly University (AFU) principles which offer a guide for institutional commitment to age-diversity that can be realized through institutional goals, aims, and initiatives (DCU, n.d., Talmage, Mark, Slowely, & Knopf, 2016). Because of the non-prescriptive nature of these principles, universities endorsing them have opportunities to forge varied paths in the unified goal of age-friendliness. Presenters will share lessons learned from development of the AgeAlive collaborative hub to advance age-friendly research and community-based projects at Michigan State University, the value of cross-campus partnerships at the University of Hartford, the critical support provided by the Adult Learner Programs and Services office at Northern Kentucky University, and the intentional alignment of AFU efforts with administrative priorities at Eastern Michigan University, and journey from focusing on programs to embedding age-friendly practices throughout the institution at Arizona's State University. Through the diverse paths these presenters used to obtain support for the AFU principles at their respective universities, participants who are just beginning their AFU journeys will learn actionable strategies for increasing age-friendliness at their own institutions.

### STRATEGIES TO GENERATE ENDORSEMENT ON CAMPUS: ALIGNING AFU PRINCIPLES WITH UNIVERSITY STRATEGIC PLANS AND GOALS

Cassandra Barragan, Sarah Walsh, and Andrea Zakrajsek, *Eastern Michigan University, Ypsilanti, Michigan, United States*

Over the course of two semesters, Aging Studies Program faculty affiliates at Eastern Michigan University generated interest and support in the pursuit of applying for the AFU designation. During this time, we learned that when the AFU principles were aligned with university strategic plans and goals of various academic units, we were overwhelmingly endorsed at all levels of the university. By using the AFU principles as foundation for our efforts, we approached stakeholders with a value-added perspective. This approach ensured that when we did receive endorsement, we also maintained the integrity of the AFU principles while also speaking to administrative and departmental concerns, such as enrollment, intergenerational programs, and community engagement. We will discuss strategies to build interest at all levels of the university when pursuing or building your AFU designation and provide ways to identify relevant stakeholders to build capacity while seeking endorsement for joining the AFU global network.

### CROSS-CAMPUS COLLABORATION

Claudia Oakes, *University of Hartford, West Hartford, Connecticut, United States*

This presentation will describe collaborative efforts on the campus of a mid-sized, private university to carry out activities consistent with the Age-Friendly University philosophy. In one program, staff from Career Services and a faculty member from the Department of Health Science coordinated with the President's College (a continuing education program for adult learners), the Emeriti Association (a group of retired faculty members), and alumni to offer mock interviews for students preparing for graduate school. In another program, steps were taken to coordinate with the office of Diversity, Equity and Inclusion to address Ageism in the Workplace. The presentation will conclude with advice for identifying allies across campus and fostering support for the AFU principles.

### CAPITALIZING ON SHARED INTERESTS: ACHIEVING AGE-FRIENDLY UNIVERSITY ENDORSEMENT THROUGH PARTNERSHIP AND ADVOCACY

Katherina Terhune, Allyson Graf, Suk-Hee Kim, Amy Danzo, and Sara Conwell, *Northern Kentucky University, Highland Heights, Kentucky, United States*

Response to the lifelong educational needs and interests of the older adult population calls for new opportunities and innovative practices in teaching, research, and community engagement that universities need to be prepared to offer their students and community. This presentation provides information from an Age-Friendly University (AFU) that currently has no gerontology degree or certificate program, highlighting various campus wide programs and initiatives aligned with the AFU principles. In particular, collaborative strategies involving faculty and the Adult Learner Programs and Services to help promote campus buy-in and AFU endorsement will be emphasized. Practical strategies will be discussed to assist junior and senior level faculty and other university personnel wanting to achieve AFU endorsement at their institution of higher education in order to shape age-friendly programs and practices.

### CONNECTING AGE-FRIENDLY UNIVERSITY PRINCIPLES TO UNDERGRADUATE EDUCATION BUILDS CAMPUS-WIDE BUY-IN

Clare Luz, *Michigan State University, East Lansing, Michigan, United States*

Michigan State University became an Age-Friendly University (AFU) in December 2019. The path to campus-wide buy-in started years earlier by AgeAlive, a program established to serve as MSU's central aging hub and to promote wellbeing throughout life. University approval and awareness of AgeAlive and what it means to be an AFU remains a key goal and crucial to sustainability. Central to strategic planning is ensuring that AgeAlive and MSU missions and values are aligned, visibility remains high, and outcomes are tangible and substantial. This presentation provides concrete examples of initiatives that meet these criteria and are building university support including a program that matches students to isolated seniors at risk for loneliness and multi-university research to help college students caring for older family members succeed as students and caregivers. Recommendations for developing and executing a strategic plan will be described and of interest to others on their own AFU journey.

### AGE-FRIENDLY INITIATIVES AT ARIZONA STATE UNIVERSITY: BUILDING TRANSFORMATIVE LEARNING SYSTEMS FOR ALL

Richard Knopf,[1] Craig Talmage,[2] and Abby Baker,[1]
*1. Arizona State University, Phoenix, Arizona, United States, 2. Hobart and William Smith Colleges, Geneva, Arizona, United States*

The AFU global initiative begin with a collaborative agreement by Arizona State University (ASU), Dublin City University (DCU), and the University of Strathclyde in 2012. The vision was to spur other universities to be fully inclusive of all generations. In 2013, ASU and DCU created a Trans-Atlantic Catalyst Fund to spur AFU initiatives at each institution, with an early focus on research in on dementia, technologies for "aging in place," challenges of caregiving, healthy and active aging, and retirement community design. Since then, ASU has embedded Age Friendly practices throughout the institution via creation of a new foundational charter (success is "measured not by whom ASU excludes, but by whom ASU includes"), creation of ASU-wide Center for Innovation in Healthy and Resilient Aging, a re-purposed Osher Lifelong Institute, construction of the Mirabella community (twenty-story gateway for older adults into ASU), new inter-generational learning platforms, and implementation of universal learning systems.

### SESSION 5040 (SYMPOSIUM)

### CREATIVE INTERVENTIONS: INCREASING ENROLLMENT, SERVING STUDENTS & COMMUNITY, REAFFIRMING GERONTOLOGY EDUCATION

Chair: Jan Abushakrah
Discussant: Mike Faber

The session will focus on innovative ways to increase student enrollment, better meet the needs of students and the communities served, and raise awareness of the crucial role of gerontology education in addressing the needs of a rapidly aging society. A panel of Community College and University gerontology professionals will share the innovative ways that they are working to address the three focus areas of this symposium. We will also include opportunities for discussion with participants about their experiences with and ideas for addressing these issues. Community College Interest Group Sponsored Symposium.

### INCREASING OUTREACH AND EXPANDING INCLUSION: COMMUNITY-BASED EDUCATIONAL GERONTOLOGY

Jennifer Sasser, *Human Development & Family Sciences, Oregon State University, Portland, Oregon, United States*

Gerontologists have the opportunity to step into an increasingly significant role as public educators who convene gatherings focused on expanding aging awareness and literacy, inter-generational inquiry and collaboration, and age inclusion, equity and justice. The purpose of this presentation is to share creative design principles and keen takeaways from several ongoing community-based educational interventions connected to these themes. As well, we will discuss the role such public-facing initiatives might play in making a compelling case for the importance of supporting and participating in various kinds of formal educational pathways in the field of aging. Part of a symposium sponsored by the Community College Interest Group.

### THINKING OUTSIDE THE (ENROLLMENT) BOX: "WHO, DISGUISED AS A PUBLIC EDUCATOR, . . ."

Roger Anunsen, *Portland Community College, Portland, Oregon, United States*

"Thank you, I really enjoyed your talk. I understand you teach college classes and I'm wondering how . . ." That's what can happen when a "College Educator" steps off campus, steps into the community. That's the enrollment superpower of a "Public Educator." We'll track a decade of innovative off-campus educational presentations strategically positioned to target new students, lead to cognitive-enhancing programs with adult residential living communities, and, importantly, help reset the role and relevancy of today's gerontology faculty. Examples will include service club meetings, public agency, business and non-profit training retreats, and residential community staff training. Examples include "This is Your Brain on . . ." events such as Music, Loneliness, Sleep, Volunteering, Sports Fandom, Quilting, Golf and, of course, TasteAerobics featuring Brain-Healthy Chocolate. We'll conclude with an innovative enrollment and awareness-raising project in a small city west of Portland that kicked off with a 6-part Aging Education Series. Part of a symposium sponsored by the Community College Interest Group.

### STRATEGIES TO OVERCOME ENROLLMENT BARRIERS

Phyllis Cummins, *Miami University, Oxford, Ohio, United States*

The importance of postsecondary education for economic vitality and individual opportunity has received increased focus with over 40 states embracing postsecondary attainment goals for their populations in alignment with Lumina Foundation's goal that 60% of individuals ages 25-64 have a recognized credential by 2025. Credentials that both meet the needs of an aging society and move the country towards achieving attainment goals are widely available at community colleges. Community colleges are not only important sources of training and education for adult learners, they also work closely with employers to meet their workforce needs. However, adult learners face many barriers to college enrollment, including poor pre-enrollment advising and lack of understanding of financial aid options. Examples of successful strategies to facilitate enrollment will be discussed, including navigators who serve as holistic advisors and work with students from pre-enrollment to graduation, and also guide the prospective student through the financial aid process. Part of a symposium sponsored by the Community College Interest Group.

### CONTINUOUS PROGRAM EVALUATION TO FOSTER AN ENVIRONMENT THAT LEADS TO STUDENT ENGAGEMENT

Janice Velazquez, *East Los Angeles College, Los Angeles, California, United States*

This presentation explores aspects related to the Gerontology Health Certificate of Achievement program and the progression of their Enrollment Management Plan. It discusses how external forces have influenced changes to the plan during the last five years. Some of the strategies to be addressed are the incorporation of adult education courses to the career path; interdisciplinary collaborations with Health, Theater and Psychology Departments; partnerships with organizations such as American Association Family and Consumer Sciences; Association Gerontology in Higher Education, and private donors. The presenter also covers innovative teaching modalities such as laboratories, research, student club, and online teaching as an attempt to accommodate students' needs. Lastly, it discusses the incorporation of workforce development data and how it is influenced by national and institutional policies and practices—ultimately creating a healthy environment to promote enrollment growth by strengthening student's retention and completion. Part of a symposium sponsored by the Community College Interest Group.

### SESSION 5045 (SYMPOSIUM)

### CULTURAL DIMENSIONS, UNIQUE CHALLENGES, AND INTERVENTIONS FOR DEMENTIA CARE IN THE ASIAN CONTEXT

Chair: Kyong Hee Chee
Discussant: Farida Ejaz

Asians in and outside of Asia are facing a rapidly-growing need for dementia care in familial, institutional, or community settings. This multidisciplinary symposium addresses issues of formal and informal dementia care in the Asian cultural context to suggest novel, culturally-appropriate interventions for education and practice. Presenters in this symposium will specifically speak to cultural dimensions, challenges, and approaches involved with caring for persons living with dementia (PLWD) in Singapore and South Korea, as well as their Japanese/Japanese-American counterparts. Malhotra and colleagues will present their qualitative study from Singapore on 26 familial care partners' preference for life-extending interventions for persons with severe dementia, such as intravenous antibiotics, tube feeding, and cardiopulmonary resuscitation. Lee and Chee will then examine how the occupational identities of 303 long-term care workers are associated with their practice of human rights for PLWD in South Korea. Next, Yen and Mayen-Cho will explain a video project that they developed at Alzheimer's Los Angeles to reach out to the Japanese American community – they conducted and filmed in-depth interviews with 7 Japanese/Japanese American family care partners of PLWD. Finally, Park will examine the emergent themes in the narratives of these Japanese/Japanese American interviewees. She will also demonstrate the relevance of the life-course perspective in developing a template for designing similar interventions to serve other ethnic communities. As a discussant, Ejaz will highlight versatility in interventions for dementia care among Asians and Asian Americans. She will also discuss broader implications of the findings within and beyond the Asian context. Aging Among Asians Interest Group Sponsored Symposium.

### SINGAPOREAN DEMENTIA CAREGIVERS' PREFERENCE FOR CARE RECIPIENTS' LIFE-EXTENDING INTERVENTIONS

Chetna Malhotra,[1] Hazirah Mohamad,[1] Truls Østbye,[2] Kathryn Pollak,[3] Bharathi Balasundaram,[4] Sungwon Yoon,[1] Rahul Malhotra,[1] and PISCES study Group PISCES study Group,[1] *1. Duke-NUS Medical School, Singapore, Singapore, 2. Duke University, Durham, North Carolina, United States, 3. Duke University School of Medicine, Durham, North Carolina, United States, 4. Changi General Hospital, Singapore, Singapore*

Family caregivers of older adults with severe dementia are decisive in use of potentially life-extending interventions for their care recipients. We conducted in-depth interviews with 26 caregivers of community-dwelling older adults with severe dementia in Singapore to assess their preferences for intravenous (IV) antibiotics for a life threatening infection, tube feeding, and cardiopulmonary resuscitation (CPR), and reasons thereof. Most caregivers' (77%) end-of-life care goal was 'no life extension'. Yet, 80%, 60% and 45% preferred IV antibiotics, tube feeding and CPR, respectively, as they: 1) perceived letting go by withholding interventions as unethical, 2) felt they had no choice, deferring to health care providers (HCPs), 3) wanted to alleviate suffering, and 4) desired trying minimally invasive (and potentially withdrawable) interventions. There was discordance between caregivers' end-of-life care goal and preferences for life-extending interventions. HCPs can suggest intervention options that concur with caregivers' end-of-life care goal, and use a shared decision-making approach. Part of a symposium sponsored by the Aging Among Asians Interest Group.

### LONG-TERM CARE WORKERS IN SOUTH KOREA AND THEIR PROTECTION OF HUMAN RIGHTS OF PERSONS LIVING WITH DEMENTIA

Hyun Ji Lee,[1] and Kyong Hee Chee,[2] *1. Daegu Catholic University, Daegu, Republic of Korea, 2. Texas State University, San Marcos, Texas, United States*

South Korea has included dementia care as part of their Long-Term Care (LTC) Insurance Program since 2014. Thus, LTC workers are increasingly engaged in dementia care. The purpose of this paper is to elucidate the role of the LTC workers' occupational identity in their consciousness of human rights as well as their practice of human rights protection. Data were collected in 2018 from 303 LTC workers employed by 12 LTC facilities who completed questionnaires. Their mean age was 53.6 years. We measured occupational identity with a 15-item scale (a=.772), consciousness of human rights for persons living with dementia (PLWD) with a 27-item scale (a=.916), and practice of human rights for PLWD with a 26-item scale (a=.956). Results show that occupational identity was positively associated with consciousness of human rights ($r=.296$, $p<.01$) and practice of human rights ($r=.295$, $p<.01$). The findings have implications for protecting the human rights of PLWD. Part of a symposium sponsored by the Aging Among Asians Interest Group.

### FACING THE JOURNEY TOGETHER: USING DIGITAL MEDIA TO SUPPORT DEMENTIA CARE AMONG ASIAN AMERICANS

Angelica Yeh,[1] and Marie Mayen-Cho,[2] *1. Alzheimer's Los Angeles, San Gabriel, California, United States, 2. Alzheimer's Los Angeles, Los Angeles, California, United States*

Asians and Pacific Islanders (APIs) in the United States have limited access to dementia care information that is linguistically and culturally appropriate. Alzheimer's Los Angeles created "Faces of Caregiving", a video project available with English/Japanese subtitles, documenting in-depth interviews with 7 Japanese/Japanese-American familial care partners of individuals living with dementia. It touched on the personal yet universal aspects of each journey embedded in a particular family context. The 5 video profiles were subsequently shown at 3 community sites to attendees comprised of mostly older-adult APIs. Among 85 attendee responses, approximately 90% stated they were more likely to seek out information on and support for Alzheimer's disease, felt more open to talking about the disease, and were more likely to advocate and raise awareness for the disease. This program could be replicated for other API communities, allowing individuals to learn more effectively from a peer-to-peer experience in a culturally familiar setting. Part of a symposium sponsored by the Aging Among Asians Interest Group.

### LIFE COURSE THEMES IN JAPANESE AMERICANS' DEMENTIA CARE PROFILES

Juyoung Park,[1] Kyong Hee Chee,[2] and Angelica Yeh,[3] *1. University of Southern California, Los Angeles, California, United States, 2. Texas State University, San Marcos, Texas, United States, 3. Alzheimer's Los Angeles, San Gabriel, California, United States*

The "Faces of Caregiving" video project highlights the social and cultural aspects of dementia care as shared by seven Japanese Americans. We performed a content analysis of the narratives in the videos, and prominent themes included stigma, gender, resilience, and values placed on family and community. We also applied the life course perspective in examining each care trajectory and found the principles of historical time, linked lives, timing, and human agency to be not only relevant but also useful for a comprehensive understanding of the shared and unique care experiences of Japanese Americans. These results have significant practical implications in that they inform a conceptual template for developing similar video projects for other Asian ethnic groups in the United States. The video project can be replicated within an appropriate cultural context, and such a culturally sensitive approach can help better meet diverse ethnic minorities' unmet needs for information and education. Part of a symposium sponsored by the Aging Among Asians Interest Group.

### SESSION 5050 (SYMPOSIUM)

### EVALUATION RESEARCH FOR INTERGENERATIONAL PROGRAMS: RIGOROUS METHODS, BEST PRACTICES, AND CHALLENGES

Chair: Skye Leedahl
Discussant: Amy Eisenstein

Conducting evaluative research on intergenerational programs is key to understanding if they are functioning as intended. Research on program impact is also critical for prioritization and decision-making in an increasingly competitive market with many programming choices. Implementation studies can help researchers identify needed modifications for replication or introduction to new populations, which is particularly important as universities and communities work to become Age-Friendly. There is growing demand by educational entities (e.g., universities, high schools) and organizations that serve older adults (e.g., senior centers, adult day services, lifelong learning institutes, and residential programs) to identify rigorous methods that can be used to analyze outcomes for students and older adults who take part. This symposium focuses on evaluation methods for intergenerational programs and highlights diverse examples of how researchers have evaluated their programs. The presenters will discuss best practices and challenges to conducting research on these programs as well as the findings of the studies. The first paper will discuss the creation of an intergenerational contact measure. The second paper will describe how an intergenerational classroom was examined using data from instructors and students. The third paper will detail how a quasi-experimental design was used to examine outcomes for an intergenerational program on older adult participants. The fourth paper will discuss how data was gathered from various stakeholders to examine the impacts of an intergenerational classroom. Amy Eisenstein, Senior Program Officer at RRF Foundation for Aging will serve as the discussant. Intergenerational Learning, Research, and Community Engagement Interest Group Sponsored Symposium.

### DEVELOPMENT OF A MEASURE OF INTERGENERATIONAL CONTACT: IMPLICATIONS FOR EVALUATING INTERGENERATIONAL PROGRAMS

Shelbie Turner,[1] Shannon Jarrott,[2] Nicole Mullican,[1] and Karen Hooker,[3] *1. Oregon State University, Corvallis, Oregon, United States, 2. The Ohio State University, Columbus, Ohio, United States, 3. BSS, Corvallis, Oregon, United States*

Evaluating intergenerational programs often involves measuring the extent to which programs actually strengthen both the quantity and quality of intergenerational contact, which is associated with attitudes towards aging, social connection, and anxiety about aging. However, a surprisingly limited number of reliable, valid measures of intergenerational contact exist. Available measures are either explicit to familial intergenerational contact, are environment specific (e.g. workplace intergenerational contact), or are un-validated. In our presentation we will describe progress on our team's efforts to develop an expert-informed reliable, valid measure of intergenerational contact that can be used widely to evaluate intergenerational programs. Specifically, we will share findings from a Delphi-style expert panel review used to develop the measure. The panel – 14 intergenerational research and practice experts – iteratively reviewed the measure, offering feedback on improving its relevance and practicality. We close with examples of how the measure can be used to evaluate intergenerational programs. Part of a symposium sponsored by Intergenerational Learning, Research, and Community Engagement Interest Group.

### EVALUATING AGE-FRIENDLY INTERGENERATIONAL CLASSROOMS: INSIGHTS FROM INSTRUCTORS AND STUDENTS

Joann Montepare, *Lasell University, Newton, Massachusetts, United States*

Age-friendly University (AFU) campuses are reshaping how we think about teaching and learning in higher education. In particular, intergenerational classrooms are on the rise as shifting age demographics call for institutions to create new opportunities for older learners and encourage intergenerational exchange. Age diverse classrooms have distinctive needs and dynamics that instructors, and students, will need to learn how to navigate. This presentation will describe outcomes of one AFU institution's attempt to identify the challenges and triumphs of intergenerational classrooms through facilitated instructor and student reflections in different classrooms over the course of several semesters. Recommendations will be offered for enhancing intergenerational exchange in classrooms across disciplines, as well as evaluating attitudes, logistics, and learning outcomes. Part of a symposium sponsored by Intergenerational Learning, Research, and Community Engagement Interest Group.

### USING A QUASI-EXPERIMENTAL STUDY TO EXAMINE OUTCOMES FOR OLDER ADULT INTERGENERATIONAL PROGRAM PARTICIPANTS

Skye Leedahl, Melanie Brasher, and Erica Estus, *University of Rhode Island, Kingston, Rhode Island, United States*

To more rigorously examine the University of Rhode Island Cyber-Seniors Program, we conducted a quasi-experimental study to examine if older adult senior center participants ($n=25$) improved scores on social and technological measures compared to a sample of senior center participants ($n=25$) who did not take part in the program. Findings showed that participants improved on technology measures compared to the non-participants, including searching and finding information about goods & services, obtaining information from public authorities or services, seeking health information, sending or receiving emails, and participating in online social networks ($p<.05$). However, participants did not change on social measures. There is either a need to identify better social measures to understand the social benefits of taking part, or to bolster the program to aid in helping older adults alleviate isolation and loneliness. Information on best practices and challenges for gathering outcomes from older participants will be discussed. Part of a symposium sponsored by Intergenerational Learning, Research, and Community Engagement Interest Group.

### IMPORTANT CONSIDERATIONS IN DEVELOPING AND EVALUATING INTERGENERATIONAL SERVICE-LEARNING PROGRAMS

Laura Donorfio, *University of Connecticut, Waterbury, Connecticut, United States*

Very often, intergenerational programs measure their success by collecting feedback primarily from the students participating in the program. Critical stakeholders such as the adults/older adults, administrators, and service-learning personnel can be overlooked, as well as the impact of the various classroom activities and tools used. Each fall semester over a five-year period, an undergraduate adulthood and aging intergenerational service-learning course was offered, measuring and building on the successes and challenges from the previous year. No matter how much thought goes into planning a class, unexpected variables naturally unfold over time. This presentation will highlight a best practices model developed over a five-year period with feedback collected from key constituents. Engaging different generations in a purposeful service-learning experience is complex and intended outcomes are not guaranteed. Understanding the key variables and the needs of all those involved through feedback and measurement, can help ensure a more meaningful educational experience. Part of a symposium sponsored by Intergenerational Learning, Research, and Community Engagement Interest Group.

### SESSION 5055 (SYMPOSIUM)

### GSA AND AGHE: 75 YEARS OF LEADING INNOVATIVE, INTERDISCIPLINARY, INTERGENERATIONAL, AND ENGAGING EDUCATION

Chair: Judith Howe
Discussant: Marie Bernard

The AGHE Symposium will highlight the many ways that education has advanced interdisciplinary work, innovative programs, intergenerational services and community engagement in the field of aging over the last decades. Presenters will reflect on how these educational modalities can inform the future of gerontology education. Educators have innovated over decades to develop a field characterized by a diversity of programs, including internships, applied learning experiences in research, policy and practice, and leadership. The history of AGHE and interdisciplinary education are linked, with AGHE serving as an incubator for the advancement of change in this area. Over the course of the last five decades, funding from federal and foundation initiatives have resulted in multi-disciplinary university based centers, interprofessional team training programs, and workforce development initiatives. These efforts have improved the health and well-being of older adults and contributed to the development of new collaborative care systems. AGHE members have also long been involved in intergenerational teaching and learning strategies, including service-learning and community activities. Intergenerational work in the field of aging has helped to shape the Age-Friendly University initiative which calls for colleges and universities to promote intergenerational learning through reciprocal sharing of expertise among learners of all ages. Finally, the AGHE community has long promoted engaged learning with opportunities for students to interact with community partners through programs such as community-based research and clinical experiences and local policy efforts. The ideas presented in the symposium will lead to reflection on how AGHE can expand its value as GSA's educational arm.

### AGHE AS AN INCUBATOR FOR INTERDISCIPLINARY EDUCATION

Thomas Teasdale,[1] Judith Howe,[2] and Carol Rogers,[3]
*1. University of Oklahoma Health Sciences Hudson College of Public Health, Oklahoma City, Oklahoma,*

*United States, 2. Icahn School of Medicine at Mount Sinai, Bronx, New York, United States, 3. University of Oklahoma Health Sciences Center, Reynolds Center for Geriatric Nursing Excellence, Oklahoma City, Oklahoma, United States*

For several decades, the history of interdisciplinary education and the development of AGHE initiatives have been closely linked. The need to educate colleagues on methods and benefits of interdisciplinary/ interprofessional cooperation toward service and research of aging has never waned. In this presentation we (a) highlight how AGHE has performed as a potent incubator for progress in this area and (b) use a few examples to illustrate how notable resulting efforts have improved geriatric care. For example, early and significant infusion of federal funds for gerontology training programs supported multi-disciplinary university-based centers, the Veterans Health Administration created interprofessional geriatric training programs, foundations such as John A. Hartford and Josiah Macy founded team training and interprofessional education programs, and the Health Resources and Services Administration funded Geriatric Education Centers and Geriatric Workforce Enhancement Programs. Efforts to advance interdisciplinary/ interprofessional education have been fruitful and AGHE's role as an incubator continues to evolve.

### WHY ENGAGEMENT MATTERS TO GERONTOLOGICAL STUDENT LEARNING

Christine Fruhauf,[1] Allyson Brothers,[1] Nicole Ehrhart,[1] Blake Naughton,[1] and Sue Schneider,[2] *1. Colorado State University, Fort Collins, Colorado, United States, 2. Larimer County Extension, Fort Collins, Colorado, United States*

Learning opportunities beyond the classroom create lasting and positive effects on students' academic and professional growth. When faculty members participate in engaged scholarship in their local communities, out-of-classroom learning opportunities for students likewise expand. In the context of aging, the array of opportunities for students to engage with community partners is vast, from interactive course assignments to research opportunities, clinical experience, local policy efforts, and more. This paper builds on previous theoretical and empirical work from scholars (including AGHE and GSA Fellows) in the science of engagement, and integrates case examples of campus-community relationships from our collective years in the academy. Examples will showcase how engaged teaching, engaged research, and engaged service collectively informs and enriches our students' experiences. In the spirit of the 2020 conference theme, we will discuss opportunities for university-community engagement as a way to strengthen and inspire current and future gerontological pedagogy.

### WHY INTERGENERATIONAL CONNECTIONS MATTER IN HIGHER EDUCATION

Joann Montepare,[1] and Skye Leedahl,[2] *1. Lasell University, Newton, Massachusetts, United States, 2. University of Rhode Island, Kingston, Rhode Island, United States*

The Academy for Gerontology in Higher Education (AGHE) has a long and rich history of fostering the commitment of higher education to the field of aging through education, research, and public service. To this end, AGHE members have made innovative and effective use of intergenerational strategies. This presentation will highlight these efforts gleaned from AGHE's official journal Gerontology and Geriatrics Education and Intergenerational Learning, Research, and Community Engagement interest group (ILRCE), with special attention to intergenerational classroom exchange, service-learning and community activities, and socially-focused undertakings. Contributions to the evaluation of intergenerational programs will also be noted. The presentation will conclude with a discussion of how this work is now helping to shape the Age-Friendly University (AFU) initiative which calls for institutions of higher education to promote intergenerational learning to facilitate the reciprocal sharing of expertise between learners of all ages.

### INNOVATION IN GERONTOLOGY EDUCATION: AN ADVENTURE OF GRAND IDEAS AND GOOD INTENTIONS

Dana Bradley, *University of Maryland Baltimore County, Baltimore, Maryland, United States*

Gerontology educators continually innovated over multiple decades to create a field characterized by its diversity of programs with a variety of structures to meet a wide range of educational experiences. This paper explores the rich history of how innovations emerged and shares insights on how these grand ideas became broadly accepted practice. Since early 1970's 6 distinct innovations emerge with sticking power, including convergence upon a core intellectual foundation; support of interdisciplinary education; offering internships or practicums; integration of gerontological training with opportunities provided by community/organizational partners, research affiliates, and alumni to foster applied learning experiences in research, policy, and practice; and, development of leadership across and among generations through the Emerging Scholar & Professional Organization (ESPO).

### SESSION 5060 (SYMPOSIUM)

### GWEP PARTNERSHIPS WITH COMMUNITY-BASED ORGANIZATIONS TO ADVANCE AGE-FRIENDLY HEALTH SYSTEMS

Chair: Leland Waters
Discussant: Nina Tumosa

From the onset of the Health Resources and Services Administration's Geriatrics Workforce Enhancement Program (GWEP), project initiatives have been developed to collaborate with Area Agencies on Aging and other community-based organizations (CBOs) serving the older adult community to improve health outcomes. The current GWEP funding cycle includes an initiative to further the principles of Age-Friendly Health Systems. Having built relationships within the aging services community, GWEPs are well positioned to deliver educational interventions framed around the 4M pillars of age-friendly healthcare. Three GWEPs are working with CBOs to implement and test interventions that address the interactive 4Ms. One GWEP has created an Age Friendly Community Education series in conjunction with their CBO partners in a low-income urban setting. Another GWEP has secured age friendly designation for its teams and worked with a coalition of CBOs to obtain both age and dementia-friendly designations for its home city of

Springfield, MA. A third GWEP is implementing age-friendly practice workflows in programs that connect primary care and community-based organizations to address specific needs of an underserved older population. By integrating CBOs within the age-friendly health system framework, we can better create sustainable partnerships with health systems to improve health outcomes for our elders.

### HOW GWEPS ARE IMPACTING AGE FRIENDLY AND DEMENTIA-FRIENDLY COMMUNITIES: BAYSTATE HEALTH

Maura Brennan, *Baystate Health, Northampton, Massachusetts, United States*

Baystate has collaborated with Community Based Organizations (CBOs) to secure designation for Springfield, MA as both age and dementia friendly. We worked together so our city could be recognized as the first in the nation which was age and dementia friendly and also had an age-friendly health system within it. Baystate joined a Springfield coalition of CBOs; with the assistance of the Massachusetts Healthy Aging Coalition, AARP, State and local Elder Affairs, the Massachusetts Councils on Aging and the Institute for Healthcare Improvement, we secured and celebrated all three recognitions at a public forum in June 2019. The event was attended by the Mayor, Baystate Health and local Elder Affairs leaders, the press and other stakeholders as well as older adults from the community. Along with ongoing efforts to improve transportation and housing, access to age-friendly health care is now also an additional area of focus for the coalition.

### AGE-FRIENDLY COMMUNITY EDUCATION: FOSTERING HEALTH BEHAVIOR CHANGE IN A MEDICALLY UNDERSERVED COMMUNITY

Katherine Thompson, *University of Chicago Medicine, Chicago, Illinois, United States*

The University of Chicago GWEP, SHARE Network (Supporting Healthy Aging Resources and Education), is located on the South Side of Chicago, an urban medically underserved community. SHARE Network partners with a variety of community based organizations (CBOs) to support Age Friendly care through an Age Friendly Community Education Curriculum. In a one year period, 28 events were held in 13 CBO sites. Each of these events addressed the 4Ms of Age Friendly care with a specific content focus on Mentation (5 events), Mobility (5), Medications (4), and What Matters (10), and 4 events focused on multiple domains. There were 458 attendees, 290 completed surveys (63% response rate). Eighty-eight percent of participants planned to make a healthy behavior change after participation. Age Friendly Community Education can complement health system transformation to lead to health behavior change for community-dwelling older adults in medically underserved communities.

### PARTNERSHIPS CONNECTING HEALTHCARE AND COMMUNITY-BASED ORGANIZATIONS IN VIRGINIA

Patricia Slattum,[1] Pamela Parsons,[2] Mary Rubino,[3] and Leland Waters,[1] *1. Virginia Commonwealth University, Richmond, Virginia, United States, 2. Virginia Commonwealth, Richmond, Virginia, United States, 3. Family and Community Medicine, Eastern Virginia Medical School, Norfolk, Virginia, United States*

The Virginia Geriatric Education Center (VGEC)'s Geriatrics Workforce Enhancement Program (GWEP) partners with two programs, Senior Strong at Eastern Virginia Medical School in Norfolk, VA and the Richmond Health and Wellness Program at Virginia Commonwealth University in Richmond VA to support their age-friendly initiatives. These programs enhance primary care for an older population experiencing adverse social determinants of health by providing screening around the 4Ms pillars of age-friendly healthcare and connecting participants with healthcare and community-based organizations. These programs offer a rich learning environment for interprofessional students. The VGEC GWEP strengthens these programs by developing faculty and student training in collaboration with the programs and facilitating program participation in the GWEP-CC Age-Friendly Action Community to develop and refine age-friendly practice workflows, referral pathways and documentation.

### AGE-FRIENDLY INITIATIVES AND PRIMARY CARE IN RURAL ARKANSAS

Robin McAtee,[1] and Leah Tobey,[2] *1. UAMS, Little Rock, Arkansas, United States, 2. University of Arkansas for Medical Sciences, Little Rock, Arkansas, United States*

The Arkansas Geriatric Education Collaborative (AGEC)'s Geriatric Workforce Enhancement Program is partnering with a plethora of community based organizations (CBO) and with ARcare, an Arkansas federally qualified healthcare clinic network, to implement the 4Ms of age-friendly care in rural clinics. Baseline clinical data related to the Age-Friendly 4M Framework has been gathered and quality improvement projects initiated to improve the outcomes. Initiatives to improve depression and cognitive screenings are addressing Mentation; fall prevention screens and the offering of fall prevention programs have been added for Mobility; high risk medication screens and chronic pain educational programs are being implemented to address Medications; and finally, Medicare Annual Wellness Visits is the cornerstone to improve what Matters to older adults. A campaign that involves partnered CBOs to address health literacy and increase involvement in evidence-based programs is also helping to drive improvements in age-friendly care in rural Arkansas.

### SESSION 5065 (SYMPOSIUM)

### INNOVATIONS IN GERONTOLOGY EDUCATION: A MULTIFACETED APPROACH

Chair: Melissa O'Connor
Discussant: Melissa O'Connor

By 2030, older adults are projected to outnumber children. This growth among older adults presents both opportunities and challenges on how to care for this unprecedented older adult population. In 2010, there were 7.2 family caregivers for every person 80 and older; this is expected to fall to 3 by 2050. Many older adults are at risk of becoming an elder orphan - someone aging alone with no family available to address their caregiving needs. This underscores the urgent need for the education of nurses who are prepared to care for older adults with complex social and health needs, many of whom are suffering from three to five chronic conditions. This symposium highlights cutting-edge research designed to transform nursing education to better prepare students to advance the health of older adults. Our first presentation describes ageism among undergraduate students and innovative curricula to positively influence their preference for working with older adults. Next, the groundbreaking tool-kit that encourages self-reflection on beliefs about aging and reframes how students view older adults. Third, the qualitative results of a collaborative learning experience designed to introduce students earlier and more often to older adults. Finally, findings from a student-led study investigating the unique needs of caregivers of older adults with diabetes highlights the importance of funding students to conduct research related to older adults. Our symposium ends with a discussion of how these approaches are transforming nursing education to adequately prepare nurses to improve the health and health care of older adults.

### CURRICULA INNOVATION TO POSITIVELY INFLUENCE PREFERENCE FOR WORKING WITH OLDER ADULTS: A REVIEW OF THE LITERATURE

Christine Brewer, *Villanova University, Villanova, Pennsylvania, United States*

Few nursing students show preference in working with older adults. The purpose of this study was to review the U.S. nursing education evidence-based literature to determine curricula innovation to positively influence preference for working with older adults. CINAHL, Medline, Ovid Emcare, PsychInfo, and PubMed databases were searched for relevant U.S studies published between 2009 and 2020 using the search terms “nursing students”, “geriatrics OR gerontology OR older adults OR elderly OR aging”, “career OR work”, and “choice OR preference OR attitude”. Nine studies were eligible for inclusion. Nursing education may play a role in influencing how students perceive and prefer to work with older adults. Promising interventions include stand-alone gerontology courses, intergenerational service-learning experiences, and clinical experiences with community dwelling older adults. More evidence-based research with larger sample sizes are needed to determine effective nursing education interventions to improve nursing students’ attitude and preference for working with older adults.

### IMPLEMENTING A POSITIVE LEARNING EXPERIENCE RELATED TO OLDER ADULTS FOR UNDERGRADUATES: A TOOLKIT

Ann Scheve, and Elizabeth Bruderle, *Villanova University, Villanova, Pennsylvania, United States*

Undergraduate nursing students are frequently exposed to older adults in the clinical setting, where they assess and manage their diseases and its consequences. But that is not enough! To support healthy aging, students need positive intergenerational learning experiences with older adults to discover the gifts of aging early in their curriculum. The goal of these experiences is to help students reflect on their thoughts about aging and reframe how they view older adults. During this presentation we will provide a tool kit based on our experience incorporating positive intergenerational learning early in our curriculum, offer practical guidelines and share constructive feedback.

### PROVIDING EXPERIENCE FOR UNDERGRADUATE NURSING STUDENTS TO CARE FOR OLDER ADULTS: A QUALITATIVE STUDY

Christine Pariseault, Christina Whitehouse, and Melissa O’Connor, *Villanova University, Villanova, Pennsylvania, United States*

Care of the older adult can be complex and frequently influenced by ageism. Nursing students do not have the frequent opportunity to provide care for older adults. The purpose of this pilot study was to expose sophomore nursing students to older adults earlier and more often in the undergraduate curriculum by providing a unique clinical experience at St. Thomas of Villanova Monastery, a residential facility for retired Augustinian priests. This study examines the experience of students’ participation in this clinical experience. Qualitative content analysis of 12 student logs was conducted. Themes that emerged included: age-related changes, environmental considerations, psychosocial needs and changes, and consideration of gerontology as a career choice and existing bias. Students gained a valuable understanding of the unique age-related changes that older adults are experiencing. Early experiences are vital in the curriculum and provide enhanced engagement in gerontology.

### ENGAGEMENT AND MENTORSHIP OF UNDERGRADUATE STUDENTS IN AGING RESEARCH

Alyssa Danner, Christina Whitehouse, and Melissa O’Connor, *Villanova University, Villanova, Pennsylvania, United States*

Education and mentorship of undergraduate students extends beyond the classroom and clinical setting into research. This presentation will discuss the experience of the student and research mentor as well as provide findings from an important qualitative research study. Through a university funded grant, a student nurse partnered with faculty to investigate the needs and experience of caregivers of recently hospitalized older adults with diabetes. Caregivers play a vital role in caring for older adults, often they do not receive the education necessary to achieve optimal health outcomes. Qualitative interviews of 20 caregivers were conducted. The main themes that emerged from the data were: the role itself, challenges, preparation, and additional comorbid diagnoses. These findings provide an understanding of experience, tasks, and needs of caregivers for older adults with diabetes. This research experience provided exposure and education that is essential to developing future aging research scientists.

### SESSION 5070 (SYMPOSIUM)

### OLDER ADULTS AS TEACHING ALLIES: OPPORTUNITIES FOR AGE-FRIENDLY UNIVERSITY INNOVATION

Chair: Joann Montepare
Co-Chair: Kimberly Farah

Changing age demographics are reshaping societies and challenging institutions of higher education to consider how they can respond to aging populations through new approaches to teaching, research, and community engagement. As well, institutions are facing a range of challenges as they look to respond to the contemporary needs of traditional-aged students. The pioneering Age-Friendly University (AFU)

initiative, endorsed by GSA's Academy for Gerontology in Higher Education (AGHE), offers a framework within which institutions can begin to address these issues through more age-friendly programs, practices, and partnerships. This symposium will feature AFU advocates discussing innovate ways in which older adults can serve as teaching allies and support the educational mission of higher education. Farah (Lasell University) will discuss how older adults can engage in diverse teaching and learning activities with examples as crime scenario developers in a forensics class, conversation partners in an international oral communication class, and professional interviewers in an internship skills class. Kaye (University of Maine) will discuss how older adults can serve as citizen scientists performing critical functions in participatory research and in community-based test-beds and co-design laboratories. Ermer (Montclair State University) will discuss how older adults can engage students in discourse as guest speakers and panel participants in classes across the curriculum. Manoogian (Western Oregon University) will describe the innovative ways that older adult students (for credit or audit) mentor and engage younger student peers in course activities as well as increase their own understanding of the aging process.

### AGE-FRIENDLY TEACHING AND LEARNING: NEW ROLES FOR OLDER ADULTS ACROSS THE CURRICULUM

Kimberly Farah, and Joann Montepare, *Lasell University, Newton, Massachusetts, United States*

The pioneering Age-Friendly University (AFU) framework, with its set of ten guiding principles, advocates for enabling older adults to participate fully in educational activities that promote positive and healthy aging. In addition, the AFU principles call attention to bringing younger and older learners together around educational goals, and engaging learners in collaborative classroom experiences that facilitate the reciprocal sharing of expertise between learners of all ages. Implied, but not articulated, in these principles is the idea that older adults' expertise, skills, and talents can also be tapped to support classroom learning goals and extend teaching strategies. This presentation will show how older adults can serve as valuable educational allies in classrooms across the curriculum with examples of crime scenario developers in a forensics class, conversation partners in an international oral communication class, and professional interviewers in an internship skills class. Evidence will argue that these roles enhance student learning.

### OLDER ADULTS AS CITIZEN SCIENTISTS AND POLICY AND PLANNING VOICES IN HIGHER EDUCATION

Lenard Kaye,[1] and Sarah Burby,[2] *1. University of Maine, Bangor, Maine, United States 2. University of Maine School of Social Work, Orono, Maine, United States*

The University of Maine is embarking on achieving AFU status. In addition to maximizing older adult participation in all facets of campus life (education, recreation, culture, etc.), their presence in nontraditional sectors of university activity will be emphasized. Building on the principles of community-based, participatory research, focal points of UMaine's AFU strategy will be to ensure that age-specific, engagement mechanisms are created and maintained that ensure older citizens play an influential role in guiding and interpreting academic research and development and curricula innovation across multiple professions and disciplines. Using a state-wide, older adult research registry, and co-design, community test-beds in partnership with continuing care retirement communities, older adults will serve as citizen scientists. Other empowerment strategies for maximizing elder voice include expanding the number of departments that incorporate life span perspectives in their mission statements and expanding the number of older adult advisory bodies that inform university policy and practice.

### OLDER ADULT LEARNERS' CONTRIBUTIONS TO ENHANCING AGE-FRIENDLY PRACTICES WITHIN THE CLASSROOM

Margaret Manoogian, *Western Oregon University, Monmouth, Oregon, United States*

Interest in college courses that focus on aging attract a variety of students. For older adults, the decision to take college courses may be fueled by a desire to engage in life-long learning, finish degrees that have been long desired, or retool and change careers. Older adults 65 years and older living in Oregon may audit courses for free in four-year public universities. Older auditors and degree-seeking students often work closely with faculty in providing support on course projects and sharing developmental insights that stimulate discussions about aging pathways. This presentation outlines the ways in which older adults contribute to educational conversations, mentor younger learners, and enhance classroom/university environments. How older adult learners have provided support in workshops, aided students in career decision-making, connected students to community resources, and provided contacts for intergenerational community activities will also be highlighted.

### WITH EXPOSURE COMES FAMILIARITY: OLDER ADULTS' CONTRIBUTIONS TO THE AGE-FRIENDLY CLASSROOM

Ashley Ermer, *Montclair State University, Montclair, New Jersey, United States*

Prior research has documented the benefits of intergenerational contact for both younger and older adults (e.g., Christian, Turner, Holt, Larkin, & Cotler, 2014). This presentation will focus on how older adults have contributed to students' classroom experiences in an upper-level, undergraduate aging policy course. In this course, older adults have previously served as guest speakers, sharing their knowledge about the creation of nonprofit organizations, and their experiences aging within the family. Older adults have also participated in an activity addressing ageism with students from the aging policy course. Feedback from students has largely been positive, with over half of students during stating that they had a better understanding of older adults, that they learned from the experience, and that viewed the intergenerational contact as helpful. Ways in which this model can be extended to other academic disciplines will also be explored.

### SESSION 5075 (SYMPOSIUM)

### STEERING AN AGE-FRIENDLY UNIVERSITY (AFU) INITIATIVE: INSIGHTS FROM DIRECTORS OF AGING CENTERS

Chair: Joann Montepare
Co-Chair: Kimberly Farah

The pioneering Age-Friendly University (AFU) initiative, endorsed in 2016 by GSA's Academy for Gerontology in Higher Education (AGHE), calls for institutions of higher education to respond to shifting demographics and the needs of our aging populations through more age-friendly programs, practices, and partnerships. Over 65 institutions have joined the AFU network and adopted the 10 AFU guiding principles. This symposium will feature leaders at AFU campuses representing centers on aging and gerontology programs who will discuss why their institution joined the initiative, their age-friendly campus vision, and their recommendations for mounting an AFU initiative. AFU Director Charness (Florida State University) will describe launching an AFU initiative from the perspective of a state university, with a focus on critical discussion points, the concerns of upper administration, and the timeline for completion of the process. AFU Director Porter (University of Manitoba) will discuss how the Centre on Aging has been using its mandate as a research centre to advance the age-friendly movement through research, knowledge mobilization, training, and partnership initiatives. AFU Director Schumacher (University of Maryland Baltimore County) will talk about how the AFU framework has served as a platform for new and constructive synergies among gerontology programs, centers on aging, health systems, and higher education. AFU Director Gugliucci (University of New England) will discuss strategies for leading health professions education programs within an AFU framework, with special attention to AGHE resources available to support these efforts. Directors of Aging Centers Interest Group Sponsored Symposium.

### A STATE UNIVERSITY PERSPECTIVE ON BECOMING AN AGE-FRIENDLY UNIVERSITY

Neil Charness, *Florida State University, Tallahassee, Florida, United States*

I describe the process of FSU becoming an Age-Friendly University (AFU). After hearing a presentation on AFU at the Age Directors meeting at GSA by Joann Montepare in November 2016, I judged that many of the ten principles of an AFU were already in operation at FSU. So, I decided to confer in December with VP for Faculty Development and Advancement, Janet Kistner, about how to move the AFU application process forward. I generated a draft document and circulated it to major units dealing with aging on campus: the Institute for Successful Longevity, the Pepper Institute on Aging and Public Policy, and the Pepper Center. Eventually, a one-page version of the longer document was introduced at an FSU Cabinet meeting and approved in April 2017. I detail some of the critical discussion points, the concerns of upper administration, and the timeline for completion of the process at FSU. Part of a symposium sponsored by Directors of Aging Centers Interest Group.

### LEADING AGE-FRIENDLY UNIVERSITY INITIATIVES AT A RESEARCH INTENSIVE UNIVERSITY IN MANITOBA, CANADA

Michelle Porter, *University of Manitoba, Winnipeg, Manitoba, Canada*

When Dublin City University introduced the Age-Friendly University (AFU) principles in 2012, the Centre on Aging at the University of Manitoba had been fully immersed in age-friendly cities research, so it was a natural for it to champion the University becoming more age-friendly. As a university-wide research centre, at a research intensive university, the Centre on Aging has built on its strengths to advance the AFU movement. Related to its goals on research, knowledge mobilization, training, and partnerships, it has been able to work with its many Research Affiliates, students and community/university partners to tackle new AFU initiatives. Recent activities have included: an awareness raising workshop/showcase, discussions with Research Affiliates about potential new degree course offerings, an environmental scan of post-secondary inter-generational possibilities, a home-sharing project, workshop development on older learners in the classroom, and provincial community workshops and consultations on communicating about aging and healthy aging. Part of a symposium sponsored by Directors of Aging Centers Interest Group.

### THE AGE-FRIENDLY UNIVERSITY UMBRELLA: NEW SYNERGIES FOR GERONTOLOGY PROGRAMS, AGING CENTERS, AND HEALTH SYSTEMS

John Schumacher, *University of Maryland, Baltimore County (UMBC), Baltimore, Maryland, United States*

Higher education has a tentative relationship with gerontology programs and research centers. Despite demographic shifts, academic gerontology serves a modest student body and attracts limited research/scholarly interest. Our new Age Friendly University (AFU) designation served to motivate our university leaders and faculty to re-imagine how aging issues are fundamental to our mission framed by: 1) Interdisciplinarity; 2) Intergenerational relations; and 3) Inclusiveness of the life course. Additionally, the AFU initiative energized the relationships among our health professions schools, academic departments, and research center to collaborate on grant opportunities. Our AFU also started collaborating with a local health system seeking Age Friendly Health System status. Finally, our AFU designation raised visibility with our three local county governments who had just received Age Friendly Community status. Our AFU efforts have served as an inclusive umbrella to attract new, interested partners in our shared effort to improve the lives of older adults. Part of a symposium sponsored by Directors of Aging Centers Interest Group.

### AGE-FRIENDLY UNIVERSITY INITIATIVE APPLIED TO HEALTH PROFESSIONS EDUCATION PROGRAMS

Marilyn Gugliucci, *University of New England College of Osteopathic Medicine, Biddeford, Maine, United States*

Opportunities for health professions (HP) education programs to make a mark in the Age Friendly University (AFU) initiative abound. Key approaches are introduced, and insights for mounting these efforts are discussed, for HP education programs based on the AFU global network initiative and the Academy for Gerontology in Higher Education (AGHE). Higher Education Institutions (HEIs) that offer HP education, have various options to establish and enhance gerontology/geriatrics competence and confidence for their students. AFU guiding principles applicable to HP education allow health gerontology faculty to be catalysts to promote and integrate the AFU guiding principles within their program's existing curriculum. This can contribute to their institution's readiness to apply for the AFU designation, advance the institution's age friendliness, and/or set up the program to apply for the AGHE Program of Merit (POM) for Health Professions Programs. All prepare future HP professions providers to improve older adult health care. Part of a symposium sponsored by Directors of Aging Centers Interest Group.

### SESSION 5080 (SYMPOSIUM)

### SUPPORTING PHD STUDENTS TO BECOME FACULTY IN GERONTOLOGICAL SOCIAL WORK: AGESW'S FELLOWSHIP PROGRAM

Chair: Rebecca Mauldin
Discussant: James Lubben

In the United States, the field of social work faces a critical shortage of students and faculty with expertise in gerontology needed to meet the growing needs of an aging society. To help recruit, train, and retain aging-related social work practitioners, researchers, and educators, the Association for Gerontological Education in Social Work (AGESW) created the Pre-Dissertation Fellowship Program in 2010. AGESW provides leadership in the areas of gerontological social work education, research, and policy and its PDFP was designed to support doctoral students in their education and future careers. In this 10th anniversary year of the PDFP, this symposium presents multiple perspectives of PDFP program evaluation. The first paper uses qualitative data from eight years of PDFP evaluations to identify types of professional skills attained through the program and areas of professional development missing from PDFP fellows' home doctoral programs. The second paper uses quantitative data from a retrospective survey administered to PDFP alumni to describe their perspectives on the effects of the program. The third paper uses data from a retrospective survey of three cohorts of PDFP alumni to demonstrate the use of social network analysis for program evaluation. The fourth and final paper uses an idiographic approach to explain benefits of the PDFP from the perspectives of early stage scholars who participated in the program. Overall, the symposium provides evidence that suggests the effectiveness of the PDFP in building professional networks, mentoring doctoral students, and teaching academic skills and discusses using the PDFP model in other gerontological fields.

### THE AGESW PRE-DISSERTATION FELLOWS PROGRAM: PARTICIPANTS' PERCEPTIONS OF MENTORSHIP AND TRAINING FOR ACADEMIA

Noelle Fields,[1] Allison Gibson,[2] Stephanie Wladkowski,[3] Cara Wallace,[4] and Abigail Latimer,[5] *1. The University of Texas at Arlington, Arlington, Texas, United States, 2. University of Kentucky, College of Social Work, Lexington, Kentucky, United States, 3. Eastern MIchigan University, Ypsilanti, Michigan, United States, 4. Saint Louis University, St. Louis, Missouri, United States, 5. University of Kentucky College of Social Work, Lexington, Kentucky, United States*

Good mentoring is key for doctoral student success. In 2010, AGESW began offering the Pre-Dissertation Fellows Program (PDFP) to enhance social work doctoral students' professional development and skillset for academia. The purpose of this study was to examine student participants' perceptions of the PDFP in its role to providing mentorship and training for an academic position. This qualitative study examined eight cohorts (2010-2018) of the AGESW PDFP (N=85). Using thematic analysis, responses identified a number of aspects of professional development gained, gratitude for the training, an appreciation for candid advice received, and areas of professional development they felt they were lacking within their doctoral training. Findings bolster support for structured programs and professional development that supplement doctoral education in a student's first two years. Implications for doctoral education, mentorship training, and avenues to enhance the AGESW pre-dissertation program will be discussed

### THE IMPACTS OF A MENTORING PROGRAM ON THE DEVELOPMENT OF GERONTOLOGICAL SOCIAL WORK FACULTY

Nancy Kusmaul,[1] Stephanie Wladkowski,[2] Allison Gibson,[3] Rebecca Mauldin,[4] Jennifer Greenfield,[5] and Noelle Fields,[6] *1. UMBC, Baltimore, Maryland, United States, 2. Eastern MIchigan University, Ypsilanti, Michigan, United States, 3. University of Kentucky, College of Social Work, Lexington, Kentucky, United States, 4. University of Texas at Arlington, Arlington, Texas, United States, 5. University of Denver, Denver, Colorado, United States, 6. The University of Texas at Arlington, Arlington, Texas, United States*

The John A. Hartford Foundation and the Association for Gerontology Education in Social Work (AGESW) have worked to develop gerontological social work faculty to address the needs of older adults. This presentation will discuss the role of AGESW's Pre-Dissertation Fellows Program in the development of social work doctoral students. All participants from the PDFP's 2010-2016 cohorts received a 38-question online survey via email exploring the program's impacts on their academic career in teaching, research, mentoring, and support. Forty-five respondents, representing all six cohorts, completed the survey. More than half said the PDFP contributed to their ability to publish research (64.4%, n = 29), grow their professional network (86.7%, n = 39), and teach (55.5%, n = 25). Doctoral programs provided different experiences: mentoring, methodological training, professional development, and peer support. Results suggest the PDFP supplements students' doctoral programs by connecting students to each other and to national leaders.

### EVALUATING PROFESSIONAL NETWORKS IN A GERONTOLOGICAL PRE-DISSERTATION INITIATIVE USING SOCIAL NETWORK ANALYSIS

Erin Murphy,[1] Rebecca Mauldin,[2] Jennifer Greenfield,[3] Nancy Kusmaul,[4] Noelle Fields,[5] Stephanie Wladkowski,[6]

and Allison Gibson,[7] *1. UT Arlington, School of Social Work, Arlington, Texas, United States, 2. University of Texas at Arlington, Arlington, Texas, United States, 3. University of Denver, Denver, Colorado, United States, 4. UMBC, Baltimore, Maryland, United States, 5. The University of Texas at Arlington, Arlington, Texas, United States, 6. Eastern MIchigan University, Ypsilanti, Michigan, United States, 7. University of Kentucky, College of Social Work, Lexington, Kentucky, United States*

Professional networks are critical for PhD students and early career faculty, yet there is scant research on the development of their professional networks. Social network analysis is a useful approach to describe the development of professional networks. This methodological paper explains its use and benefits, using a social network analysis of alumni from the first three cohorts of the Association of Gerontological Education in Social Work (AGESW)'s Pre-Dissertation Fellowship Program (PDFP) as an example. We present results, challenges, and recommendations. Alumni (n = 12) reported meeting an average of 20 scholars (SD = 13.2) through AGESW. These professional relationships led to collaborations on conference presentations and manuscripts as well as opportunities to leverage the relationships for future professional needs. Suggested applications of social network analysis for program evaluation, such as co-author and citation networks, are also presented with a focus on training programs designed to support robust professional network development.

### REFLECTIONS AND PROSPECTS OF THE GERONTOLOGICAL TRAINING AND EDUCATION

Kathy Lee,[1] Tyrone Hamler,[2] and Cara Wallace,[3]
*1. University of Texas at Arlington, Arlington, Texas, United States, 2. Case Western Reserve University, Cleveland, Ohio, United States, 3. Saint Louis University, St. Louis, Missouri, United States*

The demand for professional training, mentorship, and research in the field of aging is expected to increase remarkably. Recent statistics indicate less than 8% of social work students nationwide specialize in gerontology; however, a significant amount of social work graduates, regardless of their specialization at school, serve older adults in various social and health care settings. As a panel, former fellows present experiences as participants of the AGESW Pre-Dissertation Fellows Program. The program helps students comprehend basic principles of doctoral education and develop strong professional networks with other gerontology-focused colleagues and mentors across the country. Doctoral students are also trained to be competent working with older adults through research, teaching, and professional development. Many fellows move into faculty positions and their accomplishments are varied and impressive. It is difficult to separate these from the education, connections, mentorship, and support received from the AGESW network and through participation in this program.

### SESSION 5085 (SYMPOSIUM)

### POLICY SERIES: THE ROLE OF A STAKEHOLDER MEMBER GROUP IN SHAPING GERIATRIC POLICY: THE NATIONAL ASSOCIATION FOR GERIATRIC EDUCATION

Chair: Leland Waters
Discussant: Brian Lindberg

The National Association for Geriatric Education (NAGE) is a non-profit membership organization representing Geriatric Workforce Enhancement Programs (GWEPs), Geriatric Academic Career Awardees (GACAs) and other programs that provide education and training to health professionals in the areas of geriatrics and gerontology. Our work includes faculty training and fellowships, continuing education, and hands on experiences in the clinical setting. One of our priorities is to educate policy makers and the public about the need for health care professionals to receive geriatrics education so they will better serve the expanding older population. One of our goals is to provide a mechanism for policy development and dissemination to external audiences regarding the mission, goals and impact of geriatric education programs. Our policy objectives include providing guidance to the United States Public Health Service and other organizations in the development of programs to enhance the education of health care practitioners and others. Another objective is to educate Congress about necessary priorities in geriatric education. We serve as a voice for the goals and interests of the nation's GWEPs, GACAs, and other groups providing education in geriatrics and gerontology. This symposium will first describe how geriatric educators inform policy. Then a historical perspective of how NAGE has influenced aging policy is provided. Recent efforts to increase funding for geriatric education will be shared, followed by future directions in policymaking.

### THE CONTRIBUTION OF GERIATRIC EDUCATORS TO PUBLIC POLICY

Anna Faul,[1] Jennifer Severence,[2] and Leland Waters,[3]
*1. University of Louisville Trager Institute, Louisville, Kentucky, United States, 2. University of North Texas, Fort Worth, Texas, United States, 3. Virginia Commonwealth University, Richmond, Virginia, United States*

Despite the current pressure to reduce state and federal spending, policymakers must find ways to address the challenges of a growing population of older adults with complex health care problems. There is an increased need for the health professions workforce to have collaborative care skills and geriatric clinical competencies. Therefore, programs like the Geriatric Workforce Enhancement Program (GWEP) and the Geriatric Academic Career Awards (GACA) are important in strengthening the workforce and supporting policy development that addresses increased demands on the health care system. In 2019, the Bureau of Health Professions, under the Health Resources and Services Administration, provided 48 GWEP awards and 26 GACA awards in 37 states and 2 territories. These programs play an important advocacy role to improve on and expand geriatric education. This symposium provides an overview of these programs and their role in advancing geriatric care and in shaping policy.

### THE DEVELOPMENT OF A STAKEHOLDER MEMBER ORGANIZATION TO ADVOCATE FOR GERIATRICS EDUCATION

Leland Waters,[1] and Elyse Perweiler,[2] *1. Virginia Commonwealth University, Richmond, Virginia, United States, 2. Rowan University School of Osteopathic Medicine, Stratford, New Jersey, United States*

The National Association of Geriatric Education Centers organization was established in 1990, to promote interdisciplinary geriatric education and to provide a unified voice for Geriatric Education Centers (GECs). In 2005, the GECs voted to form two non-profit organizations due to restrictions related to lobbying activities. An umbrella organization was created, the National Association for Geriatric Education, that includes all geriatric related education programs, and maintain a lobbyist in Washington DC to protect the GECs interests. It was a pivotal time, as we had a year (2006) without federal funding that summarily dismantled the DHHS-HRSA geriatrics programs, including the entire GEC network, the geriatric fellowship program, and Geriatric Academic Career Awards. This resulted in a GEC-wide and national geriatrics movement that succeeded in restoring the geriatrics line item in the President's budget. Our advocacy efforts not only had the line item restored, but obtained an increase in funding for geriatrics.

### AN UPDATE ON RECENT GERIATRIC EDUCATION ADVOCACY EFFORTS

Catherine Carrico,[1] and Katherine Bennett,[2] *1. University of Wyoming, Laramie, Wyoming, United States, 2. University of Washington, Seattle, Washington, United States*

The National Association for Geriatric Education (NAGE) has maintained consistent education and advocacy efforts since 2006. In recent years NAGE has implemented formal and grassroots advocacy strategies. At the federal level NAGE has increased collaboration with other aging advocacy organizations and coalitions. At the request of Congress, NAGE leadership and stakeholders have testified before Congress and regularly submit testimony to the House and Senate. NAGE staff maintain strong working relationships with congressional staff. Strategies for effective grassroots education and advocacy have been taught to members, and membership has mobilized to educate elected officials about the essential work of the Geriatric Workforce Enhancement Programs across the country. This presentation will provide a thorough review of NAGE's advocacy work over the past 4 years.

### CURRENT AND FUTURE GERIATRICS EDUCATION POLICY INITIATIVES

Marla Berg-Weger,[1] and Katherine Bennett,[2] *1. Saint Louis University, St. Louis, Missouri, United States, 2. University of Washington, Seattle, Washington, United States*

Current and future NAGE policy-related activities will be the focus of this presentation. The Geriatric Academic Career Awards (GACAs), which support the career development of junior faculty clinician educators in geriatrics, were reinstituted by HRSA in 2019 after a 13-year absence. We will discuss the role of this award in the broader context of geriatrics education and GWEPs, how GACA awardees have been integrated into NAGE, and the need for expansion of the GACA program to support both the GWEP and geriatric education pipelines. Areas for future NAGE engagement will be focused on advocacy efforts to support: permanent GWEP reauthorization by Congress; expanding current level of $40.737 million to $51 million to enable HRSA to increase the number of GWEPS to further extend their reach; increasing funding for GACA awardees; and strengthening the synergies between the GACA and GWEP programs to support development of future GWEP leadership.

### SESSION 5090 (SYMPOSIUM)

### WHY AGE MATTERS TO HIGHER EDUCATION: AGE-FRIENDLY TOOLS AND TECHNIQUES FOR CULTURE CHANGE

Chair: David Burdick
Co-Chair: Karen Rose
Discussant: Dana Bradley

Momentum is growing for the Age-Friendly University Network as proponents, primarily gerontology educators, have successfully encouraged university presidents to sign nonbinding pledged to become more age-friendly in programs and policies, endorsing 10 Age-Friendly University Principles. While this trend is inspiring, more is needed to fully achieve benefits for universities, students, communities, and older adults. Four presentations discuss innovative ways of deepening university commitment, weaving the principles into the fabric of the university. The first paper describes thematic content analysis from five focus groups with admissions and career services staff at Washington University in St. Louis and the recommendations that emerged for the provision of programs and services for post-traditional students. The second paper describes efforts to utilize community-impact internships and community partnerships to build support for Age-Friendly University initiatives at Central Connecticut State University, particularly in the context of the university's recent Carnegie Foundation Engaged Campus designation. The third paper describes how Drexel University became Philadelphia's first Age-Friendly University and current efforts in the Drexel College of Nursing and Heatlh Care Profession's AgeWell Collaboratory to convene university-wide leadership for an AFU Steering Committee working on four mission-driven efforts to ensure AFU sustainability. The fourth paper describes steps taken by AFU proponents at Western Oregon State University to gain endorsement from university leadership and community, including mapping the 10 AFU Principles to the university's strategic plan, faculty senate endorsement, and survey/interview results of older community members' use of the university, which collectively have enhanced deeper and broader campus buy-in of AFU.

### MOVING TOWARD AGE-DIVERSE UNIVERSITIES: PERSPECTIVES OF ADMISSION AND CAREER SERVICE PROFESSIONALS

Nancy Morrow-Howell,[1] Natalie Galucia,[1] Emma Swinford,[2] and Tanner Meyer,[2] *1. Washington University in St. Louis, St. Louis, Missouri, United States, 2. Washington University, St. Louis, Missouri, United States*

As age diversity in universities increases in response to demographic shifts, changes in educational practices, programs and policies are needed. To inform these transformations, this research focuses on opportunities and challenges of increasing age-diversity. We conducted 5 focus groups and included 31 professional staff at Washington University in St Louis who work in admissions and career services. A thematic content analysis revealed themes in two main categories: challenges of serving non-traditionally aged students (fitting in, career concerns, acclimating to learning environment and technology, ROI, and ageism) and benefits of older students (intentional students, experienced students, and classroom diversity). Recommendations emerged, including affinity groups, social opportunities which include families, community engagement for job placement, financial aid, targeted outreach to older students, academic flexibility, and administrative support. Findings will be used to advocate for increasing age-diversity among students at Washington University and other institutions in the Age-Friendly Global Network..

### USING COMMUNITY-IMPACT INTERNSHIPS TO BROADEN AFU COMMITMENT ON CAMPUS

Andrea June, and Carrie Andreoletti, *Central Connecticut State University, New Britain, Connecticut, United States*

Ongoing efforts to support Age Friendly University (AFU) principles must be campus-wide and include commitment from upper administration, faculty, staff, and students. This presentation will describe the leveraging of a new community-based collective partnership to develop a student internship model at Central Connecticut State University (CCSU) that could be used across campus to create a deeper investment in the AFU initiative. We will discuss the valuable collaboration with the Connecticut Healthy Living Collective, an emerging hub of state, regional and local agencies and organizations dedicated to healthy aging in communities throughout our state. The presenters will describe the process of establishing the Matter of Balance internship in psychological science with the support of our dean, knowing that it would eventually serve the broader AFU mission. We will highlight the value this intentional effort offers at multiple levels and how it might be replicated on other AFU campuses.

### DREXEL UNIVERSITY'S AGE-FRIENDLY STRATEGY: STARTING WITH PURPOSEFUL AND EXISTING ENGAGEMENT MECHANISMS

Katherine Clark,[1] Laura Gitlin,[1]
Rose Ann DiMaria-Ghalili,[2] and Elizabeth Yutzey,[3]
*1. Drexel University, Philadelphia, Pennsylvania, United States, 2. Drexel University, Jenkintown, Pennsylvania, United States, 3. Drexel University, College of Nursing and Health Professions, Philadelphia, Pennsylvania, United States*

In 2019, Drexel University became the first Age-Friendly University (AFU) in Philadelphia, Pennsylvania. The College of Nursing and Health Profession's AgeWell Collaboratory (a center without walls that aims to disrupt the healthcare system's traditional approach to aging) is leading the Age-friendly Drexel Steering Committee, which is composed of leadership throughout the university. The Collaboratory purposefully connected the committee to four key mission-driven efforts in order to ensure sustainability: 1)Strategic planning, both at the university and college level 2)The institution's research agenda 3)Existing programming and work groups (ie. The University Committee on Accessibility and the School of Professional Development and Institutional Advancement's efforts to engage more older adults),4) Community engagement efforts, such as the City of Philadelphia's Age-friendly initiative and the university's community outreach hub, the Dornsife Center for Neighborhood Partnerships. This presentation will discuss how to leverage the AFU movement through mission-driven efforts among senior leadership, faculty, staff and students.

### MAKING THE CASE AND LAYING THE GROUNDWORK FOR INTEGRATING AGE-FRIENDLY PRACTICES INTO A UNIVERSITY STRATEGIC PLAN

Melissa Cannon, *Western Oregon University, Monmouth, Oregon, United States*

A crucial first step in preparing to become an Age-Friendly University (AFU) is seeking endorsement from the campus community and leadership. This presentation describes the mapping of the AFU principles to the strategic plan and initiatives of Western Oregon University, leading to endorsement by its faculty senate, and highlights a study of the older community members' use of the university, laying the groundwork for advancing age-friendliness on campus. Data were collected through surveys (N=46), interviews (N=9), and photovoice method (N=7) with older adults, and data were analyzed using SPSS, team coding, and intensive group discussion to develop categories and themes. Themes emerged related to how the college campus is used by older adults, the need to promote lifelong learning to the community, and the need to address accessibility issues in order to be more age-friendly, providing helpful insight to other institutions of higher education seeking to join the AFU network.

### SESSION 5100 (SYMPOSIUM)

### FROM AD HOC TO ASSOCIATION TO ACADEMY: DEVELOPMENTAL MILESTONES OVER 48 YEARS FOR AGHE

Chair: Lisa Hollis-Sawyer
Discussant: Lydia Manning

The Academy for Gerontology in Higher Education began as the Ad Hoc Committee on the Development of Gerontology Resources in 1972. The next year the name Association for Gerontology in Higher Education (AGHE) was selected. In 1974 AGHE held a general meeting of its membership in conjunction with the annual meeting of the Gerontological Society of America in Portland Oregon. The first annual meeting was held in 1975 in Madison, Wisconsin. The relationship with GSA has evolved over the last four decades leading to the present Academy. The first presentation will focus on the issues of gerontology and higher education that emerged in the early years and continue to be a challenge today. The second presentation will look at the future of AGHE in its new relationship with GSA. The third presentation will focus on the Academy in the present discussing current challenges facing academic gerontology in today's world including the development of AGHE's basic-competency guidelines and the emergence of measuring student learning outcomes and their role in program review and evaluation. The fourth presentation will examine the evolving discussion of standards in evaluating gerontological and geriatric programming. Geriatric Education Interest Group Sponsored Symposium.

### THE DEVELOPMENT OF AGHE AS A FORCE IN GERONTOLOGY EDUCATION

Harvey Sterns, *The University of Akron, Akron, Ohio, United States*

This presentation will focus on the history and development of AGHE beginning in 1972. The issues and concerns of early leaders will be reviewed base on articles, books and early publications of the association. There are core issues of gerontology education that continue to be challenges to the present. Significant contributions to these early years will be highlighted. Part of a symposium sponsored by the Geriatric Education Interest Group.

### FROM ASSOCIATION TO ACADEMY: EMBRACING NEW OPPORTUNITIES

David Burdick, *Stockton University, Port Republic, New Jersey, United States*

This presentation will explore the transition from Association to Academy. This transition occurred as GSA governance was also changing, presenting significant challenges on several fronts. While some of these challenges will be considered, the presentation will primarily focus on AGHE's many emerging opportunities and the steps various AGHE committees are taking to enhance the Academy's role and relevance and fulfill its mission. The Advancement Committee's project to assess educational programs across GSA sections, the Age-Friendly University initiative, Ageism First Aide, and other innovative programs moving forward will briefly be noted. Part of a symposium sponsored by the Geriatric Education Interest Group.

### THE EMERGENCE OF AGHE'S BASIC-COMPETENCIES GUIDELINES IN GERONTOLOGY

Robert Maiden, *Alfred University, Alfred, New York, United States*

This presentation will explore the development of AGHE's major contemporary contribution: AGHE's basic-competency guidelines and their role in program review and program enhancement. The presenter will describe in detail the steps in creating objective student learning outcomes and will explore how meeting these competencies improve instruction, self-reflection, program analysis and faculty discussion and ultimately gerontology programs. These guidelines and policies embody a living entity that is always evolving. Future iterations will be anticipated and discussed. Part of a symposium sponsored by the Geriatric Education Interest Group.

### AN EVOLUTION OF THE STANDARDS TO ENHANCE GERONTOLOGICAL AND GERIATRIC EDUCATION

Tamar Shovali,[1] Karen Kopera-Frye,[2] and Joann Montepare,[3] *1. Eckerd College, St Petersburg, Florida, United States, 2. New Mexico State University, Las Cruces, New Mexico, United States, 3. Lasell University, Newton, Massachusetts, United States*

This presentation will focus on AGHE's evolving discussions on gerontological and geriatric programming. The earliest discussion resulted in development of the first edition of the standards and guidelines for programs in 1989. In 2014, the AGHE Academic Program Development Committee (APDC) created new core competencies for gerontological education. Currently, members from both the AGHE APDC and Advancement Subcommittees are collaborating on revising the AGHE Standards and Guidelines (6th edition, 2015) to align with competencies. This presentation will discuss the new and improved version (draft available in November 2020). First, presenters will review points regarding how this update will aid in Accreditation for Gerontology Education Council and Program of Merit self-study program reviews. Secondly, the presenters will explain how the revision will increase gerontological education program integrity. Lastly, the presenters will explain how this update will be consistent with the trend across institutions utilizing these competencies in gerontological and geriatric education. Part of a symposium sponsored by the Geriatric Education Interest Group.

### SESSION 5105 (SYMPOSIUM)

### OLDER LEARNERS IN HIGHER EDUCATION: AGE-FRIENDLY UNIVERSITY MODELS IN AND BEYOND THE CLASSROOM (A COLLABORATIVE SYMPOSIUM BETWEEN THE AFU AND INTERGENERATIONAL LEARNING, RESEARCH AND COMMUNITY ENGAGEMENT INTEREST GROUPS)

Chair: Skye Leedahl

The Age-Friendly University (AFU) movement is specifically targeting one group of adult learners who are less represented within higher education -- individuals considered "older adults," with five of the ten principles focused on promoting educational opportunities for older adult learners. However, there is less understanding within higher education for how to ensure inclusivity of this group. Importantly, some universities across the country have identified promising strategies for engaging older adult learners within higher education classrooms and supporting them beyond the classroom. As this intergenerational learning model continues to grow, there is much to learn from those who have begun efforts to appropriately utilize and engage older adult learners. This symposium will highlight examples from universities that have identified ways to create age-diverse programs within the university setting. The first paper will begin by discussing intergenerational learning opportunities for utilizing older adult learners in innovative ways to enhance university student experiences, and the second paper will specifically highlight successful activities used in a university class to engage older and younger adult learners. The third paper will examine ways in which a university and Osher Lifelong Learning Institute work together and promote research opportunities for both generations. The fourth paper will discuss research conducted to investigate how intergenerational classroom experiences are shaped by older adults. The fifth paper will describe the use of technology training workshops to promote service learning for university students and those in a retirement community. This would be a collaborative symposium between the AFU and ILRCE Interest Groups.

### ENGAGING OLDER ADULT LEARNERS THROUGH INTERGENERATIONAL LEARNING OPPORTUNITIES

Skye Leedahl,[1] Phillip Clark,[1] and Beth Leconte,[2] *1. University of Rhode Island, Kingston, Rhode Island, United States, 2. University of Rhode Island Osher Lifelong Learning Institute, Kingston, Rhode Island, United States*

The University of Rhode Island became a part of the AFU network in 2018, and much of our rationale for joining the network was based on our strengths and growing interest in intergenerational programs and learning. The URI Osher Lifelong Learning Institute (OLLI) currently has over 1,300 members, and a large aspect of their strategic plan and current efforts are focused on increasing intergenerational learning opportunities due to interest and successes in these areas. Some of the successful strategies that have been used include an intergenerational classroom of OLLI member and university students designed within a traditional college class, a matching program where students are partnered with OLLI members, intergenerational service learning opportunities for students to engage with OLLI members in different ways, and question and answer sessions with OLLI members within college classes. This presentation will highlight these efforts, lessons learned, and efforts to track participation and outcomes.

### USING TECHNOLOGY TO CONNECT THE GENERATIONS AND UNDERSTAND LIFELONG LEARNING

Laura Donorfio, and Brian Chapman, *University of Connecticut, Waterbury, Connecticut, United States*

The University of Connecticut (UConn) has a thriving Osher Lifelong Learning Institute (OLLI), which has existed for over a decade on one of their regional campuses (700+ members). Intergenerational classes are utilized, but connecting UConn students with OLLI members outside of these classes in an effective, meaningful way is a challenge. A successful model developed within an adulthood and aging class to connect the generations outside of the classroom utilizes technology as a bridge. The two most successful activities will be highlighted. The first is a “technology clinic,” which requires students to pair up with OLLI members to assist them with technological needs. The second requires students to create a podcast by interviewing an OLLI member on the importance of lifelong learning, which is uploaded to the campus OLLI website. Implications for both generations, bi-directional affective change, and inclusivity of older learners in the classroom and beyond will be discussed.

### LIFELONG LEARNERS’ CONTRIBUTIONS TO THE CAMPUS COMMUNITY

Barbara White, *California State University, Long Beach, Huntington Beach, California, United States*

The 2,000 member Osher Lifelong Learning Institute (OLLI) at California State University, Long Beach offers non-credit classes to adults 50 and older. We have an ongoing strategic goal (2014, 2019) to “increase our University and student involvement.” Integration into the campus community includes members volunteering as participants in survey and participatory faculty and student research related to biopsychosocial aspects of aging, faculty/student data collection in selected OLLI classes, vetting students to teach OLLI classes, acting as resources for professors/students in product development related to aging, guest lecturing in University courses, and providing internships for students at OLLI. Collaborations have led to multiple faculty/student publications and presentations. We also endow an annual award for graduate students to support their research/projects related to aging. In return, we request and provide students the opportunity to present their results to our members. Outreach strategies will be discussed.

### OLDER ADULTS AS COVERT OPERATIVES IN A FIRST-YEAR UNDERGRADUATE COURSE ON AGING

Brian Carpenter,[1] Meghan McDarby,[2] Natalie Galucia,[1] and Nancy Morrow-Howell,[1] *1. Washington University in St. Louis, St. Louis, Missouri, United States, 2. Washington University in St. Louis, Saint Louis, Missouri, United States*

Age-friendly university programs are increasing in number, yet little research has evaluated how older adults shape classroom experiences. This pilot study tested one method for analyzing intergenerational classroom dynamics. Two small-group discussion sections for an introductory class on aging included older adults (n = 3 per section) and undergraduates (n = 15 per section). Class sessions on four topics (health, sexuality, housing, relationships) were video recorded. Overall, older adults spoke proportionally more during class discussions than would have been expected by chance alone. They participated most during the session about sexuality and least in the section that addressed relationships. Specific contributions from older adults included reflections on class activities, topic-specific personal anecdotes, and reactions to younger students. Research methods to investigate intergenerational learning are emerging, and this study provides one preliminary approach. We discuss additional ideas to bring empirical rigor to this emerging field of study.

### AN INTERGENERATIONAL SERVICE-LEARNING MODEL FOR YOUNGER AND OLDER LEARNERS: LINKING COMMUNITY AND CLASSROOM

Tamar Shovali, *Eckerd College, St Petersburg, Florida, United States*

The intergenerational reflective service-learning program titled Mentor Up is intentionally integrated within gerontology education. Mentor Up, a series of one-on-one technology training workshops, connects younger with older learners in the community while building on course content. Groups of students are paired with the Academy of Senior Professionals at Eckerd College (ASPEC) members, a continuing education organization whose 300+ members are committed to lifelong learning. Members are fully integrated into the course, serving as mentors for students. These class sessions support students in planning for Mentor Up service at a local CCRC and promote positive contact between students and retired community members. ASPEC members also participate with students in a significant reflective component concluding the course – in which students and older learners work together to write a script, record, and present a video essay addressing aging stereotypes. Considerations for planning, classroom integration, community connection, and project reflections will be discussed.

### SESSION 5300 (SYMPOSIUM)

### ACCEPTANCE AND UPTAKE OF TECHNOLOGY AMONG OLDER ADULTS: FINDINGS FROM CREATE

Chair: Sara Czaja

Discussant: Bo Xie

Technology is ubiquitous in everyday life and technology use is essential to independent living. Technology applications are deployed in most settings (e.g., healthcare, work) and also changing how we communicate, and access information and services. Despite increases in technology uptake among older people, an age-related digital divide remains. Drawing from research conducted by the Center for Research and Education on Aging and Technology Enhancement (CREATE), this symposium will discuss the acceptance and uptake of technology among older adults and factors influencing technology adoption. S. Czaja will discuss recent technology trends and how they vary according to technology type and subgroups of older adults. Based on CREATE findings, she will also discuss age group and cohort differences in interest in and comfort with technology. W. Boot will present findings from a study that examined adherence to a technology-based cognitive training program and how individual difference factors shaped adherence. N. Charness will present findings regarding whether advanced driver assistance systems (ADAS) can improve older adult driving performance, and older adults' acceptance and perceptions of value of ADAS systems. M. Harris will discuss health related technology interventions and how integration of technology acceptance and behavior change models can provide insights for the design of health behavior interventions aimed at older adults. J. Sharit will provide findings from a study, which examined the willingness of older adults to adopt a variety of technologies and factors influencing willingness to adopt. Bo Xie will lead a discussion of these issues and outline areas for future research.

### OLDER ADULTS' ADHERENCE TO TECHNOLOGY-BASED INTERVENTION: THE ROLE OF MESSAGING AND INDIVIDUAL DIFFERENCES

Walter Boot,[1] Nelson Roque,[2] Erin Harrell,[3] and Neil Charness,[1] *1. Florida State University, Tallahassee, Florida, United States, 2. Pennsylvania State University, University Park, Pennsylvania, United States, 3. University of Alabama, Tuscaloosa, Tuscaloosa, Alabama, United States*

Adherence to health behaviors is often poor, including adherence to at-home technology-based interventions. This study (N=120) explored adherence to a cognitive training intervention delivered via computer tablet, assessed adherence over a 4.5 month period, explored how individual difference factors shaped adherence, and tested the efficacy of message framing manipulations (positive vs. negative framing) in boosting adherence. Individual difference factors predicted adherence, including variations in self-efficacy and belief in the efficacy of cognitive training. Overall message framing had little impact. However, during the final portion of the study in which participants were asked to play as much or as little as they wanted instead of following a schedule, participants who received positively framed messages engaged with the intervention more. Implications for predicting and boosting adherence to home delivered technology-based interventions will be discussed.

### ACCEPTANCE OF TRANSPORTATION TECHNOLOGIES BY AGING ADULTS

Neil Charness,[1] Dustin Souders,[2] Ryan Best,[3] Nelson Roque,[4] JongSung Yoon,[5] and Cary Stothart,[6] *1. Florida State University, Tallahassee, Florida, United States, 2. Purdue University, West Lafayette, Indiana, United States, 3. Rand Corporation, Santa Monica, California, United States, 4. Pennsylvania State University, University Park, Pennsylvania, United States, 5. University of South Dakota, Vermillion, South Dakota, United States, 6. U.S. Army Research Institute for the Behavioral and Social Sciences., Fort Leavenworth, Kansas, United States*

Older adults are at greater risk of death and serious injury in transportation crashes which have been increasing in older adult cohorts relative to younger cohorts. Can technology provide a safer road environment? Even if technology can mitigate crash risk, is it acceptable to older road users? We outline the results from several studies that tested 1) whether advanced driver assistance systems (ADAS) can improve older adult driving performance, 2) older adults' acceptance of ADAS and Autonomous Vehicle (AV) systems, and 3) perceptions of value for ADAS systems, particularly for blind-spot detection systems. We found that collision avoidance warning systems improved older adult simulator driving performance, but not lane departure warning systems. In a young to middle-aged sample the factor "concern with AV" showed age effects with older drivers less favorable. Older drivers, however, valued an active blind spot detection system more than younger drivers.

### INTEGRATING TECHNOLOGY ACCEPTANCE AND BEHAVIOR CHANGE THEORIES TO GUIDE TECHNOLOGY INTERVENTIONS

Maurita Harris, and Wendy Rogers, *University of Illinois at Urbana-Champaign, Champaign, Illinois, United States*

Technology interventions are commonly proposed as an effective means to support health self-management in older adults. For such interventions to be successful, we must identify individuals who are willing to adopt and adhere to these technologies. The general Technology Acceptance Model (TAM; Davis 1989) has been widely used to predict intentions to adopt technology in a variety of contexts. Likewise, the Theory of Planned Behavior (Azjen, 1991) has long been used to provide insights about health behaviors. These theories share three common stages: attitudes, behavior intentions, and acceptance. However, neither perspective provides insight into continued utilization of a technology tool (i.e., long-term adoption). Our goal is to integrate these models with the Transtheoretic Model of Behavior Change (Prochaska & Velicer, 1997) to provide insights that can help design technological interventions for older adults who want to change a health behavior and maintain that change over time.

### OLDER ADULTS' WILLINGNESS TO ADOPT TECHNOLOGIES

Joseph Sharit,[1] Sara Czaja,[2] and Jerad Moxley,[2] *1. University of Miami, Miami, Florida, United States, 2. Weill Cornell Medicine, New York, New York, United States*

Willingness to adopt technology is an important precursor to technology adoption. This talk will present findings from a study which examined 187 older adults' willingness to adopt a variety of mobile technologies supporting domains such as transportation, health/wellness, and lifelong learning. Participants aged 65 years and older, including 144 females, were presented with Power Point slides describing each of five technologies, and subsequently rated each technology on their willingness to adopt it as well as on the technology's perceived value, the perceived mental effort required to learn it, confidence in one's ability to learn it, the degree of help available from family/friends for help learning it, and privacy concerns. Other measures, including self-assessment of skills, technology readiness, technology skills, and cognitive abilities, were also collected. Interrelationships among these and other study variables will be presented as a basis for a model for predicting older adults' willingness to adopt these technologies.

### SESSION 5305 (SYMPOSIUM)

### ADVANCING THE STUDY OF SUBJECTIVE AGE: MORE SERIOUSLY CONSIDERING GENDER

Chair: Shelbie Turner
Co-Chair: Karen Hooker
Discussant: Toni Calasanti

Socially-cued age expectations inform people's subjective age - that is, how old they feel relative to their chronological age. Age-graded expectations are widely considered to be gendered, yet gender has not often been empirically examined as the scholarship on subjective age has developed. Because subjective age shapes the experiences a person has becoming and being an older adult, and is an important correlate of later life health, more seriously considering gender's influence on subjective age is crucial to better understanding gender differences in older adults' well-being. In our symposium we bring gender to the center of subjective age scholarship. Barrett, Michael, & Noblitt begin by establishing that subjective age research should portray gender as a social-level, rather than individual-level, characteristic. As a complement, Turner, Settersten, and Hooker illustrate how gender has or has not been included in the four theoretical domains of subjective age (self-perceptions of aging, old age stereotypes, age identity, and awareness of age related change), and offer insights into how gender might be included in future studies on each domain. We then shift to two papers presenting new empirical analyses on the role gender plays in subjective age. Kornadt shares how men and women's commitment to certain social roles differentially informs their subjective age, while Settersten, Day, and Hagestad turn attention to a double standard of aging for women and men with evidence across Europe. Discussant Toni Calasanti closes by offering thoughts on the future of subjective age and gender scholarship, including considering gender beyond the binary.

### CONSIDERING GENDER WITHIN THE FOUR THEORETICAL DOMAINS OF SUBJECTIVE AGE

Shelbie Turner,[1] Richard Settersten,[1] and Karen Hooker,[2]
*1. Oregon State University, Corvallis, Oregon, United States, 2. BSS, Corvallis, Oregon, United States*

The broad construct of subjective age is informed by four theoretical domains – self-perceptions of aging, old age stereotypes, age identity, and awareness of age related change (Kotter-Gruhn, Kornadt, & Stephan, 2016). Each of the theoretical domains is distinct yet interconnected, and analyzing how gender operates within each yields a more nuanced understanding of gender's influence on subjective age. In our presentation, we will offer a review of researchers' consideration of gender in studies of each subjective age theoretical domain, describing (1) how gender has and has not been included, (2) key findings when gender has been included, and (3) insights into how researchers might better include – or even center – gender when studying each domain. In so doing, we highlight the contributions of past scholarship on gender and subjective age and offer insights for future studies on the topic.

### PERSONAL AGING IS POLITICAL: A FEMINIST PERSPECTIVE ON SUBJECTIVE AGING

Anne Barrett, Cherish Michael, and Jessica Noblitt,
*Florida State University, Tallahassee, Florida, United States*

An extensive literature examines subjective aging – a construct encompassing many aspects of individuals' views of aging, such as age identity, aging anxiety, awareness of aging, and views of life stages. A factor receiving attention within this research is gender, with studies revealing much about gender differences not only in subjective aging but also its health and behavioral consequences. However, we argue that the literature is limited by its focus on gender as an individual-level characteristic – rather than a profoundly social element emerging within interactions, pervading institutions, and constituting a system of inequality that intersects with others, including age. Addressing this limitation, our chapter applies a feminist perspective to the study of subjective aging. This perspective draws into focus the implications for subjective aging of gender's social embeddedness and provides an illustration of the interconnection between the personal and political spheres.

### SOCIAL ROLES, SUBJECTIVE AGE, AND GENDER: EXPLORING THE LINKS IN LATER LIFE

Anna Kornadt, *University of Luxembourg, Esch-sur-Alzette, Diekirch, Luxembourg*

Subjective age (SA) is strongly linked to positive developmental outcomes and successful aging. The social roles people assume are supposed to impact SA, since they incorporate age-graded social experiences and age-stereotypic role expectations. Social roles are also strongly gendered, providing the opportunity to understand gender-specific processes of SA. This study investigates a broad range of social roles and their relation to older men and women's SA in later life. N = 285 participants aged 50 to 86 years (Mage = 65.04, SD = 8.88) reported on 19 social roles and their SA. Higher commitment to social roles of continued development and engagement was related to a younger subjective age, above and beyond sociodemographic variables, physical and mental health, but only for younger men. Commitment to family roles was related to a younger subjective age only for older men. Implications for the gender-specific understanding of antecedents of SA are discussed.

### THE DOUBLE STANDARD OF AGING FOR MEN AND WOMEN: EVIDENCE FROM ACROSS EUROPE, 2006–2018

Richard Settersten,[1] Jack Day,[2] and Gunhild Hagestad,[3]
*1. Oregon State University, Corvallis, Oregon, United States, 2. SUNY-Oneonta, Oneonta, New York, United States, 3. Agder University & Northwestern University, Kristiansand, Vest-Agder, Norway*

Is there a "double standard" (i.e., a harsher judgment) in the perceived ages at which women and men reach old age, and have these judgments changed over time? We use European Social Survey data from 23 countries in 2006 and newly released data from 16 of those countries in 2018. In both 2006 and 2018, men typically assign women substantially earlier ages than women themselves do. In some places, however, men also give themselves lower ages than women give them. With respect to when women become old, the differential views of men and women are persistent. So is the fact that women differentiate less between the sexes¬–though men differentiate less in 2018 relative to 2006. We use multilevel modeling to examine variation explained by both individual characteristics and country indicators of demographic and policy contexts. Findings underscore the significance of the double standard in cultural constructions of aging.

### SESSION 5315 (SYMPOSIUM)

### AGING AND DECISION MAKING: SOCIO-EMOTIONAL AND CONTEXTUAL FACTORS

Chair: JoNell Strough
Co-Chair: Corinna Loeckenhoff
Discussant: Susan Charles

Maintaining sound decision-making skills in later life is a key concern in the face of population aging. The four presentations in this symposium highlight the importance of considering socio-emotional and contextual factors when investigating adult age differences in decision making. Together, they show that features of decision contexts such as the way information is presented, along with social relationships and emotional responses, have distinct implications for understanding age effects in decision-related processing and outcomes. Drawing from fuzzy trace theory, Nolte, Löckenhoff and Reyna showed that gist-based ("good," "extremely poor") versus verbatim information (exact numbers) was differentially appealing to younger and older adults, with older adults seeking more gist information than verbatim information. Young and Mikels investigated older and younger adults' integral emotional responses to a behavioral risk-taking task. Younger adults experienced more anger and less contentment than older adults. These emotions differentially predicted risk taking in the two groups. Seaman, Christensen, Senn, Cooper, and Cassidy found age differences in learning about the trustworthiness of social partners. Older adults showed less learning relative to younger adults and invested less with trustworthy partners and more with untrustworthy partners. Smith, Strough, Parker and Bruine de Bruin found that older age, perceiving better decision-making ability than age peers, and perceiving declines in ability over time, were associated with lesser preferences for making decisions with others. In her discussion, Charles will integrate these findings with existing research on aging and decision making and offers directions for future research.

### THE INFLUENCE OF VERBATIM VERSUS GIST FORMATTING ON YOUNGER AND OLDER ADULTS' INFORMATION ACQUISITION

Julia Nolte, Corinna Loeckenhoff, and Valerie Reyna, *Cornell University, Ithaca, New York, United States*

It is well-established that pre-decisional information seeking decreases with age (Mata & Nunes, 2010). However, it is still unknown whether age differences in information acquisition are influenced by the type of information provided. Fuzzy-trace theory suggests that decision makers prefer gist-based over verbatim-based processing, and that this preference increases across the lifespan. Therefore, we hypothesized that age differences arise when presenting participants with verbatim details (such as exact numbers) but not gist information (such as "extremely poor" or "good"). In a lab-based experiment, 68 younger adults and 66 older adults completed a gist-based and a verbatim-based search task before making health insurance choices. Younger and older adults reviewed similar amounts of information in either condition. In line with Fuzzy-trace theory, however, older adults sought more information when presented with gist rather than verbatim information. The role of age-associated covariates and implications for decision-making will be discussed.

### AGE DIFFERENCES IN DISCRETE EMOTIONAL STATES DURING RISK TAKING

Nathaniel Young, and Joseph Mikels, *DePaul University, Chicago, Illinois, United States*

Emotions often guide risk-taking. For example, anger tends to lead to increased risk-taking. However, older and younger adults differ in their emotional experiences: older adults tend to report more positive emotions, fewer experiences of anger, and relatively similar or increased experiences of sadness relative to younger adults. As such, differences in emotional experience may manifest in the integral emotional responses of older and younger adults as they take risks. The current work examined the discrete integral emotional responses of older and younger adults as they completed the Balloon Analogue Risk Task (BART). For the BART, participants completed 40 trials. Prior to each trial, participants reported how much anger, sadness, contentment, and excitement they felt. The results indicate that younger adults experienced more anger and less contentment than older adults in response to the BART. Importantly though, age differences also emerged in how discrete emotions predicted subsequent risk-taking.

### OPTIMIZING AND COMPENSATORY FUNCTIONS OF SOCIAL DECISION-MAKING PREFERENCES

Kelly Smith,[1] JoNell Strough,[1] Andrew Parker,[2] and Wandi Bruine de Bruin,[3] *1. West Virginia University, Morgantown, West Virginia, United States, 2. RAND, Pittsburgh, Pennsylvania, United States, 3. University of Southern California, Los Angeles, California, United States*

When making decisions, older people may prefer to work with others to optimize their performance or to compensate for declines in decision-making ability. Using participants from RAND's American Life Panel (N=1075, Mage = 53.49, we investigated associations among self-reported preferences to make decisions alone and with others, perceived ability to make decisions (compared to age peers and over time), and perceived benefits of aging for decision-making. Older age and perceiving better decision-making abilities relative to peers were associated with greater preferences to make decisions alone and lesser preferences to make decisions with others. Greater preferences for making decisions with others were associated with perceiving improvements in decision-making ability over time and more positive beliefs about aging and decision making. Women were more likely than men to report preferring to make decisions with others. We discuss optimizing and compensatory functions of social preferences for decision making.

### SOCIAL ASSOCIATIVE LEARNING AND TRUST FORMATION ACROSS ADULTHOOD

Kendra Seaman,[1] Alexander Christensen,[2] Katherine Senn,[2] Jessica Cooper,[3] and Brittany Cassidy,[2] *1. The University of Texas at Dallas, Dallas, Texas, United States, 2. University of North Carolina - Greensboro, Greensboro, North Carolina, United States, 3. Emory University, Atlanta, Georgia, United States*

Trust is a key component of social interaction. Older adults, however, often exhibit excessive trust relative to younger adults. One explanation is that older adults may learn to trust differently than younger adults. Here, we report a study examining how younger (N=36) and older adults (N=37) learn to trust over time. Participants completed a classic iterative trust game with three partners (15 trials each). Younger and older adults shared similar amounts but there were differences in how they shared that money. Compared to younger adults, older adults invested more with untrustworthy partners and less with trustworthy partners. As a group, older adults displayed less learning than younger adults and computational modeling suggests that older adults used different learning strategies. These findings suggest that older adults attend to and learn from social cues differently from younger adults. Neuroimaging results focused on reward processing will also be discussed.

### SESSION 5320 (SYMPOSIUM)

### AGING AND GAMING: THE SCIENCE AND PROMISE OF TECHNOLOGY-BASED LEISURE ACTIVITIES AND INTERVENTIONS

Chair: Chantal Kerssens

Much research has focused on technology to support older adults in basic and instrumental activities of daily living. Much less is known about technology supports of hobbies and leisure later in life. Physical, cognitive and social activities potentially delay the onset and progression of disease, including dementia. Older adults are interested in digital games, applications (apps) and social technologies, and will use technology provided their needs, preferences and goals are met. Moreover, video games can be designed to promote satisfying social experiences between players of differing capabilities. More work, however, is needed to understand older adults' interactions and engagement with game-based interventions. This symposium presents cutting-edge research findings and design recommendations for technology-based leisure activities and interventions in older adults. Yow et al. present data from a large, touch-screen dual language intervention program with cognitive training tools aimed at slowing down the rate of cognitive decline in older adults with dementia. Boot et al. present longitudinal data from the Center for Research and Education on Aging and Technology Enhancement (CREATE) with a focus on leisure and videogames; Freed et al. present older adults' attitudes and experiences with an exercise videogame (exergame). Lin et al. discuss the early effects of an exergame involving real-world physical activity on activity, social contact and stress levels in dementia caregivers. Kerssens et al. discuss the creation and testing of an adapted, accessible version of beloved board games for people with mild cognitive impairment (MCI) and a care partner without MCI. Technology and Aging Interest Group Sponsored Symposium.

### RETHINKING AND CO-DESIGN OF BELOVED BOARD GAMES FOR PEOPLE WITH MILD COGNITIVE IMPAIRMENT AND THEIR CARE PARTNERS

Chantal Kerssens,[1] Maribeth Gandy,[1] Kara Cohen,[1] Laura Levy,[1] Cecile Janssens,[2] Tracy Mitzner,[1] Molly Perkins,[2] and Suzette Binford,[3] *1. Georgia Institute of Technology, Atlanta, Georgia, United States, 2. Emory University, Atlanta, Georgia, United States, 3. Emory Brain Health Center, Atlanta, Georgia, United States*

Mild cognitive impairment (MCI) affects millions of older Americans and progression to dementia is common. Although people with MCI may experience impairments, they are often highly verbal, able, and eager to uphold beloved routines. Moreover, many seek opportunities to stay active, physically and mentally, to support their brain health. Some forms of cognitive training and social engagement potentially delay the onset and progression of disease, including dementia. This 12-month project used mixed methods to co-design and test an accessible version of well-known board games for people with MCI and a care partner without MCI. The overall goal was to foster a meaningful, joyous, social activity for players with differing capabilities using adapted game mechanics to create a compelling experience for both players. Coping strategies of care partners were studied to learn ways to foster positive interactions. Findings inform recommendations for game design and clinical interventions. Part of a symposium sponsored by Technology and Aging Interest Group.

### GAMING TECHNOLOGY TO SUPPORT LEISURE ACTIVITIES AMONG OLDER ADULTS

Walter Boot, *Florida State University, Tallahassee, Florida, United States*

There has been a great deal of research on technology to support older adults in their performance of Activities and Instrumental Activities of Daily Living. There has been substantially less research, however, on exploring technology solutions that support hobbies and leisure. This is unfortunate, as quality of life and well-being are determined by more than just one's ability to manage everyday tasks. An overview will be presented of research the Center for Research and Education on Aging and Technology Enhancement (CREATE) has conducted over two decades with the goals of understanding and supporting older adults' performance of technology-based leisure activities. Many of these studies have involved videogaming, where there exists a substantial age-related digital divide. CREATE has evaluated older adults' attitudes and game experiences through survey and research studies and has even recorded longitudinal gameplay. How these findings can be applied to support technology-based leisure activities will be expanded upon. Part of a symposium sponsored by Technology and Aging Interest Group.

### FEASIBILITY AND ENJOYMENT OF EXERCISE VIDEO GAMES IN OLDER ADULTS

Sara Freed,[1] Briana Sprague,[2] and Lesley Ross,[3] *1. Penn State University, University Park, Pennsylvania, United States, 2. University of Pittsburgh, Pittsburgh, Pennsylvania, United States, 3. The Pennsylvania State University, University Park, Pennsylvania, United States*

Interventions using exercise video games, or exergames, have shown short-term cognitive and physical benefits to older adults, though long-term effects are less promising. Enjoyment of exergames may promote exergame use after the intervention period, though little work has examined older adults' views of exergames before and after gameplay experience. We invited 20 older adults between 65 and 84 years of age (M=73.30, SD=5.95) to play two Xbox Kinect games, Just Dance and Kinect Sports Rivals, for twenty minutes. In our presentation, we will present qualitative and quantitative findings of this pilot study, including findings that older adults reported that they were not likely to play similar exergames in the future and that they did not find the exergames to be more fun compared to other ways of exercising. We will discuss implications for game design and research relevant to game developers, manufacturers, and researchers. Part of a symposium sponsored by Technology and Aging Interest Group.

### A NOVEL TOUCH-SCREEN, DUAL-LANGUAGE INTERVENTION PROGRAM FOR OLDER ADULTS

W. Quin Yow, Hui-Ching Chen, Tharshini Lokanathan, Attila Achenbach, and Lucienne Blessing, *Singapore University of Technology & Design, Singapore, Singapore*

Although cognitive training in healthy older adults (OA) has been controversial, specific and isolated cognitive skills such as semantic memory can be improved with appropriate designs. Semantic memory has been considered as a clinical marker for cognitive decline in dementia. The current study, as part of a larger touch-screen dual-language intervention program with cognitive training tools, aims to slow down the rate of cognitive decline in OA with dementia (OwD). A set of neuropsychological tests was conducted before and after the training program. After 24 training sessions over 8-12 weeks, OwD (11 females, 1 male, mean=85.8yo) improved significantly in their verbal working memory (Rey Auditory Verbal Learning Test; RAVLT) while performance of the cognitive-healthy OA (5 females, 3 males, mean=76.3yo) remained the same post-intervention. Our findings suggest that touch-screen technology can help OwD improve their semantic memory. The strengths and limitations of our game design and intervention will be discussed. Part of a symposium sponsored by Technology and Aging Interest Group.

### GO&GROW: A SOCIAL EXERGAME TO PROMOTE WELL-BEING AMONG DEMENTIA CAREGIVERS

Xin Yao Lin,[1] Herman Saksono,[2] Elizabeth Stowell,[2] Margie Lachman,[1] Carmen Castaneda-Sceppa,[2] and Andrea Parker,[3] *1. Brandeis University, Waltham, Massachusetts, United States, 2. Northeastern University, Boston, Massachusetts, United States, 3. Georgia Institute of Technology, Atlanta, Georgia, United States*

Dementia caregivers are at risk for poor health outcomes due to high stress and little time for health-promoting behaviors. The current study examined whether Go&Grow, an exergame (digital game in which play involved real-world physical activity), could increase caregivers' physical activity and social contact and reduce their stress. Go&Grow allows participants to grow flowers virtually as they increase their physical activity. Participants can also interact with others on Go&Grow. Go&Grow was piloted with 18 dementia caregivers (ages 22-70) over a six-week period. Multilevel modeling results showed that weeks with more Go&Grow usage were associated with more steps and more social contact. Days with more social contacts on Go&Grow were associated with more physical activity. Participants reported they were able to manage their distress better at posttest compared to the pretest. The discussion will highlight study limitations as well as implications for technology-based game and intervention design for caregiver well-being. Part of a symposium sponsored by Technology and Aging Interest Group.

### SESSION 5325 (SYMPOSIUM)

### AGING EXPERIENCES AROUND THE WORLD: LOCAL FINDINGS AND GLOBAL INSIGHTS FROM POPULATION SURVEYS OF AGING

Chair: Jennifer Ailshire
Discussant: Jinkook Lee

International comparisons of the aging experience offer a unique opportunity to understand how much the aging process varies according to differences in social environments. Despite the potential knowledge gained from global comparisons of health and aging, conducting cross-national comparisons can be challenging. The HRS-family of surveys, which have been harmonized within the Gateway to Global Aging, provides remarkable opportunities for cross-national comparative analysis. The papers in this session use harmonized data to compare the aging experience across different social dimensions in multiple countries from around the world, including examinations of: 1) the role of social engagement among married couples in cognitive function in Mexico and the United States; 2) the importance of work histories and macro-economic policies on later life health in England and Europe; 3) gender differences in the receipt of informal care in the U.S., Korea, and China; 4) the association between sensory impairment and disability-free life expectancy in England and the U.S.; and 5) end-of-life care arrangements and health care utilization in the context of different health systems across multiple countries.

### HEALTHCARE SYSTEMS AND VARIATION IN HEALTHCARE UTILIZATION AT THE END OF LIFE ACROSS COUNTRIES

Jennifer Ailshire,[1] Cristian herrar,[2] and Margarita Maria Osuna,[3] *1. University of Southern California, Los Angeles, California, United States, 2. Organisation for Economic Cooperation and Development OECD, Paris, Ile-de-France, France, 3. University of Southern California Leonard Davis School of Gerontology, Los Angeles, California, United States*

With rapid population ageing, providing better end-of-life care (EOLC) is becoming a source of social demand and financial pressure for public and private budgets in many countries. This paper uses data from harmonized end-of-life interviews in the HRS family of studies to assesses variation in health care utilization across different income groups and how they differ across different health care systems. Hospital stay did not vary across health care systems, but nursing home stays were lower in countries with either national or statist social health insurance systems. Hospice use was low in all countries, but particularly in national and social health insurance systems. Lower income was associated with greater use of nursing homes in both the private and social health care systems. Low income was also associated with greater use of hospice in national health service, but lower use in social health service.

### VISION AND HEARING IMPAIRMENTS IN RELATION TO DISABILITY-FREE LIFE EXPECTANCY IN PEOPLE FROM ENGLAND AND THE UNITED STATES

Paola Zaninotto,[1] Giorgio Di Gessa,[2] and Jenny Head,[3] *1. University College London, London, United Kingdom, 2. University College London, London, England, United Kingdom, 3. UCL, London, England, United Kingdom*

Both hearing and vision impairments are some of the most common deficits experienced by older adults. We examined the impact of self-reported vision and hearing impairments on disability-free life expectancy (DFLE). We used harmonized data from the Gateway to Global Aging Data from the US Health and Retirement Study (HRS) and the English Longitudinal Study of Ageing (ELSA). We used discrete-time multistate life table models to estimate disability-free life expectancy by sex, age and country. In both countries and at all ages either vision or hearing impairment was associated with shorter DFLE compared to those who reported no impairments. Reporting both vision and hearing impairments reduced DFLE. For example, at the age of 50, men and women with both vision and hearing impairments could expect to live up to 12 fewer years free from disability compared with men and women with no impairments, similar results were found in both countries.

### ADVERSE EMPLOYMENT HISTORIES AND LATER HEALTH FUNCTIONING: EUROPEAN FINDINGS BASED ON LIFE HISTORIES FROM SHARE AND ELSA

Christian Deindl,[1] and Morten Wahrendorf,[2] *1. Heinrich-Heine-Universität Düsseldorf, Duesseldorf, Nordrhein-Westfalen, Germany, 2. Centre for Health and Society, Institute of Medical Sociology, Medical Faculty, University of Düsseldorf, Duesseldorf, Nordrhein-Westfalen, Germany*

We investigate associations between adverse employment histories over time and health functioning in later life, and explore moderation by national labor market policies. Harmonized life history data come from two studies, SHARE and ELSA, with health beyond age 50 (men= 11,621; women= 10,999). Adverse employment histories consist of precarious, discontinued and disadvantaged careers between age 25 and 50, and we use depressive symptoms, grip strength and verbal memory as outcomes. Adverse employment histories are associated with poor health functioning later in life, especially repeated periods of unemployment, involuntary job losses, weak labor market ties and disadvantaged occupational positions. We find no variations of the associations by national labor market policies. Our study highlights the need to improve working conditions at early career stages. Despite the importance in shaping employment histories, the role of national policies in modifying the impact of employment on health is less clear.

### GENDER MATTERS IN THE RECEIPT OF INFORMAL CARE IN LATER LIFE: A CROSS-NATIONAL COMPARISON ACROSS THE USA, KOREA, AND CHINA

Minyoung Kwak,[1] BoRin Kim,[2] hyunjoo Lee,[1] and Jiaan Zhang,[2] *1. Daegu University, Gyeongsan, Republic of Korea, 2. University of New Hampshire, Durham, New Hampshire, United States, 3. Fudan University, Shanghai, China*

This study compares patterns of gender difference in the receipt of informal care among community-dwelling older adults across the United States, Korea, and China. Data came from the 2014 HRS, the 2014 KLoSA, and the 2015 CHARLS. Logistic regression models were used to predict the receipt of informal care by gender. We also examined how the effects of health and living arrangement on the receipt of informal care differ depending on gender. In the United States and China, older women were more likely to receive informal care than men. However, older Korean women were less likely to receive informal care than men. The effects of health and living arrangement on the use of informal care were moderated by gender in different ways across countries. Discussions include implications for practice and policy to reduce the gender gap in the receipt of informal care.

### SOCIAL ENGAGEMENT AND COGNITIVE FUNCTION OF OLDER ADULTS IN MEXICO AND THE UNITED STATES

Bret Howrey, Jaqueline Avila, Brian Downer, and Rebeca Wong, *The University of Texas Medical Branch at Galveston, Galveston, Texas, United States*

Social engagement is linked to better cognition, but it is unclear if the social engagement of husbands and wives influences their own cognition as well as each other's cognition in two very different country contexts. Data on married couples come from the 2001 Mexican Health and Aging Study (MHAS) and the 2000 Health and Retirement Study (HRS), with follow-up cognition measured in 2012. Structural equation models (SEM) were used to test the actor-partner interdependence model on the association of social engagement with cognition. In Mexico wives' social engagement benefited their own cognition as well as their husbands', but husband's social engagement was unrelated to cognition. In the U.S. both wives' and husbands' social engagement benefited their own cognition, but not each other's. Results suggest asymmetric patterns of actor-partner interdependence in Mexico, possibly reflecting more traditional social roles of women and co-dependence within couples, but more independence within U.S. couples.

### SESSION 5330 (SYMPOSIUM)

### AGING IN CONTEXT: THE IMPORTANCE OF COMPARATIVE STUDIES FOR UNDERSTANDING THE LIVES OF OLDER ADULTS

Chair: Allen Glicksman
Co-Chair: Lauren Ring
Discussant: Norah Keating

Access and use patterns of both formal and informal services for older migrants are often examined in a dyadic framework where one group of older persons (or their caregivers) is studied in relation to their use of a program or service. A comparative approach, that might also examine the reasons that some persons may (or may not) use a service, may yield important findings that place the dyadic studies within a larger social and policy context. By using a comparative approach, we can also consider influences of the culture of origin for older adults and their caregivers, as well as the policies and programs offered in the destination country. The four papers on this panel explore these issues. The first paper will frame the discussion, and the remaining three will focus on informal care, formal care, and the point of contact between aging services professionals and older immigrants. The first paper (Torres) takes a broad look at social exclusion mechanisms that bar access to services due to racism in the host societies. The second presentation (Diederich, et. al.) examines how place of origin can influence caregiving behavior. The third paper, (Thiamwong) examines a single program that is used to serve multiple ethnic minority/immigrant groups. Finally, (Ring et. al.,) will examine trust or its absence in the attitudes of older migrants toward use of formal aging services in two migrant populations. The four papers also demonstrate how different research methods (qualitative, quantitative, scoping review) can be used to illuminate these issues. International Aging and Migration Interest Group Sponsored Symposium.

### TRUST AND LANGUAGE AS PREDICTORS OF SERVICE USE

Lauren Ring,[1] Allen Glicksman,[1] Michael Liebman,[2] and Misha Rodriguez,[3] *1. Philadelphia Corporation for Aging, Philadelphia, Pennsylvania, United States, 2. IPQ Analytics, LLC, Kennett Square, Pennsylvania, United States, 3. Asociación Puertorriqueños en Marcha, Philadelphia, Pennsylvania, United States*

Research on older migrants often starts with a set of assumptions- including the importance of language as a barrier to care. A comparative approach allows us to examine these assumptions as they impact access to services for older migrants. Our study compared two groups of older migrants – Mandarin speaking Chinese and Spanish speakers from Puerto Rico. Through a series of focus groups we learned that although language can be a barrier to service access, the more important element in reducing disparities for older migrants is the level of trust between older adult and provider. For the older Chinese participants, the presence of a native speaker whom they trust is contrasted with a lack of trusted native Spanish speakers available to Puerto Rican elders, who must often rely on translators from various providers. We will use this example to help explain the differences in service use by these two communities. Part of a symposium sponsored by the International Aging and Migration Interest Group.

### SOCIAL EXCLUSION IN HEALTH AND SOCIAL SERVICES OFFERED TO MINORITY PATIENTS: DO RACIALIZATION AND RACISM PLAY ROLES?

Sandra Torres, *Uppsala University, Uppsala, Sweden*

Cultural and ethnic differences stemming from migration are a source of social exclusion in old age. This topic is of concern in part because an increased migration flow coupled with growing anti-immigrants sentiments in much of the Western world can ignite social exclusion mechanisms even when unintended. Given these trends, we ask whether racism figures in research on ethno-cultural and racial older minorities. Thus, based on a scoping review of peer-reviewed articles published between 1998-2017 (n=336), this presentation asks if, and how, racialization and racism inform this research. In answering these questions, this presentation will argue that the role that racism plays as a social exclusion mechanism that affects older ethnic and racial minorities needs to be studied in a systematic fashion. Part of a symposium sponsored by the International Aging and Migration Interest Group.

### THE IMPORTANCE OF CULTURAL TRAITS IN CHILDREN'S WILLINGNESS TO PROVIDE INFORMAL CARE TO A PARENT

Freya Diederich,[1] Hans-Helmut König,[2] and Christian Brettschneider,[3] *1. University Medical Center Hamburg-Eppendorf, Hamburg Center for Health Economics, Hamburg, Germany, 2. University Medical Center Hamburg-Eppendorf, Hamburg Center for Health Economics, Hamburg, Hamburg, Germany, 3. University Medical Center Hamburg-Eppendorf, Hamburg, Hamburg, Germany*

The likelihood that a child will provide informal care to a parent varies across countries and between social groups within countries. We highlight the importance of cultural traits in children's value of informal care and their willingness to provide informal care to a parent. We initially construct a cultural measure of the strength of family ties at the country level using data from the World Values Survey. Then, we use a sample of second-generation immigrants from the German Family Panel (N=1,041) and regress their value of informal care on the strength of family ties that prevails in their parents' country of origin. Immigrants who have origins in countries with strong family ties are significantly more likely to report a high value of informal care. Finally, we show that children who report a high value of informal care are significantly more likely to provide informal care to a parent in need. Part of a symposium sponsored by the International Aging and Migration Interest Group.

### BUILDING TRUST IN ETHNICALLY DIVERSE OLDER ADULTS USING TECHNOLOGY-BASED PHYSIO-FEEDBACK

Ladda Thiamwong, *College of Nursing, University of Central Florida, Orlando, Florida, United States*

There is no research exploring how to build trust in the context of falls risk assessment and prevention. This study describes strategies to build trust in ethnically diverse older adults using technology-based physio-feedback from two studies. The technology includes a portable BTrackS balance plate and BTrackS Balance Software running on a computer device. Participants were provided instant playback showing their static balance performance with a scale from 0 to 100. Sixty-seven community-dwelling older adults participated in the first study, and 41 of them (61.2%) participated again in the second study using the same procedures. 70% were women, 43% were immigrants, 34% Hispanics, 15% African Americans, and 9% Asians. Three reasons for participation were reported: 1) specific objective feedback on the test results that supported by technology, 2) ability to record changes over time; and 3) ability to access the fall risk technology-based test at a place of their convenience. Part of a symposium sponsored by the International Aging and Migration Interest Group.

### SESSION 5335 (SYMPOSIUM)

### AGING IN CONTEXT: THE PAST, PRESENT, AND FUTURE OF RESEARCH ON OLDER ADULTS' SOCIAL TIES

Chair: Christina Marini
Co-Chair: Stephanie Wilson
Discussant: Katherine Fiori

This symposium will examine positive and negative aspects of older adults' relationships and their impacts on health and well-being. We will begin by reviewing the past decade of research on family gerontology. Seidel's meta-analysis of 995 articles will identify prominent theories and methods, as well as remaining research gaps. The subsequent presentations provide current, cutting-edge research. Marini examines how associations between rumination and sleep unfold within a social context. The findings highlight how spousal support protects older adults' sleep quality from rumination, whereas support from family and friends is vulnerable to rumination. Using an actor-partner approach, Novak investigates the dynamics of support and control on health among older gay couples. Results reveal the benefits of support and risks of control for partners' diet quality and depression. Ermer adopts a dyadic perspective to examine links between self-perceptions of aging and inflammation. Results highlight how wives' inflammation is sensitive to husbands' aging perceptions, particularly if marital strain is low. Finally, Wilson characterizes age-graded patterns of relationship narratives and their protective effects on emotional well-being. The findings demonstrate how older-adult couples' narratives are less self- and present-focused, which helps explain protective linkages between age and negative mood. The symposium will conclude with remarks from discussant Katherine Fiori, a GSA Fellow and internationally recognized scholar on older adults' social networks. She will synthesize the research and put forth her new theory about the importance of peripheral ties in later life to help direct the future of research on older adults within a social context.

### LINKS BETWEEN RUMINATION AND SLEEP QUALITY AMONG OLDER ADULTS: AN EXAMINATION OF THE ROLE OF SOCIAL SUPPORT

Christina Marini,[1] Stephanie Wilson,[2] Suyoung Nah,[3] Lynn Martire,[4] and Martin Sliwinski,[5] *1. Adelphi University, Garden City, New York, United States, 2. Southern Methodist University, Dallas, Texas, United States, 3. The Pennsylvania State University, State College, Pennsylvania, United States, 4. The Pennsylvania State University, University Park, Pennsylvania, United States, 5. Penn State University, University Park, Pennsylvania, United States*

Rumination is a maladaptive coping strategy that gives rise to and sustains stress. Individuals who ruminate more, therefore, tend to sleep more poorly. Studies of rumination and sleep often neglect the role of social context. Social support may buffer the degree to which rumination predicts poorer sleep quality. Further, individuals with more support may ruminate less, resulting in better sleep quality. Finally, rumination may also erode social support, resulting in poorer sleep quality. The current study tested these three hypotheses within a sample of 131 partnered older adults. We examined support from spouses and friends/family separately. Findings indicated that spousal (not family/friend) support buffered the negative association between rumination and sleep quality. Neither type of support predicted rumination; however, rumination predicted lower levels of family/friend (not spousal) support. Thus, spousal support protects older adults' sleep quality from rumination, and support from their peripheral ties may be more vulnerable to rumination.

### THE STORY OF US: OLDER AND YOUNGER COUPLES' LANGUAGE AND EMOTIONAL RESPONSES TO JOINTLY TOLD RELATIONSHIP NARRATIVES

Stephanie Wilson,[1] William Malarkey,[2] and Janice Kiecolt-Glaser,[2] *1. Southern Methodist University, Dallas, Texas, United States, 2. The Ohio State University College Of Medicine, columbus, Ohio, United States*

Social-emotional well-being is said to improve with age, but evidence for age differences in couples' behavior and emotions—studied primarily during marital conflict—has been mixed. Characteristics of jointly told relationship stories predict marital quality among newlyweds and long-married couples alike, yet younger and older couples' accounts have never been compared. To examine age differences in couples' emotional responses and in their I/we-talk, emotion word use, and immediacy (i.e., self-focused, present-tense style), 42 married couples ages 22–77 recounted their relationship's history then rated the discussion and their moods. Compared to younger couples, older couples used more we than I language, more positive than negative words, and less immediacy. Partners in older pairs shared more similar language patterns. In turn, lower immediacy mediated links between older age and less negative mood, and explained husbands' more positive appraisals. Indeed, relationship accounts reveal novel insights into age differences in marriage and well-being.

### PERCEPTIONS OF AGING AND INFLAMMATION IN THE CONTEXT OF OLDER ADULT MARRIED COUPLES

Ashley Ermer,[1] and Dikla Segel-Karpas,[2] *1. Montclair State University, Montclair, New Jersey, United States, 2. University of Haifa, Haifa, Hefa, Israel*

The current study takes a dyadic perspective to understand how self-perceptions of aging are associated with C-reactive protein, an inflammation marker, among older adult married couples. The potential moderating role of marital support and strain are also examined. Respondents include 668 married couples who participated in the 2014 wave of the Health and Retirement Study. Actor-Partner Interdependence Models were conducted in Mplus. Age, functional limitations, income, and race served as covariates. Husbands' greater positive perceptions of aging were significantly associated with their own lower levels of inflammation. Husbands' greater positive perceptions of aging were significantly associated with lower levels of inflammation for women who reported lower levels of marital strain; this was not the case for women who reported higher levels of marital negativity. This study exemplifies how relationship factors are necessary to consider when examining age perceptions and health among marrieds.

### DECADE REVIEW OF SOCIAL SUPPORT IN FAMILY GERONTOLOGY: METHODS, THEORIES, AND DIRECTIONS FOR FUTURE RESEARCH

Amber Seidel,[1] Jeremy Yorgason,[2] Áine Humble,[3] and Roxanne Humphris,[1] *1. Penn State York, York, Pennsylvania, United States, 2. Brigham Young University, provo, Utah, United States, 3. Mount Saint Vincent University, Halifax, Nova Scotia, Canada*

Social support has a rich foundation in family gerontology benefiting older adults, such as bolstering mental health, decreasing social isolation, and connecting them in families and communities. This study draws from a decade review of 995 empirical articles focusing on family gerontology from 13 of the top journals in gerontology, family, and relationships. Of the 995 articles, 86 were coded as social support or social network. Of these 86 articles, less than 3% included dyadic analyses, 95% were quantitative with just over 30% longitudinal. Over 30 different theories were identified with many building their work off multiple theories. Two key theories appear in over 30% of the articles, social support and socioemotional selectivity. Only 4% did not cite any theoretical framework. Overall, this presentation will review the methods, theories, and key findings of the social network/social support subtheme of a decade review of family gerontology and identify related gaps.

### HEALTH-RELATED SUPPORT AND CONTROL ON POOR DIET IN MIXED-WEIGHT, OLDER GAY MARRIED COUPLES

Joshua Novak,[1] Stephanie Wilson,[2] Terry Peak,[3] Julie Gast,[3] and Maya Miyairi-Steel,[3] *1. Auburn University, Auburn, Alabama, United States, 2. Southern Methodist University, Dallas, Texas, United States, 3. Utah State University, Logan, Utah, United States*

The health support and control literature in same sex couples suggests partners take turns (known as health behavior work). However, research has not accounted for mixed-weight (lighter partner (LP) and heavier partner (HP)) status in these relationships nor investigated domain-specific health behavior work (i.e., dietary habits) on outcomes. We examined an actor-partner interdependence mediation model of 224 mixed-weight, older gay married couples (mean age: 52) and controlled for education, relationship satisfaction and length, frequency of eating meals together, and income. Nutrition-specific eating encouragement and discouragement was not associated with poor dietary behaviors for either partner. More frequent couples' reports of disagreements about nutrition was associated poorer diet for both the HP and LP (through their own higher depressive symptoms). Interestingly, the higher LP's health support was associated with better LP's diet (through lower depressive symptoms), and higher HP's health control was associated with poorer LP's diet (through lower depressive symptoms).

### SESSION 5340 (SYMPOSIUM)

### AGING IN RURAL COMMUNITIES: ADVOCACY DOES MATTER

Chair: Lisa Wiese
Co-Chair: Lyn Holley
Discussant: M. Aaron Guest

Disparities in health care information and access often experienced by rural residents exacerbate health risks of older adults. The purpose of this symposium is to highlight efforts in varied rural settings to develop new information that contributes to identifying, assessing, and addressing the causes of these disparities, thus informing and strengthening advocacy and intervention. Mi and Ma address an unmet need for the description of challenges related to the extensive migration of Chinese rural workers and their families (46 million increase since 2011) that has exacerbated disparity of Social Security access. They describe barriers to establishing eligibility of these newly urban families for social services as they change from rural farmers to urban householders. Asman and Sisofo discuss barriers to working successfully with an alliance of diverse city, county, and state organizations to meet perceived needs of three categories of rural older adults: 1)active but denies aging, 2)adapting to aging and achieving satisfaction; and 3)dependent aging. Using a novel measure to determine true needs of their rural older communities resulted in benchmarks, which the alliance used in creating their strategic plan. Ford and colleagues tested a church-based program in a rural African American setting to facilitate chronic disease management. They tested the efficacy of utilizing online resources in underserved settings. Wiese and Williams investigated stakeholder perceptions/knowledge regarding dementia at six diverse rural sites. Although 71% of participants believed heart disease and poor diet increased dementia risk and were willing to be screened, only 15% had been tested for memory loss.

### HEALTH JUSTICE AS THE CATALYST FOR IMPROVING HEALTH IN UNDERSERVED SETTINGS

Lisa Wiese,[1] and Ishan Williams,[2] *1. Florida Atlantic University, Boca Raton, Florida, United States, 2. University of Virginia, Charlottesville, Virginia, United States*

Older adults face an increased incidence of Alzheimer's disease and related dementias (ADRD) due to higher prevalence of risk factors such as lack of awareness of ADRD and reduced access to care. Furthermore, African American and Hispanic communities, which often reside in rural areas, face 1.5-2 times the risk of ADRD. The purpose of this research was to address this disparity. We conducted three studies investigating stakeholder perceptions about screening and ADRD knowledge from six diverse rural sites. Survey results revealed that regardless of rurality and education, most rural (71%) older adults believed heart disease and poor diet increased ADRD risk and were willing to be screened. Yet only 15% had been tested for memory loss by their provider. Rurality was the only significant predictor of willingness to participate in screening. It is important to maintain a focus on pursuing health justice among rural older adults facing multiple health disparities.

### SOCIAL SECURITY ACCESS CHALLENGES FOR CHINESE FARMERS MIGRATING TO URBAN SETTINGS

Hong Mi, and  Qiyini Ma, *Zhejiang University, Hangzhou, Zhejiang, China*

In 2018, China had 564 million rural people, accounting for 40% of the total population. However in 2018, the total number of rural workers immigrating to urban areas related to economic need reached 288 million. This represents an increase of 46 million since 2011. The Chinese government piloted a new rural insurance as an answer to pushing urbanization forward to support rural immigrants. Consequently since 2009, the number of urban and rural residents participating in the basic old-age insurance has been kept above 500 million, making it the largest basic old-age insurance system, covering the largest number of persons in the world. Due to this insurance, an estimated 515 million people were lifted out of poverty, of which 27.41 million were older adults. Challenges for the Chinese government include transforming the Chinese situation of absolute poverty to relative poverty, and improving the living standard of older adults.

### UTILIZING A COMMUNITY-BASED PARTNERSHIP TO INCREASE HEALTH PROMOTION AMONG RURAL OLDER ADULTS

Cassandra Ford,[1]  Martha Crowther,[1]  Keri Barron,[2] and Mary Ann Kelley,[2] *1. The University of Alabama, Tuscaloosa, Alabama, United States, 2. The University of Alabama Capstone College of Nursing, Tuscaloosa, Alabama, United States*

Older adults living in rural communities often encounter health disparities related to chronic conditions and access to care. In an effort to address these disparities, a community-based program focused on health promotion and education was implemented with community dwelling, rural, African American older adults. We partnered with a local church to provide education regarding health promotion, chronic disease self-management, and support for senior group members (n=32). Program materials were accessed online in order to facilitate sustainability and potential program expansion. Key findings from a qualitative content analysis indicated the majority of participants reported improved engagement in chronic disease management and health promotion activities (i.e., following a healthy diet, monitoring blood pressure, taking medications as prescribed), incorporating health behaviors to prevent the development of co-morbid conditions, and increased client-provider communication. Implications for further research will be discussed and key elements of program implementation such as a continued partnership will be explored.

### ADVOCACY THROUGH PARTNERSHIPS: DATA, DEMOGRAPHICS, AND DECISIONS

Briana Sisofo,[1] and Anne Asman,[2] *1. University of Utah, Park City, Utah, United States, 2. University of Utah, Salt Lake City, Utah, United States*

The Summit County Aging Alliance (SCAA) in Park City, Utah is representative of state government, county and city government(s), private citizens, land developers, Senior Center attendees, national and local associations, non-profit support organizations, home health agencies, colleges and universities, recreation centers, hospital administrators and area associations on aging. The focus of the Alliance has been to provide a forum for critical listening and discussion. Data from a survey to determine the 'real' needs and vision of the older adult community provided perspective from more than 100 older adults representing diverse ethnic and socio-economic backgrounds. This work is now providing a benchmark from which both the city and county governments in Summit County are creating their strategic plans, and the Alliance has become the official voice of the community's older adults.

### SESSION 5345 (SYMPOSIUM)

### APPLYING INNOVATIVE QUALITATIVE METHODS TO UNDERSTAND THE PERSPECTIVE OF VULNERABLE AND DIVERSE POPULATIONS

Chair: Martina Roes
Discussant: Chaya Koren

The application of a variety of innovative qualitative research methods and analysis as well as the possibilities it offers for the selected population to share their personal experience is the main focus of this symposium. We present the relationships between design, methodological approaches and themes of interest for the people who participate in research. The presenters used designs such as ethnographic field research or phenomenological designs. The used photos taken and analyzed by the participants; using photo-voice and tabletop exercise. All speakers will present their designs and methods linked to a specific research theme. Using examples of recent and highly innovative research practices which meaningfully challenge taken-for-granted assumptions in social science and care research, to open new ground for other ways of thinking about doing research in these fields. Goal for the discussion is a critical reflection of the designs and methods used and to provide take away messages

### ETHNOGRAPHIC METHODS, MUSIC, AND AGING

Amanda Couve,[1] and Joseph Kotarba,[2] *1. Encompass Health - Hospice, San Antonio, Texas, United States, 2. Texas State University, San Marcos, Texas, United States*

Qualitative methods are proving to be important tools for studying the multi-faceted experience of living with aging, with a focus on the arts. Ethnographic methods are productive ways to discover and examine music in everyday life. Systematically studying the normal and often taken-for-granted ways aging adults experience music, in the full range of settings where they can be found, can abide by the first rule of translational science research: to design and conduct research in order to facilitate the efficient and timely development and application of clinical and caring interventions. This presentation will review a series of ethnographic studies of music experiences in residential facilities, dementia respite groups, family, and hospice. We will suggest ways to apply findings from these studies to enlighten volunteer hospice workers' protocols for care.

### USE OF PHOTO ELICITATION WITHIN PHENOMENOLOGY: DEPICTING LIVED EXPERIENCE OF OLDER LEARNERS IN AN AFU

Andrea Zakrajsek, *Eastern Michigan University, Ypsilanti, Michigan, United States*

While the use of photographs is an emerging data generation method within phenomenology (Plunkett, Leipert & Ray, 2013; Shulze, 2007), research that incorporates photo elicitation to inform the understanding of the lived experience is limited. This presentation will describe the use of photo elicitation within a phenomenological approach to explore the lived experience of older learners in higher education. After an initial interview, six participants aged 50 years and older shared photographs that that they chose to depict experiences of being student at a regional comprehensive university in the Midwest. Photographs served as mode for participant reflection of their experiences and guided a second interview. Interpretative Phenomenological Analysis (Smith et al., 2009) with photographs and verbatim transcripts ensued and resulting findings included: complicated sense of belonging and community development and access. Implications for use and analysis of photographs within qualitative research will be discussed.

### COMMUNITY-BASED PARTICIPATORY RESEARCH STRATEGIES TO COLLECT, ANALYZE, AND DISSEMINATE QUALITATIVE DATA

Roma Hanks,[1] Hosik Min,[1] Shoon Lio,[1] Brandon Moss,[1] Sarah Wraight,[1] and Denise Lewis,[2] *1. University of South Alabama, Mobile, Alabama, United States, 2. University of Georgia, Athens, Georgia, United States*

Project 1: Community Health Advocates participated in a photovoice project that evolved into an invited installation at the Alabama Contemporary Art Center, giving statewide exposure to health issues in communities in South Alabama that had been identified as those with the highest health disparities. The context of the project was a multigenerational approach to community health advocacy. Project 2: This project focused on employing CBPR methods in Cambodian and Laotian immigrant communities in South Alabama to discover barriers to disaster preparedness, response, and recovery and to better understand the intersections of culture, spirituality, and social justice along the path to community empowerment and resiliency. Community engagement in the development of emergency plans is typically at the organization-to-organization level. This paper analyzes multi-generational, multi-disciplinary, multi-method approaches using qualitative data to build effective strategies for advocacy through community engagement in research.

### SESSION 5450 (SYMPOSIUM)

### APPROACHES FOR MODELING OF INTENSIVE LONGITUDINAL DATA TO UNDERSTAND COGNITIVE AGING

Chair: Karra Harrington
Co-Chair: Nelson Roque
Discussant: Jacqueline Mogle

Understanding age-related change in cognition and identification of pathological changes requires sensitive and valid measurement of cognitive performance across time. Technological advances, such as ambulatory assessment of cognition using smartphones, have enabled intensive longitudinal methods where data is collected with many measurements over time. Our research group has developed novel ambulatory assessments that provide reliable, sensitive, and ecologically valid measurement of cognition across multiple timescales; from momentary changes to change across years. This symposium will present a spectrum of approaches to analysis of intensive longitudinal data that can inform models of cognitive aging. All three presentations will draw on data from measurement burst studies that apply our ambulatory cognitive assessment methods in community-based samples (i.e., systematically recruited in the Bronx, New York). For each measurement burst, participants undergo assessment consisting of brief surveys and cognitive tests via smartphone, up to 7 times per day across 14 days. Oravecz et al. will discuss the application of a Bayesian multilevel implementation of the double exponential model to account for retest effects while quantifying change in peak cognitive performance across time. Kang et al., will demonstrate a growth curve modeling approach for assessing the effects of between-person variables (i.e., loneliness) on change in cognition across measurement bursts. Harrington et al., will demonstrate a model-based cluster analysis approach, leveraging ambulatory assessments of subjective and objective cognitive function to unpack latent groups as a function of age and loneliness. Measurement, Statistics, and Research Design Interest Group Sponsored Symposium.

### LONELINESS AND PROFILES OF OBJECTIVE AND SUBJECTIVE MEMORY DURING MIDLIFE

Karra Harrington,[1] Nelson Roque,[1] Ruixue Zhaoyang,[2] Jacqueline Mogle,[1] and Martin Sliwinski,[3] *1. Pennsylvania State University, University Park, Pennsylvania, United States, 2. The Pennsylvania State University, University*

*Park, Pennsylvania, United States, 3. Penn State University, University Park, Pennsylvania, United States*

Loneliness is a risk factor for dementia, however it's relationship with cognitive health during midlife is unclear. We evaluated whether loneliness was associated with profiles of objective and subjective memory in younger and middle-aged adults. Participants (aged 25 to 64 years) underwent an initial loneliness assessment, followed by 14-days of momentary (5 per day) cognitive assessments (objective memory) and daily ratings of memory (subjective memory). Cluster analysis was conducted using person-level means of objective and subjective memory. Three clusters were identified: (1) highest objective and subjective memory (9%); (2) lowest subjective but not objective memory (84%); (3) lowest objective but not subjective memory (7%).There was a trend for higher levels of loneliness in Cluster 2 relative to Clusters 1 and 3. Results suggest that loneliness is more closely related with subjective than objective memory during midlife and are informative for development of interventions targeting cognitive health. Part of a symposium sponsored by the Measurement, Statistics, and Research Design Interest Group.

### LONELINESS AND COGNITIVE FUNCTIONING OVER TIME: USING AMBULATORY COGNITIVE ASSESSMENT

Jee-eun Kang,[1] Karra Harrington,[1] and Martin Sliwinski,[2]
*1. Pennsylvania State University, University Park, Pennsylvania, United States, 2. Penn State University, University Park, Pennsylvania, United States*

Loneliness has been investigated as a risk factor for cognitive health, but results were inconsistent. This study used three measurement bursts of ambulatory cognitive assessment to determine whether loneliness affects longitudinal changes in cognitive functioning in daily life. At each burst, participants performed cognitive assessment five times a day for 14 days. 138 adults (Mage=49.4) who completed all three bursts were included in this study.

**Growth curve modeling showed that, on average, scores of cognitive functioning were improved across a 2 year period (p<.001). The chronic lonely group (in the highest tertile at all 3 bursts) showed less improvement in scores compared to non-lonely people (p<.01), although there was no difference in cognitive functioning at the baseline between two groups. This study indicates that we need a repeated measurement of cognitive functioning and longitudinal approach to detect the effect of chronic loneliness on the rate of cognitive change. Part of a symposium sponsored by the Measurement, Statistics, and Research Design Interest Group.**

### BAYESIAN MODELING OF COGNITIVE IMPAIRMENT IN THE PRESENCE OF RETEST EFFECTS

Zita Oravecz,[1] Nelson Roque,[2] and Martin Sliwinski,[3]
*1. Pennsylvania State University, State College, United States, 2. Pennsylvania State University, University Park, Pennsylvania, United States, 3. Penn State University, University Park, Pennsylvania, United States*

Diagnosing the early onset of neuropathologies, such as mild cognitive impairment (MCI), requires repeated evaluation of cognitive skills several times per year -- a measurement design known as a "burst design." Detecting the often subtle cognitive decline in the presence of retest effects requires careful statistical modeling. The double exponential model offers a modeling framework to account for retest gains across measurement bursts, as well as warm-up effects within a burst, while quantifying change across bursts in peak performance. This talk highlights how a Bayesian multilevel implementation of the double exponential model allows for flexible extensions of this framework in terms of accommodating different timescales (nesting) and person-level predictors and drawing intuitive inferences on cognitive change with Bayesian posterior probabilities. We will use reaction time data to show how individual differences in asymptotic performance and change can be related to predictors such as age and MCI status. Part of a symposium sponsored by the Measurement, Statistics, and Research Design Interest Group.

### SESSION 5455 (SYMPOSIUM)

### ARTS-BASED INTERVENTIONS FOR DEMENTIA CARE: EAST MEETS WEST SYMPOSIUM

Chair: Fei Sun
Co-Chair: Angel Duncan
Discussant: Nancy Hooyman

This East Meets West symposium presents evidence of arts-based interventions in dementia care in different societal settings, focusing on the U.S.A. and China, where live about one-third of the world's total estimated 49 million dementia population. The first study from Kansas in the U.S. outlined the varieties of arts being applied in dementia care and recommended dementia care, inter-professional teams, to involve those professionals in arts and humanities. The second paper, based upon secondary national representative data, examined the association with arts-related hobbies and cognition status among Chinese older adults. The authors called for more research to shed light on the underlying mechanisms between arts and cognition. The third paper discussed two arts-based clinical trials on persons with dementia (PWD) at different stages living in Hong Kong. It found that dancing body movement therapy improved behavioral and emotional outcomes among those at the mild dementia stage. In contrast, music and movement worked better for those at the moderate dementia stage. The fourth study reviewed the effectiveness of body movement therapies for PWD, using an example of the Wheelchair-bound Senior Elastic Band for older adults with disability and dementia. The last study examined the effectiveness of a program that used museum tours to empower, educate, and inspire PWD. One discussant will share lessons learned across studies, and the other discussant from AARP Global Council on Brain Health will speak to the effects of music relating to the AARP 2020 consensus report on music and the brain health.

### WHY ARTS MATTER TO PEOPLE AND FAMILIES LIVING WITH DEMENTIA: INTERPROFESSIONAL HEALTH HUMANITIES COLLABORATION

Teri Kennedy, *The University of Kansas Medical Center, Kansas City, Kansas, United States*

This presentation will share examples of arts-based and creative interventions serving people and their families living with dementia representing evidence-based and promising practices in the United States. Such interventions offer effective non-pharmacological approaches to dementia care including use of the visual arts (e.g., drawings, paintings, sculpture) and performing arts (e.g., music, theatre); literature and writing including reminiscence, biographical approaches, and life story work; photography and Photovoice; and dance and movement as intervention modalities. Current evidence will be presented that demonstrates the effectiveness of arts-based interventions as a form of psycho-social and self-care to alleviate the effects of dementia and enhance the quality of life. Recommendations for future research will be discussed. Strategies will be proposed to develop interprofessional health humanities networks between universities, healthcare systems, libraries, museums, and the arts community to collaborate on the creation of arts-based programs in communities currently without the benefit of such programs.

### THE RELATION BETWEEN COGNITION AND INTERESTS IN ARTS AMONG CHINESE OLDER ADULTS

Xia Li, Yuan Fang, Qi Qiu, and Shixing Qian, *Shanghai Mental Health Center, Shanghai, China*

This study aims to examine the association between arts related interests and cognition among older Chinese. Data were drawn from 3,243 participants (Mage=71.1, SD=7.8) in the China Longitudinal Aging Study collected in 2011. About 54.4% were female, the average education was eighth grade, 560 interested in music, 86 interested in drawing, and 69 interested in both. Those interested in music or drawing were more likely to enjoy tea and exercise like Taichi, and less likely to smoke or drink ($p < 0.01$). Those interested in both reported best cognitive function, and those interested in music or drawing had better cognitive function than those without these interests ($p< 0.01$). However, the difference in cognition between those interested in music and those without diminished after education was controlled. The effect of arts hobbies in cognition among older Chinese remains to be further examined within the context of education and associated lifestyle factors.

### THE HOLISTIC IMPACT OF USING ARTS-BASED INTERVENTION FOR ELDERLY WITH DEMENTIA

Rainbow Tin Hun HO, *The University of Hong Kong, Hong Kong*

The use of creative arts on supporting elderly with dementia has been becoming popular due to its safe and engaging process. This non-pharmacological approach can complement with other treatment methods to support elderly with dementia on various aspects, including physical, cognitive and social functioning. In our randomized controlled trial on dance movement therapy (DMT) for 204 community dwelling elders with mild dementia, we found DMT could significantly reduce the level of depression, loneliness and negative mood ($\beta$=0.33-0.42, $p<.01$), and also the diurnal cortisol slope ($\beta$ =0.30, $p<.01$); while in another trial on 73 elderly with moderate dementia, we found music and movement could help reduce the behavioral and psychological symptoms such as agitation ($\beta$ = -0.41, $p<.01$), aberrant motor behavior ($\beta$=-1.02, $p<.01$), and dysphonia ($\beta$=-0.61, $p<.05$). The present presentation aims to share with the audience our practical experiences, the research procedures as well as the findings of the projects.

### THE ART OF BODY MOVEMENT: HEALTH IMPACT ON OLDER ADULTS WITH DISABILITY AND DEMENTIA

Kuei-Min Chen, *Kaohsiung Medical University, Kaohsiung City, Taiwan (Republic of China)*

Body-movement is an art form of self-expression and health promotion. This presentation will provide an overview of studies that employ the use of “movement activities” or “exercise” as non-pharmacological modalities to improve activities of daily living and functional fitness (e.g., cardiopulmonary function, body flexibility, range of joint motion, and muscle strength and endurance) of older adults with disability. These studies have also been shown to decrease depression state and behavior symptoms of older adults with dementia in long-term care facilities. Moreover, the Wheelchair-bound Senior Elastic Band (WSEB) exercise program will be described that are of relevance to the older adults with disability and dementia.

### ASCERTAINING CONSCIOUSNESS IN IDENTITY FORMATION: BEST MEDICAL PRACTICES IN DEMENTIA CARE

Angel Duncan, *Albertus Magnus College, New Haven, Connecticut, United States*

This session identifies common misconceptions about identity for persons living with Alzheimer’s disease and related dementias (ADRD). Going beyond diagnostic brain imaging and neurocognitive testing, case studies and research in creativity from around the United States highlights consciousness of persons living with ADRD. Reviewing and discussing artworks is aimed to set dialogue in the question of where memory deposits emerge when engaged in creativity. Through art therapy techniques, this type of self-expression may provide new avenues in treatment for dementia care. Exploring the arts from those with Mild Cognitive Impairment to late stage Alzheimer’s and other forms of dementia, such as frontotemporal dementia, consciousness seems to remain intact despite neural death. This session aims to discourage poor spending allocations and establishing meaningful care. From clinical research trials to creativity of self-expression, the importance of why the arts and sciences matter are demonstrated as effective modalities that enhance quality of life.

### SESSION 5465 (SYMPOSIUM)

### BECOMING MORE OPEN: INCREASING RIGOR AND REPRODUCIBILITY OF GERONTOLOGICAL SCIENCE

Chair: Brad Taylor
Co-Chair: Lewina Lee
Discussant: Derek Isaacowitz

“Open science” refers to a collection of practices with the broad goal of increasing the transparency and reproducibility of scholarly research. The purpose of this symposium is to illustrate the application of open science practices to gerontological research and address barriers to adopting such practices. Drawing from his experiences in leading a research network that conducts integrative analysis of longitudinal aging studies (IALSA), Hofer will describe challenges posed by multiple sources of heterogeneity in conducting coordinated analyses, and ways of handling these challenges to maximize reproducibility. Next, Mroczek will illustrate these issues by providing two examples of coordinated analyses. This talk will highlight design features that promote openness and transparency in conducting research on longitudinal data. Third, Lodi-Smith will provide practical guidance and examples on preregistering complex projects, strategies for transparently reporting deviations from preregistrations, considerations in sharing sensitive data, and tips on transparent documentation of analysis code. She will also emphasize the pedagogical value of preregistration. Finally, Seaman will describe ongoing efforts to establish open science practices as the default in her laboratory, with the goal of providing a model for both junior and more established researchers wanting to build transparency into their research practices. Discussant Isaacowitz, editor for the Journal of Gerontology, Series B: Psychological Sciences, will evaluate the presentations from the lens of how journals can encourage more transparent and replicable scientific practices.

### OPENNESS AND TRANSPARENCY PROMOTION WITH EXISTING LONGITUDINAL DATA: A WORKED EXAMPLE OF A COORDINATED ANALYSIS

Daniel Mroczek,[1] Eileen Graham,[1] and Emily Willroth,[2]
*1. Northwestern University, Chicago, Illinois, United States, 2. Northwestern University Feinberg School of Medicine, Evanston, Illinois, United States*

The application of openness and transparency principles is challenging when using existing or ongoing long-term longitudinal data. One technique that promotes replicability and also is consistent with openness and transparency principles is coordinated analysis. Such analyses, especially when done with a large number of extant longitudinal datasets, tend to draw upon values of data sharing, revelation of code and scripts, and pre-registration. Thus coordinated analyses often provide good examples of how multiple transparency and openness values can come together. We will demonstrate this by presenting two recent large-scale coordinated analyses. One was a 15-study investigation of personality and mortality risk (Graham et al., 2017). The second is a new 16-study investigation of personality trajectories (Graham et al., under revision). We show how multi-study designs are congruent with open science and transparency ideas in the context of longitudinal and other secondary data.

### ESTABLISHING THE REPLICABILITY AND GENERALIZABILITY OF MULTI-STUDY LONGITUDINAL RESEARCH

Scott Hofer, *University of Victoria, Victoria, British Columbia, Canada*

Replication and cross-validation of research findings across independent longitudinal studies is essential for a cumulative science. However, the interplay between harmonization, replication, and generalizability of results across interdisciplinary longitudinal studies can present remarkable challenges. Careful interpretation of multistudy results must include consideration of the age, birth cohort, health, and education of individuals in the sample, measurements, the number and spacing of assessments, and rates of response and attrition. Placed in a broader historical (or future) context, we must consider the representativeness of population sampling, historical period, and analytic method in understanding the replicability and generalizability of findings. In a multistudy context, harmonization can be considered at levels of research question, statistical models, and measurements and can minimize some sources of cross-study variability. I will discuss the challenges and benefits of harmonization and the coordinated analysis approach used by the IALSA research network to achieve results from multi-study integrative research.

### APPLIED OPEN SCIENCE FOR SECONDARY DATA ANALYSIS AND META-ANALYSIS

Jennifer Lodi-Smith, *Canisius College, Buffalo, New York, United States*

This talk will provide guidance on the practicalities of open science for secondary data analysis and meta-analyses. Example studies will provide practical considerations for preregistering complex projects, insights into strategies for transparently reporting deviations from preregistrations, advice on deciding when and how to share sensitive data, and tips on transparent documentation of analysis code. Examples will be drawn from an ongoing meta-analysis of the relationship between self-concept clarity and self-esteem (https://osf.io/sa2bx/), the Rochester Adult Longitudinal Study (https://osf.io/ya4ph/), and the Aging and Autism Study (https://osf.io/g9c3e/). The pedagogical value of preregistration will be emphasized throughout the talk.

### MAKING OPEN SCIENCE THE DEFAULT: CREATING LAB PRACTICES TO PROMOTE TRANSPARENCY

Kendra Seaman, *The University of Texas at Dallas, Dallas, Texas, United States*

Many factors disincentivize researchers, particularly junior faculty members, from implementing open science practices. One way to make open science less burdensome is integrate open science methods with existing procedures. I will describe my ongoing efforts to establish open science practices as the default in my laboratory. These strategies include (1) creating and updating a lab manual to set expectations for openness, (2) articulating a standard operating procedure for creating, preregistering, and managing a new project, (3) establishing clear organizational structures for data, code, and data products, and (4) training lab members on the use of these and other open science tools like GitHub. These strategies provide a model for both junior researchers starting a lab and more established researchers who want to build transparency into their research practices. Ultimately, implementing open science methods will improve lab workflows and improves the overall quality of our science.

### SESSION 5475 (SYMPOSIUM)

### BENEVOLENT AGEISM: EXPLORING ITS BOUNDARY CONDITIONS, GENERALIZABILITY, AND CORRELATES

Chair: Toni Bisconti
Co-Chair: Jennifer Sublett
Discussant: Alison Chasteen

Ageism is one of the few prejudices that is still socially condoned (Nelson, 2016). Given the aging population and the impact of internalizing ageist thoughts, this construct needs to be at the forefront for scientific examination. The long-term effects of ageism, particularly negative self-perceptions, lead to negative health and cognitive outcomes (Chasteen et al., 2015; Levy et al., 2002). One of the intricate components of ageism, however, is that it is often "benevolent". Cuddy and colleagues developed the Stereotype Content Model (SCM) to describe how individuals are categorized based on varying degrees of warmth and competence. Unlike many devalued members of society who are viewed as low on both, older adults are viewed as having high warmth and low competence, leading to more overaccommodative treatment. The goal of the present symposium is to overview the ways in which researchers have dissected this more nuanced type of ageism. Specifically, two of the presenters will cover some of the boundary conditions of understanding age-based stereotypes and their malleability, examining them across ages and across genders. Additionally, one of our presenters will overview the validation of the Ambivalent Ageism Scale on a Chinese sample, lending support to its generalizability. Finally, our last presenter will overview the relationship between benevolent ageism and self-compassion to predict metamemory, given the pervasive stereotype that older adults suffer from severe cognitive decline. Themes and implications of these presentations will be discussed.

### BENEVOLENT AGEISM'S RELATIONSHIP TO SELF-COMPASSION AND META-MEMORY IN OLDER ADULTS

Jennifer Sublett,[1] and Toni Bisconti,[2] *1. University of Akron, Akron, Ohio, United States, 2. Univesity of Akron, Akron, Ohio, United States*

Benevolence directed towards older adults can cross the line between respect and overaccommodation that undermines their physical and cognitive capabilities (Mehrotra & Wagner, 2009); however, little research has examined the subtleties of the influence of benevolent ageism on older adults' ratings of their own functioning. Because stereotypes about older adults include the decline of mental abilities, this study examined whether their (N= 155) experiences with benevolent ageism, or overaccommodative offers of assistance and protection, influenced their own appraisals of memory abilities through their feelings of self-compassion. Older adults with fewer benevolent ageist experiences had higher rates of self-compassion, which in turn translated into better evaluations of their memory abilities. Future research should consider the potential pernicious influences that benevolent ageism has on older adults' self-evaluations and performance, consider self-compassion as a buffer in these relationships, and test whether these relationships have downstream consequences on well-being outcomes.

### THE MODIFIABILITY OF THE STEREOTYPE CONTENT MODEL TOWARD OLDER ADULT MEN AND WOMEN

Michael Vale Toni Bisconti and Jennifer Sublett, *University of Akron, Akron, Ohio, United States*

Older adults are often stereotyped in a paternalistic manner (warm, but incompetent), deserving of assistance regardless of their need. We have examined the veracity and malleability of this paternalistic stereotype using an experimental vignette with both male and female targets. Younger adults (N = 717) deemed it more necessary and appropriate to offer unnecessary help to older adults in a grocery store scenario. Additionally, competence was malleable for both older adult male and female targets if the older adults denied the offer of help. Interestingly, older women were viewed as warm, which did not change as a function of their response, whereas older men were initially viewed as colder, but their warmth ratings increased. In light of these findings, we will discuss the intersection of age and gender when considering the malleability of the warmth and competence dimensions of the paternalistic older adult stereotype.

### THE LINKS BETWEEN AGEISM AND THE AGE-BASED DOUBLE STANDARD

Changrui Li,[1] Sarah Barber,[1] and Gene Brewer,[2] *1. Georgia State University, Atlanta, Georgia, United States, 2. Arizona State University, Tempe, Arizona, United States*

There is an age-based double standard in how we evaluate memory failures by younger and older adults. Whereas younger adults' forgetfulness is attributed to lack of effort or attention, older adults' forgetfulness is attributed to lack of ability. Our goal was to replicate this phenomenon, and evaluate its links to benevolent and hostile ageism. To do so, we used a vignette paradigm in which younger and older participants read about a target person (who was a younger or older woman) who left a store without paying for a ring (which varied in price). Results showed that participants were more likely to attribute this to poor memory abilities when the target was an older adult. They were also more lenient in their ascribed punishments for the older adult targets. In addition, reading about an older adult target's mistake was associated with subsequently higher endorsement of benevolent, but not hostile, ageist attitudes.

### VALIDATION OF THE AMBIVALENT AGEISM SCALE IN CHINA

Xin Zhang, *Peking University, Beijing, China*

Two studies were conducted to validate the Ambivalent Ageism Scale in China. In the first study, 474 Chinese adults (18-58) were asked to take the Chinese version of the AAS. EFA exhibited a similar factor solution as the original study, with high internal consistency and construct validity. Moreover, in a second study, 372 Chinese adults (18-85) took the AAS and provided their estimations of the similarities between their current and their past/future self via the SIC. Results indicated that all three factors of the SIC positively related to hostile ageism, whereas succession and identity positively related to benevolent ageism and consumption negatively related to it. Additionally, past self-continuity was positively associated with hostile ageism, and future self-continuity was negatively associated with it, but neither form was associated with benevolent ageism. These results further validate the AAS in China and also provide evidence for the uniqueness of benevolent ageism.

### SESSION 5480 (SYMPOSIUM)

### BUILDING NATIONAL CAPACITY FOR DEMENTIA CAREGIVING RESEARCH: THE NIA EDWARD R. ROYBAL CENTERS

Chair: Karina Davidson
Co-Chair: Lisa Onken

More than 5.8 million Americans are currently living with Alzheimer's disease and related dementias, and they are cared for by over 16 million people at an estimated annual cost of $290 billion. The need for innovative, evidence-based interventions to support these patients and caregivers is critical to addressing this problem. The goal of the NIA Edward R. Roybal Centers for Translational Research in the Behavioral and Social Sciences of Aging is to translate and integrate basic behavioral and social research findings into interventions aimed at innovatively improving both the lives of older people and the capacity of institutions to adapt to societal aging. The Roybal Centers will develop research within the conceptual framework of the multidirectional and translational NIH Stage Model to produce these implementable, principle-driven behavioral interventions. The program has now funded new Centers, and each has a unique focus. Areas of concentration include promoting caregiving mastery (Emory University), integrating the use of technology in care support to improve assessments and interventions in care provision (Oregon Health & Science University), developing behavioral interventions to reduce isolation and promote social connectedness in caregivers (University of Rochester), promoting health in racial/ethnic minorities (University of Illinois at Chicago), and using insights from data science and behavioral economics to improve palliative care delivery and long-term support facilities for persons with dementia and their caregivers (University of Pennsylvania). Center leaders will present an overview of their cutting-edge, early-stage research projects and discuss implications for improving care of caregivers and patients.

### BUILDING NATIONAL CAPACITY FOR DEMENTIA CAREGIVING RESEARCH: THE NIA EDWARD R. ROYBAL CENTERS

Karina Davidson,[1] Susan Hughes,[2] Jeffrey Kaye,[3] Scott Halpern,[4] Kenneth Hepburn,[5] and Kathi Heffner,[6]
*1. Northwell Health, New York, New York, United States, 2. University of Illinois at Chicago, Chicago, Illinois, United States, 3. Layton Alzheimer's Disease Center, Portland, Oregon, United States, 4. University of Pennsylvania, Philadelphia, Pennsylvania, United States, 5. Emory University, Atlanta, Georgia, United States, 6. University of Rochester Medical Center, Rochester, New York, United States*

The NIA Edward R. Roybal Centers for Translational Research in the Behavioral and Social Sciences of Aging aim to translate and integrate basic behavioral and social research findings into principle-driven interventions aimed at innovatively improving both the lives of older people and the capacity of institutions to adapt to societal aging. Newly funded Centers focus on interventions to promote caregiving mastery, integrate the use of technology in care support to improve assessments and interventions in care provision, develop behavioral interventions to reduce isolation and promote social connectedness in caregivers, promote health in racial/ethnic minorities, and apply insights from data science and behavioral economics to improve palliative care delivery and long-term support facilities for persons with dementia and their caregivers. Center leaders will present an overview of their cutting-edge, early-stage research projects based on the NIH Stage model conceptual framework and discuss implications for improving care of caregivers and patients.

### EDWARD R. ROYBAL CENTERS FOR TRANSLATIONAL RESEARCH IN THE BEHAVIORAL AND SOCIAL SCIENCES OF AGING

Lisa Onken, *National Institute on Aging, Bethesda, Maryland, United States*

The goal of the Roybal Center program is the translation and integration of basic behavioral and social research findings into interventions to improve older people 's lives and the capacity of institutions to adapt to societal aging. Roybal Centers are structured within the conceptual framework of the multidirectional, translational NIH Stage Model to produce potent and implementable principle-driven behavioral interventions. The NIA's Division of Behavioral and Social Research currently supports thirteen Roybal Centers, five of which have a primary focus on issues related to dementia care and caregiving support. Each Dementia Care Center has a unique focus that addresses issues such as social isolation, caregiving mastery, community-based resources and racial/ethnic minority health promotion. Additionally, a focus on the utilization of data science and behavioral economics related to palliative care, as well as a focus on the application of technology to improve assessments and interventions complete the scope of research endeavors.

### SESSION 5485 (SYMPOSIUM)

### CAREGIVER-CENTERED COMMUNICATION: ENGAGING FAMILY CAREGIVERS

Chair: George Demiris
Discussant: Karen Hirschman

Older adults are often relying on a family member or other informal caregiver (friend or other) to jointly navigate the health care system and cope with the ramifications of serious illness; thus, the patient-caregiver dyad becomes the unit of care. The caregiving role becomes crucial in cases where patients are facing a condition that limits their cognitive and functional abilities and caregivers are called to act as proxy decision makers for significant treatment and symptom management decisions. Caregivers often report that they feel isolated and overwhelmed, and in some cases experience significant barriers in communicating with health care providers. It is important that clinicians communicate in a way that acknowledges and addresses caregivers' preferences, needs and perspectives. Caregiver centered communication can facilitate a more effective adaptation throughout the illness course with better adherence to recommended treatment plans and greater satisfaction with care for both patients and families, as well as a more comprehensive response to their psychosocial needs. While health care organizations often aim to increase caregiver engagement and involvement in care processes, there is a lack of tools or strategies not only to more actively engage caregivers but also to assess how ongoing approaches perform in terms of facilitating meaningful and inclusive communication. This symposium will review existing tools and a new instrument to measure caregiver centered communication, challenges and opportunities in measuring the quality of communication with caregivers and highlight empirical data of communication quality in various health care settings including home care and hospice.

### CAREGIVER-CENTERED COMMUNICATION: A NEW TOOL FOR ASSESSMENT OF THE QUALITY OF COMMUNICATION WITH FAMILY CAREGIVERS

George Demiris, *University of Pennsylvania, Philadelphia, Pennsylvania, United States*

Improved communication with caregivers can lead to reduced caregiver anxiety and burden, improved quality of life, and better coping during a stressful time. Even though caregiver communication with the health care team is essential in gerontology, we are lacking standardized instruments to assess quality of communication. We describe the initial development and testing of the Caregiver Centered Communication Questionnaire (CCCQ). The questionnaire has 30 items with 5 subscales: exchange of information, fostering health relationships with team/provider, recognizing and responding to emotions, managing care and decision making. We conducted a cross-sectional survey of 115 family caregivers of older adults in home care and hospice. Cronbach's $\alpha$ for the scale was 0.97. Internal consistencies of subscales were high, ranging from 0.82 to 0.93. Preliminary testing indicates the potential of CCCQ in assessing engagement and quality of communication; further testing is required.

### NOT JUST SITTING BY: FAMILY CAREGIVERS' PERSPECTIVES ON TRIADIC COMMUNICATION

Karla Washington, *University of Missouri School of Medicine, Columbia, Missouri, United States*

Most studies of communication in gerontology and palliative care focus on dyadic communication. In reality, encounters in palliative oncology very often involve three or more people: a healthcare professional, a patient, and a family caregiver. This triadic communication differs in important ways from communication involving only two parties. In this secondary analysis of qualitative data collected during a randomized controlled trial of a psychosocial intervention for family caregivers receiving palliative oncology services, researchers explored family caregivers' (n = 63) perspectives on triadic communication encounters involving themselves, the patient and one or more clinicians. Family caregivers tended to appreciate clinicians' efforts to involve them in communication, rather than regarding them as "just sitting by" the patient. Many perceived that their own wishes regarding information provision were often ignored and reported that their own coping and wellbeing were not often assessed outside of encounters with the specialist palliative care team.

### DIGITAL TOOLS TO ENHANCE CAREGIVER-CENTERED COMMUNICATION

Debra Parker Oliver, *University of Missouri, Columbia, Missouri, United States*

While it is recognized that caregiver engagement can improve processes and outcomes of care in gerontology, there are barriers to caregiver centered communication, including limited resources for health systems to devote services specifically to families, geographic distance and lack of time. Digital tools such as social media platforms and video-conferencing introduce opportunities for remote and often asynchronous communication. In this presentation, we discuss findings from two randomized clinical trials that explored digital tools to empower family caregivers. In the first we examined ways to use video-conferencing to enable family caregivers to become virtual team members during hospice interdisciplinary teams, and in the second trial we examine the use of secret Facebook groups to meet informational and emotional needs of family caregivers during episodes of care that are often linked to increased social isolation and loneliness. We discuss challenges and opportunities in designing digital tools to facilitate caregiver engagement and empowerment.

### SESSION 5490 (SYMPOSIUM)

### CAREGIVING TOGETHER: THE RELATIONSHIP BETWEEN PAID AND FAMILY CAREGIVERS IN THE HOME

Chair: Jennifer Reckrey
Discussant: Robyn Stone

Family caregivers provide the lion's share of care that allows older adults with functional impairment to remain living at home. Yet as care needs grow, many older adults and their families turn to paid caregivers (e.g. home health aides, personal care attendants, and other direct care workers) to provide additional support. While evidence suggests that paid and family caregivers work together to provide increasingly complex care at home, research that describes this important collaboration is limited. In this symposium, we present innovative and interdisciplinary research that highlights the overlap between family caregiving and long-term care workforce research. We begin by presenting two studies that focus on populations where paid caregivers may have outsized impact on family caregivers: Reckrey et al report that receipt of 20+ hours of paid caregiving per week was associated with less caregiver strain among family caregivers of those with advanced dementia and Falzarano et al report that home care hours mediated the association between caregiver stressors and negative effects of caregiving among long-distance family caregivers. Franzosa et al then describe home health aides' perceptions of relationship dynamics as aides and family members negotiate care tasks in the home. Finally, Gallopyn et al explore scenarios where paid and family caregiver roles blur (e.g., family caregivers receiving payment for providing care, paid caregivers with extensive experience as family caregivers). Taken together, these studies describe critical ways paid and family caregiver experiences are intertwined and highlight the importance of ongoing research about this collaboration.

### WE TREAT THEM BETTER THAN THE FAMILY THEY HAVE: NEGOTIATING AIDE-FAMILY RELATIONSHIPS IN THE HOME CARE SETTING

Emily Franzosa,[1] and Emma Tsui,[2] *1. James J. Peters VA Medical Center, Brooklyn, New York, United States, 2. CUNY Graduate School of Public Health & Health Policy, New York, New York, United States*

Unpaid and paid care in the home are closely intertwined, but a lack of outside supervision and support often forces family and non-family caregivers to negotiate care tasks and boundaries alone, leading to role conflict and role ambiguity. This analysis drew on two qualitative studies of home health aides (S1 n = 27, S2 n =26) to explore 1) aides' perception of their caretaking role; 2) aides' experiences co-producing care with family members; and 3) factors affecting these relationships. Data were analyzed through grounded theory and discourse analysis. We identified three relationship dynamics between aides and family members: independent, where aides and families provided care separately; competitive, where aides and families struggled over control of care tasks; and carative, where aides considered family part of the unit of care. We propose strategies for employer agencies to better support paid and unpaid caregivers in negotiating boundaries and co-producing care.

### USE OF HOME CARE SERVICES REDUCES CAREGIVING-RELATED STRAIN IN LONG-DISTANCE CAREGIVERS

Francesca Falzarano,[1] Verena Cimarolli,[2] Kathrin Boerner,[3] Amy Horowitz,[4] and Karen Siedlecki,[4] *1. Weill Cornell Medicine, New York, New York, United States, 2. LeadingAge, Washington, District of Columbia, United States, 3. University of Massachusetts Boston, Boston, Massachusetts, United States, 4. Fordham University, New York, New York, United States*

Long-distance caregivers (LDCs) comprise a growing yet relatively understudied segment of the caregiving population. The current study (N=166) investigated the mediating role of home care hours on the association between primary caregiving stressors (care recipient [CR] levels of cognitive and functional impairment) and LDCs' perceived strain (perceived interference of caregiving with other family responsibilities and work). Results from path analyses showed that home care hours fully mediated the negative effect of CRs' functional impairment on family interference and the negative effect of CRs' cognitive impairment on work interference. Further, the negative effect of CRs' cognitive impairment on family interference was partially mediated by home care hours. Thus, a mediating role of home care service hours in the associations between objective stressors and LDC strain was established for both family and work domains. Findings highlight the potential of home care services in alleviating strain in LDCs of community-dwelling CRs.

### FORMAL AND INFORMAL CAREGIVING: IMPLICATIONS FOR PAID FAMILY CAREGIVERS UNDER THE CONSUMER-DIRECTED MODEL

Naomi Gallopyn,[1] Kathrin Boerner,[2] and Lisa Iezzoni,[1] *1. Massachusetts General Hospital, Boston, Massachusetts, United States, 2. University of Massachusetts Boston, Boston, Massachusetts, United States*

The case of paid family caregiving under the consumer-directed model introduces unique complexities to patient care, especially in the presence of other formal caregiving. The purpose of this study was to address the interface of paid and family caregiving by exploring scenarios where the two types of caregiving overlap or are merged into one role. Participants were 20 formal caregivers and 21 persons with significant disability, recruited with a purposive sampling approach. In-depth, qualitative interviews were conducted with caregivers and patients to triangulate information from different perspectives. Thematic analysis uncovered pathways into formal caregiving that involved a history of family caregiving, transitions from informal family caregiving to formal family caregiving under the consumer-directed model, and challenges that emerged when formal and informal caregivers co-exist in the same environment. Implications for patient and caregiver experiences in care contexts where the boundaries between paid and unpaid care are blurred are discussed.

### DEMENTIA CAREGIVING: THE ASSOCIATION BETWEEN FAMILY CAREGIVING STRAIN AND RECEIPT OF PAID CARE

Jennifer Reckrey,[1] Evan Bollens-Lund,[1] Emma Tsui,[2] Kathrin Boerner,[3] and Katherine Ornstein,[1] *1. Icahn School of Medicine at Mount Sinai, New York, New York, United States, 2. CUNY Graduate School of Public Health & Health Policy, New York, New York, United States, 3. University of Massachusetts Boston, Boston, Massachusetts, United States*

family caregivers. Using data from three waves (2011, 2015, 2017) of the National Health and Aging Trends Study (NHATS) linked to the National Study of Caregiving (NSOC), we identified family caregivers of those with advanced dementia and compared caregiving strain among those with zero, <20, and 20+ hours/week paid care. Family caregivers of those who received 20+ hours (26% of the sample) reported less caregiver strain (mean score 3.27 vs 4.15, p=0.04) and less frequently reported having more to do than they could handle (46.1% vs 67.9%, p=0.01) or not having enough time for themselves (46.8% vs 72.2%, p<0.01). The association persisted in a multivariable model. These results support the conceptualization of dynamic and potentially multidirectional relationships between paid and family caregivers and suggest that paid caregivers impact both those receiving care and their families.

### SESSION 5500 (SYMPOSIUM)

### CHALLENGES AND INSIGHTS: PSYCHOSOCIAL STRESSORS, PHYSIOLOGY, AND HEALTH DISPARITIES

Chair: Julie Ober Allen
Discussant: Briana Mezuk

The persistence of health disparities demonstrates the need for more comprehensive research to better understand key methodological approaches and intervention leverage points among the complex relationships between psychosocial, environmental, and biological factors influencing patterns of health among older adults. Growing empirical evidence implicates differential exposure to chronic stressors that are rooted in the social environment and related social inequities in premature aging and development of chronic diseases among socially marginalized groups. This, in turn, leads to health disparities within the older adult population. Mechanisms linking stressors and health, however, remain poorly understood. This symposium assembles research examining challenges associated with measuring exposure to stressors, relationships between stressors and health outcomes, and the insight provided by stress-based frameworks for understanding mechanisms of health disparities. Allen and colleagues use comprehensive measures of exposure to stressors to identify stressor characteristics most closely associated with diurnal cortisol dysregulation and Black-White disparities, which are risk factors for adverse cardiometabolic health outcomes. Kalesnikava and colleagues will introduce methods for exploring relationships between self-reported stress and another cortisol stress biomarker—cortisol reactivity to an acute stress event. Byrd and colleagues round out the symposium with a presentation on the directionality of relationships between perceived stress and depressive symptoms contributing to health disparities among Blacks. Discussant Jackson will explore implications of these studies for more nuanced research related to mechanisms of health disparities and for more targeted approaches to the prevention of health disparities among older adults.

### MECHANISMS OF CARDIOMETABOLIC DISPARITIES: STRESSOR CHARACTERISTICS LINKED TO HPA-AXIS DYSREGULATION

Julie Ober Allen,[1] Briana Mezuk,[2] Tomohiro Ko,[2] Jane Rafferty,[3] Jamie Abelson,[3] Viktoryia Kalesnikava,[4] and James Jackson,[4] *1. University of Oklahoma, Ann Arbor, Michigan, United States, 2. University of Michigan School of Public Health, Ann Arbor, Michigan, United States, 3. University of Michigan ISR, Ann Arbor, Michigan, United States, 4. University of Michigan, Ann Arbor, Michigan, United States*

Diurnal cortisol slopes are stress-sensitive HPA-axis biomarkers implicated in cardiometabolic health outcomes and disparities. This study used two longitudinal cohort studies (CREATE and TRIAD) with harmonized variables to comprehensively examine what types of exposure to stressors are most salient for cortisol dysregulation in later life, and whether the characteristics of stressor exposure accounts for Black-White disparities in cortisol dysregulation (merged sample N=209, 65% male, mean age 61, 17% Black). Black participants reported greater stressor exposure than Whites along some dimensions (e.g., # recent major stressors, appraised severity of lifetime stressors, all p<.02) but comparable exposure in others (e.g., # of lifetime stressors and life domains). Stressor exposure measures that captured psychological components (i.e., appraised severity, psychological distress) and pervasiveness (i.e., # life domains with major stressors) were more closely related to cortisol dysregulation than more objective measures (e.g., # of recent /lifetime stressors). Everyday discrimination was associated with racial disparities.

### DO DEPRESSIVE SYMPTOMS SHAPE BLACKS' PERCEPTIONS OF STRESS OVER TIME?

DeAnnah Byrd,[1] Roland Thorpe,[2] and Keith Whitfield,[1] *1. Wayne State University, Detroit, Michigan, United States, 2. Johns Hopkins Bloomberg School of Public Health, Baltimore, Maryland, United States*

The established association between stress and depression is typically examined only in one direction and cross-sectionally. Data from the Baltimore Study of Black Aging-Patterns of Cognitive Aging was used to longitudinally examine the bi-directional relationships between (1) stress-depression and (2) depression-stress, and age as a modifier. The sample consisted of 602 community-dwelling Blacks, aged 48-92 years at baseline and 450 at follow-up 33 months later. While the stress-depression relationship was non-significant; the depression-stress was (b= 0.236, p< 0.000) and this association varied by age with the impact of baseline depression on changes in stress greatest among Blacks in their 60's versus those in their 50's (b= 0.257, p= 0.002), controlling for model covariates. Findings highlight the importance of depression in shaping Blacks' perception of stress over time. Future work should continue to identify stress and mental health risk factors that contribute to poor health and health disparities in older Blacks.

### WHICH CONTEXTUAL FEATURES PREDICT PHYSIOLOGICAL STRESS REACTIVITY IN MIDLIFE TO LATE LIFE?

Viktoryia Kalesnikava, *University of Michigan, Ann Arbor, Michigan, United States*

Chronic stress creates vulnerability to adverse mental and physical health outcomes in later life. While claims about the negative effects of stress on health are primarily based on self-report, it is unclear how subjective stress measures (chronic or perceived stress) and other environmental or individual characteristics (neighborhood, social and health behaviors) relate to physiological stress response. This study examines which contextual features contribute to differences in physiological stress reactivity among adults at risk of type II diabetes (Richmond Stress and Sugar Study, n=125, aged 40-70). Psycho-social stress was induced via Trier Social Stress Test. Using advanced selection methods, we simultaneously explore multiple predictors and illustrate how different sets of risk and protective factors contribute to normal or abnormal stress reactivity profiles. Preliminary results suggest that the top five important predictors are education, contact with friends, perceived stress, ruminative coping, and sedentary behavior. Implications for research and targeted interventions are discussed.

### SESSION 5505 (SYMPOSIUM)

### COGNITIVE AGING IN THE UNITED STATES AND MEXICO

Chair: Sunshine Rote
Co-Chair: Jacqueline Angel
Discussant: Fernando Torres-Gil

The Latino population is rapidly aging, with the number of adults 65 and older expected to increase by more than six times to 17.5 million by 2050. Mexico's population is also aging and will increase by 227 percent over the next 25 years. We focus on the consequences of rising longevity and increasing numbers of older Latinos living with dementia both in the U.S. and in Mexico. Providing cost-effective and appropriate services to aging Latinos with dementia will require a clear understanding of the intra-diversity among this group in different social and national circumstances. The purpose of this symposium is the understand how migration between and within countries and other social and health factors (e.g., diabetes) impact risk for cognitive impairment and dementia using three national datasets: the HRS, MHAS, and HEPESE. Four paper presentations and one discussant will examine several thematic issues as they relate to cognitive aging for Latinos, including: (1) cross-national estimates of dementia prevalence in Mexico and the U.S.; (2) the healthy immigrant effect and health convergence hypothesis for cognitive impairment for Latinos in the U.S. and Mexico; and (3) implications of these trends for long-term care service needs for Latinos living with dementia in the U.S. and Mexico. The resulting discussion will provide new empirical and theoretical insights on the determinants of cognitive aging for this population. It will also inform debates and aid in implementing innovative strategies and solutions to mitigate risk for impairment and improve dementia care for older Latinos.

### AGE OF MIGRATION AND COGNITIVE LIFE EXPECTANCIES AMONG OLDER LATINOS: EVIDENCE FROM THE HEALTH AND RETIREMENT STUDY

Marc Garcia, *University of Nebraska, Lincoln, Nebraska, United States*

This study used data from the Health and Retirement Study (1998-2014) to estimate Sullivan-based life tables of cognitively intact, cognitively impaired/no dementia (CIND), and dementia life expectancies by nativity, age of migration, and sex for older Latinos residing in the United States. Results show foreign-born Latinos, regardless of age of migration or sex, spend a greater number of years after age 50 with CIND compared to U.S.-born Latinos. Furthermore, we document an advantage in total life expectancy and cognitively intact life expectancy among mid-life immigrant men relative to their U.S.-born counterparts. The robust relationship between nativity, age of migration, and cognitive health suggests that the foreign-born may place particularly serious burdens on families and the government. This issue merits special attention in the development of community-based long-term care programs to appropriately target the specific needs of different subgroups of older Latinos who are entering into their last decades of life.

### RURAL AND URBAN DWELLING ACROSS THE LIFE-COURSE AND LATE-LIFE COGNITIVE ABILITY IN MEXICO

Joseph Saenz, *University of Southern California, Los Angeles, California, United States*

BACKGROUND: Research has consistently suggested urban dwelling in late adulthood is associated with better cognitive ability. Whether early life rural/urban dwelling and its interaction with late-life rural/urban dwelling relate with late-life cognitive ability in the context of Mexico is not well understood. METHOD: Data comes from the 2003 Mexican Health and Aging Study. Early life rural/urban was assessed as respondents' reports of growing up in an urban/rural area. Current rural/urban was assessed by locality size (greater/fewer than 100,000 residents). RESULTS: Both early life and current rural residence were associated with poorer cognitive ability independent of education, literacy, early life SES and health, income/wealth, healthcare access, health, and health behaviors. Compared to individuals who always lived in rural areas, rural to urban migration was associated with better cognitive ability. DISCUSSION: In addition to current rural/urban dwelling, researchers should consider where individuals lived in early life and migration across the life-course.

### DIABETES, DISABILITY, AND DEMENTIA RISK: RESULTS FROM THE HEPESE

Elizabeth Vasquez,[1] meghana Gadgil,[2] Weihu Zhang,[3] and Jacqueline L Angel,[4] *1. University at Albany (SUNY), Rensselaer, New York, United States, 2. Dell Medical School - University of Texas, Austin, Texas, United States, 3. Albany University, Rensselaer, New York, United States, 4. The University of Texas at Austin, LBJ School of Public Affairs, austin, Texas, United States*

The relationship between cognitive function, diabetes and disability among the oldest-old remains largely unexplored, particularly in the Latino population. This study examines dementia risk and diabetes status in a Mexican-origin older adult sample. The data are drawn from eight waves (1993 -2013) of the Hispanic Established Populations for the Epidemiologic Study of the Elderly (HEPESE; N=3,039, mean age at baseline=73.6 (±6.8)). We use multivariable Cox proportional hazards models to predict the relation between diabetes and time to incident dementia (MMSE<24, 1+ IADL), with risk adjustment for age of migration, socioeconomic status, acculturation, and health. Diabetes prevalence at baseline was 27.8 %. Diabetes was associated with a higher risk of developing dementia (HR)=1.22, p<0.001). Foreign-born older adults who migrated at ages 20- 49 had a higher survival probability of being dementia-free (HR=0.84, p=0.001). Our results further highlight the importance of evaluating differences in the cognitive outcomes of Mexican origin older adults.

### LIKELY DEMENTIA AND ITS IMPLICATION FOR SUPPORT AMONG THE OLDEST-OLD IN MEXICO AND THE UNITED STATES

Mariana Lopez-Ortega,[1] Silvia Mejia,[2] Emma Aguila,[3] Luis Gutiérrez-Robledo,[4] William Vega,[5] Flavia Andrade,[6] Stephanie Grasso,[7] and Jacqueline Angel,[8], *1. National Institute of Geriatrics, Mexico, Mexico City, Mexico, 2. El Colegio De La Frontera Norte, Tijuana, Baja California, Mexico, 3. University of Southern California, Los Angeles, California, United States, 4. The National Institute of Geriatrics, Mexico City, Distrito Federal, Mexico, 5. Florida International University, Miami, Florida, United States, 6. University of Illinois, Urbana, Illinois, United States, 7. University of Texas at Austin, Austin, Texas, United States, 8. The University of Texas at Austin, Austin, Texas, United States*

This study examines sources of vulnerabilities to dementia in low resource populations in two specific contexts—Mexico and the United States. Data are drawn from comparable waves of the Mexican Health and Aging Study (MHAS) and the Hispanic Established Populations for the Epidemiologic Study of the Elderly (H-EPESE) in 2012, which include representative samples of the oldest-old (82 and over), the fastest growing segment of the populations worldwide. Likely dementia prevalence is 30.9% (±0.46SD) for Mexicans in Mexico and 36.3% (±0.48SD) for Mexicans in the U.S. Odds of likely dementia in both populations were increased by age, living in extended households, depressive symptoms, and Seguro Popular and Medicaid receipt. Being female and having comorbid cardiovascular conditions were also associated with likely dementia but only for older Mexicans. There is a need to strengthen the caregiving capacity of memory care services in low resource communities in Mexico and the U.S.

### SESSION 5510 (SYMPOSIUM)

### COMMUNICATION AND LANGUAGE: WHY AGE MATTERS

Chair: Alison Chasteen
Co-Chair: Sali Tagliamonte

In line with this year's 75th anniversary theme, we will show why aging matters for communication and language. Specifically, in this symposium we will show how aging affects communication and language across a variety of social contexts, social roles, and cognitive abilities. Pabst & Tagliamonte discuss the effects of aging on language use by examining an individual's daily diary entries over 30 years, including the onset and progression of dementia. Saunders considers language and communication in the context of social interaction among persons with dementia living in a long-term care setting. Savundranayagam et al. test the efficacy of a communication intervention for personal support workers who work with persons with dementia. Chasteen & Tagliamonte consider how ageism is communicated to middle-aged and older adults in everyday life. Taken together, these presentations will provide a multidimensional lens to understanding language and communication in later life.

### AGEIST COMMUNICATION EXPERIENCED BY MIDDLE-AGED AND OLDER ADULTS

Alison Chasteen, and Sali Tagliamonte, *University of Toronto, Toronto, Ontario, Canada*

Despite the prevalence of ageism, relatively little is known about specific aspects of ageism experiences. In the present study, middle-aged and older adults described their experiences of ageism as part of an interview. Their descriptions were coded for the domain in which the ageist act occurred, the perpetrator of the act, and what type of ageist act occurred. A majority of participants indicated that they had been the target of an ageist act, with those acts occurring most commonly in the public sphere. Consistent with this finding, perpetrators of ageist acts were most often strangers. Ageist comments most often involved age-based social or physical assumptions about the target. Together, these findings suggest that there is a lack of norms prohibiting ageist communication in public spheres.

### A DIARY-BASED STUDY OF LANGUAGE USE DURING COGNITIVE DECLINE

Katharina Pabst, and Sali Tagliamonte, *University of Toronto, Toronto, Ontario, Canada*

Although the effects of dementia on language have been examined in a variety of ways, there is relatively little data available showing longitudinal effects. In the present study, we examined the effect of cognitive decline on an individual's language use over time (from age 60-91) by assessing daily diary entries. We focused on a linguistic phenomenon commonly associated with diaries and other informal registers, i.e. subject omission, as in "∅ Made cranberry muffins." Quantitative analysis revealed a drastic decline in the use of this feature, which is correlated with the stages of the individual's cognitive decline. We argue that the patterns reflect a systematic reduction in complexity that affects a writing style based on literacy and acquired at a later stage than more basic writing. This corroborates previous findings, showing later-acquired linguistic features are disproportionately affected in cognitive decline and highlights the importance of linguistic analysis in aging research.

### SOCIOLINGUISTIC MARKERS OF FRIENDSHIP AMONG PERSONS WITH DEMENTIA IN LONG-TERM CARE

Pamela Saunders, *Georgetown University, Washington, District of Columbia, United States*

Sociolinguistics and discourse analysis provide tools through which to examine how friendship is socially constructed through language and communication. Research on social isolation and loneliness reveals the importance of social interaction on the psychological and physical health of older adults. Given that linguistic, communicative, and functional abilities decline as dementia progresses, it is challenging to identify markers of friendship. The Friendship Project is an ethnographic study of social interaction among persons with dementia living in a long-term care setting. The data are from transcripts and field-notes of social interactions among residents with a range of cognitive impairments over a six-month time period. Results reveal that persons with dementia employ specific linguistic features such as narrative, evaluation, evidentials, and pronominal reference to make meaning and create relationships over time. Practical implications will be discussed.

### IMPACT OF BE EPIC ON PERSON-CENTERED COMMUNICATION

Marie Savundranayagam,[1] Kristine Williams,[2] Shalane Basque,[1] Joseph Orange,[1] Marita Kloseck,[1] Karen Johnson,[3] and Vicki Schmall,[4] *1. Western University, London, Ontario, Canada, 2. University of Kansas, Lawrence, Kansas, United States, 3. McCormick Dementia Services, London, Ontario, Canada, 4. Aging Concerns, West Linn, Oregon, United States*

This study assessed the impact of Be EPIC, a dementia-focused, person-centered communication intervention for personal support workers (PSWs). Video-recorded conversations between PSWs and simulated persons with dementia assessed whether Be EPIC participants (n=13) (versus a wait-list control group, n=8) reported a greater proportion of person-centered communication utterances (recognition, negotiation, facilitation, validation). We used linear mixed model analysis to investigate if Be EPIC influenced PSWs' person-centered communication. Group (Be EPIC versus control group), time (pre-, post-, and 3-months) and their interaction were included in the model. There was a significant group by time interaction. Follow-up tests showed that participants who took Be EPIC showed significant increases in person-centered utterances from pre- to post-training and pre-training to 3 months later. Participants in the control group showed no changes in person-centered communication. These findings show that Be EPIC enhanced person-centered communication, which is essential for quality of care.

### SESSION 5515 (SYMPOSIUM)

### CONTEMPORARY APPROACHES TO THE STUDY OF COGNITIVE AGING

Chair: Gizem Hueluer
Discussant: Elizabeth A L Stine-Morrow

Cognitive aging research is gaining societal and practical importance because of population aging. Current research is focused on describing age differences and age-related changes in cognitive performance, understanding potential causes underlying these differences and changes, and identifying factors that promote maintenance of cognitive functioning in old age. The goal of this research group is to showcase new developments in research studying age differences in cognitive performance and longitudinal cognitive change in the second half of life. Hülür et al. examine associations between midlife occupational factors and trajectories of cognitive change using data from the German Interdisciplinary Longitudinal Study of Adult Development and Aging (ILSE). Luo et al. use 12-year longitudinal data from 499 older participants in ILSE to study bidirectional associations between social relationships and cognitive performance. Small et al. examine the correspondence between objective and subjective cognitive performance, and measures of fatigue and depressed mood in experience sampling data from breast cancer survivors. Haas et al. compare laboratory and at-home online assessments of cognitive status and prospective memory over the adult lifespan and evaluate the quality of self-administered tests. The discussion by Elizabeth Stine-Morrow will focus on how these approaches contribute to our understanding of processes of cognitive aging and how they can be utilized to promote maintenance of cognitive functioning in old age.

### THE ROLE OF MIDLIFE OCCUPATIONAL FACTORS FOR TRAJECTORIES OF COGNITIVE CHANGE

Gizem Hueluer,[1] Jelena Siebert,[2] and Hans-Werner Wahl,[3] *1. University of South Florida, Tampa, Florida, United States, 2. Heidelberg University, Heidelberg, Baden-Wurttemberg, Germany, 3. University of Heidelberg, Heidelberg, Baden-Wurttemberg, Germany*

Cognitively enriching environments are usually related to higher levels of cognitive performance, while associations with longitudinal change are less clear. In the present study, we used 20-year longitudinal data from the German Interdisciplinary Longitudinal Study of Adult Development and Aging (ILSE) to examine the role of occupational factors for longitudinal trajectories of cognitive function in midlife. To do so, we used data from 374 participants in the ILSE midlife cohort (born in 1950-52; mean age at baseline = 44 years; 44 % women). Our findings showed that cognitively enriching work environments were associated with higher levels of cognitive function at baseline; however, these associations were not independent of control variables including education. There was no evidence that enriching work environments were related to the maintenance of cognitive abilities. In sum, our findings are in line with notions of "preserved differentiation". We discuss potential mechanisms underlying these findings.

### BIDIRECTIONAL LONGITUDINAL ASSOCIATIONS BETWEEN COGNITIVE ABILITIES AND SOCIAL RELATIONSHIPS IN OLD AGE

Minxia Luo,[1] Peter Edelsbrunner,[2] Jelena Siebert,[3] Mike Martin,[1] and Damaris Aschwanden,[4] *1. University of Zurich, Zurich, Zurich, Switzerland, 2. ETH Zurich, Zurich, Zurich, Switzerland, 3. Heidelberg University, Heidelberg, Baden-Wurttemberg, Germany, 4. Florida State University, Tallahassee, Florida, United States*

Individuals' social connections can both shape and be shaped by cognitive abilities in aging process. This study examined bidirectional longitudinal associations between cognitive abilities and social relationships using 12-year longitudinal data (3 waves) from 499 German older adults who were born between year 1930 and 1932. Cognitive abilities were assessed as a latent construct consisting of five cognitive tests, i.e., picture completion, block design, information, similarities, and word finding. Social relationships were assessed by the self-reported number of free time partners and scales of perceived social relationships. Using a random-intercept cross-lagged panel model, we focused on within-person causal relations. Results showed that higher cognitive abilities predicted higher number of free time partners over four years and that more positive perceived social relationships predicted higher cognitive abilities at four-year follow-up. In sum, the bidirectional longitudinal associations indicate social relationships and cognitive abilities mutually maintain each other in old age.

### UNDERSTANDING COGNITIVE COMPLAINTS AMONG BREAST CANCER SURVIVORS

Brent Small,[1] Heather Jim,[2] Sarah Eisel,[2] and Stacey Scott,[3] *1. University of South Florida, Tampa, Florida, United States, 2. Moffitt Cancer Center, Tampa, Florida, United States, 3. Stony Brook University, Stony Brook, New York, United States*

Cancer and its treatment can induce accelerated aging changes in physiological and behavioral processes. In studies of cancer associated cognitive decline, subjective reports of cognitive impairment are often many times greater than performance deficits on objective tests of neurocognitive functioning. In an Ecological Momentary Assessment study of 47 breast cancer patients (M age = 53.3 years), subjective ratings of cognitive performance and the occurrence of memory lapses assessed at the end of day were predicted by cognitive performance and ratings of fatigue and depressed mood throughout the day. Results indicated that poorer subjective cognition was significantly associated with elevated fatigue throughout the day. Slower processing speed, elevated ratings of fatigue, and depressed mood throughout the day were associated with a greater likelihood of memory lapses. Subjective ratings of cognitive deficits are related to objective performance, as well as common quality of life decrements among cancer survivors.

### ONLINE ASSESSMENT OF COGNITIVE FUNCTIONING: A RELIABLE ALTERNATIVE TO LABORATORY TESTING?

Maximilian Haas,[1] Sascha Zuber,[2] David Framorando,[3] Elissa El Khawli,[4] Susanne Scheibe,[4] and Matthias Kliegel,[2] *1. University of Geneva, Geneva, Switzerland, 2. University of Geneva, Geneva, Geneve, Switzerland, 3. University of Queensland, St Lucia, Queensland, Australia, 4. University of Groningen, Groningen, Groningen, Netherlands*

As the population ages, risks for cognitive decline threaten independence and quality of life for older adults. Classically, psychological assessment tools to evaluate cognitive functioning are administered in face-to-face laboratory sessions, which is time- and resource-consuming. With the aim of reducing such costs, the present study set out to develop and validate two new online tools, allowing a rapid assessment of general cognitive abilities and of prospective memory. We collected data from 250 participants equally spread across the adult lifespan (aged 18 – 86). Results suggest that performance assessed via these newly developed online tools is comparable to performance in face-to-face laboratory settings. Our findings thereby indicate that these online tools can reliably measure cognitive functioning across the lifespan at a reduced cost, which may help detect individuals at risk of developing age-related cognitive disorders.

### SESSION 5525 (SYMPOSIUM)

### CONTEXTUAL AND SOCIAL CHARACTERISTICS RELEVANT TO HEALTH AND WELL-BEING IN AFRICAN AMERICAN AND WHITE ADULT POPULATIONS

Chair: Angela Sardina
Co-Chair: Adrienne Aiken-Morgan
Discussant: Alyssa Gamaldo

With the burgeoning older adult population, there will be an increased demand for neighborhood and housing developments conducive to the interests and needs of older adults from diverse backgrounds of varying health and functional status. Several initiatives have sought to develop age-friendly neighborhoods, which focused on improving access and affordability of community resources. However, limited effort has focused on physical and social attributes of immediate housing environments, particularly amongst lower-income older adults. The need for affordable and usable housing developments for older adults that provide greater opportunities for social engagement, social services, and convenience to neighborhood resources (e.g., grocery stores, healthcare) will continue to rise. The objectives of the proposed symposium are the following: (1) to explore the physical and social attributes of older and low-income residents' housing and their surrounding community; and (2) discuss how older and low-income residents' housing and community resources relates to their health and well-being. This symposium will include presentations from three pilot investigations that highlight relevant subjective and objective contextual metrics related to health and well-being in underserved older populations. Tan and colleagues explored the role of well-being (i.e., purpose in life) in the relationships among sociodemographics, health, housing and community resources. Sardina and colleagues explored perceived leisure barriers and their relationship to sociodemographic, health, and psychosocial characteristics. Aiken-Morgan and colleagues examined associations between neighborhood socioeconomic disadvantage and health status among low-income African American older adults. Wright and colleagues explored associations between neighborhood disadvantage, brain health, and neurocognitive function in cognitively normal older adults.

### POSITIVE ROLE OF PURPOSE IN LIFE IN HEALTH OUTCOMES AND PERSPECTIVES ON ENVIRONMENT

Shyuan Ching Tan,[1] Alyssa Gamaldo,[2] and Angela Sardina,[3] *1. Penn State University, State College, Pennsylvania, United States, 2. Penn State, University Park, Pennsylvania, United States, 3. University of North Carolina Wilmington, Wilmington, North Carolina, United States*

Having a sense of purpose directs behaviors, hence, purpose in life (PIL) can be a useful indicator/moderator of healthy mental and physical behaviors and outcomes. As such, purpose in life, particularly in lower income older adults, might encourage meaningful engagement in activities and life that lead to positive health. Thirty-nine residents (M=68.01, SD=10.26) of affordable housing for older adults in Wilmington, NC and State College, PA were surveyed on demographics, mental health, well-being (i.e., PIL), health behaviors, and their perceptions on immediate housing and the community resources. Findings suggest that for higher educated, younger and Black older adults, PIL moderates or protects against negative mental outcomes ($p<.05$). For higher educated older adults, PIL moderates or encourages positive perception of job opportunities in the community ($p<.05$) and healthier behaviors ($p<.05$). More research is needed to understand how environment interacts with PIL to promote healthy behaviors and outcomes.

### LEISURE BARRIERS AMONG OLDER ADULTS IN LOW-INCOME HOUSING: DEMOGRAPHIC, HEALTH, AND CONTEXTUAL CORRELATES

Angela Sardina,[1] Shyuan Ching Tan,[2] and Alyssa Gamaldo,[3] *1. University of North Carolina Wilmington, Wilmington, North Carolina, United States, 2. Penn State University, State College, Pennsylvania, United States, 3. Penn State, University Park, Pennsylvania, United States*

Despite increased research pertaining to the physical, cognitive, and psychosocial benefits of leisure engagement, few studies have explored leisure barriers experienced by older adults residing in subsidized housing, and how these barriers relate to sociodemographic, health, and psychosocial characteristics. Thirty-nine Black and White residents (M=68.01, SD=10.26) from two subsidized housing communities (Wilmington, NC and State College, PA) were surveyed as part of the Tailoring Environments for Active Life Engagement study. Findings indicated that lack of available activities and low awareness of activities, limited social connections, and transportation were the most common barriers identified. Additionally, individuals with lesser years of education and poorer quality of education, worse mental and physical health, poorer cognitive function, as well as those experiencing loneliness and social isolation reported significantly more leisure barriers (ps $<.05$). More research is needed that examines micro-, meso-, and macro-level factors associated with leisure participation for older low-income housing residents.

### NEIGHBORHOOD SOCIOECONOMIC DISADVANTAGE AND HEALTH STATUS AMONG AFRICAN AMERICANS LIVING IN LOW-INCOME HOUSING

Adrienne Aiken-Morgan,[1] Dextiny McCain,[2] Karon Phillips,[3] and Keith Whitfield,[4] *1. North Carolina A&T State University, Greensboro, North Carolina, United States, 2. North Carolina A&T State University, Kernersville, North Carolina, United States, 3. UMBC, Baltimore, Maryland, United States, 4. Wayne State University, Detroit, Michigan, United States*

Research has shown the importance of social determinants of health in explaining racial/ethnic disparities in many health outcomes; however, less attention has been given to within-group differences in social determinants of health among low-income African American older adults. The Physical and Cognitive Health Pilot Study (n=50) was utilized to examine associations between level of neighborhood socioeconomic disadvantage and self-reported health in African American older adults living in public housing in Durham, NC and Annapolis, MD. Results from ANOVA showed that Durham participants living in more disadvantaged neighborhoods had statistically significantly worse cardiovascular health, higher depression symptoms, worse sleep quality, and higher alcohol use (p=.05) than Annapolis participants living in a more resource-rich neighborhood. These findings suggest that among low-income African American elders, greater neighborhood/state socioeconomic disadvantage is associated with worse health status. Future research should consider neighborhood context as an essential variable when assessing health status among aging African Americans.

### NEIGHBORHOOD DISADVANTAGE, BRAIN, AND NEUROCOGNITIVE FUNCTION: RELATIONS AMONG COGNITIVELY NORMAL OLDER ADULTS

Regina Wright,[1] Desiree Bygrave,[1] Shari Waldstein,[2] Christos Davatzikos,[3] and Guray Erus,[3] *1. University of Delaware, Newark, Delaware, United States, 2. University of Maryland Baltimore County, Baltimore, Maryland, United States, 3. University of Pennsylvania, Philadelphia, Pennsylvania, United States*

Neighborhood disadvantage has been linked to poor health; however, research has not adequately examined its influence, across multiple domains of disadvantage, on neurocognitive function and underlying brain health in older adults. Thus, the objective was to examine associations between neighborhood disadvantage, brain health, and neurocognitive performance, and examine age and sex as effect modifiers. The analysis included 136 older adults with a mean age of 68.04y who underwent neuropsychological and psychosocial testing and 3T cranial magnetic resonance imaging. Neighborhood disadvantage was characterized using the Area Deprivation Index (ADI). Multivariable regression analysis, adjusted for age, sex, education, and depression, showed that greater ADI score (greater disadvantage) was associated with poorer working memory performance (p<.05) and lower hippocampal volumes (p<.05), with no evidence of effect modification. Results suggest that greater neighborhood disadvantage may play a role in working memory and brain structure, which are vital to quality of life in older adulthood.

### SESSION 5530 (SYMPOSIUM)

### CONTRIBUTORS TO AND CONSEQUENCES OF POOR SLEEP IN OLDER ADULTS: BIOPSYCHOSOCIAL PERSPECTIVES

Chair: Adam Spira
Discussant: Katie Stone

Sleep is a significant contributor to health and wellbeing across the lifespan, especially in later life. Poor sleep is common among older adults and can be both a risk factor for and consequence of numerous physical and mental health-related outcomes. In this symposium, we will present novel results from four studies that will advance understanding of the biological, psychological, and social factors that may contribute to or result from poor sleep in older adults. Specifically, Study 1 will present findings tying objectively measured sleep to performance on cognitive tasks administered using ecological momentary assessment (EMA) in the day-to-day lives of older adults with or without mild cognitive impairment (MCI). Study 2 will examine associations of personality dimensions and facets with insomnia symptoms in well-functioning older adults. Study 3 will examine psychological pathways linking parent-child relationships to subjective and objective sleep characteristics among older parents. Finally, study 4 will examine use patterns of cannabis for the treatment of sleep problems in older adults, and the ways in which this might differ from patients using cannabis for other reasons (e.g., pain). Together, this symposium will highlight novel links of an array of factors with sleep health in the aging population and their implications for prevention. Sleep, Circadian Rhythms and Aging Interest Group Sponsored Symposium.

### ACTIGRAPHIC MEASURES OF SLEEP QUALITY ASSOCIATED WITH AMBULATORY COGNITIVE PERFORMANCE IN OLDER ADULTS

Orfeu Buxton,[1] Ruixue Zhaoyang,[2] June Jiao,[1] Rachel Zimmerman,[1] Martin Sliwinski,[3] and Carol Derby,[4] *1. Pennsylvania State University, University Park, Pennsylvania, United States, 2. The Pennsylvania State University, University Park, Pennsylvania, United States, 3. Penn State University, University Park, Pennsylvania, United States, 4. Albert Einstein College of Medicine, Bronx, New York, United States*

Few longitudinal studies link objectively assessed sleep and cognitive performance in ecologically-valid environments. Participants enrolled in the community-based Einstein Aging Study cohort (n=224, M[age]=77.17). Wake after sleep onset (WASO) was associated with worse cognitive performance with and without MCI (2-week smartphone-based EMA, six assessments/day). Models include age, gender, ethnicity, education, learning effects, sleep duration, WASO*MCI interaction (p=0.015 for Symbol Match; p=0.002 for Color Shape). Associations were stronger among those with MCI, thirty minutes more nightly WASO predicted a 500ms longer Symbol Match response time (p<0.0001), 5.05% higher Color Dot error proportion (p=0.002), 0.184 points lower Color Shape accuracy (p<0.0001). In those without MCI, WASO was associated with worse cognition: thirty minutes more nightly WASO predicted +166.7ms Symbol Match response time (p=0.03) and -0.06 points Color Shape accuracy (p=0.013). Actigraphic sleep quality associated with ambulatory cognitive performance (and worse with MCI status) suggests targets for prevention/mitigation of cognitive decline. Part of a symposium sponsored by the Sleep, Circadian Rhythms and Aging Interest Group.

### PERSONALITY AND INSOMNIA SYMPTOMS IN OLDER ADULTS: THE BALTIMORE LONGITUDINAL STUDY OF AGING

Darlynn Rojo-Wissar,[1] Amal Wanigatunga,[1] Eleanor Simonsick,[2] Antonio Terracciano,[3] Jennifer Schrack,[1] Sharmin Hossain,[2] Paul Costa Jr.,[4] and Adam Spira,[1], *1. Johns Hopkins Bloomberg School of Public Health, Baltimore, Maryland, United States, 2. National Institute on Aging, Bethesda, Maryland, United States, 3. Florida State University College of*

*Medicine, Tallahassee, Florida, United States, 4. Duke University School of Medicine, Durham, North Carolina, United States*

Personality and disturbed sleep are tied to medical morbidity in older adults. We examined associations of personality dimensions and facets from the five-factor model with reports of insomnia symptoms in 1,069 well-functioning older adults 60-97 (SD=8.64) years (51% women) from the Baltimore Longitudinal Study of Aging. Personality was assessed by the Revised NEO Personality Inventory, and insomnia symptoms measured by the Women's Health Initiative Insomnia Rating Scale. Adjusting for demographics and depressive symptoms, higher neuroticism (B=0.05, SE=-0.01, p<.001) and lower conscientiousness (B=-0.03, SE=-0.01, p<.05) were associated with greater insomnia severity. Although openness, extraversion and agreeableness were not associated with insomnia, a facet of each was. Higher scores on the "positive emotions" facet of extraversion (B =-0.03, SE=-0.01, p<.05) "ideas" facet of openness (B=-0.03, SE=-0.01, p<.05) and altruism facet of agreeableness (B=-0.03, SE=-0.01, p<.05) were associated with lower insomnia severity. Sleep disturbances may partially mediate personality's influence on health. Part of a symposium sponsored by the Sleep, Circadian Rhythms and Aging Interest Group.

### PSYCHOLOGICAL PATHWAYS LINKING PARENT-CHILD RELATIONSHIPS TO SUBJECTIVE AND OBJECTIVE SLEEP AMONG OLDER ADULTS

Haowei Wang,[1] Kyungmin Kim,[1] Jeffrey Burr,[1] and Bei Wu,[2] *1. University of Massachusetts Boston, Boston, Massachusetts, United States, 2. New York University, New York, New York, United States*

This study investigated whether older adults' relationships with their children were associated with their self-reported subjective sleep quality and actigraphy-measured objective sleep characteristics, as well as whether depressive symptoms and loneliness mediated the association between these parent-child relationships and sleep. Data were taken from the second wave of the National Social life, Health, and Aging Project, in which 569 respondents (age 57-85) participated in the sleep module, along with the social network module that provided relationship information for participants and their children. Results from structural equation modeling showed that (1) parents' closeness with children was associated with better objective sleep (i.e., fragmentation of sleep and percent sleep), (2) more frequent contact with children was related to better subjective sleep quality, (3) depressive symptoms and loneliness were associated with worse subjective sleep quality, and (4) less closeness with children were related to worse subjective sleep quality via older adults' depressive symptoms. Part of a symposium sponsored by the Sleep, Circadian Rhythms and Aging Interest Group.

### USE OF CANNABIS FOR SLEEP: PATTERNS OF USE AMONG A SAMPLE OF PATIENTS IN A GERIATRICS CLINIC

Christopher Kaufmann,[1] Kevin Yang,[2] Atul Malhotra,[2] Khai Nguyen,[2] Reva Nafsu,[2] and Alison Moore,[2] *1. University of California San Diego, San Diego, California, United States, 2. University of California San Diego, La Jolla, California, United States*

Cannabis may be useful for treatment of sleep problems in older adults. Little is known about use patterns of cannabis for sleep. We conducted a clinic-based anonymous survey in a geriatrics clinic in La Jolla, CA. Among n=568 surveys, 10% reported recent use (within past 6 months). Among recent users, 30% reported using cannabis for sleep, most finding it helpful. Compared to cannabis users for other conditions, cannabis users for sleep were more likely to use THC containing products (62.5% vs. 32.1%), use cannabis on a daily or weekly basis (76.2% vs. 43.2%), use vape pens (29.2% vs. 6.9%), and to obtain cannabis via a delivery service (33.3% vs. 14.0%) (all p's<0.05). Only 40% reported their doctor knew about their cannabis use. Our findings suggest sleep is a common reason for using cannabis. Future research should assess how use patterns can result in effective treatment for sleep in older adults. Part of a symposium sponsored by the Sleep, Circadian Rhythms and Aging Interest Group.

### SESSION 5535 (SYMPOSIUM)

### COPING MECHANISMS AND RESILIENCE IN LATER LIFE: THE ROLE OF FAMILY AND FRIENDSHIP TIES IN DIVERSE SETTINGS

Chair: Feinian Chen
Co-Chair: Rashmi Gupta
Discussant: Zhenmei Zhang

The papers in this symposium explore different aspects of social ties and how they act as critical coping mechanisms in the face of negative circumstances in later life. Using data from diverse settings, including China, Singapore, and the U.S., these papers underscore the importance of strong family and friendship ties, as they offer older adults with strong protection against social isolation and adverse health outcomes. Gupta and Pilai explore the similarity and differences in coping strategies/resilience among a diverse group of 30 U.S. older adults. Results point to the saliency of support from friends, regardless of race/ethnicity. Visaria addresses the relationship between the expression of loneliness and objective measures of social networks among older adults in Singapore. The findings shed light on how meaningful companionship and desired social connection offer powerful buffers against isolation in later life. Ruan and Chen explore which types of social ties offer the strongest protection when Chinese older adults are coping with the aftermath of negative life events. Findings point to the need to look beyond filial obligations and to consider the interplay among various forms of social support, including family, friends and the broader community. Zhang et al. examine the role of family and friendship ties in a rural Chinese community where many older adults were left behind by migrant children. The results suggest that those who are isolated from friends experience more depressive symptoms while those with close-knit friendship ties are the most resilient.

### COMPARISON OF COPING STRATEGIES AND RESILIENCE AMONG WHITE, NON-WHITE, AND ASIAN OLDER ADULTS IN SAN FRANCISCO

Rashmi Gupta,[1] and Vijayan Pillai,[2] *1. San Francisco State University, Daly City, California, United States, 2. University of Texas at Arlington, Arlington, Texas, United States*

There has been a substantial increase in research on coping strategies/resilience in later life in the last decade (Moos, et al., 2006). However studies exploring the differences in coping strategies/resilience in later life among various ethnic groups such as Whites, non-Whites and Asians are few. The purpose of this study is to explore the similarities and differences in coping strategies/resilience among a diverse group of 30 older adults age 65 and above. A purposive sample of older adults was recruited from a local senior center and fitness program in the West coast. A questionnaire was developed with open ended questions. We conducted face to face interviews with seniors. Results indicated that there were similarities in terms of coping/resilience strategies among various ethnic groups in that they relied primarily on friends for support. The sample respondents were living in their own homes and were physically active, despite health issues.

### CHANGES IN SOCIAL NETWORKS AS A RESPONSE TO LONELINESS AMONG OLDER ADULTS

Abhijit Visaria, Pildoo Sung, and Angelique WM Chan, *Duke-NUS Medical School, Singapore, Singapore*

The expression of loneliness suggests perceived social isolation and the lack of meaningful, rewarding and desired social connections. In this paper we seek to estimate whether loneliness among older adults is related to subsequent changes in two attributes of their social networks, i.e. the size and frequency of contact with network members. We use the Panel on Health and Ageing of Singaporean Elderly, a nationally representative study of older adults aged 60 years and older, conducted in 2009 (N=4990) with two follow-up waves in 2011 and 2015, and measure loneliness in terms of relational connectedness, i.e. feeling a lack of companionship, and social connectedness, i.e. feeling left out. Our preliminary analysis shows that loneliness is associated with a subsequent reduction in the size but not strength of social networks. We further propose to assess the causal relationship using cross-lagged panel analysis, thereby accounting for the possibility of a reciprocal relationship.

### NEGATIVE LIFE EVENTS, SOCIAL TIES, AND DEPRESSIVE SYMPTOMS FOR OLDER ADULTS IN CHINA

Hangqing Ruan,[1] and Feinian Chen,[2] *1. University of Maryland, Hyattsville, Maryland, United States, 2. University of Maryland, College Park, College Park, Maryland, United States*

Negative life events are considered important risk factors of depression among older adults. An overwhelming amount of literature suggests that individuals with the most supportive social relations tend to make a better recovery from stressful life events. As for which types of ties matter the most, whether being family, relatives, friends or the broader community, existing literature is much less consistent and has documented varying effects across different contexts. This study is set in China, which traditionally relies on family systems and filial obligations for old-age support. Using two waves of data from China Longitudinal Aging Social Survey, we examine the protective effect of different types of social relations on depressive symptoms, including those who are living in the household, children who live close by or far away, as well as their ties with family, relatives, and friends.

### SOCIAL INTEGRATION AND DEPRESSION OF OLDER ADULTS IN RURAL CHINA: DO FAMILY OR FRIENDSHIP TIES MAKE A DIFFERENCE?

Dan Zhang,[1] Zhiyong Lin,[2] and Shuzhuo Li,[3] *1. Xi'an Jiaotong University, college park, Maryland, United States, 2. University of Texas, Austin, Austin, Texas, United States, 3. Xi'an Jiaotong University, Xi'an, Shaanxi, China*

Despite increasing acknowledgement that social integration/isolation is an important determinant of health in later life, relevant evidence for older adults in less developed social contexts is still limited. Data derived from 2015 and 2018 waves of a longitudinal study of 976 older adults, aged 60 and older, living in rural areas of Anhui Province, China. We analyzed how the level of social integration/isolation (measured as family and friendship ties) impacted depressive symptoms of older adults. Our results showed that more than half of older adults in our sample were either isolated from family or friends. Further analysis demonstrated that older people who were isolated from friends were more depressed in comparison with those who were closely integrated into friendship ties, while no such association was found in relation to family ties. Assessments of social integration among older adults should account for both family and friendship ties.

### SESSION 5540 (SYMPOSIUM)

### DISCRIMINATION, STRESS, AND HEALTH ACROSS THE LIFE COURSE

Chair: Roland Thorpe
Discussant: Carl Hill

There is a paucity of research that seeks to understand why race disparities in health across the life course remain elusive. Two such explanations that have been garnering attention is stress and discrimination. This symposium contains papers seeking to address the impact of discrimination or stress on African American health or health disparities across the life course. First, Nguyen and colleagues examine 1) the associations between discrimination and objective and subjective social isolation and 2) how these associations vary by age in using data from the National Survey of American Life. Discrimination was positively associated with being subjectively isolated from friends only and family only. This relationship varied by age. Discrimination did not predict objective isolation. Second, Brown examines evidence of the black-white paradox in anxiety and depressive symptoms among older adults using data from 6,019 adults ages 52+ from the 2006 HRS. After adjusting for socioeconomic factors, everyday discrimination, chronic conditions, and chronic stress, there are no black-white differences in anxiety and depressive symptoms. Third, Cobb and colleagues investigate the joint consequences of multiple dimensions of perceived discrimination on mortality risk using mortality data from the 2006-2016 HRS. The authors report the number of attributed reasons for everyday discrimination is a particularly salient risk factor for mortality in later life. This collection of papers provides insights into how discrimination or stress impacts African American health or health disparities in middle to late life.

### DISCRIMINATION AND SOCIAL ISOLATION AMONG AFRICAN AMERICANS ACROSS THE LIFESPAN

Ann Nguyen,[1] and Harry Taylor,[2] *1. Case Western Reserve University, Cleveland, Ohio, United States, 2. Duke University Medical Center, Durham, North Carolina, United States*

Social isolation is associated with a wide range of health problems, including early mortality. However, little is known about the risk factors for social isolation specifically among African Americans. This study examined 1) the associations between discrimination and objective and subjective social isolation and 2) how these associations vary by age in a nationally representative sample of African American adults from the National Survey of American Life (N=3570). Multinomial logistic regression analyses indicated that discrimination was positively associated with being subjectively isolated from friends only and family only. Discrimination did not predict objective isolation. A significant interaction revealed that the association between discrimination and subjective isolation from friends only varied by age, with older adults being most vulnerable to the effects of discrimination. These findings argue for a more nuanced and systematic investigation of the detrimental effects of discrimination on older African Americans' social relationships, especially perceptions of relationships.

### THE BLACK-WHITE MENTAL HEALTH PARADOX AMONG OLDER ADULTS: EVIDENCE FROM THE HEALTH AND RETIREMENT STUDY

Lauren Brown, *University of Michigan, Ann Arbor, Michigan, United States*

Most studies of middle-aged adults find blacks have higher levels of psychological distress compared to whites but have lower risk of common psychiatric disorders. For instance, there is evidence of lower rates of depressive and anxiety disorders among blacks relative to whites despite large disparities in stress, discrimination and physical health in midlife—commonly referred to as the black-white mental health paradox. We examine evidence of the black-white paradox in anxiety and depressive symptoms among older adults. Data come from 6,019 adults ages 52+ from the 2006 Health and Retirement Study. Unadjusted models show older blacks report more anxiety and depressive symptoms than whites. After adjusting for socioeconomic factors, everyday discrimination, chronic conditions, and chronic stress, there are no black-white differences in anxiety and depressive symptoms. Findings suggest the black-white mental health paradox only extends into older adulthood for blacks living under similar stress and health landscapes as whites.

### MULTIPLE DIMENSIONS OF PERCEIVED DISCRIMINATION, RACE-ETHNICITY, AND MORTALITY RISK AMONG OLDER ADULTS

Ryon Cobb, *University of Georgia, North Richland Hills, Texas, United States*

The present study utilized data from the Health and Retirement Study (N=12,988) to investigate the joint consequences of multiple dimensions of perceived discrimination on mortality risk. Perceived discrimination is based on responses from the 2006/2008 HRS waves and included everyday discrimination, the number of attributed reasons for everyday discrimination, and major lifetime discrimination. Vital status was obtained from the National Death Index and reports from key household informants (spanning 2006–2016). Cox proportional hazard models were used to estimate the risk of mortality. During the observation period, 3,494 deaths occurred. Only the number of attributed reasons for discrimination predicted mortality risk when all discrimination measures were estimated in the same model (Hazard Ratio [HR]=1.09; 95%, Confidence Interval [CI]=1.05 - 1.14), holding all else constant. Overall, the number of attributed reasons for everyday discrimination is a particularly salient risk factor for mortality in later life.

### SESSION 5545 (SYMPOSIUM)

### DIVERSE APPROACHES TO ASSESSING WHAT MATTERS TO OLDER ADULTS

Chair: Erin Emery-Tiburcio
Discussant: Robyn Golden

Asking older adults What Matters to them and assuring that care plans are aligned with these preferences is the cornerstone of an Age-Friendly Health System (AFHS). Health systems have struggled to identify clear ways to ask this question and meaningfully utilize the responses. Both simple and complex options for addressing this challenge have been developed at Rush University Medical Center. At Rush, nurses began asking every inpatient What Matters and placing the response on the white board in the patient's room. Results of this practice include increased awareness of staff and significant increases in patient satisfaction. Qualitative analysis of responses yields increased awareness of patterns that the hospital can more systematically address. The Rush Center for Excellence in Aging hosts Schaalman Senior Voices, in which older adults from diverse backgrounds are given the unique opportunity to offer their perspectives on life, health and aging related to "What Matters" to them. The films have been used effectively to stimulate conversations among older adults and families in the community and in health professions courses, and with health systems executives. The Rush College of Medicine has integrated AFHS training into communication skills for medical students. Faculty introduce the 4Ms and demonstrate methods for having What Matters (WM) conversations. Students then practice WM conversations with simulated patients; some have had the opportunity to practice with real patients in preceptorships. Implications for the health system and community will be discussed as Rush builds an Age-Friendly Health Community.

### ASKING WHAT MATTERS IS WHAT MATTERS TO HOSPITALIZED OLDER ADULTS

Vikki Rompala, Erin Emery-Tiburcio, and Carline Guerrier, *Rush University Medical Center, Chicago, Illinois, United States*

The 4Ms of an Age-Friendly Health System place What Matters at the center of optimal care for older adults. Nurses at Rush have asked every medical inpatient What Matters early in their hospital stay since May, 2018. Responses were recorded in tablet software and on patient room white boards. What Matters responses recorded electronically were stratified by age and ethnicity. Qualitative data analysis of responses (n=660) was conducted using In-Vivo software by three raters. Themes in responses include: going home; comfort, including pain control and breathing more easily; effective staff/patient communication; compassionate care; and mobility. Patient satisfaction data for the first year showed an average 2.6% increase in satisfaction in nurses listening to the patient, and average 3.6% increase in satisfaction in nurses explaining things in an understandable way. Both increases were statistically significant. Implications of this practice for health systems improving age-friendly care will be discussed.

### SCHAALMAN SENIOR VOICES: TRANSITIONING FROM "WHAT'S THE MATTER?" TO "WHAT MATTERS?"

Grisel Rodriguez-Morales,[1] Erin Emery-Tiburcio,[1] Robyn Golden,[2] Marc Fenton,[1] and Jasmine Chandy,[1]
*1. Rush University Medical Center, Chicago, Illinois, United States, 2. Rush University Medical Center, CHICAGO, Illinois, United States*

The 4Ms of an Age-Friendly Health System start with What Matters to the older adult. A unique method for asking that question is through film. Schaalman Senior Voices (SSV) films older adults talking about What Matters to them, and uses the films to stimulate discussion about later life with older adults in the community (n=264), with health care professions students learning to listen to older adults (n=1250), and health system executives considering implementation of the Age-Friendly Health Systems (AFHS) initiative (n=100). SSV has completed longer professional films interviewing 12 older adults. Using a mobile platform, SSV has filmed 50 older adults in the community and at health events. Outcomes of film discussions will be presented, including inspiration for older adults having conversations with family and physicians about What Matters, health care students effectively using skills in asking What Matters to enhance the care they provide, and executives considering AFHS implementation.

### TEACHING MEDICAL STUDENTS HOW TO ASK OLDER ADULTS WHAT MATTERS USING SIMULATED PATIENTS

Laurin Mack, Jamie Cvengros, and Erin Emery-Tiburcio, *Rush University Medical Center, Chicago, Illinois, United States*

It is vital the workforce is prepared to meet the medical needs of our aging population. Asking older adults What Matters is an important aspect of excellence in clinical care. During a small group session in a two-year communication skills course, second year medical students (N=149) at Rush were taught how to ask What Matters as part of the 4Ms. Students then completed a video recorded Communication Skills Lab with a simulated older adult patient as they practiced how to discuss What Matters. Students then met with their instructors in individual feedback sessions to review the video and discuss strengths and areas for improvement in communicating with older adults. Students then completed a Clinical Skills Assessment for formal testing of their communication skills with older adults. Outcomes of the summative assessment will be presented and recommendations for integrating 4Ms into existing medical school and allied health curriculum will be discussed.

### SESSION 5550 (SYMPOSIUM)

### DOES THE SHARING OF RESOURCES IMPACT HEALTH AMONG MARRIED COUPLES? NEW FINDINGS FROM DYADIC MODELS

Chair: Shuangshuang Wang
Co-Chair: Kyungmin Kim
Discussant: Karen Lyons

As married couples aging together, their health behaviors and outcomes could be shaped by both one's own and the spouse's characteristics. Using dyadic datasets, speakers in this symposium explored the interdependence nature of marital relations by identifying the mechanisms of how shared resources or strains affect spouses' physical and mental health outcomes among married couples. Wang, Kim, and Burr identified distinct types of personality configurations among older couples using the Health and Retirement Study, and examined how personality compatibilities could buffer negative effects of adverse life events on older couples' mental health. Using data from the National Social Life, Health, and Aging Project, Proulx, Skoblow, and Han further investigated the associations between marital quality and mental health among caregiving dyads, with a special focus on a comparison of different caregiving groups (spouse, child, others). From a physical health perspective, Wilson and Novak presented the dynamic behind relationship quality, joint health behaviors, health problems, health satisfaction, and health similarity between spouses. Finally, Kim, Jang, Park, and Chiriboga focused on couple contexts for acculturation among older Korean immigrants in the U.S., and examined how each spousal acculturation level affects healthcare utilization and difficulties in health service use. Focusing on married couples, this symposium showcases the interplay of family experiences, health behaviors, and relational dynamics of both spouses in shaping their health, and highlights the benefits of dyadic approaches. Speakers and our discussant, Dr. Karen Lyons, will discuss implications for social program design and future research.

### OLDER COUPLES COPING WITH ADVERSE LIFE EVENTS: THE IMPORTANCE OF DYADIC PERSONALITY PROFILES

Shuangshuang Wang,[1] Kyungmin Kim,[2] and Jeffrey Burr,[2]
*1. Shandong University, Jinan, Shandong, China, 2. University of Massachusetts Boston, Boston, Massachusetts, United States*

Personality can be an important resource as older couples cope with adverse life events. Analyzing 4,893 older couples from the Health and Retirement Study, this study examined how one's own and spouse's adverse life events (health decline, job exit, loss of wealth, family member's death) occurring in the past two years are associated with changes in depressive symptoms. We further examined the moderating effects for this association of six dyadic personality profiles (combinations of spouses' positive and negative personality characteristics). We found significant actor and partner effects of health decline for increases in both spouses' depressive symptoms, and significant actor effects of a family death for husbands' increased depressive symptoms. For wives, having positive personality profiles buffered negative effects of one's own health decline and spouses' family death, whereas having negative profiles intensified negative effects of husbands' job exit and loss of wealth on the depressive symptoms for both spouses.

### MARITAL QUALITY AND MENTAL HEALTH AMONG CAREGIVING DYADS

Christine Proulx,[1] Hanamori Skoblow,[1] and Sae Hwang Han,[2] *1. University of Missouri, Columbia, Missouri, United States, 2. University of Texas at Austin, Austin, Texas, United States*

We examined whether the associations between marital quality and mental health were equally strong among dyads in which one spouse was providing care to a spouse (n = 155), parent (n = 61), or another adult (n = 176). Using Wave 2 of the NSHAP and actor-partner interdependence (APIM) models, we found significant differences (p=.004) among groups. Marital quality was negatively associated with one's own depressive symptoms (b=-1.29) for husbands in the spousal caregiver group, whereas marital quality was negatively associated with one's own depressive symptoms for wives in both the parental (b=-1.27) and other adult (b=-1.96) caregiver groups. The only partner effect was the negative association between wives' marital quality and husbands' depressive symptoms (b=-2.59) among dyads in which one spouse was a parental caregiver. These results point to the importance of understanding the context of caregiving when examining the protective effect of spouses' marital quality on mental health.

### COUPLES' JOINT HEALTH BEHAVIORS PREDICT BETTER HEALTH AND STRONGER RESEMBLANCE BETWEEN PARTNERS

Stephanie Wilson,[1] and Joshua Novak,[2] *1. Southern Methodist University, Dallas, Texas, United States, 2. Auburn University, Auburn, Alabama, United States*

Satisfying marriages pose benefits and possible risks to health. Indeed, high-quality relationships boost emotional resources and encourage healthy behaviors. However, stress and its adverse health effects also spread more easily in close relationships. To examine the relevance of joint health behaviors for health indicators and partners' health similarity, 227 couples age 23-84 reported their frequency of co-sleeping, exercising together, and sharing meals; relationship satisfaction; health satisfaction; recent medical visits; and health problems. Happier couples shared more joint health behaviors than unhappier counterparts. In turn, joint health behaviors predicted greater health satisfaction and more similar rates of health problems between partners. In particular, exercising together predicted greater health satisfaction, fewer health problems, and greater health similarity. Controlling for diet, sedentariness, and sleep, findings revealed that joint health behaviors—a characteristic of happy relationships—are linked to not only better health and greater health satisfaction, but also greater health similarity between partners.

### ACCULTURATION AND HEALTHCARE UTILIZATION AMONG OLDER KOREAN IMMIGRANTS: A DYADIC STUDY OF MARRIED COUPLES

Kyungmin Kim,[1] Yuri Jang,[2] Nan Sook Park,[3] and David Chiriboga,[3] *1. University of Massachusetts Boston, Boston, Massachusetts, United States, 2. University of Southern California, Los Angeles, California, United States, 3. University of South Florida, Tampa, Florida, United States*

Research has focused on the socioeconomic/cultural characteristics of individuals to address health disparities among immigrant populations. Dyadic studies of acculturation and healthcare utilization among older immigrants are rare. Using data from 263 older Korean immigrant couples in the U.S. (Mean_age = 74.75 for husbands; 71.03 for wives), this study examined how each spousal acculturation levels (e.g., English proficiency, familiarity with American culture) are associated with healthcare utilization (e.g., usual source of care, medical checkup) and difficulty in using health services, controlling for sociodemographic characteristics. Overall, husbands showed higher levels of acculturation than their wives, but there was also substantial similarity between spouses (ICC = .58). For healthcare utilization, one's own acculturation (actor effect) was significant only for wives, but spouse's acculturation (partner effect) was significant only for husbands. For difficulty in health service use, one's own acculturation was significant for both spouses, but spouse's acculturation was significant only for husbands.

### SESSION 5555 (SYMPOSIUM)

### EAST MEETS WEST: DEVELOPING SUPPORT SYSTEMS TO MEET THE DIVERSE NEEDS OF OLDER ADULTS IN THE UNITED STATES AND IN CHINA

Chair: Bei Wu
Co-Chair: Jiehua Lu

With the rapid growth of the aging population around the world, developing support systems for older adults has become increasingly important. It is crucial for researchers , educators, policy makers to share their experience and knowledge to initiate innovative and supportive programs and services that will meet the challenges of the aging population. The East meets West Forum is a platform that researchers from the Gerontological Society of America and the Chinese Association for Gerontology and Geriatrics established in 2017. Previously, the East meets West Forum focused on the issues of the long-term care (LTC) workforce, LTC services, and programs for older adults in the U.S and in China. In this session, we include four presentations (two from the U.S. and two from China) that focus on a broader area of support systems, beyond LTC, that would meet the diverse needs of older adults from housing, wellness visits, family caregiving system, to end of life care. More specially, it includes: 1) expand housing services for low-income older adults; 2) strengthen family support systems and promote intergenerational support; 3) develop a comprehensive program for early detection and treatment of dementia at primary care settings; and 4) examine diversity in the family care patterns for the oldest old. This session provides opportunities for aging researchers/educators from two countries to share their knowledge and experience on developing supportive systems for older adults and their families. It also provides policy discussions on improving health and family caregiver support services in these two countries.

### SCREENING FOR COGNITIVE FUNCTION IN PRIMARY CARE

Tara Cortes,[1] and Liz Seidel,[2] *1. New York University, New York, New York, United States, 2. NYU Rory Meyers College of Nursing, New York, New York, United States*

A comprehensive program for early detection and treatment of Alzheimer's and related dementias requires a systematic process for provider education, assessment of cognitive function and referral to appropriate resources when indicated. This discussion will focus on the implementation of cognitive screening into primary care. Over 200 staff across 11 primary care sites were trained on screening for dementia in the annual wellness visit. In the following 6 months nearly 2000 annual wellness visits were conducted. The annual wellness visit was designed to include a cognitive screen. 83% of the patients were asked the cognitive screen questions and 13% responded “yes” to the question. Further testing was offered to that group and 71% of those who had responded “yes” agreed to be tested with a picture memory screen. 32% scored less than 5 on that screen and were referred to gero-psychiatry, geriatricians and community based organizations for caregiver support services.

### EXPANDING HOUSING WITH SERVICES IN THE UNITED STATES: THE CASE OF THE RIGHT CARE, RIGHT PLACE, RIGHT TIME PROGRAM

Edward Miller, Pamela Nadash, Elizabeth Simpson, and Marc Cohen, *University of Massachusetts Boston, Boston, Massachusetts, United States*

Older people living in congregate environments are obvious beneficiaries of supportive services. The potential for prevention is clear, particularly among low-income elders living in subsidized housing; it is this group that is at high risk for significant healthcare and other costs, and it is this group that suffers considerably from a fragmented healthcare system. The purpose of this presentation is to illustrate the potential of housing with services, drawing from evaluation of The Right Care, Right Place, Right Time (R3) initiative (R3) located in the Greater Boston area. The R3 program consists of two on-site wellness teams, including a wellness nurse and wellness coordinator. Each team is responsible for about 200 participants across two housing sites. Evaluation findings highlight the potential of housing with services for improving the health, quality of life, and access to health-related services and supports among seniors living independently in affordable housing, while reducing healthcare costs.

### A CHALLENGE FROM LONGEVITY: STUDY ON THE CARE PROVIDER OF THE CHINESE OLDEST-OLD AT THE END OF LIFE

Wenjuan Zhang, *Renmin University of China, Beijing, China*

The study aimed to investigate the changing pattern of care for the oldest-old at the end of life in China. Data were used from the Chinese Longitudinal Longevity survey from 1998 to2018. The results indicate significant changes in the care providers of older adults at the end of life from 1998 to 2018: grandchildren played an increasing important role in providing care to the oldest-old in China. The study highlights an important role of grandchildren who replace children as main caregivers. Longevity changes the traditional pattern of end-of-life care, and increase the diversity in the pattern of care for the Oldest-old, due to different life course of families compared with general aging families, and the limited role of formal social support system in providing end-of-life care for the oldest-old. We discuss implications in the context of increasing population of the oldest-old, and its challenges to formal social support system.

### FAMILY CHANGES AND CHINA’S ELDERLY SUPPORT SYSTEM

Zhan Hu, and Ke Shen, *1. Fudan University, Shanghai, China*

Population aging has become the norm in China. Improving or reconstructing the elderly support system is therefore essential. While the government and institutional support has been improving and enhancing in China, family remains the most important source of elderly support for a long period in the future. Based on data from censuses and national surveys (1982-2015), we examine recent changes in household size and structure across ages from an individual life course perspective, to reveal the complexities and ambiguities behind the nationwide household change. Our findings suggest that multi-generational co-residence is a major vehicle to accommodate the needs of family members at critical stages of life. These salient features of family change in China call for new family-oriented policies, including pragmatic incentives to strengthen intergenerational solidarity, essential support for family caregivers, and diversified community services to care for frail elders.

### SESSION 5560 (SYMPOSIUM)

### ECONOMIC AND SOCIAL INFLUENCES ON LATER-LIFE WELL-BEING: NEW EVIDENCE FROM THE NLSY79

Chair: Deborah Carr
Discussant: Stephanie Burge

The National Longitudinal Survey of Youth 1979 (NLSY79) provides unprecedented opportunities for understanding how work, family, and socioeconomic characteristics over a 40-year period shape the well-being of older adults. The large sample enables explorations of race, gender, and socioeconomic differences in these processes. The five papers in this symposium exploit the rich life course data of NLSY79 to understand two key outcomes: health, and work/economic arrangements as adults approach their retirement years. Harrati and Heburn document the impacts of unemployment trajectories on physical and mental health, highlighting gender differences in these processes. Wolfe investigates the long-term health consequences of significant economic setbacks over the life course (shocks), taking into account those risk factors (selection) that render one vulnerable to such shocks. Jang and Tang document the negative impacts of informal caregiving on later-life physical health, yet their subgroup analyses detect positive impacts for African-Americans. Aughinbaugh delineates women’s time spent in caregiving over the life course, and discusses the implications for later-life economic well-being of both early life childcare and later-life coresidential caregiving. Walsemann, Fisk and Ailshire examine the role that parents and grandparents play in paying for their offspring’s college education, with careful attention to gender and socioeconomic differences in these patterns. Taken together, these papers underscore the importance of adopting an intersectional approach to understanding later-life well-being, and reveal the complex ways that paid work, unpaid work (caregiving), and inter-transfers (via tuition payment) bear on the health and financial stability of adults transitioning into their retirement years.

### GENDER DIFFERENCES IN THE LIFE COURSE EFFECTS OF UNEMPLOYMENT ON MID- AND LATER-LIFE HEALTH

AMAL HARRATI,[1] and Peter Heburn,[2] *1. Stanford University, Berkeley, California, United States, 2. Princeton University, Princeton, New Jersey, United States*

There is substantial evidence that unemployment is associated with adverse health. Given different lifetime employment patterns, these effects may differ between men and women. However, current studies often only characterize unemployment as a one-time shock, and measure the effects on health shortly thereafter. Using unique data available from The National Longitudinal Study of Youth 1979, we characterize employment trajectories for a nationally-representative sample of American men and women for every week of their lives between the ages of 18 and 50 years old. We then explore associations between unemployment and a number of health conditions including cancer, hypertension, diabetes, and depression at age 50--when the onset of chronic health conditions often begins—to examine the cumulative effects of unemployment over the life course on later-life health. We find that men and women have different patterns of lifetime unemployment and that these patterns have strong associations with poorer health at age 50.

### WHO'S HURT MOST BY ECONOMIC SHOCK? EXPLORING HETEROGENEITY IN THE HEALTH-RELATED EFFECTS OF WEALTH LOSS

Joseph Wolfe, *University of Alabama at Birmingham, Birmingham, Alabama, United States*

Prior research finds evidence of an effect of negative economic shocks on health, but this growing area has not fully investigated variation in this effect. A large number of people from diverse backgrounds experience a substantial financial setback of some type, and differences related to one's gender, race/ethnicity, and socioeconomic status (SES) may influence the consequences of economic shocks on one's life such that the health-related effects of shocks vary systematically in the U.S. population. Thus, this study aims to identify the effects of multiple economic shocks on health in middle adulthood, and whether the effects of shocks on health vary by one's underlying propensity to experience the shock. The analysis uses newly developed statistical techniques from causal inference literature and over twenty-five years of biographical information from the NLSY-79. Results from the analysis help shed light on important variation in the association between negative economic shocks and health.

### PARENT AND CHILD FACTORS THAT PREDICT WHO HELPS YOUNG ADULT CHILDREN PAY FOR COLLEGE

Katrina Walsemann,[1] Calley Fisk,[1] and Jennifer Ailshire,[2] *1. University of South Carolina, Columbia, South Carolina, United States, 2. University of Southern California, Los Angeles, California, United States*

In recent decades, the cost of higher education has exceeded the pace of inflation while wages have stagnated or declined. As such, young adult children may increasingly look to their parents and other family members, including grandparents, to help them pay for college. We use data from the National Longitudinal Survey of Youth 1979 to determine who financially contributes to a young adult child's college education, restricting our sample to mid-life parents with at least one biological child who attended a 2-year or 4-year college and completed the college expenditures module in 2014 (n=3,525). For each college-going child, parents reported who paid for the student's tuition – student, parents, grandparents, other family members, or a combination of these. Using multinomial logistic regression, we will estimate who paid for college as a function of parents' social and economic characteristics when the child was 16 and the child's gender and birth order.

### INFORMAL CAREGIVING AND HEALTH IN MIDDLE AND LATE ADULTHOOD

Joy Bohyun Jang, and Sandra Tang, *University of Michigan, Ann Arbor, Michigan, United States*

With average life expectancy increasing, informal caregiving has become increasingly common among aging families. Much of the research in this area suggests that informal caregiving is associated with negative psychological and physical health outcomes for the caregiver. However, there is an emerging body of work indicating that these negative associations may be overstated, and that the associations may vary by gender and race. Using NLSY79 who completed Age 50 Health Module (n=7,844), we will examine how informal caregiving is associated with health in mid/late adulthood and how the association varies by race and gender of the caregiver. Preliminary results showed that caregivers (10.6% of the sample) were less likely to report good health than non-caregivers (OR=0.43, $p<0.001$) but African-American caregivers were more likely to report good health than other racial groups (OR=1.43, $p<0.05$). Our findings will contribute to better understanding of the role of informal caregiving in older adults' health.

### THE RELATIONSHIP BETWEEN FEMALE LABOR SUPPLY AND CAREGIVING OVER THE LIFE CYCLE

Alison Aughinbaugh, *BLS, Washington, District of Columbia, United States*

I examine the effects of caring for others on female labor supply over the life-cycle using a fixed effect model. The data come from the National Longitudinal Survey of Youth 1979 (NLSY79), which collects information about the care of each child during his first three years and the care provided to household members during a woman's 50s. The NLSY79 data show that women's labor supply drops around the time a child is born and then rises, with over 50 percent working by time their children reach age 2. In addition, these data show that during their 50s, about 9 percent of women provide care to someone living in their household and that these female caregivers spend about 40 hours per week providing care. Time spent in caregiving may affect time in the labor force, and hence the ability to invest in a career and accumulate work experience and wage growth.

### SESSION 5565 (SYMPOSIUM)

### ECONOMIC INEQUALITY OVER THE LIFE COURSE: CAUSES AND EFFECTS OF CUMULATIVE DIS/ADVANTAGE PROCESSES

Chair: Jessica Kelley
Co-Chair: Stephen Crystal
Discussant: Jessica Kelley

Economic inequality has grown rapidly in all age groups in the past several decades. In each successive cohort, the wealth gap grows for young people and seems to accelerate faster over the life course. While rising inequality has taken its toll on Baby Boomers, we have become acutely aware of the increasing economic pressures across the entire life course (work precarity; student loans) that will manifest in the greatest degree of inequality in older adulthood seen to date. This session explores the forces that have shaped the degree of inequality among current older adults and are setting the stage for future cohorts of older adults. Presenters will explore several aspects of this issue: the growing state of the "risk retirement," the impact of income inequality on later-life wealth and health, the structural racism written into economic policies intended to help Americans accumulate wealth and maintain health, and the market disadvantage for GED recipients compared to high school diploma recipients.

### RACISM AND CUMULATIVE DIS/ADVANTAGE IN HEALTHCARE ACCESS: IMPLICATIONS FOR THE LIFE COURSE

Dale Dannefer, *CWRU, Cleveland, Ohio, United States*

Despite its origins in the study of race in America in Gunnar Myrdal's American Dilemma, research on cumulative dis/advantage (CDA) and the life course has paid little attention to the significance of racism in the overall production and patterning of CDA. Building on recent work that has reviewed the life-course implications of the inscribing of racist interests in social policy, this paper explores the life-course implications of race bias in another domain, specifically the domain of medical diagnosis, where algorithm formulas have been shown to disadvantage black patients based on economic and other parameters. Even with training, experimental evidence comparing human and AI diagnostics have demonstrated that despite improvements, residual racism is evident in differential diagnoses. We consider the life-course implications of this and similar race-based differentials in organizational decision-making as a component in systems of cumulating dis/advantage.

### EQUIVALENT PATHWAYS? COMPARISON OF JOB HISTORY FOR HIGH SCHOOL GRADUATES VERSUS GED RECIPIENTS

Wenxuan Huang, *CASE WESTERN RESERVE UNIVERSITY, Cleveland, Ohio, United States*

Successful integration into the paid labor market serves as a critical milestone to adulthood. Yet, this school-to-work transition has become harder to reach due to the increasing precarity in the youth labor market. Using data from the NLSY97, this study compares the job histories of young adults whose terminal education credentials are high school diploma versus GED. I conducted sequence analysis of school-to-work states from age 16 to 30 between these two education groups. Findings show that GED holders are more likely to be exposed to enduring negative labor force status (e.g., periods of unemployment) than the high school graduates. Over half of the GED recipients experience precarious early career characterized by interruptions and long-term inactivity. Despite being "equivalent" to a high school diploma, the GED diploma does not translate into the same opportunity structure as the high school degree, launching a cumulative disadvantage process in the early life course.

### WEALTH, HEALTH, AND DISABILITY GAPS AT MIDLIFE: IMPLICATIONS FOR DISPARATE RETIREMENT OUTCOME

Stephen Crystal, *Rutgers University, New Brunswick, New Jersey, United States*

Late-life economic outcomes in coming years will be strongly shaped by the impact of economic and health experience for cohorts now at midlife. These cohorts have experienced lagging and increasingly disparate wealth accumulation, along with challenges to health and earning potential that augur highly disparate retirement futures. For example, analyses of Survey of Consumer Finances data indicate that in 2016, members of Generation X had mean assets that were only 39% those of the boomers in that year. Increasing risk of "deaths of despair" among individuals in midlife have been accompanied by increases in disability that threaten the ability of those members of these cohorts who are not in the educationally advantaged minority to achieve secure retirement futures, particularly in the context of increasing employment precarity. This presentation will review recent findings on midlife wealth and health inequality, implications for retirement futures, and policy choices facing a new Presidential administration.

### SESSION 5570 (SYMPOSIUM)

### ESPO/ BEHAVIORAL AND SOCIAL SCIENCES SECTION SYMPOSIUM: PROMOTING BEHAVIORS THAT SUPPORT HEALTHY AGING

Chair: Elliane Irani
Co-Chair: Briana Sprague
Discussant: Luke Stoeckel

Maintaining healthy behaviors has been linked to positive emotional and physical health outcomes. Older adults are at a greater risk for functional decline and can benefit from the protective effects of health behaviors. The purpose of this symposium is to present and highlight: (1) innovative research linking health behaviors and health outcomes among older adults, and (2) work of emerging scholars in the Behavioral and Social Sciences (BSS) section. The papers highlight findings from descriptive studies and randomized trials testing behavioral health interventions. O'Brien and Hess describe patterns of engagement in health-promoting activities and highlight mediating and moderator factors. Fausto and colleagues report on physical activity and cognitive health benefits of a multi-level intervention focused on heart and brain health for older African American residents of public and subsidized housing. Still and colleagues assess the efficacy of a multi-component technology-based intervention on hypertension self-management in African American older adults. Nehrkorn-Bailey and colleagues report on the pilot testing of AgingPLUS, an intervention targeting attitudinal and motivational barriers to physical activity and highlight improvements in grip strength and blood pressure. Lastly, Wierenga and colleagues test an emotion regulation intervention following a cardiac event and highlight the intervention's potential efficacy in improving mental health and physical activity. These papers underscore the importance of promoting healthy behaviors in older adults and the need for large-scale interventions that support healthy aging.

As discussant, Atienza will assess the strengths and limitations of these papers, and consider how emerging scholars can contribute to the field.

### TITLE

Elliane Irani, *Case Western Reserve University, Ohio, United States*

abstract

### EXAMINING THE IMPACT OF AGING-RELATED MINDSETS AND MOTIVATION ON ACTIVITY ENGAGEMENT IN OLDER ADULTS

Erica O'Brien,[1] and Thomas Hess,[2] *1. Pennsylvania State University, University Park, Pennsylvania, United States, 2. North Carolina State University, Raleigh, North Carolina, United States*

This study examined short- and long-term patterns of engagement in health-promoting activities due to implicit beliefs about cognitive aging (mindsets) and Need for Cognition (NFC; motivation) in older adults. Prior research suggests higher NFC and growth-oriented mindsets bolster participation by enhancing perceived benefits and minimizing perceived costs of engagement. Survey responses across three bursts of an ongoing longitudinal study (N=678-725 observations) were collected from 148 people aged between 64 and 81 and subjected to three-level multilevel analyses. Results show naturally-occurring, weekly variations in NFC and mindsets that also contribute to short-term variation in activity frequency, diversity, and selectivity. Additionally, NFC and age significantly mediated and moderated the effect of mindsets on some outcomes, respectively. Initial findings highlight the value of taking a dynamic approach and using Selective Engagement Theory to understand activity maintenance. They may also inform efforts to develop interventions that promote healthful behaviors in later life.

### INCREASING PHYSICAL ACTIVITY AND REDUCING DEMENTIA RISK IN OLDER AFRICAN AMERICAN RESIDENTS OF PUBLIC HOUSING

Bernadette Fausto,[1] Paul Duberstein,[2] Shou-En Lu,[2] and Mark Gluck,[1] *1. Rutgers University, Newark, New Jersey, United States, 2. Rutgers University School of Public Health, Piscataway, New Jersey, United States*

Older African Americans—especially those with lower income and those living in urban public housing—have a greater risk of Alzheimer's disease (AD) compared to the general population. Inadequate levels of physical activity and aerobic exercise are thought to be among the probable causes for increased AD risk. Based on our preliminary data, we hypothesize that a cluster-randomized multi-level intervention in low-income public housing, focused on heart and brain health, can produce participant-level increases in physical activity among participants enrolled in an aerobic exercise class after six months (primary outcome) that are maintained at one year, as well as housing-level changes in attitudes and beliefs about physical activity and exercise participation among housing residents, both exposed and not exposed to the participant-level intervention as well as participant-level improvements in cognition and brain health evincive of decreased risk for AD.

### USING TECHNOLOGY TO SUPPORT SELF-MANAGING HYPERTENSION IN AFRICAN AMERICANS

Carolyn Still,[1] Phuong Dang,[2] and Abdus Sattar,[1] *1. Case Western Reserve University, Cleveland, Ohio, United States, 2. Case Western Reserve University, Frances Payne Bolton School of Nursing, Richmond Heights, Ohio, United States*

The purpose of this study was to examine the effects of a community and technology-based intervention to support self-managing hypertension in African American (AA). Sixty AA with hypertension were randomly assigned to Coachman (a technology-based intervention) or Enhanced Usual Care. COACHMAN is comprised of blood pressure (BP) monitoring with study issued monitor, six-weeks of web-based education, training to use a medication management application, and nurse counseling. Data were collected on contextual factors (demographics, perceived social support), process factors (hypertension knowledge, self-efficacy, technology use/adoption), and proximal health behaviors (medication adherence, diet, exercise) at baseline, and 8 and 12 weeks. While mean difference in BP reduction was not statistically significant, we found that half of the subjects randomized to the intervention group had an average systolic BP reduction of 13.5 mmHg that we would regard as clinically significant. Interventions that incorporate mHealth can support self-managing hypertension in AA, and improve BP.

### IMPROVING HAND-GRIP STRENGTH AND BLOOD PRESSURE IN ADULTS: RESULTS FROM AN AGINGPLUS PILOT STUDY

Abigail Nehrkorn-Bailey, Garrett Forsyth, Barry Braun, Kimberly Burke, and Manfred Diehl, *Colorado State University, Fort Collins, Colorado, United States*

Based on adult inactivity, a new intervention named AgingPLUS was created, targeting motivational barriers to physical activity. Data come from a pilot study ($N = 116$), with 56 participants randomized to the AgingPLUS group (Mage = 63.52 years, $SD = 7.89$ years), and 60 randomized to the active control group (Mage = 63.06 years, $SD = 8.30$ years). Multi-group linear growth curve analyses examined improvements in hand-grip strength and blood pressure from pretest (Week 0) to immediate (Week 4) and delayed posttest (Week 8). Findings showed that only participants in the AgingPLUS group had significant improvements in hand-grip strength for the right ($B = 1.34$, $p < .001$) and left hand ($B = 1.73$, $p < .001$), as well as significant reductions in systolic ($B = -3.28$, $p < .05$) and diastolic blood pressure ($B = -1.92$, $p < .01$). These findings provide support for the efficacy of AgingPLUS.

### EMOTION REGULATION TRAINING MAY IMPROVE STRESS, DEPRESSION, ANXIETY, AND PHYSICAL ACTIVITY

Kelly Wierenga,[1] David Fresco,[2] Megan Alder,[3] and Shirley Moore,[4] *1. Indiana University, Indianapolis, Indiana, United States, 2. University of Michigan, Ann Arbor, Michigan, United States, 3. Case Western Reserve University, Cleveland, Ohio, United States, 4. Case Western Reserve University, Shaker Heights, Ohio, United States*

The purpose of this two-arm randomized controlled pilot study was to assess initial efficacy of the theoretically-based

RENEwS intervention, designed to improve emotion regulation and thereby decrease depression and anxiety and increase moderate to vigorous physical activity (MVPA) following a cardiac event. Participants (n=30, 83% men) recruited from cardiac rehabilitation were randomized to five weekly 1-hour sessions of RENEwS intervention or active control. Although this trial was not powered for confirmatory efficacy (p's > .02, but many greater than .05), RENEwS participants evidenced an advantage over Control participants in terms of reductions in stress (Cohen's f = .47), depression symptoms (Cohen's f = .34), anxiety symptoms (Cohen's f = .40) but only modest improvements in MVPA from baseline to 5 months (Cohen's f = .08). Findings support potential efficacy and testing RENEwS in a larger sample.

### SESSION 5580 (SYMPOSIUM)

### EXPLORING LONGITUDINAL LINKS AND MECHANISMS CONNECTING VIEWS ON AGING WITH HEALTH IN LATER LIFE

Chair: Allyson Brothers
Co-Chair: Susanne Wurm
Discussant: Liat Ayalon

A robust body of evidence points to the importance of views on aging (VoA) in influencing developmental outcomes in later life. Now that this connection has been well-established, research has turned to examine mechanisms, processes, and pathways by which VoA and health are interconnected throughout adult development. The presentations in this symposium use longitudinal datasets to address the pressing question of how VoA and health are associated over time. Timescales range from 6-months post-hospital stay, to 9 year longitudinal follow-up. Presentations will provide evidence concerning both physical and mental health conditions and symptoms (e.g., Brothers et al; Schönstein et al.). Furthermore, the studies focus on various aging outcomes, ranging from functional ability (Brothers et al.) to recovery from hospital stays (Blawert et al.), to sensory loss (Wettstein et al.), to specific medical conditions including depression, back pain, rheumatism (Schönstein et al.). Three of the talks provide evidence for the effects of VoA on health, and one presents evidence of specific health conditions as longitudinal predictors of later VoA. Thus, an important question that this cohesive set of talks will raise is the issue of bi-directionality, by which VoA affect health, and vice versa. Concluding the symposium, the discussant will share insights about how these presentations contribute to the current understanding of mechanisms of the associations between VoA and health, and the role of VoA for ageism. She will additionally provide an overview of new areas for future research that can help to advance the understanding of these complex interactions.

### EXAMINING THE INTERPLAY OF GENERALIZED AND PERSONAL VIEWS ON AGING ON PHYSICAL AND MENTAL HEALTH ACROSS 2.5 YEARS

Allyson Brothers,[1] Anna Kornadt,[2] Abigail Nehrkorn-Bailey,[1] Hans-Werner Wahl,[3] and Manfred Diehl,[1]
*1. Colorado State University, Fort Collins, Colorado, United States, 2. University of Luxembourg, Esch-sur-Alzette, Diekirch, Luxembourg, 3. University of Heidelberg, Heidelberg, Baden-Wurttemberg, Germany*

It remains unknown how distinct types of views on aging (VoA) are related to one another, and to aging outcomes. We used a latent-variable structural equation model to test the hypothesis that generalized views on aging (assessed as Age Stereotypes (AS)) would influence personal views on aging (assessed as Self-Perceptions of Aging (SPA)), which in-turn would influence later physical and mental health. Data came from a longitudinal survey on VoA (N= 537, MageT1 = 64.13, age rangeT1 = 40-98). As expected, SPA mediated the effect of AS on physical (loss-SPA: $\beta$ = .23, p< .001; gain-SPA: $\beta$ = .06, p< .001; R2 = .62) and mental health (loss-SPA: $\beta$ = .13, p< .001; gain-SPA: $\beta$ = .03, p< .01, ; R2 = .31). Congruent with theoretical assumptions, our findings provide empirical support for a directional pathway by which generalized views on aging affect health outcomes via personal views of aging.

### THE ROLE OF SELF-PERCEPTIONS OF AGING FOR RECOVERY OF OLDER ADULTS AFTER A HOSPITAL STAY

Anne Blawert,[1] Ellen Freiberger,[2] and Susanne Wurm,[1]
*1. University Medicine Greifswald, Greifswald, Mecklenburg-Vorpommern, Germany, 2. Institute of Biomedicine of Aging, Friedrich-Alexander-University Erlangen-Nurnberg, Nurnberg, Bayern, Germany*

For older adults, a hospital stay can lead to loss of physical function and frailty. It is therefore important to investigate factors for recovery after hospitalization. Recent studies suggest negative self-perceptions of aging (SPA) as a potential risk factor in the context of serious health events. This ongoing longitudinal study investigates how negative SPA might contribute to worse physical recovery (assessed with the Short Physical Performance Battery) after hospital stay in a sample of 244 German adults aged 75 to 96. Preliminary mediation analysis based on available data of the first 50 participants indicate that negative SPA is related to increased fear of falling after 6 months, which predicts worse physical function one year after hospitalization (indirect effect: B = -0.70, SE = 0.41, p = .09). The results stress the importance of SPA for health recovery in old age and introduce fear of falling as a psychological pathway.

### THE ROLE OF SUBJECTIVE AGE VIEWS FOR 9-YEAR CHANGE IN SELF-REPORTED PROBLEMS WITH VISION AND HEARING

Markus Wettstein,[1] Hans-Werner Wahl,[2] and Svenja Spuling,[3] *1. German Centre of Gerontology, Berlin, Germany, 2. University of Heidelberg, Heidelberg, Baden-Wurttemberg, Germany, 3. German Centre of Gerontology, Berlin, Berlin, Germany*

We examined the role of subjective age views (subjective age; attitudes toward own aging [ATOA]; aging-related cognitions, comprising continuous growth, social loss, and physical decline) for changes in self-reported problems with vision and hearing over up to 9 years. A subsample of the German Ageing Survey (2,499 adults aged 60-85 years at baseline) was investigated. Controlling for gender, age, education, self-rated health, and region of residence (West vs. East Germany), a younger subjective age at baseline predicted less steep increase in vision problems among individuals who were chronologically older at baseline. More favorable ATOA scores were associated with less increase in hearing problems. Higher scores on continuous growth went along with less increase in hearing problems, whereas higher social loss scores were associated with a steeper increase in vision problems. Several associations increased with advancing age. Our findings suggest that subjective age views indeed predict late-life changes in sensory problems.

### ARE DIFFERENT DISEASES IN OLD AGE CONNECTED WITH DIFFERENT ATTITUDES TOWARD OWN AGING AND SUBJECTIVE AGE?

Anton Schönstein,[1] Hans-Werner Wahl,[1] Michael Denkinger,[2] Dhayana Dallmeier,[3] Dietrich Rothenbacher,[4] Jochen Klenk,[5] and Anke Bahrmann,[6] *1. University of Heidelberg, Heidelberg, Baden-Wurttemberg, Germany, 2. Agaplesion Bethesda Clinic, Ulm, Baden-Wurttemberg, Germany, 3. University of Ulm, AGAPLESION Bethesda Clinic, Geriatric Center Ulm/Alb-Donau, Ulm, Baden-Wurttemberg, Germany, 4. Ulm University, Ulm, Baden-Wurttemberg, Germany, 5. Institute of Epidemiology and Medical Biometry, Ulm University, Ulm, Baden-Wurttemberg, Germany, 6. Heidelberg University Hospital, Heidelberg, Baden-Wurttemberg, Germany*

Subjective views on aging (VoA; e.g., subjective age, attitude toward own aging "ATOA") are regarded as important biopsychosocial markers of aging but their antecedents are not entirely clear. Besides general risk factors (depression, cognition, activities of daily living), we compared multiple disease groups to establish connections between specific morbidities and risk for negative VoA. Data was drawn from the ActiFE-Ulm study for which a representative sample of community-dwelling older people (65-90 years) was recruited. Follow-ups were conducted 7.7 years (median) after recruitment (T2; N=526). Self-reported depression at T1 was the strongest general risk-factor for negative VoA at follow-up (both subjective age and ATOA). Back pain predicted negative ATOA, whereas rheumatism was associated to both negative ATOA and older subjective age. We conclude that diseases are differentially associated with VoA. Further, mental health problems such as depression seem to be of higher importance for VoA as compared to other factors.

### SESSION 5585 (SYMPOSIUM)

### FACTORS ASSOCIATED WITH AGING BIOMARKERS: FINDINGS FROM THE NATIONAL HEALTH AND AGING TRENDS STUDY

Chair: Laura Samuel
Co-Chair: Thomas KM Cudjoe
Discussant: Eileen Crimmins

Few studies have examined associations between socioeconomic, psychosocial and environmental characteristics with biological markers of aging among nationally representative older adult samples. This symposium will present results from four studies that examine the associations between 1) socioeconomic factors (i.e. financial strain and income to poverty ratio), 2) environmental characteristics (i.e. home disorder, street block disorder and community social cohesion) 3) social isolation (i.e. household size and social network),and 4) subjective well-being (i.e. positive affect, self-realization and personal mastery) as they relate to biomarkers of aging (hemoglobin A1c, IL-6, high-sensitivity CRP, and cytomegalovirus). Biomarker samples were obtained in 2017 via dried blood spots from 4,648 (88%) of the 5,265 self-responding participants of the National Health and Aging Trends Study (NHATS). NHATS is an ongoing study that conducts annual in-home interviews, which recruited a nationally representative cohort of Medicare beneficiaries aged 65+ residing in the contiguous United States in 2011 and replenished the sample in 2015. All analyses for the four studies presented in this symposium adjusted for demographic and socioeconomic characteristics and other potential confounders. Sampling weights were applied to account for study design and non-response so that inferences can be generalized to US adults aged ≥67 in 2017. Sessions of this symposium will highlight the socioeconomic, psychosocial and environmental characteristics that are associated with aging biomarkers. These results have clinical, policy and public health implications. These results can inform the development of interventions and policies aimed at improving biologic aging across the lifespan and reducing disparities in biologic aging.

### FINANCIAL RESOURCES AND BIOMARKERS OF AGING IN THE NATIONAL HEALTH AND AGING TRENDS STUDY

Laura Samuel,[1] Laken Roberts,[2] Danielle Boyce,[2] Melissa Hladek,[1] Sarah LaFave,[1] and Sarah Szanton,[1] *1. Johns Hopkins University, Baltimore, Maryland, United States, 2. Johns Hopkins University School of Nursing, Baltimore, Maryland, United States*

Lower income and financial strain (i.e. difficulty making ends meet) are associated with worse aging biomarkers, but evidence among nationally representative samples is limited. This cross-sectional study tested whether income to poverty ratio (analyzed separately for those <500% vs. ≥500% poverty threshold) and financial strain are associated with biomarkers of aging among NHATS participants aged ≥65 years (n=4,648), adjusting for age, race/ethnicity, gender, smoking, BMI, and diabetes diagnosis for hemoglobin A1c. Sampling weights were applied. Among those with incomes <500% poverty, higher income was associated with lower hemoglobin A1c (b= -0.0196, p=0.007), CMV (b= -0.0689, p<0.001) and CRP (b= -0.0428, p=0.012). Among those with incomes ≥500%, higher income was associated with lower IL-6 (b= -0.0001, p=0.023) and lower CMV (b= -0.0001, p<0.001). Financial strain was not associated with biomarkers. Income is more strongly associated with biomarkers among the lower income group, calling for special attention to this vulnerable population.

### GETTING UNDER THE SKIN: THE ASSOCIATION BETWEEN SOCIAL ISOLATION AND INFLAMMATORY MARKERS

Thomas KM Cudjoe,[1] carl latkin,[2] David Roth,[3] Roland Thorpe, Jr.,[3] and Cynthia Boyd,[1] *1. Johns Hopkins University School of Medicine, Baltimore, Maryland, United States, 2. Johns Hopkins Bloomberg School of Public Health, Baltimore, Maryland, United States, 3. Johns Hopkins University, Baltimore, Maryland, United States*

Social isolation is a risk factor for morbidity and mortality comparable to well-established risk factors including smoking, hypertension, and a sedentary lifestyle. Specific mechanisms that connect social isolation to important health outcomes remain unclear. We examine the cross-sectional relationship between social isolation and two biological markers: Interleukin-6 (IL-6) and C-Reactive Protein (CRP) in a nationally representative population of community dwelling older adults (IL-6: n=4336, CRP: n=4178) from the National Health Aging Trends Study in 2017. Adjusting for age, gender, race, income, tobacco use, body mass index, and multiple chronic conditions, we found that social isolation compared to no social isolation was associated with higher levels of IL-6 ($p = 0.043$) and CRP ($p = 0.038$). These results suggest that investigating inflammatory pathways between social isolation and morbidity and mortality is important.

### THE HOME, BLOCK, AND COMMUNITY ENVIRONMENTS AND BIOMARKERS OF AGING IN THE NATIONAL HEALTH AND AGING TRENDS STUDY

Laken Roberts,[1] Laura Samuel,[2] Danielle Boyce,[1] Melissa Hladek,[2] Sarah LaFave,[2] and Sarah Szanton,[2]
*1. Johns Hopkins University School of Nursing, Baltimore, Maryland, United States, 2. Johns Hopkins University, Baltimore, Maryland, United States*

Prior studies have linked household and community conditions to the health and functioning of older adults. However, few studies have investigated associations between household, block, and community environmental conditions with biomarkers of aging. This study used NHATS Round 7 (2017) data on 3,283 community-dwelling older adults to test cross-sectional associations between interior and exterior household disorder, block disorder, community social cohesion, and four biomarkers: C-reactive protein, hemoglobin A1c, cytomegalovirus, and interleukin-6. Survey-weighted models adjusted for age, sex, race/ethnicity, income, education, homeownership, housing type, and metropolitan area; HbA1c was stratified by diabetes diagnosis. Greater interior household disorder was associated with higher IL-6 ($\beta=0.06$, $SE=0.025$, $p=0.014$) and, among diabetics, greater block disorder was associated with higher HbA1c ($\beta=0.11$, $SE=0.05$, $p=0.046$). These results link home and block environmental characteristics with biomarkers of aging, suggesting that modifiable aspects of older adults' living environments may be related to disease and disability risk via physiologic dysregulation.

### GREATER SUBJECTIVE WELL-BEING ASSOCIATED WITH LOWER INFLAMMATORY PROTEINS IN AN OLDER ADULT SAMPLE FROM THE NHATS

Melissa Hladek,[1] Shang-En Chung,[1] Thomas KM Cudjoe,[2] Laura Samuel,[1] Sarah Szanton,[1] and David Roth,[1] *1. Johns Hopkins University, Baltimore, Maryland, United States, 2. Johns Hopkins University School of Medicine, Baltimore, Maryland, United States*

Subjective well-being (SWB), comprised of cognitive and affective evaluations of life, is associated with better health outcomes and lower mortality, but mechanisms are poorly understood. We examine the associations between SWB and its subscales with two biomarkers: Interleukin-6 (IL-6) and C-Reactive Protein (CRP), both common inflammatory indicators associated with mortality and increased cardiovascular disease. Dried blood spot data collected from 4,648 older adults NHATS participants in 2017 was used. After adjustment for age, sex, race/ethnicity, education, tobacco, body mass index and chronic disease, we found greater SWB and greater scores on subscales including positive affect, self-realization and personal mastery were all significantly associated with decreased IL-6 and CRP. Conversely, increases in negative affect was significantly associated with increased IL-6 and CRP values. This study adds evidence of a potential mechanistic mind-body connection pathway.

### SESSION 5590 (SYMPOSIUM)

### FACTORS THAT PROMOTE VULNERABILITY VERSUS RESILIENCE AMONG OLDER DISASTER SURVIVORS

Chair: Judith Robertson Phillips
Discussant: Rachel Pruchno

Catastrophic environmental events, such as floods and hurricanes, are associated with widespread destruction and loss of life. Older adults, who often have health challenges and medical co-morbidities, appear to be at greater risk for adverse post-disaster outcomes, such as depression, worry, and medical uncertainty, than younger adults impacted by the same disaster. Researchers, therefore, are interested in identifying factors that tend to bring about vulnerability and adverse outcomes for older adults during and after a disaster, as well as factors that may generate resilience and psychological well-being for older adults. The purpose of this symposium is to present research based on multiple disaster experiences and to examine factors associated with vulnerability and resilience. The first two speakers introduce empirical findings from research that addresses flooding, the 2016 Baton Rouge flooding and the frequent flooding of coastal Louisiana brought about by coastal erosion. The next two presenters explore successful VA and Non-VA Home-Based programs during and after the 2017 Hurricane Maria in Puerto Rico and during the 2017 Atlantic hurricane season. The final speaker highlights the impact of lifetime trauma on the recovery of older adults following a natural disaster. Collectively, these presenters will provide evidence of how lifetime adversity is a factor promoting vulnerability for older adults after a disaster. Critically, they will also examine how age, disaster preparedness, intense patient tracking, VA support networks, and community resources and programs are protective factors generating resilience post-disaster for older adult populations.

### DISASTER STRESSORS AND PSYCHOLOGICAL WELL-BEING AFTER A FLOOD

Katie E Cherry, Alyssa De Vito, Matthew Calamia, and Emily Elliott, *Louisiana State University, Baton Rouge, Louisiana, United States*

Hurricanes and floods have mental health consequences for younger and older adults alike. In August of 2016, historic flooding in Baton Rouge, Louisiana resulted in billions of dollars in damages. In this study, we compared 223 mostly middle-aged and older adults on mental health indicators. The majority of the sample ($n = 137$) were non-coastal residents and the remainder ($n = 86$) were former coastal residents who had permanently relocated inland after catastrophic losses in the 2005 Hurricanes Katrina and Rita. Multiple regressions confirmed elevations in symptoms of depression and post-traumatic stress for both adults with flood damage in 2016 and also those who doubly flooded in 2016 and 2005. Age had a protective effect for symptoms of depression and worry. Prior lifetime trauma was a risk factor for depression. Implications of these data for understanding age-related vulnerabilities after multiple disasters are discussed with suggestions to strengthen post-disaster resilience.

### AGING IN HIGH-RISK COASTAL REGIONS: EXAMINING SOCIAL INFRASTRUCTURE NEEDS OF OLDER ADULTS LIVING IN THE GULF COAST

Alexis Merdjanoff, *New York University, New York, New York, United States*

This study examines how coastal erosion, flooding susceptibility, and extreme hazard risk in Louisiana communities shape decisions to age in place. The decision to age in place is not only related to one's physical health and cognitive capabilities but strongly connected to neighborhood cohesion, sociocultural contexts, economic resources, familiarity with surroundings, and a sense of security. However, research on the types of individual and community resources that older adults need in order to successfully age in environmentally vulnerable communities is exceptionally sparse. Using in-depth interview data collected from older adults (n=20) living in coastal Louisiana parishes, this research aims: 1) to gain a deeper understanding of how coastal erosion and frequent flooding influence the decision to age in place; 2) to compile evidence as to how coastal communities can create resources that promote resilience, despite environmental risk; and 3) to use this evidence to increase awareness and enhance policy discussions on coastal adaptation.

### YOU CAN'T JUST HOPE FOR THE BEST: VA AND NON-VA HOME-BASED LONG-TERM CARE IN PUERTO RICO FOLLOWING HURRICANE MARIA

Leah Haverhals, *Department of Veterans Affairs, Denver, Colorado, United States*

This research describes how home-based long-term care settings in Puerto Rico, connected to the United States Department of Veterans Affairs (VA) and in non-VA settings, prepared for and secured the safety and wellbeing of elderly and disabled persons during and after Hurricane Maria, which struck Puerto Rico on September 20, 2017. I collected data via in-person interviews, home visits, and field observations between January-March 2019. Guided by a social vulnerability and health model, I interviewed a multitude of people connected to and/or caring for elderly and disabled populations in these settings. Results emphasize importance of disaster preparedness, incorporating lessons learned from hardships, and how Puerto Rico's colonial status and economic realities influenced recovery. VA's interconnected nature provided a stronger support network compared to non-VA settings that were often independently or family run. Regardless of setting, the resilience and collaborative spirit of Puerto Ricans proved instrumental in recovery and disaster management.

### PREPAREDNESS AND RESPONSE ACTIVITIES OF THE VA HOME-BASED PRIMARY CARE PROGRAM AROUND THE FALL 2017 HURRICANE SEASON

Tamar Wyte-Lake,[1] Claudia Der-Martirosian,[2] and Aram Dobalian,[2] *1. US Department of Veterans Affairs, North Hills, California, United States, 2. Veterans Emergency Management Evaluation Center, North Hills, California, United States*

Individuals aged seventy-five or older, who often present with multiple comorbidities and decreased functional status, typically prefer to age in their homes. Additionally, as in-home medical equipment evolves, more medically vulnerable individuals can receive care at home. Concomitantly, large-scale natural disasters disproportionally affect both the medically complex and the older old, two patient groups responsible for most medical surge after a disaster. To understand how to ameliorate this surge, we examined the activities of the nine US Department of Veterans Affairs Home-Based Primary Care programs during the 2017 Atlantic Hurricane Season. These and similar programs under Medicare connect the homebound to the healthcare community. Study findings support early implementation of preparedness procedures and intense post-Hurricane patient tracking as a means of limiting reductions in care and preventing significant disruptions to patient health. Engaging with home-based primary care programs during disasters is central to bolstering community resilience for these at-risk populations.

### TRAUMA AND ADVERSITY: FACTORS IMPACTING VULNERABILITY AND RESILIENCE AMONG OLDER DISASTER SURVIVORS

Molly Davis,[1] and Nikki Bellamy,[2] *1. George Mason University, Fairfax, Virginia, United States, 2. Center for Mental Health Services (CMHS), Substance Abuse and Mental Health Services Administration (SAMHSA), Rockville, Maryland, United States*

Most would agree that older adults represent a highly vulnerable group prior to, during and post disaster. Age-related vulnerabilities often lead into an increased risk for traumatic experiences and post-traumatic stress symptoms after a disaster. Trauma informed principles offer a possible way to reduce the vulnerability of older adults after a disaster. For example, utilizing the trauma informed question "what has happened to you" shifts the focus from a deficit approach and allows for a deeper understanding of the impact of traumatic life experiences on current functioning and reaction to the disaster. It is this understanding of trauma's impact that may have a role in how older adult disaster survivors view, respond, and recover after a natural disaster (Seery et.al. 2010; Iacoviello & Charney, 2014). In addition, understanding the role of lifetime adversity provides critical insights for disaster planning, reducing vulnerability and promoting resilience among older disaster survivors.

### SESSION 5595 (SYMPOSIUM)

### FINDINGS FROM THE VIRGINIA COGNITIVE AGING PROJECT: INDIVIDUAL DIFFERENCES, WELL-BEING, AND COGNITION

Chair: Karen Siedlecki

The Virginia Cognitive Aging Project (VCAP) is a cross-sectional and longitudinal study of cognitive functioning in a large sample of healthy community-dwelling adults between the ages of18-99 years (Salthouse,2009). Data are collected on several domains of cognitive functioning and subjective ratings of cognition, as well as a myriad of individual difference characteristics including self-reports of physical activity, cognitive activity, social support, personality, well-being, and affective measures. This symposium focuses on findings from VCAP that examine cross-sectional and longitudinal links between individual difference characteristics, indicators of well-being, and objective and subjective cognition. These topics include the cross-sectional assessment of >5,000 participants on the mediating role of Need for Cognition on the relationship between cognition and well-being (Yazdani & Siedlecki) and the relationship between social support and ratings of subjective cognition (Mueller & Minahan). Jung uses cross-lagged analyses to assess temporal relationships between physical and cognitive activity and cognition. Falzarano et al. present findings regarding the longitudinal relationship between subjective and objective measures of cognition. Finally, Minahan and Siedlecki present findings examining the temporal relationship between ratings of loneliness and depression over time. The symposium provides insights into the complex role of individual differences characteristics and cognitive functioning across the adult lifespan.

### NEED FOR COGNITION PARTIALLY MEDIATES THE RELATIONSHIP BETWEEN COGNITION AND SUBJECTIVE WELL-BEING

Neshat Yazdani,[1] and Karen Siedlecki,[2] *1. Fordham University, Bronx, New York, United States, 2. Fordham University, New York, New York, United States*

Aspects of cognitive functioning have been linked to measures of subjective well-being both cross-sectionally (Jones et al., 2003) and over time (Enkvist et al., 2013) but the mechanisms underlying this relationship remain unclear. One potential mechanism may be individuals' need for cognition, or the dispositional tendency to enjoy and engage in effortful cognitive activities (Cacioppo & Petty, 1982). Analyses were conducted to examine need for cognition as a mediator of the relationship between five domains of cognition (episodic memory, processing speed, reasoning, spatial visualization, and vocabulary) and four dimensions of subjective well-being (life satisfaction, positive affect, negative affect, and depressive symptomatology) cross-sectionally in a large sample of healthy adults between the ages of 18-99. Results indicate that need for cognition partially mediates the relationship between all five domains of cognition and life satisfaction, negative affect, and depressive symptomatology, but does not mediate the relationship between cognition and positive affect.

### THE IMPACT OF SOCIAL SUPPORT ON SUBJECTIVE COGNITION ACROSS ADULTHOOD

Annalee Mueller,[1] Jillian Minahan,[1] and Karen Siedlecki,[2] *1. Fordham University, Bronx, New York, United States, 2. Fordham University, New York, New York, United States*

Increased age is associated with declines in objective cognition (OC). A related but distinct construct is subjective cognition (SC), which is an individual's self-appraisal of their OC. Research shows that SC impairment is an important precursor to declines in OC (Sánchez-Benavidez et al., 2018). Research has also demonstrated a positive relationship between OC and social support (SS) across adulthood (La Fleur & Salthouse, 2017), but there is limited research on the relationship between SC and SS. Participants (N = 1,873; age range 18-99) from the Virginia Cognitive Aging Project completed assessments of multiple domains of SC, OC, and SS. Results from the current study showed a consistent, significant association between negative interactions with others and poorer SC (Betas ranged from -.077 to .103, $p < .05$), beyond the influence of sociodemographic, well-being, and health factors. Our findings suggest that negative interactions may adversely impact one's self-appraisal of cognitive functioning.

### TEMPORAL RELATIONSHIP BETWEEN ACTIVITY ENGAGEMENT AND COGNITION

Seojung Jung,[1] and Karen Siedlecki,[2] *1. SUNY College at Old Westbury, Old Westbury, New York, United States, 2. Fordham University, New York, New York, United States*

Previous studies have shown that activity engagement is related to cognitive function. However, few studies have examined the temporal order between activity engagement and various domains of cognition. Using data from the Virginia Cognitive Aging Project (baseline N =5430, Mage =51.28, SD =18.12), we examined the temporal relationships between engagement in physical and cognitive activity and different cognitive domains (reasoning, spatial visualization, episodic memory, processing speed, vocabulary) after controlling for age, education, self-rated health and depression. Cross-lagged panel analyses indicate that very few of the temporal relationships between activity level and cognition were significant except higher levels of cognitive activity significantly predicted better future processing speed, but not the reverse. Findings suggest the importance of engaging in cognitively stimulating activities, which help adults preserve processing speed over time. This study also highlights the importance of longitudinal design on various domains of cognition to help develop domain-specific interventions.

### INVESTIGATING THE LONGITUDINAL RELATIONSHIP BETWEEN SUBJECTIVE COGNITIVE COMPLAINTS AND OBJECTIVE COGNITION

Francesca Falzarano,[1] Karen Siedlecki,[2] and Jillian Minahan,[3] *1. Weill Cornell Medicine, New York, New York, United States, 2. Fordham University, New York, New York, United States, 3. Fordham University, Bronx, New York, United States*

Research examining the relationship between subjective cognitive complaints and objective cognitive performance has been mixed. Despite the lack of clear evidence demonstrating an association, subjective cognitive complaints are used as a criterion for the diagnosis of mild cognitive impairment and is considered a risk factor for Alzheimer's disease. Cross-lagged panel analyses were used in the current study to examine the longitudinal relationships between subjective cognitive complaints (using the Memory Functioning Questionnaire) and objective cognition (e.g., reasoning, memory, spatial visualization, processing speed, and vocabulary) in healthy adults over 60 from the Virginia Cognitive Aging Project (N=441). Results indicated that objective and subjective cognition were only weakly related but that objective cognition is a stronger predictor of subjective cognitive complaints then vice versa. Although subjective cognitive complaints may be an early indicator of pathological aging, results indicate that subjective cognitive complaints may not be a valid predictor of objective cognitive decline.

### A CROSS-LAGGED PANEL ANALYSIS OF THE RELATIONSHIP BETWEEN LONELINESS AND DEPRESSION

Jillian Minahan,[1] and Karen Siedlecki,[2] *1. Fordham University, Bronx, New York, United States, 2. Fordham University, New York, New York, United States*

Loneliness and depression have similar psychological features but are theoretically and statistically distinct (Hawkley et al., 2008). Most research has examined a unidirectional relationship between loneliness and depression (Cacioppo et al., 2006). Cacioppo et al. (2010), however, found that loneliness predicted subsequent depression, but depression did not predict subsequent loneliness. We extended this work by examining the reciprocal relationship between loneliness and depression in 1,560 healthy adults (ages ranging from 18-95 years at baseline) from the Virginia Cognitive Aging Project using cross-lagged panel analysis across three time points. Depression more strongly predicted subsequent loneliness ($\beta$s= 0.16 and 0.23, $p < .05$) than vice versa. These findings are consistent with the cognitive discrepancy theory, suggesting that affective processing, like depression, can negatively impact one's perception of their social environment (Burholt & Scharf, 2014). Our findings highlight the need for increased longitudinal research examining the relationships among indices of well-being across adulthood.

### SESSION 5600 (SYMPOSIUM)

### FORGOTTEN VARIABLES IN GERONTOLOGICAL EVALUATION OF RELIGIOSITY AND SPIRITUALITY

Chair: Alex Bishop
Discussant: Rosemary Blieszner

Despite the fact that religiosity and spirituality are commonly cited as essential elements of physical and mental health functioning in old and very old age, the conceptualization of religiosity and spirituality continues to present a quandary in contemporary gerontological inquiry and assessment. Identification of variables that best capture and truly define religious and spiritual constructs has remained relatively inconclusive over the past few decades. Recently, there has been a renewed interest among gerontologists to identify underlying religious and spiritual variables using alternative qualitative and quantitative methods of evaluation. Such methods have been used to further disentangle and understand the uniqueness of religion and spirituality as variables for studying human aging. This symposium will be used to communicate empirical results, methodological procedures, and conceptual insights concerning the identification of understudied and under-represented variables often absent within modern gerontological inquiry of religion and spirituality. Four presentations representing qualitative and quantitative studies will be used to report key religious and spiritual variables stemming from the context of personal narrative, oral storytelling, self-improvement behavior, and the disposition to seek forgiveness. Recommendations pertaining to future gerontological inquiry in the science of religion and spirituality, as well as applications within geriatric and gerontological practice will be highlighted and discussed.

### FORGOTTEN VARIABLES IN GERONTOLOGICAL EVALUATION OF RELIGIOSITY AND SPIRITUALITY

Nadia Firdausya,[1] Alex Bishop,[2] and Tanya Finchum,[3] *1. Oklahoma State University, stillwater, Oklahoma, United States, 2. Oklahoma State University, Stillwater, Oklahoma, United States, 3. Oklahoma Oral History Research Program; Oklahoma State University, Stillwater, Oklahoma, United States*

Data for this study originated from the Oklahoma 100 Life Project. The purpose of this investigation was to explore oral history storytelling surrounding centenarian religious practices in childhood. Thematic content analysis of oral narratives revealed three prominent themes surrounding the transmission, accessibility, and socialization of religion. Reflection upon the intergenerational transmission of religion, one centenarian recounted if her mother didn't go to church then, "the kids didn't go." Another centenarian noted the influence of rural opportunity structure stating, "Out there in the cotton patch, we didn't have no church." Finally, one centenarian participant highlighted value of social value of church during childhood by exclaiming, "We went to church all the time. . .we didn't know what it was to stay home from church." Findings will be used to further highlight how assessment of early life experiences is essential to gaining a fuller understanding of religiosity in human longevity.

### DESIRE FOR SPIRITUAL GROWTH: A NEGLECTED OUTCOME VARIABLE

Julie Hicks Patrick,[1] Alexandria Ebert,[1] and Amy Knepple Carney,[2] *1. West Virginia University, Morgantown, West Virginia, United States, 2. University of Wisconsin-Oshkosh, Oshkosh, Wisconsin, United States*

Social support facilitates reaching health-related goals, but has rarely been examined in relation to achieving religious/spiritual (R/S) goals. Using data from 300+ adults (M age = 40.3, range 18 to 87 yrs) , we examine the prevalence of R/S goals and the influence of age and social interactions on reaching these goals. Multinomial logistic regressions showed that adults who did not have a goal to be more religious/spiritual reported fewer positive interactions, fewer negative interactions, and were younger than those who continued to work toward their R/S goals. Those who had the intention to become more R/S but were not working toward it were younger than those who persisted. The importance of R/S goals are discussed within the context of other self-improvement goals. Unique aspects of these goals and the ways in which social interactions support achieving these goals are highlighted.

### FORGOTTEN VARIABLES AND INCARCERATED MALES

George Randall,[1] and Alex Bishop,[2] *1. Sam Houston State University, Huntsville, Texas, United States, 2. Oklahoma State University, Stillwater, Oklahoma, United States*

Religiosity tends to mitigate mental health challenges for incarcerated males. Further, negative life events experienced during childhood tend to exacerbate young adult challenges, resulting in incarceration. The current study, based on Koenig (2015) and the Developmental Adaptation Model (DAM; Martin & Martin, 2002), used self-report data from 261 older male inmates, ages 45-82 (M=57.59; SD =8.41) to test a path analytic model regressing religiosity (public religious attendance and private activities) on to known correlates and antecedents. Nested model testing found that the association between self-reported life events experienced as a child and later life religiosity was mediated by forgiveness of self and social provisions, controlling for age, race, and education (IE = -.014; p=.03, one-tail). Distal and proximal assessments influenced religiosity in this sample. Future research will want to explore assessing religiosity and possible therapeutic interventions relative to childhood difficulties and forgiveness of self for incarcerated males.

### EXPLORING SPIRITUALITY AS A FORGOTTEN VARIABLE IN THE CONTEXTS OF COMMUNITIES OF FAITH AND EDUCATION

Lydia Manning, *Concordia University Chicago, River Forest, Illinois, United States*

The purpose of this investigation was to describe the lived experience of aging in communities of faith, more specifically how older adults experienced social support. Semi-structured interviews were conducted with 15 older adults ranging in age from 63 to 84 years of age. Grounded Theory coding techniques were applied to identify themes. Findings include people feeling supported by community, connections, and beliefs, but also failed regarding opportunities for elder ministry or other types of worship. Participants expressed wanting more intentional opportunities for engagement. Many participants described the desire for an "expert" in their communities of faith. We consider the culture of support and how communities of faith may need to reconsider traditional ways of meeting the needs of people as they age. Furthermore, we explore the creation of educational tools that may allow for the emergence of an aging specialist in communities of faith.

### SESSION 5605 (SYMPOSIUM)

### GEROPSYCHOLOGY TRAINING INNOVATIONS: UNDERSTANDING AGEISM AND ADULTISM IN STUDENTS AND NEW TRAINING INITIATIVES

Chair: Katherine King
Co-Chair: Kirsten Graham

In order to address the geropsychology workforce shortage, educators are trying to better understand the attitudes, interests, and needs of prospective students as well as expand educational opportunities and specialty competence. This symposium reports on several recent and ongoing research projects and educational initiatives which can together inform future efforts to recruit students into the field. King et al. discuss how concerns about adultist oppression might keep young adults from entering the field. This study describes a new adultism measure which positively correlates with ageism and negatively correlates with both experience and interest in working with older adults. Together with Strong and Graham's study looking at ageism among North American and non-North American students, these studies remind us of the continued effort needed to better understand and combat age-related oppression of individuals at both ends of the lifespan. Two studies share specific educational initiatives addressing areas of essential but undervalues areas of training for geropsychologists. O'Malley and Graham report on the integration of health policy training into undergraduate education, while Jacobs et al. describing a new performance-based evaluation tool for training in the assessment of decision-making capacity. Postgraduate specialization continues to be crucial in establishing and growing the discipline of geropsychology. Mlinac and Smith report on the development of a mentorship program for professionals on the path to board certification.

### THE HIDDEN COST OF AGE OPPRESSION: ADULTISM, AGEISM, AND INTEREST IN SERVING OLDER ADULTS

Katherine King,[1] Kirsten Graham,[2] Briana Reid,[3] Molly Church,[3] and Juan Rosario, Jr.,[3] *1. William James College, Dedham, Massachusetts, United States, 2. Southern Utah University, Cedar City, Utah, United States, 3. William James College, Newton, Massachusetts, United States*

Adultism is an underappreciated influence on young adults' career choices and a hidden contributing factor to the geropsychology workforce shortage. This study reports on the development of an Adultist Concerns scale and its correlations with several factors relevant to careers in aging. Clinical psychology doctoral students (n = 109) completed the new scale along with measures of ageism, training interests, and experience working with older adults. The Adultist Concerns scale had strong internal consistency ($\alpha$ = .952) and factor loadings .853 and .929. Females scored significantly higher than males (p = .003). There were significant positive correlations between Adultist Concerns and both overall ageist behaviors (p = .002) and negative ageist behaviors specifically (p = .002). Adultist concerns were significantly negatively correlated with age (p = .000), interest in working with older adults (p = .003), and experience with this population (p = .043).

### AGEISM ACROSS CULTURES AND INTEREST IN GEROPSYCHOLOGY AMONG INTERNATIONAL STUDENTS

Jessica Strong,[1] and Kirsten Graham,[2] *1. University of Prince Edward Island, Charlottetown, Prince Edward Island, Canada, 2. Southern Utah University, Cedar City, Utah, United States*

Ageist attitudes are concerning when considering who will enter the geriatric workforce. The impact of ageism on intent to work with older adults (OAs) between North American and non-North American individuals is unclear. We collected data from N=186 students (n=153 N. American, n=33 non-N. American), examining ageist attitudes and intent to work with OAs. We found significant differences between groups in ageist attitudes; North American students had more positive views of aging (M=88.64, SE=0.72) than non-North American students (M=85.33, SE = 1.42; t (167) = 2.04, p = 0.04, d=0.39), but there were no differences between groups for intent to work with OAs (t (174) = 0.09, p = 0.93). Ageist attitudes predicted intent to work with OAs for North American students only ($F(2, 112) = 8.82$, $p < 0.001$, $R2 = 0.14$). We discuss implications of ageism and intent to work with OAs from a cross-cultural lens.

### CAPACITY ASSESSMENT TRAINING AND COMPETENCY EVALUATION TOOL

Lindsey Jacobs,[1] Patricia Bamonti,[1] Jessica Strong,[2] Kyle Page,[3] Barry Edelstein,[4] Rebecca Allen,[5] and Shane Bush,[6] *1. VA Boston Healthcare System, Brockton, Massachusetts, United States, 2. University of Prince Edward Island, Charlottetown, Prince Edward Island, Canada, 3. Edward Hines, Jr. VA Hospital, Hines, Illinois, United States, 4. WVU, Morgantown, West Virginia, United States, 5. Alabama Research Institute on Aging, Tuscaloosa, Alabama, United States, 6. University of Alabama, Tuscaloosa, Alabama, United States*

Given the complex interplay of ethical, clinical, and legal factors, evaluating capacities in older adults is an important competency for geropsychologists. However, the amount of quality of training in this area varies, and geropsychology trainees report less confidence in their capacity evaluation skills. To date, only the Pikes Peak Self-Assessment Tool includes items measuring competency and growth in decisional capacity evaluations. However, it is a broad self-report measure assessing general geropsychology competencies. We developed a performance-based measure of decision-making capacity evaluations, the "Capacity Assessment Training and Competency Evaluation Tool (CATCET)." Using the ABA/APA Assessment of Older Adults with Diminished Capacity as a guide, expert panels created two clinical cases across 5 capacity domains. This presentation will discuss the creation of the CATCET, its application as a training and evaluation tool, and initial performance data among psychology graduate students, intern, and fellows across settings.

### INTEGRATING HEALTH POLICY EDUCATION INTO AN UNDERGRADUATE ADULT DEVELOPMENT AND AGING PSYCHOLOGY COURSE

Kelly O Malley,[1] and Kirsten Graham,[2] *1. New England GRECC, VA Boston, Boston, Massachusetts, United States, 2. Southern Utah University, Cedar City, Utah, United States*

Active engagement in health policy by psychologists is vital to the well-being of the aging population; however, few feel prepared to engage in policy making or know how to get involved. A novel policy curriculum was developed and integrated into an undergraduate psychology course. N = 34 students completed assessments of policy knowledge and assignments designed to increase their skills, knowledge, and critical thinking about health policy. Students reported strong beliefs that psychological research can impact health policies and a desire to understand how to use research to inform policy; however, they reported less understanding of how policy is made. Preliminary evidence suggests students are interested in applying psychological research to policy processes; however, they do not know how to get involved. Policy education was easily integrated into the course, and further study is needed to determine students' future engagement in health policy and change health policy skills.

### GEROPSYCHOLOGY WORKFORCE DEVELOPMENT: MENTORING AND OUTREACH BY THE AMERICAN BOARD OF GEROPSYCHOLOGY

Michelle Mlinac,[1] and Heather Smith,[2] *1. VA Boston Healthcare System, Jamaica Plain, Massachusetts, United States, 2. Milwaukee VA Medical Center, Milwaukee, Wisconsin, United States*

To build workforce capacity and increase access to geropsychology services across the country, the American Board of Geropsychology (ABGERO) is engaged in efforts to promote competence in the specialty of Geropsychology. ABGERO developed a mentoring program to encourage psychologists to pursue board certification by demonstrating knowledge, skills, and abilities in delivering professional services to older adults. Mentors provide support around exam preparation, develop learning plans for psychologists new to the specialty, and help mentees consolidate their professional identities as geropsychologists. Candidates receiving mentorship include early career psychologists who completed geropsychology fellowships, mid-late career geropsychologists who seek board certification to be generative to the field, and psychologists looking to build expertise in geropsychology. For this latter group, clinical consultation groups were also created. Currently, 20 geropsychologists mentor 41 psychologists and 2 graduate students. Two geropsychologists have provided weekly consultation to 15 psychologists. Future implications for mentoring within geropsychology will be discussed.

### SESSION 5615 (SYMPOSIUM)

### HOUSING AND LIFE COURSE TRANSITIONS IN LATER LIFE: THE ROLE OF HOUSING, PLACE, AND SENSE OF HOME IN PERIODS OF UNCERTAINTY

Chair: Anna Wanka
Co-Chair: Steven Schmidt
Discussant: Richard Settersten

Housing is central factor for health and well-being in later life. Many countries have implemented ageing in place policies, but they tend to neglect the dynamic nature and heterogeneity of the ageing process. Housing needs change as people grow older, and experience different transitions across their life courses. Studies have demonstrated relationships between housing and health and wellbeing in later life on the one hand and life transitions and health and wellbeing in later life on the other hand. However, research on life transitions in combination with objective and perceived housing in relation to indicators of good ageing is scarce. Hence, the symposium aims to explore the dynamic relationship between housing and life transitions and how this relationship impacts health, well-being, functioning, and social/neighborhood participation along the process of ageing. First, Anna Wanka and Frank Oswald investigate how older adults' relationship to their home is interlinked with life-course transitions and social exclusion, presenting case studies from three countries. Maya Kylén explores the meaning of home and health dynamics throughout the retirement transition among the 'younger old' in Sweden. Kieran Walsh asks how 'sense of home' interrelates with risks entailed in the transitions of bereavement, dementia on-set and forced migration. Finally, Helen Barrie discusses the transition to homelessness based on the HILDA survey to identify the profile(s) of older people at risk of homelessness in Australia. Finally, Richard A. Settersten will discuss the four contributions.

### THE RELATIONSHIP BETWEEN HOUSING, LIFE-COURSE TRANSITIONS, AND OLD AGE SOCIAL EXCLUSION: A CROSS-COUNTRY ANALYSIS

Anna Wanka,[1] Urbaniak Anna,[2] Kieran Walsh,[3] and Frank Oswald,[4] *1. Goethe University Frankfurt am Main, Frankfurt am Main, Hessen, Germany, 2. University of Vienna, Vienna, Wien, Austria, 3. Irish Centre for Social Gerontology, National University of Ireland Galway, Galway, Galway, Ireland, 4. Interdisciplinary Ageing Research, Frankfurt, Hessen, Germany*

The international literature presents growing evidence of the impact of life transitions in older age on experiences of social exclusion, and place in general and as well as housing in particular potentially play a mediating role in this interrelation. However, the specific mechanisms through which the older adult place relationship mediates exclusionary outcomes of life-course transitions remains poorly understood in the study of ageing. This contribution investigates how older adults' relationship to their home is interlinked with life-course transitions and old-age social exclusion. To do so, we present case studies from three different countries (Germany, Ireland and Poland), focusing on the individual experiences of retirement and bereavement, and analyze them by drawing on the concepts of the person-environment exchange processes of agency and belonging. Finally, we draw conclusions about how spatial agency and belonging can protect and empower older people at critical junctures in their lives.

### TRANSITIONING INTO RETIREMENT AND THE MEANING OF HOME: A QUALITATIVE STUDY AMONG THE YOUNGER OLD IN SWEDEN

Maya Kylén,[1] Charlotte Löfqvist,[2] Maria Haak,[2] and Susanne Iwarsson,[2] *1. CASE Lund University, Lund, Skane Lan, Sweden, 2. Lund University, Lund, Skane Lan, Sweden*

Housing is the main spatial context for aging, important for well-being, a sense of identity and independence in daily life. Yet, as people grow older housing needs change and knowledge about how people reason about their future home when they enter retirement age is lacking. This qualitative study presents findings that explored meaning of home and health dynamics in the present and in a projected future among community-living people aged 67 – 70 years. Findings suggest that the home becomes progressively important after retirement. Not only the immediate home environment but also local neighborhoods influence perceptions about home. Home brings emotional and social benefits but also worries about how to cope with complex home ambivalence when reflecting upon future housing arrangements. The findings highlight the importance of considering perceived aspects of home and could be used to raise awareness among policymakers, housing authorities and professionals involved in housing-related counselling.

### HOUSING AND ENVIRONMENTAL UNCERTAINTY IN CONSTRUCTIONS OF EXCLUSION ARISING FROM CRITICAL LIFE-COURSE RUPTURES

Kieran Walsh,[1] anna Urbaniak,[1] and Bridin Carroll,[2] *1. Irish Centre for Social Gerontology, National University of Ireland Galway, Galway, Galway, Ireland, 2. NUI Galway, Galway, Galway, Ireland*

There is growing recognition that the older adult life course can involve critical transitions that function as significant sources of adversity, and ruptures in life trajectories. While knowledge about how these ruptures generate multidimensional disadvantage remains underdeveloped, less is known about how they are spatially constituted and how their processes and outcomes may be mediated by older peoples' relationship with place. Utilizing a 'sense of home' as a conceptual orientation, this paper explores the role of place in social exclusion arising from life-course ruptures. Focusing on bereavement, dementia on-set and forced migration, it draws data from 45 life-course interviews. Place (e.g. home environment and the wider community) was involved in three ways: as a component of the rupture; as a life domain where people experience exclusion; and as a mediator of exclusionary processes. Circularity is observed, with perceived environmental uncertainty intensifying effects of rupture-related exclusion, further contributing to that uncertainty.

### LIFE-COURSE TRANSITIONS TO PRECARIOUS HOUSING IN OLDER AGE

Helen Barrie,[1] Debbie Faulkner,[2] and Laurence Lester,[2] *1. University of South Australia, Adelaide, South Australia, Australia, 2. The Australian Alliance for Social Enterprise, University of South Australia, Adelaide, South Australia, Australia*

Home is central to health and wellbeing; yet the changing nature of work, household dynamics and especially housing markets, with scant policy attention and action around this, means low-middle income households are struggling in many countries. In Australia, while older people are considered to be at less risk because of higher levels of home ownership, there is a growing body of evidence about the living situations of older people who have not attained or retained home ownership over the life course and have limited wealth and savings moving into later life. This paper presents the findings of multivariate regression modelling using HILDA, a national longitudinal panel survey, to identify the profile(s) of older people at risk of homelessness in Australia. The data makes it clear a range of structural and individual factors across the life course are increasingly impacting on the ability to live a good life in older age.

### IMPACT OF AGE VIEWS ON COGNITION: EXPERIMENTAL, LONGITUDINAL, AND ECOLOGICAL MOMENTARY FINDINGS

Hans-Werner Wahl,[1] Becca Levy,[2] Brad Meisner,[3] Andrea Gröppel-Klein,[4] Deidre Robertson,[5] Serena Sabatini,[6] and Anna Lücke,[7] *1. University of Heidelberg, Heidelberg, Baden-Wurttemberg, Germany, 2. Yale School of Public Health, Woodbridge, Connecticut, United States, 3. York University, North York, Ontario, Canada, 4. University of Saarland, Saarbrücken, Saarland, Germany, 5. Economic and Social Research Institute, Dublin, Ireland, 6. University of Exeter, Exeter, England, United Kingdom, 7. Heidelberg University, Heidelberg, Baden-Wurttemberg, Germany*

Research on the impact of age views on cognition has seen a strong momentum in recent years, fitting the stereotype embodiment theory prediction that the stereotypes taken in from a culture can impact older persons' cognition. These studies utilize experimental, longitudinal, and ecological momentary assessments (EMA), as well as a wide reach of cognitive outcomes. This symposium starts with two experimental studies. One demonstrates that negative age stereotypes reduce cognitive processing in older consumers (Gröppel-Klein et al.). A second study strives to better understand the pathway by which age stereotypes influence cognitive outcomes by focusing on dysregulation of reward-seeking behaviors and the downregulation of the dopaminergic system (Robertson et al.). We next explore two longitudinal studies that reveal differential relations among views of aging and various cognitive indicators. The first study found that older persons with more positive age beliefs are less likely to develop dementia even in a high-risk gene subpopulation of older adults (Levy et al.). The second study examined the association between awareness of age-related changes and cognitive scores (Sabatini et al.) Finally, Lücke et al. examine in their EMA study with 6 measurement occasions per day across 7 days that such a fine-tuned seems not to clearly support a linkage among subjective age and working memory for which beginning but not consistent evidence has been reported previously. Brad Meisner will discuss contributions in the light of meta-analytic finding revealing that older persons' negative age stereotypes can impair whereas their positive age stereotypes can improve cognitive performance.

### IMPACT OF AGE STEREOTYPES ON OLDER ADULTS' COGNITIVE PERFORMANCE: AN EXPERIMENTAL VIEW ON AGING CONSUMERS

Andrea Gröppel-Klein, *University of Saarland, Saarbrücken, Saarland, Germany*

The "contamination hypothesis" (Rothermund and Brandtstädter, 2003; Levy, 2003) assumes that negative external stereotypes significantly influence the cognitive and functional well-being of older people. Negative stereotypes also play an important role in consumer decision-making and responses to sales talks. Two surveys in home environments, using a snowball-system, with subjects randomly assigned to the different conditions in a 2x2-design (age stereotype x time pressure, study 1: n=151, Mage=65, study 2: n=122, Mage=68) show that older consumers, primed with negative age stereotypes, are less effective in correctly evaluating the value-for-money-ratios of different offers), especially when they perceive time pressure (=moderator). Self-efficacy is identified as a significant mediator, explaining the impact of stereotypes on performance. Contamination via "talking down" to older consumers also occurs in sales talks. In a "vignette" experiment, patronizing (vs. neutral) communication impairs the cognitive performance (measured via recall of information) of older consumers (n=86, Mage=69,) and leads to dissatisfaction.

### LINKING NEGATIVE VIEWS OF AGING TO COGNITIVE FUNCTION: THE DOPAMINERGIC THEORY

Deidre Robertson,[1] and Arun Bokde,[2] *1. Economic and Social Research Institute, Dublin, Ireland, 2. Trinity College Dublin, Dublin, Dublin, Ireland*

Negative views on aging are known to impair cognition, in both the short and long term, but it is not fully understood why. This paper proposes that negative views impair cognition through dysregulation of reward-seeking behaviours and downregulation of the dopaminergic system. In a preliminary study to test this, 70 older adults were exposed to implicit positive and negative views on aging in a within-subject design carried out over 2 weeks, and then played a computerised game that involved deciding whether to expend effort to gain a reward. Over 216 trials, when participants were exposed to negative views, they were less likely to choose the effort-for-reward option. This effect was amplified at higher levels of effort. The paper will discuss how the dopaminergic theory ties into stereotype embodiment and other theories, and why it may explain previous findings that negative views of aging impair specific cognitive functions more than others.

### POSITIVE AGE BELIEFS LEAD TO LOWER DEMENTIA RISK EVEN AMONG OLDER PERSONS WITH HIGH-RISK GENE

Becca Levy,[1] Martin Slade,[2] Robert Pietrzak,[3] and Luigi Ferrucci,[4] *1. Yale School of Public Health, Woodbridge, Connecticut, United States, 2. Yale University, New Haven, Connecticut, United States, 3. Yale, New Haven, Connecticut, United States, 4. National Institute on Aging, Bethesda, Maryland, United States*

One of the strongest risk factors for dementia is the ε4 variant of the APOE gene. Yet, many who carry it never develop dementia. The current study examined whether positive age beliefs that are acquired from the culture may reduce the risk of developing dementia among older individuals, including those who are APOE ε4 carriers. The cohort consisted of 4,765 Health and Retirement Study participants who were aged 60 or older and dementia-free at baseline. As predicted, in the total sample those with positive age beliefs at baseline were significantly less likely to develop dementia, after adjusting for relevant covariates. Among those with APOE ε4, those with positive age beliefs were 49.8% less likely to develop dementia than those with negative age beliefs. The results of this study suggest that positive age beliefs, which are modifiable and have been found to reduce stress, can act as a protective factor, even for older individuals at high risk of dementia.

### HIGHER AWARENESS OF POSITIVE AND NEGATIVE AGE-RELATED CHANGES RELATE TO LOWER OBJECTIVE COGNITIVE SCORES

Serena Sabatini,[1] Obioha Ukoumunne,[1] Clive Ballard,[1] Kaarin Anstey,[2] Manfred Diehl,[3] Allyson Brothers,[3] Hans-Werner Wahl,[4] and Linda Clare,[1] *1. University of Exeter, Exeter, England, United Kingdom, 2. University of New South Wales, Sydney, Australia, 3. Colorado State University, Fort Collins, Colorado, United States, 4. University of Heidelberg, Heidelberg, Baden-Wurttemberg, Germany*

Existing evidence suggests that individuals' subjective experience of cognitive decline may be a risk state for dementia. However, whether self-awareness of positive changes confer cognitive protection is unknown. We examined the extent to which awareness of positive (AARC gains) and negative (AARC losses) age-related changes explains variability in objective cognitive performance in a sample of 6,231 UK residents (Mean age= 66.1 years, 75.9% women) without cognitive impairment. We tested a structural equation model with AARC gains and losses as predictors of cognitive performance and depressive symptoms as a mediator of the association of AARC losses with cognitive performance. The model fit the data well. The correlation between AARC gains and losses was negligible, yet higher levels of both AARC gains and losses predicted poorer cognitive scores. Hence, higher AARC gains did not confer cognitive protection. This unexpected pattern of results underscores the complexity of mapping individuals' awareness onto objective outcomes.

### EXAMINING THE RELATION AMONG SUBJECTIVE AGE AND WORKING MEMORY IN OLD AGE ON A HIGH-FREQUENCY BASIS ACROSS 7 DAYS

Anna Lücke,[1] Jelena Siebert,[1] Oliver Schilling,[1] Denis Gerstorf,[2] Ute Kunzmann,[3] Anna Kornadt,[4] David Weiss,[5] and Hans-Werner Wahl,[6] *1. Heidelberg University, Heidelberg, Baden-Wurttemberg, Germany, 2. Humboldt University Berlin, Berlin, Berlin, Germany, 3. University of Leipzig, Leipzig, Sachsen, Germany, 4. University of Luxembourg, Esch-sur-Alzette, Diekirch, Luxembourg, 5. Leipzig University, Leipzig, Sachsen, Germany, 6. University of Heidelberg, Heidelberg, Baden-Wurttemberg, Germany*

While increasing longitudinal evidence suggests that negative age views accelerate cognitive decline and increase dementia risk, we know little about such co-variance dynamics on a daily basis. We make use of subjective age and working memory performance data obtained six times a day over seven consecutive days as people went about their daily routines from 123 young-old (aged 66-69 years, 47.2% women) and 42 old-old (aged 86-90 years, 55.8% women) adults. Notably, multilevel models revealed considerably-sized short-term intra-individual variation of subjective age and working memory within days and these short-term within-day fluctuations in subjective age and working memory were coupled as expected. Hence, increased subjective age went along with lowered working memory confirming previous research. However, the respective between-day associations appeared reversed. Given this evidence of correlated short-term variability, we also discuss implications of different change dynamics that might explain moment-to-moment versus day-to-day associations between subjective age and working memory.

### SESSION 5625 (SYMPOSIUM)

### IMPROVING DETECTION OF COGNITIVE DECLINE AND IMPAIRMENT USING EVERYDAY BEHAVIORAL AND SOCIAL MARKERS

Chair: Ruixue Zhaoyang
Co-Chair: Eric Cerino
Discussant: Stacey Scott

Early detection of cognitive decline and mild cognitive impairment (MCI) during the pre-symptomatic phase of Alzheimer's disease is particularly important for maximizing effectiveness of clinical trials and efficiency of resource allocation. However, it is difficult to distinguish early signs of decline and impairment from normative aging, especially with biomarkers and clinical-based assessments that are expensive and challenging to apply widely. Ambulatory assessments in naturalistic settings provide opportunities to capture everyday markers of cognitive decline and offer cost-effective tools for sensitive, early detection of transitions to MCI in community-dwelling older adults. In this symposium, we present four studies that use ecological momentary assessment (EMA) data from the Einstein Aging Study to showcase how everyday markers of behavioral and social functioning assessed up to six times a day for 14 consecutive days can facilitate early detection of cognitive difficulties. Zhaoyang et al. examine whether older adults with intact cognition versus MCI differ in patterns of daily social interactions. Hyun et al. investigate how the diversity of daily activities is associated with ambulatory cognitive deficits. Cerino et al. compare the sensitivity of everyday markers of stress versus global trait-based stress measures to detect MCI. Roque et al. use completion time from EMA surveys as a reliable and unobtrusive way to measure cognition and distinguish those with and without MCI. Stacey Scott will integrate insights gained from these four papers, and discuss the opportunities and challenges faced when combining ambulatory assessments of everyday markers with traditional methods to better detect cognitive decline and impairment.

### THE ROLE OF DAILY SOCIAL INTERACTIONS IN DETECTING MILD COGNITIVE IMPAIRMENT

Ruixue Zhaoyang,[1] Stacey Scott,[2] Eric Cerino,[3] and Martin Sliwinski,[4] *1. The Pennsylvania State University, University Park, Pennsylvania, United States, 2. Stony Brook University, Stony Brook, New York, United States, 3. Pennsylvania State University, University Park, Pennsylvania, United States, 4. Penn State University, University Park, Pennsylvania, United States*

Social relationships play an important role in cognitive health and aging. However, it is unclear how older adult's cognitive function affects their everyday social interactions, especially for those with mild cognitive impairment (MCI). This study examined whether older adults with intact cognition vs. MCI differed in their daily social interactions. Community-dwelling older adults from the Einstein Aging Study (N=244, 70-91 yrs) reported their social interactions five times daily for 14 consecutive days using smartphones. Compared to those with normal cognitive function, older adults with MCI reported less frequent positive social interactions (p=0.012) and in-person social activities (p=0.006) on a daily basis. These two groups, however, did not show significant differences in their social relationships assessed by a conventional global questionnaire. The results support that, relative to global social relationships, daily social interactions are more sensitive, ecologically valid social markers that can facilitate the early detection of MCI.

### EVALUATING DAILY VERSUS GLOBAL STRESS APPRAISALS' SENSITIVITY TO MILD COGNITIVE IMPAIRMENT

Eric Cerino,[1] Stacey Scott,[2] Ruixue Zhaoyang,[3] Richard Lipton,[4] and Martin Sliwinski,[5] *1. Pennsylvania State University, University Park, Pennsylvania, United States, 2. Stony Brook University, Stony Brook, New York, United States, 3. The Pennsylvania State University, University Park, Pennsylvania, United States, 4. Albert Einstein College of Medicine, Bronx, New York,*

*United States, 5. Penn State University, University Park, Pennsylvania, United States*

Stress is an important correlate of cognitive aging that manifests in everyday life. Infrequent trait-based stress measures may not be as sensitive to mild cognitive impairment (MCI) as ecological momentary assessments (EMA). We compared EMA to global trait-based stress measures in discriminating MCI. A sample of 248 adults from the Einstein Aging Study (Mage=77.33 years, SD=5.04; 68 with MCI) were prompted to report whether a stressor occurred and to rate the severity up to four times daily for 14 days. Global perceived stress and neuroticism were assessed at baseline. Although MCI status was unrelated to stressor frequency (p>.05), individuals with MCI appraised their daily stressors as more severe than cognitively intact participants (p=.03). No MCI-related differences emerged on global stress or neuroticism assessments (ps>.05). Results suggest everyday stress markers may be more sensitive to differentiating MCI than global assessments and point toward their utility for early identification of pathological declines.

### QUESTIONNAIRE-BASED EVERYDAY REACTION TIME: RELIABLE, VALID, AND UNOBTRUSIVE MEASURE OF COGNITION

Nelson Roque,[1] Martin Sliwinski,[2] and Nilam Ram,[1] *1. Pennsylvania State University, University Park, Pennsylvania, United States, 2. Penn State University, University Park, Pennsylvania, United States*

Experience sampling paradigms provide new opportunity for early identification of mild cognitive impairment (MCI). We investigated two research questions: (1) is time to complete a repeatedly administered survey (i.e., questionnaire-based everyday reaction time, q*bert) a reliable and valid measure of cognition? (2) does this measure distinguish MCI status? To answer these questions, we leveraged ecological momentary assessment (EMA) data from the Einstein Aging Study, where older adults (N=240) completed six daily surveys and cognitive assessments on smartphones over 14 days. Q*bert had good between-person reliability after two days (~11 EMAs) and excellent reliability from three to fourteen days. Q*bert moderately correlated with ambulatory cognitive measures of processing speed and memory binding (p's < .001) and was significantly slower in those with MCI (p < .001). We propose q*bert as a reliable, valid, and unobtrusive measure of cognition when ambulatory cognitive assessments are not feasible.

### ACTIVITY DIVERSITY IN DETECTING AMBULATORY COGNITIVE DEFICITS

Jinshil Hyun,[1] Soomi Lee,[2] Mindy Katz,[1] Stacey Scott,[3] Martin Sliwinski,[4] and Richard Lipton,[1] *1. Albert Einstein College of Medicine, Bronx, New York, United States, 2. University of South Florida, Tampa, Florida, United States, 3. Stony Brook University, Stony Brook, New York, United States, 4. Penn State University, University Park, Pennsylvania, United States*

We examined whether participating in various daily activities (i.e., activity diversity) is associated with cognitive deficits measured by smartphone-based tests. Older adults (n=235; 70-91yrs) completed surveys 6 times/day for 14 days reporting on participation in 11 different activities (e.g., volunteering, physical activity), followed by brief ambulatory processing speed (PS) and working memory (WM) cognitive assessments. Activity diversity score was calculated using Shannon's (1948) entropy method. Individuals' average ambulatory PS and WM across all assessments were categorized into tertiles (i.e., high/mid/low performance). Results from multinomial logistic regression suggested that a 1SD increase in activity diversity was associated with increased odds of being in the high (better) vs. low performance tertile in PS (OR=2.1, 95%CI=[1.21, 3.51], p=.008). Activity diversity was not associated with WM. Given that cognitive deficits in PS occur earlier in the cognitive impairment process, activity diversity may be a sensitive marker for detecting very early stages of impairment.

### SESSION 5630 (SYMPOSIUM)

### INNOVATIVE APPROACHES TO INCLUSIVE DESIGN FOR TECHNOLOGY TO SUPPORT AGING WITH DISABILITY: EXAMPLES FROM TECHSAGE

Chair: Tracy Mitzner
Discussant: Anne Ordway

Technology research and development often exclude older adults with disabilities from participating in the design process. As a result, technologies may not be useful or usable by older adults with diverse abilities. This symposium, featuring projects at the TechSAge Rehabilitation Engineering Research Center, highlights ongoing efforts toward inclusive design, representing unique approaches to engage older adults with disabilities and their stakeholders in the research and development of technology supports. First, Mitzner et al., will describe the development of an online, group Tai Chi intervention, and the integral involvement of older adults with mobility disabilities, the exercise program developers, and technology partner in all steps of the process. Exploring the potential of voice-activated assistants, like Amazon Alexa, to support health management activities of older adults with mobility disabilities, Kadlyak et al. will present findings from a needs assessment of the target population and user testing in the lab and home environments. Koon et al. will present findings from a subject matter expert interview study with caregivers and medical professionals designed to identify the scope of activity challenges among people aging with long-term mobility and sensory disabilities that should be explored in more depth through our future interview study with the target population. Sanford et al., will describe a student design competition and hackathon that incorporates immersive experiences with people aging with disabilities to inspire innovative design concepts that respond to the needs of real people. NIDILRR Project Officer, Anne Ordway, will serve as the discussant.

### USING INCLUSIVE DESIGN TO DEVELOP A TELE–TAI CHI INTERVENTION FOR OLDER ADULTS WITH LONG-TERM MOBILITY DISABILITY

Tracy Mitzner,[1] Elena Remillard,[2] Kara Cohen,[2] and Jordan Chen,[1] *1. Georgia Institute of Technology, Atlanta, Georgia, United States, 2. Georgia Institute Of Technology, Atlanta, Georgia, United States*

Tele-technologies may be able to increase access to evidence-based exercise interventions for adults aging with long-term mobility disabilities. This population experiences substantial barriers in attending such programs in person, including lack of transportation to classes, inaccessible buildings where classes are held, and lack of appropriate modifications offered for this population of older adults. It is critical to overcome such barriers to ensure this population has an opportunity to receive the benefits of evidence-based programs. In this study we are translating an in-person evidence-based tai chi intervention, Tai Chi for Arthritis, to an online platform using videoconferencing software for those aging with long-term mobility disabilities. We will describe our approach of including users from the target population and industry representatives (videoconferencing software developer, Tai Chi for Arthritis program developer as well as local master trainer) in the adaptation of the intervention and present the key findings from doing so.

### DIGITAL HOME ASSISTANT HEALTH APPLICATIONS FOR OLDER ADULTS WITH LONG-TERM MOBILITY DISABILITIES

Travis Kadylak,[1] Roshanak Khaleghi,[1] Kenneth Blocker,[1] Saahithya Gowrishankar,[1] Widya Ramadhani,[2] Lyndsie Koon,[3] Ramavarapu Sreenivas,[1] and Wendy Rogers,[1], *1. University of Illinois at Urbana-Champaign, Champaign, Illinois, United States, 2. University of Illinois at Urbana-Champaign, Urbana, Illinois, United States, 3. University of Kansas, Lawrence, Kansas, United States*

The aim of the current study was to evaluate the feasibility, usability, safety, and efficacy of digital home assistant health applications (e.g., meditation applications, medication reminders, hydration management) for older adults with mobility disabilities. We used a multi-pronged approach. First, we compiled, categorized, and assessed a list of commercially available health applications compatible with Amazon Alexa devices. We reviewed data from the National Health and Aging Trends Study and the ACCESS study to identify challenges that older adults with mobility disabilities face within the home. We also reviewed the literature on the acceptance and use of digital home assistant health applications by older adults. Lastly, we conducted user testing in a laboratory and in a home-simulation environment to assess usability of different health applications. Our results provide guidance for the implementation of digital home assistant health applications to support older adults with long-term mobility disabilities.

### IDENTIFYING ACTIVITY SUPPORT NEEDS FOR INDIVIDUALS AGING WITH DISABILITY: SUBJECT MATTER EXPERT INTERVIEWS

Lyndsie Koon,[1] Megan Bayles,[2] Elena Remillard,[3] and Wendy Rogers,[4] *1. University of Kansas, Lawrence, Kansas, United States, 2. University of Illinois, Champaign, Illinois, United States, 3. Georgia Institute Of Technology, Atlanta, Georgia, United States, 4. University of Illinois at Urbana-Champaign, Champaign, Illinois, United States*

Technology designed to support aging-in-place for people with long-term disabilities begins with understanding the specific tasks that need support, and individual abilities, preferences, cultural practices, and privacy concerns. Such understanding is best achieved through a multi-method approach that includes direct, detailed assessments of representative users as well as individuals who work with or care for them. Our target users are people who identify as having a sensory or mobility impairment prior to the age of 50, including individuals aging with multiple sclerosis, late-onset hearing loss, and late-onset vision loss. In the present study, we are interviewing Subject Matter Experts (SMEs) to identify the scope of the challenges that should be explored in more depth. The SMEs include caregivers and medical professionals to identify challenges that the target populations experience in their everyday activities, advice about research adaptations, and recruitment ideas.

### CREATING INCLUSIVE DESIGN EXPERIENCES THROUGH ENGAGING SENIORS WITH DISABILITIES IN STUDENT HACKATHONS

Jon Sanford, *Georgia Tech, Atlanta, Georgia, United States*

Although voice-activated assistants (e.g., Amazon Alexa) have become smarter, faster, more personalized, and more ubiquitous, little is known about their potential to promote aging in place for people with disabilities. Partnering with Amazon's Alexa team, a 2-month long design competition and hackathon was conducted to inspire college students to develop innovative voice-activated solutions to support successful aging with disabilities. This presentation will cover the specific inclusive experiences used to immerse student teams in the daily lives of the target population to ensure that design solutions responded to real needs of real people in real environments. These included: lectures on current research findings about the everyday needs and challenges of the target users as well as universal design approaches to solving those problems; a survey of individuals currently using voice-activated assistants to understand their benefits and potential uses; and providing target with Alexa-enabled devices and embedding them into the hackathon teams.

### SESSION 5635 (SYMPOSIUM)

### INTERDISCLIPLINARY PERSPECTIVES ON AGING AT HOME ALONE WITH NEUROCOGNITIVE IMPAIRMENT

Chair: Laura Girling
Discussant: Patrick Doyle

Nearly 48 million individuals worldwide have a neurocognitive disorder with projections estimating that as many as 75 million may be afflicted by 2050.Although approximations vary, a substantial portion of those affected live in the community alone, accounting for up to one-third of cases. The true proportion of persons with neurocognitive disorders living alone in the community may be underestimated as dementias are often underdiagnosed and underreported. As the baby boom generation ages and trends towards nuclear families, geographic dispersion of families, and fewer children continue, the number of live-alone persons with neurocognitive impairment is anticipated to rise; creating increased potential for difficult, ambiguous circumstances involving the rights and needs of this population. Despite these trends, available information about this population remains limited. This symposium represents papers from social gerontology, bioethics, and policy; offering unique, but complimentary perspectives on live-alone persons with neurocognitive impairment. The four papers explore 1) how non-traditional & absent support networks impact one's ability to live alone with dementia [NIA funded], 2) social isolation and vulnerabilities of living alone with dementia [NIA-funded], 3) how bioethics can inform gerontological dementia research [NIA bioethics supplement], and 4) exploration of how law enforcement and adult protective services policies influence the precarity of living alone with dementia. Together, these papers illuminate the importance of actively including live-alone persons with dementia into research and assessing this overlooked vulnerable population from multiple research perspectives (social science, policy, bioethics).

### ADULTS WITH NEUROCOGNITIVE DISORDERS WHO RESIDE ALONE: EXPLORATION OF NONTRADITIONAL AND ABSENT SUPPORT SYSTEMS

Laura Girling,[1] and Kate de Medeiros,[2] *1. University of Maryland, Baltimore County, Baltimore, Maryland, United States, 2. Miami University, Oxford, Ohio, United States*

Research steadily demonstrates that family functions as the central component in the provision of care for persons with neurocognitive disorders. While it is clear family plays a critical role in the lives of adults with neurocognitive disorders, overlooked is the subpopulation who reside alone, but have no identifiable family to provide care. To address this gap, data were drawn from an interview-based NIA-funded study that focused on community-dwelling live-alone persons with dementia. Subanalyses were conducted on the interviews and field notes of live-alone adults with neurocognitive disorders who had no identifiable family (N=19) and their collaterals (e.g., neighbor, N=20). Using data-derived coding in ATLAS.ti., several themes emerged including transient informal care, consequential peripheral ties, and strained/traumatic nuclear relations. Themes will be discussed in detail. The present study expands the limited information on community-dwelling persons with dementia, providing a lens for understanding the complex intersection of aging and non-traditional/absent support networks.

### I'M VERY CAUTIOUS ABOUT WHO I LET INTO MY WORLD: SOCIAL VULNERABILITY FOR PEOPLE LIVING ALONE WITH DEMENTIA

Kate de Medeiros,[1] and Laura Girling,[2] *1. Miami University, Oxford, Ohio, United States, 2. University of Maryland, Baltimore County, Baltimore, Maryland, United States*

Living alone with Alzheimer's and related dementias (ADRD) can have many risks including social vulnerability that leads to loneliness. This paper reports findings from 9 people living alone with ADRD who completed in-depth, face-to-face interviews as part of a larger, NIA-sponsored study. Narrative data were analyzed using ATLAS. ti. Thematic findings revealed that although participants received supports (e.g., financial, meal preparation) from others, they lacked opportunities to participate in meaningful engagements with people of their choice (e.g., a friend who lives too far away, a son who is busy). In addition to loneliness resulting from lack of control over their social networks, many also reported that personal changes (e.g., difficulties eating) made them hesitant to seek social engagements. Overall, this paper underscores the need for social programs that extend beyond health-related outcomes and instead speak to subjective wellbeing and social connectivity for this population.

### ADULT PROTECTIVE SERVICES AND LAW ENFORCEMENT: DOES IT ADD OR SUBTRACT TO THE PRECARITY OF LIVING ALONE WITH DEMENTIA?

Michael Splaine, *Splaine Consulting, Columbia, Maryland, United States*

In 2014, more than 12.5 million people age 65+ lived alone in the U.S. Of these, approximately one third had a cognitive impairment. Although protective services may identify risks to such individuals, they may not have a full understanding of the notion of precarity, or the looming uncertainty regarding space and place, that solo dwellers experience. This presentation explores the tension between the intentions of protective services and the experience of precarity for persons living alone. More specifically, persons living alone with dementia participating in online groups and community events report feelings of risk of loss of autonomy and rights if their status becomes known. The presenter will review these impressions against current police and adult protective services policies and standard practices.

### WHAT THINKING LIKE A BIOETHICIST CAN BRING TO DEMENTIA RESEARCH

Nancy Berlinger,[1] Kate de Medeiros,[2] and Laura Girling,[3] *1. The Hastings Center, Garrison, New York, United States, 2. Miami University, Oxford, Ohio, United States, 3. University of Maryland, Baltimore County, Baltimore, Maryland, United States*

Bioethics is an interdisciplinary field that uses critical and empirical tools to explore and make recommendations concerning uncertainty about duties to others, including socially marginalized populations. In the context of social science or biomedical research involving people living alone with dementia, practical challenges in conducting research with capacity-impaired participants have ethical dimensions concerning informed consent and other aspects of research conduct. The underrepresentation in dementia research of the voices and perspectives of people living at home with dementia raises normative questions. Using data from a recent National Institute on Aging bioethics supplemental grant, this paper explores how thinking like a bioethicist can strengthen gerontological research. This paper examines areas such as precarity of housing, poverty and social interactions from a bioethicist's critical analysis/perspective and provides a framework for others to apply to their own research.

### ADULT DEVELOPMENT AND AGING IN HISTORICAL CONTEXT

Denis Gerstorf,[1] Johanna Drewelies,[2] Sandra Duezel,[3] Hans-Werner Wahl,[4] Corinna Löckenhoff,[5] Ilja Demuth,[6] and Nilam Ram,[7] *1. Humboldt University Berlin, Berlin, Berlin, Germany, 2. Humboldt University Berlin, Berlin, Germany, 3. Max Planck Institute for Human Development, Berlin, Berlin, Germany, 4. University of Heidelberg, Heidelberg, Baden-Wurttemberg, Germany,*

*5. Cornell University, Ithaca, New York, United States, 6. Charité – Universitätsmedizin Berlin, Berlin, Berlin, Germany, 7. Pennsylvania State University, University Park, Pennsylvania, United States*

Human development is shaped by socio-cultural contexts and the historical changes therein. Empirical reports suggest that old age has gotten "younger", both on behavioral measures and in people's perception. Here, we move one step further and shed light on key quality of life facets not yet well understood. We compare matched cohorts (each n = 250, Mage = 77) assessed 25 years apart in the Berlin Aging Studies (1990–93 vs. 2017–18). Extending the evidence to personality, older adults today are on average lower on neuroticism and higher on openness than their age peers in the past. Qualifying the picture, no evidence emerged for historical changes across four indices of perceptions of aging. Rounding out the picture, we also observed that older adults today perceive more time pressure than age peers 25 years ago. We discuss implications of this nuanced picture of historical changes among older adults.

### HISTORICAL CHANGES IN AGING TRAJECTORIES OF TWO ASPECTS OF SELF-PERCEPTIONS OF AGING

Oliver Huxhold,[1] Svenja Spuling,[1] and Susanne Wurm,[2] *1. German Centre of Gerontology, Berlin, Berlin, Germany, 2. University Medicine Greifswald, Greifswald, Mecklenburg-Vorpommern, Germany*

In recent years many studies have shown that adults with more positive self-perceptions of aging (SPA) increase their likelihood of aging healthily. Other studies have documented historical changes in individual resources and contextual conditions associated with aging. We explored how these historical changes are reflected in birth-cohort differences in aging trajectories of two aspects of SPA – viewing aging as ongoing development or as increasing physical losses. Using large-scale cohort-sequential data assessed across 21 years (N ≈ 19,000), the analyses modeled birth-cohort differences in aging trajectories of SPA from 40 to 85 years of age. The results illustrated differential birth-cohort differences: Later-born cohorts may experience more potential for ongoing development with advancing age than earlier-born cohorts. However, later-born cohorts seem to view their own aging as more negative than earlier-born cohorts during their early forties but may associate their aging less with physical losses after the age of fifty.

### IS LONELINESS AFTER WIDOWHOOD LESS PREVALENT IN RECENT TIMES THAN DECADES AGO?

Bianca Suanet,[1] and Theo van Tilburg,[2] *1. Vrije Universiteit Amsterdam, Amsterdam, Noord-Holland, Netherlands, 2. Vrije Universiteit, Amsterdam, Noord-Holland, Netherlands*

Due to increased longevity, widowhood occurs much later in life. When comparing over decades, we hypothesize that the increase in loneliness after losing the spouse at an early age is currently higher due to the lack of role models and knowledge of what helps in this situation, and that the recovery of loneliness after widowhood is faster because the widowed get more network attention in that exceptional situation. We analyze data from the Longitudinal Aging Study Amsterdam on 232 widowers and 468 widows with an average of four observations before and four after widowhood, spread over 21 years. The younger and recent widowed had a greater increase in loneliness, but also better recovery than the older widowed and those widowed years ago. The loneliness of widowed people has decreased regardless of age at the event, indicating the greater potential for bouncing back from this life event in today's society.

### SESSION 5650 (SYMPOSIUM)

### ISSUES RELATED TO LATE-LIFE SEXUALITY: SEX IN LONG-TERM CARE

Chair: Rachael Spalding
Discussant: Peter Lichtenberg

Despite surrounding social stigma and stereotypes of the "asexual older adult," older adults, including those residing in long-term care facilities, indicate that expressing their sexuality continues to be important to them (Doll, 2013). This presentation will feature presentations regarding recent research and perspectives relevant to late-life sexuality with a focus on how issues of sexual expression may particularly emerge in long-term care settings. Dr. Maggie Syme will present findings from mixed-methods, consumer-based approaches that elucidate how current and future long-term care residents view late-life sexuality, with a focus on the practical applications of these findings to inform facility administration and policies. Ethical and legal issues surrounding sexuality in long-term care will be discussed by Dr. Pamela Teaster, who will present ethical models that can translate into potential best-practice recommendations and strategies. Rachael Spalding will discuss the paucity of psychometrically sound assessment tools for measuring attitudes towards late-life sexuality and discuss their development of such a measure. Finally, Dr. Lilanta Bradley and Dr. Pamela Payne-Foster will present a framework for sexual agency in late-life and identify relevant gaps in the literature regarding gender, ethnicity/race, and geographical differences. Ultimately, this presentation will offer a forum for lively discussion among attendees regarding these pertinent topics.

### ATTITUDES TOWARD SEXUAL BEHAVIORS IN LONG-TERM CARE: DEVELOPMENT OF AN ASSESSMENT TOOL

Rachael Spalding,[1] Emma Katz,[1] and Barry Edelstein,[2] *1. West Virginia University, Morgantown, West Virginia, United States, 2. WVU, Morgantown, West Virginia, United States*

Most older adults living in long-term care settings (LTCs) indicate that expressing their sexuality is important to them (Doll, 2013). Little is known about the general public's attitudes towards sexual behaviors in LTCs. Attitudes of LTC residents' family members are particularly important, as family members are most likely to visit residents and to care about their quality of life. Family members' attitudes could in turn inform facility policies and management. We will present preliminary data from a series of qualitative interviews with community-dwelling adults regarding their attitudes. We will discuss how these data are being used to inform current work on a measure of attitudes toward sexual behavior in LTCs.

### USING ETHICS AND LAW TO ADDRESS SEXUALITY IN LONG-TERM CARE

Pamela Teaster, *Virginia Tech, BLACKSBURG, Virginia, United States*

Issues concerning the sexuality of older adults in long-term care have long presented an array of thorny conundrums for long-term care staff and administrators, for friends and family members, and, most importantly, for the residents themselves. At any one time, more than two million older adults reside in nursing homes or assisted living facilities, and the predominant trope of people who are helpless, demented, and asexual is at once inaccurate and unfair. Moreover, the important need for sexual intimacy remains a life-long, even when persons have worsening dementia and chronic illness. This presentation uses bona fide, de-identified case examples as a starting point for consideration. Drawing upon the extant literature, as well as best practices, this presentation applies ethical principalism (i.e., autonomy, beneficence, nonmaleficence, justice) as well as current law to recommend practices and approaches to sexuality in long-term care.

### A RESIDENT-CENTERED VIEW OF SEXUAL EXPRESSION IN LONG-TERM CARE

Maggie Syme,[1] Tracy Cohn,[2] and Allyson Graf,[3] *1. Kansas State University, Manhattan, Kansas, United States, 2. Radford University, Radford, Virginia, United States, 3. Northern Kentucky University, Highland Heights, Kentucky, United States*

Contrary to societal beliefs, older adults residing in nursing homes continue to have sexual interests. However, the right and need to engage in intimate relationships are often ignored in nursing homes, erring on the side of safety and prohibiting intimacy. This is due to ageist sexual stigma, under-educated providers, complexities of care, and fear of retaliation. Nursing homes are not attending to resident preference/choice when it comes to sexuality and intimacy, which ignores the consumer of services and is wholly inconsistent with person-centered care. However, in order to craft resident-centered/informed policies and practices, we must understand the needs and preferences of the resident/consumer. The following presentation will highlight findings from three separate mixed-methods studies that address the consumer, or how now and future residents understand and evaluate intimacy and sexuality in long-term care. We will address potential challenges, areas for intervention, and a unique consumer-oriented solution— sexual advance directives.

### AN EXPLORATION OF SEXUAL AGENCY IN THE AGING POPULATION

Lilanta Bradley, *University of Alabama, Tuscaloosa, Alabama, United States*

There are some unique social and environmental challenges to geriatric sexual well-being. This presentation explores sexual agency in the aging population with a special emphasis on barriers and facilitators of sexual agency in long-term care settings. We will situate late-life sexuality literature with Cense's (2019) theory of sexual agency. Her four components of moral agency, embodied agency, narrative agency, and bonded agency will be used to examine the older person's perceptions, beliefs, and behaviors associated with sexual autonomy from a macro and micro lens. This framework uses assertions that agency is built on self-concepts from the individual and relational level; reflects power dynamics and social inequalities; and requires strategic negotiation of social norms, policies, and relationships to shape one's autonomy and sexual self perceptions. We will also discuss gaps in the literature regarding gender, race/ethnicity, and geographical differences that can impact an older person's sexual agency and overall sexual well-being.

### SESSION 5655 (SYMPOSIUM)

### KINLESSNESS AND SOCIAL ISOLATION AT THE END OF LIFE: CROSS-NATIONAL COMPARISONS

Chair: Christine Mair
Co-Chair: Katherine Ornstein
Discussant: Feinian Chen

Nearly all older adults cross-nationally rely on close family members to facilitate care at the end-of-life, but recent demographic shifts toward declining fertility and marriage rates have yielded an unprecedented increase in older adults who lack traditional family ties ("kinless"). "Kinless" older adults may be at risk for social isolation, lack of caregiving options, and poorer end-of-life outcomes, and these risks may be buffered or exacerbated by social and economic resources as well as country context. Because the potential impact of "kinlessness" on end-of-life outcomes cross-nationally is unknown and virtually unstudied, this symposium composed of overlapping, interdisciplinary research teams investigate if and how "kinlessness" is associated with end-of-life outcomes cross-nationally. Mudrazija and Ornstein analyze a sample of older European adults (SHARE) to predict location of death (e.g., home deaths) by family size, living alone, and size of social network. Plick, Ankuda, and Ornstein examine a sample of older American decedents (HRS) to predict end-of-life outcomes (e.g., hours of care received, location of death). Mair, Ornstein, Aldridge, and Thygesen explore associations between family structure and multiple end-of-life outcomes (e.g., hospitalizations, ICU visits, medical treatment) among the population of decedents aged 50+ from Denmark. Finally, Mair, Ornstein, Calvo, Donoso, and Medina explore family structure, social isolation, loneliness, and end-of-life outcomes among a sample of older adults from 20 countries (Gateway to Global Aging). These papers will be discussed by Feinian Chen, a family sociologist and social demographer who specializes in cross-national comparisons of older adults' family structures, country context, and health.

### KINLESSNESS AT DEATH: EXAMINING END-OF-LIFE OUTCOMES WITH DANISH REGISTRY DATA

Christine Mair,[1] Katherine Ornstein,[2] Melissa Aldridge,[2] and Lau Thygesen,[3] *1. University of Maryland, Baltimore County, Baltimore, Maryland, United States, 2. Icahn School of Medicine at Mount Sinai, New York, New York, United States, 3. University of Southern Denmark, Copenhagen, Hovedstaden, Denmark*

Demographic changes that lead to "kinlessness" such as low fertility and low marriage rates are not a recent phenomenon for the countries of Northern Europe, such as Denmark. Characterized by small family sizes, high individualism, and a highly formalized healthcare system that is less dependent on family caregivers, Denmark presents a useful case study for the analysis of end-of-life outcomes among the "kinless." We analyze the population of decedents aged 50 and older (N=175,755) using Danish civil registry data. Approximately 15% of those who died in Denmark had no living partner and no living child. Danish decedents' family structures are associated with multiple end-of-life outcomes, including number of hospitalizations, ICU visits, and use of specific medical treatments—but not always in the direction hypothesized. Denmark's highly formalized and individualized healthcare system may offer insight regarding healthcare reform in countries that have yet to complete the second demographic transition.

### KINLESSNESS AT THE END OF LIFE IN THE UNITED STATES: IMPLICATIONS FOR PLACE OF DEATH, AND QUALITY OF LIFE AMONG OLDER ADULTS

Katherine Ornstein, Natalie Plick, and Claire Ankuda,
*Icahn School of Medicine at Mount Sinai, New York, New York, United States*

We used the Health and Retirement Study, a large nationally representative study of U.S. older adults from 2002-2015, to identify decedents and assess quality of EOL care by availability of kin. 7.9% of participants were kinless at EOL (no adult children or spouses), reflecting national estimates of 1,027,600 older adults. Those who were kinless at EOL were more likely to be female (61.2% vs 51.5%), from the lowest wealth quartile (53.6% vs 35.6%), and less likely to be white and non-Hispanic (75.6% vs 81.8%). Among the community-dwelling population, individuals with kin received 2.4 times as much hours of help from informal caregivers per month, compared to those without kin. We did not observe differences in rates of hospital death by kin status in adjusted models. More work is needed to assess any unmet needs in the EOL period for kinless older adults, especially as healthcare moves towards increased in-home supports.

### SOCIAL NETWORKS AND DYING AT HOME: ANALYSIS OF OUTCOMES FROM 13 COUNTRIES USING SHARE

Stipica Mudrazija,[1] and Katherine Ornstein,[2]
*1. Urban Institute, Washington, District of Columbia, United States, 2. Icahn School of Medicine at Mount Sinai, New York, New York, United States*

Social networks are critical for end-of-life decision-making and hands on support and may also impact end-of-life outcomes including location of death. Yet we fail to consider these factors in the context of cultural values and variation in healthcare system financing and resources, e.g., availability of palliative care. Using SHARE, we examined the following factors for 6,391 decedents across 13 countries interviewed in the last year of life: family size, living alone, and size and characteristics of social networks. We compared these characteristics cross-nationally for persons dying at home as opposed to in hospital and other formal settings. While individuals with larger social networks are more likely to die at home, we find a cross-national gradient of higher unmet healthcare needs resulting in overall more home-based deaths. Our findings suggest that individual factors such as family availability must be considered in the context of country-level factors when examining quality end-of-life indicators.

### KINLESSNESS, LONELINESS, AND END OF LIFE: A CROSS-NATIONAL COMPARISON OF 20 COUNTRIES

Esteban Calvo,[1] Christine Mair,[2] Katherine Ornstein,[3] Rosario Donoso,[1] and José Medina,[1]
*1. Universidad Mayor, Santiago, Region Metropolitana, Chile, 2. University of Maryland, Baltimore County, Baltimore, Maryland, United States, 3. Icahn School of Medicine at Mount Sinai, New York, New York, United States*

Countries across the globe are experiencing declining rates of fertility and marriage, which present a distinct challenge for older adults' social integration, well-being, and end-of-life care. However, older adults who are "alone" (e.g., no partner, no child) may not be lonely, and end-of-life risks faced by "kinless" older adults likely vary significantly by country context. Using harmonized, cross-national data from 20 countries (United States (HRS), England (ELSA), and European Union (SHARE)), we examine associations between family structure, loneliness, and end-of-life outcomes. Although "kinless" family structures are associated with greater loneliness in the pooled sample, the percent of "kinless" who report no signs of loneliness ranges from 7% (Greece) to 56% (Denmark). Family structure is associated with various end-of-life outcomes, and these associations vary by country—likely reflecting differences in healthcare structure. We discuss distinctions between "being alone," "being lonely," and "being without care" in light of cross-national variation.

### SESSION 5665 (SYMPOSIUM)

### MARITAL EFFECTS IN LATER LIFE: DYADIC APPROACHES AND GENDER DIFFERENCES

Chair: Jeffrey Stokes
Discussant: Deborah Carr

Marriage is a dyadic system, within which the characteristics and experiences of each partner can have implications for both. Moreover, gender of both spouses may impact these dyadic influences. The five papers comprising this symposium all take a dyadic approach to studying midlife and older couples, and how their effects on one another may vary by gender. Donnelly examines the consequences of precarious work among midlife couples, finding heightened risks for marital strain and divorce, depending on which gender spouse is exposed to precarious work. Garcia also analyzes gender differences – in this case, how the gender of a woman's spouse may affect associations between daily marital strain and sleep quality, with only women married to men showing adverse sleep outcomes. Polenick and colleagues study the long-term repercussions of chronic condition discordance, finding that both individual-level and couple-level discordance had impacts for husbands' and wives' physical activity. Gallagher and Stokes focus on cognitive functioning within dyads, revealing gendered effects: Wives' poorer cognitive functioning was associated with their own (better) marital quality, while husbands' poorer cognitive functioning was associated with wives' (worse) marital quality. Lastly, Stokes and Barooah examine longitudinal dyadic associations between loneliness and vascular health, finding that own and partner's baseline loneliness were associated with increased HbA1c levels only in the context of inferior marital support. Carr will assess the strengths and limitations of these papers, and discuss the contributions these studies can make to the field and to future research on marital effects and gender in later life.

### LONELINESS, MARITAL QUALITY, AND VASCULAR HEALTH AMONG OLDER U.S. COUPLES: A LONGITUDINAL DYADIC STUDY

Jeffrey Stokes, and Adrita Barooah, *University of Massachusetts Boston, Boston, Massachusetts, United States*

Loneliness is a contributor to later life declines in health, including vascular health. Importantly, loneliness is not restricted to those who lack close social ties: More than one-third of married U.S. older adults experience loneliness, and having a lonely spouse increases the likelihood of experiencing loneliness oneself. Thus, over time loneliness in either spouse may lead to worse health for both spouses. Using longitudinal dyadic data from the Health and Retirement Study (2008-2014), we estimated multilevel lagged dependent variable models to examine implications of both partners' loneliness at baseline for each spouse's HbA1c four years later. Findings revealed that effects of both partners' loneliness were contingent upon marital quality: Own and partner's loneliness led to increases in HbA1c when perceived marital support was low, but this was attenuated at higher levels of marital support. These results extend prior research concerning loneliness and vascular health, and loneliness as a relational experience.

### COGNITIVE FUNCTIONING, GENDER, AND MARITAL QUALITY AMONG OLDER MARRIED COUPLES: A DYADIC APPROACH

Elizabeth Gallagher,[1] and Jeffrey Stokes,[2] *1. University of Massachusetts Boston, Kingston, Massachusetts, United States, 2. University of Massachusetts Boston, Boston, Massachusetts, United States*

Older spouses influence one another in myriad ways, and dyadic effects of marital quality on health and well-being have been well-established. However, little attention has been paid to dyadic implications of cognitive functioning, including for spouses' perceptions of the relationship itself. This study examines associations of older husbands' and wives' cognitive functioning with both partners' reports of four marital quality outcomes. Structural equation modeling analyzed data from 1,414 opposite-sex couples drawn from the 2016 wave of the Health and Retirement Study. Findings revealed that (a) wives' poorer cognitive functioning was associated with wives' reporting greater closeness and higher ratings of enjoying time with a spouse, whereas (b) husbands' poorer cognitive functioning was associated with wives' reporting greater marital strain, lower marital support, lower closeness, and lower ratings of enjoying time with a spouse. This suggests that cognitive functioning/impairment has dyadic consequences for marital quality, which are highly gendered.

### PRECARIOUS WORK, MARITAL QUALITY, AND DIVORCE: A GENDERED DYADIC ANALYSIS OF AGING COUPLES

Rachel Donnelly, *Vanderbilt University, Nashville, Tennessee, United States*

Precarious work – work that is unstable and insecure – is often stressful and may contribute to marital strain and dissolution among midlife adults. However, prior research has not considered how precarious work spills over to spouses. Using longitudinal dyadic data of midlife couples from the Health and Retirement Study, I examine whether having a spouse in precarious work is associated with marital strain and dissolution, with attention to differences by gender. I find that indicators of precarious work (job insecurity, schedule variability) are associated with a heightened risk of marital strain and divorce in midlife. These patterns depend on the gender of the spouse experiencing precarious work. Understanding the implications of precarious work for marriage is important because poor marital quality and divorce hasten health declines at older ages. Thus, this study suggests that precarious work may be a risk factor for divorce and poor health among aging adults.

### CHRONIC CONDITION DISCORDANCE AND PHYSICAL ACTIVITY: LONGITUDINAL ASSOCIATIONS AMONG OLDER COUPLES

Courtney Polenick,[1] Kira Birditt,[1] Angela Turkelson,[1] and Helen Kales,[2] *1. University of Michigan, Ann Arbor, Michigan, United States, 2. UC Davis, Sacramento, California, United States*

Chronic condition discordance (i.e., the extent that two or more conditions have non-overlapping self-management requirements) is detrimental for functional health but little is known about mechanisms accounting for these associations. We examined links between chronic condition discordance at both the individual level and the couple level (i.e., between spouses) and physical activity over time. Participants included 1,095 couples from five waves (2006-2014) of the Health and Retirement Study. Dyadic growth curve models showed that greater individual-level discordance was associated with lower baseline physical activity among individuals and their partners. When husbands had greater individual-level discordance, wives and husbands had faster declines in physical activity. The findings highlight the importance of considering both members of a couple when examining the implications of chronic illness for physical activity in middle and later life.

### DAILY MARITAL STRAIN AND SLEEP IN SAME-SEX AND DIFFERENT-SEX COUPLES

Michael Garcia, *University of Texas at Austin, Austin, Texas, United States*

Marital strain has consistently been linked to many indicators of daily health and well-being, including sleep. Prior studies show that, on days when marital strain is higher, women in different-sex couples experience poorer sleep outcomes. However, this work has not yet considered whether and how these relationships differ for men and women in same-sex couples. Using 10 days of dyadic diary data from 756 midlife U.S. men and women in 378 gay, lesbian, and heterosexual marriages, we examine the associations of daily marital strain with sleep quality and duration and consider whether these relationships differ across union type. Results suggest that increased marital strain is associated with poorer sleep quality and shorter sleep duration, but only for women married to men. These findings underscore the importance of including same-sex couples when exploring linkages between marital dynamics and health, especially when considering how gender impacts these processes.

### SESSION 5670 (SYMPOSIUM)

### MEMORIES OF LOST LOVED ONES

Chair: Emily Mroz
Co-Chair: Susan Bluck
Co-Chair: Deborah Carr

The death of a loved one is a challenging but also normative occurrence in later life (e.g., Thomson et al, 2018). Experiencing the death of others typically increases with age, so personal reaction to loss becomes an ongoing process (Harrop et al., 2016). When adults lose someone, the deceased person is often 'gone but not forgotten.' That is, they are remembered over time (Klass & Steffen, 2017). The way one remembers their lost loved one's life and their death (e.g., Mroz et al., 2019) may influence post-loss emotional adjustment and personal views. This symposium brings together Psychology and Sociology researchers with data from Germany, the US, and China whose work elucidates the complex relation between loss and memory: we identify how remembering lost loved ones relates to both adaptive and difficult outcomes. In this symposium, Wolf et al. examine beneficial and harmful ways of using autobiographical memories after a personal loss. Mroz and Bluck identify how grief responses in older adult widows lead to functional use of memories from the very end of the spouse's life. Fu and Idler focus on the directive function of autobiographical memory, examining how memory for end-of-life experience with loved ones influences current choices for aggressive end-of-life care. Bolkan and Weaver examine how early life experiences with loss influence later personal views and advance care planning. Our Discussant, Debby Carr, integrates these talks to elucidate how remembering loss experiences relate to not only current grief, but also to people's preparations for the future.

### MALADAPTIVE USE OF AUTOBIOGRAPHICAL MEMORY BY BEREAVED INDIVIDUALS ACROSS ADULTHOOD

Tabea Wolf,[1] Veronika Strack,[2] and Susan Bluck,[3] *1. Ulm University, Ulm, Baden-Wurttemberg, Germany, 2. Ulm University Hospital, Ulm, Baden-Wurttemberg, Germany, 3. University of Florida, Gainesville, Florida, United States*

Remembering one's personal past can serve adaptive psychosocial functions (Bluck, Alea, & Demiray, 2010). Autobiographical remembering has been related to well-being in older age but little research has focused on grief. We address this issue in two studies grounded in the model of reminiscence and health in older adulthood (Cappeliez & O'Rourke, 2006). Participants (aged 18 - 91) completed the Reminiscence Functions Scale and the Inventory of Complicated Grief. Regression analyses show that negative self-related use of memories, but not positive use, is associated with experiencing more grief. Sharing memories with others (pro-social function) is indirectly linked to grief, as mediated by negative self-related uses. These patterns held for autobiographical recall in general (Study 1; N = 51) and when specifically remembering the deceased person (Study 2; N = 49). How adaptively individuals remember their personal past appears linked to the experience of grief, sometimes even years after the loss.

### REMEMBERING THE DYING DAYS: OLDER ADULTS' FINAL MEMORIES FROM THE LOSS OF A SPOUSE

Emily Mroz, and Susan Bluck, *University of Florida, Gainesville, Florida, United States*

Memories from the very end of the life of a deceased spouse (i.e., their dying days) are frequently carried with the bereaved as major markers in their own life stories. The current study identifies functions of these memories. Older adults (age 70-96; N = 53) told two memories from their spouse's dying days, then self-rated them for serving directive, social-bonding and self-continuity functions (TALE; Bluck & Alea, 2011). Those who found their loss more incomprehensible (ISLES; Holland, 2015) reported using these memories for directive (i.e., guidance of behaviors) and self-continuity (i.e., maintenance of a sense of self) functions more frequently (ps < 0.05). This relation was, however, mediated by older adults' current grief (ICG; Prigerson et al., 1995). Incomprehensibility of the loss of a spouse appears to lead to intense grieving, prompting individuals to draw on memories from the loss to maintain a sense of self and direct their future.

### AUTOBIOGRAPHICAL MEMORY AND END-OF-LIFE TREATMENT PREFERENCES IN CHINA

Yao Fu, and Ellen Idler, *Emory University, Atlanta, Georgia, United States*

In this mixed-methods study of religious/cultural beliefs and end-of-life treatment preferences in China, we surveyed 1,085 mainland Chinese people aged 18 or above online. We assessed the effects of past experience with dying people they have known and their own end-of-life treatment preferences in two hypothetical terminal illness vignettes. We found that respondents who knew or visited someone at the end of their lives were somewhat less likely to choose aggressive treatment for themselves in a lung cancer scenario (25% compared to 33%, p=.013). However, there was less difference in an Alzheimer's disease scenario, with a choice to use a gastric feeding tube or not (39% compared to 42%, p=.262). Open-ended responses indicate that people refer to these past experiences as a reference in making end-of-life decisions for themselves. This study provides empirical evidence that autobiographical memory has a directive function that individuals call on to inform future behaviors.

### MEMORIES OF A GRANDPARENT'S DEATH: PREPARATION FOR FUTURE LOSSES

Cory Bolkan,[1] and Raven Weaver,[2] *1. Washington State University, Vancouver, Washington, United States, 2. Washington State University, Pullman, Washington, United States*

Experiences of death in early life may result in identity-defining memories that last a lifetime. Autobiographical memories serve psychosocial functions, acting as guides for future behavior. Understanding early death experiences may thus inform lifelong personal views about death, dying, and bereavement. We queried 50 adults (ages 19 – 67 years) using a structured set of questions to recall and write about their earliest and most significant losses. The narratives were qualitatively analyzed using the constant comparative method associated with grounded theory. Results indicated a grandparent's death was the most frequently reported significant loss, reflecting the value of intergenerational relationships and the long-lasting impact of grandparent death. Themes also emerged concerning participants' reports of the benefits of actively remembering and reflecting on loss, as well as learning from others' losses, which further deepened their views of death. These findings highlight how early memories of death, including one's grandparents, can have lifelong impact.

### SESSION 5675 (SYMPOSIUM)

### MICRO- AND MACRO-LEVEL TIME ASSOCIATIONS: HOW DAILY LIFE SETTLES INTO LONGER-TERM PROFILES

Chair: Raquael Joiner
Co-Chair: Niccole Nelson
Discussant: Stacey Scott

Over a decade ago, Ram and Gerstorf (2009) proposed a descriptive framework to unite the study of intraindividual variability, operating at the micro-level timescale (e.g., minutes, days), and intraindividual change, operating at the macro-level time scale (e.g., years, decades). Since this proposal, several aging theories have incorporated a micro-level time component in their conceptualizations of longer-term aging processes. Furthermore, technological advancements have eased difficulties associated with data-collection at micro-level timescales, leading to an upsurgence of empirical investigations of dynamic characteristics and dynamic processes. This session presents theoretical, quantitative, and qualitative research aimed at better understanding the associations between micro- and macro-level time. More specifically, 1) Nelson et al. present their novel theoretical framework linking micro-level time emotion regulatory processes to intraindividual trajectories of cognitive functioning, 2) Joiner and colleagues present a quantitative study assessing the association between daily emotion-dynamics and yearly trajectories of depressive symptomatology, 3) Bergeman et al. present a quantitative study of daily risk and resilience in relation to trajectories of health and well-being, and 4) Bouklas and colleagues present a qualitative study linking individuals' daily routines and behaviors to their general life outlooks. The quantitative and qualitative studies are based on available data from the The Notre Dame Study of Health & Well-Being, a 10-year, nested-longitudinal study that incorporates yearly questionnaires, five 56-day measurement bursts, and interview data. Discussant Stacey Scott will synthesize the presentations with Ram and Gerstorf's framework and encourage researchers to integrate shorter- and longer-term timescales into their theoretical and empirical work on aging.

### DEPRESSION DYNAMICS ACROSS A DECADE: DAILY AFFECTIVE EXPERIENCE AND YEARLY DEPRESSIVE SYMPTOMATOLOGY

Raquael Joiner,[1] C. S. Bergeman,[2] Lijuan Wang,[2] Guangjian Zhang,[2] and Kristin Valentino,[2] *1. University of Notre Dame, Mishawaka, Indiana, United States, 2. University of Notre Dame, Notre Dame, Indiana, United States*

Recent conceptualizations of depression and supporting empirical work suggests that elevations and allievations of depressive symptoms can be understood from a dynamic systems perspective. Specifically, depression is proposed to result from strong-feedback loops in a system comprised of highly interdependent component parts (e.g., affect states). Supporting this perspective, individual differences in emotional interia and strong connections across emotions at micro-level timescales have been consistently associated with individual differences in depressive symptomatology such that individuals with greater emotional inertia and cross-emotion relations show higher levels of depressive symptoms. Importantly, however, individual differences do not necessarily translate to intraindividual change. The present study explores whether emotional connectivity at the daily timescale differs within individuals across a ten-year span and how these associations relate to intraindividual changes in depressive symptomatology. The results of these individual-level analyses will help further a dynamic systems perspective of depression and help inform clinical interventions for depression.

### COGNITIVE ENRICHMENT THROUGH EMOTION REGULATION: A MODEL OF SUCCESSFUL COGNITIVE AGING

Niccole Nelson, Cindy Bergeman, and Nathan Rose, *University of Notre Dame, Notre Dame, Indiana, United States*

Cognitive Enrichment Through Emotion Regulation (CENTER) is a theoretical framework of development that emphasizes the role of individuals finding their proverbial "centers" in shaping their cognitive aging trajectories. Within the CENTER framework, dynamic interactions between emotion regulatory processes that occur in real time (i.e., micro-level time), and global psychological well-being that develops over several years (i.e., macro-level time), aid in the optimization of cognitive aging. Indeed, by successfully regulating emotional reactions to stress in real time, which is supported by global psychological well-being, individuals will minimize their accumulation of allostatic load across the lifespan. Such minimization of allostatic load is key to optimizing cognitive aging through emotion regulation under the CENTER framework. CENTER will be motivated by fusing research on cognitive aging, emotion regulation, stress-and-coping, allostatic load, and psychological well-being.

### A LONGITUDINAL RESERVOIR MODEL OF STRESS DISSIPATION AND THE INFLUENCES OF CONCOMITANT PERCEPTIONS OF CONTROL

C. S. Bergeman,[1] Raquael Joiner,[2] Niccole Nelson,[1] and Pascal Deboeck,[3] *1. University of Notre Dame, Notre Dame, Indiana, United States, 2. University of Notre Dame, Mishawaka, Indiana, United States, 3. University of Utah, Salt Lake city, Utah, United States*

To characterize the stress regulation system, we use a reservoir to reflect how much stress an individual "holds" over time. Factors affecting what is contained in a stress reservoir are incoming stress (Input), accumulation/dissipation (Strdiss), and actions taken to discharge stress (e.g., Control). At the within person level, time-varying control predicts better Strdiss ($\beta$= -0.03±0.01, p <.001), even when controlling for between person differences (e.g., age, neuroticism) and between and within person impacts of Input. Thus, control reflects an important stress dissipation tool. Further analyses indicated a significant 2-way interaction between time-varying effects of Input and Control ($\beta$= 0.14±0.03, p <.0001) and Strdiss and Control ($\beta$= 0.60±0.18, p <.001) on self-reported health and a significant 3-way time-varying interaction of Input, Strdiss and Control on depression ($\beta$= -0.173±0.07, p <.012). Studies of this type move beyond the static assessments of risk and resilience to a more dynamic one.

### A QUALITATIVE ANALYSIS OF THE RELATIONSHIP BETWEEN OLDER ADULTS' DAILY LIVES AND LIFE OUTLOOK

Isabella Bouklas,[1] Giancarlo Pasquini,[1] Renee Gilbert,[1] Cindy Bergeman,[2] and Stacey Scott,[1] *1. Stony Brook University, Stony Brook, New York, United States, 2. University of Notre Dame, Notre Dame, Indiana, United States*

Leading theories of adult development suggest age-related changes in one's life perspective and changes in one's priorities are reflected in daily behavior. The present study explored how older adults understand their current lives through a qualitative study of midwestern Americans. Twenty-four participants (Mage= 69.53 years; age range=63-78 years) from the Notre Dame Study of Health & Well-Being (Whitehead & Bergeman, 2014) completed semi-structured interviews in which they were asked about turning points across their lives. Inductive analysis using the constant comparative method (Maykut & Morehouse, 1994) resulted in 10 life-domains based on common descriptions across participants. These domains represented the ways in which participants understood their identities over the course of their lives, as well as their organization and use of time and space in daily life. Participants' descriptions of both general life outlook and daily life informed one another, revealing the dialectical relationship between micro-level behaviors and macro-level attitudes.

### SESSION 5680 (SYMPOSIUM)

### NEEDS OF AND SERVICES FOR GRANDPARENTS RAISING GRANDCHILDREN: REGIONAL, NATIONAL, AND INTERNATIONAL PERSPECTIVES

Chair: Youjung Lee
Discussant: Deborah Whitley

Grandparents raising grandchildren build strong foundations for their grandchildren. Despite grandparents' significant contributions to their grandchildren's future and society in general, there is a limited understanding of the unique needs and service utilization of grandparents raising grandchildren in various contexts. This symposium is focused on the needs of and services for the grandparent population at the regional, national, and international levels. Stucki will present findings from an examination of types and locally available services for grandparents raising grandchildren in Appalachia by sub-region. Musil and colleagues will discuss the service need utilization and unmet service needs of a nationwide sample of 284 grandmothers living with/ raising grandchildren and the relationships between service use/need and resilience, resourcefulness, perceived stress, reward, and appraisals of their current living environment for themselves and their grandchildren. Lastly, Lee will describe research findings from her comparative transnational research on needs and experiences of grandparents raising grandchildren in Malawi (n=29), South Korea (n=23), and the U.S. (n=23). Unique needs and cultural interpretation of intergenerational caregiving in each country will be presented. The symposium discussion will address diverse needs of grandparents raising grandchildren and strategies to meet those needs at regional, national, and international levels. Grandparents as Caregivers Interest Group Sponsored Symposium.

### SERVICE NEED UTILIZATION AND UNMET SERVICE NEEDS OF GRANDMOTHERS LIVING WITH OR RAISING GRANDCHILDREN

Carol Musil, McKenzie Wallace, and Alexandra Jeanblanc, *Case Western Reserve University, Cleveland, Ohio, United States*

This study explores the service need utilization and unmet service needs of a nationwide sample of 284 grandmothers living with/raising grandchildren, and the relationships with service use/need, perceived stress, reward, and appraisals of their current living environment for themselves and their grandchildren. Participants were asked whether they currently used, had unmet need for, or did not need 25 different support services, including babysitting, financial assistance, legal assistance, family therapy/communication, among others. Overall, 89.5% (N=255) were receiving at least 1 service (mean = 3.4, range 0-18), and 89.1% (N=253) reported having at least 1 unmet service need (7.4, range 0-23). Receiving services was positively correlated with psychosocial resources, but not with appraisals of stress, reward, or living situation. Unmet service needs were inversely correlated with psychosocial resources, reward, and appraisals of living situation. Implications of these varying patterns will be discussed. Part of a symposium sponsored by the Grandparents as Caregivers Interest Group.

### GRANDPARENTS RAISING GRANDCHILDREN IN APPALACHIA: AN EXAMINATION OF AVAILABLE SERVICES

Bradford Stucki, *Virginia Tech, Blacksburg, Virginia, United States*

Previous research indicates that higher numbers of grandparents raising grandchildren live in Appalachia, relative to the rest of the United States. These grandparents may have diverse needs that could benefit from services. When grandparents cannot access needed services, their well-being can be negatively affected. Using the "2017 GrandFacts: State Fact Sheets for Grandfamilies" for the 13 states defined as being part of Appalachia by the Appalachian Regional Commission, this study examined the types and availability of local services by Appalachian sub-region. Excluding state and federal public benefits, most common service types were emotional support, information and referral, financial assistance, and education. Least common service types included grandchild special health needs, legal services, and early childhood intervention. For service availability, four of the five Appalachian sub-regions had no services in over 65% of their counties. South and North Central Appalachia regions had no services in over 90% of their counties. Part of a symposium sponsored by the Grandparents as Caregivers Interest Group.

### DIVERSE NEEDS OF GRANDPARENTS RAISING GRANDCHILDREN: QUALITATIVE RESEARCH IN MALAWI, SOUTH KOREA, AND THE UNITED STATES

Youjung Lee, *Binghamton University, State University of New York, Binghamton, New York, United States*

Despite custodial grandparents' significant contributions to their grandchildren's healthy development, unique needs of older adults often remain unmet with a limited cultural understanding of intergenerational caregiving. Using a phenomenological approach, interviews and focus groups were conducted with 75 custodial grandparents in Malawi (n=29), South Korea (n=23), and the U.S. (n=23). Malawian grandparents presented financial and physical hardships; however, they experienced strong support from community. Korean grandparents reported similar needs as Malawian grandparents while additionally experiencing cultural biases toward grandparent-headed families (maternal grandparenting and adult child's divorce). The U.S. grandparents disclosed increased needs for social support as well as family trauma with intergenerational impacts. The increase in custodial grandparent population across the world and findings from this comparative transnational research highlight the need for development of a model for culturally responsive practice with grandparent-headed families in a global context. Part of a symposium sponsored by the Grandparents as Caregivers Interest Group.

### SESSION 5690 (SYMPOSIUM)

### NEW PERSPECTIVES ON LONELINESS: FROM MICRO QUALITATIVE TO MACRO QUANTITATIVE CROSS-NATIONAL COMPARISONS

Chair: Marja Aartsen

Longitudinal research revealed a number of micro-level drivers of loneliness, such as widowhood, exclusion from the wider society, ill health and migrant status, but a number of questions are still unanswered. For example, the prevalence of loneliness varies substantially across countries, but we do not know precisely what causes these differences. It may be due to differences in the composition of the populations, it may also be caused by macro-level drivers, or by variations in the impact of risk factors between countries. For example, losing a spouse may be loneliness provoking in countries where living with a partner is the norm, but less so in countries where living alone is more valued. Also how early childhood and events over the life course affect the level of loneliness in later life is still under-researched. The aim of our symposium is to address this gap by presenting different perspectives on loneliness and social isolation. The first presenter interprets five-year follow-up information from qualitative interviews with a life course perspective. The second investigates the role of trust as factor producing social integration, which leads to variations in loneliness. The third compares and discusses loneliness in three different continents, based on an ecological model of contexts. The forth presenter critically discusses ways to measure loneliness in societies that are culturally distinct from western cultures. The last presenter discusses the dynamics between loneliness and material deprivation in Europe. The findings provide a new lens through which we can understand loneliness and inform about effective prevention.

### EARLY-LIFE CIRCUMSTANCES AND LATE-LIFE LONELINESS: FINDINGS FROM IN-DEPTH INTERVIEWS

Elisa Tiilikainen, *University of Eastern Finland, Kuopio, Finland*

This case study examines loneliness from the perspective of two older men, who were interviewed three times as part of a five-year qualitative longitudinal study on loneliness in later life. Both interviewees self-identified as feeling lonely "often" or "all the time" and had experienced loneliness also in previous life phases. The interviews revealed trajectories of long-term loneliness which were impacted by life events and circumstances in early life, childhood and youth. Two critical experiences were identified: childhood bereavement and sexual abuse. These factors contributed to emotional insecurities and impacted the ways the interviewees perceived their selves and their relations with others. The acknowledgement of past life experiences is important for the theoretical and conceptual understanding of loneliness and the development of different intervention strategies. However, more longitudinal analysis is needed on the cumulative disadvantages making people vulnerable to long-term loneliness.

### LONELINESS AND TRUST: AN EAST-WEST COMPARISON IN EUROPE

Marja Aartsen,[1] and Gražina Rapolienė,[2] *1. OsloMet Oslo Metropolitan University, Oslo, Norway, 2. Lithuanian Social Research Centre, Vilnius, Vilniaus Apskritis, Lithuania*

Loneliness in later life is two times more prevalent in Eastern and Southern European countries than in Northern and Western European countries. One explanation that is put forth is the difference in expectations about social relations. We examine a not often evaluated role of trust in society as factor contributing to the country differences in loneliness. We adopt the trust-as-antecedent model of social integration, and assume that social integration is associated with loneliness. We use data of respondents aged 65 and over participating in the European Social Survey and conduct a latent factors path analysis to examine the effect of trust in the system and trust in people on social capital and loneliness. Loneliness is two times more prevalent in Eastern Europe than the rest of Europe (26% vs 10%), levels of trust are substantially lower in Eastern European countries, which in turn is associated with higher levels of loneliness.

### CONTEXTS THAT EXCLUDE: LONELINESS IN NORTH AMERICA, EUROPE, AND AFRICA

Norah Keating,[1] and Vera Roos,[2] *1. Swansea University, Summerland, British Columbia, Canada, 2. Optentia Research Focus Area, North-West University, Vanderbijlpark, North-West, South Africa*

Loneliness is a key indicator of exclusion from social relations. Its prevalence among older people is of increasing global concern given the powerful impact of loneliness on health and wellbeing. To date, empirical and theoretical advancements in understanding loneliness have been focused on the micro contexts of networks of social relationships surrounding older people. Yet these relationships occur within broader socio-cultural, political and geographical settings that may help us better understand the experiences and manifestations of loneliness. In this presentation we draw on our research on loneliness on three continents. Findings from Canada show how ageing in a foreign land can truncate social connections. Research across Europe reveals differences in values and expectations about social connections. South African findings illustrate the long-term impact of dislocation resulting from political ideologies. We draw together these findings to create an ecological model of contexts that may lead to cumulative disadvantage in social relations.

### CO-CREATING CULTURALLY NUANCED MEASURES OF LONELINESS WITH MĀORI ELDERS

Charles Waldegrave,[1] Chris Cunningham,[2] Catherine Love,[1] and Giang Nguyen,[1] *1. Family Centre Social Policy Research Unit, Lower Hutt, Wellington, New Zealand, 2. Research Centre for Maori Health and Development, Massey University, Wellington, Wellington, New Zealand*

The growing international evidence on the impacts of social isolation and loneliness has profound implications for positive health status and wellbeing (Holt-Lunstad et. al. 2015). This raises important questions about how we measure loneliness, particularly in extended family cultures where loneliness may be experienced differently from western more individualistic cultures. In this research, key questions around loneliness and social isolation were co-created with Māori Elders and responses were compared with a standard international loneliness scale (De Jong Gierveld Loneliness Scale) to help identify universal aspects of loneliness and Māori specific aspects. The results demonstrated significant correlations between the co-created questions and the international scale. However, they also demonstrated substantial expressions of loneliness among older Māori that are not captured by standard scales showing the need for Māori specific scales which are more inclusive of their cultural norms and can provide more precise data for constructive policy making and service provision.

### TWO DIMENSIONS OF SOCIAL EXCLUSION: ECONOMIC DEPRIVATION AND DYNAMICS OF LONELINESS DURING LATER LIFE IN EUROPE

Michal Myck,[1] Charles Waldegrave,[2] and Lena Dahlberg,[3] *1. Centre for Economic Analysis, CenEA, Szczecin, Zachodniopomorskie, Poland, 2. Family Centre Social Policy Research Unit, Lower Hutt, Wellington, New Zealand, 3. Aging Research Center, Karolinska Institutet, Solna, Stockholms Lan, Sweden*

We contribute to the discussion on social exclusion by examining the relationship between material conditions and loneliness in a sample of individuals aged 50+ in the Survey of Health Ageing and Retirement in Europe (SHARE). In its 5th wave, the survey was extended to include specific items related to economic and social deprivation. We use this extended information on material conditions and examine how it correlates with the level and dynamics of a composite loneliness measure at the time of wave 5 and between wave 5 and 6 of the survey (undertaken in 2013 and 2015, respectively). In order to isolate the effect of material deprivation on loneliness, regression analyses include an extensive set of control variables. The analyses show a strong and significant relationship between material deprivation and both the level of loneliness and deterioration in the loneliness status.

### SESSION 5695 (SYMPOSIUM)

### OLDER LGBTQ WOMEN MATTER: AN EXAMINATION OF DISTINCTIVE HISTORIES, END-OF-LIFE NEEDS, AND SUPPORT

Chair: Korijna Valenti

LGBTQ (lesbian, gay, bisexual, transgender, and queer) aging research has reached a point at which in depth examination of the heterogenous experiences of identity, social life, and health is needed in order to better understand the distinct life courses and service needs of diverse subgroups. Older LGBTQ women have diverse life experiences and areas of need in terms of professional, social, and health-related supports and systems. As emerging literature addresses the depth of these distinctions, issues of identity development, social isolation, social networks, and end of life (EOL) planning and expectations require further study as well as the use of varied methods of data collection and analysis to enhance empirical understanding. This symposium includes findings from three studies. In a qualitative study, Valenti and colleagues examined experiences of LGB women over60 who had recently lost a spouse or partner and explored issues related to preparation and expectations at the EOL. In a discourse analysis of personal aging narratives published in the Bi Women's Quarterly newsletter (including poetry, personal reflections, and short stories), Jen and colleagues examined how bisexuality is experienced and constructed through language use in context. Rowan and colleagues provide insights from interprofessional research with a specific focus on lesbian women in later life. All three presentations in this symposium illustrate how language, individual preferences and needs, and social support issues meet to inform the needs of LGBTQ older women as well as providing implications for future aging research, theory development, and interventions in practice.

### WE'RE LESBIAN PIONEERS- WE CAN MANIFEST GOOD DEATH: EOL EXPERIENCES OF OLDER LGB WOMEN WHO HAVE LOST A SPOUSE OR PARTNER

Korijna Valenti,[1] and Leah Janssen,[2] *1. Miami University, Oxford, Ohio, United States, 2. Miami University, Hamilton, Ohio, United States*

Preparing for end-of-life (EOL) and identifying support systems are ways older lesbian, gay, and bisexual women (LGB) women can assert agency over EOL. This paper presents qualitative data from a sample of older LGB women 60+ who have lost a spouse or partner. Thematic analysis revealed four main categories of concern: 1) advance care documents and wills; 2) interactions with healthcare professionals; 3) discussions about EOL; and 4) social network support. The work also analyzed participants' discussions of how they have changed since losing a loved one and how they now view preparation for EOL. Findings reveal the need for better communication between healthcare professionals and LGB women, better understanding of care options (e.g. hospice, palliative care) and deeper EOL conversations among loved ones. This work critically engages queer gerontology, providing an important foundation of how to better understand how older LGB women perceive needs and preferences for their EOL.

### COURAGE TO COBBLE SOMETHING NEW: CREATIVE REPRESENTATIONS OF BISEXUALITY AND AGING

Sarah Jen,[1] and Rebecca Jones,[2] *1. University of Kansas, Lawrence, Kansas, United States, 2. Open University, Milton Keynes, England, United Kingdom*

There are few cultural representations or scripts available for LGBTQ aging. Among bisexual and otherwise non-monosexual (bi+) women, stereotypes of hypersexuality exclude older adults while the contrasting experience of invisibility obscures the existence of bi+ aging. In this discourse analysis, we examined three issues of the Bi Women Quarterly (BWQ) newsletter published between 2014-2019 which were devoted to the intersection of aging and bisexuality. Data include 42 narratives, personal reflections, interviews, poems, letters, advice columns, and photos which were analyzed to identify linguistic tools, visual imagery, and broader discourses used to construct and convey the meaning and experience of bisexual aging. Themes include: 1) lacking a "blueprint" for bisexual lives, 2) significant "turning points," 3) intergenerational (dis)connections across history, and 4) life-long patterns of discovery and disclosure. More visible and diverse narratives for bisexual aging might better enable bi+ individuals to envision and effectively plan for their own aging futures.

### IMPROVING RESPECT AND DIGNITY OF OLDER LESBIAN ADULTS: INTERPROFESSIONAL APPROACHES FOR SPECIALIZED CARE

Noell Rowan, Stephanie Smith, Tamatha Arms, and Kris Hohn, *University of North Carolina Wilmington, Wilmington, North Carolina, United States*

Interprofessional research pertaining to LGBTQ older adult cultural sensitivity training for social workers and nurses is often missing in the empirical literature. Members of the LGBTQ communities become increasingly vulnerable to health disparities as they age and treating clients with respect and dignity is at the forefront of this study. Students and faculty engaged in an interprofessional simulation project with older members of the LGBTQ community to learn health knowledge and applied assessment and brief intervention skills. Quantitative findings (N=58; 23 social work; 35 nursing) indicated increased student health knowledge. Reflection and qualitative findings are included with four primary themes: (a) bias of health care providers, (b) access to quality care, (c) specific health care needs, and (d) health risks of LGBTQ older adults. Specific emphasis is given to reflection and insight of the older lesbian participants about access to care, recognition of significant relationships, and marriage equality.

### SESSION 5700 (SYMPOSIUM)

### ON THE LEADING EDGE OF LONGEVITY: LESSONS FROM THE MIT AGELAB 85+ LIFESTYLE LEADERS

Chair: Julie Miller
Discussant: Lisa D'Ambrosio

The 85+ population is the fastest-growing age segment in the United States. Understanding attitudinal and behavioral trends among adults ages 85 and over is of increasing importance to researchers and practitioners alike. This symposium will present findings drawing on mixed methods across multiple domains of research with the Lifestyle Leaders, a bimonthly panel study of adults ages 85 and older at the MIT AgeLab, which began in September 2015. Each presentation in this symposium will highlight a different focus of research conducted with the Lifestyle Leaders, ultimately offering insights about physical health, social engagement, and civic engagement at age 85 and beyond. The first presentation in this symposium will frame Lifestyle Leaders' resources and how these resources affect their perceptions of risks, their worries, and their well-being. Diving deeper, the second presentation will center on how Lifestyle Leaders perceive and utilize different transportation options and how these options have changed for them over time. The third presentation will focus on civic engagement and Lifestyle Leaders' perspectives on technology as enabling or inhibiting their civic engagement and participation. The fourth presentation will examine Lifestyle Leaders' attitudes toward medication, medication management, and technology-enabled medication management devices. This symposium will deepen attendees' understandings of the attitudes and experiences of active octogenarians and nonagenarians.

### HOW ACTIVE OLDER ADULTS MANAGE THEIR MOBILITY NEEDS: TRANSPORTATION INSIGHTS FROM A PANEL OF ADULTS AGES 85+

Lisa D'Ambrosio,[1] John Rudnik,[1] Chaiwoo Lee,[1] Taylor Patskanick,[1] and Julie Miller,[2] *1. MIT, Cambridge, Massachusetts, United States, 2. MIT AgeLab, Cambridge, Massachusetts, United States*

Research suggests that adults experience tremendous transportation challenges at ages 85 and over. As mobility has been tied to overall health and wellbeing, the implications are dire. In this study, the Lifestyle Leaders were surveyed to understand their experiences with different modes of transportation. Responses to a questionnaire (N = 18) and focus groups (N = 18) indicate that many of the Lifestyle Leaders are still driving and report satisfaction with their ability to get around. However, over the past 10 years, many participants have changed their attitudes toward driving and transportation in general. Some panelists cite issues of physical discomfort, increased time and energy spent planning trips, and an increased sense of dependence. Additional data suggest that decision making processes for selecting transportation modes have changed over time. This presentation will discuss policy and practice implications for older adults, caregivers, and transportation professionals.

### HOW THE LIFESTYLE LEADERS VIEW AND MANAGE MEDICATIONS

Taylor Patskanick,[1] and Julie Miller,[2] *1. MIT, Cambridge, Massachusetts, United States, 2. MIT AgeLab, Cambridge, Massachusetts, United States*

Medication management is an ongoing consideration for adults ages 85 and older, their caregivers, and healthcare providers. When asked about their attitudes and behaviors regarding medication management, over 73% of the Lifestyle Leaders reported taking 3+ prescription medications daily and managing their own medication regimes. 61.9% of participants had taken over-the-counter, non-prescription medication for pain over the past five years. When asked why some participants didn't currently take prescription medications to manage pain, the most frequently-reported responses were: "I don't feel that my pain warrants a prescription medication," (19%, n=8), "I don't want to deal with the side effects," and "I don't trust drug companies," (9.5%, n=4, respectively). The Lifestyle Leaders reported they would be most likely to go to the internet (over their local pharmacist) to ask for advice about their medication(s). Meanwhile, 39% of Lifestyle Leaders would trust a robot to manage their medication(s) for them.

### INTERNAL AND EXTERNAL RESOURCES OF THE LIFESTYLE LEADERS

Martina Raue,[1] Lisa D'Ambrosio,[2] Taylor Patskanick,[2] John Rudnik,[2] Adam Felts,[3] and Julie Miller,[3]
*1. Massachusetts Institute of Technology, Cambridge, Massachusetts, United States, 2. MIT, Cambridge, Massachusetts, United States, 3. MIT AgeLab, Cambridge, Massachusetts, United States*

With older age, people experience declines in resources and face new challenges. The goal of this study was to understand how resource decline affects the oldest olds' well-being, but also to learn who they trust and where they go for advice in areas such as health, finances, and technology. This sample of 30 participants between the ages of 85 and 95 was generally resource-rich, scoring highest on self-esteem and optimism and lowest on mastery. Self-esteem and optimism correlated with financial resources, indicating a significant role of finances in this rather wealthy sample. Well-being was predicted by self-esteem and physical health. Presumably, their high levels of self-esteem compensate for the loss of other resources among the oldest old. The majority of lifestyle leaders trust in other people, and while friends and family are very important sources of advice, searching online was equally often mentioned as a source when looking for advice.

### AN EXPLORATORY STUDY OF CIVIC ENGAGEMENT AND PARTICIPATION AMONG A PANEL OF ADULTS AGES 85+

John Rudnik,[1] Taylor Patskanick,[1] Julie Miller,[2] Lisa D'Ambrosio,[1] and Joseph Coughlin,[1] *1. MIT, Cambridge, Massachusetts, United States, 2. MIT AgeLab, Cambridge, Massachusetts, United States*

The past twenty years have seen a surge in public attention devoted to increasing civic participation opportunities for older adults in the United States. At the same time, technology has transformed the way that information related to political and social issues is shared. A relatively small body of research has explored how older adults use technology-mediated platforms for political participation. In this study, the 85+ Lifestyle Leaders were surveyed to understand their experiences of civic engagement and participation. Responses to a questionnaire (N = 24) and focus groups (N = 22) indicate that participants are interested in and feel knowledgeable about social and political issues, but some forms of participation have decreased. Findings also suggest that there are opportunities for technology to facilitate engagement with and participation in social and political issues, for adults ages 85 and over. Issues of equity and accessibility will be highlighted in this presentation.

### SESSION 5710 (SYMPOSIUM)

### PATTERNS AND PREDICTORS OF ADRD AMONG RACIAL AND ETHNIC MINORITY GROUPS

Chair: Laura Zahodne
Discussant: Cerise Elliott

This symposium addresses issues surrounding Alzheimer's disease and related dementia (ADRD) across multiple racial/ethnic minority groups, including African Americans, Latinos, and Arab Americans. Using US national data, Kindratt and colleagues challenge the universality of the healthy migrant effect by comparing patterns of cognitive disability across US- and foreign-born Arab Americans. Arab Americans represent an increasingly visible ethnic minority group whose unique history has the potential to clarify knowledge about socio-cultural influences on ADRD. Also using US national data, Garcia and colleagues examine within-group heterogeneity among Latinos. They conclude that the number of years and proportion of life spent with and without subjective cognitive impairment differ as a function of ancestry and nativity. Using data from two local communities, Diminich and colleagues investigate mechanisms underlying ADRD risk among Latinos by considering both stress responding and plasma-based AD biomarkers as predictors of Latino cognitive health. Lee and colleagues focus on social relationships and cognitive aging in a diverse, national cohort. They suggest that the quality of social support from social network members may uniquely affect the cognitive functioning of African Americans older adults. Finally, Cerise Elliott from the National Institute on Aging (NIA) will offer perspectives on how racial/ethnic minority group focused research can advance NIA's goals related to understanding and eliminating ADRD inequalities. In total, this symposium highlights the need to disaggregate racial/ethnic groups, as well as the importance of incorporating both individual and contextual factors in order to fully understand patterns of ADRD risk and resilience.

### HETEROGENEITY IN SELF-REPORTED COGNITIVE IMPAIRMENT LIFE EXPECTANCIES AMONG OLDER LATINO SUBGROUPS IN THE UNITED STATES

Marc Garcia,[1] Kasim Ortiz,[2] Chi-Tsun Chui,[3] Brian Downer,[4] and David Warner,[5] *1. University of Nebraska, Lincoln, Nebraska, United States, 2. University of New Mexico, Albuquerque, New Mexico, United States, 3. Academia Sinica, Taiwan, Taipei, Taiwan (Republic of China), 4. The University of Texas Medical Branch at Galveston, Galveston, Texas, United States, 5. University of Alabama at Birmingham, Birmingham, Alabama, United States*

Research suggests the prevalence of cognitive impairment among older U.S. Latinos is increasing relative to the older U.S. population which has experienced and overall decline. This has been attributed in part to extended longevity, lower educational attainment, and a higher prevalence of diabetes and cardiovascular disease among older Latinos. However, less is known regarding the number of years and proportion of late life spent with self-reported cognitive impairment among this rapidly aging group and whether cognitive impairment life expectancy outcomes vary across U.S. Latino subgroups by country of origin. To fill this gap, our investigation uses data from the National Health Interview Survey (1997-2015) to estimate Sullivan-based life tables of cognitively normal and cognitively impaired life expectancies for adults 50 years and older. Results indicate significant heterogeneity among Latinos, with island-born Puerto Rican women spending the most years, and foreign-born Cuban men the fewest years lived with self-reported cognitive impairment.

### EMOTIONAL FLEXIBILITY, BIOMARKERS, AND COGNITIVE RESILIENCE IN LATINO ADULTS AT MIDLIFE

Erica Diminich, *Stony Brook University, Stony Brook, New York, United States*

A growing body of evidence underscores the important role of emotional responding and emotional flexibility in healthy adaptation. Considerable research further demonstrates that being flexible in how one copes and regulates emotions when faced with stressful events is paramount for healthy aging. However, the adaptive benefits of emotional flexibility have not been studied in Latina/o's, despite converging evidence indicating that Latina/o's report greater symptoms of depression and anxiety in the context of exposure to potentially traumatic events and stress. We demonstrate across two studies, how the ability to regulate emotional responses is associated with cognitive health in a community-based population of Latinos and a cohort of Latino responders from the World Trade Center attacks on 9/11. Furthermore, given that individual differences in emotional flexibility predict cognitive decline, we present data examining the utility of plasma biomarkers of pre-clinical Alzheimer's disease and neuropathy as diagnostic screeners of cognitive functioning and health.

### COGNITIVE DISABILITY AMONG ARAB AMERICANS BY NATIVITY STATUS: LACK OF EVIDENCE FOR THE HEALTHY MIGRANT EFFECT

Tiffany Kindratt,[1] Florence Dallo,[2] and Laura Zahodne,[3]
*1. University of Texas at Arlington, Arlington, Texas, United States, 2. Oakland University, Rochester, Michigan, United States, 3. University of Michigan, Ann Arbor, Michigan, United States*

Limited research exists on cognitive disabilities among foreign-born adults, particularly non-Hispanic Arab Americans. We analyzed 10 years (2008-2017) of data from the American Community Survey (ACS) Public Use Microdata Samples (PUMS) (n=5,011,469; ages >45 years). In US-born adults, the age- and sex-adjusted prevalence of cognitive disability among non-Hispanic Arab Americans was 5.3%, which was lower than non-Hispanic whites (6.5%), blacks (10.8%), and Hispanics (10.0%). Among foreign-born adults, the prevalence of cognitive disability was highest, 7.3%, for non-Hispanic Arab Americans compared to all other racial and ethnic groups. Among foreign-born adults, non-Hispanic Arab Americans had 1.24 times greater odds (95% CI=1.12, 1.37) of having a cognitive disability compared to foreign-born non-Hispanic whites. This is the first study to examine cognitive disabilities among US- and foreign-born Arab Americans. More research is needed to better understand factors that may contribute to the increased prevalence of cognitive disabilities for foreign-born adults.

### RELATIONSHIP QUALITY AND COGNITION ACROSS RACE AND ETHNICITY

Ji Hyun Lee,[1] Indira Turney,[2] Reza Amini,[3]
Benjamin Katz,[4] Kristine Ajrouch,[5] and Toni Antonucci,[6]
*1. Florida State University College of Medicine, Tallahassee, Florida, United States, 2. Columbia University Medical Center, New York, New York, United States, 3. The University of Michigan-Flint, Flint, Michigan, United States, 4. Virginia Tech, Blacksburg, Virginia, United States, 5. Eastern Michigan University, Ypsilanti, Michigan, United States, 6. University of Michigan (UM), Ann Arbor, Michigan, United States*

Quality of social relations have increasingly been recognized as an important factor in cognitive health in later adulthood. Less is known about the association of relationship quality with executive functioning (EF) and memory; and whether the links differ by race/ethnicity. In this paper, we investigated the associations between positive and negative quality of relationship with spouse, children, family, and friends with EF and memory across non-Hispanic Black, Hispanic, and non-Hispanic Whites. Participants are drawn from Health and Retirement Study Harmonized Cognitive Assessment Protocol (N = 2,678). Independent of network size and contact, relationship quality with family was linked to EF. Racial differences were found such that negative relationship quality with children was protective of EF for blacks. Relationship qualities were not associated with memory. These findings indicate that examining quality of relationship with distinct relationships may be essential for understanding the association with cognition, especially in the context of race/ethnicity.

### SESSION 5715 (SYMPOSIUM)

### PROSOCIAL BEHAVIOR AND CONTRIBUTORS TO OLDER ADULTS' WELL-BEING IN DAILY LIFE

Chair: Jeanne Nakamura
Co-Chair: Dwight Tse

This symposium reports findings from a national experience-sampling study of 165 older adults (mean age=71, range=60-88 years) who are heavily involved in prosocial activity, contributing to their communities as leaders or high-commitment volunteers in social-purpose organizations. Gerontological research has linked prosocial activity to a set of positive outcomes for the older adults who engage in it (e.g., better health and reduced mortality), including global measures of well-being such as life satisfaction. However, little is known about the contributors to these individuals' momentary well-being. Four presentations address this gap in knowledge. In the first presentation, Dwight Tse revisits the concept of successful aging as a within-person variable with day-to-day variations. Experience sampling data revealed great variations in successful aging indicators, and successful aging was associated with better well-being as hypothesized. In the second presentation, Ajit Mann extends beyond general control beliefs and explores the diversity in distribution of control beliefs across various daily activities (e.g., active leisure, prosocial activity, socializing, etc.), in addition to investigating the relationship between later life control diversity and subjective well-being. In the third presentation, Kelsey Finley explores Aristotle's concept of the golden mean (an ideal state between deficiency and excess) for the number of hours spent in prosocial work on momentary well-being outcomes. In the fourth presentation, Jeanne Nakamura examines the relationship between global meaning in life and meaningful engagement in the moment, illuminating variation in this relationship through a comparison of the prosocial leaders and high-commitment volunteers.

### MOMENTARY SUCCESSFUL AGING INDICATORS AND THEIR SHORT-TERM AND LONG-TERM COVARIATES

Dwight Tse,[1] Kelsey Finley,[2] and Katherine Vrooman,[2] *1. The Chinese University of Hong Kong, Hong Kong, China, 2. Claremont Graduate University, Claremont, California, United States*

Studies on successful aging have conceptualized it as a between-person construct, meaning that people's aging process is seen as more or less successful than others' across contexts. This study examines within-person, moment-to-moment successful aging indicators, such as (absence of) physical pain, good physical and cognitive functioning, and active engagement in social and productive activities, and their relations to one-time well-being indicators (affective balance, psychological needs satisfaction, meaning in life, and satisfaction with life) at the time and one year after. Multilevel modeling on experience sampling data revealed that successful aging varied substantially (over 50% total variances) within-person and was positively associated with well-being. This study illustrates the utility of momentary successful aging indicators and advocates for a more nuanced understanding besides a simple classification of older adults undergoing "successful" or "normal" aging processes.

### THE DYNAMICS OF CONTROL: INVESTIGATING THE EFFECT OF CONTROL DIVERSITY ON SUBJECTIVE WELL-BEING IN LATER LIFE

Ajit Mann, and Jeanne Nakamura, *Claremont Graduate University, Claremont, California, United States*

Recent advancements in research on control beliefs have enabled the assessment of inter-individual differences in its intra-individual variability via a measure of control diversity. However, past research has focused on control diversity in relation to daily stressors. Among a sample of prosocial exemplars, this experience sampling study investigates control diversity across general daily activities and explores its relationship with subjective well-being. Participants indicated the activity they were primarily involved in, the extent to which they felt in control over the situation, and levels of positive and negative affect each time they were signaled; satisfaction with life was measured using a one-time survey at the end of the study. Results from multilevel structural equation modeling suggested that control diversity did not significantly predict positive or negative affect, or satisfaction with life. The study points toward the lack of association between control diversity and subjective well-being and highlights future directions for research.

### IDEAL LEVELS OF PROSOCIAL INVOLVEMENT IN RELATION TO MOMENTARY AFFECT AND EUDAIMONIA: EXPLORING THE GOLDEN MEAN

Kelsey Finley,[1] Maria Axner,[1] Katherine Vrooman,[1] and Dwight Tse,[2] *1. Claremont Graduate University, Claremont, California, United States, 2. The Chinese University of Hong Kong, Hong Kong, China*

Aristotle believed that happiness and success result from cultivating virtue at the mean between deficiency and excess - the golden mean. Some evidence suggests there is a golden mean of hours spent volunteering, where well-being benefits are maximized. Our study examined potential linear and nonlinear functions in the amount of time spent in prosocial work (PSW) in a day on the outcomes of eudaimonia, high arousal negative affect, and high arousal positive affect in a sample of high-commitment volunteers and prosocial leaders. In addition to nonlinear functions, interactions were explored. For example, we found that those who spend less time per week in PSW experience less negative affect when they spend more hours per day in PSW; however, those who spend more time per week in PSW experience more negative affect when they spend more hours per day in PSW. Additional findings for positive affect and eudaimonia will be discussed.

### MEANING IN LIFE AND MEANINGFUL ENGAGEMENT IN THE MOMENT: LIVES OF PROSOCIAL LEADERS AND VOLUNTEERS

Jeanne Nakamura, and Ajit Mann, *Claremont Graduate University, Claremont, California, United States*

Recent years have seen growing interest in older adults' sense of meaning in life, a core dimension of eudaimonic well-being throughout adulthood that has been associated in later life with reduced morbidity and mortality. Currently, the relations between this global sense of meaning in life and the experience of meaningful engagement in the moment remain largely unexplored, particularly in later lives that are distinguished by high levels of meaning. Multilevel analysis of ESM data from prosocial leaders and volunteers indicated that feelings of meaningful engagement fluctuated in daily life for both groups, even while questionnaire data showed that global sense of meaning in life was high. Examining basic sources of fulfillment (e.g., sense of relatedness) revealed type of involvement (leadership vs. volunteerism) affected the source(s) of fulfillment that connected meaning at the global and momentary levels. Implications for theory, research, and applied work on meaning and prosocial commitment are discussed.

### AGING IN AN INCREASINGLY TECHNOLOGY-BASED SOCIETY: PAST, PRESENT, AND FUTURE

Walter Boot, *Florida State University, Tallahassee, Florida, United States*

While it took over 70 years for the telephone to reach 50% of U.S. households, it took only 14 years for the cellphone, and 6 years for the MP3 player. Aging takes place in the context of a rapidly evolving technological landscape. What are the implications of such radical and rapid changes for how we age? And how can existing and emerging technologies help support aging adults' health, wellbeing, social connectivity, and cognition? This talk will explore these issues, starting with the "digital divide" between younger and older adults, the reasons for this divide, and interventions to close the gap. The potential of emerging technologies to support older adults will be summarized, as well as potential pitfalls in the design and implementation of these technologies. The talk will conclude with speculation on the future of the digital divide and whether it can ever be entirely eliminated.

### NEW FRONTIERS IN CAREGIVING RESEARCH: BIOPSYCHOSOCIAL PERSPECTIVES AND INTERVENTIONS

Jyoti Savla,[1] Karen Roberto,[1] and Mamta Sapra,[2]
*1. Virginia Tech, Blacksburg, Virginia, United States,*
*2. Salem VA Medical Center, Salem, Virginia, United States*

Although families embrace the opportunity to care for a loved one, caregiving is stressful and takes a toll on the caregiver's health and well-being. Earlier studies of stress and coping among family caregivers focused on psychological outcomes and emotional well-being. In the last decade, stress researchers have broadened their focus to include biomarkers and health outcomes. Data from two studies of caregivers of persons with memory loss will be used to discuss two new frontiers of caregiving research. First, a daily-diary study will be used to identify the mechanism by which stress disrupts the physiological processes and proliferates into serious psychopathology and pre-clinical and clinical health conditions. Second, a mindfulness-based psychoeducational intervention study will be utilized to identify malleable factors that can be harnessed to lower stress and improve the well-being of family caregivers. Next steps for caregiving research in the context of demographic and technological trends will be discussed.

### SUCCESSFUL ADAPTATION IN THE CONTEXT OF CARE ENVIRONMENTS: PROMISE AND CHALLENGE FROM A CAREER IN GEROPSYCHOLOGY

Suzanne Meeks, *University of Louisville, Louisville, Kentucky, United States*

My first clinical exposure to older adults was in a psychiatric hospital, to people with life-long severe mental illnesses; I was drawn to their perseverance. The older people we see clinically interact with care environments that may or may not effectively use patients' personal histories. I have studied the affective experiences of older people in care environments--the mental health system and long-term care--to improve mental health care and well-being. Solutions seem obvious, and difficult. Theory and abundant empirical research tell us that environments can support adaptation through nurturing strengths, offering compensatory tools, and acknowledging losses. My work on depression is a simple example of how this can work in psychotherapy. Yet implementing simple solutions means overcoming barriers of training, resources, and institutional inertia. The promise is understanding age as the dynamic representation of a life span; the challenge is making this understanding work for older people.

### WHY AGE MATTERS IN HIGHER EDUCATION

Joann Montepare, *Lasell University, Newton, Massachusetts, United States*

At the core of behavioral and social scientists' work in the aging field is informing and supporting the well-being of individuals and their communities. With the shift in age demographics and the aging of our populations, broadening educational efforts are more important than ever. However, advancing knowledge about aging and creating age inclusive educational opportunities has been a challenge in higher education, reflecting its historical, age-segregated structure, among other things. The pioneering Age-Friendly University (AFU) initiative, recently endorsed by GSA's Academy for Gerontology in Higher Education (AGHE), offers a valuable set of guiding principles that institutions in higher education can use to assess the extent to which their programs and practices are age inclusive, as well as identify gaps and opportunities. This presentation will discuss how the time has come for scientists to help shape more age-friendly institutions, and what they can look like in the years to come.

### SESSION 5725 (SYMPOSIUM)

### RURAL AGING: MULTIDISCIPLINARY, MULTINATIONAL INNOVATIONS THAT SUPPORT NEW APPROACHES TO ADDRESS UNMET NEEDS

Chair: Roger O'Sullivan
Co-Chair: Lyn Holley
Discussant: Megan Bond

The meaning of aging in rural areas is not well explored in the literature. To address this gap each presenter in this multidisciplinary and international group of researchers will address different components of rural ageing. An exact definition of "rural" is essential to forming, implementing or evaluating policies and programs impacting rural populations; however there is no universal definition. Cohen introduces definitional issues, and underscores the heterogeneity and regional variability of "rurality" and how such factors drive rural-urban disparities. Pendergrast, an early career researcher, reports results of in-depth semi-structured interviews that examined institutional influences of social networks on health of rural older adults, with specific focus on support services. Leavey describes lessons learned from the activities of PLACE-EE, a transnational partnership of public health agencies, local authorities, academics and ICT experts dedicated to improving the quality of life for older people. He describes the impacts on relationships of a program of community engagement and intergenerational exchange between younger and older citizens that used an assets-based approach. Żurek explores the potential for reward structures that can be embedded in 'social games' to motivate older adults to exercise in the context of research conducted in the rural US (Appalachia) and urban Poland (Krakow), and suggests a new direction that can inform services intended to improve health and happiness of rural adults. Our discussant will reflect on the major themes that emerge from these multidisciplinary perspectives, especially the potential for intersection of rural community-based innovations and learning from different regions of the world. Rural Aging Interest Group Sponsored Symposium.

### SOCIAL GAMES: THE INTERSECTION OF ESTABLISHED REWARD STRUCTURES AND EXERCISE FOR OLDER RURAL ADULTS

Lyn Holley, *University of Nebraska Omaha, Omaha, Nebraska, United States*

A high percentage of older adults engages with games; however research about impacts of gaming on older adults is focused almost exclusively on games intended to strengthen cognitive health or slow age-related cognitive decline, very little relates gaming to exercise. Rural –dwelling older adults are underrepresented in this as in other health-related research. Nonetheless, recent developments that explore effectiveness of embedding positive health behaviors (including exercise) in established games such as Bingo hold great promise for addressing the endemic social isolation and chronically debilitating conditions often associated with rural aging. Using recent research with a challenging population group (rural Appalachian older adults) as an anchor for discussion, this paper describes the state of the art of such interventions and, through a multinational lens, finds that these suggest a new direction for gaming design that could enhance effectiveness of the use of gaming to improve health and happiness of rural adults. Part of a symposium sponsored by the Rural Aging Interest Group.

### DEFINING RURALITY: STRUCTURAL RELATIONSHIPS, REGIONAL DIFFERENCES, AND APPLICATIONS FOR RESEARCH WITH OLDER ADULTS

Steven Cohen, Julia McIlmail, and Mary Greaney, *University of Rhode Island, Kingston, Rhode Island, United States*

There is no universal definition of rurality due to the heterogeneity in what makes a place "rural" or "urban". This study explored how elements of rurality are related to each other, and how the elements that define rurality vary by region. Data were abstracted for all 1948 non-metropolitan counties in the contiguous 48 states on rurality. K-means cluster analyses (k=4-8) were conducted to examine classification structures among component variables examining regional differences. In the South region, the majority (51.2%) were "Type 2" counties: low population size and density but higher urbanized population. The Midwest had a majority of "Type 3" counties (56.4%): intermediate for population size and density, but higher distances to metro areas. These exploratory findings underscore the heterogeneity and regional variability in rurality and how those measures are structurally related to each other, and essential to understanding those factors that truly drive rural-urban health disparities for older adults. Part of a symposium sponsored by the Rural Aging Interest Group.

### HOW AREA AGENCIES ON AGING CONTRIBUTE TO SOCIAL CONNECTION FOR OLDER ADULTS IN RURAL AMERICA

Claire Pendergrast, *Syracuse University, Syracuse, New York, United States*

Social ecological models of health identify intrapersonal, interpersonal, institutional, community, and policy-level contexts as social factors influencing individual and population health outcomes. However how institutions such as Area Agencies on Aging (AAA) shape rural older adults' social networks and influence health is little explored. This research examines institutional influences of social networks for rural older adults, particularly the social connections resulting from their AAA services and programs. AAAs are local social service organizations that coordinate home- and community-based supports. Our 2020 case study of a rural AAA in upstate New York involved in-depth semi-structured interviews with AAA staff, volunteers and participants included key themes related to older adults' social networks, social wellbeing, and physical and mental health. Our findings have both theoretical implications for rural community social structure as experienced by older adults, and practical implications to build AAA's capacity to address social isolation for rural older adults. Part of a symposium sponsored by the Rural Aging Interest Group.

### SESSION 5730 (SYMPOSIUM)

### SENSORY HEALTH AND SOCIAL WELL-BEING

Chair: Corinna Tanner
Co-Chair: Jeremy Yorgason
Discussant: Joshua Ehrlich

Scientific inquiry into the psychological and social issues surrounding age-related sensory impairments has focused on ways in which the conditions are a catalyst for negative outcomes. However, investigating the patterns associated with negative life events can offer guidance on ways to circumvent or mitigate negative outcomes, and even to foster and facilitate the positive outcomes of growth and thriving. This symposium will present findings from individual studies that describe how social well-being among older adults with sensory impairments can be protected, by assessing social isolation as a point of intervention to maintain cognitive function and to promote post-traumatic growth, and by understanding the unique social considerations relevant to Hispanic older adults, and to improve the physical safety of older drivers with sensory and cognitive impairment by reducing exposure-adjusted motor vehicle crash risk. Authors will present both cross sectional and longitudinal, population based data, and will explore patterns and relationships between known variables associated with sensory impairments including depression, cognitive processing, cognitive functioning, social network, social isolation, driving patterns, and posttraumatic growth. Findings from the Longitudinal Research on Aging Drivers (LongROAD) study, the National Health and Aging Trends Study (NHATS), and data from a mixed methods study, underscore the reality that the negative outcomes associated with age related sensory impairments are not necessarily imminent and that there may be multiple intervention points to optimize the social well-being of older adults with sensory impairments.

### THE POSITIVE LEGACY OF VISION LOSS: PATHWAYS TO POSTTRAUMATIC GROWTH

Corinna Tanner,[1] Michael Caserta,[2] Jia-Wen Guo,[2] Margaret Clayton,[3] Paul Bernstein,[2] and Julia Kleinschmidt,[3] *1. Brigham Young University, Provo, Utah, United States, 2. University of Utah, Salt Lake City, Utah, United States, 3. University of utah, Salt Lake City, Utah, United States*

This mixed method study describes posttraumatic growth (PTG) accruing form experience with vision loss caused by severe age related macular degeneration (AMD) and explores relationships between depression, social support, and cognitive processing, on the path to PTG. Research describing the psychological and social issues surrounding AMD has focused on negative outcomes. However, learning from highly challenging experiences, such as vision loss, can offer benefits. In this study, these included an increased sense of personal strength, increased spirituality, and empathy for others (all domains of PTG). 89 participants with severe vision loss (mean age = 85.3 years, age range = 74–98 years) completed the interviewer-administered composite questionnaire, which identified elements of Tedeschi and Calhoun's model of PTG. Relationships between variables were examined using path analysis. Findings were contextualized with data from 15 qualitative interviews. Findings underscored the importance of supportive others and deliberate cognitive processing in the path to PTG.

### SOCIAL ISOLATION AS A MECHANISM LINKING SENSORY IMPAIRMENT WITH COGNITIVE FUNCTIONING

Jeremy Yorgason,[1] Corinna Trujillo Tanner,[2] Stephanie Richardson,[2] Allison Burch,[2] Brian Stagg,[3] and Melanie Hill,[4] *1. Brigham Young University, provo, Utah, United States, 2. Brigham Young University, Provo, Utah, United States, 3. University of Utah, Salt Lake City, Utah, United States, 4. Brigham Young University, Orem, Utah, United States*

Hearing and vision loss have been linked with cognitive decline in older adults. There may be various pathways through which sensory impairments impact cognitive functioning. Sensory impairments may lead individuals to be less socially connected, which may impact cognitive functioning due to less cognitive stimulation. As such, sensory impairments and social isolation may cascade to negatively impact cognitive functioning. Using data from 8,334 individuals aged 65-90+ in waves 6, 7, and 8 of the NHATS study, we estimated a longitudinal mediation structural equation model. Findings indicate that both self-reported vision and hearing impairment in wave 6 of NHATS were linked to concurrent cognitive functioning through social isolation. Only hearing impairment demonstrated longitudinal impact through social isolation across 2 and 3 waves. Findings suggest that medical professionals working with older adults with vision or hearing impairment should assess social isolation, as a point of intervention to maintain cognitive function.

### SENSORY IMPAIRMENT AND SOCIAL ISOLATION: IMPLICATIONS FOR THE HISPANIC POPULATION

Stephanie Richardson,[1] Corinna Tanner,[1] and Jeremy Yorgason,[2] *1. Brigham Young University, Provo, Utah, United States, 2. Brigham Young University, provo, Utah, United States*

Although the likelihood of developing a disability increases with age among all demographics, older adults of hispanic origin are more likely to experience vision and hearing impairment than both their white and black non-hispanic counterparts. Both hearing impairment and vision impairment are known risk factors for social isolation, yet little research has examined this association in Hispanic populations. Using data from 472 Hispanic and 5,186 White participants of the NHATS study, we examined 8-year trajectories of social isolation, along with how sensory impairment was associated with initial levels and change over time. Findings suggest that sensory impairments are linked with steeper increases over time among White participants. Among Hispanics vision and hearing impairments were linked with higher initial levels of social isolation, yet no associations were found across time. It may be that Hispanic older adults maintain social connections across time despite potentially isolating sensory impairments.

### EXPLORING HOW HEARING, VISION, AND COGNITION AFFECT OLDER ADULTS' DRIVING EXPOSURE PATTERNS

Jonathon Vivoda,[1] Lisa Molnar,[2] David Eby,[2] Jennifer Zakrajsek,[2] Nicole Zanier,[2] Carolyn DiGuiseppi,[3] Guohua Li,[4] and David Strogatz,[5] *1. Miami University, Oxford, Ohio, United States, 2. University of Michigan, Ann Arbor, Michigan, United States, 3. Colorado School of Public Health, Aurora, Colorado, United States, 4. Mailman School of Public Health, Columbia University, New York, New York, United States, 5. Bassett Healthcare Network, Cooperstown, New York, United States*

Better information is needed about how declines in sensory and cognitive function affect older drivers. This study assessed how hearing loss affects engagement in four challenging driving patterns. Data from the AAA Longitudinal Research on Aging Drivers study was used, including objectively-measured driving; three measures of hearing: reported hearing aid use, self-rated hearing, and the Whisper Test; visual acuity (Tumbling E); and cognition (Trail Making B). Failing the Whisper Test in both ears was related to significantly lower percentage of trips (%trips) at night, on freeways, and during rush hour, but a higher %trips >15 miles. Hearing aid use and self-rated hearing were not associated with any driving differences. Worse vision was related to a lower %trips >15 miles, while worse cognition was associated with a lower %trips at night, on freeways, and during rush hour. The Whisper Test interacted with cognition for rush hour trips.

### SESSION 5735 (SYMPOSIUM)

### SERVICES THAT MATTER FOR AGING IN PLACE: RESEARCH ON THE IMPACT AND PROMISE OF ADULT DAY CENTERS

Chair: Tina Sadarangani
Discussant: Holly Dabelko-Schoeny

Adult day service centers (ADCs) in the United States are increasingly recognized as an important source of community-based long-term care for older adults. However, awareness, widespread utilization, reimbursements, and access to ADCs have been limited by a lack of evidence on ADCs' impact. In this interdisciplinary symposium, we explore current research taking place in the realm of adult day services to understand the reach and impact of ADCs. We begin by examining the most current center-level and user-level data from the National Center for Health Statistics, and demonstrate how these data can be used to inform research and policy. We subsequently evaluate survey data from the National Adult Day Services Association that captures clinical data being collected in ADCs (N=250) surrounding users' clinical outcomes. We then explore the effectiveness of four interventions on ADC users' health and functional status: board games, cognitive behavioral therapy, aromatherapy and dance. Finally, we examine the association between adult day services use by African American persons with dementia and depressive symptoms in their caregivers. Our findings suggest that ADCs serve a complex population with high rates of poverty and chronic conditions, but ADCs can have a meaningful impact on users' health and well-being by leveraging innovative programming. We conclude by discussing how standardization of data collection efforts could enable researchers and policymakers to evaluate ADCs' impact and target funding towards services that maximizes users' health and well-being.

### EVIDENCE FOR THE NEED TO STANDARDIZE DATA COLLECTION ON PATIENT OUTCOMES ACROSS ADULT DAY CENTERS IN THE UNITED STATES

Tina Sadarangani,[1] William Zagorski,[2] and Lydia Missaelides,[3] *1. New York University, New York, New York, United States, 2. American Senior Care Centers, Nashville, Tennessee, United States, 3. California Association for Adult Day Services, Sacramento, California, United States*

Researchers' ability to measure the impact of adult day centers (ADCs) on participants' health has been hampered by a lack of large-scale data. We examined categories of data ADCs across the United States are collecting related to patients' health and health outcomes with the idea of developing a future national cohort of centers. We distributed an electronic survey to ADCs in 50-states on current data collection efforts. Forty states were represented (N=250). Only 32% of ADCs collect patient level data for research and analysis. Vital signs, nutritional risk, falls, and activities of daily living data were most commonly collected. However, validated assessment tools were used in less than 50% of cases. Researchers' ability to pool data on clinical outcomes among ADC users is limited by lack of data collection and use of uniform outcome measures across ADCs. Standardizing data collection is critical to strengthening ADC programs and demonstrating their effectiveness.

### ADVANCING ADULT DAY SERVICES RESEARCH: THE 2016 AND 2018 NATIONAL STUDY OF LONG-TERM CARE PROVIDERS

Jessica Lendon,[1] Vincent Rome,[2] Christine Caffrey,[1] Priyanka Singh,[1] and Manisha Sengupta,[1] *1. National Center for Health Statistics, Hyattsville, Maryland, United States, 2. Centers for Disease Control and Prevention/ National Center for Health Statistics, Hyattsville, Maryland, United States*

This presentation demonstrates how researchers can leverage data from the 2018 redesign and new content from the forthcoming NSLTCP survey of adult day services centers (ADSC) conducted by National Center for Health Statistics. For the first time, NSLTCP data will allow analyses at the services-user level. New policy-relevant topics about centers and ADSC participants include reasons for hospitalization, medication use, patient-centered and end-of-life care, staffing turnover, and unmet needs. Additionally, the presentation highlights latest findings from the 2016 survey. About 53% of centers were primarily medical model. Almost 79% of participants in medical model centers used Medicaid, compared to 51% in social model centers. About 4% of participants had at least one 90-day hospitalization. 40% of participants had difficulty bathing. The most prevalent chronic conditions were hypertension (50%), arthritis (38%), and diabetes (31%). Nearly 40% of participants had an advance directive. Findings are contextualized within the broader understanding of ADSCs.

### ADULT DAY SERVICES AS A PLATFORM FOR INNOVATION: MOVING BEYOND SIMPLY ATTENDANCE

Keith Anderson, *University of Texas at Arlington, Arlington, Texas, United States*

At the most basic level, adult day services (ADS) provide a congregate environment for participants and respite for caregivers. Researchers often evaluate the impact of ADS on participants and caregivers in terms of attendance; however, what happens in ADS (e.g., specific programs and interventions) may be equally or even more important than simply attendance. In this presentation, we review four recent innovative studies conducted in the ADS setting with participants: (a) a board game intervention to improve cognitive functioning; (b) a cognitive behavioral intervention to improve sleep; (c) an aromatherapy intervention to address behavioral issues; and (d) a dance and movement intervention to stimulate physical activity. While these interventions had varying levels of effectiveness, they do support a growing body of evidence that ADS can serve as a platform for innovation and suggest that attendance may be simply one facet of the overall ADS experience.

### ADULT DAY SERVICE USE DECREASES LIKELIHOOD OF DEPRESSIVE SYMPTOMS AMONG BLACK DEMENTIA CAREGIVERS

Lauren Parker,[1] and Laura Gitlin,[2] *1. Johns Hopkins Medicine, Baltimore, Maryland, United States, 2. Drexel University, Philadelphia, Pennsylvania, United States*

Black Americans are more likely than others to age with Alzheimer's Disease (AD) in the community and rely on family members for support. Despite reported positive aspects of caregiving, Black caregivers report greater need for daytime respite and caregiving support. Little is known regarding the health-promoting benefits of daytime respite, like adult day services (ADS), among Black caregivers. Using a sample of 190 Philadelphia-area Black caregivers for community-living persons with dementia, pooled from two behavioral intervention trials: Advancing Caregiver Training and Care of Persons with Dementia in their Environments, the study examined the association between ADS use and depressive symptoms. About 36% of the caregivers used adult day services for their family member with AD. Controlling for demographic variables, social support, self-rated health, religious coping, caregiver burden, and number of years caregiving Black caregivers who utilized ADS had lower depressive symptoms ($\beta = -1.60$, $p<.05$) relative not using ADS.

### SESSION 5740 (SYMPOSIUM)

### SLEEP CHARACTERISTICS, NEIGHBORHOOD FACTORS, FUNCTION, MOOD, AND WELL-BEING IN OLDER ADULTS WITH DEMENTIA

Chair: Miranda McPhillips
Discussant: Nancy Hodgson

The number of people with dementia is increasing worldwide. Circadian rhythm disorders and sleep problems are very common in this population and can have profound effects on well-being. Healthy Patterns Clinical Trial (NCT03682185) is a home-based activity intervention designed to improve circadian rhythm disorders and quality of life in people with dementia and their family caregivers. This symposium is designed to discuss the relationship between sleep characteristics and neighborhood environment, function, and psychological well-being in people with dementia. All analyses in this session were conducted on baseline data from participants enrolled in the Healthy Patterns Clinical Trial. We enrolled 170 individuals (67% female), aged (73.35 ± 8.74) with mean Clinical Dementia Rating (CDR) scores of (0.74 ± 0.51). Session 1 describes the role of neighborhood factors as influencing factors affecting sleep. Session 2 focuses on the relationship between sleep and mood. Session 3 focuses on the relationship between sleep and function. Session 4 focuses on the relationship between sleep and quality of life. Implications for future research and intervention development for people with dementia will be discussed.

### RELATIONSHIP BETWEEN SLEEP AND FUNCTION IN OLDER ADULTS WITH MILD COGNITIVE IMPAIRMENT

Miranda McPhillips,[1] Junxin Li,[2] Darina Petrovsky,[1] and Nancy Hodgson,[3], *1. University of Pennsylvania, Philadelphia, Pennsylvania, United States, 2. Johns Hopkins School of Nursing, Baltimore, Maryland, United States, 3. University of Pennsylvania, Cherry Hill, New Jersey, United States*

Our objective was to examine relationships between sleep characteristics and function in community-dwelling older adults with cognitive impairment. Sleep measures included actigraphy (total sleep time, wake after sleep onset, efficiency, awakenings), Pittsburgh Sleep Quality Index, Epworth Sleepiness Scale. Promis Physical Function Short Form and Promis Item Bank Social were used to measure physical function and social activity. We used Spearman's correlation and multivariate linear regression. In bivariate analyses, physical function was significantly related to daytime sleepiness, wake after sleep onset and awakenings; social activity was significantly related to sleep quality, daytime sleepiness, total sleep time, wake after sleep onset and number of awakenings. Controlling for cognition and age, sleep quality was independently associated with physical function ($\beta$= -0.80; p= 0.002). Relationships between sleep and social activity did not remain significant in multivariate analyses. Preliminary results suggest subjective sleep quality is most related to physical function.

### INFLUENCE OF NEIGHBORHOOD FACTORS ON SLEEP IN HOME DWELLING MULTIETHNIC OLDER ADULTS WITH DEMENTIA

Adriana Perez,[1] Augestine Boateng,[1] Sonia Talwar,[1] and Nancy Hodgson,[2] *1. University of Pennsylvania, Philadelphia, Pennsylvania, United States, 2. University of Pennsylvania, Cherry Hill, New Jersey, United States*

Current scientific paradigms inadequately capture complex clinical, behavioral, and sociocultural factors impacting health and well-being in persons living with dementia (PLWD). The purpose of this study was to identify differences in individual and neighborhood-level factors contributing to sleep among multi-ethnic PLWD. Wrist actigraphy measured objective sleep characteristics. Subjective sleep was assessed using the PROMIS sleep measure. GIS mapping analyzed neighborhood-level factors (walkability, green space, crime index, density). Walkability was significantly associated with subjective sleep (p.006) controlling for age and dementia stage. Number of night awakenings was significantly associated with density, crime and housing value (p<.001). PLWD in neighborhoods with higher population density, annual crime, low median home and low walkability would benefit from interventions targeting unsupportive neighborhood environments to improve sleep.

### HOW IS SLEEP RELATED TO ANXIETY OR DEPRESSION IN OLDER ADULTS WITH DEMENTIA?

Yeji Hwang,[1] and Nancy Hodgson,[2] *1. University of Pennsylvania, Philadelphia, Pennsylvania, United States, 2. University of Pennsylvania, Cherry Hill, New Jersey, United States*

Anxiety and depression are one of the most distressing symptoms for the family caregivers. Little is known about the relationship between sleep impairments and anxiety/depression in this population and how objective and subjective sleep measures differ in relation to anxiety. This study was designed to examine the relationship between sleep impairments and anxiety/depression in people with dementia, using both subjective and objective sleep measures. Among the 170 study participants, 50% (n=85) reported to have anxiety/depression. In univariate logistic regression analyses on anxiety/depression, adjusting for dementia stage, people with more subjective sleep impairment had higher odds of having anxiety/depression (OR=1.111; 95% CI: 1.020-1.211, p=0.016) and people with poorer subjective sleep quality had higher odds of having anxiety/depression (OR=1.702; 95% CI: 1.046-2.769, p=0.032). Objective sleep measures from actigraphy did not show any significant relationships to anxiety/depression. The results suggest that subjective sleep measures are closely related to anxiety/depression in this population.

### RELATIONSHIP BETWEEN SLEEP AND QUALITY OF LIFE IN OLDER ADULTS WITH MILD COGNITIVE IMPAIRMENT

Darina Petrovsky,[1] Miranda Varasse,[1] and Nancy Hodgson,[2] *1. University of Pennsylvania, Philadelphia, Pennsylvania, United States, 2. University of Pennsylvania, Cherry Hill, New Jersey, United States*

Our objective was to examine the independent relationship between sleep characteristics and quality of life (QOL) in community-dwelling older adults with cognitive impairment. Objective sleep variables were derived from actigraphy and included total sleep time, wake after sleep onset (WASO), efficiency, and number of awakenings. Subjective sleep quality was measured using Pittsburgh Sleep Quality Index and daytime sleepiness was measured with the Epworth Sleepiness Scale. Caregiver reported QOL-AD was used for QOL. Analyses included Spearman's correlation and multivariate linear regression. In bivariate analyses, QOL was significantly related to clinical dementia rating scale, sex, depression, daytime sleepiness, sleep quality, WASO, and number of awakenings. Controlling for depression, daytime sleepiness remained independently associated with QOL ($\beta$= -0.24; p= 0.03). In addition, number of awakenings trended towards significance ($\beta$= -0.13; p= 0.07). Results suggest daytime sleepiness and awakenings are associated with QOL in this population.

### SESSION 5745 (SYMPOSIUM)

### SOCIAL CONTEXTS OF SUBJECTIVE AGING PERCEPTIONS

Chair: Ella Cohn-Schwartz
Co-Chair: Liat Ayalon

The way adults perceive their aging process is an important predictor of later life outcomes, including mental and physical health. Despite the importance of living a socially active life in old age, the inter-connections of individuals' perceptions of aging with their social lives and behaviors are not well-understood. This symposium addresses questions of how the social environment and social behaviors are related to subjective aging perceptions, including subjective age and self-perceptions of aging. Two papers examine self-perceptions of aging in the context of couple relations. Mejía and colleagues focus on married older adults' shared beliefs about aging, showing that within older couples, beliefs about aging are shaped in part through partners' co-experience of each other's biological aging. Kim and colleagues also examine couples, finding evidence that changes across time, as well as average differences in individual characteristics, may affect self-perceptions of married/partnered men and women differently. The final two papers examine the interplay between chronological age and perceptions of aging. Weiss and Weiss examine the social conditions and consequences of subjective age across the life span in the work domain, demonstrating that feeling relatively older among young adults and younger among older adults predicts proactive behaviors such as speaking up. Cohn-Schwartz and colleagues investigate the bi-directional temporal associations of adults' self-perceptions of aging and the age composition of their social networks. The symposium concludes with summarizing remarks from the discussant who will suggest possible directions for future research on the social contexts of the perceived experience of aging.

### THE LONGITUDINAL ASSOCIATIONS OF SELF-PERCEPTIONS OF AGING AND THE AGE COMPOSITION OF SOCIAL NETWORKS

Ella Cohn-Schwartz,[1] Markus Schafer,[2] and Liat Ayalon,[3] *1. Ben Gurion University, Haifa, Israel, 2. University of Toronto, Toronto, Ontario, Canada, 3. Bar-Ilan University, Ramay Gan, HaMerkaz, Israel*

Relying on the age segregation theory (limited contact between the generations), this study examined the temporal associations between the age composition of one's social ties and one's self-perceptions of aging (SPA). Data came from the 2014 and 2017 waves of the German Ageing Survey (DEAS). Age composition of the network was assessed as the number of kin and non-kin in the social network who are either five years older or five years younger than the respondent. A latent change score model assessed the bidirectional associations. Adults who had younger social network members, both kin and non-kin, had better SPA three years later. A positive SPA at baseline also predicted a higher number of younger non-kin relationships over time. These results stress the role of SPA in adults' social network as well as the role of the age of social network members in shaping adults' SPA.

### WHEN AND WHY DOES SUBJECTIVE AGE BOOST COMPETENCE AND PROACTIVE WORK BEHAVIOR?

David Weiss, *Leipzig University, Leipzig, Sachsen, Germany*

Subjective age bias suggests that middle-aged and older adults feel relative younger, whereas adolescents and young adults often feel older than their chronological age. However, we still know very little about its social conditions and consequences across the life span particularly within the work domain. Across three studies (correlational, experimental, and field: Ns = 650, 16-85 years), we show that feeling older (among younger adults) and younger (among older adults) is triggered by undesirable age stereotypes concerning competence and status of young and later adulthood and desirable age stereotypes of midlife. We further demonstrate that feeling relatively older among young adults and younger among older adults increases individuals' self-perceived competence at work and predicts proactive behavior such as speaking up. We discuss subjective age bias as socially-mediated phenomenon and how it affects behavior at work across the life span.

### CONCURRENT AND ENDURING ALIGNMENT OF MARRIED PARTNERS' SHARED BELIEFS AND MARKERS OF AGING

Shannon Mejia,[1] Hannah Giasson,[2] Jacqui Smith,[3] and Rich Gonzalez,[3] *1. University of Illinois at Urbana-Champaign, Champaign, Illinois, United States, 2. Stanford University, Stanford, California, United States, 3. University of Michigan, Ann Arbor, Michigan, United States*

Beliefs about aging are grounded in social experience. This study considers the extent to which married older adults' shared beliefs about aging and markers of aging maintain a concurrent and enduring association with their partners' beliefs and markers of aging. Data from the 2010/2012 and 2014/2016 waves of the Health and Retirement Study provided measures of husbands' and wives' (3,779 couples) positive and negative beliefs about aging and internal (Cystatin C) and external (grip strength) markers of aging. Latent dyadic models parsed beliefs and markers into partners' individual and shared variance. Longitudinal analysis showed concurrent associations between shared beliefs and markers of aging to be stable over four years. Meanwhile, the enduring processes that connect beliefs and markers over time were best characterized as bidirectional. The study provides evidence that, within older couples, beliefs about aging are shaped in part through partners' co-experience of each other's biological aging.

### MARRIED OR PARTNERED ADULTS' SELF-PERCEPTIONS OF AGING IN LATER LIFE: THE CONTEXT OF GENDER

Yijung Kim,[1] Kyungmin Kim,[1] Shevaun Neupert,[2] and Kathrin Boerner,[1] *1. University of Massachusetts Boston, Boston, Massachusetts, United States, 2. North Carolina State University, Raleigh, North Carolina, United States*

Marriage or other types of partnerships are consequential for health in later life, but its association to self-perceptions of aging remains a relatively unexplored area of research. This study used three waves of panel data from the Health and Retirement Study (N = 4,315) to examine how changes in the health status and relationship quality over time contribute to self-perceptions of aging for married/partnered men and women. Multilevel models showed that women demonstrated more positive self-perceptions of aging than men, but there was no gender difference in how self-perceptions of aging became more negative over time. The findings on the main and moderating effects of health and relationship quality give evidence that changes across time, as well as average differences in individual characteristics, may affect self-perceptions of married/partnered men and women differently. The context in which gender shapes key aspects of life contributes to self-perceptions of aging in later life.

### SESSION 5750 (SYMPOSIUM)

### SOCIAL PARTICIPATION, SOCIAL SUPPORT, AND SOCIAL POLICY AMONG OLDER ADULTS

Chair: Linda Waite

The social world is closely linked to other dimensions of health, including physical health and illness, physical functioning, cognition and emotional well-being, and these links may change across generations and may depend on social and policy context. The papers in this symposium focus on these links. Carr examines the associations between productive engagement in later life and perceptions of social support and interactions with friends and family. She finds that volunteer engagement is associated with greater perceived social support and interaction with friends and family but not with support from spouse. Waite, Duvoisin and Kotwal measure changes in social participation between the Silent Generation cohort, born between1938 and1947, and the Baby Boom cohort, born from1948-1958. They find find that the gender differences shown in the Silent Generation cohort are reduced among those born during the Baby Boom. Azar examines the moderating role of social policy, particularly defamilization, on the link between loneliness and health, using data from30 European countries and the U.S. Choi compares marital and partnership status, social support and strain in Silent Generation vs. Baby Boom cohorts. Her results suggest that those born during the Baby Boom are embedded in looser social relationships compared to their older counterparts. Together, these papers point to the importance of considering various dimensions of social life, gender, and context, including historical time and the life cycle, in understanding how the social world acts to affect well-being.

### COHORT DIFFERENCES IN SOCIAL PARTICIPATION IN THE NATIONAL SOCIAL LIFE, HEALTH, AND AGING PROJECT

Linda Waite,[1] Rebeccah Duvoisin,[2] and Ashwin Kotwal,[3]
*1. University of Chicago, Chicago, Illinois, United States, 2. NORC at the University of Chicago, Chicago, Illinois, United States, 3. University of California, San Francisco, San Francisco, California, United States*

Has American society become more socially disconnected as Robert Putnam argues in Bowling Alone? Claude Fischer disputes this contention with evidence that Americans remain about as connected to friends and family as in the past. We address this debate with data for older adults from the National Social Life, Health and Aging Study, collected in 2005, 2010, and 2015. We compare social participation as reported at ages 57 to 68 for members of the Silent Generation cohort vs the Baby Boom cohort. We find that the gender gap in social participation evident for the Silent Generation does not exist at all for younger Baby Boomers, only appearing after age 62. These same cohort differences appear for participation in religious services and organized groups. This suggest that the gendered separation of social roles that characterized older generations is becoming less pronounced, with implications for social support and social isolation.

### SESSION 5755 (SYMPOSIUM)

### SOCIAL PROCESSES IN LATE-LIFE SUICIDE

Chair: Elizabeth Necka

Suicide is the tenth leading cause of death in the United States, and social isolation – either real or perceived – is one of the primary risk factors for a suicidal attempt (Calati et al.,2019). Late adulthood is characterized by both rapid increases in both social isolation (Cornwell,2011) and loneliness (i.e., perceived social isolation; Hawkley, Wroblewski, Kaiser, Luhmann, & Schumm,2019), which enhance risk of mental disorders (Santini et al.,2020), as well as by suicide rates that are higher than in any other age group (SAMSHA,2017). What are the mechanisms by which social isolation confers risk (and social connection confers resilience) to suicidal thoughts and behaviors in aging, and what promising interventions exist for addressing social impediments in older adulthood? What barriers exist to providing services to socially isolated older adults contemplating suicide, and what are the public health implications of social isolation and suicide in late life? This symposium will feature talks on the role of social motivation and empathy in the development of (or resilience to) suicidal ideation in older adults, on interventions that draw upon the Interpersonal Theory of Suicide and utilize social engagement and digital 'mHealth' services to reduce late-life social isolation, depression, and suicidal ideation, and on National Institute of Mental Health funding priorities and efforts to address suicide. After attending this session, participants will be able to articulate mechanisms by which social isolation confers risk for suicide in older adulthood and to identify opportunities and obstacles for effective intervention implementation.

### THE NATIONAL INSTITUTE OF MENTAL HEALTH: RESEARCH AGENDA AND PRIORITIES IN GERIATRICS AND AGING

Elizabeth Necka, *National Institute of Mental Health, Rockville, Maryland, United States*

The Geriatrics and Aging Processes Research Branch of the National Institute of Mental Health (NIMH) supports research on the etiology, pathophysiology, and trajectory of late life mental disorders. The branch encourages research using neuroscience, cognitive and affective science, and social and behavioral science to translate basic and preclinical research to clinical research. The branch prioritizes research that investigates neuropsychiatric disorders of aging, how they interact with neurodevelopment/neurodegeneration, and how to assess, treat, and prevent them. Of particular interest is research on social isolation and suicide. Suicide prevention research is an urgent priority: NIMH's portfolio includes projects aimed at identifying those at risk for suicide, understanding causes of suicide risk, developing suicide prevention interventions, and testing the effectiveness of these interventions and services in real-world settings. In this talk, a NIMH program official will discuss the NIMH research agenda in the domain of late-life mental illness, social isolation, and suicide.

### SOCIAL DECISION MAKING AND SUICIDAL BEHAVIOR IN LATE-LIFE DEPRESSION

Katalin Szanto, *University of Pittsburgh, Pittsburgh, Pennsylvania, United States*

Social motivations to engage in suicide in late life frequently include interpersonal problems and escape from perceived defeat. To describe decision making patterns that may contribute to the catastrophic decision to take one's life, we used behavioral experiments and assessed cognitive abilities and personality traits. We found that neuroticism, low extraversion, and low conscientiousness characterize older adults who contemplate suicide and those with low-lethality suicide attempts. Employing a novel version of the Ultimatum Game, we measured empathy's moderating response to social conflict. We found that older suicide attempters were less influenced by empathy scenarios, indicating that a failure to integrate others' emotions into decisions may undermine social deterrents to suicide. To simulate social status loss, we used a newly developed, competitive task (rigged toward primarily losing outcomes) paired with performance ranking. We found that suicide attempters, especially those with narcissistic traits, engaged in more excessive compensatory behaviors than older non-attempters.

### NEUROBIOLOGY INFORMED, MOBILE TECHNOLOGY AUGMENTED INTERVENTIONS FOR LATE-LIFE DEPRESSION AND SUICIDALITY

George Alexopoulos, *Weill Medical College of Cornell University, White Plains, New York, United States*

Depression is a major risk factor of suicide in late life. Evidence based psychotherapies for late-life depression are underutilized, mainly because of their complexity. In response, we created "Engage", an innovative streamlined psychotherapy that relies on neurobiology findings to identify core behavioral pathology of late-life depression and targets it with simple cognitive-behavioral strategies of known efficacy, co-designed with community therapists so that its interventions can be mastered my community based clinicians. We demonstrated that "Engage" is non-inferior to the evidence based Problem Solving Therapy and documented that behavioral activation precedes improvement of depression. We have also shown that activities with important others have the strongest behavioral activation value. "Engage" served as a model for three novel, mobile technology augmented psychotherapies that address the clinical and psychosocial context of distinct populations of depressed older adults. Our interventions and mobile apps provide interventions for emotion regulation as a way of reducing suicidality.

### THE HOPE PROJECT (HELPING OLDER PEOPLE ENGAGE): RELEVANCE TO UPSTREAM SUICIDE PREVENTION

Kim Van Orden,[1] Yeates Conwell,[2] Ben Chapman,[1] Geoff Williams,[1] Siilvia Sörensen,[3] and Jody Rowe,[4]
*1. University of Rochester School of Medicine and Dentistry, Rochester, New York, United States, 2. University of Rochester, Rochester, New York, United States, 3. URMC, Rochester, New York, United States, 4. Lifespan of the Greater Rochester Area, Rochester, New York, United States*

The HOPE Project is an ongoing RCT testing whether Senior Corps volunteering for lonely older adults (age 60+) leads to reduced loneliness and improved quality of life—outcomes associated with suicide in later life. We have randomly assigned 130 participants to 12-months of volunteering or active control. We will describe the trial as well as baseline characteristics of participants that may predict non-compliance with volunteering/control. We found no difference between conditions nor demographic characteristics (age, gender) on non-compliance. Participants demonstrated wide variability in depression at baseline (PROMIS t-score range 38.9 to 71.4) and 18% reported suicide ideation; neither were associated with compliance (p>.20). These preliminary findings indicate that those with more severe mental health symptoms were equally willing/able to engage in volunteering as those without depression and suicide ideation. Volunteering is a highly scalable intervention (given nationwide Senior Corps infrastructure) that may function as upstream suicide prevention.

### SESSION 5760 (SYMPOSIUM)

### SOUNDS OF HEALTHY AGING: ASSESSING EVERYDAY COGNITIVE ACTIVITY FROM REAL-LIFE AUDIO DATA

Chair: Burcu Demiray
Co-Chair: Minxia Luo
Discussant: Matthew Grilli

The healthy aging model of the World Health Organization (2015) highlights the value of assessing and monitoring everyday activities in understanding health in old age. This symposium includes four studies that used the Electronically Activated Recorder (EAR), a portable recording device that periodically collects sound snippets in everyday life, to assess various real-life cognitive activities in the context of healthy aging. The four studies collected over 100,000 sound snippets (30-seconds long) over a few days from young and older adults in the US and Switzerland. Participants' speech in the sound snippets were transcribed and coded for different cognitive activity information. Specifically, Haas and Kliegel have investigated the "prospective memory paradox" by examining the commonality and differences in utterances about retrospective and prospective memory failure in young and older adults' everyday conversations. Demiray and colleagues investigated the relation between autobiographical memory functions and conversation types in young and older adults in relation to well-being. Luo and colleagues have identified the compensatory function of real-world contexts in cognitive aging: Their study showed that older adults benefited from talking with their spouse in producing complex grammatical structures. Finally, Polsinelli and colleagues found robust associations between language markers (e.g., prepositions, more numbers) and executive functions, highlighting the potential use of spontaneous speech in predicting cognitive status in healthy older adults. Finally, Prof. Matthew Grilli will serve as a discussant and provide an integrative discussion of the papers, informed by his extensive work on clinical and cognitive neuroscience of memory in relation to real-life contexts.

### FUNCTIONS OF REAL-LIFE CONVERSATIONAL TIME TRAVEL IN THE CONTEXT OF HEALTHY AGING

Burcu Demiray, Minxia Luo, and Mike Martin, *University of Zurich, Zurich, Zurich, Switzerland*

Using smartphone sensing in real life, we examined conversational time travel (i.e., talking about the personal past versus future), its functions and relation with positive affect (i.e., laughing behavior). We used the Electronically Activated Recorder (audio recorder that periodically records snippets of ambient sounds and speech) and collected a random sample of over 30,000 sound snippets (30 seconds long) from 61 young and 48 healthy older adults across four days. We transcribed and manually coded participants' speech. Multilevel models conducted in R showed that individuals tended to talk about their past with more social functions (e.g., give advice), whereas talked about their future for more directive purposes (e.g., planning). Age group differences were minimal. We also found that individuals laughed two times more while talking about their past than their future. Results are discussed in relation to the functions of mental/conversational time travel in the context of healthy aging.

### REAL-WORLD LANGUAGE USE WITH FAMILIAR VERSUS UNFAMILIAR INTERLOCUTORS IN YOUNG AND OLDER ADULTS

Minxia Luo, Rudolf Debelak, Gerold Schneider, Mike Martin, and Burcu Demiray, *University of Zurich, Zurich, Zurich, Switzerland*

Real-world contexts may compensate for age-related changes in language production. We compared age effects on vocabulary richness (i.e., entropy) and grammatical complexity (i.e., clause length) in conversations with familiar interlocutors (i.e., significant other, friends, family members) versus with strangers. We collected thousands of 30-seconds speech samples from 61 young and 48 healthy older adults across four days using a portable audio recording device — the Electronically Activated Recorder (EAR). Bayesian multilevel analyses showed that participants used richer vocabulary and more complex grammar with familiar interlocutors than strangers. Young adults used richer vocabulary than older adults. Furthermore, older adults produced equally complex grammar with the significant other as young adults did, but simpler grammar with friends and family members. We found no age group differences in grammatical complexity with strangers (lacking statistical power). In sum, familiarity with the significant other may benefit older adults in producing complex grammar in real-world conversations.

### MIND YOUR LANGUAGE: NEW INSIGHTS ON MEMORY AND COGNITIVE AGING RESEARCH THROUGH REAL-LIFE METHODS

Maximilian Haas,[1] Alexandra Hering,[2] and Matthias Kliegel,[2] *1. University of Geneva, Geneva, Switzerland, 2. University of Geneva, Geneva, Geneve, Switzerland*

In the past decades, the so-called "age - prospective memory paradox"– a phenomenon comparing prospective memory (PM) performance in and outside the lab – has challenged the classical assumption that older adults necessarily evidence a marked decline in PM functioning. In our study, we want to extend established methods for measuring memory through arising technologies, such as the Electronically Activated Recorder (EAR; Mehl, 2017). Over the course of three days, 60 younger adults (18-32 years) and 45 older adults (60-82 years) completed an ambulatory assessment with the EAR in order to detect spontaneous speech production related to memory and memory failures. Results reveal that younger and older adults do not differ in the total number of utterances related to different facets of memory and cognition. However, when it comes to failures, older adults talk significantly less about PM failures than younger adults. Possible explanations for these findings will be discussed.

### NATURAL, EVERYDAY LANGUAGE USE PROVIDES A WINDOW INTO THE INTEGRITY OF OLDER ADULTS' COGNITIVE FUNCTIONING

Angelina Polsinelli,[1] Suzanne Moseley,[2] Matthew Grilli,[3] Elizabeth Glisky,[3] and Matthias Mehl,[4] *1. Indiana University School of Medicine, Indianapolis, Indiana, United States, 2. MN Epilepsy Group, St Paul, Minnesota, United States, 3. University of Arizona, Tucson, Arizona, United States, 4. University of arizona, Tucson, Arizona, United States*

Language use during structured clinical tasks predicts pathological cognitive aging. However, structured tasks reflect only a narrow band of potential communication contexts, which limits the ability to capture cognitive processes manifested in language use under more natural conditions (i.e., minimal constraints). The Electronically Activated Recorder (EAR) makes it possible to sample language from the full ecology of individuals' interactions. As interactions are cognitively complex, language use in everyday life might be especially sensitive to the integrity of higher-order cognitive processes, including executive functions (EF). Using the EAR and a standard EF battery, we show that EF, particularly working memory, is reflected in analytic (e.g. articles and prepositions), complex (e.g. longer words), and specific (e.g. more numbers) language. The EAR provides first evidence that the words used in daily life reflect the integrity of EF and that reliance on less complex language could reflect WM variability among cognitively healthy adults.

### SESSION 5765 (SYMPOSIUM)

### STRESS, TRAUMA, AND RESILIENCE AMONG U.S. ASIAN OLDER ADULTS: FINDINGS FROM THE RUTGERS ASIAN RCMAR

Chair: XinQi Dong
Co-Chair: Melissa Simon
Discussant: Bei Wu

U.S. Asians are the fastest growing group of older adults in the nation, increasing by 68% from 2000-2018. However, research on the psychological wellbeing of this population is limited. Drawing on the research of Rutgers Asian RCMAR Scientists, this symposium will address the impacts of stress, trauma and resilience on the psychological wellbeing of diverse groups of U.S. Asian older adults. Session 1 will assess the prevalence of psychological distress among older LGBT and non-LGBT U.S. Asian older adults, and the role of discrimination in medical care and intimate violence on psychological distress. Session 2 will take a mixed-methods approach to examining caregiver burden and depressive symptoms of Chinese American spouses and adult-children who provided care for their spouse or parents with dementia. Session 3 will explore the risk and protective factors for the mental health of sexual minority U.S. Asian older adults using data from the Research Program on Genes, Environment and Health. Session 4 will identify different patterns of coping repertoires of older immigrants, based on a combination of individual, family, and community coping resources, and the optimal coping repertoire that is associated with the best psychological outcomes. In summation, this symposium describes the psychological wellbeing of diverse groups of U.S. Asian older adults, including sexual minority, caregiver and immigrant groups. The symposium addresses both risk factors and the protective factors and coping mechanisms that mediate and mitigate psychological wellbeing and aims to inform interventions to improve psychological wellbeing outcomes in U.S. Asian older adults.

### PSYCHOLOGICAL DISTRESS AMONG OLDER LGBT AND NON-LGBT ASIAN AMERICANS: THE INFLUENCE OF MINORITY STRESS

Chien-Ching Li,[1] Alicia Matthews,[2] and XinQi Dong,[3]
*1. Rush University, Chicago, Illinois, United States, 2. University of Illinois at Chicago, Chicago, Illinois, United States, 3. Rutgers University, New Brunswick, New Jersey, United States*

Emerging data from epidemiological studies have confirmed elevated prevalence rates for mental health conditions among the lesbian, gay, bisexual and transgender (LGBT) populations. An estimated 2.8% of Asian Americans identify as LGBT and 26% of Asian LGBT are 40 years or older. This study analyzed the California Health Interview Survey to examine differences in psychological distress between LGBT and non-LGBT older Asian Americans, and further evaluated the role of discrimination in medical care and intimate violence on psychological distress. Regression results showed older LGBT Asians had a higher psychological distress score compared to non-LGBT Asians. After adjusting for discrimination or violence, this association no longer existed. Experiencing discrimination in medical care and intimate violence were associated with higher levels of psychological stress. This study increases our knowledge of mental health among older Asian LGBT, enhancing our ability to design culturally-targeted and trauma-informed psychosocial interventions to improve outcomes in this population.

### ARE SPOUSES MORE LIKELY TO BE DEPRESSED THAN ADULT CHILDREN IN DEMENTIA CARE? A MIXED-METHODS STUDY

Jinyu Liu, *Weatherhead East Asian institute, New York City, New York, United States*

Using a mixed-methods approach, this study examines caregiver burden and depressive symptoms of Chinese American spouses and adult-children who provided care for their spouse or parents with dementia. Quantitative data were collected from a questionnaire-based survey in 124 Chinese caregivers in New York City and narrative data were gathered from in-depth interviews with 27 of these caregivers. The results of linear regression show that there was no difference in objective burden (caring tasks) between spousal and adult-child caregivers, but spousal caregivers reported significantly higher levels of subjective burden and depressive symptoms. Based on the structural equation modeling, it was found that subjective burden significantly mediated the association between being a spousal caregiver on depressive symptoms. The narrative data show that, compared to the adult-child caregivers, spousal caregivers were more likely to express their worries about the sequence of death (what will happen if they die earlier than their care receiver?).

### EXPLORING THE RISK AND PROTECTIVE FACTORS FOR THE MENTAL HEALTH OF SEXUAL MINORITY ASIAN AMERICANS

Jason Flatt,[1] Rachel Whitmer,[2] and Paola Gilsanz,[3]
*1. University of Nevada, Las Vegas, School of Public Health, Las Vegas, California, United States, 2. University of California Davis, Davis, California, United States, 3. Kaiser Permanente, Oakland, California, United States*

This study characterizes the mental health of Asian American older adults (aged 60+) who identify as sexual minorities (SM or lesbian, gay, bisexual) and compare to their non-Asian American and non-SM counterparts. Data were from the Research Program on Genes, Environment and Health (Aged 60+; N=185,478), a representative sample of healthcare members from Northern California. It includes SM (N=447) and heterosexual/non-SM (N=15,772) older adults who identify as Asian American (Chinese, Japanese, Filipino, and South Asian) and non-Asian American SM (N=3,890). Rates of dementia, anxiety, and PTSD were similar for both SM and non-SM Asian Americans. However, older lesbian and gay Asian Americans were more likely to have a depression diagnosis (30% vs. 18%, p=0.002) compared to non-SM. Overall, mental health outcomes were lower for Asian American SM compared to non-Asian American SM. We discuss need for understanding protective factors for mental health and implications for future interventions.

### ADVERSITY, COPING REPERTOIRE, AND PSYCHOLOGICAL WELL-BEING OF CHINESE OLDER IMMIGRANTS IN THE UNITED STATES

Man Guo,[1] Yi Wang,[1] Jinyu Liu,[2] Meredith Stensland,[3] and XinQi Dong,[4] *1. University of Iowa, Iowa City, Iowa, United States, 2. Weatherhead East Asian institute, New York City, New York, United States, 3. The University of Texas at Austin, Austin, Texas, United States, 4. Rutgers University, New Brunswick, New Jersey, United States*

Using data from 2,923 Chinese older immigrants in Chicago, this study aims to identify different patterns of coping repertoires of older immigrants, based on a combination of individual, family and community coping resources, and the optimal coping repertoire that is associated with the best psychological outcomes. The results of Latent Class Analysis revealed four types of coping repertoires: low-resource (43%), spouse-oriented (32%), community-oriented (15%), and multi-source coping repertoire (10%). Overall, immigrants who had multi-source coping repertoire reported the best psychological outcomes. However, the influence of coping repertoires varied based on specific adversities. Having community-oriented coping repertoire was more protective for widowed immigrants, whereas spouse- or community-oriented coping repertoire was more protective for those with poor health. For less-acculturated older immigrants, having community-oriented coping appears most beneficial to their well-being; and for older immigrants who perceived low filial support from their children, having multi-source coping was associated with better psychological well-being.

### SESSION 5770 (SYMPOSIUM)

### SUICIDE PREVENTION IN OLDER ADULTS: EVIDENCE-BASED APPROACHES FOR CARE

Chair: Luming Li

Suicide in older adults is a major public health concern. Data of suicide rates of older adults from the Centers for Disease Control suggests that suicide is more frequent in older adults and warrants further examination of treatment and public health prevention approaches. Risk factors for suicide in the elderly include functional disability, multiple chronic physical conditions, and social isolation. Several advances have been made in healthcare policy to address practical, evidence-based approaches to preventing suicide and treating behavioral health conditions such as depression, including collaborative care and the Zero Suicide model. This symposium will focus on reviewing the epidemiology and evidence-based approaches for suicide prevention and mental health treatment for older adults. In this presentation, the presenters describe the current trends in suicide rate in older adults in the United States, indicate risk factors (both modifiable and non-modifiable), and present about the collaborative care and Zero Suicide models. Speakers will emphasize the role of these two models in suicide prevention and population-based behavioral healthcare. The presenters will also highlight examples of policy changes and provide recommendations for regulators and hospital systems to adopt these evidence-based models of care for caring for older adults at risk for suicide.

### EPIDEMIOLOGY OF SUICIDE IN OLDER ADULTS

Jane Pearson, *National Institute of Mental Health, ROCKVILLE, Maryland, United States*

This individual symposium abstract will focus on the epidemiology of suicide in older adults, with particular focus on risk factors, changing demographics, and population shifts with the baby-boomers aging. Epidemiologically, older men aged 75 and older have a suicide rate of 39.7 deaths per 100,000 in 2017, compared to the general population of 14.0 deaths per 100,000. Risk factors for suicide in older adults include functional disability, multiple chronic physical conditions, and social isolation. In addition, older adults often face stressors such as relationship issues, life crises (loss of spouse), and social factors (employment and financial challenges, housing stress, and legal issues). Limited mobility, physical and mental health conditions, and lack of social support can affect healthcare access and utilization. Many older adults do not routinely seek behavioral health treatment, with reported under-detection of mental health conditions such as depression, substance use disorders, and suicidal ideation.

### EVIDENCE-BASED APPROACHES TO SUICIDE IN OLDER ADULTS—ZERO SUICIDE

Luming Li, *Yale School of Medicine, New Haven, United States*

This individual symposium abstract will focus on evidence-based approaches to suicide in older adults, with particular focus on the Zero Suicide Model. Zero Suicide Model is a framework that applies seven essential elements of suicide care (Lead, Train, Identify, Engage, Treat, Transition, Improve). The model provides a systematic approach for quality improvement for suicide prevention and offers implementation strategies for “real-world” clinical settings using the Assess, Intervene, and Monitor for Suicide Prevention (AIM-SP) program for suicide-safer care. The authors will describe implementation of Zero Suicide in general healthcare settings that care for older adults, including health systems and outpatient clinics. The authors will also describe the value of Zero Suicide other settings such as long-term care centers, where older adults are cared for. In addition, the authors will describe future directions for research in the Zero Suicide Model and additional opportunities in public policy for suicide prevention.

### EVIDENCE-BASED APPROACHES TO SUICIDE IN OLDER ADULTS—COLLABORATIVE CARE

Michael Schoenbaum, *National Institute of Mental Health, Bethesda, Maryland, United States*

This individual symposium abstract will focus another evidence-based approach to mental health treatment and in older adults, the collaborative care model. Collaborative care is a consultation-based approach in primary care that has been described with multiple clinical trials, with significant benefit for access and treatment. The Prevention of Suicide in Primary Care Elderly: Collaborative Trial (PROSPECT) using the collaborative care model found that those older adults receiving the intervention had a higher utilization of mental health treatment (psychotherapy and/or antidepressant treatment) and had a 2.2 times greater decline in suicidal ideation over 24 months. The authors will describe the utility of using the collaborative care model on the identification of suicidal ideation and subsequent mental health treatment for older adults. The authors will also share about challenges and successes related to collaborative care implementation in healthcare settings for older adults, and relevant policy and financing components for the model.

### SESSION 5775 (SYMPOSIUM)

### SUPPORT, HEALTH, ACTIVITIES, RESOURCES, & EDUCATION (SHARE): NEW PROGRAM INNOVATIONS FOR EARLY-STAGE FAMILIES

Chair: Carol Whitlatch
Co-Chair: Steven Zarit
Discussant: Steven Zarit

Persons living with a recent diagnosis of dementia experience great uncertainty and stress as they and their families try to adjust to the new reality of their lives and futures. One fruitful strategy for intervening with these families is to include both the person living with dementia and their family care partner in the program. Although dyadic approaches are rare among early-stage programs, promising examples exist. The SHARE Program (Support, Health, Activities, Resources, and Education) is one exception where dyadic materials address: 1) current and long- term needs of care partners, and 2) how the family can develop a realistic plan of care based on their care values and preferences. This symposium describes the development and positive outcomes of the original SHARE intervention and the promising adaptations that expand how and to whom the intervention is delivered. Presentations explore: 1) the original SHARE for Dementia program and strategies for expanding its reach into chronic conditions populations (Orsulic-Jeras & Whitlatch), 2) a group version translated into Spanish (“EPIC: Early-stage Partners in Care,” Dr. Coon), and 3) the development of a remote needs assessment and unobtrusive in-home monitoring technology platform that guides care planning and helps to maintain independence (“SHARE-sense,” Dr. Miller). Discussion will focus on the challenges, unique solutions, and positive outcomes when adapting SHARE to different settings and populations (Dr. Zarit).

### ADAPTING THE SHARE PROGRAM FOR USE WITH FAMILIES FACING THE CHALLENGES OF CHRONIC ILLNESS

Silvia Orsulic-Jeras, and Carol Whitlatch, *Benjamin Rose Institute on Aging, Cleveland, Ohio, United States*

Advances in diagnostic procedures have helped to make diagnosing Alzheimer's disease and other dementias more accurate and to occur earlier in the disease progression. For persons living with dementia and their family care partners, finding programs that meet their needs for support post diagnosis can be challenging. Likewise, for persons with chronic conditions, few programs exist which help care dyads to create a manageable plan of care that addresses each person's concerns and fears. SHARE, (Support, Health, Activities, Resources, and Education), originally designed for dementia care partners, has shown positive outcomes for both members of the care partnership. This presentation describes the development of the six-session SHARE intervention, its implementation in community settings, and its current standing as an evidence-based program and product that has been commercialized. Discussion will also focus on adapting SHARE for use with chronic illness families, highlighting revisions to program procedures, materials, recruitment, and evaluation.

### DEVELOPMENT OF A REMOTE ASSESSMENT AND DYNAMIC RESPONSE INTERVENTION FOR DEMENTIA-RELATED CARE NEEDS

Lyndsey Miller,[1] Christina Reynolds,[2] Carol Whitlatch,[3] Joel Steele,[4] and Jeffrey Kaye,[5] *1. Oregon Health and Science University, Portland, Oregon, United States, 2. Oregon Health & Science University, Portland, Oregon, United States, 3. Benjamin Rose Institute on Aging, Cleveland, Ohio, United States, 4. Portland State University, Portland, Oregon, United States, 5. Layton Alzheimer's Disease Center, Portland, Oregon, United States*

Unmet dementia-related care needs are highly prevalent, and are detrimental to the care dyad's health and well-being, safety, and ability to age in place. The goal of this study was to develop an ecologically-valid needs assessment and integrate it with aspects of the SHARE intervention to inform values-based care planning. Using digital behavioral data collected via an actigraphy watch and multimodal sensors installed in the homes of 76 older adult couples with and without dementia, we created a prototype of the objective measures informing READyR: time spent together or separate as a dyad, exits from the home, sleep habits, physical activity, daily weight, driving habits, and medication taking behavior. These digital behavioral data were then mapped onto care values (e.g. safety, avoiding burden & autonomy) to create a values-based needs assessment protocol that is tailored to the individual care dyad. Discussion will focus on future testing and applications of READyR.

### TRANSLATING A GROUP-BASED DYADIC INTERVENTION FOR EARLY-STAGE PEOPLE AND THEIR CARE PARTNERS FOR SPANISH SPEAKERS

DAVID Coon, Marielysse Cortes, Abigail Gomez, Berta Carbajal, Maria Gonzalez, Lourdes Cordova, and Zenya Weatherall, *Arizona State University, Phoenix, Arizona, United States*

This presentation focuses on the development of and recruitment for a Spanish language version of EPIC, a group-based dyadic intervention for people with early-stage dementia (EPs) and their care partners (CPs). EPIC involves activities for the entire group together, separate EP or CP groups, and one individualized session for each dyad. EPIC builds on components of SHARE combined with strategies from other psychoeducational interventions (e.g., skills-training, care values clarification, care planning). Findings from 6 focus groups with Latino participants were used to help with cultural responsiveness such as fluent Spanish/bilingual staff, testimonials from Latino families affected by dementia, and minimization of "threatening" language for memory loss. Participants also provided suggestions for recruitment for Spanish speakers such as engaging promotores, using social media across generations, recruiting through Spanish language health classes, and recognizing the impact of the political environment. The presentation will review both recruitment successes and challenges using these approaches.

### SESSION 5780 (SYMPOSIUM)

### THE COMPLEX ROLE OF DISCRETE EMOTIONS IN SUCCESSFUL AGING

Chair: Jeremy Hamm
Co-Chair: Carsten Wrosch

Research shows that emotions play an important role in successful aging. However, previous studies have largely focused on the implications of dimensional indicators of emotion, such as positive and negative affect. This approach may fail to capture important distinctions between discrete emotions such as sadness, loneliness, calmness, and empathy that could become more or less adaptive with age. The present studies adopt a discrete emotion perspective to examine age-related changes in the consequences of different positive and negative emotions for successful aging. Drawing from an evolutionary-functionalist perspective, Haase, Wu, Verstaen, and Levenson investigate whether sadness becomes more salient and adaptive in old age using a multi-method approach. Lee, Lay, Mahmood, Graf, and Hoppmann address the seemingly contradictory consequences of loneliness by examining how state- and trait-loneliness interact to predict older adults' prosocial behaviors. Hamm, Wrosch, Barlow, and Kunzmann use two studies to examine the diverging salience and 10-year health consequences of discrete positive emotions posited to motivate rest and recovery (calmness) or pursuit of novelty and stimulation (excitement). Barlow and Mauss study the co-occurrence of discrete emotions and their age-dependent associations with well-being using an adult lifespan sample. Finally, Wieck, Katzorreck, Gerstorf, Schilling, Lücke, and Kunzmann examine lifespan changes in the adaptive function of empathy by assessing the extent to which empathic accuracy protects against stress-reactivity as people age. This symposium thus integrates new research on the role of discrete positive and negative emotions and will contribute to a deeper understanding of the complex relationships between emotions and successful aging.

### A TALE OF TWO EMOTIONS: THE DIVERGING SALIENCE AND HEALTH CONSEQUENCES OF CALMNESS AND EXCITEMENT IN OLD AGE

Jeremy Hamm,[1] Carsten Wrosch,[2] Meaghan Barlow,[3] and Ute Kunzmann,[4] *1. North Dakota State University, Fargo, North Dakota, United States, 2. Concordia University,*

*Montreal, Quebec, Canada, 3. University of California, Berkeley, Berkeley, California, United States, 4. University of Leipzig, Leipzig, Sachsen, Germany*

Using two studies, we examined the late life prevalence and health consequences of discrete positive emotions posited to motivate rest and recovery (calmness) or pursuit of novelty and stimulation (excitement). Study 1 assessed the salience of these discrete emotions in older adults (n=73, Mage=73) relative to younger adults (n=73, Mage=23) over a one-week period. Multilevel models showed that older (vs. younger) adults reported higher calmness and lower excitement. Study 2 examined the longitudinal health consequences of calmness and excitement in old age (n=336, Mage=75), as moderated by perceived control. Multilevel growth models showed that calmness, but not excitement, buffered against 10-year declines in psychological well-being (perceived stress, depressive symptoms) and physical health (physical symptoms, chronic conditions) for older adults with low perceived control. Results suggest that positive emotions with disparate motivational functions become more (calmness) or less (excitement) salient and have diverging implications for health in old age.

### THE BRIGHT SIDES OF SADNESS IN LATE LIFE

Claudia Haase,[1] Deborah Wu,[2] Sandy Lwi,[3] Alice Verstaen,[3] and Robert Levenson,[3] *1. Northwestern University, Evanston, Illinois, United States, 2. University of Massachusetts Amherst, Amherst, Massachusetts, United States, 3. University of California, Berkeley, Berkeley, California, United States*

Sadness is often thought of as unpleasant and dysfunctional. Yet, evolutionary-functionalist approaches and discrete emotional aging frameworks suggest that sadness is an emotion that helps us deal with loss and thus may become particularly salient and adaptive in late life. This talk presents findings from a multi-study, multi-method research program using age-diverse samples and experimental and longitudinal study designs. Findings show (1) intact or elevated levels of sadness responding in late life (i.e., higher sadness expressions in response to distressing film clips; higher coherence between sad facial expressions and autonomic physiology in response to film clips depicting loss; stability in sadness behaviors in marital conflict interactions). Moreover, (2) higher levels of sadness responding are linked to adaptive outcomes in late life (i.e., higher social connectedness, higher compensatory control strategies) with some effects generalizing across age groups (i.e., links between sadness coherence and well-being). Implications for future research are discussed.

### LONELINESS AND SOCIAL ENGAGEMENT: THE UNIQUE ROLES OF STATE AND TRAIT LONELINESS FOR DAILY PROSOCIAL BEHAVIORS

Yeeun Lee,[1] Jennifer Lay,[2] Atiya Mahmood,[3] Peter Graf,[1] and Christiane Hoppmann,[1], *1. University of British Columbia, Vancouver, British Columbia, Canada, 2. Chinese University of Hong Kong, Hong Kong, China, 3. Simon Fraser University, Vancouver, British Columbia, Canada*

Loneliness is a distressing yet adaptive emotional experience that alerts us to socially re-engage. However, loneliness can also lead to social withdrawal and isolation. To reconcile the seemingly contradictory consequences of loneliness, we unpack the timing of the underlying processes by distinguishing between the roles of state loneliness (i.e., daily variations in loneliness) and trait loneliness (i.e., person-average loneliness) in predicting social re-engagement. Using ten days of electronic daily assessments from 95 older adults (M age = 67.0 years; 64.2% women), initial findings indicate that trait loneliness moderates time-varying associations between state loneliness and prosocial behavior: On days of elevated state loneliness, older adults low in trait loneliness report increases in prosocial behavior, whereas older adults high in trait loneliness show decreases in prosocial behavior. Findings suggest that transient loneliness may motivate older adults to actively re-engage with others; chronic loneliness may undermine such adaptive responses.

### DISCRETE EMOTION NETWORKS ACROSS THE LIFESPAN: IMPLICATIONS FOR WELL-BEING

Meaghan Barlow,[1] and Iris Mauss,[2] *1. University of California, Berkeley, Berkeley, California, United States, 2. Univerity of California, Berkeley, Berkeley, California, United States*

Research examining the age-related trajectories and consequences of emotional complexity has largely lumped emotions into broad categories. The present study utilized network analyses to quantify the co-occurrence of discrete emotions and their associations with well-being across the lifespan in a sample of 156 females (aged 23-79). Participants completed assessments of 8 emotions (i.e., sad, angry, anxious, lonely, happy, excited, proud, and calm) for 16 days, and completed measures of psychological and physical well-being at a 4-month follow-up. While certain emotions were found to co-occur at similar rates across the lifespan (e.g. sad-anxious), other emotion pairs become more (e.g. sad-calm) or less (e.g. sad-angry) frequent with age. Additionally, specific emotion pairs were differentially associated with indicators of well-being across the lifespan, while controlling for mean levels of these emotions. These findings point to the importance of considering the co-occurrence of distinct emotions and potential pathways towards successful aging.

### EMPATHIC ACCURACY: HELPFUL TO AVOID NEGATIVE AFFECT IN OLD AGE?

Cornelia Wieck,[1] Martin Katzorreck,[2] Denis Gerstorf,[3] Oliver Schilling,[4] Anna Jori Lücke,[4] and Ute Kunzmann,[2] *1. Leipzig University, Leipzig, Sachsen, Germany, 2. University of Leipzig, Leipzig, Sachsen, Germany, 3. Humboldt University Berlin, Berlin, Berlin, Germany, 4. Heidelberg University, Heidelberg, Baden-Wurttemberg, Germany*

Past work suggests age-related declines in empathic accuracy and that these declines may put older people at risk for heightened stress reactivity and low affective well-being. We addressed these questions using data from the fourth wave of the Interdisciplinary Longitudinal Study of Aging (ILSE). To assess empathic accuracy, the young-old (N=115, Mage=63.4, SDage=1.13) and old-old (N=31, Mage=82.3, SDage=.87) participants of ILSE watched six film clips of individuals, who thought-aloud about an emotional autobiographical event, and were asked to rate each individual's emotions. Subsequently, participants watched a film about Alzheimer's disease and their subjective and cardiovascular stress reactions were assessed. Empathic accuracy was lower in old-old, as compared with young-old, individuals. Furthermore, empathic accuracy was only associated with low levels of stress reactivity among young-old but not old-old individuals. This suggests that empathic accuracy is not only compromised in very old age, but also appears to be of lower adaptive utility.

### SESSION 5785 (SYMPOSIUM)

### THE CRITICAL HELP-SEEKING ROLE OF FAMILY, FRIENDS, AND NEIGHBORS IN THE LIVES OF ELDER ABUSE VICTIMS

Chair: Marlene Stum
Discussant: David Burnes

Elder abuse prevention and intervention is a complex puzzle. We focus on examining the typically invisible role, experience, and impact of nonabusing family, friends, and neighbors, or "concerned persons" in stopping elder abuse. Given the reality that most elder abuse goes unreported and unaddressed, it seems essential to understand if and how concerned persons can play a role in help-seeking for older victims, and to also understand the needs and issues faced by concerned persons as a consequence. First. Breckman presents evidence of the significant distress concerned person's experience from knowing about elder abuse and trying to assist victims, and shares experience developing and implementing the first Elder Abuse Helpline for Concerned Persons in the U.S. Second, Fraga Dominguez et.al. present an important international perspective highlighting findings about concerned persons as users of a UK elder abuse helpline, their profile, the impact of helping, and variables relating to help-seeking. Third, Stum shares findings from a qualitative study of elder family financial exploitation related to what concerned family members were trying to accomplish by getting involved (motivating goals) and the resulting continuum of outcomes. Fourth, Kilaberia also explores the help-seeking experiences of concerned family members in elder family financial exploitation situations, specifically the range of tasks involved, and the impacts on the concerned family member's individual health and well- The discussion led by Burnes will focus on understanding contributions of the research presented given the current state of the field, and offer suggestions for future research and intervention directions.

### WHEN CONCERNED FAMILY MEMBERS INTERVENE IN ELDER FAMILY FINANCIAL EXPLOITATION: GOALS AND OUTCOMES

Marlene Stum, *University of Minnesota, Twin Cities, Saint Paul, Minnesota, United States*

The role and experience of non-abusing concerned family members (CFMs) in elder family financial exploitation (EFFE) is largely unexplored. This paper examines the experience of "trying to do the right thing," focusing on what CFM's were trying to accomplish (motivating goals), and resulting outcomes utilizing data from a qualitative study of 28 CFMs (primarily female adult children of an older victim, and siblings of primary perpetrator). Five common goals appear to be motivating CFM involvement, driven by a priority to ensuring the victim's quality of life, as well as the desire to honor and respect the victim's wishes, protect the victim's financial well-being, preserve family relationships, and deal with the perpetrator(s). CFM help-seeking resulted in a wide range of outcomes, from making a difference by connecting victims to supportive services and interrupting the financial exploitation, to mixed results, and in other cases frustration with no desirable outcomes.

### WHEN HELPING HURTS: NONABUSING FAMILY, FRIENDS, AND NEIGHBORS IN THE LIVES OF ELDER MISTREATMENT VICTIMS

Risa Breckman,[1] David Burnes,[2] Sarah Ross,[3] Philip Marshall,[4] J. Jill Suitor,[5] Mark Lachs,[6] and Karl Pillemer,[3] *1. Weill Cornell Medicine/ NYC Elder Abuse Center, New York, New York, United States, 2. University of Toronto, Toronto, Ontario, Canada, 3. Cornell University, Ithaca, New York, United States, 4. Historic Preservation Program, Roger Williams University, Bristol, Rhode Island, United States, 5. Purdue University, West Lafayette, Indiana, United States, 6. Weill Cornell Medicine, New York City, New York, United States*

Research conducted by the NYC Elder Abuse Center (NYCEAC) at Weill Cornell Medicine and colleagues found that concerned persons experience significant distress knowing about elder abuse and trying to assist victims. Data will be presented from a nationally representative survey which included items on concerned persons in elder abuse. Thirty-one percent of all respondents reported that they had a relative or friend who experienced elder abuse; of these, 61% had attempted to help the victim and over 80% reported the experience is very or extremely stressful (2017). By both knowing about and becoming involved in elder abuse situations, concerned persons experience significant emotional and practical problems and often need professional help. NYCEAC's Elder Abuse Helpline for Concerned Persons is the first of its kind in the country. The Helpline's services and structure will be explained, and possibilities for replication in other locations will be explored.

### EXPLORING THE HELP-SEEKING EXPERIENCE OF CONCERNED PERSONS: FINDINGS FROM AN ELDER ABUSE UK HELPLINE

Silvia Fraga Dominguez,[1] Jennifer Storey,[2] and Emily Glorney,[3] *1. Royal Holloway, University of London, Egham, United Kingdom, 2. University of Kent, Canterbury, England, United Kingdom, 3. Royal Holloway, University of London, Egham, England, United Kingdom*

Despite their potential role in elder abuse cases, knowledge about concerned persons outside of North America is scarce. This paper will discuss findings from a study focusing on concerned persons in the UK, by addressing their profile, the impact of helping, and several variables relating to help-seeking. Researchers used secondary data from a charity's helpline, encompassing a year of recorded cases (N = 1623). Concerned persons (n = 1352) were often related to the victim (80%) and/or perpetrator (59%). In 43% of cases, they reported impact as a result of their awareness of the situation or supporting the victim. This impact was thematically analysed and ranged widely in terms of severity, from slight worry to being subjected to the perpetrator's homicide threats, and it often affected the person's mental health or financial situation. Concerned persons reported substantial barriers to action relating to the access to and responses from formal services.

### CONCERNED FAMILY MEMBERS' HELP-SEEKING IN ELDER FAMILY FINANCIAL EXPLOITATION

Tina Kilaberia,[1] and Marlene Stum,[2] *1. UC Davis Health, Sacramento, California, United States, 2. University of Minnesota, Twin Cities, Saint Paul, Minnesota, United States*

This paper examines non-perpetrator family members' experience of trying to help when faced with elder family financial exploitation. Utilizing data from a qualitative study of 28 Concerned Family Members (CFMs) who were primarily adult children of older victims, findings provide evidence of the critical role CFMs play in helping the victims. Six help-seeking tasks are identified, including gathering evidence, learning new systems, and taking on money management roles. CFMs often put the victim's health and well-being before their own, becoming secondary victims in the process. CFMs experienced a wide range of costs to their individual health and well-being, including physical, emotional, psychological, social and financial dimensions (e.g. stress, depression, inability to sleep, isolation, harassment, threats to personal safety, physical abuse, attorney and court costs, time off work). Findings have implications for supporting CFMs and addressing practical and health-related needs as secondary victims.

### SESSION 5790 (SYMPOSIUM)

### THE IMPACT OF FUNCTION FOCUSED CARE FROM ACUTE CARE TO HOME CARE AND NURSING HOMES

Chair: Silke Metzelthin
Co-Chair: Sandra Zwakhalen
Discussant: Barbara Resnick

Functional decline in older adults often lead towards acute or long-term care. In practice, caregivers often focus on completion of care tasks and of prevention of injuries from falls. This task based, safety approach inadvertently results in fewer opportunities for older adults to be actively involved in activities. Further deconditioning and functional decline are common consequences of this inactivity. To prevent or postpone these consequences Function Focused Care (FFC) was developed meaning that caregivers adapt their level of assistance to the capabilities of older adults and stimulate them to do as much as possible by themselves. FFC was first implemented in institutionalized long-term care in the US, but has spread rapidly to other settings (e.g. acute care), target groups (e.g. people with dementia) and countries (e.g. the Netherlands). During this symposium, four presenters from the US and the Netherlands talk about the impact of FFC. The first presentation is about the results of a stepped wedge cluster trial showing a tendency to improve activities of daily living and mobility. The second presentation is about a FFC training program. FFC was feasible to implement in home care and professionals experienced positive changes in knowledge, attitude, skills and support. The next presenter reports about significant improvements regarding time spent in physical activity and a decrease in resistiveness to care in a cluster randomized controlled trial among nursing home residents with dementia. The fourth speaker presents the content and first results of a training program to implement FFC in nursing homes. Nursing Care of Older Adults Interest Group Sponsored Symposium

### KEEP MOVING TOWARD RECOVERY! FUNCTION-FOCUSED CARE IN HOSPITALIZED STROKE AND GERIATRIC PATIENTS

Janneke de Man-van Ginkel,[1] Carolien Verstraten,[1] Marieke Schuurmans,[2] Silke Metzelthin,[3] Johannes Reitsma,[1] and Lisette Schoonhoven,[2] *1. Julius Center for Health Sciences and Primary Care, UMC Utrecht, Utrecht, Utrecht, Netherlands, 2. University Medical Center Utrecht, Utrecht, Utrecht, Netherlands, 3. Maastricht University, Maastricht, Limburg, Netherlands*

Many hospitalized patients experience decline in functional status. Function Focused Care (FFC) has demonstrated to improve patients' functional status in long-term care. In a stepped wedge cluster trial in 893 hospitalized geriatric and stroke patients, we investigated the effectiveness of FFC compared to usual care (FFC: n=427, UC: n=466) on ADL and mobility. We measured the Barthel Index and the Elderly Mobility Scale, and analysed using a mixed-model multilevel method. At discharge, 3 month and 6 months, the mean difference (MD) was in favour of FFC, although at none of the time points the level of significance was reached: the MD for ADL was respectively: 0.79 (95%CI: -0.98-2.56), 0.43 (95%CI: 0.10-1.79), and 0.57 (95%CI: -1.34- 2.48). For mobility, the MD was respectively 0.89 (95%CI: -1.01-2.80), 0.78 (95%CI: -1.18; 2.75), and 1.09 (95%CI: -0.88-3.07). Although the results are inconclusive, FFC shows a tendency to improve ADL and mobility in hospitalized patients. Part of a symposium sponsored by Nursing Care of Older Adults Interest Group.

### A TRAINING PROGRAM FOR PROFESSIONALS TO ENCOURAGE INDEPENDENCE OF HOME-LIVING OLDER ADULTS: A PROCESS EVALUATION

Teuni Rooijackers,[1] G.A. Zijlstra,[1] Erik van Rossum,[2] Ruth G. Vogel,[1] Marja Veenstra,[3] Gertrudis I.J. Kempen,[1] and Silke Metzelthin,[1] *1. Maastricht University, Maastricht, Limburg, Netherlands, 2. Zuyd Hogeschool, Heerlen, Limburg, Netherlands, 3. Burgerkracht Libmurg, Sittard, Limburg, Netherlands*

Stay Active at Home (SAaH) was developed to change homecare professionals' behavior towards encouraging older adults' independence in daily activities. This mixed-methods study evaluated SAaH regarding implementation, mechanisms of impact, and context. SAaH was implemented in five Dutch homecare teams (162 professionals). Quantitative data were collected from all professionals, and five focus groups with 23 professionals and 4 interventionists were performed. Data were analyzed using descriptive statistics and qualitative content analysis. SAaH was feasible to implement. Professionals visited on average 73% of the programme meetings. They reported positive changes in their knowledge, attitude, and skills, and perceived social and organizational support regarding the new way of working. The extent to which professionals applied SAaH in practice varied. SAaH was easier to apply among new clients. Perceived barriers were time pressure and staff shortages, and people's resistance to change. Tailoring the intervention to professionals' needs and wishes could improve their compliance. Part of a symposium sponsored by Nursing Care of Older Adults Interest Group.

### FUNCTION-FOCUSED CARE IN THE DUTCH NURSING HOME SETTING: PAST AND CURRENT PRACTICES

Stan Vluggen,[1] Silke Metzelthin,[1] Janneke de Man-van Ginkel,[2] Getty Huisman - de Waal,[3] and Sandra Zwakhalen,[1] *1. Maastricht University, Maastricht, Limburg, Netherlands, 2. Julius Center for Health Sciences and Primary Care, UMC Utrecht, Utrecht, Utrecht, Netherlands, 3. Radboud University Medical Center, Nijmegen, Gelderland, Netherlands*

In recent years, Function Focused Care (FFC) interventions have been developed and implemented in Dutch home care, nursing homes and acute care. These interventions aimed to train nursing staff to adapt their level of support to the capabilities of elderly and to maintain/optimize their self-reliance and physical functioning. After synthesizing knowledge and experiences from the existing FFC-interventions, an advanced FFC-intervention was developed for application in long-term care: 'SELF'. SELF comprises seven interactive sessions, is theoretically grounded, primarily focuses on behavior change in nursing staff, is tailored to the ward's needs, is multidisciplinary in nature, and focuses on all care interactions. Currently, SELF is pilot-tested in one psycho-geriatric ward with 17 nursing staff members. Questionnaires on outcome-expectations, self-efficacy and intention to stimulate elderly are assessed at baseline and after 3 months. Field notes and interviews are used to assess its feasibility and acceptability. Experiences will guide the further refinement of 'SELF'. Part of a symposium sponsored by Nursing Care of Older Adults Interest Group.

### OPTIMIZING PHYSICAL ACTIVITY AND DECREASING CARE-RESISTANT BEHAVIORS AMONG NURSING HOME RESIDENTS WITH DEMENTIA

Elizabeth Galik,[1] Barbara Resnick,[2] Erin Vigne,[2] and Sarah Holmes,[3] *1. University of Maryland School of Nursing, Ellicott City, Maryland, United States, 2. University of Maryland School of Nursing, Baltimore, Maryland, United States, 3. University of Maryland School of Pharmacy, Baltimore, Maryland, United States*

The purpose of this study was to test the effectiveness of the Function and Behavior Focused Care (FBFC) intervention on function, physical activity and behavioral symptoms among nursing home residents with dementia. This study was a clustered, randomized controlled trial with a repeated measures design in 12 nursing homes. The participants (N=336) were 82.6 (SD=10.1) years of age, mostly female and were moderate to severely cognitively impaired (MMSE of 7.8, SD=5.1). There were a statistically significant improvements in time spent in light, moderate and overall physical activity based on actigraphy and a decrease in resistiveness to care at 4 months among participants in the treatment group. There was no change in mood, agitation, and the use of psychotropic medications. This study provides some support for the use of the FBFC intervention to increase time spent in physical activity and decrease resistive behaviors during care among nursing home residents with dementia. Part of a symposium sponsored by Nursing Care of Older Adults Interest Group.

### SESSION 5795 (SYMPOSIUM)

### THE ROLE OF PEER SPECIALISTS IN ENGAGING AGING ADULTS IN RECOVERY AND WELLNESS BEHAVIORS

Chair: Amanda Peeples
Discussant: Laura Wray

Peer specialists are individuals who have a lived experience with a mental health condition and who have received formal training to provide peer support and services to others. This symposium will examine the role of peer specialists in supporting aging adults' recovery, quality of life, health behaviors, and health outcomes. Peer specialists have increasingly been integrated as formal service providers in health care systems in a wide variety of settings. In the Veterans Health Administration (VHA), over 1,110 Veterans work as peer specialists across the country. Findings from both VHA and community studies will be presented. First, Amanda Peeples will present findings from a mixed-methods evaluation study on the implementation of peer specialists to primary care teams in the VHA. Next, Anjana Muralidharan will present on the role of peer specialists in promoting health and wellness among aging veterans with serious mental illness using data from three VHA studies on peer-led interventions. Finally, Karen Fortuna will present on PeerTECH, a digital peer support self-management intervention that teaches older adults with mental health conditions how to co-manage psychiatric illness and chronic health conditions. Together, these three presentations will explore the ways in which "peerness" is defined in different populations and in diverse contexts. Laura Wray, geropsychologist and Executive Director of the VHA Center for Integrated Healthcare, will serve as discussant. She will comment on the role of peer specialists in supporting the recovery and wellness of aging persons in VHA and beyond.

### PEERS IN PRIMARY CARE: HOW PEER SPECIALISTS SUPPORT AGING VETERANS

Amanda Peeples,[1] Anjana Muralidharan,[2] Lorrianne Kuykendall,[1] Richard Goldberg,[3] and Matthew Chinman,[4] *1. US Department of Veterans Affairs, Baltimore, Maryland, United States, 2. VA Capitol Healthcare Network, Baltimore, Maryland, United States, 3. Veterans Affairs Capitol Healthcare Network, Baltimore, Maryland, United States, 4. US Department of Veterans Affairs, Pittsburgh, Pennsylvania, United States*

Most of the more than 1,100 peer specialists ("peers") in the Veterans Health Administration (VHA) work in mental healthcare settings. These peers provide a variety of services to Veterans such as facilitating groups, teaching recovery and coping skills, connecting Veterans with VHA and community services, and helping Veterans navigate VHA care. In 2014 the White House issued an Executive Action mandating the reassignment of peers from mental health to primary care settings in 25 VHA locations nationwide. This paper presents qualitative findings from a mixed-methods study evaluating this implementation of peers in VHA primary care. We found that peers assisted aging Veterans in primary care through activities such as providing health coaching, facilitating health education groups, connecting Veterans with services, and providing general peer support. Findings are drawn from semi-structured interviews with 27 peers, 25 supervisors, and

10 Veterans who received services from the peers in primary care.

### VA PEER PROVIDERS SUPPORTING PHYSICAL HEALTH AND WELLNESS AMONG AGING VETERANS WITH MENTAL HEALTH CONDITIONS

Anjana Muralidharan,[1] Amanda Peeples,[2] Alicia Lucksted,[1] and Richard Goldberg,[3] *1. VA Capitol Healthcare Network, Baltimore, Maryland, United States, 2. US Department of Veterans Affairs, Baltimore, Maryland, United States, 3. Veterans Affairs Capitol Healthcare Network, Baltimore, Maryland, United States*

There is a growing evidence base for the utility of peers in supporting physical health outcomes among aging Veterans with mental illness. This talk will consider two questions: (1) what does it mean to be a "peer" when the focus is improving physical health, and (2) how does peer support promote health behavior change? In considering these questions, select peer-delivered interventions recently or currently being tested in the VA will be discussed. Data from qualitative interviews (N=16; ages 47-75) from a recent RCT of Living Well, a peer co-facilitated group intervention promoting illness self-management, will be presented. These data shed light on the peer role, especially the role of peer self-disclosure in promoting group cohesion, social learning, self-efficacy, and health behavior change. Notably, when physical health is the focus, participants relate to peer providers across diverse characteristics, and not necessarily based on a shared lived experience of mental illness.

### OLDER PEER-DELIVERED AND SMARTPHONE-SUPPORTED INTEGRATED MEDICAL AND PSYCHIATRIC SELF-MANAGEMENT INTERVENTION

Karen Fortuna, *Dartmouth College, Concord, New Hampshire, United States*

PeerTECH is older peer-delivered and technology-support integrated medical and psychiatric self-management intervention developed by older adult peer support specialists. Older adult peer support specialists are older adults with a lived experience of a mental health condition, who are trained and accredited to provide support services to others with similar conditions. A pre/post trial by our group has shown PeerTECH is associated with statistically significant improvements in self-efficacy for managing chronic disease and psychiatric self-management skills. This presentation will discuss the feasibility and potential effectiveness of a 3-month pre/post study with older adults with SMI. We will discuss the potential effectiveness of PeerTECH with older adults with SMI related to loneliness, distress, and medical and psychiatric self-management. in conclusion, we will discuss the main and interactive effects of loneliness and factors linked to mortality.

### SESSION 5805 (SYMPOSIUM)

### TRANSLATING BEHAVIORAL INTERVENTIONS: IT IS MORE THAN JUST LANGUAGE

Chair: Katherine Marx
Co-Chair: Laura Gitlin

In the United States, over 5 million people are living with Alzheimer's disease or a related dementia. Providing care are an estimated 16 million unpaid caregivers and millions of paid caregivers. Neuro-psychologic symptoms (NPS) such as agitation, aggression, depression, rejection of care, and apathy are almost universal in persons living with dementia (PLwD). Caring for NPS often leads to poor physical, mental and financial outcomes. There have been hundreds of non-pharmacologic interventions tested and found efficacious to help caregivers with NPS and daily care challenges. However, very few of these interventions have been widely adopted in different languages and settings. One promising intervention used in various countries is the Tailored Activity Program (TAP). TAP, delivered by occupational therapists, customizes activities to PLwD's current capabilities and prior roles and interests and instructs caregivers in their use. This session will examine TAP's reach and how it has been translated and adapted. First, Ms. Sokha Koeuth will present modifications needed to the program to facilitate widespread dissemination including placing training in the program online and virtual. The next two presentations will discuss adaptations to TAP in different countries and cultures; Dr. Marcia Novielli will present TAP-Brazil, and Dr. Jean Gajardo Jauregui will present TAP-Chile. Finally, Dr. Katherine Marx will examine the adaptations needed to place TAP into a long-term care setting with both family and paid caregivers. These papers highlight the cross-cultural adaptations that need to be considered in taking a program from research to different real world clinical and community-based settings. Behavioral Interventions for Older Adults Interest Group Sponsored Symposium.

### ADAPTATIONS FOR TAP IN A LONG-TERM CARE SETTING

Katherine Marx,[1] Lauren Parker,[2] and Laura Gitlin,[3] *1. Johns Hopkins University, Baltimore, Maryland, United States, 2. Johns Hopkins Medicine, Baltimore, Maryland, United States, 3. Drexel University, Philadelphia, Pennsylvania, United States*

One of the most difficult aspects of caring for people living with dementia is managing neuropsychologic symptoms and functional decline. Although there are hundreds of efficacious non-pharmacologic interventions tested in homes, few are adapted for and tested in long-term care. The purpose of this pilot study was to identify the adaptations needed for the Tailored Activity Program (TAP) to make it feasible and acceptable in a long-term care facility. TAP provides tailored activities matched to interests and abilities to address dementia-related clinical symptoms. Two sites, under the umbrella of one company, participated. A total of five persons living with dementia, their family caregivers, two direct care staff and an interventionist participated, and occupational therapist who contracts with the site on a regular basis. Adaptations included shorter sessions and changes to forms to fit with workflows and documentation. Additional considerations challenging implementation of TAP included staff turn-over and training. Part of a symposium sponsored by the Behavioral Interventions for Older Adults Interest Group.

### SCALING AN EVIDENCE-BASED PROGRAM: THE CASE OF THE TAILORED ACTIVITY PROGRAM

Sokha Koeuth,[1] Katherine Marx,[2] Laura Gitlin,[1] and Catherine Piersol,[3] *1. Drexel University, Philadelphia, Pennsylvania, United States, 2. Johns Hopkins University,*

*Baltimore, Maryland, United States, 3. Thomas Jefferson University, Ardmore, Pennsylvania, United States*

The Tailored Activity Program (TAP) is a proven program delivered primarily by occupational therapists addressing dementia-related clinical symptoms including caregiver well-being. Although used in 9 countries including the United States, scaling and widespread dissemination is challenging. We discuss key revisions to TAP to facilitate dissemination including matching assessments to those used in different practice settings, translation of materials into different languages, providing worksheets to help trainees adapt TAP to local contexts and a training/certification online experience using story board, an interactive media integrated onto the Blackboard learn management system, to provide on-demand training modules. The learning platform allows learners to engage with others, preview modules and share experiences. Revisions enable greater flexibility for program adaptation yet adherence to its core principles. With over 150 trainees, we use REAIM to evaluate effectiveness of modifications and to understand implications for its reach. Part of a symposium sponsored by the Behavioral Interventions for Older Adults Interest Group.

### CROSS-CULTURAL ADAPTATION OF THE TAILORED ACTIVITY PROGRAM FOR BRAZILIAN PORTUGUESE

Marcia Novielli, *University of Sao Paulo, Santos, Sao Paulo, Brazil*

Brazil lacks an Occupational Therapy methodology of action, justifying the cross-cultural adaptation of TAP. Objectives were to adapt TAP reference materials to the Brazilian culture and evaluation of the applicability of the Portuguese version by perceptions of Occupational Therapists (OT) and family caregivers. The methodology used translation, back translation, evaluation of semantic, idiomatic, conceptual and cultural equivalences and pretest of materials for production in Portuguese. The OT applied the translated version and evaluated its applicability. Caregivers evaluated the social impact of the adapted program. The cross-cultural adaptation process adapted the entire materials program to Portuguese culture. The OT perception is a need to include one session to guide caregivers and to modify the cognitive assessment used. The caregivers pointed out that TAP helps them in understanding and caring for the elderly with dementia. The TAP-BR has been adapted to the Brazilian culture. Part of a symposium sponsored by the Behavioral Interventions for Older Adults Interest Group.

### CULTURAL ADAPTATION OF THE HOME-BASED TAILORED ACTIVITY PROGRAM IN CHILE

Jean Gajardo,[1] Jose Aravena,[2] Ignacia Navarrete,[3] Andrea Slachevsky,[4] and Laura Gitlin,[5] *1. Universidad San Sebastián, Santiago, Chile, 2. School of Public Health, Yale University, New Haven, Connecticut, United States, 3. Universidad de Chile, Santiago, Region Metropolitana, Chile, 4. Universidad de chile, Santiago, Region Metropolitana, Chile, 5. Drexel University, Philadelphia, Pennsylvania, United States*

Chile is currently implementing policies addressing dementia care with efforts to translate evidence-based programs towards culturally sensitive models of care. This study describes the cultural adaptation of the Tailored Activity Program (TAP). A complementary mixed-method design was performed following the 4-phase Dynamic Adaptation Process (DAP) model by Aarons et al, 2012. Ten dyads (family caregivers and people with dementia) completed a regular 8-session home-based TAP intervention during 2017-2018. Qualitative data was collected through interviews and observation with caregivers, and weekly follow-up and a focus group with provider occupational therapists. Quantitative data in pilot testing was obtained through assessments at baseline and after intervention. The TAP was well accepted by family caregivers, and sociocultural adaptations on content, context, target level, and training were identified. Significant reduction of frequency and severity of neuropsychiatric symptoms in individuals with dementia was found, and caregivers reported reduction of depressive symptoms, improved perceived well-being & self-confidence. Part of a symposium sponsored by the Behavioral Interventions for Older Adults Interest Group.

### SESSION 5810 (SYMPOSIUM)

### UNDERSTANDING HEALTH CONCERNS OF OLDER ADULTS: HEALTH PERSONALITY, HEALTH ACTIVATION, AND WELL-BEING

Chair: Peter Martin

Individuals display different levels of concern about their health. These overall concerns may be a result of health personality dispositions based on the five-factor model of personality. They include health neuroticism, health extraversion, health openness, health agreeableness, and health conscientiousness. Furthermore, whether older adults take active care of their health and how they view their overall physical and emotional well-being may depend on these health personality dispositions. This symposium sheds light on the association between health personality, resilience, activation, and well-being. The first presentation provides an overview of our health personality conceptual model and summarizes measurement properties of the Health Personality Assessment. The second presentation highlights demographic differences in health personality. Gender, age, marital status, and regional differences in health personality are reported. The third presentation links health personality with levels of health activation and resilience. Direct and indirect effects of health personality on resilience and health activation are presented. Finally, we highlight results about the relationship of health personality with physical and emotional well-being in later life. All five health personality dispositions directly related to physical and mental health. Our discussion emphasizes practical implications for health practitioners and outlines future research on health personality and outcomes.

### HEALTH PERSONALITY AND WELL-BEING: AN OVERVIEW

Peter Martin, Joseph Kim, Rotem Arieli, and Nicholas Cone, *Iowa State University, Ames, Iowa, United States*

It is well established that there are interindividual differences in many areas of well-being. Based on previous segmentation research and work connecting personality traits to health outcomes, we developed the health personality segmentation model. The health personality segmentation model takes a person-centered approach to provide optimal health care based on segmenting individuals through health personality factors. The presentation provides an overview of health personality, defined as a set of individual dispositions that are directly related to health. We introduce the Health Personality Assessment based on our health personality segmentation model, and we link health personality with resilience, activation, as well as with physical and emotional well-being. Future research should evaluate the usefulness of this assessment in applied healthcare settings.

### DEMOGRAPHIC DIFFERENCES IN HEALTH PERSONALITY

Nicholas Cone, *Iowa State University, Ames, Iowa, United States*

The purpose of this study was to explore demographic differences in health personality. Data consisted of 3,907 participants, 65 years and older. Multivariate analysis of variance with post-hoc testing revealed that women had higher health neuroticism scores than men, but men had higher health extraversion scores than women. Those married reported higher health agreeableness than those not married and young-old participants had higher health extraversion and health openness compared to other age groups. Regional differences included Midwest participants reporting higher health openness but lower health conscientiousness scores when compared to participants from other regions. There were also significant interactions. For example, individuals from geographic areas with predominately White Midwest residents were significantly higher on health neuroticism when compared to Northwest, South, and West regions. The results are helpful for healthcare providers who can tailor intervention approaches to specific populations.

### HEALTH PERSONALITY, RESILIENCE, AND ACTIVATION

Joseph Kim, *Iowa State University, Ames, Iowa, United States*

The purpose of this study was to identify associations among health personality, resilience, and health activation. Participants included 3907 older adults, 65 and older. Latent variable structural equation modeling with bootstrap sampling estimation was conducted. Significant direct paths were observed between health personality factors and resilience, and in turn, resilience to health activation. The results indicate that older adults who were more health conscientious, more extraverted, and less neurotic about their health were also more resilient. Also, older adults who were more resilient were more health activated. Lastly, older adults who were more health conscientious and more agreeable about their health were more health activated. Resilience had a negative indirect effect on health activation through health neuroticism and health extraversion, and a positive indirect effect through health conscientiousness. Healthcare practitioners should target older adults based on health personality and resilience levels and develop interventions to increase health activation.

### HEALTH PERSONALITY, PHYSICAL AND EMOTIONAL WELL-BEING

Rotem Arieli, *Iowa State University, Ames, Iowa, United States*

The purpose of this study was to identify paths from health personality to outcomes of physical and emotional health. Data included 3,907 participants, 65 and older. Latent path models conducted in Mplus resulted in several significant pathways. Health neuroticism, health extraversion, and health openness negatively predicted both outcomes of physical and emotional health significantly. This negative association indicates an inverse relationship, meaning the more worried older adults were about their health, the lower their self-rating of physical and emotional health. Health agreeableness was negatively predictive of physical health, but not emotional health. Health conscientiousness had a significant positive association with both physical and emotional health, indicating that the more conscientious participants were about their health, the better their physical and emotional health. This study's findings can be translated to targeted intervention programs for emotional and physical health outcomes benefiting older adults.

### SESSION 5815 (SYMPOSIUM)

### UNDERSTANDING MENTAL HEALTH, VULNERABILITIES, AND COPING IN OLDER KOREANS AND OLDER KOREAN AMERICANS

Chair: Nan Sook Park Co-Chair: David Chiriboga
Discussant: Barbara Yee

Although significant progress has been made in understanding mental health issues, racial/ethnic minorities are disadvantaged in terms of knowledge, attitude/stigma toward mental illness, and access to treatment. Older Koreans and Korean Americans are high-risk groups with great prevalence of stigma and limited access to mental services. The two groups share similarities as well as differences. For example, Older Korean Americans, a first-generation immigrant group, tend to share traditional values and beliefs with older Koreans. However, differences in social and cultural contexts, availability of social networks and resources, and access to health care systems present unique challenges and strategies. The purpose of this symposium is to enhance the understanding of critical issues in mental health among older Koreans and Korean Americans and identify challenges and strategies to promote mental health and well-being. Five studies conducted in Korea and the US will explore a variety of personal, social, and cultural factors related with mental health, based on quantitative and qualitative approaches. The diverse topics cover the mediating role of self-esteem in the relationship of stigma to emotional well-being, the effect of stress and coping on well-being, loneliness and negative family interactions, the relationship of life stressors and social capital on mental distress among older Korean Americans compared with other older Asian Americans, and community leaders' attitude toward depression. The issues of vulnerabilities and resources will be discussed from the cultural perspectives as well as implications for future research and practice.

### PERCEIVED ELDERLY STIGMA AND EMOTIONAL WELL-BEING AMONG KOREAN OLDER ADULTS: THE MEDIATING ROLE OF SELF-ESTEEM

Eun Ha Namkung,[1] and Soondool Chung,[2] *1. Brandeis University, Waltham, Massachusetts, United States, 2. Ewha Womans University, Seoul, Republic of Korea*

This study examined whether self-esteem mediates the association between perceived elderly stigma and emotional well-being (loneliness and emotional isolation) among Korean older adults, and how these processes differ by marital status. Using the 2018 Age Integration Survey, a cross-sectional national survey of adults in Korea, we analyzed data from 266 older adults aged 60 and older. Older adults who perceived greater elderly stigma reported higher levels of loneliness and emotional isolation. Self-esteem played a significant indirect role in the association between perceived elderly stigma and the two emotional well-being outcomes. Moderated mediation analyses further revealed significant differences by marital status; self-esteem was a more powerful mechanism among unmarried older adults relative to their married counterparts. The findings suggest that efforts to minimize public and internalized stigmatization of older adults and to improve their own self-esteem may be critical for their emotional well-being.

### THE EFFECT OF OLDER ADULTS' RELATIONAL STRESS, ENVIRONMENTAL STRESS, AND COPING ON WELL-BEING

Meeryoung Kim, *Daegu University, Gyeongsan, Republic of Korea*

This study used the stress process model for analyzing older adults' stressors and coping resources, which compares relational and environmental stressors (Pearlin et al., 1990). Additionally, the effect of coping abilities and social support on well-being was compared. This study used the 5th wave data of KReIS (Korean Retirement and Income Studies) that were collected in 2014. The sample included 4,072 older Korean adults aged 60 and older. Relational and environmental stressors were used as the independent variables. Social support and coping were used as coping resources. For the dependent variable, life satisfaction and perceived health were used. Since the stress model is a process model, hierarchical multiple regression was used. Environmental stressors had a significant effect on reducing life satisfaction. Relational and environmental stressors had significant negative effects on perceived health. Both coping and social support had a significant effect on both life satisfaction and perceived health.

### CORRELATES OF LONELINESS IN OLDER KOREAN AMERICANS: INTERACTIVE EFFECTS OF NEGATIVE FAMILY INTERACTIONS

Nan Sook Park,[1] Yuri Jang,[2] David Chiriboga,[1] and Soondool Chung,[3] *1. University of South Florida, Tampa, Florida, United States, 2. University of Southern California, Los Angeles, California, United States, 3. Ewha Womans University, Seoul, Republic of Korea*

This study examined factors affecting the feelings of loneliness among older Korean Americans. Data were drawn from a survey with older Korean Americans aged 60 or over (N = 2,150) in five states (California, New York, Texas, Hawaii, and Florida), conducted during 2017–2018. In hierarchical multiple regression models, loneliness was regressed on five blocks of variables: (1) demographic/health (age, gender, education, financial status, chronic conditions, and physical disabilities); (2) immigration-related (length of stay in the U.S., and acculturation); (3) social engagement (having meals alone, family network, friend network, activity participation, and community engagement); (4) negative family interactions; and (5) interactions of negative family interactions with social engagement variables. A significant interaction was found in the relationship between friend network and negative family interactions: the impact of negative family interactions on loneliness was buffered by friend network. Implications of findings were discussed regarding working with older immigrants with limited social networks.

### LIFE STRESSORS, SOCIAL CAPITAL, AND MENTAL DISTRESS IN MULTIETHNIC GROUPS OF OLDER ASIAN AMERICANS

Yong Ju Cho,[1] Audrey Hai,[2] and Yuri Jang,[1] *1. University of Southern California, Los Angeles, California, United States, 2. University of Texas at Austin, Austin, Texas, United States*

Using data from older participants from the 2015 Asian American Quality of Life survey (n = 533), the present study assessed direct and interactive effects of life stressors and social capital. The sample includes diverse Asian ethnic groups (Chinese, Asian Indian, Korean, Vietnamese, and others). Among all groups, high levels of mental distress were found in Koreans and Vietnamese. In the multivariate analyses, Korean ethnicity (compared to Chinese) was found to be a significant predictor to mental distress. As significant risk factors to mental distress, all stressor variables accounted for 9% of the variance of mental distress. Social capital variables explained the variance by 4%. None of the interaction terms reached statistical significance. Findings confirmed the negative effects of stressors and the positive effects of social resources across older Asian Americans. However, it was interesting to note that ethnic variations disappeared when stressors and social capitals were taken into considerations.

### EXPLORING COMMUNITY LEADERS' UNDERSTANDING AND ATTITUDES TOWARD DEPRESSION AMONG OLDER KOREAN AMERICANS

Eunhye Kim, *Augusta University, Augusta, Georgia, United States*

This study explores community leaders' understanding and attitudes toward depression among older Korean Americans. The community leaders who provide services for older adults play a significant role because these leaders typically engage with the target population by providing services. A qualitative method was applied for this study. A total of 12 community leaders were interviewed for 60 minutes using the semi-structured interview guide. The constant comparative approach was employed to analyze data using NVivo software. The findings indicated that the community leaders were aware of the severity of the issues, but lacked knowledge regarding depression and had stereotypes against depression. Those leaders identified negative personality issues of people with depression and desired to avoid discussing depression with older adults. The results suggest that interventions are essential to support community leaders and empower them to take actions to provide services to promote understanding of depression in older adults.

### SESSION 5820 (SYMPOSIUM)

### UNDERSTANDING THE RELATION BETWEEN SLEEP AND HEALTH IN ADULTHOOD: DAILY EXPERIENCES TO LONG-TERM HEALTH OUTCOMES

Chair: Hye Won Chai
Co-Chair: David Almeida
Discussant: Soomi Lee

An increasing number of studies evinces the significant role of sleep in health outcomes including physical symptoms, cardiometabolic functioning, and chronic health conditions. To further advance the field's knowledge on sleep and health in adulthood, it is necessary to have an integrative understanding of this topic that pulls together short-term determinants to long-term health consequences of sleep. As such, this symposium places diverse aspects of sleep across multiple contexts, ranging from predictors and consequences of sleep in daily life to the role of sleep in long-term changes in health across adulthood. The first paper by Lee and colleagues examines the role of daily positive and negative events as precursors of concurrent and next-day sleep duration. The second paper by Joo and colleagues addresses the moderating role of nightly sleep duration in the association between daily stressor severity and intensity of headaches. The third paper by Chai and colleagues explores how the association between daily emotional well-being and cardiometabolic syndrome differs by sleep deficiency and by age. The fourth paper by Sin and colleagues focuses on daily affective vulnerability to short sleep duration as a risk factor for developing chronic conditions 10 years later. The final paper by Reither and colleagues assesses within-person changes and between-person differences in restorative sleep and their associations with body mass trajectories across 15 years. The discussant, Dr. Soomi Lee, will integrate key points from the studies, discuss the utilization of diverse measurements of sleep, and address considerations for future research on sleep and health.

### THE RELATIONSHIP BETWEEN EXPERIENCES OF DAILY EVENTS AND SLEEP DURATION IN ADULTHOOD

Sun Ah Lee,[1] Susanna Joo,[1] Hye Won Chai,[2] Hey Jung Jun,[1] and David Almeida[2] *1. Yonsei University, Seoul, Republic of Korea, 2. The Pennsylvania State University, University Park, Pennsylvania, United States*

This study aimed to examine how stressors and positive events are related to sleep duration in daily life and whether these associations differed by age. The second wave of National Study of Daily Experiences of Midlife in the United States study was used (N=1,851). Reports of daily events was coded as two categorical variables indicating experiences of concurrent and previous-day daily events: experiencing both stressors and positive events, only stressors, only positive events, and neither (reference). Results from multilevel analysis showed that experiencing an event, either a stressor or a positive event, was associated with shorter amount of sleep the same day compared to a non-event day. In particular, sleep duration was shorter when individuals experienced stressors compared to when they only reported positive events the same day. There were no age differences in these associations. Findings suggest that stressors exert a stronger influence on daily sleep than positive events.

### WHEN STRESS GETS ON YOUR NERVES: ASSOCIATIONS AMONG STRESS SEVERITY, SLEEP, AND HEADACHE IN DAILY LIFE

Susanna Joo,[1] Sun Ah Lee,[1] Hye Won Chai,[2] Hey Jung Jun,[1] and David Almeida,[2] *1. Yonsei University, Seoul, Republic of Korea, 2. The Pennsylvania State University, University Park, Pennsylvania, United States*

The present study aims to examine whether duration of sleep moderates the relationship between daily stressor severity and headache severity and test age differences in these associations. We used the second wave of the Midlife in the United States (MIDUS) study and its subproject, the National Study of Daily Experiences (N=1,590). Stress severity was measured based on the respondents' perception of reported stressors, and headache severity was indicative of subjective rating of the symptom. Multilevel analysis results showed significant interaction effects between concurrent-day stressor severity and duration of sleep. Specifically, stressor severity was associated with more severe headache when people slept for shorter amount of time the same day. There were no differences by age in these associations. These results suggest that shorter-than-usual sleep may aggravate the intensity of headache on stressful days in daily life.

### DAILY EMOTIONAL WELL-BEING AND CARDIOMETABOLIC SYNDROME IN ADULTHOOD: THE ROLE OF SLEEP DEFICIENCY

Hye Won Chai,[1] Susanna Joo,[2] Sun Ah Lee,[2] and David Almeida[1] *1. The Pennsylvania State University, University Park, Pennsylvania, United States, 2. Yonsei University, Seoul, Republic of Korea*

Previous studies note daily emotional well-being and sleep duration as significant correlates of cardiovascular health including cardiometabolic syndrome. However, not much is known about the interactive effects of emotional well-being and sleep. Expanding upon current research, this study examined whether sleep deficiency, defined as having on average <7 hours of sleep a day, moderated the associations between daily emotional well-being and cardiometabolic syndrome. Data was drawn from the Midlife in the United States Biomarker Project and the National Study of Daily Experiences (N = 1,163; Mean age = 53.3). Results showed significant interaction effects- higher negative affect was associated with worse cardiometabolic syndrome and higher positive affect was associated with better cardiometabolic syndrome only among those with deficient sleep. Such association was stronger for older adults compared to young adults. These findings suggest that individuals with insufficient sleep are more affected by health-related benefits and costs of daily emotional experiences.

### AFFECTIVE VULNERABILITY TO SHORT SLEEP PREDICTS 10-YEAR CHANGES IN CHRONIC HEALTH CONDITIONS

Nancy Sin,[1] Jonathan Rush,[2] Orfeu Buxton,[3] and David Almeida,[3] *1. University of British Columbia, Vancouver, British Columbia, Canada, 2. University of Victoria, Victoria, British Columbia, Canada, 3. Pennsylvania State University, University Park, Pennsylvania, United States*

We examined daily affective vulnerability to short sleep (i.e., individual differences in the extent that sleeping ≤6h predicts next-day affect) as a risk factor for developing chronic conditions 10 years later. Participants (N=1945, ages 35-85, 57% women) from the National Study of Daily Experiences reported sleep duration and affect in daily diary telephone interviews. Chronic conditions were assessed with a 39-item checklist (e.g., arthritis, hypertension, diabetes). Multilevel structural equation models revealed that individuals with heightened negative affect following short sleep had an increased number of chronic conditions after 10 years (Est.=1.20, SE=.48, p<.01). Positive affective vulnerability (i.e., greater declines in positive affect following shorter sleep vs. longer sleep) was marginally associated with 10-year chronic conditions (Est.=-.72, SE=.40, p=.07). Adding to the well-established connections between sleep duration and well-being across adulthood, these findings suggest that affective vulnerability to short sleep represents a unique risk factor for long-term health as people age.

### POLYSOMNOGRAPHIC INDICATORS OF RESTORATIVE SLEEP AND BODY MASS TRAJECTORIES IN THE WISCONSIN SLEEP COHORT STUDY

Eric Reither,[1] Jodi Barnet,[2] Mari Palta,[2] Yin Liu,[1] Erika Hagen,[2] and Paul Peppard,[2] *1. Utah State University, Logan, Utah, United States, 2. University of Wisconsin-Madison, Madison, Wisconsin, United States*

Previous research suggests that reductions in restorative sleep—i.e., slow-wave (N3) and rapid-eye movement (REM) sleep—are associated with weight gain and obesity in mid-to-late life. This study extends prior work by examining how within-person changes and between-person differences in restorative sleep are associated with body mass trajectories among participants in the Wisconsin Sleep Cohort Study (WSCS). We used data from 4,862 polysomnographic sleep studies and physical exams collected from 1,187 WSCS participants over an average follow-up duration of 15 years. For both men and women, we found that (1) below-average N3 and REM sleep is associated with above-average BMI, and (2) within-person loss of N3 and REM sleep is associated with larger gains in BMI, particularly between ages 30-50. Our findings highlight the importance of restorative sleep in mid-to-late life, suggesting that future clinical treatments and public health policies will benefit from heightened attention to sleep quality.

### SESSION 5830 (SYMPOSIUM)

### VETERAN STATUS MATTERS! LIFE COURSE PERSPECTIVES ON THE HEALTH AND WELL-BEING OF AGING VETERANS

Chair: Avron Spiro

Military service during early life can result in exposure to traumatic events that can reverberate throughout life. Although much attention is focused on the negative effects of military service, many veterans report positive effects. These papers explore life course effects of military service on veterans' health and well-being. Three used national US longitudinal cohorts (HRS, MIDUS); two sampled veterans from Oregon or from Korea. Three compared veterans to non-veterans; two examined veterans only. Cheng and colleagues found that veterans in HRS are more likely to be risk-averse than non-veterans. Risk aversion matters because it determines how people make decisions and predicts a wide array of health and economic outcomes. Kurth and colleagues examined Oregon veterans from several wars, finding PTSD symptoms were highest among Vietnam combat veterans, the oldest cohort; there were no differences among non-combat veterans. Piazza and colleagues examined in MIDUS the impact of veteran status on cortisol, a stress biomarker, finding older veterans more likely had non-normative patterns than did younger or non-veterans. Lee and colleagues studied patterns of mental health among Korean Vietnam veterans, identifying two patterns as 'normal' and 'resilient' encompassing half the sample; these veterans demonstrated positive outcomes of military service. Frochen and colleagues compared depression trajectories between veterans and non-veterans in HRS, finding veterans had less depression than non-veterans, but among veterans, trajectories varied based on extent of service. in sum, these papers demonstrate that military service can have positive as well as negative effects on veterans' health and well-being in later life. Aging Veterans: Effects of Military Service across the Life Course Interest Group Sponsored Symposium.

### RISK AVERSION AMONG MALE OLDER ADULTS: DOES VETERAN STATUS MATTER?

Kent Jason Cheng,[1] Scott Landes,[2] and Janet Wilmoth,[1] *1. Syracuse University, Syracuse, New York, United States, 2. Aging Studies Institute – Syracuse University, Syracuse, New York, United States*

Risk aversion determines how people make decisions and is known to predict a wide array of economic outcomes. This study assessed whether there are veteran status differences in risk aversion utilizing the Health and Retirement Study. Risk aversion is based on hypothetical financial gambles (N=2,121; 2006 wave) and self-reported risk attitudes on selected topics (N= 4,980; pooled 2014 and 2016 waves of the Leave-Behind Survey). Results from multivariate analyses reveal that veterans were more likely to be risk averse than nonveterans in financial matters, occupation, and health, but veteran status is not statistically significant in explaining risk taking in driving and leisure, and sport risk. Further research is needed to discern the role of military service-related experiences in determining levels of risk aversion among veterans and the extent to which risk aversion accounts for veteran status differences in later-life economic outcomes. Part of a symposium sponsored by the Aging Veterans: Effects of Military Service across the Life Course Interest Group.

### PTSD SYMPTOMS AMONG VIETNAM, PERSIAN GULF, AND POST-9/11 COMBAT VETERANS: FINDINGS FROM VALOR

Maria Kurth, Carolyn Aldwin, and Richard Settersten, *Oregon State University, Corvallis, Oregon, United States*

Much is known about the mental health of combat Vietnam Veterans, but less is known about Persian Gulf and post-9/11 veterans and how they compare to those from earlier eras. Using data from an online survey of Oregon veterans, we examine how PTSD symptoms differ by combat exposure across these three cohorts. The sample (N=167, Mage=57.86, SD=12.09), was largely composed of White

(88%), male (69%) Veterans. Most served in the Persian Gulf (41%), followed by Vietnam (36%) and post-9/11 (23%) eras. ANCOVAs showed significant cohort differences in PTSD, after controlling for severity of combat exposure and demographics (age, gender, education, income) ($F(2, 157) = 4.24$, $p < .05$). Post-9/11 veterans had significantly lower PTSD symptom severity than Vietnam-era veterans but were comparable to Persian Gulf. There were no cohort differences for noncombat veterans. Future research should investigate why Vietnam veterans continue to have worse mental health than younger veteran cohorts. Part of a symposium sponsored by the Aging Veterans: Effects of Military Service across the Life Course Interest Group.

### DIURNAL CORTISOL PROFILES AND VETERAN STATUS

Jennifer Piazza,[1] Scott Landes,[2] and Natalia Dmitrieva,[3] *1. California State University, Fullerton, Fullerton, California, United States, 2. Aging Studies Institute – Syracuse University, Syracuse, New York, United States, 3. Northern Arizona University, Flagstaff, Arizona, United States*

Although veterans typically have better health and lower mortality risk than nonveterans earlier in life, a crossover occurs in later life, resulting in comparatively poorer health and higher mortality risk in older age. Alterations in biomarkers of physical health could be an initial indicator of this crossover. The current study uses data from the second wave of the Midlife Development in the United States (MIDUS) survey. Previously validated cortisol profiles (Dmitrieva et al. 2013) were used to categorize participants ($N = 1101$, age range: 33-88) into normative and non-normative groups, that were examined in relation to veteran status. Results revealed a significant age by veteran interaction ($\beta = .0331$, $p = .038$), indicating that older veterans were less likely to belong to the normative profile group than non-veterans and younger veterans. Results demonstrate the need to explore underlying physiological mechanisms linking military service with poorer health in later life. Part of a symposium sponsored by the Aging Veterans: Effects of Military Service across the Life Course Interest Group.

### CLASSIFICATION OF MENTAL HEALTH AMONG KOREAN VIETNAM WAR VETERANS: A LATENT PROFILE ANALYSIS

Hyunyup Lee,[1] Carolyn Aldwin,[2] Sungrok Kang,[3] and Xyle Ku,[3] *1. Korea Military Academy, Seoul, Republic of Korea, 2. Oregon State University, Corvallis, Oregon, United States, 3. Korea Military Academy, Seoul, Seoul-t'ukpyolsi, Republic of Korea*

We investigated the dimensional structure of mental health among aging Korean Veterans using latent profile analysis (LPA) on posttraumatic stress disorder symptoms (PTSD), late onset stress symptomology (LOSS), and psychosocial well-being (PWB). The Korean Vietnam War Veterans Study consists of 367 men (Mage=72, SD=2.66). LPA identified five classes of mental health as best fitting the data. Most men were in the normal (38%) and moderate distress (31%) groups, while smaller proportions were in the low affect (13%) and severe distress (7%) groups. The resilient group (12%) had low PTSD, medium LOSS, and high PWB, and were highest on optimism, positive appraisals of military service, and social support. Negative and positive aspects of mental health outcomes were on separate dimensions rather than on a single bipolar dimension. Service providers should attempt to both reduce Veterans' negative psychological symptoms and increase psychosocial well-being. Part of a symposium sponsored by the Aging Veterans: Effects of Military Service across the Life Course Interest Group.

### VETERAN STATUS AND OLD AGE DEPRESSION: A LIFE COURSE EPIDEMIOLOGY STUDY

Stephen Frochen,[1] Connor Sheehan,[2] and Jennifer Ailshire[1] *1. University of Southern California, Los Angeles, California, United States, 2. Arizona State University, Tempe, Arizona, United States*

Military service and exposure to war may influence the development of depression, leading to disparities in the condition among veterans and non-veterans. This study included 10,512 older men from the 1996 to 2014 waves of the Health and Retirement Study (HRS). We estimated Center for Epidemiology Depression (CESD) score trajectories among veterans and non-veterans and veterans of different war cohorts in growth curve models, controlling for early, mid, and late life characteristics. CESD score trajectories were lower among veterans and war veterans than non-veterans and non-war veterans, respectively. The highest levels of depression were among war veterans who served in more than one war. Veterans demonstrated lower levels of depression than non-veterans, calling into question the health advantage of veterans and selection mechanisms into the military and out of HRS. Multiple war veterans showed the highest levels of depression, representing the greatest mental health threat to veterans in the study. Part of a symposium sponsored by the Aging Veterans: Effects of Military Service across the Life Course Interest Group.

### SESSION 5835 (SYMPOSIUM)

### WHAT SAVES THE DAY?: IDENTIFYING PROTECTIVE FACTORS THAT BUFFER THE NEGATIVE EFFECTS OF DAILY STRESSORS

Chair: Joanna Hong
Co-Chair: Meng Huo
Discussant: Christiane Hoppmann

Older adults frequently experience daily stressors and are at increased risk of adverse health consequences. Emerging studies have focused on factors that may buffer older adults' well-being and strengthen their resilience to stress. This symposium adds to this burgeoning literature and presents studies that identify a wide range of such stress-buffering factors, including individual characteristics and daily social and emotional experiences. Huo et al. considered older adults' empathy and found that more empathic individuals tended to use constructive strategies but not destructive strategies when coping with interpersonal tensions. When daily tensions occurred, more empathic individuals were also better at maintaining their mood. Kim et al. showed that older adults' marital status influenced their irritable encounters with grown children. Although irritable encounters were associated with increased daily negative mood, this link varied by parents' marital status. Hong et al. assessed the link between daily stressors and negative affect among older adults with varying levels of loneliness. They found that positive interpersonal encounters were particularly protective for lonely older adults' daily affect. Leger et al. further examined the buffering effects of trait level and same-day positive emotions during times of stress. Higher trait and same-day levels of positive emotions reduced negative emotions on the day following a stressor. Together, findings might inform future interventions aimed at increasing the daily experience of older adults. Dr. Hoppmann will serve as the Discussant and summarize the theoretical and methodological contributions of these studies. She will discuss challenges in this field and directions for future research.

### LONELINESS AND DAILY STRESSORS: THE PROTECTIVE ROLE OF DAILY POSITIVE EVENTS

Joanna Hong,[1] Susan Charles,[2] Nancy Sin,[3] and David Almeida,[4] *1. University of California, Irvine, Irvine, California, United States, 2. BSS, Irvine, California, United States, 3. University of British Columbia, Vancouver, British Columbia, Canada, 4. Pennsylvania State University, University Park, Pennsylvania, United States*

Lonely individuals are particularly vulnerable to daily stressors. Yet, less is known about the protective role of daily positive social events on days lonely individuals experience a stressor. The current study examined whether experiencing a positive social event on the same day as a stressor helps lonely individuals maintain their daily emotional well-being. Participants from the Midlife in the United States Survey II and the National Study of Daily Experiences II reported their trait-levels of loneliness. A subset of the participants (N=1,730) also completed eight days of daily interviews and reported experiences of stressful events, positive social events, and emotions. On days lonely individuals reported experiencing a stressor, experiencing a positive social event was associated with a less increase in daily negative affect. However, this buffering effect did not generalize to non-lonely individuals. Results highlight a protective asset that might be important for helping lonely individuals maintain daily well-being.

### EMPATHY AND COPING: OLDER ADULTS' SOCIAL ENCOUNTERS AND MOOD THROUGHOUT THE DAY

Meng Huo,[1] Yee To Ng,[2] Kira Birditt,[3] and Karen Fingerman,[2] *1. University of California, Davis, Davis, California, United States, 2. The University of Texas at Austin, Austin, Texas, United States, 3. University of Michigan, Ann Arbor, Michigan, United States*

Scholars have proposed empathy as a key characteristic of strong social ties, but little is known about the role empathy plays when tensions occur in these ties. We examined whether older adults' empathy was associated with their (a) coping strategies for interpersonal tensions, and (b) momentary mood when tensions occurred throughout the day. Data were from the Daily Experiences and Well-being Study. Older adults (n = 302) rated empathy, reported avoidant, constructive, and destructive coping strategies, and indicated tensions and mood every 3 hours each day over 5 to 6 days. More empathic older adults used constructive strategies more often and destructive strategies less often than less empathic older adults. Interpersonal tensions were associated with reduced positive mood throughout the day, but this link was attenuated by older adults' empathy. This study advances our understanding of empathy and social experiences in later life with a focus on the negative moments.

### IRRITATED AND FEELING BETTER? AGING PARENTS' MARITAL STATUS AND DAILY INTERACTION WITH GROWN CHILDREN

Yijung Kim,[1] Kyungmin Kim,[1] and Karen Fingerman,[2] *1. University of Massachusetts Boston, Boston, Massachusetts, United States, 2. The University of Texas at Austin, Austin, Texas, United States*

Aging parents' marital status shapes their ties to family members, but less is known about its link to their daily mood and interaction with grown children. This study examined married, widowed, or divorced/separated aging parents (N = 203, Mage = 79.80) from the Family Exchanges Study, who completed a 7-day daily diary on their daily mood (positive, negative) and interactions (any contact, irritable, enjoyable interaction) with the grown children (N = 771, Mage = 53.20). Findings from multilevel models indicated that widowed parents were more likely to report irritable interactions with their grown children than the married ones. Furthermore, married and widowed parents tended to report more negative mood, whereas separated parents tended to report less negative mood on days they had irritable interactions with grown children. This study highlights the centrality of aging parents' daily interaction with grown children and suggests that the dynamics of family composition warrant attention.

### POSITIVE AFFECT AND NEGATIVE EMOTIONAL RESPONSES TO DAILY STRESSORS

Kate Leger,[1] Susan Charles,[2] and David Almeida,[3] *1. University of Kentucky, Lexington, Kentucky, United States, 2. BSS, Irvine, California, United States, 3. Pennsylvania State University, University Park, Pennsylvania, United States*

Positive affect is beneficial for regulating negative emotional responses to stressful events. Yet, few studies have examined if positive affect may attenuate negative affect the following day. We examined how both trait positive affect and state positive affect are associated with next day stressor-related negative emotions. Participants (N = 1,588) from the National Study of Daily Experiences II (NSDE II) and the Midlife in the United States survey (MIDUS II) answered questions about stressors and emotion across eight days. People high on trait positive affect reported less negative affect the day following a stressor. On days when people experienced a stressor and higher than average state positive affect, they experienced less negative emotion the following day. This held true regardless of whether people were high or low on trait positive affect. Positive affect can help explain both who and when people will have attenuated emotional responses to stressful events.

### SESSION 5840 (SYMPOSIUM)

### WHEN THE DAY GOES AWRY: FAMILIES CARING FOR PERSONS WITH DEMENTIA

Chair: Karen Roberto
Co-Chair: Jyoti Savla
Discussant: Steven Zarit

The daily lives of family caregivers of persons with dementia (PwD) often require that they manage multiple competing demands in a context of unpredictability. Memory and behavior changes associated with dementia can cause PwD to act in random and irrational ways that create stress and influence all aspects of caregivers' everyday life. Supportive others, including informal helpers and formal service professionals, should provide relief to primary caregivers; however, help may not alleviate caregiver stress and can sometimes compound the burden of care. This symposium draws on daily diary surveys and face-to-face interviews to focus on four aspects of managing everyday care of PwD among family caregivers in rural areas. Brandy Renee McCann explores how caregivers' vigilance on behalf of PwD care quality interacts with service use. Karen Roberto examines the ways in which caregivers manage PwD resistance to help, including their use of forceful care strategies. Rosemary Blieszner focuses on competing caregiver roles and demands that may contribute to or alleviate caregiver stress. Tina Savla addresses the unexpected, and often hidden, challenges involved in using formal services. Collectively, the four presentations provide in-depth insight into the complicated daily lives of families coping with dementia and the ways in which they meet the demands of full-time caregiving under often difficult and challenging circumstances. Discussant Steve Zarit considers the efficacy of these management strategies for various aspects of everyday care and offers suggestions for future research and person-centered programs and interventions to reduce health disparities among caregivers in rural areas.

### EXPLORING THE RELATIONSHIP BETWEEN DAY-TO-DAY CARE AND CAREGIVER VIGILANCE

Brandy Renee McCann,[1] Karen Roberto,[1] Rosemary Blieszner,[1] Tina Savla,[2] Emily Hoyt,[2] and Aubrey Knight,[3], *1. Virginia Tech, Blacksburg, Virginia, United States, 2. Virginia Polytechnic Institute and State University, Blacksburg, Virginia, United States, 3. Virginia Tech Carilion School of Medicine, Roanoke, Virginia, United States*

A demanding aspect of caregiving for a relative with dementia is need for constant vigilance of their behavior and well-being. Vigilance is associated with higher quality of care, but can take a toll on caregivers who have few opportunities for respite. Home- and community-based services have the potential to offer caregivers relief from constant vigilance. Using in-depth interviews with 30 rural caregivers, we found that service use did not necessarily provide relief from constant vigilance. Caregivers typically needed to monitor aspects of home- and community-based services and care facilities such as scheduling and quality of care from CNAs. In contrast, some caregivers found respite from constant vigilance when they used formal services—typically in extreme situations such as when a husband with dementia became violent and moved temporarily to a long-term care facility. Findings connect caregiver needs and concerns related to vigilance with availability, quality, and use of formal services.

### IT HAS NOT BEEN A GOOD DAY: MANAGING DIFFICULT BEHAVIORS OF RELATIVES WITH DEMENTIA

Karen Roberto,[1] Brandy Renee McCann,[1] Tina Savla,[2] Emily Hoyt,[2] Rosemary Blieszner,[1] and Aubrey Knight,[3] *1. Virginia Tech, Blacksburg, Virginia, United States, 2. Virginia Polytechnic Institute and State University, Blacksburg, Virginia, United States, 3. Virginia Tech Carilion School of Medicine, Roanoke, Virginia, United States*

Most family caregivers provide appropriate care and a supportive environment for their older relatives with dementia (PwD), yet the stress and strain associated with caregiving can trigger potentially harmful responses. Although much has been written about dealing with memory problems, researchers know less about how caregivers cope with difficult behaviors such as hallucinations, violent outbursts, or refusing food, medicine, or bathing. Interviews with 30 relatives providing care to community-dwelling PwD in rural Virginia revealed that caregivers typically used four behavior management strategies: reasoning with PwD; redirecting PwD's attention; forceful actions, such as shouting at PwD; and withdrawing from interactions. Forceful management strategies and withdrawing from interactions were usually employed after reasoning and redirection failed to elicit desired behavior. Understanding whether caregivers' expectations of PwD's capacities are realistic, and why and when caregivers use various behavior management strategies, can help service providers develop appropriate educational interventions for frustrated caregivers.

### DO COMPETING DEMANDS ELEVATE CAREGIVER CHALLENGES?

Rosemary Blieszner,[1] Tina Savla,[2] Karen Roberto,[1] Brandy Renee McCann,[1] Aubrey Knight,[3] and Emily Hoyt,[2], *1. Virginia Tech, Blacksburg, Virginia, United States, 2. Virginia Polytechnic Institute and State University, Blacksburg, Virginia, United States, 3. Virginia Tech Carilion School of Medicine, Roanoke, Virginia, United States*

Family members caring for relatives with dementia simultaneously hold other roles and face other everyday challenges related to employment, finances, interactions with others, and the like. Using the Pearlin stress process model as a foundation, we evaluated contributions of secondary role and intrapsychic stressors such as health, relationship, and financial worries, and role captivity and overload, to 157 rural caregivers' morale and well-being. Whereas family conflict and role overload contributed significantly to higher agitation, money worries, poor health, and role captivity interfered with positive mental health ($p<.05$). Nevertheless, some caregivers characterized employment and volunteering as respite from caregiving and appreciated informal help even when family ties were complicated. Findings highlight complex responses to dementia-related caregiving and point to the need for a range of resources and support deployed creatively to assist caregivers in places such as rural Appalachia where geography, underfunding, and low income limit access to services.

### ALL IS GOOD, BUT NOT REALLY: DAILY REPORTS OF SATISFACTION WITH HOME- AND COMMUNITY-BASED SERVICES

Jyoti Savla,[1] Karen Roberto,[1] Aubrey Knight,[2] Rosemary Blieszner,[1] Brandy Renee McCann,[1] and Emily Hoyt,[3] *1. Virginia Tech, Blacksburg, Virginia,*

*United States, 2. Virginia Tech Carilion School of Medicine, Roanoke, Virginia, United States, 3. Virginia Polytechnic Institute and State University, Blacksburg, Virginia, United States*

An extensive body of literature documents correlates of and barriers to health service use, yet much less is known about satisfaction with home- and community-based services for persons with dementia (PwD). Daily diary data from 122 rural caregivers (CG) of PwD (814 daily diaries) were used to assess everyday service use experiences. At the last diary interview, CG identified areas where service use expectations were and were not being met. CGs reported problems with services used on fewer than 5% of study days (e.g., service provider was delayed because of car trouble). In contrast, 82% of CG identified areas where service expectations were not being met. Their most common concerns were lack of control over service availability and lack of adequate training among service providers. Recommendations for alternative ways for capturing service use satisfaction will be offered, and implications for theory and practice will be discussed.

### SESSION 5845 (SYMPOSIUM)

### WHY ARE INEQUALITIES IN DISABILITY-FREE LIFE EXPECTANCY BY SOCIOECONOMIC POSITION WIDENING?

Chair: Carol Jagger

Life expectancy has increased over previous decades, but several countries are seeing widening inequalities in disability-free life expectancy (DFLE) by socioeconomic position (SEP). In this symposium we address three unanswered questions.1. Do DFLE trends differ for SEP groups, and which of the underlying transitions (incidence, recovery, death when disability-free, death when already disabled) explains the differences?2. Do DFLE trends by SEP depend on when in the life-course SEP is measured (early life - education, mid-life - occupational status or late-life - material disadvantage)?3. How much does multimorbidity contribute to differing trends in DFLE by SEP, since multi-morbidity is more prevalent in low SEP groups? To answer these questions, we use unique longitudinal studies of older people across different generations in two countries: the UK (Cognitive Function and Ageing Studies – CFAS I and II) and Australia (Household, Income and Labour Dynamics in Australia – HILDA). The first presentation sets the scene with findings from a systematic review of worldwide trends in life and healthy life expectancy by SEP. Presentations two and three examine the first question using DFLE at age65 by SEP defined by late-life disadvantage in CFAS (1991-2011), followed by HILDA (2001-2017). The fourth presentation investigates the effect of different life-course measures of SEP using HILDA. The final presentation from CFAS examines the third question. This symposium increases our understanding of how and why inequalities in DFLE by SEP are changing with the goal of achieving healthy ageing for all.

### INEQUALITIES IN TRENDS IN HEALTHY AND DISABILITY-FREE LIFE EXPECTANCIES: A SYSTEMATIC REVIEW

Gemma Spiers,[1] Fiona Beyer,[2] Dawn Craig,[2] Barbara Hanratty,[1] and Carol Jagger,[1] *1. Newcastle University, Newcastle upon Tyne, England, United Kingdom, 2. Population Health Sciences Institute, Newcastle University, Newcastle upon Tyne, England, United Kingdom*

To update previous reviews, we searched Medline, Embase, Scopus and the Office for National Statistics (ONS) website for studies and reports published after 2016 that describe trends in healthy life expectancy, active life expectancy or disability-free life expectancy (DFLE) in the UK and other OECD high-income countries. We focus here on studies reporting inequalities by socioeconomic position (SEP) in these trends. There was mixed evidence of educational and area-level deprivation inequalities in trends in DFLE, with four studies indicating that educational inequalities were widening in European countries. No studies were identified that examined inequalities in disability-free life expectancy trends in the UK. All studies were based on cross-sectional data from multiple time points or longitudinal panel studies. We discuss the size of inequalities in DFLE between SEP groups and the limitations of previous studies.

### TRENDS IN HEALTH EXPECTANCIES BY LATE-LIFE DISADVANTAGE: THE COGNITIVE FUNCTION AND AGEING STUDIES

Holly Bennett,[1] Andrew Kingston,[1] Gemma Spiers,[1] Louise Robinson,[1] Clare Bambra,[1] Carol Brayne,[2] Fiona Matthews,[1] and Carol Jagger,[1] *1. Newcastle University, Newcastle upon Tyne, England, United Kingdom, 2. Cambridge Institute of Public Health, University of Cambridge, Cambridge, England, United Kingdom*

To understand how and why disability-free life expectancy (DFLE) trends differ by socioeconomic position (SEP) we use longitudinal data from the Cognitive Function and Ageing Studies (CFAS I: 1991; CFAS II: 2011), with two year follow up. Disability was defined as difficulty in activities of daily living, and SEP as area-level deprivation. Between 1991 and 2011, men aged 65 gained more in life expectancy (LE) than DFLE, with the greatest gain in DFLE for the most advantaged and in disability years for the most disadvantaged. The most advantaged men experienced a 60% reduction in the risk of death when disability-free, 30% reduction in incident disability, and 80% increase in recovery. The most disadvantaged experienced a 30% reduction of death but from disability. Women overall, and in the most advantaged groups, gained similar years of LE and DFLE to men but due to a 30% reduction in incident disability only.

### AREA-LEVEL DISADVANTAGE AND CHANGES IN DISABILITY-FREE LIFE EXPECTANCY FOR OLDER AUSTRALIANS

Richard Tawiah,[1] Kaarin Anstey,[2] Carol Jagger,[3] and Kim Kiely,[4] *1. University of New South Wales, Bankstown,*

*New South Wales, Australia, 2. University of New South Wales, Sydney, Australia, 3. Newcastle University, Newcastle upon Tyne, England, United Kingdom, 4. University of New South Wales, Sydney, New South Wales, Australia*

We report the first analysis of inequalities in Disability-Free Life Expectancy (DFLE) trends for Australia, based on two cohorts of the nationally representative Household Income and Labour Dynamics in Australia survey. Each cohort was aged 45+ at baseline with 7-years of annual follow-up (Older cohort: 2001-2007, n=6363; Younger cohort: 2011-2017, n=8197). Disability was defined by a Global Activity Limitation Indicator, and socioeconomic position (SEP) by an area-level index of disadvantage. Compared to men in high advantage areas, men residing in low advantage areas experienced smaller gains in life expectancy (3.0 vs 4.6 years at age 65), DFLE (0.6 vs 1.8 years) and years with disability (2.4 vs 2.8 years). In contrast, for women in low advantage areas all years gained in life expectancy (2.6 years) were years with disability, whereas women in high advantage areas experienced gains in DFLE (1.7 years) and even more years with disability (2.7 years).

### SOCIOECONOMIC POSITION ACROSS THE LIFE COURSE AND TRENDS IN DISABILITY-FREE LIFE EXPECTANCY IN AUSTRALIA

Kim Kiely,[1] Richard Tawiah,[2] Carol Jagger,[3] and Kaarin Anstey,[4] *1. University of New South Wales, Sydney, New South Wales, Australia, 2. University of New South Wales, Bankstown, New South Wales, Australia, 3. Newcastle University, Newcastle upon Tyne, England, United Kingdom, 4. University of New South Wales, Sydney, Australia*

There has been little investigation of how life-course social mobility is linked to Disability-Free Life Expectancy (DFLE). We report novel analysis of the HILDA survey examining how DFLE trends differs by three markers of socio-economic position (SEP): early-life (educational attainment), midlife (occupational level), and late-life (area-disadvantage). All women, irrespective of their educational level, gained years with disability (Age 65: Low education=1.5 and High education=2.5 years). Similar results were obtained by level of occupation, but women with low occupation showed small declines in LE (-0.8 years), all being losses in DFLE. Only women in the most advantaged areas gained DFLE. For men, higher levels of any marker of SEP were associated with DFLE gains that were larger than, or comparable to, gains in years lived with disability, although lower education was associated only with gains in years lived with disability. DFLE trends differ by SEP marker more in women than men.

### MULTIMORBIDITY, DISADVANTAGE, AND TRENDS IN DISABILITY-FREE LIFE EXPECTANCY: THE CFAS STUDIES

Andrew Kingston,[1] Holly Bennett,[1] Louise Robinson,[1] Lynne Corner,[1] Carol Brayne,[2] Fiona Matthews,[1] and Carol Jagger,[1] *1. Newcastle University, Newcastle upon Tyne, United Kingdom, 2. Cambridge Institute of Public Health, University of Cambridge, Cambridge, England, United Kingdom*

The combined contribution of multi-morbidity and socio-economic position (SEP) to trends in disability free life expectancy (DFLE) is unknown. We use longitudinal data from the Cognitive Function and Ageing Studies (CFAS I: 1991; CFAS II: 2011), with two year follow up. Disability was defined as difficulty in activities of daily living, and SEP as area-level deprivation. Multi-morbidity was constructed from nine self-reported health conditions and categorised as 0-1, 2-3, 4+ diseases. In 1991 and 2011, shorter total and disability-free years were associated with greater multi-morbidity. Between 1991 and 2011, gains in life expectancy and DFLE were observed at all levels of multi-morbidity, the greatest gain in DFLE being 4 years for men with 0-1 diseases. As multi-morbidity is more prevalent in more disadvantaged groups, further analyses will investigate whether SEP differences remain at all levels of multi-morbidity.

### SESSION 5850 (SYMPOSIUM)

### WHY BIOPSYCHOSOCIAL DETERMINANTS MATTER: IS AGE JUST A NUMBER?

Chair: Andrew Steptoe

Aging is a physiological and dynamic process enduring time, which is influenced by various underlying mechanisms occurring within the biological, psychological and social spheres. We investigated biopsychosocial determinants of subsequent health and mortality in the English Longitudinal Study of Ageing (ELSA) (Steptoe). Cognitive impairment and dementia, particularly Alzheimer's disease (AD), represent significant challenges to individuals, families and healthcare. We found an indication of socioeconomic differentials influencing the mediating biological and psychological pathways in relation to subsequent cognitive health, which was ascertained with a latent g factor across various cognitive domains in the Harmonised Cognitive Assessment Protocol in ELSA (Cadar). We also identified an interplay between socioeconomic markers and genetic factors influencing the time of dementia and Alzheimer's disease (AD) diagnosis in individuals from the English Longitudinal Study of Ageing, particularly in those with a polygenetic predisposition to AD (Ajnakina). Within the same cohort, we found that participants who transitioned into a single household due to divorce or bereavement had a higher risk of mortality (Abell). The adverse health outcomes associated with loneliness are well documented, but less is known in terms of hospitalization and accessing health care. In the Healthy Ageing in Scotland (HAGIS), we found an increased hospitalization for older individuals reporting higher loneliness (Douglas); and various loneliness patterns in relation to age, gender, marital status and socioeconomic status in participants from first wave of the Northern Ireland Cohort for the Longitudinal Study of Ageing (NICOLA) (Neville). Our findings highlight the imperative need for policy interventions and tailored strategies.

### OVERVIEW OF THE ENGLISH LONGITUDINAL STUDY OF AGEING

Andrew Steptoe, *University College London, London, England, United Kingdom*

The English Longitudinal Study of Ageing (ELSA) is a unique and rich resource of information on the health, social, wellbeing and economic circumstances of the English population aged 50 and older. The current sample contains data from up to nine waves of data collection covering a period of seventeen years (2002-2019). The multidisciplinary and longitudinal nature of the data allows the examination of complex relationships and causal processes. The survey data are designed to investigate a broad set of topics to help understand the ageing process. These include predictors of well-being, health trajectories, disability and healthy life expectancy, the determinants of economic position in older age, the links between socioeconomic status, physical health and functioning, cognition and mental health, the nature and timing of retirement and post-retirement labor market activity, household and family structure, social networks and social supports; patterns, determinants and consequences of social, civic and cultural participation.

### BIOPSYCHOSOCIAL DETERMINANTS OF COGNITIVE HEALTH IN LATER LIFE: THE HARMONISED COGNITIVE ASSESSMENT PROTOCOL

Dorina Cadar,[1] Jessica Abell,[1] Carol Brayne,[2] G. David Batty,[1] David Llewellyn,[3] and Andrew Steptoe,[1] *1. University College London, London, England, United Kingdom, 2. Cambridge Institute of Public Health, University of Cambridge, Cambridge, England, United Kingdom, 3. University of Exeter Medical School, Exeter, England, United Kingdom*

Despite strong evidence for a socioeconomic gradient in many health outcomes, including cognition, substantial gaps remain in understanding these disparities. We investigated the biopsychosocial mechanisms underlying the associations between socioeconomic status (SES) and later-life cognitive health using the Harmonised Cognitive Assessment Protocol (HCAP), a sub-study of the English Longitudinal Study of Ageing (ELSA) which comprises of 1,273 ELSA participants aged 65+. A latent g factor was derived using 12 tests covering a broad range of cognitive domains (memory, language, executive function, and psychomotor speed). We estimated direct and indirect pathways between SES indicators, Apolipoprotein E, inflammatory markers, chronic conditions, and depression. We found that higher education was associated with better cognition, while wealth was not. Increased depressive symptoms were linked with lower cognition, while prior inflammation was indirectly associated with cognition via depressive symptoms and chronic conditions, supporting evidence for a psychosocial role in the context of a socioeconomic gradient.

### INTERPLAY BETWEEN SOCIOECONOMIC MARKERS AND GENETIC PREDISPOSITION TO THE TIME TO DEMENTIA ONSET

Olesya Ajnakina, Dorina Cadar, and Andrew Steptoe, *University College London, London, England, United Kingdom*

Identifying interplay between socio-economic markers (education and financial resources) and genetic factors influencing time of dementia and Alzheimer's disease (AD) diagnosis is of central relevance for the development of preventative strategies. Using 7039 individuals aged >50 years from the English Longitudinal Study of Ageing, 320 (4.6%) of whom developed dementia over the 10-year follow-up, we investigated interactions between polygenic score for AD (AD-PGS) and socioeconomic markers on the timing of dementia or AD diagnosis. One standard deviation increase in AD-PGS was associated with an accelerated time to dementia onset by 4.8 months. Interactions between AD-PGS and lower wealth accelerated time to AD diagnosis by 24 months; an interaction between AD-PGS and years of schooling in decelerating time to AD by 3.0 months suggests education serves as protective mechanisms against AD diagnosis. Socioeconomic markers are important factors influencing time to dementia and AD, particularly in those with polygenetic predisposition to AD.

### LIVING ALONE TRANSITIONS AND MORTALITY IN OLDER MEN AND WOMEN

Jessica Abell, and Andrew Steptoe, *University College London, London, England, United Kingdom*

Living alone has been established as a risk factor for mortality, with biopsychosocial mechanisms suggested as plausible. However, it is unclear whether this is due to health selection. We analysed data from 4,888 individuals who participated in both wave 2 (2004-2005) and wave 4 (2008-2009) of the English Longitudinal Study of Ageing. Mortality status was ascertained from linked mortality register data. An association was found between living alone at wave four and mortality (HR: 1·20, 95% CI 1·04–1·38) in a model adjusted for a range of factors. We also found that participants who transitioned into a solo household due to divorce or bereavement had a higher risk of mortality (HR: 1·34, 95% CI 1·01–1·79). Transitioning into a solo household is also associated with mortality and the underlying reason for this transition was found to be important.

### LONELINESS AND PATTERNS OF HEALTH SERVICE USAGE: EVIDENCE FROM HEALTHY AGEING IN SCOTLAND

Elaine Douglas, and David Bell, *University of Stirling, Stirling, Scotland, United Kingdom*

Loneliness is associated with poorer health status and health outcomes. Yet, little is known about how loneliness in ageing populations is associated with health service usage. Loneliness (UCLA-3) was measured in older people in Scotland (Healthy Ageing in Scotland, HAGIS, n = 1,057). We analysed socio-demographic, perceived health, and health behaviour characteristics using descriptive statistics and logistic regression. The survey data (HAGIS, 2016/17) were linked to retrospective administrative health data to investigate patterns of health service usage (from 2005), such as the number of hospital visits and mean length of stay, and their associated costs. Two-part models were used to highlight variation i) in those who had ever vs never been admitted to hospital, and ii) between those who had been admitted. Our results highlight the variation in hospital service usage in those experiencing loneliness and opens discussion on the implications for older people and hospital services.

### LONELINESS AND SOCIAL ISOLATION AMONG OLDER PEOPLE IN NORTHERN IRELAND

Charlotte Neville,[1] and Paula Devine,[2] *1. Centre for Public Health, Queen's University Belfast, Belfast, Northern Ireland, United Kingdom, 2. Queen's University Belfast, Belfast, Northern Ireland, United Kingdom*

Loneliness and social isolation are increasingly recognised as being public health concernsparticularly in older people. Social isolation can be defined as the lack of social connections, whilst loneliness is a more subjective concept and relates to negative feelings about a lack of connections. This research explores the patterns of loneliness and social isolation of over 5,000 people aged 50 years or over living in Northern Ireland who participated in the first wave of the NICOLA study (Northern Ireland Cohort for the Longitudinal Study of Ageing). Data were obtained by computer-assisted personal interviews and self-completion questionnaires. We focused on loneliness and social interaction, in relation to key demographic and socio-economic variables including age, gender and marital status. Key findings were that loneliness patterns varied according to gender, age, income, health and living circumstances. Future waves of NICOLA will help to longitudinally explore the effects of transition on loneliness and social isolation.

### SESSION 5855 (SYMPOSIUM)

### WHY CREATIVITY MATTERS TO AGING AND HEALTH

Chair: Gay Hanna
Co-Chair: Pamela Saunders
Discussant: Marie Bernard

As GSA turns 75, it is an appropriate time to review the history of the creative aging movement. This symposium explores the research, policy and practice of creative aging - past and present, starting in the 1970's through the efforts of pioneering leaders in the aging, humanities and arts in conjunction with growing support from the newly established National Endowment for the Arts and related aging and health service systems. The foundational research by Gene Cohen, MD PHD and others at the turn of the 21st Century will be described in terms of its building the science to utilize the humanities and arts to scaffold policy and practices that promote the potential of aging through creative expression rather than the pervasive view of aging as a time of loss. Moving towards strength-based approaches to further the development of overall health including brain reserve, physical fitness and social networks, creative aging collaborations will be highlighted as the future of this initiative. Case studies of joint research projects between state departments of both aging and arts in partnership with Universities will demonstration contemporary practices to address major aging issues around isolation, loneliness and caring for the care giver.

### CREATIVITY'S IMPACT ON AGING AND HEALTH, 1970–2010

Wendy Miller, *Create Therapy Institute, Kensington, Maryland, United States*

In the 1970, practitioners in the arts, aging, health, social work and allied therapeutic professions became pioneers in a newly formed movement of creative aging. Robert Butler's "Why Survive? Growing Old in America" called for rethinking aging and the services needed to support the growing aging population. Barriers to restrictions and the lower expectations around how older people could live in their communities began to change. Opportunities for engagement in the humanities and the arts began to be tailored to support older people's life style and to accommodate the sharing of their life experiences in new ways through partnerships with schools and senior centers. The rise of these practices promoted the first multi-year study of arts engagement impact on the health on older adults and a wave of publications and new research studies followed. These were the foundation for this strength-based initiative across the spectrum of aging.

### BUILDING THE SCIENCE: CURRENT STUDIES ON THE IMPACT OF ARTS ENGAGEMENT ON THE HEALTH OF OLDER ADULTS

Marie Bernard,[1] and Sunil Iyengar,[2] *1. National Institutes of Health, Bethesda, Maryland, United States, 2. National Endowment for the Arts, Washington, District of Columbia, United States*

Nearly a decade ago, a federal interagency task force on the arts and human development was launched as the result of a research summit held by the National Endowment for the Arts and the U.S. Department of Health and Human Services to investigate the arts' relationships to health and well-being across the lifespan. Soon afterward, the National Institute on Aging partnered with the Arts Endowment and the National Academy of Sciences to identify research recommendations to benefit healthy aging and the treatment of neurodegenerative diseases in older-adult populations. While this session will revisit some of those findings, it also will share more recent advances in biomedical and behavioral research being conducted by a growing network of "Sound Health" researchers at the nexus of neuroscience, music, and health==with direct implications for the future of research on the arts and aging.

### CREATIVE AGING COLLABORATIONS: NEW DIRECTIONS IN SOLVING CRITICAL SOCIETAL AGING AND HEALTH PROBLEMS

Jamie Dunlap,[1] Steven Horner,[2] and Catherine Richmond-Cullen,[3] *1. Pennsylvania Council on the Arts, Harrisburg, Pennsylvania, United States, 2. Pennsylvania Department of Aging, Harrisburg, Pennsylvania, United States, 3. NeuroLEARN, LLC, Moosic, Pennsylvania, United States*

From the grassroots development of creative aging research to broad base community collaborations that address the issues of today and tomorrow, this presentation will illustrate how state departments of aging and state arts agencies can combine to partnership with research universities to demonstrate the efficacy of the arts to mediate problems of loneliness, isolation and caregiver stress. This case study will show how resources can be expanded and effective practices established through community based research to find ways to build healthy and engaging communities that serve to break down the barriers of isolation and promote social networks.

### SESSION 5860 (SYMPOSIUM)

### WHY INCREASED FOCUS ON AGING WITH DISABILITY MATTERS TO GERONTOLOGY RESEARCH, POLICY, AND PRACTICE

Chair: Margaret Campbell
Discussant: Matthew Janicki

This symposium elaborates on the theme of the 2020 conference, "Why Age Matters", to include aging with disability.

We ask: "can an increased focus on aging with disability within gerontological research, policy, and practice advance our knowledge of disablement across the life cycle and improve our design and implementation of health and social service interventions'? Five experts will address this from differing perspectives (including gerontology and rehabilitation). One presentation draws on national/ regional data to illustrate the changing demographics of aging and disability and highlights the health consequences of aging with- and aging into, long-term physical disabilities. A second uses data from a mixed methods study to demonstrate the unique challenges experienced by adults aging with spinal cord injury with a focus on the impact of specific environmental barriers and facilitators to maintain health and participation in social roles. A third covers three reports on data from a scoping review to document the exclusion of middle-aged and older adults with disabilities from behavioral clinical trials and describes how translational research strategies can be used to help close this gap. A fourth presents examples of how technologies, such as videoconferencing and voice activation, are being used to deliver and enhance existing EB interventions to improve health, physical activity, and participation for individuals aging with mobility impairments. The last one draws on research and scholarly work from both gerontology and rehabilitation to highlight the co-occurring issues of ageism and ableism and describes how reducing ableism is central to successfully reframing aging. Lifelong Disabilities Interest Group Sponsored Symposium.

### CHANGING DEMOGRAPHICS OF AGING AND DISABILITY: IMPLICATIONS FOR ADVANCING KNOWLEDGE OF DISABLEMENT AND LIFE COURSE

Margaret Campbell, *Campbell & Associates Consulting, Grapeview, Washington, United States*

Increased survivorship and longevity have resulted in dramatic improvements in life quality for people with significant disabilities and impairments. However, the fields of rehabilitation and gerontology have tended to divide this phenomenon into people aged under 65 aging with lifelong and early onset disabilities, and those aged 65 plus who are aging into late onset disability. But for both groups, increased survivorship also translates into more years living with comorbidities associated with the underlying condition and increased risk for premature onset and higher rates of age-related chronic conditions. Despite these widely acknowledged trends, we have no national data systems that estimate the overall prevalence of the 'aging with long-term disability' population and monitor its status. Acknowledged is that the lack of national data and reliance on chronological age undermines our knowledge of the disablement experience across the life course and the needs for services and supports associated with diverse trajectories. Part of a symposium sponsored by the Lifelong Disabilities Interest Group.

### HOW BARRIERS AND FACILITATORS IN THE COMMUNITY ENVIRONMENT SHAPE OPPORTUNITIES FOR HEALTHY AGING WITH DISABILITY

Philippa Clarke, Martin Forchheimer, Lynn Charara, Ellen Wolgat, Michelle Meade, and Denise Tate, *University of Michigan, Ann Arbor, Michigan, United States*

Due to advances in medical care and technology the average age of people living with early-acquired spinal cord injury (SCI) is increasing. Approximately 40% of adults with SCI are over age 65. However, the cumulative effects of living with a SCI for many years make aging with SCI different from those "aging into disability". For example, unstable employment histories and the premature onset of secondary health conditions can create unique challenges for adults aging with SCI. Barriers and facilitators in the community environment play an important role for their ability to maintain health, engage in society, and participate in social roles. Data from a mixed methods study of ~200 adults (age 45+) aging with SCI, will be presented to demonstrate the impact of specific environmental barriers and facilitators and to stress the importance of understanding the complex dynamics of person-environment fit to fully support adults aging with and into disability. Part of a symposium sponsored by the Lifelong Disabilities Interest Group.

### UNDERREPRESENTATION OF ADULTS AND OLDER ADULTS WITH DISABILITIES IN BEHAVIORAL CLINICAL TRIALS: A SCOPING REVIEW

Susan Stark,[1] Marian Keglovits,[2] and Sandra ESPÍN TELLO,[3] *1. Washington University in St. Louis, St. Louis, Missouri, United States, 2. Washington University School of Medicine, St. Louis, Missouri, United States, 3. University of the Basque Country (EHU/UPV), San Sebastián, Galicia, Spain*

A lack of evidence-based interventions for people aging with long-term physical disabilities exists. To examine the exclusion of people with disabilities in behavioral clinical trials, a scoping review was conducted. ClinicalTrials.gov was searched for interventional behavioral studies from the United States completed from 2008–2018, with results focused on adults (18–64) and older adults (65+). In total, 158 clinical trials were included. In 129 articles, health conditions were excluded 697 times. Seventy-one clinical trials excluded at least one health condition with strong justification, 11 with poor justification, and 115 without justification. There is strong evidence that people with disabilities are excluded from behavioral clinical trials, often without justification. To help close this gap, our presentation will discuss how translational research strategies, focused on adapting existing EB behavioral trials, can be used to increase the availability of interventions that address the needs of individuals aging with and into long-term disabilities. Part of a symposium sponsored by the Lifelong Disabilities Interest Group.

### LEVERAGING OPPORTUNITIES TO ADVANCE THE POTENTIAL OF TECHNOLOGY TO SUPPORT INDEPENDENCE AND AGING IN PLACE

Tracy Mitzner, *Georgia Institute of Technology, Atlanta, Georgia, United States*

Technology holds great potential to support those aging with and into disability. Research and development efforts in the aging space (aging into disability) have traditionally focused on improving health conditions, whereas those in the disability space (aging with disability) have primarily focused on supporting activity and participation. Bridging these perspectives and approaches adds rich context to guide the development and evaluation of technology interventions.

Examples of technology interventions that support activity and participation and thereby improve health outcomes for adults aging with mobility disabilities show the need for bridging. The Telewellness research study used videoconferencing to deliver an evidence-based tai chi intervention to small groups. The Digital Assistant study explored the potential of the Amazon Echo to support controlling the home environment, engaging in physical activity, interacting with others, and managing health. Both projects offer credence to the value of supporting adults aging-in-place with wide range of capabilities and limitations. Part of a symposium sponsored by the Lifelong Disabilities Interest Group.

### TRANSFORMING CULTURAL IDEAS OF AGING AND DISABILITY TO IMPROVE POLICY AND PRACTICE

Anne Ordway, *U.S. Department of Health and Human Services, Washington, District of Columbia, United States*

Aging and disability are normative processes that extend across the lifespan. However, ageism and ableism are incorporated into many of our practices, programs, and policies—devaluing the lives of older adults and people aging with disabilities and ultimately preventing their full participation in society. Ageism and ableism are closely connected. For example, both systems identify impairment as an individual and social liability. As recent studies have demonstrated, this has real world implications for the quantity and quality of health care requested, delivered, and received by both older adults and people with disabilities. In this session, we discuss the connections between these two forms of oppression and present recent work by researchers in both fields and the FrameWorks Institute that shows how to transform our cultural ideas of aging and disability and development more inclusive policies and services. Part of a symposium sponsored by the Lifelong Disabilities Interest Group.

### SESSION 5865 (SYMPOSIUM)

### DESIGN, UTILIZATION, AND IMPACT OF SMART VOICE INTERVENTIONS IN OLDER ADULTS

Chair: Megan Huisingh-Scheetz
Co-Chair: Louise Hawkley
Discussant: Shelia Cotten

Smart voice (voice-first) devices such as Amazon Alexa or Google Home devices use speech as the primary input method and employ artificial intelligence to “act” on spoken commands. Smart voice devices are thought to reduce technology use barriers for older adults because older users can utilize a skill they already have (talking) rather than learning a new skill (typing). Therefore, smart voice may be a promising technology vehicle for delivering social and functional resources and for assessing health in the home of older adults and their caregivers. However, very little clinical research has been conducted to understand the unique design considerations, utilization, potential impact, and limitations of smart voice applications in older adults. This symposium will 1) describe research methods and results of user-driven design of smart voice materials and programming in older adults; 2) quantify the utilization and potential impact of “EngAGE,” a smart voice-based program delivering socially-motivated exercise, on functional outcomes and summarize perceived benefits and challenges to use among older adult and caregiver participants following a feasibility study; and 3) detail key findings and policy implications following pilots of “Community Hub,” a data collection voice application to detect social isolation, and “Social Check-in,” programs leveraging smart voice to aid in older adult socialization.

### ENGAGE VIA ALEXA FOR OLDER ADULTS AND CAREGIVERS: DESIGN, UTILIZATION, AND IMPACT OF SOCIALLY MOTIVATED EXERCISE

Megan Huisingh-Scheetz,[1] Roscoe Nicholson,[2] Chelsea Smith,[2] Saira Shervani,[2] Yadira Montoya,[3] and Louise Hawkley,[3] *1. University of Chicago, Riverside, Illinois, United States, 2. University of Chicago, Chicago, Illinois, United States, 3. NORC at the University of Chicago, Chicago, Illinois, United States*

EngAGE is a technology-based program leveraging Alexa that encourages older adult (OA) activity and socialization from home while empowering caregivers to support them. EngAGE delivers daily, in-home, NIA Go4Life exercise routines with instructions, pictures and music via Alexa Echo Shows or Fire Tablets to OAs. Caregivers use EngAGE to view scheduled exercises, follow progress, and send encouraging messages that are read aloud to OAs by Alexa. We will discuss the strategic co-design of EngAGE with OAs and caregivers and the utilization and functional impact of EngAGE over a 12-week feasibility and usability study (n=10 OA + caregiver pairs). Preliminary analyses revealed improvement in upper (mean grip strength change = +1.3 kg, paired t-test p=0.34) and lower (5-repeated chair stand time change = -2.3 seconds, paired t-test p=0.02) body strength. Discussion of focus group data will cover themes of perceived benefits, user experience, drivers/barriers to usage and desired features for EngAGE.

### DEVELOPING DIGITAL HOME ASSISTANT USER GUIDES FOR OLDER ADULTS WITH AND WITHOUT LONG-TERM MOBILITY DISABILITIES

Travis Kadylak,[1] Kenneth Blocker,[1] Widya Ramadhani,[2] Lyndsie Koon,[3] Roshanak Khaleghi,[1] Chris Kovac,[1] Ramavarapu Sreenivas,[1] and Wendy Rogers,[1] *1. University of Illinois at Urbana-Champaign, Champaign, Illinois, United States, 2. University of Illinois at Urbana-Champaign, Urbana, Illinois, United States, 3. University of Kansas, Lawrence, Kansas, United States*

The aim of the current study was to understand how to integrate digital home assistant technologies and smart appliances into older adults’ homes by developing supportive user guides that facilitate adoption and continued use. We conducted a series of interviews among older adults, with and without mobility disabilities, to understand their attitudes towards digital assistants and to identify needs for instructional support and user guides. Subsequently, we developed and tested specific user guide modules for older adults aimed at addressing the identified concerns and desired instructional support. Specifically, we developed and field-tested user guides for the initial Amazon Echo device setup, basic device use (e.g., playing music and checking the weather), and separate modules for other domestic use cases (e.g., how to pair an Alexa enabled device with smart lights or appliances). Our results provide guidance for implementation of smart voice technologies to support older adults with long-term mobility disabilities.

### VOICE FIRST AS A PLATFORM FOR ADDRESSING AND MEASURING SOCIAL ISOLATION

Ryan Elza, *AARP Foundation, Washington, District of Columbia, United States*

Voice First solutions offer the opportunity to promote social connectedness and independence. AARP Foundation has been successful in deploying Voice First devices (Amazon Alexa, Google Home Assistant) in senior living and affordable housing communities and developing Voice First solutions through its Connect2Affect Connected Communities program. Connected Communities empowers older adults through voice first solutions to help them obtain the social support resources needed to stay socially connected, remain independent and age in place. AARP Foundation has developed three Voice First solutions 1) Community Hub, a voice application that encourages participation in group activities by voice-enabling a community's calendar of events and allowing older adults to discover, register and receive reminders for events, 2) A data collection voice application that administers the 10-item version of the Duke Social Support Index (DSSI) at regular intervals to assess an older adults' condition of isolation, 3) Social Check-in, a guided risk assessment for social isolation that helps older adult understand their individual risk factors.

### SESSION 5870 (SYMPOSIUM)

### THE SCIENCE OF AGEISM: TWO STEPS FORWARD, ONE STEP BACK

Chair: Jerin Lee
Co-Chair: Natalie Shook

The past two decades have been marked by a rapidly aging population in the U.S. (U.S. Census Bureau, 2018), making prejudicial attitudes toward older adults (i.e., ageism) and the impact of such attitudes more relevant. As such, ageism researchers have worked tirelessly to not only understand this normalized and insidious form of bias, but also develop efforts to combat it. This symposium will feature four ageism researchers who will showcase both the growing pains and novel contributions of ageism research, ranging from the impact of ageism on psychological health to ageism interventions to issues related to the measurement of ageism. Specifically, Dr. Ayalon will present findings regarding difficulties with the assessment of exposure to ageism and the consequences of ageism for psychological well-being. Dr. Horhota will share research demonstrating challenges associated with confronting ageism. Dr. Levy will present a model showcasing factors associated with the reduction of ageism. Ms. Lee will discuss research findings examining the construct validity of several ageism measures. These talks highlight theoretical and real-world implications associated with the complex nature of ageism, providing important directions for enriching ageism research going forward.

### WHAT IS AGEISM? DO OUR MEASURES TELL US?

Jerin Lee,[1] Jenna Wilson,[2] Alexandria Ebert,[2] and Natalie Shook,[1] *1. University of Connecticut, Storrs, Connecticut, United States, 2. West Virginia University, Morgantown, West Virginia, United States*

Ageism has been operationalized in a number of ways over the past half century, and several measures of ageism have been developed to reflect these conceptualizations. This presentation evaluates the extent to which existing ageism measures capture similar or different aspects of ageism. We examined the construct validity of several ageism measures in a sample of 473 undergraduate students (Mage = 19.5 years). Participants completed an online survey of ageism measures, as well as measures of convergent (e.g., aging semantic differentials, attitudes toward various age groups) and discriminant validity (e.g., social dominance orientation, attitudes toward other social groups, sexism). Ageism measures were generally positively associated with one another, though correlations were mostly weak to moderate in magnitude. There was also weak evidence of convergent and discriminant validity. These findings demonstrate conceptual problems with current ageism measures as they do not appear to reflect a common construct.

### CHALLENGES ASSOCIATED WITH THE ASSESSMENT OF EXPOSURE TO AGEISM

Liat Ayalon, *Bar-Ilan University, Ramay Gan, HaMerkaz, Israel*

Ageism is defined as stereotypes, prejudice and discrimination towards people of any age. Ageism can be both positive and negative. In order for an individual to report exposure to ageism, several steps should occur: the individual has to notice the events, interpret them as ageist and report exposure to ageism. Any of these steps may go awry along the way. This presentation uses data from the European Social Survey and the Health and Retirement Study to illustrate the importance of item placement, item phrasing and respondent's mood in responding to items concerning perceived exposure to ageism. A strong priming effect demonstrated a gap between reports of perceived exposure within the ageism module (33.7%) vs. reports within the neutral context (1.1%). A cross-lagged analyses revealed that one's depressive symptoms are predictive of perceived exposure to ageism and not the other way around. Findings illustrate the importance of the context effect.

### THE CHALLENGE OF CONFRONTING AGEISM: IMPRESSIONS OF TARGETS AND BYSTANDERS WHO INTERVENE

Michelle Horhota,[1] Alison Chasteen,[2] and Monika Schindwolf,[1] *1. Furman University, Greenville, South Carolina, United States, 2. University of Toronto, Toronto, Ontario, Canada*

Does confronting ageism come with a cost? Benevolent ageism is viewed as more appropriate than hostile ageism which may lead to negative consequences for individuals who confront it. We examined whether impression costs are mitigated or exacerbated by the style of confrontation (moderate or strong) and the person who confronts (the target or a bystander). Young and older participants read a vignette and rated the target, perpetrator and bystander on warmth, competence, and the acceptability of each character's actions. Participants rated targets who confronted more negatively than bystanders who confronted, and preferred moderate over strong confrontation. In addition, participants thought the perpetrator would be less likely to exhibit prejudicial behaviors again if the older target confronted the action rather than the bystander. This demonstrates the challenge that older adults face; confronting results in a negative impression of them but may be more effective in preventing ageist actions in the future.

### REDUCING AGEISM: PEACE (POSITIVE EDUCATION ABOUT AGING AND CONTACT EXPERIENCES) MODEL

Sheri Levy,[1] Ashley Lytle,[2] Jamie Macdonald,[1] and MaryBeth Apriceno,[1] *1. Stony Brook University, Stony Brook, New York, United States, 2. Stevens Institute of Technology, Hoboken, New Jersey, United States*

Drawing on interdisciplinary theorizing and research, the PEACE (Positive Education about Aging and Contact Experiences) model points to two interrelated factors that reduce ageism: providing education about aging and positive intergenerational contact experiences with older adults (Levy 2016). Evidence supporting the model will be discussed including a semester-long pre-posttest intervention with undergraduates (non-gerontology course) who learned about aging and had face-to-face and Instagram contact with older adults (Lytle, Nowacek, & Levy, 2020), brief online pre-post test experimental-control group studies with undergraduates and a community sample who learned about aging and positive intergenerational contact experiences (Lytle & Levy, 2017), and an online experimental-control group study with undergraduates who viewed brief videos addressing PEACE model components (Lytle, Macdonald, Apriceno, & Levy, under review). Across studies, PEACE model interventions promoted increased aging knowledge as well as reduced negative stereotyping of older adults, aging anxiety, and concerns about aging. Future directions will be discussed.

### SESSION 5875 (SYMPOSIUM)

### BREAKING THE SILENCE ASSOCIATED WITH DEATH AND DYING: NEW DIRECTIONS IN END-OF-LIFE RESEARCH

Chair: Sara Stemen
Discussant: Peter Lichtenberg

Conversations surrounding end-of-life care and bereavement continue to remain relatively silenced within gerontology and the general population. The purpose of this symposium is to break the silence associated with death, dying, and bereavement by sharing emerging perspectives and interventions related to end-of-life experiences. This symposium features four presentations that examine bereavement and end-of-life care from the viewpoints of individuals, families, practitioners, and researchers. Carr provides a comprehensive overview of the current state of research regarding death, dying, and bereavement - mapping out how current technological and demographic shifts have changed the nature of end-of-life experiences. Stemen presents an illustrative case study that examines how cause of death (e.g., chronic illness, suicide) shapes grief and subsequent social relationships for surviving individuals. Utz explores conversations that occur between families and professionals embedded within the hospice system, showcasing reactions from families who experienced live discharge from hospice services. Last, Ogle sheds light on the roles taken on by state tested nursing assistants (STNAs) in end-of-life care as well as the training and education they receive and need on end-of-life issues. Lichtenberg, our discussant, will tie these emerging perspectives together in order to initiate an important dialogue with attendees regarding the actions needed to break the silence associated with death and dying so that we can better serve individuals, families, and professionals.

### EXPLORING CAUSE OF DEATH IN SOCIAL CONVOY MEMBERSHIP: THE CASE OF PAULINE

Sara Stemen, Kate de Medeiros, and M. Elise Radina, *Miami University, Oxford, Ohio, United States*

People receive support from a fluid convoy of individuals. Historically, convoy membership has been limited to meaningful, living persons. However, research incorporating the continuing bonds model suggests that individuals who have died can also be convoy members as relationships can be preserved through pictures, memories, and after death communication experiences. Building on this idea, this presentation uses a qualitative case study to explore whether (and if so, how) continuing bond relationships are influenced by the way that individuals die. Pauline, a 67 year-old widow, compares the "natural" deaths of her sister and father-in-law to the suicide of her husband. Careful readings of her interview transcript reveal that the unexpected way that her husband died became a salient part of her identity and the way she connects with others. Consequently, this case study provides insights for researchers who may consider cause of death as a potential contributing factor to convoy membership.

### END OF LIFE: A NEW LIFE COURSE STAGE FOR OLDER ADULTS AND THEIR FAMILIES?

Deborah Carr, *Boston University, Boston, Massachusetts, United States*

Over the past two centuries, death has transitioned from an unexpected and uncontrollable event to a protracted process that requires individuals and families to make difficult decisions regarding where and under what conditions one will die. This new life course stage, spanning the period from diagnosis to death, provides older adults and their families an opportunity to prepare for difficult medical decisions, yet also may be a time marked by suffering and conflict. In this paper, I provide an overview of the technological, demographic, and legal context of end-of-life in the 21st century, and its implications for the quality of life for dying patients and their families. I underscore that historical shifts have created a context in which the quality of one's end-of-life experiences and autonomy are stratified by race and socioeconomic status, creating challenges for older adults and their loved ones. I highlight implications for research, policy, and practice.

### WORKSHOPS TO TEACH STATE-TESTED NURSING ASSISTANTS ABOUT END-OF-LIFE CARE

Kimberly Ogle, *Miami University, Oxford, Ohio, United States*

Given that almost 25 percent of U.S. deaths occur annually in long-term care facilities (U.S. Census Bureau, 2017), it's imperative that frontline workers are given training and support they need to deliver good, person-centered care at the end of life. Inadequate end-of-life (EOL) care may lead to unrelieved suffering and undignified deaths (Bukki, Neuhaus, Paa, 2016). Furthermore, nursing staffs have knowledge gaps and low confidence regarding end-of-life care and they may underestimate its complexity (Pfister, Markett & Muller, 2013). With the growing population of older adults, improving end-of-life care in long- term care facilities needs to be a priority. This research explored the needs of State Tested Nursing Assistants (STNAs) working in long-term care and their knowledge regarding EOL care. Based on the findings of this research, workshops were developed to better educate the STNAs regarding care of the dying and to enhance the EOL care for long term residents.

### LIVE DISCHARGE FROM HOSPICE: EXPERIENCES OF PATIENTS, FAMILIES, AND STAFF

Rebecca Utz,[1] Margaret Clayton,[2] and Katherine Supiano,[1]
*1. University of Utah, Salt Lake City, Utah, United States,*
*2. University of utah, Salt Lake City, Utah, United States*

Hospice services can provide patient stabilization and improved quality of life, making the patient ineligible for continued hospice. Using focus groups and in-depth interviews, we explored how patients, families, and hospice staff experience, anticipate, and cope with "live discharge" from hospice. Nurses and administrators tried to prepare families for the possibility and unsettling reality of live discharge, yet the aides who worked most closely with families remained unaware of regulations dictating an impending live discharge. Reactions of families ranged from excitement, when patient was selected for clinical trial, to more common expressions of frustration, as they lost access to the staff, coordinated services, and medications only covered under the Medicare hospice benefit. Families also reported relationship stressors and decisional uncertainty associated with longer than expected patient life. Families still needed and wanted hospice services, with most agreeing they would return to hospice after the patient declined and became eligible again.

### SESSION 5885 (SYMPOSIUM)

### IN SICKNESS AND IN HEALTH: DYADIC PERSPECTIVES ON HEALTH AND WELL-BEING AMONG OLDER COUPLES

Chair: Courtney Polenick

This session will incorporate a variety of dyadic methods to explore the multifaceted ways that older spouses shape their own and their partners' health and well-being. First, Dr. Karen Lyons will consider the roles of communication, collaborative decision-making, and social support in shaping the mental health of couples managing chronic pain. Her comparative dyadic analysis highlights the value of collaborative illness management in optimizing couples' mental health. Dr. Courtney Polenick will then describe how chronic condition discordance (i.e., the extent to which two or more conditions have non-overlapping self-management requirements) within individuals and between spouses is linked to perceived control among couples over an8-year period. This study reveals that more complex patterns of chronic conditions within couples have particularly detrimental implications for women's perceptions of control over their own health and other life domains. Next, Dr. Kira Birditt will examine the long-term effects of spouses' similar drinking patterns (i.e., concordance). Although drinking concordance may enhance marital satisfaction, she will explain how it can have enduring negative consequences for cardiovascular health among middle-aged men. Dr. Joan Monin will then explain the short-term benefits of laughter for blood pressure among couples during lab-based spousal support interactions. Finally, Dr. Amy Rauer will discuss how spouses react to one another's health-related support attempts using in-depth qualitative interviews conducted with both members of the couple. Taken together, these studies underscore the importance of evaluating dynamic short-term and long-term health-related influences among couples in middle and later life. Dyadic Research on Health and Illness Across the Adult Lifespan Interest Group Sponsored Symposium.

### THE ROLES OF COMMUNICATION, COLLABORATION, AND SOCIAL SUPPORT ON DYADIC MENTAL HEALTH IN THE CONTEXT OF CHRONIC PAIN

Karen Lyons, *Boston College, Chestnut Hill, Massachusetts, United States*

Drawing on the Theory of Dyadic Illness Management, the study examined the roles of communication, collaborative decision-making and social support on the mental health of 177 couples living with chronic pain. Couples ranged in age from 26-81 years of age; mean age for partner with chronic pain = 55.01 (SD=11.53) and partner without chronic pain = 57.45 (SD=12.50). Using multilevel modeling and controlling for pain severity, pain interference, time since diagnosis, age, shared activities and relationship quality, communication and collaborative decision-making played significant roles in predicting mental health of both members of the couple. Comparative dyadic analysis showed that couples with optimal dyadic mental health had significantly better communication, less concealment, greater collaboration and greater levels of support than couples with poor or incongruent dyadic mental health. Discussion will center on the roles of collaborative illness management behaviors in optimizing dyadic mental health in the context of chronic illness. Part of a symposium sponsored by Dyadic Research on Health and Illness Across the Adult Lifespan Interest Group.

### INDIVIDUAL-LEVEL AND COUPLE-LEVEL CHRONIC CONDITION DISCORDANCE: LONGITUDINAL LINKS TO PERCEIVED CONTROL

Courtney Polenick,[1] Kira Birditt,[1] Angela Turkelson,[1] Sadie Shattuck,[1] and Helen Kales,[2] *1. University of Michigan, Ann Arbor, Michigan, United States, 2. UC Davis, Sacramento, California, United States*

Chronic condition discordance (i.e., the extent that two or more conditions have non-overlapping self-management requirements) has adverse mental health implications but little is known about mechanisms accounting for these links. We considered how chronic condition discordance at the individual level and the couple level (i.e., between spouses) was associated with perceived control among 2,676 couples from three waves (2006, 2010, and 2014) of the Health and Retirement Study. Dyadic growth curve models revealed that when wives had greater individual-level discordance, they reported lower initial health-related control and personal mastery and steeper reductions in personal mastery. When husbands had greater individual-level discordance, they reported lower initial health-related control and faster declines in health-related control and personal mastery, and wives had faster declines in personal mastery. When there was greater couple-level discordance, wives reported lower initial health-related control. Targeting increases in perceived control among older couples managing complex conditions may be beneficial. Part of a symposium sponsored by Dyadic Research on Health and Illness Across the Adult Lifespan Interest Group.

### ALCOHOL CONSUMPTION AND BLOOD PRESSURE AMONG OLDER COUPLES: NEGATIVE IMPLICATIONS OF DRINKING CONCORDANCE

Kira Birditt,[1] Angela Turkelson,[1] Courtney Polenick,[1] James Cranford,[1] and Frederic Blow,[2] *1. University of Michigan, Ann Arbor, Michigan, United States, 2. University of Michigan Medical School, Ann Arbor, Michigan, United States*

Couples who are similar in their drinking behaviors (i.e., concordance) often report better quality and longer marriages. These patterns of drinking may have negative implications for blood pressure, however, as individuals age. The present study examined the effects of alcohol use on blood pressure among couples and whether the associations varied by age. Participants included 2311 married/cohabiting couples (4487 individuals; aged 52 to 92) followed from 2006 to 2014 in the Health and Retirement Study who reported their average drinks per week and had their blood pressure measured. Multilevel models revealed older men (aged 60+) who drank more had higher blood pressure irrespective of wives drinking whereas for younger men the link between their own drinking and increased blood pressure was greater when wives drank more. Although concordance may be beneficial for marriage, there may be a downside to drinking concordance for health especially among middle aged men. Part of a symposium sponsored by Dyadic Research on Health and Illness Across the Adult Lifespan Interest Group.

### LAUGHTER AND SHORT-TERM BLOOD PRESSURE REACTIVITY IN SPOUSAL SUPPORT INTERACTIONS

Joan Monin,[1] Jennifer Tomlinson,[2] and Brooke Feeney,[3]
*1. Yale University, New Haven, Connecticut, United States, 2. Colgate University, Hamilton, New York, United States, 3. Carnegie Mellon University, Pittsburgh, Pennsylvania, United States*

Individual effects of laughter in reducing stress are well-documented. However, no research has examined dyadic associations between laughter and blood pressure in spousal support interactions. This study examined the hypotheses that individual and shared laughter would be associated with lower blood pressure and distress during a support interaction for both the "support-seeker" and the "support-provider". Two hundred and seventy-one older adult couples were video-recorded and their blood pressure was monitored during a baseline, a discussion about the support-seeker's greatest fear related to aging, and while playing a game in the laboratory. Both spouses reported their distress after the support interaction. Laughter was coded by trained observers. According to the Actor Partner Interdependence Models, the more the support-seeker laughed, the lower the support-provider's systolic blood pressure was during the support interaction (partner effect). Also, laughter was associated with less distress for both spouses during the support interaction (actor effects). Part of a symposium sponsored by Dyadic Research on Health and Illness Across the Adult Lifespan Interest Group.

### OLDER ADULTS' RECEPTIVITY TO SPOUSAL HEALTH SUPPORT: A QUALITATIVE ANALYSIS

Amy Rauer,[1] Ruth Palan Lopez,[1] and Ashley Pigg,[2]
*1. The University of Tennessee, Knoxville, Knoxville, Tennessee, United States, 2. University of Tennessee, Knoxville, Knoxville, Tennessee, United States*

Romantic partners play a powerful role in promoting each other's well-being, particularly later in life. Although there is a robust literature documenting how spouses tend to respond to each other's health issues (e.g., support, control), there has been a surprising dearth of studies examining how receptive spouses are to these overtures. To reveal how older couples react to each other's health support attempts, we used a qualitative approach to analyze transcripts of 64 older, married, heterosexual couples observed discussing each other's health issues. Thematic analysis revealed older couples responded to spouses' health support strategies in four different ways: 1) listening (individuals actively listened); 2) accepting (individuals welcomed spousal efforts); 3) deflecting (individuals redirected conversation away from health); and 4) rejecting (individuals dismissed and refused spousal intervention). Given the rise in late-life caregiving, identifying the very different responses that are evoked when partners intervene in each other's health warrants further investigation. Part of a symposium sponsored by Dyadic Research on Health and Illness Across the Adult Lifespan Interest Group.

### SESSION 5890 (SYMPOSIUM)

### ENHANCING HEALTH FOR THE GROWING NUMBER OF OLDER CANCER SURVIVORS: DESIGNING INNOVATIVE BEHAVIORAL INTERVENTIONS

Chair: Heather Derry
Co-Chair: Claire Conley
Discussant: Kelly Trevino

By 2040, there will be an estimated 26.1 million cancer survivors in the United States, with 73% over age 65. Compared to younger survivors and those without cancer, older adult cancer survivors have an elevated comorbidity burden. Lifestyle interventions can play a key role in preventing and managing chronic health conditions and promoting quality of life during and after cancer treatment. Yet, behavioral interventions for maximizing health are under-utilized in older adults with cancer. At times, older adults may have unique needs that require tailoring to increase accessibility, optimization, and uptake of behavioral interventions.

**This symposium will showcase innovative approaches for enhancing health among older adult cancer survivors during and after cancer treatment. Dr. Bluethmann will discuss design considerations for**

**using geriatric assessment in an ongoing exercise trial to manage side effects of aromatase inhibitors. Dr. Gell will present data on older survivors' preferences regarding text messaging to support physical activity maintenance from an intervention study. Dr. Leach will discuss the use of technology to facilitate lifestyle change in older cancer survivors, presenting data on older adults' user preferences and benefits from an eHealth tool. The discussant, Dr. Trevino, will summarize how these interventions can be leveraged to promote engagement in managing older survivors' health and to inform next steps in intervention development. Collectively, this multidisciplinary group of speakers will provide practical information and "lessons learned" from designing behavioral and technology-based interventions, and highlight the promise that these approaches hold for improving quality of life in aging cancer survivors. Cancer and Aging Interest Group Sponsored Symposium.**

### OVERVIEW OF REJOIN: A CLINICAL TRIAL TO USE EXERCISE TO RELIEVE JOINT PAIN IN OLDER BREAST CANCER SURVIVORS

Shirley Bluethmann,[1] Cristina Truica,[2] Nancy Olsen,[1] Christopher Sciamanna,[1] Heidi Klepin,[3] Vernon Chinchilli,[1] and Kathryn Schmitz[1] *1. Penn State College of Medicine, Hershey, Pennsylvania, United States, 2. Penn State Cancer Institute, Hershey, Pennsylvania, United States, 3. Wake Forest School of Medicine, Winston-Salem, North Carolina, United States*

Aromatase Inhibitors (AIs) are recommended for survival in hormone-sensitive breast cancer survivors, yet are underutilized, especially in older survivors. Joint pain is a prevalent AI-related symptom that is associated with low adherence. The aims for this randomized clinical trial (n=76) are to: 1) adapt a self-management (exercise + education) intervention for older survivors planning to take AIs; 2) Test the effect of a pilot intervention on arthralgia; and 3) Test its effect on AI medication adherence behaviors. We will adapt a program for seniors that includes bi-weekly 60-minute sessions of supervised exercise plus 30 minutes of education. The 16-week program includes: 8-weeks in person plus 8-weeks at home with phone counseling. We will conduct geriatric plus baseline assessment of exercise, joint pain, and AI adherence (repeated 4, 6 and 12 months). More research with geriatric survivors is required to address treatment needs and to promote survival. Part of a symposium sponsored by the Cancer and Aging Interest Group.

### AGE-RELATED ENGAGEMENT AND OUTCOMES IN A CANCER SURVIVOR SELF-MANAGEMENT EHEALTH PROGRAM

Corinne Leach,[1] Shawna Hudson,[2] Michael Diefenbach,[3] Sicha Chantaprasopsuk,[1] and Catherine Alfano,[1]
*1. American Cancer Society, Atlanta, Georgia, United States, 2. Rutgers Robert Wood Johnson Medical School, New Brunswick, New Jersey, United States, 3. Northwell Health, Manhassett, New York, United States*

While eHealth programs equip survivors with tools at where and when they need, their benefit to and engagement patterns among older adults are less known. Data come from the Springboard Beyond Cancer RCT, a cancer survivor self-management program (N=176; 88 control, 88 intervention arm) and the corresponding qualitative evaluation/user testing (N=40). Younger survivors, but not older, preferred socially interactive and personalized long-in features which enable greater tailoring of the program. However, the older survivors who did enroll in the RCT were equally as likely as their younger counterparts to engage with one or more aspects of program. Health self-efficacy improvement from baseline to 3 months was significant among younger participants in the intervention ($p<.05$) but not the control arm ($p=.54$) ($d=.20$) and marginally significant among older survivors (age 60+) in intervention ($p=.06$) but not the control arm ($p=.58$) ($d=.28$). Results suggest that the program may benefit survivors regardless of age. Part of a symposium sponsored by the Cancer and Aging Interest Group.

### CANCER SURVIVOR PERSPECTIVES ON TEXT MESSAGES IN A TECHNOLOGY-BASED PHYSICAL ACTIVITY INTERVENTION

Nancy Gell,[1] and Christine Vatovec,[2] *1. The University of Vermont, Burlington, Vermont, United States, 2. University of Vermont, Burlington, Vermont, United States*

Messaging through mobile apps and texts are a common feature of technology-based physical activity interventions. We aimed to examine perspectives of mid-life and older cancer survivors on message content, timing, and two-way communication. We conducted qualitative interviews with 14 participants (Mean 59.9 years, Range 52-79) who completed a remotely delivered intervention that included text messages to support physical activity. After transcription, the interviews were coded and analyzed thematically using inductive, directed content analysis. Themes related to content preferences included personalization, accountability, perspective on the 'bigger picture,' and acknowledgement of PA-related achievements. Random timing for receipt was considered acceptable whereas interest in responding to messages was highly variable. Older cancer survivors' preferences for highly personalized messages are an important consideration in designing technology-based physical activity interventions. A combination of accountability, relation to personal factors, and acknowledgement of goal attainment need to be considered in efforts to scale interventions. Part of a symposium sponsored by the Cancer and Aging Interest Group.

### SESSION 5895 (SYMPOSIUM)

### WHERE WE LIVE MATTERS: RESIDENTIAL INFLUENCES ON HEALTH AND WELL-BEING

Chair: Yee To Ng
Co-Chair: Elizabeth Munoz
Discussant: Markus Schafer

Growing evidence indicates that residential contexts are implicated in the health and well-being of older adults. Operationalization of these contexts varies and includes psychosocial, physical and socioeconomic neighborhoods, and more proximal contexts (e.g., home environment). We aim to bring together a diverse set of papers focused on the living environment to understand how contextual factors are associated with individual outcomes. Muñoz and colleagues applied a lifespan perspective by evaluating associations between current and childhood neighborhood perceptions on cognitive health. Their results indicated that the association between perceived neighborhoods and cognition in adulthood was moderated by childhood neighborhoods. García and Ailshire contextualized the types of neighborhoods in which older Latinos live and how these influenced diabetes risk. They identified neighborhood clusters characterized by racial/ethnic and socioeconomic compositions and found that predominantly Latino neighborhoods with low SES were more likely to have diabetes compared to other neighborhood clusters. This symposium will also focus on the more proximal environment. Lee and Ailshire examined the neighborhood and home environment and found that the home's proximity to green space and level of clutter within the home was associated with increased fall risks in older adults. Fingerman and colleagues coded older adults' living spaces and found that personality type was associated with room conditions. Altogether, the presentations highlight the relevance of context measured across multiple levels of analyses and dimensions of well-being outcomes in aging individuals. Dr. Markus Schafer will provide a discussion of these findings and address the challenges and opportunities for future research.

### THE IMPACT OF PERCEIVED NEIGHBORHOODS IN CHILDHOOD AND ADULTHOOD ON DAILY COGNITIVE FUNCTION

Elizabeth Munoz,[1] Giancarlo Pasquini,[2] Alexa Allan,[3] Jacqueline Mogle,[3] Martin Sliwinski,[4] and Stacey Scott,[2]
*1. The University of Texas at Austin, Austin, United States, 2. Stony Brook University, Stony Brook, New York, United States, 3. Pennsylvania State University, University Park, Pennsylvania, United States, 4. Penn State University, University Park, Pennsylvania, United States*

Neighborhood experiences may have a cumulative effect on cognitive health outcomes across the lifespan. We investigated independent and combined associations between current and retrospective childhood neighborhoods and daily cognitive function in 209 adults (Mage=47.07; range: 25-65) who participated in a 14-day ecological momentary assessment study. Participants reported perceptions of their current neighborhood and the neighborhood they lived in at age 5; including neighborhood cohesion, safety, violence, and physical conditions. Greater current neighborhood violence was independently associated with poorer spatial working memory. Childhood neighborhood violence was not significantly associated with performance. The interaction between current and childhood neighborhood ratings was significant: individuals who reported greater childhood neighborhood violence, but lower current violence had better performance than those who experienced consistently high or low neighborhood violence. Effects for other neighborhood domains were not significant. Results indicate that neighborhood influences on cognition in adulthood may be moderated by childhood neighborhood experiences.

### ¿DÓNDE VIVEN LOS LATINOS? THE TYPES OF NEIGHBORHOODS OLDER LATINOS LIVE IN AND THEIR IMPLICATIONS FOR DIABETES

Catherine García, and Jennifer Ailshire, *University of Southern California, Los Angeles, California, United States*

The spatial distribution of Latinos in U.S. neighborhoods is highly patterned due to a complex set of social, cultural, and economic forces, which leads to the differential distribution of and exposure of resources and opportunities across space. However, less is known about the types of neighborhoods older Latinos live in and how it impacts their health. Using census tracts from the year 2000, we employed a latent class analysis to explore how social and socioeconomic characteristics cluster together to create distinct neighborhood typologies. These typologies were then combined with data from the 2006-2016 Health and Retirement Study (n=3,047). We found that Latinos were more likely to live in predominantly Latino neighborhoods with low socioeconomic status (SES) (40%), multiracial neighborhoods with moderate SES (27%), and predominantly white neighborhoods with moderate SES (15%). Latinos living in predominantly Latino neighborhoods with low SES were more likely to have diabetes than other neighborhood typologies.

### NEIGHBORHOOD AND HOUSING CONDITIONS AND RISK OF FALLS

Haena Lee, and Jennifer Ailshire, *University of Southern California, Los Angeles, California, United States*

Physical conditions of living environments can impact risk of falls, however, prior work has focused typically on one domain at a time—either neighborhood or home, capturing limited environmental boundaries of older adults. We extend prior work by considering both neighborhood and home as important residential contexts and examine their impact on the onset of falls over time. Data are drawn from two waves of the Health and Retirement Study (2012 and 2016; N = 2,244). We used interviewers' report on outdoor and indoor dwelling conditions to assess environmental risk factors. One-third of respondents reported at least one fall years later. The presence of green space such as a park near the housing unit and clutter in the home appears to increase the risk of falls over a four-year period. This finding suggests that falls in old age may be determined by a combination of outdoor and indoor risk factors.

### LIFE IN THE LIVING ROOM: AN EXPRESSION OF PERSONALITY IN LATE LIFE?

Karen Fingerman, Yee To Ng, and Shiyang Zhang, *The University of Texas at Austin, Austin, Texas, United States*

Recent research has found that micro environments (i.e., residential spaces) provide information about psychological processes in young adults. This study examined older adults' living spaces. Participants (N = 171) from the Daily Experiences and Well-being Study completed measures of personality. Interviewers took 3 to 4 photographs of the room where participant spends the most time. Independent coders rated the rooms for 33 features (e.g., cleanliness, light, clutter) on a 5 point scale. Regression analyses revealed that personality was associated in anticipated ways. For example, conscientiousness was associated with rooms in better condition, neuroticism with less brightness, and by contrast, extraversion with greater brightness and more color. Discussion focuses on bidirectional emotional aspects of home environments that may contribute to well-being. Macro environments (e.g., neighborhoods) shape late life well-being, but understanding micro environments where older adults spend the majority of their time also may contribute to advances in the field.

### SESSION 5900 (SYMPOSIUM)

### DIVERSITY MATTERS FOR HEALTH: ACTIVITY DIVERSITY, EMODIVERSITY, STRESSOR DIVERSITY, AND HAPPYVERSITY

Chair: Soomi Lee
Co-Chair: Emily Urban-Wojcik
Discussant: David Almeida

Aging theories suggest that diversity of experiences relates to social integration, cognitive reserve, and more psychological resources, all of which are important for successful aging. However, age-related declines may contribute to a monotonous daily life. Emerging studies suggest that activity diversity and positive emotional diversity are associated with better health; yet, we still know little about how a variety of diverse lifestyle indicators are associated with health in adulthood. This symposium showcases contemporary endeavors towards understanding how multiple diversity indicators of adult lifestyle relate to health. Paper 1 examines activity diversity (breadth and evenness of daily activity participation) and tests its associations with overall and nightly sleep health. Paper 2 investigates the relationship between activity diversity and hippocampus volume. Paper 3 examines whether there are age-related differences in the extent to which positive emodiversity attenuates the association between stress and depressive symptoms. Paper 4 examines stressor diversity and how it is associated with blood pressure across age and SES. Paper 5 introduces a novel concept of happyversity (diversity in life satisfaction across different domains) and tests its association with terminal decline. These papers use different project datasets that include different populations of middle-aged and older adults, such as the Midlife in the United States Study, Intraindividual Study of Affect, Health and Interpersonal Behavior, Study of Health and Interactions in the Natural Environment, and German Socioeconomic Panel Study. At the end of these presentations, Dr. Almeida will discuss their theoretical and methodological contributions, and consider challenges and opportunities for future research.

### BIDIRECTIONAL ASSOCIATIONS BETWEEN ACTIVITY DIVERSITY AND SLEEP

Soomi Lee,[1] Susan Charles,[2] and David Almeida,[3]
*1. University of South Florida, Tampa, Florida, United States, 2. BSS, Irvine, California, United States, 3. Pennsylvania State University, University Park, Pennsylvania, United States*

Activity diversity is important for psychological well-being and cognitive functioning. Yet, little is known about the relationship between activity diversity and sleep. This study examined how overall and nightly sleep health are associated with activity diversity. Participants (N=1841) from the Midlife in the United States Study II provided activity data for 8 days. We constructed overall and daily activity diversity scores. A composite score of overall sleep health across 8 dimensions and nightly sleep duration were measured. Analyses adjusted for sociodemographics, total activity time, and positive/negative affect. Participants with poorer sleep health overall had a lower activity diversity. On days following nights with short (<6hrs) or long (>8hrs) sleep duration, participants engaged in fewer-than-usual activities. Conversely, fewer daily activities also predicted long (but not short) sleep duration. Our results suggest cyclical associations between poor sleep health and activity diversity day-to-day, which, may accumulate over time to form a bidirectional relationship.

### THE RELATIONSHIP BETWEEN HIPPOCAMPAL VOLUME AND ACTIVITY DIVERSITY

Emily Urban-Wojcik,[1] Soomi Lee,[2] Susan Charles,[3] David Almeida,[4] Richard Davidson,[1] and Stacey Schaefer,[1]
*1. University of Wisconsin-Madison, Madison, Wisconsin, United States, 2. University of South Florida, Tampa, Florida, United States, 3. BSS, Irvine, California, United States, 4. Pennsylvania State University, University Park, Pennsylvania, United States*

The hippocampus, implicated in learning, memory, and spatial navigation, is one of the few brain structures that demonstrates neurogenesis across the lifespan. Hippocampal volume (HV), then, may be a marker of exposure to and engagement with novel events and environments, which may in turn be related to cognitive functioning. The present study examined the relationship between HV and activity diversity (AD), which characterizes the range and evenness of participation in daily activities. In 52 participants who completed the daily-diary and neuroscience projects of the Midlife in the United States Refresher study, greater levels of AD across an 8-day period were related to greater HV averaged across the left and right hemispheres when adjusting for overall brain volume, total activity time, time between projects, and relevant sociodemographic variables, b=1128mm3, $t(43)=2.54$, $p=.015$. These findings may point to a mechanism through which AD has been related to better cognitive and mental health outcomes.

### POSITIVE EMODIVERSITY BUFFERS THE ASSOCIATION BETWEEN STRESS AND DEPRESSIVE SYMPTOMS

Lizbeth Benson,[1] and Anthony Ong,[2] *1. The Pennsylvania State University, University Park, Pennsylvania, United States, 2. Cornell University, Ithaca, New York, United States*

Intensive measurements of individuals' experiences allow for identifying patterns of functioning that may be markers of resilience, and whether such patterns differ across the life span. Using 8 daily diary reports collected in the second burst of the National Study of Daily Experiences (NSDE, n=848, age 34-84; 55%female), we examined whether positive emodiversity (Shannon's entropy) attenuated the association between cumulative stressor exposure and depressive symptoms, and age-related differences therein. Results indicated age moderated the extent to which positive emodiversity attenuated the association between stress and depressive symptoms ($b=0.11$, $p < .05$). The attenuated association was strongest for younger adults with higher positive emodiversity, compared to those with lower positive emodiversity. For older adults, the association between stress and depressive symptoms was relatively similar regardless of their positive emodiversity. Implications pertain to for whom and in what contexts specific types of dynamic emotion experiences may promote optimal functioning and resilience.

### HIGH STRESSOR EXPOSURE AND LOW STRESSOR DIVERSITY ARE LINKED TO HIGHER BLOOD PRESSURE ACROSS AGE

Rachel Koffer, Kristina Dickman, and Thomas Kamarck, *University of Pittsburgh, Pittsburgh, Pennsylvania, United States*

Stress exposure is linked to elevated blood pressure, which increases risk for cardiovascular disease (Spruill, 2010; WHO, 2013). Stress exposure may be especially harmful when concentrated in one particular domain (i.e., low stressor diversity) (Koffer, et al., 2016). Using a diversity index, we test whether high stressor exposure and low stressor diversity is associated with high resting blood pressure. Participants (N=391, aged 40-64 years) completed four days of hourly self-report of stressful experiences (e.g., work task demand, non-work task demand, arguments, interpersonal tension), with clinic blood pressure separately assessed. Linear regression results indicate older adults experienced lower stressor diversity ($B = -0.003$, $p = .003$). Further, higher stressor exposure with lower stressor diversity related to higher diastolic blood pressure ($B= -7.21$, $p=.046$). Experiencing high stress concentrated in one domain may increase risk of high blood pressure. We discuss how low stressor diversity may help explain age-related risk of cardiovascular disease.

### SESSION 5910 (PAPER)

### FALLS, MUSCLE STRENGTH, AND CARING FOR OLDER POPULATIONS: PRESENTER DISCUSSION

### ASSOCIATIONS OF CYTOMEGALOVIRUS AND MUSCLE WEAKNESS IN OLDER ADULTS: RESULTS FROM THE HEALTH AND RETIREMENT STUDY

Kate Duchowny,[1] and Grace Noppert,[2] *1. University of California, San Francisco, San Francisco, California, United States, 2. University of North Carolina at Chapel Hill, Chapel Hill, North Carolina, United States*

Muscle weakness, measured by grip strength, is associated with disability, physical functioning and mortality; however, the sociobiologic underpinnings of muscle weakness are poorly understood. Immune response to cytomegalovirus (CMV), as measured by immunoglobulin G (IgG) antibody levels, is often used as a marker of immunosenescence with higher antibody levels suggesting accelerated immunosenescence. Previous research has shown immune response to CMV across the life course is socially patterned with disadvantaged groups having higher antibody levels across the life course. Thus, the primary objective of this study was to examine associations between CMV IgG antibodies and grip strength in a nationally representative cohort of older adults, and the role of social factors in these associations. Using data from the Health and Retirement Study (N= 4,251; Mean Age= 67.4 years), we employed gender-stratified weighted linear regression models to estimate the mean difference in grip strength associated with tertiles of CMV IgG response controlling for age, race/ethnicity (White, Black and Hispanic) and educational attainment. For men, higher IgG antibody levels were associated with decreased mean grip strength. For example, men in the highest level of IgG antibody had a grip strength of 37.67 kg (95% CI: 36.30, 39.05) compared to men seronegative to CMV with a grip strength of 38.96 kg (95% CI: 38.18, 39.74). For women, there was little difference in grip strength by CMV IgG response. Our findings suggest accelerated immunosenescence may be an important predictor of reduced grip strength in older adults, and critical to understanding social disparities in grip strength.

### FAMILISM AND ITS IMPACT ON YOUNGER AFRICAN-AMERICAN INFORMAL FAMILY CAREGIVER ROLE STRAIN AND DECISION-MAKING

Ivana Alexander, *University of Maryland Baltimore, Baltimore, Maryland, United States*

Informal family caregivers of older adults are the life's blood of the long-term services and supports (LTSS) system in the United States, providing an estimated $470 billion in unpaid care each year. This care is disproportionately provided by racial and ethnic minority families, where systemic economic disparities make it impossible to afford formal care in many cases. Adding to this are the cultural expectations or familism values that often influence attitudes and beliefs about caregiving. These expectations and values contribute to the emotional, psychological, financial, and professional strain experienced by caregivers. They also impact the caregiver-healthcare provider dyad as well as the caregiver-patient-healthcare provider triad, where shared decision-making is an essential component of providing LTSS for family members. This presentation applies an auto-enthographic lens to the author's personal experiences as a younger African-American female caregiver of an elderly, terminally ill family member and as a medical social worker to highlight the gaps that exist between familism values, the shifting reality of family caregiving, and available resources and addresses related implications for the future of long-term care in a family-centered context.

### THE BURDEN OF SERIOUS FALLS AMONG OLDER ADULTS: EVIDENCE FROM HOSPITALIZATIONS IN PHILADELPHIA, PA

Megan Todd, Kevin Pressley, and Meagan Pharis, *Philadelphia Department of Public Health, Philadelphia, Pennsylvania, United States*

Falls among older adults cause acute injury, are associated with subsequent mortality, and cost billions of dollars in medical expenses each year. However, research on falls is lacking compared to other causes of morbidity. In our rapidly aging world, a better understanding of the populations at greatest risk is urgently needed. In this paper, we used 2018 data on every inpatient hospitalization in Philadelphia and from the American Community Survey to estimate the prevalence of serious falls among older adults (60+) by age, sex, and race/ethnicity. We further assessed the relationship between age, sex, and race/ethnicity and fall outcomes (length of hospital stay (LOS), total medical charges) with linear regression models. In 2018, the rate of falls serious enough to warrant a hospital stay in Philadelphians aged 60+ was 243 per 10,000. This rate increased dramatically with age, from 116 per 10,000 (60-64) to 649 per 10,000 (85+). Men were at higher risk than women for each 5-year age group except those top-coded at 85+. Compared to white older adults, black older adults had greater risk at younger ages (60-69) and lower risk at older ages (70+). In linear models we found that charges and LOS decreased with increasing age. Both charges and LOS were higher for men than women. Hispanic patients had significantly higher charges than non-Hispanic patients, despite having similar lengths of stay. Future work will attempt to explain differences in charges and LOS by examining mortality, discharge location (e.g., home, hospice, rehab), and co-morbidities.

### SESSION 5915 (PAPER)

### HEALTH, WELL-BEING AND PSYCHOLOGICAL AGING

### CHALLENGES USING TECHNOLOGY IN A HEALTH COACHING INTERVENTION, EARLY LESSONS LEARNED

Barbara Riegel,[1] Karen Hirschman,[2] Gladys Thomas,[3] Brooke Shepard,[4] Tracie Walser,[1] Michael Stawnychy,[5] Kathryn Bowles,[5] and Lydia Garcia,[6] *1. University of Pennsylvania, Philadelphia, Pennsylvania, United States, 2. NewCourtland Center for Transitions and Health, Philadelphia, Pennsylvania, United States, 3. University Of Pennsylvania School of Nursing, Philadelphia, Pennsylvania, United States, 4. University of Pennsylvania, Lincoln, United States, 5. University of Pennsylvania School of Nursing, Philadelphia, Pennsylvania, United States, 6. University of Pennsylvania, Oakland, California, United States*

Technology provides opportunities to engage with those who are too busy, overwhelmed, or distant for face-to-face interventions, such as lay caregivers of adults with heart failure. While testing the efficacy of virtual health coaching for caregivers on stress and self-care we have encountered challenges in implementing the intervention and learned early lessons. Caregivers (n=250) enrolled into a randomized controlled trial receive a Samsung Galaxy tablet with Internet access. Half receive health information by tablet (control) and half receive 10 live virtual health coaching sessions plus health information all by tablet (intervention). Tablets are configured to allow access to preselected websites and a Vidyo conference room for the intervention group. Completing the first 6 months of enrollment, 36 caregivers have enrolled (34% black, all female, 74% spouses, mean age 56.4 years, 40% employed full-time, mean 9.3 hours spent caregiving daily), with 18 randomized to intervention. Only 2 of the 79 assessed for eligibility reported discomfort with technology. Yet, one early challenge is difficulty connecting with the health coach via Vidyo. Another is the uncertain nature of the patient's condition, which frequently precludes the caregivers' attendance at prescheduled sessions. Although tablets were anticipated to facilitate the intervention, in some cases, these two challenges interact to further accentuate caregiver stress. Alternatives such as FaceTime and plain old telephone contact suffice. We found that flexibility in intervention delivery is essential for such a real-world intervention. Contributing factors and implications of these challenges for future caregiving research and practice will be discussed.

### CHARACTERISTICS OF THE TRUE BELIEVER IN LATER LIFE

Shannon Jones, Austin Prusak, Rennae Wigton, and Andrew Futterman, *Loyola University Maryland, Baltimore, Maryland, United States*

This study describes the nature, causes, and outcomes of steadfast, unquestioned "true" belief in later life (Hoffer, 1950). According to some theorists, such "true" belief develops from more childish, extrinsically-motivated, compartmentalized beliefs and behaviors to become more mature, intrinsically-motivated, comprehensive and integrated beliefs and behaviors (e.g., Allport, 1950). Research, however, is equivocal regarding the validity of this position. From "grounded-theory" analysis of a sample of 278 semi-structured interviews of older adults from six New England states and New York (aged 55-101 years-old.), we demonstrate the need for a more nuanced definition of a "true" belief as a form of religiousness without commitment to rigid orthodoxy. For example, a sizable segment of this sample changed religious denominations over the course of their lives without ever doubting the presence of a diety (i.e., a God, a Higher Power, etc.), but who dramatically changed the way they expressed this belief. This prompts a reconceptualization of "true" belief by Hoffer and a more nuanced understanding of religious development than implied by Allport, one that more adequately accounts for individual differences in life experiences, personality, religious upbringing, and religious cultural expectations. We discuss these findings in light of recent research by Wink and Dillon (2002, 2008).

### DOUBLE CONSCIOUSNESS: EXPLAINING RACIAL PARADOX IN LATER LIFE PSYCHOLOGICAL WELL-BEING

Timothy Goler,[1] and Tirth Bhatta,[2] *1. Norfolk State University, Newport News, Virginia, United States,*

2. *University of Las Vegas Nevada, Las Vegas, Nevada, United States*

A substantial number of studies have documented paradoxical findings when examining race differences in later life psychological well-being. Despite experiencing significant structural disadvantages, Black older adults have been found to report significantly higher overall life satisfaction and lower depressive symptoms than White adults. This study relies on double consciousness framework which allows us to understand why satisfaction with material conditions (e.g., domain-specific life satisfaction) among Black older adults could differ from their evaluation of overall well-being (e.g., overall life satisfaction). Based on a survey of successful aging (n=409 aged 60 years or older) conducted by the Elderly Care Research Center (ECRC) in Cleveland, Ohio, we examined race differences in coping resources, and their role in shaping overall life satisfaction, domain-specific life satisfaction, and depressive symptoms. Findings show that Blacks on average have a higher likelihood of experiencing recent negative life events than their White counterparts. Despite adverse life circumstances, Blacks older adults expressed significantly higher overall life satisfaction than Whites. They, however, reported significantly lower domain-specific life satisfaction than their White counterparts. The differences in depressive symptoms between Black and White older adults was not statistically significant. The race differences in overall life satisfaction was explained by religiosity, religious coping, and social support. Education, income, and adverse life events were found to contribute to such differences in domain-specific life satisfaction. Our findings underscore the need to consider the unique role of racialized life course circumstances and coping resources in shaping disparities in later life psychological well-being.

### SUCCESSFUL AGING: RE-CONCEPTUALIZED

Alexander Eustice-Corwin, Rachel Missell, and Silvia Sörensen, *University of Rochester, Rochester, New York, United States*

A review of the aging literature yields 105 operational definitions of “successful aging” in use. These theoretical discrepancies have caused some investigators to question the utility of the concept (Cosco et al., 2014). Investigators who remain committed to the concept acknowledge its conceptual messiness, but have found no consensus for resolution. We propose a revised concept of successful aging, combining a life-course perspective (Rowe & Kahn, 2015) with a neo-Aristotelian theoretical framework. Such a framework justifies certain changes to the definition of “successful aging;” it situates our concept of successful aging within a broader view of human development, is more inclusive, and suggests empirically adequate research questions. Specifically, conceptualizing “successful aging” along neo-Aristotelian lines means defining it as the maintenance of proper human functioning across the life-course and into late adulthood. For Aristotle, proper human functioning entails realizing one’s potential as a “rational social animal,” with rational implying goal-oriented thinking, means-ends reasoning, other forms of instrumental rationality (not excluding emotionality). Social suggests active engagement in a community, within the limits of an individuals’ comfort and ability. These two criteria determine “success” in older age. Recent research on successful aging reveals that absence of disease and disability does not appear to be a constituent of “successful aging.” Therefore, physical health is neither necessary nor sufficient for “success.” Our re-conceptualization of “successful aging” could be tested using confirmatory factor analysis, with social and reasoning/problem-solving factors loading onto a second order Successful Aging factor. This understanding allows for greater empowerment of older adults.

### SESSION 5920 (SYMPOSIUM)

### THE POTENTIAL OF ARTIFICIAL INTELLIGENCE, MACHINE LEARNING, AND NOVEL ANALYTIC METHODS TO PROMOTE SUCCESSFUL AGING

Chair: Walter Boot

The Gerontological Society of America is celebrating its75th anniversary and in those75 years the world has undergone an amazing technological revolution. During this period, computers transformed from systems that once filled entire rooms to much more powerful devices that fit in our pockets. We have seen the introduction of wireless technologies, augmented and virtual reality, smart home devices, autonomous vehicles, and much more. This session focuses on a new technological advance that has the potential to support the health, wellbeing, and independence of older adults and caregivers: artificial intelligence (AI). This session will present applications of AI, Machine Learning (ML), and other novel analytic methods and how they have the potential to impact the lives of older adults in a variety of context. As AI is increasingly being involved in workplace hiring, the first talk focuses on older adults’ attitudes toward the role of AI in this decision making process. Next, novel ML approaches applied to social media are discussed in terms of understanding the needs of Alzheimer’s caregivers. Next, ML techniques are discussed in terms of developing biomarkers that can be applied in diagnosis and assessment of therapeutic responses by detecting mood, which may have important implications for older adults living with dementia. Then, the potential role of AI is discussed in terms of developing reminder systems to promote older adults’ adherence to technology-based health activities. Finally, novel analytic approaches are discussed in terms of harnessing digital metrics to detect the risk of cognitive decline.

### AI-ASSISTED HIRING PROCESS AND OLDER WORKERS: AN EXPLORATORY STUDY

Moon Choi, *KAIST, Daejeon, Taejon-jikhalsi, Republic of Korea*

An increasing number of companies have adopted an artificial intelligence (AI) hiring system such as resume screening and/or AI-powered video interview. This study explored older adults’ attitudes toward AI and their intentions to apply for jobs based on AI-assisted hiring process. Data came from an online survey in South Korea (N=123). Results showed an acceptable reliability of the scale of attitudes toward AI (Cronbach’s a = 0.72) and indicated no statistically significant differences in the attitudes toward AI across age groups ($F(4,118) = 0.41$, $p = .80$). However, a one-year increase in age was associated with a 5.8% decrease in the likelihood of decisions to apply for jobs based on AI-assisted hiring process after accounting for attitudes toward AI as well as socioeconomic status. The findings raise a concern about older workers' constraints in job mobility as well as early retirement due to technological change in the hiring process.

### DETECTING ADRD CAREGIVERS' INFORMATION WANTS IN SOCIAL MEDIA: A MACHINE LEARNING–AIDED APPROACH

Bo Xie,[1] Zhendong Wang,[2] Ning Zou,[2] Zhimeng Luo,[2] Robin Hilsabeck,[3] and Alyssa Aguirre,[4] *1. The University of Texas at Austin, Austin, Texas, United States, 2. University of Pittsburgh, Pittsburgh, Pennsylvania, United States, 3. University of Texas, Austin, Texas, United States, 4. Dell Medical School - University of Texas, Austin, Texas, United States*

ADRD caregivers increasingly use social media to meet their health information wants (HIW). Machine learning (ML) tools may help understand caregivers' HIW as expressed via social media. This pilot study explored a collaborative, iterative process between domain experts and ML tools to identify ADRD caregivers' HIW from social media data. The HIW-ADRD framework was adapted from an existing HIW framework. Through multiple rounds of iteration between the experts and the ML tools, the framework was expanded to include 11 types of health information. Each type included corresponding keywords developed through a hybrid approach that included keywords from both the theoretical constructs (top-down) and caregivers' posts (bottom-up). These keywords were then used to enhance the ML tools' ability to code 106 recent posts extracted from an ADRD social media group in March 2020. When compared with expert coding results, ML tools accurately predicted 56% of HIW. Further work is underway.

### CLASSIFICATION OF AGGRESSIVE BEHAVIORS BASED ON SEMG FEATURE EXTRACTION AND MACHINE LEARNING ALGORITHM

Youngjun Kim, Uchechukuwu David, and Yeonsik Noh, *University of Massachusetts Amherst, Amherst, Massachusetts, United States*

New surface electromyography (sEMG) feature extraction approach combined with Empirical Mode Decomposition (EMD) and Dispersion Entropy (DisEn) is proposed for classifying aggressive and normal behaviors from sEMG data. In this study, we used the sEMG physical action dataset from the UC Irvine Machine Learning repository. The raw sEMG was decomposed with EMD to obtain a set of Intrinsic Mode Functions (IMF). The IMF, which includes the most discriminant feature for each action, was selected based on the analysis by Hibert Transform (HT) in the time-frequency domain. Next, the DisEn of the selected IMF was calculated as a corresponding feature. Finally, the DisEn value was tested using five different classifiers, such as LDA, Quadratic DA, k-NN, SVM, and Extreme Learning Machine (ELM) for the classification task. Among these ML algorithms, we achieved classification accuracy, sensitivity, and specificity with ELM as 98.44%, 100%, and 96.72%, respectively.

### AN INTRODUCTION TO THE ADHERENCE PROMOTION WITH PERSON-CENTERED TECHNOLOGY PROJECT

Michael Dieciuc, Dawn Carr, Zhe He, Shayok Chakraborty, Neil Charness, Mia Lustria, Ankita Singh, and Walter Boot, *Florida State University, Tallahassee, Florida, United States*

The massive potential of cognitive training and longitudinal cognitive assessment to detect and prevent age-related cognitive decline and dementia will not be realized unless individuals are willing and able to engage with these protocols for an extended period of time. Unfortunately, similar to other health behaviors, adherence to home-based assessment and training is frequently poor. Addressing the gap between potential and realized benefits is an urgent goal as the population ages. APPT investigates these and related issues within samples of older adults with and without cognitive impairment. Ultimately, two randomized controlled trials will test whether an adaptive, tailored, and integrated technology-based adherence support system can boost adherence, with the ultimate goal being the early detection and treatment of age-related cognitive decline and dementia. Initial algorithm development and application to existing datasets will be presented that will inform the design of a smart reminder system that will later be assessed.

### EARLY DIFFERENCES IN COGNITION ASSOCIATED WITH FAMILIAL LONGEVITY AND APOE GENOTYPE USING DIGITAL TECHNOLOGY

Stacy Andersen,[1] Mengtian Du,[2] Nicole Roth,[1] Mary Wojczynski,[3] Bharat Thyagarajan,[4] Thomas Perls,[1] Stephanie Cosentino,[5] and Paola Sebastiani,[6] *1. Boston University School of Medicine, Boston, Massachusetts, United States, 2. Boston University School of Public Health, Boston, Massachusetts, United States, 3. Washington University School of Medicine, St Louis, Missouri, United States, 4. University of Minnesota, Minneapolis, Minnesota, United States, 5. Columbia University Medical Center, New York, New York, United States, 6. Boston University, Boston, Massachusetts, United States*

Merging digital technologies with neuropsychological testing allows for collection of novel metrics that may reveal early, subtle differences in cognitive functioning. We examined whether digital pen metrics from the Clock Drawing Test (CDT) differentiate healthy agers (i.e., individuals with familial longevity) from spouses and individuals by APOE genotype. We used generalized estimating equations adjusted for sociodemographics, familial longevity, and APOE genotype. Among 1974 participants with correct clocks (mean age 71±10 years), familial longevity was associated with better cognitive processing (i.e., shorter latencies/thinking time before cognitively demanding components) whereas the e4 allele was associated with smaller clock diameter and longer latencies. The e2 allele was negatively associated with total time and latencies. Therefore, digital metrics captured differences in cognitive processing among individuals with correct clocks and thus may be more sensitive than traditional scores. Additionally, familial longevity may confer cognitive advantages that are distinct from the risk/protection afforded by APOE genotype.

### SESSION 5925 (SYMPOSIUM)

### GERONTOLOGY AT THE INTERSECTION OF RELIGION AND FAMILIES: HONORING THE LEGACY OF VERN BENGTSON

Chair: Alex Bishop
Co-Chair: Merril Silverstein
Discussant: Monika Ardelt

The topic of religion and spirituality in later life has received intermittent but regular attention in the field of social and behavioral gerontology over the past few decades. To the extent that religion and spirituality have been linked to better health, improved well-being, and harmonious family functioning has renewed interest in this area of scholarly inquiry. Along with these positive outcomes, religion has also been examined as the basis for family conflict, as well in terms of its inverse in the transmission of secularity across generations. This symposium will communicate empirical results, theoretical insights, and conceptual developments inspired by the career contributions of Dr. Vern Bengtson, whose landmark studies have enriched the field of gerontology in the areas of intergenerational solidarity, spirituality in later life, the transmission of religion across generations, and life-course approaches to the study of family relationships. Five eminent scholars whose work touches on these areas will be represented in this symposium. The paper by Linda George compares intergenerational religious socialization and moral development. The paper by Robert Taylor and Linda Chatters examines the role of supportive church and family networks among older African-Americans. The paper by Ellen Idler addresses aging and religious in a context of secularization. The paper by Andy Achenbaum considers spiritual dimensions of friendship and meanings of aging. The paper by Merril Silverstein integrates intergenerational and temporal continuity in religious practice and identity. The discussant Monika Ardelt has contributed important scholarly work in the areas of religion, spirituality, and wisdom in later life. Religion, Spirituality and Aging Interest Group Sponsored Symposium.

### THE SPIRITUAL DIMENSIONS OF FRIENDSHIP

Andy Achenbaum, *Texas Medical Center, Houston, Texas, United States*

It was the spiritual dimensions of my friendship with Vern Bengtson that I treasure most. Vern was always willing to discuss the dark sides of himself and to listen to my spiritual pain. His empowering way of advancing the meanings of aging were a spiritual gift. This presentation will address the value of spiritual friendship in human aging. Part of a symposium sponsored by the Religion, Spirituality and Aging Interest Group.

### RELIGION AND AGING IN A CONTEXT OF SECULARIZATION

Ellen Idler, *Emory University, Atlanta, Georgia, United States*

Religion and aging has been a persistent topic of interest to gerontologists, notably Vern Bengtson over his long career. It is increasingly obvious that this research has taken place against a decades-long backdrop of declining religious attendance, with each successive cohort showing lower levels of participation. Data come from the Health and Retirement Study, a representative sample of the noninstitutionalized US population (N=20,091), ages 24-107. We examined the patterns of religious involvement during the period 2004-2014 stratified by five age groups, 24-49, 50-64, 65-74, 75-89, 90+. Attending religious services has an age-graded pattern; each older cohort has a higher level of religiosity than the one following it, with the exception of those 90. Patterns for other measures of religious involvement are less dramatic but similar in direction. Lower levels of religious participation in younger cohorts imply a smaller proportion for whom these protective social ties are available. Part of a symposium sponsored by the Religion, Spirituality and Aging Interest Group.

### TRACING THE RELIGIOUS LIFE COURSE: INTERGENERATIONAL SOURCES OF LATER LIFE RELIGIOSITY

Merril Silverstein,[1] Woosang Hwang,[1] and Joseph Blankholm,[2] *1. Syracuse University, Syracuse, New York, United States, 2. University of California, Santa Barbara, Santa Barbara, California, United States*

The development of religiosity in later life has its origins in earlier phases of the life course, yet few studies have investigated the contribution of early forms of religious exposure to religious beliefs and behaviors in old age. This investigation uses multigenerational data from the Longitudinal Study of Generations taken from 385 baby-boom children age 16-26 and their parents, linked to religious orientations of these children in midlife and old age. Relying on the "chains of risk" perspective, we found that parental religious intensity in 1971 strengthened their children's behavioral and cognitive religiosity in later life through their indirect effects on children's early and midlife religiosity. Our results demonstrate both intergenerational and life course forms of stability in religious belief and practice. Evidence suggests that parental influence creates religious momentum in their children that carries from adolescence/young adulthood through the unfolding of human lives into old age. Part of a symposium sponsored by the Religion, Spirituality and Aging Interest Group.

### RELIGIOUS SOCIALIZATION AND MORAL DEVELOPMENT: HOW SIMILAR? HOW DIFFERENT?

Linda George, *Duke University, Durham, North Carolina, United States*

Among Vern Bengtson's contributions to research on aging, religion, and the family was the finding of strong patterns of intergenerational religious socialization within families. Professor Bengtson and colleagues published at least one book and a dozen journal articles documenting the strong evidence of intergenerational religious socialization, although they also documented specific variations of this general pattern. More recently, social scientists in the culture and cognition tradition have focused on moral development. Most of this research is based on studies of adolescents and emerging adults and concludes that families influence morality at these life stages, but that the effects of peers are even stronger. Some of this research explicitly links morality to religion; most does not. This paper compares research on religious socialization and morality, focusing on similarities and differences in findings, the potential of each tradition to inform and advance the other, and how that can be accomplished. Part of a symposium sponsored by the Religion, Spirituality and Aging Interest Group.

### CHURCH AND FAMILY INFORMAL SOCIAL SUPPORT NETWORKS OF AFRICAN AMERICANS

Robert Taylor, and Linda Chatters, *University of Michigan, Ann Arbor, Michigan, United States*

Social support networks are an integral component of an individual's life. This presentation investigates the complementary roles of family and church members as sources of informal social support among African Americans. The analysis utilizes the African American sub-sample of the National Survey of American Life. A pattern variable was constructed that describes four types of church and family networks: 1) received support from both family and church members, 2) received support from family members only, 3) received support from church members only, and 4) never received support from family nor church members. Overall the findings indicated 1) the majority of African Americans received support from both groups, 2) a small group of respondents were socially isolated in that they did not receive assistance from either family or church members, 3) for some African Americans who were estranged from their family members, church members were an alternative source of social support. Part of a symposium sponsored by the Religion, Spirituality and Aging Interest Group.

### SESSION 5930 (SYMPOSIUM)

### LONGITUDINAL EFFECTS OF BECOMING A FAMILY CAREGIVER: THE CAREGIVING TRANSITIONS STUDY

Chair: David Roth
Discussant: Steven Zarit

Taking on caregiving responsibilities for older adult family members with disabilities is often considered to be a highly stressful experience that may adversely affect the health of caregivers. However, the vast majority of studies in this area compare existing samples of caregivers with questionably matched non-caregiving controls. In this symposium, we will present findings for a population-based sample of persons who became family caregivers while participating in a longitudinal epidemiological study. Changes in health and well-being are compared between these caregivers and non-caregiving control participants who were matched on multiple demographic and pre-caregiving health history variables. All persons enrolled as caregivers were providing sustained and substantial caregiving assistance. Presentations will include 1) a descriptive overview of the screening, eligibility, and enrollment methods used to construct these unique, population-based samples; 2) comparisons of within-person changes on measures of self-reported health and well-being for dementia and non-dementia caregivers; 3) changes in the caregivers' social networks, social engagement, and leisure time activities; 4) comparisons of longitudinal changes on circulating inflammatory biomarkers (e.g., IL-6, CRP, TNF alpha receptor 1) and cellular aging (telomere length); and 5) examinations of individual differences in caregiver outcomes using a stress process model. Becoming a family caregiver can be stressful, but the opportunity to help a loved one and the related feelings of purpose and deepening family connections may also promote resilience and enhance health. These questions are far from resolved, and rigorous, prospective, population-based studies like the Caregiving Transitions Study promise to provide compelling new insights.

### ENROLLING INCIDENT CAREGIVERS AND MATCHED CONTROLS FROM A NATIONWIDE EPIDEMIOLOGICAL STUDY

David Roth,[1] William Haley,[2] Orla Sheehan,[3] J David Rhodes,[4] and Virginia Howard,[4] *1. Johns Hopkins University, Baltimore, Maryland, United States, 2. University of South Florida, Tampa, Florida, United States, 3. Johns Hopkins University School of Medicine, Baltimore, Maryland, United States, 4. University of Alabama at Birmingham, Birmingham, Alabama, United States*

Participants in the national Reasons for Geographic and Racial Differences in Stroke (REGARDS) study were asked about family caregiving responsibilities at enrollment (2003-2007). Among the 88% of participants who were not caregivers at enrollment, 1,229 reported becoming caregivers before a follow-up interview 12 years later. The Caregiving Transitions Study screened these participants and enrolled 251 as incident caregivers. All reported 5 or more hours of care per week, provided assistance with at least one ADL or IADL, and were caregivers for at least 3 months before a 2nd blood sample was obtained in the REGARDS study. A total of 251 noncaregiving control participants who reported no caregiving responsibilities over this 12-year period were also enrolled. Each control was matched to a caregiver on age (+ 5 years), sex, race, other demographics, and baseline (pre-caregiving) health variables. Descriptive analyses confirm the unique comparability of the samples compared to previous caregiving studies.

### SELF-REPORTED HEALTH AND WELL-BEING ACROSS HETEROGENEOUS GROUPS OF CAREGIVERS

Orla Sheehan, and Jin Huang, *Johns Hopkins University School of Medicine, Baltimore, Maryland, United States*

Using the Caregiving Transitions Study (CTS) we compared the effects of caregiving on self-reported health and well-being in caregivers reporting providing dementia care, different levels of strain and amount of care provided. Caregivers (n-251) were 65% female, 36% African American and had a mean age of 71.8 years. A quarter of CGs reported being under a lot of strain and 47% provided care for persons with dementia. Dementia CGs (n=117) provided more hours of care per week (49.7 versus 37.7, p=0.001), more commonly reported high strain (36.8% versus 15.7%, p<0.03) and were more than twice as likely as non-dementia caregivers to report that caregiving interfered with taking care of their own health (33.9% versus 15.4%, p=0.003). Additional results will be reported on how these factors of dementia caregiving, level of strain, and hours of care affect well-being including perceived stress, treatment burden, depressive symptoms and health-related quality of life.

### CHANGES IN SOCIAL ENGAGEMENT FOR INCIDENT CAREGIVERS COMPARED TO CONTROLS

Chelsea Liu,[1] Marcela Blinka,[2] Chanee Fabius,[3] and Virginia Howard,[4] *1. Johns Hopkins University, Baltimore, Maryland, United States, 2. Johns Hopkins University, School of Medicine, Baltimore, Maryland, United States, 3. Johns Hopkins Bloomberg School of Public Health, Baltimore, Maryland, United States, 4. University of Alabama at Birmingham, Birmingham, Alabama, United States*

Maintaining social engagement is important for the health and well-being of older adults who become caregivers. We assessed the association between incident caregiving and leisure satisfaction as well as the 10-year change in social network size among 245 incident caregivers and 248 matched controls. Multiple linear regression analyses were used to adjust for age, gender, race, education level, income, and geographic region. Compared to controls, incident caregivers had significantly lower levels of leisure satisfaction ($p<0.01$) and greater declines in total social network size ($p<0.01$). Incident caregivers and controls did not differ on the change in the number of social network members contacted monthly. Among incident caregivers, dementia caregivers and spouse caregivers had lower leisure satisfaction compared to non-dementia caregivers and non-spouse caregivers, respectively, but no differences were found on social network measures. Future studies should further examine social engagement among caregivers and its influence on their health outcomes.

### THE TRANSITION TO FAMILY CAREGIVING: DOES IT AFFECT BIOMARKERS OF AGING?

Shang-En Chung,[1] David Roth,[1] John Bentley,[2] and Jeremy Walston,[1] *1. Johns Hopkins University, Baltimore, Maryland, United States, 2. University of Mississippi, Oxford, Mississippi, United States*

Blood samples were collected from participants in the REGARDS study on two separate occasions. No participants in the Caregiving Transitions Study were caregivers at the first blood draw, but 251 became caregivers before the second blood draw 9 years later. These caregivers were matched with 251 noncaregiving controls. Six circulating biomarkers of inflammation (e.g., CRP, IL-6, TNFR1) and a measure of cellular aging (leukocyte telomere length) were assessed at both blood draws. All biomarkers except CRP showed overall aging effects ($ps < 0.001$). Caregivers had a small but significantly greater increase in TNFR1 levels ($p = 0.03$) than controls, but no significant differential changes were found on the other 5 inflammatory biomarkers or on telomere length. Preliminary findings from latent variable models indicated good model fit and found caregivers to be 0.27 SDs lower than controls on a latent construct of inhibitory, regulatory feedback of systemic inflammation ($p = 0.03$).

### INDIVIDUAL DIFFERENCES IN CAREGIVING: APPLICATION OF A STRESS PROCESS MODEL

William Haley, and Joanne Elayoubi, *University of South Florida, Tampa, Florida, United States*

Stress process models propose individual differences in caregiver outcomes depending on background characteristics and primary caregiving stressors, and resilience factors including stress appraisals, and internal and external resources. This paper will examine individual differences in the effects of the transition to caregiving on indicators of well-being and biomarkers of inflammation. Completed analyses show that, contrary to previous findings from cross-sectional studies, changes in well-being after caregiving generally do not differ by caregiver race, gender, age, or relationship category (spouse, adult child, others). Additional analyses examine the relationship of primary caregiving stressors (e.g. ADL and behavioral problems), stress appraisals (e.g., perceived stressfulness of ADL and IADL problems, perceived benefits of caregiving), and personality with changes in well-being and inflammation after the transition to caregiving. The lack of differences on most biomarker measures suggests that caregivers show substantial resilience in the face of significant, chronic caregiving stress.

### SESSION 5935 (SYMPOSIUM)

### LATE-LIFE COGNITION AND DEMENTIA IN INDIA: NEW INSIGHTS FROM LASI-DAD

Chair: Jinkook Lee

With more than1.35 billion people, India, the second-most populous country in the world, is soon to experience rapid aging of its population. By2050, India's older population is projected to reach320 million (about the current size of the entire U.S. population). In this session we introduce the Longitudinal Aging Study in India – Diagnostic Assessment of Dementia (LASI-DAD), a new cohort study designed to advance dementia research to better understand late-life cognition, cognitive aging, cognitive impairment, and dementia, as well as their risk and protective factors. LASI is a prospective, multi-purpose population survey of older adults aged45 and older, representative of the entire country and of each state (N~72,000). LASI-DAD is an in-depth study of late-life cognition and dementia, drawing a sub-sample of older adults aged60 and older from LASI (N~4,300). It administered the Harmonized Cognitive Assessment Protocol (HCAP), which consists of a pair of in-person interviews, one with the target respondent and one with an informant nominated by the respondent. The respondent interview includes a neuropsychological test battery designed to measure a range of key cognitive domains affected by cognitive aging and Alzheimer's Diseases. We organize the session to showcase LASI-DAD. Specifically, the session consists of four papers, including: (1) the introduction of the design and methodology, (2) the latent structure of neuropsychological test results, (3) the investigation of the relationship between visual impairment and cognition, and (4) the examination of female disadvantage in dementia and its association with cross-state variations in gender inequality.

### GENDER INEQUALITY AND LATE-LIFE COGNITION

Jinkook Lee,[1] Marco Angrisani,[1] and Urvashi Jain,[2] *1. University of Southern California, Los Angeles, California, United States, 2. University of South Alabama, Mobile, Alabama, United States*

Indian women's disadvantage in cognition has been documented in several studies, additionally noting geographic variations. The gender gap in late-life cognition could be a manifestation of gender discrimination. Using national data from LASI-DAD together with information drawn from administrative data, we construct a state-level composite index of gender inequality, following the UNDP definition. We investigate and find strong evidence that cross-state differences in gender inequality are significantly associated with the gender gap in cognition. Women in the most discriminating state (Bihar) perform significantly worse than men (-0.21 s.d.), after controlling for key risk factors such as age and education. The gender gap in the least discriminating state (Kerala) is much smaller (-0.10 s.d.). We also find that gender inequality is strongly associated with education, early marriage, labor force participation, and social activities. This has important implications for public health policy aimed at reducing the risk of cognitive impairment and dementia.

### DESIGN AND METHODOLOGY OF THE LONGITUDINAL AGING STUDY IN INDIA—DIAGNOSTIC ASSESSMENT OF DEMENTIA

Jinkook Lee,[1] Pranali Khobragade,[2] Joyita Banerjee,[3] and Sandy Chien,[2] *1. University of Southern California, Los Angeles, California, United States, 2. University of southern california, Los Angeles, California, United States, 3. All India Institute of Medical Sciences, New Delhi, Delhi, India*

Longitudinal Aging Study in India is a nationally representative survey of the health, economic, and social wellbeing of the Indian population aged 45 and older. LASI-DAD is an in-depth study of late-life cognition and dementia, drawing a sub-sample of over 4,000 LASI respondents aged 60 or older. Respondents underwent a battery of cognitive tests, while their informants were interviewed about their cognitive and health conditions. A common set of cognitive tests was selected to enable international comparisons, and additional cognitive tests suitable for illiterate and innumerate populations were also selected. Rich data on risk factors of dementia were collected through health examinations, venous blood assays, and genotyping. The response rate was 82.9%, varying across sex, education, and urbanicity. LASI-DAD provides an opportunity to study late-life cognition and dementia and their risk factors in the older population in India and to gain further insights through cross-country analysis.

### MEASUREMENT AND STRUCTURE OF COGNITION IN THE LONGITUDINAL AGING STUDY IN INDIA—DIAGNOSTIC ASSESSMENT OF DEMENTIA

Alden Gross,[1] Pranali Khobragade,[2] Erik Meijer,[2] and Judith Saxton,[2] *1. Johns Hopkins Bloomberg School of Public Health, Baltimore, Maryland, United States, 2. University of southern california, Los Angeles, California, United States*

We tested whether a complex model of human cognitive abilities based on Cattell-Horn-Carroll (CHC) theory, developed in English-speaking samples, adequately describes correlations among tests in the Longitudinal Aging Study in India-Diagnostic Assessment of Dementia (LASI-DAD) (N=3,224). Tests in the neuropsychological battery were chosen for their appropriateness for measuring cognition in older adults in India and suitability for co-calibration with the core LASI survey (N=72,000). We evaluated the factor structure and its conformity with a classical CHC factor model incorporating measurement models for general cognition, 5 broad domains (orientation, executive functioning, language/fluency, memory, visuospatial), and 5 narrow domains (abstract reasoning, attention/speed, immediate memory, delayed memory, recognition memory) of cognitive performance. Model fit was adequate (RMSEA:0.051; CFI:0.916; SRMR:0.060). We demonstrated configural factorial invariance of a cognitive battery in the Indian LASI-DAD using CHC theory. Broad domain factors may be used to rank individuals with respect to cognitive performance and classify cognitive impairment.

### VISION AND COGNITION: FINDINGS FROM THE LONGITUDINAL AGING STUDY IN INDIA—DIAGNOSTIC ASSESSMENT OF DEMENTIA

Joshua Ehrlich,[1] Sandy Chien,[2] and Jinkook Lee,[3]
*1. University of Michigan, Ann Arbor, Michigan, United States, 2. University of southern california, Los Angeles, California, United States, 3. University of Southern California, Los Angeles, California, United States*

Vision impairment (VI) is associated with cognitive decline and dementia, however little research has been conducted in India. Using data from LASI-DAD, linear models tested the association of VI (better-seeing eye <20/60) with cognitive domains including orientation, learning/memory, language, attention, and total cognition. Models were adjusted for age, sex, geographic, and socioeconomic characteristics. VI was significantly associated with lower orientation ($\beta$=-0.47, p<.01), learning/memory ($\beta$=-4.6, p<.01), attention ($\beta$=-1.6, p<.01), and total cognition ($\beta$=-8.4, p<.01), but not language ($\beta$=-0.14, p<.1) scores. The association of VI with cognitive measures did not vary by sex. For each measure, VI was equivalent to 5-13 years of cognitive aging. In summary, VI is associated with poorer performance in most cognitive domains among older Indian adults. Longitudinal data are needed to determine directionality and causality. Since >80% of VI in India is treatable, poor vision may represent a modifiable risk factor for cognitive decline and dementia.

### SESSION 5940 (SYMPOSIUM)

### USING VIDEO TELEHEALTH TO SUPPORT FAMILY CAREGIVERS OF PEOPLE WITH DEMENTIA

Co-Chair: Joleen Sussman
Co-Chair: Lauren Moo
Discussant: Michele Karel

In 2030, predictions indicate that dementia will affect 75 million people worldwide and increase to 132 million by 2050. Persons' with dementia (PWD) associated behavioral changes are highly correlated with caregiver burden. Caregivers of PWD commonly report concerns regarding personal and home safety, meaningful activities, advance care planning, and evaluation and diagnosis of dementia of the PWD. Further, caregivers' emotional response to PWD challenging behavior has greater influence than the actual behavior on decisions to place PWD in a nursing home. Caregiver intervention reduces behavioral and psychological symptoms in the PWD, the caregiver's emotional distress from these symptoms, and cost to healthcare systems. Yet, one in four dementia caregivers are not receiving dementia support services. Difficulty attending in person clinic-based appointments may be one barrier to caregivers engaging in treatment. This symposium highlights telehealth approaches, by various disciplines (Geriatrician, Neurologist, Geriatric Psychiatric, Geropsychologist, and Occupational Therapist), across urban and rural settings to address caregiver needs and improved access to care. The first presentation will focus on education of rural caregivers of PWD and increased connection to services (Sussman et al). The second presentation will focus on Video to home dementia visits for caregivers (Gately & Moo). The third study will focus on rural tele dementia caregiver support groups and effects on caregiver burden (Rossi et al). The final study will describe co-occurring caregiver and PWD telehealth groups (Thielke & Fredrickson).

### TELEHEALTH PSYCHOLOGICAL INTERVENTIONS FOR RURAL CAREGIVERS: IMPROVING CARE TO PERSONS WITH DEMENTIA

Joleen Sussman, Nikhil Banerjee, and James Winslow, *Veterans Affairs, Aurora, Colorado, United States*

Geropsychologists are well-suited to assess dementia, assist caregivers in understand the disease and associated behavioral changes and ways to cope with their loved one's disease. However, majority of these services are offered during in-person clinic appointments in urban settings. This study aims to describe the utilization and potential benefits of providing dementia education telehealth services to Veterans and their families residing in rural mountain and plain areas of Colorado. Psychological intervention was provided via telehealth from the primary VA hospital to small community clinics or to Veterans homes via video mobile application. The present study provides demographics of participants who elect this service and discusses how these challenge ageism and other biases relate to technology use. Further, we examined how engagement in this intervention may impact utilization of geriatric and extended care services as well as use of primary care, emergency room visits, and use of anti-psychotic medications.

### TELEGROUP VISITS FOR CAREGIVERS AND PATIENTS WITH DEMENTIA

Stephen Thielke,[1] and Kris Fredrickson,[2] *1. VA Puget Sound Health Care System, Seattle, Washington, United States, 2. Veterans Affairs, Seattle, Washington, United States*

Group visits have shown promise for caregiver support and medical management of patients with dementia. In-person visits can be challenging to schedule and complete, particularly in rural areas where there are few specialists. We describe our experience with using telehealth modalities to hold group dementia visits. For the last four years, we have held telegroup appointments with Veterans with dementia and their caregivers. A geriatric psychiatrist and geriatric social worker appear from the main facility, and the Veterans and caregivers are at remote sites. Participants have actively engaged. They have expressed that the structure allows them to support and be supported by peers, and to have frequent contact with care providers. This has led to improved care metrics. The technology requirements are minimal. We discuss the advantages of this approach, including flexibility and maximizing use of specialist resources. We address challenges to scaling up such programs.

### IN-HOME VIDEO TELEHEALTH FOR CAREGIVERS AND VETERANS WITH DEMENTIA

Megan Gately, and Lauren Moo, *Bedford VA Medical Center, Bedford, Massachusetts, United States*

Home Video Telehealth offers a unique opportunity to support already burdened caregivers of persons with dementia. Veterans Health Administration, through the MISSION Act, is increasingly using video telehealth to provide "care at the right time and in the right place." Little is known about the benefits and challenges of using video telehealth for in-home caregiver support. We present findings from our seven years offering in-home dementia management to caregivers of Veterans with dementia, that includes supporting caregivers through supportive listening, tips for communication and safety strategies, and recommendations regarding non-pharmacologic management of behaviors. Perceived benefits of in-home video telehealth include an 'in vivo' perspective of the family's natural context and remediating barriers to care such as decreased mobility. Perceived challenges include dealing with technology and privacy concerns. By describing considerations for in-home video telehealth to a clinical population with unique care needs, we inform broader application of a promising technology.

### INTEGRATING CAREGIVER EDUCATION AND SUPPORT INTO MULTIDISCIPLINARY VIDEO VISITS

Michelle Rossi,[1] Lauren Jost,[2] Ina Engel,[2] Carol Dolbee,[2] and Keisha Ward,[3] *1. VA Pittsburgh Healthcare System, Pittsburgh, Pennsylvania, United States, 2. VA Pittsburgh Healthcare System, pittsburgh, Pennsylvania, United States, 3. University of Pittsburgh, pittsburgh, Pennsylvania, United States*

The TeleDementia Clinic is an interdisciplinary longitudinal telehealth dementia clinic providing care to rural Veterans in Western Pennsylvania with cognitive decline. The TeleDementia Caregiver Support group uses telehealth to provide caregiver support and education to those caregivers with the highest levels of caregiver burden in this population. The support group has caregivers participating in the session at different rural clinics while a multidisciplinary team of professional (geropsychologist, geriatrician, nurse practitioner and others) are located at an urban VA medical center. All can interact via video telehealth connection. Each session provides a short educational session on caregiving topics that then provides a springboard for caregiver discussion about their own experiences. The multidisciplinary clinician team lend their expertise to the education and support of caregivers. Both quantitative and qualitative analysis of effectiveness of this model will be discussed.

### SESSION 5945 (SYMPOSIUM)

### ACHIEVING HEALTH EQUITY FOR OLDER ADULTS THROUGH STATE-OF-THE-ART INNOVATIONS

Chair: Karen Fortuna
Co-Chair: John Batsis
Discussant: Daniel Jimenez

As health indicators such as life expectancy have improved for many older adults, some older adults experience a disproportionate amount of preventable disease, death, and disability. The causes of health disparities among older adults are multidimensional in that disparities are due to multiple, interacting factors such as socioeconomic status, disability status, geographic location, and race/ethnicity. Achieving health equity in late-life requires innovative strategies to address interconnected environmental, sociocultural, behavioral and biological factors that impede opportunities to achieve optimal health and quality of life. This symposium will present state-of-the-art innovations and strategies employed among socially disadvantaged racial, ethnic, and other population groups, and communities. We will discuss innovations in the workforce enhancements with older adult peer support specialists and community health workers, community engagement techniques in program design, and digital solutions aimed at addressing multiple dimensions of health in older adults.

### OLDER ADULT PEER SPECIALISTS' ROLE IN REDUCING LONELINESS AMONG PEOPLE WITH MENTAL HEALTH CONDITIONS

Karen Fortuna,[1] George Mois,[2] Jessica Brooks,[3] Amanda Myers,[4] and Cynthia Bianco,[1] *1. Dartmouth College, Concord, New Hampshire, United States, 2. University of Georgia, Lawrenceville, Georgia, United States, 3. Columbia University, New York, New York, United States, 4. Rivier University, Hudson, Massachusetts, United States*

PeerTECH is a peer-delivered and technology-support integrated medical and psychiatric self-management intervention developed by peers. A pre/post trial by our group has shown PeerTECH is associated with statistically significant improvements in self-efficacy for managing chronic disease and psychiatric self-management skills. This presentation will discuss the feasibility and potential effectiveness of using ecological momentary assessments (EMA) with older adults with mental health conditions to allow us to recognize early signs of loneliness and intervene as early as possible in real-world settings. EMA involves repeated sampling of an individual's behaviors and experiences in real time, real-world environments on the smartphone application. Then, we will discuss the main and interactive effects of loneliness and factors linked to mortality. In conclusion, we will discuss potential effectiveness of PeerTECH with older adults with SMI.

### TELEHEALTH-DELIVERY OF A MULTICOMPONENT OBESITY INTERVENTION IN OLDER, RURAL ADULTS WITH OBESITY

John Batsis,[1] Curtis Petersen,[1] Rima Al-Nimr,[1] Tyler Gooding,[2] Summer Cook,[3] and Todd Mackenzie,[1] *1. Dartmouth College, Lebanon, New Hampshire, United States, 2. Dartmouth Hitchcock Medical Center, Lebanon, New Hampshire, United States, 3. University of New Hampshire, Durham, New Hampshire, United States*

Older, rural residents with obesity aged ≥65 years have reduced access to health promotion programs due to geography. We conducted a 26-week intervention of 24 older obese adults (BMI≥30kg/m2) in a geographically isolated area in Northern New England. The telemedicine delivered intervention consisted of individual, weekly, dietitian visits focusing on caloric restriction, and twice-weekly physical therapist-led group strength training classes. Participants' age was 73.4±4.4years (79% female); pre-post assessments consisted of bioelectrical impedance-based body composition, functional measures, and satisfaction questionnaires. Feasibility was high (50% enrolled, 85% completed). Weight decreased 4.5±3.8kg (4.5±0.5%; 48% achieving ≥5%), 30s sit-to-stand improved (+3.8±4.1repetitions), as did 6-minute walk, +76.2±70.1m (all p<0.001). Appendicular mass did not change (+0.20±2.3kg); % body and visceral fat both decreased (-1.8±2.8% [p=0.009], -1.2±2.7L [p=0.025]). Participants endorsed telemedicine (96%); 78% preferred a home-based study. Satisfaction was high (4.2/5) and only 17% faced difficulties. Despite geography, this intervention holds promise in improving physical function.

### PREVENTING CARDIOMETABOLIC DISEASE IN HIV-INFECTED LATINO MEN: THE HAPPY OLDER LATINOS HEALTH PROMOTION STUDY

Daniel Jimenez, *University of Miami, Coral Gables, Florida, United States*

Older Latinos living with HIV have been disproportionately affected by the epidemic and experience compounded health disparities that have deepened over time. Eighteen Latinos living with HIV with a mean age of 60.3 years (SD=6.4) were enrolled in the Happy Older Latinos are Active (HOLA), a community health worker-led, multicomponent, health promotion intervention. Participants were assessed at three time points on measures of cardiometabolic risk and psychosocial functioning. We evaluated the feasibility of recruitment, retention, acceptability, and implementation of HOLA. In 4 months, we met our enrollment target with <5% of eligible participants refusing participation. Participants attended over 70% of sessions and 1 participant was lost to follow up. These results indicate that HOLA is an innovative health promotion program that is uniquely tailored to address the multiple concerns that are prevalent in this community (cardiometabolic risk, psychological distress) in a nonstigmatizing and culturally acceptable manner.

### DIGITAL TECHNOLOGIES AS A MEANS TO REDUCING MENTAL HEALTH DISPARITIES: THE ROLE OF ETHNICITY, SES, AND GEOGRAPHY

Giyeon Kim, *Chung-Ang University, Seoul, Republic of Korea*

This presentation discusses the importance of using digital technologies on reducing mental health disparities among older adults from diverse backgrounds. This talk primarily focuses on the role of ethnicity, socioeconomic status and geography. First, the speaker presents the current status of digital technology use among older adults and how different levels of digital technology use affect mental health disparities by ethnicity, SES, and place of residence. Second, the speaker introduces a recently funded government project on developing an IoT-based home system (Internet of Things) to screen mild cognitive function for Korean older adults. Lastly, the speaker discusses potential implications, as well as directions for future research on using digital technologies to reduce mental health disparities among diverse populations.

### SESSION 5950 (SYMPOSIUM)

### CARDIOVASCULAR HEALTH, FUNCTIONAL DISABILITY, AND LEISURE ACTIVITIES PROFILES IN RELATION TO MENTAL AGING

Chair: Dorina Cadar
Co-Chair: Celine De Looze
Discussant: Christine McGarrigle

We investigated cardiovascular health, functional disability and leisure activities profiles independently and in relation to cognitive decline and dementia in high and low-medium income countries using data from the English Longitudinal Study of Ageing, Irish Longitudinal Study on Ageing and Brazilian Bambui Cohort Study of Aging. Functional loss among older Brazilians has shown a hierarchical sequence over the 15-year follow-up, with the highest incidence in functional disability reported for dressing, followed by getting out of bed, bathing/showering, walking across a room, using the toilet and eating (de Oliveira). Using the Life's Simple 7, an ideal cardiovascular health scoring system evaluating the muscular strength, mobility and physiological stress, we showed a reliable prediction of cognitive trajectories in a representative sample of Irish individuals (De Looze). Within the same cohort, we report discrepancies between men and women in functional decline driven by domestic tasks, rather than determining differential cognitive trajectories (McGarrigle). In an English representative sample, we found that participants with an increasing number of functional impairments over almost a decade were more likely to be classified with subsequent dementia compared with those with no impairments and this may imply a more comprehensive ascertainment during the prodromal stage of dementia (Cadar). In contrast, a reduced risk of dementia was found for individuals with higher levels of engagement in cognitively stimulating activities, that may preserve cognitive reserve until later in life (Almeida). Identifying factors that influence cognitive aging and dementia risk in a multifactorial perspective is critical toward developing adequate intervention and treatment.

### FUNCTIONAL IMPAIRMENTS AND DEMENTIA INCIDENCE IN THE ENGLISH LONGITUDINAL STUDY OF AGEING

Dorina Cadar,[1] David Llewellyn,[2] G. David Batty,[1] and Andrew Steptoe,[1] *1. University College London, London, England, United Kingdom, 2. University of Exeter Medical School, Exeter, England, United Kingdom*

Functional disability might be related to an increased risk of dementia or could represent a prodromal stage. We examined the occurrence of functional impairments over eight years follow-up and their association with dementia incidence in 1,666 participants aged 65+ from the English Longitudinal Study of Ageing. Growth models with distal outcome were used to examine whether different trajectories of functional abilities (activities of daily living (ADL); and instrumental activities of daily living (IADL)) between 2002/03 and 2010/11, were associated with dementia incidence four years later (2014/15). Participants with an increasing number of functional impairments (Class III), were more likely to be classified with subsequent dementia compared with those with no impairments (Class I). An increased risk was also observed for individuals with raised levels of impairments (Class II). We found IADLs more sensitive than ADLs, and this may imply a more comprehensive ascertainment during the prodromal stage of dementia.

### CARDIOVASCULAR PROFILES FOR PHENOTYPING COGNITIVE TRAJECTORIES IN THE IRISH LONGITUDINAL STUDY ON AGEING

Celine De Looze,[1] Wilby Williamson,[2] Naiara Demnitz,[3] and Rose Anne Kenny,[4] *1. Trinity College Dublin, Dublin, Ireland, 2. The Irish Longitudinal Study on Ageing (TILDA), dublin, Dublin, Ireland, 3. Global Brain Health Institute, Dublin, Dublin, Ireland, 4. The Irish Longitudinal Study on Ageing (TILDA), Dublin, Dublin, Ireland*

Cardiovascular risk factors are increasingly recognized as modifiable risks for cognitive decline and dementia in later life. The Life's Simple 7, an ideal CV health scoring system, has been recently put forward as a tool for the promotion of brain health. This study aims to evaluate the LS7 as risk prediction tool for cognitive decline trajectories (MMSE, immediate/delayed recall and verbal fluency) in 2,739 adults aged ≥50 years from TILDA. We investigate if indices of muscular strength (grip strength), mobility (Time Up and Go, walking speed) and physiological stress (e.g. orthostatic blood pressure and heart rate recovery) as add-ons to the LS7 improves prediction of cognitive trajectories; and, in a subcohort, we assess CV health score in association with multimodal brain measures. Identifying the factors that influence the onset and trajectory of cognitive decline in a multifactorial perspective is critical toward lowering dementia risks and developing adequate intervention and treatment.

### GENDER-SPECIFIC DETERMINANTS OF INSTRUMENTAL ACTIVITIES AND ASSOCIATIONS WITH COGNITIVE DECLINE IN TILDA

Christine McGarrigle,[1] Orna A Donoghue,[2] Joanna Orr,[2] and Rose Anne Kenny,[2] *1. Trinity College Dublin, Dublin, Ireland, 2. The Irish Longitudinal Study on Ageing (TILDA), Dublin, Dublin, Ireland*

Detecting functional decline is critical in recognizing clinical progression into mild cognitive impairment and dementia as people age. This study aimed to investigate differences in Instrumental Activities of Daily Living (IADL) and associations with cognition by gender. Data are from five waves of TILDA, a nationally representative cohort of 8,175 aged ≥50 years, measured every 2 years between 2009 and 2018. IADL assessed were preparing a hot meal, doing household chores, shopping for groceries, making telephone calls, taking medications, and managing money. Cognition was assessed using the Mini-Mental-State Examination (MMSE). Latent class analysis and growth mixture modelling techniques were used to identify IADL and cognitive latent classes and examine change trajectories. IADL trajectories differed by gender and were associated with cognitive decline. Functional decline discrepancies between men and women may be driven by domestic tasks within IADL which are associated with social gender roles, rather than determining differential cognitive trajectories.

### HIERARCHY OF LOSS IN FUNCTIONAL CAPABILITY: FINDINGS FROM A 15-YEAR FOLLOW-UP IN A BRAZILIAN AGING COHORT

Cesar de Oliveira,[1] Dorina Cadar,[1] Jane Biddulph,[1] Juliana Vaz de Melo Mambrini,[2] Fabiola Bof de Andrad,[2] and Maria Lima-Costa,[3] *1. University College London, London, England, United Kingdom, 2. Oswaldo Cruz Foundation, Belo Horizonte, Minas Gerais, Brazil, 3. Oswaldo Cruz Foundation, Belo Horizonte, Mato Grosso, Brazil*

Functional loss among older adults is known to follow a hierarchical sequence, but little is known about this in low-middle income countries. We examined longitudinally the hierarchy of loss in physical functioning in 1,602 older Brazilian adults (aged 60+) from the Bambui Cohort Study of Aging. Functional loss was ascertained using self-reported difficulties in six basic activities of daily living (BADL). The incidence for each BADL limitation was assessed using survival analysis while controlling for the competing risk of death. Over the 15-year follow-up, the incidence in BADL disability rate was highest for dressing, followed by getting out of bed, bathing/showering, walking across a room, using the toilet or eating. The findings from this 15-year follow-up Brazilian aging cohort support a hierarchical pattern of disability in activities of daily living based on both prevalence and incidence of disabilities over time, and they suggest gender differences in the incidence of disability.

### THE ROLE OF LEISURE ACTIVITIES AS MARKERS OF COGNITIVE RESERVE ON DEMENTIA RISK IN ENGLISH OLDER ADULTS

Pamela Almeida Meza, Andrew Steptoe, and Dorina Cadar, *University College London, London, England, United Kingdom*

We examined the frequency of participation in cognitive and social type of leisure activities in association with dementia risk over 15 years of follow-up in 12,280 participants aged 50+ from the English Longitudinal Study of Ageing. Cox proportional hazards regression models were used to estimate the hazard of dementia in relation to the cognitive and social type of leisure activities as well as their interactions with sex and marital status. Medium and higher levels of engagement in cognitive leisure activities were associated with a lower risk of dementia. An analysis of the social type of leisure activities showed a similar pattern with protection for higher levels of engagement in a model adjusted for sex and marital status but further explained by wealth. This study shows a reduced risk of dementia for individuals with higher levels of engagement in cognitively stimulating activities, that may preserve cognitive reserve until later in life.

### SESSION 5955 (SYMPOSIUM)

### NO DECISION ABOUT ME WITHOUT ME: INTERNATIONAL VIEWS ON PROVIDING CARE AGAINST SOMEONE'S WILL

Chair: Michel Bleijlevens
Co-Chair: Jan Hamers
Discussant: Elizabeth Capezuti

People with cognitive impairment may experience the care provided by their caregiver(s) as unnecessary or undesirable, which is expressed by behaviors such as resisting the efforts of a caregiver or preventing the caregiver to perform or assist with ADL such as bathing, dressing and toileting. This can lead to stress, agitation and aggression for both the care recipient and the caregiver, and places the caregiver in a complex dilemma. Should the caregiver force hygiene or respect the person's autonomy to refuse care? It is difficult for caregivers to find a balance between quality of care and safety, while accepting the person's autonomy. Caregivers in long-term care often feel the necessity to provide care against the will of people with a cognitive impairment, including physical restraints, psychotropic medication, and non-consensual care. This symposium provides an international perspective with presenters from Belgium, Switzerland, Germany, and the Netherlands. The first presenter explores the use and factors associated with physical restraint, psychotropic medication, and non-consensual care in people with dementia receiving home care in the Netherlands and Belgium. Second, a presenter from Switzerland focuses on the prevalence of restraint use in nursing homes. The third presenter introduces an Advance Care Planning intervention from Germany that aims to ensure that personal wishes in home care are followed. The last presenter assesses the feasibility of an approach to prevent and reduce care against a person's will in the Netherlands. To conclude, our discussant will integrate these insights and draw conclusion for policy, practice and further research. Systems Research in Long-Term Care Interest Group Sponsored Symposium

### INVOLUNTARY TREATMENT IN DEMENTIA CARE AT HOME: RESULTS FROM THE NETHERLANDS AND BELGIUM

Vincent Moermans,[1] Angela Mengelers,[2] Michel Bleijlevens,[2] Hilde Verbeek,[2] Frans Tan,[3] Elizabeth Capezuti,[4] Koen Milisen,[5] and Jan Hamers,[2] *1. Maastricht University, Riemst, Limburg, Belgium, 2. Maastricht University, Maastricht, Limburg, Netherlands, 3. Care and Public Health Research Institute, Maastricht University, Maastricht, Limburg, Netherlands, 4. Hunter College of CUNY, New York, New York, United States, 5. KU Leuven, LEUVEN, Vlaams-Brabant, Belgium*

Most PwD remain living at home. Due to complex care needs this can result in an increased risk for care provided against the wishes of the client and/or to which the client resists, referred to as involuntary treatment. This study explores the use and factors associated with involuntary treatment in PwD receiving home care in the Netherlands and Belgium. A secondary data analysis of two cross-sectional surveys (n=844 persons) showed that more than half of the PwD (51%) living at home received involuntary treatment (Belgium 68% and the Netherlands 45%). Non-consensual care (83%) was the most common, followed by psychotropic medication (41%) and physical restraints (18%). Involuntary treatment was associated with living alone, greater ADL dependency, lower cognitive ability, higher family caregiver burden and receiving home care in Belgium versus the Netherlands. In order to provide person-centered care, it is important to study ways to prevent involuntary treatment in PwD. Part of a symposium sponsored by Systems Research in Long-Term Care Interest Group.

### PHYSICAL RESTRAINT USE IN SWISS NURSING HOMES: TWO NEW NATIONAL QUALITY INDICATORS

Lauriane Favez,[1] Franziska Zúñiga,[2] Catherine Blatter,[1] Narayan Sharma,[1] and Michael Simon,[1] *1. University of Basel, Basel, Basel-Stadt, Switzerland, 2. Basel University, Basel, Basel-Stadt, Switzerland*

Quality indicators are used in nursing homes to assess physical restraint use. Switzerland introduced two publicly reported indicators measuring the use of 1) bedrails and 2) trunk fixation or seating that prevent standing up. Whether these indicators show good between-provider variability is unknown. The study aimed to measure the prevalence of physical restraint use and assess their between-provider variability using a cross-sectional, multicentre study of a convenience sample of nursing homes. The between-provider variability of the indicators was assessed with intraclass correlation 1 and with caterpillar plots based on Empirical Bayes estimates. We included 11,412 residents from 152 nursing homes. Prevalence rates were 13.5% (n=1'433) for bedrails and 3.6% (n=411) for trunk fixation / seating that prevent standing up. For the first indicator, intraclass correlation 1 was 0.245 (95%-CI 0.197-0.286), for the second 0.343 (95%-CI 0.235-0.405). The two indicators showed good between-provider variability and can be recommended for public reporting. Part of a symposium sponsored by Systems Research in Long-Term Care Interest Group.

### ADVANCE CARE PLANNING FOR CARE-DEPENDENT OLDER PERSONS LIVING AT HOME: A CLUSTER-RANDOMIZED CONTROLLED TRIAL

Sascha Köpke,[1] Katharina Silies,[2] Rieke Schnakenberg,[3] Änne Kirchner,[4] Juliane Köberlein-Neu,[5] Gabriele Meyer,[4] and Falk Hoffmann,[6] *1. University of Cologne, Cologne, Germany, 2. University of Lübeck, Lübeck, Schleswig-Holstein, Germany, 3. University of Oldenburg, Oldenburg, Niedersachsen, Germany, 4. Martin Luther University Halle-Wittenberg, Halle (Saale), Sachsen-Anhalt, Germany, 5. Bergische Universität Wuppertal, Wuppertal, Nordrhein-Westfalen, Germany, 6. Carl von Ossietzky University Oldenburg, Oldenburg, Niedersachsen, Germany*

Advance Care Planning (ACP) for care-dependent older persons living at home is an important part of care, but remains difficult to implement, mostly due to access barriers. The aim of this trial is to increase patient activation, family communication and surrogate designation through an ACP-intervention delivered by trained nurses to care-dependent clients in their homes. The intervention is evaluated in a cluster-randomised controlled trial in Germany (DRKS00016886). Primary outcome is patient activation (PAM-13); secondary outcomes cover institutionalisation, ACP-engagement and prevalence of ACP-documents. 28 home care services (HCS) with 20 trained nurses and about 340 participants have been included. First results show that patients and caregivers judged the topic and the discussion with trusted persons as important and seized the opportunity for communication. In conclusion, established relationships can be built upon to ensure access to ACP and thus to avoid involuntary treatment in situations of decisional incapacity. Part of a symposium sponsored by Systems Research in Long-Term Care Interest Group.

### PREVENTION AND REDUCTION OF CARE AGAINST SOMEONE'S WILL IN COGNITIVELY IMPAIRED PEOPLE AT HOME: A FEASIBILITY STUDY

Angela Mengelers,[1] Michel Bleijlevens,[1] Hilde Verbeek,[1] Vincent Moermans,[2] Elizabeth Capezuti,[3] and Jan Hamers,[1] *1. Maastricht University, Maastricht, Limburg, Netherlands, 2. Maastricht University, Riemst, Limburg, Belgium, 3. Hunter College of CUNY, New York, New York, United States*

Sometimes care is provided to a cognitively impaired person against the person's will, referred to as involuntary treatment. An intervention (PRITAH) was developed to prevent and reduce involuntary treatment comprising 4 components: client-centered care policy, workshops, coaching on the job by a specialized nurse and the use of alternative interventions. A feasibility study was conducted including 30 professional caregivers. Feasibility was assessed by attendance lists (reach), a logbook (dose delivered and fidelity), evaluation questionnaires and focus group interviews (dose received, satisfaction & barriers). The workshops and coach were positively evaluated and the average attendance rate was 73%. Participants gained more awareness and knowledge and received practical tips and advice to prevent involuntary treatment. Implementation of the intervention was feasible with minor deviations from protocol. Recommendations for improvement included more emphasis on involvement of family caregivers and general practitioners and development of an extensive guideline to comply with the policy. Part of a symposium sponsored by Systems Research in Long-Term Care Interest Group.

### SESSION 6000 (SYMPOSIUM)

### ACHIEVING HEALTHCARE EQUITY: DISPARITIES IN DIAGNOSIS, QUALITY OF CARE, AND OUTCOMES AMONG PEOPLE WITH DEMENTIA

Chair: Maricruz Rivera-Hernandez
Co-Chair: Amit Kumar
Discussant: Amit Kumar

Alzheimer's disease and Related Dementia (ADRD) is a significant public health problem and improving the quality and efficiency of care for older adults with ADRD is a national priority. Approximately five million older adults in the United States, including 50% of nursing home residents and 20% of community-dwelling elderly, have ADRD or probable dementia. Although, the number of minorities affected by ADRD growing at an alarming rate, the diagnosis of ADRD and supportive care for this condition are more likely to be delayed among racial/ethnic minority groups. Given the need to ensure equity of care among racial and ethnic groups, there is a pressing need to understand disparities in diagnosis, access and quality of care among racial and ethnic groups with ADRD, specifically using nationally representative data. This symposium will feature four presentations that provide novel insight regarding racial disparities among people with ADRD in the community-, institution-based post-acute, and long-term settings. Individual presentations will describe 1) racial and ethnic differences in risk and protective factors of dementia and cognitive impairment without dementia; 2) racial and ethnic disparities in high-quality home health use among persons with dementia;

3) Within- and between-nursing homes racial and ethnic disparities in resident's outcomes for people with ADRD; and 4) racial differences in transition to post-acute care and rehab utilization following hip fracture related hospitalization in patients with ADRD. Finally, there will be a discussion regarding policy and clinical implications, as well directions for future research.

### RACIAL AND ETHNIC DIFFERENCES IN RISK AND PROTECTIVE FACTORS OF DEMENTIA AND CIND IN THE UNITED STATES

Nasim Ferdows,[1] and Maria Aranda,[2] *1. USC, Los Angeles, California, United States, 2. University of Southern California, Los Angeles, California, United States*

Recent population-based studies have shown declines in dementia prevalence in high-income countries, suggesting that improved population cardiovascular health and rising levels of education in the past 25 year were associated with reduction of dementia risks. However, in the US, there are variations in educational attainment, prevalence and management of chronic diseases, and behaviors associated with poor cardiovascular health among racial and ethnic groups. We performed a retrospective analysis of 3,495 older individuals (65+) in 2016 who participated in Harmonized Cognitive Assessment Protocol (HCAP) subsample of the Health and Retirement Study (HRS), to examine racial/ethnic differences in risk and protective factors associated with dementia and cognitive impairment. Linking HCAP to HRS, we traced individuals back to 2000 and created a longitudinal data of HCAP population (2000-2016). We found that racial/ethnic differences in risk and in protective factors throughout the life-course were associated with racial and ethnic disparities in dementia prevalence.

### RACIAL AND ETHNIC DISPARITIES IN HIGH-QUALITY HOME HEALTH USE AMONG PERSONS WITH DEMENTIA

Shekinah Fashaw, and Kali Thomas, *Brown University, Providence, Rhode Island, United States*

Prior research suggests minorities and racially-diverse neighborhoods have decreased access to high-quality hospitals, physicians, and nursing homes. It is not clear how this varies for persons with dementia (PWD) and home health agencies (HHAs). With the Medicare enrollment file, linked to the home health OASIS, the American Community Survey, and Home Health Compare, we examine the influence of individual's race/ethnicity, as well as the racial/ethnic composition of neighborhoods, on the likelihood of high quality HHA use among PWD in 2016. Minority PWD receiving home health are significantly less likely to use high-quality HHAs than their white counterparts (33% vs 39%, respectively). PWD using HHA in predominantly minority neighborhoods are less likely to use high-quality HHAs compared to PWD in predominantly white neighborhoods (31% vs 40%, respectively). This study is the first to examine racial disparities in the use of HH for PWD. Policy and practice implications will be discussed.

### RACIAL AND ETHNIC DISPARITIES IN QUALITY MEASURES FOR PEOPLE WITH ALZHEIMER'S DISEASE AND RELATED DEMENTIAS

Maricruz Rivera-Hernandez,[1] and Amit Kumar,[2] *1. Brown University School of Public Health, Providence, Rhode Island, United States, 2. Northern Arizona University, Flagstaff, Arizona, United States*

Over 50% of nursing home residents have ADRD. Prior research has shown strong evidence that, compare to non-Hispanic White, African-Americans are more likely to admitted in low performing nursing homes characterized by severe deficiencies understaffing and poor care. However, it is unknown whether these experiences are similar for Hispanics with ADRD. We conducted a cross-sectional study using 2016 data from Medicare- certified providers. Our cohort included 1,425,220 short- (30%) and long-term (70%) beneficiaries with ADRD. Approximately 81% of residents were White, 13% African Americans, and 6% Hispanics. African Americans and Hispanics have lower rates of seasonal influenza vaccination (IV). However, they were also less likely to report pain, pressure ulcers and antipsychotic use compared to white residents. After including the facility fixed-effects in the model, it appears that these disparities are mostly due to between-facility level differences.

### RACIAL DIFFERENCES IN POST-ACUTE TRANSITION AFTER HIP FRACTURE IN MEDICARE PATIENTS WITH ADRD

Indrakshi Roy,[1] Amol Karmarkar,[2] Amit Kumar,[3] Meghan Warren,[3] Patricia Pohl,[4] Stefany Shaibi,[3] Maricruz Rivera-Hernandez,[5] and James Rudolph,[6] *1. Northern Arizona University, flagstaff, Arizona, United States, 2. Virginia Commonwealth University Health, Richmond, Virginia, United States, 3. Northern Arizona University, Flagstaff, Arizona, United States, 4. Northern Arizona University, Phoenix, Arizona, United States, 5. Brown University School of Public Health, Providence, Rhode Island, United States, 6. U.S. Department of Veterans Affairs Medical Center, Providence, Rhode Island, United States*

The incidence of hip fractures in patients with Alzheimer's disease and related dementias (ADRD) is 2.7 times higher than it is in those without ADRD. However, there are no standardized post-acute transition models for patients with ADRD after hip fracture. Additionally, there is a lack of knowledge on how post-acute transitions vary by race/ethnicity. Using 100% Medicare data (2016-2017) for 120,179 older adults with ADRD, we conduct multinomial logistic regression, to examine the association between race and post-acute discharge locations (proportion discharged to skilled nursing facility [SNF], inpatient rehabilitation facility [IRF], and Home with Home Health Care [HHC]), after accounting for patient characteristics. Compared to non-Hispanic Whites, Hispanics have a significantly lower odds ratio for discharge to HHC 0.62 (95%CI=0.53-0.73), IRF 0.44 (CI=0.39-0.51), and SNF 0.26 (CI=0.23-0.30). Improving care in patients with ADRD and reducing racial and ethnic disparities in quality of care and health outcomes will be discussed.

### SESSION 6005 (SYMPOSIUM)

### ADDRESSING OLDER ADULT SOCIAL ISOLATION IN VARIOUS SETTINGS WITH UNIQUE CONFLATING TRAJECTORIES OF CARE MODELS

Chair: Sharon Bowland

No matter the age, social isolation is common among older adults due to a number of factors. In looking at social isolation through the framework of the International Classification of Functioning, Disability and Health (ICF) model, the biopsychosocial factors intersect. Care models will be explored that involve the ICF model multi-disciplinary concepts of body functions, activities, participation and environmental factors. Developing more programs to counter isolation is critical for the health of older persons. (1) The experience of vulnerability may be overcome by banding together through assisting others or building a peer support network. (2) A holistic perspective is needed in promoting interventions that support functionality. Regular programming with body awareness and cognitive reflection is enjoyed by institutionalized older adults. (3) The role of social action and social justice in reducing social isolation is part of training social work students about the importance of culture and advocacy. (4) Collected ethnographic data found that the practice of remembrance reduces social isolation regardless of the program. Gardening and storytelling were found to be opportunities to reduce social isolation. (5) With unexpected longevity in individuals with hemophilia due to scientific advances, researchers also found shame, fear, and coping through social isolation to avoid social assumptions of health status. Care models are being explored to support this cohort. To conclude, Dr. Sharon Bowland will summarize our abstracts and discuss how they revolve around the ICF model of care that can be applied to the important social determinant of health area of social isolation.

### FACING FEAR THROUGH MAKING CONNECTIONS AS WE AGE

Becky Knight,[1] Amanda Noah,[1] and Sharon Bowland,[2] *1. University of North Texas, Denton, Texas, United States, 2. University of Tennessee, Knoxville, Denton, Texas, United States*

Older residents often experience disruption in family relationships and social networks due to isolation once they move into public housing communities. While the need for social contact continues, the opportunity may diminish. Older African-American women living in one housing community attempted to cope with their personal safety fears through social isolation (Bowland, 2015). Women in the study (N = 25) were survivors of one or more forms of interpersonal trauma, including childhood abuse, sexual assault and domestic violence. In contrast, a few women in the same sample spontaneously formed social networks within the community. This qualitative investigation will examine how the women adapted to an isolating and unsafe environment by developing social connections. As a social determinant of health, developing healthy social networks inside a housing community can reduce fear and improve well-being and quality of life for low-income older residents who are aging-in-place.

### ENGAGING MIND, BODY, AND SPIRIT: LESS ISOLATION THROUGH MORE STIMULATION WITH YUMMY TIME

Connie Corley,[1] and Spring Groove,[2] *1. Fielding Graduate University, San Gabriel, California, United States, 2. Y.U.M.M.Y. Time, Los Angeles, California, United States*

Loneliness and social isolation in community-residing older adults is drawing increasing attention. Yet the needs of nursing home/assisted living residents is critical as well. Engaging professionally-trained musicians who are certified yoga instructors in providing a structured, yet flexible, program offered regularly in such settings is an innovation meriting examining what is observed as a profound experience for both older adults and care providers. Y.U.M.M.Y. Time (through music, meditation and yoga) is a paradigm created by Spring Groove (Gross) offering a unique intersection between the performance arts and the contemplative arts. We are studying the benefits of this approach combining sensory stimulation with inner engagement, with the goal to expand into community settings. As staff members note: “Although the ingredients are simple, the results are profound.” “The program is very enriching, full of great music moments, yoga and meditation to relax our residents. It is very much enjoyed by everyone!”

### A SOCIAL JUSTICE FIELD EDUCATION MODEL FOR ADDRESSING ISOLATION AND OTHER SOCIAL DETERMINANTS OF BEHAVIORAL HEALTH

Karen Bullock, Kim Stansbury, and David Fitzpatrick, *NC State University, Raleigh, North Carolina, United States*

Behavioral health and substance use disorders are significant problems among older adults who are experiencing homelessness. This presentation describes the components of a social work field education model for preparing practitioners to work effectively in community-based agencies using the Hartford Practicum Partnership in Aging Educational (HPPAE) model. A planned and systematic transition from one field setting to another with the goal of giving students a range of practice experiences with various service delivery systems to address social isolation and other social determinants of health. This exploration of demographic and social action factors that may impede or foster success among community-dwelling older adults offer insight and guidance for constructing a conceptual and theoretical model a social justice framework in geriatric social work practice and research.

### EXPERIENCES OF SOCIAL WITHDRAWAL: WHY AGING MATTERS AMONG INDIVIDUALS WITH HEMOPHILIA FACING UNEXPECTED LONGEVITY

Tam Perry,[1] Sara Schwartz,[2] Dana Francis,[3] and Charles Kaplan,[2] *1. Wayne State University, Detroit, Michigan, United States, 2. University of Southern California, Los Angeles, California, United States, 3. University of California San Francisco, San Francisco, California, United States*

For the first time, individuals with hemophilia are living beyond their 30s and 40s. This cohort, many of whom had been dependent on donated blood and contracted HIV and hepatitis, face unique challenges as they age with hemophilia and other conditions. Little is known about the experiences and health needs of these adults over the age of 40. Scientific advances have changed the life course of individuals who received earlier treatment modalities. Today, a person with hemophilia who has received preventative synthetic treatment can look forward to a long, healthy and active life. Semi-structured telephone interviews (n=32) were conducted with long-term survivors, family members, and professionals who supported these families. Findings include shame, fear and coping through social withdrawal in order to hide their hemophilia to avoid assumptions of HIV status. Analyzing unexpected longevity and why age matters for this historically isolated cohort, we explore appropriate care models.

### COULD PRACTICES OF REMEMBRANCE REDUCE SOCIAL ISOLATION IN LATE LIFE? ETHNOGRAPHIC EVIDENCE FROM POLAND AND DETROIT

Jessica Robbins, *Wayne State University, Detroit, Michigan, United States*

Because social isolation can have negative effects on older adults' wellbeing, programs that reduce social isolation have potential to improve older adults' wellbeing. One presumed aspect of these programs' significance is the social connection occurring through the programs themselves. However, drawing on ethnographic data collected in Poland and Detroit, this presentation argues that practices of remembrance, in which older adults connect with deceased kin and loved ones, may offer possibilities for reducing social isolation. In Poland, older adults engage in practices of storytelling in which they remember deceased kin and lost homes and homelands. In Detroit, Michigan, older African Americans who garden remember their deceased kin and friends through the practice of gardening itself. This presentation presents a cross-cultural analysis of how older adults' practices of remembrance may offer opportunities to reduce social isolation—even for older adults who live alone—by connecting to meaningful relations, times, and places.

### SESSION 6010 (SYMPOSIUM)

### ADDRESSING THE NEEDS OF PERSONS LIVING WITH DEMENTIA ACROSS CARE SETTINGS (A COLLABORATIVE SYMPOSIUM BETWEEN THE ASSISTED LIVING AND RESEARCH IN QUALITY CARE INTEREST GROUPS)

Chair: Howard Degenholtz
Co-Chair: Candace Kemp
Discussant: Doug Pace

Understanding the needs of persons living with dementia is critical to promoting quality of life and care. Co-sponsored by the Assisted Living (AL) and Research in Quality Care Interest Groups, this symposium includes four papers that present new ways to use claims data, qualitative data, benchmarking data, and intervention data to promote well-being for persons living with dementia. First, Degenholtz and Van Cleve use Pennsylvania Medicaid data from 2014-2016 to examine the provision of personal care to persons with and without dementia. They find that those living with dementia received more personal care per day across levels of physical disability and discuss implications for home and community based services policy. Next, Kemp et al. investigate meaningful engagement among assisted living residents with dementia using qualitative data collected over a one-year period in four diverse care communities. Findings show a range of engagement experiences and point to the influence of key resident, care partner, and care community influences. Third, Morgan et al. use data from a statewide probability sample of nursing home staff (n=438) to identify barriers and facilitators to person-centered care. Findings show key barriers to delivering person-centered care, including a lack of staff empowerment practices and low use of consistent assignment. Last, Zimmerman et al. present an evidence-based program, "Mouth Care Without a Battle" developed in nursing homes. Using data from over 2,000 assisted living stakeholders, they situate their findings within implementation science and the NIH Stage Model and make their findings transferable regardless of focus or setting.

### EXAMINING MEANINGFUL ENGAGEMENT AMONG ASSISTED LIVING RESIDENTS WITH DEMENTIA

Candace Kemp,[1] Alexis Bender,[2] Elisabeth Burgess,[1] Joy Ciofi,[1] Fayron Epps,[2] Jennifer Morgan,[1] and Molly Perkins,[2] *1. Georgia State University, Atlanta, Georgia, United States, 2. Emory University, Atlanta, Georgia, United States*

Across care setting, lack of meaningful activity is identified by persons living with dementia and their care partners as a persistent and significant unmet care need. Here, we examine the opportunities for and experiences with meaningful engagement among assisted living residents with dementia using data from our 5-year NIA-funded qualitative study (R01AG062310). Guided by grounded theory method, the study involves in-depth interviews, participant observation, and resident record review collected over a one-year period in four diverse assisted living communities. Analysis of these data shows a range of engagement opportunities and experiences within and across care communities and point to the influence of resident (e.g. cognitive and physical function, interests, relationships), care partner (e.g., dementia knowledge, strategies, availability), and care community (e.g., size, location, resources) factors. We conclude by offering suggestions for policy and practice and for future research aimed at improving the quality of life of persons living with dementia.

### HOME AND COMMUNITY BASED SERVICES FOR PEOPLE WITH DEMENTIA

Howard Degenholtz,[1] and Raymond Van Cleve,[2]
*1. University of Pittsburgh, Pittsburgh, Pennsylvania, United States, 2. U.S. Department of Veterans Affairs, West Haven, Connecticut, United States*

Home and Community Based Services has grown as an alternative to nursing homes over the past 30 years. While there are extensive data on nursing home staffing, there is a dearth of similar information about Medicaid financed home care. We use data from 2014-2016 Pennsylvania Medicaid to examine personal care for elderly with and without dementia across the range of physical disability. One challenge is that even though physical function can be measured in terms of discrete tasks (i.e., limitation in bathing, dressing, toileting), analysis of the amount of care people receive has to take into account the combination of dementia and combinations of ADL limitations. We found that older adults with dementia receive one hour more of personal care per day at the lowest level of disability and 1.5 hours at the highest level. The increased need for caregiving hours should be incorporated into policies that guide HCBS programs.

### PERSON-CENTERED DEMENTIA CARE: WORKFORCE AND ORGANIZATIONAL SUPPORTS IN NURSING HOMES

Jennifer Morgan, Elisabeth Burgess, and Yun-Zih Chen, *Georgia State University, Atlanta, Georgia, United States*

Person-centered care that supports choice and autonomy of residents is foundational to implementing high quality care for long-term care residents including those living with dementia. Organizational change that supports person-centered approaches requires leadership engagement and a stable, well-qualified dementia-capable workforce. This paper uses survey data from a statewide probability sample of nursing homes and their staff (N=438) to identify barriers and facilitators to person-centered care. Findings show key barriers to delivering person-centered care, including a lack of staff empowerment practices and irregular use of consistent assignment. While most organizations are implementing some person-centered resident care practices, few have moved beyond predominantly institutional practices. Staff stability and retention also remain key barriers to person-centered care. Facilitators include adoption of evidence-based and inclusive quality improvement strategies. The paper will discuss implications for state, community and organizational strategies for improving staff empowerment, integrating dementia care competencies, promoting culture change and increasing leadership engagement.

### DEMENTIA CARE ACROSS LONG-TERM SETTINGS: SIMILAR BUT NOT EQUAL

Sheryl Zimmerman,[1] Christine Kistler,[1] Jessica Scott,[1] Kimberly Ward,[1] Robin Zeigler,[2] Louise Sullivan,[3] and Sarah Tomlinson,[2] *1. University of North Carolina at Chapel Hill, Chapel Hill, North Carolina, United States, 2. North Carolina Department of Public Health and Human Services, Raleigh, North Carolina, United States, 3. Salve Regina University, Newport, North Carolina, United States*

Nursing homes and assisted living (AL) communities are similar but not equal, and addressing the needs of residents with dementia differs across settings. It is important to appreciate that both settings are complex adaptive systems; as such, care intended to have widespread impact must be mindful of stakeholders, understand existing practices, and be pragmatic. This session will present an evidence-based program developed in nursing homes – Mouth Care Without a Battle, which teaches staff to provide daily, personalized mouth care to persons with cognitive and physical impairment – and considerations relevant for implementation in AL. Using data from more than 2,000 AL trainees and also AL administrators, supervisors, residents, family members, and trainers, it will situate findings in the context of implementation science and the NIH Stage Model, thereby making them applicable to any dementia care practice regardless its focus and the setting in which it is to be used.

### SESSION 6015 (SYMPOSIUM)

### ADULT PROTECTIVE SERVICES' INTERVENTION ON ELDER ABUSE: USING STANDARDIZED MEASURES FOR OUTCOMES

Chair: Pi-Ju Liu
Co-Chair: Kendon Conrad
Discussant: Kathleen Wilber

Adult Protective Services (APS) investigates and substantiates vulnerable adult abuse, neglect, and exploitation (ANE) cases. The frontline social service agency also refers or provides needed services to ANE victims. Outcomes of APS has been scarce, with definitions of outcomes varying from study to study. Using a pretest-posttest design, we partnered with San Francisco and Napa APS to measure changes of ANE harm from case investigation (pretest) to case closure (posttest) using standardized measures called the Identification, Services, and Outcomes (ISO) Matrix. Forty-five APS supervisors and caseworkers used the ISO Matrix on 2,063 cases during the six-month pilot demonstration. Dr. Pi-Ju (Marian) Liu will examine findings on changes of ANE harm and APS services that effectively decreased ANE harm. Responding to 2020's Annual Scientific Meeting theme "Turning 75: Why Age Matters", ANE harm and APS services will be compared between younger APS clients age 18-64 and older ones above the age of 65. Dr. Zachary Hass will discuss allegation, abuse severity assessment, services provided, and outcomes across racial and language groups. Dr. Kendon Conrad will present reliability and validity of the ISO Matrix and a shorter version useful for APS practice. Ms Sara Stratton will review unusual cases with outlier ISO Matrix scores to inform researchers' implementation and practitioners' use of standardized measures. Dr. Kathleen Wilber, our discussant, will reflect on the use of standardized measures in APS and its impact on both practice and research based on the four presentations.

### AGE DIFFERENCES IN ELDER ABUSE HARM AND EFFECTIVE SERVICES OFFERED BY ADULT PROTECTIVE SERVICES

Pi-Ju Liu,[1] Zachary Hass,[1] Karen Conrad,[2] Sara Stratton,[3] and Kendon Conrad,[4] *1. Purdue University, West Lafayette, Indiana, United States, 2. University of Illinos at Chicago, Chicago, Illinois, United States, 3. San Francisco Adult Protective Services, San Francisco, California, United States, 4. University of Illinois at Chicago, Chicago, Illinois, United States*

In this study, abuse, exploitation, and neglect (ANE) harm was measured by type of abuse using standardized items from the Identification, Services, and Outcomes (ISO) Matrix before Adult Protective Services (APS) interventions (pretest) and after APS interventions (posttest). Change scores from 1,472 older adults (average age 78-year-old; 57% female) and 591 younger adults (average age 53-year-old; 46% female) served by APS during the six months showed reduction of harm using posttest minus pretest. Nonetheless, older adult's financial abuse harm (pretest=2.2, posttest=1.5) was higher than younger adults' (pretest=1.5, posttest=1.2), while young adults scored higher in harm on all other types of abuse. Effective interventions differ by age group and by type of abuse, and will be discussed in detail. Results demonstrate the importance to consider vulnerable adult's age and the etiology of abuse before implementing the services needed to effectively address ANE harm.

### BALANCING PRACTICALITY AND VALIDITY OF ELDER ABUSE MEASURES: DATA FROM ADULT PROTECTIVE SERVICES INVESTIGATIONS

Kendon Conrad,[1] Pi-Ju Liu,[2] Zachary Hass,[2] and Karen Conrad,[3] *1. University of Illinois at Chicago, Chicago, Illinois, United States, 2. Purdue University, West Lafayette, Indiana, United States, 3. University of Illinos at Chicago, Chicago, Illinois, United States*

Research in Adult Protective Services suffers from the problems of small sample size, low completion rates, and poor data quality. To address the "small sample size" issue, data were generated on 1,472 elder abuse cases over six months in California using the Identification, Services and Outcomes (ISO) Matrix. The problem of poor data quality was addressed by testing psychometric properties of Short-Forms on each type of abuse. Good reliability and predictive validity were found for all measures except those that were very rare. Finally, even shorter Mini-Forms were developed and tested in order to contribute to improved completion rates. Mini-Form results were mixed, and some will require further research on their reliability and validity, as well as examination of their ability to improve completion rates. Ongoing adoption by two California counties and the State of Montana demonstrate sustainability and increasing sample sizes using the ISO Matrix for research and practice.

### ALLEGATION ASSESSMENT AND OUTCOME DIFFERENCES ACROSS RACIAL GROUPS FROM STANDARDIZED ADULT PROTECTIVE SERVICES DATA

Zachary Hass,[1] Pi-Ju (Marian) Liu,[1] Karen Conrad,[2] Sara Stratton,[3] and Kendon Conrad,[4] *1. Purdue University, West Lafayette, Indiana, United States, 2. University of Illinos at Chicago, Chicago, Illinois, United States, 3. San Francisco Adult Protective Services, San Francisco, California, United States, 4. University of Illinois at Chicago, Chicago, Illinois, United States*

Objective assessment is an important tool for Adult Protective Services (APS) in supporting a diverse population. Out of the 1472 APS' clients, aged 65 and over, assisted during the study period, 39% identified as a non-white race and 30% did not speak English. Providing services to this vulnerable population is made even more difficult by the need to provide culturally appropriate services. In this work, we present on differences in types of abuse alleged, abuse severity assessment, services provided, and preliminary outcomes across racial and language groups. For example, for this population, clients identifying as black race had the highest allegation rates of neglect (16%) and financial abuse (27%), Asians of physical (15%) and emotional abuse (24%), and other races of self-neglect (56%) and isolation (9%). Standardized data collection permits tracking such patterns and objective assessment tools help to avoid systemic bias in aiding this vulnerable population.

### UNDERSTANDING PRACTICE THROUGH RESEARCH

Sara Stratton,[1] Pi-Ju (Marian) Liu,[2] Kendon Conrad,[3] and Zachary Hass,[2] *1. San Francisco Adult Protective Services, San Francisco, California, United States, 2. Purdue University, West Lafayette, Indiana, United States, 3. University of Illinois at Chicago, Chicago, Illinois, United States*

Substantiation of abuse, neglect, and exploitation (ANE) data collected during the Identification, Services, and Outcomes (ISO) Matrix pilot included some outlier scores warranting further investigation. To examine outliers and other unexpected findings in greater detail, we completed case reviews and caseworker interviews to explore why outlier cases did not follow the expected patterns of substantiation decisions. Cases were selected on a random basis from those with self-neglect outlier scores. Several factors were found to have contributed to the extreme scores in all three categories of confirmed, inconclusive, and unfounded abuse. In this study, no valid ISO Matrix scores were found that contradicted the substantiation decision. Instead, analysis revealed that most of the errors occurred: 1) in application of the intended procedures showing need for further training, and 2) some needed changes to the procedures of the ISO Matrix based on feedback from caseworkers.

### SESSION 6020 (SYMPOSIUM)

### AGE ALSO MATTERS FOR GERONTOLOGISTS: PERSONAL AND PROFESSIONAL PERSPECTIVES ON GROWING OLDER

Chair: Phillip Clark
Discussant: Margaret Perkinson

Gerontology is a unique field of scientific inquiry, because it embodies both professional and personal dimensions of experience and poses questions for its researchers. How does our work help us understand our own personal experience of aging? How does the reality of growing older change our teaching and research? As gerontologists, we embody two narratives of the aging experience, one academic and professional (with its dependence on theory and scientific research), the other intimately personal (with its own lived experience and practical insight acquired over the life course). How this dynamic unfolds is as personal as each of us as individuals, and embodies our own disciplinary backgrounds; yet collectively it has implications for how we approach an understanding of what it means to grow old. This symposium explores different facets of this dynamic from four perspectives of different individuals and differing disciplines. The first paper assesses the limitations of both quantitative and qualitative research paradigms in revealing the deeply idiosyncratic nature of personal aging. The second develops the metaphor of "double agent of aging" to characterize the two narratives of professional and personal aging. The third uses Erikson's theory of psychosocial development to weave together the professional, practical, and personal dimensions of gerontology. Finally, the last develops the metaphor of arcs and stages in conceptualizing a gerontological career. The symposium concludes with recommendations for the integration of theoretical, practical, and personal insights into teaching, research, and service in a way that embraces, enhances, and extends the field of gerontology.

### THROUGH THE GERONTOLOGICAL LOOKING GLASS: PERSPECTIVES FROM LATER LIFE

Edward Ansello, *Virginia Commonwealth University, Richmond, Virginia, United States*

Do dominant gerontological research and education models help us understand our own aging? "(These) seemingly made no impact on my expectations for my own late life" (Cohen, 2017). The gerontological looking glass tends to favor research and teaching that identify large patterns, populations, and research cell sizes of "sufficient" numbers to produce data about external, descriptive assessments; and are compliant with nomothetic ideology: general, universal, and consistent. Even qualitative data rely on numbers of respondents to identify themes. In contrast, idiographic ideology emphasizes the individual and unique. While not meant to yield general findings, neither does it produce the incompleteness of descriptive statistical approaches, i.e., trying to capture the external persona. As human aging is both inherently universal and profoundly individual, one is likely to appreciate idiographic gerontology with awareness of one's own aging. Various complementary perspectives—including humanistic gerontology, positive and conscious aging, and the curriculum palette—are relevant.

### THE SELF AS SUBJECT AND OBJECT: REFLECTIONS ON BEING A DOUBLE AGENT OF AGING

Phillip Clark, *University of Rhode Island, Kingston, Rhode Island, United States*

Being both a gerontologist and an older adult places one in a double narrative: one is the scientific story and academic life of research and education, the other is the personal experience of living through this phase of life. These two narratives become intertwined in a way that both enriches and poses challenges. This paper explores what being a "double agent of aging" means as an individual and a scholar, and offers some insights for the field of gerontology. These implications include: (1) the growing importance of values and life wisdom, (2) health behavior hypocrisy, and (3) the experience of ageing and marginalization. The implications for the field of gerontology include: (1) valuing the voices of older adults in our research and teaching, (2) being more active in confronting ageism in our own institutions, and (3) acknowledging the limitations of studying aging as a younger adult.

### SCIENCE AND SELF: AGING AS WOVEN

Helen Kivnick, *University of Minnesota, St. Paul, Minnesota, United States*

Gerontology is a field both scientific and practice-based. Aging, the subject of this field, is an experience in which all human beings participate. But scientific pillars of objectivity, quantifiability, control, and external validity have long mitigated against gerontological scholars effectively moving back and forth between professional scholarship and practice, on the one hand, and personal experience, on the other. Qualitative research approaches, informed by the humanities and arts, utilize alternative ways of knowing that, when added to positivistic science, enable us to construct a body of gerontological knowledge that is robust and useful, and that also incorporates wisdom. Aging, wisdom, and integrality—these all matter. Although often mischaracterized, Erikson's theory of healthy psychosocial development throughout the life-cycle (Erikson, Erikson, & Kivnick, 1986) weaves these constructs together in ways that can meaningfully inform professional and personal experiences of gerontology. This presentation illustrates one aging gerontologist's engagement with such weaving.

### ARCS AND STAGES: RETROSPECTIVELY CONCEPTUALIZING A GERONTOLOGICAL CAREER

Kenneth Hepburn, *Emory University, Atlanta, Georgia, United States*

The arc of a gerontologist's career is that of a "work in progress," work unlikely to be completed. Early efforts might develop interdisciplinary collaborations and establish the principles and mechanisms of a central line of work and inquiry. Mid-stage work may entail expansion and adaptation of preliminary efforts and identification of exciting areas of exploration that both fit within the gerontologist's overall thematic trajectory and extend beyond the reasonable scope of pursuit. In late stage, the most pressing concern is to sustain, but not constrain, the work's trajectory. This may best be accomplished by identifying and supporting students and early career researchers who are passionate about the work and who are likely to move it forward and expand it in their own unique and divergent ways. Emeriti gerontologists may seek to remain generatively engaged in ways that both contribute to and let go of the continuing arc of the work.

### SESSION 6025 (SYMPOSIUM)

### AGE MATTERS: THE IMPACT OF AGEISM ON HEALTH, COGNITIVE IMPAIRMENT, WORK LIVES, AND ANTI-AGEIST INTERVENTIONS

Chair: Philip Rozario
Discussant: Nancy Morrow-Howell

Age is a social constructions. The treatment of people on the basis of their age, imposes serious psycho-emotional, social and economic costs on society and older people. The experiences of ageism may be exacerbated when other forms of acute and chronic forms of oppression are experienced due to racism, sexism, etc. The first paper looks at the impact of ageism on older people's health. Their systematic review of studies showed that ageism detrimentally and consistently impacted older individuals in 11 health domains, with the prevalence of significant findings increasing over time. Informed by NIA's Health Disparities Framework, the second paper examines the relationships between discrimination and protective factors on cognitive functioning. Their analyses of the Health and Retirement Survey data reveal, among other things, that everyday experiences of ageism significantly worsen older adults cognitive functioning. Using survey data of adults throughout the life-span, our third presenter examines how multiple identities (such as age and gender) influence employees' perspectives on workplace fairness in hypothetical situations. The findings are informative to human resource departments in ensuring workplace fairness within the context of a multigenerational workforce. The final paper examines two efforts to disrupt ageism, specifically interventions that target students in an intergenerational program and staff members of senior living communities. Results from these evaluations point to an increase in positive attitudes among students and a reduction in ageist behaviors among staff members. All presenters will discuss policy, practice and research implications of their studies.

### AGEISM AND ITS IMPACT ON OLDER PERSONS' HEALTH

Becca Levy, *Yale School of Public Health, Woodbridge, Connecticut, United States*

Ageism has been called a silent epidemic. The extent to which ageism impacts the health of older persons in different countries was not well understood. In this presentation we will focus on the reach of ageism including negative age beliefs, on older individuals' health. In an exhaustive systematic review, we found that ageism influenced older individuals in 45 countries and 11 health domains, with the prevalence of significant findings increasing over time ($p < .001$). In this presentation, we will also explore the mechanism by which this impact occurs and steps that can be taken to address this epidemic.

### DISCRIMINATION AND PROTECTIVE FACTORS TO COGNITIVE HEALTH: TESTING NIA'S HEALTH DISPARITIES FRAMEWORK

Ernest Gonzales,[1] Cliff Whetung,[2] Jane Lee,[3] and Yi Wang,[4] *1. New York University, New York, New York, United States, 2. New York University, New York, New York, United States, 3. University of Hawai'i, Myron B. Thompson School of Social Work, Honolulu, Hawaii, United States, 4. University of Iowa, Iowa City, Iowa, United States*

Cognitive impairment is a worldwide epidemic. Informed by NIA's Health Disparities Framework, this study investigated interpersonal, behavioral, and sociocultural risk and protective factors associated with cognitive health trajectories. Mixed models examined factors associated with cognitive health with data from the Health and Retirement Study among Whites, Blacks, and Hispanics (2008-2014, N=4,511). A majority of respondents who experienced everyday discrimination attributed it to ageism among this racially and ethnically diverse sample. Stratified mixed models of everyday discrimination by attribution (racism or ageism) revealed worse cognitive functioning. Major lifetime discrimination was not statistically associated with cognitive functioning. Economic factors (education, income, assets) and religious activity protected cognitive functioning and were particularly salient for Blacks and Hispanics. Strategies that bolster individual resilience as well as social policies that address discrimination and structural inequities will likely reduce health disparities and improve population health.

### PERCEPTIONS OF WORKPLACE FAIRNESS IN THE CONTEXT OF AGE AND INTERSECTIONALITY

Cal Halvorsen,[1] Marcie Pitt-Catsouphes,[2] and Indrani Saran,[2] *1. Boston College, Chestnut Hill, Massachusetts, United States, 2. Boston College School of Social Work, Chestnut Hill, Massachusetts, United States*

While scholars have focused on workplace fairness (often called organizational justice) for quite some time, the context of diversity—in its many forms—has rarely been included in this conversation. This presentation will review concepts related to workplace fairness, describing how the context of diversity may influence perceptions of it. We then will present the results of a recent survey of 609 respondents aged 18 to 70 with a focus on how holding diverse attributes (e.g., age, gender, and their intersectionality) may shape perceptions of workplace fairness and diversity. Overall, we found that the perceptions of workplace fairness and diversity are similar by age and gender, with a few notable differences (e.g., older respondents value interpersonal justice the most, such as their opinions being considered, and younger respondents see workplace diversity the most positively). These results can inform scholarship and discussions on human resource practices and environmental change in organizations.

### APPROACHES TO ANTI-AGEISM INTERVENTIONS

Tracey Gendron, and Jennifer Inker, *Virginia Commonwealth University, Richmond, Virginia, United States*

Ageism, a multidimensional construct, is also understood as a relational process whereby perceptions and behaviors toward older individuals by younger individuals not only damage the self-esteem of elders, but also create a hostile environment for their own future social interactions and their own future self-development as elders. Anti-ageism interventions have the hefty task of improving attitudes and behaviors toward aging within all of these contexts. This presentation will discuss findings from two different anti-ageism interventions both designed to mitigate the negative impacts of ageism. Results from a study on an intergenerational arts-based program found that after participation students demonstrated a positive change in their attitudes toward older adults. Findings from a video-based ageism intervention among a sample of 265 staff members in 15 senior living communities demonstrated decreased internalized aging anxiety as well as decreased ageist behaviors directly after the training and at three month post follow-up. Given the complex and systemic nature of ageism, diversity is necessary in scope and type of intervention in order to reach the broadest audience.

### SESSION 6030 (SYMPOSIUM)

### AN INTERNATIONAL PERSPECTIVE: THE ROLE OF TRANSPORTATION IN SUPPORTING COMMUNITY MOBILITY NEEDS OF OLDER ADULTS

Chair: Anne Dickerson
Discussant: Brenda Vrkljan

This international symposium brings together leading scholars in occupational therapy research whose shared aim is to support community mobility in older adulthood. in this session, five groups of researchers will share their collective and individual research outcomes supporting continued community mobility of older adults, especially when driving is no longer an option. The first presentation will be their collective international, cross sectional study of 247 older adults from seven countries. This study compared community destinations, methods of transportation and factors that influence community mobility. Each presentation that follows will highlight unique studies with the overarching goal of improving mobility options for older adults to "age in place of choice." Our discussant will summarize the potential for expanding this research by engaging the audience through interactive questions, as to where local and global opportunities for innovations that support community mobility will be raised.

### AN INTERNATIONAL COMPARISON OF THE COMMUNITY MOBILITY PATTERNS OF OLDER ADULTS

Lizette Swanepoel,[1] Isabelle Gelinas,[2] and Barbara Mazer,[3] *1. Stellenbosch University, Cape Town, Western Cape, South Africa, 2. McGill University, Montreal, Quebec, Canada, 3. McGill University School of Physical and Occupational Therapy, Montreal, Quebec, Canada*

Community mobility in older adults is important for maintaining health, quality of life and social participation. Globally, older adults who are non-drivers, access their community through various modes of transport to maintain community mobility. This international cross-sectional cohort study (n=246) explored the mobility patterns of older adults and examined their access to out-of-home activities and health related quality of life in seven countries. Quality of life was determined using EQ-5D-5L and was generally high among all participants. Findings from the study indicate that a complex myriad of factors influence safe transport mobility in older adults. Results suggested that inclement weather and place of residence negatively impacted access to out-of-home activities, yet these factors but did not increase use of public transport. Given the complexity of transportation use and mobility patterns in older adults, an individualised approach may be necessary to keep older adults connected to their out-of-home activities.

### A PILOT STUDY USING THE LIFE SPACE ASSESSMENT AMONG COMMUNITY-DWELLING OLDER ADULTS IN IRELAND

Tadhg Stapleton, *Trinity College Dublin, Dublin, Dublin, Ireland*

Forty older adults (27, 67.5% female), mean age 74.3 years (range 65- 89), current drivers 31 (77.5%), 23 (57.5%) rural dwellers and 17 (42.5%) urban dwellers participated in the survey. Median LSA score was 74 (range 27-102). All participants were readily accessing life spaces within and immediately outside their own home. Over half (n=23, 57.5%) accessed spaces in their neighbourhood on a daily basis. Decreased frequency of access to spaces outside of local neighbourhood and town was noted. No significant difference in LSA scores between genders (P=0.549), current driving status (P=0.235), but urban dwellers had significantly higher LSA scores than rural dwellers (P=0.024). Spearman correlations found statistically significant negative correlation between age and LSA scores (rho= -0.445, P=0.004), and significant positive correlation between LSA and Euroqol (EQ) VAS scores (rho=0.405, P=0.010). Findings are limited by the small sample size but highlight decreasing frequency of wider community participation with increasing age.

### COMPARISON OF USA AND AUSTRALIAN MOBILITY DEVICE USERS' AND AMBULANT BUS USERS' VIEWS OF RESTRAINTS ON PUBLIC BUSES

Carolyn Unsworth, *Central Queensland University, Melbourne, Victoria, Australia*

Many older people use powered wheelchairs and mobility scooters to access the community on buses but have increased injury risk if the mobility device tips or slides. Wheelchair tie-down and occupant restraint systems (WTORS) are mandated on USA transit buses, and their introduction investigated in Australia. This study examined the views of mobility device and ambulant bus users in the USA and Australia on WTORS. A Qualtrics survey with 448 respondents showed strong support for WTORS use and found the most important factors underpinning use were Safety, Comfort, and Transit time. US research indicates dwell time while fitting WTORS is 4 minutes, and participants reported 5.65(SD3.06) minutes is acceptable. There was no difference in USA and Australian participants who have slid or tipped in their device, despite being restrained in the USA: X2(1,n=220)=.053,p=.53, phi-.016). This research suggests all bus users are supportive of WTORs, but their effectiveness requires investigation.

### PLACES VISITED, MAINTAINED, OR ABANDONED OUTSIDE HOME: A COMMUNITY MOBILITY NEED OF OLDER ADULTS WITH DEMENTIA

Isabel Margot-Cattin,[1] Sophie Gaber,[2] Nicolas Kuhne,[3] Camilla Malinowski,[4] and Louise Nygard,[5] *1. University of Applied Sciences and Arts of Western Switzerland (HES-SO), Lausanne, Vaud, Switzerland, 2. Karolinska Institutet, Stockholm, Stockholm Lans, Sweden, 3. Uiversity of Applied Sciences and Arts of Western Switzerland (HES-SO), Lausanne, Vaud, Switzerland, 4. Karolinska Institutet, Huddinge, Stockholms Lan, Sweden, 5. Karolinska Institute, Stockholm, Stockholms Lan, Sweden*

For older adults to "age in place", they need to keep engaged and mobile in their communities, whatever their health condition. The impact of age and cognitive decline on community mobility is a growing problem in Europe and worldwide. Engaging in occupations outside home implies being able to get to those places where activities are performed. Yet little is known regarding the types of places visited, maintained or abandoned for older adults with/without dementia. This study addresses community mobility needs through the places people visit, maintain or abandon. People with and without dementia, aged 55+, were interviewed using the Participation in ACTivities and Places OUTside the Home (ACT-OUT) questionnaire across Switzerland (n=70), Sweden (n=69) and the UK (n=128). Results show that people with dementia experience a higher rate of abandonment for more places than regular older adults. Insights about driving cessation and access to travel passes will be presented.

### SESSION 6035 (SYMPOSIUM)

### APPLIED SCHOLARSHIP IN LGBTQ AGING: IMPLICATIONS FOR POLICY AND PRACTICE

Chair: Austin Oswald
Discussant: Vanessa Fabbre

Lesbian, gay, bisexual, transgender, and queer (LGBTQ) elders have shown considerable strength while aging in a society predicated on heteronormative and binary expectations for gender and sexuality. The life trajectories of LGBTQ older adults are shaped by discrimination and stigmatization, and the embodied resistance that comes with demanding their full participation and recognition in society. This symposium highlights the innovative scholarship of emerging scholars in the field of LGBTQ aging who are engaging in diverse substantive and methodological investigations. The first study takes a comparative cohort approach to explore differences in stressors and depressive symptomatology between younger and older sexual minorities, highlighting the significance of cohort effects among LGBTQ people. The second paper uses data from the Health and Retirement Study to examine anticipated nursing home placement needs between LGB and heterosexual adults with suggestions to better prepare aging service networks. The third describes the influence of state legislature mandating LGBTQ-sensitivity training by examining differences in provider baseline knowledge and attitudes toward LGBTQ older adults in two states, one mandating LGBTQ-sensitivity training and one not. The final paper highlights findings from a multi-methods study that explores how long-term care workers, managers, and administrators respond when staff, visitors, or residents challenge LGBTQ rights for religious and moral reasons. Although substantively and methodologically varied, these studies all demonstrate the importance of applied scholarship that builds knowledge in support of policies and practices that promote equity among LGBTQ individuals across the life course. Rainbow Research Group Interest Group Sponsored Symposium.

### EVALUATING THE IMPACT OF STATE POLICY INTERVENTIONS FOR LGBT AGING: COMPARATIVE CASE STUDY OF NEW YORK AND CALIFORNIA

Austin Oswald,[1] Nancy Giunta,[2] Tim Johnston,[3] and Sherrill Wayland,[4] *1. The Graduate Center, City University of New York, Long Island City, New York, United States, 2. Silberman School of Social Work, Hunter College, New York, New York, United States, 3. sAGE, New York, New York, United States, 4. SAGE, New York, New York, United States*

The majority of aging network service providers are unprepared to deliver targeted services to lesbian, gay, bisexual, or transgender (LGBT) older adults. In 2017, California legislature mandated ongoing LGBT sensitivity training for congregate living providers. Services and Advocacy for GLBT Elders (SAGE) developed a specialized training, Creating Inclusive Communities, for congregate living staff to learn the unique needs of LGBT elders. This secondary data analysis compared pre-test knowledge and attitudes of training participants in two states, one mandating LGBT aging sensitivity training (California, N=328) and one without the mandate (New York, N=622). Preliminary results show that prior to receiving training, California participants demonstrate significantly less knowledge of LGBT aging issues compared to New York participants; t(948)=-3.808, p<.001. Attitudinal differences were also demonstrated. These results suggest that laws mandating LGBT sensitivity training may help reach providers with greater training needs. Policy and practice implications will be discussed. Part of a symposium sponsored by Rainbow Research Group Interest Group.

### WHY AGE MATTERS: THE IMPORTANCE OF HISTORICAL CONTEXT IN THE STUDY OF MINORITY STRESS IN SEXUAL MINORITIES

Michael Vale,[1] and Toni Bisconti,[2] *1. University of Akron, Akron, Ohio, United States, 2. Univesity of Akron, Akron, Ohio, United States*

Currently, there is support that links experiences of stigma/discrimination, known as minority stress, with depressive symptomatology in older sexual minorities (OSM). Yet, the context of cohort is ignored, despite OSM having been exposed to greater stigma across their lifetime. The current project explores how cohort informs the minority stress process by comparing three minority stressors and their relationships with depressive symptomatology across a younger (18-39, N=129) and older sample (40-80, N=104). It was found that OSM had significantly less overall stress, had different significant links between each stressor and depressive symptomatology, and significantly used social support to buffer stress contrasting their younger counterparts. These results highlight the need to consider cohort and discover why OSM are more resilient to minority stress. Considering the role of cohort improves aging and minority stress theories, is useful for professionals improving well-being in OSM, and informs our understanding of the lived experiences of OSM. Part of a symposium sponsored by Rainbow Research Group Interest Group.

### FUTURE NURSING HOME PLACEMENT, HEALTH, AND RELATIONSHIPS BY SEXUAL ORIENTATION: FINDINGS FROM THE HRS

Mekiayla Singleton, Zach Gassoumis, and Susan Enguidanos, *University of Southern California, Los Angeles, California, United States*

This study compares LGB and heterosexual adults on anticipated need for future nursing home (NH) placement and factors that influence NH placement. Using data from the 2016 HRS, we found a trend toward higher anticipated NH placement among LGB adults (M=34.0, SD=29.2) as compared to heterosexual adults (M=26.9, SD=27.3; p=0.05). Compared with LGB respondents (n=137), heterosexual adults (n=3,469) were more likely to have living child/children (37% vs. 82%, p<.001). Although there was no statistical difference in marital status between LGB (50% married/partnered) and heterosexual adults (65% married/partnered; p=.06), married/partnered LGB respondents were more likely to be very/quite close to their spouse/partner (99% vs. 90%, p<0.01) compared to heterosexual respondents. There was no difference in number of health conditions between groups. This study suggests that LGB adults may have higher rates of anticipated need for NH, thus NHs must ensure their workforce is educated and prepared to support this growing population. Part of a symposium sponsored by Rainbow Research Group Interest Group.

### NAVIGATING RELIGIOUS AND MORAL EXEMPTIONS IN SERVING LGBTQ RESIDENTS IN LONG-TERM CARE FACILITIES

Angela Perone, *University of Michigan, Ypsilanti, Michigan, United States*

Almost immediately after the U.S. Supreme Court mandated marriage equality, a surge in policies carved out exceptions to LGBTQ rights for religious or moral reasons. This study examines how long-term care workers, managers, and administrators respond when staff, visitors, or residents challenge LGBTQ rights based on these exemptions. Building on street-level bureaucracy theory, this study employs an advanced multi-method qualitative design with semi-structured staff interviews (n=85), content analysis of facility policies (n=110), and observation of two facilities. Data analysis revealed three key themes: visibility, bodily autonomy/respect, and safety. While nearly all workers expressed universal concern for LGBTQ rights, direct care workers and managers wavered when religious or moral exemptions arose in serving transgender residents. Workers invoked facility policies regarding gender and bodily care to justify differential treatment. This research provides new knowledge and guidance for workers, facilities, and policymakers and theoretical contributions to street-level bureaucracy. Part of a symposium sponsored by Rainbow Research Group Interest Group.

### SESSION 6040 (SYMPOSIUM)

### ARTS, NARRATIVE, AND CREATIVITY: ENHANCING CONNECTION, COMMUNICATION, AND HEALTH

Chair: Jacqueline Eaton

Older adults will comprise17% of the world's population by2050. Meeting the unique challenges of an aging society requires innovative solutions to enhance the aging experience, reduce the pressures placed on the current infrastructure, and inspire future generations to see that aging matters. Shifts in multi and transdisciplinary approaches to research in aging have contributed to an increasing surge in the development and evaluation of arts-based strategies to find innovative solutions to the challenges associated with aging. This symposium examines the use of creative expression as a tool to enhance older adult health and quality of life through five different approaches targeting1) workforce training,2) mental health self-support,3) perceptions of peer loss and death in long-term care and,4) social activity for persons with dementia. Cox and Maguire enhance undergraduate gerontology courses with guided autobiography, portfolios, and creative collaging processes. Eaton et al. report findings from a study testing the use of arts-based caregiving techniques by certified nursing assistants to improve resident care. Fortuna and Brooks describe the use of narrative and storytelling to share lived experiences of aging with a mental health condition while promoting age-related self-management skills development. Olson presents findings from her work using expressive arts to explore perceptions of death and peer loss by residents in long-term care. Finally, Mittelman and Harris share outcomes from the program, A Place For Us, which provides creative expression opportunities for persons with dementia.

### INCORPORATING GUIDED AUTOBIOGRAPHY, PORTFOLIOS, AND SOULCOLLAGE INTO UNDERGRADUATE GERONTOLOGY COURSES

Lisa Cox, and Gina Maguire, *Stockton University, Galloway, New Jersey, United States*

Although undergraduates don't eagerly choose gerontology classes first, a hefty number of students have enjoyed content they have explored as they were enrolled in "Aging and Spirituality" and "Therapeutic Arts with Older Adults" courses. The ten universal life themes that help people tell their stories, through the empirically validated methodology of Guided Autobiography (GAB) are incorporated in classes designed for an inter-generational group (older adults and students). Student assembled portfolios created from activities connected to 'the arts' and spiritual literacy have enhanced the acquisition of gerontological competencies. Also, through the use of SoulCollage® (developed in the late 1980s by Seena B. Frost, M. Div.) students of all ages, with and without artistic abilities, use a creative collaging process to get in touch with their ego integrity—the eighth stage of development conceptualized by Erik Erikson. Over three years, students have grown in their knowledge of aging and artistic processes.

### THE DEVELOPMENT AND TESTING OF KNACC: KNOWLEDGEABLE NURSING ASSISTANTS AS CREATIVE CAREGIVERS

Jacqueline Eaton,[1] Kristin Cloyes,[2] Brooke Paulsen,[1] Connie Madden,[1] and Lee Ellington,[1] *1. University of Utah, Salt Lake City, Utah, United States, 2. University of Utah, Portland, Oregon, United States*

Nursing assistants (NAs) provide 80% of direct care in long-term care settings, yet are seldom viewed as skilled professionals. Empowering NAs is linked to improved resident outcomes. In this study, we collaborate with NAs to adapt and test the feasibility and acceptability of arts-based creative caregiving techniques (CCG) for use in long-term care. We held a series of focus groups (n=14) to adapt, refine, and enhance usability. We then evaluated implementation in two waves of testing (n=8). Those working in memory care units were more likely to use all techniques, while those working in rehabilitation were more hesitant to implement. Participants reported using CCG to distract upset residents. Family members were excited about implementation, and NAs not participating wanted to learn CCG. Nursing assistants have the potential to become experts in creative caregiving but may require in-depth training to improve use.

### HEALING WITH THE ARTS: OLDER ADULT RECOVERY NARRATIVES

Karen Fortuna,[1] and Jessica Brooks,[2] *1. Dartmouth College, Concord, New Hampshire, United States, 2. Columbia University, New York, New York, United States*

Recovery narratives are an effective healing art medium that support individual development by virtue of the shared human experience transmitted through each story. Older adult recovery narratives are unique as they share their experiences of aging with a mental health conditions to support others with similar difficulties. Older adult recovery narratives offer encouragement of self-determination in late life, share lived experience of aging with a mental health condition, and promote age-related self-management skills development. The RecoverYdia smartphone app provides an online community for older adults with a lived experience of a mental health condition. RecoverYdia subscribers can search through hundreds of relevant videos and find the storyteller who tells the viewer's story, prompts them to reach out for help, and eventually inspires them to help others. This presentation will discuss the state of evidence regarding the evidence for recovery narratives across the globe and offer a RecoverYdia technology demonstration.

### HONORING CHOICE IN GRIEF THROUGH EXPRESSIVE ARTS WITH LONG-TERM CARE RESIDENTS

Michelle Olson, *Concordia University Chicago, Fishkill, New York, United States*

Death in long-term residential care homes is a common occurrence, yet it is often taboo and strongly avoided. Staff and residents often express deep connections to one another in these settings, but when death occurs, there is often little to no support, training or space to share these feelings. This session will discuss the findings of Dr. Olson's multicase, arts-based research from the elder voices of those who face these losses. Perceptions such as disenfranchised grief and ageism were revealed in this study as well as positive expressions such as love, kindness and acceptance. The shared findings will include poetry and artwork that was created within this research study. Utilizing the creative arts can assist in the expression of these complex and abstract human emotions, instill a sense of comfort and community and empower honor these lives and friendships.

### A PLACE FOR US: A UNIQUE EXPERIENCE THAT PROVIDES A CREATIVE AND SOCIAL EXPERIENCE FOR PEOPLE WITH DEMENTIA

Mary Mittelman,[1] and Amy Harris,[2] *1. NYU School of Medicine, New York, New York, United States, 2. NYU School of Medicine, NEW YORK, New York, United States*

A Place for Us meets weekly in community locations for a half day and is run by a recreation therapist who is also an artist, offering an opportunity to connect with others through participation in creative projects. We have found that collaborating in creating a piece of art, such as a collage provides a context for socializing. Since the program began in 2017, we provided this opportunity to 83 caregivers. The program has broad appeal and has included both men (54%) and women (46%) and people from many racial and ethnic backgrounds: 62.3% white, 13 percent African American, 7% Latino and 5% Asian. Our experience suggests that Interacting through collaborative projects utilizing what has been called, "The creative brain," offers people with dementia an opportunity to feel at ease in this social setting. A Place for Us allows people to interact socially while engaging in a pleasant and normative activity.

### SESSION 6045 (SYMPOSIUM)

### ASSISTED LIVING CARE FOR SPECIAL POPULATIONS

Chair: Philip Sloane
Discussant: Sheryl Zimmerman

Assisted living (AL) is a notable provider of residential long-term care for older adults; there are almost twice as many AL communities as nursing homes, and they provide care to more than 800,000 older adults. As AL has evolved, it has come to serve more individuals with cognitive, mental, and health care needs. For example, 70% of residents have sleep disturbances, 42% have moderate/severe dementia, and mortality rates average 14% annually. Care needs include those for behaviors such as agitation, serious mental illness, and at the end-of-life. However, not all AL communities provide similar care. This symposium will use national data and data from a seven state study of 250 AL communities to focus on four populations receiving care in AL: persons with dementia, serious mental illness, sleep disturbances, and on hospice. The first speaker will discuss how AL staff conceive of and respond to behavioral expressions of persons with dementia; the second will focus on the use of psychosocial/environmental practices for persons with dementia in AL. The third speaker will discuss the growing proportion of persons with serious mental illness in AL and related implications for care. The fourth presenter will address the high use of melatonin in AL, as well as resident- and community-level correlates of melatonin prescribing. The final speaker will examine hospice use in AL and how it varies based on community characteristics. These findings related to care and care needs for four key populations have important implications for practice, policy, and future research.

### HOW ASSISTED LIVING STAFF CONCEIVE OF DEMENTIA CARE

Debra Dobbs,[1] Sheryl Zimmerman,[2] Stephanie Miller,[3] Paula Carder,[4] Anna Beeber,[2] Jennifer Hodgkinson,[3] and Julia Thorp,[3] *1. University of South Florida, Tampa, Florida, United States, 2. University of North Carolina at Chapel Hill, Chapel Hill, North Carolina, United States, 3. Cecil G. Sheps Center for Health Services Research, Chapel Hill, North Carolina, United States, 4. Portland State University, Portland, Oregon, United States*

For those who provide care to the more than 40% of persons with dementia in assisted living (AL) communities, behavioral expressions (BEs) can be challenging. The objective of this mixed-methods study was to understand how AL staff conceive of BEs and what strategies they use to address them. Staff from 250 AL communities in seven states were asked to describe one successful and unsuccessful case of care. A conceptual model related to antecedents, behaviors, and consequences was developed and expanded to include staff strategies and outcomes of care; organizational characteristics associated with care practices were examined. Anxiety/restlessness, combativeness and resistance to care were the most prevalent BEs. Medical interventions (e.g., inpatient psychiatric assessment, medication management) were used in two-thirds of cases. Person-centered care was used more often in successful cases. Respondents in dementia-only communities identified antecedents to BEs more often than those in other communities.

### IMPLEMENTING PSYCHOSOCIAL AND ENVIRONMENTAL PRACTICES FOR PERSONS WITH DEMENTIA IN ASSISTED LIVING

Lindsay Schwartz,[1] Sheryl Zimmerman,[2] Christopher Wretman,[2] Lindsay Prizer,[3] and Philip Sloane,[2] *1. American Health Care Association/ National Center for Assisted Living, Pittsboro, North Carolina, United States, 2. University of North Carolina at Chapel Hill, Chapel Hill, North Carolina, United States, 3. Emory University School of Medicine, Atlanta, Georgia, United States*

Assisted living (AL) provides care for a large proportion of residents with dementia. Coincident with the increased focus on reducing off-label use of antipsychotics for people with dementia, providers are encouraged to turn to non-pharmacological practices to address behavioral expressions. This analysis used data from 250 AL communities in seven states, and examined familiarity, use, and practicality of twelve evidence-based practices, including music, pets, and social contact. Although a high percentage of staff reported familiarity and use of some of the practices, interviews with staff indicated that administration was not always consistent with evidence on implementation. Familiarity, use and practicality were associated with AL communities that had more residents with dementia, training on antipsychotics and non-pharmacological practices, policies on gradual dose reduction of psychotropics, and leadership that supported use of practices (p<.05 to <.001). Opportunities and barriers to implementation of these practices will be discussed.

### SERIOUS MENTAL ILLNESS AND DEMENTIA IN ASSISTED LIVING

Cassandra Hua,[1] Portia Cornell,[2] and Kali Thomas,[3] *1. Brown University School of Public Health, Providence, Rhode Island, United States, 2. Providence VA Medical Center, Providence, Rhode Island, United States, 3. Brown University, Providence, Rhode Island, United States*

Little is known about trends in the prevalence of serious mental illness (SMI) and Alzheimer's disease and Related Dementias (ADRD) in assisted living (AL). We summarize changes in the prevalence of SMI and ADRD in larger AL settings (25+ beds) from 2008-2017 using Medicare claims data. We compare these changes to nursing home (NH) and community rates of SMI and ADRD. We also examine state variability in SMI and ADRD in AL in 2017. The prevalence of SMI in AL increased 37%, from 7.8% in 2008 to 10.7% in 2017; ADRD prevalence increased 34%, from 27% to 36.4%. Over time, NHs exhibited the greatest increases in SMI (53%), followed by AL (37%) and the community cohorts (27%). Increases in ADRD were highest in AL. Rates of SMI in AL ranged from 3.5% in Wyoming to 28.7% in New York. We discuss implications for future research and policy.

### MELATONIN USE FOR SLEEP IN ASSISTED LIVING

Punita Peketi,[1] Sheryl Zimmerman,[2] Stephanie Miller,[3] Christopher Wretman,[2] John Preisser,[2] and Philip Sloane,[2] *1. Northeast Ohio Medical University, Twinsburg, Ohio, United States, 2. University of North Carolina at Chapel Hill, Chapel Hill, North Carolina, United States, 3. Cecil G. Sheps Center for Health Services Research, Chapel Hill, North Carolina, United States*

Sleep problems are common among residents of assisted living (AL) communities, and in other settings, melatonin is used to promote sleep. However, melatonin use in AL is unknown; because it may have side-effects, this knowledge gap is concerning. To address this question, data were collected across 250 AL communities in seven states; analyses used weights whereby data were scaled to represent the entirety of the states. The majority of communities prescribed melatonin (82%), albeit to a minority of residents (9%). Prescribing was more common for those with anxiety, sleep-wake disorders, dementia, and various behaviors, in communities that had more staff and more favorable non-pharmacological attitudes (p<.05). Dosages varied from 0-45 mg and co-prescribing was common. This study is the first to examine melatonin prescribing in AL; use may be appropriate if, for example, it is a replacement for hypnotics. The variation suggests practices may be modifiable; further research is needed.

### WHICH ASSISTED LIVING COMMUNITIES PROVIDE HOSPICE?

Lindsay Prizer,[1] Sheryl Zimmerman,[2] Christopher Wretman,[2] John Preisser,[2] Kali Thomas,[3] and Philip Sloane,[2] *1. Emory University School of Medicine, Atlanta, Georgia, United States, 2. University of North Carolina at Chapel Hill, Chapel Hill, North Carolina, United States, 3. Brown University, Providence, Rhode Island, United States*

Assisted living (AL) communities have become a common site for end-of-life and hospice care. However, AL is highly variable, meaning that hospice use is likely to be variable as well. This study explored the association between AL community characteristics and their residents' use of hospice. A stratified random sample of 250 AL communities in seven states was recruited. Community-level data were obtained from interviews with AL administrators, and resident-level case-mix data were abstracted from charts. Survey-weighted regressions examined the relationship between community characteristics and hospice use. Having residents on hospice was associated with being for-profit (86% vs. 51%), larger (48 vs. 31 beds), newer (16 vs. 37 years), having weekly primary care provider visits (44% vs. 26%), having more residents with dementia (50% vs. 35%) and fewer on Medicaid (4% vs. 11%), and having more lenient discharge policies. Data suggest there may be some disparity in hospice provision in AL.

### SESSION 6050 (SYMPOSIUM)

### BANE OR BOON? THE ROLE OF SPIRITUALITY, RELIGION, AND WELL-BEING IN LATER LIFE ACROSS DIVERSE OLDER POPULATIONS

Chair: Holly Nelson-Becker

Older adults tend to be religiously-affiliated to a greater extent than any other generational cohort (ARDA,2018; Koenig, King & Carson,2012; George et al.,2013;

Nelson-Becker,2018). However, their level of engagement varies across cultural and national contexts. Complex life course trajectories related to spirituality and religion mean that these domains often interface with both challenges and a search for well-being. Individuals may align with spiritual and/or religious values to a greater or lesser extent at different periods in their lives becoming more spiritual/religious, less spiritual/religious, or differently so. These papers address diverse perspectives on spirituality, religion, and well-being using samples primarily from the UK, Europe, the US, and Canada. The first paper by Christina Victor sets context by comparing the role of religion, and spirituality in well-being across three separate older adult data sets, touching on loneliness and dementia. Holly Nelson-Becker discusses results from an online international survey of older persons who walked the ancient Camino de Santiago pilgrimage regarding their motivations and learning from the experience. Michael Thomas considers the complex role of spirituality and sexuality for older LGB couples who may choose to remain in or leave their religious faith as they integrate expanding views. Keith Anderson explores perceptions of belief changes among religious and spiritual older adults across the life course. Together, these papers will address benefit and harm from formal religious practice and the advancing roles of spirituality as well as nonspirituality (the "nones") in global societies.

### OLDER ADULTS WALKING THE CAMINO DE SANTIAGO PILGRIMAGE: MOTIVATIONS, TRANSFORMATIONS, AND SPIRITUAL PERSPECTIVES

Holly Nelson-Becker,[1] Joe Pickard,[2] Gina Aitch,[3] and Alyssa Buettner,[3] *1. Brunel University London, Uxbridge, England, United Kingdom, 2. University of Missouri-St. Louis, St. Louis, Missouri, United States, 3. University of Missouri-St. Louis, st. louis, Missouri, United States*

This mixed-method study describes reasons that older people chose to complete the Camino de Santiago pilgrimage route in Spain and their assessment of how they were changed by the experience. The study is framed in Maslow's (1988) self-actualization and Tornstam's (2005) concept of gerotranscendence. We analyzed a subset of 121 participants age 65 and over who completed an online survey. Motivation included five themes: gratitude and transitions, cultural or historical curiosity. relationships, challenge and adventure, and spirituality. Transformations since their return involved greater strength, self-understanding, peace, desire to live lightly and to integrate their experience. Older individuals who walked the Camino have done so for a variety of reasons. Spiritual reasons may be more difficult to disclose. Half responded in the open-ended question, but a later spirituality question added many more respondents. Older people envision many forms of benefit to walking the pilgrimage and find growth in the experience.

### RELIGION, SPIRITUALITY, AND WELL-BEING: AN UNDEREXPLORED ASPECT OF GROWING OLDER IN THE UNITED KINGDOM?

Christina Victor,[1] Ruth Lamont,[2] Isla Rippon,[1] and Linda Clare,[2] *1. Brunel University London, Uxbridge, England, United Kingdom, 2. University of Exeter, Exeter, England, United Kingdom*

There is a rich literature from The United States looking at the importance of religion and spirituality in the lives of older adults where it is positively linked with wellbeing. Despite the increased interest in wellbeing in the UK comparatively little interest has been show in the role of religion and spirituality in promoting wellbeing including quality of life, life satisfaction and loneliness. In this paper we explore these issues using three data sets: the European Social Survey (ESS), the English Longitudinal Study of Ageing (ELSA) and the IDEAL cohort of people with dementia and their carers to examine (a) the variation in religious practice by older adults, those aged 50+, across Europe; (b) the epidemiology of religious practice among older adults within England and (c) using both ELSA and IDEAL consider the relationship between religion and wellbeing in later life.

### SPIRITUALITY AND SEXUALITY: NOT NECESSARILY A BINARY CHOICE FOR LGBTQ+ PEOPLE

Michael Thomas, *Brunel University London, Uxbridge, England, United Kingdom*

This paper reports on a qualitative study on the impact of marriage and civil partnerships for lesbian, gay and bisexual (LGB) couples. Drawing on data from 50 dyad interviews in the UK, US and Canada, the paper investigates the ways in which couples make sense of spirituality in the context of a stigmatised sexuality. For some, the task of arranging a wedding or civil partnership ceremony provided a powerful reminder of their exclusion from mainstream religious denominations. This sense of stigma (Goffman, 1963) was also present in later life, when the lack of social esteem granted to same-sex relationships gave rise to a sense of disenfranchised grief (Doka, 1989). Whereas some participants tended to frame sexuality and spirituality as a kind of binary choice, others resisted this marginalisation from religious and spiritual activities, even if this meant finding a personal sense of spirituality beyond the confines of organised religion.

### SPIRITUAL AND RELIGIOUS JOURNEYS OVER THE LIFECOURSE: CHALLENGING STEREOTYPES

Keith Anderson,[1] Cynthia Garthwait,[2] and Laurel Vielle,[2] *1. University of Texas at Arlington, Arlington, Texas, United States, 2. University of Montana, Missoula, Montana, United States*

Spiritual and religious beliefs often evolve across the lifecourse and tend to be influenced by experience, time, and maturity. Despite this evolution, stereotypes persist of older adults as being inflexible in their views and resistant to new or alternative beliefs. To explore this notion, researchers surveyed a convenience sample of 152 older adults (n = 152) who identified as spiritual/religious. Over three-quarters (77.0%) reported that their relationship with God or a higher power had grown closer over time. Most reported becoming "more liberal" (32.2%), "more conservative" (23.0%), or "more moderate" (17.8%) in religious perspective over time. Almost three-quarters of participants (73.7%) reported that they were "more accepting" of other religions", yet also "more certain" of their own beliefs (74.3%) in later life. These and other results challenge the stereotype of older adults as “set in their ways”, even in terms of spiritual and religious beliefs.

### SESSION 6055 (SYMPOSIUM)

### BRIDGING THE GAP IN QUALITY AMONG HIGH MEDICAID NURSING HOMES: THE ROLE OF MANAGEMENT AND COMMUNITY FACTORS

Chair: Robert Weech-Maldonado

Nursing home quality has been a matter of long-standing policy interest at the federal and state level, as it concerns the health and well-being of one of our most vulnerable populations. Mor et al. (2004) described the nursing home industry as a ‘two-tiered’ system, with the lower-tier nursing homes operating in a resource-constrained environment given their high proportion of Medicaid residents (85% or higher). Medicaid is the largest payer of nursing homes but its reimbursement rates typically lag Medicare as well as private pay. Lower tier facilities are characterized by lower professional staffing and occupancy rates, and worse quality. Such facilities have a higher proportion of minority residents and are generally located in communities with significant proportions of poor and minority residents, exacerbating the existing disparities in nursing home care. However, there are performance variations among high Medicaid nursing homes, with some facilities performing significantly better than others on both quality and financial performance. What may explain the superior performance of certain nursing homes that are operating in a similarly resource-constrained environment? Factors, such as management resources and environmental resource availability, may be the critically important differentiators. The purpose of this symposium is to examine the organizational/management and community factors that may be associated with high-performance among a similar group of resource-constrained nursing homes. Using survey, secondary, and qualitative data analysis, this symposium will explore the role that culture change, leadership style, human resource management practices, knowledge management, and community factors can have on nursing home performance.

### CULTURE CHANGE AND QUALITY STAR RATINGS IN HIGH MEDICAID NURSING HOMES: DOES TIME OF ADOPTION MAKE A DIFFERENCE?

Latarsha Chisholm,[1] Akbar Ghiasi,[2] Justin Lord,[3] and Robert Weech-Maldonado,[4] *1. University of Central Florida, Orlando, Florida, United States, 2. University of Incarnate Word, San Antonio, Texas, United States, 3. LSU-Shreveport, Shreveport, Louisiana, United States, 4. UAB, Birmingham, Alabama, United States*

Racial/ethnic disparities have been well documented in long-term care literature. Culture change is a movement to transition nursing homes to more home-like environments to improve the quality of care for all residents. The purpose of this study was to examine how the involvement of culture change initiatives among high Medicaid facilities was associated with nursing home quality. The study relied on both survey and secondary nursing home data for the years 2017-2018. The sample included high Medicaid facilities. The final model consisted of an ordinal logistic regression. High-Medicaid nursing homes with six or more years in culture change initiatives had higher odds of having a higher star rating, while facilities with one year or less had significantly lower odds of having a higher star rating. Culture change initiatives may require some time to effectively implement, but these initiatives are potential mechanisms to improve quality in high Medicaid nursing homes.

### THE IMPACT OF LEADERSHIP STYLES ON QUALITY AND FINANCIAL PERFORMANCE IN HIGH MEDICAID NURSING HOMES

Justin Lord,[1] Akbar Ghiasi,[2] Ganisher Davlyatov,[3] and Robert Weech-Maldonado,[4] *1. LSU-Shreveport, Shreveport, Louisiana, United States, 2. University of Incarnate Word, San Antonio, Texas, United States, 3. University of Alabama at Birmingham, Birmingham, Alabama, United States, 4. UAB, Birmingham, Alabama, United States*

This study examined the association between leadership styles (autocrat, consultative autocrat, consensus manager, and shareholder manager) and resident quality and financial performance in under-resourced nursing homes. Survey data from 391 Directors of Nursing were merged with secondary data from LTCFocus, Area Health Resource File, Medicare Cost Reports, and Nursing Home Compare. Two multivariate regressions were used to model the relationship between leadership styles and the dependent variables: nursing home star ratings (1-5) and operating margin. The independent variables were composite scores for leadership styles, while control variables included organizational and county-level factors. Results show that compared to autocratic leadership, the consultative autocrat (solicits feedback but has total authority) was associated with lower quality ($p < 0.05$), while the consensus manager (delegates authority to the group) was associated with lower profit margin ($p < 0.05$). Under-resourced facilities need to recognize tradeoffs of different decision making styles for performance.

### IS IT WHAT YOU HAVE OR WHAT YOU DO WITH IT? STAFFING, HUMAN RESOURCE MANAGEMENT, AND KNOWLEDGE MANAGEMENT PRACTICES

Larry Hearld,[1] Akbar Ghiasi,[2] Ganisher Davlyatov,[1] and Robert Weech-Maldonado,[3] *1. University of Alabama at Birmingham, Birmingham, Alabama, United States, 2. University of Incarnate Word, San Antonio, Texas, United States, 3. UAB, Birmingham, Alabama, United States*

Research in the nursing home industry shows more robust knowledge management activities are associated with the adoption of patient-centered, culture change initiatives among high Medicaid nursing homes. These findings are notable because they highlight the important role that knowledge management activities may play for improving quality of care in under-resourced nursing homes. They also raise important questions about the conditions that may support or hinder the use of these activities. Using survey responses from 393 nursing home administrators, we empirically examined whether two components of human resources – staffing levels and HRM practices – are associated with the level of knowledge management activities in high Medicaid census nursing homes. More robust HRM practices were associated with greater levels of knowledge management activities, as well as three separate domains of knowledge management

(acquisition, dissemination, and responsiveness). Staffing levels, in contrast, were not significantly associated with the level of knowledge management activities.

### SESSION 6060 (SYMPOSIUM)

### POLICY SERIES: BUILDING MOMENTUM FOR A NEW FUTURE IN POLITICS AND AGING: EXAMINING ECONOMICS, VALUES, LANGUAGE, AND CARE

Chair: Michael Lepore
Discussant: Jean Accius

Coinciding with the 2020 presidential election, the 75th anniversary of the Gerontological Society of America arrives amid the contentious creation of a new future for politics and aging. Increasing inequality, spreading disinformation, and mounting despotism are escalating threats to constitutional democracy, but at the same time other social changes are promoting the development of a more thoroughly caring, intergenerationally just, and robustly democratic society. At the crux of this societal transformation, relentless political inertia on core aging issues, like the role of government in the care and support of older adults, continues to inhibit meaningful change in federal policy, dampening the potential for older Americans to achieve desired future states, like living well despite advanced age or disability. This session examines major contemporary trends at the intersection of politics and aging in the United States. Papers address the economics and demographics of aging, drawing attention to increasing federal spending on older adults, decreasing availability of caregivers, and geographic clustering of older people; changes in the age of the electorate, intergenerational political values, and the growing politically polarization of American society; the tendency for federal initiatives to fail to support caregivers, for reasons of policy history, policy traits, and mass public features, like the political isolation of informal caregivers; and the role of linguistic and metaphorical practices in shaping our experiences and views of aging. Discussion addresses opportunities for the country to become more age-friendly while also sustaining democratic institutions and national unity.

### LEARNING LESSONS FROM LONG-TERM CARE POLICY FINANCING EFFORTS IN THE UNITED STATES

Michael Lepore, *LiveWell Alliance, Southington, Connecticut, United States*

A decades-long policy impasse has crippled our national capacity to finance long-term care (LTC) sufficiently or equitably, leaving large swaths of the US population at risk of going broke paying privately for LTC or having unmet LTC needs, while also draining state and federal budgets. By reviewing past LTC financing policy efforts—from the passage of the Social Security Act and the enactment of Medicaid and Medicare, to the LTC financing proposals advanced by 2020 presidential candidates—the political interplay of budgetary concerns in government spending and social justice concerns regarding access to care emerged as a primary LTC policymaking issue. Establishing national consensus on the prioritization of these fiscal and social justice concerns, and their respective values, could help lawmakers craft policy capable of generating the political will needed to overcome political gridlock. Clarifying how LTC benefits would be paid for appears to be a relatively straightforward technical task in comparison.

### THREE TRENDS SHAPING THE POLITICS OF AGING IN AMERICA

Nora Super, *Milken Institute, Washington, District of Columbia, United States*

The demographic bulge created by the baby boom generation has shaped American politics since they came of age in the 1960s. Over the next decade, aging issues will become more relevant as the oldest boomers reach 84 and the youngest boomers will be eligible for Medicare. This paper highlights three converging trends that will shape United States politics; including increased spending on “entitlement” programs like Social Security and Medicare, growing mismatch in caregiving need and supply, and the heightened concentration of older adults in certain geographic areas. The next decade will see not only extraordinary demographic change but also unprecedented advances in technology and medicine, and cultural and societal shifts that were once unimaginable.

### THE MEANING OF AGE OR GENERATIONAL DIFFERENCES IN U.S. POLITICAL VALUES AND PRIORITIES

Judith Gonyea, and Robert Hudson, *Boston University, Boston, Massachusetts, United States*

Nations globally are facing the fiscal consequences of being aging societies, including the redistribution of wealth resources across sectors that influence generational relations (i.e., healthcare, education, public pensions). Political differences or clashes between youth and older adults is not a new phenomenon. However, questions are being raised about whether current political systems, governing structures, and social trends are eroding generational solidarity which traditionally has a role in promoting equity and protecting vulnerable individuals from rapid social change. Reflecting on the 2020 national election results and political opinion surveys, we explore the meaning of age or generational differences in political attitudes in an increasingly partisan society. We suggest that the use of a generational location or habitus lens, which focuses on the distinct sociohistorical realities (i.e., different reference points, systems of aspiration, sets of anxieties) that shape age groups and their interrelations, may offer insights into current political debates and divides.

### POLITICAL IMPEDIMENTS TO AGING IN PLACE: THE EXAMPLE OF INFORMAL CAREGIVING POLICY

Jacqueline Chattopadhyay, *UNC Charlotte, Charlotte, North Carolina, United States*

Most Americans prefer to “age-in-place” as long as possible, but to do so often need overlapping resources—one of which is help from “formal” or “informal” caregivers (family and friends). Family and friends often want to provide care for as long as safely possible. However, informal caregiving can pose financial and physical risks to the caregiver that—as many scholars have noted—public policy in the U.S. does relatively little to mitigate. This policy shortfall also hurts care recipients since the risks that informal caregivers face can prematurely curtail their ability to provide care. Why does policy in the U.S. not better support informal caregivers? By synthesizing family caregiving research and political science research that has addressed long-term care, this paper surveys nine factors in the political system that may help answer this question. Four emanate from policy history. Three concern the mass public. Two vary at the policy level.

### REFRAMING AGING IN CONTEMPORARY POLITICS: BUILDING MOMENTUM

Patricia D'Antonio, *The Gerontological Society of America, Washington, District of Columbia, United States*

Changing American culture is challenging and changing attitudes and behaviors around the universal experience of aging especially so. Unless the field of advocates who care about aging issues cultivates a more visible, more informed conversation on older people, it will remain difficult to advance the systemic changes needed to adjust to a society with increased and increasing longevity. Advocates will need to be vigilant to avoid cueing negative attitudes towards aging and aging policies. The Reframing Aging Initiative is a long-term, social change endeavor designed to improve the public's understanding of what aging means and the many contributions older people bring to society. Using evidence-based research, the initiative seeks to teach advocates how to tell an effective story about aging that will promote positive perceptions of aging and reduce ageism. The time to change the conversation is now.

### SESSION 6065 (SYMPOSIUM)

### CAREGIVING IN THE U.S. 2020: WHAT DOES THE LATEST EDITION OF THIS SURVEY TELL US ABOUT THEIR CONTRIBUTIONS AND NEEDS?

Chair: Gabriela Prudencio
Discussant: Heather Young

Family and friends comprise the most basic unit of any society. For individuals who take on the responsibility of caring for another person through sickness or disability, it can often be challenging to see beyond the individual experience. Yet in the aggregate, family caregivers—whether they be families of kin or families of choice—are woven into the fabric of America's health, social, economic, and long-term services and supports (LTSS) systems. As the country continues to age, the need to support caregivers as the cornerstone of society will only become more important. A national profile of family caregivers first emerged from the 1997 Caregiving in the U.S. study. Related studies were conducted in 2004, 2009, and 2015 by the NAC in collaboration with AARP. Caregiving in the U.S. 2020 presents a portrait of unpaid family caregivers today. A nationally representative survey (n=1,499), it replicates the methodology used in 2015. Therefore, during this symposium, AARP and NAC will present trend data from 2015 in comparison to 2020, and explore key subgroup differences. The presentation will cover prevalence, demographic characteristics, intensity and duration of care, the well-being of caregivers, the financial impact of family caregiving on caregivers themselves, and the degree to which technology supports caregivers today.

### THE 2020 PORTRAIT OF AMERICAN CAREGIVERS

Regina Shih, *RAND Corporation, Arlington, Virginia, United States*

The prevalence of caregiving for an adult or child with special needs has increased significantly in the past five years (from 18.2% to over 21.3%), driven by an increase in the prevalence of caring for a family member or friend aged 50 and older. At the same time, care recipients have greater health and functional needs that necessitate care from others in comparison to 2015. These new 2020 data from the Caregiving in the US Survey by the National Alliance for Caregiving suggests that not only are more American adults taking on the role of caregiver, but they are doing so for increasingly complex care situations. This paper addresses the prevalence of caregiving including the demographics of family caregivers, relationship between the caregiver and the care recipient, health conditions of the care recipient, and living situations of care recipients and their caregivers.

### THE UNIQUE CHALLENGES AND CONTRIBUTIONS OF DIVERSE CAREGIVERS

Rita Choula, *AARP, Washington, District of Columbia, United States*

Caregiving in the U.S. 2020 oversampled African Americans, Hispanics, Asians, and people over the age of 75. Six in ten caregivers report being non-Hispanic white, 17% are Hispanic, 14% non-Hispanic African-American or black, 5% Asian/Pacific Islander, and 3% some other race or ethnicity, including multiracial. The session will emphasize the unique context of diverse caregivers, including African American, Hispanic, Asian, and LGBT+ caregivers. The session will begin by discussing the portrait of the typical caregiver of each of these groups. It will follow with a discussion of the challenges facing diverse caregivers in the aggregate and the opportunities to recognize and support them across settings.

### CAREGIVER HEALTH AND WELL-BEING, AND FINANCIAL STRAIN

Susan Reinhard,[1] and Lynn Feinberg,[2] *1. AARP, Washington, District of Columbia, United States, 2. AARP Public Policy Institute, Washington, District of Columbia, United States*

Family caregivers often face key challenges when caring for a relative or close friend with health or functional needs. This paper presents findings from Caregiving in the U.S. 2020 on the impact of caregiving on the physical health and well-being of family caregivers and the financial impacts of family care. The data suggest that the caregiver's own health has declined, with 1 in 5 (21%) saying they are in fair to poor health themselves, up from 17 percent in 2015. Nearly 1 in 4 (23%) feel caregiving has made their health worse. Nearly 4 in 10 (38%) family caregivers of adults experience a moderate to a high degree of financial strain from providing care. Forty-five percent have experienced at least one financial impact (e.g., stopped savings, debt, ability to pay bills, and ability to afford necessary expenses, like food). These findings reveal that because family caregiving today is more complex, costly, and stressful than in the past, action is needed to recognize and support family caregivers in the U.S adequately.

### CAREGIVER NEEDS: NAVIGATING SERVICES, TECHNOLOGY, AND INFORMATION

C. Grace Whiting, *National Alliance for Caregiving, Washington, District of Columbia, United States*

Data suggests that caregivers may be taking on this role without adequate and affordable services and supports in place. Few caregivers report access to paid help and find it difficult to navigate a healthcare system that is complex and that changes continuously. Caregivers find it challenging to coordinate their recipients' care across various providers, and the lack of affordable services make it difficult to be a caregiver today. While many caregivers rely on health care professionals (such as doctors, nurses, or social workers) as a source of information about providing care, few caregivers report having conversations with them about what they need to care for their recipient or to support their well-being. In this section, the presenter will discuss the needs of caregivers (respite, transportation, information, and training), focusing on policy and programmatic solutions geared to improving the well-being of caregivers and those under their care.

### SESSION 6070 (SYMPOSIUM)

### CHALLENGES FOR PREVENTION OF ELDER ABUSE IN JAPAN: VIEWPOINTS FROM LEGAL AND PRACTICE SETTINGS

Chair: Cynthia Thomas
Discussant: Tsuann Kuo

This symposium begins by fist comparing features of the Japanese elder abuse prevention law to 3 other abuse prevention laws - child abuse, domestic abuse and abuse against people with disabilities. It highlights amendments that have been made to the child abuse and domestic abuse prevention laws since their enactment based on implementation experience, drawing contrast to the lack of amendments made to the elder abuse prevention law despite its three-year requirement for amendment review. Overall strengths and weaknesses of the elder abuse law are discussed including needed areas of revision. Second, a case study of nearly two decades of elder abuse prevention activities in Matsudo-city are presented and the subsequent enactment in April 2020 of a new regulation of the city is discussed. In addition, interim outcomes of a related research study in Matsudo-city on elder abuse involving non-caregivers for elderly people that will expand understanding of abuse prevention efforts are presented. Third, how can we abolish elder abuse in institutional settings will be discussed by analyzing elder abuse case studies. Finally, the most updated research outcomes conducted in in-home care service settings in 2020 will be presented. Its preliminary analyses revealed out of 1,417 responses, 2.9% were found to be ”body restraints.” Such abusive actions are less likely to be detected by others. The symposium concludes by assessing challenges for preventing elder abuse in policy and practice.

### CHALLENGES FOR ELDER ABUSE PREVENTION LAW

Noriko Tsukada,[1] and Asako Katsumata,[2], *1. Nihon University, Tokyo, Tokyo, Japan, 2. Rehabilitation Institution, Chiba, Tokyo, Japan*

This paper compares components of four abuse prevention laws in Japan, including elder abuse, child abuse and domestic abuse, and abuse for people with disabilities and delineates major strengths and weaknesses of the Elder Abuse Prevention Law in comparison to the other three. Based on this analysis, this paper recommends improvements in the elder abuse prevention law given the success of the related abuse laws. Despite the requirement of re-evaluation every 3 years, no amendments have been made to the elder abuse prevention law, while amendments have been made to the child abuse and domestic abuse prevention laws, based on outcome data and implementation experience. Identified needed revisions include provisions of protection orders and temporary shelters to protect elder victims from abusers at the time abuse is reported.

### CHALLENGES FOR PRIVATE LTC SERVICE PROVIDERS FOR PREVENTING ELDER ABUSE

Naoki Ikeda,[1] and Noriko Tsukada,[2] *1. Kamihonnmachi Law Office, Tennouji-Ku, Osaka, Japan, 2. Nihon University, Tokyo, Tokyo, Japan*

his paper aims to identify factors that differentiate long-term care (LTC) service providers into the two categories: those who are successfully growing and those who are going out of business. During the past 5 years, about one out of 100 private LTC service providers has gone bankrupt in part due to issues of client abuse. This paper uses case studies to demonstrate differences between LTC service providers who have histories of elder abuse and those that do not. Business traits such as mission, client trust, and quality of LTC workers is considered along with implementation of abuse prevention practices including management approaches, oversight of workers, and atmosphere where LTC workers have their working conditions and concerns addressed, which in turn enhances workers satisfactions, accordingly, yielding better quality of care provisions.

### EFFORTS FOR CONSTRUCTION OF MORE EFFECTIVE PREVENTION AND MORE INCLUSIVE PROTECTION SYSTEMS IN MATSUDO CITY

Tadashi Wada,[1] Hitoshi suda,[2] and Kana Sato,[3] *1. Irahara Primary Care Hospital, Matsudo City, Chiba, Japan, 2. Seitoku University, Matsudo, Chiba, Japan, 3. Teikyo University of Science, Adachi, Tokyo, Japan*

Matsudo City is preparing to implement an ordinance so that 3 protective services for children, older persons, and people with disabilities can work together for efficient prevention. Supporting pregnant women is sometimes effective for prevention of future child abuse. Supporting people with disabilities is often prevention of future neglect caused by them. Supporting perpetrators and victims of domestic violence is effective to prevent future elder abuse by interruption of abusive behaviors. We expect to prevent abuses by affiliation of various protective and supportive services. According to the Elder Abuse Prevention Law, elder abuse is defined as those inflicted by caregivers. Therefore, there is a big problem that abuses by non-caregivers are excluded from the coverage of the law. We are currently investigating abuses by non-caregivers to clarify the proportion of excluded cases among those reported to Elder Protective Services. I would like to talk about preliminary result of our investigation.

### PHYSICAL RESTRAINT OF OLDER ADULTS AT HOME: PRELIMINARY ANALYSIS OF A PROSPECTIVE COHORT STUDY

Asa Inagaki, Ayumi Igarashi,
Maiko Noguchi-Watanabe, Mariko Sakka,
Chie Fukui, Taisuke Yasaka, Masumi Shinohara, and
Noriko Yamamoto-Mitani, *The University of Tokyo, Tokyo, Japan*

Our study aimed to explore the prevalence and factors of physical restraints among frail to dependent older adults living at home. We conducted an online survey to ask about the physical/mental conditions, demographics, service utilization, and physical restraints of community-dwelling older adults. Either home care nurse or care managers who were responsible for the older adult answered the survey that were conducted at baseline and one month later. We obtained data from 1,278 individuals. Physical restraint was reported for 53 (4.1%) participants. Multiple logistic regression revealed the factors associated with physical restraints at home: having been restrained at baseline, having pneumonia or heart failure, receiving home bathing, or using rental assistive devices were associated with physical restraints at one month. The findings could be used to promote discussion about which services prevent physical restraints and what we should do to support clients and their family to stay at home safely.

### GETTING INVOLVED WITH POLICY AS A GRADUATE STUDENT

Haley Gallo, *University of Southern California, Los Angeles, California, United States*

The third speaker is University of Southern California PhD candidate Haley Gallo. Haley will discuss strategies for connecting research to policy as a graduate student, as well as aging-policy internship opportunities for graduate students, including the GSA's Greg O'Neill Policy Internship. Haley's research focuses on policies that promote the goals of the Age-Friendly Cities and Communities initiative. She is passionate about including older adults—particularly those from groups who are traditionally left out—in the development of research and policy that affects people of all ages.

### SESSION 6075 (SYMPOSIUM)

### POLICY SERIES: ESPO/ SOCIAL RESEARCH, POLICY, AND PRACTICE SECTION SYMPOSIUM: CONNECTING AGING RESEARCH TO POLICY: INSIGHTS AND STRATEGIES FOR EARLY-CAREER RESEARCHERS

Chair: Claire Pendergrast
Co-Chair: Jennifer May

A wide range of policy issues, from healthcare to transportation to social insurance, influence health and wellbeing for older adults. Gerontologists have the opportunity to get involved with policy at any scale, from the organizational or community level to local, state, or federal policy. This symposium brings together a diverse panel of emerging and established academics to discuss strategies for early career researchers to understand and participate in aging policy activities. Panelists will discuss opportunities for researchers to contribute their expertise to policy discussions, and will share their own experiences and perspectives on participating in the policy process. Specific topics covered will include aging policy internship opportunities for graduate students, academic involvement with advocacy efforts to promote healthcare access to older adults, strategies for designing and conducting impactful and policy research, approaches to collaboration with diverse stakeholders to connect research to policy, and strategies for communicating policy-relevant research findings to general public and policy audiences. This symposium will reflect the SRPP's strong commitment to connecting research to policy and practice, and will provide early career scholars with strategies to connect their own research to policy in order to inform decision-making and improve health and quality of life for older adults.

### ADVOCACY AS AN ACADEMIC AND NURSE PRACTITIONER

Lori Martin-Plank, *University of Arizona, Pipersville, Pennsylvania, United States*

The first speaker is Dr. Lori Martin-Plank, an established academic at the University of Arizona, College of Nursing. Dr. Martin-Plank will provide her experiences in advocating for older adults in Pennsylvania and nationally through professional organizations, meeting with coalition partners to promote access to care for vulnerable older adults in rural areas by promoting full practice authority for nurse practitioners, and advocating for full home health authority for nurse practitioners. Dr. Martin-Plank will share how she is active in advocacy and policy at the local, state and federal levels, and how to build a presence and relationship with legislators on The Hill and State Capitol. Dr. Martin-Plank is a family, gerontological, and mental health nurse practitioner, practicing in Pennsylvania, New Jersey, and Arizona.

### POLICY-RELEVANT RESEARCH AND MEDIA COMMUNICATION

Pamela Herd, *Georgetown University, Washington, District of Columbia, United States*

The second speaker is Dr. Pamela Herd, Professor of Public Policy at Georgetown University. Dr. Herd will discuss her approach to conducting innovative and impactful policy-relevant research, as well as her experience communicating research to policymakers and the public through op-eds and other forms of media. Dr. Herd's research focuses on inequality and how it intersects with health, aging, and policy. She also has expertise in survey methods and administration. Her most recent book, Administrative Burden, was reviewed in the New York Review of Books. She has also published editorials in venues such as the New York Times and the Washington Post, as well as podcasts, including the Weeds, produced by Vox media.

### INVOLVEMENT IN STATE AND FEDERAL AGING POLICY

Erica Solway, *University of Michigan, Ann Arbor, Michigan, United States*

The fourth speaker is Dr. Erica Solway. Dr. Solway will discuss her experience working in policy at the federal level as a Health and Aging Policy Fellow and a policy advisor with the U.S. Senate Committee on Health, Education, Labor, and Pensions Subcommittee on Primary Health and Aging and in her current involvement in policy-relevant research at the state and national level at the University of Michigan Institute for Healthcare Policy & Innovation. Dr. Solway is a senior project manager forthe evaluation of the Healthy Michigan Plan, Michigan's Medicaid expansion program, and is also an associate director of the University of Michigan National Poll on Healthy Aging.

### PARTNERSHIPS TO CONNECT AGING RESEARCH TO POLICY

Kali Thomas, *Brown University, Providence, Rhode Island, United States*

The fifth speaker is Dr. Kali Thomas. Dr. Thomas will discuss her experience working in partnership with government and non-profit organizational stakeholders to conduct aging research that informs policy and practice. Dr. Thomas is an Associate Professor at the Brown School of Public Health and research health science specialist at the Providence VA Medical Center, where her research focuses on identifying ways to improve the quality of life of older adults needing long-term services and supports (LTSS) through applied health services research.

### SESSION 6080 (SYMPOSIUM)

### DEVELOPING AGE-FRIENDLY COMMUNITIES: COMPARATIVE RESEARCH ON THE ROLE OF SOCIAL INFRASTRUCTURE AND INCLUSION

Chair: Chris Phillipson
Co-Chair: Jarmin Yeh
Discussant: Chris Phillipson

Aging experiences are often framed by the forms in which communities take shape. In turn, the material forms of communities provide the conditions in which aging experiences and processes are produced. These multidirectional pathways of influence between micro-macro contexts comprise an assemblage of networks that usually go unnoticed, except on breakdown. Applying Leigh-Star's (1999) charge to study these networks as infrastructure, we examine their "invisible work" in the ecological analysis of aging in community. This symposium presents research using community-embedded methods to illuminate how paradoxical forms of transparent and opaque infrastructure facilitate and mediate interactions between people, things, and the environmental shapes that hold these interactions. Yarker's comparative case analysis of two Manchester neighborhoods highlight the critical importance of social infrastructure in supporting the diversity of relations needed to develop age-friendly communities and new forms of community action amongst older people. Grove's qualitative and geo-spatial approaches demonstrate how planned and unplanned social interactions, and variety of social infrastructural forms, contribute positively and negatively to ageing well for older people in Dublin. Simon-Rusinowitz's three-part intervention addresses unmet needs of older Baltimore residents in a low-income community; finding, in part-one, that housing is a platform for providing health and social services needed to age successfully. Yeh compares the conceptual frames undergirding age-friendly community initiatives with expert knowledges and lay perspectives of older San Franciscans, revealing tension between the rational dreams and material realities of aging in the community. These presentations contribute to gerontological discourse, providing new insights for policy and practice considerations.

### DEVELOPING AGE-FRIENDLY PROGRAMS IN GREATER MANCHESTER, UK: UNDERSTANDING THE ROLE OF SOCIAL INFRASTRUCTURE

Sophie Yarker,[1] and Chris Phillipson,[2] *1. University of Manchester, University of Manchester, United Kingdom, 2. Manchester Institute for Collaborative Research into Ageing, Manchester, United Kingdom*

This paper considers the critical role of social infrastructure in building age-friendly communities. Drawing on two neighbourhood projects, the paper explores the benefits which different types of social connections bring for older people and the types of spaces in which these connections are produced. It provides support for the importance of 'natural neighbourhood networks'(Gardner, 2011) by demonstrating how everyday encounters help promote informal networks of support. Following Klinenberg's (2018) analysis of the importance of social infrastructure, the paper argues that the decline of local high streets, closure of libraries, and cuts to the maintenance of green spaces, reduce opportunities for face-to-face social interactions. The paper presents findings from two studies illustrating the importance of social infrastructure in supporting new forms of community action amongst older people. The paper concludes that that the value of social interactions that occur in everyday mundane spaces needs greater emphasis in public policy.

### EXPLORING THE INFLUENCE OF SOCIAL INFRASTRUCTURE ON EXPERIENCES OF AGING IN PLACE IN DUBLIN, IRELAND

Hannah Grove, *Maynooth University, Maynooth, Kildare, Ireland*

This paper presents results from a PhD project that has carried out interviews, mapping exercises and 'go-along' interviews with thirty-four people aged sixty-six and over, in an inner city and suburban study area within Dublin, Ireland. A key objective of this research was to identify places, activities and interactions of most importance to older people. Through qualitative and spatial data collection and analysis techniques, different types of 'social infrastructure' that individuals value and engage with, as part of their daily lives, are identified and presented using annotated maps. The interactions and types of activities that occur in these places, and why they are important, are also discussed. Results demonstrate the benefits of high quality social infrastructure (and the challenges associated with a lack of social infrastructure) through participant's own experiences, and highlight the vital role this plays in supporting older people to age well in place.

### SHINING LIGHT AND CREATING SOLUTIONS: HEALTH AND SOCIAL NEEDS OF OLDER AFFORDABLE HOUSING RESIDENTS

Lori Simon-Rusinowitz,[1] C. Daniel Mullins,[2] Karen Morales,[3] Rodney Elliott,[3] and Constance Raab,[4] *1. School of Public Health, University of Maryland, College Park, Maryland, United States, 2. Pharmaceutical Health Services Research (PHSR) Department, Baltimore, Maryland, United States, 3. The PATIENTS Program, University of Maryland School of Pharmacy, Baltimore, Maryland, United States, 4. Center on Aging, College Park, Maryland, United States*

Aging within a community requires access to health and social services. This project lays the groundwork for an innovative, three-part health and social services intervention intended to improve the health and well-being of older affordable housing residents in a low-income, vulnerable Baltimore neighborhood. We will report on the first part, an assessment of residents' unmet health and social service needs and their ideas for meeting these needs. With guidance from a community advisory group of older residents (a key program component), we are conducting structured interviews with 50 elders to identify residents' needs and interests. These findings will inform the next project segments: Part 2- exploring how the Village model (in which neighbors identify and offer needed services to help their neighbors age within a community) can be adapted for an affordable housing setting, and Part 3- adapting an evidence-based housing-plus-services model to meet older residents' unmet needs.

### DO AGE-FRIENDLY COMMUNITY INITIATIVES FORM A BIOPOLITICAL PARADIGM?

Jarmin Yeh,[1] Pat Fox,[1] David Vlahov,[2] and Howard Pinderhughes,[1] *1. University of California, San Francisco, San Francisco, California, United States, 2. Yale University, Orange, Connecticut, United States*

Biopolitical paradigms are frameworks that specify how concerns about health and the body are made the simultaneous focus of biomedicine and state policy (Foucault 1984; Rose 2007). As aging and urbanization trends converge, developing "age-friendly community initiatives" (AFCIs) has become a global movement and important policy area. To prompt critical questioning, situational analysis was used as a theory-methods package to compare AFCI conceptual frames with perspectives of thirteen AFCI experts and seventeen older San Franciscans. Preliminary analysis suggests AFCIs form a biopolitical paradigm because they not only seek to rework boundaries between bodies and environments, they operate as modes of individual and population governance for the sake of health; yet, struggle to find ways to preserve the inclusion of older people in the ongoing social system. Understanding how AFCIs place social and physical environments squarely in view within the biomedical arena of the gerontological gaze has theoretical and policy implications.

### SESSION 6090 (SYMPOSIUM)

### ECONOMIC UNCERTAINTIES IN RETIREMENT: RISK FACTORS, STRATEGIES, AND RESILIENCE

Chair: Christine Bishop
Discussant: Karen Zurlo

Even with forethought and planning, a lot can threaten economic wellbeing in the years ahead for older adults retiring at typical retirement ages. Although results for any individual cannot be predicted with certainty, some risks are quantifiable: for example, mortality/ longevity and disability risks are reasonably well-defined. Risk of dementia is not so well understood, and may be changing. Financial risk might be seen as manageable, but older adults relying on retirement income sources can be especially vulnerable to unprecedented shocks to the general economy. We consider four aspects of this dilemma. First, older adults retiring with outstanding debts may have difficulty weathering financial shocks. Our first presentation provides up-to-date information about trends in indebtedness at older ages, especially focusing on newly salient types of indebtedness: medical and student loan debt, and debt incurred to smooth finances in the recent recession. Stewardship of finances during retirement can be a challenging personal management undertaking. Our second presentation will consider how dementia can complicate this process. Protection against outliving one's resources is more complex and costlier in the era of defined contribution retirement accounts. Our third presentation will discuss strategies to combine retirement assets, including Social Security claiming, to hedge longevity risk. Finally, needs for long-term services and supports may be met with either paid or informal (family) care, or both, but cannot be predicted with certainty. Our fourth presentation examines the long-term impacts on families due to the difficulty in insuring against this risk. Economics of Aging Interest Group Sponsored Symposium.

### FINANCIAL SECURITY AT OLDER AGES

Stipica Mudrazija, and Barbara Butrica, *Urban Institute, Washington, District of Columbia, United States*

As Americans have become increasingly indebted over time, understanding the factors influencing indebtedness is increasingly important and can help policymakers design policies that discourage debt, thus enhancing private retirement savings so that retirees become less dependent on Social Security benefits. The paper uses data from a major credit bureau of a nationally representative 2 percent random sample from more than 250 million consumer records to examine the financial health and indebtedness of older adults. The data cover the years 2010 through 2018 and follow the same consumers over time. They are supplemented with the zip-code level information from the American Community Survey on neighborhood characteristics, including racial and ethnic composition, education, median income, and poverty. Our preliminary findings suggest that, while the proportion of older adults in serious financial distress is not substantial, their indebtedness is increasing and the growth of unsecured debt (e.g., student debt) has been especially fast. Part of a symposium sponsored by the Economics of Aging Interest Group.

### THE FINANCIAL PRESENTATION OF ALZHEIMER'S DISEASE AND RELATED DEMENTIAS

Lauren Nicholas, *Johns Hopkins University, Baltimore, United States*

Dementia, a currently incurable degenerative cognitive disease, represents a major threat to financial stability. Early signs of dementia can include difficulties managing money and forgetting to pay bills, raising concerns about the implications of pre-clinical disease for financial well-being. We linked Medicare claims data to 20 years of consumer credit data for more than 80,000 older Americans living in single households to study the financial presentation of Alzheimer's Disease and Related Dementias. Using non-parametric regression models, we find elevated rates of payment delinquency, subprime credit, and withdrawal from use of credit products up to 6 years before dementia is clinically diagnosed. Similar patterns did not appear with a number of placebo acute and chronic health conditions, suggesting that the adverse financial events are unique to dementia and do not occur with other acute or chronic illnesses. Part of a symposium sponsored by the Economics of Aging Interest Group.

### ADDRESSING LONGEVITY RISK: HOW BEST TO ANNUITIZE DEFINED CONTRIBUTION ASSETS?

Alicia Munnell,[1] Gal Wettstein,[2] and Wenliang Hou,[2]
*1. Boston College, Chestnut Hill, Massachusetts, United States, 2. Center for Retirement Research at Boston College, Chestnut Hill, Massachusetts, United States*

Unlike defined benefit pensions, 401(k) plans provide little guidance on how to turn accumulated assets into income. The key risk that retirees face is outliving their assets. Insurance against such risk is available through several routes, including immediate annuities, deferred annuities, and additional Social Security through delayed claiming. Under this Social Security bridge option, participants would tap their 401(k) for payments equal to their Social Security to delay claiming. This paper compares these three options in simulations against a baseline in which no assets are used to obtain lifetime income. In each option, assets not allocated to purchasing lifetime income are consumed following the Required Minimum Distribution rules. The analysis finds that, when market and health shocks are included alongside longevity uncertainty, the Social Security bridge option is generally the best for households with median wealth. Wealthier households can benefit from combining the bridge option with a deferred annuity. Part of a symposium sponsored by the Economics of Aging Interest Group.

### FAMILY SPILLOVERS AND LONG-TERM CARE INSURANCE

Norma Coe,[1] Courtney Van Houtven,[2] and Gopi Goda,[3]
*1. University of Pennsylvania, Philadelphia, Pennsylvania, United States, 2. US Department of Veterans Affairs, Durham, North Carolina, United States, 3. Stanford University, Stanford, Colorado, United States*

We examine how the presence of long-term care insurance (LTCI) spills over to family outcomes, including informal care, co-residence, and labor supply of adult children. We instrument for long-term care insurance with changes in state tax policies to address the endogeneity of LTCI coverage. We find that for tax-filing families in the top third of the income distribution, LTCI coverage leads to a 50 percent reduction the parents' perceptions of the willingness of people to care for them in the future, including their adult children. We also find that LTCI causes changes in the residential decisions of adult children, with lower co-residence rates and higher likelihood of living within 10 miles of the parent. We also find small decreases in part-time work among adult children. Our findings provide empirical support for the presence of family spillovers of LTCI on the economic behaviors of family members. Part of a symposium sponsored by the Economics of Aging Interest Group.

### SESSION 6100 (SYMPOSIUM)

### ENGAGING AND EDUCATING OLDER ADULTS AND CAREGIVERS IN AGE-FRIENDLY HEALTHCARE

Chair: Erin Emery-Tiburcio
Discussant: Rani Snyder

As the Age-Friendly Health System initiative moves across the US and around the world, not only do health system staff require education about the 4Ms, but older adults, caregivers, and families need education. Engaging and empowering the community about the 4Ms can improve communication, clarify and improve adherence to treatment plans, and improve patient satisfaction. Many methods for engaging the community in age-friendly care are currently in development. Initiated by Health Resources and Services Administration (HRSA)-funded Geriatric Workforce Enhancement Programs (GWEPs), Community Catalyst is leading the co-design of Age-Friendly Health System materials with older adults and caregivers. Testing these materials across the country in diverse populations of older adults and caregivers will yield open-source documents for local adaptation. Rush University Medical Center is testing a method for identifying, engaging, educating, and providing health services for family caregivers of older adults. This unique program integrates with the Age-Friendly Health System efforts in addressing all 4Ms for caregivers. The Bronx Health Corps (BHC) was created by the New York University Hartford Institute of Geriatric Nursing to educate older adults in the community about health and health behaviors. BHC developed a method for engaging and educating older adults that is replicable in other communities. Baylor College of Medicine adapted and tested the Patient Priorities Care model to educate primary care providers about how to engage older adults in conversations about What Matters to them. Central to the Age-Friendly movement, John A. Hartford Foundation leadership will discuss the implications of this important work.

### CO-DESIGNING AGE-FRIENDLY HEALTH SYSTEM MATERIALS WITH OLDER ADULTS AND CAREGIVERS

Erin Emery-Tiburcio,[1] Carol Regan,[2] Judith Hilman,[3] and Michelle Newman,[1] *1. Rush University Medical Center, Chicago, Illinois, United States, 2. Community Catalyst, Boston, Massachusetts, United States, 3. Policy Catalyst, Salt Lake City, Utah, United States*

Achieving better care for older adults requires that they advocate for their own health needs. As health systems become Age-Friendly, education for older adults about the 4Ms is essential. Community Catalyst (CC) is working with older adults at six GWEPs to co-design materials explaining the interconnected 4Ms. In November 2019, CC hosted two focus groups (n=17) with older adults from Rush University Medical Center to understand their views on health care and introduce the 4M concepts. Key themes included the importance of being listened to, of each M and how they are connected, and for materials with graphics and personal stories. Based on this information, prototype educational materials were developed and three additional groups were held in February 2020 (n=30, including family caregivers) to solicit feedback on content and design. The co-design process, evaluation methods, and implications of including older adults in educational design will be discussed.

### HELPING CAREGIVERS TO BE READY, WILLING, ABLE, AND HEALTHY

Robyn Golden,[1] Vikki Rompala,[2] and Ellen Carbonell,[2]
*1. Rush University Medical Center, CHICAGO, Illinois, United States, 2. Rush University Medical Center, Chicago, Illinois, United States*

Given extended life expectancy, family caregivers are needed to provide care for older adults at home. Research has documented the deleterious effects of caregiving on mental and physical health for many. The Caregiver Health and Well-Being Initiative is a systems approach to support family caregivers of older adults by standardizing processes for identifying caregivers in ambulatory and inpatient settings, assessing caregivers' needs, and providing relevant services and resources, including caregiver health services, a Teach Back clinic for skills development, and extended goals of care conversations. N=104 caregivers have completed assessments. Participants reported the following at baseline: depressive symptoms (54%); anxiety symptoms (69%); health being affected by the care situation (59%); financial situation decreasing (58%); feeling torn between demands of their family and demands of care (63%); and some feelings of burden from caregiving (97%). Intervention components will be discussed along with a larger systems change framework for implementation.

### DEVELOPING A VOLUNTEER EDUCATION INITIATIVE FOR OLDER ADULTS' HEALTH

Liz Seidel,[1] Tara Cortes,[2] and Cinnamon St. John,[3]
*1. NYU Rory Meyers College of Nursing, New York, New York, United States, 2. New York University, New York, New York, United States, 3. Hartford Institute for Geriatric Nursing, New York, New York, United States*

Older adults need sufficient information to make healthy decisions and be active participants in their healthcare. Yet there is often a lack of information available. The Bronx Health Corps (BHC) was created to meet this need by providing older adults with usable knowledge on managing health conditions and promoting healthy behaviors in community-based settings. The BHC trained 175 volunteers, educated 2,065 older adults, with a total attendance of >5,000. Steps of creating a volunteer education initiative will be presented with qualitative and quantitative data utilization in implementation of the program. Focus groups with older adults noted challenges in addressing community health needs and the importance of reaching outside of healthcare settings to address the health of the community. Focus groups with Spanish speaking older adults and caregivers expanded knowledge on their attitudes toward the 4Ms and their ability to use that knowledge in interacting with their providers.

### EMBEDDING PATIENT PRIORITIES CARE IN AN AGE-FRIENDLY HEALTH SYSTEM

Aanand Naik,[1] Lea Kiefer,[2] Angela Catic,[3] and Lillian Dindo,[3] *1. Center for Innovations in Quality, Effectiveness, and Safety (iQuest), Houston, Texas, United States, 2. Michael E. DeBakey VA Medical Center, Houston, Texas, United States, 3. Baylor College of Medicine, Houston, Texas, United States*

Background: Patient Priorities Care (PPC) is an innovative approach to improving care for older adults with multiple morbidities. We developed a PPC training program for healthcare professionals and describe preliminary results. Methods: We implemented PPC in a geriatrics clinic. 20 staff and trainees participated on 1) how to identify patient priorities, 2) documentation in the electronic health record (EHR), and 3) strategies to align care with priorities; and received case-based audit and feedback. Results: 250 patients participated in PPC encounters. The EHR template was subsequently integrated within an Age Friendly Health System (AFHS) note. Clinicians have integrated this AFHS template for all encounters. Conclusion: PPC is a feasible approach to the care of older adults with multiple morbidities following a structured clinician training program. PPC can be effectively incorporated into the "Matter Most" component of AFHS.

### SESSION 6110 (SYMPOSIUM)

### ENRICHING FUTURE RESEARCH: THE POWER OF QUALITATIVE METHODS IN CANCER-RELATED HEALTH SERVICES RESEARCH

Chair: Sean Halpin

The ever-changing landscape of cancer care for older adults—with novel treatments, increasing survival rates, and growing population diversity—makes effective cancer care delivery increasingly complex. Qualitative research is uniquely poised to make sense of this complexity and shape potential interventions and their implementation. While the potential power of qualitative methods in cancer-related health services research and implementation science is great, as recognized in a recent National Cancer Institute report, the range of qualitative methods can make identifying and applying the most appropriate method(s) challenging. To meet this challenge, this symposium will bring together researchers across disciplines to report on three qualitative techniques and how each was applied in cancer research with older adults. Halpin will present on the use of applied conversation analysis to study medical education delivery to patients with multiple myeloma. The method is particularly well-suited to investigate health education and communicative efficacy. Carrion will discuss in-depth qualitative interviews that were conducted to understand the cancer beliefs and attitudes of older Latinx adults. The interviews, conducted in Spanish, offer an opportunity to consider how qualitative methods are key to illuminating the experiences of underrepresented populations. Seaman will report on the multiple qualitative methods used, including questionnaires, interviews, and site observations, to document survivorship care practices among head and neck cancer programs. The triangulation of qualitative methods allowed for an unparalleled understanding of guideline implementation and program variation. Exploring a range of methods, the presentations make a powerful argument for qualitative methods in cancer-related health services research.

### USING APPLIED CONVERSATION ANALYSIS IN MEDICAL EDUCATION

Sean Halpin,[1] Kathryn Roulston,[2] and Michael Konomos,[3]
*1. University of Georgia, Decatur, Georgia, United States, 2. University of Georgia, Athens, Georgia, United States, 3. Emory University, Atlanta, Georgia, United States*

Successful implementation of patient medical education is contingent on the communication strategies used by nurses, patients, and caregivers. Applied conversation analysis (A-CA) is a sociological and linguistic technique aimed at understanding how interaction is accomplished. In this demonstration of A-CA, the authors draw on an 18-month iterative-formative evaluation of patient education that precedes autologous stem cell transplant for persons diagnosed with multiple myeloma (N=70), a type of cancer which disproportionately impacts older adults. In this study, patients and caregivers received supplemental education videos before their formal education session with a nurse coordinator. Using A-CA, we examined how nurses, patients, and caregivers orient toward the videos; including demonstrated knowledge by patients and caregivers. Nurses justified repeating topics from the videos. Through a focus on the function that language plays in sequences of interaction, it may be possible to determine strategies for improving patient education, and, consequently positively impact patient care..

### HONORING OLDER LATINO IMMIGRANTS' PERSPECTIVES ON CANCER: INFORMING THE FUTURE

Iraida Carrion,[1] Malinee Neelamegam,[2] and Jane Roberts,[3]
*1. University of South Florida, Tampa, Florida, United States, 2. Yale School of Public Health, New Haven, Connecticut, United States, 3. University of South Florida, Sarasota, Florida, United States*

Cancer is the leading cause of death among Latinos in the U.S. Approximately 32.2% of Latinas and 44.1% of Latinos aged 60 years or older have a lifetime probability of developing invasive cancer (ACS, 2018), with lower survival rates for most cancers even when allowing for age and stage distribution. There is some evidence that older Latino/Latina immigrants lack knowledge about cancer treatment options and are often adversely impacted by healthcare inequities regarding cancer treatment and care options. This study compared the cancer beliefs and attitudes of 58 Latinos and 110 Latinas with a mean age of 67.9 years who reside in the Greater Tampa Bay area. Recruitment occurred in community-based settings, and interviews were conducted in Spanish and transcribed into English. The qualitative methods of constant comparison and thematic analysis will be presented along with the results related to diagnosis, medical decisions, finances, death, and family.

### TRIANGULATING QUALITATIVE METHODS TO EVALUATE SURVIVORSHIP CARE

Aaron Seaman,[1] Seyedehtanaz Saeidzadeh,[2] Emily chasco,[3] Sangil Lee,[3] Nicholas Kendell,[3] Heather Reisinger,[3] and Nitin Pagedar,[3] *1. Carver College of Medicine, University of Iowa, Iowa City, Iowa, United States, 2. College of Nursing, University of Iowa, Iowa City, Iowa, United States, 3. College of Medicine, University of Iowa, Iowa City, Iowa, United States*

With 16.9 million survivors in the US, survivorship is an increasingly important aspect of oncologic care. As the number increases, we need to provide evidence-based, standardized survivorship care, yet the evidence base is lacking and guidelines are variably implemented. This multi-sited study documented the survivorship care practices of five head and neck cancer (HNC) programs in order to identify survivorship care practices, provider preferences, practice variability, and the facilitators and barriers to effective survivorship care implementation. To ensure rich, contextual data, the study utilized multiple qualitative methods: 1) program characteristics questionnaire; 2) semi-structured interviews with providers involved in treatment and survivorship care, 3) on-site observation and clinic workflow mapping, and 4) collection of survivorship materials. Triangulating data collection provided evidence of potentially promising HNC survivorship care practices, aligned with the vision of comprehensive survivorship care, that can be used to evaluate practices and develop interventions on a larger scale.

### SESSION 6115 (SYMPOSIUM)

### EXPERIENCES OF RACIAL DISCRIMINATION AMONG COMMUNITY-DWELLING OLDER ADULTS OF COLOR AND NURSING HOME STAFF OF COLOR

Chair: Manka Nkimbeng
Co-Chair: Sarah LaFave
Discussant: Sarah Szanton

This symposium will present results of three qualitative studies that explored experiences of racial discrimination among older adults and their professional caregivers. Our first study reports on interviews conducted with older community-dwelling African American adults about their perceptions of and experiences with structural racial discrimination. Participants reported exposure that has accumulated over the life course and across contexts, including through limited access to educational and employment opportunities and disproportionate exposure to neighborhood violence and unhealthy products. Our second study reports findings from interviews conducted with older community-dwelling African immigrants about their experiences with acculturation and racial discrimination. Older African immigrants reported several types of discrimination, had developed unique strategies to cope with perceived discrimination and described how it impacted domains of health and wellbeing. Our third study reports on experiences with discrimination among U.S-born and immigrant staff of color caring for residents in high minority proportion nursing homes. Findings indicate that although staff of color are valued for the diversity they contribute to the workforce, they are also exposed to discriminatory events. In addition, tensions exist between U.S. and non-U.S born staff of color. In this symposium, we will discuss differences in experiences between U.S.-born and immigrant participants, levels of discrimination experienced (interpersonal, institutional, structural), and discrimination across contexts. We will also discuss intersectionality of race, ethnicity, immigrant status, language, gender, age, and class in relation to experiences of discrimination. We will reflect on the clinical and policy implications of our findings.

### OLDER AFRICAN AMERICANS' PERSPECTIVES ON EXPOSURE TO STRUCTURAL DISCRIMINATION ACROSS CONTEXTS AND THE LIFE COURSE

Sarah LaFave, Sarah Szanton, and Roland Thorpe, Jr., *Johns Hopkins University, Baltimore, Maryland, United States*

This presentation reports on findings from the first phase of a mixed methods study aimed at developing an instrument to assess older African Americans' exposure to structural racial discrimination. We conducted semi-structured interviews with older African Americans about their perspectives on and exposure to structural discrimination. Participants (n=20) were community-dwelling African Americans aged fifty and older in Baltimore, MD. Participants described exposure to structural discrimination that had accumulated across the life course and across the contexts of education, employment, healthcare services, criminal justice system, neighborhood factors, media and marketing of unhealthy products, environmental toxin exposures, and income, credit and wealth. In the next phase of the study, we will incorporate these findings into the development of instrument items. Developing and testing a tool to assess exposure to discrimination beyond the interpersonal level is an important step in identifying solutions to mitigate the contribute of discrimination to racial health disparities.

### OLDER AFRICAN IMMIGRANTS' EXPERIENCES OF DISCRIMINATION IN THE UNITED STATES

Manka Nkimbeng,[1] Janiece Taylor,[2] Laken Roberts,[3] Peter Winch,[4] Yvonne commodore-mensah,[2] Roland Thorpe, Jr.,[5] Hae-Ra Han,[3] and Sarah Szanton,[5] *1. University of Minnesota, Minneapolis, Minnesota, United States, 2. Johns Hopkins School of Nursing, Baltimore, Maryland, United States, 3. Johns Hopkins University School of Nursing, Baltimore, Maryland, United States, 4. Johns Hopkins Bloomberg School of Public Health, Baltimore, Maryland, United States, 5. Johns Hopkins University, Baltimore, Maryland, United States*

Discrimination is implicated in the disproportionate burden of disease and health disparities in racial/ethnic minorities. This qualitative descriptive study explored the experiences of discrimination and its impact on the health of older African immigrants. Semi-structured interviews were conducted with 15 participants. Three main themes and six sub-themes were identified. These included: 1) types of discrimination: a) accent-based, b) unfair treatment during routine activities, c) experience with police and other systems; 2) costs of discrimination; 3) surviving and thriving with discrimination: a) "blind eye to it", b) reacting to it, c) avoiding it. These themes describe common forms of discrimination that these older adults have experienced, current strategies used to deal with discrimination, and the impact of discrimination on the wellbeing of this sample. To improve the emotional and mental health of older African immigrants, providers serving them should assess for perceived discrimination, and refer participants with any concerns for treatment.

### RACE, CLASS, AND THE NURSING HOME WORKFORCE: EXPERIENCES OF STAFF OF COLOR IN HIGH-MINORITY-PROPORTION NURSING HOMES

Odichinma Akosionu,[1] Janette Dill,[1] Manka Nkimbeng,[2] Tricia Skarphol,[2] and Tetyana Shippee,[2] *1. University of Minnesota - School of Public Health, Minneapolis, Minnesota, United States, 2. University of Minnesota, Minneapolis, Minnesota, United States*

The long-term services and supports workforce is an important part of delivering quality care for nursing home (NH) residents – and increasingly includes staff who are from diverse communities. Our study captured staff (n=61) perspectives on resident quality of care and quality of life through semi-structured interviews, using thematic analysis in six Minnesota high proportion minority NHs. Findings show that although staff of color are valued for the diversity they contribute to the workforce, and the culturally sensitive care they provide, they are also exposed to discriminatory events. In addition, tensions exist between U.S. and non-U.S. born staff of color in NHs. Overall, staff of color who are lower ranked may feel less empowered. Research is needed to explore the impact of negative and discriminatory exposures on staff wellbeing and related outcomes in addition to the direct and indirect impact on the quality of care delivered to NH residents.

### SESSION 6120 (SYMPOSIUM)

### FAMILY CAREGIVER ASSESSMENTS: WHAT HAVE WE LEARNED FROM ASSESSMENTS ACROSS POPULATIONS AND HEALTH CONDITIONS?

Chair: Gabriela Prudencio
Discussant: Laura Gitlin

This symposium will bring together research on assessments of family caregivers for older individuals with different health conditions and discuss the components of effective assessments. Comprehensive characterizations of caregivers are essential due to an increase in the demand on caregivers, and how intense care contexts contribute to the caregiver's decline in health and diminished capacity to provide quality care. According to Caregiving in the U.S. 2020 (n=1,400), only 13% of caregivers were asked by healthcare professionals what they needed to take care of themselves. Peer-reviewed studies have reported that caregivers are often reluctant to self-identify and to ask for the help that they need for themselves and those in their care. Since supports to caregivers have historically relied on this self-identification, the first presentation (Grace Whiting) will focus on the work NAC has done to build pathways between caregivers and supportive services to increase availability, accessibility, and patient-centeredness. The second presentation (Esther Friedman) will identify and discuss the barriers to fully incorporating family caregivers into the health care team, as well as the solutions for removing barriers. The next two presentations, respectively, will focus on characterizing the prevalence, burden, and unmet needs of caregivers of cancer patients (Erin Kent), and the unmet needs of families of adults with intellectual and development disabilities (Tamar Heller). The final presentation will explore caregiver readiness in dementia care using the Tailored Activity Program (TAP) and how TAP interventionists can use readiness scores to determine caregiver's capacity (Katherine Marx).

### BARRIERS AND FACILITATORS TO INTEGRATING FAMILY CAREGIVERS INTO THE HEALTHCARE TEAM

Esther Friedman, and Patricia Tong, *RAND, santa monica, California, United States*

Recent changes in health care practices - shorter hospital stays, increased complexity of disease management, and greater management of chronic illnesses at home - have left family members increasingly responsible for medical tasks. Although these family caregivers represent the vital front line of care, they remain mostly excluded from the formal health care team. Based on interviews with multi-sector stakeholders representing patients/caregivers, providers, and payers, we identified several barriers to fully incorporating family caregivers into the health care team. They fall under five key themes: identification of caregivers; communication and information; time and resources; trust and cultural barriers; and Medicaid coverage. We discuss these barriers and potential solutions that could be undertaken by different stakeholder groups to improve family caregiver integration into the care team.

### CARING FOR OLDER PATIENTS WITH CANCER: ESTIMATES OF THE PREVALENCE, BURDEN, AND UNMET NEEDS OF CAREGIVERS IN THE UNITED STATES

Erin Kent, *University of North Carolina at Chapel Hill, Chapel Hill, North Carolina, United States*

In 2020, ~1.8 million Americans are expected to be newly diagnosed with cancer, with approximately 70% of cases diagnosed over the age of 65. Cancer can have a ripple effect, impacting not just patients themselves, but their family caregivers. This presentation will provide an overview of the estimates of the number of family caregivers caring for individuals with cancer in the US, focusing on older patients, from several population-based data sources: Caregiving in the US 2020, the Health Information National Trends Survey (HINTS, 2017-2019), the Behavioral Risk Factors Surveillance System (BRFSS, 2015-2019), and the National Health and Aging Trends (NHATS) Survey. The presentation will compare features of the data sources to give a comprehensive picture of the state of cancer caregiving. In addition, the presentation will highlight what is known about the experiences of cancer caregivers, including caregiving characteristics, burden, unmet needs, and ideas for improving support for family caregivers.

### UNMET NEEDS OF FAMILIES OF ADULTS WITH INTELLECTUAL AND DEVELOPMENT DISABILITIES ON WAITING LISTS

Tamar Heller, *University of Illinois, Chicago, Illinois, United States*

Most adults with intellectual and developmental disabilities live at home with their aging parents. Given the large waiting lists for residential and home-based services, families face many unmet service and support needs. The author will present results of a study that examined the impact of a Medicaid waiver program that provided either home-based or residential placements to 444 families of adults with IDD who were living at home at baseline through surveys at baseline and two years later. Families who did not receive the waiver services still had high unmet needs for person-centered planning training, networking with other families, respite, advocacy services, assistive technology, and home modifications at follow up. Regardless of services received, class members from minority backgrounds had more unmet needs than white class members, indicating the need for more targeted efforts to reach minority families.

### WHERE TO BEGIN: ASSESSING CAREGIVER READINESS

Katherine Marx,[1] and Laura Gitlin,[2] *1. Johns Hopkins University, Baltimore, Maryland, United States, 2. Drexel University, Philadelphia, Pennsylvania, United States*

There have been many interventions targeting family caregivers for people living with dementia (PLwD) that have been found to have efficacy and effectiveness. However, very few of these interventions have been widely adopted. One reason may be that clinicians and other professionals working with caregivers are unsure of what intervention to use. One measure that may help with where to begin is Caregiver Readiness. This presentation will provide case studies on the use of the Caregiver Readiness Scale in one intervention, the Tailored Activity Program (TAP). We will highlight three cases that have low, moderate and high readiness scores and how the TAP interventionists use the readiness scores to tailor the intervention to match the caregiver's need. Knowing a caregiver's readiness to receive an intervention may help clinicians and providers identify where to being in helping the caregiver.

### SESSION 6125 (SYMPOSIUM)

### HOUSING, AFFORDABILITY, AND COMMUNITY-BASED AGING

Chair: Samara Scheckler

The house acts as both an environment of care and a vehicle to financially potentiate long-term community-based support. While housing can empower a diverse set of options for a person-centered aging process, inadequate housing can also impede healthy aging in the community. This symposium teases out the nodes where housing acts to benefit or limit safe community-based aging. The first paper in this symposium, Homeownership Among Older Adults, describes typologies of older adult homeownership and sensitively highlights trends, disparities and important considerations of homeownership in later life. The next two papers take these older adults and explores situations where their housing acts as an asset or as a burden. Identifying Cost Burdened Older Adults acknowledges that housing cost burdens look different for older adults than younger cohorts. A more precise definition of older adult housing cost burden is proposed to help researchers and policymakers better synthesize the complex relationships between older adult housing and their long-term care decisions. The Long-Term Care Financing Challenge then explores the role of home equity in expanding the community-based long-term care choice set for older adults. This paper demonstrates benefits (both realized and unrealized) in home equity and suggests policy implications moving forward. Finally, Cardiometabolic Risk Among Older Renters and Homeowners disentangles the relationship between housing and health by demonstrating health disparities that are associated with housing tenure, conditions and affordability. Taken together, this symposium explores the complex and multidirectional relationships between housing, long-term care and older adult health.

### IDENTIFYING COST-BURDENED OLDER ADULTS: WHAT IS THE BEST MEASURE?

Samara Scheckler,[1] Sarah Mawhorter,[2] Jennifer Molinsky,[1] Alex Hermann,[1] and Whitney Airgood-Obrycki,[1]
*1. Harvard University, Cambridge, Massachusetts, United States, 2. University of Southern California, Syracuse, New York, United States*

While affordable housing typically describes housing costs that fall within 30 percent of total income, older adult spending systematically differs from younger cohorts. For instance, budgets may skew away from mortgages and towards home modifications while medical or personal care expenses can drive monthly costs. This research uses the Health and Retirement Study (HRS) to explore various older adult housing cost burden measures and identify their relative advantages. Measures of cost burden are applied to HRS respondents and different cost burdened groups are defined. The welfare of each group is then assessed using metrics such as unmet need and caregiver stress. Findings suggest that traditional cost burden measures identify many vulnerable older adults. However, other measures of cost burden can highlight older adults who are disproportionately impacted by medical cost. This research should help professionals better align the metric that defines their target population members with their policy area.

### HOMEOWNERSHIP AMONG OLDER ADULTS: SOURCE OF STABILITY OR STRESS?

Jennifer Molinsky,[1] and Christopher Herbert,[2] *1. Harvard University, Cambridge, Massachusetts, United States, 2. Harvard university, Cambridge, Massachusetts, United States*

For older adults, homeownership can be an important source of housing stability and personal wealth that can be tapped in later life, including for long-term care. Nonetheless, owning a home is not without physical and financial challenges for aging households, and many owners are reluctant to take advantage of housing equity later in life. This paper reviews the conditions of older homeowners to assess the degree to which owning a home is—or is not—associated with financial security and housing stability. We review trends in homeowning among older adults, including differences by race/ethnicity and income. We then describe the extent of housing affordability challenges among homeowners, the degree of mortgage indebtedness, and the extent and use of housing equity. Finally, we examine issues related to housing quality and accessibility. The paper concludes with a discussion of policy implications.

### THE LONG-TERM CARE FINANCING CHALLENGE: WHAT ROLE FOR HOME EQUITY?

Jane Tavares,[1] Marc Cohen,[2] and Lauren Popham,[3]
*1. University of Massachusetts Boston, Florence, Massachusetts, United States, 2. University of Massachusetts Boston, Boston, Massachusetts, United States, 3. National Council on Aging, Arlington, Virginia, United States*

With increasing longevity comes the need for support in activities of daily living. This growing demand for long-term services and supports (LTSS) threatens older adult financial security. About 25 million adults aged 65 and older will have a significant LTSS need that will cost up to $140,000 out-of-pocket plus an estimated $120,000 from public payers like Medicaid; the total public and private costs of care exceeding $250,000. Home equity, the most valuable financial asset owned by Americans, offers one solution to this financing challenge. While seniors boast $7 trillion in collective home equity, opportunities to annuitize this asset vary and the distributions of home ownership and value are highly skewed. Using the 2016 wave of the HRS, this paper explores the relationship between socio-demographic/health characteristics and the annuitized home value. We determine which individuals with LTSS needs might benefit from the annuitization of home equity and to what extent.

### CARDIOMETABOLIC RISK AMONG OLDER RENTERS AND HOMEOWNERS

Sarah Mawhorter,[1] Jennifer Ailshire,[2] and Eileen Crimmins,[2] *1. University of Southern California, Syracuse, New York, United States, 2. University of Southern California, Los Angeles, California, United States*

Researchers consistently find adverse long-term health outcomes among renters as compared with homeowners, yet more proximal health measures are needed to understand whether there is a direct link between tenure and health. In this paper, we compare cardiometabolic risk (CMR) levels among older renters and homeowners, and ask whether this health disparity can be explained by socioeconomic differences between renters and homeowners, or poor housing conditions for renters. Using Health and Retirement Study 2010/2012 biomarker data for adults aged 50-84 (N=10,480), we measure CMR by a scale of C-reactive protein, hemoglobin A1C, cholesterol, heart rate, blood pressure, and waist circumference metrics. We find that renters have greater CMR, even accounting for socioeconomic characteristics and health behaviors. Certain housing and neighborhood conditions, such as perceived safety, are associated with CMR. These results suggest potential pathways through which homeownership confers health advantages over renting.

### SESSION 6130 (SYMPOSIUM)

### IMPLEMENTING NON-PHARMACOLOGIC INTERVENTIONS FOR PEOPLE LIVING WITH DEMENTIA IN LONG-TERM CARE SETTINGS

Chair: Emily Ihara

Person-centered dementia care is a best practice recommendation by the Alzheimer's Association, and non-pharmacologic interventions that emphasize well-being and quality of life as defined by the individual are important to preserve personhood. Non-pharmacologic, person-centered interventions have been shown to effectively address various neuropsychiatric symptoms, commonly known as behavioral and psychological symptoms in dementia (BPSD), which include a wide range of behaviors such as verbal or physical aggression, agitation, wandering, and pacing. Interventions that are focused on an individual's holistic needs and preferences can stimulate positive emotions and behavior regardless of the stage of dementia. Person-centered care emphasizes a social model of care, rather than a medical model, by focusing on an individual's emotional needs and care preferences that are consistent with their previous lifestyle. This symposium explores four different non-pharmacologic interventions for individuals living with dementia and discusses challenges and best practices for implementation in long-term care settings. For example, a best practice includes "buy-in" from facility staff who ultimately are responsible for implementing interventions that follow a social care model. A challenge found includes creating consistency and adherence to non-pharmacologic interventions so they are sustained over time, potentially replacing additional doses of medication. Symposium presenters will discuss the Mason Music & Memory Initiative (M3I), the Alzheimer's Poetry Project, Birdsong, and TimeSlips, which are all interventions that are relatively low-cost and easy to implement by non-specialists. Strategies for intergenerational programming and adaptability of these programs to different contexts will also be discussed.

### CREATING STORIES USING TIMESLIPS WITH PEOPLE LIVING WITH DEMENTIA

Emily Ihara,[1] Kendall Barrett,[1] Catherine Tompkins,[1] Megumi Inoue,[1] and Kari Hanson,[2] *1. George Mason University, Fairfax, Virginia, United States, 2. TimeSlips, Milwaukee, Wisconsin, United States*

TimeSlips is an award-winning, non-pharmacologic, creative storytelling intervention designed specifically for individuals living with dementia by Anne Basting. As part of the NextGen pilot program, George Mason University implemented TimeSlips at a dementia-specific adult day health care center with undergraduate and graduate students trained as facilitators. They worked with two different populations living with ADRD – an early-stage group and a moderate- to late-stage group. Students who participated reflected upon their experiences and noted that they learned so much about the importance of open-ended questions and accepting even one-word answers. One student stated: "This makes so much sense, as I have noticed participants take these open-ended questions and deliver beautifully unexpected and clever responses." The implementation was different for these two groups, and our analysis of the content and process indicate that consistency and person-centeredness are key to participants' expression of pride and joy in their accomplishments.

### THE IMPLEMENTATION OF THE MUSIC AND MEMORY INTERVENTION IN NURSING FACILITIES IN VIRGINIA

Megumi Inoue,[1] Emily Ihara,[1] Catherine Tompkins,[1] Kristen Suthers Rumrill,[1] Kendall Barrett,[1] Kathryn McNeil,[1] and Christi Clark,[2] *1. George Mason University, Fairfax, Virginia, United States, 2. Harpeth Consultant Advisory Group, Franklin, Tennessee, United States*

The Music & Memory intervention is a person-centered, non-pharmacologic intervention for people living with dementia. It is considered a personalized music intervention because the playlist comprises music genres that the individual prefers. Music has shown positive effects on behavioral and psychological symptoms of dementia. However, the findings from previous studies are often based on small sample sizes, insufficient descriptions of treatment fidelity, and lack of randomization. To address these issues, we began a randomized controlled trial implementing the Music & Memory intervention in nursing facilities in Virginia with support from the Centers for Medicare and Medicaid (CMS) Civil Money Penalty. We will present our research strategies as well as preliminary results suggesting that nursing facilities with higher occupancy rates are more likely to participate in this type of a program. In addition, study participants with dementia show positive emotional and behavioral reactions when listening to their favorite music.

### POETRY BRINGS OUT THE BEST IN US: STRUCTURED POETRY-BASED GROUP ACTIVITY IN LONG-TERM CARE

Daniel Kaplan,[1] and Gary Glazner,[2] *1. Adelphi University, Cortlandt Manor, New York, United States, 2. The Alzheimer's Poetry Project, New York, New York, United States*

The Alzheimer's Poetry Project has a proven track record at over 500 facilities in 35 states and six countries, serving over 50,000 people worldwide, and has the ability to bring high-quality creative arts programming to people living with dementia in long-term care. We describe how volunteers and recreation and care staff can be trained to deliver the intervention, offering direct benefits for participants and indirect benefits when modeled in the presence of providers and family members. Basic validation techniques are easily learned and incorporated into diverse dementia care strategies. As activity programs based in creative arts help to support self-expression among participants and serve as a vehicle for generating feelings of self-efficacy, we explain how such activities are well suited to fostering the person-centered goals of dementia care programming. Clinicians and other transdisciplinary care providers are encouraged to use and teach validation-focused arts interventions with persons living with dementia.

### THE IMPLEMENTATION OF BIRDSONG: A NON-PHARMACOLOGICAL INTERVENTION FOR PERSONS WITH DEMENTIA

Catherine Tompkins,[1] Joan Thomas,[2] Christina Cole,[2] and Mei Qiu,[1] *1. George Mason University, Fairfax, Virginia, United States, 2. Birmingham Green, Manassas, Virginia, United States*

Birdsong is a person-centered, nonpharmacological intervention for people with dementia. Using a touch-screen tablet, residents with dementia are stimulated with content customized based on individual interests such as sports, videos, music or brain games. The increased stimulation is expected to redirect negative behaviors, decrease the use of anti-psychotic medications and increase positive emotions. Birdsong is being implemented in a 100% Medicaid funded, long-term care facility. Two 10-week experimental design studies were implemented with 39 residents. Preliminary findings suggest a lack of independence with the tablets among the residents, but positive stimulation of emotions and redirection of negative behaviors with assistance of staff or volunteers. A team of practitioners, faculty and student researchers continue to examine the intervention, its implementation and effects on this population by examining the Minimum Data Set (MDS data) and observations of the residents as they utilize Birdsong.

### SESSION 6135 (SYMPOSIUM)

### IMPROVING COMMUNICATION FOR OLDER ADULTS DURING DISASTER EVENTS

Chair: Allison Gibson
Co-Chair: Nancy Kusmaul
Discussant: Lisa Brown

Management of any disaster event (i.e., hurricanes, flooding, wildfires, viral pandemic) is a complicated task. An important and frequently overlooked aspect of efficient disaster response is effective information exchange among emergency managers, aging and social service providers, and those impacted by the disaster. Breakdowns in communication are regularly cited as failures in actual emergency situations. Older adults have unique challenges to effective communication during a disaster event. Further, messaging platforms may be inaccessible to older adults. Given the lack of data collected on the role of effective communication for older adults in a disaster event, we developed this symposium to highlight lessons learned for facilitating disaster communication among older community members and discuss how policy can further disaster communication efforts. The first presentation highlights the advantages of using targeted marketing communication channels to encourage older adults to prepare and recover from a disaster, as well as the value of collaborative partnerships in this communication. The second presentation introduces results from research collected from older adults on their use of social media to stay informed before and after Hurricane Matthew. The third presentation explores whether assisted living communities in Florida's affected counties evacuated or sheltered in place in the context of emergency management communications concerning evacuation. The fourth presentation highlights the experiences of older persons in Houston's communication post-Hurricane Harvey from an aging service provider's perspective. The final presentation discusses disaster literacy and how this model can be used to enhance outcomes at all phases of a disaster. Disasters and Older Adults Interest Group Sponsored Symposium.

### EFFECTIVE COMMUNICATIONS TECHNIQUES FOR COMMUNITIES TO REACH OLDER ADULTS IN A NATURAL DISASTER

Patricia Fletcher, *Federal Emergency Management Agency, Washington, District of Columbia, United States*

Framing timely messages during an emergency is often underestimated but is vitally essential and necessary, especially for the older population. While there is existing research on disaster and older adults, there is a gap in the literature that focuses on a 21st-century communication model that reaches at-risk populations. This presentation explores how practitioners can inspire change in the delivery of an emergency message to communities. Appropriately designed health communications will provide extensive knowledge about the health crisis and improve public health literacy among older adults in disaster preparedness, response, and recovery, creating messages that influence necessary behavior change during the emergency. Additionally, recognizing how collaborative partnerships with Federal, Local, State government, trusted relief agencies, and community groups benefit from designing an organizational structure to disseminate information to the community. Part of a symposium sponsored by Disasters and Older Adults Interest Group.

### OLDER PERSONS' USE OF SOCIAL MEDIA FOR COMMUNICATION DURING HURRICANE MATTHEW

Allison Gibson,[1] Nancy Kusmaul,[2] and Ethan Engelhardt,[1] *1. University of Kentucky, College of Social Work, Lexington, Kentucky, United States, 2. UMBC, Baltimore, Maryland, United States*

Older persons are frequently identified as more vulnerable during natural disasters due to age-related changes and chronic conditions. Over the last decade, the use of social media has grown, even among the older adult population. While many communities and organizations have utilized social media as a platform to communicate news and information about natural disasters among the public, little is known regarding how older adults utilize social media to plan, evacuate, and recovery from natural disaster events. This study examined the experiences of 23 older adults (n=23) use of social media following Hurricane Matthew. Individuals were able to speak about what they perceived as helpful in the information communicated, and recommendations they had for how such communications could be improved. The presentation will conclude with recommendations on how individuals helping with evacuation and recovery can utilize social media as a communication resource for older adults during disasters. Part of a symposium sponsored by Disasters and Older Adults Interest Group.

### EVACUATION OR SHELTER IN PLACE IN ASSISTED LIVING: ADHERENCE TO EMERGENCY EVACUATION ORDERS FOR HURRICANE DORIAN

Lindsay Peterson,[1] Joseph June,[2] Nazmus Sakib,[1] and Kathryn Hyer,[1] *1. University of South Florida, Tampa, Florida, United States, 2. School of Aging Studies, University of South Florida, Tampa, Florida, United States*

Hurricane Dorian devastated parts of the Bahamas in 2019 with 185-mph winds. As it moved toward Florida, the state went on alert. This paper examines whether assisted living communities (ALCs) in affected counties evacuated or sheltered in place in the context of emergency management communications concerning evacuation. In 16 coastal counties, 66 ALCs were under mandatory evacuation orders, but 12 sheltered in place. Of 603 ALCs not under orders, 17 evacuated. Seven ALCs evacuated contrary to orders in one county, which issued a mandatory order Sept. 1, delayed it to Sept. 2 as Dorian weakened, and lifted it Sept. 4. Interviews with ALC administrators and emergency managers will be used to understand more about their decisions. Given prior findings that evacuation (versus sheltering in place) increases the mortality and morbidity risk of long-term care (LTC) residents, this research suggests a need for clearer LTC evacuation standards and communication. Part of a symposium sponsored by Disasters and Older Adults Interest Group.

### A TALE OF TWO DISASTERS

Tammy Mermelstein, *University of Houston - Downtown, Houston, Texas, United States*

Preparing for or experiencing a disaster is never easy, but how leaders communicate with older adults can ease a situation or make it exponentially worse. This case study describes two disasters in the same city: Hurricane Harvey and the 2018 Houston Texas Ice Storm and the variation in messaging provided to and regarding older adults. For example, during Hurricane Harvey, the primary pre-disaster message was self-preparedness. During the storm, messages were also about individual survival. Statements such as "do not [climb into your attic] unless you have an ax or means to break through," generated additional fear for older adults and loved ones. Yet, when an ice storm paralyzed Houston a few months later, public messaging had a strong "check on your elderly neighbors" component. This talk will explore how messaging for these events impacted older adults through traditional and social media analysis, and describe how social media platforms assisted people with rescue and recovery. Part of a symposium sponsored by Disasters and Older Adults Interest Group.

### DISASTER LITERACY

Lisa Brown,[1] and Lindsay Peterson,[2] *1. Palo Alto University, Palo Alto, California, United States, 2. University of South Florida, Tampa, Florida, United States*

People who plan ahead typically fare better during the response and recovery phases of a disaster. However, problems arise when the needs, wants, abilities, and resources of vulnerable people are not adequately considered. The lack of alignment between the literacy demands of existing materials and the literacy skills of many vulnerable subgroups limits their ability to understand and effectively use potentially life-saving information. Existing health literacy models that have demonstrated effectiveness in changing health behaviors and improving outcomes is a first step to reducing disaster-related morbidity and mortality in low resource and low literacy areas. This presentation will 1) describe how inter-disciplinary collaborations can be used to address this public health issue, 2) explain how health literacy techniques can be applied when developing disaster materials, and 3) present research data on a social marketing campaign to improved disaster preparedness of older adults. Part of a symposium sponsored by Disasters and Older Adults Interest Group.

### SESSION 6140 (SYMPOSIUM)

### IN THE RED: DEBT, FINANCIAL WELL-BEING, AND RETIREMENT PREPAREDNESS AMONG OLDER ADULTS

Chair: Julie Miller
Discussant: Julie Miller

Over the past twenty years, total debt for Americans ages 70 and over has increased more relative to all other ages group- by 543% according to the Federal Reserve Bank of New York (2019). Higher rates of debt among older adults have been attributed to a range of factors including expanded access to consumer credit, the capability for people to borrow from 401(k) plans, increases in costs of living, and limited financial literacy, among others. Related research of adults nearing retirement age who carry debt points to delayed retirement timing, lower levels of retirement savings, and higher risk and rates of bankruptcy. This symposium introduces timely investigations of retirement planning and economic security among older adults with debt. The first presentation will provide an overview of the impact of financial hardship on health among older adults in the United States over a recent ten-year period. The second presentation will focus on over-indebtedness among pre-retirees. The third presentation will examine the role of safety net services and borrowing from retirement plans among older adults with debt, particularly among older adults of color. The fourth and final presentation will focus on student loan debt as a hurdle to near-term and long-term financial security for older women in particular. A discussant will comment on how, together, the aforementioned papers contribute to our understanding of economic wellbeing and retirement preparedness in this era of increasing longevity. The session will integrate policy and programming implications for gerontological professionals.

### THE LONG-TERM EFFECTS OF FINANCIAL HARDSHIP ON HEALTH: PRE AND POST THE GREAT RECESSION

Gillian Marshall, *University of Washington, Seattle, Washington, United States*

Purpose: Now 10 years after the Great Recession of 2018, we examined the impact and long-term health outcomes impacting adults (≥ 50 years). Methods: Data from the Health and Retirement Survey (sample n=5,160), was used to examine how changes in financial hardship pre-post the Great Recession of 2008 impacted the likelihood of developing new chronic conditions in 2016. Results: Preliminary results suggest that reduced medication use during the recession was significantly correlated with a higher likelihood of developing arthritis, lung disease, psychological conditions, depression, and greater deterioration of mental and physical health relative to an absence of reduced medication use in 2006 and 2010 (1). Conclusion: These findings underscore the adverse influences of increased financial hardships that impact medication use during recessionary periods on long term health and wellbeing of older adults. They also provide evidence of deleterious effects on health of difficulty paying bills throughout the study period.

### EMERGING RETIREMENT CRISIS: WHEN SOCIAL SECURITY IS NOT ENOUGH

David Miller, *Case Western Reserve University, Cleveland, Ohio, United States*

Evidence suggests a growing retirement crisis in the United States among older adults placing many of them at risk of falling into poverty. While Social Security provides income assistance to retirees, the average monthly benefit is $1,300. Among older adults nearing or in retirement, the use of public assistance programs is increasing. Using data collected by the Associated Press-NORC Center for Public Affairs Research we examine retirement preparedness, borrowing from retirement plans, and use of social welfare programs. Findings indicate increased borrowing from retirement plans due to debt, significant differences in racial and gender groups accessing and receiving services among those 75 and older. Increasing rates of unpreparedness for retirement exist among older Americans, particularly among adults of color. An increase in the use of safety net services among older adults is occurring concurrently with severe funding reductions in social welfare programming.

### REPAYING STUDENT LOAN DEBT: IMPLICATIONS FOR OLDER WOMEN'S FINANCIAL WELL-BEING AND RETIREMENT PLANNING

Julie Miller,[1] Samantha Brady,[1] Alexa Balmuth,[1] Lisa D'Ambrosio,[2] and Joseph Coughlin,[2] *1. MIT AgeLab, Cambridge, Massachusetts, United States, 2. MIT, Cambridge, Massachusetts, United States*

Previous research demonstrates that student loan debt disproportionately affects women and is being repaid increasingly by older adults. Utilizing data collected through a mixed methods study at the MIT AgeLab (involving focus groups and a large national survey), we examine the impacts of repaying student loans on older women's financial wellbeing. Findings focus on multiple areas, including the timing in which older women repaying student loans were able to pay down other debts, change jobs, get married, and pursue further education. Analysis also revealed that student loans impacted the amount that 63% of older women in the study were able to contribute to their retirement savings. Findings from this study suggest that repaying student loan debt for oneself and/or a family member can compromise older women's near-term and longer-term financial being. Policy reforms are needed to support older women repaying student loans.

### SESSION 6145 (SYMPOSIUM)

### INTERPROFESSIONAL COLLABORATION TO ADDRESS ELDER ABUSE, NEGLECT, AND EXPLOITATION WITH ADULT PROTECTIVE SERVICES

Chair: Pi-Ju Liu
Discussant: Bridget Penhale

Adult Protective Services (APS) is responsible for investigating reports of abuse, exploitation, and neglect among vulnerable adults. Additionally, APS also refers or provides needed services to victims. Though APS also takes the lead position in investigation and service planning, determining harm and designing person-centered remedies often require other professionals to be prepared to address these elder justice issues with APS. Afterall, adult safeguarding is everybody's business. This symposium includes four presentations on researchers' findings working with APS and other professionals. Dr. Burnes will compare outcomes on usual APS care (APS only) versus enhanced APS care (APS plus advocate who are case managers). Dr. Pi-Ju (Marian) Liu will examine nurses' role in working in and with APS, focusing on how their job responsibilities are different from other nurses and APS social workers. Dr. Jason Burnett will present an innovative referral portal linking banks and other financial firms directly to APS. Lastly, Dr. Zachary Gassoumis will appraise the multidisciplinary teams across the country, including professionals involved, and provide input on what makes a team work well together. Following the four presentations, Dr. Bridget Penhale will open up the discussion regarding protective agencies' operations and collaboration with other agencies, focusing on the comparison between protective agencies in the United States and European countries. Abuse, Neglect and Exploitation of Elderly People Interest Group Sponsored Symposium.

### EVALUATING AN INTEGRATED APS–ELDER ADVOCATE INTERVENTION MODEL IN THE STATE OF MAINE

David Burnes,[1] Marie-Therese Connolly,[2] Patricia Kimball,[3] Stuart Lewis,[4] and Erin Salvo,[5] *1. University of Toronto, Toronto, Ontario, Canada, 2. University of Southern California Leonard Davis School of Gerontology, Washington, District of Columbia, United States, 3. Elder Abuse Institute Of Maine, Brunswick, Maine, United States, 4. Hofstra/Northwell Health, New York City, New York, United States, 5. Maine Adult Protective Services, Augusta, Maine, United States*

Despite recommendations to include a distinct intervention phase in Adult Protective Services (APS), most APS programs close cases following investigation/substantiation phases without engaging in a defined intervention phase. This study implements and evaluates a novel APS service planning/intervention model in the state of Maine. Using an experimental efficacy trial design with stratified random sampling at the level of Maine APS offices, this study compares standard APS care with an enhanced/integrated APS intervention model involving "elder advocates". Advocates were trained in motivational interviewing, supported decision-making, teaming, restorative justice, and goal attainment scaling to develop capacity to work with both the older adult victim and perpetrator and to strengthen the family and social systems surrounding the victim-perpetrator dyad. This presentation will present results on the efficacy of this integrated APS/elder advocate model and discuss the challenges and successes in conducting elder abuse intervention research in collaboration with APS and APS clients. Part of a symposium sponsored by Abuse, Neglect and Exploitation of Elderly People Interest Group.

### NURSES WORKING IN AND WITH ADULT PROTECTIVE SERVICES: AGENCY CHARACTERISTICS AND JOB RESPONSIBILITIES

Pi-Ju Liu,[1] and Jessica Hernandez Chilatra,[2] *1. Purdue University, West Lafayette, Indiana, United States, 2. Universidad Nacional de Colombia, Bogotá, Distrito Capital de Bogota, Colombia*

The majority of Adult Protective Services (APS) workforce is staffed by social workers, though some agencies have recognized the need to address clients' medical needs such as wounds, injuries, nutrition issues, hydration issues, premature death and more. Using survey data from the National Adult Protective Services Association (NAPSA), we analyzed 99 nurses' responses on their role in working in/with APS to help abused, neglect, and exploited adults. Out of the 99 nurses, 65 were direct employees of APS, and 61 did not report directly to a nurse supervisor. Forty-nine nurses carry a caseload like social workers, and 27 carry a caseload in conjunction with social workers. The most common services nurses provide are home visits, evaluations of clients and their medications, and client education. Qualitative data revealed the benefits of having nurses on staff, including assessing medical needs, preventing medical emergencies, providing holistic care, and navigating the healthcare system. Part of a symposium sponsored by Abuse, Neglect and Exploitation of Elderly People Interest Group.

### A DIRECT REPORTING PLATFORM FOR FINANCIAL EXPLOITATION: FROM BANKS TO APS

Jason Burnett,[1] Sally Reisch,[2] and Geoffrey Rogers,[2]
*1. McGovern Medical School at UTHealth, Houston, Texas, United States, 2. Hunter College, New York, New York, United States*

Despite encouraging changes in regulations and practices, opportunities remain to strengthen the collaboration between Financial Services Institutions (FSI) and Financial Exploitation (FE) investigative agencies such as Adult Protective Services (APS). A major barrier to these collaborations is timely and effective information exchange, between the agencies, for maximizing client protection. Often times, the need for more information from the agencies involved delays or mitigates the provision of financial protection to the client/victim. Through the U.S. Office for Victims of Crime, funding has been provided to develop a single reporting platform for enhancing communication and collaboration between FSI and APS. The helpful platform provides an innovative conduit for providing timely and effective information exchange between FSI and APS agencies to better serve and protect older adults against FE. This presentation will discuss the platform development, the lessons learned, preliminary data and future research. Part of a symposium sponsored by Abuse, Neglect and Exploitation of Elderly People Interest Group.

### ELDER ABUSE MULTIDISCIPLINARY TEAMS: DESCRIBING AND CLASSIFYING A KEY COLLABORATIVE RESOURCE FOR APS WORKERS

Zachary Gassoumis, Gerson Galdamez, Julia Rowan, and Kathleen Wilber, *University of Southern California, Los Angeles, California, United States*

Elder abuse multidisciplinary teams (MDTs) are a key resource when APS workers address their most complex cases. MDTs promote coordination and information sharing, and provide access to highly specialized input and problem-solving from legal, health, social service, and financial fields. This paper characterizes the range of elder abuse MDTs across the U.S. We identified 324 MDTs in the U.S., which most frequently addressed cases of financial exploitation (90.8%), physical abuse (83.6%) and neglect (81.6%). Based on a follow-up survey, latent class analysis was used to determine closeness of a subset (n=91) to the elder abuse forensic center model, which has received much evaluation and policy attention. Twenty-six showed strong similarity to forensic centers, with 24 others showing partial similarity. Coupled with observations from site visits to 4 teams, findings can guide the development and evaluation of elder abuse MDTs to foster better interdisciplinary collaboration for APS workers. Part of a symposium sponsored by Abuse, Neglect and Exploitation of Elderly People Interest Group.

### SESSION 6150 (SYMPOSIUM)

### INTERSECTIONS OF GENDER AND COGNITION IN OLDER ADULTS

Chair: Shana Stites
Co-Chair: Jason Flatt
Discussant: Carol Derby

The National Institutes of Health (NIH) is committed to supporting rigorous science that advances what is understood about the influences of sex and gender in health and disease in order to inform the development of prevention strategies and treatment interventions. In research on aging and Alzheimer's disease, sex/gender disparities in key outcomes are common. But, much of this research hinges on asking a single question: Is the patient or research participant male or female, man or woman? This practice offers few options for disambiguating sociocultural effects associated with gender from those related to biologic sex. It also assumes that self-reports are a suitable proxy for social phenotypes and that a dichotomous variable adequately captures the wide-range of sociocultural effects attributable to gender. The premise of this symposium is to evaluate how gender interacts with cognitive outcomes in order to advance measurement. This symposium will review evidence from five distinct lines of research on associations between gender and cognition for individuals and for individual's interactions with their family members: (1) effects of normative shifts in American education on cognition in older adults; (2) hospitalization as a risk factor for cognitive decline in racially diverse American men and women; (3) caregivers who identify as sexual and gender minorities (SGM or LGBTQ+) and care for persons with dementia; (4) correlates of cognitive function in SGM older adults; and (5) differences in adults' cognition based on childhood exposure to women's social empowerment in 30+ Organisation for Economic Co-operation and Development (OECD) countries.

### THE COGENT3 STUDY: EXAMINING GENDER'S IMPACT ON EDUCATION AND COGNITION TRENDS IN THREE AMERICAN GENERATIONS

Shana Stites,[1] Hannah Cao,[1] Jeanine Gill,[1] Kristin Harkins,[1] Jonathan Rubright,[2] and Jason Flatt,[3]
*1. University of Pennsylvania, Philadelphia, Pennsylvania, United States, 2. National Board of Medical Examiners, Philadelphia, South Carolina, United States, 3. University of Nevada, Las Vegas, School of Public Health, Las Vegas, California, United States*

How older adults protect their cognitive health, reduce their risk for cognitive decline, and manage cognitive changes vary for men and women. To advance what is known about these differences and to promote inclusion of sexual and gender minorities in research, we are developing an empirically-informed research framework for studying gender effects in aging and Alzheimer's research. In this presentation, we describe the framework informing our approach and present results from analyses of gender effects in The Health and Retirement Study that examine gender differences in the associations observed between education and cognitive measures in older adults. Our findings show gender's effects on education vary in direction and magnitude as gender norms changed over time. Although college education serves as a factor protective against cognitive decline, characteristics of who achieves a four-year college degree change over time. We discuss the implications of our results for aging and Alzheimer's disease research.

### COGNITIVE FUNCTION IN SEXUAL AND GENDER MINORITY OLDER ADULTS

Jason Flatt,[1] Samantha John,[2] and Paula Frew,[3]
*1. University of Nevada, Las Vegas, School of Public Health, Las Vegas, California, United States, 2. University of Nevada, Las Vegas, Las Vegas, California, United States, 3. University of Nevada, Las Vegas, School of Public Health, Las Vegas, Nevada, United States*

Nearly 3.5 million sexual and gender minority (SGM) adults aged 60+ in the U.S. identify as lesbian, gay, bisexual, transgender, and/or queer. We recruited over 50 diverse SGM older adults from the community to better understand correlates of their cognitive function. The Telephone Interview for Cognitive Status, an 11-item screening test of global cognition was used over the phone or in-person. We will describe relationships among cognition and several sociodemographic and health variables (age, sex assigned at birth, SGM identities, race/ethnicity, and health). Past research has highlighted higher rates of perceived memory problems among lesbian, bisexual and transgender adults compared to both gay men and heterosexual men and women. These rates were also higher among those who identify as women. We highlight implications for researching gender identity and cognition in late life, such as the influence of gender roles on cognition and the assessment of gender expression and related constructs.

### SEXUAL AND GENDER MINORITY CAREGIVERS OF PEOPLE WITH DEMENTIA AND THEIR CARE RECIPIENTS

Joel Anderson,[1] Jason Flatt,[2] Jennifer Jabson Tree,[3] Alden Gross,[4] and Karen Rose,[5] *1. University of Tennessee-Knoxville, Knoxville, Tennessee, United States, 2. University of Nevada, Las Vegas, School of Public Health, Las Vegas, California, United States, 3. University of Tennessee, Knoxville, Tennessee, United States, 4. Johns Hopkins Bloomberg School of Public Health, Baltimore, Maryland, United States, 5. Ohio State University, Colombus, Ohio, United States*

Little is known about the unique experiences of sexual and gender minority (SGM) caregivers of people with dementia or their care recipients. We used an electronic survey to assess psychosocial measures within this caregiving population, including measures related to the care recipient. The majority of caregivers (N=285) were gay men (62%). Most respondents were white (80%), with a quarter identifying as Latinx. The majority of caregivers were a spouse/partner (59.3%) and were providing care for someone who identified as LGB (70%), with 20% caring for someone transgender. Half of care recipients did not have an advance directive. The majority of care recipients needed assistance with ≥5 instrumental activities of daily living (83%) and ≥1basic activities of daily living (74%). This study is the first to provide data regarding the unique needs of SGM caregivers of someone with dementia and will support the development of targeted interventions for this population.

### GENDER DIFFERENCES IN 30-DAY RE-HOSPITALIZATION AMONG MEDICARE BENEFICIARIES WITH ALZHEIMER'S AND DEMENTIA

Andrea Gilmore-Bykovskyi, *University of Wisconsin-Madison, Madison, Wisconsin, United States*

Hospitalization is associated with accelerated cognitive decline for persons with Alzheimer's disease and related dementia (ADRD), which disproportionately impacts women. Persons with ADRD are also at higher risk for 30-day rehospitalization, which may compound the impact of hospitalization-related exposures that precipitate decline. Evidence surrounding the intersections between gender and rehospitalization risk among diverse, representative populations with ADRD are lacking. This retrospective cohort study used a 100% national sample of Medicare beneficiaries with a diagnosis of ADRD and qualifying index hospitalization in 2014 (n= 1,033,144 unique beneficiaries and 1,672,238 unique stays). The primary outcome was rate of 30-day rehospitalization by gender and race. Within each racial group, men have higher rehospitalization rates than women: 2.6% higher among white men, 1.7% among African American men, and 2.6% higher among other racial/ethnic minorities. Findings highlight the importance of elucidating mechanisms underlying gender differences in hospital utilization and subsequent impact on cognitive decline.

### WOMEN'S SOCIAL EMPOWERMENT AND GENDER DIFFERENCES IN ADULTS' COGNITIVE COMPETENCES

Daniela Weber, *Vienna University of Economics and Business, Vienna, Wien, Austria*

Female social empowerment has the potential to enhance women's cognitive abilities. Our previous work investigating the role of gender equity in education and improved living conditions during early adulthood suggest that European women gain more from societal improvements over time than their male counterparts. This study extends this work by investigating the association between women's social empowerment during childhood and gender differences in adults' cognition for more than 30 OECD countries. We analyze established cognitive competence measures in literacy and numeracy with mixed effect models using the national survey data PIAAC collected within three rounds in 2011, 2014, and 2017. Our preliminary findings suggest that gender equity factors associated with women's empowerment, are more beneficial for women's cognitive key competences than men's. High cognitive competences are particularly relevant at advanced age to enable an independent life and long economic activity.

### SESSION 6155 (SYMPOSIUM)

### LEARNING WHILE DOING: COMMUNITY-ENGAGED ACTION RESEARCH WITH AGE-FRIENDLY COMMUNITY LEADERS

Chair: Althea Pestine-Stevens
Co-Chair: Emily Greenfield
Discussant: Stephanie Firestone

Age-Friendly Community Initiatives (AFCIs) are expanding throughout the United States to make social and built environments within local communities more responsive to population aging. With over 450 initiatives affiliated with the AARP Network of Age-Friendly Communities, Cities, and States (125 of which began in 2019-early 2020), rapid growth on the ground necessitates that theory and research develop alongside practice innovations. This symposium showcases the intersection of cutting-edge scholarship with community-based efforts to generate knowledge of community change processes that is immediately actionable by community leaders. Collectively, these papers emphasize the benefits of action research and developmental evaluation in community gerontology towards building the theories of age-friendly change that will set the stage for outcomes research. The first paper will present on work with 83 AFCIs in rural Maine involving interviews with organizational leaders to inform which types of supports could stimulate age-friendly changes to communities' built and social environments. The second paper will share a mixed-methods approach used to develop a global toolkit for dementia-friendly communities. The third presenter will describe the collaborative development and utilization of social network analysis to help age-friendly leaders plan their work, while simultaneously advancing research on variation in AFCI implementation. The final paper will present an evaluative framework that identifies roles and outcome measures for collective impact at the intersection of public health and age-friendly communities.

### UNIVERSITY-COMMUNITY PARTNERSHIPS TO DEVELOP A MUTUALLY BENEFICIAL TOOL TO MEASURE AGE-FRIENDLY COLLABORATION

Althea Pestine-Stevens,[1] and Emily Greenfield,[2] *1. Rutgers–New Brunswick, New Brunswick, New Jersey, United States, 2. Rutgers, The State University of New Jersey, New Brunswick, New Jersey, United States*

Age-friendly community initiatives (AFCIs) are frequently described as community collaborations; the AARP program model encourages diverse stakeholder engagement to achieve its aims of improving the social and built environments for long lives. However, little is known empirically about how AFCIs function as collaborations and how these relationships and activities lead to community changes. This paper presents how we developed a social network analysis tool to measure collaboration in AFCIs, which emerged from multi-year, university-community partnerships on AFCIs in western New York and northern New Jersey. Iterative processes, including inductive analysis of qualitative interviews and facilitated meetings with local AFCI work-groups, provided opportunities to create survey items on collaboration with meaning specific to AFCIs. We describe this tool's application as part of a survey of AFCI stakeholders, demonstrating how findings contribute both to advancing knowledge on AFCIs in general and directly informing the efforts of AFCI actors on the ground.

### BUILDING SUSTAINABLE RURAL AGE-FRIENDLY COMMUNITIES: GRASSROOTS PERSPECTIVES

Patricia Oh, *AARP, Bowdoinham, Maine, United States*

Joining the AARP Network of Age-Friendly States and Communities does not make a community age-friendly; the age-friendly team must cultivate community engagement, develop collaborations with diverse stakeholders, mobilize resources, and document achievements. Little research describes the tools age-friendly rural communities use to effect change and develop sustainability. Thematic content analysis of 67 interviews conducted between December 09, 2018 and January 24, 2020 with age-friendly leaders in rural Maine communities suggested that peer-to-peer networking, privileging local knowledge, engaging local and regional partners, technical advice from a trusted source, and fun were among the tools used to move age-friendly rural work forward.

### THE PROCESS OF DEVELOPING A WHO DEMENTIA-FRIENDLY COMMUNITY GLOBAL TOOLKIT: INPUT FROM MULTIPLE STAKEHOLDERS

Fei Sun,[1] katrin Seeher,[2] Stéfanie Fréel,[2] and Tarun Dua,[2] *1. Michigan State University, East Lansing, Michigan, United States, 2. WHO, Geneva, Geneve, Switzerland*

This study examined the process that the Department of Mental Health and Substance Abuse of WHO used to develop a global toolkit for dementia friendly initiatives (DFI). Data were collected through a mix-method approach consisting of individual interviews of 20 DFI leaders, four focus group interviews of persons living with dementia (PWD), three group interviews of professionals, and an online survey of 129 participants from 46 countries. Data from multiple sources were examined. The meaning of DFIs centered on the needs of PWD, multi-sector collaboration, and physical and social environmental changes. Over 70% participants in the survey reported their DFIs targeted PWD and included PWD as important partners. The EASTT model can be used to summarize DFI strategies including Education, Advocacy, Support, Training and Transforming environment. Countries advanced in DFI tended to focus on enhancing professional capacity and environmental adaptation, while countries launching DFI appeared to prioritize dementia awareness campaigns.

### EVALUATING COLLECTIVE IMPACT FOR HEALTHY AGING AT THE INTERSECTION OF PUBLIC HEALTH AND AGE-FRIENDLY COMMUNITIES

Kathy Black,[1] and Kathryn Hyer,[2] *1. University of South Florida, Sarasota-Manatee, Sarasota, Florida, United States, 2. University of South Florida, Tampa, Florida, United States*

There is mounting interest in promoting - and evaluating efforts that improve healthy aging in age-friendly communities. Additionally, there is increasing recognition that multisectoral engagement beyond the aging network is needed to maximize impact and sustainability. Within the context of collective impact, this paper reviews a framework that explicates public health activities in collaboration across a range of stakeholders in age-friendly communities. Metrics demonstrating evidence of five categorical roles, processes and outcomes will be presented including: 1) Connecting and Convening; 2) Coordinating; 3) Collecting and Disseminating Data; 4) Communicating; and 5) Complementing and Supplementing. Examples that illustrate evidence vis-à-vis the components and phases of collective impact will be presented.

### SESSION 6160 (SYMPOSIUM)

### LESSONS LEARNED IN RESEARCH TO ADDRESS WICKED PROBLEMS FACING THE CARE OF OLDER ADULTS ACROSS THE CARE CONTINUUM

Chair: Debra Dobbs
Discussant: Sheryl Zimmerman

A wicked problem, by definition, has innumerable causes, is tough to describe, and doesn't necessarily have a right answer. This describes today's health care for older adults across the long-term care continuum. Our interdisciplinary group has almost as many years (n=70) of experience as GSA conducting research in community-based groups across the continuum of care from skilled nursing facilities, assisted living communities, adult day care, to independent living. In this symposium we will discuss lessons learned in recruitment, intervention delivery, and unexpected outcomes. Peterson will discuss lessons learned in using large data sets to derive actionable information on staff license mix and SNF complaints. Dobbs will discuss the utility of using hospice nurses to train ALC nurses in delivering palliative care. Lee will discuss lessons in engaging direct care workers in their need for sleep. Meng will discuss learning to embrace an unexpected finding that friendships developed in a dementia family caregiver music and mindfulness intervention were as meaningful as positive health outcomes. Buck will discuss lessons learned in recruiting a hidden group – informal caregivers with complicated grief. Finally, Zimmerman, as expert long-term services and support discussant, will pull the pieces together across the studies to facilitate discussion. Enrich your future research related to addressing wicked problems in health care for older adults by learning from our experiences.

### THE INTEGRAL ROLE OF HOSPICE NURSE EDUCATORS IN A PALLIATIVE CARE EDUCATION PROGRAM FOR NURSES IN ASSISTED LIVING

Debra Dobbs, Hongdao Meng, William Haley, Harleah Buck, Kathryn Hyer, Maureen Templeman, and Carlyn Vogel, *University of South Florida, Tampa, Florida, United States*

Persons with dementia (PWD) are increasingly cared for in assisted living (AL) settings with an annual mortality rate of close to 20%. Palliative care (PC) for PWD in ALs can improve end-of-life care. From May, 2019 to February, 2020 a 4-week PC education in AL (PCEAL) program for nurses who provide care to PWD, facilitated by hospice nurses in Florida, was tested in a sample of nurses (N=20) in a cluster randomized trial (9 ALs, 4 treatment/5 control). We examined if PC knowledge increased from pre to post-intervention using a validated measure (Thompson, 2011). All intervention nurses (N=10) completed all four sessions of the PCEAL. While the baseline score was lower for the intervention group compared to the control group, the intervention group improved (M=2.20 to 2.37) compared to the control group (M=2.83 to 2.75) post-intervention. Two-month booster sessions indicate nurses have integrated PC care learned in the PCEAL program.

### LESSONS LEARNED IN A COMMUNITY-BASED, RANDOMIZED CONTROLLED TRIAL OF A COMPLICATED GRIEF INTERVENTION

Harleah Buck,[1] Paula Cairns,[1] Nnadozie Emechebe,[1] Diego Hernandez,[1] Tina Mason,[1] Jesse Bell,[2] Kevin Kip,[1] and Cindy Tofthagen,[3] *1. University of South Florida, Tampa, Florida, United States, 2. University of south florida, Tampa, Florida, United States, 3. Mayo Clinic, Jacksonville, Florida, United States*

Complicated grief (CG), severe, prolonged (>12 months) grieving, disproportionately affects older adults. A prospective two-group, waitlisted RCT examined whether four sessions of Accelerated Resolution Therapy (ART) was effective in informal caregivers by comparing pre-to-post ART changes and investigating variation in treatment response by baseline CG levels. Inclusion: ≥60 years, Inventory of Complicated Grief >25. Paired t-tests of mean (SD) differences compared pre- to post-ART; pre-ART to 8-week follow-up, and post-ART to 8-week follow-up; then stratified by median baseline level of CG. Mean (SD) age of 54 participants was 68.7 (7.2) years, 85% female, and 93% white. Significantly greater CG reduction (-22.8 (10.3)) vs. waitlist (-4.3 (6.0)) was found. Within-participant effect sizes from baseline to 8-weeks post treatment were 1.96 (95% CI: 1.45, 2.47; $p<0.0001$). Treatment effects did not substantially differ by baseline levels. Lesson learned was that it was possible to successfully recruit and treat CG in the community.

### THE ASSOCIATION OF NURSING HOME STAFFING LEVELS WITH CONSUMER COMPLAINTS

Lindsay Peterson,[1] John Bowblis,[2] Dylan Jester,[1] and Kathryn Hyer,[1] *1. University of South Florida, Tampa, Florida, United States, 2. Miami University, Oxford, Ohio, United States*

Nursing homes (NH) are inspected annually, however, residents and others can file complaints any time. Complaints are critical to NH oversight. Another important quality factor is staffing. Our objective was to examine the association of complaints and staffing levels in a 2017 sample of 14,194 freestanding NHs. We used federal data on NH complaints, quality, staffing, and other characteristics. The outcomes were having received at least one complaint (or not) and numbers of complaints. Using logit and negative binomial regression, controlling for facility and resident characteristics, we found greater registered nurse, nursing assistant, and social services staffing were associated with fewer complaints. Interestingly, licensed practical nurse (LPN) staffing was associated with a higher likelihood of receiving a complaint. Results are consistent with literature on nurse staffing and quality. LPN results raise questions about substituting LPNs for RNs. The social services results show social services staffing may be important for quality.

### BRIEF MINDFULNESS INTERVENTION FOR FAMILY CAREGIVERS OF PERSONS WITH DEMENTIA: A PILOT STUDY

Hongdao Meng,[1] Debra Dobbs,[1] Lindsay Peterson,[1] Punam Risal,[1] Britney Veal,[2] and William Monaco,[1]
*1. University of South Florida, Tampa, Florida, United States, 2. University of South Florida, Winter Haven, Florida, United States*

Family caregivers (FCGs) play a crucial role in helping persons with dementia age-in-place. This pilot study is designed to test a group-based mindfulness intervention for FCGs to reduce stress and enhance wellbeing. We recruited two groups of FCGs (n=7), one from an assisted living community and another from an adult day service center. We used a mixed-methods pre-post study design. Quantitative outcome measures included stress, sleep quality, and quality of life. We conducted focus group interviews to gain an in-depth understanding of the intervention acceptability. Participants reported that they benefited from participating in the intervention sessions, and were more aware of their reactions to stressors. FCGs indicated that the mindfulness practice assignments were acceptable and feasible. An unexpected finding was that the majority of the participants intended to continue with informal meetings after the pilot study. Implications of the intervention benefits for FCGs in the community will be discussed.

### PREVALENCE OF INSOMNIA SYMPTOMS AND NEED FOR AN INTERVENTION AMONG DIRECT-CARE WORKERS IN LONG-TERM CARE

Soomi Lee, Taylor Vigoureux, Kathryn Hyer, and Brent Small, *University of South Florida, Tampa, Florida, United States*

This study examined the prevalence of insomnia symptoms among direct-care workers at an assisted-living community and their perceived need for a sleep intervention. Thirty-five participants reported their main sleep-related concerns, willingness to participate in a sleep intervention, and preferred delivery forms/content of the intervention. They also reported nightly sleep characteristics via ecological momentary assessment (EMA) for 2 weeks. 80% reported any sleep-related concern; insomnia-related concern was most prevalent (57%). This was also evident in their EMA reports of waking up in the middle of the night or early morning for 72% of the days. Most (66%) expressed interest in participating in a sleep intervention either online or in group sessions. Mindfulness strategies were most preferred, followed by cognitive-behavioral therapy, and sleep hygiene education. The high prevalence of insomnia symptoms in direct-care workers needs to be addressed by future interventions for their well-being as well as for the quality of care.

### SESSION 6165 (SYMPOSIUM)

### LEST DEATH DO THEM PART: PROTECTION OF OLDER WOMEN FROM INTIMATE PARTNER INJURY AND FATALITY

Chair: Janet Wilson
Discussant: Lazelle Benefield

Protecting and preventing older women from fatal and near fatal violence by current or former intimate partners are public health and international human rights concerns. Older women are most often killed ("femicide"), injured in the home by a spouse, partner, x-partner, or family member. Older women experience physical, psychological, sexual abuse, neglect, and financial exploitation. Wherein there is more research on risk factors, study of protections lags behind. Through three very different studies, risks and protections will be explored with national and international policy implications for each.

### OLDER ADULT FATALITY REVIEWS: A POWERFUL TOOL TO IMPROVE POLICIES, PROTECTIONS, AND SERVICES

Janet Wilson,[1] and Kimethria Jackson,[2] *1. University of Oklahoma Health Sciences Center, Reynolds Center for Geriatric Nursing Excellence, Oklahoma City, Oklahoma, United States, 2. University of Oklahoma Health Sciences Center, Oklahoma City, Oklahoma, United States*

Older adult maltreatment is a serious problem that can hasten mortality and cause fatality. Child and Domestic Violence Fatality Review teams have made a major impact to improve protections, services, and system responses for victims. There are over 1300 child fatality review teams in 50 US states and 200 domestic violence fatality teams in 45 states. Older adult fatality reviews, in contrast, have not proliferated across the country, missing opportunities to bring interdisciplinary expertise together to resolve policy, protection, and services needed to prevent older adult premature deaths due to violence and abuse. Method: A mini mock review of a fatality of an older adult woman will demonstrate the possibilities of beginning Older Adult Fatality Review Board as recommended by the American Bar Association. Conclusion: Interdisciplinary older adult fatality review teams have been an underutilized tool to make changes in older adult mistreatment protection services and systems.

### INJURY OF AFRICAN AMERICAN WOMEN BY FINANCIAL EXPLOITATION

Kimethria Jackson,[1] Patsy Smith,[1] and Janet Wilson,[2]
*1. University of Oklahoma Health Sciences Center, Oklahoma City, Oklahoma, United States, 2. University of Oklahoma Health Sciences Center, Reynolds Center for Geriatric Nursing Excellence, Oklahoma City, Oklahoma, United States*

Financial exploitation (FE) is one of the most common forms of older adult mistreatments. The World Bank defines FE as financial violence and the World Health Organization describes FE as financial or material abuse. Both international organizations recognize that FE causes deprivation/neglect leading to physical and emotional injury to victims. Older African Americans (OAA) are disproportionately affected by FE, impacting their health and welfare. A qualitative phenomenological study explored the lived experiences of FE among OAA. A Community Based Participatory Research approach was used to partner with a predominately African American-faith based community. Participant recruitment (n=12) was through community sponsored seminars that included verbal presentations, group discussions; individual surveys, Older Adult Financial Exploitation Measure (OAFEM). OAFEM data was analyzed to identify risks. Analysis of the interviews included Open and Axial coding, and data categorization.

Using NVivo, Stevick-Colaizzi-Keen analysis as modified by Moustakas, five themes emerged.

### HOME HEALTH REGISTERED NURSES' EXPERIENCES WITH FINANCIAL EXPLOITATION OF OLDER WOMEN WITH COGNITIVE IMPAIRMENT

Carol Rogers,[1] Lisa DeSpain,[2] and Janet Wilson,[1]
*1. University of Oklahoma Health Sciences Center, Reynolds Center for Geriatric Nursing Excellence, Oklahoma City, Oklahoma, United States, 2. University of Oklahoma Health Sciences Center, Oklahoma City, Oklahoma, United States*

Older adults diagnosed with cognitive impairment (CI) who live at home are at high risk for FE due to dependence on caregivers and diminishing cognitive and financial capacities. Health care providers are mandated reporters for elder abuse, that includes financial exploitation (FE), one of the seven types of older adult maltreatments. Twenty Home Health Care Nurses (HHRN) of older adults in Oklahoma were interviewed to discover their understanding and experiences with FE. Transcripts were analyzed by conventional content analysis. Line-by-line codes were generated inductively and codes were grouped into categories and themes until data saturation was reached. Five themes emerged: Red Flags, Familiar Offenders, Dire Consequences, Barriers/Facilitators, Doing Better. Conclusions: HHRNs are an untapped resource to provide suggestions for improvements of FE detection/reporting of older adults with CI and to help formulate policies, procedures, strategies to improve coordination and communication among healthcare, law enforcement, and social service systems.

### SESSION 6170 (SYMPOSIUM)

### LET'S STOP SPINNING OUR WHEELS: STRENGTHENING THE CASE FOR COMMUNITY-ENGAGED TRANSPORTATION SOLUTIONS

Chair: Holly Dabelko-Schoeny
Co-Chair: Noelle Fields
Discussant: Nina Silverstein

On average in the United States, older adults outlive their ability to drive by seven years. Having safe, affordable and accessible alternative transportation options is critical to supporting the well-being of older adults and their ability to age in community. This symposium will provide evidence for utilizing community-engaged research strategies with diverse populations to identify the opportunities and barriers for the development and utilization of alternative transportation. The presentations will include up-to-date examples of innovative alternative transportation solutions and evaluation data. The first presentation will illustrate how community-based participatory research (CBPR) strategies were used to develop, use and evaluate alternative transportation options including walking, biking, fixed route busing, senior circulator, ride sharing and transit training in an age-friendly community. Data were collected through mapping the built environment, an electronic daily transportation diary app called "MyAmble" on tablets, walk audits and focus groups. The second presentation uses an environmental justice (EJ) framework to present the findings of a qualitative interpretive meta-synthesis (QIMS) about older adults' experiences with outdoor spaces and buildings and transportation as part of the World Health Organization's age-friendly network assessment. The third paper explores the barriers and facilitators of transportation among diverse older adults (English, Nepali, Khmer, Somali, Russian and Mandarin Chinese) using Rapid and Rigorous Qualitative Data Analysis (RADaR) technique. The final paper examines the challenges of rural transportation services supported by senior centers. The symposium will conclude with a critical reflection on the empirical contributions needed to advance scholarship on alternative transportation for older adults.

### USING COMMUNITY-BASED RESEARCH STRATEGIES IN AGE-FRIENDLY COMMUNITIES TO SOLVE MOBILITY CHALLENGES

Holly Dabelko-Schoeny,[1] Noelle Fields,[2] Katie White,[3] Marisa Sheldon,[3] Sarah Robinson,[4] Ian Murphy,[1] and Claire Jennings,[1] *1. The Ohio State University, Columbus, Ohio, United States, 2. The University of Texas at Arlington, Arlington, Texas, United States, 3. Age Friendly Columbus, Columbus, Ohio, United States, 4. University of Texas at Arlington, Arlington, Texas, United States*

Aging is linked to an increased risk of disability. Disabilities that limit major life activities such as seeing, walking, and motor skills impact a person's ability to drive a car. Low utilization of alternative transportation by older adults may put them at risk for social isolation. The purpose of this paper is to illustrate how community-based participatory research (CBPR) was used to engage older residents in the development of alternative transportation options in a metropolitan county in the Midwestern U.S. Older residents worked as co-investigators to develop, use and evaluate alternative transportation options including walking, biking, fixed route busing, senior circulator, ride sharing, and transit training. Data were collected through mapping the built environment, an electronic daily transportation diary app called "MyAmble" on tablets, walk audits and focus groups. CBPR approaches led by interdisciplinary teams resulted in community engagement and more equitable strategies for transportation planning and utilization.

### OUTDOOR SPACES, TRANSPORTATION, AND ENVIRONMENTAL JUSTICE: A QUALITATIVE INTERPRETIVE META-SYNTHESIS

Noelle Fields,[1] Kristen Ravi,[2] and Holly Dabelko-Schoeny,[3]
*1. The University of Texas at Arlington, Arlington, Texas, United States, 2. University of Texas at Arlington, Arlington, Texas, United States, 3. The Ohio State University, Columbus, Ohio, United States*

Age-friendly environments aim to promote healthy and active aging by building and maintaining capacity across the life course and allow people who have a loss of capacity to continue engaging in activities that they value. Age-friendly community assessments are being conducted worldwide. This qualitative interpretive meta-synthesis (QIMS) aims to create a rich description of older adults' experiences with outdoor spaces and buildings as well as transportation as part of an age-friendly assessment. The themes that emerged regarding older adults' experiences with outdoor space and buildings included 1) accessibility and 2) appropriate infrastructure. Regarding transportation, the theme of accessibility included subthemes of 1) availability and 2) affordability. Further reduction indicated that age-friendliness can be conceptualized as environmental justice (EJ). The three areas of EJ including distributional justice, procedural justice, and recognition provide a helpful framework for social workers and their interdisciplinary partners to systematically document and evaluate their age-friendly community efforts.

### WE WANT TO GO, BUT THERE ARE NO OPTIONS: FACILITATORS AND BARRIERS OF TRANSPORTATION AMONG DIVERSE OLDER ADULTS

Quichang Cao,[1] Arati Maleku,[1] Katie White,[2] Basar Ozbilen,[1] and Holly Dabelko-Schoeny,[1] *1. The Ohio State University, Columbus, Ohio, United States, 2. Age Friendly Columbus, Columbus, Ohio, United States*

Transportation plays an important role in social connectedness and quality of life among older adults. Despite the increasing number of diverse older adults in the U.S., few studies have explored the barriers and facilitators of transportation among this group. We conducted eight 90-minute focus groups in six languages (English, Nepali, Khmer, Somali, Russian and Mandarin) with older volunteers (N=70) in a large Midwestern metropolitan city. Using the Rapid and Rigorous Qualitative Data Analysis (RADaR) technique, four transportation determinants emerged: (1) Service: affordability, accessibility, availability, and reliability, lack of options, and service coordination (2) Built environment: safety and walkability; (3) Social environment: language barriers and lack of information, neighborhood cohesion; and (4) Individual determinants: ability to drive, walk, and family support system. Results reveal the interconnectedness of multi-level determinants and the need for a systematic approach to improve transportation access among diverse older adults.

### IDENTIFYING THE TRANSPORTATION NEEDS OF A RURAL AGING COMMUNITY: FINDINGS FROM A COMMUNITY ASSESSMENT

Vivian Miller, Jordan Wilfong, Melissa Burek, and Logan Lanson, *Bowling Green State University, Bowling Green, Ohio, United States*

Community senior centers are tasked with providing aging adults services and programs, congregate meals, and transportation, set forth by the Older Americans Act. The overall function of senior centers is especially critical for rural communities, as rural communities are home to a greater proportion of older adults compared to metropolitan and low-density urban areas. To assess the current needs of the aging population in rural Northwest, OH, a total of 9 focus groups were held (N=45) as part of a larger mixed-methodological study. Through this work, older adults identified limitations of the current senior center transportation. A lack of transportation and accessibility of current transportation were noted as barriers to full senior center use. Findings from this study confirm disparities in rural transportation; a lack of transportation is a major obstacle to program completion. Recommendations address the unique challenges and needs for transportation services for in rural communities are presented.

### SESSION 6180 (SYMPOSIUM)

### PATHWAYS TO AND CONSEQUENCES OF SURROGATE DECISION-MAKING FOR OLDER ADULTS

Chair: Pamela Teaster
Co-Chair: Cory Bolkan
Discussant: Shawn Meyers

With a burgeoning aging population, there is a growing need for surrogate decision makers, yet oversight of and guidance for them remains inadequate. People needing surrogate decision makers are an especially vulnerable population because they rely on others for care and/or are unable to advocate for themselves. Their vulnerability leaves them susceptible to elder abuse (e.g., physical, sexual, psychological abuse; active and passive neglect; financial exploitation), which affects approximately five million older Americans each year. Personal, financial, and societal impacts can be devastating and are estimated to cost billions annually. The issue of abuse, neglect and exploitation by surrogates has been highly visible nationally, evidence indicates that some surrogate decision makers perpetrate abuse. One purpose of this symposium is to discuss ways in which surrogates do and do not make decisions for older adults. Ramsey-Klawsnik and Burnett present data at the systemic level to illustrate how self-neglect sequelae can result in placement under surrogate decision-making authority of either well-intended or opportunistic others. Bolkan, Teaster, Ramsey-Klawsnik, and Gerow present findings from a six-state study on surrogate decision maker victims and perpetrators who were substantiated in Adult Protective Services cases. Zhao, Katz, and Teaster show, using a survey of M-Turk participants, how a general population makes and is comfortable with surrogate decisions. Discussant Shawn Meyers will pull together the findings by exploring their translation to judicial best practices for making determinations regarding surrogate decision makers and the effects of their decisions on the surrogate as well as collaterals.

### ABUSE OF VULNERABLE OLDER ADULTS BY DESIGNATED SURROGATE DECISION MAKERS

Cory Bolkan,[1] Pamela Teaster,[2] Holly Ramsey-Klawsnik,[3] and Kenneth Gerow,[4] *1. Washington State University, Vancouver, Washington, United States, 2. Virginia Tech, BLACKSBURG, Virginia, United States, 3. Self-Employed, Canton, Massachusetts, United States, 4. University of Wyoming, laramie, Wyoming, United States*

Abuse perpetrated by designated surrogates has become highly visible nationally, yet no reliable data exist on its nature or extent. Because vulnerable older adults needing surrogate decision makers typically rely upon others for care, they may be unable to advocate for themselves and are susceptible to abuse. We prospectively gathered Adult Protective Services (APS) data from six geographically diverse counties on over 400 substantiated cases of abuse by perpetrators (53% non-surrogates; 47% documented or claimed surrogate) of vulnerable adults 65+ living in community settings. Most perpetrators (85%) were designated power of attorney, while approximately 8% were guardians, and 7% were representative payees; most perpetrators were family members. Polyabuse occurred frequently. Almost 25% of cases involved a prior substantiated APS report. This presentation highlights how surrogates perpetuate abuse and outcomes on older adult victims. Our findings inform practice and policy for better prevention, detection, investigation, and intervention in these challenging cases.

### SELF-NEGLECT: ONE PATHWAY TO SURROGATE DECISION-MAKING

Holly Ramsey-Klawsnik,[1] and Jason Burnett,[2],
*1. Self-Employed, Canton, Massachusetts, United States,*
*2. McGovern Medical School at UTHealth, Houston, Texas, United States*

Self-neglect, the most frequently reported allegation to Adult Protective Services (APS), involves profoundly harmful behaviors often due to functional or cognitive limitations, health problems, and insufficient resources that result in older adults insufficiently meeting their basic needs. Outcomes include high risk of illness, hospitalization and readmission, hospice and nursing home use, early mortality, and placement under surrogate decision-making authority of either well-intended or opportunistic others. APS staff are charged with assessing self-neglect and intervening to reduce client danger. A nationwide APS survey revealed program policies, procedures, resources, and needs affecting the client welfare. For example, 92% of APS programs have provisions for seeking guardianship for self-neglecting individuals, in 25% of programs staff serve as court-appointed guardians, and a wide variety of tools are used within APS programs to assess clients' mental capacity. Key study findings, implications, and recommendations will be presented.

### A SCENARIO-BASED INVESTIGATION OF SURROGATE DECISION MAKING FOR OLDER ADULTS

Yuxin Zhao,[1] Benjamin Katz,[2] and Pamela Teaster,[3]
*1. Booz Allen Hamilton, Bethesda, Maryland, United States,*
*2. Virginia Tech, Blacksburg, Virginia, United States,*
*3. Virginia Tech, BLACKSBURG, Virginia, United States*

Surrogate decisions involve complex, challenging choices; surrogate decision-makers make treatment decisions for approximately 40% of hospitalized adults and 70% of older adults, and up to 95% of critically ill adults of any age. The purpose of our study was to understand how people make decisions for others and how surrogate decision making is linked to people's cognition, self-efficacy, and demographics, especially differences in acute (e.g., health and medical care, financial management, and end of life) versus general scenarios (spending time with family, contacting an insurance company on behalf of a family member). Participants were recruited through Amazon's Mechanical Turk. We collected data from 290 adult participants aged 18 years or older. On average, people reported a higher level of confidence in general versus acute scenario. The differences of confidence in scenario-based surrogate decision-making links to decision-makers' cognition, self-efficacy, the experience of decision-making, the experience of caregiving, and demographic factors.

### SESSION 6185 (SYMPOSIUM)

### PERCEPTION VERSUS REALITY: SUBJECTIVE AND OBJECTIVE NEIGHBORHOOD CHARACTERISTICS AND COGNITIVE FUNCTION IN REGARDS

Chair: Jessica Finlay
Co-Chair: Philippa Clarke
Discussant: Lisa Barnes

Does the world shrink as we age? The neighborhood captures a spatial area someone inhabits and moves through on a daily basis. It reflects a balance between internal perceptions and abilities, and the external environment which may enable or restrict participation in everyday life. We frequently hear that older adults have shrinking neighborhoods given declining functional mobility. This is associated with declines in physical and cognitive functioning, depression, poorer quality of life, and mortality. Knowledge of the interplay between objective and subjective neighborhood measurement remains limited. This symposium will explore these linked yet distinct constructs based on secondary data analyses of the Reasons for Geographic and Racial Differences in Stroke (REGARDS) study, a racially diverse sample of 30,000+ aging Americans. Finlay investigates how someone's perceived neighborhood size (in number of blocks) varies by individual and geographic characteristics including age, cognitive function, self-rated health, and urban/rural context. Esposito's analyses focus on neighborhood size in relation to race and residential segregation. Clarke compares subjective perceptions of neighborhood parks and safety from crime to objective indicators, and examines variations by health and cognitive status. Barnes will critically consider implications for how older adults interpret and engage with their surrounding environments. The symposium questions the validity of neighborhood-based metrics to reflect the perspectives and experiences of older residents, particularly those navigating cognitive decline. It informs policy-making efforts to improve physical neighborhood environments and social community contexts, which are critical to the health and well-being of older adults aging in place.

### MY NEIGHBORHOOD IS FUZZY, NOT HARD AND FAST: A MIXED-METHODS STUDY OF NEIGHBORHOOD SIZE AMONG AGING AMERICANS

Jessica Finlay, Joy Bohyun Jang, Michael Esposito, Sandra Tang, Anam Khan, and Philippa Clarke, *University of Michigan, Ann Arbor, Michigan, United States*

In this exploratory sequential mixed-methods study, interviews with 125 adults aged 55-92 living in the Minneapolis (Minnesota) metropolitan area suggest that neighborhood boundaries are "fuzzy". Qualitative analysis of neighborhood perceptions identified race, mobility, driving status, social connections, housing insecurity, land use, urbanicity, and crime as key themes. Over 8,000 REGARDS participants (mean age 72) indicated how many blocks composed their neighborhoods (mean=9.9, SD=35.4). Linear regression models showed that being over the age of 85, white, less educated, lower income, less physically and cognitively healthy, and living outside of a metropolitan area significantly predicted smaller self-reported neighborhood sizes. Further, there was significant variation among participants residing in the same areas as other respondents. The mixed-methods results indicate that neighborhoods are fluid and dependent on a mix of personal and geographic factors. Findings inform the scale of environmental audits, place-based interventions, and community outreach programs targeting older adults.

### BLACK-WHITE INEQUITIES IN PERCEPTION OF ACCESS TO NEIGHBORHOOD RESOURCES AMONG OLDER ADULTS

Michael Esposito, Dominique Sylvers, Philippa Clarke, Jessica Finlay, Joy Bohyun Jang, and Sandra Tang, *University of Michigan, Ann Arbor, Michigan, United States*

Drawing on insights from critical race scholarship, this study examines how access to neighborhood resources varies among black and white older adults. Using Bayesian multi-level models, we estimate how evaluations of one's neighborhood environment (e.g., perceived access to parks) varies by race, conditional on objective environmental measures (e.g., park area in one's neighborhood). Results suggest that within the same spatial areas, individuals occupying marginalized statuses are less likely to perceive their neighborhoods as providing sufficient/accessible collective goods. Conditional on living in tracts with equal public park infrastructure, for instance, black respondents are 15% [95-CI: 8%, 22%] more likely to describe their neighborhoods as "lacking accessible parks." Results suggest that these inequities are further exacerbated by race-related structural features (e.g., residential segregation) and other markers of welfare and marginalization (e.g., cognitive function). Overall, findings suggest that access to neighborhood-resources—and the benefits they confer—are fashioned by broader systems of power and inequality.

### PARKS AND SAFETY: HOW COGNITIVE DECLINE MAY IMPACT ACCESS AND PERCEPTION

Philippa Clarke, Michael Esposito, Joy Bohyun Jang, Sandra Tang, Anam Khan, Dominique Sylvers, and Jessica Finlay, *University of Michigan, Ann Arbor, Michigan, United States*

Older adults' perceptions of the presence and quality of neighborhood resources provide important information about the potential benefit of those resources but are not necessarily concordant with the actual physical resources available in that environment. There is debate about whether subjective perceptions of local context are more important for individual behavior and well-being than objective indicators of resources. However, little research has examined how cognitive function is related to differences in the perceived availability and quality of neighborhood resources among older adults. We found that subjective reports of neighborhood safety and adequacy of parks were positively associated with objective measures of property crime and park density. Cognitive function was associated with higher subjective neighborhood evaluations, but adults with lower cognitive function reported more discordance between objective and subjective measures of neighborhood resources. These findings inform how neighborhood resources may have different consequences for older adults experiencing cognitive decline.

### SESSION 6190 (SYMPOSIUM)

### PERSON-CENTERED HEALTH CARE: AN APPROACH THAT INTEGRATES ACUTE AND LONG-TERM CARE

Chair: Yuchi Young
Discussant: Barbara Resnick

The world population is aging. The proportion of the population over 60 will nearly double from 12% in 2015 to 22% in 2050. Global life expectancy has more than doubled from 31 years in 1900 to 72.6 years in 2019. The need for long-term care (LTC) services is expanding with the same rapidity. A comprehensive response is needed to address the needs of older adults. Learning from health systems in other countries enables health systems to incorporate best long-term care practices to fit each country and its culture.

**This symposium aims to compare long-term care policies and services in Taiwan, Singapore, and the USA where significant growth in aging populations is evidenced. In 2025, the aging population will be 20% in Taiwan, 20% in Singapore and 18 % in the USA. In the case of Taiwan, it has moved from aging society status to aged society, and to super-aged society in 27 years. Such accelerated rate of aging in Taiwan is unparalleled when compared to European countries and the United States. In response to this dramatic change, Taiwan has passed long-term care legislation that expands services to care for older adults, and developed person-centered health care that integrates acute and long-term care services. Some preliminary results related to access, care and patterns of utilization will be shared in the symposium. International Comparisons of Healthy Aging Interest Group Sponsored Symposium.**

### IMPACT OF HEALTH SYSTEMS ON LIFE EXPECTANCY IN TAIWAN, SINGAPORE, AND THE USA: SIMILARITIES AND DIFFERENCES

Ya-Mei Chen,[1] Yuchi Young,[2] and Patrick Schumacher,[3] *1. National Taiwan University, Taipei, Taiwan (Republic of China), 2. SUNY at Albany, Rensselaer, New York, United States, 3. University at Albany State University of New York, Rensselaer, New York, United States*

Like Singapore, Taiwan has universal health care and universal long-term care (LTC). The USA has neither. Those who need LTC in the US pay out of pocket, buy private LTC insurance or spend down to qualify for Medicaid; there are inherent issues related to cost and quality. For example, nursing home care costs over US$100,000/year and only about 8% of the population has private LTC insurance. Many people become impoverished or struggle financially to qualify for Medicaid. Conversely, Taiwan has universal LTC insurance that offers comprehensive services for all, but it is relatively new and its impact remains to be seen. Similarities and differences related to LTC quality indicators such as functional independence, and the NCQA quality measures (e.g., effectiveness of care, access/availability) in Taiwan, Singapore and the US will be presented. The results of this comparison can inform policy makers and stakeholders leading to best practices. Part of a symposium sponsored by International Comparisons of Healthy Aging Interest Group.

### DISABILITY AND LONG-TERM CARE IN SINGAPORE

Cynthia Chen Huijun, and Ngee Choon CHIA, *National University of Singapore, Singapore, Singapore*

Public systems for long term care (LTC) redistribute resources between generations. Population aging is one of the most significant transformations in the 21st century, where the number of older persons aged 60 years and above is expected to double by 2050, rising to 2.1 billion. We used the Future Elderly Model (FEM) to project the impact of population aging in Singapore up to the year 2050. The FEM is a dynamic economic and demographic microsimulation model. By 2050, the total number of older adults with potential limitation in activities of daily living (ADL) was projected to increase to 275 thousand (18.9%). With the increasing prevalence of disability and chronic diseases, older adults might not have sufficient savings to meet future needs sustainably, despite the expansion of disability insurance from ElderShield to CareShield Life. Lessons and best practices for LTC could be transferred from our experiences to other aging cities globally. Part of a symposium sponsored by International Comparisons of Healthy Aging Interest Group.

### DISPARITIES IN THE ACCESS TO DIFFERENT TYPES OF LONG-TERM CARE SERVICES IN TAIWAN

Ya-Mei Chen,[1] Yuchi Young,[2] Shih-Cyuan Wu,[3] Hsiu-Hsi Chen,[1] Yen-Po Yeh,[4] Wan-Yu Chiu,[1] and Kuo-Piao Chung,[5] *1. National Taiwan University, Taipei, Taiwan (Republic of China), 2. SUNY at Albany, Rensselaer, New York, United States, 3. National Taiwan University, Taipei, Taipei, Taiwan, 4. Changhua County Public Health Bureau, Changhua, Taiwan (Republic of China), 5. Institute of Health Policy and Management, National Taiwan University, Taipei, Taipei, Taiwan*

Taiwan implemented version 1.0 of its 10-Year Long-Term Care Plan (TLTCP 1.0) in 2007, and version 2.0 (TLTCP 2.0) in 2017. The aim was to develop a system of home- and community-based services (HCBS) for long-term care (LTC). This study assessed and compared disparities in access to LTC services using one county's database that claims made by 14,051 older adults who applied for LTC services from TLTCP 1.0 (n = 5,025) and TLTCP 2.0 (n = 9,026). We assessed LTC disparities related to five sociodemographic factors (age, gender, living status, urbanization, income status) and seven types of LTC services. Older adults who lived in a city were more likely to use multiple HCBS and transportation services. Older adults who lived alone were more likely to use home care and meal services but not other types of LTC services. All disparities in service use increased from TLTCP 1.0 to TLTCP 2.0. Part of a symposium sponsored by International Comparisons of Healthy Aging Interest Group.

### WHO ARE THE HEAVY USERS OF ACUTE AND LONG-TERM CARE SERVICES IN CHANGHUA COUNTY, TAIWAN?

Yuchi Young,[1] Ye-Mei Chen,[2] Kuo-Piao Chen,[2] Hsiu-Hsi Chen,[2] Wei-Jung Chang,[2] Wan-Hsiang Hsu,[3] and Yen-Po Yeh,[4] *1. SUNY at Albany, Rensselaer, New York, United States, 2. National Taiwan University, Taipei, Taiwan (Republic of China), 3. New York State Department of Health, Albany, New York, United States, 4. Changhua County Public Health Bureau, Changhua, Taiwan (Republic of China)*

Identification of heavy health care users among community-dwelling older adults can lead to strategic care planning and management that positively impacts health care use and costs. This study aims to identify risk factors associated with heavy users of acute care and long-term care (LTC) in Changhua County, Taiwan. Study participants (n=8,090) included residents (age 65+) of Changhua County. Data were collected from 4/1/2017-10/26/2018. Linked hospitalization and LTC use information was provided by the Changhua County Public Health Bureau. Hospitalization was grouped into 1 vs. 2+. Univariate and multivariate logistic models will be used to address the study aim. Preliminary results show the average age was 81 years ranging from 65-105. Of this population, 56% are female, and 10% live alone. The average length of hospital stay was 9.7 days. Risk factors associated with heavy health care users identified in multivariate analysis will be presented and intervention strategies will be discussed. Part of a symposium sponsored by International Comparisons of Healthy Aging Interest Group.

### PERSON-CENTERED HEALTH CARE: AN APPROACH THAT INTEGRATES ACUTE AND LONG-TERM CARE

Yuchi Young,[1] Ye-Mei Chen,[2] Kuo-Piao Chung,[3] Patrick Schumacher,[4] Hsiu-Hsi Chen,[2] and Yen-Po Yeh,[5] *1. SUNY at Albany, Rensselaer, New York, United States, 2. National Taiwan University, Taipei, Taiwan (Republic of China), 3. Institute of Health Policy and Management, National Taiwan University, Taipei, Taipei, Taiwan, 4. University at Albany State University of New York, Rensselaer, New York, United States, 5. Changhua County Public Health Bureau, Changhua, Taiwan (Republic of China)*

The concept of person-centered health care (PCHC) shifts from the individual's diagnosis to the holistic health and individual social needs. PCHC is defined by WHO as "empowering people to take charge of their own health rather than being passive recipients of services." This care strategy is based on the belief that if an individual is an equal partner in care planning and care decision making process, and that his/her view, input, and experiences can help improve overall health outcomes. PCHC requires a reconfiguration of the health care system. A vertical or/and horizontal integration of a variety of healthcare settings is necessary. A proposed 5-year PCHC pilot study in Taiwan's Changhua county aims to reduce unnecessary hospitalizations by 15% in 4 years. Year one will focus on alignment of health care providers, baseline data collection, and assessment plans development. PCHC intervention will start in years two to five. Part of a symposium sponsored by International Comparisons of Healthy Aging Interest Group.

### SESSION 6200 (SYMPOSIUM)

### PSYCHOACTIVE PRESCRIBING IN ASSISTED LIVING

Chair: Sheryl Zimmerman
Discussant: Philip Sloane

More than 800,000 older adults reside in almost 30,000 assisted living (AL) communities across the country, 42% of who have moderate or severe dementia. As many as 97%

of persons with dementia display symptoms of distress such as agitation or anxiety, and 69% of AL communities provide medications to treat this distress. Unfortunately, the psychoactive medications that are prescribed are often ineffective, contraindicated, and may cause serious adverse events including mortality. Especially concerning is the use of antipsychotic medications, which carry a black-box warning for persons with dementia. This symposium will present data from a seven state study of 250 AL communities and the 13,600 individuals who reside there. The first speaker will discuss the prevalence of psychoactive prescribing in AL overall and by medication type, and community characteristics that relate to use (e.g., staffing, resident case-mix). The second presentation will focus on the use of pro re nata (PRN) psychotropic medications, to examine the extent to which use is situational. The third speaker will address the use of off-label antipsychotic medications, and typologies of AL communities that differentiate use. The fourth speaker will discuss the prevalence of potential antipsychotic side-effects and adverse events, and also family member knowledge of medication use. The final speaker will compare the use of antipsychotic and antianxiety prescribing in proximate AL communities and nursing homes, to examine the extent to which local prescribing patterns may influence use. All five presentations of this symposium convey important issues for practice, policy, and future research.

### ANTIPSYCHOTIC AND PSYCHOTROPIC PRESCRIBING IN ASSISTED LIVING

Sheryl Zimmerman,[1] Philip Sloane,[1] Christopher Wretman,[1] Stephanie Miller,[2] and John Preisser,[1] *1. University of North Carolina at Chapel Hill, Chapel Hill, North Carolina, United States, 2. Cecil G. Sheps Center for Health Services Research, Chapel Hill, North Carolina, United States*

The Center for Medicare & Medicaid Services has brought national attention to potentially inappropriate antipsychotic prescribing for residents with dementia in nursing homes, but no such attention has been paid to prescribing in assisted living (AL), despite the fact that more than 40% of AL residents have moderate/severe dementia. This study examined antipsychotic and other psychotropic prescribing in AL using a seven-state sample of 250 communities, and examined correlates of prescribing; analyses used weights based on probability proportional to bed size to derive state-level estimates. Overall, 19% of residents were taking an antipsychotic medication (27% of those with dementia); corresponding numbers for antidepressants were 46% (54%) and for anxiolytics/hypnotics were 24% (28%; both p <.001). By way of example, communities that did not have an RN or LPN were more likely to prescribe antipsychotics (34% vs. 17%; p<.001). The discussion will highlight next steps regarding practice, policy, and research.

### PRO RE NATA USE OF PSYCHOTROPIC MEDICATIONS IN ASSISTED LIVING

Paula Carder,[1] Sheryl Zimmerman,[2] Christopher Wretman,[2] Sarah Dys,[3] and Philip Sloane,[2] *1. Portland State University, Portland, Oregon, United States, 2. University of North Carolina at Chapel Hill, Chapel Hill, North Carolina, United States, 3. Institute on Aging, Portland, Oregon, United States*

This study examined the use of pro re nata (PRN, or as needed) psychotropic medications among assisted living (AL) residents. We examined prescriptions and administrations, and compared use based on dementia diagnosis. Data sources included interviews with administrators of 250 AL communities in 7 states and medication administration record review for the prior 7 days; analyses were weighted to the entire state. The percent of all residents prescribed a PRN psychotropic medication was 10.3%. However, residents with a dementia diagnosis were twice as likely to have a PRN psychotropic prescription (15.2% versus 7.2%; p<.001). The majority of psychotropic medications prescribed and administered were for anxiolytics/hypnotics rather than antipsychotics. Additional resident-level factors significantly associated with higher PRN prescribing included psychiatric diagnosis, incontinence, hospice use, confusion/disorientation, and agitation. We summarize these and other findings in the context of state regulatory requirements for staffing, chemical restraints, and dementia care.

### OFF-LABEL ANTIPSYCHOTIC PRESCRIBING FOR PERSONS WITH DEMENTIA IN ASSISTED LIVING

Christopher Wretman, Sheryl Zimmerman, Philip Sloane, and John Preisser, *University of North Carolina at Chapel Hill, Chapel Hill, North Carolina, United States*

Off-label antipsychotic use is contra-indicated for persons with dementia due to lack of efficacy and FDA black box warnings. Yet, residents in assisted living (AL), including those with dementia, are frequently prescribed such medications. If distinct typologies of AL communities exist based on prescribing rates, it might be possible to reduce use. Toward that end, data from 247 AL communities in seven states were analyzed to discern potential unobserved latent classes that differentiate prescribing levels. Results using finite mixture modeling determined a 5-class solution best fit the data, with class-level prescribing rates ranging from 16.9% of residents with dementia to 27.4% (Mean = 18.9%). Bivariate tests found differences across classes by variables related to community structure, medication processes, and resident-case mix (e.g., frequent formal pharmacy review was more likely in communities with higher prescribing). Typologies are useful to identify differences and care and may be useful for quality improvement.

### POTENTIAL ANTIPSYCHOTIC SIDE EFFECTS AND ADVERSE EVENTS OF ASSISTED LIVING RESIDENTS WITH DEMENTIA

Anna Beeber,[1] Sheryl Zimmerman,[1] Christopher Wretman,[1] Stephanie Miller,[2] Kush Patel,[3] and Philip Sloane,[1] *1. University of North Carolina at Chapel Hill, Chapel Hill, North Carolina, United States, 2. Cecil G. Sheps Center for Health Services Research, Chapel Hill, North Carolina, United States, 3. Northeast Ohio Medical University, Rootstown, Ohio, United States*

This presentation provides the findings from a descriptive study examining the potential adverse events and side effects of antipsychotic medications prescribed to assisted living (AL) residents with dementia drawn from interviews with family members and chart review on 238 AL residents, from 91 AL communities in seven US states. We found that 85% of family reported that medication had been administered for agitation or aggression, 93% of the sample experienced at least one potential side effect, and 19% experienced five or more. The most common potential side effects were neurologic/psychological effects (89% of residents), and somnolence during the day (81%). Six percent of the sample experienced at least one potential adverse event. This work implies a need for caution when prescribing antipsychotics to older adults with dementia in AL. Medication management efforts should extend to monitoring AL residents for potential side effects and adverse events from specific psychoactive medications.

### LOCAL NURSING HOME PRESCRIBING PATTERNS AND PSYCHOACTIVE PRESCRIBING IN ASSISTED LIVING

Kali Thomas,[1] Christopher Wretman,[2] Philip Sloane,[2] Anna Beeber,[2] Paula Carder,[3] Lindsay Schwartz,[4] Sheryl Zimmerman,[2] and Johanna Silbersack,[5] *1. Brown University, Providence, Rhode Island, United States, 2. University of North Carolina at Chapel Hill, Chapel Hill, North Carolina, United States, 3. Portland State University, Portland, Oregon, United States, 4. American Health Care Association/ National Center for Assisted Living, Pittsboro, North Carolina, United States, 5. University of North Carolina at Chapel Hill*

Because prescribing practices in long-term care settings may reflect regional influences, we examined how potentially inappropriate antipsychotic and antianxiety medication prescribing in assisted living (AL) compared to prescribing in nursing homes (NHs) based on their proximity, using generalized linear models adjusting for facility characteristics and state fixed effects. Data were derived from a seven state sample of AL communities and data for the same seven states drawn from publicly available data reported on the Nursing Home Compare website. In adjusted analyses, AL rates of antipsychotic use were not associated with the rates in the nearest or farthest NHs. However, AL communities that were affiliated with a NH had lower rates of potentially inappropriate antipsychotic use (b=–0.27[95%CI=–0.50,–0.04]). In a separate model, antianxiety medication prescribing rates in AL were significantly associated with neighboring NHs' rates of prescribing (b=2.65[95%CI=1.00,4.29]). Findings suggest efforts to change prescribing in NHs may influence prescribing in AL.

### SESSION 6205 (SYMPOSIUM)

### REFRAMING AGING-IN-THE-RIGHT-PLACE FOR HOUSING INSECURE OLDER ADULTS

Chair: Sarah Canham
Co-Chair: Mineko Wada
Discussant: Stephen Golant

Amidst rising costs of housing and changing personal needs, considerations of the availability of appropriate and accessible housing are becoming increasingly salient for older adults. While it has been widely acknowledged that older adults would prefer to age-in-place, recent reframing of this trend promotes the ideal as aging-in-the-right-place. This symposium will provide an updated understanding of how to support older adults' ability to age-in-the-right-place, regardless of income or physical, mental, or social status. Presenters include international and interdisciplinary researchers representing perspectives from gerontology, social work, community planning, and health sciences. The symposium will begin with Wada examining resilience scholarship, with a focus on older people who are experiencing homelessness, which has been largely neglected. In the next presentation, Humphries will outline distinct, senior-specific needs and shelter/housing solutions for newly and chronically homeless older adults. Following, Canham will describe promising practices of shelter/housing to support aging-in-the-right-place for older people experiencing homelessness in Montréal, Calgary, and Vancouver identified through an environmental scan. Extending these efforts to an international scale, Mahmood will outline findings from a scoping review of supportive shelter/housing options, supports, and interventions. A final presentation will report on how community development practices implemented by a not-for-profit affordable housing provider promote older tenants' food security and social support needs. Stephen Golant, a leading expert on housing, geography, and long-term needs on older adults, will discuss implications of these studies for policy and practice for supporting housing insecure older adults while advancing scholarship on aging-in-the-right-place for this marginalized population. Environmental Gerontology Interest Group Sponsored Symposium.

### ADVANCING THE CONCEPT OF RESILIENCE FOR OLDER PEOPLE WHO ARE EXPERIENCING HOMELESSNESS

Mineko Wada,[1] Sarah Canham,[2] and Mei Lan Fang,[3]
*1. STAR Institute, Vancouver, British Columbia, Canada, 2. University of Utah, Salt Lake City, Utah, United States, 3. University of Dundee, Dundee, Scotland, United Kingdom*

Current conceptualizations of resilience have overlooked the lived expertise of older people experiencing homelessness (OPEH) – individuals who have much insight to offer in terms of progressing notions on how people 'stand up' to adversity and 'bounce back' to a state of physical and psychological homeostasis across the life course. Drawing from extant literature and data from a community-engaged research project, which interviewed 40 participants and examined the health supports needed for individuals experiencing homelessness upon hospital discharge, we provide a comparison of resilience among homeless individuals generally and resilience among OPEH. Based on narratives of significant adversity experienced by OPEH in Vancouver, Canada, we offer a critical analysis of 'resilience in ecological context' that identifies unique characteristics of resilience at micro, meso, exo, and macro system levels. We discuss how our conceptual model of resilience pertinent to OPEH can be used to shape research, policy, and practice. Part of a symposium sponsored by the Environmental Gerontology Interest Group.

### CONCEPTUALIZING THE SHELTER AND HOUSING NEEDS AND SOLUTIONS OF OLDER PEOPLE EXPERIENCING HOMELESSNESS

Sarah Canham,[1] and Joe Humphries,[2], *1. University of Utah, Salt Lake City, Utah, United States, 2. Simon Fraser University, Vancouver, British Columbia, Canada*

Newly and chronically homeless older adults have unique pathways into homelessness and distinct physical, mental, and social needs. Using a five-step process, we conducted a scoping review of primary research to investigate the needs and solutions for sheltering/housing older people experiencing homelessness (OPEH). Thematic analysis of data from 19 sources revealed 1) shelter/housing needs and challenges of newly vs. chronically homeless older adults; 2) existing shelter/housing solutions addressing the needs of OPEH, including Housing First, permanent supportive housing, and multiservice homelessness intervention programs; and 3) outcomes of rehousing OPEH. Following, we developed a conceptual model which outlines how unique health and psychosocial needs of newly and chronically homeless older adults can be met through appropriately-designed shelter/housing solutions with individualized levels of senior-specific support. Future shelter/housing initiatives and strategies should use a rights-based approach and prioritize matching diverse OPEH needs to appropriate shelter/housing options that will support their ability to age-in-the-right-place. Part of a symposium sponsored by the Environmental Gerontology Interest Group.

### UNCOVERING PROMISING PRACTICES FOR SUPPORTING OLDER PEOPLE EXPERIENCING HOMELESSNESS

Sarah Canham,[1] Joe Humphries,[2] Victoria Burns,[3] Tamara Sussman,[4] and Christine Walsh,[3] *1. University of Utah, Salt Lake City, Utah, United States, 2. Simon Fraser University, Vancouver, British Columbia, Canada, 3. University of Calgary, Calgary, Alberta, Canada, 4. McGill University, Montreal, Ontario, Canada*

Montréal, Calgary, and Vancouver have seen a dramatic increase in homelessness among adults aged 50+. In order to identify 'promising practices' that promote aging-in-the-right-place for older people experiencing homelessness (OPEH) in Montréal, Calgary, and Vancouver, we conducted an environmental scan and three World Café workshops with 99 service providers and OPEH. We identified 53 promising practices managed or operated by 42 providers which we categorized across a shelter/housing continuum: 1) Emergency/transitional/temporary shelter/housing; 2) Independent housing with offsite supports; 3) Supported independent housing with onsite, non-medical supports; 4) Permanent supportive housing with onsite medical support and/or specialized services; 5) Long-term care; and 6) Palliative care/hospice. Study findings provide a template for existing solutions to the diverse shelter/housing needs of OPEH and insight into the gaps in shelter/housing and services that would support OPEH to age-in-the-right place. Policy and practice implications for scaling promising practices will be discussed. Part of a symposium sponsored by the Environmental Gerontology Interest Group.

### SHELTER AND HOUSING OPTIONS, SUPPORTS, AND INTERVENTIONS FOR OLDER PEOPLE EXPERIENCING HOMELESSNESS

Atiya Mahmood,[1] Joe Humphries,[1] Piper Moore,[2] Victoria Burns,[3] and Sarah Canham,[4] *1. Simon Fraser University, Vancouver, British Columbia, Canada, 2. College of Social Work, The University of Utah, Salt Lake City, Utah, United States, 3. University of Calgary, Calgary, Alberta, Canada, 4. University of Utah, Salt Lake City, Utah, United States*

While older people experiencing homelessness (OPEH) can have life histories of homelessness or experience homelessness for the first time in later life, understandings of shelter/housing models that meet diverse needs of this population are limited. We conducted a scoping review of the international literature on shelter/housing models available to support OPEH. Through an iterative process of reading and rereading 24 sources (published 1999-2019), findings were organized into 5 categories of shelter/housing models that have been developed to support OPEH: 1) Permanent supportive housing (PSH), including PSH delivered through Housing First, 2) Transitional housing, 3) Shelter settings with medical supports, 4) Drop-in centers, and 5) Case management and outreach. Findings expand our understanding of how a continuum of shelter/housing options are needed to support distinct health and housing needs of diverse OPEH. Policy and practice implications related to integrating health and social care to support OPEH to age-in-the-right-place will be discussed. Part of a symposium sponsored by the Environmental Gerontology Interest Group.

### AFFORDABLE RENTAL HOUSING AND OLDER ADULTS: A CASE STUDY FOR COMMUNITY DEVELOPMENT INITIATIVES

Muhammad Qureshi,[1] Atiya Mahmood,[1] Ghazaleh Akbarnejad,[1] Rahil Adeli,[1] and Dana Sharon,[2] *1. Simon Fraser University, Vancouver, British Columbia, Canada, 2. Brightside Community Homes Foundation, Vancouver, British Columbia, Canada*

Older adults living in rental housing with limited income are at-risk for experiencing life-course disruptions, including social isolation, loneliness and homelessness. To address these needs, one Vancouver-based affordable housing provider for older adults has implemented community development initiatives (CDIs), including resident-led community gardens, workshops, and social events. Based on data from three focus groups, resulting in a total of fifteen participants, this study explored the efficacy of five different CDIs in supporting residents' social connection and sense of community. Findings revealed that CDIs contribute to increased social engagement and inclusion of older adults living in affordable rental housing. In particular, resident-led community gardens were identified as the most impactful CDI in supporting both social engagement and inclusion, with the added benefit of addressing resident's food insecurity. We discuss how CDIs can be implemented in various housing settings for low-income older adults as an effective method of supporting aging-in-the-right place. Part of a symposium sponsored by the Environmental Gerontology Interest Group.

### SESSION 6210 (SYMPOSIUM)

### RESEARCH TO STRENGTHEN POLICY, PRACTICE, AND ADVOCACY ON HOUSING FOR AGING SOCIETIES

Chair: Emily Greenfield
Discussant: Nancy Berlinger

Population aging alongside other global trends—such as urbanization, widening economic inequality, and climate change—accelerate the need for systematic efforts to improve housing for diverse individuals, families, and communities as they age. This symposium features gerontological research explicitly designed to advance policy, practice, and advocacy on aging and housing. The first presentation demonstrates the use of data from U.S. surveys to better characterize the nature of current and future challenges in access to affordable, accessible, and safe housing for older adults. The second paper presents findings from a mixed-methods action research project in Toronto involving tenants living in properties managed by the second largest senior housing provider in North America. The paper indicates how findings on facilitators and threats to aging in place directly inform policy implementation on integrated services in Toronto. The third paper presents findings from a longitudinal, in-depth interview study with leaders of age-friendly community initiatives in suburban New Jersey, demonstrating the simultaneous challenges and opportunities of embedding housing advocacy at the local level within broader age-friendly community change efforts. The fourth paper presents three case studies based on interviews with key stakeholders involved with anti-displacement housing preservation and public housing organizing in New York City, highlighting the often invisible work of older, lower income, African American women at the center of advocacy efforts to preserve affordable housing. Guided by interdisciplinary critical work on ethical responses to population aging, the discussant will integrate themes from the papers to propose a framework for research, policy, practice, and advocacy.

### CAN THE NATION'S HOUSING SUPPORT A POPULATION SEEKING TO AGE IN PLACE?

Jennifer Molinsky, *Harvard University, Cambridge, Massachusetts, United States*

While surveys report that most older adults wish to "age in place," the nation's current housing and neighborhoods fall short on several dimensions needed to support independence and health in later life. Drawing from national data (including the American Housing Survey, American Community Survey, Health and Retirement Survey, and Survey of Consumer Finances), we describe the current housing and living situations of older adults and key challenges they face in securing affordable, accessible housing while also securing supportive services. We identify three challenges: the unaffordability of housing, which causes budgetary tradeoffs in healthcare spending; a lack of accessibility features in homes and neighborhoods, which can limit independence and safety, and the low-density location of much of the US housing stock (including that inhabited by older adults), where service delivery is difficult and the potential for isolation is high. We conclude with an overview of the policy implications of these challenges.

### PROMOTING AGING IN PLACE IN SOCIAL HOUSING

Christine Sheppard,[1] Tam Perry,[2] Andrea Austen,[3] and Sander Hitzig,[4] *1. Sunnybrook Research Institute, Toronto, Ontario, Canada, 2. Wayne State University, Detroit, Michigan, United States, 3. City of Toronto, Toronto, Ontario, Canada, 4. Sunnybrook Hospital, North York, Ontario, Canada*

As cities around the globe plan for current and future older cohorts, there is a need to explore innovative housing models to help older adults age in place. This paper presents findings from an action-research academic/community partnership on a new service model at Toronto Community Housing, the second largest social housing landlord in North America and home to 27,000 older adults. As Toronto works to improve delivery of housing/support services, more knowledge was needed to understand the inadequate and inconsistent delivery of services to tenants. Interviews/focus groups with older tenants and service providers (N=116) identified challenges related to unit condition (e.g., pest control) and tenancy management (e.g., arrears), and that the fragmentation of housing and health services negatively impacts older tenants' abilities to access supports and age in place. The presentation will conclude with discussion of planning and policy decision making approaches relevant to both Canadian and American contexts.

### OLDER WOMEN CAUSING A RUCKUS: GENTRIFICATION, DISPLACEMENT, AND TENANT ADVOCACY

H. Shellae Versey, *Wesleyan University, Middletown, Connecticut, United States*

Gentrification is a process through which lower-income neighborhoods experience large-scale investments and an influx of wealthier residents, often displacing lower-income residents. The restructuring of neighborhoods for newer, wealthier residents can compromise belonging, place attachment, and security for existing residents. This study explores resistance to displacement through tenant advocacy and organizing in New York City. This research specifically focuses on the efforts of older, lower-income, African American women, who are most at risk for eviction and housing stability, and yet are at the center of advocacy efforts to preserve affordable, low-income housing. In three case studies, we interview key stakeholders invested in anti-displacement housing preservation, eviction resistance, and public housing organizing to highlight the often invisible work taking place from within socially vulnerable communities. Implications for policy and future directions for applied research are discussed.

### SESSION 6215 (SYMPOSIUM)

### SERVICE DEVELOPMENT AND IMPLEMENTATION: LEARNING FROM DIVERSE CASE EXAMPLES

Chair: Liat Ayalon
Co-Chair: Mary Wyman
Discussant: Anne Martin-Matthews

The development of health and social services should be based on a thorough needs assessment with all stakeholders, followed by ongoing monitoring of implementation and subsequent short and long term outcomes. Relying on four different service models, this symposium reviews their evaluation processes and summarizes the main lessons learned, in order to inform future efforts. Wyman and colleagues outline efforts to develop culturally-sensitive dementia care services for American Indian and Alaska Native older adults and caregivers using qualitative interviews and community-based participatory research methods. Findings offer guidance for culturally-tailored implementation of services. Shepherd-Banigan et al. report on a quantitative needs assessment of over 1,500 caregivers of older Veterans enrolled in Veterans Administration healthcare. Providing care to individuals with co-morbid conditions predicted higher levels of distress and burden among caregivers, pointing to likely benefit from additional caregiver-focused support programs. The paper by Gum and co-authors moves to assessment of outcomes by examining mortality and other outcomes among older adults screened by an Area Agency on Aging and either receiving or waiting for services. Finally, the paper by Ayalon and Shinan-Altman use service evaluation to demonstrate the importance of needs assessment and the gap between the vision of service developers and real life constraints. The included papers discuss the value of various methodologies, illustrating the important role that assessment and evaluation play in service development and implementation for older adults and caregivers.

### LESSONS FROM AN EVALUATION OF A TRAINING PROGRAM OF PAID ELDERCARE WORKERS

Liat Ayalon, *Bar-Ilan University, Ramay Gan, HaMerkaz, Israel*

The present study is based on a three-year evaluation of an Israeli training program for local paid elder care workers, called "community care." Interviews were conducted with all stakeholders involved in the program, including program developers, facilitators, funders, trainees, dropouts, employers, and older care recipients. Qualitative thematic analysis was used, supplemented by quantitative data concerning the program's inputs, outputs and outcomes. The program had multiple strengths, including a substantial funding stream and a highly skilled and committed team. Yet, out of 130 participants (in the 7 training programs evaluated), only 94 completed the program and 31 were later employed as care workers. Three main challenges to the efficacy of the training program were identified. The findings stress the importance of adequately conducting the appropriate needs assessment prior to embarking on a new social program and illustrate the tension between an ideal prototype and real-life constraints.

### PERSPECTIVES ON DEMENTIA SERVICE USE AND FAMILY CAREGIVING AMONG THE ONEIDA NATION OF WISCONSIN

Mary Wyman,[1] Nickolas Lambrou,[1] Debra Miller,[2] Sunshine Wheelock,[3] Florence Petri,[4] Marlene Summers,[1] Carey Gleason,[5] and Dorothy Edwards,[1] *1. University of Wisconsin, Madison, Wisconsin, United States, 2. Oneida Comprehensive Health Division, Madison, Wisconsin, United States, 3. Oneida Elder Services, Madison, Wisconsin, United States, 4. Oneida Nation Commission on Aging, Madison, Wisconsin, United States, 5. University of Wisconsin School of Medicine and Public Health, Madison, Wisconsin, United States*

Prevalence of dementia among American Indian/Alaska Natives (AI/AN) is higher than in white populations, and AI/AN communities experience dementia care service gaps. This study explored perspectives within AI/AN communities regarding dementia, the family caregiver role, and home and community-based service use. Using tenets of Community-Based Participatory Research, qualitative interviews and a brief survey were conducted with 22 members of the Oneida Nation of Wisconsin (mean age 71 years, 73% female). Of the sample, 63.6% identified as a past or current family caregiver for a loved one with dementia. Awareness of services varied; 82% were aware of memory cafes, 75% knew of the caregiver support group, and 43% were familiar with dementia care specialist services. Thematic analysis revealed shared values of involving the family and community in dementia care, and offer guidance to support greater engagement in services. Implications for culturally-tailored service provision within AI/AN communities are discussed.

### FAMILY CAREGIVERS OF VETERANS EXPERIENCE HIGH BURDEN, MENTAL HEALTH DISTRESS, AND FINANCIAL STRAIN

Megan Shepherd-Banigan,[1] Sophia Sherman,[2] Jennifer Lindquist,[2] Katherine Miller,[2] Matthew Tucker,[2] Valerie Smith,[3] and Courtney Van Houtven,[4] *1. Duke University School of Medicine, Durham, North Carolina, United States, 2. US Department of Veterans Affairs HSR&D, Durham, North Carolina, United States, 3. U.S. Department of Veterans Affairs HSR&D, Durham, North Carolina, United States, 4. US Department of Veterans Affairs, Durham, North Carolina, United States*

We describe the caregiving experiences and needs of family caregivers of older Veterans enrolled in the U.S. Department of Veterans Affairs (VA). We conducted telephone surveys with 1,509 caregivers to assess caregiver health and well-being. Caregivers were primarily female, <50 years old, white, and the Veterans' spouse. Veterans had substantial functional limitations and required care for multiple conditions, commonly, mental illness, dementia, and heart disease. On average, caregivers provided care for 9.6 hours per day and 6.7 days per week. Burden and depressive symptoms were above clinical thresholds with average scores of 21.8 (Zarit burden) and 11.5 (CES-D 10). Levels of perceived loneliness and financial strain were high. As this population needs emotional support, respite care services, social engagement, and training to care for aging Veterans, the expansion of enhanced caregiver services and supports to this population (expected in 2020) through the VA Mission Act of 2018 will be beneficial.

### MORTALITY AND HEALTH OUTCOMES FOR OLDER ADULTS SCREENED BY AN AREA AGENCY ON AGING OVER A 4.5-YEAR PERIOD

Amber Gum,[1] Lawrence Schonfeld,[1] Kevin Kip,[1] Mary Goldsworthy,[1] Jesse Bell,[2] Kyaien Conner,[1] Ohad Green,[3] and Katie Parkinson,[4] *1. University of South Florida, Tampa, Florida, United States, 2. University of south florida, Tampa, Florida, United States, 3. University of Oxford, Oxford, England, United Kingdom, 4. Senior Connection Center, Inc., Tampa, Florida, United States*

Area Agencies on Aging (AAA) screen older adults and oversee delivery of a wide range of home- and community-based services (HCBS). We examined the assessment process, services, and mortality and health outcomes for older adults screened by an Area Agency on Aging in west-central Florida. Most were self/family referred (78.9%). Using data from July 2013-December 2018, 23,225 older adults were screened.

Individuals had an average of 2.6 years follow-up in the dataset, during which time 63.6% received additional assessments: follow-up screening (50.6%), comprehensive assessment for enrollment in HCBS (35.7%), or assessments for congregate meals or other services (13.7%). Results revealed differences in mortality: 22.5% of clients receiving services died compared to 32.1% of clients prioritized as lower risk and on waiting lists for services. Long-term care placement and functional decline outcomes also will be reviewed, along with implications for service delivery and managing waitlists.

### SESSION 6220 (SYMPOSIUM)

### SOCIAL AND PHYSICAL CONTEXTS OF LONG-TERM SERVICES AND SUPPORTS

Chair: Chanee Fabius
Discussant: Philippa Clarke

In the coming years, inevitably growing numbers of older populations will yield more older Americans with extensive medical and long-term care needs. This will lead to an increasing need for long-term services and supports (LTSS) to assist older adults with routine daily activities (e.g., bathing, dressing, medication management). There is a growing interest in understanding how social and physical environments contribute to health outcomes and the provision of services and resources for older persons with disabilities requiring assistance from LTSS. Decisions about care and subsequent experiences are likely a result of factors that extend beyond personal preference or individual factors, such as neighborhood quality, housing context, and living situations (i.e., homebound status) among community-dwelling older adults. Given population aging and the shift of LTSS from nursing homes toward community settings, there is a pressing need for more information about contextual factors that might help better develop supports for vulnerable older adults. This symposium will feature four presentations that provide novel insight regarding social and physical contextual factors contributing to LTSS. Presentations leverage data from the National Health and Aging Trends Study (NHATS), a nationally representative survey of Medicare beneficiaries aged 65 and older, and will describe: 1) associations between individual and home environment risk-factors, neighborhood-level social deprivation, and falls; 2) the relationship between neighborhood-level social deprivation and caregiving intensity (number of hours of caregiving per week) among community-dwelling older adults; 3) associations between living in single-family vs. multi-unit housing and social networks; and 4) community tenure among homebound older adults.

### NEIGHBORHOOD SOCIAL DEPRIVATION AND CAREGIVING INTENSITY FOR COMMUNITY-DWELLING OLDER ADULTS WITH DISABILITIES

Chanee Fabius, John Mulcahy, Safiyyah Okoye, and Jennifer Wolff, *Johns Hopkins Bloomberg School of Public Health, Baltimore, Maryland, United States*

There is growing interest in the role of "place" in the provision of long-term services and supports (LTSS) for older adults with disabilities, who receive ~16 billion hours of care per year from family and unpaid caregivers, but information is lacking. Using data from the 2015 National Health and Aging Trends Study (NHATS) linked to census-tract-level information from the American Community Survey, we described the association between caregiving intensity (hours of care received per week) and neighborhood social deprivation among N=2125 community-dwelling older adults with disabilities. Individuals receiving 40 hours or more of help per week had greater levels of functional impairment and dementia, and more often lived in neighborhoods at the highest quartile of social deprivation compared to those receiving fewer than 20 hours of care (26.8% vs. 21.7%, respectively). Findings have policy implications for targeting LTSS strategies toward addressing inequities in social determinants of health for vulnerable populations.

### HOME AND NEIGHBORHOOD CONTEXT AND FALL RISK AMONG OLDER AMERICANS

Safiyyah Okoye, John Mulcahy, Chanee Fabius, and Jennifer Wolff, *Johns Hopkins Bloomberg School of Public Health, Baltimore, Maryland, United States*

Falls result from complex interactions between individuals and their environment and are the leading cause of injuries among older adults. A nascent literature demonstrates an association between neighborhood characteristics and falls. However, available evidence is from small, nonrepresentative samples and generally focuses on individual, home, or neighborhood risk-factors rather than the contribution of all three. We link information from N=6,489 community-dwelling participants in the 2015 National Health and Aging Trends Study with the Social Deprivation Index (SDI), which yields a census-tract-level score of socioeconomic disadvantage, to assess associations between home and neighborhood context and falls in the previous year. Household financial strain was associated with a 31% increased risk of falling, and indoor trip hazards with a 14% increased risk, after adjusting for individual factors and neighborhood SDI (all $p < 0.05$). Findings reflect the interplay between home and neighborhood context and fall-risk, and can inform community-based fall-prevention interventions.

### HOW LONG DO OLDER ADULTS REMAIN HOMEBOUND IN THE COMMUNITY? IMPLICATIONS FOR LONG-TERM SERVICES AND SUPPORT SYSTEMS

Katherine Ornstein, Jennifer Reckrey, Evan Bollens-Lund, Katelyn Ferreira, Mohammed Husain, Shelley Liu, and Albert Siu, *Icahn School of Medicine at Mount Sinai, New York, New York, United States*

A large and growing population of older adults with multimorbidity, cognitive impairment, and functional disability live in the community but are homebound (never/rarely leave home). While homebound status is associated with decreased access to medical services and poor health outcomes, it is unclear how long individuals remain homebound. We used the National Health and Aging Trends Study (NHATS), a nationally representative sample of Medicare beneficiaries age 65 and over, with survey weighting to assess duration of homebound status in the community. Among the incident homebound in 2016 (n=253) , only 28% remained homebound after 1 year. 21% died, 18% were recovered, and one-third left the home but still reported difficulty. As the locus of long-term care shifts from nursing homes to the community and models of care expand to serve the needs of the homebound, it is critical that we better understand the heterogeneity and transitions of the homebound population.

### HOUSING CONTEXT AND SOCIAL NETWORKS AMONG LOWER INCOME OLDER ADULTS

Noah Webster, *University of Michigan, Ann Arbor, Michigan, United States*

Housing context among lower income older adults plays an important role in shaping access to resources and ultimately well-being. We know very little about how housing influences access to social resources. This study examines the association between housing context (multi-unit vs. free-standing homes) and network structure among a U.S. nationally representative sample of independent living, lower income (<$15,000 in past year) adults age 65+ from the National Health and Aging Trends Study (N=1,795). Regression analyses indicate the housing-networks link is moderated by martial/partner status and gender. Among those married/living with a partner, living in multi-unit buildings (compared to free-standing homes) is associated with larger networks (i.e., more people to talk with about important things). Among women, living in multi-unit buildings was associated with more friends and neighbors in one's network. Findings highlight variation in access to social resources across housing contexts. Findings should inform policy aimed at reducing social isolation.

### SESSION 6225 (SYMPOSIUM)

### SOCIAL EXCLUSION AND MATERIAL DISADVANTAGE: HOUSING, POVERTY, AND LIVING STANDARDS IMPACTS

Chair: Charles Waldegrave
Discussant: Chris Phillipson

SRPP 2020 Ollie Randall Symposium Award Winner. Many of the detrimental effects of material disadvantage on the lives of citizens have been well understood by public health and social scientists, and post-World War II social protection polices were designed to mitigate the negative impacts of them. As the numbers of older people increase proportionally to the rest of the population in most countries, less is known about the exclusionary impacts of material disadvantage and the roles housing, poverty and living standards play on the health, well-being and social connections of their lives. This symposium draws together research emanating out of four countries Norway, Poland, Ireland and New Zealand that are part of the European COST Action 15122 Reducing Old-Age Social Exclusion: Collaborations in Research and Policy (ROSEnet). The papers present contemporary results of specific health well-being and social impacts of material disadvantage in the four quite different countries and assesses them through the lens of social exclusion. As the growing international evidence during the last decade has highlighted the negative health and well-being impacts of loneliness and social disconnection (Holt-Lunstad et. al. 2015), the role of housing, poverty and living standards has in creating social exclusion is less well known. This research analyses the subjective and objective experiences of material disadvantage and quantifies their exclusionary impacts on well-being (e.g. quality of life, loneliness), health functioning (mental and physical) and their challenges to macro-structures (e.g. government policies, social protection).

### CO-CREATED RESEARCH ASSESSING THE EXCLUSIONARY IMPACTS OF LOW LIVING STANDARDS ON OLDER PEOPLE

Charles Waldegrave,[1] Chris Cunningham,[2] Catherine Love,[1] and Giang Nguyen,[1] *1. Family Centre Social Policy Research Unit, Lower Hutt, Wellington, New Zealand, 2. Research Centre for Maori Health and Development, Massey University, Wellington, Wellington, New Zealand*

The aim of this research is to identify the impacts of material resources such as income, assets, housing and living standards on quality of life, health status and social relations. Amartya Sen's capabilities approach has formed the conceptual basis of the theoretical framework. This paper will report on the results of co-created research with older Māori in New Zealand aged 50 years and older. Objective measures of income, wealth, housing and living standards are compared with a range of scales including overall wellbeing and subjective health status and co-created scales of indigenous loneliness. The results demonstrate significant relationships between material resources and quality of life, health status and other social relations indicators. They quantify the impact material resources have on key indicators of social relations and social exclusion which enables informed and targeted policy interventions for social inclusion.

### MATERIAL CONDITIONS AND WELL-BEING IN OLD AGE: THE ROLE OF CONTEXTUAL FACTORS AT REGIONAL LEVEL

Michal Myck,[1] Martina Brandt,[2] Claudius Garten,[3] Monika Oczkowska,[1] and Alina Schmitz,[3] *1. Centre for Economic Analysis, CenEA, Szczecin, Zachodniopomorskie, Poland, 2. TU Dortmund, Dortmund, Nordrhein-Westfalen, Germany, 3. TU Dortmund, Institute for Sociology, Dortmund, Nordrhein-Westfalen, Germany*

In our Beethoven project "Age-well" we examine the role of regional contextual factors for the relationship between individual wellbeing and material conditions over time, using a unique combination of individual and regional level longitudinal data. The analyses consider a broad range of regional, community-level indicators for two countries, Germany and Poland, both characterised by rapid population ageing and significant regional variation in the standard of living. These variables, including local indicators of economic conditions and public services, are combined with detailed individual-level information on wellbeing from the Survey of Health, Ageing and Retirement in Europe (SHARE). This data match allows us to study the degree to which regional contexts affect the relationship between individual material conditions and wellbeing in later life. Local public services are shown to mediate the importance of individual level resources for wellbeing confirming an important channel through which public policy can improve welfare of older people.

### EXCLUSION FROM SOCIAL RELATIONS AND LONELINESS: INDIVIDUAL AND COUNTRY-LEVEL CHANGES

Marja Aartsen,[1] Deborah Morgan,[2] Lena Dahlberg,[3] Charles Waldegrave,[4] Sarmitė Mikulionienė,[5] Gražina Rapolienė,[5] and Giovanni Lamura,[6] *1. OsloMet Oslo Metropolitan University, Oslo, Norway, 2. Swansea University Wales, Swansea, Wales, United Kingdom,*

*3. Aging Research Center, Karolinska Institutet, Solna, Stockholms Lan, Sweden, 4. Family Centre Social Policy Research Unit, Lower Hutt, Wellington, New Zealand, 5. Lithuanian Social Research Centre, Vilnius, Vilniaus Apskritis, Lithuania, 6. Centre for Socio-Economic Research on Ageing, Ancona, Marche, Italy*

Social isolation and loneliness have profound implications for quality of life and health and welfare budgets, but interventions to reduce loneliness are limited effective. The aim of this study is to examine the often-ignored impact of macro-level drivers of loneliness, in addition to micro-level drivers by adopting a cross-national perspective. We use longitudinal data from 2013 and 2015 from the Survey of Health, Aging, and Retirement in Europe (SHARE), combined with macro-level data from additional sources. Our study confirms that key micro-level drivers of loneliness are gender, health and partnership status, frequency of contact with children and changes therein. Macro level drivers are level of safety in the neighbourhood, and poverty and social deprivation of a society. In order to understand and reduce loneliness we require not just a focus on individual risk factors, behaviours and expectations, but also on macro-level factors that are associated with exclusion from social relations.

### MATERIAL DISADVANTAGE AND POSITIVE SUBJECTIVE HEALTH OF OLDER HOMELESS AND OLDER IRISH TRAVELERS

Bridin Carroll,[1] and Kieran Walsh,[2] *1. NUI Galway, Galway, Galway, Ireland, 2. Irish Centre for Social Gerontology, National University of Ireland Galway, Galway, Galway, Ireland*

Focusing on older Irish Travellers and older homeless people (OTOH) as two marginalised sub-sections of the older population, this paper investigates life-course and structural forms of material disadvantage, and its implications for positive health and accessing community care in older age. With growing interest in strengthening home care structures for older people, it is critical to interrogate the relevance of these structures for those who experience environmental uncertainty in later life, and possess significant trajectories of disadvantage. The analysis draws on 50 life-course interviews with OTOH aged between 50-72 years. The findings illustrate significant life-course experiences of material and multi-faceted forms of disadvantage, including stigma and discrimination, with implications across health and social lives. Housing deprivation was a multi-factorial player, causing certain physical illnesses, hindering some health treatments, and contributing to precarious conditions and sense of self worth. Findings are discussed in relation to flexible models of home care delivery.

### SESSION 6230 (SYMPOSIUM)

### SOCIAL ISOLATION AND LONELINESS IN OLDER ADULTS: A NATIONAL ACADEMIES OF SCIENCES, ENGINEERING, AND MEDICINE REPORT

Chair: Colleen Galambos
Discussant: James Lubben

Social isolation and loneliness (SIL) are serious yet underappreciated public health risks for many older adults (AARP, 2018a). Strong evidence suggests that, for older adults, social isolation and loneliness are associated with an increased likelihood of early death, dementia, heart disease, and more (AARP, 2018b, Holt-Lunstad and Smith, 2016). While all ages may experience SIL, older adults are at increased risk because they are more likely to face predisposing factors such as living alone, the loss of family or friends, chronic illness, and sensory impairments. Health care providers may be in the best position to identify older individuals who are at highest risk for SIL – individuals for whom the health care system may be the only point of contact with their broader community. The National Academies of Sciences, Engineering, and Medicine (NASEM) developed a consensus study report on this issue. This symposium presents the study recommendations. Dr. Holt-Lunstad examines the recommendations to develop a more robust evidence base for effective assessment, prevention, and intervention strategies for social isolation and loneliness. Dr. Galambos examines the recommendations to translate current research into health care practices and to improve awareness of the health and medical impacts of SIL. Dr. Lustig examines the recommendations to strengthen ongoing education and training and to strengthen ties between the health care system and community-based resources. Dr. Demiris examines the role of technology across all of these recommendations. Loneliness and Social Isolation Interest Group Sponsored Symposium

### SOCIAL ISOLATION AND LONELINESS: TRANSLATING CURRENT RESEARCH INTO HEALTH CARE PRACTICES AND IMPROVING AWARENESS

Colleen Galambos, *University of Wisconsin Milwaukee Helen Bader School of Social Welfare, Milwaukee, Wisconsin, United States*

This paper examines the evidence to support the need to translate current research into health care practices about social isolation and loneliness (SIL) among older adults. The health care system may be in the best position to identify those at highest risk—namely, older adults, whose only interactions are with members of the health care system. This paper reviews recommendations related to periodic assessments, including the use of validated tools to identify those at highest risk. Through this identification, clinicians and health care researchers may be able to use these findings to better target meaningful clinical and public health interventions. Additionally, a critical step toward preventing, mitigating, or eliminating negative health impacts will be to improve awareness about the problem and impact of SIL within the older adult population. This paper reviews recommendations for improving overall awareness by including SIL in national health strategies and public campaigns. Part of a symposium sponsored by Loneliness and Social Isolation Interest Group.

### SOCIAL ISOLATION AND LONELINESS: DEVELOP A MORE ROBUST EVIDENCE BASE

Holt-Lunstad Julianne, *Brigham Young University, Provo, Utah, United States*

This paper reviews the evidence base for the health impacts of SIL on older adults, the risk factors, and the potential moderators and mediators of those relationships. Substantial evidence indicates that SIL are associated with physical, cognitive, and psychological morbidity; health-related behaviors; and health-related quality of life. Social isolation in particular is associated with a significantly increased risk of premature mortality from all causes. The evidence base for interventions in the clinical setting is also reviewed. Finally, the report's specific recommendations are discussed, including the need for basic science research, increased funding, key elements in the design and evaluation of interventions, and gap areas (including trends among current younger adults and approaches for specific understudied groups of at-risk older adults). Part of a symposium sponsored by Loneliness and Social Isolation Interest Group.

### SOCIAL ISOLATION AND LONELINESS: STRENGTHENING EDUCATION AND TRAINING

Tracy Lustig, *The National Academies of Sciences, Engineering, and Medicine, Washington, District of Columbia, United States*

This paper reviews recommendations for a variety of opportunities to improve education and training of the health care workforce on the health impacts of SIL and clinical approaches for assessment as well as testing different approaches for such education and training. The fifth goal of the NASEM study is to "strengthen ties between the health care system and community-based networks and resources." Similar to other social determinants of health, addressing SIL will require coordinated efforts among a variety of stakeholders. This paper reviews recommendations for improving coordination, including team-based care and promotion of tailored community-based services, as well as the creation of a centralized repository for new evidence and best practices. Finally, connections between this NASEM study and the 2019 report Integrating Social Care into the Delivery of Health Care are discussed. Part of a symposium sponsored by Loneliness and Social Isolation Interest Group.

### SOCIAL ISOLATION AND LONELINESS: THE PROS AND CONS OF TECHNOLOGICAL INTERVENTIONS

George Demiris, *University of Pennsylvania, Philadelphia, Pennsylvania, United States*

A variety of technologies have been proposed, tested, and used to reduce social isolation and loneliness [SIL]. Furthermore, information technology may offer a way to detect or predict patterns of SIL. In this paper, the range of technology tools for assessment (e.g., passive monitoring, tracking data patterns, electronic health records) and intervention (e.g., social robots, social media, virtual reality) are reviewed for evidence of impact, and consideration is given for related ethical issues. The role of technology in relation to the report's recommendations is discussed, including assessment and testing of new technological interventions for their potential benefits and harms, consideration of contextual issues such as broadband access, and the role of technology in education and training. Finally, gap areas of research are explored, such as the impact of the use of technology among current younger adults as they age. Part of a symposium sponsored by Loneliness and Social Isolation Interest Group.

### SESSION 6235 (SYMPOSIUM)

### SOMETHING OLD, SOMETHING NEW: THE MODERNIZATION OF THE OLDER AMERICANS ACT

Chair: Lauren Bangerter
Co-Chair: Beth Prusaczyk
Discussant: Brian Kaskie

The Older Americans Act (OAA) is the foremost federal law focused on the wellbeing of aging adults in the US. Since its conception 1965, the OAA has sought to optimize the lives of aging Americans, with emphasis on low-income adults, through programs that promote nutrition, transportation, support caregivers, offer employment, and combat elder abuse. This symposium will explore the modernization of the 2020 OAA, which was last reauthorized in 2016. Presentations 1 and 2 will focus on important updates to the definitions used throughout OAA (Title I). Presentation 3 will cover several noteworthy changes to improving grants for states and community programs on aging's (Title II). Presentation 4 will provide additional context to amendments made to modernize activities for health, independence, and longevity (Title III) prioritize senior Community Service Employment Programs (Title IV) and enhance grants for Native Americans (Title V). Presentation 5 explores the modernizing allotments for vulnerable elder rights protection activities and other programs (Title IV) included changes in funding and home and community-based best practices and elder justice activities. Collectively, these presentations will provide an overview of the key changes in the reauthorization of the OAA. This work will allow GSA attendees to understand the specific efforts to modernization this critical legislation to better serve the aging US population.

### TITLE 1 CHANGES: BRINGING THE OLDER AMERICANS ACT DEFINITIONS TO 2020

Thomas Eagen, *University of Washington, Washington, District of Columbia, United States*

This session will review the changes to Title I of the Reauthorization of the Older Americans Act. This title provides important updates and modernization to the definitions used throughout the Older Americans Act. Some definitions were modified such as adding the prevention of sexually transmitted diseases, chronic pain management, and screening for suicide risk to evidence-based health promotion programs under the definition of disease prevention and health promotion services. Other changes include services to provide screening for fall-related injuries, services that are responses to public health emergencies, and services to prevent and address negative health effects associated with social isolation. Title I also includes a series of important including access to person-centered services and coordinating services provided by aging and disability resource centers. Several other changes to Title I will be discussed.

### TITLE I: MODERNIZING DEFINITIONS AND PROGRAMS UNDER THE ADMINISTRATION ON AGING

Marie Gualtieri, *Washington, District of Columbia, United States*

The recent reauthorization of the Older Americans Act adds language and definitions to current issues facing the aging population. Specifically, Title I includes definitions related to program adaptation and coordination, workforce and long-term care issues, nutrition and social isolation, as well as family caregivers. Different from the last authorization, these definitions span beyond the individual experience to include other entities impacted by an aging society, such as the workforce and families. Overall, the Title I reauthorization seeks to modernize policy to reflect the current influx of the older adult population and its consequences.

### TITLE II: IMPROVING GRANTS FOR STATES AND COMMUNITY PROGRAMS ON AGING

Jamie Kuhne, *Department of Veterans Affairs, Worthington, Ohio, United States*

There are several noteworthy changes to the Improving Grants for States and Community Programs on Aging's portion of the OAA. The reauthorization adds language requiring data collection on the needs of older adults and specifying additional populations on whom to focus outreach efforts, such as survivors of the Holocaust. The reauthorization also expands what States may fund with Supportive Services grants, adding screening for social isolation and traumatic brain injuries. The Act goes on extend Stated the option of funding programs to address both of these issues. The Act also requires the Assistant Secretary to study the supply and demand of home delivered and congregant meals and make recommendations to address the gap. Finally, there is expansion of the section on Caregiving, including a definition of caregiver assessment and the removal of a limit on funds States can use on support services to family caregivers.

### AMENDMENTS MADE TO THE OLDER AMERICANS ACT TITLES III, IV, AND V

Lieke van Heumen, *University of Illinois at Chicago, Chicago, Illinois, United States*

This presentation discusses amendments made to the Older Americans Act titles III, IV and V through the most recent reauthorization. Title III reauthorizes Title IV programs, Title IV reauthorizes title V programs and Title V reauthorizes title VI programs. The reauthorizations each include a seven percent increase in fiscal year 2020 and a six percent increase per year for the next four fiscal years. New in title III are an amendment that allows projects that address traumatic brain injury among older adults to be included in grant programs, an amendment that improves an existing transportation grant program and an amendment that improves an existing grant program for multigenerational collaboration. Additionally, existing falls prevention and chronic disease self-management programs are codified within title III. New in title IV is an amendment that allows eligible previously incarcerated individuals to be considered a prioritized population for the Senior Community Service Employment Program.

### TITLE VI: MODERNIZING ALLOTMENTS FOR VULNERABLE ELDER RIGHTS PROTECTION ACTIVITIES AND OTHER PROGRAMS

Taneika Duhaney, *Health and Aging Policy Fellowship, Springfield, Virginia, United States*

Of the many changes to the OAA, the Modernizing Allotments for Vulnerable Elder Rights Protection Activities and Other Programs section included notable changes. This bill specifies that Title VII programs will receive a 7% increase in 2020 and a 6% increase in the following four fiscal years. It extends the Supporting Grandparents Raising Grandchildren Act for an additional year. The reauthorization ensures that ombudsman representatives can be reimbursed for costs incurred through their services. The Act requires that the Government Accountability Office study federal programs for home modification assistance for older adults and individuals with disabilities. The Act directs the Administrator of the Administration for Community Living to continue the 2017 requirement of disseminating and soliciting feedback on the Principles for Person-directed Services and Supports during Serious Illness. This reauthorization updates home and community-based best practices; and elder justice activities, including community outreach and education to bolster community partnerships.

### SESSION 6240 (SYMPOSIUM)

### STATE VARIABILITY IN ASSISTED LIVING REGULATIONS, ACCESS, AND OUTCOMES FOR PERSONS WITH DEMENTIA

Chair: Kali Thomas

Approximately one million individuals, an estimated40% with a diagnosis of Alzheimer's disease-related dementias (ADRD), reside in assisted living (AL); yet, little is known about their experience or the quality of care provided in AL. Unlike other forms of long-term care (LTC), the licensing, operating, and enforcement requirements for AL falls to the states, which vary dramatically in their regulatory approaches. The overall objective of this symposium is to examine states' AL regulatory environments and understand if and how access to AL and the health outcomes of AL residents with ADRD are impacted by states' regulatory decisions. Presenters will highlight the state variability in the regulation, access, and outcomes of AL residents with ADRD. The first presentation will describe the within and between state differences in regulatory approaches as it relates to dementia care. The second presentation will describe the variation in Medicaid financing of services in AL and its potential impact on access to AL within those states. The third will present geographic disparities in access to specialized dementia care in AL. The fourth presentation will characterize differences in emergency department utilization among AL residents with ADRD across states. Finally, the fifth presenter will report on the effect of establishing or increasing state staffing requirements on outcomes of AL residents with ADRD. Results will ultimately inform policy-makers, organizational leaders, and clinicians as they seek the most effective ways to ensure equal access to AL and optimal outcomes for residents with ADRD. Assisted Living Interest Group Sponsored Symposium.

### VARIATION IN ASSISTED LIVING REGULATIONS WITHIN AND ACROSS STATES

Paula Carder,[1] Lindsey Smith,[2] Taylor Bucy,[2] Jaclyn Winfree,[1] Wenhan Zhang,[3] and Kali Thomas,[3]
*1. Portland State University, Portland, Oregon, United States, 2. PSU School of Public Health, Portland, Oregon, United States, 3. Brown University, Providence, Rhode Island, United States*

Assisted living (AL) regulations have been long recognized as being highly variable across states. A new approach developed by our team, Health Services Regulatory Analysis, allows for a more granular identification of within-state variation in AL regulation. We identified 172 licensing classifications from the 50 states and DC representing 58 primary license types, 48 sub-types, and 66 designations that can modify a primary or sub-license. Over two-thirds (72%)

of dementia-specific classifications require that all staff receive initial dementia training, compared to only one-third (33%) of general AL classifications. This trend is similarly reflected in cognitive-screening requirements, present in 67% of dementia-specific classifications and 42% of general AL classifications. Regulatory theory describes how licensing agencies respond to various forces and values. Within-state AL regulatory variation reflects a combination of oversight mandates, population-specific needs (e.g., people with dementia), historic policies, and provider influence, with implications for consumers, policy-makers and researchers. Part of a symposium sponsored by Assisted Living Interest Group.

### THE INTERSECTION OF MEDICAID AND ASSISTED LIVING FOR RESIDENTS WITH DEMENTIA

Brian Kaskie,[1] Seamus Taylor,[1] and Lili Xu,[2] *1. University of Iowa, Iowa City, Iowa, United States, 2. University of Iowa, University City, Iowa, United States*

Medicaid has increasingly offered coverage to persons residing in assisted living (AL). However, the scope of coverage across states is unknown. We sourced 2019 state administrative regulations specific to Medicaid and AL and determined forty-five (45) states link Medicaid with AL. Twenty-seven (27) do so as part of their state plan, 32 use a §1915(c) waiver, and 11 use a §1115 waiver. Forty-four states limit Medicaid coverage to a specific population, 16 limit coverage to those with a diagnosed disability, and 1 state limits coverage to a specific geographic region. In addition, 33 states provide payment for room and board with 28 states upholding a payment cap. In regards to services, 13 states reimburse a limited range of services while 32 offer a more expansive range of services. As Medicaid programs have extended coverage to residents of AL, researchers must now consider the impact on AL access and residents' outcomes. Part of a symposium sponsored by Assisted Living Interest Group.

### GEOGRAPHIC DISPARITIES IN ACCESS TO SPECIALIZED DEMENTIA CARE

Portia Cornell,[1] Wenhan Zhang,[2] Lindsey Smith,[3] Shekinah Fashaw,[2] and Kali Thomas,[2] *1. Providence VA Medical Center, Providence, Rhode Island, United States, 2. Brown University, Providence, Rhode Island, United States, 3. PSU School of Public Health, Portland, Oregon, United States*

With novel, previously undescribed data on the availability of dementia-specific assisted living communities (ALs), we analyzed variation among counties in the availability of this important service for persons with dementia. In twenty-one states, we identified 6,961 ALs (16%) with a dementia-specific license/certification. Counties with at least one AL providing dementia-specific care had substantially higher college attainment versus counties that had at least one AL, but no dementia-specific beds: 25% versus 18% (p<0.01). Counties with dementia care also had significantly greater median incomes ($54,000 vs. $46,400), and home values ($159,000 vs. $113,000), lower poverty rates (13.7 percent vs. 16.3 percent), and lower proportions of Black residents (7.8 percent vs. 8.7 percent). Our findings are suggestive of a mismatch in need and availability of residential care options for older adults with ADRD that are also low-income or racial/ethnic minorities. Part of a symposium sponsored by Assisted Living Interest Group.

### STATE VARIABILITY IN EMERGENCY DEPARTMENT VISITS AMONG ASSISTED LIVING RESIDENTS WITH DEMENTIA

Cassandra Hua,[1] Wenhan Zhang,[2] Portia Cornell,[3] Momotazur Rahman,[1] David Dosa,[4] and Kali Thomas,[2] *1. Brown University School of Public Health, Providence, Rhode Island, United States, 2. Brown University, Providence, Rhode Island, United States, 3. Providence VA Medical Center, Providence, Rhode Island, United States, 4. Brown University, Barrington, Rhode Island, United States*

Emergency department (ED) visits are associated with poor outcomes; however, state variation in ED use among assisted living (AL) residents is not well understood. Using 2017 Medicare data, we identified a cohort of 88,880 beneficiaries with dementia residing in larger ALs (25+ beds) and calculated risk-adjusted rates of all-cause and injury-related ED use per 100 person years, by state, adjusting for demographics and chronic conditions. Risk-adjusted state rates of all-cause ED visits ranged from 129.5 visits/100 person-years (95%CI=114.6,148.2) in New Mexico to 246.1 visits/100 person-years (95%CI= 224.9,274.8) in Rhode Island. The risk-adjusted rate of injury-related ED visits ranged from 91.4 visits/100 person-years (95%CI=83.0,101.4) in New Mexico to 135.9 visits/100 person-years (95%CI=126.9,146.6) in Montana. Potential reasons for these state variations will be discussed. Part of a symposium sponsored by Assisted Living Interest Group.

### THE IMPACT OF CHANGES IN DIRECT CARE STAFFING POLICIES AND OUTCOMES FOR ASSISTED LIVING RESIDENTS WITH DEMENTIA

Kali Thomas,[1] Portia Cornell,[2] Wenhan Zhang,[1] Paula Carder,[3] Lindsey Smith,[4] Cassandra Hua,[5] and Momotazur Rahman,[5] *1. Brown University, Providence, Rhode Island, United States, 2. Providence VA Medical Center, Providence, Rhode Island, United States, 3. Portland State University, Portland, Oregon, United States, 4. PSU School of Public Health, Portland, Oregon, United States, 5. Brown University School of Public Health, Providence, Rhode Island, United States*

We identified a cohort of 410,413 Medicare beneficiaries residing in 10,623 large (25+bed) assisted living (AL) communities between 2007 and 2017. We conducted linear probability models with a difference-in-difference framework to examine the association between hospitalization and changes in regulations pertaining to staff training (model 1) and staffing levels (model 2), adjusting for time trends, resident characteristics, and state-license fixed effects. During this 11-year period, six states changed their staff training requirements and two states introduced/increased direct care staffing levels. A change in regulations related to staffing levels was associated with a reduction in the probability of hospitalization during the month of -0.0056 percentage points (95%CI=-0.008,-0.003). A change in regulations related to staff training was associated with a reduction in the probability of hospitalization during the month of -0.0035 percentage points (95%CI=-0.006,-0.002). The policy effects represent clinically important differences of approximately 21% in the mean monthly hospitalization rate. Part of a symposium sponsored by Assisted Living Interest Group.

### SESSION 6245 (SYMPOSIUM)

### STEPPING INTO THE BREACH OF FEDERAL INACTION: REFORMING THE FINANCING OF LONG-TERM SERVICES AND SUPPORTS

Chair: Marc Cohen
Co-Chair: Edward Miller
Discussant: Judith Feder

The growing need for long-term services and supports (LTSS) poses significant challenges to both individuals and government. For the current cohort of adults aged 65 years and over, it is estimated that the total cost of LTSS (including the value of care provided by family members) will exceed $6.3 trillion. These staggering national costs are driven by the large and rapidly growing number of individuals in need, their longer life expectancies, significant caregiver shortages, and the high costs of care. Neither private health insurance nor Medicare cover these expenses; Medicaid serves as a safety net for individuals once they have impoverished themselves. With limited to no retirement savings, most older adults cannot cover this liability. Moreover, few people have private long-term care insurance to cover this risk and the private market for such insurance is troubled. Thus, the ability to pay for LTSS is sharply bifurcated: people must be either be rich enough to pay out-of-pocket, or poor enough to qualify for Medicaid. However, even Medicaid-eligible individuals may not be able access needed care due to the waiting lists common in Medicaid. The purpose of this symposium is to document the continuing failure to tackle this problem by the federal government, and to review state and national proposals and initiatives aimed at filling the void left by federal inaction. Marc Cohen and Edward Miller will serve as presenters and chair and co-chair, respectively; Eileen Tell will also present; Judy Feder will serve as the discussant.

### FINANCING LONG-TERM SERVICES AND SUPPORTS: OPPORTUNITIES FOR OVERCOMING HISTORICAL ROADBLOCKS TO REFORM

Edward Miller, Pamela Nadash, and Marc Cohen, *University of Massachusetts Boston, Boston, Massachusetts, United States*

This presentation documents the continuing failure to tackle the problem of financing long-term services and supports (LTSS)—a failure most recently seen in the only national legislation ever enacted to comprehensively address LTSS costs: the Community Living Assistance Services and Supports (CLASS) Act. The CLASS Act was included in the Affordable Care Act, but was repealed in 2013. Subsequently, policy experts and some Democrats have made proposals for addressing the LTSS financing crisis. Moreover, significant government action is taking place at the state level, both to relieve financial and emotional burdens on LTSS recipients and their families and to ease pressure on state budgets. Lessons from these initiatives could serve as opportunities for learning how to overcome roadblocks to successful policy development, adoption, and implementation across states and for traversing the policy and political tradeoffs should a policy window open once again for addressing the problem of LTSS financing nationally.

### LEARNING FROM NEW STATE INITIATIVES IN FINANCING LONG-TERM SERVICES AND SUPPORTS

Marc Cohen,[1] Eiileen Tell,[2] and Bonnie Albright,[1]
*1. University of Massachusetts Boston, Boston,*

*Massachusetts, United States, 2. ET Consulting, LLC, Boston, Massachusetts, United States*

A number of states are taking concrete action on long-term services and supports (LTSS) financing which provides an opportunity to learn about the reasons why they are doing so, to identify the coalitions that have come together in support of such actions, and to understand factors associated with the choice of particular strategies and approaches. The purpose of the presentation is to report on a comparative qualitative study across six leading-edge states—Washington State, Hawaii, Maine, Minnesota, California, and Michigan—in order to describe their activities and identify and analyze commonalities and differences in their specific approaches and programs. An overarching goal is to help state officials, consumer advocates, and interested LTSS providers understand the strategies and approaches that other states—who may be further along in their development—are taking in this area so that they might have insights into strategies that might be a fit for their state.

### INTEGRATION AND IMPLEMENTATION CHALLENGES OF STATE-BASED LONG-TERM SERVICES AND SUPPORTS FINANCING PROGRAMS

Eiileen Tell,[1] Marc Cohen,[2] Ben Veghte,[3] and Alexandra Bradley,[4] *1. ET Consulting, LLC, Boston, Massachusetts, United States, 2. University of Massachusetts Boston, Boston, Massachusetts, United States, 3. Caring Across Generations, washington, District of Columbia, United States, 4. National Academy of Social Insurance, Washington, District of Columbia, United States*

Several states are in the process of developing or implementing programs that use a social insurance approach to financing long-term services and supports (LTSS). The National Academy of Social Insurance developed a roadmap that highlights key design parameters for states to consider in crafting a program. It also outlines a range of vetted approaches states could adopt, and describes the building blocks and tradeoffs associated with a range of options. States that establish a new LTSS program will need to make decisions about the integration of the program with other payers and benefit programs as well as address concrete implementation challenges. This presentation summarizes these issues including coordination of benefits with other payers, Federal Medicaid funding issues, integration of LTSS and medical care, and challenges around program startup, administration and monitoring as well as evaluation and modification. Addressing such issues is critical to the success of these type of programs.

### SESSION 6250 (SYMPOSIUM)

### THE 2019 NATIONAL NURSING HOME SOCIAL SERVICES DIRECTORS STUDY

Chair: Mercedes Bern-Klug
Discussant: Robin Bonifas

Psychosocial and emotional needs of nursing home (NH) residents can take the back seat to physical needs in NHs that do not employ staff with the appropriate educational and experience level to anticipate, identify and address these important issues. When present, professional licensed social workers can enhance the NH's ability to address psychosocial issues. However, federal guidelines do not require licensed social workers in NHs, nor does it collect data that would reveal the extent to which licensed social workers are present. With financial support from the RRF Foundation for Aging the NNHSSD Study was undertaken in 2019 to build understanding of departmental staffing characteristics and involvement of the department in key activities and processes. The 924 respondents from around the country also answered questions about training needs, barriers to addressing resident needs, compensation, and job satisfaction. Findings reveal that about half of the nation's NH social services directors have earned a bachelors or master's degree in social work, and about half are licensed (although not all degreed social workers are licensed and not all licensed social workers have earned a social work degree). Half of all social services departments employ only one staff member, one-third have two staff members, and 9% have three staff. About 90% report enjoying their job, with over half reporting they are thriving (not just surviving) in their job. Respondents provided feedback that can be used to strengthen the role of the department and its ability to identity and address resident psychosocial needs.

### FIRST NATIONAL DATA REPORTING NURSING HOME SOCIAL SERVICES DIRECTORS' TRAINING INTERESTS AND NEEDS

Mercedes Bern-Klug, and Elizabeth Cordes, *University of Iowa, Iowa City, Iowa, United States*

A national sample of 924 social services directors reported training needs in three ways. First, their level of interest in 14 topics, second by how much preparation time needed to provide one-on-one training to a colleague about 27 difference issues/tasks (no time needed, up to 2 hours, up to 10 hours, not able to do), and third, by indicating their top training priority. At least 2/3s reported interest in each of the 14 topics with 86% interested in common mental health and psychosocial challenges, and 86% in the psychosocial needs of persons with dementia. Education, social work licensure, and characteristics of the nursing home explained some of the variation, e.g. respondents with a social work degree reported higher interest in more training in trauma-informed care and culturally competent care. Dementia was by far the highest training priority, followed by better understanding of regulations, behavioral health issues, and trauma-informed care.

### BARRIERS TO MEETING THE SOCIAL AND EMOTIONAL NEEDS OF NURSING HOME RESIDENTS

Amy Restorick Roberts,[1] and Mercedes Bern-Klug,[2]
*1. Miami University, Oxford, Ohio, United States,*
*2. University of Iowa, Iowa City, Iowa, United States*

Presenters will describe barriers to psychosocial care and identify which factors increase the odds of experiencing a psychosocial care barrier. Reported major barriers include: insufficient number of nurse aide staff (31%), having to do things other people could do (29%), lack of resources to provide residents with opportunities to leave the nursing home on outings (25%), pressure to admit and discharge post/sub-acute patients takes time away from attending to the social and emotional needs of long stay residents (23%), and not enough social service staff for the number of residents (21%). With data from the 2019 National Nursing Home Social Services Directors Survey, a series of logistic regressions found that significant predictors varied by specific barrier, although devoting more time to short-term residents predicted a greater likelihood of reporting a major barrier in four of the five outcomes. Strategies to address these structural and contextual factors will be discussed.

### SOCIAL WORK TRAINING AND READINESS FOR TRAUMA-INFORMED CARE IN NURSING HOMES

Nancy Kusmaul,[1] and Todd Becker,[2] *1. UMBC, Baltimore, Maryland, United States, 2. University of Maryland, Baltimore, Baltimore, Maryland, United States*

Most adults have experienced traumatic events (SAMHSA, 2017). Late-life traumas may compound upon trauma histories (Maschi, et al., 2013), accentuating the risks confronting older adults. Per CMS' updated Requirements for Participation, nursing homes (NHs) must implement trauma-informed care (TIC) approaches, effective November 2019. Many NHs do not staff Masters of Social Work (MSWs), despite their expertise in providing mental health care. Notwithstanding, employed MSWs feel unprepared to help their NHs implement TIC. This presentation discusses findings from a national survey of NH social service directors (N = 932). Results showed 71% (n = 650) reported moderate to strong interest in TIC training. A Kruskal-Wallis H test revealed a statistically significant difference in TIC training interest $\chi2(1) = 43.690$, $p < .001$, such that MSWs reported higher interest (M = 486.47) than non-MSWs (M = 375.23). There was no difference between those with and without a Bachelor of Social Work.

### THE CONNECTION OF SOCIAL SERVICES TO CARE TRANSITIONS AND DISCHARGE PLANNING IN NURSING HOME SETTINGS

Colleen Galambos, Laura Rollin, and Eric Engelbart, *University of Wisconsin Milwaukee Helen Bader School of Social Welfare, Milwaukee, Wisconsin, United States*

Care transitions are critical junctures in the healthcare delivery process. Effective transitions and discharge planning reduce the need for subsequent transfers between healthcare settings (Boutwell et al., 2015). Understanding social services (SS) involvement in these processes is important due to its key role in their success (Fabbre et al., 2011). Facility characteristics from 924 nursing homes were evaluated in relation to SS involvement in care transitions and discharge planning. Chi-square tests indicate associations between SS involvement and level of engagement of SS expertise by the nursing home administrator (p=.004), medical director (p=.002), nursing staff (p=.003), community physicians (p=.049), and family members (p<.001). An association between SS involvement and freestanding SS departments was also observed. Results suggest the level of SS involvement in care transitions and discharge planning relates to structural (i.e. SS positioning within the facility) and relational (i.e. perceptions and utilization of SS designees by key facility leadership) factors.

### SESSION 6255 (SYMPOSIUM)

### THE AGING ENTERPRISE: A 40-YEAR RETROSPECTIVE

Chair: Larry Polivka
Discussant: Carroll Estes

Dr. Carroll Estes has long been recognized as one of our most influential social gerontologists beginning with the publication of the Aging Enterprise over 40 years ago. This book quickly achieved iconic status among gerontologists and other social scientists as one of the founding texts in critical gerontology, which Dr. Estes has played a leading role in developing with numerous publications over the course of her illustrious career. The panelists will focus on Dr. Estes' application of the theoretical frameworks offered by the social construction of reality and the political economy of aging to a critique of federal and state policies designed to improve the quality of life of older Americans. Many of the programs and policies included in Dr. Estes' critique are still in place, including the Older Americans Act and the nonprofit aging network. On the other hand, much about the aging enterprise has changed since 1979. The panelists, Drs. Chris Phillipson, Pamela Herd and Larry Polivka, will discuss the value of and challenges to these theoretical and empirical perspectives within the current contemporary neoliberal political economy that has gradually displaced the welfare state capitalism of the postwar period. As this shift has occurred in the political economy, a neoliberal policy agenda featuring for-profit privatization of public services, including aging services, has become dominant at the federal and state levels. Dr. Estes will respond to the panelists' presentations and discuss the future of critical gerontology. Women's Issues Interest Group Sponsored Symposium.

### THE POLITICAL ECONOMY OF AGING SERVICES IN THE UNITED STATES SINCE THE AGING ENTERPRISE

Larry Polivka, *Florida State University, Tallahassee, Florida, United States*

The Aging Enterprise was the first book length analysis of the origins and operations of the aging services system that grew out of the 1963 Older Americans Act and provided the first state system of community-based services. The book also featured an original critique of the aging services system based on an application of the theoretical perspectives that were new to social gerontology. This presentation will use one of these perspectives, the political economy of aging in the U.S., to chart changes in aging services systems since the publication of The Aging Enterprise. The presentation will show that Estes' pioneering work on the application of the political economy perspective on aging policies and programs in the US, is more relevant to our understanding of these programs than it was 40 years ago, by describing and analyzing the increasing control of for profit corporations over the aging services systems. Part of a symposium sponsored by the Women's Issues Interest Group.

### REASSESSING THE AGING ENTERPRISE: NEW PERSPECTIVES ON THE SOCIAL CONSTRUCTION OF LATER LIFE

Chris Phillipson, *Manchester Institute for Collaborative Research into Ageing, Manchester, England, United Kingdom*

Four decades on from the publication of 'The Ageing Enterprise', this paper provides a critical review of the relationship between social theory and social policies for later life. To what extent do current theoretical perspectives in gerontology bear the influence of ideas laid out in that pioneering book? How has the 'ageing enterprise' fared given the dominant ideology of neo-liberalism and the precarious lives faced by people moving through the life course? The paper considers these questions in the context of globalization processes, and the imposition of austerity policies. The paper will consider the continuing importance of 'The Ageing Enterprise' by reviewing three main themes: first, assessing the changing relationship between the state and social policy; second, through examining current perspectives within critical gerontology; third, highlighting new forms of empowerment developing amongst older people, and the relationship of these to the values and ideas expressed in 'The Ageing Enterprise'. Part of a symposium sponsored by the Women's Issues Interest Group.

### PRIVATIZING THE WELFARE STATE

Pamela Herd, *Georgetown University, Washington, District of Columbia, United States*

The growth of the private sector in the Medicare and Medicaid programs is a sea change, leading many to argue that the old age welfare state is effectively becoming privatized. I examine these trends, but focus on the consequences for how older adults experience their interactions with government. In particular, I examine how privatization increases administrative burden for beneficiaries. Older adults must navigate hundreds of choices, leading to significant confusion. Most fail to pick policies that maximize their benefits and reduce their cost. This confusion harms beneficiaries. They end up with suboptimal coverage, with increased out of pocket costs and decreased access to care. The confusion, however, generates profits for insurers. Part of a symposium sponsored by the Women's Issues Interest Group.

### SESSION 6260 (SYMPOSIUM)

### THE BOLD PUBLIC HEALTH RESPONSE TO ALZHEIMER'S DISEASE AND RELATED DEMENTIAS: PAST, PRESENT, AND FUTURE

Chair: Lisa McGuire
Co-Chair: Nia Reed

Achieving meaningful progress against Alzheimer's disease and related dementias (ADRD) requires an urgent public health response. Since 2005, the Alzheimer's Association, Centers for Disease Control and Prevention (CDC), and other public health partners collaborated on the Healthy Brain Initiative (HBI). HBI seeks to advance public health awareness of and action on ADRD as a public health issue. The HBI Road Map Series, State and Local Public Health Partnerships to Address Dementia: The 2018–2023 Road Map (S&L RM) and Road Map for Indian Country, provide the public health with concrete steps to respond to the growing burden of ADRD in communities, consistent with the aim of the Building Our Largest Dementia (BOLD) Infrastructure for Alzheimer's Act (P.L. 115-406). This series of RMs for state, local, and tribal public health provide flexible menus of actions to address cognitive health, including ADRD, and support for dementia caregivers with population-based approaches. The purpose of this session is to illustrate public health's role with ADRD—past, present, and future. An overview of the evolution of HBI RM series will be provided (McGuire) and preliminary evaluation data of 24 actions from S&L RM will be presented

(French, Shean, Beatty). Holt, Reed, & McGuire will share lessons learned and next steps from CDC's Regional Action Institutes designed to facilitate implementation of the RM series across the US. The intersection between the RM series and Age-Friendly Public Health Systems will be described as a starting point for public health (Wolfe, French, Shean).

### THE EVOLUTION OF THE HEALTHY BRAIN INITIATIVE

Lisa McGuire, *Centers for Disease Control and Prevention, Atlanta, Georgia, United States*

The Healthy Brain Initiative (HBI) seeks to advance public health awareness of and action on ADRD as a public health issue. The HBI Road Map Series, State and Local Public Health Partnerships to Address Dementia: The 2018–2023 Road Map (S&L RM) and Road Map for Indian Country (RMIC), provide the public health with concrete steps to respond to the growing burden of ADRD in communities, consistent with the aim of the Building Our Largest Dementia (BOLD) Infrastructure for Alzheimer's Act (P.L. 115-406). This series of RMs for state, local, and tribal public health provide flexible menus of actions to address cognitive health, including ADRD, and support for dementia caregivers with population-based approaches. This session will describe how the initiative evolved over the past 15 years including policy and implementation success stories.

### GETTING THE SHOW ON THE ROAD! STATES AMP UP A LIFE COURSE APPROACH TO ALZHEIMER'S

John Shean,[1] Molly French,[1] and Carissa Beatty,[2]
*1. Alzheimer's Association, Washington, District of Columbia, United States, 2. Emory Centers For Training And Technical Assistance, atlanta, Georgia, United States*

Alzheimer's and other dementias are chronic conditions that disproportionately impact some populations. The dementia continuum spans decades, so life-course approaches by state health departments (SHDs) are crucial. SHDs' implementation of the Healthy Brain Initiative Road Map (RM) represents policy/system/environmental actions to improve population-level dementia outcomes. METHODS. Alzheimer's Association uses online reporting by affiliate chapters to monitor SHDs' RM implementation. Association staff reviewed data and calculated frequencies by RM action and issue areas. Documentation from SHDs and national partners are supplementary data. RESULTS. Preliminary results from July-December 2019 indicate 34 SHDs were implementing 60 RM actions. Interventions clustered in risk reduction, early detection, workforce competencies, surveillance, and emergency preparedness. RM implementation lagged on access to community supports, attention to caregiving, and workforce supply analyses. A majority of states had multi-faceted initiatives. IMPLICATIONS. SHDs are deploying life-course approaches for dementia. SHD leaders must build towards a full response by expanding RM implementation.

### CDC HEALTHY BRAIN INITIATIVE ACTION INSTITUTES: INNOVATIVE PLANNING AND LESSONS LEARNED

Heidi Holt, *Centers for Disease Control and Prevention (CDC), Atlanta, Georgia, United States*

This presentation will demonstrate an innovative strategic planning effort, coined Action Institutes (AI's), which are designed to promote the implementation of CDC's "The Healthy Brain Initiative's State and Local Public Health Partnerships to Address Dementia" and the "Road Map for Indian Country." Both of these documents outline how the champions of public health and their partners can create a statewide effort to promote brain health, increase early diagnosis, address cognitive impairment for individuals living in the community, and help meet the needs of care partners. The purpose of these 1-2-day AI's is to familiarize leaders with the topic, encourage their adoption into current priority setting, and guide participants in creating action plans. The CDC's Alzheimer's Disease and Healthy Aging Program is conducting a series of these AIs, which are made possible through partnerships with the Association of State and Territorial Health Officials and the National Indian Health Board.

### START AT THE CENTER: AGE-FRIENDLY PUBLIC HEALTH SYSTEMS AND HEALTHY BRAIN INITIATIVE FRAMEWORKS

Megan Wolfe,[1] Molly French,[2] and John Shean,[2] *1. Trust for America's Health, Washington, District of Columbia, United States, 2. Alzheimer's Association, Washington, District of Columbia, United States*

SIGNIFICANCE. Older adults can contribute wisdom, skills, and time to communities. The public health sector has unique capabilities for creating conditions that promote health, foster community connections, and quality of life. METHODS. Two frameworks provide public health (PH) with core strategies to improve outcomes for all older adults. The Framework for Creating an Age-Friendly Public Health System (AFPHS) supports the PH role, as demonstrated by 37 of Florida's 67 county health departments that are piloting the AFPHS Framework. The Healthy Brain Initiative's (HBI) State and Local Public Health Partnerships to Address Dementia is a framework for action used by PH to promote cognitive health, improve care for cognitive impairment, and increase caregiving supports. Both frameworks call for utilizing regional data and cross-sector partnerships. IMPLICATIONS. PH can contribute to community-wide initiatives to promote well-being and community connections for older adults. Cross-sector partnerships can start by using available tools and planning guides.

### SESSION 6265 (SYMPOSIUM)

### POLICY SERIES: THE ELDER JUSTICE ACT, NURSING HOME REFORM, AND FUNDING LEGISLATION

Chair: Brian Lindberg

This session will provide updates on major federal efforts to address elder abuse, neglect, and exploitation, including strategies for prevention, intervention, services, and prosecution. Congress has been working on both reauthorizing the Elder Justice Act and policies to address poor long-term care facility quality issues, and this panel will provide an update on those efforts and what lies ahead in2021. The panel will include elder justice and nursing home advocates and congressional staff.

### FRAMING THE ISSUE

Brian Lindberg, *GSA, Washington, District of Columbia, United States*

GSA's Public Policy Advisor will lead a discussion of legislative activities related to elder abuse, neglect, and exploitation during the past year. The panel will examine successes and failures and what may still be possible in the lame duck session and the next Congress.

### THE ELDER JUSTICE COALITION AND REAUTHORIZING THE ELDER JUSTICE ACT

Robert Blancato, *Matz Blancato & Associates, Washington, District of Columbia, United States*

The presentation and dialogue will focus on the elder justice advocacy community and their work to secure reauthorization of the Elder Justice Act, which was originally passed as part of the ACA. Both grassroots and DC-based efforts have been employed to move this and other legislation forward and to secure much needed funding for the programs designed to end elder abuse.

### ELDER JUSTICE AND NURSING HOME REFORMS 2020

Lori Smetanka, *The National Consumer Voice for Quality Long-Term Care, Washington, District of Columbia, United States*

The presentation will provide perspective on the current state of care quality in long-term care facilities and the legislative discussions and actions of the 116th Congress.

### SESSION 6270 (SYMPOSIUM)

### THE EXPERIENCE OF WORKING FAMILY CAREGIVERS: IMPLICATIONS FOR A NATIONAL PAID LEAVE POLICY

Chair: Tiffany Washington
Discussant: Lynn Friss Feinberg

Family caregivers provide the bulk of unpaid care to older adults. The typical family caregiver is a 49-year-old woman who works full time and simultaneously provides an average of 24 hours of care per week for an older relative. Unfortunately, their caregiving duties places them at risk for lost wages and termination due to frequent interruptions at work, especially in the absence of a national paid family leave policy. It is possible that such a policy could mitigate these risks; however, the United States is the only developed nation that lacks a national paid family leave policy for all workers. This symposium will highlight the psychosocial, economic, and health issues experienced by working caregivers, and conclude by linking presenters' findings to implications for a national paid leave policy. To start, presenter one will describe findings from a scoping review on workplace experiences of female family caregivers. Next, presenter two will describe findings from a systematic review to explore predictors of the adoption and implementation of state-level paid family leave policies. Presenter three's study examines interest in supportive services among working and non-working Black caregivers in the Deep South. Presenter four will describe factors associated with healthcare utilization of working caregivers using data from the Regional Healthcare Partnership – Region 17 Health Assessment Survey. The final presenter, HHS Advisory Council to Support Grandparents Raising Grandchildren co-chair, will describe development of policy initiatives to identify, coordinate, and promote information, resources, and best practices for working grandparents raising grandchildren.

### FACTORS ASSOCIATED WITH HEALTH CARE UTILIZATION AMONG WORKING CAREGIVERS

Tiffany Washington,[1] Eunhye Kim,[2] George Mois,[3] and Matthew Smith,[4] *1. University of Georgia, Athens, Georgia, United States, 2. Augusta University, Augusta, Georgia, United States, 3. University of Georgia, Lawrenceville, Georgia, United States, 4. Texas A&M University, College Station, Texas, United States*

This study examined factors associated with health care utilization among working family caregivers using data from the 2013 Regional Healthcare Partnership – Region 17 Health Assessment survey. Anderson's Behavioral Model of Health Services Use guided the selection of variables. Chi-square or t-test were computed to compare statistically significant differences between caregivers who did and did not utilize health care, and logistic regression was employed to identify factors associated with health care utilization. Of the 220 working caregivers, 41.8% put off their health care primarily because they could not miss work. Age, days pain limited activity, and days not having enough rest or sleep were associated with health care utilization. Paid family leave could mitigate the challenge of simultaneously managing employment responsibilities, caregiving tasks, and health needs of working family caregivers.

### WORKING WHILE CAREGIVING: A SCOPING REVIEW OF THE EXPERIENCES OF EMPLOYED FEMALE CAREGIVERS

Jessica King McLaughlin, *University of Denver, Denver, Colorado, United States*

The negative consequences of caregiving disproportionately impact women, as women make up the majority of informal caregivers and spend more time doing caregiving duties than men. The competing demands inherent in being a caregiver while working can increase levels of stress and burden that these individuals may experience. However, the nuances of the challenges of working female caregivers are still not salient. This review synthesizes the extant literature on working female caregivers to clarify the dilemmas they face and factors that impact their ordeals. Thirty studies on working female caregivers met inclusion criteria. Findings indicate common themes that emerged across the studies. These themes indicate the need to more comprehensively support the multifaceted nature of working female caregivers' experiences in the workplace and outside of work. Furthermore, this review identifies areas in which informed policy can have a meaningful impact on working female caregivers' lives.

### BEYOND FMLA: MAKING THE CASE FOR STATE-LEVEL PAID FAMILY LEAVE, THE PATH TO POLICY ADOPTION

Eugenie Stephenson, *University of Maryland, Baltimore County, Baltimore, Maryland, United States*

The Family Medical Leave Act (FMLA) was established in 1993, and while limited in scope, it is the only national policy in place to support individuals that need to take time away from work to care for a sick family member. Paid Family Leave, a state-level mechanism of FMLA, creates a safety net to support these workers. Despite its potential impact, such programs currently exist in only 6 states and the District of Columbia. Further, even within these states, individuals are often unaware of the policy's existence. A systematic review of peer reviewed literature from 1994-2019 explores the predictors of the adoption and implementation of state-level Paid Family Leave and public awareness of Paid Family Leave programs in these states. This presentation will explore these findings and their implications on future state level adoption, implementation and awareness of Paid Family Leave.

### INTEREST IN SUPPORTIVE SERVICES AMONG BLACK WORKING AND NON-WORKING CAREGIVERS IN THE DEEP SOUTH

Olivio Clay,[1] Fayron Epps,[2] Cathy Scott,[3] Fawn Cothran,[4] and Ishan Williams,[5] *1. University of Alabama at Birmingham, Birmingham, Alabama, United States, 2. Emory University, Atlanta, Georgia, United States, 3. University of Tennessee at Chattanooga, Chattanooga, Tennessee, United States, 4. UC Davis Betty Irene Moore School of Nursing, Sacramento, California, United States, 5. University of Virginia, Charlottesville, Virginia, United States*

The current investigation provides information on supportive services caregivers said they would be interested in if they were made available. Participants were recruited from the Birmingham, AL metro area and received a $25 gift card for completing a telephone interview. Of the 38 caregivers enrolled, 18 (47.37%) reported being currently employed and working an average of 33.92 hours per week (range = 5 – 60). Participants were caring for individuals with average scores on the AD8 Dementia Screening Scale of 7.3 and 10.4 on the Clinical Dementia Scale Sum of Boxes (above the cutoffs for probable dementia). Thirty-three caregivers (87%) reported being interested in at least one of the services listed with differences observed for services that would be preferred by working vs. non-working caregivers. These data will be utilized to provide initial support for a multicomponent intervention that may be effective in reducing negative outcomes within Black caregivers.

### FEDERAL ADVISORY COUNCILS ESTABLISHED TO ASSIST WORKING CAREGIVING FAMILIES

Ethlyn McQueen Gibson, *Hampton University, Hampton, Virginia, United States*

This presentation will describe the legislative charge of two advisory councils established in 2018 through the Recognize, Assist, Include, Support and Engage Family Caregivers Act and the Supporting Grandparents Raising Grandchildren Act to support working family caregivers. The advisory councils are charged with 1) providing recommendations on effective models of family caregiving 2) improving coordination across federal government programs; and 3) identifying, coordinating, and promoting information, resources, and 4) best practices for working grandparents raising grandchildren, while maintaining their own physical, mental, and emotional well-being. The experiences of working family caregivers will be the foundation for these recommendations that are being developed over the next two years to present to Congress. These recommendations will support the development and execution of a national family caregiving strategy.

### SESSION 6275 (SYMPOSIUM)

### POLICY SERIES: THE IMPACT OF AGEISM ON HEALTH AND EFFORTS TO ADDRESS IT THROUGH REFRAMING AGING

Chair: Erica Solway

Ageism can manifest in a variety of ways including in institutional policies and practices, interpersonal interactions, older adults' internalization of ageist stereotypes, and through the media. While major incidents of age-based discrimination and their negative effects on health are more recognized, older adults also experience less momentous, but still harmful ageism in their day-to-day lives. These types of everyday ageism are also harmful to health. This symposium will highlight recent research from the University of Michigan National Poll on Healthy Aging on self-reported experiences of everyday ageism among a nationally representative sample of U.S. adults age50-80 and its connections to physical and mental health outcomes. Next, another researcher also using data from the National Poll on Healthy Aging will explore the prevalence of positive attributes of aging, its relationships to health, and ways in which it may buffer older adults from the adverse health consequences of ageism. Then speakers will highlight the Reframing Aging initiative and the ways in which GSA and other organizations are leading a national effort to address ageism through education and dissemination of research-based resources. This session will feature research, education, and advocacy efforts and will focus on the policy implications of efforts to better understand the prevalence of and strategies to counter the effects of ageism.

### POSITIVE ATTRIBUTES OF AGING AND CONNECTIONS TO HEALTH: EVIDENCE FROM THE NATIONAL POLL ON HEALTHY AGING

Erica Solway,[1] Julie Ober Allen,[1] Matthias Kirch,[1] Dianne Singer,[1] Jeffrey Kullgren,[1] and Preeti Malani,[2] *1. University of Michigan, Ann Arbor, Michigan, United States, 2. University of Michigan Medical School, Ann Arbor, Michigan, United States*

This study explored the prevalence of positive attributes of aging among older adults in a nationally representative sample age 50-80 ((N=2,048, 52% female, 71% White). Nearly 70% of older adults reported that people sought their guidance because of their wisdom and experience. Older adults reported that, as they have gotten older, they have become more comfortable with themselves (88%), have a strong sense of purpose (80%), feel more positively about aging (67%), and have found their life to be better than they had thought it would be (65%). Over half (52%) of those who said their lives were better than they thought reported very good or excellent physical health. Among those who disagreed, only one out of four (25%) reported very good/excellent physical health; similar results were found for mental health (48% vs. 22%). This session will describe positive attributes of aging and their association to physical and mental health.

### EVERYDAY AGEISM AND HEALTH: EVIDENCE FROM THE NATIONAL POLL ON HEALTHY AGING

Julie Ober Allen,[1] Erica Solway,[2] Matthias Kirch,[2] Dianne Singer,[2] Jeffrey Kullgren,[2] and Preeti Malani,[3] *1. University of Oklahoma, Ann Arbor, Michigan, United States, 2. University of Michigan, Ann Arbor, Michigan, United States, 3. University of Michigan Medical School, Ann Arbor, Michigan, United States*

This study examined the prevalence of everyday ageism, routine types of age-based discrimination, prejudice, and stereotyping that older adults encounter in their daily lives, and its relationships with health in a nationally representative sample age 50-80 (N=2,048, 52% female, 71% White). Nearly all older adults said they sometimes or often experienced everyday ageism (96% age 65-80, 92% age 50-64). The most common types were beliefs that health problems were an inevitable part of getting older (78%), hearing jokes about aging/older people (61%), and seeing material suggesting that older adults were unattractive/undesirable (38%). Those reporting more experiences with everyday ageism (>3 types) were less likely than those reporting fewer types to have excellent/very good physical health (31% vs. 50%); similar results were found for mental health (60% vs. 80%). This poll documented the ubiquity of minor, but not inconsequential, everyday ageism reported by older adults and its potential ramifications for health.

### REFRAMING AGING: A GENERATION'S WORK

Patricia D'Antonio, *The Gerontological Society of America, Washington, District of Columbia, United States*

The Reframing Aging initiative, led by GSA on behalf of the Leaders of Aging Organizations, is a long-term, grant-funded social change endeavor designed to improve – or "reframe" the public's understanding of what aging means and the many contributions older people bring to society. Using evidence-based research, the initiative seeks to teach advocates how to tell an effective story about aging that will promote positive perceptions of aging and reduce ageism. This session will explore GSA's efforts to address ageism through Reframing Aging and the policy implications of this important initiative and will include lessons learned from experts who are utilizing reframing aging in their teaching and practice.

### SESSION 6280 (SYMPOSIUM)

### THE IMPACT OF INTERSECTIONAL IDENTITIES ON OLDER PEOPLE WITH HIV

Chair: Mark Brennan-Ing
Discussant: Charles Emlet

Kimberlé Crenshaw introduced the term "intersectionality" in the late 1980s to highlight the experience discrimination and marginalization of Black and African-American women originating from the confluence of their racial/ethnic and gender identities. Since that time the focus on intersectionality has broadened to consider other communities and individuals who may have multiple stigmatized and discredited identities, including older people with HIV (PWH). For example, Porter and Brennan-Ing described the "Five Corners" model as the intersection of ageism, racism, classism, sexism, and HIV stigma for older transgender and gender non-conforming PWH. HIV disproportionately affects marginalized communities (e.g., racial/ethnic and sexual minorities). Thus, for older PWH it is important to consider how HIV stigma may intersect with other marginalized identities and impact physical and psychological well-being. The first paper in this session examines how the intersection of HIV serostatus, gay identity, and age complicates identity disclosure, leading to social isolation and interference with care planning. The second paper describes how intersectional identities among older PWH interfere with access to mental health services in a population that is disproportionately affected by depression and PTSD. Our third paper examines the role of race, education, and behavioral health in neurocognitive functioning among a diverse sample of older HIV+ gay and bisexual men. Our last paper examines neurocognitive functioning among older Latinx PWH, finding that sexual and gender minorities were at greater risk for impairment. Implications of these findings for research and programming that accounts for the effects of intersectionality among older PWH will be discussed.

### LOOKING AHEAD: INTERSECTING INFLUENCES ON OLDER GAY MEN LIVING WITH HIV

Brian de Vries,[1] Gloria Gutman,[2] Tamara Sussman,[3] Shari Brotman,[3] and Denis Dube,[3] *1. San Francisco State University, San Francisco, California, United States, 2. Simon Fraser University, Vancouver, British Columbia, Canada, 3. McGill University, Montreal, Quebec, Canada*

Older HIV-positive gay men live at the intersection of multiple inequalities—with cascading effects on their present and future lives. This qualitative study explored how they plan for their future, with a focus on Advance Care Planning—the process of reflecting/communicating preferences and values for future health and end-of-life care. Seven French-speaking gay men aged 55+ in Montreal, Canada participated in a focus group that was audio-recorded, transcribed and thematically analyzed in four steps. Findings suggest the intersection of sexual orientation and HIV-positive status exacerbated self-disclosure issues; the further addition of age led to preoccupation with day-to-day living and rendered these men vulnerable to social isolation. These tensions not only interfered with their capacities to talk about future care, but also created barriers to thinking about future care. These findings describe the multiple layers and compounding consequences of inequality among older gay men living with HIV.

### INTERSECTIONAL STIGMA AND BARRIERS TO MENTAL HEALTH AMONG OLDER ADULTS WITH HIV IN SAN FRANCISCO

Paul Nash,[1] Mark Brennan-Ing,[2] Tonya Taylor,[3] and Stephen Karpiak,[4] *1. University of Southern California, Los Angeles, California, United States, 2. Hunter College, CUNY, New York, New York, United States, 3. SUNY Downstate Health Sciences University, Brooklyn, New York, United States, 4. ACRIA Center on HIV & Aging at GMHC, new York, New York, United States*

For older adults with HIV, forms of privilege and oppression (racism, poverty, limited access to quality education, and inequalities in criminal justice system) intersect with stigmatized social identities (immigrant status, non-cisgender identity, sexual orientation, depression, and addiction) that may increase cumulative burden of psychological distress, contribute to poor clinical outcomes, and create disparities in health care utilization. Using survey and focus group data from the San Francisco ROAH 2.0 (Research on Older Adults with HIV) site, we explored how layered intersectional identities (minority affiliation, gender and sexual orientation), life experiences (immigration, trauma) and forms of systemic oppression (poverty, low educational attainment, and incarceration) impact the utilization of mental health supportive services. Immigrants, minority women, and heterosexual men had higher burdens of depression compared to their white counterparts. Similarly, inhabiting multiple stigmatized identities resulted in both low and variable levels of mental health care utilization, suggesting need for targeted intervention efforts.

### THE INTERSECTION OF MENTAL HEALTH, SELF-REPORTED NEUROCOGNITIVE FUNCTIONING, AND EDUCATION IN OLDER HIV+ GAY MEN

Kristen Krause, *Rutgers University, Piscataway, New Jersey, United States*

Older people living with HIV/AIDS (PLWHA) face different mental and neurocognitive challenges related to their health and well-being. Using data from a cross-sectional study (n=250) on older (age 50-69) gay men living with HIV/AIDS in NYC, this study examined the multi-level associations between self-reported neurocognitive functioning, mental health, and key sociodemographics (age, race/ethnicity, financial situation, and education). Findings suggest those who have higher self-reported neurocognitive functioning have higher levels of education, better self-rated health, and lower levels of PTSD and depression (p<0.01). Differences were not observed based on race/ethnicity, financial situation, and age. The overall findings demonstrate educational differences in self-reported cognitive functioning among older HIV+ gay men and highlight the importance of enhancing interventions and policies to promote better cognitive and mental health outcomes. More research is warranted to understand the intersection of education and cognitive performance among other sub-groups of PLWHA to understand whether these findings are consistent.

### INTERSECTIONALITY AND COGNITIVE IMPAIRMENT RISK IN OLDER PERSONS WITH HIV: AGE, ETHNICITY, AND LGBT STATUS

Monica Rivera Mindt, Micah Savin, Angela Summers, Jordan Stiver, and Alex Slaughter, *Fordham University, New York, New York, United States*

The Latinx population is disproportionately affected by HIV-infection and older Latinx persons living with HIV (PLWH) are at greater risk for neurocognitive impairment (NCI). However, no studies have examined whether intersectionality (including Lesbian Gay Bisexual Transgender [LGBT] status) increases NCI risk. This study investigated whether LGBT status increases NCI risk in 126 PLWH (Ages 19-73 years; 74% Male; 66% Latinx, 34% NHW) who completed a comprehensive NC battery. Domain average T-scores were based on demographically-corrected norms. Multiple regressions revealed that after accounting for covariates (cocaine use, premorbid IQ) and other dimensions of intersectionality (age, ethnicity), LGBT status significantly contributed to NCI risk in attention/working memory (B=-4.50, p=.01) and executive functioning (trend-level; B=-3.67, p=.06). LGBT status, a key dimension of intersectionality, should be considered in NC assessment of PLWH. Future research is needed to identify factors (e.g., discrimination) that may confer increased NCI risk in this population.

### SESSION 6285 (SYMPOSIUM)

### THE IN-CARE PROJECT: SOCIOECONOMIC INEQUALITIES IN CARE USE AND PROVISION ACROSS COUNTRIES AND OVER TIME

Chair: Marjolein Broese van Groenou

Ageing societies and recent reforms to long-term care (LTC) in many European countries are likely to make informal care by kin and nonkin increasingly critical for fulfilling the care needs of older people. To date, it is unknown whether informal care falls disproportionately on disadvantaged populations. The IN-CARE project (a collaboration of Dutch, UK and German research teams; http://in-care.fk12.tu-dortmund.de/) examines if and how LTC reforms exacerbate existing social disparities in care use and provision in older age. To this end, this project compares the socioeconomic status (SES) gradient in formal and informal care across Europe and over time. A particular effort is made to include macro-level indicators of LTC systems in cross-level analyses across countries. The first paper presented in this symposium by the UK team studied SES-inequality in care receipt across European nations with different care systems; the second paper presented by the German team studied the same question but now among caregivers, the third paper provides the analyses for caregivers in Japan, and the fourth paper by the Dutch team studies SES-inequalities in care use within the Netherlands over time (1995-2015). The symposium will start off with a short description of the IN-CARE project (2019-2022). Tine Rostgaard agreed to be our discussant.

### SOCIOECONOMIC INEQUALITIES IN CARE RECEIPT AT OLDER AGES: A COMPARATIVE EUROPEAN STUDY

Ginevra Floridi,[1] Ludovico Carrino,[2] and Karen Glaser,[2]
*1. King's College London, London, United Kingdom,*
*2. King's College London, London, England, United Kingdom*

This study is among the first to investigate whether and how socioeconomic inequalities in the receipt of formal and/or informal care by disabled older adults vary across long-term care (LTC) systems. We link data from the SHARE survey with LTC system indicators for 136 regions in 12 European countries in 2015. Using multinomial multilevel models with cross-level interactions, we test whether and how income and wealth gradients in the receipt of only informal, only formal, and mixed care vary with the number of beds in residential LTC facilities across regions. We find pro-rich inequalities in the receipt of formal and mixed care only in regions with low or intermediate numbers of LTC beds, and no inequalities in regions with greater availability of residential beds. Our findings suggest that the presence of extensive formal LTC services may lead to a fairer distribution of formal and mixed care use across socioeconomic groups.

### SOCIOECONOMIC INEQUALITIES IN INFORMAL CARE PROVISION AND ITS CONSEQUENCES FOR WELL-BEING ACROSS EUROPE

Nekehia Quashie,[1] Judith Kaschowitz,[2] Christian Deindl,[3] and Martina Brandt,[2] *1. TU Dortmund, Dortmund, Germany, 2. TU Dortmund, Dortmund, Nordrhein-Westfalen, Germany, 3. Heinrich-Heine-Universität Düsseldorf, Duesseldorf, Nordrhein-Westfalen, Germany*

We assess socioeconomic inequalities in informal care provision and its consequences for the wellbeing of informal caregivers. The literature states that a lower socio economic status (SES) is linked to a higher probability to give care (at higher intensities) which then leads to a higher caregiving burden. People with lower SES additionally have fewer resources to alleviate caregiving pressures. Thus, they are likely to experience decreased wellbeing compared to those with higher SES. Our analyses based on data from SHARE and ELSA confirm, that individuals with lower SES are indeed more likely to provide care all over Europe. They also report a lower wellbeing than people with higher SES, even if controlling for further important influences. In the next step we investigate longitudinally, if taking over care responsibilities leads to a wellbeing decline and if this decline is more pronounced for people with lower SES.

### SOCIOECONOMIC STATUS AND LONG-TERM CARE PROVISION TO PARENTS IN JAPAN

Yoko Ibuka,[1] and Yui Ohtsu,[2] *1. Keio University, Tokyo, Japan, 2. National Institute of Population and Social Security Research, Tokyo, Tokyo, Japan*

Socioeconomic status (SES) is generating considerable interest in terms of health of individuals, but how it is associated with long-term care has not been established yet. We study the relationship between SES and long-term care provision to parents among the Japanese adults using JSTAR. We use the following six measures of SES for the analysis: income, asset, expenditure, living condition, housing condition and education. We find a greater probability of care provision to parents among those in higher SES categories for some SES measures, compared to the lowest category. However, after considering the survival probability of parents, the relationship is reversed and the probability of care provision is found to be greater among lower SES individuals. The association is more pronounced among males. The association is likely to be partly mediated by care needs of parents. These results suggest a higher burden of care disproportionately falls in low SES individuals.

### SOCIOECONOMIC INEQUALITY IN LONG-TERM CARE: A COMPARISON OF THREE TIME PERIODS IN THE NETHERLANDS

Jens Abbing,[1] Bianca Suanet,[2] and Marjolein Broese van Groenou,[3] *1. Vrije Universiteit, Amsterdam, Netherlands, 2. Vrije Universiteit Amsterdam, Amsterdam, Noord-Holland, Netherlands, 3. VU University, Amsterdam, Noord-Holland, Netherlands*

This study aims at investigating to what extent inequalities in the use of formal, informal and privately paid care have changed over time. Data from the Longitudinal Aging Study Amsterdam (LASA) was used from three points in time (1995, 2005 and 2015) that capture distinct periods in the recent development of the Dutch long-term care system. In particular, the reforms of 2007 and 2015 might have impacted care uses. All participants (N = 1810) were living at home and between the age of 75 and 85 at measurement. The results indicate that, adjusted for health and partner status, formal, informal and privately paid care have decreased over time. Socioeconomic differences in informal care use have increased over time, but no change was found for privately paid or formal care use. These findings suggest that changes in the LTC system and long-term care resources in particular benefit lower socioeconomic groups.

### SESSION 6290 (SYMPOSIUM)

### POLICY SERIES: THE OLDER AMERICANS ACT REAUTHORIZATION: MAJOR SUCCESSES AND SHORTCOMINGS

Chair: Brian Lindberg

This session provides an update on the2020 Older Americans Act (OAA) reauthorization process and passage of the new law "Supporting Older Americans Act of2020." Presenters will provide insights on both the content of the new law and the process that led to the bicameral, bipartisan legislation. Speakers will include representatives of the aging network and other stakeholders, who will discuss key changes to the OAA and how they may be implemented over the next five years.

### FRAMING THE ISSUE

Brian Lindberg, *GSA, Washington, District of Columbia, United States*

The GSA Public Policy Advisor will facilitate a discussion about the 2020 reauthorization of the Older Americans Act with key stakeholders from Washington, DC. Also, the presentation will include perspective on GSA's active role in policy development and the legislative process.

### THE AGING NETWORK AND THE OLDER AMERICANS ACT REAUTHORIZATION

Amy Gotwals, *National Association of Area Agencies on Aging, Washington, District of Columbia, United States*

This presentation will cover a range of OAA reauthorization proposals - successes and what was not possible this year. As a leader in the aging network, n4a played a major role in the legislative process, and the discussion will include top priorities for improving the ability of the network to serve older adults with community-based supports and services.

### ADVANCING STATES THROUGH THE OAA REAUTHORIZATION

Damon Terzaghi, *ADvancing States, Arlington, Virginia, United States*

States are major policymakers and funders of community-based services for older Americans and ADvancing States played a significant role in supporting the OAA authorization this year. This presentation will provide the states' perspective regarding funding, services, research and demonstrations, and more.

### NEW PIECES FOR THE OLDER AMERICANS ACT PUZZLE

Andrew MacPherson, *Healthsperien, Washington, District of Columbia, United States*

With the passage of the new "Supporting Older Americans Act of 2020," Congress acknowledged and address several under appreciated issues -the growing social isolation and loneliness of older adults, and the significant role that the aging network does and can play in providing supportive services to older adults with advanced illness (also known as serious illness). This presentation will provide information on these and other pieces of the OAA puzzle.

### SESSION 6295 (SYMPOSIUM)

### THE ROAD TO IMPROVED ASSISTED LIVING QUALITY: STATE EFFORTS AND NEW METRICS

Chair: Tetyana Shippee
Discussant: Lindsay Schwartz

Assisted living (AL), a senior housing option that combines housing, support services, and health care, is recognized as one of the fastest-growing components of the long term care industry. AL is also a relatively expensive service, whether it's paid for privately or publicly. Also, an increasing proportion of AL residents have diagnoses of Alzheimer's and related dementias. However, little is known about AL quality, in part due to lack of measures. Quality of AL matters to residents, their families, and policy makers because AL is not only about the experience of receiving specific services, but about a place that many will call home. Concerns have been surfacing regarding the quality of AL, including poor staffing, inadequate teamwork, and poor management, which can negatively impact resident well-being and result in abuse and neglect in some cases. This symposium will feature four presentations that will review efforts from two states that have been actively developing measures to address AL quality: Oregon and Minnesota. We focus on new legislation in both states to address AL quality, the new metrics being adopted, and preliminary results. Individual presentations will describe 1) Oregon's new quality measures for AL ;2) Oregon's use of Resident VIEW project, including measures of person-centered care from the perspectives of residents; 3) Minnesota's development of AL report card, and 4) development and results from MN pilot surveys of resident quality of life and family satisfaction in all licensed ALs in the state. Policy implications for other states and researchers will be discussed.

### IDENTIFYING DOMAINS OF ASSISTED LIVING QUALITY FOR MINNESOTA'S ASSISTED LIVING REPORT CARD

Tetyana Shippee,[1] Odichinma Akosionu,[2] Tricia Skarphol,[1] and Timothy Beebe,[1] *1. University of Minnesota, Minneapolis, Minnesota, United States, 2. University of Minnesota - School of Public Health, Minneapolis, Minnesota, United States*

Concerns around assisted living (AL) quality have prompted the 2019 passage of the MN legislature, which provided funding for the development of an Assisted Living Report Card. We present results from the first two phases of this project. The first phase involved a national literature review of quality measures and technical advisory panels to understand the types of domains and indicators for AL quality that are measured. Nine quality domains were identified. The second phase focused on state-wide stakeholder engagement to determine priority rankings for nine AL quality domains and indicators identified. Quality of life, staff quality and resident safety were the top three domains across all stakeholder groups. The state will implement surveys of AL resident quality of life and family satisfaction as mandated by the legislature, but findings indicate that other aspects of quality such as staff-related measures and resident safety, are also important to address.

### IMPLEMENTING OREGON'S NEW QUALITY MEASUREMENT PROGRAM FOR ASSISTED LIVING COMMUNITIES

Paula Carder,[1] Ann McQueen,[2] and Ozcan Tunalilar,[1]
*1. Portland State University, Portland, Oregon, United States, 2. Oregon Department Human Services, Salem, Oregon, United States*

Oregon legislation requires assisted living (AL) communities to report selected quality measures to the state licensing agency. The Quality Measurement Program (QMP) includes five metrics that assess different areas of resident safety and wellbeing: falls, antipsychotic medication use, staff training, staff retention, and resident satisfaction. This paper describes findings based on our 2019 survey of AL communities and offers suggestions for stakeholders interested in public reporting and quality metrics. Assisted living providers reported 28% of AL residents and 38% of memory care (MC) residents fell at least once in the prior 90 days, with 39% and 45% reporting an injury, respectively. Antipsychotic medication use was 20% among AL and 44% among MC residents. These findings and the survey methods used to collect them, combined with stakeholder and state agency staff participation, informed the current QMP approach. We describe how to collect meaningful quality metrics within the AL context.

### QUALITY IN ASSISTED LIVING: DOES IT LIE IN THE EYES OF THE BEHOLDER?

Diana White, Tunalilar Ozcan, Serena Hasworth, and Jaclyn Winfree, *Portland State University, Portland, Oregon, United States*

Quality is defined in multiple ways and by different stakeholders (e.g., residents, regulators, informed observers). Using a two-stage stratified sampling strategy, we collected data from N=241 residents living in 31 assisted living and residential care communities (AL/RC) in Oregon. Residents rated their overall satisfaction and satisfaction with the AL/RC as a place to live and to receive care. Each interviewer completed a facility profile summarizing their observations about the setting, including quality of staff-resident interactions and physical environment. Residents and interviewers were also asked whether they would recommend the community to others. Finally, we used deficiency citations given during regular inspections by the licensing agency to proxy regulatory perspective. Results show that perceived quality varied by stakeholder (e.g., residents' assessments differed from deficiency citations). Given this variation, findings suggest that efforts to make quality indicators publicly available should include multiple measures and perspectives, especially residents.

### RESIDENT QUALITY OF LIFE AND FAMILY SATISFACTION: DEVELOPING MEASURES FOR MINNESOTA ASSISTED LIVING REPORT CARD

Muriel Wheatley,[1] Valerie Cooke,[2] and Tetyana Shippee,[3]
*1. Vital Research, Los Angeles, California, United States, 2. Minnesota Department of Human Services, St. Paul, Minnesota, United States, 3. University of Minnesota, Minneapolis, Minnesota, United States*

The 2019 Minnesota Legislature requested the Department of Human Services (DHS) and Minnesota Board on Aging to develop and administer a report card for assisted living (AL), including conducting annual resident and family surveys in Minnesota AL settings. This presentation includes the perspectives of representatives from MN DHS and Vital Research, as well as the University of Minnesota team who worked together to develop survey items, carry out the cognitive testing, and conduct analyses. Survey items were developed from published literature and existing tools on assisted living quality and underwent testing with MN stakeholders and cognitive testing with MN AL residents. Pilot testing assessed any further changes that needed to take place for resident and family satisfaction with AL quality (n=400). Presenters will share lessons learned with implementing the new tools and different aspects of the of the report card development and implementation process as well as the survey findings.

### SESSION 6300 (SYMPOSIUM)

### THROUGH THEIR LENS: USING VISUAL METHODS TO ILLUMINATE THE LIVED EXPERIENCES OF DIVERSE OLDER ADULTS

Chair: Jarmin Yeh
Discussant: Tam Perry

Visual methods, like photovoice and photo-elicitation, have attracted modest attention in gerontological inquiry with diverse and vulnerable community-dwelling older adults. Visual methods are based on the idea of inserting images, produced by informants or not, into research interviews, allowing informants to be the experts of knowledge and meaning-making while the researcher becomes the student. The empowerment of informants as subject-collaborators in the research process is a distinctive feature of visual methods. Benefits include revealing unique insights into diverse phenomena by evoking elements of human consciousness, feelings, and memories that words may not easily express and surveys may not easily capture. This symposium presents qualitative research using visual methods to illuminate the lived experiences, voices, and perspectives of diverse and vulnerable older adults living in New Jersey, Connecticut, and California. Reyes' research critiques how the operationalization of mainstream notions of civic participation becomes exclusionary and provides a more inclusive understanding of how civic participation is enacted and performed through the practices of Latinx and African American older adults living in New Jersey. Versey's research with homeless older adults subverts the attention often focused within cities by interrogating the meaning of place with informants whose needs and desires are often overlooked or obscured by residing in a small, rural town in central Connecticut. Yeh's research on aging in place inequalities chronicles the everyday lives of housed and unhoused older San Franciscans to reveal their tactics for negotiating a moving tension between the daily interiority of identity and contingencies of a changing environment. Qualitative Research Interest Group Sponsored Symposium.

### NEGOTIATING THE GERONTOLOGICAL UNCANNY AND PASSING TACTICS FOR AGING IN PLACE

Jarmin Yeh,[1] Pat Fox,[1] David Vlahov,[2] and Howard Pinderhughes,[1] *1. University of California, San Francisco, San Francisco, California, United States, 2. Yale University, Orange, Connecticut, United States*

Insisting people are equally worthy of aging in place is a radical but challenging idea. Scenes of homelessness texture the contemporary city, manifesting broader issues of inequality. This qualitative study explored material with twenty-two housed and unhoused San Franciscans born in 1950 or earlier, who participated in semi-structured in-depth interviews and chronicled their everyday lives using disposable cameras. Theoretical work on social and spatial practices was drawn upon to help understand their aging in place experiences. In interpreting themes, informants elucidated a moving tension between the daily interiority of identity and the negotiation of a changing environment that produces a sensation characterized as the uncanny. The vicissitudes of life and precariousness of their positionalities exposed tactics for "passing" as creative forms of resistance to their expulsion from society. Utilizing visual methods helped reveal assumptions and contributed to gerontological discourse and critical theorizations about aging in place inequalities. Part of a symposium sponsored by the Qualitative Research Interest Group.

### OLDER LATINX AND AFRICAN AMERICANS' EXPERIENCES OF CIVIC PARTICIPATION

Laurent Reyes, *Rutgers, The State University of New Jersey, Highland Park, New Jersey, United States*

By 2030 Latinx and African Americans are expected to be the largest non-White groups of older adults. In the past 20 years, older adults' civic participation has received considerable attention. However, until now most scholarship has focused on formal volunteerism and voting, activities that remain inaccessible to many marginalized groups. As a consequence, other civic activities are going unrecognized. The aim of this study is to understand how civic participation is experienced throughout the lives of 24 African American and Latinx adults 60+ living in New Jersey. Because civic participation is a concept that has many names and meanings depending on culture, language, and history I employ photo-elicitation techniques followed by in-depth interviews to understand civic participation through participants' lens. Findings from this study can serve to improve conceptualizations and measurements of civic participation for future studies and inform efforts to strengthen civic participation among these populations. Part of a symposium sponsored by the Qualitative Research Interest Group.

### HOMELESS ON MAIN STREET: USING PHOTOVOICE TO HIGHLIGHT OLDER ADULTS AMONG THE HIDDEN HOMELESS

H. Shellae Versey, *Wesleyan University, Middletown, Connecticut, United States*

Homelessness is a reality for a growing number of Americans living in small towns and rural areas. However, unlike in cities, housing instability may be less visible. Using a photo-elicitation method (i.e., Photovoice), this study explores the meaning of place and obscured visibility to currently and formerly homeless older adults living in a small town in central Connecticut.

**Participants (N = 27) were recruited from a local service agency, given cameras and asked to photograph areas around town that were meaningful to them. Photographs were developed and followed by in-person, semi-structured interviews with participants in which photos and experiences during the project were discussed. Primary themes included belonging, generativity, social isolation, and place-making as meaning-making. The study culminated in a community photography exhibition in which photographs from the project were displayed in public spaces around town. Implications for community-based interventions to reach homeless groups in rural areas are discussed. Part of a symposium sponsored by the Qualitative Research Interest Group.**

### SESSION 6305 (SYMPOSIUM)

### TRANSFORMING GERONTOLOGICAL RESEARCH BY MEANINGFULLY ENGAGING PERSONS LIVING WITH DEMENTIA

Chair: Michael Lepore
Discussant: Richard Fortinsky

Whereas persons living with dementia have commonly been subjects of gerontological research, participation of persons with dementia in designing and conducting studies and in scientific research meetings has been rare in the United States. In recent years, person-centered research models have arisen which give persons with dementia and their caregivers core roles in the research enterprise. As "co-researchers" with academic/professional researchers, persons with dementia and their caregivers are engaged in all aspects of the research enterprise, jointly developing research questions and study designs, collecting and analyzing data, planning research meetings, and disseminating results. International studies have shown that conducting research in collaboration with the population that is being studied has potential to enhance the quality and appropriateness of research and has been identified as an essential component of studies examining the effectiveness of different approaches to care. This session spotlights innovative advances in gerontological research that meaningfully engages persons with dementia. First, the engagement of persons with dementia in scientific meetings is addressed drawing on the examples of the 2017 and 2020 National Research Summits on Care Services and Supports for Persons with Dementia and their Caregivers (i.e., Summits). Next, a study using a patient engagement framework for caregivers and individuals with mild cognitive impairment living at home is discussed. Finally, the Empowering Partnerships program, which prepares researchers, persons with dementia, and care partners to collaborate in conducting research is reviewed. Outcomes and challenges of these innovations are examined, and the need for academic/professional researcher roles to evolve is discussed.

### RESEARCH ENGAGEMENT WITH PERSONS LIVING WITH DEMENTIA: PUTTING LESSONS LEARNED INTO PRACTICE

Lori Frank, *RAND Corporation, Arlington, Virginia, United States*

In the US few research initiatives actively engage persons living with dementia (PLWD) as partners in the research. The 2017 Summit actively engaged multiple types of stakeholder groups, including one for Persons Living with Dementia (PLWD), and was the first large-scale US research meeting to actively engage PLWD in planning and conduct of the meeting. The PLWD conducted a self-evaluation of their work that yielded best practices, meeting the need for guidelines for engaging with PLWD. The 2020 Summit presented the opportunity to test best practices. Some were implemented by the group conveners, like use of video-enabled meetings. Others were implemented with the PLWD, including decisions about governance structure for the group. The use of learnings from the first Summit in engaging with PLWD in the following Summit supported refinement of some engagement practices, yielding a list of recommendations for future work.

### ELICITING CARE MANAGEMENT PREFERENCES WITH ENGAGED CAREGIVERS AND INDIVIDUALS WITH MILD COGNITIVE IMPAIRMENT

Tabassum Majid, *University of Maryland, Baltimore County, Baltimore, United States*

The goal of this study was to engage individuals with mild cognitive impairment (MCI) in developing a survey instrument that assists in the selection and prioritization of meaningful treatment outcomes in health care decision making. A multi-stakeholder advisory panel was engaged throughout the first qualitative phase of the study. 15 in-depth, guided interviews were conducted with caregiver-care recipient dyads who had been recently diagnosed with MCI. In addition to questions about meaningful outcomes, individuals were asked about quality of life and the severity of their symptoms using the Functional Attainment Staging Tool. 14 thematic concepts identified in an earlier, non-engagement-based caregiver study were all endorsed as relevant to the dyads interviewed. However, participants included 6 more thematic concepts, many of which were focused on social and community factors relevant to health care decision making. Engaging patients and caregivers allowed for focused dialogue within the interviews on shared treatment goals.

### EMPOWERING PARTNERSHIPS AMONG RESEARCHERS, PERSONS LIVING WITH DEMENTIA, AND CAREGIVERS

Heidi Gil,[1] Stephani Shivers,[2] Bob Savage,[3] Geri Taylor,[4] Jim Taylor,[4] Erica DeFrancesco,[5] Maria O'Connell,[6] and Michael Lepore,[7] *1. LiveWell Alliance, LiveWell Alliance, Connecticut, United States, 2. LiveWell Alliance, Plantsville, Connecticut, United States, 3. Dementia Peer Coalition, Plantsville, Connecticut, United States, 4. The Taylors, New York City, New York, United States, 5. LiveWell Dementia Specialists, Plantsville, Connecticut, United States, 6. Yale School of Medicine, New Haven, Connecticut, United States, 7. LiveWell Alliance, Southington, Connecticut, United States*

Conducting research collaboratively with persons living with dementia (PLWD) requires preparation. To enhance the capacity of PLWD to be engaged in research, we collaboratively developed and deployed a replicable two-day training workshop for PLWD, care partners, and professional/academic researchers. An assessment of strengths, values and experiences, and an introductory education session, served as experiential warmup activities for PLWD prior to the two-day workshop. Twelve PLWD, nine care partners, and 14 professional/academic researchers participated in the workshop. Participants identified 16 topics as highly important for research and prioritized four topics for immediate study design. Studies addressing these topics were collaboratively designed by PLWD, care partners, and professional researchers during day two of the workshop. Lessons learned from the workshop, including key preparation steps for collaboration—such as securing minimally distracting collaboration environments—are summarized, and access to a free toolkit that supports workshop replication is provided.

### SESSION 6310 (SYMPOSIUM)

### TRANSFORMING PRIMARY CARE PRACTICE INTO AGE-FRIENDLY HEALTH SYSTEMS

Chair: Marla Berg-Weger
Co-Chair: Erin Emery-Tiburcio
Discussant: Nina Tumosa

Transforming primary care practice to improve the health of older adults is a major focus of the Geriatric Workforce Enhancement Program (GWEP). Using the 4Ms of an Age-Friendly Health System (What Matters, Mentation, Medication, and Mobility) as a framework, presenters will describe their GWEP's ongoing development, training, and evaluation initiatives aimed at increasing providers' knowledge and practice skills, and improving older adult's health outcomes. These initiatives are creating increased professional competencies in geriatric care that will: 1) help older adults maximize their health and wellbeing, and 2) better support caregivers and families. In this symposium, presenters from three GWEPs, Pennsylvania State University, Rush University Medical Center, and Saint Louis University will describe AFHS initiatives with rural and urban primary care partner sites. Educational and programmatic initiatives and strategies that map onto the 4Ms that will be discussed including geriatric assessment, dementia-focused interventions, falls prevention, opioid assessment, and caregiver well-being and support. Outcomes in older adult's health and functional status will be discussed. Presenters will highlight the importance of building AFHS in primary care and strategies to bridge this framework into the community.

### SAINT LOUIS UNIVERSITY PRIMARY CARE AGE-FRIENDLY HEALTH SYSTEM INITIATIVE IN RURAL AND URBAN COMMUNITIES

Marla Berg-Weger,[1] and John Morley,[2] *1. Saint Louis University, St. Louis, Missouri, United States, 2. Saint Louis University School of Medicine, Saint Louis, Missouri, United States*

The Saint Louis University GWEP partners with a rural Missouri critical access hospital and an urban Federally Qualified Health Center to support their transformations into an Age-Friendly Health System in their community. With an emphasis on the 4Ms, programmatic innovations include development and training of: 1) electronic health records integration of the Rapid Geriatric Assessment (RGA) for patients 65+ years old; 2) RGA-based protocol for Medicare Annual Wellness Visits (MAWV); 3) Interprofessional health care team approach; 4) Evidence-based or Evidence-Informed treatment interventions, including Cognitive Stimulation Therapy (CST), exercise and strengthening program, caregiver support, and Circle of Friends, an intervention for loneliness and social isolation. Outcomes will be presented which suggest increased assessment practices and improvement in functional and cognitive status. Successes and lessons learned regarding strategies to develop an Age-Friendly Health System in two different primary care settings will be discussed.

### CATCH-ON AGE-FRIENDLY HEALTH SYSTEM IMPLEMENTATION ACROSS VARIED GEOGRAPHIC AND SYSTEM SETTINGS

Erin Emery-Tiburcio,[1] Robyn Golden,[2] and Michelle Newman,[1] *1. Rush University Medical Center, Chicago, Illinois, United States, 2. Rush University Medical Center, CHICAGO, Illinois, United States*

GWEP program goals include transformation of clinical training environments into integrated geriatrics and primary care systems to become Age-Friendly Health Systems. CATCH-ON, the collaborative GWEP led by Rush University Medical Center, is working with primary care health systems in four highly varied geographic and system settings to achieve this goal. Each of these clinic systems has unique successes and challenges in developing and implementing workflow protocols; engaging providers, patients, families, and community-based organizations in the development of Age-Friendly Health Communities; and modifying the electronic health record to document assessing and acting on 4Ms. Clinic team members participate in monthly Learning Community sessions to learn about and reinforce the 4Ms, along with practical recommendations for implementing with patients. This session will focus on CATCH-ON's process for implementing the 4Ms in various practice settings, lessons learned in implementation across large and small health systems, and opportunities to bridge AFHS with the community.

### AGE-FRIENDLY CARE, PA: A COLLABORATIVE FOR AGE- AND DEMENTIA-FRIENDLY CARE

Erica Husser,[1] Donna Fick,[2] Judith Hupcey,[3] and Jenny Knecht Fredo, PA,[3] *1. Penn State, Spring Mills, Pennsylvania, United States, 2. Penn State, University Park, Pennsylvania, United States, 3. Penn State College of Nursing, University Park, Pennsylvania, United States*

The Penn State College of Nursing (including the Center for Geriatric Nursing Excellence [CGNE] and Center for Nursing Research [CNR]) has partnered with the Primary Health Network (PHN) to work collaboratively toward implementing the 4Ms framework of an Age-Friendly Health System at PHN's primary care sites in Pennsylvania. PHN is the largest FQHC in the state and spans 16 counties in PA and 2 in Ohio. Twenty of PHN's PA primary care sites are in rural counties, and both rural and urban sites serve older adult populations with major health disparities. Connecting primary care practices with local community resources and programs is an important step in serving rural populations, and we have used the ECHO model to help facilitate connections. This session will focus on how we are using the ECHO model to engage our partners in a collaborative learning environment.

### SESSION 6315 (SYMPOSIUM)

### UNDERSTANDING DRIVING AS AN EVERYDAY ACTIVITY: INTERNATIONAL PERSPECTIVES

Chair: Anne Dickerson
Co-Chair: Isabelle Gelinas
Discussant: Moon Choi

This international symposium brings together leading occupational therapy researchers from around the world with a shared focus is on evaluating and improving the driving performance of older adults to decrease their crash risk and facilitate their community participation. In this session, five groups of international scholars will share their collective and individual research outcomes for driving as a means of community mobility. The first presentation will outline their collective international, cross-sectional study of 247 older adults from seven countries where the impact of driving on out-of-home mobility was compared. Each presentation that follows will then present results from innovative studies of ways in which to assess and address fitness to drive in older adulthood. Our discussant will summarize the potential expansion of the current work and engage the audience through interactive questions. Transportation and Aging Interest Group Sponsored Symposium.

### INTERNATIONAL COMPARISON OF OLDER DRIVERS AND NON-DRIVERS FOR ACTIVITY ACCESS AND QUALITY OF LIFE

Cilla Harries,[1] Carolyn Unsworth,[2] Brenda Vrkljan,[3] and Anne Dickerson,[4] *1. Kingston University and St George's Joint Faculty, London, England, United Kingdom, 2. Central Queensland University, Melbourne, Victoria, Australia, 3. McMaster University, Hamilton, Ontario, Canada, 4. East Carolina University, Greenville, North Carolina, United States*

Driving is crucial for many people to age in place, as it is the most convenient option, and somtimes the only option for personal transport. This international, cross-sectional, cohort study of older adults (n=246) compared drivers' and non-drivers' quality of life and levels of community participation. Following ethical approval, data were collected across seven countries. The EQ-5D-5L was used to measure health related quality of life and a modified version of the Participation in Activities and Places Outside the Home (ACT-OUT) was used to measure community participation (T-ACT-OUT). Drivers accessed more out-of-home activities than non-drivers, suggesting higher community participation among this group. Health related quality of life was generally high among all participants, but slightly higher for drivers (U=3186, z=-2.78, p=.005, r=0.18). These findings resonate with recent evidence, which suggests supporting older drivers to continue to drive for as long as possible provides critical access to their communities (O'Neill et al., 2019). Part of a symposium sponsored by Transportation and Aging Interest Group.

### IMPROVING THE DRIVING OF COMMUNITY-DWELLING OLDER CANADIANS: A RANDOMIZED CONTROLLED TRIAL

Brenda Vrkljan,[1] Ruheena Sangrar,[1] Lauren Griffith,[1] Lori Letts,[1] and Michelle Porter,[2] *1. McMaster University, Hamilton, Ontario, Canada, 2. University of Manitoba, Winnipeg, Manitoba, Canada*

Older Canadians, similar to aging drivers in many other countries, want to drive, need to drive, and live in communities where driving is both valued and necessary for out-of-home participation. Many community-dwelling seniors are medically fit-to-drive, yet their collision risk remains higher than most other age groups, which some have attributed to their propensity to drive shorter distances in high-traffic areas (Antin et al., 2017). In this randomized controlled trial, the effect of a customized video-based older driver training program on behind-the-wheel performance was captured using the latest technology for an on-road evaluation. Results indicated the mean reduction in number of driving errors [mean (95% CI)=-12.0(-16.5, -7.6),p<0.001] favoured the intervention group where their change between baseline and 4-week follow-up was statistically significant [mean(95% CI)=-10.3(-13.8, -6.8),p<0.001], but not for the control group [mean (95% CI)=1.7(-0.08, 4.2), p>0.05]. Our novel, video-based approach that provided individualized feedback improved driving performance for older drivers. Part of a symposium sponsored by Transportation and Aging Interest Group.

### USE OF A DRIVING SIMULATOR TO FACILITATE OLDER PEOPLE TO RETURN TO DRIVING

Carolyn Unsworth,[1] Megan White,[2] and Natasha Lannin,[3] *1. Central Queensland University, Melbourne, Victoria, Australia, 2. Alfred Health - Caulfield Hospital, Caulfield, Victoria, Australia, 3. Monash University, Melbourne, Victoria, Australia*

Driving simulators are a relatively underutilized therapy tool that provide an opportunity for older drivers with a range of health-related problems to participate in simulated driving scenarios in a low cost and safe environment. The aim of this paper is to (i) describe the use of a Forum 8 driving simulator prior to a driver assessment, (ii) detail the story-boarding technique used to develop and grade driving scenes to enable older drivers to increase confidence, practice using vehicle modifications such as a spinner knob (e.g. for one-handed driving following stroke), and train specific skills including visual scanning and attention, and (iii) present five case studies to identify the strengths and limitations of incorporating the simulator into therapy programs with older drivers. of simulator use. The establishment and use of a driving simulator in a rehabilitation unit highlights both the challenges and benefits of using this kind of technology in practice. Part of a symposium sponsored by Transportation and Aging Interest Group.

### USING A PERFORMANCE BASED ASSESSMENT TOOL OF EVERYDAY IADL TO DETERMINE DRIVING RISK IN OLDER ADULTS

Anne Dickerson, *East Carolina University, Greenville, North Carolina, United States*

For older adults living in rural/suburban communities, driving is often their only means of transportation. Although considered safe drivers, drivers older than 70 years have higher crash rates with fatality rates amplified due to the increased frailty/fragility. However, research evidence clearly indicates that cognitive factors contribute to driving impairment in older adults. Occupational therapists, as experts in observation of functional performance, use the Assessment of Motor and Process Skills (AMPS), for measurement of performance in everyday activities using two scales (motor and process). Previously demonstrated as a sensitive tool for cognitive changes, this presentation will summarize the research outcomes between older adults with cognitive impairment (N=57+) and without (N=53) who completed a comprehensive driving evaluation. Analysis of the two samples using receiving operating curves suggests the AMPS has potentially excellent specificity and sensitivity, specifically AUC = 0.826(0.73-0.92) for motor, AUC = 0.909(0.84-0.98) for process, and AUC = 0.936(0.88-0.99) together. Part of a symposium sponsored by Transportation and Aging Interest Group.

### EVALUATING OLDER DRIVERS IN THEIR EVERYDAY DRIVING ENVIRONMENTS

Isabelle Gelinas,[1] Barbara Mazer,[2] Yu-Ting Chen,[1] Brenda Vrkljan,[3] Shawn Marshall,[4] Judith Charlton,[5] and Sjaanie Koppel,[5] *1. McGill University, Montreal, Quebec, Canada, 2. McGill University School of Physical and Occupational Therapy, Montreal, Quebec, Canada, 3. McMaster University, Hamilton, Ontario, Canada, 4. University of Ottawa, Ottawa, Ontario, Canada, 5. Monash University Accident Research Centre, Clayton, Victoria, Australia*

Developing tools that accurately detect at-risk driving behaviors is a public-health priority. There is a need for a measure that accurately assesses older drivers' level of competence on familiar roadways. The objective of this presentation is to describe the development of the procedures and scoring of a new approach, the Electronic Driving Observation Schedule (eDOS), to observe everyday driving in the community. The eDOS was used to record and compare the driving environment and performance of older drivers and low-risk younger drivers during their everyday driving. Older (n=160, >74y) and younger (n=60, 35-64y) drivers completed a 20-30-minute drive from their home to destinations of their choice. Older drivers drove on simpler routes with fewer intersections and lane changes. Both groups made few driving errors, which were mostly low-risk. Younger drivers tended to demonstrate poor driving habits (not signaling, speeding, poor lane position) and compliance with road rules. Part of a symposium sponsored by Transportation and Aging Interest Group.

### SESSION 6320 (SYMPOSIUM)

### USING SUBJECTIVE AND OBJECTIVE MEASURES OF FINANCIAL SITUATION TO IMPROVE PROGRAM OUTCOMES FOR OLDER ADULTS

Chair: Hector Ortiz
Co-Chair: Jan Mutchler

Researchers in the field of aging rely on various measures of financial security to assess the needs of older adults and the outcomes of interventions. Recently, subjective measures have gained attention among researchers and organizations that serve older adults. This symposium brings together researchers from academia, government, and the non-profit sector to discuss the relationship between subjective financial well-being and objective financial situation. The first project describes the relationship between the Consumer Financial Protection Bureau's Financial Well-being Score and the Elder Index. The CFPB Financial Well-Being Score provides a standardized and validated measure of a person's sense of financial security and freedom in the present and future. The Elder Index provides a measure of older adults' income against the average income needed for adults age 65 or older to live independently in their communities. The second project discusses the findings of a study into the changes in outcomes among older adults assisted through the National Council on Aging's Benefits Enrollments Centers. The third project describes the overall findings and changes in financial well-being among SCSEP participants who attended a series of financial education workshops offered through the Benjamin Rose Institute on Aging's subsidiary Empowering and Strengthening Ohio's People. Together, the studies show that both measures are, on average, strongly correlated and predictive of a range of factual experiences such as material hardship and financial stress. The studies, however, show that subjective measures may help identify and target underlying behavioral and attitudinal factors that influence people's satisfaction with their economic situation.

### EXPLORING THE RELATIONSHIP BETWEEN THE CFPB FINANCIAL WELL-BEING SCORE AND THE ELDER INDEX

Jan Mutchler, *University of Massachusetts Boston, Boston, Massachusetts, United States*

The Elder Index is a cost of living indicator that measures the income older adults need to meet their living expenses while staying independent in the community, calculated on a county-by-county basis for the United States. Analyses based on the Elder Index show that a large segment of the age 65+ population has incomes below the Index, reflecting a level of insecurity that is considerably higher than suggested by the poverty rate. Moreover, comparison of the Elder Index to household income illustrates differences across states in the extent to which incomes cover the cost of necessary expenditures. In this paper we explore how cost of living contributes to subjective financial security among older people, as measured by the CFPB Financial Well-Being Score, using a data match of the Understanding America Study with the Elder Index. Results document this association, offering insight to spatial patterns of financial insecurity in later life.

### EMPOWERING THROUGH EDUCATION: UNDERSTANDING FINANCIAL WELL-BEING AMONG LOW-INCOME OLDER ADULTS

Sara Powers,[1] and Sonya Edwards,[2] *1. Benjamin Rose Institute on Aging, Cleveland, Ohio, United States, 2. Empowering and Strengthening Ohio's People, Cleveland, Ohio, United States*

Recognizing the need for and importance of financial capability education among low-income older adults, the non-profit agency, Empowering and Strengthening Ohio's People (ESOP), implemented an 11-part financial education workshop series with a sample of older adults enrolled in the Senior Community Service Employment Program (SCSEP). Over the course of 10-months, participants attended workshops that covered an array of topics (e.g., budgeting and goal setting, avoiding financial exploitation) and completed a variety of measures aimed at understanding their financial situation and well-being (e.g., CFPB Financial Well-Being Scale, Financial Shocks, Material Hardship, Financial Skill). Results indicated that financial well-being scores significantly increased from the beginning of the workshop series (M=54.49, SD=7.23) to the end (M=58.10, SD=8.10); t(48) = 3.66, p=.001. Discussion will offer insights into the subjective financial experiences of low-income older adults who were actively trying to seek and gain permanent employment to improve their financial situation.

### THE IMPACT OF PUBLIC BENEFITS ON LOW-INCOME OLDER ADULTS' FINANCIAL WELL-BEING

Lauren Popham, *National Council on Aging, Arlington, Virginia, United States*

Nineteen percent (13 million) older adults have incomes below 150% of the Federal Poverty Level, leaving them with limited means to afford basic living expenses. Public benefits can help bridge the gap allowing older adults to afford food, home energy, and health expenses. There are studies demonstrating the positive health outcomes associated with public benefits in older adults. It remains unclear how benefits may also improve subjective measures of well-being in older adults. To examine this question, baseline measures of well-being including the CFPB Financial Well-Being Scale were administered to older adults before they enrolled in benefits and again six months after receiving benefits to examine changes in well-being as a result of accessing benefits to help ease some of their financial burdens. Results revealed that older adults experience subjective, psychological improvements from benefits. These findings have implications for the social and behavioral determinants of health in older adults.

### SESSION 6325 (SYMPOSIUM)

### USING IMPLEMENTATION RESEARCH TO PROMOTE THE EXPANSION AND SUSTAINABILITY OF INTERGENERATIONAL PROGRAMS

Chair: Leslie Hasche

Implementation studies provide guidance on how to reduce the gap between empirically-supported interventions and routine care. For intergenerational programs that bring older adults and other generations together to promote social connection, improve health and well being, and to reduce ageism, the role of implementation science is rarely studied. Intergenerational programs have a long-standing role in social service and housing contexts, yet the quality of the evidence and sustainability of these programs is often in question. This symposium aims to demonstrate how implementation studies can identify available evidence and influential contextual factors to examine issues of adoption, fidelity, and sustainability of intergenerational programs. First, a scoping review of the available evidence on best practices of intergenerational programs will help highlight fidelity issues. Second, an environmental scan for intergenerational housing will highlight how contextual factors may impact the adoption and spread of intergenerational programs. Third, a pre-implementation study delivering multi-modal best practices training to local community sites will share indicators of the feasibility of training staff to implement evidence-based intergenerational practices. Finally, an evaluation of a community collaborative of organizations implementing intergenerational programs will highlight the process by which organizations develop and sustain partnerships. The chair will summarize how the studies' methodological approaches incorporate implementation science and outcomes. Implications for both future research on organizational context, funding, and implementation strategies, as well as for practice settings will be named. The discussion will identify implementation gaps that will need to be overcome to expand and sustain intergenerational programs.

### IMPLEMENTATION OF EVIDENCE-BASED PRACTICES IN INTERGENERATIONAL PROGRAMMING: A SCOPING REVIEW

Shannon Jarrott, Rachel Scrivano, Nancy Mendoza, and Cherrie Park, *The Ohio State University, Columbus, Ohio, United States*

Intergenerational programs facilitate mutually beneficial interactions between youth and older adults, achieving an array of outcomes. With few exceptions, implementation factors rarely figure into outcome analyses, though researchers frequently gather data on factors influencing outcomes. The resulting practice-evidence gap may deter wide-spread adoption of intergenerational programming. We conducted a scoping review of 35 peer-reviewed articles (2000-2019) to map key concepts and evidence sources of empirically-supported practices impacting intergenerational program outcomes. A scoping review is appropriate when an area is complex, like intergenerational programs that incorporate diverse participants, content, and goals. Primarily qualitative studies involved programs equally likely to involve young, school-age, or post-secondary age youth and independent or frail older adults. Half had sample sizes under 50, frequently measuring both age groups. Implementation practices included co-learning and sharing personal stories. Program content (e.g., technology) and greater exposure amplified outcomes. Rigorous implementation research is needed to advance evidence-based intergenerational practice.

### CONNECTING GENERATIONS IN SENIOR HOUSING: PROMISING PRACTICES AND CHALLENGES

Nancy Henkin, *Generations United, Merion, Pennsylvania, United States*

Senior housing can be an ideal platform for high quality intergenerational programming. Often older adults who move to congregate housing settings experience feelings of loneliness and a loss of purpose. Creating long term partnerships with educational and youth-serving organizations can help senior housing providers expand residents' social networks and create meaningful civic engagement opportunities. A 3-year national initiative involving an environmental scan of intergenerational practice in senior housing communities, the development of a toolkit for senior housing providers, and the piloting of intergenerational partnerships and programs in six national housing communities was conducted by Generations United and Leading Age and supported by the Retirement Research Foundation. Promising practices, challenges, and lessons learned from this initiative will be shared and strategies for "scaling" this work will be discussed.

### IMPLEMENTING IG BEST PRACTICES IN COMMUNITY-BASED SETTINGS: A PRE-IMPLEMENTATION STUDY

Lisa Juckett,[1] Shannon Jarrott,[1] Jill Naar,[2] Rachel Scivano,[1] and Alicia Bunger,[3] *1. The Ohio State University, Columbus, Ohio, United States, 2. Appalachian State University, Boone, North Carolina, United States, 3. Ohio State University, Columbus, Ohio, United States*

Programs that intentionally engage unrelated young and old persons often lead to mutual benefits; however, specific implementation strategies that support the use of evidence-based intergenerational programming in community settings are understudied. With strong demand for training resources among intergenerational program providers, this pilot study examined how a multifaceted training strategy facilitated the implementation of 14 distinct evidence-based intergenerational best practices. Intergenerational programming was implemented with nine staff from two small community sites using three implementation strategies including educational meetings, ongoing consultation, and routine practice reminders. Observational analysis of video recorded intergenerational program sessions indicated that staff adopted an average of 81.7% of intergenerational best practices suggesting the feasibility of implementing IG in community settings. Findings yield valuable insight that can inform training refinements, and selection of strategies for improving implementation. Next steps include aligning specific practices with participant outcomes.

### A PROCESS EVALUATION ON THE IMPLEMENTATION OF CROSS-ORGANIZATIONAL INTERGENERATIONAL PARTNERSHIPS

Carson De Fries,[1] Andrew Steward,[1] Rachel Fix,[2] and Leslie Hasche,[1] *1. University of Denver, Denver, Colorado, United States, 2. University of Denver - Graduate School of Social Work, Denver, Colorado, United States*

A collaborative group of organizations in the rocky mountain region of the U.S. implemented sixteen intergenerational programs with varying themes (i.e. mentorship, music and art therapy, baking, life stories, etc.). This two-year study used survey data and a focus group to understand successful approaches and challenges to implementing cross-organizational partnerships between adult-serving and youth-serving organizations. We utilized qualitative data through open-ended comments on program evaluations and a focus group discussion with leaders of seven community organizations. Questions in the focus group prompted participants to describe the process of initiating, developing, and sustaining partnerships. The three main themes were: the benefits of shared organizational values, outlining programmatic roles and expectations, and an ability to cope with negative partnership experiences. Insights about successful approaches to implementing cross-organizational partnerships, as well as the challenges of developing and sustaining such partnerships may be beneficial in similar settings.

### SESSION 6330 (SYMPOSIUM)

### VULNERABLE OLDER ADULTS IN DISASTERS: EFFECTS OF HURRICANE IRMA ON NURSING HOMES AND ASSISTED LIVING

Chair: Lindsay Peterson
Co-Chair: David Dosa
Discussant: Patricia D'Antonio

Preparedness of residents in long-term care (LTC) in the face of hurricane emergencies is a contested and largely unanswered question. Our prior work involving the U.S. Gulf Coast hurricanes of 2005-08 showed that exposure to various storms on nursing home (NH) residents resulted in significantly more deaths than reported by health care officials. This work also highlighted that evacuation of NH residents, compared to sheltering in place, was independently associated with morbidity and mortality. Hurricane Irma struck Florida on Sept. 10, 2017, prompting the evacuation of thousands of NH and assisted living community (ALC) residents. This symposium will discuss the effects of Hurricane Irma on vulnerable older adults residing in NHs and ALCs using mixed quantitative and qualitative methodologies. The first presentation will discuss morbidity and mortality of NH residents exposed to Hurricane Irma and will stratify by long stay/short stay status and hospice enrollment. The second presentation will discuss improvements and continued barriers to NH preparedness based on interviews with 30 administrators following Hurricane Irma. Using a novel methodology to identify residents of ALCs using secondary data sources, the third presentation will document AL resident morbidity and mortality risk following Hurricane Irma. The final presentation will highlight results of interviews with 70 stakeholders from small and large ALCs concerning the hurricane experiences of residents, including those with dementia. This symposium offers a multi-faceted view of a disaster's effects on LTC residents across Florida, including novel data from the NH environment and lesser-examined ALCs.

### MORTALITY AND MORBIDITY AMONG NURSING HOME RESIDENTS EXPOSED TO HURRICANE IRMA

David Dosa,[1] Julianne Skarha,[2] Lindsay Peterson,[3] Dylan Jester,[3] Nazmus Sakib,[3] Jessica Ogarek,[4] Ross Andel,[3] and Kathryn Hyer,[3] *1. Brown University, Barrington, Rhode Island, United States, 2. Brown University, Providence, Rhode Island, United States, 3. University of South Florida, Tampa, Florida, United States, 4. Brown University, PROVIDENCE, Rhode Island, United States*

We combined Medicare claims and nursing home (NH) administrative data to determine the mortality and morbidity effect of Hurricane Irma on nursing home residents. We utilized the Centers for Medicare and Medicaid Services (CMS) Standard Analytical Files (SAFs) combined with the Minimum Data Set (MDS) to create an exposure cohort of NH residents residing in Florida facilities immediately prior to Hurricane Irma's landfall on September 10, 2017. We created a control group of residents who resided in the same NHs over the same dates in 2015, a year when there were no hurricanes. Outcome variables included 30/90-day mortality and first hospitalizations post storm. Compared to the control, an additional 260 more NH deaths were identified at 30 days and 429 more deaths at 90 days. Long stay residents (≥100 days) were at particular risk for mortality compared to short stay residents (<100 days). Hospitalization was also markedly increased.

### CHALLENGES OF CARING FOR AN ACUTE POPULATION IN A DISASTER

Lindsay Peterson,[1] Kathryn Hyer,[1] David Dosa,[2] Joseph June,[3] and Debra Dobbs,[1] *1. University of South Florida, Tampa, Florida, United States, 2. Brown University, Barrington, Rhode Island, United States, 3. School of Aging Studies, University of South Florida, Tampa, Florida, United States*

The U.S. Gulf Coast hurricanes of 2004-08 led to research and policy reports highlighting the need for more emergency preparation among nursing homes (NH). In 2016, the federal government issued final rules requiring Medicaid and Medicare providers to develop comprehensive preparedness plans. The state of Florida previously imposed its own long-term care (LTC) preparedness requirements. Hurricane Irma tested the readiness of LTC facilities that care for disabled and vulnerable residents. This research examined the experiences of NHs (N=30) affected by the hurricane through qualitative interviews with administrative staff. Research team members analyzed the transcripts, identified codes, and met to reach consensus on themes. Three major themes emerged, 1) managing the unexpected, including last-minute evacuation orders, 2) caring for vulnerable residents amid the crisis, and 3) the struggle of maintaining staff. Results suggest LTC preparation has increased but long-standing problems continue, including conflicts with emergency management priorities.

### HURRICANE IRMA'S IMPACT ON ASSISTED LIVING RESIDENTS' RATES OF HOSPITALIZATION, NURSING HOME PLACEMENT, AND MORTALITY

Cassandra Hua,[1] Kathryn Hyer,[2] Wenhan Zhang,[3] Jessica Ogarek,[4] and David Dosa,[5] *1. Brown University School of Public Health, Providence, Rhode Island, United States, 2. University of South Florida, Tampa, Florida, United States, 3. Brown University, Providence, Rhode Island, United States, 4. Brown University, PROVIDENCE, Rhode Island, United States, 5. Brown University, Barrington, Rhode Island, United States*

Little is known about the impact of hurricanes on the large and increasingly vulnerable population residing in assisted living communities (ALs). The objective of this paper was to leverage a novel methodology to identify Medicare beneficiaries residing in Florida ALs and determine their outcomes associated with Hurricane Irma in 2017. With Medicare enrollment records, claims, and the nursing home Minimum Data Set, we identified a cohort of AL residents in 2015 (n=45,505) and 2017 (n=42,306) and compared their rates of 30-day hospitalization, nursing home placement, and mortality in the two years. AL residents in 2017 had a 10% increase in their 30-day hospitalization rates (3.96 in 2015, 4.34 in 2017), 16% increase in their 30-day nursing home placement rates (1.61 in 2015, 1.87 in 2017), and 22% increase in their 30-day mortality (0.54 in 2015, 0.66 in 2017). Findings suggest Florida AL residents experienced adverse outcomes following Hurricane Irma.

### EXPERIENCES OF ASSISTED LIVING COMMUNITIES AFFECTED BY HURRICANE IRMA: LEADERSHIP, LESSONS LEARNED

Kathryn Hyer,[1] Lindsay Peterson,[1] David Dosa,[2] Joseph June,[3] and Debra Dobbs,[1] *1. University of South Florida, Tampa, Florida, United States, 2. Brown University, Barrington, Rhode Island, United States, 3. School of Aging Studies, University of South Florida, Tampa, Florida, United States*

Little is known about the effects of disasters on assisted living community (ALC) residents. This is a concern given the growth of the AL industry and the increasing numbers of AL residents with functional limitations and chronic health conditions. This research examined the experiences of AL administrative staff to better understand the impact of Hurricane Irma. Qualitative interviews were conducted with representatives of ALCs across Florida (N=70), and transcripts were analyzed using Atlas.ti version 8. Research team members met regularly to reach consensus on codes, identifying five major themes across the interviews, 1) planning and preparation, 2) leadership, including plan execution and managing the unexpected, 3) effects/consequences of the storm, including effect on residents and staff, 4) lessons learned, and 5) electrical power. Results provide a broad view of ALC preparedness, how it varies across different types of ALCs and implications for resident wellbeing and future emergency planning.

### SESSION 6335 (SYMPOSIUM)

### WHY DO AGE AND BIAS MATTER? HISTORICAL, SOCIAL, AND BIOLOGICAL IMPACTS ACROSS GENERATIONS OF LGBTQ OLDER ADULTS

Chair: Charles Emlet
Co-Chair: Karen Fredriksen Goldsen

We do not live in a stagnant world, politically, historically or socially - neither are the life experiences of lesbian, gay, bisexual, and transgender (LGBTQ) older adults. Their physical, psychological and social lives are impacted by their present and past historical contexts and generational identity, as well as the sociopolitical environment, social pressures, support and experiences of biases. This symposium draws upon data from Aging with Pride: National Health, Aging and Sexuality/Gender Study, the first longitudinal study of LGBTQ older adults in the United States, in order to examine how environmental, interpersonal and intrapersonal structures impact the lives of the three oldest generations of LGBTQ adults. Emlet and colleagues examine the generational and historical forces across the life course among 205 transgender older adults in order to illuminate how historical context and gender influences social life and physical health. Prasad and colleagues explore differences between various constructs of bias including lifetime discrimination, victimization, everyday discrimination and microaggressions on LGBTQ older adults and how those experiences impact depression, general health and quality of life. Lastly, Hoy-Ellis and colleagues explore how life course predictors including interpersonal violence, legal marriage, and identity management are associated with allostatic load (AL) in a sample of 317 LGBTQ older adults who range in age from 50-97. We will address the intersectionality of generational identity, experiences of bias and biomarkers, as well as the implications for policy and practice.

### HISTORICAL AND GENERATIONAL FORCES AND GENDER DIFFERENCES IN THE IRIDESCENT LIFE COURSE OF TRANSGENDER OLDER ADULTS

Charles Emlet,[1] Karen Fredriksen Goldsen,[2] Vanessa Fabbre,[3] Hailey Jung,[2] and Hyun Kim,[2] *1. University of Washington, Tacoma, Tacoma, Washington, United States, 2. University of Washington, Seattle, Washington, United States, 3. Washington University in St. Louis, St. Louis, Missouri, United States*

Shifting historical, political, and social times have impacted transgender older adults in experiences and development. Examining how gender identities interact with life events within a historical context elucidates differences by generations and gender. Using a transgender subsample (n=205) from the National Health, Aging, and Sexuality/Gender Study (N=2,450), (logistic) weighted regressions with sociodemographic adjustment were performed to examine generational differences and gender interaction in key life experiences in identity, work, kin, bias, community, and health/well-being. Silenced (born 1935-1949, 18%), than Pride (1950-1964, 79%), Generation disclosed identity later, is less visible, more likely retired and veteran, more likely for lifetime opposite-sex marriage and divorce. Significant gender interactions exist in community engagement, kin relationships, and physical health. Despite small sample size, Invisible Generation (1914-1934, 3%) has unique differences. Findings show complexity of generational differences among transgender older adults, understanding of what helps better serve them as they age with their accumulated life experiences.

### TYPES OF INTERPERSONAL BIAS: MEASUREMENT AND THEIR ASSOCIATION WITH HEALTH AMONG LGBTQ OLDER ADULTS

Anyah Prasad,[1] Karen Fredriksen Goldsen,[2] Hyun Kim,[2] and Hailey Jung,[2] *1. University of Massachusetts Boston, Boston, Massachusetts, United States, 2. University of Washington, Seattle, Washington, United States*

Increasing diversity and rapidly evolving sociopolitical context is changing the face of bias experiences in contemporary America. Accordingly, literature has evolved into four unique ways of describing bias, namely lifetime discrimination and victimization, everyday discrimination, and microaggressions. However, there is less conceptual clarity on the way these constructs are operationalized, measured, and their relationship to one another and health outcomes. In this paper, we discuss the measurement of these four constructs and report their prevalence among a national sample of LGBTQ older adults (N=2,450). The correlation between these constructs varied between 0.36 to 0.63, which shows that despite considerable overlap they are measuring distinct aspects of bias experiences. Each of the constructs had a significant association with depression, general health and quality of life. When the four constructs were entered into a regression model, everyday discrimination (due to sexual and gender identity and other factors) emerged as the strongest predictor.

### LIFE COURSE PREDICTORS OF ALLOSTATIC LOAD AMONG LGBTQ OLDER ADULTS

Charles Hoy-Ellis,[1] Hyun Kim,[2] and Karen Fredriksen Goldsen,[2] *1. University of Utah, Salt Lake City, Utah, United States, 2. University of Washington, Seattle, Washington, United States*

LGBTQ older adults are at significantly increased risk for poor mental and physical health, likely consequential to lifelong bias. Allostatic load (AL), the net effect of "wear and tear" on the body resulting from repeated, chronic over-activation of the psychophysiological stress response system. Utilizing the Health Equity Promotion Model, the aim of this study was to test potential life course predictors of AL, including interpersonal violence, legal marriage, and identity management in a sample of LGBTQ adults 50 to 97 years of age (n=317). Results from a series of hierarchical linear regression models showed that adult physical abuse and late identity disclosure for those who had been in an opposite-sex marriage predicted higher AL in this sample of LGBTQ older adults, indicating need for increased research on bias over the life course as contributory to AL and biopsychosocial dysfunction among LGBTQ older adults.

### SESSION 6500 (SYMPOSIUM)

### THE FUTURE OF AGING RESEARCH

Chair: Stephen Helfand
Co-Chair: Jackson Taylor

We are at a particularly propitious time in the history of the biology of aging and the science of health and humanity.

It is a time for us to "Honor the Past, Enrich the Future". Not long ago the study of the biology of aging was exclusively one of description of what happened with age—how do organisms, including humans, change with age at the level of proteins, cells, tissues and physiology. The identification of major genetic factors that substantially increase life span in model organisms ushered in the advent of a highly exploratory period focusing on the molecular, cellular and genetics mechanisms of aging. The convergence of many different streams of basic and clinical research have brought us to today, where we stand on the cusp of new environmental, molecular genetic and pharmacological breakthroughs in the biology of aging that presage new interventions that promise a healthier lifespan. The presentations in this Presidential Symposium will be from four Early Career Investigators presenting their own pioneering research in some of the most important areas of research in the biology of aging.

### MECHANISMS OF LIFESPAN EXTENSION BY SIRT6 IN DROSOPHILA MELANOGASTER

Jackson Taylor, Jason Wood, Matthew Finn, Julianna Liu, Evan Mizerak, Sarah Gordon, Corinne Hutfilz, and Stephen Helfand, *Brown University, Providence, Rhode Island, United States*

Sirt6 is a multifunctional enzyme that regulates numerous cellular processes connected to longevity. Overexpressing Sirt6 extends lifespan in mice, but the underlying cellular mechanisms are unclear. Here, we used the powerful genetic tools and short lifespan of Drosophila melanogaster to better understand the precise mechanisms by which Sirt6 regulates longevity. Sirt6 OE in flies produces robust extension of median lifespan in both sexes. Molecular and biochemical analyses reveal that Sirt6 OE reduces expression of genes involved in protein synthesis, including many Myc target genes, via epigenetic regulation. We will further discuss our findings on the connection between Sirt6, Myc, and the molecular regulation of protein synthesis and lifespan, as well as additional Sirt6 longevity mechanisms we identified, including autophagy and silencing of transposable elements.

### NEW COMPUTATIONAL APPROACHES TO AGING RESEARCH

Morgan E. Levine, *Yale University School of Medicine, New Haven, Connecticut, United States*

Aging is associated with numerous changes at all levels of biological organization. Harnessing this information to develop measures that accurately and reliably quantify the biological aging process will require systems biology approaches. This talk will illustrate how epigenetic data can be integrated with cellular, physiological, proteomic, and clinical data to model age-related changes that propagate up the levels—finally manifesting as age-related disease or death. I will also describe how network modeling and machine learning approaches (linear and non-linear) can be used to identify causal features in aging from which to generate novel biomarkers. Given the complexity of the biological aging process, modeling of systems dynamics over time will both lead to the development of better biomarkers of aging, and also inform our conceptualization of how alterations at the molecular level propagate up levels of organization to eventually influence morbidity and mortality risk.

### EPIGENETICS, HEREDITY, AND AGING

Eric Greer, *Boston Children's Hospital/Harvard Medical School, Boston, Massachusetts, United States*

Longevity has long been shown to be regulated by genetic and environmental factors. We recently showed that longevity in the nematode Caenorhabditis elegans can also be regulated by the transmission of epigenetic information. We have shown that several chromatin modifying enzymes have a transgenerational non-Mendellian effect in worms on the longevity of their descendants. I will discuss some of our recent work attempting to decipher how epigenetic modifications can regulate complex phenotypes, including longevity, and how this non-genetic information can be transmitted across generations.

### SEX-DIMORPHISM IN AGING GENE REGULATION: ARE WE MISSING HALF OF THE PICTURE?

Bérénice Benayoun, Ryan Lu, Nirmal Sampathkumar, and Min Hoo Kim, *University of Southern California, Los Angeles, California, United States*

The existence of human supercentenarians reveals a surprising predictor for exceptional longevity: being female. Not only are 33 out of 34 living supercentenarians women, women are also more resistant to most diseases responsible for age-related morbidity in the US. However, because most molecular aging studies generally opt to use only one sex, sex-driven differences in aging remain poorly understood. A key compartment that can actively respond to sex-specific inputs throughout life is the immune system. Indeed, the majority of age-related diseases share common inflammatory mechanisms, a phenomenon described as "inflamm-aging". Macrophages play an important role in the inflammatory response throughout life, and are considered major mediators of this phenomenon. Thus, to unbiasedly dissect sex differences in immune aging, we generated 'omics' data from 4 and 20 months old female and male mice. Intriguingly, we found that transcriptional aging in primary macrophage populations varies strongly between sexes, with up to 20-fold more aging changes in female vs. male cells. Pathways specifically downregulated in females with aging included lysosome, inflammation and phagolysosome. We confirmed experimentally that metabolic preferences of macrophages are indeed directly modulated in this context (e.g. glycolytic preference for male-derived cells). Our results support the notion that there are functional differences in aging trajectories in the immune system of female vs. male mice. Our research could provide new insights into the molecular underpinnings of sex-dimorphism in aging and disease.

### SESSION 6505 (SYMPOSIUM)

### METABOLISM AND AGING: NEW APPROACHES TO NUTRITION AND DIET IN AGING

Chair: Blanka Rogina

Aging is associated with a functional decline in metabolic, physiological, proliferative, and tissue homeostasis leading to deterioration at an organismal level and increases risk for disease and death. Genetic, pharmacological and nutritional interventions have been successfully used to preserve metabolic health, which leads to preserved healthspan and extended longevity. This symposium will discuss new approaches to nutrition and diet and mechanisms underlying interventions such as calorie restriction and genetic CR. We will also discuss species-specific metabolic mechanisms based on longitudinal studies in mice, monkeys and humans.

### THE EFFECTS OF INDY ON FLY METABOLISM

Blanka Rogina,[1] Kavitha Kannan,[1] Dushyant Mishra,[1] Jacob Macro,[1] Danielle Lesperance,[2] Nichole Broderick,[2] Shivani Padhi,[2] and Aaron Rosenbloom-Snow,[1]
*1. UConn Health, Farmington, Connecticut, United States, 2. University of Connecticut, Storrs, Connecticut, United States*

The Indy (I'm not dead yet) gene encodes a plasma membrane citrate transporter in Drosophila. INDY reduction affects metabolism and extends longevity of flies and worms. In flies, INDY is predominantly expressed in the midgut, fat body and oenocytes, tissues with a key role in metabolism. We hypothesize that INDY reduction in the midgut regulates citrate levels leading to metabolic changes that preserve intestinal stem cell (ISC) homeostasis and slows aging by modifying Insulin/Insulin-like signaling (IIS), which is a key nutrient sensing pathway. Our second goal was to examine the role of JAK/STAT signaling pathway, which activates epithelial renewal in the gut, in response to aging-related stressors. We hypothesize that Indy reduction has effects on the microbiome, preventing bacterial overgrowth and altering community diversity, leading to extended longevity in a JAK/STAT-mediated fashion. Our data suggest that effects of Indy reduction is mediated by reduced IIS and JAK/STAT pathways

### SOURCES AND IMPORTANCE OF MITOCHONDRIAL NAD

Joseph Baur,[1] Timothy Luongo,[2] Jared Eller,[3] Mu-Jie Lu,[4] Caroline Perry,[2] and Lulu Cambronne,[3] *1. Perelman School of Medicine at the University of Pennsylvania, Philadelphia, Pennsylvania, United States, 2. University of Pennsylvania, Philadelphia, Pennsylvania, United States, 3. University of Texas at Austin, Austin, Texas, United States, 4. UT Austin, Austin, Texas, United States*

Nicotinamide adenine dinucleotide (NAD) levels fall with age or disease, and rise with exercise or caloric restriction. Moreover, the demonstration that supplemental NAD precursors drive beneficial effects in rodent models has driven a resurgence in interest in the basic biology of this molecule. Although NAD is present in the mitochondrial matrix and critical to the function of the organelle, the source of mitochondrial NAD has been debated. We recently used isotopic labeling to demonstrate that direct uptake of intact NAD is one mechanism by which mitochondria are able to obtain this nucleotide. Here, we show that this activity is sufficient to restore respiratory capacity in NAD-deficient isolated mitochondria, and identify SLC25A51 as a carrier that can mediate the transport of NAD across mitochondrial membranes. Understanding the compartment-specific regulation of NAD will be crucial to understanding how cells and tissues adapt their metabolism to changes in NAD availability. Funding: DK098656 to J.A.B., GM126897 to L.A.C.

### ROLE OF REDUCED PROTEIN INTAKE IN METABOLIC AND HEALTHSPAN BENEFITS OF PLANT-BASED DIETS

James Mitchell,[1] Michael MacArthur,[2] and Sarah Mitchell,[2]
*1. ETH Zurich, Zurich, Massachusetts, Switzerland, 2. Harvard T.H. Chan School of Public Health, Boston, Massachusetts, United States*

Plant-based nutrition consisting of vegetarian/vegan dietary patterns are positively associated with metabolic fitness and inversely associated with risk of cardiovascular disease, diabetes and all-cause mortality. The nutritional and molecular basis of such benefits remains unclear. Here we considered the potential contribution of protein quality (amino acid profiles) and quantity to plant-based nutritional benefits. To this end, we investigated whether individuals adhering to plant-based diets consume a different AA profile, and used isocaloric diets in controlled rodent studies with modulation of both AA composition and total protein amount using crystalline AA vs. naturally-sourced protein ingredients. We found surprisingly few differences between AA profiles of vegans vs. omnivores, but large effects of total protein independent of source in rodent studies, strongly suggesting a major effect of total protein rather than AA composition in health benefits of plant-based diets. Mechanistically, we discuss the role of reduced protein intake on glucose and lipid homeostasis.

### LONGITUDINAL FASTING BLOOD GLUCOSE TRENDS AND MORTALITY RISK IN MICE DIFFERS FROM THAT OF NON-HUMAN PRIMATES AND HUMANS

Rafael de Cabo, Dushani Palliyaguru, Eric Shiroma, John Nam, SLAM Investigators, and Luigi Ferrucci,
*National Institute on Aging, Bethesda, Maryland, United States*

Longitudinal studies in humans have led to the development of strong predictors of outcomes of health, disease and mortality. Translation from model organisms to human has been faced with species-specific regulation of metabolic function and challenged by the lack of longitudinal studies addressing trajectories of change that can be used, as in humans to predict outcomes. Here we compare longitudinal predictors of health and mortality of three major metabolic indices among mice, non-human primates and humans. Longitudinal fasting blood glucose, body weight and body composition over the lifespan were compared across species, mice, Rhesus monkeys and humans. Survival analysis was conducted to calculate the risk of death for subjects with highest and lowest quartiles of fasting blood glucose. We will present data highlighting species-specific mechanisms of glucose homeostasis over the lifespan and its association with mortality.

### SESSION 6510 (SYMPOSIUM)

### COGNITIVE AGING: NEW INSIGHTS INTO HOW OUR BRAINS AGE

Chair: Coleen Murphy
Discussant: Saul Villeda

Maintaining quality of life with age is as important as slowing human aging. Slowing cognitive decline will be a critical aspect of keeping older adults healthy as we extend lifespan. To that end, identifying the molecular regulators of neuronal health, including learning and memory, in model systems that can later be applied to humans is an important step. In this symposium, we will hear about work on cognitive decline and its prevention being done in a variety of model systems.

### USING C. ELEGANS TO FIND GENES, MECHANISMS, AND DRUGS TO SLOW COGNITIVE DECLINE

Coleen Murphy, *Princeton University, Princeton, New Jersey, United States*

Cognitive decline is one of the most important losses of human quality of life with age. To find new treatments that might slow these declines, it is useful to have short-lived, rapidly aging models in which to identify possible treatments. We found that C. elegans also lose their ability to learn and remember with age, along with other neuronal functions (axon regeneration, motility, etc.), and these declines are staved off by reduction in the insulin signaling pathway, which also extends lifespan. Both the mechanisms that regulate neuronal functions and the mechanisms that slow decline are well conserved evolutionarily, suggesting that we can use worms to identify new genes and compounds that might slow decline. Additionally, we have developed new C. elegans models of Parkinson's and Alzheimer's diseases, and through genetic, genomic, and metabolic studies we have found new approaches that may slow these declines.

### REGENERATION AND COGNITION IN AGING

Saul Villeda, *University of California San Francisco, San Francisco, California, United States*

### RETROTRANSPOSONS AND BRAIN AGING

Joshua Dubnau, *Stony Book School of Medicine, Stony Brook, New York, United States*

### SYSTEMS GENETICS OF COGNITIVE AGING

Adam Hudgins, *Columbia University Irving Medical Center, New York, New York, United States*

### SESSION 6515 (SYMPOSIUM)

### AGING AND NEURODEGENERATION: NEW APPROACHES TO NEURODEGENERATION AND AGING

Chair: Nancy Bonini

Model organisms like C. elegans and Drosophila are powerful systems to help dissect molecular genetic aspects of complex biological processes. Here we will present a number of biological questions and approaches that have been used to study questions of neural integrity and healthful aging using these systems.

### MODULATION OF THE AGING DROSOPHILA BRAIN AND SUSCEPTIBILITY TO NEURODEGENERATION

Nancy Bonini, *University of Pennsylvania, Philadelphia, Pennsylvania, United States*

Human neurodegenerative diseases, like amyotrophic lateral sclerosis, Alzheimer's disease and frontotemporal dementia, are late-onset progressive neurodegenerative disorders for which few cures or treatments are available. To develop new insight, we have been using the model organism Drosophila. We can re-create disease-associated protein toxicity in the fly brain, and then take advantage of the powerful molecular genetic approaches to define pathways and mechanisms. The fly is allowing us to reveal mechanistic understanding that we can then extend to the human condition. These studies have highlighted multiple novel pathways involved in longterm integrity of the brain. Among these processes, we have defined epigenetic pathways critical for longterm brain health. These pathways typically converge on stress pathway players, highlighting that boosting the ability of the nervous system to combat stress may help promote healthful brain aging and resistance to degenerative disease pathways.

### NEURONS PUTTING OUT THE TRASH: A NOVEL FACET OF PROTEOSTASIS AND MITOCHONDRIAL QUALITY CONTROL

Monica Driscoll, *Rutgers University, Piscataway, New Jersey, United States*

Toxicity of misfolded proteins and mitochondrial dysfunction are pivotal factors that promote age-associated functional neuronal decline and neurodegenerative disease across species. Although these neurotoxic challenges have long been considered to be cell-intrinsic, considerable evidence now supports that misfolded human disease proteins originating in one neuron can appear in neighboring cells, a phenomenon proposed to promote pathology spread in human neurodegenerative disease. We have found that C. elegans adult neurons that express aggregating proteins can extrude large (~5μM) membrane-surrounded vesicles that can include the aggregated proteins and damaged mitochondria. We speculate that throwing out the trash may correspond to a conserved mechanism that constitutes a fundamental, but formerly unrecognized, branch of neuronal proteostasis and mitochondrial quality control. I will discuss our current understanding of the mechanisms of neuronal trash elimination.

### NEURAL CONTROL OF HEALTHY AGING IN DROSOPHILA THROUGH PERCEPTIVE EXPERIENCES

Scott Pletcher, *University of Michigan, Ann Arbor, Michigan, United States*

We will describe how neurosensory circuits relate information about nutrition, danger, and mates to initiate rapid changes health and aging, including evolutionarily conserved neural signaling pathways and molecules that extend organism lifespan. These findings provoke the notion that aging may be considered a complex behavior that is acutely malleable, susceptible to sensory influences, and strictly controlled by coordinated sets of neurons.

### FUNCTIONAL MODULATORS OF RESILIENCE TO AGE-ASSOCIATED DOPAMINERGIC NEURODEGENERATION IN C. ELEGANS

Guy Caldwell, *The University of Alabama, Tuscaloosa, Alabama, United States*

The societal costs of neurodegenerative diseases demand a sense of urgency and innovative strategies toward therapeutic intervention. We have exploited the experimental attributes of roundworm, C. elegans, as a model to expedite discovery of genetic and chemical modifiers of the age-dependent progressive neurodegeneration of dopaminergic neurons commonly associated with Parkinson's disease (PD). Our model of transgenic nematodes overexpressing the misfolding-prone familial PD gene product, alpha-synuclein, has proven prescient in illuminating key aspects of PD pathology, including vesicular trafficking, autophagy, stress response, lipid dynamics, as well as the first evidence of endolysosomal function in effecting dopaminergic neurodegeneration. Subsequent chemical-genetic analyses reveal a mechanism whereby dopamine transport intersects with epigenetic control of gene expression through proteins functioning in endocytic regulation to modulate neuroprotection. We theorize this reflects a "tunable" nexus of gene-by-environmental regulation where dopamine levels, which impact alpha-synuclein misfolding, are coordinately controlled with epigenetic response to mediate resilience to age-dependent neurodegeneration.

### SESSION 6520 (SYMPOSIUM)

### MICROBIOME: LIVING WELL AND THRIVING WITH YOUR BACTERIA

Chair: Meng Wang
Co-Chair: William Ludington

This symposium aims to invite speakers who are the leading experts in the field of microbiome and longevity, and select short talks from abstracts submitted by young investigators, postdoctoral fellows and graduate students. We will discuss new progress in this exciting research area and raise new questions for future studies. Confirmed speakers include Evgeny Nudler from NYU, Will Ludington from Carnegie, Paul O'Toole from University College Cork, Ireland, and Meng Wang from Baylor College of Medicine.

### MICROBIOME-MITOCHONDRIA COMMUNICATION IN THE REGULATION OF HOST LONGEVITY

Meng Wang, *Baylor College of Medicine/HHMI, Houston, Texas, United States*

Mitochondria are ancient relatives of bacteria in eukaryotic cells and dynamically interconnected through organelle fusion and fission. Given the close relationship between bacteria and eukaryotic mitochondria during evolution, my group is interested in understanding the critical role of their communication in regulating host's longevity. We have conducted genome-scale screens to decipher how bacterial genetic composition impacts host longevity, leading to the discovery of specific bacteria-secreted metabolites that fine-tune the mitochondrial fusion-fission balance and consequently promotes longevity across different host species. We have further developed optogenetic approaches to manipulate bacterial gene expression and metabolite production inside the gut of live organisms, in order to investigate the microbiome-mitochondria communication in time and space. Our studies demonstrate a novel mode of signaling communication between bacteria and mitochondria, reveal its vital impacts on host healthy aging, and provide new methods to decipher the spatiotemporal relationship between the microbiome and the host.

### INTER-SPECIES INTERACTIONS IN THE FLY GUT MICROBIOME SHAPE AGING

William Ludington, *Carnegie Institution, Baltimore, Maryland, United States*

Gut bacteria affect key aspects of host fitness, including fecundity and lifespan. However, it is unclear to what extent individual species versus complex interactions drive host fitness. We dissected the natural microbiome of Drosophila melanogaster and revealed that interactions between bacteria shape host fitness through life history tradeoffs. Empirically, we made germ-free flies and colonized them with each possible combination of the five core species of bacteria. We measured the microbiome and fly fitness traits including reproduction and lifespan. Notably, flies that reproduced more died sooner. Removing bacteria after reproduction extended lifespan in most cases, suggesting an indirect tradeoff. However, in certain cases, antibiotics did not extend lifespan, indicating a metabolic memory of the microbiome. Overall, complex interactions within the microbiome had significant effects on host fitness. We suggest that model systems with reduced complexity will be instrumental in elucidating mechanisms of microbiome-host interactions.

### GENOMICS AND ECOLOGY OF GASTROINTESTINAL BACTERIA IN HUMAN HEALTHSPAN

Paul O'Toole

### BACTERIAL TOXIC OXIDANTS IN HEALTHY AGING AND DISEASE

Evgeny Nudler

### SESSION 6525 (SYMPOSIUM)

### PHYSIOLOGY AND AGING: NEW UNDERSTANDING OF ORGANS THAT AFFECT THE PHYSIOLOGY OF AGING

Chair: Daniela Drummond-Barbosa

As organisms age, many changes occur to their physiology, which in turn impact the function of multiple tissues. It is therefore critical to investigate the fundamental mechanisms of how endocrine organs shape our physiology, and how changes in our physiology affect stem cell lineages, which generate new cells for maintenance and repair of tissues/organs throughout life. This symposium will highlight the research in the laboratories of Dr. Gerard Karsenty (Columbia University) on the multiple endocrine functions of bone, of Dr. Nicholas Buchon (Cornell University) on the role of host-microbe interactions in intestinal homeostasis, of Dr. Jane Hubbard (NYU/Skirball Institute) on the physiological control of the germline, and of Dr. Daniela Drummond-Barbosa (Johns Hopkins University) on how diet and adipocyte factors regulate oogenesis. As research by these and other groups illustrate, the complex physiological regulation of tissue/organ maintenance and function is not only a fascinating biological problem, but it also has implications for many diseases and other conditions that are tightly linked to our endocrine state, including aging.

### STEM CELLS, DIET, AND PHYSIOLOGY

Daniela Drummond-Barbosa, *Johns Hopkins Bloomberg School of Public Health, Baltimore, Maryland, United States*

Nutrient availability, stresses, and aging affect tissue stem cells in multicellular organisms; yet, the underlying physiological mechanisms in vivo remains largely unexplored. Dr. Drummond-Barbosa pioneered using Drosophila to study the physiology of tissue stem cell regulation. Her laboratory played a major role in delineating how diet, brain insulin-like peptides, and the TOR nutrient sensor control the germline stem cell (GSC) lineage. They also discovered that adipocyte-specific disruption of amino acid transport, other nutrient signaling, and metabolic pathways causes distinct germline phenotypes. They also showed that nuclear receptors act in multiple tissues to affect the GSC lineage through direct and indirect mechanisms. More recently, her group has been exploring how other physiological stresses affect the GSC lineage. Her group's studies point to extensive communication between the brain, adipocytes, hepatocyte-like cells, and the germline, and underscore the complexity of the physiological network that modulates stem cell lineage behavior.

### THE IMPACT OF BONE ON THE BIOLOGY OF AGING

Gerard Karsenty, *Columbia University, New York, New York, United States*

We hypothesized that bone may secrete hormones that regulate energy metabolism and reproduction. Testing this hypothesis revealed that the osteoblast-specific secreted protein osteocalcin is a hormone regulating glucose homeostasis and male fertility by signaling through a GPCR, Gprc6a, expressed in pancreatic β bells and Leydig cells of the testes. The systematic exploration of osteocalcin biology, revealed that it regulates an unexpectedly large spectrum of physiological functions in the brain and peripheral organs and that it has most features of an antigeromic molecule. As will be presented at the meeting, this body of work suggests that harnessing osteocalcin for therapeutic purposes may be beneficial in the treatment of age-related diseases such as depression, age-related memory loss and the decline in muscle function seen in sarcopenia.

### AGING GERMLINE STEM CELLS IN C. ELEGANS

E. Jane Hubbard, *Skirball/NYU, New York, New York, United States*

Failure to maintain stem cells with age is associated with conditions such as tissue degeneration and increased susceptibility to tissue damage. We use the C. elegans germline stem cell system as a model to study stem cell aging. This system combines a well-established model for aging with an accessible stem cell system, providing a unique opportunity to understand how aging influences stem cell dynamics. The germline stem/progenitor pool in in C. elegans becomes depleted over time. At the cellular level, aging influences both the size of the stem cell pool and the proliferation rate of stem cells. The flux of differentiated cells also affects how aging impacts the pool. This depletion is partially alleviated in mutants with reduced insulin/IGF-like signaling via inhibition of the transcription factor DAF-16/FOXO. In this role, DAF-16 does not act in the germ line, and its anatomical requirements are different from its previously described roles in larval germline proliferation, dauer control, and lifespan regulation. We found that DAF-16/FOXO is required in certain somatic cells in the proximal part of the reproductive system to regulate the stem cell pool. We also find that the degree to which various age-defying perturbations affect lifespan does not correlate with their effect on germline stem cell maintenance. We are investigating additional aspects of aging germline stem cells using this system.

### HOSTMICROBE GENETIC NETWORK INTERACTIONS GOVERN THE RESPONSE TO MICROBES

Nicolas Buchon, *Cornell University, Ithaca, New York, United States*

### SESSION 6530 (SYMPOSIUM)

### CIRCADIAN RHYTHMS, SLEEP, AND AGING: NEW VISIONS ON THE CLOCK, SLEEP, AND AGING

Chair: Amita Sehgal

The symposium will highlight the reciprocal relationship between aging and sleep and circadian rhythms. Circadian rhythms and sleep deteriorate with age, but at the same time, disruptions in these processes can contribute too aging. Presenters will show how work in model organisms is providing insight into this relationship

### MOLECULAR AND CELLULAR NETWORKS THAT DRIVE SLEEP

Amita Sehgal, *University of Pennsylvania, Philadelphia, Pennsylvania, United States*

### CIRCADIAN REGULATION OF MITOCHONDRIAL UNCOUPLING AND LIFESPAN

Matthew Ulgherait, *CUMC, New York, New York, United States*

Because old age is associated with defects in circadian rhythm, loss of circadian regulation is thought to be pathogenic and contribute to mortality. We show instead that loss of specific circadian clock components Period (Per) and Timeless (Tim) in male Drosophila significantly extends lifespan. This lifespan extension is not mediated by canonical diet-restriction longevity pathways, but is due to altered cellular respiration via increased mitochondrial uncoupling. Lifespan extension of per mutants depends on mitochondrial uncoupling in the intestine. Moreover, up-regulated uncoupling protein UCP4C in intestinal stem cells and enteroblasts is sufficient to extend lifespan and preserve proliferative homeostasis in the gut with age. Consistent with inducing a metabolic state that prevents over-proliferation, mitochondrial uncoupling drugs also extend lifespan and inhibit intestinal stem cell overproliferation due to aging or even tumorigenesis. These results demonstrate that circadian-regulated intestinal mitochondrial uncoupling controls longevity in Drosophila and suggest a new potential anti-aging therapeutic target.

### AGE-RELATED CHANGES IN CELLULAR PROTECTIVE PATHWAYS IN SLEEP DEPRIVATION

Nirinjini Naidoo, *University of Pennsylvania, Philadelphia, Pennsylvania, United States*

### SESSION 6535 (SYMPOSIUM)

### SIRTUINS: NEW ROLES FOR SIRTUINS IN AGING AND DISEASE

Chair: Katrin Chua

Sirtuins are NAD+-dependent enzymes related to yeast silent information regulator-2, a histone deacetylase that prevents premature aging through chromatin silencing. Mammalian sirtuins, SIRT1-SIRT7, have key functions that protect against diverse aging-related pathologic states, from cancer to metabolic and neurodegenerative disease. In addition to chromatin regulation, mammalian sirtuins also govern myriad cellular processes through a growing list of enzymatic activities and substrates. This session will highlight new discoveries and directions in Sirtuin research.

### LINKING EPIGENETICS, METABOLISM AND CANCER: LESSONS FROM SIRT6

Raul Mostoslavsky, *Mass General Cancer Center, Boston, Massachusetts, United States*

In recent years, chromatin regulators have emerged as key modulators in cancer. We discovered that the mammalian histone deacetylase SIRT6 is a key chromatin factor, modulating expression of metabolic, developmental, and ribosomal protein genes. In recent studies, we identified a loss of function mutation in SIRT6 behind a human syndrome of perinatal lethality. In the context of cancer, we found SIRT6 to act as a robust tumor suppressor, by modulating glucose metabolism. As Otto Warburg described decades ago, cancer cells exhibit glycolytic metabolism, where pyruvate, instead of contributing to ATP production in the mitochondria, is converted to lactate even under normoxia conditions. We found SIRT6 as the first chromatin factor in charge of suppressing the Warburg effect in colon and skin cancer. At the cellular level, SIRT6 inactivation leads to increased cellular glucose uptake, higher lactate production and decreased mitochondrial activity. Our results indicate that SIRT6 directly regulates expression of several key glycolytic and ribosomal genes, co-repressing Hif1a and Myc, respectively, and acting as a histone H3 lysine9 (H3K9) and lysine 56 (H3K56) deacetylase. Notably, we recently identified SIRT6 as the first deacetylase to specifically inhibit transcriptional elongation, rather than initiation, in its targets. Strikingly, we determined in new studies that such glycolytic switch provides an advantage even at the early initiating cancer stem cells stage, in what we identified as the cell-of-origin for the Warburg effect. In addition, we found SIRT6 to act as a robust tumor suppressor in the context of pancreatic cancer. However, in this case, SIRT6 did not influence metabolism, but rather silenced expression of the developmental gene Lin28b, in this way protecting against aggressive undifferentiated pancreatic adenocarcinoma. Our studies highlight the important role epigenetic factors, such as SIRT6, play in protecting against tumor progression by providing “epigenetic plasticity”, inhibiting adaptive responses in transformed cells.

### NOVEL ROLES FOR THE SIRTUIN DEACYLASE SIRT5 IN NORMAL PHYSIOLOGY AND IN CANCER

David Lombard, *University of Michigan, Ann Arbor, Michigan, United States*

Sirtuins are NAD+-dependent deacylases that regulate diverse cellular processes such as metabolic homeostasis and genomic integrity. Mammals possess seven sirtuin family members, SIRT1-SIRT7, that display diverse subcellular localization patterns, catalytic activities, protein targets, and biological functions. Three sirtuins, SIRT3, SIRT4, and SIRT5, are primarily located in the mitochondrial matrix. SIRT5 is a very inefficient deacetylase, instead removing negatively charged post-translational modifications (succinyl, glutaryl, and malonyl groups) from lysines of its target proteins, in mitochondria and throughout the cell. SIRT5 plays only modest known roles in normal physiology, with its major functions occurring in the heart under stress conditions. In contrast, in specific cancer types, including melanoma, we have identified a major pro-survival role for SIRT5. We have traced this function of SIRT5 to novel roles for this protein in regulating chromatin biology. New insights into mechanisms of SIRT5 action in cancer, and in normal myocardium, will be discussed.

### CHROMATIN REGULATION AND GENOME MAINTENANCE BY MAMMALIAN SIRT7

Joonseok Cho, *Stanford School of Medicine, Stanford, California, United States*

Members of the Sirtuin family of enzymes are important regulators of genomic stability, stress responses, and metabolic programs that impact on human physiology, aging, and age-related disease processes. We previously showed that the mammalian Sirtuins SIRT6 and SIRT7 have high-selectivity histone deacetylase activities at chromatin, and inactivation of SIRT6 or SIRT7 results in dysregulated histone acetylation states and gene expression programs, with pathological consequences at the cellular and whole organism levels. Recently, we have been exploring novel functions of SIRT6 and SIRT7 in silencing of heterochromatic regions of the genome, the deregulation of which has been linked to aging and cancer biology. We found that pericentric heterochromatin silencing by SIRT6 prevents acute cellular senescence that is triggered by pathologic pericentric transcripts. We also uncovered a second novel trigger of human cellular senescence, ribosomal DNA instability in nucleoli, and we showed that SIRT7 guards against senescence induced by this instability. In our studies, a long-term focus has been identifying substrates of SIRT6 and SIRT7 and their roles in aging and disease pathways. In new work, are studying a novel physiologic substrate of SIRT7 at chromatin, H3K36Ac, and we are characterizing the genomic landscape of this SIRT7-dependent deacetylation target, and its downstream chromatin and nuclear signaling mechanisms. We will also discuss mechanistic insights into the functions of SIRT6 and SIRT7 from new proteomic, cellular, and mouse model studies.

### SESSION 6540 (SYMPOSIUM)

### SENESCENCE AND SENOLYTICS: STATE OF THE ART ON CELLULAR SENESCENCE, SENOLYTICS, AND HEALTHSPAN

Chair: Judith Campisi

Cellular senescence is a cell fate decision that is made by many mammalian cell types in response to damage, stress or certain physiological signals. Senescent cells arrest proliferation, essentially permanently, and develop a complex multi-component senescence-associated secretory phenotype (SASP). Recent studies using human and rodent cells, tissue samples and transgenic mouse models have defined a causal role for senescent cells, acting largely through the SASP, in a surprisingly large and diverse number of age-related diseases. Subsequently, several synthetic and natural compounds have been identified that have the ability to selectively kill senescent, but not non-senescent, cells. These compounds, termed senolytics, are now of intense interest to both basic research groups and biotechnology companies because they hold promise for postponing, ameliorating or, in some cases, reversing certain age-related pathologies. A related group of compounds, termed senomorphics, hold similar promise and act by selectively suppressing certain modules of the SASP. This symposium will feature presentations on some of the latest developments in the fields of cellular senescence and the SASP and how these cellular responses affect organismal health span. The symposium will particularly emphasize recent findings on the identification and activities of senolytic and senomorphic agents that have the potential to significantly extend the health span of mammalian organisms.

### CELLULAR SENESCENCE AND MAMMALIAN HEALTHSPAN

Judith Campisi, *The Buck Institute for Age Research, Novato, California, United States*

Cellular senescence is a complex cell fate, often induced by stress or damage, that can be beneficial or deleterious, depending on the physiological context and age of the organism. A prominent feature of senescent cells is a multi-faceted senescence-associated secretory phenotype (SASP), which includes growth factors, cytokine and chemokines, growth factors, proteases, bioactive lipids and metabolites. Senescent cells increase with age in most, if not all, mammalian tissues. Through the use of transgenic mouse models, senescent cells are now known to causally drive numerous age-related pathologies, largely through the SASP. Eliminating senescent cells, genetically or through the use of senolytic/senomorphic agents, can improve the health span, at least in mice, and hold promise for extension to humans in the near future.

### UNCOVERING THE MOLECULAR UNDERPINNINGS OF OXIDATIVE STRESS-INDUCED SENESCENCE

Melissa Carpenter, Laura Corrales-Diaz Pomatto, Jonathan Kato, Sarah Wong, Oye Bosompra, Michel Bernier, and Rafael de Cabo, *National Institute on Aging, Bethesda, Maryland, United States*

The aging process is sexually dimorphic, with males having higher occurrence rates of cancer and facing a greater risk of mortality. Sexual dimorphism in the response to cellular damage may account for distinct phenotypic changes with age as they relate to the accumulation of cellular damage leading to cancer. Cellular senescence triggers permanent cell cycle arrest in order to protect against malignant growth. However, organismal senescence increases with age and is associated with the release of pro-inflammatory signals (cytokines, chemokines, and proteases) known as the 'senescence-associated-secretory-phenotype' (SASP) that, if unchecked, accelerates tissue damage and creates a microenvironment ripe for cancer development. In this study, we hypothesized that sexual disparities in mortality and cancer prevalence stems from differences in the rate of accumulation of senescent cells in mice. Male and female C57BL/6J mice were fed ad libitum or subjected to 30% calorie restriction, a nutritional intervention known to delay the onset of various cancers and prevent senescent cell accumulation. Primary skin fibroblasts were collected longitudinally to allow measurement of cell proliferation, wound healing and the release of SASP factors. The results indicate that when compared to males, fibroblasts of CR-fed females showed significant improvements in cell growth rate, wound healing and SASP markers vs. AL controls. Work is underway to determine how sex influences cellular protective pathways. Thus, like other cell processes, cellular senescence is unequal between males and females and CR delays the emergence of the senescence phenotype.

### DNA DAMAGE, CELLULAR SENESCENCE IN HEALTH AND DISEASE

Laura Neidernhofer

### TARGETING CELLULAR SENESCENCE IN CANCER, HEALTH AND DISEASE

Daohong Zhou

### SESSION 6545 (SYMPOSIUM)

### EPIGENETICS: THE MANY FACES OF EPIGENETICS IN AGING

Chair: Shelley Berger

### EPIGENETICS OF HUMAN NEURODEGENERATION

Shelley Berger, *University of Pennsylvania - Perelman School of Me*

### EPIGENETICS AND AGING IN KILLIFISH

Anne Brunet, *Stanford School of Medicine, Stanford, California, United States*

### DNA DAMAGE: EPIGENETICS, AGING AND REJUVENATION

David Sinclair, *Paul F. Glenn Center for Biology of Aging Research at Harvard Medical School, Boston, Massachusetts, United States*

### SESSION 6550 (SYMPOSIUM)

### STEM CELLS: THE FUTURE PROMISE OF STEM CELLS IN HEALTHSPAN

Chair: Ashley Webb

Tissue specific stem cells are critical for maintenance and repair of tissues and organs throughout life. However, during aging, the functionality of stem cells declines, contributing to tissue decline. This symposium will focus on the mechanisms underlying stem cell aging in various compartments, including muscle, brain and the hematopoietic system.

### GENETIC, EPIGENETIC, AND METABOLIC MAINTENANCE OF NEURAL STEM CELLS DURING AGING

Ashley Webb, *Brown University, Providence, Rhode Island, United States*

Tight metabolic regulation is essential to maintain stem cell homeostasis and support healthy aging. With age, metabolic alterations cause neural stem cell (NSC) dysfunction and are associated with a decline in neurogenesis, but the underlying mechanisms are not known. Aged stem cells display defects in the autophagy-lysosomal pathway that may disrupt mitochondrial dynamics, resulting in metabolic disruptions that alter self-renewal and differentiation potential. We have used genomic and functional approaches to investigate the metabolic mechanisms that support NSCs throughout aging. We found that mitochondrial and mitophagy gene networks as well as mitophagy dynamics are differentially regulated between the quiescent and activated states and become dysregulated with age. This work provides new insight into the metabolic regulation of NSCs and may lead to strategies to enhance neurogenesis in the context of aging and neurodegenerative disease.

### INTERVENTIONS TO DRIVE IMPROVED FUNCTIONAL POTENTIAL OF AGED OR DYSFUNCTIONAL HEMATOPOIETIC STEM CELLS

Isabel Beerman,[1] Le Zong,[2] Mayuri Tanaka-Yano,[3] Hagai Yanai,[2] and Ferda Tekan-Turhan,[2], *1. NIA, Baltimore, Maryland, United States, 2. NIA / NIH, Baltimore, Maryland, United States, 3. NIA/ NIH, Baltimore, Maryland, United States*

Stem cell dysfunction is a hallmark of aging, associated with the decline of physical and cognitive abilities of humans and other mammals. Therefore, it has become an active area of research within the aging and stem cell fields, and various techniques have been employed to mitigate the decline of stem cell function both in vitro and in vivo. We have examined changes in the hematopoietic system after interventions and show modest, but positive effects on the aged system as well as the aged stem cells

### STEM CELLS IN TISSUE MAINTENANCE AND REPAIR

Amy Wagers, *Harvard University, Cambridge, Massachusetts, United States*

### SESSION 6555 (SYMPOSIUM)

### MODEL ORGANISMS: GRANDEUR IN THE DIVERSITY OF AGING ORGANISMS

Chair: Anne Brunet

Aging is a complex process that converts vigorous and healthy individuals into frail and decrepit ones, with increased susceptibility to a constellation of diseases. Human aging is influenced by many factors, including genetics, environment, lifestyle, sex, and socio-economic status. While aspects of aging can be studied directly in humans, discovering the causative factors that modulate this process often requires interventions and modeling. Traditional models will likely continue to provide a wealth of translatable information. Studying 'extremophiles' has exciting potential for providing new concepts that could be implemented for lifespan regulation. The development of new experimental models uniquely tailored to aging studies is also an essential step. This symposium will discuss African killifish, planarian, naked mole rats, and domestic dogs as new models for aging and exceptional longevity and rejuvenation. The iteration between new models and humans could be particularly helpful in delineating strategies to promote healthy aging and extend the disease-free portion of life.

### DEVELOPMENT OF THE AFRICAN KILLIFISH AS A NEW MODEL TO STUDY AGING AND SUSPENDED ANIMATION

Anne Brunet, *Stanford School of Medicine, Stanford, California, United States*

We have pioneered a new model organism for aging research, the naturally short-lived African killifish Nothobranchius furzeri. The African killifish lives in ephemeral pools of water in Africa, and has evolved a short life cycle adapted to this habitat. Its embryos can also resist drought until the next wet season in a state of 'suspended life'. In laboratory conditions, the African killifish has a maximal lifespan of about 4-6 months, and is, so far, the shortest-lived vertebrate that can be bred in captivity. We have successfully transformed this natural short-lived vertebrate into a usable model organism for aging research, including de novo assembly of the genome and CRISPR-Cas9 mediated genome-editing. Our goal is to use this model to discover new principles underlying aging, longevity, and 'suspended animation' in vertebrates.

### EXTREME LONGEVITY MECHANISMS IN THE NAKED MOLE RAT

Shelley Buffenstein

### COMPANION DOGS TO TEST LONGEVITY INTERVENTIONS

Matt Kaeberlein, *University of Washington, Seattle, Washington, United States*

### PLANARIA: A MODEL FOR IMMORTALITY

Alejandro Sanchez Alvarado

### SESSION 6560 (SYMPOSIUM)

### REGULATION OF AUTOPHAGY IN AGING AND DISEASE

Chair: Malene Hansen

The cytosolic recycling process of autophagy plays an important role in many age-related diseases and has been directly linked to aging, including in the nematode C. elegans where autophagy appears beneficially induced in many conserved longevity models. As a critical process to ensure cellular homeostasis, autophagy is regulated at multiple levels, yet it remains a challenge in the field to understand how the regulation of autophagy is integrated at the cellular and molecular level to ensure health- and lifespan benefits. I will here discuss our progress on understanding the different molecular mechanisms employed by cells and organisms to regulate autophagy in response to stressors such as aging and disease.

### REGULATION OF AUTOPHAGY IN AGING AND DISEASE

Malene Hansen, *Sanford Burnham Prebys Medical Discovery Institute, La Jolla, California, United States*

The cytosolic recycling process of autophagy plays an important role in many age-related diseases and has been directly linked to aging, including in the nematode C. elegans where autophagy appears beneficially induced in many conserved longevity models. As a critical process to ensure cellular homeostasis, autophagy is regulated at multiple levels, yet it remains a challenge in the field to understand how the regulation of autophagy is integrated at the cellular and molecular level to ensure health- and lifespan benefits. I will here discuss our progress on understanding the different molecular mechanisms employed by cells and organisms to regulate autophagy in response to stressors such as aging and disease.

### DISCOVERY OF NOVEL REGULATORS OF AUTOPHAGY IN ANIMALS

Eric Baehrecke, *UMass Medical School, Worcester, Massachusetts, United States*

The clearance of organelles by autophagy is important for cell health, and defects in this process have been associated with age-associated degenerative disorders. Ubiquitination of proteins enables their recognition by cargo receptors that facilitate the delivery of both protein aggregates and organelles to forming autophagosomes for degradation. We have investigated developmentally programmed autophagy to identify novel regulators of organelle clearance. We identified an Atg7-independent autophagy program that is required for cell size reduction and clearance of mitochondria. We have used this system to screen for new factors regulate ubiquitin-dependent autophagy and clearance of organelles. We screened a collection of putative ubiquitin binding domain encoding genes, and identified the novel gene Vps13D. Vps13D is an essential gene that is necessary for autophagy, mitochondrial size, mitochondrial clearance, and is associated with human movement disorders. We have used genetics and biochemistry to identify factors that link Vps13D, mitochondrial biology and autophagy.

### AUTOPHAGY AND IMMUNITY

Vojo Deretic

### LYSOSOMES AND CANCER

Rushika Perera

### SESSION 7000 (SYMPOSIUM)

### 3D TEAM CARE MANAGEMENT TRIAL FOR COGNITIVELY VULNERABLE OLDER ADULTS: WHO PARTICIPATES AND HOW DOES THE TEAM WORK?

Chair: Richard Fortinsky Discussant: Caroline Stephens

Community-dwelling older adults often experience cognitive symptoms, and three common conditions that contribute to changes in cognition are dementia, depression and delirium. Despite the clinical inter-connectedness among these medical conditions, hereafter referred to collectively as cognitive vulnerability, little is known about the potential for success of clinical interventions that simultaneously address these conditions. From the perspective of older adults with cognitive vulnerability and their families, hospital admissions and emergency department (ED) visits are disorienting and often lead to declines in functional capacity and well-being, and significant family distress, threatening continued independent living. In this Symposium, we present details about an ongoing clinical trial testing a novel in-home, multidisciplinary team care management intervention for older adults with cognitive vulnerability and their families. This care management intervention led by nurse practitioners, called the3D Team care model, aims to help reduce ED visits and hospitalizations and achieve other health-related outcomes. The first presentation will provide study background and design features as well as characteristics of study participants. The next two presentations by the3D Team nurse practitioners will provide details about how the multidisciplinary team works, and how each team member provides interventions intended to address risk factors for adverse health outcomes. The fourth presentation by the3D Team community health educator will explain how needs related to social determinants of health are addressed. The Discussant will place this clinical trial within the broader context of multidisciplinary team care for older adults with cognitive vulnerability led by nurse practitioners trained in geropsychiatry.

### 3D TEAM CARE MANAGEMENT TRIAL: STUDY DESIGN FEATURES AND PARTICIPANT CHARACTERISTICS

Richard Fortinsky, *University of Connecticut, Farmington, Connecticut, United States*

This clinical trial was designed in partnership with a Medicare Advantage (MA) plan, with the goal of comparing two care management approaches for its MA policyholders age >65 living with cognitive vulnerability. To test the efficacy of the 3D Team, we are using a randomized design, with the comparison group receiving telephonic care management currently offered to MA members. In this presentation, detailed aspects of the study design, characteristics of the study population, and patterns of 3D team referrals will be explained. To date, 390 older adults and 306 informal caregivers are enrolled in the trial toward a recruitment goal of 576 older adults and 380 caregivers. Among older adults, 40% have depression only, 23% have dementia only, and the rest have more than one of these conditions and/or delirium. Most common referrals by 3D Team nurse practitioners are to the community health educator and to physical and occupational therapy.

### NURSE PRACTITIONER'S ROLE AS 3D TEAM LEADER

Kathleen Obuchon, *UConn Health, Farmington, Connecticut, United States*

The nurse practitioner's (NP) clinical activities during the 12-month intervention period include 4 monthly in-home visits and 8 monthly telephone contacts. This presentation will detail the clinical assessments and activities conducted during the initial home visit, and how subsequent home visit activities and interventions are structured for older adults and their informal caregivers depending on whether older adults have dementia, depression, and/or recent delirium. Because the potential for medication-related problems is a critical concern for older adults with cognitive vulnerability, this presentation also will detail how the NP works with the 3D Team pharmacist to determine potential inappropriate medications through a review and reconciliation process, and how the NP and pharmacist summarize these results and correspond accordingly with the older adult's primary care physician. Finally, this presentation will explain how the NP manages communication among members of the 3D Team who provide interventions to the same older adult and caregiver.

### NURSE PRACTITIONER'S CLINICAL TRIGGERS FOR REFERRAL TO OTHER 3D TEAM MEMBERS

Shawn Ladda, *UConn Health, Farmington, Connecticut, United States*

This presentation features how 3D Team nurse practitioners (NP) use results of clinical assessments to determine whether older adults and caregivers enrolled in the study are referred to other Team members; these assessment results are called "clinical triggers". Other team members who receive referrals based on NP-generated clinical triggers include: Licensed Clinical Social Workers, who deliver Problem Solving Therapy to older adults with significant depressive symptoms; Occupational Therapists, who deliver an evidence-based dementia care intervention; Physical Therapists, who deliver an adapted Otago exercise program; Registered Dietician, who provides nutrition and dietary instruction; and Community Health Educator, who provides community resource information to address social determinants of health. All clinical triggers will be detailed in this presentation, along with a description of each intervention delivered by other team members except the Community Health Educator. Case studies will be presented to illustrate how study participants receive multiple interventions from the 3D Team.

### COMMUNITY HEALTH EDUCATOR'S ROLE ON THE 3D TEAM

Alba Santiago, *UConn Health, Farmington, Connecticut, United States*

As a member of the 3D Team, the bilingual, bicultural Community Health Educator (CHE) addresses needs expressed by study participants related to social determinants of health. Clinical triggers generated by nurse practitioners (NPs) that lead to CHE referral include: social isolation and loneliness; lack of transportation access; lack of resources to sustain nutritional adequacy, purchase medications, and purchase assistive devices; and cultural and linguistic barriers that lead to lack of knowledge about community resources. To date, 50% of study participants randomized to receive 3D Team care have triggered referral to the CHE. In this presentation, the team CHE will provide details on the frequency of different needs expressed by study participants, how she utilizes an ever-growing community resource directory, and specific types of information and guidance she provides to address their expressed needs. Case studies will help illustrate ways in which CHE services have successfully provided assistance to study participants.

### SESSION 7005 (SYMPOSIUM)

### ADVANCING ALZHEIMER'S DISEASE CARE AND SERVICES AMONG RACIAL AND ETHNIC MINORITIES

Chair: Lenora Smith
Discussant: Roland Thorpe, Jr.

Research shows consistent and adverse disparities among racial and ethnic minorities compared to non-Hispanic Whites in the prevalence and incidence of Alzheimer's disease, mortality, participation in clinical trials, use of medications and other interventions, health care expenditures, and quality-of-life outcomes. The literature suggests numerous underlying causes, including factors related to measurement of the disease, genetics, socioeconomic factors, cultural differences, lack of culturally competent interventions, and discrimination in services and care. Although these disparities are well known, little is known about the effectiveness of various strategies to address these differences within the context of Alzheimer's disease services and care. This symposium aims to contribute to this knowledge. The first presentation examines the role of race with marital status and risk for dementia using data from the Health and Retirement Study. Results suggest differences for unmarried White and unmarried older adults of color, which can inform dementia care services. The second presentation highlights the opportunities and challenges of facilitating cognitive impairment screenings among African American congregations. The third presentation introduces attitudes about brain donation among African American research participants and suggestions to increase involvement. The symposium concludes with a presentation on hearing care disparities in dementia with practical recommendations on how to close this gap in hearing care. The findings from these papers contribute significantly to the impact of ethnoracial differences in dementia and the need to include more diverse populations in ADRD research to promote equity. Alzheimer's Disease Research Interest Group Sponsored Symposium.

### MARITAL STATUS AND RISK OF DEMENTIA: DOES RACE MATTER?

Zhenmei Zhang,[1] Hui Liu,[2] and Seung-won Choi,[3]
*1. Michigan State University, Okemos, Michigan, United States, 2. Michigan State University, EAST LANSING, Michigan, United States, 3. Texas Tech University, Lubbock, Texas, United States*

Previous research has shown that unmarried individuals (i.e., divorced, widowed, and never married) had a higher risk of dementia than their married counterparts. However, few studies examined whether the link between marital status and dementia varies by race. To fill the gap, we used data from the Health and Retirement Longitudinal Study (2000-2014) and analyzed 15,379 respondents (13,278 non-Hispanic whites and 2,101 non-Hispanic blacks) ages 50 and older in 2000 who had no dementia. Discrete-time event history models were estimated. Our preliminary analysis showed that marital status was significantly associated with the odds of dementia for both whites and blacks. Furthermore, the associations between unmarried status (i.e., cohabiting, widowed, and never married) and dementia were stronger among blacks than whites. The effect of divorce on odds of dementia did not differ by race. The results were robust after controlling for socioeconomic status, health and lifestyle factors, and social engagements. Part of a symposium sponsored by the Alzheimer's Disease Research Interest Group.

### SCREENING FOR COGNITIVE IMPAIRMENT IN AFRICAN AMERICAN CONGREGATIONS

Fayron Epps, *Emory University, Atlanta, Georgia, United States*

Having access to memory screenings is pivotal to early detection in the African American (AA) community. The purpose of this report is to describe the willingness and perceived barriers of AA congregants to participate in memory screenings. Out of 283 attendees to dementia-related church forums, 26% (n = 73) of the attendees participated in private memory screenings. The majority of the participants were female (88%, n = 64). Under half of the participants (37%, n = 27) scored below normal with 81% (n =22) being female. Several attendees declined the opportunity to have their memory screened for various reasons. These results support how women are disproportionately affected by cognitive impairment. Another alarming point was the low participation in memory screenings of event attendees. This report is important because it raises awareness of the need within the AA community, who are at a higher risk for memory loss, to receive screening. Part of a symposium sponsored by the Alzheimer's Disease Research Interest Group.

### ATTITUDES ABOUT BRAIN DONATION AMONG AFRICAN AMERICAN RESEARCH PARTICIPANTS

Deborah Dyslin,[1] Sara Dunlop,[1] Brenda Aldridge,[2] Robin Tillotson,[3] and Darby Morhardt,[1] *1. Northwestern University, Chicago, Illinois, United States, 2. SouthEast (Atlas) Regional Senior Center, Chicago, Illinois, United States, 3. Chicago Department of Family & Support Services, Chicago, Illinois, United States*

Alzheimer's and related dementias (ADRD) disproportionately affect the African American community. Brain donation, a crucial part of translational research, is less common among African American research participants compared to White research participants at Alzheimer's Disease Research Centers (ADRCs) across the US. Existing literature suggests three categories of contributory factors for African Americans: concerns and misconceptions about brain research and brain donation; religious beliefs; and the role of the family. Existing knowledge of community interventions is limited. We conducted seven focus groups, stratified by brain donation intent and cognitive status, to capture the perspectives of African American research participants. Qualitative content analysis reveal the following contributory themes: personal connection to memory loss or dementia; altruism; spirituality/religion; historical and current racism in health care and research; trauma and objectification; trust; representation; understanding the purpose and process of brain donation; and fluidity in decision-making. Future research will explore trauma-informed and culturally responsive interventions. Part of a symposium sponsored by the Alzheimer's Disease Research Interest Group.

### HEARING CARE DISPARITIES IN DEMENTIA: ACCESS AND USABILITY IN THE COMING ERA OF OTC HEARING AIDS

Carrie Nieman,[1] Jennifer Deal,[2] Sara Czaja,[3] and Esther Oh,[1] *1. Johns Hopkins University School of Medicine, Baltimore, Maryland, United States, 2. Johns Hopkins Bloomberg School of Public Health, Baltimore, Maryland, United States, 3. Weill Cornell Medicine, New York, New York, United States*

Age-related hearing loss is highly prevalent among persons with dementia (PwDs) and is associated with an increased risk of neuropsychiatric symptoms. However, few use hearing aids and disparities exist. PwDs and, in particular, minority older adults, have some of the lowest rates of hearing aid use. Recent federal legislation created the designation of over-the-counter hearing aids, which will debut by 2020-2021, and represents an opportunity to advance accessibility. This presentation will share estimates of hearing aid use among community-dwelling PwDs from two cohorts, where hearing aid use ranges from 7-11% among African Americans versus 33-45% among whites. To explore this gap, the presentation will share findings from semi-structured interviews with care partners of PwDs and hearing loss around barriers and facilitators of hearing care, including device usability. With growing understanding of sensory health, a changing hearing care landscape represents a critical opening to increase access to hearing care for PwDs. Part of a symposium sponsored by the Alzheimer's Disease Research Interest Group.

### SESSION 7010 (SYMPOSIUM)

### ADVERSE OUTCOMES, POLYSUBSTANCE USE, AND POLYPHARMACY IN OLDER VETERANS

Chair: Amy Byers

This session will provide information about adverse health outcomes, including suicide, suicide attempts and unintended death, that may be related to polysubstance use and polypharmacy in older adults, particularly older veterans. It will further provide information that will help support late-life suicide prevention and intervention efforts. Older veterans (age50 and older) have the highest number of lives lost to suicide, make up majority of the veteran population, and are highly likely to experience conditions (e.g., chronic pain, sleep disorders, musculoskeletal) associated with commonly prescribed medications that are potential markers for suicide risk (hereafter referred to as "high-risk" drug categories), including benzodiazepines, sedative-hypnotics, opioids, antidepressants, antipsychotics, and antiepileptics. The research presented in this session will highlight important patterns in high-risk drug prescribing and use and related outcomes in late life. The presentations will underscore various groups of older veterans that may be important to consider and, yet, neglected in the polysubstance and polypharmacy and suicide prevention conversation, including those who recently attempted suicide, veterans with late-life posttraumatic stress disorder (PTSD), and older veterans transitioning from prison to community. Drs. Maust and Morin will present findings on prescribing and use of high-risk medications in late life, including an overview of trends in polypharmacy and associations with suicide attempts. Drs. Byers and Barry will speak about older veterans with PTSD and those with a history of incarceration, with information on suicide, unintended death by overdose, and substance use disorder-related emergency department visits and hospitalizations, emphasizing importance of care transition models for prevention. Aging, Alcohol and Addictions Interest Group Sponsored Symposium.

### PTSD, RISK OF SUICIDE, AND UNINTENDED DEATH BY OVERDOSE IN LATE LIFE

Amy Byers,[1] Yixia Li,[2] Shira Maguen,[1] and Thomas Neylan,[1] *1. University of California, San Francisco / San Francisco VA Health Care System, San Francisco, California, United States, 2. Northern California Institute for Research and Education, San Francisco, California, United States*

Little is known about late-life posttraumatic stress disorder (PTSD) and risk of suicide and apparent accidental death by overdose. We studied 488,044 older veterans (50 and older) with PTSD and propensity-matched comparison group without PTSD (n=488,044), seen in VA 2012-2013 followed to 12/31/2016. There were 5,693 non-fatal and fatal suicide attempts for those with PTSD and 4,310 for those without PTSD (approximately 20% fatal for each group). Those with PTSD had nearly 2-fold increased risk of any attempt [HR=1.55 (95% CI=1.49-1.62)]. While results specific to death by suicide were non-significant, impact of PTSD on method of death (e.g., drug overdose, firearms, hanging) was significant only for drug overdose [HR=1.58 (95% CI=1.22-2.03)]. Intentional and unintentional death by narcotics and such drugs as non-opioid analgesics and autonomic nervous system drugs were most highly associated with late-life PTSD. This study provides important implications for late-life suicide prevention related to PTSD and cause-specific drugs. Part of a symposium sponsored by the Aging, Alcohol and Addictions Interest Group.

### RISING CNS POLYPHARMACY AMONG OLDER ADULTS IN THE UNITED STATES

Donovan Maust, *University of Michigan, Ann Arbor, Michigan, United States*

CNS-polypharmacy is defined by the AGS Beers Criteria as using 3 or more individual medications from the following classes: antidepressants, antipsychotics, benzodiazepines, other sedative/hypnotics, opioids, antiepileptics. Dr. Maust will review data suggesting that such prescribing has increased among older adults, along with the data suggesting there are associated harms. In addition, he will review recent evidence from the Department of Veterans Affairs, which suggests that older Veterans who use both Medicare and the VA system for medical care are at higher risk of potentially inappropriate CNS-active prescribing. Part of a symposium sponsored by the Aging, Alcohol and Addictions Interest Group.

### HIGH-RISK MEDICATION USE AND POLYPHARMACY IN OLDER VETERANS WHO ATTEMPT SUICIDE

Ruth Morin,[1] Yixia Li,[2] Michael Steinman,[3] Ilse Wiechers,[4] and Amy Byers,[3] *1. John D. Dingell VA Medical Center, Detroit, Michigan, United States, 2. Northern California Institute for Research and Education, San Francisco, California, United States, 3. University of California, San Francisco / San Francisco VA Health Care System, San Francisco, California, United States, 4. Office of Mental Health and Suicide Prevention, Department of Veterans Affairs, Menlo Park, California, United States*

Late-life veteran suicide is a public health concern, and may overlap with recent high-risk medication use. We identified use in the 6 months prior to attempt and assessed salient risk factors. 13,872 veterans aged 50 years and older that attempted suicide were compared with demographically-matched controls utilizing VHA healthcare in a similar time period. Medications potentially related to suicide risk were included. Other variables were psychiatric and medical diagnoses, fatality of attempt and means. Compared with controls, veterans who attempted were nearly 3 times more likely to have been prescribed benzodiazepines and opioids, even when controlling for other diagnoses. Those taking 3 or more high-risk medications were between 7 and 11 times more likely to attempt than controls, with a higher risk of death particularly by drug overdose. These findings begin to uncover the complex contribution of prescription medications and polypharmacy to late-life veteran suicide, with implications for prevention. Part of a symposium sponsored by the Aging, Alcohol and Addictions Interest Group.

### SUBSTANCE USE DISORDER–RELATED HOSPITALIZATIONS AND ED USE IN THOSE RETURNING TO COMMUNITY FROM PRISON IN LATER LIFE

Lisa Barry,[1] David Steffens,[2] Kenneth Covinsky,[3] Yeates Conwell,[4] and Amy Byers,[5], *1. UConn Center on Aging, Farmington, Connecticut, United States, 2. University of Connecticut School of Medicine, Farmington, Connecticut, United States, 3. University of California San Francisco, San Francisco, California, United States, 4. University of Rochester, Rochester, New York, United States, 5. University of California, San Francisco / San Francisco VA Health Care System, San Francisco, California, United States*

High rates of substance use disorders (SUDs) in persons age 50 and older are concerning. Those reentering the community in later life after incarceration are especially at risk. We determined if later-life prison release increases risk of SUD-related hospitalizations and ED visits in a national sample of veterans (N=7,671) released from prison between 2012 to 2014 and matched never-incarcerated controls (N=7,671). Later-life prison release was associated with increased risk of any SUD-related hospitalization/ED visit (2907.1 vs. 465.0 per 100,000/year; adjusted HR=2.67; 95% CI, 2.11-3.36) and 3-fold risk of hospitalizations/ED visits due to alcohol use disorder (1955.4.1 vs. 282.6 per 100,000/year; adjusted HR=3.04; 95% CI, 2.24-4.13) and drug use disorder

(1586.1 vs. 252.0 per 100,000/year; adjusted HR=3.09; 95% CI, 2.23-4.30). Those reentering the community in later life after prison are at higher risk of experiencing SUD-related hospitalizations or ED visits. Prevention and intervention efforts targeting later-life prison-to-community care transitions are needed. Part of a symposium sponsored by the Aging, Alcohol and Addictions Interest Group.

### SESSION 7015 (SYMPOSIUM)

### BEYOND SEX: GENDER, LGBTQ, AND ALZHEIMER'S DISEASE AND RELATED DEMENTIAS

Chair: C. Elizabeth Shaaban
Discussant: Michelle Mielke

Sex and gender are important sources of variation in Alzheimer's disease and related dementias (ADRD) and associated caregiving. Women comprise 2/3 of ADRD cases and the majority of ADRD caregivers. Sex encompasses biological differences due to sex chromosomes, reproductive tract, and hormones, while gender constitutes socioculturally constructed psychosocial aspects of sex. Several lines of research have begun to interrogate sex differences, but less is known about the relation of gender and lesbian, gay, bisexual, transgender, and/or queer (LGBTQ) status with ADRD. In this symposium featuring both trainees and faculty we highlight novel research addressing these factors from multiple perspectives. Two presentations address how psychosocial characteristics and their strengths of association with brain health may vary by gender. C. Elizabeth Shaaban presents analyses testing whether gendered psychosocial factors explain sex differences in white matter hyperintensities, a neuroimaging marker of cerebral small vessel disease and risk factor for ADRD. Justina Avila-Rieger presents results testing region of birth-based spatial patterning of dementia risk among Black men and women. Next, Jason Flatt presents prevalence estimates of subjective memory problems and dementia and describe factors associated with dementia among LGBTQ older adults. Finally, gender may also impact perceptions of individuals with dementia. Shana Stites explores gender differences in AD stigma and discuss implications for who is willing to be an AD caregiver. Michelle Mielke, an expert in sex and gender differences in neurodegenerative and age-associated diseases will facilitate conversation about these results and place them in the context of current sex and gender-based ADRD research.

### DO GENDERED PSYCHOSOCIAL FACTORS EXPLAIN SEX DIFFERENCES IN WHITE MATTER HYPERINTENSITIES?

C. Elizabeth Shaaban,[1] Caterina Rosano,[1] Heather Shattuck-Heidorn,[2] Sara Godina,[3] Minjie Wu,[1] and Andrea Rosso,[4] *1. University of Pittsburgh, Pittsburgh, Pennsylvania, United States, 2. Women and Gender Studies Program, University of Southern Maine, Portland, Maine, United States, 3. University of Pittsburgh Graduate School of Public Health, Pittsburgh, Pennsylvania, United States, 4. School of Public Health, University of Pittsburgh, Pittsburgh, Pennsylvania, United States*

Women have a greater burden than men of white matter hyperintensities (WMH), a marker of cerebral small vessel disease (cSVD). Psychosocial factors including education, household income, neighborhood socioeconomic status (nSES), happiness, and depression may differ by gender and could explain women's higher burden of WMH. In a cohort of older adults (N=250, median age=82, 58% women, 39% Black), we found that women had lower education, household income, nSES and were less happy and more depressed. Race stratified Spearman correlations showed women had greater whole brain WMH volume in white participants only (white: rho=0.23, p=0.004; Black: rho=-0.05, p=0.64). In partial Spearman correlations, education, happiness, and depression attenuated but did not fully explain the relationship when added individually or all together to the model for whites (fully adjusted rho=0.19, p=0.03). Gendered psychosocial factors may partially explain sex differences in WMH; interventions targeting these factors may reduce cSVD burden, particularly in white women.

### DIFFERENTIAL ASSOCIATION OF GEOGRAPHICAL REGION OF BIRTH WITH DEMENTIA RISK ACROSS BLACK WOMEN AND MEN

Justina Avila-Rieger,[1] Audrey Murchland,[2] Nika Seblova,[3] Maria Glymour,[4] Adam Brickman,[5] Nicole Schupf,[3] Richard Mayeux,[5] and Jennifer Manly,[5] *1. University of New Mexico, New York, New York, United States, 2. University of California, San Francisco, San Francisco, California, United States, 3. Columbia University Medical Center, New York, New York, United States, 4. University of California, San Francisco, California, United States, 5. Columbia University, New York, New York, United States*

Risk of dementia is both racially and spatially patterned. Less is known about sex/gender differences in pathways linking birth place to late-life cognitive outcomes in older non-Latino Blacks. The 1464 Black men and women included in these analyses were Northern Manhattan residents. Cox regressions revealed that Stroke-Belt South (SB) and Non-Stroke-Belt South (NSB) birth was associated with a higher dementia risk, adjusted for birth year, childhood SES, and risk of death. Compared to Northern-born (NB) men, SB men had the highest risk, followed by NSB women and SB women, while NSB men and NB women had a similar risk to NB men. The higher risk for SB men and NSB women remained after adjusting for education, adult income, and CVD burden. Future work should identify why birth in the SB is uniquely detrimental for cognitive health among Black men, while birth in NSB has the strongest impact on Black women.

### THE EPIDEMIOLOGY OF DEMENTIA IN LGBTQ OLDER ADULTS

Jason Flatt, *University of Nevada, Las Vegas, School of Public Health, Las Vegas, California, United States*

Over 3 million or more adults aged 60 + live in the US who identify as lesbian, gay, bisexual, transgender, and/or queer (LGBTQ). Less is known about dementia risk in LGBTQ older adults. We will discuss dementia risk and related risk factors among LGBTQ adults from multiple population-based and cohort studies. We found higher rates of subjective memory problems among lesbian, bisexual and transgender adults compared to both gay men and heterosexual men and women. Using medical record data, 8% (343) of LGB adults aged 60+ were diagnosed with dementia. They were more likely to identify as male (63% vs. 44%), had a higher education level (college degree+ 63% vs.

40%) and were younger than their non-LGB counterparts. These findings highlight dementia risk and related problems among LGBTQ older adults. Future studies are needed to better understand dementia risk and recruiting, screening and improving dementia-related outcomes in LGBTQ older adults.

### GENDER DIFFERENCES IN HOW THE AMERICAN PUBLIC REACTS TO A PERSON WITH MILD-STAGE DEMENTIA

Shana Stites,[1] Emily Largent,[1] Jeanine Gill,[1] Rebecca Johnson,[2] Kristin Harkins,[1] and Jason Karlawish,[1]
*1. University of Pennsylvania, Philadelphia, Pennsylvania, United States, 2. Princeton University, Princeton, New Jersey, United States*

Many studies show that caregivers for those with Alzheimer's Disease (AD) are disproportionately female, but few studies have investigated how public attitudes influence this gender disparity. We analyzed secondary data from an experimental study of public reactions to AD dementia. Analysis included 944 respondents who read a vignette about a man with mild stage dementia and completed a modified Family Stigma in Alzheimer's Disease Scale (FS-ADS), which assesses 7 domains of stigma. Multivariable ordered logistic regression compared men and women on FS-ADS ratings. Women were less likely than men to endorse stronger negative aesthetic attributions (OR=0.75) and negative feelings (OR=0.76) and more likely to endorse stronger feelings of pity (OR=1.33; all $p<0.05$). No other differences were observed in FS-ADS domains (all $p>0.05$). The findings offer insights into relationships between gender and AD stigma, which may influence who is willing to become a caregiver for persons with AD and related dementias.

### SESSION 7020 (SYMPOSIUM)

### BREATHING WELL ACROSS THE LIFESPAN: PULMONARY AGING AND GEROSCIENCE-TARGETED THERAPIES

Chair: Jason Sanders

Excellent pulmonary function is one of the strongest predictors of longevity across animal models and human populations. Unfortunately, none of the major age-associated pulmonary diseases – obstructive lung disease, pulmonary fibrosis, and increased susceptibility to pneumonia – have strongly effective disease modifying therapies. There is growing evidence that normal age-associated decline in pulmonary function and major age-associated pulmonary diseases are linked to the hallmarks of aging including senescence, nutrient signaling dysregulation, mitochondrial dysfunction, and telomere disorders. This presents opportunities for collaboration between gerontologists and pulmonologists to unravel age-associated developmental mechanisms and design novel treatments. In this symposium, leaders in pulmonary aging research will present novel data on links between aging and pulmonary health and geroscience-based interventions under study. Dr. Sanders will provide an overview of the scientific and clinical space and present epidemiologic associations between aging biomarkers, early pulmonary fibrosis, and mortality. Dr. Le Saux will discuss senescence and specifically how eicosanoid biology may explain organ-specific patterns of senescence-associated fibrosis. Dr. Thannickal will discuss age-associated perturbations in metabolism and mitochondrial function and targeting these pathways to improve lung function and treat pulmonary diseases. Dr. Newton will discuss mechanisms and clinical applications of telomere biology to pulmonary aging. Symposium attendees will (1) be poised to generate collaborations between gerontologists and pulmonologists to address existing knowledge gaps in mechanisms of pulmonary aging, and (2) develop a better understanding of translational opportunities to design geroscience-based diagnostics and therapeutics to improve pulmonary health with aging.

### ASSOCIATIONS BETWEEN AGING-RELATED BIOMARKERS, INTERSTITIAL LUNG ABNORMALITIES, AND MORTALITY

Jason Sanders, *Brigham And Women's Hospital, Wellesley, Massachusetts, United States*

Interstitial lung abnormalities (ILA) exist in ~10% of adults >50 and associate with increased morbidity/mortality. Their pathobiology is poorly understood; age is the strongest risk factor. In the Framingham Heart Study, we determined associations between ILA and 10 blood biomarkers previously robustly associated with aging and mortality. Odds of ILA increased directly with ln-transformed GDF15 (OR [95% CI] = 3.20 [1.74-5.91], p=0.0002), TNF-αRII (2.41 [1.34-4.34], p=0.003), IL6 (1.76 [1.39-2.22], p<0.0001), insulin (1.56 [1.11-2.20], p=0.01), and CRP (1.53 [1.27-1.84], p<0.0001). Causal analysis showed GDF15 (p=0.008), TNF-αRII (p=0.004), and IL6 (p<0.0001) mediate the age effect on ILA. In adjusted survival models, only higher ln(GDF15) and ln(TNF-αRII) were associated with mortality (HR [95% CI] = 4.3 [2.3-8.1], p<0.0001 and 2.9 [1.5-5.8], p=0.002). GDF15 results were replicated in the COPDGene Study. These results suggest aging biomarkers may help risk stratify adults with ILA, and unmeasured ILA may confound prior associations between biomarkers and mortality.

### SENESCENCE AND ITS ROLE IN FIBROSIS

Claude Le Saux, *University of California San Francisco, San Francisco, California, United States*

The presence of senescent cells (epithelial and mesenchymal) in fibrotic organs has been well established. Removal of senescent cells in animal models of fibrosis indicate an overall beneficial effect. The general consensus is that the senescent cells contribute to the fibrotic phenotype by the secretion of factors, mainly cytokines and chemokines. We recently demonstrated that senescent cells can also secreted eicosanoids. These lipids are implicated in the pathogenesis of fibrosis in multiple organs. Prostaglandins, especially PGE2, are generally regarded as anti-fibrotic, whereas leukotrienes are thought to be pro-fibrotic. Recent studies indicate that the senescence-associated secretory profile is a dynamic process and its composition is cell, tissue, and time-dependent. In this session I will discuss how senescent cells from specific origin have the potential to regulate fibro-genesis and its resolution by switching their eicosanoid profile expression over time. These findings have important implications for emerging senolytic drugs, which have the potential to provide novel therapeutic benefits for the treatment of fibrosis.

### ENERGY SENSING PATHWAYS IN AGING AND CHRONIC LUNG DISEASE

Victor Thannickal, *University of Alabama, Birmingham, Alabama, United States*

The cause-effect relationships between the various "hallmarks of aging" and chronic lung disease are not well understood. We have determined overlapping pathways involving deregulated nutrient sensing, mitochondrial dysfunction, and cellular senescence that may contribute to the evolution of chronic lung disease. In particular, I will discuss alterations in energy/metabolic sensing pathways and mitochondrial dysfunction as pathobiological mechanisms that may explain the age-related increased susceptibility to the development and progression of idiopathic pulmonary fibrosis (IPF), a disease of pulmonary aging. I will then broaden the discussion to include the potential role of these biologic alterations in other chronic lung disease which burden older adults.

### TELOMERE DYSFUNCTION AND AGING: INSIGHTS FROM PULMONARY FIBROSIS

Chad Newton, *University of Texas Southwestern, Dallas, Texas, United States*

Telomeres are specialized genomic elements located at the ends of chromosomes that protect protein-encoding DNA from progressive loss during cellular replication. Telomeres shorten with age; therefore, telomere dysfunction is of particular relevance for understanding age-related disease mechanisms. Telomere disorders produce multisystem degenerative organ dysfunction that largely resembles aging phenotypes, including pulmonary fibrosis (PF), emphysema, cirrhosis, bone marry dysfunction, immunosenescence, and premature hair graying. The degree of telomere shortening influences age of disease onset, involved organs, and disease severity. Notably, the most common manifestation of telomere biology disorders is PF. Subsequently, PF is a model for translating telomere biology into clinical practice. I will discuss the genetics of telomere dysfunction and the clinical manifestations that overlap with age-related phenotypes, focusing on PF. Next, I will discuss how short leukocyte telomere length is an informative prognostic, and potentially theragnostic, biomarker in patients with a range of PF subtypes.

### SESSION 7025 (SYMPOSIUM)

### CAREGIVING ARRANGEMENTS AND HEALTH OUTCOMES OF CHINESE OLDER ADULTS WITH DISABILITY IN CROSS-NATIONAL SETTINGS

Chair: Jing Wang
Co-Chair: Bei Wu

This symposium focuses on the wellbeing of older adults with disability/cognitive impairment and their family caregivers. More specifically, it aims to understand how familly support, community resources utilization, internal migration, and immigrant status impact older adults' caregiving arrangement, health outcomes and end-of-life preferences and family caregivers' caregiving burden in China and the U.S. The first study explored how perceived spousal relationships and support impact dyadic experiences of living with cognitive impairment through a person-centered care lens during a three-year period. The second presentation examined the association between adult children's support and the trajectories of depressive symptom level among Chinese older adults with disabilities. The third investigated how family relationship and immigrant status matter in advanced care planning (ACP) engagement and end-of-life preferences over burial plan among US-born and foreign-born older Chinese Americans living in Honolulu, Hawaii. The fourth study study explored family caregivers' caregiving burden for community-dwelling patients with dementia and its associated factors. The last study conducted an inventory of longitudinal aging survey datasets to stimulate research on intersection of migration and caregiving arrangement. It paved the way to use existing high-quality datasets to examine the significant impact of massive rural-to-urban migration on caregiving arrangement among Chinese older adults. This symposium presents empirical evidence of the impact of family, migration and culture-related factors on caregiving arrangement and health outcomes of Chinese older adults. The presenters emphasize the importance of providing family-centered care and design culturally sensitive interventions to improve the health outcomes of older adults.

### EXISTING DATASETS TO STUDY THE IMPACT OF INTERNAL MIGRATION ON CAREGIVING ARRANGEMENTS

Hanzhang Xu,[1] Yaolin Pei,[2] Matthew Dupre,[3] and Bei Wu,[2] *1. Duke University School of Medicine, Durham, North Carolina, United States, 2. New York University, New York, New York, United States, 3. Duke University, Durham, North Carolina, United States*

Massive rural-to-urban migration in China has a significant impact on caregiving arrangements among Chinese older adults. To stimulate research on the intersection of migration and caregiving, we conducted an inventory of longitudinal aging survey datasets that included older adults from mainland China. Large public available datasets that included measures related to migration and caregiving were searched and reviewed for eligibility. Key characteristics of each dataset, including study design, sample size, and measures, were extracted. Seven eligible datasets were identified, and five included national representative samples. Measures for migration varied across datasets. Some datasets included information on the migration history of older adults, whereas others focused on the migration of adult children. Similarly, caregiving was measured using different questions in each dataset. Caregiving activities were assessed with regard to their type, source, and amount. High-quality datasets exist to support research on migration and caregiving arrangements among Chinese older adults.

### ADVANCE CARE PLANNING ENGAGEMENT AND END-OF-LIFE PREFERENCE AMONG OLDER CHINESE AMERICANS

Yaolin Pei,[1] Wei Zhang,[2] and Bei Wu,[1] *1. New York University, New York, New York, United States, 2. University of Hawaii at Manoa, Honolulu, Hawaii, United States*

The study aimed to examine how immigrant status and family relationships are associated with advanced care planning (ACP) engagement and end-of-life (EOL) preference over burial plan among older Chinese Americans, the largest subgroup of Asian Americans. Logistic regressions were used to analyze data from a survey of 430 older Chinese Americans aged 55 and older living in a Honolulu, Hawai'i. Results show that U.S.-born Chinese Americans were more likely to engage in ACP, including willingness thought of EOL care, and discussion about EOL care, and having preference over burial plan, than the foreign-born Chinese American. Family cohesion was not associated with ACP engagement and EOL preference over burial plan. Family conflict increased the possibility of ACP engagement, indicated by willingness thought of ACP, willingness discussion on ACP, and EOL preference over burial plan. The culturally sensitive educational intervention is needed to increase ACP engagement among older Chinese Americans.

### ADULT CHILDREN'S SUPPORT AND TRAJECTORY OF DEPRESSIVE SYMPTOMS AMONG DISABLED CHINESE OLDER ADULTS

Jianyun Wang,[1] Renyao Zhong,[1] Yaolin Pei,[2] and Bei Wu,[2] *1. East China Normal University, Shanghai, China, 2. New York University, New York, New York, United States*

This study aimed to examine the trajectory of depressive symptoms among Chinese older adults with disabilities and the role of adult children's support in predicting trajectory classes of depressive symptoms. Data were drawn from three waves of the China Health and Retirement Longitudinal Study (2011-2015). The sample included 1420 disabled older adults age 60+ at the baseline and completed all three waves of the data. Growth mixture model shows two-class depressive symptoms trajectories: the higher risk group (25.49%) and the lower risk group (74.51%). Logistic regression results showed that respondents who received a longer term of adult children's instrumental support were more likely to be classified in a higher risk group after controlling the covariates (OR=1.184, $p<0.05$), while financial support and the frequency of contacts were not associated with the increased level of depressive symptoms. The policy implications were also discussed in this study.

### DYADIC EXPERIENCES OF LIVING WITH COGNITIVE IMPAIRMENT THROUGH A 3-YEAR LONGITUDINAL QUALITATIVE STUDY

Jing Wang,[1] Bei Wu,[2] Eleanor McConnell,[3] Ding Ding,[4] and Kirsten Corazzini,[5] *1. Fudan University, Nantong, Shanghai, China, 2. New York University, New York, New York, United States, 3. Duke University Medical Center, Durham, North Carolina, United States, 4. Fudan University Affiliated Huashan Hospital, Shanghai, China, 5. University of Maryland, Baltimore, Maryland, United States*

The fastest growth of population living with cognitive impairment takes place in China. The estimated prevalence of cognitive impairment among older adults in China is between 13% and 20%. This study focused on persons with cognitive impairment (PWCI) and their spousal care partners to explore how spousal relationships impact dyadic experiences of living with cognitive impairment through a person-centered care lens. We conducted a longitudinal qualitative study of 10 dyads of PWCI and their care partners over three years with three data collection time points. Our findings suggest that the complexity of changing experience of living with cognitive impairment is interpreted in the dynamic nature of their spousal relationship and relationship with others, patterns of communication, daily activities and care during the extended period of cognitive decline. It is crucial to help them nurture the belief that there is a significant meaning in the journey of living with cognitive impairment.

### CAREGIVER BURDEN OF FAMILY CAREGIVERS OF PATIENTS WITH DEMENTIA IN A SHANGHAI COMMUNITY

Juan Li,[1] Zhijian Liu,[2] and You YIn,[3] *1. Naval Medical University, Shanghai, China, 2. Naval medical University, Shanghai, China, 3. Changzheng Hospital, affiliated to Naval Medical University, Shanghai*

This study explored the level of and factors related to Chinese dementia family caregiver burden. We recruited 109 dyads of patients with dementia and their family caregivers from communities in Shanghai, China. Caregiver burden was assessed by Caregiver Burden Inventory. We used multivariate linear regression model to investigate factors associated with caregiver burden, including patients' socio-demographic information, cognitive function, ADL, neuropsychiatric symptoms, caregivers' depression, coping strategy, social support and community service utilization. The level of caregiver burden was moderate (65.92±16.74). The patients' cognitive function and social support were negatively associated with caregiver burden ($\beta$=-0.850, $p < 0.01$, $\beta$=-1.787, $p$=0.044, respectively). Caregivers' depression and community service utilization were positively associated with caregiver burden ($\beta$=2.109, $p < 0.01$,$\beta$=1.215, $p < 0.01$, respectively). These findings suggest enhancing social support and reducing depression of caregivers may have the potential of decreasing caregiver burden.

### SESSION 7030 (SYMPOSIUM)

### CHANGING CULTURE AROUND SERIOUS ILLNESS: THE VA LIFE-SUSTAINING TREATMENT DECISIONS INITIATIVE

Chair: Joan Carpenter
Discussant: Robert Burke

Discussing and documenting goals of care and life-sustaining treatment decisions with seriously ill patients is a widely endorsed practice by healthcare and professional organizations. In 2018, The Veterans Health Administration (VA) initiated a new national policy to standardize such practices, the Life Sustaining Treatment Decisions Initiative (LSTDI), which included a coordinated set of evidence-based strategies and practice standards for conducting, documenting, and supporting high-quality goals of care conversations (GoCCs); staff training to enhance skills in conducting, documenting, and supporting GoCCs; standardized, durable electronic health record tools for documenting patients' goals and preferences; and monitoring and information technology tools to support implementation and improvement. In this symposium, we will describe the first 20 months of implementing the LSTDI across the VA, the largest integrated healthcare system in the US. The first paper will focus on the factors associated with documentation of a GoCC and treatment preferences. The second paper will present findings describing facilitators and barriers to implementing the LSTDI and identifying factors that promote high rates of LSTDI documentation. The third paper examines patient level outcomes associated with a documented goal of comfort care, specifically the odds of receipt of hospice/palliative care, hospitalization, or ICU admission. This symposium will provide attendees with important information regarding a wide range of individual and system strategies to enhance the care of seriously ill older adults by engaging patients with serious illness in GoCCs and documenting their preferences for treatment in durable, easily accessible notes and orders.

### VA'S LIFE-SUSTAINING TREATMENT DECISIONS INITIATIVE: FIRST 20 MONTHS OF NATIONAL IMPLEMENTATION

Mary Ersek,[1] Winifred Scott,[2] Joan Carpenter,[3] Jennifer Kononowech,[4] Ciarian Phibbs,[5] Jennifer Cohen,[6] Mary Beth Foglia,[7] and Cari Levy,[8] *1. Department of Veterans Affairs, Philadelphia, Pennsylvania, United States, 2. VA Palo Alto Healthcare System, Palo Alto, California, United States, 3. Department of Veterans Affairs, Berlin, Maryland, United States, 4. VA Ann Arbor Healthcare System, Ann Arbor, Michigan, United States, 5. VA Palo Alto Healthcare System, Menlo Park, California, United States, 6. VA Puget Sound Health Care System, Seattle, Washington, United States, 7. VA - National Center for Ethics in Health Care, Seattle, Washington, United States, 8. VA Eastern Colorado Health Care System, Aurora, Colorado, United States*

This retrospective observational study describes the first 20 months of implementing the Life-Sustaining Treatment Decisions Initiative. We examined patient and facility characteristics associated with life sustaining treatment (LST) orders template completion, including the association between template completion and the Care Assessment Need (CAN) score, which quantifies Veterans' risk of hospitalization and mortality. As of February 29, 2020, over 274,200 Veterans received at least one goal of care conversation and LST preferences documented on a template. Eighty-two percent of deceased Veterans with the highest risk of hospitalization or mortality had an LST note and order documented prior to their death. Factors that predicted a greater likelihood of LST template completion included higher CAN score, older age, nursing home stay, and being white non-Hispanic. Findings suggest that clinicians are engaging older, sicker veterans in goals of care conversations. Research is needed to understand potential disparities in LST template completion.

### BEST PRACTICES IN IMPLEMENTING THE VA'S LIFE-SUSTAINING TREATMENT DECISIONS INITIATIVE

Leah Haverhals,[1] Jennifer Kononowech,[2] Courtney Bauers,[3] Chelsea Manheim,[4] and Cari Levy,[5] *1. Department of Veterans Affairs, Denver, Colorado, United States, 2. VA Ann Arbor Healthcare System, Ann Arbor, Michigan, United States, 3. Denver-Seattle Center of Innovation for Veteran-Centered and Value-Driven Care, Aurora, Colorado, United States, 4. Rocky Mountain Regional Medical Center, Aurora, Colorado, United States, 5. VA Eastern Colorado Health Care System, Aurora, Colorado, United States*

To study use and completion of Life Sustaining Treatment (LST) templates across the Department of Veterans Affairs (VA) healthcare system, we designed a qualitative study to interview VA sites we identified who had high rates of LST template completion between July 1, 2018 (the official implementation start date) and March 2019. We then conducted site visits with two VA sites and phone interviews with nine other VA site to better describe facilitators and barriers to implementation of this new practice and identified factors influencing high rates of LST documentation completion. Researchers who conducted the interviews analyzed interview data through applying the Consolidated Framework for Implementation Research. From these efforts, we identified best practices used, including the importance of a formally appointed implementation leader, gaining leadership support, and engaging staff through education. We will share these recommendations with VA sites across the healthcare system to improve LST documentation.

### HONORING PREFERENCES—THE CONSISTENCY BETWEEN END-OF-LIFE CARE AND A VETERAN'S GOAL FOR COMFORT CARE

Joan Carpenter,[1] Winifred Scott,[2] Mary Ersek,[3] Cari Levy,[4] Jennifer Cohen,[5] Mary Beth Foglia,[6] Ciarian Phibbs,[7] and Susan Miller,[8] *1. Department of Veterans Affairs, Berlin, Maryland, United States, 2. VA Palo Alto Healthcare System, Palo Alto, California, United States, 3. Department of Veterans Affairs, Philadelphia, Pennsylvania, United States, 4. VA Eastern Colorado Health Care System, Aurora, Colorado, United States, 5. VA Puget Sound Health Care System, Seattle, Washington, United States, 6. VA - National Center for Ethics in Health Care, Seattle, Washington, United States, 7. VA Palo Alto Healthcare System, Menlo Park, California, United States, 8. Brown University, Providence, Rhode Island, United States*

This study examined the alignment between Veterans' end-of-life care and a Life-Sustaining Treatment (LST) goal "to be comfortable." It includes Veterans with VA inpatient or community living center stays overlapping July 2018--January 2019, with a LST template documented by January 31, 2019, and who died by April 30, 2019 (N = 18,163). Using VA and Medicare data, we found 80% of decedents with a comfort care goal received hospice and 57% a palliative care consult (compared to 57% and 46%, respectively, of decedents without a comfort care goal). Using multivariate logistic regression, a comfort care goal was associated with significantly lower odds of EOL hospital or ICU use. In the last 30 days of life, Veterans with a comfort care goal had 43% lower odds (AOR 0.57; 95% CI: 0.51, 0.64) of hospitalization and 46% lower odds of ICU use (AOR 0.54; 95% CI: 0.48, 0.61).

### SESSION 7035 (SYMPOSIUM)

### CHARACTERIZING PHYSICAL RESILIENCE IN OLDER ADULTS: INSIGHTS FROM SECONDARY ANALYSES IN THREE CLINICAL GROUPS

Chair: Brian Buta
Discussant: Anne Newman

Resilience is defined as the ability of a physiologic system to recover from a stressor that has pushed the system into a state far from its original state of equilibrium. The level of resilience can be understood by whether or not the system's essential identity and function are retained following the stressor. The study of resilience in older adults has potential to provide clinically relevant insights into our understanding of who will or will not recover when encountering a stressful medical procedure, especially those common to older patients. The main Study of Physical Resilience and Aging (SPRING) at Johns Hopkins includes prospective data collection of determinants, phenotypes, surrogates, dynamic stimulation measures, and outcomes of resiliency among older persons undergoing knee replacement surgery, or the initiation of hemodialysis, or bone marrow transplantation. SPRING also includes analyses of existing data sources to inform these prospective studies. This symposium briefly presents the conceptual framework and design of SPRING, and focuses on the results of secondary analyses from three existing data sets that mirror the ongoing stressor studies: FORCE-TKR (knee/joint replacement, N=9006), CHOICE (dialysis initiation, N=487), and a database of patients undergoing treatment for hematologic malignancies (bone marrow transplantation, N=1011). For each clinical population, we present results on phenotypic and/or biomarker trajectories, as well as the factors associated with resilience phenotypes and how these are predictive of clinical outcomes. These analyses display the utility of resilience phenotypes for predicting risk of adverse outcomes and complement the new data being collected in our main study.

### RESILIENCE IN INCIDENT HEMODIALYSIS: CHARACTERIZATION AND OUTCOME PREDICTION

Karen Bandeen-Roche,[1] Jiafeng Zhu,[2] Deidra Crews,[3] Mara McAdams-DeMarco,[1] Brian Buta,[3] Ravi Varadhan,[3] and Jeremy Walston,[3] *1. Johns Hopkins Bloomberg School of Public Health, Baltimore, Maryland, United States, 2. School of Public Health, Johns Hopkins University, Baltimore, Maryland, United States, 3. Johns Hopkins University, Baltimore, Maryland, United States*

The Resiliency in Dialysis Initiation (ReDI) Study aims to develop physical resilience signatures in older adults initiating hemodialysis. Study design—comprising a pilot, confirmatory study, and secondary data analyses—will be presented. So also will a method for characterizing resilience phenotypes—using mixed-model analysis of SF-36 subscale trajectories—among participants of age 55 and older who had undergone hemodialysis in the Choices for Healthy Outcomes in Caring for ESRD study (n=485). Analyses revealed stable, improving, and declining phenotypes. In Cox models, both baseline phenotypic status and trajectory type predicted mortality after adjusting for age, CVD status, and CHF (global Wald test for trajectory type P-value=0.020 for vitality; 0.030 for general health). These analyses evidence usefulness of resilience phenotypes as markers of adverse outcome risk and foreshadow application to novel ReDI data.

### RISK FACTORS FOR POOR RECOVERY AFTER TOTAL KNEE REPLACEMENT AMONG OLDER ADULTS IN THE FORCE-TJR COHORT

Thomas Laskow,[1] Jiafeng Zhu,[2] Brian Buta,[1] Frederick Sieber,[1] Karen Bandeen-Roche,[3] Jeremy Walston,[1] Patricia Franklin,[4] and Ravi Varadhan,[1] *1. Johns Hopkins University, Baltimore, Maryland, United States, 2. School of Public Health, Johns Hopkins University, Baltimore, Maryland, United States, 3. Johns Hopkins Bloomberg School of Public Health, Baltimore, Maryland, United States, 4. Northwestern University Feinberg School of Medicine, Chicago, Illinois, United States*

Total knee replacement (TKR) is a common procedure in older adults with broad variability in outcomes. We sought to identify factors that contribute to resilient outcomes in 7,239 older adults (age 60 or older) who underwent TKR in the TJR-FORCE, a prospective registry of total joint replacement. Outcomes utilized were bodily pain and physical component score (PCS) from the Short Form 36 Health Survey (SF-36), at pre-op, 1-year, and 2-year post-procedure. Participants were grouped according to their outcome trajectories as “improving”, “worsening”, “variable,” or “stable.” Multinomial regression (with 4 outcome categories) was used to evaluate demographic risk factors (age, gender, BMI, marital status, education, smoking history, comorbidity count, household income). Older age, larger comorbidity count, low-income, smoking, and being unmarried were significant risk factors for poor recovery (not “improving”) in terms of bodily pain and physical component score. Next steps include evaluating risk factors for resilience outcomes in prospective studies.

### PREDICTING SURVIVAL AFTER BONE MARROW TRANSPLANT USING TIME SERIES OF ROUTINE LABORATORY BIOMARKERS

Ravi Varadhan, Bohao Tang, Hua-Ling Tsai, and Phil Imus, *Johns Hopkins University, Baltimore, Maryland, United States*

Bone marrow transplant (BMT) is a curative therapy for patients with hematologic malignancies. However, there is still a high rate of relapse and mortality after BMT. It would be tremendously valuable if we can identify older adults at high-risk for mortality using readily available information. A number of biomarkers are routinely collected during follow-up for clinical care, but this information is seldom used in prediction models. We examined the data from 1011 patients who had BMT at Johns Hopkins between 2013 and 2019. There were 364 death over a median follow-up of 431 days. We considered 4 biomarkers: albumin, hemoglobin, lymphocytes count, and platelets. Biomarker data from one week pre-BMT to 8 weeks post-BMT was used for prediction using a random survival forest model. The model performed quite well and had a 5-fold cross-validated c-index of 0.733 (95%CI: 0.724-0.739). Routine laboratory biomarkers can help identify poorly resilient older BMT patients.

### SESSION 7045 (SYMPOSIUM)

### CULTURAL AND INSTITUTIONAL CONSIDERATIONS IN ADVANCE CARE PLANNING IN LONG-TERM CARE SETTINGS

Chair: Gloria Gutman
Co-Chair: Brian de Vries

Advance Care Planning (ACP) is a process that supports individual's understanding and sharing of personal values, life goals, and preferences regarding future medical care, so that they obtain care consistent with these during serious and chronic illness. While ACP is important for all, it is especially so for people who fall outside traditional, western, heteronormative contexts (e.g. who belong to ethnic, racial and/or sexual/gender minorities). This symposium draws from research conducted by the Diversity Access Team [part of a national project iCAN-ACP Improving Advance Care Planning for Frail Elderly Canadians]. The first paper presents results from focus groups conducted with loved ones of South Asian, Chinese and Lesbian, Gay, Bisexual, and Transgender (LGBT) older adults living in care homes; issues identified as barriers include starting ACP conversations too late ("my husband has severe dementia"), lack of consideration of cultural traditions and, in the case of LGBT older adults, their non-family support networks. The second paper draws from focus groups with care home staff, implicating their own training as a barrier to assisting residents/families with ACP as well as resident, family, institutional and cultural influences. A third paper reports on an educational intervention designed to increase staff understanding of ACP and comfort in assisting residents/families with ACP. The fourth paper reports feedback received on two ACP planning tools, reflecting the importance of minority group representation in visuals and text. Together, these papers underscore the importance of taking culture into consideration in framing and discussions of fostering ACP among minority populations.

### CULTURAL AND INSTITUTIONAL ISSUES IN ACP FOR CARE HOME RESIDENTS: PERSPECTIVES OF LOVED ONES

Brian de Vries,[1] Gloria Gutman,[2] Helen Kwan,[3] Katrina Jang,[3] Shimae Soheilipour,[2] Avantika Vashisht,[4] and Taranjot Kaur,[4] *1. San Francisco State University, San Francisco, California, United States, 2. Simon Fraser University, Vancouver, British Columbia, Canada, 3. Gerontology Research Centre, Vancouver, British Columbia, Canada, 4. Simon Fraser University Gerontology Research Centre, Vancouver, British Columbia, Canada*

Focus groups were held with family/decision makers of residents in an exclusively Chinese (EC; N=7) and a multi-ethnic (ME; N=8) care home, as well as South Asian (SA; n = 5) and lesbian, gay, bisexual and transgender caregivers (LGBT; n = 5) who had/have a loved one in a care home. Shared themes across groups included the role of the care home in Advance Care Planning (ACP) discussions, the timing of such discussions (i.e., at admission), and the extent to which another person was available and appropriate for such discussions. Issues unique to groups included superstition and the equation of ACP with funeral planning (EC), family history and regrets about not having planned (ME), gender differences and the need for education about ACP (SA) and the absence of traditional family among LGBT older adults. These themes highlight the challenges in ACP among diverse populations and the need for targeted interventions.

### INSTITUTIONAL AND CULTURAL BARRIERS TO ACP: STAFF PERSPECTIVES

Gloria Gutman,[1] Brian de Vries,[2] Helen Kwan,[3] Katrina Jang,[3] and Shimae Soheilipour,[1] *1. Simon Fraser University, Vancouver, British Columbia, Canada, 2. San Francisco State University, San Francisco, California, United States, 3. Gerontology Research Centre, Vancouver, British Columbia, Canada*

This study explored staff knowledge and engagement in assisting residents/families with ACP. Focus groups were conducted at two long-term care homes, one Exclusively Chinese (EC; n = 25); one Multi-Ethnic (ME; n = 41). In each, separate focus groups were held with registered staff, care aides, and support staff who also completed brief surveys providing socio-demographic data and information about their training and experience with ACP. Perceived barriers to engagement in ACP included limited knowledge and inadequate training in facilitating ACP, cognitive impairment of residents, language barriers, lack of openness to discussing ACP, family expectations and misinformation. EC staff also considered cultural and religious beliefs as one of the main barriers to engaging in ACP both for residents and families; staff at ME focused more on timing and the role of family. Support staff and care aides did not perceive ACP as within their scope of practice, deferring to nurses.

### EDUCATIONAL INTERVENTION FOR CARE HOME STAFF TO ENCOURAGE RESIDENT ACP

Shimae Soheilipour,[1] Gloria Gutman,[1] Brian de Vries,[2] Helen Kwan,[3] and Katrina Jang,[3] *1. Simon Fraser University, Vancouver, British Columbia, Canada, 2. San Francisco State University, San Francisco, California, United States, 3. Gerontology Research Centre, Vancouver, British Columbia, Canada*

Twenty-five Multi-Ethnic (ME) and 21 Exclusively Chinese (EC) care home staff participated in an education intervention focused on helping staff assist residents/families with goals of care conversations and other aspects of ACP in a culturally sensitive way. Surveys were completed at baseline and 6-8 week follow-up. Staff reported that the education session increased their ACP knowledge and provided strategies to help them initiate relevant ACP conversations. Registered staff (nurses, social worker, physician) demonstrated significantly higher levels of confidence to participate in and assist residents/families with goals of care conversations after compared to before training.

### CREATING INCLUSIVE ADVANCE CARE PLANNING TOOLS

Robert Beringer,[1] Gloria Gutman,[2] Brian de Vries,[3] Helen Kwan,[4] Katrina Jang,[4] Avantika Vashisht,[5] and Taranjot Kaur,[5] *1. University of Victoria, Victoria, British Columbia, Canada, 2. Simon Fraser University, Vancouver, British Columbia, Canada, 3. San Francisco State University, San Francisco, California, United States, 4. Gerontology Research Centre, Vancouver, British*

*Columbia, Canada, 5. Simon Fraser University Gerontology Research Centre, Vancouver, British Columbia, Canada*

The cultural appropriateness of two ACP tools, My Wishes, My Care, and Conversation Starter Kit, are examined. In development by the BC Centre for Palliative Care, My Wishes, My Care was reviewed with: LGBT Non-Metropolitan Older Adults (6 focus groups, N=32); English/Chinese Speaking Older Adults (1 focus group, N=9); and English/Punjabi or Hindi Speaking South Asian Older Adults (1 focus group, N=5). The Conversation Starter Kit, developed by Ariadne Labs, was reviewed with focus groups of people whose loved one was/is in a Care Home: one LGBT group (N = 5); one South Asian group (N = 5). General feedback on both tools included: concerns over level of understanding required to use the tool; the role of family in ACP planning; cultural inclusivity (e.g., images and text) and design elements. The feedback will inform revision of these tools and assist organizations in developing inclusive ACP planning guides or educational materials.

### SESSION 7055 (SYMPOSIUM)

### DIGITAL INTERVENTIONS TO PROMOTE HEALTH AND WELL-BEING OF OLDER ADULTS

Chair: Gloria Gutman
Co-Chair: Marcia Shade

The population of older adults is on the rise and so is their adoption of technology. According to the Pew Research Center, growing numbers of older adults are using smartphones, tablets, and the internet. Technology can also be an innovative and accessible way to deliver interventions that promote positive health outcomes. But implementing and evaluating digital interventions, especially those targeted to cognitively frail or otherwise vulnerable older adults, is not easy. The first presentation in this international interdisciplinary symposium sets the stage for discussing some of the barriers encountered. Specifically, it addresses the issue of compliance of adults with Mild Cognitive Impairment (MCI) while using internet-based cognitive behavioral therapy for sleep disturbance. The second presentation described a digital tool to screen and interview older adults in a hospital emergency department who may be experiencing mistreatment. This is followed by a study of use of voice assistant reminders to help aging adults adhere to daily completion of a diary to self-monitor pain symptoms. The fourth presentation describes a feasibility study of a digital screen-based device to calm long-term care home residents exhibiting disruptive Behavioral and Psychological Symptoms of Dementia. Symposium speakers and participants will be encouraged to dialogue on how some of the implementation and evaluation barriers described might be overcome or avoided as well as to share success stories from their own research on digital interventions for older adults living at home, in congregate housing or in hospital settings.

### FEASIBILITY STUDY OF A DIGITAL SCREEN-BASED CALMING DEVICE FOR MANAGING BPSD DURING CARE

Gloria Gutman,[1] Avantika Vashisht,[2] Taranjot Kaur,[2] Ryan Churchill,[2] Amir Moztarzadeh,[2] and Mojgan Karbakhsh,[2] *1. Simon Fraser University, Vancouver, British Columbia, Canada, 2. Simon Fraser University Gerontology Research Centre, Vancouver, British Columbia, Canada*

MindfulGarden (MG) is a digital device resembling a flat screen TV, with touchless sensors that react to voice and motion. In this study 13 long-term care home residents aged 74-100 exhibiting Behavioural and Psychological Symptoms of Dementia (BPSD) were randomized to treatment and control groups. On days 1-3 the treatment group received usual care plus exposure to MG during morning and evening care - events well documented to be problematic for residents and care staff; controls received usual care only. On day 4 both groups were exposed to MG with verbal cueing. A 26-item checklist was used to record frequency and types of disruptive BPSD exhibited; care duration was recorded in minutes. There was a trend toward reduction of BPSD and duration of care during morning care. Findings suggest that verbal cueing may be important for successful implementation of MG in calming residents with dementia during routine care.

### HEY GOOGLE, REMIND ME TO WRITE IN MY DIARY: VOICE ASSISTANTS FOR DAILY PAIN MONITORING

Marcia Shade,[1] Rasila Soumana Hama,[1] Kyle Rector,[2] and Kevin Kupzyk,[1] *1. University of Nebraska Medical Center, Omaha, Nebraska, United States, 2. University of Iowa, Iowa city, Iowa, United States*

Diaries can be important tools to document and communicate pain symptoms. Diary-based assessments can be prone to poor adherence and limitations with biased recall. One strategy to help adherence is to use voice assistant reminders. A sample of 15 community dwelling aging adults used the Google Assistant for reminders to complete pain self-management tasks. One task was a reminder created to write daily in a pain diary. Within the diary, participants could document a change in pain, pain severity, average and worst amount of pain, and pain relief. At follow-up, it was noted that participants adhered to writing most pain characteristics in the diaries, but there were times that each question was not answered. Participant's pain characteristics varied, which could be a helpful assessment to communicate to health providers for pain management. This feasibility study is preliminary support that voice assistants may help with the daily pain diary completion.

### DIGITAL HEALTH TOOL TO ASSIST OLDER ADULTS IN SELF-DISCLOSING ELDER MISTREATMENT

Fuad Abujarad,[1] Esther Choo,[2] Michael Pantalon,[1] Karen Jubanyik,[1] James Dziura,[1] Chelsea Edwards,[3] Gail D'Onofrio,[1] and Thomas Gill,[1] *1. Yale University, New Haven, Connecticut, United States, 2. Oregon Health & Science University, Portland, Oregon, United States, 3. Yale University School of Medicine, New Haven, Connecticut, United States*

Elder Mistreatment (EM) is a global health issue that continues to be under detected and inadequately addressed in healthcare settings. In this symposium, we will describe how we built and currently use the VOICES digital health tool to screen for EM. The tool is designed exclusively with older adults in mind and runs on iPads to be used in the emergency department. VOICES screens, educates, uses motivational interviewing to facilitate self-disclosure of EM, and refers patients for in-person screening. The tool includes multimedia components such as videos, audio and animations designed to educate and enhance screening for EM. Patients who screen positive are then guided through an automated Brief Negotiated Interview (BNI) that uses motivational interviewing to help them self-identify and self-disclose. Our tool will help identify cases of EM that otherwise may go undetected and overcomes major challenges associated with traditional methods of screening.

### USE OF TECHNOLOGY IN AN INTERNET-DELIVERED INTERVENTION FOR OLDER ADULTS WITH MILD COGNITIVE IMPAIRMENT

Meghan Mattos,[1] Laura Barnes,[1] Eric Davis,[1]
Carol Manning,[1] Mark Quigg,[2] and Lee Ritterband,[2]
*1. University of Virginia, Charlottesville, Virginia, United States, 2. University of Virginia, School of Medicine, Charlottesville, Virginia, United States*

Internet-based interventions using technology can promote access to treatment and reduce participant burden for sleep disorders. However, preliminary studies examining technology use and compliance in older adults with mild cognitive impairment (MCI) are needed prior to undertaking large-scale interventions. Older adults with MCI were recruited from hospital-based memory and sleep disorders clinics and enrolled in a single-arm intervention pilot study. An Internet-delivered cognitive behavioral therapy for insomnia program collected daily sleep diary data and delivered the automated intervention over nine weeks. Sleep diaries and wrist-worn actigraphs collected sleep data for 14 days, pre- and post-intervention. Descriptive statistics for participant technology use are presented. We have recruited 12 subjects with MCI. Most subjects with MCI accessed the intervention program daily; however, actiwatch compliance varied. Incorporating technology for intervention delivery and data collection in this population is promising, and future work should consider using reminders with wearable technology to increase compliance.

### SESSION 7060 (SYMPOSIUM)

### DOPAMINERGIC SIGNALING: DRIVER OF MOBILITY RESILIENCE AND MODERATOR OF RESPONSE TO PHARMACOTHERAPY

Chair: Caterina Rosano
Co-Chair: Andrea Rosso
Discussant: Nicolaas Bohnen

Although effective mobility is the end result of the functional capacity of multiple systems, some adults appear resilient and maintain higher mobility later in life. Epidemiological studies suggest up to 20% of adults aged 65 and older maintain fast walking speed in the presence of multi-system impairments. What could be the reason for such mobility resilience? In this symposium, we focus on the brain dopaminergic signaling as a potential driver of mobility resilience. We examine the genetic val158met polymorphism of catechol-O-methyltransferase (COMT), an enzyme regulating dopaminergic signaling in the brain. The Met allele results in higher tonic dopamine levels; the Val allele results in lower tonic dopamine levels. The influence of COMT polymorphism on mobility is known for Parkinson's disease (PD) and neurodegenerative conditions, but it not clear for asymptomatic adults aged 65 living in the community. We present data from the Cardiovascular Health Study, the Health Aging Body Composition Study, and several neuropharmachological studies with extensive information on PD ascertainment, medications, and health characteristics. We test the hypothesis that the Met allele of the COMT gene is associated with a) lower fall risk (Rosso), b) slower decline in gait reserve (Sprague), c) less gait slowing due to frailty (Mannon), d) more favorable response to pharmacotherapy in older adults without PD (Bohnen). Our results suggest a unique modulatory capacity of dopaminergic-related genes that may favor mobility resilience and predict better response to therapy. Our data provide clues for novel targeted interventions to delay physical disability in older adults.

### ANTICHOLINERGIC MEDICATION USE, DOPAMINERGIC GENOTYPE, AND RECURRENT FALLS

Andrea Rosso,[1] Xiaonan Zhu,[2] Zachary Marcum,[3]
Nico Bohnen,[4] Briana Sprague,[5] and Caterina Rosano,[5]
*1. School of Public Health, University of Pittsburgh, Pittsburgh, Pennsylvania, United States, 2. University of Pittsburgh Graduate School of Public Health, Pittsburgh, Pennsylvania, United States, 3. University of Washington, Seattle, Washington, United States, 4. University of Michigan, Ann Arbor, Michigan, United States, 5. University of Pittsburgh, Pittsburgh, Pennsylvania, United States*

Anticholinergic medications (A-chol) increase risk for falls; higher dopaminergic signaling may provide resilience to these effects. In 2489 older adults (mean age=74; 52% women) with 10 years of data on medication use, falls, and dopaminergic genotype (catechol-O-methyltransferase (COMT)), we assessed the association of A-chol use with recurrent falls (≥2) over the subsequent 12 months using generalized estimating equations. Effect modification by COMT (met/met, higher dopamine signaling, n=473 vs val carriers, lower dopamine signaling, n=2016) was tested; analyses were then stratified by COMT and adjusted for demographics and A-chol use indicators. During follow-up, 843 people reported recurrent falls. A-chol use doubled the odds of recurrent falls (OR [95%CI]=2.13[1.74, 2.60]), with a suggested effect modification by COMT (p=0.1). The association was present in val carriers (adjusted OR [95%CI]=1.93[1.36, 2.73]) but not in met/met (adjusted OR [95%CI]=1.30[0.53, 3.22]). Higher dopaminergic signaling may provide protection against the effects of A-chol use on fall risk.

### DOPAMINE-RELATED GENOTYPE PREDICTS TRAJECTORIES OF GAIT RESERVE IN OLDER ADULTS

Briana Sprague,[1] Andrea Rosso,[2] Xiaonan Zhu,[3] and
Caterina Rosano,[1] *1. University of Pittsburgh, Pittsburgh, Pennsylvania, United States, 2. School of Public Health, University of Pittsburgh, Pittsburgh, Pennsylvania, United States, 3. University of Pittsburgh Graduate School of Public Health, Pittsburgh, Pennsylvania, United States*

The capacity to increase one's gait speed is critical for maintaining safe community ambulation. There is limited work on the longitudinal changes in this capacity and its predictors. Because lower dopamine is associated with lower task adaptation and motivation, we hypothesized that lower dopamine would predict more decline in rapid gait speed. Catechol-O-methyltransferase (COMT) polymorphism and at least 3 repeated rapid and usual pace gait speed assessments were obtained over 10 years in 1,261 older adults (mean age=75.2, 867 White, 659 women). Linear mixed models computed person-specific rapid and usual pace gait speed trajectories. Regression models adjusted for usual gait trajectory tested whether COMT predicted rapid gait trajectory; covariates included, demographic, psychological, cognitive, and physical factors. Val/Val carriers (lower dopamine) declined more in rapid gait compared to Met/Met carriers (higher dopamine; adjusted b=-.002, SE=.001, p=.042). Modifying dopamine may positively influence the ability to maintain rapid gait over time.

### CATECHOL-O-METHYLTRANSFERASE GENOTYPE, FRAILTY, AND GAIT SPEED: THE CARDIOVASCULAR HEALTH STUDY

Shannon Mance,[1] Andrea Rosso,[2] Joshua Bis,[3] Stephanie Studenski,[4] Nico Bohnen,[5] and Caterina Rosano,[4] *1. University of Pittsburgh Graduate School of Public Health, Pittsburgh, Pennsylvania, United States, 2. School of Public Health, University of Pittsburgh, Pittsburgh, Pennsylvania, United States, 3. University of Washington, Seattle, Washington, United States, 4. University of Pittsburgh, Pittsburgh, Pennsylvania, United States, 5. University of Michigan, Ann Arbor, Michigan, United States*

The association of COMT with gait speed varies across studies; frailty, a condition increasing vulnerability to stressors, may influence this association. Cross-sectional data was obtained in 3,744 participants (71 years, 82% white, 39% male) for gait speed, frailty (Fried definition), and COMT. Multivariable regression models of COMT predicting gait were adjusted for demographics, chronic conditions, and locomotor factors. Interactions of COMT by frailty and by race predicting gait speed were p=0.03 and p=0.02, respectively. Compared to Met/Met, the Val/Val group walked marginally more slowly in the full cohort (0.87 vs 0.89 m/sec, p=0.2); differences were significant for those with frailty (n=220, 0.55 vs 0.63m/sec, p=0.03), but not for those with moderate (n=1691, 0.81 vs 0.81m/sec, p=0.9), or no frailty (n=1833, 0.98 vs 0.97 m/sec, p=0.7). Associations were similar by race, but significant for whites only. Studies should assess the influence of dopaminergic signaling on gait slowing due to frailty.

### DOPAMINERGIC VULNERABILITY AND MOTOR IMPAIRMENTS IN OLDER ADULTS: NEED FOR TARGETED PHARMACOTHERAPY

Nicolaas Bohnen, *University of Michigan, Ann Arbor, Michigan, United States*

Motor impairments, including slow walking, are common in older age in the absence of prototypical Parkinson's disease (PD). The etiology of such disturbances is heterogeneous and multi-system in nature. Dopamine is a key neurotransmitter involving motor, cognitive and behavioral circuitry. Normal aging is associated with substantial dopaminergic losses in the brain. Dopaminergic vulnerability of aging can be augmented by genotypic changes that may further compromise dopaminergic signaling, such as polymorphisms in the COMT gene. These observations may augur dopaminergic pharmacotherapy studies to treat gait disturbances in older adults. Prior dopaminergic therapy studies have shown overall limited effects in non-PD older adults. A major limitation of these studies is the non-targeted selection calling for personalized medicine approaches. Recent dopaminergic treatment studies targeting specific sub-groups of non-PD older adults conducted by us and others will be discussed.

### SESSION 7065 (SYMPOSIUM)

### DYADS IN DEMENTIA CARE: AN INTERNATIONAL PERSPECTIVE ON RESEARCH CHALLENGES AND OPPORTUNITIES

Chair: Marie Boltz
Co-Chair: Karin Wolf-Ostermann
Discussant: Katie Maslow

Dementia poses a societal challenge that is life-changing not only for persons with dementia (PWD) but also for family members and friends (informal carers) directly involved in the care arrangement. Informal carers (IC) have typically poorer outcomes in terms of well-being, quality of life (QoL), health status, and use of health care resources. Dyads of PWD and IC living with dementia are characterized by strong reciprocal relationships and complex living contexts. Therefore, research should investigate home based dementia caregiving from a dyadic perspective to yield interventions that support the PWD, the IC, and the unit as a whole. However, it is an ongoing challenge to investigate dyadic needs and preferences in daily practice and develop effective interventions. Challenges are related to incomplete understanding of dyadic characteristics, attitudes and beliefs within the dyad, as well as how to adapt research approach to engage and retain the dyad in research. This international symposium will therefore address these issues. The first presentation will describe a typology of dementia care dyad characteristics and needs in Germany. The second presentation will examine the challenges and opportunities associated with recruiting and retaining dementia dyads. The third presentation will explore ethical challenges posed in communication with dyads and possible solutions for the researcher. The final presentation reports on the Meeting Centre Support Program as an example of an effective psychosocial intervention employing research strategies that transcend cultural barriers. Our discussant, Katie Maslow, will synthesize the presentations and lead a discussion of future directions for policy and practice.

### WHO CARES FOR WHOM AND IS THAT IMPORTANT? A TYPOLOGY OF DEMENTIA CARE DYADS AND HEALTH-RELATED SUPPORT MEASURES

Karin Wolf-Ostermann,[1] Henrik Wiegelmann,[2] Liane Schirra-Weirich,[3] Lisa-Marie Verhaert,[4] Werner Brannath,[2] and Farhad Arzideh,[2] *1. University of Bremen, Bremen, Germany, 2. University of Bremen, Bremen, Bremen, Germany, 3. Catholic University of Applied Sciences, Köln, Nordrhein-Westfalen, Germany, 4. Catholic University of Applied Sciences, Koln, Nordrhein-Westfalen, Germany*

In Germany, about 70% of all care-dependent persons are community dwelling. In dementia care arrangements you will commonly find a primary informal caregiver (IC)

taking on the decisive role in providing care and support for the person with dementia (PwD). This study aimed to develop a typology of dyads based on typical characteristics of the PwD, the IC and their relationship and to gain a better understanding of home-based dementia care arrangements. A latent class analysis was used to detect different dyad types based on personal, social, care and disease characteristics of 551 dyads of CGs and PwD living at home. A 6-class model was identified. The classes could be differentiated based on IC-PwD key characteristics (gender, age, relationship, living situation, occupation). There are significant differences with regard to observed outcomes. The verification of different types of dyads strengthens the need to develop tailored dyad-centred interventions in dementia care

### THE CHALLENGES OF RECRUITING AND RETAINING DYADS OF HOSPITALIZED PERSONS WITH DEMENTIA AND THEIR FAMILY CARERS

Marie Boltz,[1] and Ashley Kuzmik,[2] *1. Penn State University, University Park, Pennsylvania, United States, 2. Pennsylvania State University, University Park, Pennsylvania, United States*

Persons with dementia (PWD) have high rates of hospitalization, and along with their family caregivers (FCGs), commonly experience negative hospital experiences and outcomes. The recruitment and retention challenges encountered in an ongoing cluster randomized clinical trial in PWDs and FCGs are described. The trial tests the efficacy of a nurse-FCG partnership model that aims to improve: 1) the physical and cognitive recovery in hospitalized PWD, and 2) FCG preparedness and anxiety. Recruitment and retention challenges, identified in team meetings and extracted from team documentation,. include factors in the hospital environment, the PWD, and FCGs. Strategies that address these challenges include careful pre-planning and preparation with the site, strong communication with dyads, and honoring preferences for communication. The recruitment and retention of acutely ill older adults with dementia and FCGs can pose a challenge to investigators and threaten the validity of findings. Recruitment and retention strategies that help improve validity are described

### TO LIE OR NOT TO LIE: THE VIEWS OF PEOPLE WITH DEMENTIA AND THEIR CARERS

Dympna Casey,[1] Una Lynch,[2] Adeline Cooney,[3] Catherine Houghton,[4] Siobhan Smyth,[5] Fionnuala Jordan,[5] and Heike Felzman,[6]
*1. National University of Ireland Galway, Galway, Ireland, 2. Sonista, Banbridge, Northern Ireland, United Kingdom, 3. Maynooth University, Maynooth, Galway, Ireland, 4. National University of Ireland Galway, Galway, Galway, Ireland, 5. School of Nursing & Midwifery, National University of Ireland Galway, Galway, Galway, Ireland, 6. School of Humanities, National University of Ireland Galway, Galway, Galway, Ireland*

In Ireland over 36,000 people with dementia live at home cared for by informal carers. Yet often these carers do not know how to deal with cognitive symptoms, including repeated questions wherein 'truthful'responses cause distress. Carers face a dilemma, do they avoid, distract or 'correct' the person and tell the 'truth', or lie? This paper explores the concept of lying from the perspective of the carer and person with dementia. A descriptive qualitative methodology was used. Focus groups with a purposive sample of people with memory problems (n = 14) and carers (n = 18) were conducted. The results found that deliberate lying with the intention to deceive was deemed unacceptable by all. However, in certain circumstances telling a 'good lie' or 'white lie' was considered acceptable when the carer knew the person and the intention behind the 'lie' was to mitigate the distress of the person with dementia.

### THE MEETING CENTRE SUPPORT PROGRAMME: INTERNATIONAL EVALUATION OF A DYADIC INTERVENTION IN DEMENTIA

Dorota Szcześniak,[1] Katarzyna Lion,[2] Franka Meiland,[3] Dawn Brooker,[4] Elisabetta Farina,[5] Rabih Chattat,[6] Rose-Marie Dröes,[7] and Joanna Rymaszewska,[8]
*1. Wroclaw Medicla University, Wrocław, Dolnoslaskie, Poland, 2. Menzies Health Institute Queensland Griffith University, Nathan, Queensland, Australia, 3. University Medical Centers, VU University Medical Center, Amsterdam, Noord-Holland, Netherlands, 4. Association for Dementia Studies, University of Worcester, Worcester, England, United Kingdom, 5. IRCCS Fondazione Don Carlo Gnocchi, Milan, Lombardia, Italy, 6. University of Bologna, Bologna, Emilia-Romagna, Italy, 7. Amsterdam Public Health research institute, Amsterdam, Noord-Holland, Netherlands, 8. Wroclaw Medical University, Wrocław, Dolnoslaskie, Poland*

In Europe, 10 million people are living with dementia. Most of them reside in their own home, cared for by their loved ones. As a consequence, there is a great need to provide both, people with dementia and their carers, tailored support. The Dutch Meeting Centres Support Programme (MCSP), adaptively implemented in three European countries within the JPND-MEETINGDEM project, is an excellent example of an effective dyadic psychosocial intervention, which seems to have no cultural barriers. The mixed-methods analysis showed that participant-dyads reported great satisfaction with MCSP. People with dementia experienced improvement of their quality of life, motivation and ability to participate in everyday activities, as well as improvement in their relationship with family members. Carers felt less burdened and highly appreciated the emotional and social support they received. Repeated user evaluation shows that this dyadic support effectively helps people with dementia and their families better deal with dementia.

### SESSION 7070 (SYMPOSIUM)

### EFFECT OF VITAMIN D SUPPLEMENTATION ON FALLS, PHYSICAL FUNCTION, AND ACTIVITY: RESULTS FROM THE STURDY TRIAL

Chair: Jennifer Schrack
Co-Chair: Lawrence Appel
Discussant: Lewis Lipsitz

Each year, 2.8 million older adults are treated for falls, with over 800,000 hospitalized. Evidence suggests vitamin D supplementation might reduce the risk of falls, potentially through improvements in skeletal muscle function; however, results are inconsistent. In 2013 the NIA issued a request for applications to assess the efficacy and dose-response of vitamin D supplementation for fall prevention across a range of doses and serum 25(OH)D concentrations, resulting in the funding of STURDY (Study To Understand Fall Reduction and Vitamin D in You). STURDY was a seamless dose-finding and confirmatory, double-masked, response adaptive Bayesian randomized trial designed to find the best dose of vitamin D supplementation for fall prevention. Participants (n=688, ≥70 years with serum 25(OH)D of 10-29 ng/mL) were randomized to 200 (control), 1000 , 2000, or 4000 IU/day of vitamin D3.The first participant was randomized on 10/30/2015 and data collection ended on 5/31/2019. The primary outcome was time to first fall or death, and the secondary outcome was gait speed. Dr. Appel will present the main findings of the effect of vitamin D supplementation on time to first fall. Dr. Wanigatunga will present a more detailed analysis of the effect of vitamin D supplementation on fall characteristics, including indoor vs. outdoor falls, consequential falls, and repeat fall risk. Dr. Guralnik will present the effect of vitamin D supplementation on physical functioning, including gait speed, SPPB, 6-minute walk, and TUG performance. Dr. Schrack will present the effect of vitamin D supplementation on objectively measured physical activity.

### DESIGN AND MAIN RESULTS OF STURDY: A RANDOMIZED CLINICAL TRIAL OF FOUR VITAMIN D3 DOSES TO PREVENT FALLS IN OLDER ADULTS

Lawrence Appel,[1] Jennifer Schrack,[2] Erin Michos,[3] Christine Mitchell,[2] Stephen Juraschek,[4] Edgar Miller,[3] and David Roth,[5] *1. Johns Hopkins University School of Medicine, Baltimore, Maryland, United States, 2. Johns Hopkins Bloomberg School of Public Health, Baltimore, Maryland, United States, 3. Johns Hopkins School of Medicine, Baltimore, Maryland, United States, 4. Harvard Medical School, Boston, Massachusetts, United States, 5. Johns Hopkins University, Baltimore, Maryland, United States*

STURDY was a Bayesian, response-adaptive trial with dose-finding and confirmatory stages. Participants (n=688; ≥70years with serum 25(OH)D of 10-29ng/mL) were randomized to 200 (control), 1000, 2000, or 4000 IU/day of vitamin D3. The primary outcome was time to first fall or death over 2 years. During dose-finding, the best non-control dose was determined to be 1000IU/day based on higher primary outcome event rates in the 2000 and 4000IU/day doses than the 1000IU/day dose (posterior probability of being best dose=0.90; hazard ratios[HR] were 1.86 [95%CI: 1.16-2.97] and 1.68 [95%CI: 1.05-2.69], respectively). Participants were then switched from other non-control doses to 1000IU/day, and event rates did not differ between the pooled higher doses and control groups (HR=1.02, P=0.84). There was no heterogeneity by baseline 25(OHD). In conclusion, high-dose vitamin D supplementation ≥1000IU/day did not prevent falls. Whether vitamin D doses >2000IU/day increase the risk of falls is uncertain.

### VITAMIN D SUPPLEMENTATION ON DETAILED FALL CHARACTERISTICS

Amal Wanigatunga,[1] Alice Sternberg,[2] Amanda Blackford,[3] Yurun Cai,[1] Jennifer Schrack,[1] Edgar Miller,[4] David Roth,[5] and Lawrence Appel,[6] *1. Johns Hopkins Bloomberg School of Public Health, Baltimore, Maryland, United States, 2. Johns Hopkins School of Public Health, Baltimore, Maryland, United States, 3. Johns Hopkins School Of Medicine, Baltimore, Maryland, United States, 4. Johns Hopkins School of Medicine, Baltimore, Maryland, United States, 5. Johns Hopkins University, Baltimore, Maryland, United States, 6. Johns Hopkins University School of Medicine, Baltimore, Maryland, United States*

Evidence suggests Vitamin D supplementation may reduce fall risk in older adults, but effects on fall location and severity are less well described. We used STURDY trial data to examine whether Vitamin D supplementation reduces indoor, outdoor, “consequential” (falls resulting in injury or medical care), and repeat fall risk. Participants (77[SD=5.4] years; 44% women) were randomized to receive 200 (n=339) or 1000IU/day (n=349) of vitamin D3. Indoor, outdoor and consequential fall rates were similar between the ≥1000IU/day and 200IU/day groups (rate ratio [RR]:1.22, 95%CI:0.96-1.55; RR:0.85, 95%CI:0.65-1.10; and RR:1.16, 95%CI:0.93-1.45, respectively) during follow-up. The proportion of repeat fallers was similar between ≥1000IU/day versus 200IU/day groups over 3 months (7.8%[27/346] versus 6.5%[22/336], p=0.22), 6 months (18.8%(60/319) versus 16.2%(51/315), p=0.40), 12 months (29.9%(81/271) versus 31.2%(84/269), p=0.78) and 24 months (48.2%(66/137) versus 49.6%(66/133), p=0.90). In conclusion, Vitamin D supplementation ≥1000IU/day did not reduce indoor, outdoor, consequential or repeat fall risk.

### VITAMIN D SUPPLEMENTATION AND CHANGE IN OBJECTIVELY MEASURED PHYSICAL PERFORMANCE

Jack Guralnik,[1] Jennifer Schrack,[2] Amal Wanigatunga,[2] Alice Sternberg,[3] Christine Mitchell,[2] and Lawrence Appel,[4] *1. University of Maryland School of Medicine, Baltimore, Maryland, United States, 2. Johns Hopkins Bloomberg School of Public Health, Baltimore, Maryland, United States, 3. Johns Hopkins School of Public Health, Baltimore, Maryland, United States, 4. Johns Hopkins University School of Medicine, Baltimore, Maryland, United States*

Change in usual gait speed (GS) was a prespecified secondary outcome in STURDY and additional physical performance measures were also assessed. Over 12 months, gait speed declined slightly in both the 200IU/d (control) and the intervention groups receiving 1000 or more IU/d, with identical declines in both groups. However, at month 24 GS declined 0.09 m/s from baseline in controls and 0.03 m/s in the intervention group (p=0.03). Subgroup analyses of persons receiving just 1000IU/d throughout the trial compared to controls did not reach significance (p=0.07). Neither SPPB, balance, chair stand tests, or 6-minute walk test showed differences between controls and intervention at any follow-up point. The timed up and go (TUG) test showed a significantly greater improvement in the intervention group at 2 months (p=0.004) but not 12 or 24 months (p=0.17 and 0.79, respectively). Effects of long-term vitamin D supplementation on physical functioning remain unclear.

### EFFECTS OF DAILY VITAMIN D SUPPLEMENTATION ON OBJECTIVELY MEASURED PHYSICAL ACTIVITY: RESULTS FROM THE STURDY TRIAL

Jennifer Schrack,[1] Jacek Urbanek,[2] Amal Wanigatunga,[1] Stephen Juraschek,[3] Christine Mitchell,[1] Erin Michos,[4] David Roth,[2] and Lawrence Appel,[5] *1. Johns Hopkins Bloomberg School of Public Health, Baltimore, Maryland, United States, 2. Johns Hopkins University, Baltimore, Maryland, United States, 3. Harvard Medical School, Boston, Massachusetts, United States, 4. Johns Hopkins School of Medicine, Baltimore, Maryland, United States, 5. Johns Hopkins University School of Medicine, Baltimore, Maryland, United States*

Cross-sectional evidence suggests older adults with higher serum vitamin D are more physically active, but whether long-term vitamin D supplementation attenuates age-related declines in physical activity (PA) is undefined. We examined the association between vitamin D supplementation and daily PA in 639 STURDY participants (aged 77 (5.4) years; 44% women) over up to 24-months. Participants were randomized to receive 200 (n=275), 1000 (n=168), 2000 (n=59), or 4000 (n=63) IU/day of vitamin D3. PA was measured using the Actigraph Link wrist-worn accelerometer 24 hours/day for 7-days at baseline, 3, 12, and 24 months. In linear mixed models adjusted for baseline PA level, total daily PA appeared to decline ($\beta$=-43.3 counts, p=0.06) annually for all groups and there was no difference by vitamin D3 dose (p for group*time =0.14). These results suggest daily vitamin D supplementation has no effect on quantities of daily PA.

### SESSION 7075 (SYMPOSIUM)

### EFFECTS OF A NOVEL APPROACH TO REDUCING COSTLY HOSPITALIZATIONS OF NURSING HOME RESIDENTS

Chair: Ruth Tappen
Co-Chair: David Wolf
Discussant: Karen Southard

The reduction of preventable hospitalizations from long term care facilities has been identified by CMS as an important measure of quality, both in terms of resident outcomes and nursing home performance. As many as one-quarter of individuals admitted to nursing homes from acute care are rehospitalized within the month placing them at high risk for increased falls, delirium, skin breakdown, nosocomial infection and the like and costing an estimate $4.3 billion annually. To address this well documented threat to care quality, CMS has imposed significant penalties for excessive readmissions on facilities with high rehospitalization rates. An important contributor to these preventable readmissions is resident and family insistence on the transfer. Early efforts to reduce potentially preventable hospitalization of nursing home residents focused on developing systems to identify and respond to acute changes in condition before hospitalization becomes necessary. Reports from facility staff, however, brought to our attention the additional problem of resident and family insistence on transfer despite provider recommendations to the contrary. A series of funded studies to understand this problem, develop a solution and test the effectiveness of this solution will be reported by an interdisciplinary team. We begin with description of the development and clinical trial of Go to the Hospital or Stay Here?, an evidence-based, patient-centered decision aid, with individual residents and families, followed by a pilot test of facility-wide implementation; then an eight state regional dissemination supported by CMS and participating states and finally a discussion of best practices for effective implementation of the Guide.

### GO TO THE HOSPITAL OR STAY HERE?: A RANDOMIZED CLINICAL TRIAL

Sarah Worch,[1] and Ruth Tappen,[2] *1. The Ohio State University, Columbus, Ohio, United States, 2. Florida Atlantic University, Boca Raton, Florida, United States*

With the exception of guides for making end of life choices, there are very few if any patient decision aids created for residents of long-term care facilities. Further, only half of patient decision aids produced for any purpose have actually been field tested with patients and even fewer have been evaluated by providers other than the developers of the decision aid. Development of Go to the Hospital or Stay Here? was based on expert experience combined with extensive input from over 270 long-term care residents, their families and their caregivers. The initial clinical trial of this decision aid is reported in this presentation. Increased knowledge, reduced decisional conflict, increased preference for care in the nursing home when possible and a high rating of the helpfulness of the Guide were found in those who received the Guide (n=97) compared to those who did not (n=95).

### PILOT STUDY TO IMPLEMENT A RESIDENT AND FAMILY DECISION GUIDE

David Wolf,[1] and Janet Sopcheck,[2] *1. Bellarmine University, Louisville, Kentucky, United States, 2. Florida Atlantic University, Boca Raton, Florida, United States*

In this first stage of an 8 state initiative designed to assist nursing homes in reducing unnecessary hospital readmissions, 16 nursing homes were identified and invited by CMS and state agency advisors to participate in the pilot study of the effects of intervention (use of the Guide). Selected facilities received an online orientation to the project and onsite visit from project team leadership prior to launch. Pre and post implementation data were uploaded to a secure section of the project website by the facilities. Three facilities withdrew due to change in top management and a fourth facility provided incomplete data resulting in data for analysis from 12 pilot facilities. Results show the average reduction in readmissions was 31.2% for the project period as compared with the 3-month pre-project period. This presentation will include facility reports of the effect of Guide use on resident and family decision making.

### REGIONAL WORKSHOPS TO WIDELY DISSEMINATE AN INNOVATIVE PRACTICE

Debra Hain, and Armiel Suriaga, *Florida Atlantic University, Boca Raton, Florida, United States*

The second stage of the CMS Region IV project involved a series of 20 half-day workshops and five follow-up webinars to introduce Go to the Hospital or Stay Here?, the training resources available (12 videos, 5 case studies and Best Practices summary), and strategies for implementation. Altogether 1124 representatives from long-term care facilities, state agencies, ombudsman groups and hospitals attended these interactive workshops. Polled participants reported that resident and family insistence on transfer to the hospital was a major barrier to successful reduction of acute care transfers for them. Those who implemented the Guide facility-wide reported an average 28% reduction in transfers. Primary barriers to facility-wide implementation were lack of leadership, making a choice to absorb the cost of financial penalties and failure to distinguish the goal of Guide use from those of existing transfer reduction programs. The facilities found the workshops helpful preparation for effective implementation of the Guide.

### UTILIZING IMPLEMENTATION SCIENCE TO ACCELERATE ADOPTION OF A PROGRAM TO PREVENT AVOIDABLE TRANSFERS

Karen Southard,[1] and Suzie Kaye,[2] *1. The Carolinas Center for Medical Excellence (CCME), Columbia, South Carolina, United States, 2. Florida Atlantic University, Boca Raton, Florida, United States*

The research team combined their long-term care management experience with feedback from the 16 pilot nursing homes and several hundred workshop and webinar participants' reports to identify the most effective strategies for implementation of the Guide and smaller Trifold. The learning framework employed to develop these Best Practices was directed to two distinct audiences 1) the licensed staff of the facility and 2) the residents of the facilities and their families. Critical elements identified included: when and how to distribute the Guide; how the Guide can be used to support difficult conversations e.g. about end of life choices; and effective and efficient ways to train staff. The framework for discussion during the symposium will be achievement of organizational readiness and the relevance of the four E's of implementation science as it applies to long term care organizations.

### SESSION 7080 (SYMPOSIUM)

### ENGAGING PHARMACISTS IN INTERPROFESSIONAL PROGRAMS TO SUPPORT OLDER RESIDENTS OF HUD-ASSISTED HOUSING

Chair: Patricia Slattum
Discussant: Elvin Price

Medications are one of the pillar 4Ms of an age-friendly healthcare system. Ensuring that benefits of a medication regimen outweigh the risks and that medications are not contributing to meaningful outcomes such as functional or cognitive decline is a challenge for our healthcare system, particularly for older persons experiencing adverse social determinants of health, health disparities or uncoordinated care. More fully engaging pharmacists in the older person's health care team is one strategy to improve clinical, humanistic and economic outcomes. This workshop reports findings on medication management capacity among older residents of five low-income apartment buildings in urban Richmond, VA, indicating that this population experiences challenges in managing medications as measured by MedMaIDE. The second presentation describes a pilot partnership between an independent community pharmacy and a local area agency on aging to provide medication coaching to residents of six affordable housing buildings in Seattle, WA, demonstrating feasibility of engaging pharmacists in the healthcare team and resulting in an average of five interventions per resident. The third presentation describes an interprofessional educational intervention based in two low-income apartment buildings for older persons in underserved West Baltimore, MD involving pharmacy students, to better prepare pharmacists to engage in interprofessional team care and meet the needs of a low income culturally diverse older adult population. Discussion will focus on barriers and opportunities to more fully engage pharmacists to support urban residents of low-income housing buildings to optimize medication outcomes and reduce medication-related harm.

### MEDICATION MANAGEMENT CAPACITY AMONG OLDER RESIDENTS OF LOW-INCOME APARTMENT BUILDINGS

Amal Badawoud,[1] Pamela Parsons,[2] Juan Lu,[3] Emily Peron,[4] Teresa Salgado,[4] and Patricia Slattum,[5] *1. Princess Nourah Bint Abdulrahman University, Riyadh, Saudi Arabia, 2. Virginia Commonwealth, Richmond, Virginia, United States, 3. Division of Epidemiology, Virginia Commonwealth University School of Medicine, richmond, Virginia, United States, 4. Virginia Commonwealth University School of Pharmacy, Richmond, Virginia, United States, 5. Virginia Commonwealth University, Richmond, Virginia, United States*

MMC is an essential component of safe and independent living. This cross-sectional study was designed to assess MMC among low-income older persons residing in HUD-subsidized housing, located at one of five apartment buildings in urban Richmond, VA. Medication Management Instrument for Deficiencies in the Elderly (MedMaIDE) was used to measure MMC during individual, face-to-face interviews. Of the 107 participants, 89% were African-American with an average age of 68.5 years (±7.2), an average of 4.9 (±2.9) comorbidities and using approximately 8 (±4.1) medications on a regular basis. The mean deficit in MMC was 3 (±2.0). The most difficult skill was naming all of the medications (69.2%) followed by stating the indication (46.7%) and knowing how or when all of the medications should be taken or identifying existing refills (38.3%). Many older residents of low-income housing experience challenges in managing medications.

### A PHARMACIST-LED MEDICATION COACHING PROGRAM SERVING SENIOR RESIDENTS OF LOW-INCOME HOUSING

Jennifer Bacci,[1] Joshua Akers,[2] Katie Mahan,[3] Geoffrey Meer,[4] Jeffrey Kinter,[5] and Anna Shields,[6] *1. University of Washington, Seattle, Washington, United States, 2. Premera, Mountlake Terrace, Washington, United States, 3. Kelley-Ross Pharmacy, Seattle, Washington, United States, 4. Kelley-Ross Pharmacy Group, Seattle, Washington, United States, 5. Kaiser Permanente,*

*Olympia, Washington, United States, 6. Kaiser Permanente Washington, Seattle, Washington, United States*

In 2015, one independent community pharmacy partnered with the local Area Agency on Aging to provide medication coaching to low-income, culturally diverse, older adults living in 6 affordable housing buildings in the Seattle area. A pilot was conducted during the 2015-2016 fiscal year to determine the need for and feasibility of the service. Process outcomes, including patient and service demographics, medication-related problems, and pharmacist interventions, were captured via the pharmacists' patient care documentation. Pharmacists had 34 total visits with 17 unique residents who were taking an average of 8.1 medications. Pharmacists identified 97 medication-related problems, averaging 5.7 problems per resident, and performed 88 interventions, averaging 5.2 interventions per resident. The findings of this pilot demonstrated the needs and feasibility of implementing pharmacists' services within a housing organization structure and has resulted in the continuation and growth of the program.

### PREPARING PHARMACY STUDENTS FOR PRACTICE TO SUPPORT UNDERSERVED OLDER PERSONS

Daniel Mansour, *University of Maryland School of Pharmacy, Baltimore, Maryland, United States*

Interprofessional collaboration is needed to ensure high quality care. Effective programs teaching necessary team-based care skills are under investigation. This study evaluated the Aging in Place program, an interprofessional practice experience (IPE), in preparing pharmacy students for practice with underserved older persons. The Assessment of Interprofessional Team Collaboration Scale (AITCS) and Team Decision Making Questionnaire (TCMQ) were administered to students before and after their experience. The number of disciplines represented, campuses involved, resident sessions, resident interactions, and health screenings performed were documented. Overall, AITCS and TDMQ scores improved after participation in the program. Since the program's inception, there have been 7 disciplines represented, 2 campuses involved, 125 student participants, 2 housing buildings, 90 resident sessions, and 370 health screenings performed. The Aging in Place program has grown and shown that an IPE program is feasible to better prepare pharmacy students for collaborative care with older residents of affordable housing buildings.

### SESSION 7085 (SYMPOSIUM)

### FOCUS ON FUNCTION IN HOSPITALIZED PERSONS WITH DEMENTIA: THE IMPACT OF HOSPITAL-ACQUIRED COMPLICATIONS

Chair: Marie Boltz
Discussant: Lorraine Mion

Persons with dementia (PWD) are two-three times more likely to be hospitalized as persons without dementia and comprise one fourth of hospitalized older adults. Hospitalization often has a dramatic impact upon the health and disposition of the older PWD. They are at increased risk for hospital acquired complications (HAC) such as functional decline, behavioral symptoms of distress, and delirium, all of which contribute to increased disability, mortality, and long-term nursing home stays. Despite the unprecedented number of PWD admitted to acute care, little attention has focused on their specialized needs and HAC, and how they impact functional recovery. The purpose of this symposium is to describe the incidence of common HACs, and factors that influence their occurrence and presentation in PWD. Utilizing baseline findings from the Family-centered Function-focused Care (Fam-FFC) trial, the presentations will address this objective and discuss the ramifications for functional and cognitive post-acute recovery in PWD. The first presentation will describe the incidence and pharmacologic management of pain in PWD, and its association with common HACs. The second presentation will describe physical activity in PWD on medical units and the validity of the Motionwatch8 actigraphy. The third session will describe differences in common HACs between white and black PWD. The final presentation will examine function-focused goals developed in collaboration with family caregivers and patients, and the functional outcomes associated with goal attainment. Our discussant, Dr. Lorraine Mion, will synthesize the research findings and lead a discussion of future directions for policy and practice in dementia-capable acute care.

### ENGAGING FAMILIES IN FUNCTION-FOCUSED CARE: GOAL ATTAINMENT AND ASSOCIATED OUTCOMES

Marie Boltz,[1] Joanne Roman Jones,[2] and Robin Hermann,[3] *1. Penn State University, University Park, Pennsylvania, United States, 2. Pennsylvania State University, State College, Pennsylvania, United States, 3. Hospital of the University of Pennsylvania, Philadelphia, Pennsylvania, United States*

Partnering with families to develop function-focused plans for hospitalized persons with dementia (PWD) improves both the hospital experience and patient outcomes. This secondary analysis included patients enrolled in the intervention arm of the on-going Family-centered Function-focused Care (Fam-FFC).study. This study examined the goals co-established by family caregivers, PWD, and nurses to prevent hospital-acquired complications and promote functional and cognitive recovery. The influence of goal attainment upon delirium and physical function at discharge was also examined. The majority of patients (N=162) were female (65%), black (53%) with a mean age of 82.7 (SD= 8.2). Goal attainment ranged from -2 to 2; mean = -0.24 (SD= 0.75). The goals (N=432) represent three main areas: mobility, self-care, and cognitive stimulation. Controlling for age and admission function, goal attainment was associated with less discharge delirium (F=3.2, p = .022) but not discharge function. Results support the contribution of function-focused care to improving delirium outcomes.

### PAIN INCIDENCE, TREATMENT, AND ASSOCIATED SYMPTOMS IN HOSPITALIZED PERSONS WITH DEMENTIA

Barbara Resnick,[1] and Marie Boltz,[2] *1. University of Maryland School of Nursing, Baltimore, Maryland, United States, 2. Penn State University, University Park, Pennsylvania, United States*

A better understanding of the relationships between pain and other syndromes in hospitalized persons with dementia (PWD) will help establish pain as a critical symptom to target. Secondary analyses from the Fam- FFC study describe the incidence and pharmacologic management of pain, and its association with physical function, delirium, and behavioral and psychological symptoms of dementia (BPSD). The sample (N=299) was mostly female (62%), non-Hispanic (98%), and Black (53%), with a mean age of 81.6 (SD=8.5); 166 (56%) received pain medication, whereas 40% (n=43) of 108 individuals who demonstrated pain did not receive analgesics. Regression analyses showed that, controlling for age, gender, cognition, and comorbidities, pain was associated with function (t= -.3.2, p=.001), delirium (t =5.0, p < .000), and BPSD severity (t = 2.3, p=.023). Findings suggest pain may be undertreated in hospitalized PWD but should be considered to optimize function, decrease delirium, and prevent or decrease BPSD.

### PHYSICAL ACTIVITY IN HOSPITALIZED PERSONS WITH DEMENTIA: FEASIBILITY AND VALIDITY OF THE MOTIONWATCH 8

Ashley Kuzmik,[1] Barbara Resnick,[2] and Marie Boltz,[2]
*1. Pennsylvania State University, University Park, Pennsylvania, United States, 2. University of Maryland School of Nursing, Baltimore, Maryland, United States*

Interventions to prevent functional decline in hospitalized persons with dementia (PWD) require objective measures of physical activity (PA). This secondary analysis described PA using MotionWatch 8 actigraphy and considered the feasibility and validity of the MotionWatch 8 in hospitalized PWD. In the first 320 PWD enrolled in the Fam-FFC study, 261 agreed to wear a MotionWatch for 24 hours within 48 hours of admission. Minutes were recorded in sedentary ( $\bar{x}$ =1767.35, SD= 1327.43), low ($\bar{x}$ = 202.52, SD=127.78), moderate ($\bar{x}$ =7.93, SD=25.80 ), and vigorous activity ( $\bar{x}$ = .85, SD=4.50). Controlling for age, gender, race and comorbidity, counts of activity were significantly associated with ADL function (t =4.3, p <.001). Sedentary (t =-3.9, p<.001), low (t =2.8, p =.006), and moderate (t =3.0, p =.003) activity, but not vigorous activity were significantly associated with ADL function. MotionWatch 8 appears feasible and valid when evaluating PA among hospitalized PWD.

### A COMPARISON OF SYMPTOMS IN HOSPITALIZED AFRICAN AMERICAN AND WHITE PERSONS WITH DEMENTIA

Elizabeth Galik,[1] Boltz Marie,[2] Rachel Arendacs,[3] and Ashley Kuzmik,[4] *1. University of Maryland School of Nursing, Ellicott City, Maryland, United States, 2. Penn State, University Park, Pennsylvania, United States, 3. Penn State University College of Nursing, State College, Pennsylvania, United States, 4. Pennsylvania State University, University Park, Pennsylvania, United States*

There exist significant race disparities in the prevalence of dementia, with black persons with dementia (PWD) showing higher co-morbidity and more frequent hospitalizations, yet little is known how clinical presentations compare. This study compared physical function, delirium, depressive symptoms, and behavioral and psychological symptoms of distress (BPSD) in black and white PWDs when hospitalized. A multivariate analysis of covariance showed that, controlling for age, gender, cognitive status, and comorbidities, black PWD had more delirium (mean= 3.8, SD= 2.9) as compared to white PWDs (mean=2.4, SD= 2.2, F=4.8, p =.029). Additionally, black PWD had more depressive symptoms (mean= 11.7, SD= 6.7) as compared to white PWD (mean = 9.0, SD= 5.2, F=6.6, p =.011), and less improvement in functional status admission to discharge (mean =12.4, SD= 18.9) as compared to white PWD (mean=17.8, SD=18.8, F=12.3, p=.001). There were no differences in BPSD. Continued research examining factors influencing differences in race cohorts is warranted.

### SESSION 7090 (SYMPOSIUM)

### LARGE-SCALE MEASUREMENTS OF PHYSICAL ACTIVITY WITH WEARABLE DEVICES: AN INTERNATIONAL PERSPECTIVE

Chair: Jacek Urbanek
Co-Chair: Jennifer Schrack
Discussant: David Roth

In recent years the popularity and application of both research- and consumer-grade wearable physical (PA) activity monitors have witnessed substantial growth in large observational studies and clinical trials. For example, the NHANES and UKBiobank, have collected accelerometry data on thousands of participants contributing to the reputation of wearable technology overall as well as in aging-oriented research. As a result, more aging-focused studies including the Baltimore Longitudinal Study of Aging, Maastricht Study, Finnish Retirement and Aging study, and the National Health and Aging Trends Study, along with clinical trials have introduced accelerometry protocols into their design. The symposium focuses on challenges in the implementation of the objective measurements of PA into large studies on older adults. We will discuss the design of successful projects held and/or completed in the United States and Europe including: (1) types of devices, (2) size of datasets, (3) steps necessary for the successful device implementation, (4) data management and (5) statistical analyses. We will also present primary, PA-related findings in each study, together with funded or planned follow-up work. Collectively, these presentations will improve understanding of the technology and effort necessary for the successful application of objective PA monitoring and the resulting data analysis, providing a better context for investigators in the field of aging who want to introduce wearable devices into existing and upcoming research. The discussion will focus on the future of these technologies in the context of geriatric medicine and gerontology and the consequent steps essential for their best utilization and further expansion.

### THIGH-WORN ACCELEROMETER DATA IN THE MAASTRICHT STUDY

Annemarie Koster, *Maastricht University, Maastricht, Limburg, Netherlands*

This study describes the use of thigh-worn accelerometers to collect high-quality data on sedentary time and physical activity in the Maastricht Study, a prospective cohort study in the Netherlands. Data have been collected in 9000 participants, aged 40-75 years, 49% women, and 25% has type 2 diabetes by design. All participants were asked to wear an activPAL accelerometer for 7 days. Percentage sedentary, standing and stepping time of waking time were calculated. Participants had on average 6.4 valid days (≥14h of monitoring) and 90% wore the device >4 days. Men spent significantly more time sedentary than women (63.3±10.3% versus 57.0±10.1%); standing and stepping time were significantly higher in women (30.1±7.9%; 12.9±4.4%) than in men (24.7±7.4%; 12.1±4.6%). Sedentary time significantly increased with increasing age while standing time decreased; no clear age-gradient in stepping time was found.

### MULTI-SENSOR MEASUREMENT OF PHYSICAL BEHAVIOR IN THE FINNISH RETIREMENT AND AGING STUDY

Sari Stenholm, *University of Turku, Turku, Finland*

Retirement is a life transition that is accompanied by changes in time use and removal of work-related exposures. The Finnish Retirement and Aging study (FIREA) was established in 2013 to examine changes in 24-hour physical behavior, lifestyle factors, health and functioning by following aging workers annually from full-time work to retirement. FIREA activity substudy includes 1200 participants (mean age 63.2 years, 85% women) and their physical behavior has been measured annually with multiple accelerometers (wrist ActiGraph, thigh Axivity and hip SenseDoc including GPS). The mean number of measurement days is seven at each measurement wave with a valid wake wear time 16.0 hours before retirement and 15.6 hours after retirement. Currently, information is available from 3-5 measurement waves. Transition to retirement induced changes in 24-h physical behavior towards increased sleep and less physical activity among women, especially in those retiring from manual occupations.

### SESSION 7095 (SYMPOSIUM)

### NUTRITIONAL MEDIATORS OF CELLULAR DECLINE AND MITOCHONDRIAL DYSFUNCTION IN OLDER ADULTS

Chair: Roger Fielding

Aging is the primary risk factor for progressive loss of function, onset of disease, and increased vulnerability to negative health-related outcomes. These clinical manifestations arise from a decline in mitochondrial and metabolic processes considered the hallmarks of aging. Collectively, these changes can be defined as age associated cellular decline (AACD) and are often associated with signs and symptoms such as fatigue, reduced strength and low physical activity. This symposium will explore mechanisms, clinical signs, and emerging nutritional interventions for AACD. Dr. Feige's presentation will serve as an introduction by highlighting mechanisms underlying functional declines in skeletal muscle with aging. He will discuss the Multi-Ethnic Molecular determinants of Sarcopenia (MEMOSA) study, which found impaired mitochondrial bioenergetic capacity in skeletal muscle of older adults with sarcopenia compared to age-matched controls, and identified mitochondrial function as a key target for intervention. Dr. Guralnik will discuss the connection between cellular changes and clinical manifestations of AACD. He will report on an expert consensus study group which developed an initial framework to identify self-reported symptoms and observable signs of AACD in adults over50 years. Lastly, Dr. Singh will discuss the evidence for nutritional interventions to address sources of AACD, focusing on those targeting mitochondrial dysfunction. Recent research on dietary interventions with urolithin A (an activator of mitophagy) and nicotinamide riboside (an NAD+ booster) will be reviewed. Overall, this symposium will highlight key mechanisms and clinical signs of AACD, and the potential for novel nutrition interventions to support cellular function and healthy aging.

### EARLY DETECTION OF AGE-ASSOCIATED CELLULAR DECLINE: REPORT OF AN EXPERT CONSENSUS

Jack Guralnik,[1] Matteo Cesari,[2] Ariel Beresniak,[3] Leocadio Rodriguez-Manas,[4] and Antonio Cherubini,[5]
*1. University of Maryland School of Medicine, Baltimore, Maryland, United States, 2. University of Milan, Milan, Lombardia, Italy, 3. Data Mining International, Geneva, Geneve, Switzerland, 4. University Hospital of Getafe, Getafe, Madrid, Spain, 5. INRCA, Ancona, Marche, Italy*

Cellular processes often decline with age and cells lose their ability to function optimally, which may lead to organ-specific dysfunction and the development of systemic age-related diseases. The cellular hallmarks of aging are associated with clinical signs and symptoms and can be termed Age Associated Cellular Decline. An expert consensus study group was convened to provide an initial framework for the development of a tool for adults over 50 years old, which identifies self-reported symptoms and observable signs likely to be early and/or surrogate markers of age associated cellular decline. A total of 16 potential early signs and symptoms of age associated cellular decline were identified and need to be validated in further research.

### NUTRITIONAL STRATEGIES TO COUNTERACT MITOCHONDRIAL DYSFUNCTION AND NAD+ DEFICIENCY IN HUMAN SARCOPENIA

Jerome Feige, *Nestlé Research, Lausanne, Switzerland*

The causes of impaired skeletal muscle mass and strength during aging are well-studied in healthy populations. Less is known on pathological age-related muscle wasting and weakness termed sarcopenia, which directly impacts physical autonomy and survival. We compared genome-wide transcriptional changes of sarcopenia versus age-matched controls in muscle biopsies from 119 older men of different ethnicity. Individuals with sarcopenia demonstrate a prominent transcriptional signature of mitochondrial bioenergetic dysfunction in skeletal muscle, with low PGC-1α/ERRα signalling, and downregulation of oxidative phosphorylation and mitochondrial proteostasis genes. These changes translate functionally into fewer mitochondria, reduced bioenergetic activity, and NAD+ deficiency in sarcopenic muscle. Our results point to mitochondrial homeostasis as a key mediator of pathological muscle aging. Novel nutritional solutions enhancing muscle strength and performance by enhancing mitochondrial function are being tested clinically and will be reviewed. These include activating mitophagy with Urolithin A or restoring NAD+ levels via tryptophane/kynurenine or with nicotinamide riboside.

### EMERGING NUTRITIONAL INTERVENTIONS FOR AGE-ASSOCIATED CELLULAR DECLINE

Anurag Singh, *Amazentis SA, Lausanne, Vaud, Switzerland*

Aging is associated with a progressive decline in cellular health leading to dysfunction in organs with a high metabolic demand. A key feature of age associated cellular decline is impairment of mitochondrial quality control pathways such as mitophagy. Deficits in optimal functioning of these pathways results in a compromise in cellular bioenergetics that ultimately leads to mitochondrial dysfunction. Promising nutritional interventions have emerged that boost mitochondrial health such as nicotinamide riboside (vitamin B3 precursor) and Urolithin A (gut metabolite of compounds found in pomegranates), that act via different mechanisms of action to improve overall mitochondrial health. Recent literature on the evidence behind these interventions will be presented and discussed during this symposium. We will also share recent clinical evidence from double-blind placebo-controlled studies with Urolithin A. Our results suggest that nutritional interventions such as Urolithin A are promising approaches that can be employed to manage age associated cellular decline.

### SESSION 7100 (SYMPOSIUM)

### SUPPORTING PEOPLE WITH DEMENTIA IN THE COMMUNITY: OCCUPATIONAL THERAPY SERVICE AND REIMBURSEMENT INNOVATIONS

Chair: Michael Lepore
Co-Chair: Erin Long
Discussant: Richard Fortinsky

The wide range of services needed to support a safe and quality life among people living with dementia at home is growing and extends beyond the bounds of traditional reimbursement models. Within the context of a health care system that is not designed to reimburse for these types of services, federal grants from the Alzheimer's Disease Programs Initiative (ADPI) funded by the Administration for Community Living (ACL) have supported the delivery of home- and community-based services (HCBS) for people with dementia and their care partners with a pragmatic emphasis on sustainability, such as establishing successful reimbursement pathways. Drawing lessons from ACL's ADPI program and from the Health Resources & Services Administration Geriatric Workforce Enhancement Program and Geriatrics Academic Career Award program, this symposium examines opportunities and strategies for providing services to people living with dementia in the community and highlights occupational therapy as a valuable dementia care service that has potential for sustainable delivery and opportunities for professional expansion. Papers address needed workforce development for delivering HCBS to diverse populations living with dementia and examine occupational therapy roles in delivering HCBS to persons with dementia and their care partners. Additionally, papers examine the implementation and outcomes of evidence-based occupational therapy and interprofessional interventions for persons living with dementia in the community. Reimbursement mechanisms for occupational therapy services delivered to people with dementia in the community are described. Discussion addresses how these innovative interventions and reimbursement mechanisms align with the recent surge of National Institute on Aging funding for pragmatic trials.

### OCCUPATIONAL THERAPY FOR PEOPLE LIVING WITH DEMENTIA IN THE COMMUNITY: WORKFORCE DEVELOPMENT OPPORTUNITIES

Michael Lepore,[1] Erin Long,[2] and Joan Weiss,[3] *1. LiveWell Alliance, Southington, Connecticut, United States, 2. Administration for Community Living, Washington, District of Columbia, United States, 3. Health Resources and Services Administration (HRSA), Washington, District of Columbia, United States*

Establishing a workforce capable of delivering evidence- and team-based HCBS for the growing population impacted by dementia is of growing public health importance. Occupational therapy (OT), in collaboration with other disciplines, offers promise for supporting people with dementia to live well at home. However, neither uptake of team-based programs for people with dementia nor availability of providers who work in teams to support people with dementia are well understood. We reviewed information from three federal programs to improve understanding of team-based workforce development needs and opportunities. Findings indicate that interprofessional evidence-based interventions for people with dementia are increasingly implemented but geographically limited and development of the OT workforce's dementia capability is nascent in interprofessional/interdisciplinary training. OT is a key profession delivering evidence-based HCBS for people with dementia, but substantial opportunity exists for workforce development, including education, training, financing, recruitment, retention, care coordination, and translation and implementation of effective care.

### POST-DIAGNOSTIC SUPPORT AND OCCUPATIONAL THERAPY PROGRAM FOR COMMUNITY-BASED DEMENTIA SERVICES

Stephani Shivers,[1] Julie Robison,[2] Erica DeFrancesco,[3] Caroline Kate Keefe,[3] Deidre Sommerer,[3] Christine Bailey,[4] and Alis Ohlheiser,[5] *1. LiveWell Alliance, Plantsville, Connecticut, United States, 2. University of Connecticut, Farmington, Connecticut, United States, 3. LiveWell Dementia Specialists, Plantsville, Connecticut, United States, 4. Center on Aging | UConn Health, Farmington, Connecticut, United States, 5. UConn Health, Farmington, Connecticut, United States*

Service gaps and the absence of a clear-cut care/symptom management pathway for people recently diagnosed with dementia and their family carepartners motivated LiveWell Dementia Specialists to implement a multi-service post diagnostic support program including three occupational therapy (OT) interventions. Program services include an education series on 'Resilient Living with Dementia', family coaching and topical education sessions, and OT services including Care of Persons with Dementia in their Environments (COPE), Skills2Care®, and Home Based Memory Rehabilitation. Program services promote adoption of adaptive strategies and action steps to increase carepartner capacity and enhance quality of life among people with dementia. Participants complete assessments at baseline, program completion, and 4- and/or 10-month follow-up. Carepartners show improvements in dementia knowledge (mean baseline score = 24.6, 4-month = 26.0) and preparedness for caregiving (mean baseline score = 18.1, 4-month = 21.9). Program elements and adaptations of COPE for real world practice are discussed.

### MEASURING IMPACT AND SUSTAINABILITY OF EVIDENCE-BASED DEMENTIA CARE TREATMENT IN THE COMMUNITY

Jill Cigliana,[1] Kari Burch,[1] Nancy Mueller,[2] and Smriti Bajracharya,[2] *1. Memory Care Home Solutions, St. Louis, Missouri, United States, 2. Washington University in St. Louis Brown School, St. Louis, Missouri, United States*

Through a collaborative agreement with the Administration for Community Living, Memory Care Home Solutions translated the evidence-based program (EBP) Care of Persons with Dementia in their Environments (COPE) into community practice as a covered benefit through Medicare and commercial health insurance for people living with dementia. While many studies have demonstrated the efficacy of family-centered behavioral interventions for this population, few of these interventions have been translated from academic studies into successful community practice. This session will describe the process of translating an EBP into community practice utilizing social work and occupational therapy interventionists with a diverse population of people living in both urban and rural areas of Missouri and Illinois, including modifications required and lessons learned in rural service delivery. Attendees will gain an understanding of the translation process, differential treatment outcomes for people living with dementia and care partners and Medicare billing mechanisms for sustainability.

### OCCUPATIONAL THERAPY INNOVATIONS IN HOME AND COMMUNITY PRACTICE FOR PEOPLE LIVING WITH DEMENTIA

Catherine Piersol,[1] and Scott Trudeau,[2] *1. Thomas Jefferson University, Ardmore, Pennsylvania, United States, 2. American Occupational Therapy Association, Bethesda, Maryland, United States*

Most Americans with dementia live at home and families are commonly responsible for overseeing the daily activities of the person with dementia. Families require support, education, and skill-building to manage caregiving responsibilities. Occupational therapist are ideally suited to teach care partners tailored strategies for obtaining the “just right fit” between the capacities of the person and the demands of the environment, thus reducing behavioral symptoms, optimizing function and safety and enhancing well-being. Care of Persons with Dementia in their Environments (COPE) is an evidence-based intervention designed to address these outcomes. Over three phases, the occupational therapist employs a problem-solving method to identify strategies that address caregiver-reported difficulties related to managing daily activities, behavioral challenges and other caregiver concerns. This presentation describes the development and implementation of COPE highlighting the distinct approaches of occupational therapy in delivering home- and community-based services to persons living with dementia and their care partners.

### SESSION 7105 (SYMPOSIUM)

### THE AGING BRAIN: CROSSROAD OF NORMAL AGING AND DEMENTIA

Chair: Qu Tian
Discussant: Luigi Ferrucci

The functional consequences of the aging brain include several aspects of physical and cognitive decline that ultimately cause loss of mobility and dementia. Although in certain individuals, the cognitive and physical correlates of the aging brain occur in parallel, others show decline in one of these functional parameters. Underlying mechanism of this complex process that lead to different manifestations is not well understood. Proposed mechanisms include brain structural changes, tau pathology, specific white matter degeneration, metabolic derangement mostly including lipids metabolism and others. This symposium aims to address the complexity of the aging brain by showcasing studies that span the continuum from normal aging to dementia using data from the Baltimore Longitudinal Study of Aging (BLSA). The wealth of neuroimaging and phenotype data from the BLSA provides the unique opportunity to investigate neural substrates and predictors of aging phenotype, and mechanisms of age-related neurodegeneration and pathology, such as dementia, and loss of mobility. First, we identify multimodal neuroimaging predictors of important aging phenotypes of gait decline (Sargent/Tian) and memory decline (Bilgel). Second, using advanced quantitative MRI technology, we investigate underlying mechanisms of age-related white matter degeneration through potential oligodendrocyte metabolism (Bouhrara). Third, we demonstrate unique cognitive and neuroimaging profiles of dual memory and gait decline (Tian) and neural substrates for bile acids, the primary cholesterol breakdown products (Varma) in relation to dementia. We seek to generate discussions of mechanisms of the aging brain that connect the age-related phenotypes, such as decline of mobility and cognition, to the development of dementia.

### COGNITIVE AND NEUROIMAGING PROFILES OF INDIVIDUALS WITH DUAL DECLINE IN MEMORY AND GAIT

Qu Tian,[1] Susan Resnick,[1] Christos Davatzikos,[2] Stephanie Studenski,[3] and Luigi Ferrucci,[1] *1. National Institute on Aging, Bethesda, Maryland, United States, 2. University of Pennsylvania, Philadelphia, Pennsylvania, United States, 3. University of Pittsburgh, Pittsburgh, Pennsylvania, United States*

Across 6 aging cohorts we showed that dual decline in memory and gait speed was associated with increased risk of dementia compared to memory or gait decline only. We now characterize dual decliners. Using longitudinal BLSA data, we examined associations of phenotypic groups with changes in cognition, depressive symptoms, and brain volumes in areas important for cognitive (dorsolateral prefrontal, medial temporal) and motor functions (precentral gyrus,striatum,tha lamus,anterior cingulate cortex) using linear mixed effects models (usual agers=reference), adjusting for covariates. Compared to usual agers, dual decliners had faster decline in card rotation score, greater increase in CES-D, and greater atrophy in thalamus and anterior cingulate cortex. Rates of change in these parameters did not differ among the other three groups. Dual decliners experience faster decline in visuospatial ability, greater atrophy in selected motor areas, and greater increase in depressive symptoms, suggesting potential mechanisms underlying increased dementia risk in dual decliners.

### MULTIMODAL NEUROIMAGING PREDICTORS OF GAIT DECLINE

Lana Sargent,[1] Qu Tian,[2] Mike Nalls,[2] Andrew Singleton,[2] Stephanie Studenski,[3] Susan Resnick,[2] and Luigi Ferrucci[2]
*1. Virginia Commonwealth University School of Nursing, Richmond, Virginia, United States, 2. National Institute on Aging, Bethesda, Maryland, United States, 3. University of Pittsburgh, Pittsburgh, Pennsylvania, United States*

Prior studies suggesting associations between cortical brain areas and gait speed has been largely cross-sectional and limited to one modality neuroimaging. Using machine learning from 506 cognitively normal BLSA participants aged 55+ who had repeated measures of brain volumes, diffusion tensor imaging (DTI), and gait speed, we examined multimodal neuroimaging predictors of gait decline, accounting for demographics, body composition, and grip strength. Significant predictors of gait decline included changes in volumes and DTI measures of gray matter in selected frontal, parietal, temporal, and subcortical areas, as well as white matter changes in both fractional anisotropy and diffusivity of tracts connecting frontal areas to subcortical motor areas. This predictive model highlights the importance of atrophy and microstructural deterioration in selected frontal and subcortical motor areas in predicting gait speed decline.

### MEDIAL TEMPORAL TAU PATHOLOGY IS ASSOCIATED WITH VERBAL MEMORY

Murat Bilgel,[1] Jacob Ziontz,[2] Andrea Shafer,[1] Luigi Ferrucci,[1] Dean Wong,[2] and Susan Resnick,[1]
*1. National Institute on Aging, Bethesda, Maryland, United States, 2. Johns Hopkins University School of Medicine, Baltimore, Maryland, United States*

Medial temporal tau pathology is frequently observed in individuals over 70 regardless of cognitive status. To understand the link between tau and cognitive performance, we evaluated tau pathology using 18F-flortaucipir positron emission tomography among 95 cognitively normal participants from the Baltimore Longitudinal Study of Aging. We examined tau levels in early Braak regions (entorhinal cortex and hippocampus) in relation to verbal episodic memory performance concurrent with and prior to the tau scan using linear mixed effects models adjusted for age, sex, amyloid status, and time from PET scan. Higher hippocampal tau burden had a trend-level association with lower concurrent memory performance ($p=0.05$). Greater tau pathology in the hippocampus and entorhinal cortex was associated with steeper decline in memory performance prior to tau scan ($p=0.013$ and $0.026$, respectively). These findings suggest that therapeutic interventions targeting tau pathology may need to be administered early among cognitively normal individuals to prevent memory decline.

### CEREBRAL MICROSTRUCTURE IN AGING USING ADVANCED QUANTITATIVE MRI

Mustapha Bouhrara, Nikkita Khattar, Richard Kim, Wenshu Qian, Joseph Alisch, Luigi Ferrucci, Susan Resnick, and Richard Spencer, *National Institute on Aging, Bethesda, Maryland, United States*

White matter (WM) maintenance through oligodendrocyte metabolism is an energy-intensive process. Myelin homeostasis is sensitive to hypoxia, ischemia, or hypoperfusion. Besides substrate delivery, adequate cerebral blood flow (CBF) is crucial for removal of metabolic byproducts such as iron. While some data show decreased myelin content and CBF with aging, the association between CBF and myelination is unknown. Further, breakdown of oligodendrocyte and iron may potentiate myelin loss through oxidative mechanisms. Whether iron deposition is related to myelination is unclear. Using advanced MRI methodology, we investigated associations between CBF deficits, myelin loss, and iron deposition in cognitively normal individuals. We found significant association between i) CBF deficits and myelin loss (N=67,age24-88), ii) myelin loss and iron accumulation (N=92,age21-94), and iii) CBF deficits and iron accumulation (N=35,age22-88) in critical brain structures. This work may lay the foundation for further investigations of age-related WM degeneration and markers to differentiate normal from abnormal alterations.

### BILE ACIDS IN DEMENTIA: BRAIN AMYLOID, WHITE MATTER LESIONS, AND ATROPHY

Vijay Varma,[1] Youjin Wang,[2] Yang An,[1] Sudhir Varma,[3] Murat Bilgel,[1] Jimit Doshi,[4] Cristina Legido-Quigley,[5] and Madhav Thambisetty,[6] *1. National Institute on Aging, Bethesda, Maryland, United States, 2. NCI, Bethesda, Maryland, United States, 3. HiThru Analytics, Laurel, Maryland, United States, 4. UPenn, Philadelphia, Pennsylvania, United States, 5. King's College London, London, England, United Kingdom, 6. NIA, Baltimore, Maryland, United States*

While Alzheimer's disease (AD) and vascular dementia (VaD) may be accelerated by hypercholesterolemia, the mechanisms underlying this association is unclear. Using a novel, 3-step study design we examined the role of cholesterol catabolism in dementia by testing whether 1) the synthesis of the primary cholesterol breakdown products (bile acids (BA)) were associated with neuroimaging markers of dementia; 2) pharmacological modulation of BAs alters dementia risk; and 3) brain BA concentrations and gene expression were associated with AD. We found that higher serum concentrations of BAs are associated with lower brain amyloid deposition, slower WML accumulation, and slower brain atrophy in males. Opposite effects were observed in females. Modulation of BA levels alters risk of incident VaD in males. Altered brain BA signaling at the metabolite and gene expression levels occurs in AD. Dysregulation of peripheral cholesterol catabolism and BA synthesis may impact dementia pathogenesis through signaling pathways in the brain.

### SESSION 7110 (SYMPOSIUM)

### ENGAGING UNDERREPRESENTED CAREGIVING COMMUNITIES IN DEMENTIA RESEARCH

Chair: Andrea Gilmore-Bykovskyi
Discussant: Ishan Williams

Family and friend caregivers of persons with dementia from underrepresented and traditionally underserved backgrounds are significantly underrepresented in dementia and caregiving research despite heightened disease risk, poorer outcomes, and disproportionate use of services within these populations. Efforts to develop and disseminate methods that foster greater inclusion of underrepresented caregiving populations in research are essential to ensuring that culturally specific understandings, priorities, and needs of these groups are systematically understood and addressed. In this symposium, we present a variety of studies that illustrate successful efforts to include dementia caregivers from underrepresented backgrounds in research. Two presentations focus on African American caregivers, one on caregivers residing in highly under-resourced areas, one on Latino caregivers, and one on sexual and gender minority (SGM) caregivers. The first presentation describes a capacity building approach through African American faith communities to develop a research registry and address informational needs regarding dementia. The second presentation focuses on eliciting African American caregivers experiences of crisis events. Presentation three describes a coalitional, community-informed approach to engaging caregivers in highly under-resourced areas to investigate experiences with post-acute care. The fourth presentation describes a community-network approach to implementing a text-message based support intervention among Latino caregivers; and the fifth presentation illustrates the utility of digital methods for engaging SGM dementia caregivers. Collectively, these presentations demonstrate a variety of approaches to engaging dementia caregivers from underrepresented and traditionally underserved backgrounds in research that are specific to individual communities and local contexts – as well as the findings that result from these efforts.

### ENGAGING DEMENTIA CAREGIVERS IN UNDER-RESOURCED AREAS TO EXAMINE DISPARITIES IN POST-ACUTE CARE QUALITY AND ACCESS

Andrea Gilmore-Bykovskyi,[1] and Laura Block,[2]
*1. University of Wisconsin-Madison, Madison, Wisconsin, United States, 2. University of Wisconsin-Madison School of Nursing, Madison, Wisconsin, United States*

Dementia disproportionately impacts racial/ethnic minorities and individuals from under-resourced environments, yet these groups are under-represented in research. For caregivers, managing dementia often involves navigating frequent post-acute care (PAC) transitions. Despite evidence of segregation-associated disparities in PAC access and quality, the perspectives of caregivers from under-resourced areas regarding these disparities and how they are experienced, are poorly understood. We engaged a coalitional, community-informed approach to engaging caregivers in highly under-resourced areas to elicit experiences surrounding PAC through semi-structured interviews (N=25; 65% African American; 25% White; 88% female). Data were analyzed using thematic analysis. Caregivers spontaneously connected issues in PAC quality to racial/ethnic disparities and discrimination citing differences in geographic availability, financial barriers, eligibility, access to information, and transportation. To mitigate these challenges caregivers remained highly involved in the care recipient's PAC, describing the need to continue to "advocate" and "supervise." Collectively, these perspectives can help inform future, targeted policy interventions.

### LEVERAGING DIGITAL METHODS TO ENGAGE SEXUAL AND GENDER MINORITY CAREGIVERS OF PEOPLE WITH DEMENTIA

Joel Anderson,[1] Jason Flatt,[2] Jennifer Jabson Tree,[3] Alden Gross,[4] and Karen Rose,[5] *1. University of Tennessee-Knoxville, Knoxville, Tennessee, United States, 2. University of Nevada, Las Vegas, School of Public Health, Las Vegas, California, United States, 3. University of Tennessee, Knoxville, Tennessee, United States, 4. Johns Hopkins Bloomberg School of Public Health, Baltimore, Maryland, United States, 5. Ohio State University, Colombus, Ohio, United States*

Digital methods are a way to engage marginalized populations, such as sexual and gender minority (SGM) adults. No study to date has leveraged these methods to engage SGM caregivers of people with dementia. We used digital methods to access SGM caregivers of people with dementia in our study of psychosocial measures of caregiving for recruitment and data collection. Posts on social media and online registries targeted SGM caregivers. The study landing page received 2201 views; 285 caregivers completed the survey. Participants learned of the study most frequently from Facebook (45%). The sample was 84% white, with gay (52%), lesbian (32%), bisexual (11%), and other sexual orientations (5%) and transgender (17%) caregivers represented. While we exceeded goals for inclusion of Latinx (26%) and Native American (4%) caregivers, the number of African American SGM caregivers was lower than projected (7%). Digital methods are effective for engaging SGM caregivers of people with dementia.

### PARTNERING WITH AFRICAN AMERICAN CHURCHES TO SUPPORT FAMILIES AFFECTED BY DEMENTIA THROUGH RESEARCH

Fayron Epps, *Emory University, Atlanta, Georgia, United States*

African Americans (AA) are disproportionately impacted by dementia when compared to the non-Hispanic white population, yet are significantly underrepresented in research. Often times, families in the AA community turn to their church for help when in distress. Recognizing that churches are frequently the cornerstone of AA communities, they are an ideal setting for health promotion, research, and education. However, many AA churches do not have the resources to support their congregants affected by dementia. To build capacity within churches to address brain health promotion and facilitate research access/participation, we partnered with 6 predominantly AA churches in the metropolitan Atlanta area to facilitate research and develop dementia-related programs. While stakeholders were initially reluctant, continual engagement with senior faith leaders helped to facilitate the successful development of a research registry of congregants interested in participating in faith-based and clinical research and establishment of new programs to congregants around brain health and dementia.

### CUIDATXT: A TEXT MESSAGE DEMENTIA-CAREGIVER INTERVENTION FOR LATINOS

Jaime Perales Puchalt, *University of Kansas, Fairway, Kansas, United States*

Latino family caregivers of people with dementia have low access to caregiver support. Text messaging holds potential to dramatically enhance the reach of caregiver support interventions among Latinos. This presentation will describe the CuidaTXT Project, with a special emphasis on approach to recruitment and community engagement to achieve the objectives of designing and testing the first dementia caregiver-support text message intervention for Latinos. Based on the Stress Process Framework, CuidaTXT incorporates social support and coping components including AD education, problem-solving skills training, social network support, care management and referral to community resources via tailored two-way messaging. Engagement in CuidaTXT benefited from multi-source recruitment efforts in the Latino community-network built over a three-year period. The network is comprised of senior, religious and community centers, the local media, clinics, a Latino registry and a dementia health navigation service. This presentation will describe processes for assembling and engaging the network for CuidaTXT.

### EXAMINING HOW AFRICAN AMERICAN FAMILY DEMENTIA CAREGIVERS CONCEPTUALIZE AND MANAGE CRISIS EVENTS

Quinton Cotton,[1] Laura Block,[2] Jennifer Morgan,[3] and Andrea Gilmore-Bykovskyi,[1] *1. University of Wisconsin-Madison, Madison, Wisconsin, United States, 2. University of Wisconsin-Madison School of Nursing, Madison, Wisconsin, United States, 3. Georgia State University, Atlanta, Georgia, United States*

African American (AA) family dementia caregivers report high unmet needs, which often culminate in crisis – an unplanned stressful situation requiring immediate decision. However, perspectives from AA caregivers regarding crisis are lacking. To gain insight into caregivers' conceptualization and experiences of crisis, we used community/coalitional-based recruitment of AA caregivers to conduct semi-structured interviews with 34 AA caregivers which were analyzed using thematic analysis (N=34, 94% female, 56% ages 65 to 74). AA caregivers largely perceived crisis as stressful events, a normal part of caregiving and viewed management of these events as routine. Crisis was characterized as ongoing, lengthy or emergent, sometimes necessitating external support (.e.g. hospitalization). Caregivers managed crisis by increasing caregiving work, de-prioritizing their own health and needs, involving family and friends, and accessing emotional support through neighborhood connections. These perspectives can inform future culturally-tailored interventions that are responsive to AA strengths, values, and help seeking preferences.

### SESSION 7115 (SYMPOSIUM)

### FIGS STUDY OUTCOMES: DISENTANGLING RELATIONSHIPS BETWEEN VISION LOSS, THE ENVIRONMENT, PHYSICAL ACTIVITY, AND FALLS

Chair: Pradeep Ramulu
Co-Chair: Laura Gitlin
Discussant: Jennifer Schrack

The longitudinal relationships between various aspects of mobility (with each other and with vision loss) are important to understand for healthy aging. The Falls in Glaucoma Study (FIGS) was a three-year longitudinal study conducted in persons with a range of visual field damage from glaucoma (from normal visual fields to severe visual field damage) and evaluated several aspects of mobility: physical function (gait and balance), physical activity (annual accelerometer trials), fall rates (prospectively-collected falls calendars), environmental features (an in-home assessment), and fear of falling. In this symposium, we present data demonstrating that: (1) physical activity is altered by visual field damage - lowering the overall amount of physical activity, and also resulting in more fragmented activity (i.e. shorter activity bouts); (2) specific home environmental features, such as better lighting, are associated with lower rates of falls within the home; (3) specific gait and balance features increase the risk of falling, but do not explain the association between visual field damage and a higher rate of falls; (4) injurious falls, but not non-injurious falls, lead to future reductions in physical activity; and (5) worsening of fear of falling (FoF) leads to either a higher rate of falls (at low FoF levels) or decreases in physical activity (at higher FoF levels). Study findings will educate the audience about the types of mobility problems found in persons with visual field damage, potential methods to prevent falls in older adults, and factors likely to predict future mobility deficits in older adults.

### IMPACT OF FEAR OF FALLING ON FUTURE FALLS AND CHANGES IN PHYSICAL ACTIVITY IN OLDER ADULTS WITH GLAUCOMA

Pradeep Ramulu,[1] Jian-Yu E,[2] Aleksandra Mihailovic,[3] Pei-Lun Kuo,[4] Sheila West,[1] Laura Gitlin,[5] Tianjing Li,[6] and Jennifer Schrack,[7] *1. Johns Hopkins School of Medicine, Baltimore, Maryland, United States, 2. Johns hopkins Bloomberg School of Public Health, Baltimore, Maryland, United States, 3. Johns Hopkins University/ Wilmer Eye Institute, Baltimore, Maryland, United States, 4. National Institute on Aging, Bethesda, Maryland, United States, 5. Drexel University, Philadelphia, Pennsylvania, United States, 6. University of Colorado, Arora, Colorado, United States, 7. Johns Hopkins Bloomberg School of Public Health, Baltimore, Maryland, United States*

To understand how Fear of falling (FoF) alters mobility, FoF was evaluated annually in 243 older adults (median age=70) with varying degrees of visual field loss from glaucoma, and Rasch-analyzed FoF scores associated with the likelihood of falling in the following year (judged by prospective calendar data) and changes in physical activity (Judged by annual accelerometer trials). At lower FoF levels, each one-unit worsening in FoF was associated with a 2.73-fold higher odds of reporting a fall in the next year (95% CI:1.55,4.81) but not with average daily steps taken

(p = 0.44). At higher FoF levels, inter-year changes in FoF were not significantly associated with a fall in the next year (p = 0.78); but were associated with 407 fewer daily steps taken per one-unit change in FoF (95% CI:-743,-71). FoF is an important driver of mobility; the specific aspects of mobility affected varies by the degree of FoF.

### COMPARING LONGITUDINAL CHANGES IN PHYSICAL ACTIVITY AND FEAR OF FALLING IN NON-FALLERS, FALLERS, AND INJURIOUS FALLERS

Jian-Yu E,[1] Jennifer Schrack,[2] Aleksandra Mihailovic,[3] Tianjing Li,[4] David Friedman,[5] Sheila West,[6] Laura Gitlin,[7] and Pradeep Ramulu,[6] *1. Johns hopkins Bloomberg School of Public Health, Baltimore, Maryland, United States, 2. Johns Hopkins Bloomberg School of Public Health, Baltimore, Maryland, United States, 3. Johns Hopkins University/Wilmer Eye Institute, Baltimore, Maryland, United States, 4. University of Colorado, Arora, Colorado, United States, 5. Massachusetts Eye and Ear, Harvard Medical School, Boston, Massachusetts, United States, 6. Johns Hopkins School of Medicine, Baltimore, Maryland, United States, 7. Drexel University, Philadelphia, Pennsylvania, United States*

Older adults with visual impairments experience a higher risk of falling, and are more vulnerable to associated adverse health consequences than those with normal vision. We investigated the onset and magnitude of declines in physical activity (PA), and corresponding changes in self-reported fear of falling (FoF) in 234 visually impaired persons (mean age=69.8, 48.7% women) over three years. PA was measured using the Actical hip-worn accelerometer and falls were reported using a calendar. In fully adjusted linear models, PA declined 426 steps/year (p<0.01) and 15.1 active minutes/year (p<0.01) among injurious fallers compared to non-fallers; PA did not change among non-injurious fallers. No longitudinal declines in FoF scores were observed. Among visually impaired older adults, an injurious fall contributed to subsequent declines in activity, although FoF remained unchanged. Further longitudinal research is warranted to better understand how different groups respond to falls, either by behavioral changes and/or changes in FoF.

### PATTERNS OF DAILY PHYSICAL ACTIVITY ACROSS THE SPECTRUM OF VISUAL FIELD DAMAGE IN GLAUCOMA PATIENTS

Jennifer Schrack,[1] Jian-Yu E,[2] Aleksandra Mihailovic,[3] Amal Wanigatunga,[1] Sheila West,[4] Laura Gitlin,[5] Tianjing Li,[6] and Pradeep Ramulu,[4] *1. Johns Hopkins Bloomberg School of Public Health, Baltimore, Maryland, United States, 2. Johns hopkins Bloomberg School of Public Health, Baltimore, Maryland, United States, 3. Johns Hopkins University/Wilmer Eye Institute, Baltimore, Maryland, United States, 4. Johns Hopkins School of Medicine, Baltimore, Maryland, United States, 5. Drexel University, Philadelphia, Pennsylvania, United States, 6. University of Colorado, Arora, Colorado, United States*

Visual impairment (VI) has been linked with low physical activity (PA) and higher risk of poor function with aging, yet how VI affects daily patterns of physical activity (PA) is undefined. We assessed the association between visual field (VF) damage and: (i) activity fragmentation and (ii) diurnal patterns of PA in 237 glaucoma patients (mean age=70.6, 51.5% men). PA was measured using the Actical hip-worn accelerometer and VF damage was defined by average VF sensitivity. In adjusted linear models, each 5-dB decrement in IVF sensitivity was associated with 16.3 fewer active minutes/day and 2% higher activity fragmentation (both p<.05). In time-of-day analyses, those with lower IVF sensitivity were less active, but this did not vary by time-of-day. Older adults with worse VF damage demonstrate shorter, more fragmented bouts of PA throughout the day, which may reflect lower physiologic functioning. Future work should examine the temporality of this association.

### THE RELATIONSHIP BETWEEN THE HOME ENVIRONMENT AND FALLS FOR OLDER ADULTS WITH VISUAL IMPAIRMENTS

Bonnielin Swenor,[1] Aleksandra Mihailovic,[2] and Pradeep Ramulu,[3] *1. Johns Hopkins University, Baltimore, Maryland, United States, 2. Johns Hopkins University/Wilmer Eye Institute, Baltimore, Maryland, United States, 3. Johns Hopkins School of Medicine, Baltimore, Maryland, United States*

The home environment and features of the home have been identified as important risk factors for falls, and may pose particular risk for older adults with visual impairments given difficulty with hazard perception. We used data from 245 participants in the Falls in Glaucoma Study [mean age: 71 years, mean follow-up: 31 months] with homes graded using our previously validated Home Environment Assessment for the Visually Impaired (HEAVI), which quantifies the number of in-home fall-related hazards and found that neither the number of hazards nor the percentage of hazardous items were associated falls/year. However, each 10-fold increase in lighting was associated with a 35% lower rate of falls/year (RR=0.65, 95%CI=0.46 to 0.92) and there was a 50% reduction in falls/year when lighting was at or above 30 footcandles (minimum lighting level recommended by the Engineering Society of North America) compared to lighting <30 footcandles (RR=0.50, 95%CI=0.26 to 0.96).

### GAIT AND BALANCE AS PREDICTORS OR MEDIATORS OF FALLS IN GLAUCOMA

Aleksandra Mihailovic,[1] Regina De Luna,[2] Sheila West,[3] David Friedman,[4] Laura Gitlin,[5] and Pradeep Ramulu,[3] *1. Johns Hopkins University/Wilmer Eye Institute, Baltimore, Maryland, United States, 2. Wilmer Eye Institute/Glaucoma Center of Excellence, Baltimore, Maryland, United States, 3. Johns Hopkins School of Medicine, Baltimore, Maryland, United States, 4. Massachusetts Eye and Ear, Harvard Medical School, Boston, Massachusetts, United States, 5. Drexel University, Philadelphia, Pennsylvania, United States*

Balance and gait are modifiable targets for falls prevention and may play an important role in preventing falls in older visually impaired individuals. Balance and gait were objectively evaluated in the 239 Falls in Glaucoma Study participants (average age=70.5, 22% with moderate-severe visual field (VF) damage). Greater sway, more time in double support and greater swing time variability were associated with higher fall rates, while higher gait velocity and faster cadence were associated with lower rates (p<0.05 for all). Neither gait nor balance mediated the relationship between VF damage and fall rates, with VF damage remaining an independent predictor of fall in models including gait and/or balance features (RR =1.36 to 1.48, p-value= <0.001 to 0.005). While balance and gait measures are associated with fall rates, they do not explain why persons with greater VF damage fall more frequently, suggesting the importance of other factors such as hazard perception.

### SESSION 7120 (SYMPOSIUM)

### FITNESS MATTERS AS WE AGE: A CELEBRATION OF THE VA GEROFIT PROGRAM

Chair: Miriam Morey
Discussant: Cathy Lee

In recognition of the GSA's 75th Anniversary "Why Age Matters" we celebrate the 7th anniversary of the Gerofit dissemination initiative. Gerofit is an exercise and health promotion program for older Veterans that has been declared a Veterans Health Administration (VA) "Best Practice" and been disseminated to 17 VA's across the country. Over 7000 Veterans have participated in Gerofit initiated programs and have reported robust outcomes including improved quality of life, physical and mental health, and high levels of satisfaction with the programs. For this symposium, we focus on newly acquired program outcomes that emphasize the importance of fitness as we age. The first paper compares hospitalization and emergency room visits between individuals participating in Gerofit for 12 months compared to a matched control group. The second paper describes four-year trajectories of physical performance to highlight the impact of becoming fit over expected normative trajectories. The third paper examines outcomes of a home-based geriatric walking clinic. The fourth paper describes the impact of exercise adherence on chronic pain. The fifth paper describes changes in medication utilization compared to a matched control group following 12-months of supervised exercise. These papers highlight the importance of fitness as a contributor to overall health during the aging process and celebrates that fitness matters, no matter when you start!

### DOES GEROFIT EXERCISE REDUCE VETERANS' USE OF EMERGENCY DEPARTMENT AND INPATIENT CARE?

Lauren Abbate,[1] Jiejin Li,[2] Peter Veazie,[2] Orna Intrator,[3] Cathy Lee,[4] Leslie Katzel,[5] Megan Pearson,[6] and Miriam Morey,[7] *1. Rocky Mountain Regional VA Medical Center, Aurora, Colorado, United States, 2. Geriatrics and Extended Care Data and Analysis Center (GECDAC), Canandaigua, New York, United States, 3. University of Rochester School of Medicine and Dentistry, Rochester, New York, United States, 4. VA Greater Los Angeles, Los Angeles, California, United States, 5. Geriatric Research, Education, and Clinical Center, VA Maryland Health Care System, Baltimore, Maryland, United States, 6. Veteran Affairs Health Care System, Durham, North Carolina, United States, 7. Veterans Health Administration, Durham, North Carolina, United States*

Little is known about the relationship between exercise and health care utilization in older adults. This study examined hospitalizations/emergency Department (ED) visits in the 12 months prior to and during 12 months of active Gerofit participation (across 5 sites). Data were compared for each outcome to a propensity matched nearest neighbor sample from the same site [Mean, 95% CI]. Of the 226 Veterans who were active in the program for ≥12 months and enrolled in VA and Traditional Medicare for 12 months prior to Gerofit participation, hospitalizations/ED visits were greater prior to (15.3%/42.0%) than during (6.8%/37.1%) Gerofit participation. Gerofit participants were 8% less likely to have a hospitalization in the 12 months following enrollment than controls [-0.08 (-0.14, -0.02)] but no between-group differences in ED use [-0.00 (-0.11, 0.10)] were observed. Participation in Gerofit may reduce hospitalizations, but its impact on ED use is inconclusive.

### TRANSLATING EXERCISE BENEFITS OVER TELEHEALTH

Kalpana Padala,[1] Shelly Lensing,[2] Tammy Evans,[3] Megan Pearson,[4] Miriam Morey,[5] and Prasad Padala,[6] *1. Central Arkansas Veterans Healthcare System, North Little Rock, Arkansas, United States, 2. University of Arkansas for Medical Sciences, Little Rock, Arkansas, United States, 3. GRECC, Central Arkansas Veterans Healthcare System, North Little Rock, Arkansas, United States, 4. Veteran Affairs Health Care System, Durham, North Carolina, United States, 5. Veterans Health Administration, Durham, North Carolina, United States, 6. Central Arkansas Veterans Healthcare System, Little Rock, Arkansas, United States*

Background: Gerofit-Geriatric walking clinic is a home-based program that helps older veterans engage in regular walking via different platforms to improve access. The objective was to compare the outcomes of face-to-face visits to telehealth visits. Methods: Older Veterans (N=646) and walking-buddies (N=154) were seen either face-to-face or via telehealth at baseline, 2-, and 6-months. The primary intervention, pedometer feedback and motivational phone calls were delivered remotely. Results: Demographic data were similar in both Veteran groups and 47% were seen via telehealth. Compared to face-to-face, a higher proportion in the telehealth group had walking buddies (10% vs. 27%; p<0.001), received exercise counseling (75% vs. 95%; p=0.001), and reported perceived barriers (40% vs. 67%; p=0.004). There were statistically significant improvements in step-counts at 2- and 6-months compared to baseline (57% and 99% improvement; p<0.01) with no significant between-group differences. Conclusion: Tailored activity promotion programs via telehealth are effective in reaching older veterans.

### CREATING AND KEEPING EXERCISE GAINS INTO YOUR 70S, 80S AND 90S

Kenneth Manning,[1] Teresa Kopp,[2] Kris ann Oursler,[3] Michelle McDonald,[4] Miriam Morey,[5] Katherine Hall,[6] Cathy Lee,[7] and Jamie Giffuni,[8] *1. Durham VA Health Care System, Durham, North Carolina, United States, 2. Canandaigua VA Medical Center, Canandaigua, New York, United States, 3. Salem VA Medical Center, Salem, Virginia, United States, 4. VA Pacific Island Healthcare System, Honolulu, Hawaii, United States, 5. Veterans Health Administration, Durham, North*

*Carolina, United States, 6. Duke University, Veterans Health Administration, Durham, North Carolina, United States, 7. VA Greater Los Angeles, Los Angeles, California, United States, 8. Baltimore VA Healthcare System, Baltimore, Maryland, United States*

Background: Gerofit, an exercise program for older Veterans, is undergoing national dissemination (17 sites in 6 years). Four sites have accrued 4-year functional outcomes (gait speed, 8-foot-up-and-go, 30-second chair stand, and six-minute walk). Methods: Functional assessments were administered quarterly in first year and annually thereafter. Individuals with baseline and at least two follow up measures were included for analysis (n=587). Means were gathered across each timepoint. Results: Mean values for functional assessments from baseline to 4 year were as follows: gait speed m/s- 1.04, 1.12, 1.13, 1.13, 1.09, 1.07, 1.13; 8 ft-up-and-go seconds- 7.6, 6.82, 6.69, 6.69, 7.29, 7.29, 7.51; 30 second chair stands 11.88, 14.06, 14.72, 14.89, 14.69, 14.71, 14.96; and six-minute-walk yards- 499, 532, 541, 544, 531, 530, 556. All follow up measures were significantly improved over baseline (P<.01) and superior to normative age-related decline. Implications: Results indicate that exercise promotes compression of morbidity and improved functional health.

### GEROFIT DECREASES MEDICATION USE AMONG OLDER VETERANS

Orna Intrator,[1] Jiejin Li,[2] Peter Veazie,[2] and Sara Wesgate,[3] *1. University of Rochester School of Medicine and Dentistry, Rochester, New York, United States, 2. Geriatrics and Extended Care Data and Analysis Center (GECDAC), Canandaigua, New York, United States, 3. VA Medical Center, Canandaigua, New York, United States*

We examined whether Veterans enrolled in Gerofit for at least 12 months between 10/2012-3/2017 saw reductions in their medication utilization (5 sites, 226 Veterans). VA outpatient pharmacy data was used to identify medications used 12-months prior, and 12-months following Gerofit enrollment. Seven drug classes were identified (cardiovascular, diabetic, lipid, mental health, opioids, vitamins, other medications). Nearest-neighbor propensity-matched analyses was conducted with exact match on number of baseline medications and site. At baseline, Gerofit participants were taking, on average, 11.6 medications (1.7 cardiovascular, 0.6 diabetes, 0.7 lipid lowering, 0.7 mental health, 0.5 opioids, 0.6 vitamins, 7.0 other). At 12-month follow-up, Gerofit patients were taking fewer medications: any (-2.7 [-4.4, -1.0]); cardiovascular (-0.5 [-0.7, -0.3]); diabetes (-0.7 [-1.2, -0.2]); mental health (-0.9 [-1.5, -0.2]); and other (-2.4 [-3.5, -1.4]) compared to matched comparisons. No significant differences at 12-months were found for lipid lowering, opioids, or vitamins. Conclusion: Gerofit participation reduced medication use.

### OLDER VETERANS WITH CHRONIC PAIN WHO PERSIST IN GROUP EXERCISE SHOW IMPROVED MOBILITY

Odessa Addison,[1] Jamie Giffuni,[2] Megan Kelly,[3] Beth Hogans,[3] Leslie Katzel,[3] and Miriam Morey,[4] *1. VA Maryland Healthcare System, Baltimore, Maryland, United States, 2. Baltimore VA Healthcare System, Baltimore, Maryland, United States, 3. Geriatric Research, Education, and Clinical Center, VA Maryland Health Care System, Baltimore, Maryland, United States, 4. Veterans Health Administration, Durham, North Carolina, United States*

Approximately 50% of older adults receiving care at VA are Veterans with chronic pain (V-CP). While physical activity can reduce chronic pain and increase mobility, little is known about group exercise (GE) effects for V-CP. We hypothesized that attrition may limit GE effectiveness. In this study, we retrospectively compared program attrition and participant mobility at three-months of Gerofit GE. At baseline, Older V-CP (N=21) had lower mobility assessment scores (gait speed, 6MW, chair stands, TUG: 1.04 m/s, 443 yd, 11, 9.35s vs. 1.09 m/s, 463 yd, 12, 8.19s respectively) compared to unaffected Veterans. Three-month attrition was higher for older V-CP (54% vs 39%). For those completing three months GE, gains in mobility were similar. We conclude that Veterans with chronic pain are less likely to persist in group exercise but those who persist benefit much like those without pain. Further study is needed to understand successful exercise adherence.

### SESSION 7125 (SYMPOSIUM)

### FOOD INSECURITY IN THE UNITED STATES: IDENTIFICATION, TREATMENT, AND IMPLICATIONS

Co-Chair: Rose Ann DiMaria-Ghalili
Co-Chair: Connie Bales
Discussant: Julie Locher

Food insecurity is an under-recognized geriatric syndrome that has extensive implications in the overall health and well-being of older adults. Understanding the impact of food insecurity in older adults is a first step in identifying at-risk populations and provides a framework for potential interventions in both hospital and community-based settings. This symposium will provide an overview of current prevalence rates of food insecurity using large population-based datasets. We will present a summary indicator that expands measurement to include the functional and social support limitations (e.g., community disability, social isolation, frailty, and being homebound), which disproportionately impact older adults, and in turn their rate and experience of food insecurity and inadequate food access. We will illustrate using an example of at-risk seniors the association between sarcopenia, the age-related loss of muscle mass and function, with rates of food security in the United States. The translational aspect of the symposium will then focus on identification of psychosocial and environmental risk factors including food insecurity in older veterans preparing for surgery within the Veterans Affairs Perioperative Optimization of Senior Health clinic. Gaining insights into the importance of food insecurity will lay the foundation for an intervention for food insecurity in the deep south. Our discussant will provide an overview of the implications of these results from a public health standpoint. By highlighting the importance of food insecurity, such data can potentially become a framework to allow policy makers to expand nutritional programs as a line of defense against hunger in this high-risk population.

### BROADENING THE CONCEPTION OF FOOD INSECURITY AMONG OLDER ADULTS: DEVELOPMENT OF A SUMMARY INDICATOR IN THE NHATS

Emma Tucher,[1] Tamra Keeney,[1] Alicia Cohen,[2] and Kali Thomas,[1] *1. Brown University, Providence, Rhode Island, United States, 2. Providence VA Medical Center, Providence, Rhode Island, United States*

Comprehensive measurement of food insecurity among older adults has focused on financial barriers to food access. Using data from the community-dwelling older adults in Round 5 of the U.S. National Health and Aging Trends Study (n=7,070), we constructed a summary indicator of food insecurity and inadequate food access incorporating items related to food scarcity within three domains: functional, social support, and financial limitations. We analyzed the construct validity of the summary indicator for known biopsychosocial factors associated with food insecurity among older adults. In 2015, 4.3% of community-dwelling older adults, approximately 1,673,775 million people, were characterized as food insecure or lacking adequate food access. Multivariable regression models identified that being homebound, frail, and experiencing community disability were associated with food insecurity and inadequate food access. These findings indicate food insecurity and inadequate food access among older adults is associated with functional and mobility characteristics and not isolated to financial barriers.

### ASSOCIATION OF FOOD INSECURITY AND SARCOPENIA: THE NATIONAL HEALTH AND NUTRITION EXAMINATION SURVEYS

John Batsis,[1] Curtis Petersen,[1] and Tyler Gooding,[2] *1. Dartmouth College, Lebanon, New Hampshire, United States, 2. Dartmouth Hitchcock Medical Center, Lebanon, New Hampshire, United States*

Understanding the association between food insecurity and sarcopenia can inform policies that address healthcare disparities. We identified 2,965 subjects aged ≥60 years with grip strength and food security data from the National Health and Nutrition Examination Surveys 2011-14. Sarcopenia was defined using grip strength <26kg for men and <16kg for women, and food security with the 18-item US Household Food Security Survey. Logistic regression evaluated the association of food insecurity (referent = full security) with sarcopenia, adjusting for age, sex, marital status, race/ethnicity, education, body mass index, smoking, and comorbidities. Mean age (% female) was 76.6 (60.9%) and 68.9 years (53.4%) with and without sarcopenia, respectively. Sarcopenia prevalence was 8.4%. Rates of full food security were higher in those without sarcopenia (90% vs. 84.2%;p<0.001). Food insecurity was strongly associated with sarcopenia (OR 1.79 [1.18, 2.72]), suggesting a need for both longitudinal and interventional studies to target these disparities.

### NUTRITIONAL VULNERABILITY: THE COMPLEXITY OF PREPARING OLDER VETERANS FOR SURGERY

Kathyrn Starr,[1] Nancy Loyack,[2] Shelley McDonald,[3] Mitchell Heflin,[4] Sandhya Lagoo-deenadayalan,[1] and Connie Bales,[1] *1. Duke University School of Medicine, Durham, North Carolina, United States, 2. Durham VA Medical Center, Durham, North Carolina, United States, 3. Duke University, Durham, North Carolina, United States, 4. Duke University Medical Center, Durham, North Carolina, United States*

Poor nutritional status leads to postoperative complications, infections, poor healing, and increased mortality, creating a high-risk situation for older adults undergoing surgery. Psychosocial and environmental factors, including socioeconomic disadvantage, disability, social isolation and depression, are known precipitators of nutritional risk. However, these potentially modifiable concerns are rarely taken into consideration preoperatively. In 736 older Veterans preparing for surgery, we found 42% reported it was hard/somewhat hard to pay for basic needs, 6% reported sometimes/often times not having enough to eat, 24% reported living along, 47% reported needing assistance with 1 or more IADLs, and 42% reported a history of depression. Findings from older, Veterans, illustrate the prevalence of psychosocial and environmental risk factors prior to surgery. Best practices for identifying these factors, the importance of an interprofessional team for intervening, and specific resources that can be utilized throughout the perioperative period to improve outcomes will be presented.

### CHOICE FOOD PANTRIES IN THE DEEP SOUTH: A NOVEL APPROACH TO ADDRESSING FOOD INSECURITY IN OLDER ADULTS

David Buys, Masey Smith, and Erin King, *Mississippi State University, Mississippi State, Mississippi, United States*

Older adults in the deep south are living with high food insecurity rates; this is exacerbated by challenges with rural-living, like transportation limitations and no grocery stores. To address this, we must increase emergency food assistance offerings and adopt best practices for food pantries including choice food pantry approaches, which empowers clients with some autonomy in choosing the foods they receive as part of their pantry distribution. Coalitions in eight income-limited, aging, rural Mississippi Delta counties received support from a Centers for Disease Control and Prevention Grant to enhance the food-related infrastructure in their communities through technical assistance and economic investments. A detailed process evaluation was conducted on this effort. Each coalition adopted food pantry-related policies like adding new food pantries and adapting their existing food pantries with the choice model. Both aging volunteers and clients indicated positive outcomes from the process of adding pantries and adapting existing ones.

### SESSION 7130 (SYMPOSIUM)

### IMPLEMENTING EFFECTIVE, EVIDENCE-BASED OLDER ADULT FALL PREVENTION

Chair: Gwen Bergen

Over one in four older adults (65 years and older) in the US reports falling annually with estimated medical costs of $50 billion. Evidence-based strategies exist that can reduce falls with one of the most promising being multifactorial, clinically-based initiatives such as the Centers for Disease Control and Prevention's STEADI (Stopping Elderly Accidents, Deaths, and Injuries) Initiative. STEADI includes three core components for health care providers: screen for risk factors, assess modifiable factors, and intervene to reduce falls with evidence-based strategies. Barriers to implementation include competing patient demands and limited time during patient visits. Efficient, effective implementation of clinical fall prevention is important to increase the use of multifactorial interventions. In addition, understanding older adult attitudes about the preventability of falls is needed to increase patient adherence to prescribed interventions. This symposium will cover:1. Background data on older adult falls over time,2. Description of an initial implementation of STEADI in an outpatient, Southeastern clinical practice including lessons learned,3. Attitudes of older adults toward fall prevention with implications for health promotion,4. Process evaluation of an ongoing implementation of STEADI in New York State with lessons learned. Understanding practical methods of implementing the three core components of fall prevention into practice supports wider dissemination of evidence-based fall prevention, while understanding patient attitudes toward falls informs the design of health promotion approaches to increase patient uptake of prescribed interventions. Wider dissemination and increased patient adherence in combination can reduce older adult falls and their associated medical costs.

### IMPLICATIONS OF OLDER ADULT ATTITUDES TOWARD THE PREVENTABILITY OF FALLS FOR HEALTH PROMOTION

Gwen Bergen, Janice Mark, and Ankita Henry, *Centers for Disease Control and Prevention, Atlanta, Georgia, United States*

Older adults' behavioral stage of change for adopting fall prevention interventions, and their use of evidence-based interventions are not well understood. A survey was administered to older adults (65 years+) (n=1063) to understand their stage of change and fall prevention behaviors. Descriptive statistics were calculated and logistic regression conducted to determine factors most related to stage. The distribution of subjects by stage was precontemplation (17%), contemplation (2%), preparation (5%), action (15%), and maintenance (61%). The strongest variable related to being in an action stage (preparation, action, maintenance) was screening positively for fall risk (Risk Ratio: 8.7, 95% CI: 5.4, 14.1). The most common preventive actions for those in an action stage were taking Vitamin D (37%), and having vision tested (30%). Older adults at risk for a fall are ready to take action to prevent falls; health promotion should focus on increasing knowledge and use of different evidence-based interventions.

### CHANGES IN THE AGE-ADJUSTED RATE OF OLDER ADULTS DYING FROM A FALL AND REPORTING A FALL AND FALL INJURY, 2012–2018

Elizabeth Burns,[1] Ramakrishna Kakara,[2] Briana Moreland,[3] and Ankita Henry,[1] *1. Centers for Disease Control and Prevention, Atlanta, Georgia, United States, 2. CDC, Atlanta, Georgia, United States, 3. Synergy America Inc., Atlanta, Georgia, United States*

Falls are a leading cause of injury among older men and women (≥65 years) in the United States. Vital Statistics and Behavioral Risk Factor Surveillance System data were analyzed to determine the age-adjusted fall death rate, the rates of older adults reporting a fall and fall injury, and associated trends. The fall death rate increased 16% from 55.3/100,000 in 2012 to 64.4/100,000 in 2018 (p≤0.05). Like the rates in 2012, the rate of falls reported in 2018 was 713/1000 older adults and the rate of fall injuries reported was 171/1000 older adults. When assessing the rates of older adults reporting a fall or fall injury by sex, the rates among men increased from 2012 to 2016 from 637/1000 to 773/1000 (21% increase, p≤0.05) for falls and from 120/1000 to 153/1000 (28% increase, p≤0.05) for fall injuries. Understanding how these data change over time can inform targeted interventions to reduce falls.

### AN EFFECTIVENESS TRIAL OF STEADI IN AN OUTPATIENT, PRIMARY CARE PRACTICE

David Rein,[1] Madeleine Hackney,[2] Michele Dougherty,[3] Camille Vaughan,[2] Laurie Imhof,[4] Theodore Johnson II,[5] and The Emory Clinic at St. Josephs STEADI Implementation Group,[6] *1. NORC, Atlanta, Georgia, United States, 2. Emory University, Atlanta, Georgia, United States, 3. NORC at the University of Chicago, Bethesda, Georgia, United States, 4. NORC at the University of Chicago, Chicago, Illinois, United States, 5. Emory University School of Medicine, Atlanta, Georgia, United States, 6. Emory at Saint Joseph's-Primary Care, Atlanta, Georgia, United States*

The STEADI Options trial uses a randomized, controlled-trial design to assess the effectiveness and cost-effectiveness of the STEADI Initiative . Beginning March, 2020, we will randomize 3,000 adults ≥ 65 years of age at risk for falls seen in an Emory Clinic primary care practice to: (1) full STEADI; (2) a STEADI-derived gait, balance, and strength assessment with physical therapy referrals; (3) a STEADI-derived medication review and management; or (4) usual care. This presentation will discuss decisions made by the study team to facilitate implementation of STEADI including electronically conducting screening prior to the date of encounter, the use of dedicated nursing staff to conduct assessments, implementation of strength, balance, orthostatic hypotension, and vision testing, methods to facilitate medication review, and communication of assessment information to providers. The results from this study will be used to estimate the impact of STEADI on falls, service utilization, and costs over one year.

### STEADI IN PRIMARY CARE: A PROCESS EVALUATION OF THE NEW YORK STATE IMPLEMENTATION

Yvonne Johnston,[1] and Chelsea Reome-Nedlik,[2] *1. Binghamton University, Binghamton, New York, United States, 2. Broome County Health Department, Binghamton, New York, United States*

This session presents findings from a STEADI process evaluation that was conducted within a primary care setting in New York State. This process evaluation used mixed methods including quantitative analysis of surveys with clinic staff as well as qualitative methods such as intercept interviews with healthcare providers and clinic staff, and structured interviews with key stakeholders. The RE-AIM framework guided development of the process evaluation tools. The process evaluation was conducted over a 2-month period approximately 18 months post-implementation. Facilitators included: (a) Adoption - physician champion and administrative support; (b) Implementation - wellness coordinators, preparation and training, and organizational quality measures; (c) Maintenance - feedback from patients, local and national recognition, and impact on fall-related outcomes. Barriers included: (a) Adoption – organizational priorities and complexity of electronic health records; (b) Implementation – resistance to change and competing patient care demands; (c) Maintenance - staff turnover and follow through of referrals.

### SESSION 7135 (SYMPOSIUM)

### INNOVATIVE INTERVENTIONS: CARING FOR PERSONS WITH ALZHEIMER'S DISEASE AND THEIR CAREGIVERS

Chair: Stacy Andersen
Co-Chair: Allison Gibson
Discussant: Katherine Marx

There are 5.8 million Americans living with Alzheimer's disease and more than 16 million Americans providing unpaid care for people with AD and related dementias. Since a treatment that can slow or stop progression of this disease has yet to be discovered, novel interventions are sorely needed to maintain cognitive function and quality of life among individuals with dementia, improve the health and well-being of caregivers, and provide assistance in caregiving duties. This symposium addresses novel interventions in the dementia care continuum ranging from social and leisure activities for improving cognition to incorporation of emerging technologies to assist with caregiving and provides recommendations and priorities for future studies. The first presentation introduces evidence that participation in an intergenerational choir can improve cognition, social connectedness, and quality of life among people with dementia and their caregivers. The second presentation systematically assesses recent randomized controlled trials of computerized cognitive training (i.e., brain games) aimed at improving cognitive function among individuals with cognitive impairment or dementia. The third presentation examines the evidence that interventions employing artificial intelligence such as robots may improve care for persons with Alzheimer's disease and caregivers' quality of life and provides suggestions for future studies to better assess the efficacy of these interventions. The session concludes with a presentation on a survey method used to build consensus among a panel of experts across academia and industry which identified the emerging technologies that are expected to become the most prevalent in dementia care and provides recommendations for limiting associated risks.

### ONE SONG, MANY VOICES: DEMENTIA AND THE POWER OF MUSIC

Debra Sheets,[1] Stuart MacDonald,[2] and Andre Smith,[2] *1. University of Victoria, Victoria, British Columbia, Canada, 2. University of Victoria, Victoria, British Columbia, Canada*

Choral singing is a novel approach to reduce dementia stigma and social isolation while offering participants a sense of purpose, joy and social connection. The pervasiveness of stigma surrounding dementia remains one of the biggest barriers to living life with dignity following a diagnosis (Alzheimer Society of Canada, 2018). This paper examines how a social inclusion model of dementia care involving an intergenerational choir for people living with dementia, their care partners and high school students can reduce stigma and foster social connections. Multiple methodologies are used to investigate the effects of choir participation on cognition, stress levels, social connections, stigma, and quality of life. Results demonstrate the positive impact of choir participation and indicate that this socially inclusive intervention offers an effective, non-pharmacological alternative for older adults living with dementia in the community. Discussion focuses on the importance of instituting meaningful and engaging dementia-friendly activities at the community level.

### BRAIN GAMES FOR DEMENTIA: DO THEY HELP?

Patricia Heyn,[1] Pallavi Sood,[2] Hannes Devos,[3] Ahmed Negm,[4] and Sandra Kletzel,[5], *1. University of Colorado Anschutz Medical Campus, Aurora, Colorado, United States, 2. University of Florida, Gainesville, Florida, United States, 3. University of Kansas Medical Center, Kansas City, Kansas, United States, 4. University of Alberta, Edmonton, Alberta, Canada, 5. Edward Hines, Jr. VA Hospital, Hines, Illinois, United States*

Brain Gaming (BG) Interventions have been shown to improve the cognitive function of older adults with cognitive impairments (CIs). However, rigorous evaluation supporting BG effectiveness is needed. Thus, we used meta-analysis to evaluate the effectiveness of BG. Several search databases (i.e. Pubmed) were used to identify relevant randomized controlled trials (RCTs). Cochrane RoB tool evaluated risk of bias. The main outcome was the composite score of cognitive function. Inverse-variance random effects model was used to compare the pooled standardized mean difference (SMD) across studies. A total of 16 RCTs included 909 participants. The RCTs varied in sample size, gaming platform, training prescription, and cognition. The meta-analysis showed no significant effects of BG on overall cognitive function (pooled SMD = 0.08, 95% CI [-0.24 – 0.41], p = 0.61, I2 = 77%. However, due to high heterogeneity, we cannot confidently refute that BG is an effective cognitive training approach.

### ARTIFICIAL INTELLIGENCE FOR ADRD CAREGIVERS: A SYSTEMATIC LITERATURE REVIEW

Bo Xie,[1] Cui Tao,[2] Juan Li,[3] Robin Hilsabeck,[4] and Alyssa Aguirre,[5] *1. The University of Texas at Austin, Austin, Texas, United States, 2. School of Biomedical Informatics, The University of Texas Health Science Center at Houston, Houston, Texas, United States, 3. Naval Medical University, Shanghai, China, 4. University of Texas, Austin, Texas, United States, 5. Dell Medical School - University of Texas, Austin, Texas, United States*

Artificial intelligence (AI) may improve the care for persons with Alzheimer's disease and related dementias (ADRD) and caregivers' quality of life. To examine existing research on this topic, we searched 5 publication databases using keywords related to Alzheimer's, dementia, caregiver, and artificial intelligence, and found 113 relevant results. We then screened the titles, abstracts, and full texts, and excluded studies not including family caregivers, not involving providing information to users, screening/diagnosis of dementia, caregiver anxiety/burnout, or lacking empirical data. Thirty results remained. These studies included technologies such as robots to facilitate social engagement and assist with daily activities. Most were qualitative or cross-sectional with small samples. These studies generally reported positive outcomes in favor of AI tools but levels of evidence were low. These findings suggest the need for systematic designs that can generate high levels of evidence for the feasibility and efficacy of AI-based interventions for ADRD caregivers.

### A DELPHI STUDY ON DEMENTIA CARE TECHNOLOGY USE AND HOW TO MITIGATE RISKS: DESIGN TO IMPLEMENTATION

Clara Berridge,[1] and George Demiris,[2]
*1. University of Washington, Seattle, Washington, United States, 2. University of Pennsylvania, Philadelphia, Pennsylvania, United States*

What information is needed to navigate person-centered technology use in dementia care? The threefold aim of this Delphi study was to learn which technologies will be most prevalent in dementia care in 5 years, understand benefits and risks, and identify specific options to mitigate risks. Twenty-one interdisciplinary domain experts from academia and industry in aging and technology in the U.S. and Canada participated, an 84% response rate. Technologies rated most likely to cause value tensions were also predicted to be most prevalent and will be described, along with the identified risks. Suggestions to mitigate the risks are categorized as follows: intervene during design; make specific technical choices; build in choice and control; require data transparency; place restrictions on data use and ensure security; enable informed consent; and proactively educate users. The specific recommendations that are relevant to designers, clinicians, researchers, ethicists, and policy makers will be presented and discussed.

### SESSION 7140 (SYMPOSIUM)

### LESSONS LEARNED FROM THE FUNCTION AND BEHAVIOR FOCUSED CARE TRIAL: FROM EFFICACY TO IMPLEMENTATION

Chair: Elizabeth Galik
Discussant: Marie Boltz

Long term care residents with dementia are sedentary, experience rapid functional decline, and frequently exhibit behavioral and psychological symptoms of dementia. Our prior cluster, randomized controlled trial among 336 residents with moderate to severe dementia in 12 nursing homes demonstrated that it is possible to increase time spent in physical activity and decrease resistiveness to care through a theory based intervention, Function and Behavior Focused Care (FBFC). FBFC is based on the Social Ecological Model and Social Cognitive Theory and focuses on having long term care staff cue, model, and assist residents with dementia to engage in physical activity and perform functional tasks. Learning from prior work, it was noted that future implementation of FBFC would benefit from de-implementing inaccurate care practices, such as restricting resident mobility and providing custodial care and also by engaging a full stakeholder team in intervention activities. Additionally, there were measurement issues, such as the use of actigraphy with a sedentary, cognitively impaired population, and the need to assess the quality of care interactions between residents and staff. This symposium will review lessons learned from the FBFC trial and will discuss 1) facilitators and barriers to the implementation of the FBFC intervention within long term care settings; 2) measurement opportunities and challenges with a cognitively impaired long term care population; and 3) adaptation of the FBFC intervention to be appropriate for a dissemination and implementation trial that incorporates the Synthesis Model of De-Adoption and the Evidence Integration Triangle implementation strategy.

### MEASUREMENT OPPORTUNITIES AND CHALLENGES WITH LONG-TERM CARE RESIDENTS WITH DEMENTIA

Sarah Holmes,[1] Elizabeth Galik,[2] and Barbara Resnick,[3]
*1. University of Maryland School of Pharmacy, Baltimore, Maryland, United States, 2. University of Maryland School of Nursing, Ellicott City, Maryland, United States, 3. University of Maryland School of Nursing, Baltimore, Maryland, United States*

Measuring clinical outcomes among older adults with moderate to severe dementia present practical and psychometric challenges. Proxy report surveys, direct observations, chart abstraction, and objective measures, such as actigraphy are frequently used with this population. The purpose of this paper is to describe issues related to measurement in the FBFC trial. Challenges to actigraphy included the resistance of residents and removal of devices, inability to detect limited movement and/or the type of activity performed. The combined use of the three measures to optimize understanding of physical activity will be addressed. Behavioral outcome measures included the Cornell Scale for Depression in Dementia, the Cohen Mansfield Agitation Inventory, and the Resistiveness to Care Scale. Despite the use of these multiple measures, limited behaviors were identified due to timing of assessments and items included within the measures. Optimal ways in which to capture behavioral symptoms will be reviewed.

### FACILITATORS AND BARRIERS TO THE IMPLEMENTATION OF FUNCTION AND BEHAVIOR FOCUSED CARE

Erin Vigne,[1] Elizabeth Galik,[2] and Barbara Resnick,[1]
*1. University of Maryland School of Nursing, Baltimore, Maryland, United States, 2. University of Maryland School of Nursing, Ellicott City, Maryland, United States*

Function and Behavior Focused Care (FBFC) is a two tiered intervention based on the Social Ecological Model and Social Cognitive Theory that focuses on training long term care staff to cue, model, and assist residents with dementia to engage in physical activity and perform functional tasks. FBFC was implemented by a Research Nurse and a facility based Champion for 12 months and involved a four step approach: (1) Environment and Policy Assessments; (2) Staff Education; (3) Establishing Resident Care Plans; and (4) Mentoring and Motivating of Staff and Residents. Facilitators of implementation included administrative support, an identified champion, and supportive environments and policies. Challenges and barriers included the fear of falls, fear of behavioral symptom exacerbation, competing priorities, and lack of facility specific goals. Innovative approaches to overcoming these barriers will be reviewed.

### IMPLEMENTATION OF FUNCTION AND BEHAVIOR FOCUSED CARE

Barbara Resnick,[1] and Elizabeth Galik,[2] *1. University of Maryland School of Nursing, Baltimore, Maryland, United States, 2. University of Maryland School of Nursing, Ellicott City, Maryland, United States*

There are many challenges to engaging long term care residents with dementia in physical and functional activities. Resident factors include age, comorbidities, cognitive impairment, motivation, sedation and polypharmacy. Facility factors include the environment, policies and culture within the setting such as a focus on safety versus function. Commonly used non-evidence based interventions include discouraging residents from walking by repeatedly telling them to sit down, and limiting recreational activities to seated positions. De-implementation to remove inaccurate care practices and implementation approaches are needed to facilitate implementation of Function and Behavior Focused Care. This paper will describe the use of the Synthesis Model for the Process of De-Adoption and the Evidence Integration Triangle implementation strategy in the development of the Function and Behavior Focused Care intervention to alter behavior among staff and cognitively impaired residents and optimize function and physical activity in long term care.

### SESSION 7145 (SYMPOSIUM)

### LEVERAGING EXISTING DATA FROM CMS-LINKED COHORT STUDIES FOR THE ADVANCEMENT AND TRANSLATION OF FRAILTY RESEARCH

Chair: Qian-Li Xue
Co-Chair: Kristine Ensrud
Discussant: Shari Lin

As population aging is accelerating rapidly, there is growing concern on how to best provide patient-centered care for the most vulnerable. Establishing a predictable and affordable cost structure for healthcare services is key to improving quality, accessibility, and affordability. One such effort is the “frailty” adjustment model implemented by the Centers for Medicare & Medicaid Services (CMS) that adjusts payments to a Medicare managed care organization based on functional impairment of its beneficiaries. Earlier studies demonstrated added value of this frailty adjuster for prediction of Medicare expenditures independent of the diagnosis-based risk adjustment. However, we hypothesize that further improvement is possible by implementing more rigorous frailty assessment rather than relying on self-report of ADL difficulties as used for the frailty adjuster. This is supported by the consensus and clinical observations that neither multimorbidity nor disability alone is sufficient for frailty identification. This symposium consists of four talks that leverage data from three CMS-linked cohort studies to investigate the utility of assessment of the frailty phenotype for predicting healthcare utilization and costs. Talk 1 and 2 use data from the NHATS cohort to assess healthcare utilization by frailty status in the general population and the homebound subset. Talk 3 and 4 use data from the MrOS study and the SOF study to investigate the impact of frailty phenotype on healthcare costs. Taken together, their findings highlight the potential of incorporating phenotypic frailty assessment into CMS risk adjustment to improve the planning and management of care for frail older adults.

### PHYSICAL FRAILTY, COGNITIVE IMPAIRMENT, AND HEALTHCARE UTILIZATION IN LINKED COHORT AND CLAIMS DATA

Brian Buta,[1] Orla Sheehan,[2] Shang-En Chung,[1] Marcela Blinka,[3] and Qian-Li Xue,[4] *1. Johns Hopkins University, Baltimore, Maryland, United States, 2. Johns Hopkins University School of Medicine, Baltimore, Maryland, United States, 3. Johns Hopkins University, School of Medicine, Baltimore, Maryland, United States, 4. Johns Hopkins School of Medicine, Baltimore, Maryland, United States*

Accurate prediction of healthcare utilization is an important issue for Medicare managed care organizations. We hypothesized that physical frailty and cognitive impairment increase the risk of healthcare utilization in older adults receiving Medicare coverage, independent of age and multimorbidity. We used the marginal means/rates model to investigate the association between baseline cognitive impairment with/without frailty (using the physical frailty phenotype), vs. frailty alone, in NHATS and the number of incident non-ER-related hospitalizations and emergency room (ER) visits within 12 months in linked Medicare claims data (N=3,915). After covariate adjustment, physical frailty alone was predictive of both non-ER-related hospitalizations (HR=1.77; p=0.012) and ER visits (HR=1.75; p<0.001). Cognitive impairment with or without frailty was only associated with ER visits (HR=1.53, p=0.002; HR=1.30, p=0.001). Our findings support the value of physical frailty and cognitive impairment assessment above and beyond multimorbidity to improve the prediction of care utilization for vulnerable subgroups of Medicare beneficiaries.

### ARE ALL HOMEBOUND OLDER ADULTS FRAIL?

Orla Sheehan,[1] Karen Bandeen-Roche,[2] Christine Ritchie,[3] Shang-En Chung,[4] Jeremy Walston,[4] David Roth,[4] and Bruce Leff,[1] *1. Johns Hopkins University School of Medicine, Baltimore, Maryland, United States, 2. Johns Hopkins Bloomberg School of Public Health, Baltimore, Maryland, United States, 3. UCSF, San Francisco, California, United States, 4. Johns Hopkins University, Baltimore, Maryland, United States*

Seven million adults in the United States are homebound and suffer the negative, powerful synergies of multiple chronic conditions, functional impairment, social stressors, and limited social capital. The prevalence of frailty in this vulnerable homebound population is unknown. Using representative data from the National Health and Aging Trends study (NHATS) study linked to Medicare claims (n=4756) we sought to assess the prevalence of frailty in the homebound population (n=361). Among the homebound, 68.5% met the frailty criteria compared to 12.3% of the non-homebound population. The frail homebound had lower educational attainment, were more likely to live alone, self-reported poorer health and more chronic physical and mental health conditions than the non-frail homebound (p<0.05 for all). Frail homebound older adults utilized more health services utilization than non-frail homebound and were twice as likely to be hospitalized (49.8% versus 28.0%, p=0.004).

### FRAILTY PHENOTYPE AND HEALTHCARE COSTS IN OLDER COMMUNITY-DWELLING MEN: THE MROS STUDY

Kristine Ensrud, Allyson Kats, Lisa Lisa Langsetmo, Tien Vo, and John Schousboe, *University of Minnesota, Minneapolis, Minnesota, United States*

To determine the association of the frailty phenotype with subsequent healthcare costs, we studied 1514 men (mean age 79.3 years) participating in the 2007-2009 exam linked with their Medicare claims data. The frailty phenotype (5 components) was categorized as robust, pre-frail or frail. Multimorbidity and a frailty indicator (approximating the deficit accumulation index) were derived from claims data. Functional limitations were assessed by asking about difficulty performing 5 IADL. Total direct healthcare costs were ascertained during 36 months following the exam. Mean annualized costs (2018 dollars) was $5707 among robust, $8964 among pre-frail and $20,027 among frail men. Compared with robust, pre-frailty and frailty were each associated with higher costs after accounting for demographics, multimorbidity, functional limitations and the frailty indicator (cost ratio 1.18 [1.02-1.36] among pre-frail and 1.87 [1.47-2.39] among frail). Findings suggest that assessment of the phenotype may improve identification of individuals at increased risk of costly care.

### FRAILTY PHENOTYPE AND HEALTHCARE COSTS IN WOMEN IN LATE LIFE: THE SOF STUDY

Lisa Langsetmo,[1] Allyson Kats,[1] John Schousboe,[2] Tien Vo,[1] Brent Taylor,[1] and Kristine Ensrud,[1]
*1. University of Minnesota, Minneapolis, Minnesota, United States, 2. University of Minnesota, St Louis Park, Minnesota, United States*

We used data from 1324 women (mean age 83) at the 2002-2004 exam linked with their Medicare claims to determine the association of the frailty phenotype with healthcare costs. The frailty phenotype was categorized as robust, pre-frail or frail. Multimorbidity and a frailty indicator (approximating the deficit accumulation index) were derived from claims. Functional limitations were assessed by asking about difficulty performing IADL. Total direct healthcare costs were ascertained during 36 months following the exam. Compared with robust, pre-frailty and frailty were associated with higher costs after accounting for demographics, multimorbidity, functional limitations and the frailty indicator (cost ratio 1.37 [1.10-1.71] among pre-frail and 1.63 [1.28-2.08] among frail). Discrimination of high-cost (top decile) women was improved by adding the phenotype and functional limitations to a model containing demographics and the claims-based measures. Findings suggest that assessment of the phenotype may improve identification of individuals at higher risk of costly care.

### SESSION 7150 (SYMPOSIUM)

### ESPO/ HEALTH SCIENCES SECTION SYMPOSIUM: LEVERAGING OLDER ADULTS' PERCEPTIONS OF CHRONIC ILLNESS TO IMPROVE CARE

Chair: Anyah Prasad
Co-Chair: Brianna Morgan
Discussant: Mary Naylor

In advanced years of life comorbidity of chronic illnesses is a common phenomenon. While chronic illnesses have been documented to impact overall quality of life, morbidity, and mortality, older adults may develop psychological resources in the years of coping and managing their illnesses. These psychological resources can be influenced by individual perceptions and context as people age, and include concepts such as psychological resilience, inner strength, subjective age, and illness perception. This symposium brings together emerging health science scholars' work on exploring and leveraging these psychological resources to improve care. Amy Ketcham will present a systematic review of psychological resilience and depression in adults with cardiac disease. Brianna Morgan will present a concept analysis exploring the nature of inner strength in people aging with serious illness and cognitive impairment. Anyah Prasad will present results examining the association between chronic illnesses and subjective age and discuss its clinical relevance. Eleanor Rivera will explore illness perception phenotypes in a longitudinal cohort study of older adults with chronic kidney disease. Together, the perspectives shared in this symposium improve understanding of and indicate ways to move toward person-centered and contextual clinical models of care in the management of chronic illnesses among older adults. In addition, the discussant will engage in a dynamic conversation about psychological resources in later life and the role these projects have played in advancing the presenters along their academic trajectories.

### OLDER ADULTS' SUBJECTIVE AGE AS A POTENTIAL PSYCHOLOGICAL RESOURCE IN CLINICAL MANAGEMENT OF CHRONIC ILLNESSES

Anyah Prasad,[1] Nidya Velasco Roldan,[1] Meghan Hendricksen,[2] and Natalie Shellito,[1]
*1. University of Massachusetts Boston, Boston, Massachusetts, United States, 2. University of Massachusetts, Boston, Boston, Massachusetts, United States*

Subject age is predictive of future morbidity and mortality and can be potentially viewed as a psychological resource. However, there seems to be a reciprocal relationship between subjective age and health. In a series of analyses, we demonstrated that various measures of health status such as number of chronic illnesses, self-rated health and sensory impairment have an adverse association with older adults' subjective age. Specifically, chronic illnesses seem to have a period effect and age effect. Living with chronic illness over a period of time seems to attenuate its association with subjective age. Similarly, the association between chronic illnesses and subjective age gets weaker with increase in older adults' chronological age. Therefore, asking those living with chronic health conditions and specifically younger older adults about their subjective age and providing appropriate resources, counseling and reassurance about chronic illness management may prevent the downstream negative health effects of increased subjective age.

### CHARACTERIZING ILLNESS PERCEPTION PHENOTYPES IN OLDER ADULTS WITH CHRONIC KIDNEY DISEASE

Eleanor Rivera,[1] Karen Hirschman,[2] and Raymond Townsend,[1] *1. University of Pennsylvania, Philadelphia, Pennsylvania, United States, 2. NewCourtland Center for Transitions and Health, Philadelphia, Pennsylvania, United States*

An individual's understanding of their chronic illness (illness perception) is a psychological resource that has an impact on coping and self-management behaviors. Our previous study identified illness perception phenotypes (overall patterns of illness perceptions) in a sample of older adults with heart failure, COPD, and chronic kidney disease. These phenotypes were associated with perceived self-management ability (patient activation) and recent hospitalizations. To further characterize the illness perception phenotypes we focused on older adults with chronic kidney disease, analyzing illness perception data along with potential covariates from the multi-center longitudinal Chronic Renal Insufficiency Cohort study (CRIC). Covariates include sociodemographics, disease parameters, personality type, disease knowledge, and treatment adherence. While personality type was associated with illness perception phenotype, disease knowledge and treatment adherence were not. We have also conducted qualitative analyses of in-depth interviews. These results will inform the development of a pilot intervention incorporating illness perception information into the clinical setting.

### EXPLORING INNER STRENGTH IN PEOPLE AGING WITH SERIOUS ILLNESS AND COGNITIVE IMPAIRMENT

Brianna Morgan, *University of Pennsylvania, Philadelphia, Pennsylvania, United States*

Inner strength is a psychological resource that supports people as they move through challenging life events. Understanding how people living with serious illness conceptualize inner strength is key in informing person-centered healthcare. This concept analysis used dimensional analysis methods to explore the nature of inner strength in people aging with serious illness to define a situation specific theory. 1212 abstracts and articles from the published literature were reviewed. 30 articles were included and analyzed as data sources. The resulting explanatory matrix conceptualized inner strength in people as an inward and outward process of "meeting me" – an authentic version of oneself – in the context of shifting health. Of note, while other serious illnesses were represented, no literature focused on dementia. Understanding inner strength in people with dementia is the next step for further inquiry. A pilot study exploring feasibility and informing further research is planned.

### ASSOCIATION OF PSYCHOLOGICAL RESILIENCE AND DEPRESSIVE SYMPTOMS IN ADULTS WITH CARDIAC DISEASE

Amy Ketcham, Austin Matus, and Barbara Riegel, *University of Pennsylvania, Philadelphia, Pennsylvania, United States*

Depressive symptoms predict hospitalization and mortality in adults with cardiac disease. Psychological resilience may protect against depressive symptoms, but benefits are not yet conclusive. We conducted a systematic review to examine the association between resilience and depressive symptoms in adults with cardiac disease. Seven databases were searched from inception to December 2019 using the search terms "cardiac disease," "depressive symptoms," "depression," and "resilience." The 623 articles identified were narrowed through title, abstract and full-text review leaving 13 studies for final analysis. Resilience and depressive symptoms were inversely related in 10 of 13 studies. The three studies with poor quality sampling techniques or significant loss to follow-up found null results. The major gap identified in the literature was poor understanding of the longitudinal pattern between resilience and depressive symptoms. If the direction of causality functions as expected in longitudinal research, optimizing resilience could help attenuate depressive symptoms.

### SESSION 7155 (SYMPOSIUM)

### LIFELONG ENGAGEMENT: THE ROLE OF COGNITIVE RESERVE AND PHYSICAL HEALTH IN VERY LATE LIFE

Chair: Peter Martin
Co-Chair: Bradley Willcox
Discussant: D. Craig Willcox

At the end of a very long life, older adults often experience a significant decline in cognitive function. However, there are older adults who have maintained high levels of cognition and physical health. The purpose of this symposium is to illuminate interdisciplinary findings of cognitive engagement with late-life benefits of cognitive functioning and physical health. Components of cognitive reserve include sociodemographic variables (e.g., education, occupational complexity and responsibility), psychosocial variables (e.g., engaged life style and activity) and physical and genetic reserve (e.g., strength, APOE4). Based on three major research studies (the Japanese SONIC study; the Honolulu Asia Aging Study, HAAS; and the Georgia Centenarian Study, GCS), we highlight important aspects of building cognitive reserve and the implications for cognitive and physical health. The first presentation evaluates the importance of work complexity as a predictor of cognitive and physical health among participants of the SONIC study. Multiple group analyses yielded strong associations of occupational complexity with cognitive functioning for men. The second presentation reports logistic regression findings from the HAAS including education, strength and genetic markers, as well as mental health and their relatedness to cognitive abilities and physical health. The final presentation evaluates a structural equation model from the GCS, highlighting the interrelationship of cognitive reserve components (i.e., education, occupational responsibility, engaged lifestyle, social support, and activity) with cognitive and physical health in very late life. We will summarize and integrate the findings for their theoretical and practical implications and provide future directions.

### COGNITIVE RESERVE AND WELL-BEING AMONG CENTENARIANS

Peter Martin,[1] Gina Lee,[1] Yasuyuki Gondo,[2] and Leonard Poon,[3] *1. Iowa State University, Ames, Iowa, United States, 2. Osaka University, Suita, Osaka, Japan, 3. University of Georgia, Athens, Georgia, United States*

The purpose of this study was to assess cognitive reserve among centenarians and to evaluate whether reserve variables explain significantly more variance in cognitive functioning than education alone. Centenarians from the Georgia Centenarians Study were included. Results indicate that education, activity, social engagement, and engaged lifestyle significantly related to cognitive functioning. After adding cognitive reserve variables, the effect of education on cognitive functioning diminished. The overall model fit well, X2 (df=6) = 12.35, p = .06; CFI = .97, RMSEA = .067. Education indirectly related to cognitive functioning through occupational responsibility and engaged lifestyle. Social engagement directly related to cognitive functioning but also indirectly through activity levels. The four direct predictors (i.e., education, social engagement, activity, and engaged lifestyle) explained 33 percent of the variance in cognitive functioning. The results provide important information for cognitive reserve theories with implications for physical health and interventions that build cognitive reserves.

### RISK AND PROTECTIVE FACTORS FOR COGNITIVE RESERVE IN OLDEST-OLD JAPANESE AMERICANS

Bradley Willcox,[1] Randy Chen,[2] Tim Donlon,[3] D. Craig Willcox,[4] Richard Allsopp,[3] and Kamal Masaki,[2] *1. Kuakini Health Systems, Honolulu, Hawaii, United States, 2. Kuakini Medical Center, Honolulu, Hawaii, United States, 3. University of Hawaii, Honolulu, Hawaii, United States, 4. Okinawa International University, Urasoe, Okinawa, Japan*

3,734 Japanese-American male oldest-old (aged 85+ years), from the Kuakini Honolulu Asia Aging Study, were assessed for prevalent cognitive impairment (CI). CI was defined as scoring <74 on the 100-point Cognitive Abilities Screening Instrument (CASI; 80% sensitivity, 90% specificity for dementia). CASI tests from 1991 (Exam 4) to 2012 (Exam 12) identified 1496 cases of CI (i.e. low cognitive reserve) and 1222 non-CI controls (mean diagnosis age: 85.7 ± 5.3; range 71-100 years). Baseline risk factors were compared between groups, adjusted for age at onset of CI or last CASI. Step-wise conditional logistic regression was used to assess risk for CI. Among other factors, education (0.88; 0.85-0.9, p<0.0001), hand-grip strength (0.98; 0.97-0.99, p=0.03), and height (0.97; 0.96-0.99, p=0.002) had protective effects; whereas APOE4 carriage (1.31; 1.04-1.63, p=0.02) and depression (1.4; 1.06-1.96, p=0.02) were risk factors for CI. The implications of these data for cognitive and physical health will be discussed.

### OCCUPATIONAL COMPLEXITY AND LATE-LIFE GLOBAL COGNITION: THE SONIC STUDY

Yoshiko Ishioka,[1] Yasuyuki Gondo,[2] Yukie Masui,[3] Takeshi Nakagawa,[4] Hiroki Inagaki,[3] Madoka Ogawa,[3] Saori Yasumoto,[2] and Tatsuro Ishizaki,[3] *1. Keio University, Minato-ku, Kanagawa, Japan, 2. Osaka University, Suita, Osaka, Japan, 3. Tokyo Metropolitan Institute of Gerontology, Itabashi-ku, Tokyo, Japan, 4. National Center for Geriatrics and Gerontology, Ōbu, Aichi, Japan*

Studies have reported that work exposure to cognitively demanding environments predicted the level of late-life cognitive abilities. To date, whether or not such a relationship between work complexity and cognitive function is maintained in very old adults remains unknown. In the present study, we examined how the associations between lifetime work's complexity and global cognition vary by age groups (70s, 80s, and 90s). To this end, we used data from 2754 Japanese community-dwelling participants in the SONIC Project. Specifically, we tested multiple group path models comparing the models based on differences in age and gender. The effects of work complexities on global cognition were found for male septuagenarians and octogenarians, having controlled for the variables related to education. The relationships between them were marginally significant for male nonagenarians. Based on the analysis, we discuss the maintenance of cognitive reserve and implications for cognitive and physical health in very old ages.

### SESSION 7160 (SYMPOSIUM)

### MECHANISMS OF RACIAL AND ETHNIC DIFFERENCES IN COGNITIVE AGING

Chair: Andrea Rosso
Discussant: Jennifer Manly

Racial and ethnic disparities in age-related cognitive function and dementia risk in the US are well recognized. However, the psychosocial drivers of these disparities and underlying mechanisms are less well studied. This symposium will highlight novel research regarding our current understanding of racial/ethnic differences in brain and cognitive aging and the underlying mechanisms of the disparities. Frist, two papers will describe results regarding racial/ethnic differences in cognitive function and brain aging markers. Few studies have assessed racial/ethnic differences in cognitive function across age groups. Indira Turney will utilize data from a multigenerational study to explore how age impacts racial/ethnic differences in cognitive function. Underlying brain mechanisms of racial/ethnic differences in cognitive outcomes are also not well defined. Sara Godina will present a systematic review of racial/ethnic differences in structural markers of brain aging and neuropathology. Second, three papers will explore how various risk factors may explain the racial/ethnic disparities in cognitive outcomes. Melissa Lamar will demonstrate the differential associations of various blood pressure indicators with cognitive change among black older adults. B. Gwen Windham will present data from two studies that illustrate differential effects of common risk factors by race and region, highlighting inherent difficulties in race-place disparity research. Finally, Laura Zahodne will present results on how psychosocial factors, beyond socioeconomic status, contribute to racial/ethnic disparities in cognitive function. Jen Manly will lead a discussion on the implications of these results for the future of dementia prevention efforts for an increasingly diverse older US population.

### DOES RACE OR ETHNICITY MODIFY THE IMPACT OF AGE ON COGNITION IN MIDDLE- AND OLDER-AGED ADULTS?

Indira Turney,[1] Miguel Arce Rentería,[2] Patrick Lao,[3] Adam Brickman,[2] and Jennifer Manly,[2] *1. Columbia University Medical Center, New York, New York, United States, 2. Columbia University, New York, New York, United States, 3. Columbia University Medical Center, new york, New York, United States*

We compared verbal list learning, verbal memory, animal fluency, and letter fluency in 1407 education-matched participants from two community-based, intergenerational studies of cognitive aging and dementia. WHICAP participants are sampled from Medicare-eligible people aged 65+ and the Offspring cohort includes their middle-aged children. WHICAP participants (n=1218) were 72.1±6.5 years old and Offspring participants (n=189) were 53.7±8.4 years old at baseline. WHICAP participants had lower scores on most cognitive measures than Offspring participants; however, these differences were not uniform across race/ethnicity. Compared to non-Hispanic Whites, non-Hispanic Blacks in WHICAP had disproportionately lower scores on letter fluency compared to their offspring. On delayed verbal memory, non-Hispanic White and Hispanic offspring obtained higher scores than the parent generation – but among Blacks, memory scores were relatively low regardless of cohort. Racial disparities in cognition are apparent in both mid- and late-life and may be amplified in older age, particularly in Blacks.

### RACIAL AND ETHNIC DISPARITIES IN STRUCTURAL MEASURES OF BRAIN AGING: A SYSTEMATIC REVIEW

Sara Godina,[1] Indira Turney,[2] Laura Zahodne,[3] Danielle Beatty Moody,[4] Kacie Deters,[5] Jennifer Manly,[6] and Andrea Rosso,[7] *1. University of Pittsburgh Graduate School of Public Health, Pittsburgh, Pennsylvania, United States, 2. Columbia University Medical Center, New York, New York, United States, 3. University of Michigan, Ann Arbor, Michigan, United States, 4. University of Maryland Baltimore County, Baltimore, Maryland, United States, 5. Stanford University, Palo Alto, California, United States, 6. Columbia University, New York, New York, United States, 7. School of Public Health, University of Pittsburgh, Pittsburgh, Pennsylvania, United States*

Examining the neural correlates underlying racial differences in cognitive functioning is necessary to help clarify mechanisms and to improve the generalizability of findings related to brain-behavior relationships. This systematic review aimed to determine whether there are racial differences in structural markers of brain aging among community-dwelling, neurologically healthy adults aged 18 and older. We identified studies (n=5,399) from searches in Ovid Medline, Ovid PsychINFO, and Elsevier EMBASE published until February 13, 2020. We included original articles that examined structural markers of brain aging and neuropathology across individuals from more than one race or ethnicity. These measures included magnetic resonance imaging, positron emission tomography amyloid and tau, cerebrospinal fluid amyloid, tau, and neurofilament light chain and computed tomography. Two reviewers independently screened full-text articles. We will present ongoing results and discuss the current state of knowledge, quality of existing studies, and gaps in the literature and highlight potentially key next steps.

### RELATIONSHIP OF INDICATORS OF CARDIOVASCULAR HEALTH WITH CHANGE IN COGNITION IN OLDER BLACKS

Melissa Lamar,[1] Debra Fleischman,[2] Sue Leurgans,[3] Konstantinos Arfanakis,[2] and Lisa Barnes,[1] *1. Rush University Medical Center, Chicago, Illinois, United States, 2. Rush Alzheimer's Disease Center, Chicago, Illinois, United States, 3. Rush Alzheimer's Disease Center, Chicago, Illinois, United States*

Older Blacks perform more poorly on cognitive testing than older Whites. Increased prevalence of cardiovascular disease risk factors (CVD-RFs) among Blacks compared to Whites contribute to these disparities. We investigated whether white matter hyperintensities (WMHs) in late-life, a consequence of mid-life CVD-RFs, modify the association between late-life CVD-RFs and cognition in 167 Blacks (age~75yrs; non-demented at baseline). Participants were evaluated for blood pressure (BP) markers of cardiovascular health (systolic/diastolic BP, pulse pressure, mean arterial pressure (MAP) hypertension), WMH volumes adjusted for intracranial volume, and cognition (global and domain-specific at baseline and annually, ~8yrs). Adjusted models revealed differential associations between BP markers and baseline cognition; diastolic BP and MAP predicted faster decline in episodic memory. Hypertension was not significant in any model; however, when adding WMH, the hypertension*WMH interaction was significant for baseline cognition. Cognition among older Blacks is a complex function of BP markers of cardiovascular health and WMH.

### RACE AND REGIONAL DIFFERENCES IN RISK FACTORS FOR COGNITIVE IMPAIRMENT FROM A COMBINED COHORT ANALYSIS

B. Gwen Windham,[1] Michael Griswold,[2] James Henegan,[1] David Knopman,[3] Seth Lirette,[1] Michelle Mielke,[3] Ron Petersen,[3] and Thomas Mosley,[1] *1. University of Mississippi Medical Center, Jackson, Mississippi, United States, 2. The University of Mississippi Medical Center, Jackson, Mississippi, United States, 3. Mayo Clinic, Rochester, Minnesota, United States*

Few studies examined race-regional influences on mild cognitive impairment (MCI) and dementia risk factors. We examined 14 risk factors in 5 race-regions, e.g. cross-sectional age- and sex-adjusted relative prevalence ratios (RPR) from multinomial regression comparing associations of low education (<12yrs) with MCI and dementia (versus normal cognition) across 3 race-region groups, average age 76yrs: whites in the Mayo Clinic Study of Aging[MCSA, Olmsted County, MN—MCSA-MN-Whites); Atherosclerosis Risk in Communities(ARIC) whites in suburban Minneapolis, MN—ARIC-MN-Whites); ARIC-African Americans in Jackson, MS—ARIC-MS-AA. Among 3,787 MCSA-Whites, low education exerted a 2-fold risk for MCI, RPR=2.09(95%CI: 1.57,2.78). Conversely, low education was not a supported MCI risk factor for ARIC-MN-Whites (n=1,901—RPR=0.63(0.31,1.28) or ARIC-MS-AA, (n=1,416—RPR=.0.81 (0.60,1.10)), with substantially differential race-region effects. Low education RPRs for dementia also differed by race-region: RPR=4.43(2.68,7.31-MCSA-MN-W), RPR=0.70 (0.16,2.99-ARIC-MN-Whites), and RPR=2.54 (1.74,3.72-ARIC-MS-AA). Understanding why risk factors differ by race and region likely requires diverse samples and harmonized methods using culturally-appropriate assessments.

### SOCIOECONOMIC AND PSYCHOSOCIAL MECHANISMS UNDERLYING RACIAL AND ETHNIC DISPARITIES IN COGNITION AMONG OLDER ADULTS

Laura Zahodne,[1] Neika Sharifian,[1] A. Zarina Kraal,[1] Emily Morris,[1] Afsara Zaheed,[1] Ketlyne Sol,[1] Jennifer Manly,[2] and Adam Brickman,[2] *1. University of Michigan, Ann Arbor, Michigan, United States, 2. Columbia University, New York, New York, United States*

Racial/ethnic disparities in cognitive aging are only partly attributable to socioeconomic indicators. Emerging literature highlights psychosocial factors, such as related constructs of discrimination and perceived control. Using data from 1,463 older adults (51% Hispanic, 27% non-Hispanic Black, 22% non-Hispanic White) in the Washington Heights-Inwood Columbia Aging Project, cross-sectional mediation models quantified separate indirect effects of Black race and Hispanic ethnicity on global cognitive composite scores. Socioeconomic status explained approximately 50% of Black-White and Hispanic-White disparities in cognition. Perceived control explained an additional 5-8%. Discrimination was not associated with cognition. Significant racial/ethnic disparities remained after accounting for the included socioeconomic and psychosocial factors, indicating that future studies should consider additional potential mediators. Lower perceived control, which likely reflects chronic exposure to interpersonal and institutional marginalization, may be a particularly salient psychosocial risk factor for poorer cognitive aging among certain racial/ethnic minority groups.

### SESSION 7165 (SYMPOSIUM)

### MORE ENERGY AND LESS FATIGUE: IMPLICATIONS FOR SUCCESSFUL BRAIN AGING

Chair: Caterina Rosano
Co-Chair: Nancy Glynn

Higher energy and lower fatigue are intuitively important to live independent, active lives. However, little is known about the relation between brain health, energy, and fatigue in older adults. From a neurobiological standpoint, energy and fatigue appear to rely on distinct, albeit overlapping brain networks, but most evidence is from patients with neurodegenerative conditions. These relations have not been fully examined in community older adults. In this symposium, we first present an overview of the neurobiology underlying fatigability and energy states. We will then present original unpublished data on brain health, fatigability, and energy from four well-established epidemiological studies of aging: Osteoporotic Fractures in Men Study (MrOS), Long Life Family Study (LLFS), Baltimore Longitudinal Study of Aging (BLSA), and the Health Aging Body Composition Study (Health ABC). Specifically, Ms. Allen will explore whether personality traits are related to perceived mental fatigability in MrOS. Using LLFS data, Ms. Gmelin will examine whether perceived physical fatigability is associated with global cognition, verbal fluency, memory and psychomotor speed. Dr. Schrack will share BLSA data showing cross-sectional and longitudinal associations between lower walking efficiency and reduced brain volumes. Dr. Tian will evaluate the neuroimaging signature of perceived energy levels in Health ABC. Taken together, our data indicate that higher energy and lower fatigability likely reflect overlapping but distinct aspects of brain health. The long-term effects of promoting energy and lowering fatigability on dementia should be further studied.

### GREATER PERCEIVED PHYSICAL FATIGABILITY IS ASSOCIATED WITH LOWER COGNITION: THE LONG LIFE FAMILY STUDY

Theresa Gmelin,[1] Andrea Rosso,[2] Stacy Andersen,[3] Stephanie Cosentino,[4] Mary Wojczynski,[5] Kaare Christensen,[6] Robert Boudreau,[1] and Nancy Glynn,[1] *1. University of Pittsburgh, Pittsburgh, Pennsylvania, United States, 2. School of Public Health, University of Pittsburgh, Pittsburgh, Pennsylvania, United States, 3. Boston University School of Medicine, Boston, Massachusetts, United States, 4. Columbia University Medical Center, New York, New York, United States, 5. Washington University School of Medicine, St Louis, Missouri, United States, 6. University of Southern Denmark, Odense C, Syddanmark, Denmark*

Greater perceived physical fatigability is associated with physical functional decline, but few studies have examined its relation with cognition. Adults ≥60 (mean±SD age 73.7±10.5, 54.7% female, 99.6% white) from the Long Life Family Study (n=2355) completed the Pittsburgh Fatigability Scale (PFS, 0-50, higher=greater fatigability) and a neurocognitive examination. Generalized estimating equations were used to account for family structure. Covariates included age, sex, field center, depressive symptoms (Center for Epidemiological Studies-Depression), education, and self-reported health. Each 1-point greater PFS was associated with lower: (1) global cognition (Mini-Mental Status Exam; $\beta$=-0.36,p<.0001), (2) verbal fluency (phonemic: $\beta$=-0.09,p=.029 and semantic: $\beta$=-0.14,p<.0001), (3) memory (Hopkins Verbal Learning Test-Revised: $\beta$=-0.06,p=.037), and (4) psychomotor speed (Digit Symbol Substitution Test: $\beta$=-0.10,p<.0001), after covariate adjustment. Greater perceived physical fatigability was significantly associated with lower memory and cognitive function in older adults, and may represent a promising new biomarker of biological aging reflecting declining brain reserve, resilience, and neurodegeneration.

### THE RELATIONSHIP BETWEEN PERSONALITY AND PERCEIVED MENTAL FATIGABILITY

Hannah Allen,[1] Theresa Gmelin,[2] Stephen Smagula,[2] Robert Boudreau,[2] Jane Cauley,[2] and Nancy Glynn,[2] *1. University of Pittsburgh Graduate School of Public Health, Pittsburgh, Pennsylvania, United States, 2. University of Pittsburgh, Pittsburgh, Pennsylvania, United States*

Several personality traits are known to be protective against global fatigue, however perceived mental fatigability (PMF, Pittsburgh Fatigability Scale 0-50) specifically measures an individual's susceptibility to cognitive tiredness and is associated with mobility decline. We assessed whether optimism, conscientiousness, goal reengagement and goal disengagement contributed to greater PMF in 1,812 men (mean±SD age 84.4±4.2 years, 90.4% white) in the Osteoporotic Fractures in Men Study 4th visit (2014-2016). Covariates included demographic, psychological/behavioral factors, health conditions, physical activity and function. Prevalence of higher PMF (score ≥13) was 25% (n=448). In a covariate-adjusted regression model, each SD lower conscientiousness and lower optimism were associated with 0.93 and 0.61 SDs greater PMF, each p<0.01. Goal disengagement and goal reengagement were not associated with PMF. These findings warrant further investigation into how personality traits may help clinicians design targeted and effective interventions to reduce fatigability, and consequently lower the risk of adverse aging-related health outcomes.

### CHANGES IN SELF-REPORTED ENERGY AND BRAIN VOLUMES

Qu Tian,[1] Andrea Rosso,[2] Nancy Glynn,[2] Xiaonan Zhu,[2] and Caterina Rosano,[2] *1. National Institute on Aging, Bethesda, Maryland, United States, 2. School of Public Health, University of Pittsburgh, Pittsburgh, Pennsylvania, United States*

The brain demands and consumes more energy than any other organ. Lower perceived energy may indicate compromised brain health. Little empirical data exists on the association between perceived energy and brain structure. Neuroimaging was obtained in 300 participants (mean age=83±3 y/o, 40% blacks, 57%women) with repeated self-reported energy measures(scale0-10) in the past decade. Energy decline was computed as rate of change by linear mixed models(-0.06/year). Associations of energy decline with volumes of cognitive (dorsolateral prefrontal cortex, hippocampus) and motor (precentral gyrus, putamen, caudate) areas were examined using linear regression, adjusted for demographics and total gray matter atrophy. A steeper decline in energy was associated with smaller volumes of right putamen (p=0.013) and caudate (p=0.043), a trend in right precentral gyrus (p=0.085), but not in prefrontal cortex or hippocampus. Declining energy by self-report may indicate atrophy localized in subcortical motor areas. Studies to identify the mechanisms underlying these associations are warranted.

### THE LONGITUDINAL ASSOCIATION OF WALKING EFFICIENCY WITH BRAIN VOLUMES IN COMMUNITY-DWELLING OLDER ADULTS

Jennifer Schrack,[1] Fangyu Liu,[1] Amal Wanigatunga,[1] Yang An,[2] Christos Davatzikos,[3] Eleanor Simonsick,[2] Luigi Ferrucci,[2] and Susan Resnick[2] *1. Johns Hopkins Bloomberg School of Public Health, Baltimore, Maryland, United States, 2. National Institute on Aging, Bethesda, Maryland, United States, 3. University of Pennsylvania, Philadelphia, Pennsylvania, United States*

Walking efficiency (WE) predicts mobility decline and is linked with higher fatigability. Fatigability is associated with cognitive decline and reduced brain volumes (BV), but the link between WE and BV is undefined. We examined associations between WE and BV in 860 participants of the BLSA (mean age 66.4(14.4) years, 54.5% women). WE was assessed during 2.5-minutes of usual-paced walking using indirect calorimetry and standardized per meter (ml/kg/m). BV measures were derived using MRI scans and an automated multi-atlas region-of-interest approach. In linear mixed models adjusted for demographics, education, BMI, intracranial volume, and cognitive status, lower baseline WE was associated with lower total, white, and gray matter, primarily in the frontal and temporal lobes (all p<0.05). Longitudinally, declining WE was associated with increasing ventricular and decreasing hippocampal volumes over follow-up (all p<0.01). Findings suggest rising age-related inefficiencies may reflect underlying brain atrophy and serve as a novel indicator for future interventions.

### SESSION 7170 (SYMPOSIUM)

### MUSIC AND BRAIN HEALTH: RECOMMENDATIONS FROM THE GLOBAL COUNCIL ON BRAIN HEALTH, AN AARP COLLABORATIVE

Chair: Sarah Lock

Music is a complex auditory stimulus that resonates on a physiological, psychological, and spiritual level for people around the world. This symposium will provide highlights from the Global Council on Brain Health consensus report aimed at helping the public to understand the potential that music holds for supporting and enriching brain health. The Global Council on Brain Health (GCBH) is an independent collaborative of scientists, clinicians, scholars, and policy experts convened by AARP to provide evidence-based advice on what people and professionals can do to maintain and improve brain health. The Council translates scientific research into actionable recommendations for the public that will help drive behavior change in individuals across communities and cultures. Issue specialists from around the world were brought together to build consensus, issue recommendations, and offer practical tips. Moreover, we will feature research from our issue experts and provide an overview of the impact of music participation on older adults, including those with dementia. Data from surveys fielded by AARP research, developed in consultation with the GCBH, will also be featured. In sum, this presentation will highlight the work of the Council at the forefront of this international effort to translate advancements in brain health research to the wider public, with an emphasis on individuals aged50 and older.

### GLOBAL COUNCIL ON BRAIN HEALTH: CONSENSUS AND RECOMMENDATIONS FOR PROMOTING BRAIN HEALTH THROUGH MUSIC ENGAGEMENT

Jacobo Mintzer, *Roper St. Francis Healthcare, Charleston, South Carolina, United States*

This presentation will summarize the points of consensus reached by the experts and describes the major points of discussion that led to their recommendations for men and women age 50 and older. The GCBH considered questions surrounding what happens in the brain when listening to music as well as creating music. Topics such as music therapy and the clinical dimensions of introducing music into caring for patients will be addressed. Alongside the consensus points, recommendations and practical tips will be presented. Moreover, we will also identify gaps in our knowledge that currently exist in the evidence base surrounding music and brain health.

### THE EFFECT OF MUSIC THERAPY ON COGNITIVE FUNCTIONING AMONG OLDER ADULTS

Kuei-Min Chen, *Kaohsiung Medical University, Kaohsiung City, Taiwan (Republic of China)*

Music creates and fosters connection and interrelationships between individuals and encourages social interaction. Indeed, community-based interventions are a powerful way to engage older people. This presentation will provide an overview of music therapy and the impact it can have on the cognitive functioning of older people. Examples of music therapy interventions in the community can be found in nursing homes, hospitals, or communities. Differences in receptive (passive) music therapy and active music therapy will be discussed along with the impact these therapies may have on individuals experiencing cognitive decline. Moreover, studies of music therapy combined with other activities, such as exercise or art will be discussed. This research will be presented within the context of the recommendations put forth by the Global Council on Brain Health aimed at adults aged 50+.

### BENEFITS OF THE UNFORGETTABLES: A CHORUS FOR PEOPLE WITH DEMENTIA TOGETHER WITH THEIR FAMILY CAREGIVERS

Mary Mittelman, *NYU School of Medicine, New York, New York, United States*

The Unforgettables was founded in 2011 for people with dementia and their family caregivers. We hypothesized that singing and rehearsing together would providing an opportunity for people in the early and moderate stages of dementia and their family caregivers to share a normative, stimulating and social activity. Pilot study results showed that quality of life and communication with the other member of the dyad improved for people with dementia; quality of life and, social support, communication and self- esteem improved for caregivers. Moreover, people with dementia learn new songs for every performance, suggesting that this activity may slow cognitive decline. The chorus continues to rehearse and perform, and now has approximately 100 members in two locations in NYC. These findings support the recommendations of the Global Council on Brain Health by underscoring the many benefits of music performance in enhancing social engagement and providing joy to participants and the community.

### SESSION 7175 (SYMPOSIUM)

### NEW EVIDENCE ON THE BIOBEHAVIORAL MECHANISMS OF CHRONIC PAIN IN AGING AFRICAN AMERICANS

Chair: Staja Booker

African American older adults are living longer with chronic pain, which presents a huge personal and societal burden. A growing group of scholars are now devoted to accurately and precisely characterizing and phenotyping the experience of pain in aging using within-group and advanced methodological designs to elucidate the biopsychosocial-behavioral responses to pain. In this symposium, five dynamic presenters present new evidence on mechanisms of pain in older African-Americans. Dr. Roach's investigation reveals the effect of genetic alterations of sickle cell disease (SCD) on stress-related pain in younger and older adults; this scientific inquiry is especially important because there is little research on SCD in aging. Next, Dr. Terry, extends these findings by exploring the association between psychosocial factors such as experiences of discrimination, pain catastrophizing, and perceived stress on neural (brain) responses via magnetic resonance imaging. From a clinical perspective, Dr. Booker reports on the first-ever model of intra-racial differences in movement-evoked pain in older African-Americans with knee osteoarthritis and healthy controls. Our final two presenters use a translational approach to identify how older African-Americans cope with chronic pain. Dr. Robinson-Lane's study highlights the unique experience and predictors of coping, adaptation, and self-management of chronic pain in Black dementia caregivers. Finally, Dr. Cobb's research from a large cross-sectional study correlates social, behavioral, and health factors with opioid and psychotropic use in economically disadvantaged older African-Americans. This symposium offers novel ways of understanding social determinants of pain and assisting African-Americans and their caregivers to manage complex chronic pain in later life.

### A PRELIMINARY MEASUREMENT AND UNDERSTANDING OF MOVEMENT-EVOKED PAIN IN AFRICAN AMERICAN ELDER ADULTS

Staja Booker, *The University of Florida, Gainesville, Florida, United States*

Osteoarthritis (OA) is a principal cause of disabling knee pain, and movement is a known exacerbator of pain in African Americans (AAs). Still, research has neglected to understand the relationship between pain with movement and its impact on function and mobility. Our previous study found significantly higher movement-evoked pain between AAs and White American (WAs). Therefore, this case-control observational study investigated inter-racial and intra-racial differences in movement-evoked pain in AAs and WAs (N= 28) who were 55-78 years-of-age (M= 65.75, SD= 6.23).

We measured pain intensity (0-10) pre/ante/post multiple performance-based functional activities; we report preliminary results for 7-meter GAITRite® walk and Stair climbs. Pain intensity was higher before and after the 7-meter walk and stair climbs in AAs, although not significantly different than WAs. We will conduct additional statistical tests for the remaining functional activities to identify potential differences and ethnic-specific factors that distinguish movement-evoked pain and function by race.

### CAREGIVING, PAIN, AND DEMENTIA: EVALUATING THE ROLE OF COPING AND ADAPTATION

Sheria Robinson-Lane, and Xingyu Zhang, *University of Michigan, Ann Arbor, Michigan, United States*

Pain is a stressor that can negatively influence quality of life for caregivers. Dementia caregivers have an increased risk for stress-related health outcomes including death. Few studies have examined the relationships between pain and coping-related outcomes for dementia caregivers. In the present study, Black family caregivers (n=56) completed a survey inclusive multiple health and coping measures. In addition to descriptive statistics, Pearson's correlation coefficients were completed. 33% of caregivers experienced moderate to severe pain. The majority of participants with pain (72%) also had hypertension and were obese (69%). Pain intensity was significantly correlated with anxiety (p=.001). Effective coping and adaptation was correlated with perceived social support (p=.002) and perceived positive aspects of caregiving (p=.0.027). The primary coping strategies used by caregivers with chronic pain included spiritual coping, information gathering, reliance on past experiences, and maximizing resource use. Improving pain outcomes for caregivers may benefit both caregivers and persons with dementia.

### UNCOVERING THE INFLUENCE OF PSYCHOSOCIAL FACTORS ON PAIN-RELATED BRAIN RESPONSES IN OLDER ADULTS WITH CHRONIC PAIN

Ellen Terry,[1] Staja Booker,[2] Keesha Roach,[1] Sharon Cobb,[3] and Sheria Robinson-Lane,[4] *1. University of Florida, Gainesville, Florida, United States, 2. The University of Florida, Gainesville, Florida, United States, 3. Charles R. Drew University of Medicine & Science, Los Angeles, California, United States, 4. University of Michigan, Ann Arbor, Michigan, United States*

Psychosocial factors such as experiences of discrimination, pain catastrophizing and perceived stress are associated with poor osteoarthritis-related pain and disability outcomes across sex and ethnic/race groups. However, the mechanisms that mediate these psychosocial factors and knee osteoarthritis outcomes across race and sex are unclear. A cross-sectional correlational design identified the associations between everyday discrimination and clinical pain, disability and functional performance among 188 non-Hispanic Black (NHB) and non-Hispanic White (NHW) persons with knee osteoarthritis. In a serial mediated model, perceived stress and pain catastrophizing mediated the relationship between discrimination and osteoarthritis-related outcome variables in female participants. Using magnetic resonance imaging, findings suggest that experiences of discrimination differentially affect structural brain regions based on both race/ethnicity and sex in older adults with knee osteoarthritis. Given this, we are also currently investigating the extent to which pain catastrophizing on pain-related brain structure differs across race/ethnic groups in older adults with knee osteoarthritis.

### AVPR1A AND STRESS IN ADULTS WITH SICKLE CELL DISEASE–RELATED CHRONIC PAIN

Keesha Roach,[1] Brenda Dyal,[1] Srikar Chamala,[1] Yingwei Yao,[1] Roger Fillingim,[2] Zajie Wang,[3] Robert Molokie,[3] and Diana Wilkie,[1] *1. University of Florida, Gainesville, Florida, United States, 2. University of Florida, Pain Research and Intervention Center of Excellence, Gainesville, Florida, United States, 3. University of Illinois at Chicago, Chicago, Illinois, United States*

Purpose: Emotional stress is a known pain trigger in patients with sickle cell disease (SCD). The Arginine vasopressin receptor 1A gene (AVPR1A), SNP rs10877969, is associated with acute pain and stress-related pain. Our study investigated the association between AVPR1A genotype with stress and age in adults with SCD pain. Methods: 169 participants with SCD and chronic pain (100% African descent; mean age 36.4 ± 11.6 years [range =18-74 years]) completed the Perceived Stress Questionnaire. The SNP was evaluated as the imputed score was R2>0.8. ANOVA compared stress by genotype and age. Findings: Mean stress scores were significantly lower (p<0.05) for the older adults (0.35 ± 0.18) than the younger adults (0.41 ± 0.17). Mean stress scores were not significantly different by genotype for younger or older adults. Discussion: The rs10877969 genotype frequency was not different by age. In contrast to prior research, there was no association between genotype and stress.

### CORRELATES OF PAIN, OPIOID USE, AND PSYCHOTROPIC MEDICATIONS AMONG OLDER AFRICAN AMERICANS

Sharon Cobb, Mohsen Bazargan, and Shervin Assari, *Charles R. Drew University of Medicine & Science, Los Angeles, California, United States*

Unrecognized and undertreated pain among older African Americans (AAs) is well-documented. This study explored the link between social, behavioral, and health correlates of pain and psychotropic as well as opioid-based medications in a sample of underserved 740 AA older adults. Almost 16% and 17% of participants used at least one psychotropic and opioid-based medications, respectively. Of those who use opioid-based medications, 73% used opioids only, 28% used opioids and at least one psychotropic medication. Use of opioid or psychotropic medications were associated with increased polypharmacy. Multivariate analysis showed different types of pain are predictors of opioid use, however, depressive symptoms and level of pain were associated with use of psychotropic medication. Moreover, the relationship of pain and psychotropic medications warrants more study due to emerging mental health crisis. These findings underscore the need for optimal concurrent management of pain and mental health for older AAs with potential inappropriate medication use.

### SESSION 7180 (SYMPOSIUM)

### PALLIATIVE CARE WITH DEMENTIA AND FAMILY CAREGIVER INVOLVEMENT (A COLLABORATIVE SYMPOSIUM BETWEEN THE HOSPICE, PALLIATIVE, AND END-OF-LIFE CARE AND NURSING CARE OF OLDER ADULTS INTEREST GROUPS)

Chair: Jenny van der Steen
Co-Chair: Christopher Johnson Discussant:
Sheryl Zimmerman

This collaborative symposium offered by the Hospice, Palliative, and End-of-Life Care and Nursing Care of Older Adults Interest Groups addresses palliative care including advance care planning considerations for family caregivers and persons with dementia. Family caregivers may need information about palliative and end-of-life care that is specific to the person, the situation or the stage of dementia. This symposium shows information needs also differ by country and setting. Conversations about symptoms, and about current and end-of-life treatment preferences need support from healthcare professionals. The symposium shows results of a study on video recordings with end-of-life preferences and how, as a stand-alone, they may not inform palliative care practice, and integration of information sources for advance care planning is needed. We will also show that a question prompt list with examples of questions to encourage family caregivers to ask healthcare professionals can and should have different contents for different countries as the content reflects socio-cultural differences. In more studies, participants clearly neede information on the disease trajectory and available services. Such needs go beyond need for information on pain and other symptoms, as family caregivers often appreciate opportunities for social activities for persons with dementia. A decision aid study shows that persons with dementia and family caregivers can participate in advance care planning conversations when supported by the right tools. We argue that local client participation is important when developing tools. Overall, the symposium highlights the need for tailored tools to support face-to-face conversations with all stakeholders to encourage person-centred caregiving.

### DEVELOPING COUNTRY-SPECIFIC QUESTION PROMPT LISTS ABOUT DEMENTIA PALLIATIVE CARE FOR FAMILY CAREGIVERS

Laura Bavelaar,[1] Jenny van der Steen,[1] Maria Nicula,[2] Sophie Morris,[3] Sharon Kaasalainen,[4] Wilco Achterberg,[1] and Kevin Brazil,[3] *1. Leiden University Medical Center, Leiden, Zuid-Holland, Netherlands, 2. McMaster University, School of Nursing, Hamilton, Ontario, Canada, 3. Queen's University Belfast, School of Nursing and Midwifery, Belfast, Northern Ireland, United Kingdom, 4. McMaster University, Hamilton, Ontario, Canada*

We aimed to develop question prompt lists for family caregivers of nursing home residents with advanced dementia to augment advance care planning conversations. In the context of a joint European-Canadian study, we used standardized nominal group methods to create country-specific lists of questions. (Bereaved) family caregivers of persons with dementia read an information booklet about end-of-life care for people with dementia, and generated questions to ask healthcare professionals. They also marked the most important questions from pre-selected questions from other lists. In the Netherlands, 20 participants contributed to a question prompt list of 24 questions that gravitated towards questions about terminating life and the responsibilities of physicians and family involved in decision making. In Canada, 4 participants came up with a question prompt list of 15 questions, related mostly to staff-family communication, with some the same as selected in the Netherlands. Data from the other countries will be presented too.

### ME & MY WISHES VIDEOS: CONGRUENCE OF END-OF-LIFE PREFERENCES BETWEEN RESIDENTS WITH DEMENTIA, FAMILY, AND STAFF

Sarah Neller,[1] Gail Towsley,[1] and Bob Wong,[2] *1. University of Utah, Salt Lake City, Utah, United States, 2. University of Utah College of Nursing, Salt Lake City, Utah, United States*

Me & My Wishes are person-centered videos of long term care residents (ages 65-95) living with dementia discussing their preferences for care including end-of-life (EOL) medical intervention. We evaluated the congruence of six EOL treatment preferences between the residents' personal videos, medical records (e.g. advance directive), and surveys of family (n= 49) and staff (n=37; 118 responses) knowledge of their preferences. Results were highly discordant. Treatments with the most discordance when comparing videos to comparison groups were IV fluids (medical record, 57.1%) and life support (family, 69.4%; staff, 82.2%). Residents reported EOL treatments were considered acceptable if they were temporary, would relieve suffering, or enabled a return to baseline health. These caveats may lead to discordance if they are not conveyed to family or staff. Our findings highlight the need for conversations among residents living with dementia and their caregivers to improve understanding, congruence and adherence of resident EOL preferences.

### IMPACT OF PAIN AND DISCOMFORT ON THE LIVES OF COGNITIVELY IMPAIRED OLDER ADULTS AND THEIR INFORMAL CAREGIVERS

Hyunjin Noh, *The University of Alabama, Tuscaloosa, Alabama, United States*

This qualitative study explored the impact of pain and discomfort on the lives of cognitively impaired older adults and their caregivers from the caregiver perspective. Forty-three individuals of age 19+, who identified themselves as primary caregiver to a chronically or seriously ill older adult (age 50+) with cognitive impairment, such as Alzheimer's Disease and Related Dementia, were recruited at various community settings. Individual, face-to-face interviews were conducted to ask participants how they thought their care-recipient's pain and discomfort affected the care-recipient's and the caregiver's life respectively. Inductive, thematic analysis of interview transcripts revealed several key themes: compromised mobility, limited social interaction or activities, and depressive symptoms in both care-recipients and caregivers; aggravated cognitive decline in care-recipients; and poorer physical health in caregivers. Participants wanted more information on the disease trajectory and available services, particularly home-based therapies and social activities for care-recipients, which provides future program/practice implications.

### PERSPECTIVES OF PERSONS WITH DEMENTIA, FAMILY, AND HEALTHCARE PROVIDERS ON AN ADVANCE CARE PLANNING DECISION AID

Lisa Geshell,[1] and Jung Kwak,[2] *1. The University of Texas at Austin, Austin, Texas, United States, 2. University of Texas at Austin, Austin, Texas, United States*

Few evidence-based interventions to engage persons living with dementia in advance care planning (ACP) exist. We developed and explored the acceptability and appropriateness of an ACP decision aid for PLWD/family caregiver dyads living in the U.S. We conducted a mixed-methods study with 10 persons with dementia-caregiver dyads, and 4 healthcare providers. Content analysis and descriptive analysis were conducted. Major and subthemes included: (1) the role of caregivers during ACP discussion: clarifier, guide, and proxy; (2) decisional needs of persons with dementia for ACP: lack of knowledge and decisional conflict; and (3) perceptions of the decision aid: lack of clarity between advance directive types, a need to remove any questions that may be conceptually different, but appear similar to users, and need to include summary of decision-support needs. Findings provide implications for how healthcare providers can use a decision aid to facilitate ACP conversations with persons with dementia and caregivers.

### SESSION 7185 (SYMPOSIUM)

### PARTNERSHIPS MATTER IN SUCCESSFUL AGING: COLLABORATIONS THAT PUT RESEARCH INTO PRACTICE ACROSS THE COMMUNITY

Chair: Tina Sadarangani
Discussant: Bei Wu

Healthy aging begins and ends in the community with age friendly health systems and communities. In order to promote healthy aging and help older adults make healthy choices we must engage them as partners in healthcare with access to appropriate information and resources. This symposium will examine four community based studies that engage stakeholders to improve care quality through evidence-based interventions for older adults. These programs are all novel as they seek to engage stakeholders, perform pragmatic interventions, and improve outcomes in non-academic, community-based settings which are often overlooked. Promoting healthy behavior through peer coaching, using early detection and treatment to impact cognitive decline in social day care programs, evaluating the feasibility of screening for palliative care in assisted living and implementing evidence-based dementia care in hospice settings are all explored. Analysis of these initiatives showed improvements in perceived-health, reductions in unnecessary healthcare utilization, and improvements in the physical and emotional health of caregivers, and positive changes in health behaviors. Our discussion will underscore the importance of engaging key stakeholders in study design and implementation to yield better outcomes. Community engagement is an essential part of facilitating aging-in-place, and findings illustrate that this can be achieved through innovative collaborations between researchers and community-based organizations across a variety of settings.

### ADAPTING THE KAER FRAMEWORK TO SUPPORT EARLY DIAGNOSIS AND TREATMENT OF DEMENTIA IN ASIAN AMERICANS

Tina Sadarangani,[1] Jennifer Zanowiak,[2] Janet Pan,[3] Vanessa Salcedo,[2] Stella Yi,[3] Simona Kwon,[3] Joshua Chodosh,[4] and Chau Trinh-Shevrin,[2] *1. New York University, New York, New York, United States, 2. NYU Grossman School of Medicine, New York, New York, United States, 3. NYU School of Medicine, New York, New York, United States, 4. New York University Grossman School of Medicine, New York, New York, United States*

Asian Americans (AAs) are frequently diagnosed with dementia in advanced disease stages and have difficulty accessing services. The NYU Center for the Study of Asian American Health, set out to culturally adapt The Kickstart-Assess-Evaluate-Refer (KAER) framework to support earlier detection of dementia in AA communities. Working with Bangladeshi, Chinese, and Korean senior centers, we used a participatory action approach to assess cultural relevancy, usability and acceptability of KAER to improve timely diagnosis and access to care. We found that community-based organizations (CBOs), not physicians, were often “first responders” in identifying and managing dementia. However, CBO staff felt unprepared to “Kickstart” discussions, found certain KAER questions were not culturally appropriate, and encountered barriers in communicating their concerns to physicians. Adaptations to KAER can maximize its impact and reach in AA communities. Suggestions include group education, as opposed to individualized screening, and stronger linkages between physicians and CBOs to ensure care continuity.

### PARTNERING WITH HOSPICES TO DEVELOP, TEST, AND IMPLEMENT ALIVIADO DEMENTIA CARE IN A 25-SITE PRAGMATIC TRIAL

Abraham Brody,[1] Tina Sadarangani,[2] Alycia Bristol,[1] Tessa Jones,[3] Kimberly Convery,[1] Donna McCabe,[1] Aditi Durga,[4] and Shih-Yin Lin,[1] *1. NYU Rory Meyers College of Nursing, New York, New York, United States, 2. New York University, New York, New York, United States, 3. NYU Rory Meyers College of Nursing, New york, New York, United States, 4. NYU Rory Meyers College of nursing, New York, New York, United States*

Almost half of hospice patients currently have a diagnosis of dementia. However, few pragmatic, evidence-based, programs have been developed and tested in this setting overall, and targeting persons living with dementia and their caregivers specifically. Furthermore, limited academic-hospice research partnership exist. We thus undertook efforts to develop and test Aliviado Dementia Care-Hospice Edition, a complex multi-modal quality improvement intervention, as part of the HAS-QOL Trial, a 2-phase, 25 site embedded pragmatic clinical trial, through partnership and engagement with hospice and caregiver stakeholders. We discuss methods for engagement and barriers to implementation. Stakeholders assisted with alignment of the intervention to their needs and how to flexibly implement, substantially strengthening the intervention. They also provided important input regarding how to sustain the intervention through technological, mentorship and communication support mechanisms. Barriers found include the nature of implementing in limited resource settings with multiple competing priorities and little research infrastructure.

### IMPACTING HEALTH BEHAVIORS OF OLDER ADULTS THROUGH A VOLUNTEER-LED HEALTH LITERACY PROGRAM

Tara Cortes,[1] Liz Seidel,[2] and Cinnamon St. John,[3]
*1. New York University, New York, New York, United States, 2. NYU Rory Meyers College of Nursing, New York, New York, United States, 3. Hartford Institute for Geriatric Nursing, New York, New York, United States*

A community based, peer volunteer-driven educational program was developed to empower older adults to improve health behaviors through health literacy. A community needs assessment determined the curricula which included classes on chronic disease management and issues impacting older adult health. Volunteers were recruited and trained on the curricula by health educators using a train-the-trainer module. 173 volunteers convened 302 workshops, and trained 2,065 unique older adults who account for 5000 attendees. Using a pretest posttest design, an outcome evaluation found statistically significant increases in knowledge among older adult workshop attendees on 6 of the 7 health topics ($p<.01$). Eighty-three percent of attendees reported positive health behavior changes at 3-month follow-up. The most commonly cited changes were increased physical activity and improved nutrition. Program challenges included volunteer scheduling and retention. Building a sustainable community-based, peer volunteer-driven health education program for older adults will be discussed.

### IDENTIFYING PALLIATIVE CARE NEEDS IN ASSISTED LIVING

Daniel David,[1] Abraham Brody,[2] Tina Sadarangani,[1] Bei Wu,[1] and Tara Cortez,[3] *1. New York University, New York, New York, United States, 2. NYU Rory Meyers College of Nursing, New York, New York, United States, 3. NYU, New York, New York, United States*

Many residents of Assisted Living (AL) confront serious illness and therefore might benefit from greater access to Palliative Care Services to improve quality of life. We surveyed resident records and AL nursing staff to identify patients in need of Palliative Care. Preliminary findings showed that nurses predicted 23% would not be alive and 49% would no longer live in AL. A majority of residents were over the age of 90, yet 30% did not have a reported code status. These findings suggest that a substantial portion of AL residents may have unmet needs with respect to palliative care. Future interventions are needed to support advance care planning conversations and make palliative care more accessible to this population.

### SESSION 7190 (SYMPOSIUM)

### PATIENT ENGAGEMENT IN RESEARCH DESIGN AND IMPLEMENTATION: MOVING FROM INFANCY TO ADOLESCENCE

Chair: Carol Geary
Co-Chair: Martina Roes
Discussant: Katherine Abbott

Patient (also referred to as User or Older Adult) engaged research is a unique approach to research design, implementation, and dissemination. The practice of engaged research, in its broader sense, involves service recipients, caregivers, clinicians, and other stakeholders in prioritization of research questions, approach, and practice translation to ensure strong stewardship of funds, valid and reliable methods, and practical application. Patient engaged research also aligns with tenets of person-centered care in the inclusive nature and the expectation that the practice will improve the processes and the outcomes of research. Likewise, GSA's focus on diversity of thought through interdisciplinary work strongly aligns with inclusion of patient and stakeholder voices in the performance of interdisciplinary research. Although patient and stakeholder engagement in research is a new approach for many, globally, funders increasingly require evidence of "meaningful" engagement in project proposals. In the United States, the Patient-Centered Outcomes Research Institute (PCORI) is well-known for funding engaged research and the National Institute on Aging (NIA) has demonstrated use of "collaborative" methods central to patient engagement within the IMPACT Collaboratory. Patient engagement literature, while growing, does not yet provide adequate guidance for replication of current or development of new approaches to patient engagement in research. Therefore, the purpose of this symposium is to frame patient engagement through a historic lens (Roes) and discuss the ethics of engagement (O'Sullivan). In addition, we will share program outcomes from three patient engagement programs: the Leading Age LTSS Center, Rural Patient & Stakeholder Engagement, and Sages in Every Setting. Patient/Person Engagement in Research Interest Group Sponsored Symposium.

### OUTCOMES OF JUST-IN-TIME PATIENT AND STAKEHOLDER ENGAGEMENT AT 1 YEAR

Carol Geary,[1] June Eilers,[2] Melissa Leon,[3] James McClay,[4] and Paul Estabrooks,[5] *1. University of Nebraska Medical Center, Omaha, Nebraska, United States, 2. UNMC, Omaha, Nebraska, United States, 3. UNMC Center for Advanced Surgical Technology, Omaha, Nebraska, United States, 4. UNMC College of Medicine, Department of Emergency Medicine, Omaha, Nebraska, United States, 5. University of Nebraska Medical Center College of Public Health, Omaha, Nebraska, United States*

The University of Nebraska Medical Center's Rural Patient and Stakeholder Engagement in Research project used a novel approach to patient engagement in research:

just-in-time engagement. In this approach, patients were identified and matched with researchers with shared research interests without preemptive patient and stakeholder education and training. Ten research teams initiated engaged research design between August and October of 2019. In this presentation, we will share process and outcomes measures of the just-in-time approach: measures of ongoing engagement at 6 and 9 months; evidence of research productivity, and patient and stakeholder satisfaction with their research involvement. We will also address ethical challenges, including challenges to engaging a diverse population. Part of a symposium sponsored by the Patient/Person Engagement in Research Interest Group.

### DEFINING THE PARTICIPATORY ASPECT IN HEALTH AND DEMENTIA CARE RESEARCH

Martina Roes, *German Center for Neurodegenerative Diseases (DZNE), Witten, Germany*

In the last decade, the terms 'engagement', 'involvement', 'patient-public involvement (PPI)' and/or 'participatory research' have been increasingly used in literature. However, it seems that each author has their own definition of the terms and there is not consensus on what constitutes engagement, involvement or participation in health care research. In a broad sense, it seems that PPI can be understood as a co-design strategy, but the terms are not used consistently. Definitions of participation in research tend toward describing the methodological aspect of research rather than mapping appropriate use of methods. Representation of the diversity of society is also challenging and involvement of people with dementia is often neglected. To improve participatory research and outcomes, it is valuable to understand applied methods. Therefore, we conducted a meta-analysis of literature reviews that analyzed methodological concepts and application of these participatory concepts. This presentation will provide key results of this review. Part of a symposium sponsored by the Patient/Person Engagement in Research Interest Group.

### APPLYING ENGAGEMENT FRAMEWORKS TO CASE SCENARIOS: A RETROSPECTIVE ANALYSIS

Erin McGaffigan, *Gerontology Institute, North Reading, United States*

The LeadingAge LTSS Center @UMass Boston has worked with the Patient-Centered Outcomes Research Institute (PCORI), service recipients, researchers, and providers since 2017 to understand the obstacles and creative solutions for meaningful and effective engagement in research. The LTSS Center's work has been informed by multiple engagement frameworks, including Dr. McGaffigan's 2011 PAE Attention Framework, the result of a multi-state, multi-site research study examining the factors to user engagement in Cash & Counseling programs. In this presentation, we will apply the PAE Attention Framework (2011), the PCORI Engagement Rubric (2014), and the NHS INVOLVE Framework (2015) to three research study case scenarios to understand the strategies used, outcomes realized, and factors influencing engagement success. Lessons learned from each and their application to future research will be discussed, including ethical considerations. Part of a symposium sponsored by the Patient/Person Engagement in Research Interest Group.

### PRACTICAL, METHODOLOGICAL, AND ETHICAL CONSIDERATIONS OF USER INVOLVEMENT

Roger O'Sullivan, *Institute of Public Health in Ireland, Belfast & Dublin, Northern Ireland, Ireland*

Traditionally, older people were seen simply as 'subjects' of research but increasingly the case for utilizing the knowledge and insights of older people in planning research, policy, and services is gaining momentum. This presentation explores the nature of user involvement, focusing not just on the practical and methodological considerations, but the ethical aspects, which are often under-discussed. Prof O'Sullivan argues that the increasing focus on user involvement is welcomed but we need to understand and debate how it can be appropriate, meaningful, and beneficial for all involved. Using desk research as well as the results of a web-based survey and semi-structured interviews with researchers, government, and NGO representatives, Prof O'Sullivan will set out the practical as well as the ethical aspects of user involvement. He will recommend how researchers, funders, professional bodies and older people's organizations and older people themselves can best advance the user involvement agenda. Part of a symposium sponsored by the Patient/Person Engagement in Research Interest Group.

### DEVELOPMENTAL EVALUATION OF AN ESTABLISHED ADVISORY BOARD

Rachel Lessem, *CJE SeniorLife, Chicago, Illinois, United States*

CJE SeniorLife developed the PCORI-funded Bureau of Sages, a research advisory board of nursing home community members and older adults, who live at home and receive long term services and supports (LTSS). Bureau members share experiences, build knowledge, and develop skills for working together to provide voice to the direction, design, and implementation of aging research. The PCORI-funded Sages in Every Setting project developed four additional bureaus, expanding and diversifying a pool of researchers, providers, and older adults living with LTSS who have the knowledge, skills, and opportunity to collaborate in research. Evaluation results reveal that the Sage Model is translatable into new settings; partners were able to adapt the provided resources; researchers receive useful input from bureaus to improve their work; and, participation in a research advisory board is a benefit to its members. In addition, we will share challenges to establishing and expanding the Sage Model. Part of a symposium sponsored by the Patient/Person Engagement in Research Interest Group.

### SESSION 7195 (SYMPOSIUM)

### PHYSICAL ACTIVITY AND HEALTH: DOES THE PATTERN MATTER?

Chair: Annemarie Koster
Co-Chair: Sari Stenholm
Discussant: Paul Gardiner

It is well-known that physical activity is key in the prevention of many diseases and disability in old age. Much less is, however, known about the pattern of activity in relation to health. While there are differences in how people spread their activity and sedentary behavior over the day or over the week, we don't know which activity pattern of most beneficial for health. This symposium focuses on patterns of physical activity and sedentary behavior and health in five different studies with accelerometry data in Europe and the USA. Dr. Rosenberg will show how sedentary behavior patterns are associated with various health outcomes in the Adult Changes in Thought (ACT) study. Using data from The Maastricht Study, Dr. Vandercappellen will present how weekly activity patterns, in particular comparing regularly actives to weekend warriors, are associated with arterial stiffness. Dr. Shiroma will show how patterns of physical activity and sedentary behavior, taking the volume, intensity, and frequency of sessions into account, are associated with mortality in the Women's Health Study. Dr. Caserotti will present the association between physical activity fragmentation and physical function in the SITLESS Study. Dr. Stenholm will present data from the Finish Retirement and Aging Study, using latent class trajectory analyses to identify daily activity patterns and how these patterns are associated with health-related physical fitness. Taken together, this symposium will provide insight into different ways patterns of activity can be operationalized using accelerometer data and if the patterns of activity and sedentary behavior are associated with health.

### PATTERNS OF SEDENTARY BEHAVIOR IN A COMMUNITY-BASED COHORT OF OLDER ADULTS

Dori Rosenberg,[1] Rod Walker,[2]
MIkael Anne Greenwood-Hickman,[2] KatieRose Richmire,[2]
John Bellettiere,[3] David Wing,[3] and Andrea LaCroix,[3]
*1. Kaiser Permanente Washington, Seattle, Washington, United States, 2. Kaiser Permanente Washington Health Research Institute, Seattle, Washington, United States, 3. University of California San Diego, La Jolla, California, United States*

Few epidemiologic studies have examined device-measured sitting patterns by demographics and health status. The Adult Changes in Thought (ACT) study is an on-going epidemiologic study of adults age ≥65 years. We conducted a sub-study that added a thigh-worn activPAL device and sleep logs for 7 days to the measurement protocol. A total of 997 had valid wear time (≥4 days with 10-20 hours of data per day) and covariate data. activPAL sedentary pattern measures included number of sitting bouts lasting 30 minutes or more and mean sitting bout duration. On average, participants (56% female, 57% > age 75, 89% non-Hispanic white) sat in bouts lasting 17 minutes (SD = 12) and had 5.9 (SD = 1.7) bouts of sitting lasting 30 minutes or more. Participants who were older, were male, had obesity, had worse self-rated health, had depression, and had difficulty walking had longer sitting bouts and more prolonged bouts.

### ASSOCIATION OF THE AMOUNT AND PATTERN OF PHYSICAL ACTIVITY WITH ARTERIAL STIFFNESS: THE MAASTRICHT STUDY

Evelien Vandercappellen,[1] Ronald Henry,[2]
Coen Stehouwer,[2] and Annemarie Koster,[3] *1. Maastricht University Medical Center, Maastricht, Netherlands, 2. Maastricht University Medical Center+, Maastricht, Limburg, Netherlands, 3. Maastricht University, Maastricht, Limburg, Netherlands*

We examined the associations of the amount and the pattern of higher intensity physical activity with arterial stiffness. Data from The Maastricht Study (n=1699; mean age: 60±8 years, 49.4% women, 26.9% type 2 diabetes (T2DM)) were used. Arterial stiffness was assessed by carotid-to-femoral pulse wave velocity (cfPWV). The amount (hours/day) and pattern of higher intensity physical activity were assessed with the activPAL3®. Activity groups were: inactive (<75min/week), insufficiently active (75-150min/week), weekend warrior (>150min/week in ≤2 sessions), and regularly active (>150min/week in ≥3 sessions). After full adjustment, higher intensity physical activity was associated with lower cfPWV (amount: -0.35[-0.65;-0.05], insufficiently active: -0.33[-0.55;-0.11]; weekend warrior: -0.38[-0.64;-0.12] and regularly active: -0.46[-0.71;-0.21] (reference: inactive)). These associations were stronger in those with T2DM. Participating in higher intensity physical activity was associated with lower cfPWV, regardless of the weekly pattern, and may be an important strategy to reduce CVD risk, particularly in T2DM.

### EXAMINING THE WHEN AND HOW OF PHYSICAL ACTIVITY PATTERNS IN OLDER ADULTS: THE WOMEN'S HEALTH STUDY

Eric Shiroma, *National Institute on Aging, Bethesda, Maryland, United States*

Using data from the Women's Health Study (N = 17,708, mean age = 72 years), we investigated the relative importance of physical activity volume, intensity, frequency of sessions, and session duration. We calculated the mortality hazard ratio for varying patterns and combinations of physical activity intensities and sedentary behavior. In separate analyses, we compared participants who engaged in activity regularly throughout the week to those who focused on one or two days a week, and the relative importance of session frequency and duration. These characteristics of activity patterns and their impact on health may help design interventions and public policy.

### THE IMPACT OF FRAGMENTED PHYSICAL ACTIVITY ON PHYSICAL FUNCTION IN EUROPEAN OLDER ADULTS IN THE SITLESS STUDY

Paolo Caserotti,[1] Mark Tully,[2] Mathias Skjodt,[3]
Nicole Blackburn,[4] Maria Gine-Garriga,[5]
Laura Coll Planas,[6] Michael Denkinger,[7] and
Jason Wilson,[2] *1. University of Southern Denmark,*

*Odense M, Syddanmark, Denmark, 2. Ulster University, Newtownabbey, Northern Ireland, United Kingdom, 3. University of Southern Denmark, Odense, Syddanmark, Denmark, 4. Ulster University, Belfast, Northern Ireland, United Kingdom, 5. Ramon Llull University, Barcelona, Catalonia, Spain, 6. Fundacio Salut i Envelliment UAB, Barcelona, Catalonia, Spain, 7. Agaplesion Bethesda Clinic, Ulm, Baden-Wurttemberg, Germany*

Patterns of physical activity (PA) may be associated with physical function independently of total volume. The study aim was to explore associations of PA fragmentation (PAF) and function in ≥65-year-old European adults (SITLESS study: n=1360). The ActiGraph wGT3X+ accelerometer was worn for seven consecutive days at the dominant hip. PAF was assessed as the ratio of the number of ≥10-second PA bouts divided by an individual's total minutes in PA. Physical function was assessed using the 2-minute maximum walk test (2MWT) and short physical performance battery test (SPPB). Multiple linear regression was utilized adjusting for relevant covariates. Lower PA fragmentation was significantly (p<0.01) associated with longer 2MWT distances and better SPPB scores. The model explained 54% and 41% of the variance in the 2MWT distance and in SPPB score, respectively. Increased PAF seems associated with reduced physical function; independent of sedentary behavior and numerous important health and socio-demographic covariates.

### DAILY PHYSICAL ACTIVITY PATTERNS AND THEIR ASSOCIATION WITH HEALTH-RELATED PHYSICAL FITNESS AMONG AGING WORKERS

Sari Stenholm,[1] Anna Pulakka,[2] Tuija Leskinen,[3] Jaana Pentti,[4] Olli Heinonen,[3] Annemarie Koster,[5] and Jussi Vahtera,[3] *1. University of Turku, Turku, Finland, 2. National Institute for Health and welfare, Helsinki, Uusimaa, Finland, 3. University of Turku, Turku, Varsinais-Suomi, Finland, 4. University of Helsinki, Helsinki, Uusimaa, Finland, 5. Maastricht University, Maastricht, Limburg, Netherlands*

This study aimed to identify accelerometer measured daily physical activity patterns and to examine how they associate with health-related physical fitness among 258 participants (mean age 62.4 years, SD 1.0) from the Finnish Retirement and Aging Study. Wrist-worn ActiGraph accelerometer was used and health-related physical fitness measures included body composition, cardiorespiratory fitness and muscular fitness. Based on latent class trajectory analysis, six different patterns of daily physical activity was identified on workdays and two on days off. Having low activity throughout the workday was associated with poorest health-related physical fitness, whereas a combination of low or moderate activity during working hours and increase of activity level in the evening was associated with most favorable body composition and better physical fitness compared to the other trajectories. In conclusion, a large variation in the workday physical activity patterns and health-related physical fitness was observed among aging workers.

### SESSION 7200 (SYMPOSIUM)

### PREFRONTAL CORTEX CONTROL OF WALKING: FUNCTIONAL NEAR-INFRARED SPECTROSCOPY AND BEYOND

Chair: Andrea Rosso
Discussant: Roee Holtzer

Cognitive control of walking may change with aging and is associated with poorer mobility and greater fall risk. The prefrontal cortex function is important for cognitive control of walking, and functional near-infrared spectroscopy (fNIRS) provides the primary means for assessing prefrontal activation during walking. Growing interest in fNIRS to assess cognitive control of walking has led to advancements in the methodologies for processing and analyzing the data, a greater sophistication of experimental protocols and participant samples, and implementation within intervention studies. These advancements will be highlighted in five presentations from an international group of researchers at the forefront of the field. First, Meltem Izzetoglu will provide direct comparisons of various data processing methodologies, demonstrating comparability across approaches. Three talks will demonstrate the range of applications of fNIRS to studying walking in older adults. Nemin Chen will present data on task-related patterns of prefrontal activation across walking tasks in relation to performance, cognitive function, and structural brain health. Sarah Fraser will present results from stair climbing, a critical task for daily function which also presents a fall risk. Inbal Maidan will examine how individual differences affect prefrontal activity during walking across older adults, younger adults, and patients with Parkinson's disease or multiple sclerosis. Finally, David Clark will demonstrate the use of fNIRS in assessing outcomes from an intervention that combined walking with non-invasive frontal brain stimulation. Roee Holtzer will lead a discussion of the results and the future of fNIRS in assessing cognitive control of walking in older adults.

### ASSESSMENT OF FNIRS PROCESSING METHODS ON ACTIVE WALKING DATA: FINDINGS AND IMPLICATIONS FOR FUTURE RESEARCH

Meltem Izzetoglu,[1] and Roee Holtzer,[2] *1. Villanova University, Villanova, Pennsylvania, United States, 2. Yeshiva University, Bronx, New York, United States*

Functional near infrared spectroscopy (fNIRS) studies utilized a limited and inconsistent number of processing algorithms to assess the prefrontal activity during active walking. To address this critical limitation, we have reanalyzed our large dataset of older adults (n=83) who underwent single and dual-task walking (STW and DTW) protocol by applying different hemodynamic conversion parameters and movement and physiological artifact removal methods. Linear mixed effect model results indicated significant increases in oxygenated-hemoglobin (HbO2) with expected decline in deoxygenated-hemoglobin (Hb) from STW to DTW (range of effect sizes: 0.59 to 0.64 for HbO2, 0.18

to 0.32 for Hb) irrespective of the methods used. In addition, intraclass correlations suggested excellent reliability across methods and task conditions (HbO2 range=0.982 to 0.996; Hb range=0.883 to 0.984). These findings support fNIRS as a robust approach for measuring prefrontal activity in older adults during walking and emphasize the importance for establishing explicit guidelines/principles for fNIRS processing.

### PATTERNS OF PREFRONTAL ACTIVATION AND PERFORMANCE DURING WALKING TASKS AMONG OLDER ADULTS

Nemin Chen,[1] Theodore Huppert,[2] Robert Krafty,[2] and Andrea Rosso,[3] *1. University of pittsburgh, Pittsburgh, Pennsylvania, United States, 2. University of Pittsburgh, Pittsburgh, Pennsylvania, United States, 3. School of Public Health, University of Pittsburgh, Pittsburgh, Pennsylvania, United States*

Differences in prefrontal cortex (PFC) control of walking in older age likely arise from changes in neural capacity and compensation. PFC activation by changes in oxygenated hemoglobin from functional near infra-red spectroscopy was examined in 29 older adults (mean age=76). Tasks included standing with cognitive challenge and walking with and without cognitive challenge on even and uneven surfaces. Three PFC activation-performance patterns were identified using K-means clustering: 1) low activation during walking tasks and high activation during standing cognitive task, with the best performance in terms of walking speed and cognitive performance (n=10); 2) low activation on all tasks, with the lowest performance (n=15); 3) high activation during walking and low activation during cognitive, with intermediate performance (n=5). Associations of patterns with cognitive function and structural neuroimaging were explored, with results informing interpretation of functional changes of PFC during aging process, including compensatory mechanisms for primary network impairment.

### USING FNIRS TO CAPTURE CEREBRAL OXYGENATION IN OLDER ADULTS NAVIGATING STAIRS

Sarah Fraser,[1] Talia Salzman,[1] Hyejun Kim,[1] Hawazin Badawi,[1] Diana Tobon Vallejo,[2] Yves Lajoie,[1] Lara Pilutti,[1] and John Farrell III,[1] *1. University of Ottawa, Ottawa, Ontario, Canada, 2. Universidad de Medellin, Medellin, Antioquia, Colombia*

Navigating stairs is a complex motor activity and while it provides health benefits it can also increase the risk of falls in older adults (OA). The prefrontal cortex (PFC) contributions to stairclimbing (with or without a cognitive task) remain unknown. Using functional near infra-red spectroscopy (fNIRS) and wireless insoles, this study evaluated cerebral oxygenation changes (ΔHbO2) in the PFC, gait parameters (speed) and cognitive performance (reaction time(RT)/accuracy) during stair ascent and descent in single (SMup/SMdown) and dual task (DTup/DTdown) conditions. OAs navigated stairs with or without a simple reaction time task. Participants had longer RTs in DTup ($p < .001$) and DTdown ($p < .001$) in comparison to standing, with no significant differences in accuracy or walk speed. ΔHbO2 was significantly different ($p = .003$) between SMdown and DTdown. Findings suggest that despite the simplicity of the cognitive task, dual-tasking on stairs resulted in increased cerebral oxygenation and slowed cognitive responses.

### OVERLAP, COMMONALITY, DISPARITY, AND VARIABILITY OF FRONTAL LOBE ACTIVATION IN AGING AND NEURODEGENERATION

Inbal Maidan,[1] Hagar Bernad-Elazari,[2] Roni Hacham,[2] Jeffrey Hausdorff,[2] and Anat Mirelman,[2] *1. Tel Aviv Sourasky Medical Center, Tel Aviv, Israel, 2. Tel Aviv Sourasky Medical Center, Tel Aviv, Tel Aviv, Israel*

Recent work suggests that the prefrontal cortex is recruited during complex walking as a form of cognitive compensation to maintain performance in aging and neurodegenerative diseases. Evidence from fNIRS studies is accumulating on different patient groups demonstrating the utility of this method and its sensitivity to neural dysfunction. However a direct comparison that explores the specificity of prefrontal activation patterns has not been conducted. This process is essential towards implementing the use of fNIRS at the individual level. Data collected from four different cohorts; young adults, older adults, PD patients at different stages of the disease, and patients with Multiple-Sclerosis during challenging tasks will be presented. Overlap, commonality, disparity and variability between groups and conditions will be presented and modifiers and moderators that can affect individual performance will be discussed. Understanding individual differences in fNIRS response will enhance data interpretation and promote translation of this technology to clinical care applications.

### FNIRS OUTCOMES FOR A PILOT CLINICAL TRIAL COMBINING FRONTAL TDCS WITH WALKING REHABILITATION IN OLDER ADULTS

David Clark,[1] Sudeshna Chatterjee,[1] Jared Skinner,[2] paige Lysne,[3] Samuel Wu,[4] Ronald Cohen,[4] Dorian Rose,[1] and Adam Woods,[5] *1. University of Florida, Gainesville, Florida, United States, 2. Appalachian State University, Boone, North Carolina, United States, 3. Aging and Geriatric Research, Gainesville, Florida, United States, 4. University Of Florida, Gainesville, Florida, United States, 5. Clinical and Health Psychology, Gainesville, Florida, United States*

This pilot study assessed a novel intervention to enhance both walking and executive function in older adults. The primary hypothesis was that eighteen sessions of frontal lobe tDCS combined with walking rehabilitation would be feasible, safe, and show preliminary efficacy. Eighteen participants were randomized to one of three intervention groups: active tDCS and rehabilitation with complex walking tasks (Active/Complex); sham tDCS and rehabilitation with complex walking tasks (Sham/Complex); or sham tDCS and rehabilitation with typical walking (Sham/Typical). Outcome measures included multiple tests of walking function, executive function, and prefrontal activity during walking as measured by functional near infrared spectroscopy (fNIRS).

Of the three groups, the Active/Complex group demonstrated the broadest improvements across outcome measures including for prefrontal activity. The functional range of prefrontal activity in this group was increased considerably, as conceptualized by the Compensation Related Utilization of Neural Circuits Hypothesis. Frontal tDCS is a promising adjuvant to walking rehabilitation.

### SESSION 7205 (SYMPOSIUM)

### PREVENTING AND MANAGING CHRONIC CONDITIONS IN OLDER ADULTS: LESSONS LEARNED FROM THE NHATS AND NSOC 2017

Chair: Rose Ann DiMaria-Ghalili
Discussant: Cheryl Monturo

According to the Centers for Disease Control, 85% of older adults have at least one chronic health condition, and 60% percent have at least two chronic conditions. This symposium explores strategies to prevent and manage chronic conditions (nutritional status, medication management, wound care, and physical function) using data from the 2017 National Health and Aging Trends Study (NHATS) and corresponding National Study on Caregiving (NSOC). The datasets include a nationally representative sample of US older adults (NHATS) and their caregivers (NSOC). In addition to survey questions, the 2017 NHATS cohort submitted dried blood samples which include inflammatory biomarkers (hs-C reactive protein [hsCRP] and interleukin-6 [IL-6]). All individual presentations report on weighted data from the analysis to more accurately reflect the US population. In the first paper, DiMaria-Ghalili explores the prevalence of and factors related to malnutrition in community-dwelling and residential living older adults. In the second paper Coates examines the extent to which source of purchased medications impacts the occurrence of self-reported medication mistakes and hospitalizations in community-dwelling participants who managed medications independently. In the third paper, Hathaway compares the socio-demographic, nutrition, and inflammatory profile of older adults with and without wounds. In the fourth paper, Sefcik examines the relationship between the frequency of community-dwelling older adults going outside and physical function. Collectively, findings provide insight into the experiences of vulnerable older adults with chronic conditions informed from the NHATS and NSOC datasets. The symposium will conclude with a discussion by Monturo on implications for research, policy and practice.

### PREVALENCE OF MALNUTRITION IN A NATIONAL SAMPLE OF OLDER ADULTS RESIDING IN COMMUNITY OR RESIDENTIAL CARE: NHATS 2017

Rose Ann DiMaria-Ghalili, Janeway Granche,
Martha Coates, Zachary Hathaway, and Justine Sefcik,
*Drexel University, Philadelphia, Pennsylvania, United States*

The national prevalence of malnutrition in older adults living in the community and residential care (non-nursing home) is not known. We determined the prevalence of malnutrition (Mini-Nutritional Assessment) in a representative sample (N=4472) living in the community (95%) or residential care (5%), and examined known nutrition risk factors (inflammation [hsCRP, IL-6], socio-demographic variables). The majority (68%) were nourished, 26% were at risk, and 6% were malnourished. Those living in residential care vs community were more likely to be malnourished (12% vs 5%, respectively p<.01). Compared to nourished group, those with malnutrition were more likely to have hsCRP greater than median (1.36) (OR = 1.45 [95% CI 1.01-1.92]) and those at nutritional risk were more likely to have IL-6 greater than median (4.22) (OR=1.34 [95% CI 1.09-1.63]). Malnourished older adults were more likely to be older, female, live alone, report worse self-reported health, and use Meals on Wheels (p <.05).

### SOURCE OF PURCHASED MEDICATIONS AND ITS IMPACT ON MEDICATION MISTAKES AND HOSPITALIZATIONS: THE NHATS 2017

Martha Coates, Janeway Granche, and
Rose Ann DiMaria-Ghalili, *Drexel University, Philadelphia, Pennsylvania, United States*

Older adults self-administer prescribed medication regimens to treat chronic diseases which can lead to mismanagement, medication related harm and hospitalizations. We examined the extent to which source of purchased medications influenced the occurrence of self-reported medication mistakes and hospitalizations in community-dwelling participants who managed medications independently (N= 3899). The majority (65%) picked-up medications, 18% had medications delivered, and 17% used both (picked-up and delivery). Compared to those picking up their medications, those using delivery only were less likely to have a hospital stay (OR=0.691 [95% CI 0.507-0.943]) and no difference in odds of medication mistakes (OR=1.051 [95% CI 0.764-1.445]), while those using both methods were more likely to report hospital stays (OR=1.429 [95% CI 1.106-1.846]) and medication mistakes (OR = 1.576[95% CI 1.078-2.304]). Older adults who picked-up medications from a local pharmacy and had medications delivered were more likely to report medication mistakes and hospitalizations.

### ASSESSING OLDER ADULTS' FREQUENCY OF GOING OUTSIDE AND PHYSICAL PERFORMANCE: NHATS 2017

Justine Sefcik, Janeway Granche, Martha Coates,
Zachary Hathaway, and Rose Ann DiMaria-Ghalili, *Drexel University, Philadelphia, Pennsylvania, United States*

Little is known about community-dwelling older adults' outdoor activity and the relationship between physical function and frequency of going outside. Using the 2017 NHATS (N = 4,465), we looked at self-reported outdoor frequency (Likert scale: every day to once a week or less) and the Short Physical Performance Battery (SPPB; participants completed five different physical activities to measure physical performance; total scores ranged from 0, not attempted, to 12, the best). A logistic model comparing community-dwelling older adults going out most days (18.3%), some days (10.3%), or rarely/never (3.4%) to those going out every day found ORs of 0.85, 0.70, and 0.58 respectively (all p<0.0001) for a one-unit increase in SPPB score. Interdisciplinary teams can use findings to assess disabled community-dwelling older adults' frequency of going outdoors. Implications for interventions to assist with increasing times leaving the home (e.g. mobility devices, caregiver assistance) will be discussed.

### PROFILE OF OLDER ADULTS WHO RECEIVED WOUND CARE BY A FAMILY CAREGIVER: THE NHATS-NSOC 2017

Zachary Hathaway, Janeway Granche, Martha Coates, Justine Sefcik, Michael Neidrauer, Peter Lewin, and Rose Ann DiMaria-Ghalili, *Drexel University, Philadelphia, Pennsylvania, United States*

A gap in knowledge exists related to the socio-demographic and health characteristics of older adults receiving wound care from a family caregiver in the home. We created a cohort (N=992) of older adults from NHATS who lived in the community or residential care (non-nursing home) and had a family caregiver complete the NSOC question "provides help with skin care related to wounds or sores". Approximately one third (32%) of these older adults received wound care from a family caregiver. These older adults were more likely to be men, live with others, have lower levels of physical function, be malnourished (OR = 1.63 [95% CI = 1.02-2.60]), and have inflammation (hsCRP > median 1.89), P < .05. These findings can inform the needs of older adults receiving wound care from a family caregiver and lead to development of additional supports for caregivers (e.g., multi-component interventions).

### SESSION 7210 (SYMPOSIUM)

### RESERVE AND HEALTHY AGING

Chair: Cynthia Felix
Co-Chair: Briana Sprague

In line with the GSA 2020 Annual Scientific Meeting theme of "Turning 75: Why Age Matters", our symposium highlights the fact healthy aging is relevant to maintaining reserve- be it brain/cognitive reserve or physiological reserve. Even among older adults 75 or older, continuing to practice healthy aging habits, helps with reserve. In this symposium, Drs. Felix and Carlson discuss how positive neuroplastic processes such as social engagement and social volunteering may aid in brain/cognitive reserve. Dr. Lin discusses how negative neuroplastic processes such as hearing loss may hamper the same. The "use-it-or-lose-it" hypothesis may be a common pathway in effecting brain reserve, regardless of whether the inputs are social or sensory stimuli. Physiological reserve is also important in aging, and Dr. Sprague talks about energy and frailty, with frailty being an accelerated decline of physiological reserve. While the studies presented are from older adult populations, reserve often takes a lifetime of effort to build and maintain. The symposium speakers present several hypotheses such as brain reserve, cognitive reserve, cognitive load, information degradation, sensory deprivation and frailty. An application of these concepts, would help older adults practice aging habits that promote reserve, into advanced old age, at individual and community levels. Brain Interest Group Sponsored Symposium

### GREATER SOCIAL ENGAGEMENT AND GREATER GRAY MATTER MICROSTRUCTURAL INTEGRITY OF AGING ADULTS

Cynthia Felix,[1] Caterina Rosano,[1] Xiaonan Zhu,[1] Jason Flatt,[2] and Andrea Rosso,[1] *1. University of Pittsburgh, Pittsburgh, Pennsylvania, United States, 2. University of Nevada, Las Vegas, Las Vegas, Nevada, United States*

Social engagement reflects habitual social roles in aging adults and may protect against dementia. Cross-sectional associations of social engagement (SE) index with gray matter (GM) microstructure was studied in regions of interest relevant to social cognition among community-dwelling older adults [n=293, mean age: 82.8 years (SD: 2.8), 43% males] using linear regression models. Greater SE was significantly related to lower mean diffusivity (MD) (greater GM microstructural integrity) [shown as standardized estimate (p-value)] in: left middle frontal gyrus-orbital part: -0.168 (0.005), left caudate nucleus: -0.141 (0.02), left temporal pole-middle temporal gyrus: -0.136 (0.03), right middle frontal gyrus: -0.160 (0.006), right superior frontal gyrus-orbital part: -0.187 (0.002), right middle frontal gyrus, orbital part: -0.124 (0.04), adjusted for demographic attributes. Associations were robust to adjustment for hearing or ADL difficulty. Findings were generally stronger in females than in males. Social engagement may prevent GM integrity loss and build brain reserve in dementia-related regions. Part of a symposium sponsored by Brain Interest Group.

### SELF-REPORTED ENERGY TRAJECTORIES PREDICT ADVERSE HEALTH OUTCOMES IN OLDER ADULTS

Briana Sprague,[1] Xiaonan Zhu,[2] Rebecca Ehrenkranz,[1] Qu Tian,[3] Theresa Gmelin,[1] Nancy Glynn,[1] Andrea Rosso,[4] and Caterina Rosano,[1] *1. University of Pittsburgh, Pittsburgh, Pennsylvania, United States, 2. University of Pittsburgh Graduate School of Public Health, Pittsburgh, Pennsylvania, United States, 3. National Institute on Aging, Bethesda, Maryland, United States, 4. School of Public Health, University of Pittsburgh, Pittsburgh, Pennsylvania, United States*

Declining energy may indicate homeostatic dysregulation and predict adverse health outcomes. We hypothesized that declining energy would predict greater frailty (1-10), greater mortality, and faster mood (CES-D) and cognition (3MS)

decline over time. This observational cohort studies included 2,443 older adults (mean age=74.6, 62.5% White, 47.8% men) from the Health ABC Study with up to eight years of data. Energy was assessed using a single-item question about prior month's energy (baseline mean=6.7, SD=1.7, range=0–10, lower=less energy). We used linear mixed models to create energy change scores (mean=-.07 points/year, SD=.05, range=-0.32-0.21, negative=decreased energy). In regression models adjusting for baseline outcome performance and energy and demographics, declining energy predicted greater frailty (β=-2.72, 95%CI = -3.39,-2.06), greater mortality (hazard ratio=.07, p<.001), and faster CES-D (β=-.93, 95%CI=-1.10,-0.75) but not 3MS decline. Energy changes are easy to assess and predict clinically-relevant outcomes. Future work should consider mechanisms of declining energy on disability-related outcomes. Part of a symposium sponsored by Brain Interest Group.

### SOCIAL VOLUNTEERING IN AGING ADULTS INCREASES REGIONS OF THE AMYGDALA AND CORRELATES WITH ENHANCED GENERATIVITY

Michelle Carlson, *Johns Hopkins Bloomberg School of Public Health, Baltimore, Maryland, United States*

The Brain Health Study (BHS) of the Baltimore Experience Corps Trial (BECT) examined whether a randomized, controlled trial of an intergenerational social volunteer program, entitled Experience Corps, increased subregions of the amygdala related to socioemotional memory and risk for Alzheimer's disease in aging adults. We further assessed functional correlates of these intervention-related changes and changes in aging adults' developmental need to be generative, or, to give back to the well-being of others. The BHS simultaneously randomized 112 men and women (59 intervention; 53 control) within BECT to evaluate intervention impact on biomarkers of brain health at baseline and annual follow-ups during the two-year trial. Intention-to-treat analyses revealed program-specific increases in the shape of the centromedial and basomedial regions of the left amygdala (p's≤0.05 adjusted), which were correlated with increases in generativity (p's =0.06). Meaningful social engagement buffered amygdalar declines important to preservation of emotionally salient memory and risk for dementia. Part of a symposium sponsored by Brain Interest Group.

### HEARING LOSS IN OLDER ADULTS: IMPLICATIONS FOR COGNITIVE LOAD AND BRAIN STRUCTURE AND FUNCTION

Frank Lin, *Johns Hopkins University School of Medicine, Baltimore, Maryland, United States*

Age-related hearing loss is prevalent in two-thirds of older adults and reflects progressive impairments in cochlear function leading to impoverished peripheral neural encoding of sound. Research has demonstrated the broader implications of hearing loss for the health and functioning of older adults, particularly with respect to brain aging and dementia. This presentation will summarize current epidemiological and neuroimaging evidence for how hearing loss in older adults affects cognitive load and brain structure/function and relate this contemporary research with previous psychological studies proposing 'information degradation' and 'sensory deprivation' hypotheses of how hearing may affect cognitive function. Finally, the design of an ongoing NIA-funded randomized controlled trial (ACHIEVE- Aging and Cognitive Health Evaluation in Elders) that will determine if hearing treatment reduces the risk of cognitive decline, dementia, and brain aging in adults will be discussed. Part of a symposium sponsored by Brain Interest Group.

### SESSION 7215 (SYMPOSIUM)

### SCALING GERIATRIC AND TELEMEDICINE CARE FOR OLDER ADULTS IN RURAL AREAS THROUGH CLINICAL STRATEGIES AND TRAINING

Chair: William Hung
Co-Chair: Becky Powers
Discussant: Stuti Dang

Telemedicine, the use of electronic information and communication technologies to deliver care, has grown substantially over the past few years, potentially benefiting older adults who have difficulty accessing and traveling to care locations. Given that providers and interprofessional staff with training in geriatric medicine often practice in urban rather than rural areas, older adults' access to quality geriatric care is limited. Prior experiences with telemedicine adoption for geriatric team consultation, though limited in scope, were well accepted by older adults and demonstrated benefits such as identifying and meeting care needs for older adults. Bringing geriatric team care to large regions across the country requires further consideration of population needs, local contexts and training and enhancement of an interprofessional workforce to deliver geriatric care through telemedicine. The Veteran healthcare system has been a pioneer in telemedicine care and considers the use of telemedicine necessary for all providers in its system. This symposium aims to discuss approaches to identify and target older adults who may benefit from geriatric consultation, how care delivery is scaled through identifying common approaches and local adaptations, what the important elements are for providers and teams to deliver care effectively for the older adult population, especially those with multiple complex chronic conditions and functional limitations, and considerations for training the next generation of providers to provide care for older adults with complex conditions, particularly in rural areas with limited access.

### GRECC CONNECT INCREASES ACCESS TO GERIATRIC SPECIALTY CARE FOR RURAL, OLDER VETERANS WITH COMPLEX CARE NEEDS

Kathryn Nearing,[1] Stuti Dang,[2] Eileen Dryden,[3] Laura Kernan,[4] Lauren Moo,[5] and Camilla Pimentel,[6] *1. University of Colorado Anschutz Medical Campus, Aurora, Colorado, United States, 2. Miami VA Healthcare System, Miami, Florida, United States, 3. Center for*

*Health Care Implementation Research (CHOIR), Bedford, Maryland, United States, 4. Center for Health Care Implementation Research (CHOIR), Newburyport, Maryland, United States, 5. Bedford VA Medical Center, Bedford, Massachusetts, United States, 6. Edith Nourse Rogers Memorial Veterans Hospital, Center for Heallthcare Organization and Implementation research, Bedford, Massachusetts, United States*

A higher percentage of Veterans in rural areas are older, have multiple chronic conditions and select the VA for healthcare. To address the needs of rural older Veterans with complex needs, GRECC Connect hubs use case finding approaches combined with regular outreach and education to VA community-based outpatient clinic (CBOC) providers serving rural Veterans and caregivers. Alignment of GRECC Connect services with needs of providers and patients promotes establishment of therapeutic alliances in caring for medically complex older Veterans. After identifying high risk, high need patients, hubs use the following strategies to increase access to geriatric specialty care through telehealth modalities: 1) Co-management of patients through e-consultation and telehuddles (GRECC Connect interprofessional geriatric specialty care teams extend support to CBOC providers); 2) Clinical video telehealth to CBOCs and Video on Demand to Veteran homes (to reduce travel burden); and, 3) Tele-group visits (especially for behavioral health and caregiver support).

### SPREADING TELEHEALTH FOR OLDER ADULTS IN RURAL AREAS THROUGH NETWORK OF GERIATRIC INTERPROFESSIONAL TEAMS

Steven Barczi,[1] Megan Gately,[2] Lauren Welch,[3] Kathryn Nearing,[4] Stephen Thielke,[5] Camilla Pimentel,[6] Laura Previll,[7] and Eileen Dryden,[8] *1. University of Wisconsin, Madison; William S. Middleton Memorial Veterans Hospital, Madison, Wisconsin, United States, 2. Bedford VA Medical Center, Bedford, Massachusetts, United States, 3. William S Middleton VAMC GRECC, Madison, Wisconsin, United States, 4. University of Colorado Anschutz Medical Campus, Aurora, Colorado, United States, 5. VA Puget Sound Health Care System, Seattle, Washington, United States, 6. Edith Nourse Rogers Memorial Veterans Hospital, Center for Healthcare Organization and Implementation research, Bedford, Massachusetts, United States, 7. Duke University School of Medicine, Durham, North Carolina, United States, 8. Center for Health Care Implementation Research (CHOIR), Bedford, Maryland, United States*

Older adults living in rural areas have limited access to geriatrics interprofessional team care. In the Veteran healthcare system, geriatric teams such as geriatricians, nursing professionals, social workers, pharmacists and psychologists, located in urban areas link up with rural clinics to provide geriatric consultation remotely through clinical video telehealth and other means in the project GRECC Connect. Since its inception in 2014, the service has now grown to 16 geriatric teams offering consultation to over 100 clinic sites serving older rural Veterans. GRECC Connect delivered over 2,000 consultations in 2019, meeting complex care needs by identifying and linking geriatric services and management to patients with geriatric syndromes. The network of established geriatric teams, local champions and a shared Electronic Health Record facilitated the spread, while ongoing effort to build and maintain relationships between consultants and local rural provider teams and other community based services are important for ongoing success.

### TELEHEALTH COMPETENCIES FOR INTERPROFESSIONAL TEAMS CARING FOR OLDER ADULTS AND CARE PARTNERS

Becky Powers,[1] Kathryn Nearing,[2] Studi Dang,[3] William Hung,[4] and Hillary Lum,[5] *1. South Texas Veterans Health Care System, San Antonio, Texas, United States, 2. University of Colorado Anschutz Medical Campus, Aurora, Colorado, United States, 3. Miami GRECC, Miami, Florida, United States, 4. Icahn School of Medicine at Mount Sinai; James J Peters VA Medical Center, Bronx, New York, United States, 5. VA Eastern Colorado GRECC, Aurora, Colorado, United States*

Providing interprofessional geriatric care via telehealth is a unique clinical skillset that differs from providing face-to-face care. The lack of clear guidance on telehealth best practices for providing care to older adults and their care partners has created a systems-based practice educational gap. For several years, GRECC Connect has provided interprofessional telehealth visits to older adults, frequently training interprofessional learners in the process. Using our interprofessional telehealth expertise, the GRECC Connect Education Workgroup created telehealth competencies for the delivery of care to older adults and care partners for interprofessional learners. Competencies incorporate key telehealth, interprofessional and geriatric domains, and were informed by diverse stakeholders within the Veterans Health Administration. During this symposium, comments will be solicited from attendees. Once finalized, these competencies will drive the development of robust curricula and evaluation measures aimed at training the next generation of interprofessional providers to expertly care for older adults via telehealth.

### MEDICAL STUDENT'S PERCEPTION OF DEMENTIA ASSESSMENT AND MANAGEMENT AMONG RURAL VETERANS

Prasad Padala,[1] Jessica Stovall,[2] Matthew Kern,[2] Jeremy Curtis,[2] Eugenia Boozer,[2] Shelly Lensing,[3] and Kalpana Padala,[2] *1. Central Arkansas Veterans Healthcare System, Little Rock, Arkansas, United States, 2. Central Arkansas Veterans Healthcare System, North Little Rock, Arkansas, United States, 3. University of Arkansas for Medical Sciences, Little Rock, Arkansas, United States*

Background: Rural Veterans rely on their caregivers, case managers and primary care providers for dementia management. Providers of such patients need to work closely with caregivers, know the local dementia resources and be comfortable with the multiple facets of dementia assessment and management. Unfortunately, medical students are not particularly well trained in these aspects and huge knowledge gaps exist. The goal was to study the impact of a multi-component, experiential, brief curriculum on attitudes of dementia care. Methods: 108 medical students participated in a curriculum including didactics, clinical, and team-based learning followed by pre-post assessments. Results: Student's perception of their ability to assess multiple facets of dementia such as behaviors, caregiver burden, and cognition improved significantly (p<0.001). Students' perception of the role of social worker improved significantly (p=0.002). Conclusion: An interdisciplinary curriculum, improved medical students' perception of their ability to assess for dementia in a cohort of predominantly rural Veterans.

### SESSION 7220 (SYMPOSIUM)

### SENSORY LOSS AND THE HEALTHCARE SYSTEM: OUTCOMES AND NAVIGATION

Chair: Nicholas Reed
Discussant: Charlotte Yeh

Communication is fundamental to patient-centered care. However, sensory impairment limits communication among older adults. Specifically, hearing impairment strains communication via degraded auditory encoding while vision impairment distresses ability to read and interpret visual cues. The presence of dual sensory impairment, defined as concurrent hearing and vision impairment, may exacerbate these effects. The potential consequence s of age-related sensory loss on health care interactions and outcomes are beginning to surface in epidemiologic studies demonstrating poorer patient-provider communication, higher medical expenditures, increased risk of 30-day readmission, and longer length of stay when compared to individuals without sensory loss. Importantly, these associations may be amenable to intervention via sensory aids; however, uptake to sensory care is low. Notably, less than 20% of persons with hearing impairment have hearing aids and over 55% of Medicare Beneficiaries with reported vision problems have not had an eye examination in the prior year. Affordability and access may contribute to lack of sensory care uptake as Medicare explicitly excludes coverage of vision and hearing services. In this symposium, we will review current and new evidence for whether sensory loss affects health care outcomes, including satisfaction with care, incident delirium during hospitalization, navigation of Medicare, and present data on how persons with sensory loss are more likely to delay their care independent of cost and insurance factors suggesting fundamental changes in health care system interaction. We will place these results within the context of current national quality care and policy initiatives and review methods to address sensory loss.

### HEARING LOSS AND HELP-SEEKING BEHAVIOR

Nicholas Reed, *Johns Hopkins University, Baltimore, Maryland, United States*

Hearing Loss (HL) is common among older adults and is associated with poor health care quality outcomes include 30-day readmissions, length of stay, poorer satisfaction, and increased medical expenditures. These associations may manifest in changes in help-seeking behaviour. In the 2015 Current Medicare Beneficiary Study (MCBS) (n=10848; weighted sample=46.3 million), participants reported whether they knowingly had avoided seeking care in the past year and self-reported HL was measured as degree of trouble (none, a little, or a lot) hearing when using a hearing aid if applicable. In a model adjusted for demographic, socioeconomic, and health factors, those with a little trouble (OR= 1.612; 95% CI= 1.334-1.947; P<0.001) and a lot of trouble hearing (OR= 2.011; 95% CI= 1.443-2.801; P<0.001) had 61.2% and 101.1% higher odds of avoiding health care over the past year relative to participants with no trouble hearing. Future work should examine whether hearing care modifies this association.

### SENSORY LOSS AND DELIRIUM AMONG MEDICARE BENEFICIARIES

Emmanuel Garcia Morales,[1] and Nicholas Reed,[2] *1. Johns Hopkins Bloomberg School of Public Health, Baltimore, Maryland, United States, 2. Johns Hopkins University, Baltimore, Maryland, United States*

Sensory impairment is prevalent among older adults and may increase risk for delirium via mechanisms including sensory deprivation and poor communication which may result in confusion and agitation. In the Medicare Current Beneficiary Study (MCBS), delirium was measured using a validated algorithm of claims data. Sensory impairment was defined as any self-reported trouble hearing or seeing, with the use of aids, and was categorized as no impairment, hearing impairment only (HI), vision impairment only (VI), and dual sensory impairment (DSI). Among, 3,240 hospitalized participants in 2016-2017, 346 (10.7%) experienced delirium. In a model adjusted for socio-demographic and health characteristics, those with HI only, VI only, and DSI had 0.84 (95% CI: 0.6-1.3), 1.1 (95% CI 0.7-1.7), and 1.5 (95% CI 1.0-2.1) times the odds of experiencing delirium compared to those without sensory impairment. Future research should focus on mechanisms underlying association and determine the impact of treatment of sensory loss.

### UNDERSTANDING MEDICARE WITH HEARING LOSS

Amber Willink, *The University of Sydney, Sydney, New South Wales, Australia*

Medicare has become an increasingly complex program to navigate with numerous choices available to beneficiaries with important implications on their financial exposure and access to care. While research has identified poor health literacy as a barrier to understanding Medicare, little information is available on the experience of individuals with hearing loss. Using the Medicare Current Beneficiary Survey (2016), a nationally-representative sample of 10,841 beneficiaries, we examined if difficulty understanding Medicare was associated with reported trouble hearing, while controlling for socio-demographic and health literacy factors. Compared to no trouble, Medicare beneficiaries with a little or a lot of trouble hearing had 44% (95% CI OR:1.34-1.55) and 63% (95% CI OR: 1.44-1.83) increased odds of reporting greater difficulty with understanding Medicare. The existing tools to support Medicare beneficiaries understand and navigate the program must evolve to meet the needs of those with hearing loss- a highly prevalent condition among Medicare beneficiaries.

### SATISFACTION WITH HEALTH CARE BY DUAL SENSORY IMPAIRMENT STATUS

Lama Assi,[1] Ahmed Shakarchi,[1] Bonnielin Swenor,[2] and Nicholas Reed,[2] *1. Johns Hopkins Wilmer Eye Institute, Baltimore, Maryland, United States, 2. Johns Hopkins University, Baltimore, Maryland, United States*

Sensory impairment is a barrier to patient-provider communication and access to care, which may impact satisfaction with care. Satisfaction with the quality of care received in the past year was assessed in the 2017 Medicare Current Beneficiary Survey (weighted sample=53,905,182 Medicare beneficiaries). Self-reported sensory impairment was categorized as no sensory impairment, hearing impairment (HI)-only, vision impairment (VI)-only, and dual sensory impairment (DSI) – concurrent HI and VI. In a model adjusted for sociodemographic characteristics and health determinants, having DSI was associated with higher odds of dissatisfaction with the quality of care received (Odds Ratio [OR]=1.53, 95%Confidence Interval [CI]=1.14-2.06) relative to no sensory impairment; however, having HI-only or VI-only were not (OR=1.33, 95%CI=1.94-1.89, and OR=1.32, 95%CI=0.95-1.93, respectively). These findings have implications for healthcare providers as Medicare shifts to value-based reimbursement. Moreover, previous work that singularly focused on HI or VI alone may have failed to recognize the compounded effect of DSI.

### ADDRESSING SENSORY IMPAIRMENT IN THE ICU: CRITICALLY IMPORTANT FOR IMPROVING OUTCOMES

Lauren Ferrante, *Yale School of Medicine, New Haven, Connecticut, United States*

Millions of older adults are admitted to an intensive care unit (ICU) every year. Many of these patients have preexisting impairment in hearing or vision, but sensory impairment is rarely addressed in the ICU. This talk will present recent work evaluating the association of sensory impairment with functional outcomes among older ICU patients, and discuss barriers to and potential strategies for addressing sensory impairment in the ICU.

### SESSION 7225 (SYMPOSIUM)

### SMART WEARABLES IN THE LENS OF AGING: RESULTS FROM THE ROAMM STUDY

Chair: Mamoun Mardini
Co-Chair: Todd Manini
Discussant: Jennifer Schrack

Continuous, long-term monitoring with remote capabilities using wearable technology is ideal for capturing information about patient/participant symptoms synced to sensor-based information. The Real-time Online Assessment and Mobility Monitor (ROAMM) is a smartwatch framework configured to collect data in free-living settings from both sensor-based (location and movement) and responses to symptom notifications through a visual display. The symposium presents the overall framework and preliminary findings from a demonstration study in older adults with knee osteoarthritis. Karnati will present the general framework of ROAMM explaining the data flow from the smartwatch to end users (clinicians and research). He will highlight components in the design that makes the framework unique and highly flexible to serve different studies with different research questions. Rouzaud evaluated satisfaction, usability and compliance wearing a smartwatch and using the ROAMM app. Participants were compliant to ecological prompts about pain, fatigue and mood three times a day (82.5% compliance rate). Additionally, > 70% reported being satisfied with the function/usability and comfort with using ROAMM and wearing the smartwatch. Mardini examined the temporal relationship between ecological pain and derived life-space mobility features from Global Positioning System coordinates. Results suggested that higher level of knee pain in older adults was associated with lower life-space mobility. Manini examined physician perception towards an electronic health record (EHR) graphical interface of top ranked patient attributes of pain, falls, hydration and mobility patterns. Results indicated a relatively high level of usability of the EHR interface depicting smartwatch data.

### A SMARTWATCH-BASED FRAMEWORK FOR REAL-TIME AND ONLINE ASSESSMENT AND MOBILITY MONITORING

Yashaswi Karnati,[1] Rohith reddy Kessi reddy,[1] Aseem Thakkar,[1] Matthew McConnell,[1] Tonatiuh Mendoza,[1] Mamoun Mardini,[1] Todd Manini,[2] and Sanjay Ranka,[1] *1. University of Florida, Gainesville, Florida, United States, 2. The University of Florida, Gainesville, Florida, United States*

Pervasive computing is changing the monitoring landscape for patients to communicate their healthcare information in real-time to clinicians and researchers. We developed a framework based on a smartwatch application allowing researchers to execute a study that is customized to their needs. The application is configured to collect patient generated data in remote settings from both sensor-based (location and movement) and user-reported health factors through the visual display. For example, data are used to investigate concurrent symptoms and mobility patterns in free-living conditions. To support the collection and analysis of this data in a robust and scalable fashion, we have developed an event driven, serverless computing platform using Amazon cloud services. This system also allows multiple campaigns to run concurrently each under the auspices of a different researcher. The framework is ideal for harnessing and scaling the utilization of smart wearable devices in research and clinical settings.

### PHYSICIAN SUITABILITY OF AN EHR INTERFACE FOR DEPICTING ECOLOGICAL SYMPTOMS DERIVED FROM A SMARTWATCH

Todd Manini,[1] Jordan Alpert,[2] Tonatiuh Mendoza,[3] Satya Prabhakar,[4] Laurence Solberg,[3] and Parisa Rashidi,[3] *1. The University of Florida, Gainesville, Florida,*

*United States, 2. University of Florida, gainesville, Florida, United States, 3. University of Florida, Gainesville, Florida, United States, 4. ANSYS Inc, Canonsburg, Pennsylvania, United States*

Physicians desire an electronic health record (EHR) interface to visualize data from mobile devices in rapid and unobtrusive manner. We developed an EHR interface after performing semi-structured qualitative interviews with 12 physicians. These interviews led to creating an EHR graphical interface that allowed seamless viewing of the top ranked patient attributes of pain, falls, hydration and mobility patterns. Physicians then evaluated the interface using the International Standard ISO 9241, Part 110— a survey to evaluate the design of human computer interactions. The median response for each of domains were as follows: suitability = 18.5 out of 24 (range: 9-24); conformity=34 out of 40 (range: 16-40); self-descriptiveness=25 out of 32 (range: 13-31); controllability=19.5 out of 24 (range: 8-23); error tolerance=5 out of 8 (range: 1-8). The median total score of 111.5 out of 140 (range: 88-128) indicated a relatively high level of usability of the EHR interface depicting smartwatch data.

### THE TEMPORAL RELATIONSHIP BETWEEN ECOLOGICAL PAIN AND LIFE-SPACE MOBILITY IN OLDER ADULTS WITH KNEE OSTEOARTHRITIS

Mamoun Mardini,[1] Subhash Nerella,[1] Matin Kheirkhahan,[2] Sanjay Ranka,[1] Roger Fillingim,[3] Erta Cenko,[1] Parisa Rashidi,[1] and Todd Manini,[4]
*1. University of Florida, Gainesville, Florida, United States, 2. Google, Mountain View, California, United States, 3. University of Florida, Pain Research and Intervention Center of Excellence, Gainesville, Florida, United States, 4. The University of Florida, Gainesville, Florida, United States*

Older adults who experience pain are thought to have lower life-space mobility (spatial size and frequency of interaction with the surrounding environment). However, there is significant day-to-day variability in pain experiences that offer insights into consequences on life-space mobility that aren't understood. This study examined the temporal association between ecological pain and metrics from Global Positioning System in older adults with symptomatic knee osteoarthritis. Participants (n=19, 73.1+/- 4.8 yrs, 68.4% female) wore a smartwatch for an average period of 13.16 (+/-2.94) days. Participants were prompted in their free-living environment about their pain intensity (range 0-10) at random times in the morning, afternoon and evening. Results suggest that higher level of knee pain in older adults was associated with a 6.4 (2.81, p=0.02) fewer miles traveled per day – indicating a lower life-space mobility. A custom designed smartwatch is effective at simultaneously collecting rich information about ecological pain and life-space mobility.

### OLDER ADULTS' SATISFACTION AND COMPLIANCE OF SMARTWATCHES PROVIDING ECOLOGICAL MOMENTARY

Charlotte Laborde,[1] Erta Cenko,[2] Mamoun Mardini,[2] Sanjay Ranka,[2] Parisa Rashidi,[2] and Todd Manini,[3]
*1. Institute on aging, gainesville, Florida, United States, 2. University of Florida, Gainesville, Florida, United States, 3. The University of Florida, Gainesville, Florida, United States*

The objective of this study was to evaluate satisfaction, usability and compliance using the Real-Time, Online Assessment and Mobility Monitor (ROAMM) application designed for smartwatches. Twenty-eight older adults with knee osteoarthritis (66-88 years old) wore a Samsung Gear S3 smartwatch with ROAMM. While wearing the watch for 13.16 (±2.94) days, participants gave ecological momentary assessments (EMA) of pain intensity, mood and fatigue at random times in the morning, afternoon and evening. EMA compliance rate was 82.5%. Satisfaction ratings were high with 73% reporting being satisfied with the function, 78% reported being satisfied with the comfort and 73% reported as likely to use ROAMM on a daily basis for a one-year research study. Participants reported to reduce watch size and weight, style and increase battery life. Older adults were compliant to ecological assessment of their symptoms and reported positive usability and satisfaction of wearing a smartwatch and ROAMM app.

### SESSION 7230 (SYMPOSIUM)

### SOCIAL AND BEHAVIORAL FACTORS, ORAL HEALTH, AND DENTAL CARE UTILIZATION

Chair: Bei Wu
Co-Chair: Stephen Shuman
Discussant: Elisa Ghezzi

Oral health status and dental care utilization is strongly associated with social and behavioral factors and health outcomes. The five papers in this symposium address how several of these factors affect oral health and dental care among diverse groups of older adults. Using data from the Health and Retirement Study, the first paper examined the impact of early childhood disadvantages on oral health in later life among adults age 51 and above in the U.S. The second paper used large-scale epidemiological data that addressed the relationship between acculturation and subsequent oral health problems. It also tested the moderating role of neighborhood disorder in such a relationship among older Chinese Americans. The third paper demonstrated the importance of examining different pathways among foreign-born and native-born Chinese older adults with regard to offspring's support on their oral health outcomes. While increasing evidence shows that cognitive function is associated with oral health, limited studies have been conducted to examine the impact of cognitive impairment, e.g., Alzheimer's Disease (AD) and related dementias (RD), on dental care utilization and costs in older adults. The fourth paper aimed to address this knowledge gap. Results showed that AD and RD had different impacts on different types of dental care utilization and costs. The fifth paper further displayed that individuals with cognitive impairment face a significant challenge in handling dental-related medications. This symposium provides policy and clinical implications on improving oral health and dental care utilization among older adults in the U.S. Oral Health Interest Group Sponsored Symposium.

### EARLY LIFE CONDITIONS, ADULTHOOD EXPERIENCES, AND EDENTULISM AT OLDER AGES

Haena Lee,[1] and Linda Waite,[2] *1. University of Southern California, Los Angeles, California, United States, 2. University of Chicago, Chicago, Illinois, United States*

The role of childhood in shaping overall adult health has been well documented, especially for physical and mental health, but much less is known about the impact of early disadvantage on oral health in later life. Using data from the 2006 and 2012 Health and Retirement Study, we investigate the link between childhood financial and psychosocial adversity and edentulism over a six-year period among U.S. adults aged 51 and older. We find that those growing up with parents with fewer resources face higher risks of having lost all their tooth at baseline and during the follow-up. Adulthood socioeconomic status and health behaviors are strongly associated with the risk of edentulism, net of childhood conditions. However, the effect of low parental resources on the onset of edentulism persists when accounting for these life course factors. Part of a symposium sponsored by the Oral Health Interest Group.

### ACCULTURATION AND SUBSEQUENT ORAL HEALTH PROBLEMS AMONG OLDER CHINESE AMERICANS: THE ROLE OF NEIGHBORHOOD DISORDER

Weiyu Mao,[1] Bei Wu,[2] Iris Chi,[3] Wei Yang,[1] and XinQi Dong,[4] *1. University of Nevada, Reno, Reno, Nevada, United States, 2. New York University, New York, New York, United States, 3. University of Southern California, Los Angeles, California, United States, 4. Rutgers University, New Brunswick, New Jersey, United States*

To further understand social, cultural, and personal predictors of oral health outcomes, this study addressed the relationship between acculturation and subsequent oral health problems and tested the moderating role of neighborhood disorder in such a relationship among older Chinese Americans. The working sample included 2,706 foreign-born community-dwelling older Chinese Americans aged 60 years or older who participated in the Population Study of Chinese Elderly in Chicago at the baseline and the first follow-up. Stepwise Poisson regression using lagged dependent variable was conducted. Behavioral acculturation was protective against subsequent oral health problems. Residence in Chinatown was associated with an increase in the risk of subsequent oral health problems. The relationship between behavioral acculturation and subsequent oral health problems varied by levels of neighborhood disorder. To reduce oral health-related disease burdens, it is important to consider the role of acculturation and the neighborhood on subsequent oral health problems in practice and policy. Part of a symposium sponsored by the Oral Health Interest Group.

### ORAL HEALTH, IMMIGRANT STATUS, AND ADULT CHILDREN'S SUPPORT AMONG CHINESE AMERICAN OLDER ADULTS

Nan Jiang,[1] Bei Wu,[2] and Wei Zhang,[3] *1. National University of Singapore, Singapore, New York, Singapore, 2. New York University, New York, New York, United States, 3. University of Hawaii at Manoa, Honolulu, Hawaii, United States*

Adult children play an important role in older immigrants' health outcomes. Research has indicated that older adults may benefit from adult children caregivers' support for oral health. However, little is known about children's support for improving oral health for older immigrants. Using the 2018 survey of 430 Chinese older adults age 55 and older in Honolulu, Hawai'i, we examine the associations among immigrant status, adult children's support and perceived oral health for Chinese American older adults. Emotional support from adult children protects the self-rated oral health for the immigrant group, while financial support is linked to fewer oral health problems among the US-born group. Therefore, the current study underlines the importance of investigating different pathways among foreign-born and native-born Chinese older adults with regard to children's support on their oral health outcomes. Part of a symposium sponsored by the Oral Health Interest Group.

### THE IMPACT OF ALZHEIMER'S DISEASE AND RELATED DEMENTIAS ON DENTAL CARE UTILIZATION AND COSTS IN OLDER ADULTS

Sam Li,[1] Isaac Donkor,[2] Liang Hong,[1] Kevin Lu,[3] and Bei Wu,[4] *1. University of Tennessee Health Science Center, Memphis, Tennessee, United States, 2. Vanderbilt University, Nashville, Tennessee, United States, 3. University of South Carolina, Columbia, South Carolina, United States, 4. New York University, New York, New York, United States*

There is limited information on the impact of cognition function on dental care utilization and costs. This study used the Medicare current beneficiaries survey in 2016 and included 4,268 participants 65+. Dental care utilization and costs were measured by self-report and included preventive and treatment events. Negative binomial regression and generalized linear regression were used to examine the impact of Alzheimer's disease (AD) and related dementia (RD) on dental care utilization and costs. We found that AD was not associated with dental care utilization, but RD was associated with a lower number of total treatment dental care visits (IRR: 0.60; 95% CI: 0.37~0.98). RD was not associated with dental care costs, but AD was associated with higher total dental care costs (estimate: 1.08; 95% CI: 0.14~2.01) and higher out-of-pocket costs (estimate: 1.25; 95% CI: 0.17~2.32). AD and RD had different impacts on different types of dental care utilization and costs. Part of a symposium sponsored by the Oral Health Interest Group.

### HOW COGNITIVE IMPAIRMENT AFFECTS MEDICATION MANAGEMENT IN DENTAL SETTINGS

Xi Chen,[1] Jirakate Madiloggovit,,[1] Lisa Jacobson,[2] Daniel Tranel,[3] Carissa Comnick,[4] and Xianjin Xie,[1] *1. University of Iowa, Iowa City, Iowa, United States, 2. University of Iowa Hospitals & Clinics, Iowa City, Iowa, United States, 3. University of Iowa Hospitals & Clinics, iowa City, Iowa, United States, 4. University of Iowa, Iowa city, Iowa, United States*

Mishandling medications is commonly seen in persons with dementia, which can lead to poor treatment outcome and serious complications. Whether individuals with cognitive impairment can appropriately manage dental-related medication remains unknown, raising a liability concern for dentists who fail to recognize the patients at risk for mishandling their medications. To address this concern, we conducted a study with 51 participants with various cognitive impairment to describe their ability to handle dental-related medications. After cognitive assessment, participants were asked to set up an antibiotics pill-box and use a mouthwash as instructed, and their performance were scored. Number and type of prompts given to facilitate task completion were also documented. Mishandling of dental-related medications was common in participants with cognitive impairment. As expected, participants with poor cognition needed more assistance on handling their medications. Dentists should be aware of this concern and take it into consideration when treatment plan for these individuals. Part of a symposium sponsored by the Oral Health Interest Group.

### SESSION 7235 (SYMPOSIUM)

### TECHNOLOGY-BASED APPLICATIONS FOR THE ASSESSMENT AND MANAGEMENT OF ALZHEIMER'S DISEASE AND RELATED DISORDERS

Chair: Lauren Massimo
Discussant: George Demiris

Over the last decade, technological advances have made it possible for people to access health information right at their fingertips. Indeed, technology-based applications (apps) have been developed to tackle a wide range of health-related issues, including Alzheimer's disease and related disorders (ADRD). As such, there is a critical need to develop and test effective dementia apps that can ultimately be converted in to scalable programs. In this interdisciplinary symposium, we will discuss the development and testing of novel apps for the assessment and management of ADRD. The first session will discuss the development of an online money management credit card task to assess cognitively vulnerable older adults who are at risk for making poor financial decisions. The second session will describe the development and psychometric testing properties of a mobile app to detect the earliest features of preclinical Alzheimer's disease. The third session will highlight findings from a study testing a smartphone-based prompting app to improve everyday task completion in persons with mild cognitive impairment and mild dementia. The last session will share findings from a study testing a mobile app to increase goal-directed behavior and reduce apathy in persons with dementia. Together, these presentations describe how technology-based applications can be used to assess and manage cognitive and behavioral symptoms of ADRD.

### DEVELOPMENT OF THE ONLINE MONEY MANAGEMENT CREDIT CARD TASK

Preeti Sunderaraman,[1] Ziqian Dong,[2] Santhoshkumar Sampath,[2] Silvia Chapman,[3] Jillian Joyce,[1] Yaakov Stern,[1] and Stephanie Cosentino,[4] *1. Columbia University, New York, New York, United States, 2. New York Institute of Technology, New York, New York, United States, 3. Columbia University, New York, Pennsylvania, United States, 4. Columbia University Medical Center, New York, New York, United States*

Older adults (OAs), a wealthy but vulnerable segment of our population, are at risk to make compromised financial decisions. Evidence suggests that OAs increasingly use technology to perform everyday financial transactions, such as to manage their credit card statements. However, current tools are lacking in terms of assessing how older adults navigate and handle the online financial milieu. We will discuss the development of a novel, simulated online money management (OMM) credit card statement task. OMM examines OAs performance on several indices including reaction time, nature and frequency of errors, and their ability to comprehend and trouble shoot problems. Psychometric properties related to the reliability and validity will be discussed. Ultimately, by examining the longitudinal performance of OMM in OAs, we can better characterize the natural course of OMM. Such an approach will enable clinicians to accurately and objectively examine OMM and identify those at risk for making financial errors.

### DETECTION OF ALZHEIMER'S DISEASE-RELATED COGNITIVE CHANGE WITH THE MOBILE COGNITIVE APP PERFORMANCE PLATFORM

Dawn Mechanic-Hamilton, Sean Lydon, Alexander Miller, Kimberly Halberstadter, Jacqueline Lane, Sandhitsu Das, and David Wolk, *University of Pennsylvania, Philadelphia, Pennsylvania, United States*

This study investigates the psychometric properties of the mobile cognitive app performance platform (mCAPP), designed to detect memory changes associated with preclinical Alzheimer's Disease (AD). The mCAPP memory task includes learning and matching hidden card pairs and incorporates increasing memory load, pattern separation features, and spatial memory. Participants included 30 older adults with normal cognition. They completed the mCAPP, paper and pencil neuropsychological tests and a subset completed a high-resolution structural MRI. The majority of participants found the difficulty level of the mCAPP game to be "just right". Accuracy on the mCAPP correlated with performance on memory and executive measures, while speed of performance on the mCAPP correlated with performance on attention and executive function measures. Longer trial duration correlated with measures of the parahippocampal cortex. The relationship of mCAPP variables with molecular biomarkers, at-home and burst testing, and development of additional cognitive measures will also be discussed.

### SMARTPROMPT REMINDER APPLICATION IMPROVES EVERYDAY TASK COMPLETION AND REDUCES INEFFICIENT BEHAVIORS

Hackett Katherine, Sarah Lehman, Ross Drivers, Matthew Ambrogi, Likhon Gomes, Chiu Tan, and

Tania Giovannetti, *Temple University, Philadelphia, Pennsylvania, United States*

The SmartPrompt phone-based reminder application was designed according to neuropsychological theory and pilot testing to facilitate everyday functioning. A laboratory-based pilot of ten participants with MCI and mild dementia showed significantly greater task completion with significantly fewer checking behaviors when using the SmartPrompt versus a control condition. Younger individuals and those who engaged in more checking behaviors completed more tasks in the control condition, but these relations were not significant when using the SmartPrompt. After 15 minutes of training, caregivers achieved near perfect scores on a SmartPrompt configuration quiz. Participant and caregiver usability ratings were strong, even though participants reported relatively low computer proficiency and neutral/unfavorable attitudes towards technology. Piloting informed modifications of the SmartPrompt to enhance personalization (e.g., customized alarms/rewards) and improved human-computer-interaction for in-home testing. Preliminary in-home test data on individually-owned smartphones and conclusions regarding barriers and facilitators to the effectiveness of the modified SmartPrompt will be discussed.

### ACTIVIDAILY: TURNING APATHY INTO ACTION IN NEURODEGENERATIVE DISEASE

Lauren Massimo, Sean Lydon, Alexander Miller, Katya Rascovsky, and Dawn Mechanic-Hamilton, *University of Pennsylvania, Philadelphia, Pennsylvania, United States*

Impairment of goal-directed behavior (GDB), often labeled apathy, is a common behavioral symptom in dementia. ActiviDaily is a novel mobile app that engages both patients and caregivers to increase GDB to improve everyday function. ActiviDaily targets key components of GDB (motivation, planning and initiation) and individualizes patient goals. Pilot testing in twelve patient/caregiver dyads occurred over 4 weeks of app use. Measures of behavior, everyday functioning, and psychological distress were assessed in a pre-post design. Goal Attainment Scaling (GAS) was used to establish individualized goals and measure progress on a standard scale. GAS showed that 79% of participants' goals were met at or above expectations. Caregiver depression and stress were significantly reduced. There was also a reduction in ratings of patient apathy. ActiviDaily is an innovative intervention that individualizes treatment of apathy and has the potential to increase independence in day-to-day life and decrease caregiver burden.

### CAN WE END THE AGE-OLD PROBLEM OF PRESSURE INJURIES?

Tracey Yap, *Duke University, Chapel Hill, North Carolina, United States*

A pressure injury/ulcer (PrI) is a localized area of injured skin and tissue usually over a bony prominence and one of the highest priority problems identified in U.S. health care's federal quality initiatives; approximately 26.8 billion is spent for treatment each year. The problem is accentuated for nursing home residents who are often immobile/bedridden. Currently, resident repositioning/movement by nursing staff every 2-hours is the cornerstone of prevention care. Successful interventions must be nurse-led and designed to facilitate the prevention care of nursing staff on the front lines. My research focuses on integrating movement into everyday care for institutionalized older adults and is advancing the science of PrI prevention through testing of cueing interventions for nursing staff to improve the care delivery. My goals for innovating preventive care include enhancing our understanding of nursing subcultures' influence on care outcomes and leveraging emerging technology to enhance the care team's collaborative efforts.

### NAVIGATING THE TREACHEROUS WATERS OF GERIATRIC COMPLEXITY AND HETEROGENEITY WITH THE HELP OF TEAM SCIENCE

George Kuchel, *University of Connecticut, Farmington, Connecticut, United States*

Multifactorial complexity and heterogeneity challenge the care of older adults and research into the pathophysiology of common geriatric syndromes. Multicomponent interventions matching intervention components with individual risk factors are grounded in precision medicine by ensuring that interventions may be offered to those who will more likely benefit, sparing expense and side effects for those who will not. Nonetheless, the development of mechanism-guided interventions has been hampered by failure to identify single mechanisms for effective targeting within this multifactorial complexity, a problem worsened by historical barriers between research disciplines and silos. Geroscience-guided interventions target biological hallmarks of aging representing mechanisms that geriatric syndromes share with aging. We will present examples of multidisciplinary bench-to-bedside translational science seeking to transform the care of common geriatric conditions as diverse as frailty, voiding disorders and immunization against influenza and pneumococcal infections via geroscience-guided therapies applied with a greater emphasis on heterogeneity of aging and targeting.

### DEPRESCRIBING IN OLDER ADULTS: IS EVIDENCE FOR CONTINUED MEDICATION USE GENERALIZABLE BEYOND AGE 75?

Joshua Niznik, *University of North Carolina at Chapel Hill, Chapel Hill, North Carolina, United States*

Older adults over the age of 75 are severely underrepresented in many of the clinical trials used to justify the continued use of medications for chronic disease prevention in advanced age. The gaps in evidence in this population have fueled an interest in research to better understand the potential benefits and harms associated with the continued use of medications with uncertain benefit in advanced age. Deprescribing, the intentional reduction or discontinuation of medications, has recently gained traction as an important component of the prescribing process, but raises questions about the safety of stopping medications. This presentation will provide an overview of the evolution of deprescribing research and how this has shaped my career as a geriatric health services researcher. Specifically, I will address early studies that defined the field, challenges and opportunities for studying deprescribing in older adults, and future directions and priorities in deprescribing research.

### A PATIENT WORK LENS TO UNDERSTANDING AND SUPPORTING HEALTH CARE AT HOME

Nicole Werner, *University of Wisconsin-Madison, Madison, Wisconsin, United States*

A majority of healthcare today occurs in private homes and is performed by older adults and their informal caregivers. However, only recently have we begun to understand more about the healthcare work of older adults and their caregivers and the home as a healthcare delivery system. Work science allows us to conceptualize the healthcare activities of older adults and their caregivers as a type of health-related work that has been conceptualized under the broad term, patient work. Applying a patient work lens can offer a unique perspective to addressing the persistent and pervasive challenges faced by older adults and their caregivers as they manage their healthcare at home and across healthcare settings. I will describe the historical development of the patient work approach and highlight recent efforts to use a patient work approach to understand and support the work of older adults and their caregivers providing care in the home.

### GEROSCIENCE BRINGS NEW FOCUS ON THE LIFE-COURSE APPROACH TO HEALTH IN HUMANS

Luigi Ferrucci, *National Institute on Aging, Bethesda, Maryland, United States*

The geroscience paradigm stands on two assumptions: 1. Major changes in health are caused by accelerated aging; 2. The rate of aging can be modified by interventions with significant effects on health span. These simple statements change dramatically the portrayal of aging research in medicine. In the past, aging was considered fixed and irreversible, and the study of aging a speculative science, closer to anthropology than to physiology. However, if biological aging is the root cause of chronic disease and if the rate of aging can be changed, then the study of aging becomes the most important branch of medical research. The last twenty years of research support this view and suggest that the rate of aging is set during early development but is modifiable by appropriate interventions. These findings suggest that the architecture of disease development and subsequent loss of function may be more easily influenced by early interventions.

### SESSION 7240 (SYMPOSIUM)

### THE PAST, PRESENT, AND FUTURE OF HEALTH SCIENCES RESEARCH

Chair: Cynthia Brown
Co-Chair: John Batsis

The health sciences have experienced an evolution in research over the past 75 years, moving along the translational spectrum from bench to bedside to the community. This transformation has impacted health outcomes for older adults at a global and public health level. Expansion in geroscience and implementation science research has changed the lens through which we view how aging occurs, precision management of disease, and has allowed the integration of tested interventions into healthcare systems. This Presidential symposium will showcase investigators, ranging from junior to senior researchers, who will share where their science began, how their research has built on the past, provide insights into their own work, and share their perspectives on the continuing trajectory of their scientific work.

### SESSION 7245 (SYMPOSIUM)

### TRANSITIONAL CARE MANAGEMENT: EVIDENCE FOR NOVEL IMPLEMENTATION MODELS AND REHABILITATION IMPLICATIONS

Chair: Margaret Danilovich
Discussant: Margaret Danilovich

The transition between healthcare settings is a complex process presenting challenges for effective and consistent communication between older adults, their caregivers, and healthcare providers. These challenges often result in adverse health events and re-hospitalizations. Further, once transitioned to home, older adults often need ongoing care management and support and evidence for models remains unclear as to the precise parameters of supports needed for comprehensive care. This symposium will provide an overview of the evidence for both interdisciplinary care management models and transitional care programs, present the implementation of a care management program for low income older adults at one social service agency, and provide evidence-based tools for older adult functional assessment and decision-making for transitional care. The speakers will present new tools from the American Physical Therapy Association home health toolbox that promote patient-centered health care decision-making to facilitate successful transitions that reduce resource use and hospital readmission. The speakers will also discuss the implementation of a care management program for older adults in a care gap (having too much income for Medicaid home and community-based services, but still <200% of the federal poverty line). An implementation framework for the needs assessment will be highlighted and 1-year program outcomes will be presented. Attendees will learn strategies for interprofessional collaboration, enhanced communication, and advocacy within the interprofessional team to facilitate improved care management and transitional services for older adults.

### EVIDENCE FOR A NOVEL CARE MANAGEMENT PROGRAM FOR LOW-INCOME OLDER ADULTS

Rachel Lessem,[1] and Margaret Danilovich,[2] *1. CJE SeniorLife, Chicago, Illinois, United States, 2. CJE SeniorLife, Evanston, Illinois, United States*

The purpose of this study was to evaluate the implementation and effectiveness of a novel care management program for low income older adults in Chicago. Older adults (n=200) who had annual income below $31,225 but about the state level for home and community based services were received care management. Program participants completed a battery of assessments (UCLA Loneliness Scale, single item Quality of Life and Physical Health scales, and Nutritional assessment) at initial assessment and 1-year follow-up. We also conducted interviews with clients and care managers. We used a t-test to evaluate participant outcomes and coded qualitative data to identify themes. Results showed no significant differences between baseline and 1 year follow-up indicating that this care management program kept participants stable. Only 5 of 200 (2.5%) of clients transitioned to a nursing home. This study contributes important results on a novel program to sustain vulnerable older adults in the community.

### IMPLEMENTATION OF A CARE MANAGEMENT PROGRAM FOR LOW-INCOME OLDER ADULTS

Anna Schloen, and Helen Grimaldi, *CJE SeniorLife, Chicago, Illinois, United States*

The focus of this session will be on the implementation of a new care management program in Chicago for low income older adults. Framed by the Exploration, Installation, Initial Implementation, and Full Implementation framework, the presenter will first discuss the process and outcomes of a needs assessment which informed the program. The presenter will share strategies and lessons learned from getting the program off the ground and initial lessons learned which informed the full program as it is operated today. In 2 years, the program has grown from 1 FTE to 4.5 FTE and to 250 clients. The presenter will provide strategies for managing growth while maintaining quality care. Finally, the presenter will provide information on the program's collaboration with a researcher to enhance evidence-based service delivery within the care management program. Participants will learn specific strategies they can take back to their own communities to implement care management programs.

### THE ROLE OF REHABILITATION IN REDUCING RESOURCE USE AND LOWERING HOSPITAL READMISSION THROUGH DISCHARGE PLANNING

Kenneth Miller, *UNT Health Science Center, Fort Worth, Texas, United States*

The transitions between medical settings, the community and back again is a complex and intimidating process for patients, families and caregivers. These transitions are vulnerable points where planning is key and must begin at the initial examination with rehabilitation providers (PTs/OTs,SLPs). These providers are key members of the healthcare team to facilitate effective transition management. In this session, attendees will learn the critical factors rehabilitation providers use to evaluate patients in order to facilitate successful care transitions. An overview of the indications for rehabilitation referral will be presented, as well as evidence for effective rehabilitation strategies. The speaker will present tools from the American Physical Therapy Association Home Health Toolbox and outline a decision-making process for care transitions based on the individual, caregivers, and health care providers to achieve successful transitions that reduce resource use and hospital readmission rates. Attendees will learn strategies to facilitate inter-professional collaboration, communication, and advocacy.

### SESSION 7250 (SYMPOSIUM)

### UNDERSTANDING THE SENSORY LOSS–COGNITIVE FUNCTION RELATIONSHIP IN OLDER ADULTS: BIOMARKER OR CAUSAL RISK FACTOR?

Chair: Jennifer Deal
Discussant: Heather Whitson

Sensory impairment in older adults is common, over 55% of Americans 60 years and older have either hearing or vision impairment, and it is linked to accelerated cognitive decline and increased risk of incident dementia in population-based observational studies. However, whether sensory impairment is a marker or a cause of cognitive decline and dementia is unknown. Both sensory impairment and cognitive decline/dementia may be caused by a common underlying pathology (e.g., microvascular disease), or sensory impairment may be a marker of dementia-related pathological changes in the brain. Alternatively, causal mechanisms include increased cognitive load, changes brain structure/function, depression, social isolation and/or reduced activity. This session will investigate the role of sensory impairment in cognitive decline and dementia in older adults and discuss the ramifications of these different possibilities for risk prediction and stratification, and potentially, for disease prevention. The co-occurrence of multiple sensory deficits will be described, and the potential utility of the use of retinal signs as predictive markers for cognitive decline/dementia will be discussed. We will also describe current evidence for both non-causal and causal relationships between sensory impairment and cognition with a focus on hearing impairment. Finally, we will describe the relationship of dual sensory (both hearing and vision) impairment on cognitive performance and dementia in a biracial population-based study.

### PREVALENCE OF MULTIPLE SENSORY DEFICITS IN OLDER ADULTS IN BLSA AND ARIC STUDIES

Nicole Armstrong,[1] Jennifer Deal,[2] Hang Wang,[3] Jennifer Schrack,[2] Qu Tian,[1] Eleanor Simonsick,[1] Yuri Agrawal,[4] and Frank Lin,[4] *1. National Institute on Aging, Bethesda, Maryland, United States, 2. Johns Hopkins Bloomberg School of Public Health, Baltimore, Maryland, United States, 3. Johns Hopkins School of Medicine, Baltimore, Maryland, United States, 4. Johns Hopkins University School of Medicine, Baltimore, Maryland, United States*

Individual sensory deficits have been associated with adverse outcomes, including dementia, in older adults. Using data from the Baltimore Longitudinal Study of Aging (BLSA) (N=259) and Atherosclerosis Risk in Communities Study (ARIC) (N=962), we examined the prevalence of one, two, or three sensory deficits (hearing, vision, and olfaction) among older adults ≥70 years. Any hearing loss was the most prevalent sensory deficit (70-79 year-olds: 41.3% [BLSA] and

51.2% [ARIC]; ≥80 year-olds: 82.6% [BLSA] and 74.2% [ARIC]), followed by vision loss and olfactory loss. Hearing and vision impairments were more prevalent than hearing and olfactory losses as well as vision and olfactory losses in both age groups and studies There were few people with deficits in all three senses (70-79 year-olds: 3.3% [BLSA] and 2.0% [ARIC]; ≥80 year-olds: 5.8% [BLSA] and 7.4% [ARIC]). Further research should investigate the potential impact of multisensory impairments on older adults.

### USING GENETIC INFORMATION TO EXPLORE WHETHER PRECLINICAL ALZHEIMER'S DISEASE AFFECTS HEARING DIFFICULTY

Willa Brenowitz,[1] Teresa Filshtein,2 Kristine Yaffe,[3] Stefan Walter,[4] Thomas Hoffmann,[5] Eric Jorgenson,[6] Rachel Whitmer,[7] and Maria Glymour,[8] *1. University of California, San Francisco, San Francisco, California, United States, 223. andMe, Mountain View, California, United States, 3. UCSF Weill Institute for Neurosciences, San Francisco, California, United States, 4. Rey Juan Carlos University, Madrid, Madrid, Spain, 5. UCSF, San Francisco, California, United States, 6. Kaiser Permanente, Oakland, California, United States, 7. University of California Davis, Davis, California, United States, 8. University of California, San Francisco, California, United States*

Underlying AD-related neurodegeneration or shared risk factors may influence hearing loss; in an innovative approach we tested whether genetic risk for AD also influences functional hearing loss. We studied 401,084 UK Biobank participants aged 40-70, with Caucasian genetic ancestry, and enrolled 2007-2010. Participants self-reported hearing difficulty and were followed for AD diagnosis until 2018. A genetic risk score for AD (AD-GRS) was calculated as a weighted sum of 23 AD risk variants. In age-, sex-, and genetic ancestry- adjusted models higher AD-GRS was associated with problem hearing in ages 60+(OR= 1.03; 95%CI:1.00, 1.05), but not ages <60 (p>0.05). Using the AD-GRS as an instrumental variable for AD diagnosis, we estimated that incipient AD increased probability of difficulty hearing at enrollment by 45% (95%CI: 1%, 93%). Higher AD-GRS was associated with slightly higher odds of hearing difficulty in older adults. Genetics that predispose for AD also influence late-life hearing difficulty.

### RETINAL MICROVASCULAR HEALTH: CAN THE EYE TELL US ABOUT THE BRAIN?

Alison Abraham,[1] Xinxing Guo,[1] Xiangrong Kong,[1] A. Richey Sharrett,[2] Brandon Lujan,[3] David Huang,[3] and Pradeep Ramulu,[4] *1. Johns Hopkins University School of Medicine, Baltimore, Maryland, United States, 2. Johns Hopkins University, Baltimore, Maryland, United States, 3. Casey Eye Institute, Oregon Health and Science University, Portland, Oregon, United States, 4. Johns Hopkins School of Medicine, Baltimore, Maryland, United States*

Cognitive impairment caused by Alzheimer's and other dementias is linked to vascular damage in the brain. Early microvascular changes in the brain are difficult to detect but in the retina, they can potentially be seen using optical coherence tomographic angiography. In cross-sectional analysis, associations between retinal vessel density (VD) and cognitive performance were assessed by regression analysis of cognitive function z-scores (for global function as well as language, memory and executive function domains) on VD, controlling for age, race and education. Among 177 participants without dementia (50% black; 67% female; mean age 78 years [range: 71-93 years]), the mean (SD) superficial vascular complex VD was 48.0% (7.2). Among the 191 eyes without eye disease, there were no significant associations of VD with global or domain specific cognitive function. Early changes in the eye related to systemic disease processes may not currently be detectable in healthy older adults with imaging data.

### HEARING LOSS AND COGNITIVE DECLINE: CAUSAL EVIDENCE

Justin Golub,[1] Rahul Sharma,[2] Alex Chern,[3] Brady Rippon,[3] Adam Ciarlegio,[4] Nicole Schupf,[5] Adam Brickman,[3] and José Luchsinger,[3] *1. NewYork-Presbyterian Hospital/Columbia University Irving Medical Center, New York City, New York, United States, 2. Columbia University Irving Medical Center, New York, New York, United States, 3. Columbia University, New York, New York, United States, 4. George Washington University, Washington, District of Columbia, United States, 5. Columbia University Medical Center, New York, New York, United States*

Age-related hearing loss (HL) has recently been associated with cognitive decline, dementia, and changes in brain structure. HL has near universal prevalence in later life (involving 80% of those over 80 years old) and is rarely treated (under 20% in the same age group). Dementia is also common in later life, carrying staggering societal implications, including a worldwide cost of over $605 billion/year. Research establishing an association between HL and cognition has included cross-sectional and prospective studies. New data show a link not only in the earliest stages of HL but also in hearing ranges still considered normal. The current evidence for an independent association between HL and impaired cognition, including a possible causal connection, will be reviewed. Plausible mechanistic pathways will be discussed with an emphasis on brain imaging biomarkers of dementia. Finally, future avenues for research and policy change will be proposed.

### DUAL SENSORY IMPAIRMENT AND COGNITIVE PERFORMANCE, MCI, AND DEMENTIA IN OLDER ADULTS: THE ARIC NEUROCOGNITIVE STUDY

Jennifer Deal,[1] Junghyun Park,[2] Nicholas Reed,[3] Alison Abraham,[4] Frank Lin,[4] B. Gwen Windham,[5] and A. Richey Sharrett,[3] *1. Johns Hopkins Bloomberg School of Public Health, Baltimore, Maryland, United States, 2. New York University, New York City, New York, United States, 3. Johns Hopkins University, Baltimore, Maryland, United States, 4. Johns Hopkins University School of Medicine, Baltimore, Maryland, United States, 5. University of Mississippi Medical Center, Jackson, Mississippi, United States*

Dual sensory impairment (DSI) affects 11.3% of adults aged ≥80 years. Hearing and vision impairments are each associated with cognitive decline and dementia, but DSI's impact is unknown. All-cause dementia and mild cognitive impairment (MCI) were adjudicated using longitudinal cognitive information. Ten neurocognitive tests were summarized using latent variable methods. Hearing was measured using pure tone better-ear thresholds (0.5-4 kHz) and vision with better-eye presenting distance visual acuity and/or contrast sensitivity. In 881 adults (79±4 years, 44% black, 64% female), DSI (vs. no hearing or vision impairment) was cross-sectionally associated with -0.17 standard deviations (SD) [95% confidence interval (CI): -0.32, -0.02] lower global cognitive score and an 87% increased odds (95% CI: 1.01, 3.45) of combined MCI/dementia, after full adjustment for demographic and clinical factors. Future longitudinal research should elucidate the mechanism underlying this association to determine if treatment can delay cognitive decline and MCI/dementia in older adults.

### SESSION 7255 (SYMPOSIUM)

### VACCINATION TO PROMOTE HEALTHY AGING: THE FIVE WS

Chair: Leonard Friedland
Discussant: Leonard Friedland

This symposium addresses the role of vaccination to promote healthy aging and the process of developing and maintaining the functional ability that enables wellbeing in older age. Adults age 65 and over are at increased risk of certain infectious diseases due to immunosenescence. Therefore, immunization of older adults against targeted infectious diseases, including pertussis, shingles, influenza, and pneumococcal disease, can help to reduce morbidity and premature mortality. Vaccines in development to protect against additional infectious diseases causing significant morbidity and mortality in older adults, such as respiratory syncytial virus, can further promote healthy aging. The population of older adults in the US is projected to grow significantly over the next 30 years, with a corresponding increase in the incidence and economic costs of vaccine-preventable diseases. Immunization of older adults is a proven, cost-effective strategy that is critical for reducing the public health impact and societal costs in an aging US population. Implementation of evidence-based recommended vaccines for older adults presents challenges, including financial barriers, addressing disparities and inequities in health care delivery for older adults, and overcoming vaccine hesitancy. We plan to review these topics and present data we have generated to support the value of vaccination in adults age 65 and over. Health Behavior Change Interest Group Sponsored Symposium.

### VACCINATION IN OLDER ADULTS: THE WHO AND WHAT

Leonard Friedland, *GSK Vaccines, Philadelphia, Pennsylvania, United States*

Aging brings increased impact of infectious disease in terms of hospitalization, morbidity, and mortality. This increased susceptibility to infection results from immunosenescence, age-related changes in the immune system, anatomic and functional changes, and environmental exposure to infections. Adults age 65 and over are at increased risk of pertussis, shingles, influenza and pneumococcal disease, and evidence-based recommendations for vaccination are protect older adults against these diseases. Underlying medical conditions including end stage renal disease, chronic lung, heart and liver disease, diabetes and immunocompromised place adults age 65 and over at increased risk of infectious diseases, therefore evidence-based vaccine recommendations in older adults with additional risk factors are in place to protect against varicella, hepatitis A and B, meningococcal meningitidis and Haemophilus influenzae type b. Investigational vaccines are developed to protect against infectious diseases causing significant morbidity and mortality in older adults, for example, respiratory syncytial virus, to further promote healthy aging. Part of a symposium sponsored by the Health Behavior Change Interest Group.

### VACCINATION IN OLDER ADULTS: THE WHEN AND WHERE

Sara Poston, *GSK Vaccines, Philadelphia, Pennsylvania, United States*

Despite the well-understood benefits of vaccination in older adults, national rates still fall below public health targets, especially among certain racial and ethnic groups. Recent scholarship examining healthcare use patterns in adults revealed that health care providers miss several opportunities to provide vaccination during regular healthcare encounters, including Medicare annual wellness visits. Several barriers to older adult vaccination have been identified, including lack of patient and provider understanding of the importance of vaccination, financial barriers to vaccines covered under Medicare Part D, and patient hesitancy about the safety and effectiveness of vaccines. Strategies to address these barriers will be discussed, including the use of national quality measures to strengthen incentives for adult vaccination. Part of a symposium sponsored by the Health Behavior Change Interest Group.

### THE VALUE OF VACCINATION IN OLDER ADULTS: THE WHY

Philip Buck, *GSK Vaccines, Philadelphia, Pennsylvania, United States*

The incidence of vaccine-preventable diseases remains high among older adults in the US, despite longstanding immunization recommendations, and is projected to increase as the population ages. The impact of US population aging on the burden of four vaccine-preventable diseases (influenza, pneumococcal disease, shingles, and pertussis) was modeled over a 30-year time horizon, with cumulative direct and indirect costs increasing from $378 billion over 10 years to

$1.28 trillion over 30 years. Compared to current levels of vaccination coverage, increasing coverage was predicted to avert over 33 million cases of disease and greater than $96 billion in disease-associated costs, with a corresponding increase in vaccination costs of approximately $83 billion over the entire 30-year time period. Specific examples of cost-effectiveness analyses that assess the epidemiologic and economic impact of vaccination against shingles and pertussis in older adults will be discussed. Part of a symposium sponsored by the Health Behavior Change Interest Group.

### SESSION 7260 (SYMPOSIUM)

### WHO WORLD REPORTS ON HEARING AND VISION: MEETING THE GROWING NEEDS OF OLDER ADULTS

Chair: Carrie Nieman
Co-Chair: Bonnielin Swenor
Discussant: Charlotte Yeh

The World Health Organization's (WHO) World Report on Vision was released in October 2019 and the World Report on Hearing debuted at the World Health Assembly in May 2020. The Reports recognize the fundamental nature of sensory health in the health and well-being of individuals and societies and outline the significant and growing burden of hearing loss and vision impairment across the life course. Together the efforts call for more affordable, accessible, and integrated care to foster sensory health for all and represent a major opportunity to advance vision and hearing care as public health priorities nationally and internationally. With the largest burden of sensory impairment among older adults, this symposium will focus on applying the findings and recommendations of the Reports to gerontology. The first presentations will provide an overview of the World Reports on Vision and Hearing from members of the core working groups involved in the Reports and discuss them within the context of the WHO's Decade of Healthy Aging. The second set of presentations will feature the latest findings related to sensory health from the Global Burden of Disease Study. In moving toward action, task sharing is a critical theme that runs throughout the Reports and we will cover the application of task sharing to hearing care as an example of applying public health principles to advance sensory health. As we advocate for improving the lives of older adults, sensory health remains a significant component with great potential for progress and collaboration.

### WHO WORLD REPORT ON HEARING: IMPLICATIONS FOR THE UNITED STATES AND THE WHO DECADE OF HEALTHY AGING

Carrie Nieman, *Johns Hopkins University School of Medicine, Baltimore, Maryland, United States*

The past 5 years have seen incredible advances in approaching hearing loss as a major public health issue. National efforts include the 2015 President' Council of Advisors on Science and Technology and the National Academies of Science, Engineering, & Medicine's 2016 Commission on Hearing Health Care for Adults, which led to the 2017 OTC hearing aid legislation and the expected debut of OTC hearing aids in 2020-2021. The World Report on Hearing amplifies these efforts. This presentation will cover the role of the Report in the context of the rapidly evolving hearing care landscape in the US and how the Report's call for affordable, accessible hearing care fit within current national efforts focused on older adults. Finally, the WHO recognized 2020-2030 as the Decade of Healthy Aging. We will discuss how the World Report on Hearing integrates with broader efforts to support healthy aging locally and globally.

### TASK SHARING IN HEARING CARE: PUTTING PRINCIPLES INTO PRACTICE TO ADVANCE ACCESS TO HEARING CARE

Nicole Marrone,[1] Aileen Wong,[1] Maia Ingram,[2] Rosie Piper,[3] Scott Carvajal,[1] and Sonia Colina,[1]
*1. University of Arizona, Tucson, Arizona, United States, 2. Arizona Prevention Research Center, Tucson, Arizona, United States, 3. Mariposa Community Health Center, Nogales, Arizona, United States*

Task sharing, through models such as community health workers (CHWs), is considered an efficacious and cost-effective approach to extending access, addressing disparities, and building capacity. Increasingly, task sharing is recognized as a promising approach within sensory health. This session will share results from an NIH-funded trial of a first-in-kind CHW-delivered intervention along the U.S.-Mexico border. Trained CHWs provided a 5-week group aural rehabilitation program that included education and counseling on age-related hearing loss. A total of 136 Spanish-speaking older adults with hearing loss were randomized. Those in the immediate treatment group reported significantly greater use of communication strategies post-intervention, which was maintained over 1 year. Participants were more likely to report taking action on their hearing at 6 months (OR:1.56, p=0.001) and 1 year (OR:1.82, p=0.001). Building upon lessons learned, including post-intervention focus groups, the presentation will share guiding principles on the application of task sharing to support sensory health.

### WORLD HEALTH REPORT ON VISION: AGING IMPLICATIONS FOR GLOBAL VISION AND EYE HEALTH

Bonnielin Swenor,[1] Varshini Varadaraj,[1] Moon Jeong Lee,[2] Heather Whitson,[3] and Pradeep Ramulu,[4] *1. Johns Hopkins University, Baltimore, Maryland, United States, 2. The Wilmer Eye Institute, Johns Hopkins University School of Medicine; Department of Epidemiology, Johns Hopkins Bloomberg School of Public Health, Baltimore, Maryland, United States, 3. Duke University, Durham, North Carolina, United States, 4. Johns Hopkins School of Medicine, Baltimore, Maryland, United States*

In 2019, the World Health Organization World Report on Vision estimated that that 2.2 billion people have a vision impairment, of which almost half could have been prevented or is yet to be addressed. As the global population ages and the prevalence of visual impairment increases, inequities in eye care and the downstream health and aging consequences of vision loss will become magnified. This session will: (1) provide key information regarding the burden of eye disease and visual impairment among older adults worldwide; (2) outline a framework created to conceptualize the aging and long-term health implications of vision loss, and (3) discuss the global public health challenges to eye care and to maximizing health for older adults with visual impairments.

### UPDATES ON HEARING FROM THE GLOBAL BURDEN OF DISEASE STUDY

Lydia Haile,[1] Aislyn Orji,[2] Paul Briant,[2] Jaimie Adelson,[1] Adrian Davis,[3] and Theo Vos,[2], *1. University of Washington, Seattle, Washington, United States, 2. Institute for Health Metrics and Evaluation, Seattle, Washington, United States, 3. Imperial College London, London, England, United Kingdom*

Hearing loss is estimated annually as part of the Global Burden of Disease study. In 2019, input data came from 308 surveys in 77 countries. Hearing loss was measured by taking the pure-tone average of audiometric thresholds ranging from .5– 4 kHz. We ran severity-specific prevalence models, adjusting the severity for hearing aid use down one level, and separately estimating hearing loss due to meningitis, otitis media, congenital abnormalities, and age-related and other factors. In 2019, 1.5 billion (95% UI=1.5–1.6) people experienced hearing loss, primarily due to age and other factors. Of those with hearing loss, 74% (UI=71–77%) had mild hearing loss and 7% (UI=6–9%) used hearing aids. Globally, age-related hearing loss was the third-ranked cause of Years Lived with Disability after low back pain and migraine. Due to ageing, the hearing loss burden has increased over time, indicating a greater need for hearing healthcare services world-wide.

### UPDATES ON VISION FROM THE GLOBAL BURDEN OF DISEASE STUDY

Jaimie Adelson,[1] Paul Briant,[2] Seth Flaxman,[3] Hugh Taylor,[4] Serge Resnikoff,[5] Nikolas Reinig,[2] Rupert Bourne,[6] and Theo Vos,[2] *1. University of Washington, Seattle, Washington, United States, 2. Institute for Health Metrics and Evaluation, Seattle, Washington, United States, 3. Imperial College London, London, England, United Kingdom, 4. Melbourne School of Population Health, Melbourne, Victoria, Australia, 5. Brien Holden Vision Institute, Sydney, New South Wales, Australia, 6. Vision and Eye Research Institute, Cambridge, England, United Kingdom*

The Global Burden of Disease study estimates the prevalence of vision loss by WHO severity group, and the distribution of vision loss due to underlying causes such as cataract, glaucoma, and uncorrected refractive error. Data sources include national and local surveys, and scientific literature, with over 500 sources in 113 countries. Cause-specific prevalence estimates were proportionally fit into estimates of total vision loss by severity. Globally, 821.9 million (95% UI=682.2–970.4) people experienced moderate or worse distance vision loss or near vision loss in 2019 (age-standardized prevalence = 0.10, UI=0.08–0.12). Of these, 41.9 million (UI=36.4–46.8) were blind. The majority of Years Lived with Disability due to vision loss occurred in people with cataract and uncorrected refractive error. Vision loss numbers will continue to increase due to aging populations. Main causes of vision loss are correctable and, therefore, should be major targets of interventions.

### SESSION 7500 (SYMPOSIUM)

### POLICY SERIES: ILLUMINATING THE INTERSECTION BETWEEN EMPLOYERS AND EXPERIENCED EMPLOYEES: CURRENT RESEARCH AND POLICY DIRECTIONS

Chair: Brian Kaskie

Population aging, resulting from the combination of longer life expectancy and lower birthrates, has widespread implications for both employees and employers across the United States. By2040, age55-plus workers are projected to account for more than25 percent of America's workforce. Yet, even though we have gained a better understanding of the needs and preferences of aging workers, we know far less about the organizations which employ them. This symposium presents the latest research concerning: age discrimination in the workplace, intergenerational workplace arrangements, work ability and performance, and the increasingly varied pathways older persons are taking from work to retirement. We also examine how employers have addressed these issues, and consider why one type of employer may be more likely to adopt and implement a policy or program supporting older workers. Panelists then discuss policy alternatives that may increase and expand current employer efforts to support experienced employees.

### IS WORKING LONGER THE SOLUTION TO AN AGING SOCIETY?

Phyllis Moen, *University of Minnesota, Minneapolis, Minnesota, United States*

Population aging, together with increases in life expectancy, women's employment, dual-earner households, and unraveling safety nets, are transforming the demography of work and retirement in the later life course. A seemingly obvious policy solution is to encourage Boomers and those following to work longer, postponing retirementt. And yet there is insufficient understanding of factors that predict ongoing older adult participation in paid work. The simple work/retirement dichotomy no longer fits the experience of growing numbers of Americans. Drawing on census data, I show six such pathways differing by age, gender, and race/ethnicity. Needed is policy development recognizing the new realities of different and unequal pathways. The challenge is recognizing disparities in capacities and constraints among our aging population, along with the need to change the way we work, opening up flexible options in order to enable working longer.

### THE IMPORTANCE OF BRIDGE JOBS IN AMERICA TODAY

Kevin Cahill, *Boston College, Boston, Iowa, United States*

Gradual retirement, consisting of phased retirement, bridge employment, and reentry, has been a persistent feature of older Americans' work decisions for decades. This persistence is remarkable in light of the many changes to the retirement landscape that have taken place since the mid-1980s, including changes to Social Security incentives, private-sector pensions, and macroeconomic volatility. Our paper addresses two topics related to these retirement transitions. First, using 26 years of data from the Health and Retirement Study (HRS), we quantify the prevalence of bridge employment among the initial group of HRS respondents. For the first time, bridge job prevalence can be calculated for a large cohort of older Americans who have completed their retirement transitions. Second, we comment on what these findings mean for policymakers, with respect to income inequality later in life, barriers to retirement pathways, and public-private sector differences, and what role public employers might be able to play.

### WHY RETIREMENT AND AGE DISCRIMINATION POLICIES NEED TO CONSIDER THE INTERSECTIONAL EXPERIENCES OF OLDER WOMEN

Patrick Button,[1] Theodore Figinski,[2] Joanne Song McLaughlin,[3] and Ian Burn,[4] *1. Tulane University, New Orleans, Louisiana, United States, 2. United States Department of the Treasury, Washington, District of Columbia, United States, 3. SUNY Buffalo, Buffalo, New York, United States, 4. University of Liverpool, Liverpool, England, United Kingdom*

We summarize how older women face intersectional experiences that affect their retirement security. These include differential trends in aging, life expectancy, labor supply, work history, retirement savings, and poverty at old age. We also highlight research showing that older women experience significantly more age discrimination than older men. affecting the ability for older women to improve their retirement security by working longer. We demonstrate through examples that these differential trends and intersectional experiences of older women have important policy implications. We provide examples of how Social Security policies, such as increases in the full benefit retirement age and changes to the retirement earnings test, have differential effects on older women. We also discuss how age and gender employment discrimination law fails to protect older women from intersectional sex-plus-age discrimination. We conclude by urging policymakers to consider how older women experience different challenges and how policy should consider their unique experiences.

### HOW DO EMPLOYERS SPAN THE GENERATIONS?

Cort Rudolph, *Saint Louis University, St Louis, Iowa, United States*

The concepts of "generations" and "generational differences" are nearly ubiquitous phenomena. Organizational policy makers often face questions about the purported influence of generations and differences assumed to exist between them for a variety of issues. Given the sheer volume of work dedicated to the topic, both academic and in the popular press, it can be difficult to parse correct from incorrect answers to such questions. In the present article, we pose and answer 10 common questions about generations and generational differences in an attempt to "clear the air" about common thinking, (mis)conceptions, and myths surrounding these phenomena. We offer this work with the goal of clarifying the truth about generations and generational differences, with an eye toward helping organizational policy makers navigate the complexities of these issues and making better decisions about the (assumed) role that generations play across different contexts.

### EMPLOYER PERCEPTIONS AND RESPONSES TO AN AGING WORKFORCE: DIFFERENCES BY INDUSTRY, OCCUPATION, AND SIZE

Robert Clark, *North Carolina State University, Raleigh, North Carolina, United States*

Employers are facing significant demographic changes in their workforce as the US population continues to age and older workers seek to delay retirement until later ages. The challenges associated with workforce aging vary substantially across the economy and vary by industry, occupation, and size of the organization. This analysis highlights the findings from employer surveys and workshops of senior HR leaders. The article summarizes the findings from our three-year research project including whether employers perceive that workforce aging is a challenge to their organization and if so, what changes in compensation and working conditions are being made to accommodate the changes in age structure

### SESSION 7501 (SYMPOSIUM)

### RESPONDING TO THE PANDEMIC: AN UPDATE FROM THE GSA COVID-19 TASK FORCE

Chair: Kathryn Hyer
Co-Chair: Elizabeth Sobczyk

The GSA COVID-19 Task Force was convened in March 2020 to address the needs of GSA members and older adults related to the COVID-19 pandemic. This interdisciplinary group of members represents physicians, nurses, social workers, psychologists, educators, and researchers with expertise in disaster preparedness, caregiving, trauma, HIV, vaccination, home health, long-term care, social isolation, motivation, and risk communication with older adults. In this symposium, Task Force members will share how they have advanced the areas of research, education, and practice related to the pandemic. Speakers will discuss the process of developing a COVID-19 research agenda and how that is moving forward. They will also share how Task Force members representing AGHE have been supporting members through listening sessions and the development of materials to assist with the transition to online teaching. Finally, speakers will provide updates on the several fact sheets, webinars, and a decision aid that have been developed to support GSA members and their work with older adults.

### GSA COVID-19 TASK FORCE: DEVELOPING A RESEARCH AGENDA

Barbara Resnick,[1] and Sheryl Zimmerman,[2] *1. University of Maryland School of Nursing, Baltimore, Maryland, United States, 2. University of North Carolina at Chapel Hill, Chapel Hill, North Carolina, United States*

GSA COVID-19 Task Force members worked with organizational leaders and special interest groups to ascertain areas where further research about COVID-19 and older adults would be helpful. The responses were compiled into a single document to create a research agenda. Processes to share the agenda with federal agencies and partners will be shared.

### GSA COVID-19 TASK FORCE: SUPPORTING THOSE IN PRACTICE

Lisa Brown, *Palo Alto, California, United States*

The GSA COVID-19 Task Force developed multiple materials to assist older adults, those who care for them, and policy makers in understanding a variety of topics, including aging and immunity, ageism, the process of developing a vaccine, and how to social distance. Members of the group also undertook development of a decision aid on whether to engage in activities outside the home. The process of developing the decision aid according to Ottawa Research Institute guidelines will be explained.

### GSA COVID-19 TASK FORCE: SUPPORTING TRANSITIONS TO ONLINE TEACHING

Joann Montepare,[1] and Judith Howe,[2] *1. Lasell University, Newton, Massachusetts, United States, 2. Icahn School of Medicine at Mount Sinai, Bronx, New York, United States*

GSA COVID-19 Task Force members collaborated with AGHE to develop multiple teaching briefs and biblio briefs on various topics to support faculty who had to adjust to online teaching quickly. Task Force members will review those documents, as well as the AGHE listening session to identify where educators and students need additional support.

### SESSION 7510 (SYMPOSIUM)

### THE STUDY OF SENSORY AGING IN EXISTING COHORTS OF OLDER ADULTS

Chair: Joshua Ehrlich

Sensory impairments in later life are associated with numerous adverse health outcomes, including falls, depression, cognitive decline and dementia, and loss of independence. While population-based cohort studies offer a wealth of data for studying health and aging, these studies have not been widely leveraged to investigate the epidemiology of sensory impairment and the effect of sensory impairments on health outcomes and well-being in older adults. This symposium will provide attendees with examples from presenters' own research using sensory function data from large-scale and population-based cohort studies. Presentations will also describe available sensory data and opportunities to improve measures of sensory function in cohort studies. Finally, presenters will focus on methodological considerations when studying the effect of sensory impairment on health outcomes, trends, and trajectories in older adults using data from cohort studies. The symposium will include presentations on vision, hearing, olfaction, and nesting of a randomized controlled trial within an existing cohort study. Participants will gain a clear understanding of the importance of measuring sensory function; methods for integrating sensory measures into existing cohorts; and the original results of analyses that have used sensory data from existing cohorts to study health and well-being in older adults. Sensory Health Interest Group Sponsored Symposium.

### THE SENSE OF SMELL AND AGING: WHAT WE HAVE LEARNED FROM POPULATION STUDIES

Jayant Pinto, *University of Chicago, Chicago, Illinois, United States*

Decline of the sense of smell with age causes a marked impact on older adults, markedly reducing quality of life. Olfactory dysfunction impairs nutrition, decreases the ability to experience pleasure, and results in depression, among other burdens. Large-scale population studies have identified impaired olfaction as a key heath indicator that predicts the development of decreased physical and mental health, reduced physical activity, weight loss, mild cognitive impairment and dementia, and mortality itself. These data have been generated via analyses of data from several aging cohorts, including the National Social Life, Health, and Aging Project (NSHAP); the Beaver Dam cohort; the Atherosclerosis Risk in Communities project; the Rush Memory and Aging Project; the Health, Aging, and Body Composition project; the Washington Heights/Inwood Columbia Aging Project; among others. In this presentation, we will review the close connection between olfaction, health, aging, including discussion of insights from these studies. We will also discuss emerging data from NSHAP on the effects of sensory function on cognition, mental health, and social interaction, which demonstrate that sensory function plays a vital role in the lives of older adults. Part of a symposium sponsored by Sensory Health Interest Group.

### HEARING LOSS AND FRAILTY AMONG OLDER ADULTS: THE ARIC NEUROCOGNITIVE STUDY

Nicholas Reed,[1] Emmanuel Garcia-Morales,[2] Priya Palta,[3] Frank Lin,[4] Josef Coresh,[2] Jennifer Schrack,[2] and Jennifer Deal,[2] *1. Johns Hopkins University, Baltimore, Maryland, United States, 2. Johns Hopkins Bloomberg School of Public Health, Baltimore, Maryland, United States, 3. Columbia University Irving Medical Center, New York, New York, United States, 4. Johns Hopkins University School of Medicine, Baltimore, Maryland, United States*

Hearing Loss (HL) is common among older adults and is associated with factors (e.g., walking speed and social isolation) that may mediate an association with frailty. In the Atherosclerosis Risk in Communities (ARIC) study, frailty was defined as a composite variable (unintentional weight loss, energy expenditure, walking speed, low energy, and grip strength) while HL was measured using pure-tone audiometry. Among, 3179 participants in 2015-2017, 251 (7.9%) were frail. In a model adjusted for demographic and clinical risk factors, mild HL (n=1263; Odds Ratio[OR]=1.42; 95%Confidence Interval[CI]=1.01-2.01) and moderate HL (n=854; OR=1.67; 95%CI=1.09-2.55) were associated with higher odds of frailty relative to those without HL (n=1063). Among participants who completed an ARIC visit 2-years later, the odds of developing frailty tended to be higher among those with mild (OR=1.46; 95%CI=0.91-2.33) and moderate HL (OR=1.43; 95%CI=0.77-2.67). Future research should focus on mechanisms underlying association and determine the impact of treatment of HL. Part of a symposium sponsored by Sensory Health Interest Group.

### VISION MEASURES IN AGING COHORTS: GAPS AND OPPORTUNITIES

Varshini Varadaraj,[1] Ahmed Shakarchi,[2] Lama Assi,[2] and Bonnielin Swenor,[1], *1. Johns Hopkins University, Baltimore, Maryland, United States, 2. Johns Hopkins Wilmer Eye Institute, Baltimore, Maryland, United States*

There is wide variation in the methods used to assess vision across existing aging cohort studies. While subjective vision measures are valuable in capturing individuals' perspectives, objective criteria are often needed to fully characterize visual impairment in older adults. In addition, visual function extends beyond acuity; contrast sensitivity, visual fields, and depth perception are known to impact daily functioning, and are needed to comprehensively assess visual function. Broader uptake of subjective and objective vision measures, and harmonization of vision measures across datasets is needed to robustly examine the role of visual impairment on aging and health outcomes. In this presentation, we will draw on work using data from the National Health and Aging Trends Study, Health and Retirement Study, and Health, Aging and Body Composition Study, among others, to illustrate uses of vision data in aging cohort studies, and examine the gaps and opportunities for improvement in vision measures. Part of a symposium sponsored by Sensory Health Interest Group.

### THE ACHIEVE TRIAL: LESSONS LEARNED FROM NESTING A RANDOMIZED CONTROLLED TRIAL WITHIN AN OBSERVATIONAL COHORT STUDY

Jennifer Deal,[1] Nicholas Reed,[2] David Couper,[3] Kathleen Hayden,[4] Thomas Mosley,[5] Jim Pankow,[6] Frank Lin,[7] and Josef Coresh,[1] *1. Johns Hopkins Bloomberg School of Public Health, Baltimore, Maryland, United States, 2. Johns Hopkins University, Baltimore, Maryland, United States, 3. University of North Carolina at Chapel Hill, Chapel Hill, North Carolina, United States, 4. Wake Forest School of Medicine, Winston-Salem, North Carolina, United States, 5. University of Mississippi Medical Center, Jackson, Mississippi, United States, 6. University of Minnesota, Minneapolis, Minnesota, United States, 7. Johns Hopkins University School of Medicine, Baltimore, Maryland, United States*

Hearing impairment in older adults is linked to accelerated cognitive decline and a 94% increased risk of incident dementia in population-based observational studies. Whether hearing treatment can delay cognitive decline is unknown but could have substantial clinical and public health impact. The NIH-funded ACHIEVE randomized controlled trial of 977 older adults aged 70-84 years with untreated mild-to-moderate hearing loss, is testing the efficacy of hearing treatment versus health education on cognitive decline over 3 years in community-dwelling older adults (Clinicaltrials.gov Identifier: NCT03243422.) This presentation will describe lessons learned from ACHIEVE's unique study design. ACHIEVE is nested within a large, well-characterized multicenter observational study, the Atherosclerosis Risk in Communities Study. Such nesting within an observational study maximizes both operational and scientific efficiency. With trial results expected in 2022, this presentation will focus on the benefits gained in design and recruitment/retention, including dedicated study staff, well-established protocols, and established study staff-participant relationships. Part of a symposium sponsored by Sensory Health Interest Group.

### VISION IMPAIRMENT AMONG LATE MID-LIFE WOMEN: MICHIGAN STUDY OF WOMEN'S HEALTH ACROSS THE NATION

Carrie Karvonen-Gutierrez,[1] Navasuja Kumar,[1] Sayoko Moroi,[2] David Musch,[1] Sarah Dougherty Wood,[1] Joshua Ehrlich,[1] and Michelle Hood,[3], *1. University of Michigan, Ann Arbor, Michigan, United States, 2. The Ohio State University College of Medicine, Columbus, Ohio, United States, 3. University of Michigan School of Public Health, Ann Arbor, Michigan, United States*

There are few data on mid-life sensory health and its contribution to health and disability outcomes. The Study of Women's Health Across the Nation—Michigan site (N=252 women, mean age=66.0 years) collected comprehensive data regarding ocular health to estimate the burden and correlates of vision impairment (VI) and unmet need for vision correction in this population. The prevalence of VI based on presenting and best-corrected visual acuity was 11.1% (95% CI 7.5,15.7) and 2.8% (95% CI 1.1,5.6), respectively. Thus, 75.0% of presenting VI was correctable, yet only 29.0% of women wore an appropriate eyeglass prescription. Black women and those with greater financial strain or less education were more likely to have presenting VI, but there were no differences in best-corrected VI. This presentation will include a discussion of the contributions of SWAN to the study of sensory aging, as well as methodological considerations for research from a mid-life aging cohort. Part of a symposium sponsored by Sensory Health Interest Group.

### SESSION 7515 (SYMPOSIUM)

### AAA RESEARCH METHODOLOGY I: OVERCOMING LINGUISTIC AND CULTURAL BARRIERS IN AGING RESEARCH AMONG ASIANS

Chair: Wenjun Li
Discussant: Su-I Hou

Asians are the largest and the fastest growing segment of the world population. Asian immigrants are the second largest immigrant population in the U.S. However, their age-related social and health issues are understudied. Because studies on older Asians are often scattered geographically and small-sized and study instruments are usually inconsistent in context, language and culture, it is difficult to synthesize findings from different studies on Asians. Little data exist to support health promotion, policy evaluation and clinical practice in this population. To advance research into aging among Asians, a fundamental step is to create content-relevant, linguistically and culturally appropriate research instruments, and encourage use of these consistent and comparable instruments across studies. This symposium brings together four abstracts that report the development and adaptation of linguistically and culturally appropriate survey instruments for health and behavioral studies in older Asians. The topics range from development of new scales for generative concern and acts in older Singaporeans, assessment of appropriateness of health literacy measurements among East Asian older adults, validation of Health Aging Instrument in Southeastern Asian older adults. Based on the experiences in these studies, the group will discuss the need and strategies to develop an international network to promote resource sharing and research collaborations across geographic boundaries and disciplinary divisions. By bringing together the isolated but talented Asian health researchers, the new network may accelerate the growth of research on Aging Among Asians. This symposium is a collaborative effort of the Aging Among Asians Interest Group.

### DEVELOPING RESEARCH AND EDUCATION ON AGING AMONG ASIANS THROUGH INTERNATIONAL NETWORKS

Wenjun Li, *University of Massachusetts Medical School, Worcester, Massachusetts, United States*

Asians are the largest and the fastest growing segment of the world population. However, age-related social and health issues are understudied among them. Studies on older Asians are often small-sized and scattered geographically, and use instruments that are inconsistent in context, language and culture. Findings from different studies on Asians are difficult to synthesize to support health promotion, clinical practice and policy evaluations. To advance research into aging among Asians, a fundamental step is to create content-relevant, linguistically and culturally appropriate research instruments, and encourage use of these consistent and comparable instruments across studies. In this interactive session, the author will lead the group to discuss the need, feasibility and strategies for developing an international network to promote resource sharing and research collaborations across country borders and disciplines. By bringing the isolated but passionate Asian aging researchers together, this network will accelerate the growth of research on Aging Among Asians.

### DEVELOPMENT OF SCALES FOR GENERATIVE CONCERN AND GENERATIVE ACTS AMONG OLDER SINGAPOREANS

Rahul Malhotra,[1] Ad Maulod,[1] June May Ling Lee,[1] Grand Hak-Land Cheng,[2] Si Yinn Lu,[3] Leng Leng Thang,[4] and Angelique WM Chan,[1] *1. Duke-NUS Medical School, Singapore, Singapore, 2. The Open University of Hong Kong, Hong Kong, China, 3. Mailman School of Public Health, Columbia University, New York, New York, United States, 4. National University of Singapore, Singapore, Singapore*

Generativity (concern in establishing and guiding the next generation) at older ages is increasingly relevant with population ageing and realization of older people as a resource for younger generations. Generativity encompasses two aspects, concern (attitudes/motivations for generativity) and acts (activities to enact/achieve generativity). Existing scales for generative concern and acts pertain to Western populations, limiting their valid measurement in Asian populations. We conducted 12 focus group discussions with 103 older adults in Singapore, to inform a conceptual model of generativity. A striking finding was the family-centric focus of generativity. It led to the development of content-validated scales for generative concern (38-items; e.g. I am concerned that younger people are too pampered) and generative acts (56-items; e.g. In past 3 months, how many times did you teach younger people right from wrong), in English, Mandarin, Malay and Tamil. Future work will establish their structural, convergent/divergent and predictive validity, and reliability.

### ASSESSING HEALTH LITERACY AMONG EAST ASIAN OLDER POPULATIONS

Taka Yamashita, *University of Maryland, Baltimore, Maryland, United States*

Health literacy assessment tools that are designed and validated specifically for East Asian older adults are limited. This session reviews the existing health literacy assessment tools and discusses possible applications in East Asian older adults. As an alternative to the population-level health literacy assessment, the general literacy measures and assessment approach in the Program for International Assessment of Adult Competencies (PIAAC) data are discussed. As a case demonstration, the PIAAC literacy measures in Japan and Korea are summarized and evaluated for the applicability in aging and health research with East Asian older adults.

### VALIDITY AND RELIABILITY OF THE HEALTHY AGING INSTRUMENT IN OLDER ADULTS WITH AND WITHOUT FEAR OF FALLING

Ladda Thiamwong, *College of Nursing, University of Central Florida, Orlando, Florida, United States*

An instrument to measure healthy aging has not been validated in older adults with and without fear of falling, expressing a methodological gap that limits research in this field. This cross-sectional study aimed to validate the 35-item Healthy Aging Instrument (HAI) in adults aged 60 years and over. 389 community-dwelling older adults from two provinces in Thailand were recruited. The mean age of participants was 70.4 years, and 63% were women. The HAI showed good construct validity and internal reliability in the total group and subgroups with respect to levels of fear of falling. All correlations among the HAI, Fall Risk Assessment Test, Fall Efficacy Scale-International, and full tandem stance test were significant. While Western cultures value the expression of thoughts and feelings, it is not particularly encouraged in Asian cultures. The HAI demonstrates good psychometric properties and provides the basis for measuring healthy aging in Asian older adults.

### SESSION 7520 (SYMPOSIUM)

### CHANGING SAFETY NET HEALTHCARE SYSTEMS TO AGE-FRIENDLY HEALTHCARE SYSTEMS: LESSONS LEARNED

Chair: Anna Faul
Discussant: Nina Tumosa

Federally Qualified Health Centers (FQHC) are important safety net providers in disadvantaged communities. As outpatient clinics in these areas, they qualify for specific reimbursement systems under Medicare and Medicaid. Age-friendly health care is an urgent need to be able to provide quality healthcare to more than 46 million Americans age 65 and older, with that number projected to double to more than 98 million by 2060. Friendly healthcare systems require a focus on the 4Ms framework and is focused on improving the health of people at every life stage and in every community across the country. The 4Ms are as follows: 1) What Matters: Aligning care with each older adult's specific health outcome goals and care preferences; 2) Medications: If medications are necessary, use age-friendly medications that do not interfere with What Matters, Mentation or Mobility; 3) Mentation: Prevent, identify, treat and manage depression, dementia and delirium across settings of care and 4) Mobility: Ensure that older adults move safely every day in order to maintain function and do What Matters. An age-friendly health system is one in which every older adult's care is guided by these evidence-based practices (the 4Ms), where the care causes no harms, and where the care is consistent with what matters to older adults and their families. In this symposium five Geriatric Workforce Enhancement Programs at five diverse universities will share their experiences with supporting FQHC in their areas to become age-friendly healthcare systems. The unique lessons learned at these different sites will be shared.

### IMPLEMENTING THE 4MS IN A RURAL FEDERALLY QUALIFIED HEALTH CENTER: LESSONS LEARNED

Anna Faul,[1] Joseph D'Ambrosio,[1] Pamela Yankeelov,[2] and Sam Cotton,[2] *1. University of Louisville Trager Institute, Louisville, Kentucky, United States, 2. University of Louisville, Louisville, Kentucky, United States*

The University of Louisville GWEP has partnered with Mountain Comprehensive Care, a FQHC with 9 practices in 5 rural counties in KY, to infuse the 4 M's of age-friendly health care systems into their daily practices, namely what matters most, medication management, mentation and mobility. Many lessons were learned during this infusion period, specifically related to cultural and rural barriers that make some of these principles very difficult to implement. Specifically, what matters most to older adults in these very poor rural areas may not be the safest way to proceed; medication management may be difficult to do due to the extent of opioid addictions in these areas, mentation has many challenges related to isolation and lack of understanding of dementia and mobility issues are complicated due to the many home barriers to fall safety environments. These lessons will be discussed in this part of the symposium presentation.

### CHALLENGES OF IMPLEMENTING THE 4MS IN FEDERALLY QUALIFIED HEALTH CENTERS AND PRIVATE ACADEMIC MEDICAL CENTERS

Bonnie Olsen, Theresa Sivers-Teixeira, and Kelly Sadamitsu, *Keck School of Medicine of USC, Alhambra, California, United States*

As our country increases our capacity to provide quality interprofessional geriatric medical care to older adults, we find challenges that are unique to the setting. Many older adults receive care in Federally Qualified Healthcare Centers where implementing Age-Friendly Care is critical. Implementing the 4M's in this setting has specific challenges including lack of geriatric trained staff, staff turnover, leadership engagement and financial sustainability. When implementing Age-Friendly Care within a large-scale complex academic health system, equally difficult challenges surface. With less nimble infrastructures, changes in one service area can have a domino effect and create larger barriers in other parts of the system. Instituting the 4M's in an academic health system environment requires a careful strategic approach and support from many areas of organizational leadership. This talk will focus on strategies to anticipate and adapt implementation plans to best address barriers that are unique to the setting.

### VALUE OF ACADEMIC AND COMMUNITY PARTNERSHIPS IN DEVELOPING AGE-FRIENDLY HEALTHCARE SYSTEMS ACROSS TWO COUNTIES

Roopali Gupta,[1] Phillip Greiner,[2] Jennifer Reichstadt,[3] and Kellie Scott,[4] *1. University of California, San Diego, San Diego, California, United States, 2. San Diego State University, San Diego, California, United States, 3. University of California San Diego, San Diego, California, United States, 4. University of California San diego, San Diego, California, United States*

The San Diego Imperial Geriatric Education Center is comprised of a robust partnership between two academic institutions, two County Area Agencies on Aging, three Federally Qualified Health Centers (FQHCs), three Programs of All-Inclusive Care for the Elderly (PACE), and several local community organizations providing older adult services across two large, diverse counties. Guiding the implementation of Age-Friendly and Dementia-Friendly Healthcare at our partner FQHC and PACE sites, and a large academic institution, has allowed us to review the similarities and unique qualities of each organization. We identified a common theme that providers would benefit from improved awareness and access to community resources (e.g., cognitive assessment and other ADRD (Alzheimer's Disease and Related Dementias) support services), regardless of the specific health care system. The value of academic and community partnerships and the development of an infrastructure for information sharing and linking resources will be highlighted during this symposium presentation.

### DEVELOPMENT OF AGE-FRIENDLY HEALTH SYSTEMS IN CULTURALLY DIVERSE SETTINGS

Jung-Ah Lee,[1] Lisa Gibbs,[2] Camille Fitzpatrick,[3] and Neika Saville,[2] *1. University of California, Irvine, Irvine, California, United States, 2. UCI Health ACO, Irvine, California, United States, 3. UC Irvine School of Nursing, Irvine, California, United States*

UC Irvine (UCI) GWEP partnerships were designed to address a startling increase in impoverished older adults and the disproportionate medical and social determinants of health faced by Latino and Asian communities. All partner clinics primarily serve racial/ethnic minorities, including Hispanic/Latino or Asian minorities (83-88%), with >90% below the 200% poverty threshold. Our major FQHCs include the academic UCI Family Health Center, Vietnamese and Korean community Services. We established an FQHC Geriatric Clinic at UCI with the 4 M's paradigm (i.e., matter, mobility, medication, mentation) integrated into the clinic through the Medicare Annual Wellness Exam. We are disseminating this model to our smaller community sites, along with 4 M presentations. Roughly 5-7% of patients in FQHCs are over 65, allowing leadership time to prepare a foundation for increased growth. Furthermore, we have learned that each FQHC's cultural setting and infrastructure is unique and requires a customized approach.

### SESSION 7525 (SYMPOSIUM)

### OPPORTUNITIES AND CHALLENGES WITH CREATIVE COMMUNITY PARTNERSHIPS

Chair: Melinda Heinz

Arts programming can address chronic conditions prevalent among older adults. An overview of an implementation of the revised nationwide Opening Minds through Arts (OMA) program anchors the symposium. The paper reports an implementation by an area university and an eldercare facility to recruit and train student volunteers to collaborate with persons living with dementia, and create paintings for a public exhibition. The Arts for a Lifetime Program used bi-weekly student led programming in a long-term care community; the paper includes an overview of materials used throughout the program and reports resident preferences for materials. A report of results of an ethnographic investigation of the impact of creative aging fine arts programs on older adults adds information about how participation might influence the older person's self-esteem or perceptions of aging. The presentation about creation of music modules investigates the potential of music therapy for the promotion of healing for older adults managing pain. The final paper describes the methodology lessons learned from ARTmail, a community engaged study of the benefits of a structured participatory arts program for older adults with memory symptoms or cognitive impairment. Presentations in this symposium inform identification and development of opportunities to create and engage in meaningful experiences with older adults.

### EVALUATION OF AN EARLY IMPLEMENTATION OF THE REVISED OPENING MINDS THROUGH ART PROGRAM

Christopher Kelly, Lyn Holley, and Stephen Fogle, *University of Nebraska Omaha, Omaha, Nebraska, United States*

The established international program Opening Minds through Art (OMA) has been revised; this presentation reports evaluation of an early implementation with Gerontology university students and Alzheimer's nursing home patients. Ten patients were paired with student volunteers meeting once a week for eight weeks to co-create original artwork in structured one-hour sessions. Before and after art creation each volunteer recorded personal feelings and their partner's mood and satisfaction. Findings indicate the revised program is satisfying for patients and improves their mood. Families seeing the art expressed surprise and appreciation regarding patient creative capacity. Analysis of data indicates positive outcomes for student volunteers and Alzheimer's patients. Student volunteer reflections link program participation with expanded knowledge, insight, and especially empathy for Alzheimer's patients and their families. The current study contributes to robust support in the literature for efficacy of arts programming for student learning and the morale of Alzheimer's patients and their families.

### ART FOR A LIFETIME

Alexis Schramel, *Upper Iowa University, Monona, Iowa, United States*

Art for a lifetime was a bi-weekly programming opportunity in a long-term care (LTC) community taught by students and faculty. We predicted that 1) Resident physical and mental abilities may influence art-making preferences and 2) residents would be concerned about their perceived lack of creativity. Findings revealed that residents with arthritis preferred working with larger forms (e.g., collage and sculpture) and residents with dementia preferred working with bright, colorful materials. Private one-on-one sessions were beneficial for increasing resident confidence and for working on individual projects. Programming also allowed for increased social opportunities among residents, offering occasions to reflect on life experiences. Overall, facilitators of art programming need to quickly adjust and adapt programming based on resident abilities and preferences. Expanding art programming to other long-term care facilities is important for providing increased opportunities for autonomy and decision making, areas that often become more limited when living in LTC.

### IT'S LATE, BUT EVERYTHING COMES NEXT: CREATIVE AGING IN THE MODERN UNITED STATES

Shayna Gleason, *University of Massachusetts, Boston, Boston, Massachusetts, United States*

The emergence of "creative aging" programs, or fine arts programs exclusively for older adults, invites analysis of these new institutions and their influence on the social elements of the aging process. While past studies have demonstrated the cognitive and health-related benefits of arts participation in old age, little research has examined how participation might influence the older person's self-esteem or perceptions of aging. The present study draws on ethnographic methods including participant observation at eight creative aging programs, six semi-structured in-depth interviews with teaching artists leading these programs, and content analysis of paintings and vignettes made by participants. The results show the observed creative aging programs to have a unique, cyclical pattern of discourse characterized alternately by older adults' recurring self-deprecation and the affirming responses of instructors. This pattern of interaction renders such programs sites for the contestation of negative popular discourses around aging.

### WE'RE IN THIS TOGETHER, BUT . . . : COMMUNITY-ENGAGED RESEARCH PROCESS OF AN ARTS INTERVENTION WITH OLDER ADULTS

Sudha Sudha,[1] and Lia Miller,[2], *1. UNC Greensboro, Greensboro, North Carolina, United States, 2. Creative Aging Network NC, Greensboro, North Carolina, United States*

Community engaged (CE) methods are used in health-related research, but few discuss methodological aspects among older adults. We describe the methodology and lessons learned from a CE study of whether ARTmail, a structured participatory arts program, benefited older adults aged 60+ with memory symptoms / cognitive impairment (MS/CI). Our study, conducted in 2015-2017 with support from the NEA, was a partnership between a non-profit organization in North Carolina that provides creative programming for older adults with varied abilities, and researchers in an area University. Older adults with MS/CI symptoms receiving care in area communities were recruited into either the art intervention or a control group, in collaboration with community staff. We reflect on the CE research process with a partnership among community organizations, researchers, care staff, and older adults. Asymmetries in priorities, resources, and decision-making power are described, and implications for the research process and findings are discussed.

### MUSIC IS HEALING: DEVELOPING A MUSIC MODULE FOR A PAIN MANAGEMENT INTERVENTION FOR AFRICAN AMERICAN OLDER ADULTS

Mary Janevic,[1] Sheria Robinson-Lane,[2] Afton Hassett,[2] and Rebecca Courser,[1] *1. University of Michigan School of Public Health, Ann Arbor, Michigan, United States, 2. University of Michigan, Ann Arbor, Michigan, United States*

Music has a known analgesic effect. Our multidisciplinary team is developing a music-focused module for Positive STEPS, a pain self-management intervention based on principles of positive psychology. The priority population is African American older adults with disabling chronic pain. Positive STEPS is delivered via website and phone calls from community health workers. To inform program design, we conducted two focus groups with older adults in Detroit (n=16; 100% female and African American; 75% age 70+). All participants said they would enjoy using music to cope with pain. Content analysis revealed the following themes regarding music for pain management: it elicits positive memories, reduces stress, motivates exercise and daily activities, and promotes relaxation. Participants offered ideas for music-focused activities, including learning about unfamiliar genres and using music for meditation/relaxation. Findings will inform the design of a new music module, to be pilot-tested for its effect on participant engagement and pain-related outcomes.

### SESSION 7530 (SYMPOSIUM)

### AAA RESEARCH METHODOLOGY II: INNOVATIVE OBSERVATIONAL AND INTERVENTION STUDIES

Chair: Wenjun Li
Discussant: Vivian Lou

Asians are the largest and the fastest growing segment of the world population, and the second largest immigrant population in the U.S. A high proportion of Asians live in developing countries or are immigrants to developed countries. Studies on age-related social and health issues of Asians have lagged. To advance research into aging among Asians living in the U.S. and elsewhere in the world, sound methodologies can be adopted from those well-developed in other settings while novel methodologies are to be developed to meet the unique needs of Asian studies. This symposium brings together five abstracts that address a variety of innovative methodological issues in social and health studies among Asian older adults. The topics range from use of wearable devices to measure travel patterns, life space and physical activity in late-life Asian immigrants in the U.S.; use of Ecological Momentary Assessment method to examine influences of social contact satisfaction on neighborhood affect in Chinese in mainland China; use of multilingual age-friendly touch-screen device for cognitive intervention in Singapore; use of GIS to evaluate potential influences of accessibility to neighborhood living resources on attitudes towards aging among the oldest old in Shanghai; and use of a multi-phase complex mixed methods to evaluate a Chinese cancer screening program in Taiwan. Together, these studies showed that innovative methods can be adapted for context relevance and linguistic and cultural appropriateness, and successfully executed in studies on aging among Asians. This symposium is a collaborative effort of the Aging Among Asians Interest Group.

### SPACE USE, PHYSICAL ACTIVITY, SLEEP QUALITY AND SELF-RATED HEALTH AMONG LATE-LIFE ASIAN IMMIGRANTS

Wenjun Li,[1] Elizabeth Proctor-Gray,[1] Linda Churchill,[1] Jie Cheng,[1] Kevin Kane,[1] Rachel Siden,[1] and

Scott Crouter,[2], *1. University of Massachusetts Medical School, Worcester, Massachusetts, United States, 2. The University of Tennessee, Knoxville, Tennessee, United States*

Little is known about the health and health behaviors of non-English Speaking late-life Asian immigrants, which is attributable to language and cultural barriers to participate research. In our Healthy Aging and Neighborhood Study, we collected objective measures of space and time use, location- and time-specific physical activities using accelerometer and Global Positioning System devices, and self-reported sleep quality and health. We obtained 3,915 person-days of accelerometer readings from 511 participants including 43 Asians. Compared to non-Hispanic Whites, Asians had worse self-rated health and poorer vision, more medical conditions and physical limitations as measured by ADL and IADL. Asians had higher daily step counts overall and at home; high proportions of steps on sidewalk or street and senior centers, lower in fee-based outdoor recreational areas, less in restaurant and vehicle. Sleep quality did not differ significantly between Asians and Whites. Risk profiles differed between Asians and Whites, which warrants further investigation.

### MIXED METHODS EVALUATION - A CHINESE CANCER SCREENING PROGRAM

Su-I Hou, *University of Central Florida, Orlando, Florida, United States*

This paper introduces the rapidly growing modern mixed methods research (MMR) and its application in a Chinese cancer screening program. While some previous researchers have incorporated quantitative and qualitative data in research, recent mixed methods developments have provided significant clarity that can guide those new to the MMR field. Understanding the context for using MMR and examining a complex mixed methods evaluation study in Taiwan can help illustrate opportunities for and application of mixed methods in Asians. The Taiwan Cervical Cancer Screening Education Program is used as an exemplar of a multi-phase complex mixed methods evaluation study showcasing various MMR designs. These include an exploratory sequential design to develop culturally sensitive study instrument, iterative concurrent and sequential mixed methods for intervention mapping, and an embedded mixed methods evaluation design to assess impact. Visual diagrams are introduced to facilitate communication of mixed methods design procedures and products in each phase.

### ECOLOGICAL MOMENTARY ASSESSMENT OF SOCIAL CONTACT SATISFACTION AND MOMENTARY AFFECT IN CHINESE OLDER ADULTS

Huiying Liu,[1] and Vivian Lou,[2], *1. Central South University, Changsha, Hunan, China, 2. The University of Hong Kong, Hong Kong, China*

Previous researches have examined the influences of satisfaction with social relations on affective well-being on a long time-frame among older adults. Using the Ecological Momentary Assessment (EMA) method, we examined within-person changes in social contact satisfaction in relation to momentary affect on short time-scales in 78 community-dwelling older Chinese. Each participant provided up to 7 EMA surveys per day during a one-week period and reported his/her satisfaction, positive affect (PA) and negative affect (NA). Multilevel modelling was used for testing the within-person concurrent and time-lagged relations of social interactions and affect. Higher satisfaction was concurrently associated with more PA and less NA. The satisfaction of prior contact was predictive of greater low-arousal PA during the next contact. Prior NA was predictive of lower satisfaction at the next contact. Such dynamic interplays of social and affective experiences should be considered to support the maintenance of affective well-beings in older Chinese.

### OLDER ADULTS-FRIENDLY MULTI-MODAL TOUCH-SCREEN PLATFORM MEASURES FOR BILINGUAL COGNITIVE INTERVENTIONS

W. Quin Yow, Tharshini Lokanathan, and Hui-Ching Chen, *Singapore University of Technology & Design, Singapore, Singapore*

There is an increasing interest in using touch-screen devices to conduct cognitive training and collect measurements of cognitive performance. However, older adults often have concerns such as anxiety about using these systems and poor comprehension of language instructions (Czaja & Lee, 2007). Given that Singapore is a multilingual society, we examined the deployment of an age-friendly multi-modal touch-screen platform (a game-based application on a tablet) in a cognitive intervention research. After modification of the platform to include features such as simplified instructions, multi-level prompts with a local accent, and four different instructional languages (including local dialects), participants were less reliant on the researchers and reported fewer difficulties in comprehending the instructions. The integrity and reliability of the data collected improved as a result. In sum, multilingual age-friendly touch-screen platform can be a novel yet effective method to study cognitive interventions in the Asian older adult populations.

### ASSOCIATIONS OF LIVING ENVIRONMENT ATTRIBUTES WITH ATTITUDES TOWARDS AGING AMONG OLDER ADULTS IN SHANGHAI CHINA

Minzhi Ye,[1] and Lin Chen,[2] *1. Benjamin Rose Institute on Aging, Cleveland, Ohio, United States, 2. Fudan University, Shanghai, China*

This study examined the relationships between several attributes of living environment and attitudes towards aging among persons of age 75 years and older in Shanghai, China. The data were collected using self-administered surveys in a study of successful aging in 2018. Respondents included 139 persons of age 75 years and older living in 12 neighborhoods in Shanghai. Attitudes towards aging were measured by indices of happiness, time use and worry about the future. Using BaiduMap and GIS ArcPro, we quantified accessibility to neighborhood living resources as numbers of recreational facilities, medical facilities, parks, and grocery stores located within 500 meters of each respondent's home. The analysis found that more positive attitudes towards aging were correlated with less depressive symptom as measured by CES-D and greater numbers of neighborhood resources near home, especially greater number of grocery stores near home. In conclusion, accessibility to neighborhood resources may influence attitudes towards aging.

### SESSION 7535 (SYMPOSIUM)

### FOSTERING MULTIDISCIPLINARY SOLUTIONS IN AGING: THE RESEARCH CENTERS COLLABORATIVE NETWORK

Chair: Stephen Kritchevsky
Co-Chair: Odette van der Willik
Discussant: Basil Eldadah

The important problems facing older adults will not be solved through the methods of a single discipline. In recognition of this, the NIA funded the Research Centers Collaborative Network (RCCN) to build collaborations between scientists from the 6 NIA-sponsored center programs: Alzheimer's Disease Research Centers, Centers on the Demography and Economics of Aging, Claude D. Pepper Older Americans Independence Centers, Nathan Shock Centers of Excellence in the Basic Biology of Aging, Resource Centers for Minority Aging Research, and Roybal Centers for Translational Research on Aging. RCCN's central premise is that researchers from different disciplines are most likely to collaborate when they are addressing similar problems. To foster collaboration the RCCN has convened 5 workshops on: 1. achieving and sustaining behavior change in older adults; 2. sex and gender in aging research; 3. reserve and resilience; 4. life course perspectives on aging; and 5. promoting the inclusion of older adults in clinical research. After each Workshop the RCCN awards pilot funds related to the theme. This symposium will review key learnings from the workshops and present work of four RCCN pilot teams from the first two workshops which focused on changing and sustaining behavior change in older adults, and sex and gender differences in aging. Dr. Hughes will discuss the value of interdisciplinary research to maintain behavior change while Dr. Lee will discuss social incentives to improve mobility postoperatively. Dr. Stites will discuss cognition and gender trends, while Dr. Ware will discuss sex differences in genetic effects.

### THE COGENT3 STUDY: COGNITION AND GENDER TRENDS IN THREE AMERICAN GENERATIONS

Shana Stites, *University of Pennsylvania, Philadelphia, Pennsylvania, United States*

Many studies find gender differences in how older adults' report on their memory, perform on cognitive testing, and manage functional impairments that can accompany cognitive impairment. Thus, understanding gender's effects in aging and Alzheimer's research is key for advancing methods to prevent, slow, manage, and diagnosis cognitive impairment. Our study, CoGenT3 – The study of Cognition and Gender in Three Generations – seeks to disambiguate the effects of gender on cognition in order to inform a conceptual model, guide innovations in measurement, and support future study. To accomplish this ambitious goal, we have gathered an interdisciplinary team with expertise in psychology, cognition, sexual and gender minorities, library science, measurement, quantitative methods, qualitative methods, and gender and women's studies. The team benefits from the intersections of expertise in being able to build new research ideas, gain novel insights, and evaluate a wide-range of actions and re-actions but this novelty can also raise challenges.

### INTERDISCIPLINARY PERSPECTIVES ON MAINTENANCE OF HEALTH BEHAVIOR CHANGE

Jaime Hughes,[1] Janet Bettger,[1] Susan Hughes,[2] and Mina Raj,[3] *1. Duke University School of Medicine, Durham, North Carolina, United States, 2. University of Illinois at Chicago, Chicago, Illinois, United States, 3. University of Michigan, Ann Arbor, Michigan, United States*

Modifying health behaviors can be difficult, especially for older adults who are challenged by multiple chronic conditions, reduced functional and/or cognitive capacity, and limited social support. Although much attention has been given to the theories, skills, and resources behind initiating and achieving behavior change, less work has focused on maintenance of health behaviors over time. This presentation will showcase pilot research inspired by RCCN's first workshop, Achieving and Sustaining Behavior Change. Specifically, this pilot brings together an interdisciplinary team of behavioral scientists and health services researchers working at the intersection of intervention science and implementation science to better understand the construct of maintenance and discuss emerging methods for intervention development and evaluation. The presentation will utilize physical activity as an example behavior to demonstrate the value of interdisciplinary research, including recommendations on how some of the six NIA research centers can make unique contributions to understanding health behavior maintenance.

### SOCIAL INCENTIVES TO IMPROVE MOBILITY AMONG ELDERLY PATIENTS AFTER RADICAL CYSTECTOMY

Daniel Lee,[1] Kenneth Covinsky,[2] and S. Ryan Greysen,[3], *1. University of Pennsylvania Health System, Philadelphia, Pennsylvania, United States, 2. University of California San Francisco, San Francisco, California, United States, 3. University of Pennsylvania, Philadelphia, Pennsylvania, United States*

Radical cystectomy is a complex surgery that disproportionately affects elderly patients and is associated with high morbidity and mortality rates. We proposed a randomized control trial to improve mobility using gamification and social incentives while hospitalized, and immediately after surgery at home. Social incentives may be particularly useful in this population as the majority of cystectomy patients rely on a primary caregiver. There have been multiple challenges in this interdisciplinary work to utilize innovative technologies. This would represent one of the first use of gamification and behavioral economic principles in postoperative patients, so there are questions about how patients would respond to gamification while recovering postoperatively. There were challenges in patient selection and interdisciplinary collaboration to consider, especially in an elderly population that may not have ready mobile access. By collaborating across disciplines, we hope to make significant improvements in postoperative outcomes for elderly patients by utilizing innovative interventions.

### SEX DIFFERENCES IN GENETIC EFFECTS USING TWO GENE AGGREGATION TECHNIQUES: DEPRESSION AND COGNITION

Erin Ware,[1] Lauren Schmitz,[2] and Arianna Gard,[1]
*1. University of Michigan, Ann Arbor, Michigan, United States, 2. University of Wisconsin, Madison, Wisconsin, United States*

The state of science has increasingly valued interdisciplinary training and research. As an early-stage investigator, interdisciplinary research, such as that supported by the Research Centers Collaborative Network through the National Institute on Aging, provides valuable opportunities to connect researchers across disciplinary boundaries. This program encourages researchers use their diverse training experiences to tackle difficult questions in aging research and forge scholarly relationship that advance the state of science through creative problem-solving and a spectrum of interdisciplinary perspectives. This presentation describes a recently funded project to examine sex differences in aging outcomes using genetics, depression, and impaired cognition with polygenic and gene-region aggregation techniques. We will highlight the benefits and opportunities of the Research Centers Collaborative Network pilot grant to this highly interdisciplinary work, co-lead by early-stage investigators.

### SESSION 7540 (SYMPOSIUM)

### FROM THE NATHAN SHOCK CENTERS COORDINATING CENTER: BIOLOGY OF AGING FOR NONBIOLOGISTS

Chair: Steven Austad

Advances in the biology of aging that promise to dramatically extend the healthy length of human life are now appearing at a breathtaking rate. At the same time, baseless claims of research breakthroughs in maintaining youth have exploded on electronic media. It often difficult for people outside basic aging research to keep track of what is real, what is imaginary, progress. This symposium brings together four scientists working in some of the most exciting research areas to update attendees in terms that will be understandable to anyone. Our speakers were carefully selected both for their scientific achievements and for their outstanding skill as science communicators. Matt Kaeberlein (University of Washington) will discuss some of the most exciting emerging pharmacological interventions that delay or prevent multiple features of aging. Nathan LeBrasseur (Mayo Clinic) will update us on a specific type of pharmacological intervention – senolytics – which target aged, nonfunctioning cells distributed throughout the body to broadly improve and extend health. Morgan Levine (Yale) will discuss how newly discovered molecular clocks give us more accurate information on our real biological age than does our birth certificate and will explain the research significance of these clocks. Melissa Harris (UAB) will update us on progress in stem cell biology. The promise of stem cells to treat and possibly reverse multiple aspects of aging are real, but perhaps no area of research has been more abused by hyperbolic claims. Dr. Harris will separate the real hope from the false hype.

### PHARMACOLOGICAL INTERVENTIONS THAT TARGET BIOLOGICAL AGING

Matt Kaeberlein, *University of Washington, Seattle, Washington, United States*

There is a high level of interest in drugs that may delay or even reverse the functional declines and disease risks that accompany biological aging. Several interventions have been shown to improve age-related outcomes and increase lifespan in laboratory animals by targeting the hallmarks of aging. A number of these small molecules are being clinically evaluated for age-related indications, including mTOR inhibitors such as rapamycin, the anti-diabetic drug metformin, and senescent-cell clearing senolytics. Others are being marketed to consumers outside of the federal regulatory process as “anti-aging” natural products with little information about safety or efficacy. Here I will provide an overview of the current state of “anti-aging drugs” with an emphasis on potential mechanisms of action and evaluation of the existing pre-clinical and clinical data.

### TARGETING SENESCENT CELLS TO COUNTER AGING AND AGING-RELATED CONDITIONS

Nathan LeBrasseur, *Mayo Clinic, Rochester, Minnesota, United States*

In response to various forms of age-associated damage, cells can enter a state of senescence. Senescent cells can compromise the health and function of a tissue, and their accumulation with advancing age is believed to contribute to age-related diseases and geriatric syndromes. In preclinical models (i.e., mice), selective elimination of senescent cells through either genetic approaches or a new class of pharmacological agents, termed “senolytics”, has been show to effectively delay, prevent, or reverse the onset and/or progression of pulmonary disease, osteoporosis, atherosclerosis, diabetes, cognitive decline, and several other conditions. Thus, considerable efforts are underway to optimize pharmacological strategies and test their effectiveness in human populations. This seminar will highlight the state-of-the-science of senolytic drugs, and the opportunities and challenges for early phase clinical trials in humans.

### AGING CLOCKS

Morgan Levine, *Yale University School of Medicine, New Haven, Connecticut, United States*

While chronological age is arguably the strongest risk factor for death, disease, and disability, same-aged individuals remain heterogeneous in their susceptibilities to these various outcomes. One explanation is that chronological age is an imperfect proxy of the degree of biological aging an individual has undergone. Thus, defining measurable estimates of ‘biological age’ (in contrast to chronological age) has become a major initiative in Geroscience research. Such biomarkers of aging, or ‘aging clocks’ will 1) help identify underlying mechanisms of aging, 2) enable identification of at-risk individuals prior to disease onset, and 3) provide outcomes to assess efficacy of interventions. In this session, I will describe the various aging clocks, how they were developed, and what they track. I will also describe how aging clocks can facilitate research both within and outside of the biological sciences.

### THE HYPE AND HOPE OF STEM CELL REJUVENATION

Melissa Harris, *University of Alabama, Birmingham, Birmingham, Alabama, United States*

Adult stems play a major role in keeping our bodies in good working order by replenishing tissue as it wears out, and by providing new cells in the event of injury or damage. Unfortunately, the biological process of aging does not discriminate, and all cells, including our stem cells fall prey to getting older. Failing stem cells contribute to characteristics of aging and degenerative disease, and regenerative medicine research has focused on identifying ways in which stem cells can be used or rejuvenated to alleviate the symptoms of old age. Stem cell rejuvenation, however, has also inspired companies to provide services for harvesting and storing young stem cells with little real idea of how these stem cells might be used or their efficacy. In this seminar we will review what is known about the way in which stem cells fail, and the hype and hope surrounding stem cell rejuvenation.

### SESSION 7545 (SYMPOSIUM)

### IMMIGRANT OLDER ADULTS: IS DIVERSITY A CHALLENGE FOR A GLOBAL INITIATIVE?

Chair: Noriko Toyokawa
Discussant: Vivian Lou

Although the global community has discussed needs for establishing international standards of health care for immigrant older adults for decades, it is challenging for policy makers to consider international standards that could meet diverse needs for older adults from various migrant groups. The purpose of this symposium is to discuss challenges and possible strategies to develop global standards to protect immigrant older adults. There will be four presentations on the topic of various needs of older adults from different migrant groups. Noriko Toyokawa will present a study in diversity in parents' expectations on filial piety among immigrant older adults from different racial/ethnic groups in the Southern California. Weiyu Mao and her colleagues will present their study in the perceived neighborhood cohesion as a protective factor for older Chinese immigrants' oral health. Allen Glicksman and his colleagues will report the diversity across migrant groups and State Policies that create a challenge in using finding to establish global standards for best practices with older migrants based on a series studies on Mandarin speaking Chinese and Puerto Rican older immigrants. Finally, Mika Marumoto will suggest the 'reframing of aging initiative' as a possible means of leading the way of cultivating transformative solutions. Vivian Lou will comment on each presentation, discuss common themes among the presented studies, and address future research directions. With the audience, the presenters will discuss challenges in dealing with diversity issues and suggestions for a global initiative to protect human rights and health care accessibilities for immigrant older adults.

### WHO WILL TAKE CARE OF ME?: OLDER PARENTS' PRACTICE AND PLANS FOR THEIR FUTURE

Noriko Toyokawa, *California State University San Marcos, San Marcos, California, United States*

The current study investigated older parents' practices and plans to support their own autonomous lives with/without children's living assistance and how these parents negotiate autonomous lives with their adult children among three racial/ethnic groups. The study participants were older parents who resided in the Southern California who were recruited through local senior centers (N = 15, Mage = 75, SD = 8.7; Caucasians 47%, Mexicans 40%, Filipinos 13%). Content analysis revealed that Caucasian American parents supported their autonomous lives by using friend networks and professional caregivers rather than depending on their children. Parents with Mexican origin emphasized that their children well took care of them and there were no conflicts with their children. Filipino parents planned to migrate to the Philippines where professional services were affordable so that they would not rely on their children's care. Similarities and differences in older parents' need among different racial/ethnic groups will be discussed.

### REFRAMING AGING IN THE SDGS ERA: THE ROLE OF NONPROFITS IN HELPING OLDER PERSONS AND HOLDING GOVERNMENTS TO ACCOUNT

Mika Maromoto, *HelpAge International, Washington, District of Columbia, United States*

With five years gone since the adoption of the UN Agenda for Sustainable Development, the global community is moving far too slowly toward achieving its 2030 objectives for older persons. Achieving proper rights and wellbeing for older persons requires strategic and concerted action. The WHO Decade of Healthy Ageing initiative is one useful step toward defining the diverse physical, psychological, social and healthcare needs of older persons. But government leadership remains weak, and the nonprofit sector plays the key role spearheading advocacy efforts toward the SDG motto of "leave no one behind." The urgent needs of older persons – whether fighting ageism, upholding the rights of people living with dementia, or promoting lifelong learning and contributions to society - can best be met by societies that "reframe aging" and craft creative and innovative solutions. This presentation showcases NGOs that are leading the way in cultivating such transformative solutions.

### BUILDING GLOBAL PERSPECTIVES IN A DIVERSE WORLD

Allen Glicksman,[1] Lauren Ring,[1] Michael Liebman,[2] and Misha Rodriguez,[3] *1. Philadelphia Corporation for Aging, Philadelphia, Pennsylvania, United States, 2. IPQ Analytics, LLC, Kennett Square, Pennsylvania, United States, 3. Asociación Puertorriqueños en Marcha, Philadelphia, Pennsylvania, United States*

There is a growing emphasis on diversity and its impact on health care. Our research has focused on migrants who are Mandarin speaking Chinese and Puerto Rican. Through a series of focus groups conducted in their native languages we discovered that barriers to service access differ across the two groups. In addition, many of these services are administered by individual states rather than the federal government. The diversity across migrant groups and state policies creates a challenge in using findings to establish national or international standards for best practices with older migrants. How then can we apply lessons about diversity and the impact of programs on a national and international scale? We propose the use of Social Interaction Modeling as a method for understanding patterns of behavior at both national and international levels, while preserving the unique character of each migrant group and the context within which they live.

### NEIGHBORHOOD COHESION, ACCULTURATION, AND ORAL HEALTH PROBLEMS AMONG OLDER CHINESE AMERICAN IMMIGRANTS

Weiyu Mao,[1] Bei Wu,[2] Iris Chi,[3] Wei Yang,[1] and XinQi Dong,[4] *1. University of Nevada, Reno, Reno, Nevada, United States, 2. New York University, New York, New York, United States, 3. University of Southern California, Los Angeles, California, United States, 4. Rutgers University, New Brunswick, New Jersey, United States*

The influences of neighborhood characteristics remain understudied in relation to oral health, especially within the context of immigration. Acculturation exerts influences on the oral health of immigrants. This study investigated the relationship between neighborhood cohesion and oral health problems among older Chinese American immigrants and examined the moderating role of acculturation in such a relationship. The working sample included 3,157 older Chinese American immigrants aged 60 years or older from the baseline of the Population Study of Chinese Elderly in Chicago. Stepwise logistic regression models with interaction terms were conducted. Individuals experiencing higher levels of neighborhood cohesion reported a lower likelihood of having oral health problems. The protective effect of neighborhood cohesion against having oral health problems was stronger when individuals resided in ethnic enclaves such as Chinatown. To promote optimal oral health, interventions need to account for individuals' perceptions and levels of integration into their neighborhoods and communities.

### SESSION 7550 (SYMPOSIUM)

### USER INVOLVEMENT IN RESEARCH ON AGING AND HEALTH: CREATING KNOWLEDGE AND TECHNOLOGIES WITH OLDER ADULTS

Chair: Sofi Fristedt
Co-Chair: Anna Wanka
Discussant: Neil Charness

Although, user involvement is largely recognized as instrumental when developing relevant knowledge, services as well as products - aging populations are still likely to be sparsely involved in such processes. Surprisingly, many gerontechnologies are still developed based on a technological perspective rather than a gerontological perspective. Consequently, age-related changes as well as needs, actual use or perceptions of older adults are disregarded or neglected. Similar problems apply to public and private environments with potentially negative implications on accessibility. The present symposium includes four presentations that address user involvement, by capturing older adults' and aging populations' use as well as perceptions of emerging technologies, successful development of gerontechnologies, and a multigenerational mass-experiment on housing accessibility in later life. The first study from Germany captures the everyday situation of smartphone use as well as aspects of user experience, affect and social context among older adults. The second study addresses perceptions and attitudes of three generations in Sweden related to continuous technological advancement of products intended to support active and healthy aging. The third presentation will describe the iterative development process of the 2020 mass-experiment – the Housing Experiment -- involving older adults, stakeholders in the housing sector, teachers and pupils in Sweden. The fourth presentation from Canada explores the benefits, challenges, and solutions to support older adult engagement in research that leads to the successful development of technologies for and with older adults. Finally, our discussant will further elaborate on the respective study findings and summarize the symposium.

### USER ATTITUDES TOWARD RESEARCH AND DEVELOPMENT OF TECHNOLOGIES: PERSPECTIVES FROM THREE GENERATIONS

Steven Schmidt,[1] Sofi Fristedt,[2] Charlotte Löfqvist,[1] and Susanne Iwarsson,[1] *1. Lund University, Lund, Skane Lan, Sweden, 2. Jonkoping University, Lund, Jonkopings Lan, Sweden*

New technologies are being touted as solutions to many societal challenges not least of which are ageing and health. However, the rapid development of new technologies is proceeding with little input from older adults. This presentation highlights the perceptions and attitudes of three age cohorts related to the continuous technological advancement of products intended to support active and healthy aging. Participants were 30-39 (n= 639), 50-59 (n=703), 70-79 (n=779) years-old randomly sampled from the Swedish population registry. Results showed both similarities and difference across generations. For example, 24%-35% of older adults would like to use home monitoring devices (e.g. fall sensors, smart home devices) to support active and healthy aging, compared to 35%-56% of younger groups. More than 82% of all groups highlighted the importance of involving intended users in the development process. Results can be used to support the needs and desires of current older adults and future generations.

### THE HOUSING EXPERIMENT: CITIZEN SCIENCE ENGAGING OLDER ADULTS AND SCHOOL PUPILS TO ASSESS HOUSING ACCESSIBILITY

Susanne Iwarsson,[1] Fredrik Brounéus,[2] Knut mårtensson,[3] and Marianne Granbom,[4] *1. Lund University, Lund, Skane Lan, Sweden, 2. Vetenskap & Allmänhet, Stockholm, Stockholms Lan, Sweden, 3. miThings, Lund, Skane Lan, Sweden, 4. Lund University, Lund, Sweden*

Citizen science is gaining momentum as an approach in many scientific fields. However, it is scarcely used in aging research. Since 2009 in Sweden, Public & Science (NGO) has coordinated an annual mass-experiment where thousands of school pupils have collected data that would have been impossible for researchers to collect on their own. Designed as a cross-generational endeavour, the 2020 mass-experiment, the Housing Experiment, is based on scientific methodology for data collection on housing accessibility. The aim of this presentation is to describe the iterative development process involving older adults, stakeholders in the housing sector, teachers and pupils. We will present the app and instructions developed and piloted for data collection, as well as usability and interrater reliability results. With media attention already in the planning phases, this citizen science initiative has the potential to both generate a unique dataset and increase engagement in housing matters concerning the aging population.

### ENGAGING OLDER ADULTS IN TECHNOLOGY RESEARCH: PERSPECTIVES ON BARRIERS AND FACILITATORS USING A CASE STUDY APPROACH

Susan Kirkland, *Dalhousie University, Halifax, Nova Scotia, Canada*

The aim of OA-INVOLVE is to provide recommendations to support older adult engagement in research that leads to the successful development of technologies for and with older adults. To support this aim we conducted a longitudinal case study project in which we interviewed eight AGE-WELL research teams conducting technology projects to explore the benefits, challenges, and solutions for meaningful engagement. Members of the OA-INVOLVE Older Adult Research Partner Group (OARPG) were involved in all aspects of the project. Findings from the case studies provided important insights regarding structural, contextual, and individual factors that enable and constrain active involvement of older adults. Many projects reported developing unique "workarounds" in order to move beyond involving older adults as participants to involving them as advisors and decision makers. Researchers identified that they often lack the skills, training and resources to engage older adults in a meaningful way and could benefit from capacity building.

### PURPOSES OF SMARTPHONE USE IN LATER LIFE: USER PERSPECTIVES ON DIGITAL DAILY LIFE IN GERMANY

Friedrich Wolf,[1] and Frank Oswald,[2], *1. Interdisciplinary Ageing Research (IAW), Frankfurt, Hessen, Germany, 2. Interdisciplinary Ageing Research, Frankfurt, Hessen, Germany*

Smartphones affect everyday life of people across the lifespan with increasing relevance in later life. Although half of older adults in Germany (65+) use smartphones and the numbers of users are growing, only few studies address the integration of older adult's smartphone use in everyday life as well as its potential effects on social context and wellbeing, which is been done in this study. Data are drawn from surveying the devices of 35 older adults (age 61-77 years; 60% women), allowing to objectively monitor real time smartphone use over the course of a regular week. In addition a semi-standardized ambulatory assessment procedure is administered four times a day to capture the everyday situation of smartphone use as well as aspects of user experience, affect and social context. Preliminary findings show inter- and intra-individual differences in everyday smartphone use in relation to individual differences in social context and wellbeing.

### SESSION 7555 (SYMPOSIUM)

### BIAS AND MISSING DATA: REPRESENTATION OF OLDER ADULTS WITH SENSORY IMPAIRMENT IN EPIDEMIOLOGIC STUDIES

Chair: Bonnielin Swenor
Co-Chair: Alden Gross

Diverse research populations are necessary to maximize the generalizability of results. Differences between people, including age, gender, racial or ethnic origin may impact observed associations in epidemiologic studies or influence the efficacy and safety of interventions in controlled trials. For these reasons, the diversity of research study populations is critical to public health and well-being. While there have been concerted efforts to examine and enhance the representation of older adults in research studies, not all subgroups of these populations, including those with sensory impairments, have been considered. Approximately 55% of adults 60 years and older have a vision and/or hearing impairment, yet despite this high prevalence, little attention has been paid to determining if or how sensory impairment affects research study participation or measurement of outcomes. This symposium aims to address these gaps, with a focus on how vision and hearing impairments may be associated with bias and missing data in epidemiologic studies of cognition and cognitive aging. The objective of this session is to highlight ongoing work in this novel area, and spark discussion and collaborations to catalyze this area of research.

### IMPACT OF HEARING IMPAIRMENT ON MISSINGNESS OF COGNITIVE TEST SCORES IN THE ARIC STUDY

Jennifer Deal,[1] Alden Gross,[1] Alison Abraham,[2] A. Richey Sharrett,[3] Nicholas Reed,[3] Thomas Mosley,[4] Frank Lin,[2] and Bonnielin Swenor,[3] *1. Johns Hopkins Bloomberg School of Public Health, Baltimore, Maryland, United States, 2. Johns Hopkins University School of Medicine, Baltimore, Maryland, United States, 3. Johns Hopkins University, Baltimore, Maryland, United States, 4. University of Mississippi Medical Center, Jackson, Mississippi, United States*

Despite its high prevalence, the impact of hearing impairment on completion of cognitive tests, many of which rely on auditory input to access test material, has not been described. We investigated if hearing impairment is associated with missing scores in 3602 adults (72-94 years, 23% black, 60% female). Cognition was measured using 10 neurocognitive tests. Pure tone better-ear hearing thresholds (0.5-4 kHz) were averaged and categorized. ≥Moderate hearing impairment (versus none) was associated with greater missingness on two auditory tests: Logical Memory (prevalence ratio [PR]:1.40, 95% confidence interval [CI]:1.01,1.70) and Digits Backwards (PR:1.35, 95% CI:1.00,1.82); and the non-auditory Trail Making Test Part B (PR:1.48, 95% CI:1.24,1.77). Compared to models using complete cognitive data, models that imputed missing scores showed stronger associations of hearing impairment with poor cognitive performance. Older adults with HI are less likely to complete cognitive testing, resulting in biased estimates of the hearing impairment-cognitive performance relationship.

### ASSESSMENT OF HEARING OR VISION IMPAIRMENT IN STUDIES COLLECTING COGNITIVE DATA IN OLDER ADULTS

Chelsea Liu,[1] Lama Assi,[2] Niranjani Nagarajan,[3] Danielle Powell,[1] Kening Jiang,[4] Dillan Villavisanis,[5] Bonnielin Swenor,[1] and Jennifer Deal,[6] *1. Johns Hopkins University, Baltimore, Maryland, United States, 2. Johns Hopkins Wilmer Eye Institute, Baltimore, Maryland, United States, 3. Johns Hopkins University School of Medicine, Baltimore, Maryland, United States, 4. Johns Hopkins School of Public Health, Baltimore, Maryland, United States, 5. Icahn School of Medicine at Mount Sinai, new York, New York, United States, 6. Johns Hopkins Bloomberg School of Public Health, Baltimore, Maryland, United States*

Despite the high prevalence of sensory impairment in older adults, there are no standard practices for its consideration in cognitive studies. We conducted a systematic review to identify and survey prospective cohort studies collecting cognitive data in older adults in order to determine whether and how hearing and vision were considered. Among 81 cohorts that responded, 30 (37%) objectively assessed hearing, with audiometry as the most frequently-used method; 61 (75%) used patient-report and 12 (15%) used provider-report to subjectively assess hearing. Forty-one (51%) cohorts objectively assessed vision, half of which measured distance or near visual acuity; 55 (68%) used patient-report and 10 (12%) used provider-report to subjectively assess vision. Nineteen (23%) cohorts offered hearing accommodations and 30 (37%) offered vision accommodations during cognitive testing. Findings indicate variation in methods used to assess hearing and vision as well as in accommodation practices that could impact estimates of cognition among older adults.

### WHAT AM I MEASURING? DISENTANGLING COGNITIVE STATUS FROM SENSORY IMPAIRMENT

Emma Nichols,[1] Bonnielin Swenor,[2] Jennifer Deal,[1] Alison Abraham,[3] Michelle Carlson,[1] Pradeep Ramulu,[3] Susan Resnick,[4] and Alden Gross[1] *1. Johns Hopkins Bloomberg School of Public Health, Baltimore, Maryland, United States, 2. Johns Hopkins University, Baltimore, Maryland, United States, 3. Johns Hopkins School of Medicine, Baltimore, Maryland, United States, 4. National Institute on Aging, Bethesda, Maryland, United States*

Some studies have shown sensory impairment is associated with impaired cognitive test performance, however tests rely to varying degrees on hearing and vision. We hypothesized scores for cognitive tests whose administration depends on vision or hearing are biased among those with vision or hearing impairment, respectively, after controlling for underlying cognitive performance. We used item response theory methods to test for differential item functioning (DIF) by hearing and vision impairment in the Baltimore Longitudinal Study of Aging (BLSA) and the Atherosclerosis Risk in Communities study (ARIC). We identified DIF by hearing impairment for tests that do not rely on hearing and DIF by vision impairment for tests that do not rely on vision. We found no clinically meaningful differences between unadjusted and DIF-adjusted measures of cognition. Our finding that DIF by sensory domain was independent of administration modality suggests cognitive load may play a larger role than previously acknowledged.

### WHO'S IN AND WHO'S OUT? SELECTION BIAS IN AGING RESEARCH

Elizabeth Rose Mayeda,[1] Eleanor Hayes-Larson,[1] and Hailey Banack,[2], *1. University of California Los Angeles, Los Angeles, California, United States, 2. University of Buffalo, Buffalo, New York, United States*

Selection bias presents a major threat to both internal and external validity in aging research. "Selection bias" refers to sample selection processes that lead to statistical associations in the study sample that are biased estimates of causal effects in the population of interest. These processes can lead to: (1) results that do not generalize to the population of interest (threat to external validity) or (2) biased effect estimates (associations that do not represent causal effects for any population, including the people in the sample; a threat to internal validity). In this presentation, we give an overview of selection bias in aging research. We will describe processes that can give rise to selection bias, highlight why they are particularly pervasive in this field, and present several examples of selection bias in aging research. We end with a brief summary of strategies to prevent and correct for selection bias in aging research.

### SESSION 7560 (SYMPOSIUM)

### TECHNOLOGY USE OF OLDER ADULTS AND CAREGIVERS: DISCOVERIES AND OPPORTUNITIES FOR IMPROVEMENT

Chair: Justine Sefcik
Discussant: Jina Huh-Yoo

Innovative technology can improve the lives of older adults, including those diagnosed with dementia, and their caregivers. Yet a lack of careful attention to preferences and needs of end-users and continuous updates to resources could leave consumers without a valuable user experience. This symposium will cover exemplar cases of innovative technologies, available resources, and current research. The first presentation will discuss virtual reality used among older adults with dementia and the opportunities to further explore it's use as an intervention. The second presentation will share the process of seeking stakeholders' preferences in the design specifications for a socially assistive robot and how the perspectives shaped the development of the Quori robot. The third presentation will focus on detailing the Information Quality Framework for Online Dementia Care Resources. The fourth presentation will discuss the implications of a systematic review that revealed researchers are reporting on all older adults within a category of 65 and older, thus failing to present variance among different older age cohorts. These presentations will all conclude with a discussion on opportunities for improvement in the respective areas.

### VIRTUAL REALITY USE AMONG PERSONS WITH DEMENTIA: AN INTEGRATIVE REVIEW

Justine Sefcik,[1] Darina Petrovsky,[2] Pamela Cacchione,[2] Sungho Oh,[2] and George Demiris,[2] *1. Drexel University, Philadelphia, Pennsylvania, United States, 2. University of Pennsylvania, Philadelphia, Pennsylvania, United States*

It is not well understood how virtual reality (VR) is currently used by older adults who have cognitive deficits due to dementia. The aim of this integrative review was to examine and report on published research exploring VR use among older adults with dementia. We searched 3 data bases for publications and used Whittemore and Knafl's methodology for data extraction. Out of 122 articles we identified 24 that met our inclusion criteria, 15 published in 2012 and later. Most articles (12) used VR for assessment, and the others used VR for cognitive training (5) and as an intervention (3) (i.e., for exercise). Sample sizes were 30 or fewer persons with dementia. There is heterogeneity in the types of VR equipment, experiences, and foci of assessment through VR use. We identify opportunities to further explore VR as an intervention for persons with dementia to improve quality of life.

### AD

Justine Sefcik, *Drexel University, Philadelphia, Pennsylvania, United States*

ad

### ENGAGING END USERS IN DESIGNING SYSTEMS AND HARDWARE FOR A SOCIALLY ASSISTIVE ROBOT

Pamela Cacchione,[1] Caio Mucchiani,[1] Kristine Lima,[1] Ross Mead,[2] Mark Yim,[1] and Michelle Johnson,[1]
*1. University of Pennsylvania, Philadelphia, Pennsylvania, United States, 2. Semio AI, Venice, California, United States*

Development of low-cost robots to assist older adults requires the input of end users: older adults, paid caregivers and clinicians. This study builds on prior work focused on the task investigation and deployment of mobile robots in a Program of All-inclusive Care for the Elderly. We identified hydration, walking and reaching as tasks appropriate for the robot and helpful to the older adults. In this study we investigated the design specifications for a socially assistive robot to perform the above tasks. Through focus groups of clinicians, older adults and paid caregivers we sought preferences on the design specifications. Using conventional content analysis, the following four themes emerged: the robot must be polite and personable; science fiction or alien like; depends on the need of the older adult; and multifaceted to meet the needs of older adults. These themes were used in the design and deployment of the Quori robot.

### INFORMATION QUALITY ASSESSMENT FRAMEWORK FOR ONLINE DEMENTIA CARE RESOURCES

Diva Smriti,[1] Rose Ann DiMaria-Ghalili,[2] Laura Gitlin,[1] Aleksandra Sarcevic,[3] Erjia Yan,[3] and Jina Huh-Yoo,[1]
*1. Drexel University, Philadelphia, Pennsylvania, United States, 2. Drexel University, Jenkintown, Pennsylvania, United States, 3. Information Science, Drexel University, Philadelphia, Pennsylvania, United States*

Persons with dementia and caregivers can benefit from online resources. The quality and accessibility of these resources, however, can vary. We present work on the Information Quality Framework for Online Dementia Care Resources. To develop the framework, we first empirically examine resources being retrieved with query terms developed with a medical librarian. Searching one of the possible keyword combinations related to living with dementia on Google "Alzheimer AND financial planning" returned 18,900,000 results. Among the top 13 results on the first page of the search results, six were websites of government or non-profit organizations, four were for-profit companies, and three were advertisements. Out of eight unique organizations and companies, two provided support through online communities, but only one is active. The next steps include developing systematic ways to evaluate the credibility and accuracy of these resources, and search and test broader topics of dementia care resources online.

### OLDER ADULTS AND TECHNOLOGY USE: A SYSTEMATIC LITERATURE REVIEW

Hyung Wook Choi,[1] Rose Ann DiMaria-Ghalili,[2] Mat Kelly,[1] Alexander Poole,[1] Erjia Yan,[3] and Jina Huh-Yoo,[1] *1. Drexel University, Philadelphia, Pennsylvania, United States, 2. Drexel University, Jenkintown, Pennsylvania, United States, 3. Information Science, Drexel University, Philadelphia, Pennsylvania, United States*

Researchers are increasingly interested in leveraging technology to support the physical and mental well-being of older adults. We systematically reviewed previous scholars' criteria for sampling older adult populations, focusing on age cohorts (namely adults over 65) and their use of internet and smart technologies. We iteratively developed keyword combinations that represent older adults and technology from the retrieved literature. Between 2011 and 2020, 70 systematic reviews were identified, 26 of which met our inclusion criteria for full review. Most important, not one of the 26 papers used a sample population classification more fine-grained than "65 and older." A knowledge gap thus exists; researchers lack a nuanced understanding of differences within this extraordinarily broad age-range. Demographics that we propose to analyze empirically include not only finer measures of age (e.g., 65-70 or 71-75, as opposed to "65 and older"), but also those age groups' attitudes toward and capacity for technology use.

### SESSION 7565 (SYMPOSIUM)

### FINDINGS FROM THE UNDERSTANDING PATTERNS OF HEALTHY AGING AMONG MEN WHO HAVE SEX WITH MEN PROJECT

Chair: Mark Brennan-Ing
Co-Chair: Michael Plankey
Discussant: Deborah Gustafson

In 1984, the Multicenter AIDS Cohort Study (MACS) was started to identify factors in the HIV epidemic related to disease risk and treatment progression among gay, bisexual, and other men who have sex with men (MSM) in four urban areas in the US: Baltimore, MD/Washington, D.C.; Chicago, IL; Pittsburgh, PA, and Los Angeles, CA. MACS participants complete biannual study visits involving HIV testing, biometric screenings, and psychosocial data collection. In 2015 a MACS sub-study, the Understanding Patterns of Healthy Aging among MSM Project (HAMSM), was started to better understand resiliencies promoting well-being among MSM age 40 and older, including those with HIV. HAMSM has helped us to understand aging trajectories among MSM, and provides a unique combination of physiological and psychosocial data that can inform efforts to support MSM in healthy aging. This symposium will present emerging findings from the HAMSM study. Our first paper examines the relationships between psychological connection to the gay community (PSOC) and developmental regulatory strategies associated with health behaviors and more positive self-appraisals. The second paper examines how PSOC is related to HIV risk reduction behaviors, and if there are differences in such behaviors based on HIV status. Our third paper considers how self-perceptions of aging (age discrepancy, aging satisfaction) are related to frailty and frailty transitions, and if these relationships differ by HIV status. The final paper examines the relationship of social support to frailty among MSM by HIV status. Implications of these findings for research, policy, and programs targeting MSM will be discussed.

### CONNECTION TO THE GAY COMMUNITY AND SELF-APPRAISALS AMONG OLDER MEN WHO HAVE SEX WITH MEN

Mark Brennan-Ing,[1] Michael Plankey,[2] Sabina Haberlen,[3] Steven Meanley,[4] Andre Brown,[5] Deanna Ware,[2] James Egan,[5] and Mackey Friedman,[5], *1. Hunter College, CUNY, New York, New York, United States, 2. Georgetown University Medical Center, Washington, District of Columbia, United States, 3. Johns Hopkins University, Baltimore, Maryland, United States, 4. University of Pennsylvania, Philadelphia, Pennsylvania, United States, 5. University of Pittsburgh, Pittsburgh, Pennsylvania, United States*

Men who have sex with men (MSM) report greater body dissatisfaction compared with heterosexual men, which increases with age. This may result from internalized gay community values regarding ideal physiques and youth. Using structural equation modeling, we examined these relationships among 1,118 MSM men age 40 and older from the Healthy Aging Study (M age=59.9 years/50.1% HIV+/69.8% non-Hispanic White). We hypothesized gay community attachment would be related to self-appraisals (body dissatisfaction/internalized ageism), and that this relationship would be mediated by developmental regulation strategies (physical activity/exercise intentions). The model fit the data well (RMSEA = .048, 90% CI 0.017-0.079). Contrary to our hypothesis, connection to the gay community was related to positive self-appraisals (-.40, p<.001), with significant indirect effects via regulation strategies (-.12, p<.002). Thus, gay community connections are related to positive self-appraisals in older GB men and enhance strategies supporting physical and psychological health. Implications for practice will be discussed.

### PSYCHOLOGICAL SENSE OF COMMUNITY AND THE USE OF CONDOMS AND PREP AMONG A SAMPLE OF AGING BLACK MSM

Andre Brown,[1] Mark Brennan-Ing,[2] Steven Meanley,[3] Sabina Haberlen,[4] Deanna Ware,[5] James Egan,[1] Mackey Friedman,[1] and Michael Plankey,[5], *1. University of Pittsburgh, Pittsburgh, Pennsylvania, United States, 2. Hunter College, CUNY, New York, New York, United States, 3. University of Pennsylvania, Philadelphia, Pennsylvania, United States, 4. Johns Hopkins University, Baltimore, Maryland, United States, 5. Georgetown University Medical Center, Washington, District of Columbia, United States*

Psychological sense of community (PSOC) in Black men who have sex with men (BMSM) may facilitate condom and pre-exposure prophylaxis (PrEP) use to prevent HIV transmission. Understanding BMSM's PSOC contribution to HIV risk reduction may inform HIV prevention efforts for this population, that is disproportionately affected by HIV. Adjusted for sociodemographic characteristics and HIV status, we conducted logistic regressions to test the association between PSOC and condom use among aging BMSM (n=176). Multivariate analyses exhibited no association between PSOC and condom use (AOR= 0.994, 95% CI= 0.942, 1.049). HIV+ participants had higher condom use odds compared to HIV- participants (AOR= 4.031, 95% CI= 1.723, 9.426). A sub-analysis of HIV- participants (n=61), showed no associated between PSOC and PrEP use (AOR= 1.002, 95% CI= 0.904, 1.112). These results have implications for secondary HIV prevention and future research on alternative aspects of social support that may increase BMSM's HIV risk reduction behaviors.

### SELF-PERCEPTION OF AGING AND FRAILTY PHENOTYPE AMONG HIV+ AND HIV- OLDER MEN

Michael Plankey,[1] Karen Nieves-Lugo,[2] Deanna Ware,[1] Sabina Haberlen,[3] Keri Althoff,[3] James Egan,[4] Andre Brown,[4] and Mackey Friedman,[4] *1. Georgetown University Medical Center, Washington, District of Columbia, United States, 2. National Institutes of Health, Bethesda, Maryland, United States, 3. Johns Hopkins University, Baltimore, Maryland, United States, 4. University of Pittsburgh, Pittsburgh, Pennsylvania, United States*

Self-perception of aging is an important predictor of health. We examined the relationship of self-perception of aging (age discrepancy and aging satisfaction) with frailty phenotype between HIV+ and HIV- men in the Multicenter AIDS Cohort Study. 499 HIV+ and 549 HIV- men were included in the analytic sample (median age 61 years IQR 56-66 years). Frailty status was based on the Fried frailty phenotype and measured at semi-annual study visits beginning 3/2015 or 9/2015, 3/2016 and 3/2019. Baseline frailty was: HIV- 8.9%; HIV+ 13.9%. Low aging satisfaction and feeling older was positively associated with remaining frail (ORs: 6.64;95%CI:3.88-11.38; 5.68; 95%CI:3.06-10.56) or transitioning between non-frail and frail states (ORs: 2.72;95%CI:1.56-4.74; 2.50; 95%CI:1.11-5.64),), over a 3-year period. There was no statistically significant difference by HIV status. Assessment of self-perception of aging may be useful in the setting of frailty evaluation among HIV+ and HIV- men.

### SOCIAL SUPPORT AND FRAILTY IN HIV POSITIVE AND NEGATIVE MEN WHO HAVE SEX WITH MEN

Laura Graf,[1] Mackey Friedman,[2] Steven Meanley,[3] James Egan,[2] Andre Brown,[2] Deanna Ware,[4] Michael Plankey,[4] and Sabina Haberlen,[5] *1. Johns Hopkins Bloomberg School of Public Health, Brooklyn, New York, United States, 2. University of Pittsburgh, Pittsburgh,*

*Pennsylvania, United States, 3. University of Pennsylvania, Philadelphia, Pennsylvania, United States, 4. Georgetown University Medical Center, Washington, District of Columbia, United States, 5. Johns Hopkins University, Baltimore, Maryland, United States*

Social support is linked to a myriad of positive health outcomes, yet little is understood about its potential role on frailty development among older men who have sex with men (MSM). We evaluated data from 929 MSM aged 40-81 years enrolled in the MACS Health Aging sub-study. Social support (Social Provisions Scale[SPS-24]; range 24-96) was high, though slightly lower among the HIV-positive versus HIV-negative men (median: 80 vs. 82, p=0.12). Each SD increase in social support associated with a 21% decrease in incident frailty (Fried phenotype), independent of age, race, and education (aIRR=0.79, IQR[0.65, 0.97]), though attenuated after adjustment for depressive symptoms. This protective association was observed to be strongest among HIV-positive MSM. High social support is a strength among older MSM, which associates with positive frailty outcomes. Assessing and strengthening social support systems may have potential as a psychosocial component of frailty interventions.

### SESSION 7570 (SYMPOSIUM)

### WHY AGE AND AGING RESEARCH MATTERS: A CHAT WITH NIA SENIOR LEADERSHIP

Chair: Marie Bernard

The National Institute on Aging (NIA) at the National Institutes of Health, Department of Health and Human Services, is the federally designated lead agency on aging research, and has supported significant research on aging as a life-long process. In the last five years, NIA experienced a tripling of its budget. Although much of this funding is targeted to Alzheimer's disease (AD) and AD related dementias (ADRD) research, there was an increase in funds allocated to non-AD research in keeping with the overall growth of NIH. This symposium will provide a forum for exploration of the implications of the budget increases for the general research community. It will involve NIA's senior staff discussing research priorities and programs supported by the Institute. A question-and-answer session will follow brief introductory remarks on current funding and future priorities and research directions of NIA.

### DIVISION OF EXTRAMURAL ACTIVITIES

Kenneth Santora, *National Institute on Aging, Bethesda, Maryland, United States*

Dr. Santora will discuss research priorities for the Division of Extramural Activities. He and his team will in addition be available for small group discussions

### UPDATE ON NIH INCLUSION ACROSS THE LIFESPAN

Marie Bernard, *National Institutes of Health, Bethesda, Maryland, United States*

The National Institute on Aging (NIA) at the National Institutes of Health, Department of Health and Human Services, is the federally designated lead agency on aging research, and has supported significant research on aging as a life-long process. In the last five years, NIA experienced a tripling of its budget. Although much of this funding is targeted to Alzheimer's disease (AD) and AD related dementias (ADRD) research, there was an increase in funds allocated to non-AD research in keeping with the overall growth of NIH. This symposium will provide a forum for exploration of the implications of the budget increases for the general research community. It will involve NIA's senior staff discussing research priorities and programs supported by the Institute. A question-and-answer session will follow brief introductory remarks on current funding and future priorities and research directions of NIA.

### FUNDING OPPORTUNITIES AT THE NATIONAL INSTITUTES ON AGING, NIH

Richard Hodes, *National Institute on Aging, National Institutes of Health, Bethesda, Maryland, United States*

### SESSION 7575 (SYMPOSIUM)

### EXPLORING AGING MASCULINITIES FROM DIFFERENT DISCIPLINES AND PERSPECTIVES

Chair: Liat Ayalon
Co-Chair: Josep Armengol
Discussant: Michael Kimmel

Traditionally, gerontology research has been relatively genderless. When the intersection of age and gender was explored, this was done primarily by focusing on the experiences of older women. Much less is known about the experiences of older men. The present symposium brings together work from the humanities and the social sciences in order to explore societal images and personal experiences of aging men. The paper by Maierhofer and Ratzenböck provides a theoretical outlook on this intersection from the humanities perspective, followed by empirical applications from the social sciences. Next, Armengol uses contemporary American literature to challenge the traditional stereotype of decline in sexuality and masculinity. The paper by Ni Leime & O'Neill examines stereotypes of aging masculinities, but this time from the perspective of older men as the audience who react to their portrayal in visual culture. Finally, Ayalon and Gweyrtz-Meydan present ethical dilemmas faced by physicians who treat older men's sexuality in light of active marketing campaigns of the pharmaceutical industry, which advocate for a model of successful aging and ongoing sexual intercourse. The discussant, Kimmel, will conceptualize the four papers by stressing the different types of information that can be obtained via different methods of inquiry. The complementary information provided by the different papers and the integration of methods and findings from the humanities with the social sciences will be discussed.

### PHYSICIANS' MORAL DILEMMAS IN THE AGE OF VIAGRA

Liat Ayalon, *Bar-Ilan University, Ramay Gan, HaMerkaz, Israel*

Oral phosphodiesterase5 inhibitors (PDE5i; e.g., Viagra®) have become the first line of treatment of erectile dysfunction in men. Relying on interviews with 38 physicians, this study explored moral dilemmas associated with the prescription of PDE5i to older men. Moral dilemmas at the micro level concerned the interest of the patients to receive medical treatment, even when this was counter-indicated. At the meso level, physicians expressed their concerns about the impact of PDE5i on their patients' partners. At the macro level, physicians discussed the substantial contribution of the pharmaceutical industry to the education of patients and physicians about pharmacological treatments for sexual problems. Physicians reported no moral concerns about industry involvement and reported only the benefits associated with it. The study raises moral issues associated with the treatment of erectile dysfunction. As such, it stresses the importance of facilitating a bio-psycho-social approach to treat sexual dysfunctions.

### THEORIZING CULTURAL AND SOCIAL INTERSECTIONS OF AGE AND GENDER: THE APPROACH OF ANOCRITICISM

Roberta Maierhofer, *University of Graz, Graz, Steiermark, Austria*

This contribution discusses empirical applications of the approach of 'anocriticism' in interdisciplinary gerontological research. Despite the connection in terms of epistemology and ontology, the intersection of gender and age has been mostly ignored, privileging works focusing either on age or gender (Calasanti & Slevin 2001:27; Denninger & Schütze 2017:7). Age/ing Studies, however, would not have been established as a field without the theoretical and methodological approaches of feminist theory (Maierhofer (2019:2). Anocriticism was originally developed in order to investigate cultural representations of age/ing (Maierhofer 2003, 2004b, 2004a, 2007, 2012), but has recently been taken up in social sciences (Ratzenböck 2016a, 2016b, 2017a, 2017b; Gales and Loos 2020, forthcoming) in order to draw attention to four dimensions: (a) age and aging's collective cultural construction and relation to gender, (b) the individual dimension of aging, (c) people's interpretative power and narrative performance, and (d) age/ing's potential for resistance and change.

### MASCULINITIES AND AGING IN IRELAND

Aine Ni Leime, *NUI Galway, Galway, Galway, Ireland*

There is a need for research to gain understanding of the social and cultural constructions of ageing masculinities that, as Gullette emphasises, operate together to construct a 'culture of decline'. This presentation explores how cultural images of older men inform constructions of ageing and lived realities in Ireland. It draws on the Irish findings from a cross-national, inter-disciplinary project conducted in 2019 investigating older men's perceptions of how they are represented in film and advertising. It applies innovative narrative and thematic analysis to data from four focus group discussions, interviews and reflective diaries, to explore participants' (Irish men aged 65+) reactions to the portrayal of older men in TV and film. Stereotypes identified included older men as conservative, grumpy, sad, streetwise, trickster, or action hero. Thematic analysis identified themes including men's identification with their jobs; their diminishing roles in the family; and old age as a matter of perception.

### SEX AND TEXT: QUEERING OLDER MEN'S SEXUALITY IN CONTEMPORARY U.S. FICTION

Josep Armengol, *Universidad de Castilla-La Mancha, Ciudad Real, Castilla-La Mancha, Spain*

This paper will explore the representation of men's aging experiences in contemporary U.S. fiction. While most gender-ed approaches to aging have focused on women, which has contributed to the cultural invisibility of older men, this study focuses on men's aging experiences as men, thus challenging the inverse correlation between masculinity and aging. To do so, the study draws on a selected number of contemporary U.S. male-authored fictional works, which question the widely-held assumption that aging is a lesser concern for men, or that men and women's aging experiences may be simply defined as opposed. The literary corpus includes male authors from different backgrounds so as to illustrate how (self-)representations of aging men vary according not only to gender but also class (Richard Ford), race (Ernest Gaines), and sexual orientation (Edmund White), amongst other factors. The presentation will thus end up challenging the conventional equation of men's aging processes with (sexual) decline, exemplifying their plurality as well as irreducible contradictions.

### SESSION 7580 (SYMPOSIUM)

### MAINTENANCE OF HEALTH BEHAVIORS: EXPLORING THE CONSTRUCT AND ITS IMPLICATIONS ON RESEARCH, EDUCATION, AND PRACTICE

Chair: Jaime Hughes

Modifying health behaviors, including diet, physical activity, sleep, and/or medication adherence, can have a range of positive effects on older adults' overall health, function, and well-being. Although many evidence-based programs exist to support the initiation of health behavior changes, few address longterm maintenance. Emerging research suggests initiation and maintenance are distinct constructs, each requiring unique skills. Furthermore, maintaining health behaviors depends upon health promotion programs that are sustained, or continually delivered with high fidelity, at community and population levels. The objective of this symposium is to present findings from a series of research projects designed to investigate the concept of behavior change maintenance. Activities were supported by NIA Research Centers Collaborative Network (RCCN) funding and included community listening sessions plus an interdisciplinary think tank of national thought leaders. This symposium will begin with an overview of health behavior change, including the rationale for studying maintenance as a critical yet overlooked phase of successful behavior change (J. Hughes). A proposed conceptual model of maintenance will then be discussed, including constructs distinguishing maintenance from initiation (Raj). These introductory presentations will be followed by a discussion of multi-level barriers and facilitators related to maintenance on individual, community, and population levels (S. Hughes). The session will close with implications for research, education, and practice (Bettger).

### THE IMPORTANCE OF STUDYING HEALTH BEHAVIOR IN OLDER ADULTS

Jaime Hughes,[1] Susan Hughes,[2] Mina Raj,[3] and Janet Bettger,[1], *1. Duke University School of Medicine, Durham, North Carolina, United States, 2. University of Illinois at Chicago, Chicago, Illinois, United States, 3. University of Michigan, Ann Arbor, Michigan, United States*

Behavior change is an inherent aspect of routine geriatric care. However, most research and clinical programs emphasis how to initiate behavior change with less emphasis placed on skills and strategies to maintain behaviors over time, including after an intervention has concluded. This presentation will provide an introduction to the symposium, including a review of prior work and our rationale for studying the critical yet overlooked construct of maintenance in older adults. Several key considerations in our work include the impact of multiple chronic conditions, declines in cognitive and functional capacity over time, changes in environmental context and/or social support, and sustainability of community and population-level programs and services.

### DEFINING THE CONSTRUCT OF HEALTH BEHAVIOR MAINTENANCE

Mina Raj,[1] Janet Bettger,[2] Susan Hughes,[3] and Jaime Hughes,[2], *1. University of Michigan, Ann Arbor, Michigan, United States, 2. Duke University School of Medicine, Durham, North Carolina, United States, 3. University of Illinois at Chicago, Chicago, Illinois, United States*

Prior research on health behavior maintenance has proposed several key constructs that distinguish this concept from initiation. Initiation is thought to depend upon action self-efficacy, goal setting, and outcome expectations. Maintenance, on the other hand, depends upon self-regulation, relapse prevention, recovery self-efficacy, and satisfaction with original outcome expectations. Although much prior research has focused on cognitive components of maintenance, there has been little attention to how higher-order cognitive processing and decision-making may be challenging for some older adults. This presentation will discuss a proposed conceptual model of health behavior maintenance, specifically as this construct applies to older adults. Special consideration will be given to how both normative and non-normative age-related changes (e.g., physical, cognitive, psychosocial) impact maintenance and how such changes might influence older adults' goals and outcome expectations. Finally, individual-level maintenance will be discussed within a larger context of program sustainability at community and population levels.

### MULTI-LEVEL RESOURCES TO SUPPORT HEALTH BEHAVIOR MAINTENANCE

Susan Hughes,[1] Janet Bettger,[2] Mina Raj,[3] and Jaime Hughes,[2] *1. University of Illinois at Chicago, Chicago, Illinois, United States, 2. Duke University School of Medicine, Durham, North Carolina, United States, 3. University of Michigan, Ann Arbor, Michigan, United States*

Although behavior change is largely thought to occur on the individual level, maintenance of health behaviors also depends upon factors at the community and population levels. As discussed in earlier presentations, health behaviors can be influenced by social support, environmental context, and population-level policies. For example, maintaining a physical activity regimen is made easier when an older adult has access to a safe environment, support of family and friends, and/or ongoing access to a program that is continuously offered, or sustained, at a local community center. This presentation will focus on the importance of discussing sustainability when considering long-term maintenance of health behaviors. Specifically, this presentation will use examples from the evidence-based Fit & Strong! program, now being offered in 32 states, to explore sustainability of programs at the participant and community level and will provide an overview of barriers and facilitators faced by community organizations.

### IMPLICATIONS AND RECOMMENDATIONS FOR RESEARCH, EDUCATION, AND PRACTICE

Janet Bettger,[1] Susan Hughes,[2] Mina Raj,[3] and Jaime Hughes,[1] *1. Duke University School of Medicine, Durham, North Carolina, United States, 2. University of Illinois at Chicago, Chicago, Illinois, United States, 3. University of Michigan, Ann Arbor, Michigan, United States*

Throughout this symposium, we have advocated for increased attention to how the chronicity and complexity of older adults' health might impact their ability to maintain health behaviors over time. This final presentation will explore some implications of this chronicity and complexity on research study designs, formal education and professional training, and routine clinical practice. For example, existing research designs and funding mechanisms such as tightly controlled randomized controlled trials within a five-year grant may not permit researchers to study behaviors over an extended period of time and to do so within the real-world environments and social contexts in which older adults live. In contrast, integrating principles of community-engaged and participatory research, adaptive designs, pragmatic trials, and implementation science may encourage researchers to design for widespread dissemination. This session will close with recommendations to enhance educational initiatives and professional development to promote cross-disciplinary teams.

### SESSION 7585 (SYMPOSIUM)

### WHY AGE AND AGING RESEARCH MATTERS—FOR EARLY-CAREER RESEARCHERS

Chair: Marie Bernard

The National Institute on Aging (NIA) at the National Institutes of Health, Department of Health and Human Services, supports biomedical and behavioral research with a life-span focus. There is attention to understanding basic processes of aging, improving prevention and treatment of diseases and conditions common in later years, improving the health of older persons, as well as a focus on Alzheimer's disease and related dementias. The NIA also supports the training and career development of scientists focusing on aging research and the development of research resources. The symposium, meant for junior faculty and emerging scholars, will provide an update on the latest research findings from the NIA followed by a brief update on funding mechanisms. An opportunity is provided to meet and consult with NIA extramural staff.

### OVERVIEW OF THE NATIONAL INSTITUTE ON AGING

Richard Hodes, *National Institutes of Health, Bethesda, Maryland, United States*

Dr. Hodes will provide an overview of National Institute on Aging research priorities and funding availability.

### HOW TO GET A GRANT FROM THE NATIONAL INSTITUTE ON AGING

Kenneth Santora, *National Institute on Aging, Bethesda, Maryland, United States*

Dr. Santora will provide an overview of grant mechanisms available at the National Institute on Aging, with an emphasis on mechanisms of value to early career researchers.

### SESSION 7590 (SYMPOSIUM)

### INNOVATIVE APPROACHES TO UNDERSTANDING RESILIENCE

Chair: B. Gwen Windham
Co-Chair: Michael Griswold

Physical resilience, generally defined as the ability to recover or resist functional decline following a stressor, holds promise for clinicians to identify patients who are likely to benefit from a planned stressor (such as an elective procedure) or deteriorate after an unplanned stressor (such as heart failure). This symposium will present innovative research approaches that enhance understanding of the constructs of resilience from human and animal models. The symposium will include presentations from leading experts addressing issues in the study of resilience including choices of definitions, study design, comparator groups, and considerations of underlying latent factors. Data from population-based cohort studies and animal models will be presented to inform the discussions. This interdisciplinary symposium will address recommendations for advancing resilience knowledge through elucidating biologic, clinical and environmental influences and optimizing definitions, designs, and analyses. Epidemiology of Aging Interest Group Sponsored Symposium.

### CONCEPTUALIZING RESILIENCE: IMPACTS OF ADVERSE STRESSOR SEVERITY, CHRONICITY, AND RELEVANCE

Michael Griswold,[1] B. Gwen Windham,[2] James Henegan,[2] Karen Bandeen-Roche,[3] Matthew Mcmullan,[2] Anna Kucharska-Newton,[4] Priya Palta,[5] and Thomas Mosley,[2] *1. The University of Mississippi Medical Center, Jackson, Mississippi, United States, 2. University of Mississippi Medical Center, Jackson, Mississippi, United States, 3. Johns Hopkins Bloomberg School of Public Health, Baltimore, Maryland, United States, 4. Gillings School of Global Public Health, University of North Carolina at Chapel Hill, Chapel Hill, North Carolina, United States, 5. Columbia University Irving Medical Center, New York, New York, United States*

Guidance on frameworks for physical resilience, resistance and reserve are underdeveloped. We examined different "physical resilience" characterizations within n=6,538 Atherosclerosis Risk in Communities Study participants (median age: 75 years), followed for 5+ years. Fifteen illustrative clinical, lifestyle and social stressors, each having varying levels of severity, chronicity and relevance, were paired with six functional outcome measure trajectories. Contrasts were made against four fundamental comparator groups (including those without stressors). Particular pairings of stressors and functional measures substantially impacted resilience classifications and related determinants. For example, 5-year recurring robustness (0/5 frailty indicators) was only 12% for participants after Heart Failure (HF), versus 47% with no HF event; relative-risk: RR=0.26 (95%CI: 0.15,0.44). Conversely, recurring robustness was 43% after reporting low social support versus 51% with adequate support; RR=0.87(0.73,1.02). We highlight major components that impacted resilience determinations and outline a broad conceptual framework to help optimize physical resilience assessment and aid future research. Part of a symposium sponsored by Epidemiology of Aging Interest Group.

### THE MEASUREMENT OF PHYSICAL RESILIENCIES: CONCEPTUALIZATION, STUDY DESIGN, AND EARLY DATA

Karen Bandeen-Roche,[1] Ravi Varadhan,[2] Brian Buta,[2] and Jeremy Walston,[2] *1. Johns Hopkins Bloomberg School of Public Health, Baltimore, Maryland, United States, 2. Johns Hopkins University, Baltimore, Maryland, United States*

This talk presents the conceptual framework, study design, and pilot data for the Study of Physical Resilience and agING (SPRING). SPRING aims to develop signatures of physical resilience in older adults who will undergo clinical stressors; validate the signatures' ability to distinguish those who will respond resiliently; and characterize underlying physio-biological determinants. The underlying physiology is conceptualized as a dynamical system, and resilience, as a property thereof. The SPRING pilot has assessed n=77 older adults before, during and repeatedly after experiencing knee replacement surgery, incident hemodialysis, or bone marrow transplant; a confirmatory study evaluating n=100 older adults per each of these stressors is underway. Resilience signatures grounded in dynamical data from multiple stress-response assessments will be presented. So also will resilience phenotypes—longitudinal functional trajectories over the study visits: These show considerable heterogeneity within and across stressors. If successful, our study will open the way for interventions to bolster resiliency. Part of a symposium sponsored by Epidemiology of Aging Interest Group.

### LATENT CLASS TRAJECTORY AND GROWTH MIXTURE MODELS IN THE STUDY OF PHYSICAL RESILIENCE

Carl Pieper,[1] Jane Pendergast,[2] and Megan Neely,[2] *1. Center on Aging and Human Development, DUMC, Durham, North Carolina, United States, 2. Duke University, Durham, North Carolina, United States*

After a stressor, individuals may experience different trajectories of function and recovery. One potential explanation for this variation is differing trajectories may be indicators of differing classes or levels of resilience to the stressor. Latent Class Trajectory (LCTA) and Growth Mixture models (GMM) are two similar approaches used to discover the number and types of trajectories in a study population. Class membership may determine the shape and level of recovery, which may be predicted by individual characteristics. In this talk, we present some insights to using these models to successfully identify the number of classes of trajectories, membership of trajectory classes, and the functional form of the trajectory. We will identify methods for deciding class enumeration, indices for assessing fit quality, and, importantly, the importance of proper model specification. Real life and simulated examples will be shown to compare and contrast differences between GMM and LCTA results. Part of a symposium sponsored by Epidemiology of Aging Interest Group.

### PHYSICAL RESILIENCE AS A DETERMINANT OF HEALTHSPAN AND LIFESPAN IN MICE

Nathan LeBrasseur, *Mayo Clinic, Rochester, Minnesota, United States*

Dynamic measures of physical resilience—the ability to resist and recover from a challenge—may be informative of biological age far prior to overt manifestations such as age-related diseases and geriatric syndromes (i.e., frailty). If true, physical resilience at younger or middle ages may be predictive of future healthspan and lifespan, and provide a unique paradigm in which interventions targeting the fundamental biology of aging can be tested. This seminar will discuss research on the development of clinically-relevant measures of physical resilience in mice, including anesthesia, surgery, and cytotoxic drugs. It will further highlight how these measures compare between young, middle-aged, and older mice, and how mid-life resilience relates to later-life healthspan. Finally, it will provide insight into whether interventions targeting the biology of aging can modify physical resilience in mice. Part of a symposium sponsored by Epidemiology of Aging Interest Group.

### IMPORTANCE OF WILL IN RESILIENCE AND RECOVERY: FINDINGS FROM THE BLSA

Eleanor Simonsick,[1] Michael Griswold,[2] and B. Gwen Windham,[3] *1. National Institute on Aging, Bethesda, Maryland, United States, 2. The University of Mississippi Medical Center, Jackson, Mississippi, United States, 3. University of Mississippi Medical Center, Jackson, Mississippi, United States*

Using data from 743 initially well-functioning men and women (49.5%) aged ≥60 in the Baltimore Longitudinal Study of Aging we interrogated the association between physical reserve operationalized as fast 400m walk performance scaled from 0 to 4 and psychological reserve ("will") operationalized as personal mastery (high versus not) and likelihood of recovery from a decline of ≥2 points in reported walking ability. Of the 35% who declined 1-2 years post study baseline, 54% recovered 1-2 years later and 45% did not. Controlling for age, sex, race and initial walking ability, for each increment in reserve, likelihood of recovery was 43% greater (95% confidence interval (1.10, 1.85); p=.007). This association was most pronounced in women (odds ratio=1.84; 95% CI (1.19, 2.86; p=.006). Personal mastery showed no association with likelihood of recovery. Continuing work will further explore alternative operationalizations of "will". Part of a symposium sponsored by Epidemiology of Aging Interest Group.

### SESSION 7600 (SYMPOSIUM)

### VOICES IN MOTION: RESULTS FROM A COMMUNITY CHOIR INTERVENTION TO PROMOTE LIVING WELL WITH DEMENTIA

Chair: Debra Sheets
Co-Chair: Stuart MacDonald
Discussant: Theresa Allison

Dementia is recognized as a global public health priority because of the significant impact it has on individuals, families and society. The numbers of people living with dementia worldwide are currently estimated at 35.6 million; this will double by 2030 and more than triple by 2050. Given the lack of a medical cure for dementia, lifestyle interventions to complement existing treatment are urgently needed to support living well with this disease. Engagement in the arts is a novel intervention which is relatively low cost and engaging. This study examines the effect of participation in an intergenerational choir on psychosocial and cognitive function for persons with dementia (PwD). Participants (n = 32), in partnership with their family caregivers and local high school students, sang in a professionally conducted choir for as many as three seasons (~ 12 weeks long) spanning up to 18 months of follow-up. Assessments of psychosocial, physiological, and cognitive function were completed every four to six weeks as part of an intensive repeated measures design. Taken as a whole, the symposium papers indicate that this novel lifestyle intervention offers an effective non- pharmacological alternative approach for older adults with dementia. Choir participation has important and significant impacts on psycho-social well-being and quality of life. Discussion focuses on policy implications and the need for community-based programs that reflect a social model for dementia and support living well by through engaging and meaningful activities

### RAISING OUR VOICES: THE IMPACT OF A DEMENTIA CHOIR ON WELL-BEING AND QUALITY OF LIFE

Debra Sheets,[1] Stuart MacDonald,[2] and Andre Smith,[2] *1. University of Victoria, Victoria, British Columbia, Canada, 2. University of Victoria, Victoria, British Columbia, Canada*

Stigma represents one of the biggest barriers to living well with dementia following diagnosis. Social isolation is common as roles, friendships and opportunities to participate in the broader community disappear. An intergenerational dementia choir is a joyful activity that offers opportunities for learning, friendships and purposeful engagement towards common goals (e.g., regular social engagement, public concerts at season's end). Data collection involved surveys and interviews with 32 dyads comprised of persons with dementia (PwD) and caregivers, as well as focus groups with 29 high school students. Results illustrate the development of a choir community across weeks of participation with far reaching benefits. Both caregivers and PwD experienced reductions in health risks and improvements in quality of life. Students' understanding of dementia became more positive over time and new friendships developed. The discussion focuses on the need for meaningful and inclusive community activities for PwD and their caregivers.

### THE MAGIC OF MUSIC FOR IMPROVING PSYCHOLOGICAL HEALTH FOR THOSE WITH DEMENTIA AND THEIR CAREGIVERS

Stuart MacDonald,[1] Debra Sheets,[2] and Andre Smith,[1] *1. University of Victoria, Victoria, British Columbia, Canada, 2. University of Victoria, Victoria, British Columbia, Canada*

Arts-based interventions for person's with dementia and their caregivers represent an inexpensive, non-invasive, and non-pharmacological intervention with the potential to improve psychological function as well as reduce healthcare costs. The paper presents an overview of Voices in Motion (ViM), and the impact of this social-cognitive intervention on changes in psychological function for those with dementia and their caregivers (current n=26 dyads). Choir rehearsals were held on a weekly basis, and included a social discussion component. A range of outcomes (neuropsychological and physiological function, neural activation) were assessed using an intensive repeated measures design that facilitates both between- and within-person analyses, including nuanced evaluation of whether psychological function improves post intervention relative to an individual's personal average, yielding a conservative within-person test of the benefits of intervention. Discussion focuses on the promise of such interventions for mitigating dementia symptoms and facilitating the psychological health of caregivers.

### SYSTEMATIC CHANGES IN GAIT PURSUANT TO CHOIR PARTICIPATION FOR CAREGIVERS AND PERSONS WITH DEMENTIA

Michael Willden,[1] Stuart MacDonald,[1] Debra Sheets,[2] and Andre Smith,[1] *1. University of Victoria, Victoria, British Columbia, Canada, 2. University of Victoria, Victoria, British Columbia, Canada*

Choir interventions confer psychological benefits for persons with dementia (PwD) and their caregivers. However, less is known about whether physiological function also exhibits improvements pursuant to such social-cognitive interventions. The present study, based upon a subsample of the Voices in Motion (ViM) project, explored whether participation in an intergenerational choir results in systematic improvements in gait velocity (indexed using a GAITRite computerized walkway) for both informal caregivers (n=14; 71.4% female) and PwD (n=14; 64.3% female). Longitudinal burst data from the first of three cohorts spanning 4 assessments over 3.5 months was analysed using multilevel modeling. Whereas caregivers exhibited significant improvements ($p<.05$) in gait velocity, PwD showed no improvement. Ongoing analyses are exploring additional cohorts, and whether improvements in gait dynamically covary with reductions in comorbidities (e.g., neuropsychological function, caregiver burden, depressive affect). These results underscore the potential of choir for facilitating both psychosocial and physiological function for caregivers and PwD.

### SESSION 7605 (SYMPOSIUM)

### MAPPING THE POSSIBLE FUTURES OF TECHNOLOGY AND CAREGIVING

Chair: Joseph Coughlin
Discussant: Terry Fulmer

As people age, more individuals are expected to need health and caregiver services; it is therefore anticipated that families will face greater demands and carry an increasing burden. Frontier technologies – such as artificial intelligence, sensors and naturalistic user interfaces – are increasingly entering people's lives, and may be leveraged to support family caregivers. These innovations may make it possible to provide comprehensive care to loved ones while reducing caregiver burden. Similarly, professional caregivers, from social workers to clinicians, may benefit from new devices and services that enable them to provide care more effectively and even proactively prevent a health event. However, challenges around technology maturity, potential risks and skepticism may interfere with a successful implementation. This session reports on research that explores how evolving technologies may shape the future of caregiving. A panel of 41 experts from related disciplines – engineering, data science, medicine, nursing, public policy, business, aging services and more – participated in a modified Delphi study. Across two rounds of data collection, the expert panel provided their opinions and insights about how various technologies and related systems may impact family and professional caregivers by the year 2030. This symposium will present findings from the expert panel, including: how different caregiving tasks and activities may be impacted by new technologies, and what the benefits and barriers may be; possible impacts of new technologies on related topics including elder abuse and fraud, service delivery, and labor and education; and possible future scenarios and expert predictions for 2030.

### FUTURE CAREGIVING SCENARIOS: EFFECTS OF TECHNOLOGY INNOVATION AND ADOPTION

John Rudnik, Lisa D'Ambrosio, Chaiwoo Lee, and Joseph Coughlin, *MIT, Cambridge, Massachusetts, United States*

Based on the expert panel's response to the first round of the study, four scenarios that describe possible futures for the intersection of caregiving and technology were created. These scenarios differ along two dimensions – focus of technology deployment and adoption promotion – along which stakeholders' decisions make a crucial difference for outcomes. These four scenarios describing possibilities for the year 2030 will be illustrated during this presentation, along with results from the second round of the study, which include the expert panel's evaluations of their likelihood and impacts on caregiving.

### SOCIETAL IMPACTS AND BROADER IMPLICATIONS OF USING TECHNOLOGIES FOR CAREGIVING

Lisa D'Ambrosio, John Rudnik, and Joseph Coughlin, *MIT, Cambridge, Massachusetts, United States*

Advances and greater adoption of new technologies may have impacts beyond day-to-day caregiving. This presentation will explore additional implications related to the future of caregiving and technology, including their potential effects on elder abuse, financial fraud, career and labor, social isolation and more. Opinions from the expert panel – which pointed to various possibilities, benefits and challenges – will be shared during this presentation.

### USING TECHNOLOGIES TO ADDRESS CAREGIVING CHALLENGES

Chaiwoo Lee, John Rudnik, and Joseph Coughlin, *MIT, Cambridge, Massachusetts, United States*

As the caregiver ratio declines, technology will play an increasingly important role in supporting formal and informal caregivers. This presentation will report on the particular effects that frontier technologies may have on various tasks associated with caregiving, including assisting with basic Activities of Daily Living (ADLs) and Instrumental Activities of Daily Living (IADLs). The expert panel predicted that different technologies and new products will have varied effects on caregiving tasks, and that some tasks may be more impacted than others. Some of the key opportunities and barriers to integrating technologies into various tasks of caregiving will be discussed.

### SESSION 7610 (SYMPOSIUM)

### PRESIDENTIAL SYMPOSIUM: INTERDISCIPLINARY PERSPECTIVES FROM EARLY-CAREER SCHOLARS ON WHY AGE MATTERS

Chair: Darina Petrovsky
Co-Chair: Danielle Waldron
Discussant: Katie Sakel

The 2020 ESPO Presidential Symposium features interdisciplinary perspectives and recent scientific advances made by early career researchers from each of the GSA scientific sections. They provide examples of how their work is addressing ways age matters. The first paper by Justice (Biological Sciences) will present the latest geroscience research. The second presentation will explore how age influences end-of-life care (Health Sciences, Starr). The third presentation will focus on age as a predictor of kidney function decline (Behavioral and Social Sciences, Surachman). The fourth presentation will explore how age shapes older adults' resilience (Academy for Gerontology in Higher Education, Bouchard). The fifth presentation will examine opioid use in older adults (Social Research, Policy, and Practice, Jansen). Additionally, early career scholars will share information about their research trajectories and future directions within their disciplines. Given the diverse nature of these presentations, attendees will be exposed to varying strategies, methodologies, and tools that are employed across disciplines. The symposium concludes with a discussion on ways to identify synergies across different fields and promote strategies for successful cross-disciplinary collaboration.

### THE TRANSLATIONAL GEROSCIENCE NETWORK: SUPPORTING A NEW PARADIGM TO ALLEVIATE AGE-RELATED CHRONIC DISEASE

Jamie Justice,[1] Stephen Kritchevsky,[1] George Kuchel,[2] and James Kirkland,[3] *1. Wake Forest School of Medicine, Winston-Salem, North Carolina, United States, 2. University of Connecticut, Farmington, Connecticut, United States, 3. Mayo Clinic, Rochester, Minnesota, United States*

Aging is the leading risk factor for many chronic diseases. Through traditional approaches to drug development and treatment focus on treating one disease at a time, the geroscience hypothesis posits that by targeting fundamental aging processes one could alleviate multiple age-related diseases. Now several geroscience-guided interventions are at the point of entering human clinical trials. To accelerate testing of this important hypothesis, an interdisciplinary Translational Geroscience Network (TGN; R33 AG061456) has recently been established. The TGN is a new national resource of aging research centers committed to working together toward complementary, small-scale, proof-of-concept "use case" clinical studies. One such pilot will be highlighted: a translational trial of senolytics, or drugs targeting the biological aging process cellular senescence in patients with idiopathic pulmonary fibrosis. The promise of geroscience provides another reason "why age matters": by studying the basic biology of aging, we may open novel therapeutic opportunities for challenging age-related diseases.

### PATIENT RISK FACTOR PROFILES ASSOCIATED WITH TIMING OF GOALS-OF-CARE CONSULTATION BEFORE DEATH

Lauren Starr,[1] Connie Ulrich,[1] Paul Junker,[2] Liming Huang,[3] Nina O'Connor,[2] and Salimah Meghani,[1] *1. University of Pennsylvania School of Nursing, Philadelphia, Pennsylvania, United States, 2. University of Pennsylvania Health System, Philadelphia, Pennsylvania, United States, 3. University of Pennsylvania, Philadelphia, Pennsylvania, United States*

Early palliative care consultation to discuss goals-of-care ("PCC") benefits seriously ill patients. To identify risk factor profiles associated with inpatient PCC timing before death, we conducted a secondary analysis of seriously ill adults who had PCC at a high-acuity hospital and died 2014-2016. Of 1,141 patients, 54% had PCC "close to death" (0-14 days before death); 26% had PCC 15-60 days before death; 21% had PCC >60 days before death (median 13 days). Classification and Regression Tree modeling showed Hispanic or "Other" race/ethnicity intensive care patients with extreme illness severity (85%) were most likely to have PCC close to death, with age <46 or >75 increasing probability (98%). Among age groups, the highest proportion of patients with PCC close to death was >75 years. Complex variable interactions associated with PCC timing suggests we need a systematic process for initiating PCC earlier and effective primary palliative training for providers across settings.

### LIFE COURSE PATHWAYS FROM CHILDHOOD SES TO AGE-RELATED DECLINES IN KIDNEY FUNCTION ACROSS ADULTHOOD

Agus Surachman,[1] Alexis Santos,[2] Jonathan Daw,[1] Lacy Alexander,[1] Christopher Coe,[3] and David Almeida,[2] *1. The Pennsylvania State University, University Park, Pennsylvania, United States, 2. Pennsylvania State University, University Park, Pennsylvania, United States, 3. University of Wisconsin - Madison, Madison, Wisconsin, United States*

Age is a strong predictor of declines in kidney function across adulthood. Using data from 2,045 adults (ages 25-84) in the Midlife in the United States (MIDUS) study, we examined the life course pathways through which low parental education, through adult SES and body mass index (BMI), was associated with faster age-related declines in kidney function. Kidney function declines by 0.8 mL/min/1.73 m2 per year across adulthood. Lower parental education, through adult SES and BMI, was associated with higher kidney function among younger adults (Est = -1.61, SE = 0.62, 95%CI = -2.62, -0.60), but lower kidney function among older adults (Est = 0.93, SE = 0.51, 95%CI = 0.11, 1.79). The impact of early socioeconomic adversity on kidney function is initiated by kidney hyperfiltration in early adulthood and followed by faster declines and development into disease state in later adulthood.

### RESILIENT IDENTITY: HOW AGING MATTERS IN EXPLORING RESILIENCE

Lauren Bouchard,[1] and Lydia Manning,[2] *1. Concordia University Chicago, Chicago, Illinois, United States, 2. Concordia University Chicago, River Forest, Illinois, United States*

Defining resilience is complex given its multidimensional and contextualized nature within the gerontological literature. The construct has been described as a trait, state, and process, and less often, as a cultivated identity. Older adults are key in the understanding of resilience from their own point view as experts of their experiences with adversity. This presentation focuses upon the findings of qualitative research utilizing grounded theory methodology, which explores the way aging may shape "resilient identity." Given the varying challenges across the life course, resilient identity may be highly dependent on individual and environmental context. This talk also focuses on who determines the definition of resilience as well as the nature of such an identity. Educational implications regarding key factors, management strategies, and protective practices are also discussed.

### DISPARITIES IN OPIOID USE FOR PAIN CONTROL AMONG OLDER ADULTS: THE ROLE OF SOCIAL FACTORS

Taylor Jansen, *University of Massachusetts Boston, Boston, Massachusetts, United States*

Over 50% of older adults (65+ years old) suffer from pain, and an estimated 25% of older adults use prescription opioids to treat their pain. Older adults are physiologically vulnerable to the effects of opioids; yet, they are prescribed more than all other age groups. This study used the Health and Retirement Study 2016 Core dataset (N=3,916) to analyze the moderation effect of social support on the association between pain and prescription opioid use in people aged 65+ using logistical regression analysis. Results show that older adults with severe pain were more likely to use prescription opioids (OR= 4.84) after considering covariates. Higher perceived social support was associated with higher likelihood of prescription opioid use for severe pain (OR=1.53). Older adults are at greater risk of pain and social isolation compared to younger age groups, making them a vulnerable group to consider as policy makers tackle this nationwide epidemic.

### SESSION 7615 (SYMPOSIUM)

### SOCIOECONOMIC STATUS, HEALTH BEHAVIORS, AND ORAL HEALTH OF OLDER ADULTS IN CHINA

Chair: Yaolin Pei
Co-Chair: Xi Chen
Discussant: Michele Saunders

Many Chinese older adults suffer from oral health diseases and problems due to low oral health literacy, limited dental coverage and lack of dental care services for this segment of the population in China. However, so few studies have been conducted to examine social and behavior factors related to oral health among Chinese older adults. This symposium examines how socioeconomic status (SES) and health behaviors are associated with oral health among Chinese older adults. The first paper used the Nanjing Centenarians Study to examine the association between health behaviors and oral health among Chinese centenarians. The results showed that health behaviors were associated with self-rated oral health and edentulism. Using the Chinese Longitudinal Healthy Longevity Survey, the second one employed an 'after death' approach to examine risk factors for orofacial pain symptoms at the end of life among Chinese older adults. The third paper investigated the association between SES and tooth loss among middle-aged and older adults in ten cities of China. SES played a stronger role in tooth retention for non-migrants and migrants with high education vs those migrants with low education. The last paper examined the association between health behaviors and retention of teeth among Chinese older adults using data from the Chinese 4th National Oral health Survey. This symposium provides empirical evidence on the current status of oral health and health behaviors at the national level, and also suggests that is critical to improve oral health education and access to dental care.

### RISK FACTORS OF OROFACIAL PAIN SYMPTOMS AMONG OLDER ADULTS AT THE END OF LIFE

Yaolin Pei, and Bei Wu, *New York University, New York, New York, United States*

The aims of this study were to examine the prevalence of orofacial pain symptoms in Chinese older adults at the end of life, and to investigate risk factors related to orofacial pain. The sample derived from the Chinese Longitudinal Healthy Longevity Survey (CLHLS), a national respresentative sample of the oldest-old. The results showed that the 6-month prevalence of pain when chewing or biting at the end of life was 11.1%, and the rate was 5% for jaw joint pain/facial pain. Lower SES, smokers, and having chronic diseases were associated with having orofacial symptoms. Unexpectedly, the results revealed that dentate older adults (retain at least one natural tooth) who brushed their teeth more often were more likely to have orofacial symptoms. Older adults have poor oral health, particularly at the end of their life. This study highlights the importance of improving oral health for vulnerable older adults.

### HEALTH BEHAVIORS AND ORAL HEALTH AMONG CENTENARIANS IN NANJING, CHINA: A CROSS-SECTIONAL STUDY

Xin Xu,[1] Yuan Zhao,[1] Danan Gu,[2] Yaolin Pei,[3] and Bei Wu,[3] *1. Nanjing Normal University, Nanjing, Jiangsu, China, 2. United Nations, New York, New York, United States, 3. New York University, New York, New York, United States*

The aim of this study was to examine the association between health behaviors and oral health among Chinese centenarians. Data from the Nanjing Centenarians Study (NCS) in China was used (N=185, Mean age = 102). Oral health status was measured by self-reported oral health and edentulous status. Results from ordinal regression and logistic regression models indicated that centenarians who were male, smoking, normal weight (18.5-24.9kg/m2), participated in more activities, and brushed teeth more frequently were more likely to report better oral health. Those who ate fruits daily and brushed teeth more frequently were more likely to be dentate. The association between frequent toothbrushing and oral health was stronger for those who had some formal education and were living with family members. Our study demonstrated the significance of health behaviors on oral health in very old age, and the importance of lifestyle on healthy aging.

### SOCIAL STRATIFICATION AND TOOTH LOSS AMONG MIGRANT AND NON-MIGRANT MIDDLE-AGED AND OLDER ADULTS IN CHINA

Xiaomin Qu,[1] Bei Wu,[2] Jiaojiao Yu,[3] and Haidong Zhang,[4] *1. East China University of Political Science and Law, Shanghai, China, 2. New York University, New York, New York, United States, 3. School of History and Ethnology, Qiannan Normal University for Nationalities, Duyun, Guizhou, China, 4. School of Sociology and Political Science, Shanghai University, Shanghai, China*

This study investigated the association between socioeconomic status (SES) and tooth loss in middle-aged and older adults by migrant status. The sample included 2,390 participants aged 45-65 from the 2017 Urbanization and New Migrant Survey conducted from 10 cities in China. Results from the negative binomial regression and the marginal effect analysis showed that education, income, and residence in a developed city were negatively associated with tooth loss for non-migrants and migrants with high levels of education. These associations were not found to be significant for migrants with low education levels. The findings suggest that SES plays a more significant role in tooth retention for migrants with higher education levels compared to those with lower education levels. These results may largely be due to different levels of health literacy and unequal access to dental care services. Tailored intervention needs to be target migrant populations with low SES.

### TOOTH RETENTION AND HEALTH BEHAVIORS: FINDINGS FROM THE 4TH CHINESE NATIONAL ORAL HEALTH SURVEY

Xiaoyan Ou,[1] Liwei Zeng,[2] Yixuan Zeng,[2] Yaolin Pei,[3] and Bei Wu,[3] *1. Nanchang University, Nanchang, Jiangxi, China, 2. Affiliated Stomatological Hospital of Nanchang University, Nanchang, Jiangxi, China, 3. New York University, New York, New York, United States*

This study aimed to investigate the association between tooth retention and health behaviors among Chinese older adults. Data was used from the Chinese 4th National Oral Health Survey, a national representative sample. The sample included 9054 older adults age 55-74. The control variables included age, gender, residence, region, education level, occupation, periodontal health, self-reported oral health, and self-reported general health. Logistic regression models shows that older adults who used tooth picks (OR=3.37, 95% CI: 2.94-3.85), dental floss (OR=1.93, 95%CI: 1.05-3.53), and toothpaste (OR=3.89, 95%CI: 3.14-4.83), and never smoked (OR=1.43 95%CI: 1.20-1.70) were more likely to retain more than 20 natural teeth; while having dental visit had the opposite association (OR = 0.45, 95% CI: 0.39-052). Unexpectedly, this study did not find frequency of toothbrushing was associated with tooth retention. This study suggest that improving oral hygiene and preventive dental care are key for good oral health. .

### SESSION 7620 (SYMPOSIUM)

### BRIDGING THE PAST AND THE FUTURE: WHY AGE MATTERS IN BEHAVIORAL HEALTH TRAINING

Chair: Rebecca Allen
Co-Chair: Keisha Carden

This symposium presents data from three applied clinical research projects that involve intergenerational interaction as one component of effective treatment. The first paper describes learner outcomes in an intergenerational art therapy and reminiscence program provided in an adult day service facility. Results show that, in comparison with students in a didactic psychology of aging course or an introductory psychology course, learners in the experiential learning course demonstrated increased empathy, as well as better attitudes toward and increased interest in working with persons with dementia (PWD). The second paper focuses on observed outcomes for older PWD participants in this art therapy and reminiscence program, showing that intergenerational communication engagements exceed engagements with art. Mixed method data across time indicated that PWD benefitted from the treatment, facilitated by undergraduate student learners. The third paper focuses on cultural humility and the importance of racial diversity in providers conducting behavioral health screening in an integrated geriatric primary care clinic. Training issues and behavioral health outcomes regarding assessment of cognitive status, cultural mistrust, and test validity are considered. The fourth and final paper considers how intergenerational dynamics facilitated group cohesion and allowed for increased normalization of common challenges in mindfulness practice. Training issues for graduate student therapists are described. Consideration of level of behavioral health integration in each site, treatment efficacy, and the impact of intergenerational relationships are the foci of discussion. This symposium will show why age matters in behavioral health training.

### WHY AGE MATTERS: A MIXED-METHODS ANALYSIS OF INTERGENERATIONAL CONTACT WITH OLDER ADULTS WITH DEMENTIA

Keisha Carden,[1] Sarah Letang,[2] Jaimie Choi,[2] Daniel Potts,[3] and Rebecca Allen,[4], *1. University of Alabama, Tuscaloosa, Alabama, United States, 2. The University of Alabama, Tuscaloosa, Alabama, United States, 3. Tuscaloosa VA Medical Center, Tuscaloosa, Alabama, United States, 4. Alabama Research Institute on Aging, Tuscaloosa, Alabama, United States*

This integrated mixed methods analysis examined outcomes for students enrolled in an experiential course (BATL), a didactic psychology of aging course, and introductory psychology courses. Students in the experiential course showed increased empathy, $F(2, 345) = 29.058$, $p = .000$ ($M = 47.52$, $SD = .75$), improved attitudes towards persons with dementia (PWDs), $F(2,355) = 8.98$, $p < .0001$ ($M = 14.25$, $SD = .36$), and greater increased interest in working with older adults, $F(2,361) = 30.228$, $p=.000$ compared to the other courses. A qualitative phase II follow-up explanations model (Creswell et. al., 2003) of participants' journals using a hermeneutic coding process was employed to explore underlying mechanisms for such changes. These identified underlying mechanisms have significant implications for increasing interest and ability among students to work effectively with older adults with and without cognitive impairment. Further, this information yields insights for addressing workforce shortages in geriatric care.

### BRINGING ART TO LIFE: MEASURING ENGAGEMENT THROUGH ART, CONNECTION, AND DIGNITY

Candice Reel,[1] Rebecca Allen,[2] Bailey Lanai,[1] Sami Sneed,[1] and Daniel Potts,[3] *1. The University of Alabama, Tuscaloosa, Alabama, United States, 2. Alabama Research Institute on Aging, Tuscaloosa, Alabama, United States, 3. Tuscaloosa VA Medical Center, Tuscaloosa, Alabama, United States*

The BATL program aims to improve the quality of life for community-dwelling people living with dementia (PWD) through art therapy, intergenerational contact, and life story preservation. This study utilized a modified ENGAGE measure (Hartmann et. al, 2017) to evaluate ethnographic transcripts recording activity and social engagement over time (N=100). A five-member analysis team independently coded the observation transcripts for engagement and qualitative themes. Results reveal average communication engagements ($M=28.30$, $SD=13.36$) significantly exceed the average art engagements ($M=9.86$, $SD=5.56$) over time $t(98) = 20.85$, $p<0.001$). Emergent themes included reminiscence, intergenerational communication, and creating art. These mixed method results suggest the intergenerational contact and reminiscence portions of BATL were fundamental features in comparison with art-making. More studies are needed to determine if the BATL intervention 1) has greater comparative effectiveness than typical programs in adult service centers, and 2) is scalable nationally to enhance the quality of life in PWD.

### ADDRESSING RACIAL COMPATIBILITY BETWEEN TRAINEES AND MINORITY OLDER ADULTS IN APPLIED CARE SETTINGS

Danielle McDuffie, *The University of Alabama, Tuscaloosa, Alabama, United States*

This talk explores the importance of a racially diverse graduate student interacting in an applied care setting with minority older adults in the Southeast relative to assessment of cognitive status and in the context of cultural mistrust and misdiagnosis. ~250 older adults (16% AA) in an applied care setting were administered a cognitive screener as part of a larger research battery. An independent samples t-test was conducted to assess differences in mean cognitive status. Non-Hispanic Whites (NHW) ($M=76.8$ years) were found to be more intact than AAs ($M=73.27$) on measures of cognitive status (21.32 vs. 14.72, $t=4.976$, $p=.0001$). Implications highlight that cognitive screeners have often been found to lack sensitivity in groups of marginalized older adults. Having an AA graduate student in these settings could be a way of mitigating the effects of culturally incompatible screening tools and bridging the gap between research and practice for AA older adults.

### ASSESSING AND HARNESSING THE POWER OF INTERGENERATIONAL INTERACTIONS IN MINDFULNESS-BASED TREATMENT GROUPS

Deanna Dragan,[1] Andrea Newman,[1] Calia Torres,[2] Keisha Carden,[3] Sarah Letang,[1] Danielle McDuffie,[1] Andrew Bontemps,[1] and Rebecca Allen,[4] *1. The University of Alabama, Tuscaloosa, Alabama, United States, 2. The University of Alabama, tuscaloosa, Alabama, United States, 3. University of Alabama, Tuscaloosa, Alabama, United States, 4. Alabama Research Institute on Aging, Tuscaloosa, Alabama, United States*

Mindfulness-based interventions (MBIs) in individual and group formats, have been shown to be effective for a variety of psychological disorders. Due to the promising evidence supporting the wide applicability of mindfulness skills, graduate student therapists were trained to deliver groups that attracted diverse individuals across the lifespan. In these groups, therapists noted how intergenerational dynamics facilitated group cohesion and allowed for increased normalization of common challenges related to practicing mindfulness skills. Therapists' prior training on cohort differences and treatment recommendations for older adults served as an important foundation to navigating these group interactions. Barriers to simultaneously collecting data and delivering intervention components were noted by the student therapists. Future research and therapist training gaps in knowledge related to effectively facilitating intergenerational groups were identified.

### SESSION 7625 (SYMPOSIUM)

### ARTS-BASED PROGRAMS FOR OLDER ADULTS FOR THEIR COGNITION, IDENTITY, COMMUNICATION, DIGNITY, AND MORE

Chair: Kyong Hee Chee
Discussant: Seoyoun Kim

This symposium takes an interdisciplinary perspective in order to address psychological and behavioral benefits of various arts-based programs for older adults including persons living with dementia. Presenters in this symposium specialize in diverse disciplines including psychology, social work, cognitive neuroscience, education, and sociology. Using novel approaches and various research methods, the presenters will speak to the specific outcomes of arts-based interventions. The positive outcomes include: 1) improved cognitive health rediscovered identities among cognitively normal older adults; and 2) better communication and reduced agitation for persons with dementia. The first two presentations concern cognitively intact participants. Brown will present a mixed-methods study that examined cognitive benefits and differential experiences of 11 older adults who participated in a 12-week, arts-based intervention. Chow will identify the themes in the 'Tree of Life' drawings of 144 Hong Kong Chinese older adults, who re-authored their sense of self transcending life challenges. Next, Mohan will discuss results from an analysis of communication exchanges among 6 older participants in a 6-week, creative group storytelling program (TimeSlips) offered in a memory care community. Halpin-Healy will explain the research-based practices used in museum programming (Arts & Minds) for persons with dementia and their care partners. She will summarize the assessments of the programs that have served approximately 500 participants over a decade. As a discussant, Kim will summarize common threads that lead to effective arts-based interventions for older adults regardless of their cognitive status. She will also highlight implications regarding the benefits of arts-based interventions in late life development.

### I'M NOT CREATIVE IN THAT WAY: OLDER ADULTS' DIFFERENTIAL EXPERIENCES OF AN ARTS-BASED INTERVENTION

Colette Brown,[1] Andrea Chirino,[2] Cristina Cortez,[3] and Cassandra Gearhart,[4] *1. University of California, Irvine, Irvine, California, United States, 2. Chapman University, Orange, California, United States, 3. University of Southern California, Los Angeles, California, United States, 4. University of Texas at Austin, Austin, Texas, United States*

A recent 12-week intervention study revealed that making conceptual art is linked to improved cognitive health among community-dwelling older adults (Brown et al., 2020). Unknown, however, is whether the intervention experience differed for participants who exhibited more versus less improvement. This pilot study examined 163 excerpts from semi-structured interviews with cognitively normal, older adult participants (N = 11, Mean age = 72.82). Using thematic analysis and data displays on Dedoose, key themes were distilled regarding intervention acceptability. Participants exhibiting less cognitive improvement more often mentioned personally connecting to topics of dementia and aging through art, but more often mentioned scheduling conflicts. Conversely, participants exhibiting greater cognitive improvement more often mentioned experiencing intellectual enrichment, but feeling insecure about their art capabilities. Novel art activities may be personally meaningful and cognitively stimulating for some participants, but emotionally frustrating for others. Future work should explore ways to optimize arts-based interventions for older participants.

### REDISCOVERY OF OLDER ADULTS' LIFE WISDOM: APPLICATION OF NARRATIVE THERAPY USING A TREE-OF-LIFE METAPHOR

Chow Oi Esther, *City University of Hong Kong, Kowloon, China*

Using narrative therapy with a "Tree of Life" metaphor, practitioners facilitated 144 Hong Kong Chinese older adults through 24 4-session groups to rediscover their preferred identity and celebrate their lives. Thematic analysis of their 'Tree of Life' drawings revealed five themes: 1) Insights about their abilities, intentions and problem-solving capabilities; 2) A sense of purpose and commitment to their preferred identities; 3) Realization of personal values and beliefs; 4) Reconnecting sense of agency and hope; and 5) Cherishing life as a journey to celebrate their wealth of wisdom for common good. Despite confronting life challenges, participants re-author their sense of self consistent to their preferred identity, and renew purpose of life through drawing and dialectic narrative conversations. These findings depict a deeper understanding of older adults' lives, and have significant theoretical and practical implications for health and social care professions to advance a constructive perspective to late life development.

### IMPROVING SOCIAL COMMUNICATION WITH TIMESLIPS AMONG PERSONS LIVING WITH DEMENTIA: RESULTS FROM A PILOT STUDY

Ranjini Mohan,[1] Kyong Hee Chee,[2] Seoyoun Kim,[2] and Olga Gerhart,[2] *1. Texas State University, Round Rock, Texas, United States, 2. Texas State University, San Marcos, Texas, United States*

Persons living with dementia (PLWD) often display declining communication and conversational skills, leading to reduced quality of life and social isolation. Given limited evidence for interventions targeting conversation skills in PLWD, we examined the transcripts of a creative group storytelling program, TimeSlips, offered in a memory care community in Central Texas. We analyzed communicative exchanges using modified rules of the 'Conversational Act Profile' (Fey, 1986). Utterances were coded for each participant's interaction 1) with the facilitator and 2) with other participants. Preliminary evidence for six participants across six sessions is reported here. While the number and type of participant-to-facilitator utterances did not show a specific pattern, five of the six participants showed increased participant-to-participant communication across sessions. These findings suggest that a creative storytelling program such as TimeSlips may promote greater communication among PLWD, thereby improving social connectedness amongst them and possibly, their quality of life.

### BEST PRACTICES IN MUSEUM PROGRAMMING FOR PEOPLE WITH DEMENTIA AND THEIR CARE PARTNERS

Carolyn Halpin-Healy,[1] and James Noble,[2], *1. Arts & Minds, New York, New York, United States, 2. Columbia University Medical Center, New York, New York, United States*

As more people are living longer with dementia, museums can play an important public health role to support individuals, families, and communities. Arts & Minds programs for people with dementia and their care partners are designed according to principles of person-centered care and adult learning theory. The approach builds on research and evaluation to provide contact with art, which has been shown to reduce apathy and soothe agitation in persons with dementia and also to reduce caregiver stress. According to our observation- and feedback-based assessments of more than 500 program participants over the past 10 years, the processes of collective looking and art making specifically address symptoms of dementia and support human dignity. This presentation addresses the growing research base on the benefits of museum programming, and the ways in which research and evaluation support best practices in museum education to address the challenge of living well with dementia.

### SESSION 7630 (SYMPOSIUM)

### FROM MICRO TO MACRO: EXPLORING PREFERENCE-BASED PERSON CENTERED CARE FROM MULTIPLE PERSPECTIVES

Chair: Katherine Abbott
Discussant: Kristine Williams

Advancing our knowledge related to honoring nursing home resident preferences is a cornerstone of person-centered care (PCC). While there are multiple approaches to providing PCC, we focus on resident preferences as assessed via the Preferences for Everyday Living Inventory (PELI). The PELI is an evidenced-based, validated instrument that can be used to enhance the delivery of PCC. In this symposium, we explore the perspectives of a variety of stakeholders including nursing home residents, staff, and the impact of preference-based care on provider level regulatory outcomes. First, we present a comparative study of preference importance among n=317 African America and White nursing home residents that found more similarities than differences between the two groups. Second, a content analysis of the responses from n=196 interviews with nursing home residents details the barriers and facilitators connected to their levels of satisfaction with their preferences being fulfilled. Third, perspectives from n=27 direct care workers explore the concept of pervasive risk avoidance to the delivery of PCC. Fourth, systems-level practices, such as shift assignments and provider schedules are identified as barriers to successfully fulfilling resident preferences from the perspectives of n=19 staff within assisted living. Our final presentation utilizes a fixed-effects panel regression analysis with n=551 Ohio nursing home providers to explore the impact of PELI use on regulatory outcomes such as substantiated complaints and deficiency scores reported in the CMS Nursing Home Compare data. Discussant Dr. Kristi Williams will integrate findings, highlighting implications for policy, practice, and future directions. Research in Quality of Care Interest Group Sponsored Symposium.

### PATTERNS OF PREFERENCE IMPORTANCE RATINGS AMONG AFRICAN-AMERICAN AND WHITE NURSING HOME RESIDENTS

Nytasia Hicks,[1] Katherine Abbott,[1] Allison Heid,[2] Kendall Leser,[1] and Kimberly Van Haitsma,[3] *1. Miami University, Oxford, Ohio, United States, 2. Ardmore, Pennsylvania, United States, 3. Penn State University, University Park, Pennsylvania, United States*

Background: The Preferences for Everyday Living Inventory (PELI) was developed to assess the psychosocial preferences of older adults receiving home care (PELI-HC) and then revised for nursing home residents (PELI-NH). While the PELI-HC has been tested to identify patterns in preference ratings by race, the PELI-NH has not. We sought to explore whether the PELI-NH tool captures differences in preference ratings of African-American and White NH residents. Methods: Preference assessment interviews were conducted with NH residents (n = 317). Analysis via a Mann-Whitney U test, results show that 46 of 72 (63.88%) a preference importance items were not statistically different between African-American and White NH residents. Additionally, African-Americans reported greater importance than White older adult NH residents in 26 of 72 (36%) preference importance items. Conclusion/Implications: It appears that the PELI-NH can test group differences in preference importance among African-American and White NH residents; implications for practice will be discussed. Part of a symposium sponsored by the Research in Quality of Care Interest Group.

### I'M COMPLETELY SATISFIED BUT…: BARRIERS AND FACILITATORS TO FULFILLING NURSING HOME RESIDENTS' PREFERENCES

Caroline Madrigal,[1] Liza Behrens,[2] Vanessa Burshnic,[3] Julie Murpy,[4] Katherine Abbott,[5] and Kimberly Van Haitsma,[6] *1. Providence VA Medical Center, Providence, Rhode Island, United States, 2. University of Pennsylvania School of Nursing, Philadelphia, Pennsylvania, United States, 3. Durham VA Health Care System, Durham, North Carolina, United States, 4. Philadelphia, Pennsylvania, United States, 5. Miami University, Oxford, Ohio, United States, 6. Penn State University, University Park, Pennsylvania, United States*

The US federal government mandates nursing homes (NHs) provide preference-based, person-centered care for residents. Yet only 2% of NHs successfully implement person-centered care. The Care Preference Assessment of Satisfaction (ComPASS) is a 16-item tool that facilitates residents' preference fulfillment (i.e., satisfaction with quality of daily care and activities). This study examines barriers/facilitators to preference fulfillment from the perspective of residents who completed ComPASS. Secondary data analysis from an R21 (NR011334-01) provided qualitative data from

196 residents across 28 NHs on why residents were satisfied (or not) with the fulfillment of their individual preferences. Most residents were female (70.4%) and white (80.1%) with a range of cognitive/physical abilities. Content analysis revealed six thematic codes classifying barriers/facilitators to preference fulfillment: resident agency, values, and physical characteristics; social support systems; staff competence; communication success; built environment; access to resources. Discussion will include implications for ameliorating barriers to preference fulfillment while meeting government mandates. Part of a symposium sponsored by the Research in Quality of Care Interest Group.

### RISKY PREFERENCES: NURSING STAFF PERCEPTIONS OF RISKS MAY IMPEDE DELIVERY OF PERSON-CENTERED CARE

Liza Behrens,[1] Kimberly Van Haitsma,[2] Ann Kolanowski,[3] Marie Boltz,[2] Mark Sciegaj,[2] Katherine Abbott,[4] and Caroline Madrigal,[5], *1. University of Pennsylvania School of Nursing, Philadelphia, Pennsylvania, United States, 2. Penn State University, University Park, Pennsylvania, United States, 3. PSU, University Park, Pennsylvania, United States, 4. Miami University, Oxford, Ohio, United States, 5. Providence VA Medical Center, Providence, Rhode Island, United States*

Nursing home (NH) staff perceptions of risks to residents' health and safety is a major barrier to honoring resident preferences, the cornerstone of person-centered care delivery. This study examined direct-care nursing staff perceptions of risk (possibilities for harm or loss) associated with honoring residents' preferences for everyday living and care activities. Participants (N=27) were mostly female (85%), had more than 3 years of experience (74%), and worked in NHs experiencing 6-12 health citations. Content analysis of 12 focus groups indicated nursing staff perceptions of risks may impede delivery of PCC. This is supported by the overarching theme: pervasive risk avoidance; and sub-themes of: staff values, supports for risk-taking, and challenges to honoring preferences. Findings will be discussed considering a newly modified risk engagement framework meant to understand and inform the clinical management of older adult preferences perceived to carry risks. Opportunities for future research will be discussed (e.g. measurement development). Part of a symposium sponsored by the Research in Quality of Care Interest Group.

### STAFF BARRIERS TO FULFILLING ASSISTED LIVING RESIDENT DAILY CARE AND ACTIVITY PREFERENCES

Tonya Roberts,[1] Jillian Parks,[2] Ella Greenhalgh,[2] Josephine Hansen,[2] Olivia Wheelis,[2] and Sara Wilke,[2] *1. University of Wisconsin-Madison, Madison, Wisconsin, United States, 2. University of Wisconsin, Madison, Wisconsin, United States*

Person-centered care (PCC), or delivery of care consistent with preferences, has been associated with improved care and quality of life for residents in long-term care (LTC). However, research has shown PCC has not been universally adopted. While general implementation barriers have been identified, little research has focused on barriers to meeting specific types of resident daily care and activity preferences. The purpose of this study was to describe LTC staff barriers to fulfilling specific types of resident preferences. A descriptive, qualitative study with 19 assisted living staff from nursing, dietary, and activities was conducted. Semi-structured interviews focused on identifying work system barriers to meeting specific types of resident preferences were analyzed using thematic analysis. Findings suggest shift assignments, staffing challenges, and facility schedules influence staff ability to meet certain types of preferences. The results suggest innovative design of shift schedules and assignments may help staff meet certain types of preferences. Part of a symposium sponsored by the Research in Quality of Care Interest Group.

### THE IMPACT OF PREFERENCE-BASED, PERSON-CENTERED CARE ON REGULATORY OUTCOMES

Xiao Qiu,[1] Katherine Abbott,[1] John Bowblis,[1] and Kimberly Van Haitsma,[2] *1. Miami University, Oxford, Ohio, United States, 2. Penn State University, University Park, Pennsylvania, United States*

The Preferences for Everyday Living Inventory (PELI) was mandated as a pay for performance indicator by the Ohio Department of Medicaid in 2015. This study explored the impacts of PELI implementation on regulatory outcomes in 2017.

The level of PELI implementation from n=551 Ohio nursing home providers between 2015 and 2017 were linked with Centers for Medicare and Medicaid Services Nursing Home Compare data. Fixed effects panel regression analyses assessed the effects of time-varying PELI implementation on 2015-2017 regulatory outcomes that could be correlated with quality of life including fines, substantiated complaints, health scores, deficiency counts and deficiency scores.

Results show a significant increase in substantiated complaints among providers that were slow adopters of the PELI. Overall, the extent of PELI implementation was not associated with regulatory outcomes.

The use of the PELI may not impact substantiated complaints suggesting further research is needed to identify person-centered outcomes of interest. Part of a symposium sponsored by the Research in Quality of Care Interest Group.

### SESSION 7635 (SYMPOSIUM)

### MEANINGFUL AGING IN THE FACE OF VULNERABILITY: PERSPECTIVES FROM THE HUMANITIES AND ARTS

Chair: Kate de Medeiros
Discussant: Desmond O'Neill

This symposium interprets GSA's 2020 leading conference theme, "Why Age Matters", as touching upon fundamental existential questions about the meaning of old age. Although meanings of aging have always been implicitly present in a variety of disciplinary gerontological studies, scholars from the humanities and arts have traditionally taken the lead in the field to provide thorough reflections and analyses about what makes later life meaningful. In this symposium, we aim to present a selection of perspectives from the humanities and arts that explore how meaningful aging can be realized in circumstances of the increasing vulnerability that inevitably accompanies old age. First, Hanne Laceulle uses a practice-theoretical philosophical framework to argue that the common assumption that vulnerability constitutes a threat to meaningfulness deserves to be nuanced, because meaning can also occur in the process of integrating vulnerability in one's life. Second, Theresa Allison, Jennie Gubner and Alexander Smith show how vulnerable older adults living with dementia and their caregivers seek meaning in daily life, adapting meaningful activities to circumstances of increasing vulnerability. Third, Kate de Medeiros and Ulla Kriebernegg discuss how a dialogue between facts and fictions, narrative and literary gerontology, can contribute in seeing vulnerability as a form of resistance. Fourth, Margaret Perkinson illustrates the power of visual images as elicitors of reflections on meaning among the older inhabitants of a Guatemalan village. Documenting villagers' own perspectives through PhotoVoice methodology underscores the fundamental importance of taking first-person perspectives into account when studying meaning.

### MEANING AND LATE-LIFE VULNERABILITY: A PHILOSOPHICAL PRACTICE-BASED APPROACH

Hanne Laceulle, *University of Humanistic Studies, Utrecht, Netherlands*

The quest for meaning constitutes one of the fundamental human existential motivations. At first glance, late life vulnerability appears to pose a threat to meaningfulness. Sources of meaning associated with certain activities or relations may seem less accessible once vulnerabilities, caused by social losses or diminishing health, start to dominate people's daily life experience. On the other hand, some theorists have argued that it is particularly in the confrontation with our human vulnerability that the need for meaningfulness is most urgently felt. This paper presents a philosophical-conceptual exploration of meaning as a dynamic, context-dependent social practice. Contrasting theoretical approaches that analyse meaningfulness and/or meaninglessness in terms of the presence or absence of certain components or qualities, a practice-based approach enhances our sensivitity for concrete ways in which people manage to find or create meaning in situations of vulnerability, thereby integrating this vulnerability in their life narratives and sense of self.

### MEANINGFUL ACTIVITIES AMONG VULNERABLE OLDER ADULTS LIVING WITH DEMENTIA

Theresa Allison,[1] Jennie Gubner,[2] and Alexander Smith,[3]
*1. UCSF Division of Geriatrics/San Francisco VA Medical Center, San Francisco, California, United States,*
*2. University of Arizona, Tucson, Arizona, United States,*
*3. University of California San Francisco Division of Geriatrics, San Francisco, California, United States*

This paper examines self-identified meaningful activities in the daily lives of 21 vulnerable older adults living with dementia and the people who care for them at home (dyads). Using ethnographic observation and interviews, we asked the dyads to identify which aspects of daily life were most meaningful and how these activities changed as dementia progressed. Results ranged from pleasure-seeking activities like cigarette smoking and eating, to spiritual or mindfulness activities like hymn-singing, prayer and tai chi. Dyads identified specific examples of the ways in which meaningful activities and meaning-making both persisted and adapted throughout the progression of dementia. Using these identifiable moments of meaning-making as a starting point for inquiry, we explore underlying questions of how to adapt to dementia progression while retaining meaning in relationships.

### FACTS AND FICTIONS: RETHINKING VULNERABILITY AND RESISTANCE IN LATE-LIFE NARRATIVES AND WHY AGE MATTERS

Ulla Kriebernegg,[1] and Kate de Medeiros,[2] *1. University of Graz, Graz, Steiermark, Austria, 2. Miami University, Oxford, Ohio, United States*

Narrative gerontology examines the experience of aging through life stories and other first-person accounts. Literary gerontology explores cultural narratives (e.g., novels, films) that link us to our own aging through stories of others, real or imagined. Our paper focuses on narrative constructions of vulnerability, resistance, subjectivity and agency in life stories, interviews and fictional texts (e.g., Margaret Atwood's short story "Torching the Dusties.") It considers how aspects of vulnerability are embedded in stories and what they reveal about the cultural construction of age and aging or what makes us vulnerable as we age. Overall, our paper highlights the socio-cultural construction of vulnerability in narratives related to age and aging, focusing on the representation of vulnerability as a form of resistance and position of strength.

### COMMUNICATING GUATEMALAN VILLAGERS' EXPERIENCE OF AGING THROUGH PHOTOGRAPHY OF LOCAL AGING ADVOCATES

Margaret Perkinson, *University of Hawaii, Honolulu, Hawaii, United States*

What does it mean to grow old in a remote Guatemalan village? How do locals perceive the experience and meaning of later life, and how can a non-local access those perceptions? Volunteers from the village church's committee on aging learned PhotoVoice methodology to document townspeople's experiences of aging. Team members reassembled to discuss reasons for selecting a particular subject, how the photos' contents related to their own lives, what the images said about aging in their village, and possible actions to consider in response. Qualitative analysis of the discussions revealed perceptions and meanings attached to family relationships, living environments, work and leisure activities, and health issues of their older subjects. The images evoked exchanges, commentary, and reflections among group members, creating an "eliciting context" in which processes of negotiated, shared meaning-making regarding later life emerged.

### SESSION 7640 (SYMPOSIUM)

### GUT MICROBIOME IN OLDER MEN: FINDINGS FROM THE OSTEOPOROTIC FRACTURES IN MEN STUDY

Chair: Michelle Shardell
Co-Chair: Lisa Langsetmo
Discussant: Ryan Demmer

There is great interest in identifying determinants and health consequences of the human gut microbiota, the dynamic population of microorganisms living in the human digestive tract. However, the role of gut microbiota in the health of older adults has received considerably less attention than that among younger or middle-aged adults. Findings among younger age groups are not necessarily generalizable to older adults due to differences in lifestyle, health conditions, and medication usage. Therefore, understanding the role of gut microbial communities in aging-related phenotypes is an emerging gerontology research priority. To fill this significant knowledge gap, the Osteoporotic Fractures in Men (MrOS) Study Microbiome Ancillary Study was conducted in 2014-2016, coinciding with the parent study's 4th clinic visit. A total of 912 men with mean aged 84.2 (standard deviation=4.0) years provided fecal samples, and 16S ribosomal RNA target gene sequencing was used to characterize the gut microbiota composition. In this symposium, we present findings on the first research projects completed with these data. Dr. Lisa Langsetmo will characterize the association between objectively measured physical activity and the composition of gut microbiota. Dr. James Shikany will present dietary patterns, another lifestyle determinant of gut microbiota. Dr. Deborah Kado will focus on a specific micronutrient, vitamin D, and its metabolites as another factor that may influence the gut microbiota in older men. Lastly, Dr. Michelle Shardell will overview the analytical challenges of microbiome research and illustrate an approach to quantify the potential role of gut microbiota composition and weight in older men.

### COMPARING ANALYTICAL METHODS FOR GUT MICROBIOME AND AGING: GUT MICROBIOTA AND BODY WEIGHT IN THE MROS

Michelle Shardell,[1] Neeta Parimi,[2] Lisa Langsetmo,[3] Eric Orwoll,[4] James Shikany,[5] Deborah Kado,[6] and Peggy Cawthon,[2] *1. University of Maryland School of Medicine, Baltimore, Maryland, United States, 2. California Pacific Medical Center, San Francisco, California, United States, 3. University of Minnesota, Minneapolis, Minnesota, United States, 4. Oregon Health & Science University, Portland, Oregon, United States, 5. University of Alabama at Birmingham, Birmingham, Alabama, United States, 6. University of California, San Diego, La Jolla, California, United States*

Gut microbiome datasets comprise microbial taxa relative abundances that necessarily sum to 1; analysis ignoring this feature may produce misleading results. We assessed 163 genera from the first batch of Microbiome Ancillary Study (n=530) stool samples and examined associations between microbiota and body weight. We compared conventional Bayesian linear regression (BLR) and network analysis to their compositional counterparts, adjusting for past weight and other covariates. Conventional BLR identified Roseburia and Dialister (positive association) and Coprococcus-1 (negative association) after multiple comparisons adjustment(P<.0125). No conventional network module was associated with weight. Using compositional BLR, men with higher Coprococcus-2 and Acidaminococcus had higher weight, whereas men with higher Coprococcus-1 and Ruminococcus-1 had lower weight (P<.05), but findings were non-significant after multiple comparisons adjustment. Two compositional network modules with respective hub taxa Blautia and Faecalibacterium were associated with weight(P<.01). Findings depended on analytical workflow; compositional analysis is advocated to appropriately handle the sum-to-1 constraint.

### THE ASSOCIATIONS BETWEEN PHYSICAL ACTIVITY AND GUT MICROBIOTA AMONG OLDER COMMUNITY-DWELLING MEN

Lisa Langsetmo,[1] Abigail Johnson,[1] Ryan Demmer,[1] Kristine Ensrud,[1] Eric Orwoll,[2] and James Shikany,[3] *1. University of Minnesota, Minneapolis, Minnesota, United States, 2. Oregon Health & Science University, Portland, Oregon, United States, 3. University of Alabama at Birmingham, Birmingham, Alabama, United States*

We determined the relationship between objectively measured physical activity (PA) and the gut microbiome among community-dwelling older men from the Osteoporotic Fractures in Men (MrOS) cohort participants at Visit 4 (2014-16). Eligible men (n=373, mean age 84 y) included participants with 5-day activity assessment and stool samples analyzed for 16S marker genes. Armband data together with sex, height, and weight were used to estimate total steps and energy expenditure. We used linear regression analysis, principal coordinate analysis, zero-inflated Gaussian models to assess association between PA and α-diversity, β-diversity, and specific taxa, respectively, with adjustments for age, race, BMI, clinical center, library size, and number of chronic conditions. There was a slight association between PA and β-diversity but no association with α-diversity. After multivariate adjustment, those who had higher step counts vs lower step counts had higher relative abundance of Cetobacterium and lower relative abundance of Coprobacillus, Adlercreutzia, Erysipelotrichaceae CC-115.

### ASSOCIATIONS OF DIETARY PATTERNS WITH THE GUT MICROBIOTA IN OLDER, COMMUNITY-DWELLING MEN

James Shikany,[1] Ryan Demmer,[2] Abigail Johnson,[2] Katie Meyer,[3] Kristine Ensrud,[2] Eric Orwoll,[4] Deborah Kado,[5] and Lisa Langsetmo,[2] *1. University of Alabama at Birmingham, Birmingham, Alabama, United States, 2. University of Minnesota, Minneapolis, Minnesota, United States, 3. University of North Carolina at Chapel Hill, Chapel Hill, North Carolina, United States, 4. Oregon Health & Science University, Portland, Oregon, United States, 5. University of California, San Diego, La Jolla, California, United States*

We investigated associations of dietary patterns with composition and diversity of the gut bacterial microbiota in 517 community-dwelling older men (mean age 84.3 y) who were participants in the Osteoporotic Fractures in Men

(MrOS) study. Eligible participants provided a stool sample and completed a food frequency questionnaire at the MrOS Visit 4 in 2014-2016. Dietary patterns were derived by factor analysis. 16S rRNA target gene sequencing was performed. Linear regression and PERMANOVA considered variation in alpha and beta-diversity by dietary pattern, and metagenomeSeq assessed taxonomic variation by dietary pattern. In multivariable-adjusted models, greater adherence to the Western pattern was positively associated certain taxa, including Alistipes, Desulfovibrio, Dorea, Eubacterium, and Ruminococcus, while greater adherence to the prudent pattern was positively associated with certain taxa, including Faecalibacterium, Lachnospira, and Paraprevotella. Dietary patterns were not associated with measures of alpha diversity; beta diversity measures were significantly associated with both Western and prudent patterns.

### VITAMIN D METABOLITES AND THE GUT MICROBIOME IN OLDER MEN

Deborah Kado,[1] Robert Thomas,[1] Lingjing Jiang,[1] John Adams,[2] Rob Knight,[1] and Eric Orwoll,[3] *1. University of California, San Diego, La Jolla, California, United States, 2. UCLA, Los Angeles, California, United States, 3. Oregon Health & Science University, Portland, Oregon, United States*

We examined the bidirectional impact of vitamin D on the composition and diversity of the gut microbiome in 567 MrOS men. Vitamin D metabolites were measured using LC-MSMS and stool sub-operational taxonomic units defined from 16S ribosomal RNA sequencing data using Deblur and Greengenes 13.8. Men's mean serum level of 25(OH)D was in the sufficient range. Faith's Phylogenetic Diversity and non-redundant covariate analyses revealed that 1,25(OH)2D explained 5% of variance in α-diversity; the other non-redundant covariates of site, race, recent antibiotic and antidepressant use explained another 6%. In β-diversity analyses using unweighted UniFrac, 1,25(OH)2D was the strongest factor assessed, explaining 2%. Random forest plot analyses identified 12 taxa, 6 in the phylum Firmicutes, positively associated with either 1,25(OH)2D and/or [1,25(OH)2D/25(OH)D] activation ratio. Higher levels of the active 1,25(OH)2D, but not 25(OH)D, were associated with butyrate producing bacteria. Men with favorable vitamin D activation profiles also had greater gut microbial diversity.

### SESSION 7645 (SYMPOSIUM)

### MULTIDIMENSIONAL BENEFITS OF WEIGHT MANAGEMENT IN OLD AGE: THE MOBILITY AND VITALITY LIFESTYLE PROGRAM

Chair: Steven Albert
Co-Chair: Elizabeth Venditti
Discussant: Barbara Nicklas

The high prevalence of overweight or obesity in older adults is a public health concern because obesity is associated with risk of mobility disability. The benefits of brief community-based lifestyle interventions that promote modest weight loss and increased physical activity are unclear. We assessed the impact of a 13-month lifestyle intervention, the Mobility and Vitality Lifestyle Program (MOVE UP), delivered by community health workers (CHW), on a variety of outcomes, including weight loss, performance-based lower extremity function (Short Physical Performance Battery, SPPB), activity, diet, and health-related quality of life (CDC U48 DP005001). The 32-session behavioral weight management intervention enrolled 303 community-dwelling adults (90.4% of those eligible), who were followed for 12 months (2015-19). Participants completed the program at 26 sites led by 22 CHWs. Participants were age (sd) 67.7 (4.1) and were mostly female (87%). 22.7% were racial minorities. The mean (sd) BMI at baseline was 34.7 (4.7). Median weight loss in the sample was 5% of baseline body weight. SPPB total scores improved by +0.31 units (p < .006), gait speed by +0.04 m/sec (p < .0001), and time to complete chair stands by -0.95 sec (p < .0001). Presenters will assess the effect of MOVE UP on activity, diet, fatigue, and health-related quality of life. A final paper examines implementation of MOVE UP and how site and CHW factors affected outcomes. Findings suggest that promoting healthier eating, weight loss, and physical activity in a community setting is an effective strategy for reducing risk of disability in older adults.

### WEIGHT LOSS IMPROVES HRQOL PHYSICAL FUNCTION AND VITALITY MORE IN BLACKS THAN WHITES

Robert Boudreau, Elizabeth Venditti, Michelle Danielson, Nancy Glynn, John Jakicic, Anne Newman, and Steven Albert, *University of Pittsburgh, Pittsburgh, Pennsylvania, United States*

Participant-reported outcomes are important. Prior MOVE UP reports show ≥5% weight loss was not significantly associated with depressive symptoms but was associated with positive SPPB physical function and the Physical Component Score of the SF-36 HRQOL scale. We examined the SF-36 subscales that showed, a priori, clinically meaningful +5.0-point increases over 13 months, the change in subscales per 5% weight loss, and variability by race. Among all participants (n =240) several subscales show significant pre-post changes [mean (SD)] but only Vitality [+5.6 (15.4)] and Physical Function [+5.0 (16.7)] meet the criterion. Blacks (n = 60) compared to Whites (n = 172) had higher baseline scores on these subscales, were less likely to lose ≥5% (31.7% vs. 59.9%), but mixed regression models indicate that those who did demonstrated a larger change on Vitality (+5.2; p<0.048) than Whites (+3.1; p<0.0003). Studying weight loss and HRQOL associations in larger minority samples is needed.

### PERCEIVED PHYSICAL FATIGABILITY IMPROVES AFTER A WEIGHT MANAGEMENT INTERVENTION

Jessica Graves, Theresa Gmelin, Robert Boudreau, Steven Albert, Anne Newman, Elizabeth Venditti, and Nancy Glynn, *University of Pittsburgh, Pittsburgh, Pennsylvania, United States*

The effects of a weight loss and physical activity (PA) intervention on improving perceived physical fatigability are unknown. We examined this question in a subset (n=79) of older adults who are obese enrolled in the 13-month Mobility and Vitality Lifestyle Program (mean□SD age 68.8±4.2 years, 83.5% female, 26.6% African American, body mass index 34.6±4.3 kg/m2). Accelerometer-assessed PA (mean/day vector magnitude) was measured with a wrist-worn triaxial GT3X+ ActiGraph for 7 full days. Perceived physical fatigability was measured using the 10-item self-administered Pittsburgh Fatigability Scale (PFS, 0-50; lower score= less fatigability). Baseline PFS was 18.7±8.5 with 69.6% having higher fatigability (PFS ≥15). At 13-months, PFS decreased by 15% (2.8 points) to 15.9±8.4 (p<0.01) and prevalence of higher fatigability declined to 60.8%. Concurrently, participants lost 6.2% of their body weight and PA increased by 2.4%. A lifestyle intervention may be effective at reducing fatigability, an important component in the age-related disablement pathway.

### QUALITY OF DIET AMONG COMMUNITY-DWELLING ELDERS PARTICIPATING IN THE MOBILITY AND VITALITY LIFESTYLE PROGRAM

Jessica Cheng, *University of Pittsburgh, Pittsburgh, Pennsylvania, United States*

Dietary choices play an important role in disease prevention both through its effect on weight and independent of it. Improving diet can be an effective means of disease prevention among older adults. Participants (n=303) were recruited from the Allegheny County, PA area and received nutritional education in group sessions led by trained community health workers over one year. Diet quality was captured at baseline and final endpoint (either 9 or 13 months) using the Rate Your Plate (RYP) instrument for assessing healthfulness of diet and includes 24 items that can be summed to generate a total quality score. The mean RYP diet quality score improved from baseline (RYP=50.87) to endpoint (RYP=54.85) (p<.001). Over the course of the intervention, 30.9% of participants made enough improvement in diet to move to a better RYP category. A community-based group intervention for older adults was effective in inducing improvements in diet quality.

### IMPLEMENTATION OF MOVE UP AND EFFECT ON OUTCOMES

Kieffer Lori, Linda Semler, Jennifer King, Brandi Boak, Elizabeth Venditti, Anne Newman, and Steven Albert, *University of Pittsburgh, Pittsburgh, Pennsylvania, United States*

The MOVE UP behavioral activation program, consisting of 32 sessions over 12 months, was delivered by trained community health workers (CHWs) at 26 sites. 300 participants completed a mean of 21.5 sessions. Change in body weight was associated with site attendance: among 9 sites with mean attendance < 70%, participants lost a mean of 5.3%; among 12 sites with 70-80% attendance, 5.6%; and among 5 sites with > 80% attendance, 9.2%. Completion of activity and diet logs followed a similar pattern (34.9%, 56.2%, and 72.7%, respectively), as did retention for 13-month outcome assessment (70%, 85%, and 88%, respectively). CHWs at the high-performing sites were more likely to have prior or current employment in weight management and fitness (90% vs. 41.7% and 44.4%), but did not differ in education, age, race, or employment by sites. CHW experience, not sociodemographics, affected outcomes.

### SESSION 7650 (SYMPOSIUM)

### TOWARDS A GREATER CONTEXTUALIZATION OF MUSIC AND THE ARTS IN AGING AND COGNITIVE DISORDERS

Chair: Desmond O'Neill

There is an increasing prominence of arts and cultural interventions related to aging and cognitive disorders in the scholarly literature and at gerontological conferences. However, the mechanisms of the salience and relevance of aesthetics, culture and leisure in the lives of older people remains unclear. One aspect which has emerged is that of aesthetic deprivation and its consequences for well-being. This symposium aims to provide perspectives from a range of researchers involved in programs of research and implementation to try to contextualize and better understand the perspectives of older people, arts practitioners and therapists the place and context of arts, culture and leisure in optimal aging.

### TOWARD A GREATER CONTEXTUALIZATION OF MUSIC AND THE ARTS IN AGING AND COGNITIVE DISORDERS

Sophie Lee,[1] Hilary Moss,[2] and Desmond O'Neill,[3]
*1. University of Limerick, Limerick, Limerick, Ireland, 2. Irish World Academy of Music and Dance, Limerick, Limerick, Ireland, 3. Trinity College Dublin, Dublin, Dublin, Ireland*

Research suggests that group music-making can improve well-being and cognitive function in people with dementia and their family carers. The importance of the music facilitator's role is recognised. However, empirical studies rarely capture their experiences and perspectives. Semi-structured interviews were conducted with three music therapists and three community musicians with specialisms in dementia care. The interviews sought to gain a detailed understanding of their work with people with dementia. Interpretative Phenomenological Analysis revealed eight super-ordinate themes: (1) benefits of music-making for people with dementia; (2) challenges of working with people with dementia; (3) involving family carers; (4) musical content; (5) impact of the facilitator; (6) developing field of Arts and Health; (7) work as a privilege; and (8) potential for misuse of music. This study provides a useful basis from which to further develop concepts for the amelioration of people living with dementia and their families.

### COMMUNITY ARTS PROGRAMMING SUPPORTS PERSONAL GROWTH AND WELL-BEING IN OLDER ADULTS

Niyati Dhokai, and Jatin Ambegaonkar, *George Mason University, Manassas, Virginia, United States*

Our research study examined how taking part in the arts compared to control affects older adults' health and well-being. 64 older adults took part in dance, music, or control workshops 2 times/week for 10 weeks. We examined participants' psychological health, social engagement, and personal growth outcomes using mixed methods during pre- and post-workshop assessments. Focus group and arts survey results revealed that participants felt ownership of new skills learned and felt engaged. Participants, especially for those in arts workshops, described having increased self-perception of creative skills resulting in a sense of personal growth, which occurred despite mind/body challenges experienced during workshops including musculoskeletal challenges, hearing impairments, and challenges retaining new information. Our observations provide avenues for future researchers to create programming that empowers older adults, as well as to utilize the participants' ongoing feedback to create participant-empowered adaptations that transcends mind/body limitations through accessible pedagogical methods.

### ARTS ENGAGEMENT PROGRAMS IMPROVES HEALTH IN COMMUNITY-DWELLING OLDER ADULTS

Jatin Ambegaonkar, and Niyati Dhokai, *George Mason University, Manassas, Virginia, United States*

We examined how different arts engagement programs compared to control affect health in community-dwelling older adults. 64 adults(71.3 + 4.6years; Dance n=23, Music n=17, Control, n=24) took part in free Dance(Ballroom), Music(Ukulele), or Control(Active social conversation) sessions 2 times/week for 10 weeks. We assessed cognition(Montreal-Cognitive-Assessment-MoCA), physical(Short-Physical-Performance-Battery-SPPB), and Health-Related Quality-of-Life(HRQoL-SF-20) 3 times: (1) before(pre), (2) at the end of 10 weeks(post-1) and (3) 1-month after intervention(post-2). Separate 3(Time)x3(Group) ANOVAs and Bonferroni-pairwise-comparisons examined changes across groups and time(p<.05). Participants' physical health improved equally across groups(p=.4) and over time(p<.001), specifically from pre(10.5 + 1.4) to post-1(10.7 + 1.3; p=.002), and pre to-post-2(11.3 + 1.0;p<.001). Participants' cognition improved equally across groups(p=.6) and over time(p<.001) from pre(26.3 + 2.8) to post-1(27.3 + 2.5; p=.002), and pre-to-post-2(27.5 + 2.5;p=.001). Participants' HRQoL remained similar over time(p=.6) and across groups(p=.7). Overall, participants' health improved after taking part in arts engagement and social conversation programs. Study findings offer insights about successful implementation of arts-engaged programs in community-dwelling older adults.

### ARTZ @ JEFFERSON: HOW ARTS-BASED EXPERIENCES SUPPORT PEOPLE WITH DEMENTIA AS MENTORS AND AID IN DIGNITY PRESERVATION

Susan Shifrin,[1] Florence Gelo,[2] and Anne Mitchell,[3]
*1. ARTZPhilly, Philadelphia, Pennsylvania, United States, 2. Drexel University, Philadelphia, Pennsylvania, United States, 3. Thomas Jefferson University, Philadelphia, Pennsylvania, United States*

ARTZ @ Jefferson positions people with dementia and care partners as authorities about their lived experiences; arts-based experiences assist them in communicating with and mentoring health-professions students about those lived experiences. Since Spring 2016, over 100 students have been mentored by people with dementia and their care partners. Their first encounters take place in museum galleries, through facilitated conversations about works of art. Over the next six to eight weeks, students and mentors build relationships through group meetings and individual conversations. Post course surveys demonstrated that nearly 100% of students indicated their increased ability to value listening and listen to others, enhanced the healthcare provider/patient relationship, and prioritized patients' life experiences. The majority of mentors noted that student interactions added to their quality of life, social engagement and sense of purpose. Preliminary outcomes suggest that arts-based experiences establish mutual respect and empathy between people with dementia and students.

### GROUP MUSIC INTERVENTION FOR PERSONS WITH DEMENTIA IN ASSISTED LIVING: A PILOT STUDY

Hongdao Meng,[1] Jennifer Bugos,[1] Debra Dobbs,[1] Soomi Lee,[1] Punam Risal,[1] Britney Veal,[2] and William Patterson,[3] *1. University of South Florida, Tampa, Florida, United States, 2. University of South Florida, Winter Haven, Florida, United States, 3. Digital Media Innovations and eLearning Enterprises, Tampa, Florida, United States*

Dementia is the third leading diagnosis among US residents in assisted living communities (ALCs), and agitation is a major challenge for residents, families, and staff. While music interventions in nursing homes and the community have generated promising findings, little evidence of acceptability or efficacy data are available in ALCs. This pilot study tested the acceptability and preliminary efficacy of a staff-led group music intervention among ALC residents with dementia (n=19). We used a mixed-methods pre-post study design. The primary outcome measure was the Cohen-Mansfield Agitation Inventory-Short Form (CMAI-SF), and facilitator focus group interviews were conducted to gain additional insight into intervention acceptability and facilitators/barriers to implementation among activity staff. Seventeen (89.5%) participants completed the intervention with a 77% overall session attendance rate. Results suggest that the intervention is well-received by management, family, and activity staff, Implications for intervention design, efficacy testing, and contextual factors related to implementation will be discussed.

### SESSION 7655 (SYMPOSIUM)

### A NEW LENS ON PHYSICAL ACTIVITY PROMOTION: CAN TECHNOLOGY BOOST EXERCISE PRESCRIPTION AND ADHERENCE?

Chair: Patricia Heyn
Discussant: Amber Watts

Adherence to exercise prescription for older adults (OAs) is a significant problem and can have a detrimental effect on key health outcomes. Exercise adherence for OAs is a multifactorial problem encompassing many factors affecting adherence such as socioeconomic status, education, physical fitness, and mental and health status. Improving exercise adherence could have a significant impact on longevity, quality of life, and health care costs. This symposium brings multiple perspectives to closely examine promising technology approaches, both in the form of models and programs. We will also discuss gaps regarding adherence to physical activity (PA) and exercise prescription for OA and the application of current publicly available technologies to boost PA adherence and compliance accordingly to the U.S. Department of Health and Human Services national standards for promoting health and preventing disease. The symposium includes five novel presentations addressing several key factors related to successful implementation of technology approaches to exercise program delivery and adherence for OAs. In addition, we will have one presentation highlighting the key factors that impact exercise prescription, compliance, and adherence. The speakers will present and address important components related to technology use with the goal to increase older adult's PA participation. The exercise programs will target key areas affecting older adult's health such as cognitive function, falls, obesity, gender, environments, and self-efficacy. Technology user-usability perspective will be presented. Current challenges and recommendations for future research will be comprehensively discussed to properly address the exercise adherence and compliance needs of our OA populations.

### PROMOTING ADHERENCE TO EXERCISE PRESCRIPTION USING MOBILE HEALTH TECHNOLOGY: LESSONS LEARNED FROM OLDER ADULTS

Zvinka Zlatar, and Camille Nebeker, *University of California, San Diego, La Jolla, California, United States*

We will present the implementation approach used to evaluate a novel mobile health (mHealth) exercise intervention designed to improve older adult's fitness and brain health. A randomized controlled trial design evaluated if the use of mHealth strategies improved walking speed in real world environments to help participants achieve current physical activity guidelines. Cognitively healthy older adults were randomized to a 12-week, unsupervised, exercise prescription condition or to a healthy aging education condition. Participants used a heart rate tracker programmed to provide haptic feedback when they fell outside of their prescribed heart rate target during an exercise session. Participant's perceptions regarding the mHealth technology device, as well as their feedback regarding the privacy of their data when using mHealth devices will be presented. Recommendations to improve the design of future mHealth exercise trials will be discussed.

### TECHNOLOGY IN EXERCISE INTERVENTIONS FOR OLDER WOMEN: ACCEPTABILITY, ADHERENCE, AND SPECIAL CONSIDERATIONS

Shannon Halloway, *Rush University, Chicago, Illinois, United States*

Women aged 65 years and older participate in less moderate-vigorous physical activity (PA) than men of this age group, which increases the risk for a myriad of chronic health problems. Interventions that utilize a lifestyle approach to increase PA in everyday life are preferred by women compared to structured exercise, but long-term adherence is a challenge. Technology (e.g., wearables, social media, computers) can be efficiently leveraged as motivational tools in lifestyle PA interventions. However, the unique needs of older women must be considered. Thus, the purpose is to examine the: (a) types of technology that were successfully integrated into existing PA intervention studies designed for older women; (b) acceptability and adherence to technological approaches by older women; and (c) additional considerations needed for special populations, including older women with chronic health problems. The efficiency and scalability of technological approaches with clinical and public health implications will also be discussed.

### PHYSIO-FEEDBACK AND EXERCISE PROGRAM (PEER) FOR SHIFTING MALADAPTIVE FALL RISK APPRAISAL

Ladda Thiamwong, *College of Nursing, University of Central Florida, Orlando, Florida, United States*

We aimed to examine the effectiveness of Physio-feEdback and Exercise pRogram (PEER) for shifting maladaptive to adaptive fall risk appraisal and determine the feedback and acceptability of the program. Forty-one older adults were assigned to either PEER intervention or attention control (AC) group. The 8-week PEER intervention consists of a visual physio-feedback, cognitive reframing, and combined group and home-based exercise led by a trained peer coach. The AC group read fall prevention brochures and continued their normal activities. BTrackS Balance Test and Fall Efficacy Scale were measured from pre- to post-intervention. About 11% of participants in the PEER group had positive shifting but none in AC group. Up to 32% of the participants in AC had negative shifting while 5.3% in the PEER group. PEER intervention facilitates a shift from maladaptive to adaptive fall risk appraisal. PEER group reported significant decreases in fall risk and high acceptability of the program.

### THE EFFECT OF A PERSONALIZED AUTOMATED EXERCISE GUIDANCE SYSTEM ON THE IMPROVEMENT OF OBESITY

Yeonsik Noh,[1] Jae-Seung Chang,[2] Ki Chon,[3] Hyungro Yoon,[2] In Cheol Jeong,[4] and In Deok Kong[2], *1. University of Massachusetts Amherst, Amherst, Massachusetts, United States, 2. Yonsei University Wonju College of Medicine, Wonju, Republic of Korea, 3. University of Connecticut, Storrs, Connecticut, United States, 4. Icahn School of Medicine at Mount Sinai, New York, New York, United States*

This study introduces a personalized automated exercise guidance system for the efficient reduction of obesity. The proposed system was composed of wearable biometric devices and exercise-machine control systems connected with the integrated database server. It was designed for providing customized exercise prescription (intensity, repetition, frequency, etc.) according to ACSM guidelines for obesity based on real-time biosignals feedback and an individual's exercise capacity. Nineteen subjects participated in evaluating the proposed system, and we found that the risk factors (body composition, hemodynamics, blood enzymes, and exercise variables) related to obesity were statistically significantly improved. Other exercise prescriptions for chronic diseases and symptoms will be able to adapt to the proposed system so that we believe it would be substantially helpful to improve geriatric diseases and symptoms by using technology-driven exercise.

### TECHNOLOGY, YOGA, AND HALLWAY WALKS TO MAINTAIN FUNCTION AND SELF-EFFICACY DURING THE HOME-BOUND MONTHS OF WINTER

Kim LeBard-Rankila, *University of Wisconsin Superior, Superior, Wisconsin, United States*

Winter months present individuals with a unique challenge, decreased daily movement that can lead to decreased physical and emotional health. Snow and ice cause hazardous walkways and driving conditions that can lead to falls and decreased travel out of the home. For those that live in states that have four seasons, when spring comes many of them have spent a large majority of their daily lives indoors for 3-5 months. This isolation can cause decreased daily movement, aggravate medical conditions, challenge healthy eating habits, and cause social isolation. Participants for this study live in a 32-apartment building, which all occupants are independent with daily living skills and are 60 years or older. The study assessed if motivational texting, yoga in the apartment's community center, and short hall-way walks can help maintain or increase self-efficacy. Flexibility, hand-grip strength, and perceived quality of life were assessed.

### SESSION 7660 (SYMPOSIUM)

### INSIGHTS GAINED FROM DEVELOPING ACADEMIC-COMMUNITY PARTNERSHIPS FOR MINORITY AGING, COMMUNITY-ENGAGED RESEARCH

Chair: Ronica Rooks
Discussant: Peter Lichtenberg

Increasingly community-engaged research, characterized by collaborations between researchers and community partners, is recognized as an important part of translating research into improved health outcomes and reduced health disparities for community participants. Training community participants to engage in some or all aspects of this research, particularly focusing on racial and ethnic minority older adults, highlights the need to understand its opportunities and challenges. With this symposium we will discuss and reflect on community-engaged and community-based participatory research approaches to community-academic partnerships with minority older adults. The first presentation addresses recruitment, retention, and training of a community advisory board of older African Americans in Michigan. The second presentation addresses a health education outreach and engagement program to improve health outcomes among older African Americans in California. The third presentation combines community engagement with survey design methods for research with older Native Hawaiian and Pacific Islander adults to improve data collection and health outcomes in this U.S. population. The final presentation examines partnerships between a hospital memory clinic, meal delivery service, research university, and low-income health clinic to improve caregiver and dementia patient outcomes for minority older adults. The symposium discussant will address opportunities, challenges, and implications of community-academic partnerships promoting minority aging.

### BUILDING AND SUSTAINING A COMMUNITY ADVISORY BOARD OF AFRICAN AMERICAN OLDER ADULTS FOR VOLUNTEER RESEARCH

Jamie Mitchell,[1] Tam Perry,[2] Vicki Johnson-Lawrence,[3] and Vanessa Rorai,[4] *1. University of Michigan, Ann Arbor, Michigan, United States, 2. Wayne State University, Detroit, Michigan, United States, 3. Michigan State University-Flint, Flint, Michigan, United States, 4. Healthier Black Elders Center, Detroit, Michigan, United States*

Older African Americans' (AA) participation in health-related research is severely limited; they are not involved in sufficient numbers and for sufficient duration to ensure the applicability of advancements in medical and behavioral health. This research participation gap exacerbates older AAs vulnerability to poor health outcomes and disparities. The Michigan Center for Urban African American Aging Research employs a progressive community-based participatory model that utilizes a structured community advisory board (CAB) of older AAs in metro Detroit to oversee the research recruitment and retention of fellow AA older adult research participants. CAB members are provided ongoing training on social and behavioral health research, supported in acting as a consultancy to outside researchers where they can be compensated for their expertise and engagement, and empowered as gatekeepers of a participant research registry of over 1000 AA older adults. This model has broad potential for advancing community engaged research with AA older adults.

### ADVOCATES FOR AFRICAN AMERICAN ELDERS: ENGAGING OLDER ADULTS IN EDUCATION AND RESEARCH

Karen Lincoln, *University of Southern California, Los Angeles, California, United States*

Advocates for African American Elders (AAAE) is an outreach and engagement program at the University of Southern California, providing culturally competent health education for older African Americans throughout Los Angeles County (LAC). Founded in 2012 to address racial disparities in health outcomes, AAAE partners with community-based agencies, government, and health plans to address the persistent growing needs of older African Americans. AAAE educates and disseminates information about healthcare policies and resources through fact sheets, educational forums, and the AAAE website. It collaborates with local healthcare providers to improve outreach, education, and care, and assesses service needs and resources via surveys in LAC. The program engages in community-based participatory research to provide real-world solutions for improving health outcomes and build community research capacity. This presentation will highlight AAAE's community-based approach to raising awareness, increasing knowledge and access to healthcare resources, and improving health outcomes for older African Americans and their families.

### COMMUNITY-ENGAGED RESEARCH OF OLDER PACIFIC ISLANDER ADULTS: RESULTS FROM THE PIHS AND THE NHPI NHIS

Sela Panapasa, *University of Michigan, University of Michigan, Michigan, United States*

Despite the well-established need to measure and address the growing issue of disparities among older minority populations, little is known about the prevalence and correlates of disability, morbidity, and mortality among older US Pacific Islander adults. This paper discusses culturally appropriate approaches for conducting evidence-based research on a representative probability sample of older Native Hawaiian and Pacific Islander adults interviewed as part of the Pacific Islander Health Study and Native Hawaiian and Pacific Islander National Health Interview Survey. Important strategies that increase response rates and respondent participation when engaging this multi-ethnic and culturally diverse special population in research are highlighted. The model describes successful methodologies that combine CBPR approaches of community engagement with more traditional survey design methods. The findings from this work emphasizes the importance of representative data on hard-to-survey populations to illustrate granular differences in health outcomes within underrepresented populations that are not reflected in national health surveys.

### WORKING WITH COMMUNITIES TO SUPPORT MINORITY CAREGIVERS: THE PRISMA HEALTH REACH EXPANSION PROJECT

Mindi Spencer,[1] Maggi Miller,[1] Diana Jahries,[2] and James Davis,[2] *1. University Of South Carolina, Columbia, South Carolina, United States, 2. Center for Success in Aging, PRISMA Health, Greenville, South Carolina, United States*

In 2019, the NIH presented the results of its two-year visioning process to advance the science of minority health and health disparities. The PRISMA Health REACH Expansion (PH-REACH E) is an innovative, community-academic partnership between a hospital memory clinic, meal delivery service, research university, and low-income health clinic. The purpose is to: 1) increase the dementia capability of community-based programs, 2) offer caregivers of persons with dementia the REACH intervention, and 3) identify and connect racial minority and/or rural residents with services to promote health and well-being in older adulthood. This presentation will detail the PH-REACH E framework and present program results, which include improved caregiver outcomes (e.g., reduced burden, increased self-efficacy, reduced depression) and enhanced dementia capability (e.g., increased dementia knowledge) of partner organizations. This program illustrates some key recommendations of the NIH – community engagement in intervention adaptation, multisectoral collaboration, and promoting systems-level change to reduce health disparities.

### SESSION 7665 (SYMPOSIUM)

### THE GEOMETRIC FRAMEWORK FOR NUTRITION: NEW INSIGHTS INTO HOW WHAT WE EAT AFFECTS HOW WE AGE

Chair: Alan Cohen
Co-Chair: David Raubenheimer

The geometric framework for nutrition (GFN) is an approach to understanding the effect of nutrition considering multiple nutrients simultaneously. Originally developed in experimental studies of insects to model how nutritional needs evolve depending on ecological context, and since extended to many taxa including non-human primates in the wild, the technique is increasingly applied to understand human health and aging. Here, we invite four varied talks showcasing the flexibility and potential of this approach from the basic biology of aging to observational human studies and clinical trials. D. Raubenheimer will give an overview of the method, its history, and its applications in aging and human health. D. Wahl will present results showing how GFN can help develop diets that recapitulate caloric restriction and its effects on brain aging. S. Das will show how GFN can be used to improve the feasibility of caloric restriction in humans without compromising its effects. Finally, A. Cohen will present results showing how GFN can be deployed in an epidemiological context and used to characterize complex interactions among large numbers of nutrients in determining health. Together, these results show that a simplistic conception of nutrition as calories is far from sufficient to understand its effects on health and aging. Evolution has shaped the nutritional needs of each species for its environment, with appropriate levels of flexibility. GFN provides an approach to capture the relevant nuance, with the results presented at this symposium but scratching the surface. Nutrition Interest Group Sponsored Symposium.

### NUTRIENT INTAKE PATTERNS PREDICT HOMEOSTATIC DYSREGULATION IN AN OLDER QUEBEC POPULATION

Alan Cohen,[1] Alistair Senior,[2] Véronique Legault,[3] David Raubenheimer,[4] Stephen Simpson,[2] Nancy Presse,[5] Pierrette Gaudreau,[6] and David Le Couteur,[2], *1. Universite de Sherbrooke, St-Denis-de-Brompton, Quebec, Canada, 2. University of Sydney, Camperdown, New South Wales, Australia, 3. Université de Sherbrooke, Sherbrooke, Quebec, Canada, 4. University of Sydney, Sydney, New South Wales, Australia, 5. University of Sherbrooke, Sherbrooke, Quebec, Canada, 6. University of Montreal, Montreal, Quebec, Canada*

The geometric framework for nutrition has largely been applied to macronutrients in experimental settings. Here, we utilize the framework to examine both macro and micronutrient intake patterns in observational human data. We used nutritional intake patterns (3x 24h recall per visit) of 1754 older Quebecers from the NuAge cohort to predict multi-system homeostatic dysregulation scores calculated from 30 biomarkers. Intermediate intake of both macro- and micronutrients was generally associated with lower dysregulation scores (i.e., better health). Furthermore, there were often nutrient-nutrient interactions, such that the optimal level of one nutrient depends on the intake level of others. However, higher protein intake was generally associated with better health, and results varied substantially across different dysregulation systems. Accordingly, even though nutrition does have important effects on health trajectories during aging, it will be challenging to arrive at population-level recommendations to fine-tune nutrient intake patterns to optimize health beyond "everything in moderation." Part of a symposium sponsored by the Nutrition Interest Group.

### NUTRITIONAL ECOLOGY, NUTRITIONAL GEOMETRY, AND AGING RESEARCH

David Raubenheimer,[1] Stephen Simpson,[2] and David Le Couteur,[2], *1. University of Sydney, Sydney, New South Wales, Australia, 2. University of Sydney, Camperdown, New South Wales, Australia*

Substantial advances have been made in understanding both evolutionary and mechanistic aspects of biological ageing, but the two areas remain poorly integrated. I suggest that a greater emphasis on ecology can help to integrate evolutionary and mechanistic research on ageing, by providing insight into the interface between biological mechanisms and the environments in which they evolved. Among the most salient aspects of the environment relevant to ageing is nutrition. And yet in the bulk of ageing research nutrition is coarsely represented as dietary restriction or caloric restriction, without consideration for which components of the diet or which energetic substrates are driving the observed effects. I show how a method developed in nutritional ecology, called the nutritional geometry framework, can help to understand the nutritional interactions of animals with their environments, by explicitly distinguishing the roles of calories, individual nutrients and nutrient balance. Part of a symposium sponsored by the Nutrition Interest Group.

### COMPARING THE EFFECTS OF LOW-PROTEIN AND HIGH-CARBOHYDRATE DIETS AND CALORIC RESTRICTION ON BRAIN AGING IN MICE

Devin Wahl,[1] David Raubenheimer,[2] Rafael de Cabo,[3] David Sinclair,[4] Stephen Simpson,[5] Samantha Solon-Biet,[6] and David Le Couteur,[5] *1. Colorado State University, Fort Collins, Colorado, United States, 2. University of Sydney, Sydney, New South Wales, Australia, 3. National Institute on Aging, Bethesda, Maryland, United States, 4. Paul F. Glenn Center for Biology of Aging Research at Harvard Medical School, Boston, Massachusetts, United States, 5. University of Sydney, Camperdown, New South Wales, Australia, 6. University of Sydney, Sydney, New South Wales, Australia*

The Geometric Framework for Nutrition (GFN) has revealed that ad-libitum low-protein, high-carbohydrate (LPHC) diets improve cardiometabolic health and extend lifespan in rodents, but it is not known whether these diets are also beneficial for brain health. Here, we utilized previous results from GFN studies and compared hippocampus biology and memory in mice subjected to 20% calorie restriction (CR) or provided ad-libitum access to several LPHC diets. RNA expression in the hippocampus of 15-month-old mice were similar between mice fed CR and LPHC diets. Nutrient-sensing proteins, including SIRT1, MTOR, and PGC1-alpha, were also influenced by diet; however, the effects varied by sex. CR and LPHC diets were associated with increased dendritic spines in dentate gyrus neurons. Mice fed CR and LPHC diets had modest improvements in the Barnes maze spatial recognition memory paradigm and novel object recognition test. LPHC diets recapitulate some of the benefits of CR on brain aging. Part of a symposium sponsored by the Nutrition Interest Group.

### DIET COMPOSITION, ADHERENCE TO CALORIE RESTRICTION, AND CARDIOMETABOLIC DISEASE RISK MODIFICATION

Sai Krupa Das,[1] Alistair Senior,[2] Rachel Silver,[3] Cheryl Gilhooly,[3] and David Le Couteur,[2] *1. Tufts University, Boston, Massachusetts, United States, 2. University of Sydney, Camperdown, New South Wales, Australia, 3. Jean Mayer USDA Human Nutrition Research Center on Aging at Tufts University, Boston, Massachusetts, United States*

Calorie restriction (CR) is a promising strategy to attenuate age-related disease risk. Higher protein diets enhance satiety but may also impair metabolic health and accelerate aging. The effect of higher protein intake on adherence to CR and cardiometabolic markers of healthspan remains unknown. We used the Geometric Framework for Nutrition to examine the association between diet composition and 1) CR adherence; and 2) cardiometabolic risk factors during a 2-year intervention. The CR group consumed higher percentage energy from protein and lower fat at 12 months compared to baseline (logit % protein=0.1; 95% CI=0.05, 0.15; logit % fat= -0.12; CI=-0.29, -0.18). Higher protein intake over the 2-year intervention was associated with higher adherence to CR. No effect of diet composition on cardiometabolic risk factors was observed. These findings suggest that dietary protein plays a critical role in adherence to CR with no adverse effects on cardiometabolic markers of healthspan. Part of a symposium sponsored by the Nutrition Interest Group.

### SESSION 7670 (SYMPOSIUM)

### THE INTERSECTIONALITY OF AGEISM

Chair: Paul Nash
Co-Chair: Tonya Taylor
Discussant: Becca Levy

Ageism is unlike any other form of prejudice in that, all things being equal, we are all at risk of experiencing it as we age. Whilst this stands true, many communities experience discrimination in other domains such as gender, race, sexuality and disability. As these communities age they are likely to experience cumulative or intersectional disadvantage due to multiple stigmatized group memberships. Where it is understood that there are negative health (cognition, gait speed, longevity, cardiovascular) and psychological (depression, stress, anxiety) outcomes associated with each form of discrimination, the often focused research is less than conclusive regarding the consequences of intersectional discrimination. Using both qualitative and quantitative methodologies from across the US, this symposium explores the impacts of ageism with regard to HIV status, disability status, race/ethnicity and LGBT identification. Exploring the intersections of ageism, each paper will highlight the challenges faced by the respective communities as well as some of the ways to address this using policy, practice and highlighting where additional research would be needed to fully understand the phenomena. Finally the discussant will summarise and collate ideas for a concluding panel discussion.

### AGEISM IN HIV RESEARCH

Tonya Taylor, *SUNY Downstate Health Sciences University, Brooklyn, New York, United States*

Sex and sexuality are important determinants of health and wellbeing across the life course. The desire and capacity for sexual intimacy and pleasure among older adults are neglected areas of research due to ageist assumptions that they no longer engage in sexual activity. These assumptions are most pronounced in HIV research, where we aggressively studied intimate details of sexual behaviors of people living with HIV until they became "old." Interest in the sexual behaviors among older adults with HIV has waned in HIV prevention, suggesting an inherent ageism within the field. We will discuss emerging new HIV and STI risks for older adults, declining trends in gerosexuality funding, HIV media campaigns targeted for older adults, and new evidence that suggest that interventions that engage older adults with HIV in conversations about sexual health, menopause, and erectile dysfunction may be an effective strategy for promoting overall successful aging

### AGEISM AND PEOPLE WITH EARLY-ONSET DISABILITY

Patricia Heyn, *University of Colorado Anschutz Medical Campus, Aurora, Colorado, United States*

People with disabilities are underrepresented in health research and usually they are explicitly excluded from research participation. There are no longitudinal studies that follows cohorts with disabilities to learn about their healthspan and aging processes. Thus, we know very little about how people with early onset disability age, and the medical conditions and health risk factors contributing to the "accelerated aging" phenomena commonly observed in this population. Our research group has been following a cohort group of individuals with pediatric onset disability since 2013. We collected pediatric and adult data from 70 persons with cerebral palsy and we evaluated key health outcomes as well as their access to care. In this session, we will present some of the key findings from our longitudinal study. We will discuss future research and key areas that needs to be addressed for us to properly move forward this urgent unmet area of aging research.

### CRITICAL INTERSECTIONALITY, AGEISM, AND INTERGENERATIONAL SUPPORTS FOREGROUNDING BLACK SGL AND GAY OLDER MEN

Angela Perone,[1] Beth Glover Reed,[2] and Danae Ross,[2]
*1. University of Michigan, Ypsilanti, Michigan, United States, 2. University of Michigan, Ann Arbor, Michigan, United States*

Using critical intersectionality frameworks, this project foregrounds how Black same-gender-loving (SGL), gay, and bisexual older men navigate complexities of interacting positionalities (e.g. race, gender, sexual orientation, HIV-status, and class). This study employs and further develops intracategorical and intercategorical analytic methods with data from eight focus groups, conducted as part of a larger collaborative project in Detroit. Data from two intragroup focus groups with Black same-gender-loving older men and six subsequent intergroup focus groups with Black and white lesbian, gay, bisexual, SGL, and queer participants of various ages revealed concerns and responses to barriers and facilitators for intergenerational support and intergenerational transfer of knowledge. Building on intersectionality frameworks of power, this research provides new insights from a vastly underrepresented and understudied community about how shifting contexts shape how experiences of oppression like racism, ageism, and homophobia interact and reveal potential opportunities for intergenerational supports moving forward.

### RELATION ORIENTATION AND AGEISM: A CROSS-CULTURAL COMPARISON BETWEEN CHINESE AND AMERICANS

Xin Zhang, *Peking University, Beijing, China*

Attitudes toward older adults were negatively associated with ageism. However, whether this association is universal or cultural specific remained unknown. On the basis of well-documented cultural difference in relation orientation between westerners and easterners, this study aimed to investigate whether participants of different cultural background would show different association between ageism and attitudes toward close vs. non-close older adults in a sample of 211 Chinese (Mean age = 33.27) and 241 American (Mean age = 34.56) younger adults. Multiple regressions were conducted, and as expected, attitudes toward older adults (of different relation orientation) were found to be associated with ageism differently in two cultures. For American participants, attitudes toward both close and non-close older adults significantly correlated with ageism, while only attitudes toward close older adults were significant predictors of ageism in Chinese sample. This result had important implications for understanding and intervening ageism with people of different culture background.

### SESSION 7675 (SYMPOSIUM)

### HIV AND AGING: TRACKING THE EPIDEMIC VIA THE 30-YEAR HISTORY OF GSA'S HIV INTEREST GROUP

Chair: Charles Emlet

The HIV and Aging Interest Group at GSA begin30 years ago. At that time, Highly Active Antiretroviral Treatments (HAART) did not exist. The Ryan White CARE act was newly enacted and older adults living with HIV/AIDS were primarily infected in later life. HIV was considered a life threatening disease. This Interest Group‘s existence has paralleled the epidemic including its challenges, innovations and rewards. The number of sessions focusing on HIV and aging has grown at GSA, with shifting emphasis toward longevity, quality of life and comorbidity. This symposium, celebrating the theme of “Turning75: Why age Matters” calls upon past and current IG conveners, including the founding convener, to review the history of the HIV and Aging interest group. Tepper discusses the group’s origins and the historical context of HIV as an emerging issue for older people. Emlet will discuss the changes in HIV care and treatment during the early2000s and how these advances were reflected in the interest group and GSA sessions. Brennan-Ing reviews significant changes between2012 through2015, when the proportion of older adults with HIV was projected to surpass50%, and the NIH Office of AIDS Working Group on HIV and Aging published its first set of recommendations. Finally, Taylor and colleagues brings us to the IG’s current activities including the importance of HIV Cohort studies and national policy highlights such as the National HIV/AIDS and Aging Awareness Day. The symposium provides a thoughtful account of the importance of this interest group. HIV, AIDS and Older Adults Interest Group Sponsored Symposium.

### THE BACKGROUND AND RATIONALE BEHIND THE ESTABLISHMENT OF THE FIRST AIDS AND THE ELDERLY GSA INTEREST GROUP

Lynn Tepper, *CDM, New York City, New York, United States*

Although HIV became known during the decade of the 1980’s, it was not until 1987 that the WHO launched the Global Program on AIDS to raise awareness, generate evidence-based policies, and provide technical and financial support to conduct research, promote NGO participation, and promote the rights of people living with HIV. It was then also that robust educational and prevention initiatives began to take place. At this time, a Columbia University study, led by Dr. Lynn Tepper, a gerontologist, initiated a study to see if the older population fully understood the disease and the practice of prevention behaviors, as they were not a specific target for AIDS education and prevention. This led to the establishment of the AIDS and the Elderly Interest Group, sponsored by GSA, in an attempt to know more about the impact of AIDS in the older population. Part of a symposium sponsored by the HIV, AIDS and Older Adults Interest Group.

### HIV AND OLDER ADULTS: EVOLVING INTO A MANAGEABLE CONDITION

Charles Emlet, *University of Washington, Tacoma, Tacoma, Washington, United States*

I was fortunate to serve at the HIV and Aging Interest Convener from 2000 until 2008 and co-convener from 2009 until 2012. During this period, we began to see the full and positive impact of Highly Active Antiretroviral Treatments creating highly sought longevity for older adults. During that time, the numbers of people over the age of 50, living with HIV, climbed from an estimated 81,103 to 330,843. HIV was identified by President Clinton as a national security threat while the CDC unveiled a new plan for HIV prevention. In 2006 the CDC released revised HIV testing recommendations for those 18-64 years of age to include opt out testing, stopping short of recommending HIV testing for older people. HIV sessions at GSA’s Annual Scientific meeting continued to broaden and evolve. Part of a symposium sponsored by the HIV, AIDS and Older Adults Interest Group.

### HIV AND AGING COMES INTO THE SPOTLIGHT: 2012 TO 2015

Mark Brennan-Ing, *Hunter College, CUNY, New York, New York, United States*

I had the privilege of serving as Principal- and Co-Convener of the HIV, AIDS and Older Adults Special Interest Group (SIG) for four years (2012 through 2015). During this era, when the proportion of U.S. older adults with HIV was projected to surpass 50%, we witnessed a number of milestones including the NIH Office of AIDS Working Group on HIV and Aging recommendations for critical research focus publication (JAIDS, 2012), the first CDC Surveillance Report on people 50 and older with HIV (2013), and the first UNAIDS report on HIV and aging (2013). During this period, the SIG was very successful in raising awareness about HIV and aging through numerous GSA presentations. Topics covered ranged from sexual health, to cognitive function, psychological well-being, social isolation, successful aging, and resilience. These presentations highlighted research findings that have been critical in developing interventions and shaping policy initiatives to support this growing population. Part of a symposium sponsored by the HIV, AIDS and Older Adults Interest Group.

### HIV AND AGING COMES INTO THE SPOTLIGHT: 2013 TO NOW

Tonya Taylor, *SUNY Downstate Health Sciences University, Brooklyn, New York, United States*

I have had the pleasure of serving as Co-Convener of the HIV, AIDS and Older Adults Special Interest Group (SIG) for seven years (2013 to now). During this time, we witnessed an increase in NIH funding opportunities, most notable was the Multidisciplinary Studies in HIV/AIDS and Aging FOA. NIH also renewed its support for two prominent longitudinal HIV cohort studies (now knowns as the Combined Cohort Study) because of their unmatched ability to provide insight on the effects of HIV infection and aging. Individuals older than 50 years of age are now included in AIDS and HIV prevention clinical trials. And, in 2008 the AIDS Institute launched the first National HIV/AIDS and Aging Awareness Day (Sept 18th). During this period, the SIG continued to raise awareness about HIV and aging through sponsored symposia and Webinars, and participants for the SIG participated in the first HIV and aging GSA Momentum Discussion. Part of a symposium sponsored by the HIV, AIDS and Older Adults Interest Group.

### SESSION 7680 (SYMPOSIUM)

### EVIDENCE-BASED EXERCISE INTERVENTIONS AND FITNESS TECHNOLOGIES FOR OLDER ADULTS WITH INTACT OR IMPAIRED COGNITION

Chair: Patricia Heyn
Co-Chair: Stacy Andersen
Discussant: Walter Boot

Effective exercise training and regular physical activity (PA) practice are important for healthy aging and are key modalities for chronic disease management. Approaches that enhance exercise prescriptions and adherence for older adults should be innovative and based on evidence. Thus, the exercise prescription should be tailored accordingly to the person's health status, functional capacity, and living arrangements. To properly achieve the desirable health outcomes, the PA should be performed at higher intensity, greater frequency, and longer duration. National PA Guidelines recommend at least 150 minutes of moderate-intensity aerobic activity or 75 minutes of vigorous-intensity aerobic activity and at least two days of muscle-strengthening activities per week. Each prescribed modality (aerobic exercise, strength training, flexibility/stretching exercises, and balance training) has unique benefits. Therefore, it is critical to investigate and integrate novel techniques and tools to ensure adherence to the recommended PA guidelines. One of the presentations in this symposium, will highlight the research that has been evaluating for the last 15 years the effectiveness of the exercise randomized trial for older adults with/out cognitive impairments. The second presentation will introduce a novel technology paradigm designed to improve guidelines for exercise training as well as our understanding of how technology may motivate exercise behavior for older adults. The last presentation will discuss the design and methodological flaws that impact the outcomes of the exercise intervention by addressing the interpretations of non-findings and key factors affecting the desirable outcomes.

### EVIDENCE-BASED EXERCISE RECOMMENDATIONS FOR OLDER ADULTS WITH COGNITIVE IMPAIRMENTS

Patricia Heyn,[1] Alex Tagawa,[2] Ahmed Negm,[3] and Pallavi Sood,[4], *1. University of Colorado Anschutz Medical Campus, Aurora, Colorado, United States, 2. Children's Hospital Colorado, Aurora, Colorado, United States, 3. University of Alberta, Edmonton, Alberta, Canada, 4. University of Florida, Gainesville, Florida, United States*

Since the publishing of our meta-analysis evaluating the effects of randomized exercise trials on cognitive function of Older Adults with Cognitive Impairments (OAwCIs) (Heyn et al 2004), several meta-analysis reviews were published addressing similar question. We currently appraised this evidence and preliminary synthesis of twelve, well-designed meta-analysis reports resulted in 193 RCTs and 15,614 participants over the age of 65 years old diagnosed with MCI or Alzheimer's disease (AD). Exercise prescription paradigms averaged 156 minutes per week for 20-week. The combined cognitive function outcome mean effect size was medium; 0.67 (0.06-1.34 95% CI). Grounded in this unique umbrella study results, sustained and prolonged exercise training might provide an effective intervention for the maintenance or enhancement of cognitive function for MCI and AD. This comprehensive meta-analysis umbrella offers valuable and strong exercise recommendations for OAwCIs. This study results will be of great significance to professionals involved in the care of OAwCIs.

### DEVELOPING AN EXERGAME INTERVENTION TO PREVENT ALZHEIMER'S DISEASE

Fang Yu,[1] Dereck Salisbury,[2] and Tom Plocher,[3] *1. Arizona State University, Minneapolis, Minnesota, United States, 2. University of Minnesota, Minneapolis, Minnesota, United States, 3. Moai Technologies, Maple Grove, Minnesota, United States*

Delaying the onset of Alzheimer's Disease by five years could save the U.S. ~$89 billion by 2030. Aerobic exercise and cognitive training are two promising interventions for AD prevention and the two together may have a synergistic cognitive effect than either alone. The purposes of this study were to develop an integrated virtual-reality cognitive training (VRCT) and cycling intervention known as exergame and test its feasibility in older adults with subjective cognitive decline (SCD). The VRCT included grocery shopping from a list, flower shopping from a list, dinnerware sorting, book sorting, and postage estimation. Twelve participants enrolled in the 1-month program (12 sessions) achieved 81.2% session adherence and 81.4% adherence to the exercise prescription. The exergame was well accepted by 75% of the participants and 100% were satisfied with the exergame quality and delivery. To conclude, exergame is a flexible intervention that is feasible for older adults with SCD.

### SELF-DETERMINATION THROUGH TECHNOLOGY: UNDERSTANDING PHYSICAL ACTIVITY ENGAGEMENT FOR OLDER ADULTS

Lyndsie Koon,[1] Sean Mullen,[2] and Wendy Rogers,[3], *1. University of Kansas, Lawrence, Kansas, United States, 2. University of Illinois at Urbana-Champaign, Urbana, Illinois, United States, 3. University of Illinois at Urbana-Champaign, Champaign, Illinois, United States*

Despite the known health benefits of exercise, only 30% of older adults (65-75 years) and 18.5% (85 years+) meet the recommendations for exercise. Barriers include difficulty accessing facilities, and lack of motivation and social support. Research results indicate that exercise adoption and adherence is higher among older adults when basic psychological needs are met. Technologies (e.g., exergames, activity trackers) have the potential to satisfy the three basic needs as indicated by the Self-Determination Theory. Technology may satisfy a user's need for autonomy by offering different activities to choose from (biking versus resistance training) or intensity and duration options. They may promote competence by allowing for individualized goal setting and tracking. Technologies have the potential to promote relatedness through virtual instruction, subsequently removing the accessibility barrier. The application of this theory provides design guidelines for exercise technologies and a greater understanding of how technology may motivate exercise behavior for older adults

### LESSONS LEARNED FROM NONSIGNIFICANT FINDINGS IN EXERCISE WITH INDIVIDUALS WITH DEMENTIA

Nicole Dawson,[1] and Heather Menne,[2] *1. University of Central Florida, Orlando, Florida, United States, 2. RTI International, Washington, District of Columbia, United States*

The National Institute on Aging recognizes the importance of identifying promising non-pharmacological interventions (NPI) to promote health in individuals with Alzheimer's disease and related dementias. Several systematic reviews have been completed investigating exercise in this population resulting in mixed evidence regarding efficacy across functional domains. It is critical to investigate the methodological factors from the original interventions for a true understanding of these findings as to not outright dismiss exercise as beneficial. One example is Ohio's replication of Reducing Disability in Alzheimer's Disease (n=508), which resulted in no significant improvements in physical performance for individuals with dementia ((gait speed (p=.81), balance (p=.82), functional reach (p=.58)). In this investigation, along with many others, researchers were not guided by key principles of exercise science leading to critical intervention design and methodological flaws. Thus, exercise interventions for individuals with dementia need to include interpretations of non-findings and report key factors affecting the outcomes.

### SESSION 7685 (SYMPOSIUM)

### CAUSES AND MECHANISMS UNDERLYING DISPARITIES AND TIME TRENDS IN ALZHEIMER'S DISEASE AND RELATED DEMENTIAS

Chair: Igor Akushevich
Co-Chair: Carl Hill
Discussant: Konstantin Arbeev

The objective of the Symposium is to make progress in understanding the causes and mechanisms of health-related disparities in Alzheimer's disease, related dementias and other prominent age-related diseases. Topics will cover a range of academic and administrative topics including: i) partitioning analysis of disparities and time trends in Alzheimer's Disease and Related Dementia; ii) a structural model approach to ethnic disparities in dementia and its assessment; iii) traumatic brain injury and dementia in the Medicare population: differences in genotype and phenotype-related risk between veteran and non-veteran subsets; iv) geographic disparities in county-level prevalence of Alzheimer's disease across the United States; and v) the role of comorbidities in the geographic disparities of AD/ADRD mortality. A focus will be made on evaluating patterns of race/ethnicity and geographic health disparities as well as changes in time trends in AD/ADRD prevalence and mortality; identifying the causes and describing the mechanisms of these respective processes, and demonstrating how they can be identified in studies using established administrative data resources such as Medicare claims databases; and demonstrating how innovative analytic approaches such as partitioning analyses, structural model approaches, and methods of latent data analyses can be used in conjunction with empirical and regression approaches to uncover previously overlooked or understudied aspects in this area of research. Analyses of such increasingly available large health datasets provides an opportunity to obtain nationally representative results based on individual-level measures that reflect the real care-related and epidemiological processes generating disparities and time trends in AD/ADRD health outcomes.

### PARTITIONING ANALYSIS OF DISPARITIES AND TIME TRENDS IN ALZHEIMER'S DISEASE AND RELATED DEMENTIA

Igor Akushevich, *Duke University, Durham, North Carolina, United States*

This study uses Medicare data to i) identify age patterns, time trends, and race/geography-related disparities in prevalence and mortality of AD/ADRD; ii) apply partitioning methodology to separate out trends in causal components including incidence and survival, and iii) expand the method for analysis of disparities to identify the magnitude and trends in causal components of race-related disparities in AD/ADRD. Analysis shows that the trend in AD/ADRD incidence explains up to 70% of the observed changes in AD prevalence and mortality and is the main contributor to the differences in race-specific prevalence (higher for African Americans (AA)). Differences in race-specific incidence explain up to 80% of the disparity while 20% are due to difference in survival. This indicates that for AAs, incidence is worse but survival is better. This is confirmed by direct evaluations of hazard ratios: AD incidence HR for AA is 1.50(1.46,1.54) and 0.93(0.91,0.96) for survival after AD diagnosis.

### A STRUCTURAL MODEL APPROACH TO ETHNIC DISPARITIES IN DEMENTIA AND ITS ASSESSMENT

Donald Royall, *UTHSCSA, San Antonio, Texas, United States*

Ethnicity complicates the assessment of dementia and its biomarkers in the Texas Alzheimer's Research and Care Consortium (TARCC). Its effect can be mitigated by the construction of latent variables in a structural equation model (SEM) framework. We have developed a dementia-specific phenotype, i.e. "δ" (for "dementia) by that approach. δ provides a continuously distributed dementia severity measure that may be resistant to ethnicity effects. We propose to test the impact of Mexican-American (MA) ethnicity in TARCC data [N = 3502; MA = 1313; Non-Hispanic Whites (NHW) = 2189]. Significant structural associations between observed cognitive performance, δ and δ's serum protein biomarkers will be tested for ethnicity effects by CHI SQ differences across ethnicity stratified models. Observed clinical variables can be impacted by demographic effects. Those can lead to presumed disparities in clinical outcomes or biomarkers. Latent variables have potential to mitigate demographic effects, refute some perceived disparities and confirm others.

### TRAUMATIC BRAIN INJURY AND DEMENTIA IN MEDICARE POPULATION: DIFFERENCES IN RISK BETWEEN VETERANS AND NON-VETERANS

Arseniy Yashkin, *Duke University, Durham, North Carolina, United States*

The aim of this study was to assess differences in the effect of traumatic brain injury (TBI) on the onset of Alzheimer's disease (AD) and other dementias between veteran and non-veteran respondents of the Health and Retirement Study as well as to measure the sensitivity of these differences to the introduction of controls for groups of demographic, medical co-morbidity and polygenic risk scores reflecting AD hallmarks. Using the Fine-Gray proportional hazards model we found that TBI was a strong predictor of dementia in community dwelling residents age 65+: for AD associated risk was 181% [Hazard Ratio (HR): 2.81; CI:2.05-3.86] sample-wide and 142% [HR: 2.42; CI:1.31-2.46] in veteran males. Effect magnitude decreased with the addition of risk-related control variables but remained associated with significantly increased risk. Large differences in risk were observed between veteran and non-veteran males for AD, vascular dementia, senile dementia, and dementia with Lewy Bodies

### GEOGRAPHIC DISPARITIES IN COUNTY-LEVEL PREVALENCE OF ALZHEIMER'S DISEASE ACROSS THE UNITED STATES

Mackenzie Fowler, Michael Crowe, and Richard Kennedy, *University of Alabama at Birmingham, Birmingham, Alabama, United States*

While national and state estimates of the prevalence and incidence of AD are available, estimates across finer geographic regions offer an opportunity to tailor programs to the needs of the local population. Previously, we estimated prevalence and incidence of AD at the county level across the continental United States and found that estimated prevalence of AD varied more than threefold across counties, predominantly in the Southeastern and Midwestern United States. We also observed "islands" of low AD within regions with high AD, and vice versa. We update these findings by examining changes in projected prevalence of AD over time, and comparing projected prevalence of AD to prevalence of AD diagnoses in Medicare. We also examine regional variation in provider specialty patterns and racial differences across counties as possible explanatory factors. Understanding small-area geographic disparities in prevalence will be critical for addressing practice variation in the prevention and diagnosis of dementia.

### GEOGRAPHIC DISPARITIES OF COMORBIDITIES IN MORTALITY OF PATIENTS WITH ALZHEIMER'S DISEASE

Julia Kravchenko, *Duke University, Durham, North Carolina, United States*

Comorbidities can contribute to the gap in Alzheimer's disease (AD) mortality between the East and West coast U.S. Using Multiple-Cause-of-Death and 5%-Medicare data, we analyzed age-adjusted (65+) mortality rates from AD in two Health and Human Services (HHS) areas with opposed mortality patterns in 2010-2018: 150.9±0.6/100,000 in HHS2 (NJ,NY) and 363.1±1.5/100,000 in HHS10 (AK,ID,OR,WA). Co-existing diabetes, heart failure, cerebrovascular, digestive, and kidney diseases significantly contributed to this gap, while contribution of heart diseases reduced its magnitude. An unexpectedly strong effect (higher rate in HH10 by a factor of 3-5) was identified for symptoms/signs that are not from identified specific diseases, life-threatening injures/falls and other external causes that are common among patients with AD. We concluded that although contributions of comorbidities with well-developed treatment guidelines (e.g., heart disease) to geographic disparities in AD mortality were small, the disparities can be generated by unexpected comorbidities including diseases with poorly defined conditions.

### SESSION 7690 (SYMPOSIUM)

### COMPLEMENTARY AND INTEGRATED STUDIES OF LONGEVITY AND HEALTHY AGING

Chair: Steven Cummings
Co-Chair: Thomas Perls
Discussant: Evan Hadley

Five NIH-funded studies, the Long Life Family Study (LLFS, U19), the Longevity Consortium (LC, U19), Longevity Genomics (U24), and Protective Omics Profiles in Centenarians (UH2) work together to triangulate on mechanisms of extreme longevity and healthy aging with the ultimate goal of discovering predictors and targetable pathways. Linkage analyses by LLFS identified extremely strong genetic linkage peaks for cross-sectional as well as longitudinal trajectory rates-of-change phenotypes. Deep sequencing suggests these peaks are driven by rare, protective variants in selected pedigrees. In cross-species studies (UH2, LC), genomics, metabolomics and proteomics are used to exploit many-fold variances in natural life spans to discover protective mechanisms that explain some of these differences. Proteome analysis reveal several longevity-related proteins such as Cip1/p21, FOXO3, TOP2A, AKT1, RICTOR, INSR and SIRT6 harboring post translational modification sites that preferentially appear in short- versus long-lived species. The U24 effort developed a tool using genetically-mediated gene expression to prioritize genes for longevity translational efforts. We found that BLOC1S1 was associated with longevity and protection from atrial fibrillation and hearing loss without being associated with adverse events. This novel target is undergoing functional characterization. A proteomic assay (4,131 proteins, Somascan) annotated by genome-wide association study results in a total of 1,797 centenarians and 3,685 controls divided into independent discovery and replication sets, discovered significant and replicated over-expression (thus, pro-longevity) of BIRC2 and under-expression of APOB in carriers of the APOE ε-2 allele. A novel protein signature of rs2184061 (CDKN2a/CDKN2B in chromosome 9) was also associated with slower aging.

### THE LONG LIFE FAMILY STUDY: SEQUENCING EXCEPTIONAL PEDIGREES FOR RARE PROTECTIVE VARIANTS

Michael Province,[1] Kaare Christensen,[2] Stephanie Consentino,[3] Joseph Lee,[3] Anne Newman,[4] Thomas Perls,[5] Bharat Thyagarajan,[6] and Joseph Zmuda,[4]
*1. Washington University School of Medicine, SAINT LOUIS, Missouri, United States, 2. University of Southern Denmark, Odense C, Syddanmark, Denmark, 3. Columbia University, New York, New York, United States, 4. University of Pittsburgh, Pittsburgh, Pennsylvania, United States, 5. Boston University School of Medicine, Boston,*

*Massachusetts, United States, 6. University of Minnesota, Minneapolis, Minnesota, United States*

The Long Life Family Study (LLFS) has longitudinally measured key aging phenotypes on 4,953 participants (539 pedigrees) in the USA and Denmark selected for exceptional familial longevity. On average, both generations of the LLFS sample are healthier than average for their age/sex, for many phenotypes. However, the pedigrees are heterogeneous, with different families showing familial clustering of protection for different phenotypes. Linkage analyses identified extremely strong genetic linkage peaks for many cross-sectional as well as longitudinal trajectory rates of change phenotypes. These peaks are NOT explained by GWAS SNPs (either measured or imputed). Pedigree specific HLODs and preliminary deep sequencing suggests that these peaks are driven by rare, protective variants running in selected pedigrees. Whole Genome Sequencing, a third longitudinal visit, and extensive OMICs (transcriptomics, epigenomics, metabolomics and proteomics) will help us resolve the mechanisms behind these protective genetically linked variants, and could illuminate new biology and enable new therapeutics.

### OMICS OF LONG-LIVED MAMMALS AND LINKS TO HUMAN CENTENARIANS

Gregory Tombline,[1] Jonathan Gigas,[1] Matthew Simon,[1] Yousin Suh,[2] Andrei Seluanov,[1] and Vera Gorbunova,[1] *1. University of Rochester, Rochester, New York, United States, 2. Columbia University, New York, New York, United States*

Mammalian species differ up to 100-fold in their aging rates and maximum lifespans. Long-lived mammals appear to possess traits that extend lifespan and healthspan. Pro-longevity mechanisms are complex traits afforded by connections between metabolism and protein functions that are unlikely to be predicted by genomic approaches alone. Thus, metabolomics and proteomics studies are required to understand the mechanisms of longevity. Sirtuin 6 will be presented as an example of a protein that evolved enhanced enzymatic function in long-lived species and also demonstrates enhanced activity and unique alleles in human centenarians. Proteome analysis reveal several longevity related proteins such as Cip1/p21, FOXO3, TOP2A, AKT1, RICTOR, INSR and SIRT6 harboring PTM sites that preferentially appear in either short- or long-lived species. The prospects of enhancing life expectancy and healthspan of humans by altering metabolism and proteoforms with drugs that mimic changes observed in long-lived species will be discussed.

### IDENTIFYING TRANSLATIONAL LONGEVITY TARGETS WITH GENETICALLY MEDIATED TRANSCRIPTOME-WIDE ASSOCIATION STUDIES

Daniel Evans,[1] Steven Cummings,[1] and Nicholas Schork,[2], *1. California Pacific Medical Center, San Francisco, California, United States, 2. Translational Genomics Research Institute, Phoenix, Arizona, United States*

We hypothesized that trait associations with genetically mediated gene expression could be used to screen for genes that are good candidates for translational studies of longevity. We compiled a collection of genetically-mediated transcriptome-wide association studies using 33 traits and outcomes from large-scale, publicly-available GWAS meta-analysis results. The traits/outcomes were grouped within eight categories (aging, anthropometric, cardiovascular, inflammation, lung function, metabolic, musculoskeletal, and neurological). To test the utility of this approach, we examined trait associations with the drug target of statins, and we correctly identified known therapeutic effects and adverse events of statins. Specifically addressing the hypothesis, we examined a collection of candidate longevity-associated genes and identified one gene associated with lifespan that appears to also be associated with protection from atrial fibrillation and hearing impairment without being associated with adverse events. This screening approach can be used to prioritize gene targets for longevity translational efforts.

### EFFECT OF LONGEVITY GENETIC VARIANTS ON THE MOLECULAR AGING RATE

Paola Sebastiani,[1] Anastasia Gurinovich,[1] Zeyuan Song,[1] William Zhang,[2] Stefano Monti,[1] Sofiya Millman,[2] Nir Barzilai,[2] and Thomas Perls,[3], *1. Boston University, Boston, Massachusetts, United States, 2. Albert Einstein College of Medicine, Bronx, New York, United States, 3. Boston University School of Medicine, Boston, Massachusetts, United States*

We conducted a genome-wide association study of 1317 centenarians from the New England Centenarian Study and 2885 controls using >9M genetic variants. The most significantly associated variants were correlated to 4131 serum proteins in 224 study participants. The genetic and protein associations were replicated in a genome-wide association study of 480 centenarians and ~800 controls of Ashkenazy Jewish descent and a proteomic scan of approximately 1000 participants of the same study. The analysis replicated a protein signature associated with APOE genotypes and confirmed strong overexpression of BIRC2 ($p < 5E-16$) and underexpression of APOB in carriers of the APOE2 allele ($p< 0.05$). The analysis also discovered and replicated associations between longevity variants and slower changes of protein biomarkers of aging, including a novel protein signature of rs2184061 (CDKN2a/CDKN2B in chromosome 9). The analyses show that longevity variants correlate with proteome signatures that could be manipulated to discover healthy aging targets.

### SESSION 7695 (SYMPOSIUM)

### POLICY SERIES: INTERDISCIPLINARY PUBLIC POLICY DISCUSSION

Chair: Brian Lindberg
Discussant: Linda Harootyan

Aging and health care public policy in Washington can be driven and influenced by the work of GSA researchers, educators, and practitioners from across the nation. This session will examine and explore public policy priorities from an interdisciplinary perspective and consider opportunities for communicating these policies with key policymakers. This session is an interdisciplinary look at policy issues in aging with the speakers representing views from the six sections of GSA. This session, organized by the GSA Public Policy Committee, will provide both GSA section leadership and attendees an opportunity to have an open dialogue on important public policy issues of significance in the field of aging. The session discussant will help to facilitate a robust discussion of the presentations by speakers. Organized by the GSA Public Policy Committee, this dialogue will benefit the work of the Committee in 2021.

### PUBLIC POLICY ADVISORY COMMITTEE PERSPECTIVE

Linda Harootyan, *Harootyan*
*2. , LLC, Wilmington, North Carolina, United States*

### SOCIAL RESEARCH, POLICY AND PRACTICE SECTION PERSPECTIVE

Bob Harootyan, *Harootyan*
*2. , LLC, Wilmington, North Carolina, United States*

### ACADEMY OF GERONTOLOGY IN HIGHER EDUCATION PERSPECTIVE

Dana Bradley, *University of Maryland Baltimore County, Baltimore, Maryland, United States*

### BEHAVIORAL AND SOCIAL SCIENCES SECTION PERSPECTIVE

Eileen Crimmins, *University of Southern California, Los Angeles, California, United States*

### BIOLOGICAL SCIENCES SECTION PERSPECTIVE

Matt Kaeberlein, *University of Washington, Seattle, Washington, United States*

### HEALTH SCIENCES SECTION PERSPECTIVE

George Kuchel, *University of Connecticut, Farmington, Connecticut, United States*

### EMERGING SCHOLAR AND PROFESSIONAL ORGANIZATION PERSPECTIVE

Darina Petrovsky, *University of Pennsylvania, Philadelphia, Pennsylvania, United States*

### SESSION 7700 (SYMPOSIUM)

### BEHAVIORAL HEALTH AND LEARNER-CENTERED OUTCOMES IN GERIATRIC PRIMARY CARE OR COMMUNITY SETTINGS

Co-Chair: Rebecca Allen
Co-Chair: Anne Halli-Tierney

This symposium presents data from interdisciplinary behavioral health training and research conducted in primary geriatrics care or community settings in the Deep South. The first paper describes mixed-method learner-centered outcomes from interprofessional education case sessions. Survey and qualitative data revealed the most important experiential learning derived from collaboration, problem solving, and learning about various disciplines' professional roles. The second paper presents longitudinal patient cognitive outcome data from the primary care, outpatient geriatrics clinic in which most of these interprofessional learners learn. Results show that only 26.2% of patients had scores indicating cognitive functioning within normal limits; 32.6% had scores indicative of mild neurocognitive disorder, and 41.2% had scores indicative of dementia. Over 30% of patients reported clinically significant levels of depression or anxiety, and 16.5% of patients reported at least one indicator of hazardous alcohol use at their baseline assessment. The third paper demonstrates that psychological inflexibility is associated with depression and anxiety at baseline, and that symptoms of depression and anxiety do not change one year later. The fourth and final paper considers the impact of hearing loss on quality of life in community-dwelling older adults. Effect size calculations indicated that adults with hearing loss who lived in the most rural regions of Alabama, had lower reported QOL scores than their counterparts who had hearing within normal limits This symposium will show why age matters in behavioral health training. Mental Health Practice and Aging Interest Group Sponsored Symposium.

### BEHAVIORAL HEALTH SCREENING IN GERIATRIC PRIMARY CARE IN THE DEEP SOUTH

Rebecca Allen,[1] Anne Halli-Tierney,[2] Dana Carroll,[3] Amy Albright,[4] Deanna Dragan,[2] Gregg Bell,[5] and Brian Cox,[1], *1. Alabama Research Institute on Aging, Tuscaloosa, Alabama, United States, 2. The University of Alabama, Tuscaloosa, Alabama, United States, 3. Auburn University Harrison School of Pharmacy, Tuscaloosa, Alabama, United States, 4. University of Alabama, Northport, Alabama, United States, 5. The University of Alabama College of Community Health Sciences, Tuscaloosa, Alabama, United States*

Behavioral health screening by interprofessional teams practicing in outpatient geriatric primary care improves identification of patient cognitive functioning and emotional needs. On average, geriatrics clinic patients who consented to participate in research (N = 209; 74% women; 16.6% African American) were 76.7 years old. Patients had an average of 5.83 medical diagnoses. Only 26.2% of patients had scores indicating cognitive functioning within normal limits; 32.6% had scores indicative of mild neurocognitive disorder, and 41.2% had scores indicative of dementia at their baseline visit. Over 30% of patients reported clinically significant levels of depression or anxiety, and 16.5% of patients reported at least one indicator of hazardous alcohol use. Five-year longitudinal data analysis reveals multiple patient profiles. Behavioral health screening in primary geriatrics clinic care may help identify patient cognitive and emotional needs across time. Part of a symposium sponsored by the Mental Health Practice and Aging Interest Group.

### INTERPROFESSIONAL EDUCATION THROUGH CASE STUDY: LEARNERS' ATTITUDES ON INTERPROFESSIONAL EDUCATION

Anne Halli-Tierney,[1] Rebecca Allen,[2] Dana Carroll,[3] and Robert McKinney,[1] *1. The University of Alabama, Tuscaloosa, Alabama, United States, 2. Alabama Research Institute on Aging, Tuscaloosa, Alabama, United States, 3. Auburn University Harrison School of Pharmacy, Tuscaloosa, Alabama, United States*

Interprofessional education case sessions allow learners to apply discipline-specific knowledge to real-life scenarios through thorough facilitated discussion of a patient case.

Our interprofessional case discussion was implemented for learners to develop care plans for complex geriatric patients; learners have intentional time to learn with, from and about each other's roles in geriatric care. All learners receive the case and work through it from their discipline's perspective, then join a facilitated group discussion to develop collaborative care plans. Participants were surveyed using the ICAS and qualitative comments about perceptions of interprofessional learning, and most interprofessional (medicine, pharmacy, psychology and social work) learners found the sessions to be educational. Themes emerging from qualitative analysis about what was most educational were "different professional approaches", "professional roles", "collaboration" and "problem solving". Typically, learners were unable to identify "least educational" components to the activity. Overall feedback from learners aligns with the goals of interprofessional education. Part of a symposium sponsored by the Mental Health Practice and Aging Interest Group.

### GERIATRICS CLINIC PRIMARY CARE: SCREENING FOR MENTAL HEALTH AND PSYCHOLOGICAL FLEXIBILITY

Monica Scicolone, *The University of Alabama, Tuscaloosa, Alabama, United States*

At baseline assessment, average level of symptoms for both depression (M = 3.75) and anxiety are low (M = 1.65). OLS regression analyses at baseline revealed psychological inflexibility ($\beta$ = .474, p < .001) and anxiety symptoms ($\beta$ = .334, p < .001) were significantly associated with depressive symptoms, and psychological inflexibility ($\beta$ = .331, p = .002) and depression symptoms ($\beta$ = .381, p < .001) were significantly associated with anxiety symptoms. These associations remained at year two in data collection. As of March 2019, there are 88 individuals with time two data for depressive symptoms (M = 2.99), and 79 individuals with time two data for anxiety symptoms (M = 1.34). Repeated measures ANOVA revealed that neither mean scores for depression nor anxiety changed significantly from time one to time two. As data collection continues into year three, multilevel modeling will be used to analyze longitudinal data. Part of a symposium sponsored by the Mental Health Practice and Aging Interest Group.

### GEOGRAPHICAL RESIDENCY AND QUALITY OF LIFE IN ADULTS WITH AND WITHOUT HEARING LOSS

Marcia Hay-McCutcheon,[1] and Xin Yang,[2], *1. Audiology, Tuscaloosa, Alabama, United States, 2. The University of Alabama, Tuscaloosa, Alabama, United States*

There is an increased interest in the impact that hearing loss has on general well-being, including overall quality of life (QOL). The Quality of Life Inventory (QOLI), the Charlson Comorbidity Index and an Accessibility to Health Care questionnaire were administered to 108 participants. For adults with hearing loss who did not have access to hearing health care, lower QOL scores were reported compared to those with access to hearing health care, but this finding was not significant. Effect size calculations indicated that adults with hearing loss who lived in the most rural regions of Alabama, had lower reported QOL scores than their counterparts who had hearing within normal limits. Finally, those with higher incomes, who were older, and who had fewer physical disorders reported higher QOL compared to those with lower incomes, were younger, and who had more physical ailments. Part of a symposium sponsored by the Mental Health Practice and Aging Interest Group.

### SESSION 7705 (SYMPOSIUM)

### HETEROGENEITY OF AGING: IMPLICATIONS FOR TEAM CARE AND TEAM SCIENCE

Chair: George Kuchel
Co-Chair: Richard Fortinsky
Discussant: Luigi Ferrucci

Increasing heterogeneity with aging is a deeply held belief in gerontology often used to combat generalizations and ageist stereotypes regarding older adults. Nevertheless, the vast majority of published studies do not report or discuss variability in their findings with aging, instead focusing on average differences between age groups. Yet, when data diversity is examined, most studies do find increased heterogeneity with aging across all domains – biological, immunological, behavioral, social, clinical, and population. Although heterogeneity has been described across the aging literature, including most GSA journals, little or no effort has been made to define and better understand the very nature of heterogeneity as a conserved feature of aging evident across all of its varied dimensions. It is well established that multidisciplinary team-based approaches are essential to clinical care of older adults, to research efforts in aging, and to the training of future generations of scientists, clinicians, educators and others in the aging field. Over the last 75 years, GSA has been a leading and unique vehicle for the development of multidisciplinary and interdisciplinary dialogue and collaborations involving its six membership sections. This symposium will provide a unique opportunity to begin a multidisciplinary dialogue designed to engage the broader GSA community in determining shared, as well as distinct, features of heterogeneity as they are manifested in terms of biology, immunology, behavioral and social considerations, and clinical and population issues, with ultimate impact on health policy and practice.

### BIOLOGICAL HETEROGENEITY

Blanka Rogina, *UConn Health, Farmington, Connecticut, United States*

Studies of aging in invertebrates, mammalian animal models and humans have demonstrated increasing heterogeneity with aging in terms of varied facets of biological aging. In addition to growing heterogeneity, aging is also associated with qualitative and quantitative changes involving DNA methylation captured in epigenetic clocks of aging which seek to predict chronological and biological aging. Increased heterogeneity with aging is also evident in terms of posttranslational histone modification, gene expression, somatic clonal expansion, and increased degree of tissue mosaicism. Senescent cells accumulating with aging demonstrate significant heterogeneity. For example, while most studies targeting senescent cells have focused on cells expressing p16 (CDKN2A), not all p16-positive cells are senescent and not all senescent cells express p16. Further studies are needed to better define heterogeneity involving other hallmarks of aging and to also explore associations between heterogeneity involving these biological measures with clinical manifestations or outcomes.

### IMMUNOLOGICAL HETEROGENEITY

Laura Haynes, *UConn Health, Farmington, Connecticut, United States*

Ease of access to circulating peripheral blood cells (PBMCs) can offer unique insights into human immune function, as well as responses to vaccination and infection. Nevertheless, PBMC heterogeneity has been under-appreciated since results obtained from mixed populations may reflect changes in subset abundance as opposed to true age-related changes involving a specific subset. Technological advances have allowed for the examination of age-related heterogeneity with regards to systemic cytokine levels, immune cell frequencies and chemokine receptor expression by peripheral lymphocytes. Furthermore, introduction of sex as a variable in the examination of human PBMCs adds additional dimorphism to the study of aging and immunity including differences in epigenetic modifications, levels of pro-inflammatory activity and adaptive immunity.

### BEHAVIORAL AND SOCIAL CONSIDERATIONS

Lisa Barry, *UConn Center on Aging, Farmington, Connecticut, United States*

Cognitive, behavioral and social dimensions also demonstrate increasing heterogeneity with aging. For example, a longitudinal study of over 1,000 clergy revealed increasing heterogeneity in cognitive function and rate of decline with aging. Moreover, studies of individuals with probable Alzheimer's disease have shown heterogeneity in terms of clinical manifestations and rates of cognitive decline. Older adults also demonstrate greater heterogeneity in mood, anxiety, and the nature and patterns of symptoms over time. Heterogeneity of overall health status increases with aging, as does reported quality of life. Health and Retirement Study (HRS) data have shown that low socioeconomic status or being an underrepresented minority are both associated with greater intra-individual variability in health status in old age, with greatest differences seen in Hispanics. Finally, early life adversity can contribute to heterogeneity of multidimensional health trajectories even in late life.

### CLINICAL CONSIDERATIONS

George Kuchel, *University of Connecticut, Farmington, Connecticut, United States*

Varied physiological functions demonstrate increased heterogeneity with aging. Variability in force exertion and motor performance is higher in old age, with increased step-to-step gait variability indicating greater risk of falls and cognitive decline. Even in healthy older adults, renal function may show no change, slight decline, or marked decline. In contrast, heart rate variability declines with age, with decreased complexity and a higher risk of cardiac events. The risk of death, disease and disability varies among individuals with increasing heterogeneity with aging. As a result, frailty has been conceptualized as both as a phenotype and an accumulation deficit index, offering strong predictive validity when seeking to understand the heterogeneity of aging from the perspective of risk of mortality and physiologic dysregulation across different systems. Physical resilience defined as ability to maintain or restore function following exposure to stressors also demonstrates increased heterogeneity with aging.

### POPULATION AND HEALTH POLICY CONSIDERATIONS

Julie Robison, *University of Connecticut, Farmington, Connecticut, United States*

The risk of death, disease, disability, hospitalization, institutionalization and high health care costs varies among individuals with increasing heterogeneity associated with aging. Frailty, physical performance measures, self-reported measures and multimorbidity all represent measures that are useful in helping to better define such heterogeneity at the level of populations and to ultimately define such risk in individuals. These higher risk individuals account for a growing proportion of this nation's health care costs, with continued increases over time that appear unsustainable in the long term. Therefore, efforts to better define the nature of such heterogeneity of risk and improved targeting, with the goals of improving outcomes and reducing costs, are essential. A closely related challenge is to effectively translate proven clinical and health system interventions from the world of research to that of health policy and real-world clinical practice via pragmatic trials.

### SESSION 7710 (SYMPOSIUM)

### REGARDS: A CASE STUDY IN AGING AND DISPARITIES RESEARCH, MENTORING, AND DATA SHARING

Chair: Virginia Howard
Co-Chair: Jennifer Manly
Discussant: Maria Glymour

Investigators in the NIH-funded REGARDS (REasons for Geographic and Racial Differences in Stroke) project have taken a novel approach to break the paradigm of epidemiologic studies limited to clinic-based convenience samples, by developing a national cohort of 30,239 black and white participants recruited from communities across all lower 48 US states, including 1,855 of the 3,033 counties. Mean age at enrollment (Jan 2003-Oct 2007) was 65.3 years. The four initial aims were to further understanding of: 1) geographic and racial differences in stroke risk factors; 2) geographic and racial differences in stroke incidence and mortality; 3) association of stroke risk factors and stroke risk (incidence and mortality) focusing on effect modification by race or region; and 4) establishment of a repository of serum, plasma, urine and DNA for use in future studies. When the grant was awarded, the study goals were broadened to include longitudinal remote assessment of cognitive function. A second in-home visit was completed May 2013-Dec 2016 including measures of functional status. The cohort is in its 17th year of follow-up. We will detail recruitment and enrollment methods, characteristics of the cohort and status, with brief overview of the biological, medical, psychosocial, environmental, and contextual data collected in the parent study. Speakers will discuss in more detail the stroke and cognitive data, ancillary studies focused on caregiver and heart disease outcomes, and provide examples of national and international mentoring that has leveraged REGARDS data. Finally, we will describe opportunities for additional data sharing and new ancillary studies.

### DESIGN OF REGARDS: A NATIONAL COHORT OF BLACK AND WHITE ADULTS TO STUDY DISPARITIES IN STROKE AND COGNITIVE FUNCTION

Virginia Howard,[1] Mary Cushman,[2] Virginia Wadley,[1] Jennifer Manly,[3] Suzanne Judd,[1] and George Howard,[1]
*1. University of Alabama at Birmingham, Birmingham, Alabama, United States, 2. University of Vermont, Colchester, Vermont, United States, 3. Columbia University, New York, New York, United States*

The REGARDS study enrolled 30,239 whites and blacks aged >45 from 2003 – 2007, with oversampling of blacks and residents of the Stroke Belt. Potential participants were mailed a letter/brochure followed by telephone call. After verbal consent, telephone interview assessed cardiovascular health and cognitive function. In a home visit, measurements of risk factors, biological samples, EKG, written consent were obtained; during the in-home visit, self-administrated questionnaires were left to be completed and returned. Participants are followed for hospitalizations via telephone at 6-month intervals. Annually and biennially, brief and more comprehensive assessments of global cognitive function are conducted. Medical records for suspected strokes are collected with adjudication by stroke experts. A 2nd in-home and telephone assessment was conducted 2013-2016, approximately 10 years after baseline. This presentation will describe the methodological details of REGARDS, progress on the specific aims of the current grant, and establish the context for the remaining presentations.

### REGARDS COGNITIVE ASSESSMENT AND APPROACHES TO DEFINING COGNITIVE IMPAIRMENT AND CHANGE IN COGNITIVE FUNCTION

Jennifer Manly,[1] Frederick Unverzagt,[2] Leslie McClure,[3] Suzanne Judd,[4] J David Rhodes,[4] and George Howard,[4]
*1. Columbia University, New York, New York, United States, 2. Indiana University School of Medicine, Indianapolis, Indiana, United States, 3. Drexel University, Philadelphia, Pennsylvania, United States, 4. University of Alabama at Birmingham, Birmingham, Alabama, United States*

Since 2003, REGARDS participants have taken part in telephone-based cognitive assessments. Global cognitive status is assessed annually with the Six-item Screener. Between 2006 and 2009, measures of learning and memory (CERAD Word List) and language/executive function (Animal and Letter Fluency) were implemented, and are administered biennially. A Brain Health Substudy, conducting in-home clinical examinations of neuropsychological, neurological, and functional status among 1000 participants, is underway to validate telephone assessments and estimate prevalence of VCID in REGARDS. Approaches to defining incident cognitive impairment and cognitive change, including definitions employed for case/cohort studies using stored blood samples, will be described. We will discuss psychometric and methodological considerations for characterization of risks for cognitive impairment across race and region, as well as longitudinal trajectories of cognitive function.

### USING REGARDS TO STUDY FAMILY CAREGIVER WELL-BEING, HEALTH, AND MORTALITY

David Roth,[1] and William Haley,[2], *1. Johns Hopkins University, Baltimore, Maryland, United States, 2. University of South Florida, Tampa, Florida, United States*

The REGARDS study has provided a unique opportunity to study both disease-specific (stroke) and broader samples of family caregivers, and to examine the effect of transitions to caregiving over time. Using REGARDS has afforded many advantages over conventional caregiving research, including the availability of biomarker and mortality data, a large sample of non-caregiving controls who can be carefully matched to caregivers, and ability to track onset of caregiving over time. Our findings illustrate the complex nature of caregiving-related effects. While caregiving leads to worse psychological well-being, we have found minimal physical health decreases and reduced mortality rates compared to matched non-caregiving samples. These findings have policy implications and have challenged the conventional beliefs about caregiving based on previous studies of convenience samples. Diverse students and junior faculty members from multiple universities have also gained experience and contributed to high impact papers from this work.

### ADDING HEART DISEASE OUTCOMES TO REGARDS: THE REGARDS MYOCARDIAL INFARCTION ANCILLARY STUDY

Raegan Durant,[1] Emily Levitan,[2] Paul Muntner,[2] Todd Brown,[2] and Monika M Safford,[3], *1. University of Alabama at Birmingham, BIRMINGHAM, Alabama, United States, 2. University of Alabama at Birmingham, Birmingham, Alabama, United States, 3. Weill Cornell Medical College, New York, New York, United States*

The REGARDS-MI ancillary study provided new outcomes of heart disease events and adjudicated cause of death. A primary focus has been disparities in and risk factors for coronary artery disease. We demonstrated that compared to White men, Black men have a higher risk of fatal coronary heart disease (CHD) but a lower risk of non-fatal CHD. Ongoing work is investigating potential reasons for this. We have investigated the role of CHD in aging including the relationship between heart failure and cognitive function and the association of MI with functional status. The REGARDS-MI study has served as a platform for mentoring trainees and early stage investigators, many from underrepresented groups, and provided data to a large number of investigators to purse research in CHD. To date, REGARDS-MI has contributed to nearly 200 publications and spawned additional ancillary studies. This presentation will highlight some of these publications and other research in progress.

### OPPORTUNITIES FOR MORE AGING AND DISPARITIES RESEARCH, MENTORING, AND DATA SHARING WITH REGARDS

Suzanne Judd,[1] Virginia Howard,[1] Mary Cushman,[2] Jennifer Manly,[3] and George Howard,[1], *1. University of*

*Alabama at Birmingham, Birmingham, Alabama, United States, 2. University of Vermont, Colchester, Vermont, United States, 3. Columbia University, New York, New York, United States*

One of the benefits of this large, national cohort study with black and white participants is the opportunity to involve a breadth of researchers in the science. REGARDS actively solicits and engages early career and minority investigators to lead or participate in manuscripts as well as ancillary studies. REGARDS has provided opportunities for >175 ancillary studies, including those that enrich existing outcomes, provide new outcomes, assess new exposures, link with other national data, support extended analyses, and assess genetic associations. Over 70% of the 500+ publications to date have a lead author who was not a funded REGARDS investigator. In this presentation, we will discuss some of the innovative ancillary studies and high-impact manuscripts that have grown out of REGARDS, the processes for developing an ancillary study/manuscript, and the procedures for obtaining REGARDS data. We will describe opportunities for mentored research for junior investigators, as well as independent research projects.

### SESSION 7715 (SYMPOSIUM)

### REVERSING COGNITIVE DECLINE IN AGING: REVERSIBLE MECHANISTIC DEFECTS AND A NOVEL NUTRITIONAL INTERVENTION

Chair: Rajagopal Sekhar
Co-Chair: George Taffet

Aging is the biggest risk factor for cognitive-decline and Alzheimer's disease (AD), but underlying mechanisms are not well-understood and interventions are lacking. Cognitive-decline in AD has been associated with deficiency of glutathione, (the most abundant, intracellular, antioxidant protein), elevated oxidative-stress, insulin-resistance and increased inflammation. We identified and reported that glutathione-deficiency and oxidative-stress in older-adults occur due to decreased availability of precursor amino-acids glycine and cysteine, and can be corrected with GlyNAC (a combination of glycine and the cysteine precursor N-acetylcysteine). We hypothesized that cognitive decline in older-adults is linked to glutathione-deficiency, mitochondrial-dysfunction, oxidative-stress, insulin-resistance, and inflammation. The first abstract discusses the rationale and findings of an open-label clinical trial: compared to young-humans, older-adults had cognitive-decline, glutathione-deficiency, mitochondrial-dysfunction, abnormal glucose-metabolism and insulin-resistance, oxidative-stress, endothelial-dysfunction and inflammation. These defects were improved/reversed by supplementing GlyNAC for 24-weeks, but benefits receded on stopping GlyNAC for 12-weeks. The second abstract presents a study in 8 young (20-weeks old) and 16 aged (90-weeks old) wild-type male C57BL/6J mice where we found that aged-mice had naturally-occurring cognitive-impairment, and brain defects in glutathione-deficiency, oxidative-stress, glucose-transport, mitochondrial glucose-oxidation, insulin-resistance, endoplasmic-reticulum stress, autophagy, mitophagy, inflammation, senescence, genomic and telomere damage. Aged-mice received either GlyNAC or isonitrogenous-placebo supplementation for 8-weeks, and only GlyNAC-fed mice improved cognition and brain defects. Collectively these data highlights the discovery of novel and reversible mechanistic defects in older-adults and aged-mice with naturally-occurring cognitive-decline, and identifies that supplementing GlyNAC can improve brain-health and cognition. These findings could have important implications for reversing cognitive-decline in older-adults, and AD.

### REVERSING COGNITIVE-DECLINE IN OLDER ADULTS IN AN OPEN-LABEL CLINICAL TRIAL: NOVEL MECHANISMS AND THE ROLE OF GLYNAC

Chun Liu, Rajagopal Sekhar, Premranjan Kumar, Charles Minard, and Shaji Chacko, *Baylor College of Medicine, Houston, Texas, United States*

Age-associated cognitive-decline is an important risk factor for Alzheimer's disease, but interventions are lacking. We conducted an open-label trial to test our hypotheses on whether: (1) compared to 8 healthy young adults (25y), 8 'healthy' older adults (74y) have cognitive decline, decreased glucose availability for the brain due to mitochondrial dysfunction, elevated insulin-resistance, oxidative-stress and elevated inflammation; (2) supplementing glycine and N-acetylcysteine (GlyNAC) for 24-weeks corrects deficiency of the endogenous-antioxidant Glutathione and improves these defects, and thereby cognition; (3) stopping GlyNAC supplementation for 12-weeks results in a decline in accrued benefits. Outcome measures included cognitive testing (Montreal cognitive assessment; trail-making tests; verbal-fluency tests; digital-symbol substitution-test), mitochondrial fuel-oxidation, RBC-Glutathione concentrations, plasma oxidative-stress, insulin-resistance and inflammation, and tracer-studies to measure glucose metabolism. Results validated our hypotheses and showed that GlyNAC-supplementation corrected these defects and improved cognition. This trial suggests that supplementing GlyNAC may be important for improving/preventing age-associated cognitive-decline in older adults.

### REVERSING MITOCHONDRIAL, METABOLIC AND MOLECULAR DEFECTS IN THE BRAIN IMPROVES COGNITION IN AGED MICE

Rajagopal Sekhar, and Premranjan Kumar, *Baylor College of Medicine, Houston, Texas, United States*

Age-associated cognitive-decline is a risk factor for Alzheimer's disease (AD), but mechanisms are not well understood, and interventions are lacking. Rodent studies on AD have not led to therapeutic breakthroughs for cognitively-impaired humans. In an open-label trial in older-adults we found that supplementing GlyNAC (glutathione precursors glycine and N-acetylcysteine) improved cognitive-decline, defects in whole-body mitochondrial-function, and systemic insulin-resistance, oxidative-stress, and inflammation. We hypothesized that aged-mice will have similar defects in the brain, and studied male C57BL/6J mice as follows: young-mice (20w) were compared to two-groups of aged-mice (90-weeks) receiving either GlyNAC or isonitrogenous-placebo diets for 8-weeks. GlyNAC-supplementation improved cognition, and the following measures in the brain: glutathione-concentrations, glucose-transporters in blood-brain-barrier and neurons, mitochondrial glucose-oxidation, oxidative-stress, endoplasmic-reticulum stress, autophagy, mitophagy, inflammation, senescence, genomic and telomere damage. These data provide mechanistic insights into the novel and beneficial role of GlyNAC supplementation to reverse cognitive-decline in aging, and holds promise for human AD.

### SESSION 7720 (SYMPOSIUM)

### HOW TO PUBLISH: PREPARING MANUSCRIPTS FOR CLARITY, TRANSPARENCY, SCHOLARLY INTEGRITY, AND SUCCESS

Chair: Suzanne Meeks

The GSA publications team sponsors this annual symposium to assist prospective authors to successfully publish their gerontological scholarship in GSA's high impact and influential journals. The first part of the session will include five brief presentations from the Editors-in-chief of Journals of Gerontology-Series B, Social and Psychological Sciences, The Gerontologist, and Innovation in Aging, plus one of GSA's managing editors. We will integrate practical tips with principles of publication ethics and scholarly integrity. The topics will be as follows: (1) preparing your manuscript, including how to choose the right journal; (2) strong and ethical scholarly writing for multidisciplinary audiences; (3) transparency, documentation, and Open Science; (4) successfully responding to reviews; and (5) working with Scholar One. Following these presentations, we will hold round table discussions with editors from the GSA journals portfolio. At these roundtables, editors will answer questions related to the podium presentations and other questions specific to each journal. Intended audiences include emerging and international scholars, and authors interested in learning more about best practices and tips for getting their scholarly work published.

### HOW TO SUCCESSFULLY CHOOSE A JOURNAL

Laura Sands, *Virginia Tech, Blacksburg, Virginia, United States*

Article submissions to The Gerontological Society of America's high impact scientific journals continue to increase each year which has led to editors becoming more selective about which articles are accepted for publication. The purpose of this session is to describe how to efficiently and successfully navigate the process of determining which journal is the best fit for your manuscript. Objectives of the workshop include guidance in: (1) understanding the differences in scope and features of each journal; (2) determining which journal is most appropriate for the topic and methods of your manuscript; (3) appreciating how to use journals' Instructions to Authors to your benefit, and (3) conveying the scientific contribution of your article to the journal.

### STRONG AND ETHICAL SCHOLARLY WRITING FOR MULTIDISCIPLINARY AUDIENCES

Suzanne Meeks, *University of Louisville, Louisville, Kentucky, United States*

This presentation will emphasize the importance of plain, good writing. Editors of high impact journals read 10 or more manuscripts per week, and are under pressure to reject 80-90% of them. Regardless of scholarly quality, if the point and contribution are not clear in a quick scan of the paper, it likely will not be reviewed favorably. I will provide tips for strong scientific writing that are commonly violated in manuscript submissions, and provide references for additional writing support. I will also discuss some common publication ethics issues that arise during the review process, including author contributions and embedding your scholarship in the context of prior work.

### TRANSPARENCY, DOCUMENTATION, AND OPEN SCIENCE

Derek Isaacowitz, *Northeastern University, Boston, Massachusetts, United States*

Some GSA journals are especially interested in promoting transparency and open science practices, reflecting how some subdisciplines in aging are moving toward open science practices faster than others. In this talk, I will consider the transparency and open science practices that seem most relevant to aging researchers, such as preregistration, open data, open materials and code, sample size justification and analytic tools for considering null effects. I will also discuss potential challenges to implementing these practices as well as reasons why it is important to do so despite these challenges. The focus will be on pragmatic suggestions for researchers planning and conducting studies now that they hope to publish later.

### RESPONDING TO REVIEWERS

Deborah Carr, *Boston University, Boston, Massachusetts, United States*

An invitation to "revise and resubmit" one's manuscript, especially for a highly competitive journal, is an important achievement. However, it is not a guarantee that a revised manuscript will ultimately be accepted for publication. I will provide insights into what differentiates a "major" versus "minor" R&R invitation, will provide background on how editors and reviewers arrive at these decisions, and will provide detailed tips for crafting a revision memo and revised manuscript that has an excellent chance of being accepted for publication (while staying within a journal's word count limits). I will also offer suggestions for navigating revisions when reviewers offer discrepant feedback, or make suggestions that may not ultimately enhance the quality or impact of the manuscript.

### GSA MANAGING EDITORS' PERSPECTIVE ON SUBMISSION DOS AND DON'TS

Kathleen Jackson, and Karen Jung, *The Gerontological Society of America, Washington, District of Columbia, United States*

In this presentation, the managing editors of GSA's peer-reviewed journals will discuss how the editorial offices operate and their roles in the publishing process. The topics will include how to navigate the ScholarOne submission system, why it is important to read the Instructions to Authors, and how authors can work with the editorial offices to increase the visibility and impact of their published articles.

### SESSION 7725 (SYMPOSIUM)

### ESPO AND BUTLER-WILLIAMS SYMPOSIUM: RACE, ETHNICITY, AND OVERCOMING BARRIERS TO UNDERSTAND WHY AGING MATTERS

Chair: Roland Thorpe, Jr.
Co-Chair: Jamie Justice

The NIA's Butler-Williams Scholars Program and GSA's Emerging Scholars and Professional Organization are united in providing career development opportunities for early career scholars in a manner that promotes leadership, diversity, and inclusivity. This provides a foundation to develop a network of next generation of scientists, clinicians, and policy makers capable of shaping health in aging. Among the chief concerns of our aging population are disparities in health associated with race/ethnicity, experience, sociocultural and socioeconomic factors, as well as access to and communications regarding health care. GSA's early career professionals and alumni of the prestigious NIA Butler-Williams Scholars Program have tackled these issues directly and the scientific scholarship that results is astounding in its breadth and depth. Dr. Glenna Brewster (Butler-Williams class of 2018) will discuss new findings from a study of African American caregivers of persons living with dementia. Dr. Candace Brown, Ph.D. (Butler-Williams class of 2017), will present on overcoming social and environmental barriers to exercise among older adults. Dr. Joseph Saenz (Butler-Williams class of 2017), will present current work disparities in cognition function in the older Mexican population. The final speaker, Dr. Sarah Forrester (Butler-Williams class of 2019) will explore new perspectives on health equity in physiological dysregulation and aging. In sum, the featured talks by rising stars in aging research deepen our understanding of the influence of race, ethnicity, and overcoming barriers to understand 'why aging matters' across our diverse aging populations.

### HEALTH-RELATED QUALITY OF LIFE FACTORS IN AFRICAN AMERICAN CAREGIVERS OF PERSONS WITH DEMENTIA

Glenna Brewster, *Emory University, Atlanta, Georgia, United States*

African American caregivers of persons living with dementia have a high prevalence of caregiving compared to other caregiving groups. The goal of this study was to explore health-related quality of life factors in a sample (n=25) of African American caregivers with a body mass index of more than Caregivers were female (87%). Caregivers were an average age of 63.2 (±6) years, had 16.6 (±2) years of education, and were providing care for 4.3 (±4) years. Care-recipients were on average 79.1 (±11) years and mothers (52.2%). 74% of the caregivers reported good-to-excellent quality of life. Caregivers reported low anxiety and depression but high perceived stress and strain. Higher anxiety and depression were correlated with greater strain and lower quality of life. Healthcare providers need to examine specific situational caregiving factors that can affect African American caregiver outcomes; since, their stress and strain may not reflect in their response to psychological questionnaires.

### LONG-TERM ADHERENCE TO PHYSICAL ACTIVITY AMONG OLDER VETERANS

Candace Brown, *University of North Carolina at Charlotte, Charlotte, North Carolina, United States*

The benefits of physical activity (PA) are well-established and it is recommended that older adults achieve at least 150 to 300 minutes of moderate intensity PA and strengthening activities weekly. However, only 54.0% and 23.2% of older adults achieve these recommendations for endurance and strengthening (respectively), and 48% dropout within the first 6-months. Most PA research focuses on the 6-month initiation phase leaving a gap regarding long-term adherence. We explored predictors of long-term adherence (>2-years) to PA from 97participants at 6-month follow-up and yearly surveys. Variables examined included age, race, gender, body mass index (BMI), and self-reported comorbidities, symptoms, physical function, and barrier-specific self-efficacy scale (α-level 0.05). Lower BMI (29.1±5.1 versus 31.6±6.5, p=0.047) and higher self-efficacy to overcome environmental barriers (p=0.016) and social isolation (p=0.05) were associated with long-term adherence. Self-efficacy to overcome environmental and social barriers should be addressed to promote long-term adherence to exercise among older adults

### LITERACY AND COGNITIVE FUNCTION IN THE CONTEXT OF MARRIAGE: A STUDY OF OLDER MEXICAN COUPLES

Joseph Saenz, *University of Southern California, Los Angeles, California, United States*

Many studies have reported that literacy is associated with favorable cognitive outcomes in late-life. Few have evaluated whether the cognitive benefits of literacy extend to a spouse's cognitive ability. Among married husband-wife dyads from the 2012 Mexican Health and Aging Study (n=4,078 dyads), literacy was assessed as self-reported ability to read and write. General cognitive ability was assessed using performance across several cognitive domains. Approximately 11% and 15% of husbands and wives, respectively, could not read or write. For both husbands and wives, both own literacy, and having a spouse who could read and write were independently associated with better cognitive ability even after accounting for both partners' education. Literacy may represent an important form of capital that may be beneficial to preserve cognitive function among older adults. Benefits of spousal literacy may operate by facilitating access to resources such as information and cognitive stimulation.

### PHYSIOLOGICAL DYSREGULATION AND AGING: IMPLICATIONS FOR HEALTH EQUITY

Sarah Forrester,[1] Catarina Kiefe,[1] and Roland Thorpe, Jr.,[2], *1. University of Massachusetts Medical School, Worcester, Massachusetts, United States, 2. Johns Hopkins University, Baltimore, Maryland, United States*

Social determinants of health (SDOH) are a major public health issue that affect the magnitude and prognosis of many diseases in the U.S., including cardiovascular disease and cognitive disorders of aging. These associations are especially deleterious among minority persons in the US due to consistently unfavorable social determinants in this population including socioeconomic position, residential segregation, and quality of education. Physiological dysregulation measures the "true global state" of an individual that includes, but goes beyond, chronological age, reflects the health burden produced, in part, by SDOH, and is associated with the development and prognosis of disease. A better understanding of how and when physiological dysregulation occurs may allow us to prevent or reverse dysregulation among the most vulnerable aging populations leading to improved health equity in cardiovascular and cognitive outcomes.

### SESSION 7750 (SYMPOSIUM)

### HEALTH AMONG SEXUAL MINORITY OLDER ADULTS: INDIVIDUAL DIFFERENCES AND IMPLICATIONS FOR IMPROVING HEALTH

Chair: Britney Wardecker
Co-Chair: Cara Exten

The number of sexual minority (SM) older adults is increasing rapidly, yet this population continues to be underrepresented in research (Fredriksen-Goldsen & Kim, 2017) and experiences significant disparities in health and health care access (Fredriksen-Goldsen, 2016; Wallace et al., 2011). In the current symposium, we analyze data from U.S. national probability samples of middle-aged and older adults (MIDUS, HRS, NESARC-III) to consider how age-related concerns and challenges may be experienced differently by SM individuals compared to their heterosexual counterparts. This symposium includes novel methods and statistical tools, such as daily diary assessments, multilevel modeling, and time-varying effects models. Individual presentations evaluate how: (1) SM women, compared to heterosexual women, may respond differently to menopause through norms and values surrounding womanhood; (2) midlife and older SM individuals use alcohol and cigarettes more frequently across a typical week than their heterosexual counterparts, though their substance use may not be tied to common triggers (e.g., negative mood, stress); (3) despite bisexual older adults reporting more health problems compared to lesbian and gay counterparts, they are less prepared for health concerns and crises (e.g., reporting a lower number of valid wills); and (4) the prevalence of depression and anxiety varies across age, such that older SM adults—especially women—are particularly vulnerable to psychological health problems. These presentations collectively examine complex issues facing older SM adults while emphasizing individual differences (i.e., women's concerns, bisexual people's issues). We discuss challenges in researching this growing at-risk population, and we highlight areas of future research and intervention.

### ADVANCE CARE PLANS FOR SEXUAL MINORITY OLDER ADULTS: DISPARITIES ACROSS LESBIAN, GAY, AND BISEXUAL INDIVIDUALS

Britney Wardecker, and Jes Matsick, *The Pennsylvania State University, University Park, Pennsylvania, United States*

To ensure preference-based care, health providers advise individuals to establish advance care plans (e.g., power of attorney, valid will; NIA, 2018). Though important for all individuals to prepare for declining health, it is critical for bisexual older adults to establish plans given they evidence more health problems compared to heterosexual, lesbian, and gay counterparts (Fredriksen-Goldsen et al., 2016). To assess factors related to having advance care plans, we used data from sexual minority older adults (n=158) in the Health and Retirement Study (2016). Bisexual individuals reported a lower number of valid wills than lesbian and gay participants, $t(152)=3.80$, $p<.001$. This finding is particularly alarming given bisexual people are the numeric majority of sexual minorities and experience elevated health problems in older age. We will discuss mechanisms of disparities, the need for research on end-of-life care for sexual minorities, and implications for improving healthcare providers role in facilitating care plans.

### MAJOR DEPRESSIVE EPISODE AND GENERALIZED ANXIETY DISORDER AMONG SEXUAL MINORITY ADULTS IN THE UNITED STATES

Cara Exten, and Anna Salomaa, *Penn State University, University Park, Pennsylvania, United States*

Sexual minority (SM) health disparities constitute a serious public health concern, and disparities in mental health are among the most striking. Among a nationally-representative sample (NESARC-III) of US adults (aged 18-66), we used time-varying effects models (TVEM) to estimate the age-varying prevalences of past year major depressive episode (pyMDE) and generalized anxiety disorder (pyGAD) by SM status and biological sex. pyMDE and pyGAD were most common among SM women, followed by SM men, heterosexual women, and heterosexual men. pyMDE was highest among SM women and SM men in early adulthood with a second peak in the mid-50s (women)and around age 40 (men). pyGAD was highest among SM women aged 54-60 and among SM men aged 30-33. Our findings reveal that older adulthood may be a time of increased risk for pyMDE and pyGAD among SM women. Future work should explore factors that contribute to this increased risk.

### SEXUAL ORIENTATION, FEMININITY, AND ATTITUDES TOWARD MENSTRUATION AMONG WOMEN: IMPLICATIONS FOR MENOPAUSE

Jes Matsick, Mary Kruk, and Britney Wardecker, *The Pennsylvania State University, University Park, Pennsylvania, United States*

Women differ in how they psychologically respond to the end of menstruation and onset of menopause (Lorber & Moore, 2002); however, little empirical evidence exists for understanding how sexual orientation and gendered dynamics contribute to menopausal experiences. Do women's attitudes toward the cessation of menstruation vary by sexual orientation? Using data from the Midlife in the U.S. Study (MIDUS, Wave 2; N=2,951), we test how sexual orientation relates to attitudes toward menstruation cessation through norms and values surrounding womanhood (i.e., "traditional femininity concerns," such as worries about fertility and attractiveness). Cisgender heterosexual women, compared to sexual minority women, expressed greater regret of menstrual periods ending, and heterosexual women's heightened concerns about traditional femininity mediated the association between sexual orientation and regret, $b=-.09$, 95%CI [-.176,-.008]. This research yields implications for understanding aging stigma and women's health, and we discuss how menopause may be differently experienced by women based on sexual orientation.

### DAILY ALCOHOL AND CIGARETTE USE AMONG SEXUAL MINORITY ADULTS: MODERATING ROLE OF DAILY STRESS AND DAILY WELL-BEING

Agus Surachman,[1] Britney Wardecker,[1] Cara Exten,[2] Jes Matsick,[1] and David Almeida,[3] *1. The Pennsylvania State University, University Park, Pennsylvania, United*

States, 2. Penn State University, University Park, Pennsylvania, United States, 3. Pennsylvania State University, University Park, Pennsylvania, United States

The goal of this study was two-fold: 1) to investigate whether gay, lesbian, and bisexual (GLB) adults, compared to heterosexual adults, used alcohol and cigarettes daily to a greater extent, and 2) to test the moderating role of daily stress and well-being on the association between GLB status and alcohol and cigarette use. We analyzed data from 3,421 adults (GLB = 98; age range = 20-83 years) who completed an 8-day daily diary protocol as part of the Midlife in the U.S. Study (MIDUS). Compared to heterosexual adults, GLB participants reported greater daily alcohol and cigarette use. However, among GLB individuals, more negative affect was associated with less daily alcohol use and people who reported more stressor days and physical symptoms across the week consumed less alcohol. We will discuss how daily affect, stress, and substance use may function differently among GLB people in middle and older adulthood.

### SESSION 7800 (SYMPOSIUM)

### DESIGNING TECHNOLOGY FOR AGING AND DISABILITY

Chair: Shelia Cotten

Technology has massive potential to improve the lives of older adults in terms of their health, wellbeing, quality of life, and independence. However, benefits will not be realized unless these technologies are designed considering the needs, abilities, and attitudes of the diverse population of older adults. This is especially true when we consider older adults experiencing physical, sensory, or cognitive impairments that influence their ability to adopt and use technology-based solutions. This symposium highlights a variety of approaches to using technology to support older adults living with disability, and important design considerations. The first talk will highlight the important role technology can play in helping persons with cognitive and /or physical impairment in the workplace. The next talk outlines a framework and methodology designed to provide older adults with hearing or visual impairments the capability to use and adapt digital health tools. This is followed by a discussion of a research agenda to use technology to help older adults experiencing disability as a result of cognitive impairment participate in their community. Then, there will be a discussion of how digital health technologies, when considering their unique needs and abilities, can support older adults with cognitive impairment and dementia. The final talk focuses on the intersection of technology, cognitive impairment, and leisure, and explores engagement with digital games by older adults with and without dementia. Common themes that emerge and future directions will be highlighted.

### WORKPLACE ADAPTIVE TECHNOLOGIES FOR WORKING ADULTS WITH COGNITIVE AND PHYSICAL DISABILITIES

Patricia Heyn, *University of Colorado Anschutz Medical Campus, Aurora, Colorado, United States*

Individuals with disabilities usually have difficulty in finding and maintaining employment prospects and thus, they are extremely underrepresented in the workforce. These challenges are even greater when the person has both cognitive and physical disabilities. While there is evidence supporting the benefits of employing individuals with disabilities in the workforce, employers are usually unprepared to hire individuals with disabilities. They are also concerned that the work productivity may be impacted by the employee with a disability. Thus, technology can play an important role in helping a person with cognitive and /or physical impairment work on tasks that require memorization and assembly performance. We will present a mobile technology system that was planned and piloted with working adults with physical and cognitive impairments. Founded on our pilot study, mobile technologies hold the potential to help people with disabilities to perform jobs that require memorization as well as systematic assembly tasks.

### BUILDING DIGITAL HEALTH TOOLS FOR OLDER ADULTS WITH DISABILITIES

Fuad Abujarad,[1] Sarah Swierenga,[2] and Chelsea Edwards,[3] *1. Yale University, New Haven, Connecticut, United States, 2. Michigan State University, East Lansing, Michigan, United States, 3. Yale University School of Medicine, New Haven, Connecticut, United States*

Adults with disabilities are more likely to be excluded from digital health research compared to those without. In this symposium, we will describe how user experience (UX) research can be used to successfully develop digital health tools that are both usable and acceptable by older adults with physical disabilities. Our framework and methodology are designed to provide older adults, age 60+ with hearing or visual impairments, the capability to use and adapt digital health tools (including tools that run on mobile devices like smart phones and tablets). Our approach includes one-on-one sessions to evaluate the ease of use and usefulness of digital health for older adults, including 6 blind, 6 deaf, 6 low vision, 6 hard of hearing. This approach allows us to increase the scope of the research participants and, eventually, end users to include adults with disabilities that we may not normally include in research study design.

### INTRODUCING THE ENHANCE (ENHANCING NEUROCOGNITIVE HEALTH, ABILITIES, NETWORKS, & COMMUNITY ENGAGEMENT) CENTER

Walter Boot,[1] Sara Czaja,[2] Wendy Rogers,[3] and Neil Charness,[1] *1. Florida State University, Tallahassee, Florida, United States, 2. Weill Cornell Medicine, New York, New York, United States, 3. University of Illinois at Urbana-Champaign, Champaign, Illinois, United States*

Cognitive impairment (CI) refers to changes in cognition that result in difficulties remembering, learning new things, concentrating, making decisions important to everyday life, responding to environmental demands, or understanding social cues, and these difficulties can result in disability (limiting one or more major life activities). Existing and emerging technology applications hold promise for providing everyday support for older adults with CI, promoting independence and community living. However, these solutions will only be viable if they consider the needs, preferences, and abilities of older adults experiencing cognitive impairment. This talk introduces a new center, funded by the National Institute on

Disability, Living, and Rehabilitation Research (NIDILRR), with the aim of supporting older adults with CI (MCI, CI as a result of traumatic brain injury or stroke) through adaptive and individualized technology solutions.

### STRATEGIES TO IMPROVE HEALTH TECHNOLOGIES FOR OLDER ADULTS WITH COGNITIVE IMPAIRMENT AND DEMENTIA

Stacey Schepens Niemiec, Elissa Lee, Jeanine Blanchard, and Adam Strizich, *University of Southern California, Los Angeles, California, United States*

Technology may improve health self-management of older people with cognitive deficits, yet these individuals may have unique needs influencing its utility. This study's purpose was to gather current strategies described in the literature and by expert stakeholders for utilizing digital health technologies in older adults with cognitive impairment (CI) or dementia. We conducted a rapid literature review to identify articles that featured digital health technology use in persons with CI/dementia. Additionally, we conducted interviews (n=12) with expert stakeholders who were identified through online academic, professional, and community organization biographies and snowball referral. Qualitative-based thematic analysis was used to identify emergent themes from selected literature and transcribed interviews. Recommended strategies addressed instructional methods (e.g., reducing distractions), technology adaptations (e.g., simplified interface), care partner involvement, and dosage/exposure. Findings are applicable to development of technology-driven interventions and products, with the aim of improving the effectiveness of such technology for older people with CI/dementia.

### PREDICTING ENGAGEMENT WHILE PLAYING COMPUTER GAMES IN OLDER ADULTS WITH AND WITHOUT DEMENTIA

Antonio Miguel-Cruz,[1]
Daniel Alejandro Quiroga-Torrez,[2]
Adriana Maria Rios-Rincon,[1] Christine Daum,[1]
Ruby De Jesus,[1] Lili Liu,[3] and Eleni Stroulia,[1]
*1. University of Alberta, Edmonton, Alberta, Canada, 2. Universidad del Rosario, Bogotá, Distrito Capital de Bogota, Colombia, 3. University of Waterloo, Waterloo, Ontario, Canada*

The purpose of this study was to determine what factors predict the level of engagement of older adults, with and without dementia, while playing computer games. Fourteen older adults with and without dementia (60%/40%) played a computer game over 16 sessions, each for 30 minutes. Variables included participants' demographics, game-play data and environmental factors. Mixed fixed model for longitudinal data analysis design was used to determine how these variables predicted engagement. Five variables predicted engagement at a statistically significant level: Participant's performance (B1=+0.16, p<0.03), age (B2=+0.20, p<0.00), previous experience with computer games (B3=+1.021, p<0.02), positive emotions (B4=+0.16, p<0.00), and distractions (noise) during gameplay (B5=-1.07, p<0.05). Cognitive impairment and general health status were correlated with engagement, but these correlations were not statistically significant. Previous experience using computer games and distractions during gameplay were the most important predictors of engagement while older adults with and without dementia played computer games.

### SESSION 7805 (PAPER)

### DEMENTIA, COGNITIVE IMPAIRMENT, AND MENTAL ILLNESS: PRESENTER DISCUSSION

### BARRIERS AND FACILITATORS FOR OLDER ADULTS WITH SERIOUS MENTAL ILLNESS TO UTILIZING MEDICAID SMARTPHONE SERVICES

Amanda Myers,[1] and Karen Fortuna,[2] *1. Rivier University, Hudson, Massachusetts, United States, 2. Dartmouth College, Concord, New Hampshire, United States*

As increasingly more older adults in the general population utilize smartphones to access health services, the digital divide between older adults with serious mental illness (SMI) and the general older adult population continues to widen. The purpose of this study was to examine older adult peer support specialists' and older people with SMI's perspectives of barriers and facilitators to utilizing Medicaid Safelink smartphone services. Data from two focus groups and five semi-structured interviews from older adult peer support specialists (N=10) and older adults with SMI (N=15) were analyzed using the Consolidated Framework for Implementation Science Research. A mixed methods convergent design integrated quantitative with qualitative data. Older adults with SMI (N= 15) had a mean age of 55 years and were mainly women (70%) and White (100%). Certified peer specialists (N= 10) had a mean age of 52 years (age range 45-67) and were mainly female (75%), 66% identified as White, and 33% identified as African American. Four themes that were identified across different aspects of barriers included technology knowledge, technology adoption, design features (i.e., smartphone size, option to increase font sizes, multi-modal capacity, navigational architecture, 508 compliance), and Safelink policies and procedures. Facilitators included free and continuous services, access to technical support, and smartphone capabilities to enable healthcare communications and facilitate the delivery of services. Improving upon the themes identified as barriers to utilizing Safelink may promote a continuum of care for older adults with SMI, closing the gap of services that occurs between in-person therapy and other interventions.

### SIBLING DEATH IN THE FAMILY AND DEMENTIA RISK

Hyungmin Cha,[1] Patricia Thomas,[2] and Debra Umberson,[3] *1. The University of Texas at Austin, Austin, Texas, United States, 2. Purdue University, West Lafayette, Indiana, United States, 3. University of Texas at Austin, Austin, Texas, United States*

Growing evidence points to the role of stress in contributing to dementia risk, and experiencing the death of a family member is a particularly stressful life event. Sibling relationships are typically life-long relationships and the death of a sibling is likely to be a stressful event in the life course; however, there is little research illuminating the possible consequences of sibling loss for dementia risk. This study considers whether experiencing the death of a sibling before midlife is associated with subsequent dementia risk and how such losses, which are more common for Black and Hispanic than for White populations, may add to racial/ethnic disparities in dementia risk. We use discrete-time event history models to predict dementia incidence among 9,590 non-Hispanic white, 1,669 non-Hispanic black, and 1,109 Hispanic respondents from the Health and Retirement Study, 2000-2014. Losing a sibling during the observation period is associated with increased risk for later dementia. The death of a sibling is robust to the inclusion of a variety of biosocial factors that contribute to subsequent dementia risk. The death of a sibling is a life course event with consequences that appear to increase dementia risk for Black and Hispanic older adults, and this increased risk is explained by biosocial processes likely activated by bereavement. However, Black and Hispanic Americans are further disadvantaged in that they are more likely than White Americans to experience the death of a sibling, and such losses add to the already substantial racial disadvantage in dementia risk.

### THE PRICE OF PRIVILEGE: HELPING MOM DIE AT HOME

Renee Beard, *College of the Holy Cross, Worcester, Massachusetts, United States*

Americans overwhelmingly wish to age in place and many explicitly want to die at home. Yet, the anemic welfare state means that only the most fortunate among us are able to achieve that goal. A disproportionate burden of care falls squarely to families, which are smaller and more geographically spread out than ever before. Carers too often wind up in environments that are far from conducive, namely being older and perhaps frail themselves or younger and perhaps with small children of their own. Drawing on an autoethnographic study of my mother's final years and a case study analysis of one innovative home care agency, this project examines the individual and organizational factors that allow one family to grant their family member's wish to die at home. Grounded theory methods revealed facilitators including presence of a home-based long term care insurance policy, geographic mobility, and access to a democratically-oriented home care organization. Barriers, of course, include lack of access to long term care insurance and a daughter who lives in a progressive state with a waiver for Home and Community Based Services. While the privilege of access underscores the social determinants of aging, this case study reveals some important features that suggest how senior social services could be. Even for the "ideal type" presented here, the many trials and tribulations of aiding a loved one to die at home relate to the untenable nature of doing it all in a context whereby social services are fragmented and driven by financial incentives.

### THE QUALITY OF INTERACTIONS BETWEEN STAFF AND RESIDENTS WITH COGNITIVE IMPAIRMENT IN NURSING HOMES

Anju Paudel,[1] Barbara Resnick,[2] and Elizabeth Galik,[3]
*1. University of Maryland, Glen Burnie, Maryland, United States, 2. University of Maryland School of Nursing, Baltimore, Maryland, United States, 3. University of Maryland School of Nursing, Ellicott City, Maryland, United States*

Background: Positive and effective staff–resident interactions are imperative to adequately assess and meet the needs of cognitively impaired residents in nursing homes and optimize their quality of life. Purpose: The purpose of this study was to quantify, describe, and analyze the interaction between staff and cognitively impaired residents in nursing homes, using the Quality of Interaction Schedule (QuIS). Specifically, the following aims were addressed— Aim 1: To quantify and describe the quality of interactions between staff and cognitively impaired residents in nursing homes. Aim 2: To analyze whether the quality of staff–resident interactions vary by resident cognitive status (moderate vs severe) and interaction characteristics (interaction location, interaction situation, interpersonal distance, type of staff, and resident level of participation). Method: This descriptive analysis utilized baseline data from the first 2 cohorts in a randomized clinical trial including 341 residents from 35 nursing homes. Results: Five hundred fifty-six staff–resident interactions were evaluated; majority were positive (n = 466, 83.8%) and the remaining were either neutral (n = 60, 10.8%) or negative (n = 30, 5.4%). The quality of interactions varied by interaction location, interpersonal distance, and resident participation. Conclusion: This study provides some current descriptive information about the quality of staff-resident interactions in nursing homes and the interaction characteristics that might impact these interactions. Future research should focus on decreasing the negative/neutral interactions and explore staff characteristics (e.g., gender, level of experience) and facility factors (e.g., size, ownership) that might influence the quality of interactions.

### SESSION 7810 (PAPER)

### DEPRESSION AND DEMENTIA AMONG OLDER ADULTS: PRESENTER DISCUSSION

### GLOBAL REACH OF CONCEPTUAL MODELS USED IN AGEISM AND DEMENTIA STUDIES: A SCOPING REVIEW

Atiqur Rahman, and Lars-Christer Hyden, *Linkoping University, Norrköping, Ostergotlands Lan, Sweden*

Older adults are discriminated against at different social levels- called ageism and the people living with dementia (PlwD) not only face ageism due to being elder but also encounter prejudices, negative attitudes and discrimination because they live with dementia. The purpose of this review is to highlight the different conceptual and methodological approaches used and the groups being investigated as well as the findings from studies in the combined research field of ageism and dementia. Five scientific databases were used within a timeframe between 2009 and 2018. A total number of 98 articles (quantitative, qualitative, and mixed methods) were included and analyzed based on (i) the conceptual frameworks, (ii) methodological approaches, (iii) groups studied, and (iv) findings. The result emerged six themes (i) stereotypes and attitudes in the general population; (ii) stereotypes and attitudes of themselves; (iii) stereotypes and attitudes among staff; (iv) stereotypes and attitudes as part of culture; (v) stereotypes and attitudes of family members; and (vi) miscellaneous. The principal findings identified lower education, lack of knowledge and less information about dementia as the main contributing factors to manifest stereotypes, attitudes, and ageism in society toward PlwD that leads to stigma. Moreover, this review found few studies were based on explicit conceptual frameworks but rather on descriptive understandings of relations between attitudes. There is a domination of the quantitative approach where the target populations were mostly from Western regions. The review suggests that a more conceptually coherent approach would allow for a better cumulative building of knowledge.

### PREDICTING DEPRESSION IN DEMENTIA CAREGIVERS: DO RELIGIOUS/SPIRITUAL STRUGGLES PLAY A ROLE?

Elizabeth MacDougall, *Bridgewater College, Bridgewater, Virginia, United States*

Using a stress process framework model, this study is the first to comprehensively examine the role that religious/spiritual struggles play in the lives of informal dementia caregivers. A convenience sample of 156 informal dementia caregivers completed a scale measuring six domains of religious/spiritual struggles, as well as other measures of primary stressors, background/contextual variables, and mental health outcome (depression). Overall levels of religious/spiritual struggle were low, but 26 percent of the sample were classified as possible cases of clinically significant religious/spiritual struggle for at least one of the six domains. Of this group, 49 percent acknowledged struggles with ultimate meaning. Religious/spiritual struggles predicted greater self-reported depression over and above number of care recipient problem behaviors (primary stressor), caregiver sex, and caregiver personality (i.e., emotional stability). Although no individual domain of religious/spiritual struggle emerged as most salient, caregivers reported significantly more ultimate meaning struggles than demonic or interpersonal struggles. These findings support the growing body of research suggesting that religious/spiritual struggles serve as a secondary stressor, adding predictive power to background/contextual factors and to primary stressors for informal dementia caregiver mental health outcomes. Further research in this area may advance efforts to better equip both secular and religious professionals to provide evidence-based counsel to informal dementia caregivers.

### RESEARCH LITERATURE ON THE INTERSECTION OF DEMENTIA, SPIRITUALITY, AND PALLIATIVE CARE: A SCOPING REVIEW

Jennifer Palmer,[1] Alyssa Smith,[2] Sara Paasche-Orlow,[3] and George Fitchett,[4] *1. Hebrew SeniorLife, Boston, Massachusetts, United States, 2. Independently Employed, Bethesda, Maryland, United States, 3. Hebrew SeniorLife, Roslindale, Massachusetts, United States, 4. Rush University Medical Center, Chicago, Illinois, United States*

Dementia marks an increasingly prevalent terminal illness for which palliative care, including spiritual care, could improve quality of life. Research gaps exist in understanding the intersection of dementia, spirituality, and palliative care. Thus, we conducted the first scoping review examining the nature and breadth of peer-reviewed studies across these three topics. The scoping review followed methods from The Joanna Briggs Institute Reviewers' Manual (2015). We developed a priori a scoping review protocol outlining the Population, Concept and Context for study, data sources, search strategy, inclusion/exclusion criteria, and procedure for screening, extracting, and analyzing data. The final sample consisted of 19 studies with the following themes: Characterizing Spiritual Needs, Preferences, and Resources; Characterizing Palliative or Spiritual Care; Predicting Provision of Spiritual Care; and Assessing Spiritual Care Interventions. Eighteen studies were published in the past decade, and eleven were based in Europe. The majority of studies focused on long-term care settings, grouped stages of dementia or did not specify dementia stage, and investigated interventions indirectly related to spiritual palliation. Many studies were limited in sample size and in generalizability / transferability and used less sophisticated research designs. Accordingly, research across dementia, spirituality, and palliative care needs to examine distinct stages of dementia; hospital-, home- and community-based settings; and formal spiritual care interventions (e.g., administered by chaplains) and needs to utilize rigorous study designs (e.g., randomized clinical trials). Such research could advance practice and policy that enhance quality of life for tens of millions of persons with dementia and their family members worldwide.

### SESSION 9050 (SYMPOSIUM)

### CLARK TIBBITTS AWARD LECTURE

Chair: Cynthia Hancock

The Clark Tibbitts Award lecture will feature an address by the 2020 award recipient, Jan Abusharkrah, PhD, FAGHE. AGHE's Clark Tibbitts Award was established in 1980 and named for an architect of the field of gerontological education. The award is given each year to an individual or organization that has made an outstanding contribution to the advancement of gerontology and geriatrics education.

### AT 75: COMING OF AGING WITH GSA

Jan Abushakrah, *Portland Community College, Portland, Oregon, United States*

This lecture reflects on the highlights of my journey to become a gerontologist: •Joining AGHE in 1998 as PCC explored the development of the first and only Gerontology associate degree in Oregon •Collaborating with community and academic partners to create an applied and evolving curriculum •Empowering older, encore students to translate their experience and compassion into professional careers that embodied their passion •Mentoring hundreds of students to harness their creativity and engage in bold innovation to transform aging lives •Grappling with how to measure and ensure student learning in a way that would make a difference in aging lives •Participating in the working group that developed the Gerontology Education Competencies and currently serving on the founding Board of Governors of the Accreditation for Gerontology Education Council.

### SESSION 9055 (SYMPOSIUM)

### HIRAM J. FRIEDSAM MENTORSHIP AWARD LECTURE

Chair: Cynthia Hancock

The Hiram J. Friedsam Award lecture will feature an address by the 2020 award recipient, Karen Kopera-Frye,

PhD, MPA, FGSA, FAGHE. Hiram J. Friedsam was the professor, co-founder, and director of the Center for Studies in Aging and dean of the School of Community Service at the University of Northern Texas. Dr. Friedsam was an outstanding teacher, researcher, colleague, and mentor to students, faculty, and administrators, as well as a past president of AGHE. The purpose of this award is to recognize those who emulate Dr. Friedsam's excellence in mentorship.

### MENTORING ACROSS ACADEMIA AND COMMUNITIES: A HOLISTIC APPROACH INVOLVING NEEDS OF THE MENTEE

Karen Kopera-Frye, *New Mexico State University, Las Cruces, New Mexico, United States*

What is mentoring? Mentoring is defined as a professional relationship in which an experienced person assists another in developing specific skills and knowledge enhancing the mentee's professional and personal growth. Mentoring benefits include: Knowledge transfer, creating a mentoring culture, and challenging the mentee to move beyond their comfort zone. Carmel and Paul (2015) describe self-selected mentoring as a process in which a mentee identifies a potential mentor based on similarities in interests and need. Findings indicated mentees experienced opportunities in career advancement, expanded thinking, scholarly confidence, facilitation of a collaborative culture, and understanding the importance of goal setting. Mentoring as a health promotion or intervention strategy has become widespread in communities. Two mentoring approaches will be discussed: a traditional format with students and faculty in academia, another utilizing an intergenerational approach with Latinx and Native American families. Recommendations will be discussed in terms of underlying core similarities across the venues.

### SESSION 9100 (SYMPOSIUM)

### KENT AND KLEEMEIER AWARD LECTURE AND PRESENTATIONS

Chair: Debra Dobbs

The Donald P. Kent Award lecture will feature an address by the 2019 Kent Award recipient, Terry Fulmer, PhD, of The John A. Hartford Foundation. The Kent Award is given annually to a member of The Gerontological Society of America who best exemplifies the highest standards of professional leadership in gerontology through teaching, service, and interpretation of gerontology to the larger society. The Robert W. Kleemeier Award lecture will feature an address by the 2019 Kleemeier Award recipient, Steven Zarit of Pennsylvania State University. The Kleemeier Award is given annually to a member of The Gerontological Society of America in recognition for outstanding research in the field of gerontology

### CREATING AGE-FRIENDLY HEALTH SYSTEMS: AGE MATTERS

Terry Fulmer, *The John A. Hartford Foundation, New York, New York, United States*

Since 2015, The John A. Hartford Foundation has been funding strategies to create Age-Friendly Health Systems (AFHS). Led by the Institute of Healthcare Improvement, in partnership with the American Hospital Association and the Catholic Health Association, the AFHS movement is rapidly growing, with participation in all 50 states from over 450 sites, including the full continuum of care settings. Partnerships with private and public entities are accelerating the work. As one example, the Health Resources and Services Administration has embedded AFHS principles into the Geriatrics Workforce Enhancement Program. This Kent Lecture will focus on the genesis and trajectory of the AFHS social movement and discuss how the effort will lead to an age-friendly ecosystem that transcends boundaries and cultures and leads to a common framework for the way we approach care, caregiving and communities for optimizing the lives and wellbeing of all older adults.

### REFLECTIONS ON A CAREER IN RESEARCH AND EDUCATION

Steven Zarit, *Penn State University, State College, Pennsylvania, United States*

This presentation will focus on three issues that were important in my career: family caregiving, international collaborations, and mentorship. Having spent considerable time studying family caregiving, I will highlight what I consider are fundamental issues that characterize family care and provide a necessary foundation for generating strong research questions. I will also suggest new directions for improving design and evaluation of interventions. International collaborations can help us broaden our understanding of aging and on care of older people. I had the good fortune to work with a great research team in Sweden. I will describe research we did on functioning and cognition among the oldest old. I also had the opportunity to see first-hand high-quality programs for older people that showed we can do better than accept mediocrity as the norm in care. Finally, I will discuss the importance of mentorship and bringing forward the next generation of researchers.

### SESSION 9150 (SYMPOSIUM)

### M. POWELL LAWTON AWARD PRESENTATION

Chair: Debra Dobbs

"The lecture will be given by the2019 recipient, Barbara Resnick, PhD, CRNP, FGSA of the University of Maryland. The session will also include the presentation of the2020 Lawton Award to recipient Sara J. Czaja, PhD, FGSA. The M. Powell Lawton Award is presented annually to an individual who has made outstanding contributions from applied research that has benefited older people and their care. The Lawton Award is generously funded by the Polisher Research Institute of the Madlyn and Leonard Abramson Center for Jewish Life."

### M. POWELL LAWTON AWARD PRESENTATION

Barbara Resnick, *University of Maryland School of Nursing, Baltimore, Maryland, United States*

Dr. Lawton focused his life work, starting in the 1960s, on improving the lives of older adults, particularly those with dementia. He did this at a time when care was custodial at best. He was innovative and initiated new thoughts about how to best care for individuals in institutional settings.

He raised our awareness about the importance of the environment and the value of individualized care. We build on that work today. The focus of this 2019 Powell Lawton award presentation is on sharing my lifetime work, starting in 1972, on helping older adults, particularly those who are institutionalized and may have cognitive changes, maintain their functional ability and increase time in physical activity. Along with Dr. Lawton's experiences, I and the teams I have worked with, have met challenges ranging from the use of Houdini vests to bed alarms and wheelchairs, to the persistent call out by caregivers to older adults-"don't get up you might fall." I will share information, resources and data from over a 20 year period developing and testing Function Focused Care, an approach to care that helps caregivers optimize function and increase physical activity among institutionalized older adults. Further, the process for how to disseminate and implement Function Focused Care across multiple settings and how to be a resilient researcher will also be reviewed. Participants will be able to take the findings and resources from this presentation and implement this approach into their own settings of care.

### SESSION 9200 (SYMPOSIUM)

### IRVING S. WRIGHT AWARD OF DISTINCTION LECTURE

Chair: Nir Barzilai
Co-Chair: Stephanie Lederman

The Irving S. Wright Award of Distinction Lecture will feature an address by the 2020 recipient James L. Kirkland, MD, PhD. This award is given by the American Federation for Aging Research, Inc.

### AGING, SENESCENT CELLS, AND SENOLYTIC DRUGS: THE PATH TO TRANSLATION

James Kirkland, *Mayo Clinic, Rochester, Minnesota, United States*

Senescent cells (SC) accumulate with aging and at causal sites of multiple chronic disorders and diseases, including those accounting for the bulk of morbidity, mortality, and health expenditures. SC do not replicate. Some SC release factors that cause tissue dysfunction, the senescence-associated secretory phenotype (SASP). Transplanting small numbers of SC into younger mice to above a critical threshold leads to frailty, early onset of multiple age-related diseases, and premature death. A report in 2004 showing caloric restriction causes both healthspan extension and delayed SC accumulation prompted us to begin efforts to discover senolytic drugs, agents that selectively eliminate SC. We used a hypothesis-driven, mechanism-based strategy to discover senolytics, reasoning that senescent cell anti-apoptotic pathways (SCAPs) exist that defend SC against their own SASP, allowing them to survive, despite killing neighboring cells. Senolytics cause SC apoptosis by transiently disabling these SCAPs. Because SC take weeks to re-accumulate, senolytics can be administered intermittently – a "hit-and-run" approach. Senolytics delay, prevent, or alleviate frailty and cardiovascular, neuropsychiatric, liver, kidney, musculoskeletal, lung, eye, hematological, metabolic, and skin disorders as well as complications of organ transplantation, radiation, and cancer treatment in pre-clinical models. As anticipated for agents targeting the fundamental aging mechanisms that are "root cause" contributors to multiple disorders, potential uses of senolytics are protean, potentially alleviating over 40 conditions in preclinical studies, opening a new route for treating age-related dysfunction and diseases. We review the discovery of senolytics and potential strategies for translation into the clinic. Early trials indicate effectiveness of senolytics in humans.

### SESSION 9205 (SYMPOSIUM)

### VINCENT CRISTAFALO AWARD LECTURE

Chair: Nir Barzilai
Co-Chair: Stephanie Lederman

The Vincent Cristofalo Rising Star Award in Aging Research lecture will feature an address by the 2020 recipient, Sean P. Curran, PhD. This award is given by the American Federation for Aging Research, Inc

### DIET-BASED STRATEGIES, INFORMED BY GENETICS, TO IMPROVE HEALTHSPAN

Sean Curran, *Leonard Davis School of Gerontology, University of Southern California, Los Angeles, California, United States*

Diet is one of the most variable aspects of life history, as most individuals have a large diversity of choices, varying in the type and amount that they ingest. In the short-term, diet can affect metabolism and energy levels. However, in the long run, the net deficiency or excess of calories from diet can influence the progression and severity of age-related diseases. An old and yet still debated question is: how do specific dietary choices impact health- and lifespan? It is clear that genetics can play a critical role - perhaps just as important as diet choices. For example, poor diet in combination with genetic susceptibility can lead to metabolic disorders, such as obesity and type 2 diabetes. We have identified the existence of diet-gene pairs, where the consequence of mutating a specific gene is only realized on specific diets. Although only a handful of these diet-gene pairs have been characterized, there are potentially thousands of such interactions, which may explain the variability in the rates of aging in humans and the incidence and severity of age-related diseases.

### SESSION 9250 (SYMPOSIUM)

### MARGRET M. AND PAUL B. BALTES AWARD

Chair: Merril Silverstein

The lecture will be given by the2019 Baltes Award recipient, Allison Bielak, PhD, FGSA, of Colorado State University. The recipient of the2020 Baltes Award is William J. Chopik, PhD. The Margret M. and Paul B. Baltes Foundation Award in Behavioral and Social Gerontology recognizes outstanding early-career contributions in behavioral and social gerontology. The award is generously funded by the Margret M. and Paul B. Baltes Foundation.

### NEW PERSPECTIVES ON PREDICTING AND MODIFYING COGNITION IN OLDER ADULTHOOD

Allison Bielak, *Colorado State University, Fort Collins, Colorado, United States*

The underlying theme of my research is to advance our understanding of cognitive aging through early detection of cognitive decline and the promotion of positive lifestyle factors. I will present my past and recent work that uses new perspectives to predict and modify cognition with older age, specifically focusing on intraindividual variability in cognitive speed, and lifestyle activity engagement. This includes examining the utility of variability in cognitive speed, usually a predictor of decline, instead as a predictor of cognitive plasticity following activity interventions. Further, the association between activity engagement and later cognitive ability and dementia risk is more complex than first appears. I will present research that looks at critical issues about the activity-cognition relation from a different perspective, including unique frameworks investigating the ideal way to measure activity, and the duration of how long past activity can potentially impact cognition. Finally, I will discuss my future research directions.

### SESSION 9300 (SYMPOSIUM)

### MAXWELL A. POLLACK AWARD PRESENTATION AND LECTURE

Chair: Bob Harootyan

The lecture will feature an address by the2019 Pollack Award recipient, Karen Lincoln, PhD, FGSA of the University of Southern California. The2020 Pollack Award recipient is Karl Pillemer, PhD, FGSA. The Maxwell A. Pollack Award for Productive Aging Award recognizes instances of practice informed by research and analysis, research that has directly improved policy or practice, and distinction in bridging the worlds of research and practice. Supported by The New York Community Trust.

### BEST OF BOTH WORLDS: BRIDGING RESEARCH AND PRACTICE TO ACHIEVE HEALTH EQUITY

Karen Lincoln, *University of Southern California, Los Angeles, California, United States*

Health inequity is linked to societal and social determinants that constrain health promoting opportunities for older adults. From birth to death, many older adults contend with the prevailing social and health effects of discrimination in employment, education, and housing. As a consequence, middle-aged Black Americans are experiencing accelerated aging, and living with and dying from preventable chronic health conditions typically diagnosed at older ages. Despite these conditions, there is much variation in health outcomes among older Black Americans. Professor Karen Lincoln will share her approach to research that explores heterogeneity within the Black American population to discover factors that promote healthy aging. She will describe her unique outreach and health education program that serves older adults in underserved communities, and her roles as an aging advocate and public scholar. She will discuss how these experiences led to the development and testing of an innovative intervention to reduce Alzheimer's disease disparities.

### SESSION 9350 (SYMPOSIUM)

### BUILDING THE EVIDENCE FOR HOSPITAL MOBILITY

Low mobility in the hospital, defined as mobility limited to bed rest or bed to chair transfers, is associated with high rates of functional decline, nursing home placement, and death even after adjusting for illness severity and comorbidity. This lecture will describe the gradual of building of evidence for both the adverse outcomes and potential solutions at both an individual and a health system level to address the challenge of low mobility.

### SESSION 9350 (SYMPOSIUM)

### JOSEPH T. FREEMAN AWARD LECTURE

The Joseph T. Freeman Award lecture will feature an address by the2020 Freeman Award recipient Cynthia Brown, MD, MSPH. The Joseph T. Freeman Award is a lectureship in geriatrics awarded to a prominent physician in the field of aging, both in research and practice. The award was established in1977 through a bequest from a patient's estate as a tribute to Dr. Joseph T. Freeman.

### SESSION 9355 (SYMPOSIUM)

### EXCELLENCE IN REHABILITATION AWARD

The Excellence in Rehabilitation of Aging Persons Award lecture will feature an address by the 2020 Excellence in Rehabilitation Award recipient Kenneth J. Ottenbacher, PhD, OTR. The Excellence in Rehabilitation of Aging Persons Award is designed to acknowledge outstanding contributions in the field of the rehabilitation of aging individuals.

### REHABILITATION OF AGING PERSONS: THE PAST, THE PRESENT & THE PROMISE

Kenneth Ottenbacher, *The University of Texas Medical Branch at Galveston, Galveston, Texas, United States*

The 2020 Excellence in Rehabilitation of Aging Persons Award presentation will address my efforts over the past 35 years related to research methods, functional status, mobility, and self-care. Studies conducted in the past 25 years on disability and recovery in older adults with an emphasis on minority health will be presented. Research examining rehabilitation outcomes related to health care reform including the Improving Medicare Post-Acute Care Transformation (IMPACT) Act, and using Medicare files, will be described. The role of Data Science and Discovery, as defined by the NIH and related to rehabilitation in older adults, will also be presented.

### SESSION 9450 (SYMPOSIUM)

### DISTINGUISHED FACULTY AWARD LECTURES

Chair: Cynthia Hancock

The Distinguished Faculty Award lecture will feature an address by2020 recipient Mary W. Carter, PhD. The

Distinguished Faculty Award acknowledges individuals whose teaching stands out as exemplary, innovative, or influential-- or any combination thereof.

### HOW COVID FLIPPED THE CLASSROOM: LEARNING FROM THE NEXT GENERATION OF GERONTOLOGISTS

Mary Carter, *Towson University, Towson, Maryland, United States*

Covid laid bare the structural inequities and systemic challenges facing LTC, both in terms of risk and neglect. Lost in the daily tally of new cases and mortality rates are the stories of essential workers who have neither degree nor stature in the field, yet who fulfill an awesome responsibility—caring for older adults no longer visible behind locked doors. Suddenly, these full-time students and part-time workers found themselves employed in one of the most dangerous occupations in the country—providing direct care in nursing homes and assisted living communities. This award lecture highlights their voices and lessons learned.

### SESSION 9455 (SYMPOSIUM)

### RISING STAR EARLY-CAREER FACULTY AWARD LECTURE

Chair: Cynthia Hancock

The Rising Star Early-Career Faculty Award lecture will feature an address by2020 recipient Laurinda Reynolds, MA. The Rising Star Early-Career Faculty Award acknowledges new faculty whose teaching and leadership stand out as influential and innovative.

### DECONSTRUCTING AGEISM

Laurinda Reynolds, *American River College, Sacramento, California, United States*

Fifty years of ageism research has identified the psychosocial subconstructs and consequences of ageism and produced over 30 published definitions. However, some educators still define ageism as age discrimination or prejudice against one age group by another age group. This oversimplification provides camouflage for insidious ageism, messages that are not recognized as harmful by their source or by the target of the ageism message. This type of ageism message cultivates implicit fears of aging and negative self-perceptions, and because they are not recognized, they undermine efforts to reframe aging. This presentation deconstructs ageism to make this extremely complex phenomenon clear without oversimplifying its nature or understating its consequences. It differentiates ageism from youthism through their developmental processes, persistence, and consequences. Knowledge will be integrated and synthesized across disciplines using a biopsychosocial lens. Examples of insidious ageism are presented to increase one's capability to recognize and avoid insidious ageism.

### SESSION 10000 (LATE BREAKING POSTER)

### ADULT PROTECTION AND ELDER ABUSE

### ESTIMATING RISK OF ELDER ABUSE: INTERGENERATIONAL COMMUNICATION/ EMPATHY, CENSUS-BASED ASSESSMENT

Heng Wu, and Lyn Holley, *The University of Nebraska at Omaha, Omaha, Nebraska, United States*

COVID highlights the importance of accurate estimates of the risk of elder abuse to guide prevention. Reliability of data is problematic; reports have issues with consistency of definitions, time periods, and stigma. This paper demonstrates the use of US Census data to estimate risk of elder abuse by mapping generational incongruities between care-givers and care-receivers that invite dissonance (Cohen, 2011). Using the 2014-2018 Nebraska Public Use Microdata and 5-year American Community Survey, this research identifies and profiles personal care aides/nursing aides for institutionalized persons (65+). Data reveal generation gaps in age, education, and race between care-receivers and care-givers that are geographically comparable in the three most populous counties (the Big-3), but different for the 90 rural counties. In Nebraska's Big-3 Counties the difference in education between care-givers (ages 25+) and care-receivers (65+) is considerable; 42.9% of Big-3 care-givers have some college, while 43.6% of care-receivers have only high school. Intergenerational differences in education are greater in Nebraska's 90 rural counties than in the Big-3; 41.2% of rural care-givers have some college; 46.1% of rural care-receivers have only high school. Racial intergenerational differences are greater in Big-3 than in rural counties; 90.3% of care-receivers and only 62.3% of care-givers are non-Hispanic White. For rural Nebraskans, intergenerational differences in race are smaller, 97.2% of care-receivers and 79.4% of care-givers are non-Hispanic White. This type of analysis can be used to identify geographic settings where elder abuse is most likely to happen and guide the development of preventive measures that mitigate elder abuse.

### MULTIDISCIPLINARY HEALTHCARE PROVIDERS' PERSPECTIVES ON MANAGING SUSPECTED ELDER ABUSE IN THE HEALTHCARE SETTING

Lena Makaroun,[1] Gloria Klima,[2] Michele Nichols,[1] Keri Rodriguez,[2] Ann O'Hare,[3] and Ann-Marie Rosland,[1] *1. University of Pittsburgh School of Medicine, Pittsburgh, Pennsylvania, United States, 2. VA Pittsburgh Healthcare System Center for Health Equity Research and Promotion, Pittsburgh, Pennsylvania, United States, 3. University of Washington School of Medicine, Seattle, Washington, United States*

Elder abuse (EA) is common and has devastating health consequences, yet is rarely detected by healthcare professionals. Veterans are at high risk for EA, and the VA has unique resources (e.g., comprehensive social work services) that can help address EA in the healthcare setting. This qualitative study aimed to assess perceived barriers and facilitators to detecting, reporting, intervening on and monitoring EA for VA providers. Providers from two VA facilities were recruited to participate in a one-on-one semi-structured interview. Transcripts of audio-recorded interviews were analyzed using thematic content analysis. Participants (n=22) were 82% female, age 33-64 years, had 4-25 years practicing in VA, and varied in discipline (e.g., nurse, physician, social worker) and practice setting (e.g., emergency department, geriatrics, primary care). For detecting EA, patient and caregiver cognitive impairment were frequently cited barriers, while an interdisciplinary team approach and ability to do home visits were noted facilitators. Common challenges with reporting EA to adult protective services (APS) were perceived lack of APS follow up and discrepancies in VA provider and APS investigator findings. While removing a patient from an unsafe living situation was a frequently cited successful intervention, providers also expressed feeling conflicted when infringing on patient autonomy. Poor communication with APS, patient loss to follow up, and caregiver interference made monitoring EA cases more difficult; intensive case management and in-home services facilitated monitoring. In conclusion, healthcare professionals see interdisciplinary care, in-home care, and better coordination with APS as key facilitators to managing suspected EA in the healthcare setting.

### PERCEIVED SOCIAL SUPPORT AND HELP-SEEKING AMONG U.S. CHINESE OLDER ADULTS WITH ELDER MISTREATMENT

Ying-Yu Chao,[1] Dexia Kong,[2] and XinQi Dong,[2]
*1. Rutgers School of Nursing, Newark, New Jersey, United States, 2. Rutgers University, New Brunswick, New Jersey, United States*

Background/Purpose: Older immigrants are at risk of experiencing marginalization and social exclusion. Traditional Chinese culture values could deeply influence the older Chinese immigrants' perceptions regarding mistreatment and motivating them to seek help. This study aimed to examine the associations between perceived social support and informal/formal help-seeking intentions and behaviors among U.S. Chinese older adults experiencing elder mistreatment. Methods: Data derived from the Population Study of Chinese Elderly in Chicago (PINE). Independent variables were positive and negative perceived social support. Dependent variables were informal/formal help-seeking intentions and behaviors. Descriptive statistics and logistic regression analyses were performed. Results: A total of 423 participants experienced elder mistreatment (mean age: 72.4 ±7.88 years old). The most common informal help-seeking sources were adult children, followed by partner, and friends/neighbors/colleagues. The most common sources of formal help-seeking were community social services organizations and the legal criminal justice system. After controlling for covariates, positive perceived social support was associated with informal help-seeking intentions (OR=1.14, 95% CI: 1.05-1.24, $p < .01$) and behaviors (OR=1.12, 95% CI: 1.04-1.22, $p < .01$). However, the associations between perceived social support and formal help-seeking intentions and behaviors were not significant. Conclusions & Implications: Further research is needed to examine the mediating effects of cultural values on the relationship between perceived social support and help-seeking among mistreated older Chinese immigrants. In addition, additional studies are needed to identify impede or facilitate factors of informal/formal elder mistreatment help-seeking. Prevention and intervention programs should incorporate valuable cultural insight to improve help-seeking among this population.

### PERCEPTIONS OF MANDATORY REPORTING AMONG FAMILY PHYSICIANS TO PROTECT VICTIMS OF ELDER ABUSE IN MONGOLIA

Ariunsanaa Bagaajav, *The Graduate Center, CUNY, New York, New York, United States*

Every year, approximately 10% of older adults worldwide experience elder abuse (EA), a serious violation of human rights. A wide range of consequences of EA have been identified including increased vulnerability to morbidity and mortality. Some countries have regulations to protect victims of EA either under stand-alone EA laws or relevant laws such as mandatory reporting. As of 2016, family physicians (FPs) in Mongolia became mandatory reporters of domestic violence alongside other human service workers under the newly amended Law to Combat Domestic Violence. Evidence shows that health providers who report abuse cases have greater awareness of their mandatory obligations to report. No rigorous study has explored the extent to which FPs in Mongolia know about mandatory reporting and their perceptions about its effectiveness to help victims of EA. An exploratory qualitative study was conducted. Through purposive sampling, 15 FPs participated in semi-structured in-depth interviews. The data collection took place over Skype. All interviews were recorded, transcribed verbatim and analyzed using thematic analysis. Surprisingly, all participants were aware of their mandatory obligations to report cases of domestic violence including EA. However, the study revealed that FPs were doubtful that potential victims of EA would be effectively protected under the new regulation due to the public perception that services and legal resources are designed primarily for women and children. Addressing this misconception is critical for ensuring that resources are appropriately utilized for potential victims of EA as well.

### SUCCESSFUL INFORMAL HELP-SEEKING AND RESOLUTION OF ELDER FAMILY FINANCIAL EXPLOITATION: A CASE STUDY

Tina Kilaberia,[1] and Marlene Stum,[2] *1. UC Davis Health, Sacramento, California, United States, 2. University of Minnesota, Twin Cities, Saint Paul, Minnesota, United States*

Effective interventions are needed to address elder family financial exploitation (EFFE), one of the most prevalent types of elder abuse globally. This poster examines the unique and critical help-seeking role informal family support can play when faced with EFFE. We present a holistic case study that offers an understanding of one family's successful help-seeking and resolution of the EFFE situation. The family drew on internal family and community supports and did not seek formal elder abuse services. The case stood out as unique relative to 23 family's help-seeking attempts in a larger study of the meaning and experience of EFFE from the perspective of concerned family members (non-abusing/non-victims). The case summary and analysis are based on an in-depth interview narrative reflecting the subjective experience of a concerned family member who was directly involved in the EFFE situation (in-law relative to both the older victim and the perpetrator). Study findings reveal 5 interwoven themes related to help-seeking processes and outcomes: 1) honoring the victim's wishes, 2) providing support and accountability for perpetrator, 3) restoring family relationships and functioning, 4) maintaining internal (family-based) control, and 5) engaging in family problem solving processes. The family's help-seeking demonstrated three distinctive features: a) embracing their informal social support role, b) the interdependence of family members, and c) restorative justice principles. The findings raise questions about broadening the scope and continuum of EFFE intervention research and practices to recognize and support informal social intervention.

### TRAJECTORY OF DEPRESSION AMONG VICTIMS OF ELDER MISTREATMENT

Gabriella Dong, and Mengting Li, *Rutgers University, New Brunswick, New Jersey, United States*

Individuals exposed to elder mistreatment are affected in dissimilar ways. Most existing studies are cross-sectional and fail to capture the change in mental health of older adults with exposure to elder mistreatment. This study aims to examine depression trajectories of elder mistreatment victims and identify protective factors. Data were drawn from the two-wave Population Study of Chinese Elderly in Chicago (PINE) with 725 participants who reported elder mistreatment at the baseline. Depression was measured by Patient Health Questionnaire-9. Self-mastery was assessed by the Pearlin Mastery Scale. Conscientiousness was evaluated by the NEO Five-Factor Inventory. Multinomial logistic regression was used while controlling demographic factors and recurrence of elder mistreatment. We identified four depression trajectories among elder mistreatment victims: chronic (9.61%), delayed (6.27%), recovery resilience (21.17%), and resistance resilience (62.95%). The chronic group was showing severe depression in both waves. The delayed group experienced a delayed reaction with increasing depression over time. The depression level of the recovery resilience group bounced back from elder mistreatment. The resistance resilience group exhibited low depression over time. Elder mistreatment victims with increasing self-mastery were more likely to be in recovery resilience group than in chronic group (RRR=1.05, 95%CI=1.02-1.09). In addition, elder mistreatment victims with increasing conscientiousness were less likely to be in delayed group than in resistance resilience group (RRR=0.96, 95%CI=0.92-1.00). Healthcare providers and social service agents could focus on elder mistreatment victims with chronic and delayed depression trajectories. Interventions could promote mental health of elder mistreatment victims through improving self-mastery and conscientiousness.

### SESSION 10100 (LATE BREAKING POSTER)

### AGEISM

### A PILOT STUDY ON RELATIONSHIPS OF THE WORKPLACE INTERGENERATIONAL CLIMATE SCALE WITH OTHER AGEISM AND SEXISM SCALES

Sangkyung Bae, and Moon Choi, *KAIST, Daejeon, Taejon-jikhalsi, Republic of Korea*

Due to the demographic changes such as longevity and low birthrates, the proportion of workers over 55 years old is expected to rise more than twice in the coming years. As the age diversity in the workplace is increasing, ageism needs more attention in every context. This study aims to explore relationships of the workplace intergenerational climate scale with other ageism and sexism scales in workplace and non-workplace contexts. Data came from a pilot online survey conducted in South Korea in December 2019 (N=74; average age=46.8 years old ranged from 20 to 76), and the data was analyzed using a series of ANOVAs and logistic regressions. The results showed that chronological age did not have a linear relationship with ageist attitudes in the workplace although relatively younger groups tend to have more ageist attitudes compared to their counterparts. In particular, those in their thirties were most reluctant to work with different generations. Conspicuously, negative attitudes towards working with different generations in the workplace were statistically significantly related to ageist attitudes towards older adults in non-workplace contexts as well as sexist attitudes in the workplace. The findings imply that prejudice and stereotypes towards different age and gender groups in workplace and non-workplace contexts might be intertwined, and interventions reducing ageism in the workplace might also have positive impacts on alleviating other types of ageism and sexism while promoting diversity.

### ASKING YOUNG ADULTS ABOUT THEIR ATTITUDES TOWARD AND KNOWLEDGE OF SEX IN LATER LIFE

Iulia Fratila, and Liza Berdychevsky, *University of Illinois at Urbana-Champaign, Champaign, Illinois, United States*

Sexual expression is a lifelong need related to health and wellbeing. However, older adults' sexuality is often neglected and stigmatized due to societal ageist stereotypes portraying them as asexual. Although baby boomers' generation resists such portrayals, societal acceptance of sexuality in later life is slow to materialize. The purpose of this study was to explore this acceptance among young adults while focusing on three research questions: (1) How much do young adults know about older adults' sexuality and how do they feel about it? (2) Do young adults' knowledge and views of later-life sexuality vary by gender? (3) Do young adults' views of later-life sexuality vary based on their general attitudes toward sexuality? Data collection included online and intercept survey methods. The sample (N=270) was young (M=21.58 years, SD=4.32) and included 149 women and 113 men. Results revealed that young adults had medium levels of knowledge, yet rather permissive/open-minded attitudes regarding later-life sexuality. Higher levels of knowledge were unrelated to more permissive attitudes. Independent samples t-test revealed no differences by gender in young adults' knowledge and attitudes. However, multiple regression results indicated that general liberal attitudes toward sexuality ($\beta$=.772, t=17.867, p=.000) and viewing sex as leisure activity ($\beta$=.147, t=3.338, p=.001) are significant predictors of having more permissive/open-minded attitudes toward older adults' sexuality (R2=.557, F(3,266)=111.390, p=.000). These findings suggest that socio-psychological (rather than cognitive and demographic) factors drive the acceptance of later-life sexuality among young adults. This study offers valuable insights for knowledge, practice, and advocacy concerning older adults' sexuality.

### DOES AGING IDENTITY MODERATE THE IMPACT OF EXPERIENCES WITH FAMILIAL AGEISM ON WELL-BEING?

Emily Kinkade, *North Dakota State University, Fargo, North Dakota, United States*

This study begins to investigate the effects of ageism in the family context. The current literature has documented the negative impacts that negative stereotypes and negative perceptions of aging have on older adults' health, mortality, and well-being (Levy, 1996; Levy, 2003). However, the majority of extant research on ageism focuses on age discrimination in the workplace and in healthcare despite the majority of peoples' time being spent in the family context. Therefore examining experiences of ageism sourced from family members merits study. Walker, Bisconti and Kinkade (in preparation) found evidence that the experience of ageism within the family context varies from the workplace context. Past research has demonstrated that older adults who identify as being older and adapt to the changes that arise with aging are associated with higher levels of self-esteem (Whitbourne, Sneed, & Skultety, 2002; Weinberger & Whitbourne, 2010). It is hypothesized that age identification will serve as a moderator for the relationship between familial ageism and well-being. Participants completed a set of questionnaires measuring experiences with familial ageism, depression, self-esteem, and ego strength. Experiences of familial ageism correlated with the well-being outcome variables in the predicted direction. Age identity moderated the relationship between familial ageism and depression and ego strength, such that participants who identified as being younger or identified as their age reported lower depression scores and higher ego strength scores. These findings suggest that age identity may serve as a buffer against the negative impacts that experiences of familial ageism has on well-being.

### INTERSECTIONAL DISCRIMINATION ATTRIBUTIONS AND HEALTH OUTCOME AMONG AMERICAN OLDER ADULTS

Peiyi Lu,[1] Dexia Kong,[2] Mack Shelley,[1] and Joan Davitt,[3]
*1. Iowa State University, Ames, Iowa, United States, 2. Rutgers University, New Brunswick, New Jersey, United States, 3. University of Maryland, Baltimore, Baltimore, Maryland, United States*

Discrimination has been consistently documented to relate to adverse health outcomes. However, most existing research focused on a single discrimination attribution (e.g. ageism). Few studies considered multifaceted discrimination attributions. Guided by an intersectionality framework, this study examined intersectional discrimination attributions and their associations with health outcomes. Older respondents (aged >50) from the Health and Retirement Study in 2014-2015 were included in the analysis (n=6,286). Their experiences and self-perceived reasons (age, gender, sexual orientation, race, national origin, religion, financial status, weight, physical appearance, disability, and others) for everyday discrimination were examined. Latent class analysis was employed to ascertain the profiles of subgroups characterized by their intersectional discrimination attributions. Regression models examined the correlates of the class memberships and the associations with health outcomes. Six classes were identified: class 1 (54.52% of the sample) had no/minimal discrimination experience; Class 2 (21.89%) experienced primarily ageism; class 3 (8.81%) reported discrimination based on age/gender/national origin/race; class 4 (7.99%) attributed discrimination to financial/other reasons; class 5 (5.87%) experienced discrimination based on age/weight/physical appearance/disability; and class 6 (0.92%) perceived discrimination from almost every aspect. Intersectional discrimination attributions were associated with poorer self-rated health, and greater levels of depressive symptoms and loneliness. The associations between intersectional discrimination and cognition were not statistically significant. This study found multiple marginalized identities co-occur and compound to contribute to perceived everyday discrimination among American older adults. Those experiencing discrimination due to multiple reasons warrant particular attention. Results underscore the utility of an intersectional approach in understanding discrimination in later life.

### JUDGING OLDER ADULT SPEAKERS: ONLY YOUNG ADULTS LOW IN AGEISM NOTICE THE NUANCES

Jennifer Turner,[1] Renee Hayslip,[2] and Jennifer Stanley,[2]
*1. Pennsylvania State University, State College, Pennsylvania, United States, 2. University of Akron, Akron, Ohio, United States*

Ageism negatively impacts hiring and electability success, as well as intergenerational relationships (Levy,2003;2009). The current study sought to examine whether personality cues influenced performance ratings of older adult (OA) speakers whose behavior had been modified by an embodiment intervention (i.e., "power posing"). One-hundred-and-three young adults (YA; Mage=19.6, SD=2.06; 49.5% women) rated the performance of 9 OA speakers performing 5-minute campaign speeches, and reported the cues that influenced their ratings. Two independent raters coded the cues (i.e., introversion and extroversion; coded by two independent raters, $\kappa$ = .72 [moderate-to-substantial interrater reliability; Chen, 2019; McHugh, 2012]). Participants also completed the Refined-Aging Semantic Differential (Polizzi & Millikin, 2002) as a measure of ageism endorsement. Greater ageism was associated with lower performance ratings (F(1,101)=15.97, p<.001, R2=.14); performance was reduced by .12, 95%CIs[-.018,-.006] for each additional point of ageism endorsement. Next, we investigated whether personality cues would modify this relationship using Hayes PROCESS Model 1 (2018). A significant interaction emerged between ageism and introverted cues (b=.015, p=.05, 95%CIs[.006,.023]), suggesting that greater perceived introversion was negatively associated with performance ratings. Additionally, individuals lower in ageism were more likely to calibrate their performance judgments based on the pose condition of the speaker, with participants lower in ageism exhibiting greater variability across pose conditions (Ms=3.96-4.37), than individuals higher in ageism (Ms=4.21-4.31), suggesting that individuals lower in ageism were attending to nuances in the speech (e.g., pose influence), while higher ageist individuals relied more heavily on age-related judgments.

### PERCEIVED EVERYDAY AGE DISCRIMINATION AND DEPRESSIVE SYMPTOMS:THE MODERATING EFFECT OF SOCIAL ENVIRONMENT

Min Kyoung Park, and Christine Mair, *University of Maryland, Baltimore County, Baltimore, Maryland, United States*

Approximately 30% of men and women in the United States have experienced age discrimination (Rippon, Zaninotto, & Steptoe, 2015). Experiencing age discrimination may lead to increased risk of depressive symptoms among older adults. Although positive social environments are known to buffer depressive symptoms, it is unknown to what extent a positive social environment may buffer the association between age discrimination and depressive symptoms for older adults in the US. The purpose of this study is to examine the association between perceived age discrimination and depressive symptoms among older adults, and to explore whether this association varies by two aspects of the social environment: social support and neighborhood environment. We explore this topic with data on 5,439 adults aged 50 and older in a sample drawn from the Psychosocial Module of the Health and Retirement Study (HRS, 2016 wave). Our results show a clear association between age discrimination and increased risk of depressive symptoms, net of a range of covariates. Older adults who receive more positive social support and rate their neighborhood environment more positively also report lower depressive symptoms. Finally, we find statistically significant interactions between age discrimination and both measures of the social environment, which suggest that social support and a positive neighborhood environment may buffer the negative impact of age discrimination on depressive symptoms. We discuss these findings in light of the prevalence of age discrimination in the US and cross-nationally, and consider potential mechanisms for improving the social environment of older adults, particularly in the post-COVID era.

### SESSION 10110 (LATE BREAKING POSTER)

### AGING IN PLACE

### AGING IN PLACE AMONG ADULTS WITH TRAUMATIC PERSONAL EXPERIENCES AND THE COMMUNITY'S ROLE: A SCOPING REVIEW

Ramona Danielson,[1] and Susan Ray-Degges,[2] *1. North Dakota State University, Fargo, North Dakota, United States, 2. Interior Design Educators Council (IDEC), Fargo, North Dakota, United States*

We propose that trauma history is important context for understanding the adaptive capacity of aging adults to achieve a suitable person-environment fit. We conducted a scoping review using bibliographic databases to identify studies focused on aging in place, vulnerability and traumatic personal experiences, and aging adults' maladaptive behaviors. Our review showed little research directly exploring the connection between trauma-related needs and aging in place and limited research about the community's role in supporting those needs. Literature about the impacts of trauma revealed that trauma could stem from adverse childhood experiences (e.g., abuse, neglect, household dysfunction) and adult adverse experiences (e.g., domestic violence, substance use disorder, military trauma). Adults who have experienced trauma may have increased physiological (e.g., sensory sensitivities) and psychological (e.g., post-traumatic stress disorder) conditions. Sensory sensitivities in the home environment—including sounds (e.g., air conditioners), air quality (e.g., pollutants), and lighting (e.g., flickering lights)—can substantially decrease quality-of-life. Strong emotional reactions can make interpersonal relationships, such as those with a landlord or neighbors, difficult and increase the likelihood of eviction. Aging adults with trauma histories also experience greater poverty rates, increasing the likelihood of substandard housing (e.g., mold, rats). Because the housing environment is a significant aspect of overall well-being, challenges to aging in place for adults with trauma histories warrants further research, as does the role of community supports in mitigating housing-related stressors. Examples may include trauma-informed approaches in universal design, the availability of safe affordable housing, community education, and funding for intermediaries who can support aging adults.

### CAPABLE TRANSITIONS: A HOME HEALTH AGENCY-BASED INTERVENTION TO OPTIMIZE THE SNF-TO-HOME TRANSITION

Rachel Missell,[1] Sarah Szanton,[2] Thomas Caprio,[1] Kobi Nathan,[3] and Adam Simning,[4] *1. University of Rochester, Rochester, New York, United States, 2. Johns Hopkins University, Baltimore, Maryland, United States, 3. St. John Fisher College, Rochester, New York, United States, 4. University of Rochester Medical Center, Rochester, New York, United States*

Community Aging in Place-Advancing Better Living for Elders (CAPABLE) consists of an interprofessional team of a registered nurse (RN), occupational therapist (OT), and handyworker that delivers an in-home client-specific package of interventions to optimize function. CAPABLE aims to reduce functional impairment, home hazards, and acute medical services use and is being widely disseminated. To expand CAPABLE to older adults transitioning from the skilled nursing facility (SNF) to home, we developed CAPABLE Transitions, which makes several important modifications to CAPABLE. First, CAPABLE Transitions will be implemented within a Medicare-certified home health agency (CHHA) and delivered to CHHA clients. Second, it will be delivered to CHHA clients with and without dementia. Adding urgency to CAPABLE Transitions' development, including persons with dementia has the potential to decrease high utilization of services and meet care transition needs. Third, it includes an initial RN care transition visit. Fourth, its services are more intensely delivered at the beginning of the intervention, shortly after SNF discharge. Beginning in the fall of 2020, CAPABLE Transitions will be tested in a feasibility study of 60 older adults discharged from post-acute SNF care to CHHA services in Rochester, NY. We have designed this 3-year feasibility study to consist of yearly recruitment waves that will enable us to iteratively assess and refine the intervention. Following this study, we hope to test CAPABLE Transitions' effect on improving home time, quality of life, and the use of acute medical services in order to assist older adults in aging in place.

### CHALLENGES AND SUPPORTS TO AGING IN PLACE IN A GENTRIFYING CONTEXT

Manish Kumar,[1] and Laura Richman,[2] *1. Duke University, Durham, North Carolina, United States, 2. Duke University School of Medicine, Menlo Park, California, United States*

Neighborhoods play a central role in healthy aging, with changes to neighborhoods having a profound impact on older adults' ability to age in place. Using gentrification as an indicator of neighborhood change and applying the theoretical framework of the Environmental Press model (Lawton and Nahemow, 1973), this study examined the relationship between changing environments, affordable housing, and environmental attributes that support and hinder the health and well-being of older adults. A qualitative, case-study approach was used to interview low-income, majority Black older adults in a gentrifying area of Washington DC. 32 individuals (16 in non-profit and 16 in for-profit affordable housing) aged 55 and older participated in semi-structured interviews on perceptions of gentrification, neighborhood change, and challenges and supports to aging in place. Transcripts were then analyzed using the framework method of analysis. Although participants generally reported that gentrification improved their neighborhood's built environment, many attributed it to a decline in social capital. Affordable housing provided an ability to age in place, though participants expressed uncertainty over their long-term ability to age in the context of continuing change. These findings suggest that while the physical changes accompanying gentrification may support older adults' ability to age in place, its detrimental impact on social capital further increases their risk for social isolation. While affordable housing may enable older adults to age in place, fostering a greater sense of permanence and well-being will require additional policies that both increase accessibility to the physical amenities provided by gentrification and preserve older adults' social capital.

### ENGAGING STAKEHOLDERS TO IMPLEMENT AN AGING IN PLACE INTERVENTION THROUGH AN AREA AGENCY ON AGING

Pamela Toto,[1] Caylee Yanes,[1] Molly Ennis,[1] and Beth Fields,[2], *1. University of Pittsburgh, Pittsburgh, Pennsylvania, United States, 2. University of Wisconsin–Madison, Madison, Wisconsin, United States*

Community Aging in Place, Advancing Better Living for Elders (CAPABLE) is an interdisciplinary evidence-based intervention for reducing disability, reducing cost, and promoting aging in place for older adults who qualify for Medicaid services. However, implementation of CAPABLE through existing community services and with different older adult populations may require adaptations based on stakeholder input. The purpose of this qualitative study was to adapt CAPABLE for implementation through an Area Agency on Aging (AAA) targeting older adults with disability but who do not meet thresholds for Medicaid services. Data collection occurred with stakeholders from a single AAA. Two, 60-minute focus groups were conducted with frontline providers (n=7) and administrators (n=7). Thematic analysis of the data were completed using NVivo 12 Pro. Stakeholders described three themes to consider for implementing CAPABLE in their AAA: screening and referral process, eligibility, and team meetings. Frontline providers recognized the need to allow care managers "to decide who would be most appropriate for the program because they have a better understanding of the person, their family, and home environment." Administrators supported expanding eligibility requirements, "Could we offer the program to someone even if they were recently hospitalized?" All stakeholders expressed that an initial interdisciplinary team meeting could be beneficial for goal setting, "If the team and consumer could be together for the first meeting that could save time and help generate ideas for goals." These perspectives affirm the importance of early stakeholder engagement in adapting to adopt evidence-based programs into "real-world" settings.

### HOW DOES ADULT CHILDREN MIGRATION EFFECTS OLDER ADULTS AGING IN PLACE? COMMUNITY AND INDIVIDUAL LEVEL INTERVENTIONS

Shantha Balaswamy,[1] and Rufus Singh,[2], *1. The Ohio State University, Columbus, Ohio, United States, 2. Madras School of Social Work, Chennai, Tamil Nadu, India*

In India, the increasing emigration by adult children for job opportunity and socio-economic betterment has left vulnerable older adults (OA) to age in place. Almost 'one of out of ten households in Tamil Nadu state has one or more (e) migrants' (TN Migration Survey 2015), leaving behind OA who traditionally depend on family as a major source of support.

There are few studies on impact of immigration of male adult on wife and children who are left behind, but hardly any research on OA who stay behind. This study aims to understand the social, emotional, economic, and health experiences of OA in one of the districts that has the highest rates of out migration. Data was collected using in-person interviews with 304 randomly selected OA whose adult child had migrated to another country. Nearly half of OA live alone (49.7%), have one or more chronic diseases (60%), require support for meeting their ADLs (90%), and half receive no financial support from adult child (50%) contrary to expectations of providing care for OA in this culture. Findings also reveal that persons over the age of 70 exhibit greater coping skills despite lower functional health, smaller social network, and less financial resources. A significant relationship exists between frequency of visits by adult child and reported levels of anxiety and depression. Implication discussions explore solutions at individual, community and policy levels to address vulnerabilities of the older population who are left behind in villages and towns of India due the globalization related mobility.

### IMPROVING AGING IN PLACE FOR OLDER ADULTS WITH LOW INCOMES: PERSPECTIVES OF HOME HEALTH AIDES

Momana Jahan,[1] Claudia Hentschel,[1] Sonia Pandit,[1] Mona Akhiary,[1] Tiffany Wisdom-Goulbourne,[1] Barbara Resnick,[2] Carolina Reid,[3] and Rebecca Brown,[1] *1. University of Pennsylvania, Philadelphia, Pennsylvania, United States, 2. University of Maryland, Baltimore, Maryland, United States, 3. University of California, Berkeley, Berkeley, California, United States*

Older adults with low incomes experience disproportionate rates of cognitive and functional impairment and an elevated risk for nursing home admission. Home health aides (HHAs) can help older adults to age in place by optimizing function and engaging them in routine physical activity. Despite this potential role, little is known about HHAs' perspectives on how to facilitate aging in place for this population. We conducted 6 focus groups with 21 English-speaking and 10 Spanish-speaking HHAs working in Philadelphia and New Jersey. Transcripts were analyzed using qualitative thematic analysis. HHAs described wearing multiple hats and pushing the boundaries of their role as a HHA to provide a "comfortable and safe" environment through nursing and emotional support. Many HHAs shared that they serve as surrogate family, often spending more time in clients' homes than family members or other healthcare providers. This unique position provides HHAs with valuable insights into clients' changing health which allows them to detect early warning signs of clients' functional and cognitive decline, including falls, depression, and confusion. HHAs noted several factors that worsened clients' decline including a lack of adaptive equipment, social isolation, and limited HHA input into clients' care plans. They also pointed to factors that facilitated clients' aging in place, including utilization of community-based services, family support, and communication between healthcare team members. Our findings suggest that HHAs have important insights into improving aging in place for older adults with low incomes and should be incorporated into care planning and intervention delivery.

### INCLUDING CAREGIVERS IN THE COMMUNITY AGING IN PLACE, ADVANCING BETTER LIVING FOR ELDERS PROGRAM

Beth Fields,[1] Caylee Yanes,[2] Molly Ennis,[2] and Pamela Toto,[2], *1. University of Wisconsin–Madison, Madison, Wisconsin, United States, 2. University of Pittsburgh, Pittsburgh, Pennsylvania, United States*

Older adults frequently turn to informal caregivers for support to age independently in their home as long as possible. Yet, many evidence-based programs designed to support aging in place do not include caregivers, including the well-known Community Aging in Place, Advancing Better Living for Elders (CAPABLE) program. The purpose of this qualitative study was to adapt CAPABLE to include caregivers using a grounded theory approach. Data collection occurred with stakeholders from an Area Agency on Aging (AAA) in Pennsylvania. Two, 60-minute focus groups were conducted with frontline providers (n=7) and administrators (n=7). Eight, 30-minute individual interviews were conducted with AAA consumers including older adults and their caregivers. Constant comparative analysis of the data were completed using NVivo 12 Pro. Stakeholders described three considerations for adapting CAPABLE to include caregivers: older adult preference and caregiver willingness, clear guidelines and expectations, and hands-on training. Older adults and caregivers recognized the need to "allow them to decide when and why family should be involved in the program." Frontline providers and administrators explained the importance of "determining whether older adults and caregivers should have shared or separate goals for the program." All stakeholders expressed that including caregivers would "reaffirm the hands-on training like fall prevention." These perspectives shed light on how and why to include caregivers in CAPABLE. Information gleaned from this study may help researchers think about ways in which to adapt other evidence-based programs to include caregivers, and help healthcare providers target and support caregivers in the delivery of their services.

### NOT ALL HOME CARE IS CREATED EQUAL: THE RELATIONSHIP BETWEEN SOURCES OF CARE AND RECIPIENTS' PERCEIVED CONTROL

Molly Wylie, *University of Massachusetts Boston, Boston, Massachusetts, United States*

Perceived control is an important psychological resource for middle-aged and older adults. Aging in place may help foster feelings of control and autonomy, yet many community-dwelling older adults must rely on others for physical assistance. Little is known about psychological reactions to receiving this support. This study investigated how receiving home care from different sources was associated with two facets of perceived control (mastery and perceived constraints) among adults with varying levels of physical disability. Data was drawn from the 2012 and 2014 waves of the Health and Retirement Study. Community-dwelling adults aged 50 years and older receiving help for at least one activity of daily living (ADL) impairment (N = 884) reported their relationship to each respective caregiver (formal professional and/or informal family or friend), level of ADL impairment, and ratings of perceived control. Ordinary least squares (OLS) regression was used to examine the association between type of support and perceived control and the moderating effect of physical disability on that relationship. Receiving formal or a combination of formal and informal support was related to perceptions of greater control over one's life, but only in terms of mastery. The level of one's ADL impairment did not have a moderating effect on the relationship between support type and perceived control. Findings suggested that the type of instrumental support adults receive in their home has implications for specific facets of perceived control. Those receiving formal or mixed support had perceptions of greater mastery than those only receiving informal support.

### OUT-OF-POCKET EXPENSES RELATED TO AGING IN PLACE FOR OLDER PEOPLE EXPERIENCING FRAILTY: A SCOPING REVIEW

Elaine Moody,[1] Rebecca Ganann,[2] Marilyn Macdonald,[1] Lori Weeks,[1] Liz Orr,[2] Keisha Jefferies,[1] Jenny Ploeg,[2] and Ruth Martin Misener,[1] *1. Dalhousie University, Halifax, Nova Scotia, Canada, 2. McMaster University, Hamilton, Ontario, Canada*

Supporting older people to live in the community as they experience health and functional changes has become a priority for policy makers, health system leaders and community members, including many older people themselves. Aging-in-place has been promoted as a way to support the sustainability of health care systems and limit health care and societal costs. However, the expenses borne by individuals and caregivers to support older people to age-in-place when experiencing changes in health and functional ability are often not considered in health care literature and policy. We conducted a scoping review using Joanna Briggs Institute methodology to explore the out-of-pocket expenses for people with frailty living in the community. We included research and policy papers on community-dwelling people over 60 and experiencing frailty. Findings about financial out-of-pocket expenses were extracted. A total of 9669 sources were screened by two reviewers and 42 sources were included. The sources were from 17 countries, most from the US, and had various designs, including 14 qualitative designs, 15 cross sectional, 11 other quantitative and 2 policy discussions. The sources most often reported expenses related to home care (16), medication (12), housekeeping (10), transportation (8), and medical equipment (6). Gaps in the body of literature include lack of a consistent measure of out-of-pocket expenses and cost considerations of co-housing programs. The context—including policy, community and personal—was particularly important to the experience of out-of-pocket expenses for people with frailty, and further research is needed to expand on this knowledge.

### SPATIO-TEMPORAL GAIT VARIABLE DIFFERENCES BETWEEN INDEPENDENT AND ASSISTED LIVING OLDER ADULTS

Christopher Nightingale, Jennifer McNulty, and Stephen Butterfield, *University of Maine, Orono, Maine, United States*

As the US population continues to age, it is important to gain a better understanding of factors that differ between independently living older adults and those that require assisted living. Accidental falls contribute to decreased quality of life for many older adults, and considerable research has been conducted examining measures of fall-risk and potential interventions. In this pilot study, we investigated fall-risk via assessment of spatio-temporal variables associated with gait in independent and assisted-living older adults aged 65+. Two gender-matched groups of participants were tested on seven gait variables (step length, stride length, contact time, contact phase, foot flat, propulsive phase, and average speed) utilizing Opto-Gait technology. Each group included sixteen participants. Independent living participants were recruited through word of mouth and flyers at local senior centers. Assisted living participants all resided in one local health care facility and were recruited via word of mouth and flyers posted in the facility. A one way ANOVA with an alpha level of .05 revealed significant differences between groups in contact time, foot flat, propulsive phase, and average speed. The independent living group performed better on each variable. These findings indicate that older adults living in assisted living facilities may be at much greater risk of accidental fall than their independent living counterparts. While this study identified several key factors related to instability among old adults, additional investigation is warranted to further examine the relationships among gait, fall-risk, and differentiators between independently living and assisted living older adults in these variables.

### SUPPORTIVE BUILT ENVIRONMENT FOR ACTIVE AGING IN KOREA'S HEALTHY CITY

Eunju Hwang,[1] Chungwen Hsu,[1] Jinwook Jeong,[2] Nancy Brossoie,[1] and Kimin Song,[2] *1. Virginia Tech, Blacksburg, Virginia, United States, 2. Hanyang University, Seoul, Seoul-t'ukpyolsi, Republic of Korea*

Many small and medium-size towns in South Korea experience an increasing number of older adults growing older. This medium-size mountain city has been a member of WHO's Healthy City Alliances and we investigated the impacts of built environment (BE) in the community on active aging. The purpose of the survey was to identify the BE affecting the residents' physical activity level and quality of life. The survey data (N=630) were collected from the residents aged 40 years and over in May 2019 from a mountain city near a national park in Northeast part of Korea. The average age of the participants was 67.41 years of age and most wanted to stay in their community. Our descriptive data analysis show that the following items are perceived very important to address in creating more supportive environment: benches in public spaces; safe pathways and public transportation station; well-maintained sidewalks; affordable and accessible housing options. Regression models were also developed to examine the impacts of BE on activity level and quality of life. BE factors included the features related to outdoor spaces and buildings, public transportation, streetscape and housing, and found a significant relation between the participants' activity level and public transportation in addition to demographic factors such as age and health status. Regarding the quality of life, the participants' perception on outdoor spaces and buildings and safe public spaces were positively related to quality of life. The study showed the importance of supportive and age-friendly environments for active living.

### SESSION 10120 (LATE BREAKING POSTER)

### ALZHEIMER'S DISEASE AND RELATED DEMENTIAS

### A COMMUNITY-BASED WORKSHOP ON ADDRESSING DEMENTIA-RELATED STIGMA: FIRST INSIGHTS FROM A RURAL COMMUNITY

Juanita Bacsu,[1] Marc Viger,[2] Shanthi Johnson,[3] and Megan O'Connell,[2] *1. University of Regina, Regina, Saskatchewan, Canada, 2. University of Saskatchewan, Saskatoon, Saskatchewan, Canada, 3. University of Alberta, Edmonton, Alberta, Canada*

Dementia-related stigma can delay early dementia diagnosis and lead to social isolation, depression, and suicide. Despite this knowledge, few studies identify strategies to reduce dementia-related stigma. This late-breaker poster begins to address this gap by showcasing the educational components of a community-based workshop to share study findings on reducing dementia-related stigma in rural communities. Guided by solutions-focused theory, semi-structured interviews were conducted with 18 seniors including family members, friends, caregivers and people affected by dementia and other forms of cognitive impairment in rural Saskatchewan, Canada. A focus group was conducted with 7 rural community leaders. The interview and focus group transcripts were analyzed using thematic analysis. Based on the interview and focus group findings, educational components of the workshop included: a dementia definition, different dementia types, warning signs/ symptoms, risk reduction strategies, and information on dementia-related stigma and myths. Several strategies to reduce stigma were identified ranging from hosting intergenerational programs to inviting guest speakers with dementia. This study was found to be beneficial for improving knowledge, attitudes, comfort levels, and awareness of dementia. Additional research is needed to develop, implement, and evaluate interventions to reduce dementia-related stigma in different cultures and contexts.

### AGING ACROSS THE LIFE COURSE: RESEARCH COLLECTIONS AVAILABLE FROM THE NATIONAL ARCHIVE OF COMPUTERIZED DATA ON AGING

James McNally, *University of Michigan, Ann Arbor, Michigan, United States*

The creation and maintenance of sustainable data archives can be challenging, but it offers clear advantages. Properly curated data can be used by multiple researchers, testing a variety of hypotheses, and increasing the return on investment to the expensive process of data collection. Having an internally managed archival system also provides greater control and autonomy in the equitable distribution of data resources. This process ensures all researchers will have full use of the data for original research, teaching, and new directions once the data leaves the control of the local investigator's control. This poster reviews the advantages of having a local strategy geared toward the preservation and sharing of gerontological research data. Using the National Archive of Computerized Data on Aging (NACDA) as a working example, the poster offers an overview of collections at NACDA. Using our metadata tools and variable search database, NACDA can identify studies in its collections that examine aspects of aging and health among adults during their lifecourse. Many of the studies are longitudinal or repeat measure cross-sectional studies. We are also able to identify studies that focus on aging that are not maintained by NACDA but which are available to interested researchers. This poster focuses on newly released work by NACDA that has compared to major aging studies (NHATS and NSHAP) and organized them into quasi-harmonized files for download and analysis by the gerontological research community. These recently available enhancements will allow researchers to obtain user-friendly comparative data for these complex studies.

### ASSOCIATION BETWEEN LATE-LIFE HYPERCHOLESTEROLEMIA AND PROGRESSION OF DEMENTIA SEVERITY AMONG OLDER ADULTS

Mo-kyung Sin,[1] Yan Cheng,[2] Ali Ahmed,[2] and Edward Zamrini,[2], *1. Seattle University, Seattle, Washington, United States, 2. George Washington University, Washington, District of Columbia, United States*

Progression of dementia severity varies widely by individuals and multiple factors might influence the progression. The aim of this study was to examine the relationship between late-life hypercholesterolemia and progression of dementia severity in older adults. We used prospectively collected longitudinal data from 2,686 adults aged ≥65 years in the National Alzheimer's Coordinating Center. Progression of dementia severity was measured using both Clinical Dementia Rating (CDR) - Sum of Boxes (SOB) and Global scores. Kaplan Meier curves were plotted to estimate the association between hypercholesterolemia and progression of dementia severity. We also conducted multivariate Cox regression models to estimate the association of hypercholesterolemia with the outcomes adjusting for age, gender, race, ethnicity, marital status, living status, education, smoking, heart failure, atrial fibrillation, blood pressure, and diabetes. Hypercholesterolemia had significant association with CDR-SOB ≥ 1 point increase (unadjusted HR, 1.23; 95% CI, 1.13-1.35; p<0.001; adjusted HR, 1.17; 95% CI, 1.07-1.28; p<0.001). In addition, hypercholesterolemia had significant association with CDR-Global ≥ 0.5 point increase (unadjusted HR, 1.14; 95% CI, 1.04-1.25; p<0.001; adjusted HR, 1.11; 95% CI, 1.01-1.22 p=0.036). If these findings can be replicated in future studies, future studies need to examine if proper management of cholesterol may reduce the risk of Alzheimer's dementia in late-life older adults.

### CHANGE IN BODY MASS INDEX IS ASSOCIATED WITH CHANGE IN COGNITION IN OLDER ADULTS

Joy Douglas,[1] Kristi Crowe-White,[2] Amy Ellis,[1] Chuong Bui,[1] Saroja Voruganti,[3] and Kristine Yaffe,[4] *1. The University of Alabama, Tuscaloosa, Alabama, United States, 2. University of Alabama, Tuscaloosa, Alabama, United States, 3. University of North Carolina at Chapel Hill, Kannapolis, North Carolina, United States, 4. UCSF Weill Institute for Neurosciences, San Francisco, California, United States*

Background: Alzheimer's disease and related dementias affect one in ten Americans age 65y and older. Considering the rapid growth of the aging population, identifying modifiable risk factors for cognitive decline is a public health priority. Although weight change later in life is common, its impact on cognition is unclear. The objective of this study was to examine the relationship between change in body mass index (BMI) and cognition among older adults. Methods: The Health, Aging, and Body Composition Study was a prospective study of community-dwelling adults ages 70-79y at baseline (n=3,075; 49% males, 42% African-American). Using baseline and year 10 visit data, we evaluated change in BMI and change in cognition measured by the Modified Mini-Mental Status Exam (3MS) using a linear mixed model. Change in 3MS scores were regressed on changes in time-varying BMI after controlling for blood pressure, glucose, cholesterol, race, education, biological sex, and APOE genotype. Results: At baseline, average BMI was 27.4 (n=3075) and average 3MS was 90.1 (n=3061). At year 10, average BMI was 27.1 (n=1600) and average 3MS was 88.6 (n=1598). Higher BMI was associated with less cognitive decline (ceteris paribus). This finding suggests that weight gain is associated with cognitive maintenance. The effect of an increase in BMI was largest for those underweight at baseline. Conclusion: Among underweight older adults, an increase in BMI may be desirable for maintaining cognition. Although more research is needed, these findings suggest the need for interventions to prevent unintentional weight loss among older adults.

### CLINICAL CORRELATION OF CEREBROSPINAL FLUID TOTAL TAU LEVELS AND MMSE SCORE IN A MEMORY CLINIC.

Neil Tangal, Neeraj Tangal, Anil Nair, and Malini Nair, *Alzheimer's Disease Center, Quincy, Massachusetts, United States*

Tau protein levels in cerebrospinal fluid are a biomarker of Alzheimer's disease. We correlated MMSE severity to CSF tau levels in a large memory clinic sample. We retrospectively analyzed data from patients attending a memory clinic in the south shore of Boston from 2010 to 2020, and had a lumbar puncture to obtain CSF Tau levels. We compiled cognitive screen data from MMSE scores. Univariate analyses used Spearman correlation as data were non-normal. A multivariate model was created including covariates of age, sex, and race. 965 patients attended the memory clinic from 2010 to 2020. 711 had available MMSE scores. 129 subjects had lumbar punctures and available CSF tau levels. Univariate analyses showed that cognition as measured by MMSE total was not correlated to total tau levels in the CSF (rho=-0.07, p>0.05), but caucasian race was inversely associated with CSF tau levels (rho= -0.217, p<0.05). In a multivariate model, tau levels in the CSF were not associated with MMSE, race, gender, or age. In a large memory clinic sample, CSF tau levels did not correlate to MMSE scores, age, race or gender.

### DEMYSTIFYING GRIEF IN THE DEMENTIA DIVIDE: A CASE FOR GRIEF THERAPY IN DEMENTIA CARE

Adrienne Ione, *Silver Linings Integrative Health, LLC, Tacoma, Washington, United States*

The results of emotional and psychological losses overlap with behavioral and psychological symptoms of dementia (BPSD) in the neuronal and structural changes in the brain. The aim of this current study was to explore the magnifying effects of COVID-19 on exacerbating residual losses, illuminating a case for using grief therapy to moderate BPSD. A case control study was conducted with people ages 65 and greater, with an established diagnosis of dementia prior to March 2020. Compared with an active control group - participants without a current dementia diagnosis who self-reported mild cognitive shifts and who also received active grief-informed therapies – offer supporting evidence of a strong factor of efficacy for including grief therapy in services offered to people living with dementia. Evidence of a continued point improvement on both the brief grief questionnaire and inventory of complicated grief, as well as decreased severity of items on NPI-Q corroborate this therapeutic recommendation. Now more than ever – as people across the globe who are diagnosed with dementia face uncertain ramifications of previous grief episodes, ones that have potentially been reignited by the flames of COVID-19 – therapists must foster safe spaces informed by novel therapeutic grief approaches. In any just society, emphasis on therapeutic techniques that allow participants to ventilate their feelings and fears, as well as promote movement along a continuum from isolation to intimacy, must prevail. People exhibiting BPSD should not be excluded from such treatments.

### DEVELOPMENT AND PILOT OF A NEWLY DEVELOPED TOOL FOR ASSESSING RESEARCH PARTICIPATION FOR INDIVIDUALS WITH DEMENTIA

Katherine Judge, Morgan Minyo, Amanda MacNeil, Kaitlyn Lucas, and Claire Grant, *Cleveland State University, Cleveland, Ohio, United States*

Research supports the inclusion of individuals with mild to moderate dementia (IWDs) as study participants in providing reliable and valid self-report information about their illness experience. However, no clear guidelines or tools exist for determining study eligibility with many studies relying on brief cognitive measures (e.g., MMSE). The literature suggests not all individuals with mild/moderate dementia can participate and some individuals with severe symptoms of dementia can participate. This study piloted a new measure designed to assess whether IWDs can participate in self-report data protocols. The measure consists of 10 questions that assess relatively in-tact cognitive processes hypothesized for successful participation. Example questions include: "What is your favorite holiday?" and "Give an example of a sad occasion/event". Questions are scored as 'correct' or 'incorrect' and summed for a total score. To examine the descriptive characteristics of the measure, IWDs (n=18) completed the measure along with the MMSE and, for some IWDs (n=12), several self-report measures. Scores on the new measure ranged from 0-10, with a M=7.61; SD=2.75. MMSE scores ranged from 2-22, with a M=13.39; SD=6.47. A significant correlation (r = .86, p< .001) was found with the MMSE, indicating a high degree of relatedness but not complete construct overlap. Results also highlight the variability of the measure, with incorrect responses ranging from 3 to 6 across participants. Additional properties of the measure will be discussed along with highlighting how study findings fit with recommendations from 2020 NIA Research Summit on Dementia Care and next-steps in refinement and testing.

### DOES ANXIETY AFFECT PERFORMANCE ON ATTENTION TASK (DIGIT SPAN FORWARD) ON THE MOCA TEST? A CLINICAL CORRELATION STUDY

Rima Patel,[1] and Anil Nair,[2], *1. The Alz Center, Burlington, Massachusetts, United States, 2. Alzheimer's Disease Center, Quincy, Massachusetts, United States*

It is unknown if anxiety affects performance on Digit span forward (DSF) in memory clinic patients. We performed a retrospective chart review of memory clinic patients in the south shore of Boston from 2010 to 2020. We correlated anxiety screen data (GAD7) to Digit Span Forward (DSF) scores obtained from the MoCA. As the data were not normal, we performed univariate analyses with Spearman correlation. A multivariate regression model estimated the relationship of DSF to covariates of GAD7, age, sex, and race. We hypothesized a negative correlation between anxiety levels scored by GAD7 and DSF. H0: Digit span forward DSF ~ GAD7+Age+Sex+Race. A chart review found 965 patients attending the memory clinic between 2010 to 2020 had analyzable data. 433 patients with available DSF and 737 had available GAD7. The patients were 58.7% female and 84.7% caucasian. The mean age was 70.1±14.4, DSF 0.8±0.4 and GAD 5.6±5.7. DSF correlated significantly to race (ρ=-0.25, p=<0.001), but not to gender (ρ=0.05, p=0.149), age (ρ=-0.04, p=0.3), or GAD7 (ρ=-0.018, p=0.71). There was no significant association of DSF to race, age, gender or GAD7 on the multivariate model. In memory clinic subjects there exists no correlation between anxiety levels scored by GAD7 and DSF performance.

### EARNING THE TRUST OF AFRICAN AMERICAN COMMUNITIES TO INCREASE REPRESENTATION IN DEMENTIA RESEARCH

Elena Portacolone,[1] Nynikka Palmer,[1] Peter Lichtenberg,[2] Catherine Waters,[1] Carl Hill,[3] Sahru Keiser,[1] and Julene K. Johnson,[1], *1. University of California San Francisco, San Francisco, California, United States, 2. Wayne State University, Detroit, Michigan, United States, 3. Alzheimer's Association, Chicago, Illinois, United States*

Black/African American populations are underrepresented as participants in dementia research. A major barrier to participation of African American older adults in dementia research is a tendency to distrust research institutions owing to a legacy of racism. Building on the Ford framework, the objective of our study was to examine factors that influence participation in dementia research among African American older adults and caregivers, with an emphasis on understanding factors related to trust. Data were collected from 10 focus groups with African American older adults (n=91), 5 focus groups with caregivers (n=44), and interviews with administrators of community-based organizations (n=11), and meetings with our Community Advisory Board. Inductive/deductive content analysis was used to identify themes. The results identified an overall tension between distrust of researchers and a compelling desire to engage in dementia research. This overarching theme was supported by six themes that provided insights about the multiple layers of distrust, as well as expectations about the appropriate conduct of researchers and academic institutions. Strong commitment to the community was identified as a priority. The findings suggest that a paradigm shift is needed to increase the representation of African Americans in dementia research. In this new paradigm, earning the trust of African American communities becomes a systemic endeavor, with academic, state and national institutions deeply committed to earning the trust of African American communities and guiding researchers in this endeavor. The findings also generated actionable recommendations to help improve representation of African American older adults in dementia research.

### GUIDELINES FOR USING TELE-TECHNOLOGY TO DELIVER MIND-BODY INTERVENTIONS FOR PEOPLE WITH MILD COGNITIVE IMPAIRMENT

Kara Cohen, Tracy Mitzner, and Patricia Griffiths, *Georgia Institute of Technology, Atlanta, Georgia, United States*

Individuals with Mild Cognitive Impairment (MCI) may have limited access to intervention programs that support their mental and physical health. The COVID-19 pandemic has put them at an even greater risk of not having access to such programs. While there is currently no cure, there is growing evidence that intervention programs may attenuate the progression from MCI to dementia, particularly those which 1) have potential to reduce the level of cardiovascular risk factors, 2) employ cognitively stimulating activities, and 3) create opportunities for social interaction (Petersen, Lopez, Armstrong et al., 2018; Wayne, Yeh, & Mehta, 2018; Mortimer, Ding, Borenstein et al., 2012). Many mind-body interventions, such as tai chi, yoga, and mindfulness classes, contain these three elements and have been shown to benefit individuals diagnosed with MCI, including improving cognition (e.g., Wells, Kerr, Wolkin, et al. 2013; Yang, 2016). Tele-technology (i.e., technology that supports communication between people who are not co-located) can aid in overcoming the logistical barriers by bringing instructors and interventions to these individuals to help them stay engaged and attend activities more frequently from the comfort and convenience of their home. We will present recent findings from a user study with 8 stakeholders (4 subject matter experts, 2 individuals with MCI, 2 care partners) to assess barriers and facilitators to using tele-technology to bring instruction of mind-body interventions to individuals diagnosed with MCI. This poster will present guidelines for delivering such interventions based on our findings from the user study, including safety and training protocols.

### INFLUENCES OF DEMENTIA ON LONG-TERM SURGICAL OUTCOMES IN OLDER ADULTS AFTER HIP FRACTURE

Tingzhong (Michelle) Xue,[1] Eleanor McConnell,[2] Chiyoung Lee,[1] Hideyo Tsumura,[3] Sijia Wei,[3] and Wei Pan,[1] *1. Duke University, Durham, North Carolina, United States, 2. Durham, North Carolina, United States, 3. Duke University School of Nursing, Durham, North Carolina, United States*

Older adults with dementia are more prone to have adverse health outcomes following hip fracture surgery. However, individuals with dementia and hip fracture are older and have more co-morbidities; these baseline differences can bias estimates of the influence of dementia. This study aims to investigate how dementia influences disposition, mortality rates and readmission rates at 365 days after hip surgery in older adults over age 65, after accounting for baseline factors such as socioeconomic status, health behaviors, co-morbidities, and type of hip fracture repair. A cohort of 1172 patients who had hip fracture surgery between October 2015 and December 2018 was extracted from electronic health records; among those, 376 had a diagnosis of dementia. Inverse probability of treatment weighting using propensity scores method was used to reduce the influence of factors that may confound the relationship between dementia status and hip surgery outcomes. Logistic regression was applied to estimate influences on discharge disposition and Cox proportional hazards model for one-year mortality. To account for competing risk of death, a Fine and Gray regression model was used to calculate subdistribution hazard ratios of readmission. Disparities in long-term surgical outcomes in patients with dementia were found. Results show that dementia was a significant predictor for being discharged to facilities (OR=1.92, 95% CI 1.09, 3.39, p=.025), death (HR=1.98, 95% CI 1.50-2.62, p<.0001) and being readmitted within one year (HR=1.31, 95% CI 1.15-1.50, p<.0001). These findings call for more efforts in developing effective multidisciplinary perioperative assessments and rehabilitation for patients with dementia.

### INTERVENTIONS TO REDUCE STIGMA OF DEMENTIA: FIRST INSIGHTS FROM A RURAL COMMUNITY-BASED PARTICIPATORY STUDY

Juanita Bacsu,[1] Shanthi Johnson,[2] Megan O'Connell,[3] and Marc Viger,[3], *1. University of Regina, Regina, Saskatchewan, Canada, 2. University of Alberta, Edmonton, Alberta, Canada, 3. University of Saskatchewan, Saskatoon, Saskatchewan, Canada*

Age is the greatest risk factor for dementia, and the number of rural older adults is rising. Although dementia-related stigma is widely documented, few studies focus on ways to reduce stigma, especially within rural communities. This late breaker presentation aims to: 1) explore the contributing factors of dementia-related stigma in rural communities; and 2) identify interventions to reduce stigma of dementia in rural communities. Drawing on a community-based participatory approach, data were collected through semi-structured interviews with 18 older adults, and a focus group with 7 community leaders in rural Saskatchewan, Canada. Thematic analysis was used to identify key themes and patterns within the data. Contributing factors of dementia-related stigma ranged from fear to lack of dementia knowledge. Several anti-stigma interventions were identified including: forming support groups; hosting educational workshops; inviting guest speakers with dementia; talking openly about dementia; learning more about dementia; asking questions; sharing your lived-experiences; being inclusive; developing inter-generational programs; and avoiding assumptions and hurtful jokes. As the rural population ages, there is a growing need for interventions, programs, and policies to address stigma of dementia. Engaging in rural partnerships and collaborative research is essential to developing community-informed strategies to reduce dementia-related stigma and improve the quality of life for people with dementia.

### LIFESTYLE INTERVENTIONS FOR PERSONS WITH DEMENTIA: SINGING YOUR WAY TOWARD WELLNESS

Cynthia McDowell, Sebastian Santana, André Smith, Debra Sheets, and Stuart MacDonald, *University of Victoria, Victoria, British Columbia, Canada*

Arts-based interventions represent an inexpensive, non-pharmacological, and non-invasive approach to help mitigate negative symptoms and improve quality of life for persons with dementia (PwD). The present study examined whether a social singing intervention can modulate patterns of cognitive change and whether select biopsychosocial indicators exhibit concomitant within-person time-varying covariation. Participants with dementia (n=32, mean age=79.6 years; 53% female) engaged weekly in the Voices in Motion project, an intergenerational, social-cognitive choral intervention spanning up to 18 months and 9 individual assessments. The Mini Mental State Examination (MMSE), gait velocity, and positive and negative affect were assessed using an intensive repeated-measures design, with multilevel models of change employed to disaggregate both between- and within-person effects. Across months of the social intervention, several significant within-person time-varying associations were observed; on occasions when a given individual performed one unit faster on gait velocity (p<.05) or one unit lower on negative affect (p<.01), relative to their personal average, there were corresponding improvements in cognitive function. Notably, in contrast, MMSE change remained relatively stable over the course of the 18-month intervention (-0.105, p=0.12), with little between-subject variability in rates of change. These findings imply that, within-persons, reducing comorbidities associated with dementia (e.g., elevated negative affect and its corresponding impact on cognitive resource competition) through participation in a lifestyle intervention may facilitate increases in cognitive, physiological, and psychological function. Implications are discussed with regard to the merits of invoking virtual lifestyle interventions for socially isolated individuals (e.g., PwD and those in residential care).

### PAIN INTENSITY AND UNPLEASANTNESS IN PEOPLE WITH VASCULAR DEMENTIA: A CROSS SECTIONAL STUDY

Angela Humbel,[1] R. Cowan,[2] Mary Dietrich,[3] Wm. Larkin Iversen,[1] Sebastian Atalla,[1] Karen Moss,[1] Sydney Englehart,[1] and Todd Monroe,[1], *1. The Ohio State University, Columbus, Ohio, United States, 2. The University of Tennessee Health Science Center, Memphis, Tennessee, United States, 3. Vanderbilt University, Nashville, Tennessee, United States*

Pain is a multidimensional sensory and affective experience. People with Vascular Dementia (VaD) experience pain more intensely and have negative emotional responses. Further investigation is needed to understand the neurobiology of pain in VaD. We used experimental thermal pain in a cross-sectional design to determine if adults (age>64) with probable VaD experience increased pain intensity and increased pain unpleasantness during "mild" and "moderate" thermal pain. The final sample included 46 sex- and age-matched adults (23 VaD; 12 female) and controls (23 cognitively intact; 12 female) with an average age of

76.5 years (SD=7.5). Participants reported no daily analgesic use. We used a thermode placed on the thenar eminence to assess temperatures perceived as mild and moderate pain (°C) followed by unpleasantness ratings (0-20 scale). We assessed cognition and depression with the Mini-Mental State Exam (MMSE) and the Geriatric Depression Scale. After controlling for depression, and relative to controls, there was no statistically significant difference in the temperature at which people with VaD perceived mild or moderate pain (p = .086; Cohen's d: mild=0.55, moderate=0.27). However, there was a statistically significant effect of VaD status on pain unpleasantness (p = .003). People with VaD reported mild and moderate pain as more unpleasant than controls (Cohen's d = 0.79 and 0.60, respectively). Findings support previous work that people with VaD are at risk of experiencing more pain. Assessing pain intensity and affect can avoid under-treated pain in those with VaD.

### PRE-STROKE DEMENTIA AND IN-HOSPITAL OUTCOMES IN THE CHINESE STROKE CENTER ALLIANCE

Chelsea Liu,[1] Hong-Qiu Gu,[2] Xin Yang,[2] Chun-Juan Wang,[2] Kai-Xuan Yang,[2] Zi-Xiao Li,[2] and Yong-Jun Wang,[2], *1. Harvard School of Public Health, Boston, Massachusetts, United States, 2. Beijing Tiantan Hospital, Beijing, China*

Little is known about the prevalence of pre-stroke dementia in China and whether this group is at higher risk of adverse in-hospital outcomes. We aimed to understand this association using data from the Chinese Stroke Center Alliance. Multivariable logistic regressions were conducted to assess the association between pre-stroke dementia status and ambulation at day 2, in-hospital mortality, and in-hospital complications. Covariates included age, sex, medical history, history of smoking, history of alcohol use, medication history (antiplatelet drugs, lipid-lowering drugs), stroke severity (measured by the National Institute of Health Stroke Scale), whether IV tPA was administered within 4.5 hours, and whether the patient received deep vein thrombosis prophylaxis as needed. Odds ratios and 95% confidence intervals were presented for the adjusted models. In the final analytic sample of 559,070 ischemic stroke patients with no prior stroke history enrolled across 1476 hospitals, 1511 (0.3%) had pre-stroke dementia, and they were older and more likely to be female. Patients with pre-stroke dementia had lower odds of ambulating at day 2, higher odds of having any complications and higher odds of in-hospital mortality compared to those without pre-stroke dementia, despite little difference in treatment received. Our findings may be explained by communication barriers experienced by patients with pre-stroke dementia that limited their ability to advocate for their own care needs. Further research is needed to determine whether a different care pathway or additional attention from clinicians is necessary for patients with pre-stroke dementia.

### RESEARCH PARTICIPANT INTEREST IN ALZHEIMER'S DISEASE BIOMARKER DISCLOSURE

Claire Erickson,[1] Nathaniel Chin,[1] Carey Gleason,[2] Sterling Johnson,[1] and Lindsay Clark,[1] *1. University of Wisconsin-Madison, Madison, Wisconsin, United States, 2. University of Wisconsin School of Medicine and Public Health, Madison, Wisconsin, United States*

Researchers can characterize the pathophysiological progression of Alzheimer's disease (AD) even in the absence of symptoms. As we better understand the role of biomarker accumulation in the clinical manifestation of AD, disclosing personal biomarker information will become increasingly relevant. Yet, interest and preferences for AD biomarker disclosure are not well understood. We developed a 30-minute phone survey to gather information from Black and white participants on likelihood to enroll in biomarker disclosure studies, reasons for enrolling, and potential outcomes following a hypothetical positive result. Data were collected from cognitively healthy participants (n=334, mean age=64.8±7.7, 45% Black) enrolled in the Wisconsin Alzheimer's Disease Research Center or Wisconsin Registry for Alzheimer's Prevention. 49.7% of participants were very or extremely likely to enroll in an AD biomarker disclosure study. This result varied by biomarker method, with about half the sample very or extremely likely to enroll in PET scan disclosure (45.5%), fewer likely to enroll in cerebrospinal fluid disclosure (32.2%), and a majority likely to enroll in blood-based biomarker disclosure (86.2%). The most important reasons for learning biomarker results included informing lifestyle changes to help prevent dementia (82.9% responded very or extremely important) and knowing more about personal AD risk (69.1% responded very or extremely important). These results suggest that as biomarker collection method burden decreases, willingness to participate in a biomarker disclosure study increases. Further, personal dementia prevention and risk are a strong motivator for learning biomarker results. Moving forward, these results may inform AD biomarker protocol development.

### SEX-SPECIFIC BDNF AND APOE ε4 GENOTYPE INTERACTIONS ON WHITE MATTER HYPERINTENSITY VOLUME

Brandon Pitts, *University of Kansas, Olathe, Kansas, United States*

The apolipoprotein E (APOE) gene is the strongest genetic risk factor for late-onset Alzheimer's disease (AD). One variant of the brain-derived neurotrophic factor (BDNF) gene also confers higher risk of AD. APOE and BDNF genotypes may have interactive effects on AD pathology. The aim of this study was to determine whether APOE and BDNF genotype differentially impact white matter hyperintensity volume (WMHV). We used data from 212 cognitively unimpaired individuals from the Alzheimer's Disease Neuroimaging Initiative (ADNI) We used generalized linear models to predict WMHV from BDNF genotype and an interaction with APOE. Sex, age, education, vascular risk, and imaging-based amyloid load (Florbetapir SUVR) were used as covariates. . WMHV was derived by using the Lesion Segmentation Toolbox (LST) in SPM12 with a threshold of k = 0.15. We used the Hachinski Ischemic Scale to measure for vascular risk. We found no significant interaction of BDNF-APOE on WMHV (β = 0.40, 95% CI: (-0.39, 1.20), p = 0.32). In sex-stratified analyses the BNDF-APOE interaction was significantly associated with WMHV in males (β = 1.14, 95% CI:(0.17, 2.11), p = 0.02), but not in females (β = -0.37, 95% CI: (-1.47, 0.76), p = 0.50). For males, carriers of both BDNF Met and APOE ε4 alleles had the highest WMHV. Sex-specific differences in BDNF expression may be related to hormonal differences, and BDNF genotype might underlie these differences. Given the continued interest in using BDNF as a treatment target for AD, understanding complex genotype interactions may aid in future therapeutic studies.

### THE ROLE OF GEOGRAPHICAL DIFFERENCES AND DISCRIMINATION IN THE COGNITIVE HEALTH OF OLDER BLACKS

Kimson Johnson,[1] Ketlyne Sol,[1] Briana Sprague,[2] Tamara Cadet,[3] Elizabeth Munoz,[4] and Noah Webster,[1]
*1. University of Michigan, Ann Arbor, Michigan, United States, 2. University of Pittsburgh, Pittsburgh, Pennsylvania, United States, 3. Simmons University, Boston, Massachusetts, United States, 4. The University of Texas at Austin, Austin, United States*

Where you live in old age matters in the United States in terms of access to health care and potential exposure to discrimination. This is especially important for non-Hispanic Blacks. Everyday experiences of discrimination are known to “get under the skin” but to date few studies have examined if associations between discrimination and cognitive health differ by region and urbanicity. This study aims to fill this gap. Data are from the 2012 and 2014 waves of the Health and Retirement Study (HRS) (N =2,347). Non-Hispanic Blacks over age 51 with complete data on the Everyday Discrimination Scale in either 2012 or 2014 and cognition, residential location were included in the analysis. Regression models included covariates for age, gender, education and examined interactive effects of everyday discrimination, region, and urbanicity on episodic memory in a nationally representative sample of non-Hispanic older Black adults in the U.S. Descriptive analyses revealed that the majority lived in Southern regions and in non-urban areas. Respondents also reported experiencing discrimination a few times a year. Regression analyses indicated that experiencing more everyday discrimination was significantly associated with lower episodic memory when living in urban areas. Among Blacks, the discrimination-episodic memory link did not significantly vary across U.S. regional contexts. Findings highlight the importance of socio-environmental factors in shaping how stressful experiences such as everyday discrimination are linked to cognitive health in later life. Future research should focus on the development of focused upstream interventions that are responsive to racial disparities in cognitive health in urban areas.

### UNMET NEEDS AND BEHAVIORAL EXPRESSIONS: THE PERSPECTIVE OF INDIVIDUALS WITH MILD TO MODERATE DEMENTIA

Morgan Minyo, and Katherine Judge, *Cleveland State University, Cleveland, Ohio, United States*

Research examining the illness experience from the perspective of individuals with dementia (IWDs) has gained increased attention. Previous studies suggest many IWDs experience unmet needs and behaviors that can impact their well-being. Most research has assessed these constructs using caregiver proxy-reports, resulting in underestimation of the amount and type of unmet needs. Additionally, no published studies have assessed self-reported behavioral expressions. As part of a larger study conducted in a memory care unit, the current pilot study examined the reliability of these measures along with examining how IWD’s experienced unmet needs, behaviors, and behavioral distress. Results found that individuals with mild to moderate dementia (n=12) were able to provide reliable self-report data about their own unmet needs (∝=.96), unmet need-related distress (∝=.98), and behavioral expressions (∝=.91). Nearly all participants identified at least one unmet need (74.8%). The highest reported unmet needs concerned health information (75.0%) and findings/arranging services (41.7%). The least reported unmet needs concerned daily living activities (16.7%) and legal/financial services (33.2%). The highest unmet needs related-distress concerned emotional support (41.7%) and the least distressing concerned daily living activities (16.7%). The most frequently reported behaviors included agitation (66.7%) and complaining/criticizing things (58.3%) while the least reported behaviors were refusing to be left alone (8.3%) and yelling/swearing (8.3%). The highest behavioral distress reported was agitation (58.3%) while the least was wandering (8.3%). Discussion will highlight how these results fit within current literature, recommendations for future research in exploring these constructs using self-report methodology, and how well-being outcomes may be impacted.

### SESSION 10130 (LATE BREAKING POSTER)

### ASSESSMENT

### AGE DIFFERENCES IN EXECUTIVE CONTROL ASSESSED WITH A ROBOTIC OBJECT HIT AND AVOID TEST OF RAPID MOTOR DECISION MAKING

Alexandra Watral, and Kevin Trewartha, *Michigan Technological University, Houghton, Michigan, United States*

Motor decision-making processes are required for many standard neuropsychological tasks, including the Trail Making Test (TMT), that aim to assess cognitive functioning in older adults. However, in their standard formats, it is difficult to isolate the relative contributions of sensorimotor and cognitive processes to performance on these neuropsychological tasks. Recently developed clinical tasks use a robotic manipulandum to assess both motor and cognitive aspects of rapid motor decision making in an object hit (OH) and object hit and avoid (OHA) task. We administered the OH and OHA tasks to 77 healthy younger adults and 59 healthy older adults to assess age differences in the motor and cognitive measures of performance. We administered the TMT parts A and B to assess the extent to which OHA performance is associated with executive functioning in particular. The results indicate that after controlling for hand speed, older adults performed worse on the OH and OHA tasks than younger adults, performance declines were far greater in the OHA task, and the global performance measures, which have been associated with cognitive status, were more sensitive to age differences than motor measures of performance. Those global measures of performance were also associated with measures of executive functioning on the TMT task. These findings provide evidence that rapid motor decision making tasks are sensitive to declines in executive control in aging. They also provide a way to isolate cognitive declines from declines in sensorimotor processes that are likely a contributing factor to age differences in neuropsychological test performance.

### ASSESSING SLEEP QUALITY IN OLDER ADULTS: A COMPARISON OF THREE MEASUREMENT APPROACHES

Amy Berkley,[1] and Patricia Carter,[2], *1. University of Kansas, Kansas City, Kansas, United States, 2. Capstone College of Nursing, The University of Alabama, Tuscaloosa, Alabama, United States*

Discrepancies between subjective and objective sleep measures have been reported for some time; however, it is critical to consider the implications of inaccurate or incomplete sleep assessment for frail older adults who are struggling to maintain independence. To compare sleep assessment methods, we collected objective sleep measurements (via wrist actigraphy), subjective measures via self-report sleep surveys (Pittsburgh Sleep Quality Index; Insomnia Severity Index, Sleep Hygiene Index), and qualitative data through semi-structured audio-recorded interviews, from 8 older adults who self-reported sleep problems while living in a retirement community in southwestern US. Participants' objective sleep (Total Sleep Time, Sleep Onset Latency, Wake After Sleep Onset, and Sleep Efficiency) and qualitative narratives were congruent, but self-report measures failed to capture several unique sleep problems identified in the sample. Disordered sleep in older adults has been linked to increased incidence of falls, depression and anxiety, cognitive impairment, institutionalization, and mortality, but traditional sleep assessment instruments, designed for the general adult population, fail to capture many of the experiences and causes that are unique to older adults. functioning. A sleep assessment tool designed to measure older people's sleep experiences could provide more accurate and sensitive data.

### PRIMARY CARE PROVIDER ATTITUDES AND PERCEPTIONS OF ROUTINE COGNITIVE SCREENING IN OLDER ADULTS

Giovanna Potenza, and Meghan Mattos, *University of Virginia, Charlottesville, Virginia, United States*

The 2011 Annual Wellness Visit (AWV) Medicare benefit includes a cognitive screening component intended to improve screening of older adults. However, available literature only presents physician perspectives on cognitive screening prior to 2011. The purpose of this study was to explore primary care provider (PCP) attitudes and perceptions of cognitive screening in older adults. An Internet-based survey link was sent to Virginia professional organizations and clinics to distribute to PCPs serving older adults. Likert scale, multiple choice, and free response questions were used to understand current attitudes, perceptions, and practices. The sample (N=39) was comprised of 26 nurse practitioners (NPs), 9 physicians, and four who did not disclose role. Most participants were aware of the AWV (n=31, 88.6%) and agreed that early detection "promotes earlier diagnosis and access to resources" (mean ± standard deviation,1.58±0.69). However, less than half of NPs screened annually (n=10/26, 38.5%) and even less conducted screening during an AWV (n=7/26, 26.9%). About half of MDs conducted cognitive screening during an AWV (n=5/9, 55.6%). Although NPs screened less, they more strongly agreed that screening should occur annually (1.92±1.15 vs. 2.67±1.23) and "additional training would improve [screening] ability" (2.04±1.0 vs. 3.22±1.20). Also, few NPs independently managed impairment (n=5/26, 19.2%) compared to MDs (n=5/9, 55.6%). Our findings demonstrate that NPs screen less and feel less prepared to conduct cognitive screenings. It is important to provide additional resources and training for all PCPs, but especially NPs who are rapidly entering primary care to help improve identification and management of cognitive impairment.

### PSYCHOMETRIC PROPERTIES OF THE PHQ-9 AND CESD-R DEPRESSION MEASURES WITH OLDER ADULTS

Emma Katz, Rachael Spalding, and Barry Edelstein, *West Virginia University, Morgantown, West Virginia, United States*

Our understanding of older adult depression has been impeded by the paucity of assessment instruments with validity evidence for older adults. Therefore, measures of depression that were initially developed for use with younger adults are commonly used with older adults as well, such as the Center for Epidemiological Studies Depression Scale-Revised (CESD-R; Eaton et al., 2004) and Patient Health Questionnaire-9 (PHQ-9; Kroenke, Spitzer, & Williams, 2001). The CESD-R (Jiang et al., 2019; Van Dam & Earleywine, 2011) and PHQ-9 (Indu et al., 2018; Levis, Benedetti, & Thombs, 2019) have strong psychometric support for their use with young adults, and are two frequently used depression measures. In light of age-related differences in the experience and presentation of depression (e.g., Balsamo, et al., 2015; Fiske, Wetherell, & Gatz, 2009; Hybels, Laderman, & Blazer, 2012; Wuthrich, Johnco & Wetherell, 2015), the present study examined the psychometric properties of these instruments with older adults. Two-hundred-and-seventy-seven older adults (ages 65 and older) completed an online survey including the PHQ-9, CESD-R, and instruments measuring several other constructs with anticipated relations to depression (anxiety, general depression, positive and negative affect, self-esteem, personality traits, and satisfaction with life). The relation between the two depression scales and measures of the other constructs were examined. Both the PHQ-9 and CESD-R evidenced good internal consistency reliability (a = .82 and .83, respectively) and strong correlations in anticipated directions with many of the related constructs. These results support the use of the CESD-R and PHQ-9 with community-dwelling older adults.

### SESSION 10140 (LATE BREAKING POSTER)

### ATTITUDES ABOUT AGING

### ADAPTING TO HEALTH CHANGE: AGING BIAS, SENSITIVITY, AND INTERPROFESSIONALISM IN AN AGING SENSITIVITY TRAINING

Keith Kleszynski,[1] Lee Jennings,[1] Thomas Teasdale,[2] Carrie Ciro,[3] Carol Rogers,[4] Tina Peterson,[5] Keith Swanson,[3] and Beavers Kay,[3] *1. University of Oklahoma Health Sciences Center, Oklahoma City, Oklahoma, United States, 2. University of Oklahoma Health Sciences Hudson College of Public Health, Oklahoma City, Oklahoma, United States, 3. University of OKlahoma Health Sciences Center, Oklahoma City, Oklahoma, United States, 4. University of Oklahoma*

*Health Sciences Center, Reynolds Center for Geriatric Nursing Excellence, Oklahoma City, Oklahoma, United States, 5. University of Oklahoma, Norman, Oklahoma, United States*

Adapting to Health Change, a 2-hour aging sensitivity simulation to increase student sensitivity to age-related changes, was delivered to 148 health professions students in March 2020. Five small group stations (hearing loss, neuropathy and dexterity, vision loss and medication management, language and cognitive impairment, and mobility and balance) were facilitated by older adult volunteers and interdisciplinary faculty. Students completed (pre-post) Aging IQ Quiz, Aging Attitudes Assessment, Inter-professional Attitudes Scale (IPAS) Teamwork, Roles, and Responsibilities and Interprofessional Biases subscales, and an overall satisfaction rating. There was a statistically significant but small change in perceived knowledge of aging (mean Aging IQ score changed -0.025 points, p = 0.03). We also found a post-training increase in negative bias (mean Aging Attitudes Assessment score changed +2.68 points, p = 0.0001). While there was no change in attitudes about interprofessional education, this wasattributed to high baseline scores, suggesting students already valued interprofessional interactions. Multiple high satisfaction scores were encouraging. Ninety-four percent agreed that the simulation increased their understanding of age-related changes, while 97% indicated facilitators provided useful insights into the experiences of aging. A high majority (89%) felt they would be better health care providers for older patients than they would have been without the experience. This exercise to increase student empathy about age-related disabilities was well received, but did not achieve the usual intended aims. Simulation content should "re-aim" beyond empathy to teach new knowledge, highlight positive aspects of aging and professional care provision, and reinforce interprofessional roles toward wellness for older adults.

### RELATIONSHIP WITH GRANDPARENTS AND YOUNG ADULTS' ATTITUDES ABOUT AGING AND FUTURE-ORIENTED TENDENCIES

Tianyuan Li, and Pok Man Siu, *Education University of Hong Kong, Hong Kong, China*

It is important but always challenging to restrain from immediate temptations and behave conscientiously for long-term goals. Constructive interactions with older adults may promote young adults' positive attitudes about aging. With a brighter later adulthood in mind, young adults may then demonstrate a higher level of future-oriented tendency in their behaviors. The current study recruited 371 college students (Mage = 22.43, SDage = 2.88; 203 females) from Hong Kong. They completed an online survey about their interaction with the closest grandparent, attitudes about aging, and measures related to future-oriented tendencies (i.e., consideration of future consequences, healthy lifestyle, and impulsiveness). Parental intimacy and demographic information were assessed as potential covariates. Supporting the hypotheses, more interaction with grandparents was related to more positive attitudes about aging, $\beta$ = .40, SE = .11, p < .001. More positive attitudes about aging was then related to more consideration of future consequences, $\beta$ = .14, SE = .07, p = .03, healthier lifestyle, $\beta$ = .16, SE = .06, p = .005, and less impulsivity, $\beta$ = -.10, SE = .03, p < .001. Bootstrapping tests for the indirect effects from interaction with grandparents to the future-oriented outcomes through positive attitudes about aging were all significant as well. Although the current cross-sectional data could not confirm the causal links among the variables, the results provide some initial insight on how older adults can foster a long-term orientation in younger generations and contribute to the sustainable development of our societies through constructive intergenerational interactions.

### SELF-PERCEPTIONS OF AGING, COGNITIVE FUNCTION, AND PHYSICAL ACTIVITY

Caitlin Connelly, *University of Massachusetts Boston, Boston, Massachusetts, United States*

Stereotype embodiment theory suggests that internalized aging stereotypes will influence subsequent physical and cognitive health for older adults. This is proposed to occur through behavioral, physiological, and psychological pathways. Guided by stereotype embodiment theory, this study examined the how self-perceptions of aging are associated with cognitive function and the mediating role of physical activity as a behavioral pathway. The sample consists of 7,990 community-dwelling older adults age 65 from the Health and Retirement Study. Cross-sectional data analyses were conducted using bivariate and multivariate linear regression. Positive self-perceptions of aging were significantly associated with better cognitive function. Physical activity partially mediated the association between self-perceptions of aging and cognitive function. Findings suggest that self-perceptions of aging are important for cognitive function and physical activity may help to explain this relationship. Self-perceptions of aging may serve a possible intervention point to increase physical activity engagement and improve cognitive function.

### SENIOR ADULTS: DOES BODY IMAGE MATTER?

Keri Larsen,[1] Myia Graves,[1] Ashley Bowers,[1] Valerie Saba,[2] and Lauren Himel,[3] *1. Southeastern Louisiana University, Hammond, Louisiana, United States, 2. Southeastern Louisiana University, Loranger, Louisiana, United States, 3. Southeastern Louisiana University, Prairieville, Louisiana, United States*

Through the theoretical framework of the Social Comparison Theory, the current study will examine general attitudes and perceptions of body image in senior adults who are currently participating in organized recreational activities. Participants between the ages of 50 years of age and older participating in organized recreational programs in the Southeast will be administered the Sociocultural Attitudes Toward Appearance Scale (SATAQ) to measure participants' body image as influenced by general media, athletic and sport figures, as well as pressure to conform to the media ideal. The Figure Rating Scale will be administered, and is composed of nine drawings of bodies that progressively increase in size from very thin to overweight. Pearson product moment coefficient of correlation will be used to determine the association of scores between the SATAQ and Figure Rating Scale.

### SESSION 10150 (LATE BREAKING POSTER)

### BIOBEHAVIORAL HEALTH

### PHYSICAL ACTIVITY ADHERENCE RATES IN OLDER KIDNEY TRANSPLANT RECIPIENTS: A PILOT RANDOMIZED CONTROLLED TRIAL

Tara O'Brien, Karen Rose, and Alai Tan, *The Ohio State University, Columbus, Ohio, United States*

Daily walking activities are associated with improving cardiovascular and well-being in older kidney transplant recipients. Multicomponent interventions using technology and goal setting holds promise for sustaining daily walking activity among this population. The purpose of this randomized controlled trial pilot study was to evaluate the feasibility of a multicomponent intervention called SystemCHANGE™ + activity tracker for daily walking activity in older (age 60 and over) kidney recipients from baseline to 12 months. The intervention group implement a personal-system solution and wore a mobile activity tracker daily for 12 months. The attention-control group received educational information on healthy living as a transplant recipient and was asked to wear a mobile activity tracker daily for 12 months. Participants were randomized 1:1 to the intervention or control group. The sample consisted of 53 participants (n = 27 intervention, and n = 26 control). At the 12-month follow-up visit, the total study attrition rate was 23%. The adherence rates at 12 months were 96.5% in the intervention group and 80.8% in the attention- control group. The intervention group increased their steps from baseline to 12 months by 334 steps per day. The attention-control group demonstrated a decrease in steps by 563 steps per day. We found a mean difference of 1041± 2440 (Cohen's d = 0.43) in daily steps between the groups from baseline to 12 months. The data suggests SystemCHANGE™ in combination with activity trackers may be feasible for older kidney transplant recipients to enhance and sustain physical activity with daily walking.

### SEDENTARY BEHAVIOR, BRAIN-DERIVED NEUROTROPHIC FACTOR (BDNF), AND BRAIN STRUCTURE IN MIDLIFE: A BRAIN MRI STUDY

Xuan Zhang,[1] Denise Gaughan,[1] Chengxuan Qiu,[2] Osorio Meirelles,[1] and Lenore Launer,[1], *1. National Institute on Aging, Bethesda, Maryland, United States, 2. Karolinska Institutet, Solna, Sweden*

Long sedentary time (ST) is associated with poor brain health but the underlying mechanisms are unclear. Studies suggest exercise increases BDNF levels, and that low BDNF levels are associated with cognitive impairment. Limited population-based studies have examined associations among sedentary behavior (SB), BDNF, and brain structures. Here we explore the mediation and interaction effect of BDNF in the association of SB to brain measures. We included 612 participants from the MRI sub-study of the Coronary Artery Risk Development in Young Adults who had plasma BDNF and SB data at the Year 25 examination. SB was estimated by self-reported average ST hours/day spent sitting while watching television, using computers, and riding transportation. Outcome measures were total and selected brain volumes in cubic centimeters (cc). ST was categorized into quartiles. We used general linear regression to examine the following associations, adjusting for age, sex, race, and intracranial volume: Interactions between BDNF and ST on MRI; ST and MRI; ST and BDNF; BDNF and MRI; and ST, BDNF, and MRI. People in the upper 25%ile ST (>8.4 hours/day) had a decreased TB volume of 12.2 cc (p=0.01) compared to the lower 25%ile (<4.3 hours/day). Neither ST nor brain measures were associated with BDNF (p>0.05). Instead, BDNF interacted with ST for TB and WM (p < 0.03): The difference of brain volumes between the upper and lower 25%ile decreased with increasing BDNF levels. Accordingly, higher BDNF levels may protect brain function in the middle-aged and potentially older populations with a sedentary lifestyle.

### SEEING THE PAST THROUGH ROSE-COLORED GLASSES? AGE DIFFERENCES IN RECOUNTING A DIFFICULT MEMORY

Arya Jones,[1] Stephanie Wilson,[2] M. Rosie Shrout,[3] and Janice Kiecolt-Glaser,[4], *1. Rice University, Southlake, Texas, United States, 2. Southern Methodist University, Dallas, Texas, United States, 3. Institute for Behavioral Medicine Research, Columbus, Ohio, United States, 4. The Ohio State University College Of Medicine, columbus, Ohio, United States*

According to socioemotional aging theories, people better regulate their emotions in older age by reframing stressors and focusing on the positive aspects of difficult experiences. However, empirical results have been mixed. To address this gap, we examined age differences in the language use and cardiovascular reactivity of 188 adults (mean age=56, range=40-86) who relived an upsetting memory from their past. Consistent with theory, results revealed that older adults used significantly fewer negative emotion words and, among the negative emotions, marginally fewer words of anger, to describe their upsetting memory. Notably, however, there were no age differences in the expression of positive emotion or sadness. Controlling for education and cognitive function, greater expression of anger was associated with heightened systolic blood pressure (SBP) reactivity among older adults, not middle-aged individuals. Despite their expression of less negative emotion, older adults' heart rate variability (HRV) dipped lower during disclosure than did middle-aged adults'. However, among those who used more positive emotion, sadness, and/or cognitive processing words, older adults no longer showed lower HRV than middle-aged participants. Overall, these results provide some evidence of positivity bias among older adults even when asked to recount a distressing personal memory, although this trend was not consistent for the expression of sadness or positive emotion. Further, cardiovascular responses appear more clearly tied to older adults' level of engagement and emotional focus compared to their middle-aged counterparts'.

### THE AGE-DEPENDENT RELATIONSHIP BETWEEN VASCULAR RISK FACTORS AND TRAJECTORIES OF DEPRESSED MOOD

Maria Blöchl,[1] Lina Schaare,[2] Ute Kunzmann,[3] and Steffen Nestler,[4], *1. University of Münster, Leipzig, Germany, 2. Max Planck Institute for Human Cognitive*

*and Brain Sciences, Leipzig, Germany, 3. University of Leipzig, Leipzig, Sachsen, Germany, 4. University of Münster, Münster, Nordrhein-Westfalen, Germany*

Cardiovascular risk factors (CVRFs) have been linked to depression, but it is still unclear whether this association becomes stronger or weaker from mid- to later life. Thus, our main aim was to investigate the influence of age on the associations between CRVFs and trajectories of depressed mood. Our sample included 6835 individuals (aged 52–89 years) from the English Longitudinal Study of Ageing (ELSA), who were free of manifest vascular disease at baseline and had bi-yearly measurements of depressed mood over ten years. A composite score incorporated the presence of five CVRFs: hypertension, diabetes, smoking, obesity, and hypercholesterolemia. We used second-order latent growth models to examine the effect of CVRFs, age, and their interaction on levels and changes in depressed mood over time. Our results revealed that baseline CVRFs were associated with higher levels of depressed mood. This association decreased with age and was stronger in midlife compared to later life. CVRFs were not related to changes in depressed mood, indicating that these differences remained stable over time. These findings suggest that CVRFs in midlife, but less so in older age, predict stable differences in depressed mood. They are consistent with reports on the importance of CVRFs in midlife and may support the idea that prevention of vascular burden in this age period may be critical to maintain mental health.

### SESSION 10160 (LATE BREAKING POSTER)

### BIOLOGY OF AGING

### 2-DEOXY-D-GLUCOSE-(2-DG) PREVENTS PATHOGEN DRIVEN ACUTE INFLAMMATION AND ASSOCIATED TOXICITY

Sanjay Pandey, Vandana Anang,[1] Saurabh Singh,[2] Anant Narayan Bhatt,[3] K Natarajan,[1] and BS Dwarakanath,[4], *1. Dr. B.R. Ambedkar Center for Biomedical Research, University of Delhi, Delhi, Delhi, India, 2. Albert Einstein College of Medicine, Bronx, New York, United States, 3. Institute of Nuclear Medicine and Allied Sciences, Delhi, Delhi, India, 4. Shanghai Proton and Heavy Ion Center of Fudan University Shanghai Cancer Center, Delhi, Delhi, India*

Pathogen-associated molecular patterns (PAMPs) associated with viral and bacterial infections trigger multiple inflammatory pathways which may result in oxidative stress driven toxicity, tissue fibrosis organ dysfunction and ageing. Inflammatory events need high energy demands and predominantly depends on the glycolysis. Thus, energy metabolism of the inflammatory events can be targeted to reducing the magnitude of the PAMPs driven inflammation and preventing tissue toxicity. Here we propose that 2-DG, a glycolytic inhibitor, and a potential Energy Restriction Mimetic agent (ERMA) can modulate inflammatory events and can prevent the development of acute as well as chronic pathology. For this study we induced LPS (bacterial PAMP) induced endotoxemia in mice which models infection associated inflammatory acute inflammatory events, tissue damage and organ dysfunction. 2-DG fed mice (0.4% w/v in drinking water) showed reduced LPS driven oxidative stress and capillary damage in lungs. Administration of 2-DG also reduced LPS induced spike in inflammatory cytokines (TNF, IL6 and IL1β) in the BALF and serum. Lungs of 2-DG fed mice showed lesser infiltration of inflammatory cells and reduced inflammatory signaling activation. 2-DG also downregulated the ex-vivo and in-vivo migration of the PMNCs. Furthermore, 2-DG also reduced the activation of the macrophage cells (RAW264.7) which was seen with reduction and the glycolysis and increased mitochondrial functions. Our data suggest that 2-DG administration as ERMA in drinking water can prevent pathogenic exposure driven inflammatory events which may prevent acute as well as chronic inflammatory disorders.

### A DNA DAMAGE RESPONSE-INDEPENDENT MECHANISM FOR TELOMERE SHORTENING-ELICITED AGE-RELATED PATHOLOGIES

Amanda Stock,[1] Kun Wang,[1] Chongkui Sun,[1] Chengyu Liu,[2] Yi Gong,[1] and Yie Liu,[1], *1. National Institute on Aging, Bethesda, Maryland, United States, 2. National Heart, Lung, and Blood Institute, Bethesda, Maryland, United States*

Telomere attrition is associated with telomeropathies and age-related pathologies. In telomeropathies, telomere uncapping induces a DNA damage response (DDR) that drives apoptosis or senescence. However, a defined mechanism by which telomere attrition contributes to other age-related pathologies has not been determined. Telomere integrity is maintained by shelterin, a six-protein complex. Rap1 is the only shelterin member that is not essential for telomere capping but engages non-telomeric DNA and regulates gene transcription. We hypothesized that non-telomeric Rap1 accumulation could contribute to age-related pathologies in a DDR-independent manner. To test this, we used CRISPR/Cas9 editing to generate a Rap1 mutant mouse model in which Rap1 at telomeres is prevented, leaving only non-telomeric Rap1. Indirect immunostaining showed no differences in telomere dysfunction-induced DDR foci in Rap1 mutant compared to wild-type primary fibroblasts. Cell fractionation/western blotting of fibroblasts from Rap1 mutants demonstrated decreased Rap1 expression and Rap1 re-localization off telomeres, which mimics the same alteration of Rap1 in human cells with telomere attrition. Rap1 mutant mice exhibited increased body weight and altered metabolic and immune-response transcripts in various tissues, indicating that altered transcription could account for some of the observed phenotypes related to telomere attrition. In conclusion, telomere shortening may facilitate non-telomeric Rap1, which alters gene transcription and drives metabolic and immune dysfunction in a DDR-independent manner.

### A NARRATIVE REVIEW ON THE RELATIONSHIP BETWEEN FEMALE REPRODUCTIVE FACTORS AND LONGEVITY

Christy Costanian,[1] Raymond Farah,[2] Sola Bahous,[1] and Abla Sibai,[3] *1. Lebanese American University, Beirut, Lebanon, 2. Lebanese University, Beirut, Lebanon, 3. American University of Beirut, Beirut, Lebanon*

This review presents findings on the role of female reproductive factors on longevity.A comprehensive systematic literature search was conducted using four electronic databases: OVID Medline, Web of Science, PubMed and Google Scholar from inception until May 2020 and restricted to English language articles that tackle the relationship between reproductive factors and longevity in its various definitions. Our search yielded a total of 306 articles. After screening based on the eligibility criteria,37 articles were included for review. The majority of studies were prospective and conducted in Western populations. The most consistent findings were between parity and increased longevity. The role of ages at menarche and menopause, premature menopause, as well as reproductive lifespan on longevity were not conclusive. Whether gender of offspring is related to maternal longevity is yet to be fully elucidated.Variations in findings are in the majority due to differentials in the definition of longevity as an outcome. Further longitudinal studies based in developing countries are needed to examine reproductive factors related to longevity.

### A ROBOTIC SYSTEM FOR HIGH-THROUGHPUT AUTOMATED LIFESPAN AND PHENOTYPING ANALYSIS IN C. ELEGANS

Elena Vayndorf, Jason Pitt, Judy Wu, Emily Chang, Richard Nguyen, Ashley Liang, Renee Ruan, and Matt Kaeberlein, *University of Washington, Seattle, Washington, United States*

A goal of gerontology-related research is to develop therapies to improve the healthy period of life by understanding and targeting the molecular hallmarks of biological aging. Much progress has been made toward understanding the genetic and biochemical nature of these hallmarks through studies using simple invertebrate model organisms, such as the nematode Caenorhabditis elegans. Over the past decade, the identification of potential genetic and pharmacological modifiers of lifespan and age-related pathologies in C. elegans and other model organisms has yielded fruitful leads for follow-up investigation. However, such studies are typically time- consuming and labor-intensive. The goal of our work is to automate tasks that require frequent, repeated observations and hours of manual labor to collect and analyze lifespan, motility, and other behavioral data in C. elegans and other nematode models. The advent of affordable high-quality digital cameras, robotics systems, and 3D printers, as well as the decreasing financial and computational costs of image storage and processing, have allowed us to automate data capture and analysis on a large scale. To this end, our group recently developed a tool, we call the WormBot, consisting of an unbiased, high-throughput, automated robotic system and corresponding software, to perform genetic and pharmacological quantification of lifespan and health measures in C. elegans and related nematode species. We will report updates recently made to this system, including significant improvements to hardware, and present screening results from proteasome stimulator drugs known to reduce the accumulation of proteotoxic proteins linked to neurodegenerative diseases and aging.

### ACARBOSE SUPPRESSES SYMPTOMS OF MITOCHONDRIAL DISEASE IN A MOUSE MODEL OF LEIGH SYNDROME.

Alessandro Bitto, Anthony Grillo, and Matt Kaeberlein, *University of Washington, Seattle, Washington, United States*

Mitochondrial diseases are pathologies characterized by impairment in mitochondrial function. Mitochondrial dysfunction is also a hallmark of the aging process. Rapamycin, a drug that increases lifespan and reduces the incidence of age-related pathologies in multiple models, increases survival and reduces the impact of neurological symptoms in a mouse model lacking the complex I subunit Ndufs4. Here we show that acarbose, another drug that extends lifespan in mice, suppresses symptoms of disease and improves survival of Ndufs4-/- mice. Unlike rapamycin, acarbose rescues disease phenotypes independently of mTOR inhibition. Furthermore, rapamycin and acarbose have additive effects on clasping and maximum lifespan in Ndufs4-/- mice. Acarbose rescues mitochondrial disease independently of glycolytic flux and Sirt3 activity by potentially remodeling the microbiome. This study provides the first evidence that the microbiome may rescue severe mitochondrial disease and proof of principle that biological aging and mitochondrial disorders are driven by common mechanisms.

### AGE, SEX AND CEREBRAL MICROBLEED EFFECTS ON WHITE MATTER DEGRADATION AFTER TRAUMATIC BRAIN INJURY

Andrei Irimia, Ammar Dharani, Van Ngo, David Robles, and Kenneth Rostowsky, *University of Southern California, Los Angeles, California, United States*

Mild traumatic brain injury (mTBI) affects white matter (WM) integrity and accelerates neurodegeneration. This study assesses the effects of age, sex, and cerebral microbleed (CMB) load as predictors of WM integrity in 70 subjects aged 18-77 imaged acutely and ~6 months after mTBI using diffusion tensor imaging (DTI). Two-tensor unscented Kalman tractography was used to segment and cluster 73 WM structures and to map changes in their mean fractional anisotropy (FA), a surrogate measure of WM integrity. Dimensionality reduction of mean FA feature vectors was implemented using principal component (PC) analysis, and two prominent PCs were used as responses in a multivariate analysis of covariance. Acutely and chronically, older age was significantly associated with lower FA ($F2,65 = 8.7$, $p < .001$, $\eta 2 = 0.2$; $F2,65 = 12.3$, $p < .001$, $\eta 2 = 0.3$, respectively), notably in the corpus callosum and in dorsolateral temporal structures, confirming older adults' WM vulnerability to mTBI. Chronically, sex was associated with mean FA ($F2,65 = 5.0$, $p = 0.01$, $\eta 2 = 0.1$), indicating males' greater susceptibility to WM degradation. Acutely, a significant association was observed between CMB load and mean FA ($F2,65 = 5.1$, $p = 0.009$, $\eta 2 = 0.1$), suggesting that CMBs reflect the acute severity of diffuse axonal injury. Together, these findings indicate that older age, male sex, and CMB load are risk factors for WM degeneration. Future research should examine how sex- and age-mediated WM degradation lead to cognitive decline and connectome degeneration after mTBI.

### AMYLOID BETA-42 LEVELS IN COMPANION DOG BRAINS CORRELATE WITH AGE AND COGNITIVE FUNCTION

Silvan Urfer,[1] Martin Darvas,[1] Dirk Keene,[1] Kálman Czeibert,[2] Enikő Kubinyi,[2] Sára Sándor,[2] Franco Guscetti,[3] and Matt Kaeberlein,[1], *1. University of Washington, Seattle, Washington, United States, 2. Etövös Loránd University, Budapest, Budapest, Hungary, 3. University of Zurich, Zurich, Zurich, Switzerland*

The privately owned companion dog is an increasingly important model in aging research because it shares the human environment, is exposed to similar environmental risk factors, receives comparable medical care, and develops many of the same age-related pathologies. One such pathology is Canine Cognitive Dysfunction (CCD), which shares many of the clinical features of human Alzheimer's Disease (AD), including progressive loss of cognitive function, loss of normal sleep patterns, increased anxiety, and aimless wandering. Amyloid-beta 42 (Aβ42) plaques similar to these found in humans with AD are known to naturally occur in the brains of aged dogs, making them an intriguing potential model for AD in humans. As part of the Dog Aging Project (www.dogagingproject.org), we studied frozen samples taken from the frontal cortex, medial temporal cortex, entorhinal cortex, and hippocampus of n=24 companion dogs of various ages that were euthanized for unrelated health reasons and donated by their owners. Brains were removed and frozen within 4 hours post mortem. Using a novel quantitative Luminex assay, we found a significant correlation between age and Aβ42 levels in all of these brain regions, as well as a significant correlation between Aβ42 levels and cognitive function scores as measured by the Canine Cognitive Dysfunction Scale. We will now investigate histopathology in the same dogs and brain regions, and investigate whether we can also measure Tau and pTau in these samples using Luminex and mass spectrometry.

### DEVELOPMENT AND VALIDATION OF TWO COMPOSITE AGING MEASURES USING CLINICAL BIOMARKERS IN THE CHINESE POPULATION

Zuyun Liu, *School of Public Health and the Second Affiliated Hospital, Zhejiang University School of Medicine, Hangzhou, China*

Quantifying aging is crucial for addressing aging and related issues. This study aimed to: 1) develop two composite aging measures in the Chinese population using two recent advanced algorithms (the Klemera and Doubal method and Mahalanobis distance); and 2) validate the two measures by examining their associations with mortality and disease counts. Based on data from the China Nutrition and Health Survey 2009 wave (N=8,119, aged 20-79 years, 53.5% women), a nationwide prospective cohort study of the Chinese population, we developed Klemera and Doubal method-biological age (KDM-BA) and physiological dysregulation (PD, derived from Mahalanobis distance) using 12 routine clinical biomarkers. For the validation analysis, we used Cox proportional hazard regression models (for mortality) and linear, Poisson, and logistic regression models (for disease counts) to examine the associations. We replicated the validation analysis in the China Health and Retirement Longitudinal Study (CHARLS, N=9,304, aged 45-99 years, 53.4% women). We found that both aging measures were predictive of mortality after accounting for age and gender (KDM-BA, per one-year, HR=1.14, 95%CI=1.08, 1.19; PD, per one-SD, HR=1.50, 95%CI=1.33, 1.69). With few exceptions, these mortality predictions were robust across stratifications by age, gender, education, and health behaviors. The two aging measures were associated with disease counts both cross-sectionally and longitudinally. These results were generally replicable in CHARLS although four biomarkers were not available. In summary, we successfully developed and validated two composite aging measures––KDM-BA and PD, which have great potentials for applications in early identifications and preventions of aging and aging related diseases in China.

### EFFECTS OF ANGIOTENSIN (1-7) EXPRESSING LACTOBACILLUS AND EXERCISE ON GUT-BRAIN AXIS IN AGED RATS

Yi Sun,[1] Youfeng Yang,[1] Anisha Banerjee,[1] Amrisha Verma,[2] Qiuhong Li,[2] Christy Carter,[1] and Thomas Buford,[1] *1. University of Alabama at Birmingham, Birmingham, Alabama, United States, 2. University of Florida, Gainesville, Florida, United States*

Aging is associated with gut dysbiosis – a condition linked with altered central nervous system function ("gut-brain axis"). Age-related health benefits have been ascribed to the renin-angiotensin system, mediated partially via the angiotensin(1-7) axis. Research has shown exercise altering gut microbiota composition and function. This study explored the effects of a genetically modified probiotic expressing angiotensin (1-7) and exercise on the gut-brain axis. Sixty-two male F344/BN rats were randomized at 24-months-old to receive oral gavage of angiotensin (1-7) Lactobacillus paracasei (LP) or LP-A, wide-type LP, or control 3-times/week for 12 weeks; with or without exercise. Rats in exercise groups were walking on a treadmill 10-minutes/day for 5-days/week. Microbiome taxonomic analysis of fecal samples post intervention was performed via 16S-based PCR. A battery of behavior tests were performed before and after the intervention. PCoA revealed that groups differed in the overall fecal microbiota community structure by weighted UniFrac (p=0.034). Indices of alpha-diversity, including Shannon (p=0.02) and Simpson (p=0.019) indexes, displayed LP-A groups with more diverse microbiome than controls. Grip strength (p=0.042) and exercise tolerance (p<0.001) tests showed exercise groups had more muscle strength and aerobic capacity than the sedentary groups. Exercise groups had more lean mass (p=0.019); both LP-A and LP groups had lower fat mass compared to the controls in body composition measurement. LP-A travelled longer distance in the central area than the control in the open field test showing reduced anxiety (p=0.029). Therefore, we conclude LP-A and exercise have distinct and possibly overlapping beneficial effects on the gut-brain axis.

### ENDURANCE EXERCISE TRAINING MODES TO IMPROVE PHYSICAL FUNCTION IN OLDER MICE: HIIT VS. VWR

Ted Graber, Chris Byrd, Emily Seguin, Anna Seguin, Alyssa Fennel, and Nainika Nadigama, *East Carolina University, Greenville, North Carolina, United States*

With age, we experience a progressive loss of physical function. Exercise is a promising therapy to preserve muscle health and functional aptitude. Animal models are needed to examine the underlying molecular mechanisms at the intersection of aging, exercise, and functional decline. In this study, we compare the efficacy of two types of individualized endurance exercise training in older C57BL/6 mice (26-months old at completion): HIIT (high intensity interval training on a treadmill, n=10) and VWR (voluntary wheel running, n=8). We hypothesized that while both exercises would improve function, HIIT would promote more extensive adaptation. For four months the VWR mice ran 4 days/week and the HIIT group ran 3x/week. We determined function pre/post-training by utilizing our composite scoring system, the Comprehensive Functional Assessment Battery (CFAB). CFAB consists of the following: treadmill running (endurance), rotarod (overall motor function), wheel running (volitional exercise rate/activity), grip strength (fore-limb strength), and inverted cling (overall strength/endurance). EchoMRI determined body composition. After training, we found significant CFAB improvement (repeated measures t-test, $p<0.05$) in both exercise groups, specifically including: rotarod (+37%, HIIT and VWR); treadmill (+61% VWR; +58% HIIT), grip strength (+20% VWR), body mass (-17% VWR, -10% HIIT), and fat percentage (-44% VWR, -20% HIIT). Contrary to our hypothesis, HIIT did not improve function more than VWR, though we suspect increasing training intensity would improve response. Thus, future studies will need to address defining HIIT dose response and optimal training volume for older mice. We conclude that our models will be useful for future mechanistic investigations.

### IMPACT OF RADIOTHERAPY ON DAILY FUNCTION AMONG OLDER ADULTS LIVING WITH ADVANCED CANCER

Anthony Nehlsen, Parul Agarwal, Madhu Mazumdar, and Kavita Dharmarajan, *Mount Sinai School of Medicine, New York, New York, United States*

Radiation therapy (RT) improves quality of life and symptomatic burden for patients with advanced malignancies. However, RT can also confer toxicity and little is known about the contribution of geriatric conditions to RT-related outcomes in older adults. This study aims to examine changes in daily function among RT patients at 1 and 6 months following RT. We reviewed charts of 137 patients who underwent RT with intent to improve daily functioning. ADL and IADL scores ranging from 0-6 and 0-8, respectively were collected at baseline, 1-, and 6-months post-RT. Latent class analysis of baseline ADL and IADL was conducted to categorize patients into two classes (high and low deficit). Latent transition analysis was used to examine transitions at each time point. One-hundred seventy courses of RT were identified; 99 were fully evaluable. Median age was 66 years. For ADL and IADL, at baseline 28.9% and 28.3% were classified as high deficit and 71.1%, 71.3% as low deficit respectively; 2% and 7% of low deficit patients had the potential to move to high deficit group at 1 month; and 20% and 13% had the potential to have the same movement from 1 to 6 months. All patients classified as high deficit for both measures at 1 month remained so at 6 months. ADL and IADL functioning may be useful in describing changes in daily function after and identifying groups of patients who may benefit from additional supportive geriatric and/or palliative care interventions.

### INFLUENCE OF AGING, MACRONUTRIENT COMPOSITION AND TIME-RESTRICTED FEEDING ON THE RAT GUT MICROBIOME

Abbi Hernandez,[1] Sara Burke,[2] Thomas Buford,[1] and Christy Carter,[1] *1. University of Alabama at Birmingham, Birmingham, Alabama, United States, 2. University of Florida, Gainesville, Florida, United States*

Declining health and cognition are hallmarks of advanced age that reduce both the quality and length of the lifespan. While caloric restriction has been highlighted as a strategy for increasing healthspan, time-restricted feeding and changes in dietary macronutrient composition may be more feasible alternatives with similar health outcomes. Furthermore, age-related changes in gut microbiome composition may reciprocally interact with several physiological systems – providing a good target for future therapeutic interventions. To begin to investigate the potential utility of a ketogenic (high fat, low carbohydrate) diet and/or time-restricted feeding, fully mature young (5 mo) and older (22 mo) adult male Fischer Brown Norway Hybrid rats were placed on a time-restricted feeding regimen of a ketogenic or micronutrient and calorically matched control diet for 7 months. A third group of rats was permitted to eat standard chow ad libitum. Fecal samples collected at the conclusion of the study were submitted for 16S microbiome analysis, which revealed significant differences across age and diet groups, as well as across feeding paradigms. Beta diversity analysis demonstrated distinct microbiome composition across the three diet groups regardless of age. Furthermore, diet group significantly impacted abundance in expression of several microbiota at the phylum level, including Verrucomicrobia, Cyanobacteria, Actinobacteria and Patescibacteria, though age did not. Verrucomicrobia was significantly increased ($p=0.02$) and Actinobacteria and Patescibacteria ($p<0.01$) were significantly decreased in animals fed in a time-restricted fashion. These results indicate the value of both altered macronutrient composition and altered feeding methodology for therapeutic interventions targeting the gut microbiome.

### MUSCLE MITOCHONDRIAL DNA COPY NUMBER, DELETION MUTATION FREQUENCY, AND PHYSICAL PERFORMANCE IN OLDER ADULTS

Jonathan Wanagat,[1] Allen Herbst,[2] Steven Prior,[3] Judd Aiken,[4] Debbie McKenzie,[4] NIanjun liu,[5] Xiwei Chen,[5] and David Allison,[5] *1. UCLA, Los Angeles, California, United States, 2. University of Alberta, Edmonton, Alberta, Canada, 3. University of Maryland School of Medicine, Baltimore, United States, 4. University of Alberta-Edmonton, Edmonton, Canada, 5. Indiana University, Bloomington, Indiana, United States*

Mitochondrial DNA (mtDNA) quantity and quality influence hallmarks of aging – mitochondrial dysfunction and genomic instability. The interactions between mtDNA quantity and quality and physical performance have not been extensively examined in humans. The aim of this study was to test the interactions between skeletal muscle mtDNA copy number, mtDNA deletion mutation frequency, and physical performance measures in older adults. Total DNA was isolated from muscle biopsies and used for quantitation of mtDNA copy number and mutation frequency by digital PCR. The biopsies were obtained from a cross-sectional cohort of 53 adults aged 50 to 86 years. Before the biopsy, physical performance measures were collected. MtDNA deletions increased exponentially with advancing age. On average, mtDNA deletion frequency increased 18-fold between 50 and 80, with a trend toward lower deletion frequency in females. MtDNA deletion frequency predicted declines in VO2 max, where 4.7% of the variation in VO2 max was explained by mtDNA deletion frequency. MtDNA copy number was negatively correlated with age and mtDNA deletion frequency, but positively correlated with lean mass. There was a trend to lower mtDNA deletion frequency in females, consistent with increased longevity in females. Larger studies may better delineate sex effects. These data are consistent with a role for mitochondrial function and genome integrity in the maintenance of physical performance with age. Analyses of mtDNA quality and quantity in longitudinal studies could extend our understanding of the importance of mitochondria in human aging and longevity.

### PICK YOUR POISON CAREFULLY: HOW ALCOHOL CONSUMPTION AND SERUM BIOMARKERS INFLUENCE BODY FAT – A UK BIOBANK STUDY

Brittany Larsen,[1] Brandon Klinedinst,[1] Scott Le,[1] Colleen Pappas,[1] Nathan Meier,[2] Ye-Lim Lim,[3] Tovah Wolf,[4] and Auriel Willette,[1] *1. Iowa State University, Ames, Iowa, United States, 2. Concordia University Irvine, Irvine, California, United States, 3. Virginia Polytechnic Institute, Blacksburg, Virginia, United States, 4. Western Carolina University, Cullowhee, North Carolina, United States*

Aging is characterized by physiological alterations in body composition, such as increased visceral adiposity accumulation and bone loss. Alcohol consumption is thought to partially drive these associations, but findings have been mixed. To clarify inconsistent findings, different types of alcohol--beer, liquor, and wine--may show different association patterns with body composition. Our longitudinal U.K. Biobank study leveraged 1,874 White British participants (aged 40-79 years; 58.9% male). Participants self-reported demographic, alcohol and dietary consumption patterns, and lifestyle factors using a touchscreen questionnaire. Anthropometrics and serum for proteomics were collected and body composition was obtained via dual-energy X-ray absorptiometry (DEXA). Structural equation modeling was used to probe direct and indirect associations between adiposity and bone, alcohol types, and cardiometabolic biomarkers. Over a mean duration of four years, greater consumption of beer and liquor were significantly associated with more visceral adiposity ($\beta$=.069, p<.001 and $\beta$=.014, p<.001, respectively); these associations were driven by dyslipidemia and insulin resistance. In contrast, greater red wine consumption predicted less adipose mass ($\beta$=-.023, p<.001), and this association was mediated by reduced inflammation and higher high-density lipoproteins (HDL) cholesterol. White wine consumption did not influence visceral adiposity but did predict greater bone mineral density (BMD) ($\beta$=.051, p=.003). Taken together, these data suggest that beer and liquor may drive the "empty calorie" hypothesis related to adipogenesis, while red wine may be protective due to anti-inflammatory and eulipidemic effects. Furthermore, white wine may benefit bone mineral density in older adults.

### PROTEIN KINASE C DOWNREGULATION UPON RAPAMYCIN TREATMENT ATTENUATES NEUROINFLAMMATION AND MITOCHONDRIAL DISEASE

Anthony Grillo,[1] Alessandro Bitto,[2] and Matt Kaeberlein,[2] *1. University of Washington, Issaquah, Washington, United States, 2. University of Washington, Seattle, Washington, United States*

Mitochondrial dysfunction causes many poorly understood diseases, such as Leigh Syndrome, that are often caused by dysfunctions in proteins involved in the electron transport chain. My lab previously reported mTOR is pathologically involved in the neurodegenerative phenotype and premature death of mice missing the Complex I subunit Ndufs4 (Ndufs4-/- mice). We discovered treatment with rapamycin extends lifespan, reduces neuroinflammation, and attenuates the neurodegenerative phenotype in these mice, although the mechanisms remain unclear. Rapamycin-treated Ndufs4-/- mice exhibited decreased activation of the mTORC1 pathway. It also deactivated the mTORC2 pathway. We observed that phosphorylation of the canonical protein kinase C (PKC) isoforms (PKC-$\alpha$, -$\beta$, and -$\gamma$) decreased more than any other kinases, leading us to hypothesize its deactivation contributes to the observed lifespan extension. To test this, we treated Ndufs4-/- mice with three different PKC inhibitors: the pan-PKC inhibitors GO6983 and GF109203X, and the PKC-$\beta$ specific inhibitor ruboxistaurin. Similar to rapamycin, all three drugs were able to significantly delay the onset of neurological symptoms (i.e. clasping) and increase survival. We also observed that PKC-$\beta$ inhibition reduced skin inflammation to suppress the hair loss phenotype displayed by Ndufs4-/- mice at weaning. We further discovered PKC-$\beta$ inhibition reduces neuroinflammation by deactivating the NF-kB inflammatory pathway. These results suggest that mTORC2 may play a critical role in the etiology of mitochondrial diseases such as Leigh Syndrome.

### STATISTICAL ESTIMATION OF ACCELERATED BIOLOGICAL BRAIN AGING AFTER MILD TRAUMATIC BRAIN INJURY IN OLDER ADULTS

Andrei Irimia, Jun Kim, Shania Wang, Hyung Jun Lee, Van Ngo, Sean Mahoney, and David Robles,*University of Southern California, Los Angeles, California, United States*

Estimating biological brain age (BA) has the potential of identifying individuals at relatively high risk for accelerated neurodegeneration. This study compares the brain's chronological age (CA) to its BA and reveals the BA rate of change after mild traumatic brain injury (mTBI) in an aging cohort. Using T1-weighted magnetic resonance imaging (MRI) volumes and cortical thickness, volume, surface area, and Gaussian curvature obtained using FreeSurfer software; we formulated a multivariate linear regression to determine the rate of BA increase associated with mTBI. 95 TBI patients (age in years (y): $\mu = 41$ y, $\sigma = 17$ y; range = 18 to 83) were compared to 462 healthy controls (HCs) (age: $\mu = 69$ y, $\sigma = 18$ y; range = 25 to 95) over a 6-month time period following mTBI. Across the initial ~6 months following injury, patients' BAs increased by ~3.0 ± 1.2 years due to their mTBIs alone, i.e., above and beyond typical brain aging. The superior temporal and parahippocampal gyri, two structures involved in memory formation and retrieval, exhibited the fastest rates of TBI-related BA. In both hemispheres, the volume of the hippocampus decreased (left: $\mu$=0.28%, $\sigma$=4.40%; right: $\mu$=0.12%, $\sigma$=4.84%). These findings illustrate BA estimation techniques' potential to identify TBI patients with accelerated neurodegeneration, whose rate is strongly associated with the risk for dementia and other aging-related neurological conditions.

### TESTING CELLULAR PROLIFERATION RESPONSES TO OXIDATIVE, GLUCOCORTICOID AND METABOLIC CHALLENGES IN THE BABOON

Daniel Adekunbi,[1] Cun Li,[1] Peter Nathanielsz,[2] and Adam Salmon,[3] *1. University of Wyoming, San Antonio, Texas, United States, 2. University of Wyoming, Laramie, Wyoming, United States, 3. University of Texas Health at San Antonio, San Antonio, Texas, United States*

Aging is associated with progressive loss of cellular homeostasis which results from intrinsic and extrinsic challenges. Testing how such challenges relate to the aging process is often limited by the available model systems. We use primary cells (fibroblasts) isolated from baboons as a non-human primate cellular model to address how stressful challenges affect resilience. Using a real-time live-cell imaging system, we characterized a protocol for testing the effects of pro-oxidant compounds (e.g hydrogen peroxide ($H_2O_2$), paraquat and thapsigargin), dexamethasone and low glucose environment on cellular proliferation in fibroblasts derived from baboons across the life-course. The inhibitory effect of $H_2O_2$ (50 and 100µM), an oxidative stress, on cell proliferation was dose-dependent, with a higher impact in old males (age 14.52-14.80 years; average life span 21 years) compared to young males (6.35-6.39 years). Exposure to a different oxidative stress, paraquat (100 and 200µM) tended to reduce cell proliferation rate with age in males but not females. Inhibitory effects of thapsigargin, an endoplasmic reticulum stress inducer, on cell proliferation were dependent on challenge duration (2 vs 24h), concentration (0.1 and 1µM) and donor age, with greater resilience in young males than young females (4.33-6.70 years). Dexamethasone (100 and 500µM), a glucocorticoid, reduced cell proliferation dose-dependently, with older males exhibiting more resilience than females. In response to low glucose (1mM), cell proliferation reduced with age. Donor's chronological age and sex are important variables in cellular response to challenge compounds faced during aging, which will guide our on-going studies on the cellular transcriptome and proteome.

### SESSION 10170 (LATE BREAKING POSTER)

### CHRONIC DISEASE MANAGEMENT

### A MOBILE APP FOR TRACKING NON-MOTOR SYMPTOMS OF PEOPLE WITH PARKINSON'S DISEASE: A USABILITY STUDY

JuHee Lee, Yujin Suh, and Yielin Kim, *Yonsei University, Seoul, Republic of Korea*

Smart phone-based technology for people with Parkinson's disease has been developed worldwide. Unmonitored non-motor symptoms decrease quality of life of people with Parkinson's disease, so the needs for technology to manage non-motor symptoms are increasing. The technology is needed to detect subtle changes in non-motor symptoms by healthcare professional. There is no mobile app which manage comprehensive symptoms of Parkinson's disease including non-motor symptoms. It is necessary to develop a new tracking system that can effectively manage non-motor symptoms as well as motor symptoms of Parkinson's disease. We developed a prototype of mobile app for Android smartphones, with cooperation with Mazelone company. we also have shaped functions for monitoring of motor symptoms and medication adherence. It also provided a section for caregivers to use on behalf of people with Parkinson's disease who have difficulty to use app due to hand tremor. Through Delphi technique, we obtained content validity from eight medical and nursing experts on the contents of the application. We provided regular telephone counseling to improve and encourage their app usage. Fifteen participants used the app for 6 weeks. To evaluate usability of mobile app, we provided constructed questionnaire and conducted individual telephone interview. A mobile app for tracking non-motor symptoms demonstrated high usability and satisfaction. We learned lessons about facilitators and barriers when implementing an app such as perception and acceptance of mobile technology. The mobile app will improve continuum of care. Future studies need to improve the contents and refine technical approach for people with Parkinson's disease.

### DYNAMICS OF MULTIMORBIDITY RESILIENCE AND HEALTH OUTCOMES OVER TIME IN COMMUNITY-RESIDING OLDER ADULTS

Yingzhi Xu,[1] Zahra Rahmaty,[2] Eleanor McConnell,[3] Tingzhong (Michelle) Xue,[1] Bada Kang,[3] and Kirsten Corazzini,[2] *1. Duke University, Durham, North Carolina, United States, 2. University of Maryland School*

*of Nursing, Baltimore, Maryland, United States, 3. Duke University School of Nursing, Durham, North Carolina, United States*

Multimorbidity resilience may mitigate the adverse effects of multiple chronic diseases on older adults' health. Wister et al.'s (2018) multimorbidity resilience index was developed and tested in a cross-sectional sample of older adults in Canada. Building on these findings, we examined the reciprocal relationships of resilience on outcomes to test these potentially mitigating effects in a community-based, U.S. sample of older adults over time. The study sample includes 1,054 older adults from waves 2 and 3 of the National Social Life, Health, and Aging Project (NSHAP) study (Waite et al 2020). Wister et al.'s (2018) index was mapped to NSHAP measures, and reciprocal relationships of multimorbidity resilience and health outcomes over a 5-year period was tested using structural equation modeling (SEM). Results indicated significant effects of multimorbidity resilience on self-rated physical health and pain. Interestingly, a better functional resilience at baseline conferred better self-rated physical health at follow-up, while better psychological resilience predicted lower pain level. By contrast, the influence of health outcomes on any domain of multimorbidity resilience was not detectable at all, supporting the direction of these associations from resilience to outcomes. The study systematically investigated the dynamic hypotheses between multimorbidity resilience and health outcomes. That is, whether they are determinants or consequences, or both. Our findings suggest multimorbidity resilience predicts subsequent 5-year change in health outcomes, especially self-rated physical health and pain level, but not vice versa, strengthening the evidence of the importance of resilience in the health of older adults.

### REVIEW OF TYPE-2 DIABETES MELLITUS ILLNESS PERCEPTIONS AND ASSOCIATIONS WITH ILLNESS MANAGEMENT

Samuel Hatala, and Cleveland Shields, *Purdue University, West Lafayette, Indiana, United States*

Objective: Determine illness perceptions associated most frequently with measures of type-2 diabetes mellitus (T2DM) maintenance. We measured illness perceptions using the Illness Perception Questionnaire (IPQ) and variants (IPQ-Revised and Brief IPQ) Design: Review of literature from publication of IPQ to September 2020. Searched for articles utilizing IPQ but no other models of illness perception and studying T2DM Main Outcome Measures: Glucose control (measured by HbA1c levels), adherence to medications, and adherence to diet, exercise, and other lifestyle recommendations Results: Symptom attribution and fear of consequences are frequently associated with worse T2DM management and sense of control of illness progression and positive emotional valence are frequently associated with better T2DM management. Other subscales have less frequent but generally positive associations with the exceptions of recurring thoughts about T2DM duration, which had a negative association with management, and understanding the causes of T2DM, which had no associations at all. Other reviews found similar associations and highlighted a need for more general T2DM education. Conclusion: Future T2DM management interventions should promote sense of control over illness progression and positive emotional valence and provide education regarding symptoms to expect. Interventions should also consider managing patient fear, which has many associations with worse management.

### SOCIAL WELL-BEING AND CHRONIC DISEASE CONDITION AMONG OLDER ADULTS

Jungkyung Min, Idethia Harvey, and Yuchen Yeh, *Texas A&M University, College Station, Texas, United States*

Aging is characterized by the decline in physical health, functional status, and loss of social roles and relationships that can challenge the quality of life. A protective factor that moderates the impact of these phenomena is psychological (e.g., social) well-being. Despite the high prevalence of chronic conditions among older adults, research exploring the relationship between social well-being and chronic disease is sparse. The study aims were to investigate the relationship between social well-being among older adults (N = 1,251, R = 65 – 92 years) who participated in the National Survey of Mid-life in the United States (MIDUS 3). This study used variables for the MIDUS 3 study to test a structural equation model to examine the hypothesized relationships between social well-being, chronic conditions, life satisfaction, self-esteem, active coping, optimism, and religious coping. The findings indicate that perceived control, self-esteem, active coping, optimism, and religious coping were statistically significant for the participants' social well-being ($\beta$ =.29, p <.001, $\beta$ =.16, p<.001, $\beta$ =.08, p<.05, $\beta$ =.35, p<.001, and $\beta$ =.07, p<.05, respectively). However, life satisfaction was not significantly associate with social well-being ($\beta$ =.04, p >.05). For individuals' diagnosed with more than one chronic condition, perceived control, self-esteem, and optimism statistically significant impact their social well-being ($\beta$ = .33, p < .001, $\beta$ =.17, p < .001, and $\beta$ =.33, p < .001, respectively). Findings suggested that multiple chronic conditions influence social well-being. Chronic disease management programs may be useful in increasing social well-being among individuals with multiple chronic conditions.

### SESSION 10180 (LATE BREAKING POSTER)

### COGNITION

### CAN LIGHT PHYSICAL ACTIVITY IMPROVE COGNITION AMONG OLDER ADULTS? A SCOPING REVIEW

Emily Erlenbach, Edward McAuley, and Neha Gothe, *University of Illinois at Urbana-Champaign, Urbana, Illinois, United States*

Although the physical and cognitive benefits of moderate-vigorous intensity physical activity (MVPA) for older adults is well documented, this population often faces age-related functional and physical limitations impeding recommended MVPA participation. Recently, there has been a surge of interest in the independent health benefits of light-intensity physical activity (LPA) and its association with morbidity and mortality risk. LPA is the most common form of activity among older adults and its potential to combat cognitive aging needs to be investigated. The purpose of this scoping review was to catalog existing evidence on the association between device-based or technologically measured LPA and cognition among healthy older adults, identify trends in the literature, and pinpoint future areas of research. Six electronic databases were searched between January and August 2020. Eighteen published studies met the inclusion criteria: one acute exercise study, two randomized control trials (RCTs), twelve cross-sectional studies, and three longitudinal studies. Overall, n=9 studies (n=1 RCT, n=7 cross-sectional, and n=1 longitudinal) reported a significant, positive relationship between LPA and one or more cognitive outcomes including memory, attention, executive function and global cognition (MMSE/MOCA). These heterogeneous findings can largely be attributed to the diverse study designs, inconsistent definitions of LPA and numerous assessments used to test the cognitive domains. Collectively, these findings suggest LPA may be a potential lifestyle intervention to improve cognition among healthy older adults. However, the inconsistent approaches used among these studies suggests a more concerted, unified scientific approach and rigorous methodology are needed to further understand the LPA-cognition relationship.

### DAILY STRESSORS DEGRADE PERCEIVED COGNITIVE ABILITIES IN HEALTHCARE PROFESSIONALS

Rhitik Joshi Taylor Drury, and Soomi Lee, *University of South Florida, Tampa, Florida, United States*

Stress negatively impacts cognitive functioning. Less is known about whether daily stress is associated with perceived cognitive abilities in healthcare workers who require mental sharpness and attention to provide high-quality patient care. We examined daily associations between stressors and perceived cognitive abilities in nurses and whether the associations differed between workday vs. non-workday. Using 14-day smartphone-based ecological momentary assessment, 61 inpatient nurses at a U.S. cancer hospital reported the frequency and severity of daily stressors (e.g., arguments, accidents). Each day, participants subjectively evaluated their mental focus, memory, and attention. Multilevel modeling examined the within- and between-person associations of daily stressors with cognitive abilities adjusting for sociodemographics, work shift, and workday. Nurses reported experiencing stressors once every other day. More stressors were associated with poorer cognitive abilities. At the between-person level, those with more frequent or severe stressors reported poorer mental focus (B=-22.4, p<.01; B=-0.35, p<.01, respectively), worse memory (B=-24.35, p<.01; B=-0.37, p<.01, respectively), and lower attention (B=-25.47, p<.05; B=-0.40, p<.01, respectively). At the within-person level, on days with more frequent or severe stressors, participants reported poorer mental focus (B=-2.05, p<.05; B=-.03, p<.05, respectively) and lower attention (B=-1.95, p<.05; B=-.04, p<.01, respectively). Some of the between-person associations were more apparent on workdays; those with more stressors reported poorer mental focus and lower attention on workdays than on non-workdays. Nurses' perceived cognitive abilities at work vary by daily stressors. Disconnecting the linkage between stressors and perceived cognition may help improve work performance in nurses who may encounter frequent stressors at work.

### DEPRESSION COMPLICATES THE RELATIONSHIP BETWEEN SUBJECTIVE MEMORY COMPLAINTS AND MEMORY IN OLDER ADULTS

Kristi Wisniewski, Eun Young Choi, Christopher Beam, and Elizabeth Zelinski, *University of Southern California, Los Angeles, California, United States*

Subjective memory complaints (SMCs) are self-reported changes in memory performance and may indicate prospective dementia. Cognitive function and depressive symptoms are negatively correlated; however, depression's influence on the relationship between SMCs and cognition is unclear. Data were drawn from the Long Beach Longitudinal Study, a three-panel study of community-dwelling older adults from southern California who were assessed every three years. The present study included participants ages 54-89 who completed a 20-item immediate recall task at each wave and the Memory Functioning Questionnaire Frequency of Forgetting (FOF) subscale, a measure of SMC, on 2+ occasions from 1994-2014 (n=788; Mage=73 years at baseline, Meduc=15 years). Higher FOF scores indicated fewer memory complaints. Depression was measured as the average Geriatric Depression Scale score across occasions. Bivariate linear latent growth models over age adjusted for age, sex, and education (covariates associated with cognition). The SMC and recall slopes were positively correlated (parameter estimate=1.40; p<0.05). After controlling for depression, the slope-slope correlation was not significant (parameter estimate=0.83; p=0.127). However, after removing cognitively-relevant items from the Geriatric Depression Scale then adjusting for depression, the SMC slope-recall slope correlation trended toward significance (parameter estimate=0.99; p=0.070) suggesting that the cognitively relevant items were confounding the slope-slope correlation. These findings indicate that depression impacts the relationship between SMC and objective memory over age; therefore, depression may limit the clinical utility of SMCs as a cognitive screening measure. Future studies should assess depression using scales devoid of cognitive items to differentiate its influence on subjective and objective memory performance.

### EARLY DETECTION OF COGNITIVE IMPAIRMENT BY NATURAL LANGUAGE AMONG OUTPATIENTS AND COMMUNITY DWELLERS

Eri Kiyoshige,[1] Mai Kabayama,[1] Yasushi Takeya,[1] Yoichi Takami,[1] Shuko Takeda,[1] Yasuyuki Gondo,[2] Hiromi Rakugi,[1] and Kei Kamide,[1] *1. Osaka University Graduate School of Medicine, Suita, Osaka, Japan, 2. Osaka University, Suita, Osaka, Japan*

Background: Although early detection of cognitive decline has a significant relation to improving the quality of life of dementia patients, this early detection has been difficult due to requires of neuropsychological tests which people generally take when they notice their cognitive impairment. The timing of patients' notice was reported to be worse cognitive decline already, thus, we aimed to determine if cognitive impairment from a short interview by using Natural Language Processing approach. Methods: The present study used cross-sectional analysis among elderly outpatients and community-dwelling elderly from Septuagenarians, Octogenarians, Nonagenarians Investigation with Centenarians (SONIC) study. Cognitive decline was assessed by Telephone Interview of Cognitive

Status for Japanese (TICS-J) and modeled as a binary outcome (cut-off <33 points). Natural language data was collected by semistructured interviews about health conditions and cognitive orientation in space, time, and place. We used an open-source text segmentation library to parse natural language text into bag-of-words and term frequency-inverse document frequency (TF-IDF) representations. Results: There were 38 (19.9%) outpatients and 153 (80.1%) community dwellers, and 60 (31.4%) participants were defined as cognitive impairment. The maximized TF-IDF score was 0.49 in cognitive orientation in time questions. In this question, participants without cognitive impairment could not calculate the score. There were no significant differences in TF-IDF scores between participants with and without cognitive impairment. Conclusions: Elderly without cognitive impairment might not have an episode about cognitive orientation in time, and this may help for early detection of cognitive impairment

### EXAMINING COGNITIVE FUNCTION AND SELF-ESTEEM OF MIDDLE AGED AND OLDER ADULTS

Daniela Catarino, and Colin Adams, *University of Colorado Colorado Springs, Colorado Springs, Colorado, United States*

Research shows that self-esteem and well-being have strong correlations to cognitive abilities. People with high self-esteem, compared to those with a low self-esteem, tend to evaluate themselves as more favorable after both high and low performance. However, less research has been conducted on self confidence among the older population and how this can potentially negatively or positively influence the aging process. The purpose of the study was to see if there is an effect of age on cognitive function. The second aim was to see if there is an effect of cognitive function on self-esteem. The first hypothesis was that middle-aged adults would exhibit higher cognitive functioning than older adults. The second hypothesis was that those with lower cognition would exhibit lower self-esteem. A secondary analysis of data from the National Social Life, Health, and Aging Project (NSHAP) was performed on 60 randomly selected individuals from a total of 3,005 participants. A 2 x 2 chi square test revealed that the younger group (63%) compared to the older group (37%) were significantly more likely to exhibit perfect cognitive functions (versus not), $\chi^2$ (1) = 4.27, p < .05. A One-Way ANOVA revealed no significant main effect of cognitive function on self-esteem, F(1, 58) = 2.97, p = .09. This suggests that cognitive functions are more likely to decline as one ages but cognitive function alone might not strongly influence self-esteem. Future research should aim to understand under what conditions confidence influences cognitive function to promote healthy interventions for successful aging.

### NEUROPSYCHIATRIC SYMPTOMS BY COGNITIVE STATUS FOR MEXICAN-AMERICANS AGED 85 AND OLDER

Lan Vu, *University of Texas Medical Branch at Galveston, Galveston, Texas, United States*

Older adults with dementia and cognitive impairment often experience neuropsychiatric symptoms (NPS). Few studies have investigated the presence of NPS among older Mexican-American adults. Our objective was to describe the NPS of Mexican-Americans 85 years and older according to cognitive status. Data came from wave 9 (conducted in 2016) of the Hispanic Established Populations for the Epidemiological Study of the Elderly. The final sample consisted of 381 care recipients aged 85 years and older, along with their caregivers. The 12-item Neuropsychiatric Inventory (NPI-12) was administered to measure behavioral and psychiatric symptoms among the care recipients. Cognitive impairment was defined as a score of 18 or less on the Mini Mental Status Exam (MMSE). Care recipients with a diagnosis of dementia as reported by the caregiver were also classified as cognitively impaired. Overall, 259 (68.0%) participants had one or more NPS. Logistic regression models were used to estimate the average marginal effect (range = -1 to 1) of cognitive impairment on NPS, controlling for care-recipient characteristics. Approximately 87% of care recipients with cognitive impairment had at least one NPS compared to 55.8% of those without cognitive impairment (p<0.01). The predicted probability of having one or more NPI symptoms was 0.25 percentage points (95% CI=0.14-0.35) higher for participants with cognitive impairment than those without. NPS are present in the majority of older Mexican American adults, particularly in those with cognitive impairment. Future research could also investigate sociodemographic correlates of NPS.

### OLDER AND YOUNGER ADULTS REAPPRAISE NEGATIVE LIFE EVENTS IN DIFFERENT WAYS

Lucas Hamilton, and Eric Allard, *Cleveland State University, Cleveland, Ohio, United States*

Past reappraisal studies have been equivocal regarding age and reappraisal efficacy potentially due to the use of laboratory-generated stimuli. We examined reappraisal in a more self-relevant context: negative autobiographical events. 49 younger adults (YA) and 47 older adults (OA) generated 50 negative memories and provided negativity, positivity, and vividness ratings. One to two weeks later, participants underwent the reappraisal task during which physiological data were collected. Participants implemented one of three instructions for 30 seconds: remember naturally, increase negative reactions, or decrease negative reactions via a “positivizing” tactic. Each instruction was provided for 10 unique memories with negativity, positivity, and vividness ratings collected after each trial. 2 (Age; YA, OA) × 3 (Instruction; Remember, Increase, Decrease) mixed ANOVAs uncovered no differences in negativity or vividness ratings before reappraisal. However, OAs rated all memories more positively than YAs. This age difference persisted after reappraisal; however, OAs rated all memories more negatively and vividly than YAs, although both decreased compared to pre-reappraisal levels. Cardiorespiratory data were tested via 2 × 3 mixed ANOVAs, uncovering only a main effect of age on average heart rate. A multilevel model revealed significant variability in the time-course of pupillary responses. 2 × 3 mixed ANOVAs illustrated that reappraisal brought about faster and more frequent spikes in pupil diameter, particularly for OAs. We conclude that OAs and YAs may achieve reappraisal in different ways. Contrary to strict hedonic orientations, OAs simultaneously maintain higher negativity and positivity than YAs challenging existing propositions regarding age-related prioritization of hedonic goals.

### RACIAL/ETHNIC/GENDER-BASED DISPARITIES IN HEALTH TRAJECTORIES AMONG AMERICAN OLDER ADULTS

Dexia Kong,[1] Peiyi Lu,[2] Joan Davitt,[3] and Mack Shelley,[2]
*1. Rutgers University, New Brunswick, New Jersey, United States, 2. Iowa State University, Ames, Iowa, United States, 3. University of Maryland, Baltimore, Baltimore, Maryland, United States*

Numerous studies have examined racial/ethnic- or gender-based disparities in health. However, few examined health outcomes based on a combination of individuals' race, ethnicity, and gender. Guided by an intersectionality framework, this study explores racial/ethnic/gender-based differences in older adults' health trajectories over a ten-year period. Longitudinal data from the Health and Retirement Study (2004-2014) were used (n=16,654). Older adults (65+) were stratified into six groups based on their race, ethnicity, and gender, including (1) Non-Hispanic (NH) White Men; (2) NH White Women; (3) NH Black Men; (4) NH Black Women; (5) Hispanic Men; and (6) Hispanic Women. Growth curve models were used to examine the trajectories of three health indicators over time, including cognitive function, physical function (i.e. the sum of activities of daily living and instrumental activities of daily living), and depressive symptoms. The results indicated that NH White men and women outperformed racial/ethnic minority groups in cognition and physical function trajectories. Females in all racial/ethnic groups had more depressive symptoms but better cognition than their male counterparts. Hispanic women reported the most depressive symptoms. Hispanic women and NH Black women had the poorest physical function. NH Black men/women had the lowest cognition. Study findings highlighted the utility of an intersectionality framework in understanding health disparities in later life. Multiple social identities intersect with each other and generate protective and/or risk effects on cognitive, mental, and physical health status. Multilevel intervention strategies are warranted to close the health equity gap among various marginalized population groups.

### THE ASSOCIATION BETWEEN ENVIRONMENTAL FACTORS, RACE, AND COGNITIVE STATUS

Ethan Siu Leung Cheung, and Ada Mui, *Columbia University, New York, New York, United States*

Based on the data from National Social Life, Health and Aging Project, Wave 3, this study examined two research questions: what is the role of race in predicting cognitive status? and what are predictors of cognitive status between white and black older adults? Cognitive status was assessed using the 18-item survey-adapted Montreal Cognitive Assessment. Using the ecological framework, correlates of cognitive status were conceptualized in three levels of environments: micro- (personal health), meso- (social relationship), and macro-environments (community characteristics). Hierarchical regressions analyses were employed. Findings indicated that 83% of the sample (n= 2,829) were whites and the mean age was 72.95. Bivariate analyses suggested significant racial differences in cognitive status, marital status, income, education, health, social relationship, and community characteristics. Additive and interactive models showed that race had an independent effect as well as joint effects with the three levels of environments in explaining cognitive status. Parallel regression analyses for each racial group were undertaken and models were significant ($P < .0001$). In two separate models, common predictors for better cognition included being younger, more educated, fewer IADL impairments, and less depression. For older whites, unique correlates for better cognition were being female, higher income, sense of control in life, safer community, and neighbor relations. The only unique correlate for older blacks to have better cognition was community cohesion. Results provided insights on racial differences in cognition experienced among community-dwelling older Americans, and emphasized the need for social programs that promote race-sensitive, age-friendly communities to protect against cognitive decline.

### THE EFFECT OF MIDLIFE CARDIOVASCULAR RISK FACTORS ON COGNITIVE FUNCTION NEARLY HALF A DECADE LATER

Teresa Warren,[1] Shandell Pahlen,[2] Hong Xian,[3]
Jennifer De Anda,[1] William Kremen,[4] and Carol Franz,[4]
*1. San Diego State University, La Jolla, California, United States, 2. University of California, Riverside, Riverside, California, United States, 3. Saint Louis University, St. Louis, Missouri, United States, 4. University of California San Diego, San Diego, California, United States*

Several cardiovascular risk factors (CVRFs) have been associated with poor cognitive function. However, few studies have examined these factors longitudinally during midlife. We hypothesized that more midlife CVRFs would predict worse cognitive function approximately six years later. Participants were 886 men who participated in waves 2 and 3 of the Vietnam Era Twin Study of Aging. The American Heart Association's "Life's Simple 7" index was used to measure CVRFs. CVRFs were assessed at mean age 61 (range 55-66) and included smoking, physical activity, diet, body mass index, cholesterol, glucose, and blood pressure. Each factor was coded on a 3-point scale (0-2), ranging from poor to ideal status. These scores were then used to create a composite CVRF index (0-14). We examined several cognitive domains assessed at mean age 67 (range 61-73): abstract reasoning, episodic memory, processing speed, executive function, working memory, general verbal fluency, and semantic fluency. Analyses were adjusted for ethnicity, and education, and mean age 61. In the generalized estimating equation models, there were significant main effects indicating that the CVRF index at mean age 61 significantly predicted cognitive function at mean age 67 in episodic memory, 95% CI [.01, .08], $p = .01$, processing speed, 95% CI [.02, .09], $p = .01$, and executive function, 95% CI [.00, .06] ], $p = .03$. The CVRF index did not predict cognitive function in the other cognitive domains. These results suggest that poor cardiovascular health in late midlife may exacerbate cognitive decline.

### THE INFLUENCE OF MENTAL, PHYSICAL, AND SOCIAL ACTIVITY ON EPISODIC MEMORY OF AMERICANS AGED 50 AND OLDER

Ayse Malatyali, Shirley Gordon, John Morris, Lisa Wiese, and Christine Williams, *Florida Atlantic University, Boca Raton, Florida, United States*

The aim of this study was to describe the relationship between mental, physical, and social activity and episodic memory (EM) and further examine whether the relationship between these activities and EM is moderated by each activity type and demographics such as gender, marital status, and race. Participants were 3,903 cognitively intact persons who completed the 2016 Health and Retirement Study core interview and the 2015 Consumptions and Activities Mail Survey. Preliminary results showed no significant relationship between EM and mental, physical, and social activity. Mental activity significantly moderated the relationship between physical activity and EM, revealing a positive association between physical activity and EM for participants reporting low mental activity levels and a negative but non-significant association for participants with high mental activity scores. Although women demonstrated higher EM scores than men, there was a negative association between mental activity and EM for women. A significant reduction of EM performance was observed in participants who were divorced and widowed, compared to participants who were married. There was a positive relationship between physical activity and EM only for participants who were widowed. Furthermore, race significantly related to EM: African Americans had lower EM scores than Whites/Caucasians, but no significant association was found for the other race groups. Effect sizes were small throughout the analyses. Results emphasize the underlying relationship between different activity types and demographics in terms of EM performance. Further investigation is needed to explore changes over time in this relationship for persons who are cognitively intact or impaired.

### UNDERSTANDING SUBJECTIVE COMPARISON OF MEMORY TO PEERS: A BIOPSYCHOSOCIAL APPROACH

Allison Walden, Anna Robertson, and Madeline Lag, *University of Colorado Colorado Springs, Colorado Springs, Colorado, United States*

Identifying factors that contribute to subjective reports of memory decline may have important implications for interpretation of objective memory performance. Research suggests that subjective memory decline is influenced by affective symptoms such as anxiety and stress that are subsequently associated with poorer performance on neurocognitive tests. Personality is an additional factor, as evidence demonstrates that neuroticism may predict subjective memory decline. More research is needed to understand how factors such as demographics, personality traits, and subjective health impact subjective memory. The present study applies a comprehensive, biopsychosocial approach to identify constructs that predict how individuals view subjective memory decline compared to others their age. Researchers used a national database, Midlife in the United States (MIDUS), due to its comprehensive and diverse sample (N = 3,291). Predictor variables pertained to demographics, personality traits, as well as physical and mental health self-reports, and the outcome variable captured subjective memory relative to one's peers. A three-step hierarchical regression model indicated that demographic variables, personality traits, and self-evaluated health significantly contributed to 26.7% of the variance of self-evaluated memory compared to others their age, $F(35, 2836) = 29.48$, $p < .001$, with each block incrementally adding to the significance of the findings. Results suggest that subjective evaluation of physical health, personality traits, and demographic variables uniquely influence subjective memory decline relative to others of the same age. Awareness of how biopsychosocial factors may contribute to affective symptoms may have implications for the interpretation of neuropsychological testing results.

### VISION AND COGNITIVE FUNCTION IN THE MID- TO LATER-LIFE TRANSITION: THE STUDY OF WOMEN'S HEALTH ACROSS THE NATION

Ajay Kolli,[1] Michelle Hood,[2] Carrie Karvonen-Gutierrez,[1] Sayoko Moroi,[3] Joshua Ehrlich,[1] Brenda Gillespie,[1] Sarah Wood,[1] and David Musch,[1] *1. University of Michigan, Ann Arbor, Michigan, United States, 2. University of Michigan School of Public Health, Ann Arbor, Michigan, United States, 3. The Ohio State University College of Medicine, Columbus, Ohio, United States*

In this analysis of data from the Study of Women Across the Nation (SWAN), a longitudinal cohort study of mid-life women, we sought to describe the relationship between vision impairment (VI) during mid-life and future CF during the mid- to later-life transition period. Presenting visual acuity was assessed at baseline, between 1996-2000, using a Titmus occupational screener. "Mild" and "moderate to severe" VI were respectively categorized as visual acuity <20/40 and <20/60 in the better seeing eye. CF was measured at near-annual visits from 2003-2015 using the East Boston Memory Test immediate (EBMTi) and delayed (EBMTd) recall and Digit Span Backwards (DSB). Linear mixed models with a random intercept and slope for age were constructed to detect associations between vision and CF, adjusting for race, education, financial strain, alcohol use, and tobacco use. The sample included 341 women aged 42-53 years (mean: 46.6) at baseline with up to 16 years of follow-up. The prevalence of VA <20/40 and <20/60 was 12.9% and 7.6%, respectively. While mild VI was associated with lower EBMTi ($\beta$= -0.46, p=0.034), EBMTd ($\beta$= -0.49, p=0.031), and DSB ($\beta$= -0.78, p= 0.015) scores in unadjusted models, these relationships were not statistically significant after adjustment for covariates. After adjusting for covariates, baseline moderate to severe VI was associated with worse EBMTi ($\beta$= -0.87, p=0.001) and EBMTd ($\beta$= -0.52 p=0.039) scores. Altogether, moderate to severe VI during mid-life was associated with lower verbal episodic memory scores (EMBTi/d) over a 16-year period representing the transition from mid-life to later adulthood.

### SESSION 10190 (LATE BREAKING POSTER)

### COGNITIVE IMPAIRMENT

### A LONGITUDINAL ANALYSIS OF THE ASSOCIATION BETWEEN SUBCLINICAL HEARING LOSS AND COGNITION

Alexandria Irace,[1] Nicole Armstrong,[2] Jennifer Deal,[3] Alexander Chern,[1] Luigi Ferrucci,[4] Frank Lin,[5] Susan Resnick,[4] and Justin Golub[1], *1. NewYork-Presbyterian Hospital/Columbia University Irving Medical Center, New York, New York, United States, 2 Brown University Warren Alpert Medical School, Providence,*

*Rhode Island, United States, 3. Johns Hopkins Bloomberg School of Public Health, Baltimore, Maryland, United States, 4. National Institute on Aging, Bethesda, Maryland, United States, 5. Johns Hopkins University School of Medicine, Baltimore, Maryland, United States*

Several studies have demonstrated that age-related hearing loss (defined as >25 dB pure tone average [PTA]) is longitudinally associated with worse cognition. We aimed to investigate whether subclinical hearing loss (SCHL), or imperfect hearing traditionally categorized as normal (PTA ≤25 dB), may be similarly linked to cognitive decline. Subjects included cognitively normal adults ≥50 years old in the Baltimore Longitudinal Study of Aging with PTA ≤25 dB measured between January 1991 - September 1994 who had repeated cognitive assessments from January 1991 - November 2019 (n=263). The exposure was hearing based on the better ear PTA. The outcomes were standardized test scores in the following domains: learning/memory, mental status, executive function, visuospatial ability, and language. Multivariable linear-mixed effects models with random intercepts and slopes and unstructured variance-covariance structure were used to model the association between hearing and change in cognition over time, adjusting for baseline age, sex, years of education, and race. Mean age was 68.3 years (standard deviation [SD]=8.9) and follow-up ranged from 0-27.7 years (mean=12.5, SD=7.9). A 10-dB worsening in hearing was longitudinally associated with an annual decline of 0.016 SDs (95% confidence interval [CI]: 0.0002, 0.033) in California Verbal Learning Test (CVLT) short-delayed recall, 0.019 SDs (95% CI: 0.002, 0.036) in CVLT long-delayed recall, and 0.017 SDs (95% CI: 0.006, 0.028) in letter fluency after covariate adjustment. Poorer hearing among those with SCHL was associated with steeper declines in memory and verbal fluency scores. This relationship may begin at earlier levels of hearing loss than previously recognized.

### CLINICAL INTERVENTIONS FOR HIV-ASSOCIATED NEUROCOGNITIVE DISORDER (HAND): A SYSTEMATIC REVIEW

Hannah Mitchell, Erin Robinson, and Allison Donehower, *University of Missouri Columbia, Columbia, Missouri, United States*

Despite the widespread use of antiretroviral therapy, HIV-associated neurocognitive disorder (HAND) continues to be one of the most common central nervous system complications of human immunodeficiency virus type 1 (HIV-1). The severity and prevalence of HAND underscores the need for safe, effective therapies to mitigate or eliminate the impacts of the disorder to improve the quality of life of individuals living with HAND. The current study conducted a systematic review of the literature regarding experimental studies of clinical therapeutic interventions for HAND. An electronic search of four databases (PsycINFO, SCOPUS, Ovid MEDLINE, and CINAHL) initially returned 4,280 articles, 31 of which met the inclusion criteria for this study. Articles were selected for inclusion based on several criteria, including the use of a clinical experimental study design and measurement of neuropsychological performance. A large number of studies were excluded due to utilizing observational or cross-sectional designs, relevance, or for otherwise not meeting inclusion criteria. The results of this review revealed 31 articles that investigated both pharmaceutical and cognitive therapies for HAND. Pharmaceutical interventions range from common antiretroviral therapies to novel drug classes with various mechanisms of action. Importantly, this review revealed a number of limitations present in the greater body of HAND research including inconsistencies among methods of diagnosis of HAND and study design, which ultimately make comparisons across studies difficult. This review presents the current evidence that exists regarding therapies for HAND and broadly discusses trends, limitations, and gaps in the literature.

### COGNITIVE IMPAIRMENT AND DECLINE IN PHYSICAL FUNCTION AMONG OLDER MEXICAN AMERICANS OVER 20 YEARS

Amy Givan, and Soham Al Snih, *The University of Texas Medical Branch at Galveston, Galveston, Texas, United States*

The aim of this study was to examine cognitive function as a predictor of physical function decline over a 20-year follow-up period among older Mexican Americans who were non-disabled and were able to complete the Short Physical Performance Battery (SPPB) at baseline. The sample consisted of 2,232 Hispanics 65 years and older from the Hispanic Established Population for the Epidemiological Study of the Elderly. Measures included socio-demographics, self-reported medical conditions, body mass index (BMI), disability, depressive symptoms, limitations in activities of daily living, Mini-Mental State Examination (MMSE), and SPPB. General linear mixed models were used to estimate changes in SPPB over time as a function of MMSE. At baseline, 11% of the participants had cognitive impairment (MMSE < 21) and the average SPPB score for those with and without cognitive impairment was 7.16 + 2.75 and 7.81 + 2.36, respectively. Mixed model analysis showed that those with cognitive impairment (MMSE < 21) experienced a decline in the SPPB of 0.34 points per year (Standard Error = 0.09, p-value = 0.0002) after controlling for all covariates. Other significant predictor factors of decline in the SPPB were older age, depressive symptoms, diabetes, any assistance with activities of daily living, having had a hip fracture, and high BMI. Cognitive impairment predicted decline in physical function among older Mexican Americans who were non-disabled at baseline. These findings underscore the need of developing interventions to maintain cognitive and physical function to delay or prevent disability in this underserved population with high rates of disability.

### EARLY INDICATORS OF COGNITIVE DECLINE IN ONLINE FINANCIALLY ACTIVE OLDER ADULTS

Kimberly Grzesek Nora Mattek, Nicole Sharma, and Katherine Wild, *Oregon Health & Science University, Portland, Oregon, United States*

As access to computers and use of technology becomes more common in older adults, the incidence of online financial fraud increases. Law enforcement officials and fraud experts predict this trend to continue as aging baby boomers increasingly become targets. One reason this population might be at risk for financial fraud is due to subtle, undetected decline in cognitive abilities that have been associated with decline in financial capacity. Many cases of incipient cognitive decline go undetected. Further, those with early cognitive decline often have poor insight to its potential impact on daily functioning. Assessment of Activities of Daily Living Skills (ADLs) is paramount to determine early decline in daily activities. The ORCATECH Life Lab was designed to evaluate subtle neurological and other health changes and their relation to changes in daily functioning. Older adults participating in the Life Lab complete annual ADL and neurocognitive assessments. Additionally, 97 participants completed an online technology questionnaire where 64 participants reported participating in online financial activity. Results revealed that within the online financially active group, some assistance in ADL's was required. However, inconsistencies in ADL change over time highlight the challenges of screening for early signs of mild cognitive impairment (MCI) in patients that fall between normal cognition and MCI. In-home information technology may help overcome these challenges. Defining subtle changes in ADLs is a crucial step to enable early diagnosis of neurocognitive disorders and assist health care providers in improving disease management and to prevent incidents of financial fraud in this vulnerable population.

### EXAMINING THE INTERSECTION OF COGNITIVE & PHYSICAL FUNCTION IN THE BRAIN NETWORKS AND MOBILITY FUNCTION (BNET) STUDY

Elizabeth Handing, Michael Miller, Haiying Chen, Laura Baker, and Stephen Kritchevsky, *Wake Forest School of Medicine, Winston-Salem, North Carolina, United States*

Cognitive function and physical function are associated however less is known about task complexity and how individual tasks relate to one another. This project seeks to describe the relationship between cognition and physical function measures across 22 tasks ranging in task complexity and difficulty. Data are from the baseline visits of a new longitudinal study, Brain Networks and Mobility Function (B-NET) Study, mean age: 76.0±4.2 years; 55% women, and 90% Caucasian. We hypothesize there would be a set of "complex" tasks that would intersect both cognitive and physical function abilities such as the Four Square Step Test or Dual Task. We conducted principal components analysis on data from the first 110 participants to describe what factors could be identified across cognition and physical function measures. Seven factors, explaining 73% of the variability, were identified: 1) a complex physical function (postural sway on foam, expanded Short Physical Performance Battery, 400 meter walk, Four Square Step Test, Dual Task), 2) physical strength (grip strength and leg press), 3) visual recall (Brief Visuospatial Memory Test-immediate and delayed), 4) Craft story recall (immediate and delayed), 5) global cognition & fluency (MoCA, category and word fluency) 6) auditory recall (Auditory Verbal Learning Test- immediate and delayed), and 7) executive function (Trail Making Test A & B). We did not identify factors that intersected both physical and cognitive tasks. These results may help to inform measurement selection in future studies that seek to evaluate components of function among older adults.

### LONGITUDINAL DAILY LIVING LIMITATIONS AND COGNITIVE STATUS: RESULTS FROM THE 1998-2016 HEALTH AND RETIREMENT STUDY

Benson Wu,[1] Mohammad Usama Toseef,[2] Wassim Tarraf,[2] Ariana Stickel,[3] Sonya Kaur,[4] Alberto Ramos,[4] and Hector Gonzalez[1] *1. University of California, San Diego School of Medicine, La Jolla, California, United States, 2. Wayne State University, Detroit, Michigan, United States, 3. Shiley-Marcos Alzheimer's Disease Research Center, La Jolla, California, United States, 4. University of Miami Miller School of Medicine, Miami, Florida, United States*

Data increasingly points to midlife health and modifiable risk factors as critical targets for improving older-age health outcomes and mitigating potential cognitive impairment and disease. We used biennial Health and Retirement Study data (1998-2016) collected on adults ages 50-64 years who did not meet criteria for dementia at baseline and who remained living by 2016 (unweighted-n=4,803). Cognitive status was defined using Langa-Weir criteria: Normal, Cognitively Impaired Not Dementia (CIND), and Dementia. We examined how 18-year patterns in activities of daily living (ADLs) and instrumental activities of daily living (IADLs) predicted cognitive status in 2016. We used latent class analysis to extract longitudinal phenotypes of activities limitations, followed by survey multinomial logistic regressions to examine their associations with cognitive status and test for race/ethnic modifications. We identified three groups of functional impairment: (1) gradually increasing (15.7%), (2) stable elevated (5.6%), and (3) minimal dysfunction (78.7%). After covariates adjustment, both the gradual and stable elevated impairment groups (vs. minimal) had substantially higher relative risk ratios (RRR) for dementia (RRR=5.71[3.89;8.39] and RRR=7.87[4.23,14.64]) and CIND (RRR=2.21 [1.69,2.88] and RRR=1.92[1.16;3.17]). We detected modifications by race/ethnicity such that Hispanics with stable elevated impairment had a higher probability of dementia compared to their White counterparts. The results varied for Blacks and did not significantly differ from Whites. Data-driven methods may improve our understanding of heterogeneous functional impairment patterns among late middle-aged adults and allow for tailored ADRD prevention strategies. Focused risk-based interventions can yield important public health savings and reductions in structural, social, and individual health burdens.

### PREVALENCE OF MILD COGNITIVE IMPAIRMENT IN LATIN AMERICA AND THE CARIBBEAN: A SYSTEMATIC REVIEW

Fabiana Ribeiro,[1] Ana Carolina Teixeira-Santos,[2] and Anja Leist[1], *1. University of Luxembourg, Esch-sur-Alzette, Luxembourg, 2. University of Minho, Braga, Portugal*

Background. The population of Latin America and the Caribbean (LAC) is ageing rapidly, presenting the highest prevalence rates of dementia in the world. In this context, mild cognitive impairment (MCI) is an intermediate condition between normal ageing and dementia. However, very few studies verified the prevalence of MCI in LAC countries; earlier global systematic reviews only considered prevalence reports published in English language. Method. We conducted a systematic review to evaluate the prevalence of MCI in LAC countries and to explore the factors associated with MCI (i.e., age, gender, and education). A database search was conducted in February 2020 using PubMed, Web of Science, Scopus, Lilacs, SciELO, and EMBASE, for population-or community-based studies with MCI data for countries in LAC, published in English, Spanish, or Portuguese language. From k=2,168 identified and k=1,684 screened studies, only articles were selected that included subjects with a precise diagnosis of MCI. The studies were qualitatively assessed using the JBI critical appraisal checklist for studies reporting prevalence data tool. Results. A total of nine studies met the criteria, published between 2007 and 2019, including a total of 17,812 participants in nine countries Brazil, Mexico, Argentina, Colombia, Peru, Cuba, Dominican Republic, Venezuela, and Costa Rica. Estimates for MCI prevalence ranged from 1.2% to 34%, with most estimates between 1.2% and 6.45%. Estimates differed by age group, gender, and educational level. Discussion. This is the first systematic review of the prevalence of MCI in LAC countries, considering only high-quality studies adopting rigorous diagnostic criteria.

### THE BAND TEST: IMPROVING RELIABILITY AND BALANCE TESTING FOR PEOPLE WITH NEUROCOGNITIVE IMPAIRMENTS: A PILOT STUDY

Evelyn Hahn,[1] Melissa Moore,[1] Lindsay Neirman,[1] Stephanie Arcadia,[2] Stacy-Jo Krasa,[1] and Tabassum Majid,[3]
*1. Functional Pathways, Sykesville, United States, 2. Sykesville, Maryland, United States,*
*3. University of Maryland, Baltimore County, Baltimore, United States*

People with neurocognitive impairments have a higher risk of falls compared to other older adults and require specific cues for evaluation. Additional options for balance testing is necessary to improve reliability and assessment of fall risk. This study established the efficacy of the novel Balance Assessment for Neurocognitive Deficits (BAND) in order to improve measurement of fall risk for people with neurocognitive impairments. The BAND was analyzed for construct validity and reliability through comparison with the Berg Balance Scale (BBS). Older adults with neurocognitive impairments (n=15) in subacute and long-term settings performed BAND and BBS assessments during therapy. Clinicians determined ambulation assistance, fall risk, and time. Calculation of intraclass correlation coefficients (ICCs), standard error of measurement (SEM), and minimal detectable change (MDC95) values was completed. Corresponding ICC values were 0.985 (95% confidence interval (95% CI), 0.956-0.995) for test-retest reliability and 0.995 (95% CI, 0.985-0.998) for inter-rater reliability. Other values included SEM=0.79 and MDC95=2.18. A linear-regression graph including Pearson's coefficient (r) demonstrated validity through comparing BAND and BBS and showed a strong correlation (r=0.94, 95% CI, 0.825-0.98). A Bland-Altman plot was created to assess agreement between clinicians, and the mean difference was 0.2667 with 95% limits of agreement (-0.897 to 1.430). The BAND demonstrated excellent reliability and agreement for clinicians providing the test. Further research is necessary to compare the BAND with additional assessments and to demonstrate the utility in expanded populations including the community.

### WITHIN-GUIDELINE ALCOHOL CONSUMPTION PROTECTS AGAINST DEMENTIA?: OFFSET EFFECT OF HISTORY OF DRINKING PROBLEMS

Penny Brennan, *University of California, San Francisco, San Francisco, California, United States*

Research on the prospective relationship between older adults' alcohol consumption and their subsequent risk of dementia and cognitive impairment, no dementia (CIND) has been limited by inconsistent definitions of "moderate" drinking, use of short follow-ups, and an exclusive focus on either amounts of alcohol, or history of drinking problems, as predictors. To overcome these limitations we analyzed a longitudinal, 18-year Health and Retirement Study cohort (n=4,421) to determine how older adults' baseline membership in one of six drinking categories (Non-Drinker, Without and With a History of Drinking Problems (HDP); Within-Guideline Drinker, Without and With a HDP; and Outside-Guideline Drinker, Without and With a HDP) predicted dementia and CIND 18 years later. Among participants with No HDP, 12.6% of Non-Drinkers, 5.2% of Within-Guideline Drinkers, and 8.8% of Outside-Guideline Drinkers were classified as having dementia at the 18-year follow-up; among participants With HDP, 14.1% of Non-Drinkers, 8.9 % of Within-Guideline Drinkers, and 6.9% of Outside-Guideline Drinkers were classified with dementia. Being a baseline Within-Guideline Drinker with No HDP reduced the likelihood of dementia 18 years later by 45%, independent of baseline demographic and health characteristics; being a baseline Within-Guideline Drinker With a HDP reduced the likelihood of dementia by only 13% (n.s.). Similar patterns obtained for the effects of baseline drinking group membership on likelihood of CIND at follow-up. These findings suggest that consuming alcohol at levels within validated guidelines for low-risk drinking may protect against dementia and CIND, but only among older adults who have no history of drinking problems.

### SESSION 10200 (LATE BREAKING POSTER)

### COMMUNICATION AND LANGUAGE

### AN ASSESSMENT OF ATTENDEE EXPERIENCES WITH A WORKSHOP TO REFRAME AGING-RELATED COMMUNICATIONS

Stephanie Chesser, and Michelle Porter, *University of Manitoba, Winnipeg, Manitoba, Canada*

In 2018, the Centre on Aging at the University of Manitoba adapted the FrameWorks Institute's Gaining Momentum toolkit into an interactive workshop that was delivered to aging-related stakeholders (e.g., health professionals, educators, researchers, advocates, older persons) across the province of Manitoba, Canada. The purpose of this study was to complete a qualitative assessment of attendee experiences with the workshop and its impact, if any, on their aging-related communication behaviours. Study participants completed two telephone interviews—one approximately two weeks and one approximately three months post-workshop—about their workshop experiences, motivations for attending, and short and long-term communication goal setting. Through the interviews, most participants expressed positive experiences with the workshop content, as well as an enjoyment of its interactive elements (e.g., self-introduction activity, reframing short and long form aging-related communications, group feedback). Several also shared specific examples of how workshop content was influencing their short/long-term aging communication strategies. Some participants, however, also identified ways that local culture could impact the interpretation of and, thus, potential success of workshop language and/or framing recommendations in specific communities. Overall, the findings from this study suggest that the Gaining Momentum workshop was a valuable experience for attendees that inspired critical assessment of, and changes to, the ways they communicated about aging in their professional and personal lives. Future research is warranted to explore the ways its content could be adapted to better meet the unique communication considerations within the province of Manitoba (e.g., cultural, geographic, and language-related implications).

### AN ASSESSMENT OF ATTENDEE EXPERIENCES WITH A WORKSHOP TO REFRAME AGING-RELATED COMMUNICATIONS

Stephanie Chesser, and Michelle Porter, *University of Manitoba, Winnipeg, Manitoba, Canada*

The Centre on Aging at the University of Manitoba adapted the FrameWorks Institute's Gaining Momentum toolkit into an interactive workshop that was delivered to aging-related stakeholders (e.g., health professionals, educators, researchers, advocates, older persons) across the province of Manitoba, Canada. The purpose of this study was to complete a qualitative assessment of attendee experiences with the workshop and its impact, if any, on their aging-related communication behaviors. Study participants recruited from four communities (two urban, two rural) completed two telephone interviews—one approximately two weeks and one approximately three months post-workshop—about their experiences, motivations for attending, and short and long-term communication goal setting. Through the interviews, most participants expressed positive experiences with the workshop content, as well as an enjoyment of its interactive elements (e.g., self-introduction activity, reframing short and long form aging-related communications, group feedback). Several also shared specific examples of how workshop content was influencing their short/long-term aging communication strategies. Some participants, however, also identified ways that local culture could impact the interpretation of and, thus, potential success of workshop language and/or framing recommendations in specific communities. Overall, the findings from this study suggest that the Gaining Momentum workshop was a valuable experience for attendees that inspired critical assessment of, and changes to, the ways they communicated about aging in their professional and personal lives. Future research is warranted to explore the ways its content could be adapted to better meet the unique communication considerations within the province of Manitoba (e.g., cultural, geographic, and language-related implications).

### COMMUNICATION IN HOME CARE: THE EXPERIENCES OF FORMAL CAREGIVERS IN COMMUNICATING WITH PERSONS LIVING WITH DEMENTIA

Pabiththa Kamalraj, Marie Savundranayagam, J.B. Orange, and Marita Kloseck, *Western University, London, Ontario, Canada*

There is limited literature on formal caregivers' communication with persons living with dementia (PLWD) in home settings. Most research comes from studies of long-term care home settings or informal home care contexts. Yet, there are expected needs and rising demands for formal caregiver support within home care. The aim of this study was to understand better the lived experiences of personal support workers (PSWs) regarding their communication with PLWD in home settings. A hermeneutic phenomenological approach guided this research. Semi-structured interviews were conducted with 15 PSW participants. Three major themes were identified through thematic analysis: (1) challenged by dementia-related impairments; (2) valuing communication in care; and (3) home is a personal space. PSWs experienced difficulties in their communication with PLWD despite recognizing the importance of communication in providing optimal home care. This suggests that while PSWs possess good intentions, they do not possess the skills necessary to ensure effective interactions. Dementia-specific education and training are recommended to improve PSWs' communication skills and to enhance quality of care. Findings highlight further the uniqueness of the personal home space itself on PSWs experiences with communication. Aspects of the home care environment can enable, but also complicate, successful communication between PSWs and PLWD. Consequently, findings also have implications for family members of PLWD and home care employers regarding optimizing practice and improving care.

### THE USE OF TAG QUESTIONS IN PERSON-CENTERED COMMUNICATION

Shalane Basque,[1] Marie Savundranayagam,[1] Maren Kimura,[1] and Kristine Williams[2] *1. Western University, London, Ontario, Canada, 2. University of Kansas, Lawrence, Kansas, United States*

Tag questions are imperative, declarative, exclamative or interrogative statements that have been modified to include a question (e.g.., It is hot out, isn't it?). Tag questions have been characterized as elderspeak because it suggests an expected response from the person with dementia, thus limiting his/her ability to make a decision independently. However, tag questions serve multiple functions in conversation. There is limited research on the multidimensional nature of tag questions in conversations between formal caregivers and their clients with dementia. Accordingly, the purpose of this study was to investigate the functions of tag questions used by formal caregivers in utterances coded as person-centered. Conversations (N= 87) between formal caregivers and a simulated person with dementia were video-recorded during a 5-minute care interaction involving morning care. Caregivers' utterances were coded for the use of the following types of person-centered communication: recognition, negotiation, facilitation, and validation. During secondary data analysis, the person-centered utterances were analyzed for the use and function of tag questions. Conversational analyses revealed two broad functions of tag questions: gather information and facilitate a desired action. Tag questions used to gather information included the following specific functions: acknowledge response, establish common ground, state fact or opinion, initiate topic, conversational joking, state what is being done and questions. Tag questions that facilitated a desired reaction included the following specific functions: offers, advice and suggestions and requests and commands. Findings from the current study reveal that tag questions are not exclusively elderspeak and can be used to illicit conversation.

### SESSION 10210 (LATE BREAKING POSTER)

### DEPRESSION AND ANXIETY

### AN ANALYSIS OF THE IMPACT OF SEXUAL ORIENTATION ON LONGITUDINAL DEPRESSION

Anna Thompson, and Britney Wardecker, *The Pennsylvania State University, University Park, Pennsylvania, United States*

Research suggests that mental health and well-being improve as we age, and this trend is dubbed "the paradox of aging" (Charles & Carstensen, 2010). However, little is known about whether this trend happens for individuals who may experience lifelong disadvantage, such as those who identify as lesbian, gay, or bisexual. We used data from the Midlife in the United States Study (MIDUS) to examine lesbian/gay, bisexual, and heterosexual adults' changes in depression from 1995 to 2014. Participants identified as lesbian/gay ($n = 46$), bisexual ($n = 37$), and heterosexual ($n = 3030$) and 45.1% identified as female. Participants' ages ranged from 20-74 years ($M = 45.61$, $SD = 11.41$) in 1995 and 39-93 years ($M = 63.64$, $SD = 11.35$) in 2014. We analyzed our data using a repeated measures ANOVA and our results indicate that depression decreased on average from 1995 to 2014 for heterosexual [Wilk's Lamda = .996, $F (1, 3029) = 12.23$, $p < .001$] and lesbian/gay adults [Wilk's Lamda = .848, $F (1, 45) = 8.08$, $p = .007$]. However, bisexual adults did not experience this decrease in depression [Wilk's Lamda = .990, $F (1, 36) = 0.36$, $p = .550$] and their depression remained relatively stable. Our results are consistent with previous studies that indicate bisexuals experience poorer mental health when compared to lesbian/gay and heterosexual adults (Bostwick, Hughes, & Everett, 2015). The current research highlights depression as a condition that may not decrease universally over time. We discuss implications for bisexuals' health and well-being.

### ANXIETY AND DEPRESSION AS PREDICTIVE FACTORS OF QUALITY OF LIFE IN OLD AGE: A LONGITUDINAL ANALYSIS

Oscar Ribeiro,[1] Lia Araújo,[2] Laetitia Teixeira,[3] Carmen Rodríguez-Blázquez,[4] and Maria Joao Forjaz,[4] *1. University of Aveiro, Aveiro, Portugal, 2. Instituto Politécnico de Viseu, Viseu, Portugal, 3 Institute of Biomedical Sciences Abel Salazar | Center for Health Technology and Services Research (CINTESIS), University of Porto, Porto, Portugal, 4. Carlos III Health Institute, Madrid, Spain*

Research on the effect of age in quality of life (QoL) is controversial, and doubts remain if this effect is continuous and if it disappears when controlling for other factors. Most studies have verified the association of health-related factors, such as poor physical health, functional impairment, and depression with lower scores of QoL in old age. But studies that analyse such an association in non-clinical samples through longitudinal analysis and including anxiety as a mental health factor remain very rare. This study relies on data from two waves from the Survey of Health, Ageing and Retirement in Europe (SHARE) and focuses on a representative sample of individuals aged 50+ from Portugal. It comprises 1782 participants (baseline), of which 1201 were reassessed. The mean age was 64.8 years (sd=9.3 years) and 55.4% were female. Linear Mixed Effects models were performed to identify potential factors associated with changes of QoL across age. According with the final model (pseudo-$R2$ equal to 0.421), a significant effect of age as time-dependent factor was verified, observing a decline of QoL with increasing age. Years of education, depression and anxiety at baseline were significantly associated with QoL. Thus, the effect of age on quality of life remains significant even when other factors are considered. The influence of mental health on QoL reinforces the importance of intervening in both depressive and anxiety symptoms in old age.

### LIFE REVIEW ON PSYCHO-SPIRITUAL OUTCOMES AMONG OLDER ADULTS WITH LIFE-THREATENING ILLNESS: A SYSTEMATIC REVIEW

Mandong Liu,[1] Ying Wang,[2] and Iris Chi,[1] *1. University of Southern California, Los Angeles, California, United States, 2. Lanzhou University, Lanzhou, Gansu, China*

Life review has been widely conducted to help older adults enhance well-being and cope with burdens. Spirituality is an important part of an older adult's overall well-being, especially for those with life-threatening illnesses. However, there is no review study examining the effectiveness of life review interventions on psycho-spiritual outcomes among this population. The aim of the study was to examine the effectiveness of life review on psycho-spiritual well-being among older adults with life-threatening illnesses. A systematic review with meta-analysis consistent with the recommendations of the Cochrane Collaboration was conducted. Database searches included PubMed, PsycINFO, the Cochrane Library, the Campbell Library, EBSCO, CNKI, and the Airiti Library up to March 2020. Grey literature and reference lists from relevant articles were also searched and reviewed. 34 studies were included in the systematic review and 33 were included in the meta-analysis for outcomes of anxiety, depression, and quality of life. Other psycho-spiritual outcome measures that were not included in the meta-analysis due to the small number of studies included mood, apathy, life satisfaction, meaning in life, spiritual well-being, and some multi-dimensional instruments. The studies greatly varied in program design, content, format, length, interventionist, and more. The meta-analyses demonstrated standardized mean differences in favor of life review compared with the control with small to moderate effect sizes. The reviewers call for including more psycho-spiritual well-being measures among interventions for older adults with life-limiting illnesses, as well as studies with adequate sample size and rigorous designs in future research.

### POST-HOSPITAL CARE INTENSITY MODIFIES THE RELATIONSHIP BETWEEN DEPRESSIVE SYMPTOMS AND HOSPITAL READMISSIONS

Leah Abrams,[1] and Geoff Hoffman,[2] *1. University of Michigan School of Public Health, Ann Arbor, Michigan, United States, 2. University of Michigan School of Nursing, Ann Arbor, Michigan, United States*

The objective of this study was to examine whether more structured post-acute care reduces the deleterious impact of depressive symptoms on older adults' post-hospital outcomes (30-day hospital readmissions, 30-day falls, 1-year falls, and 1-year mortality). The sample comprised 23,485 eligible index hospitalizations from 7,151 unique fee-for-service Medicare beneficiaries from Health and Retirement Study linked to Medicare claims from 2000 to 2014. Depressive symptoms were measured using the eight-item Center for Epidemiologic Scale - Depression. We ran multinomial probit models regressing post-hospital setting on depressive symptoms while adjusting for sociodemographic factors, socioeconomic factors, family support, and health status. Then, we ran adjusted logistic regression models of each outcome while interacting depressive symptoms with post-acute care setting. We found that 62% of hospitalizations were routine discharges home, 17% were discharged to home health, and 21% were discharged to a Skilled Nursing Facilities (SNF). When adjusting for sociodemographic and socioeconomic factors, each increasing depressive symptom was associated with a half percentage point higher probability of referral to home health and 1.6 percentage points higher probability of discharge to SNFs, driven by differences in family support and health status. Rehabilitation in SNFs, compared to routine discharges home, reduced the positive association between depressive symptoms and 30-day readmissions (OR=0.95, p=0.027). However, post-acute care settings did not modify the association of depressive symptoms with falls or mortality. Considering depressive symptoms in discharge decisions, above and beyond their associations with family support or health status, may help hospitals avoid readmissions but may not improve health and functioning.

### TYPES OF INSOMNIA MEDIATE THE RELATION BETWEEN ANXIETY AND DEPRESSIVE SYMPTOMS LONGITUDINALLY IN LATE LIFE

Courtney Bolstad, and Michael Nadorff, *Mississippi State University, Mississippi State, Mississippi, United States*

The current study examined onset and maintenance insomnia as mediators of the relation between anxiety and depressive symptoms over a three-year period in a sample of older adults. We hypothesized that anxiety symptoms at timepoint one would significantly predict depressive symptoms at timepoint four, while controlling for depressive symptoms at timepoint one. We also hypothesized that this effect would be significantly reduced when adding onset and maintenance insomnia at timepoint two and three, respectively, as mediators. Participants included 3,484 older adults, ages 66 to 103 (M = 77, SD = 7), included in the National Health and Aging Trends Study who completed measures of types of insomnia, anxiety, and depressive symptoms at four different timepoints (2012 through 2015). The model showed a significant direct effect of anxiety on depressive symptoms at timepoint four, independent of baseline depressive symptoms. The relation was mediated by onset and maintenance insomnia, though a significant direct effect remained. Therefore, types of insomnia contribute to the development of depressive symptoms in older adults with anxiety symptoms over time, even when controlling for baseline depressive symptoms. Further, onset and maintenance insomnia are unique outcomes and predictors of anxiety and depressive symptoms, respectively, even when controlling for baseline depressive symptoms. Our findings provide a foundation for future intervention research with clinical samples that control for confounding variables in further elucidating the development and change in depressive symptoms among older adults with anxiety through onset and maintenance insomnia.

### VIRTUAL REALITY THERAPY TAKES GREENSPACE TO OLDER NURSING HOME RESIDENTS: A PILOT STUDY

Jody Blankenship, *University of Missouri - Columbia, Columbia, Missouri, United States*

Nature-based virtual reality (VR) interventions can offer a safe adjunct approach for managing depression and anxiety, but applications of this innovative technology have not been studied in the nursing home (NH) setting. Pleasant events like those generated with VR may provide a psychological time-out from the ongoing stress associated with living in an NH, and thereby, may facilitate adaptive coping. This study, conducted between January and February of 2020, aimed to pilot test the effects of ViRT-Ta-GO: Virtual Reality Therapy Takes Greenspace to Older NH residents on the severity of depressive and anxiety symptoms among NH residents aged 60 years and older and obtain estimates of effect sizes on outcome measures. Using a quasi-experimental, one-group, pretest-posttest design, nine cognitively intact NH residents (Mage = 81, SD = 10.23) were recruited from two Pennsylvania NHs. The outcome measures included the Patient Health Questionnaire-9, Geriatric Anxiety Inventory, and 10 open-ended questions. Residents participated individually in three 10-minute VR sessions per week for four weeks. There were no adverse effects. Participant attrition and self-report indicated that ViRT-Ta-GO was feasible and acceptable. At posttest, a Wilcoxon signed rank test showed a significant decrease in anxiety symptoms (z = -2.02, p = .04) with a large effect size (r = -.48). Nonsignificant trend improvements in depression were observed (p = .08), pretest to posttest. The 4-week VR intervention was effective in reducing symptoms of anxiety. Preliminary findings demonstrate utility within an NH population, suggesting it will be advantageous to evaluate ViRT-Ta-GO through controlled clinical trials.

### SESSION 10220 (LATE BREAKING POSTER)

### EDUCATION AND TRAINING

### EMBEDDING AGE-FRIENDLY CARE USING THE 4MS FRAMEWORK IN THE CONVENIENT CARE SETTING

Sherry Greenberg,[1] Lilia Pino,[2] Mary McCormack,[2] Evelyn Duffy,[3] Elizabeth Zimmermann,[3] Robin Hughes,[3] Anne Pohnert,[2] and Mary Dolansky,[3] *1. Seton Hall University College of Nursing, South Orange, New Jersey, United States, 2. CVS Health Minute Clinic, Woonsocket, Rhode Island, United States, 3. Case Western Reserve University, Cleveland, Ohio, United States*

This late-breaker presentation highlights the implementation science plan for Age-Friendly care in CVS Health MinuteClinic's convenient care clinics. This project, a partnership between MinuteClinic, Case Western Reserve University Frances Payne Bolton School of Nursing, and the Institute for Healthcare Improvement, funded by The John A. Hartford Foundation, increases the number of care providers trained in the provision of Age-Friendly care using the 4Ms Framework: What Matters, Medication, Mentation, and Mobility. The implementation team learned the MinuteClinic usual processes, then developed strategies for successful Age-Friendly care implementation. Data from 21 nurse practitioner, 5 patient interviews, educator focus groups and 14 site visits revealed time, resources, and perceived value as barriers to implementation. Most reported increased knowledge and willingness to change practice. Based on the Plan-Do-Study-Act change process data, the implementation team developed solutions that addressed gaps leading to the development of practice-based tools for successful Age-Friendly care project adoption and implementation.

### INFLUENCE OF PROVIDER CHARACTERISTICS ON GERIATRIC MENTAL HEALTH WORKFORCE TRAINING NEEDS

Travis Mitchell,[1] Kristen Sorocco,[1] Mary Wyman,[2] Andrea Vincent,[3] Laura Wray,[4] and Lindsey Slaughter,[5] *1. University of Oklahoma Health Sciences Center, Oklahoma City, Oklahoma, United States, 2. University of Wisconsin, Madison, Wisconsin, United States, 3. University of Oklahoma, Norman, Oklahoma, United States, 4. Veterans Health Administration, Buffalo, New York, United States, 5. Richmond VAMC, Richmond, Virginia, United States*

Approximately half of VA users are 65 years of age or older, with a substantial subset having complex and interacting medical, neurocognitive, and behavioral disorders. The goal of the present study was to assess knowledge and training gaps of VA mental health staff using a Web-based training needs assessment developed specifically for VA personnel. Provider characteristics, confidence in working with older adults with and without cognitive disorder, and geriatric training needs were assessed. VA psychologists, psychiatrists, social workers, nurses, and peer support specialists (N=3313) were invited to participate via email. Respondents were 489 mental health providers, a response rate of 13.8 percent. Respondents reported less confidence in treating and assessing older adults with cognitive disorders then older adults in general. This did not differ by any of the examined provider characteristics, including age, gender, or professional experience. Training need endorsement was high across most of the training categories; however, a few differences according to provider characteristics were noted. Providers under the age of 50 endorsed a greater need for training in psychotherapy with older adults with cognitive disorder ($p = .02$). Female providers endorsed a greater need for training on providing psychoeducation about cognitive disorder, such as dementia, to older adults and their families ($p = .02$). In sum, VA MH providers in general reported strong interest in a wide range of geriatric MH training topics, indicating a need for universal geriatric-related staff education. For some topics, endorsing geriatric expertise predicted stronger interest in training.

### LICENSED NURSE/CERTIFIED NURSE AIDE COLLABORATION IN THE CARE OF NURSING HOME RESIDENTS

Cynthia Beynon, *University of Utah, Bountiful, Utah, United States*

This mixed-method study explores how nursing team collaboration is perceived and experienced in four nursing homes (NHs) in the Western United States. Licensed nurse (LN) and certified nurse aide (CNA) participants completed two survey tools to assess their perception of collaboration and teamwork in their current work environment. The LNs and CNAs were paired and interviewed both individually and as a caregiving pair to explore the lived experience of collaboration in the care of NH residents. Quantitative survey results were analyzed using IBM® SPSS Version 25, and participants reported a collaborative working environment with equally strong ratings in the following categories: partnership, cooperation, and coordination; they agreed with statements reflective of teamwork including team structure, leadership, situation monitoring, mutual support, and communication. No significant difference was found between LN and CNA responses or between team members in any of the four participating facilities. Qualitative survey data were loaded in NVivo12 and analyzed using a thematic analysis approach. The findings revealed five primary themes, including essential elements in successful team collaboration—perspective, coworker connection, communication, and mutual support—and ways teamwork and collaboration impact resident care.

### SUPPORTING RELATIONSHIPS, PROVIDING PRESENCE: ARTS-INFORMED APPROACHES TO LTC STAFF DEMENTIA CARE LITERACY

Kelly O'Neil,[1] Marco Redden,[2] and Katie Aubrecht[2] *1. St. Francis Xavier University, Antigonish, Nova Scotia, Canada, 2. Mount Saint Vincent University, Halifax, Nova Scotia, Canada*

Early findings and insights are shared from an interpretive analysis of interviews with 15 leaders in arts-based approaches in dementia care. This was conducted as part of a larger project that has the goal of operationalizing 'good' literacy in social and relational care in long-term care (LTC). Interviews aimed to identify promising directions in arts-based approaches in education and training for LTC staff provincially, nationally, and internationally, and understand how they contribute to 'good' mental health and dementia literacy. Participants were recruited using a purposive snowball sampling method and semi-structured interview guide. Interviews were conducted in-person, via telephone and Zoom, and digitally recorded. Arts-making enhances mental health and dementia literacy of LTC staff by: 1) supporting relationships by generating trust and collaboration among persons living with mental health conditions and/or dementia, arts facilitators, and family members; 2) creating alternative communication spaces that allow people to see themselves and be seen by others from new perspectives; 3) fostering an artistic sensibility that: encourages imagination and empathy, brings spontaneity and playfulness to interactions, and disrupts restrictive expectations entrenched in typical caregiver/care receiver relationships. 'Good' literacy involves a relational and spatial awareness which manifests in the form of an artistic sensibility. Arts-based approaches can be used to enhance quality care by capacitating staff in the art of being open and curious, nimble and flexible, in how they know and make connections on an interpersonal level, in the moment.

### SESSION 10230 (LATE BREAKING POSTER)

### END-OF-LIFE

### POWER DYNAMICS IN A TRIAD RELATIONSHIP: ADVANCE CARE PLANNING FOR OLDER ADULTS WITH DEMENTIA IN CHINA

Yifan Lou,[1] Jinyu Liu,[1] and Bei Wu,[2] *1. Columbia University, New York, New York, United States, 2. New York University, New York, New York, United States*

Objective: Primary family caregiver (CG), other family members (FM), and medical professionals (MP) play important roles in medical decision-making for older adults with dementia, who often have lost the capacity to make decisions on their own. Power dynamics within the CG-FM-MP triad relationship determine the process and outcome of the decision-making. Guided by Rahl's relational power model, this study is among the first to understand the experiences of advance care planning among Chinese. Method: This study includes a total of 25 primary CGs or FMs and 5 MPs from 3 neurology departments. Hybrid grounded theory method was used to analyze the preliminary data we had so far. Based on the dimensions of power, we analyzed the power base, means, and scope of each agent in each interview to determine the power comparability. Results: Three types of triadic power relations were categorized: 1) shared-power with shared-decision, in which three agents shared the power of decision-making and CG as the lawful decision-maker makes the final decisions; 2) balanced-power with reversed-patriarchal decisions, in which FM's power is over both CG and MP and become the actual decision-maker; and 3) unbalanced power with conflicting decisions, in which neither CG and FM has absolute power over each other and MP becomes the actual decision-maker implicitly. Conclusion: The study provides a framework for researchers and practitioners to understand the ACP process for Chinese older adults, which helps develop intervention strategies to improve surrogates' ACP knowledge and reduce potential conflicts during the stressful process for the population.

### RACIAL DIFFERENCES IN THE IMPACT OF SUBJECTIVE LIFE EXPECTANCY ON ADVANCE CARE PLANNING

Yifan Lou,[1] and Deborah Carr,[2] *1. Columbia University, New York, New York, United States, 2. Boston University, Boston, Massachusetts, United States*

The need for advance care planning (ACP) is heightened during the COVID-19 pandemic, especially for older Blacks and Latinx persons who are at a disproportionate risk of death from both infectious and chronic disease. A potentially important yet underexplored explanation for well-documented racial disparities in ACP is subjective life expectancy (SLE), which may impel or impede ACP. Using Health and Retirement Study data (n=7484), we examined the extent to which perceived chances of living another 10 years (100, 51-99, 50, 1-49, or 0 percent) predict three aspects of ACP (living will (LW), durable power of attorney for health care designations (DPAHC), and discussions). We use logistic regression models to predict the odds of each ACP behavior, adjusted for sociodemographic, health, and depressive symptoms. We found modest evidence that SLE predicts ACP behaviors. Persons who are 100% certain they will be alive in ten years are less likely (OR = .68 and .71, respectively) whereas those with pessimistic survival prospects are more likely (OR = 1.23 and 1.15, respectively) to have a LW and a DPAHC, relative to those with modest perceived survival. However, upon closer inspection, these patterns hold only for those whose LW specify aggressive measures versus no LW. We found no race differences for formal aspects of planning (LW, DPAHC) although we did detect differences for informal discussions. Blacks with pessimistic survival expectations are more likely to have discussions, whereas Latinos are less likely relative to whites. We discuss implications for policies and practices to increase ACP rates.

### REASONS FOR REFUSING HOME-BASED PALLIATIVE CARE: A QUALITATIVE STUDY OF PATIENTS AND CAREGIVERS

Valeria Cardenas, YuJun Zhu, Anna Rahman, and Susan Enguidanos, *University of Southern California, Los Angeles, California, United States*

Despite some insurance plans now paying for home-based palliative care, recent reports have suggested that coverage for palliative care may be insufficient to expanding patient access to home-based palliative care. Research has yet to explore palliative care barriers from the perspective of palliative care-eligible patients and their caregivers. To identify patients and caregivers' perceived barriers to home-based palliative care and their recommendations for overcoming these barriers, we conducted a qualitative study using semi-structured individual interviews. Participants (patients, proxies, and their caregivers) who were eligible for a randomized controlled trial of home-based palliative care were interviewed via telephone. Our interview protocol elicited participants' perspectives on home-based palliative care services; positive and negative aspects of the program explanation; and suggestions for improving messaging around home-based palliative care. Researchers used grounded theory to identify the themes within the transcripts. Two researchers independently coded the transcripts and then met to compare coding and reconciled discrepancies until 100% consensus was reached. Identified themes related to home-based palliative care referral barriers included reluctance to have home visits, timing, lack of palliative care knowledge, misconceptions of palliative care, and patients' self-perceived health condition (not sick enough for palliative care). Themes related to recommendations for overcoming these barriers included preferring a palliative care referral from healthcare providers or from insurance company and clearer presentation of palliative care service. Findings reinforce the need for additional palliative care education among patients with serious illness and the importance of delivering the information from a trusted source.

### SPATIAL ANALYSIS OF HEALTHCARE OFFER AND REQUEST FOR OLDER PEOPLE AGED 65 YEARS AND OVER IN QUEBEC

Juliette Duc,[1] Sébastien Barbat-Artigas,[2] and Delphine Bosson-Rieutort,[1] *1. School of Public Health, University of Montréal, Amiens, France, 2. INESSS, Montréal, Canada*

With years, the health-needs of an individual become numerous and more complex, resulting in the requirement of an even more appropriate offer of health services. However, it is known that different factors make the services and the request of the population to health care unequally, especially the interregional variations. These are represented by a gap between the offer of healthcare and the need of the populations. The aim of this study was 1) to map the relationship between the location of the healthcare services in Quebec and population aged 65 years and over, and 2) to identify the characteristics related to the geographic variations in access to healthcare. We used data from "statcan.gc.ca", "donneesquebec.ca" and "msss.gouv.qc.ca" regarding the facilities, their capacity, their services, and the populations' characteristics. Analyses were performed on QGIS and R software. As expected, our results showed that there is a gap between the healthcare needs and the services: older people need a large amount of diverse services which are not always provided by secluded areas. Moreover, it also appeared that the deprivation index is related to the offer of health care. As this project takes part in a global project studying the health care trajectories of older people in Quebec using their administrative health databases, those findings will help better understand the impact of the geographic factors for the interregional variations of healthcare.

### SESSION 10240 (LATE BREAKING POSTER)

### EPIDEMIOLOGY

### IMPACT OF AGE ON COMMUNITY DIABETES PREVENTION PROGRAM ATTENDANCE AND WEIGHT LOSS GOALS

Jenna Napoleone, Elsa Strotmeyer, Rachel Miller, Susan Devaraj, Bonny Rockette-Wagner, Vincent Arena, Elizabeth Venditti, and Andrea Kriska, *University of Pittsburgh, Pittsburgh, Pennsylvania, United States*

The Diabetes Prevention Program (DPP) lifestyle intervention demonstrated that meeting the weight loss (WL) and activity goals prevents/delays type 2 diabetes. Older DPP participants, 60-85 years, reduced the risk of developing diabetes by 71% versus 58% in those <60 years. Currently, community translated DPP-based lifestyle interventions including Group Lifestyle Balance (DPP-GLB), are reimbursed by Medicare for overweight/obese older adults with prediabetes. This effort examined the impact of age group (60-65: reference, 66-70, ≥71 years) on both DPP-GLB maintenance session attendance (months 7-12) and achieving the 5% WL goals at 6- and 12-months. Data were combined from two identical 12-month DPP-GLB intervention trials involving overweight/obese adults with prediabetes and/or metabolic syndrome. Participants ≥60 years attending ≥4 sessions (months 0-6), with complete data on session attendance and WL were included (n=145; age=68.7 + 5.8 years, range 60-88; 79% women). Participants aged 66-70 years (N=46) were more likely to meet the 6-month 5% WL goal (67.4%) vs. 60-65 years (N=51; 45.1%; p=0.03). Participants aged 66-70 (69.6%) and ≥71 years (N=48; 60.4%) were more likely to meet the 12-month WL goal vs. 60-65 years (35.3%; 66-70: p=0.0007; ≥71: p=0.01). Maintenance attendance did not vary by age group with approximately 30% of each group attending ≥4 of 6 maintenance sessions (p=0.55). In conclusion, adults 66+ vs. 60-65 years more successfully met the clinically meaningful 5% WL goals at 6 and 12 months. With nationwide implementation of community-based "real-world" DPP-GLB lifestyle interventions, better understanding of program success across older adult age groups will enhance program reach and effectiveness.

### INPATIENT DIAGNOSIS OF DELIRIUM AND ENCEPHALOPATHY: CODING TRENDS IN 2011-2018

Jeffrey Franks, Jami Anderson, Richard Kennedy, and Huifeng Yun, *University of Alabama at Birmingham, Birmingham, Alabama, United States*

Physicians have long debated the diagnosis of acute confusional states as delirium or encephalopathy, often based on specialty. Recently, CMS assigned a lower severity to the nonspecific behavioral diagnosis of delirium than for the pathophysiological diagnosis of encephalopathy, potentially exacerbating these disagreements. Therefore, we sought to evaluate trends in these two diagnoses among hospitalized adults. Using 2011-2018 IBM MarketScan datasets, we identified delirium/encephalopathy patients who were ≥ 18 years and enrolled with medical and pharmacy coverage for each calendar year. Delirium/encephalopathy were defined using validated ICD-9/10 codes among hospitalized patients. We identified the physician specialties associated with the hospitalization and comorbidities using ICD9/10 inpatient/outpatient diagnosis codes within one year prior to the diagnosis of delirium or encephalopathy. Log-binomial models were used to evaluate the trends adjusting for age, gender, insurance and comorbidities. We identified 10,418 delirium and 87,393 encephalopathy hospitalized patients in 2011-2018. Of these patients, the total number of patients with either diagnosis increased, but the proportion of patients with delirium for each year decreased from 20% in 2011 to 9% in 2018. During the 8 years, neurologists and internists increased their use of both diagnoses, whereas psychiatrists only increased for delirium. Patients with encephalopathy are more likely to be older, female, and have more comorbidities. These shifts in diagnosis complicate the study of delirium and encephalopathy, and can lead to erroneous conclusions about trends in the incidence and prevalence of these disorders unless properly understood.

### METABOLOMIC SIGNATURES OF HIGH RED BLOOD CELL DISTRIBUTION WIDTH

Abigail Corkum, Qu Tian, and Luigi Ferrucci, *1. National Institute of Aging, Baltimore, Maryland, United States*

Red blood cell distribution width (RDW) describes the amount of variation in blood cell volume and size and increases with age. Higher RDW predicts all-cause mortality, metabolic syndrome, diabetes, and markers of glycemic control, such as glycosylated hemoglobin. However, mechanisms that connect high RDW with these health outcomes are unknown. Thus, identification of high risk in these patients cannot be addressed. This study aims to identify metabolites and pathways that are associated with high levels of RDW in community-dwelling older adults. Using data from the Baltimore Longitudinal Study of Aging, we identified 1,004 cognitively normal participants (mean age: 67.1±13, 48% women, 26% black) with concurrent data on RDW and comprehensive targeted plasma metabolites by Biocrates p500. Participants were grouped into RDW quartiles (Q1:14%). Associations of metabolites with quartiles of RDW were examined using multivariable linear regression with Q1 being the reference group. Models were adjusted for age, sex, and race. Compared to Q1, Q4 had higher concentrations of SM(OH)C14:1, PC ae C30:2, and hypoxanthine, and lower concentrations of DHEAS, Cortisol, Tryptophan, and Hex2Cer(d/18:1/24:0) (all $p<0.01$). These metabolites are critical components of sphingolipid metabolism and steroid hormone biosynthesis pathways. Elevated RDW was associated with metabolites derived from classes of hormones, amino acids, ceramides, sphingomyelins, PCs, and nucleobases. Individuals with elevated RDW (i.e. ≥14%) may have disrupted sphingolipid metabolism and steroid hormone biosynthesis. These pathways can be targeted for prevention.

### TRAJECTORIES OF HEALTH CHANGES IN OLDER ADULTS WITH CHRONIC HEPATITIS B INFECTION

I-Chien Wu,[1] Chang-Hua Chen,[2] Yu-Wen Yang,[2] Yen-Tze Liu,[2] Yen-Feng Chiu,[1] Chih-Cheng Hsu,[1] Shu-Chun Chuang,[1] and Chao A. Hsiung[1] *1. National Health Research Institutes, Taiwan, Zhunan, Miaoli County, Taiwan (Republic of China), 2. Changhua Christian Hospital, Changhua City, Taiwan, Changhua, Taiwan (Republic of China)*

Despite the increasing burden of chronic hepatitis B (CHB) in aging populations, little is known about the course of health-related quality of life (HRQoL) changes during older adulthood in CHB patients. We aimed to assess individual-level longitudinal HRQoL changes in older patients with CHB and to examine their correlates. A 5-year prospective cohort study was conducted in 503 hepatitis B surface antigen-positive community-dwelling adults aged 55 years or older. Participants underwent comprehensive assessments at baseline and serial measurement of HRQoL using the short-form (12) health survey version 2. Of these participants, 82.7% remained in good physical health throughout the study period, whereas 9.1% had declining physical health and 8.2% were in poor physical health. We likewise identified three trajectories of mental health changes ("good mental health" [86.9%], "declining mental health" [6.8%], and "poor mental health" [6.4%]). Three baseline characteristics were independently associated with a lower likelihood of remaining physically or mentally healthy during older adulthood: sarcopenic obesity (odds ratio [OR] with 95% confidence interval [95% CI] of 7.5[2.8-20.5] for poor physical health, 3.1 [1.1-8.4] for declining physical health, 4.3 [1.4-13.0] for poor mental health), higher number of metabolic abnormalities (OR [95% CI] of 3.6 [1.6-8.0] for poor physical health) and depressed mood (OR [95% CI] of 21.7 [5.8-81.0] for poor physical health, 5.3 [1.4-19.9] for declining physical health, 83.1 [19.7-350.2] for poor mental health, 13.6 [2.9-64.8] for declining mental health). In conclusion, we demonstrated the heterogeneity and nonlinearity of HRQoL changes and their associations with variations in specific extrahepatic organs/systems.

### SESSION 10250 (LATE BREAKING POSTER)

### FALLS

### EXPLORATION OF BARRIERS AND FACILITATORS FOR DEPRESCRIBING OPIOIDS AND BENZODIAZEPINES TO REDUCE OLDER ADULT FALLS

Ellen Roberts,[1] Cristine Henage,[1] Lori Armistead,[2] Tamera Hughes,[2] Joshua Niznik,[1] Courtney Schlusser,[3] Jan Busby-Whitehead,[1] and Stefanie Ferreri,[2] *1. University of North Carolina School of Medicine, Chapel Hill, North Carolina, United States, 2. University of North Carolina Eshelman School of Pharmacy, Chapel Hill, North Carolina, United States, 3. University of North Carolina Gillings School of Global Public Health, Chapel Hill, North Carolina, United States*

As part of a randomized control trial for deprescribing opioids and benzodiazepines (BZD) to reduce falls (funded by Centers for Disease Control), we conducted a virtual focus group and surveys to evaluate opioid and BZD prescribing practices among healthcare providers in four primary care clinics in North Carolina. Survey and focus group questions measured providers' confidence in their abilities to weigh benefits and harms of opioids and/or BZDs in older adults; determine alternative interventions; create a safe dosing plan; and incorporate patient preferences. A validated pre-intervention survey, adapted from a survey by the Canadian Deprescribing Network, was administered to providers in control and intervention clinics (n=29). Providers expressed high confidence in their abilities to weigh risks and benefits of deprescribing opioids and BZDs, but low confidence in deprescribing under impeding circumstances (e.g. when not the original prescriber or when there is no evidence to inform them). Results were similar across opioids and BZDs. A focus group was conducted among seven providers from the two intervention clinics. Barriers to deprescribing identified included patient resistance, lack of knowledge of deprescribing best practices, and lack of time to discuss deprescribing during regular clinic visits. Providers also expressed concerns about deprescribing medications initiated by or managed by other prescribers. Key facilitators of deprescribing included patient trust in physician, patients being agreeable to reduce medications, and use of gradual tapering rather than abrupt discontinuation. Barriers and facilitators were subsequently used to optimize training and provider resources for the deprescribing intervention, which is currently being implemented.

### FEAR OF FALLS & QUALITY OF LIFE IN COMMUNITY-DWELLING OLDER ADULTS: IS DIABETES AN EFFECT MODIFIER?

Christina Whitehouse, Janell Mensinger, and Michelle McKay, *Villanova University, Villanova, Pennsylvania, United States*

Prior research suggests that diabetes is a risk factor for falling. Moreover, older adults with diabetes are more likely to experience hip fractures when compared to older adults without diabetes. Research has also shown a relationship between fear of falling and quality of life. This study aimed to examine the moderating role of diabetes in the relationship between fear of falling (FoF) and quality of life (QoL) among older adults in a program for all-inclusive care for the elderly (PACE). This was a retrospective single cohort study that included 84 older adults in a PACE program located in the Northeastern United States. Participants were 55 years of age or older (M=70.33; SD=6.46) and cognitively intact. Diabetes diagnosis (n=46) was determined according to chart review. Fear of Falling was assessed with the Falls Efficacy Scale-International, and the Short Form 12v2 (SF-12v2) was used to measure the quality of life, including physical and mental domains. Hayes' Process Macro was used to conduct moderation analyses (model 1) controlling race, gender, age, and comorbidities. Alpha was set at .10 to account for low power to detect interaction effects with small groups. Our results indicate the interaction between diabetes status and FoF was significant for mental QoL (p=.09) and not significant for physical QoL (p=.99). The association between FoF with lower mental QoL was stronger for patients with diabetes than for patients without diabetes; this finding was not replicated for physical QoL. Regardless of diabetes status, physical QoL significantly decreased as FoF increased.

### IMPLEMENTATION OF AN OPIOID AND BENZODIAZEPINE DEPRESCRIBING PROGRAM FOR OLDER ADULTS IN AN OUTPATIENT SETTING

Lori Armistead,[1] Jan Busby-Whitehead,[2] Stefanie Ferreri,[1] Cristine Henage,[2] Tamera Hughes,[1] Jack McBride,[2] Ellen Roberts,[2] and Ben Urick,[1] *1. University of North Carolina Eshelman School of Pharmacy, Chapel Hill, North Carolina, United States, 2. University of North Carolina School of Medicine, Chapel Hill, North Carolina, United States*

The United States spends $50 billion each year on 2.8 million injuries and 800,000 hospitalizations older adults (age 65 years and older) incur as the result of falls. Chronic use of central nervous system (CNS)-active medications, such as opioid and/or benzodiazepine (BZD) medications, increases the risk of falls and falls-related injuries in this older adult population. This Centers for Disease Control and Prevention (CDC)-funded randomized control trial uses electronic health record (EHR) data from primary care outpatient clinics to identify older adult patients at risk for falls due to chronic opioid or BZD use. The primary program aim is to test the efficacy of a targeted consultant pharmacist service to reduce the dose burden of these medications in the targeted population. Impact of this intervention on the risk of falls in this population will also be assessed. Licensed clinical pharmacists will review at-risk patients' medical records weekly and make recommendations through the EHR to primary care providers for opioid or BZD dose adjustments, alternate medications, and/or adjunctive therapies to support deprescribing for approximately 1265 patients in the first two cohorts of intervention clinics. One thousand three hundred eighty four patients in the control clinics will receive usual care. Outcome measures will include reduction or discontinuation of opioids and BZDs and falls risk reduction as measured by the Stop Elderly Accidents, Death and Injuries (STEADI) Questionnaire. Primary care provider adoption of pharmacists' recommendations and satisfaction with the consult service will also be reported.

### INVESTIGATING FALLS AND REHABILITATION USE IN OLDER WHEELCHAIR USERS FROM THE NATIONAL HEALTH AND AGING TRENDS STUDY

Qiong Nie, Laura Rice, Jacob Sosnoff, and Wendy Rogers, *University of Illinois at Urbana-Champaign, Champaign, Illinois, United States*

Falling is a serious health concern among older wheelchair users as falling may result in functional impairment, morbidity, and mortality. Researchers have examined prevention strategies of falls. However, little is known about the prevalence of falls and rehabilitation service use among wheelchair users over age 65. We analyzed a population-based data set – the National Health and Aging Trends Study– to describe trends over time of wheelchair use among older adults who completed a sample interview in this study; we further identified 417 community-dwelling older wheelchair users in wave 5, described prevalence of falls and rehabilitation service use among them and investigated indicators of fall-related rehabilitation service utilization using multinomial logistic regression. The results indicated that there was an increasing trend of wheelchair use from 2011 (7.2%) to 2019 (10.6%). Falling was a common problem (56.1%); however, only 12.1% received rehabilitation service addressing the problem of falls. Around half of rehabilitation service users reported that their functioning improved, but the effect was not lasting. Age (74-79 years old: odds ratio [OR] =2.72, 95% confidence interval [CI], 1.01-7.33; 80-84 years old: OR= 2.93, 95% CI, 1.19-7.25) and balance or coordination problem (OR= 4.70, 95% CI, 1.84-12.06) were associated with receiving fall-related rehabilitation. These data suggest that we need to understand more about the underuse of the rehabilitation services, ensure that older wheelchair users are equally utilizing the services to prevent falling and improve the long-lasting effect of the services.

### SESSION 10260 (LATE BREAKING POSTER)

### FAMILY CAREGIVING

### ANTECEDENTS TO CAREGIVING DIFFERENCES IN BLACK AND WHITE FAMILIES

Kristie Wood,[1] Yee To Ng,[1] Meng Huo,[2] and Karen Fingerman,[1] *1. University of Texas, Austin, Austin, Texas, United States, 2. University of California, Davis, Davis, California, United States*

Researchers have observed racial differences in midlife adults' caregiving for aging parents. Black adults typically provide more parental caregiving and report greater rewards in doing so. We asked whether Black and White young adults differ in their support to midlife parents, and furthermore, whether this support differs based on parental gender. We also examined cultural beliefs and rewards of providing support underlying racial differences in support to parents. Black and White young adults from the Family Exchanges Study II (2013; aged 18–30 years; n=114 Black and, n=358 White) reported support provided to parents, and beliefs and rewards associated with support. We assessed 6 types of support (emotional, advice, listening to talk, socializing, practical, and financial) to each parent rated 1 = once a year or less often to 8 = everyday. Multilinear models revealed that Black young adults gave significantly more support to parents than White offspring, and these racial differences were mediated by filial obligation beliefs. Compared to White young adults, Black offspring provided more frequent support to their mothers and reported that it was more rewarding, and endorsed more negative relationship quality with mothers than with fathers. Research has shown that involvement correlates with conflict, which may underlie these findings. Further, compared to White offspring, Black young adults show significantly more behaviors that lead to caregiving in later life, and there are nuanced gender differences within Black parent-child relationships, which may need to be better understood to support Black caregivers.

### DEMENTIA CAREGIVERS PERSPECTIVES OF CAREGIVING, BURDEN, EMERGENCIES, AND EMERGENCY PREPAREDNESS

Nicole Crawford,[1] Bruce Levin,[1] Gum Amber,[1] Dinorah Martinez-Tyson,[1] Makeba Huntington-Symons,[2] and Anthony Masys[1] *1. University of South Florida, Tampa, Florida, United States, 2. Alzheimer's Association, Clearwater, Florida, United States*

Limited research has been conducted regarding the perspectives of caregivers of persons with dementia (PWD) regarding preparing for emergencies. The purpose of this study was to understand dementia caregivers' perceptions of burden, emergencies, and emergency preparedness through the caregiving lens. Fourteen semi-structured interviews were conducted with a purposive sample of dementia caregivers who reside in Hillsborough County, Florida, to gain participants' perspectives. Data were analyzed using the thematic analysis method. Caregivers discussed emergencies in terms of medical issues, mostly related to the PWD's health, as well as natural disasters such as hurricanes. Caregivers discussed their perspectives of caregiving and burden as centered around their roles and experiences with the PWD, including ways in which they would deal with emergencies such as natural disasters. Caregivers reported that they were physically prepared for emergencies (i.e., hurricanes) but not mentally prepared for coping with or helping their loved ones cope with the stress of an emergency related event. The interviews presented opportunities to raise awareness of emergency preparedness resources and provide information specific to each caregivers' situation. The results suggest the caregiver role may be essential to mitigating the adverse effects of emergencies on PWDs. Recommendations for practice include providing person-centered care, individualized emergency planning, and interprofessional collaboration.

### DIFFERENCE IN PARENT-CHILD CARE EXPERIENCE BY DEMOGRAPHIC CHARACTERISTICS: FOCUSING ON STRESS, NEEDS, AND MEANING

Ayako Baba, *Kanazawa University, Tokyo, Japan*

OBJECTIVE: In super aging society, unpaid caregivers play important roles. Therefore, they should be supported to realize the lives they want. However, it is unclear what caregivers' particular needs are under different conditions. This study aimed to explore the difference in forms of stress and needs of caregivers by demographic characteristics. METHOD: Adult children who were caring for or had cared for their parents at home under the Japanese long-term care insurance system (n=653) completed the three open-ended questions concerning caregiving stress, unmet needs, and meaning, combined with a demographic survey. Data were analyzed using text mining approaches such as correspondence analysis and co-occurrence network analysis, which illustrated differences in description according to demographic variables. RESULT: 1) Caregivers of fathers felt stressed when rebuked by care-recipients, whereas daughter caregivers of mothers felt stressed in coping with care-recipient dementia and complaint, and balancing caregiving with parenting. 2) Caregivers in economic difficulty needed instrumental and financial support, whereas others needed flexible support and accessible consultation services. 3) Caregivers living with care-recipients found meaning in experience and repaying parents for raising them, whereas caregivers living away found meaning in improved parent-child relationships and images of care-recipients. Caregivers of persons with dementia found meanings in sharing time of tranquility with care-recipients. CONCLUSION: The study revealed differences in caregiving experience by care dyads, economic situation, living arrangement, and dementia etc. These results suggest that family caregivers should be provided with services to help find meanings in work and reduce stress levels, according to caregiving conditions.

### SPOUSES' EMOTION REGULATION SKILLS AND PERCEIVED PARTNER EMOTIONAL SUPPORT IN THE CONTEXT OF EARLY STAGE DEMENTIA

Amanda Piechota,[1] and Joan Monin,[2] *1. Yale School of Public Health, New Haven, Connecticut, United States, 2. Yale University, New Haven, Connecticut, United States*

Within older adult marriages, spouses often help regulate one another's emotions, especially in times of stress (Monin, 2016). Although research shows that better emotion regulation skills are associated with better social skills (Lopes, 2005), less is known about whether better emotion regulation skills in one spouse are associated with greater perceived relationship quality from their partner's point of view. Further, very little is known about these dynamics in the context of early stage dementia in which both spouses may be feeling more stress over time and need support from one another. In the present study, we hypothesized that (a) one spouse's greater emotion regulation skills would be associated with their own greater perceived emotional support from their partner and (b) their partner would also report greater perceived emotional support. We made the same hypothesis for both the person with dementia and their spouse without dementia. We used self-report baseline data from an intervention study of 45 older adult married couples (N=90) where one spouse has dementia. Spouses completed questionnaires that measured their emotional regulation habits (DERS; Gratz & Roemer, 2004) and perceived emotional support from their partner (Feeney & Collins, 2000). Results from the actor partner interdependence model showed that when both partners had high emotional regulation skills there were the highest levels of perceived partner support for each dyad member (B=-.02, SE=.01, t(38.83)=-2.37, p=.023). Findings suggest couple-focused interventions to enhance emotion regulation skills are important for maintaining relationship quality in the early stages of dementia.

### THE ENDURING STRENGTH OF BIOLOGY AND GENDER: CARE FOR AGING PARENTS AMONG ADULT CHILDREN

Sarah Patterson,[1] Robert Schoeni,[1] Vicki Freedman,[1] and Judith Seltzer,[2] *1. University of Michigan, Ann Arbor, Michigan, United States, 2. University of California Los Angeles, Los Angeles, California, United States*

Family complexity in the form of step-relationships are increasing across cohorts. Filial obligation, or the social norm that adult children should care for aging parents, are generally weaker in stepfamilies. Further, gender continues to be a main axis of stratification of who provides care within families. Taken together, we test whether biological versus step ties, the gender of the adult child, and the interaction between these two factors are associated with helping aging parents (ages 65 and older) with functional or health limitation based care needs. We use Round 5 (2015) of the National Health and Aging Trends Study. Results illustrate the enduring strength of both biological and gendered ties, with biological daughters being the most likely to help an aging parent, followed by biological sons, step-daughters, and lastly step-sons. This pattern holds even when we control for important characteristics of both the adult child and the care receiver. As families become more complex, these findings could mean that gaps in unmet care needs will emerge, especially for older adults who only have stepchildren.

### THE GENERATIONAL IMPACT ON MEANING MAKING AND WELL-BEING OF ADULT CHILDREN CAREGIVERS IN DEMENTIA CAREGIVING

Vivian Lou,[1] Daniel W. L. Lai,[2] Daniel Fu-Keung Wong,[1] Doris Yu,[1] Shuangzhou Chen,[1] and Reynold Leung,[1] *1. The University of Hong Kong, Hong Kong, China, 2. Hong Kong Baptist University, Hong Kong, China*

Children caregivers contributed significantly to care and support dementia parents globally. In the caregiving journey, making sense of providing care plays significant role in their caregiving journey. In an ageing society such as Hong Kong, different generations of children caregivers take up dementia caregiver roles. We hypothesized that from studying baby boomers (BB, born in 1946-1964) and generation X (GX, born in 1965-1980), generations have impacts on their meaning making and well-being outcomes. 601 Caregivers completed a paper or online battery of questionnaires on burden (ZBI-4), mental well-being (PHQ-9), caregiving factors (ADL, IADL, caregiving hours, Positive Aspect of Caregiving; PAC) and the meaning making factors (Finding Meaning Through Caregiving; FMTC). Results showed that significant difference between caregivers from two generations. GX have significantly lower meaning made, measured by PAC affirming self and enriching life, as well as FMTC provisional meaning. While they spent less caregiving hours for the more independent care recipients, they suffered from higher burden, higher FMTC loss/powerless and worse psychological well-being (PHQ). The findings demonstrated generation X caregiver suffered from lower level of the meaning made and worse psychological wellbeing outcomes than BB caregivers. Future caregiver studies should take generational effect into account and services shall be provided in a generation-responsive approach.

### SESSION 10270 (LATE BREAKING POSTER)

### FRAILTY

### COGNITIVE FRAILTY IN RELATION TO ADVERSE HEALTH OUTCOMES INDEPENDENT OF MULTIMORBIDITY IN CHINESE OLDER ADULTS

Zuyun Liu,[1] Chen Chen,[2] Juyoung Park,[3] Chenkai Wu,[4] Qian-Li Xue,[5] George Agogo,[6] Ling Han,[7] and Zunyou Wu,[2], *1. School of Public Health and the Second Affiliated Hospital, Zhejiang University School of Medicine, Hangzhou, China, 2. Chinese Center for Disease Control and Prevention, Beijing, China, 3. Boca Raton, Florida, United States, 4. Duke Kunshan University, Kunshan, Jiangsu, China, 5. Johns Hopkins School of Medicine, Baltimore, Maryland, United States, 6. Centers for Disease Control and Prevention, Nairobi, Kenya, 7. Yale School of Medicine, New Haven, Connecticut, United States*

Cognitive frailty was proposed in 2013 by an (I.A.N.A./I.A.G.G.) international consensus group; however, little is known about its status and associations with adverse health outcomes in China. The objectives of this study were to evaluate: 1) the associations of cognitive frailty with various health outcomes including disability, hospitalization, and death; 2) whether the associations differed by multimorbidity in Chinese older adults. We included 5113 Chinese older adults (aged 60+ years) who had baseline (2011 wave) cognition and physical frailty assessments and follow-up for 4 years from the China Health and Retirement Longitudinal Study. We found that about 16.0% had cognitive impairment; 6.7% had physical frailty; and 1.6% met criteria for cognitive frailty (having both cognitive impairment and physical frailty). Both cognitive impairment (odds ratios (ORs) range: 1.41 to 2.11) and physical frailty (ORs range: 1.51 to 2.43) were independently associated with basic activities of daily living (BADL), instrumental ADL (IADL), mobility disability, hospitalization, and death among participants without that corresponding outcome at baseline, even after accounting for covariates. Relative to participants who had normal cognition and were nonfrail, those with cognitive frailty had the highest risk for IADL disability (OR=3.40, 95% CI, 1.23–9.40) and death (OR=3.89, 95% CI, 2.25–6.47). We did not find significant interaction effects between cognitive frailty and multimorbidity (P for interactions>0.05). Overall, cognitive frailty was associated with disability and death, independent of multimorbidity. This highlights the importance of assessing cognitive frailty in the community to promote primary and secondary preventions for healthy aging.

### DIMENSIONS OF SOCIAL ISOLATION AND WEIGHT LOSS AMONG OLDER MEN AND WOMEN IN S. KOREA

Youngjoon Bae, and Mark Pachucki, *University of Massachusetts Amherst, Amherst, Massachusetts, United States*

Older men who live alone are typically believed to be highly susceptible to malnutrition. However, recent studies report their living alone status is associated with frailty negatively and with Type 2 diabetes positively. Meanwhile, older women who live alone are believed to be less susceptible to malnutrition, but qualitative research point out their high likelihood of malnutrition. There is little literature to explain these paradoxes. To evaluate this gap in understanding of how a metabolic process may be shaped by social context, this study examines whether different aspects of social isolation among older men and women (living alone, social contact, loneliness) are associated with adverse weight loss, as well as with indicators of meal frequency and body weight. For this, a data set comprised of 6,680 older adults from the Korean Longitudinal Study of Aging surveyed every two years from 2006 to 2018 was analyzed using panel regression models. Among older men, living alone was negatively associated with logged body weight even when considering loneliness but not associated with meal frequency and 5kg or more weight loss. Among older women, living alone was not associated with logged body weight but associated with fewer meals and 5kg or more weight loss. The association disappeared when considering loneliness. Unexpectedly, social contact was not significant to prevent adverse weight loss.

### FRAILTY & DEPRESSION: MEDIATORS OF FEAR OF FALLING AND QUALITY OF LIFE IN OLDER ADULTS

Michelle McKay, Christina Whitehouse, and Janell Mensinger, *Villanova University, Villanova, Pennsylvania, United States*

Fear of falling is a known predictor for decreased health-related quality of life (QoL) in older adults, including among high risk frail community-dwelling older adults with multiple comorbidities including depression. The study aimed to examine the sequential explanatory roles of frailty and depressive symptoms in the relationship between fear of falling (FoF) and QoL in a program for all-inclusive care for the elderly (PACE). This was a retrospective single cohort study that included 84 older adults in a PACE program located in the Northeastern United States. Participants were cognitively intact older adults ≥55 years (M=70.33; SD=6.46). FoF was assessed with the Falls Efficacy Scale-International; frailty was measured with the Edmonton Frail Scale; QoL was measured with the Short Form 12v2 which includes both physical and mental domains. Using the Process Macro (model 6) in SPSS, path models were constructed hypothesizing frailty and depressive symptoms as serial mediators of the relationship between FoF and QoL while controlling for race, gender, age and comorbidities. Frailty and depressive symptoms serially mediated the FoF and mental QoL relationship (Indirect Effect = -0.10; 95% CI= -0.19, -0.03). Serial mediation effects of frailty and depressive symptoms were not replicated for the association between FoF and physical QoL (Indirect Effect = 0.00; 95% CI= -0.04, 0.05). Understanding the roles of frailty and depressive symptoms in explaining the association between FoF and mental health-related QoL can delineate targeted areas for intervention development not typically considered when attempting to reduce the influence of FoF on QoL in older adults.

### THE RECIPROCAL CAUSAL RELATIONSHIP BETWEEN FRAILTY AND PHYSICAL ACTIVITY AMONG KOREAN OLDER ADULTS BY AGE GROUPS

Hae Sagong, and Ju Young Yoon, *Seoul National University, Seoul, Republic of Korea*

Among associated factors to frailty, physical activity is a highly recommended intervention that prevents the risk factors of the frailty. However, most of the older adults are lack of sufficient physical activity to obtain health-related benefits. The purpose of this study is to investigate the reciprocal relationship between frailty and physical activity among Korean older adults by age groups of middle-old (70-79) and oldest-old (more than 80) between two years using cross-lagged panel analysis. This study is a secondary data analysis of the Korean Frailty and Aging Cohort Study (KFACS) and a total of 1,092 participants were included. Frailty was measured by the FRAIL scale and physical activity was measured by the International Physical Activity Questionnaire (IPAQ). As for the result, in the middle-old group, frailty and high PA had significant reciprocal causal relationships while moderate PA with frailty had no significant relationship reference to low PA. In short, frailty was associated with less high PA, and high PA predicts less frailty after two years. In the oldest-old group, surprisingly, there was no reciprocal causal relationship between frailty and any level of PA reference to low PA which means PA has no effects on frailty and vice versa. This can be explained by the ceiling effect or overestimation of the physical activity. Therefore, further studies on the relationship between frailty and physical activity of the oldest-old population are needed. Also, specific physical activity guidelines and effective measurement of physical activity for older adults by age segment should be developed.

### SESSION 10280 (LATE BREAKING POSTER)

### HEALTH AND SOCIAL SERVICES

### A SCOPING REVIEW OF SOCIAL HOUSING MODELS FOR OLDER ADULTS

Christine Sheppard,[1] Matthew Yau,[2] Carol Kwon,[3] Jorge Rios,[1] Andrea Austen,[4] and Sander Hitzig,[5]
*1. Sunnybrook Research Institute, Toronto, Ontario, Canada, 2. University of Toronto, University of Toronto, Ontario, Canada, 3. University of Toronto, Toronto, Ontario, Canada, 4. City of Toronto, Toronto, Ontario, Canada, 5. Sunnybrook Hospital, North York, Ontario, Canada*

Access to affordable housing is a rising concern for many older adults, and government-sponsored social housing programs are one approach to support low-income older adult renters; however, these housing models are limited in availability and may not all be well-suited to support aging in place. To better understand how to promote the physical, mental and social wellbeing of older tenants in social housing, this scoping review mapped relevant literature to examine: 1) the characteristics of older adults in social housing; and 2) social housing service models and policies. Seven peer reviewed databases were searched for relevant articles, which were screened by two independent reviewers. A total of 140 articles met the inclusion criteria. Studies were predominately from the US and Canada; spanning over five decades of research, with publications surging in the 1980's and in the 2010's. Almost all studies reported on the sociodemographic and health characteristics of older tenants, and two thirds presented findings on social housing service models, including policies, staff positions and training, and access to on-site support services. This review points to a high level of vulnerability among older adult tenants living in social housing and highlights the importance of co-locating support services in social housing buildings, with dedicated tenant-support staff to identify vulnerable tenants and link them to these services. There is an acute need for more research on key issues related to housing retention, such as eviction prevention, in order to identify opportunities for social housing landlords to help older tenants age in place.

### AN INTERNATIONAL ENVIRONMENTAL SCAN OF SOCIAL HOUSING FOR OLDER ADULTS

Sander Hitzig,[1] Christine Sheppard,[2] Ariana Holt,[3] Andrea Austen,[4] and Miller Glenn,[5] *1. Sunnybrook Hospital, North York, Ontario, Canada, 2. Sunnybrook Research Institute, Toronto, Ontario, Canada, 3. Canadian Urban Institute, Toronto, Ontario, Canada, 4. City of Toronto, Toronto, Ontario, Canada, 5. Canadian Urban Institute, Toronto, Canada*

The City of Toronto is creating a standalone housing corporation to focus on the specific needs of low-income older adults living in social housing. A key focus of this new corporation will be to provide housing, health and community support services needed to optimize older adult tenants' ability to maintain their tenancy and age in place with dignity and in comfort. To support this work, we conducted an environmental scan of service delivery models that connect low-income older adults living in social housing with health and support services. Desktop research was undertaken to identify relevant programs. For each model, key details were extracted including housing type, services offered, provider information, rent structure and funding sources. The scan identified 34 examples of social housing programs for older adults run by public, private and non-profit agencies across Canada, the United States and Europe that integrated health and supportive services. Successful models were those that understood the needs of tenants and developed collaborative partnerships with health and social service providers to create flexible place-based programs. A common challenge across jurisdictions was privacy legislation that made it difficult to share health and tenancy data with program partners. The presence of on-site staff that focused on building trust and community among tenants was considered key for identifying tenants requiring additional supports in order to age in place. These insights offer important considerations on how integrated supportive housing service models promote housing stability and support better health and wellbeing among older adults residing in social housing.

### DELIVERY OF COMMUNITY SUPPORT SERVICES FOR OLDER ADULTS IN SOCIAL HOUSING

Matthew Yau,[1] Christine Sheppard,[2] Jocelyn Charles,[3] Andrea Austen,[4] and Sander Hitzig,[5] *1. University of Toronto, University of Toronto, Ontario, Canada, 2. Sunnybrook Research Institute, Toronto, Ontario, Canada, 3. Sunnybrook Health Sciences, Toronto, Canada, 4. City of Toronto, Toronto, Ontario, Canada, 5. Sunnybrook Hospital, North York, Ontario, Canada*

Community support services are an integral component of aging in place. In social housing, older adult tenants struggle to access these services due to the siloed nature of housing and health services. This study aims to describe the relationship between community support services and social housing for older adults and examine ways to optimize delivery. Data on government-funded community support services delivered to 74 seniors' social housing buildings in Toronto, Ontario was analyzed. Neighbourhood profile data for each building was also collected, and correlational analyses were used to examine the link between neighbourhood characteristics and service delivery. Fifty-six community agencies provided 5,976 units of services across 17 service categories, most commonly mental health supports, case management and congregate dining. On average, each building was supported by nine agencies that provided 80 units of service across 10 service categories. Buildings in neighbourhoods with a higher proportion of low-income older adults had more agencies providing on-site services ($r = .275$, $p < .05$), while those in neighbourhoods with more immigrants ($r = -.417$, $p < .01$), non-English speakers ($r = -.325$, $p < .01$), and visible minorities ($r = -.381$, $p < .01$) received fewer services. Findings point to a lack of coordination between service providers, with multiple agencies offering duplicative services within the same building. Vulnerable seniors from equity-seeking groups, including those who do not speak English and recent immigrants, may be excluded from many services, and future service delivery for seniors should strive to address disparities in availability and access.

### RECRUITMENT AND RETENTION STRATEGIES OF CANCER PATIENTS AND THEIR CAREGIVERS IN FAMILY-BASED INTERVENTION STUDIES

Ting Guan,[1] Yousef Qan'ir,[1] Ahrang Jung,[1] Shenmeng Xu,[2] Eno Idiagbonya,[3] and Lixin Song,[3]
*1. University of North Carolina at Chapel Hill, Chapel Hill, North Carolina, United States, 2. University of North Carolina at Chapel Hill, Chapel hill, United States, 3. University of North Carolina at Chapel Hill, Chapel hill, North Carolina, United States*

Family-based psychosocial behavioral interventions (PBIs) that target both the cancer patients and their caregivers may more effectively help them with self-care and improve quality of life; however, family-based PBIs often face unique challenges during study implementation. This systematic review aimed to a) examine the recruitment and retention rates of cancer patients and their caregivers in clinical trials testing family-based PBIs; and b) explore the recruitment and retention strategies. We systematically searched five electronic databases to identify randomized controlled trials that tested family-based psychosocial or behavioral interventions among adult patients with cancer and their adult family caregivers. Our searches yielded 48 studies. The average recruitment rates of patients and caregivers were 56.8% (SD=31.8%; range=8-100%) and 54.5% (SD=32.4%; range=8-100%), respectively. The majority of the studies have focused on white and female patients and caregivers. The average retention rate at end of follow-up times was 69.1%. Only 13 studies reported retention strategies, including providing money/gift cards upon returning of each follow-up survey or study completion, and excluding advanced cancer patients. Reasons for attrition, i.e., dropping out of studies, were classified as: health-related (e.g., death, illness, psychological distress), intervention-related (e.g., intervention does not meet expectation, frustration with group allocation, intervention burden) and other reasons (e.g. lack of time, unable to establish contact). Recruitment and retention of patients and caregivers in family-based PBI are integral to the success of interventions. Researchers need to incorporate effective strategies for optimizing recruitment and retention at the planning stage of their studies.

### USING MEDICARE DATA TO INFORM INTERVENTION AND CARE DELIVERY FOR THE MOST EXPENSIVE PATIENTS

Sam Amodeo,[1] Henrik Kowalkowski,[2] Halley Brantley,[3] Lauren Bangerter,[3] Nicholas Jones,[4] and David Cook,[5]
*1. UnitedHealth Group, Eagan, Minnesota, United States, 2. UnitedHealth Group, Minneapolis, Minnesota, United States, 3. UnitedHealth Group, Minnetonka, Minnesota, United States, 4. Brown University School of Public Health, Providence, Rhode Island, United States, 5. United Health Group, Edina, Minnesota, United States*

Older adults with high medical spend require tailored interventions and care delivery models to meet their complex needs. Segmenting high-spend patients is a promising approach to designing such interventions. In this study we explored patient spend across 4 years (2016-2019) using claims from 799,205 patients continuously enrolled in UnitedHealth Group Medicare Advantage (mean age=73.7; S.E.=0.01). Patients with healthcare spend in the top decile were segmented into three subgroups: catastrophic, persistent, and semi-persistent. Catastrophic patients had more acute events (acute myocardial infarction and hip/pelvic fracture) driving their cost. Persistent patients were younger (mean age=67.8; S.E.=0.06) and had significantly more medications. Semi-persistent patients were older (mean age=76.6; S.E.=0.04) and had significantly more chronic conditions and frailty, indicating their cost was driven by medical complexity. These subgroups displayed different temporal stability in their healthcare costs over time. Each year, 79-81% of the catastrophic group dropped out of the top decile. In contrast, nearly 72% of the persistent group remained in the top decile whereas only 37% of the semi-persistent group remained year over year. As the global population continues to age, it will be necessary to design interventions and care delivery models that address the complex needs of older adults in the high-spend patient population. Our study suggests that segmenting high-spend patients into potentially actionable subgroups is an important first step in achieving these goals.

### SESSION 10290 (LATE BREAKING POSTER)

### HEALTH CARE

### 1-YEAR REHOSPITALIZATION AND MORTALITY RATES IN GERIATRIC PATIENTS AFTER ACUTE HOSPITALIZATION

Sun Paek, Elena Volpi, and Rachel Deer, *The University of Texas Medical Branch at Galveston, Galveston, Texas, United States*

Hospitalization for an acute illness often leads to accelerated sarcopenia, debility, and loss of independence in geriatric patients. These post-hospitalization effects can further deteriorate health and lead to rehospitalizations and sometimes death. To improve health outcomes in these patients, it is important to determine predictive factors for increased readmission and mortality rates. Data regarding readmission and mortality within 1 year post-discharge were collected from the PACE and GRAMS studies (NCT02203656, NCT02990533) conducted at UTMB Galveston (Jan 2014 - Mar 2019). Readmission and mortality rates were analyzed to find associations with total days hospitalized and total number of readmissions within 1 year, demographics (age, highest education level, BMI, percent fat, baseline SPPB score), and interventions (exercise with protein supplementation, exercise with placebo, protein supplementation, placebo supplementation, and testosterone injection). Of the 175 subjects, 63.4% were rehospitalized and 9.7% died within 1 year. We found that patients who were rehospitalized within a year of their initial hospitalization were over 4 times more likely to die that year than patients who were not rehospitalized. Patients who died within one year had twice the amount of hospitalization within the year and spent three times the number of days hospitalized. Of the interventions studied, a single testosterone injection at discharge from the index admission appeared the most effective to reduce rehospitalizations.

### A GERIATRICS TELEHEALTH CURRICULUM: A WORKSHOP FOR MEDICAL STUDENTS ON "TELE-SKILLS" USING STANDARDIZED PATIENTS

Lindsay Wilson,[1] Ross Powell,[1] Megan Foster,[1] Kathleen Vogel,[1] Casey Kelley,[1] Cristine Henage,[2] Ellen Roberts,[2] and Jan Busby-Whitehead[2] *1. University of North Carolina, Chapel Hill, North Carolina, United States, 2. University of North Carolina School of Medicine, Chapel Hill, North Carolina, United States*

Background: Telemedicine allows for interprofessional care of geriatric patients and allows older adults to access healthcare from their homes. The coronavirus pandemic has prompted a rapid shift to telemedicine. In 2016-2017, only 58% of medical schools in the US offered telemedicine curricula. Thus, a large gap in medical education has emerged. There are specific skills needed to ensure students' "webside" manner is comparable to their bedside manner. This curriculum was created to train medical students in geriatric-sensitive telemedicine using standardized patients (SP). Methods: A didactic detailing geriatric interviewing preceded the SP encounter. Students were assigned roles for the SP encounter as follows: A) Set agenda, elicit questions, triage problems, perform a history, ensure appropriate lighting and audio B) Perform a geriatric review of systems and reconcile medications C) Present an assessment and plan to the preceptor D) Relay the plan to the SP E) Provide feedback. Students were given pre- and post-surveys to assess their comfort using telemedicine and caring for SP's >65 years old. Results: Seventeen participants were surveyed (pre-survey=17, post-survey=10). Fifty-nine percent of participants reported no prior experience with telemedicine. Participants reported statistically significant increases in comfort using telemedicine ($p=0.022$), using telemedicine for patients >65 years old ($p<0.001$), interviewing patients >65 years old over telemedicine ($p=0.007$), managing patients over telemedicine ($p=0.040$), and managing patients >65 years old over telemedicine ($p=0.001$) after completing the curriculum. Discussion: This virtual curriculum improved medical student comfort with geriatric care and telemedicine and highlights the need for telemedicine curricula in medical schools.

### DYADIC AGREEMENT IN THE PERCEPTIONS OF PATIENT DISABILITY BETWEEN THE STROKE PATIENT AND REHABILITATION THERAPIST

Naoki Takashi Michael McCarthy,[1] Rie Suzuki,[2] Kakuya Ogahara,[3] Masako Kihara,[4] Masahiro Kihara,[4] and Takeo Nakayama,[4] *1. Northern Arizona University, Flagstaff, Arizona, United States, 2. University of Michigan-Flint, Flint, Michigan, United States, 3. Kanagawa University of Human Services, Yokosuka city, Kanagawa, Japan, 4. Kyoto University, Kyoto City, Kyoto, Japan*

Research supports that an agreement about the consequences of the illness within related parties is critical for optimal patient outcomes. This study aimed to explore the association between patient QOL and the degree of agreement in the perceptions of patient disability within the stroke patient-rehabilitation therapist dyad. A cross-sectional study was conducted in Japan from March 2019 to February 2020. A total of 81 dyads consisting of a male stroke patient living at home and the therapist in charge of the eligible patient participated. Patient QOL was measured using the WHOQOL BREF. Perceptions of patient disability were measured using the 12-item WHO Disability Assessment Schedule 2.0 (DAS). DAS scores of patients and therapists were classified into two (high, low) and three (high, medium, low) categories, respectively, and six patterns of agreement on patient ability were created to use in the analysis. Generalized estimating equations were used to examine multivariable associations between the degree of agreement within dyad and WHOQOL scores in patients. Results suggested that when the patient appraised himself as having a low disability, the degree of patient-therapist disagreement was negatively associated with patient QOL. When the patient appraised himself as having a high disability, his QOL was lower, regardless of the degree of agreement. Disagreements in the perception of disability between patients and therapists can worsen patient QOL, especially when the patient perceives himself as having a low disability.

### IMPROVEMENT IN ADLS DURING A NURSING HOME STAY FOR OLDER ADULTS WITH STROKE, JOINT REPLACEMENT OR HIP FRACTURE

Warona Mathuba, Brian Downer, and Rachel Deer, *The University of Texas Medical Branch at Galveston, Galveston, Texas, United States*

Regularly assessing the health and function of older adults who are in the hospital is important for preventing poor outcomes. Such information may also be useful in post-acute care settings, such as skilled nursing facilities (SNFs) to identify older adults who are high risk for poor outcomes. This study had two objectives: Map items from the Acute Care for the Elderly (ACE) Tracker to items from the Minimum Data Set (MDS). (2) Examine the association between ACE Tracker items and improvement in activities of daily living (ADLs) during a SNF stay. We identified Medicare fee-for-service beneficiaries admitted to a SNF within 3 days of hospital discharge for a hip fracture (n=118,790), joint replacement (n=245,845), or stroke (n=64,153). Items from the ACE Tracker were matched to patients' first MDS assessment. The first and last MDS assessments were used to calculate a total score for self-performance on seven ADLs. Multivariable logistic regression models were used to identify patient characteristics associated with the odds for improvement in ADL function. Severe ADL limitations at admission and greater hours of physical and occupational therapy were associated with significantly higher odds of ADL improvement. Cognitive impairment, vision limitations, indwelling catheters, and unhealed pressure ulcers were associated with significantly lower odds of ADL improvement. The characteristics associated with improved ADL function were similar between patients with joint replacement, hip fracture, and stroke. Many of the health and functional characteristics routinely measured in hospital settings are also collected in SNFs and are associated with improvement in ADL function.

### UNPLANNED RE-HOSPITALIZATION AMONG OLDER PERSONS RESULTS IN LOSS OF THEIR INDEPENDENCE

Brandon Qualls,[1] Hiwot Seyoum,[2] Tammy Walker,[2] and Mary Carey,[1] *1. University of Rochester Medical Center,*

*Rochester, New York, United States, 2. Highland Hospital, Rochester, New York, United States*

The Centers for Medicare & Medicaid Services defines a hospital readmission as an inpatient stay that begins within 30 days of the discharge date of an index admission, to the same or a different hospital. The aims of the study were to analyze the recurrent readmissions of older persons admitted to a community hospital with diagnoses of: Chronic Obstructive Pulmonary Disease (COPD), Pneumonia (PNA), and Congestive Heart Failure (CHF). Based on the results, we will develop additional strategies that can be used to reduce the rate for hospital readmission for older patients. A retrospective chart review of hospitalizations was conducted. Among 30 readmissions, the mean age was 79.5±14. The index disposition was distributed among three destinations: self-care (27%), home health organization (40%), and to skilled nursing home (33%). Most of the readmissions were for CHF (27%), COPD (10%), and PNA (13%), the only other large category include respiratory failure (10%). The readmission disposition was different from the index disposition: self-care (7%), home health organization (47%), and to skilled nursing home (20%). After hospital readmission within 30 days, older persons were more likely to be discharged to home health care organization than self-care, p<0.05. Actions that can be taken by hospitals to reduce 30-day readmissions to maintain older person independence include: clinical readiness of patients for discharge, proper infection prevention techniques, reconciling medications, good communication, and adequate patient education. This study reports that that older persons are at higher risk for unplanned hospital re-admissions and often lose their independence.

### SESSION 10300 (LATE BREAKING POSTER)

### HEALTH PROMOTION

### ATTITUDES TOWARD AGING, PHYSICAL ACTIVITY, AND FUNCTIONAL LIMITATIONS TEN YEARS LATER IN MIDDLE AND OLDER ADULTS

Yen Chen, Carole Holahan, and Charles Holahan, *The University of Texas at Austin, Austin, Texas, United States*

Positive attitudes toward aging have been associated with better functional health (Bryant et al., 2012; Levy, Slade, & Kasl, 2002; Sargent-Cox, Anstey, & Luszcz, 2012). The current study examined the mediational role of regular leisure-time physical activity (LTPA) in this association among middle-aged and older adults. Participants were 2,209 adults ranging in age from 40 to 75 at baseline (M = 56.19; 51.2% women) from the second and the third waves of the Survey of Midlife Development in the United States (MIDUS). The mediation model was tested in Mplus 7.4. The model tested the direct association and indirect association through LTPA of attitudes toward aging in MIDUS 2 with functional limitations in MIDUS 3 ten years later. Attitudes toward aging were measured as a latent variable with two indicators (subjective age and future health expectancies). Age, sex, race/ethnicity, education, marital status, and number of chronic conditions were included as covariates. Individuals with more positive attitudes toward aging reported less functional limitations ten years later ($\beta = -.43$, $p < .001$). Further, more positive attitudes toward aging related to higher levels of LTPA ($\beta = .20$, $p < .001$), which in turn predicted less functional limitations ten years later ($\beta = -.10$, $p < .01$). The indirect effect of attitudes toward aging on functional limitations through LTPA was statistically significant (indirect effect = -.02, $p < .01$). Positive attitudes toward aging operating through higher levels of LTPA may play an important role in functional health among middle-aged and older individuals.

### BLUE ZONE REGION OLDER ADULTS MITIGATE ACES AND OTHER CHRONIC STRESS EXPOSURES

Fatimah Alramadhan,[1] Manisa Bhuyan,[2] and Rhonda Spencer,[1] *1. Loma Linda University, Loma Linda, California, United States, 2. Texas State Health Department, Huntington Beach, California, United States*

Adverse Childhood Experiences (ACEs) has been linked to many negative health outcomes, including shortened lifespan. ACEs are prevalent in the US, where it is estimated that one in ten have experienced at least three ACEs. Knowing the adverse ACEs impact, it is important to understand mitigating factors, leading to healthier and longer life. Loma Linda, CA has been identified as a longevity hotspot region (LHR) and may provide insight into reducing impact of chronic stressors. Our purpose to interview LHR older adults regarding their childhood experiences to inform chronic disease prevention framework. We conducted a qualitative study with LHR community members. Childhood exposures and practices assessed using semi-structured key informant interviews (KIs), with questions informed by ACE International Questionnaire and supplemented with lifestyle and resiliency factor (RFs) questions. Data were audio recorded and transcribed. Integrative grounded theory methods guided coding and theming. Participants included 26 community members (14 aged 100 or older and 12 aged between 90 and 99) who reported numerous chronic stressors including ACEs and environmental exposures (i.e. household economic depression). Correlation analysis revealed overlapping of negative experiences. Several domains of protective themes (i.e. guiding presence, spirituality)—were identified in childhood, with potential to dampen chronic systemic inflammation—and many themes maintained across their life. Our findings show that even among older adults who are 90 or older there is a high prevalence of ACEs and stressors; however, many lifestyle protective factors were practiced by them, which may offset damage of stressors and positively influence health and longevity.

### OLDER ADULTS' VACCINE HESITANCY: PSYCHOSOCIAL PREDICTORS OF INFLUENZA, PNEUMOCOCCAL, & SHINGLES VACCINE UPTAKE.

Louise Brown Nicholls, Allyson Gallant, Nicola Cogan, Susan Rasmussen, David Young, and Lynn Williams, *University of Strathclyde, Glasgow, United Kingdom*

Influenza, pneumococcal disease, and shingles are more prevalent in older people, with this group having an increased risk of developing severe illnesses and complications. These illnesses are preventable via vaccination, but uptake of these vaccines is low and decreasing year-on-year. However, little research has focused on understanding the reasons behind vaccine hesitancy in older adults. We implemented a cross-sectional survey to determine the self-reported vaccination behaviours of 372 UK-based adults aged 65-92 years. We assessed previous uptake and future intention to receive the influenza, pneumococcal, and shingles vaccines. Participants also self-reported their health and socio-demographic data, and completed two scales measuring the psychological factors associated with vaccination behaviour (5C and VAX scales). Self-reported daily functioning, cognitive ability, and social support were also assessed. Considerably more participants had received the influenza vaccine in the last 12 months (83.6%), relative to having ever received the pneumococcal (60.2%) and shingles vaccines (58.9%). Multivariate logistic regression analyses showed that a lower sense of collective responsibility independently predicted lack of uptake of all three vaccines in this population. Greater calculation of the disease/vaccination risk and preference for natural immunity also predicted not getting the influenza vaccine. For both the pneumococcal and shingles vaccines, concerns about profiteering predicted lack of uptake. Therefore, more understanding of vaccine benefits and disease risks may be required for these vaccines. Additional qualitative data generally supported these findings, which can contribute to future intervention development and research targeted at more diverse groups (e.g. older adults with cognitive impairments).

### SCREENING OLDER ADULTS FOR HEARING LOSS IN PRIMARY CARE: INSIGHTS OF PATIENTS, PROVIDERS, AND STAFF

Mina Silberberg,[1] Anisha Singh,[1] Janet Bettger,[2] Rowena Dolor,[1] Sherri Smith,[3] and Howard Francis,[1] *1. Duke University, Durham, North Carolina, United States, 2. Duke University School of Medicine, Durham, North Carolina, United States, 3. Duke University Medical Center, Durham, North Carolina, United States*

Over one-third of older adults have a disabling hearing loss, with potentially severe implications for well-being. Hearing screening is not routine in primary care (PC) and patients are relied upon to report hearing concerns. We compared outcomes of three approaches to linking telephone-based screening with PC (providing information at PC visit, encouraging at visit, or completing at visit). This poster presents results of focus groups/interviews with providers and staff from participating clinics (n= 35), study enrollees who completed screening and were referred for diagnosis (n=14 ), and enrollees who did not complete screening (n=12). Results show that most patients had prior hearing concerns they had not reported to their PC. Patients forgot or were resistant to completing screening at home. Negative attitudes towards admitting hearing loss and using hearing aids were common; experiences of family and friends influenced many patient attitudes, both negative and positive. PC personnel wish to help, but are challenged by lack of time, space, and reimbursement for screening, and loathe to screen when specialty care and hearing aids are costly. Study results indicate that relying on patients to report hearing concerns is inadequate. Integration of hearing screening into PC would be helped by strengthening reimbursement for screening, specialty care, and hearing aids, and education of both providers and patients on other available treatments for hearing loss. Patients also require education on hearing aid technology. There is a need to address stigma associated with hearing loss, taking into consideration the influence of family and friends on attitudes.

### SESSION 10310 (LATE BREAKING POSTER)

### LONG TERM CARE

### IS HEALTH INFORMATION EXCHANGE USE BY HOSPITALS AND HOME HEALTH AGENCIES ASSOCIATED WITH LOWER READMISSION RATES?

Christine Jones,[1] Jacob Thomas,[2] Marisa Roczen,[2] Kate Ytell,[2] and Mark Gritz,[2] *1. Rocky Mountain Regional VA Medical Center, Aurora, Colorado, United States, 2. University of Colorado, Aurora, Colorado, United States*

For older adults transitioning from the hospital to home health agencies (HHAs), clinical information exchange is key for optimal transitional care. Hospital and HHA participation in regional health information exchanges (HIEs) could address fragmented communication and improve patient outcomes. We examined differences in characteristics and outcomes for patients with either Medicare or Medicare Advantage (MA) insurance who transitioned from hospitals to HHAs based on HIE participation with 2014-2018 data from the Colorado All Payer Claims Database. We performed analyses including chi square and t tests to compare patient characteristics and 30-day readmission rates for high versus lower HIE use, determined by HIE participation (+) and non-participation (-) among HHAs and hospitals: High HIE use dyads (Hospital+/HHA+) were compared to lower HIE use dyads (Hospital+/HHA-, Hospital-/HHA+, Hospital-/HHA-). We identified 57,998 care transitions from 123 acute care hospitals to 71 HHAs. On average, patients were 75 years old, had a three day hospital length of stay, over half were female (58%), 82% had Medicare and 18% had MA insurance. Although most characteristics were similar between high versus lower HIE use dyads, high HIE use dyads had a higher proportion of Medicare patients compared to the lower HIE use dyads (85% vs 79%, $p < 0.001$). Thirty-day readmissions were 12.4% for care transitions that occurred among high HIE use dyads (n=27,784) compared to 12.8% among lower HIE use dyads (n=32,929, p=0.102). For adults transitioning from hospitals to HHAs among high HIE use dyads, a trend toward lower 30-day readmission rates was identified.

### MINDFULNESS INTERVENTION BENEFITS OLDER ADULTS RECEIVING REHABILITATION SERVICES IN LONG TERM CARE

Katarina Felsted, Katherine Supiano, Trinh Mai, and Anthony Muradas, *University of Utah, Salt Lake City, Utah, United States*

Research literature includes preliminary examination of mindfulness in rehabilitation settings; however, further investigation is warranted. Some of the strongest findings to date are adaptation improvements such as self-efficacy, increased quality of life, and decreased stress. The purpose and aims of this pilot feasibility and acceptability study were to develop, administer, and evaluate a modified mindfulness program for older adults in rehabilitation in long term care, and to measure self-efficacy, quality of life, and perceived stress. Nine residents 65+ were recruited. Inclusion criteria for participants included residents receiving any type of therapy (e.g., physical, occupational, speech), an anticipated length of stay inclusive of the intervention treatment period, and cognitive capacity to participate. A mindfulness intervention was developed by the research team and administered by a CITI trained, qualified mindfulness instructor. As this is a pilot study, no control group was used. This study proved both feasible and acceptable. All eligible participants consented; both attendance and retention percentages were above the 75% standard (78% and 89%, respectively), and the Meaningful Activities Scale rating=82.4, indicating strong acceptability. Statistical results values for the Health-Related Quality of Life (V=153, p< 0.001), Bandura's Self Efficacy Questionnaire (V=153, p< 0.001), and Cohen's Perceived Stress Scale (V=152, p< 0.001) were all statistically significant. These preliminary research findings will inform a larger pragmatic trial testing preliminary effectiveness of the intervention in this population in quality of life, self-efficacy and stress reduction. While this study began prior to the COVID-19 pandemic, its findings are now even more relevant to gerontology.

### SEQUENTIAL DEPENDENCIES IN SOLID AND FLUID INTAKE IN NURSING HOME RESIDENTS WITH DEMENTIA: A MULTISTATE MODEL

WEN LIU,[1] Kristine Williams,[2] and Yong Chen,[1]
*1. University of Iowa, Iowa City, Iowa, United States, 2. University of Kansas, Lawrence, Kansas, United States*

Nursing home (NH) residents with dementia commonly experience low food intake leading to negative consequences. While multilevel factors influence intake, evidence is lacking on how intake is sequentially associated. This study examined the temporal association between previous and current solid and fluid intake in NH residents with dementia. We analyzed 160 mealtime videos involving 27 residents and 36 staff (53 dyads) in 9 NHs. The dependent variable was the current intake state (fluid, solid, no-intake). Independent variables included the prior intake state, technique of current intake state (resident-initiated, staff-facilitated), duration between previous and current intakes. Covariates included resident and staff characteristics. Two-way interactions of duration and technique with the prior intake state, and resident comorbidity and dementia severity were examined using Multinomial Logit Models. Interactions were significant for technique by comorbidity, technique by dementia severity, technique by prior fluid and solid intake, and duration by prior fluid intake. Successful previous intake increased odds of current solid and fluid intake. Staff-facilitation (vs. resident-initiation) reduced odds of solid and fluid intake for residents with moderately severe (vs. severe) dementia. Higher morbidity decreased odds of solid intake (vs. no-intake) for staff-facilitated intake. Resident with severe dementia had smaller odds of solid and fluid intake for resident-initiated intake. Longer duration increased odds of transition from liquid to solid intake. Findings supported strong sequential dependencies in intake, indicating the promise of intervening behaviorally to modify transitions to successful intake during mealtime. Findings inform the development and implementation of innovative mealtime assistance programs to promote intake.

### UNDERSTANDING PATIENT AND CLINICIAN PERSPECTIVES OF ANTIBIOTIC USE FOR THE TREATMENT OF UTIS

Susan Hannum,[1] Ja Alah-Ai Heughan,[1] Ayse Gurses,[2] Morgan Katz,[2] and Jonathan Zenilman,[2] *1. Johns Hopkins Bloomberg School of Public Health, Baltimore, Maryland, United States, 2. Johns Hopkins School of Medicine, Baltimore, Maryland, United States*

Background. Multidrug resistant organisms are highly prevalent in post-acute long-term care [LTC] and skilled nursing facilities [SNF], driven by overdiagnosis of urinary tract infections [UTI] and overuse of antibiotics, despite clinical guidelines for UTI management. Using the Systems Engineering Initiative for Patient Safety [SEIPS] framework to understand sociotechnical work systems within LTC/SNFs, we are conducting mixed methodological research to examine work systems gaps that may require structural modification to ensure appropriate prescribing behaviors. Methods. To begin this research, we conducted semi-structured interviews with residents, caregivers, and clinical staff at three LTC/SNF locations. Resident and caregiver interviews queried knowledge, attitudes, and beliefs about UTIs and antibiotics, previous use, and communication with clinical staff. Clinical staff interviews queried procedures for diagnosing UTIs, prescribing decisions, communication with residents/caregivers, and resident/caregiver demand. Findings. Resident/caregiver interviews highlighted three common themes: (1) doctors have the right to deny antibiotics, but communication about decisions is critical; (2) trust doctors' knowledge and use of objective testing for decision-making; (3) want detailed explanations and education about antibiotics, including potential side effects. Clinical staff described: (1) caregiver as the primary barrier, even with education about antibiotics; (2) using a general protocol for diagnosis, but also prior knowledge and experience with the resident; (3) importance of educating and communicating with residents/caregivers about antibiotic treatment, prescribing recommendations, or side effects. Conclusions. Our study highlights a gap in communication and workflow between residents, caregivers, and clinical staff that may be amendable to improved interventions that decrease inappropriate prescribing of antibiotics for this population.

### SESSION 10320 (LATE BREAKING POSTER)

### MINORITY & DIVERSE POPULATIONS

### CAREGIVER'S RELATIONSHIP TYPE AND RACE/ETHNIC GROUP COMPARISONS IN A COMMUNITY-BASED CAREGIVER SUPPORT PROGRAM

Jinmyoung Cho,[1] Donald Smith,[2] and Alan Stevens,[1]
*1. Baylor Scott & White Health, Temple, Texas, United States, 2. United Way's Area Agency on Aging of Tarrant County, Fort Worth, Texas, United States*

In the past decades, a number of evidence-based interventions has been implemented to improve caregiver outcomes; however, limited attention to specific subgroups (e.g., racial/ethnic groups) still exists in dementia caregiver support interventions in community settings. The purpose of this study is to examine the effect of caregiver's race/ethnicity and relationship type to the care-recipient on the quality of life among caregivers. This study included 354 informal caregivers enrolled in REACH-TX, a community-based caregiver program, provided by the Alzheimer's Association North Central Texas Chapter's from November 2011 to June 2019. Propensity score matching extracted a subset of participants who enrolled in REACH-TX to balance caregivers from three race/ethnic groups (White: 171; African American: 103; Hispanic: 80). Five dimensions of quality of life (depression, caregiving burden, social support, self-care, and problem behaviors) were assessed at baseline and 6-month follow-up among spousal and adult-child caregivers by race/ethnic group. Generalized linear models showed that significant improvement on the five domains of quality of life among White child caregivers, whereas neither spousal nor child caregivers in African American group showed improvement on any domains after adjusting covariates (age, gender, risk levels, number of therapeutic contacts), These findings indicate that the responses to intervention components vary by race/ethnic group and relationship type. Advancing tailored dementia caregiver interventions to specific subgroup needs and unique context, especially for non-White caregivers, is needed to maximize the benefit of community resources and support for diverse caregivers.

### CHILD'S DEVELOPMENTAL DISABILITIES AND PARENTAL WELL-BEING IN MIDLIFE AND OLD AGE: DOES RACE MATTER?

Juha Lee, Manjing Gao, and Chioun Lee, *University of California, Riverside, Riverside, California, United States*

Having a child with developmental disabilities (DD) compromises parents' health and well-being. We have little knowledge on whether the association is robust to the presence of exposure-outcome confounders and how it varies by race. Guided by life-course perspectives, we evaluate (1) the association between having a child with DD and parental well-being and (2) racial disparities in the likelihood of having a child with DD (differential exposure), and/or the effect of having a child with DD on parental well-being (differential vulnerabilities). We advance prior studies by including a wide array of parent's early-life adversities (ELAs, e.g., poverty and abuse), which may link the predictor to the outcome. Using the core, Refresher, and Milwaukee samples from Midlife in the United States (N=9,640, 25% non-Whites), we conducted regression analysis with race as a moderator. Compared to having a healthy child, parents having a child with DD reported lower well-being even after controlling for ELAs. While the likelihood of having a child with DD (around 10%) is similar for both non-Hispanic Whites and African Americans, African American parents are more adversely affected by having a child with DD across most of the eudaimonic well-being indicators (i.e., autonomy, self-acceptance, positive relationships with others, personal growth, environmental mastery). The later-life well-being of racial minorities is disproportionally affected by having a child with DD. Future research avenues include identifying life-course pathways that contribute to this differential vulnerability.

### EXPLORATIONS OF ALASKA NATIVE URBAN ELDERSHIP

Steffi Kim, *University of Alaska Anchorage, Anchorage, Alaska, United States*

Successful aging in Alaska Native people or "Eldership" is a state that is embedded within a cultural, relational, and generational context (Boyd, 2018; Lewis, 2011; Wexler, 2014). Eldership has been described as a developing and nuanced personal quality shaped by individual, relational, and contextual influences (Kim, 2020). Within the cultural and traditional understanding of Alaska Native people, the concept of Eldership is analogous to the Western concept of successful aging. With increasing numbers of outmigration from rural community members (Driscoll et al., 2010), this study investigated the impact of relocation from a rural traditional community to an urban Western community and its effect on the Elder's perception of "Eldership." This study considered the broader impact of the multi-systemic and socio-ecological context of minority and majority culture, dominant culture, and its implications for successful aging and identity (Grandbois & Sanders, 2009; Kirmayer et al., 2011). Gee's discourse analysis was used for the systematic interpretative study of 25 Elder interviews regarding their use of language describing their lived experiences, including three identified discursive patterns: cultural discourse, psychological discourse, and Elder identity discourse. Based on the study findings, Elders' experiences are impacted by socioeconomical and cultural differences encountered during relocation shifting the cultural frame of Alaska Native "Eldership" according to rural or urban contexts.

### FACILITATING MENTAL HEALTH SERVICE USE FOR OLDER CHINESE IMMIGRANTS: A CASE STUDY OF COMMUNITY-BASED ORGANIZATIONS

Yuanyuan Hu, and Qingwen Xu, *New York University, New York, New York, United States*

New York City has the largest older Chinese population of any city in the United States. Older Chinese adults in New York City often meet significant stress to fulfill their needs, and mental health issues are common among this population (Mui, 1996; Dong, 2012). Despite the high prevalence, Asian Americans have the lowest rates of mental health services use compared to other ethnic groups (Abe-Kim et al., 2007). Additional to wide disparities in mental health access, older immigrants experience additional factors that affect their decision making to use mental health services. Limited knowledge exists about community-based organizations facilitating mental health services use for this population. This study aimed to fill this gap by case study approach and conducted a qualitative analysis of data collected as part of a study that investigated the resilience of the Chinese communities in New York City in the context of aging and immigration. Data from five community-based organizations serving this population were examined, through reading agency history and program introduction, visiting agency location and observing its operation, and interviewing the agency staff and program directors. Data collected were integrated, synthesized, and analyzed. Findings represent organizational staff's perceptions of the mental health issues among older Chinese immigrants, needs and accessibility of mental health services, and facilitation of access and utilization of services by screening, education and referral. The qualitative results address individual help-seeking behavior and pattern, organizational response to and coordination of mental health needs, and capacity building on the community level.

### IF I'M WORRYING ABOUT GETTING WELL, HOW CAN I GET WELL? DECISION-MAKING AMONG AFRICAN AMERICANS WITH ADVANCED CKD.

Tyrone Hamler,[1] Megan Schmidt-Sane,[2] Anne Huml,[3] Mirela Dobre,[4] and Donna Taylor,[4] *1. Case Western Reserve University, Cleveland, Ohio, United States, 2. University of Sussex, Brighton, England, United Kingdom, 3. Cleveland Clinic, Cleveland, Ohio, United States, 4. University Hospital, Cleveland, Ohio, United States*

Objectives: Chronic kidney disease (CKD) is an emerging major public health concern in the United States. Shared decision-making (SDM) has gained attention as an important area of inquiry in chronic kidney disease research. Few studies focus on shared decision-making or preferences of older African Americans during the advanced stages of CKD before the initiation of dialysis. The objective of this study was to understand decision-making preferences and shared decision-making among older African Americans with advanced chronic kidney disease who have yet to start dialysis. Methods: Data were collected from an outpatient clinic sample of older African Americans ≥ 55 years old (N = 10) diagnosed with advanced CKD. Participants were administered a survey with open-ended questions related to shared decision-making, CKD healthcare and general healthcare preferences (both open-ended and closed-ended questions), and participant characteristics. A thematic analysis framework was applied to identify themes and patterns in the data. Results: Several themes emerged in regard to shared decision-making and patient preferences including: complexity of CKD management, uncertainty of prognosis, barriers and facilitators to CKD self-management and SDM, diagnosis and dialysis information, elements of SDM, and the structural and social context of SDM related to racial inequities. Discussion: Participants identified a nuanced understanding of the concerns related to managing CKD. The complex and ever-changing nature of CKD was emphasized as participants discussed how they perceived their care needs. This study provides implications for social work practice, healthcare policy and interprofessional collaboration in the care of older African Americans.

### MINORITY STATUS AND READMISSIONS: A MODERATED MEDIATION MODEL OF CAREGIVERS' HEALTH LITERACY

Nosaiba Rayan-Gharra, Nurit Gur-Yaish, and Ksenya Shulyaev, *University of Haifa, Haifa, Israel*

Family caregivers help patients to understand informa¬tion during clinical encounters. Less is known about factors that may affect family caregiver's Ensuring and Explaining Medical Care (EEMC) during hospitalization and its impact on improved health outcomes. This study examined whether EEMC during hospitalization mediated the association between minority status of patients and 30-day-readmissions, and whether levels of Health Literacy (HL) of caregivers moderated this mediated association. A prospective cohort study of 517 internal medicine patients, Hebrew (general population, coded as 0) and Russian, or Arabic native speakers (minority status, coded as 1), at a tertiary medical center in central Israel, who were accompanied by an informal caregiver. EEMC and HL were patients' self-reported. 30-day-readmissions were retrieved from the healthcare organization. Logistic regression indicated that minority status was not associated with 30-day readmission when the mediator ICEEMC was not included (B=0.98; p>0.05). However, moderated mediation analysis indicated significant direct (B=-1.08; p=0.003) and indirect effect of minority status on readmission through high ICEEMC during hospitalization among patients who had informal caregivers with high HL level (Mediated effect (ME)=−0.62; CI= -1.07 to -0.29) but not among ones with low HL level (ME= 0.37; CI=-0.24 to 1.06). These findings suggest that caregivers' high HL may be an essential factor in improving EEMC among minorities. Identifying informal caregivers with high HL level at time of admission to the hospital, and encouraging their involvement during patients' hospital stay, might be a useful strategy to improve transitions and reducing 30-day readmission, especially among minority patients.

### SUCCESSFUL AGING AMONG OLDER HISPANICS

Marlen Garcia,[1] Alejandra Morlett Paredes,[2] Dilip Jeste,[2] Alison Moore,[2] and Maria J. Marquine,[2] *1. San Diego State University, San Ysidro, California, United States, 2. University of California, San Diego, La Jolla, California, United States*

Successful Aging has been defined as the absence of objective physical, cognitive, and social difficulties. More recently, self-rated successful aging (SRSA) has been recognized as an important outcome in its own right. The purpose of this study was to assess SRSA and its correlates among older Hispanics/Latinos. Seventy-four Hispanic/Latino adults age 50+ (31.9% primarily Spanish-speaking; 62.5% women, mean age=69.6±12.2, mean years of education=14.3±3.3) completed a measure of SRSA (scaled from 1 [lowest] to 10 [highest]), and self-report measures of hypothesized correlates, including culturally-relevant factors (language use, acculturation, fatalism, familism, perceived discrimination and frame of reference), as well as physical (perception of physical health and physical performance), cognitive (perception of cognitive problems), and psychosocial correlates (social functioning and resilience). Fifty-five percent of the participants reported SRSA of 8 or above (mean=7.99±, range: 3-10). Factors that were significantly associated with SRSA in univariable models, were entered into a multiple linear regression on SRSA. The final multivariable model explained 58.5% of the variance on SRSA (F(3,54)=27.8, p<.001) and showed that social functioning (B=.21; p=.031), resilience (B=.34; p=.002), and perception of physical health (scaled from 1 [highest] to 5 [lowest]), (B=-.43; p<.001) were independent predictors of SRSA. Culturally-relevant factors were not independently associated with SRSA in the multivariable model. While future longitudinal studies would be better suited to address causality, the present cross-sectional findings indicate psychosocial correlates of SRSA are as important as physical correlates among older Latinos. Future studies might examine whether culturally relevant factors modify these associations.

### TRAJECTORIES IN HEALTH IN LATE MIDLIFE: DISPARITIES PERSIST ACROSS RACIAL/ETHNIC GROUPS IN US

Adit Doza, Gail Jensen Summers, and Wassim Tarraf, *Wayne State University, Detroit, Michigan, United States*

Introduction: Recent research has revealed that during late midlife, Hispanics experience a lower mortality relative to non-Hispanic Whites (Whites), whereas non-Hispanic Blacks (Blacks) experience a higher mortality relative to Whites. Much less is known about whether there are also racial/ethnic disparities in patterns of how overall health changes during this period, and if so, whether personal lifestyle or economic characteristics that can explain it. We examine these important issues. Methods: Longitudinal and nationally representative data on adults ages 50-64 from the Health and Retirement Study are used for our analysis. Latent class discrete time and growth curve modeling are implemented to identify different types of "trajectories" in self-rated health and mortality across racial/ethnic groups and three distinct time periods, 1998-2004, 2004-2010, and 2010-2016. Additionally, multinomial logit models are estimated to determine whether Hispanics or Blacks experience different trajectory profiles than Whites do, and how different characteristics at baseline affect one's own trajectory. Results: We identify five types of trajectories: "Poor-Health with Rapid Mortality," "Sustained Poor-Health," "Moderate-Health Back and Forth," "Good-Health with Rapid Mortality," and "Sustained Good-Health." These specific types persist even after controlling for multiple health determinants. Discussion: We find significant and persistent differences across racial/ethnic groups in how health changes during late midlife. After controlling for a wide range of health determinants, Blacks and Hispanics are less likely than Whites to experience "Sustained Good-Health" and more likely to experience "Sustained Poor-Health" across all three time periods. Blacks are also consistently more likely to experience "Poor-Health with Rapid Mortality."

### SESSION 10330 (LATE BREAKING POSTER)

### MOBILITY/DISABILITY

### BRAIN RESILIENCE: THE EFFECT OF WHITE MATTER DISEASE ON BRAIN NETWORKS IN COGNITIVELY NORMAL OLDER ADULTS

Blake Neyland, Christina Hugenschmidt, Samuel Lockhart, Laura Baker, Suzanne Craft, Paul Laurienti, and Stephen Kritchevsky, *Wake Forest School of Medicine, Winston-Salem, North Carolina, United States*

Brain pathologies are increasingly understood to confer mobility risk, but the malleability of functional brain networks may be a mechanism for mobility reserve. In particular, white matter hyperintensities (WMH) are strongly associated with mobility and alter functional network connectivity. To assess the potential role of brain networks as a mechanism of mobility reserve, 116 participants with MRI from the Brain Networks and Mobility Function (B-NET) were categorized into 4 groups based on median splits of SPPB scores and WMH burden: Expected Healthy (EH: low WMH, SPPB>10, N=45), Expected Impaired (EI: high WMH, SPPB10, N=24), Unexpected Impaired (EI: low WMH, SPPB<10, N=10) and Unexpected Unhealthy (UH: low WMH, SPPB<10, N=37). Functional brain networks were calculated using graph theory methods and white matter hyperintensities were quantified with the Lesion Segmentation Toolbox (LST) in SPM12. Somatomotor cortex community structure (SMC-CS) was similar between UH and EH with both having higher consistency than EI and UI. However, UH displayed a unique increase in second-order connections between the motor cortex and the anterior cingulate. It is possible that this increase in connections is a signal of higher reserve or resilience in UH participants and may indicate a mechanism of compensation in regards to mobility function and advanced WMH burden. These data suggest functional brain networks may be a mechanism for mobility resilience in older adults at mobility risk due to WMH burden.

### DIABETES, DISABILITY AND MORTALITY IN VERY OLD MEXICAN AMERICANS

Caroline Kelly, Kyriakos S. Markides, and Phillip Cantu, *The University of Texas Medical Branch at Galveston, Galveston, Texas, United States*

This study examines the relationship between diabetes, disability and mortality for very old Mexican American, a rapidly growing and understudied segment of the population. Data comes from Wave 7 of the Hispanic EPESE which surveyed 1078 Mexican Americans ≥80 years old, living in the Southwestern United States. Measures included self-reported physician diagnosis diabetes, ADL disability, demographic characteristics, and other self-reported comorbidities. Severe diabetes was defined as being diagnosed more than 10 years ago and/or taking insulin. Diabetes and mortality were studied over a five-year follow-up. Participants with diabetes are more likely to be disabled, 55% prevalence compared to 46% in non-diabetics. Even when controlling for demographic characteristics and comorbidities, the predicted probability of ADL disability in diabetics is 54% compared to 45% in non-diabetics. Logistic regression analysis showed an odds ratio (OR) of 1.51, 95% confidence interval [CI], 1.14-2.02 of any ADL disability among diabetics, controlling for demographic characteristics and comorbidities. Stroke and arthritis were significantly associated with ADL disability as well, OR 2.59, [1.6-4.2] and 1.53 [1.15-2.04] respectively. Diabetics had greater odds of mortality, OR 1.25, than non-diabetics, and among diabetics, severity was predictive of mortality OR 1.38. Gender was an insignificant predictor of mortality. Diabetes prevalence in the very old Mexican American population is increasing and is associated with increased disability and mortality. Increased awareness of the prevalence of diabetes in this population and thus the increased potential of disability is needed to decrease the burden of disease and increase quality of life.

### FEAR OF FALLING AND WALKING QUALITY: WHAT YOUR WALKING REVEALS

Anisha Suri,[1] Andrea Rosso,[2] Jessie VanSwearingen,[3] Gelsy Torres-Oviedo,[1] Leslie Coffman,[3] Mark Redfern,[1] Jennifer Brach,[3] and Ervin Sejdic,[1] *1. Swanson School of Engineering, University of Pittsburgh, Pittsburgh, Pennsylvania, United States, 2. School of Public Health, University of Pittsburgh, Pittsburgh, Pennsylvania, United States, 3. School of Health and Rehabilitation Sciences, University of Pittsburgh, Pittsburgh, Pennsylvania, United States*

Fear of Falling (FOF) is common among community-dwelling older adults and is associated with increased fall-risk. In this cross-sectional study we examined the relationships between FOF and factors associated with fall-risk such as gait quality, cognition, and walking-confidence. Using baseline data from older adult participants in a randomized exercise trial (N=232; age 77±6; 65% females; 40% reported FOF), we quantified the following outcome measure for (1) gait quality: harmonic ratio (smoothness) and time-frequency spatiotemporal variables from triaxial accelerometry during 6 minute walk; gait speed, step-time CoV (variability) and walk-ratio (step-length/cadence) on an instrumented walkway; (2) cognition: Trails A and B (3) walking-confidence: Gait efficacy Scale. Mann Whitney U-tests indicated individuals without FOF had better gait quality ($p<0.05$): greater smoothness (2.38±.58 vs 1.14±.73), speed (1.10±.15 vs 1.04±.17 m/s) and walk-ratio (.56±.07 vs .53±.08 cm/steps/min), lower step-time CoV (3.72±1.24 vs 4.17±1.66), and greater walking-confidence (89±11 vs 79±13). A random forest classifier predicted FOF with 64% (gait only) and 70% (additional variables: cognition, walking-confidence) accuracy; Gini-index based ranking indicated gait quality (smoothness vertical (V) direction, walking speed) were consistently important variables. Linear Support Vector Machine learning yielded accuracies of 60% (only gait) and 68% (with additional measures): smoothness V, mediolateral frequency bandwidth, gait speed among top 4 ranked variables in both models, and walking-confidence in the additional measures model; smoothness-V the highest weighted coefficient (-0.52). Based on these findings, interventions targeted for gait quality and walking-confidence may be important to overcome FOF and reduce fall risks.

### PERSONAL ACTIVITIES, SOCIAL PARTICIPATION AND FUNCTIONAL DISABILITY IN AGED LIVING ALONE WITH FALL EXPERIENCE

SuJung Jung, and Sunghee Tak, *Seoul National University, Seoul, Republic of Korea*

Functional disability leads to limitations in the older adults' personal activities and social participation. The purpose of this study was to examine the International Classification of Functioning Disability and Health(so called ICF) model in which personal activities and social participation influence functional disability in older adults who live alone and have experienced falls. The study used a secondary data analysis of the 2017 National Survey of Older Koreans. A total of 501 study participants met the inclusion criteria. The results of multiple linear regression indicated that gender and the number of acquaintances were significantly related to the functional disability of social participation while overnutrition, depressed symptom and cognitive dysfunction were related to the functional disability of personal activities. Lastly, poor muscle strength, old age and economic status were predictors of the functional disabilities of both personal activities and social participation. The findings of the study revealed that it is important to comprehensively evaluate not only personal activities but also social participation of older adults who live alone and have experienced falls. In addition, the ICF model may be useful in the development of intervention programs for preventing functional disability in the population.

### SESSION 10340 (LATE BREAKING POSTER)

### NUTRITION | OBESITY & EATING DISORDERS

### ASSOCIATIONS BETWEEN DEPRESSIVE SYMPTOMS, APPETITE AND FOOD INTAKE AMONG HOSPITALIZED OLDER ADULTS

Amos Rogozinski, and Anna Zisberg, *University of Haifa, Haifa, Israel, United States*

Inadequate food intake is common among hospitalized older adults and is linked to negative hospitalization outcomes, including functional decline and mortality. Depression is a well-established risk factor in inadequate food intake in the community but its role in food consumption during hospitalization is poorly studied. To examine the associations between depressive symptoms, appetite and the quantity of food consumed by older inpatients, we conducted a secondary data analysis of 724 hospitalized adults aged 69 to 95 using a prospective cohort dataset: Hospitalization Process Effects on Functional Outcomes and Recovery. Depression was evaluated with Tucker's Short Zung Instrument at time of admission. Food intake and appetite were examined daily for three consecutive days, using self-reports of food consumed at breakfast, lunch and supper, based on the nDay Express Questionnaire. Approximately 40% of respondents reported eating half or less than half of each meal. The risk of depression was prevalent among a third of respondents, 54% of whom were identified at high risk of depression. The association between depression and inadequate food intake was found to be negative [$F(2,716)=9.00$ ,$p=0.000$ ,$\eta2=0.025$]. Low appetite was significantly linked to reduced food consumption [$\beta=-0.39$, $t=-12.06$, $p=0.000$] and made a considerable contribution to the explained variance of food consumed [F-change (1,717)=145.41 , $p=0.000$]. Finally, decreased appetite partially mediated the association between depressive symptoms and food intake during hospitalization (B=-0.001, UCI=-0.001; LCI=-0.002). These findings contribute to the understanding of inadequate food intake during hospitalization and indicate the importance of screening for depression among hospitalized older adults.

### BODY MASS INDEX AND 12-YEAR MORTALITY AMONG OLDER MEXICAN AMERICANS AGED 75 YEARS AND OLDER

Reshma Jadhav, Kyriakos S. Markides, and Soham Al Snih, *The University of Texas Medical Branch at Galveston, Galveston, United States*

The objective of this study was to examine the effect of body mass index (BMI) on 12-year mortality among older Mexican Americans aged 75 years and older. Data are from wave 5 (N=1,367) of the Hispanic Established Population for the Epidemiologic Study of the Elderly (2004/2005-2016). Measures included socio-demographics, self-reported medical conditions, body mass index (BMI), disability, Mini-Mental-State-Examination (MMSE), short physical performance battery (SPPB), high depressive symptoms, and falls. BMI was classified as underweight (< 18.5), normal weight (18.5 to < 25), overweight (25 to < 30), obesity type I (30 to <35), and morbid obesity (≥ 35).Cox proportional hazards regression analysis was performed to estimate the hazard ratio of 12-year mortality as a function of BMI categories at baseline. The average of the sample was 81.2 years, 17.2% were underweight, 22.7% were normal weight, 27.4% were overweight, 31.9% were obesity type I, and 38.6% were morbid obesity. Mexican Americans aged ≥75 years with overweight or obesity Type I had a reduced hazard ratio (HR) of death (HR=0.80, 95% CI=0.68-0.93 and HR=0.73, 95% CI=0.60-0.88, respectively) over 12-years of follow-up. The HR of death for underweight and morbid obesity participants was 1.78 (95% CI=1.13-2.80) and 1.03 (95% CI=0.79-1.36), respectively. Female participants and those with high scores in the MMSE and SPPB had decreased risk of death. This study confirmed the protective effect of overweight and obesity on mortality seen in this population at a younger age, which might have implications when treating older adults with overweight and obesity.

### DIET QUALITY AND BIOLOGICAL RISK IN A NATIONAL SAMPLE OF OLDER AMERICANS

Yeon Jin Choi, Jennifer Ailshire, Jung Ki Kim, and Eileen Crimmins, *University of Southern California, Los Angeles, California, United States*

Biomarkers are sensitive to current health status and capture aspects of health that may precede the development of disease and other health problems. Using comprehensive measures of biological risk, this study aims to investigate the relationship between intake of individual dietary components, overall diet quality, and biological dysregulation. For the analysis, we used nutrition and biomarker data from 3,641 older adults (over age 50) in the Health and Retirement Study. Eleven out of 13 individual dietary components were associated with lower biological risk. After controlling for SES, health behaviors, and access to health care, a high intake of fruits, greens and beans, whole grains, seafood and plant proteins, and fatty acids and a low intake of sodium and saturated fat were still associated with lower biological risk. Respondents with poor/suboptimal quality diet had higher biological risk than those with good quality diet. After controlling for SES, health behaviors, and access to health care, respondents with poor/suboptimal quality diet continued to exhibit higher biological risk than those with good quality diet, though the differences in biological risk were reduced. Findings from this study emphasize the importance of healthy eating in improving health of older adults. Encouraging intake of fruits, greens and beans, whole grains, seafood and plant proteins, and fatty acids, while limiting consumption of sodium and saturated fat would improve overall diet quality and contribute to the prevention of chronic diseases and morbidity.

### DIETARY ADMINISTRATION OF 2-DEOXY-D-GLUCOSE REDUCES HIGH FAT DIET INDUCED OBESITY AND META-INFLAMMATION IN MICE

Sanjay Pandey,[1] Diksha Joshi[1] Saurabh Singh,[2] Sushma Ray,[1] Anant Narayan Bhatt,[1] K Natarajan,[3] and BS Dwarakanath,[4] *1. Institute of Nuclear Medicine and Allied Sciences, Delhi, Delhi, India, 2. Albert Einstein College of Medicine, Bronx, New York, United States, 3. Dr. B.R. Ambedkar Center for Biomedical Research, University of Delhi, Delhi, Delhi, India, 4. Shanghai Proton and Heavy Ion Center of Fudan University Shanghai Cancer Center, Delhi, Delhi, India*

Obesity is a major risk factor for type 2 diabetes, NAFLD, chronic diseases and cancer. Insulin resistance, oxidative stress, high ectopic lipid levels and meta-inflammation are the mechanisms proposed to play a leading role in the morbidity associated with obesity. Energy restriction mimetics (ERMAs) has also been shown earlier to reduce the scale and the severity of these disorders by mimicking the physiological effects of the Energy Restriction. In present study we propose that the use of 2-DG as ERMA can be effective in regulating the High Fat Diet (HFD) induced obesity. Effect of 2-DG (0.4% w/v in drinking water) on the HFD and Insulin Resistance (IR). HFD induced change in body weight, adipose tissue mass, and ectopic lipid levels was assessed as the measure of obesity.IR and glucose levels were also estimated to evaluate the effect of 2-DG on the insulin sensitivity in HFD mice. 2-DG significantly altered HFD induced increase in the mice body weight, epididymal White Adipose Tissue (WAT) and liver weight. 2-DG fed mice also showed reduced lipid levels in serum and liver. Furthermore, 2-DG also reduced the oxidative damage in the liver with concomitant increase in enzymatic (SOD and Catalase) and non-enzymatic (reduced Glutathione) antioxidant levels. 2-DG fed mice also showed reduced levels of Leptin, IL-6 and TGF-β which are early drivers of the etiology of the metabolic diseases. Our results suggest that 2-DG as ERMA can prevent obesity and etiology of associated disorders. However, more relevant models are needed to further strengthen these observation

### EARLY-LIFE STRESS, DEPRESSIVE SYMPTOMS, AND INFLAMMATION: THE ROLE OF SOCIAL FACTORS

Julia Nakamura,[1] Eric Kim,[1] Kelly Rentscher,[2] and Kate Kuhlman,[3] *1. University of British Columbia, Vancouver, British Columbia, Canada, 2. University of California, Los Angeles, Los Angeles, California, United States, 3. University of California, Irvine, Irvine, California, United States*

Early-life stress (ELS) is associated with elevated risk of adverse psychological (e.g., depression) and physical health outcomes (chronic diseases driven by inflammation) in older adulthood. We evaluated whether four social factors buffered the ELS-depressive symptoms and ELS-inflammation associations. Data were from 3,416 adults (58.28% female; Mage=68.41; SDage=10.24) who participated in the 2006 wave of the Health and Retirement Study, a nationally representative sample of older adults in the United States. We used hierarchical regression analyses to first test the main effects of ELS on depressive symptoms and inflammation (high-sensitivity C-reactive protein). We then assessed whether four social factors (perceived support, frequency of social contact, network size, and volunteer activity) moderated the ELS-depressive symptoms and ELS-inflammation relationships. We found a small positive association between ELS and depressive symptoms (B=0.17, SE=0.05, p=.002), which was moderated by social contact and perceived support. Specifically, ELS was only associated with elevated depressive symptoms for participants with limited social contact (B=0.24, SE=0.07, p<.001) and low perceived support (B=0.24, SE=0.07, p<.001). These associations remained after accounting for potential confounders (age, body-mass index, adulthood stress, and marital status). ELS was not associated with inflammation, and no social factors moderated the ELS-inflammation link. Increased social contact and perceived support may be protective for individuals at an elevated risk of developing depressive symptoms as a result of ELS. Future interventions may benefit from leveraging these social factors to improve quality of life in adults with ELS.

### FOOD AND NUTRIENT INTAKE AND DIET QUALITY AMONG OLDER AMERICANS

Yeon Jin Choi, Eileen Crimmins, Jung Ki Kim, and Jennifer Ailshire, *University of Southern California, Los Angeles, California, United States*

A suboptimal diet and nutritional deficiencies can have important influences on health with significant impact among older adults. This study aims to assess the presence of suboptimal dietary intake among older Americans and identify risk and protective factors influencing diet quality. For this study, data from a nationally representative sample of 5,614 community-dwelling older adults over age 54 in the Health and Retirement Study – Health Care and Nutrition Survey were used. Descriptive analyses were conducted to assess average intake of 17 food groups and nutrients and the percentage of respondents who consumed an optimal amount of food and nutrients. Differences in diet quality by sociodemographic, psychosocial, environmental, and geographic factors were assessed using chi-square and OLS regression was used to identify risk and protective factors for good quality diet. Overall, only 10.7% of respondents had a good quality diet (HEI score 81 and above); the majority had diets considered poor or needing improvement. Less than 50% of respondents met dietary guidelines and nutritional goals for most individual food groups and nutrients. Respondents with low socioeconomic status, fewer psychosocial resources, and those who had limited access to healthy food outlets were more likely to have a diet of suboptimal quality. Efforts to remove identified barriers that put older adults at risk for poor nutrition and to provide resources that increase access to healthy food should be made to encourage healthy eating and enhance diet quality.

### SESSION 10350 (LATE BREAKING POSTER)

### PAIN MANAGEMENT

### RACIAL DIFFERENCES OF ATTITUDES TOWARDS MEDICAL MARIJUANA USE AMONG AMERICAN OLDER ADULTS

Kedong Ding,[1] and Yifan Lou,[2] *1. University of Chicago School of Social Service Administration, Chicago, Illinois, United States, 2. Columbia University, New York, New York, United States*

Background: Extensive evidence documented that medical marijuana (MM) could be used for pain management with fewer side effects. Although MM legalization remains controversial as it is perceived to be a “gateway drug”, more states are striving for MM legalization and calling for health insurance coverage. Racial difference in the use of opioids for pain management is well-documented, which may also exist in the use of MM. This study explored an understudied topic on how people from different racial backgrounds perceive MM and its behind mechanism. Method: Data is from Health and Retirement Study Wave 2018 who answered the special modules on MM (n=1340). The attitude is proxied by two dichotomous measures on whether they think MM is acceptable and would lead to hard drug. Logistic regressions were used to evaluate the relationships between attitude and race, adjusted for sociodemographic, health and mental health, and MM knowledge. Moderating effects of diseases and socioeconomic-status (SES) were tested using interaction terms. Results: Older adults who hold positive attitudes towards MM are more likely to be younger, high SES, using opioids. Hispanics and Blacks are more negative towards MM relative to White counterparts. Blacks with cancer (OR=.30) are less likely to believe MM is acceptable, whereas blacks with arthritis are more likely to accept MM (OR=2.08) compared to Whites. Hispanic females (OR=.26) are more likely to oppose MM while Hispanics with higher education background (OR=4.3) are more likely to hold positive attitudes. Implication: The results may guide development of future guidelines on MM prescription.

### STRESSORS AND PAIN OVER THE LATE-LIFE COURSE: FINDINGS FROM TWO PARENT LONGITUDINAL STUDIES OF AGING AND HEALTH

Penny Brennan, *University of California, San Francisco, San Francisco, California, United States*

With this study we sought to determine how older adults’ stressors influence their levels and rates of change in pain during the late-life span. We harmonized repeated measures data from two parent longitudinal studies of aging and health, Longitudinal Late-Life Health (LLLH; n=1,1884) and the Health and Retirement Study (HRS; n=7,703), to determine how participants’ stressor levels in the domains of finances, spouse, children, extended family, and friends, and in stressors overall, influenced their average levels and rates of change in painful conditions, pain severity, and pain interference over 13-year (LLLH) and 8-year (HRS) intervals. Participants' within-person stressor levels declined somewhat, whereas their number of painful conditions, pain severity, pain interference, and prescription painkiller use increased steadily, over these intervals. In both the LLLH and HRS samples, participants who experienced higher average stressor levels over the 13- and 8-year intervals had more numerous painful conditions and higher pain severity over these intervals. In the HRS sample, they also experienced higher levels of pain interference. These effects occurred independent of the demographic characteristics of age, gender, and race. In general, participants' stressor levels did not influence rates of increase in their pain. Gender and race had some moderating effects on associations between stressors and pain, but these occurred only within certain specific stressor and pain domains. These findings demonstrate an association between stressors and pain across the late-life course. Further research is needed to determine the mediating mechanisms that account for this association and the moderating factors that affect its strength.

### THE IMPORTANCE OF PERSON CENTERED COACHING ON PHYSICAL ACTIVITY IN OLDER ADULTS

Lenny Chiang-Hanisko, Sue Graves, Jean Joseph-Rendel, David Newman, and Debroah D'Avolio, *Florida Atlantic University, Boca Raton, Florida, United States*

Osteoarthritis (OA) is the most common form of arthritis and imposes a substantial social and economic burden on American society. Evidence suggests that physical exercise provides health-related benefits to reduced osteoarthritis pain, stress and depression. However, exercise alone may not be sufficient to make necessary lifestyle changes to reduce OA pain. This study aimed to determine the effectiveness of Person Centered Coaching (PCC) and Health Education (HE) on physical activity of exercising older adults with OA pain. 14 exercising older adults were included in this experimental, randomized, pre and posttest study. Age ranged from 69 -88 years (mean = 77) with most participants identifing as non-Hispanic whites, all currently retired and 71% female. Participants completed OA Diary, 3 PROMIS short-form instruments (Pain Interference, Perceived Stress and Instrumental Support) and Personal Growth Initiative Scale. Data was collected over six weeks to include baseline and post-intervention measurements using repeated measures analysis. The result indicated the PCC treatment group had lower scores on pain interference and perceived stress compared to the HE group. The PCC group also had increased higher level on personal growth and instrumental support compared to the HE group. The results suggest that additional interventions are needed to enhance exercise effectiveness in reducing pain, inflammation and stress. The intervention (PCC), has the potential to provide a positive impact on OA outcomes for exercising older adults by increasing self-knowledge and self-monitoring of OA pain, as well as facilitating persistence with exercise.

### THE SUNDAY EFFECT: HOW CHURCH ATTENDANCE IS RELATED TO DAILY PAIN AND AFFECT IN OLDER ADULTS WITH OSTEOARTHRITIS

Shelley Condon,[1] Katherine Cheesman,[1] Dylan Smith,[2] and Patricia Parmelee[2], *1. The University of Alabama, Tuscaloosa, Alabama, United States, 2. Stony Brook University, Stony Brook, New York, New York, United States*

Attending church is related to elevated mood (Law & Sbarra, 2009), greater social connectivity (Obst & Tham, 2009), and purpose in life (Robbins & Francis, 2000). More research is needed on how these relationships function among older adults, especially those living with chronic pain. The current study aimed to explore the effects of attending church on Sunday on the daily pain and affect of older adults with arthritis pain. Using a subset of 185 participants living in Alabama from the Everyday Quality of Life (EQUAL) project, the current study utilized multilevel modeling to examine (1) the main effects of church attendance and day of week (Sunday vs other) on daily pain and affect (positive and negative), and (2) the interaction [Sunday] effect of church attendance and day of week on those outcomes, controlling for sociodemographic variables (i.e., employment status, sex, race, and age). Preliminary covariate analyses revealed that church attendance was higher among participants who were unemployed, female, African American, and older. For the multilevel models predicting daily positive and negative affect, significant main effects were found for day of week and church attendance; however, the interaction effect was not significant. Interestingly, no main or interaction effects were found for the models predicting daily pain. Thus, while church attendance and day of week significantly predicted daily ratings of positive and negative affect, there was no support for the Sunday Effect on those outcomes. Implications and ideas for future research are discussed. (R01-AG041655, P. Parmelee & D. Smith, Co-PIs).

### SESSION 10360 (LATE BREAKING POSTER)

### RESEARCH METHODS AND ISSUES: QUANTITATIVE

### EMOTIONAL COMPLEXITY IN DAILY LIFE: ON THE ROLE OF EMOTIONAL DYNAMICS, AGE, & CULTURE

Yoonseok Choi,[1] Jennifer Lay,[2] Minjie Lu,[3] Helene Fung,[4] and Christiane Hoppmann,[1] *1. University of British Columbia, Vancouver, British Columbia, Canada, 2. University of Exeter, Exeter, England, United Kingdom, 3. Sun Yat-Sen University, Guangzhou, China, 4. The Chinese University of Hong Kong, Sha Tin, China*

Emotional complexity is a construct that has attracted significant interest in the aging literature. It often refers to two aspects — the co-occurrence of positive and negative emotions and emotion differentiation (experiencing emotions with specificity). Emotional complexity is thought to increase with aging. However, recent research points to inconsistent results showing a positive relationship between age and emotional complexity, non-significant associations and even negative relationships. The present study seeks to address this inconsistency in findings by examining three possible sources: 1) different indicators of emotional complexity, 2) age differences in emotional dynamics (individual differences in means & variability of momentary positive & negative emotions), and 3) differences in cultural backgrounds. Community-dwelling adults from Vancouver (96 older adults, 51 young adults; 56% of Asian heritage, 30% of Caucasian heritage, and others 14%) and in Hong Kong (56 older adults, 59 young adults; 100% Asian heritage) completed approximately 30 ecological momentary assessments over a 10-day period assessing their current emotional experiences. When the mean and variability of emotional experiences were controlled for, most emotional complexity measures showed a negative relationship with age indicating that older adults displayed lower emotional complexity compared to young adults. This pattern was consistent across participants of Asian and Caucasian heritage. Additional analyses will explore the link between different emotional complexity measures and well-being indicators. Our findings point to the need to provide a more nuanced perspective on the correlates and consequences of emotional complexity in old age.

### FORECASTING INDIVIDUAL AGING TRAJECTORIES AND SURVIVAL WITH AN INTERPRETABLE NETWORK MODEL

Spencer Farrell, Arnold Mitnitski, Kenneth Rockwood, and Andrew Rutenberg, *Dalhousie University, Halifax, Nova Scotia, Canada*

We have built a computational model of individual aging trajectories of health and survival, containing physical, functional, and biological variables, conditioned on demographic, lifestyle, and medical background information. We combine techniques of modern machine learning with a network approach, where the health variables are coupled by an interaction network within a stochastic dynamical system. The resulting model is scalable to large longitudinal data sets, is predictive of individual high-dimensional health trajectories and survival, and infers an interpretable network of interactions between the health variables. The interaction network gives us the ability to identify which interactions between variables are used by the model, demonstrating that realistic physiological connections are inferred. We use English Longitudinal Study of Aging (ELSA) data to train our model and show that it performs better than standard linear models for health outcomes and survival, while also revealing the relevant interactions. Our model can be used to generate synthetic individuals that age realistically from input data at baseline, as well as the ability to probe future aging outcomes given an arbitrary initial health state.

### SOCIAL CHARACTERISTICS, HEALTH AND MORTALITY AMONG MALE CENTENARIANS USING VETERANS AFFAIRS (VA) HEALTHCARE

Lien Quach David Gagnon,[1] Elizabeth Dugan,[2] Kelly Cho,[3] Michael Gaziano,[3] Avron Spiro,[3] Rachel Ward,[3] and Jane Driver,[3] *1. Boston University School of Public Health, Boston, Massachusetts, United States, 2. University of Massachusetts Boston, Boston, Massachusetts, United States, 3. VA Boston Healthcare System, Boston, Massachusetts, United States*

Studying health and mortality among centenarian Veterans is critical to understanding the limit of the male human life span and Veterans' extraordinary model of successful aging. The majority of VA users are male, but little is known about social characteristics and health among male centenarians in general. We investigated the annual mortality rate of male centenarian Veterans seeking care from the VA and identified social characteristics and health conditions that influenced the risk of mortality. This longitudinal study used VA Electronic Health Record (EHR) data from 1997 – 2012 (n=1858). Dates of death were obtained from the EHR, aggregated by the Corporate Data Warehouse from multiple sources. Independent variables included age, race, marital status, and periods of military service. Health conditions consisted of cancer, congestive heart failure (CHF), diabetes, chronic renal disease, chronic pulmonary disease, peripheral vascular disease, dementia, myocardial infarction, liver diseases. The mean age was 100.4 (range: 100-115), 76% were white and 49% married. The average annual mortality rate was 32 per 100 person-years. The annual mortality rate was stable and not affected by race, but did differ by marital status. Divorced or separated centenarians had a 21% higher rate of death than married centenarians [Hazard Ratio (HR):1.21, 95% CI 1.07 - 1.36]. A diagnosis of dementia increased the mortality risk by 37% (HR: 1.37, 95% CI: 1.04 - 1.81) and CHF by 37% (HR: 1.37; 95% CI: 1.13 - 1.66). Providers should consider prevalent health conditions, as well as marital status, in managing care of centenarian veterans.

### THE FRAILTY INDEX, MISSING DATA, AND IMPUTATION

Glen Pridham, and Andrew Rutenberg, *Dalhousie University, Halifax, Nova Scotia, Canada*

The frailty index (FI) is a summary measure of health during aging that is defined by the average number of 'things wrong', i.e. health deficits, across a sundry of lab, clinical, and questionnaire measurements. Missing data are ubiquitous in aging studies. Although the FI appears to have robust predictive power—even when ignoring missing data, there has not been a systematic study of the consequences of imputation when used in the principle investigation. We investigated the standard imputation methodology, multiple imputation using chained equations (MICE), and other missing data methods, in terms of prediction of mortality and statistical power using the 2003/04 and 2005/06 NHANES datasets. When we masked known data completely at random, we observed that available case analysis incorrectly estimated the true variance of the FI leading to potential problems in hypothesis testing, whereas imputation helped mitigate this effect. We also observed that the default imputation methods from MICE showed a significant increase in FI relative to the ground truth together with a decrease in predictive power, hence we suggest other options when performing imputation with NHANES. The underlying missing mechanism in NHANES is not random and appears to be important, for example survival curve analysis showed that the top half of patients with the most missing data died significantly younger than the bottom half.

### SESSION 10370 (LATE BREAKING POSTER)

### SOCIAL DETERMINANTS OF HEALTH AND AGING (SDOH)

### A COMPARISON OF COGNITIVE FUNCTION IN PINE STUDY TO HEALTH AND RETIREMENT STUDY & CHARLS STUDY

Charu Verma, Mailun Zhang, Mengting Li, Stephanie Bergren, and XinQi Dong, *Rutgers University, New Brunswick, New Jersey, United States*

Healthy immigrant theory assumes that immigrants have better health than natives, while it remains unclear whether this theory applies to older Chinese Americans with respect to cognitive health. The objective of this study was to estimate the differences in cognitive function between the US and Chinese older adults. PINE (n=3,157) (2011-2013) with Chinese older adults was compared to HRS study (2010-2011) (n=22,034) and HRS sister CHARLS (n=17,708) (2010-2011). Cognitive function was assessed by episodic memory, working memory, executive function, and MMSE. Cognition impairment was defined by 1.5 standard deviations below mean of z scores. After 1:1 matched samples determined by propensity score and verified by McNemar and Cohen's Kappa test, conditional logistic regression model with demographic variables controlled was used to assess the differences between the three studies. Multivariable analyses results showed that participants from PINE are 5.667 (OR= 5.667, 95% CI 3.893-8.248, $p < .001$) times more likely to have cognitive impairment in comparison to HRS's and 0.166 (OR= 0.166, 95% CI 0.136-0.203, $p < .001$) times less likely in comparison to CHARLS's. Result demonstrated that Chinese older adults living in the US have a higher likelihood of cognitive impairment in comparison to the US older adults but less likely than those living in China. Healthy immigrant theory is partially supported in Chinese Americans. Immigrants with better cognitive health are likely to migrate to the US, but limited social engagement in the receiving communities might be a risk factor for cognitive function, leading to worse cognitive health than natives.

### ADULT PERSONAL NETWORK CHARACTERISTICS ASSOCIATED WITH EARLY LIFE ADVERSITY

Janae Briggs, and Stephanie Child, *University of California, Berkeley, Berkeley, United States*

Early life adversity (ELA) is associated with poor health through social and economic pathways. ELA also shapes cognitive and emotional development, including self-perception, social attachment and mental well-being. As such, ELA may shape later life health through social relationships, yet few studies have examined these associations. Data from the UC Berkeley Social Network Study were used to examine ELA measured retrospectively and current personal network characteristics among young (21-30 years) and older adults (50-70 years). ELA was operationalized as a summary of six experiences occurring before age 18 (e.g., parents' divorce/separation, violence/drug use in the home, etc.). Personal network characteristics included objective measures, such as the number of ties who provide or receive various types of support, and subjective assessments about the adequacy of support received. Multivariate regression models adjusted for gender, race/ethnicity, and level of education. Among young adults, ELA was associated with more ties who rely upon the ego for support (b=0.15, 95% CI: 0.02, 0.28, p=0.02). Among older adults, ELA was associated with more ties named as either an advisor (b=0.14, 95% CI: 0.04, 0.21, p=0.02) or difficult/demanding (b=0.12, 95% CI: 0.04, 0.21, $p<0.01$). Furthermore, ELA was associated with less confidence in family support available (b= -0.09, 95% CI: -0.16, -0.03, $p<0.01$) and fewer emotionally close family members (b= -0.18, 95% CI: -0.32, -0.03, p=0.02) among older adults. In conclusion, clear differences emerged in network characteristics by exposure to ELA, particularly among older adults. The findings highlight potential pathways through which ELA patterns later life health.

### DEFINING THE PERSONAL DETERMINANTS OF HEALTH FOR OLDER ADULTS

Stephanie MacLeod,[1] Sandra Kraemer,[2] Annette Fellows,[2] Laurie Albright,[2] Joann Ruiz,[2] Michael McGinn,[1] James Schaeffer,[1] and Charlotte Yeh,[3] *1. Optum, Ann Arbor, Michigan, United States, 2. UnitedHealthcare, Minneapolis, Minnesota, United States, 3. AARP Services, Inc, Dedham, Massachusetts, United States*

Social Determinants of Health (SDOH) are defined as the broad community-scale conditions and factors shaping daily life. Meanwhile, less is known about the personal, individual-level characteristics impacting health outcomes. These have not been defined as a construct, leaving a gap in overall understanding of the resources that shape healthy and successful aging. Our primary purpose is to propose and define a new construct encompassing critical personal resources to be known as the Personal Determinants of Health (PDOH), built on resilience as a key strength later in life and supported by factors that buffer challenges and declining health. A literature search was conducted to streamline the scope of this review, with key terms determined to identify relevant publications; common databases and resources were utilized. Search strategies failed to identify a standard definition for Personal Determinants of Health as a construct, nor does this term exist regarding applied initiatives with older adults. Thus, a clear opportunity exists to establish PDOH as a unique construct. Despite evidence that individual characteristics impact late-life health outcomes, these key personal resources have not been established as a separate construct. Thus, we propose to define PDOH with a foundation of resilience supported by selected personal resources. Establishing this new construct will be critical in designing initiatives to support older adults and improve their health outcomes.

### POLITICAL CONTEXT IS ASSOCIATED WITH EXTENT OF EVERYDAY PHYSIOLOGICAL SYNCHRONY IN OLDER COUPLES

Theresa Pauly,[1] Karolina Kolodziejczak,[2] Johanna Drewelies,[3] Nilam Ram,[4] Denis Gerstorf,[2] and Christiane Hoppmann,[5] *1. University of Zurich, Zurich, Switzerland, 2. Humboldt University Berlin, Berlin, Berlin, Germany, 3. Humboldt University Berlin, Berlin, Germany, 4. Stanford University, Stanford, California, United States,*

5. University of British Columbia, Vancouver, British Columbia, Canada

Social units such as couples exist within a broader societal and cultural context. Characteristics of this macro-level context may indirectly shape couple dynamics by influencing opportunities and motivation for interdependence, e.g. through legislation and prevalent norms/values. The current study investigates the association between political context (left-right political spectrum) and physiological linkage (cortisol synchrony) in older couples' daily lives. Older adult couples (N = 162) aged 56 to 89 years (M age = 72.3 years) and residing in Germany provided salivary cortisol samples 7 times daily for a 7-day period. Political context in which dyads lived was quantified with respect to where the federal state of residence was located on the left-right political spectrum using voting data from the 2017 federal election. Links between macro-context and extent of cortisol synchrony were examined using multilevel models, controlling for differences in diurnal rhythm, sex, age, body mass index, and individual-level political orientation. On average, there was evidence of synchrony in fluctuations in partners' cortisol (b = 0.08, SE = 0.02, p < .001). The extent of cortisol synchrony was moderated by macro-context, such that couples living in a federal state placed further right on the left-right political spectrum exhibited greater cortisol synchrony (b = 0.03, SE = 0.01, p = .010). This new evidence provides a foundation for theorizing about and investigating how specific mechanisms contributing to political context, including family values, gender role attitudes, and laws supporting gender equality contribute to interpersonal linkages of physiological stress responses in daily life.

### SOCIAL ADVERSITY IMPACTS ON THE RHESUS MACAQUE IMMUNE SYSTEM RESEMBLE THOSE OF AGING

Mitchell Sanchez-Rosado,[1] Noah Snyder-Mackler,[2] Lauren Brent,[3] James Higham,[4] Clare Kimock,[4] Marina Watowich,[5] Melissa Pavez-Fox,[3] and Petraleigh Pantoja-Maldonado,[1] *1. University of Puerto Rico Medical Sciences Campus, San Juan, Puerto Rico, 2. Arizona State University, Tempe, Arizona, United States, 3. University of Exeter, Exeter, United Kingdom, 4. New York University, New York, New York, United States, 5. University of Washington, Seattle, Washington, United States*

Social adversity can impact immune function and is associated with increased morbidity and mortality. Many of these immune-related changes resemble the effects of the natural aging process. To date, little is known about the effects of social adversity on the immune system change across the lifetime. Here, we investigated how aging and social adversity (measured as social status) impact immune cell proportions. We performed flow cytometry on peripheral whole blood from a population of free-ranging rhesus macaques to quantify changes on immune cell proportions across the lifespan (n=99) and across different social statuses (n=53). Overall, we found that high adversity recapitulated the effects of aging. We found significant shared decreases in CD3+/CD4+ T cell proportions and corresponding increases in CD3+/CD8+ T cell proportions between individuals of older ages and low social status. These findings suggest that social adversity has similar effects to aging on T cell proportions, possibly affecting the T cell component of the immune response. In contrast, CD3+/CD4+/CD25+ T regulatory cell proportions increased with age, which is typical of normal aging. Contrary to our expectations, these cells were less abundant in low status individuals, indicating some overall regulatory immune deficits specific to lower status individuals. CD3+/CD8+/CD25+ T regulatory cells, which contribute to self-tolerance, were higher in high status individuals, suggesting that overall primary immune regulatory cells can be affected by social adversity and impair the regulation of inflammation. We provide evidence that social adversity alters immune cell proportions, implicating it in the development of inflammatory and/or aging-related diseases.

### THE LONG-TERM EFFECT OF JOB LOSS ON MORTALITY

Qian Song and Emily Lim, *University of Massachusetts Boston, Boston, Massachusetts, United States*

The associations between job loss and health were established. However, these associations were usually biased estimates of causality, as individuals with adverse health were self-selected into job loss. To address such selectivity, we take advantage of the case of massive layoffs of State-Owned Enterprise (SOE) workers in urban China, which was driven by policy change rather than individual characteristics and mimicked a quasi-experiment. We employ China Health and Nutrition Survey (1991-2015) that span more than two decades and survival analysis to analyze the effect of job loss on individual mortality. Our analyses with 3,494 SOE workers show that over the past twenty years, job loss has accelerated mortality (hazard ratio = 1.50; p<0.05) for SOE workers in urban China. Further, these accelerated moralities occurred mostly for workers who lost their jobs between 30-39 (hazard ratio = 2.52, p<0.05) and 40-50 (hazard ratio = 1.77, p<0.1) years old, highlighting the adverse consequences of job loss for the young and middle-aged workers. Our analyses established the causality of job loss and mortality over an extended time, and identified the most vulnerable groups to job loss for policies to target to in an effort to improving the well-being of workers later in life.

### THE ROLE OF COMMUNITIES OF RESIDENCE ON OLDER MEXICAN ADULTS' LIKELIHOOD OF HIGH DEPRESSIVE SYMPTOMS

Paige Downer and Rebeca Wong, *The University of Texas Medical Branch at Galveston, Galveston, Texas, United States*

Exposure to population- and individual-level poverty, poor health, and negative life-events contributes to overall life adversity that may increase older adults risk of depression. We hypothesize that increased neighborhood-level multidimensional poverty and decreased proportions of within-neighborhood health promoting socio-demographic characteristics will be positively associated with depression among older Mexican adults. This study uses data from Wave 1 (2001) and Wave 3 (2012) of the Mexican Health and Aging Study (MHAS). Wave 1 will be used for information for socio-demographic characteristics, including gender, female-headed households, rural settings, and employment status. Wave 3 will be used for information on self-reported depressive symptoms. Information for neighborhood characteristics will come from the 2000 Mexican Census that has been linked with the 2001 MHAS wave. Older Mexican adult's exposure to multidimensional poverty at the locality (city/town) level will be measured by the proportion of the population aged 15 and older with low education; the proportion of the population with low access to health care services; the percentage of homes with inferior construction materials; and the proportion of the population without sewage and running water. A multivariable logistic regression will be used to model the association between older Mexican adult's neighborhood and community characteristics in 2001 on depression in 2012. The expected findings will inform government policies to increase access to affordable housing, the availability of health care services, educational and employment opportunities, and public infrastructures such as transportation, water, and sanitation services, and energy services to reduce mental health burden.

### SESSION 10380 (LATE BREAKING POSTER)

### SOCIAL ISOLATION AND LONELINESS

### LONELINESS, ISOLATION AND LIVING ALONE ASSOCIATE WITH PSYCHOLOGICAL WELL-BEING AMONG THE OLDER ADULTS IN TAIPEI

Hui-chuan Hsu, *Taipei Medical University, Taipei, Taiwan (Republic of China)*

Purpose: The purpose of this study was to examine the clustering of loneliness, isolation, and living alone, the risk factors and the associations with psychological wellbeing. Methods: The data were from the 2019 Taipei City Senior Citizen Condition Survey collected by face-to-face interviews, which included aged 60 and above community-based and institution-based samples. The completed sample was 3,853 persons. Loneliness, isolation, and living arrangement were analysed by cluster analysis to define the Loneliness-Isolation-Living Alone clusters. Multinomial logistic regression was used to examine the related factors to LIL clusters. Results: Four clusters of the older adults were identified and named as following: Connected (44.1%), Alone /Institutionalized (9.2%); Lonely (10.7%); and Isolated (22.0%). Compared with the Connected cluster, the Alone/ Institutionalized cluster was more likely to have higher education, more IADL difficulties, more diseases , lower economic satisfaction, more likely to be males, having no spouse, and no children; the Lonely cluster was more likely to poor self-rated health, lower financial satisfaction, feeling less age-friendliness, more likely to be older, female, and no spouse; the Isolated cluster was more likely to have lower education, reported poorer self-rated health, lower economic satisfaction, and being older. The Alone/Institutionalized cluster and the Lonely cluster had higher depressive symptoms; the Alone/Institutionalized, Lonely, and Isolated clusters reported lower life satisfaction and had higher risks of cognitive impairment. Discussion: Loneliness, isolation, and living alone jointly associate with psychological health and well-being. High risk older populations may need social care and encourage social participation to promote health and wellbeing.

### OWNERS' PERCEPTIONS OF PET INFLUENCE: RELATION TO HEALTH OUTCOMES & PET ATTACHMENT IN COMMUNITY-LIVING OLDER ADULTS

Erika Friedmann,[1] Nancy Gee,[2] Eleanor Simonsick,[3] Barbara Resnick,[1] Erik Barr,[1] Melissa Kitner-Triolo,[3] and Alisha Hackney[1], *1. University of Maryland School of Nursing, Baltimore, Maryland, United States, 2. Virginia Commonwealth University, Richmond, Virginia, United States, 3. National Institute on Aging, Bethesda, Maryland, United States*

Pet ownership (PO) has been linked to better health outcomes in older adults, particularly those with chronic health conditions. It is suggested that pets influence their owners lives both by encouraging social interaction and by interfering with owners' willingness or ability to seek care for themselves. We use data from 6 questions about the positive and negative influence of pets on community dwelling older adults' administered to pet owners (N=223, age >=50 years) in the Baltimore Longitudinal Study of Aging. We use principal components analysis (oblique rotation) to extract dimensions of owner's perceptions of pet influences (PPI) and examine the relationship of these dimensions to owners' cognitive, physical functional, and psychological status. Three dimensions of PPI include: fiscal/health challenges (F1: 3 items, alpha=0.70), wellness promotion (F2: 2 items, alpha=0.80); and reason for social/travel constraints (F3: 1 item). In regression analysis with all factors entered simultaneously, after controlling for age, higher magnitude of F1 significantly independently predicted poor physical quality of life (p=.0007), greater perceived stress (p=0.041), and lower happiness (p=0.014); F2 did not independently predict any health outcome; higher F3 significantly independently predicted lower emotional vitality (p=0.048). Controlling for age, all three factors were independent predictors of pet attachment (p's=0.001, 0.010, 0.047, respectively). F1 and F3 were positively and F2 was negatively correlated with attachment. PPI was associated with owners' physical and mental health. Perhaps older adults with higher attachment to pets are more likely to keep them despite higher challenges.

### SENSORY LOSS AND COGNITIVE DECLINE AMONG OLDER ADULTS: AN ANALYSIS OF MEDIATION AND MODERATION EFFECTS OF LONELINESS

Shaoqing Ge,[1] Wei Pan,[2] Bei Wu,[3] Brenda Plassman,[2] XinQi Dong,[4] and Eleanor McConnell[2], *1. University of Washington School of Nursing, Seattle, Washington, United States, 2. Duke University, Durham, North Carolina, United States, 3. New York University, New York, New York, United States, 4. Rutgers University, New Brunswick, New Jersey, United States*

Multiple studies have reported that hearing and vision loss are linked to cognitive decline. Yet little is known about factors that may influence the association between sensory loss and cognitive decline. This study examined if loneliness mediates or moderates the impact of sensory loss on cognitive decline as individuals age. This was a longitudinal study using data (N = 243) from the Health and Retirement Study (HRS) (2006 – 2014) and its supplement: The Aging, Demographics, and Memory Study (ADAMS) (Wave C). Hearing loss was defined by an inability to hear pure-tone stimuli of 25 dB at frequencies between 0.5 – 4.0 kHz in either ear. Vision loss was defined as having corrected binocular vision worse than 20/40. Loneliness was measured by the 3-item UCLA Loneliness Scale. Longitudinal parallel-process (LPP) analysis was conducted at a significance level of $\alpha = .05$ (one-tailed). Loneliness moderated but did not mediate the associations between vision loss and the rate of cognitive decline (standardized $\beta = -.108$, $p < .05$). No moderation or mediation effect of loneliness was found for the association between hearing loss and cognitive decline. Both hearing and vision loss were significantly associated with increased severity of loneliness. Vision loss combined with an elevated level of loneliness may produce a more synergistic, deleterious impact on older adults' cognitive function than vision loss alone. This study highlights the importance of promoting a healthy social and psychological status for older adults with vision loss.

### THE EFFECT OF TECHNOLOGY USE IN REDUCING SOCIAL ISOLATION OR LONELINESS IN OLDER ADULTS: A SYSTEMATIC REVIEW

Yoon Chung Kim,[1] Gail Kohn,[2] Carenado Davis,[3] and Pamela Saunders,[1] *1. Georgetown University, Washington, District of Columbia, United States, 2. Age-Friendly DC, Washington, District of Columbia, United States, 3. Wake Technical Community College, Raleigh, North Carolina, United States*

The US population over 65 is projected to increase to 21% by 2050. Given mobility issues arising from health concerns, economic status changes, loss of friends and partners, older people are at a higher risk for social isolation and loneliness. Since the declaration of national emergency for COVID-19 on March 13, 2020, many older adults have not been able to connect with others in traditional ways. Instead, activities and contacts have been facilitated virtually via videoconferencing or phone calls to maintain physical and social distance. Amidst COVID-19, the transition to using technologies to connect socially and reduce loneliness has been a critical factor in preventing social isolation and loneliness. Identifying effective strategies involving the use of technology, designing new ways to deliver services virtually, and developing educational programs to promote technology is vitally necessary. This systematic review explored the relationship between technology use and social isolation or loneliness, and examined interventions that reduced social isolation in older adults. A unified strategy was used to systematically search seven databases (MEDLINE, PubMed, Embase, CINAHL, Web of Science, PsycInfo, and AgeLine) to examine qualitative and quantitative studies published in English between 2010 and 2020. Preliminary results indicate that technology can alleviate social isolation and loneliness in older adults despite some mixed results. The findings of this study will provide a foundation for policymakers and practitioners to shape policies and design programs that help older adults to alleviate social isolation and loneliness, particularly amidst the COVID-19 pandemic.

### THE ROLE OF MEASUREMENTS TOOLS IN THE ASSOCIATION BETWEEN LONELINESS AND COGNITIVE DECLINE

Ji Hyang Cheon, *University of Maryland, Baltimore, Maryland, United States*

Previous findings on the association between loneliness and cognitive decline are mixed, with some studies indicating a significant association and others finding no statistical relationship. Since studies have used various inequivalent measurement tools, this might explain inconsistent findings on the relationship between loneliness and cognitive decline. This systematic review aims to 1) summarize the relationship between loneliness and cognitive decline and 2) examine whether the association varies depending on the measurement tools. This review's inclusion criteria were studies with key terms loneliness as a predictor and global cognitive function as an outcome, peer-reviewed articles, written in English, involving community-dwelling older adults of ages 65 and older, and published since August 2013. Six out of ten studies showed that the relationship between loneliness and cognitive decline was statistically significant. Three reciprocal studies found that cognitive decline was a predictor of loneliness. Those studies that had nonsignificant findings used a single question to measure loneliness. Studies using the 23 item MMSE also reported non-significant association, whereas those using the 30 item MMSE had significant results. The findings indicate a bidirectional relationship derived from the studies with inconsistent results and the reciprocal studies and the potential role of measurement tools on the association's change. This poster will conclude with the research implication that a systematic review of reciprocal studies is needed to clarify the bidirectional relationship of variables. Comparison studies of various measurement tools are needed to confirm the role of measurement tools on the result.

### SESSION 10390 (LATE BREAKING POSTER)

### TECHNOLOGY

### A PILOT STUDY OF AN INNOVATIVE MODEL OF CARE DELIVERY: SERVICE ROBOT FOR OLDER PEOPLE WITH DEMENTIA IN A RURAL COMMUNITY

Kuan-Yuan Wang, Cheng-Sheng Chen, Hui-Mei Chen, and I-Te Chen, *Kaohsiung Medical University Hospital, Kaohsiung Medical University, Kaohsiung, Taiwan (Republic of China)*

Background: The telehealth approach offers enhanced service delivery for older patients living in rural areas. Purpose: We conducted a pilot study to evaluate the feasibility of using the Zenbo robot to improve the quality of care of elderly individuals with dementia. Methodology: In this study, we developed a digital solution on service robots and smart devices, which can leverage the capacity of the user-friendly interactive interface and digital dialog system. A group of eleven volunteered older adults was selected for this study. To assess the likability and acceptance of the Zenbo, we conducted a one-on-one (robot vs human) pilot study in our long-term care stations. Each participant engaged in conversational interactions for five consecutive days and completed a survey of 12 questions about the experiences they had with the Zenbo, at the beginning and the end of the study respectively. Results: Subjects with lower GDS-15 scores have more positive attitudes toward the robot before the intervention. 27% of older adults had an increase in positive attitudes toward the interaction with the Zenbo robot. With the aid of teleoperated mobile robotic systems at home, the innovative service model can be achieved through telecommunication between primary health professionals or caregivers at remote locations and psychiatrists at the medical center to make the seamless care environment real. Conclusion: The IoT technologies can be used to assist physicians in switching from a hospital-centered model of care to a home-based service for older people with dementia. It merits more future clinical trials and usability tests.

### ACCEPTANCE COMMITMENT THERAPY INTERVENTION FOR CUSTODIAL GRANDFAMILIES

Manuela E. Faulhaber, Amie Zarling, and Jeongeun Lee, *Iowa State University, Ames, Iowa, United States*

Millions of American children under the age of 18 are being cared for by their grandparents and without the presence of the biological parents. The number of custodial grandfamilies has significantly increased over the last five years. Recent studies have shown that custodial grandparents (CPGs) are often facing specific challenges in life, such as lower emotional well-being, higher parenting burden and stress related to this unique situation. Despite these findings, few interventions take a strengths based approach to improve their mental health and resilience. We describe our efforts to address these issues by proposing intervention anchored in the Acceptance and Commitment Therapy (ACT), emphasizing the importance of acceptance of challenging circumstances outside of one's control and promoting resilience among participants. The program consists of a web based ACT program with online coaching meetings, six common core sessions and six separate sessions for each age group over a time period of six months. This program is unique in the sense that it utilizes both individual and group session techniques to facilitate the learning process. Main active ingredients of this program are to promote effective coping strategies, to reduce parenting stress among grandparents and to increase life skills (i.e., decision-making, proactivity) among grandchildren. We are hypothesizing that participating in the ACT program will help CGPs to improve self-efficacy, emotional well-being, higher self-confidence, social competence, lower depressive symptoms, and parenting distress, thereby leading to positive outcomes such as improved mental health and higher resilience.

### BRIDGING THE DIGITAL DIVIDE FOR VULNERABLE PATIENT TELEHEALTH

Diab Ali,[1] Monica Gillie,[1] and Kathy Jo Carstarphen,[2] *1. University of Queensland - Ochsner Clinical School, New Orleans, Louisiana, United States, 2. Ochsner Health, New Orleans, Louisiana, United States*

In New Orleans, Louisiana, 61% of advanced age patients have low health literacy, 50% report financial insecurity, and more than one-third have high hospital readmission rates and medical complexity scores. This population has been disproportionately impacted by the COVID-19 pandemic and the system-wide transition to remote, virtual visits. While COVID-19 has accelerated needs for impactful remote care and research, this has been impeded by low telehealth literacy and structural barriers, such as lack of internet and devices. There are major gaps in the literature regarding telemedicine services in geriatric and complex populations and efficacy rates are variable in these populations. There is currently no telehealth literacy screening tool designed for identifying patients with barriers requiring additional interventions to succeed, no uniform understanding of the factors affecting use or how to increase engagement, and telehealth models requiring fluent use of technology for older adults have been met with poor rates of completed visits and associated harm. Following a dedicated 358.5 hours to training 309 geriatric, complex patients in telemedicine using our pre-existing telehealth model, averaging 4.78 hours per patient, only 18.8% of these patients were subsequently able to connect to the provider virtually. Here we describe lessons learned and tools developed from the Ochsner MedVantage Network Innovation project, including the development of a telehealth literacy screening tool, the adaptation and provision of simplified, user-friendly tablets, and a randomized control trial to determine if increased accessibility to telehealth leads to improved healthcare outcomes, such as decreased hospital admissions and emergency department utilization.

### INTERGENERATIONAL CONNECTIONS THROUGH TECHNOLOGY: INSIGHTS FROM THE TECHNOLOGY USE IN LATER LIFE MULTI-SITE STUDY

Shannon Freeman,[1] Hannah R. Marston,[2] Charles Musselwhite,[3] Janna Olynick,[1] Rebecca Genoe,[4] Cory Kulczycki,[4] and Beibei Xiong,[1] *1. University of Northern British Columbia, Prince George, British Columbia, Canada, 2. Open University UK, Milton Keynes, England, United Kingdom, 3. Swansea University, Swansea, Wales, United Kingdom, 4. University of Regina, Regina, Saskatchewan, Canada*

With enhanced challenges to maintain social connections especially during times of social distancing due to the COVID-19 pandemic, the need for technology solutions grow. Technologies have become interwoven into the daily lives for many older adults. The Technology Use in Later Life (TILL) study investigated how the perceptions and use of technology both can foster new and leverage existing intergenerational relationships. Through a mixed methods study engaging older adults aged 70 years of age and greater across rural and urban sites in Canada and the UK (N=37), participants described how the interconnection between technology and intergenerational relationships was an integral component to social connectedness with others. Through a qualitative descriptive approach, it was noted that older adults leveraged intergenerational relationships with family and friends to adjust to new technologies and to remain connected to adult children and grandchildren especially when there is high geographic separation between them. Especially during times of COVID-19, younger family members can play an important role to introduce and teach older adults how to use, technologies such as digital devices, computers, and social networking sites. Participants emphasized the benefits of intergenerational connections to adopt and use technology in later life noting flexibility and willingness to overcome barriers to technology adoption and remain connected across the generations. The adoption and uptake of technologies may continue as viable options during times of social distancing to support older persons to remain independent, age in place, in both age-friendly cities and across rural geographies during and post COVID-19.

### SOCIAL ASSISTIVE ROBOTS FOR ASSISTING ACTIVITY PROFESSIONALS

Jason Zamer,[1] Anne Adams,[1] Jenay Beer,[2] Xian Wu,[2] and Jane Komsky[1], *1. SimpleC, LLC, Atlanta, Georgia, United States, 2. University of Georgia, Athens, Georgia, United States*

Activity Professionals have high expectations for creating engaging and active resident social programming. A socially assistive robot (SAR) specifically designed for community-based settings has the potential to improve social programming. A SAR is suitable for engagement during times with social contact is restricted, such as COVID-19, other infectious outbreaks, weak immune system, or inability to move. We conducted an online survey to determine how a SAR can best support the responsibilities of Activity Professionals. Activity Professionals (N=19) completed the online questionnaire. Respondents (aged M=48.00, SD=12.87; 95% female, 100% native English speakers, 68% White/Caucasian, 21% Black/African American) were highly educated/experienced: 68% had a Bachelor's degree or above, and 53% had 10-35 years of experience. Respondents worked in Independent Living (68%), Assisted Living (37%), Memory Care (26%), Skilled Nursing (21%), or Personal Care (11%). Respondents rated their job as very demanding (8 out of 10). Differences existed in terms of physical and temporal demands. Job satisfaction was high (average 8 out of 10; SD= 2). Respondents reported enjoyment in preparing, personalizing, and running activities. Least preferred was gathering residents for activities. Respondents wanted more help, but it depended on the task. Qualitative data analysis showed that help was desired for motivating residents to join activities, group communication, and resident devices. A SAR, equipped with the ability to reach every resident's living quarter, has the potential to provide group communication, deliver engagement programs, and motivate residents to join events, providing Activity Professionals more time to engage with residents for more personal interaction.

### TRACING THE PAST: RENEWING LIFE NARRATIVES THROUGH ROBOTS

Anna Ueda,[1] and Hideyuki Takahashi,[2] *1. St. Cloud State University, Minneapolis, Minnesota, United States, 2. Osaka University, Toyonaka, Osaka, Japan*

The study explores the effectiveness and efficacy of using robotics in clinical settings to facilitate Life Review. Life Review is a process in which subjects retrospectively analyze major life events with a conversation partner in order to find meaning and to synthesize a narrative. In this experiment, Life Review was conducted with 5 elderly subjects and two types of partners: a human and a robot. The partners utilized a set of trigger questions to review past events with their subjects. Two sequences of Life Review, each comprising four sessions, were completed. Four sessions involved a human partner, and four involved a robot partner. The recorded correspondences in Life Review were transcribed, and the utterances of the participants with the two partners were compared and analyzed qualitatively. This preliminary study was the first attempt to explore the benefits of conducting Life Review with robotic conversation partners. The results showcased distinct differences between a human partner and a robotic partner. Specifically, subjects in sessions with a human partner showed stronger awareness of generational gaps between the human partner rather than the robotic partner. In contrast, sessions with a robotic partner included more universally transmissive values. The outcome suggests Life Review with robots can potentially provide elderly patients greater safety and comfort in telling their unique life narratives. The usage of robotic partners in Life Review provides a promising and novel research area into improving and re-imagining mental health access and outcomes for patients.

### USABILITY OF TELEMEDICINE IN RELATION TO ACCEPTABILITY, PSYCHOSOCIAL IMPACT, AND FUTURE USE

Katja Prevodnik, Simona Hvalič-Touzery, Vesna Dolničar, Maja Škafar, and Andraž Petrovčič, *University of Ljubljana, Ljubljana, Slovenia*

This poster presents the results of an intervention study exploring how engagement in telemedicine at home affects chronic patients' perceptions of usability and acceptability of the employed equipment, perceptions of its psychosocial impact, and intention of future use in the context of population aging. A purposively selected sample of 103 patients (mean age: 58 years) with chronic conditions (diabetes and/or hypertension) recruited in a community health center in Slovenia tested a home telemedicine system (TMS). After three months of utilization, an assessment of the relative importance of the usability and acceptance of TMS as factors influencing the patients' self-reported psychosocial perception of TMS and intention of future use was performed based on a proposed structural equation model explaining these interdependencies. The results confirmed four of eight tested hypotheses. Notably, the intensity of TMS use was found to affect the evaluation of its usability, the perception of its psychosocial impact, and the intent of future use. Usability was found to be the main factor directly influencing acceptability, perception of psychosocial impact and intent for future use, whereas acceptability did not significantly affect either the perception of the psychosocial impact of TMS or the intent of future use.

### SESSION 10400 (LATE BREAKING POSTER)

### CROSS CULTURAL

### ATTITUDES TOWARD OLDER ADULTS: A CROSS-CULTURAL APPROACH ACROSS RESIDENTIAL TOWNS IN SINGAPORE

Dylan Lien, Clara G.H. Chan, and W. Quin Yow, *Singapore University of Technology and Design, Singapore, Singapore*

Past research identified several variables that affect individuals' attitudes toward older adults (OA), such as age and experience living with OA. However, the effect of environmental variables on these attitudes, such as ethnic-culture and proportion of OA living in their neighborhood, remain unclear. Additionally, most previous studies sampled specific populations (e.g., undergraduate students), limiting generalizability. To address these limitations, we modified the Kogan's Attitudes Toward Old People Scale (Yen et al., 2008) and included it in a large-scale cross-sectional survey conducted among adult residents of three residential towns (TP, JE, PG) in multiracial Singapore. The towns varied in the proportion of OA in residence, with TP having the largest proportion, PG the smallest, and JE in between. In total, 3134 respondents completed the survey via interview. Exploratory factor analysis identified two factors: Appreciation and Prejudice. Multiple linear regressions revealed main effects of age and ethnicity, qualified by interactions of age with town, and age with ethnicity. Specifically, respondents from JE showed greater increase in Appreciation with age compared to those from TP (t=-2.04, p=.003) and PG (t=-2.95, p=.042). There were also increases in Appreciation with age among participants of three ethnicities (Chinese, Malay, Others; t=-3.95, p<.001; no increase with age for Indian participants). Separately, there was a main effect of age on Prejudice, where Prejudice increased with age (t=4.21, p<.001). Detailed analysis will be presented to elucidate the role of environmental variables on attitudes toward OA.

### MUSIC INTERVENTIONS FOR OLDER ADULTS IN CLINICAL TRIALS AND RESEARCH STUDIES ACROSS CULTURES AND SETTINGS

Tara Rose, Elyse Manzo, Katherine Erickson, and Joshua Valenzuela, *University of Southern California, Los Angeles, California, United States*

Music interventions and music therapy have become more common globally as nonpharmacological treatment options for memory loss, pain management, reduction of behavioral and psychological symptoms, and increased quality of life. Knowledge of multiethnic interventions is important when creating evidence-based programs within culturally diverse countries, such as the U.S. The purpose of this systematic review is to analyze music interventions for older adults across the globe to better understand emerging best practices. A review of all trials registered at clinicaltrials.gov and registries in the WHO Registry Network containing the key words "music therapy" were included, regardless of intervention type. Of the 627 studies generated, 449 met the eligibility criteria, with 11% enrolling only older adults and 89% enrolling older adults along with other age groups. Studies were conducted in 6 continents, 48 countries (23% in the U.S.), and in 23 languages. Music interventions for specific medical conditions (64%) or medical procedures (24%) were the primary foci in studies. While studies crossed multiple continents, less than 2% referenced ethnicity or culture in the study details. Detailed data on intervention types, demographics, measures, settings, and methodology will be presented. Results suggest that best practices in music therapy are being developed world-wide for the multitude of health challenges faced by older adults and demonstrate the diversity of music interventions in both medical and community settings. Information from this review can be used to improve the implementation of music intervention programs and may be particularly beneficial in countries with diverse multicultural populations.

### MUSIC THERAPY CLINICAL TRIALS IN CROSS-CULTURAL SETTINGS

Tara Rose, *University of Southern California, Los Angeles, California, United States*

Music therapy in clinical trials has shown efficacy as a nonpharmacological intervention for multiple medical conditions and procedures. Every culture has music and virtually everyone on this globe enjoys music suggesting the universality of music therapy. However, in the US, most music therapy clinical trials participants are English-speaking Caucasians. That narrow pool limits our understanding of the benefits of music in an ethnically and culturally heterogeneous nation. This study looks to the international clinical trials for lessons and information that can advance U.S. studies by expanding the methodology and clinical reach to benefit a more extensive population of patients. A review of 449 studies in 48 countries from clinical trials registries supports an effort to expand music therapy studies and interventions by incorporating a cross-cultural perspective. Researchers and clinicians using international resources can increase their understanding and capacity. Globally, many standardized measures have been translated, including self-report measures of behavioral and mental health, pain, sleep, medical conditions, and symptom severity used for outcome measures, as well as music therapy measures and intervention checklists. Scientifically accepted physiological outcome measures have shown the benefits of music interventions for older adults regardless of cultural or ethnic differences. For example, neuroimaging research supports the clinically derived notion that music can address needs of people with dementia. The future will require new standards for multicultural research. To expand studies and methodologies, we need to include more diverse populations. This paper proposes that to do that, we must look to the global scientific community.

### SESSION 10500 (LATE BREAKING POSTER)

### COVID-19 PANDEMIC

### "DOING THE IMPOSSIBLE WITH THE INADEQUATE": COVID-19 RESPONSE IN U.S. ASSISTED LIVING SETTINGS

Sarah Dys,[1] Jaclyn Winfree,[2] Paula Carder,[2] Kaylin Dugle,[2] Sheryl Zimmerman,[3] and Kali Thomas,[4] *1. Institute on Aging, Portland, Oregon, United States, 2. Portland State University, Portland, Oregon, United States, 3. University of North Carolina at Chapel Hill, Chapel Hill, North Carolina, United States, 4. Brown University, Providence, Rhode Island, United States*

The COVID-19 pandemic has disproportionately affected long-term care operators, staff, residents and their families; although much attention has been given to nursing homes, largely lost in the discourse are assisted living, residential care, and dementia care (AL) communities. As part of a broader, ongoing study assessing states' AL regulations regarding medical and mental health care for residents with Alzheimer's and related dementias (ADRD), stakeholders across the United States were recruited in July and August 2020 for semi-structured interviews to provide their perspectives on AL policies and practices specific to COVID-19 response. Stakeholders (n=32) consisted of state healthcare and trade association representatives, clinical practitioners, operators, researchers, and dementia care experts experienced in AL-related operational, healthcare, and regulatory affairs. Using thematic analysis, we describe several emerging topics regarding the opportunities, challenges, and innovations of responding to COVID-19 within the unique context of AL. States' public health responses to COVID-19 lacked an understanding of the broader long-term care system, especially AL's scope and purpose, workforce, capacity to implement infection control practices and policies, and unintended consequences of social isolation for older adults, specifically residents living in dementia care units. Despite these challenges, stakeholders described opportunities to expand telehealth infrastructure, communication and collaboration across states and among operators, and several innovations to mitigate the effects of social isolation. It is imperative for policymakers to understand the nuances of the AL context and design regulations and public health responses grounded in a whole-person perspective and in partnership with operators during, and beyond, pandemic circumstances.

### "WHEN YOU ARE WORKING IN THIS ENVIRONMENT, YOU'RE MORE LIKELY TO GET SICK": MAPPING CARE RELATIONSHIPS IN LTC

Andreina Marquez de la Plata Gregor,[1] Katie Aubrecht,[1] Tamara Daly,[2] Ivy Bourgeault,[3] Susan Braedley,[4] Prince Owusu,[4] Pat Armstrong,[2] and Hugh Armstrong,[4]
*1. St. Francis Xavier University, Antigonish, Nova Scotia, Canada, 2. York University, Toronto, Ontario, Canada, 3. University of Ottawa, Ottawa, Ontario, Canada, 4. Carleton University, Ottawa, Ontario, Canada*

The pandemic has shone a light on problems within the long-term care (LTC) sector. As was true prior to COVID-19, many of the present issues in LTC can be traced to challenging working conditions, such as persistent understaffing of care workers. Working short-staffed means rushing through care, while only satisfying the most basic bodily needs of the resident. This presentation shares early findings from a thematic analysis of interviews conducted with seven care workers as part of the "Mapping Care Relationships" stream of the Seniors –Adding Life to Years (SALTY) project, a pan-Canadian research program that maps how promising approaches to care relationships are organized and experienced in LTC. The purpose of the analysis was to understand how short-staffing is affecting the formation and preservation of meaningful staff-resident relations, and what the impact is on quality of care. Two overarching themes emerged: 1) a relationship between time and work-place illness, injury and violence; 2) a relationship between care worker autonomy and resident quality of care. When working conditions do not support workers in voicing and/or addressing challenges they experience in the workplace, whether this results from understaffing or hierarchical power structures, care workers' ability to deliver even basic care is jeopardized, and resident and worker health and wellness are placed at risk. Themes are discussed in the context of COVID-19 in light of responses to outbreaks in LTC that have reduced the availability of care workers, family visitors and volunteers, and emphasized top-down and even militarized approaches to care management.

### #BOOMERREMOVER: COVID-19, AGEISM, AND THE INTERGENERATIONAL TWITTER RESPONSE

Antonius Skipper,[1] and Daniel Rose[2], *1. Georgia State University, Atlanta, Georgia, United States, 2. Winston-Salem State University, Winston-Salem, North Carolina, United States*

In March 2020, COVID-19 was declared a pandemic and frequently presented as a virus primarily affecting older adults. News headlines led with statements such as, "Coronavirus deaths are so far mostly older men" (Ramzy, 2020). Although later determined inaccurate, this perspective contributed to openly ageist views and exchanges from people around the world. On the social media platform of Twitter, #BoomerRemover was used as a hashtag to express views related to older adults, and particularly baby boomers, as the primary targets of COVID-19. This study uses qualitative methods to analyze the messages of Twitter users that discuss COVID-19 with the use of the hashtag #BoomerRemover. A total of 1,875 tweets posted in English and including the hashtag "#BoomerRemover" from March 16, 2020 to March 30, 2020 were analyzed. Analytic methods employed an open coding procedure consistent with grounded theory and Numeric Content Analysis (Marks, 2015). Salient themes include: (1) COVID-19 is Politically Driven (2) There's a Real Intergenerational Divide, (3) Young People are Dying Too, and (4) #BoomerRemover is Simply Disrespectful. Findings suggest that only about a fourth of #BoomerRemover tweets could be considered ageist, and the large majority of tweets using the hashtag were related to politics and elections. Further, several of those using the #BoomerRemover hashtag to defend older adults were inadvertently causing it to remain relevant (trend) as a Twitter topic. This study recognizes the importance of considering Twitter – primarily composed of young adults – as a place where intergenerational attitudes vis-à-vis COVID-19 may be expressed.

### A NOVEL SURVEY PLATFORM IN THE AGE OF COVID-19 TO INCREASE ACCURACY AND ADOPTABILITY WHILE REDUCING SELECTION BIAS.

Dinesh Mendhe, Stephanie Bergren, and XinQi Dong, *Rutgers University, New Brunswick, New Jersey, United States*

Given the ongoing COVID-19 pandemic, secure and distanced data collection platforms are critical for reaching vulnerable populations. Commonly used electronic data collection systems lack a myriad of critical features, including a modern technology stack, new data encryption and security standards, study workflows, and reporting algorithms. Moreover, these systems do not have multilingual mapping functionalities of survey and consent forms. All of these components ultimately increase selection bias while simultaneously reducing the security and quality of the response data. In order to directly address the aforementioned issues, we have developed a multilingual and highly secure data management platform. Our application is built using stable, tested, and modular programming frameworks and design patterns targeted at accommodating intricately complex structures of polyglot mapping, large volume of data, encryption and granular user authorization. The statistical accuracy along with the multilingual mapping are the core highlights of this system. The multilingual function of this platform has the ability to eliminate selection biases while creating a well-balanced cross-section of society. Modern survey design workflows and validation checks ultimately prevent data loss and help reduce data collection errors. The platform design was initiated in April 1, 2020 and has been pilot tested for use in multilingual populations. The currently active application version of the system is capable of supporting in-person and telephone interviews, emailing survey links to every registered participant, building family tree architecture, and online consent management. This platform also has built-in report functionality. Additional features are being explored to improve study coordination and monitoring.

### A SURVEY OF OLDER ADULTS' PERSPECTIVES OF IN-PERSON AND VIRTUAL PARKINSON'S-SPECIFIC EXERCISE CLASSES

Holly Bennett, Jennifer Vincenzo, and Chris Oholendt, *University of Arkansas for Medical Sciences, Little Rock, Arkansas, United States*

Due to COVID-19, many health/wellness programs transitioned from in-person to virtual. This mixed methods study aims to explore older adults with Parkinson's disease (PD) perceptions of in-person versus virtual Parkinson's-specific exercise classes. Attitudes, perceptions, and experiences were determined through focus groups (n=9; Male=4; aged 75 years) among older adults with PD and an online survey (n=23; Male=14; aged 74 years). Eighteen respondents attended both in-person and virtually (n=18; Male=9). Four respondents only attended in-person, citing reasons such as difficulty with computer access to virtual classes, limited internet, easier accessibility to in-person classes, and physical injury preventing attendance to any classes. Respondents who participated in both delivery methods preferred virtual classes. Time, convenience, comfort at home, and not having to navigate transportation barriers supported participants' preference for virtual classes. The majority of respondents indicated their fatigue and mental health were either unchanged or improved. Eighty-nine percent reported improved mobility since attending either class; specifically, in balance (n=8), flexibility (n=7), and coordination (n=3). Older adults with PD who attended both classes had minimal difficulty with computer usage and accessing the virtual program with only one participant reporting difficulty transitioning from in-person to virtual classes. Seventy percent stated they would enjoy a combination of on-site and virtual programming. Eighty-nine percent and seventy-seven perfect felt the virtual-based program was safe and beneficial, respectively. Participants who transitioned from an in-person to a virtual exercise program for people with Parkinson's disease felt the program was safe, effective, and improved or prevented declines in their mobility.

### AGE DIFFERENCES IN PREFERENCES FOR FEAR-ENHANCING VERSUS FEAR-REDUCING NEWS IN A GLOBAL PANDEMIC

Anthony Villalba, Jennifer Stanley, Jennifer Turner, and Michael Vale, *The University of Akron, Akron, Ohio, United States*

Older adults (OA) prefer positive over negative information in a lab setting, compared to young adults (i.e., positivity effects; YA). The extent to which OA avoid negative events or information relevant for their health and safety is not clear. We first investigated age differences in preferences for fear-enhancing versus fear-reducing news articles during the Ebola Outbreak of 2014. We built upon this pilot study to further investigate this research question during the COVID-19 pandemic. In this study, 164 YA (18-30 years) and 171 OA (60-80 years) responded to an online survey about their preferences, feelings, and behaviors related to the COVID-19 pandemic across 13-days during the initial peak of the pandemic in the United States. Both YA and OA preferred to read positive over negative news about the coronavirus, but OA were even more likely than YA to prefer the positive news article. No age differences in the fear of contraction were found, but OA engaged in more health-protective behaviors compared to YA. Additionally, media engagement was related to fear for both age groups, with social media engagement, specifically, emerging as a key moderating factor for protective behavior change. Although OA may not fear or seek out negative information related to a health concern; they still engage in more protective health behaviors compared to YA. In this study, positivity effects are shown to exist within a health-related event, but OA appeared to still attend to enough negative information about COVID-19 to avoid impairing their health protective behaviors.

### AGE DIFFERENCES IN THE CONSUMPTION AND AVOIDANCE OF COVID-19 INFORMATION

Stephanie Deng, Julia Nolte, and Corinna Loeckenhoff, *Cornell University, Ithaca, New York, United States*

Staying informed about COVID-19 is crucial to maintaining public health. Although older adults are at increased risk of complications, recent data (Global Web Index, 2020) suggest that they are less likely to seek out information about COVID-19. This is consistent with prior evidence indicating that information seeking is negatively associated with age (Mata & Nunes, 2010). However, it remains unclear whether older adults merely fail to seek information or intentionally avoid information. In response, we examined whether age is associated with general information seeking and deliberate information avoidance in the wake of the COVID-19 pandemic. Based on previous work indicating age-related shifts in motivational priorities (Carstensen, 2006) we also examined whether avoidance motives differ by age. In a pre-registered online study, an adult lifespan sample (N=500, Mage=49.90, 51% female, 67% non-Hispanic White) completed self-report measures of media consumption, information avoidance, and avoidance motives with respect to the COVID-19 pandemic. In addition, we measured behavioral information avoidance by allowing participants to opt out of receiving valid COVID-19 information. As predicted, age was associated with decreased media consumption (p<.001) and higher information avoidance about COVID-19 on the behavioral measure (p<.01). Self-reported information avoidance, in contrast, was highest among younger adults (p<.05). Further, with the exception of concerns about trustworthiness, older adults were less likely than younger adults to endorse various information avoidance motives (ps<.05). Thus, although information seeking is lower and behavioral information avoidance about COVID-19 is higher in later life, this cannot be traced to explicit intentions or select motives.

### AGE DOES NOT PREDICT MORTALITY IN HOSPITALIZED COVID-19+ OLDER ADULTS: RETHINKING RESOURCE ALLOCATION BASED ON AGE

Liron Sinvani,[1] Allison Marziliano,[1] Alex Makhnevich,[1] Meng Zhang,[2] Maria Carney,[3] Michael Diefenbach,[4] Karina Davidson,[5] and Edith Burns,[1] *1. Zucker School of Medicine at Hofstra/Northwell, Manhasset, New York, United States, 2. Feinstein Institute, Hofstra Northwell School of Medicine, Manhasset, New York, United States, 3. Northwell Health, New Hyde Park, New York, United States, 4. Northwell Health, Manhassett, New York, United States, 5. Northwell Health, New York, New York, United States*

Older adults are disproportionately affected by the coronavirus (COVID-19) pandemic. While age has been used to guide resource allocation based on studies implicating age as the main risk factor for COVID-19-related mortality, most did not account for critical factors such as baseline functional and cognitive status, or life-sustaining treatment preferences. The objective of this study was to determine whether age is independently associated with mortality in older adults hospitalized with COVID-19. We conducted a retrospective cohort study of adults age 65+ with confirmed COVID-19 hospitalized in the greater NY metropolitan area between 3/1/20-4/20/20. Primary outcome was in-hospital mortality, with age as the primary predictor. Multivariate logistic regression was used to evaluate association between age and in-hospital mortality after controlling for demographics, severity of acute illness, comorbidities, and baseline function, cognition, and life-sustaining treatment preferences. 4,969 patients were included, average age 77.3, 56.0% male, 46.8% White, 20.8% African American, 15.1% Hispanic. Common comorbidities included hypertension (61.1%), and diabetes (36.8%); average number of comorbidities was 3.4 (SD 2.8) and 13.0% had dementia. 20.8% arrived from a facility and 5.7% had early do-not-resuscitate orders. On arrival, the Modified Early Warning System score was 4.2 (SD 1.7) and 79.6% required oxygen therapy. 35.3% of patients expired. In multivariate analysis, age was not independently associated with mortality (p = .173). Functional status, multi-morbidity, life-sustaining treatment preferences, and illness severity, not age, were associated with mortality among older adults hospitalized with COVID-19, suggesting age should not be used as the main indicator to guide resource allocation.

### AGE VARIATIONS IN PERCEIVED COVID-19 THREATS, NEGATIVE IMPACTS, AND ASSOCIATIONS WITH WELL-BEING

Tara Gruenewald,[1] Anthony Ong,[2] and Danielle Zahn,[1] *1. Chapman University, Orange, California, United States, 2. Cornell University, Ithaca, New York, United States*

The COVID-19 pandemic represents an unprecedented threat to individual and public health, psychosocial, and economic well-being, although COVID-19 threats and impacts may vary by age and other demographic characteristics. Although greater age is a risk factor for greater COVID-19 disease severity, we know little about the association between age and perceived and experienced COVID-19 threats and their association to well-being. These associations were examined in an ongoing 3-wave investigation of over 1,700 U.S. adults (age 18-89; 53.1% female). Wave 1 analyses indicate no significant age variation in perceived threat of COVID-19 infection, with older and younger individuals reporting similar levels of COVID-19 infection threat. However, greater age was associated with lower perceived negative impact on financial and needed resources (r=-.10**), lower perceptions of COVID-19 induced harm to mental well-being (r=-.17**), and more favorable well-being profiles. Greater perceived COVID-19 threat and negative impact on resources and well-being were linked to greater feelings of stress (β's=.45 to .68***), loneliness (β's=.24 to .49***), social well-being (β's=-.19 to -.36***), and poor sleep quality (β's=.34 to .51***). These associations did not vary with age with the exception that older individuals showed stronger links between COVID-19 threat and impacts and poorer sleep quality. Ongoing analyses are examining whether these associations persist over time. Despite older adults' greater risk of COVID-19 disease severity and mortality, older age did not appear to be linked to greater perceived COVID-19 threat or impacts, nor linkages to ill-being, with the possible exception of potential greater vulnerability to poor sleep quality.

### AGEISM IN COVID-19-RELATED MEDIA COVERAGE: EXAMINING PUBLICATIONS DURING THE FIRST MONTH OF THE PANDEMIC

Mijin Jeong,[1] Sarah Jen,[2] Hyun Kang,[2] and Michael Riquino,[2] *1. University of Kansas School of Social Welfare, Lawrence, Kansas, United States, 2. University of Kansas, Lawrence, Kansas, United States*

The media has consistently described older adults as the population most vulnerable to COVID-19. Anti-ageism critics have taken issue with the oft-repeated statement that "only" older adults are at risk, a construction that dismisses and devalues the nuances within this population. The purpose of this study was to analyze instances of ageism in national media sources during the first month of the COVID-19 pandemic. A systematic search returned 287 articles concerning older adults and COVID-19 published in four major newspapers in the United States—USA Today, The New York Times, Los Angeles Times, and The Washington Post—between March 11 and April 10, 2020. Combining the strengths of content analysis and critical discourse analysis, we deductively and inductively reviewed the articles for patterns related to implicit and explicit forms of ageism. While ageism was rarely discussed explicitly, ageist bias was evident in implicit reporting patterns, such as frequent use of the phrase the elderly, which was often paired with statements describing older adults as vulnerable. Infection and death rates among older adults, as well as institutionalized care practices, were among the most commonly reported topics, providing a limited portrait of aging during the pandemic. While some authors utilized a survivor narrative by portraying older adults as having survived hardships, this construction implicitly places blame on those unable to do so. Older adults, when quoted directly, produced more complex and nuanced narratives of aging during the COVID-19 pandemic. Such narratives can combat societal ageism and promote self-determination and -definition.

### ALCOHOL USE AND MENTAL HEALTH AMONG OLDER AMERICAN ADULTS DURING THE EARLY MONTHS OF THE COVID-19 PANDEMIC

Marisa Eastman, Lindsay Kobayashi, and Jessica Finlay, *University of Michigan, Ann Arbor, Michigan, United States*

We investigated the association of self-reported changes in alcohol consumption with the prevalence of anxiety, depression, and loneliness in the early months of the COVID-19 pandemic among middle-aged and older US adults. Between April and May 2020, 6,938 US adults aged 55+ completed online questionnaires in the COVID-19 Coping Study, a national cohort study of older adults' mental health and well-being. Multinomial logistic regression estimated self-reported changes in the frequency of alcohol consumption relative to before the pandemic, according to anxiety (5-item Beck Anxiety Inventory), depression (8-item Center for Epidemiologic Studies Depression Scale), and loneliness (3-item UCLA Loneliness Scale). All models were population-weighted and adjusted for confounders. Nearly half (46%) of adults reported drinking 1-7 drinks/week prior to the pandemic, 12% reported drinking 8+ drinks/week, and 42% reported not drinking. One in five adults (21%) reported a change in their alcohol consumption since the start of the pandemic, while 38% indicated they were drinking the same amount, and 42% reported not drinking alcohol. Older adults who screened positive for each of anxiety, depression, and loneliness reported drinking more than usual (OR=1.92; 95% CI: 1.92–1.93 for anxiety; OR=2.67; 95% CI: 2.67–2.68 for depression; OR=2.46; 95% CI: 2.45–2.46 for loneliness), compared to drinking the same as before the pandemic. These results demonstrate potentially negative changes in alcohol intake among middle-aged and older adults experiencing mental health symptomology during the early months of the COVID-19 pandemic.

### AN ACTION RESEARCH METHOD FOR GENERATING HUMAN-CENTERED COVID-19 CAREGIVING INTERVENTIONS

Natalie Leonard,[1] Alicia Carmichael,[1] Jeannette Jackson,[1] Linde Huang,[1] Aalap Doshi,[1] Amanda Leggett,[2] Sheria Robinson-Lane,[1] and RIchard Gonzalez,[1]
*1. University of Michigan, Ann Arbor, Michigan, United States, 2. The University of Michigan, Ypsilanti, Michigan, United States*

Caregiving for post-intensive care COVID-19 patients is an important determinant of successful recovery, including the reduced likelihood of ICU readmission. With possible ICU readmissions coinciding with a second wave of the pandemic, researchers and clinicians at the University of Michigan sought to develop a patient and caregiver-informed intervention that was remote, accessible, and could be immediately delivered. The resulting study, Health Enhanced by Adjusting and Recovering Together, reinforces these imperatives common in action research frameworks. Action research, emerging itself from a tumultuous time (1930s-40s) paralleling the COVID-19 pandemic, is a pragmatic research approach with the explicit goal of resolving a community problem or enacting social change--and doing so quickly. Here, we demonstrate a unique method for rapid intervention development that intertwines elements of (a) Human-centered design, for the purpose of a people-focused outlook, (b) Action research, for the purpose of rapid intervention, and (c) Traditional qualitative analysis, for the purpose of knowledge creation. The result of this combined method is an efficiently developed intervention that, while imperfect, is a user-centered, contextually-relevant viable product that can be quickly disseminated, tested, and further refined. The method presented is timely and relevant to other clinical and research teams addressing caregiving during the COVID-19 pandemic.

### BEREAVEMENT AND PHYSIOLOGICAL DYSREGULATIONS IN AFRICAN AMERICAN ADULTS

Jieun Song,[1] and Marsha Mailick,[2] *1. Waisman center, Madison, Wisconsin, United States, 2. University of Wisconsin - Madison, Madison, Wisconsin, United States*

This study uses data from National Survey of Midlife in the U.S. (MIDUS) to examine the effect of bereavement on physiological dysregulations in African American adults, with moderating effects of gender. Models were estimated using data from 210 Non-Hispanic African American respondents who participated in MIDUS 2 (M2: 2004-2005) and the biomarker data collection (2004-2009). We analyzed data from two groups, respondents who experienced the death of an individual(s) close to them, either family or friends (97 women, 40 men) and respondents who did not experience any deaths of close individuals during the same period (46 women, 27 men), controlling for age, education, marital status, prior family bereavement, number of negative life events since M2, and physical health prior to bereavement. Physiological dysregulations were assessed for 7 systems: HPA axis, glucose metabolism, lipids metabolism, sympathetic system, parasympathetic system, inflammation, and cardiovascular functioning. The results show that African American men and women who experienced bereavement were at higher risk of dysregulation of glucose metabolism (assessed by HbA1c, HOMA-IR, and fasting glucose) than the non-bereaved, even after adjusting prior diabetes diagnosis. In addition, African American women (but not men) who experienced recent bereavement were at higher risk of dysregulation of HPA axis functioning (assessed by urinary cortisol and blood DHEA-S) than their counterparts. The other physiological systems were not significantly associated with bereavement experience in African American adults. The findings suggest that bereavement has adverse impacts on health in African American adults via dysregulations in glucose metabolism and HPA axis functioning.

### BRIEF TELEPHONE INTERVENTION FOR CAREGIVERS OF MEDICALLY COMPLEX PATIENTS DURING COVID-19

Mary Stevens-Carr,[1] Kevin Sethi,[2] Channing Cochran,[2] Margaret Bencomo-Rivera,[3] and Janice Marceaux,[2]
*1. South Texas Veteran Healthcare System, San Antonio, Texas, United States, 2. South Texas Veterans Health Care System, San Antonio, Texas, United States, 3. STVHCS, San Antonio, Texas, United States*

The VA Home Based Primary Care (HBPC) program consists of an interdisciplinary team, including psychology, serving veterans with complex medical conditions who are supported by live-in caregiver(s). HBPC psychologists may work with caregivers to address caregiver stress. Some veterans enrolled in HBPC attend Adult Day Care (ADC) programs, allowing respite for caregivers. At the onset of COVID19 pandemic, ADC centers closed to minimize spread of the virus. The authors identified these caregivers to be at high risk for burnout and sought to develop a protocol to assist these caregivers via telephone and evaluate outcomes. PreCOVID-19 caregiver stress was known via a 4-item Zarit Caregiver Burden annual screening (Bédard et al., 2001). Following ADC closures, caregivers of veterans enrolled in ADC programs were contacted and re-administered the Zarit to determine impact of COVID-19 on caregiver stress. Caregivers of veterans not attending ADC were also contacted for comparison. Contacted caregivers were provided a brief CBT-based intervention via telephone, and post-intervention Zarit screening was administered after two weeks. Ultimately, 4 ADC caregivers and 4 non-ADC caregivers were contacted and provided with services before ADC centers reopened. Statistical analysis via mixed model ANOVA did not yield significant results, likely due to small sample size, although there was a large effect size (η_p^2 =.566). ADC caregivers generally reported increased stress from baseline following ADC closure and reduced stress following provision of intervention. The authors will present caregiver feedback about aspects of telephone intervention that were helpful, and not helpful, as well as authors impression.

### CHALLENGES, BARRIERS, STRATEGIES IMPLEMENTING AN ORAL HYGIENE PROGRAM IN ASSISTED LIVING FACILITIES POST COVID-19

Kim Attanasi and Victoria Raveis, *New York University, Maplewood, New Jersey, United States*

[Objective] Almost 8% of the U.S. population, 65 and older, reside in long term care facilities with limited delivery of essential dental care to prevent and manage oral health disease. By 2050, this population is expected to increase by 1.6 billion. Multiple bi-directional connections exist between oral disease and overall health. [Methods] Faculty from the Dental Hygiene Department, New York University College of Dentistry conducted an extensive outreach effort and randomly selected assisted living facilities. Facilities were offered the opportunity to receive at no-cost, a dental hygiene-led, educational, preventive oral health program delivered virtually to their residents as a community service. Incentives discussed. [Results] Twenty-one facilities were contacted, 17 (94.4%) had no oral healthcare program; one had an oral health component. In 13 (72%), the concierge functioned as gatekeeper, unwilling to transfer calls or deliver messages. In five (28%), calls were directed to the activity coordinator. Feasibility concerns and uncertainty about oral health service necessity and resident safety were voiced. Two facilities mentioned familiarity with dental hygiene professionals. Strategic changes in outreach resulted in successfully engaging with facility administrators. Strategies included identifying directors with familiarity or experience with dental hygiene profession, establishing a portfolio and utilizing technology that facilitate incorporating COVID-19 protocols. [Conclusions] Efforts to initiate a dental hygiene-led virtual oral health program encountered gatekeeper challenges. Although facility activity coordinators acknowledged benefits for their population, they were not final decision-makers. It was necessary to implement strategies that facilitated discussing the virtual oral hygiene program directly with the facility's executive leadership.

### CHANGES IN FAMILY CAREGIVER ROLES AND INTERACTIONS DURING THE COVID-19 PANDEMIC

Rajean Moone,[1]
Elizabeth Lightfoot Kamal Abdi Suleiman,[2]
Courtney Kutzler,[3] Jacob Otis,[3] Kenneth Turck,[3] and Heejung Yun,[3] *1. University of Minnesota, Woodbury, Minnesota, United States, 2. University of Pennsylvania, St. Paul, Minnesota, United States, 3. University of Minnesota, St. Paul, Minnesota, United States*

Family caregivers provide the majority of support for older adults and people with disabilities in the U.S. The onset of the COVID-19 pandemic forced radical changes in duties and relationships between family caregivers and care recipients. These changes can be attributed to fears of virus transmission as well as federal, state and local government mitigation strategies resulting in social distancing and quarantining limiting caregiving interactions. This qualitative investigation conducted 55 Zoom interviews over summer 2020 with family caregivers to explore their changing roles and duties during the pandemic. Researchers utilized a semi-structured interview guide to explore caregiver experiences with COVID-19. The average age of the caregiver participants was 59 and the average age of the care recipients for whom they provided care was 74. All participants provided unpaid care for family members. Interviews were conducted in English (n=40), Spanish (n=5), Somali (n=5) and Korean (n=5). Care recipients resided in a facility (nursing home, memory care, ICF-DD, or other assisted living) (70%) with the caregiver (20%), and in a separate independent setting (10%). Data from each interview were coded into themes by two researchers. Themes that emerged from the analyses included concerns about care recipient mental and physical health deterioration, lack of communication from formal providers, change in relationships with other family members, and future concerns. Implications for additional research and practice are included.

### CHANGES IN PHYSICAL ACTIVITY DURING COVID-19 PANDEMIC IN URBAN AND RURAL OLDER VETERANS AND THEIR COHABITANTS

Chao-Yi Wu,[1] Rachel Wall,[2] Zachary Beattie,[1] Nora Mattek,[1] Jeffrey Kaye,[3] Hiroko Dodge,[4] and Lisa Silbert,[1] *1. Oregon Health & Science University, Portland, Oregon, United States, 2. Portland Veterans Affairs Medical Center, Portland, Oregon, United States, 3. Layton Alzheimer's Disease Center, Portland, Oregon,*

*United States, 4. Pregon Health & Science University, Portland, Oregon, United States*

Background: The COVID-19 pandemic has changed the health security of older adults. Few have examined how older US veterans have reacted and coped with the COVID-19 pandemic. We aimed to identify changes in physical health and their differential impact by rurality of older veterans. Method: Participants were veterans (aged ≥ 62 years) and their cohabitants, living in the Pacific Northwest, enrolled in the Collaborative Aging Research using Technology (CART) initiative. Daily step counts via actigraphy were collected from January 1st to July 8th, 2020. COVID-19 time periods were determined by stay-at-home orders issued on March 13th, 2020. Generalized estimating equation models were used to examine changes in physical activities associated with COVID-19 time periods and rurality indicated by the rural-urban commuting area score. Results: A total of 102 participants were included in the analysis (mean age = 71.0 years, 56% male, 32 living in urban areas). Daily average step counts were 2318 and 3012 before and after COVID-19 stay-at-home orders (t=4.85, p<.001). After controlling for covariates, participants living in large rural (β=.26, p=.03) and small/isolated areas (β=.23, p=.02) walked more than those living in urban areas after COVID-19 stay-at-home orders. Conclusion: Older adults cope differently during the COVID-19 pandemic based on rurality, with those living in large rural and small/isolated rural areas have an increased physical activity. Reasons for increased step counts (e.g., mood, visitors, size of the house) require further investigation. This result demonstrates the potential utility of real-world monitoring to objectively inform interventions for COVID-related secondary health changes.

### COMMUNITY ENVIRONMENT AND COVID-19 RELATED STRESS AMONG OLDER ADULTS WITH DISABILITIES IN TAIWAN

Meng-Hsuan Yu,[1] and Shiau-Fang Chao,[2] *1. National Taiwan University, Taipei City, Taipei, Taiwan (Republic of China), 2. National Taiwan University, Taipei City, Taiwan (Republic of China)*

The outbreak of the COVID-19 pandemic at the beginning of 2020 forced many countries to implement social distancing policies such as the suspension of activities and gathering. Taiwan is the leading country which took active epidemic prevention measures in local communities, including closing the community centers and programs for older adults. Older adults with chronic health conditions are particularly vulnerable to the COVID-19 pandemic because they have disproportionally been affected by it. This study examined the associations between community environment and COVID-19 related stress of community-dwelling older adults with disabilities. Data were collected from a sample of 547 community-dwelling older adults aged 65 and over with disabilities in Taiwan between April and July, 2020. Multiple Regression Analysis was applied to test the hypothesized relationships. The analytic results suggested that: First, participants who were younger and with better cognitive functioning had higher levels of COVID-19 related stress. Second, as the confirmed case number dropped by month, participants interviewed in the later months expressed lower levels of COVID-19 related stress. Third, older adults who perceived more obstacles in their environment reported higher levels of COVID-19 related stress. In conclusion, although restrictions during the pandemic is inevitable to secure the safety of the public, programs should be designed for older adults with disabilities to remove the obstacles and to make information, policies and services more accessible in the communities to mitigate their COVID-19 related stress.

### COMPARING THE PROTECTIVE VALUE OF HUMAN AND PET SOCIAL SUPPORT ON WELL-BEING OF OLDER ADULTS DURING COVID-19

Juliet Sobering,[1] and Lisa Brown,[2] *1. Palo Alto University, San Jose, California, United States, 2. Palo Alto University, Palo Alto, California, United States*

Older adults are vulnerable to particular risk factors that contribute to lower well-being and poorer functioning. With the COVID-19 pandemic, the importance of social support has been highlighted in media reports because of its well-known beneficial effects on overall well-being. However, as adults age, social networks, contacts, and activities naturally decrease. These age-related losses are often difficult, if not impossible, to replace. Pets have recently been recognized as a valuable source of social support for many older adults, providing both physical and psychological benefits through mutual connection and behavioral activation. Previous studies have examined how human social support or pet social support enhance older adults' well-being (i.e., positive emotions, engagement, relationships, accomplishment, and meaning). However, there is a gap in our scientific knowledge as previous research has not evaluated if pet social support can serve as a protective factor in the absence of adequate human social support. Current analyses, with 141 older adult participants, suggests that pet owners with a positive attachment to their pet experience higher well-being as pets serve as a coping resource that protects against common life stressors. Similar to human social support, pet social support appears to be a protective factor that also promotes and fosters a sense of well-being in older adults. Support in late life is especially important for families and agencies to be attuned to, especially during a global pandemic.

### CONVERTING PHYSICAL FUNCTION TESTING TO THE REMOTE SETTING: ADAPTING OUR RESEARCH PROTOCOL DURING COVID-19

Kerri Winters-Stone,[1] Colin Lipps,[2] Carolyn Guidarelli,[3] and Pablo Herrera-Fuentes,[2] *1. Oregon Health & Science University, Portland, Oregon, United States, 2. Oregon Health & Science University, portland, Oregon, United States, 3. Oregon Health & Science University, PORTLAND, Oregon, United States*

Objective measurement of physical function can be a more sensitive predictor of future disability and changes over time than self-report measures. However, objective measures require in-person assessments which can limit their use in hard-to-reach populations. During the COVID-19 pandemic, laboratory assessment of physical functioning in our two large randomized controlled exercise trials in older adults with cancer was temporarily suspended. We adapted testing protocols for administering the short physical performance battery (PPB), including chair stand (CS; sec) and 4m usual walk (4MW; m/s) tests, and timed-up-and go (TUG; sec) tests of physical functioning for remote assessment by video conferencing technology. We report on interim assessments of inter and intra-rater reliability and validity against newly resumed in-person tests. Repeat assessments were conducted on the same participant 1-2 weeks apart, by the same assessor (intra-rater reliability) or by another assessor (inter-rater reliability) for remote visits and by the same assessor for in-person tests (validity). Pearson-product moment correlations are provided for PPB, CS, 4MW and TUG, in that order. Intra-rater reliability values are: 0.97, 0.88, 0.90, and 0.96; Inter-rater reliability values are: 0.79, 0.14, 0.40, and 0.99; Validity values (no TUG) are: 0.66, 0.81, 0.78. Reliability of conducting remote PPB by video is very high when using the same assessor, while TUG reliability is high within and between assessors. Remote PPB administration has a good correlation with in-person visits, but the two modes of delivery should not yet be used interchangeably in longitudinal studies until sources of error can be determined and minimized.

### COPING WITH THE IMPACT OF COVID-19 SAFETY RECOMMENDATIONS: THE IMPORTANCE OF PETS

Kerstin Emerson,[1] Deborah Kim,[1] George Mois,[2] and Jenay Beer,[3] *1. University of Georgia, Athens, Georgia, United States, 2. University of Georgia, Lawrenceville, Georgia, United States, 3. Institute of Gerontology, Athens, Georgia, United States*

The COVID-19 pandemic is disproportionately affecting older adults. Initial research suggests that older adults are experiencing negative emotions and stress during social distancing and self-quarantining. Consistently, literature shows that pet ownership can mitigate some negative emotions, including stress and loneliness. Our goal was to explore the impact of pets on older adults who were restricting their social interactions during the first three to five weeks of the COVID-19 pandemic. The study was based on web-based survey data from 833 persons aged 60 and older in the United States. The sample age of respondents ranged from 60-85, with a majority (80.2%) female and White (95%). About 56% of the sample reported that they owned at least one pet. Respondents with pets did not significantly differ in stress or loneliness measures compared to those without pets. A majority of respondents reported engaging in more distance communication compared to before the pandemic. However, respondents with pets were less likely to report an increase in the following technology-mediated forms of communication: emails, texts, computers, and telephone (all p-values < .05) compared to those without pets. There was no significant difference in other coping mechanisms (e.g. exercise, alcohol intake) by pet ownership. These results suggest that respondents did not rely on pets to reduce stress or loneliness. However, pets may fill other communication/connection needs, and older adults may then rely less on communication with other people. Research on coping with the impact of Covid-19 has implications for interventions, including specific interventions for pet ownership.

### COVID IMPACT AND RESPONSE IN RURAL AGE-FRIENDLY COMMUNITIES

Patricia Oh,[1] David Wihry,[2] and Jennifer Crittenden,[2] *1. AARP, Bowdoinham, Maine, United States, 2. University of Maine, Bangor, Maine, United States*

Maine is an age-friendly state with 72 communities enrolled in the AARP Network of Age-Friendly States and Communities and another 25+ using a similar approach to engage residents and local organizations in community planning and development that enhances the health and well-being of older people. The majority (83%, n=60) of Maine's age-friendly communities have fewer than 10,000 residents and only two have populations over 20,000. Before the pandemic, rural Mainers struggled to find safe and convenient transportation options, afford healthy food, access primary and secondary medical care, and participate in activities to stay physically fit and socially connected. Age-friendly community efforts attempted to address those issues by building on community strengths and assets to develop local solutions. The challenges of rural aging were amplified by the pandemic and reliance on older community leaders and volunteers, who were seen as an at-risk population and thus strongly encouraged to avoid social contact. This study offers a narrative exploration of how rural age-friendly communities responded to COVID-19. The leaders of 71 age-friendly communities were contacted by phone, email, or Zoom between May 15 and August 30. Thirty-nine of the communities (54.9%) actively partnered with residents and organizations to address needs during the pandemic. Identified themes described the challenges faced in developing an effective response and new opportunities to expand the age-friendly work in the community. Suggestions for developing new partnerships between age-friendly initiatives and local organizations were highlighted. A key practice implication is the vital importance of intergenerational collaborations in rural areas.

### COVID TRANSITIONS: CHARACTERISTICS OF OLDER ADULTS WHO ENGAGE OR DO NOT ENGAGE IN VIRTUAL EXERCISE

Catalin Mateas,[1] Janet Bettger,[2] Stephen Jennings,[3] Kenneth Manning,[1] Katherine Hall,[4] Megan Pearson,[5] and Miriam Morey,[6] *1. Durham VA Health Care System, Durham, North Carolina, United States, 2. Duke University School of Medicine, Durham, North Carolina, United States, 3. Durham VA Geriatric Research, Education, and Clinical Center (GRECC), Veterans Affairs Health Care System, Durham, North Carolina, United States, 4. Duke University, Veterans Health Administration, Durham, North Carolina, United States, 5. Veteran Affairs Health Care System, Durham, North Carolina, United States, 6. Veterans Health Administration, Durham, North Carolina, United States*

Background. Exercise is a crucial component of maintaining good health in older individuals. The COVID-19 stay-at-home orders forced Veterans actively engaged in facility-based exercise to stop attending in-person group exercise programs like Gerofit. Objective. To compare the characteristics of Veterans who enrolled (E) or declined enrollment (DE) in the transition from a facility-based exercise program, Gerofit, to a virtual Gerofit-to-Home (GTH) program. Methods. Gerofit is a supervised exercise "VA Best Practice" program for older Veterans implemented at 17 VA medical centers around the country. At the time of COVID-19 mandated closures, 1149 Veterans were actively engaged in facility-based programs and invited to attend GTH classes. Comparisons between those enrolling and those declining enrollment were performed by t-tests. Results. Three hundred and eight of 1149 (27%) Veterans made the transition to telehealth delivered classes, with several sites having enrolled participants aged in their mid-nineties. Age was not associated with GTH adoption rates (74.0 vs. 74.7, p=not significant for E vs. NE). Body mass index (31.3 vs. 30.5 kg/m2, $p<0.05$), gait speed (1.19 vs. 1.12 m/s, $p<0.001$), arm curls (20.8 vs. 19.5, $p<0.001$), and chair stands (14.7 vs. 13.2, $p<0.05$) were higher in individuals actively participating in GTH compared to those that never enrolled. Conclusions. Some older adults can adopt a virtual approach to group-based exercise, demonstrating its feasibility. Further research is needed to improve GTH implementation for lower functioning individuals. Virtual group-based exercise could reduce negative health effects associated with isolation due to lack of in-person exercise.

### COVID-19 AND ASSOCIATIONS WITH FRAILTY AND MULTIMORBIDITY: A PROSPECTIVE ANALYSIS OF UK BIOBANK PARTICIPANTS

Stephen Woolford,[1] Stefania D'Angelo,[1] Elizabeth Curtis,[1] Camille Parsons,[1] Elaine Dennison,[1] Harnish Patel,[2] Cyrus Cooper,[1] and Nicholas Harvey,[1] *1. MRC Lifecourse Epidemiology Unit, Southampton, England, United Kingdom, 2. University Hospital Southampton NHS Foundation Trust, Southampton, England, United Kingdom*

Frailty and multimorbidity, which are more prevalent in older people, have been suggested as risk factors for severe COVID-19 disease. We investigated whether frailty and multimorbidity were associated with risk of hospitalisation with COVID-19 in the UK Biobank. 502,640 participants aged 40-69 years at baseline (54-79 years at COVID-19 testing) were recruited across UK 2006-10. A modified assessment of frailty using Fried's classification was generated from baseline data. COVID-19 test results (England) were available 16/03/2020-01/06/2020, mostly taken in hospital settings. Logistic regression was used to discern associations between frailty, multimorbidity and COVID-19 diagnoses, adjusting for sex, age, BMI, ethnicity, education, smoking and number of comorbidity groupings, comparing COVID-19 positive, COVID-19 negative and non-tested groups. 4,510 participants were tested for COVID-19 (positive=1,326, negative=3,184). 497,996 participants were not tested. Compared to the non-tested group, after adjustment, COVID-19 positive participants were more likely to be frail (OR=1.4 [95%CI=1.1, 1.8]), report slow walking speed (OR=1.3 [1.1, 1.6]), report two or more falls in the past year (OR=1.3 [1.0, 1.5]) and be multimorbid (≥4 comorbidity groupings vs 0-1: OR=1.9 [1.5, 2.3]). However, similar strength of associations were apparent when comparing COVID-19 negative and non-tested groups. Furthermore, frailty and multimorbidity were not associated with COVID-19 diagnoses, when comparing COVID-19 positive and COVID-19 negative participants. In conclusion, frailty and multimorbidity do not appear to aid risk stratification, in terms of a positive versus negative results of COVID-19 testing. Investigation of the prognostic value of these markers for adverse clinical sequelae following COVID-19 disease is urgently needed.

### COVID-19 BEREAVEMENT: CONSIDERATIONS FOR FAMILIES, PSYCHOLOGISTS, AND STUDENT TRAINEES

Jacqueline Hogan,[1] and Lisa Richards,[2] *1. University of Massachusetts Boston, Boston, Massachusetts, United States, 2. Bedford VA Medical Center, Bedford, Massachusetts, United States*

During the peak of COVID-19, inflicted individuals died unexpectedly and in isolation. These circumstances deprived families of opportunities to say goodbye or memorialize the death of their loved one in alignment with their values and cultural heritage (e.g., wakes/vigils, funerals, shiva, washing, shrouding, military honors). Unable to hold hands, have final conversations, or develop treatment plans with providers, bereaved families experienced compounded losses. Concurrent quarantine hindered their engagement in coping strategies. COVID-19 bereavement increases the risk for complicated grief, which escalates the risk of physical and mental health problems, suicide, drug abuse, and family discord (Shear, 2015, 2020). While death, grief, and mourning are normal life experiences, traumatic and sudden death during a global pandemic is a new domain and the voices of those left behind are under-represented in social discourse. Simultaneously, psychologists and trainees quickly became last responders. COVID-19 presented a constellation of clinical challenges. Practitioners provided care during a time of political and racial tension, civil unrest, school closures, health and financial insecurity, and a collective loss of normalcy. Additionally, COVID-19 cast a spotlight on ageist attitudes and critical need for increased representation of older adults in training curricula. These issues echo the call to embrace aging as a valued aspect of diversity, and to strengthen psychology's workforce in the areas of training, practice, research and advocacy for aging adults (Hoge, et al., 2015). This poster will explore the impact of COVID-19 bereavement on families and practitioners, promote advocacy efforts, and offer tangible training recommendations for psychology programs.

### COVID-19 PANDEMIC LOCKDOWN RESPONSES FROM AN EMOTIONAL PERSPECTIVE: FAMILY FUNCTION AS A DIFFERENTIAL PATTERN

Javier Lopez,[1] Gema Perez-Rojo,[2] Cristina Noriega,[2] Cristina Velasco,[2] Isabel Carretero,[2] Patricia López-Frutos,[2] and Leyre Galarraga,[2] *1. Universidad San Pablo CEU, Boadilla del Monte, Spain, 2. Universidad San Pablo-CEU, Alcorcón, Spain*

Family can be an essential resource at times of loss or vital crisis. Loneliness and isolation in older adults might have serious negative consequences for their mental health. For this reason, this research aims to analyze the role of family function in the anxiety and depression experienced by older adults during the COVID-19 crisis. Participants were 882 Spanish community-dwelling adults over 60 years of age. Sociodemographic characteristics, characteristics related to the coronavirus, self-perceived health, family function, avoidance, depression and anxiety were analyzed. Data suggest a buffering effect of family function on anxiety and depression during the pandemic. Furthermore, being unmarried or a female, greater fear of COVID-19, worse self-perceived health, greater avoidance, and worse family function were associated with higher levels of anxiety. Likewise, greater fear of COVID-19, poorer self-perceived health, greater avoidance, and poorer family function, were associated with greater depression. These results point out that family dysfunction is a predisposing factor for the development of the emotional problems of anxiety and depression in older people in potentially stressful and loss situations.

### COVID-19 SOCIAL DISTANCING MEASURES AND LONELINESS AMONG OLDER ADULTS

Eun Young Choi,[1] Matthew Farina,[2] Qiao Wu,[1] and Jennifer Ailshire[1], *1. University of Southern California, Los Angeles, Los Angeles, California, United States, 2. University of Texas at Austin, Austin, Texas, United States*

In response to the COVID-19 pandemic, older adults are advised to follow social distancing measures to prevent infection. However, such measures may increase the risk of loneliness. The current study aimed to investigate (1) whether social distancing measures, particularly limiting close social interactions, are associated with loneliness among older adults, and (2) whether the association between social distancing measures and loneliness is moderated by sociodemographic characteristics. Data were from the fourth wave (April 29 to May 26, 2020) of the nationally representative Understanding America Study (UAS) COVID-19 Survey. We used data on adults 50 years or older (N = 3,283). Multivariate logistic regression models of loneliness were examined to test the independent effects of social distancing measures and their interaction with sociodemographic characteristics on loneliness. Four indicators of social distancing measures were considered: (a) avoiding public spaces, gatherings, or crowds, (b) canceling or postponing social activities, (c) social visits, (d) close contact (within 6 feet) with others. Cancelling or postponing social activities and avoiding close contact with other people were associated with 36% and 41% greater odds of loneliness, respectively. Furthermore, males and non-Hispanic Whites who had no close contact with others had a significantly greater probability of reporting loneliness than those who had contact. Our findings emphasize the heterogeneous nature of COVID-19 related experiences across subpopulations of older adults and call special attention to vulnerable groups that may be more impacted by the challenge of COVID-19 social distancing.

### COVID-19 SWEPT NURSING HOMES IN THE UNITED STATES

Lei Yu, *Miami University, Oxford, Ohio, United States*

Nursing Homes experienced a colossal loss impacted by the Covid-19 pandemic in the United States. This study applied Covid-19 Nursing Home Dataset (updated on 08/16/2020) released by Data.CMS.gov to explore possible factors behind the death s at nursing homes. The results indicated 2.55 residents died per week at a nursing home averagely. Besides, the absence of nursing staff, aides, clinical physicians, PPE supplies contributes to more deaths at nursing homes. Lastly, the number of positive COVID-19 cases of nursing home staff positively associate with the number of total deaths of residents ( R=0.65). These findings provide more pieces of evidence for nursing home administrators and policymakers to make adjustments to help nursing home residents better cope with challenges caused by the pandemic; however, this dataset is not the final data for the pandmic is not over. Also, the dataset covers few demographic information (gender, race, ethinicty and so on) ; therefore, researchers could explore the relationship between the demographic features and COVID-19 deaths at nursing homes.

### COVID-19 TRANSITION: DOES VIRTUAL EXERCISE MAINTAIN PHYSICAL FUNCTION?

Stephen Jennings,[1] Kenneth Manning,[2] Megan Pearson,[3] Catalin Mateas,[2] Katherine Hall,[4] Janet Bettger,[5] and Miriam Morey,[6] *1. Durham VA Geriatric Research, Education, and Clinical Center (GRECC), Veterans Affairs Health Care System, Durham, North Carolina, United States, 2. Durham VA Health Care System, Durham, North Carolina, United States, 3. Veteran Affairs Health Care System, Durham, North Carolina, United States, 4. Duke University, Veterans Health Administration, Durham, North Carolina, United States, 5. Duke University School of Medicine, Durham, North Carolina, United States, 6. Veterans Health Administration, Durham, North Carolina, United States*

Background: In March 2020, COVID-19 mandates to restrict face to face exercise and group based gatherings were enacted. These mandates were enforced within most states in the US. Gerofit, a facility-based exercise program for older Veterans in Durham, NC, transitioned to remote virtual exercise instruction to accommodate continuity of care. Objectives: To explore whether remote virtual exercise (RVE) can sustain physical function within individuals previously participating in onsite face to face exercise (OFF). Methods: Physical function assessments performed during OFF were compared with assessments conducted remotely over virtual platform. Assessments included the 30-second arm curl, the 30-second chair stand, time to complete five chair stands, and either 6-minute walk or 2-minute step test. All assessments for RVE were completed via a remote virtual platform. Only participants enrolled in both OFF and home based RVE with functional assessments within 6-months of pre and post COVID-19 transition were compared. Descriptive comparisons, opposed to statistical, were reported due to the limited sample size. Results: Fourteen OFF Gerofit participants were reassessed remotely within the first 6-months of transitioning to RVE (12 male, 2 female, mean age 73.1, mean body mass index 31.5). Functional assessments between OFF versus RVE were arm curls (21.0 vs 20.4 repetitions), chair stands (15.0 vs 17.5 repetitions), and time to 5 chair stands (9.0 vs 8.4 seconds). Cardiovascular function, reported in normalized percentiles (46.4%tile vs 58.9%tile) Conclusion: Among older Veterans engaged in regular structured exercise, physical function was preserved with transition to virtual exercise.

### COVID-19 WORRIES AND BEHAVIOR CHANGES IN OLDER AND YOUNGER MEN AND WOMEN

Sarah Barber, and Hyunji Kim, *Georgia State University, Atlanta, Georgia, United States*

The case fatality rate of COVID-19 is higher among older than younger adults, and is also higher among men than women. However, worry, which is a key motivator of behavioral health changes, occurs less frequently for older than younger adults, and less frequently for men than women. Building on this, we tested whether older adults – and particularly older men -- would report the least amount of COVID-19 worry and also fewer COVID-19 behavior changes. To do so, from March 23-31, 2020, we administered an online questionnaire assessing COVID-19 perceptions, worries, and behavior changes. Participants were a convenience sample of United States residents, who were community-dwelling younger adults (18-35) or older adults (65 to 81). Analyses included 146 younger adults (68 men, 78 women) and 156 older adults (82 men, 74 women). Participants was predominately White, living in suburban/urban areas, and had completed some college. Our results showed that during the early phase of the outbreak in the United States, older adults perceived the risks of COVID-19 to be higher than did younger adults (e.g., thought COVID-19 was different than the flu). Despite this, older men were comparatively less worried about COVID-19 than their younger counterparts. Compared to the other participants, older men had also implemented the fewest behavior changes, such as wearing a mask. These tesults suggest that interventions are needed to increase COVID-19 behavior changes in older men. These results also highlight the importance of understanding emotional-responses to COVID-19, as these are predictive of their behavioral responses.

### COVID-19, SOCIAL ISOLATION, AND LONELINESS IN OLDER ADULTS: LEVERAGING EXERCISE TO AGE IN PLACE STUDY

Meghan Reddy,[1] Celina Shirazipour,[2] and Allison Mays,[2] *1. David Geffen School of Medicine at UCLA, Los Angeles, California, United States, 2. Cedars-Sinai Medical Center, West Hollywood, California, United States*

Social isolation and loneliness are associated with morbidity and mortality and highly prevalent in older adults. Older adults, a high-risk group for developing serious complications from COVID-19, are asked to shelter-in-place limiting physical interactions. We aimed to determine the effect of the COVID-19 pandemic on social isolation and loneliness among community-dwelling older adults previously enrolled in in-person exercise classes in the Leveraging Exercise to Age in Place (LEAP) study before March 19th, 2020 when California started shelter-in-place. We conducted a pre-post analysis of cognitively intact participants (n=59) >50 years, who had social connectedness, loneliness, and demographic data collected pre- and post-COVID shelter-in-place. Participants' social connectedness was measured via the 11-question Duke Social Support Index (DSSI) and loneliness via the 3-question UCLA Loneliness Scale (UCLA 3). Participants had an average (±SD) baseline DSSI of 27.2 (± 3.5) and UCLA 3 of 4.8 (± 1.7) and were an average of 76.6 ± 9.2 years, 81% female, 63% white, 29% widowed, 42% living alone, 27% acting as caregivers, and 44% were diagnosed with 3 or more chronic health conditions. We completed post-assessments on average 61 ± 29 days after the start of shelter-in-place. Results of the paired t-tests indicated no statistically significant difference in social connectedness and loneliness pre- and post-shelter-in-place. Reasons for lack of observed change include: limitations of a small sample size, possible protective factors from enrollment in the LEAP program, or insufficient time at post-assessment to develop changes in loneliness and social isolation. Repeated assessments are needed throughout the pandemic.

### COVID-19-RELATED CHANGES IN POTENTIAL RISK FACTORS FOR ELDER MISTREATMENT REPORTED BY CAREGIVERS OF OLDER ADULTS

Lena Makaroun,[1] Scott Beach,[2] Tony Rosen,[3] and Ann-Marie Rosland,[1] *1. University of Pittsburgh School of Medicine, Pittsburgh, Pennsylvania, United States, 2. University of Pittsburgh, Pittsburgh, Pennsylvania, United States, 3. Weill Cornell Medicine / NewYork-Presbyterian Hospital, New York, New York, United States*

In previous studies, caregiver (CG) stress, substance use, poor physical health, poor mental health, financial problems, and social isolation have been associated with increased risk of elder mistreatment (EM) for older care recipients (CR). This study aimed to assess how the COVID-19 pandemic has impacted these CG-related risk factors for EM in a community sample of CGs. A non-probability sample of 433 CGs caring for adult CRs age ≥60 years with physical (76%), cognitive (34%) and mental health (14%) conditions completed a survey on COVID-19 impacts in April-May 2020. CGs had mean age 61 (range 21 – 91), were 75% female and 92% non-Hispanic White. Over 40% of CGs reported doing worse financially since COVID-19. Compared to before COVID-19, 15% reported drinking more alcohol and 64% reported somewhat or greatly increased feelings of social isolation and loneliness. CGs reported that COVID-19 had made caregiving more physically (18.7%), emotionally (48.5%) and financially (14.5%) difficult, interfered with their own healthcare (19%), and led to family conflict over caring for CR (13.2%). Younger CGs (age <65) and those with annual income <$50,000 were more likely to report negative COVID-19 impacts. This study suggests CGs of older adults may be experiencing increased stress, alcohol use, social isolation and negative impacts on their own health and financial situation. Healthcare and social service providers should assess for these EM risk-factors in caregivers and connect them and their care recipients with resources and services to address these stressors to reduce risk of EM during the COVID-19 pandemic.

### COVID-19-RELATED FREE TELEPHONE CONSULTATIONS BY PUBLIC HEALTH NURSES

Yuka Sumikawa,[1] Chikako Honda,[1] Kyoko Yoshioka-Maeda,[2] Riho Iwasaki-Motegi,[1] and Noriko Yamamoto-Mitani,[2], *1. Graduate School of Medicine, The University of Tokyo, Tokyo, Japan, 2. National Institute of Public Health, Wako City, Japan*

Public health centers are located in each municipality in Japan and are responsible for infectious disease control including COVID-19. Public health nurses (PHNs) are stationed at the centers and work at the forefront, covering a variety of services from individual consultations to hospital escort for those tested positive. Starting January, PHNs at A city (population approx. 210,000) established a free telephone consultation hotline for COVID-19. This study aims to review the PHNs' telephone consultations during the first wave of COVID-19. The number of calls were aggregated weekly and their time-trend was examined. The study was approved by the University of Tokyo Ethics Review Board. During the first wave between January and May, there were 3,242 calls, with the highest number of calls (n=491/week) in the second week of April. At this point the regular PHNs were not enough to meet the hightened needs of consultations and PHNs from other departments were temporalily transferred for support. The number of consultation calls fluctuated weekly. The increase of calls seemed to preceed the increase of positive cases by one week. We consider that the call may be an initial action of those who suspected possible infection, and the consultation by the PHN might have led them to proper clinic visits and PCR testing. Telephone consultation is an easy tool to use for general public, especially older persons. Having health professionals respond directly to calls may have had the advantage of providing appropriate guidance for infection control and PCR testing and mental support.

### DAILY AFFECTIVE EXPERIENCES ACROSS THE LIFESPAN DURING THE COVID-19 OUTBREAK

Da Jiang, and Shuang Liu, *Education University of Hong Kong, Hong Kong, China*

Objectives Older adults are considered one of the most vulnerable groups to COVID-19. However, previous studies on emotion and aging have found that older adults report better well-being than younger adults in global survey and daily report. To better understand older adults' well-being during the COVID-19 outbreak, we examined age differences in daily affective experiences in this study. Method A total of participants from mainland China aged 18 to 85 were recruited to participate in the 14-day daily diary study, after a pretest. Their trait affect and demographic information were measured in the pretest. Their daily affect and stress levels were measured in the daily assessments. Results We found that older adults reported a higher level of low arousal positive affect (e.g., calm) and lower levels of high arousal negative affect (HAN; e.g., anxiety), low arousal negative affect (LAN; e.g., dullness), and perceived stress related to COVID-19 in daily life, compared to younger adults. Discussion These results provide initial evidence of daily affective well-being across different age groups in adulthood during the COVID-19 outbreak. Such information is important for developing interventions to promote better well-being during the COVID-19 outbreak.

### DAILY STRESS PROCESSES IN A PANDEMIC: THE EFFECTS OF AFFECT, WORRY, AND AGE

Niccole Nelson, and Cindy Bergeman, *University of Notre Dame, Notre Dame, Indiana, United States*

On March 13th, 2020, the World Health Organization declared a novel coronavirus, COVID-19, a pandemic. Given the day-to-day behavioral changes necessitated by this global threat, the current study examined daily stress reactivity and its potential moderators during the COVID-19 pandemic. Two-level, multilevel modeling was used to examine the daily relationship between perceived stress and negative affect, as well as the moderating effects of daily positive affect, average pandemic worry, and age, on this process. Participants included 349 individuals from the young adult, midlife, and later-life cohorts of the Notre Dame Study of Health & Well-being who completed a 28-day, daily diary study amidst the COVID-19 pandemic (NDHWB; Age Range = 26-89). Individuals were affectively reactive to perceived stress during the COVID-19 pandemic, experiencing higher negative affect on days of higher perceived stress. Regarding moderators, older individuals were less stress reactive than younger individuals, and the extent of individuals' pandemic worry exacerbated their stress reactivity. Furthermore, daily positive affect buffered daily stress reactivity, regardless of pandemic worry and age. In sum, individuals who were younger or more worried about the pandemic tended to be more stress reactive than older or less worried individuals. Furthermore, daily positive affect buffered stress reactivity, and this buffering effect did not depend on age or the extent to which individuals were worried about the pandemic. Thus, mobilizing positive affect during the COVID-19 pandemic may be a promising avenue for intervention in daily stress processes.

### DEMENTIA CARE PROVIDERS' DELIVERY OF FAMILY CAREGIVER SUPPORT DURING COVID-19

Emma Quach,[1] Emily Franzosa,[2] Rachael Scali,[3] Lauren Moo,[4] and Christine Hartmann,[5] *1. Holden, Massachusetts, United States, 2. James J. Peters VA Medical Center, Bronx, New York, United States, 3. Bedford VA Research Corporation, Inc., Belmont, Massachusetts, United States, 4. Bedford VA Medical Center, Bedford, Massachusetts, United States, 5. Edith Nourse Rogers Memorial Veterans Hospital, Bedford, Massachusetts, United States*

Family caregiver support is a cornerstone of dementia care. Yet the transition to virtual care during COVID raised questions about the ability of dementia care teams to maintain caregiver support services. We surveyed Veterans Affairs clinicians about dementia caregiver support delivery following the COVID surge in the six New England states. 38 out of 68 (55%) clinicians from 6 states responded in June and July 2020. We found: 1) Clinicians continued providing the same types of support services for family caregivers before and after COVID, with over 50% of providers interacting with caregivers daily or multiple times per week. The most prevalent services were caregiver needs assessments, information and referrals, and assistance with accessing services. Two-thirds reported continuing to offer caregiver skills training and counseling, including peer support groups. 2) Caregiver support modality changed, most frequently through the combined use of phone and video, followed by only phone, and rarely, by only video. 3) Providers indicated that phone, more than video, increased to replace in-person interactions, because of multiple factors: caregivers (who continued to call for support on an as-needed basis but declined video encounters), providers (who began to provide group support via phone), and service factors (ad hoc versus scheduled encounters). Results suggest clinicians continued providing caregiver support despite suspension of in-person interactions, but future research is needed to assess the impacts of caregiver support delivery mostly by phone and factors underlying the limited use of video in delivering caregiver support.

### DEMENTIA CARE UNDER COVID-19 AND INFECTIOUS DISEASE PANDEMIC RESTRICTIONS

Kelly Bradbury,[1] Elaine Moody,[1] Katie Aubrecht,[2] Meaghan Sim,[1] and Melissa Rothfus,[1] *1. Dalhousie University, Halifax, Nova Scotia, Canada, 2. St. Francis Xavier University, Antigonish, Nova Scotia, Canada*

Emergency measures including social distancing and program restrictions during COVID-19 has reduced supports for people living with dementia and family/friend caregivers in the community. Consequently, these reductions in dementia services and resources have added to existing challenges and (in)equities for this stigmatized population. The objectives of this study were to identify how community-based resources and services for people with dementia and their caregivers are impacted by public health emergency measures enacted during COVID-19 and other infectious pandemics and secondly, use an intersectional health equity perspective to explore how supports for people and families living with dementia are affected by social determinants of health. A scoping review using JBI methodology was conducted. Academic databases searched included Embase, Medline, CINAHL and PAIS. Grey literature was searched using the CADTH tool. English articles published after 2000 in high-income countries were included. Data was extracted by two reviewers using an adaptation of the Health Equity Impact Assessment tool to explore factors related to health equity. Findings included articles discussing the COVID-19 pandemic (N=15). Most alterations to dementia services included switching to telehealth platforms with some advantages/disadvantages of this method discussed. Limited information on how different populations experienced service changes was identified and more research is needed to address issues of (in)equities for people living with dementia and their caregivers during public health emergencies. Information on how health emergency responses affects dementia services and their users will provide important information on resources for current and future efforts to analyze and assess their impacts.

### DEVELOPMENT OF SERUM ANTIBODIES TO SARS-COV-2 AND EVIDENCE OF IMMUNITY IN OLDER ADULTS CONVALESCING FROM COVID-19

Rebecca Caffrey,[1] James Wright,[2] Nicole Waltrip,[1] and David Bostwick,[1] *1. Granger Genetics, N Chesterfield, Virginia, United States, 2. Canterbury Rehabilitation and Healthcare, Richmond, Virginia, United States*

The presence and magnitude of SARS-CoV-2 IgG, IgM, and Neutralizing Antibody (NAbs) response was investigated in study cohort comprised of 58 volunteers who survived COVID-19 in a Virginia LTC facility. All subjects were confirmed positive by PCR nasal swab at least once and blood samples were drawn a minimum of 14 days post symptom onset or first positive COVID-19 test. The cohort was split between LTC residents (n=32, mean age 77.8 yrs, age range 48-97), and the LTC staff (n=26, mean age 41.3 yrs, range 23-61); the age difference between groups was statistically significant (P<0.001). Serum IgG measurement was quantitative over 5 orders of magnitude (0.6-1250µg/mL) and IgM was qualitative, measured with sandwich ELISA; NAbs were measured with surrogate virus neutralization assay (sVNT) competition ELISA (both Genscript). The convalescent older adult LTC patients were fully immuno-competent and showed no significant difference in IgG, IgM, or NAbs compared to the younger staff group. All older adults developed NAbs and were positive for either IgG, IgM, or both. All study participants were then grouped by age range and IgG, IgM, and NAbs compared between the following groups: ≤ 50 yrs old(n=20), 51-60(n=6), 61-70(n=8), 71-80(n=14), 81-90(n=6), and ≥91(n=4). There was no significant difference in immune response parameters between the age groups. Furthermore, repeat testing at 3 months on a subset of participants showed that NAbs, IgG and IgM persist. We conclude that development of competent immune response was age-independent, and that presence of NAbs in serum suggests older adults may develop true immunity.

### DIFFERENTIAL IMPACT OF THE COVID-19 PANDEMIC ON STUDENTS, FACULTY AND STAFF AT A FLORIDA HBCU

Felicia Wheaton, Matilda Johnson, and Thometta Cozart, *Bethune-Cookman University, Daytona Beach, Florida, United States*

The COVID-19 pandemic has caused major disruption to society, including education, the economy, daily life, etc. To understand the impact of the pandemic on both short-term and anticipated long-term mental and physical health, as well as potential period and cohort differences, surveys were emailed to all students, faculty and staff at a Florida HBCU. The survey included the GAD-7 anxiety scale, PHQ-9 scale of depression severity and the UCLA Revised Loneliness Scale (3rd revision), as well as questions about the pandemic's impact on physical and mental wellbeing in the month of April and long-term physical and mental health. Although loneliness did not differ among groups, students reported the highest levels of moderate/severe depression (46.6%), followed by faculty (21.1%) and staff (6.9%). Students also reported the highest levels of moderate/severe anxiety (48.6%) compared with faculty (29.4%) and staff (12.1%). Students were more likely to say the pandemic moderately or very much impacted their overall physical and mental wellbeing in April. However, faculty were more likely to report that their long-term physical and mental health would be somewhat/greatly affected, followed by students, and then staff. Staff reported the highest levels of optimism about the future, followed by students and faculty. Taken together, these findings indicate substantial differences in the perceived impact of the COVID-19 pandemic.

### DIGITAL TECHNOLOGIES TO ENHANCE SOCIAL CONNECTEDNESS IN LONG-TERM CARE FACILITIES DURING COVID-19: A REVIEW

Shannon Freeman, Aderonke Abgoji, Alanna Koopmans, and Christopher Ross, *University of Northern British Columbia, Prince George, British Columbia, Canada*

A consequence of the strict visitor restrictions implemented by many Long-term Care Facilities (LTCFs), during the COVID-19 pandemic, was the exacerbation of loneliness and social isolation felt by older adult residents. While there had been a shift by some persons to utilize digital solutions to mitigate the effects of the imposed social isolation, many facilities did not have sufficient information regarding available solutions to implement institutional strategies to support social connectedness through digital solutions. To support our partners in evidence-based policy-making we conducted a scoping review to identify existing virtual technology solutions, apps, and platforms feasible to promote social connectedness among persons residing in a long-term care facility context during times of lockdown such as experienced during the COVID-19 pandemic. Initial identification of relevant literature involved a combination of keywords and subject headings searches within 5 databases (PubMed, CINAHL EBSCO, PsychINFO EBSCO, Embase OVIDSP, and Web of Science ISI). DistillerSR was used to screen, chart and summarize the data. There is growth in the availability of technologies focused on promoting health and well-being in later life for persons in long-term care facilities however a gap remains in widespread uptake. We will describe the breadth of technologies identified in this review and discuss how they vary in utility in smaller scale facilities common in rural areas. Of the technologies that can be used to mitigate the impacts of social isolation felt by long-term care residents, many "solutions" depend on stable highspeed internet, which remains a challenge in rural and northern areas.

### DISRUPTIONS TO CARE AND THE USE OF TELEHEALTH AMONG OLDER ADULTS DURING THE COVID-19 PANDEMIC

Beth Prusaczyk,[1] Brian Carpenter,[2] Nancy Morrow-Howell,[2] and Eric Lenze,[1] *1. Washington University School of Medicine in St. Louis, Saint Louis, Missouri, United States, 2. Washington University in St. Louis, St. Louis, Missouri, United States*

Reports emerged early in the 2020 COVID-19 pandemic that older adults were foregoing non-COVID19-related care, due to fears of contracting the virus during appointments and because of healthcare providers' reduced operations. Beginning in July, 2020, we explored the impact of the pandemic on disruptions to care and older adults' use of telehealth. Preliminary results from 53 older adults aged 66 to 93 (mean: 72.6) found that many older adults experienced disruptions in their care, ranging from 30-50% depending on the type of care. The most commonly disrupted care types were mental health and rehabilitation care (occupational, physical, or speech therapy), with 50% of older adults reporting disruptions to mental health and 50% to rehabilitation care. The most common reason for the disruptions was closed care providers' offices. Similar results were found for primary care (46% reporting disruptions), dental care (44%), and vision care (30%), with between 62-71% citing closed offices as a reason for the disruption. The use of telehealth among the sample was high (44%), and the majority (83%) of these older adults reported never having used it previously. All who used it reported being very or somewhat comfortable with the technology, and 83% said they would use it again even if in-person care was available. These findings suggest the pandemic has had a significant impact on older adults' care and that the expansion of telehealth could be increase access to care during and after the pandemic.

### DUAL CAREGIVERS OF RELATIVES WITH DEMENTIA IN RURAL VIRGINIA: THE ADDED STRESS OF COVID-19

Emily Hoyt,[1] Jyoti Savla,[1] Karen Roberto,[1] Aubrey L. Knight,[2] Rosemary Blieszner,[1] and Brandy R. McCann,[1] *1. Virginia Tech, Blacksburg, Virginia, United States, 2. Carilion Clinic Center for Healthy Aging, Roanoke, Virginia, United States*

Family caregivers often find themselves "sandwiched" between caring for an older relative with dementia (PWD) and another person. Serving in a dual caregiving role presents unique challenges and has consequences for caregivers' physical and mental health. Seven daily diary interviews with 46 dual dementia caregivers assessed their daily stressors and informal and formal supports. Results showed that dementia caregivers who also cared for another older relative reported poorer physical health and used more community-based services to care for the PwD. Conversely, dementia caregivers who also cared for younger relatives reported greater secondary stressors, lower family support, and use of fewer community-based services to care for the PwD. Since the COVID-19 pandemic began, two telephone interviews were conducted with 15 dual dementia caregivers. Caregivers were asked in-depth questions about how the pandemic had impacted their caregiving responsibilities, mental health, and use of community-based services. Guided by stress process and behavior models, a thematic analysis of dual caregivers' interviews revealed that caregivers had less time for themselves, engaged in self-care activities less often, and felt their social life had suffered. Many of the caregivers reported feeling exhausted, stressed, and had more things to do than they could handle. Of the eight caregivers that used services before COVID-19, six experienced a change in services including loss of services, different workers, or self-selected cancellation of services. Discussion focuses on challenges dual dementia caregivers face and the added stressors they experienced during the COVID-19 pandemic.

### EFFECTS OF COVID-19 PANDEMIC ON THE LIVED EXPERIENCE OF DIVERSE OLDER ADULTS LIVING ALONE WITH COGNITIVE IMPAIRMENT

Elena Portacolone,[1] Anna Chodos,[1] Jodi halpern,[2] Kenneth Covinsky,[1] Sahru Keiser,[3] Jennifer Fung,[1] Elizabeth Rivera,[1] and Julene K. Johnson,[1] *1. University of California San Francisco, San Francisco, California, United States, 2. University of California Berkeley, Berkeley, California, United States, 3. UCSF, San Francisco, California, United States*

Background and Objectives: Even before the COVID-19 pandemic, older adults with cognitive impairment living alone (an estimated 4,3 million individuals in the United States) were at high risk for negative health outcomes. There is an urgent need to learn how this population is managing during the pandemic. Research Design and Methods: This is a qualitative study of 24 adults aged 55 and over living alone with cognitive impairment from diverse racial/ethnic backgrounds. Participants' lived experiences during the pandemic were elicited via 59 ethnographic interviews conducted over the phone either in English, Spanish, or Cantonese. Using a qualitative content analysis approach, interview transcripts and fieldnotes were analyzed to identify codes and themes. Results: Qualitative analysis of transcripts revealed five themes: 1) fear generated by the pandemic; 2) distress stemming from feeling extremely isolated; 3) belief in misinformation, 4) strategies for coping during the pandemic; and 5) the importance of access to essential services. Discussion and Implications: This pandemic put a spotlight on the precarity and unmet needs of older adults living alone with cognitive impairment living. Findings underscore the need to expand access to home care aides and mental health services for this population.

### EVALUATING SERVICE-LEARNING WITH OLDER ADULTS ON UNDERGRADUATE COLLEGE STUDENTS DURING THE COVID-19 PANDEMIC

Sharon Merkin, *UCLA Geffen School of Medicine, Los Angeles, California, United States*

Introduction: Students in the Frontiers in Human Aging course at UCLA participate in service-learning (SL) with older adults. In 2020, completion of SL coincided with the outbreak of the novel coronavirus disease (COVID-19) pandemic. We evaluated the impact of SL on student attitudes on aging and community service in the context of the pandemic. Methods: Students were assigned to senior residential and daycare programs for 18-20 hours of SL. A retrospective pretest-posttest survey asked about attitudes and interests before and after SL and how the COVID-19 pandemic affected these perceptions; 73 (of 103) students responded.

Mean differences before and after SL were tested and differences were assessed within groups reporting COVID-19 effects. Results: SL improved students' attitudes and ability to engage with older adults, knowledge about aging concepts, interest in future work with older adults, attitudes on community service, social well-being and feelings of usefulness ($p<0.001$). There was no significant change in overall anxiety about aging ($p=0.1$), however, students showed increased anxiety about losing independence and finances when older ($p<0.05$). At least 50% of students reported that the COVID-19 pandemic increased their awareness of needs of older adults (81.9%) and decreased connection to their peers (50.7%); the impact of SL remained unchanged by these effects. Conclusion: Despite the overall benefits of SL, increased anxiety about aspects of aging suggests the need to address these concerns. While the COVID-19 pandemic did not seem to affect the impact of SL, this event did seem to influence perceptions about aging and social integration.

### EVALUATION OF THE VA MEDICAL FOSTER HOME PROGRAM: FACTORS IMPORTANT FOR EXPANSION AND SUSTAINABILITY DURING COVID19

Nelly Solorzano,[1] Chelsea Manheim,[2] Leah Haverhals,[3] and Cari Levy,[4] *1. Denver Research Institute, Aurora, Colorado, United States, 2. Rocky Mountain Regional Medical Center, Aurora, Colorado, United States, 3. Department of Veterans Affairs, Denver, Colorado, United States, 4. VA Eastern Colorado Health Care System, Aurora, Colorado, United States*

In 2020, the Center of Innovation for Veteran Centered and Value Driven Care (COIN) continued its monitoring and evaluation of the Veterans Administration (VA) Medical Foster Home (MFH) programs expansion into rural areas. Veterans in MFHs are provided 24/7 care by VA trained and supervised community caregivers and primary care by VA Home Based Primary Care (HBPC) teams. One year after the three-year (2017-2019) expansion funds stopped, COIN continued monitoring remaining programs. Objectives were to understand factors critical for program expansion and sustainability and the impact of COVID-19. Phone interviews were conducted with sixteen coordinators from seventeen programs. A thematic analysis approach was used to address the evaluation objectives using transcript data. Findings showed factors important to program sustainability were: 1) Program fit (finding caregivers in the community); and 2) Local VA facility support (staffing, adaptation, and local leadership support). COVID prompted losing some caregivers and prevented others from joining. Program staffing was not impacted as many program activities ceased. Recreational therapists (RTs) were significant to maintaining Veterans well-being and reducing social isolation through virtual activities. COVID required coordinators transition their supervision of MFHs to new virtual environments and HBPC to increase telehealth to new levels. Local leadership became important to monitoring local conditions and providing support to programs. The evaluation: 1) Found factors important to program sustainability were also critical to keeping programs operational during the pandemic; and 2) Stimulated future research on the suitability of MFH programs to meet challenges to resurgences of COVID or other national emergencies.

### EVERYDAY REMEMBERING DURING A GLOBAL PANDEMIC IN CARING DYADS

Emily Lustig,[1] Alysha Naran,[2] Ann Pearman,[1] and Christopher Hertzog,[1] *1. Georgia Institute of Technology, Atlanta, Georgia, United States, 2. Georgia Institute of Technology, Atlanta, United States*

During the COVID-19 global pandemic people's lived experiences and day-to-day lives have been tremendously impacted. This impact is believed to be more severe in people with a memory-impaired partner at home. As part of an ongoing cognitive intervention project with the Emory-Georgia Tech Cognitive Empowerment Program (CEP), we conducted interviews with dyads (one person with diagnosed amnestic mild cognitive impairment (aMCI) and one person, in this case, a spouse who is an identified care partner). To address the COVID-19 pandemic, we supplemented the existing interview about everyday cognition with several questions about the dyadic experience during the pandemic. To date, we have conducted 5 qualitative interviews with dyads. Preliminary results indicate that the COVID-19 pandemic has created additional everyday challenges and cognitive burden for care partners of people diagnosed with aMCI. Some of these challenges include the need to manage pandemic precautionary behaviors, such as mask wearing and maintaining social distancing, for both themselves and the care recipient. In contrast, some aspects of everyday remembering among these dyads have improved (e.g. more advance planning of things like grocery shopping and outings). The results of these interviews will provide additional unique insights into the everyday cognitive challenges of the pandemic on caregivers and persons with aMCI.

### EXAMINING THE IMPACT OF THE COVID-19 PANDEMIC ON PSYCHOSOCIAL OUTCOMES ACROSS AGE: A STRESS AND COPING FRAMEWORK

Jillian Minahan,[1] Francesca Falzarano,[2] Neshat Yazdani,[3] and Karen Siedlecki,[4] *1. Fordham University, New Providence, New Jersey, United States, 2. Weill Cornell Medicine, New York, New York, United States, 3. Fordham University, Bronx, New York, United States, 4. Fordham University, New York, New York, United States*

The emergence of COVID-19 and the measures implemented to curb its spread are anticipated to have long-term implications for mental health. Older adults may be at increased risk for adverse mental health outcomes as opportunities to remain socially connected have diminished. Further research is needed to better understand the impact of pandemic-related stress on mental health. Utilizing the stress and coping framework, the purpose of this study is three-fold: 1) to examine the influences of COVID-19-related stress on depression, anxiety, and loneliness, 2) to assess the mediating role of coping style and social support, and 3) to investigate whether these relationships vary across age. Participants ($N = 1,318$) between the ages of 18-92 years completed an online survey, assessing pandemic-related stress, mental health, social support, coping, and their experiences with social distancing, during the initial implementation of social distancing measures in the United States. Stress, social support, and coping style were related to psychosocial outcomes. Results suggested that avoidant coping mediated the relationship between pandemic-related stress and psychosocial outcomes, particularly depression.

Avoidant coping more strongly mediated the relationship between stress and depression in younger adults compared to older adults. Results were consistent with the stress and coping framework and recent work highlighting the older adults' resilience during the COVID-19 pandemic. Findings highlight the associations between positive coping behaviors and psychosocial well-being and indicate that older adults may use unique adaptive mechanisms to preserve well-being during the COVID-19 pandemic.

### EXPLORATION OF COVID-19-BASED CHANGES TO CAREGIVER BURDEN AND CAREGIVING INTENSITY AMONG INFORMAL CAREGIVERS

Steven Cohen,[1] Zachary Kunicki,[2] Megan Drohan,[1] and Mary Greaney,[1] *1. University of Rhode Island, Kingston, Rhode Island, United States, 2. Brown University, Providence, Rhode Island, United States*

Individuals providing unpaid care of assistance to family members and friends (e.g. informal caregivers), may have been uniquely impacted by the COVID-19 pandemic. Research is needed to examine the pandemic's effect on informal caregivers' caregiving intensity and burden. Therefore, this cross-sectional study was conducted to explore self-reported changes in caregiver intensity (CI) and caregiver burden (CB) due to the pandemic to identify factors associated with changes in responsibilities and burdens. In June 2020, informal caregivers providing care to someone aged 50+ (n=835) reported their current and pre-pandemic caregiving intensity and burden. Data were collected via Amazon's Mechanical Turk. Chi-square tests were used to examine bivariate associations between pandemic time (pre vs. post) differences in CI and CB. Multinomial regression was used to assess multivariate predictors of changes to CI and CB due to COVID-19. Results showed a significant U-shaped association between initial CB and CB change due to COVID-19. Higher levels of initial CB were associated with both a significant decrease in CB during COVID-19 (OR 1.33, 95%CI 1.06-1.67), and a significant increase in CB during COVID-19 (OR 1.22, 95%CI 1.05-1.43). There were no significant associations between initial CB and changes in CI due to COVID-19, although older caregivers were more likely to experience a decrease in CB due to caregiving (OR 1.02, 95%CI 1.00-1.05). These mixed results suggest that caregivers with high initial CB experienced the most extreme changes to CB due to COVID-19. Future planned analyses will focus on understanding the potential drivers behind these unexpected results.

### EXPLORING THE IMPACT OF AGE-RELATED COVID-19 MESSAGING ON INTERNALIZED AGEISM IN OLDER ADULTHOOD

Ruheena Sangrar, Stephanie Chesser, and Michelle Porter, *University of Manitoba, Winnipeg, Manitoba, Canada*

Public health messages during the COVID-19 pandemic have indicated a higher risk for older people and/or those who have multiple health conditions. Subsequent societal discourse, however, has at times arguably protested the full protection and treatment of older people from COVID-19, potentially contributing to internalized ageism. To date, how older people interpret age-related pandemic messaging and discourse has not been explored. This study examined older adults' perspectives of age-related COVID-19 messaging and societal discourse, as well as their perceptions of vulnerability, using a social constructionism framework. Adults age 65 to 89 years participated in semi-structured interviews about their thoughts and experiences with ongoing pandemic-related public messaging. Preliminary analysis suggests that participant perspectives of COVID-19 messaging are situated along a continuum of concern associated with contracting the virus. While some, for example, describe minimal concern, others express being fearful. Individual perceptions of safety appear to be informed, in part, by the presence or absence of an underlying health condition. Individual approaches to media criticism and consumption, personal risk-taking thresholds, financial stability, and social connectedness also appear to influence how the participants perceive pandemic-related messaging. Findings suggest the framing of COVID-19 and pandemic protocols, as well as the media's sensationalization of age-related issues, can impact older peoples' perceived vulnerability of contracting the virus. Future research is needed to understand the long-term implications of ongoing pandemic-related messaging on older adults' experiences of aging, as well as the consequences such messaging could pose to for their health and social behaviors.

### FACILITY CHARACTERISTICS AND QUALITY DEFICIENCIES OF FLORIDA NURSING HOMES WITH COVID-19 CASES AND DEATHS

Xiaochuan Wang and Courtney Wilson, *University of Central Florida, Orlando, Florida, United States*

The Coronavirus disease 2019 (COVID-19) has been disproportionately affecting nursing homes throughout the United States, resulting elevated risk for COVID-19 morbidity and mortality to nursing home residents. Given the high percentage of aging population, large number of nursing homes, and staggering surge of COVID-19 cases in Florida, it's critical to understand factors that may affect Florida nursing homes' vulnerability to the COVID-19 pandemic. Using Nursing Home COVID-19 Dataset as of July 26, 2020 obtained through Centers for Medicare and Medicaid Services (CMS), and Provider Info Dataset and Health Deficiencies Dataset available through CMS Nursing Home Compare data, we constructed a database of Florida nursing facilities with confirmed COVID-19 cases and deaths, with corresponding facility characteristics and quality deficiencies. We examined the facility characteristics (e.g. facility size, ownership state, chain affiliation, staffing level) and quality deficiencies (e.g. infection control deficiencies) of Florida nursing homes with and without publicly reported COVID-19 cases and deaths. Results indicated that, as of July 26, 2020, 73.3% and 40.8% of Florida nursing homes had resident COVID-19 cases and death, respectively (N=701). Findings also suggested that Florida nursing homes of large facility size, chain affiliated, and for profit, were significantly more likely to have documented resident COVID-19 cases (p<.05). Larger facility size (120 beds or more), staff shortage, and having prior infection control deficiency citation, were significantly related to the odds of having resident COVID-19 deaths (p<.05). Policy and practice implications and future research directions will be addressed to better protect the at-risk nursing home residents.

### FACTORS ASSOCIATED WITH LONELINESS DURING THE COVID-19 PANDEMIC AMONG OLDER ADULTS WITH CHRONIC CONDITIONS

Courtney Polenick,[1] Emily Perbix,[1] Shreya Salwi,[1] Donovan Maust,[1] Kira Birditt,[1] and Jessica Brooks,[2]

1. University of Michigan, Ann Arbor, Michigan, United States, 2. Columbia University, New York, New York, United States

Social distancing related to the COVID-19 pandemic may heighten loneliness among older adults, especially those with chronic conditions that increase risk for severe illness from the coronavirus. Little is known, however, about potential risk and protective factors linked to loneliness during the pandemic. In the present study, we examined factors associated with loneliness in a U.S. sample of adults aged 50 and older with at least one chronic condition. Participants included 701 adults aged 50 to 94 (M = 64.57 years, SD = 8.84) who were recruited over 8 consecutive weeks between May 14 and July 9, 2020 to complete an anonymous online survey. We estimated a series of multiple linear regressions to determine how sociodemographic characteristics, health characteristics, stress related to COVID-19, and social resources were independently associated with loneliness during the pandemic. Two-thirds of participants reported moderate to severe loneliness. The fully adjusted regression model showed that being a person of color, having a spouse or cohabiting partner, and reporting more emotional support were linked to lower levels of loneliness. Higher anxiety symptoms, more worry about being infected with COVID-19, and greater perceived financial strain because of COVID-19 were associated with higher levels of loneliness. These findings pinpoint potential targets for interventions to improve and maintain the well-being of a particularly vulnerable subgroup of older adults during the COVID-19 pandemic and in future public health crises.

### FAMILY CAREGIVERS ON THE FRONTLINE: CHALLENGES OF PROVIDING CARE TO POST-ICU COVID-19 PATIENTS

Amanda Leggett,[1] Alicia Carmichael,[2] Natalie Leonard,[2] Sheria Robinson-Lane,[2] Sophia Li,[2] Grace Oxford,[2] Maren Wisniewski,[2] and RIchard Gonzalez,[2]
1. The University of Michigan, Ypsilanti, Michigan, United States, 2. University of Michigan, Ann Arbor, Michigan, United States

Family caregivers are essential care providers helping to ensure the sometimes complicated recovery of recently hospitalized COVID-19 patients. COVID-19 caregivers face pandemic-specific challenges such as not being at patient bedside throughout the hospital stay and managing social distancing post-discharge. The current study aims to explore the unique experiences of family caregivers of Intensive Care Unit (ICU) COVID-19 patients. In-depth qualitative interviews were conducted by web conference with 13 dyads of adults who were in an ICU for COVID-19 between March and August 2020 and their primary caregiver (n=26). Participants were interviewed about the care recipient's hospitalization and recovery journey, supports received, challenges experienced, and gaps in the system of care. Thematic qualitative analysis was conducted utilizing Watkins' (2017) rigorous and accelerated data reduction (RADaR) technique. Caregivers played a critical role in patient admission, discharge, and recovery. Themes of caregiving challenges included self-management of COVID-19 infection, knowledge deficits of available resources and post-discharge care needs, post-infection stigma, separation guilt, deprioritized self-care, financial challenges, and lengthy recoveries with some ongoing health needs. While receipt of emotional support was considered an advantage, some caregivers expressed contact fatigue. Understanding how COVID caregivers experience illness management across the recovery journey can aid our understanding of the COVID caregiving process and identify intervention targets to improve overall health and well-being of the care dyad.

### FIRESIDE CHATS DURING COVID-19: CAREGIVER COMMUNITY-BASED EDUCATION

Kara Dassel, Rand Rupper, Jorie Butler, Jacqueline Telonidis, Catherine Witt, and Linda Edelman, University of Utah, Salt Lake City, Utah, United States

The Utah Geriatric Education Consortium (UGEC) provides education about Age-Friendly Health Care and Dementia-Friendly Communities to both informal and professional caregivers. As such, we have collaborated with our community partners to hold 12 "Fireside Chats" (2 in person, and 10 virtually due to COVID-19) between December 2019 and September 2020. Our expert speakers and panelists have given presentations on a variety of topics, specifically focused on coping with COVID-19, such as physical, music, and arts-based activities to do in the home, advance care planning, local services and supports, resiliency, and mindfulness. A total of 463 participants attended the Fireside Chats. A total of 169 attendees completed evaluations regarding the programs (a completion rate of 37%). Attendees were primarily White (86%), non-Hispanic (95%), well educated (86% had a college degree or higher), about half of the group were community caregivers (55%), while the other attendees were primarily from health professional backgrounds (e.g., nursing, social work, physical therapy). We obtained evaluation data in seven domains based on a 5-point Likert scale (1=strongly disagree to 5=strongly agree). The mean level of agreement in the seven following domains were: satisfaction (M=4.68/SD=.53), effectiveness (M=4.72/SD=.52), met stated goals (M=4.70/SD=.53), met educational needs (M=4.64/SD=.6), will improve the care they provide (M=4.57/SD=.70), included useful examples (M=4.59/SD=.66), and was better than similar trainings (M=4.23/SD=.86). These results along with steady attendance of our "Fireside Chats" demonstrate the need and satisfaction with our community-based education based on improving geriatric care practices within the community and in long-term services and support programs.

### FORWARD-FOCUSED COPING PREDICTS BETTER MENTAL HEALTH OUTCOMES IN OLDER ADULTS DURING COVID-19 PANDEMIC

Leslie Jordan,[1] John Woodard,[2] Gabriel Pena,[1] Naomi Arnold-Nedimala,[1] Junyeon Won,[1] Daniel Callow,[1] and J. Carson Smith,[1] 1. University of Maryland, College Park, College Park, Maryland, United States, 2. Wayne State University, Detroit, Michigan, United States

Psychosocial stressors associated with the COVID-19 pandemic may increase risk of depression and anxiety in the general population. Older adults may be especially vulnerable to these psychosocial stressors and their impact on mental health outcomes. Consequently, there is an urgent need to identify protective factors for older adults. The purpose of the present study is to determine the relative contribution of coping flexibility and two distinct coping strategies, forward-focused and trauma-focused, on negative affect in older adults during the COVID-19 pandemic. Data were collected using an online survey, including questions about demographic information, coping, depression, and anxiety. Participants aged 50 and over were included in our analyses of depression (N = 800) and anxiety (N = 638). Results indicate that both higher coping flexibility and higher forward-focused coping predict lower depression and lower anxiety. In contrast, higher trauma-focused coping predicts slightly higher depressive symptoms but is not a significant predictor of anxiety. Our findings suggest that higher forward-focused coping may serve as a protective factor in older adults during the pandemic and, therefore, may be an effective treatment target for mental health interventions.

### FRAILTY AND COVID 19 INFECTION IN HOSPITALIZED VETERANS: A CASE SERIES

Marlena Fernandez,[1] Valerie Hart,[2] Alma Diaz,[1] Lorena Burton,[1] Victor Cevallos,[1] and Jorge Ruiz,[1]
*1. Miami VA Healthcare System, Miami, Florida, United States, 2. University of Miami / Jackson Health System, Miami, Florida, United States*

Frailty, a clinical syndrome characterized by vulnerability to stressors resulting from loss of physiological reserve across multiple systems. In patients with COVID 19 infection, the presence of frailty may place older adults at higher risk for poor clinical outcomes including hospitalizations and mortality. The aim of this case-series study was to describe the characteristics of patients with frailty and COVID-19 who were hospitalized at a VA Medical Center. A VA Frailty Index (VA-FI) was generated at baseline as a proportion of variables from electronic health records. The VA-FI categorized Veterans into non-frail (FI<.21) and frail (FI≥.21). We calculated the VA-FI for Veterans admitted at the time of COVID-19 admission date. We compared the characteristics of frail and non-frail Veterans. A total of 137 veterans were admitted, 96.3% (n=132) were male, mean age 66.81 (SD=13.87) years, and 65.7% (n=90) were frail. When comparing Veterans who were frail versus non-frail, there were no differences in age, race, ethnicity, BMI, rates of cardiopulmonary resuscitation, ICU admissions, use of vasopressors or length of stay. There were significant differences in rates of intubation (frail n=10, vs. non-frail n=0), p = 0.018 and non-invasive respiratory support (frail n=9 vs non-frail n=0), p=.025. There were 13 and 7 readmissions in the frail and non-frail groups respectively. Eleven Veterans died during hospitalization, all of whom were frail. Frailty is associated with poor clinical outcomes in hospitalized Veterans with COVID 19 infection. Recognition of frailty may help to optimize the management of COVID 19 related complications.

### FRONT-LINE EXPERIENCES AND PERSPECTIVES OF OLDER AND YOUNGER REGISTERED NURSES DURING THE COVID-19 PANDEMIC

Victoria Raveis,[1] Nancy VanDevanter,[2] Christine Kovner,[3] Gary Yu,[3] Laura Jean Ridge,[4] and Kimberly Glassman,[5]
*1. New York University, Maplewood, New Jersey, United States, 2. NYU Rory Meyers College of Nursing, New York, New York, United States, 3. Rory Meyers College of Nursing, New York, New York, United States, 4. University of Michigan, Ann Arbor, Michigan, United States, 5. New York University, NY, New York, United States*

Having an experienced and trained healthcare workforce available during public health emergencies, such as the COVID-19 pandemic, is critical. While all healthcare workers are at risk of contracting COVID-19, older workers are at increased risk of serious or fatal illness. This investigation explores the front-line experiences and perspectives of registered nurses (RNs) at a major New York City medical center during the COVID-19 pandemic, focusing on two age cohorts: older (50+) and younger (20-49) RNs. An anonymous internet-based survey was sent to all RN's employed at the center. Data collection was initiated in May 2020, following the initial surge in NYC's COVID-19 related hospitalizations and deaths; 1,483 surveys were completed. This investigation found that in comparison to younger RNs (n=1,067), older RNs' (n=416) psychosocial well-being was significantly better -- fewer depression, anxiety, and PTSD symptoms (p<.001). They were less stressed caring for COVID-19 patients (p <.001) and less worried about work-related exposure risk (p<.001). They also reported higher job satisfaction (p<.001), less work-home stress (p<.001), a higher commitment to choosing the nursing profession (p<.001), were more confident in the profession (p<.001) and to meeting its' expectations (p<.001). Overall, although older RNs represent a smaller proportion of the nursing workforce, their collective expertise and clinical experience in healthcare delivery are significant. The older RN workforce is a seasoned resource to draw upon during public health emergencies and a valuable role model for younger RNs, particularly given their continued adherence to nursing, during this COVID-19 time of uncertainty and personal health risk.

### FRONT-LINE PERSPECTIVES FROM AGING SERVICES PROVIDERS ON THE COVID-19 PANDEMIC AND OLDER COMMUNITY RESIDENTS

Victoria Raveis,[1] Anderson Torres,[2] Jane Arce-Bello,[3] Donna Atmore-Dolly,[4] and Allison Nickerson,[5] *1. New York University, Maplewood, New Jersey, United States, 2. R.A.I.N. Total Care, Inc., Bronx, New York, United States, 3. Regional Aid for Interim Needs, Inc., Bronx, New York, United States, 4. Allen Community Non-profit Programs, Jamaica, New York, United States, 5. LiveOn NY, New York, New York, United States*

Older urban residents' daily lives have been broadly impacted by the COVID-19 pandemic. Rapid acceleration of COVID-19 cases in mid-March 2020, forced the shuttering of senior centers in New York City's (NYC) five boroughs. Center programming was suspended, including congregate meals for older adults. As centers, a focal point for social interaction, closed, older community residents experienced social isolation, compounded further by social distancing. Key informant interviews with the leadership of community-based multi-service agencies, provided frontline perspectives on essential programmatic services NYC's oldest residents needed, challenges encountered, and strategies implemented to adjust to issues emerging from the pandemic. Alternative meal delivery plans were devised by the City and aging services agencies initiated wellness outreach calls and also facilitated access to technological resources to maintain social connections. Various advocacy and policy-relevant insights emerged from these experiences. Specifically, a priority in preparedness planning should be on solutions addressing the social isolation precipitated by the pandemic that older adults experienced. Technology may help. Devoting more programming to activities enabling older adults to utilize accessible technological resources is key. Efforts implemented during the pandemic have demonstrated that with preparation and access to resources, virtual communication is a feasible option for the older population. With limited access to alternative neighborhood food sources, devising a contingency plan for adequate food delivery services to replace congregate meals also merits attention. Overall, the pandemic's widespread impact highlights the importance of creating an infrastructure that builds upon older persons' strengths, while addressing their vulnerabilities, enabling them to thrive.

### FUNCTIONAL STATUS, MEANINGS OF LIFE ACTIVITIES, AND COVID-19 RELATED DISRUPTIONS AMONG DISABLED OLDER ADULTS

Meng-Hsuan Yu,[1] and Shiau-Fang Chao,[2] *1. National Taiwan University, Taipei City, Taipei, Taiwan (Republic of China), 2. National Taiwan University, Taipei City, Taiwan (Republic of China)*

Participating in meaningful activities has been proven beneficial to the well-being of disabled older adults. However, social distancing policies and restrictions on public activities have been implemented since the outbreak of COVID-19 at the beginning of 2020 in Taiwan. These restrictions not only prevent older individuals from performing meaningful activities but also have actual impacts on their daily life. This study aims to elucidate the intervening role of meanings of life activities on the relationship between functional status and COVID-19 disruptions. Data were collected from a sample of 526 community-dwelling older adults with disabilities in Taiwan between April and July, 2020. Utilizing Multiple Regression Analysis, the research findings were as follows. First, participants with better functional status experienced more COVID-19 related disruptions to their daily routine. In the meantime, they also valued their life activities as more meaningful than those with worse functional status. Second, higher levels of meanings in performing life activities also positively related to COVID-19 disruptions. Third, meanings of life activities fully mediated the relationship between functional status and COVID-19 disruptions. That is, disabled older individuals with better functional status may experience more COVID-19 related disruptions because their accessibility to meaningful activities was limited. Since preventive approaches to control the spread are necessary during COVID-19 epidemic, efforts should be made to sustain meaningful life activities participation among disabled older adults. Based on the findings of this study, this would be especially critical to the well-being of more capable older individuals with disabilities during the pandemic.

### HOW AGE AFFECTS PERSONAL AND SOCIAL REACTIONS TO COVID-19: RESULTS FROM THE NATIONAL UNDERSTANDING AMERICA SURVEY

Jung Ki Kim,[1] and Eileen Crimmins,[2] *1. University of Southern California, Los Angeles, United States, 2. University of Southern California, Los Angeles, California, United States*

The pandemic of COVID-19 has had tremendous impact on Americans' lives including their personal and social behaviors. While everyone is affected in some way by the pandemic, older persons have been far more likely to suffer the most severe health consequences. For this reason, how people have responded to the COVID-19 outbreak may differ by age. Using a nationally representative sample from the Understanding America Study (UAS), we examined differentials in behavioral responses to COVID-19 by age and how they change over time. At the beginning of the pandemic (March, 2020), older people were less likely than younger ones to engage in preventive behaviors. As the pandemic progressed, however, older people have adopted healthy behavioral changes more than younger people, such that about two months after the pandemic started, older people were more likely to comply with suggested and regulated behaviors including practicing better hygiene, quarantining, and social distancing. Even when considering other potential influences on behavioral responses, older age was significantly related to performing more preventive behaviors, and gender, racial/ethnic minority status, perceived risk for infection and dying and political orientation were also found to be related to people's behavioral responses.

### HOW ARE INFORMAL CAREGIVERS ADAPTING TO COVID19? PRELIMINARY RESULTS OF AN ONLINE SURVEY.

Ranak Trivedi,[1] Madhuvanthi Suresh,[2] Rashmi Risbud,[3] Marika Humber,[4] Josephine Jacobs,[5] and Samuel Thomas,[6] *1. VA Palo Alto/Stanford University, Menlo Park, California, United States, 2. Georgia Regional Hospital-Atlanta, Atlanta, Georgia, United States, 3. VA Palo Alto Health Care System, Palo Alto, California, United States, 4. VA Palo Alto Health Care System, Menlo Park, California, United States, 5. Palo Alto Veterans Health Administration, Menlo Park, California, United States, 6. Stanford University School of Medicine, Salt Lake City, Utah, United States*

COVID19 may disrupt informal caregivers' (CG) ability to support their care recipients (CR) but little is known how caregivers adapt. A 10-minute, anonymous, online survey with no geographic restrictions was fielded April-August 2020. Two screening items ensured that the respondents were at least 18y and self-identified as a CG. This English-language survey assessed: sociodemographics; reactions to COVID19; changes in CG responsibilities and abilities; depression (Patient Health Questionnaire, PHQ-2); CG burden (Zarit Burden Inventory, ZBI-4); and anxiety (Generalized Anxiety Disorder, GAD-2). Univariate analyses determined the proportion of those who screened positive on PHQ-2 (cutoff=3), GAD-2 (cutoff=3), and ZBI-4 (cutoff=8). Of the 314 respondents, 74% lived in USA; 73.5% of caregivers and 48.2% CR were women. While 63.4% were married, only 28% cared for their spouse. CG mainly cared for adults (83%), and reported that 75.0% of their CR had 2+ conditions. 49.6% CG provided >20h of care/wk. Since COVID19, 53% reported an increase in CG responsibilities; 28.0% noted a decrease in income. Many CG screened positive on the ZBI-4 (48.4%), GAD-2 (30.9%), and PHQ-2 (26.8%). 74% worried about contracting COVID19 at least some of the time. 35.0% noted limits to performing all caregiving tasks when they (N=34) or their CR (N=57)

were asked to self-isolate/quarantine. 163 (51.9%) CG noted spending less time with their CR, of which 46.4% used alternate means (e.g., telephone calls). Preliminary results show that a plurality of CG had changes in their responsibilities and abilities during COVID19. A sizable proportion also reported poor well-being.

### IMPACT OF ADULT DAY SERVICE CENTER CLOSURES IN THE TIME OF COVID-19

Paayal Vora,[1] Lydia Missaelides,[2] Chau Trinh-Shevrin,[3] and Tina Sadarangani,[4] *1. Northeast Ohio Medical University (NEOMED), Marion, Ohio, United States, 2. California Association for Adult Day Services, Sacramento, California, United States, 3. NYU School of Medicine, New York, New York, United States, 4. New York University, New York, New York, United States*

Adult Day Service Centers (ADCs) are a form of community-based long-term care that address frail older adults' health and social needs. Due to their congregate nature and participants' compromised health, many ADCs have been forced to temporarily shutter during the COVID-19 pandemic. It is unknown how closures have impacted service delivery at ADCs. Guided by the Resiliency Framework, we (1) explore methods employed by ADCs during the pandemic to meet participant/caregiver needs and (2) determine how/whether these methods have mitigated the negative effects of ADC closures on participants and their caregivers. Both virtual focus groups and one-on-one semi-structured qualitative interviews were conducted with ADC staff members (n=20) across the United States. Preliminary results showed precipitous declines in physical, cognitive, and mental health of participants, as well as increased caregiver strain, particularly among dementia caregivers. However, ADCs found creative solutions to care for participants despite restrictions, creating, in their words, "centers without walls." Staff developed virtual programs (e.g. support groups, music and exercise therapy) and conducted "door-step" visits to support productive engagement and combat loneliness. Telehealth supported care coordination and identification of emergent clinical problems. Results suggest that despite innumerable COVID-19-related challenges, ADCs demonstrated resilience and creativity in managing participants' needs, fostered a sense of purpose, and provided caregiver respite. Further research on the effectiveness of remote programming and advocacy for sustainable reimbursement by payers is needed to ensure that ADCs can continue to provide older adults and their families with meaningful support as the pandemic wears on.

### IMPACT OF COVID-19 PANDEMIC ON SENIOR-TO-SENIOR SOCIAL ENGAGEMENT ACTIVITIES AT A COMMUNITY SPACE IN JAPAN

Neo Kazembe,[1] Hongjik Kim,[1] Cong Wang,[1] Shuichiro Higuma,[1] Yerim Yang,[1] Taeeun Kim,[1] Ryogo Ogino,[2] and Mai Takase[1] *1. The University of Tokyo, Tokyo, Japan, 2. Saga University, Saga, Japan*

A local community space in Japan, Chiki Kastudokan, has been hosting several senior-to-senior social activities for community-dwelling older adults with support from the University of Tokyo. The activities are aimed at fostering communication and social engagement through exercise, music, cafe, and craft. After the emergence of COVID-19 pandemic, these activities have been disrupted. We conducted a questionnaire survey between June and July 2020 to assess how the pandemic has affected 26 activities and identify support needs that can enable their continuity amid the pandemic (response rate: 77%). First, all 26 activities were suspended by the onset of COVID-19. Hosts of 8 (40%) activities devised alternative ways to engage participants at home. For instance, hosts of craft activities arranged and sent to participants crafting kits. Limited capacity in using online platforms like Skype or Zoom prevented virtual interaction of hosts and participants. Notably, hosts were anxious towards resuming activities amid the pandemic. They worried about their health (50%), of being criticized by friends and family(25%), and of infections spreading among participants (85%). Hosts wanted strategies to prevent infections during activities, and easy to understand infection prevention guidelines for participants. These findings reveal that COVID-19 pandemic has negatively affected senior-to-senior activities at Chiki Kastudokan. The University of Tokyo could help hosts identify effective infection prevention strategies to use when hosting activities amid the pandemic. It could also provide ICT training to hosts and participants to enhance their capacity in using online platforms in case of future waves of COVID-19.

### IMPACT OF THE 2020 COVID-19 PANDEMIC ON OLDER ADULTS' PERCEIVED SEDENTARY BEHAVIOR AND PHYSICAL ACTIVITY

MIkael Anne Greenwood-Hickman,[1] Jacklyn Dahlquist,[1] Julie Cooper,[1] and Dori Rosenberg,[2] *1. Kaiser Permanente Washington Health Research Institute, Seattle, Washington, United States, 2. Kaiser Permanente Washington, Seattle, Washington, United States*

Starting in March 2019, stay-at-home orders meant to control the spread of the COVID-19 pandemic have limited movement, activities, and services in Washington State. For older adults, who are the most sedentary age group, we hypothesized that these public health measures encouraged increased sedentary behavior (SB) and reduced physical activity (PA) levels. To explore this, we conducted 25 semi-structured interviews with a sub-sample of participants in an ongoing sedentary behavior reduction intervention. Interviews were recorded and transcribed, and an iterative coding process was used to extract key themes related SB changes, PA changes, and other impacts of COVID-19 social distancing measures. Most participants reported an increase in SB due to limitations on leaving their home, canceled activities, increased free time in which to pursue indoor hobbies traditionally done in a seated posture (reading, sewing, tv, etc.), and feelings of depression or lack of motivation. Some participants suggested that these restrictions also led to a decrease in their PA and exercise levels due to cancelled fitness classes, loss of social support around exercise routines, and fear of leaving the house. However, other participants reported that the social distancing measures have allowed them to increase their PA levels, giving them more time to walk outdoors or pursue active outdoor hobbies like tennis, gardening, or home improvement tasks. In conclusion, the COVID-19 pandemic and associated social distancing measures have had varying impacts on participant perceived SB and PA levels. It is unclear how these changes will be maintained when pandemic restrictions are lifted.

### IMPACT OF THE COVID-19 PANDEMIC ON FAMILY CAREGIVERS

Tina Kilaberia,[1] Janice Bell,[2] Kristen Bettega,[3] Jennifer Mongoven,[4] Kathleen Kelly,[5] and Heather Young,[2] *1. UC Davis Health, Sacramento, California, United States, 2. University of California Davis, Sacramento, California, United States, 3. Betty Irene Moore School of Nursing at UC Davis, Sacramento, California, United States, 4. University of California Davis Health, Sacramento, United States, 5. Family Caregiver Alliance, San Francisco, California, United States*

The COVID-19 pandemic has affected human life in unprecedented ways. Lives of older persons and their families have been especially adversely affected. Eleven Caregiver Resource Centers (CRCs) support family caregivers across the state of California, providing services such as assessment, counseling and respite. This presentation is part of a larger evaluation study and addresses the impact of the COVID-19 pandemic on family caregivers and systems supporting them. We interviewed directors, clinical staff and family consultants (n=35) from CRCs across diverse communities and geography in California, conducting semi-structured focus group interviews by Zoom. Questions explored perceptions of staff about effects on caregivers and implications for systems of support for caregivers. Interviews were transcribed and analyzed using the Dedoose software. Caregiver effects included increased isolation, higher stress, loss of support, neglected health needs, and accelerated technology adoption. Caregivers at particular risk were those facing multiple demands and experiencing compromised resources under the pandemic, such as closure of adult day care. System effects included challenges with hastened virtual delivery, disruption in services, and new opportunities to serve clients virtually. Community resources, such as internet connectivity, exacerbated disparities for family caregivers. We make recommendations to mitigate these challenges including technology platforms to support service delivery and education, training and preparedness for both caregivers and providers. These recommendations are relevant to the ongoing COVID-19 pandemic and adaptable to other crisis situations such as natural disasters.

### IMPLEMENTATION OF REMOTE INTERVENTIONS IN OLDER ADULTS: LESSONS LEARNED DURING COVID-19 ISOLATION

Jamie Rincker,[1] Jessica Wallis,[2] Angela Fruik,[2] Alyssa King,[2] Kenlyn Young,[2] Thoma Tucker,[2] Connie Bales,[2] and Kathryn Porter Starr,[2] *1. Duke University, Durham, North Carolina, United States, 2. Duke University School of Medicine, Durham, North Carolina, United States*

Recommendations for older adults to socially isolate during the COVID-19 pandemic will have lasting impacts on body weight and physical activity. Due to the pandemic, two in-person RCT weight-loss interventions in obese older adults with prediabetes, Veterans Achieving Weight Loss and Optimizing Resilience-Using Protein (VALOR-UP, n=12) and the Egg-Supplemented Pre-Diabetes Intervention Trial (EGGSPDITE, n=7), were converted to remote formats and weekly nutrition (EGGSPDITE and VALOR-UP) and exercise (VALOR-UP only) classes were delivered using synchronous videoconference technology (Webex); classes were accessed via tablet/desktop/laptop or smart phone. Steps taken to transition participants to remote formats included technology training, implementation of staff tech-support, and delivery of nutrition education, tablets, scales, and exercise bands. The time to successfully transition participants was 1 week for early adopters (n=10) and up to 4 weeks for those with significant technology barriers (n=9); their difficulties included internet access, camera and microphone access and use, and electronic submission of weight and food records. Even with these challenges, in the first 3 months of remote delivery, participant dropout rate was low (10.5%, n=2), attendance was high (87.6% nutrition class (n=19); 76.4% exercise class (VALOR-UP, n=12)), and weight loss was successful (>2.5% loss (n=13); >5% loss (n=8)), showing that lifestyle interventions can be successfully adapted for remote delivery. Remote interventions also have potential for use in non-pandemic times to reach underserved populations who often have high drop-out rates due to caretaker roles, transportation limitations, and work schedules. These barriers were significantly reduced using a virtual intervention platform.

### INFLUENCE OF COVID-19 ON DEMENTIA CAREGIVERS LIVING IN COMMUNITY

Yeji Hwang,[1] Anjali Rajpara,[1] Sonia Talwar,[1] LaShauna Connell,[1] Katie Ramirez,[1] and Nancy Hodgson,[2] *1. University of Pennsylvania, Philadelphia, Pennsylvania, United States, 2. University of Pennsylvania, Cherry Hill, New Jersey, United States*

Community-residing persons living with dementia (PLWD) are a high-risk population for the Coronavirus Disease 2019 (COVID-19) because of advanced age, comorbidities, and impaired cognition. While family caregivers report additional anxiety symptoms by caring for PLWD at home, little is known about their caregiving experience during the current COVID-19 pandemic. The purpose of this study is to examine the influence of the COVID-19 on dementia caregivers. Study participants were recruited from parent study, the Healthy Patterns Clinical Trial (Hodgson; R01NR015226). Data were collected from June 13th to August 28th, 2020 via semi-structured telephone interviews. Thirty-four dementia caregivers participated in this study. Mean caregiver age was 59.2±12.7. Over 70% of the study participant reported worry about spreading COVID-19 to the PLWD and 41.2% reported that they have taken on additional caregiving duties for others in their family or network since COVID-19. In this sample, 61.8% of the dementia caregivers reported one or more anxiety symptoms. Independent t-tests were conducted to compare dementia caregivers who reported anxiety symptoms and those who did not report anxiety symptoms. Anxiety symptoms in dementia caregivers were related to poor physical function of their care recipients (p=0.036). However, anxiety symptoms of dementia caregivers were not related to either cognitive level (p=0.11) or number of neuropsychiatric symptoms of their care recipients (p=0.366). Health care professionals should be alert to the anxiety and concerns expressed by dementia caregivers in order to maintain and improve the well-being of both caregivers and their care recipients during this unprecedented pandemic.

### LEARNING TOGETHER DURING A PANDEMIC LOCKDOWN: CONNECTING OLDER MENTORS WITH NURSING STUDENTS

Alison Phinney,[1] and Frances Affleck,[2] *1. University Of British Columbia, Vancouver, British Columbia, Canada, 2. University of British Columbia, Vancouver, British Columbia, Canada*

Nursing education tends to focus on complex clinical issues affecting older adults who are acutely ill or in long-term care. This creates challenges for educators wanting to expose students to a greater range of experience, including realities of healthy aging. Opportunities to do things differently were presented when an established undergraduate nursing course on complex aging care underwent significant adjustment in the early months of the COVID-19 pandemic. As the course was condensed and moved online and clinical sites closed, invitations were extended to community-dwelling older people who wanted to "help teach nursing students about aging". The response was overwhelming; over nine days, 118 people (ages 65-94) volunteered to be mentors. Through weekly online/ phone conversations, each person guided their assigned student to learn about diverse experiences of aging. Post-survey results showed the impact of these conversations. Over 90% of mentors felt they had contributed in a meaningful way to student learning and would do it again and recommend it to others. 85% of students felt it was a meaningful experience, offering comments like: "I am more mindful of my assumptions now" and "I learned to approach interactions with older adults as a collaboration; we have so much to give each other". These results provide a needed counterpoint to the predominant COVID discourse of older people as "isolated, helpless, and needy". Students came to understand that older people were also "engaged, active, and contributing" and identified how this had changed their view of aging. Implications for nursing education are explored.

### LIVING ALONE DURING COVID-19: SOCIAL CONTACT AND EMOTIONAL WELL-BEING AMONG OLDER ADULTS

Yee To Ng,[1] Karen Fingerman,[1] Shiyang Zhang,[1] Katherine Britt,[1] Gianna Colera,[2] Kira Birditt,[3] and Susan Charles,[4] *1. The University of Texas at Austin, Austin, Texas, United States, 2. Texas State University, Austin, Texas, United States, 3. University of Michigan, Ann Arbor, Michigan, United States, 4. BSS, Irvine, California, United States*

The COVID-19 outbreak and the associated physical distancing measures dramatically altered the social world for most older adults, but people who live alone may have been disproportionately affected. The current study examined how living alone was related to social contact and emotional well-being among older adults during the pandemic. Adults (N = 226) aged 69+ completed a brief survey assessing their living situation, social contact with different social partners (in person, by phone, electronically), and emotions during the morning, afternoon and evening the prior day. Older adults who live alone were less likely to see others in person or to receive or provide help, and reported less positive emotion the prior day than those who lived with others. Living alone was associated with more positive emotions concurrent with in-person contact. In contrast, phone contact was related to higher levels of negate affect among those living alone, but not among those who live with others. Findings suggest older adults who live alone may be more reactive to social contact during the COVID 19 outbreak than older adults who reside with others. In-person contact appears to confer distinct benefits not available via telephone contact, suggesting that possible interventions during the pandemic may work best with safe forms of in-person contact.

### LONELINESS AMONG RURAL AND UNDERSERVED OLDER ADULTS DURING THE COVID-19 PANDEMIC

Lindsay Wilkinson,[1] Julie Masters,[2] Christopher Kelly,[1] Miechelle McKelvey,[3] Ladan Ghazi Saidi,[3] and Toni Hill,[4] *1. University of Nebraska Omaha, Omaha, Nebraska, United States, 2. University of Nebraska Omaha, Lincoln, Nebraska, United States, 3. University of Nebraska Kearney, Kearney, Nebraska, United States, 4. University of Nebraska - Kearney, Kearney, Nebraska, United States*

During the COVID-19 pandemic, older adults are among the most vulnerable populations to the medical complications of COVID-19; however, they are also deeply affected by the unintended consequences of social distancing and sheltering in place. Social distancing effectively mitigates the spread of COVID-19, but this practice can also lead to social isolation and loneliness. Drawing on a sample of adults age 60 or older receiving Meals on Wheels/Grab and Go Meals in the state of Nebraska, this study investigates loneliness among rural and underserved older adults during the COVID-19 pandemic. Surveys were distributed to 3725 meal recipients across Nebraska's eight Area Agencies on Aging in July 2020 (response rate = 50%), and a stratified random subsample was selected for preliminary analysis (N = 240). Logistic regression models were used to estimate the effects of COVID-19 and its associated safety precautions on loneliness. The findings reveal that 1 in 10 older adults have not left their home in over a month, and 38 percent feel lonelier due to the impact of COVID-19. Older adults who engaged in more community activities before the pandemic, reported leaving their home less, and experienced a longer absence of social interaction since the pandemic all had significantly increased odds of feeling lonelier in the COVID-19 era. Longer duration of sheltering in place was marginally associated with increased loneliness. The findings from this study show the consequences of social distancing on rural and underserved older adults, which calls for coordinated intervention.

### LONELINESS DURING COVID-19: DOES LIVING SITUATION OR ABILITY TO ACCESS INFORMATION ABOUT SOCIAL ACTIVITIES MATTER?

Patti Parker,[1] Verena Menec,[2] and Nancy Newall,[3] *1. University of Alberta, Edmonton, Alberta, Canada, 2. University of Manitoba, Winnipeg, Manitoba, Canada, 3. Brandon University, Brandon, Manitoba, Canada*

Social isolation is deleterious for both mental and physical health (Coyle & Dugan, 2012; Hawkley et al., 2006). Conversely, social participation has mental and physical health benefits (Novek et al., 2013). In light of the current Covid-19 pandemic requiring social distancing, the present study examined whether living situation and ability to access information about social activities are associated with older adults' loneliness during the pandemic. Specifically, we surveyed ninety-one adults aged 60 years or older in May and June of 2020, at a time when social distancing measures were still in place. We tested whether their living situation and having access to information about social activities was associated with loneliness. OLS regression analyses revealed living alone was associated with higher loneliness (b = .43, p = .050); and having access to information about social activities was associated with lower loneliness (b = -.18, p = .027) amidst the pandemic. The analyses controlled for participants' age, gender, and education. Our findings highlight that during Covid-19, older adults' living situation and access to information about social activities matter and may impact their social behavior. Thus, at this difficult time, it is recommended organizations that offer social activities find creative ways to reach those living alone who will benefit most from having access to such activities.

### LONG-TERM CARE REGISTERED DIETITIANS' INITIAL RESPONSE TO THE COVID-19 PANDEMIC

Julie Beitel, and Allison Cammer, *University of Saskatchewan, Saskatoon, Saskatchewan, Canada*

At the outset of the global pandemic, long-term care (LTC) homes in Canada were captured in media reports as the centre of Canada's COVID-19 epidemic. An estimated 80% of all COVID-19 deaths in Canada were associated with LTC outbreaks as of May 25, 2020. Infection control measures have swiftly changed the environment in many LTC homes for residents, workers, loved ones, and other supports. Registered Dietitians (RDs) are among the many care professionals working in LTC affected by these changes. The aim of this qualitative study was to examine the roles of RDs in supporting LTC residents during the initial phases of the pandemic. RDs faced remote practice, redeployment to address pandemic priorities, or cohorting to a sole practice site, yet were responsible for resident nutritional health. In-depth, web-based, semi-structured interviews with thirteen RDs working in LTC in a prairie province of Canada were used to explore the changes to work, challenges faced, impact on residents, and innovations in practice. The findings from this study capture nutrition and wellness-related implications of the COVID-19 pandemic within LTC homes. Examining the initial response of LTC RDs to the COVID-19 pandemic can help in planning for opportunities to support or enhance delivery of nutrition care in LTC homes, both in the context of the ongoing pandemic as well as future practice.

### MATERIAL HARDSHIP, MENTAL HEALTH, AND PARENTING STRESS AMONG GRANDPARENT KINSHIP PROVIDERS IN COVID-19

Yanfeng Xu,[1] Qi Wu,[2] Sue Levkoff,[1] and Merav Jedwab,[3] *1. University of South Carolina, Columbia, South Carolina, United States, 2. Arizona State University, Phoenix, Arizona, United States, 3. Hadassah Academic College, Jerusalem Israel, Israel*

The COVID 19 pandemic has exposed the vulnerability of many families, including grandparent kinship families, to deal with a health/economic crisis. The fear of COVID-19 plus stay-at-home orders have increased individuals' psychological distress. Moreover, school closures and homeschooling further increased parenting stress among caregivers. This study examined the relationship between material hardship and parenting stress among grandparent kinship providers and assessed grandparents' mental health as a potential mediator to this relationship during the COVID-19 pandemic in the United States. Grandparent kinship providers (N=362) that took primary care of their grandchildren participated in a cross-sectional survey via Qualtrics Panels in June 2020 in the United States. Descriptive and bivariate analyses, binary logistic regression, and mediation analyses were conducted using STATA 15.0. Experiencing material hardship (OR = 1.67, p < 0.001) was significantly associated with higher odds of parenting stress among grandparent kinship providers, and grandparents' mental health (indirect effect = 0.11, 95% CI [0.01, 0.25]) partially mediated this association. Addressing material and mental health needs among grandparent kinship providers is critical to decreasing their parenting stress.

### MENTAL HEALTH IMPACT OF THE CONFINEMENT MEASURES DURING THE COVID-19 PANDEMIC

Claire Pauly,[1] Valerie Schröder,[2] Laure Pauly,[3] Rejko Krüger,[4] and Anja Leist,[1] *1. University of Luxembourg, Esch-sur-Alzette, Luxembourg, 2. Luxembourg Centre for Systems Biomedicine, Belvaux, Luxembourg, 3. Clinical and Experimental Neuroscience, Luxembourg Centre for Systems Biomedicine, University of Luxembourg, Esch-Belval, Luxembourg, Esch-sur-Alzette Belval, Luxembourg, 4. Luxembourg Institute of Health, Strassen, Luxembourg*

Background. Mid-March 2020, with exponentially increasing COVID-19 infections, Luxembourg closed schools and businesses, and declared a crisis (état de crise) to implement confinement measures, including orders to not leave the home unless to fulfill essential needs. The psychological consequences of these policy responses to the pandemic on older people, considered a high-risk group, were unknown at the time. The aim of this study was to use the nationally representative CON-VINCE study that assessed mental health at the height of the confinement measures mid-April 2020, to assess the psychological impact of quarantine on older adults. Method. A total of 451 participants aged 60+ years (55.0% male) filled in the CES-D, the GAD-7 and the 3-item loneliness scale that measured depressive symptoms, level of anxiety, and feelings of social isolation. Results. Mean age was 67.7 years (SD 5.5), average number of school years were 13.1 (SD 3.6). The participants were mainly of Luxembourgish nationality (87.8%), and a majority (69.8%) was married. Clinically relevant depression scores were present in 13.1%, generalized anxiety in 1.8%, and self-perceived isolation in 16.9% of participants. Number of depressive symptoms was associated with perceived isolation (p<0.001) and current exercise levels (p=0.02). Discussion. The rate of older adults with clinically relevant impaired mental health was similar to pre-pandemic levels in Luxembourg, suggesting that negative mental health consequences of the confinement measures were largely absent. Possible explanations are that confinement was considered a universal experience, and that the healthcare system remained functional, unlike in other countries at the time.

### MODERN UBASUTE: PUBLIC DISCOURSE AND SENTIMENT ABOUT OLDER ADULTS AND COVID19 USING MACHINE LEARNING

Xiaoling Xiang,[1] Xuan Lu,[1] Alex Halavanau,[2] Jia Xue,[3] Yihang Sun,[1] Patrick Ho Lam Lai,[4] and Zhenke Wu,[1] *1. University of Michigan, Ann Arbor, Michigan,*

*United States, 2. Stanford University, Menlo Park, California, United States, 3. University of Toronto, Toronto, Canada, 4. University of Michigan-Ann Arbor, Ypsilanti, Michigan, United States*

This study examined public discourse and sentiment on social media regarding older adults in COVID-19. Twitter data (N=82,893) related to both older adults and COVID-19 and dated from January 23rd to May 20th, 2020, were analyzed. Classification of tweets involved supervised machine learning. Latent Dirichlet Allocation was used to identify dominant themes in public discourse using, accompanied by a qualitative thematic analysis. Sentiment analysis was conducted based on the NRC Emotion Lexicon. The most common category in the coded tweets was "personal opinions" (66.2%), followed by "informative" (24.7%), "jokes/ridicule" (4.8%), and "personal experiences" (4.3%). More than one in ten (11.5%) tweets implied that the life of older adults is less valuable or downplayed the pandemic because it mostly harms older adults. A small proportion (4.6%) explicitly supported the idea of just isolating older adults. Almost three-quarters (72.9%) within "jokes/ridicule" targeted older adults, half of which were "death jokes." The daily average of ageist content was 18%, with the highest of 52.8% on March 11th, 2020. We extracted 14 themes, such as perceptions of lockdown and risk. A bivariate Granger causality test suggested that informative tweets regarding at-risk populations increased the prevalence of tweets that downplayed the pandemic. The COVID-19 pandemic has exposed and intensified ageism in our society. Information about COVID-19 on Twitter influenced public perceptions of risk and acceptable ways of controlling the pandemic. Public education on the risk of severe illness is needed to correct misperceptions.

### NIMBLE GERONTOLOGICAL INTERPROFESSIONAL EDUCATION DURING A PANDEMIC

Megan Thomas Hebdon,[1] Christina Wilson,[2] Katherine Bernier Carney,[2] Jacqueline Telonidis,[2] and Sue Chase-Cantarini,[2] *1. University of Utah, Blacksburg, Virginia, United States, 2. University of Utah, Salt Lake City, Utah, United States*

To improve communication and collaboration among health professionals, interprofessional education (IPE) experiences have been offered to students through the Utah Geriatric Education Consortium (UGEC) with the support of long-term care (LTC) partners since 2017. The COVID-19 pandemic presented a unique challenge in delivering in-person IPE training. Here we describe adaptations and student outcomes with our Spring/Summer 2020 training sessions. Students (n=46) from health profession programs were recruited and enrolled in the sessions. A LTC partner helped plan two-hour remote training sessions to introduce students to current issues and health care team member roles in LTC. Moderated small group discussions regarding the 4 Ms Framework and a patient case were completed using virtual breakout rooms. A shared virtual document was used to guide discussions and record insights. Student participants (n=46) were primarily White (85%), female (70%), and enrolled in physical therapy (28%), nutrition (33%), and medicine (15%) programs. Thirty-one students completed post-course satisfaction surveys with Likert-scale and open-ended questions. Most students who completed the survey agreed or strongly agreed that the course was effective (85%) and engaging (81%), and will improve care (88%). Positive course aspects included: comprehensive information with speaker experiences and use of 4 Ms; course structure with moderated small groups; and interprofessional collaboration with common goals and multiple perspectives. Despite the challenges of COVID-19, an IPE experience was effectively delivered using video conferencing technology, community collaboration, and moderated small group discussions. The successes of this IPE delivery model will enhance engagement and accessibility of future gerontological workforce training.

### NURSING HOME SOCIAL WORKERS PERCEPTIONS OF PREPAREDNESS AND COPING FOR COVID-19

Vivian Miller,[1] Noelle Fields,[2] Nancy Kusmaul,[3] Keith Anderson,[4] and Christy Maxwell,[4] *1. Bowling Green State University, Bowling Green, Ohio, United States, 2. The University of Texas at Arlington, Arlington, Texas, United States, 3. UMBC, Baltimore, Maryland, United States, 4. University of Texas at Arlington, Arlington, Texas, United States*

Social work has a long history of responding to the needs of vulnerable populations during times of crisis and disaster. Social workers are working at the front lines responding to the current COVID-19 pandemic in a variety of health care practice settings, including nursing homes, however it is unclear how social workers perceive their preparedness during this time. This study employed a cross-sectional survey to nursing home social workers via social media on feelings of preparedness for COVID-19, what has been most professionally helpful for social workers during these times in their role in COVID-19, as well as demographic questions. Demographic data were analyzed using SPSS and qualitative data were analyzed using the rigorous and accelerated data reduction (RADaR) technique. Data are based on a sample of 63 (N=63) nursing home social workers. Findings revealed that while some social workers felt prepared for the coronavirus, many respondents stated that they were unprepared to meet the demands and challenges they were facing. Moreover, participants shared that professional support was critically important to get through COVID-19. These findings are important, as social workers are tasked with ensuring each resident attains their highest level of psychosocial well-being, which can be achieved only when nursing home staff are supported. Findings from the present study suggest that additional support for nursing home staff ought to include peer mentoring and mutual support. Additionally, improved leadership across health care settings is worth assessing.

### OBESITY INTERVENTION IMPROVES OUTCOMES IN SOCIALLY ISOLATED OLDER ADULTS: PROGRESS DESPITE A PANDEMIC

Kathryn Porter Starr,[1] Michael Borack,[2] Marshall Miller,[3] Angela Fruik,[1] Jamie Rincker,[2] Jessica Wallis,[1] Amir Hakim,[1] and Connie Bales,[1] *1. Duke University School of Medicine, Durham, North Carolina, United States, 2. Duke University, Durham, North Carolina, United States, 3. Duke University, Efland, North Carolina, United States*

The extended social isolation necessitated by the COVID-19 pandemic will likely have a prolonged negative impact on the health of community-dwelling older adults. We studied the potential to counteract these negative effects, examining the before and after measurements of participants in two obesity intervention studies that were converted from in-person to remote delivery, due to COVID-19. The Veterans Achieving Weight Loss and Optimizing Resilience-Using Protein study (VALOR-UP; n=9) and the Egg-Supplemented Pre-Diabetes Intervention Trial (EGGSPDITE; n=6) enrolled obese (BMI = 34.2±3.8 kg/m2) older adults (age = 71.5±4.5 yrs; 80% male; 47% black) with prediabetes (fasting blood glucose 100-125 mg/dL and/or HbA1c of 5.7-6.4%). Participants followed a hypocaloric diet, attended weekly support groups, and weighed themselves weekly; VALOR-UP participants also attended a weekly exercise class delivered remotely. Outcomes were assessed at baseline (pre-COVID) and the end of a 4-month period coinciding with stay-at-home orders. Between baseline and 4 months, calorie intakes (3-day diet record) decreased by 402.1±529.3 kcal/day ($p<0.05$) and body weight by 6% (6.1±3.4 kg; $p<0.0001$). Physical function (Short Physical Performance Battery) improved by 1.1±1.4 units ($p<0.01$) and Short Form Health Survey (SF-36) physical and mental composite quality of life remained stable ($p=0.63$ and $p=0.48$, respectively). Thus, despite COVID-19 circumstances, most participants were able to benefit from an intervention to improve function and reduce obesity. The somewhat surprising findings for this small cohort offer promise not only for future lifestyle interventions during COVID-19 isolation but also for other isolated populations, including home-bound older adults.

### OLDER ADULTS' ATTITUDES TOWARD VIRTUAL VOLUNTEERING DURING THE COVID-19 PANDEMIC

Peter Sun,[1] Nancy Morrow-Howell,[2] Elizabeth Pawloski,[3] and Emma Swinford,[4] *1. Washington University in St. Louis, Saint Louis, Missouri, United States, 2. Washington University in St. Louis, St. Louis, Missouri, United States, 3. Oasis Institute, St. Louis, Missouri, United States, 4. University of Missouri-Kansas City, Kansas City, Missouri, United States*

This study explored older adults' attitudes toward virtual volunteering during the COVID-19 pandemic. A 22-item survey was administered to 229 volunteers who previously worked with children through the Oasis Intergenerational Tutoring program in St. Louis. Questions focused on technology use patterns and attitudes toward virtual volunteering. Most respondents have used a computer, a smartphone, and the Internet before at home (90.3%), but 22.8% of respondents feel uncomfortable or very uncomfortable when using the Internet. Video conferencing software such as Zoom or Skype was not used before by 14.0% of the respondents, the top reasons being because they prefer other forms of communication (48.4%) or find it too difficult to keep up with technology (19.4%). If tutoring becomes virtual-only, 60.6% of the participants responded they were somewhat likely or very likely to participate, with significant variation by school districts ($X2 = 21.92$, $p < .05$, Cramer's $V = 0.33$) ranging from 42.6% to 96.0% (Bonferroni post hoc $p < .05$). Tutors from school districts that were less likely to tutor virtually had lower levels of education and higher levels of discomfort when using the Internet. The respondents also voiced that while virtual tutoring may eliminate barriers to in-person tutoring, such as commuting to schools and inclement weather, they were concerned about establishing a personal connection with their students online. These findings suggest that tutors anticipate both benefits and challenges with virtual volunteering and efforts to engage older adults virtually should factor in prior use of technology and variations by geography.

### OLDER AGE IS ASSOCIATED WITH MORE POSITIVE REFRAMING OF MEMORIES FROM THE SPRING PHASE OF THE COVID-19 PANDEMIC

Sandry Garcia,[1] Jaclyn Ford,[2] Eric Fields,[3] Tony Cunningham,[2] and Elizabeth Kensinger,[2] *1. Boston College, Brookline, Massachusetts, United States, 2. Boston College, Chestnut Hill, Massachusetts, United States, 3. Boston College, Chestnut Hill, United States*

Older adults often focus on positive aspects of past events, showing resiliency that runs counter to negative stereotypes of aging. We asked whether older adults would retain this positive focus even during the COVID-19 pandemic that puts older adults at particular health risk. We examined how age would relate to the experience of affect during the spring phase of the pandemic and also how it would affect their memories of that spring phase. We predicted that when reflecting on the spring phase of the pandemic, older age would be associated with an increased tendency to focus on "silver linings" and a decreased focus on negative aspects. Furthermore, we explored whether focusing on those "silver linings" would be driven by (i) more positive experienced affect during the spring-phase, or (ii) by a memory-specific effect that would persist after controlling for experienced affect. As predicted, older age was associated with increased focus on "silver linings" such as feelings of hope that the measures would reduce disease spread ($p <.001$) or memories of the community coming together ($p <.001$). These effects remained even when controlling for older adults' more-positive affect during the spring phase of the pandemic (community coming together, $p=.03$, and feeling hope, $p=.008$). These results suggest that older adults remain resilient during the ongoing pandemic. Compared to younger adults, older adults experience greater affective well-being in the moment and also benefit from a memory-specific mechanism allowing them to view the ongoing negative event through a rosier lens.

### ONLINE SOCIAL ENVIRONMENT IMPACTS FEELINGS OF SOCIAL CONNECTION IN OLDER ADULTS DURING THE COVID-19 PANDEMIC

Brittany Derynda,[1] Mary Goodyear,[2] Jade Kushner,[3] and Nicole Cook,[4] *1. Nova Southeastern University Dr. Kiran C Patel College of Osteopathic Medicine, Clearwater, Florida, United States, 2. Nova Southeastern University Dr. Kiran C Patel College of Osteopathic Medicine, Fort Lauderdale, Florida, United States, 3. College of Psychology, Fort Lauderdale, Florida, United States, 4. Nova Southeastern University, Davie, Florida, United States*

Evidence suggests that nearly one-third of older adults experience loneliness and/or social isolation; an increase in these rates during the current pandemic is anticipated. The Lifelong Learning Institute (LLI) at Nova Southeastern University (NSU) in South Florida has worked to engage seniors in fun learning activities and social opportunities since 1977. When "stay at home" orders went into effect in March, 2020 the LLI moved to online program delivery via Zoom. To understand the implementation of zoom among LLI members, NSU students, researchers and LLI member advisory committee developed a cross-sectional research study using an online survey that was administered to LLI members three months post-zoom implementation in May, 2020. Results among the 127 responders demonstrated that a majority of members were not comfortable using zoom (57%) especially the chat, reactions or camera features. More than 80% of responders did report that zoom helped them keep their spirits up. Respondents had specific feedback to improve Zoom programming including Youtube videos on use, retraining, training on features (e.g. chat, camera, reaction), closed captioning, program reminders and links sent out more frequently and within 30 minutes of start time. There were also several comments about internet connectivity, identifying opportunities for router and internet plan education. Finally, respondents noted new opportunities to enhance virtual programming including engaging speakers from across the nation and world. In summary, direct feedback from seniors on how to improve the online social and learning environment is pivotal to improving experience, programming and social connection during COVID-19.

### PRECAUTIONARY MEASURES AGAINST COVID-19 IN RESIDENTIAL AND COMMUNITY-BASED FACILITIES: SINGAPORE'S RESPONSE

Vanessa Yong, *Ministry of Health, Singapore, Singapore*

The COVID-19 pandemic has disproportionately infected older adults who are also at higher risks of developing severe health complications and having case fatality if infected. In particular, older adults in residential and community-based facilities are at greater risks of contracting COVID-19, in part due to close proximities and frequent human interactions in these facilities. In Singapore, the Ministry of Health and its implementation agency, the Agency for Integrated Care (AIC), together with long-term care service providers, have jointly developed a number of precautionary measures against COVID-19. This paper defines and describes Singapore's response in terms of the 'ABC' of COVID-19 safety measures for facilities, namely, safe Access, Behaviors, Compounds (i.e. spatial surroundings). These included infection prevention and control measures, access to PPE, distancing and zoning measures, suspension of visitors, alternative accommodation for long-term care workers, and testing to monitor people with long-term care needs and care workers. Incident response teams to support facilities providers in responding to COVID-19 infections were also swiftly set up. The outreach arm of AIC, the Silver Generation Office, further provided information and services support to older people during the pandemic. We share these measures as a set of practices and learning points for other countries undergoing the current pandemic and for future references.

### RECOMBINANT SARS-COV-2 SPIKE PROTEIN MEDIATES GLYCOLYTIC AND INFLAMMATORY ACTIVATION IN HUMAN MONOCYTES

Brandt Pence, *University of Memphis, Memphis, Tennessee, United States*

Severe acute respiratory syndrome coronavirus-2 (SARS-CoV-2) causes coronavirus disease 2019 (COVID-19) in part through cytokine storm. Metabolic reprogramming in immune cells mediates inflammation, and recent evidence suggests SARS-CoV-2 activates glycolysis in monocytes to facilitate cytokine production. In this study I investigated the ability of the spike protein (subunit 1) from SARS-CoV-2 to cause glycolytic reprogramming and inflammatory activation in isolated human monocytes. Primary human monocytes were isolated from healthy young donors (N=4) by immunomagnetic negative selection and stimulated with recombinant SARS-CoV-2 spike protein subunit 1 (rS1) for 6 hr. Glycolysis was monitored by assessing extracellular acidification using a Seahorse assay. Supernatants and cell lysates were subsequently processed for gene and protein expression assays by qPCR and ELISA respectively. Treatment of monocytes with rS1 at 10 nM and 30 nM led to significant upregulation of glycolysis, as well as a substantial increase in gene and protein expression of interleukin-6. Mouse bone marrow-derived macrophages did not display enhanced glycolysis when stimulated with rS1, suggesting a specific interaction of the protein with the ACE2 receptor, rather than a general inflammatory response caused by contamination with endotoxin or similar. Glycolytic activation in monocytes in response to rS1 suggests that immunometabolic modulators, including common geroprotectors such as rapamycin and metformin, may have efficacy in treating COVID-19.

### RESILIENCE TO LONELINESS AMONGST OLDER ADULTS LIVING IN THE BRONX, NY, DURING THE COVID-19 PANDEMIC

Karra Harrington,[1] Ruixue Zhaoyang,[2] Stacey Scott,[3] Jennifer Graham-Engeland,[2] and Martin Sliwinski,[4]
*1. Pennsylvania State University, University Park, Pennsylvania, United States, 2. The Pennsylvania State University, University Park, Pennsylvania, United States, 3. Stony Brook University, Stony Brook, New York, United States, 4. Penn State University, University Park, Pennsylvania, United States*

Older adults may be particularly vulnerable to experiencing loneliness as a result of stay-at-home and social distancing orders during the COVID-19 pandemic. This study evaluated change in loneliness following the COVID-19 outbreak, using a longitudinal design and a validated loneliness measure, in a well-characterized sample who are at heightened risk for COVID-19 due to both age and location. The study included n = 226 older adults aged 70-90 years old, living in the Bronx, New York City, who had completed the 3-item Loneliness Scale prior to and during the peak of the COVID-19 outbreak in New York City. There was no evidence of significant increases in mean loneliness from pre- to post-COVID-19 outbreak. Multiple regression analyses were used to identify risk and protective factors for change in loneliness during the COVID-19 outbreak, adjusting for pre-outbreak loneliness. Living alone, higher levels of education, greater worry about contracting the coronavirus, and limiting of daily exercise activities were risk factors for greater loneliness after the outbreak. In contrast, Black race, older age, greater social support and frequent social interactions via video call, all related to lower levels of loneliness after the outbreak.The outcomes of this study demonstrate substantial resilience among older adults to loneliness in response to the COVID-19 pandemic, and highlight key risk and protective factors that may play an important role in individual differences in loneliness as pandemic-driven isolation continues.

### RISK AND PROTECTIVE FACTORS ASSOCIATED WITH CUSTODIAL GRANDPARENTS' PSYCHOLOGICAL DISTRESS IN COVID-19

Yanfeng Xu,[1] Merav Jedwab,[2] Qi Wu,[3] Sue Levkoff,[1] and Ling Xu,[4] *1. University of South Carolina, Columbia, South Carolina, United States, 2. Hadassah Academic College, Jerusalem Israel, Israel, 3. Arizona State University, Phoenix, Arizona, United States, 4. The University of Texas at Arlington, Arlington, Texas, United States*

The fear and anxiety of COVID-19 and its related policy measures have increased individuals' psychological distress. The objective of this study was to examine relationships between material hardship, parenting stress, social support, and resilience and custodial grandparents' psychological distress during the COVID-19 pandemic and further investigate the moderating role of kinship license status. A cross-sectional survey was administered to collect data from custodial grandparents (N = 362) in the United States. T-tests, chi-square tests, and logistic regression models were conducted using STATA 15.0. Results indicated that material hardship (OR = 1.77, $p < 0.001$) was associated with higher odds of psychological distress, whereas custodial grandparents' resilience (OR = 0.08, $p < 0.001$) and social support (OR = 0.39, $p < 0.001$) were associated with lower odds of experiencing psychological distress. Increased parenting stress in COVID-19 was not significantly associated with psychological distress. Kinship license status moderated the relationships between social support (OR = 0.23, $p < 0.05$), resilience (OR = 5.06, $p < 0.05$) and psychological distress. To address custodial grandparents' psychological distress, more allocated emergency funds and tailored financial services should be provided to meet material needs, and interventions with a focus on resilience and social support are particularly needed. Although licensed custodial grandparents were more likely to experience psychological distress due to their pre-existing vulnerability than unlicensed counterparts, parallel services should be provided to all kinship caregivers.

### RISK FACTORS FOR GENERALIZED ANXIETY IN OLDER AFRICAN AMERICANS DURING THE COVID-19 PANDEMIC

Dwana Bass,[1] Sophie Hanna,[2] Sarah Shair,[3] Loraine DiCerbo,[4] Bruno Giordani,[5] and Voyko Kavcic,[2] *1. Wayne State University, Sterling Heights, Michigan, United States, 2. Wayne State University, Detroit, Michigan, United States, 3. University of Michigan, Detroit, Michigan, United States, 4. Wayne State University, Clinton Township, Michigan, United States, 5. University of Michigan, Ann Arbor, Michigan, United States*

The COVID-19 pandemic has created worldwide uncertainty and heightened fear and worry, elevating the potential for anxiety. We examined environmental, sociodemographic, and behavioral risk factors predicting generalized anxiety among older African Americans in a large metropolitan area during the COVID-19 outbreak. Sixty African American participants (92% female) age 65 and older were recruited from the Wayne State University Institute of Gerontology Healthier Black Elders Center. In addition to initial demographic questions, the scale (5-point Likert scale from "Not At All" to "A Great Deal") assessed a range of everyday concerns, such as time spent indoors; family relationships; economic problems; restricted freedom of movement; and access to food, cleaning supplies, medical care, and personal protective equipment during the COVID-19 pandemic. Participants also completed a brief generalized anxiety screener, GAD-7. Of the 60 participants, 5 reported they were COVID-19 infected and their responses were not used for the analyses. Older Black Americans reported that during the pandemic, they were most affected by: Illness in Family (53%), Death in Family (35%) and Isolation (75%). Significant risk factors for anxiety were: Missing Close Ones, Annoyance, Sense of Safety, Media Coverage, and Time Indoors (explaining 58% of variance). The current study highlights everyday risk factors for anxiety in the context of the coronavirus pandemic among city-dwelling African Americans. Identified factors are common concerns that may be ameliorated with reasonable interventions. More research is needed in order to fully understand the scope and correlates of anxiety during these challenging times.

### SAFE EXERCISE AT HOME WEBSITE: KEEPING OLDER PEOPLE ACTIVE DURING COVID-19

Anne-Marie Hill,[1] Cathy Said,[2] Cathie Sherrington,[3] Keith Hill,[4] Frances Batchelor,[5] Courtney West,[3] Shylie Mackintosh,[6] and Michele Callisaya,[7] *1. Curtin University, Perth, Western Australia, Australia, 2. University of Melbourne, Melbourne, Victoria, Australia, 3. University of Sydney, Sydney, New South Wales, Australia, 4. Monash University, Melbourne, Victoria, Australia, 5. The University of Melbourne, Melbourne, Victoria, Australia, 6. University of South Australia, Adelaide, South Australia, Australia, 7. Monash University, Frankston, Australia*

Background Physical distancing restrictions, including recommendations to 'stay home' during the COVID-19 pandemic has restricted exercise opportunities for older adults. Our group of academic and clinical physiotherapists and a communications expert identified the need to support safe exercise at home to minimise the impact of these restrictions. The project aimed to develop an online, publicly available resource to support older adults to exercise at home. We met virtually and: a) developed home exercise programs for people at three levels of functional ability; b) developed simple advice about exercising safely, exercise intensity, and staying motivated; and c) reviewed and selected online exercise programs and resources for consumers and health professionals to access. Examples of older adults keeping active during the pandemic were sourced to provide motivation. Website content was made available to download and print to increase accessibility. Modifications were made after consumer and international advisor feedback, the website was endorsed by the Australian Physiotherapy Association. Content is updated as restrictions are modified and in response to feedback received. Google Analytics was used to evaluate website usage. The website was launched on 5 May 2020, 35 days after the group's initial meeting. In the first 9 weeks of website availability 20,608 users accessed the website, with 14.6% being returning users. There were 27,513 sessions and 74,927 page views. Most users were from Australia (80.74%), followed by Denmark (5.15%) and USA (3.85%) We present this example of the benefits of time-critical collaboration to facilitate rapid translation of evidence into practice.

### SHIFTS IN OLDER ADULTS' SOCIAL SUPPORT DURING EARLY MONTHS OF THE COVID-19 PANDEMIC

Heather Fuller, Andrea Huseth-Zosel, Emily Sturn, Shawn Carlson, Brittany Hofmann, Emily Kinkade, and Bryce Van Vleet, *North Dakota State University, Fargo, North Dakota, United States*

In recent months, older adults have faced great health risks due to COVID-19, yet social distancing measures may also heighten risks to their social well-being. This mixed methods study explores changes in older adults' social support satisfaction and interpersonal connections across the first few months of the COVID-19 pandemic. A Midwestern sample of 70 older adults aged 70-97 completed two phone interviews (April and June 2020) about their experiences with social distancing due to COVID-19. At both timepoints participants rated their satisfaction with social support and responded to open-ended questions about their interpersonal interactions, communication, and support in current daily life. Mean social support satisfaction significantly increased between the two interviews. Ensuing analysis of qualitative responses suggested this shift could reflect psychological adjustment to the circumstances or adaptation in methods of interpersonal connection over time. Emergent themes included: 1) increased family support and strain, 2) adaptable and flexible friendships, 3) isolation fatigue, and 4) communication through technology. Evaluation of change over time indicated divergent and shifting perceptions of social support as the pandemic endures. Findings suggest nuanced and diverse social support experiences amongst older adults, yet general patterns of initial shock to social support systems that subsided or adapted over time. Future research should build upon these findings to better understand older adults' social support needs and seek to explore ways to best foster social connections during instances of forced social isolation or societal crises such as the current and ongoing global COVID-19 pandemic.

### SOCIAL ACTIVITIES IN COMMUNITY SETTINGS: IMPACT OF COVID-19 AND TECHNOLOGY SOLUTIONS

Anne Adams,[1] Jenay Beer,[2] Xian Wu,[3] Jane Komsky,[1] and Jason Zamer,[1] *1. SimpleC, LLC, Atlanta, Georgia, United States, 2. Institute of Gerontology, Athens, Georgia, United States, 3. University of Georgia, Athen, Georgia, United States*

COVID-19 has created challenges for staff in promoting resident activity. To better understand the pandemic-related challenges that Activity Professionals are facing, we asked COVID-19 specific questions as part of a larger survey. The overall survey focused on identifying challenges and potential technology solutions (e.g., socially assistive robots) to assist Activity Professionals in their job duties. Activity Professionals (N=19) completed the online questionnaire. Respondents (aged M=48.00, SD=12.87; 95% female, 100% native English speakers, 68% White/Caucasian, 21% Black/African American) were highly educated/experienced: 68% had a Bachelor's degree or above, and 53% had 10-35 years of experience. Respondents worked in Independent Living (68%), Assisted Living (37%), Memory Care (26%), Skilled Nursing (21%), or Personal Care (11%). All Activity Professionals reported impact by COVID-19, as follows: 1) Cancelled activities: Group activities/gatherings; hosting outside entertainment; fewer volunteers, vendors, paid sources. 2) New restrictions: Number of people in elevators, rooms; no contact with residents. 3) Unexpected new tasks: More 1:1 meetings; video conferencing; additional phone calls with residents, staff, and families; ordering groceries online. Daily duties changed significantly with less help and limited availability of technology. 4) Effect: Fewer activities and new delivery models (online, TV). Concerns about potential negative effects on residents while trying to meet the creativity challenge: "This has caused lots of "out of the box" thinking for ways to engage residents and keep things upbeat despite the challenges." Results illustrate the breadth of challenges that staff are facing, some of which can be addressed by technology.

### SOCIAL DISTANCING WITH OLDER ADULTS: COMPARING PRE- AND POST-PANDEMIC PREFERENCES IN CHILDREN

Juliana Lapp,[1] Jenny Bauer,[1] Lea Scholz,[1] Sonja Steltmann,[1] Marit Lange,[1] Lena-Emilia Schenker,[1] and Jennifer Bellingtier,[2] *1. Friedrich Schiller University Jena, Jena, Germany, 2. Friedrich Schiller University Jena, Jena, Thuringen, Germany*

The Covid-19 pandemic has made age more salient, and the media has included numerous ageist messages (Bronwen, 2020), included messages aimed at children (e.g., "stay home to protect grandma and grandpa!"). When the pandemic reached Germany in March, we halted data collection on a project assessing ageism in children ages 4 to 8. In July, the situation had improved and testing resumed following hygiene protocols. We report findings from a simulated-behavioral measure where 45 children were asked to plan a party. One task involved asking the children to place pictures of ten party guests, plus themselves, around two party tables. We then assessed how many seats away the younger and older adult guests were placed, on average, from the child (i.e., social distance). Although, we anticipated that the pandemic might lead children to further distance themselves from older adults, our results, thus far, indicate similarity between pre- and post-pandemic preferences. At both occasions older adult guests were seated, on average, one seat further away from the child then younger adult guests. The guest chosen to sit closest to the child was younger on 88% of occasions, whereas the guest chosen to sit farthest away from the child was older on 64% of occasions. Preference for younger adults was confirmed in a second task where children selected teammates for a game. On average, children's teams consisted of 70% younger guests versus 30% older guests. Findings indicate a social preference for younger, versus older, adults in children irrespective of the pandemic.

### SOCIAL ISOLATION, LONELINESS AND WELL-BEING IN THE COVID-19 CRISIS: A LOOK AT NURSING HOME RESIDENTS IN LUXEMBOURG

Isabelle Albert,[1] Martine Hoffmann,[2] Anna Kornadt,[3] Elke Murdock,[1] and Josepha Nell,[1] *1. University of Luxembourg, Esch-sur-Alzette, Luxembourg, 2. RBS Center fier Altersfroen, Itzig, Luxembourg, 3. University of Luxembourg, Esch-sur-Alzette, Diekirch, Luxembourg*

During the COVID-19 crisis, older adults, in particular those with underlying health conditions, were at a special risk for severe illness and mortality, and efforts were made to shield them from exposure to the virus. While measures of physical distancing and reduction of in-person contacts were necessary to prevent contraction, they hit residents of care settings particularly hard since visits from family and friends were banned and the risk for loneliness and social isolation increased. In the present study, we therefore gave the voice to nursing home residents and focused on their perceived loneliness and subjective well-being during the crisis. We were both interested in difficulties but also in personal resources and resilience factors that might protect older adults from negative mental health outcomes and help to maintain subjective well-being. A sample of N = 76 residents in care homes in the Grand Duchy of Luxembourg were interviewed by use of a standardized questionnaire during July and August 2020. Participants reported on their loneliness and life satisfaction during the crisis, on their self-regulatory strategies as well as on personal and social resources (e.g. self-efficacy, generativity, social support). Data will be analyzed by use of regression analysis to predict loneliness and well-being by risk and protective factors. Results will be discussed applying a life-span developmental and systemic perspective to understand the mutual interplay of individual, social and institutional resources to mitigate negative side effects of protective measures on care home residents.

### SUCCESSFUL TRANSITIONS TO REMOTE ASSESSMENTS AND TECHNOLOGY INSTALLATIONS DUE TO COVID-19: THE I-CONECT PROJECT

Khoa Nguyen,[1] Mattie McDonald,[1] Colton Scavone,[1] Nora Mattek,[1] Jeffrey Kaye,[2] and Hiroko Dodge,[3] *1. Oregon Health & Science University, Portland, Oregon, United States, 2. Layton Alzheimer's Disease Center, Portland, Oregon, United States, 3. Pregon Health & Science University, Portland, Oregon, United States*

I-CONECT is a randomized controlled clinical trial to examine the impact of social interaction delivered via video-chat on cognitive function (clinicaltrials.gov number: NCT02871921, project website: www.I-CONECT.org ). We aimed to enroll 320 community-dwelling socially isolated older adults (age >=75 years). The recruitment of participants has started in 2018 and was ongoing when COVID-19 pandemic began. Video chat and telephone-based social interaction interventions did not change during COVID-19. However, new recruitment and cognitive assessments, which require in-person contact and deployment and retrieval of video chat devices in participant homes, were suspended due to the nature of our study population (i.e., older age, higher likelihood of comorbidities). Recently we were able to successfully switch to complete remote assessments including 1) telephone-based cognitive assessments using T-COG (Telephone Cognitive Assessment battery), and 2) contactless delivery of our study devices (Chrome books and electronic pill boxes) for subject self-installation. Our creative approach to self-installations includes color coded pictures and an easy-to-follow installation manual, accompanied by remote instruction and support via telephone. This poster introduces our remote assessment and installation protocol and participant and technical support team feedback regarding this new contactless protocol. This presentation provides useful guidance for future studies considering completely remote assessment and telemedicine approaches.

### SUPPORTING OLDER ADULTS AND CAREGIVERS THROUGH INTEGRATIVE SERVICE LEARNING DURING COVID

Keith Chan,[1] Sarah LaFave,[2] Maggie Ratnayake,[3] Christina Marsack-Topolewski,[4] Jillian Graves,[4] Diane Fenske,[4] and Shay Lukas,[5] *1. Hunter College, City University of New York, New York, New York, United States, 2. Lori's Hands, Inc, Newark, Delaware, United States, 3. Lori's Hands, Inc., Newark, Delaware, United States, 4. Eastern Michigan University, Ypsilanti, Michigan, United States, 5. University of Delaware, Newark, Delaware, United States*

There is a growing population of older adults who are living longer and acquiring chronic illness and disabilities, making it difficult for them to complete everyday activities and age in place. More than 2 million of these older adults are homebound and 5 million need help leaving their homes. They experience social isolation, food insecurity, and lack of connection to community resources which has intensified since the pandemic. Integrative service learning models can provide home-based support to older adults while offering valuable, hands-on learning experiences for students. This study examined findings for a community-based program which trained university students to provide practical home-based support for older adults and their caregivers. Data was collected for 109 older adults who were connected with student trainees. Students provided services with groceries, companionship, and help accessing needed services. Findings from t-test results using the UCLA Loneliness Scale indicated that older adults reported less loneliness after engagement with students (mean difference = 6.15, t = 3.14, df = 82, p < 0.01). Qualitative process data suggested that older adults benefited from services and a connection to their assigned students prior to and during the pandemic. Student trainees reported that the experience enriched their learning and reaffirmed their commitment to working with older adults. Community-based service learning can address home-based needs of older adults and their caregivers and enhance learning opportunities for students. Policies and practice can support a pipeline of geriatric health professionals through innovative service learning models to benefit older adults, caregivers and students.

### THE FIRST COVID-19 STAY-AT-HOME RESTRICTIONS: AN INTERGENERATIONAL COMPARISON OF THE IMPACTS

Anne Glass, and Lauretta Lawlor, *University of North Carolina Wilmington, Wilmington, North Carolina, United States*

"Social distancing" and stay-at-home orders were enacted in many states in response to the spread of COVID-19. We sought to understand intergenerational differences in the impact of the initial COVID-19 restrictions on interactions, loneliness, and stress. Data was collected via online survey from individuals ages 18 and above during the period April 7-May 8, 2020. The predominantly female, White, and well-educated sample (n = 962) included 245 younger adults (YAs) ages 18-34, 374 middle-aged adults (MAs) ages 35-64, and 343 older adults (OAs) ages 65+, with overall mean age 51.67 (SD=20.257; range 18-96). Before the restrictions, 41% of these OAs infrequently/never interacted face-to-face with children, increasing to 74% after restrictions. Three quarters (77%) of YAs reported seeing OAs less often, but 42% reported increasing their connections with OAs via technology. About a third of MAs (35%) and OAs (36%) were lonely, compared to 48% of YAs (p = .003), and a higher percentage of YAs (57%) reported being "more lonely" now, compared to MAs (36%) and OAs (41%). OAs reported the least stress; 42% reported low/very low levels of stress compared to YAs (9%) and MAs (20%). All generations most often identified "being able to go places" as the thing they missed most, but it increased significantly with age (p < .001), from YAs (32%), MAs (37%), to OAs (44%). More YAs (20%) than OAs (7%) reported missing "structure to their day." Results of these intergenerational comparisons suggest the resilience of older adults is helping them during the current pandemic.

### THE IMPACT OF COVID-19 ON INFORMAL CAREGIVERS' HEALTH BEHAVIORS

Mary Greaney,[1] Zachary Kunicki,[2] Megan Drohan,[1] and Steven Cohen,[1] *1. University of Rhode Island, Kingston, Rhode Island, United States, 2. Brown University, Providence, Rhode Island, United States*

The population of older adults aged 65+ in the US is projected to increase from 15% to 21% in the next 30 years. Aging in place provides cost-savings and familiarity to the older adult, but often requires informal caregivers. Informal caregivers, individuals who provide unpaid care of assistance to family members and friends may have been uniquely impacted by the COVID-19 pandemic and shelter-at-home orders. Research is needed to examine how the pandemic impacted caregivers' caregiving responsibilities and health behaviors (e.g., physical activity, sedentary time, fruit and vegetable intake, snacking, etc.) as this information will be invaluable to determine if health promotion interventions are needed for informal caregivers. Self-reported data were gathered from informal caregivers providing care to someone aged 50+ (n=835) through Amazon's Mechanical Turk. Respondents reported their current and pre-pandemic health behaviors and demographics. Chi-square tests were used to examine bivariate associations between pandemic time (pre vs. post) and each examined behavior. The analysis identified some positive health behavior changes due to the pandemic: caregivers felt since the pandemic they ate more fruits and vegetables (p < .001), walked more, exercised more (p < .001), increased amounts of sleep (p < .001), and higher sleep quality (p < .001). However, respondents also had more screen time (p < .001) and sedentary time (p < .001). Future planned analyses will focus on examining whether these changes were consistent across all sociodemographic subgroups of caregivers and whether they persist after the pandemic recedes.

### THE IMPACT OF COVID-19 SAFETY RECOMMENDATIONS ON ADULTS AGE 60 AND OLDER: A QUALITATIVE STUDY

Kerstin Emerson,[1] Deborah Kim,[1] George Mois,[2] and Jenay Beer,[3] *1. University of Georgia, Athens, Georgia, United States, 2. University of Georgia, Lawrenceville, Georgia, United States, 3. Institute of Gerontology, Athens, Georgia, United States*

In March 2020, the United States Centers for Disease Control (CDC) began recommending social distancing and sheltering in place, in particular for older adults. This resulted in many older adults staying at home for long periods of time in relative isolation. Because there is little prior evidence of the emotional impact that this has on older adults, we conducted an exploratory qualitative study on how older adults felt during the first three to five weeks of the CDC recommendations. We fielded a web-based cross-sectional survey. Our analytic sample consisted of 673 respondents aged 60 and older who respondent to the prompt: "How are you feeling during this time of social distancing?". We used a thematic bottom-up qualitative analysis, via MAXQDA, to analyze segments into general affect codes and detailed emotion subcodes, as well as coping mechanisms. Results showed that while many older adults reported neutral (9%) or positive (9%) affect, a larger proportion reported negative affect (42%) or reported mixed affect (35%). The most common negative emotions mentioned were anxiety and loneliness/boredom, while the most common positive emotions mentioned were optimism and feeling grateful. The most common coping mechanisms reported by participants included making life adjustments, keeping busy, prayer/spirituality, and mediation/mindfulness. This study provides an initial understanding into how older adults experienced and coped with the first stages of restricting social interactions. If social distancing continues to be a recommended disease-containment strategy, information about how older adults are coping can be critical for public health interventions. Implications will be discussed.

### THE IMPACT OF THE COVID-19 PANDEMIC ON VILLAGES: RESULTS OF A NATIONAL SURVEY

Natalie Galucia,[1] Nancy Morrow-Howell,[1] Peter Sun,[2] Tanner Meyer,[3] and Ying Li,[2] *1. Washington University in St. Louis, St. Louis, Missouri, United States, 2. Washington University in St. Louis, Saint Louis, Missouri, United States, 3. Washington University, St. Louis, Missouri, United States*

This study, launched in June 2020, documents the impact of the COVID-19 pandemic on Villages nationally. Villages are non-profit, membership-based organizations that provide support from volunteers and social connections to enable aging in place. We distributed on-line surveys to the leaders of the 287 Villages in the national network to capture the effects of the pandemic on organizational operations, membership recruitment, service provision, and member well-being. A 40% response rate (n=116) was obtained. A majority of Villages reported that the pandemic greatly affected the organization, with the top concerns being: 1) membership recruitment, 2) the health and well-being of members and volunteers and 3) connecting with their members outside of normal in-person events. Over half of the respondents reported that the mental health of members had declined; and there were high levels of disruption to usual health care. New member recruitment efforts were thwarted and most Villages lost revenue. About 70% offered virtual programming but, in general, participation in these on-line events dropped. From the survey respondents' perspective, the value of the Village to members and their family increased (48%) or remained the same (22%). New opportunities emerged that may be continued post-pandemic: new meal and medicine delivery volunteer services, more on-line communication and telephone reassurance, and new family and community connections. Findings indicate a wide range of experiences during the pandemic, with variation stemming from age of the Village and size of membership. The study informs the sustainability and growth efforts of Villages during and after the pandemic.

### THE SOCIAL WORKER IN THE NURSING HOME: A SYSTEMATIC REVIEW OF THE LITERATURE FROM 2010 TO 2020

Vivian Miller,[1] Tyrone Hamler,[2] Susanny Beltran,[3] and Jacquelyn Burns,[1] *1. Bowling Green State University, Bowling Green, Ohio, United States, 2. Case Western Reserve University, Cleveland, Ohio, United States, 3. University of Central FLorida, Orlando, Florida, United States*

Social workers have an integral role in health care and a long-standing history in nursing homes. The novel pandemic, COVID-19, has disproportionately adversely affected nursing home residents, bringing to the fore the importance of practice in this setting. However, the role of the nursing home social worker is often ambiguous. The objective of this study is to (1) identify existing research studies that discuss the role of social work and nursing facilities, (2) synthesize findings to determine what is most often reported in the literature, (3) present recommendations for practice and implications for research and policy change. This rapid review used the PICO framework and PRISMA guidelines to systematically search for articles published in English between 2010 and 2020 across 11 databases. A final sample of 23 articles met inclusion criteria. Relevant details were extracted from these articles, and the following 3 categories emerged as broadly describing the literature: (1) elements of social work practice in NHs, (2) social worker responsibilities, (3) policy impacts on practice. Findings highlight that the literature on NH social workers is limited and outdated, despite the 2016 federal rule requiring increased consideration for resident quality of life, quality of care, and behavioral health, all features of social work practice. The nursing home social work role warrants greater attention, with an emphasis on how to support residents during the current pandemic to ensure the development and implementation of person-centered care. The literature points to research priorities including involving families and residents in data collection efforts.

### UNDERSTANDING CONTRIBUTORS TO QUALITY OF CARE IN LONG-TERM CARE AND CHANGES UNDER COVID-19

Sara Luck, and Katie Aubrecht, *St. Francis Xavier University, Antigonish, Nova Scotia, Canada*

Nursing home facilities are responsible for providing care for some of the most vulnerable groups in society, including the elderly and those with chronic medical conditions. In times of crisis, such as COVID-19 or other pandemics, the delivery of 'regular' care can be significantly impacted. In relation to COVID-19, there is an insufficient supply of personal protective equipment (PPE) to care for residents, as PPE not only protects care staff but also residents. Nursing homes across the United States and Canada have also taken protective measures to maximize the safety of residents by banning visitors, stopping all group activities, and increasing infection control measures. This presentation shares a research protocol and early findings from a study investigating the impact of COVID-19 on quality of care in residential long-term care (LTC) in the Canadian province of New Brunswick. This study used a qualitative description design to explore what contributes to quality of care for residents living in long-term care, and how this could change in times of crisis from the perspective of long-term care staff. Interviews were conducted with a broad range of staff at one LTC home. A semi-structured interview guide and approach to thematic analysis was framed by a social ecological perspective, making it possible to include the individual and proximal social influences as well as community, organizations, and policy influencers. Insights gained will improve the understanding of quality of care, as well as potential barriers and facilitators to care during times of crisis.

### UNDERSTANDING OLDER ADULTS' TECHNOLOGY USE DURING COVID-19 TO SUPPORT HEALTH AND CONNECTION

Shani Bardach,[1] Elizabeth Rhodus,[2] Kelly Parsons,[1] and Allison Gibson,[3] *1. University of Kentucky, Lexington, Kentucky, United States, 2. University of Kentucky, Richmond, Kentucky, United States, 3. University of Kentucky, College of Social Work, Lexington, Kentucky, United States*

The COVID-19 crisis has disrupted everyday life as individuals, especially older adults, are encouraged to distance to reduce virus transmission. Remote strategies for connection may ameliorate risks for social isolation, however, older adults' adoption of such strategies remains unknown. This study involved in-depth semi-structured interviews with 30 older adults (ages 68-94) regarding adaptations to the call for social distancing and use of technology. From a socioemotional selectivity theory lens, findings demonstrate respondents' positive views of technology, expressing value in the context of supporting emotionally important relationships and goals, e.g. social connection and entertainment, rather than knowledge acquisition. Surprisingly, most respondents were uninterested in technology training. This may be consistent with diminished future time perspectives; several participants referenced their advanced age to explain disinterest in learning new technology and most seemed to have developed a level of technology use that met their needs. Technology resistance was consistent with a focus on emotionally meaningful goals; several respondents conveyed disinterest in social media due to perceived intrusiveness and others indicated a lack of interest in telemedicine and health portals due to the perceived loss in valuable human contact. Personal challenges with social distancing generally reflected limitations in safe human interactions, e.g. a desire for hugs or missing the spontaneity of social get-togethers, which remained emotionally meaningful but outside the scope of how technology could support wellbeing. These results suggest that focusing on older adults' emotional goals, and highlighting how technology can support their achievement, may support meaningful use to promote health and connection.

### UNDERSTANDING THE RELATIONSHIP BETWEEN AGE AND INFORMATION-SEEKING IN THE CONTEXT OF COVID-19

Li Chu,[1] Theresa Pauly,[2] Elizabeth Zambrano Garza,[3] Denis Gerstorf,[4] and Christiane Hoppmann,[3] *1. The Chinese University of Hong Kong, Shatin, New Territories, China, 2. University of Zurich, Vancouver, British Columbia, Canada, 3. University of British Columbia, Vancouver, British Columbia, Canada, 4. Humboldt University Berlin, Berlin, Berlin, Germany*

Socioemotional selectivity theory proposes that older adults engage in less information-seeking than younger adults as future time perspective becomes more limited and expansive goals are prioritized less. However, gathering information is crucial in emergencies like the COVID-19 pandemic, especially for older adults, who are particularly vulnerable to the virus. This study aims to better understand the association between age and information-seeking patterns during the current pandemic. Two hundred and sixty-six participants (age range = 18 – 84, Mage = 38.86, female = 77.06%, received postsecondary education = 83.08%, born in Canada = 73.68%) completed an online study between May and August 2020. We found that older age was associated with more information-seeking time ($b = .45$, $SE = .16$, $p < .001$). We then investigated whether perceived worries of getting COVID-19 might provide insights into this association. Findings point to a partial mediation with a significant direct effect ($b = .37$, $SE = .16$, $p = .02$, 95% bootstrap CI=[.07, .68]), a marginally significant indirect effect ($b = .08$, $SE = .04$, $p = .06$, 95% bootstrap CI=[-.003, .18]) and a significant total effect ($b = .46$, $SE = .16$, $p < .001$, 95% bootstrap CI=[.14, .77]). That is, older adults engaged in more information-seeking than younger adults in contexts in which information-seeking was personally relevant as indicated by perceived worries. These findings shed light on key correlates of information-seeking in older adulthood and highlight the importance for government and health organizations to make suitable information accessible for older adults.

### UNSUNG HEROES: CAREGIVER EXPERIENCES DURING COVID 19

Aaron Mata,[1] Melody Mickens,[2] Kara Dassel,[3] and Rebecca Utz,[3] *1. University of Utah, Layton, Utah, United States, 2. University of Utah, Richmond, Virginia, United States, 3. University of Utah, Salt Lake City, Utah, United States*

Objectives: COVID-19 has spared no age group but is proving to be most dangerous to the elderly and individuals with more vulnerable immune systems. COVID-19 represents a unique challenge to caregivers above and beyond the ordinary stresses they experience and many informal caregivers currently providing care have not planned for the pandemic and its effect on access to health care services, respite resources, and support groups, etc. The purpose of our study is to assess COVID-19's impact on the mental health and wellness of informal caregivers. Method: An interdisciplinary collaborative of caregiving scholars created a 47-item web-based anonymous survey with quantitative and qualitative questions to assess caregiving experiences during COVID-19. Participants were recruited through convenience sampling using social media and flyers distributed by local/national organizations. Results: Participants (N=96) reported an average age of 50 years old and were predominately adult children caring for parents (45%) with neurological (40%) and chronic medical conditions (25%). Only 4% cared for individuals diagnosed with COVID 19. Nearly all reported significant changes in caregiving that included increased time spent caregiving for care recipients and other family members, greater stress and worry, fewer breaks for self-care, increased isolation, and decreased access to social/emotional supports and respite services. Discussion: Unplanned changes to caregiving during COVID 19 have created an acute need for research, clinical care guidelines, and policy promoting virtual respite care, social support for caregivers, and more readily available community based resources that promote caregivers' physical and emotional wellbeing without requiring them to leave their homes.

### VIEWS ON AGING AND WELL-BEING IN THE COVID CRISIS – A LONGITUDINAL STUDY IN LUXEMBOURG

Anna Kornadt,[1] Martine Hoffmann,[2] Elke Murdock,[3] Josepha Nell,[3] and Isabelle Albert,[3] *1. University of Luxembourg, Esch-sur-Alzette, Diekirch, Luxembourg, 2. RBS Center fier Altersfroen, Itzig, Luxembourg, 3. University of Luxembourg, Esch-sur-Alzette, Luxembourg*

During the Covid-Crisis, stereotypes of older adults as helpless and vulnerable were spread, and intergenerational conflict was stirred more or less openly. We thus focused on perceived ageism during the crisis and its effects on well-being and health of older adults. Since views on aging are multifaceted and can be both, risk and resource for individual development, we assessed people's self-perceptions of aging (SPA) as social loss, continued growth and physical decline and subjective age (SA). We hypothesized that people with SPA of social loss and physical decline would be more susceptible to negative effects of perceived ageism, whereas those with SPA of continued growth and younger SA would be less affected. NT1 = 611 community-dwelling adults aged 60 – 98 (Mage = 69.92 years) were recruited in June 2020 online and via phone in Luxembourg. In September 2020, participants will be contacted again for a follow-up. Analyses with cross-sectional data show that participants who felt more discriminated reported lower life satisfaction after the onset of the crisis ($r = -.35$) and worse subjective health ($r = -.14$). SPA of social loss and higher SA increased the negative effect of ageism on well-being (beta = -.57) and subjective health (beta = -.53), respectively. Our results point to mid- and long-term consequences of age discriminatory and stereotype-based crisis communication for the well-being of older adults and the importance of individual SPA in critical situations.

### VIRTUAL CONNECTION BETWEEN OLDER PEOPLE WITH DEMENTIA AND FAMILIES DURING COVID-19 PANDEMIC

Lillian Hung, *University of British Columbia, Vancouver, British Columbia, Canada*

People staying in hospitals need more support to cope with the lockdown and visitor restriction during the COVID-19 pandemic, especially for older people with cognitive or physical impairment. Everyday technology such as a touchscreen tablet has great potential to support person-centred care. We aimed to support the adoption of tablets for hospitalized people with dementia to connect with families and friends. A patient-oriented research approach was employed to co-produce the toolkit. We are a transdisciplinary team, including a medical student, physicians, nurses, patients, and family partners. We facilitated staff focus groups (n=3), and conducted stakeholders' interviews (n=4) to gain a more comprehensive understanding of users' needs. The sample included ten patients, ten family members, 40 staff members, nurses, care workers, physicians, and unit clerks (n=40). The Consolidated Framework for Implementation Research (CFIR) guided the research design and qualitative analysis. A toolkit was developed based on participants' perspectives on what needs to be in place to support successful adoption. We developed a mobile tablet with one mechanical arm and one leg on wheels. Participants reported impacts: (a) it puts a smile on the patient's face, (b) it alleviates anxiety and worries on both sides, and (c) it reduces responsive behaviors. The conceptual framework CFIR provides helpful guidance in identifying barriers to implementation. Working with users including patient and family partners to explore possible solutions was key to our success. Future research should engage patient and family partners to seek proactive strategies to address obstacles to advance the science of technology implementation.

### VOICES FROM THE FRONTLINES IN LONGTERM CARE DURING COVID-19: NARRATIVES OF DIRECT CARE WORKERS

Christin Wolf, Andrea Freidus, and Dena Shenk, *University of North Carolina at Charlotte, Charlotte, North Carolina, United States*

Our study draws from the narratives of 30 staff caring for residents in congregate care communities in central North Carolina from June-September 2020. It is part of phase 2 of an on-going 3-phase rapid qualitative appraisal of workers providing longterm care to older adults with the purpose of disseminating findings to key stakeholders to inform policy, programming, and funding decisions. The 3-phase project involves semi-structured interviews with 60+ participants that were videorecorded using a web-based platform. We report on the emotional and visceral experiences of these direct care workers providing care during the pandemic. We organize the data into four affect categories: fear/anxiety, sadness/grief, anger/frustration, and trauma/stress. The 30 participants include nurses, activities staff, med techs, CNAs, housekeepers, dining staff, chaplains and administrators at nursing homes, assisted living communities, memory care units and continuing care retirement communities. We amplify the voices of these formal caregivers in order to demonstrate how their sensorial and emotive experiences can speak to the human suffering they bore witness to, the underlying ageism that permeates our culture, and the social hierarchy that devalues their labor and their worth as they serve on the frontlines during this unprecedented global pandemic.

### WORKING CONDITIONS SUPPORTING PERSON-CENTERED CARE OF PERSONS LIVING WITH DEMENTIA IN LONG-TERM CARE HOMES

Marie Savundranayagam,[1] Susan Docherty-Skippen,[2] and Shalane Basque,[1] *1. Western University, London, Ontario, Canada, 2. Sam Katz Community Health and Aging Research Unit, London, Ontario, Canada*

The COVID-19 pandemic has underscored the importance of person-centered dementia care and working conditions that support such care in long-term care (LTC) home settings. Personal support workers (PSWs), known also as certified nursing assistants, provide the most direct formal care for persons living with dementia. However, little is known about the working conditions that enable person-centered care. Accordingly, the purpose of this study was to examine the working conditions and the impact of those conditions on PSWs in LTC homes. PSWs (N=39) employed at one of five LTC homes in southwestern Ontario, Canada participated in a series of one-hour focus groups before, during, and after Be-EPIC, a person-centred communication training program for formal caregivers of persons living with dementia. Using an interpretive description investigative framework, textual data from focus group conversation transcripts were open-coded into categories. Overarching themes were interpreted inductively. Study credibility was enhanced through investigator triangulation. Three themes emerged related to working conditions of PSWs: dementia care is complex, lack of trained staff to provide person-centered dementia care, and residents' families are not situated in the residents' care circle. Four themes emerged related to the impact of current working conditions of PSWs: occupational burnout, poor resident care, frustrated and disengaged families, and PSWs leave their role. The findings offer opportunities for employers to ameliorate working conditions to support person-centered care. We conclude with specific workplace recommendations that respond to the complexity of dementia care and the associated occupational stresses PSWs experience in the current LTC environment.

### NATIONAL EARLY WARNING SCORES AND COVID-19 DEATHS IN CARE HOMES: AN ECOLOGICAL TIME SERIES STUDY

Daniel Stow,[1] Robert Barker,[2] Fiona Matthews,[3] and Barbara Hanratty,[3] *1. Institute of Health and Society, Newcastle upon Tyne, United Kingdom, 2. Newcastle University, Newcastle upon Tyne, United Kingdom, 3. Newcastle University, Newcastle upon Tyne, England, United Kingdom*

Tracking COVID-19 infections in the care home population has been challenging, because of the limited availability of testing and varied disease presentation. We consider whether National Early Warning Scores (NEWS/NEWS2) could contribute to COVID-19 surveillance in care homes. We analysed NEWS measurements from care homes in England (December 2019 to May 2020). We estimated pre-COVID (baseline) levels for NEWS and NEWS components using 80th and 20th centile scores for measurements before March 2020. We used time-series to compare the proportion of above-baseline NEWS to area-matched reports of registered deaths in care home residents from the Office for National Statistics We analysed 29,656 anonymised NEWS from 6,464 people in 480 care home units across 46 local authority areas. From March 23rd to May 20th, there were 5,753 deaths (1,532 involving COVID-19, 4,221 other causes) in corresponding geographical areas. A rise in the proportion of above-baseline NEWS was observed from

March 16th 2020. The proportion of above-baseline oxygen saturation, respiratory rate and temperature measurements also increased approximately two weeks before peaks in deaths. We conclude that NEWS could contribute to disease surveillance in care homes during the COVID-19 pandemic. Oxygen saturation, respiratory rate and temperature could be prioritised as they appear to signal rise in mortality almost as well as total NEWS. This study reinforces the need to collate data from care homes, to monitor and protect residents' health. Further work using individual level outcome data is needed to evaluate the role of NEWS in the early detection of resident illness.

### SESSION 10550 (SYMPOSIUM)

### COVID-19 PANDEMIC: BEHAVIORAL AND SOCIAL SCIENCES SECTION

Chair: Tamara Baker

### AAA RESEARCH METHODOLOGY I: OVERCOMING LINGUISTIC AND CULTURAL BARRIERS IN AGING RESEARCH AMONG ASIANS: PRESENTER DISCUSSION

MacKenzie Hughes,[1] Clara Coblenz,[1] Emily Smith,[2] Shevaun Neupert,[2] and Ann Pearman,[1] *1. Georgia Institute of Technology, Atlanta, Georgia, United States, 2. North Carolina State University, Raleigh, North Carolina, United States*

Slowing the spread of COVID-19 depends on public adherence to precautionary actions, such as wearing masks. The Health Belief Model (Rosenstock, 1974) suggests the likelihood of using precautionary measures depends on perceived susceptibility, the severity of a disease, and whether effective measures can be taken to reduce the perceived threat of a disease. This daily diary study focused on identifying predictors of daily precautionary behavior in older persons. Between April 1 and June 26, 2020, 261 adults ages 55-79 (M = 64.29, SD = 5.20) completed up to 30 consecutive days of online diaries. We examined whether perceived risk, COVID-19 knowledge, fake news beliefs, information seeking, disruption to routine, in-person interactions, and leaving the house predicted the number of daily precautions participants engaged in. Multilevel modeling was used to examine within-person fluctuations in precautions as well as change in precautions from one day to the next. People who reported higher education, scored higher on the COVID-19 knowledge quiz, had lower fake news beliefs, and perceived a higher risk of contracting COVID-19 endorsed more precautions. At the daily level, increases in the number of in-person interactions, leaving home, and disruption to daily routine were each associated with decreases in precautionary behaviors. Concurrent day and lagged models showed significant interactions between information seeking and perceived risk, suggesting increases in information seeking are related to increases in precautions for those who consider their risk to be low. Findings highlight potentially intervenable factors that influence older adults' daily decision making related to precautionary actions.

### AGE DIFFERENCES IN COVID-19 STRESS AND SOCIAL TIES DURING THE COVID-19 PANDEMIC: IMPLICATIONS FOR WELL-BEING

Kira Birditt,[1] Angela Turkelson,[1] Karen Fingerman,[2] Courtney Polenick,[1] and Akari Oya,[1] *1. University of Michigan, Ann Arbor, Michigan, United States, 2. The University of Texas at Austin, Austin, Texas, United States*

The experience of the COVID-19 (coronavirus) pandemic and its implications for well-being may vary widely by age group across the adult lifespan. The purpose of this study was to examine age differences in stress related to the pandemic and social ties, and whether those experiences are linked to well-being. Participants included a total of 645 adults (43% women) ages 18 to 97 (M = 50.8; SD = 17.7) from the May 2020 nationally representative Survey of Consumers. Participants reported the extent to which they felt stress related to the pandemic in the last month as well as social isolation, negative relationship quality, positive relationship quality, and frequent depression, anxiety and rumination in the past week. Results showed that older people reported less COVID-19 related stress, less social isolation, and lower negative relationship quality than younger people. Greater stress, social isolation, and negative relationship quality were associated with poorer well-being and greater social isolation, and negative quality ties exacerbated the effects of stress on well-being. Although many researchers have indicated that older adults may be more vulnerable to COVID-19 related stress and social isolation, this study indicates that young adults may be relatively more vulnerable. Because isolation and negative relationship quality appear to exacerbate the effects of stress, reducing social isolation and negative relations are potential targets for intervention.

### BEST PRACTICES FOR MEASURING THE SOCIAL, BEHAVIORAL, AND ECONOMIC IMPACT OF COVID 19 USING SECONDARY DATA

James McNally, and Kathryn Lavender, *University of Michigan, Ann Arbor, Michigan, United States*

This presentation reviews best practices for using data resources from the NACDA Program on Aging, its projects, and its collaborating partners for measuring the impact of epidemics. The report summarizes resources to identify measures of well-being, social connectedness, and other constructs to measure the social and behavioral effects of the COVID-19 epidemic on population health outcomes. The report suggests data resources to identify pre-crisis measures of social distancing, social networks, consumer confidence, unemployment, and the use of social media. The COVID-19 pandemic presents research challenges for how we measure governmental, community, and population responses to a crisis. COVID-19 represents a unique case because it is a new virus with a novel impact profile and a long latency period. We offer examples of variables and concepts found across multiple studies and multiple years that can provide baseline information on pre-COVID-19 social behaviors using studies managed and distributed by NACDA and ICPSR. This report also provides guidance on how to search for specific topics using the ICPSR generated search tools. Finally, we offer information on how to obtain these data for research purposes. Our hope is this information will be valuable in identifying key baseline measures of social behavior, health, and employment before the COVID-19 epidemic, and this information can allow us to evaluate challenges and recovery once the crisis has passed. Both NACDA and ICPSR remain available 24 hours a day, 365 days a year, to assist researchers in locating and obtaining data related to their research.

### DO PERCEPTIONS OF OWN AND SPOUSE SURVIVAL AFFECT ADVANCE CARE PLANNING?

Deborah Carr,[1] and Yifan Lou,[2] *1. Boston University, Boston, Massachusetts, United States, 2. Columbia University, New York, New York, United States*

COVID-19 has intensified the need for advance care planning (ACP), or formal preparations for end-of-life care, prior to the time such decisions are required. We propose that older adults' perceived chances of survival, and one's perceptions of whether they will outlive their spouse may be powerful motivators of ACP. Using data from the Wisconsin Longitudinal Study (WLS, n=4908, M age = 65), we examine the extent to which: (a) one's perceived 10- and 20-year survival and (b) projections of dying before, after, or at the same time as one's spouse affect three aspects of ACP (living will, durable power of attorney for health care designations (DPAHC), and discussions). Multivariate analyses are adjusted for health, demographics, socioeconomic characteristics, and death anxiety. In the full sample, women who perceived a high likelihood of 20-year survival were less likely (OR=.604, p < .05) whereas their male counterparts were more likely (OR = 1.4, p <.01) to name a DPAHC (relative to those who perceived a medium likelihood of survival). Among married persons only (n=3860), people who perceive that they will pre-decease their spouse are 1.5 times as likely to name their spouse as DPAHC (vs. no DPAHC), but are no more likely to name a different person to the role (relative to those who perceive that they and spouse will die at the same time). Practitioner and family conversations about older patients' projected survival and how it shapes decision making are especially important as COVID-19 may require rapid decisions about end-of-life treatments.

### INCREASED BLUE MOOD AND LONELINESS DURING COVID-19 PANDEMIC IN A VETERAN COHORT

Nora Mattek,[1] Rachel Wall,[2] Zachary Beattie,[1] Chao-Yi Wu,[1] Jeffrey Kaye,[3] Hiroko Dodge,[1] and Lisa Silbert,[1] *1. Oregon Health & Science University, Portland, Oregon, United States, 2. Portland Veterans Affairs Medical Center, Portland, Oregon, United States, 3. Layton Alzheimer's Disease Center, Portland, Oregon, United States*

Older Veterans are at especially high risk of depression and social isolation due to COVID-19 stay-at-home orders and necessary safety precautions. We aimed to objectively measure differences in mood reports before and after COVID-19 stay-at-home orders in rural older Veterans. Participants age > 62 were enrolled in the Collaborative Aging Research using Technology (CART) initiative, a NIH and VA HSRD funded multi-site study examining the feasibility of unobtrusive remote sensing and monitoring of physical, cognitive, and health-related activities. The VA CART site consists of Pacific Northwest Veteran volunteers and their cohabitants. Weekly online health forms including questions about blue mood and loneliness were collected January – July 2020. A COVID stay-at-home order was instituted March 13 2020. Generalized estimating equations (GEE) with logit link was used to investigate differences in mood reports pre- and post- stay-at-home orders. 100 older volunteers completed 2441 health reports (mean age 71.2 years, 41% female, 19% single). Thirty-five percent were urban, 34% large rural, and 31% small rural using rural-urban commuting area (RUCA) scores urban: 1-3; large rural 4-6; small rural 7-10. After adjusting for covariates, incidence of blue mood and loneliness reports were significantly higher after stay-at-home orders (OR=4.4, p<0.0001 and OR=7.2, p<0.0001 respectively). Results varied by rurality with large rural volunteers showing the largest increases. Real-world monitoring of weekly health reports may identify those at greatest risk for depression and social isolation in older Veterans and their cohabitants and is of particular relevance in rural settings, where access to specialty care is limited.

### SESSION 10560 (SYMPOSIUM)

### COVID-19 PANDEMIC: HEALTH SCIENCES SECTION

### AN EXPLORATION OF ONLINE EXERCISE PARTICIPATION OF OLDER MANITOBANS DURING THE COVID-19 PANDEMIC

Michelle Porter, Mikyung Lee, Ruth Barclay, Stephen Cornish, Nicole Dunn, Jacquie Ripat, Kathryn Sibley, and Sandra Webber, *University of Manitoba, Winnipeg, Manitoba, Canada*

During the COVID-19 pandemic, in-person exercise programs for older people were temporarily closed, and some were replaced with online exercise. We explored the online exercise experiences of older people in Manitoba, Canada, using an online survey. We recruited a convenience sample (≥ 65 years), primarily through community organizations, and 745 people (57.5% female) consented. About 38.2% reported participating in online exercise during the pandemic. Most used pre-recorded classes (80.4%), from their local community (79.7%), and YouTube was the most used platform (57.4%). Almost all (82.7%) found the classes had the right variety and intensity. Of those who had participated in online exercise, 67.0% said they would participate in an online exercise class outside of a pandemic time. Participants like the following aspects better about online exercise: no transportation arrangements, it doesn't matter what they wear, no travel time, and they like to exercise without others seeing them. However, they also miss being with and socializing with others, and they reported feeling unsafe when the instructor cannot see them. Of those who did not participate online, several reasons were given: they prefer to exercise with others in the same room, they prefer to exercise with an instructor directly present, no appropriate device, and their internet is not reliable. Many also provided examples of future circumstances when they might participate online, including: when the weather is not conducive to outdoor exercise, and if they can overcome technical issues. Lessons learned from this study can help those delivering online exercise in the future.

### PREDICTORS OF HOSPITALIZATION IN VETERANS WITH COVID 19 INFECTION

Jorge Ruiz, Sergio Ruiz, Marlena Fernandez, Victor Cevallos, Yaseen Jumani, Manasa Morisetti, Sahar Hasanain, and Michael Mintzer, *Miami VA Healthcare System, Miami, Florida, United States*

Frailty, a clinical syndrome characterized by vulnerability to stressors resulting from a loss of physiological reserve across multiple systems, is a common condition that affects older adults. In patients with COVID 19 infection, frailty may place older adults at higher risk for poor clinical outcomes including hospitalizations. The aim of this study was to determine independent variables associated with all-cause hospitalization in Veterans with COVID 19 infection. This was a cross-sectional study nested within a retrospective cohort study of Veterans who contracted COVID 19 as detected by PCR. A VA Frailty Index (VA-FI) was generated at baseline as a proportion of morbidity, function, sensory loss, cognition and mood and other variables from electronic health records. The VA-FI categorized Veterans into non-frail (FI<.21) and frail (FI≥.21). At the time or after the COVID 19 testing, hospitalization data was aggregated, and proposed variables were subjected to stepwise backward logistic regression. Likelihood ratios were obtained, and those with p<0.05 were identified as independent risk factors for hospitalization. A total of 2621 Veterans with COVID 19 infection were identified during the study period with a total of 650 hospitalizations. Mean age was 54.99 (SD=16.71) years, 59.80% White, 87.60% male, 25.10% frail. The independent factors associated with hospitalization upon backward logistic regression were age (OR=1.049, 95%CI:1.041-1.057), frailty (OR=2.350, 95%CI: 1.797-3.074), African American race (OR=1.467, 95%CI:1.188-1.812), schizophrenia (OR=0.401, 95%CI:0.235-0.686), and substance abuse (OR=0.602, 95%CI:0.476-0.762). In veterans with COVID 19 infection, age, frailty, race (African American), schizophrenia, and substance abuse were predictors of all-cause hospitalization.

### TECHNOLOGY ADOPTION AMONG SENIORS DURING COVID-19 PANDEMIC IMPACTS MENTAL HEALTH AND FEELINGS OF COMPANIONSHIP

Brittany Derynda,[1] Mary Goodyear,[2] Jade Kushner,[3] and Nicole Cook,[4] *1. Nova Southeastern University Dr. Kiran C Patel College of Osteopathic Medicine, Clearwater, Florida, United States, 2. Nova Southeastern University Dr. Kiran C Patel College of Osteopathic Medicine, Fort Lauderdale, Florida, United States, 3. College of Psychology, Fort Lauderdale, Florida, United States, 4. Nova Southeastern University, Davie, Florida, United States*

Social isolation and lack of companionship, exacerbated by COVID-19 "stay at home" orders, has been an ongoing concern among seniors in the US. Among other strategies, Lifelong Learning Institutes (LLIs) were created to support continuing education for older adults. These programs bring seniors together to encourage engagement through lectures, art and fitness classes in a common space. LLI in South Florida adapted to COVID-19 "stay at home" orders by moving all programming online in March 2020. In May 2020 LLI members, faculty and students designed a research study to understand the experience of LLI members with social isolation and companionship prior to, and during, "stay at home" orders. Responses included 127 members (mean age 75.5). Respondents reported significantly lower social isolation (p<.01) and lack of companionship (p<.01) as a result of "stay at home". Interestingly, social isolation had no significant explanatory variables. However, significant results (p<.05) showed that seniors who isolated alone were 6.7 times more likely to lack companionship compared to those who isolated with a friend or spouse; seniors who reported they are not tech savvy were 8.3 times more likely to lack companionship compared to those who reported they are tech savvy; and that for every additional day of poor mental health respondents had a 1.15 higher odds of lacking companionship. These results underscore the importance of technology adoption among seniors during times of social isolation and the positive impact this can have on companionship and mental health.

### SESSION 10570 (SYMPOSIUM)

### COVID-19 PANDEMIC: SOCIAL RESEARCH, POLICY, AND PRACTICE SECTION

### "WE ARE SCAPEGOATS": FRONT-LINE NURSING HOME STAFF EXPERIENCES DURING THE COVID-19 PANDEMIC

Elizabeth White,[1] Terrie Wetle,[2] Ann Reddy,[3] and Rosa Baier,[4] *1. Brown University School of Public Health, Providence, Rhode Island, United States, 2. Brown University, School of Public Health, Providence, Rhode Island, United States, 3. Brown University, Providence, Rhode Island, United States, 4. Center for Long-Term Care Quality & Innovation, Providence, Rhode Island, United States*

The COVID-19 pandemic is an unprecedented challenge for nursing homes, where staff have faced rapidly evolving circumstances to care for a vulnerable resident population. To document these healthcare professionals' experiences during the pandemic, we used social media and professional networks to disseminate an electronic survey with closed- and open-ended questions to a convenience sample of long-term care staff from May 11 through June 4, 2020. Four investigators identified themes from qualitative responses for 152 nursing home staff respondents from 32 states. Key themes included: constraints on personal protective equipment (PPE) and testing; burdensome regulations and guidance; concern for self, family, and residents; workforce burnout; organizational communication and teamwork; and public lack of recognition. Respondents described ongoing constraints on testing, and reliance on crisis standards for extended use and reuse of PPE. Administrators discussed implementing sometimes confusing or contradictory guidance from numerous agencies. Direct-care staff expressed fears of infecting themselves and their families, and expressed empathy and concern for their residents. They described burnout due to increased workloads and the emotional burden of caring for residents facing isolation, illness, and death. Respondents cited the presence or lack of organizational communication and teamwork as factors influencing their ability to work under challenging circumstances. They also described the demoralizing impact of negative media coverage of nursing homes, contrasting this with the heroic public recognition given to hospital staff. These challenges added significant burden to an already strained workforce and are likely to contribute to increased burnout, turnover, and staff shortages in the long-term.

### EARLY LEARNING FROM THE HEALTHY END OF LIFE PROJECT (HELP) OTTAWA IN THE CONTEXT OF COVID-19

Pamela Grassau,[1] Hayley Miloff,[2] Emily Davison,[1] Arne Stinchcombe,[2] David Kenneth Wright,[3] and Roanne Thomas,[3] *1. Carleton University, Ottawa, Ontario, Canada, 2. Brock University, St. Catharines, Ontario, Canada, 3. University of Ottawa, Ottawa, Ontario, Canada*

Healthy End of Life Project (HELP) Ottawa is a community-based participatory research initiative which is based in four community sites in Ottawa, Ontario (Canada) (2 community health centers, and 2 faith communities). Focused on the needs of people who are frail, living with advanced illness, and their caregivers, including the needs of people who are bereaved, HELP Ottawa strives to, 1) strengthen informal and community social networks, organizational cultures and linkages across local health and social care services; 2)create a community culture that supports people to build social care networks to be able to ask for and accept help, and, 3)mobilize and prepare community members to be confident and capable of offering and providing help to people in their communities. Unfolding within the context of COVID-19, each HELP Ottawa site has found ways to mobilize, adapt and respond to lockdowns, quarantines, increased isolation and altered needs and services. Drawing on 89 initial consultations, followed by 111 interviews and 16 focus group participants (n=164), qualitative findings speak to the heightened grief and fear experienced within each site during the COVID-19 pandemic, and the multiple costs of severed ‘essential’ links. Critically highlighted is the need to build and sustain social supports and connection through everyday and local means while also integrating technology and online communication. Further apparent are the critical questions that need to be asked about how compassionate communities, and communities at large, can prepare for and respond to current and future waves of COVID-19.

### HEALTH EQUITY IMPACTS OF COVID-19 POLICIES ON DEMENTIA-RELEVANT COMMUNITY SERVICES: A SGBA+ POLICY SCAN

Katie Aubrecht,[1] Rosanne Burke,[2] Jacqueline Gahagan,[3] Laura Dowling,[1] Christine Kelly Mary Jean Hande,[2] Susan Hardie,[4] and Janice Keefe,[5] *1. St. Francis Xavier University, Antigonish, Nova Scotia, Canada, 2. Mount Saint Vincent University, Halifax, Nova Scotia, Canada, 3. Dalhousie University, Halifax, Nova Scotia, Canada, 4. Evaince (Canadian Centre on Disability Studies), Ottawa, Nova Scotia, Canada, 5. Mount Saint Vincent University, Halifax, Alberta, Canada*

This presentation shares the methodology and early findings from a policy scan conducted to understand and assess the impact of COVID-19 policies on dementia care in the community for diverse populations in the province of Nova Scotia, Canada. The scan provided baseline information on: 1) Provincial legislative and regulatory policies related to dementia care in the community; 2) Orders and legislation enacted in response to COVID-19 that potentially impact those policies. Information was obtained from publicly accessible databases and government websites. Searches were also conducted using Google. 135 Acts were collected and reviewed. A specific aim of the scan was to generate knowledge about the impact of these layered policies in the context of a public health crisis from the perspective of local socially and geographically marginalized communities. A Sex and Gender Based Analysis Plus analytical approach was used to assess potential health equity impacts of COVID-19 policies on dementia care in the community. Information was organized using an adapted Health Equity Impact Assessment tool and Systems Health Equity Lens. Strengths and limitations of the approach and tools are discussed.

### VIRTUAL FUNERALS DURING COVID-19 AND BEYOND

Ivy Muturi, Shannon Freeman, and Davina Banner-Lukaris, *University of Northern British Columbia, Prince George, British Columbia, Canada*

Physical distancing measures and the restrictions on large group gatherings following the COVID-19 pandemic have left many to consider alternative approaches to commemorating the death of a loved one. Advancements in information technologies and the availability of affordable electronic devices have brought forth the ability to use virtual platforms to host funeral services for those unable to be with their loved ones. The aim of this study was to identify existing and potential online platforms for hosting a virtual funeral, explore the safety considerations of hosting a service during the pandemic and share the experiences of individuals who have previously hosted a virtual service. To conduct the research, an environmental scan was undertaken searching academic, grey literature and online websites. The results showed that there are currently several online platforms made specifically for virtual services and many free public platforms that can be used. Death services must ensure staff who are in direct contact with the deceased have proper Personal Protective Equipment and companies must adhere to regulations regarding group gatherings, screening for symptoms and physical distancing. Some individuals expressed having a positive experience, stating that the virtual service felt more intimate, while others expressed difficulty in navigating the technology, particularly the older adult attendees. Virtual funeral services may prove to be a practical and safer alternative during COVID-19 and may provide some comfort for those facing such challenging times. By examining existing platforms and their use, an opportunity exists to generate recommendations for additional supports, particularly for older adults.
